# Genetic diversity and selection of Tibetan sheep breeds revealed by whole-genome resequencing

**DOI:** 10.5713/ab.22.0432

**Published:** 2023-05-02

**Authors:** Dehong Tian, Buying Han, Xue Li, Dehui Liu, Baicheng Zhou, Chunchuan Zhao, Nan Zhang, Lei Wang, Quanbang Pei, Kai Zhao

**Affiliations:** 1 Key Laboratory of Adaptation and Evolution of Plateau Biota, Qinghai Provincial Key Laboratory of Animal Ecological Genomics, Northwest Institute of Plateau Biology, Chinese Academy of Sciences, Xining 810008, Qinghai, China; 2 Graduate School, University of Chinese Academy of Sciences, Beijing 100049, China; 3 Key Laboratory of Adaptation and Evolution of Plateau Biota, Northwest Institute of Plateau Biology, Chinese Academy of Sciences, Xining 810001, China; 4 General Station of Animal Husbandry of Qinghai Province, Xining 810001, Qinghai, China; 5 Qinghai Conservation and Utilization Center of Livestock and Poultry Genetic Resources, Xining 810000, Qinghai, China; 6 Qinghai Sheep Breeding and Promotion Service Center, Gangcha 812300, Qinghai, China

**Keywords:** Haplotypes, Hypoxic Adaptability, Indigenous Breeds, Production, Selective Sweep

## Abstract

**Objective:**

This study aimed to elucidate the underlying gene regions responsible for productive, phenotypic or adaptive traits in different ecological types of Tibetan sheep and the discovery of important genes encoding valuable traits.

**Methods:**

We used whole-genome resequencing to explore the genetic relationships, phylogenetic tree, and population genetic structure analysis. In addition, we identified 28 representative Tibetan sheep single-nucleotide polymorphisms (SNPs) and genomic selective sweep regions with different traits in Tibetan sheep by fixation index (Fst) and the nucleotide diversity (θπ) ratio.

**Results:**

The genetic relationships analysis showed that each breed partitioned into its own clades and had close genetic relationships. We also identified many potential breed-specific selective sweep regions, including genes associated with hypoxic adaptability (*MTOR*, *TRHDE*, *PDK1*, *PTPN9*, *TMTC2*, *SOX9*, *EPAS1*, *PDGFD*, *SOCS3*, *TGFBR3*), coat color (*MITF*, *MC1R*, *ERCC2*, *TCF25*, *ITCH*, *TYR*, *RALY*, *KIT*), wool traits (*COL4A2*, *ERC2*, *NOTCH2*, *ROCK1*, *FGF5*, *SOX9*), and horn phenotypes (*RXFP2*). In particular, a horn-related gene, *RXFP2*, showed the four most significantly associated SNP loci (g. 29481646 A>G, g. 29469024 T>C, g. 29462010 C>T, g. 29461968 C>T) and haplotypes.

**Conclusion:**

This finding demonstrates the potential for genetic markers in future molecular breeding programs to improve selection for horn phenotypes. The results will facilitate the understanding of the genetic basis of production and adaptive unique traits in Chinese indigenous Tibetan sheep taxa and offer a reference for the molecular breeding of Tibetan sheep.

## INTRODUCTION

Tibetan sheep are one of the three coarse wool sheep breeds in China and are mainly distributed across Qinghai, Tibet, Gansu Sichuan provinces and their adjacent high altitude cold areas. It has the unique characteristics of cold zone breed resources in the Qinghai-Tibet Plateau and is the dominant breed and valuable gene pool in the Qinghai-Tibet Plateau. Previous investigations have indicated that Tibetan sheep initially stepwise spread onto the Qinghai–Tibetan Plateau from its northeastern part 3,100 years ago following the expansions of the Di-Qiang people and then spread further from the southwest to the center 1,300 years ago [1,2].

Since then, Tibetan sheep have been raised to provide fur and meat products for nomads, playing an important role in agriculture, economics, culture and even religion [3] as well as being crucial to long-term human settlement at high altitudes. The spread of Tibetan sheep on the Qinghai-Tibet Plateau represents an important event of human occupation of the plateau in the late Holocene [1]. Tibetan sheep mainly live in the region of the Qinghai-Tibet Plateau with a nomadic culture. Due to natural geographical isolation and the rare invasion of exotic species, Tibetan sheep have rich genetic resources and particular local characteristics, with 16 indigenous populations of approximately 2.3 million distributed over 2.5 million km^2^ on the Qinghai-Tibet Plateau [4]. Under the influence of geographical distribution and artificial selection, Tibetan sheep can be divided into three ecological groups, namely, plateau, mountain-valley and Oula types.

Tibetan sheep have formed some unique breeds with valuable genetic resources in the process of adapting to the local environment by the different needs and preferences of human beings, such as Speckled and Zeku sheep. These indigenous sheep breeds have rich particular morphological features (e.g., horn morphology, ear size, tail length, coat color, etc.), disease resistance, reproductive performance and environmental adaptability. Tibetan sheep breeds in different regions left different genomic selection marks on the genome, and the frequency of these imprinted fragments increased in the process of Tibetan sheep domestication generation by generation and eventually became fixed in some Tibetan domestic breeds, which are valuable materials for researchers to study. However, as a result of genetic drift and a lack of systematic breeding and conservation measures, indigenous groups are facing the crisis of population reduction and loss of excellent genetic resources. Indigenous sheep breeds have relatively small numbers, and it is evident that they have declined steadily over the past two decades [5]. Such a great reduction could result in the loss of unique genetic variation in sheep populations. However, little is known about the genetic basis of the rich phenotypic diversity of different local groups of Chinese native sheep breeds.

Hence, we collected and newly sequenced whole genome resequencing data of six representative Tibetan sheep breeds. By analysing single nucleotide polymorphisms, genetic variant annotation and selected signals, candidate genes related to phenotypic traits were identified using selective sweep mapping, which revealed the potential genetic basis of genetic diversity and some valuable genetic resource footprints of Tibetan sheep with natural selection advantages.

## MATERIALS AND METHODS

### Animal care

The study was conducted according to the guidelines of the Institutional Animal Care and Use Committee of Institute of Animal Science and Veterinary Medicine, Chinese Academy of Sciences (IACUC2021311).

### Animals and whole-genome sequencing

**Figure 1:**
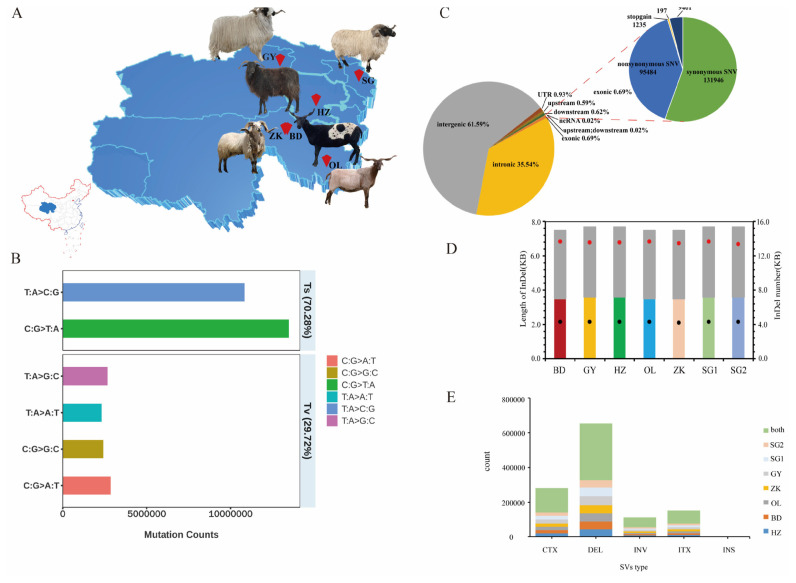
Geographic distribution and genetic diversity analyses. (A) Geographic distribution of six Tibetan sheep breeds in Qinghai. The map was generated using Adobe Illustrator software. (B) Mutation spectrum analysis. The colored bars represent the number of six SNP mutations and the proportion of Ts and Tv in the genome. Ts, transition; Tv, transversion. (C) Functional classification of the detected SNPs. (D) The length and number of indels in six Tibetan sheep breeds. Black and red dots represent the number of insertions and indels, respectively. Colored and gray pillars represent the lengths of insertions and indels, respectively. (E) The number of different types of SVs. DEL, deletions; INS, insertions; INV, inversions; CTX, interchromosomal translocation; ITX, intrachromosomal translocation; SNP, single-nucleotide polymorphism.

We sampled ear tissues from 28 female sheep of six geographical and phenotypic representative Tibetan sheep breeds in Qinghai Province. The sample information, such as species names, codes, sampling sites and altitude, longitude and latitude, and phenotype/features, is shown (Figure 1A; Supplementary Table S1). All tissue samples were preserved in 95% alcohol and stored at −80°C for later genomic analysis. Total genomic DNA was extracted from samples, and at least 3 μg genomic DNA was used to construct paired-end libraries of 2×150 bp using paired-end sequencing. These libraries were sequenced using the Illumina NovaSeq6000 at Personalbio (Shanghai, China).

### Read processing and variant calling

FASTP Toolkit v0.18.0 was used for quality control of the raw reads according to three stringent filtering standards: i) removing reads with ≥10% unidentified nucleotides (N); ii) removing reads with>50% bases having Phred quality scores of ≤20; and iii) removing reads aligned to the barcode adapter. The Burrows–Wheeler Aligner was used to align the clean reads from each sample against the reference genome (https://www.ncbi.nlm.nih.gov/genome/?term=Ovis%20aries) with the settings ‘mem 4 -k 32 -M’, where −k is the minimum seed length and −M is an option used to mark shorter split alignment hits as secondary alignments [6]. Variant calling was performed for all samples using GATK’s Unified Genotyper [7]. Single-nucleotide polymorphisms (SNPs) and InDels were filtered using GATK’s Variant Filtration with proper standards (-Window 4, -filter “QD<2.0 || FS>60.0 || MQ<40.0, -G_filter “GQ<20”), and those exhibiting segregation distortion or sequencing errors were discarded [8]. To determine the physical positions of each SNP, the software tool ANNOVAR [9] was used to align and annotate SNPs or InDels. structural variation (SV) types included translocations, inversions and insertion events, and SVs were determined by the software breakdancer (Max1.1.2.) [10]. Copy number variants (CNVs) were classified by CNVnator (0.3.2) [11].

### Population genetic analyses

The resulting SNP-only dataset was analysed using a maximum likelihood algorithm. A phylogenetic tree was constructed using PHYML 3.0 [12] software and fast Tree based on the selected best nucleotide substitution model of generalized time reversible. Node support was estimated with 1,000 bootstrap replicates. The SNP dataset with minor allele frequency (MAF) <0.05 removed was used to perform principal component analysis using GCTA to estimate the variance explained by genome-wide SNPs. Structure 2.3.4 [13] was used to estimate the population genetic structure, which implies the genetic ancestry of each sample, with the Bayesian method.

### Selected regions analysis

The distribution of θπ ratios (θπ, A/θπ, B) and *F*st values are calculated in 20-kb sliding windows in 5-kb steps. The index of nucleotide diversity π values were calculated using the formula: $\pi =\Sigma_{ij}^{q}\;x_{i}x_{j}d_{ij}$, where *x_i_* and *x_j_* represents the frequency of sequence i and j between two populations, *d_ij_* represents the number of base differences, the weighted population pairwise *F*st values were calculated using the formula $F_{st}=\frac{\pi between-\pi within}{\pi between}$, where πwithin represents the average number of individual differentially paired bases within population, π between represents the average number of individual differentially paired bases between populations. To detect regions with significant signatures of selective sweep, we considered the distribution of the θπ ratios (θπ, A/θπ, B) and *F*st values. We selected windows simultaneously with significantly low and high θπ ratios (the 5% left and right tails, respectively) and significantly high *F*st values (the 5% right tail) of the empirical distribution as regions with strong selective sweep signals along the genome, which should harbor genes that underwent a selective sweep. To detect genome selection signatures and SNPs related to important phenotypic traits, both the *F*st and θπ in the top 5% were considered to be potentially positively selected regions.

### Enrichment analysis of selected candidate genes

Gene ontology and Kyoto encyclopedia of genes and genomes (KEGG) [14,15] enrichment analyses for all candidate genes were performed with DAVID 6.8 [16], and KEGG orthology annotation in the Pathway database were used to uncover the biological functional pathways. The calculated p value was subjected to false discovery rate (FDR) correction, with FDR ≤0.05 as a threshold. Pathways meeting this condition were defined as significantly enriched pathways in genes.

### Sanger sequencing validation and detection of selective loci

To confirm the SNPs detected in exons of the genes identified by sweep analysis, 50 hornless and 50 horned sheep were selected, and primers were designed for Sanger sequencing (Supplementary Table S17) using Primer3 v0.4.0 (1) [17] and SNaPshot minisequencing.

## RESULTS

### Sequencing, mapping and genetic variation

Whole-genome sequencing of 28 samples generated a total of 15.84 billion paired-end raw reads with a 400-bp insert size, and stringent quality filtering yielded 15.26 billion reads at a total of 560× effective sequence depth for the subsequent analyses. Clean reads were aligned to the reference genome with a coverage rate of 99.39% and mapping rate of 99.91% (Supplementary Table S2). The transition-to-transversion average ratio (ts/tv) was 2.36 (Figure 1B; Supplementary Table S3). A total of 34.57 million SNPs were obtained, and used for subsequent analyses. Most of the high-quality SNPs (61.59%) were present in intergenic regions with T/C and A/G replacement, with only 0.69% located in exonic regions. The remaining SNPs were located upstream (0.59%) and downstream (0.62%) of the open reading frame in introns (35.54%) and untranslated regions (3%). Exons contained 0.69% of the total SNPs, with 95,484 nonsynonymous SNPs and 131,946 synonymous SNPs, which resulted in a nonsynonymous/synonymous ratio of 0.724 (Figure 1C; Supplementary Table S4). In addition, an average of 885,612 insertions and 1,010,337 deletions were identified for six indigenous sheep (Figure 1D; Supplementary Table S5). Most indels were located in intergenic regions (Supplementary Figure S1,S2) and belonged to frameshift deletion/insertion, which were enriched for sizes that were multiples of three lengths (3n). We detected 7,105, 7,865, 7,612, 7,079, 7,036, 7,430, and 7,039 CNVs after quality control on a per sample and on a per CNV basis in Qinghai black Tibetan sheep (HZ), Qumaari Speckled sheep (BD), Plateau Tibetan sheep (GY), Oula sheep (OL), Zeku sheep (ZK), Valley Tibetan sheep1 (SG1), and Valley Tibetan sheep2 (SG2), respectively (Supplementary Table S6). A total of 601,986 reliable SVs were detected. Among these, 1,609 were insertions, 327,658 were deletions, 55,889 were inversions, and 216,830 were chromosomal translocations (Figure 1E; Supplementary Table S7).

### Population genetic structure

**Figure 2:**
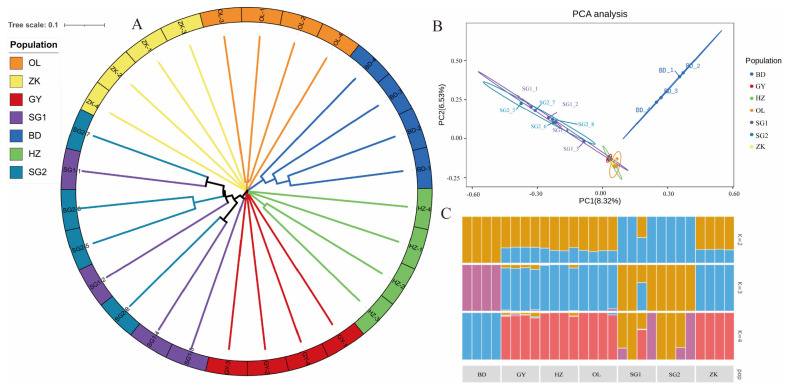
Population genetic analysis. (A) Plots of principal components 1 and 2 for the 28 individuals. (B) Neighbor-joining tree constructed from single-nucleotide polymorphism data among six sheep populations. (C) Genetic structure analysis of samples using Admixture, with changing ancestral populations from K = 2 to K = 4.

To explore the relationships among the Tibetan sheep breeds under investigation, we constructed a phylogenetic tree by using the weighted method. The resulting traditional→ rectangular type of neighbor-joining tree supported the evidence of separations occurring between breeds, with each breed partitioned into its own clades (Figure 2A). However, hornless traits did not cause genetic differentiation within varieties showed that GY, HZ, OL, and ZK were genetically clustered tightly at an intermediate position, while parts of BD and SG were separated from them (Figure 2B). To further estimate the proportion of common ancestry among breeds, we performed a population structure analysis for a range of K (K = 2 to 4) (Figure 2C). At K = 2, SG showed strong genetic differentiation from the other groups. At K = 3, BD tended to be separated from the main population in another direction, while other breeds were distributed across the two remaining clusters. When K = 4, SG showed evidence of admixture, while GY, HZ, and OL were much closer to ZK.

### Genome-wide selective sweep study

#### Hypoxic adaptability

**Figure 3:**
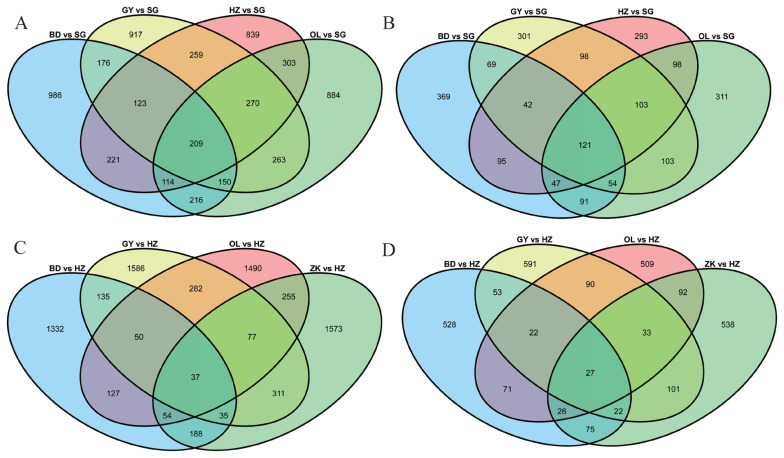
Venn diagrams of common selected regions for hypoxic adaptability (A) and corresponding genes (B) among different comparisons. Venn diagrams of common selected regions for coat color (C) and corresponding genes (D) among different comparisons.

Tibetan sheep breeds are well adaptable to the high-altitude stress of the environment, and they are also an excellent model for rapid adaptation to extreme environments. Genomic regions with a high *F*st and nucleotide diversity ratio (θπ) were identified relative to hypoxic adaptability. We detected 2,279, 2,464, 2,460, and 2,517 potentially positive selection regions identified for BD (*F*st>0.29, θπ>2.10), GY (*F*st>0.19, θπ>2.40), HZ (*F*st>0.20, θπ>2.35), and OL (*F*st>0.21, θπ>2.33) (Supplementary Table S8), corresponding to 888, 891, 897, and 928 candidate genes on breed-specific selection events, respectively, while 209 identical selective regions (121 co-selection genes) were shared by all four breeds (Figure 3A, 3B; Supplementary Table S12). These shared genes were selected across the four breeds through responses that critically involve the mammalian target of rapamycin (*MTOR*) under plateau environments. Comparison with genomic regions around the *MTOR* locus showed a higher level of population differentiation (*F*st_(BD vs SG)_ = 0.38, *F*st_(GY vs SG)_ = 0.27, *F*st_(HZ vs SG)_ = 0.29, *F*st_(OL vs SG)_ = 0.29; Supplementary Table S8). Several positively selected genes associated with hypoxic adaptability were found in one or more groups, including thyrotropin-releasing hormone degrading enzyme (*TRHDE*) in ZK, GY, OL, and BD, pyruvate dehydrogenase kinase 1 (*PDK1*) in OL and ZK, protein tyrosine phosphatase non-receptor type 9 (*PTPN9*) in SG1, transmembrane O-mannosyltransferase targeting cadherins 2 (*TMTC2*) in GY and BD, SRY-box transcription factor 9 (*SOX9*) in OL and BD, endothelial PAS domain protein 1 (*EPAS1*) in GY and OL, platelet-derived growth factor D (*PDGFD*) in OL, suppressor of cytokine signaling 3 (*SOCS3*) in BD and OL, and transforming growth factor beta receptor 3 (*TGFBR3*) in HZ.

Kyoto encyclopedia of genes and genomes enrichment analyses were performed on the highlighted candidate genes. There were 15, 22, 16, and 14 significant KEGG terms (p<0.05) for BD, GY, HZ, and OL, respectively (Supplementary Table S13). Genes selected by at least three breeds were enriched in significantly enriched pathways, including dopaminergic synapse, glutamatergic synapse, and phospholipase D signaling pathways. This indicates that the adaptation to the hypoxic environment of Tibetan sheep is regulated by certain pathways involving some important genes, such as *PDGFD*, *MTOR*, adenylate cyclase 5 (*ADCY5*), homer scaffold protein 1 (*HOMER1*), and protein phosphatase 2 regulatory subunit Bbeta (*PPP2R2B*), indicating functional importance.

#### Coat color

Considering the physical characteristics of the coat color, we utilized the broader approach of comparing the BD, ZK, GY, and OL breeds (control) to apparently coat color different HZ (black Tibetan sheep) (case). There were 2,016, 2,639, 2,483, and 2,628 selective regions identified for BD (*F*st>0.24, θπ<0.35), ZK (*F*st>0.15, θπ<0.50), GY (*F*st>0.14, θπ<0.54), and OL (*F*st>0.16, θπ<0.49) (Supplementary Table S9), corresponding to 824, 914, 939, and 870 candidate genes for breed-specific selection events, respectively, while 37 identical selective regions (27 co-selection genes) were shared by all four breeds (Figure 3C, 3D; Supplementary Table S12). These shared genes were selected through responses that critically involve the microphthalmia-associated transcription factor (*MITF*). Comparison with genomic regions around the *MITF* locus showed a higher level of population differentiation (*F*st_(BD vs HZ)_ = 0.48, *F*st_(GY vs HZ)_ = 0.44, *F*st_(OL vs HZ)_ = 0.41, *F*st_(ZK vs HZ)_ = 0.37; Supplementary Table S9), suggesting that a strong selective sweep occurred in these genes. Several genes associated with hypoxic adaptability appeared to be targets of positive selection, including melanocortin 1 receptor (*MC1R*) in HZ, excision repair cross-complementing 2 (*ERCC2*) in HZ, transcription factor 25 (*TCF25*) in BD and OL, itchy E3 ubiquitin protein ligase (*ITCH*) in HZ and BD, tyrosinase (*TYR*) in GY, ZK and SG1, RALY heterogeneous nuclear ribonucleoprotein (*RALY*) in OL, and KIT proto-oncogene receptor tyrosine kinase (*KIT*) in ZK.

KEGG pathway analysis showed that 16, 41, 36, and 14 terms were significantly enriched (p<0.05) for BD, GY, OL, and ZK relative to HZ, respectively (Supplementary Table S14). Genes selected by at least three breeds were enriched in significantly enriched pathways, including melanogenesis, cAMP signaling pathway, calcium signaling pathway, and glutamatergic synapse.

#### Wool traits

Tibetan carpet wool is not only a protective material against environmental changes but also an important economic trait. We employed a comprehensive approach by comparing the long-haired fiber breeds of HZ, ZK, and GY (cases) with the short-haired fiber breed of OL (controls), and we detected 2,663, 2,797, and 2,563 selective regions identified for HZ (*F*st>0.16, θπ>1.95), ZK (*F*st>0.29, θπ>2.10), GY (*F*st>0.14, θπ>1.81) (Supplementary Table S10), corresponding to 886, 885, and 941 candidate genes, respectively, on breed-specific selection events, while 96 identical selective regions (50 co-selection genes) were shared by all three breeds (Supplementary Figure S3, S4; Supplementary Table S12). Several shared genes within these regions may be related to wool fiber traits, including collagen type IV alpha 2 chain (*COL4A2*) (*F*st_(GY vs OL)_ = 0.17, *F*st_(HZ vs OL)_ = 0.20, *F*st_(ZK vs OL)_ = 0.21; Supplementary Table S2), and ELKS/RAB6-interacting/CAST family member 2 (*ERC2*) (*F*st_(GY vs OL)_ = 0.15, *F*st_(HZ vs OL)_ = 0.25, *F*st_(ZK vs OL)_ = 0.24; Supplementary Table S10). Several genes associated with wool traits showed clear evidence of positive selection, including notch receptor 2 (*NOTCH2*) in ZK and GY, Rho associated coiled-coil containing protein kinase 1 (*ROCK1*) in GY, fibroblast growth factor 5 (*FGF5*) in GY and HZ, and *SOX9* in BD and OL.

KEGG pathway analysis showed that 32, 15, and 37 terms were significantly enriched (p<0.05) for GY, ZK, and HZ relative to OL, respectively (Supplementary Table S15). Common selected genes were closely related to extracellular matrix -receptor interactions, focal adhesion, the relaxin signaling pathway, and the cancer pathway. These findings suggest that wool length-related genes are pervasive targets of positive selection because of their critical role in carpet wool breed selection.

#### Horn phenotypes

Tibetan sheep are the dominant breed with an excellent gene pool on the Qinghai-Tibet Plateau, and some rams and ewes showed no horns. Selective sweeps across the sheep genome in three horned populations, SG2, ZK, and OL, were detected by comparison with polled SG1. There were 2,598, 2,768, and 2,691 selective regions identified for SG2 (*F*st>0.13, θπ<0.57), ZK (*F*st>0.17, θπ<0.49), and OL (*F*st>0.19, θπ<0.48) (Supplementary Table S11), corresponding to 895, 927, and 976 candidate genes in breed-specific selection events, respectively, while 40 identical selective regions (30 co-selection genes) were shared by all three breeds (Supplementary Figure S5, S6; Supplementary Table S12). Relaxin family peptide receptor 2 (*RXFP2*) gene (*F*st_(ZK vs SG1)_ = 0.22, *F*st_(OL vs SG1)_ = 0.35, *F*st_(SG2 vs SG1)_ = 0.22; Supplementary Table S11) within shared regions may be related to the hornless phenotype.

KEGG pathway analysis showed that 34, 16, and 25 terms were significantly enriched (p<0.05) for ZK, OL, and SG2 relative to SG1, respectively (Supplementary Table S16). The *RXFP2* gene was significantly enriched in the Relaxin signaling pathway, which may also be the difference between the polled and horned phenotypes.

### A horn-related locus RXFP2 underlies selection in six sheep breeds

**Figure 4:**
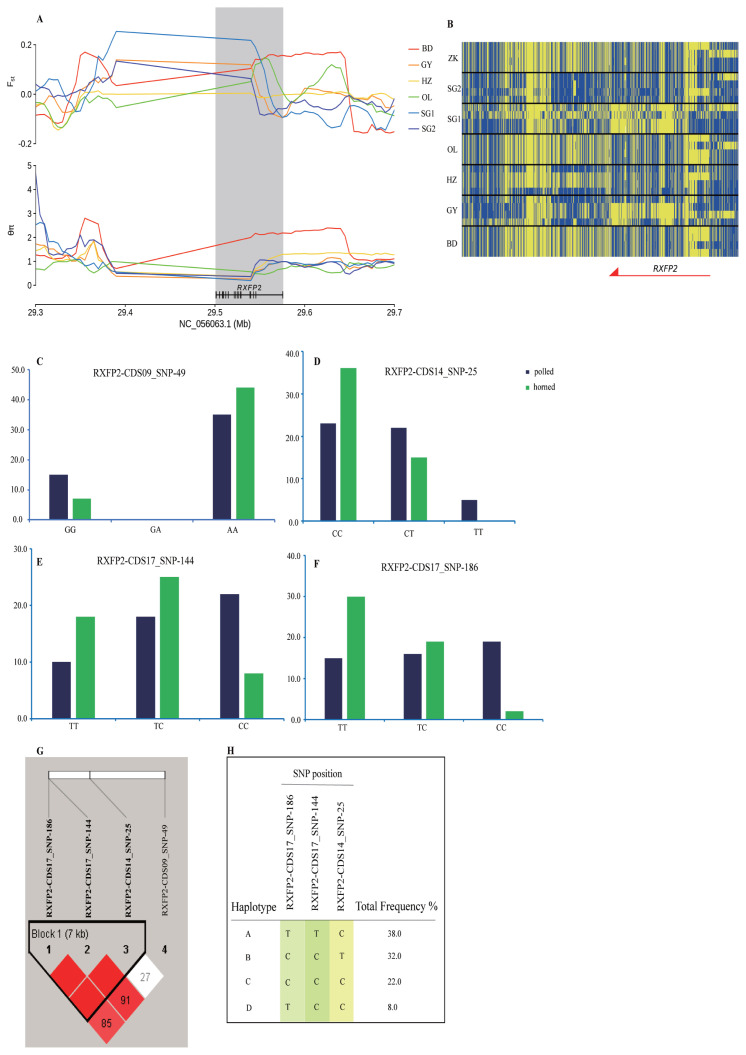
Characterization of selection signals around the horn-related gene *RXFP2* gene locus and correlation of horn phenotypes. (A) Fst and θπ statistics plotted over an approximately 400-kb region surrounding *RXFP2*. (B) Haplotypic distributions among 28 sheep of a local region of *RXFP2* (chromosome 10: 29,450,000 to 29,600,000 bp). The allele consistent with the reference genome is indicated in yellow, and the derived allele is indicated in blue. (C) Genotype frequency at locus RXFP2-CDS09_SNP-49. (D) Genotype frequency at locus RXFP2-CDS09_SNP-25. (E) Genotype frequency at locus RXFP2-CDS09_SNP-144. (F) Genotype frequency at locus RXFP2-CDS09_SNP-186. (G) 7-kb LD block. (H) Four haplotypes within the sampled sheep population. *RXFP2*, relaxin family peptide receptor 2.

We investigated selected *RXFP2* gene-adjacent regions in the genomic region of the six Tibetan sheep breeds at chromosome 10 spanning a 400 kb region. The sweep region exhibits a higher differentiation (*F*st_(IZK vs SG1)_ = 0.20, *F*st_(IOL vs SG1)_ = 0.11) and a lower heterozygosity (θπ_ZK_/θπ_SG1_ = 0.22, θπ_OL_/θπ_SG1_ = 0.27) (Figure 4A, 4B). The *RXFP2* gene was selected for further analysis of the association between the polymorphic loci and horned phenotype by Sanger sequencing and SNaPshot minisequencing. The four most significantly associated frequencies of SNPs for the horned phenotype were located on chromosome 10 (g. 29481646 A>G, p = 0.048, g. 29469024 T>C, p = 0.010, g. 29462010 C>T, p = 0.007, g. 29461968 C>T, p = 0.074 E3; Figure 4C, 4D, 4E, 4F; Supplementary Table S18) within the *RXFP2* gene. Four haplotypes incorporating three SNPs were identified within the 7 Kb LD block, which included the abovementioned SNPs on chromosome 10 (Figure 4G, 4H). Of the associated haplotypes, three were observed at a higher frequency in the polled population. Two nonsynonymous mutations cause amino acid sequence changes in the translated protein (Supplementary Table S18).

## DISCUSSION

Native sheep breeds were the dominant breed with a gene pool exhibiting a rich diversity of phenotypes and production traits [2]. The phenotypic polymorphism of Tibetan sheep is largely the result of natural and artificial selection. Due to the rise and development of human civilization, different domestic animal groups were subjected to strong artificial selection and show a high degree of phenotypic polymorphism. However, few reports have focused on the comparison of different taxa of landraces at the genomic level. In the present study, the genetic variation of different groups of Tibetan sheep was analysed. These animals were separated into various local breeds, within which distinctive traits were observed. Our analyses revealed the selection signatures that help provide potential genomic evidence linking indigenous sheep breeds with their unique morphological traits.

The six indigenous sheep breeds used in this study are widely distributed and have their own distinctive traits. Due to living in cold environments of the highlands, GY, HZ, ZK, and SG sheep are covered with long and thick wool fiber, whereas the wool of BD and OL sheep is thick and short and has almost no textile value. The coat color of wool fibers is mainly white but also black and brown. Interestingly, BD sheep coat color is mainly dark brown and tan, and there are brown and black brown spots on the white rump (Figure 1).

Unique genomic features and precise molecular regulatory elements ensure the adaptation of plateau species to high-altitude environments. Many intensive studies have been performed, such as studies on the mechanisms underlying the adaptive evolution of yaks [18], insights into the rapid adaptation of local sheep to extreme environments [19], associations between CNV variation and high-altitude adaptation of Chaidamu cattle [20], high-altitude adaptation in the Tibetan horse [21], and high-altitude Tibetan semiwild wheat [22]. Genome-wide association studies and selective sweep tests using mathematical approaches to pinpoint candidate causal genes and variants for related traits have been proven to be an effective strategy [23]. We conducted a comprehensive study to identify the selection characteristics of Tibetan sheep in different regions and found some genetic variations associated with hypoxic adaptive performance. Mechanistic target of rapamycin (*MTOR*) controls biomass accumulation and metabolism by modulating key cellular processes [24]. Hypoxia-inducible factor 1α (HiF1α) translation is heavily influenced by *MTOR*, and activating mutations in *MTOR* increase HiF1α expression to influence the hypoxia-induced transcriptional landscape [25–27]. Pairwise comparison of genetic differentiation between sheep inhabiting high-altitude and low-altitude environments identified selection signatures in the *MTOR* genes that have been associated with high-altitude adaptation [28]. The *PDGFD* has been found to be a hypoxia-inducible gene. Under hypoxic conditions, HIF1α can induce the expression of *PDGFD* and then increase the protein level and activity of HIF1α, which plays a key role in promoting cell growth [29]. Moreover, *SOCS3*, *TGFBR3* [30], *PDK1* [19], and *EPAS1* [30] genes involved in hypoxic adaptation were positively selected. Tibetan sheep have stable genetic performance in adapting to hypoxic environments, which can induce a series of adaptive genomic footprints that play an important role in the response to extreme high-altitude stress.

The highly diversified coat of domesticated animals largely reflects the differences in people’s preferences, or because some special color was linked to domestication traits, including docile, birth rate and growth rate and may have become fixed. In the local taxa of Tibetan sheep, there are black, white, brown, color and other phenotypes, among which white is more common. The *MITF* is an activator of tyrosinase family genes and melanin biosynthesis, as it connects melanogenesis with other signaling pathways [31]. *MITF* plays a key role in the modulation of hair pigmentation in mammals. Studies have shown that the black coat of Tibetan sheep is related to high *MITF* expression in hair follicles, and both *MITF* mRNA and protein levels were significantly higher in both solid-black skin tissue and black-spot tissue than in white-color tissue [32]. These genes were also associated with coat color, such as *MC1R*, *TCF25* [33], *RALY* [34], *KIT* [35], and *TYR* [36]. The fleece of carpet or coarse wool sheep is dominated by long and thick medullated wool developed from primary wool follicles [37], and hair follicle development is essential for the genetic basis of sheep production traits. *COL4A2* is a key molecule and a key gene involved in collagen fibrillogenesis [38]. *COL4A2* may regulate secondary hair follicle growth [39]. *ERC2* is involved in neurotransmitter release by binding cytomatrix at the active zone (CAZ) proteins [40] and was shown to be a significant feature related to behavior evolution in domestic animals [41,42]. *NOTCH2* is involved in hair follicle cycling and participates in multiple wingless-type (WNT), mitogen-activated protein kinase (MAPK), and notch signaling pathways [43]. *NOTCH2* is involved in hair follicle morphogenesis and skin development, and it may regulate cashmere fineness [44].

To reduce injury risk to the handlers and other farm animals, reduce economic losses, and for animal welfare reasons, efforts are taken to breed hornless sheep. More and more artificial interventions are involved in breeding genetically hornless sheep in recent years. The *RXFP2* locus not only controls horn type but is also is a QTL with a major contribution to heritable variation in horn size in normal-horned males [45,46]. One study found that an 1,833-bp genomic insertion located in the 3’-UTR of *RXFP2* was associated with polledness [47]. Several novel mutations were identified in ovine *RXFP2*, which is a prime candidate gene for horn type and size indigenous sheep breeds in China, a synonymous mutation, p. P375 (c.1125A>G) is regarded as an indicator for the presence and absence of horns in Tan sheep [48]. To date, causal mutation(s) of the horn phenotype have not been definitively identified. Although *RXFP2* has been successfully mapped to the genome, there are some differences in *RXFP2* association among different breeds, and the complex and variable results indicate that the inheritance of hornless phenotype genes is complicated. In these studies, mapped loci only explain a small portion of the overall genetic variation. Any causal sites for polledness in ruminants warrant additional experimentation.

## CONCLUSION

Our experimental strategy relies on whole-genome resequencing to detect potentially selective scanning regions that include novel genes and important pathways associated with hypoxic adaptation, coat color, wool, and hornless traits. These results provide new ideas for the formation of the unique production and adaptation characteristics of Tibetan native sheep breeds and provide a scientific basis for the genetic basis of phenotypic variation diversity.

## SUPPLEMENTARY MATERIAL

**Supplementary Table S1.** Sample information of six indigenous Tibetan sheep breeds in Qinghai

**Supplementary Table S2.** Sequencing reads, alignment statistics and mean genome-wide coverage of each sample

**Supplementary Table S3.** The result statistics of population SNP in each sample

**Supplementary Table S4.** SNP annotation

**Supplementary Table S5.** The number and length of InDels in each breed

**Supplementary Table S6.** The number of CNV

**Supplementary Table S7.** The number of SV

**Supplementary Table S8.** Putative selection regions associated with hypoxic adaptation among different breeds

**Supplementary Table S9.** Putative selection regions associated with coat color among different breeds

**Supplementary Table S10.** Putative selection regions associated with wool traits among different breeds

**Supplementary Table S11.** Putative selection regions associated with horn phenotypes among different breeds

**Supplementary Table S12.** Lists of putative co-selected genes among different breeds

**Supplementary Table S13.** KEGG analysis of putative selected genes compared with SG2 breed

**Supplementary Table S14.** KEGG analysis of putative selected genes compared with HZ breed

**Supplementary Table S15.** KEGG analysis of putative selected genes compared with OL breed

**Supplementary Table S16.** KEGG analysis of putative selected genes compared with SG1 breed

**Supplementary Table S17.** Primer information of Sanger sequencing

**Supplementary Table S18.** Mutations in exons of gene

**Supplementary Figure S1.** The pie plot shows annotated SNPs.

**Supplementary Figure S2.** The pie plot shows annotated SNPs at exonic regions.

**Supplementary Figure S3.** Venn diagrams of common selected regions for wool traits.

**Supplementary Figure S4.** Venn diagrams of common selected genes for wool traits.

**Supplementary Figure S5.** Venn diagrams of common selected regions for horn phenotypes.

**Supplementary Figure S6.** Venn diagrams of common selected genes for horn phenotypes.
