# Supplementary material for: Genetic diversity and selection of Tibetan sheep breeds revealed by whole-genome resequencing
Source: Anim Biosci. 2023 May 2;36(7):991–1002. doi: 10.5713/ab.22.0432 (PMC10330983; doi:10.5713/ab.22.0432)
Supplement: Supplementary file 1 [file ab-22-0432-Supplementary-Table-1.pdf]

Supplementary Table1. Sample information of six indigenous Tibetan sheep breeds in Qinghai

Species	Breed	Code	Sample Site	Altitude(m)	Latitude(^{0} N)	Longitude(^{0} E)	Phenotype/Feature
	Grassland	GY	Tianjun County, Haixi Mongolian and Tibetan Autonomous Prefecture	3352	37.18	99.12	Fiber(carpet wool), Horned
	Valley	SG1	Huzhu County, Haidong City	2913	36.71	102.1	Fiber(carpet wool), Horned
	Valley	SG2	Huzhu County, Haidong City	2913	36.71	102.1	Fiber(carpet wool), Hornless
Ovis aries	Black	HZ	Hainan Tibetan Autonomous Prefecture Guinan County	2918	36.29	100.63	Black coated(black fur), Horned
	Qumaari Speckled	BD	Qumaari Village, Zeku County, Huangnan Tibetan Autonomous Prefecture	3704	34.96	101.84	Meat, higher body weight, speckled, Horned
	Zeku	ZK	Zeku County, Huangnan Tibetan Autonomous Prefecture	3662	35.04	101.47	Meat, Horned
	Oula	OL	Henan County, Huangnan Prefecture	3538	34.74	101.61	Meat, higher body weight, tawny coated, Horned, high fertility
