# Supplementary material for: Genetic diversity and selection of Tibetan sheep breeds revealed by whole-genome resequencing
Source: Anim Biosci. 2023 May 2;36(7):991–1002. doi: 10.5713/ab.22.0432 (PMC10330983; doi:10.5713/ab.22.0432)
Supplement: Supplementary file 2 [file ab-22-0432-Supplementary-Table-2.pdf]

Supplementary Table2. Sequencing reads, alignment statistics and mean genome-wide coverage of each sample

Breed	Pool ID	Raw Data				Clean Data		
		Reads_Num	Total_Bases(bp)	Proportion of Raw Q20 (%)	Proportion of Raw Q30 (%)	Clean Reads (M)	Proportion of Clean Base (%)	Clean Base (Gb)
	GY_1	611551140	91732671000	97.11	92.4	586598100	95.92%	86557178866
Grassland	GY_2	624776106	93716415900	96.92	91.96	596719088	95.51%	88011605642
	GY_3	565390970	84808645500	97.14	92.36	543421054	96.11%	80220709964
	GY_4	648807520	97321128000	96.95	92.07	619877196	95.54%	91409966698
	OL_1	611049336	91657400400	97.89	94.24	592184980	96.91%	87738649083
Oula	OL_2	534141524	80121228600	97.72	93.86	516153874	96.63%	76411552331
	OL_3	507820734	76173110100	97.61	93.44	492742584	97.03%	72916335934
	OL_4	513851334	77077700100	97.24	92.78	490647718	95.48%	72475857863
	HZ_1	520325678	78048851700	97.1	92.35	498794148	95.86%	73653755404
Black	HZ_2	611350244	91702536600	97.81	94.22	595669022	97.43%	88129289790
	HZ_3	618848128	92827219200	97.72	94	602207762	97.31%	89059897622
	HZ_4	589211150	88381672500	97.75	94.06	573649464	97.36%	84853357312
	ZK_1	472016560	70802484000	97.17	92.7	449397280	95.21%	66345789487
Zeku	ZK_2	601406180	90210927000	97.18	92.54	577830774	96.08%	85282206854
	ZK_3	538868474	80830271100	97.24	92.62	518329742	96.19%	76559153760
	ZK_4	601758146	90263721900	97.03	92.2	575845380	95.69%	84974710819
	BD_1	511305860	76695879000	97.29	92.68	491844456	96.19%	72688438411
maari Speck	BD_2	498914288	74837143200	97.2	92.53	479030396	96.01%	70769383105
	BD_3	499894628	74984194200	97.17	92.42	480411684	96.10%	70946741709
	BD_4	502276640	75341496000	97.49	93.15	485115508	96.58%	71739959579
	SG_1	578532698	86779904700	96.86	91.93	552264714	95.46%	81379892384
	SG_2	567632722	85144908300	96.91	92.06	542169336	95.51%	79885209347
	SG_3	585761800	87864270000	97.06	92.39	560696422	95.72%	82683116106
Valley	SG_4	529377242	79406586300	96.74	91.56	506989760	95.77%	74667678966
	SG_5	652807604	97921140600	97.82	94.21	636246682	97.46%	94146995050
	SG_6	610474484	91571172600	97.79	94.15	594608310	97.40%	87965598794
	SG_7	566156954	84923543100	97.84	94.23	551868986	97.48%	81676831865
	SG_8	565482238	84822335700	97.8	93.92	549377954	97.15%	81352337197
Total/Mean		15839790382	2375968557300	97.34	92.97	15260692374	96.32%	2254502199942

Proportion of Clean Reads (%)	Mapped_reads (bp)	Mapping rate (%)	Average Sequencing depth	Coverage_at_least_4X	Coverage_at_least_10X
94.36%	587461676	99.94%	22.56	99.47%	97.86%
93.91%	597660137	99.94%	23.08	99.48%	98.13%
94.59%	544238054	99.94%	20.93	99.44%	97.24%
93.93%	620864948	99.94%	22.96	99.47%	97.70%
95.72%	592232626	99.81%	21.51	99.45%	97.14%
95.37%	515857832	99.74%	18.64	99.24%	92.74%
95.72%	493039223	99.84%	16.75	99.22%	88.83%
94.03%	491055518	99.87%	21.15	99.42%	97.06%
94.37%	499335606	99.90%	20.07	99.39%	96.41%
96.10%	596440193	99.92%	18.13	99.36%	92.98%
95.94%	602903426	99.91%	18.41	99.37%	93.17%
96.01%	574386540	99.92%	17.89	99.36%	93.22%
93.71%	450540692	99.93%	19.39	99.38%	93.94%
94.54%	578895333	99.94%	22.40	99.45%	96.45%
94.72%	518785859	99.83%	20.18	99.27%	93.89%
94.14%	576764885	99.94%	22.27	99.46%	97.79%
94.77%	492521122	99.93%	19.91	99.36%	96.22%
94.56%	479780003	99.94%	19.07	99.32%	95.42%
94.62%	481110521	99.94%	18.88	99.36%	94.91%
95.22%	485846994	99.94%	19.86	99.39%	96.50%
93.78%	553004701	99.93%	20.61	99.43%	97.10%
93.82%	542566237	99.86%	20.41	99.42%	96.75%
94.10%	561385733	99.92%	20.62	99.44%	96.98%
94.03%	507704794	99.93%	20.69	99.43%	97.12%
96.15%	636894182	99.90%	19.07	99.41%	95.06%
96.06%	595391543	99.93%	18.38	99.38%	93.79%
96.18%	552700015	99.94%	16.98	99.35%	91.25%
95.91%	549993912	99.90%	19.94	99.41%	96.41%
94.87%	15279362305	99.91%	20.03	99.39%	95.43%
