# Supplementary material for: Genetic diversity and selection of Tibetan sheep breeds revealed by whole-genome resequencing
Source: Anim Biosci. 2023 May 2;36(7):991–1002. doi: 10.5713/ab.22.0432 (PMC10330983; doi:10.5713/ab.22.0432)
Supplement: Supplementary file 3 [file ab-22-0432-Supplementary-Table-3.pdf]

Supplementary Table3. The result statistics of population SNP in each sample

Sample	HOM_REF	HET	UNKNOWN	HOM_ALT	Ts	Tv	Ts/Tv
BD_1	20565561	7934061	102652	5965623	9788814	4110870	2.3812
BD_2	20761217	7490779	101282	6214619	9652529	4052869	2.3817
BD_3	20018397	9052376	86762	5410362	10185823	4276915	2.3816
BD_4	19994397	9087198	84343	5401959	10201384	4287773	2.3792
GY_1	20013554	9180202	73275	5300866	10195714	4285354	2.3792
GY_2	19935826	9296480	73103	5262488	10247568	4311400	2.3769
GY_3	19949988	9234000	83613	5300296	10235532	4298764	2.381
GY_4	19958631	9251223	72170	5285873	10236973	4300123	2.3806
HZ_1	20071614	8988526	80959	5426798	10150465	4264859	2.38
HZ_2	19919623	9240368	78447	5329459	10259020	4310807	2.3798
HZ_3	19980781	9155042	73565	5358509	10219341	4294210	2.3798
HZ_4	19893854	9349644	80268	5244131	10275069	4318706	2.3792
OL_1	20006915	9092175	77489	5391318	10195136	4288357	2.3774
OL_2	20049210	9013736	101783	5403168	10154053	4262851	2.382
OL_3	19979781	9135396	94418	5358302	10207028	4286670	2.3811
OL_4	20025354	9069749	81312	5391482	10183575	4277656	2.3806
ZK_1	20126420	8916970	77722	5446785	10116287	4247468	2.3817
ZK_2	20177359	8846203	69878	5474457	10084057	4236603	2.3802
ZK_3	20080295	8912131	71205	5504266	10147361	4269036	2.377
ZK_4	20023492	9096701	70858	5376846	10192554	4280993	2.3809
SG_1	19989416	9390613	80940	5106928	10208594	4288947	2.3802
SG_2	20146916	9039418	79182	5302381	10097374	4244425	2.379
SG_3	19864891	9649577	73491	4979938	10302751	4326764	2.3812
SG_4	20025433	9175629	80911	5285924	10181124	4280429	2.3785
SG_5	19953610	9467585	76812	5069890	10234997	4302478	2.3789
SG_6	19852319	9598640	80719	5036219	10303579	4331280	2.3789
SG_7	20060110	9202533	71608	5233646	10164843	4271336	2.3798
SG_8	20026033	9123738	92103	5326023	10171182	4278579	2.3772
