# Supplementary material for: Genetic diversity and selection of Tibetan sheep breeds revealed by whole-genome resequencing
Source: Anim Biosci. 2023 May 2;36(7):991–1002. doi: 10.5713/ab.22.0432 (PMC10330983; doi:10.5713/ab.22.0432)
Supplement: Supplementary file 4 [file ab-22-0432-Supplementary-Table-4.pdf]

Supplementary Table4. SNP annotation

Type	Number	Percentage
exonic total	238323	0.69
synonymous SNV	131946	0.38
nonsynonymous SNV	95484	0.28
stopgain	1235	0
stoploss	197	0
unknown	9461	0.03
splicing	788	0
ncRNA total	6434	0.02
ncRNA_exonic	3021	0.01
ncRNA_splicing	6	0
ncRNA_exonic;splicing	0	0
ncRNA_intronic	3407	0.01
intronic	12286032	35.54
intergenic	21289559	61.59
UTR5	102440	0.3
UTR3	220607	0.63
UTR5;UTR3	391	0
upstream	204148	0.59
downstream	212755	0.62
upstream;downstream	6420	0.02
Total	34567897	100
