# Supplementary material for: Genetic diversity and selection of Tibetan sheep breeds revealed by whole-genome resequencing
Source: Anim Biosci. 2023 May 2;36(7):991–1002. doi: 10.5713/ab.22.0432 (PMC10330983; doi:10.5713/ab.22.0432)
Supplement: Supplementary file 5 [file ab-22-0432-Supplementary-Table-5.pdf]

Supplementary Table5.The number and length of InDels in each breed

BD				GY				HZ			
Type	Length	Count	Percent(%)	Type	Length	Count	Percent(%)	Type	Length	Count	Percent(%)
Deletion	-44	199	0.01081993	Deletion	-58	24	0.001259952	Deletion	-68	3	0.00015833
Deletion	-34	584	0.03175297	Deletion	-23	1507	0.079114464	Deletion	-68	1	5.24E-05
Deletion	-48	124	0.00674207	Deletion	-42	267	0.014016962	Deletion	-68	2	0.0001049
Deletion	-14	6956	0.37820833	Deletion	-29	753	0.039530983	Deletion	-68	2	0.00010477
Deletion	-12	9989	0.54311716	Deletion	-65	5	0.00026249	Deletion	-67	2	0.00010555
Deletion	-20	2970	0.16148343	Deletion	-3	99389	5.21772227	Deletion	-67	1	5.24E-05
Deletion	-54	64	0.00347978	Deletion	-39	323	0.016956849	Deletion	-67	1	5.25E-05
Deletion	-61	15	0.00081557	Deletion	-18	4257	0.223483924	Deletion	-67	1	5.24E-05
Deletion	-50	83	0.00451284	Deletion	-68	2	0.000104996	Deletion	-66	3	0.00015833
Deletion	-16	5208	0.2831669	Deletion	-10	14694	0.771405397	Deletion	-66	3	0.00015736
Deletion	-59	20	0.00108743	Deletion	-8	20501	1.076261198	Deletion	-66	1	5.24E-05
Deletion	-27	994	0.0540453	Deletion	-32	723	0.037956043	Deletion	-65	2	0.00010555
Deletion	-38	387	0.02104178	Deletion	-5	42240	2.217514903	Deletion	-65	4	0.00020961
Deletion	-7	17632	0.95867873	Deletion	-16	5423	0.284696575	Deletion	-65	4	0.00020981
Deletion	-1	423788	23.0419998	Deletion	-60	19	0.000997462	Deletion	-65	5	0.00026191
Deletion	-49	68	0.00369726	Deletion	-54	71	0.003727357	Deletion	-64	5	0.00026388
Deletion	-22	2452	0.13331898	Deletion	-14	7248	0.380505398	Deletion	-64	6	0.00031441
Deletion	-41	176	0.00956939	Deletion	-26	1468	0.077067043	Deletion	-64	5	0.00026226
Deletion	-32	711	0.03865815	Deletion	-38	388	0.020369218	Deletion	-64	4	0.00020953
Deletion	-9	13495	0.73374373	Deletion	-1	439000	23.04661559	Deletion	-63	4	0.0002111
Deletion	-2	168114	9.14061455	Deletion	-41	210	0.011024577	Deletion	-63	5	0.00026201
Deletion	-45	152	0.00826447	Deletion	-31	634	0.033283723	Deletion	-63	6	0.00031471
Deletion	-57	23	0.00125055	Deletion	-66	3	0.000157494	Deletion	-63	4	0.00020953
Deletion	-28	1266	0.06883435	Deletion	-22	2546	0.133659871	Deletion	-62	5	0.00026388
Deletion	-66	2	0.00010874	Deletion	-48	110	0.005774778	Deletion	-62	6	0.00031441
Deletion	-10	14083	0.76571419	Deletion	-15	4529	0.237763376	Deletion	-62	6	0.00031471
Deletion	-46	149	0.00810136	Deletion	-4	82498	4.330978799	Deletion	-62	8	0.00041906
Deletion	-18	4194	0.22803418	Deletion	-28	1273	0.066829935	Deletion	-61	16	0.00084441
Deletion	-60	24	0.00130492	Deletion	-30	1042	0.054702901	Deletion	-61	17	0.00089084
Deletion	-30	965	0.05246852	Deletion	-33	493	0.025881507	Deletion	-61	18	0.00094413
Deletion	-47	99	0.00538278	Deletion	-17	3187	0.167311079	Deletion	-61	11	0.00057621
Deletion	-62	12	0.00065246	Deletion	-27	988	0.05186801	Deletion	-60	19	0.00100274
Deletion	-56	45	0.00244672	Deletion	-43	163	0.008557172	Deletion	-60	22	0.00115285
Deletion	-67	1	5.44E-05	Deletion	-61	16	0.000839968	Deletion	-60	19	0.00099658
Deletion	-37	305	0.01658332	Deletion	-20	3173	0.166576108	Deletion	-60	23	0.0012048
Deletion	-11	7989	0.43437411	Deletion	-21	1942	0.101951088	Deletion	-59	14	0.00073886
Deletion	-36	501	0.02724013	Deletion	-50	101	0.005302297	Deletion	-59	14	0.00073363
Deletion	-40	366	0.01989998	Deletion	-51	79	0.004147341	Deletion	-59	19	0.00099658
Deletion	-19	2272	0.1235321	Deletion	-13	6289	0.33015983	Deletion	-59	14	0.00073336
Deletion	-63	5	0.00027186	Deletion	-36	501	0.026301491	Deletion	-58	28	0.00147772
Deletion	-33	494	0.02685953	Deletion	-35	396	0.020789202	Deletion	-58	21	0.00110045

Deletion	-35	407	0.02212921	Deletion	-40	361	0.018951773	Deletion	-58	26	0.00136374
Deletion	-52	57	0.00309918	Deletion	-19	2390	0.125470185	Deletion	-58	24	0.00125719
Deletion	-58	31	0.00168552	Deletion	-34	612	0.032128767	Deletion	-57	23	0.00121384
Deletion	-26	1468	0.0798174	Deletion	-62	6	0.000314988	Deletion	-57	20	0.00104805
Deletion	-24	1982	0.10776436	Deletion	-12	10478	0.550073891	Deletion	-57	22	0.00115394
Deletion	-29	731	0.03974558	Deletion	-46	148	0.007769702	Deletion	-57	22	0.00115242
Deletion	-25	1137	0.06182042	Deletion	-56	42	0.002204915	Deletion	-56	40	0.00211103
Deletion	-17	2973	0.16164654	Deletion	-55	52	0.002729895	Deletion	-56	39	0.00204369
Deletion	-8	19657	1.06878107	Deletion	-49	77	0.004042345	Deletion	-56	42	0.00220297
Deletion	-53	54	0.00293606	Deletion	-47	109	0.00572228	Deletion	-56	43	0.00225246
Deletion	-3	95132	5.17247191	Deletion	-37	315	0.016536865	Deletion	-55	49	0.00258601
Deletion	-68	2	0.00010874	Deletion	-11	8210	0.43100846	Deletion	-55	51	0.00267252
Deletion	-64	6	0.00032623	Deletion	-64	8	0.000419984	Deletion	-55	57	0.00298975
Deletion	-5	40898	2.22368663	Deletion	-45	170	0.008924658	Deletion	-55	48	0.00251437
Deletion	-65	6	0.00032623	Deletion	-52	59	0.003097381	Deletion	-54	57	0.00300822
Deletion	-55	53	0.00288169	Deletion	-2	174446	9.158063559	Deletion	-54	65	0.00340615
Deletion	-4	79547	4.3250917	Deletion	-53	59	0.003097381	Deletion	-54	66	0.00346181
Deletion	-31	601	0.03267729	Deletion	-24	2033	0.106728404	Deletion	-54	55	0.00288105
Deletion	-42	251	0.01364725	Deletion	-7	18323	0.961920586	Deletion	-53	52	0.00274434
Deletion	-23	1411	0.07671822	Deletion	-59	20	0.00104996	Deletion	-53	58	0.00303933
Deletion	-6	32010	1.74043251	Deletion	-6	33129	1.739205758	Deletion	-53	52	0.00272749
Deletion	-15	4346	0.23629865	Deletion	-63	5	0.00026249	Deletion	-53	57	0.00298582
Deletion	-51	83	0.00451284	Deletion	-57	21	0.001102458	Deletion	-52	60	0.00316654
Deletion	-21	1883	0.10238158	Deletion	-25	1205	0.063260072	Deletion	-52	65	0.00340615
Deletion	-13	6082	0.33068761	Deletion	-44	180	0.009449637	Deletion	-52	66	0.00346181
Deletion	-39	272	0.01478905	Deletion	-9	13982	0.734026832	Deletion	-52	57	0.00298582
Deletion	-43	178	0.00967813	Deletion	-57	19	0.000993144	Deletion	-51	72	0.00379985
Deletion	-19	2239	0.1232213	Deletion	-65	6	0.000313624	Deletion	-51	68	0.00356336
Deletion	-46	154	0.00847525	Deletion	-60	24	0.001254497	Deletion	-51	76	0.00398633
Deletion	-5	40388	2.22271631	Deletion	-15	4562	0.238459017	Deletion	-51	67	0.00350964
Deletion	-29	735	0.04045005	Deletion	-9	13983	0.730901456	Deletion	-50	97	0.00511925
Deletion	-12	9822	0.54054471	Deletion	-36	520	0.027180774	Deletion	-50	91	0.00476861
Deletion	-3	93730	5.15834405	Deletion	-25	1187	0.062045343	Deletion	-50	87	0.0045633
Deletion	-48	120	0.00660409	Deletion	-62	9	0.000470436	Deletion	-50	89	0.00466207
Deletion	-45	141	0.0077598	Deletion	-67	1	5.23E-05	Deletion	-49	67	0.00353597
Deletion	-2	165638	9.11573446	Deletion	-18	4324	0.226018586	Deletion	-49	68	0.00356336
Deletion	-67	2	0.00011007	Deletion	-64	8	0.000418166	Deletion	-49	67	0.00351427
Deletion	-20	2980	0.16400155	Deletion	-66	1	5.23E-05	Deletion	-49	73	0.00382394
Deletion	-50	85	0.0046779	Deletion	-43	182	0.009513271	Deletion	-48	123	0.00649142
Deletion	-27	992	0.0545938	Deletion	-35	395	0.020646934	Deletion	-48	126	0.00660269
Deletion	-36	502	0.02762711	Deletion	-37	304	0.015890298	Deletion	-48	124	0.00650401
Deletion	-64	6	0.0003302	Deletion	-5	42556	2.224432693	Deletion	-48	120	0.00628593
Deletion	-47	104	0.00572354	Deletion	-4	82595	4.317299987	Deletion	-47	111	0.00585811
Deletion	-55	51	0.00280674	Deletion	-52	66	0.003449867	Deletion	-47	104	0.00544984

Deletion	-40	339	0.01865655	Deletion	-14	7215	0.377133233	Deletion	-47	117	0.00613685
Deletion	-53	46	0.00253157	Deletion	-55	61	0.003188514	Deletion	-47	100	0.00523828
Deletion	-22	2443	0.13444825	Deletion	-27	1031	0.053891111	Deletion	-46	140	0.0073886
Deletion	-10	13884	0.76409313	Deletion	-42	265	0.01385174	Deletion	-46	149	0.00780794
Deletion	-44	198	0.01089675	Deletion	-11	8287	0.433167443	Deletion	-46	145	0.0076055
Deletion	-30	966	0.05316292	Deletion	-19	2391	0.124979288	Deletion	-46	150	0.00785741
Deletion	-14	6884	0.37885459	Deletion	-30	992	0.051852553	Deletion	-45	152	0.00802191
Deletion	-41	163	0.00897055	Deletion	-10	14707	0.768745456	Deletion	-45	143	0.00749353
Deletion	-32	684	0.03764331	Deletion	-29	715	0.037373564	Deletion	-45	149	0.00781531
Deletion	-42	265	0.01458403	Deletion	-51	78	0.004077116	Deletion	-45	156	0.00817171
Deletion	-52	58	0.00319198	Deletion	-63	4	0.000209083	Deletion	-44	189	0.00997461
Deletion	-43	169	0.00930076	Deletion	-31	651	0.034028238	Deletion	-44	193	0.01011364
Deletion	-16	5175	0.28480135	Deletion	-58	28	0.00146358	Deletion	-44	206	0.01080506
Deletion	-33	462	0.02542574	Deletion	-22	2595	0.135642514	Deletion	-44	176	0.00921937
Deletion	-57	19	0.00104565	Deletion	-32	711	0.037164481	Deletion	-43	174	0.00918298
Deletion	-49	74	0.00407252	Deletion	-7	18475	0.965701523	Deletion	-43	158	0.00827956
Deletion	-13	5989	0.32959909	Deletion	-24	2050	0.107154973	Deletion	-43	161	0.00844473
Deletion	-51	73	0.00401749	Deletion	-47	94	0.004913448	Deletion	-43	165	0.00864316
Deletion	-35	356	0.01959213	Deletion	-13	6386	0.333800808	Deletion	-42	269	0.01419667
Deletion	-58	31	0.00170606	Deletion	-17	3146	0.16444368	Deletion	-42	254	0.01331019
Deletion	-54	58	0.00319198	Deletion	-54	71	0.003711221	Deletion	-42	266	0.01395216
Deletion	-37	303	0.01667533	Deletion	-2	175225	9.159136634	Deletion	-42	245	0.01283378
Deletion	-61	18	0.00099061	Deletion	-38	413	0.021587807	Deletion	-41	196	0.01034405
Deletion	-62	9	0.00049531	Deletion	-59	14	0.00073179	Deletion	-41	206	0.01079487
Deletion	-8	19499	1.07310947	Deletion	-12	10423	0.544817698	Deletion	-41	193	0.01012318
Deletion	-1	418740	23.0449694	Deletion	-45	147	0.007683796	Deletion	-41	205	0.01073847
Deletion	-26	1433	0.07886383	Deletion	-56	47	0.002456724	Deletion	-40	359	0.01894649
Deletion	-38	359	0.01975723	Deletion	-23	1481	0.077412934	Deletion	-40	371	0.01944126
Deletion	-11	7787	0.42855036	Deletion	-26	1552	0.081124155	Deletion	-40	367	0.01924978
Deletion	-21	1844	0.10148284	Deletion	-40	346	0.018085669	Deletion	-40	337	0.01765299
Deletion	-60	23	0.00126578	Deletion	-33	514	0.026867149	Deletion	-39	294	0.01551607
Deletion	-68	1	5.50E-05	Deletion	-8	20543	1.073797368	Deletion	-39	280	0.01467265
Deletion	-66	4	0.00022014	Deletion	-48	120	0.006272486	Deletion	-39	284	0.01489629
Deletion	-4	78309	4.30966354	Deletion	-46	159	0.008311044	Deletion	-39	284	0.0148767
Deletion	-28	1230	0.06769191	Deletion	-44	191	0.009983707	Deletion	-38	396	0.02089919
Deletion	-23	1416	0.07792825	Deletion	-1	441386	23.07156332	Deletion	-38	368	0.01928405
Deletion	-7	17404	0.95781308	Deletion	-6	33387	1.745162476	Deletion	-38	390	0.02045617
Deletion	-34	581	0.0319748	Deletion	-61	16	0.000836331	Deletion	-38	378	0.01980068
Deletion	-59	21	0.00115572	Deletion	-53	53	0.002770348	Deletion	-37	322	0.01699379
Deletion	-39	282	0.01551961	Deletion	-41	198	0.010349602	Deletion	-37	315	0.01650673
Deletion	-56	38	0.00209129	Deletion	-20	3159	0.165123199	Deletion	-37	297	0.01557816
Deletion	-18	4080	0.22453903	Deletion	-28	1315	0.068735995	Deletion	-37	309	0.01618627
Deletion	-24	1899	0.10450971	Deletion	-3	99790	5.216094991	Deletion	-36	494	0.02607122
Deletion	-17	2939	0.16174515	Deletion	-49	73	0.003815762	Deletion	-36	523	0.02740641

Deletion	-15	4227	0.23262904	Deletion	-21	1949	0.10187563	Deletion	-36	521	0.02732735
Deletion	-65	6	0.0003302	Deletion	-39	300	0.015681216	Deletion	-36	497	0.02603423
Deletion	-31	576	0.03169963	Deletion	-50	93	0.004861177	Deletion	-35	367	0.0193687
Deletion	-25	1141	0.06279388	Deletion	-68	2	0.000104541	Deletion	-35	383	0.02007008
Deletion	-9	13301	0.73200826	Deletion	-16	5398	0.282157338	Deletion	-35	372	0.01951204
Deletion	-6	31874	1.75415617	Deletion	-34	626	0.03272147	Deletion	-35	368	0.01927686
Deletion	-63	6	0.0003302	Deletion	-66	1	5.24E-05	Deletion	-34	603	0.03182377
Deletion	-1	438076	23.0639635	Deletion	-12	10455	0.548162829	Deletion	-34	588	0.03081256
Deletion	-66	3	0.00015794	Deletion	-31	628	0.032926471	Deletion	-34	581	0.03047445
Deletion	-22	2502	0.13172609	Deletion	-39	283	0.014837884	Deletion	-34	567	0.02970103
Deletion	-9	13913	0.73249601	Deletion	-22	2609	0.136791661	Deletion	-33	481	0.02538513
Deletion	-18	4207	0.22149146	Deletion	-51	70	0.003670148	Deletion	-33	479	0.0251007
Deletion	-44	191	0.01005583	Deletion	-32	755	0.039585168	Deletion	-33	499	0.02617341
Deletion	-65	5	0.00026324	Deletion	-59	16	0.000838891	Deletion	-33	484	0.02535326
Deletion	-59	19	0.00100032	Deletion	-36	511	0.026792081	Deletion	-32	676	0.0356764
Deletion	-46	151	0.0079499	Deletion	-1	440230	23.08156118	Deletion	-32	703	0.03683882
Deletion	-16	5464	0.2876704	Deletion	-64	6	0.000314584	Deletion	-32	688	0.03608679
Deletion	-40	366	0.01926928	Deletion	-6	33268	1.744264083	Deletion	-32	728	0.03813465
Deletion	-2	173497	9.13432481	Deletion	-61	12	0.000629168	Deletion	-31	615	0.03245708
Deletion	-53	49	0.00257977	Deletion	-67	1	5.24E-05	Deletion	-31	606	0.0317558
Deletion	-15	4515	0.23770714	Deletion	-26	1457	0.07639151	Deletion	-31	611	0.032048
Deletion	-35	403	0.02121727	Deletion	-38	389	0.020395537	Deletion	-31	619	0.03242493
Deletion	-27	1031	0.05428041	Deletion	-10	14738	0.77272346	Deletion	-30	1014	0.0535146
Deletion	-12	10330	0.5438571	Deletion	-19	2364	0.123946143	Deletion	-30	1014	0.05313594
Deletion	-68	2	0.0001053	Deletion	-68	3	0.000157292	Deletion	-30	1008	0.05287134
Deletion	-31	646	0.03401081	Deletion	-28	1310	0.068684199	Deletion	-30	982	0.05143987
Deletion	-32	723	0.03806473	Deletion	-57	23	0.001205906	Deletion	-29	731	0.03857907
Deletion	-67	1	5.26E-05	Deletion	-20	3144	0.164842079	Deletion	-29	752	0.03940653
Deletion	-38	377	0.01984841	Deletion	-29	747	0.039165723	Deletion	-29	752	0.0394437
Deletion	-26	1504	0.07918307	Deletion	-46	151	0.007917034	Deletion	-29	741	0.03881563
Deletion	-57	25	0.00131621	Deletion	-33	490	0.025691036	Deletion	-28	1257	0.06633911
Deletion	-48	140	0.00737076	Deletion	-3	99526	5.218216518	Deletion	-28	1190	0.06235874
Deletion	-56	46	0.00242182	Deletion	-47	99	0.005190638	Deletion	-28	1272	0.06671859
Deletion	-23	1429	0.07523444	Deletion	-13	6316	0.331152217	Deletion	-28	1313	0.06877856
Deletion	-64	6	0.00031589	Deletion	-14	7201	0.377553374	Deletion	-27	1025	0.05409513
Deletion	-28	1328	0.06991696	Deletion	-16	5415	0.283912168	Deletion	-27	1011	0.05297873
Deletion	-42	269	0.0141624	Deletion	-34	581	0.030462229	Deletion	-27	1023	0.05365811
Deletion	-33	493	0.02595562	Deletion	-58	29	0.00152049	Deletion	-27	1036	0.05426854
Deletion	-24	2038	0.10729727	Deletion	-52	58	0.00304098	Deletion	-26	1466	0.07736923
Deletion	-20	3174	0.16710575	Deletion	-63	5	0.000262153	Deletion	-26	1475	0.0772934
Deletion	-14	7217	0.37996289	Deletion	-24	2056	0.107797492	Deletion	-26	1501	0.07873004
Deletion	-5	42402	2.23239388	Deletion	-37	305	0.015991359	Deletion	-26	1446	0.07574547
Deletion	-45	154	0.00810784	Deletion	-23	1495	0.078383877	Deletion	-25	1201	0.06338366
Deletion	-29	774	0.0407498	Deletion	-27	1048	0.05494736	Deletion	-25	1184	0.06204433

Deletion	-7	18254	0.96104235	Deletion	-44	186	0.009752108	Deletion	-25	1211	0.06351904
Deletion	-41	192	0.01010848	Deletion	-62	9	0.000471876	Deletion	-25	1169	0.06123545
Deletion	-47	113	0.00594926	Deletion	-56	43	0.00225452	Deletion	-24	2039	0.10760973
Deletion	-13	6355	0.33458005	Deletion	-15	4469	0.234312739	Deletion	-24	2021	0.10590506
Deletion	-37	311	0.01637363	Deletion	-7	18358	0.962522545	Deletion	-24	2031	0.10652945
Deletion	-52	67	0.00352744	Deletion	-2	174920	9.171175706	Deletion	-24	2008	0.10518458
Deletion	-8	20424	1.0752892	Deletion	-48	133	0.006973281	Deletion	-23	1485	0.07837197
Deletion	-21	1942	0.10224303	Deletion	-5	42560	2.231450023	Deletion	-23	1473	0.0771886
Deletion	-58	25	0.00131621	Deletion	-65	8	0.000419445	Deletion	-23	1499	0.07862514
Deletion	-30	984	0.05180594	Deletion	-11	8299	0.435122268	Deletion	-23	1468	0.07689789
Deletion	-10	14644	0.77098193	Deletion	-53	53	0.002778826	Deletion	-22	2501	0.13199213
Deletion	-25	1191	0.06270414	Deletion	-40	344	0.018036156	Deletion	-22	2500	0.13100576
Deletion	-34	619	0.03258931	Deletion	-4	82682	4.335074032	Deletion	-22	2564	0.13448622
Deletion	-63	3	0.00015794	Deletion	-25	1182	0.061973072	Deletion	-22	2536	0.13284268
Deletion	-62	10	0.00052648	Deletion	-50	85	0.004456608	Deletion	-21	1873	0.09884896
Deletion	-61	14	0.00073708	Deletion	-41	210	0.011010444	Deletion	-21	1898	0.09945958
Deletion	-11	8149	0.42903112	Deletion	-49	82	0.004299316	Deletion	-21	1900	0.09965828
Deletion	-3	99132	5.21913282	Deletion	-17	3126	0.163898326	Deletion	-21	1944	0.10183209
Deletion	-4	82196	4.32748095	Deletion	-43	168	0.008808355	Deletion	-20	3132	0.16529362
Deletion	-43	180	0.0094767	Deletion	-8	20348	1.066859612	Deletion	-20	3129	0.16396681
Deletion	-51	79	0.00415922	Deletion	-21	1903	0.099775597	Deletion	-20	3119	0.16359693
Deletion	-17	3082	0.16226211	Deletion	-9	13975	0.732718846	Deletion	-20	3194	0.16731054
Deletion	-36	526	0.02769301	Deletion	-18	4320	0.226500566	Deletion	-19	2338	0.12338968
Deletion	-60	18	0.00094767	Deletion	-55	47	0.002464242	Deletion	-19	2391	0.12529391
Deletion	-6	33351	1.75587397	Deletion	-45	151	0.007917034	Deletion	-19	2450	0.12850673
Deletion	-54	52	0.00273771	Deletion	-60	23	0.001205906	Deletion	-19	2353	0.12325664
Deletion	-19	2387	0.12567153	Deletion	-35	378	0.0198188	Deletion	-18	4242	0.22387469
Deletion	-55	50	0.00263242	Deletion	-42	254	0.013317394	Deletion	-18	4281	0.22433427
Deletion	-50	94	0.00494894	Deletion	-30	1015	0.053217147	Deletion	-18	4223	0.22150363
Deletion	-49	78	0.00410657	Deletion	-54	67	0.003512856	Deletion	-18	4219	0.22100287
Deletion	-39	292	0.01537331	Deletion	-50	92	0.004821666	Deletion	-17	3070	0.16202152
Deletion	-27	1048	0.05502838	Deletion	-62	9	0.000471685	Deletion	-17	3137	0.16438603
Deletion	-7	18336	0.96278654	Deletion	-47	109	0.005712627	Deletion	-17	3157	0.1655901
Deletion	-67	1	5.25E-05	Deletion	-2	175186	9.181396334	Deletion	-17	3143	0.16463902
Deletion	-20	3109	0.16324735	Deletion	-38	397	0.020806539	Deletion	-16	5472	0.28878885
Deletion	-1	439544	23.0795727	Deletion	-23	1482	0.077670758	Deletion	-16	5430	0.28454452
Deletion	-30	989	0.0519304	Deletion	-54	62	0.003249384	Deletion	-16	5502	0.28858939
Deletion	-54	70	0.00367556	Deletion	-1	440014	23.06087773	Deletion	-16	5356	0.28056207
Deletion	-28	1312	0.06889049	Deletion	-24	2064	0.108173039	Deletion	-15	4458	0.23527425
Deletion	-50	84	0.00441067	Deletion	-37	308	0.016142101	Deletion	-15	4500	0.23581037
Deletion	-12	10514	0.55206902	Deletion	-27	999	0.052357009	Deletion	-15	4487	0.23535089
Deletion	-8	20541	1.07856666	Deletion	-60	16	0.000838551	Deletion	-15	4474	0.23436047
Deletion	-32	731	0.03838334	Deletion	-39	292	0.01530355	Deletion	-14	7177	0.37877149
Deletion	-15	4473	0.23486825	Deletion	-66	2	0.000104819	Deletion	-14	7195	0.37703458

Deletion	-25	1177	0.06180191	Deletion	-10	14709	0.770890132	Deletion	-14	7181	0.37665584
Deletion	-26	1489	0.0781844	Deletion	-55	43	0.002253605	Deletion	-14	7182	0.37621299
Deletion	-3	99028	5.1997614	Deletion	-21	1928	0.101045358	Deletion	-13	6199	0.32715681
Deletion	-57	20	0.00105016	Deletion	-15	4488	0.235213469	Deletion	-13	6344	0.33244022
Deletion	-11	8213	0.43124814	Deletion	-33	510	0.026728803	Deletion	-13	6275	0.32913457
Deletion	-52	62	0.0032555	Deletion	-35	421	0.022064365	Deletion	-13	6438	0.33724021
Deletion	-59	18	0.00094514	Deletion	-36	534	0.027986629	Deletion	-12	10423	0.55008154
Deletion	-24	2045	0.10737884	Deletion	-40	366	0.019181847	Deletion	-12	10400	0.54498397
Deletion	-23	1490	0.07823691	Deletion	-12	10459	0.548150105	Deletion	-12	10422	0.54665188
Deletion	-2	174305	9.1524055	Deletion	-19	2362	0.123791046	Deletion	-12	10430	0.5463522
Deletion	-37	324	0.01701259	Deletion	-32	751	0.039359473	Deletion	-11	8230	0.43434434
Deletion	-55	59	0.00309797	Deletion	-45	149	0.007809003	Deletion	-11	8398	0.44007456
Deletion	-43	177	0.00929391	Deletion	-42	267	0.013993315	Deletion	-11	8238	0.43209731
Deletion	-39	276	0.01449221	Deletion	-4	82617	4.329908902	Deletion	-11	8350	0.43739605
Deletion	-51	69	0.00362305	Deletion	-48	127	0.006655996	Deletion	-10	14515	0.76603987
Deletion	-38	412	0.02163329	Deletion	-6	33177	1.738787267	Deletion	-10	14617	0.76596449
Deletion	-49	79	0.00414813	Deletion	-7	18288	0.95846344	Deletion	-10	14634	0.76757854
Deletion	-68	2	0.00010502	Deletion	-28	1321	0.069232841	Deletion	-10	14674	0.76866463
Deletion	-66	1	5.25E-05	Deletion	-52	58	0.003039746	Deletion	-9	13978	0.73769929
Deletion	-31	620	0.03255495	Deletion	-57	20	0.001048188	Deletion	-9	14176	0.74285508
Deletion	-36	525	0.0275667	Deletion	-46	165	0.008647554	Deletion	-9	14129	0.74109042
Deletion	-18	4253	0.22331649	Deletion	-29	762	0.039935977	Deletion	-9	14050	0.73597779
Deletion	-61	11	0.00057759	Deletion	-20	3157	0.165456533	Deletion	-8	20402	1.07673065
Deletion	-21	1940	0.1018655	Deletion	-9	14102	0.739077615	Deletion	-8	20518	1.0751905
Deletion	-53	56	0.00294045	Deletion	-17	3159	0.165561352	Deletion	-8	20364	1.06812692
Deletion	-60	20	0.00105016	Deletion	-58	24	0.001257826	Deletion	-8	20467	1.07211797
Deletion	-17	3115	0.16356239	Deletion	-31	639	0.033489618	Deletion	-7	18393	0.97070419
Deletion	-19	2380	0.12496902	Deletion	-5	42329	2.218438262	Deletion	-7	18480	0.9683946
Deletion	-65	5	0.00026254	Deletion	-41	194	0.010167427	Deletion	-7	18420	0.96616077
Deletion	-56	46	0.00241537	Deletion	-44	189	0.00990538	Deletion	-7	18390	0.96331897
Deletion	-22	2538	0.13326528	Deletion	-61	12	0.000628913	Deletion	-6	33072	1.74539927
Deletion	-44	194	0.01018655	Deletion	-14	7307	0.382955619	Deletion	-6	33246	1.74216703
Deletion	-14	7167	0.37632478	Deletion	-34	596	0.031236013	Deletion	-6	33408	1.75230722
Deletion	-41	188	0.0098715	Deletion	-30	981	0.051413639	Deletion	-6	33451	1.75225573
Deletion	-47	118	0.00619594	Deletion	-56	47	0.002463243	Deletion	-5	42060	2.21974763
Deletion	-35	403	0.02116072	Deletion	-49	82	0.004297572	Deletion	-5	42106	2.20645146
Deletion	-29	750	0.03938099	Deletion	-64	8	0.000419275	Deletion	-5	42226	2.21482653
Deletion	-40	366	0.01921792	Deletion	-53	56	0.002934927	Deletion	-5	42585	2.23071987
Deletion	-64	5	0.00026254	Deletion	-68	1	5.24E-05	Deletion	-4	82359	4.34655717
Deletion	-9	14046	0.73752725	Deletion	-51	85	0.004454801	Deletion	-4	82708	4.33408985
Deletion	-5	42348	2.22360843	Deletion	-8	20528	1.075860536	Deletion	-4	82725	4.33906893
Deletion	-46	163	0.0085588	Deletion	-65	6	0.000314457	Deletion	-4	82983	4.34687864
Deletion	-58	27	0.00141772	Deletion	-26	1475	0.077303892	Deletion	-3	98510	5.19893815
Deletion	-42	259	0.01359957	Deletion	-59	18	0.00094337	Deletion	-3	99701	5.22456222

Deletion	-10	14654	0.76945211	Deletion	-63	4	0.000209638	Deletion	-3	99647	5.22665702
Deletion	-48	122	0.00640597	Deletion	-3	99270	5.202682943	Deletion	-3	99974	5.23691413
Deletion	-63	4	0.00021003	Deletion	-18	4299	0.225308089	Deletion	-2	173109	9.13595558
Deletion	-45	147	0.00771867	Deletion	-43	170	0.008909601	Deletion	-2	174940	9.16725925
Deletion	-13	6312	0.33143044	Deletion	-16	5414	0.28374459	Deletion	-2	174716	9.16415554
Deletion	-34	595	0.03124226	Deletion	-22	2607	0.136631353	Deletion	-2	174793	9.15613991
Deletion	-6	33254	1.74610076	Deletion	-13	6272	0.328711871	Deletion	-1	437858	23.10828
Deletion	-33	499	0.02620149	Deletion	-25	1237	0.06483045	Deletion	-1	440415	23.0787612
Deletion	-4	82453	4.32944144	Deletion	-11	8176	0.428499403	Deletion	-1	439467	23.0508021
Deletion	-62	9	0.00047257	Insertion	46	1	5.25E-05	Deletion	-1	440818	23.0912639
Deletion	-16	5418	0.2844883	Insertion	30	539	0.028296414	Insertion	1	500410	26.4095081
Insertion	33	318	0.01729014	Insertion	23	1180	0.061947623	Insertion	1	503878	26.4043687
Insertion	37	190	0.01033059	Insertion	1	502977	26.4052792	Insertion	1	503442	26.4064012
Insertion	24	1230	0.06687698	Insertion	45	7	0.000367486	Insertion	1	503421	26.3705818
Insertion	25	822	0.04469339	Insertion	4	52613	2.762076505	Insertion	2	131666	6.94877059
Insertion	11	5039	0.27397811	Insertion	38	173	0.009082151	Insertion	2	132288	6.93219613
Insertion	30	518	0.02816445	Insertion	26	930	0.048823126	Insertion	2	132272	6.93789454
Insertion	41	85	0.00462158	Insertion	33	331	0.017376833	Insertion	2	132229	6.92652008
Insertion	44	27	0.00146803	Insertion	8	12039	0.632023246	Insertion	3	67900	3.58347275
Insertion	32	436	0.02370598	Insertion	41	91	0.004777317	Insertion	3	68769	3.60365412
Insertion	17	2073	0.11271217	Insertion	21	1480	0.077697018	Insertion	3	68246	3.57962041
Insertion	16	3059	0.16632249	Insertion	17	2130	0.111820709	Insertion	3	68276	3.57648538
Insertion	9	8413	0.45742764	Insertion	12	6270	0.329162368	Insertion	4	52321	2.7612795
Insertion	31	407	0.02212921	Insertion	22	1581	0.082999315	Insertion	4	52550	2.75374113
Insertion	26	897	0.04877126	Insertion	44	27	0.001417446	Insertion	4	52650	2.76158331
Insertion	42	88	0.00478469	Insertion	6	19738	1.036205236	Insertion	4	52650	2.75795236
Insertion	22	1495	0.08128543	Insertion	18	2668	0.140064625	Insertion	5	25669	1.35470047
Insertion	7	10525	0.5722603	Insertion	32	454	0.023834085	Insertion	5	26168	1.37126352
Insertion	40	143	0.00777513	Insertion	15	3155	0.165631144	Insertion	5	25989	1.36316787
Insertion	38	181	0.00984125	Insertion	11	5221	0.274091982	Insertion	5	26249	1.37499509
Insertion	6	18873	1.02615379	Insertion	37	178	0.009344641	Insertion	6	19337	1.02052449
Insertion	2	127617	6.9387309	Insertion	2	131837	6.9211769	Insertion	6	19624	1.02834283
Insertion	15	3015	0.16393015	Insertion	5	26181	1.374449755	Insertion	6	19675	1.03198768
Insertion	27	730	0.03969121	Insertion	31	430	0.022574134	Insertion	6	19811	1.03775487
Insertion	45	7	0.0003806	Insertion	10	8862	0.465237147	Insertion	7	10865	0.57340842
Insertion	28	681	0.03702701	Insertion	27	741	0.038901007	Insertion	7	10924	0.57244278
Insertion	19	1676	0.09112668	Insertion	28	707	0.037116076	Insertion	7	10897	0.57156644
Insertion	8	11593	0.63032909	Insertion	9	8830	0.463557211	Insertion	7	10904	0.57118162
Insertion	20	1890	0.10276218	Insertion	34	300	0.015749396	Insertion	8	11993	0.63293945
Insertion	10	8417	0.45764513	Insertion	14	4374	0.229626188	Insertion	8	11914	0.62432106
Insertion	4	50553	2.7486437	Insertion	35	232	0.012179533	Insertion	8	11993	0.62905353
Insertion	29	506	0.02751199	Insertion	42	84	0.004409831	Insertion	8	12068	0.63215516
Insertion	35	223	0.01212485	Insertion	20	1994	0.104680983	Insertion	9	8723	0.46036278
Insertion	43	55	0.00299043	Insertion	40	134	0.00703473	Insertion	9	8773	0.45972542

Insertion	12	6060	0.32949144	Insertion	3	68130	3.576687745	Insertion	9	8887	0.46613848
Insertion	5	25306	1.37592581	Insertion	7	10930	0.57380298	Insertion	9	8705	0.45599193
Insertion	46	2	0.00010874	Insertion	19	1737	0.091189001	Insertion	10	8738	0.46115442
Insertion	14	4186	0.2275992	Insertion	43	59	0.003097381	Insertion	10	8734	0.45768173
Insertion	39	138	0.00750327	Insertion	25	879	0.046145729	Insertion	10	8830	0.46314873
Insertion	21	1459	0.07932805	Insertion	24	1275	0.066934931	Insertion	10	8855	0.46384935
Insertion	36	256	0.01391911	Insertion	16	3287	0.172560878	Insertion	11	5169	0.2727978
Insertion	23	1118	0.06078736	Insertion	13	4209	0.220964021	Insertion	11	5282	0.27678898
Insertion	3	65735	3.57411219	Insertion	36	277	0.014541942	Insertion	11	5232	0.27442742
Insertion	34	295	0.0160396	Insertion	29	504	0.026458985	Insertion	11	5243	0.27464282
Insertion	1	487998	26.5331954	Insertion	39	144	0.00755971	Insertion	12	6105	0.32219589
Insertion	18	2626	0.14277962	Insertion	36	266	0.013904011	Insertion	12	6224	0.32615195
Insertion	13	4003	0.21764921	Insertion	44	25	0.001306768	Insertion	12	6252	0.32792818
Insertion	37	195	0.01073165	Insertion	1	505425	26.41892785	Insertion	12	6226	0.32613507
Insertion	41	87	0.00478796	Insertion	16	3222	0.168416255	Insertion	13	4126	0.2177527
Insertion	21	1445	0.07952424	Insertion	45	7	0.000365895	Insertion	13	4232	0.22176656
Insertion	17	2030	0.11171918	Insertion	46	1	5.23E-05	Insertion	13	4190	0.21977273
Insertion	35	221	0.01216253	Insertion	8	12015	0.628032682	Insertion	13	4159	0.2178599
Insertion	43	54	0.00297184	Insertion	33	321	0.016778901	Insertion	14	4271	0.22540519
Insertion	27	718	0.03951447	Insertion	5	26187	1.368813303	Insertion	14	4322	0.22648276
Insertion	9	8346	0.45931441	Insertion	25	851	0.044482381	Insertion	14	4330	0.22711597
Insertion	42	86	0.00473293	Insertion	3	68284	3.569253736	Insertion	14	4261	0.22320294
Insertion	26	864	0.04754944	Insertion	26	919	0.04803679	Insertion	15	3101	0.16365757
Insertion	29	511	0.02812241	Insertion	42	86	0.004495282	Insertion	15	3127	0.16386201
Insertion	20	1910	0.10511509	Insertion	29	533	0.027860293	Insertion	15	3105	0.16286261
Insertion	34	287	0.01579478	Insertion	35	216	0.011290475	Insertion	15	3092	0.1619675
Insertion	38	174	0.00957593	Insertion	23	1167	0.060999928	Insertion	16	3183	0.16798518
Insertion	12	6001	0.3302595	Insertion	17	2144	0.11206842	Insertion	16	3110	0.16297117
Insertion	15	2987	0.16438679	Insertion	40	135	0.007056547	Insertion	16	3257	0.17083527
Insertion	10	8416	0.46316679	Insertion	27	750	0.039203039	Insertion	16	3164	0.16573906
Insertion	2	126467	6.95999463	Insertion	19	1775	0.092780525	Insertion	17	2126	0.11220122
Insertion	30	511	0.02812241	Insertion	12	6255	0.326953344	Insertion	17	2160	0.11318898
Insertion	13	3920	0.21573358	Insertion	22	1566	0.081855945	Insertion	17	2152	0.11287611
Insertion	4	50049	2.75440052	Insertion	24	1275	0.066645166	Insertion	17	2165	0.11340868
Insertion	31	407	0.02239887	Insertion	38	183	0.009565541	Insertion	18	2636	0.13911685
Insertion	6	18855	1.03766752	Insertion	20	1976	0.10328694	Insertion	18	2712	0.14211505
Insertion	22	1520	0.0836518	Insertion	18	2716	0.141967271	Insertion	18	2707	0.14198682
Insertion	33	306	0.01684043	Insertion	39	148	0.007736066	Insertion	18	2688	0.14080486
Insertion	1	482279	26.5417797	Insertion	34	297	0.015524403	Insertion	19	1762	0.09299085
Insertion	45	8	0.00044027	Insertion	4	52872	2.763657424	Insertion	19	1775	0.09301409
Insertion	19	1659	0.09130153	Insertion	13	4206	0.219850642	Insertion	19	1733	0.09089884
Insertion	46	1	5.50E-05	Insertion	6	19738	1.03171944	Insertion	19	1760	0.09219366
Insertion	5	24974	1.37442104	Insertion	32	469	0.024514967	Insertion	20	1994	0.10523483
Insertion	23	1122	0.06174823	Insertion	41	91	0.004756635	Insertion	20	1944	0.10187008

Insertion	14	4100	0.22563972	Insertion	30	526	0.027494398	Insertion	20	1981	0.10390687
Insertion	28	658	0.03621242	Insertion	2	132695	6.93606298	Insertion	20	2006	0.10507982
Insertion	3	64943	3.57407807	Insertion	43	56	0.00292716	Insertion	21	1439	0.07594429
Insertion	36	260	0.01430886	Insertion	9	8901	0.465261665	Insertion	21	1489	0.07802703
Insertion	24	1212	0.0667013	Insertion	7	10873	0.568339521	Insertion	21	1498	0.07857268
Insertion	32	448	0.02465527	Insertion	10	8885	0.464425333	Insertion	21	1467	0.07684551
Insertion	40	134	0.00737457	Insertion	11	5196	0.271598653	Insertion	22	1523	0.08037745
Insertion	11	4991	0.27467508	Insertion	37	180	0.009408729	Insertion	22	1538	0.08059475
Insertion	18	2552	0.14044696	Insertion	15	3111	0.162614205	Insertion	22	1542	0.08088056
Insertion	8	11551	0.63569862	Insertion	28	702	0.036694044	Insertion	22	1543	0.0808266
Insertion	25	819	0.04507291	Insertion	21	1481	0.077412934	Insertion	23	1162	0.06132541
Insertion	39	145	0.00797994	Insertion	14	4346	0.227168542	Insertion	23	1135	0.05947662
Insertion	7	10389	0.57174903	Insertion	31	429	0.022424138	Insertion	23	1121	0.05879838
Insertion	44	25	0.00137585	Insertion	36	269	0.014103855	Insertion	23	1157	0.06060685
Insertion	16	3044	0.16752373	Insertion	20	1988	0.104232205	Insertion	24	1287	0.06792238
Insertion	9	8756	0.46098865	Insertion	12	6257	0.328058806	Insertion	24	1295	0.06786099
Insertion	38	177	0.00931875	Insertion	3	68284	3.580177006	Insertion	24	1244	0.06524995
Insertion	21	1456	0.07665595	Insertion	39	148	0.007759742	Insertion	24	1291	0.06762614
Insertion	22	1586	0.08350023	Insertion	8	12004	0.629377962	Insertion	25	866	0.04570379
Insertion	29	520	0.02737712	Insertion	25	848	0.044461222	Insertion	25	845	0.04427995
Insertion	7	10806	0.5689177	Insertion	14	4265	0.223616878	Insertion	25	840	0.04405945
Insertion	37	194	0.01021377	Insertion	27	739	0.038746277	Insertion	25	864	0.04525871
Insertion	17	2146	0.11298329	Insertion	16	3234	0.169560841	Insertion	26	908	0.04792037
Insertion	14	4326	0.22775661	Insertion	32	459	0.024065685	Insertion	26	900	0.04716207
Insertion	45	7	0.00036854	Insertion	45	7	0.000367015	Insertion	26	886	0.04647223
Insertion	15	3077	0.16199887	Insertion	17	2151	0.112778407	Insertion	26	920	0.04819214
Insertion	19	1733	0.09123953	Insertion	38	177	0.009280232	Insertion	27	748	0.03947625
Insertion	18	2670	0.140571	Insertion	30	528	0.027683403	Insertion	27	777	0.04071659
Insertion	42	87	0.0045804	Insertion	5	26211	1.374260727	Insertion	27	753	0.03949615
Insertion	24	1255	0.06607364	Insertion	46	1	5.24E-05	Insertion	27	752	0.03939184
Insertion	12	6251	0.32910462	Insertion	6	19558	1.025439369	Insertion	28	696	0.03673192
Insertion	46	1	5.26E-05	Insertion	13	4125	0.216276582	Insertion	28	702	0.03678642
Insertion	13	4105	0.21612134	Insertion	29	524	0.02747368	Insertion	28	701	0.03676866
Insertion	26	895	0.04712024	Insertion	9	8901	0.466685542	Insertion	28	713	0.03734891
Insertion	20	2018	0.1062443	Insertion	40	132	0.006920851	Insertion	29	533	0.02812947
Insertion	11	5230	0.27535069	Insertion	44	24	0.001258336	Insertion	29	514	0.02693478
Insertion	6	19516	1.02748453	Insertion	1	503076	26.37662011	Insertion	29	533	0.02795677
Insertion	31	428	0.02253348	Insertion	11	5204	0.272849293	Insertion	29	522	0.0273438
Insertion	5	26212	1.38001765	Insertion	42	89	0.004666331	Insertion	30	522	0.02754894
Insertion	28	680	0.03580085	Insertion	21	1497	0.078488738	Insertion	30	516	0.02703959
Insertion	2	131908	6.94473401	Insertion	7	10885	0.570708024	Insertion	30	518	0.02716999
Insertion	32	472	0.02485	Insertion	34	284	0.014890315	Insertion	30	531	0.02781525
Insertion	36	263	0.01384651	Insertion	33	325	0.017039973	Insertion	31	412	0.0217436
Insertion	33	323	0.01700541	Insertion	26	945	0.049546999	Insertion	31	407	0.02132774

Insertion	43	55	0.00289566	Insertion	22	1581	0.082892916	Insertion	31	416	0.02181992
Insertion	34	318	0.01674216	Insertion	37	206	0.010800721	Insertion	31	424	0.02221029
Insertion	35	220	0.01158263	Insertion	41	93	0.004876054	Insertion	32	434	0.02290467
Insertion	44	26	0.00136886	Insertion	18	2662	0.139570488	Insertion	32	434	0.0227426
Insertion	3	68166	3.58882508	Insertion	19	1723	0.090338073	Insertion	32	432	0.02265915
Insertion	4	52398	2.75866644	Insertion	35	236	0.012373642	Insertion	32	441	0.0231008
Insertion	25	853	0.04490901	Insertion	43	58	0.00304098	Insertion	33	310	0.01636048
Insertion	23	1134	0.05970319	Insertion	10	8868	0.464955329	Insertion	33	320	0.01676874
Insertion	40	135	0.00710752	Insertion	28	699	0.03664905	Insertion	33	312	0.01636494
Insertion	39	145	0.00763401	Insertion	24	1267	0.06642968	Insertion	33	326	0.01707678
Insertion	16	3205	0.16873785	Insertion	23	1187	0.062235225	Insertion	34	306	0.01614938
Insertion	10	8667	0.45630295	Insertion	2	132238	6.933329139	Insertion	34	304	0.0159303
Insertion	1	501343	26.3948645	Insertion	4	52646	2.760265928	Insertion	34	313	0.01641739
Insertion	41	87	0.0045804	Insertion	31	428	0.022440334	Insertion	34	281	0.01471956
Insertion	30	532	0.0280089	Insertion	15	3056	0.160228178	Insertion	35	226	0.01192732
Insertion	8	11990	0.6312533	Insertion	3	68224	3.575580146	Insertion	35	233	0.01220974
Insertion	27	740	0.03895975	Insertion	22	1595	0.083593022	Insertion	35	229	0.01201144
Insertion	19	1728	0.09073381	Insertion	2	132279	6.932665428	Insertion	35	225	0.01178612
Insertion	25	873	0.04583948	Insertion	27	764	0.040040795	Insertion	36	266	0.01403835
Insertion	13	4187	0.21985096	Insertion	32	430	0.02253605	Insertion	36	270	0.01414862
Insertion	33	340	0.01785272	Insertion	8	12072	0.632686496	Insertion	36	268	0.01405706
Insertion	7	10902	0.57244213	Insertion	28	700	0.036686593	Insertion	36	265	0.01388143
Insertion	4	52510	2.75719465	Insertion	4	52662	2.75998478	Insertion	37	199	0.01050237
Insertion	41	89	0.00467321	Insertion	13	4228	0.22158702	Insertion	37	192	0.01006124
Insertion	37	192	0.01008153	Insertion	30	537	0.028143858	Insertion	37	199	0.01043789
Insertion	45	6	0.00031505	Insertion	43	54	0.002830109	Insertion	37	202	0.01058132
Insertion	43	57	0.00299296	Insertion	29	526	0.027567354	Insertion	38	169	0.0089191
Insertion	36	274	0.01438719	Insertion	24	1277	0.066926827	Insertion	38	185	0.00969443
Insertion	32	450	0.0236286	Insertion	23	1150	0.060270831	Insertion	38	176	0.0092315
Insertion	44	26	0.00136521	Insertion	39	150	0.007861413	Insertion	38	167	0.00874792
Insertion	16	3200	0.16802557	Insertion	11	5201	0.272581384	Insertion	39	150	0.00791636
Insertion	10	8777	0.46086264	Insertion	35	229	0.012001757	Insertion	39	139	0.00728392
Insertion	8	12057	0.63308886	Insertion	38	177	0.009276467	Insertion	39	149	0.00781531
Insertion	9	8793	0.46170277	Insertion	41	87	0.004559619	Insertion	39	141	0.00738597
Insertion	12	6154	0.32313418	Insertion	26	912	0.047797389	Insertion	40	138	0.00728305
Insertion	20	2025	0.10632868	Insertion	18	2685	0.140719288	Insertion	40	146	0.00765074
Insertion	42	90	0.00472572	Insertion	33	317	0.016613786	Insertion	40	140	0.00734324
Insertion	3	68330	3.58787107	Insertion	40	142	0.007442137	Insertion	40	135	0.00707167
Insertion	18	2664	0.13988129	Insertion	7	10950	0.573883129	Insertion	41	87	0.00459149
Insertion	39	143	0.00750864	Insertion	37	200	0.010481884	Insertion	41	85	0.0044542
Insertion	34	294	0.01543735	Insertion	36	267	0.013993315	Insertion	41	81	0.00424859
Insertion	31	437	0.02294599	Insertion	12	6297	0.330022106	Insertion	41	87	0.0045573
Insertion	15	3108	0.16319484	Insertion	42	85	0.004454801	Insertion	42	82	0.00432761
Insertion	17	2165	0.1136798	Insertion	14	4281	0.224364719	Insertion	42	84	0.00440179

Insertion	27	756	0.03969604
Insertion	46	1	5.25E-05
Insertion	23	1158	0.06080425
Insertion	26	905	0.04751973
Insertion	22	1576	0.08275259
Insertion	11	5185	0.27225394
Insertion	14	4316	0.22662449
Insertion	28	696	0.03654556
Insertion	21	1504	0.07897202
Insertion	38	172	0.00903137
Insertion	2	132454	6.95489353
Insertion	29	531	0.02788174
Insertion	6	19594	1.02884159
Insertion	5	26269	1.37933243
Insertion	30	540	0.02835432
Insertion	24	1242	0.06521493
Insertion	35	238	0.0124969
Insertion	40	135	0.00708858
Insertion	1	502321	26.3758669
Insertion	16	3221	0.168810736
Insertion	5	26169	1.371502064
Insertion	45	6	0.000314457
Insertion	6	19629	1.028744469
Insertion	15	3120	0.163517385
Insertion	10	8847	0.463666123
Insertion	25	850	0.044548005
Insertion	20	1962	0.102827278
Insertion	17	2130	0.111632061
Insertion	1	503847	26.40632812
Insertion	21	1480	0.077565939
Insertion	9	8840	0.463299257
Insertion	31	411	0.021540271
Insertion	19	1758	0.092135757
Insertion	44	25	0.001310235
Insertion	34	283	0.014831865
Insertion	42	88	0.00461575
Insertion	42	84	0.00440015
Insertion	43	51	0.00269156
Insertion	43	59	0.00309174
Insertion	43	55	0.00288484
Insertion	43	55	0.00288105
Insertion	44	24	0.00126662
Insertion	44	25	0.00131006
Insertion	44	27	0.0014162
Insertion	44	28	0.00146672
Insertion	45	8	0.00042221
Insertion	45	7	0.00036682
Insertion	45	7	0.00036716
Insertion	45	7	0.00036668
Insertion	46	1	5.28E-05
Insertion	46	1	5.24E-05
Insertion	46	1	5.25E-05
Insertion	46	2	0.00010477

OL				ZK				SG1		
Type	Length	Count	Percent(%)	Type	Length	Count	Percent(%)	Type	Length	Count
Deletion	-64	8	0.000420504	Deletion	-54	65	0.003450251	Deletion	-47	108
Deletion	-14	7242	0.380660832	Deletion	-1	435071	23.09390893	Deletion	-20	3151
Deletion	-35	418	0.02197131	Deletion	-13	6216	0.329950141	Deletion	-39	265
Deletion	-2	174030	9.147528937	Deletion	-50	91	0.004830351	Deletion	-24	1997
Deletion	-59	19	0.000998696	Deletion	-32	704	0.037368871	Deletion	-11	8198
Deletion	-22	2589	0.136085459	Deletion	-36	502	0.026646553	Deletion	-28	1302
Deletion	-68	2	0.000105126	Deletion	-55	57	0.003025605	Deletion	-4	82448
Deletion	-7	18313	0.962585172	Deletion	-60	17	0.000902373	Deletion	-58	31
Deletion	-50	97	0.005098605	Deletion	-30	975	0.051753762	Deletion	-35	409
Deletion	-34	602	0.031642892	Deletion	-67	2	0.000106162	Deletion	-15	4521
Deletion	-26	1482	0.077898281	Deletion	-57	19	0.001008535	Deletion	-33	482
Deletion	-3	99361	5.222706561	Deletion	-45	145	0.007696713	Deletion	-31	647
Deletion	-16	5364	0.281947625	Deletion	-21	1845	0.097934043	Deletion	-60	20
Deletion	-37	315	0.016557327	Deletion	-58	31	0.001645504	Deletion	-6	33061
Deletion	-28	1310	0.068857455	Deletion	-63	7	0.000371565	Deletion	-63	8
Deletion	-9	14023	0.737090147	Deletion	-16	5281	0.28031961	Deletion	-1	439168
Deletion	-53	45	0.002365332	Deletion	-61	12	0.000636969	Deletion	-42	259
Deletion	-1	439043	23.0773921	Deletion	-53	53	0.002813281	Deletion	-65	5
Deletion	-31	608	0.031958269	Deletion	-51	74	0.003927978	Deletion	-56	47
Deletion	-43	168	0.008830574	Deletion	-59	17	0.000902373	Deletion	-14	7121
Deletion	-18	4253	0.223550196	Deletion	-14	7092	0.376448906	Deletion	-34	611
Deletion	-66	1	5.26E-05	Deletion	-10	14328	0.760541445	Deletion	-66	1
Deletion	-36	518	0.027227604	Deletion	-65	5	0.000265404	Deletion	-57	28
Deletion	-40	365	0.019185474	Deletion	-15	4392	0.233130795	Deletion	-37	311
Deletion	-6	33154	1.742671806	Deletion	-37	314	0.016667366	Deletion	-38	409
Deletion	-4	82462	4.33444539	Deletion	-42	264	0.014013326	Deletion	-16	5349
Deletion	-13	6312	0.331777295	Deletion	-52	56	0.002972524	Deletion	-25	1213
Deletion	-5	42227	2.219575386	Deletion	-64	6	0.000318485	Deletion	-51	76
Deletion	-63	5	0.000262815	Deletion	-38	386	0.020489182	Deletion	-13	6291
Deletion	-54	68	0.00357428	Deletion	-40	357	0.018949839	Deletion	-2	174547
Deletion	-62	7	0.000367941	Deletion	-44	181	0.009607622	Deletion	-55	51
Deletion	-32	714	0.037529941	Deletion	-3	98146	5.209666435	Deletion	-67	1
Deletion	-20	3145	0.165310455	Deletion	-6	32733	1.737493239	Deletion	-50	93
Deletion	-38	407	0.021393118	Deletion	-7	18084	0.959912863	Deletion	-52	65
Deletion	-51	78	0.00409991	Deletion	-47	94	0.004989594	Deletion	-19	2391
Deletion	-49	80	0.004205035	Deletion	-11	8069	0.42830883	Deletion	-12	10449
Deletion	-41	207	0.010880529	Deletion	-18	4171	0.221399942	Deletion	-10	14692
Deletion	-48	124	0.006517805	Deletion	-12	10252	0.544184178	Deletion	-32	741
Deletion	-24	2028	0.106597648	Deletion	-46	156	0.008280602	Deletion	-26	1492
Deletion	-56	43	0.002260207	Deletion	-27	993	0.052709217	Deletion	-7	18370
Deletion	-11	8252	0.433749404	Deletion	-5	41687	2.212778561	Deletion	-21	1904

Deletion	-60	25	0.001314074	Deletion	-39	276	0.014650296	Deletion	-30	996
Deletion	-33	491	0.025808405	Deletion	-19	2368	0.125695292	Deletion	-17	3165
Deletion	-8	20576	1.081535111	Deletion	-62	9	0.000477727	Deletion	-5	42310
Deletion	-45	153	0.00804213	Deletion	-26	1428	0.075799357	Deletion	-27	1013
Deletion	-10	14646	0.76983686	Deletion	-17	3051	0.161949466	Deletion	-40	362
Deletion	-44	200	0.010512589	Deletion	-35	373	0.019799132	Deletion	-9	14068
Deletion	-12	10416	0.547495612	Deletion	-68	1	5.31E-05	Deletion	-45	148
Deletion	-58	28	0.001471762	Deletion	-48	132	0.007006663	Deletion	-22	2513
Deletion	-27	1014	0.053298824	Deletion	-24	2034	0.107966311	Deletion	-62	11
Deletion	-47	108	0.005676798	Deletion	-56	45	0.002388635	Deletion	-44	205
Deletion	-19	2390	0.125625433	Deletion	-2	172473	9.155001722	Deletion	-49	77
Deletion	-67	2	0.000105126	Deletion	-33	477	0.025319533	Deletion	-46	156
Deletion	-52	61	0.00320634	Deletion	-4	81347	4.317962377	Deletion	-36	514
Deletion	-17	3116	0.16378613	Deletion	-23	1448	0.076860972	Deletion	-68	1
Deletion	-25	1174	0.061708895	Deletion	-31	621	0.032963166	Deletion	-23	1499
Deletion	-21	1927	0.101288791	Deletion	-49	69	0.003662574	Deletion	-64	3
Deletion	-29	780	0.040999095	Deletion	-34	623	0.033069327	Deletion	-8	20423
Deletion	-57	23	0.001208948	Deletion	-29	734	0.038961294	Deletion	-29	764
Deletion	-30	986	0.051827062	Deletion	-41	201	0.010669237	Deletion	-18	4239
Deletion	-23	1513	0.079527732	Deletion	-9	13728	0.728692976	Deletion	-41	191
Deletion	-46	166	0.008725449	Deletion	-66	1	5.31E-05	Deletion	-61	13
Deletion	-55	56	0.002943525	Deletion	-8	20326	1.078919976	Deletion	-53	67
Deletion	-39	300	0.015768883	Deletion	-28	1276	0.067731078	Deletion	-59	23
Deletion	-15	4441	0.233432029	Deletion	-20	3152	0.167310625	Deletion	-43	160
Deletion	-61	18	0.000946133	Deletion	-43	159	0.008439844	Deletion	-3	99066
Deletion	-65	3	0.000157689	Deletion	-25	1169	0.062051434	Deletion	-48	134
Deletion	-42	278	0.014612498	Deletion	-22	2524	0.133975894	Deletion	-54	68
Deletion	-50	95	0.005042557	Deletion	-21	1885	0.099934844	Deletion	-15	4478
Deletion	-24	1991	0.105681369	Deletion	-57	23	0.001219364	Deletion	-19	2404
Deletion	-40	375	0.019904828	Deletion	-20	3155	0.16726495	Deletion	-22	2566
Deletion	-33	467	0.024788146	Deletion	-2	172973	9.170307529	Deletion	-7	18311
Deletion	-61	13	0.000690034	Deletion	-43	169	0.008959676	Deletion	-59	17
Deletion	-32	698	0.037049521	Deletion	-22	2540	0.134660214	Deletion	-36	509
Deletion	-65	6	0.000318477	Deletion	-13	6197	0.328539112	Deletion	-63	4
Deletion	-13	6305	0.334666515	Deletion	-61	14	0.000742222	Deletion	-30	993
Deletion	-37	290	0.015393067	Deletion	-51	65	0.003446029	Deletion	-1	435584
Deletion	-6	32695	1.735435637	Deletion	-56	47	0.002491744	Deletion	-4	82168
Deletion	-16	5271	0.279782268	Deletion	-5	41827	2.21749321	Deletion	-42	264
Deletion	-22	2498	0.132592697	Deletion	-46	156	0.00827047	Deletion	-24	2064
Deletion	-2	172470	9.154628669	Deletion	-59	17	0.000901269	Deletion	-13	6247
Deletion	-11	8082	0.428988861	Deletion	-28	1270	0.067330107	Deletion	-6	33187
Deletion	-9	13806	0.732816162	Deletion	-6	32743	1.735897391	Deletion	-21	1905
Deletion	-21	1845	0.097931756	Deletion	-49	71	0.003764124	Deletion	-52	70
Deletion	-44	182	0.009660477	Deletion	-8	20327	1.077652819	Deletion	-67	2

Deletion	-58	22	0.00116775	Deletion	-4	81720	4.332453801	Deletion	-38	388
Deletion	-10	14333	0.76078908	Deletion	-19	2350	0.124587205	Deletion	-26	1501
Deletion	-3	98465	5.226477137	Deletion	-33	506	0.026826011	Deletion	-49	72
Deletion	-51	64	0.003397091	Deletion	-65	4	0.000212063	Deletion	-44	192
Deletion	-41	189	0.010032034	Deletion	-60	20	0.001060317	Deletion	-31	642
Deletion	-47	105	0.005573352	Deletion	-37	317	0.016806019	Deletion	-28	1311
Deletion	-15	4351	0.230949089	Deletion	-48	123	0.006520947	Deletion	-8	20421
Deletion	-23	1441	0.076487621	Deletion	-53	51	0.002703807	Deletion	-37	323
Deletion	-14	7069	0.375219285	Deletion	-63	4	0.000212063	Deletion	-25	1191
Deletion	-55	45	0.002388579	Deletion	-62	8	0.000424127	Deletion	-40	360
Deletion	-62	8	0.000424636	Deletion	-18	4241	0.224840144	Deletion	-45	142
Deletion	-68	1	5.31E-05	Deletion	-16	5425	0.287610889	Deletion	-5	42212
Deletion	-27	1007	0.053451099	Deletion	-11	8120	0.430488557	Deletion	-34	614
Deletion	-56	38	0.002017023	Deletion	-34	577	0.030590135	Deletion	-10	14698
Deletion	-42	254	0.013482204	Deletion	-55	52	0.002756823	Deletion	-47	110
Deletion	-45	152	0.00806809	Deletion	-68	2	0.000106032	Deletion	-48	127
Deletion	-43	157	0.008333488	Deletion	-10	14542	0.770956231	Deletion	-33	500
Deletion	-28	1257	0.066720985	Deletion	-15	4404	0.233481725	Deletion	-14	7194
Deletion	-63	4	0.000212318	Deletion	-38	393	0.020835222	Deletion	-16	5367
Deletion	-7	18163	0.964083728	Deletion	-12	10208	0.541185614	Deletion	-17	3094
Deletion	-29	738	0.039172702	Deletion	-27	994	0.052697737	Deletion	-20	3138
Deletion	-38	388	0.020594862	Deletion	-7	18030	0.955875453	Deletion	-46	158
Deletion	-46	144	0.007643454	Deletion	-50	78	0.004135235	Deletion	-11	8099
Deletion	-57	19	0.001008511	Deletion	-9	13660	0.724196267	Deletion	-53	52
Deletion	-5	41937	2.225996767	Deletion	-66	1	5.30E-05	Deletion	-3	98882
Deletion	-54	60	0.003184773	Deletion	-41	175	0.009277771	Deletion	-27	1063
Deletion	-12	10246	0.543852991	Deletion	-40	357	0.018926652	Deletion	-65	5
Deletion	-17	3052	0.161998763	Deletion	-25	1133	0.060066938	Deletion	-51	69
Deletion	-20	3149	0.167147479	Deletion	-17	3058	0.162122415	Deletion	-9	14023
Deletion	-48	121	0.006422625	Deletion	-64	6	0.000318095	Deletion	-43	168
Deletion	-30	965	0.051221758	Deletion	-39	292	0.015480623	Deletion	-61	11
Deletion	-35	376	0.019957908	Deletion	-54	69	0.003658092	Deletion	-64	4
Deletion	-8	20219	1.073215267	Deletion	-23	1473	0.078092321	Deletion	-12	10390
Deletion	-64	6	0.000318477	Deletion	-35	394	0.020888238	Deletion	-29	742
Deletion	-1	435392	23.1104081	Deletion	-26	1507	0.079894859	Deletion	-35	410
Deletion	-60	28	0.001486227	Deletion	-44	189	0.010019992	Deletion	-50	76
Deletion	-31	615	0.032643919	Deletion	-24	2022	0.107198013	Deletion	-56	50
Deletion	-39	276	0.014649954	Deletion	-29	732	0.038807589	Deletion	-41	198
Deletion	-53	49	0.002600898	Deletion	-1	435080	23.06612824	Deletion	-39	281
Deletion	-59	18	0.000955432	Deletion	-45	157	0.008323486	Deletion	-54	68
Deletion	-18	4112	0.218263078	Deletion	-31	613	0.032498705	Deletion	-55	49
Deletion	-67	1	5.31E-05	Deletion	-47	108	0.00572571	Deletion	-66	2
Deletion	-26	1459	0.077443052	Deletion	-30	1011	0.053599006	Deletion	-23	1452
Deletion	-25	1171	0.062156144	Deletion	-32	713	0.037800288	Deletion	-62	10

Deletion	-4	81735	4.338456394	Deletion	-36	459	0.024334267	Deletion	-32	748
Deletion	-36	495	0.026274373	Deletion	-14	7098	0.376306376	Deletion	-57	23
Deletion	-66	2	0.000106159	Deletion	-52	67	0.003552061	Deletion	-18	4225
Deletion	-34	568	0.03014918	Deletion	-3	98718	5.233616915	Deletion	-2	173651
Deletion	-49	79	0.004193284	Deletion	-58	25	0.001325396	Deletion	-58	23
Deletion	-19	2336	0.123993811	Deletion	-42	274	0.014526338	Deletion	-68	2
Deletion	-52	50	0.002653977	Deletion	-57	19	0.001005797	Deletion	-60	20
Deletion	-31	596	0.031479294	Deletion	-46	141	0.007464073	Deletion	-9	14052
Deletion	-2	173216	9.148854809	Deletion	-60	16	0.000846987	Deletion	-62	5
Deletion	-15	4459	0.235513715	Deletion	-17	3085	0.163309687	Deletion	-33	487
Deletion	-62	7	0.000369723	Deletion	-68	1	5.29E-05	Deletion	-3	99410
Deletion	-67	1	5.28E-05	Deletion	-34	585	0.030967963	Deletion	-14	7240
Deletion	-61	15	0.000792264	Deletion	-66	2	0.000105873	Deletion	-22	2533
Deletion	-43	160	0.008450817	Deletion	-21	1876	0.099309229	Deletion	-64	7
Deletion	-18	4201	0.221886772	Deletion	-59	20	0.001058734	Deletion	-63	5
Deletion	-6	32976	1.741713445	Deletion	-63	6	0.00031762	Deletion	-66	3
Deletion	-33	464	0.02450737	Deletion	-2	172901	9.152806518	Deletion	-58	21
Deletion	-1	438790	23.17583827	Deletion	-67	1	5.29E-05	Deletion	-37	308
Deletion	-34	590	0.031162389	Deletion	-40	355	0.018792525	Deletion	-51	77
Deletion	-30	959	0.050652086	Deletion	-3	98015	5.188589602	Deletion	-17	3199
Deletion	-32	704	0.037183596	Deletion	-53	48	0.002540961	Deletion	-32	729
Deletion	-9	13857	0.731893596	Deletion	-61	14	0.000741114	Deletion	-42	270
Deletion	-59	20	0.001056352	Deletion	-47	114	0.006034783	Deletion	-28	1323
Deletion	-46	154	0.008133912	Deletion	-27	1021	0.05404836	Deletion	-2	175532
Deletion	-16	5279	0.278824153	Deletion	-62	6	0.00031762	Deletion	-20	3172
Deletion	-4	81894	4.3254452	Deletion	-23	1529	0.080940198	Deletion	-41	188
Deletion	-5	42024	2.219607164	Deletion	-44	174	0.009210984	Deletion	-8	20604
Deletion	-3	98595	5.207552073	Deletion	-8	20276	1.073344312	Deletion	-55	54
Deletion	-38	378	0.019965056	Deletion	-31	603	0.031920824	Deletion	-50	90
Deletion	-20	3023	0.159667629	Deletion	-10	14443	0.764564604	Deletion	-52	58
Deletion	-27	1010	0.053345784	Deletion	-19	2322	0.122918993	Deletion	-38	394
Deletion	-35	371	0.019595333	Deletion	-45	150	0.007940503	Deletion	-68	2
Deletion	-68	2	0.000105635	Deletion	-33	499	0.026415408	Deletion	-18	4323
Deletion	-64	5	0.000264088	Deletion	-52	64	0.003387948	Deletion	-59	16
Deletion	-51	69	0.003644415	Deletion	-51	76	0.004023188	Deletion	-30	1014
Deletion	-41	192	0.010140981	Deletion	-7	18257	0.966465137	Deletion	-25	1146
Deletion	-39	291	0.015369924	Deletion	-16	5351	0.283264224	Deletion	-5	42415
Deletion	-22	2539	0.134103907	Deletion	-39	292	0.015457513	Deletion	-23	1502
Deletion	-10	14428	0.76205245	Deletion	-15	4418	0.233874293	Deletion	-13	6366
Deletion	-56	45	0.002376792	Deletion	-20	3142	0.166327078	Deletion	-1	442201
Deletion	-13	6236	0.329370604	Deletion	-13	6164	0.326301753	Deletion	-65	5
Deletion	-58	20	0.001056352	Deletion	-65	3	0.00015881	Deletion	-26	1523
Deletion	-53	45	0.002376792	Deletion	-6	32734	1.732829588	Deletion	-45	153
Deletion	-24	1961	0.10357533	Deletion	-48	122	0.006458276	Deletion	-21	1916

Deletion	-12	10347	0.546503791	Deletion	-22	2536	0.134247444	Deletion	-4	82941
Deletion	-28	1280	0.067606538	Deletion	-26	1463	0.077446376	Deletion	-47	101
Deletion	-14	7067	0.373262037	Deletion	-4	81530	4.315928279	Deletion	-57	25
Deletion	-42	244	0.012887496	Deletion	-58	25	0.001323417	Deletion	-7	18305
Deletion	-29	799	0.042201269	Deletion	-38	397	0.021015866	Deletion	-43	153
Deletion	-65	4	0.00021127	Deletion	-35	382	0.020221815	Deletion	-60	19
Deletion	-66	2	0.000105635	Deletion	-25	1177	0.062306483	Deletion	-29	743
Deletion	-63	4	0.00021127	Deletion	-55	48	0.002540961	Deletion	-16	5390
Deletion	-17	3100	0.163734585	Deletion	-24	1978	0.104708771	Deletion	-36	492
Deletion	-8	20306	1.072514351	Deletion	-43	164	0.008681617	Deletion	-39	303
Deletion	-60	19	0.001003535	Deletion	-56	42	0.002223341	Deletion	-54	70
Deletion	-37	297	0.01568683	Deletion	-28	1278	0.067653089	Deletion	-35	396
Deletion	-50	90	0.004753585	Deletion	-12	10327	0.54667719	Deletion	-56	47
Deletion	-25	1172	0.061902237	Deletion	-42	270	0.014292906	Deletion	-6	33335
Deletion	-44	197	0.010405069	Deletion	-11	8270	0.437786421	Deletion	-44	198
Deletion	-54	58	0.003063421	Deletion	-1	436457	23.10458861	Deletion	-53	58
Deletion	-11	7990	0.422012689	Deletion	-36	498	0.026362471	Deletion	-12	10370
Deletion	-19	2386	0.126022813	Deletion	-37	281	0.01487521	Deletion	-27	1031
Deletion	-57	22	0.001161987	Deletion	-49	73	0.003864378	Deletion	-24	2012
Deletion	-7	18138	0.958005776	Deletion	-32	688	0.036420442	Deletion	-61	14
Deletion	-49	71	0.00375005	Deletion	-64	6	0.00031762	Deletion	-48	137
Deletion	-36	488	0.025774993	Deletion	-54	66	0.003493821	Deletion	-19	2388
Deletion	-21	1902	0.100459091	Deletion	-18	4254	0.225192676	Deletion	-34	585
Deletion	-55	54	0.002852151	Deletion	-50	99	0.005240732	Deletion	-40	358
Deletion	-47	96	0.00507049	Deletion	-29	782	0.041396491	Deletion	-10	14697
Deletion	-26	1438	0.07595172	Deletion	-14	7144	0.378179708	Deletion	-46	150
Deletion	-23	1471	0.077694702	Deletion	-5	41879	2.216935612	Deletion	-31	596
Deletion	-48	108	0.005704302	Deletion	-9	13954	0.738678563	Deletion	-67	1
Deletion	-52	54	0.002852151	Deletion	-30	991	0.052460259	Deletion	-15	4529
Deletion	-40	365	0.019278427	Deletion	-41	182	0.009634477	Deletion	-11	8258
Deletion	-45	155	0.008186729	Deletion	-35	393	0.020635879	Deletion	-49	76
Deletion	-6	32858	1.731146035	Deletion	-39	294	0.015437528	Deletion	-49	75
Deletion	-53	49	0.002581598	Deletion	-47	93	0.0048833	Deletion	-14	7114
Deletion	-19	2352	0.123916717	Deletion	-26	1505	0.07902544	Deletion	-20	3120
Deletion	-38	406	0.021390386	Deletion	-46	149	0.007823781	Deletion	-60	23
Deletion	-68	1	5.27E-05	Deletion	-68	3	0.000157526	Deletion	-30	1004
Deletion	-23	1447	0.076236177	Deletion	-59	20	0.001050172	Deletion	-53	57
Deletion	-15	4480	0.236031841	Deletion	-17	3145	0.165139542	Deletion	-19	2410
Deletion	-18	4242	0.22349265	Deletion	-6	33343	1.750794193	Deletion	-38	386
Deletion	-59	17	0.000895657	Deletion	-50	87	0.004568248	Deletion	-1	437801
Deletion	-13	6284	0.331076806	Deletion	-29	772	0.040536638	Deletion	-29	746
Deletion	-44	190	0.010010279	Deletion	-10	14677	0.770668697	Deletion	-50	91
Deletion	-55	52	0.002739655	Deletion	-43	159	0.008348867	Deletion	-63	5
Deletion	-39	301	0.015858389	Deletion	-55	51	0.002677939	Deletion	-6	33068

Deletion	-30	1003	0.052843736	Deletion	-45	155	0.008138833	Deletion	-37	311
Deletion	-58	32	0.001685942	Deletion	-56	41	0.002152853	Deletion	-9	13952
Deletion	-25	1175	0.061905673	Deletion	-58	23	0.001207698	Deletion	-17	3152
Deletion	-35	375	0.01975713	Deletion	-51	87	0.004568248	Deletion	-3	99233
Deletion	-28	1265	0.066647384	Deletion	-53	51	0.002677939	Deletion	-8	20459
Deletion	-46	157	0.008271652	Deletion	-37	303	0.015910105	Deletion	-2	174003
Deletion	-63	3	0.000158057	Deletion	-8	20574	1.080311901	Deletion	-13	6309
Deletion	-52	57	0.003003084	Deletion	-57	23	0.001207698	Deletion	-11	8262
Deletion	-12	10363	0.54598169	Deletion	-38	421	0.02210612	Deletion	-41	190
Deletion	-2	173812	9.157403207	Deletion	-19	2406	0.126335687	Deletion	-18	4245
Deletion	-42	264	0.013909019	Deletion	-61	14	0.00073512	Deletion	-47	103
Deletion	-67	1	5.27E-05	Deletion	-65	7	0.00036756	Deletion	-27	1019
Deletion	-66	3	0.000158057	Deletion	-42	284	0.014912442	Deletion	-39	290
Deletion	-60	21	0.001106399	Deletion	-40	350	0.018378009	Deletion	-46	147
Deletion	-36	477	0.025131069	Deletion	-15	4438	0.233033159	Deletion	-65	7
Deletion	-64	8	0.000421485	Deletion	-62	9	0.000472577	Deletion	-4	82303
Deletion	-51	72	0.003793369	Deletion	-3	99329	5.215626559	Deletion	-45	141
Deletion	-5	42066	2.216275765	Deletion	-22	2542	0.133476857	Deletion	-43	181
Deletion	-37	328	0.017280903	Deletion	-31	613	0.032187771	Deletion	-40	368
Deletion	-47	116	0.006111539	Deletion	-7	18316	0.961747486	Deletion	-56	47
Deletion	-61	13	0.000684914	Deletion	-63	6	0.000315052	Deletion	-68	2
Deletion	-17	3111	0.163905147	Deletion	-49	71	0.00372811	Deletion	-28	1285
Deletion	-29	732	0.038565917	Deletion	-16	5435	0.285384232	Deletion	-7	18181
Deletion	-27	1007	0.053054479	Deletion	-66	1	5.25E-05	Deletion	-16	5370
Deletion	-32	696	0.036669232	Deletion	-54	66	0.003465567	Deletion	-33	494
Deletion	-9	13996	0.737388761	Deletion	-33	483	0.025361653	Deletion	-42	261
Deletion	-4	81980	4.31917195	Deletion	-60	19	0.000997663	Deletion	-12	10276
Deletion	-50	88	0.00463634	Deletion	-52	74	0.003885636	Deletion	-57	30
Deletion	-48	139	0.007323309	Deletion	-67	2	0.000105017	Deletion	-21	1889
Deletion	-24	2003	0.105529415	Deletion	-27	1023	0.053716296	Deletion	-10	14617
Deletion	-10	14622	0.770369996	Deletion	-9	13908	0.730289585	Deletion	-34	589
Deletion	-14	7143	0.376333804	Deletion	-32	731	0.038383785	Deletion	-61	12
Deletion	-21	1884	0.099259819	Deletion	-5	42305	2.22137625	Deletion	-22	2507
Deletion	-31	596	0.031400665	Deletion	-48	125	0.006563575	Deletion	-48	114
Deletion	-20	3174	0.167224345	Deletion	-24	2024	0.106277403	Deletion	-55	46
Deletion	-62	8	0.000421485	Deletion	-36	510	0.026779385	Deletion	-25	1182
Deletion	-41	184	0.009694165	Deletion	-30	998	0.052403581	Deletion	-62	10
Deletion	-65	7	0.0003688	Deletion	-2	174629	9.16952401	Deletion	-35	413
Deletion	-57	24	0.001264456	Deletion	-41	199	0.010449211	Deletion	-64	5
Deletion	-16	5394	0.284186552	Deletion	-18	4232	0.222216388	Deletion	-58	24
Deletion	-11	8252	0.434762222	Deletion	-4	82705	4.342723621	Deletion	-31	569
Deletion	-40	345	0.018176559	Deletion	-13	6240	0.327653653	Deletion	-52	61
Deletion	-54	64	0.003371883	Deletion	-20	3168	0.166347239	Deletion	-67	1
Deletion	-56	42	0.002212799	Deletion	-44	197	0.010344194	Deletion	-32	719

Deletion	-22	2520	0.132767911	Deletion	-14	7186	0.377326787	Deletion	-23	1485
Deletion	-49	73	0.003846055	Deletion	-25	1196	0.062800284	Deletion	-24	1958
Deletion	-8	20474	1.078686588	Deletion	-1	438415	23.02055712	Deletion	-66	2
Deletion	-26	1460	0.076921091	Deletion	-21	1906	0.100081388	Deletion	-36	487
Deletion	-43	172	0.009061937	Deletion	-23	1528	0.080233138	Deletion	-15	4481
Deletion	-1	437618	23.05620139	Deletion	-28	1323	0.069468876	Deletion	-59	20
Deletion	-34	595	0.031347979	Deletion	-12	10469	0.549712515	Deletion	-54	63
Deletion	-3	99260	5.22958048	Deletion	-64	5	0.000262543	Deletion	-26	1466
Deletion	-7	18273	0.962725409	Deletion	-34	586	0.030770039	Deletion	-44	192
Deletion	-45	155	0.00816628	Deletion	-11	8161	0.428522671	Deletion	-51	71
Deletion	-33	497	0.026184782	Insertion	37	187	0.009926106	Deletion	-5	42139
Insertion	30	522	0.027437856	Insertion	29	514	0.027283522	Insertion	40	136
Insertion	24	1275	0.067017752	Insertion	8	11867	0.629909641	Insertion	5	26139
Insertion	28	704	0.037004312	Insertion	46	1	5.31E-05	Insertion	6	19563
Insertion	44	26	0.001366637	Insertion	18	2677	0.142097254	Insertion	15	3028
Insertion	31	433	0.022759754	Insertion	16	3159	0.16768219	Insertion	21	1523
Insertion	40	138	0.007253686	Insertion	25	842	0.044694018	Insertion	11	5265
Insertion	3	68030	3.575856999	Insertion	15	3077	0.163329566	Insertion	37	195
Insertion	17	2175	0.114324401	Insertion	34	287	0.015234184	Insertion	10	8785
Insertion	43	55	0.002890962	Insertion	35	234	0.012420903	Insertion	38	175
Insertion	8	12049	0.633330898	Insertion	42	84	0.004458786	Insertion	28	715
Insertion	15	3095	0.162682308	Insertion	5	25887	1.374102205	Insertion	4	52427
Insertion	35	228	0.011984351	Insertion	24	1302	0.069111178	Insertion	25	862
Insertion	16	3185	0.167412973	Insertion	40	133	0.007059744	Insertion	30	527
Insertion	6	19687	1.034806655	Insertion	39	136	0.007218986	Insertion	44	28
Insertion	20	1974	0.103759249	Insertion	43	55	0.002919443	Insertion	2	132171
Insertion	19	1774	0.093246661	Insertion	44	25	0.00132702	Insertion	20	2007
Insertion	2	131793	6.927427922	Insertion	32	430	0.022824736	Insertion	13	4122
Insertion	18	2656	0.139607176	Insertion	14	4330	0.229839786	Insertion	8	12097
Insertion	27	745	0.039159392	Insertion	31	425	0.022559332	Insertion	19	1722
Insertion	23	1155	0.060710199	Insertion	36	265	0.014066407	Insertion	23	1178
Insertion	13	4132	0.21719008	Insertion	27	739	0.039226698	Insertion	22	1590
Insertion	12	6266	0.3293594	Insertion	12	6148	0.326340648	Insertion	31	438
Insertion	5	26123	1.373101755	Insertion	33	330	0.017516658	Insertion	43	53
Insertion	38	176	0.009251078	Insertion	19	1711	0.090821218	Insertion	16	3236
Insertion	1	502009	26.38707036	Insertion	4	51984	2.759351374	Insertion	24	1260
Insertion	42	90	0.004730665	Insertion	7	10648	0.565204167	Insertion	27	754
Insertion	14	4339	0.228070609	Insertion	41	87	0.004618028	Insertion	39	141
Insertion	34	295	0.015506068	Insertion	6	19265	1.022601266	Insertion	41	90
Insertion	26	914	0.04804253	Insertion	2	130779	6.941851596	Insertion	35	230
Insertion	46	1	5.26E-05	Insertion	21	1454	0.077179457	Insertion	45	7
Insertion	21	1532	0.080526428	Insertion	3	67251	3.569735674	Insertion	3	68061
Insertion	32	421	0.022128999	Insertion	22	1539	0.081691324	Insertion	14	4282
Insertion	11	5186	0.272591421	Insertion	28	685	0.036360336	Insertion	33	333

Insertion	36	275	0.014454809	Insertion	1	498892	26.48157752	Insertion	9	8760
Insertion	7	10840	0.5697823	Insertion	38	174	0.009236056	Insertion	17	2202
Insertion	9	8780	0.461502638	Insertion	10	8638	0.458511795	Insertion	32	453
Insertion	37	193	0.010144648	Insertion	11	5125	0.272039008	Insertion	29	546
Insertion	10	8781	0.461555201	Insertion	13	4128	0.219117468	Insertion	18	2640
Insertion	45	6	0.000315378	Insertion	45	8	0.000424646	Insertion	36	264
Insertion	41	82	0.004310161	Insertion	17	2175	0.115450701	Insertion	7	10877
Insertion	33	322	0.016925268	Insertion	26	885	0.046976492	Insertion	26	889
Insertion	4	52535	2.761394201	Insertion	20	1984	0.105312272	Insertion	46	2
Insertion	22	1566	0.082313568	Insertion	9	8701	0.461855885	Insertion	34	301
Insertion	25	867	0.045572071	Insertion	23	1160	0.061573707	Insertion	12	6269
Insertion	29	522	0.027437856	Insertion	30	519	0.027548926	Insertion	1	502485
Insertion	39	149	0.007831878	Insertion	34	284	0.015056496	Insertion	42	84
Insertion	28	721	0.03827035	Insertion	13	4121	0.218478244	Insertion	34	301
Insertion	6	19169	1.017481747	Insertion	33	317	0.016806019	Insertion	30	512
Insertion	30	520	0.027601362	Insertion	45	7	0.000371111	Insertion	43	57
Insertion	34	291	0.015446147	Insertion	18	2691	0.142665604	Insertion	40	129
Insertion	9	8716	0.462641291	Insertion	17	2141	0.113506897	Insertion	5	26024
Insertion	13	4072	0.216139896	Insertion	35	219	0.011610467	Insertion	29	519
Insertion	44	24	0.001273909	Insertion	10	8697	0.461078692	Insertion	38	169
Insertion	16	3184	0.169005263	Insertion	21	1435	0.076077719	Insertion	25	830
Insertion	43	53	0.002813216	Insertion	2	130718	6.930123543	Insertion	42	78
Insertion	8	11862	0.629629531	Insertion	28	698	0.037005051	Insertion	46	1
Insertion	23	1172	0.062209224	Insertion	24	1258	0.066693917	Insertion	36	255
Insertion	33	304	0.016136181	Insertion	15	3051	0.161751304	Insertion	35	234
Insertion	31	411	0.021815692	Insertion	19	1736	0.092035485	Insertion	33	323
Insertion	21	1440	0.076434541	Insertion	1	498650	26.43634469	Insertion	31	392
Insertion	2	130143	6.90793088	Insertion	12	6230	0.330288634	Insertion	19	1767
Insertion	38	165	0.008758124	Insertion	5	25669	1.360863395	Insertion	12	6266
Insertion	32	424	0.022505726	Insertion	4	52152	2.764881677	Insertion	28	695
Insertion	15	3074	0.163166513	Insertion	44	26	0.001378412	Insertion	24	1287
Insertion	45	7	0.000371557	Insertion	42	84	0.00445333	Insertion	27	752
Insertion	22	1495	0.079353916	Insertion	23	1156	0.061286302	Insertion	20	2031
Insertion	4	52185	2.769955917	Insertion	36	266	0.014102211	Insertion	16	3208
Insertion	39	142	0.007537295	Insertion	8	11873	0.629456975	Insertion	14	4262
Insertion	17	2119	0.11247555	Insertion	11	5181	0.274675026	Insertion	3	67641
Insertion	36	266	0.014119158	Insertion	30	527	0.027939344	Insertion	8	11934
Insertion	24	1266	0.067198701	Insertion	26	912	0.048350439	Insertion	45	7
Insertion	10	8701	0.461845098	Insertion	43	59	0.003127934	Insertion	15	3110
Insertion	20	1976	0.104885176	Insertion	38	176	0.009330786	Insertion	22	1571
Insertion	18	2632	0.139705355	Insertion	29	527	0.027939344	Insertion	13	4157
Insertion	1	498416	26.45569318	Insertion	22	1552	0.082280571	Insertion	7	10871
Insertion	11	5194	0.275695143	Insertion	27	730	0.038701557	Insertion	37	195
Insertion	26	880	0.046709997	Insertion	37	198	0.010497135	Insertion	32	449

Insertion	29	536	0.028450635	Insertion	40	132	0.00699809	Insertion	6	19553
Insertion	5	25806	1.36977067	Insertion	39	136	0.007210153	Insertion	23	1170
Insertion	14	4245	0.225322657	Insertion	31	421	0.022319665	Insertion	11	5219
Insertion	41	84	0.004458682	Insertion	3	67482	3.577614383	Insertion	39	144
Insertion	37	191	0.010138193	Insertion	20	1961	0.103964047	Insertion	41	92
Insertion	40	140	0.007431136	Insertion	41	83	0.004400314	Insertion	2	130810
Insertion	7	10747	0.570445842	Insertion	7	10713	0.567958609	Insertion	26	911
Insertion	27	735	0.039013464	Insertion	32	432	0.022902839	Insertion	44	25
Insertion	3	67430	3.579153541	Insertion	25	842	0.044639331	Insertion	4	52222
Insertion	12	6083	0.322882856	Insertion	6	19466	1.032006188	Insertion	21	1490
Insertion	35	232	0.012314454	Insertion	14	4292	0.227543951	Insertion	9	8799
Insertion	25	860	0.045648406	Insertion	9	8669	0.459594249	Insertion	17	2135
Insertion	42	86	0.004564841	Insertion	16	3220	0.170710979	Insertion	18	2648
Insertion	19	1746	0.092676881	Insertion	17	2169	0.114819679	Insertion	10	8876
Insertion	16	3140	0.16584729	Insertion	31	433	0.022921586	Insertion	1	499252
Insertion	6	19333	1.021122818	Insertion	13	4193	0.221963538	Insertion	11	5274
Insertion	19	1785	0.09427943	Insertion	15	3129	0.165638901	Insertion	18	2662
Insertion	7	10842	0.572648507	Insertion	9	8728	0.462031424	Insertion	33	327
Insertion	10	8814	0.465534398	Insertion	28	684	0.036208695	Insertion	14	4300
Insertion	12	6099	0.322134592	Insertion	44	27	0.001429291	Insertion	15	3086
Insertion	26	923	0.048750652	Insertion	11	5149	0.272571013	Insertion	27	757
Insertion	33	321	0.016954452	Insertion	19	1736	0.091898093	Insertion	21	1490
Insertion	24	1303	0.068821343	Insertion	12	6194	0.327889854	Insertion	41	86
Insertion	23	1139	0.060159256	Insertion	46	2	0.000105873	Insertion	6	19515
Insertion	21	1478	0.078064425	Insertion	1	499416	26.43742963	Insertion	3	68338
Insertion	4	52246	2.759508754	Insertion	14	4244	0.224663309	Insertion	24	1249
Insertion	43	58	0.003063421	Insertion	35	224	0.011857818	Insertion	26	894
Insertion	46	1	5.28E-05	Insertion	32	442	0.023398017	Insertion	17	2162
Insertion	13	4122	0.217714181	Insertion	45	7	0.000370557	Insertion	32	447
Insertion	41	84	0.004436679	Insertion	37	192	0.010163844	Insertion	25	838
Insertion	30	514	0.027148251	Insertion	21	1480	0.0783463	Insertion	36	264
Insertion	27	738	0.038979395	Insertion	4	52126	2.759377867	Insertion	2	132784
Insertion	35	233	0.012306503	Insertion	3	67704	3.584025613	Insertion	10	8886
Insertion	44	27	0.001426075	Insertion	27	736	0.038961403	Insertion	1	506598
Insertion	40	142	0.0075001	Insertion	42	83	0.004393745	Insertion	8	12122
Insertion	42	81	0.004278226	Insertion	18	2634	0.13943524	Insertion	43	57
Insertion	15	3073	0.16230851	Insertion	38	173	0.009158047	Insertion	38	180
Insertion	8	11845	0.625624568	Insertion	34	299	0.01582807	Insertion	35	231
Insertion	25	850	0.044894967	Insertion	10	8744	0.462878411	Insertion	31	416
Insertion	37	198	0.010457886	Insertion	16	3132	0.165797711	Insertion	30	517
Insertion	17	2161	0.114138851	Insertion	6	19423	1.028189316	Insertion	44	25
Insertion	20	1934	0.102149254	Insertion	23	1162	0.061512433	Insertion	23	1145
Insertion	18	2643	0.139596938	Insertion	25	827	0.043778642	Insertion	34	310
Insertion	1	500632	26.44218479	Insertion	20	1943	0.102855987	Insertion	46	1

Insertion	3	67515	3.565980812	Insertion	36	271	0.014345843	Insertion	28	711
Insertion	45	7	0.000369723	Insertion	29	537	0.028427002	Insertion	20	1992
Insertion	11	5197	0.274493109	Insertion	26	897	0.04748421	Insertion	7	10924
Insertion	31	420	0.022183395	Insertion	43	63	0.003335011	Insertion	22	1559
Insertion	36	264	0.013943849	Insertion	2	131097	6.939841158	Insertion	42	81
Insertion	14	4231	0.2234713	Insertion	7	10855	0.574627762	Insertion	29	528
Insertion	2	131129	6.925920136	Insertion	41	85	0.004499619	Insertion	37	197
Insertion	38	174	0.009190264	Insertion	40	132	0.006987643	Insertion	16	3257
Insertion	29	519	0.027412339	Insertion	8	11843	0.626929211	Insertion	19	1763
Insertion	28	678	0.035810338	Insertion	5	25974	1.374977568	Insertion	5	26101
Insertion	34	293	0.015475559	Insertion	33	329	0.017416171	Insertion	12	6209
Insertion	9	8791	0.464319593	Insertion	22	1506	0.079722654	Insertion	40	141
Insertion	22	1527	0.080652488	Insertion	30	520	0.027527078	Insertion	4	52548
Insertion	5	25907	1.368345774	Insertion	39	148	0.00783463	Insertion	45	6
Insertion	39	152	0.008028276	Insertion	24	1271	0.067282532	Insertion	9	8711
Insertion	32	445	0.023503836	Insertion	29	530	0.027829557	Insertion	13	4193
Insertion	46	2	0.000105371	Insertion	32	443	0.023261309	Insertion	39	145
Insertion	13	4147	0.21848751	Insertion	28	687	0.036073407	Insertion	41	89
Insertion	23	1138	0.059956302	Insertion	1	503010	26.41235002	Insertion	5	26076
Insertion	41	82	0.004320226	Insertion	41	88	0.004620757	Insertion	10	8800
Insertion	19	1738	0.09156771	Insertion	25	851	0.044684817	Insertion	42	81
Insertion	37	193	0.010168336	Insertion	2	131883	6.924991467	Insertion	25	875
Insertion	20	1980	0.104317644	Insertion	35	224	0.011761926	Insertion	34	302
Insertion	43	60	0.003161141	Insertion	8	11931	0.626480086	Insertion	27	750
Insertion	7	10858	0.572061101	Insertion	9	8800	0.462075665	Insertion	8	11927
Insertion	35	220	0.011590849	Insertion	4	52876	2.776444643	Insertion	44	27
Insertion	40	139	0.007323309	Insertion	7	10878	0.571188532	Insertion	43	61
Insertion	21	1499	0.078975833	Insertion	45	7	0.00036756	Insertion	18	2710
Insertion	34	308	0.016227189	Insertion	16	3208	0.168447583	Insertion	35	235
Insertion	12	6146	0.323806182	Insertion	27	742	0.03896138	Insertion	23	1198
Insertion	6	19524	1.028635193	Insertion	20	2025	0.106329912	Insertion	20	2054
Insertion	5	26058	1.372883419	Insertion	15	3109	0.163249232	Insertion	38	172
Insertion	2	131510	6.928693622	Insertion	13	4148	0.217805666	Insertion	12	6246
Insertion	45	6	0.000316114	Insertion	19	1775	0.093202762	Insertion	40	134
Insertion	17	2115	0.111430211	Insertion	6	19686	1.033684266	Insertion	17	2154
Insertion	39	147	0.007744795	Insertion	5	26220	1.376775447	Insertion	36	264
Insertion	24	1278	0.067332298	Insertion	43	55	0.002887973	Insertion	24	1265
Insertion	15	3086	0.162588005	Insertion	33	332	0.017432855	Insertion	29	520
Insertion	11	5246	0.276389071	Insertion	44	25	0.001312715	Insertion	6	19551
Insertion	8	11949	0.629541176	Insertion	14	4308	0.226207041	Insertion	30	514
Insertion	26	914	0.04815471	Insertion	30	521	0.02735698	Insertion	28	696
Insertion	1	502177	26.45753613	Insertion	39	150	0.00787629	Insertion	31	434
Insertion	32	447	0.023550498	Insertion	22	1578	0.082858568	Insertion	22	1588
Insertion	31	424	0.022338728	Insertion	37	192	0.010081651	Insertion	9	8785

Insertion	33	309	0.016279875	Insertion	26	920	0.04830791	Insertion	15	3088
Insertion	44	27	0.001422513	Insertion	38	189	0.009924125	Insertion	7	10798
Insertion	9	8844	0.465952143	Insertion	42	89	0.004673265	Insertion	32	456
Insertion	30	511	0.026922382	Insertion	21	1499	0.078710389	Insertion	21	1497
Insertion	10	8799	0.463581288	Insertion	23	1165	0.061172517	Insertion	19	1736
Insertion	3	67970	3.581045589	Insertion	3	68256	3.584026884	Insertion	11	5135
Insertion	36	270	0.014225133	Insertion	31	410	0.021528525	Insertion	14	4307
Insertion	16	3179	0.167487773	Insertion	11	5143	0.270051721	Insertion	37	199
Insertion	29	525	0.027659981	Insertion	40	137	0.007193678	Insertion	4	52486
Insertion	27	761	0.040093802	Insertion	12	6209	0.326025887	Insertion	26	895
Insertion	42	90	0.004741711	Insertion	17	2148	0.112788469	Insertion	2	131786
Insertion	18	2650	0.139617049	Insertion	36	257	0.01349471	Insertion	45	7
Insertion	14	4276	0.225283963	Insertion	24	1276	0.067000971	Insertion	1	502525
Insertion	25	859	0.045256998	Insertion	10	8871	0.465803775	Insertion	46	2
Insertion	38	166	0.008745823	Insertion	34	298	0.015647562	Insertion	13	4110
Insertion	22	1544	0.081346688	Insertion	18	2698	0.141668198	Insertion	16	3255
Insertion	28	685	0.03608969					Insertion	39	140
Insertion	4	52315	2.756251288					Insertion	33	323
								Insertion	3	67726

	SG2			
Percent(%)	Type	Length	Count	Percent(%)
0.005673956	Deletion	-30	1016	0.053055107
0.165542908	Deletion	-4	83514	4.36106716
0.013922206	Deletion	-12	10494	0.547992418
0.104915642	Deletion	-25	1215	0.063446807
0.430695259	Deletion	-54	68	0.003550932
0.068402687	Deletion	-66	3	0.000156659
4.331539733	Deletion	-45	154	0.008041817
0.001628635	Deletion	-23	1503	0.07848605
0.02148748	Deletion	-65	3	0.000156659
0.237518086	Deletion	-48	125	0.006527449
0.025322654	Deletion	-59	15	0.000783294
0.033991197	Deletion	-15	4455	0.232638291
0.001050733	Deletion	-60	21	0.001096611
1.736913389	Deletion	-57	24	0.00125327
0.000420293	Deletion	-10	14738	0.769612374
23.07240493	Deletion	-52	70	0.003655372
0.013606986	Deletion	-27	1067	0.055718307
0.000262683	Deletion	-7	18499	0.966010266
0.002469221	Deletion	-21	1934	0.100992694
0.374113313	Deletion	-61	10	0.000522196
0.032099878	Deletion	-22	2594	0.135457626
5.25E-05	Deletion	-14	7172	0.374518927
0.001471026	Deletion	-43	170	0.008877331
0.016338891	Deletion	-53	56	0.002924297
0.02148748	Deletion	-41	203	0.010600578
0.281018412	Deletion	-39	292	0.015248121
0.063726927	Deletion	-29	783	0.040887942
0.003992784	Deletion	-37	307	0.016031415
0.330507914	Deletion	-63	5	0.000261098
9.170110442	Deletion	-24	2032	0.106110215
0.002679368	Deletion	-18	4325	0.225849743
5.25E-05	Deletion	-34	597	0.031175098
0.004885906	Deletion	-28	1272	0.066423323
0.003414881	Deletion	-62	7	0.000365537
0.125615073	Deletion	-51	75	0.00391647
0.548955204	Deletion	-35	402	0.020992277
0.771868108	Deletion	-47	102	0.005326399
0.03892964	Deletion	-33	485	0.025326503
0.078384646	Deletion	-26	1474	0.076971681
0.965097818	Deletion	-16	5417	0.28287354
0.100029736	Deletion	-67	1	5.22E-05

0.052326479	Deletion	-49	71	0.003707591
0.166278421	Deletion	-50	98	0.00511752
2.222824642	Deletion	-31	640	0.03342054
0.053219602	Deletion	-46	154	0.008041817
0.019018259	Deletion	-2	175675	9.173677147
0.739085253	Deletion	-56	49	0.00255876
0.007775421	Deletion	-5	42868	2.238549549
0.132024541	Deletion	-58	22	0.001148831
0.000577903	Deletion	-1	441082	23.03312289
0.010770008	Deletion	-13	6383	0.333317667
0.00404532	Deletion	-68	2	0.000104439
0.008195714	Deletion	-8	20601	1.075775853
0.027003826	Deletion	-36	523	0.027310848
5.25E-05	Deletion	-44	196	0.01023504
0.078752402	Deletion	-19	2404	0.125535904
0.00015761	Deletion	-42	261	0.013629314
1.072955511	Deletion	-40	358	0.018694615
0.040137982	Deletion	-6	33508	1.74977415
0.222702757	Deletion	-38	398	0.020783398
0.010034496	Deletion	-9	14269	0.745121384
0.000682976	Deletion	-3	100218	5.233343255
0.003519954	Deletion	-64	9	0.000469976
0.001208342	Deletion	-17	3163	0.165170575
0.00840586	Deletion	-11	8321	0.43451924
5.204593382	Deletion	-20	3192	0.166684944
0.007039908	Deletion	-55	56	0.002924297
0.003572491	Deletion	-32	703	0.036710374
0.236593602	Deletion	-24	2010	0.104879013
0.127014519	Deletion	-26	1484	0.077433063
0.135573734	Deletion	-3	100322	5.234662879
0.967455435	Deletion	-8	20672	1.078636301
0.000898189	Deletion	-42	250	0.013044653
0.026892841	Deletion	-61	9	0.000469608
0.000211339	Deletion	-5	42749	2.230583555
0.052464816	Deletion	-35	385	0.020088766
23.01393197	Deletion	-60	21	0.001095751
4.341318235	Deletion	-21	1909	0.099608973
0.01394835	Deletion	-52	61	0.003182895
0.109050736	Deletion	-59	21	0.001095751
0.330058113	Deletion	-46	134	0.006991934
1.753423818	Deletion	-7	18544	0.967600212
0.100650025	Deletion	-39	299	0.015601405
0.003698426	Deletion	-68	2	0.000104357
0.000105669	Deletion	-32	711	0.037098994

0.020499848	Deletion	-45	151	0.007878971
0.079304823	Deletion	-1	442020	23.06399081
0.003804095	Deletion	-37	302	0.015757941
0.010144254	Deletion	-48	122	0.006365791
0.033919851	Deletion	-41	193	0.010070472
0.069266238	Deletion	-56	46	0.002400216
1.078936565	Deletion	-23	1503	0.078424456
0.017065595	Deletion	-15	4423	0.230786008
0.062926079	Deletion	-43	162	0.008452935
0.019020477	Deletion	-6	33555	1.750853381
0.007502522	Deletion	-63	5	0.000260893
2.230256613	Deletion	-33	530	0.027654665
0.03244048	Deletion	-67	2	0.000104357
0.776563813	Deletion	-55	47	0.002452395
0.005811812	Deletion	-4	83134	4.337816868
0.006710002	Deletion	-12	10463	0.545944835
0.026417329	Deletion	-30	993	0.051813363
0.380092535	Deletion	-64	7	0.00036525
0.283563613	Deletion	-44	174	0.009079079
0.163470434	Deletion	-20	3173	0.165562741
0.165795159	Deletion	-10	14690	0.766503835
0.008347876	Deletion	-34	612	0.031933312
0.427907901	Deletion	-65	3	0.000156536
0.002747402	Deletion	-25	1193	0.062249086
5.224396721	Deletion	-40	369	0.019253908
0.056163242	Deletion	-53	56	0.002922002
0.000264173	Deletion	-18	4289	0.223794074
0.003645591	Deletion	-16	5529	0.288495555
0.740900419	Deletion	-19	2432	0.126898388
0.008876223	Deletion	-31	623	0.032507276
0.000581181	Deletion	-2	175511	9.157920661
0.000211339	Deletion	-38	390	0.020349659
0.548952104	Deletion	-9	14143	0.737962133
0.039203317	Deletion	-28	1322	0.068980127
0.02166221	Deletion	-27	1055	0.055048437
0.004015434	Deletion	-54	74	0.003861217
0.002641733	Deletion	-50	95	0.004956968
0.010461262	Deletion	-13	6376	0.332690841
0.014846539	Deletion	-29	769	0.040125354
0.003592757	Deletion	-11	8317	0.43396953
0.002588898	Deletion	-58	20	0.001043572
0.000105669	Deletion	-51	77	0.004017753
0.076715924	Deletion	-62	7	0.00036525
0.000528347	Deletion	-47	110	0.005739648

0.039520325	Deletion	-57	21	0.001095751
0.001215197	Deletion	-22	2580	0.134620823
0.223226433	Deletion	-17	3131	0.163371239
9.174791316	Deletion	-36	504	0.026298021
0.001215197	Deletion	-49	74	0.003861217
0.000105669	Deletion	-14	7260	0.378816735
0.001056693	Deletion	-42	273	0.014414144
0.734112514	Deletion	-61	15	0.000791986
0.000261213	Deletion	-62	6	0.000316794
0.025442129	Deletion	-11	8214	0.433691505
5.193433319	Deletion	-66	2	0.000105598
0.378236166	Deletion	-7	18289	0.965642066
0.132330415	Deletion	-18	4218	0.222706448
0.000365698	Deletion	-5	42222	2.229282044
0.000261213	Deletion	-46	146	0.007708663
0.000156728	Deletion	-55	45	0.002375958
0.001097094	Deletion	-3	98947	5.2243089
0.01609071	Deletion	-20	3150	0.166317049
0.004022677	Deletion	-4	82202	4.340188588
0.167123963	Deletion	-54	60	0.003167944
0.038084829	Deletion	-30	1010	0.053327054
0.014105492	Deletion	-35	386	0.020380438
0.069116913	Deletion	-41	195	0.010295817
9.170241799	Deletion	-25	1174	0.0619861
0.165713414	Deletion	-16	5402	0.285220539
0.009821602	Deletion	-27	1046	0.05522782
1.076405795	Deletion	-40	340	0.017951681
0.002821098	Deletion	-21	1860	0.098206257
0.004701831	Deletion	-29	753	0.039757695
0.003030069	Deletion	-2	173227	9.146223309
0.02058357	Deletion	-36	487	0.025713144
0.000104485	Deletion	-47	111	0.005860696
0.225844606	Deletion	-53	61	0.003220743
0.000835881	Deletion	-13	6267	0.330891729
0.05297396	Deletion	-22	2485	0.131205672
0.059869979	Deletion	-12	10393	0.548740663
2.215868365	Deletion	-63	4	0.000211196
0.078468332	Deletion	-44	177	0.009345434
0.332576164	Deletion	-10	14496	0.765375219
23.10171418	Deletion	-26	1463	0.077245029
0.000261213	Deletion	-43	159	0.008395051
0.079565425	Deletion	-38	393	0.020750032
0.007993112	Deletion	-9	13958	0.736969323
0.100096753	Deletion	-51	67	0.003537537

4.333050527	Deletion	-39	290	0.015311728
0.005276499	Deletion	-23	1473	0.07777302
0.001306064	Deletion	-52	64	0.00337914
0.95630014	Deletion	-49	78	0.004118327
0.007993112	Deletion	-37	302	0.015945317
0.000992609	Deletion	-56	41	0.002164762
0.038816225	Deletion	-59	16	0.000844785
0.281587422	Deletion	-34	575	0.030359461
0.025703342	Deletion	-1	436965	23.07134262
0.015829497	Deletion	-31	635	0.033527405
0.00365698	Deletion	-24	1986	0.104858939
0.020688055	Deletion	-14	7150	0.377513301
0.002455401	Deletion	-64	8	0.000422393
1.741505881	Deletion	-57	26	0.001372776
0.010344028	Deletion	-48	142	0.007497467
0.003030069	Deletion	-8	20318	1.072771365
0.541755392	Deletion	-45	145	0.007655864
0.053862084	Deletion	-32	713	0.037645732
0.105112039	Deletion	-58	30	0.001583972
0.000731396	Deletion	-28	1284	0.067793997
0.007157231	Deletion	-19	2405	0.126981747
0.124755244	Deletion	-17	3164	0.167056236
0.0305619	Deletion	-15	4454	0.235167027
0.018702838	Deletion	-33	458	0.024181971
0.767808968	Deletion	-6	33065	1.745801023
0.007836385	Deletion	-67	1	5.28E-05
0.031136568	Deletion	-65	5	0.000263995
5.22E-05	Deletion	-50	94	0.004963112
0.236606574	Deletion	-60	23	0.001214378
0.431419096	Deletion	-10	14536	0.765976641
0.003970435	Deletion	-16	5341	0.281444774
0.003948154	Deletion	-7	18243	0.961317547
0.374495558	Deletion	-18	4252	0.224059761
0.164243202	Deletion	-55	53	0.002792843
0.001210767	Deletion	-49	77	0.004057526
0.05285262	Deletion	-21	1899	0.100068082
0.003000597	Deletion	-20	3154	0.16620049
0.126867345	Deletion	-42	269	0.014174994
0.020319832	Deletion	-63	3	0.000158085
23.04674298	Deletion	-28	1273	0.067080921
0.039270971	Deletion	-36	510	0.026874524
0.004790427	Deletion	-43	171	0.00901087
0.00026321	Deletion	-34	590	0.031090136
1.740767374	Deletion	-44	188	0.009906687

0.016371678	Deletion	-9	13971	0.736203884
0.734461909	Deletion	-25	1159	0.061073674
0.165927748	Deletion	-29	771	0.040627958
5.223828741	Deletion	-33	497	0.026189488
1.077003741	Deletion	-23	1512	0.079675061
9.159874965	Deletion	-57	21	0.001106598
0.332118706	Deletion	-6	33029	1.740467975
0.434928633	Deletion	-45	149	0.007851577
0.01000199	Deletion	-39	265	0.013964214
0.223465511	Deletion	-22	2544	0.134056451
0.005422131	Deletion	-60	22	0.001159293
0.053642251	Deletion	-3	99012	5.217451789
0.015266195	Deletion	-2	173757	9.156150472
0.007738382	Deletion	-5	42281	2.228003465
0.000368494	Deletion	-24	2004	0.105601072
4.332598801	Deletion	-56	41	0.002160501
0.007422529	Deletion	-58	20	0.001053903
0.009528211	Deletion	-50	85	0.004479087
0.019372275	Deletion	-1	437809	23.07040915
0.002474176	Deletion	-47	104	0.005480295
0.000105284	Deletion	-27	1020	0.053749049
0.067645037	Deletion	-4	82206	4.331857167
0.957085146	Deletion	-51	70	0.00368866
0.282687819	Deletion	-14	7164	0.377508025
0.026005174	Deletion	-65	5	0.000263476
0.013739576	Deletion	-59	18	0.000948513
0.540949726	Deletion	-31	577	0.030405099
0.001579262	Deletion	-12	10341	0.544920504
0.099440836	Deletion	-52	67	0.003530575
0.769468873	Deletion	-26	1459	0.076882218
0.031006169	Deletion	-53	52	0.002740148
0.000631705	Deletion	-13	6360	0.335141128
0.131973624	Deletion	-40	360	0.018970253
0.006001194	Deletion	-62	7	0.000368866
0.002421534	Deletion	-17	3160	0.166516661
0.062222905	Deletion	-41	193	0.010170163
0.000526421	Deletion	-30	1017	0.053590963
0.021741167	Deletion	-46	137	0.007219235
0.00026321	Deletion	-19	2382	0.125519838
0.001263409	Deletion	-61	11	0.000579647
0.029953328	Deletion	-37	302	0.015913934
0.003211165	Deletion	-54	70	0.00368866
5.26E-05	Deletion	-64	7	0.000368866
0.037849635	Deletion	-15	4422	0.233017935

0.078173447	Deletion	-67	1	5.27E-05
0.103073138	Deletion	-35	392	0.020656497
0.000105284	Deletion	-48	122	0.006428808
0.025636679	Deletion	-11	8172	0.430624733
0.235889035	Deletion	-38	400	0.021078058
0.001052841	Deletion	-8	20348	1.072240829
0.003316449	Deletion	-32	704	0.037097383
0.077173248	Insertion	32	446	0.023289939
0.010107274	Insertion	19	1759	0.091854266
0.003737586	Insertion	35	239	0.012480483
2.218283427	Insertion	37	196	0.01023504
0.007144981	Insertion	34	299	0.015613659
1.373254865	Insertion	30	546	0.028511898
1.027774013	Insertion	23	1182	0.06172356
0.159080903	Insertion	10	8921	0.465850997
0.080013281	Insertion	41	89	0.004647544
0.276605335	Insertion	31	441	0.023028841
0.010244642	Insertion	18	2656	0.138695241
0.461534259	Insertion	11	5223	0.272742939
0.00919391	Insertion	36	249	0.013002679
0.037563688	Insertion	40	136	0.007101865
2.754337687	Insertion	14	4299	0.224492034
0.045286572	Insertion	26	875	0.045692145
0.027686802	Insertion	28	683	0.035665983
0.001471026	Insertion	42	86	0.004490885
6.943818383	Insertion	17	2198	0.114778667
0.105441008	Insertion	33	345	0.01801576
0.216555972	Insertion	4	52897	2.762259855
0.635535564	Insertion	7	10903	0.569350232
0.09046807	Insertion	15	3104	0.162089619
0.061888145	Insertion	46	1	5.22E-05
0.083533235	Insertion	6	19776	1.032694688
0.023011042	Insertion	1	504019	26.31966747
0.002784441	Insertion	44	27	0.001409929
0.170008521	Insertion	24	1279	0.066788861
0.066196149	Insertion	20	2013	0.105118042
0.039612616	Insertion	38	175	0.009138429
0.007407664	Insertion	12	6342	0.331176664
0.004728296	Insertion	45	6	0.000313318
0.012083424	Insertion	13	4123	0.215301385
0.000367756	Insertion	27	779	0.040679064
3.575695296	Insertion	16	3226	0.16846041
0.224961832	Insertion	22	1590	0.083029154
0.017494696	Insertion	3	68551	3.579705377

0.460220843	Insertion	21	1490	0.077807195
0.11568565	Insertion	25	885	0.046214341
0.023799092	Insertion	39	146	0.007624061
0.028684998	Insertion	29	515	0.026893091
0.138696692	Insertion	8	12100	0.631857085
0.013869669	Insertion	5	26530	1.385385824
0.57144088	Insertion	2	132825	6.936067551
0.04670506	Insertion	43	56	0.002924297
0.000105073	Insertion	9	8841	0.461673429
0.015813524	Insertion	15	3120	0.162797275
0.329352108	Insertion	3	68583	3.57856586
26.39886647	Insertion	28	682	0.035585815
0.004413077	Insertion	34	304	0.015862299
0.015903232	Insertion	10	8838	0.461154587
0.027051345	Insertion	33	330	0.017218943
0.003011576	Insertion	29	517	0.026976343
0.006815671	Insertion	38	172	0.008974722
1.374969158	Insertion	8	12139	0.633396191
0.027421188	Insertion	9	8828	0.460632801
0.008929057	Insertion	24	1281	0.066840804
0.043852767	Insertion	2	132717	6.924989069
0.004121103	Insertion	39	144	0.00751372
5.28E-05	Insertion	44	28	0.001461001
0.013472838	Insertion	19	1756	0.091625646
0.01236331	Insertion	37	204	0.010644437
0.017065595	Insertion	25	876	0.045708466
0.020711186	Insertion	31	438	0.022854233
0.093358842	Insertion	42	85	0.004435182
0.331061971	Insertion	5	26410	1.378037187
0.036720088	Insertion	14	4319	0.225359432
0.067998206	Insertion	22	1558	0.08129428
0.039731663	Insertion	18	2641	0.137803719
0.107307192	Insertion	35	234	0.012209796
0.169493585	Insertion	27	755	0.039394853
0.225181315	Insertion	40	142	0.007409363
3.573789149	Insertion	46	1	5.22E-05
0.630528817	Insertion	7	10933	0.570468783
0.000369843	Insertion	17	2182	0.113853735
0.164315789	Insertion	36	256	0.013357725
0.083003249	Insertion	30	528	0.027550308
0.219633676	Insertion	16	3206	0.167284635
0.574365575	Insertion	41	82	0.004278646
0.010302758	Insertion	13	4142	0.216123818
0.023722762	Insertion	6	19812	1.033762694

1.033076081	Insertion	32	446	0.023271662
0.061816551	Insertion	26	886	0.046230252
0.275744084	Insertion	1	505422	26.37221927
0.007608191	Insertion	12	6304	0.32893398
0.004860789	Insertion	23	1174	0.061257692
6.911301703	Insertion	20	2046	0.106757444
0.048132374	Insertion	45	6	0.000313072
0.001320866	Insertion	21	1477	0.077067812
2.759131546	Insertion	4	52976	2.764214237
0.078723641	Insertion	43	59	0.003078538
0.464892162	Insertion	11	5235	0.273155042
0.112801996	Insertion	35	241	0.012724574
0.139906176	Insertion	6	19400	1.024301825
0.468960431	Insertion	24	1247	0.065840432
26.37780902	Insertion	26	900	0.047519157
0.275527284	Insertion	13	4154	0.219327308
0.139069706	Insertion	7	10779	0.569121102
0.017083319	Insertion	34	293	0.015470125
0.224643027	Insertion	45	6	0.000316794
0.161220553	Insertion	41	83	0.004382322
0.039547621	Insertion	32	453	0.023917976
0.077841421	Insertion	18	2606	0.137594359
0.004492861	Insertion	39	138	0.007286271
1.019513643	Insertion	46	2	0.000105598
3.57015236	Insertion	21	1450	0.076558642
0.065250963	Insertion	25	864	0.045618391
0.046704852	Insertion	10	8800	0.464631756
0.112948424	Insertion	5	25995	1.372511646
0.023352426	Insertion	3	67853	3.582574831
0.043779269	Insertion	4	52272	2.759912628
0.013792037	Insertion	44	26	0.001372776
6.93697666	Insertion	31	423	0.022334004
0.464227427	Insertion	9	8742	0.46156941
26.4659786	Insertion	16	3218	0.169907385
0.633284365	Insertion	29	517	0.027297116
0.002977826	Insertion	40	140	0.007391869
0.009403662	Insertion	12	6193	0.326984598
0.012068032	Insertion	1	500225	26.41141136
0.021732907	Insertion	20	1981	0.104594944
0.027009406	Insertion	15	3089	0.163096306
0.001306064	Insertion	27	722	0.038120924
0.059817736	Insertion	17	2168	0.114468369
0.016195195	Insertion	14	4280	0.22597999
5.22E-05	Insertion	36	256	0.01351656

0.037144463	Insertion	11	5225	0.275875105
0.104067188	Insertion	19	1720	0.090814389
0.570697773	Insertion	43	55	0.002903948
0.081446158	Insertion	33	321	0.016948499
0.004231648	Insertion	28	681	0.035956162
0.027584074	Insertion	22	1538	0.081204959
0.010291785	Insertion	42	84	0.004435121
0.170154032	Insertion	30	528	0.027877905
0.092103641	Insertion	2	131357	6.935526536
1.363583171	Insertion	8	11852	0.625774496
0.324374082	Insertion	38	180	0.009503831
0.007366202	Insertion	37	177	0.009345434
2.74524227	Insertion	23	1126	0.059451745
0.000313455	Insertion	17	2165	0.114084991
0.455084978	Insertion	18	2651	0.139694832
0.219053072	Insertion	2	131619	6.935682413
0.007575172	Insertion	28	688	0.03625426
0.004685143	Insertion	42	84	0.004426392
1.372694147	Insertion	5	26071	1.37381515
0.463250057	Insertion	22	1565	0.082467903
0.004264006	Insertion	11	5166	0.272223124
0.046061795	Insertion	26	894	0.04710946
0.0158979	Insertion	29	525	0.027664952
0.039481539	Insertion	40	136	0.00716654
0.627861754	Insertion	43	58	0.003056318
0.001421335	Insertion	24	1262	0.066501274
0.003211165	Insertion	19	1743	0.091847639
0.142659961	Insertion	21	1501	0.079095414
0.012370882	Insertion	32	439	0.023133169
0.063065178	Insertion	36	261	0.013753433
0.108126775	Insertion	35	227	0.011961798
0.009054433	Insertion	31	432	0.022764303
0.328802256	Insertion	45	7	0.000368866
0.007054035	Insertion	23	1136	0.059861686
0.11339098	Insertion	20	2001	0.105442987
0.013897502	Insertion	27	747	0.039363274
0.066592196	Insertion	30	514	0.027085305
0.027373867	Insertion	6	19432	1.023972076
1.029204758	Insertion	4	52295	2.755692657
0.027058015	Insertion	41	88	0.004637173
0.036638868	Insertion	46	2	0.00010539
0.022846651	Insertion	12	6213	0.327394942
0.083595578	Insertion	3	67922	3.579159702
0.462460426	Insertion	14	4315	0.227379555

0.162558656	Insertion	7	10796	0.568896796
0.568428877	Insertion	34	305	0.01607202
0.024004776	Insertion	15	3091	0.162880696
0.078805152	Insertion	37	195	0.010275553
0.091386602	Insertion	8	11902	0.627177627
0.270316937	Insertion	44	27	0.001422769
0.226729318	Insertion	38	178	0.009379736
0.010475768	Insertion	13	4098	0.215944708
2.762970738	Insertion	25	883	0.046529814
0.047114636	Insertion	10	8736	0.460344795
6.937485458	Insertion	16	3177	0.167412479
0.000368494	Insertion	33	313	0.016493581
26.45394715	Insertion	1	501842	26.44463743
0.000105284	Insertion	9	8707	0.458816636
0.216358833	Insertion	39	141	0.007430016
0.171349879				
0.007369887				
0.017003383				
3.56523561				
