# Supplementary material for: Genetic diversity and selection of Tibetan sheep breeds revealed by whole-genome resequencing
Source: Anim Biosci. 2023 May 2;36(7):991–1002. doi: 10.5713/ab.22.0432 (PMC10330983; doi:10.5713/ab.22.0432)
Supplement: Supplementary file 6 [file ab-22-0432-Supplementary-Table-6.pdf]

Supplementary Table6.The number of CNV

Sample	deletion	duplication	Count of CNV
HZ_1	1188	682	1870
HZ_2	1045	710	1755
HZ_3	1012	732	1744
HZ_4	1033	703	1736
BD_1	1339	628	1967
BD_2	1410	677	2087
BD_3	1193	689	1882
BD_4	1286	643	1929
GY_1	1210	724	1934
GY_2	1206	664	1870
GY_3	1231	732	1963
GY_4	1162	683	1845
OL_1	1180	695	1875
OL_2	1051	695	1746
OL_3	952	665	1617
OL_4	1204	637	1841
ZK_1	989	720	1709
ZK_2	1029	817	1846
ZK_3	930	650	1580
ZK_4	1209	692	1901
SG_1	1196	662	1858
SG_2	1217	622	1839
SG_3	1169	717	1886
SG_4	1171	676	1847
SG_5	1157	688	1845
SG_6	1018	710	1728
SG_7	892	707	1599
SG_8	1168	699	1867
