# Supplementary material for: Genetic diversity and selection of Tibetan sheep breeds revealed by whole-genome resequencing
Source: Anim Biosci. 2023 May 2;36(7):991–1002. doi: 10.5713/ab.22.0432 (PMC10330983; doi:10.5713/ab.22.0432)
Supplement: Supplementary file 7 [file ab-22-0432-Supplementary-Table-7.pdf]

Supplementary Table7.The number of SV

Sample	CTX	DEL	INS	INV	ITX	Count of SV
HZ_1	5177	11594	93	1873	2584	21321
HZ_2	4980	11097	6	1847	2640	20570
HZ_3	4763	10484	34	1793	2665	19739
HZ_4	4725	10421	61	1709	2476	19392
BD_1	4832	11283	43	1746	2622	20526
BD_2	4692	10501	111	1718	2542	19564
BD_3	4799	11112	40	1799	2534	20284
BD_4	4996	11072	37	1811	2583	20499
OL_1	5067	12498	21	2137	2904	22627
OL_2	4376	11069	27	1869	2676	20017
OL_3	4254	10943	50	1549	2377	19173
OL_4	4994	12365	48	2095	2772	22274
ZK_1	4032	11110	0	3238	2658	21038
ZK_2	4934	12219	0	2443	3244	22840
ZK_3	5404	12711	111	2251	2926	23403
ZK_4	5293	13023	20	2227	3042	23605
GY_1	5775	13588	18	2187	3023	24591
GY_2	5724	12925	49	2241	3163	24102
GY_3	5286	12369	27	2117	2911	22710
GY_4	5000	12637	0	2171	3204	23012
SG_1	5403	12187	49	2059	2737	22435
SG_2	5114	11581	132	1886	2747	21460
SG_3	5369	12210	289	1971	2619	22458
SG_4	5659	12595	231	2035	2554	23074
SG_5	4802	10658	46	1762	2734	20002
SG_6	4739	10611	36	1796	2618	19800
SG_7	4655	10500	6	1709	2202	19072
SG_8	5505	12295	24	1850	2724	22398
