# Supplementary material for: Genetic diversity and selection of Tibetan sheep breeds revealed by whole-genome resequencing
Source: Anim Biosci. 2023 May 2;36(7):991–1002. doi: 10.5713/ab.22.0432 (PMC10330983; doi:10.5713/ab.22.0432)
Supplement: Supplementary file 8 [file ab-22-0432-Supplementary-Table-8.pdf]

Supplementary Table8.Putative selection regions associated with hypoxic adaptation among different breeds

BD vs SG2							GY vs SG2		
CHROM	Start	end	Pi	Fst	Region	Gene	CHROM	Start	end
NC_056068.1	1205001	1225001	10.0294	0.356387	BD	AASDHPPT;KBTBD3	NC_056057.1	30380001	30400001
NC_056077.1	14820001	14840001	2.13714	0.294611	BD	ABCC1	NC_056057.1	30385001	30405001
NC_056056.1	1.94E+08	193940001	2.38445	0.367681	BD	ABCC9	NC_056057.1	30390001	30410001
NC_056056.1	1.94E+08	193945001	2.1189	0.347875	BD	ABCC9	NC_056080.1	67170001	67190001
NC_056059.1	37170001	37190001	4.46913	0.352764	BD	ABCG2	NC_056080.1	67175001	67195001
NC_056059.1	37175001	37195001	2.92135	0.348616	BD	ABCG2	NC_056080.1	67180001	67200001
NC_056059.1	37125001	37145001	2.27849	0.347842	BD	ABCG2;PPM1K	NC_056080.1	67185001	67205001
NC_056070.1	64130001	64150001	3.24393	0.450178	BD	ACACB;UNG	NC_056054.1	203740001	203760001
NC_056070.1	64125001	64145001	4.9632	0.405495	BD	ACACB;UNG	NC_056073.1	26670001	26690001
NC_056070.1	64120001	64140001	2.38671	0.315771	BD	ACACB;UNG	NC_056073.1	26675001	26695001
NC_056054.1	1.93E+08	193025001	2.16193	0.336964	BD	ACAP2	NC_056073.1	26680001	26700001
NC_056054.1	1.93E+08	193030001	3.50064	0.328763	BD	ACAP2	NC_056073.1	26690001	26710001
NC_056065.1	60965001	60985001	2.84688	0.35444	BD	ACBD6	NC_056073.1	26685001	26705001
NC_056065.1	60970001	60990001	11.8736	0.292642	BD	ACBD6	NC_056056.1	5920001	5940001
NC_056056.1	1.17E+08	117230001	4.95959	0.387928	BD	ACSS3	NC_056068.1	82095001	82115001
NC_056056.1	1.17E+08	117235001	3.95786	0.379339	BD	ACSS3	NC_056068.1	82100001	82120001
NC_056056.1	1.17E+08	117225001	3.15127	0.304654	BD	ACSS3	NC_056068.1	82105001	82125001
NC_056058.1	66715001	66735001	2.73729	0.312833	BD	ADAM19	NC_056068.1	82090001	82110001
NC_056057.1	73740001	73760001	3.50848	0.400585	BD	ADAM22	NC_056056.1	117175001	117195001
NC_056055.1	86625001	86645001	3.49715	0.341407	BD	ADAMTSL1	NC_056056.1	117180001	117200001
NW_024599827.1	865001	885001	3.23273	0.33907	BD	ADAMTSL3	NC_056056.1	117185001	117205001
NW_024599827.1	870001	890001	2.49759	0.321849	BD	ADAMTSL3	NC_056056.1	117190001	117210001
NC_056060.1	87080001	87100001	3.45946	0.422234	BD	ADCK1	NC_056056.1	117195001	117215001
NC_056060.1	87085001	87105001	3.52444	0.421494	BD	ADCK1	NC_056056.1	117205001	117225001
NC_056060.1	87090001	87110001	2.31484	0.382871	BD	ADCK1	NC_056056.1	117210001	117230001
NC_056060.1	87075001	87095001	2.33724	0.381632	BD	ADCK1	NC_056056.1	117215001	117235001
NC_056054.1	1.2E+08	120045001	9.43229	0.494807	BD	ADCY10	NC_056056.1	117220001	117240001
NC_056054.1	1.2E+08	120050001	7.24784	0.49394	BD	ADCY10	NC_056055.1	14395001	14415001
NC_056054.1	1.2E+08	120055001	4.61901	0.482188	BD	ADCY10	NC_056058.1	66715001	66735001
NC_056054.1	1.2E+08	120060001	3.22962	0.461121	BD	ADCY10	NC_056057.1	73705001	73725001
NC_056059.1	1.03E+08	102675001	2.97156	0.423006	BD	AFF1	NC_056057.1	73710001	73730001
NC_056059.1	1.03E+08	102670001	2.6419	0.298723	BD	AFF1	NC_056057.1	73835001	73855001
NC_056080.1	87120001	87140001	2.45506	0.332609	BD	AFF2	NC_056057.1	73845001	73865001
NC_056080.1	87115001	87135001	2.38188	0.317502	BD	AFF2	NC_056057.1	73855001	73875001
NC_056080.1	87110001	87130001	2.30843	0.308042	BD	AFF2	NC_056071.1	5860001	5880001
NC_056071.1	16985001	17005001	2.15202	0.323999	BD	AGBL1	NC_056071.1	5865001	5885001
NC_056079.1	5960001	5980001	19.33	0.333027	BD	AGPAT5	NC_056071.1	5870001	5890001
NC_056055.1	1.33E+08	132655001	2.21647	0.294797	BD	AGPS	NC_056071.1	5875001	5895001
NC_056057.1	94895001	94915001	4.36304	0.326651	BD	AHCYL2	NC_056055.1	86510001	86530001
NC_056057.1	94890001	94910001	4.28054	0.311131	BD	AHCYL2	NC_056055.1	86515001	86535001
NC_056057.1	94900001	94920001	3.03891	0.302793	BD	AHCYL2	NC_056070.1	46190001	46210001
NC_056055.1	73050001	73070001	2.85648	0.41107	BD	AK3	NC_056080.1	17235001	17255001
NC_056061.1	76120001	76140001	5.66166	0.35385	BD	AKAP12	NC_056079.1	32295001	32315001
NC_056061.1	76115001	76135001	3.8617	0.328331	BD	AKAP12	NC_056059.1	102565001	102585001
NC_056071.1	15655001	15675001	2.14143	0.309902	BD	AKAP13	NC_056059.1	102630001	102650001
NC_056061.1	49600001	49620001	6.61438	0.418045	BD	AKIRIN2	NC_056059.1	102635001	102655001
NC_056061.1	49605001	49625001	6.30768	0.37918	BD	AKIRIN2	NC_056059.1	102640001	102660001
NC_056061.1	49610001	49630001	6.08696	0.338459	BD	AKIRIN2	NC_056059.1	102645001	102665001
NC_056054.1	1.71E+08	171165001	6.08952	0.475712	BD	ALCAM	NC_056059.1	102650001	102670001
NC_056054.1	1.71E+08	171170001	7.15548	0.468021	BD	ALCAM	NC_056059.1	102655001	102675001
NC_056054.1	1.71E+08	171160001	5.05782	0.458181	BD	ALCAM	NC_056059.1	102660001	102680001
NC_056054.1	1.71E+08	171175001	7.19678	0.452922	BD	ALCAM	NC_056080.1	86995001	87015001
NC_056054.1	1.71E+08	171155001	4.43673	0.437737	BD	ALCAM	NC_056080.1	87015001	87035001
NC_056054.1	1.71E+08	171180001	4.63637	0.390534	BD	ALCAM	NC_056080.1	87110001	87130001
NC_056054.1	1.71E+08	171150001	4.38824	0.386369	BD	ALCAM	NC_056080.1	87115001	87135001
NC_056054.1	1.71E+08	171185001	3.10375	0.30094	BD	ALCAM	NC_056080.1	87120001	87140001
NC_056068.1	21900001	21920001	2.42971	0.303658	BD	ALG9;C15H11orf1;FI	NC_056056.1	101370001	101390001
NC_056070.1	64135001	64155001	2.61637	0.398454	BD	ALKBH2;UNG	NC_056054.1	10845001	10865001
NC_056070.1	64145001	64165001	2.65127	0.342211	BD	ALKBH2;UNG;USP30	NC_056054.1	264610001	264630001
NC_056070.1	64140001	64160001	2.65293	0.313128	BD	ALKBH2;UNG;USP30	NC_056061.1	56735001	56755001
NC_056070.1	64155001	64175001	16.1892	0.424262	BD	ALKBH2;USP30	NC_056072.1	48750001	48770001
NC_056070.1	64150001	64170001	7.67949	0.375825	BD	ALKBH2;USP30	NC_056072.1	48745001	48765001
NC_056058.1	14740001	14760001	2.18444	0.328763	BD	ANGPTL4	NC_056055.1	247460001	247480001
NC_056078.1	15435001	15455001	5.60194	0.413864	BD	ANK3	NC_056072.1	52795001	52815001
NC_056078.1	15430001	15450001	4.49124	0.381643	BD	ANK3	NC_056072.1	52780001	52800001
NC_056078.1	15440001	15460001	4.36531	0.379779	BD	ANK3	NC_056072.1	52785001	52805001
NC_056078.1	15500001	15520001	2.75669	0.320854	BD	ANK3	NC_056080.1	124960001	124980001
NC_056078.1	15505001	15525001	2.45268	0.29984	BD	ANK3	NC_056080.1	124980001	125000001
NC_056078.1	15445001	15465001	2.54865	0.295523	BD	ANK3	NC_056080.1	125015001	125035001

NC_056060.1	11855001	11875001	2.72701	0.311709	BD	ANKDD1A	NC_056080.1	121900001	121920001
NC_056060.1	6990001	7010001	11.0891	0.296336	BD	ANKDD1B;POLK	NC_056068.1	15230001	15250001
NC_056068.1	23635001	23655001	5.34725	0.304566	BD	ANKK1	NC_056060.1	86225001	86245001
NC_056061.1	47970001	47990001	3.90696	0.313587	BD	ANKRD6	NC_056060.1	11845001	11865001
NC_056055.1	49065001	49085001	18.7985	0.308504	BD	ANKS6	NC_056060.1	11850001	11870001
NC_056055.1	49060001	49080001	4.71853	0.306327	BD	ANKS6	NC_056060.1	11855001	11875001
NC_056056.1	1.7E+08	170445001	3.80808	0.371925	BD	ANO4	NC_056068.1	23630001	23650001
NC_056056.1	1.7E+08	170440001	12.062	0.334937	BD	ANO4	NC_056068.1	23635001	23655001
NC_056074.1	20060001	20080001	6.16667	0.504217	BD	ANO5	NC_056068.1	23640001	23660001
NC_056074.1	20065001	20085001	3.60578	0.48754	BD	ANO5	NC_056068.1	23645001	23665001
NC_056074.1	20055001	20075001	5.51242	0.466613	BD	ANO5	NC_056068.1	23650001	23670001
NC_056074.1	20050001	20070001	4.6455	0.447036	BD	ANO5	NC_056068.1	23655001	23675001
NC_056074.1	20035001	20055001	3.43128	0.418587	BD	ANO5	NC_056063.1	84690001	84710001
NC_056074.1	20070001	20090001	2.10004	0.40105	BD	ANO5	NC_056063.1	84675001	84695001
NC_056074.1	20045001	20065001	2.43986	0.371126	BD	ANO5	NC_056063.1	84680001	84700001
NC_056074.1	20040001	20060001	2.225	0.310676	BD	ANO5	NC_056063.1	84685001	84705001
NC_056080.1	1425001	1445001	5.93411	0.315337	BD	ANOS1	NC_056060.1	6610001	6630001
NC_056080.1	1430001	1450001	6.67339	0.302819	BD	ANOS1	NC_056060.1	6615001	6635001
NC_056074.1	46840001	46860001	2.14471	0.334127	BD	AP2A2	NC_056060.1	6620001	6640001
NC_056074.1	46835001	46855001	2.77778	0.322396	BD	AP2A2	NC_056069.1	62770001	62790001
NC_056056.1	28785001	28805001	2.11722	0.297305	BD	APOB	NC_056069.1	62775001	62795001
NC_056056.1	28790001	28810001	2.94609	0.295876	BD	APOB	NC_056069.1	62780001	62800001
NC_056074.1	15960001	15980001	2.57783	0.37651	BD	AQP11	NC_056069.1	62785001	62805001
NC_056074.1	15965001	15985001	2.11335	0.308076	BD	AQP11	NC_056079.1	14845001	14865001
NC_056074.1	15955001	15975001	2.49347	0.3905	BD	AQP11;CLNS1A	NC_056079.1	14835001	14855001
NC_056074.1	15950001	15970001	2.44166	0.389193	BD	AQP11;CLNS1A	NC_056079.1	14840001	14860001
NC_056055.1	37705001	37725001	5.15228	0.361816	BD	AQP3;NOL6	NC_056055.1	199425001	199445001
NC_056060.1	41095001	41115001	2.97819	0.334943	BD	ARF6	NC_056055.1	199430001	199450001
NC_056070.1	10395001	10415001	3.42869	0.353769	BD	ARHGAP10	NC_056055.1	199435001	199455001
NC_056070.1	10390001	10410001	5.19577	0.335118	BD	ARHGAP10	NC_056056.1	170315001	170335001
NC_056070.1	10385001	10405001	2.65275	0.331548	BD	ARHGAP10	NC_056056.1	170320001	170340001
NC_056070.1	10400001	10420001	3.38248	0.301819	BD	ARHGAP10	NC_056056.1	170325001	170345001
NC_056076.1	40355001	40375001	3.12107	0.332311	BD	ARHGAP28	NC_056056.1	170330001	170350001
NC_056076.1	40365001	40385001	2.15683	0.298722	BD	ARHGAP28	NC_056056.1	170365001	170385001
NC_056076.1	40360001	40380001	2.41898	0.290952	BD	ARHGAP28	NC_056056.1	170370001	170390001
NC_056080.1	9680001	9700001	11.4155	0.46378	BD	ARHGAP6	NC_056056.1	170375001	170395001
NC_056080.1	9675001	9695001	4.94558	0.424336	BD	ARHGAP6	NC_056056.1	170380001	170400001
NC_056080.1	9720001	9740001	3.37127	0.415007	BD	ARHGAP6	NC_056056.1	170385001	170405001
NC_056080.1	9730001	9750001	2.82927	0.412982	BD	ARHGAP6	NC_056056.1	170555001	170575001
NC_056080.1	9725001	9745001	2.52864	0.404416	BD	ARHGAP6	NC_056056.1	141285001	141305001
NC_056080.1	9710001	9730001	3.52173	0.397896	BD	ARHGAP6	NC_056056.1	141290001	141310001
NC_056080.1	9715001	9735001	3.325	0.396154	BD	ARHGAP6	NC_056056.1	28790001	28810001
NC_056080.1	9550001	9570001	8.56801	0.391342	BD	ARHGAP6	NC_056055.1	37705001	37725001
NC_056080.1	9670001	9690001	2.22222	0.391316	BD	ARHGAP6	NC_056060.1	41095001	41115001
NC_056080.1	9335001	9355001	2.12575	0.383333	BD	ARHGAP6	NC_056080.1	9260001	9280001
NC_056080.1	9625001	9645001	2.43038	0.375152	BD	ARHGAP6	NC_056058.1	58755001	58775001
NC_056080.1	9735001	9755001	2.2093	0.352938	BD	ARHGAP6	NC_056062.1	42985001	43005001
NC_056080.1	9310001	9330001	3.06333	0.345628	BD	ARHGAP6	NC_056060.1	68490001	68510001
NC_056080.1	9585001	9605001	2.60172	0.342356	BD	ARHGAP6	NC_056056.1	188040001	188060001
NC_056080.1	9590001	9610001	2.83983	0.330296	BD	ARHGAP6	NC_056056.1	188045001	188065001
NC_056080.1	9595001	9615001	3.27419	0.316558	BD	ARHGAP6	NC_056072.1	9610001	9630001
NC_056080.1	9600001	9620001	2.67136	0.310181	BD	ARHGAP6	NC_056072.1	9615001	9635001
NC_056080.1	9545001	9565001	4.09565	0.310043	BD	ARHGAP6	NC_056079.1	6960001	6980001
NC_056080.1	9315001	9335001	2.13333	0.29321	BD	ARHGAP6	NC_056074.1	45670001	45690001
NC_056054.1	1.06E+08	105990001	3.31648	0.35843	BD	ARHGEF2;RXFP4	NC_056065.1	57750001	57770001
NC_056054.1	1.06E+08	105985001	13.6194	0.358021	BD	ARHGEF2;RXFP4	NC_056065.1	57755001	57775001
NC_056054.1	1.06E+08	106045001	2.21542	0.297381	BD	ARHGEF2;SSR2	NC_056065.1	57760001	57780001
NC_056054.1	1.01E+08	100810001	6.12063	0.365189	BD	ARNT	NC_056080.1	127080001	127100001
NC_056054.1	1.01E+08	100805001	4.28434	0.355037	BD	ARNT	NC_056067.1	7200001	7220001
NC_056054.1	1.01E+08	100815001	5.55718	0.354405	BD	ARNT	NC_056067.1	7205001	7225001
NC_056054.1	1.01E+08	100795001	3.77131	0.333374	BD	ARNT	NC_056058.1	69800001	69820001
NC_056054.1	1.01E+08	100800001	3.75532	0.324018	BD	ARNT	NC_056058.1	69805001	69825001
NC_056068.1	39040001	39060001	4.74745	0.377746	BD	ARNTL;BTBD10	NC_056058.1	69810001	69830001
NC_056068.1	39045001	39065001	2.27272	0.338572	BD	ARNTL;BTBD10	NC_056064.1	42275001	42295001
NC_056068.1	39035001	39055001	5.62173	0.312885	BD	ARNTL;BTBD10	NC_056080.1	69055001	69075001
NC_056062.1	23595001	23615001	2.19563	0.342958	BD	ASAP1	NC_056080.1	69060001	69080001
NC_056062.1	23590001	23610001	2.17369	0.297093	BD	ASAP1	NC_056080.1	69065001	69085001
NC_056055.1	2.21E+08	221450001	3.26235	0.322916	BD	ASIC4	NC_056080.1	69070001	69090001
NC_056064.1	24075001	24095001	4.36843	0.481744	BD	ASPA;SPATA22	NC_056054.1	247180001	247200001
NC_056064.1	24080001	24100001	2.1658	0.330863	BD	ASPA;SPATA22	NC_056054.1	247185001	247205001
NC_056055.1	6910001	6930001	2.30689	0.299527	BD	ASTN2	NC_056066.1	59195001	59215001
NC_056055.1	1.35E+08	135015001	3.30829	0.336234	BD	ATF2	NC_056066.1	59200001	59220001
NC_056055.1	1.35E+08	135010001	5.24818	0.315713	BD	ATF2	NC_056066.1	59205001	59225001
NC_056055.1	1.35E+08	135020001	2.98039	0.311883	BD	ATF2	NC_056066.1	59210001	59230001

NC_056055.1	1.35E+08	135000001	3.66498	0.309466	BD	ATF2	NC_056066.1	59215001	59235001
NC_056055.1	1.35E+08	135005001	4.1768	0.295426	BD	ATF2	NC_056078.1	7580001	7600001
NC_056060.1	41700001	41720001	4.83697	0.417802	BD	ATL1	NC_056078.1	7585001	7605001
NC_056060.1	41695001	41715001	4.63835	0.40332	BD	ATL1	NC_056076.1	25780001	25800001
NC_056060.1	41705001	41725001	4.42548	0.372941	BD	ATL1	NC_056076.1	25785001	25805001
NC_056060.1	41690001	41710001	2.63992	0.322904	BD	ATL1	NC_056076.1	25790001	25810001
NC_056067.1	7200001	7220001	9.20987	0.519729	BD	ATMIN;CENPN	NC_056076.1	25820001	25840001
NC_056067.1	7205001	7225001	4.10382	0.455697	BD	ATMIN;CENPN	NC_056076.1	25825001	25845001
NC_056067.1	7210001	7230001	2.35674	0.35818	BD	ATMIN;CENPN	NC_056076.1	25830001	25850001
NC_056058.1	69800001	69820001	5.23786	0.364987	BD	ATP10B	NC_056074.1	40820001	40840001
NC_056058.1	69805001	69825001	3.19513	0.361235	BD	ATP10B	NC_056074.1	40825001	40845001
NC_056058.1	69810001	69830001	2.43125	0.360637	BD	ATP10B	NC_056074.1	40810001	40830001
NC_056080.1	95530001	95550001	5.81545	0.33288	BD	ATP11C	NC_056074.1	40815001	40835001
NC_056056.1	1.62E+08	161750001	2.16788	0.396681	BD	ATP23	NC_056079.1	32455001	32475001
NC_056056.1	1.62E+08	161745001	2.10556	0.394292	BD	ATP23	NC_056059.1	23195001	23215001
NC_056056.1	1.62E+08	161735001	2.35361	0.341823	BD	ATP23	NC_056059.1	23200001	23220001
NC_056064.1	24420001	24440001	2.71134	0.477816	BD	ATP2A3;P2RX1	NC_056067.1	23620001	23640001
NC_056064.1	24415001	24435001	2.32443	0.451638	BD	ATP2A3;P2RX1	NC_056057.1	65110001	65130001
NC_056054.1	1.22E+08	122305001	3.47988	0.448261	BD	ATP5PO	NC_056067.1	52335001	52355001
NC_056054.1	1.22E+08	122310001	3.19005	0.431099	BD	ATP5PO	NC_056067.1	52340001	52360001
NC_056054.1	1.22E+08	122315001	3.15662	0.448611	BD	ATP5PO;ITSN1	NC_056054.1	70870001	70890001
NC_056071.1	54585001	54605001	2.84597	0.532062	BD	ATXN3	NC_056080.1	39390001	39410001
NC_056071.1	54580001	54600001	2.32857	0.502432	BD	ATXN3	NC_056075.1	50220001	50240001
NC_056071.1	54575001	54595001	2.24546	0.50049	BD	ATXN3	NC_056075.1	50225001	50245001
NC_056071.1	54570001	54590001	2.43988	0.454296	BD	ATXN3	NC_056075.1	50230001	50250001
NC_056071.1	54600001	54620001	2.70454	0.34833	BD	ATXN3;NDUFB1	NC_056069.1	5500001	5520001
NC_056071.1	54595001	54615001	2.26755	0.307696	BD	ATXN3;NDUFB1	NC_056054.1	69350001	69370001
NC_056066.1	59205001	59225001	5.21239	0.729278	BD	AURKA;CSTF1	NC_056054.1	69355001	69375001
NC_056066.1	59200001	59220001	4.45929	0.687639	BD	AURKA;CSTF1	NC_056060.1	33990001	34010001
NC_056066.1	59195001	59215001	2.32719	0.574545	BD	AURKA;CSTF1	NC_056060.1	34020001	34040001
NC_056066.1	59210001	59230001	3.91259	0.706025	BD	AURKA;CSTF1;FAM	NC_056063.1	20855001	20875001
NC_056077.1	30530001	30550001	2.48087	0.378398	BD	AUTS2	NC_056065.1	64915001	64935001
NC_056077.1	30535001	30555001	3.41576	0.36483	BD	AUTS2	NC_056065.1	64920001	64940001
NC_056077.1	30540001	30560001	3.86416	0.346626	BD	AUTS2	NC_056065.1	64925001	64945001
NC_056077.1	30545001	30565001	2.40586	0.307757	BD	AUTS2	NC_056065.1	64930001	64950001
NC_056074.1	40825001	40845001	5.72254	0.410358	BD	B4GAT1;BRMS1	NC_056065.1	64935001	64955001
NC_056074.1	40820001	40840001	6.47501	0.400067	BD	B4GAT1;BRMS1	NC_056065.1	65035001	65055001
NC_056074.1	40815001	40835001	2.87138	0.33751	BD	B4GAT1;BRMS1;RIN	NC_056065.1	65040001	65060001
NC_056079.1	32455001	32475001	2.54286	0.388034	BD	BAG4	NC_056065.1	65045001	65065001
NC_056066.1	35405001	35425001	2.39033	0.32917	BD	BAMBI	NC_056067.1	12355001	12375001
NC_056057.1	65110001	65130001	5.14635	0.474808	BD	BBS9	NC_056067.1	12360001	12380001
NC_056057.1	65115001	65135001	3.36878	0.422684	BD	BBS9	NC_056067.1	55360001	55380001
NC_056057.1	65105001	65125001	4.14457	0.414775	BD	BBS9	NC_056068.1	21940001	21960001
NC_056057.1	65120001	65140001	2.18574	0.315913	BD	BBS9	NC_056068.1	21945001	21965001
NC_056057.1	65100001	65120001	2.89513	0.303578	BD	BBS9	NC_056068.1	19010001	19030001
NC_056067.1	52335001	52355001	6.88449	0.352636	BD	BCAM;CBLC	NC_056068.1	15635001	15655001
NC_056080.1	18455001	18475001	2.38308	0.299855	BD	BCLAF3	NC_056068.1	15640001	15660001
NC_056080.1	1.29E+08	129150001	2.90919	0.324352	BD	BEX4	NC_056068.1	15645001	15665001
NC_056080.1	1.29E+08	129155001	2.96306	0.295606	BD	BEX4	NC_056080.1	104945001	104965001
NC_056062.1	13605001	13625001	8.23496	0.434705	BD	BOP1;MROH1	NC_056054.1	69460001	69480001
NC_056062.1	13600001	13620001	7.58683	0.419655	BD	BOP1;MROH1;SCX	NC_056054.1	69485001	69505001
NC_056062.1	13595001	13615001	4.08756	0.344555	BD	BOP1;MROH1;SCX	NC_056054.1	69490001	69510001
NC_056054.1	19475001	19495001	5.31034	0.354312	BD	BTBD19;DYNLT4;PL	NC_056054.1	69495001	69515001
NC_056054.1	69355001	69375001	2.79629	0.319519	BD	BTBD8	NC_056054.1	69500001	69520001
NC_056080.1	1.33E+08	133065001	2.66789	0.431104	BD	BTK;LOC101105982	NC_056054.1	69505001	69525001
NC_056060.1	33990001	34010001	5.11707	0.307025	BD	BUB1B	NC_056054.1	102650001	102670001
NC_056065.1	64925001	64945001	10.8593	0.472874	BD	C12H1orf21	NC_056054.1	232600001	232620001
NC_056065.1	64920001	64940001	13.2903	0.441683	BD	C12H1orf21	NC_056056.1	102170001	102190001
NC_056065.1	64930001	64950001	4.89429	0.412041	BD	C12H1orf21	NC_056059.1	25430001	25450001
NC_056065.1	64935001	64955001	3.57143	0.388845	BD	C12H1orf21	NC_056059.1	25435001	25455001
NC_056065.1	64855001	64875001	2.67476	0.351579	BD	C12H1orf21	NC_056059.1	25440001	25460001
NC_056065.1	64915001	64935001	5.22763	0.319776	BD	C12H1orf21	NC_056059.1	25445001	25465001
NC_056065.1	64940001	64960001	2.51306	0.309872	BD	C12H1orf21	NC_056059.1	103935001	103955001
NC_056065.1	64850001	64870001	2.84342	0.291474	BD	C12H1orf21	NC_056059.1	103940001	103960001
NC_056068.1	21940001	21960001	8.32789	0.444844	BD	C15H11orf52;DIXDC	NC_056068.1	25495001	25515001
NC_056068.1	21945001	21965001	3.69474	0.424458	BD	C15H11orf52;DIXDC	NC_056054.1	154775001	154795001
NC_056068.1	21935001	21955001	3.68931	0.314659	BD	C15H11orf52;DIXDC	NC_056054.1	154780001	154800001
NC_056068.1	15635001	15655001	2.24532	0.403139	BD	C15H11orf97	NC_056054.1	154785001	154805001
NC_056068.1	15640001	15660001	2.29783	0.375269	BD	C15H11orf97	NC_056054.1	154790001	154810001
NC_056068.1	15630001	15650001	2.38709	0.372015	BD	C15H11orf97	NC_056056.1	132815001	132835001
NC_056068.1	15625001	15645001	3.6189	0.370715	BD	C15H11orf97	NC_056056.1	132820001	132840001
NC_056072.1	50890001	50910001	7.10184	0.396902	BD	C19H3orf84;CCDC71	NC_056056.1	79865001	79885001
NC_056072.1	50895001	50915001	7.90982	0.400322	BD	C19H3orf84;CCDC71	NC_056056.1	79870001	79890001
NC_056072.1	50900001	50920001	7.89566	0.394188	BD	C19H3orf84;CCDC71	NC_056056.1	79875001	79895001

NC_056072.1	50885001	50905001	3.10769	0.307843	BD	C19H3orf84;KLHDC8	NC_056056.1	79880001	79900001
NC_056080.1	1.05E+08	104965001	9.26895	0.462851	BD	C1GALT1C1	NC_056056.1	79885001	79905001
NC_056080.1	1.05E+08	104960001	4.02703	0.426046	BD	C1GALT1C1	NC_056056.1	79890001	79910001
NC_056054.1	69505001	69525001	7.97115	0.424315	BD	C1H1orf146;GLMN	NC_056056.1	79895001	79915001
NC_056054.1	69500001	69520001	6.44473	0.334037	BD	C1H1orf146;GLMN	NC_056056.1	79900001	79920001
NC_056054.1	69495001	69515001	6.1845	0.313016	BD	C1H1orf146;GLMN	NC_056056.1	79925001	79945001
NC_056054.1	1.04E+08	104515001	2.34649	0.35661	BD	C1H1orf189;C1H1orf	NC_056056.1	79945001	79965001
NC_056054.1	1.14E+08	113920001	7.13445	0.465109	BD	C1H1orf226	NC_056056.1	79950001	79970001
NC_056054.1	1.14E+08	113915001	4.38095	0.430793	BD	C1H1orf226	NC_056073.1	39195001	39215001
NC_056054.1	1.14E+08	113910001	2.85219	0.387946	BD	C1H1orf226;SPATA4	NC_056073.1	39200001	39220001
NC_056074.1	41180001	41200001	4.40137	0.423037	BD	C21H11orf80	NC_056065.1	26240001	26260001
NC_056074.1	41375001	41395001	2.34627	0.329488	BD	C21H11orf86	NC_056065.1	26245001	26265001
NC_056074.1	41370001	41390001	2.61765	0.301544	BD	C21H11orf86;PC	NC_056065.1	26250001	26270001
NC_056055.1	1.19E+08	119125001	6.40116	0.411657	BD	C2H2orf88	NC_056065.1	26255001	26275001
NC_056055.1	1.19E+08	119130001	6.91176	0.40421	BD	C2H2orf88	NC_056056.1	181540001	181560001
NC_056055.1	1.19E+08	119120001	7.87584	0.396556	BD	C2H2orf88	NC_056056.1	181545001	181565001
NC_056055.1	1.19E+08	119115001	8.32025	0.395257	BD	C2H2orf88;HIBCH	NC_056056.1	181550001	181570001
NC_056055.1	1.19E+08	119110001	9.21311	0.366203	BD	C2H2orf88;HIBCH	NC_056056.1	181555001	181575001
NC_056055.1	1.19E+08	119105001	2.37201	0.310526	BD	C2H2orf88;HIBCH	NC_056080.1	40870001	40890001
NC_056055.1	64670001	64690001	8.76548	0.387096	BD	C2H9orf85	NC_056054.1	111220001	111240001
NC_056055.1	64675001	64695001	5.5826	0.329636	BD	C2H9orf85	NC_056066.1	59170001	59190001
NC_056055.1	64665001	64685001	4.51447	0.301317	BD	C2H9orf85	NC_056066.1	59175001	59195001
NC_056057.1	88550001	88570001	3.44883	0.301341	BD	CADPS2	NC_056066.1	59180001	59200001
NC_056057.1	88545001	88565001	3.48546	0.293298	BD	CADPS2	NC_056067.1	13625001	13645001
NC_056056.1	79880001	79900001	7.72109	0.72329	BD	CAMKMT	NC_056067.1	13630001	13650001
NC_056056.1	79885001	79905001	8.57928	0.654468	BD	CAMKMT	NC_056067.1	13635001	13655001
NC_056056.1	79875001	79895001	3.91135	0.600541	BD	CAMKMT	NC_056067.1	13650001	13670001
NC_056056.1	79890001	79910001	7.46584	0.598198	BD	CAMKMT	NC_056067.1	52320001	52340001
NC_056056.1	79895001	79915001	3.9106	0.523592	BD	CAMKMT	NC_056067.1	52325001	52345001
NC_056056.1	79870001	79890001	2.86477	0.482718	BD	CAMKMT	NC_056067.1	52330001	52350001
NC_056056.1	79945001	79965001	2.47834	0.459855	BD	CAMKMT	NC_056078.1	24275001	24295001
NC_056056.1	79940001	79960001	3.13031	0.403876	BD	CAMKMT	NC_056078.1	24260001	24280001
NC_056058.1	14195001	14215001	3.83165	0.349296	BD	CAMSAP3;LOC1011	NC_056078.1	24265001	24285001
NC_056080.1	40860001	40880001	2.46558	0.378833	BD	CASK;GPR34	NC_056064.1	44155001	44175001
NC_056080.1	40865001	40885001	2.51282	0.391851	BD	CASK;GPR34;GPR82	NC_056076.1	54625001	54645001
NC_056080.1	40870001	40890001	2.79736	0.372707	BD	CASK;GPR82	NC_056076.1	54630001	54650001
NC_056065.1	42175001	42195001	3.19356	0.362699	BD	CASZ1	NC_056076.1	54635001	54655001
NC_056065.1	42180001	42200001	2.69452	0.339946	BD	CASZ1	NC_056064.1	15485001	15505001
NC_056065.1	42185001	42205001	2.37389	0.317141	BD	CASZ1	NC_056064.1	15490001	15510001
NC_056067.1	13630001	13650001	4.73786	0.457249	BD	CBFA2T3	NC_056059.1	34735001	34755001
NC_056067.1	13625001	13645001	2.6134	0.404291	BD	CBFA2T3	NC_056059.1	34740001	34760001
NC_056067.1	13635001	13655001	6.7234	0.369101	BD	CBFA2T3	NC_056059.1	34895001	34915001
NC_056067.1	52330001	52350001	11.6605	0.388421	BD	CBLC	NC_056059.1	34900001	34920001
NC_056067.1	52325001	52345001	7.95798	0.373265	BD	CBLC	NC_056059.1	34905001	34925001
NC_056067.1	52320001	52340001	6.33015	0.350895	BD	CBLC	NC_056059.1	34910001	34930001
NC_056064.1	52095001	52115001	2.49275	0.354594	BD	CBX2	NC_056064.1	45985001	46005001
NC_056064.1	52100001	52120001	2.31836	0.33582	BD	CBX2	NC_056067.1	32370001	32390001
NC_056078.1	24265001	24285001	6.32172	0.318068	BD	CCAR1;TET1	NC_056076.1	60235001	60255001
NC_056078.1	24260001	24280001	7	0.303511	BD	CCAR1;TET1	NC_056066.1	55030001	55050001
NC_056056.1	2.19E+08	218650001	2.60686	0.345449	BD	CCDC134;MEI1	NC_056066.1	55035001	55055001
NC_056080.1	1.01E+08	100735001	2.43263	0.386661	BD	CCDC160	NC_056066.1	55040001	55060001
NC_056064.1	50255001	50275001	3.07235	0.295782	BD	CCDC57	NC_056078.1	38215001	38235001
NC_056064.1	50230001	50250001	2.53658	0.292958	BD	CCDC57	NC_056055.1	2975001	2995001
NC_056064.1	50265001	50285001	2.95906	0.310417	BD	CCDC57;FASN	NC_056055.1	2980001	3000001
NC_056064.1	50260001	50280001	3.6845	0.308226	BD	CCDC57;FASN	NC_056059.1	91165001	91185001
NC_056070.1	54705001	54725001	9.17647	0.341534	BD	CCDC63	NC_056059.1	91170001	91190001
NC_056070.1	54710001	54730001	6.84082	0.317164	BD	CCDC63	NC_056059.1	91175001	91195001
NC_056072.1	50905001	50925001	4.89142	0.376857	BD	CCDC71;KLHDC8B;l	NC_056059.1	91180001	91200001
NC_056072.1	50910001	50930001	3.07089	0.332878	BD	CCDC71;LOC106991	NC_056060.1	4640001	4660001
NC_056077.1	34490001	34510001	13.6979	0.302801	BD	CCL26	NC_056060.1	21540001	21560001
NC_056074.1	43275001	43295001	2.83473	0.400539	BD	CCND1	NC_056060.1	21545001	21565001
NC_056074.1	43280001	43300001	2.19772	0.302432	BD	CCND1	NC_056067.1	7195001	7215001
NC_056072.1	53005001	53025001	16.5279	0.403998	BD	CCR5	NC_056067.1	7190001	7210001
NC_056072.1	53010001	53030001	17.2611	0.394156	BD	CCR5	NC_056055.1	140890001	140910001
NC_056072.1	53000001	53020001	15.5965	0.361427	BD	CCR5	NC_056055.1	140895001	140915001
NC_056072.1	53015001	53035001	23.5969	0.400063	BD	CCR5;LOC10111770	NC_056055.1	140900001	140920001
NC_056072.1	53020001	53040001	8.84298	0.329522	BD	CCR5;LOC10111770	NC_056055.1	140905001	140925001
NC_056072.1	52990001	53010001	13.4078	0.317948	BD	CCRL2	NC_056055.1	140910001	140930001
NC_056054.1	1.28E+08	127720001	2.34449	0.342471	BD	CCT8;USP16	NC_056055.1	140915001	140935001
NC_056054.1	1.12E+08	112090001	3.00624	0.299017	BD	CD244	NC_056055.1	140920001	140940001
NC_056054.1	1.12E+08	112095001	2.9878	0.297789	BD	CD244	NC_056080.1	35435001	35455001
NC_056064.1	45925001	45945001	2.12791	0.465863	BD	CDC27	NC_056080.1	35440001	35460001
NC_056064.1	45985001	46005001	2.52355	0.33929	BD	CDC27	NC_056080.1	35445001	35465001
NC_056058.1	35790001	35810001	2.46154	0.36417	BD	CDHR2;GPRIN1	NC_056080.1	35450001	35470001

NC_056061.1	26780001	26800001	3.10207	0.412537	BD	CDK19	NC_056080.1	35455001	35475001
NC_056061.1	26795001	26815001	2.64893	0.37292	BD	CDK19	NC_056057.1	98260001	98280001
NC_056061.1	26775001	26795001	2.20793	0.330349	BD	CDK19	NC_056057.1	98270001	98290001
NC_056055.1	3070001	3090001	21.5117	0.315346	BD	CDK5RAP2	NC_056057.1	98275001	98295001
NC_056055.1	3020001	3040001	2.54054	0.312881	BD	CDK5RAP2	NC_056057.1	98280001	98300001
NC_056055.1	3075001	3095001	16.0785	0.309695	BD	CDK5RAP2	NC_056057.1	98285001	98305001
NC_056055.1	3065001	3085001	16.1835	0.308639	BD	CDK5RAP2	NC_056057.1	98290001	98310001
NC_056055.1	3080001	3100001	9.93672	0.299976	BD	CDK5RAP2	NC_056057.1	98295001	98315001
NC_056055.1	3085001	3105001	8.08275	0.295338	BD	CDK5RAP2	NC_056057.1	98300001	98320001
NC_056074.1	26775001	26795001	3.49448	0.472929	BD	CDON;VSIG10L2	NC_056057.1	98305001	98325001
NC_056074.1	26780001	26800001	2.72557	0.423974	BD	CDON;VSIG10L2	NC_056057.1	98330001	98350001
NC_056074.1	26770001	26790001	2.48303	0.397848	BD	CDON;VSIG10L2	NC_056057.1	98335001	98355001
NC_056055.1	64950001	64970001	11.2576	0.493627	BD	CEMIP2	NC_056057.1	98375001	98395001
NC_056055.1	64940001	64960001	5.20995	0.312043	BD	CEMIP2	NC_056077.1	1160001	1180001
NC_056059.1	22050001	22070001	2.17647	0.3572	BD	CENPE	NC_056068.1	24030001	24050001
NC_056067.1	7195001	7215001	9.30179	0.5243	BD	CENPN	NC_056068.1	24035001	24055001
NC_056067.1	7190001	7210001	4.22233	0.465018	BD	CENPN;LOC1011211	NC_056068.1	24025001	24045001
NC_056065.1	60575001	60595001	2.44386	0.300932	BD	CEP350	NC_056068.1	24020001	24040001
NC_056056.1	43275001	43295001	5.39655	0.319819	BD	CEP68	NC_056056.1	206070001	206090001
NC_056056.1	43255001	43275001	6.74672	0.315987	BD	CEP68	NC_056056.1	206075001	206095001
NC_056056.1	43270001	43290001	5.45652	0.293347	BD	CEP68	NC_056056.1	206080001	206100001
NC_056060.1	6895001	6915001	3.53447	0.438298	BD	CERT1	NC_056056.1	206085001	206105001
NC_056060.1	6890001	6910001	3.13536	0.428448	BD	CERT1	NC_056073.1	19130001	19150001
NC_056054.1	1.79E+08	179035001	2.15038	0.373671	BD	CFAP44	NC_056073.1	19135001	19155001
NC_056080.1	35450001	35470001	3.99167	0.422454	BD	CFAP47	NC_056073.1	19140001	19160001
NC_056080.1	35445001	35465001	4.25208	0.415058	BD	CFAP47	NC_056077.1	33500001	33520001
NC_056080.1	35455001	35475001	2.5071	0.405319	BD	CFAP47	NC_056077.1	33535001	33555001
NC_056080.1	35440001	35460001	3.72393	0.392537	BD	CFAP47	NC_056077.1	33540001	33560001
NC_056055.1	1.35E+08	135190001	3.83378	0.297097	BD	CHN1	NC_056077.1	33545001	33565001
NC_056078.1	11575001	11595001	3.59872	0.341972	BD	CHRM3	NC_056077.1	33550001	33570001
NC_056078.1	11570001	11590001	2.54023	0.32901	BD	CHRM3	NC_056077.1	33555001	33575001
NC_056072.1	7845001	7865001	2.42131	0.292617	BD	CLASP2	NC_056054.1	249490001	249510001
NC_056080.1	55520001	55540001	2.18875	0.349548	BD	CLCN5	NC_056063.1	75990001	76010001
NC_056080.1	55510001	55530001	2.31127	0.322693	BD	CLCN5	NC_056063.1	75995001	76015001
NC_056080.1	55515001	55535001	2.42194	0.308426	BD	CLCN5	NC_056063.1	76000001	76020001
NC_056056.1	2.07E+08	207240001	2.39851	0.305033	BD	CLEC4A	NC_056067.1	25430001	25450001
NC_056077.1	33500001	33520001	7.61608	0.554959	BD	CLIP2	NC_056067.1	25435001	25455001
NC_056077.1	33545001	33565001	4.03323	0.524996	BD	CLIP2	NC_056067.1	25440001	25460001
NC_056077.1	33550001	33570001	2.76311	0.479996	BD	CLIP2	NC_056067.1	25445001	25465001
NC_056077.1	33540001	33560001	7.23888	0.439236	BD	CLIP2	NC_056067.1	25450001	25470001
NC_056077.1	33535001	33555001	13.1795	0.417851	BD	CLIP2	NC_056067.1	25455001	25475001
NC_056077.1	26545001	26565001	2.33648	0.527247	BD	CLN3	NC_056075.1	23385001	23405001
NC_056077.1	26550001	26570001	2.32783	0.447446	BD	CLN3	NC_056075.1	23390001	23410001
NC_056077.1	26540001	26560001	4.15807	0.662763	BD	CLN3;LOC10111269	NC_056075.1	23395001	23415001
NC_056074.1	15945001	15965001	2.78125	0.313402	BD	CLNS1A	NC_056075.1	23410001	23430001
NC_056054.1	2.49E+08	249510001	7.48327	0.647187	BD	CLSTN2	NC_056075.1	23415001	23435001
NC_056054.1	2.49E+08	249475001	2.23549	0.543251	BD	CLSTN2	NC_056058.1	63815001	63835001
NC_056054.1	2.49E+08	249515001	2.31441	0.523059	BD	CLSTN2	NC_056058.1	63820001	63840001
NC_056061.1	15550001	15570001	5.35309	0.33803	BD	CLVS2	NC_056056.1	146160001	146180001
NC_056061.1	15545001	15565001	2.67057	0.301979	BD	CLVS2	NC_056056.1	146165001	146185001
NC_056054.1	1.65E+08	165035001	2.13528	0.337953	BD	CMSS1;FILIP1L	NC_056068.1	8965001	8985001
NC_056054.1	1.65E+08	165030001	2.49578	0.318344	BD	CMSS1;FILIP1L	NC_056068.1	9500001	9520001
NC_056075.1	23385001	23405001	4.83771	0.331376	BD	CNNM2	NC_056068.1	9505001	9525001
NC_056056.1	1.49E+08	149505001	5.63141	0.333402	BD	CNOT2	NC_056068.1	9510001	9530001
NC_056056.1	1.49E+08	149510001	5.66	0.325027	BD	CNOT2	NC_056068.1	9800001	9820001
NC_056056.1	1.49E+08	149495001	4.6884	0.322727	BD	CNOT2	NC_056057.1	112050001	112070001
NC_056056.1	1.49E+08	149450001	3.34339	0.313269	BD	CNOT2	NC_056057.1	112055001	112075001
NC_056056.1	1.49E+08	149480001	4.6265	0.312404	BD	CNOT2	NC_056057.1	50060001	50080001
NC_056056.1	1.49E+08	149515001	3.86765	0.310537	BD	CNOT2	NC_056057.1	50065001	50085001
NC_056056.1	1.49E+08	149455001	2.72821	0.310524	BD	CNOT2	NC_056057.1	50070001	50090001
NC_056056.1	1.49E+08	149445001	2.84455	0.309944	BD	CNOT2	NC_056057.1	50075001	50095001
NC_056056.1	1.49E+08	149500001	3.50635	0.307918	BD	CNOT2	NC_056057.1	50080001	50100001
NC_056056.1	1.49E+08	149470001	2.60639	0.304658	BD	CNOT2	NC_056057.1	50085001	50105001
NC_056056.1	1.49E+08	149460001	2.62654	0.300832	BD	CNOT2	NC_056062.1	4270001	4290001
NC_056056.1	1.49E+08	149490001	3.73091	0.300731	BD	CNOT2	NC_056062.1	4275001	4295001
NC_056056.1	1.49E+08	149465001	2.60763	0.292075	BD	CNOT2	NC_056062.1	4295001	4315001
NC_056056.1	1.49E+08	149485001	2.8953	0.291893	BD	CNOT2	NC_056062.1	4300001	4320001
NC_056068.1	8720001	8740001	14.8854	0.35668	BD	CNTN5	NC_056054.1	62915001	62935001
NC_056068.1	8715001	8735001	9.79084	0.339112	BD	CNTN5	NC_056055.1	120350001	120370001
NC_056068.1	8725001	8745001	12.3747	0.317527	BD	CNTN5	NC_056055.1	120355001	120375001
NC_056058.1	37060001	37080001	6.70475	0.33867	BD	COL23A1	NC_056055.1	120360001	120380001
NC_056054.1	62915001	62935001	2.19481	0.309328	BD	COL24A1	NC_056055.1	120370001	120390001
NC_056059.1	16465001	16485001	3.45539	0.351738	BD	COL25A1	NC_056055.1	120375001	120395001
NC_056059.1	16455001	16475001	5.03136	0.335293	BD	COL25A1	NC_056055.1	120380001	120400001

NC_056059.1	16460001	16480001	4.06982	0.301748	BD	COL25A1	NC_056055.1	120385001	120405001
NC_056059.1	16430001	16450001	3.32409	0.30016	BD	COL25A1	NC_056055.1	120390001	120410001
NC_056077.1	35640001	35660001	4.11985	0.368646	BD	COL26A1	NC_056063.1	84355001	84375001
NC_056077.1	35650001	35670001	4.0299	0.316632	BD	COL26A1	NC_056063.1	84360001	84380001
NC_056077.1	35635001	35655001	2.53572	0.31514	BD	COL26A1	NC_056054.1	3760001	3780001
NC_056077.1	35645001	35665001	3.70115	0.308039	BD	COL26A1	NC_056058.1	35405001	35425001
NC_056055.1	1.2E+08	120400001	8.49558	0.809906	BD	COL3A1	NC_056062.1	44560001	44580001
NC_056055.1	1.2E+08	120405001	8.15388	0.807423	BD	COL3A1	NC_056062.1	44565001	44585001
NC_056055.1	1.2E+08	120395001	7.72264	0.802796	BD	COL3A1	NC_056062.1	44570001	44590001
NC_056055.1	1.2E+08	120390001	3.05705	0.735703	BD	COL3A1	NC_056062.1	44575001	44595001
NC_056055.1	1.2E+08	120410001	2.18107	0.708724	BD	COL3A1	NC_056062.1	44580001	44600001
NC_056080.1	1.27E+08	126820001	2.22505	0.404387	BD	COL4A6	NC_056062.1	44585001	44605001
NC_056080.1	1.27E+08	126825001	2.12624	0.374449	BD	COL4A6	NC_056058.1	5855001	5875001
NC_056058.1	35320001	35340001	8.53139	0.393967	BD	COMMD10	NC_056058.1	5860001	5880001
NC_056058.1	35315001	35335001	6.11142	0.365238	BD	COMMD10	NC_056057.1	68625001	68645001
NC_056058.1	35325001	35345001	3.88109	0.360743	BD	COMMD10	NC_056057.1	68630001	68650001
NC_056058.1	35310001	35330001	3.94506	0.354232	BD	COMMD10	NC_056057.1	68645001	68665001
NC_056054.1	2.59E+08	259075001	2.11308	0.30941	BD	CPNE4	NC_056057.1	68655001	68675001
NC_056073.1	10845001	10865001	14.063	0.305131	BD	CPNE5;PPIL1	NC_056057.1	68660001	68680001
NC_056054.1	1.64E+08	163815001	2.1121	0.301612	BD	CPOX	NC_056056.1	102420001	102440001
NC_056054.1	1.64E+08	163830001	2.936	0.299541	BD	CPOX	NC_056056.1	102425001	102445001
NC_056071.1	54605001	54625001	3.45439	0.413932	BD	CPSF2;NDUFB1	NC_056056.1	102430001	102450001
NC_056071.1	54615001	54635001	2.17167	0.409838	BD	CPSF2;NDUFB1	NC_056057.1	69260001	69280001
NC_056071.1	54610001	54630001	3.48907	0.399656	BD	CPSF2;NDUFB1	NC_056069.1	4965001	4985001
NC_056057.1	68660001	68680001	6.38889	0.501877	BD	CPVL	NC_056069.1	4970001	4990001
NC_056057.1	68655001	68675001	11.8074	0.474884	BD	CPVL	NC_056069.1	4975001	4995001
NC_056057.1	68645001	68665001	27.9466	0.375644	BD	CPVL	NC_056069.1	4980001	5000001
NC_056057.1	68625001	68645001	4.31012	0.322925	BD	CPVL	NC_056066.1	47890001	47910001
NC_056056.1	1.3E+08	130135001	2.34254	0.366463	BD	CRADD	NC_056066.1	47895001	47915001
NC_056056.1	1.3E+08	130140001	2.21606	0.358921	BD	CRADD	NC_056066.1	47900001	47920001
NC_056056.1	1.3E+08	130095001	3.21477	0.297633	BD	CRADD	NC_056066.1	47905001	47925001
NC_056057.1	69025001	69045001	13.6104	0.308921	BD	CREB5	NC_056066.1	47910001	47930001
NC_056057.1	69020001	69040001	8.54763	0.306491	BD	CREB5	NC_056059.1	86190001	86210001
NC_056069.1	4970001	4990001	5.52867	0.551475	BD	CREBRF	NC_056059.1	86195001	86215001
NC_056069.1	4975001	4995001	3.56922	0.54495	BD	CREBRF	NC_056059.1	86200001	86220001
NC_056069.1	4980001	5000001	2.25743	0.527208	BD	CREBRF	NC_056059.1	86205001	86225001
NC_056069.1	4965001	4985001	3.23165	0.502049	BD	CREBRF	NC_056062.1	44185001	44205001
NC_056080.1	6765001	6785001	3.38451	0.348173	BD	CRLF2	NC_056055.1	143960001	143980001
NC_056080.1	6770001	6790001	2.5202	0.330603	BD	CRLF2	NC_056055.1	143965001	143985001
NC_056080.1	6760001	6780001	3.25426	0.307258	BD	CRLF2	NC_056055.1	144010001	144030001
NC_056064.1	17870001	17890001	5.1025	0.400397	BD	CRLF3	NC_056055.1	144015001	144035001
NC_056064.1	17875001	17895001	4.10495	0.378128	BD	CRLF3	NC_056055.1	144080001	144100001
NC_056064.1	17865001	17885001	3.89691	0.358818	BD	CRLF3	NC_056055.1	144085001	144105001
NC_056064.1	17880001	17900001	2.43724	0.31191	BD	CRLF3	NC_056055.1	144090001	144110001
NC_056066.1	47910001	47930001	7.51935	0.328426	BD	CRLS1;LRRN4	NC_056056.1	112765001	112785001
NC_056066.1	47905001	47925001	6.3112	0.297736	BD	CRLS1;LRRN4	NC_056056.1	51885001	51905001
NC_056068.1	74810001	74830001	6.42446	0.444878	BD	CRY2	NC_056056.1	51890001	51910001
NC_056068.1	74805001	74825001	5.6689	0.425466	BD	CRY2	NC_056056.1	51895001	51915001
NC_056068.1	74825001	74845001	2.60988	0.368322	BD	CRY2	NC_056069.1	61520001	61540001
NC_056068.1	74800001	74820001	2.68208	0.365449	BD	CRY2	NC_056057.1	52095001	52115001
NC_056068.1	74815001	74835001	2.52607	0.346641	BD	CRY2	NC_056057.1	52100001	52120001
NC_056068.1	74820001	74840001	2.43719	0.33661	BD	CRY2	NC_056057.1	52120001	52140001
NC_056068.1	74835001	74855001	4.20512	0.414167	BD	CRY2;MAPK8IP1	NC_056057.1	52125001	52145001
NC_056068.1	74830001	74850001	3.71186	0.40566	BD	CRY2;MAPK8IP1	NC_056057.1	52130001	52150001
NC_056068.1	74840001	74860001	2.6134	0.388647	BD	CRY2;MAPK8IP1	NC_056057.1	52135001	52155001
NC_056059.1	86200001	86220001	5.60181	0.650656	BD	CSN2	NC_056066.1	30750001	30770001
NC_056059.1	86205001	86225001	6.29659	0.556494	BD	CSN2	NC_056066.1	30755001	30775001
NC_056059.1	86195001	86215001	2.68635	0.537898	BD	CSN2	NC_056066.1	30760001	30780001
NC_056059.1	86210001	86230001	2.70799	0.334409	BD	CSN2	NC_056066.1	30765001	30785001
NC_056056.1	1.13E+08	112785001	2.14935	0.386861	BD	CSRP2	NC_056068.1	15715001	15735001
NC_056056.1	1.13E+08	112790001	2.90205	0.335833	BD	CSRP2	NC_056068.1	15720001	15740001
NC_056056.1	51875001	51895001	5.04405	0.308206	BD	CTNNA2	NC_056068.1	15725001	15745001
NC_056056.1	51870001	51890001	4.05818	0.308168	BD	CTNNA2	NC_056068.1	15730001	15750001
NC_056078.1	21660001	21680001	2.52445	0.357421	BD	CTNNA3	NC_056068.1	15735001	15755001
NC_056078.1	21670001	21690001	5.44581	0.347887	BD	CTNNA3	NC_056068.1	15740001	15760001
NC_056078.1	21665001	21685001	5.31798	0.325271	BD	CTNNA3	NC_056068.1	15745001	15765001
NC_056078.1	21680001	21700001	6.19658	0.296807	BD	CTNNA3	NC_056068.1	15755001	15775001
NC_056078.1	21675001	21695001	5.98618	0.295031	BD	CTNNA3	NC_056068.1	15760001	15780001
NC_056066.1	30660001	30680001	3.83925	0.405614	BD	CUBN	NC_056068.1	15825001	15845001
NC_056066.1	30665001	30685001	3.49171	0.371976	BD	CUBN	NC_056068.1	15830001	15850001
NC_056066.1	30655001	30675001	2.79121	0.408752	BD	CUBN;RSU1	NC_056068.1	15835001	15855001
NC_056068.1	15835001	15855001	2.85456	0.557187	BD	CWF19L2	NC_056068.1	15840001	15860001
NC_056068.1	15830001	15850001	2.88697	0.527579	BD	CWF19L2	NC_056068.1	15700001	15720001
NC_056068.1	15825001	15845001	2.33228	0.498747	BD	CWF19L2	NC_056068.1	15705001	15725001

NC_056068.1	15730001	15750001	7.05065	0.420791	BD	CWF19L2	NC_056080.1	37130001	37150001
NC_056068.1	15735001	15755001	6.19616	0.386881	BD	CWF19L2	NC_056080.1	37135001	37155001
NC_056068.1	15725001	15745001	4.19508	0.369338	BD	CWF19L2	NC_056080.1	37140001	37160001
NC_056068.1	15720001	15740001	2.6922	0.365619	BD	CWF19L2	NC_056080.1	37145001	37165001
NC_056068.1	15740001	15760001	4.92787	0.355713	BD	CWF19L2	NC_056058.1	66590001	66610001
NC_056060.1	57310001	57330001	2.39843	0.425611	BD	CYP19	NC_056058.1	66595001	66615001
NC_056056.1	1.81E+08	181345001	5.10741	0.322833	BD	CYTH4	NC_056058.1	66600001	66620001
NC_056054.1	1.31E+08	130530001	2.34283	0.346461	BD	CYYR1	NC_056056.1	218940001	218960001
NC_056063.1	46925001	46945001	2.3163	0.335303	BD	DACH1	NC_056056.1	181315001	181335001
NC_056063.1	46855001	46875001	20.4571	0.307459	BD	DACH1	NC_056056.1	181320001	181340001
NC_056059.1	25185001	25205001	2.21027	0.350441	BD	DAPP1	NC_056056.1	181325001	181345001
NC_056056.1	2445001	2465001	2.3109	0.2972	BD	DBH	NC_056063.1	47120001	47140001
NC_056067.1	14315001	14335001	2.96515	0.360458	BD	DBNDD1;GAS8	NC_056063.1	47125001	47145001
NC_056067.1	14305001	14325001	7.2381	0.352456	BD	DBNDD1;GAS8;LOC	NC_056063.1	47130001	47150001
NC_056067.1	14310001	14330001	4.62089	0.332245	BD	DBNDD1;GAS8;LOC	NC_056063.1	47135001	47155001
NC_056067.1	14295001	14315001	8.9156	0.340447	BD	DBNDD1;LOC10111	NC_056055.1	230040001	230060001
NC_056067.1	14290001	14310001	6.46024	0.337538	BD	DBNDD1;LOC10111	NC_056055.1	230045001	230065001
NC_056067.1	14300001	14320001	8.93196	0.333441	BD	DBNDD1;LOC10111	NC_056067.1	14315001	14335001
NC_056056.1	1.42E+08	141570001	2.55838	0.305519	BD	DBX2	NC_056067.1	14305001	14325001
NC_056072.1	49360001	49380001	3.95343	0.342321	BD	DCAF1	NC_056067.1	14310001	14330001
NC_056072.1	49370001	49390001	3.09392	0.327853	BD	DCAF1	NC_056067.1	14290001	14310001
NC_056072.1	49365001	49385001	3.20919	0.315631	BD	DCAF1	NC_056067.1	14295001	14315001
NC_056070.1	3340001	3360001	2.30125	0.291733	BD	DCHS2	NC_056067.1	14300001	14320001
NC_056070.1	7775001	7795001	2.60212	0.405686	BD	DCLK2	NC_056056.1	141550001	141570001
NC_056054.1	62385001	62405001	2.12283	0.313028	BD	DDAH1	NC_056070.1	3400001	3420001
NC_056054.1	62415001	62435001	2.47832	0.298216	BD	DDAH1	NC_056070.1	3405001	3425001
NC_056054.1	62425001	62445001	2.47023	0.294452	BD	DDAH1	NC_056070.1	7835001	7855001
NC_056054.1	62410001	62430001	2.3346	0.292052	BD	DDAH1	NC_056072.1	47745001	47765001
NC_056057.1	6185001	6205001	4.07927	0.302501	BD	DDC	NC_056072.1	47750001	47770001
NC_056060.1	63775001	63795001	5.18297	0.440934	BD	DDHD1	NC_056072.1	47755001	47775001
NC_056060.1	63770001	63790001	4.81088	0.419196	BD	DDHD1	NC_056072.1	47760001	47780001
NC_056060.1	63765001	63785001	4.16596	0.407067	BD	DDHD1	NC_056072.1	47765001	47785001
NC_056060.1	63780001	63800001	3.1879	0.338832	BD	DDHD1	NC_056074.1	36395001	36415001
NC_056079.1	32505001	32525001	7.48215	0.447114	BD	DDHD2;NSD3;PLPP5	NC_056079.1	32505001	32525001
NC_056054.1	1.14E+08	114210001	2.36643	0.529254	BD	DDR2	NC_056062.1	15685001	15705001
NC_056054.1	1.14E+08	114205001	2.34182	0.503056	BD	DDR2	NC_056056.1	183985001	184005001
NC_056054.1	1.14E+08	114200001	2.11201	0.479484	BD	DDR2	NC_056056.1	183990001	184010001
NC_056054.1	1.14E+08	114325001	2.5697	0.346833	BD	DDR2	NC_056056.1	183995001	184015001
NC_056054.1	1.14E+08	114295001	2.54732	0.335973	BD	DDR2	NC_056072.1	43675001	43695001
NC_056054.1	1.14E+08	114335001	2.16887	0.33233	BD	DDR2	NC_056072.1	43680001	43700001
NC_056080.1	21375001	21395001	2.64333	0.33802	BD	DDX53	NC_056062.1	94205001	94225001
NC_056068.1	29110001	29130001	3.08897	0.314861	BD	DDX6	NC_056056.1	199565001	199585001
NC_056068.1	29105001	29125001	3.14463	0.311408	BD	DDX6	NC_056068.1	75265001	75285001
NC_056068.1	29100001	29120001	2.56331	0.303039	BD	DDX6	NC_056068.1	75270001	75290001
NC_056067.1	14280001	14300001	2.83681	0.417652	BD	DEF8;LOC101113004	NC_056068.1	75275001	75295001
NC_056067.1	14250001	14270001	2.67347	0.332133	BD	DEF8;MC1R	NC_056065.1	25710001	25730001
NC_056056.1	12185001	12205001	3.90705	0.650668	BD	DENND1A	NC_056068.1	21950001	21970001
NC_056056.1	12190001	12210001	2.87276	0.643718	BD	DENND1A	NC_056068.1	21955001	21975001
NC_056056.1	12180001	12200001	2.6195	0.529849	BD	DENND1A	NC_056068.1	21995001	22015001
NC_056062.1	94205001	94225001	4.56446	0.410358	BD	DEPTOR;DSCC1	NC_056074.1	11100001	11120001
NC_056062.1	94210001	94230001	2.91986	0.317361	BD	DEPTOR;DSCC1	NC_056074.1	11105001	11125001
NC_056058.1	11110001	11130001	2.41366	0.400326	BD	DHPS;FBXW9;GNG1	NC_056074.1	11110001	11130001
NC_056058.1	11120001	11140001	2.42073	0.44339	BD	DHPS;MAN2B1;WDI	NC_056074.1	11115001	11135001
NC_056058.1	11115001	11135001	2.21554	0.407332	BD	DHPS;WDR83;WDR8	NC_056074.1	11120001	11140001
NC_056075.1	44815001	44835001	2.17459	0.352638	BD	DHX32;LOC1011161	NC_056074.1	11125001	11145001
NC_056054.1	28440001	28460001	3.68831	0.552359	BD	DIO1	NC_056080.1	63340001	63360001
NC_056054.1	28445001	28465001	11.3445	0.491865	BD	DIO1;HSPB11	NC_056076.1	38490001	38510001
NC_056065.1	25710001	25730001	2.52526	0.391506	BD	DISP1	NC_056076.1	38495001	38515001
NC_056065.1	25715001	25735001	2.66072	0.365691	BD	DISP1	NC_056080.1	30935001	30955001
NC_056068.1	21950001	21970001	2.45323	0.390215	BD	DIXDC1	NC_056080.1	30940001	30960001
NC_056068.1	21955001	21975001	2.37298	0.376527	BD	DIXDC1	NC_056080.1	30945001	30965001
NC_056068.1	21995001	22015001	2.47396	0.301363	BD	DIXDC1	NC_056080.1	30950001	30970001
NC_056074.1	11110001	11130001	2.8876	0.388793	BD	DLG2	NC_056080.1	31710001	31730001
NC_056074.1	11105001	11125001	2.73377	0.382091	BD	DLG2	NC_056080.1	31715001	31735001
NC_056074.1	11100001	11120001	2.18696	0.361268	BD	DLG2	NC_056080.1	31720001	31740001
NC_056074.1	11115001	11135001	4.61152	0.320351	BD	DLG2	NC_056080.1	32265001	32285001
NC_056080.1	30950001	30970001	3.77248	0.518824	BD	DMD	NC_056072.1	43200001	43220001
NC_056080.1	30940001	30960001	4.8979	0.508557	BD	DMD	NC_056072.1	43205001	43225001
NC_056080.1	30945001	30965001	4.34482	0.507051	BD	DMD	NC_056060.1	82250001	82270001
NC_056080.1	30955001	30975001	2.09896	0.470471	BD	DMD	NC_056057.1	63605001	63625001
NC_056080.1	32265001	32285001	2.33413	0.443009	BD	DMD	NC_056057.1	63610001	63630001
NC_056080.1	32270001	32290001	2.27572	0.404117	BD	DMD	NC_056057.1	63615001	63635001
NC_056080.1	30935001	30955001	3.34623	0.376537	BD	DMD	NC_056057.1	63620001	63640001
NC_056080.1	32315001	32335001	10.2727	0.352415	BD	DMD	NC_056057.1	63660001	63680001

NC_056080.1	32310001	32330001	4.08095	0.342054	BD	DMD	NC_056057.1	63665001	63685001
NC_056080.1	32305001	32325001	2.37187	0.328523	BD	DMD	NC_056057.1	63670001	63690001
NC_056080.1	32110001	32130001	3.34957	0.324476	BD	DMD	NC_056057.1	63685001	63705001
NC_056080.1	32115001	32135001	8.05915	0.322104	BD	DMD	NC_056062.1	82440001	82460001
NC_056064.1	30375001	30395001	11.5125	0.43023	BD	DNAH9	NC_056062.1	82445001	82465001
NC_056064.1	30360001	30380001	3.03907	0.413873	BD	DNAH9	NC_056062.1	82450001	82470001
NC_056064.1	30380001	30400001	3.55468	0.369536	BD	DNAH9	NC_056062.1	82455001	82475001
NC_056064.1	30265001	30285001	2.13681	0.318169	BD	DNAH9	NC_056062.1	82475001	82495001
NC_056064.1	30250001	30270001	2.50931	0.305881	BD	DNAH9	NC_056062.1	82485001	82505001
NC_056065.1	38735001	38755001	4.58713	0.410033	BD	DNM3	NC_056062.1	82495001	82515001
NC_056065.1	38730001	38750001	2.91947	0.369978	BD	DNM3	NC_056062.1	82500001	82520001
NC_056063.1	75325001	75345001	4.81169	0.290805	BD	DOCK9	NC_056058.1	56385001	56405001
NC_056054.1	78345001	78365001	4.42013	0.374242	BD	DPH5	NC_056068.1	27950001	27970001
NC_056054.1	78350001	78370001	3.43778	0.352157	BD	DPH5	NC_056068.1	27970001	27990001
NC_056054.1	78355001	78375001	3.37149	0.347056	BD	DPH5	NC_056068.1	27975001	27995001
NC_056054.1	78360001	78380001	2.41287	0.295208	BD	DPH5	NC_056068.1	27980001	28000001
NC_056060.1	29125001	29145001	12.4132	0.412588	BD	DPH6	NC_056068.1	27985001	28005001
NC_056060.1	29130001	29150001	5.53901	0.377053	BD	DPH6	NC_056068.1	27990001	28010001
NC_056060.1	29120001	29140001	8.96	0.36399	BD	DPH6	NC_056068.1	28005001	28025001
NC_056060.1	29115001	29135001	9.68487	0.321485	BD	DPH6	NC_056068.1	28045001	28065001
NC_056060.1	29135001	29155001	2.95996	0.321328	BD	DPH6	NC_056068.1	28050001	28070001
NC_056060.1	29110001	29130001	7.69836	0.300394	BD	DPH6	NC_056062.1	94200001	94220001
NC_056062.1	94200001	94220001	8.06635	0.442025	BD	DSCC1	NC_056061.1	21540001	21560001
NC_056062.1	94195001	94215001	6.25841	0.375764	BD	DSCC1	NC_056061.1	21555001	21575001
NC_056062.1	94190001	94210001	14.0281	0.353497	BD	DSCC1;TAF2	NC_056061.1	21560001	21580001
NC_056062.1	94180001	94200001	9.91452	0.342233	BD	DSCC1;TAF2	NC_056054.1	203775001	203795001
NC_056062.1	94185001	94205001	9.95512	0.335781	BD	DSCC1;TAF2	NC_056063.1	72370001	72390001
NC_056076.1	22585001	22605001	7.78096	0.309641	BD	DTNA	NC_056056.1	20390001	20410001
NC_056074.1	46345001	46365001	2.19991	0.32255	BD	DUSP8	NC_056056.1	20395001	20415001
NC_056076.1	49065001	49085001	2.45643	0.320444	BD	DYM	NC_056056.1	112905001	112925001
NC_056076.1	49070001	49090001	2.18624	0.319631	BD	DYM	NC_056080.1	62675001	62695001
NC_056076.1	49060001	49080001	2.25503	0.29417	BD	DYM	NC_056080.1	62680001	62700001
NC_056068.1	5145001	5165001	3.60524	0.367746	BD	DYNC2H1	NC_056080.1	62685001	62705001
NC_056068.1	5150001	5170001	4.55872	0.349282	BD	DYNC2H1	NC_056080.1	62690001	62710001
NC_056068.1	5155001	5175001	4.12364	0.318709	BD	DYNC2H1	NC_056080.1	62695001	62715001
NC_056080.1	62690001	62710001	12.1354	0.330603	BD	EDA	NC_056058.1	81560001	81580001
NC_056080.1	62685001	62705001	15.7542	0.320728	BD	EDA	NC_056058.1	81565001	81585001
NC_056080.1	62725001	62745001	6.28672	0.292966	BD	EDA	NC_056058.1	81570001	81590001
NC_056064.1	46550001	46570001	2.15201	0.551872	BD	EFCAB3	NC_056063.1	53625001	53645001
NC_056064.1	46540001	46560001	2.11728	0.52037	BD	EFCAB3	NC_056063.1	53630001	53650001
NC_056064.1	46535001	46555001	2.34305	0.504997	BD	EFCAB3	NC_056063.1	53635001	53655001
NC_056066.1	61930001	61950001	3.47961	0.397341	BD	EFCAB8;SUN5	NW_02459982'	550001	570001
NC_056066.1	61925001	61945001	2.62703	0.360955	BD	EFCAB8;SUN5	NC_056062.1	22210001	22230001
NC_056080.1	43515001	43535001	2.31786	0.317599	BD	EFHC2	NC_056062.1	22195001	22215001
NC_056080.1	43495001	43515001	12.3507	0.312973	BD	EFHC2	NC_056062.1	22200001	22220001
NC_056080.1	43450001	43470001	2.62577	0.312475	BD	EFHC2	NC_056062.1	22205001	22225001
NC_056080.1	43455001	43475001	2.58278	0.310641	BD	EFHC2	NC_056072.1	29935001	29955001
NC_056080.1	43505001	43525001	10.9619	0.307102	BD	EFHC2	NC_056072.1	29930001	29950001
NC_056080.1	43500001	43520001	11.2695	0.304886	BD	EFHC2	NC_056057.1	61555001	61575001
NC_056080.1	43510001	43530001	6.07383	0.301552	BD	EFHC2	NC_056057.1	61560001	61580001
NC_056080.1	43485001	43505001	3.80088	0.294819	BD	EFHC2	NC_056057.1	61565001	61585001
NW_024599827.1	555001	575001	3.24433	0.53722	BD	EFL1	NC_056057.1	61570001	61590001
NW_024599827.1	550001	570001	3.53402	0.518301	BD	EFL1	NC_056057.1	61575001	61595001
NW_024599827.1	620001	640001	2.47312	0.362055	BD	EFL1	NC_056057.1	61580001	61600001
NC_056062.1	22225001	22245001	6.26333	0.644181	BD	EFR3A	NC_056057.1	61600001	61620001
NC_056062.1	22230001	22250001	2.5914	0.618603	BD	EFR3A	NC_056057.1	61605001	61625001
NC_056062.1	22210001	22230001	9.94023	0.596758	BD	EFR3A	NC_056055.1	242865001	242885001
NC_056062.1	22205001	22225001	7.74194	0.595501	BD	EFR3A;OC90	NC_056080.1	103860001	103880001
NC_056062.1	22200001	22220001	6.73656	0.553462	BD	EFR3A;OC90	NC_056072.1	32615001	32635001
NC_056064.1	44305001	44325001	9.55797	0.29963	BD	EFTUD2;LOC11411€	NC_056056.1	78030001	78050001
NC_056058.1	35815001	35835001	3.69565	0.37077	BD	EIF4E1B;SNCB	NC_056060.1	1525001	1545001
NC_056058.1	35820001	35840001	3.0295	0.383322	BD	EIF4E1B;SNCB;TSPA	NC_056060.1	1530001	1550001
NC_056079.1	32320001	32340001	2.21716	0.339408	BD	EIF4EBP1	NC_056060.1	1535001	1555001
NC_056077.1	41695001	41715001	3.00625	0.31603	BD	ELFN1	NC_056055.1	14065001	14085001
NC_056077.1	41690001	41710001	2.4138	0.301841	BD	ELFN1	NC_056055.1	14070001	14090001
NC_056056.1	1.66E+08	166090001	5.2562	0.351299	BD	ELK3	NC_056055.1	14080001	14100001
NC_056056.1	1.66E+08	166095001	4.39578	0.300311	BD	ELK3	NC_056076.1	46080001	46100001
NC_056057.1	61600001	61620001	3.00011	0.685883	BD	ELMO1	NC_056076.1	46085001	46105001
NC_056057.1	61555001	61575001	5.49891	0.440746	BD	ELMO1	NC_056054.1	158330001	158350001
NC_056057.1	61560001	61580001	6.40334	0.440657	BD	ELMO1	NC_056054.1	158335001	158355001
NC_056057.1	61565001	61585001	6.79397	0.436155	BD	ELMO1	NC_056064.1	39830001	39850001
NC_056057.1	61550001	61570001	4.30726	0.431744	BD	ELMO1	NC_056064.1	39835001	39855001
NC_056057.1	61570001	61590001	5.93433	0.378642	BD	ELMO1	NC_056064.1	39825001	39845001
NC_056057.1	61575001	61595001	7.41339	0.355071	BD	ELMO1	NC_056055.1	214045001	214065001

NC_056057.1	61580001	61600001	7.72681	0.34823	BD	ELMO1	NC_056055.1	214050001	214070001
NC_056068.1	14975001	14995001	2.52381	0.337186	BD	ENDOD1	NC_056055.1	214055001	214075001
NC_056073.1	19350001	19370001	2.59376	0.302639	BD	ENPP4;ENPP5	NC_056055.1	214060001	214080001
NC_056060.1	1525001	1545001	9.61911	0.564534	BD	EPB41L4A	NC_056055.1	214065001	214085001
NC_056060.1	1530001	1550001	7.6062	0.554517	BD	EPB41L4A	NC_056055.1	214070001	214090001
NC_056060.1	1535001	1555001	5.20801	0.527502	BD	EPB41L4A	NC_056055.1	214075001	214095001
NC_056060.1	1520001	1540001	5.47191	0.527226	BD	EPB41L4A	NC_056055.1	214080001	214100001
NC_056060.1	1700001	1720001	2.18087	0.320658	BD	EPB41L4A	NC_056055.1	214280001	214300001
NC_056060.1	1695001	1715001	2.20448	0.304389	BD	EPB41L4A	NC_056055.1	214290001	214310001
NC_056055.1	2.14E+08	214315001	2.24832	0.326824	BD	ERBB4	NC_056055.1	214295001	214315001
NC_056055.1	2.14E+08	214305001	2.23645	0.316718	BD	ERBB4	NC_056056.1	213710001	213730001
NC_056055.1	2.14E+08	214310001	2.33638	0.31431	BD	ERBB4	NC_056056.1	213715001	213735001
NC_056056.1	2.14E+08	213730001	3.58	0.4937	BD	ERC1	NC_056072.1	44985001	45005001
NC_056056.1	2.14E+08	213735001	4.55147	0.466079	BD	ERC1	NC_056072.1	44990001	45010001
NC_056056.1	2.14E+08	213725001	2.20307	0.418987	BD	ERC1	NC_056072.1	44995001	45015001
NC_056056.1	2.14E+08	213890001	2.13065	0.408983	BD	ERC1	NC_056054.1	3120001	3140001
NC_056056.1	2.14E+08	213895001	7.32822	0.386965	BD	ERC1	NC_056069.1	4835001	4855001
NC_056072.1	45115001	45135001	2.86286	0.375103	BD	ERC2	NC_056069.1	4840001	4860001
NC_056072.1	45120001	45140001	2.49847	0.362464	BD	ERC2	NC_056069.1	4845001	4865001
NC_056072.1	45110001	45130001	2.2175	0.326108	BD	ERC2	NC_056069.1	4850001	4870001
NC_056064.1	48060001	48080001	5.83708	0.581676	BD	ERN1	NC_056055.1	73940001	73960001
NC_056064.1	48080001	48100001	2.65229	0.458035	BD	ERN1;TEX2	NC_056055.1	73945001	73965001
NC_056060.1	11480001	11500001	3.80061	0.561906	BD	ERO1A	NC_056055.1	73895001	73915001
NC_056060.1	11485001	11505001	2.43058	0.544512	BD	ERO1A	NC_056055.1	73900001	73920001
NC_056060.1	11475001	11495001	4.44136	0.490177	BD	ERO1A	NC_056055.1	73905001	73925001
NC_056060.1	11470001	11490001	2.25192	0.38826	BD	ERO1A	NC_056064.1	48035001	48055001
NC_056056.1	2.01E+08	200620001	2.14105	0.309414	BD	ERP27	NC_056064.1	48060001	48080001
NC_056054.1	3165001	3185001	4.42378	0.649914	BD	ESPNL;KLHL30	NC_056064.1	48070001	48090001
NC_056054.1	3160001	3180001	2.97897	0.603212	BD	ESPNL;KLHL30	NC_056060.1	11470001	11490001
NC_056054.1	3155001	3175001	2.35475	0.575683	BD	ESPNL;KLHL30	NC_056060.1	11475001	11495001
NC_056060.1	85970001	85990001	2.29801	0.47274	BD	ESRRB	NC_056060.1	11480001	11500001
NC_056060.1	85965001	85985001	2.35293	0.451994	BD	ESRRB	NC_056060.1	11485001	11505001
NC_056060.1	85915001	85935001	2.15371	0.352775	BD	ESRRB	NC_056057.1	23045001	23065001
NC_056060.1	85880001	85900001	2.15154	0.314428	BD	ESRRB	NC_056057.1	23050001	23070001
NC_056060.1	85885001	85905001	2.2871	0.300317	BD	ESRRB	NC_056054.1	201910001	201930001
NC_056060.1	79105001	79125001	5.5082	0.714931	BD	EXD2	NC_056054.1	201915001	201935001
NC_056071.1	65400001	65420001	3.78618	0.323566	BD	EXOC3L4	NC_056054.1	201920001	201940001
NC_056071.1	65395001	65415001	3.76565	0.338569	BD	EXOC3L4;LBHD2	NC_056054.1	201925001	201945001
NC_056071.1	65390001	65410001	3.363	0.312967	BD	EXOC3L4;LBHD2	NC_056054.1	201930001	201950001
NC_056071.1	65405001	65425001	5.58253	0.380777	BD	EXOC3L4;LOC10560	NC_056054.1	201935001	201955001
NC_056071.1	65410001	65430001	7.14653	0.366207	BD	EXOC3L4;LOC10560	NC_056054.1	201945001	201965001
NC_056071.1	65415001	65435001	15.3643	0.293523	BD	EXOC3L4;LOC10560	NC_056054.1	201950001	201970001
NC_056065.1	41785001	41805001	6.99487	0.365308	BD	EXOSC10	NC_056056.1	62545001	62565001
NC_056065.1	41790001	41810001	5.14143	0.341881	BD	EXOSC10	NC_056056.1	62550001	62570001
NC_056065.1	41780001	41800001	8.92776	0.377152	BD	MTOR	NC_056056.1	62580001	62600001
NC_056059.1	36660001	36680001	4.46961	0.669265	BD	FAM13A	NC_056056.1	62585001	62605001
NC_056059.1	36665001	36685001	2.4816	0.5927	BD	FAM13A	NC_056056.1	62590001	62610001
NC_056059.1	36625001	36645001	2.88958	0.487096	BD	FAM13A	NC_056056.1	62595001	62615001
NC_056059.1	37970001	37990001	6.3803	0.490797	BD	FAM184B	NC_056071.1	65400001	65420001
NC_056059.1	37935001	37955001	6.82288	0.450637	BD	FAM184B	NC_056071.1	65405001	65425001
NC_056059.1	37965001	37985001	3.44385	0.430603	BD	FAM184B	NC_056071.1	65410001	65430001
NC_056059.1	37960001	37980001	2.33269	0.373577	BD	FAM184B	NC_056071.1	65415001	65435001
NC_056059.1	37905001	37925001	3.39467	0.35724	BD	FAM184B	NC_056065.1	41790001	41810001
NC_056080.1	1.3E+08	129810001	2.54243	0.393876	BD	FAM199X	NC_056065.1	41795001	41815001
NC_056080.1	1.3E+08	129805001	3.67546	0.461165	BD	FAM199X;SLC25A53	NC_056065.1	41800001	41820001
NC_056057.1	73645001	73665001	2.15818	0.30209	BD	FAM221A;STK31	NC_056080.1	81460001	81480001
NC_056056.1	2.02E+08	202505001	2.44927	0.390841	BD	FAM234B	NC_056080.1	81465001	81485001
NC_056056.1	2.02E+08	202510001	2.17875	0.380761	BD	FAM234B	NC_056080.1	81470001	81490001
NC_056059.1	13735001	13755001	7.09999	0.424778	BD	FAM241A	NC_056059.1	36620001	36640001
NC_056059.1	13710001	13730001	8.38909	0.421491	BD	FAM241A	NC_056059.1	36625001	36645001
NC_056059.1	13730001	13750001	6.4395	0.411956	BD	FAM241A	NC_056059.1	36660001	36680001
NC_056059.1	13740001	13760001	6.04404	0.411331	BD	FAM241A	NC_056059.1	36665001	36685001
NC_056059.1	13715001	13735001	7.13674	0.403464	BD	FAM241A	NC_056059.1	36690001	36710001
NC_056059.1	13725001	13745001	6.3284	0.403281	BD	FAM241A	NC_056059.1	36695001	36715001
NC_056059.1	13720001	13740001	5.43107	0.384168	BD	FAM241A	NC_056065.1	60350001	60370001
NC_056059.1	13705001	13725001	3.40833	0.370785	BD	FAM241A	NC_056065.1	60355001	60375001
NC_056059.1	13745001	13765001	3.97746	0.369429	BD	FAM241A	NC_056065.1	60360001	60380001
NC_056054.1	2.62E+08	262305001	3.54952	0.291035	BD	FAM3B;MX2	NC_056065.1	60365001	60385001
NC_056064.1	39580001	39600001	2.16667	0.311827	BD	FBXL20;MED1	NC_056065.1	60370001	60390001
NC_056064.1	39585001	39605001	2.10779	0.29976	BD	FBXL20;MED1	NC_056058.1	91090001	91110001
NC_056069.1	57745001	57765001	2.73287	0.329875	BD	FBXL7	NC_056058.1	91095001	91115001
NC_056055.1	2760001	2780001	16.8039	0.398955	BD	FBXW2	NC_056059.1	37905001	37925001
NC_056055.1	2765001	2785001	12.7313	0.377323	BD	FBXW2	NC_056071.1	25970001	25990001
NC_056055.1	2770001	2790001	7.16279	0.345863	BD	FBXW2	NC_056072.1	52820001	52840001

NC_056070.1	5405001	5425001	11.4054	0.539754	BD	FBXW7	NC_056072.1	52825001	52845001
NC_056070.1	5400001	5420001	6.53969	0.539179	BD	FBXW7	NC_056054.1	262225001	262245001
NC_056070.1	5395001	5415001	5.28333	0.522385	BD	FBXW7	NC_056054.1	262230001	262250001
NC_056070.1	5390001	5410001	2.65392	0.466161	BD	FBXW7	NC_056054.1	262235001	262255001
NC_056070.1	5410001	5430001	4.22831	0.40004	BD	FBXW7	NC_056054.1	262240001	262260001
NC_056070.1	58040001	58060001	2.68056	0.549501	BD	FBXW8;TESC	NC_056073.1	9525001	9545001
NC_056070.1	58035001	58055001	2.96376	0.520013	BD	FBXW8;TESC	NC_056073.1	9530001	9550001
NC_056054.1	1.13E+08	112995001	5.80093	0.379907	BD	FCGR3A	NC_056073.1	9535001	9555001
NC_056054.1	1.13E+08	113000001	5.45763	0.35045	BD	FCGR3A	NC_056056.1	185950001	185970001
NC_056054.1	1.13E+08	113010001	2.12903	0.335338	BD	FCGR3A	NC_056055.1	224815001	224835001
NC_056054.1	1.13E+08	113005001	2.45865	0.323633	BD	FCGR3A	NC_056055.1	224820001	224840001
NC_056057.1	88305001	88325001	2.36652	0.310288	BD	FEZF1	NC_056070.1	44685001	44705001
NC_056063.1	77695001	77715001	5.63669	0.550799	BD	FGF14	NC_056070.1	44690001	44710001
NC_056063.1	77700001	77720001	3.61054	0.544537	BD	FGF14	NC_056070.1	44695001	44715001
NC_056063.1	77705001	77725001	2.71707	0.493161	BD	FGF14	NC_056070.1	44700001	44720001
NC_056063.1	77690001	77710001	3.00182	0.450448	BD	FGF14	NC_056070.1	44705001	44725001
NC_056063.1	77710001	77730001	2.16507	0.448362	BD	FGF14	NC_056070.1	44710001	44730001
NC_056070.1	6520001	6540001	2.25302	0.340598	BD	FHIP1A	NC_056070.1	44715001	44735001
NC_056070.1	6525001	6545001	2.14046	0.32413	BD	FHIP1A	NC_056070.1	44720001	44740001
NC_056070.1	6515001	6535001	2.1869	0.31992	BD	FHIP1A	NC_056070.1	44725001	44745001
NC_056070.1	6510001	6530001	2.34081	0.313947	BD	FHIP1A	NC_056070.1	44735001	44755001
NC_056072.1	41200001	41220001	2.32376	0.306281	BD	FHIT	NC_056067.1	12470001	12490001
NC_056080.1	99055001	99075001	4.80316	0.491931	BD	FHL1	NC_056067.1	12490001	12510001
NC_056080.1	99040001	99060001	5.73787	0.425139	BD	FHL1	NC_056067.1	12495001	12515001
NC_056080.1	99060001	99080001	2.38581	0.413335	BD	FHL1	NC_056067.1	12500001	12520001
NC_056080.1	99030001	99050001	5.09218	0.354226	BD	FHL1	NC_056069.1	3880001	3900001
NC_056080.1	99025001	99045001	2.61885	0.340206	BD	FHL1	NC_056069.1	3885001	3905001
NC_056056.1	97190001	97210001	3.5065	0.41881	BD	FHL2	NC_056069.1	3890001	3910001
NC_056056.1	97185001	97205001	6.53788	0.355911	BD	FHL2	NC_056069.1	3895001	3915001
NC_056056.1	97180001	97200001	7.144	0.332133	BD	FHL2	NC_056069.1	3900001	3920001
NC_056076.1	21110001	21130001	3.85245	0.453078	BD	FHOD3	NC_056069.1	3905001	3925001
NC_056056.1	5735001	5755001	3.54574	0.311394	BD	FIBCD1	NC_056069.1	3910001	3930001
NC_056056.1	5740001	5760001	2.87113	0.298003	BD	FIBCD1	NC_056069.1	3915001	3935001
NC_056056.1	1.34E+08	134485001	2.80006	0.331299	BD	FIGNL2	NC_056070.1	5390001	5410001
NC_056056.1	1.34E+08	134490001	2.25646	0.307004	BD	FIGNL2	NC_056070.1	5395001	5415001
NC_056073.1	27400001	27420001	2.35294	0.383731	BD	FLOT1;IER3;MDC1;T	NC_056070.1	5400001	5420001
NC_056073.1	27395001	27415001	2.50394	0.366576	BD	FLOT1;IER3;TUBB	NC_056070.1	5405001	5425001
NC_056073.1	27410001	27430001	5.38956	0.389508	BD	FLOT1;MDC1;TUBB	NC_056070.1	5410001	5430001
NC_056073.1	27405001	27425001	3.46093	0.385752	BD	FLOT1;MDC1;TUBB	NC_056070.1	58035001	58055001
NC_056080.1	87715001	87735001	3.24278	0.376434	BD	FMR1	NC_056070.1	58040001	58060001
NC_056080.1	87720001	87740001	2.68663	0.373034	BD	FMR1	NC_056054.1	112965001	112985001
NC_056060.1	75235001	75255001	2.59394	0.354709	BD	FNTB	NC_056069.1	8680001	8700001
NC_056056.1	76130001	76150001	2.93899	0.331841	BD	FOXN2	NC_056069.1	8685001	8705001
NC_056056.1	76135001	76155001	2.55262	0.314434	BD	FOXN2	NC_056069.1	8690001	8710001
NC_056072.1	30105001	30125001	2.93824	0.510832	BD	FOXP1	NC_056069.1	8695001	8715001
NC_056056.1	75470001	75490001	8.42123	0.509738	BD	FSHR	NC_056069.1	8700001	8720001
NC_056056.1	75465001	75485001	7.78908	0.480585	BD	FSHR	NC_056069.1	8705001	8725001
NC_056056.1	75475001	75495001	2.84699	0.398555	BD	FSHR	NC_056069.1	8715001	8735001
NC_056056.1	75460001	75480001	4.75875	0.393228	BD	FSHR	NC_056069.1	8720001	8740001
NC_056072.1	53295001	53315001	8.02144	0.415263	BD	FYCO1;XCR1	NC_056069.1	8735001	8755001
NC_056072.1	53300001	53320001	3.73469	0.349283	BD	FYCO1;XCR1	NC_056069.1	8745001	8765001
NC_056072.1	53290001	53310001	3.50631	0.334058	BD	FYCO1;XCR1	NC_056069.1	8750001	8770001
NC_056054.1	1.31E+08	131290001	14.5158	0.642705	BD	GABPA	NC_056069.1	8760001	8780001
NC_056054.1	1.31E+08	131285001	6.30001	0.63545	BD	GABPA	NC_056069.1	8780001	8800001
NC_056054.1	1.31E+08	131280001	3.19486	0.572157	BD	GABPA	NC_056069.1	8785001	8805001
NC_056054.1	1.31E+08	131300001	6.34417	0.474195	BD	GABPA	NC_056069.1	8790001	8810001
NC_056054.1	1.31E+08	131275001	2.21702	0.421417	BD	GABPA	NC_056069.1	8795001	8815001
NC_056080.1	83510001	83530001	8.90625	0.340983	BD	GABRA3	NC_056054.1	201315001	201335001
NC_056057.1	1.16E+08	116145001	4.86021	0.396743	BD	GALNT11;GALNTL5	NC_056055.1	220915001	220935001
NC_056060.1	79130001	79150001	2.1	0.565184	BD	GALNT16	NC_056072.1	41200001	41220001
NC_056060.1	79135001	79155001	2.72593	0.507918	BD	GALNT16	NC_056080.1	99055001	99075001
NC_056068.1	41125001	41145001	2.17883	0.509214	BD	GALNT18	NC_056072.1	43340001	43360001
NC_056057.1	1.16E+08	116135001	2.6359	0.562511	BD	GALNTL5	NC_056073.1	27400001	27420001
NC_056057.1	1.16E+08	116140001	4.25	0.518656	BD	GALNTL5	NC_056073.1	27395001	27415001
NC_056055.1	1.08E+08	107770001	4.16729	0.378935	BD	GALNTL6	NC_056073.1	27405001	27425001
NC_056055.1	1.08E+08	107850001	5.77852	0.371727	BD	GALNTL6	NC_056073.1	27410001	27430001
NC_056055.1	1.08E+08	107775001	6.79048	0.3546	BD	GALNTL6	NC_056056.1	76090001	76110001
NC_056055.1	1.08E+08	107765001	2.30551	0.353482	BD	GALNTL6	NC_056056.1	76095001	76115001
NC_056055.1	1.08E+08	107985001	2.16609	0.323446	BD	GALNTL6	NC_056060.1	99095001	99115001
NC_056055.1	1.08E+08	107980001	2.96428	0.310313	BD	GALNTL6	NC_056060.1	99120001	99140001
NC_056076.1	25020001	25040001	2.14474	0.349896	BD	GAREM1	NC_056060.1	99125001	99145001
NC_056076.1	25025001	25045001	2.46659	0.340447	BD	GAREM1	NC_056060.1	99130001	99150001
NC_056067.1	14355001	14375001	2.3624	0.386685	BD	GAS8	NC_056063.1	22240001	22260001
NC_056067.1	14320001	14340001	2.41126	0.399744	BD	GAS8;LOC10110903	NC_056072.1	30105001	30125001

NC_056067.1	7265001	7285001	5.29392	0.501949	BD	GCSH;PKD1L2	NC_056072.1	30110001	30130001
NC_056067.1	7260001	7280001	3.77083	0.470129	BD	GCSH;PKD1L2	NC_056057.1	55485001	55505001
NC_056058.1	63885001	63905001	2.77735	0.515257	BD	GEMIN5;MRPL22	NC_056057.1	55520001	55540001
NC_056058.1	63880001	63900001	2.77412	0.508616	BD	GEMIN5;MRPL22	NC_056057.1	55525001	55545001
NC_056066.1	26110001	26130001	15.4722	0.302442	BD	GJD4	NC_056057.1	55530001	55550001
NC_056054.1	2.47E+08	247335001	13.3889	0.474674	BD	GK5	NC_056066.1	27780001	27800001
NC_056054.1	2.47E+08	247340001	5.96184	0.457157	BD	GK5	NC_056066.1	27785001	27805001
NC_056054.1	2.47E+08	247345001	2.38636	0.430404	BD	GK5	NC_056066.1	27790001	27810001
NC_056054.1	2.47E+08	247330001	10.7921	0.419532	BD	GK5	NC_056066.1	27795001	27815001
NC_056057.1	80465001	80485001	2.57071	0.478175	BD	GLI3	NC_056066.1	28165001	28185001
NC_056055.1	52395001	52415001	3.88638	0.310291	BD	GLIPR2	NC_056066.1	28175001	28195001
NC_056054.1	69515001	69535001	12.755	0.61154	BD	GLMN	NC_056066.1	28180001	28200001
NC_056054.1	69510001	69530001	9.98334	0.524108	BD	GLMN	NC_056066.1	28185001	28205001
NC_056054.1	2.33E+08	232545001	6.49506	0.367339	BD	GMPS	NC_056066.1	28190001	28210001
NC_056054.1	2.33E+08	232540001	3.96946	0.343377	BD	GMPS	NC_056080.1	10735001	10755001
NC_056054.1	2.33E+08	232550001	8.46252	0.301415	BD	GMPS	NC_056054.1	31270001	31290001
NC_056055.1	1.53E+08	153205001	11.4587	0.389075	BD	GPD2	NC_056054.1	31275001	31295001
NC_056055.1	1.53E+08	153210001	9.21265	0.377228	BD	GPD2	NC_056054.1	31280001	31300001
NC_056055.1	1.53E+08	153130001	2.12622	0.366803	BD	GPD2	NC_056054.1	31285001	31305001
NC_056055.1	1.53E+08	153200001	7.32584	0.364071	BD	GPD2	NC_056054.1	31265001	31285001
NC_056055.1	1.53E+08	153195001	4.17257	0.333242	BD	GPD2	NC_056080.1	81165001	81185001
NC_056055.1	1.53E+08	153215001	4.89387	0.33318	BD	GPD2	NC_056080.1	81170001	81190001
NC_056079.1	6585001	6605001	5.28403	0.616285	BD	GPM6A	NC_056080.1	81190001	81210001
NC_056079.1	6590001	6610001	4.33032	0.615352	BD	GPM6A	NC_056054.1	131255001	131275001
NC_056079.1	6595001	6615001	3.29309	0.58782	BD	GPM6A	NC_056054.1	131260001	131280001
NC_056079.1	6580001	6600001	2.36631	0.564177	BD	GPM6A	NC_056054.1	131265001	131285001
NC_056079.1	6600001	6620001	2.76355	0.52483	BD	GPM6A	NC_056054.1	131270001	131290001
NC_056080.1	11945001	11965001	4.31932	0.403369	BD	GPM6B	NC_056054.1	131280001	131300001
NC_056080.1	11940001	11960001	8.69442	0.356708	BD	GPM6B	NC_056080.1	83505001	83525001
NC_056080.1	11935001	11955001	6.36109	0.355217	BD	GPM6B	NC_056080.1	83510001	83530001
NC_056054.1	2.18E+08	217960001	2.84245	0.389748	BD	GPR160;PHC3	NC_056058.1	70955001	70975001
NC_056054.1	2.18E+08	217965001	2.14699	0.334464	BD	GPR160;PHC3	NC_056055.1	242855001	242875001
NC_056055.1	1.45E+08	145065001	24.033	0.328012	BD	GRB14	NC_056055.1	155080001	155100001
NC_056055.1	1.45E+08	145070001	16.3448	0.323084	BD	GRB14	NC_056055.1	155085001	155105001
NC_056064.1	55525001	55545001	5.1791	0.392791	BD	GRB2	NC_056055.1	155090001	155110001
NC_056076.1	35230001	35250001	2.61237	0.298898	BD	GREB1L	NC_056055.1	155095001	155115001
NC_056068.1	2085001	2105001	43.0832	0.396508	BD	GRIA4	NC_056078.1	1795001	1815001
NC_056068.1	2080001	2100001	29.3485	0.393757	BD	GRIA4	NC_056055.1	107490001	107510001
NC_056068.1	2090001	2110001	23.9469	0.372844	BD	GRIA4	NC_056055.1	107495001	107515001
NC_056068.1	2110001	2130001	17.3429	0.364458	BD	GRIA4	NC_056070.1	45010001	45030001
NC_056068.1	2095001	2115001	18.1313	0.363985	BD	GRIA4	NC_056055.1	107910001	107930001
NC_056068.1	2155001	2175001	16.7857	0.363454	BD	GRIA4	NC_056059.1	15940001	15960001
NC_056068.1	2100001	2120001	15.5	0.350935	BD	GRIA4	NC_056059.1	15945001	15965001
NC_056068.1	2105001	2125001	13.3121	0.345509	BD	GRIA4	NC_056059.1	15925001	15945001
NC_056068.1	2160001	2180001	5.68447	0.297481	BD	GRIA4	NC_056059.1	15930001	15950001
NC_056054.1	1.27E+08	127135001	3.52519	0.371276	BD	GRIK1	NC_056059.1	15935001	15955001
NC_056054.1	1.27E+08	126850001	2.12796	0.35715	BD	GRIK1	NC_056060.1	6490001	6510001
NC_056054.1	1.27E+08	126815001	3.22368	0.33584	BD	GRIK1	NC_056067.1	7260001	7280001
NC_056054.1	1.27E+08	126830001	2.76254	0.30855	BD	GRIK1	NC_056067.1	7265001	7285001
NC_056054.1	1.27E+08	127130001	2.31484	0.30407	BD	GRIK1	NC_056054.1	95265001	95285001
NC_056055.1	21375001	21395001	3.18458	0.363254	BD	GRIN3A	NC_056067.1	34790001	34810001
NC_056055.1	21380001	21400001	2.65659	0.327054	BD	GRIN3A	NC_056067.1	34795001	34815001
NC_056055.1	21370001	21390001	2.19105	0.303875	BD	GRIN3A	NC_056067.1	34800001	34820001
NC_056056.1	1.53E+08	153045001	4.66872	0.368244	BD	GRIP1	NC_056067.1	34805001	34825001
NC_056056.1	1.53E+08	153050001	3.28712	0.326734	BD	GRIP1	NC_056069.1	32235001	32255001
NC_056056.1	1.53E+08	153040001	3.30899	0.309682	BD	GRIP1	NC_056068.1	82165001	82185001
NC_056072.1	19395001	19415001	2.25125	0.456517	BD	GRM7	NC_056057.1	80435001	80455001
NC_056072.1	19400001	19420001	2.63305	0.415584	BD	GRM7	NC_056057.1	80465001	80485001
NC_056057.1	92445001	92465001	2.56152	0.442263	BD	GRM8	NC_056057.1	80470001	80490001
NC_056057.1	92450001	92470001	2.42942	0.398955	BD	GRM8	NC_056057.1	80475001	80495001
NC_056077.1	25915001	25935001	4.59548	0.382909	BD	GSG1L	NC_056057.1	80480001	80500001
NC_056077.1	25910001	25930001	3.40262	0.345371	BD	GSG1L	NC_056054.1	69510001	69530001
NC_056077.1	25905001	25925001	3.37263	0.327816	BD	GSG1L	NC_056054.1	69515001	69535001
NC_056077.1	25920001	25940001	4.07127	0.317396	BD	GSG1L	NC_056080.1	12895001	12915001
NC_056057.1	76165001	76185001	2.20185	0.482834	BD	GTPBP10	NC_056070.1	48225001	48245001
NC_056057.1	76160001	76180001	3.1687	0.473717	BD	GTPBP10	NC_056056.1	131965001	131985001
NC_056057.1	76155001	76175001	2.56147	0.424316	BD	GTPBP10	NC_056054.1	232530001	232550001
NC_056077.1	750001	770001	11.3649	0.354358	BD	HBM;HBQ1;LOC101	NC_056072.1	48265001	48285001
NC_056077.1	755001	775001	6.41246	0.353851	BD	HBM;HBQ1;LOC101	NC_056071.1	55625001	55645001
NC_056077.1	760001	780001	3.95009	0.341466	BD	HBQ1;LOC10110879	NC_056071.1	55630001	55650001
NC_056077.1	765001	785001	2.54639	0.308706	BD	HBQ1;LUC7L	NC_056080.1	101300001	101320001
NC_056080.1	64990001	65010001	2.18447	0.354646	BD	HDAC8	NC_056080.1	101305001	101325001
NC_056057.1	79210001	79230001	5.74688	0.426664	BD	HECW1	NC_056063.1	66710001	66730001
NC_056057.1	79205001	79225001	5.93671	0.408345	BD	HECW1	NC_056063.1	66715001	66735001

NC_056057.1	79200001	79220001	4.39671	0.394208	BD	HECW1	NC_056063.1	66720001	66740001
NC_056057.1	79195001	79215001	2.92897	0.381309	BD	HECW1	NC_056063.1	68740001	68760001
NC_056057.1	79215001	79235001	2.17062	0.316914	BD	HECW1	NC_056055.1	153180001	153200001
NC_056059.1	36995001	37015001	15.7683	0.565956	BD	HERC6	NC_056055.1	153185001	153205001
NC_056059.1	37000001	37020001	17.1356	0.549693	BD	HERC6	NC_056055.1	153190001	153210001
NC_056059.1	37005001	37025001	16.0495	0.48643	BD	HERC6	NC_056079.1	6580001	6600001
NC_056061.1	12550001	12570001	2.88764	0.41942	BD	HINT3	NC_056079.1	6585001	6605001
NC_056070.1	63290001	63310001	15.1733	0.3169	BD	HNF1A	NC_056079.1	6590001	6610001
NC_056062.1	51790001	51810001	3.72096	0.349018	BD	HNF4G	NC_056079.1	6595001	6615001
NC_056062.1	51785001	51805001	3.53656	0.319378	BD	HNF4G	NC_056079.1	6600001	6620001
NC_056062.1	51780001	51800001	3.57311	0.314395	BD	HNF4G	NC_056054.1	217940001	217960001
NC_056080.1	1.01E+08	100550001	2.31332	0.310271	BD	HPRT1	NC_056080.1	70375001	70395001
NC_056080.1	1.02E+08	102205001	2.92911	0.352393	BD	HS6ST2	NC_056054.1	237515001	237535001
NC_056080.1	1.02E+08	102200001	4.00404	0.326021	BD	HS6ST2	NC_056054.1	237530001	237550001
NC_056080.1	1.02E+08	102195001	3.55839	0.293534	BD	HS6ST2	NC_056054.1	237535001	237555001
NC_056054.1	28450001	28470001	5.68724	0.481652	BD	HSPB11	NC_056054.1	237540001	237560001
NC_056054.1	28455001	28475001	3.36171	0.450203	BD	HSPB11	NC_056054.1	237510001	237530001
NC_056055.1	2.45E+08	244680001	2.70167	0.318017	BD	HSPG2	NC_056076.1	35215001	35235001
NC_056055.1	2.45E+08	244675001	2.85663	0.291282	BD	HSPG2	NC_056068.1	1640001	1660001
NC_056074.1	22120001	22140001	2.35411	0.297866	BD	HTATIP2;LOC121817	NC_056068.1	1645001	1665001
NC_056068.1	24150001	24170001	3.86227	0.365679	BD	HTR3B	NC_056068.1	2080001	2100001
NC_056067.1	39485001	39505001	4.41644	0.35803	BD	HYDIN	NC_056068.1	2085001	2105001
NC_056067.1	39480001	39500001	3.83298	0.307834	BD	HYDIN	NC_056068.1	2090001	2110001
NC_056055.1	2.42E+08	242500001	3.64227	0.376627	BD	IFNLR1	NC_056068.1	2095001	2115001
NC_056055.1	2.42E+08	242505001	7.30412	0.477587	BD	IFNLR1;IL22RA1	NC_056068.1	2105001	2125001
NC_056057.1	57490001	57510001	4.77127	0.294386	BD	IFRD1	NC_056059.1	31865001	31885001
NC_056057.1	57465001	57485001	2.14385	0.305618	BD	IFRD1;LSMEM1	NC_056059.1	31870001	31890001
NC_056068.1	28925001	28945001	7.31328	0.313694	BD	IFT46;KMT2A;TMEM	NC_056059.1	31875001	31895001
NC_056068.1	28930001	28950001	5.02013	0.324468	BD	IFT46;TMEM25;TTC3	NC_056059.1	31880001	31900001
NC_056060.1	12240001	12260001	3.96422	0.396397	BD	IGDCC3	NC_056059.1	31885001	31905001
NC_056060.1	12245001	12265001	4.89718	0.372353	BD	IGDCC3	NC_056059.1	31895001	31915001
NC_056060.1	12235001	12255001	4.83123	0.354659	BD	IGDCC3	NC_056059.1	31900001	31920001
NC_056060.1	12250001	12270001	3.74399	0.315851	BD	IGDCC3	NC_056059.1	31905001	31925001
NC_056054.1	2.37E+08	237400001	2.11136	0.317493	BD	IGSF10	NC_056059.1	31910001	31930001
NC_056074.1	23565001	23585001	2.22523	0.339514	BD	IGSF22;PTPN5	NC_056059.1	31915001	31935001
NC_056080.1	1.2E+08	119715001	8.78048	0.480057	BD	IL13RA2	NC_056059.1	31935001	31955001
NC_056080.1	1.2E+08	119720001	6.5	0.450633	BD	IL13RA2	NC_056059.1	31940001	31960001
NC_056080.1	1.2E+08	119705001	4.02695	0.443476	BD	IL13RA2	NC_056059.1	31945001	31965001
NC_056080.1	1.2E+08	119725001	2.90517	0.344025	BD	IL13RA2	NC_056059.1	31950001	31970001
NC_056056.1	99340001	99360001	2.4009	0.383945	BD	IL18R1;IL1RL1	NC_056059.1	31955001	31975001
NC_056055.1	2.42E+08	242510001	10.717	0.467903	BD	IL22RA1	NC_056059.1	32110001	32130001
NC_056055.1	74270001	74290001	12.4732	0.346327	BD	IL33	NC_056059.1	32115001	32135001
NC_056055.1	74265001	74285001	9.10828	0.331379	BD	IL33	NC_056067.1	50785001	50805001
NC_056055.1	74260001	74280001	5.67555	0.321556	BD	IL33	NC_056067.1	50795001	50815001
NC_056055.1	74255001	74275001	3.81937	0.314047	BD	IL33	NC_056055.1	21375001	21395001
NC_056068.1	61050001	61070001	2.40129	0.486764	BD	IMMP1L	NC_056055.1	21380001	21400001
NC_056057.1	58775001	58795001	3.18901	0.404117	BD	IMMP2L	NC_056056.1	152885001	152905001
NC_056057.1	58780001	58800001	3.10982	0.39746	BD	IMMP2L	NC_056074.1	6380001	6400001
NC_056057.1	58770001	58790001	3.05597	0.394656	BD	IMMP2L	NC_056074.1	6385001	6405001
NC_056057.1	59110001	59130001	3.76391	0.383654	BD	IMMP2L	NC_056072.1	19370001	19390001
NC_056057.1	59105001	59125001	2.96812	0.363637	BD	IMMP2L	NC_056072.1	19375001	19395001
NC_056057.1	58705001	58725001	3.66247	0.358357	BD	IMMP2L	NC_056072.1	19380001	19400001
NC_056057.1	59115001	59135001	4.16577	0.355858	BD	IMMP2L	NC_056057.1	92445001	92465001
NC_056057.1	58765001	58785001	2.37336	0.347386	BD	IMMP2L	NC_056057.1	92450001	92470001
NC_056057.1	58760001	58780001	2.3316	0.347157	BD	IMMP2L	NC_056057.1	92485001	92505001
NC_056057.1	59120001	59140001	6.76395	0.336153	BD	IMMP2L	NC_056057.1	92490001	92510001
NC_056057.1	58510001	58530001	2.17536	0.311046	BD	IMMP2L	NC_056057.1	92495001	92515001
NC_056057.1	58520001	58540001	3.56527	0.307629	BD	IMMP2L	NC_056057.1	92500001	92520001
NC_056057.1	58515001	58535001	2.80909	0.30577	BD	IMMP2L	NC_056067.1	11080001	11100001
NC_056057.1	58710001	58730001	3.3501	0.296693	BD	IMMP2L	NC_056077.1	25860001	25880001
NC_056057.1	58785001	58805001	2.4732	0.35015	BD	IMMP2L;LRRN3	NC_056077.1	25865001	25885001
NC_056079.1	14090001	14110001	9.46845	0.355623	BD	IRF2	NC_056055.1	165735001	165755001
NC_056079.1	14085001	14105001	12.2686	0.355441	BD	IRF2	NC_056055.1	165740001	165760001
NC_056079.1	14095001	14115001	5.41257	0.326067	BD	IRF2	NC_056055.1	165745001	165765001
NC_056079.1	14120001	14140001	3.23891	0.312595	BD	IRF2	NC_056055.1	165750001	165770001
NC_056056.1	1.89E+08	188890001	7.8421	0.376275	BD	ITPR2	NC_056079.1	25855001	25875001
NC_056056.1	1.89E+08	188895001	7.97413	0.375265	BD	ITPR2	NC_056079.1	25860001	25880001
NC_056056.1	1.89E+08	188900001	6.55986	0.372206	BD	ITPR2	NC_056079.1	25865001	25885001
NC_056056.1	1.89E+08	188905001	3.48252	0.350789	BD	ITPR2	NC_056073.1	15915001	15935001
NC_056056.1	1.89E+08	188885001	6.14852	0.338493	BD	ITPR2	NC_056073.1	15920001	15940001
NC_056056.1	1.89E+08	188880001	2.31055	0.309824	BD	ITPR2	NC_056055.1	120940001	120960001
NC_056068.1	28605001	28625001	2.68011	0.302053	BD	JAML;SCN2B	NC_056055.1	120945001	120965001
NC_056065.1	52035001	52055001	2.24297	0.347881	BD	KAZN	NC_056055.1	120995001	121015001
NC_056065.1	52040001	52060001	2.98191	0.313743	BD	KAZN	NC_056055.1	121045001	121065001

NC_056068.1	1210001	1230001	11.7936	0.369328	BD	KBTBD3	NC_056055.1	121050001	121070001
NC_056068.1	1215001	1235001	11.9841	0.366661	BD	KBTBD3	NC_056055.1	121055001	121075001
NC_056068.1	1260001	1280001	9.43665	0.355992	BD	KBTBD3	NC_056058.1	66370001	66390001
NC_056068.1	1265001	1285001	8.33745	0.351598	BD	KBTBD3	NC_056057.1	79200001	79220001
NC_056068.1	1255001	1275001	8.08593	0.349455	BD	KBTBD3	NC_056057.1	79205001	79225001
NC_056068.1	1245001	1265001	6.91525	0.337473	BD	KBTBD3	NC_056057.1	79210001	79230001
NC_056068.1	1230001	1250001	8.42487	0.336855	BD	KBTBD3	NC_056057.1	79215001	79235001
NC_056068.1	1270001	1290001	4.19717	0.292552	BD	KBTBD3	NC_056059.1	36700001	36720001
NC_056056.1	1.04E+08	104420001	3.17827	0.358635	BD	KCNIP3	NC_056057.1	40460001	40480001
NC_056056.1	1.04E+08	104415001	2.75869	0.332446	BD	KCNIP3	NC_056057.1	40465001	40485001
NC_056056.1	1.04E+08	104425001	2.68726	0.32775	BD	KCNIP3	NC_056065.1	66400001	66420001
NC_056056.1	1.04E+08	104440001	6.73189	0.426945	BD	KCNIP3;PROM2	NC_056065.1	66405001	66425001
NC_056056.1	1.04E+08	104435001	5.23077	0.408429	BD	KCNIP3;PROM2	NC_056065.1	66410001	66430001
NC_056056.1	1.04E+08	104430001	2.9142	0.337287	BD	KCNIP3;PROM2	NC_056070.1	63290001	63310001
NC_056059.1	41675001	41695001	3.13478	0.471541	BD	KCNIP4	NC_056070.1	63295001	63315001
NC_056059.1	41680001	41700001	2.23751	0.408159	BD	KCNIP4	NC_056058.1	36985001	37005001
NC_056059.1	41420001	41440001	29.9263	0.325822	BD	KCNIP4	NC_056058.1	36990001	37010001
NC_056064.1	35060001	35080001	3.48631	0.330878	BD	KCNJ12	NC_056074.1	37570001	37590001
NC_056054.1	2.09E+08	208990001	2.82892	0.364219	BD	KCNMB2	NC_056074.1	37575001	37595001
NC_056054.1	2.09E+08	208995001	3.03553	0.351904	BD	KCNMB2	NC_056060.1	10420001	10440001
NC_056074.1	45460001	45480001	17.2875	0.517639	BD	KCNQ1	NC_056064.1	9090001	9110001
NC_056074.1	45465001	45485001	10.5878	0.450907	BD	KCNQ1	NC_056064.1	9095001	9115001
NC_056074.1	45450001	45470001	2.68224	0.36446	BD	KCNQ1	NC_056064.1	9075001	9095001
NC_056074.1	45470001	45490001	13.2414	0.319098	BD	KCNQ1	NC_056064.1	9080001	9100001
NC_056080.1	44090001	44110001	10.675	0.350642	BD	KDM6A	NC_056054.1	112955001	112975001
NC_056080.1	44095001	44115001	10.391	0.332032	BD	KDM6A	NC_056054.1	112950001	112970001
NC_056080.1	44100001	44120001	9.47975	0.318155	BD	KDM6A	NC_056055.1	246730001	246750001
NC_056062.1	19530001	19550001	2.6439	0.36258	BD	KHDRBS3	NC_056075.1	41840001	41860001
NC_056062.1	19535001	19555001	2.4374	0.35286	BD	KHDRBS3	NC_056059.1	116325001	116345001
NC_056062.1	19540001	19560001	2.36052	0.34801	BD	KHDRBS3	NC_056059.1	116330001	116350001
NC_056062.1	19525001	19545001	2.36502	0.342674	BD	KHDRBS3	NC_056059.1	116335001	116355001
NC_056068.1	63020001	63040001	13.9703	0.560983	BD	KIAA1549L	NC_056059.1	116340001	116360001
NC_056068.1	63015001	63035001	6.13777	0.51654	BD	KIAA1549L	NC_056059.1	116310001	116330001
NC_056068.1	63010001	63030001	2.23956	0.398035	BD	KIAA1549L	NC_056059.1	116315001	116335001
NC_056073.1	38965001	38985001	2.42634	0.358001	BD	KIF13A	NC_056059.1	116320001	116340001
NC_056073.1	38775001	38795001	2.29859	0.353559	BD	KIF13A	NC_056074.1	46100001	46120001
NC_056073.1	38970001	38990001	2.30134	0.342902	BD	KIF13A	NC_056057.1	57475001	57495001
NC_056073.1	38780001	38800001	2.46581	0.339765	BD	KIF13A	NC_056057.1	57480001	57500001
NC_056054.1	19415001	19435001	6.58	0.302785	BD	KIF2C	NC_056057.1	57485001	57505001
NC_056054.1	19420001	19440001	3.77988	0.29344	BD	KIF2C	NC_056057.1	57490001	57510001
NC_056074.1	27665001	27685001	2.29781	0.394635	BD	KIRREL3	NC_056060.1	12235001	12255001
NC_056074.1	27670001	27690001	2.55963	0.362977	BD	KIRREL3	NC_056060.1	12240001	12260001
NC_056074.1	27660001	27680001	2.61214	0.341808	BD	KIRREL3	NC_056060.1	12245001	12265001
NC_056074.1	27615001	27635001	6.89547	0.311676	BD	KIRREL3	NC_056060.1	12250001	12270001
NC_056066.1	40130001	40150001	2.19022	0.598676	BD	KIZ	NC_056060.1	12260001	12280001
NC_056063.1	49460001	49480001	2.28022	0.359388	BD	KLF12	NC_056056.1	225310001	225330001
NC_056063.1	49455001	49475001	2.1254	0.358715	BD	KLF12	NC_056058.1	9965001	9985001
NC_056063.1	49465001	49485001	2.41862	0.345185	BD	KLF12	NC_056058.1	9960001	9980001
NC_056063.1	49470001	49490001	2.39179	0.32916	BD	KLF12	NC_056058.1	9970001	9990001
NC_056066.1	44190001	44210001	2.11299	0.358464	BD	KLF6	NC_056058.1	9975001	9995001
NC_056066.1	44185001	44205001	2.11428	0.35183	BD	KLF6	NC_056057.1	58525001	58545001
NC_056059.1	58965001	58985001	2.22337	0.299456	BD	KLHL5	NC_056057.1	58530001	58550001
NC_056059.1	58970001	58990001	2.23619	0.294824	BD	KLHL5	NC_056057.1	58535001	58555001
NC_056068.1	28845001	28865001	6.8125	0.340532	BD	KMT2A	NC_056057.1	58705001	58725001
NC_056076.1	40450001	40470001	2.41059	0.420286	BD	LAMA1	NC_056057.1	58710001	58730001
NC_056076.1	40455001	40475001	2.24646	0.414779	BD	LAMA1	NC_056057.1	58715001	58735001
NC_056076.1	40470001	40490001	2.72969	0.401956	BD	LAMA1	NC_056057.1	58720001	58740001
NC_056076.1	40465001	40485001	2.99338	0.381521	BD	LAMA1	NC_056057.1	58725001	58745001
NC_056076.1	40460001	40480001	2.69325	0.373951	BD	LAMA1	NC_056057.1	58730001	58750001
NC_056076.1	40480001	40500001	2.11043	0.304225	BD	LAMA1	NC_056057.1	58735001	58755001
NC_056061.1	54795001	54815001	2.11258	0.325198	BD	LAMA2	NC_056057.1	58740001	58760001
NC_056057.1	50645001	50665001	2.29879	0.426336	BD	LAMB4	NC_056057.1	58745001	58765001
NC_056059.1	38065001	38085001	9.53751	0.767146	BD	LCORL	NC_056057.1	58750001	58770001
NC_056059.1	38215001	38235001	2.18447	0.376163	BD	LCORL	NC_056057.1	58780001	58800001
NC_056059.1	38210001	38230001	2.18447	0.339313	BD	LCORL	NC_056057.1	58880001	58900001
NC_056059.1	38205001	38225001	2.34356	0.30054	BD	LCORL	NC_056057.1	58885001	58905001
NC_056059.1	17520001	17540001	12.057	0.304056	BD	LEF1	NC_056057.1	58890001	58910001
NC_056054.1	41245001	41265001	5.37346	0.50997	BD	LEPR	NC_056057.1	58895001	58915001
NC_056054.1	41240001	41260001	4.89913	0.488654	BD	LEPR	NC_056057.1	58900001	58920001
NC_056054.1	41235001	41255001	3.38437	0.371609	BD	LEPR	NC_056057.1	58905001	58925001
NC_056063.1	23265001	23285001	2.57111	0.294491	BD	LHFPL6	NC_056057.1	58910001	58930001
NC_056059.1	61095001	61115001	6.2711	0.516324	BD	LIMCH1	NC_056057.1	59110001	59130001
NC_056059.1	61090001	61110001	6.10909	0.516279	BD	LIMCH1	NC_056077.1	41790001	41810001
NC_056059.1	61100001	61120001	5.97692	0.506586	BD	LIMCH1	NC_056077.1	41795001	41815001

NC_056059.1	61085001	61105001	3.82413	0.444674	BD	LIMCH1	NC_056077.1	41800001	41820001
NC_056059.1	61105001	61125001	2.74157	0.431116	BD	LIMCH1	NC_056063.1	21220001	21240001
NC_056055.1	97690001	97710001	3.49712	0.327657	BD	LINGO2	NC_056054.1	191040001	191060001
NC_056062.1	4610001	4630001	2.44148	0.343341	BD	LMBRD1	NC_056054.1	191045001	191065001
NC_056062.1	4615001	4635001	2.39493	0.325285	BD	LMBRD1	NC_056070.1	8555001	8575001
NC_056056.1	1.99E+08	198905001	2.78657	0.380787	BD	LMO3	NC_056070.1	8560001	8580001
NC_056056.1	1.99E+08	198900001	3.08373	0.374336	BD	LMO3	NC_056070.1	8565001	8585001
NC_056056.1	1.99E+08	198910001	2.22357	0.311915	BD	LMO3	NC_056054.1	106485001	106505001
NC_056064.1	41330001	41350001	2.17727	0.43771	BD	LOC100526781	NC_056054.1	106490001	106510001
NC_056064.1	41325001	41345001	2.44739	0.428143	BD	LOC100526781	NC_056054.1	106495001	106515001
NC_056064.1	41335001	41355001	2.48335	0.387367	BD	LOC100526781	NC_056054.1	106500001	106520001
NC_056064.1	41320001	41340001	3.14155	0.368818	BD	LOC100526781;LOC1	NC_056054.1	106505001	106525001
NC_056064.1	41340001	41360001	2.62897	0.326203	BD	LOC100526781;V15	NC_056080.1	51165001	51185001
NC_056064.1	41310001	41330001	3.88481	0.332065	BD	LOC100526782	NC_056056.1	212905001	212925001
NC_056064.1	41315001	41335001	3.35731	0.322364	BD	LOC100526782	NC_056071.1	32205001	32225001
NC_056055.1	2.34E+08	234055001	8.77711	0.3903	BD	LOC101101962	NC_056060.1	86835001	86855001
NC_056080.1	63760001	63780001	2.39667	0.356364	BD	LOC101102537	NC_056064.1	24315001	24335001
NC_056075.1	36475001	36495001	4.3266	0.300626	BD	LOC101103610	NC_056064.1	24320001	24340001
NC_056055.1	18335001	18355001	2.32895	0.398913	BD	LOC101103627	NC_056080.1	63985001	64005001
NC_056075.1	43590001	43610001	5.84666	0.497953	BD	LOC101104360	NC_056056.1	188865001	188885001
NC_056075.1	43595001	43615001	3.42215	0.447684	BD	LOC101104360	NC_056056.1	188870001	188890001
NC_056075.1	43580001	43600001	7.21138	0.509283	BD	LOC101104360;NKX	NC_056056.1	188875001	188895001
NC_056073.1	42425001	42445001	2.66502	0.301579	BD	LOC101104613	NC_056056.1	188880001	188900001
NC_056068.1	44540001	44560001	2.18168	0.296953	BD	LOC101105275;LOC1	NC_056056.1	188885001	188905001
NC_056075.1	16190001	16210001	3.48114	0.420807	BD	LOC101105314	NC_056056.1	188975001	188995001
NC_056075.1	16185001	16205001	3.24834	0.408355	BD	LOC101105314	NC_056054.1	122400001	122420001
NC_056075.1	16180001	16200001	2.97151	0.376412	BD	LOC101105314	NC_056054.1	122405001	122425001
NC_056075.1	16175001	16195001	3.17802	0.3512	BD	LOC101105314	NC_056070.1	29180001	29200001
NC_056075.1	16195001	16215001	2.92232	0.350105	BD	LOC101105314	NC_056055.1	73350001	73370001
NC_056058.1	50155001	50175001	2.81746	0.307833	BD	LOC101105495	NC_056055.1	73380001	73400001
NC_056058.1	40550001	40570001	4.75503	0.329007	BD	LOC101105657	NC_056073.1	40885001	40905001
NC_056066.1	59330001	59350001	2.67505	0.316974	BD	LOC101105678;LOC1	NC_056064.1	41350001	41370001
NC_056060.1	25820001	25840001	42.7772	0.376484	BD	LOC101105825	NC_056064.1	41355001	41375001
NC_056060.1	25815001	25835001	15.05	0.32332	BD	LOC101105825;LOC1	NC_056054.1	278280001	278300001
NC_056074.1	35605001	35625001	2.89814	0.351268	BD	LOC101106045	NC_056054.1	278285001	278305001
NC_056074.1	35610001	35630001	4.36631	0.295749	BD	LOC101106045	NC_056054.1	278290001	278310001
NC_056074.1	35615001	35635001	5.81918	0.292463	BD	LOC101106045	NC_056054.1	278295001	278315001
NC_056074.1	36425001	36445001	3.59374	0.372464	BD	LOC101106637;TMEI	NC_056054.1	278300001	278320001
NC_056080.1	64030001	64050001	3.75943	0.717519	BD	LOC101106743	NC_056054.1	278305001	278325001
NC_056080.1	64035001	64055001	3.87612	0.604582	BD	LOC101106743	NC_056054.1	278310001	278330001
NC_056080.1	64040001	64060001	2.76992	0.373834	BD	LOC101106743	NC_056054.1	278315001	278335001
NC_056056.1	2.01E+08	200665001	2.11671	0.346744	BD	LOC101106925	NC_056054.1	278325001	278345001
NC_056056.1	2.01E+08	200670001	2.30698	0.296239	BD	LOC101106925	NC_056054.1	278330001	278350001
NC_056066.1	3495001	3515001	3.88429	0.344165	BD	LOC101109032	NC_056054.1	278335001	278355001
NC_056077.1	7640001	7660001	9.3067	0.433697	BD	LOC101109062	NC_056054.1	278340001	278360001
NC_056077.1	7635001	7655001	3.96711	0.404147	BD	LOC101109062	NC_056054.1	278345001	278365001
NC_056077.1	7630001	7650001	2.36149	0.359203	BD	LOC101109062	NC_056054.1	232105001	232125001
NC_056074.1	5035001	5055001	7.924	0.300102	BD	LOC101110373	NC_056057.1	86720001	86740001
NC_056068.1	44535001	44555001	3.02824	0.342254	BD	LOC101111047	NC_056060.1	73050001	73070001
NC_056077.1	28920001	28940001	3.13314	0.383594	BD	LOC101111335	NC_056054.1	277615001	277635001
NC_056077.1	28915001	28935001	3.20667	0.358214	BD	LOC101111335	NC_056054.1	277635001	277655001
NC_056077.1	28925001	28945001	2.23849	0.354358	BD	LOC101111335	NC_056054.1	277640001	277660001
NC_056054.1	1.08E+08	107725001	3.77638	0.381608	BD	LOC101111337;LOC1	NC_056054.1	277645001	277665001
NC_056054.1	1.08E+08	107720001	2.5188	0.355816	BD	LOC101111337;LOC1	NC_056054.1	277650001	277670001
NC_056068.1	81335001	81355001	2.23639	0.298826	BD	LOC101111486	NC_056054.1	277655001	277675001
NC_056058.1	29220001	29240001	2.37624	0.340285	BD	LOC101111543	NC_056054.1	277660001	277680001
NC_056065.1	28200001	28220001	4.2859	0.351384	BD	LOC101111566	NC_056054.1	277670001	277690001
NC_056065.1	28195001	28215001	3.34216	0.33566	BD	LOC101111566;TMEI	NC_056054.1	277675001	277695001
NC_056054.1	1.08E+08	107735001	4.18045	0.384564	BD	LOC101111854	NC_056059.1	41675001	41695001
NC_056054.1	1.08E+08	107730001	4.31428	0.364524	BD	LOC101111854	NC_056059.1	41680001	41700001
NC_056068.1	81350001	81370001	10.3025	0.363383	BD	LOC101112419	NC_056060.1	98130001	98150001
NC_056068.1	81345001	81365001	5.40323	0.348017	BD	LOC101112419	NC_056060.1	3120001	3140001
NC_056068.1	81360001	81380001	4.91405	0.30108	BD	LOC101112419	NC_056074.1	45460001	45480001
NC_056068.1	81365001	81385001	4.06294	0.2924	BD	LOC101112419	NC_056080.1	44080001	44100001
NC_056077.1	26535001	26555001	4.65218	0.698727	BD	LOC101112694	NC_056080.1	44090001	44110001
NC_056077.1	26530001	26550001	2.96176	0.62095	BD	LOC101112694	NC_056080.1	44095001	44115001
NC_056077.1	26525001	26545001	2.16487	0.553041	BD	LOC101112694	NC_056080.1	44100001	44120001
NC_056066.1	53975001	53995001	2.74285	0.307827	BD	LOC101112800	NC_056080.1	44105001	44125001
NC_056066.1	53970001	53990001	4.21312	0.293477	BD	LOC101112800	NC_056080.1	44110001	44130001
NC_056054.1	1.1E+08	109950001	12.3233	0.409822	BD	LOC101112936	NC_056080.1	44115001	44135001
NC_056054.1	1.1E+08	109955001	6.91193	0.38296	BD	LOC101112936	NC_056073.1	800001	820001
NC_056054.1	1.1E+08	109960001	2.54354	0.338722	BD	LOC101112936	NC_056073.1	805001	825001
NC_056054.1	1.1E+08	109945001	3.20573	0.330253	BD	LOC101112936;LOC1	NC_056054.1	10465001	10485001
NC_056067.1	14285001	14305001	4.38291	0.349236	BD	LOC101113004;LOC1	NC_056068.1	63040001	63060001

NC_056065.1	4870001	4890001	4.84375	0.33577	BD	LOC101114456	NC_056073.1	38790001	38810001
NC_056065.1	4900001	4920001	3.60231	0.320765	BD	LOC101114456;LOC1	NC_056073.1	38865001	38885001
NC_056065.1	4905001	4925001	3.02334	0.300375	BD	LOC101114456;LOC1	NC_056073.1	38870001	38890001
NC_056054.1	1.79E+08	178540001	3.61127	0.309804	BD	LOC101114747	NC_056073.1	38875001	38895001
NC_056054.1	23115001	23135001	2.88129	0.388091	BD	LOC101115174	NC_056073.1	38880001	38900001
NC_056054.1	23100001	23120001	3.14916	0.386285	BD	LOC101115174	NC_056073.1	38970001	38990001
NC_056054.1	23095001	23115001	3.71641	0.382437	BD	LOC101115174	NC_056074.1	27665001	27685001
NC_056054.1	23090001	23110001	2.61813	0.366996	BD	LOC101115174	NC_056063.1	49220001	49240001
NC_056054.1	23110001	23130001	3.44284	0.362036	BD	LOC101115174	NC_056063.1	49310001	49330001
NC_056054.1	23120001	23140001	2.46003	0.360629	BD	LOC101115174	NC_056063.1	49315001	49335001
NC_056054.1	23105001	23125001	3.50493	0.35082	BD	LOC101115174	NC_056063.1	49325001	49345001
NC_056067.1	62115001	62135001	5.96846	0.401715	BD	LOC101115398	NC_056063.1	49330001	49350001
NC_056068.1	46930001	46950001	4.24275	0.398567	BD	LOC101115569;LOC1	NC_056063.1	49465001	49485001
NC_056068.1	46935001	46955001	2.19925	0.403193	BD	LOC101115569;LOC1	NC_056063.1	48395001	48415001
NC_056068.1	46945001	46965001	2.29804	0.346286	BD	LOC101115824;LOC1	NC_056063.1	44570001	44590001
NC_056080.1	34140001	34160001	17.7	0.394551	BD	LOC101115943	NC_056063.1	44575001	44595001
NC_056080.1	34145001	34165001	12.6613	0.361556	BD	LOC101115943	NC_056063.1	44580001	44600001
NC_056080.1	34150001	34170001	10.812	0.345928	BD	LOC101115943	NC_056063.1	44585001	44605001
NC_056080.1	34155001	34175001	10.6339	0.335024	BD	LOC101115943	NC_056063.1	44610001	44630001
NC_056058.1	9080001	9100001	21.2296	0.373502	BD	LOC101116053	NC_056063.1	44640001	44660001
NC_056058.1	9075001	9095001	26.8532	0.355423	BD	LOC101116053	NC_056063.1	44645001	44665001
NC_056058.1	9070001	9090001	25.3178	0.332762	BD	LOC101116053	NC_056063.1	44670001	44690001
NC_056054.1	2.24E+08	223560001	2.20711	0.330622	BD	LOC101116449	NC_056063.1	44675001	44695001
NC_056062.1	65200001	65220001	2.71338	0.406379	BD	LOC101116738	NC_056063.1	44680001	44700001
NC_056062.1	65195001	65215001	3.53228	0.3853	BD	LOC101116738	NC_056063.1	44685001	44705001
NC_056062.1	65190001	65210001	2.69794	0.291714	BD	LOC101116738	NC_056063.1	44690001	44710001
NC_056067.1	53615001	53635001	2.34286	0.378463	BD	LOC101117098;STRN	NC_056063.1	44695001	44715001
NC_056080.1	9240001	9260001	9.53229	0.466922	BD	LOC101118248	NC_056063.1	44700001	44720001
NC_056080.1	9245001	9265001	4.69018	0.421503	BD	LOC101118248	NC_056063.1	44705001	44725001
NC_056080.1	9235001	9255001	15.364	0.418996	BD	LOC101118248	NC_056063.1	44710001	44730001
NC_056080.1	2800001	2820001	6.53351	0.341107	BD	LOC101118336	NC_056063.1	44715001	44735001
NC_056080.1	2795001	2815001	6.32158	0.307968	BD	LOC101118336	NC_056063.1	44720001	44740001
NC_056056.1	44585001	44605001	13.0322	0.473554	BD	LOC101119202	NC_056063.1	44725001	44745001
NC_056056.1	44590001	44610001	11.4041	0.46621	BD	LOC101119202	NC_056063.1	44750001	44770001
NC_056056.1	44580001	44600001	9.21797	0.454922	BD	LOC101119202	NC_056063.1	44755001	44775001
NC_056056.1	44575001	44595001	5.54135	0.431761	BD	LOC101119202	NC_056063.1	44760001	44780001
NC_056056.1	44595001	44615001	6.3992	0.420645	BD	LOC101119202	NC_056063.1	44765001	44785001
NC_056056.1	44560001	44580001	4.06862	0.378074	BD	LOC101119202	NC_056063.1	44770001	44790001
NC_056056.1	44565001	44585001	3.17692	0.353108	BD	LOC101119202	NC_056063.1	44775001	44795001
NC_056056.1	44600001	44620001	3.34832	0.352803	BD	LOC101119202	NC_056063.1	44780001	44800001
NC_056058.1	6835001	6855001	2.2069	0.29952	BD	LOC101119214	NC_056063.1	44785001	44805001
NC_056058.1	18605001	18625001	2.23399	0.518985	BD	LOC101119303;LOC1	NC_056063.1	44790001	44810001
NC_056058.1	18610001	18630001	2.28108	0.510709	BD	LOC101119303;LOC1	NC_056063.1	44795001	44815001
NC_056066.1	59325001	59345001	2.18449	0.31306	BD	LOC101119839	NC_056063.1	44800001	44820001
NC_056068.1	38940001	38960001	2.33404	0.305023	BD	LOC101120269;PTH	NC_056063.1	44805001	44825001
NC_056068.1	38945001	38965001	2.8532	0.302378	BD	LOC101120269;PTH	NC_056063.1	44810001	44830001
NC_056056.1	1.95E+08	194525001	2.33274	0.312866	BD	LOC101120653	NC_056063.1	44815001	44835001
NC_056056.1	1.95E+08	194530001	2.48962	0.294657	BD	LOC101120653	NC_056063.1	44820001	44840001
NC_056064.1	24045001	24065001	6.60446	0.44475	BD	LOC101120675	NC_056063.1	44825001	44845001
NC_056064.1	24040001	24060001	3.42341	0.393874	BD	LOC101120675;LOC1	NC_056063.1	44830001	44850001
NC_056064.1	24035001	24055001	2.33992	0.333924	BD	LOC101120675;LOC1	NC_056063.1	44835001	44855001
NC_056073.1	19800001	19820001	2.1913	0.327034	BD	LOC101120701	NC_056063.1	44840001	44860001
NC_056073.1	19795001	19815001	2.38495	0.312315	BD	LOC101120701	NC_056063.1	44845001	44865001
NC_056080.1	1.29E+08	129315001	2.18977	0.299147	BD	LOC101120810	NC_056063.1	44850001	44870001
NC_056068.1	53715001	53735001	7.53158	0.427234	BD	LOC101120816	NC_056063.1	44855001	44875001
NC_056068.1	53720001	53740001	7.50861	0.422321	BD	LOC101120816	NC_056063.1	44860001	44880001
NC_056068.1	53705001	53725001	6.90122	0.401844	BD	LOC101120816	NC_056063.1	44865001	44885001
NC_056068.1	53700001	53720001	6.35763	0.39326	BD	LOC101120816	NC_056063.1	44870001	44890001
NC_056068.1	53710001	53730001	5.91667	0.385486	BD	LOC101120816	NC_056063.1	44875001	44895001
NC_056068.1	53695001	53715001	5.93749	0.384403	BD	LOC101120816	NC_056063.1	44880001	44900001
NC_056068.1	53730001	53750001	7.77174	0.416383	BD	LOC101120816;LOC1	NC_056063.1	44885001	44905001
NC_056068.1	53725001	53745001	7.22786	0.415683	BD	LOC101120816;LOC1	NC_056063.1	44890001	44910001
NC_056068.1	53740001	53760001	7.9652	0.405382	BD	LOC101120816;LOC1	NC_056071.1	15975001	15995001
NC_056068.1	53735001	53755001	6.87898	0.402412	BD	LOC101120816;LOC1	NC_056071.1	15980001	16000001
NC_056064.1	24050001	24070001	3.279	0.433582	BD	LOC101120929	NC_056059.1	58965001	58985001
NC_056060.1	80105001	80125001	2.11815	0.40165	BD	LOC101121353;LOC1	NC_056059.1	58970001	58990001
NC_056074.1	41855001	41875001	4.65301	0.312178	BD	LOC101121557;NDU	NC_056059.1	58975001	58995001
NC_056074.1	41860001	41880001	2.5387	0.319424	BD	LOC101121557;NDU	NC_056064.1	27795001	27815001
NC_056058.1	39795001	39815001	3.02759	0.352283	BD	LOC101121936	NC_056070.1	68440001	68460001
NC_056058.1	39800001	39820001	6.30814	0.348577	BD	LOC101121936	NC_056070.1	68465001	68485001
NC_056054.1	1.03E+08	102645001	2.82337	0.299547	BD	LOC101121986	NC_056070.1	68470001	68490001
NC_056058.1	39805001	39825001	6.67269	0.340499	BD	LOC101122191	NC_056056.1	134180001	134200001
NC_056058.1	39810001	39830001	4.62963	0.31718	BD	LOC101122191	NC_056059.1	38065001	38085001
NC_056067.1	46620001	46640001	5.96998	0.342349	BD	LOC101122210;ZNF5	NC_056068.1	65420001	65440001

NC_056067.1	46410001	46430001	2.37036	0.380885	BD	LOC101122457	NC_056068.1	56945001	56965001
NC_056054.1	1.44E+08	143670001	3.28194	0.352068	BD	LOC101122496	NC_056060.1	9605001	9625001
NC_056054.1	1.44E+08	143675001	2.66563	0.302191	BD	LOC101122496	NC_056060.1	9610001	9630001
NC_056058.1	14200001	14220001	3.91335	0.345375	BD	LOC101122597;PCP2	NC_056060.1	9615001	9635001
NC_056058.1	14205001	14225001	3.81538	0.315792	BD	LOC101122597;PCP2	NC_056060.1	9620001	9640001
NC_056068.1	53750001	53770001	7.70229	0.404321	BD	LOC101122934	NC_056060.1	9625001	9645001
NC_056068.1	53745001	53765001	7.47335	0.40225	BD	LOC101122934	NC_056060.1	9630001	9650001
NC_056068.1	53755001	53775001	5.71951	0.381173	BD	LOC101122934	NC_056060.1	9640001	9660001
NC_056072.1	43535001	43555001	6.08	0.507193	BD	LOC101123588	NC_056057.1	47300001	47320001
NC_056072.1	43530001	43550001	6.84293	0.505544	BD	LOC101123588	NC_056057.1	47305001	47325001
NC_056072.1	43525001	43545001	5.76922	0.501424	BD	LOC101123588	NC_056057.1	47310001	47330001
NC_056072.1	43540001	43560001	5.86898	0.500753	BD	LOC101123588	NC_056057.1	47315001	47335001
NC_056072.1	43545001	43565001	5.70121	0.495009	BD	LOC101123588	NC_056068.1	56950001	56970001
NC_056072.1	43515001	43535001	4.49148	0.488329	BD	LOC101123588	NC_056068.1	56955001	56975001
NC_056072.1	43520001	43540001	3.9476	0.483407	BD	LOC101123588	NC_056055.1	97430001	97450001
NC_056072.1	43550001	43570001	3.2595	0.438928	BD	LOC101123588	NC_056055.1	97435001	97455001
NC_056072.1	43510001	43530001	4.46244	0.392154	BD	LOC101123588	NC_056055.1	97440001	97460001
NC_056072.1	43555001	43575001	2.21753	0.367186	BD	LOC101123588	NC_056055.1	97445001	97465001
NC_056072.1	43505001	43525001	3.39042	0.317278	BD	LOC101123588	NC_056055.1	97535001	97555001
NC_056064.1	54605001	54625001	11.6797	0.311743	BD	LOC105601853;RHBI	NC_056055.1	97685001	97705001
NC_056060.1	4445001	4465001	10.1219	0.37097	BD	LOC105601870	NC_056055.1	97690001	97710001
NC_056060.1	4440001	4460001	3.19566	0.306465	BD	LOC105601870	NC_056056.1	102150001	102170001
NC_056068.1	19975001	19995001	2.76405	0.326503	BD	LOC105602148;RDX	NC_056064.1	41325001	41345001
NC_056068.1	19970001	19990001	2.24711	0.322985	BD	LOC105602148;RDX	NC_056064.1	41335001	41355001
NC_056068.1	61730001	61750001	2.25	0.373952	BD	LOC105602333	NC_056064.1	41320001	41340001
NC_056074.1	45825001	45845001	2.83594	0.307194	BD	LOC105604151	NC_056064.1	41340001	41360001
NC_056074.1	45830001	45850001	3.25654	0.295038	BD	LOC105604151	NC_056064.1	41305001	41325001
NC_056080.1	57085001	57105001	2.39864	0.355641	BD	LOC105605483	NC_056064.1	41310001	41330001
NC_056071.1	65450001	65470001	6.84448	0.368694	BD	LOC105605761;LOC1	NC_056064.1	41315001	41335001
NC_056071.1	65455001	65475001	4.93342	0.325762	BD	LOC105605761;LOC1	NC_056077.1	37225001	37245001
NC_056073.1	25895001	25915001	16.3849	0.374776	BD	LOC105605990	NC_056077.1	37230001	37250001
NC_056073.1	25900001	25920001	16.2016	0.34596	BD	LOC105605990	NC_056077.1	37235001	37255001
NC_056073.1	25905001	25925001	17.6037	0.311376	BD	LOC105605990	NC_056074.1	8630001	8650001
NC_056073.1	25985001	26005001	12.9822	0.304491	BD	LOC105605990	NC_056074.1	8635001	8655001
NC_056073.1	25960001	25980001	22.1371	0.300051	BD	LOC105605990	NC_056074.1	8640001	8660001
NC_056067.1	44755001	44775001	2.89572	0.395617	BD	LOC105607568;LOC1	NC_056054.1	181375001	181395001
NC_056058.1	60960001	60980001	2.25501	0.315874	BD	LOC105615377	NC_056074.1	35190001	35210001
NC_056054.1	1.58E+08	157520001	3.74901	0.33264	BD	LOC105616630	NC_056074.1	35195001	35215001
NC_056071.1	65445001	65465001	8.05157	0.375125	BD	LOC114109056	NC_056074.1	35200001	35220001
NC_056071.1	65440001	65460001	9.08365	0.347809	BD	LOC114109056	NC_056074.1	35205001	35225001
NC_056074.1	35475001	35495001	2.1155	0.365793	BD	LOC114110058	NC_056074.1	35210001	35230001
NC_056074.1	35470001	35490001	2.29443	0.344083	BD	LOC114110058	NC_056074.1	35215001	35235001
NC_056077.1	14175001	14195001	3.82257	0.631743	BD	LOC114110664;RRN3	NC_056058.1	8120001	8140001
NC_056080.1	1.37E+08	137420001	2.58034	0.325729	BD	LOC114111709	NC_056058.1	8125001	8145001
NC_056080.1	1.37E+08	137425001	2.16025	0.291097	BD	LOC114111709	NC_056073.1	42415001	42435001
NC_056055.1	61440001	61460001	2.19789	0.293498	BD	LOC114113073	NC_056073.1	42420001	42440001
NC_056056.1	92185001	92205001	4.31137	0.48925	BD	LOC114113944	NC_056073.1	42425001	42445001
NC_056056.1	92180001	92200001	14.0107	0.416998	BD	LOC114113944	NC_056058.1	39020001	39040001
NC_056056.1	92175001	92195001	14.1602	0.379256	BD	LOC114113944;SRD5	NC_056058.1	39025001	39045001
NC_056057.1	1.21E+08	121445001	4.88119	0.3086	BD	LOC114114472	NC_056058.1	39030001	39050001
NC_056058.1	9005001	9025001	5.82265	0.432151	BD	LOC114114788	NC_056077.1	4705001	4725001
NC_056058.1	9010001	9030001	15.9901	0.329298	BD	LOC114114788	NC_056077.1	4710001	4730001
NC_056060.1	25825001	25845001	16.876	0.387269	BD	LOC114115708	NC_056077.1	4715001	4735001
NC_056060.1	25830001	25850001	6.29383	0.373688	BD	LOC114115708	NC_056055.1	89835001	89855001
NC_056060.1	25835001	25855001	4.41817	0.352773	BD	LOC114115708	NC_056080.1	19350001	19370001
NC_056060.1	25840001	25860001	3.82016	0.3186	BD	LOC114115708	NC_056064.1	49895001	49915001
NC_056064.1	23965001	23985001	2.57699	0.306545	BD	LOC114116984	NC_056058.1	50150001	50170001
NC_056066.1	35555001	35575001	4.89563	0.323975	BD	LOC114117570	NC_056058.1	50155001	50175001
NC_056067.1	44715001	44735001	3.11297	0.374792	BD	LOC114117974	NC_056058.1	50165001	50185001
NC_056067.1	44720001	44740001	3.26694	0.328307	BD	LOC114117974	NC_056058.1	50170001	50190001
NC_056067.1	44725001	44745001	3.97604	0.319462	BD	LOC114117974;LOC1	NC_056068.1	64210001	64230001
NC_056067.1	44740001	44760001	2.31336	0.308784	BD	LOC114117975	NC_056068.1	64215001	64235001
NC_056069.1	75001	95001	7.7921	0.351695	BD	LOC114118677	NC_056068.1	64220001	64240001
NC_056069.1	80001	100001	7.00001	0.343939	BD	LOC114118677	NC_056068.1	64225001	64245001
NC_056069.1	90001	110001	7.38946	0.323333	BD	LOC114118677	NC_056068.1	64230001	64250001
NC_056065.1	78425001	78445001	8.7652	0.299064	BD	LOC121816068	NC_056074.1	35405001	35425001
NC_056067.1	51565001	51585001	19.437	0.293722	BD	LOC121816525	NC_056074.1	35425001	35445001
NC_056076.1	24870001	24890001	8.57819	0.475107	BD	LOC121817702	NC_056074.1	35430001	35450001
NC_056076.1	24875001	24895001	4.10754	0.426791	BD	LOC121817702	NC_056074.1	35495001	35515001
NC_056076.1	24885001	24905001	3.16417	0.418193	BD	LOC121817702	NC_056074.1	35500001	35520001
NC_056076.1	24880001	24900001	3.39411	0.416244	BD	LOC121817702	NC_056074.1	35505001	35525001
NC_056076.1	24865001	24885001	3.43919	0.404682	BD	LOC121817702	NC_056074.1	35485001	35505001
NC_056080.1	1.31E+08	131480001	7.05861	0.32979	BD	LOC121818354	NC_056074.1	35490001	35510001
NC_056080.1	1.31E+08	131485001	2.98526	0.306564	BD	LOC121818354	NC_056074.1	35620001	35640001

NW_024599807.1	15001	35001	3.88229	0.477145	BD	LOC121818477	NC_056074.1	36425001	36445001
NW_024599807.1	20001	40001	5.59018	0.473326	BD	LOC121818477	NC_056080.1	64030001	64050001
NW_024599807.1	25001	45001	12.9055	0.467352	BD	LOC121818477	NC_056080.1	64035001	64055001
NC_056054.1	1.16E+08	116430001	2.96039	0.66878	BD	LOC121818668	NC_056080.1	116285001	116305001
NC_056054.1	1.16E+08	116425001	2.15482	0.622934	BD	LOC121818668	NC_056054.1	72195001	72215001
NC_056054.1	1.16E+08	116435001	5.58759	0.60763	BD	LOC121818668	NC_056063.1	27275001	27295001
NC_056054.1	1.16E+08	116440001	5.47018	0.522871	BD	LOC121818668	NC_056063.1	27285001	27305001
NC_056054.1	1.16E+08	116445001	2.59083	0.41621	BD	LOC121818668	NC_056054.1	199485001	199505001
NC_056056.1	1.16E+08	116445001	2.29717	0.456485	BD	LOC121819170	NC_056074.1	15575001	15595001
NC_056056.1	1.16E+08	116450001	2.40541	0.45193	BD	LOC121819170	NC_056074.1	15565001	15585001
NC_056056.1	1.16E+08	116455001	2.4554	0.415446	BD	LOC121819170	NC_056074.1	15570001	15590001
NC_056056.1	1.16E+08	116460001	2.58629	0.363364	BD	LOC121819170	NC_056074.1	5035001	5055001
NC_056056.1	1.16E+08	116465001	2.30166	0.30669	BD	LOC121819170	NC_056074.1	5040001	5060001
NC_056057.1	83400001	83420001	4.30794	0.449948	BD	LOC121819513	NC_056059.1	25330001	25350001
NC_056057.1	83405001	83425001	2.88446	0.409284	BD	LOC121819513	NC_056059.1	25335001	25355001
NC_056057.1	83395001	83415001	2.29174	0.388688	BD	LOC121819513	NC_056059.1	25340001	25360001
NC_056061.1	14970001	14990001	6.19262	0.430787	BD	LOC121820153	NC_056059.1	25345001	25365001
NC_056061.1	14975001	14995001	4.0669	0.376373	BD	LOC121820153	NC_056059.1	25350001	25370001
NC_056061.1	14965001	14985001	6.0903	0.34602	BD	LOC121820153	NC_056059.1	25355001	25375001
NC_056061.1	14980001	15000001	3.12477	0.344059	BD	LOC121820153	NC_056054.1	10840001	10860001
NC_056061.1	15045001	15065001	2.38106	0.299775	BD	LOC121820153	NC_056064.1	36700001	36720001
NC_056055.1	1.7E+08	169585001	2.73068	0.395801	BD	LRP1B	NC_056077.1	28920001	28940001
NC_056055.1	1.68E+08	168340001	2.94104	0.320996	BD	LRP1B	NC_056068.1	81335001	81355001
NC_056055.1	1.68E+08	168345001	3.19101	0.307609	BD	LRP1B	NC_056058.1	65965001	65985001
NC_056056.1	80350001	80370001	2.42396	0.478925	BD	LRPPRC	NC_056058.1	65970001	65990001
NC_056056.1	80375001	80395001	2.4312	0.46124	BD	LRPPRC	NC_056079.1	14815001	14835001
NC_056056.1	80385001	80405001	3.84031	0.459757	BD	LRPPRC	NC_056079.1	14820001	14840001
NC_056056.1	80380001	80400001	3.51175	0.456179	BD	LRPPRC	NC_056068.1	81345001	81365001
NC_056056.1	80345001	80365001	2.24888	0.413739	BD	LRPPRC	NC_056068.1	81350001	81370001
NC_056060.1	17845001	17865001	2.75274	0.382596	BD	LRRC49	NC_056067.1	14285001	14305001
NC_056060.1	17840001	17860001	2.8497	0.358662	BD	LRRC49	NC_056058.1	38450001	38470001
NC_056074.1	45950001	45970001	2.20976	0.342212	BD	LSP1;TNNT3	NC_056058.1	38455001	38475001
NC_056077.1	770001	790001	2.51984	0.33447	BD	LUC7L	NC_056077.1	19640001	19660001
NC_056077.1	785001	805001	3.18807	0.328327	BD	LUC7L	NC_056077.1	19645001	19665001
NC_056077.1	780001	800001	3.02638	0.324071	BD	LUC7L	NC_056077.1	19650001	19670001
NC_056077.1	775001	795001	2.44293	0.31847	BD	LUC7L	NC_056078.1	40935001	40955001
NC_056074.1	18080001	18100001	12.2612	0.546508	BD	LUZP2	NC_056078.1	40940001	40960001
NC_056074.1	18055001	18075001	14.243	0.543021	BD	LUZP2	NC_056078.1	40945001	40965001
NC_056074.1	18115001	18135001	11.6163	0.540661	BD	LUZP2	NC_056078.1	40950001	40970001
NC_056074.1	18125001	18145001	14.9743	0.538442	BD	LUZP2	NC_056078.1	40955001	40975001
NC_056074.1	18120001	18140001	11.72	0.528705	BD	LUZP2	NC_056078.1	40960001	40980001
NC_056074.1	18110001	18130001	9.48703	0.524826	BD	LUZP2	NC_056074.1	37580001	37600001
NC_056074.1	18050001	18070001	8.03509	0.520866	BD	LUZP2	NC_056065.1	4870001	4890001
NC_056074.1	18105001	18125001	8.53704	0.512094	BD	LUZP2	NC_056061.1	50075001	50095001
NC_056074.1	18090001	18110001	7.25892	0.510413	BD	LUZP2	NC_056077.1	34570001	34590001
NC_056074.1	18100001	18120001	7.98785	0.50981	BD	LUZP2	NC_056058.1	9015001	9035001
NC_056074.1	18085001	18105001	6.10552	0.50619	BD	LUZP2	NC_056058.1	9020001	9040001
NC_056074.1	18130001	18150001	6.97163	0.503332	BD	LUZP2	NC_056067.1	62115001	62135001
NC_056074.1	18095001	18115001	7.7026	0.502375	BD	LUZP2	NC_056058.1	9035001	9055001
NC_056074.1	18045001	18065001	4.40292	0.461479	BD	LUZP2	NC_056058.1	9040001	9060001
NC_056074.1	18135001	18155001	3.77096	0.459538	BD	LUZP2	NC_056058.1	9045001	9065001
NC_056066.1	8790001	8810001	6.38695	0.428607	BD	MACROD2	NC_056077.1	16935001	16955001
NC_056066.1	8785001	8805001	2.22928	0.36041	BD	MACROD2	NC_056077.1	16940001	16960001
NC_056066.1	9660001	9680001	4.12269	0.339168	BD	MACROD2	NC_056077.1	16945001	16965001
NC_056066.1	9655001	9675001	4.51807	0.297725	BD	MACROD2	NC_056080.1	34140001	34160001
NC_056077.1	41530001	41550001	18.0426	0.568256	BD	MAD1L1	NC_056080.1	34145001	34165001
NC_056077.1	41525001	41545001	20.801	0.567699	BD	MAD1L1	NC_056080.1	34150001	34170001
NC_056077.1	41535001	41555001	13.7903	0.562853	BD	MAD1L1	NC_056080.1	34155001	34175001
NC_056077.1	41520001	41540001	23.1921	0.561627	BD	MAD1L1	NC_056058.1	9070001	9090001
NC_056077.1	41540001	41560001	6.27878	0.523872	BD	MAD1L1	NC_056058.1	9075001	9095001
NC_056077.1	41545001	41565001	5.8537	0.500658	BD	MAD1L1	NC_056058.1	9080001	9100001
NC_056077.1	41560001	41580001	7.60133	0.480251	BD	MAD1L1	NC_056071.1	24550001	24570001
NC_056077.1	41570001	41590001	4.92655	0.474881	BD	MAD1L1	NC_056071.1	24565001	24585001
NC_056077.1	41555001	41575001	5.72537	0.468677	BD	MAD1L1	NC_056065.1	55560001	55580001
NC_056077.1	41550001	41570001	4.702	0.457591	BD	MAD1L1	NC_056065.1	55545001	55565001
NC_056077.1	41575001	41595001	4.33334	0.433056	BD	MAD1L1	NC_056065.1	55550001	55570001
NC_056077.1	41565001	41585001	4.35801	0.426272	BD	MAD1L1	NC_056065.1	55555001	55575001
NC_056077.1	41515001	41535001	3.2461	0.406827	BD	MAD1L1	NC_056059.1	8845001	8865001
NC_056077.1	41580001	41600001	4.05976	0.372407	BD	MAD1L1	NC_056059.1	8850001	8870001
NC_056077.1	41510001	41530001	2.2777	0.332935	BD	MAD1L1	NC_056059.1	8855001	8875001
NC_056070.1	18085001	18105001	2.21181	0.33758	BD	MAML3;MGST2	NC_056058.1	38970001	38990001
NC_056055.1	2.41E+08	241200001	5.58529	0.364602	BD	MAN1C1	NC_056058.1	38980001	39000001
NC_056055.1	2.41E+08	241195001	2.96905	0.322009	BD	MAN1C1	NC_056058.1	38985001	39005001
NC_056058.1	11125001	11145001	3.81915	0.415661	BD	MAN2B1;WDR83OS	NC_056058.1	39065001	39085001

NC_056056.1	99970001	99990001	5.62567	0.348043	BD	MAP4K4	NC_056067.1	53615001	53635001
NC_056056.1	99965001	99985001	5.37204	0.347237	BD	MAP4K4	NC_056056.1	165205001	165225001
NC_056056.1	99960001	99980001	4.10552	0.319557	BD	MAP4K4	NC_056056.1	165210001	165230001
NC_056056.1	99975001	99995001	5.03438	0.318176	BD	MAP4K4	NC_056056.1	165215001	165235001
NC_056056.1	99955001	99975001	2.6804	0.301365	BD	MAP4K4	NC_056074.1	16545001	16565001
NC_056074.1	38670001	38690001	3.90104	0.459163	BD	MARK2	NC_056074.1	16550001	16570001
NC_056074.1	38665001	38685001	2.48993	0.410678	BD	MARK2	NC_056056.1	104795001	104815001
NC_056074.1	38690001	38710001	5.90001	0.405982	BD	MARK2	NC_056056.1	104800001	104820001
NC_056074.1	38695001	38715001	6.5746	0.323609	BD	MARK2;RCOR2	NC_056056.1	104805001	104825001
NC_056054.1	20450001	20470001	4.98388	0.311707	BD	MAST2	NC_056056.1	104790001	104810001
NC_056080.1	20170001	20190001	5.4815	0.306587	BD	MBTPS2;YY2	NC_056074.1	16740001	16760001
NC_056067.1	14245001	14265001	2.7526	0.331831	BD	MC1R;TCF25	NC_056074.1	16745001	16765001
NC_056054.1	2.19E+08	218785001	2.70204	0.398644	BD	MECOM	NC_056074.1	16840001	16860001
NC_056054.1	2.19E+08	218790001	2.29923	0.385276	BD	MECOM	NC_056074.1	16845001	16865001
NC_056054.1	2.19E+08	218795001	2.67612	0.35472	BD	MECOM	NC_056059.1	113755001	113775001
NC_056054.1	2.38E+08	237525001	2.14286	0.302439	BD	MED12L;P2RY13	NC_056059.1	113760001	113780001
NC_056063.1	18250001	18270001	3.62296	0.305229	BD	MED4	NC_056059.1	113765001	113785001
NC_056060.1	12865001	12885001	3.51579	0.330075	BD	MEGF11	NC_056059.1	113770001	113790001
NC_056060.1	12860001	12880001	2.24235	0.293062	BD	MEGF11	NC_056059.1	113775001	113795001
NC_056056.1	2.19E+08	218645001	2.68545	0.356634	BD	MEI1	NC_056059.1	113780001	113800001
NC_056056.1	2.19E+08	218640001	2.62023	0.352143	BD	MEI1	NC_056059.1	113785001	113805001
NC_056056.1	2.19E+08	218635001	2.31087	0.333177	BD	MEI1	NC_056059.1	113790001	113810001
NC_056059.1	37510001	37530001	20.6132	0.487708	BD	MEPE	NC_056055.1	105195001	105215001
NC_056059.1	37505001	37525001	18.7057	0.47767	BD	MEPE	NC_056055.1	105200001	105220001
NC_056059.1	37500001	37520001	14.8857	0.470387	BD	MEPE	NC_056055.1	105205001	105225001
NC_056059.1	37515001	37535001	8.0956	0.42796	BD	MEPE	NC_056080.1	9235001	9255001
NC_056057.1	53105001	53125001	2.18096	0.466526	BD	MET	NC_056080.1	9240001	9260001
NC_056057.1	53100001	53120001	2.28732	0.45171	BD	MET	NC_056080.1	9245001	9265001
NC_056057.1	53095001	53115001	2.28961	0.434572	BD	MET	NC_056080.1	2750001	2770001
NC_056057.1	53110001	53130001	2.55882	0.417505	BD	MET	NC_056080.1	2755001	2775001
NC_056057.1	53115001	53135001	2.60072	0.37406	BD	MET	NC_056080.1	2760001	2780001
NC_056057.1	53120001	53140001	3.44104	0.338719	BD	MET	NC_056080.1	2765001	2785001
NC_056076.1	37335001	37355001	5.94764	0.596293	BD	METTL4	NC_056080.1	2770001	2790001
NC_056076.1	37330001	37350001	7.51498	0.541609	BD	METTL4	NC_056080.1	2785001	2805001
NC_056076.1	37325001	37345001	10.9482	0.495122	BD	METTL4	NC_056080.1	2790001	2810001
NC_056076.1	37305001	37325001	2.65493	0.466339	BD	METTL4	NC_056055.1	230080001	230100001
NC_056076.1	37320001	37340001	10.1538	0.455476	BD	METTL4	NC_056055.1	230085001	230105001
NC_056076.1	37310001	37330001	5.21705	0.432231	BD	METTL4	NC_056055.1	230115001	230135001
NC_056076.1	37315001	37335001	8.30264	0.413939	BD	METTL4	NC_056055.1	230120001	230140001
NC_056076.1	37340001	37360001	3.20469	0.508252	BD	METTL4;NDC80	NC_056062.1	14585001	14605001
NC_056076.1	37345001	37365001	2.2	0.430427	BD	METTL4;NDC80	NC_056062.1	14590001	14610001
NC_056076.1	37350001	37370001	3.17048	0.420154	BD	METTL4;NDC80	NC_056062.1	14595001	14615001
NC_056076.1	37355001	37375001	3.5234	0.409016	BD	METTL4;NDC80	NC_056080.1	36640001	36660001
NC_056055.1	1.19E+08	118865001	13.6967	0.458609	BD	MFSD6	NC_056080.1	36655001	36675001
NC_056055.1	1.19E+08	118860001	8.28752	0.430779	BD	MFSD6	NC_056080.1	36685001	36705001
NC_056055.1	1.19E+08	118895001	9.68155	0.403116	BD	MFSD6	NC_056080.1	36690001	36710001
NC_056055.1	1.19E+08	118855001	5.07977	0.366024	BD	MFSD6	NC_056055.1	224825001	224845001
NC_056070.1	18095001	18115001	3.42124	0.393748	BD	MGST2	NC_056059.1	16250001	16270001
NC_056070.1	18100001	18120001	2.90733	0.377008	BD	MGST2	NC_056066.1	36545001	36565001
NC_056070.1	18090001	18110001	2.8328	0.372542	BD	MGST2	NC_056066.1	36550001	36570001
NC_056056.1	2.15E+08	215130001	2.33334	0.307764	BD	MICAL3	NC_056068.1	53720001	53740001
NC_056056.1	2.15E+08	215125001	2.30096	0.297807	BD	MICAL3	NC_056068.1	53725001	53745001
NC_056057.1	96730001	96750001	2.22031	0.31707	BD	MKLN1	NC_056068.1	53730001	53750001
NC_056057.1	96715001	96735001	2.16006	0.307823	BD	MKLN1	NC_056068.1	46955001	46975001
NC_056057.1	96725001	96745001	2.22616	0.304531	BD	MKLN1	NC_056068.1	46960001	46980001
NC_056057.1	96735001	96755001	2.30903	0.292156	BD	MKLN1	NC_056068.1	46950001	46970001
NC_056062.1	87535001	87555001	3.22705	0.343059	BD	MMP16	NC_056074.1	41855001	41875001
NC_056062.1	87530001	87550001	2.82429	0.296432	BD	MMP16	NC_056074.1	41860001	41880001
NC_056062.1	13610001	13630001	8.24431	0.432539	BD	MROH1	NW_02459976	135001	155001
NC_056062.1	13615001	13635001	6.93082	0.414187	BD	MROH1	NW_02459976	160001	180001
NC_056062.1	13625001	13645001	3.3662	0.328829	BD	MROH1	NW_02459976	165001	185001
NC_056062.1	13630001	13650001	3.05714	0.317886	BD	MROH1	NW_02459976	145001	165001
NC_056058.1	63890001	63910001	2.67729	0.47727	BD	MRPL22	NW_02459976	150001	170001
NC_056058.1	63895001	63915001	2.40763	0.440651	BD	MRPL22	NW_02459976	155001	175001
NC_056063.1	22110001	22130001	5.10958	0.515814	BD	MRPS31	NC_056054.1	20195001	20215001
NC_056063.1	22115001	22135001	5.22886	0.484894	BD	MRPS31	NC_056060.1	21475001	21495001
NC_056063.1	22105001	22125001	6.39514	0.45588	BD	MRPS31	NC_056054.1	102620001	102640001
NC_056063.1	22120001	22140001	6.13774	0.346023	BD	MRPS31	NC_056054.1	102625001	102645001
NC_056063.1	22100001	22120001	2.2381	0.297862	BD	MRPS31	NC_056054.1	239620001	239640001
NC_056074.1	34465001	34485001	3.00265	0.355745	BD	MS4A15;MS4A18	NC_056054.1	239625001	239645001
NC_056074.1	34470001	34490001	3.70037	0.309044	BD	MS4A15;MS4A18	NC_056064.1	6100001	6120001
NC_056074.1	34460001	34480001	3.19639	0.366842	BD	MS4A18	NC_056064.1	6110001	6130001
NC_056054.1	52015001	52035001	5.03704	0.36155	BD	MSH4;RABGGTB	NC_056064.1	6115001	6135001
NC_056080.1	84925001	84945001	9.9716	0.316667	BD	MTM1	NC_056056.1	204755001	204775001

NC_056069.1	23910001	23930001	7.13291	0.32343	BD	MTREX;PLPP1	NC_056054.1	260295001	260315001
NC_056069.1	23920001	23940001	6.74997	0.306076	BD	MTREX;PLPP1	NC_056054.1	247050001	247070001
NC_056058.1	11340001	11360001	7.44933	0.481529	BD	MUC16	NC_056078.1	40965001	40985001
NC_056058.1	11345001	11365001	22.5545	0.436077	BD	MUC16	NC_056071.1	65420001	65440001
NC_056058.1	11355001	11375001	3.98652	0.377869	BD	MUC16	NC_056071.1	65425001	65445001
NC_056058.1	11335001	11355001	4.01487	0.369395	BD	MUC16	NC_056071.1	65430001	65450001
NC_056077.1	35620001	35640001	2.52174	0.434456	BD	MYL10	NC_056073.1	25975001	25995001
NC_056077.1	35625001	35645001	2.88572	0.433217	BD	MYL10	NC_056073.1	25980001	26000001
NC_056077.1	35615001	35635001	2.44265	0.416992	BD	MYL10	NC_056073.1	25985001	26005001
NC_056077.1	35610001	35630001	2.4945	0.390619	BD	MYL10	NC_056055.1	126945001	126965001
NC_056056.1	1.63E+08	162575001	2.4051	0.350228	BD	MYO1A	NC_056055.1	126950001	126970001
NC_056056.1	1.63E+08	162580001	2.16428	0.334471	BD	MYO1A	NC_056056.1	102575001	102595001
NC_056056.1	1.63E+08	162565001	2.12335	0.376711	BD	MYO1A;NEMP1	NC_056057.1	6975001	6995001
NC_056056.1	1.63E+08	162570001	2.64746	0.352304	BD	MYO1A;NEMP1	NC_056054.1	157500001	157520001
NC_056076.1	49860001	49880001	2.6097	0.508878	BD	MYO5B	NC_056080.1	79100001	79120001
NC_056076.1	49855001	49875001	3.75527	0.4523	BD	MYO5B	NC_056074.1	46270001	46290001
NC_056076.1	49850001	49870001	3.83057	0.394404	BD	MYO5B	NC_056074.1	18295001	18315001
NC_056076.1	49870001	49890001	2.28593	0.312394	BD	MYO5B	NC_056077.1	14170001	14190001
NC_056076.1	49845001	49865001	3.39811	0.304588	BD	MYO5B	NC_056077.1	14175001	14195001
NC_056076.1	37735001	37755001	2.67022	0.304879	BD	MYOM1	NC_056080.1	79045001	79065001
NC_056076.1	37750001	37770001	8.47153	0.295819	BD	MYOM1	NC_056080.1	79050001	79070001
NC_056076.1	37745001	37765001	5.69231	0.29164	BD	MYOM1	NC_056080.1	79055001	79075001
NC_056055.1	1065001	1085001	2.12892	0.31226	BD	MYT1L	NC_056080.1	70190001	70210001
NC_056054.1	2.13E+08	212690001	2.29162	0.318621	BD	NAALADL2	NC_056055.1	111030001	111050001
NC_056056.1	3420001	3440001	6.88073	0.300369	BD	NACC2	NC_056055.1	111035001	111055001
NC_056056.1	93065001	93085001	2.1425	0.35862	BD	NAGK;PAIP2B	NC_056055.1	111040001	111060001
NC_056074.1	22830001	22850001	2.7614	0.43799	BD	NAV2	NC_056055.1	111045001	111065001
NC_056056.1	23605001	23625001	6.1344	0.347229	BD	NBAS	NC_056058.1	9005001	9025001
NC_056056.1	23610001	23630001	4.36719	0.336112	BD	NBAS	NC_056058.1	9010001	9030001
NC_056056.1	23615001	23635001	3.47393	0.32242	BD	NBAS	NC_056059.1	59670001	59690001
NC_056063.1	26595001	26615001	2.94687	0.455128	BD	NBEA	NC_056059.1	59655001	59675001
NC_056063.1	26590001	26610001	3.25941	0.415847	BD	NBEA	NC_056059.1	59660001	59680001
NC_056063.1	26585001	26605001	2.94143	0.388889	BD	NBEA	NC_056059.1	59665001	59685001
NC_056063.1	26580001	26600001	2.43303	0.371111	BD	NBEA	NC_056065.1	61780001	61800001
NC_056062.1	86695001	86715001	8.37662	0.315105	BD	NBN	NC_056065.1	61785001	61805001
NC_056062.1	86705001	86725001	6.28909	0.314383	BD	NBN;OSGIN2	NC_056065.1	61790001	61810001
NC_056062.1	86700001	86720001	8.2262	0.314203	BD	NBN;OSGIN2	NC_056067.1	51575001	51595001
NC_056059.1	38035001	38055001	5.86137	0.506885	BD	NCAPG	NC_056068.1	21450001	21470001
NC_056080.1	1.27E+08	127470001	13.4895	0.29433	BD	NCBP2L	NC_056068.1	82120001	82140001
NC_056054.1	2.53E+08	252945001	4.54741	0.592257	BD	NCK1	NC_056068.1	82125001	82145001
NC_056054.1	2.53E+08	252950001	5.78173	0.539076	BD	NCK1	NC_056068.1	82130001	82150001
NC_056054.1	2.53E+08	252940001	2.28142	0.51065	BD	NCK1	NC_056068.1	82135001	82155001
NC_056054.1	2.53E+08	252955001	5.41288	0.503152	BD	NCK1;SLC35G2	NC_056068.1	82140001	82160001
NC_056054.1	2.53E+08	252960001	3.4034	0.354031	BD	NCK1;SLC35G2	NC_056068.1	82145001	82165001
NC_056055.1	1.78E+08	177535001	2.34764	0.359903	BD	NCKAP5	NC_056068.1	82150001	82170001
NC_056056.1	32225001	32245001	4.60547	0.587886	BD	NCOA1	NC_056068.1	82155001	82175001
NC_056056.1	32230001	32250001	3.75893	0.552706	BD	NCOA1	NC_056068.1	82160001	82180001
NC_056056.1	32220001	32240001	2.76041	0.522851	BD	NCOA1	NW_02459982!	1415001	1435001
NC_056070.1	51260001	51280001	2.33562	0.334143	BD	NCOR2	NW_02459982!	1440001	1460001
NC_056070.1	51275001	51295001	2.12451	0.306789	BD	NCOR2	NC_056074.1	35150001	35170001
NC_056070.1	51265001	51285001	2.30035	0.304545	BD	NCOR2	NW_02459972:	20001	40001
NC_056076.1	37360001	37380001	3.67077	0.398677	BD	NDC80	NW_02459972:	25001	45001
NC_056076.1	37365001	37385001	3.05566	0.371281	BD	NDC80	NC_056067.1	51550001	51570001
NC_056080.1	57660001	57680001	4.17295	0.295246	BD	NDUFB11	NC_056067.1	51555001	51575001
NC_056054.1	2.59E+08	259405001	2.94533	0.521978	BD	NEK11	NC_056067.1	51560001	51580001
NC_056056.1	1.42E+08	141900001	3.19209	0.446018	BD	NELL2	NC_056067.1	51565001	51585001
NC_056055.1	1.28E+08	128145001	2.33641	0.39085	BD	NEUROD1	NC_056067.1	51570001	51590001
NC_056080.1	15915001	15935001	5.77366	0.650133	BD	NHS	NC_056069.1	23810001	23830001
NC_056080.1	15910001	15930001	7.19867	0.640359	BD	NHS	NC_056069.1	23815001	23835001
NC_056080.1	15920001	15940001	2.34586	0.521835	BD	NHS	NC_056080.1	81840001	81860001
NC_056080.1	15785001	15805001	3.3795	0.492378	BD	NHS	NC_056080.1	81845001	81865001
NC_056080.1	15630001	15650001	2.51315	0.395866	BD	NHS	NC_056080.1	131460001	131480001
NC_056080.1	15770001	15790001	6.37513	0.3837	BD	NHS	NC_056080.1	131465001	131485001
NC_056080.1	15625001	15645001	2.4972	0.349781	BD	NHS	NC_056055.1	146885001	146905001
NC_056080.1	15765001	15785001	3.55047	0.324965	BD	NHS	NC_056055.1	146890001	146910001
NC_056080.1	15740001	15760001	9.38345	0.316634	BD	NHS	NC_056054.1	116405001	116425001
NC_056080.1	15735001	15755001	7.32074	0.306721	BD	NHS	NC_056054.1	116410001	116430001
NC_056055.1	2.36E+08	236150001	3.69261	0.338493	BD	NKAIN1	NC_056054.1	116415001	116435001
NC_056055.1	2.36E+08	236155001	2.49636	0.332817	BD	NKAIN1	NC_056054.1	116420001	116440001
NC_056061.1	13940001	13960001	2.39178	0.468392	BD	NKAIN2	NC_056054.1	116425001	116445001
NC_056061.1	13935001	13955001	2.49949	0.429986	BD	NKAIN2	NC_056056.1	116420001	116440001
NC_056061.1	13930001	13950001	2.45901	0.408742	BD	NKAIN2	NC_056056.1	116425001	116445001
NC_056061.1	13955001	13975001	2.43572	0.324431	BD	NKAIN2	NC_056056.1	116430001	116450001
NC_056069.1	71345001	71365001	2.201	0.333585	BD	NKD2;SLC12A7	NC_056056.1	116435001	116455001

NC_056066.1	40245001	40265001	4.43396	0.377976	BD	NKX2-4	NC_056056.1	116440001	116460001
NC_056066.1	40240001	40260001	2.95082	0.402973	BD	NKX2-4;XRN2	NC_056059.1	113740001	113760001
NC_056066.1	40235001	40255001	2.26437	0.345818	BD	NKX2-4;XRN2	NC_056059.1	116285001	116305001
NC_056054.1	2.14E+08	214025001	2.78925	0.300697	BD	NLGN1	NC_056061.1	14935001	14955001
NC_056064.1	19670001	19690001	4.18461	0.712931	BD	NLK	NC_056061.1	14940001	14960001
NC_056064.1	19675001	19695001	3.14508	0.596233	BD	NLK	NC_056061.1	14945001	14965001
NC_056064.1	19680001	19700001	2.12766	0.407887	BD	NLK	NC_056061.1	14950001	14970001
NC_056057.1	67420001	67440001	2.1586	0.350099	BD	NOD1	NC_056061.1	14955001	14975001
NC_056076.1	23495001	23515001	2.72028	0.403917	BD	NOL4	NC_056061.1	14960001	14980001
NC_056076.1	23490001	23510001	2.77492	0.390356	BD	NOL4	NC_056061.1	14965001	14985001
NC_056076.1	23485001	23505001	2.9826	0.358407	BD	NOL4	NC_056061.1	14970001	14990001
NC_056076.1	23480001	23500001	2.18058	0.301441	BD	NOL4	NC_056061.1	14975001	14995001
NC_056055.1	37700001	37720001	2.1875	0.301079	BD	NOL6	NC_056061.1	14980001	15000001
NC_056070.1	57900001	57920001	3.79794	0.340104	BD	NOS1	NC_056054.1	90335001	90355001
NC_056071.1	34520001	34540001	4.14326	0.42115	BD	NOVA1	NC_056054.1	90340001	90360001
NC_056071.1	34525001	34545001	3.34326	0.405611	BD	NOVA1	NC_056054.1	90345001	90365001
NC_056071.1	34515001	34535001	3.23766	0.380622	BD	NOVA1	NC_056070.1	7355001	7375001
NC_056071.1	34530001	34550001	2.68377	0.380228	BD	NOVA1	NC_056063.1	16860001	16880001
NC_056057.1	73210001	73230001	3.66968	0.426679	BD	NPY	NC_056063.1	16865001	16885001
NC_056057.1	73205001	73225001	3.9138	0.421013	BD	NPY	NC_056063.1	16870001	16890001
NC_056057.1	73200001	73220001	2.11742	0.334514	BD	NPY	NC_056063.1	16875001	16895001
NC_056058.1	48760001	48780001	3.03942	0.315476	BD	NRG2	NC_056063.1	16880001	16900001
NC_056058.1	48765001	48785001	2.88757	0.299379	BD	NRG2	NC_056063.1	16885001	16905001
NC_056056.1	73505001	73525001	11.6267	0.456869	BD	NRXN1	NC_056063.1	16890001	16910001
NC_056056.1	73500001	73520001	8.12682	0.452333	BD	NRXN1	NC_056063.1	16895001	16915001
NC_056056.1	73495001	73515001	3.46901	0.402734	BD	NRXN1	NC_056063.1	16900001	16920001
NC_056056.1	74070001	74090001	3.99048	0.392625	BD	NRXN1	NC_056063.1	16905001	16925001
NC_056056.1	74075001	74095001	4.3511	0.380907	BD	NRXN1	NC_056059.1	15915001	15935001
NC_056056.1	74060001	74080001	2.6096	0.37881	BD	NRXN1	NC_056056.1	162410001	162430001
NC_056056.1	74065001	74085001	2.9655	0.37837	BD	NRXN1	NC_056056.1	162395001	162415001
NC_056056.1	73595001	73615001	2.98318	0.357179	BD	NRXN1	NC_056056.1	162400001	162420001
NC_056056.1	73600001	73620001	3.67436	0.353254	BD	NRXN1	NC_056056.1	80380001	80400001
NC_056056.1	73590001	73610001	2.28938	0.322308	BD	NRXN1	NC_056056.1	80385001	80405001
NC_056056.1	74080001	74100001	4.30911	0.312961	BD	NRXN1	NC_056060.1	17840001	17860001
NC_056056.1	73605001	73625001	3.97192	0.309549	BD	NRXN1	NC_056060.1	17845001	17865001
NC_056056.1	74085001	74105001	3.85277	0.303813	BD	NRXN1	NC_056054.1	3405001	3425001
NC_056056.1	74090001	74110001	2.20398	0.300726	BD	NRXN1	NC_056054.1	3400001	3420001
NC_056074.1	39400001	39420001	12.3187	0.308004	BD	NRXN2	NC_056074.1	17640001	17660001
NC_056060.1	88690001	88710001	2.25606	0.42568	BD	NRXN3	NC_056074.1	17645001	17665001
NC_056060.1	87430001	87450001	3.50753	0.422109	BD	NRXN3	NC_056060.1	4830001	4850001
NC_056060.1	88685001	88705001	2.38087	0.397663	BD	NRXN3	NC_056062.1	36115001	36135001
NC_056060.1	88680001	88700001	2.24966	0.339063	BD	NRXN3	NC_056055.1	242860001	242880001
NC_056060.1	88015001	88035001	2.3078	0.315651	BD	NRXN3	NC_056077.1	41460001	41480001
NC_056060.1	87960001	87980001	2.22206	0.311247	BD	NRXN3	NC_056077.1	41510001	41530001
NC_056060.1	88020001	88040001	2.25115	0.309243	BD	NRXN3	NC_056077.1	41515001	41535001
NC_056060.1	88025001	88045001	2.19283	0.304785	BD	NRXN3	NC_056077.1	41520001	41540001
NC_056060.1	87955001	87975001	2.33106	0.292298	BD	NRXN3	NC_056077.1	41525001	41545001
NC_056079.1	32510001	32530001	3.54545	0.395542	BD	NSD3;PLPP5	NC_056077.1	41530001	41550001
NC_056056.1	510001	530001	4.19076	0.464795	BD	NSMF;PNPLA7	NC_056077.1	41535001	41555001
NC_056056.1	505001	525001	3.81977	0.45211	BD	NSMF;PNPLA7	NC_056077.1	41540001	41560001
NC_056056.1	500001	520001	3.88343	0.440558	BD	NSMF;PNPLA7	NC_056077.1	41545001	41565001
NC_056056.1	515001	535001	2.96347	0.408474	BD	NSMF;PNPLA7	NC_056077.1	41550001	41570001
NC_056054.1	1.59E+08	159360001	2.53383	0.295722	BD	NSUN3	NC_056077.1	41555001	41575001
NC_056075.1	23490001	23510001	14.915	0.436928	BD	NT5C2	NC_056077.1	41560001	41580001
NC_056075.1	23485001	23505001	5.61182	0.410255	BD	NT5C2	NC_056077.1	41565001	41585001
NC_056075.1	23495001	23515001	6.83151	0.38878	BD	NT5C2	NC_056077.1	41570001	41590001
NC_056075.1	23480001	23500001	2.57346	0.334341	BD	NT5C2	NC_056077.1	41575001	41595001
NC_056074.1	31985001	32005001	17.7156	0.44909	BD	NTM	NC_056077.1	41580001	41600001
NC_056074.1	32055001	32075001	31.1334	0.389433	BD	NTM	NC_056080.1	53685001	53705001
NC_056074.1	32060001	32080001	7.91905	0.369699	BD	NTM	NC_056072.1	35960001	35980001
NC_056074.1	31980001	32000001	3.57003	0.352805	BD	NTM	NC_056072.1	35965001	35985001
NC_056074.1	32065001	32085001	2.80541	0.338737	BD	NTM	NC_056072.1	35970001	35990001
NC_056071.1	18650001	18670001	5.50002	0.631478	BD	NTRK3	NC_056080.1	42840001	42860001
NC_056071.1	18635001	18655001	2.6859	0.530613	BD	NTRK3	NC_056080.1	42845001	42865001
NC_056072.1	6365001	6385001	2.15122	0.409849	BD	OSBPL10	NC_056067.1	12505001	12525001
NC_056055.1	1.32E+08	131965001	3.09608	0.575606	BD	OSBPL6	NC_056078.1	41755001	41775001
NC_056055.1	1.32E+08	131970001	2.58886	0.552052	BD	OSBPL6	NC_056064.1	45670001	45690001
NC_056056.1	1.16E+08	116400001	2.28081	0.450284	BD	OTOGL	NC_056064.1	45675001	45695001
NC_056056.1	1.16E+08	116395001	3.12366	0.434252	BD	OTOGL	NC_056064.1	45680001	45700001
NC_056056.1	1.16E+08	116385001	2.78297	0.431259	BD	OTOGL	NC_056064.1	45685001	45705001
NC_056056.1	1.16E+08	116380001	2.32777	0.426297	BD	OTOGL	NC_056074.1	38665001	38685001
NC_056056.1	1.16E+08	116390001	3.1652	0.419694	BD	OTOGL	NC_056074.1	38670001	38690001
NC_056056.1	1.16E+08	116370001	2.23465	0.418964	BD	OTOGL	NC_056074.1	38690001	38710001
NC_056056.1	1.16E+08	116375001	2.21423	0.403371	BD	OTOGL	NC_056074.1	38695001	38715001

NC_056066.1	37875001	37895001	2.89491	0.348912	BD	OVOL2	NC_056067.1	14235001	14255001
NC_056066.1	37870001	37890001	2.29037	0.339752	BD	OVOL2	NC_056067.1	14240001	14260001
NC_056066.1	37880001	37900001	3.19426	0.329359	BD	OVOL2	NC_056054.1	237505001	237525001
NC_056066.1	37885001	37905001	3.47618	0.311352	BD	OVOL2	NC_056063.1	18245001	18265001
NC_056071.1	67420001	67440001	4.71163	0.537341	BD	PACS2;TEX22	NC_056063.1	18250001	18270001
NC_056071.1	67415001	67435001	2.29724	0.36896	BD	PACS2;TEX22	NC_056058.1	66375001	66395001
NW_024599828.1	1010001	1030001	2.72285	0.302366	BD	PAG3	NC_056058.1	66380001	66400001
NW_024599828.1	1015001	1035001	3.55567	0.291703	BD	PAG3	NC_056058.1	66385001	66405001
NC_056057.1	72800001	72820001	4.20593	0.323014	BD	PALS2	NC_056059.1	37500001	37520001
NC_056057.1	72805001	72825001	3.17241	0.311679	BD	PALS2	NC_056057.1	53095001	53115001
NC_056057.1	72810001	72830001	3.4101	0.308853	BD	PALS2	NC_056057.1	53100001	53120001
NC_056057.1	72795001	72815001	3.08761	0.295009	BD	PALS2	NC_056057.1	53110001	53130001
NC_056066.1	18385001	18405001	4.28631	0.345336	BD	PARD3	NC_056057.1	53115001	53135001
NC_056066.1	18390001	18410001	7.13056	0.328585	BD	PARD3	NC_056057.1	53120001	53140001
NC_056056.1	2.21E+08	220690001	3.55313	0.421809	BD	PARVB	NC_056057.1	53125001	53145001
NC_056056.1	2.21E+08	220695001	3.55756	0.326677	BD	PARVB	NC_056057.1	53150001	53170001
NC_056056.1	1.16E+08	115695001	3.09823	0.294209	BD	PAWR	NC_056057.1	53155001	53175001
NC_056072.1	48275001	48295001	2.13227	0.531695	BD	PBRM1	NC_056057.1	53160001	53180001
NC_056072.1	48270001	48290001	2.26766	0.528787	BD	PBRM1	NC_056057.1	53165001	53185001
NC_056074.1	41290001	41310001	2.25235	0.367911	BD	PC	NC_056068.1	57680001	57700001
NC_056054.1	2.66E+08	266250001	2.66105	0.533188	BD	PCBP3	NC_056068.1	57685001	57705001
NC_056054.1	2.66E+08	266245001	2.73114	0.532713	BD	PCBP3	NC_056056.1	118410001	118430001
NC_056054.1	2.66E+08	266240001	4.4451	0.432409	BD	PCBP3	NC_056056.1	118415001	118435001
NC_056063.1	76325001	76345001	4.3896	0.362497	BD	PCCA	NC_056056.1	118420001	118440001
NC_056063.1	76320001	76340001	2.9375	0.311554	BD	PCCA	NC_056056.1	118510001	118530001
NC_056075.1	4565001	4585001	2.80991	0.292735	BD	PCDH15	NC_056056.1	118515001	118535001
NC_056058.1	93370001	93390001	2.78658	0.447458	BD	PCSK1	NC_056056.1	118520001	118540001
NC_056058.1	93365001	93385001	7.89547	0.441309	BD	PCSK1	NC_056056.1	118525001	118545001
NC_056058.1	93360001	93380001	13	0.413696	BD	PCSK1	NC_056056.1	118530001	118550001
NC_056058.1	93355001	93375001	20.9841	0.38431	BD	PCSK1	NC_056076.1	37305001	37325001
NC_056058.1	93350001	93370001	7.06845	0.304473	BD	PCSK1	NC_056076.1	37310001	37330001
NC_056080.1	22940001	22960001	2.16072	0.300595	BD	PCYT1B	NC_056076.1	37315001	37335001
NC_056057.1	66145001	66165001	11.6402	0.437306	BD	PDE1C	NC_056076.1	37320001	37340001
NC_056057.1	66155001	66175001	25.9024	0.394536	BD	PDE1C	NC_056076.1	37325001	37345001
NC_056057.1	66150001	66170001	18.0407	0.390764	BD	PDE1C	NC_056076.1	37330001	37350001
NC_056057.1	66160001	66180001	33.991	0.383641	BD	PDE1C	NC_056076.1	37335001	37355001
NC_056057.1	66165001	66185001	22.0608	0.349821	BD	PDE1C	NC_056076.1	37340001	37360001
NC_056057.1	66115001	66135001	2.53163	0.332002	BD	PDE1C	NC_056076.1	37345001	37365001
NC_056057.1	66170001	66190001	13.7231	0.326064	BD	PDE1C	NC_056076.1	37350001	37370001
NC_056057.1	66120001	66140001	2.61242	0.318736	BD	PDE1C	NC_056076.1	37355001	37375001
NC_056057.1	66105001	66125001	2.16914	0.316906	BD	PDE1C	NC_056055.1	118840001	118860001
NC_056057.1	66110001	66130001	2.12207	0.31636	BD	PDE1C	NC_056070.1	18095001	18115001
NC_056057.1	66175001	66195001	4.6313	0.306019	BD	PDE1C	NC_056070.1	18100001	18120001
NC_056068.1	37830001	37850001	2.82153	0.32014	BD	PDE3B	NC_056068.1	40135001	40155001
NC_056068.1	37825001	37845001	2.64515	0.319252	BD	PDE3B	NC_056056.1	102145001	102165001
NC_056058.1	58960001	58980001	2.82185	0.349477	BD	PDE6A;PPARGC1B	NC_056056.1	102135001	102155001
NC_056058.1	58955001	58975001	2.69882	0.343483	BD	PDE6A;PPARGC1B	NC_056056.1	102140001	102160001
NC_056058.1	58950001	58970001	2.34417	0.317008	BD	PDE6A;PPARGC1B	NC_056058.1	19280001	19300001
NC_056058.1	58995001	59015001	2.19276	0.397808	BD	PDE6A;SLC26A2	NC_056058.1	19285001	19305001
NC_056054.1	2.64E+08	263700001	4.38898	0.321849	BD	PDE9A	NC_056056.1	225375001	225395001
NC_056054.1	2.64E+08	263705001	3.71915	0.308344	BD	PDE9A	NC_056056.1	225380001	225400001
NC_056064.1	48260001	48280001	2.92308	0.337437	BD	PECAM1	NC_056056.1	225385001	225405001
NC_056060.1	66790001	66810001	6.04312	0.351289	BD	PELI2	NC_056056.1	225360001	225380001
NC_056054.1	2.08E+08	208010001	2.54472	0.33272	BD	PEX5L	NC_056056.1	225365001	225385001
NC_056054.1	2.18E+08	217950001	2.14229	0.411817	BD	PHC3	NC_056056.1	225370001	225390001
NC_056054.1	2.18E+08	217955001	2.33397	0.407408	BD	PHC3	NC_056055.1	159875001	159895001
NC_056054.1	2.18E+08	217920001	2.35556	0.32002	BD	PHC3	NC_056055.1	159880001	159900001
NC_056057.1	20075001	20095001	5.86665	0.354767	BD	PHF14	NC_056055.1	159885001	159905001
NC_056057.1	20080001	20100001	3.05982	0.295536	BD	PHF14	NC_056055.1	159890001	159910001
NC_056054.1	97085001	97105001	3.20254	0.378796	BD	PHGDH	NC_056055.1	159895001	159915001
NC_056054.1	97075001	97095001	2.59901	0.371019	BD	PHGDH	NC_056055.1	159900001	159920001
NC_056054.1	97080001	97100001	2.30694	0.350552	BD	PHGDH	NC_056056.1	225410001	225430001
NC_056054.1	97070001	97090001	2.24786	0.344288	BD	PHGDH	NC_056056.1	225415001	225435001
NC_056054.1	97090001	97110001	2.87553	0.343005	BD	PHGDH	NC_056056.1	225420001	225440001
NC_056067.1	51405001	51425001	2.21179	0.297359	BD	PHLDB3	NC_056077.1	695001	715001
NC_056059.1	45375001	45395001	3.24239	0.452282	BD	PI4K2B	NC_056064.1	43550001	43570001
NC_056059.1	45380001	45400001	3.84559	0.427003	BD	PI4K2B	NC_056056.1	62710001	62730001
NC_056059.1	45370001	45390001	3.25845	0.426749	BD	PI4K2B	NC_056065.1	55585001	55605001
NC_056059.1	45365001	45385001	2.43032	0.399237	BD	PI4K2B	NC_056065.1	55595001	55615001
NC_056059.1	45385001	45405001	2.60242	0.371711	BD	PI4K2B	NC_056065.1	55600001	55620001
NC_056059.1	45390001	45410001	2.90613	0.304272	BD	PI4K2B	NC_056054.1	52015001	52035001
NC_056076.1	14240001	14260001	15.116	0.456028	BD	PIK3C3	NC_056054.1	52020001	52040001
NC_056076.1	14235001	14255001	15.0336	0.445923	BD	PIK3C3	NC_056054.1	52025001	52045001
NC_056076.1	14230001	14250001	7.15556	0.408144	BD	PIK3C3	NC_056070.1	62815001	62835001

NC_056076.1	14225001	14245001	2.53328	0.33188	BD	PIK3C3	NC_056070.1	62790001	62810001
NC_056076.1	14285001	14305001	21.189	0.292521	BD	PIK3C3	NC_056056.1	81550001	81570001
NC_056067.1	7275001	7295001	13.3986	0.516709	BD	PKD1L2	NC_056056.1	81555001	81575001
NC_056067.1	7270001	7290001	8.33332	0.508137	BD	PKD1L2	NC_056056.1	81565001	81585001
NC_056067.1	7280001	7300001	17.752	0.46137	BD	PKD1L2	NC_056056.1	81570001	81590001
NC_056067.1	7290001	7310001	5.13702	0.378958	BD	PKD1L2	NC_056062.1	43005001	43025001
NC_056067.1	7295001	7315001	3.05106	0.306269	BD	PKD1L2	NC_056062.1	43010001	43030001
NC_056056.1	7675001	7695001	2.25227	0.319519	BD	PKN3;ZDHHC12	NC_056062.1	43015001	43035001
NC_056067.1	7900001	7920001	2.84816	0.312661	BD	PLCG2	NC_056062.1	43020001	43040001
NC_056054.1	2.16E+08	216490001	20.1802	0.516703	BD	PLD1	NC_056062.1	43025001	43045001
NC_056054.1	2.16E+08	216495001	5.99134	0.482566	BD	PLD1	NC_056060.1	74610001	74630001
NC_056054.1	2.16E+08	216485001	16.9237	0.482266	BD	PLD1	NC_056060.1	74615001	74635001
NC_056054.1	2.16E+08	216500001	3.0254	0.456552	BD	PLD1	NC_056060.1	74620001	74640001
NC_056054.1	2.16E+08	216480001	16.5664	0.432324	BD	PLD1	NC_056070.1	69085001	69105001
NC_056054.1	2.16E+08	216460001	5.94118	0.406999	BD	PLD1	NC_056070.1	69090001	69110001
NC_056054.1	2.16E+08	216465001	16.4156	0.401414	BD	PLD1	NC_056065.1	41650001	41670001
NC_056054.1	2.16E+08	216455001	3.05499	0.369704	BD	PLD1	NC_056065.1	41745001	41765001
NC_056057.1	67845001	67865001	2.17257	0.386131	BD	PLEKHA8	NC_056065.1	41750001	41770001
NC_056057.1	67840001	67860001	2.24836	0.320489	BD	PLEKHA8	NC_056065.1	41755001	41775001
NC_056057.1	67810001	67830001	2.15384	0.308206	BD	PLEKHA8	NC_056065.1	41760001	41780001
NC_056054.1	2.43E+08	243020001	12.5619	0.468636	BD	PLOD2	NC_056060.1	10910001	10930001
NC_056054.1	2.43E+08	243015001	12.7869	0.466228	BD	PLOD2	NC_056063.1	83280001	83300001
NC_056054.1	2.43E+08	243000001	11.3289	0.462391	BD	PLOD2	NC_056063.1	83285001	83305001
NC_056054.1	2.43E+08	243010001	12	0.458541	BD	PLOD2	NC_056063.1	83290001	83310001
NC_056054.1	2.43E+08	243005001	9.5767	0.453588	BD	PLOD2	NC_056063.1	83295001	83315001
NC_056054.1	2.43E+08	243025001	8.57141	0.439171	BD	PLOD2	NC_056063.1	83300001	83320001
NC_056054.1	2.43E+08	242995001	5.11881	0.384161	BD	PLOD2	NC_056063.1	83330001	83350001
NC_056054.1	2.43E+08	243030001	3.43617	0.348553	BD	PLOD2	NC_056063.1	83335001	83355001
NC_056054.1	2.43E+08	242990001	3.67404	0.340832	BD	PLOD2	NC_056056.1	162550001	162570001
NC_056054.1	2.43E+08	243035001	2.53498	0.30372	BD	PLOD2	NC_056066.1	25855001	25875001
NC_056054.1	2.43E+08	243040001	2.29063	0.298921	BD	PLOD2	NC_056066.1	25860001	25880001
NC_056069.1	23905001	23925001	9.05838	0.32948	BD	PLPP1	NC_056060.1	56275001	56295001
NC_056069.1	23840001	23860001	6.72379	0.32931	BD	PLPP1	NC_056060.1	56280001	56300001
NC_056069.1	23900001	23920001	8.01116	0.31899	BD	PLPP1	NC_056076.1	37815001	37835001
NC_056069.1	23830001	23850001	5.14284	0.318376	BD	PLPP1	NC_056076.1	37820001	37840001
NC_056069.1	23895001	23915001	9.20709	0.3177	BD	PLPP1	NC_056076.1	37860001	37880001
NC_056069.1	23890001	23910001	8.87349	0.314671	BD	PLPP1	NC_056074.1	39695001	39715001
NC_056069.1	23835001	23855001	6.32379	0.313786	BD	PLPP1	NC_056074.1	39700001	39720001
NC_056069.1	23880001	23900001	6.1544	0.305732	BD	PLPP1	NC_056054.1	212670001	212690001
NC_056069.1	23885001	23905001	7.86792	0.30553	BD	PLPP1	NC_056056.1	112090001	112110001
NC_056069.1	23825001	23845001	3.09813	0.301589	BD	PLPP1	NC_056063.1	26585001	26605001
NC_056069.1	23875001	23895001	4.59701	0.301586	BD	PLPP1	NC_056063.1	26590001	26610001
NC_056069.1	23845001	23865001	4.90001	0.296899	BD	PLPP1	NC_056063.1	26595001	26615001
NC_056069.1	23855001	23875001	4.46602	0.296164	BD	PLPP1	NC_056063.1	26600001	26620001
NC_056054.1	2.47E+08	247025001	20.4943	0.676001	BD	PLS1	NC_056068.1	23215001	23235001
NC_056054.1	2.47E+08	247030001	2.10714	0.445554	BD	PLS1	NC_056068.1	23220001	23240001
NC_056056.1	490001	510001	4.64147	0.476693	BD	PNPLA7	NC_056068.1	23225001	23245001
NC_056056.1	480001	500001	4.14542	0.454006	BD	PNPLA7	NC_056068.1	23390001	23410001
NC_056056.1	485001	505001	3.77523	0.445103	BD	PNPLA7	NC_056068.1	23395001	23415001
NC_056056.1	495001	515001	3.50772	0.438586	BD	PNPLA7	NC_056068.1	23400001	23420001
NC_056056.1	475001	495001	2.8525	0.409512	BD	PNPLA7	NC_056068.1	82050001	82070001
NC_056060.1	7100001	7120001	2.14375	0.345848	BD	POC5	NC_056068.1	82055001	82075001
NC_056060.1	6945001	6965001	30.1649	0.47946	BD	POLK	NC_056068.1	82060001	82080001
NC_056060.1	6950001	6970001	32.7619	0.478178	BD	POLK	NC_056068.1	82065001	82085001
NC_056060.1	6955001	6975001	29.8831	0.435004	BD	POLK	NC_056055.1	126815001	126835001
NC_056060.1	6960001	6980001	25.2337	0.385905	BD	POLK	NC_056055.1	233210001	233230001
NC_056058.1	58915001	58935001	3.05491	0.387128	BD	PPARGC1B	NC_056055.1	233215001	233235001
NC_056058.1	58910001	58930001	2.26866	0.375175	BD	PPARGC1B	NC_056055.1	233220001	233240001
NC_056058.1	58920001	58940001	2.46396	0.374718	BD	PPARGC1B	NC_056070.1	51240001	51260001
NC_056058.1	58925001	58945001	2.1828	0.329749	BD	PPARGC1B	NC_056070.1	51250001	51270001
NC_056056.1	1.88E+08	187750001	3.25092	0.363821	BD	PPFIBP1	NC_056070.1	51255001	51275001
NC_056056.1	1.88E+08	187755001	3.4139	0.327034	BD	PPFIBP1	NC_056070.1	51370001	51390001
NC_056073.1	10855001	10875001	27.6309	0.307317	BD	PPIL1	NC_056070.1	51375001	51395001
NC_056073.1	10850001	10870001	24.9256	0.307019	BD	PPIL1	NC_056070.1	51380001	51400001
NC_056060.1	70535001	70555001	5.56731	0.503237	BD	PPM1A	NC_056076.1	37360001	37380001
NC_056060.1	70530001	70550001	5.22831	0.497851	BD	PPM1A	NC_056076.1	37365001	37385001
NC_056060.1	70540001	70560001	3.57851	0.458339	BD	PPM1A	NC_056064.1	27800001	27820001
NC_056060.1	70525001	70545001	3.81027	0.456005	BD	PPM1A	NC_056056.1	86830001	86850001
NC_056060.1	70520001	70540001	3.89309	0.455152	BD	PPM1A	NC_056054.1	47815001	47835001
NC_056060.1	70545001	70565001	2.87314	0.421448	BD	PPM1A	NC_056054.1	47820001	47840001
NC_056060.1	70515001	70535001	3.20513	0.395743	BD	PPM1A	NC_056054.1	47825001	47845001
NC_056056.1	80095001	80115001	6.61249	0.500034	BD	PPM1B	NC_056054.1	47830001	47850001
NC_056056.1	80090001	80110001	5.82292	0.490062	BD	PPM1B	NC_056074.1	21765001	21785001
NC_056056.1	80100001	80120001	4.93082	0.466438	BD	PPM1B	NC_056074.1	21770001	21790001

NC_056056.1	80085001	80105001	4.60957	0.465045	BD	PPM1B	NC_056060.1	19715001	19735001
NC_056056.1	80105001	80125001	4.3453	0.45084	BD	PPM1B	NC_056080.1	15625001	15645001
NC_056056.1	80080001	80100001	3.30623	0.441324	BD	PPM1B	NC_056080.1	15630001	15650001
NC_056056.1	80140001	80160001	2.2886	0.387904	BD	PPM1B	NC_056080.1	15735001	15755001
NC_056056.1	80135001	80155001	2.52586	0.35673	BD	PPM1B	NC_056080.1	15740001	15760001
NC_056056.1	80110001	80130001	2.13135	0.32616	BD	PPM1B	NC_056080.1	15765001	15785001
NC_056056.1	80145001	80165001	2.45126	0.324596	BD	PPM1B	NC_056080.1	15770001	15790001
NC_056056.1	80130001	80150001	2.12889	0.316623	BD	PPM1B	NC_056080.1	15785001	15805001
NC_056056.1	80150001	80170001	3.02624	0.302243	BD	PPM1B	NC_056069.1	31600001	31620001
NC_056059.1	37110001	37130001	3.55017	0.328181	BD	PPM1K	NC_056069.1	31605001	31625001
NC_056059.1	37115001	37135001	2.3542	0.324975	BD	PPM1K	NC_056069.1	31610001	31630001
NC_056059.1	37105001	37125001	3.7458	0.318645	BD	PPM1K	NC_056069.1	31615001	31635001
NC_056074.1	36505001	36525001	2.62313	0.36826	BD	PPP1R32	NC_056061.1	13895001	13915001
NC_056074.1	36510001	36530001	2.91261	0.315192	BD	PPP1R32	NC_056061.1	13900001	13920001
NC_056058.1	55725001	55745001	2.31423	0.379701	BD	PPP2R2B	NC_056062.1	40410001	40430001
NC_056058.1	55720001	55740001	2.34328	0.360118	BD	PPP2R2B	NC_056062.1	40415001	40435001
NC_056059.1	23925001	23945001	9.51733	0.361051	BD	PPP3CA	NC_056062.1	40420001	40440001
NC_056059.1	23930001	23950001	6.06449	0.313755	BD	PPP3CA	NC_056062.1	40425001	40445001
NC_056071.1	56700001	56720001	5.71536	0.300426	BD	PPP4R4	NC_056062.1	40430001	40450001
NC_056071.1	56705001	56725001	6.00321	0.29945	BD	PPP4R4	NC_056062.1	40435001	40455001
NC_056072.1	51180001	51200001	3.47442	0.335788	BD	PRKAR2A	NC_056062.1	40470001	40490001
NC_056064.1	61905001	61925001	2.58264	0.316122	BD	PRKCA	NC_056080.1	3695001	3715001
NC_056064.1	61910001	61930001	3.42196	0.312845	BD	PRKCA	NC_056080.1	3700001	3720001
NC_056061.1	85400001	85420001	2.36235	0.310213	BD	PRKN	NC_056080.1	3705001	3725001
NC_056061.1	85395001	85415001	2.40056	0.297657	BD	PRKN	NC_056064.1	19595001	19615001
NC_056059.1	1.12E+08	112050001	8.1392	0.380631	BD	PROM1	NC_056064.1	19600001	19620001
NC_056059.1	1.12E+08	112060001	18.4758	0.366642	BD	PROM1	NC_056064.1	19670001	19690001
NC_056059.1	1.12E+08	112055001	15.4475	0.358362	BD	PROM1	NC_056064.1	19675001	19695001
NC_056059.1	1.12E+08	112045001	3.23517	0.355413	BD	PROM1	NC_056064.1	19680001	19700001
NC_056059.1	1.12E+08	112065001	14.4539	0.353387	BD	PROM1	NC_056058.1	38435001	38455001
NC_056056.1	1.04E+08	104445001	7.03201	0.429141	BD	PROM2	NC_056058.1	38445001	38465001
NC_056056.1	1.04E+08	104450001	5.2235	0.407018	BD	PROM2	NC_056067.1	36350001	36370001
NC_056056.1	1.04E+08	104455001	2.84172	0.340934	BD	PROM2	NC_056076.1	23180001	23200001
NC_056066.1	15910001	15930001	13.2578	0.297706	BD	PROSER2	NC_056076.1	23185001	23205001
NC_056056.1	1.37E+08	136955001	3.73469	0.356123	BD	PRPH	NC_056076.1	23190001	23210001
NC_056056.1	1.37E+08	136960001	4.79435	0.334459	BD	PRPH	NC_056077.1	700001	720001
NC_056077.1	41750001	41770001	3.97446	0.366803	BD	PSMG3	NC_056057.1	73205001	73225001
NC_056077.1	41755001	41775001	3.73779	0.313494	BD	PSMG3;TMEM184A	NC_056057.1	73210001	73230001
NC_056073.1	49565001	49585001	2.28463	0.394125	BD	PSMG4;SLC22A23	NC_056078.1	36140001	36160001
NC_056073.1	49570001	49590001	2.64151	0.34772	BD	PSMG4;SLC22A23	NC_056078.1	36145001	36165001
NC_056054.1	19500001	19520001	2.60588	0.299991	BD	PTCH2	NC_056078.1	36305001	36325001
NC_056055.1	13930001	13950001	13.8086	0.383544	BD	PTPN3	NC_056078.1	36310001	36330001
NC_056055.1	13935001	13955001	16.2055	0.366653	BD	PTPN3	NC_056056.1	73290001	73310001
NC_056055.1	13940001	13960001	7.53622	0.318521	BD	PTPN3	NC_056056.1	73295001	73315001
NC_056055.1	13925001	13945001	7.74644	0.317118	BD	PTPN3	NC_056056.1	73380001	73400001
NC_056072.1	39220001	39240001	2.11238	0.519721	BD	PTPRG	NC_056056.1	73385001	73405001
NC_056072.1	39215001	39235001	3.14095	0.511908	BD	PTPRG	NC_056056.1	73390001	73410001
NC_056057.1	1.21E+08	120715001	2.29875	0.3424	BD	PTPRN2	NC_056056.1	73820001	73840001
NC_056057.1	1.21E+08	120710001	2.5362	0.337497	BD	PTPRN2	NC_056056.1	73825001	73845001
NC_056072.1	50975001	50995001	3.27207	0.328307	BD	QRICH1	NC_056056.1	73830001	73850001
NC_056072.1	50980001	51000001	3.00612	0.3214	BD	QRICH1	NC_056056.1	74055001	74075001
NC_056072.1	50970001	50990001	3.70662	0.321296	BD	QRICH1	NC_056056.1	74060001	74080001
NC_056072.1	50965001	50985001	3.71132	0.312997	BD	QRICH1	NC_056056.1	74065001	74085001
NC_056064.1	55985001	56005001	6.29765	0.334536	BD	RAB37	NC_056056.1	74070001	74090001
NC_056064.1	55980001	56000001	7.20491	0.319973	BD	RAB37;SLC9A3R1	NC_056056.1	74075001	74095001
NC_056054.1	2.56E+08	256140001	2.64261	0.578593	BD	RAB6B	NC_056056.1	74080001	74100001
NC_056054.1	2.56E+08	256145001	2.1831	0.520295	BD	RAB6B	NC_056056.1	74085001	74105001
NC_056054.1	52010001	52030001	5.95569	0.366845	BD	RABGGTB	NC_056056.1	74090001	74110001
NC_056058.1	19645001	19665001	3.79724	0.47029	BD	RAD50	NC_056056.1	74095001	74115001
NC_056058.1	19560001	19580001	2.77833	0.459197	BD	RAD50	NC_056060.1	88285001	88305001
NC_056058.1	19575001	19595001	6.07326	0.435594	BD	RAD50	NC_056060.1	88290001	88310001
NC_056058.1	19585001	19605001	4.67632	0.419265	BD	RAD50	NC_056079.1	32510001	32530001
NC_056058.1	19590001	19610001	3.53164	0.400072	BD	RAD50	NC_056071.1	25975001	25995001
NC_056058.1	19635001	19655001	3.74198	0.399324	BD	RAD50	NC_056071.1	25980001	26000001
NC_056058.1	19640001	19660001	3.63557	0.398203	BD	RAD50	NC_056075.1	23480001	23500001
NC_056058.1	19570001	19590001	4.03891	0.39727	BD	RAD50	NC_056075.1	23485001	23505001
NC_056058.1	19565001	19585001	3.408	0.396412	BD	RAD50	NC_056075.1	23490001	23510001
NC_056058.1	19580001	19600001	6.17264	0.392232	BD	RAD50	NC_056075.1	23495001	23515001
NC_056058.1	19555001	19575001	2.13991	0.348541	BD	RAD50	NC_056074.1	31980001	32000001
NC_056058.1	19595001	19615001	2.72633	0.342752	BD	RAD50	NC_056074.1	31985001	32005001
NC_056060.1	78075001	78095001	4.46728	0.534045	BD	RAD51B	NC_056074.1	32055001	32075001
NC_056060.1	78070001	78090001	2.15927	0.517753	BD	RAD51B	NC_056074.1	32775001	32795001
NC_056072.1	49245001	49265001	3.15772	0.409551	BD	RAD54L2	NC_056074.1	32810001	32830001
NC_056072.1	49240001	49260001	2.99086	0.405522	BD	RAD54L2	NC_056065.1	37055001	37075001

NC_056072.1	49235001	49255001	2.73717	0.392404	BD	RAD54L2	NC_056065.1	37060001	37080001
NC_056072.1	49230001	49250001	3.22868	0.422006	BD	RAD54L2;TEX264	NC_056065.1	37065001	37085001
NC_056072.1	49220001	49240001	3.32744	0.406979	BD	RAD54L2;TEX264	NC_056065.1	37070001	37090001
NC_056072.1	49225001	49245001	2.58075	0.382967	BD	RAD54L2;TEX264	NC_056063.1	54345001	54365001
NC_056079.1	39785001	39805001	19.4058	0.463128	BD	RARB	NC_056063.1	54350001	54370001
NC_056079.1	39780001	39800001	16.7192	0.395485	BD	RARB	NC_056057.1	78480001	78500001
NC_056079.1	39775001	39795001	12.8274	0.367426	BD	RARB	NC_056057.1	78485001	78505001
NC_056079.1	39770001	39790001	7.82992	0.318901	BD	RARB	NC_056057.1	78490001	78510001
NC_056079.1	39800001	39820001	14.9068	0.298783	BD	RARB	NC_056057.1	78495001	78515001
NC_056058.1	78075001	78095001	2.46056	0.319786	BD	RARS1	NC_056074.1	31595001	31615001
NC_056065.1	59015001	59035001	2.76028	0.565159	BD	RASAL2	NC_056074.1	31600001	31620001
NC_056065.1	59010001	59030001	4.05595	0.545307	BD	RASAL2	NC_056074.1	31605001	31625001
NC_056065.1	59005001	59025001	2.65939	0.462134	BD	RASAL2	NC_056076.1	32800001	32820001
NC_056065.1	59000001	59020001	2.72169	0.456696	BD	RASAL2	NC_056076.1	32805001	32825001
NC_056065.1	58995001	59015001	2.20935	0.439389	BD	RASAL2	NC_056076.1	32810001	32830001
NC_056065.1	59295001	59315001	6.75556	0.309892	BD	RASAL2	NC_056076.1	32815001	32835001
NC_056065.1	59315001	59335001	15.9005	0.30631	BD	RASAL2	NC_056076.1	32820001	32840001
NC_056077.1	6615001	6635001	4.48233	0.310955	BD	RBFOX1	NC_056056.1	116290001	116310001
NC_056077.1	6610001	6630001	4.09422	0.305519	BD	RBFOX1	NC_056056.1	116295001	116315001
NC_056077.1	6620001	6640001	3.84906	0.294868	BD	RBFOX1	NC_056056.1	116365001	116385001
NC_056064.1	52360001	52380001	2.13359	0.29859	BD	RBFOX3	NC_056056.1	116370001	116390001
NC_056064.1	52370001	52390001	2.2012	0.294514	BD	RBFOX3	NC_056056.1	116375001	116395001
NC_056066.1	58385001	58405001	2.20174	0.327115	BD	RBM38	NC_056056.1	116395001	116415001
NC_056072.1	4470001	4490001	3.31237	0.535546	BD	RBMS3	NC_056056.1	116400001	116420001
NC_056072.1	4465001	4485001	2.24678	0.494398	BD	RBMS3	NC_056056.1	116405001	116425001
NC_056072.1	4475001	4495001	2.37603	0.466759	BD	RBMS3	NC_056056.1	116410001	116430001
NC_056072.1	4485001	4505001	2.25878	0.403245	BD	RBMS3	NC_056061.1	86400001	86420001
NC_056065.1	42780001	42800001	4.2411	0.399457	BD	RBP7	NC_056061.1	86405001	86425001
NC_056065.1	42805001	42825001	3.5287	0.35643	BD	RBP7	NC_056061.1	86410001	86430001
NC_056068.1	19965001	19985001	2.11073	0.309019	BD	RDX	NC_056061.1	86415001	86435001
NC_056055.1	52420001	52440001	12.3396	0.339139	BD	RECK	NC_056061.1	86425001	86445001
NC_056057.1	46470001	46490001	4.30195	0.352736	BD	RELN	NC_056071.1	67415001	67435001
NC_056057.1	46475001	46495001	3.02174	0.309075	BD	RELN	NC_056071.1	67420001	67440001
NC_056056.1	1E+08	100195001	2.54717	0.369086	BD	RFX8	NC_056073.1	8670001	8690001
NC_056056.1	1E+08	100140001	19.843	0.362306	BD	RFX8	NC_056055.1	248965001	248985001
NC_056056.1	1E+08	100190001	2.47642	0.361178	BD	RFX8	NC_056055.1	248970001	248990001
NC_056056.1	1E+08	100185001	3.88001	0.30215	BD	RFX8	NC_056055.1	248975001	248995001
NC_056080.1	57685001	57705001	4.68719	0.309254	BD	RGN	NC_056055.1	248980001	249000001
NC_056065.1	34775001	34795001	8.60244	0.365771	BD	RGS7	NC_056055.1	248920001	248940001
NC_056065.1	34780001	34800001	20.5009	0.336716	BD	RGS7	NC_056055.1	248925001	248945001
NC_056065.1	63050001	63070001	2.36591	0.352584	BD	RGSL1;RNASEL	NC_056055.1	248930001	248950001
NC_056077.1	34565001	34585001	2.13453	0.34548	BD	RHBDD2	NC_056055.1	248935001	248955001
NC_056062.1	73885001	73905001	3.03041	0.324371	BD	RIMS2	NC_056055.1	248940001	248960001
NC_056065.1	63060001	63080001	2.77277	0.398792	BD	RNASEL	NC_056056.1	220670001	220690001
NC_056065.1	63065001	63085001	3.36741	0.389074	BD	RNASEL	NC_056068.1	81410001	81430001
NC_056065.1	63055001	63075001	2.2919	0.363765	BD	RNASEL	NC_056068.1	81415001	81435001
NC_056064.1	51715001	51735001	9.5085	0.532938	BD	RNF213	NC_056056.1	115670001	115690001
NC_056055.1	52105001	52125001	5.14286	0.549448	BD	RNF38	NC_056056.1	115675001	115695001
NC_056055.1	52125001	52145001	4.04838	0.499302	BD	RNF38	NC_056056.1	115685001	115705001
NC_056055.1	52130001	52150001	2.91639	0.374198	BD	RNF38	NC_056072.1	48270001	48290001
NC_056060.1	47140001	47160001	4.07945	0.421722	BD	RORA	NC_056072.1	48275001	48295001
NC_056060.1	47145001	47165001	3.25895	0.409043	BD	RORA	NC_056058.1	43995001	44015001
NC_056060.1	47135001	47155001	4.73475	0.359565	BD	RORA	NC_056058.1	44000001	44020001
NC_056060.1	47130001	47150001	5.67133	0.331586	BD	RORA	NC_056058.1	44005001	44025001
NC_056060.1	47125001	47145001	7.37553	0.317247	BD	RORA	NC_056058.1	44010001	44030001
NC_056054.1	69615001	69635001	4.09001	0.342833	BD	RPAP2	NC_056063.1	76315001	76335001
NC_056054.1	69605001	69625001	5.48487	0.303534	BD	RPAP2	NC_056063.1	76320001	76340001
NC_056054.1	69610001	69630001	4.82258	0.293632	BD	RPAP2	NC_056063.1	76325001	76345001
NC_056054.1	69595001	69615001	7.01665	0.29127	BD	RPAP2	NC_056054.1	246805001	246825001
NC_056061.1	48155001	48175001	4.38829	0.301554	BD	RRAGD	NC_056054.1	246810001	246830001
NC_056077.1	14185001	14205001	7.41669	0.628476	BD	RRN3	NC_056054.1	246815001	246835001
NC_056077.1	14190001	14210001	3.39732	0.500428	BD	RRN3	NC_056055.1	127655001	127675001
NC_056077.1	14195001	14215001	3.00793	0.449556	BD	RRN3	NC_056060.1	8630001	8650001
NC_056077.1	14200001	14220001	2.79862	0.38077	BD	RRN3	NC_056059.1	59650001	59670001
NC_056077.1	14205001	14225001	3.27211	0.299666	BD	RRN3	NC_056056.1	145545001	145565001
NC_056064.1	22910001	22930001	3.43769	0.335557	BD	RTN4RL1	NC_056056.1	145550001	145570001
NC_056078.1	9160001	9180001	3.60849	0.339804	BD	RYR2	NC_056070.1	29700001	29720001
NC_056055.1	1.07E+08	107395001	2.13167	0.310808	BD	SAP30	NC_056054.1	217930001	217950001
NC_056055.1	1.07E+08	107390001	2.12308	0.294662	BD	SAP30	NC_056054.1	217935001	217955001
NC_056058.1	43765001	43785001	2.2992	0.321234	BD	SAR1B	NC_056055.1	27940001	27960001
NC_056058.1	43760001	43780001	2.11175	0.312118	BD	SAR1B	NC_056055.1	27945001	27965001
NC_056058.1	43770001	43790001	2.45313	0.311419	BD	SAR1B	NC_056058.1	36995001	37015001
NC_056080.1	22190001	22210001	2.16316	0.31455	BD	SAT1	NC_056059.1	45375001	45395001
NC_056055.1	87565001	87585001	3.34106	0.383229	BD	SAXO1	NC_056059.1	45380001	45400001

NC_056055.1	87570001	87590001	2.58576	0.35796	BD	SAXO1	NC_056075.1	17770001	17790001
NC_056068.1	42260001	42280001	5.09386	0.412739	BD	SBF2	NC_056066.1	23030001	23050001
NC_056068.1	42265001	42285001	3.34537	0.392811	BD	SBF2	NC_056067.1	7270001	7290001
NC_056068.1	42255001	42275001	3.56686	0.346157	BD	SBF2	NC_056067.1	7275001	7295001
NC_056068.1	42250001	42270001	4.26907	0.317083	BD	SBF2	NC_056067.1	7280001	7300001
NC_056068.1	42245001	42265001	3.49229	0.2985	BD	SBF2	NC_056067.1	7290001	7310001
NC_056061.1	79310001	79330001	2.82774	0.383319	BD	SCAF8	NC_056075.1	20960001	20980001
NC_056061.1	79315001	79335001	2.8425	0.359067	BD	SCAF8	NC_056056.1	182585001	182605001
NC_056061.1	79320001	79340001	3.51948	0.349891	BD	SCAF8	NC_056066.1	1185001	1205001
NC_056055.1	1.44E+08	144175001	2.11792	0.359518	BD	SCN2A	NC_056067.1	7900001	7920001
NC_056068.1	28595001	28615001	2.43253	0.363044	BD	SCN2B	NC_056067.1	7905001	7925001
NC_056068.1	28600001	28620001	2.7645	0.29576	BD	SCN2B	NC_056054.1	216460001	216480001
NC_056068.1	28575001	28595001	2.13841	0.341493	BD	SCN4B	NC_056054.1	216465001	216485001
NC_056062.1	3450001	3470001	5.08499	0.487929	BD	SDHAF4	NC_056054.1	216485001	216505001
NC_056062.1	3455001	3475001	4.70559	0.479918	BD	SDHAF4	NC_056065.1	51135001	51155001
NC_056062.1	3460001	3480001	3.83308	0.454798	BD	SDHAF4	NC_056065.1	51140001	51160001
NC_056062.1	3465001	3485001	2.99058	0.421681	BD	SDHAF4	NC_056065.1	51145001	51165001
NC_056062.1	3470001	3490001	2.74399	0.401306	BD	SDHAF4	NC_056054.1	76675001	76695001
NC_056062.1	3480001	3500001	2.48596	0.341465	BD	SDHAF4	NC_056054.1	76680001	76700001
NC_056062.1	3475001	3495001	2.23031	0.335312	BD	SDHAF4	NC_056054.1	76685001	76705001
NC_056056.1	62445001	62465001	9.89005	0.539142	BD	SEPTIN10	NC_056054.1	76690001	76710001
NC_056056.1	62440001	62460001	7.51613	0.52972	BD	SEPTIN10	NC_056054.1	247005001	247025001
NC_056056.1	62450001	62470001	7.26984	0.524642	BD	SEPTIN10	NC_056054.1	247010001	247030001
NC_056056.1	62455001	62475001	5.70069	0.500484	BD	SEPTIN10	NC_056054.1	247035001	247055001
NC_056056.1	62415001	62435001	4.46386	0.496722	BD	SEPTIN10	NC_056054.1	247040001	247060001
NC_056056.1	62425001	62445001	5.12687	0.494885	BD	SEPTIN10	NC_056054.1	247045001	247065001
NC_056056.1	62410001	62430001	5.1069	0.494314	BD	SEPTIN10	NC_056066.1	20870001	20890001
NC_056056.1	62430001	62450001	5.39251	0.474259	BD	SEPTIN10	NC_056060.1	6945001	6965001
NC_056056.1	62435001	62455001	5.42056	0.470485	BD	SEPTIN10	NC_056060.1	6950001	6970001
NC_056056.1	62420001	62440001	5.11072	0.466406	BD	SEPTIN10	NC_056060.1	6955001	6975001
NC_056056.1	62405001	62425001	5.75467	0.490675	BD	SEPTIN10;SH3RF3	NC_056063.1	54340001	54360001
NC_056056.1	62460001	62480001	3.64444	0.424754	BD	SEPTIN10;SOWAHC	NC_056056.1	117325001	117345001
NC_056056.1	95065001	95085001	3.14973	0.382543	BD	SFXN5	NC_056056.1	117330001	117350001
NC_056056.1	95070001	95090001	2.41596	0.331664	BD	SFXN5	NC_056056.1	117335001	117355001
NC_056054.1	1.14E+08	113925001	7.05556	0.460393	BD	SH2D1B	NC_056056.1	117340001	117360001
NC_056054.1	1.14E+08	113930001	3.16666	0.353555	BD	SH2D1B	NC_056056.1	117345001	117365001
NC_056080.1	18245001	18265001	8.65253	0.406098	BD	SH3KBP1	NC_056056.1	117350001	117370001
NC_056080.1	18240001	18260001	3.01869	0.391696	BD	SH3KBP1	NC_056056.1	117695001	117715001
NC_056080.1	18115001	18135001	12.0956	0.380608	BD	SH3KBP1	NC_056056.1	117700001	117720001
NC_056080.1	18120001	18140001	9.52703	0.348553	BD	SH3KBP1	NC_056056.1	117705001	117725001
NC_056080.1	18095001	18115001	3.93087	0.338941	BD	SH3KBP1	NC_056056.1	131975001	131995001
NC_056056.1	62400001	62420001	4.0964	0.460847	BD	SH3RF3	NC_056055.1	127660001	127680001
NC_056056.1	62395001	62415001	3.3764	0.425254	BD	SH3RF3	NC_056055.1	127665001	127685001
NC_056056.1	62390001	62410001	2.46923	0.357216	BD	SH3RF3	NC_056055.1	127670001	127690001
NC_056074.1	43970001	43990001	3.26997	0.323448	BD	SHANK2	NC_056055.1	127675001	127695001
NC_056074.1	43975001	43995001	2.30621	0.31727	BD	SHANK2	NC_056055.1	127680001	127700001
NC_056074.1	43965001	43985001	3.4517	0.303312	BD	SHANK2	NC_056055.1	127685001	127705001
NC_056077.1	12415001	12435001	8.32307	0.496906	BD	SHISA9	NC_056055.1	127690001	127710001
NC_056077.1	12420001	12440001	4.76232	0.46061	BD	SHISA9	NC_056055.1	127695001	127715001
NC_056077.1	12425001	12445001	2.98782	0.452186	BD	SHISA9	NC_056058.1	55720001	55740001
NC_056077.1	12410001	12430001	4.63793	0.40702	BD	SHISA9	NC_056058.1	55725001	55745001
NC_056061.1	70400001	70420001	6.71052	0.523191	BD	SHPRH	NC_056059.1	23915001	23935001
NC_056061.1	70435001	70455001	4.31236	0.517909	BD	SHPRH	NC_056059.1	23920001	23940001
NC_056061.1	70395001	70415001	6.22802	0.512921	BD	SHPRH	NC_056059.1	23925001	23945001
NC_056061.1	70405001	70425001	3.87931	0.481341	BD	SHPRH	NC_056059.1	23930001	23950001
NC_056061.1	70390001	70410001	4.16164	0.474957	BD	SHPRH	NC_056059.1	23935001	23955001
NC_056061.1	70430001	70450001	2.13878	0.452749	BD	SHPRH	NC_056064.1	43555001	43575001
NC_056061.1	70440001	70460001	2.95703	0.450799	BD	SHPRH	NC_056065.1	49110001	49130001
NC_056061.1	70365001	70385001	2.50961	0.442789	BD	SHPRH	NC_056065.1	49115001	49135001
NC_056061.1	70350001	70370001	4.93289	0.406088	BD	SHPRH	NC_056065.1	49120001	49140001
NC_056061.1	70385001	70405001	2.43809	0.405444	BD	SHPRH	NC_056065.1	49125001	49145001
NC_056061.1	70345001	70365001	3.07272	0.328588	BD	SHPRH	NC_056065.1	49135001	49155001
NC_056074.1	25595001	25615001	4.79366	0.363043	BD	SIAE;SPA17	NC_056065.1	49140001	49160001
NC_056068.1	27360001	27380001	2.1622	0.358773	BD	SIK3	NC_056065.1	66835001	66855001
NC_056068.1	27405001	27425001	2.20569	0.354821	BD	SIK3	NC_056065.1	66840001	66860001
NC_056068.1	27345001	27365001	2.13281	0.321797	BD	SIK3	NC_056064.1	61905001	61925001
NC_056068.1	27365001	27385001	2.83451	0.300349	BD	SIK3	NC_056064.1	61910001	61930001
NC_056068.1	27400001	27420001	2.99849	0.299145	BD	SIK3	NC_056064.1	61915001	61935001
NC_056057.1	70795001	70815001	14.7571	0.62285	BD	SKAP2	NC_056064.1	61920001	61940001
NC_056057.1	70800001	70820001	8.92856	0.565209	BD	SKAP2	NC_056064.1	61970001	61990001
NC_056057.1	70865001	70885001	12.889	0.529925	BD	SKAP2	NC_056064.1	61975001	61995001
NC_056057.1	70870001	70890001	12.0762	0.503122	BD	SKAP2	NC_056064.1	61980001	62000001
NC_056057.1	70875001	70895001	9.48616	0.467779	BD	SKAP2	NC_056080.1	5585001	5605001
NC_056057.1	70880001	70900001	4.92555	0.441447	BD	SKAP2	NC_056059.1	112045001	112065001

NC_056057.1	70760001	70780001	6.63158	0.3345	BD	SKAP2	NC_056056.1	136935001	136955001
NC_056056.1	43290001	43310001	4.78265	0.315678	BD	SLC1A4	NC_056056.1	136940001	136960001
NC_056066.1	46935001	46955001	2.43201	0.313405	BD	SLC23A2	NC_056058.1	30875001	30895001
NC_056066.1	46940001	46960001	3.15352	0.305972	BD	SLC23A2	NC_056060.1	52875001	52895001
NC_056066.1	46945001	46965001	3.16103	0.295245	BD	SLC23A2	NC_056060.1	52880001	52900001
NC_056055.1	87980001	88000001	2.1871	0.325342	BD	SLC24A2	NC_056060.1	52885001	52905001
NC_056055.1	87985001	88005001	2.15325	0.321625	BD	SLC24A2	NC_056071.1	29875001	29895001
NC_056055.1	88010001	88030001	2.93282	0.318698	BD	SLC24A2	NC_056071.1	29880001	29900001
NC_056055.1	88005001	88025001	2.90232	0.300544	BD	SLC24A2	NC_056071.1	29885001	29905001
NC_056055.1	88015001	88035001	2.8911	0.299667	BD	SLC24A2	NC_056069.1	9370001	9390001
NC_056055.1	88020001	88040001	2.24898	0.29636	BD	SLC24A2	NC_056069.1	9375001	9395001
NC_056064.1	55535001	55555001	3.40106	0.345875	BD	SLC25A19	NC_056069.1	9380001	9400001
NC_056065.1	43200001	43220001	2.23152	0.37684	BD	SLC25A33	NC_056069.1	9385001	9405001
NC_056080.1	1.15E+08	115035001	2.97323	0.291708	BD	SLC25A43	NC_056072.1	39215001	39235001
NC_056058.1	1.07E+08	107250001	4.72467	0.402481	BD	SLC25A46	NC_056072.1	39220001	39240001
NC_056058.1	1.07E+08	107245001	3.58105	0.375723	BD	SLC25A46	NC_056072.1	39560001	39580001
NC_056058.1	1.07E+08	107240001	2.69302	0.369473	BD	SLC25A46	NC_056057.1	48095001	48115001
NC_056058.1	1.07E+08	107255001	4.02857	0.355841	BD	SLC25A46	NC_056057.1	48100001	48120001
NC_056058.1	1.07E+08	107260001	2.97647	0.329219	BD	SLC25A46	NC_056056.1	194330001	194350001
NC_056058.1	1.07E+08	107265001	3.1548	0.302548	BD	SLC25A46	NC_056064.1	43560001	43580001
NC_056058.1	1.07E+08	107235001	2.12272	0.372611	BD	SLC25A46;TMEM232	NC_056064.1	43565001	43585001
NC_056080.1	1.3E+08	129800001	4.96729	0.49923	BD	SLC25A53	NC_056059.1	4340001	4360001
NC_056080.1	1.3E+08	129795001	3	0.436583	BD	SLC25A53	NC_056055.1	175215001	175235001
NC_056074.1	40835001	40855001	3.60938	0.355644	BD	SLC29A2	NC_056055.1	175220001	175240001
NC_056074.1	40830001	40850001	4.57778	0.354641	BD	SLC29A2	NC_056065.1	55505001	55525001
NC_056056.1	1.48E+08	147600001	22.744	0.310078	BD	SLC2A13	NC_056065.1	55520001	55540001
NC_056056.1	1.48E+08	147595001	15.2432	0.307461	BD	SLC2A13	NC_056065.1	55525001	55545001
NC_056056.1	1.48E+08	147590001	10.2412	0.302176	BD	SLC2A13	NC_056065.1	55530001	55550001
NC_056054.1	2.33E+08	232590001	4.29897	0.369783	BD	SLC33A1	NC_056065.1	55535001	55555001
NC_056054.1	2.33E+08	232585001	8.584	0.352117	BD	SLC33A1	NC_056065.1	55540001	55560001
NC_056068.1	74775001	74795001	3.60158	0.291466	BD	SLC35C1	NC_056058.1	38115001	38135001
NC_056055.1	1.48E+08	147895001	3.37195	0.368988	BD	SLC4A10	NC_056060.1	78070001	78090001
NC_056055.1	1.48E+08	147900001	3.45202	0.315909	BD	SLC4A10	NC_056060.1	78075001	78095001
NC_056055.1	1.48E+08	147890001	2.29683	0.311742	BD	SLC4A10	NC_056060.1	78095001	78115001
NC_056054.1	2.18E+08	217650001	2.58933	0.361483	BD	SLC7A14	NC_056060.1	78100001	78120001
NC_056054.1	2.18E+08	217645001	2.34615	0.341219	BD	SLC7A14	NC_056060.1	78105001	78125001
NC_056067.1	35210001	35230001	3.4319	0.365828	BD	SLC7A6	NC_056060.1	78110001	78130001
NC_056067.1	35205001	35225001	2.55098	0.320871	BD	SLC7A6	NC_056060.1	78130001	78150001
NC_056067.1	35215001	35235001	2.90704	0.314735	BD	SLC7A6	NC_056060.1	78135001	78155001
NC_056067.1	35220001	35240001	2.52706	0.29686	BD	SLC7A6;SLC7A6OS	NC_056060.1	78140001	78160001
NC_056058.1	99025001	99045001	7.10675	0.361389	BD	SLCO6A1	NC_056080.1	16080001	16100001
NC_056058.1	99020001	99040001	10.6245	0.351131	BD	SLCO6A1	NC_056080.1	16085001	16105001
NC_056058.1	99015001	99035001	5.71021	0.325496	BD	SLCO6A1	NC_056056.1	8920001	8940001
NC_056058.1	99010001	99030001	2.92155	0.294752	BD	SLCO6A1	NC_056056.1	8925001	8945001
NC_056070.1	12795001	12815001	2.7603	0.722879	BD	SMAD1	NC_056056.1	8930001	8950001
NC_056070.1	12790001	12810001	2.57804	0.719807	BD	SMAD1	NC_056056.1	8935001	8955001
NC_056055.1	70330001	70350001	2.33504	0.396692	BD	SMARCA2	NC_056062.1	90765001	90785001
NC_056055.1	70335001	70355001	2.15283	0.390688	BD	SMARCA2	NC_056079.1	39800001	39820001
NC_056055.1	70340001	70360001	2.19186	0.337533	BD	SMARCA2	NC_056065.1	58990001	59010001
NC_056055.1	66305001	66325001	3.70348	0.365412	BD	SMC5	NC_056065.1	58995001	59015001
NC_056055.1	66315001	66335001	3.66827	0.361322	BD	SMC5	NC_056065.1	59000001	59020001
NC_056055.1	66310001	66330001	3.63578	0.356168	BD	SMC5	NC_056065.1	59230001	59250001
NC_056055.1	66325001	66345001	3.20513	0.346448	BD	SMC5	NC_056076.1	33925001	33945001
NC_056055.1	66320001	66340001	3.68449	0.34569	BD	SMC5	NC_056076.1	33930001	33950001
NC_056055.1	66330001	66350001	2.52062	0.296142	BD	SMC5	NC_056077.1	6100001	6120001
NC_056080.1	20050001	20070001	2.14376	0.439893	BD	SMPX	NC_056073.1	39310001	39330001
NC_056080.1	20055001	20075001	3.39444	0.380844	BD	SMPX	NC_056066.1	58385001	58405001
NC_056080.1	20060001	20080001	3.19908	0.306641	BD	SMPX	NC_056072.1	3865001	3885001
NC_056064.1	48530001	48550001	3.21054	0.314198	BD	SMURF2	NC_056072.1	3870001	3890001
NC_056064.1	48535001	48555001	3.85453	0.311869	BD	SMURF2	NC_056072.1	4460001	4480001
NC_056064.1	48520001	48540001	2.84999	0.307149	BD	SMURF2	NC_056072.1	4465001	4485001
NC_056064.1	48525001	48545001	3.24162	0.307099	BD	SMURF2	NC_056072.1	4470001	4490001
NC_056064.1	48515001	48535001	3.40425	0.303524	BD	SMURF2	NC_056072.1	4475001	4495001
NC_056062.1	32950001	32970001	2.44163	0.293223	BD	SNAI2	NC_056072.1	4480001	4500001
NC_056059.1	35555001	35575001	4.37282	0.39121	BD	SNCA	NC_056072.1	4485001	4505001
NC_056059.1	35560001	35580001	4.68893	0.356996	BD	SNCA	NC_056071.1	29845001	29865001
NC_056059.1	35550001	35570001	3.50183	0.325243	BD	SNCA	NC_056057.1	46420001	46440001
NC_056057.1	93680001	93700001	3.69862	0.424693	BD	SND1	NC_056057.1	46465001	46485001
NC_056057.1	93675001	93695001	2.99391	0.390832	BD	SND1	NC_056057.1	46470001	46490001
NC_056057.1	93670001	93690001	2.84928	0.386541	BD	SND1	NC_056057.1	46475001	46495001
NC_056057.1	93665001	93685001	2.45024	0.364502	BD	SND1	NC_056057.1	46480001	46500001
NC_056057.1	93685001	93705001	2.46129	0.330055	BD	SND1	NC_056057.1	46485001	46505001
NC_056080.1	63765001	63785001	3.15507	0.366484	BD	SNX12	NC_056057.1	46490001	46510001
NC_056058.1	28705001	28725001	4.85091	0.313161	BD	SNX2	NC_056057.1	46495001	46515001

NC_056056.1	77590001	77610001	2.95365	0.336799	BD	SOCS5	NC_056065.1	44165001	44185001
NC_056056.1	77585001	77605001	2.42613	0.295544	BD	SOCS5	NC_056065.1	44170001	44190001
NC_056064.1	58100001	58120001	3.37132	0.293001	BD	SOX9	NC_056065.1	44175001	44195001
NC_056064.1	24070001	24090001	5.59859	0.459609	BD	SPATA22	NC_056065.1	44180001	44200001
NC_056056.1	69480001	69500001	2.37699	0.377509	BD	SPTBN1	NC_056056.1	100120001	100140001
NC_056054.1	2.27E+08	226720001	2.23473	0.345249	BD	SPTSSB	NC_056056.1	100170001	100190001
NC_056069.1	66605001	66625001	2.15776	0.323871	BD	SRD5A1	NC_056056.1	100175001	100195001
NC_056073.1	9970001	9990001	11.66	0.459371	BD	SRPK1	NC_056065.1	63075001	63095001
NC_056073.1	9965001	9985001	11.81	0.446837	BD	SRPK1	NC_056065.1	63080001	63100001
NC_056073.1	9980001	10000001	10.5	0.422453	BD	SRPK1	NC_056077.1	34565001	34585001
NC_056073.1	9930001	9950001	4.78161	0.420571	BD	SRPK1	NC_056058.1	68340001	68360001
NC_056073.1	9960001	9980001	9.7523	0.414412	BD	SRPK1	NC_056058.1	68345001	68365001
NC_056073.1	9955001	9975001	8.38617	0.379136	BD	SRPK1	NC_056058.1	68350001	68370001
NC_056073.1	9950001	9970001	8.58592	0.374616	BD	SRPK1	NC_056058.1	68355001	68375001
NC_056073.1	9925001	9945001	3.79208	0.349714	BD	SRPK1	NC_056062.1	76990001	77010001
NC_056057.1	47980001	48000001	9.10991	0.384251	BD	SRPK2	NC_056062.1	76995001	77015001
NC_056057.1	47975001	47995001	7.45712	0.373569	BD	SRPK2	NC_056062.1	77000001	77020001
NC_056057.1	47985001	48005001	4.51561	0.318482	BD	SRPK2	NC_056062.1	77005001	77025001
NC_056057.1	47970001	47990001	6.14708	0.311288	BD	SRPK2	NC_056062.1	77010001	77030001
NC_056070.1	56265001	56285001	2.13766	0.322056	BD	SRRM4	NC_056062.1	77015001	77035001
NC_056070.1	56315001	56335001	9.50345	0.306395	BD	SRRM4	NC_056062.1	77020001	77040001
NC_056076.1	31110001	31130001	3.258	0.298621	BD	SS18	NC_056054.1	147580001	147600001
NC_056058.1	79240001	79260001	2.57517	0.311517	BD	SSBP2	NC_056054.1	147745001	147765001
NC_056062.1	20995001	21015001	6.96531	0.3853	BD	ST3GAL1	NC_056054.1	147750001	147770001
NC_056062.1	21000001	21020001	4.02846	0.296854	BD	ST3GAL1	NC_056054.1	145345001	145365001
NC_056063.1	28315001	28335001	3.91916	0.360702	BD	STARD13	NC_056076.1	35475001	35495001
NC_056080.1	61620001	61640001	4.0736	0.471381	BD	STARD8	NC_056076.1	35480001	35500001
NC_056064.1	42215001	42235001	2.12963	0.341595	BD	STAT3	NC_056076.1	35485001	35505001
NC_056057.1	73970001	73990001	3.61382	0.503057	BD	STEAP4	NC_056076.1	35520001	35540001
NC_056057.1	73980001	74000001	6.17561	0.463965	BD	STEAP4	NC_056060.1	47125001	47145001
NC_056057.1	73975001	73995001	3.89397	0.451585	BD	STEAP4	NC_056055.1	104520001	104540001
NC_056057.1	73985001	74005001	11.372	0.377744	BD	STEAP4	NC_056055.1	104525001	104545001
NC_056057.1	73990001	74010001	15.0434	0.299286	BD	STEAP4	NC_056055.1	104530001	104550001
NC_056057.1	73615001	73635001	3.11282	0.382959	BD	STK31	NC_056055.1	104535001	104555001
NC_056057.1	73610001	73630001	3.64015	0.346244	BD	STK31	NC_056056.1	59350001	59370001
NC_056057.1	73585001	73605001	4.90613	0.319898	BD	STK31	NC_056056.1	59355001	59375001
NC_056057.1	73580001	73600001	4.63889	0.316563	BD	STK31	NC_056056.1	59360001	59380001
NC_056057.1	73620001	73640001	2.87309	0.293808	BD	STK31	NC_056056.1	59365001	59385001
NC_056059.1	26995001	27015001	5.03808	0.477611	BD	STPG2	NC_056056.1	59370001	59390001
NC_056059.1	26990001	27010001	4.77068	0.470209	BD	STPG2	NC_056061.1	48155001	48175001
NC_056059.1	27000001	27020001	4.35761	0.460866	BD	STPG2	NC_056059.1	15920001	15940001
NC_056059.1	27005001	27025001	3.29853	0.421708	BD	STPG2	NC_056077.1	14185001	14205001
NC_056059.1	26985001	27005001	4.35412	0.405239	BD	STPG2	NC_056077.1	14190001	14210001
NC_056059.1	27010001	27030001	2.38701	0.380065	BD	STPG2	NC_056075.1	18420001	18440001
NC_056059.1	27015001	27035001	2.52519	0.357131	BD	STPG2	NC_056064.1	22910001	22930001
NC_056059.1	27030001	27050001	11.4545	0.351975	BD	STPG2	NC_056064.1	22915001	22935001
NC_056059.1	27020001	27040001	3.01779	0.339964	BD	STPG2	NC_056054.1	143435001	143455001
NC_056059.1	27025001	27045001	3.5226	0.33516	BD	STPG2	NC_056055.1	117445001	117465001
NC_056059.1	27035001	27055001	5.81726	0.329712	BD	STPG2	NC_056055.1	117450001	117470001
NC_056059.1	26980001	27000001	4.9706	0.306955	BD	STPG2	NC_056055.1	117460001	117480001
NC_056054.1	86100001	86120001	2.33036	0.324865	BD	STXBP3	NC_056055.1	117465001	117485001
NC_056057.1	81970001	81990001	6.63879	0.502115	BD	SUGCT	NC_056055.1	117470001	117490001
NC_056057.1	82070001	82090001	3.28659	0.49279	BD	SUGCT	NC_056055.1	117475001	117495001
NC_056057.1	81985001	82005001	5.99999	0.486816	BD	SUGCT	NC_056055.1	117480001	117500001
NC_056057.1	81890001	81910001	2.90154	0.459585	BD	SUGCT	NC_056055.1	117500001	117520001
NC_056057.1	81895001	81915001	2.53062	0.45709	BD	SUGCT	NC_056055.1	117505001	117525001
NC_056057.1	82220001	82240001	2.2358	0.432801	BD	SUGCT	NC_056055.1	117510001	117530001
NC_056057.1	82075001	82095001	2.62805	0.424803	BD	SUGCT	NC_056055.1	117515001	117535001
NC_056057.1	82290001	82310001	2.52247	0.420693	BD	SUGCT	NC_056055.1	107355001	107375001
NC_056057.1	82085001	82105001	2.73054	0.420403	BD	SUGCT	NC_056055.1	107360001	107380001
NC_056057.1	82080001	82100001	2.35678	0.412376	BD	SUGCT	NC_056055.1	107350001	107370001
NC_056057.1	82235001	82255001	2.31182	0.411009	BD	SUGCT	NC_056058.1	43765001	43785001
NC_056057.1	82395001	82415001	8.45712	0.400894	BD	SUGCT	NC_056058.1	43770001	43790001
NC_056057.1	81960001	81980001	2.42722	0.37149	BD	SUGCT	NC_056071.1	29710001	29730001
NC_056057.1	81965001	81985001	3.40575	0.371442	BD	SUGCT	NC_056071.1	29715001	29735001
NC_056057.1	81885001	81905001	2.61896	0.357257	BD	SUGCT	NC_056071.1	29720001	29740001
NC_056057.1	82415001	82435001	6.36669	0.332296	BD	SUGCT	NC_056071.1	29725001	29745001
NC_056066.1	76185001	76205001	2.53219	0.308984	BD	SULF2	NC_056070.1	29140001	29160001
NC_056066.1	76180001	76200001	2.78331	0.299172	BD	SULF2	NC_056070.1	29145001	29165001
NC_056066.1	76190001	76210001	2.23149	0.295741	BD	SULF2	NC_056070.1	29150001	29170001
NC_056066.1	61935001	61955001	2.55859	0.348068	BD	SUN5	NC_056070.1	29155001	29175001
NC_056066.1	61940001	61960001	2.43173	0.33017	BD	SUN5	NC_056070.1	29160001	29180001
NC_056060.1	79480001	79500001	4.17666	0.410963	BD	SUSD6	NC_056070.1	29165001	29185001
NC_056060.1	79475001	79495001	3.53058	0.396024	BD	SUSD6	NC_056070.1	29170001	29190001

NC_056060.1	79490001	79510001	2.80426	0.382788	BD	SUSD6	NC_056070.1	29175001	29195001
NC_056060.1	79470001	79490001	2.16607	0.342193	BD	SUSD6	NC_056077.1	21525001	21545001
NC_056060.1	79495001	79515001	2.20705	0.310847	BD	SUSD6	NC_056077.1	21530001	21550001
NC_056071.1	15315001	15335001	2.32669	0.298429	BD	SV2B	NC_056062.1	3465001	3485001
NC_056071.1	15320001	15340001	2.48545	0.29583	BD	SV2B	NC_056062.1	3470001	3490001
NC_056060.1	7550001	7570001	3.83973	0.302882	BD	SV2C	NC_056056.1	62410001	62430001
NC_056060.1	7545001	7565001	3.06911	0.295484	BD	SV2C	NC_056056.1	62415001	62435001
NC_056066.1	34105001	34125001	2.11511	0.361715	BD	SVIL	NC_056056.1	62420001	62440001
NC_056068.1	42660001	42680001	3.54969	0.47868	BD	SWAP70	NC_056056.1	62425001	62445001
NC_056068.1	42665001	42685001	2.60788	0.447114	BD	SWAP70	NC_056056.1	62430001	62450001
NC_056072.1	56940001	56960001	2.60424	0.31879	BD	SYN2	NC_056056.1	62435001	62455001
NC_056058.1	59720001	59740001	16.7913	0.478541	BD	SYNPO	NC_056056.1	62440001	62460001
NC_056058.1	59725001	59745001	10.4002	0.458191	BD	SYNPO	NC_056056.1	62445001	62465001
NC_056058.1	59730001	59750001	6.43157	0.350235	BD	SYNPO	NC_056056.1	62450001	62470001
NC_056056.1	1.15E+08	115465001	2.63516	0.416057	BD	SYT1	NC_056056.1	62455001	62475001
NC_056056.1	1.82E+08	181965001	2.49447	0.369356	BD	SYT10	NC_056056.1	62405001	62425001
NC_056056.1	1.82E+08	181940001	3.79956	0.344859	BD	SYT10	NC_056056.1	62460001	62480001
NC_056056.1	1.82E+08	181935001	2.58738	0.336848	BD	SYT10	NC_056064.1	53830001	53850001
NC_056056.1	1.82E+08	181960001	2.54191	0.33084	BD	SYT10	NC_056079.1	35535001	35555001
NC_056056.1	1.82E+08	181945001	4.44828	0.322988	BD	SYT10	NC_056058.1	65505001	65525001
NC_056054.1	1.06E+08	105915001	8.36031	0.405697	BD	SYT11;YY1AP1	NC_056058.1	65510001	65530001
NC_056054.1	1.06E+08	105910001	4.04349	0.312648	BD	SYT11;YY1AP1	NC_056058.1	65515001	65535001
NC_056074.1	46030001	46050001	5.10238	0.350782	BD	SYT8	NC_056055.1	86170001	86190001
NC_056074.1	46025001	46045001	3.72544	0.300504	BD	SYT8;TNNI2	NC_056055.1	86175001	86195001
NC_056068.1	44965001	44985001	3.1726	0.364324	BD	SYT9	NC_056080.1	18220001	18240001
NC_056068.1	44960001	44980001	2.45842	0.338268	BD	SYT9	NC_056080.1	18245001	18265001
NC_056068.1	44970001	44990001	2.36613	0.328348	BD	SYT9	NC_056056.1	62395001	62415001
NC_056080.1	37245001	37265001	2.45219	0.306893	BD	SYTL5	NC_056056.1	62400001	62420001
NC_056075.1	41485001	41505001	6.85811	0.409656	BD	TACC2	NC_056077.1	12405001	12425001
NC_056075.1	41490001	41510001	4.3339	0.338469	BD	TACC2	NC_056077.1	12410001	12430001
NC_056064.1	47380001	47400001	2.21748	0.35661	BD	TANC2	NC_056077.1	12415001	12435001
NC_056067.1	35790001	35810001	17.9444	0.303488	BD	TANGO6	NC_056077.1	12420001	12440001
NC_056059.1	46660001	46680001	2.80366	0.313492	BD	TBC1D19	NC_056077.1	12425001	12445001
NC_056059.1	19330001	19350001	2.20885	0.337548	BD	TBCK	NC_056080.1	54435001	54455001
NC_056059.1	19335001	19355001	2.37314	0.309115	BD	TBCK	NC_056068.1	21640001	21660001
NC_056059.1	19340001	19360001	2.40266	0.307709	BD	TBCK	NC_056068.1	27330001	27350001
NC_056058.1	55355001	55375001	2.29167	0.36251	BD	TCERG1	NC_056068.1	27340001	27360001
NC_056060.1	51345001	51365001	2.28402	0.442586	BD	TCF12	NC_056068.1	27365001	27385001
NC_056060.1	51340001	51360001	2.23	0.409547	BD	TCF12	NC_056068.1	27370001	27390001
NC_056060.1	51350001	51370001	2.94737	0.372855	BD	TCF12	NC_056068.1	27375001	27395001
NC_056060.1	51355001	51375001	2.47619	0.362683	BD	TCF12	NC_056068.1	27380001	27400001
NC_056060.1	51510001	51530001	2.39881	0.341096	BD	TCF12	NC_056068.1	27385001	27405001
NC_056060.1	51360001	51380001	2.78632	0.311996	BD	TCF12	NC_056068.1	27390001	27410001
NC_056054.1	1.05E+08	104770001	2.1053	0.316611	BD	TDRD10	NC_056068.1	27395001	27415001
NC_056054.1	1.05E+08	104775001	2.33406	0.302456	BD	TDRD10	NC_056068.1	27400001	27420001
NC_056054.1	1.05E+08	104780001	2.10032	0.293695	BD	TDRD10	NC_056068.1	27405001	27425001
NC_056055.1	50215001	50235001	15.525	0.6304	BD	TDRD7	NC_056068.1	27415001	27435001
NC_056080.1	1.09E+08	109020001	4.59091	0.520167	BD	TENM1	NC_056068.1	27445001	27465001
NC_056080.1	1.09E+08	109025001	3.71213	0.4412	BD	TENM1	NC_056068.1	27450001	27470001
NC_056080.1	1.09E+08	108975001	2.21634	0.353137	BD	TENM1	NC_056068.1	27455001	27475001
NC_056080.1	1.09E+08	109015001	2.50344	0.322354	BD	TENM1	NC_056068.1	27460001	27480001
NC_056074.1	15000001	15020001	2.66964	0.297176	BD	TENM4	NC_056060.1	5045001	5065001
NC_056070.1	58030001	58050001	5.04325	0.347957	BD	TESC	NC_056060.1	5050001	5070001
NC_056078.1	24210001	24230001	2.66431	0.363132	BD	TET1	NC_056060.1	5070001	5090001
NC_056078.1	24205001	24225001	3.56807	0.352785	BD	TET1	NC_056060.1	5075001	5095001
NC_056078.1	24235001	24255001	2.49885	0.340336	BD	TET1	NC_056060.1	5080001	5100001
NC_056078.1	24200001	24220001	5.57778	0.335997	BD	TET1	NC_056060.1	5085001	5105001
NC_056078.1	24240001	24260001	3.88135	0.307016	BD	TET1	NC_056064.1	37955001	37975001
NC_056080.1	63555001	63575001	5.1087	0.377107	BD	TEX11	NC_056064.1	37960001	37980001
NC_056080.1	63560001	63580001	3.15556	0.341543	BD	TEX11	NC_056057.1	70705001	70725001
NC_056080.1	63550001	63570001	3.32707	0.315074	BD	TEX11	NC_056057.1	70725001	70745001
NC_056064.1	48085001	48105001	2.11507	0.444607	BD	TEX2	NC_056057.1	70750001	70770001
NC_056064.1	48160001	48180001	12.5429	0.372452	BD	TEX2	NC_056057.1	70755001	70775001
NC_056064.1	48150001	48170001	4.80052	0.363635	BD	TEX2	NC_056057.1	70760001	70780001
NC_056064.1	48145001	48165001	2.76131	0.329351	BD	TEX2	NC_056057.1	70795001	70815001
NC_056064.1	48155001	48175001	4.43379	0.313078	BD	TEX2	NC_056057.1	70800001	70820001
NC_056072.1	49205001	49225001	3.16666	0.452505	BD	TEX264	NC_056057.1	70865001	70885001
NC_056072.1	49215001	49235001	3.6	0.416202	BD	TEX264	NC_056057.1	70870001	70890001
NC_056072.1	49210001	49230001	3.20556	0.40605	BD	TEX264	NC_056057.1	70875001	70895001
NC_056065.1	59330001	59350001	2.89971	0.481084	BD	TEX35	NC_056057.1	70880001	70900001
NC_056065.1	59325001	59345001	6.59623	0.422188	BD	TEX35	NC_056056.1	199250001	199270001
NC_056065.1	59320001	59340001	12.1196	0.394037	BD	TEX35	NC_056056.1	199255001	199275001
NC_056055.1	2.33E+08	233345001	2.66793	0.33793	BD	TEX44	NC_056056.1	199260001	199280001
NC_056055.1	2.33E+08	233340001	3.01572	0.334697	BD	TEX44	NC_056080.1	66615001	66635001

NC_056055.1	2.33E+08	233335001	3.46341	0.315689	BD	TEX44	NC_056080.1	66620001	66640001
NC_056073.1	22925001	22945001	2.48808	0.300979	BD	TFAP2D	NC_056080.1	66625001	66645001
NC_056062.1	21300001	21320001	7.63692	0.425703	BD	TG	NC_056080.1	66640001	66660001
NC_056076.1	38015001	38035001	7.61687	0.320172	BD	TGIF1	NC_056061.1	84250001	84270001
NC_056074.1	45745001	45765001	2.36093	0.408394	BD	TH	NC_056061.1	84255001	84275001
NC_056056.1	80640001	80660001	12.6747	0.468493	BD	THADA	NC_056066.1	46940001	46960001
NC_056056.1	80635001	80655001	2.13816	0.41648	BD	THADA	NC_056066.1	46945001	46965001
NC_056056.1	80765001	80785001	3.00941	0.413429	BD	THADA	NC_056080.1	115015001	115035001
NC_056056.1	80930001	80950001	10.6138	0.370675	BD	THADA	NC_056074.1	40830001	40850001
NC_056056.1	80910001	80930001	21.7857	0.35442	BD	THADA	NC_056074.1	40835001	40855001
NC_056056.1	80770001	80790001	2.61952	0.342125	BD	THADA	NC_056074.1	40840001	40860001
NC_056056.1	80935001	80955001	4.62163	0.295245	BD	THADA	NC_056054.1	17380001	17400001
NC_056060.1	18475001	18495001	4.18884	0.449727	BD	THSD4	NC_056056.1	147600001	147620001
NC_056060.1	18480001	18500001	2.92606	0.423898	BD	THSD4	NC_056056.1	147605001	147625001
NC_056060.1	18470001	18490001	3.14551	0.380147	BD	THSD4	NC_056056.1	147610001	147630001
NC_056065.1	25835001	25855001	2.24614	0.313422	BD	TLR5	NC_056056.1	147615001	147635001
NC_056056.1	1.37E+08	136560001	2.50653	0.373835	BD	TMBIM6	NC_056064.1	57345001	57365001
NC_056056.1	1.37E+08	136565001	2.95541	0.325017	BD	TMBIM6	NC_056067.1	23370001	23390001
NC_056060.1	4450001	4470001	13.0662	0.380706	BD	TMED7	NC_056067.1	23375001	23395001
NC_056060.1	4460001	4480001	6.87599	0.313918	BD	TMED7	NC_056067.1	35205001	35225001
NC_056055.1	22575001	22595001	2.93112	0.373237	BD	TMEFF1	NC_056067.1	35210001	35230001
NC_056054.1	2.57E+08	257060001	2.29114	0.496682	BD	TMEM108	NC_056067.1	35215001	35235001
NC_056054.1	2.57E+08	257055001	2.67302	0.458258	BD	TMEM108	NC_056060.1	21480001	21500001
NC_056054.1	2.57E+08	257050001	2.30804	0.449432	BD	TMEM108	NC_056060.1	21485001	21505001
NC_056054.1	2.57E+08	256970001	2.36597	0.296758	BD	TMEM108	NC_056060.1	21490001	21510001
NC_056074.1	7795001	7815001	2.60961	0.316919	BD	TMEM135	NC_056067.1	42770001	42790001
NC_056056.1	45725001	45745001	11.8045	0.365839	BD	TMEM17	NC_056067.1	42775001	42795001
NC_056056.1	45730001	45750001	6.06896	0.325267	BD	TMEM17	NC_056067.1	42780001	42800001
NC_056056.1	45720001	45740001	4.76956	0.304245	BD	TMEM17	NC_056067.1	42765001	42785001
NC_056056.1	84495001	84515001	2.11727	0.44363	BD	TMEM178A	NC_056056.1	194335001	194355001
NC_056056.1	84470001	84490001	2.96357	0.359157	BD	TMEM178A	NC_056056.1	194340001	194360001
NC_056056.1	84465001	84485001	2.10169	0.309721	BD	TMEM178A	NC_056056.1	194345001	194365001
NC_056056.1	99020001	99040001	2.90387	0.336581	BD	TMEM182	NC_056077.1	16950001	16970001
NC_056056.1	99015001	99035001	3.38883	0.31313	BD	TMEM182	NC_056077.1	16955001	16975001
NC_056056.1	98985001	99005001	2.25414	0.299399	BD	TMEM182	NC_056077.1	16960001	16980001
NC_056057.1	29555001	29575001	2.39192	0.376858	BD	TMEM196	NC_056077.1	16965001	16985001
NC_056057.1	29560001	29580001	2.40631	0.34631	BD	TMEM196	NC_056077.1	16970001	16990001
NC_056077.1	28770001	28790001	3.28667	0.323411	BD	TMEM248	NC_056077.1	16975001	16995001
NC_056071.1	55620001	55640001	2.39297	0.314727	BD	TMEM251	NC_056077.1	16980001	17000001
NC_056056.1	1.68E+08	168280001	7.55921	0.369464	BD	TMPO	NC_056067.1	35950001	35970001
NC_056056.1	1.68E+08	168285001	5.80103	0.341237	BD	TMPO	NC_056067.1	35955001	35975001
NC_056056.1	1.68E+08	168275001	4.29393	0.335172	BD	TMPO	NC_056067.1	35980001	36000001
NC_056056.1	1.68E+08	168270001	3.04789	0.328375	BD	TMPO	NC_056067.1	35985001	36005001
NC_056056.1	1.68E+08	168295001	2.86096	0.325626	BD	TMPO	NC_056056.1	191340001	191360001
NC_056056.1	1.68E+08	168290001	3.54198	0.3223	BD	TMPO	NC_056056.1	191345001	191365001
NC_056056.1	1.68E+08	168305001	2.34912	0.32008	BD	TMPO	NC_056056.1	191350001	191370001
NC_056056.1	1.68E+08	168300001	2.32021	0.305322	BD	TMPO	NC_056056.1	191355001	191375001
NC_056056.1	1.19E+08	118960001	8.53076	0.334451	BD	TMTC2	NC_056056.1	191360001	191380001
NC_056056.1	1.19E+08	118970001	2.8018	0.330857	BD	TMTC2	NC_056056.1	191365001	191385001
NC_056056.1	1.19E+08	118965001	5.90051	0.318682	BD	TMTC2	NC_056056.1	191370001	191390001
NC_056056.1	1.19E+08	118955001	13.0492	0.312888	BD	TMTC2	NC_056056.1	191665001	191685001
NC_056066.1	470001	490001	7.02607	0.369264	BD	TMX4	NC_056056.1	191670001	191690001
NC_056066.1	475001	495001	3.2331	0.35777	BD	TMX4	NC_056056.1	191675001	191695001
NC_056066.1	465001	485001	6.66932	0.332962	BD	TMX4	NC_056056.1	191680001	191700001
NC_056065.1	39940001	39960001	2.56447	0.291656	BD	TNFSF18	NC_056056.1	191685001	191705001
NC_056065.1	40070001	40090001	2.25771	0.389509	BD	TNFSF4	NC_056056.1	191690001	191710001
NC_056055.1	8875001	8895001	4.47485	0.428407	BD	TNFSF8	NC_056056.1	191695001	191715001
NC_056055.1	8870001	8890001	2.1	0.364532	BD	TNFSF8	NC_056066.1	22905001	22925001
NC_056055.1	8880001	8900001	2.47716	0.354645	BD	TNFSF8	NC_056063.1	34445001	34465001
NC_056079.1	24700001	24720001	9.46154	0.427201	BD	TNKS	NC_056063.1	34465001	34485001
NC_056079.1	24695001	24715001	4.07605	0.375988	BD	TNKS	NC_056063.1	34470001	34490001
NC_056079.1	24705001	24725001	3.86973	0.331889	BD	TNKS	NC_056063.1	34475001	34495001
NC_056057.1	76610001	76630001	2.52289	0.473531	BD	TNS3	NC_056063.1	34480001	34500001
NC_056057.1	76490001	76510001	4.61958	0.427284	BD	TNS3	NC_056079.1	6990001	7010001
NC_056057.1	76495001	76515001	2.8976	0.367473	BD	TNS3	NC_056069.1	1655001	1675001
NC_056057.1	76485001	76505001	3.01579	0.358141	BD	TNS3	NC_056069.1	1660001	1680001
NC_056057.1	76470001	76490001	3.16131	0.358073	BD	TNS3	NC_056069.1	1665001	1685001
NC_056057.1	76475001	76495001	3.20468	0.344414	BD	TNS3	NC_056058.1	57115001	57135001
NC_056057.1	76465001	76485001	2.9327	0.323843	BD	TNS3	NC_056067.1	14220001	14240001
NC_056057.1	76480001	76500001	2.80153	0.308716	BD	TNS3	NC_056067.1	14225001	14245001
NC_056062.1	16435001	16455001	3.3659	0.310811	BD	TRAPPC9	NC_056059.1	117930001	117950001
NC_056056.1	1.09E+08	108570001	6.64239	0.540925	BD	TRHDE	NC_056059.1	117935001	117955001
NC_056056.1	1.09E+08	108565001	5.13636	0.515927	BD	TRHDE	NC_056059.1	37375001	37395001
NC_056056.1	1.09E+08	108560001	2.85382	0.464506	BD	TRHDE	NC_056060.1	86840001	86860001

NC_056056.1	1.09E+08	108575001	2.8072	0.459654	BD	TRHDE	NC_056070.1	56265001	56285001
NC_056056.1	1.09E+08	108535001	2.17903	0.302263	BD	TRHDE	NC_056070.1	56270001	56290001
NC_056080.1	44320001	44340001	19.2621	0.52169	BD	TRIM60	NC_056070.1	56290001	56310001
NC_056080.1	44325001	44345001	18.1918	0.470335	BD	TRIM60	NC_056070.1	56295001	56315001
NC_056080.1	44330001	44350001	10.5952	0.356003	BD	TRIM60	NC_056070.1	56300001	56320001
NC_056062.1	61965001	61985001	7.85712	0.390955	BD	TRPS1	NC_056070.1	56315001	56335001
NC_056062.1	61935001	61955001	2.96939	0.370506	BD	TRPS1	NC_056070.1	56320001	56340001
NC_056062.1	61960001	61980001	5.18584	0.36685	BD	TRPS1	NC_056070.1	56325001	56345001
NC_056062.1	61970001	61990001	4.28096	0.341281	BD	TRPS1	NC_056056.1	189385001	189405001
NC_056062.1	61940001	61960001	2.58572	0.331812	BD	TRPS1	NC_056056.1	189390001	189410001
NC_056062.1	61945001	61965001	2.7599	0.328079	BD	TRPS1	NC_056056.1	189395001	189415001
NC_056062.1	61950001	61970001	3.20158	0.327555	BD	TRPS1	NC_056056.1	189400001	189420001
NC_056062.1	61930001	61950001	3.22671	0.312487	BD	TRPS1	NC_056063.1	28315001	28335001
NC_056062.1	61975001	61995001	3.96327	0.307913	BD	TRPS1	NC_056057.1	51575001	51595001
NC_056062.1	61955001	61975001	2.80445	0.291604	BD	TRPS1	NC_056057.1	51580001	51600001
NC_056072.1	56565001	56585001	2.27133	0.31452	BD	TSEN2	NC_056057.1	73970001	73990001
NC_056058.1	35840001	35860001	2.17583	0.313663	BD	TSPAN17	NC_056057.1	73975001	73995001
NC_056058.1	35845001	35865001	3.40059	0.303374	BD	TSPAN17	NC_056057.1	73980001	74000001
NC_056059.1	26655001	26675001	3.21647	0.464145	BD	TSPAN5	NC_056057.1	73985001	74005001
NC_056059.1	26660001	26680001	2.74453	0.440274	BD	TSPAN5	NC_056057.1	73990001	74010001
NC_056059.1	26665001	26685001	2.46496	0.426427	BD	TSPAN5	NC_056057.1	73995001	74015001
NC_056059.1	26650001	26670001	2.22874	0.405423	BD	TSPAN5	NC_056057.1	74000001	74020001
NC_056080.1	38175001	38195001	4.15244	0.511617	BD	TSPAN7	NC_056057.1	73550001	73570001
NC_056080.1	38160001	38180001	8.06351	0.471467	BD	TSPAN7	NC_056057.1	73555001	73575001
NC_056080.1	38095001	38115001	4.06354	0.456558	BD	TSPAN7	NC_056057.1	73580001	73600001
NC_056080.1	38155001	38175001	6.00553	0.438962	BD	TSPAN7	NC_056057.1	73600001	73620001
NC_056080.1	38130001	38150001	4.03402	0.428982	BD	TSPAN7	NC_056057.1	73605001	73625001
NC_056080.1	38150001	38170001	2.44625	0.35339	BD	TSPAN7	NC_056057.1	73610001	73630001
NC_056080.1	38140001	38160001	2.43086	0.327862	BD	TSPAN7	NC_056079.1	13635001	13655001
NC_056080.1	38145001	38165001	2.21869	0.309126	BD	TSPAN7	NC_056079.1	13645001	13665001
NC_056058.1	92425001	92445001	3.17308	0.323262	BD	TTC37	NC_056079.1	13770001	13790001
NC_056056.1	77300001	77320001	4.36181	0.7988	BD	TTC7A	NC_056079.1	13775001	13795001
NC_056056.1	2.15E+08	215235001	10.5471	0.296903	BD	TUBA8	NC_056079.1	13780001	13800001
NC_056056.1	2.15E+08	215230001	9.1495	0.291598	BD	TUBA8	NC_056059.1	26955001	26975001
NC_056056.1	3435001	3455001	5.46454	0.482914	BD	UBAC1	NC_056059.1	26960001	26980001
NC_056079.1	41420001	41440001	2.23797	0.309134	BD	UBE2E1	NC_056059.1	26965001	26985001
NC_056055.1	1.29E+08	128940001	6.25748	0.51316	BD	UBE2E3	NC_056059.1	26970001	26990001
NC_056055.1	1.29E+08	128935001	8.61362	0.487604	BD	UBE2E3	NC_056059.1	26975001	26995001
NC_056055.1	1.29E+08	128930001	5.8184	0.408469	BD	UBE2E3	NC_056059.1	26980001	27000001
NC_056055.1	1.29E+08	128925001	4.04772	0.318172	BD	UBE2E3	NC_056059.1	26985001	27005001
NC_056062.1	50135001	50155001	4.13437	0.349533	BD	UBE2W	NC_056059.1	26990001	27010001
NC_056065.1	42645001	42665001	2.35518	0.310958	BD	UBE4B	NC_056059.1	26995001	27015001
NC_056065.1	41620001	41640001	3.08075	0.314569	BD	UBIAD1	NC_056059.1	27000001	27020001
NC_056065.1	41615001	41635001	2.86387	0.297659	BD	UBIAD1	NC_056059.1	27005001	27025001
NC_056073.1	16275001	16295001	3.32387	0.293851	BD	UBR2	NC_056059.1	27030001	27050001
NC_056059.1	29665001	29685001	2.97388	0.42903	BD	UNC5C	NC_056059.1	27035001	27055001
NC_056059.1	29660001	29680001	2.69544	0.380118	BD	UNC5C	NC_056057.1	82515001	82535001
NC_056059.1	29655001	29675001	2.24675	0.365505	BD	UNC5C	NC_056057.1	82545001	82565001
NC_056058.1	4185001	4205001	2.13997	0.343339	BD	UPF1	NC_056057.1	82550001	82570001
NC_056058.1	4170001	4190001	2.24507	0.336371	BD	UPF1	NC_056056.1	181975001	181995001
NC_056072.1	60375001	60395001	2.37123	0.360579	BD	UROC1;ZXDC	NC_056056.1	181980001	182000001
NC_056067.1	10410001	10430001	6.10736	0.319652	BD	USP10	NC_056056.1	181985001	182005001
NC_056070.1	64160001	64180001	8.48149	0.429952	BD	USP30	NC_056056.1	182005001	182025001
NC_056072.1	50830001	50850001	4.60667	0.337436	BD	USP4	NC_056056.1	182010001	182030001
NC_056072.1	50825001	50845001	4.41667	0.33656	BD	USP4	NC_056056.1	182015001	182035001
NC_056072.1	50815001	50835001	3.66831	0.318634	BD	USP4	NC_056056.1	182020001	182040001
NC_056072.1	50820001	50840001	3.20196	0.301816	BD	USP4	NC_056080.1	37525001	37545001
NC_056065.1	70075001	70095001	2.2275	0.307615	BD	VASH2	NC_056061.1	73695001	73715001
NC_056060.1	41265001	41285001	3.94026	0.293877	BD	VCPKMT	NC_056061.1	73700001	73720001
NC_056074.1	26765001	26785001	2.5211	0.365123	BD	VSIG10L2	NC_056061.1	73705001	73725001
NC_056060.1	65515001	65535001	2.1414	0.357368	BD	WDHD1	NC_056061.1	73710001	73730001
NC_056060.1	65510001	65530001	2.15283	0.350526	BD	WDHD1	NC_056059.1	57970001	57990001
NC_056075.1	40410001	40430001	9.86254	0.449245	BD	WDR11	NC_056059.1	57975001	57995001
NC_056075.1	40400001	40420001	10.2316	0.440641	BD	WDR11	NC_056059.1	57980001	58000001
NC_056075.1	40415001	40435001	7.35649	0.440155	BD	WDR11	NC_056056.1	222975001	222995001
NC_056075.1	40405001	40425001	15.1708	0.439975	BD	WDR11	NC_056056.1	222980001	223000001
NC_056075.1	40395001	40415001	8.3696	0.434965	BD	WDR11	NC_056056.1	222985001	223005001
NC_056075.1	40420001	40440001	3.02841	0.365947	BD	WDR11	NC_056056.1	223075001	223095001
NC_056075.1	40390001	40410001	4.55087	0.361032	BD	WDR11	NC_056056.1	223080001	223100001
NC_056075.1	40355001	40375001	2.10182	0.304607	BD	WDR11	NC_056061.1	17110001	17130001
NC_056075.1	40360001	40380001	2.1	0.296612	BD	WDR11	NC_056061.1	17115001	17135001
NC_056071.1	64655001	64675001	5.0801	0.38575	BD	WDR20	NC_056054.1	275150001	275170001
NC_056071.1	64660001	64680001	3.21542	0.306292	BD	WDR20	NC_056054.1	275155001	275175001
NC_056056.1	2.14E+08	213920001	12.0206	0.291937	BD	WNT5B	NC_056060.1	51350001	51370001

NC_056056.1	2.22E+08	222155001	2.2072	0.374826	BD	WNT7B	NC_056060.1	51355001	51375001
NC_056070.1	64865001	64885001	2.27947	0.303826	BD	WSCD2	NC_056060.1	51360001	51380001
NC_056080.1	7990001	8010001	2.35065	0.322549	BD	WWC3	NC_056060.1	51465001	51485001
NC_056062.1	35785001	35805001	2.14428	0.292433	BD	XKR4	NC_056060.1	51510001	51530001
NC_056079.1	5985001	6005001	14.879	0.365171	BD	XKR5	NC_056060.1	51515001	51535001
NC_056079.1	5990001	6010001	6.20053	0.318861	BD	XKR5	NC_056060.1	51520001	51540001
NC_056079.1	6015001	6035001	4.73991	0.30564	BD	XKR5;ZNF596	NC_056067.1	14230001	14250001
NC_056054.1	2.47E+08	247275001	2.23763	0.50288	BD	XRN1	NC_056056.1	57025001	57045001
NC_056054.1	2.47E+08	247280001	2.19728	0.453247	BD	XRN1	NC_056056.1	57030001	57050001
NC_056054.1	2.47E+08	247285001	2.43697	0.446893	BD	XRN1	NC_056056.1	57035001	57055001
NC_056066.1	40160001	40180001	4.06658	0.500656	BD	XRN2	NC_056056.1	28915001	28935001
NC_056066.1	40170001	40190001	3.17475	0.436806	BD	XRN2	NC_056055.1	50250001	50270001
NC_056066.1	40165001	40185001	3.36557	0.435079	BD	XRN2	NC_056080.1	108990001	109010001
NC_056066.1	40185001	40205001	2.45714	0.306817	BD	XRN2	NC_056080.1	108995001	109015001
NC_056066.1	40175001	40195001	2.26717	0.300306	BD	XRN2	NC_056080.1	109000001	109020001
NC_056068.1	6335001	6355001	3.61606	0.298639	BD	YAP1	NC_056080.1	109005001	109025001
NC_056068.1	24425001	24445001	2.46522	0.335815	BD	ZBTB16	NC_056080.1	109010001	109030001
NC_056068.1	24430001	24450001	3.18068	0.326166	BD	ZBTB16	NC_056080.1	109025001	109045001
NC_056068.1	24415001	24435001	2.11823	0.31542	BD	ZBTB16	NC_056080.1	109030001	109050001
NC_056068.1	24435001	24455001	2.67621	0.311389	BD	ZBTB16	NC_056080.1	109035001	109055001
NC_056068.1	24440001	24460001	3.08623	0.306609	BD	ZBTB16	NC_056070.1	58030001	58050001
NC_056068.1	24445001	24465001	2.68281	0.306296	BD	ZBTB16	NC_056078.1	24185001	24205001
NC_056068.1	24410001	24430001	2.11083	0.295338	BD	ZBTB16	NC_056078.1	24200001	24220001
NC_056068.1	24460001	24480001	2.59084	0.293973	BD	ZBTB16	NC_056078.1	24205001	24225001
NC_056054.1	1.8E+08	180170001	2.11704	0.344414	BD	ZBTB20	NC_056078.1	24210001	24230001
NC_056068.1	19905001	19925001	5.13556	0.353216	BD	ZC3H12C	NC_056078.1	24215001	24235001
NC_056068.1	19900001	19920001	4.76103	0.331486	BD	ZC3H12C	NC_056078.1	24230001	24250001
NC_056072.1	2925001	2945001	2.21559	0.331246	BD	ZCWPW2	NC_056078.1	24235001	24255001
NC_056056.1	1.13E+08	112750001	2.63717	0.505087	BD	ZDHHC17	NC_056078.1	24240001	24260001
NC_056056.1	1.13E+08	112745001	2.81712	0.429881	BD	ZDHHC17	NC_056078.1	24245001	24265001
NC_056056.1	1.13E+08	112740001	3.13748	0.421282	BD	ZDHHC17	NC_056078.1	24250001	24270001
NC_056056.1	1.13E+08	112735001	3.21793	0.376004	BD	ZDHHC17	NC_056080.1	63550001	63570001
NC_056056.1	1.13E+08	112670001	2.15571	0.373922	BD	ZDHHC17	NC_056080.1	63555001	63575001
NC_056056.1	1.13E+08	112665001	2.46728	0.361749	BD	ZDHHC17	NC_056080.1	63560001	63580001
NC_056056.1	1.13E+08	112760001	2.13332	0.331162	BD	ZDHHC17	NC_056071.1	67425001	67445001
NC_056056.1	1.13E+08	112710001	2.33865	0.317432	BD	ZDHHC17	NC_056057.1	53960001	53980001
NC_056056.1	1.13E+08	112730001	2.78285	0.314306	BD	ZDHHC17	NC_056057.1	53965001	53985001
NC_056066.1	33290001	33310001	24.3041	0.380784	BD	ZEB1	NC_056057.1	53970001	53990001
NC_056066.1	33295001	33315001	23.8755	0.375793	BD	ZEB1	NC_056057.1	54000001	54020001
NC_056066.1	33300001	33320001	18.9446	0.372629	BD	ZEB1	NC_056062.1	21300001	21320001
NC_056066.1	33285001	33305001	21.725	0.351645	BD	ZEB1	NC_056056.1	80640001	80660001
NC_056066.1	33305001	33325001	8.10714	0.323319	BD	ZEB1	NC_056056.1	80690001	80710001
NC_056066.1	33280001	33300001	18.1829	0.308776	BD	ZEB1	NC_056060.1	18465001	18485001
NC_056066.1	33350001	33370001	2.51327	0.292394	BD	ZEB1	NC_056060.1	18470001	18490001
NC_056067.1	65255001	65275001	2.62405	0.409739	BD	ZNF304	NC_056060.1	18475001	18495001
NC_056067.1	65260001	65280001	2.91028	0.384674	BD	ZNF304	NC_056060.1	18480001	18500001
NC_056055.1	1.03E+08	102850001	2.18587	0.330193	BD	ZNF395	NC_056054.1	124815001	124835001
NC_056067.1	17505001	17525001	3.5191	0.322741	BD	ZNF423	NC_056054.1	124820001	124840001
NC_056067.1	17500001	17520001	3.2852	0.309979	BD	ZNF423	NC_056054.1	124825001	124845001
NC_056067.1	46615001	46635001	3.35231	0.390335	BD	ZNF585B	NC_056054.1	125025001	125045001
NC_056079.1	6025001	6045001	6.23517	0.330436	BD	ZNF596	NC_056064.1	46660001	46680001
NC_056079.1	6020001	6040001	5.67594	0.328751	BD	ZNF596	NC_056064.1	46665001	46685001
NC_056079.1	6030001	6050001	4.96314	0.317092	BD	ZNF596	NC_056055.1	22590001	22610001
NC_056060.1	43715001	43735001	5.58651	0.435598	BD	ZNF609	NC_056055.1	22595001	22615001
NC_056060.1	43710001	43730001	5.67051	0.430195	BD	ZNF609	NC_056055.1	22600001	22620001
NC_056060.1	43705001	43725001	3.68633	0.416059	BD	ZNF609	NC_056056.1	142800001	142820001
NC_056060.1	43700001	43720001	2.11795	0.333497	BD	ZNF609	NC_056056.1	142805001	142825001
NC_056060.1	43720001	43740001	2.70652	0.304342	BD	ZNF609	NC_056056.1	142810001	142830001
NC_056067.1	46555001	46575001	2.14809	0.311543	BD	ZNF793	NC_056056.1	142815001	142835001
NC_056055.1	1.25E+08	124965001	2.11884	0.330371	BD	ZNF804A	NC_056056.1	142820001	142840001
NC_056070.1	12530001	12550001	2.35856	0.310891	BD	ZNF827	NC_056056.1	142825001	142845001
NC_056072.1	60380001	60400001	2.46965	0.34953	BD	ZXDC	NC_056056.1	84470001	84490001
NC_056064.1	24480001	24500001	4.67335	0.443221	BD	ZZEF1	NC_056056.1	84485001	84505001
NC_056054.1	54110001	54130001	2.4226	0.336853	BD	ZZZ3	NC_056056.1	84490001	84510001
							NC_056056.1	98990001	99010001
							NC_056056.1	99010001	99030001
							NC_056056.1	99015001	99035001
							NC_056056.1	99020001	99040001
							NC_056077.1	28770001	28790001
							NC_056077.1	28775001	28795001
							NC_056071.1	55620001	55640001
							NC_056062.1	21660001	21680001
							NC_056055.1	50080001	50100001
							NC_056068.1	28315001	28335001

NC_056068.1	28320001	28340001
NC_056068.1	28325001	28345001
NC_056068.1	28330001	28350001
NC_056068.1	28335001	28355001
NC_056068.1	28340001	28360001
NC_056056.1	119035001	119055001
NC_056056.1	119040001	119060001
NC_056056.1	119045001	119065001
NC_056055.1	8670001	8690001
NC_056065.1	40070001	40090001
NC_056055.1	8875001	8895001
NC_056055.1	8880001	8900001
NC_056069.1	8915001	8935001
NC_056069.1	8920001	8940001
NC_056065.1	66845001	66865001
NC_056062.1	16430001	16450001
NC_056062.1	16435001	16455001
NC_056056.1	108400001	108420001
NC_056056.1	108540001	108560001
NC_056056.1	108545001	108565001
NC_056056.1	108550001	108570001
NC_056056.1	108555001	108575001
NC_056080.1	44320001	44340001
NC_056080.1	44325001	44345001
NC_056080.1	44330001	44350001
NC_056080.1	44335001	44355001
NC_056062.1	61775001	61795001
NC_056080.1	127505001	127525001
NC_056080.1	127510001	127530001
NC_056080.1	127515001	127535001
NC_056056.1	102175001	102195001
NC_056056.1	102180001	102200001
NC_056056.1	102185001	102205001
NC_056056.1	102195001	102215001
NC_056056.1	102200001	102220001
NC_056056.1	102235001	102255001
NC_056056.1	102240001	102260001
NC_056056.1	102245001	102265001
NC_056056.1	102250001	102270001
NC_056056.1	102255001	102275001
NC_056056.1	102260001	102280001
NC_056056.1	102265001	102285001
NC_056056.1	102270001	102290001
NC_056059.1	26650001	26670001
NC_056059.1	26655001	26675001
NC_056059.1	26660001	26680001
NC_056059.1	26665001	26685001
NC_056080.1	38090001	38110001
NC_056080.1	38095001	38115001
NC_056080.1	38130001	38150001
NC_056080.1	38160001	38180001
NC_056080.1	38175001	38195001
NC_056074.1	37590001	37610001
NC_056060.1	85110001	85130001
NC_056060.1	85390001	85410001
NC_056056.1	225320001	225340001
NC_056055.1	33510001	33530001
NC_056055.1	33515001	33535001
NC_056055.1	13005001	13025001
NC_056055.1	13010001	13030001
NC_056055.1	13015001	13035001
NC_056055.1	13030001	13050001
NC_056055.1	13035001	13055001
NC_056055.1	81175001	81195001
NC_056062.1	50065001	50085001
NC_056062.1	50070001	50090001
NC_056062.1	50075001	50095001
NC_056062.1	50080001	50100001
NC_056062.1	50085001	50105001
NC_056062.1	50090001	50110001
NC_056062.1	50095001	50115001
NC_056062.1	50100001	50120001
NC_056065.1	42630001	42650001
NC_056065.1	42660001	42680001

NC_056065.1	41615001	41635001
NC_056065.1	41620001	41640001
NC_056065.1	11420001	11440001
NC_056065.1	11425001	11445001
NC_056065.1	11430001	11450001
NC_056055.1	53215001	53235001
NC_056071.1	55975001	55995001
NC_056065.1	18845001	18865001
NC_056065.1	18850001	18870001
NC_056054.1	29585001	29605001
NC_056080.1	40470001	40490001
NC_056061.1	73340001	73360001
NC_056061.1	68935001	68955001
NC_056061.1	68940001	68960001
NC_056061.1	68945001	68965001
NC_056064.1	41345001	41365001
NC_056080.1	82150001	82170001
NC_056072.1	55565001	55585001
NC_056072.1	55570001	55590001
NC_056057.1	83805001	83825001
NC_056057.1	83810001	83830001
NC_056057.1	83815001	83835001
NC_056080.1	116435001	116455001
NC_056080.1	116440001	116460001
NC_056080.1	116450001	116470001
NC_056080.1	116455001	116475001
NC_056080.1	116460001	116480001
NC_056075.1	21100001	21120001
NC_056056.1	92300001	92320001
NC_056056.1	92305001	92325001
NC_056062.1	35735001	35755001
NC_056062.1	35740001	35760001
NC_056055.1	49895001	49915001
NC_056054.1	247190001	247210001
NC_056054.1	247195001	247215001
NC_056054.1	247200001	247220001
NC_056054.1	247220001	247240001
NC_056054.1	247225001	247245001
NC_056054.1	247230001	247250001
NC_056054.1	247235001	247255001
NC_056054.1	247240001	247260001
NC_056054.1	247245001	247265001
NC_056054.1	247250001	247270001
NC_056054.1	247255001	247275001
NC_056054.1	247260001	247280001
NC_056054.1	247265001	247285001
NC_056076.1	36085001	36105001
NC_056060.1	8265001	8285001
NC_056060.1	8270001	8290001
NC_056060.1	8275001	8295001
NC_056068.1	24330001	24350001
NC_056068.1	24335001	24355001
NC_056068.1	24415001	24435001
NC_056068.1	24420001	24440001
NC_056068.1	24425001	24445001
NC_056068.1	24430001	24450001
NC_056068.1	24435001	24455001
NC_056068.1	24440001	24460001
NC_056068.1	24445001	24465001
NC_056068.1	24500001	24520001
NC_056068.1	24505001	24525001
NC_056074.1	33765001	33785001
NC_056074.1	33770001	33790001
NC_056074.1	33775001	33795001
NC_056074.1	33780001	33800001
NC_056074.1	33785001	33805001
NC_056074.1	33790001	33810001
NC_056074.1	33795001	33815001
NC_056068.1	19900001	19920001
NC_056068.1	19905001	19925001
NC_056072.1	2925001	2945001
NC_056056.1	112650001	112670001
NC_056056.1	112715001	112735001
NC_056056.1	112720001	112740001

NC_056056.1	112725001	112745001
NC_056056.1	112730001	112750001
NC_056060.1	86495001	86515001
NC_056062.1	71755001	71775001
NC_056062.1	71760001	71780001
NC_056074.1	38565001	38585001
NC_056074.1	38570001	38590001
NC_056080.1	57440001	57460001
NC_056067.1	39120001	39140001
NC_056067.1	65255001	65275001
NC_056067.1	65260001	65280001
NC_056055.1	130215001	130235001
NC_056055.1	130220001	130240001
NC_056080.1	57425001	57445001
NC_056067.1	17485001	17505001
NC_056066.1	33735001	33755001
NC_056066.1	33740001	33760001
NC_056066.1	33745001	33765001
NC_056066.1	33750001	33770001
NC_056066.1	33755001	33775001
NC_056077.1	37290001	37310001
NC_056062.1	57125001	57145001
NC_056067.1	1850001	1870001
NC_056067.1	1855001	1875001
NC_056067.1	1860001	1880001
NC_056067.1	1865001	1885001
NC_056060.1	13335001	13355001

Pi	Fst	Region	Gene	HZ vs SG2<br>CHROM	Start	end	Pi	Fst	Region	Gene
2.78758	0.227128	GY	ABCB5	NC_056055.1	217165001	217185001	2.5442	0.20615	HZ	ABCA12
3.48179	0.267094	GY	ABCB5	NC_056055.1	217170001	217190001	2.54794	0.217452	HZ	ABCA12
3.5414	0.209753	GY	ABCB5	NC_056057.1	30380001	30400001	2.53487	0.342098	HZ	ABCB5
2.75926	0.241556	GY	ABCB7	NC_056057.1	30385001	30405001	2.77989	0.414043	HZ	ABCB5
2.52598	0.249573	GY	ABCB7	NC_056057.1	30390001	30410001	2.83864	0.363961	HZ	ABCB5
3.04676	0.244416	GY	ABCB7	NC_056077.1	14850001	14870001	2.45011	0.214202	HZ	ABCC1
2.84172	0.221245	GY	ABCB7	NC_056067.1	16070001	16090001	3.11406	0.238945	HZ	ABCC12
2.71495	0.193572	GY	ABCF3;AP2M1	NC_056067.1	16075001	16095001	2.99737	0.216361	HZ	ABCC12
3.29385	0.201436	GY	ABHD16A;LOC101	NC_056073.1	26700001	26720001	2.87951	0.245532	HZ	ABHD16A;CSNK2B;
4.33636	0.223962	GY	ABHD16A;LOC101	NC_056073.1	26695001	26715001	3.44827	0.22439	HZ	ABHD16A;CSNK2B;
5.22158	0.217241	GY	ABHD16A;LOC101	NC_056073.1	26670001	26690001	4.79387	0.244081	HZ	ABHD16A;LOC1011
4.78431	0.220321	GY	ABHD16A;LY6G5I	NC_056073.1	26675001	26695001	5.72272	0.270309	HZ	ABHD16A;LOC1011
6.21568	0.238707	GY	ABHD16A;LY6G6I	NC_056073.1	26680001	26700001	6.61363	0.261171	HZ	ABHD16A;LOC1011
2.90955	0.193025	GY	ABL1;EXOSC2	NC_056073.1	26690001	26710001	4.5098	0.277472	HZ	ABHD16A;LY6G5B;I
3.88123	0.257036	GY	ACAD8	NC_056073.1	26685001	26705001	6.32028	0.309894	HZ	ABHD16A;LY6G6F
3.02581	0.232428	GY	ACAD8	NC_056056.1	5920001	5940001	2.53784	0.258198	HZ	ABL1;EXOSC2
3.30859	0.227082	GY	ACAD8	NC_056068.1	82095001	82115001	3.76245	0.255501	HZ	ACAD8
2.99501	0.237583	GY	ACAD8;THYN1	NC_056068.1	82100001	82120001	3.23548	0.241829	HZ	ACAD8
3.51391	0.259504	GY	ACSS3	NC_056068.1	82105001	82125001	3.86328	0.265457	HZ	ACAD8
6.16292	0.295992	GY	ACSS3	NC_056068.1	82090001	82110001	2.9227	0.253892	HZ	ACAD8;THYN1
4.34719	0.253634	GY	ACSS3	NC_056068.1	82075001	82095001	6.75481	0.416145	HZ	ACAD8;THYN1;VPS
4.15071	0.245913	GY	ACSS3	NC_056068.1	82080001	82100001	5.59744	0.37441	HZ	ACAD8;THYN1;VPS
3.36454	0.230466	GY	ACSS3	NC_056068.1	82085001	82105001	3.93548	0.323885	HZ	ACAD8;THYN1;VPS
3.26432	0.340507	GY	ACSS3	NC_056054.1	110155001	110175001	7.04573	0.238578	HZ	ACKR1
4.38131	0.395442	GY	ACSS3	NC_056054.1	110135001	110155001	4.14984	0.229507	HZ	ACKR1;CADM3
3.6954	0.371403	GY	ACSS3	NC_056054.1	110140001	110160001	4.16308	0.232206	HZ	ACKR1;CADM3
3.1381	0.238056	GY	ACSS3	NC_056054.1	110145001	110165001	5.04202	0.220242	HZ	ACKR1;CADM3
3.09894	0.217557	GY	ACTL7A;ACTL7B;	NC_056056.1	117215001	117235001	4.06705	0.219015	HZ	ACSS3
2.95551	0.216427	GY	ADAM19	NC_056057.1	73835001	73855001	3.2013	0.256191	HZ	ADAM22
4.03488	0.233633	GY	ADAM22	NC_056057.1	73840001	73860001	4.64286	0.245025	HZ	ADAM22
23.3879	0.334982	GY	ADAM22	NC_056057.1	73845001	73865001	6.34312	0.260391	HZ	ADAM22
3.37879	0.204354	GY	ADAM22	NC_056057.1	73850001	73870001	4.39841	0.213122	HZ	ADAM22
6.30881	0.195722	GY	ADAM22	NC_056057.1	73855001	73875001	3.48386	0.226116	HZ	ADAM22
3.26165	0.217008	GY	ADAM22	NC_056055.1	86510001	86530001	3.22918	0.32665	HZ	ADAMTSL1
3.8893	0.224246	GY	ADAMTS17	NC_056055.1	86515001	86535001	3.51755	0.353616	HZ	ADAMTSL1
3.69388	0.250693	GY	ADAMTS17	NC_056055.1	86610001	86630001	3.75749	0.250234	HZ	ADAMTSL1
4.01259	0.261317	GY	ADAMTS17	NC_056066.1	45785001	45805001	3.05819	0.21431	HZ	ADARB2
4.54864	0.251145	GY	ADAMTS17	NC_056066.1	45790001	45810001	4.91732	0.256804	HZ	ADARB2
3.14792	0.258932	GY	ADAMTSL1	NC_056066.1	45795001	45815001	6.40217	0.23259	HZ	ADARB2
3.48601	0.306255	GY	ADAMTSL1	NC_056066.1	45800001	45820001	7.77866	0.237838	HZ	ADARB2
2.90703	0.20349	GY	ADGRD1	NC_056066.1	45835001	45855001	2.62371	0.270706	HZ	ADARB2
2.42609	0.198404	GY	ADGRG2	NC_056066.1	45840001	45860001	3.64657	0.322791	HZ	ADARB2
8.80819	0.237516	GY	ADRB3	NC_056066.1	45845001	45865001	4.36287	0.36122	HZ	ADARB2
2.54167	0.20749	GY	AFF1	NC_056066.1	45850001	45870001	3.21221	0.314138	HZ	ADARB2
4.87166	0.234239	GY	AFF1	NC_056076.1	36175001	36195001	6.74397	0.258654	HZ	ADCYAP1
4.39829	0.218858	GY	AFF1	NC_056076.1	36180001	36200001	5.46599	0.22056	HZ	ADCYAP1
4.06402	0.265547	GY	AFF1	NC_056062.1	5290001	5310001	2.39788	0.465754	HZ	ADGRB3
3.7353	0.276052	GY	AFF1	NC_056059.1	25595001	25615001	2.7881	0.307761	HZ	ADH1C
3.15915	0.25808	GY	AFF1	NC_056059.1	25600001	25620001	4.20186	0.363719	HZ	ADH1C
3.57187	0.332584	GY	AFF1	NC_056059.1	25510001	25530001	3.23616	0.368545	HZ	ADH7;C6H4orf17
2.49015	0.242002	GY	AFF1	NC_056059.1	25515001	25535001	2.39858	0.284211	HZ	ADH7;C6H4orf17
4.60316	0.256001	GY	AFF2	NC_056059.1	25505001	25525001	2.45864	0.320307	HZ	ADH7;C6H4orf17;LO
5.29652	0.302087	GY	AFF2	NC_056080.1	87110001	87130001	2.50844	0.235224	HZ	AFF2
2.46265	0.231336	GY	AFF2	NC_056080.1	87115001	87135001	2.5534	0.26557	HZ	AFF2
2.86084	0.221304	GY	AFF2	NC_056080.1	87120001	87140001	2.91573	0.303459	HZ	AFF2
3.20786	0.192614	GY	AFF2	NC_056080.1	87130001	87150001	3.71094	0.222449	HZ	AFF2
2.49895	0.242444	GY	AFF3	NC_056071.1	16960001	16980001	3.58168	0.209031	HZ	AGBL1
3	0.19848	GY	AGO3;LOC101111	NC_056055.1	132635001	132655001	3.03839	0.225999	HZ	AGPS
4.7249	0.209055	GY	AGPAT3	NC_056061.1	49600001	49620001	6.71895	0.299888	HZ	AKIRIN2
3.1754	0.193563	GY	AKAP7	NC_056061.1	49605001	49625001	6.10651	0.246982	HZ	AKIRIN2
2.63265	0.193226	GY	ALAS1;TLR9	NC_056061.1	49610001	49630001	5.63353	0.226823	HZ	AKIRIN2
2.62462	0.192792	GY	ALAS1;TLR9;TWF	NC_056054.1	171135001	171155001	5.03481	0.206212	HZ	ALCAM
3.61206	0.194315	GY	ALDH4A1	NC_056054.1	171140001	171160001	5.61884	0.247963	HZ	ALCAM
5.0871	0.482733	GY	ALS2CL	NC_056054.1	171145001	171165001	6.21484	0.215343	HZ	ALCAM
2.62539	0.192754	GY	ALS2CL;PRSS50;T	NC_056056.1	36560001	36580001	4.26495	0.237982	HZ	ALK
5.38709	0.297062	GY	ALS2CL;TMIE	NC_056059.1	13565001	13585001	2.75682	0.351925	HZ	ALPK1
2.75806	0.513592	GY	AMMECR1	NC_056059.1	13570001	13590001	2.90047	0.29874	HZ	ALPK1
3.08805	0.507783	GY	AMMECR1	NC_056059.1	13575001	13595001	3.39894	0.256506	HZ	ALPK1
3.18301	0.416587	GY	AMMECR1	NC_056059.1	13580001	13600001	2.68346	0.218981	HZ	ALPK1

2.56303	0.284035	GY	AMOT	NC_056080.1	121900001	121920001	2.60745	0.224617	HZ	AMOT
2.42285	0.193872	GY	AMOTL1	NC_056063.1	84675001	84695001	7.42639	0.243432	HZ	ANKRD10;LOC1218
2.4	0.203018	GY	ANGEL1	NC_056063.1	84680001	84700001	13.9212	0.230029	HZ	ANKRD10;LOC1218
2.47779	0.232773	GY	ANKDD1A	NC_056063.1	84685001	84705001	16.3942	0.219131	HZ	ANKRD10;LOC1218
3.20044	0.194903	GY	ANKDD1A	NC_056076.1	33350001	33370001	2.40701	0.217055	HZ	ANKRD29
3.85184	0.226707	GY	ANKDD1A	NC_056060.1	6610001	6630001	4.04226	0.215406	HZ	ANKRD31
2.99583	0.281761	GY	ANKK1	NC_056069.1	62765001	62785001	2.46201	0.356757	HZ	ANKRD33B
5.55218	0.330485	GY	ANKK1	NC_056069.1	62770001	62790001	2.80664	0.373698	HZ	ANKRD33B
10.3586	0.338092	GY	ANKK1	NC_056069.1	62775001	62795001	3.75214	0.394062	HZ	ANKRD33B
16.8117	0.318109	GY	ANKK1	NC_056069.1	62780001	62800001	3.4881	0.432102	HZ	ANKRD33B
11.3272	0.294015	GY	ANKK1	NC_056069.1	62785001	62805001	3.48473	0.449237	HZ	ANKRD33B
7.40971	0.272675	GY	ANKK1;DRD2	NC_056069.1	62790001	62810001	2.63928	0.437372	HZ	ANKRD33B
12.7212	0.260823	GY	ANKRD10	NC_056055.1	199425001	199445001	2.62488	0.473464	HZ	ANKRD44
7.37562	0.281677	GY	ANKRD10;LOC121	NC_056055.1	199430001	199450001	2.43395	0.440748	HZ	ANKRD44
13.3779	0.306976	GY	ANKRD10;LOC121	NC_056056.1	210320001	210340001	2.44775	0.211934	HZ	ANO2
15.5289	0.297127	GY	ANKRD10;LOC121	NC_056056.1	141285001	141305001	3.37983	0.21535	HZ	ANO6
4.07802	0.214081	GY	ANKRD31	NC_056056.1	141290001	141310001	3.06611	0.213213	HZ	ANO6
6.5646	0.240608	GY	ANKRD31	NC_056067.1	38775001	38795001	3.36046	0.249449	HZ	AP1G1
3.86651	0.218416	GY	ANKRD31	NC_056074.1	15960001	15980001	3.09602	0.277585	HZ	AQP11
2.44958	0.227469	GY	ANKRD33B	NC_056074.1	15965001	15985001	2.62529	0.227831	HZ	AQP11
3.08038	0.195529	GY	ANKRD33B	NC_056074.1	15950001	15970001	3.125	0.266501	HZ	AQP11;CLNS1A
2.83961	0.227581	GY	ANKRD33B	NC_056074.1	15955001	15975001	3.08044	0.280876	HZ	AQP11;CLNS1A
2.79574	0.226143	GY	ANKRD33B	NC_056055.1	37705001	37725001	4.64467	0.256297	HZ	AQP3;NOL6
16.5763	0.260085	GY	ANKRD37;C26H4c	NC_056062.1	44360001	44380001	2.88586	0.208991	HZ	ARFGEF1
26.4881	0.265392	GY	ANKRD37;UFSP2	NC_056062.1	44370001	44390001	2.51915	0.219177	HZ	ARFGEF1
22.958	0.274701	GY	ANKRD37;UFSP2	NC_056070.1	5025001	5045001	4.97826	0.252343	HZ	ARFIP1
3.66296	0.260753	GY	ANKRD44	NC_056070.1	5030001	5050001	3	0.243699	HZ	ARFIP1
3.04115	0.241553	GY	ANKRD44	NC_056070.1	5035001	5055001	2.77035	0.247407	HZ	ARFIP1
2.83449	0.22881	GY	ANKRD44	NC_056070.1	5040001	5060001	2.45842	0.256122	HZ	ARFIP1
2.77224	0.198	GY	ANO4	NC_056068.1	20425001	20445001	5.53278	0.231891	HZ	ARHGAP20
3.36285	0.211358	GY	ANO4	NC_056064.1	31035001	31055001	3.46421	0.226293	HZ	ARHGAP44
2.7621	0.226452	GY	ANO4	NC_056064.1	31040001	31060001	3.99123	0.223037	HZ	ARHGAP44
2.4588	0.226009	GY	ANO4	NC_056064.1	31045001	31065001	4.42796	0.205417	HZ	ARHGAP44
14.2671	0.19405	GY	ANO4	NC_056080.1	9680001	9700001	8.21127	0.217348	HZ	ARHGAP6
5.054	0.257348	GY	ANO4	NC_056056.1	200585001	200605001	2.59822	0.205535	HZ	ARHGDIB;ERP27
4.31523	0.267653	GY	ANO4	NC_056057.1	108790001	108810001	2.6875	0.23359	HZ	ARHGEF5
3.17697	0.290438	GY	ANO4	NC_056056.1	140585001	140605001	6.43369	0.211531	HZ	ARID2
2.53558	0.27568	GY	ANO4	NC_056056.1	140655001	140675001	2.72579	0.224145	HZ	ARID2
2.42686	0.247465	GY	ANO4	NC_056056.1	140660001	140680001	2.37428	0.241275	HZ	ARID2
3.51933	0.20394	GY	ANO6	NC_056056.1	140570001	140590001	4.27718	0.248344	HZ	ARID2;SCAF11
3.1471	0.204426	GY	ANO6	NC_056056.1	140575001	140595001	6.8631	0.232448	HZ	ARID2;SCAF11
2.74663	0.274681	GY	APOB	NC_056056.1	140580001	140600001	7.31868	0.211931	HZ	ARID2;SCAF11
4.84263	0.227308	GY	AQP3;NOL6	NC_056060.1	68480001	68500001	2.81712	0.233767	HZ	ARMH4
2.7193	0.198903	GY	ARF6	NC_056060.1	68485001	68505001	2.84486	0.242171	HZ	ARMH4
2.76297	0.198269	GY	ARHGAP6;LOC101	NC_056060.1	68490001	68510001	3.20563	0.264535	HZ	ARMH4
2.47354	0.202652	GY	ARHGEF37	NC_056056.1	188045001	188065001	2.54933	0.247708	HZ	ARNTL2
2.54691	0.255008	GY	ARMC1	NC_056056.1	188050001	188070001	2.92509	0.244576	HZ	ARNTL2
3.17498	0.209233	GY	ARMH4	NC_056056.1	188055001	188075001	4.39671	0.236174	HZ	ARNTL2
2.82279	0.205166	GY	ARNTL2	NC_056056.1	188060001	188080001	6.05627	0.271146	HZ	ARNTL2
3.51757	0.21234	GY	ARNTL2	NC_056056.1	188065001	188085001	2.77025	0.214973	HZ	ARNTL2
2.72153	0.233293	GY	ARPP21	NC_056077.1	37460001	37480001	6.02248	0.220246	HZ	ARPC1A
2.80115	0.197706	GY	ARPP21	NC_056077.1	37465001	37485001	5.09054	0.23282	HZ	ARPC1A
3.41444	0.397186	GY	ASB5	NC_056060.1	55940001	55960001	2.82641	0.266281	HZ	ARPP19;FAM214A
2.6878	0.218115	GY	ASCL2	NC_056060.1	55975001	55995001	2.59679	0.246415	HZ	ARPP19;MYO5A
3.06647	0.214578	GY	ASTN1	NC_056056.1	200685001	200705001	3.69545	0.253847	HZ	ART4
3.50385	0.244361	GY	ASTN1	NC_056056.1	200695001	200715001	2.69908	0.21806	HZ	ART4;C3H12orf60;SM
2.51812	0.254924	GY	ASTN1	NC_056056.1	200690001	200710001	3.75299	0.266955	HZ	ART4;SMCO3
2.50246	0.284628	GY	ATG4A;COL4A6	NC_056079.1	18375001	18395001	2.61773	0.23449	HZ	ASAH1
10.9671	0.28895	GY	ATMIN;CENPN	NC_056079.1	18380001	18400001	2.95376	0.246043	HZ	ASAH1
5.07416	0.254051	GY	ATMIN;CENPN	NC_056079.1	18385001	18405001	3.30784	0.206325	HZ	ASAH1
5.74515	0.279972	GY	ATP10B	NC_056064.1	24075001	24095001	5.30995	0.211091	HZ	ASPA;SPATA22
3.50072	0.267193	GY	ATP10B	NC_056065.1	57650001	57670001	2.37736	0.211878	HZ	ASTN1
2.64791	0.267034	GY	ATP10B	NC_056056.1	135535001	135555001	2.77727	0.273376	HZ	ATF1
2.58983	0.259135	GY	ATP6V0A1	NC_056056.1	135540001	135560001	5.71911	0.242875	HZ	ATF1
2.48829	0.19574	GY	ATP7A	NC_056067.1	7200001	7220001	10.679	0.290754	HZ	ATMIN;CENPN
2.6079	0.222683	GY	ATP7A	NC_056067.1	7205001	7225001	4.87924	0.238664	HZ	ATMIN;CENPN
2.54063	0.212402	GY	ATP7A	NC_056058.1	70075001	70095001	2.60663	0.22332	HZ	ATP10B
2.4428	0.208197	GY	ATP7A	NC_056080.1	95530001	95550001	4.17597	0.23275	HZ	ATP11C
3.56937	0.227646	GY	ATR;XRN1	NC_056062.1	34850001	34870001	2.72806	0.240185	HZ	ATP6V1H
4.28714	0.23479	GY	ATR;XRN1	NC_056080.1	69100001	69120001	2.6923	0.330887	HZ	ATP7A
5.39631	0.232463	GY	AURKA;CSTF1	NC_056080.1	69105001	69125001	3.81706	0.361378	HZ	ATP7A
9.4898	0.278977	GY	AURKA;CSTF1	NC_056055.1	26230001	26250001	6.22312	0.24952	HZ	AUH
11.3632	0.288858	GY	AURKA;CSTF1	NC_056055.1	26235001	26255001	9.72389	0.279922	HZ	AUH
7.81562	0.283103	GY	AURKA;CSTF1;FA	NC_056066.1	59195001	59215001	7.48387	0.28445	HZ	AURKA;CSTF1

3.47797	0.2602	GY	AURKA;FAM210B	NC_056066.1	59200001	59220001	11.6939	0.348618	HZ	AURKA;CSTF1
2.43086	0.211107	GY	B3GALNT2	NC_056066.1	59205001	59225001	11.684	0.380438	HZ	AURKA;CSTF1
3.33839	0.203121	GY	B3GALNT2	NC_056066.1	59210001	59230001	7.61563	0.366115	HZ	AURKA;CSTF1;FAM
2.93404	0.196373	GY	B4GALT6	NC_056066.1	59215001	59235001	3.41525	0.300572	HZ	AURKA;FAM210B
3.15404	0.212157	GY	B4GALT6	NC_056077.1	30870001	30890001	2.73012	0.22324	HZ	AUTS2
3.01624	0.206531	GY	B4GALT6	NC_056074.1	40820001	40840001	5.47502	0.484413	HZ	B4GAT1;BRMS1
4.65835	0.229562	GY	B4GALT6	NC_056074.1	40825001	40845001	4.75721	0.499189	HZ	B4GAT1;BRMS1
3.39729	0.216377	GY	B4GALT6	NC_056074.1	40815001	40835001	2.63987	0.39092	HZ	B4GAT1;BRMS1;RIN
2.50896	0.228546	GY	B4GALT6	NC_056066.1	35405001	35425001	2.77696	0.262023	HZ	BAMBI
5.90626	0.418981	GY	B4GAT1;BRMS1	NC_056059.1	23195001	23215001	3.11612	0.282238	HZ	BANK1
5.08091	0.424373	GY	B4GAT1;BRMS1	NC_056059.1	23200001	23220001	2.98489	0.256421	HZ	BANK1
2.96078	0.244214	GY	B4GAT1;BRMS1;R	NC_056059.1	23205001	23225001	2.91585	0.25337	HZ	BANK1
3.17686	0.326243	GY	B4GAT1;BRMS1;R	NC_056054.1	70865001	70885001	5.40465	0.238976	HZ	BCAR3
3.3619	0.276948	GY	BAG4	NC_056080.1	39475001	39495001	2.96178	0.27442	HZ	BCOR
3.23958	0.209274	GY	BANK1	NC_056080.1	129130001	129150001	2.36202	0.209696	HZ	BEX4
2.56256	0.25783	GY	BANK1	NC_056068.1	6005001	6025001	2.59427	0.248894	HZ	BIRC2
20.5221	0.191956	GY	BBS2	NC_056068.1	6010001	6030001	3.99193	0.267058	HZ	BIRC2
5.70771	0.205839	GY	BBS9	NC_056068.1	6015001	6035001	3.66935	0.276021	HZ	BIRC2
6.78746	0.26822	GY	BCAM;CBLC	NC_056054.1	100000001	100020001	4.58621	0.23704	HZ	BOLA1;SV2A
2.61701	0.239265	GY	BCAM;CBLC	NC_056054.1	100005001	100025001	6.05724	0.227831	HZ	BOLA1;SV2A
11.5535	0.196277	GY	BCAR3	NC_056056.1	203025001	203045001	4.41122	0.389624	HZ	BORCS5
2.54756	0.234785	GY	BCOR	NC_056056.1	203030001	203050001	8.16239	0.435324	HZ	BORCS5
2.58148	0.195578	GY	BNIP3	NC_056056.1	203035001	203055001	10.5738	0.435227	HZ	BORCS5
2.93466	0.21234	GY	BNIP3	NC_056056.1	203040001	203060001	5.22014	0.383112	HZ	BORCS5
2.56105	0.19513	GY	BNIP3	NC_056056.1	203045001	203065001	2.86884	0.323513	HZ	BORCS5
3.59876	0.219603	GY	BOD1	NC_056057.1	105535001	105555001	3.10227	0.324785	HZ	BRAF
2.77012	0.251	GY	BTBD8	NC_056057.1	105540001	105560001	6.17361	0.401672	HZ	BRAF
4.10741	0.193252	GY	BTBD8	NC_056057.1	105545001	105565001	7.24348	0.418816	HZ	BRAF
4.97562	0.229775	GY	BUB1B	NC_056057.1	105550001	105570001	5.75412	0.422262	HZ	BRAF
3.60361	0.378177	GY	BUB1B	NC_056057.1	105555001	105575001	6.76891	0.423738	HZ	BRAF
2.45516	0.260645	GY	C10H13orf42	NC_056057.1	105560001	105580001	4.79977	0.422988	HZ	BRAF
7.00747	0.211625	GY	C12H1orf21	NC_056057.1	105565001	105585001	4.0626	0.43446	HZ	BRAF
20.0108	0.314594	GY	C12H1orf21	NC_056057.1	105570001	105590001	3.5123	0.475044	HZ	BRAF
14.6222	0.326262	GY	C12H1orf21	NC_056057.1	105575001	105595001	2.74092	0.479015	HZ	BRAF
6.09142	0.238563	GY	C12H1orf21	NC_056057.1	105580001	105600001	2.57211	0.528393	HZ	BRAF
4.27339	0.209094	GY	C12H1orf21	NC_056057.1	105585001	105605001	3.73005	0.498064	HZ	BRAF
10.1102	0.202988	GY	C12H1orf21	NC_056057.1	105590001	105610001	4.15231	0.426714	HZ	BRAF
13.8571	0.201186	GY	C12H1orf21	NC_056057.1	105595001	105615001	4.45697	0.407761	HZ	BRAF
6.90418	0.213175	GY	C12H1orf21	NC_056057.1	105600001	105620001	5.97394	0.293381	HZ	BRAF
2.45336	0.26081	GY	C14H16orf95	NC_056057.1	105605001	105625001	9.02723	0.215586	HZ	BRAF
2.61392	0.192306	GY	C14H16orf95	NC_056074.1	46445001	46465001	3.98852	0.31269	HZ	BRSK2
11.3714	0.228097	GY	C14H19orf73;LIN7I	NC_056074.1	46450001	46470001	3.63365	0.236863	HZ	BRSK2
11.1475	0.277279	GY	C15H11orf52;DIXD	NC_056074.1	46455001	46475001	6.9948	0.231256	HZ	BRSK2
4.67369	0.264726	GY	C15H11orf52;DIXD	NC_056074.1	46460001	46480001	11.8507	0.217476	HZ	BRSK2
3.3093	0.369616	GY	C15H11orf87	NC_056074.1	46465001	46485001	16.7652	0.221252	HZ	BRSK2
3.26532	0.239997	GY	C15H11orf97	NC_056074.1	46470001	46490001	20.9625	0.225049	HZ	BRSK2
3.09206	0.239149	GY	C15H11orf97	NC_056074.1	46430001	46450001	6.06015	0.288727	HZ	BRSK2;MOB2
2.50275	0.281736	GY	C15H11orf97	NC_056074.1	46435001	46455001	7.0695	0.281658	HZ	BRSK2;MOB2
9.31031	0.22135	GY	C1GALT1C1	NC_056074.1	46440001	46460001	6.46191	0.319769	HZ	BRSK2;MOB2
2.43513	0.193376	GY	C1H1orf146	NC_056054.1	19475001	19495001	5.71552	0.2201	HZ	BTBD19;DYNLT4;PI
3.1777	0.227364	GY	C1H1orf146	NC_056080.1	133045001	133065001	2.94525	0.233775	HZ	BTK;LOC101105982
4.43393	0.271605	GY	C1H1orf146;GLMN	NC_056067.1	12265001	12285001	2.95937	0.254108	HZ	C14H16orf95
6.92514	0.298335	GY	C1H1orf146;GLMN	NC_056067.1	12270001	12290001	5.22694	0.307624	HZ	C14H16orf95
7.32663	0.299362	GY	C1H1orf146;GLMN	NC_056067.1	12275001	12295001	7.51513	0.397481	HZ	C14H16orf95
9.50961	0.324759	GY	C1H1orf146;GLMN	NC_056067.1	12280001	12300001	2.93086	0.300074	HZ	C14H16orf95
2.46299	0.320751	GY	C1H1orf68	NC_056068.1	15625001	15645001	3.82865	0.261539	HZ	C15H11orf97
2.75341	0.201311	GY	C1H3orf33	NC_056068.1	15630001	15650001	2.82878	0.33411	HZ	C15H11orf97
6.58492	0.21792	GY	C3H2orf15;TSGA1(	NC_056068.1	15635001	15655001	2.74666	0.38399	HZ	C15H11orf97
17.783	0.31927	GY	C6H4orf17	NC_056068.1	15640001	15660001	2.78724	0.371361	HZ	C15H11orf97
5.72633	0.282454	GY	C6H4orf17	NC_056054.1	104495001	104515001	2.46491	0.315418	HZ	C1H1orf189;C1H1orf
6.018	0.301369	GY	C6H4orf17	NC_056054.1	202870001	202890001	2.49518	0.261565	HZ	C1H3orf70
3.60437	0.286361	GY	C6H4orf17	NC_056073.1	44640001	44660001	2.65861	0.24439	HZ	C20H6orf52;LOC101
2.73921	0.21137	GY	C6H4orf50	NC_056074.1	41180001	41200001	6.28571	0.205282	HZ	C21H11orf80
2.45592	0.206814	GY	C6H4orf50	NC_056056.1	102165001	102185001	3.33701	0.213965	HZ	C3H2orf15;LIPT1;TS(
3.03255	0.198088	GY	CADM1	NC_056056.1	102170001	102190001	3.93868	0.223038	HZ	C3H2orf15;TSGA10
4.77652	0.22793	GY	CADM2	NC_056056.1	95640001	95660001	4.3328	0.209227	HZ	C3H2orf78
3.09351	0.263557	GY	CADM2	NC_056059.1	25430001	25450001	16.4273	0.472966	HZ	C6H4orf17
2.64196	0.261988	GY	CADM2	NC_056059.1	25435001	25455001	5.31852	0.413084	HZ	C6H4orf17
2.51149	0.252814	GY	CADM2	NC_056059.1	25440001	25460001	5.74107	0.414029	HZ	C6H4orf17
2.69548	0.268707	GY	CALCOCO1	NC_056059.1	25445001	25465001	3.57143	0.365418	HZ	C6H4orf17
6.09134	0.315134	GY	CALCOCO1	NC_056059.1	25410001	25430001	5.30697	0.284377	HZ	C6H4orf17;TRMT10A
2.79967	0.277916	GY	CAMKMT	NC_056061.1	49930001	49950001	2.9914	0.227369	HZ	C8H6orf163;SMIM8
4.30836	0.356124	GY	CAMKMT	NC_056056.1	214555001	214575001	2.44894	0.209391	HZ	CACNA1C
6.9461	0.437686	GY	CAMKMT	NC_056056.1	216910001	216930001	2.46022	0.218395	HZ	CACNA1I

14.7925	0.506314	GY	CAMKMT	NC_056056.1	216915001	216935001	2.36659	0.227434	HZ	CACNA1I
16.2034	0.420571	GY	CAMKMT	NC_056054.1	110130001	110150001	5.53947	0.223253	HZ	CADM3
13.1118	0.401848	GY	CAMKMT	NC_056057.1	100200001	100220001	3.35224	0.205276	HZ	CALD1
6.79801	0.348777	GY	CAMKMT	NC_056057.1	100205001	100225001	2.45379	0.214404	HZ	CALD1
2.95555	0.210322	GY	CAMKMT	NC_056059.1	12175001	12195001	15.9236	0.236161	HZ	CAMK2D
2.61368	0.225137	GY	CAMKMT	NC_056056.1	79615001	79635001	3.01062	0.207506	HZ	CAMKMT
4.73105	0.264611	GY	CAMKMT	NC_056056.1	79620001	79640001	3.83455	0.286767	HZ	CAMKMT
3.03994	0.318063	GY	CAMKMT;PREPL	NC_056056.1	79625001	79645001	3.16085	0.330351	HZ	CAMKMT
2.64429	0.214827	GY	CAP2	NC_056056.1	79630001	79650001	2.85284	0.328443	HZ	CAMKMT
2.99176	0.217839	GY	CAP2	NC_056056.1	79740001	79760001	2.79581	0.223374	HZ	CAMKMT
2.60782	0.199077	GY	CAPN8	NC_056056.1	79745001	79765001	3.18432	0.21172	HZ	CAMKMT
2.47323	0.223369	GY	CAPN8	NC_056056.1	79870001	79890001	5.42606	0.218998	HZ	CAMKMT
2.46112	0.227531	GY	CAPN8	NC_056056.1	79875001	79895001	8.49036	0.273091	HZ	CAMKMT
2.43454	0.231333	GY	CAPN8	NC_056056.1	79880001	79900001	17.2403	0.34473	HZ	CAMKMT
4.11652	0.229069	GY	CARD10	NC_056056.1	79885001	79905001	18.7519	0.261275	HZ	CAMKMT
12.2672	0.363904	GY	CARD10	NC_056056.1	79890001	79910001	15.3478	0.219036	HZ	CAMKMT
27.6616	0.400523	GY	CARD10	NC_056058.1	14190001	14210001	5.61203	0.216658	HZ	CAMSAP3;LOC1011
25.023	0.405316	GY	CARD10	NC_056058.1	14180001	14200001	7.24201	0.210048	HZ	CAMSAP3;XAB2
2.86343	0.196249	GY	CASK;GPR82	NC_056073.1	39205001	39225001	6.04045	0.206001	HZ	CAP2
3.36667	0.258705	GY	CASQ1;LOC10111	NC_056080.1	40970001	40990001	2.93795	0.384353	HZ	CASK
2.50976	0.215309	GY	CASS4	NC_056066.1	59190001	59210001	4.50178	0.211776	HZ	CASS4;CSTF1
2.53885	0.199625	GY	CASS4	NC_056065.1	42185001	42205001	2.67256	0.206161	HZ	CASZ1
2.49325	0.198304	GY	CASS4;CSTF1	NC_056067.1	13630001	13650001	4.00979	0.384279	HZ	CBFA2T3
3.32989	0.197755	GY	CBFA2T3	NC_056067.1	13635001	13655001	5.59588	0.333769	HZ	CBFA2T3
6.01939	0.203173	GY	CBFA2T3	NC_056055.1	68515001	68535001	5.7195	0.240088	HZ	CBWD1
8.28723	0.2098	GY	CBFA2T3	NC_056078.1	24275001	24295001	6.51113	0.524355	HZ	CCAR1
2.5365	0.195068	GY	CBFA2T3	NC_056078.1	24280001	24300001	3.03481	0.316653	HZ	CCAR1
5.61269	0.217578	GY	CBLC	NC_056078.1	24260001	24280001	6.67621	0.430784	HZ	CCAR1;TET1
7.53781	0.265426	GY	CBLC	NC_056078.1	24265001	24285001	4.95654	0.447418	HZ	CCAR1;TET1
11.0325	0.266705	GY	CBLC	NC_056080.1	100700001	100720001	3.12061	0.508974	HZ	CCDC160
11.4111	0.260483	GY	CCAR1	NC_056080.1	100705001	100725001	2.79327	0.418771	HZ	CCDC160
9.44763	0.29978	GY	CCAR1;TET1	NC_056080.1	100710001	100730001	2.76078	0.362748	HZ	CCDC160
8.00002	0.295149	GY	CCAR1;TET1	NC_056080.1	100715001	100735001	3.35462	0.345569	HZ	CCDC160
2.51131	0.358495	GY	CCDC43;MEIOC	NC_056064.1	50165001	50185001	2.67233	0.204917	HZ	CCDC57
2.83734	0.192951	GY	CCDC68	NC_056056.1	68690001	68710001	2.44762	0.348891	HZ	CCDC88A
3.21774	0.229163	GY	CCDC68	NC_056077.1	34470001	34490001	7.21528	0.3308	HZ	CCL26
2.65567	0.24663	GY	CCDC68	NC_056059.1	34900001	34920001	8.19445	0.224203	HZ	CCSER1
4.11473	0.19249	GY	CCL1	NC_056059.1	35075001	35095001	2.58004	0.215231	HZ	CCSER1
2.77914	0.202946	GY	CCL1	NC_056059.1	35080001	35100001	2.77659	0.219854	HZ	CCSER1
3.92288	0.214061	GY	CCSER1	NC_056069.1	24130001	24150001	10.228	0.248195	HZ	CDC20B;GPX8
4.04236	0.218709	GY	CCSER1	NC_056069.1	24135001	24155001	15.0968	0.287031	HZ	CDC20B;GPX8
3.26408	0.413228	GY	CCSER1	NC_056069.1	24140001	24160001	16.6666	0.291706	HZ	CDC20B;GPX8
6.69723	0.49504	GY	CCSER1	NC_056069.1	24145001	24165001	6.0828	0.260024	HZ	CDC20B;GPX8
10.3409	0.395961	GY	CCSER1	NC_056067.1	8905001	8925001	2.51912	0.284797	HZ	CDH13
3.60587	0.283298	GY	CCSER1	NC_056067.1	8910001	8930001	2.36141	0.218206	HZ	CDH13
3.21553	0.227416	GY	CDC27	NC_056059.1	91165001	91185001	3.17979	0.507628	HZ	CDKL2
3.40071	0.195469	GY	CDH11	NC_056059.1	91170001	91190001	3.38254	0.544615	HZ	CDKL2
2.5067	0.313797	GY	CDH20	NC_056059.1	91175001	91195001	2.45714	0.520899	HZ	CDKL2
3.1728	0.213472	GY	CDH4	NC_056060.1	4640001	4660001	3.99711	0.347698	HZ	CDO1
2.89706	0.234758	GY	CDH4	NC_056056.1	86835001	86855001	2.36375	0.385527	HZ	CEBPZ;NDUFAF7;PI
2.50629	0.191996	GY	CDH4	NC_056067.1	7195001	7215001	11.0811	0.279256	HZ	CENPN
7.31417	0.217728	GY	CDHR1;LRIT2	NC_056067.1	7190001	7210001	5.1365	0.23387	HZ	CENPN;LOC1011211
7.00725	0.202226	GY	CDK5RAP2	NC_056065.1	60575001	60595001	2.45039	0.263384	HZ	CEP350
34.209	0.246699	GY	CDK5RAP2	NC_056060.1	6890001	6910001	2.98252	0.208132	HZ	CERT1
4.31515	0.218688	GY	CDKL2	NC_056060.1	6895001	6915001	4.53449	0.217978	HZ	CERT1
4.79834	0.227392	GY	CDKL2	NC_056057.1	98275001	98295001	2.66893	0.284025	HZ	CHCHD3
3.67321	0.212832	GY	CDKL2	NC_056057.1	98280001	98300001	3.07222	0.29272	HZ	CHCHD3
2.55929	0.198466	GY	CDKL2	NC_056057.1	98285001	98305001	3.27922	0.333112	HZ	CHCHD3
5.61741	0.212566	GY	CDO1	NC_056057.1	98290001	98310001	3.08261	0.247434	HZ	CHCHD3
9.62524	0.313928	GY	CEBPE;SLC7A8	NC_056057.1	98295001	98315001	3.29814	0.238706	HZ	CHCHD3
5.1927	0.225613	GY	CEBPE;SLC7A8	NC_056057.1	98300001	98320001	2.99414	0.211193	HZ	CHCHD3
10.9324	0.275931	GY	CENPN	NC_056056.1	104085001	104105001	3.71667	0.220393	HZ	CIAO1;SNRNP200;TI
4.80426	0.225005	GY	CENPN;LOC10112	NC_056056.1	104090001	104110001	6.18752	0.296825	HZ	CIAO1;SNRNP200;TI
2.83928	0.244791	GY	CERS6	NC_056056.1	104095001	104115001	6.37698	0.309275	HZ	CIAO1;TMEM127
2.93174	0.241536	GY	CERS6	NC_056055.1	135660001	135680001	2.43096	0.279261	HZ	CIR1;SP9
2.89008	0.266233	GY	CERS6	NC_056074.1	41670001	41690001	2.84884	0.209197	HZ	CLCF1
2.96074	0.243953	GY	CERS6	NC_056074.1	41675001	41695001	2.44167	0.208618	HZ	CLCF1;RAD9A
3.29744	0.238159	GY	CERS6	NC_056054.1	126335001	126355001	7.56756	0.401882	HZ	CLDN8
4.23062	0.267056	GY	CERS6	NC_056077.1	10255001	10275001	3.92396	0.231391	HZ	CLEC16A
3.06109	0.230592	GY	CERS6	NC_056077.1	10260001	10280001	2.84482	0.247966	HZ	CLEC16A
2.41682	0.232223	GY	CFAP47	NC_056077.1	10265001	10285001	2.37869	0.235814	HZ	CLEC16A
4.44599	0.26596	GY	CFAP47	NC_056056.1	206075001	206095001	2.62127	0.224809	HZ	CLEC2D
4.9877	0.303454	GY	CFAP47	NC_056056.1	206080001	206100001	3.05008	0.22688	HZ	CLEC2D;LOC101122
4.79166	0.299769	GY	CFAP47	NC_056077.1	33500001	33520001	9.63392	0.330233	HZ	CLIP2

3.38388	0.264793	GY	CFAP47	NC_056077.1	33550001	33570001	3.34565	0.243242	HZ	CLIP2
2.46634	0.286061	GY	CHCHD3	NC_056077.1	26545001	26565001	3.73346	0.279579	HZ	CLN3
2.45663	0.30183	GY	CHCHD3	NC_056077.1	26550001	26570001	3.44576	0.232558	HZ	CLN3
2.85075	0.263047	GY	CHCHD3	NC_056077.1	26540001	26560001	7.61169	0.353169	HZ	CLN3;LOC10111269
3.3037	0.302985	GY	CHCHD3	NC_056074.1	15935001	15955001	5.75699	0.34549	HZ	CLNS1A
3.4724	0.412706	GY	CHCHD3	NC_056074.1	15940001	15960001	4.12621	0.284837	HZ	CLNS1A
3.15798	0.375407	GY	CHCHD3	NC_056074.1	15945001	15965001	4.11655	0.29062	HZ	CLNS1A
3.4452	0.378972	GY	CHCHD3	NC_056074.1	15920001	15940001	8.75469	0.310244	HZ	CLNS1A;RSF1
3.32904	0.349412	GY	CHCHD3	NC_056064.1	23450001	23470001	2.40939	0.206811	HZ	CLUH;PAFAH1B1
3.64286	0.274425	GY	CHCHD3	NC_056064.1	23455001	23475001	2.60938	0.211162	HZ	CLUH;PAFAH1B1
2.40514	0.216437	GY	CHCHD3	NC_056061.1	15550001	15570001	7.46208	0.20981	HZ	CLVS2
2.65498	0.2261	GY	CHCHD3	NC_056067.1	25440001	25460001	4.31391	0.239678	HZ	CNGB1
2.67213	0.265872	GY	CHCHD3	NC_056067.1	25445001	25465001	7.22807	0.32679	HZ	CNGB1
10.3315	0.261718	GY	CIAO3;HAGHL	NC_056067.1	25450001	25470001	5.71053	0.314336	HZ	CNGB1
3.44972	0.265973	GY	CLDN25;USP28	NC_056067.1	25455001	25475001	4.57143	0.218351	HZ	CNGB1
6.68447	0.311356	GY	CLDN25;USP28	NC_056065.1	27060001	27080001	2.57993	0.430085	HZ	CNIH3
3.00145	0.304402	GY	CLDN25;USP28;ZV	NC_056072.1	7110001	7130001	2.51122	0.288531	HZ	CNOT10
2.68749	0.253168	GY	CLDN25;ZW10	NC_056058.1	63815001	63835001	3.69037	0.302153	HZ	CNOT8;FAXDC2
2.70895	0.194532	GY	CLEC2D	NC_056058.1	63820001	63840001	4.15263	0.310204	HZ	CNOT8;FAXDC2
2.54042	0.230354	GY	CLEC2D	NC_056058.1	63825001	63845001	3.69263	0.334891	HZ	CNOT8;FAXDC2
2.49071	0.235114	GY	CLEC2D;LOC1011	NC_056058.1	63830001	63850001	5.00436	0.354913	HZ	CNOT8;GEMIN5
2.60001	0.201256	GY	CLEC2D;LOC1011	NC_056058.1	63835001	63855001	6.05509	0.346906	HZ	CNOT8;GEMIN5
5.06956	0.202958	GY	CLIC5	NC_056058.1	63840001	63860001	6.09364	0.345473	HZ	CNOT8;GEMIN5
2.9093	0.214157	GY	CLIC5	NC_056056.1	146340001	146360001	2.86397	0.269332	HZ	CNTN1
2.61753	0.279929	GY	CLIC5	NC_056056.1	146345001	146365001	2.82237	0.280573	HZ	CNTN1
8.01785	0.471998	GY	CLIP2	NC_056056.1	146350001	146370001	2.37257	0.286406	HZ	CNTN1
15.7449	0.32805	GY	CLIP2	NC_056056.1	146355001	146375001	2.39621	0.238895	HZ	CNTN1
9.10194	0.309138	GY	CLIP2	NC_056056.1	146400001	146420001	4.43257	0.214006	HZ	CNTN1
5.45622	0.340054	GY	CLIP2	NC_056068.1	9800001	9820001	4.95723	0.228243	HZ	CNTN5
3.6913	0.237771	GY	CLIP2	NC_056057.1	111395001	111415001	2.88282	0.238086	HZ	CNTNAP2
2.58662	0.247045	GY	CLIP2	NC_056057.1	111400001	111420001	2.57834	0.215473	HZ	CNTNAP2
9.97027	0.19695	GY	CLSTN2	NC_056057.1	112050001	112070001	3.10491	0.245987	HZ	CNTNAP2
3.39539	0.243921	GY	CLYBL	NC_056057.1	112055001	112075001	4.51454	0.22226	HZ	CNTNAP2
4.07544	0.276693	GY	CLYBL	NC_056057.1	50065001	50085001	8.37221	0.240208	HZ	COG5
2.61782	0.292834	GY	CLYBL	NC_056057.1	50070001	50090001	11.0562	0.262526	HZ	COG5
2.63225	0.218322	GY	CNGB1	NC_056057.1	50075001	50095001	10.398	0.255728	HZ	COG5;DUS4L
3.73373	0.228005	GY	CNGB1	NC_056057.1	50080001	50100001	4.67413	0.208217	HZ	COG5;DUS4L
4.65024	0.234498	GY	CNGB1	NC_056078.1	25420001	25440001	8.7627	0.22373	HZ	COL13A1
6.86403	0.334667	GY	CNGB1	NC_056059.1	16360001	16380001	3.09075	0.205982	HZ	COL25A1
5.11842	0.339852	GY	CNGB1	NC_056059.1	16365001	16385001	3.47397	0.259402	HZ	COL25A1
3.70239	0.305207	GY	CNGB1	NC_056059.1	16370001	16390001	3.20813	0.250583	HZ	COL25A1
4.82457	0.226348	GY	CNNM2	NC_056080.1	126610001	126630001	3.34452	0.272602	HZ	COL4A5
3.91111	0.217199	GY	CNNM2	NC_056080.1	126615001	126635001	5.29735	0.27286	HZ	COL4A5
2.58607	0.199525	GY	CNNM2	NC_056080.1	126620001	126640001	4.24544	0.27295	HZ	COL4A5
3.45082	0.283333	GY	CNNM2;NT5C2	NC_056080.1	126625001	126645001	3.09819	0.255463	HZ	COL4A5
2.6591	0.226374	GY	CNNM2;NT5C2	NC_056058.1	12400001	12420001	2.69622	0.267517	HZ	COL5A3
2.84937	0.213518	GY	CNOT8;FAXDC2	NC_056058.1	12405001	12425001	2.35326	0.261614	HZ	COL5A3
2.89473	0.20578	GY	CNOT8;FAXDC2	NC_056058.1	12410001	12430001	2.63303	0.237624	HZ	COL5A3
2.63624	0.226427	GY	CNTN1	NC_056058.1	35305001	35325001	2.46481	0.218505	HZ	COMMD10
2.81011	0.209512	GY	CNTN1	NC_056058.1	35355001	35375001	2.60127	0.250097	HZ	COMMD10
3.12623	0.226865	GY	CNTN5	NC_056058.1	35360001	35380001	2.65411	0.236043	HZ	COMMD10
2.53222	0.220345	GY	CNTN5	NC_056058.1	35405001	35425001	2.46976	0.218745	HZ	COMMD10
3.00897	0.247754	GY	CNTN5	NC_056062.1	80320001	80340001	2.47334	0.237431	HZ	CPQ
2.68193	0.286149	GY	CNTN5	NC_056062.1	80325001	80345001	3.01112	0.247738	HZ	CPQ
4.80866	0.193581	GY	CNTN5	NC_056062.1	80330001	80350001	2.91521	0.285002	HZ	CPQ
2.83706	0.206835	GY	CNTNAP2	NC_056062.1	80335001	80355001	2.96722	0.304677	HZ	CPQ
4.02617	0.209473	GY	CNTNAP2	NC_056057.1	68570001	68590001	5.70293	0.265956	HZ	CPVL
5.19397	0.307448	GY	COG5	NC_056057.1	68575001	68595001	17.0173	0.316808	HZ	CPVL
8.69999	0.411813	GY	COG5	NC_056057.1	68625001	68645001	4.66455	0.251798	HZ	CPVL
11.2809	0.460326	GY	COG5	NC_056057.1	68630001	68650001	5.52513	0.261087	HZ	CPVL
10.0714	0.478737	GY	COG5;DUS4L	NC_056057.1	68635001	68655001	6.63744	0.267255	HZ	CPVL
4.56468	0.41879	GY	COG5;DUS4L	NC_056057.1	68640001	68660001	6.89063	0.278388	HZ	CPVL
2.73588	0.349227	GY	COG5;DUS4L	NC_056057.1	68645001	68665001	26.4799	0.248886	HZ	CPVL
4.75432	0.391499	GY	COL19A1	NC_056057.1	68655001	68675001	10.8696	0.238599	HZ	CPVL
6.48861	0.418359	GY	COL19A1	NC_056057.1	68660001	68680001	6.02222	0.243421	HZ	CPVL
6.2779	0.437891	GY	COL19A1	NC_056057.1	69305001	69325001	2.43022	0.249821	HZ	CREB5
2.95402	0.321247	GY	COL19A1	NC_056080.1	6765001	6785001	3.25592	0.21434	HZ	CRLF2
3.11688	0.201354	GY	COL24A1	NC_056068.1	74800001	74820001	2.93063	0.270221	HZ	CRY2
3.20613	0.220676	GY	COL3A1	NC_056068.1	74805001	74825001	6.17566	0.307353	HZ	CRY2
3.81952	0.234926	GY	COL3A1	NC_056068.1	74810001	74830001	6.83455	0.335165	HZ	CRY2
3.56694	0.251745	GY	COL3A1	NC_056068.1	74815001	74835001	2.67773	0.253337	HZ	CRY2
7.11745	0.255125	GY	COL3A1	NC_056068.1	74820001	74840001	2.59799	0.232482	HZ	CRY2
18.1679	0.311151	GY	COL3A1	NC_056068.1	74825001	74845001	2.861	0.263968	HZ	CRY2
21.0973	0.286426	GY	COL3A1	NC_056068.1	74830001	74850001	4.60454	0.238364	HZ	CRY2;MAPK8IP1

21.4039	0.2777	GY	COL3A1	NC_056079.1	3890001	3910001	2.746	0.216383	HZ	CSMD1
6.7037	0.227103	GY	COL3A1	NC_056059.1	86170001	86190001	2.56045	0.263571	HZ	CSN1S1
4.66603	0.195478	GY	COL4A1;COL4A2	NC_056059.1	86175001	86195001	2.499	0.280126	HZ	CSN1S1
10.416	0.242989	GY	COL4A1;COL4A2	NC_056059.1	86190001	86210001	2.44915	0.373613	HZ	CSN1S1;CSN2
2.73591	0.20385	GY	COL6A3	NC_056059.1	86195001	86215001	3.43092	0.440398	HZ	CSN2
4.3182	0.195016	GY	COMMD10	NC_056059.1	86200001	86220001	6.93185	0.541964	HZ	CSN2
2.40117	0.354283	GY	CPA6	NC_056059.1	86205001	86225001	7.12058	0.46922	HZ	CSN2
2.87004	0.346756	GY	CPA6	NC_056059.1	86210001	86230001	2.41296	0.305812	HZ	CSN2
3.45076	0.353776	GY	CPA6	NC_056078.1	21660001	21680001	3.0281	0.275491	HZ	CTNNA3
3.93437	0.357085	GY	CPA6	NC_056078.1	21665001	21685001	7.25876	0.21792	HZ	CTNNA3
3.30368	0.325697	GY	CPA6	NC_056069.1	61520001	61540001	8.61839	0.210333	HZ	CTNND2
2.76138	0.259403	GY	CPA6	NC_056068.1	15690001	15710001	2.53817	0.216917	HZ	CWF19L2
2.73233	0.262498	GY	CPAMD8	NC_056068.1	15720001	15740001	3.0109	0.237136	HZ	CWF19L2
2.47601	0.282667	GY	CPAMD8	NC_056068.1	15740001	15760001	4.94201	0.204824	HZ	CWF19L2
3.63925	0.261919	GY	CPVL	NC_056068.1	15745001	15765001	3.75528	0.22791	HZ	CWF19L2
3.91061	0.215365	GY	CPVL	NC_056068.1	15750001	15770001	2.97828	0.257692	HZ	CWF19L2
23.0399	0.370796	GY	CPVL	NC_056068.1	15755001	15775001	3.48606	0.26858	HZ	CWF19L2
11.118	0.463898	GY	CPVL	NC_056068.1	15760001	15780001	3.82988	0.252212	HZ	CWF19L2
6.54105	0.477674	GY	CPVL	NC_056068.1	15825001	15845001	2.41073	0.496353	HZ	CWF19L2
3.3923	0.210784	GY	CRACDL	NC_056068.1	15830001	15850001	2.93407	0.52191	HZ	CWF19L2
9.00867	0.277301	GY	CRACDL	NC_056068.1	15835001	15855001	2.88388	0.547484	HZ	CWF19L2
5.73663	0.25117	GY	CRACDL	NC_056068.1	15695001	15715001	2.57713	0.261502	HZ	CWF19L2;LOC11411
9.71242	0.20607	GY	CREB5	NC_056058.1	44705001	44725001	16.1984	0.239182	HZ	CXCL14
3.31661	0.392481	GY	CREBRF	NC_056080.1	37130001	37150001	2.51555	0.24292	HZ	CYBB
6.00638	0.43889	GY	CREBRF	NC_056080.1	37135001	37155001	4.24203	0.295236	HZ	CYBB
4.24999	0.394145	GY	CREBRF	NC_056080.1	37140001	37160001	3.7949	0.294915	HZ	CYBB
2.93069	0.367051	GY	CREBRF	NC_056080.1	37145001	37165001	2.51677	0.271923	HZ	CYBB
2.48622	0.214798	GY	CRLS1	NC_056077.1	37170001	37190001	3.04524	0.37874	HZ	CYP3A24
2.50324	0.24108	GY	CRLS1	NC_056077.1	37175001	37195001	3.44456	0.389208	HZ	CYP3A24
5.18152	0.232421	GY	CRLS1	NC_056056.1	181325001	181345001	2.57407	0.624005	HZ	CYTH4
6.37345	0.253239	GY	CRLS1;LRRN4	NC_056063.1	46715001	46735001	2.63387	0.390387	HZ	DACH1
7.51935	0.269429	GY	CRLS1;LRRN4	NC_056063.1	46720001	46740001	4.93219	0.370229	HZ	DACH1
2.44061	0.38187	GY	CSN1S1;CSN2	NC_056063.1	46725001	46745001	3.90336	0.253952	HZ	DACH1
3.42363	0.447976	GY	CSN2	NC_056063.1	46735001	46755001	3.23902	0.276371	HZ	DACH1
6.91171	0.551096	GY	CSN2	NC_056063.1	46740001	46760001	3.25167	0.311367	HZ	DACH1
7.12425	0.48516	GY	CSN2	NC_056063.1	46745001	46765001	3.4775	0.312023	HZ	DACH1
2.43085	0.194846	GY	CSPP1	NC_056063.1	46750001	46770001	4.27299	0.31084	HZ	DACH1
3.41049	0.215563	GY	CSRNP3	NC_056063.1	46755001	46775001	8.69387	0.255613	HZ	DACH1
3.59346	0.238528	GY	CSRNP3	NC_056063.1	46760001	46780001	14.7663	0.213963	HZ	DACH1
2.85498	0.274722	GY	CSRNP3	NC_056063.1	46780001	46800001	4.91974	0.267745	HZ	DACH1
3.93419	0.251326	GY	CSRNP3	NC_056063.1	46785001	46805001	2.83805	0.336008	HZ	DACH1
2.4	0.249159	GY	CSRNP3	NC_056063.1	46845001	46865001	6.96282	0.217384	HZ	DACH1
3.04992	0.317778	GY	CSRNP3	NC_056063.1	47090001	47110001	3.14433	0.328558	HZ	DACH1
4.49218	0.368818	GY	CSRNP3	NC_056063.1	47095001	47115001	2.88139	0.367152	HZ	DACH1
2.77273	0.222369	GY	CSRP2	NC_056063.1	47100001	47120001	2.58206	0.332821	HZ	DACH1
3.41291	0.251057	GY	CTNNA2	NC_056063.1	47105001	47125001	2.41096	0.312583	HZ	DACH1
4.70343	0.295503	GY	CTNNA2	NC_056055.1	230045001	230065001	8.11111	0.211174	HZ	DAW1
2.62296	0.233461	GY	CTNNA2	NC_056056.1	141550001	141570001	5.2335	0.316707	HZ	DBX2
8.27021	0.19572	GY	CTNND2	NC_056060.1	79015001	79035001	2.55084	0.26247	HZ	DCAF5
4.23636	0.19999	GY	CTTNBP2	NC_056060.1	79020001	79040001	3.66234	0.303349	HZ	DCAF5
2.44006	0.206006	GY	CTTNBP2	NC_056064.1	47605001	47625001	2.71312	0.206318	HZ	DCAF7
2.59257	0.193194	GY	CTTNBP2	NC_056068.1	60905001	60925001	3.84946	0.277426	HZ	DCDC1
2.75959	0.206964	GY	CTTNBP2	NC_056068.1	60910001	60930001	2.87207	0.251996	HZ	DCDC1
3.05764	0.227229	GY	CTTNBP2	NC_056072.1	47740001	47760001	2.41481	0.299494	HZ	DCP1A
2.85143	0.208971	GY	CTTNBP2	NC_056072.1	47745001	47765001	2.49412	0.283738	HZ	DCP1A
3.73572	0.2289	GY	CUBN	NC_056072.1	47750001	47770001	2.89286	0.350576	HZ	DCP1A
3.59151	0.247311	GY	CUBN	NC_056072.1	47755001	47775001	4.41777	0.275285	HZ	DCP1A
4.36689	0.325842	GY	CUBN	NC_056072.1	47760001	47780001	4.64285	0.216715	HZ	DCP1A
2.49507	0.207606	GY	CUBN	NC_056074.1	36395001	36415001	5.70909	0.293651	HZ	DDB1;TKFC
2.49447	0.239174	GY	CWF19L2	NC_056079.1	32505001	32525001	7.42855	0.216261	HZ	DDHD2;NSD3;PLPP5
3.36832	0.237418	GY	CWF19L2	NC_056054.1	114335001	114355001	5.71092	0.206297	HZ	DDR2;HSD17B7
4.6715	0.241527	GY	CWF19L2	NC_056054.1	114340001	114360001	18.6788	0.269138	HZ	DDR2;HSD17B7
7.52435	0.266182	GY	CWF19L2	NC_056054.1	114345001	114365001	25.0508	0.292646	HZ	DDR2;HSD17B7
6.74521	0.255809	GY	CWF19L2	NC_056066.1	60535001	60555001	3.19114	0.209116	HZ	DEFB115
5.50353	0.240704	GY	CWF19L2	NC_056062.1	94205001	94225001	4.28643	0.318646	HZ	DEPTOR;DSCC1
3.83727	0.196881	GY	CWF19L2	NC_056063.1	12675001	12695001	2.47323	0.240078	HZ	DGKH
3.50348	0.201494	GY	CWF19L2	NC_056063.1	12680001	12700001	2.64493	0.259659	HZ	DGKH
3.90344	0.207439	GY	CWF19L2	NC_056063.1	12685001	12705001	2.44329	0.233915	HZ	DGKH
3.08529	0.395394	GY	CWF19L2	NC_056068.1	75265001	75285001	3.33464	0.267221	HZ	DGKZ
3.73784	0.424754	GY	CWF19L2	NC_056068.1	75270001	75290001	4.76924	0.287444	HZ	DGKZ
3.65727	0.446891	GY	CWF19L2	NC_056068.1	75275001	75295001	5.61299	0.250642	HZ	DGKZ
2.52421	0.404647	GY	CWF19L2	NC_056068.1	75280001	75300001	5.15142	0.236251	HZ	DGKZ
2.83958	0.248181	GY	CWF19L2;LOC114	NC_056058.1	78290001	78310001	2.451	0.312988	HZ	DHFR
2.5602	0.276359	GY	CWF19L2;LOC114	NC_056058.1	78295001	78315001	2.65836	0.240346	HZ	DHFR

2.49296	0.206529	GY	CYBB	NC_056065.1	40400001	40420001	2.38193	0.207065	HZ	DHRS3
4.20968	0.239641	GY	CYBB	NC_056059.1	44685001	44705001	4.67146	0.262952	HZ	DHX15
3.7783	0.235181	GY	CYBB	NC_056059.1	44690001	44710001	4.72367	0.243031	HZ	DHX15
2.45804	0.223101	GY	CYBB	NC_056080.1	138240001	138260001	2.98732	0.453668	HZ	DIAPH2;RPA4
3.42712	0.219383	GY	CYFIP2;FNDC9	NC_056068.1	21995001	22015001	2.64099	0.338323	HZ	DIXDC1
3.63232	0.253717	GY	CYFIP2;FNDC9	NC_056074.1	11105001	11125001	2.57089	0.269649	HZ	DLG2
2.99875	0.256419	GY	CYFIP2;FNDC9	NC_056074.1	11110001	11130001	2.86093	0.268182	HZ	DLG2
7.64768	0.212911	GY	CYP2D6;LOC1141	NC_056074.1	11115001	11135001	4.18467	0.221321	HZ	DLG2
3.45123	0.264659	GY	CYTH4	NC_056080.1	63340001	63360001	2.91456	0.20937	HZ	DLG3
3.97981	0.397091	GY	CYTH4	NC_056076.1	38490001	38510001	2.92167	0.226865	HZ	DLGAP1
4.10001	0.558276	GY	CYTH4	NC_056076.1	38495001	38515001	2.93812	0.214593	HZ	DLGAP1
2.72935	0.238235	GY	DACH1	NC_056060.1	65695001	65715001	3.39389	0.225674	HZ	DLGAP5;LGALS3
3.34867	0.229832	GY	DACH1	NC_056080.1	30935001	30955001	3.67413	0.309304	HZ	DMD
3.76016	0.204699	GY	DACH1	NC_056080.1	30940001	30960001	5.63964	0.398818	HZ	DMD
3.69646	0.200346	GY	DACH1	NC_056080.1	30945001	30965001	5.03736	0.403355	HZ	DMD
3.35394	0.204008	GY	DAW1	NC_056080.1	30950001	30970001	4.42094	0.43516	HZ	DMD
7.20228	0.215951	GY	DAW1	NC_056080.1	32150001	32170001	14.9824	0.251934	HZ	DMD
2.60279	0.408557	GY	DBNDD1;GAS8	NC_056080.1	32265001	32285001	2.68497	0.26017	HZ	DMD
7.00682	0.425845	GY	DBNDD1;GAS8;LC	NC_056080.1	32270001	32290001	2.7284	0.236312	HZ	DMD
4.45055	0.380645	GY	DBNDD1;GAS8;LC	NC_056065.1	27350001	27370001	3.0095	0.231834	HZ	DNAH14
6.4477	0.394048	GY	DBNDD1;LOC1011	NC_056065.1	27355001	27375001	3.32863	0.248527	HZ	DNAH14
8.9156	0.407869	GY	DBNDD1;LOC1011	NC_056065.1	27360001	27380001	3.71717	0.281679	HZ	DNAH14
8.90476	0.402545	GY	DBNDD1;LOC1011	NC_056065.1	27365001	27385001	4.16821	0.299087	HZ	DNAH14
5.41624	0.291901	GY	DBX2	NC_056065.1	27370001	27390001	2.46054	0.234696	HZ	DNAH14
3.00303	0.254881	GY	DCHS2	NC_056064.1	30360001	30380001	3.95079	0.26369	HZ	DNAH9
3.17035	0.240079	GY	DCHS2	NC_056064.1	30375001	30395001	14.575	0.271909	HZ	DNAH9
3.70544	0.204866	GY	DCLK2	NC_056063.1	75310001	75330001	4.89903	0.26224	HZ	DOCK9
2.42353	0.295275	GY	DCP1A	NC_056063.1	75315001	75335001	4.46763	0.253401	HZ	DOCK9
3.05134	0.319488	GY	DCP1A	NC_056063.1	75320001	75340001	4.30851	0.226092	HZ	DOCK9
4.68	0.269546	GY	DCP1A	NC_056063.1	75325001	75345001	5.48376	0.246298	HZ	DOCK9
5.33333	0.29425	GY	DCP1A	NC_056063.1	75335001	75355001	3.03411	0.207177	HZ	DOCK9
2.68065	0.199708	GY	DCP1A	NC_056063.1	75340001	75360001	2.65905	0.22423	HZ	DOCK9
5.51637	0.227922	GY	DDB1;TKFC	NC_056063.1	75345001	75365001	2.99115	0.234338	HZ	DOCK9
7.7857	0.293601	GY	DDHD2;NSD3;PLP	NC_056060.1	29105001	29125001	5.34933	0.214316	HZ	DPH6
2.63636	0.208213	GY	DENND3	NC_056060.1	29110001	29130001	8.68216	0.259399	HZ	DPH6
3.11682	0.254486	GY	DENND5B	NC_056060.1	29115001	29135001	10.9454	0.286243	HZ	DPH6
3.20779	0.280274	GY	DENND5B	NC_056060.1	29120001	29140001	9.745	0.288608	HZ	DPH6
4.3964	0.3145	GY	DENND5B	NC_056060.1	29125001	29145001	12.6467	0.28444	HZ	DPH6
3.81503	0.302803	GY	DENND6A	NC_056060.1	29130001	29150001	5.45862	0.228092	HZ	DPH6
2.92972	0.255677	GY	DENND6A	NC_056057.1	63600001	63620001	2.6008	0.353988	HZ	DPY19L1
5.14937	0.235102	GY	DEPTOR;DSCC1	NC_056057.1	63605001	63625001	2.46154	0.291431	HZ	DPY19L1
4.95286	0.250917	GY	DERA	NC_056057.1	63610001	63630001	2.51835	0.21115	HZ	DPY19L1
3.40313	0.195732	GY	DGKZ	NC_056062.1	82445001	82465001	4.44701	0.239661	HZ	DPY19L4
4.77163	0.221168	GY	DGKZ	NC_056062.1	82450001	82470001	6.3367	0.330702	HZ	DPY19L4
5.51412	0.195529	GY	DGKZ	NC_056062.1	82455001	82475001	13.2927	0.381857	HZ	DPY19L4
3.68014	0.200335	GY	DISP1	NC_056062.1	82500001	82520001	2.9339	0.218147	HZ	DPY19L4
3.07433	0.239493	GY	DIXDC1	NC_056054.1	261290001	261310001	2.4987	0.209514	HZ	DSCAM
2.90827	0.228241	GY	DIXDC1	NC_056068.1	27920001	27940001	17.6691	0.255537	HZ	DSCAML1
2.78531	0.197243	GY	DIXDC1	NC_056068.1	27925001	27945001	7.60341	0.205425	HZ	DSCAML1
2.45804	0.247405	GY	DLG2	NC_056068.1	27950001	27970001	4.18406	0.208712	HZ	DSCAML1
3.09865	0.276351	GY	DLG2	NC_056068.1	28045001	28065001	3.1596	0.20533	HZ	DSCAML1
3.29979	0.289442	GY	DLG2	NC_056068.1	28055001	28075001	8.82488	0.292973	HZ	DSCAML1
5.42504	0.250408	GY	DLG2	NC_056068.1	28060001	28080001	15.7584	0.31099	HZ	DSCAML1
5.93549	0.249658	GY	DLG2	NC_056068.1	28065001	28085001	24.9347	0.339214	HZ	DSCAML1
3.93325	0.208781	GY	DLG2	NC_056068.1	28080001	28100001	11.1774	0.367587	HZ	DSCAML1
2.43216	0.277252	GY	DLG3	NC_056068.1	28085001	28105001	3.73433	0.253355	HZ	DSCAML1
2.93025	0.207784	GY	DLGAP1	NC_056062.1	94195001	94215001	5.80698	0.288309	HZ	DSCC1
2.94541	0.201777	GY	DLGAP1	NC_056062.1	94200001	94220001	7.91312	0.342035	HZ	DSCC1
4.07739	0.380771	GY	DMD	NC_056062.1	94180001	94200001	8.26428	0.27171	HZ	DSCC1;TAF2
5.65766	0.409451	GY	DMD	NC_056062.1	94185001	94205001	8.50375	0.265935	HZ	DSCC1;TAF2
5.06322	0.399338	GY	DMD	NC_056062.1	94190001	94210001	12.514	0.301688	HZ	DSCC1;TAF2
4.46032	0.436268	GY	DMD	NC_056061.1	21540001	21560001	4.29915	0.220511	HZ	DSE
2.81208	0.201465	GY	DMD	NC_056073.1	3735001	3755001	2.40525	0.21791	HZ	DST
3.04557	0.206961	GY	DMD	NC_056076.1	22580001	22600001	3.08572	0.222773	HZ	DTNA
2.59857	0.1994	GY	DMD	NC_056076.1	22585001	22605001	7.8762	0.345757	HZ	DTNA
2.7685	0.237633	GY	DMD	NC_056076.1	22590001	22610001	6.33245	0.371441	HZ	DTNA
8.4969	0.228225	GY	DNASE1L3	NC_056076.1	22595001	22615001	2.70413	0.292442	HZ	DTNA
6.5077	0.194639	GY	DNASE1L3	NC_056068.1	80980001	81000001	3.14164	0.286142	HZ	DTX4
2.43619	0.234708	GY	DPF3	NC_056068.1	80985001	81005001	2.78088	0.272956	HZ	DTX4
3.37253	0.22442	GY	DPY19L1	NC_056068.1	80990001	81010001	3.98222	0.261858	HZ	DTX4
3.16463	0.219548	GY	DPY19L1	NC_056068.1	80995001	81015001	4.8555	0.238843	HZ	DTX4
3.15297	0.255847	GY	DPY19L1	NC_056068.1	81000001	81020001	5.02026	0.232244	HZ	DTX4;LOC10110990′
3.05301	0.25115	GY	DPY19L1	NC_056068.1	81005001	81025001	5.42225	0.216246	HZ	DTX4;LOC10110990′
3.46315	0.193835	GY	DPY19L1	NC_056068.1	81010001	81030001	3.24386	0.233074	HZ	DTX4;LOC10110990′

3.77664	0.210775	GY	DPY19L1	NC_056068.1	81015001	81035001	2.89439	0.234907	HZ	DTX4;LOC10110990
4.06729	0.218904	GY	DPY19L1	NC_056068.1	80975001	80995001	3.63167	0.255756	HZ	DTX4;LOC12181673
2.42281	0.235291	GY	DPY19L1	NC_056056.1	80500001	80520001	2.448	0.240964	HZ	DYNC2LI1;PLEKHH
3.01179	0.221242	GY	DPY19L4	NC_056056.1	80505001	80525001	2.64051	0.233427	HZ	DYNC2LI1;PLEKHH
4.00993	0.276573	GY	DPY19L4	NC_056080.1	62580001	62600001	3.00358	0.218965	HZ	EDA
6.17587	0.346037	GY	DPY19L4	NC_056080.1	62665001	62685001	2.81111	0.258587	HZ	EDA
12.7439	0.386718	GY	DPY19L4	NC_056080.1	62670001	62690001	6.18855	0.418121	HZ	EDA
27.0779	0.224386	GY	DPY19L4	NC_056080.1	62675001	62695001	8.42966	0.55577	HZ	EDA
28.2563	0.254744	GY	DPY19L4	NC_056080.1	62680001	62700001	11.2759	0.541503	HZ	EDA
10.5455	0.276837	GY	DPY19L4	NC_056080.1	62685001	62705001	12.6145	0.555818	HZ	EDA
2.98275	0.247985	GY	DPY19L4	NC_056080.1	62690001	62710001	9.28647	0.523746	HZ	EDA
2.95155	0.193285	GY	DPYSL3;LOC1218	NC_056080.1	62695001	62715001	13.5766	0.420146	HZ	EDA
2.75332	0.202549	GY	DSCAML1	NC_056080.1	62730001	62750001	2.51861	0.217581	HZ	EDA
2.48765	0.245601	GY	DSCAML1	NC_056058.1	81590001	81610001	4.00735	0.244286	HZ	EDIL3
2.85355	0.243669	GY	DSCAML1	NC_056058.1	81595001	81615001	3.5157	0.291416	HZ	EDIL3
3.32734	0.273633	GY	DSCAML1	NC_056058.1	81600001	81620001	4.14391	0.336781	HZ	EDIL3
2.90965	0.28671	GY	DSCAML1	NC_056064.1	46535001	46555001	4.93204	0.276833	HZ	EFCAB3
2.7369	0.230529	GY	DSCAML1	NC_056064.1	46540001	46560001	4.1893	0.291521	HZ	EFCAB3
2.82537	0.201555	GY	DSCAML1	NC_056064.1	46545001	46565001	3.25947	0.300647	HZ	EFCAB3
3.01556	0.241089	GY	DSCAML1	NC_056064.1	46550001	46570001	3.0416	0.244493	HZ	EFCAB3
3.1579	0.260693	GY	DSCAML1	NW_024599827	550001	570001	5.37074	0.247503	HZ	EFL1
8.88932	0.243157	GY	DSCC1	NW_024599827	555001	575001	4.81612	0.221212	HZ	EFL1
5.3205	0.209193	GY	DSE	NC_056058.1	104180001	104200001	3.97841	0.28317	HZ	EFNA5
10.4709	0.287001	GY	DSE	NC_056058.1	104185001	104205001	7.87363	0.346174	HZ	EFNA5
3.66053	0.268452	GY	DSE	NC_056058.1	104190001	104210001	15.9431	0.377514	HZ	EFNA5
2.81904	0.229135	GY	DVL3;EIF2B5	NC_056058.1	104195001	104215001	15.0714	0.342446	HZ	EFNA5
3.36062	0.209948	GY	DZIP1	NC_056058.1	104200001	104220001	5.97891	0.266495	HZ	EFNA5
3.47145	0.235178	GY	E2F6	NC_056056.1	45365001	45385001	3.57327	0.233526	HZ	EHBP1
2.7476	0.243932	GY	E2F6	NC_056056.1	45370001	45390001	4.84862	0.25656	HZ	EHBP1
2.6832	0.236981	GY	E2F7	NC_056056.1	45375001	45395001	8.91334	0.290313	HZ	EHBP1
11.1863	0.254939	GY	EDA	NC_056072.1	29950001	29970001	4.41145	0.20638	HZ	EIF4E3
14.9902	0.25192	GY	EDA	NC_056072.1	29935001	29955001	8.04153	0.270134	HZ	EIF4E3;GPR27
17.2179	0.26024	GY	EDA	NC_056072.1	29940001	29960001	8.68966	0.294201	HZ	EIF4E3;GPR27
13.0677	0.244063	GY	EDA	NC_056072.1	29945001	29965001	10.7273	0.273947	HZ	EIF4E3;GPR27
15.4817	0.223608	GY	EDA	NC_056072.1	29930001	29950001	4.50335	0.218063	HZ	EIF4E3;GPR27;PROK
2.9971	0.247933	GY	EDIL3	NC_056058.1	92870001	92890001	2.47436	0.20755	HZ	ELL2
8.06371	0.29991	GY	EDIL3	NC_056057.1	61570001	61590001	5.91044	0.21505	HZ	ELMO1
7.10283	0.27884	GY	EDIL3	NC_056057.1	61575001	61595001	8.46458	0.264013	HZ	ELMO1
7.91562	0.246246	GY	EDNRB	NC_056057.1	61580001	61600001	9.6907	0.299846	HZ	ELMO1
4.30708	0.276908	GY	EDNRB	NC_056057.1	61600001	61620001	13.8801	0.254914	HZ	ELMO1
2.87373	0.288136	GY	EDNRB	NC_056080.1	103860001	103880001	2.57424	0.238715	HZ	ENOX2
5.56122	0.218221	GY	EFL1	NC_056062.1	93980001	94000001	8.4064	0.311906	HZ	ENPP2
18.0544	0.254856	GY	EFR3A	NC_056062.1	93985001	94005001	6.53873	0.262064	HZ	ENPP2
2.4515	0.191788	GY	EFR3A;OC90	NC_056062.1	93990001	94010001	3.7392	0.219265	HZ	ENPP2
13.4193	0.243389	GY	EFR3A;OC90	NC_056061.1	57145001	57165001	5.66471	0.327076	HZ	ENPP3
14.7419	0.248464	GY	EFR3A;OC90	NC_056061.1	57150001	57170001	2.40564	0.253883	HZ	ENPP3
7.19031	0.204125	GY	EIF4E3;GPR27	NC_056060.1	1520001	1540001	5.48417	0.29073	HZ	EPB41L4A
4.17226	0.212528	GY	EIF4E3;GPR27;PR	NC_056060.1	1525001	1545001	10.9977	0.326964	HZ	EPB41L4A
5.50766	0.209254	GY	ELMO1	NC_056060.1	1530001	1550001	9.78237	0.286086	HZ	EPB41L4A
6.67781	0.215618	GY	ELMO1	NC_056060.1	1535001	1555001	7.44618	0.285754	HZ	EPB41L4A
7.24623	0.248232	GY	ELMO1	NC_056055.1	160755001	160775001	3.02904	0.213671	HZ	EPC2
6.37613	0.265581	GY	ELMO1	NC_056055.1	160780001	160800001	2.92291	0.2141	HZ	EPC2
8.02364	0.298281	GY	ELMO1	NC_056077.1	36290001	36310001	6.04336	0.292507	HZ	EPO;ZAN
8.20618	0.333803	GY	ELMO1	NC_056077.1	36295001	36315001	2.84116	0.229889	HZ	EPO;ZAN
8.98191	0.328728	GY	ELMO1	NC_056055.1	214065001	214085001	22.5773	0.239848	HZ	ERBB4
2.57634	0.211184	GY	ELMO1	NC_056055.1	214070001	214090001	21.4196	0.278763	HZ	ERBB4
2.76739	0.22537	GY	ELOA;PITHD1	NC_056055.1	214075001	214095001	17.0357	0.375071	HZ	ERBB4
2.89957	0.240502	GY	ENOX2	NC_056055.1	214080001	214100001	7.04302	0.389906	HZ	ERBB4
4.02718	0.200746	GY	EOGT	NC_056055.1	214085001	214105001	3.68437	0.37044	HZ	ERBB4
4.02438	0.223788	GY	EPAS1	NC_056055.1	214090001	214110001	3.2637	0.338645	HZ	ERBB4
10.9248	0.221514	GY	EPB41L4A	NC_056055.1	214095001	214115001	2.52916	0.31694	HZ	ERBB4
9.72704	0.209681	GY	EPB41L4A	NC_056055.1	214100001	214120001	2.50108	0.324627	HZ	ERBB4
7.53795	0.229345	GY	EPB41L4A	NC_056055.1	214105001	214125001	2.43808	0.315525	HZ	ERBB4
3.72391	0.199841	GY	EPB41L4B	NC_056055.1	214110001	214130001	2.36192	0.317603	HZ	ERBB4
4.0909	0.202601	GY	EPB41L4B	NC_056056.1	213705001	213725001	2.94569	0.21329	HZ	ERC1
3.0806	0.256203	GY	EPB41L4B	NC_056056.1	213710001	213730001	4.69	0.268629	HZ	ERC1
2.93789	0.21513	GY	EPG5	NC_056056.1	213715001	213735001	5.41177	0.259496	HZ	ERC1
2.63309	0.217529	GY	EPG5	NC_056072.1	44815001	44835001	7.69379	0.247659	HZ	ERC2
4.7275	0.280174	GY	EPHA3	NC_056072.1	44820001	44840001	4.30973	0.240402	HZ	ERC2
2.43196	0.274749	GY	EPHA3	NC_056072.1	44825001	44845001	2.94817	0.212342	HZ	ERC2
2.94517	0.265653	GY	ERBB2;GRB7;MIE	NC_056072.1	44995001	45015001	2.74447	0.409333	HZ	ERC2
2.53098	0.274888	GY	ERBB2;GRB7;MIE	NC_056054.1	271440001	271460001	2.96642	0.282482	HZ	ERG
3.10393	0.230067	GY	ERBB2;MIEN1	NC_056054.1	271445001	271465001	3.10954	0.310931	HZ	ERG
3.37725	0.260969	GY	ERBB4	NC_056054.1	271450001	271470001	3.65092	0.33248	HZ	ERG

5.7713	0.303926	GY	ERBB4	NC_056054.1	271455001	271475001	2.6776	0.310548	HZ	ERG
8.8614	0.340874	GY	ERBB4	NC_056069.1	4835001	4855001	2.54944	0.293621	HZ	ERGIC1
22.4207	0.372467	GY	ERBB4	NC_056069.1	4840001	4860001	3.64893	0.300998	HZ	ERGIC1
20.5625	0.391136	GY	ERBB4	NC_056069.1	4845001	4865001	3.62903	0.24966	HZ	ERGIC1
18.8632	0.39034	GY	ERBB4	NC_056079.1	595001	615001	21.4747	0.24913	HZ	ERICH1
14.1607	0.369351	GY	ERBB4	NC_056079.1	600001	620001	21.0094	0.22679	HZ	ERICH1
5.91398	0.251799	GY	ERBB4	NC_056055.1	73940001	73960001	22.5042	0.243983	HZ	ERMP1
2.5547	0.196578	GY	ERBB4	NC_056055.1	73945001	73965001	18.4445	0.24481	HZ	ERMP1
2.52825	0.235154	GY	ERBB4	NC_056055.1	73950001	73970001	15.7967	0.206735	HZ	ERMP1
2.65178	0.199115	GY	ERBB4	NC_056060.1	11480001	11500001	9.18405	0.206465	HZ	ERO1A
3.576	0.528176	GY	ERC1	NC_056056.1	200600001	200620001	2.40506	0.233982	HZ	ERP27
4.52696	0.491414	GY	ERC1	NC_056060.1	85970001	85990001	2.41281	0.251961	HZ	ESRRB
3.12825	0.226127	GY	ERC2	NC_056060.1	85975001	85995001	3.36872	0.306616	HZ	ESRRB
4.53547	0.248047	GY	ERC2	NC_056060.1	85980001	86000001	4.35238	0.343496	HZ	ESRRB
6.50138	0.254729	GY	ERC2	NC_056060.1	85985001	86005001	6.95238	0.387578	HZ	ESRRB
2.77238	0.304162	GY	ERFE;LOC1011093	NC_056060.1	79050001	79070001	3.4163	0.268221	HZ	EXD2
2.80143	0.234178	GY	ERGIC1	NC_056060.1	79055001	79075001	4.57905	0.357623	HZ	EXD2
3.76369	0.266306	GY	ERGIC1	NC_056060.1	79060001	79080001	2.9788	0.379165	HZ	EXD2
3.54754	0.251408	GY	ERGIC1	NC_056060.1	79100001	79120001	3.98704	0.449413	HZ	EXD2
3.89611	0.191718	GY	ERGIC1	NC_056060.1	79105001	79125001	12.9836	0.452743	HZ	EXD2
22.6457	0.203037	GY	ERMP1	NC_056056.1	94245001	94265001	3.79503	0.212704	HZ	EXOC6B
17.8823	0.207076	GY	ERMP1	NC_056064.1	54900001	54920001	6.33989	0.263127	HZ	EXOC7
4.47133	0.20668	GY	ERMP1;RIC1	NC_056064.1	54910001	54930001	6.95983	0.300169	HZ	EXOC7;GALR2;ZAC
7.24491	0.204408	GY	ERMP1;RIC1	NC_056064.1	54915001	54935001	6.21646	0.249088	HZ	EXOC7;GALR2;ZAC
5.08694	0.194903	GY	ERMP1;RIC1	NC_056064.1	54905001	54925001	7.39474	0.29711	HZ	EXOC7;ZACN
4.9534	0.240331	GY	ERN1	NC_056065.1	41785001	41805001	2.41026	0.303046	HZ	EXOSC10
8.46492	0.1932	GY	ERN1	NC_056065.1	41790001	41810001	2.85858	0.334973	HZ	EXOSC10
4.51671	0.197853	GY	ERN1	NC_056065.1	41795001	41815001	2.79032	0.305912	HZ	EXOSC10
3.37558	0.267858	GY	ERO1A	NC_056065.1	41800001	41820001	3.08648	0.299068	HZ	EXOSC10
7.59568	0.32589	GY	ERO1A	NC_056065.1	41805001	41825001	2.37692	0.271711	HZ	EXOSC10
7.10123	0.356361	GY	ERO1A	NC_056080.1	81410001	81430001	5.77571	0.248858	HZ	F8
4.18512	0.320254	GY	ERO1A	NC_056080.1	81460001	81480001	2.44632	0.427479	HZ	F8
3.88373	0.221403	GY	ETV1	NC_056080.1	81465001	81485001	2.80924	0.502728	HZ	F8
2.95731	0.198397	GY	ETV1	NC_056080.1	81470001	81490001	3.22715	0.600193	HZ	F8
3.06397	0.20272	GY	ETV5	NC_056055.1	236020001	236040001	3.0328	0.292522	HZ	FABP3;ZCCHC17
2.86419	0.240884	GY	ETV5	NC_056055.1	236025001	236045001	4.25141	0.393983	HZ	FABP3;ZCCHC17
2.76676	0.251919	GY	ETV5	NC_056055.1	236030001	236050001	5.99999	0.485375	HZ	FABP3;ZCCHC17
2.61997	0.27553	GY	ETV5	NC_056055.1	236035001	236055001	6.43011	0.501471	HZ	FABP3;ZCCHC17
2.54579	0.25399	GY	ETV5	NC_056055.1	236040001	236060001	2.44578	0.421757	HZ	FABP3;ZCCHC17
2.53499	0.208941	GY	ETV5	NC_056059.1	36690001	36710001	3.22176	0.441833	HZ	FAM13A
2.58071	0.20415	GY	ETV5	NC_056059.1	36695001	36715001	3.80574	0.41963	HZ	FAM13A
2.61281	0.197251	GY	ETV5	NC_056065.1	60345001	60365001	2.83836	0.22792	HZ	FAM163A
4.15179	0.198822	GY	EVA1A	NC_056065.1	60350001	60370001	4.23032	0.281624	HZ	FAM163A
4.78004	0.199173	GY	EVA1A	NC_056065.1	60355001	60375001	9.8732	0.301205	HZ	FAM163A
3.13446	0.209202	GY	EVA1A	NC_056065.1	60360001	60380001	7.57569	0.262782	HZ	FAM163A
2.85913	0.212525	GY	EVA1A	NC_056065.1	60365001	60385001	4.85065	0.238591	HZ	FAM163A
3.18821	0.231416	GY	EVA1A	NC_056058.1	91085001	91105001	6.15866	0.236001	HZ	FAM172A
4.04194	0.204443	GY	EVA1A	NC_056058.1	91090001	91110001	6.60813	0.26364	HZ	FAM172A
3.79718	0.20755	GY	EXOC3L4	NC_056059.1	37950001	37970001	2.99212	0.207858	HZ	FAM184B
5.65371	0.300934	GY	EXOC3L4;LOC105	NC_056059.1	37955001	37975001	2.40706	0.25588	HZ	FAM184B
7.31973	0.286648	GY	EXOC3L4;LOC105	NC_056059.1	37960001	37980001	3.09671	0.304478	HZ	FAM184B
16.8701	0.290765	GY	EXOC3L4;LOC105	NC_056059.1	37965001	37985001	4.32621	0.356365	HZ	FAM184B
2.84345	0.355594	GY	EXOSC10	NC_056059.1	37970001	37990001	8.32394	0.401201	HZ	FAM184B
2.99462	0.299344	GY	EXOSC10	NC_056065.1	59810001	59830001	2.50344	0.210122	HZ	FAM20B
3.04864	0.311497	GY	EXOSC10	NC_056060.1	55910001	55930001	3.48937	0.226517	HZ	FAM214A
3.48322	0.252829	GY	F8	NC_056060.1	55915001	55935001	3.27722	0.239921	HZ	FAM214A
4.32658	0.30087	GY	F8	NC_056060.1	55920001	55940001	4.98325	0.340414	HZ	FAM214A
5.42382	0.35925	GY	F8	NC_056060.1	55925001	55945001	7.8102	0.401725	HZ	FAM214A
2.9885	0.233979	GY	FAM13A	NC_056060.1	55930001	55950001	7.82021	0.399562	HZ	FAM214A
6.50159	0.214703	GY	FAM13A	NC_056060.1	55935001	55955001	3.93259	0.329533	HZ	FAM214A
13.6133	0.226014	GY	FAM13A	NC_056056.1	202485001	202505001	3.00012	0.270716	HZ	FAM234B
6.53845	0.209709	GY	FAM13A	NC_056056.1	202490001	202510001	2.52134	0.256067	HZ	FAM234B
4.09206	0.284169	GY	FAM13A	NC_056059.1	13710001	13730001	2.68727	0.22147	HZ	FAM241A
4.0596	0.305487	GY	FAM13A	NC_056054.1	193680001	193700001	3.41042	0.23687	HZ	FAM43A
3.4446	0.246176	GY	FAM163A	NC_056054.1	193685001	193705001	2.75716	0.220005	HZ	FAM43A;LSG1
8.69741	0.29479	GY	FAM163A	NC_056070.1	44690001	44710001	6.03449	0.232404	HZ	FBRSL1
6.70413	0.327642	GY	FAM163A	NC_056070.1	44695001	44715001	5.6213	0.222642	HZ	FBRSL1
4.39931	0.348392	GY	FAM163A	NC_056070.1	44700001	44720001	4.49152	0.209013	HZ	FBRSL1
2.44432	0.264499	GY	FAM163A	NC_056070.1	44705001	44725001	5.39072	0.224016	HZ	FBRSL1
7.99144	0.228395	GY	FAM172A	NC_056070.1	44710001	44730001	5.23116	0.221811	HZ	FBRSL1
4.00114	0.19766	GY	FAM172A	NC_056070.1	44715001	44735001	5.79083	0.233867	HZ	FBRSL1
14.3155	0.224819	GY	FAM184B	NC_056070.1	44720001	44740001	6.98852	0.237637	HZ	FBRSL1
2.56408	0.2888	GY	FAM189A1;NSMC	NC_056070.1	44725001	44745001	6.23077	0.232163	HZ	FBRSL1
3.7398	0.239265	GY	FAM240A	NC_056062.1	77160001	77180001	5.95556	0.316244	HZ	FBXO43

4.01062	0.215111	GY	FAM240A	NC_056062.1	77165001	77185001	5.03439	0.320835	HZ	FBXO43
2.84894	0.257029	GY	FAM3B	NC_056062.1	77170001	77190001	2.81206	0.230149	HZ	FBXO43
3.3307	0.24005	GY	FAM3B	NC_056055.1	2760001	2780001	9.88891	0.627308	HZ	FBXW2
2.68005	0.210312	GY	FAM3B	NC_056055.1	2765001	2785001	8.47137	0.585429	HZ	FBXW2
3.25362	0.199948	GY	FAM3B	NC_056055.1	2770001	2790001	5.7093	0.486429	HZ	FBXW2
5.05251	0.288757	GY	FANCE	NC_056055.1	2775001	2795001	5.51105	0.345587	HZ	FBXW2
6.31017	0.312288	GY	FANCE	NC_056055.1	2780001	2800001	3.87352	0.239493	HZ	FBXW2
5.23486	0.273721	GY	FANCE	NC_056070.1	58035001	58055001	5.72461	0.253071	HZ	FBXW8;TESC
2.61628	0.254551	GY	FAR2	NC_056070.1	58040001	58060001	6.18749	0.332572	HZ	FBXW8;TESC
3.065	0.294948	GY	FARSB	NC_056069.1	8700001	8720001	2.57773	0.209857	HZ	FCHO2
3.64621	0.352681	GY	FARSB;LOC10112(	NC_056069.1	8705001	8725001	2.55933	0.209835	HZ	FCHO2
6.00487	0.249433	GY	FBRSL1	NC_056069.1	8710001	8730001	2.41866	0.239702	HZ	FCHO2
6.49427	0.318451	GY	FBRSL1	NC_056069.1	8715001	8735001	2.55753	0.214256	HZ	FCHO2
6.05916	0.323986	GY	FBRSL1	NC_056069.1	8785001	8805001	3.08703	0.210049	HZ	FCHO2
4.62146	0.311301	GY	FBRSL1	NC_056069.1	8790001	8810001	3.22182	0.223408	HZ	FCHO2
5.96025	0.284795	GY	FBRSL1	NC_056069.1	8795001	8815001	3.5721	0.247775	HZ	FCHO2
5.44222	0.263503	GY	FBRSL1	NC_056080.1	96465001	96485001	2.38117	0.208311	HZ	FGF13
6.06123	0.258511	GY	FBRSL1	NC_056063.1	77690001	77710001	3.88514	0.220963	HZ	FGF14
7.25864	0.270183	GY	FBRSL1	NC_056063.1	77695001	77715001	7.97799	0.275023	HZ	FGF14
6.52661	0.257508	GY	FBRSL1	NC_056063.1	77700001	77720001	5.68043	0.285442	HZ	FGF14
6.4563	0.245338	GY	FBRSL1	NC_056063.1	77705001	77725001	4.44334	0.295364	HZ	FGF14
2.46849	0.192151	GY	FBXO31	NC_056063.1	77710001	77730001	3.69191	0.284031	HZ	FGF14
2.7029	0.219048	GY	FBXO31	NC_056072.1	40960001	40980001	3.34158	0.212037	HZ	FHIT
3.87805	0.323391	GY	FBXO31;MAP1LC3	NC_056072.1	40965001	40985001	3.20452	0.210339	HZ	FHIT
3.54055	0.360842	GY	FBXO31;MAP1LC3	NC_056076.1	21005001	21025001	3.04318	0.230523	HZ	FHOD3
2.55991	0.232395	GY	FBXW11	NC_056056.1	5735001	5755001	4.51524	0.228127	HZ	FIBCD1
2.72751	0.237393	GY	FBXW11	NC_056056.1	5740001	5760001	3.5973	0.212917	HZ	FIBCD1
2.86198	0.246033	GY	FBXW11	NC_056064.1	41730001	41750001	2.36238	0.262121	HZ	FKBP10;P3H4
3.28471	0.289099	GY	FBXW11	NC_056074.1	29545001	29565001	2.43468	0.254861	HZ	FLI1
3.41942	0.312966	GY	FBXW11	NC_056074.1	29550001	29570001	2.45853	0.283698	HZ	FLI1
3.29245	0.286702	GY	FBXW11	NC_056072.1	43355001	43375001	4.19523	0.206402	HZ	FLNB
3.38877	0.278251	GY	FBXW11	NC_056073.1	27400001	27420001	2.99705	0.259503	HZ	FLOT1;IER3;MDC1;T
2.71236	0.250866	GY	FBXW11	NC_056073.1	27395001	27415001	2.38582	0.288874	HZ	FLOT1;IER3;TUBB
3.17639	0.337173	GY	FBXW7	NC_056073.1	27405001	27425001	4.08203	0.2834	HZ	FLOT1;MDC1;TUBB
5.84285	0.414238	GY	FBXW7	NC_056073.1	27410001	27430001	5.85141	0.291995	HZ	FLOT1;MDC1;TUBB
7.21693	0.430165	GY	FBXW7	NC_056080.1	87715001	87735001	3.03468	0.350099	HZ	FMR1
12.2973	0.442149	GY	FBXW7	NC_056080.1	87720001	87740001	2.42857	0.337954	HZ	FMR1
4.30365	0.401462	GY	FBXW7	NC_056056.1	76090001	76110001	4.02469	0.582077	HZ	FOXN2
5.64491	0.322135	GY	FBXW8;TESC	NC_056056.1	76095001	76115001	3.46866	0.483544	HZ	FOXN2
6.15972	0.391919	GY	FBXW8;TESC	NC_056072.1	30105001	30125001	4.34412	0.321634	HZ	FOXP1
3.7551	0.243699	GY	FCGR3A	NC_056072.1	30110001	30130001	2.81081	0.298939	HZ	FOXP1
2.42344	0.283098	GY	FCHO2	NC_056057.1	55935001	55955001	2.41355	0.21884	HZ	FOXP2
2.7264	0.347588	GY	FCHO2	NC_056057.1	55940001	55960001	4.6891	0.2505	HZ	FOXP2
3.05882	0.359203	GY	FCHO2	NC_056057.1	55945001	55965001	4.84238	0.272285	HZ	FOXP2
2.64219	0.382245	GY	FCHO2	NC_056057.1	55950001	55970001	4.42921	0.255396	HZ	FOXP2
2.53684	0.376159	GY	FCHO2	NC_056057.1	55955001	55975001	2.58547	0.228419	HZ	FOXP2
2.59225	0.374568	GY	FCHO2	NC_056079.1	18320001	18340001	2.72189	0.282788	HZ	FRG1
2.40478	0.409126	GY	FCHO2	NC_056079.1	18325001	18345001	2.93536	0.274601	HZ	FRG1
2.49494	0.381114	GY	FCHO2	NC_056079.1	18330001	18350001	3.22683	0.282188	HZ	FRG1
2.54863	0.334023	GY	FCHO2	NC_056079.1	18335001	18355001	4.51813	0.279934	HZ	FRG1
2.58478	0.317727	GY	FCHO2	NC_056079.1	18340001	18360001	4.68124	0.257224	HZ	FRG1
2.76906	0.31228	GY	FCHO2	NC_056079.1	18345001	18365001	5.05555	0.261697	HZ	FRG1
2.49828	0.320969	GY	FCHO2	NC_056066.1	27780001	27800001	13.0567	0.251497	HZ	FRMD4A
2.67729	0.347217	GY	FCHO2	NC_056066.1	27785001	27805001	25.2564	0.253247	HZ	FRMD4A
2.58785	0.359164	GY	FCHO2	NC_056066.1	27790001	27810001	8.77506	0.219793	HZ	FRMD4A
2.70395	0.350419	GY	FCHO2	NC_056063.1	29215001	29235001	2.49999	0.244409	HZ	FRY
2.90543	0.313174	GY	FCHO2	NC_056055.1	126875001	126895001	2.44274	0.216916	HZ	FRZB
2.5086	0.386771	GY	FETUB;HRG	NC_056056.1	75405001	75425001	2.42569	0.226703	HZ	FSHR
2.77054	0.210375	GY	FEV	NC_056054.1	31270001	31290001	4.61224	0.211762	HZ	FYB2
2.42652	0.287148	GY	FHIT	NC_056054.1	31275001	31295001	5.87534	0.214656	HZ	FYB2
3.10236	0.247695	GY	FHL1	NC_056054.1	31280001	31300001	9.95944	0.252946	HZ	FYB2
5.31034	0.195508	GY	FLNB	NC_056054.1	31285001	31305001	11.6908	0.26513	HZ	FYB2
3.07647	0.202266	GY	FLOT1;IER3;MDC1	NC_056072.1	53285001	53305001	2.48936	0.21982	HZ	FYCO1;XCR1
2.6483	0.20609	GY	FLOT1;IER3;TUBB	NC_056072.1	53290001	53310001	3.73109	0.25763	HZ	FYCO1;XCR1
4.04313	0.232743	GY	FLOT1;MDC1;TUE	NC_056072.1	53295001	53315001	10.4786	0.233136	HZ	FYCO1;XCR1
5.65483	0.266936	GY	FLOT1;MDC1;TUE	NC_056054.1	131250001	131270001	2.56521	0.25003	HZ	GABPA
5.06173	0.510063	GY	FOXN2	NC_056054.1	131255001	131275001	3.85938	0.37468	HZ	GABPA
3.78201	0.497264	GY	FOXN2	NC_056054.1	131260001	131280001	6.71784	0.427016	HZ	GABPA
4.2973	0.191812	GY	FOXN3	NC_056054.1	131265001	131285001	10.9265	0.400335	HZ	GABPA
2.84802	0.238468	GY	FOXN3	NC_056054.1	131270001	131290001	20.1291	0.390175	HZ	GABPA
5.87454	0.263602	GY	FOXN3	NC_056054.1	131280001	131300001	7.15348	0.300962	HZ	GABPA
3.8568	0.263853	GY	FOXN3	NC_056059.1	65530001	65550001	2.4062	0.22757	HZ	GABRA2
2.53269	0.19822	GY	FOXO1	NC_056059.1	65535001	65555001	2.48418	0.212926	HZ	GABRA2
4.54422	0.241865	GY	FOXP1	NC_056080.1	83505001	83525001	4.24206	0.220956	HZ	GABRA3

2.98431	0.208253	GY	FOXP1	NC_056080.1	83510001	83530001	7.16408	0.281067	HZ	GABRA3
8.01554	0.194613	GY	FOXP2	NC_056080.1	83525001	83545001	7.81396	0.286204	HZ	GABRA3
8.47618	0.217209	GY	FOXP2	NC_056058.1	70935001	70955001	2.36029	0.2459	HZ	GABRA6
8.38125	0.244092	GY	FOXP2	NC_056058.1	70940001	70960001	2.72808	0.258647	HZ	GABRA6
4.44774	0.192876	GY	FOXP2	NC_056058.1	70955001	70975001	2.7497	0.247718	HZ	GABRA6
12.0618	0.273434	GY	FRMD4A	NC_056058.1	63435001	63455001	3.17557	0.377426	HZ	GALNT10
23.5726	0.286252	GY	FRMD4A	NC_056058.1	63440001	63460001	7.03896	0.496827	HZ	GALNT10
8.23577	0.248403	GY	FRMD4A	NC_056058.1	63445001	63465001	7.4	0.515273	HZ	GALNT10
5.59954	0.210842	GY	FRMD4A	NC_056058.1	63450001	63470001	8.00865	0.52452	HZ	GALNT10
2.93229	0.236332	GY	FRMD4A	NC_056058.1	63455001	63475001	4.71428	0.47898	HZ	GALNT10
3.1142	0.193302	GY	FRMD4A	NC_056058.1	63460001	63480001	2.59117	0.402449	HZ	GALNT10
3.14316	0.203975	GY	FRMD4A	NC_056060.1	79130001	79150001	6.72727	0.331537	HZ	GALNT16
2.98451	0.199901	GY	FRMD4A	NC_056060.1	79135001	79155001	6.57777	0.34588	HZ	GALNT16
2.61431	0.215397	GY	FRMD4A	NC_056078.1	1790001	1810001	5.89139	0.210385	HZ	GALNT2
2.79894	0.29469	GY	FRMPD4	NC_056060.1	63225001	63245001	2.95959	0.260757	HZ	GATM
4.85714	0.256544	GY	FYB2	NC_056060.1	6490001	6510001	3.31164	0.233559	HZ	GCNT4
6.1355	0.267217	GY	FYB2	NC_056062.1	50595001	50615001	2.40849	0.208545	HZ	GDAP1
10.6396	0.285111	GY	FYB2	NC_056062.1	80970001	80990001	2.74801	0.214844	HZ	GDF6
15.4408	0.292686	GY	FYB2	NC_056058.1	63845001	63865001	6.06122	0.344097	HZ	GEMIN5
3.15844	0.23093	GY	FYB2;PRKAA2	NC_056058.1	63850001	63870001	4.41946	0.355821	HZ	GEMIN5
5.00002	0.196441	GY	GAB3	NC_056058.1	63855001	63875001	2.77037	0.361406	HZ	GEMIN5
7.86991	0.256806	GY	GAB3	NC_056054.1	260180001	260200001	3.82093	0.320075	HZ	GET1
5.71997	0.230945	GY	GAB3	NC_056054.1	260185001	260205001	6.60526	0.24678	HZ	GET1
4.91841	0.2517	GY	GABPA	NC_056054.1	260190001	260210001	9.78006	0.231392	HZ	GET1
8.58209	0.272434	GY	GABPA	NC_056054.1	260195001	260215001	13.7564	0.242373	HZ	GET1;LCA5L
13.1581	0.279785	GY	GABPA	NC_056057.1	67330001	67350001	3.35255	0.207916	HZ	GGCT
20.1923	0.30082	GY	GABPA	NC_056066.1	26125001	26145001	8.50598	0.216777	HZ	GJD4
6.82324	0.212038	GY	GABPA	NC_056054.1	247310001	247330001	25.2475	0.437134	HZ	GK5
4.68792	0.212531	GY	GABRA3	NC_056054.1	247315001	247335001	24.963	0.397693	HZ	GK5
8.21097	0.257431	GY	GABRA3	NC_056054.1	247320001	247340001	9.9542	0.32739	HZ	GK5
3.04744	0.298737	GY	GABRA6	NC_056054.1	247325001	247345001	3.80372	0.23215	HZ	GK5
2.94313	0.298397	GY	GALE;LYPLA2;PIT	NC_056057.1	80465001	80485001	7.08081	0.247665	HZ	GLI3
5.90143	0.265085	GY	GALNT13	NC_056057.1	80470001	80490001	4.09508	0.278273	HZ	GLI3
7.65174	0.281076	GY	GALNT13	NC_056057.1	80475001	80495001	4.01219	0.282277	HZ	GLI3
4.9067	0.239641	GY	GALNT13	NC_056057.1	80480001	80500001	3.27415	0.228325	HZ	GLI3
3.05078	0.203877	GY	GALNT13	NC_056057.1	80485001	80505001	2.72479	0.254248	HZ	GLI3
19.8864	0.201247	GY	GALNT2	NC_056057.1	80685001	80705001	2.95528	0.280162	HZ	GLI3
3.85582	0.292149	GY	GALNT7	NC_056058.1	92745001	92765001	2.46656	0.222736	HZ	GLRX
2.92983	0.332964	GY	GALNT7	NC_056054.1	232530001	232550001	10.3875	0.246786	HZ	GMPS
2.99643	0.235917	GY	GALNT9	NC_056077.1	1605001	1625001	3.04953	0.228203	HZ	GNPTG;UNKL
2.94389	0.297877	GY	GALNTL6	NC_056077.1	1610001	1630001	2.86812	0.21804	HZ	GNPTG;UNKL
2.75438	0.222143	GY	GAR1	NC_056054.1	20295001	20315001	3.9901	0.208059	HZ	GPBP1L1;TMEM69
2.46185	0.212611	GY	GAR1	NC_056055.1	153175001	153195001	4.28319	0.292377	HZ	GPD2
3.00919	0.234307	GY	GAR1;RRH	NC_056055.1	153180001	153200001	7.58426	0.335961	HZ	GPD2
2.90191	0.239491	GY	GAR1;RRH	NC_056055.1	153185001	153205001	11.203	0.356552	HZ	GPD2
4.10783	0.202106	GY	GAR1;RRH	NC_056055.1	153190001	153210001	8.33335	0.364748	HZ	GPD2
3.11456	0.21039	GY	GCNT4	NC_056055.1	153195001	153215001	4.52245	0.290861	HZ	GPD2
3.53907	0.275036	GY	GCSH;PKD1L2	NC_056080.1	11935001	11955001	6.54443	0.335815	HZ	GPM6B
4.82433	0.290904	GY	GCSH;PKD1L2	NC_056080.1	11940001	11960001	9.06943	0.326952	HZ	GPM6B
2.94149	0.206394	GY	GDAP2	NC_056080.1	11945001	11965001	4.90336	0.354037	HZ	GPM6B
3.06211	0.266425	GY	GFOD2	NC_056080.1	11950001	11970001	2.51905	0.287532	HZ	GPM6B
3.24573	0.28153	GY	GFOD2	NC_056056.1	6465001	6485001	3.53242	0.240814	HZ	GPR107
3.37573	0.306652	GY	GFOD2	NC_056056.1	6470001	6490001	4.43934	0.244334	HZ	GPR107
3.44805	0.294484	GY	GFOD2	NC_056056.1	6475001	6495001	4.15656	0.221445	HZ	GPR107
2.5398	0.276061	GY	GHR	NC_056056.1	6480001	6500001	3.49355	0.210136	HZ	GPR107
2.9389	0.309979	GY	GLB1L3;LOC11411	NC_056056.1	6485001	6505001	2.81863	0.207461	HZ	GPR107
2.48561	0.362496	GY	GLI3	NC_056068.1	2075001	2095001	3.09501	0.233547	HZ	GRIA4
7.49496	0.305422	GY	GLI3	NC_056068.1	2080001	2100001	28.9558	0.391698	HZ	GRIA4
3.6451	0.370847	GY	GLI3	NC_056068.1	2085001	2105001	42.3964	0.387405	HZ	GRIA4
3.27395	0.424854	GY	GLI3	NC_056068.1	2090001	2110001	23.4616	0.370055	HZ	GRIA4
2.68921	0.335542	GY	GLI3	NC_056068.1	2095001	2115001	17.4808	0.365738	HZ	GRIA4
12.8875	0.339612	GY	GLMN	NC_056068.1	2100001	2120001	14.6194	0.358955	HZ	GRIA4
17.8013	0.34546	GY	GLMN	NC_056068.1	2105001	2125001	12.5603	0.384341	HZ	GRIA4
3.52425	0.197782	GY	GLRA2	NC_056068.1	2110001	2130001	16.4857	0.408008	HZ	GRIA4
2.77976	0.203415	GY	GLT1D1	NC_056068.1	2155001	2175001	15.9286	0.487668	HZ	GRIA4
3.87439	0.216578	GY	GLYCAM1	NC_056068.1	2160001	2180001	5.63102	0.401222	HZ	GRIA4
13.65	0.19288	GY	GMPS	NC_056068.1	2165001	2185001	4.24779	0.369162	HZ	GRIA4
2.54982	0.200087	GY	GNL3;PBRM1	NC_056059.1	32110001	32130001	2.52413	0.293813	HZ	GRID2
3.31872	0.256013	GY	GON7;TMEM251	NC_056059.1	32115001	32135001	2.68149	0.294383	HZ	GRID2
2.78068	0.192405	GY	GON7;TMEM251	NC_056054.1	126785001	126805001	2.60869	0.210406	HZ	GRIK1
6.17332	0.253222	GY	GPC3	NC_056054.1	126795001	126815001	3.80827	0.243304	HZ	GRIK1
3.03418	0.302728	GY	GPC3	NC_056054.1	126830001	126850001	2.42993	0.223225	HZ	GRIK1
5.45417	0.232614	GY	GPC5	NC_056077.1	9430001	9450001	2.35355	0.269873	HZ	GRIN2A
5.0679	0.233146	GY	GPC5	NC_056057.1	92400001	92420001	4.34227	0.305559	HZ	GRM8

4.1208	0.213966	GY	GPC5	NC_056057.1	92405001	92425001	6.59174	0.346189	HZ	GRM8
3.41526	0.204033	GY	GPC6	NC_056057.1	92410001	92430001	3.73328	0.302424	HZ	GRM8
6.95505	0.200395	GY	GPD2	NC_056057.1	92415001	92435001	2.95213	0.281438	HZ	GRM8
10.188	0.211762	GY	GPD2	NC_056057.1	92445001	92465001	3.1672	0.322853	HZ	GRM8
7.66094	0.220911	GY	GPD2	NC_056057.1	92450001	92470001	3.02402	0.329885	HZ	GRM8
3.66316	0.329072	GY	GPM6A	NC_056057.1	92455001	92475001	2.54395	0.267032	HZ	GRM8
8.03254	0.325753	GY	GPM6A	NC_056057.1	92480001	92500001	3.01185	0.413307	HZ	GRM8
5.87783	0.389198	GY	GPM6A	NC_056057.1	92485001	92505001	4.39184	0.460715	HZ	GRM8
4.66613	0.378182	GY	GPM6A	NC_056057.1	92490001	92510001	5.04269	0.368157	HZ	GRM8
4.02781	0.352232	GY	GPM6A	NC_056057.1	92495001	92515001	3.70904	0.31792	HZ	GRM8
3.372	0.195722	GY	GPR160;PHC3	NC_056067.1	11080001	11100001	3.2931	0.235246	HZ	GSE1
3.15833	0.26129	GY	GPR174	NC_056067.1	11085001	11105001	2.86696	0.211823	HZ	GSE1
3.17806	0.219203	GY	GPR87;MED12L	NC_056077.1	25865001	25885001	2.70444	0.246434	HZ	GSG1L
2.86557	0.21785	GY	GPR87;MED12L	NC_056077.1	25870001	25890001	2.71429	0.207443	HZ	GSG1L
3.05112	0.224022	GY	GPR87;MED12L	NC_056077.1	26075001	26095001	7.40202	0.239034	HZ	GSG1L
2.70163	0.202219	GY	GPR87;MED12L	NC_056077.1	26080001	26100001	7.74689	0.256823	HZ	GSG1L
3.2158	0.24595	GY	GPR87;MED12L;P2	NC_056077.1	26085001	26105001	5.87877	0.274842	HZ	GSG1L
4.39896	0.192181	GY	GREB1L	NC_056077.1	26090001	26110001	5.2064	0.287335	HZ	GSG1L
2.69641	0.250449	GY	GRIA4	NC_056077.1	26095001	26115001	3.22074	0.224779	HZ	GSG1L
2.85714	0.308395	GY	GRIA4	NC_056079.1	25855001	25875001	5.53732	0.259096	HZ	GTF2E2
35.9557	0.253571	GY	GRIA4	NC_056079.1	25860001	25880001	5.08571	0.318167	HZ	GTF2E2
54.1769	0.239682	GY	GRIA4	NC_056079.1	25865001	25885001	4.54434	0.362845	HZ	GTF2E2
30.6811	0.219125	GY	GRIA4	NC_056079.1	25870001	25890001	5.19808	0.354085	HZ	GTF2E2
22.2932	0.206033	GY	GRIA4	NC_056079.1	25875001	25895001	3.75991	0.291588	HZ	GTF2E2
15.2553	0.198461	GY	GRIA4	NC_056079.1	25880001	25900001	3.16635	0.251949	HZ	GTF2E2
4.75687	0.265675	GY	GRID2	NC_056079.1	25885001	25905001	3.10734	0.230494	HZ	GTF2E2
3.44891	0.294078	GY	GRID2	NC_056057.1	76155001	76175001	2.59442	0.362005	HZ	GTPBP10
2.55587	0.293417	GY	GRID2	NC_056057.1	76160001	76180001	3.54447	0.359247	HZ	GTPBP10
2.85484	0.322193	GY	GRID2	NC_056057.1	76165001	76185001	2.93008	0.282144	HZ	GTPBP10
4.0678	0.202024	GY	GRID2	NC_056073.1	15910001	15930001	3.44964	0.226233	HZ	GUCA1ANB;LOC101
3.85312	0.207767	GY	GRID2	NC_056055.1	120985001	121005001	4.39888	0.247651	HZ	GULP1
3.51016	0.222475	GY	GRID2	NC_056055.1	120990001	121010001	6.00223	0.233022	HZ	GULP1
3.11058	0.229508	GY	GRID2	NC_056055.1	120995001	121015001	13.1154	0.311879	HZ	GULP1
3.30935	0.239993	GY	GRID2	NC_056055.1	121000001	121020001	4.16029	0.247323	HZ	GULP1
2.90595	0.208385	GY	GRID2	NC_056055.1	121030001	121050001	8.59601	0.219448	HZ	GULP1
3.27216	0.204466	GY	GRID2	NC_056055.1	121035001	121055001	7.11796	0.210638	HZ	GULP1
4.80311	0.234834	GY	GRID2	NC_056055.1	121040001	121060001	6.81996	0.219408	HZ	GULP1
9.4739	0.260099	GY	GRID2	NC_056055.1	121045001	121065001	3.91956	0.260342	HZ	GULP1
4.64296	0.243394	GY	GRID2	NC_056055.1	121050001	121070001	3.58947	0.251979	HZ	GULP1
2.52039	0.229417	GY	GRID2	NC_056055.1	121055001	121075001	3.11255	0.245204	HZ	GULP1
2.56573	0.305809	GY	GRID2	NC_056073.1	29955001	29975001	2.79721	0.293552	HZ	H1-5;LOC101102344;
2.63799	0.320334	GY	GRID2	NC_056073.1	29950001	29970001	2.63982	0.280325	HZ	H1-5;LOC101102344;
18.3984	0.222217	GY	GRIK5	NC_056057.1	28400001	28420001	2.97091	0.219286	HZ	HDAC9
17.5806	0.217721	GY	GRIK5	NC_056057.1	79200001	79220001	4.34426	0.210018	HZ	HECW1
3.23629	0.314033	GY	GRIN3A	NC_056057.1	79205001	79225001	6.1519	0.31478	HZ	HECW1
2.43602	0.262926	GY	GRIN3A	NC_056057.1	79210001	79230001	6.07054	0.37222	HZ	HECW1
11.37	0.371783	GY	GRIP1	NC_056057.1	79215001	79235001	2.5944	0.278226	HZ	HECW1
2.43296	0.194134	GY	GRM5	NC_056054.1	190290001	190310001	3.29306	0.387839	HZ	HEG1
3.22014	0.279894	GY	GRM5	NC_056075.1	15560001	15580001	3.70654	0.223216	HZ	HELLS;TBC1D12
2.55596	0.245266	GY	GRM7	NC_056075.1	15565001	15585001	3.10211	0.210644	HZ	HELLS;TBC1D12
2.71629	0.202342	GY	GRM7	NC_056059.1	36700001	36720001	5.75362	0.435846	HZ	HERC3
2.42915	0.232381	GY	GRM7	NC_056059.1	36705001	36725001	3.54684	0.286583	HZ	HERC3
3.44164	0.24274	GY	GRM8	NC_056059.1	36820001	36840001	5.44754	0.259499	HZ	HERC3
3.16217	0.247692	GY	GRM8	NC_056059.1	36825001	36845001	4.45733	0.209539	HZ	HERC3
4.57082	0.22064	GY	GRM8	NC_056059.1	36720001	36740001	2.42915	0.219657	HZ	HERC3;NAP1L5
5.71965	0.216858	GY	GRM8	NC_056059.1	36995001	37015001	20.1463	0.304672	HZ	HERC6
4.44717	0.20806	GY	GRM8	NC_056059.1	37000001	37020001	20.6067	0.311429	HZ	HERC6
2.82244	0.222082	GY	GRM8	NC_056059.1	37005001	37025001	17.7073	0.27293	HZ	HERC6
2.71336	0.422334	GY	GSE1	NC_056077.1	34450001	34470001	2.67704	0.381288	HZ	HIP1
2.41111	0.301491	GY	GSG1L	NC_056077.1	34455001	34475001	2.58026	0.382982	HZ	HIP1
2.5222	0.245729	GY	GSG1L	NC_056077.1	34460001	34480001	4.32728	0.416813	HZ	HIP1
3.77188	0.194783	GY	GTDC1	NC_056070.1	63290001	63310001	34.1599	0.228736	HZ	HNF1A
4.46026	0.211529	GY	GTDC1	NC_056070.1	63295001	63315001	32.814	0.20769	HZ	HNF1A
4.27931	0.208377	GY	GTDC1	NC_056060.1	10405001	10425001	2.42566	0.324598	HZ	HOMER1
3.93119	0.20645	GY	GTDC1	NC_056060.1	10410001	10430001	2.74023	0.377075	HZ	HOMER1
5.90299	0.209875	GY	GTF2E2	NC_056070.1	69155001	69175001	3.0841	0.338603	HZ	HORMAD2
5.70793	0.229981	GY	GTF2E2	NC_056070.1	69160001	69180001	9.27064	0.293157	HZ	HORMAD2
5.1896	0.232803	GY	GTF2E2	NC_056070.1	69165001	69185001	14.5664	0.300763	HZ	HORMAD2
3.62864	0.198862	GY	GUCA1ANB;LOC1	NC_056054.1	64005001	64025001	2.47286	0.238202	HZ	HS2ST1;SELENOF
6.39455	0.19873	GY	GUCA1B;LOC1011	NC_056054.1	114350001	114370001	8.00733	0.246151	HZ	HSD17B7
36.5074	0.243992	GY	GULP1	NC_056054.1	114355001	114375001	4.09858	0.210052	HZ	HSD17B7
16.2372	0.211847	GY	GULP1	NC_056075.1	36745001	36765001	2.42237	0.209359	HZ	HSPA12A
12.9279	0.235419	GY	GULP1	NC_056055.1	244650001	244670001	2.56152	0.253481	HZ	HSPG2
2.98038	0.352714	GY	GULP1	NC_056055.1	244655001	244675001	3.34768	0.317138	HZ	HSPG2

2.88694	0.363647	GY	GULP1	NC_056055.1	244660001	244680001	3.17671	0.320608	HZ	HSPG2
2.75826	0.389324	GY	GULP1	NC_056055.1	244665001	244685001	2.43358	0.232781	HZ	HSPG2
3.42651	0.220467	GY	HAVCR2;MED7	NC_056068.1	24150001	24170001	2.48502	0.207418	HZ	HTR3B
4.06885	0.230529	GY	HECW1	NC_056055.1	242485001	242505001	7.79636	0.307704	HZ	IFNLR1;IL22RA1
5.81434	0.347024	GY	HECW1	NC_056057.1	57480001	57500001	3.0597	0.211737	HZ	IFRD1
6.02075	0.355604	GY	HECW1	NC_056077.1	1720001	1740001	2.37297	0.270687	HZ	IFT140;TMEM204
2.46153	0.249415	GY	HECW1	NC_056068.1	28925001	28945001	5.89158	0.338041	HZ	IFT46;KMT2A;TMEM
5.98188	0.312857	GY	HERC3	NC_056068.1	28935001	28955001	2.45575	0.215077	HZ	IFT46;TMEM25
3.08932	0.237015	GY	HGF	NC_056068.1	28930001	28950001	4.35572	0.351022	HZ	IFT46;TMEM25;TTC
2.9613	0.262446	GY	HGF	NC_056060.1	12240001	12260001	2.86817	0.210973	HZ	IGDCC3
4.0172	0.250347	GY	HMCN1	NC_056074.1	45775001	45795001	3.18314	0.215382	HZ	IGF2
5.18182	0.244176	GY	HMCN1	NC_056074.1	45780001	45800001	2.76922	0.222863	HZ	IGF2
3.05069	0.193407	GY	HMCN1	NC_056059.1	73165001	73185001	2.45115	0.303327	HZ	IGFBP7
33.7465	0.293529	GY	HNF1A	NC_056059.1	73170001	73190001	3.16356	0.281401	HZ	IGFBP7
32.6407	0.275856	GY	HNF1A	NC_056059.1	73175001	73195001	2.86617	0.267432	HZ	IGFBP7
4.50251	0.272454	GY	HNRNPAB;PHYKF	NC_056064.1	39940001	39960001	3.60975	0.277392	HZ	IKZF3
4.00426	0.22794	GY	HNRNPAB;PHYKF	NC_056064.1	39950001	39970001	3.05634	0.245068	HZ	IKZF3;ZPBP2
4.84302	0.197688	GY	HNRNPUL2;LOC1(	NC_056064.1	39955001	39975001	3.44068	0.213155	HZ	IKZF3;ZPBP2
3.91796	0.218852	GY	HNRNPUL2;LOC1(	NC_056056.1	225300001	225320001	3.10046	0.23084	HZ	IL17REL
2.46064	0.219171	GY	HOMER1	NC_056056.1	225305001	225325001	3.87338	0.287326	HZ	IL17REL
6.42677	0.331352	GY	HSF5	NC_056056.1	225310001	225330001	5.61596	0.324736	HZ	IL17REL
4.49443	0.275442	GY	HSF5	NC_056056.1	99580001	99600001	4.66011	0.227911	HZ	IL1R1
5.68453	0.21113	GY	HSF5;RNF43	NC_056056.1	99585001	99605001	4.47668	0.226727	HZ	IL1R1
16	0.311783	GY	HSF5;RNF43	NC_056056.1	99590001	99610001	4.30829	0.227419	HZ	IL1R1
5.89011	0.30846	GY	HSPA6	NC_056056.1	99595001	99615001	4.2187	0.214921	HZ	IL1R1
4.01908	0.291807	GY	HSPA6;LOC10110(	NC_056056.1	99625001	99645001	14.1401	0.217616	HZ	IL1R1
2.97554	0.195259	GY	HTR6;LOC1011081	NC_056056.1	99630001	99650001	14.8925	0.207942	HZ	IL1R1
4.73685	0.308496	GY	HTRA1	NC_056055.1	242490001	242510001	11.5494	0.339883	HZ	IL22RA1
5.62254	0.413347	GY	HTT	NC_056058.1	9965001	9985001	2.46589	0.255287	HZ	IL27RA
5.30049	0.463167	GY	HTT	NC_056058.1	9970001	9990001	2.54262	0.239125	HZ	IL27RA;RLN3
4.34596	0.444517	GY	HTT	NC_056057.1	58705001	58725001	4.25693	0.310798	HZ	IMMP2L
2.98052	0.447258	GY	HTT	NC_056057.1	58710001	58730001	4.08176	0.319564	HZ	IMMP2L
2.57518	0.230674	GY	HTT;LOC12181979	NC_056057.1	58715001	58735001	4.62231	0.34298	HZ	IMMP2L
3.53449	0.34185	GY	HTT;MSANTD1	NC_056057.1	58720001	58740001	4.85156	0.356882	HZ	IMMP2L
3.56433	0.41004	GY	HTT;MSANTD1	NC_056057.1	58725001	58745001	3.38956	0.302043	HZ	IMMP2L
2.59096	0.198386	GY	IFITM10	NC_056057.1	58730001	58750001	3.38107	0.289389	HZ	IMMP2L
2.42529	0.282732	GY	IFRD1	NC_056057.1	58735001	58755001	3.09755	0.217823	HZ	IMMP2L
2.4904	0.264431	GY	IFRD1	NC_056057.1	58740001	58760001	3.22673	0.204979	HZ	IMMP2L
3.70977	0.205354	GY	IFRD1	NC_056057.1	58755001	58775001	4.48537	0.206451	HZ	IMMP2L
4.98669	0.251736	GY	IFRD1	NC_056057.1	58760001	58780001	3.8883	0.241601	HZ	IMMP2L
3.50633	0.252289	GY	IGDCC3	NC_056057.1	58765001	58785001	3.31707	0.285313	HZ	IMMP2L
2.63465	0.292455	GY	IGDCC3	NC_056057.1	58770001	58790001	4.18066	0.287345	HZ	IMMP2L
2.96915	0.235477	GY	IGDCC3	NC_056057.1	58775001	58795001	3.14286	0.264879	HZ	IMMP2L
2.568	0.292378	GY	IGDCC3	NC_056057.1	58780001	58800001	3.14486	0.229507	HZ	IMMP2L
3.10289	0.279221	GY	IGDCC3	NC_056057.1	58900001	58920001	6.61552	0.26093	HZ	IMMP2L
6.2168	0.200571	GY	IL17REL	NC_056057.1	58905001	58925001	5.26327	0.282259	HZ	IMMP2L
2.93177	0.255796	GY	IL27RA	NC_056057.1	58910001	58930001	3.8713	0.266328	HZ	IMMP2L
2.49398	0.297994	GY	IL27RA;PALM3	NC_056057.1	59110001	59130001	2.44098	0.46361	HZ	IMMP2L
3.00464	0.263667	GY	IL27RA;RLN3	NC_056057.1	59115001	59135001	3.57118	0.345728	HZ	IMMP2L
3.00106	0.239592	GY	IL27RA;RLN3	NC_056057.1	59120001	59140001	6.025	0.372551	HZ	IMMP2L
2.82714	0.238436	GY	IMMP2L	NC_056057.1	59125001	59145001	2.69123	0.382584	HZ	IMMP2L
3.28922	0.341134	GY	IMMP2L	NC_056070.1	15300001	15320001	2.38523	0.240936	HZ	INPP4B
3.35279	0.40862	GY	IMMP2L	NC_056070.1	15305001	15325001	2.40657	0.255473	HZ	INPP4B
5.66498	0.280152	GY	IMMP2L	NC_056077.1	41790001	41810001	4.39387	0.35361	HZ	INTS1;MAFK
5.03144	0.267291	GY	IMMP2L	NC_056077.1	41795001	41815001	3.86519	0.303728	HZ	INTS1;MAFK
5.10252	0.247592	GY	IMMP2L	NC_056077.1	41800001	41820001	3.2341	0.25036	HZ	INTS1;MAFK
5.02734	0.268073	GY	IMMP2L	NC_056080.1	99405001	99425001	3.25807	0.323485	HZ	INTS6L
3.46226	0.209485	GY	IMMP2L	NC_056061.1	78645001	78665001	3.80654	0.206271	HZ	IPCEF1
3.23274	0.229642	GY	IMMP2L	NC_056061.1	78650001	78670001	5.39882	0.244397	HZ	IPCEF1
3.19511	0.25587	GY	IMMP2L	NC_056061.1	78655001	78675001	6.37907	0.241538	HZ	IPCEF1
3.32219	0.218251	GY	IMMP2L	NC_056061.1	78660001	78680001	6.66574	0.239729	HZ	IPCEF1
3.75243	0.217425	GY	IMMP2L	NC_056070.1	8560001	8580001	3.78031	0.221158	HZ	IQCM
4.05456	0.212221	GY	IMMP2L	NC_056070.1	8565001	8585001	2.82875	0.247713	HZ	IQCM
3.89018	0.221653	GY	IMMP2L	NC_056054.1	106485001	106505001	3.93411	0.391376	HZ	IQGAP3
2.85541	0.221409	GY	IMMP2L	NC_056054.1	106490001	106510001	4.69919	0.479508	HZ	IQGAP3
3.3048	0.243037	GY	IMMP2L	NC_056054.1	106495001	106515001	3.0233	0.444827	HZ	IQGAP3
4.00294	0.302184	GY	IMMP2L	NC_056054.1	106500001	106520001	2.60639	0.459065	HZ	IQGAP3
7.22087	0.396855	GY	IMMP2L	NC_056080.1	51170001	51190001	2.64583	0.277351	HZ	IQSEC2
7.18546	0.449523	GY	IMMP2L	NC_056056.1	212905001	212925001	10.6353	0.281366	HZ	IQSEC3
4.62101	0.430202	GY	IMMP2L	NC_056056.1	212910001	212930001	2.84514	0.286687	HZ	IQSEC3
3.46668	0.35901	GY	IMMP2L	NC_056067.1	11380001	11400001	3.51383	0.285632	HZ	IRF8
4.06106	0.22601	GY	IMMP2L	NC_056067.1	11385001	11405001	6.7189	0.339182	HZ	IRF8
4.2779	0.217703	GY	INTS1;MAFK	NC_056067.1	11390001	11410001	7.5116	0.293949	HZ	IRF8
3.69813	0.220174	GY	INTS1;MAFK	NC_056066.1	29515001	29535001	3.38288	0.269288	HZ	ITGA8

3.3131	0.211027	GY	INTS1;MAFK	NC_056080.1	63985001	64005001	3.85898	0.399939	HZ	ITGB1BP2;NONO
4.33616	0.194587	GY	INTS6	NC_056056.1	188860001	188880001	2.60941	0.26556	HZ	ITPR2
2.66225	0.27437	GY	IQCG	NC_056056.1	188865001	188885001	5.60521	0.362751	HZ	ITPR2
2.69948	0.299429	GY	IQCG	NC_056056.1	188870001	188890001	6.57295	0.430223	HZ	ITPR2
2.64762	0.229054	GY	IQCM	NC_056056.1	188875001	188895001	6.66062	0.424941	HZ	ITPR2
3.54771	0.247112	GY	IQCM	NC_056056.1	188880001	188900001	5.69214	0.369407	HZ	ITPR2
2.81236	0.22995	GY	IQCM	NC_056056.1	188885001	188905001	3.36555	0.304853	HZ	ITPR2
4.33333	0.296097	GY	IQGAP3	NC_056056.1	188975001	188995001	2.52934	0.2311	HZ	ITPR2
5.86992	0.356029	GY	IQGAP3	NC_056056.1	188980001	189000001	3.39861	0.215343	HZ	ITPR2
3.51651	0.32851	GY	IQGAP3	NC_056056.1	188985001	189005001	4.29019	0.234904	HZ	ITPR2
3.24646	0.315216	GY	IQGAP3	NC_056056.1	188990001	189010001	4.70658	0.250527	HZ	ITPR2
2.4771	0.312357	GY	IQGAP3	NC_056056.1	188995001	189015001	10.6103	0.323717	HZ	ITPR2
2.45312	0.19856	GY	IQSEC2	NC_056056.1	189000001	189020001	11.4932	0.314488	HZ	ITPR2
12.6589	0.236652	GY	IQSEC3	NC_056056.1	189005001	189025001	5.31466	0.249954	HZ	ITPR2
3.4	0.256414	GY	ISLR2	NC_056055.1	73330001	73350001	4.25675	0.226756	HZ	JAK2
19.8589	0.222595	GY	ISM2;SPTLC2	NC_056055.1	73335001	73355001	3.89705	0.212864	HZ	JAK2
2.93065	0.207344	GY	ITGAE	NC_056055.1	73345001	73365001	5.11179	0.215031	HZ	JAK2
7.6282	0.191683	GY	ITGAE;NCBP3	NC_056055.1	73350001	73370001	9.44047	0.261172	HZ	JAK2
3.54488	0.35949	GY	ITGB1BP2;NONO	NC_056055.1	73380001	73400001	9.33094	0.296419	HZ	JAK2
5.91918	0.275492	GY	ITPR2	NC_056055.1	73385001	73405001	3.92772	0.251018	HZ	JAK2
7.21974	0.358198	GY	ITPR2	NC_056068.1	28605001	28625001	2.63306	0.234137	HZ	JAML;SCN2B
7.45367	0.37813	GY	ITPR2	NC_056060.1	84740001	84760001	11.8284	0.225522	HZ	JDP2
6.22285	0.362084	GY	ITPR2	NC_056064.1	41350001	41370001	5.25144	0.298105	HZ	K38;V15
3.49575	0.325752	GY	ITPR2	NC_056064.1	41355001	41375001	3.19925	0.260553	HZ	K38;V15
2.47496	0.205855	GY	ITPR2	NC_056064.1	41360001	41380001	2.38271	0.227816	HZ	K38;V15
2.67068	0.295088	GY	ITSN1	NC_056054.1	232105001	232125001	3.01602	0.223632	HZ	KCNAB1
2.48524	0.291354	GY	ITSN1	NC_056054.1	232110001	232130001	2.49617	0.222781	HZ	KCNAB1
12.0645	0.256807	GY	JADE1;SCLT1	NC_056056.1	104375001	104395001	2.49327	0.226016	HZ	KCNIP3
4.01191	0.731841	GY	JAK2	NC_056056.1	104385001	104405001	5.5928	0.209456	HZ	KCNIP3
4.3813	0.745413	GY	JAK2	NC_056056.1	104390001	104410001	6.93006	0.238054	HZ	KCNIP3
3.28856	0.225049	GY	JARID2	NC_056056.1	104395001	104415001	8.03695	0.236131	HZ	KCNIP3
5.06393	0.277629	GY	K38;V15	NC_056056.1	104400001	104420001	7.11701	0.268769	HZ	KCNIP3
2.88693	0.255357	GY	K38;V15	NC_056056.1	104405001	104425001	5.11577	0.268605	HZ	KCNIP3
11.304	0.204163	GY	KAT2B	NC_056056.1	104410001	104430001	4.34903	0.291922	HZ	KCNIP3;PROM2
12.1727	0.206879	GY	KAT2B	NC_056056.1	104415001	104435001	5.31794	0.405669	HZ	KCNIP3;PROM2
15.6083	0.227424	GY	KAT2B	NC_056056.1	104420001	104440001	6.86473	0.422891	HZ	KCNIP3;PROM2
19.0588	0.24927	GY	KAT2B	NC_056059.1	41675001	41695001	4.85983	0.365825	HZ	KCNIP4
20.1223	0.279781	GY	KAT2B	NC_056059.1	41680001	41700001	3.16792	0.337473	HZ	KCNIP4
6.4186	0.230498	GY	KAT2B	NC_056054.1	208970001	208990001	2.99593	0.221694	HZ	KCNMB2
4.97583	0.217391	GY	KAT2B	NC_056074.1	45450001	45470001	2.88038	0.221748	HZ	KCNQ1
4.97296	0.210236	GY	KAT2B	NC_056074.1	45460001	45480001	18.9375	0.337836	HZ	KCNQ1
5.41137	0.210599	GY	KAT2B	NC_056074.1	45465001	45485001	11.6216	0.296816	HZ	KCNQ1
10.8732	0.230693	GY	KAT2B	NC_056074.1	45470001	45490001	10.0414	0.263547	HZ	KCNQ1
12.6666	0.242016	GY	KAT2B	NC_056074.1	45475001	45495001	7.91603	0.219521	HZ	KCNQ1
12.8862	0.245342	GY	KAT2B	NC_056080.1	44090001	44110001	4.63333	0.661361	HZ	KDM6A
5.75391	0.202413	GY	KAT2B	NC_056080.1	44095001	44115001	5.57693	0.665163	HZ	KDM6A
2.9861	0.199294	GY	KCNAB1	NC_056080.1	44100001	44120001	5.50288	0.651503	HZ	KDM6A
3.66083	0.217406	GY	KCND2	NC_056080.1	44105001	44125001	3.94916	0.587774	HZ	KDM6A
2.62407	0.28267	GY	KCNH5	NC_056080.1	44110001	44130001	4.88889	0.53215	HZ	KDM6A
7.84538	0.239244	GY	KCNH8	NC_056080.1	44115001	44135001	3.18997	0.449241	HZ	KDM6A
21.9565	0.295788	GY	KCNH8	NC_056058.1	91380001	91400001	6.54476	0.211756	HZ	KIAA0825
20.6937	0.304204	GY	KCNH8	NC_056058.1	91385001	91405001	4.04805	0.21254	HZ	KIAA0825
12.4947	0.280404	GY	KCNH8	NC_056058.1	91390001	91410001	3.46051	0.208182	HZ	KIAA0825
11.5753	0.27402	GY	KCNH8	NC_056073.1	38865001	38885001	2.56035	0.282731	HZ	KIF13A
10.5021	0.271929	GY	KCNH8	NC_056073.1	38870001	38890001	2.57275	0.317936	HZ	KIF13A
13.2055	0.273968	GY	KCNH8	NC_056073.1	38880001	38900001	2.57074	0.348192	HZ	KIF13A
17.5392	0.257619	GY	KCNH8	NC_056073.1	38885001	38905001	6.45395	0.266394	HZ	KIF13A
13.9006	0.202994	GY	KCNH8	NC_056073.1	38890001	38910001	7.65886	0.264142	HZ	KIF13A
5.21832	0.225872	GY	KCNIP4	NC_056073.1	38895001	38915001	4.80322	0.250032	HZ	KIF13A
3.21331	0.2204	GY	KCNIP4	NC_056073.1	38900001	38920001	2.45739	0.239106	HZ	KIF13A
6.11402	0.198025	GY	KCNK10	NC_056073.1	38930001	38950001	2.50914	0.283321	HZ	KIF13A
3.19411	0.235506	GY	KCNN2	NC_056073.1	38935001	38955001	2.73066	0.267039	HZ	KIF13A
16.0625	0.217596	GY	KCNQ1	NC_056073.1	38940001	38960001	5.02906	0.294232	HZ	KIF13A
3.772	0.267893	GY	KDM6A	NC_056073.1	38945001	38965001	2.49828	0.207887	HZ	KIF13A
11.05	0.382825	GY	KDM6A	NC_056073.1	38965001	38985001	2.7567	0.28041	HZ	KIF13A
10.6282	0.36337	GY	KDM6A	NC_056073.1	38970001	38990001	3.13393	0.227485	HZ	KIF13A
9.38727	0.341485	GY	KDM6A	NC_056074.1	27385001	27405001	2.93584	0.208677	HZ	KIRREL3
5.86101	0.302804	GY	KDM6A	NC_056074.1	27390001	27410001	2.84738	0.222263	HZ	KIRREL3
5.93253	0.252499	GY	KDM6A	NC_056063.1	49250001	49270001	3.80296	0.240928	HZ	KLF12
3.88173	0.228144	GY	KDM6A	NC_056063.1	49255001	49275001	5.63426	0.302978	HZ	KLF12
3.69302	0.327908	GY	KHDRBS2	NC_056063.1	49260001	49280001	4.09422	0.259162	HZ	KLF12
3.59655	0.305036	GY	KHDRBS2	NC_056066.1	44180001	44200001	2.48955	0.214268	HZ	KLF6
4.58426	0.196921	GY	KIAA0319L	NC_056066.1	44185001	44205001	2.46467	0.231793	HZ	KLF6
6.08537	0.219968	GY	KIAA1549L	NC_056066.1	44190001	44210001	2.44676	0.21707	HZ	KLF6

4.82352	0.237184	GY	KIF13A	NC_056063.1	44610001	44630001	5.78178	0.242348	HZ	KLHL1
2.84669	0.204882	GY	KIF13A	NC_056063.1	44675001	44695001	3.40189	0.223988	HZ	KLHL1
2.88785	0.230231	GY	KIF13A	NC_056063.1	44680001	44700001	3.6996	0.25688	HZ	KLHL1
2.5942	0.235866	GY	KIF13A	NC_056063.1	44685001	44705001	3.09251	0.241581	HZ	KLHL1
2.66349	0.254013	GY	KIF13A	NC_056063.1	44690001	44710001	2.86808	0.221938	HZ	KLHL1
3.4442	0.192924	GY	KIF13A	NC_056063.1	44695001	44715001	3.37589	0.232581	HZ	KLHL1
2.71679	0.22017	GY	KIRREL3	NC_056063.1	44700001	44720001	6.13407	0.283775	HZ	KLHL1
2.44117	0.215893	GY	KLF12	NC_056063.1	44705001	44725001	11.2896	0.332919	HZ	KLHL1
3.50464	0.197992	GY	KLF12	NC_056063.1	44710001	44730001	14.6995	0.354664	HZ	KLHL1
4.29588	0.208262	GY	KLF12	NC_056063.1	44715001	44735001	10.8616	0.336278	HZ	KLHL1
4.55906	0.206228	GY	KLF12	NC_056063.1	44720001	44740001	6.72802	0.307643	HZ	KLHL1
3.96446	0.201683	GY	KLF12	NC_056063.1	44725001	44745001	3.22638	0.233662	HZ	KLHL1
2.40772	0.274425	GY	KLF12	NC_056063.1	44755001	44775001	3.42368	0.304566	HZ	KLHL1
9.13773	0.191791	GY	KLF5	NC_056063.1	44760001	44780001	6.09755	0.31376	HZ	KLHL1
7.09525	0.327596	GY	KLHL1	NC_056063.1	44765001	44785001	12.4255	0.303856	HZ	KLHL1
7.31092	0.336191	GY	KLHL1	NC_056063.1	44770001	44790001	15.7209	0.297101	HZ	KLHL1
7.36507	0.312012	GY	KLHL1	NC_056063.1	44775001	44795001	15.1905	0.254025	HZ	KLHL1
10.1146	0.337729	GY	KLHL1	NC_056063.1	44780001	44800001	14.8333	0.247588	HZ	KLHL1
5.81818	0.270104	GY	KLHL1	NC_056063.1	44785001	44805001	17.7815	0.269444	HZ	KLHL1
2.73302	0.224914	GY	KLHL1	NC_056063.1	44790001	44810001	17.4427	0.273849	HZ	KLHL1
3.12622	0.223065	GY	KLHL1	NC_056063.1	44795001	44815001	24.3303	0.350996	HZ	KLHL1
3.62785	0.223152	GY	KLHL1	NC_056063.1	44800001	44820001	20.9593	0.388173	HZ	KLHL1
3.96075	0.250454	GY	KLHL1	NC_056063.1	44805001	44825001	18.3225	0.444011	HZ	KLHL1
4.32613	0.262382	GY	KLHL1	NC_056063.1	44810001	44830001	23.5823	0.474703	HZ	KLHL1
3.72728	0.244466	GY	KLHL1	NC_056063.1	44815001	44835001	28.1432	0.48632	HZ	KLHL1
3.43329	0.224282	GY	KLHL1	NC_056063.1	44820001	44840001	40.578	0.49357	HZ	KLHL1
3.85961	0.23886	GY	KLHL1	NC_056063.1	44825001	44845001	47.1011	0.436167	HZ	KLHL1
6.54167	0.250708	GY	KLHL1	NC_056063.1	44830001	44850001	30.2315	0.395098	HZ	KLHL1
11.304	0.28533	GY	KLHL1	NC_056063.1	44835001	44855001	21.7775	0.303304	HZ	KLHL1
14.6256	0.285156	GY	KLHL1	NC_056063.1	44840001	44860001	23.6961	0.259666	HZ	KLHL1
10.955	0.27187	GY	KLHL1	NC_056063.1	44845001	44865001	21.6959	0.282638	HZ	KLHL1
6.8022	0.270691	GY	KLHL1	NC_056063.1	44850001	44870001	30.3943	0.303473	HZ	KLHL1
3.34506	0.250225	GY	KLHL1	NC_056063.1	44855001	44875001	32.7485	0.355876	HZ	KLHL1
2.44107	0.2618	GY	KLHL1	NC_056063.1	44860001	44880001	16.7312	0.385953	HZ	KLHL1
3.91843	0.241718	GY	KLHL1	NC_056063.1	44865001	44885001	18.3886	0.398001	HZ	KLHL1
6.56909	0.234158	GY	KLHL1	NC_056063.1	44870001	44890001	18.8036	0.42618	HZ	KLHL1
11.7943	0.250359	GY	KLHL1	NC_056063.1	44875001	44895001	16.1611	0.447196	HZ	KLHL1
13.6977	0.272508	GY	KLHL1	NC_056063.1	44880001	44900001	15.7468	0.402245	HZ	KLHL1
12.5	0.261588	GY	KLHL1	NC_056063.1	44885001	44905001	7.56011	0.251251	HZ	KLHL1
11.9524	0.274542	GY	KLHL1	NC_056063.1	45010001	45030001	2.39755	0.263703	HZ	KLHL1
15.0252	0.323392	GY	KLHL1	NC_056063.1	45045001	45065001	2.41622	0.291523	HZ	KLHL1
14.9771	0.347124	GY	KLHL1	NC_056063.1	45050001	45070001	3.51404	0.33822	HZ	KLHL1
22.9911	0.39705	GY	KLHL1	NC_056063.1	45055001	45075001	3.27581	0.324238	HZ	KLHL1
21.065	0.413071	GY	KLHL1	NC_056063.1	45060001	45080001	2.89599	0.315396	HZ	KLHL1
19.7578	0.410846	GY	KLHL1	NC_056063.1	45065001	45085001	2.4446	0.285303	HZ	KLHL1
26.0374	0.431734	GY	KLHL1	NC_056080.1	116860001	116880001	2.48606	0.303804	HZ	KLHL13
30.8173	0.449124	GY	KLHL1	NC_056068.1	28840001	28860001	2.61271	0.293338	HZ	KMT2A
45.4132	0.422418	GY	KLHL1	NC_056068.1	28845001	28865001	5.62499	0.428361	HZ	KMT2A
49.7	0.398899	GY	KLHL1	NC_056070.1	52260001	52280001	2.38982	0.23116	HZ	KMT5A
31.6635	0.354117	GY	KLHL1	NC_056070.1	52265001	52285001	3.07392	0.213264	HZ	KMT5A;SBNO1
21.0432	0.297636	GY	KLHL1	NC_056054.1	201265001	201285001	2.46884	0.219113	HZ	KNG1
25.695	0.284746	GY	KLHL1	NC_056076.1	40470001	40490001	2.75367	0.207285	HZ	LAMA1
27.7586	0.271842	GY	KLHL1	NC_056057.1	50640001	50660001	3.05796	0.361771	HZ	LAMB4
40.2921	0.269367	GY	KLHL1	NC_056057.1	50645001	50665001	4.69414	0.400728	HZ	LAMB4
45.6655	0.296762	GY	KLHL1	NC_056057.1	50650001	50670001	3.92669	0.394379	HZ	LAMB4
20.3658	0.310962	GY	KLHL1	NC_056057.1	50655001	50675001	2.75954	0.304788	HZ	LAMB4
19.8457	0.346283	GY	KLHL1	NC_056080.1	36905001	36925001	3.31659	0.293522	HZ	LANCL3
20.2262	0.382392	GY	KLHL1	NC_056056.1	27895001	27915001	3.45299	0.217489	HZ	LAPTM4A
17.3833	0.397868	GY	KLHL1	NC_056058.1	63715001	63735001	3.5566	0.206709	HZ	LARP1
15.0844	0.402787	GY	KLHL1	NC_056058.1	63725001	63745001	4.15316	0.239012	HZ	LARP1
5.98241	0.304837	GY	KLHL1	NC_056054.1	260200001	260220001	19.61	0.251721	HZ	LCA5L
3.81482	0.200931	GY	KLHL1	NC_056054.1	260205001	260225001	23.2372	0.254951	HZ	LCA5L
2.87373	0.234093	GY	KLHL25	NC_056054.1	260210001	260230001	23.6115	0.241705	HZ	LCA5L
3.11597	0.209336	GY	KLHL25	NC_056054.1	260215001	260235001	22.1095	0.232502	HZ	LCA5L
2.45395	0.308793	GY	KLHL5	NC_056054.1	260220001	260240001	22.2229	0.258416	HZ	LCA5L
2.57226	0.28247	GY	KLHL5	NC_056054.1	260225001	260245001	24.8231	0.330578	HZ	LCA5L
2.51155	0.244842	GY	KLHL5	NC_056054.1	260230001	260250001	23.1938	0.370774	HZ	LCA5L
2.67735	0.219255	GY	KRBA2;RNF222;RI	NC_056054.1	260235001	260255001	18.7178	0.361204	HZ	LCA5L
6.12396	0.199325	GY	KREMEN1	NC_056054.1	260240001	260260001	15.7021	0.357771	HZ	LCA5L
3.36589	0.249931	GY	KREMEN1	NC_056059.1	38065001	38085001	15.6625	0.638702	HZ	LCORL
2.81825	0.260798	GY	KREMEN1	NC_056059.1	112540001	112560001	3.28837	0.223454	HZ	LDB2
2.40262	0.198933	GY	KRT80	NC_056059.1	112545001	112565001	3.44022	0.287742	HZ	LDB2
18.875	0.280603	GY	LCORL	NC_056068.1	56940001	56960001	3.63385	0.440419	HZ	LGR4
2.70284	0.194217	GY	LDLRAD3	NC_056068.1	56945001	56965001	6.68302	0.504461	HZ	LGR4

8.47925	0.217737	GY	LGR4	NC_056059.1	61095001	61115001	4.83556	0.217766	HZ	LIMCH1
3.05667	0.216852	GY	LHFPL2	NC_056059.1	61100001	61120001	6.19999	0.244868	HZ	LIMCH1
4.26413	0.267755	GY	LHFPL2	NC_056059.1	61105001	61125001	3.43659	0.236577	HZ	LIMCH1
5.6337	0.318593	GY	LHFPL2	NC_056059.1	61110001	61130001	3.42568	0.216764	HZ	LIMCH1
5.49324	0.311951	GY	LHFPL2	NC_056068.1	56950001	56970001	10.8218	0.531102	HZ	LIN7C
9.04929	0.304585	GY	LHFPL2	NC_056068.1	56955001	56975001	6.32673	0.509926	HZ	LIN7C
15.7217	0.28041	GY	LHFPL2	NC_056068.1	56960001	56980001	2.9453	0.43337	HZ	LIN7C
8.68037	0.195024	GY	LHFPL2	NC_056065.1	28540001	28560001	2.51113	0.210935	HZ	LIN9
9.35417	0.267401	GY	LHFPL3	NC_056055.1	97295001	97315001	2.51698	0.314959	HZ	LINGO2
20.6489	0.300442	GY	LHFPL3	NC_056055.1	97300001	97320001	3.72964	0.35506	HZ	LINGO2
16.4715	0.295433	GY	LHFPL3	NC_056055.1	97305001	97325001	2.82622	0.327468	HZ	LINGO2
6.44953	0.249465	GY	LHFPL3	NC_056055.1	97685001	97705001	3.21657	0.339682	HZ	LINGO2
13.2012	0.233123	GY	LIN7C	NC_056055.1	97690001	97710001	3.01343	0.398931	HZ	LINGO2
7.45545	0.230261	GY	LIN7C	NC_056060.1	49850001	49870001	15.9111	0.22308	HZ	LIPC
8.28928	0.279742	GY	LINGO2	NC_056060.1	49860001	49880001	21.5071	0.239398	HZ	LIPC
6.20598	0.27029	GY	LINGO2	NC_056060.1	49865001	49885001	23.4844	0.236319	HZ	LIPC
4.05707	0.243997	GY	LINGO2	NC_056056.1	189900001	189920001	2.44559	0.314074	HZ	LMNTD1
2.88058	0.21172	GY	LINGO2	NC_056056.1	189920001	189940001	2.515	0.31866	HZ	LMNTD1
4.18626	0.245043	GY	LINGO2	NC_056056.1	189925001	189945001	2.44814	0.299794	HZ	LMNTD1
4.75371	0.204352	GY	LINGO2	NC_056064.1	41325001	41345001	2.61327	0.342075	HZ	LOC100526781
3.71593	0.200429	GY	LINGO2	NC_056064.1	41330001	41350001	2.41704	0.447181	HZ	LOC100526781
5.35884	0.221314	GY	LIPT1;MITD1	NC_056064.1	41335001	41355001	2.71858	0.43779	HZ	LOC100526781
2.50427	0.491993	GY	LOC100526781	NC_056064.1	41320001	41340001	3.31029	0.339951	HZ	LOC100526781;LOC
2.80343	0.429324	GY	LOC100526781	NC_056064.1	41340001	41360001	2.73611	0.333738	HZ	LOC100526781;V15
3.06908	0.444068	GY	LOC100526781;LO	NC_056064.1	41305001	41325001	3.88411	0.330879	HZ	LOC100526782
3.00298	0.324284	GY	LOC100526781;V1	NC_056064.1	41310001	41330001	3.94452	0.337341	HZ	LOC100526782
3.87692	0.328578	GY	LOC100526782	NC_056064.1	41315001	41335001	3.55251	0.300217	HZ	LOC100526782
3.93495	0.385858	GY	LOC100526782	NC_056057.1	89875001	89895001	3.35017	0.208816	HZ	LOC101101889
3.34633	0.405005	GY	LOC100526782	NC_056057.1	41685001	41705001	17.4246	0.208947	HZ	LOC101102230
2.50626	0.522089	GY	LOC101102366;ZS	NC_056057.1	41690001	41710001	15.2895	0.235949	HZ	LOC101102230
3.54746	0.585014	GY	LOC101102366;ZS	NC_056077.1	37230001	37250001	6.38631	0.257695	HZ	LOC101102366;ZSC
11.3459	0.583606	GY	LOC101102366;ZS	NC_056077.1	37235001	37255001	16.6415	0.289017	HZ	LOC101102366;ZSC
3.41366	0.274944	GY	LOC101102680;ME	NC_056074.1	8630001	8650001	4.29857	0.223586	HZ	LOC101102680;ME3
3.24972	0.287913	GY	LOC101102680;ME	NC_056074.1	8635001	8655001	3.23438	0.238542	HZ	LOC101102680;ME3
3.94668	0.272081	GY	LOC101102680;ME	NC_056074.1	8640001	8660001	3.9083	0.237933	HZ	LOC101102680;ME3
6.92306	0.241619	GY	LOC101104028	NW_024599828	1195001	1215001	2.35374	0.211071	HZ	LOC101103023;LOC
2.57827	0.250569	GY	LOC101104438	NW_024599828	1200001	1220001	2.42212	0.20705	HZ	LOC101103023;LOC
2.70206	0.248517	GY	LOC101104438	NW_024599828	1210001	1230001	2.46726	0.214602	HZ	LOC101103023;LOC
2.5055	0.242991	GY	LOC101104438	NW_024599828	1190001	1210001	2.57914	0.233286	HZ	LOC101103023;PAG
2.5657	0.2505	GY	LOC101104438	NW_024599828	1215001	1235001	2.53261	0.211627	HZ	LOC101103278;PAG
3.15936	0.259988	GY	LOC101104438	NW_024599828	1235001	1255001	4.20269	0.211475	HZ	LOC101103278;PAG
3.14634	0.287214	GY	LOC101104438	NW_024599828	1245001	1265001	5.98278	0.210254	HZ	LOC101103278;PAG
3.44287	0.406207	GY	LOC101104488	NC_056054.1	181375001	181395001	7.30769	0.367889	HZ	LOC101104028
4.33204	0.458072	GY	LOC101104488	NC_056054.1	181380001	181400001	3.43999	0.267324	HZ	LOC101104028
2.50289	0.265326	GY	LOC101104613	NC_056077.1	4705001	4725001	3.31014	0.229904	HZ	LOC101105047
2.70377	0.340824	GY	LOC101104613	NC_056077.1	4710001	4730001	3.29071	0.222367	HZ	LOC101105047
2.41031	0.398212	GY	LOC101104613	NC_056077.1	4715001	4735001	3.19069	0.256299	HZ	LOC101105047
2.66567	0.201486	GY	LOC101104974	NC_056076.1	37920001	37940001	10.7143	0.241769	HZ	LOC101105123
2.47644	0.215339	GY	LOC101104974	NC_056080.1	19350001	19370001	12.6063	0.240533	HZ	LOC101105138
2.41807	0.240128	GY	LOC101104974	NC_056074.1	35405001	35425001	15.2117	0.231664	HZ	LOC101105540
3.5264	0.206068	GY	LOC101105047	NC_056074.1	35425001	35445001	23.1765	0.261381	HZ	LOC101105540
3.53358	0.198513	GY	LOC101105047	NC_056074.1	35430001	35450001	26.2745	0.279573	HZ	LOC101105540
3.18206	0.231052	GY	LOC101105047	NC_056074.1	35495001	35515001	2.71663	0.204844	HZ	LOC101105788
2.66666	0.237731	GY	LOC101105135	NC_056074.1	35615001	35635001	6.38745	0.226586	HZ	LOC101106045
12.1302	0.230931	GY	LOC101105138	NC_056074.1	35620001	35640001	20.302	0.289547	HZ	LOC101106045
8.41986	0.206347	GY	LOC101105265	NC_056072.1	58985001	59005001	2.67253	0.215609	HZ	LOC101106550
2.44947	0.21942	GY	LOC101105495	NC_056072.1	58990001	59010001	4.29784	0.31	HZ	LOC101106550
2.9504	0.247706	GY	LOC101105495	NC_056072.1	58995001	59015001	4.12367	0.299542	HZ	LOC101106550
2.70691	0.230106	GY	LOC101105495	NC_056074.1	36425001	36445001	3.87856	0.275822	HZ	LOC101106637;TME
2.42397	0.214058	GY	LOC101105495	NC_056075.1	15885001	15905001	3.73319	0.21409	HZ	LOC101106641
5.44139	0.240009	GY	LOC101105523	NC_056080.1	64030001	64050001	7.84434	0.421755	HZ	LOC101106743
10.2864	0.307127	GY	LOC101105523	NC_056080.1	64035001	64055001	5.86727	0.37465	HZ	LOC101106743
14.6997	0.314994	GY	LOC101105523;PD	NC_056072.1	55745001	55765001	2.55571	0.40989	HZ	LOC101106751
14.6348	0.281843	GY	LOC101105523;PD	NC_056072.1	55750001	55770001	2.375	0.3955	HZ	LOC101106751
4.15416	0.21021	GY	LOC101105523;PD	NC_056072.1	55755001	55775001	2.47895	0.363794	HZ	LOC101106751
14.4941	0.268011	GY	LOC101105540	NC_056063.1	70640001	70660001	2.5866	0.236459	HZ	LOC101106781
20.9818	0.347316	GY	LOC101105540	NC_056063.1	70645001	70665001	4.96508	0.330291	HZ	LOC101106781
23.3447	0.357768	GY	LOC101105540	NC_056063.1	70650001	70670001	4.5649	0.320293	HZ	LOC101106781
2.54785	0.243104	GY	LOC101105788	NC_056063.1	70655001	70675001	3.46993	0.300487	HZ	LOC101106781
2.47656	0.233562	GY	LOC101105788	NC_056068.1	53620001	53640001	6.28178	0.211292	HZ	LOC101107135;LOC
2.53412	0.22159	GY	LOC101105788	NC_056055.1	52570001	52590001	7.55922	0.210971	HZ	LOC101108371;LOC
2.63564	0.197689	GY	LOC101105788;LO	NC_056055.1	52575001	52595001	4.02465	0.227247	HZ	LOC101108371;LOC
2.56709	0.238706	GY	LOC101105788;LO	NC_056068.1	48385001	48405001	2.76412	0.317421	HZ	LOC101108775
19.8437	0.198141	GY	LOC101106045	NC_056068.1	48390001	48410001	3.11592	0.329237	HZ	LOC101108775

3.73263	0.225427	GY	LOC101106637;TM	NC_056054.1	108005001	108025001	3.59101	0.297804	HZ	LOC101108789
9.29247	0.267136	GY	LOC101106743	NC_056068.1	48400001	48420001	12.5985	0.312655	HZ	LOC101109299
7.56197	0.253389	GY	LOC101106743	NC_056068.1	48405001	48425001	15.5192	0.313131	HZ	LOC101109299
6.15055	0.218213	GY	LOC101108113	NC_056068.1	48420001	48440001	6.47962	0.467793	HZ	LOC101109827;LOC
3.18454	0.344622	GY	LOC101108787	NC_056068.1	48425001	48445001	2.99138	0.39073	HZ	LOC101109827;LOC
18.411	0.238231	GY	LOC101109717	NC_056068.1	48430001	48450001	2.64436	0.374371	HZ	LOC101109827;LOC
9.91529	0.193757	GY	LOC101109717	NC_056054.1	199415001	199435001	2.45589	0.352043	HZ	LOC101110107
2.88319	0.229715	GY	LOC101110107	NC_056054.1	199420001	199440001	2.40898	0.333308	HZ	LOC101110107
11.7108	0.244232	GY	LOC101110189	NC_056054.1	199425001	199445001	2.49811	0.318547	HZ	LOC101110107
4.75748	0.193564	GY	LOC101110189;ND	NC_056058.1	39535001	39555001	2.47359	0.263105	HZ	LOC101110587
6.2381	0.260827	GY	LOC101110189;ND	NC_056059.1	72910001	72930001	4.56645	0.212997	HZ	LOC101110593
4.57154	0.270131	GY	LOC101110373	NC_056059.1	72915001	72935001	4.1741	0.225357	HZ	LOC101110593
3.62992	0.20338	GY	LOC101110373	NC_056059.1	72920001	72940001	2.75879	0.207927	HZ	LOC101110593
4.1509	0.255315	GY	LOC101110939	NC_056059.1	73035001	73055001	2.86098	0.216236	HZ	LOC101111099
2.58659	0.226753	GY	LOC101110939	NC_056059.1	73040001	73060001	2.9341	0.241925	HZ	LOC101111099
4.34102	0.26725	GY	LOC101110939	NC_056059.1	73045001	73065001	4.56831	0.237576	HZ	LOC101111099
4.10611	0.262605	GY	LOC101110939	NC_056059.1	73050001	73070001	4.87847	0.212704	HZ	LOC101111099
4.61732	0.299652	GY	LOC101110939	NC_056077.1	28910001	28930001	2.6919	0.244141	HZ	LOC101111335
3.68884	0.230916	GY	LOC101110939	NC_056077.1	28915001	28935001	3.57481	0.280801	HZ	LOC101111335
3.26591	0.198863	GY	LOC101111060	NC_056077.1	28920001	28940001	3.80026	0.321469	HZ	LOC101111335
2.8082	0.217467	GY	LOC101111300	NC_056077.1	28925001	28945001	2.72609	0.299161	HZ	LOC101111335
2.51358	0.278344	GY	LOC101111335	NC_056058.1	65970001	65990001	3.35218	0.242845	HZ	LOC101111631
2.50143	0.289239	GY	LOC101111486	NC_056059.1	73080001	73100001	5.5	0.21557	HZ	LOC101111633;LOC
2.72354	0.303933	GY	LOC101111631	NC_056059.1	73085001	73105001	6.49511	0.217954	HZ	LOC101111633;LOC
3.34384	0.290521	GY	LOC101111631	NC_056059.1	73090001	73110001	4.02547	0.219966	HZ	LOC101111633;LOC
5.86364	0.247538	GY	LOC101112284	NC_056059.1	73095001	73115001	3.14668	0.229999	HZ	LOC101111633;LOC
8.37816	0.257639	GY	LOC101112284	NC_056068.1	81345001	81365001	5.01613	0.449411	HZ	LOC101112419
5.88307	0.363755	GY	LOC101112419	NC_056068.1	81350001	81370001	8.63864	0.553498	HZ	LOC101112419
11.8739	0.331333	GY	LOC101112419	NC_056068.1	81360001	81380001	3.74228	0.435955	HZ	LOC101112419
4.18216	0.415062	GY	LOC101113004;LO	NC_056068.1	81365001	81385001	2.84622	0.434692	HZ	LOC101112419
2.68865	0.234334	GY	LOC101113495;NL	NC_056077.1	26525001	26545001	3.70271	0.316759	HZ	LOC101112694
2.40899	0.196861	GY	LOC101113495;NL	NC_056077.1	26530001	26550001	5.6	0.322708	HZ	LOC101112694
3.78096	0.307621	GY	LOC101113819	NC_056077.1	26535001	26555001	9.1739	0.352912	HZ	LOC101112694
2.68922	0.265135	GY	LOC101113819	NC_056077.1	33190001	33210001	2.64791	0.270234	HZ	LOC101112784
3.0647	0.31266	GY	LOC101113819	NC_056077.1	33195001	33215001	3.24637	0.25616	HZ	LOC101112784
2.66618	0.219163	GY	LOC101114082	NC_056077.1	33200001	33220001	3.40443	0.251898	HZ	LOC101112784
3.45068	0.251008	GY	LOC101114082	NC_056077.1	33205001	33225001	3.96167	0.207508	HZ	LOC101112784;NCF
4.1646	0.283784	GY	LOC101114082	NC_056056.1	80175001	80195001	3.51766	0.218188	HZ	LOC101112892
6.69109	0.311692	GY	LOC101114082	NC_056058.1	38450001	38470001	2.53753	0.245829	HZ	LOC101113495;NLR
9.86101	0.285231	GY	LOC101114082	NC_056077.1	19640001	19660001	3.34285	0.306622	HZ	LOC101113819
9.95539	0.281593	GY	LOC101114082;LO	NC_056077.1	19650001	19670001	2.70001	0.316231	HZ	LOC101113819
2.4232	0.197627	GY	LOC101114319;TT	NC_056078.1	40950001	40970001	4.39604	0.217571	HZ	LOC101114082
5.25263	0.206606	GY	LOC101114456	NC_056078.1	40955001	40975001	8.84407	0.271023	HZ	LOC101114082
2.44759	0.343815	GY	LOC101114528;ZN	NC_056078.1	40960001	40980001	8.8067	0.26845	HZ	LOC101114082;LOC
2.44536	0.257111	GY	LOC101115252;RH	NC_056061.1	50075001	50095001	2.84275	0.254034	HZ	LOC101114528;ZNF
20.6774	0.229651	GY	LOC101115276	NC_056068.1	24915001	24935001	2.62997	0.228534	HZ	LOC101115226
24.4559	0.21061	GY	LOC101115276	NC_056077.1	34570001	34590001	2.66191	0.206676	HZ	LOC101115252;RHB
2.85788	0.524613	GY	LOC101115398	NC_056058.1	9015001	9035001	26.4903	0.236763	HZ	LOC101115276
27.4066	0.213061	GY	LOC101115538	NC_056058.1	9020001	9040001	32.8236	0.252377	HZ	LOC101115276
26.0211	0.241394	GY	LOC101115538	NC_056058.1	9025001	9045001	38.4358	0.272312	HZ	LOC101115276;LOC
24.2581	0.264638	GY	LOC101115538	NC_056058.1	9030001	9050001	28.654	0.307722	HZ	LOC101115276;LOC
4.77038	0.235899	GY	LOC101115593;SM	NC_056067.1	62115001	62135001	3.08733	0.603165	HZ	LOC101115398
5.32087	0.241482	GY	LOC101115593;SM	NC_056058.1	9035001	9055001	33.113	0.329149	HZ	LOC101115538
4.95973	0.252819	GY	LOC101115593;SM	NC_056058.1	9040001	9060001	29.4159	0.337505	HZ	LOC101115538
15.9001	0.236923	GY	LOC101115943	NC_056058.1	9045001	9065001	24.8755	0.309904	HZ	LOC101115538
11.4597	0.21402	GY	LOC101115943	NC_056058.1	9070001	9090001	30.0655	0.257784	HZ	LOC101116053
10.9401	0.241854	GY	LOC101115943	NC_056058.1	9075001	9095001	30.0315	0.296964	HZ	LOC101116053
10.8215	0.222981	GY	LOC101115943	NC_056058.1	9080001	9100001	24.7791	0.297401	HZ	LOC101116053
28.1318	0.238517	GY	LOC101116053	NC_056071.1	24550001	24570001	11.7046	0.223409	HZ	LOC101116178
28.8811	0.267061	GY	LOC101116053	NC_056071.1	24565001	24585001	10.7588	0.258833	HZ	LOC101116178
24.4616	0.301039	GY	LOC101116053	NC_056065.1	55570001	55590001	2.70927	0.215311	HZ	LOC101116245;LOC
18.2787	0.193702	GY	LOC101116178	NC_056059.1	8845001	8865001	2.50326	0.271174	HZ	LOC101116481
20.5263	0.194188	GY	LOC101116178	NC_056059.1	8850001	8870001	3.74353	0.286975	HZ	LOC101116481
2.87754	0.273815	GY	LOC101116245	NC_056059.1	8855001	8875001	3.90957	0.265784	HZ	LOC101116481
4.05289	0.242976	GY	LOC101116245;RA	NC_056059.1	8860001	8880001	2.60413	0.212348	HZ	LOC101116481
3.42195	0.21195	GY	LOC101116245;RA	NC_056080.1	68920001	68940001	5.10569	0.26737	HZ	LOC101116886;MAG
3.17144	0.232901	GY	LOC101116245;RA	NC_056080.1	68925001	68945001	5.24463	0.230969	HZ	LOC101116886;MAG
2.6628	0.33258	GY	LOC101116481	NC_056073.1	20610001	20630001	5.6154	0.334172	HZ	LOC101117542
3.8099	0.343	GY	LOC101116481	NC_056074.1	37945001	37965001	3.38011	0.21717	HZ	LOC101118064
4.15072	0.273662	GY	LOC101116481	NC_056080.1	2750001	2770001	2.81688	0.273851	HZ	LOC101118336
2.62035	0.199803	GY	LOC101116574	NC_056080.1	2755001	2775001	8.65163	0.351753	HZ	LOC101118336
2.49485	0.198469	GY	LOC101116574	NC_056080.1	2760001	2780001	6.3297	0.311175	HZ	LOC101118336
2.58434	0.19791	GY	LOC101116574	NC_056080.1	2765001	2785001	3.38072	0.249006	HZ	LOC101118336
3.30198	0.192857	GY	LOC101117077;TR	NC_056080.1	2770001	2790001	2.7865	0.21139	HZ	LOC101118336

2.60496	0.237591	GY	LOC101117098;STI	NC_056080.1	2780001	2800001	3.96741	0.217388	HZ	LOC101118336
2.67308	0.243843	GY	LOC101117240	NC_056080.1	2785001	2805001	12.6651	0.271123	HZ	LOC101118336
3.2075	0.274248	GY	LOC101117240	NC_056080.1	2790001	2810001	9.74514	0.243218	HZ	LOC101118336
2.99615	0.273348	GY	LOC101117240	NC_056080.1	2795001	2815001	5.28393	0.230312	HZ	LOC101118336
3.02772	0.276285	GY	LOC101117547	NC_056080.1	2800001	2820001	6.61106	0.209387	HZ	LOC101118336
2.61588	0.257508	GY	LOC101117547	NC_056058.1	6715001	6735001	3.47276	0.214338	HZ	LOC101118444
4.55744	0.246951	GY	LOC101117749	NC_056055.1	230080001	230100001	8.13184	0.207492	HZ	LOC101118932
6.30123	0.208738	GY	LOC101117749	NC_056055.1	230085001	230105001	4.68101	0.211888	HZ	LOC101118932
4.58195	0.191728	GY	LOC101117749	NC_056074.1	38170001	38190001	2.97491	0.234723	HZ	LOC101119087
3.32378	0.283212	GY	LOC101117749;MT	NC_056056.1	44930001	44950001	4.53334	0.253095	HZ	LOC101119202
2.47855	0.303949	GY	LOC101117804	NC_056056.1	44935001	44955001	6.37454	0.259359	HZ	LOC101119202
2.97341	0.308684	GY	LOC101117804	NC_056056.1	44940001	44960001	5.28663	0.237367	HZ	LOC101119202
14.6539	0.283276	GY	LOC101117804	NC_056056.1	44945001	44965001	6.58875	0.240426	HZ	LOC101119202
16.9606	0.256718	GY	LOC101117804	NC_056056.1	44950001	44970001	5.99042	0.226382	HZ	LOC101119202
2.89419	0.201033	GY	LOC101117846	NC_056062.1	14585001	14605001	5.26764	0.470424	HZ	LOC101119226
2.77266	0.210797	GY	LOC101117846	NC_056062.1	14590001	14610001	9.84397	0.503192	HZ	LOC101119226
3.19194	0.223028	GY	LOC101117846	NC_056062.1	14580001	14600001	3.16839	0.399413	HZ	LOC101119226;LY6I
3.43363	0.2321	GY	LOC101117846	NC_056062.1	14595001	14615001	4.96583	0.422499	HZ	LOC101119226;LYPI
3.69925	0.23512	GY	LOC101117846	NC_056075.1	20675001	20695001	3.54595	0.278846	HZ	LOC101120033
4.28015	0.244425	GY	LOC101117846	NC_056075.1	20680001	20700001	11.5605	0.296537	HZ	LOC101120033
4.18571	0.234077	GY	LOC101117846	NC_056075.1	20685001	20705001	14.0567	0.301656	HZ	LOC101120033
3.29837	0.20817	GY	LOC101117846	NC_056075.1	20700001	20720001	7.90624	0.288095	HZ	LOC101120033
8.12942	0.341199	GY	LOC101118164	NC_056075.1	20705001	20725001	4.57323	0.263159	HZ	LOC101120033
11.7846	0.330709	GY	LOC101118164	NC_056055.1	224825001	224845001	2.53958	0.20581	HZ	LOC101120221;MOG
3.38773	0.241306	GY	LOC101118164	NC_056071.1	19420001	19440001	3.97611	0.32557	HZ	LOC101120360
15.9773	0.300194	GY	LOC101118248	NC_056071.1	19425001	19445001	6.65341	0.398216	HZ	LOC101120360;LOC
10.3205	0.275317	GY	LOC101118248	NC_056071.1	19430001	19450001	12.1554	0.43666	HZ	LOC101120360;LOC
5.14034	0.210028	GY	LOC101118248	NC_056071.1	19435001	19455001	3.5396	0.337774	HZ	LOC101120360;LOC
2.706	0.342222	GY	LOC101118336	NC_056071.1	19440001	19460001	2.4663	0.272652	HZ	LOC101120610
7.98711	0.453044	GY	LOC101118336	NC_056056.1	194505001	194525001	2.43628	0.273418	HZ	LOC101120653
6.08447	0.387671	GY	LOC101118336	NC_056056.1	194510001	194530001	2.56071	0.29376	HZ	LOC101120653
3.43883	0.301819	GY	LOC101118336	NC_056056.1	194515001	194535001	3.72821	0.268003	HZ	LOC101120653
3.02066	0.22395	GY	LOC101118336	NC_056066.1	36550001	36570001	5.0589	0.207843	HZ	LOC101120769
10.6875	0.231536	GY	LOC101118336	NC_056068.1	53695001	53715001	11.5382	0.275425	HZ	LOC101120816
7.48086	0.20851	GY	LOC101118336	NC_056068.1	53700001	53720001	9.16667	0.243701	HZ	LOC101120816
8.1219	0.242695	GY	LOC101118932	NC_056068.1	53710001	53730001	6.53571	0.229104	HZ	LOC101120816
5.01612	0.201894	GY	LOC101118932	NC_056068.1	53715001	53735001	8.18946	0.233052	HZ	LOC101120816
3.49546	0.204982	GY	LOC101118932	NC_056068.1	53720001	53740001	8.03879	0.258905	HZ	LOC101120816
2.72544	0.200443	GY	LOC101118932	NC_056068.1	53725001	53745001	7.86921	0.256818	HZ	LOC101120816;LOC
6.496	0.216093	GY	LOC101119226	NC_056068.1	53730001	53750001	8.89674	0.218754	HZ	LOC101120816;LOC
10.6598	0.257679	GY	LOC101119226	NC_056080.1	133425001	133445001	2.71597	0.235448	HZ	LOC101120992
5.34892	0.217209	GY	LOC101119226;LY	NC_056080.1	133430001	133450001	2.68622	0.2299	HZ	LOC101120992
3.52597	0.347874	GY	LOC101119283	NC_056054.1	239620001	239640001	3.33138	0.236931	HZ	LOC101121993
14.2435	0.325239	GY	LOC101119283	NC_056054.1	239625001	239645001	2.66483	0.247357	HZ	LOC101121993
2.63296	0.213805	GY	LOC101119538	NC_056064.1	6100001	6120001	2.4044	0.3616	HZ	LOC101122120
2.46954	0.222719	GY	LOC101119538;LO	NC_056064.1	6105001	6125001	2.39787	0.28201	HZ	LOC101122120
2.69062	0.358065	GY	LOC101120221;MO	NC_056064.1	6110001	6130001	3.48206	0.216116	HZ	LOC101122120
3.1773	0.261585	GY	LOC101120329;SE	NC_056058.1	49630001	49650001	2.9963	0.242665	HZ	LOC101122274;LOC
3.977	0.203709	GY	LOC101120769	NC_056072.1	52230001	52250001	2.40666	0.210209	HZ	LOC101122312
4.21239	0.192748	GY	LOC101120769	NC_056059.1	25635001	25655001	12.0033	0.238619	HZ	LOC101122865
7.94828	0.212813	GY	LOC101120816	NC_056059.1	25640001	25660001	12.0189	0.308143	HZ	LOC101122865
7.79747	0.210026	GY	LOC101120816;LO	NC_056059.1	25645001	25665001	9.53848	0.299083	HZ	LOC101122865
8.64133	0.192286	GY	LOC101120816;LO	NC_056059.1	25650001	25670001	7.67723	0.273277	HZ	LOC101122865
3.7299	0.230709	GY	LOC101121198	NC_056072.1	43495001	43515001	3.11243	0.233711	HZ	LOC101123588
2.60772	0.208948	GY	LOC101121198	NC_056072.1	43500001	43520001	4.58823	0.281778	HZ	LOC101123588
3.20253	0.209822	GY	LOC101121198;LO	NC_056072.1	43505001	43525001	4.32746	0.294745	HZ	LOC101123588
6.73771	0.20091	GY	LOC101121557;ND	NC_056072.1	43510001	43530001	5.44132	0.321889	HZ	LOC101123588
3.87676	0.19502	GY	LOC101121557;ND	NC_056072.1	43515001	43535001	4.96808	0.329964	HZ	LOC101123588
5.13904	0.293544	GY	LOC101121837	NC_056072.1	43520001	43540001	4.4738	0.319863	HZ	LOC101123588
3	0.231062	GY	LOC101121837	NC_056072.1	43525001	43545001	6.44	0.337037	HZ	LOC101123588
3.65829	0.25154	GY	LOC101121837	NC_056072.1	43530001	43550001	7.63218	0.336885	HZ	LOC101123588
3.21922	0.242543	GY	LOC101121837;LO	NC_056072.1	43535001	43555001	6.82547	0.329882	HZ	LOC101123588
3.30367	0.253644	GY	LOC101121837;LO	NC_056072.1	43540001	43560001	7.15172	0.308426	HZ	LOC101123588
3.10322	0.290214	GY	LOC101121837;LO	NC_056072.1	43545001	43565001	9.29085	0.255134	HZ	LOC101123588
4.04762	0.216821	GY	LOC101121892	NC_056072.1	43550001	43570001	6.25	0.214477	HZ	LOC101123588
2.43347	0.362715	GY	LOC101121938	NC_056055.1	46855001	46875001	2.71052	0.25169	HZ	LOC101123612
2.58866	0.209006	GY	LOC101121986	NC_056055.1	46860001	46880001	3	0.25787	HZ	LOC101123612
3.37351	0.287392	GY	LOC101121986	NC_056055.1	46865001	46885001	3.20859	0.242852	HZ	LOC101123612
2.9593	0.407763	GY	LOC101121993	NC_056054.1	247050001	247070001	5.43694	0.254318	HZ	LOC105601893;PLS1
2.51099	0.358545	GY	LOC101121993	NC_056067.1	57615001	57635001	3.3064	0.204784	HZ	LOC105601981;ZNF1
2.62014	0.408506	GY	LOC101122120	NC_056078.1	40965001	40985001	6.76224	0.249937	HZ	LOC105605095
3.00527	0.323305	GY	LOC101122120	NC_056073.1	25960001	25980001	5.99085	0.293397	HZ	LOC105605990
2.63839	0.234431	GY	LOC101122120	NC_056073.1	25965001	25985001	6.67845	0.28447	HZ	LOC105605990
2.48645	0.299767	GY	LOC101123029	NC_056073.1	25970001	25990001	11.3051	0.373498	HZ	LOC105605990

14.1024	0.202435	GY	LOC101123341	NC_056073.1	25975001	25995001	11.7532	0.384924	HZ	LOC105605990
5.61712	0.241898	GY	LOC105601893;PL	NC_056073.1	25980001	26000001	12.2863	0.388629	HZ	LOC105605990
7.52797	0.226572	GY	LOC105605095	NC_056073.1	25985001	26005001	12.9229	0.431719	HZ	LOC105605990
8.50556	0.240044	GY	LOC105605780	NC_056058.1	13685001	13705001	2.58742	0.362536	HZ	LOC105609437
9.07778	0.232837	GY	LOC105605780	NC_056080.1	86120001	86140001	3.24406	0.212469	HZ	LOC105614134
7.65847	0.195736	GY	LOC105605780	NC_056056.1	176775001	176795001	2.51429	0.291968	HZ	LOC105614892;SYN
10.3346	0.1932	GY	LOC105605990	NC_056056.1	176780001	176800001	5.7684	0.377487	HZ	LOC105614892;SYN
11.1486	0.215826	GY	LOC105605990	NC_056056.1	176785001	176805001	6.37132	0.354023	HZ	LOC105614892;SYN
12.311	0.227657	GY	LOC105605990	NC_056057.1	6975001	6995001	20.5074	0.292159	HZ	LOC105614936
3.23365	0.313691	GY	LOC105608906	NC_056077.1	14170001	14190001	2.81887	0.235701	HZ	LOC114110664
2.88145	0.317271	GY	LOC105608906	NC_056077.1	14175001	14195001	6.26345	0.280222	HZ	LOC114110664;RRN
2.45885	0.219495	GY	LOC105612510;MC	NC_056080.1	79045001	79065001	3.52246	0.361565	HZ	LOC114111496
20.0735	0.302381	GY	LOC105614936	NC_056080.1	79050001	79070001	2.66721	0.317769	HZ	LOC114111496
3.46407	0.430281	GY	LOC105616630	NC_056080.1	79055001	79075001	2.47073	0.274372	HZ	LOC114111496
2.49401	0.343908	GY	LOC106991972	NC_056055.1	111030001	111050001	6.73486	0.28447	HZ	LOC114113035
2.90264	0.215721	GY	LOC114110090	NC_056055.1	111035001	111055001	3.81406	0.262038	HZ	LOC114113035
3.50864	0.264046	GY	LOC114110167	NC_056055.1	111040001	111060001	3.19598	0.211007	HZ	LOC114113035
2.94133	0.245355	GY	LOC114110664	NC_056055.1	111045001	111065001	2.69853	0.211875	HZ	LOC114113035
6.36021	0.290831	GY	LOC114110664;RR	NC_056056.1	92190001	92210001	2.66771	0.280315	HZ	LOC114113944
4.38878	0.415848	GY	LOC114111496	NC_056058.1	9010001	9030001	33.6201	0.218372	HZ	LOC114114788
2.99143	0.408725	GY	LOC114111496	NC_056058.1	42035001	42055001	2.69016	0.220099	HZ	LOC114115009
2.51507	0.392943	GY	LOC114111496	NC_056058.1	42040001	42060001	3.05454	0.246855	HZ	LOC114115009
2.46753	0.193924	GY	LOC114111618	NC_056059.1	73100001	73120001	2.56204	0.229278	HZ	LOC114115373;POLI
7.14121	0.279952	GY	LOC114113035	NC_056060.1	64670001	64690001	2.49274	0.207482	HZ	LOC114115867
3.97069	0.27762	GY	LOC114113035	NC_056060.1	84305001	84325001	3.00545	0.213345	HZ	LOC114115884
3.17438	0.271073	GY	LOC114113035	NC_056064.1	23965001	23985001	3.09867	0.209725	HZ	LOC114116984
2.52151	0.302739	GY	LOC114113035	NC_056065.1	42625001	42645001	4.44405	0.252567	HZ	LOC114117226;UBE
7.18869	0.300992	GY	LOC114114788	NC_056065.1	43970001	43990001	2.72618	0.223536	HZ	LOC114117228;RERI
25.75	0.252657	GY	LOC114114788	NC_056065.1	55575001	55595001	3.21053	0.241474	HZ	LOC114117245;MRP
2.79522	0.217311	GY	LOC114115302;LO	NC_056067.1	62480001	62500001	5.51265	0.348877	HZ	LOC114117836
2.80427	0.244217	GY	LOC114115304;PD	NC_056068.1	82115001	82135001	2.80291	0.271222	HZ	LOC114118341
3.32082	0.265403	GY	LOC114115304;PD	NC_056068.1	82120001	82140001	4.07517	0.369635	HZ	LOC114118341
3.36432	0.260565	GY	LOC114115304;PD	NC_056068.1	82125001	82145001	4.54268	0.391192	HZ	LOC114118341
21.0902	0.226176	GY	LOC114117249	NC_056068.1	82130001	82150001	4.12427	0.35164	HZ	LOC114118341
23.528	0.23795	GY	LOC114117249	NC_056068.1	82135001	82155001	3.67065	0.271673	HZ	LOC114118341
16.7281	0.197605	GY	LOC114117249	NW_02459982	1415001	1435001	15.6032	0.206579	HZ	LOC121816021
3.65092	0.266099	GY	LOC114118001	NW_02459982	1440001	1460001	10.5471	0.217771	HZ	LOC121816021
2.6527	0.27524	GY	LOC114118245	NC_056074.1	35115001	35135001	3.33251	0.221101	HZ	LOC121816022
3.85948	0.313815	GY	LOC114118341	NC_056074.1	35150001	35170001	2.47578	0.271044	HZ	LOC121816022
4.4878	0.378849	GY	LOC114118341	NC_056065.1	78425001	78445001	8.67521	0.302621	HZ	LOC121816068
4.54913	0.406048	GY	LOC114118341	NC_056065.1	78430001	78450001	5.63876	0.236831	HZ	LOC121816068
4.78311	0.384982	GY	LOC114118341	NC_056054.1	730001	750001	2.47641	0.213689	HZ	LOC121817700;SNEI
3.70759	0.337054	GY	LOC114118341	NC_056080.1	131460001	131480001	5.88279	0.395317	HZ	LOC121818354
3.11312	0.272102	GY	LOC114118341	NC_056080.1	131465001	131485001	2.49053	0.381637	HZ	LOC121818354
3.36887	0.271887	GY	LOC114118341	NC_056054.1	116410001	116430001	7.06609	0.262004	HZ	LOC121818668
3.32885	0.282639	GY	LOC114118341	NC_056054.1	116415001	116435001	10.3811	0.306778	HZ	LOC121818668
3.62641	0.300901	GY	LOC114118341	NC_056054.1	116420001	116440001	8.12417	0.267549	HZ	LOC121818668
14.5159	0.206968	GY	LOC121816021	NC_056054.1	116425001	116445001	3.13377	0.219663	HZ	LOC121818668
10.3518	0.220993	GY	LOC121816021	NC_056061.1	14945001	14965001	2.69713	0.252359	HZ	LOC121820153
2.43727	0.199169	GY	LOC121816022	NC_056061.1	14965001	14985001	7.5351	0.228552	HZ	LOC121820153
4.36595	0.203881	GY	LOC121816038	NC_056061.1	14970001	14990001	7.03244	0.330097	HZ	LOC121820153
4.61074	0.22863	GY	LOC121816038	NC_056061.1	14975001	14995001	4.31751	0.351182	HZ	LOC121820153
6.31584	0.507456	GY	LOC121816525	NC_056061.1	14980001	15000001	3.26985	0.356378	HZ	LOC121820153
8.54871	0.485554	GY	LOC121816525	NC_056065.1	12530001	12550001	2.96846	0.209917	HZ	LOC121820762
18.8789	0.476475	GY	LOC121816525	NC_056065.1	12535001	12555001	4.48449	0.277312	HZ	LOC121820762
20.9169	0.418763	GY	LOC121816525	NC_056070.1	7355001	7375001	2.51415	0.2113	HZ	LRBA
6.89449	0.329919	GY	LOC121816525	NC_056063.1	16870001	16890001	2.61799	0.358063	HZ	LRCH1
3.33962	0.205147	GY	LOC121816765;PLI	NC_056063.1	16875001	16895001	2.64982	0.348809	HZ	LRCH1
4.31022	0.209645	GY	LOC121816765;PLI	NC_056063.1	16880001	16900001	2.74776	0.339153	HZ	LRCH1
2.82711	0.220397	GY	LOC121818145	NC_056063.1	16885001	16905001	3.2069	0.328174	HZ	LRCH1
3.1118	0.207825	GY	LOC121818145	NC_056063.1	16890001	16910001	2.68606	0.273469	HZ	LRCH1
6.39194	0.253243	GY	LOC121818354	NC_056063.1	16895001	16915001	2.65827	0.260151	HZ	LRCH1
2.97895	0.247849	GY	LOC121818354	NC_056063.1	16900001	16920001	2.58255	0.239658	HZ	LRCH1
4.05699	0.274104	GY	LOC121818515	NC_056056.1	162410001	162430001	6.24223	0.335119	HZ	LRP1
2.70434	0.192526	GY	LOC121818515	NC_056056.1	162395001	162415001	2.83327	0.299605	HZ	LRP1;NXPH4
3.44073	0.43001	GY	LOC121818668	NC_056056.1	162400001	162420001	4.18656	0.403372	HZ	LRP1;NXPH4
4.29752	0.565956	GY	LOC121818668	NC_056055.1	168320001	168340001	3.3159	0.254491	HZ	LRP1B
6.0106	0.545043	GY	LOC121818668	NC_056055.1	168325001	168345001	3.56304	0.226265	HZ	LRP1B
5.53819	0.435341	GY	LOC121818668	NC_056055.1	168375001	168395001	2.42857	0.302458	HZ	LRP1B
2.4469	0.357085	GY	LOC121818668	NC_056079.1	38700001	38720001	2.93313	0.209778	HZ	LRRC3B
2.6325	0.361907	GY	LOC121819170	NC_056062.1	86200001	86220001	3.40161	0.260061	HZ	LRRC69
2.81586	0.33175	GY	LOC121819170	NC_056062.1	86205001	86225001	5.78304	0.326542	HZ	LRRC69
2.87065	0.3173	GY	LOC121819170	NC_056062.1	86210001	86230001	8.53049	0.366554	HZ	LRRC69
2.92032	0.271396	GY	LOC121819170	NC_056062.1	86215001	86235001	10.6462	0.369661	HZ	LRRC69

2.64776	0.218026	GY	LOC121819170	NC_056062.1	86220001	86240001	6.49231	0.305998	HZ	LRRC69;OTUD6B
3.9213	0.202071	GY	LOC121819791	NC_056062.1	86225001	86245001	5.36499	0.226043	HZ	LRRC69;OTUD6B
3.21032	0.283392	GY	LOC121819792	NC_056056.1	90675001	90695001	3.65027	0.22606	HZ	LTBP1
2.50697	0.210493	GY	LOC121820153	NC_056056.1	90680001	90700001	4.22775	0.284034	HZ	LTBP1
2.4344	0.264959	GY	LOC121820153	NC_056056.1	90685001	90705001	3.25126	0.272415	HZ	LTBP1
2.93943	0.27572	GY	LOC121820153	NC_056062.1	14575001	14595001	2.43772	0.371743	HZ	LY6D
3.52018	0.249405	GY	LOC121820153	NC_056062.1	14600001	14620001	3.08953	0.36193	HZ	LYPD2;SLURP1
3.87062	0.224645	GY	LOC121820153	NC_056066.1	8350001	8370001	2.66667	0.217719	HZ	MACROD2
7.43864	0.193608	GY	LOC121820153	NC_056066.1	8355001	8375001	2.57347	0.25588	HZ	MACROD2
9.86955	0.194882	GY	LOC121820153	NC_056066.1	8360001	8380001	2.64579	0.257315	HZ	MACROD2
8.55966	0.207824	GY	LOC121820153	NC_056066.1	8365001	8385001	3.0101	0.316105	HZ	MACROD2
4.97281	0.238582	GY	LOC121820153	NC_056066.1	8370001	8390001	2.73736	0.296498	HZ	MACROD2
3.50578	0.27277	GY	LOC121820153	NC_056066.1	8375001	8395001	2.87824	0.267355	HZ	MACROD2
2.4344	0.198783	GY	LOC443456	NC_056066.1	8825001	8845001	3.62026	0.213939	HZ	MACROD2
2.53425	0.207687	GY	LOC443456	NC_056066.1	9650001	9670001	3.40733	0.286972	HZ	MACROD2
2.41114	0.200582	GY	LOC443456	NC_056066.1	9655001	9675001	6.00402	0.355729	HZ	MACROD2
2.45377	0.193946	GY	LRBA	NC_056066.1	9660001	9680001	4.22521	0.424758	HZ	MACROD2
2.48765	0.230785	GY	LRCH1	NC_056077.1	41575001	41595001	4.25514	0.206462	HZ	MAD1L1
2.81059	0.261438	GY	LRCH1	NC_056077.1	41580001	41600001	4.6016	0.233045	HZ	MAD1L1
3.7888	0.245507	GY	LRCH1	NC_056077.1	41785001	41805001	6.69444	0.366949	HZ	MAFK
3.93206	0.204349	GY	LRCH1	NC_056077.1	41765001	41785001	11.5937	0.357512	HZ	MAFK;PSMG3;TMEI
4.00446	0.198059	GY	LRCH1	NC_056057.1	44610001	44630001	2.39038	0.278441	HZ	MAGI2
4.45074	0.256312	GY	LRCH1	NC_056080.1	68895001	68915001	2.49686	0.253452	HZ	MAGT1
3.194	0.340651	GY	LRCH1	NC_056080.1	68900001	68920001	5.29861	0.310949	HZ	MAGT1
3.14331	0.343539	GY	LRCH1	NC_056080.1	68905001	68925001	5.26495	0.321646	HZ	MAGT1
2.86292	0.329034	GY	LRCH1	NC_056080.1	68915001	68935001	5.21052	0.278861	HZ	MAGT1
2.49674	0.224046	GY	LRCH1	NC_056055.1	241180001	241200001	3.26478	0.470687	HZ	MAN1C1
2.90126	0.195291	GY	LRIT3;RRH	NC_056055.1	118105001	118125001	6.69872	0.278857	HZ	MAP3K2
6.08073	0.354658	GY	LRP1	NC_056055.1	118110001	118130001	3.5498	0.240452	HZ	MAP3K2
2.71338	0.310679	GY	LRP1;NXPH4	NC_056061.1	84445001	84465001	3.18007	0.237847	HZ	MAP3K4
4.38657	0.383494	GY	LRP1;NXPH4	NC_056061.1	84450001	84470001	6.49138	0.225872	HZ	MAP3K4
2.64751	0.226376	GY	LRPPRC	NC_056056.1	99960001	99980001	3.8539	0.233559	HZ	MAP4K4
2.92147	0.206272	GY	LRPPRC	NC_056056.1	99965001	99985001	5.09842	0.256553	HZ	MAP4K4
3.49422	0.288826	GY	LRRC49	NC_056056.1	99970001	99990001	5.33969	0.257346	HZ	MAP4K4
3.34354	0.295442	GY	LRRC49	NC_056056.1	99975001	99995001	4.61886	0.237294	HZ	MAP4K4
3.48081	0.253152	GY	LRRFIP1	NC_056056.1	225555001	225575001	2.71604	0.294815	HZ	MAPK11;PLXNB2
3.63508	0.194366	GY	LRRFIP1;RBM44	NC_056076.1	50585001	50605001	7.88552	0.242192	HZ	MAPK4
2.71726	0.259245	GY	LUZP2	NC_056076.1	50590001	50610001	29.8592	0.240833	HZ	MAPK4
2.43095	0.235935	GY	LUZP2	NC_056078.1	34680001	34700001	24.6055	0.275889	HZ	MBLA;SFTPA1
2.49861	0.258385	GY	LVRN	NC_056078.1	34685001	34705001	22.1959	0.268065	HZ	MBLA;SFTPA1
2.71316	0.198966	GY	LYN	NC_056078.1	34690001	34710001	27.1199	0.234362	HZ	MBLA;SFTPA1
2.74526	0.275181	GY	LYPLA2;PITHD1	NC_056078.1	34670001	34690001	9.01026	0.224046	HZ	MBLA;SFTPD
2.39989	0.349928	GY	MAD1L1	NC_056078.1	34675001	34695001	22.9167	0.270333	HZ	MBLA;SFTPD
2.44533	0.2279	GY	MAD1L1	NC_056080.1	102470001	102490001	3.36001	0.230779	HZ	MBNL3
3.49054	0.284428	GY	MAD1L1	NC_056080.1	102490001	102510001	4.95213	0.218234	HZ	MBNL3
24.791	0.397919	GY	MAD1L1	NC_056073.1	36970001	36990001	6.59401	0.222158	HZ	MBOAT1
22.5204	0.395526	GY	MAD1L1	NC_056073.1	36975001	36995001	3.33806	0.23349	HZ	MBOAT1
19.6398	0.391335	GY	MAD1L1	NC_056059.1	37505001	37525001	20.1333	0.223377	HZ	MEPE
15.0502	0.378815	GY	MAD1L1	NC_056059.1	37510001	37530001	22.4898	0.22841	HZ	MEPE
6.85448	0.353387	GY	MAD1L1	NC_056057.1	53120001	53140001	3.62444	0.212239	HZ	MET
6.23572	0.351564	GY	MAD1L1	NC_056057.1	53125001	53145001	5.08064	0.208241	HZ	MET
4.80377	0.340958	GY	MAD1L1	NC_056076.1	37320001	37340001	11.7421	0.224839	HZ	METTL4
5.82382	0.358514	GY	MAD1L1	NC_056076.1	37325001	37345001	12.8447	0.250745	HZ	METTL4
8.03268	0.342577	GY	MAD1L1	NC_056076.1	37330001	37350001	9.52852	0.2765	HZ	METTL4
4.82716	0.292262	GY	MAD1L1	NC_056076.1	37335001	37355001	9.86604	0.279252	HZ	METTL4
5.59324	0.310768	GY	MAD1L1	NC_056076.1	37340001	37360001	5.80081	0.21582	HZ	METTL4;NDC80
4.96295	0.318657	GY	MAD1L1	NC_056076.1	37345001	37365001	3.68921	0.211194	HZ	METTL4;NDC80
4.91633	0.307285	GY	MAD1L1	NC_056076.1	37350001	37370001	4.69342	0.229701	HZ	METTL4;NDC80
3.25318	0.202213	GY	MAGED1	NC_056076.1	37355001	37375001	4.36119	0.253132	HZ	METTL4;NDC80
3.0612	0.203488	GY	MAGI1	NC_056056.1	122355001	122375001	2.6404	0.214154	HZ	MGAT4C
3.74433	0.246604	GY	MAGI1	NC_056056.1	122360001	122380001	2.41512	0.257448	HZ	MGAT4C
4.0735	0.252713	GY	MAGI1	NC_056056.1	123010001	123030001	5.02703	0.211947	HZ	MGAT4C
5.80642	0.354819	GY	MAOA	NC_056070.1	18095001	18115001	2.74543	0.547268	HZ	MGST2
2.63518	0.257278	GY	MAOA	NC_056076.1	34915001	34935001	2.97143	0.217036	HZ	MIB1
2.87306	0.312099	GY	MAP1LC3B	NC_056076.1	34920001	34940001	2.55701	0.280663	HZ	MIB1
3.4367	0.198152	GY	MAPK8	NC_056076.1	34925001	34945001	3.70748	0.289551	HZ	MIB1
2.56292	0.200789	GY	MAPT	NC_056076.1	34930001	34950001	4.03714	0.297777	HZ	MIB1
3.40336	0.249884	GY	MAPT	NC_056076.1	34935001	34955001	4.29114	0.276729	HZ	MIB1
5.02571	0.30255	GY	MAPT	NC_056076.1	34940001	34960001	4.33607	0.243981	HZ	MIB1
4.33423	0.364772	GY	MAPT	NC_056068.1	40160001	40180001	2.94679	0.207108	HZ	MICAL2
3.25503	0.270588	GY	MARK2	NC_056068.1	40165001	40185001	7.46683	0.241637	HZ	MICAL2
4.93751	0.299023	GY	MARK2	NC_056056.1	7400001	7420001	4.25518	0.363623	HZ	MIGA2;SH3GLB2
8.90833	0.302501	GY	MARK2	NC_056056.1	7405001	7425001	4.1087	0.281706	HZ	MIGA2;SH3GLB2
7.67161	0.267616	GY	MARK2;RCOR2	NC_056057.1	67025001	67045001	2.50301	0.305708	HZ	MINDY4

2.68339	0.319948	GY	MC1R;TCF25	NC_056057.1	67030001	67050001	2.52295	0.321496	HZ	MINDY4
2.85253	0.350583	GY	MC1R;TCF25	NC_056071.1	45260001	45280001	6.61267	0.225042	HZ	MIPOL1
3.05271	0.29131	GY	MED12L;P2RY13	NC_056071.1	45265001	45285001	6.25428	0.212567	HZ	MIPOL1
2.62186	0.222234	GY	MED4	NC_056071.1	45270001	45290001	4.60067	0.225662	HZ	MIPOL1
3.63024	0.24064	GY	MED4	NC_056056.1	225375001	225395001	8.66754	0.49727	HZ	MLC1
5.04347	0.265734	GY	MED7	NC_056056.1	225380001	225400001	8.91473	0.408957	HZ	MLC1;MOV10L1
4.82373	0.256381	GY	MED7	NC_056056.1	225385001	225405001	10.0121	0.259805	HZ	MLC1;MOV10L1
4.22073	0.241716	GY	MED7	NC_056056.1	225360001	225380001	26.4445	0.540417	HZ	MLC1;TTLL8
18.0683	0.202857	GY	MEPE	NC_056056.1	225365001	225385001	15.7431	0.594877	HZ	MLC1;TTLL8
2.50743	0.333488	GY	MET	NC_056056.1	225370001	225390001	9.5071	0.560145	HZ	MLC1;TTLL8
2.55492	0.334101	GY	MET	NC_056074.1	46400001	46420001	2.53594	0.221736	HZ	MOB2
2.5084	0.329481	GY	MET	NC_056074.1	46405001	46425001	3.99077	0.248008	HZ	MOB2
2.49469	0.360964	GY	MET	NC_056074.1	46410001	46430001	3.57031	0.232545	HZ	MOB2
3.22706	0.402515	GY	MET	NC_056074.1	46415001	46435001	3.45	0.250919	HZ	MOB2
5.11288	0.369223	GY	MET	NC_056074.1	46420001	46440001	3.05796	0.232135	HZ	MOB2
3.86265	0.232315	GY	MET	NC_056074.1	46425001	46445001	4.27704	0.26587	HZ	MOB2
3.84255	0.248843	GY	MET	NC_056077.1	690001	710001	6.18062	0.224528	HZ	MPG;NPRL3
3.77661	0.255355	GY	MET	NC_056077.1	695001	715001	11.1212	0.271314	HZ	MPG;NPRL3
2.47742	0.245272	GY	MET	NC_056056.1	62710001	62730001	2.49572	0.246759	HZ	MRPL19
2.68703	0.235549	GY	METTL15	NC_056063.1	22115001	22135001	13.1443	0.205024	HZ	MRPS31
2.90476	0.2205	GY	METTL15	NC_056063.1	22120001	22140001	12.4592	0.216016	HZ	MRPS31
3.06908	0.270785	GY	METTL25	NC_056063.1	22125001	22145001	12.0058	0.228803	HZ	MRPS31
4.71654	0.280874	GY	METTL25	NC_056056.1	217615001	217635001	3.24337	0.21746	HZ	MRTFA
4.31431	0.299799	GY	METTL25	NC_056056.1	217620001	217640001	8.78419	0.322705	HZ	MRTFA
2.96788	0.20256	GY	METTL25	NC_056056.1	217685001	217705001	5.57555	0.290317	HZ	MRTFA
4.65441	0.213736	GY	METTL25	NC_056074.1	34465001	34485001	3.01587	0.278467	HZ	MS4A15;MS4A18
4.39776	0.230602	GY	METTL25	NC_056074.1	34470001	34490001	3.95677	0.253557	HZ	MS4A15;MS4A18
4.03217	0.25747	GY	METTL25	NC_056074.1	34475001	34495001	4.16829	0.23379	HZ	MS4A15;MS4A18
2.58839	0.22427	GY	METTL25	NC_056074.1	34460001	34480001	2.37984	0.238473	HZ	MS4A18
4.28169	0.239683	GY	METTL4	NC_056070.1	62815001	62835001	3.29535	0.221825	HZ	MSI1
7.12791	0.277625	GY	METTL4	NC_056076.1	41740001	41760001	2.39736	0.266705	HZ	MTCL1
10.671	0.287618	GY	METTL4	NC_056060.1	74610001	74630001	2.86586	0.349693	HZ	MTHFD1
12.7243	0.337947	GY	METTL4	NC_056060.1	74615001	74635001	4.51013	0.425948	HZ	MTHFD1
13.645	0.366724	GY	METTL4	NC_056060.1	74620001	74640001	2.72563	0.367001	HZ	MTHFD1
9.43998	0.392677	GY	METTL4	NC_056065.1	41650001	41670001	3.19791	0.29301	HZ	MTOR
8.44419	0.358438	GY	METTL4	NC_056065.1	41745001	41765001	2.41616	0.275325	HZ	MTOR
4.96615	0.253001	GY	METTL4;NDC80	NC_056065.1	41750001	41770001	2.66449	0.277562	HZ	MTOR
3.21727	0.233388	GY	METTL4;NDC80	NC_056065.1	41755001	41775001	3.41463	0.283201	HZ	MTOR
4.26392	0.244875	GY	METTL4;NDC80	NC_056065.1	41760001	41780001	3.60974	0.289557	HZ	MTOR
4.14548	0.283543	GY	METTL4;NDC80	NC_056058.1	11340001	11360001	11.793	0.23785	HZ	MUC16
6.74049	0.197493	GY	MFSD6	NC_056058.1	11345001	11365001	28.2272	0.228199	HZ	MUC16
2.87363	0.454771	GY	MGST2	NC_056067.1	56305001	56325001	3.03201	0.258356	HZ	MYBPC2;POLD1;SPI
2.64554	0.394051	GY	MGST2	NC_056063.1	83290001	83310001	2.88075	0.220249	HZ	MYO16
2.50307	0.314236	GY	MICAL2	NC_056076.1	37735001	37755001	3.04625	0.262014	HZ	MYOM1
12.3416	0.27538	GY	MITD1	NC_056076.1	37740001	37760001	5.21931	0.280914	HZ	MYOM1
4.07982	0.233354	GY	MITD1;MRPL30	NC_056076.1	37745001	37765001	6.99797	0.291344	HZ	MYOM1
6.81311	0.258328	GY	MITD1;MRPL30	NC_056076.1	37750001	37770001	10.1626	0.261319	HZ	MYOM1
12.4026	0.191982	GY	MKNK2	NC_056059.1	6485001	6505001	2.42781	0.253068	HZ	MYOZ2
11.0794	0.203552	GY	MKNK2	NC_056059.1	6490001	6510001	2.39129	0.264427	HZ	MYOZ2
7.19966	0.546615	GY	MLC1	NC_056059.1	6500001	6520001	2.43313	0.247991	HZ	MYOZ2
8.3411	0.466698	GY	MLC1;MOV10L1	NC_056056.1	112085001	112105001	2.40719	0.272678	HZ	NAP1L1
11.8606	0.332465	GY	MLC1;MOV10L1	NC_056056.1	112090001	112110001	3.11963	0.287979	HZ	NAP1L1
21.0894	0.6287	GY	MLC1;TTLL8	NC_056056.1	112095001	112115001	6.4551	0.278087	HZ	NAP1L1
10.7958	0.703693	GY	MLC1;TTLL8	NC_056056.1	112100001	112120001	9.07538	0.314728	HZ	NAP1L1
6.79411	0.662409	GY	MLC1;TTLL8	NC_056056.1	112105001	112125001	13.6202	0.425879	HZ	NAP1L1
3.59531	0.222179	GY	MMADHC	NC_056056.1	112110001	112130001	13.0359	0.477133	HZ	NAP1L1
5.04303	0.254326	GY	MMADHC	NC_056056.1	112115001	112135001	12.6905	0.498209	HZ	NAP1L1
13.5	0.291152	GY	MMADHC	NC_056074.1	15105001	15125001	2.44259	0.312716	HZ	NARS2
6.99204	0.263062	GY	MMADHC	NC_056074.1	15110001	15130001	3.47706	0.360394	HZ	NARS2
4.43125	0.228891	GY	MMADHC	NC_056074.1	15115001	15135001	3.90544	0.374856	HZ	NARS2
2.43505	0.207265	GY	MMADHC	NC_056074.1	15120001	15140001	4.98744	0.391617	HZ	NARS2
21.1953	0.202264	GY	MOV10L1	NC_056074.1	15125001	15145001	5.70172	0.420258	HZ	NARS2
16.9614	0.197709	GY	MOV10L1	NC_056074.1	15130001	15150001	4.47733	0.387401	HZ	NARS2
16.099	0.195929	GY	MOV10L1	NC_056074.1	15135001	15155001	3.55285	0.321078	HZ	NARS2
12.4091	0.214877	GY	MPG;NPRL3	NC_056068.1	23220001	23240001	3.83769	0.209714	HZ	NCAM1
2.75718	0.206242	GY	MPP2;PPY;PYY	NC_056068.1	23390001	23410001	2.5114	0.272216	HZ	NCAM1
2.5113	0.21722	GY	MRPL19	NC_056068.1	23395001	23415001	2.74632	0.312705	HZ	NCAM1
2.45514	0.232272	GY	MRPS14	NC_056068.1	23400001	23420001	2.93392	0.315843	HZ	NCAM1
3.19413	0.193959	GY	MRPS14	NC_056068.1	82050001	82070001	3.23767	0.412637	HZ	NCAPD3
4.64754	0.214718	GY	MRPS14	NC_056068.1	82070001	82090001	11.3725	0.446074	HZ	NCAPD3;THYN1;VP
5.5	0.218431	GY	MSH4;RABGGTB	NC_056068.1	82055001	82075001	3.33499	0.4157	HZ	NCAPD3;VPS26B
4.2108	0.219333	GY	MSH4;RABGGTB	NC_056068.1	82060001	82080001	3.57906	0.380033	HZ	NCAPD3;VPS26B
3.60541	0.193887	GY	MSH4;RABGGTB	NC_056068.1	82065001	82085001	8.19231	0.429027	HZ	NCAPD3;VPS26B
4.02532	0.192694	GY	MSI1	NC_056059.1	38035001	38055001	7.91092	0.412574	HZ	NCAPG

3.11578	0.220111	GY	MSI1;PLA2G1B	NC_056070.1	51240001	51260001	2.78572	0.219087	HZ	NCOR2
3.3666	0.197756	GY	MTA3	NC_056070.1	51245001	51265001	2.54658	0.304681	HZ	NCOR2
4.30096	0.224312	GY	MTA3	NC_056070.1	51250001	51270001	2.68302	0.317125	HZ	NCOR2
3.59656	0.192273	GY	MTA3	NC_056070.1	51255001	51275001	2.4322	0.340766	HZ	NCOR2
2.81987	0.197851	GY	MTA3	NC_056076.1	37360001	37380001	4.09984	0.275483	HZ	NDC80
2.69539	0.258016	GY	MTFR1	NC_056076.1	37365001	37385001	3.4026	0.270424	HZ	NDC80
3.38855	0.292631	GY	MTFR1	NC_056056.1	86815001	86835001	2.99636	0.217813	HZ	NDUFAF7;PRKD3
3.88764	0.309209	GY	MTFR1	NC_056056.1	86820001	86840001	3.28314	0.229797	HZ	NDUFAF7;PRKD3
3.4813	0.307128	GY	MTFR1	NC_056056.1	86825001	86845001	4.21236	0.273848	HZ	NDUFAF7;PRKD3
2.54505	0.268003	GY	MTFR1	NC_056056.1	86830001	86850001	5.2094	0.313462	HZ	NDUFAF7;PRKD3
3.0991	0.243832	GY	MTHFD1	NC_056054.1	47500001	47520001	2.71886	0.208697	HZ	NEGR1
4.88008	0.299831	GY	MTHFD1	NC_056055.1	111530001	111550001	3.28177	0.297299	HZ	NEK1
3.07025	0.251153	GY	MTHFD1	NC_056055.1	111535001	111555001	3.55624	0.305249	HZ	NEK1
3.79533	0.203215	GY	MTMR3	NC_056055.1	111540001	111560001	3.27343	0.294279	HZ	NEK1
3.61734	0.212656	GY	MTMR3	NC_056055.1	111545001	111565001	3.0049	0.256459	HZ	NEK1
3.2257	0.231721	GY	MTOR	NC_056055.1	111575001	111595001	2.54745	0.285397	HZ	NEK1
2.50621	0.271429	GY	MTOR	NC_056055.1	111580001	111600001	3.69048	0.318577	HZ	NEK1
2.78176	0.262058	GY	MTOR	NC_056055.1	111585001	111605001	5.56452	0.346274	HZ	NEK1
3.60162	0.260343	GY	MTOR	NC_056055.1	111590001	111610001	5.05238	0.326141	HZ	NEK1
3.85366	0.25956	GY	MTOR	NC_056055.1	111595001	111615001	2.74468	0.244873	HZ	NEK1
2.76249	0.191742	GY	MTX3	NC_056079.1	39395001	39415001	2.52034	0.224941	HZ	NGLY1
2.43719	0.257118	GY	MYO16	NC_056080.1	15615001	15635001	2.54913	0.269436	HZ	NHS
2.5813	0.274481	GY	MYO16	NC_056080.1	15620001	15640001	3.05	0.270327	HZ	NHS
2.58223	0.19834	GY	MYO16	NC_056080.1	15625001	15645001	3.32962	0.250964	HZ	NHS
2.579	0.22325	GY	MYO16	NC_056080.1	15630001	15650001	3.5921	0.279981	HZ	NHS
2.7553	0.221007	GY	MYO16	NC_056080.1	15735001	15755001	6.84904	0.240758	HZ	NHS
7.64287	0.262509	GY	MYO16	NC_056080.1	15740001	15760001	10.7819	0.232478	HZ	NHS
2.55914	0.22289	GY	MYO16	NC_056080.1	15785001	15805001	4.75386	0.214092	HZ	NHS
2.46613	0.229709	GY	MYO1A;NEMP1	NC_056080.1	15910001	15930001	13.5768	0.245467	HZ	NHS
3.04074	0.258883	GY	MYO3A	NC_056080.1	15915001	15935001	10.3791	0.256978	HZ	NHS
2.4897	0.228181	GY	MYO3A	NC_056069.1	31600001	31620001	2.47113	0.20502	HZ	NIM1K
2.67992	0.208327	GY	MYO5C	NC_056055.1	236130001	236150001	3.83233	0.23866	HZ	NKAIN1
2.67896	0.206445	GY	MYO5C	NC_056055.1	236135001	236155001	2.55183	0.230527	HZ	NKAIN1
5.08585	0.215827	GY	MYOM1	NC_056062.1	40805001	40825001	3.18824	0.229924	HZ	NKAIN3
5.85715	0.266865	GY	MYOM1	NC_056062.1	40810001	40830001	4.65766	0.263077	HZ	NKAIN3
2.47271	0.232983	GY	MYOM1	NC_056062.1	40815001	40835001	5.37838	0.289076	HZ	NKAIN3
7.58306	0.209066	GY	NAALADL1;SAC3I	NC_056062.1	40820001	40840001	6.30649	0.292608	HZ	NKAIN3
5.72	0.197057	GY	NAALADL1;SAC3I	NC_056062.1	40825001	40845001	6.50358	0.253821	HZ	NKAIN3
2.89883	0.20505	GY	NAALADL2	NC_056062.1	40830001	40850001	4.45292	0.219487	HZ	NKAIN3
2.6856	0.222565	GY	NAP1L1	NC_056066.1	40245001	40265001	4.30189	0.339491	HZ	NKX2-4
3.27197	0.210383	GY	NBEA	NC_056066.1	40235001	40255001	2.37931	0.266409	HZ	NKX2-4;XRN2
3.64436	0.218905	GY	NBEA	NC_056066.1	40240001	40260001	3.21311	0.313411	HZ	NKX2-4;XRN2
3.4058	0.248538	GY	NBEA	NC_056064.1	19670001	19690001	17.0927	0.342555	HZ	NLK
2.4685	0.21958	GY	NBEA	NC_056064.1	19675001	19695001	9.06756	0.309239	HZ	NLK
2.96411	0.237769	GY	NCAM1	NC_056064.1	19680001	19700001	5.16121	0.233669	HZ	NLK
4.06228	0.259193	GY	NCAM1	NC_056058.1	38445001	38465001	2.4915	0.243907	HZ	NLRP3
3.72819	0.239056	GY	NCAM1	NC_056057.1	67365001	67385001	2.51497	0.209994	HZ	NOD1
2.63889	0.264916	GY	NCAM1	NC_056057.1	67370001	67390001	2.35494	0.291608	HZ	NOD1
2.76305	0.298884	GY	NCAM1	NC_056057.1	67380001	67400001	2.41176	0.336092	HZ	NOD1
2.9584	0.315746	GY	NCAM1	NC_056057.1	67385001	67405001	2.49601	0.33243	HZ	NOD1
3.49157	0.374016	GY	NCAPD3	NC_056057.1	67390001	67410001	2.56232	0.303917	HZ	NOD1
3.54337	0.387321	GY	NCAPD3;VPS26B	NC_056057.1	67395001	67415001	2.5315	0.257192	HZ	NOD1
3.25547	0.281204	GY	NCAPD3;VPS26B	NC_056057.1	67400001	67420001	2.40476	0.259604	HZ	NOD1
6.76923	0.204234	GY	NCAPD3;VPS26B	NC_056057.1	67405001	67425001	2.38433	0.261236	HZ	NOD1
2.69281	0.19453	GY	NCKAP1	NC_056057.1	67415001	67435001	2.56981	0.308302	HZ	NOD1
2.59744	0.205049	GY	NCL	NC_056057.1	67420001	67440001	2.91821	0.245978	HZ	NOD1
2.80182	0.22024	GY	NCL	NC_056057.1	67425001	67445001	2.76265	0.245083	HZ	NOD1
3.21409	0.213001	GY	NCL	NC_056057.1	67430001	67450001	3.06529	0.222541	HZ	NOD1
2.67857	0.255298	GY	NCOR2	NC_056057.1	67435001	67455001	2.6863	0.209661	HZ	NOD1
2.58035	0.321382	GY	NCOR2	NC_056057.1	67440001	67460001	2.42471	0.230357	HZ	NOD1
2.44492	0.318698	GY	NCOR2	NC_056076.1	23180001	23200001	20.736	0.262935	HZ	NOL4
3.0997	0.211049	GY	NCOR2	NC_056076.1	23185001	23205001	16.5532	0.27613	HZ	NOL4
4.90552	0.1934	GY	NCOR2	NC_056076.1	23490001	23510001	3.13368	0.220686	HZ	NOL4
5.28571	0.192758	GY	NCOR2	NC_056076.1	23495001	23515001	3.27028	0.229347	HZ	NOL4
4.03329	0.306524	GY	NDC80	NC_056076.1	23505001	23525001	2.54097	0.215657	HZ	NOL4
3.52134	0.266372	GY	NDC80	NC_056070.1	57900001	57920001	3.64124	0.377837	HZ	NOS1
2.78704	0.202739	GY	NDEL1;RNF222;RI	NC_056056.1	100650001	100670001	3.3541	0.22957	HZ	NPAS2
2.84402	0.26686	GY	NDUFAF7;PRKD3	NC_056056.1	100655001	100675001	3.16194	0.211577	HZ	NPAS2
2.53248	0.251603	GY	NEGR1	NC_056057.1	78530001	78550001	2.55237	0.213795	HZ	NPC1L1
4.45181	0.311198	GY	NEGR1	NC_056077.1	700001	720001	32.3473	0.262444	HZ	NPRL3
7.20003	0.29962	GY	NEGR1	NC_056057.1	73200001	73220001	2.39613	0.222273	HZ	NPY
6.56675	0.257896	GY	NEGR1	NC_056057.1	73205001	73225001	4.20504	0.292521	HZ	NPY
2.96867	0.255441	GY	NELL1	NC_056057.1	73210001	73230001	3.95019	0.3034	HZ	NPY
2.47413	0.220765	GY	NELL1	NC_056070.1	10065001	10085001	2.65889	0.28912	HZ	NR3C2

3.00917	0.272932	GY	NEO1	NC_056070.1	10070001	10090001	2.9909	0.290927	HZ	NR3C2
2.44134	0.212731	GY	NHS	NC_056060.1	99925001	99945001	2.40678	0.217616	HZ	NRDE2
3.01973	0.219303	GY	NHS	NC_056060.1	99905001	99925001	5.48107	0.223246	HZ	NRDE2;PSMC1
5.03774	0.258849	GY	NHS	NC_056060.1	99920001	99940001	4.83552	0.259563	HZ	NRDE2;PSMC1
4.75941	0.263909	GY	NHS	NC_056078.1	36145001	36165001	2.64399	0.338806	HZ	NRG3
3.6538	0.202419	GY	NHS	NC_056080.1	131600001	131620001	2.9838	0.396915	HZ	NRK
6.62432	0.222731	GY	NHS	NC_056080.1	131605001	131625001	2.38878	0.376229	HZ	NRK
4.28205	0.284979	GY	NHS	NC_056056.1	73290001	73310001	5.00944	0.387995	HZ	NRXN1
2.53811	0.264594	GY	NIM1K	NC_056056.1	73820001	73840001	2.40139	0.432567	HZ	NRXN1
2.69041	0.228249	GY	NIM1K	NC_056056.1	73825001	73845001	2.77778	0.361035	HZ	NRXN1
2.79011	0.214777	GY	NIM1K	NC_056056.1	73830001	73850001	2.74418	0.340107	HZ	NRXN1
2.54359	0.225926	GY	NIM1K	NC_056056.1	74060001	74080001	3.63041	0.25982	HZ	NRXN1
3.34687	0.211459	GY	NKAIN2	NC_056056.1	74065001	74085001	4.02991	0.265547	HZ	NRXN1
2.95701	0.207158	GY	NKAIN2	NC_056056.1	74070001	74090001	5.51044	0.29857	HZ	NRXN1
3.319	0.236666	GY	NKAIN3	NC_056056.1	74075001	74095001	5.7373	0.304881	HZ	NRXN1
5.5637	0.236549	GY	NKAIN3	NC_056056.1	74080001	74100001	5.90158	0.257546	HZ	NRXN1
4.6258	0.248213	GY	NKAIN3	NC_056056.1	74085001	74105001	4.91411	0.262457	HZ	NRXN1
3.43561	0.242132	GY	NKAIN3	NC_056056.1	74090001	74110001	2.77352	0.226627	HZ	NRXN1
2.57923	0.246526	GY	NKAIN3	NC_056056.1	500001	520001	4.18098	0.352356	HZ	NSMF;PNPLA7
2.76157	0.224272	GY	NKAIN3	NC_056056.1	505001	525001	4.21221	0.369211	HZ	NSMF;PNPLA7
4.39938	0.301207	GY	NKAIN3	NC_056056.1	510001	530001	4.3844	0.41124	HZ	NSMF;PNPLA7
8.82417	0.199962	GY	NLGN4X	NC_056056.1	515001	535001	2.92347	0.397973	HZ	NSMF;PNPLA7
36.3302	0.242424	GY	NLGN4X	NC_056075.1	23480001	23500001	2.38532	0.306479	HZ	NT5C2
19.2402	0.225052	GY	NLGN4X	NC_056075.1	23485001	23505001	5.31876	0.357307	HZ	NT5C2
2.45842	0.214164	GY	NLK	NC_056075.1	23490001	23510001	14.5229	0.37537	HZ	NT5C2
2.42481	0.241319	GY	NLK	NC_056075.1	23495001	23515001	6.67034	0.332317	HZ	NT5C2
10.0615	0.291461	GY	NLK	NC_056057.1	65330001	65350001	3.15945	0.328429	HZ	NT5C3A
7.50258	0.282835	GY	NLK	NC_056057.1	65335001	65355001	3.17832	0.351711	HZ	NT5C3A
5	0.2387	GY	NLK	NC_056057.1	65340001	65360001	2.4473	0.321956	HZ	NT5C3A
2.60372	0.195216	GY	NLRP3	NC_056073.1	39055001	39075001	2.78889	0.207649	HZ	NUP153
2.80067	0.206532	GY	NLRP3	NC_056073.1	39060001	39080001	2.72276	0.250977	HZ	NUP153
22.2574	0.221015	GY	NOB1;NQO1	NC_056060.1	55770001	55790001	3.06643	0.399323	HZ	ONECUT1
19.3583	0.266445	GY	NOL4	NC_056076.1	32775001	32795001	4.30108	0.255605	HZ	OSBPL1A
15.2815	0.283878	GY	NOL4	NC_056076.1	32780001	32800001	4.42727	0.226289	HZ	OSBPL1A
4.06072	0.208315	GY	NOL4	NC_056076.1	32785001	32805001	6.42212	0.224368	HZ	OSBPL1A
39.3579	0.238047	GY	NPRL3	NC_056076.1	32790001	32810001	8.38931	0.219619	HZ	OSBPL1A
4.1873	0.317555	GY	NPY	NC_056076.1	32795001	32815001	8.00247	0.221035	HZ	OSBPL1A
3.99403	0.317187	GY	NPY	NC_056076.1	32800001	32820001	5.90679	0.254981	HZ	OSBPL1A
2.54905	0.223724	GY	NRG3	NC_056076.1	32805001	32825001	4.15185	0.285705	HZ	OSBPL1A
2.85896	0.253214	GY	NRG3	NC_056076.1	32810001	32830001	3.16893	0.301682	HZ	OSBPL1A
2.54806	0.205511	GY	NRG3	NC_056076.1	32815001	32835001	3.26066	0.303949	HZ	OSBPL1A
2.9078	0.247297	GY	NRG3	NC_056076.1	32820001	32840001	3.61025	0.277083	HZ	OSBPL1A
6.26416	0.274548	GY	NRXN1	NC_056076.1	32825001	32845001	2.59953	0.209173	HZ	OSBPL1A
2.46617	0.211618	GY	NRXN1	NC_056055.1	131945001	131965001	4.44142	0.382934	HZ	OSBPL6
2.52631	0.225608	GY	NRXN1	NC_056055.1	131950001	131970001	4.18827	0.361595	HZ	OSBPL6
2.56431	0.260757	GY	NRXN1	NC_056071.1	67420001	67440001	4.01395	0.575665	HZ	PACS2;TEX22
3.51772	0.301488	GY	NRXN1	NC_056055.1	248915001	248935001	2.80988	0.233136	HZ	PADI4;PADI6
2.65053	0.415996	GY	NRXN1	NC_056064.1	23445001	23465001	2.41553	0.222063	HZ	PAFAH1B1
2.91769	0.382119	GY	NRXN1	NW_024599828	1010001	1030001	2.95916	0.218388	HZ	PAG3
2.87907	0.356184	GY	NRXN1	NW_024599828	1015001	1035001	3.67752	0.227904	HZ	PAG3
2.65883	0.218564	GY	NRXN1	NW_024599828	1175001	1195001	3.51534	0.210903	HZ	PAG3
3.45441	0.328309	GY	NRXN1	NW_024599828	1180001	1200001	3.69345	0.257156	HZ	PAG3
3.57268	0.333543	GY	NRXN1	NW_024599828	1185001	1205001	2.65929	0.240827	HZ	PAG3
4.3484	0.377107	GY	NRXN1	NC_056060.1	16220001	16240001	4.91095	0.214327	HZ	PAQR5
4.1763	0.403714	GY	NRXN1	NC_056056.1	115665001	115685001	2.39949	0.250384	HZ	PAWR
4.1249	0.376369	GY	NRXN1	NC_056056.1	115670001	115690001	2.78309	0.35675	HZ	PAWR
3.74642	0.377131	GY	NRXN1	NC_056056.1	115675001	115695001	2.85461	0.373486	HZ	PAWR
2.551	0.311762	GY	NRXN1	NC_056056.1	115680001	115700001	2.40035	0.333962	HZ	PAWR
2.42401	0.286499	GY	NRXN1	NC_056056.1	115685001	115705001	2.73956	0.265896	HZ	PAWR
3.36177	0.332862	GY	NRXN3	NC_056058.1	43990001	44010001	2.84502	0.232303	HZ	PCBD2
2.7072	0.339979	GY	NRXN3	NC_056058.1	43995001	44015001	8.98969	0.273208	HZ	PCBD2
3.76622	0.235693	GY	NSD3;PLPP5	NC_056058.1	44000001	44020001	14.8067	0.295668	HZ	PCBD2
4.3942	0.283243	GY	NSMCE3	NC_056058.1	44005001	44025001	8.86635	0.293108	HZ	PCBD2
4.07458	0.273893	GY	NSMCE3	NC_056058.1	44010001	44030001	3.91111	0.295512	HZ	PCBD2
2.60954	0.250898	GY	NT5C2	NC_056075.1	4560001	4580001	2.92001	0.245734	HZ	PCDH15
5.60668	0.325672	GY	NT5C2	NC_056075.1	4565001	4585001	2.91346	0.277892	HZ	PCDH15
14.4706	0.37856	GY	NT5C2	NC_056054.1	246810001	246830001	2.82228	0.217623	HZ	PCOLCE2
6.57143	0.34759	GY	NT5C2	NC_056058.1	14215001	14235001	7.73785	0.278811	HZ	PCP2;STXBP2
4.10424	0.204177	GY	NTM	NC_056068.1	27610001	27630001	3.06857	0.231876	HZ	PCSK7;SIDT2;TAGL
19.0917	0.2783	GY	NTM	NC_056068.1	27615001	27635001	3.50215	0.269541	HZ	PCSK7;SIDT2;TAGL
26.4111	0.221509	GY	NTM	NC_056055.1	73665001	73685001	2.76084	0.336007	HZ	PDCD1LG2
2.733	0.296245	GY	NTM	NC_056055.1	73670001	73690001	2.53943	0.322259	HZ	PDCD1LG2
2.60646	0.229213	GY	NTM	NC_056056.1	100870001	100890001	2.54659	0.228311	HZ	PDCL3
3.55283	0.206411	GY	NTMT2	NC_056056.1	100875001	100895001	2.50045	0.2425	HZ	PDCL3

3.91058	0.203706	GY	NTMT2	NC_056055.1	127420001	127440001	2.43069	0.241209	HZ	PDE1A
4.32543	0.207529	GY	NTMT2	NC_056055.1	127425001	127445001	2.43548	0.237206	HZ	PDE1A
3.83871	0.214503	GY	NTMT2	NC_056058.1	58995001	59015001	3.38841	0.215882	HZ	PDE6A;SLC26A2
4.47644	0.216572	GY	OBI1;POU4F1	NC_056058.1	59000001	59020001	3.36378	0.210332	HZ	PDE6A;SLC26A2
3.99532	0.210408	GY	OBI1;POU4F1	NC_056058.1	59005001	59025001	2.60827	0.212458	HZ	PDE6A;SLC26A2
12.2081	0.231211	GY	OGDH	NC_056058.1	59020001	59040001	2.62514	0.216675	HZ	PDE6A;SLC26A2
5.00407	0.250198	GY	OGDH	NC_056058.1	59030001	59050001	2.44864	0.2142	HZ	PDE6A;SLC26A2
3.26074	0.256152	GY	OGDH	NC_056060.1	8525001	8545001	2.49494	0.273843	HZ	PDE8B
2.63359	0.240302	GY	OGDH;TMED4	NC_056060.1	8535001	8555001	3.33806	0.226172	HZ	PDE8B
4.32325	0.283938	GY	OPCML	NC_056080.1	22730001	22750001	9.54288	0.237435	HZ	PDK3
4.85331	0.290565	GY	OPCML	NC_056080.1	22735001	22755001	3.33035	0.211576	HZ	PDK3
4.31345	0.280483	GY	OPCML	NC_056080.1	22800001	22820001	6.218	0.275405	HZ	PDK3
6.27573	0.20854	GY	OSBPL1A	NC_056080.1	22805001	22825001	4.7986	0.230372	HZ	PDK3
4.21481	0.235486	GY	OSBPL1A	NC_056060.1	66790001	66810001	5.78446	0.223606	HZ	PELI2
3.10912	0.257613	GY	OSBPL1A	NC_056080.1	20415001	20435001	3.49231	0.216962	HZ	PHEX
3.16126	0.253793	GY	OSBPL1A	NC_056080.1	20420001	20440001	4.80818	0.264094	HZ	PHEX
3.40646	0.213174	GY	OSBPL1A	NC_056080.1	20425001	20445001	3.85396	0.233237	HZ	PHEX
3.75544	0.24607	GY	OTOGL	NC_056080.1	20435001	20455001	2.6498	0.243256	HZ	PHEX
2.72525	0.235635	GY	OTOGL	NC_056080.1	20440001	20460001	2.80255	0.220876	HZ	PHEX
2.75458	0.318882	GY	OTOGL	NC_056080.1	20445001	20465001	2.76031	0.24443	HZ	PHEX
3.36445	0.266613	GY	OTOGL	NC_056080.1	20450001	20470001	2.59776	0.260465	HZ	PHEX
3.05452	0.338612	GY	OTOGL	NC_056080.1	20455001	20475001	2.9422	0.209298	HZ	PHEX
2.47131	0.311047	GY	OTOGL	NC_056057.1	20075001	20095001	3.345	0.282447	HZ	PHF14
2.68106	0.250073	GY	OTOGL	NC_056055.1	2670001	2690001	3.83499	0.313161	HZ	PHF19
2.64979	0.264489	GY	OTOGL	NC_056055.1	2675001	2695001	3.9086	0.282418	HZ	PHF19
2.42206	0.292575	GY	OTOGL	NC_056055.1	2680001	2700001	3.92534	0.270024	HZ	PHF19
2.65233	0.248995	GY	PACRG	NC_056055.1	2685001	2705001	4.70018	0.259244	HZ	PHF19
2.86155	0.238203	GY	PACRG	NC_056055.1	27915001	27935001	4.03354	0.270481	HZ	PHF2
2.84079	0.228495	GY	PACRG	NC_056055.1	27920001	27940001	5.41109	0.32771	HZ	PHF2
2.91647	0.214641	GY	PACRG	NC_056055.1	27925001	27945001	3.90643	0.30298	HZ	PHF2
2.91664	0.199485	GY	PACRG	NC_056055.1	27930001	27950001	3.81876	0.331268	HZ	PHF2
2.66667	0.279207	GY	PACS2;TEX22	NC_056055.1	27935001	27955001	4.51345	0.380446	HZ	PHF2
5.86046	0.358877	GY	PACS2;TEX22	NC_056055.1	27940001	27960001	4.77945	0.398859	HZ	PHF2
2.43991	0.238608	GY	PACSIN1	NC_056055.1	27945001	27965001	7.30226	0.447053	HZ	PHF2
19.4004	0.196887	GY	PADI3	NC_056055.1	27950001	27970001	6.48069	0.425858	HZ	PHF2
21.5088	0.257977	GY	PADI3	NC_056080.1	65190001	65210001	2.39886	0.254911	HZ	PHKA1
23.8975	0.288459	GY	PADI3	NC_056080.1	65195001	65215001	2.61914	0.234281	HZ	PHKA1
15.5969	0.228076	GY	PADI3	NC_056076.1	60875001	60895001	19.6723	0.208362	HZ	PIGN
8.68234	0.230154	GY	PADI4	NC_056076.1	60895001	60915001	27.7724	0.211076	HZ	PIGN
21.1863	0.304509	GY	PADI4	NC_056076.1	60900001	60920001	31.1746	0.270765	HZ	PIGN;RELCH
33.5681	0.277048	GY	PADI4	NC_056076.1	60905001	60925001	30.7833	0.30797	HZ	PIGN;RELCH
31.4769	0.261316	GY	PADI4	NC_056076.1	60915001	60935001	22.9804	0.303756	HZ	PIGN;RELCH
31.0269	0.259369	GY	PADI4	NC_056067.1	7275001	7295001	14.9301	0.23378	HZ	PKD1L2
4.79361	0.201887	GY	PARVB	NC_056067.1	7895001	7915001	2.42605	0.205316	HZ	PLCG2
2.50812	0.208373	GY	PATL1	NC_056067.1	7900001	7920001	2.62627	0.21127	HZ	PLCG2
2.58089	0.217773	GY	PATL1	NC_056054.1	216440001	216460001	6.04412	0.208819	HZ	PLD1
2.48356	0.4081	GY	PAWR	NC_056054.1	216445001	216465001	16.2468	0.285329	HZ	PLD1
2.4833	0.442216	GY	PAWR	NC_056054.1	216460001	216480001	17.0354	0.308512	HZ	PLD1
2.57473	0.294401	GY	PAWR	NC_056054.1	216465001	216485001	18.7627	0.310856	HZ	PLD1
3.16702	0.218355	GY	PBRM1	NC_056054.1	216470001	216490001	22.982	0.313666	HZ	PLD1
2.93387	0.214017	GY	PBRM1	NC_056054.1	216475001	216495001	7.02891	0.326583	HZ	PLD1
7.38143	0.602413	GY	PCBD2	NC_056054.1	216480001	216500001	3.92063	0.30687	HZ	PLD1
12.0336	0.640769	GY	PCBD2	NC_056054.1	216485001	216505001	2.52312	0.269719	HZ	PLD1
7.00921	0.61459	GY	PCBD2	NC_056056.1	196430001	196450001	2.39666	0.239203	HZ	PLEKHA5
3.05397	0.53297	GY	PCBD2	NC_056068.1	35350001	35370001	4.97015	0.223889	HZ	PLEKHA7
2.52959	0.203838	GY	PCCA	NC_056068.1	35355001	35375001	3.9675	0.219446	HZ	PLEKHA7
3.3616	0.250642	GY	PCCA	NC_056065.1	51135001	51155001	2.75785	0.27766	HZ	PLEKHM2
4.77924	0.28594	GY	PCCA	NC_056065.1	51140001	51160001	3.23354	0.27675	HZ	PLEKHM2
3.87027	0.266837	GY	PCOLCE2	NC_056065.1	51145001	51165001	2.51697	0.249649	HZ	PLEKHM2
7.88554	0.340732	GY	PCOLCE2	NC_056054.1	76280001	76300001	2.88908	0.228804	HZ	PLPPR5
28.4941	0.343839	GY	PCOLCE2	NC_056054.1	76285001	76305001	3.23729	0.264244	HZ	PLPPR5
3.03615	0.345271	GY	PDE1A	NC_056054.1	76290001	76310001	2.81129	0.364747	HZ	PLPPR5
2.56347	0.257716	GY	PDE8B;WDR41	NC_056054.1	76295001	76315001	2.43764	0.400355	HZ	PLPPR5
2.51361	0.224004	GY	PDS5A	NC_056054.1	247005001	247025001	22.1494	0.367989	HZ	PLS1
6.25607	0.496384	GY	PDZRN4	NC_056054.1	247040001	247060001	3.12663	0.218186	HZ	PLS1
5.44775	0.509898	GY	PDZRN4	NC_056054.1	247045001	247065001	3.20404	0.270871	HZ	PLS1
2.98246	0.193669	GY	PGRMC2	NC_056065.1	74575001	74595001	22.7626	0.2082	HZ	PLXNA2
2.6502	0.210048	GY	PHC3	NC_056056.1	225560001	225580001	2.5449	0.287732	HZ	PLXNB2
2.87666	0.212096	GY	PHC3	NC_056056.1	475001	495001	3.15392	0.337883	HZ	PNPLA7
5.51418	0.20445	GY	PHF2	NC_056056.1	480001	500001	4.3677	0.40408	HZ	PNPLA7
8.23152	0.193751	GY	PHF2	NC_056056.1	485001	505001	3.82899	0.413917	HZ	PNPLA7
3.93118	0.202083	GY	PHYKPL	NC_056056.1	490001	510001	4.83801	0.419874	HZ	PNPLA7
3.64292	0.211725	GY	PI4K2B	NC_056056.1	495001	515001	3.8256	0.357074	HZ	PNPLA7
4.06316	0.20746	GY	PI4K2B	NC_056060.1	7105001	7125001	2.53913	0.22311	HZ	POC5

2.77208	0.214923	GY	PIK3AP1	NC_056060.1	7110001	7130001	2.47984	0.260312	HZ	POC5
2.81781	0.196738	GY	PIP4K2A	NC_056059.1	20110001	20130001	6.73059	0.212543	HZ	PPA2
7.51961	0.301405	GY	PKD1L2	NC_056058.1	55720001	55740001	2.89093	0.245403	HZ	PPP2R2B
13.2238	0.323275	GY	PKD1L2	NC_056058.1	55725001	55745001	2.86265	0.287224	HZ	PPP2R2B
17	0.297574	GY	PKD1L2	NC_056054.1	253950001	253970001	2.3911	0.249903	HZ	PPP2R3A
5.36538	0.200526	GY	PKD1L2	NC_056059.1	23900001	23920001	4.13458	0.254035	HZ	PPP3CA
2.47429	0.231305	GY	PKD2L1	NC_056059.1	23925001	23945001	11.6971	0.250458	HZ	PPP3CA
2.40921	0.360833	GY	PKP2	NC_056059.1	23930001	23950001	8.6994	0.215248	HZ	PPP3CA
4.79973	0.194228	GY	PLCB1	NC_056056.1	176530001	176550001	4.72333	0.275378	HZ	PRDM4;PWP1
3.26562	0.200665	GY	PLCG2	NC_056056.1	176535001	176555001	3.95272	0.247666	HZ	PRDM4;PWP1
2.40573	0.21651	GY	PLCG2	NC_056059.1	4895001	4915001	2.61825	0.220511	HZ	PRDM5
26.5044	0.217572	GY	PLD1	NC_056059.1	4900001	4920001	3.83922	0.228142	HZ	PRDM5
24.7034	0.218836	GY	PLD1	NC_056059.1	4905001	4925001	3.55951	0.232906	HZ	PRDM5
2.97643	0.197042	GY	PLD1	NC_056059.1	4910001	4930001	2.96089	0.249804	HZ	PRDM5
2.70106	0.333784	GY	PLEKHM2	NC_056065.1	54290001	54310001	5.63935	0.244905	HZ	PRDX6;SLC9C2
3.22554	0.365146	GY	PLEKHM2	NC_056065.1	66835001	66855001	3.32792	0.30273	HZ	PRG4;TPR
2.47305	0.312831	GY	PLEKHM2	NC_056065.1	66840001	66860001	5.76392	0.355178	HZ	PRG4;TPR
11.1919	0.242895	GY	PLPPR4	NC_056057.1	49690001	49710001	2.80782	0.206578	HZ	PRKAR2B
16.7832	0.237107	GY	PLPPR4	NC_056056.1	78220001	78240001	4.44444	0.239476	HZ	PRKCE
21.3276	0.270855	GY	PLPPR4	NC_056056.1	78225001	78245001	4.00633	0.228099	HZ	PRKCE
17.988	0.271118	GY	PLPPR4	NC_056056.1	78230001	78250001	4.4642	0.25035	HZ	PRKCE
24.3678	0.385557	GY	PLS1	NC_056056.1	78235001	78255001	2.91002	0.216833	HZ	PRKCE
3.57773	0.19245	GY	PLS1	NC_056056.1	78605001	78625001	4.61756	0.274089	HZ	PRKCE
2.45356	0.219132	GY	PLS1	NC_056075.1	7825001	7845001	3.27991	0.295833	HZ	PRKG1
2.80156	0.274625	GY	PLS1	NC_056075.1	7830001	7850001	5.58997	0.333273	HZ	PRKG1
3.01616	0.340872	GY	PLS1	NC_056075.1	7840001	7860001	28.9047	0.401182	HZ	PRKG1
2.79661	0.240038	GY	PLXDC2	NC_056075.1	7845001	7865001	21.381	0.357726	HZ	PRKG1
40.4964	0.223225	GY	POLK	NC_056075.1	7850001	7870001	13.3433	0.267161	HZ	PRKG1
42.7043	0.224815	GY	POLK	NC_056075.1	7855001	7875001	5.77479	0.214644	HZ	PRKG1
35.1705	0.197342	GY	POLK	NC_056059.1	112025001	112045001	2.6838	0.322568	HZ	PROM1
4.99283	0.231866	GY	POU4F1	NC_056059.1	112030001	112050001	6.49085	0.518035	HZ	PROM1
2.94291	0.194741	GY	PPFIA2	NC_056059.1	112035001	112055001	12.3217	0.568567	HZ	PROM1
5.5535	0.235852	GY	PPFIA2	NC_056059.1	112040001	112060001	12.4919	0.577953	HZ	PROM1
8.69545	0.248386	GY	PPFIA2	NC_056059.1	112045001	112065001	9.46808	0.425684	HZ	PROM1
4.99835	0.265452	GY	PPFIA2	NC_056056.1	104425001	104445001	7.20197	0.426805	HZ	PROM2
4.65698	0.270322	GY	PPFIA2	NC_056056.1	104430001	104450001	5.31792	0.413833	HZ	PROM2
3.03523	0.231438	GY	PPFIA2	NC_056056.1	104435001	104455001	2.94978	0.367465	HZ	PROM2
2.56625	0.383291	GY	PPFIA2	NC_056056.1	136935001	136955001	3.7551	0.403957	HZ	PRPH
5.18943	0.319649	GY	PPFIA2	NC_056056.1	136940001	136960001	4.52823	0.440608	HZ	PRPH
4.55986	0.224595	GY	PPFIA2	NC_056058.1	31075001	31095001	2.8518	0.218297	HZ	PRR16
7.77881	0.242718	GY	PPP1R1A	NC_056060.1	52885001	52905001	3.17748	0.209109	HZ	PRTG
5.97102	0.474735	GY	PPP1R1C	NC_056077.1	41750001	41770001	4.53995	0.290968	HZ	PSMG3
6.15773	0.480239	GY	PPP1R1C	NC_056077.1	41755001	41775001	4.59063	0.302252	HZ	PSMG3;TMEM184A
7.59108	0.507259	GY	PPP1R1C	NC_056077.1	41760001	41780001	4.05351	0.271197	HZ	PSMG3;TMEM184A
6.8254	0.507562	GY	PPP1R1C	NC_056071.1	29875001	29895001	3.80662	0.318674	HZ	PSTPIP1
4.20994	0.48116	GY	PPP1R1C	NC_056071.1	29880001	29900001	4.22023	0.331931	HZ	PSTPIP1
2.79549	0.426976	GY	PPP1R1C	NC_056071.1	29885001	29905001	2.97026	0.352379	HZ	PSTPIP1
2.64386	0.424756	GY	PPP1R1C	NC_056069.1	9375001	9395001	4.37812	0.214636	HZ	PTCD2
2.46245	0.40994	GY	PPP1R1C	NC_056069.1	9380001	9400001	4.37017	0.204831	HZ	PTCD2
3.21814	0.239488	GY	PPP2R2B	NC_056054.1	19500001	19520001	2.81764	0.278962	HZ	PTCH2
3.0089	0.276192	GY	PPP2R2B	NC_056055.1	78130001	78150001	2.41942	0.217286	HZ	PTPRD
3.42013	0.213125	GY	PPP3CA	NC_056055.1	78135001	78155001	3.87393	0.260086	HZ	PTPRD
3.79933	0.293002	GY	PPP3CA	NC_056072.1	39215001	39235001	4.64936	0.317412	HZ	PTPRG
8.95594	0.37853	GY	PPP3CA	NC_056072.1	39220001	39240001	3.63027	0.303375	HZ	PTPRG
5.42193	0.34693	GY	PPP3CA	NC_056072.1	39530001	39550001	2.61589	0.214286	HZ	PTPRG
2.52214	0.267546	GY	PPP3CA	NC_056056.1	176525001	176545001	4.40316	0.270875	HZ	PWP1
3.29956	0.222435	GY	PPY;PYY	NC_056056.1	194330001	194350001	3.95282	0.220288	HZ	PYROXD1
2.98387	0.387358	GY	PRDM16	NC_056055.1	175215001	175235001	3.14634	0.234318	HZ	R3HDM1
3.80961	0.418031	GY	PRDM16	NC_056055.1	175220001	175240001	2.55827	0.210171	HZ	R3HDM1
4.14453	0.399217	GY	PRDM16	NC_056075.1	37845001	37865001	8.52468	0.210446	HZ	RAB11FIP2
2.61144	0.372858	GY	PRDM16	NC_056054.1	256125001	256145001	2.62297	0.307486	HZ	RAB6B
2.47166	0.249914	GY	PRDM16	NC_056065.1	55505001	55525001	3.58838	0.208051	HZ	RABGAP1L
2.8058	0.254193	GY	PRDM16	NC_056065.1	55510001	55530001	2.8117	0.290612	HZ	RABGAP1L
3.43992	0.226628	GY	PRG4;TPR	NC_056065.1	55515001	55535001	2.67236	0.31703	HZ	RABGAP1L
5.62764	0.26645	GY	PRG4;TPR	NC_056065.1	55520001	55540001	3.66201	0.332136	HZ	RABGAP1L
2.66109	0.344278	GY	PRKCA	NC_056065.1	55525001	55545001	5.56862	0.312801	HZ	RABGAP1L
3.49736	0.356154	GY	PRKCA	NC_056065.1	55530001	55550001	6.94884	0.281708	HZ	RABGAP1L
4.17848	0.326797	GY	PRKCA	NC_056065.1	55535001	55555001	8.36843	0.249984	HZ	RABGAP1L
2.76807	0.223709	GY	PRKCA	NC_056060.1	78070001	78090001	3.24597	0.302959	HZ	RAD51B
2.69085	0.242415	GY	PRKCA	NC_056060.1	78075001	78095001	6.67289	0.350762	HZ	RAD51B
2.84044	0.268862	GY	PRKCA	NC_056060.1	78090001	78110001	9.7	0.356652	HZ	RAD51B
2.63561	0.285863	GY	PRKCA	NC_056060.1	78095001	78115001	3.90576	0.287562	HZ	RAD51B
4.55178	0.195553	GY	PRKX	NC_056060.1	78100001	78120001	2.53358	0.218009	HZ	RAD51B
16.2978	0.206619	GY	PROM1	NC_056056.1	3830001	3850001	2.65224	0.205921	HZ	RALGDS

4.07823	0.192086	GY	PRPH	NC_056070.1	40045001	40065001	2.38182	0.339092	HZ	RAPGEF2
4.85484	0.225371	GY	PRPH	NC_056070.1	40050001	40070001	2.68583	0.32215	HZ	RAPGEF2
2.47177	0.200855	GY	PRR16	NC_056079.1	39770001	39790001	9.86394	0.25598	HZ	RARB
2.46359	0.194189	GY	PRTG	NC_056079.1	39775001	39795001	14.335	0.307338	HZ	RARB
3.14732	0.196334	GY	PRTG	NC_056079.1	39780001	39800001	17.2877	0.344047	HZ	RARB
3.31602	0.192214	GY	PRTG	NC_056079.1	39785001	39805001	21.0652	0.368795	HZ	RARB
3.57735	0.380804	GY	PSTPIP1	NC_056079.1	39800001	39820001	20.017	0.381514	HZ	RARB
3.97917	0.398688	GY	PSTPIP1	NC_056079.1	39805001	39825001	12.9181	0.347165	HZ	RARB
2.96568	0.397761	GY	PSTPIP1	NC_056079.1	39810001	39830001	14.7377	0.294433	HZ	RARB
3.13379	0.250802	GY	PTCD2	NC_056079.1	39815001	39835001	3.68571	0.211816	HZ	RARB
4.54386	0.287283	GY	PTCD2	NC_056065.1	58935001	58955001	2.67164	0.29998	HZ	RASAL2
4.48956	0.280762	GY	PTCD2	NC_056065.1	58940001	58960001	4.23418	0.329192	HZ	RASAL2
2.64809	0.216349	GY	PTCD2	NC_056065.1	58945001	58965001	4.28471	0.335491	HZ	RASAL2
7.05801	0.312406	GY	PTPRG	NC_056065.1	58950001	58970001	2.36557	0.331489	HZ	RASAL2
5.58082	0.33104	GY	PTPRG	NC_056077.1	6490001	6510001	5.08872	0.22046	HZ	RBFOX1
3.80836	0.195466	GY	PTPRG	NC_056077.1	6495001	6515001	3.82561	0.205621	HZ	RBFOX1
4.04593	0.277139	GY	PUS7	NC_056077.1	6830001	6850001	2.63892	0.212879	HZ	RBFOX1
2.77814	0.256343	GY	PUS7	NC_056077.1	6835001	6855001	3.39072	0.22804	HZ	RBFOX1
3.17022	0.264978	GY	PYROXD1	NC_056056.1	179610001	179630001	2.41797	0.216201	HZ	RBFOX2
7.27692	0.274143	GY	PYY	NC_056064.1	52520001	52540001	3.56177	0.406577	HZ	RBFOX3
6.63404	0.216834	GY	PYY	NC_056055.1	149295001	149315001	3.11683	0.245676	HZ	RBMS1
3.65415	0.232346	GY	QRFPR	NC_056072.1	4460001	4480001	2.71761	0.339053	HZ	RBMS3
2.81597	0.401771	GY	R3HDM1	NC_056072.1	4465001	4485001	3.11874	0.341888	HZ	RBMS3
2.40038	0.352594	GY	R3HDM1	NC_056072.1	4470001	4490001	4.45563	0.356746	HZ	RBMS3
2.42791	0.294637	GY	RABGAP1L	NC_056072.1	4475001	4495001	3.33853	0.325309	HZ	RBMS3
3.09777	0.390465	GY	RABGAP1L	NC_056072.1	4480001	4500001	2.8043	0.305985	HZ	RBMS3
5.31765	0.319232	GY	RABGAP1L	NC_056072.1	4485001	4505001	3.25869	0.31977	HZ	RBMS3
7.07441	0.30008	GY	RABGAP1L	NC_056071.1	29845001	29865001	8.93428	0.295235	HZ	RCN2
8.36257	0.281896	GY	RABGAP1L	NC_056055.1	52415001	52435001	3.39408	0.248344	HZ	RECK
5.84375	0.233066	GY	RABGAP1L	NC_056055.1	52420001	52440001	12.9622	0.330719	HZ	RECK
2.71336	0.243398	GY	RACK1;TRIM52	NC_056076.1	60920001	60940001	17.1053	0.280132	HZ	RELCH
3.34879	0.282718	GY	RAD51B	NC_056076.1	60925001	60945001	11.9858	0.266926	HZ	RELCH
7.07944	0.289153	GY	RAD51B	NC_056076.1	60930001	60950001	9.59355	0.256521	HZ	RELCH
4.78012	0.202226	GY	RAD51B	NC_056076.1	60935001	60955001	11.4272	0.257332	HZ	RELCH
2.903	0.251218	GY	RAD51B	NC_056076.1	60940001	60960001	9.31788	0.247157	HZ	RELCH
2.77114	0.201576	GY	RAD51B	NC_056076.1	60945001	60965001	10.5385	0.235552	HZ	RELCH
3	0.201272	GY	RAD51B	NC_056076.1	60950001	60970001	11.6407	0.230139	HZ	RELCH
3.26696	0.210337	GY	RAD51B	NC_056057.1	46470001	46490001	4.27598	0.370886	HZ	RELN
3.10708	0.247845	GY	RAD51B	NC_056057.1	46475001	46495001	2.91616	0.329508	HZ	RELN
2.41084	0.328571	GY	RAD51B	NC_056057.1	46480001	46500001	3.32774	0.32359	HZ	RELN
2.51271	0.192899	GY	RAI2	NC_056057.1	46485001	46505001	3.04256	0.284756	HZ	RELN
3.82782	0.236312	GY	RAI2	NC_056058.1	14225001	14245001	6.03507	0.295118	HZ	RETN
3.75975	0.264599	GY	RALGPS1	NC_056056.1	101870001	101890001	2.89258	0.206991	HZ	REV1
6.29488	0.324657	GY	RALGPS1	NC_056056.1	101875001	101895001	2.7282	0.2114	HZ	REV1
8.32856	0.329633	GY	RALGPS1	NC_056056.1	101880001	101900001	2.73509	0.233071	HZ	REV1
10.7614	0.337919	GY	RALGPS1	NC_056055.1	71855001	71875001	5.2247	0.229741	HZ	RFX3
2.46447	0.208232	GY	RALYL	NC_056055.1	71860001	71880001	8.01328	0.262806	HZ	RFX3
17.1695	0.198844	GY	RARB	NC_056056.1	100120001	100140001	24.2976	0.3137	HZ	RFX8
2.95067	0.218076	GY	RASAL2	NC_056056.1	100170001	100190001	2.53425	0.250078	HZ	RFX8
3.40701	0.220951	GY	RASAL2	NC_056056.1	100175001	100195001	2.76479	0.268419	HZ	RFX8
4.09021	0.197882	GY	RASAL2	NC_056056.1	100180001	100200001	2.3569	0.23809	HZ	RFX8
2.65603	0.197386	GY	RASAL2	NC_056062.1	77175001	77195001	2.57931	0.210262	HZ	RGS22
6.01676	0.201696	GY	RBBP8	NC_056060.1	81590001	81610001	3.05867	0.293699	HZ	RGS6
11.9627	0.258553	GY	RBBP8	NC_056060.1	81595001	81615001	3.84727	0.315536	HZ	RGS6
2.53958	0.247712	GY	RBFOX1	NC_056060.1	81600001	81620001	3.72372	0.311659	HZ	RGS6
3.09192	0.196776	GY	RBM24	NC_056060.1	81625001	81645001	2.52465	0.332212	HZ	RGS6
2.49458	0.214815	GY	RBM38	NC_056060.1	81630001	81650001	2.988	0.356316	HZ	RGS6
2.89013	0.220578	GY	RBMS3	NC_056060.1	81635001	81655001	3.00914	0.353386	HZ	RGS6
3.12756	0.209079	GY	RBMS3	NC_056060.1	81640001	81660001	2.7708	0.367217	HZ	RGS6
2.51984	0.35414	GY	RBMS3	NC_056065.1	34775001	34795001	5.44674	0.578728	HZ	RGS7
2.82495	0.382072	GY	RBMS3	NC_056065.1	34780001	34800001	13.8571	0.539079	HZ	RGS7
4.05948	0.407175	GY	RBMS3	NC_056058.1	92705001	92725001	5.11644	0.206138	HZ	RHOBTB3
3.0437	0.304512	GY	RBMS3	NC_056058.1	92710001	92730001	3.95891	0.245243	HZ	RHOBTB3
2.52046	0.221028	GY	RBMS3	NC_056055.1	73755001	73775001	2.85826	0.302902	HZ	RIC1
2.8426	0.218277	GY	RBMS3	NC_056055.1	73820001	73840001	2.63216	0.220098	HZ	RIC1
9.26276	0.258529	GY	RCN2	NC_056055.1	73825001	73845001	2.49663	0.231577	HZ	RIC1
2.7451	0.209896	GY	RELN	NC_056062.1	73870001	73890001	4.05681	0.241298	HZ	RIMS2
2.42934	0.2558	GY	RELN	NC_056062.1	73875001	73895001	4.67593	0.317567	HZ	RIMS2
4.51947	0.326338	GY	RELN	NC_056062.1	73880001	73900001	3.50336	0.250087	HZ	RIMS2
2.95031	0.305278	GY	RELN	NC_056062.1	73885001	73905001	3.2669	0.27508	HZ	RIMS2
3.35294	0.324159	GY	RELN	NC_056058.1	68340001	68360001	2.41783	0.340789	HZ	RNF145
3.18297	0.291858	GY	RELN	NC_056058.1	68345001	68365001	2.72184	0.293843	HZ	RNF145
2.56897	0.245194	GY	RELN	NC_056058.1	68350001	68370001	3.05289	0.314866	HZ	RNF145
2.55755	0.220249	GY	RELN	NC_056062.1	76990001	77010001	3.55314	0.272727	HZ	RNF19A

7.77457	0.202927	GY	RERE	NC_056062.1	76995001	77015001	4.46995	0.267653	HZ	RNF19A
9.3586	0.226194	GY	RERE	NC_056062.1	77000001	77020001	3.7077	0.231728	HZ	RNF19A
8.57954	0.238306	GY	RERE	NC_056055.1	52125001	52145001	4.72044	0.332043	HZ	RNF38
6.53893	0.205952	GY	RERE	NC_056055.1	52130001	52150001	2.96141	0.252083	HZ	RNF38
17.2727	0.304227	GY	RFX8	NC_056054.1	147745001	147765001	4.41215	0.272505	HZ	ROBO1
2.58448	0.21931	GY	RFX8	NC_056054.1	147750001	147770001	2.79667	0.25294	HZ	ROBO1
2.61761	0.230973	GY	RFX8	NC_056076.1	35475001	35495001	2.52966	0.267053	HZ	ROCK1
4.81818	0.193887	GY	RGS16;RNASEL	NC_056076.1	35480001	35500001	2.60325	0.247026	HZ	ROCK1
6.07074	0.20996	GY	RGS16;RNASEL	NC_056076.1	35485001	35505001	2.76959	0.274601	HZ	ROCK1
2.53246	0.229049	GY	RHBDD2	NC_056076.1	35520001	35540001	2.50799	0.286376	HZ	ROCK1
2.86491	0.226626	GY	RNF145	NC_056054.1	39205001	39225001	2.58701	0.22508	HZ	ROR1
2.94483	0.275471	GY	RNF145	NC_056060.1	47370001	47390001	2.61283	0.250032	HZ	RORA
3.28606	0.293544	GY	RNF145	NC_056080.1	37835001	37855001	4.01209	0.277224	HZ	RPGR
2.5095	0.223312	GY	RNF145	NC_056080.1	37840001	37860001	3.37226	0.273262	HZ	RPGR
3.48309	0.342929	GY	RNF19A	NC_056077.1	14185001	14205001	12.8241	0.274701	HZ	RRN3
4.56012	0.335789	GY	RNF19A	NC_056074.1	15910001	15930001	2.49236	0.212993	HZ	RSF1
4.33538	0.276662	GY	RNF19A	NC_056074.1	15915001	15935001	6.1111	0.291386	HZ	RSF1
3.51546	0.212915	GY	RNF19A	NC_056066.1	30515001	30535001	2.46879	0.2242	HZ	RSU1
3.84123	0.194404	GY	RNF19A	NC_056079.1	13565001	13585001	3.94776	0.211183	HZ	RWDD4;TRAPPC11
3.28058	0.210044	GY	RNF19A	NC_056079.1	13570001	13590001	5.35424	0.239784	HZ	RWDD4;TRAPPC11
3.81473	0.227459	GY	RNF19A	NC_056078.1	9155001	9175001	6.91667	0.277762	HZ	RYR2
2.60201	0.203705	GY	ROBO1	NC_056078.1	9160001	9180001	4.1151	0.281795	HZ	RYR2
4.68276	0.228075	GY	ROBO1	NC_056067.1	19045001	19065001	4.54357	0.279246	HZ	SALL1
3.05318	0.196262	GY	ROBO1	NC_056067.1	19050001	19070001	7.66893	0.243467	HZ	SALL1
3.04805	0.213311	GY	ROBO2	NC_056067.1	19055001	19075001	5.75171	0.225492	HZ	SALL1
2.53814	0.210464	GY	ROCK1	NC_056062.1	58795001	58815001	3.73684	0.245752	HZ	SAMD12
2.45476	0.312696	GY	ROCK1	NC_056062.1	58800001	58820001	3.31029	0.24064	HZ	SAMD12
2.6636	0.321781	GY	ROCK1	NC_056062.1	58805001	58825001	2.70158	0.249446	HZ	SAMD12
2.644	0.268332	GY	ROCK1	NC_056056.1	220605001	220625001	3.14888	0.241843	HZ	SAMM50
9.07859	0.192598	GY	RORA	NC_056058.1	43760001	43780001	2.38897	0.345135	HZ	SAR1B
7.65949	0.253827	GY	RP1L1	NC_056058.1	43765001	43785001	2.68456	0.349616	HZ	SAR1B
10.978	0.270088	GY	RP1L1	NC_056058.1	43770001	43790001	2.62109	0.352794	HZ	SAR1B
12.3788	0.283845	GY	RP1L1	NC_056056.1	140490001	140510001	3.53458	0.258677	HZ	SCAF11
6.31956	0.215228	GY	RP1L1	NC_056056.1	140495001	140515001	3.3889	0.253273	HZ	SCAF11
5.2453	0.277997	GY	RPIA	NC_056056.1	140500001	140520001	3.58918	0.277565	HZ	SCAF11
7.25869	0.293575	GY	RPIA	NC_056056.1	140505001	140525001	4.1	0.277018	HZ	SCAF11
8.08	0.2842	GY	RPIA	NC_056056.1	140510001	140530001	4.06416	0.306399	HZ	SCAF11
5.71888	0.265083	GY	RPIA	NC_056056.1	140515001	140535001	6.14529	0.286491	HZ	SCAF11
3.32267	0.204413	GY	RPIA	NC_056056.1	140520001	140540001	6.17349	0.293324	HZ	SCAF11
4.33512	0.216752	GY	RRAGD	NC_056056.1	140525001	140545001	4.57142	0.306615	HZ	SCAF11
2.71511	0.200518	GY	RRH	NC_056056.1	140530001	140550001	4.11905	0.302582	HZ	SCAF11
11.6111	0.298543	GY	RRN3	NC_056056.1	140535001	140555001	3.40411	0.347993	HZ	SCAF11
4.87947	0.209833	GY	RRN3	NC_056056.1	140540001	140560001	2.59694	0.302691	HZ	SCAF11
2.64122	0.213238	GY	RRP12	NC_056056.1	140545001	140565001	2.44845	0.303309	HZ	SCAF11
3.45289	0.327159	GY	RTN4RL1	NC_056056.1	140565001	140585001	2.92447	0.277449	HZ	SCAF11
2.99606	0.295449	GY	RTN4RL1	NC_056070.1	29140001	29160001	3.79497	0.298797	HZ	SCLT1
3.22183	0.204711	GY	SAMSN1	NC_056070.1	29145001	29165001	3.90532	0.378656	HZ	SCLT1
2.65053	0.230641	GY	SAP130	NC_056070.1	29150001	29170001	3.63117	0.32669	HZ	SCLT1
2.77339	0.244646	GY	SAP130	NC_056070.1	29155001	29175001	4.23708	0.302769	HZ	SCLT1
2.44569	0.272861	GY	SAP130	NC_056070.1	29160001	29180001	4.48074	0.254856	HZ	SCLT1
3.00803	0.341306	GY	SAP130	NC_056070.1	29165001	29185001	7.26696	0.213479	HZ	SCLT1
2.71221	0.321006	GY	SAP130	NC_056070.1	29170001	29190001	9.4964	0.218588	HZ	SCLT1
2.4133	0.304534	GY	SAP130	NC_056068.1	28595001	28615001	2.49308	0.333758	HZ	SCN2B
2.74877	0.209662	GY	SAP130	NC_056068.1	28600001	28620001	2.71807	0.241266	HZ	SCN2B
4.05411	0.222416	GY	SAP130	NC_056057.1	37510001	37530001	3.16167	0.210458	HZ	SEMA3A
2.9896	0.230528	GY	SAP130	NC_056064.1	53830001	53850001	11.2768	0.208204	HZ	SEPTIN9
2.96993	0.229683	GY	SAP130	NC_056080.1	18095001	18115001	3.75309	0.347484	HZ	SH3KBP1
2.5062	0.224022	GY	SAP130	NC_056080.1	18235001	18255001	2.81677	0.246594	HZ	SH3KBP1
2.50649	0.212484	GY	SAP30	NC_056080.1	18240001	18260001	3.42055	0.323094	HZ	SH3KBP1
2.83476	0.193963	GY	SAP30	NC_056080.1	18245001	18265001	8.89832	0.433574	HZ	SH3KBP1
2.45056	0.231958	GY	SAP30;SCRG1	NC_056069.1	4350001	4370001	2.50108	0.20622	HZ	SH3PXD2B
2.57562	0.322682	GY	SAR1B	NC_056069.1	4355001	4375001	6.3931	0.241422	HZ	SH3PXD2B
2.59179	0.329807	GY	SAR1B	NC_056069.1	4360001	4380001	3.92182	0.208715	HZ	SH3PXD2B
2.48345	0.316094	GY	SCAPER	NC_056074.1	44065001	44085001	3.17269	0.234198	HZ	SHANK2
3.24602	0.367526	GY	SCAPER	NC_056074.1	44070001	44090001	2.37007	0.243048	HZ	SHANK2
2.67718	0.350855	GY	SCAPER	NC_056077.1	12405001	12425001	2.40313	0.214505	HZ	SHISA9
3.32215	0.334028	GY	SCAPER	NC_056077.1	12410001	12430001	4.41133	0.29611	HZ	SHISA9
3.24952	0.353798	GY	SCLT1	NC_056077.1	12415001	12435001	10.7308	0.325632	HZ	SHISA9
3.12426	0.418542	GY	SCLT1	NC_056077.1	12420001	12440001	9.45217	0.269832	HZ	SHISA9
2.96662	0.366426	GY	SCLT1	NC_056077.1	12425001	12445001	6.25389	0.252765	HZ	SHISA9
3.44833	0.315724	GY	SCLT1	NC_056061.1	70365001	70385001	2.83174	0.251517	HZ	SHPRH
4.3436	0.212163	GY	SCLT1	NC_056061.1	70385001	70405001	2.76905	0.213244	HZ	SHPRH
7.64705	0.23177	GY	SCLT1	NC_056061.1	70390001	70410001	4.65479	0.271532	HZ	SHPRH
10.5288	0.248853	GY	SCLT1	NC_056061.1	70395001	70415001	6.95767	0.305874	HZ	SHPRH

13.05	0.238459	GY	SCLT1	NC_056061.1	70400001	70420001	7.42105	0.325675	HZ	SHPRH
2.68348	0.209865	GY	SCNN1B	NC_056061.1	70405001	70425001	4.27203	0.308067	HZ	SHPRH
2.63549	0.201199	GY	SCNN1B	NC_056074.1	25595001	25615001	6.42329	0.272247	HZ	SIAE;SPA17
2.90908	0.204203	GY	SDHAF4	NC_056068.1	27350001	27370001	2.692	0.238146	HZ	SIK3
3.00219	0.205	GY	SDHAF4	NC_056068.1	27355001	27375001	2.76049	0.218284	HZ	SIK3
5.24721	0.418739	GY	SEPTIN10	NC_056068.1	27360001	27380001	2.62882	0.220777	HZ	SIK3
4.66266	0.401679	GY	SEPTIN10	NC_056068.1	27400001	27420001	3.4129	0.22989	HZ	SIK3
5.06786	0.387123	GY	SEPTIN10	NC_056068.1	27405001	27425001	2.73423	0.288182	HZ	SIK3
5.20151	0.425177	GY	SEPTIN10	NC_056068.1	27415001	27435001	2.48533	0.219334	HZ	SIK3
5.32709	0.43286	GY	SEPTIN10	NC_056068.1	27445001	27465001	2.64706	0.244897	HZ	SIK3
5.14954	0.428967	GY	SEPTIN10	NC_056068.1	27450001	27470001	2.55474	0.227635	HZ	SIK3
7.82256	0.469866	GY	SEPTIN10	NC_056068.1	27455001	27475001	2.79747	0.220701	HZ	SIK3
10.1257	0.483914	GY	SEPTIN10	NC_056068.1	27460001	27480001	3.28499	0.220721	HZ	SIK3
7.60713	0.45415	GY	SEPTIN10	NC_056068.1	27490001	27510001	19.195	0.223075	HZ	SIK3
6.2449	0.420802	GY	SEPTIN10	NC_056068.1	27495001	27515001	8.22273	0.210273	HZ	SIK3
5.87616	0.424756	GY	SEPTIN10;SH3RF3	NC_056060.1	5070001	5090001	12.5897	0.21262	HZ	SIMC1
3.99682	0.356055	GY	SEPTIN10;SOWAH	NC_056060.1	5075001	5095001	10.6834	0.241745	HZ	SIMC1
8.63604	0.202832	GY	SEPTIN9	NC_056060.1	5080001	5100001	5.95824	0.220658	HZ	SIMC1
6.44404	0.281335	GY	SFRP1	NC_056064.1	37950001	37970001	3.92987	0.217877	HZ	SKAP1
3.17135	0.225815	GY	SGCD	NC_056064.1	37955001	37975001	5.10453	0.247251	HZ	SKAP1
4.32856	0.21707	GY	SGCD	NC_056064.1	37960001	37980001	7.12501	0.276705	HZ	SKAP1
3.90785	0.208641	GY	SGCD	NC_056064.1	37965001	37985001	6.09724	0.252337	HZ	SKAP1
3.82022	0.288109	GY	SH3GL2	NC_056064.1	37970001	37990001	3.39696	0.220097	HZ	SKAP1
2.43851	0.304869	GY	SH3GL2	NC_056057.1	70705001	70725001	2.53267	0.293842	HZ	SKAP2
2.71617	0.27142	GY	SH3KBP1	NC_056057.1	70725001	70745001	2.51916	0.290348	HZ	SKAP2
6.86442	0.535702	GY	SH3KBP1	NC_056057.1	70730001	70750001	2.52881	0.240185	HZ	SKAP2
3.37417	0.369532	GY	SH3RF3	NC_056057.1	70735001	70755001	2.59941	0.242843	HZ	SKAP2
4.19429	0.395221	GY	SH3RF3	NC_056057.1	70740001	70760001	2.76558	0.231973	HZ	SKAP2
2.68125	0.219443	GY	SHISA9	NC_056057.1	70745001	70765001	2.6088	0.249282	HZ	SKAP2
4.67365	0.325262	GY	SHISA9	NC_056057.1	70750001	70770001	3.4762	0.285198	HZ	SKAP2
10.5205	0.347012	GY	SHISA9	NC_056057.1	70755001	70775001	5.80789	0.311392	HZ	SKAP2
8.74784	0.262718	GY	SHISA9	NC_056057.1	70760001	70780001	9.14286	0.338859	HZ	SKAP2
5.67997	0.249969	GY	SHISA9	NC_056057.1	70795001	70815001	30.8143	0.570264	HZ	SKAP2
15.9524	0.216336	GY	SHROOM4	NC_056057.1	70800001	70820001	36.6571	0.561351	HZ	SKAP2
5.23934	0.205992	GY	SIK2	NC_056063.1	74950001	74970001	3.60408	0.310262	HZ	SLC15A1
2.4189	0.217594	GY	SIK3	NC_056063.1	74955001	74975001	6.51452	0.316729	HZ	SLC15A1
2.4975	0.354458	GY	SIK3	NC_056063.1	74960001	74980001	41.4592	0.326309	HZ	SLC15A1
3.09693	0.365978	GY	SIK3	NC_056063.1	74965001	74985001	16	0.277339	HZ	SLC15A1
5.65934	0.311581	GY	SIK3	NC_056063.1	74970001	74990001	6.77777	0.209906	HZ	SLC15A1
7.67582	0.322662	GY	SIK3	NC_056080.1	66625001	66645001	3.52543	0.261188	HZ	SLC16A2
9.15585	0.327137	GY	SIK3	NC_056080.1	66640001	66660001	4.29914	0.329296	HZ	SLC16A2
11.1104	0.28936	GY	SIK3	NC_056080.1	66645001	66665001	2.63718	0.264898	HZ	SLC16A2
17.4505	0.238491	GY	SIK3	NC_056066.1	46940001	46960001	2.98507	0.266349	HZ	SLC23A2
4.84692	0.303168	GY	SIK3	NC_056066.1	46945001	46965001	2.89871	0.258762	HZ	SLC23A2
3.33182	0.310163	GY	SIK3	NC_056055.1	87995001	88015001	4.64584	0.220391	HZ	SLC24A2
2.63813	0.314164	GY	SIK3	NC_056055.1	88000001	88020001	6.85976	0.225395	HZ	SLC24A2
2.44695	0.256466	GY	SIK3	NC_056072.1	51110001	51130001	3.34376	0.379121	HZ	SLC25A20
2.56863	0.240007	GY	SIK3	NC_056074.1	40830001	40850001	3.85556	0.427428	HZ	SLC29A2
2.43552	0.212925	GY	SIK3	NC_056074.1	40835001	40855001	2.73438	0.452372	HZ	SLC29A2
3.08438	0.238997	GY	SIK3	NC_056056.1	147560001	147580001	2.84097	0.234108	HZ	SLC2A13
3.45999	0.248275	GY	SIK3	NC_056056.1	147565001	147585001	3.96822	0.287964	HZ	SLC2A13
5.27387	0.230054	GY	SIMC1	NC_056056.1	147570001	147590001	9.8216	0.346102	HZ	SLC2A13
4.12935	0.222217	GY	SIMC1	NC_056056.1	147575001	147595001	14.7034	0.339179	HZ	SLC2A13
11.7331	0.200459	GY	SIMC1	NC_056056.1	147580001	147600001	22.0784	0.337536	HZ	SLC2A13
10.8571	0.264298	GY	SIMC1	NC_056056.1	147585001	147605001	12.2292	0.3003	HZ	SLC2A13
6.57802	0.281833	GY	SIMC1	NC_056055.1	119800001	119820001	2.36505	0.227789	HZ	SLC40A1
4.31507	0.256681	GY	SIMC1	NC_056055.1	147875001	147895001	4.63719	0.239657	HZ	SLC4A10
4.87853	0.201667	GY	SKAP1	NC_056067.1	23370001	23390001	2.57692	0.335072	HZ	SLC6A2
6.88792	0.219868	GY	SKAP1	NC_056067.1	23375001	23395001	2.45306	0.381119	HZ	SLC6A2
2.54799	0.279076	GY	SKAP2	NC_056067.1	35210001	35230001	2.80642	0.443367	HZ	SLC7A6
2.60626	0.218603	GY	SKAP2	NC_056067.1	35215001	35235001	2.3673	0.392486	HZ	SLC7A6
3.59047	0.197651	GY	SKAP2	NC_056067.1	42770001	42790001	4.29972	0.249263	HZ	SLC7A9
6.14778	0.25068	GY	SKAP2	NC_056067.1	42775001	42795001	3.75281	0.23339	HZ	SLC7A9
9.89474	0.261646	GY	SKAP2	NC_056067.1	42780001	42800001	8.67857	0.27931	HZ	SLC7A9
33.2143	0.301191	GY	SKAP2	NC_056067.1	42785001	42805001	2.58557	0.216406	HZ	SLC7A9
39.1571	0.308772	GY	SKAP2	NC_056065.1	54295001	54315001	6.17979	0.237845	HZ	SLC9C2
23.7841	0.322045	GY	SKAP2	NC_056056.1	194335001	194355001	7.12467	0.262494	HZ	SLCO1A2
16.5052	0.315466	GY	SKAP2	NC_056056.1	194340001	194360001	5.50452	0.266414	HZ	SLCO1A2
12.4737	0.308664	GY	SKAP2	NC_056056.1	194345001	194365001	3.87846	0.23773	HZ	SLCO1A2
8.00001	0.315964	GY	SKAP2	NC_056063.1	61310001	61330001	2.48526	0.268819	HZ	SLITRK6
4.67935	0.192448	GY	SLC15A5	NC_056063.1	61315001	61335001	3.62409	0.297419	HZ	SLITRK6
6.02431	0.194525	GY	SLC15A5	NC_056063.1	61320001	61340001	2.83798	0.20558	HZ	SLITRK6
4.9364	0.200288	GY	SLC15A5	NC_056070.1	12785001	12805001	5.42256	0.209556	HZ	SMAD1
2.71111	0.26511	GY	SLC16A2	NC_056070.1	12790001	12810001	6.93348	0.218437	HZ	SMAD1

2.85384	0.231366	GY	SLC16A2	NC_056070.1	12795001	12815001	6.39182	0.23402	HZ	SMAD1
3.38984	0.238229	GY	SLC16A2	NC_056080.1	51250001	51270001	2.61445	0.24284	HZ	SMC1A
3.44444	0.272646	GY	SLC16A2	NC_056080.1	51255001	51275001	2.64458	0.26236	HZ	SMC1A
3.21884	0.201004	GY	SLC22A3	NC_056055.1	66305001	66325001	3.591	0.363857	HZ	SMC5
3.45297	0.2014	GY	SLC22A3	NC_056055.1	66310001	66330001	3.44613	0.393698	HZ	SMC5
3.44562	0.211903	GY	SLC23A2	NC_056055.1	66315001	66335001	3.40865	0.413222	HZ	SMC5
3.22598	0.214039	GY	SLC23A2	NC_056055.1	66320001	66340001	3.29946	0.409271	HZ	SMC5
3.19464	0.260584	GY	SLC25A43	NC_056055.1	66325001	66345001	2.87179	0.414874	HZ	SMC5
4.23888	0.361337	GY	SLC29A2	NC_056080.1	100055001	100075001	2.90942	0.253729	HZ	SMIM10
4.08595	0.289658	GY	SLC29A2	NC_056059.1	35550001	35570001	3.5006	0.209756	HZ	SNCA
3.31631	0.194881	GY	SLC29A2	NC_056057.1	93780001	93800001	2.41983	0.229445	HZ	SND1
2.49592	0.227689	GY	SLC2A1	NC_056057.1	93790001	93810001	3.81913	0.408497	HZ	SND1
6.79658	0.208263	GY	SLC2A13	NC_056057.1	93795001	93815001	6.62384	0.48119	HZ	SND1
12.1287	0.220815	GY	SLC2A13	NC_056057.1	93800001	93820001	2.42084	0.326432	HZ	SND1
6.51539	0.206538	GY	SLC2A13	NC_056062.1	93665001	93685001	10.2789	0.299931	HZ	SNX16
5.72193	0.212778	GY	SLC2A13	NC_056062.1	93670001	93690001	5.8395	0.222891	HZ	SNX16
2.46904	0.249336	GY	SLC39A11;SSTR2	NC_056059.1	114715001	114735001	3.83194	0.248501	HZ	SORCS2
3.00699	0.211484	GY	SLC6A2	NC_056059.1	114720001	114740001	3.63619	0.256838	HZ	SORCS2
3.13673	0.243004	GY	SLC6A2	NC_056059.1	114725001	114745001	3.15551	0.240747	HZ	SORCS2
2.69257	0.193844	GY	SLC7A6	NC_056058.1	42055001	42075001	4.33334	0.229664	HZ	SOWAHA
3.66349	0.239235	GY	SLC7A6	NC_056056.1	191665001	191685001	2.62767	0.261494	HZ	SOX5
3.02415	0.193948	GY	SLC7A6	NC_056056.1	191670001	191690001	3.35533	0.282435	HZ	SOX5
3.98034	0.426838	GY	SLC7A8	NC_056056.1	191675001	191695001	4.67747	0.295226	HZ	SOX5
2.69313	0.222005	GY	SLC7A8	NC_056056.1	191680001	191700001	7.58536	0.309361	HZ	SOX5
3.5101	0.22338	GY	SLC7A8	NC_056056.1	191685001	191705001	18.3313	0.323088	HZ	SOX5
4.22466	0.342718	GY	SLC7A9	NC_056056.1	191690001	191710001	7.41984	0.2948	HZ	SOX5
3.60257	0.365472	GY	SLC7A9	NC_056056.1	191695001	191715001	3.40611	0.237382	HZ	SOX5
8.50511	0.425224	GY	SLC7A9	NC_056056.1	191990001	192010001	2.42378	0.233013	HZ	SOX5
2.39931	0.25769	GY	SLC7A9;TDRD12	NC_056056.1	191995001	192015001	2.41083	0.246102	HZ	SOX5
5.95066	0.253475	GY	SLCO1A2	NC_056064.1	24070001	24090001	5.69718	0.220764	HZ	SPATA22
4.70062	0.235786	GY	SLCO1A2	NC_056056.1	69480001	69500001	4.10224	0.211168	HZ	SPTBN1
3.51157	0.200757	GY	SLCO1A2	NC_056058.1	79025001	79045001	2.36955	0.233416	HZ	SSBP2
4.20994	0.232367	GY	SMG1	NC_056057.1	52670001	52690001	6.5	0.205161	HZ	ST7
4.46753	0.239779	GY	SMG1	NC_056057.1	52675001	52695001	4.88178	0.212068	HZ	ST7
3.79905	0.291617	GY	SMG1	NC_056057.1	52715001	52735001	2.49481	0.206536	HZ	ST7
3.3077	0.288756	GY	SMG1	NC_056057.1	52855001	52875001	19.5797	0.213049	HZ	ST7
3.25727	0.357253	GY	SMG1	NC_056057.1	52860001	52880001	19.9999	0.215585	HZ	ST7
2.92032	0.338572	GY	SMG1	NC_056057.1	52865001	52885001	11.5929	0.210689	HZ	ST7
2.69903	0.234377	GY	SMG1	NC_056063.1	28315001	28335001	4.21557	0.260382	HZ	STARD13
14.8181	0.192948	GY	SNTB2	NC_056057.1	51580001	51600001	2.42181	0.222376	HZ	STARD3NL
14.2	0.23807	GY	SNTB2	NC_056080.1	61620001	61640001	4.07868	0.209028	HZ	STARD8
6.46789	0.278151	GY	SNTB2	NC_056062.1	49835001	49855001	2.66101	0.209075	HZ	STAU2
2.64427	0.255714	GY	SNTB2	NC_056062.1	49840001	49860001	3.04824	0.232746	HZ	STAU2
2.83249	0.273703	GY	SOX5	NC_056062.1	49845001	49865001	2.97029	0.211066	HZ	STAU2
9.72639	0.225045	GY	SOX5	NC_056057.1	73980001	74000001	4.19755	0.254439	HZ	STEAP4
2.96774	0.204841	GY	SOX5	NC_056057.1	73985001	74005001	10.4831	0.372928	HZ	STEAP4
3.52256	0.242483	GY	SOX5	NC_056057.1	73990001	74010001	19.471	0.355913	HZ	STEAP4
2.72789	0.256397	GY	SOX5	NC_056057.1	73995001	74015001	4.87244	0.305833	HZ	STEAP4
2.50077	0.262362	GY	SOX5	NC_056057.1	74000001	74020001	2.86393	0.273103	HZ	STEAP4
2.48859	0.252754	GY	SOX5	NC_056063.1	74840001	74860001	4.36718	0.216743	HZ	STK24
2.75748	0.312207	GY	SOX5	NC_056063.1	74845001	74865001	5.09452	0.234104	HZ	STK24
3.55622	0.334839	GY	SOX5	NC_056079.1	13635001	13655001	14.4327	0.323173	HZ	STOX2
4.97269	0.353191	GY	SOX5	NC_056079.1	13645001	13665001	15.6818	0.31916	HZ	STOX2
8.05366	0.377023	GY	SOX5	NC_056079.1	13650001	13670001	5.06849	0.207223	HZ	STOX2
19.6265	0.391873	GY	SOX5	NC_056079.1	13770001	13790001	5.1145	0.244202	HZ	STOX2
7.82951	0.351377	GY	SOX5	NC_056079.1	13775001	13795001	6.91032	0.308963	HZ	STOX2
3.4242	0.277413	GY	SOX5	NC_056079.1	13780001	13800001	3.43985	0.36392	HZ	STOX2
3.48742	0.220125	GY	SPAG6	NC_056079.1	13785001	13805001	2.66164	0.350932	HZ	STOX2
11.268	0.289921	GY	SPATA13	NC_056058.1	14220001	14240001	7.3689	0.296224	HZ	STXBP2
32.8	0.24768	GY	SPATA13	NC_056057.1	82040001	82060001	2.41964	0.22151	HZ	SUGCT
33.7663	0.228332	GY	SPATA13	NC_056057.1	82045001	82065001	3.63925	0.355159	HZ	SUGCT
27.4905	0.219818	GY	SPATA13	NC_056057.1	82070001	82090001	4.24999	0.263266	HZ	SUGCT
12.9217	0.19474	GY	SPATA13	NC_056057.1	82075001	82095001	3.55487	0.224963	HZ	SUGCT
12.8726	0.343652	GY	SPCS3	NC_056057.1	82080001	82100001	3.23115	0.220118	HZ	SUGCT
45.6714	0.232384	GY	SPDL1	NC_056057.1	82085001	82105001	3.56288	0.212873	HZ	SUGCT
25.537	0.256767	GY	SPDL1	NC_056057.1	82395001	82415001	7.90475	0.264032	HZ	SUGCT
9.19198	0.247766	GY	SPDL1	NC_056057.1	82415001	82435001	5.62151	0.237287	HZ	SUGCT
2.42601	0.234656	GY	SPINK5	NC_056062.1	46330001	46350001	19.0714	0.252566	HZ	SULF1
2.44658	0.260763	GY	SPIRE2;TCF25	NC_056062.1	46335001	46355001	12.7302	0.301746	HZ	SULF1
2.73214	0.246862	GY	SPIRE2;TCF25	NC_056062.1	46340001	46360001	6.92414	0.440093	HZ	SULF1
7.34044	0.311612	GY	SPON2	NC_056062.1	46345001	46365001	4.52747	0.393804	HZ	SULF1
7.36701	0.269271	GY	SPON2	NC_056062.1	46350001	46370001	2.7802	0.319298	HZ	SULF1
14.0543	0.20751	GY	SPP1	NC_056062.1	46355001	46375001	2.44843	0.271062	HZ	SULF1
21.3204	0.261791	GY	SPTLC2	NC_056054.1	100010001	100030001	2.55453	0.286447	HZ	SV2A

3.57896	0.226923	GY	SRRM4	NC_056060.1	7545001	7565001	2.82385	0.262088	HZ	SV2C
2.8248	0.275683	GY	SRRM4	NC_056060.1	7550001	7570001	3.02633	0.274564	HZ	SV2C
3.77915	0.213081	GY	SRRM4	NC_056068.1	42660001	42680001	3.96895	0.242254	HZ	SWAP70
8.6078	0.236845	GY	SRRM4	NC_056068.1	42665001	42685001	2.99303	0.226258	HZ	SWAP70
25.9892	0.270575	GY	SRRM4	NC_056056.1	176790001	176810001	4.97048	0.365006	HZ	SYN3
8.99999	0.291779	GY	SRRM4	NC_056056.1	176795001	176815001	6.0787	0.356048	HZ	SYN3
4.56419	0.243437	GY	SRRM4	NC_056056.1	176835001	176855001	3.4123	0.272415	HZ	SYN3
2.92764	0.202416	GY	SRRM4	NC_056056.1	176840001	176860001	3.91666	0.301387	HZ	SYN3
4.55898	0.225153	GY	SSPN	NC_056056.1	176845001	176865001	2.91978	0.330798	HZ	SYN3
6.29592	0.230484	GY	SSPN	NC_056056.1	176900001	176920001	2.47213	0.235668	HZ	SYN3
5.85246	0.229606	GY	SSPN	NC_056056.1	176910001	176930001	2.68422	0.251729	HZ	SYN3
5.85424	0.219047	GY	SSPN	NC_056056.1	176915001	176935001	3.60725	0.272145	HZ	SYN3
3.99701	0.396061	GY	STARD13	NC_056056.1	176920001	176940001	3.53624	0.279355	HZ	SYN3
2.51418	0.238476	GY	STARD3NL	NC_056056.1	176925001	176945001	3.70297	0.286993	HZ	SYN3
2.70972	0.236695	GY	STARD3NL	NC_056061.1	76960001	76980001	2.92172	0.206512	HZ	SYNE1
4.45099	0.20121	GY	STEAP4	NC_056061.1	76965001	76985001	3.65181	0.218243	HZ	SYNE1
5.43721	0.206423	GY	STEAP4	NC_056061.1	76970001	76990001	12.2443	0.237556	HZ	SYNE1
8.97073	0.229643	GY	STEAP4	NC_056058.1	59720001	59740001	16.8545	0.208929	HZ	SYNPO
15.2609	0.311266	GY	STEAP4	NC_056056.1	115280001	115300001	2.71193	0.231552	HZ	SYT1
17.6521	0.335022	GY	STEAP4	NC_056056.1	115445001	115465001	2.72607	0.452589	HZ	SYT1
4.60519	0.341801	GY	STEAP4	NC_056061.1	82810001	82830001	6.13872	0.453039	HZ	SYTL3
2.53865	0.327817	GY	STEAP4	NC_056061.1	82825001	82845001	10.6898	0.264482	HZ	SYTL3
2.89291	0.215551	GY	STK31	NC_056062.1	94175001	94195001	6.62778	0.251794	HZ	TAF2
2.6831	0.231038	GY	STK31	NC_056066.1	6855001	6875001	5.28985	0.242158	HZ	TASP1
2.66667	0.193242	GY	STK31	NC_056066.1	6860001	6880001	17.942	0.323861	HZ	TASP1
2.51163	0.290946	GY	STK31	NC_056066.1	6865001	6885001	16.1399	0.330386	HZ	TASP1
2.52395	0.264891	GY	STK31	NC_056066.1	6870001	6890001	8.7	0.292481	HZ	TASP1
2.52273	0.255331	GY	STK31	NC_056066.1	6875001	6895001	3.62408	0.215439	HZ	TASP1
15.4904	0.261162	GY	STOX2	NC_056075.1	15525001	15545001	3.08541	0.208794	HZ	TBC1D12
17.5	0.266655	GY	STOX2	NC_056075.1	15530001	15550001	3.31614	0.218	HZ	TBC1D12
5.16031	0.210296	GY	STOX2	NC_056075.1	15535001	15555001	3.5993	0.231916	HZ	TBC1D12
7.33152	0.236944	GY	STOX2	NC_056075.1	15540001	15560001	3.92415	0.246734	HZ	TBC1D12
4.01606	0.220363	GY	STOX2	NC_056075.1	15545001	15565001	3.74658	0.244098	HZ	TBC1D12
3.13477	0.264214	GY	STPG2	NC_056075.1	15550001	15570001	3.9056	0.238177	HZ	TBC1D12
4.30632	0.303124	GY	STPG2	NC_056075.1	15555001	15575001	3.71605	0.232891	HZ	TBC1D12
7.02481	0.381311	GY	STPG2	NC_056056.1	107910001	107930001	4.14568	0.225838	HZ	TBC1D15
6.59183	0.391386	GY	STPG2	NC_056056.1	107975001	107995001	8.81821	0.25874	HZ	TBC1D15
6.21707	0.398285	GY	STPG2	NC_056056.1	107980001	108000001	10.8626	0.274206	HZ	TBC1D15
5.70589	0.403015	GY	STPG2	NC_056056.1	107985001	108005001	12.2069	0.282613	HZ	TBC1D15
4.70101	0.400256	GY	STPG2	NC_056059.1	46685001	46705001	2.6709	0.22203	HZ	TBC1D19
4.90953	0.412208	GY	STPG2	NC_056059.1	46690001	46710001	3.13576	0.267505	HZ	TBC1D19
5.23531	0.413283	GY	STPG2	NC_056054.1	275230001	275250001	2.78387	0.22487	HZ	TBC1D5
4.7	0.390915	GY	STPG2	NC_056054.1	275235001	275255001	3.05524	0.237584	HZ	TBC1D5
3.51091	0.355938	GY	STPG2	NC_056060.1	8860001	8880001	2.70814	0.207908	HZ	TBCA
5.63202	0.376964	GY	STPG2	NC_056060.1	8885001	8905001	3.46454	0.224067	HZ	TBCA
3.31124	0.238247	GY	STPG2	NC_056060.1	8910001	8930001	3.52421	0.214447	HZ	TBCA
3.04625	0.2005	GY	SUGCT	NC_056061.1	52065001	52085001	4.36666	0.242076	HZ	TBX18
3.45663	0.308381	GY	SUGCT	NC_056060.1	51240001	51260001	2.39181	0.21814	HZ	TCF12
3.99686	0.233363	GY	SUGCT	NC_056060.1	51245001	51265001	2.52	0.303155	HZ	TCF12
2.46333	0.266511	GY	SYT10	NC_056060.1	51250001	51270001	2.61905	0.322962	HZ	TCF12
2.49332	0.266862	GY	SYT10	NC_056060.1	51255001	51275001	2.41964	0.358413	HZ	TCF12
2.42708	0.25474	GY	SYT10	NC_056060.1	51315001	51335001	2.49655	0.368376	HZ	TCF12
3.6551	0.211854	GY	SYT10	NC_056060.1	51350001	51370001	3.44361	0.234113	HZ	TCF12
3.67411	0.240667	GY	SYT10	NC_056060.1	51355001	51375001	3.12698	0.223417	HZ	TCF12
3.48115	0.256323	GY	SYT10	NC_056060.1	51475001	51495001	3.18367	0.235116	HZ	TCF12
2.96638	0.250931	GY	SYT10	NC_056060.1	51480001	51500001	3.40178	0.256148	HZ	TCF12
6.37902	0.327829	GY	SYTL5	NC_056060.1	51485001	51505001	2.36735	0.300466	HZ	TCF12
2.7547	0.217577	GY	TAB2	NC_056060.1	51505001	51525001	2.3522	0.323662	HZ	TCF12
3.20229	0.243915	GY	TAB2	NC_056060.1	51510001	51530001	2.78571	0.309316	HZ	TCF12
3.13694	0.233191	GY	TAB2	NC_056060.1	51515001	51535001	3.40177	0.304648	HZ	TCF12
3.50383	0.221808	GY	TAB2	NC_056060.1	51520001	51540001	3.04676	0.319314	HZ	TCF12
4.40085	0.233358	GY	TBC1D1	NC_056056.1	57005001	57025001	2.36552	0.253972	HZ	TCF7L1
5.485	0.316801	GY	TBC1D1	NC_056056.1	57025001	57045001	3.26641	0.246812	HZ	TCF7L1
2.89482	0.267988	GY	TBC1D1	NC_056056.1	57030001	57050001	3.43167	0.313023	HZ	TCF7L1
3.36508	0.222106	GY	TBC1D22A	NC_056055.1	50215001	50235001	22.151	0.36641	HZ	TDRD7
3.36454	0.253793	GY	TBC1D22A	NC_056055.1	50250001	50270001	3.65903	0.468369	HZ	TDRD7
2.74037	0.221379	GY	TBC1D22A	NC_056059.1	80525001	80545001	3.41538	0.260202	HZ	TECRL
5.62636	0.197687	GY	TBC1D22A	NC_056059.1	80530001	80550001	5.61623	0.283863	HZ	TECRL
7.83552	0.212193	GY	TBC1D22A	NC_056059.1	80535001	80555001	6.30051	0.288466	HZ	TECRL
10.7138	0.232027	GY	TBC1D32	NC_056059.1	80540001	80560001	6.56618	0.300104	HZ	TECRL
10.9518	0.227696	GY	TBC1D32	NC_056059.1	80545001	80565001	4.32461	0.286587	HZ	TECRL
2.81204	0.29856	GY	TBC1D5	NC_056059.1	80550001	80570001	3.08678	0.266073	HZ	TECRL
4.00627	0.261414	GY	TBC1D5	NC_056080.1	109000001	109020001	7.31818	0.266859	HZ	TENM1
2.90226	0.247719	GY	TCF12	NC_056080.1	109005001	109025001	5.70454	0.218275	HZ	TENM1

2.65873	0.260287	GY	TCF12	NC_056054.1	20065001	20085001	3.67841	0.213657	HZ	TESK2
2.88888	0.203519	GY	TCF12	NC_056078.1	24185001	24205001	2.6566	0.334823	HZ	TET1
2.65476	0.240005	GY	TCF12	NC_056078.1	24200001	24220001	3.01111	0.506566	HZ	TET1
2.91667	0.331995	GY	TCF12	NC_056078.1	24230001	24250001	2.55985	0.359001	HZ	TET1
2.75892	0.328895	GY	TCF12	NC_056078.1	24235001	24255001	3.83908	0.390741	HZ	TET1
2.741	0.301663	GY	TCF12	NC_056078.1	24240001	24260001	5.20001	0.439831	HZ	TET1
2.47683	0.275199	GY	TCF25	NC_056078.1	24245001	24265001	10.4253	0.431697	HZ	TET1
3.83269	0.200552	GY	TCF7L1	NC_056078.1	24250001	24270001	12.35	0.408109	HZ	TET1
3.94453	0.214638	GY	TCF7L1	NC_056054.1	256195001	256215001	2.4515	0.311949	HZ	TF
2.44159	0.226539	GY	TCF7L1	NC_056057.1	53890001	53910001	12.0455	0.212367	HZ	TFEC
2.54515	0.269282	GY	TDRD15	NC_056057.1	53895001	53915001	12.5711	0.219588	HZ	TFEC
4.25788	0.365985	GY	TDRD7	NC_056057.1	53905001	53925001	4.84082	0.209003	HZ	TFEC
2.95361	0.204299	GY	TENM1	NC_056057.1	53960001	53980001	2.56697	0.387755	HZ	TFEC
4.33043	0.263808	GY	TENM1	NC_056057.1	53965001	53985001	6.75107	0.436124	HZ	TFEC
11.7386	0.317685	GY	TENM1	NC_056057.1	53970001	53990001	15.7305	0.387158	HZ	TFEC
7.71969	0.345334	GY	TENM1	NC_056057.1	54000001	54020001	12.7428	0.333373	HZ	TFEC
2.97035	0.382662	GY	TENM1	NC_056062.1	21300001	21320001	10.0308	0.233914	HZ	TG
2.93271	0.35749	GY	TENM1	NC_056054.1	68990001	69010001	2.63677	0.229913	HZ	TGFBR3
4.41177	0.286263	GY	TENM1	NC_056076.1	38000001	38020001	7.17425	0.294036	HZ	TGIF1
4.60097	0.253138	GY	TENM1	NC_056076.1	38005001	38025001	5.8106	0.274473	HZ	TGIF1
4.05289	0.2317	GY	TESC	NC_056076.1	38010001	38030001	5.9394	0.309035	HZ	TGIF1
4.19246	0.212141	GY	TET1	NC_056076.1	38015001	38035001	7.21429	0.382295	HZ	TGIF1
6.63888	0.315215	GY	TET1	NC_056056.1	80905001	80925001	4.61496	0.209954	HZ	THADA
4.37089	0.294397	GY	TET1	NC_056056.1	80910001	80930001	25.8286	0.234336	HZ	THADA
3.28621	0.284776	GY	TET1	NC_056056.1	80930001	80950001	12.3241	0.273963	HZ	THADA
2.49619	0.217263	GY	TET1	NC_056061.1	53185001	53205001	2.87986	0.219992	HZ	THEMIS
2.57217	0.246894	GY	TET1	NC_056061.1	53220001	53240001	2.46259	0.243554	HZ	THEMIS
3.78161	0.240744	GY	TET1	NC_056061.1	53225001	53245001	2.61943	0.340353	HZ	THEMIS
5.4339	0.246225	GY	TET1	NC_056061.1	53230001	53250001	2.67428	0.319951	HZ	THEMIS
10.8433	0.238584	GY	TET1	NC_056075.1	17685001	17705001	4.25001	0.209182	HZ	TM9SF3
12.77	0.257629	GY	TET1	NC_056075.1	17690001	17710001	7.03721	0.244858	HZ	TM9SF3
3.15436	0.41003	GY	TEX11	NC_056060.1	4460001	4480001	7.55999	0.221824	HZ	TMED7
4.89164	0.448576	GY	TEX11	NC_056055.1	22585001	22605001	2.81306	0.272336	HZ	TMEFF1
2.65099	0.428299	GY	TEX11	NC_056055.1	22590001	22610001	3.80543	0.372018	HZ	TMEFF1
3.0126	0.426974	GY	TEX22	NC_056055.1	22595001	22615001	3.70386	0.359394	HZ	TMEFF1
3.05504	0.287019	GY	TFEC	NC_056055.1	22600001	22620001	3.68307	0.350965	HZ	TMEFF1
7.51964	0.294375	GY	TFEC	NC_056055.1	22605001	22625001	3.06887	0.312833	HZ	TMEFF1
15.8087	0.275766	GY	TFEC	NC_056054.1	256915001	256935001	2.52632	0.296617	HZ	TMEM108
12	0.250606	GY	TFEC	NC_056054.1	256920001	256940001	3.2231	0.268421	HZ	TMEM108
10.04	0.19968	GY	TG	NC_056054.1	256925001	256945001	2.92157	0.301187	HZ	TMEM108
12.6747	0.290203	GY	THADA	NC_056054.1	256930001	256950001	2.52338	0.307789	HZ	TMEM108
2.7007	0.241071	GY	THADA	NC_056054.1	256935001	256955001	2.71821	0.328837	HZ	TMEM108
2.48308	0.232664	GY	THSD4	NC_056056.1	104100001	104120001	3.352	0.225064	HZ	TMEM127
3.81734	0.298519	GY	THSD4	NC_056074.1	36430001	36450001	2.48956	0.207925	HZ	TMEM138;TMEM21(
4.64807	0.341859	GY	THSD4	NC_056056.1	98990001	99010001	3.05185	0.227614	HZ	TMEM182
3.45821	0.299996	GY	THSD4	NC_056056.1	99005001	99025001	3.64093	0.236606	HZ	TMEM182
2.58863	0.216141	GY	TIAM1	NC_056056.1	99010001	99030001	2.52697	0.328892	HZ	TMEM182
3.05927	0.242929	GY	TIAM1	NC_056056.1	99015001	99035001	2.90548	0.431513	HZ	TMEM182
3.058	0.248972	GY	TIAM1	NC_056056.1	99020001	99040001	2.36569	0.459143	HZ	TMEM182
2.59108	0.195194	GY	TIAM1	NC_056080.1	86155001	86175001	3.2561	0.209037	HZ	TMEM185A
3.51524	0.202543	GY	TLK2	NC_056057.1	90050001	90070001	3.10547	0.213596	HZ	TMEM229A
5.45448	0.220131	GY	TLK2	NC_056057.1	90055001	90075001	3.32143	0.218345	HZ	TMEM229A
3.94897	0.20885	GY	TMEFF1	NC_056061.1	55470001	55490001	2.48119	0.218808	HZ	TMEM244
4.17355	0.236641	GY	TMEFF1	NC_056061.1	55475001	55495001	2.90877	0.226445	HZ	TMEM244
3.86315	0.214725	GY	TMEFF1	NC_056060.1	86540001	86560001	12.5101	0.262678	HZ	TMEM63C
3.62775	0.232663	GY	TMEM117	NC_056055.1	50080001	50100001	5.79799	0.275373	HZ	TMOD1
5.86447	0.262902	GY	TMEM117	NC_056056.1	168255001	168275001	3.58786	0.215051	HZ	TMPO
9.29293	0.239528	GY	TMEM117	NC_056056.1	168260001	168280001	6.10524	0.269346	HZ	TMPO
6.08099	0.233169	GY	TMEM117	NC_056056.1	168265001	168285001	4.69389	0.264092	HZ	TMPO
5.7339	0.218426	GY	TMEM117	NC_056056.1	168270001	168290001	3.07635	0.224586	HZ	TMPO
2.54888	0.203904	GY	TMEM117	NC_056055.1	8645001	8665001	2.48799	0.215324	HZ	TNC
3.14718	0.193053	GY	TMEM178A	NC_056055.1	8650001	8670001	4.39742	0.240143	HZ	TNC
2.45276	0.200236	GY	TMEM178A	NC_056055.1	8655001	8675001	3.52495	0.216722	HZ	TNC
2.57232	0.208328	GY	TMEM178A	NC_056065.1	40070001	40090001	2.57983	0.23383	HZ	TNFSF4
3.25025	0.235335	GY	TMEM182	NC_056055.1	8875001	8895001	4.95203	0.24684	HZ	TNFSF8
2.56676	0.236246	GY	TMEM182	NC_056055.1	8880001	8900001	2.66593	0.222999	HZ	TNFSF8
3.38023	0.321221	GY	TMEM182	NC_056058.1	60085001	60105001	2.37159	0.207155	HZ	TNIP1
2.93968	0.330775	GY	TMEM182	NC_056058.1	60090001	60110001	2.85893	0.245088	HZ	TNIP1
3.76223	0.237263	GY	TMEM248	NC_056058.1	60095001	60115001	2.8233	0.258851	HZ	TNIP1
2.67983	0.227965	GY	TMEM248	NC_056057.1	76510001	76530001	2.69966	0.223034	HZ	TNS3
3.80191	0.251741	GY	TMEM251	NC_056064.1	34355001	34375001	2.44023	0.406965	HZ	TOM1L2
2.81981	0.335504	GY	TMEM71	NC_056064.1	34360001	34380001	2.47084	0.419928	HZ	TOM1L2
5.79799	0.341878	GY	TMOD1	NC_056056.1	107990001	108010001	12.7586	0.286674	HZ	TPH2
3.8	0.46234	GY	TMPRSS13	NC_056056.1	107995001	108015001	19.2903	0.301037	HZ	TPH2

5.09795	0.410773	GY	TMPRSS13	NC_056056.1	108000001	108020001	12.3312	0.291404	HZ	TPH2
3.53025	0.293382	GY	TMPRSS13	NC_056056.1	108005001	108025001	9.56865	0.272792	HZ	TPH2
2.93634	0.291904	GY	TMPRSS13	NC_056056.1	108010001	108030001	5.30633	0.238328	HZ	TPH2
2.41547	0.23568	GY	TMPRSS13	NC_056056.1	108020001	108040001	4.99709	0.228828	HZ	TPH2
3.67414	0.204925	GY	TMPRSS13	NC_056056.1	108025001	108045001	4.12345	0.211189	HZ	TPH2
2.40567	0.290563	GY	TMTC2	NC_056056.1	108030001	108050001	4.99999	0.234809	HZ	TPH2
3.26315	0.265352	GY	TMTC2	NC_056056.1	108035001	108055001	7.2	0.259965	HZ	TPH2
2.42243	0.199531	GY	TMTC2	NC_056056.1	108040001	108060001	6.11441	0.254631	HZ	TPH2
2.59407	0.192221	GY	TNC	NC_056056.1	108045001	108065001	7.58457	0.270098	HZ	TPH2
2.52941	0.343609	GY	TNFSF4	NC_056056.1	108050001	108070001	11.1176	0.284647	HZ	TPH2
4.47485	0.475887	GY	TNFSF8	NC_056056.1	108055001	108075001	9.50944	0.278578	HZ	TPH2
2.50403	0.391858	GY	TNFSF8	NC_056056.1	108060001	108080001	9.59889	0.27795	HZ	TPH2
3.2368	0.235863	GY	TNPO1	NC_056056.1	108065001	108085001	10.4683	0.2729	HZ	TPH2
2.94565	0.20672	GY	TNPO1	NC_056056.1	108070001	108090001	6.05357	0.242978	HZ	TPH2
9.15731	0.267106	GY	TPR	NC_056056.1	108075001	108095001	5.9496	0.22993	HZ	TPH2
5.18816	0.207342	GY	TRAPPC9	NC_056057.1	108895001	108915001	5.94217	0.268774	HZ	TPK1
5.12068	0.213323	GY	TRAPPC9	NC_056057.1	108900001	108920001	7.55619	0.300469	HZ	TPK1
3.20748	0.217858	GY	TRHDE	NC_056057.1	108905001	108925001	40.4706	0.302637	HZ	TPK1
4.14277	0.208128	GY	TRHDE	NC_056057.1	108965001	108985001	19	0.275897	HZ	TPK1
7.67244	0.20986	GY	TRHDE	NC_056057.1	108970001	108990001	8.06873	0.219334	HZ	TPK1
8.53521	0.277191	GY	TRHDE	NC_056057.1	109035001	109055001	2.51918	0.235389	HZ	TPK1
3.65647	0.233279	GY	TRHDE	NC_056057.1	109040001	109060001	3.99307	0.317545	HZ	TPK1
20.7767	0.341782	GY	TRIM60	NC_056057.1	109045001	109065001	3.39143	0.233839	HZ	TPK1
18.7192	0.310624	GY	TRIM60	NC_056065.1	66845001	66865001	9.3764	0.378328	HZ	TPR
12.6667	0.317976	GY	TRIM60	NC_056065.1	66850001	66870001	3.49718	0.344131	HZ	TPR
8.53804	0.313567	GY	TRIM60	NC_056079.1	13575001	13595001	7.63999	0.270995	HZ	TRAPPC11
2.7619	0.254694	GY	TRPS1	NC_056079.1	13605001	13625001	3.21818	0.213089	HZ	TRAPPC11
2.74759	0.224603	GY	TSC22D3	NC_056062.1	16155001	16175001	2.63741	0.214648	HZ	TRAPPC9
2.73942	0.22395	GY	TSC22D3	NC_056062.1	16430001	16450001	5.26638	0.247884	HZ	TRAPPC9
3.38805	0.215489	GY	TSC22D3	NC_056062.1	16435001	16455001	5.03066	0.271086	HZ	TRAPPC9
20.2935	0.312642	GY	TSGA10	NC_056062.1	16440001	16460001	2.46429	0.268988	HZ	TRAPPC9
18.9646	0.310346	GY	TSGA10	NC_056080.1	44320001	44340001	13.6893	0.680685	HZ	TRIM60
20.8149	0.282965	GY	TSGA10	NC_056080.1	44325001	44345001	14.0617	0.630599	HZ	TRIM60
21.4285	0.216378	GY	TSGA10	NC_056080.1	44330001	44350001	9.54762	0.512176	HZ	TRIM60
8.20124	0.203218	GY	TSGA10	NC_056080.1	44335001	44355001	6.89673	0.390295	HZ	TRIM60
3.32375	0.204389	GY	TSGA10	NC_056062.1	48585001	48605001	2.96955	0.208795	HZ	TRPA1
3.91316	0.262028	GY	TSGA10	NC_056062.1	48590001	48610001	5.88286	0.302404	HZ	TRPA1
5.72696	0.306886	GY	TSGA10	NC_056062.1	48595001	48615001	7.76571	0.319858	HZ	TRPA1
5.47227	0.295856	GY	TSGA10	NC_056062.1	48600001	48620001	7.6008	0.328614	HZ	TRPA1
5.84097	0.221171	GY	TSGA10	NC_056062.1	48605001	48625001	4.21459	0.278439	HZ	TRPA1
7.56837	0.216554	GY	TSGA10	NC_056074.1	45555001	45575001	2.92019	0.242524	HZ	TRPM5;TSSC4
7.83376	0.217694	GY	TSGA10	NC_056062.1	61965001	61985001	8.69525	0.225308	HZ	TRPS1
12.4454	0.24015	GY	TSGA10	NC_056073.1	26010001	26030001	5.96669	0.28143	HZ	TSBP1
2.51011	0.240646	GY	TSPAN5	NC_056073.1	26015001	26035001	5.85483	0.286023	HZ	TSBP1
3.6236	0.285862	GY	TSPAN5	NC_056073.1	26020001	26040001	3.45941	0.243723	HZ	TSBP1
3.06328	0.285455	GY	TSPAN5	NC_056056.1	102175001	102195001	10.7717	0.264744	HZ	TSGA10
2.78505	0.274266	GY	TSPAN5	NC_056056.1	102180001	102200001	13.9557	0.286444	HZ	TSGA10
3.67026	0.272249	GY	TSPAN7	NC_056056.1	102185001	102205001	14.6593	0.292007	HZ	TSGA10
6.30434	0.348711	GY	TSPAN7	NC_056056.1	102195001	102215001	17.5535	0.299096	HZ	TSGA10
5.25851	0.330467	GY	TSPAN7	NC_056056.1	102200001	102220001	5.86068	0.263462	HZ	TSGA10
7.59524	0.275	GY	TSPAN7	NC_056057.1	86910001	86930001	3.875	0.21677	HZ	TSPAN12
5.49391	0.317703	GY	TSPAN7	NC_056057.1	86915001	86935001	2.59091	0.24116	HZ	TSPAN12
2.46429	0.222093	GY	TTC9C;ZBTB3	NC_056059.1	26650001	26670001	2.35627	0.314403	HZ	TSPAN5
2.73921	0.244204	GY	TTLL5	NC_056059.1	26655001	26675001	3.40958	0.352578	HZ	TSPAN5
2.80468	0.229755	GY	TTLL5	NC_056059.1	26660001	26680001	2.9298	0.322426	HZ	TSPAN5
16.1454	0.207765	GY	TTLL8	NC_056059.1	26665001	26685001	2.71145	0.286483	HZ	TSPAN5
2.84759	0.223869	GY	TUT7	NC_056080.1	38090001	38110001	3.0388	0.323065	HZ	TSPAN7
3.73451	0.292414	GY	TUT7	NC_056080.1	38095001	38115001	5.77257	0.388594	HZ	TSPAN7
25.5185	0.19617	GY	TXNDC8	NC_056080.1	38130001	38150001	5.80952	0.374428	HZ	TSPAN7
25.6219	0.224315	GY	TXNDC8	NC_056080.1	38135001	38155001	2.52125	0.247394	HZ	TSPAN7
24.7828	0.260451	GY	TXNDC8	NC_056080.1	38160001	38180001	7.96031	0.241547	HZ	TSPAN7
4.22465	0.270815	GY	TXNDC8	NC_056080.1	38175001	38195001	5.26219	0.284341	HZ	TSPAN7
3.22822	0.217814	GY	TXNDC8	NC_056058.1	92480001	92500001	5.42536	0.224178	HZ	TTC37
3.36027	0.204031	GY	TYRP1	NC_056058.1	92485001	92505001	3.94893	0.209619	HZ	TTC37
6.07297	0.201167	GY	UBE2W	NC_056060.1	100440001	100460001	3.21893	0.298852	HZ	TTC7B
8.59202	0.205799	GY	UBE2W	NC_056056.1	225320001	225340001	14.7839	0.340624	HZ	TTLL8
12.1062	0.212774	GY	UBE2W	NC_056079.1	20715001	20735001	6.00014	0.207748	HZ	TUSC3
16.5189	0.202036	GY	UBE2W	NC_056055.1	13000001	13020001	17.5245	0.342981	HZ	TXNDC8
15.7862	0.19278	GY	UBE2W	NC_056055.1	13005001	13025001	26.7252	0.367414	HZ	TXNDC8
18.5726	0.203484	GY	UBE2W	NC_056055.1	13010001	13030001	28.4253	0.403495	HZ	TXNDC8
16.5874	0.202695	GY	UBE2W	NC_056055.1	13015001	13035001	27.8621	0.424817	HZ	TXNDC8
13.8457	0.194494	GY	UBE2W	NC_056055.1	13030001	13050001	5.64383	0.365171	HZ	TXNDC8
3.48611	0.219476	GY	UBE4B	NC_056055.1	13035001	13055001	4.26348	0.293552	HZ	TXNDC8
2.76786	0.215274	GY	UBE4B	NC_056076.1	56300001	56320001	5.85163	0.213719	HZ	TXNL1

4.15183	0.220038	GY	UBIAD1	NC_056059.1	22380001	22400001	3.98617	0.22842	HZ	UBE2D3
4.95031	0.273455	GY	UBIAD1	NC_056065.1	42630001	42650001	3.4639	0.288459	HZ	UBE4B
19.7752	0.247298	GY	UCHL5	NC_056065.1	42635001	42655001	2.46324	0.31632	HZ	UBE4B
22.8915	0.238338	GY	UCHL5	NC_056065.1	42640001	42660001	2.5292	0.296286	HZ	UBE4B
20.375	0.199504	GY	UCHL5	NC_056065.1	42645001	42665001	2.43292	0.305965	HZ	UBE4B
2.48864	0.195791	GY	UNC13B	NC_056065.1	41620001	41640001	5.09628	0.232415	HZ	UBIAD1
3.25603	0.218117	GY	UNC79	NC_056073.1	16275001	16295001	3.89016	0.281839	HZ	UBR2
5.43751	0.438116	GY	USH2A	NC_056073.1	16320001	16340001	2.55394	0.240575	HZ	UBR2
2.64466	0.382584	GY	USH2A	NC_056073.1	16325001	16345001	2.5853	0.205804	HZ	UBR2
2.91413	0.205501	GY	USP24	NC_056065.1	11420001	11440001	19.5116	0.257738	HZ	UCHL5
3.11049	0.307745	GY	USP9X	NC_056065.1	11425001	11445001	23.3876	0.238914	HZ	UCHL5
3.18021	0.212382	GY	UST	NC_056065.1	11430001	11450001	22.5074	0.209806	HZ	UCHL5
2.60902	0.206049	GY	UTRN	NC_056072.1	14265001	14285001	2.54071	0.207207	HZ	ULK4
3.40333	0.217289	GY	UTRN	NC_056072.1	14270001	14290001	3.40867	0.22639	HZ	ULK4
3.62429	0.257922	GY	UTRN	NC_056072.1	14275001	14295001	4.82041	0.224612	HZ	ULK4
3.27595	0.254797	GY	V15	NC_056072.1	14280001	14300001	4.1166	0.217651	HZ	ULK4
3.33858	0.250986	GY	VAMP7	NC_056057.1	16640001	16660001	2.97338	0.231216	HZ	UMAD1
6.04598	0.19289	GY	VGLL4	NC_056057.1	16645001	16665001	2.73738	0.240025	HZ	UMAD1
4.50403	0.197884	GY	VGLL4	NC_056060.1	54045001	54065001	5.40407	0.212867	HZ	UNC13C
3.86	0.201672	GY	VPS41	NC_056077.1	1615001	1635001	2.9525	0.228282	HZ	UNKL
2.84905	0.216498	GY	VPS41	NC_056065.1	18840001	18860001	2.68346	0.245267	HZ	USH2A
2.62066	0.248219	GY	VPS41	NC_056065.1	18845001	18865001	6.675	0.322102	HZ	USH2A
4.65628	0.391096	GY	WDR44	NC_056065.1	18850001	18870001	2.91262	0.33772	HZ	USH2A
5.21298	0.50913	GY	WDR44	NC_056067.1	10410001	10430001	3.45764	0.205656	HZ	USP10
2.53333	0.466781	GY	WDR44	NC_056067.1	10420001	10440001	3.2447	0.224695	HZ	USP10
4.0432	0.56082	GY	WDR44	NC_056064.1	41345001	41365001	3.21612	0.275878	HZ	V15
3.16981	0.573508	GY	WDR44	NC_056080.1	13535001	13555001	2.96359	0.207696	HZ	VEGFD
2.56291	0.207769	GY	WNT8B	NC_056054.1	203005001	203025001	3.63741	0.251011	HZ	VPS8
3.34279	0.41134	GY	XDH	NC_056054.1	203010001	203030001	4.00557	0.26885	HZ	VPS8
2.41257	0.43063	GY	XDH	NC_056054.1	203015001	203035001	2.60622	0.252823	HZ	VPS8
9.28644	0.299567	GY	XKR4	NC_056057.1	6740001	6760001	2.687	0.252037	HZ	VWC2
3.07898	0.292837	GY	XKR4	NC_056071.1	64655001	64675001	4.38835	0.213516	HZ	WDR20
2.65312	0.239313	GY	XPA	NC_056080.1	116435001	116455001	4.72658	0.336424	HZ	WDR44
6.27273	0.220415	GY	XRN1	NC_056080.1	116440001	116460001	6.41667	0.345238	HZ	WDR44
7.05691	0.214569	GY	XRN1	NC_056080.1	116450001	116470001	3.19333	0.341812	HZ	WDR44
2.99405	0.219285	GY	XRN1	NC_056080.1	116455001	116475001	5.38889	0.294949	HZ	WDR44
2.41082	0.201997	GY	XRN1	NC_056080.1	116460001	116480001	4.32548	0.265249	HZ	WDR44
4.40989	0.199035	GY	XRN1	NC_056080.1	116465001	116485001	2.77663	0.216733	HZ	WDR44
4.37092	0.207288	GY	XRN1	NC_056076.1	56410001	56430001	8.54942	0.225091	HZ	WDR7
3.7676	0.206474	GY	XRN1	NC_056076.1	56415001	56435001	6.18698	0.209971	HZ	WDR7
3.79371	0.242022	GY	XRN1	NC_056070.1	64870001	64890001	2.38103	0.336587	HZ	WSCD2
3.063	0.278045	GY	XRN1	NC_056079.1	13215001	13235001	5.45001	0.218369	HZ	WWC2
2.55253	0.318299	GY	XRN1	NC_056079.1	13220001	13240001	5.86615	0.233754	HZ	WWC2
3.51486	0.336324	GY	XRN1	NC_056079.1	13225001	13245001	3.07522	0.304419	HZ	WWC2
4.1973	0.317708	GY	XRN1	NC_056079.1	13235001	13255001	2.47251	0.320206	HZ	WWC2
4.79831	0.322826	GY	XRN1	NC_056079.1	13240001	13260001	2.59782	0.2853	HZ	WWC2
2.47516	0.269633	GY	YES1	NC_056079.1	13245001	13265001	2.52016	0.227029	HZ	WWC2
2.58142	0.251293	GY	ZBED3	NC_056062.1	35715001	35735001	5.20105	0.313948	HZ	XKR4
3.29163	0.288233	GY	ZBED3	NC_056062.1	35720001	35740001	26.9722	0.408046	HZ	XKR4
11.7432	0.346431	GY	ZBED3	NC_056062.1	35735001	35755001	8.73367	0.456586	HZ	XKR4
3.4988	0.220171	GY	ZBTB16	NC_056062.1	35740001	35760001	3.43443	0.361107	HZ	XKR4
2.47395	0.193797	GY	ZBTB16	NC_056054.1	247250001	247270001	2.51361	0.310254	HZ	XRN1
2.39902	0.257797	GY	ZBTB16	NC_056054.1	247255001	247275001	3.54455	0.323479	HZ	XRN1
2.98438	0.29135	GY	ZBTB16	NC_056054.1	247260001	247280001	4.29253	0.28464	HZ	XRN1
3.75869	0.257081	GY	ZBTB16	NC_056054.1	247265001	247285001	5	0.274376	HZ	XRN1
4.18535	0.270105	GY	ZBTB16	NC_056054.1	247295001	247315001	11.9024	0.419962	HZ	XRN1
3.54185	0.262204	GY	ZBTB16	NC_056054.1	247300001	247320001	11.9444	0.474179	HZ	XRN1
3.51351	0.261354	GY	ZBTB16	NC_056077.1	36270001	36290001	6.63493	0.336279	HZ	ZAN
2.86562	0.298856	GY	ZBTB16	NC_056077.1	36280001	36300001	7.7404	0.327569	HZ	ZAN
2.84814	0.22702	GY	ZBTB16	NC_056077.1	36285001	36305001	6.44985	0.286893	HZ	ZAN
3.79537	0.25724	GY	ZBTB16	NC_056060.1	8270001	8290001	3.08382	0.341537	HZ	ZBED3
2.70212	0.264957	GY	ZBTB44	NC_056060.1	8275001	8295001	9.882	0.46865	HZ	ZBED3
2.65641	0.255647	GY	ZBTB44	NC_056074.1	33765001	33785001	2.41844	0.210345	HZ	ZBTB44
2.82243	0.25348	GY	ZBTB44	NC_056074.1	33770001	33790001	2.52821	0.230135	HZ	ZBTB44
5.49029	0.279808	GY	ZBTB44	NC_056074.1	33775001	33795001	3.01869	0.248556	HZ	ZBTB44
4.25572	0.27267	GY	ZBTB44	NC_056074.1	33780001	33800001	5.75729	0.313358	HZ	ZBTB44
3.37435	0.261716	GY	ZBTB44	NC_056074.1	33785001	33805001	4.91603	0.297239	HZ	ZBTB44
3.42029	0.267399	GY	ZBTB44	NC_056074.1	33790001	33810001	3.84031	0.281215	HZ	ZBTB44
3.56364	0.208957	GY	ZC3H12C	NC_056074.1	33795001	33815001	3.77682	0.283769	HZ	ZBTB44
3.98	0.275058	GY	ZC3H12C	NC_056072.1	2925001	2945001	2.43758	0.242871	HZ	ZCWPW2
2.41367	0.273219	GY	ZCWPW2	NC_056056.1	112705001	112725001	2.39739	0.278679	HZ	ZDHHC17
2.82699	0.197012	GY	ZDHHC17	NC_056056.1	112710001	112730001	2.47903	0.271307	HZ	ZDHHC17
3.18457	0.26637	GY	ZDHHC17	NC_056056.1	112715001	112735001	2.67153	0.240922	HZ	ZDHHC17
3.38249	0.30586	GY	ZDHHC17	NC_056056.1	112720001	112740001	2.653	0.250188	HZ	ZDHHC17

3.15964	0.302887	GY	ZDHHC17	NC_056056.1	112725001	112745001	2.50507	0.266529	HZ	ZDHHC17
3.47198	0.366275	GY	ZDHHC17	NC_056056.1	112730001	112750001	2.37167	0.275368	HZ	ZDHHC17
3.69638	0.207645	GY	ZDHHC22	NC_056060.1	86495001	86515001	2.70797	0.278013	HZ	ZDHHC22
3.51162	0.251684	GY	ZFPM2	NC_056062.1	71755001	71775001	3.48102	0.227547	HZ	ZFPM2
2.78621	0.241397	GY	ZFPM2	NC_056062.1	71760001	71780001	2.72611	0.227765	HZ	ZFPM2
3.33334	0.228218	GY	ZFTA	NC_056080.1	133495001	133515001	3.58572	0.223942	HZ	ZMAT1
3.7848	0.220188	GY	ZFTA	NC_056080.1	133500001	133520001	3.21324	0.236111	HZ	ZMAT1
2.78899	0.229785	GY	ZNF157	NC_056057.1	78395001	78415001	2.57172	0.216137	HZ	ZMIZ2
2.43582	0.235662	GY	ZNF19;ZNF23	NC_056057.1	57695001	57715001	3.46627	0.205141	HZ	ZNF277
2.88387	0.221048	GY	ZNF304	NC_056073.1	30435001	30455001	2.68127	0.271527	HZ	ZNF322
2.91713	0.209207	GY	ZNF304	NC_056073.1	30440001	30460001	3.11985	0.273543	HZ	ZNF322
2.69407	0.2087	GY	ZNF385B	NC_056067.1	17490001	17510001	3.81366	0.236693	HZ	ZNF423
2.56038	0.200493	GY	ZNF385B	NC_056067.1	17495001	17515001	3.42471	0.251123	HZ	ZNF423
3.89319	0.307937	GY	ZNF41	NC_056067.1	17500001	17520001	3.2052	0.284338	HZ	ZNF423
3.38205	0.193854	GY	ZNF423	NC_056067.1	17505001	17525001	2.9191	0.249847	HZ	ZNF423
2.82786	0.20631	GY	ZNF438	NC_056057.1	74950001	74970001	2.68354	0.218982	HZ	ZNF804B
4.07019	0.249189	GY	ZNF438	NC_056057.1	74960001	74980001	2.35321	0.23509	HZ	ZNF804B
6.54153	0.295673	GY	ZNF438	NC_056057.1	67530001	67550001	3.20078	0.207975	HZ	ZNRF2
6.06426	0.285038	GY	ZNF438	NC_056057.1	67535001	67555001	3.79919	0.263978	HZ	ZNRF2
2.97027	0.229553	GY	ZNF438	NC_056057.1	67540001	67560001	3.93674	0.295051	HZ	ZNRF2
3.01232	0.327454	GY	ZNF655	NC_056057.1	67545001	67565001	3.44245	0.305403	HZ	ZNRF2
2.87692	0.210729	GY	ZNF704	NC_056057.1	67550001	67570001	2.36678	0.257589	HZ	ZNRF2
2.93191	0.208079	GY	ZNRF1	NC_056057.1	67560001	67580001	2.57086	0.239192	HZ	ZNRF2
2.91446	0.20723	GY	ZNRF1							
3.05625	0.199513	GY	ZNRF1							
2.86584	0.208302	GY	ZNRF1							
2.53735	0.199638	GY	ZWILCH							

OL vs SG2

CHROM	Start	end	Pi	Fst	Region	Gene
NC_056059.1	37250001	37270001	2.74573	0.220624	OL	ABCG2
NC_056059.1	37255001	37275001	2.81686	0.26136	OL	ABCG2
NC_056059.1	37260001	37280001	2.87673	0.278973	OL	ABCG2
NC_056059.1	37265001	37285001	2.93722	0.219208	OL	ABCG2;PKD2
NC_056073.1	26695001	26715001	2.46896	0.227776	OL	ABHD16A;CSNK2B;LY6G5B;LY6G5C
NC_056073.1	26675001	26695001	5.13637	0.21655	OL	ABHD16A;LOC101119591;LY6G6D;LY6G6F
NC_056073.1	26680001	26700001	5.60225	0.232909	OL	ABHD16A;LOC101119591;LY6G6F
NC_056073.1	26690001	26710001	3.56863	0.287973	OL	ABHD16A;LY6G5B;LY6G5C
NC_056073.1	26685001	26705001	5.15687	0.294779	OL	ABHD16A;LY6G6F
NC_056055.1	64755001	64775001	3.14991	0.236842	OL	ABHD17B
NC_056055.1	64760001	64780001	5.20076	0.288226	OL	ABHD17B
NC_056058.1	5580001	5600001	2.3878	0.271663	OL	ABHD8;ANO8;DDA1;MRPL34
NC_056065.1	59940001	59960001	3.8614	0.381972	OL	ABL2
NC_056065.1	59945001	59965001	2.40476	0.427371	OL	ABL2
NC_056065.1	60970001	60990001	8.81611	0.277535	OL	ACBD6
NC_056054.1	110155001	110175001	6.09142	0.213054	OL	ACKR1
NC_056077.1	18760001	18780001	9.93449	0.230428	OL	ACSM1
NC_056055.1	14395001	14415001	3.16962	0.276844	OL	ACTL7A;ACTL7B;ELP1
NC_056055.1	14390001	14410001	3.35102	0.241905	OL	ACTL7A;ELP1
NC_056064.1	44220001	44240001	8.10466	0.240856	OL	ADAM11
NC_056064.1	44225001	44245001	8.09782	0.229137	OL	ADAM11
NC_056064.1	44210001	44230001	7.29937	0.235249	OL	ADAM11;DBF4B
NC_056064.1	44215001	44235001	6.81396	0.239046	OL	ADAM11;DBF4B
NC_056057.1	73705001	73725001	4.13787	0.26291	OL	ADAM22
NC_056057.1	73710001	73730001	23.9138	0.343701	OL	ADAM22
NC_056078.1	26085001	26105001	2.45353	0.225469	OL	ADAMTS14
NC_056069.1	14235001	14255001	4.23743	0.215749	OL	ADAMTS6
NC_056066.1	45790001	45810001	4.72848	0.216238	OL	ADARB2
NC_056066.1	45795001	45815001	6.78318	0.255283	OL	ADARB2
NC_056066.1	45800001	45820001	8.09905	0.249382	OL	ADARB2
NC_056066.1	45805001	45825001	7.99056	0.29041	OL	ADARB2
NC_056066.1	45810001	45830001	9.02432	0.318009	OL	ADARB2
NC_056066.1	45815001	45835001	4.77088	0.269785	OL	ADARB2
NC_056066.1	45820001	45840001	2.71827	0.223456	OL	ADARB2
NC_056060.1	87075001	87095001	2.46475	0.297386	OL	ADCK1
NC_056060.1	87080001	87100001	3.69041	0.324747	OL	ADCK1
NC_056060.1	87085001	87105001	3.60445	0.283778	OL	ADCK1
NC_056060.1	87090001	87110001	2.62969	0.226541	OL	ADCK1
NC_056054.1	120025001	120045001	8.83949	0.245974	OL	ADCY10
NC_056054.1	120030001	120050001	8.40654	0.23669	OL	ADCY10
NC_056054.1	120035001	120055001	6.53964	0.218979	OL	ADCY10
NC_056062.1	5285001	5305001	2.7311	0.247322	OL	ADGRB3
NC_056062.1	5290001	5310001	3.19903	0.226909	OL	ADGRB3
NC_056080.1	87110001	87130001	2.46265	0.258749	OL	AFF2
NC_056080.1	87115001	87135001	2.81877	0.272467	OL	AFF2
NC_056080.1	87120001	87140001	3.16293	0.288099	OL	AFF2
NC_056061.1	76115001	76135001	4.14265	0.279116	OL	AKAP12
NC_056061.1	76120001	76140001	6.65804	0.292476	OL	AKAP12
NC_056061.1	76125001	76145001	6.09887	0.310639	OL	AKAP12
NC_056056.1	211320001	211340001	2.81482	0.29273	OL	AKAP3;NDUFA9
NC_056056.1	211325001	211345001	2.46914	0.347823	OL	AKAP3;NDUFA9
NC_056057.1	10105001	10125001	5.71236	0.26655	OL	AKAP9
NC_056057.1	10110001	10130001	6.47999	0.288219	OL	AKAP9
NC_056057.1	10115001	10135001	4.46028	0.266019	OL	AKAP9
NC_056057.1	10120001	10140001	3.0404	0.244754	OL	AKAP9
NC_056057.1	10170001	10190001	3.59055	0.231679	OL	AKAP9
NC_056057.1	10175001	10195001	3.48499	0.222927	OL	AKAP9
NC_056061.1	49600001	49620001	6.29411	0.268652	OL	AKIRIN2
NC_056061.1	49605001	49625001	5.3136	0.252165	OL	AKIRIN2
NC_056061.1	49610001	49630001	4.68943	0.239635	OL	AKIRIN2
NC_056056.1	36420001	36440001	2.61478	0.290484	OL	ALK
NC_056056.1	36425001	36445001	3.4608	0.338821	OL	ALK
NC_056056.1	36430001	36450001	3.42063	0.292868	OL	ALK
NC_056056.1	36435001	36455001	2.7678	0.24401	OL	ALK
NC_056056.1	36560001	36580001	3.43163	0.329314	OL	ALK
NC_056059.1	13535001	13555001	4.17347	0.220675	OL	ALPK1
NC_056068.1	73315001	73335001	4.12329	0.261806	OL	ALX4
NC_056068.1	73320001	73340001	3.64973	0.245243	OL	ALX4
NC_056080.1	124960001	124980001	3.32257	0.456802	OL	AMMECR1

NC_056080.1	124980001	125000001	3.77985	0.408101	OL	AMMECR1
NC_056080.1	124985001	125005001	2.79245	0.360886	OL	AMMECR1
NC_056080.1	125015001	125035001	2.44445	0.576926	OL	AMMECR1
NC_056060.1	11860001	11880001	4.19691	0.212496	OL	ANKDD1A;SPG21
NC_056060.1	11865001	11885001	5.55258	0.255236	OL	ANKDD1A;SPG21
NC_056068.1	23630001	23650001	2.96536	0.252743	OL	ANKK1
NC_056068.1	23635001	23655001	5.55788	0.337226	OL	ANKK1
NC_056068.1	23640001	23660001	9.86297	0.323038	OL	ANKK1
NC_056068.1	23645001	23665001	15.3453	0.294261	OL	ANKK1
NC_056068.1	23650001	23670001	9.88971	0.261893	OL	ANKK1
NC_056060.1	6615001	6635001	3.61351	0.47059	OL	ANKRD31
NC_056077.1	4380001	4400001	4.848	0.22753	OL	ANKS3;DNAAF8;ZNF500
NC_056056.1	210345001	210365001	2.54664	0.261513	OL	ANO2
NC_056056.1	210350001	210370001	2.91361	0.25094	OL	ANO2
NC_056056.1	170390001	170410001	3.1704	0.30454	OL	ANO4
NC_056056.1	170395001	170415001	6.36644	0.24381	OL	ANO4
NC_056056.1	170420001	170440001	12.1705	0.24537	OL	ANO4
NC_056056.1	170425001	170445001	4.12627	0.230963	OL	ANO4
NC_056056.1	141280001	141300001	2.39971	0.262785	OL	ANO6
NC_056056.1	141285001	141305001	2.9916	0.4034	OL	ANO6
NC_056056.1	141290001	141310001	2.59669	0.39377	OL	ANO6
NC_056060.1	4770001	4790001	2.89382	0.270145	OL	AP3S1
NC_056056.1	28790001	28810001	2.38275	0.280005	OL	APOB
NC_056054.1	192895001	192915001	2.37829	0.315272	OL	APOD
NC_056060.1	41090001	41110001	3.65366	0.255615	OL	ARF6
NC_056060.1	41095001	41115001	3.30427	0.274081	OL	ARF6
NC_056070.1	10415001	10435001	2.54387	0.225258	OL	ARHGAP10
NC_056059.1	101165001	101185001	3.99754	0.225215	OL	ARHGAP24
NC_056080.1	9310001	9330001	2.84667	0.400511	OL	ARHGAP6
NC_056080.1	9710001	9730001	3.36231	0.247104	OL	ARHGAP6
NC_056080.1	9715001	9735001	3.125	0.258649	OL	ARHGAP6
NC_056080.1	9720001	9740001	3.13174	0.25271	OL	ARHGAP6
NC_056080.1	9730001	9750001	2.69512	0.246416	OL	ARHGAP6
NC_056054.1	105965001	105985001	15.3284	0.305157	OL	ARHGEF2;RXFP4
NC_056054.1	105970001	105990001	3.61236	0.291045	OL	ARHGEF2;RXFP4
NC_056055.1	240160001	240180001	4.24114	0.23109	OL	ARID1A
NC_056055.1	240165001	240185001	2.50575	0.230471	OL	ARID1A
NC_056060.1	68420001	68440001	2.90379	0.21915	OL	ARMH4;LOC114115623
NC_056077.1	37460001	37480001	6.31087	0.32273	OL	ARPC1A
NC_056077.1	37465001	37485001	5.38272	0.375111	OL	ARPC1A
NC_056077.1	37470001	37490001	3.34455	0.303862	OL	ARPC1A
NC_056077.1	37475001	37495001	2.6626	0.227048	OL	ARPC1A
NC_056077.1	37450001	37470001	3.69501	0.215289	OL	ARPC1A;ARPC1B
NC_056077.1	37455001	37475001	4.85949	0.27898	OL	ARPC1A;ARPC1B
NC_056072.1	9610001	9630001	2.76954	0.216993	OL	ARPP21
NC_056058.1	92490001	92510001	3.64109	0.355275	OL	ARSK;TTC37
NC_056058.1	92495001	92515001	2.41076	0.3043	OL	ARSK;TTC37
NC_056065.1	57640001	57660001	2.99723	0.227992	OL	ASTN1
NC_056065.1	57645001	57665001	3.32265	0.257486	OL	ASTN1
NC_056065.1	57650001	57670001	2.89758	0.256566	OL	ASTN1
NC_056065.1	70400001	70420001	3.48376	0.252287	OL	ATF3
NC_056065.1	70405001	70425001	3.64336	0.261565	OL	ATF3
NC_056065.1	70395001	70415001	2.80796	0.221975	OL	ATF3;FAM71A
NC_056060.1	41735001	41755001	3.07659	0.22012	OL	ATL1
NC_056060.1	41740001	41760001	2.84241	0.236552	OL	ATL1;SAV1
NC_056060.1	41745001	41765001	3.00393	0.23181	OL	ATL1;SAV1
NC_056060.1	41750001	41770001	2.94142	0.252239	OL	ATL1;SAV1
NC_056067.1	7200001	7220001	9.4609	0.214672	OL	ATMIN;CENPN
NC_056067.1	10165001	10185001	3.71662	0.221568	OL	ATP2C2
NC_056067.1	10170001	10190001	6.52019	0.277394	OL	ATP2C2
NC_056056.1	132840001	132860001	3.06144	0.267712	OL	ATP5MC2
NC_056056.1	132860001	132880001	2.44764	0.267372	OL	ATP5MC2
NC_056080.1	69095001	69115001	2.49751	0.228488	OL	ATP7A
NC_056080.1	69105001	69125001	4.29267	0.25039	OL	ATP7A
NC_056080.1	68765001	68785001	2.3516	0.277778	OL	ATRX
NC_056077.1	26470001	26490001	2.57641	0.220274	OL	ATXN2L;SH2B1;TUFM
NC_056077.1	26475001	26495001	2.45339	0.214401	OL	ATXN2L;SH2B1;TUFM
NC_056071.1	54570001	54590001	3.4914	0.300502	OL	ATXN3
NC_056071.1	54575001	54595001	3.69091	0.351806	OL	ATXN3
NC_056071.1	54580001	54600001	3.47286	0.332964	OL	ATXN3
NC_056071.1	54585001	54605001	4.49289	0.329465	OL	ATXN3
NC_056071.1	54590001	54610001	2.94542	0.265655	OL	ATXN3
NC_056071.1	54595001	54615001	3.30267	0.234054	OL	ATXN3;NDUFB1
NC_056071.1	54600001	54620001	3.99668	0.319643	OL	ATXN3;NDUFB1

NC_056057.1	65520001	65540001	2.56746	0.244375	OL	AVL9
NC_056057.1	65525001	65545001	3.15803	0.246873	OL	AVL9
NC_056057.1	65530001	65550001	2.51172	0.230487	OL	AVL9
NC_056057.1	65535001	65555001	2.47906	0.235925	OL	AVL9
NC_056080.1	63120001	63140001	3.43284	0.268908	OL	AWAT1
NC_056076.1	25820001	25840001	3.71806	0.234151	OL	B4GALT6
NC_056074.1	40820001	40840001	3.00625	0.510085	OL	B4GAT1;BRMS1
NC_056074.1	40825001	40845001	2.73988	0.523074	OL	B4GAT1;BRMS1
NC_056057.1	65100001	65120001	4.06367	0.231474	OL	BBS9
NC_056054.1	70860001	70880001	2.84189	0.348535	OL	BCAR3
NC_056054.1	70865001	70885001	7.43721	0.437013	OL	BCAR3
NC_056054.1	70870001	70890001	10.7736	0.509791	OL	BCAR3
NC_056054.1	70875001	70895001	11.6084	0.466118	OL	BCAR3
NC_056054.1	70880001	70900001	3.09011	0.329158	OL	BCAR3
NC_056080.1	18420001	18440001	2.59016	0.27869	OL	BCLAF3
NC_056080.1	18440001	18460001	2.34982	0.298792	OL	BCLAF3
NC_056080.1	18455001	18475001	2.46268	0.303822	OL	BCLAF3
NC_056080.1	18470001	18490001	2.41772	0.256526	OL	BCLAF3
NC_056080.1	18475001	18495001	2.50667	0.290948	OL	BCLAF3
NC_056080.1	39390001	39410001	2.85708	0.221726	OL	BCOR
NC_056056.1	183095001	183115001	2.49553	0.263305	OL	BICD1
NC_056056.1	203025001	203045001	4.3014	0.410413	OL	BORCS5
NC_056056.1	203030001	203050001	7.90598	0.455433	OL	BORCS5
NC_056056.1	203035001	203055001	10.3443	0.44491	OL	BORCS5
NC_056056.1	203040001	203060001	4.72014	0.42844	OL	BORCS5
NC_056074.1	46430001	46450001	5.77819	0.273643	OL	BRSK2;MOB2
NC_056074.1	46435001	46455001	6.69517	0.269268	OL	BRSK2;MOB2
NC_056074.1	46440001	46460001	6.40608	0.274167	OL	BRSK2;MOB2
NC_056063.1	20855001	20875001	2.64573	0.257593	OL	C10H13orf42
NC_056065.1	65040001	65060001	13.9694	0.252822	OL	C12H1orf21
NC_056065.1	65045001	65065001	5.97543	0.278841	OL	C12H1orf21
NC_056067.1	55360001	55380001	7.45712	0.293126	OL	C14H19orf73;LIN7B;PPFIA3
NC_056067.1	55370001	55390001	5.45188	0.213654	OL	C14H19orf73;LIN7B;PPFIA3
NC_056067.1	55355001	55375001	3.70662	0.271545	OL	C14H19orf73;LIN7B;PPFIA3;SNRNP70
NC_056068.1	21940001	21960001	11.1803	0.239323	OL	C15H11orf52;DIXDC1
NC_056068.1	19010001	19030001	3.11547	0.366213	OL	C15H11orf87
NC_056068.1	15625001	15645001	2.79024	0.221627	OL	C15H11orf97
NC_056068.1	15630001	15650001	2.96773	0.262159	OL	C15H11orf97
NC_056068.1	15635001	15655001	3.16932	0.267922	OL	C15H11orf97
NC_056068.1	15640001	15660001	3.49105	0.223837	OL	C15H11orf97
NC_056068.1	15645001	15665001	2.70465	0.264465	OL	C15H11orf97
NC_056072.1	16255001	16275001	2.51172	0.228953	OL	C19H3orf86;TCAIM
NC_056080.1	104940001	104960001	4.30502	0.283169	OL	C1GALT1C1
NC_056080.1	104945001	104965001	8.88274	0.361006	OL	C1GALT1C1
NC_056054.1	102650001	102670001	2.79527	0.268891	OL	C1H1orf68
NC_056074.1	41180001	41200001	5.64625	0.297353	OL	C21H11orf80
NC_056055.1	64660001	64680001	3.23048	0.220634	OL	C2H9orf85
NC_056055.1	64665001	64685001	4.2562	0.253524	OL	C2H9orf85
NC_056055.1	64670001	64690001	8.57981	0.329843	OL	C2H9orf85
NC_056055.1	64675001	64695001	5.16956	0.288496	OL	C2H9orf85
NC_056055.1	64680001	64700001	4.69451	0.281898	OL	C2H9orf85
NC_056055.1	64685001	64705001	2.83698	0.222533	OL	C2H9orf85
NC_056061.1	49930001	49950001	2.84318	0.402618	OL	C8H6orf163;SMIM8
NC_056057.1	39840001	39860001	5.17875	0.23868	OL	CACNA2D1
NC_056057.1	39845001	39865001	4.93622	0.217728	OL	CACNA2D1
NC_056056.1	132820001	132840001	9.93748	0.232849	OL	CALCOCO1
NC_056057.1	100200001	100220001	3.00626	0.242492	OL	CALD1
NC_056057.1	100205001	100225001	2.68456	0.226968	OL	CALD1
NC_056078.1	28970001	28990001	2.63593	0.220582	OL	CAMK2G
NC_056078.1	28975001	28995001	3.08611	0.222571	OL	CAMK2G
NC_056056.1	79865001	79885001	3.35018	0.235853	OL	CAMKMT
NC_056056.1	79870001	79890001	4.83458	0.329709	OL	CAMKMT
NC_056056.1	79875001	79895001	7.42856	0.409258	OL	CAMKMT
NC_056056.1	79880001	79900001	14.8182	0.505814	OL	CAMKMT
NC_056056.1	79885001	79905001	15.1504	0.46589	OL	CAMKMT
NC_056056.1	79890001	79910001	12.6646	0.417839	OL	CAMKMT
NC_056056.1	79895001	79915001	6.63576	0.356744	OL	CAMKMT
NC_056056.1	79900001	79920001	2.86667	0.223419	OL	CAMKMT
NC_056056.1	79945001	79965001	5.16426	0.229628	OL	CAMKMT
NC_056056.1	79950001	79970001	4.13825	0.224048	OL	CAMKMT;PREPL
NC_056056.1	79955001	79975001	2.57004	0.225882	OL	CAMKMT;PREPL
NC_056065.1	26225001	26245001	3.34712	0.377362	OL	CAPN8
NC_056065.1	26230001	26250001	5.2847	0.346981	OL	CAPN8
NC_056065.1	26235001	26255001	3.92757	0.316444	OL	CAPN8

NC_056065.1	26240001	26260001	2.6792	0.243171	OL	CAPN8
NC_056065.1	26245001	26265001	2.46133	0.213846	OL	CAPN8
NC_056065.1	42175001	42195001	3.54193	0.259139	OL	CASZ1
NC_056065.1	42180001	42200001	3.37753	0.232641	OL	CASZ1
NC_056078.1	24275001	24295001	8.75555	0.306415	OL	CCAR1
NC_056078.1	24260001	24280001	6.26667	0.305882	OL	CCAR1;TET1
NC_056078.1	24265001	24285001	5.76523	0.314423	OL	CCAR1;TET1
NC_056056.1	68690001	68710001	2.36905	0.324435	OL	CCDC88A
NC_056064.1	15485001	15505001	4.24185	0.300354	OL	CCL1
NC_056064.1	15490001	15510001	2.74949	0.288233	OL	CCL1
NC_056074.1	43270001	43290001	2.39783	0.243835	OL	CCND1
NC_056074.1	43275001	43295001	3.2887	0.26402	OL	CCND1
NC_056075.1	17260001	17280001	2.54518	0.214175	OL	CCNJ
NC_056059.1	33685001	33705001	3.45848	0.241824	OL	CCSER1
NC_056059.1	33690001	33710001	5.1978	0.244345	OL	CCSER1
NC_056059.1	33695001	33715001	4.75748	0.250156	OL	CCSER1
NC_056059.1	33700001	33720001	5.47766	0.243544	OL	CCSER1
NC_056059.1	33855001	33875001	9.05694	0.227022	OL	CCSER1
NC_056059.1	33860001	33880001	9.09704	0.224796	OL	CCSER1
NC_056059.1	34895001	34915001	2.66344	0.479934	OL	CCSER1
NC_056059.1	34900001	34920001	4.88056	0.579317	OL	CCSER1
NC_056059.1	34905001	34925001	7.78637	0.469428	OL	CCSER1
NC_056059.1	34910001	34930001	2.65293	0.368503	OL	CCSER1
NC_056074.1	34775001	34795001	3.73029	0.261471	OL	CD5
NC_056074.1	34780001	34800001	2.95636	0.217652	OL	CD5
NC_056078.1	38215001	38235001	7.68835	0.38505	OL	CDHR1;LRIT2
NC_056078.1	38220001	38240001	2.69081	0.266819	OL	CDHR1;LRIT2
NC_056061.1	26780001	26800001	3.04828	0.237726	OL	CDK19
NC_056059.1	91165001	91185001	3.88282	0.368236	OL	CDKL2
NC_056059.1	91170001	91190001	4.079	0.406731	OL	CDKL2
NC_056059.1	91175001	91195001	3.20179	0.366409	OL	CDKL2
NC_056059.1	91180001	91200001	2.49734	0.293271	OL	CDKL2
NC_056060.1	4640001	4660001	4.04349	0.329187	OL	CDO1
NC_056055.1	140890001	140910001	3.3485	0.225424	OL	CERS6
NC_056055.1	140895001	140915001	3.59169	0.226215	OL	CERS6
NC_056060.1	6895001	6915001	3.43679	0.506515	OL	CERT1
NC_056068.1	6350001	6370001	2.87925	0.269168	OL	CFAP300
NC_056074.1	42005001	42025001	3.68554	0.215917	OL	CHKA
NC_056074.1	42010001	42030001	3.87668	0.224082	OL	CHKA
NC_056074.1	42015001	42035001	3.73181	0.245937	OL	CHKA
NC_056074.1	42020001	42040001	3.33172	0.236915	OL	CHKA
NC_056078.1	11980001	12000001	28.4636	0.274541	OL	CHRM3
NC_056058.1	21930001	21950001	2.40795	0.245309	OL	CHSY3
NC_056058.1	21935001	21955001	2.42532	0.237244	OL	CHSY3
NC_056075.1	20860001	20880001	3.12107	0.368325	OL	CHUK;ERLIN1
NC_056077.1	1160001	1180001	10.5217	0.311925	OL	CIAO3;HAGHL
NC_056077.1	1165001	1185001	9.21256	0.320186	OL	CIAO3;MSLN
NC_056080.1	55435001	55455001	3.73809	0.228921	OL	CLCN5
NC_056068.1	24030001	24050001	3.94708	0.258473	OL	CLDN25;USP28
NC_056068.1	24035001	24055001	8.4385	0.27898	OL	CLDN25;USP28
NC_056068.1	24025001	24045001	3.2595	0.242462	OL	CLDN25;USP28;ZW10
NC_056068.1	24020001	24040001	2.54068	0.267684	OL	CLDN25;ZW10
NC_056077.1	10250001	10270001	2.9744	0.276738	OL	CLEC16A
NC_056077.1	10255001	10275001	3.32892	0.344058	OL	CLEC16A
NC_056056.1	206080001	206100001	2.70101	0.359779	OL	CLEC2D;LOC101122428
NC_056056.1	206085001	206105001	3.4384	0.377856	OL	CLEC2D;LOC101122428
NC_056056.1	206090001	206110001	4.2795	0.333558	OL	CLEC2D;LOC101122428
NC_056056.1	207220001	207240001	3.2756	0.228954	OL	CLEC4A
NC_056080.1	81820001	81840001	3.19424	0.259197	OL	CLIC2
NC_056080.1	81825001	81845001	2.87129	0.360215	OL	CLIC2
NC_056077.1	33500001	33520001	6.5491	0.604458	OL	CLIP2
NC_056077.1	33535001	33555001	6.80868	0.588921	OL	CLIP2
NC_056077.1	33540001	33560001	4.3488	0.563588	OL	CLIP2
NC_056077.1	33545001	33565001	3.50057	0.571178	OL	CLIP2
NC_056077.1	33550001	33570001	2.36745	0.535849	OL	CLIP2
NC_056063.1	75990001	76010001	2.5523	0.456746	OL	CLYBL
NC_056063.1	75995001	76015001	2.7306	0.511189	OL	CLYBL
NC_056060.1	10625001	10645001	2.46097	0.290547	OL	CMYA5;TENT2
NC_056067.1	25440001	25460001	2.89686	0.358407	OL	CNGB1
NC_056067.1	25445001	25465001	3.86842	0.569143	OL	CNGB1
NC_056067.1	25450001	25470001	2.79934	0.582627	OL	CNGB1
NC_056058.1	63815001	63835001	3.27614	0.212502	OL	CNOT8;FAXDC2
NC_056058.1	63820001	63840001	3.93158	0.239758	OL	CNOT8;FAXDC2
NC_056058.1	63825001	63845001	3.58196	0.261419	OL	CNOT8;FAXDC2

NC_056058.1	63830001	63850001	4.8087	0.249553	OL	CNOT8;GEMIN5
NC_056058.1	63835001	63855001	5.98727	0.229205	OL	CNOT8;GEMIN5
NC_056058.1	63840001	63860001	6.04348	0.21828	OL	CNOT8;GEMIN5
NC_056068.1	8715001	8735001	16.0448	0.230049	OL	CNTN5
NC_056068.1	9800001	9820001	3.27299	0.289707	OL	CNTN5
NC_056068.1	9805001	9825001	2.76987	0.269732	OL	CNTN5
NC_056067.1	3035001	3055001	2.77718	0.22762	OL	CNTNAP4
NC_056067.1	3040001	3060001	2.43643	0.255127	OL	CNTNAP4
NC_056067.1	3045001	3065001	2.3414	0.254394	OL	CNTNAP4
NC_056055.1	189690001	189710001	2.57965	0.306947	OL	CNTNAP5
NC_056055.1	189695001	189715001	5.09975	0.338114	OL	CNTNAP5
NC_056055.1	189700001	189720001	5.43589	0.310033	OL	CNTNAP5
NC_056055.1	189705001	189725001	2.5777	0.212639	OL	CNTNAP5
NC_056057.1	50035001	50055001	2.39648	0.23466	OL	COG5
NC_056057.1	50040001	50060001	2.43401	0.252529	OL	COG5
NC_056057.1	50045001	50065001	2.56973	0.217776	OL	COG5
NC_056063.1	22995001	23015001	2.72263	0.230568	OL	COG6
NC_056063.1	23000001	23020001	3.87339	0.277437	OL	COG6
NC_056062.1	4250001	4270001	3.11201	0.264534	OL	COL19A1
NC_056062.1	4270001	4290001	4.71337	0.22435	OL	COL19A1
NC_056062.1	4275001	4295001	6.65569	0.237064	OL	COL19A1
NC_056062.1	4295001	4315001	7.00632	0.258201	OL	COL19A1
NC_056062.1	17240001	17260001	6.78611	0.214988	OL	COL22A1
NC_056062.1	17245001	17265001	9.70351	0.377656	OL	COL22A1
NC_056058.1	37325001	37345001	2.41644	0.217077	OL	COL23A1
NC_056077.1	35635001	35655001	2.41786	0.242063	OL	COL26A1
NC_056077.1	35640001	35660001	3.92807	0.30411	OL	COL26A1
NC_056077.1	35645001	35665001	3.1839	0.277793	OL	COL26A1
NC_056077.1	35650001	35670001	3.46845	0.287444	OL	COL26A1
NC_056055.1	120365001	120385001	2.86868	0.217741	OL	COL3A1
NC_056055.1	120370001	120390001	6.91275	0.269664	OL	COL3A1
NC_056055.1	120375001	120395001	18.1241	0.290704	OL	COL3A1
NC_056055.1	120380001	120400001	19.8672	0.287521	OL	COL3A1
NC_056055.1	120385001	120405001	19.8462	0.27736	OL	COL3A1
NC_056054.1	273020001	273040001	2.67881	0.287313	OL	COL6A5
NC_056058.1	35355001	35375001	2.54944	0.213367	OL	COMMD10
NC_056058.1	35400001	35420001	3.03378	0.238876	OL	COMMD10
NC_056058.1	35405001	35425001	4.34345	0.294722	OL	COMMD10
NC_056058.1	35410001	35430001	2.67299	0.249056	OL	COMMD10
NC_056071.1	54605001	54625001	4.73172	0.365217	OL	CPSF2;NDUFB1
NC_056071.1	54610001	54630001	4.26574	0.356106	OL	CPSF2;NDUFB1
NC_056071.1	54615001	54635001	3.04216	0.358422	OL	CPSF2;NDUFB1
NC_056057.1	68565001	68585001	3.26259	0.334617	OL	CPVL
NC_056057.1	68570001	68590001	5.43005	0.441499	OL	CPVL
NC_056057.1	68575001	68595001	17.1839	0.497422	OL	CPVL
NC_056057.1	68625001	68645001	3.79747	0.383303	OL	CPVL
NC_056057.1	68630001	68650001	4.80445	0.359898	OL	CPVL
NC_056057.1	68635001	68655001	6.44446	0.338807	OL	CPVL
NC_056057.1	68640001	68660001	6.66147	0.338432	OL	CPVL
NC_056057.1	68645001	68665001	28.7599	0.368878	OL	CPVL
NC_056057.1	68655001	68675001	11.4658	0.344416	OL	CPVL
NC_056057.1	68660001	68680001	6.3074	0.321869	OL	CPVL
NC_056056.1	130150001	130170001	2.35726	0.263763	OL	CRADD
NC_056056.1	130155001	130175001	2.35284	0.247053	OL	CRADD
NC_056069.1	4965001	4985001	3.55985	0.371161	OL	CREBRF
NC_056069.1	4970001	4990001	6.81527	0.384725	OL	CREBRF
NC_056069.1	4975001	4995001	4.32307	0.398214	OL	CREBRF
NC_056069.1	4980001	5000001	2.8911	0.390162	OL	CREBRF
NC_056068.1	74800001	74820001	3.18786	0.292425	OL	CRY2
NC_056068.1	74805001	74825001	7.0135	0.331775	OL	CRY2
NC_056068.1	74810001	74830001	7.60433	0.324339	OL	CRY2
NC_056056.1	180970001	180990001	11.2973	0.214017	OL	CSF2RB
NC_056079.1	2485001	2505001	11.2022	0.221117	OL	CSMD1
NC_056079.1	3880001	3900001	2.58316	0.242937	OL	CSMD1
NC_056079.1	3885001	3905001	2.41827	0.326936	OL	CSMD1
NC_056059.1	86195001	86215001	3.6721	0.215794	OL	CSN2
NC_056059.1	86200001	86220001	7.33694	0.280393	OL	CSN2
NC_056059.1	86205001	86225001	7.21644	0.257515	OL	CSN2
NC_056076.1	850001	870001	2.49939	0.234764	OL	CTDP1
NC_056056.1	51885001	51905001	3.41472	0.271362	OL	CTNNA2
NC_056056.1	51890001	51910001	4.53718	0.327645	OL	CTNNA2
NC_056056.1	51895001	51915001	2.3726	0.312949	OL	CTNNA2
NC_056078.1	21665001	21685001	2.44737	0.258918	OL	CTNNA3
NC_056078.1	21685001	21705001	4.31396	0.328351	OL	CTNNA3

NC_056078.1	21690001	21710001	3.50944	0.303704	OL	CTNNA3
NC_056078.1	21695001	21715001	3.27791	0.296028	OL	CTNNA3
NC_056078.1	21700001	21720001	2.61131	0.30019	OL	CTNNA3
NC_056069.1	61520001	61540001	6.71589	0.245435	OL	CTNND2
NC_056069.1	61905001	61925001	2.33222	0.253383	OL	CTNND2
NC_056054.1	15530001	15550001	4.07628	0.370905	OL	CTPS1;SCMH1;SLFNL1
NC_056071.1	22960001	22980001	2.6989	0.221076	OL	CTSH;MORF4L1
NC_056057.1	52085001	52105001	2.81955	0.235982	OL	CTTNBP2
NC_056057.1	52090001	52110001	2.50117	0.269056	OL	CTTNBP2
NC_056066.1	30760001	30780001	4.72727	0.220453	OL	CUBN
NC_056055.1	130080001	130100001	3.29522	0.280787	OL	CWC22
NC_056055.1	130085001	130105001	2.98512	0.293602	OL	CWC22
NC_056055.1	130090001	130110001	2.46798	0.265212	OL	CWC22
NC_056068.1	15825001	15845001	3.19064	0.243554	OL	CWF19L2
NC_056068.1	15830001	15850001	3.83516	0.255646	OL	CWF19L2
NC_056068.1	15835001	15855001	3.79707	0.257669	OL	CWF19L2
NC_056068.1	15840001	15860001	2.75801	0.22144	OL	CWF19L2
NC_056068.1	15700001	15720001	2.81151	0.252453	OL	CWF19L2;LOC114118382
NC_056068.1	15705001	15725001	2.52921	0.303752	OL	CWF19L2;LOC114118382
NC_056068.1	15710001	15730001	2.48525	0.261827	OL	CWF19L2;LOC114118382
NC_056078.1	43890001	43910001	3.6403	0.215376	OL	CXCL12
NC_056078.1	43895001	43915001	4.97519	0.256098	OL	CXCL12
NC_056078.1	43900001	43920001	6.53465	0.266743	OL	CXCL12
NC_056078.1	43905001	43925001	4.84596	0.299146	OL	CXCL12
NC_056078.1	43910001	43930001	4.34472	0.297295	OL	CXCL12
NC_056078.1	43915001	43935001	3.95851	0.287599	OL	CXCL12
NC_056078.1	43920001	43940001	3.13862	0.278166	OL	CXCL12
NC_056058.1	66590001	66610001	2.52901	0.306731	OL	CYFIP2;FNDC9
NC_056058.1	66595001	66615001	2.82097	0.343717	OL	CYFIP2;FNDC9
NC_056058.1	66600001	66620001	2.38451	0.359985	OL	CYFIP2;FNDC9
NC_056056.1	181325001	181345001	5.0926	0.318264	OL	CYTH4
NC_056063.1	46715001	46735001	2.8833	0.284083	OL	DACH1
NC_056063.1	46720001	46740001	4.90253	0.28033	OL	DACH1
NC_056063.1	46850001	46870001	8.96538	0.273274	OL	DACH1
NC_056063.1	46855001	46875001	21.403	0.327435	OL	DACH1
NC_056063.1	46890001	46910001	4.24782	0.24081	OL	DACH1
NC_056063.1	46895001	46915001	2.37461	0.287402	OL	DACH1
NC_056063.1	46925001	46945001	2.53771	0.312299	OL	DACH1
NC_056063.1	46980001	47000001	2.59305	0.292436	OL	DACH1
NC_056063.1	46985001	47005001	2.62767	0.257674	OL	DACH1
NC_056056.1	2440001	2460001	2.40491	0.272415	OL	DBH
NC_056067.1	14315001	14335001	2.43206	0.372899	OL	DBNDD1;GAS8
NC_056067.1	14305001	14325001	6.66667	0.469199	OL	DBNDD1;GAS8;LOC101113264
NC_056067.1	14310001	14330001	4.12637	0.390756	OL	DBNDD1;GAS8;LOC101113264
NC_056067.1	14290001	14310001	6.0251	0.438866	OL	DBNDD1;LOC101113264
NC_056067.1	14295001	14315001	8.37662	0.466741	OL	DBNDD1;LOC101113264
NC_056067.1	14300001	14320001	8.36735	0.459524	OL	DBNDD1;LOC101113264
NC_056056.1	141550001	141570001	4.1396	0.402853	OL	DBX2
NC_056060.1	79020001	79040001	3.61472	0.249151	OL	DCAF5
NC_056068.1	60905001	60925001	3.68109	0.386284	OL	DCDC1
NC_056068.1	60910001	60930001	2.67722	0.374889	OL	DCDC1
NC_056070.1	7725001	7745001	2.39425	0.248098	OL	DCLK2
NC_056070.1	7730001	7750001	3.15578	0.238596	OL	DCLK2
NC_056066.1	28935001	28955001	8.03226	0.219609	OL	DCLRE1C;MEIG1
NC_056072.1	47735001	47755001	2.49893	0.221502	OL	DCP1A
NC_056072.1	47740001	47760001	2.55866	0.224318	OL	DCP1A
NC_056072.1	47745001	47765001	2.6521	0.240841	OL	DCP1A
NC_056072.1	47750001	47770001	3.01339	0.320471	OL	DCP1A
NC_056072.1	47755001	47775001	4.44889	0.250203	OL	DCP1A
NC_056057.1	6180001	6200001	3.71518	0.2592	OL	DDC
NC_056057.1	6185001	6205001	4.00422	0.301305	OL	DDC
NC_056079.1	32505001	32525001	8.14285	0.27343	OL	DDHD2;NSD3;PLPP5
NC_056054.1	114180001	114200001	2.75338	0.317914	OL	DDR2
NC_056054.1	114185001	114205001	3.10857	0.304491	OL	DDR2
NC_056054.1	114190001	114210001	3.18972	0.298742	OL	DDR2
NC_056074.1	26735001	26755001	2.34181	0.277051	OL	DDX25
NC_056074.1	26740001	26760001	2.38828	0.21836	OL	DDX25
NC_056068.1	29100001	29120001	2.59387	0.362242	OL	DDX6
NC_056068.1	29105001	29125001	3.12811	0.371187	OL	DDX6
NC_056068.1	29110001	29130001	3.0178	0.360666	OL	DDX6
NC_056068.1	29115001	29135001	2.33939	0.266849	OL	DDX6
NC_056056.1	12185001	12205001	6.88244	0.23451	OL	DENND1A
NC_056056.1	12190001	12210001	5.52419	0.257421	OL	DENND1A
NC_056056.1	12195001	12215001	3.74167	0.241523	OL	DENND1A

NC_056062.1	94215001	94235001	2.68066	0.26058	OL	DEPTOR
NC_056062.1	94205001	94225001	4.73541	0.340334	OL	DEPTOR;DSCC1
NC_056062.1	94210001	94230001	3.16239	0.323849	OL	DEPTOR;DSCC1
NC_056056.1	199565001	199585001	4.6047	0.240049	OL	DERA
NC_056068.1	75265001	75285001	3.1272	0.276591	OL	DGKZ
NC_056068.1	75270001	75290001	4.33413	0.312854	OL	DGKZ
NC_056068.1	75275001	75295001	5.07062	0.316273	OL	DGKZ
NC_056068.1	75280001	75300001	4.50157	0.302893	OL	DGKZ
NC_056068.1	75285001	75305001	4.03815	0.293957	OL	DGKZ
NC_056080.1	137580001	137600001	4.08333	0.268175	OL	DIAPH2
NC_056063.1	2925001	2945001	2.83559	0.214117	OL	DIAPH3
NC_056078.1	4090001	4110001	2.41168	0.297717	OL	DISC1
NC_056068.1	21995001	22015001	3.05097	0.246885	OL	DIXDC1
NC_056068.1	22000001	22020001	2.52517	0.255847	OL	DIXDC1
NC_056068.1	22005001	22025001	2.428	0.294956	OL	DIXDC1
NC_056079.1	22795001	22815001	4.0493	0.234529	OL	DLC1
NC_056079.1	22800001	22820001	4.12875	0.246385	OL	DLC1
NC_056074.1	10205001	10225001	3.77678	0.220995	OL	DLG2
NC_056074.1	10210001	10230001	7.32114	0.261209	OL	DLG2
NC_056074.1	10215001	10235001	6.63178	0.260015	OL	DLG2
NC_056074.1	10220001	10240001	5.53494	0.252791	OL	DLG2
NC_056074.1	11105001	11125001	3.10985	0.22236	OL	DLG2
NC_056074.1	11110001	11130001	3.36142	0.229641	OL	DLG2
NC_056076.1	38490001	38510001	2.78218	0.265943	OL	DLGAP1
NC_056076.1	38495001	38515001	2.86806	0.237163	OL	DLGAP1
NC_056076.1	38805001	38825001	2.66151	0.241055	OL	DLGAP1
NC_056080.1	31715001	31735001	2.49479	0.240522	OL	DMD
NC_056060.1	10040001	10060001	3.72545	0.352522	OL	DMGDH
NC_056062.1	21675001	21695001	2.33506	0.240658	OL	DNAAF11
NC_056062.1	21670001	21690001	2.39903	0.234379	OL	DNAAF11;TMEM71
NC_056077.1	4385001	4405001	4.48799	0.221947	OL	DNAAF8;ZNF500
NC_056077.1	4390001	4410001	7.0522	0.256052	OL	DNAAF8;ZNF500
NC_056078.1	23535001	23555001	2.3828	0.231128	OL	DNAJC12
NC_056078.1	23540001	23560001	3.76436	0.261902	OL	DNAJC12
NC_056055.1	40245001	40265001	3.26129	0.218462	OL	DOCK5
NC_056055.1	181095001	181115001	2.59417	0.246411	OL	DPP10
NC_056055.1	181100001	181120001	6.34705	0.395482	OL	DPP10
NC_056055.1	181105001	181125001	6.34821	0.406405	OL	DPP10
NC_056055.1	181110001	181130001	3.25167	0.328911	OL	DPP10
NC_056057.1	63595001	63615001	2.46195	0.221168	OL	DPY19L1
NC_056057.1	63600001	63620001	2.84085	0.320398	OL	DPY19L1
NC_056062.1	82440001	82460001	2.36873	0.381041	OL	DPY19L4
NC_056062.1	82445001	82465001	3.149	0.427731	OL	DPY19L4
NC_056062.1	82450001	82470001	4.90453	0.494466	OL	DPY19L4
NC_056062.1	82455001	82475001	9.9634	0.518348	OL	DPY19L4
NC_056062.1	82475001	82495001	23.5324	0.553694	OL	DPY19L4
NC_056062.1	82485001	82505001	26	0.517751	OL	DPY19L4
NC_056062.1	82495001	82515001	11.4647	0.31195	OL	DPY19L4
NC_056062.1	82500001	82520001	3.3908	0.221104	OL	DPY19L4
NC_056058.1	56385001	56405001	2.65652	0.289889	OL	DPYSL3;LOC121819651
NC_056062.1	94195001	94215001	6.34983	0.309285	OL	DSCC1
NC_056062.1	94200001	94220001	8.41164	0.354333	OL	DSCC1
NC_056062.1	94180001	94200001	8.73592	0.285006	OL	DSCC1;TAF2
NC_056062.1	94185001	94205001	8.96994	0.296406	OL	DSCC1;TAF2
NC_056062.1	94190001	94210001	12.2056	0.289953	OL	DSCC1;TAF2
NC_056061.1	21555001	21575001	12.3544	0.231176	OL	DSE
NC_056073.1	3735001	3755001	2.36235	0.258004	OL	DST
NC_056076.1	22585001	22605001	4.35874	0.617659	OL	DTNA
NC_056076.1	22590001	22610001	4.42021	0.613007	OL	DTNA
NC_056068.1	80980001	81000001	3.69529	0.232142	OL	DTX4
NC_056068.1	80985001	81005001	3.1623	0.261286	OL	DTX4
NC_056068.1	80990001	81010001	4.38311	0.250647	OL	DTX4
NC_056068.1	80995001	81015001	5.26795	0.234303	OL	DTX4
NC_056068.1	81000001	81020001	5.23113	0.245258	OL	DTX4;LOC101109907
NC_056068.1	81005001	81025001	5.58041	0.233256	OL	DTX4;LOC101109907
NC_056068.1	81010001	81030001	3.20853	0.277556	OL	DTX4;LOC101109907
NC_056068.1	81015001	81035001	2.88503	0.254566	OL	DTX4;LOC101109907
NC_056068.1	80955001	80975001	3.93025	0.223149	OL	DTX4;LOC121816730
NC_056068.1	80960001	80980001	4.06086	0.228163	OL	DTX4;LOC121816730
NC_056068.1	80965001	80985001	5.34662	0.237176	OL	DTX4;LOC121816730
NC_056068.1	80970001	80990001	5.64484	0.218724	OL	DTX4;LOC121816730
NC_056068.1	80975001	80995001	3.47185	0.288258	OL	DTX4;LOC121816730
NC_056067.1	35045001	35065001	3.36937	0.212766	OL	DUS2
NC_056074.1	46355001	46375001	2.53249	0.283141	OL	DUSP8

NC_056074.1	46365001	46385001	2.47476	0.28685	OL	DUSP8
NC_056056.1	80500001	80520001	3.01379	0.24409	OL	DYNC2LI1;PLEKHH2
NC_056056.1	80505001	80525001	3.20263	0.249609	OL	DYNC2LI1;PLEKHH2
NC_056054.1	191735001	191755001	3.04333	0.287573	OL	DYNLT2B;TM4SF19
NC_056054.1	187190001	187210001	2.44678	0.2257	OL	EAF2;SLC15A2
NC_056055.1	12090001	12110001	2.47786	0.255341	OL	ECPAS
NC_056055.1	12095001	12115001	2.58918	0.276431	OL	ECPAS
NC_056055.1	12100001	12120001	3.54545	0.331397	OL	ECPAS
NC_056055.1	12105001	12125001	2.54366	0.260539	OL	ECPAS
NC_056055.1	12110001	12130001	4.39485	0.387346	OL	ECPAS
NC_056055.1	12115001	12135001	3.57303	0.38689	OL	ECPAS;LOC101112627
NC_056080.1	62580001	62600001	3.05	0.221222	OL	EDA
NC_056080.1	62670001	62690001	6.28282	0.252394	OL	EDA
NC_056080.1	62675001	62695001	9.6768	0.375666	OL	EDA
NC_056080.1	62680001	62700001	13.0887	0.378113	OL	EDA
NC_056080.1	62685001	62705001	15.0894	0.413213	OL	EDA
NC_056080.1	62690001	62710001	12.1146	0.423527	OL	EDA
NC_056080.1	62695001	62715001	15.6204	0.366236	OL	EDA
NC_056080.1	62725001	62745001	9.5366	0.257997	OL	EDA
NC_056080.1	62730001	62750001	2.90425	0.25037	OL	EDA
NC_056065.1	31495001	31515001	2.46859	0.258702	OL	EFCAB2
NC_056065.1	31500001	31520001	2.58342	0.260847	OL	EFCAB2
NC_056065.1	31505001	31525001	2.60996	0.25535	OL	EFCAB2
NC_056065.1	31510001	31530001	2.51764	0.243182	OL	EFCAB2
NC_056065.1	31515001	31535001	2.38524	0.253608	OL	EFCAB2
NW_02459982	550001	570001	5.89116	0.302949	OL	EFL1
NW_02459982	555001	575001	5.27204	0.282962	OL	EFL1
NW_02459982	560001	580001	3.22824	0.235302	OL	EFL1
NW_02459982	615001	635001	2.36364	0.229837	OL	EFL1
NC_056062.1	22210001	22230001	16.2174	0.338823	OL	EFR3A
NC_056062.1	22200001	22220001	12.457	0.238907	OL	EFR3A;OC90
NC_056062.1	22205001	22225001	13.6183	0.295767	OL	EFR3A;OC90
NC_056064.1	44305001	44325001	9.94928	0.245239	OL	EFTUD2;LOC114116839
NC_056056.1	38025001	38045001	12.5322	0.348352	OL	EHD3
NC_056056.1	38035001	38055001	8.01144	0.350727	OL	EHD3
NC_056056.1	38040001	38060001	4.6345	0.275386	OL	EHD3
NC_056062.1	60720001	60740001	4.17231	0.216352	OL	EIF3H
NC_056062.1	60725001	60745001	3.09681	0.228922	OL	EIF3H
NC_056062.1	60730001	60750001	2.75283	0.258748	OL	EIF3H
NC_056072.1	29935001	29955001	6.03806	0.273253	OL	EIF4E3;GPR27
NC_056072.1	29940001	29960001	6.36017	0.244048	OL	EIF4E3;GPR27
NC_056072.1	29930001	29950001	4.4273	0.235018	OL	EIF4E3;GPR27;PROK2
NC_056077.1	41695001	41715001	3.12716	0.217489	OL	ELFN1
NC_056077.1	41625001	41645001	2.4278	0.241588	OL	ELFN1;MAD1L1
NC_056056.1	166070001	166090001	5.38842	0.238365	OL	ELK3
NC_056056.1	166080001	166100001	3.42406	0.214914	OL	ELK3
NC_056057.1	61580001	61600001	7.74225	0.21771	OL	ELMO1
NC_056057.1	61600001	61620001	8.43993	0.253462	OL	ELMO1
NC_056060.1	98475001	98495001	7.26241	0.249072	OL	EML5
NC_056060.1	98480001	98500001	13.6154	0.271889	OL	EML5
NC_056060.1	98485001	98505001	9.45276	0.260041	OL	EML5
NC_056060.1	98490001	98510001	7.45834	0.250229	OL	EML5
NC_056060.1	98495001	98515001	5.91091	0.230696	OL	EML5
NC_056062.1	93955001	93975001	6.06926	0.400943	OL	ENPP2
NC_056062.1	93980001	94000001	6.68447	0.354958	OL	ENPP2
NC_056062.1	93985001	94005001	5.68309	0.307246	OL	ENPP2
NC_056062.1	93990001	94010001	3.26721	0.285873	OL	ENPP2
NC_056073.1	19335001	19355001	6.66788	0.243529	OL	ENPP4;ENPP5
NC_056073.1	19340001	19360001	4.32741	0.220863	OL	ENPP4;ENPP5
NC_056073.1	19345001	19365001	3.39386	0.260497	OL	ENPP4;ENPP5
NC_056056.1	78020001	78040001	2.48299	0.225288	OL	EPAS1
NC_056056.1	78030001	78050001	3.48293	0.242292	OL	EPAS1
NC_056054.1	158330001	158350001	4.57	0.299296	OL	EPHA3
NC_056054.1	158335001	158355001	2.73218	0.24031	OL	EPHA3
NC_056054.1	162575001	162595001	2.88969	0.354628	OL	EPHA6
NC_056054.1	162580001	162600001	3.22286	0.393635	OL	EPHA6
NC_056054.1	162585001	162605001	4.58964	0.447333	OL	EPHA6
NC_056054.1	162590001	162610001	4.68018	0.445122	OL	EPHA6
NC_056054.1	162595001	162615001	2.4313	0.399833	OL	EPHA6
NC_056056.1	199850001	199870001	4.28438	0.280786	OL	EPS8
NC_056056.1	199855001	199875001	6.7739	0.305397	OL	EPS8
NC_056056.1	199860001	199880001	4.7871	0.300266	OL	EPS8
NC_056056.1	199865001	199885001	3.87392	0.303569	OL	EPS8
NC_056056.1	199870001	199890001	4.14576	0.221278	OL	EPS8

NC_056056.1	199875001	199895001	3.7877	0.215817	OL	EPS8
NC_056056.1	199880001	199900001	3.96153	0.214363	OL	EPS8
NC_056056.1	199885001	199905001	4.02926	0.212353	OL	EPS8
NC_056056.1	199920001	199940001	3.7205	0.214673	OL	EPS8
NC_056056.1	199925001	199945001	6.26511	0.24481	OL	EPS8
NC_056056.1	199930001	199950001	4.37291	0.231865	OL	EPS8
NC_056055.1	214045001	214065001	3.38989	0.297161	OL	ERBB4
NC_056055.1	214050001	214070001	5.76682	0.359507	OL	ERBB4
NC_056055.1	214055001	214075001	8.91576	0.419089	OL	ERBB4
NC_056055.1	214060001	214080001	22.4275	0.455662	OL	ERBB4
NC_056055.1	214065001	214085001	20.5139	0.466605	OL	ERBB4
NC_056055.1	214070001	214090001	18.7863	0.466006	OL	ERBB4
NC_056055.1	214075001	214095001	13.6607	0.48674	OL	ERBB4
NC_056055.1	214080001	214100001	4.98208	0.508915	OL	ERBB4
NC_056055.1	214085001	214105001	2.60548	0.521471	OL	ERBB4
NC_056055.1	214285001	214305001	2.34393	0.22345	OL	ERBB4
NC_056055.1	214290001	214310001	2.4458	0.235983	OL	ERBB4
NC_056055.1	214295001	214315001	2.43136	0.230128	OL	ERBB4
NC_056056.1	213710001	213730001	4.514	0.218215	OL	ERC1
NC_056056.1	213715001	213735001	5.34069	0.237694	OL	ERC1
NC_056056.1	213870001	213890001	2.48474	0.356181	OL	ERC1
NC_056056.1	213875001	213895001	8.41026	0.323385	OL	ERC1
NC_056072.1	44985001	45005001	2.37871	0.400089	OL	ERC2
NC_056072.1	44990001	45010001	3.55091	0.449877	OL	ERC2
NC_056072.1	44995001	45015001	5.30995	0.46079	OL	ERC2
NC_056072.1	45000001	45020001	3.4112	0.314184	OL	ERC2
NC_056072.1	45115001	45135001	2.66571	0.28391	OL	ERC2
NC_056072.1	45120001	45140001	2.56463	0.255044	OL	ERC2
NC_056054.1	271450001	271470001	4.30116	0.222623	OL	ERG
NC_056054.1	271530001	271550001	3.15034	0.232966	OL	ERG
NC_056054.1	271535001	271555001	3.38179	0.248179	OL	ERG
NC_056054.1	271540001	271560001	3.4115	0.224559	OL	ERG
NC_056075.1	20835001	20855001	2.38953	0.289442	OL	ERLIN1
NC_056075.1	20840001	20860001	2.82754	0.324403	OL	ERLIN1
NC_056075.1	20845001	20865001	3.09673	0.344357	OL	ERLIN1
NC_056075.1	20850001	20870001	3.70391	0.377997	OL	ERLIN1
NC_056075.1	20855001	20875001	3.56787	0.383365	OL	ERLIN1
NC_056060.1	11475001	11495001	8.01544	0.271129	OL	ERO1A
NC_056060.1	11480001	11500001	7.10429	0.318946	OL	ERO1A
NC_056060.1	11485001	11505001	3.69014	0.332386	OL	ERO1A
NC_056054.1	3155001	3175001	5.38826	0.263129	OL	ESPNL;KLHL30
NC_056054.1	3160001	3180001	6.9426	0.263912	OL	ESPNL;KLHL30
NC_056054.1	3165001	3185001	9.85417	0.275082	OL	ESPNL;KLHL30
NC_056074.1	29260001	29280001	2.96706	0.22252	OL	ETS1
NC_056074.1	29265001	29285001	3.17204	0.232686	OL	ETS1
NC_056074.1	29270001	29290001	3.51408	0.279056	OL	ETS1
NC_056071.1	65400001	65420001	3.60693	0.241439	OL	EXOC3L4
NC_056071.1	65390001	65410001	3.15239	0.240707	OL	EXOC3L4;LBHD2
NC_056071.1	65395001	65415001	3.60835	0.245837	OL	EXOC3L4;LBHD2
NC_056071.1	65405001	65425001	5.6343	0.27959	OL	EXOC3L4;LOC105605780
NC_056071.1	65410001	65430001	6.58844	0.281889	OL	EXOC3L4;LOC105605780
NC_056071.1	65415001	65435001	8.57142	0.286697	OL	EXOC3L4;LOC105605780
NC_056057.1	98755001	98775001	2.6336	0.237923	OL	EXOC4
NC_056065.1	41785001	41805001	6.57949	0.450982	OL	EXOSC10
NC_056065.1	41790001	41810001	4.86363	0.407312	OL	EXOSC10
NC_056065.1	41795001	41815001	2.93011	0.327081	OL	EXOSC10
NC_056065.1	41800001	41820001	3.09729	0.306801	OL	EXOSC10
NC_056065.1	41805001	41825001	2.52691	0.266952	OL	EXOSC10
NC_056065.1	41780001	41800001	8.36667	0.468363	OL	EXOSC10;MTOR
NC_056059.1	36620001	36640001	3.0658	0.324443	OL	FAM13A
NC_056059.1	36625001	36645001	6.64669	0.356125	OL	FAM13A
NC_056059.1	36660001	36680001	13.5196	0.342584	OL	FAM13A
NC_056059.1	36665001	36685001	6.39478	0.306874	OL	FAM13A
NC_056059.1	37970001	37990001	10.0563	0.265129	OL	FAM184B
NC_056071.1	25970001	25990001	2.39663	0.397061	OL	FAM189A1;NSMCE3
NC_056057.1	73630001	73650001	2.48326	0.214214	OL	FAM221A;STK31
NC_056057.1	73635001	73655001	2.36629	0.266824	OL	FAM221A;STK31
NC_056054.1	262285001	262305001	3.20194	0.25063	OL	FAM3B;MX2
NC_056058.1	104730001	104750001	2.59478	0.223668	OL	FBXL17
NC_056058.1	104735001	104755001	2.87393	0.215254	OL	FBXL17
NC_056058.1	104740001	104760001	3.77063	0.221583	OL	FBXL17
NC_056062.1	77160001	77180001	4.51111	0.37784	OL	FBXO43
NC_056062.1	77165001	77185001	4.47883	0.417406	OL	FBXO43
NC_056062.1	77170001	77190001	2.46928	0.327305	OL	FBXO43

NC_056055.1	2760001	2780001	13.3464	0.61342	OL	FBXW2
NC_056055.1	2765001	2785001	10.3084	0.586448	OL	FBXW2
NC_056055.1	2770001	2790001	5.6279	0.563007	OL	FBXW2
NC_056055.1	2775001	2795001	4.39502	0.474301	OL	FBXW2
NC_056055.1	2780001	2800001	3.00337	0.326844	OL	FBXW2
NC_056055.1	2785001	2805001	2.43196	0.270149	OL	FBXW2
NC_056070.1	5390001	5410001	3.1481	0.247366	OL	FBXW7
NC_056070.1	5395001	5415001	5.70952	0.294091	OL	FBXW7
NC_056070.1	5400001	5420001	6.98677	0.302729	OL	FBXW7
NC_056070.1	5405001	5425001	11.7946	0.302658	OL	FBXW7
NC_056070.1	5410001	5430001	4.34932	0.28977	OL	FBXW7
NC_056054.1	112965001	112985001	7.34693	0.224237	OL	FCGR3A
NC_056058.1	105355001	105375001	2.84787	0.322889	OL	FER
NC_056058.1	105360001	105380001	4.02466	0.338465	OL	FER
NC_056058.1	105365001	105385001	4.07587	0.25785	OL	FER
NC_056062.1	28915001	28935001	2.69697	0.232576	OL	FER1L6
NC_056063.1	77695001	77715001	7.58349	0.238187	OL	FGF14
NC_056072.1	40960001	40980001	3.33099	0.226871	OL	FHIT
NC_056073.1	27400001	27420001	3.02352	0.21557	OL	FLOT1;IER3;MDC1;TUBB
NC_056073.1	27405001	27425001	4.28142	0.215573	OL	FLOT1;MDC1;TUBB
NC_056073.1	27410001	27430001	5.8155	0.235128	OL	FLOT1;MDC1;TUBB
NC_056055.1	217355001	217375001	7.45902	0.217639	OL	FN1
NC_056055.1	217360001	217380001	3.0638	0.222394	OL	FN1
NC_056055.1	217365001	217385001	2.41685	0.242812	OL	FN1
NC_056058.1	20465001	20485001	12.0533	0.3695	OL	FNIP1;MEIKIN
NC_056058.1	20470001	20490001	4.41327	0.34021	OL	FNIP1;MEIKIN
NC_056058.1	20475001	20495001	2.98675	0.312488	OL	FNIP1;MEIKIN
NC_056058.1	20480001	20500001	2.41205	0.292114	OL	FNIP1;MEIKIN
NC_056055.1	89350001	89370001	2.5445	0.356708	OL	FOCAD
NC_056056.1	76090001	76110001	6.82715	0.25448	OL	FOXN2
NC_056056.1	76095001	76115001	4.9564	0.247778	OL	FOXN2
NC_056056.1	76100001	76120001	2.6731	0.232286	OL	FOXN2
NC_056060.1	99095001	99115001	4.06177	0.225231	OL	FOXN3
NC_056063.1	22185001	22205001	5.89646	0.235589	OL	FOXO1
NC_056063.1	22190001	22210001	23.325	0.290977	OL	FOXO1
NC_056063.1	22195001	22215001	12.3182	0.281241	OL	FOXO1
NC_056063.1	22200001	22220001	4.08795	0.224691	OL	FOXO1
NC_056057.1	55480001	55500001	4.05499	0.241391	OL	FOXP2
NC_056057.1	55485001	55505001	6.74611	0.316196	OL	FOXP2
NC_056057.1	55510001	55530001	34.4	0.225947	OL	FOXP2
NC_056057.1	55515001	55535001	35.8809	0.256911	OL	FOXP2
NC_056057.1	55520001	55540001	10.6923	0.278269	OL	FOXP2
NC_056057.1	55525001	55545001	8.41562	0.271351	OL	FOXP2
NC_056057.1	55530001	55550001	4.52614	0.216206	OL	FOXP2
NC_056057.1	55750001	55770001	2.51072	0.260517	OL	FOXP2
NC_056066.1	27700001	27720001	2.86036	0.338919	OL	FRMD4A
NC_056066.1	27705001	27725001	4.90507	0.399821	OL	FRMD4A
NC_056066.1	27710001	27730001	5.81468	0.409086	OL	FRMD4A
NC_056066.1	27740001	27760001	18.3221	0.414852	OL	FRMD4A
NC_056066.1	27745001	27765001	4.52579	0.3395	OL	FRMD4A
NC_056066.1	27780001	27800001	12.4845	0.28764	OL	FRMD4A
NC_056066.1	27785001	27805001	23.8033	0.28614	OL	FRMD4A
NC_056066.1	27790001	27810001	8.16259	0.240981	OL	FRMD4A
NC_056063.1	29215001	29235001	2.48076	0.236432	OL	FRY
NC_056072.1	53285001	53305001	2.62953	0.240376	OL	FYCO1;XCR1
NC_056072.1	53290001	53310001	3.30672	0.311111	OL	FYCO1;XCR1
NC_056072.1	53295001	53315001	7.9	0.361477	OL	FYCO1;XCR1
NC_056072.1	53300001	53320001	3.7704	0.288614	OL	FYCO1;XCR1
NC_056054.1	131255001	131275001	3.47223	0.2337	OL	GABPA
NC_056054.1	131260001	131280001	6.75031	0.308338	OL	GABPA
NC_056054.1	131265001	131285001	11.7506	0.300058	OL	GABPA
NC_056054.1	131270001	131290001	21.201	0.288239	OL	GABPA
NC_056055.1	107540001	107560001	16.5408	0.22702	OL	GALNT7
NC_056076.1	34595001	34615001	2.65841	0.254597	OL	GATA6
NC_056056.1	34540001	34560001	3.86321	0.288204	OL	GCKR
NC_056056.1	34545001	34565001	4.9524	0.298274	OL	GCKR
NC_056056.1	34550001	34570001	9.54288	0.299904	OL	GCKR
NC_056067.1	7260001	7280001	4.12761	0.217379	OL	GCSH;PKD1L2
NC_056067.1	7265001	7285001	5.71959	0.228758	OL	GCSH;PKD1L2
NC_056054.1	95265001	95285001	2.72187	0.354086	OL	GDAP2
NC_056058.1	63845001	63865001	5.9932	0.224038	OL	GEMIN5
NC_056058.1	63850001	63870001	4.36475	0.258632	OL	GEMIN5
NC_056058.1	63855001	63875001	2.79444	0.315071	OL	GEMIN5
NC_056067.1	34790001	34810001	2.63975	0.277752	OL	GFOD2

NC_056067.1	34795001	34815001	3.11428	0.253298	OL	GFOD2
NC_056067.1	34800001	34820001	3.1156	0.299741	OL	GFOD2
NC_056067.1	34805001	34825001	3.25325	0.28857	OL	GFOD2
NC_056057.1	80435001	80455001	2.96761	0.27495	OL	GLI3
NC_056057.1	80465001	80485001	6.79295	0.288094	OL	GLI3
NC_056057.1	80470001	80490001	3.23321	0.398399	OL	GLI3
NC_056057.1	80475001	80495001	3.57516	0.333577	OL	GLI3
NC_056057.1	80480001	80500001	2.46835	0.32611	OL	GLI3
NC_056057.1	80685001	80705001	2.9839	0.332963	OL	GLI3
NC_056057.1	80690001	80710001	3.32397	0.238376	OL	GLI3
NC_056057.1	80695001	80715001	2.98279	0.223104	OL	GLI3
NC_056065.1	62900001	62920001	16.6776	0.216303	OL	GLUL
NC_056068.1	80870001	80890001	6.12433	0.218945	OL	GLYATL2
NC_056077.1	4440001	4460001	3.26373	0.350054	OL	GLYR1;ROGDI
NC_056077.1	4445001	4465001	3.17352	0.33902	OL	GLYR1;ROGDI
NC_056077.1	4450001	4470001	2.69125	0.321422	OL	GLYR1;ROGDI
NC_056077.1	4475001	4495001	3.66374	0.37324	OL	GLYR1;UBN1
NC_056077.1	4480001	4500001	4.53512	0.408066	OL	GLYR1;UBN1
NC_056077.1	4485001	4505001	4.25244	0.387536	OL	GLYR1;UBN1
NC_056073.1	50585001	50605001	2.89473	0.279762	OL	GMDS
NC_056073.1	50635001	50655001	8.31657	0.364098	OL	GMDS
NC_056073.1	50640001	50660001	10.327	0.381661	OL	GMDS
NC_056073.1	50645001	50665001	13.825	0.390549	OL	GMDS
NC_056073.1	50650001	50670001	17.3875	0.397978	OL	GMDS
NC_056060.1	65075001	65095001	2.35435	0.285971	OL	GMFB
NC_056060.1	65080001	65100001	2.56041	0.306554	OL	GMFB
NC_056054.1	232505001	232525001	10.2367	0.222064	OL	GMPS
NC_056054.1	232510001	232530001	8.92558	0.215186	OL	GMPS
NC_056054.1	232515001	232535001	5.38943	0.220341	OL	GMPS
NC_056054.1	232520001	232540001	5.77098	0.220022	OL	GMPS
NC_056054.1	232525001	232545001	9.24755	0.236859	OL	GMPS
NC_056054.1	232530001	232550001	10.05	0.242134	OL	GMPS
NC_056077.1	1605001	1625001	2.84191	0.271233	OL	GNPTG;UNKL
NC_056077.1	1610001	1630001	2.76232	0.259891	OL	GNPTG;UNKL
NC_056055.1	153185001	153205001	11.2707	0.218743	OL	GPD2
NC_056055.1	153190001	153210001	8.63794	0.223746	OL	GPD2
NC_056080.1	11935001	11955001	4.9722	0.439706	OL	GPM6B
NC_056080.1	11940001	11960001	7.06945	0.445259	OL	GPM6B
NC_056080.1	11945001	11965001	4.02101	0.479446	OL	GPM6B
NC_056080.1	12035001	12055001	2.35198	0.263116	OL	GPM6B
NC_056055.1	135545001	135565001	2.44396	0.363039	OL	GPR155
NC_056055.1	135550001	135570001	3.64718	0.428146	OL	GPR155
NC_056055.1	135555001	135575001	7.132	0.502185	OL	GPR155
NC_056055.1	135560001	135580001	11.5714	0.537059	OL	GPR155
NC_056055.1	135565001	135585001	3.65492	0.400634	OL	GPR155
NC_056076.1	35215001	35235001	3.70079	0.426406	OL	GREB1L
NC_056076.1	35220001	35240001	3.08259	0.398396	OL	GREB1L
NC_056076.1	35205001	35225001	2.51013	0.36249	OL	GREB1L;LOC114110473
NC_056076.1	35210001	35230001	2.75791	0.410951	OL	GREB1L;LOC114110473
NC_056068.1	2075001	2095001	3.53196	0.329643	OL	GRIA4
NC_056068.1	2080001	2100001	34.8687	0.461045	OL	GRIA4
NC_056068.1	2085001	2105001	54.3139	0.4424	OL	GRIA4
NC_056068.1	2090001	2110001	32.9992	0.419382	OL	GRIA4
NC_056068.1	2095001	2115001	26.5744	0.378625	OL	GRIA4
NC_056068.1	2100001	2120001	26.0074	0.343354	OL	GRIA4
NC_056068.1	2105001	2125001	19.695	0.31663	OL	GRIA4
NC_056068.1	2110001	2130001	29.7143	0.23913	OL	GRIA4
NC_056059.1	32110001	32130001	2.6007	0.296308	OL	GRID2
NC_056059.1	32115001	32135001	2.7227	0.303652	OL	GRID2
NC_056059.1	32210001	32230001	3.67154	0.215816	OL	GRID2
NC_056059.1	32225001	32245001	3.45214	0.248898	OL	GRID2
NC_056059.1	32230001	32250001	5.31135	0.246053	OL	GRID2
NC_056059.1	32235001	32255001	4.83831	0.232841	OL	GRID2
NC_056059.1	32240001	32260001	5.12685	0.241169	OL	GRID2
NC_056054.1	127115001	127135001	4.84616	0.213601	OL	GRIK1
NC_056068.1	31035001	31055001	3.04736	0.223764	OL	GRIK4
NC_056068.1	31040001	31060001	6.55526	0.230097	OL	GRIK4
NC_056056.1	152885001	152905001	12.34	0.217141	OL	GRIP1
NC_056074.1	6375001	6395001	2.69746	0.223177	OL	GRM5
NC_056074.1	6380001	6400001	3.02607	0.232691	OL	GRM5
NC_056074.1	6385001	6405001	3.94614	0.298395	OL	GRM5
NC_056074.1	6390001	6410001	2.39466	0.258359	OL	GRM5
NC_056057.1	92455001	92475001	2.36263	0.218657	OL	GRM8
NC_056057.1	92480001	92500001	2.49821	0.512222	OL	GRM8

NC_056057.1	92485001	92505001	3.67321	0.570484	OL	GRM8
NC_056057.1	92490001	92510001	3.63037	0.568083	OL	GRM8
NC_056057.1	92495001	92515001	2.73966	0.51551	OL	GRM8
NC_056057.1	92950001	92970001	2.80737	0.225277	OL	GRM8
NC_056077.1	25855001	25875001	2.39761	0.21803	OL	GSG1L
NC_056077.1	25860001	25880001	2.68181	0.237679	OL	GSG1L
NC_056077.1	26075001	26095001	7.03434	0.291229	OL	GSG1L
NC_056077.1	26080001	26100001	6.9876	0.330884	OL	GSG1L
NC_056077.1	26085001	26105001	5.25887	0.355219	OL	GSG1L
NC_056077.1	26090001	26110001	4.68076	0.368813	OL	GSG1L
NC_056077.1	26095001	26115001	2.75	0.297301	OL	GSG1L
NC_056061.1	26695001	26715001	7.60499	0.216222	OL	GTF3C6
NC_056056.1	200825001	200845001	2.58506	0.277666	OL	GUCY2C
NC_056056.1	200830001	200850001	2.60056	0.282407	OL	GUCY2C
NC_056055.1	120940001	120960001	42.7352	0.259433	OL	GULP1
NC_056055.1	120945001	120965001	19.0272	0.229096	OL	GULP1
NC_056055.1	120950001	120970001	15.0146	0.311864	OL	GULP1
NC_056055.1	120955001	120975001	4.8832	0.220485	OL	GULP1
NC_056055.1	120965001	120985001	4.07424	0.217408	OL	GULP1
NC_056055.1	120975001	120995001	3.62768	0.222033	OL	GULP1
NC_056055.1	120980001	121000001	4.659	0.262806	OL	GULP1
NC_056055.1	120985001	121005001	5.42104	0.275826	OL	GULP1
NC_056055.1	120990001	121010001	7.43207	0.265867	OL	GULP1
NC_056055.1	120995001	121015001	14.0529	0.334304	OL	GULP1
NC_056055.1	121000001	121020001	5.22405	0.222341	OL	GULP1
NC_056055.1	121030001	121050001	8.35895	0.213245	OL	GULP1
NC_056055.1	121035001	121055001	6.41351	0.224817	OL	GULP1
NC_056055.1	121040001	121060001	5.72478	0.281435	OL	GULP1
NC_056055.1	121045001	121065001	3.10197	0.366205	OL	GULP1
NC_056055.1	121050001	121070001	2.88763	0.367404	OL	GULP1
NC_056055.1	121055001	121075001	2.85864	0.343209	OL	GULP1
NC_056067.1	45280001	45300001	3.18182	0.364954	OL	HAMP;MAG
NC_056067.1	45275001	45295001	3.5079	0.354119	OL	HAMP;MAG;USF2
NC_056057.1	27615001	27635001	11.5534	0.278395	OL	HDAC9
NC_056057.1	27620001	27640001	5.67337	0.25169	OL	HDAC9
NC_056057.1	79200001	79220001	3.99344	0.305029	OL	HECW1
NC_056057.1	79205001	79225001	5.39663	0.441898	OL	HECW1
NC_056057.1	79210001	79230001	5.18672	0.502176	OL	HECW1
NC_056059.1	98900001	98920001	2.74303	0.296068	OL	HELQ
NC_056064.1	62350001	62370001	2.90391	0.223439	OL	HELZ
NC_056064.1	62355001	62375001	3.24826	0.266687	OL	HELZ
NC_056064.1	62360001	62380001	3.18556	0.277352	OL	HELZ
NC_056064.1	62365001	62385001	3.1981	0.276492	OL	HELZ
NC_056064.1	62370001	62390001	2.74339	0.269252	OL	HELZ
NC_056064.1	62375001	62395001	2.85131	0.267219	OL	HELZ
NC_056064.1	62380001	62400001	2.66878	0.270159	OL	HELZ
NC_056064.1	62385001	62405001	2.43788	0.26113	OL	HELZ
NC_056064.1	62390001	62410001	2.66667	0.262561	OL	HELZ
NC_056064.1	62395001	62415001	2.64878	0.25041	OL	HELZ
NC_056064.1	62400001	62420001	2.64067	0.227715	OL	HELZ
NC_056064.1	62405001	62425001	2.90209	0.251433	OL	HELZ
NC_056064.1	62410001	62430001	2.4725	0.251165	OL	HELZ
NC_056064.1	62415001	62435001	2.50605	0.265498	OL	HELZ
NC_056064.1	62420001	62440001	2.58193	0.275411	OL	HELZ
NC_056064.1	62425001	62445001	2.6779	0.263624	OL	HELZ;LOC106991442
NC_056064.1	62430001	62450001	2.73518	0.26329	OL	HELZ;LOC106991442
NC_056064.1	62435001	62455001	2.33825	0.237347	OL	HELZ;LOC106991442
NC_056065.1	49645001	49665001	2.5129	0.221565	OL	HES5;PANK4
NC_056065.1	49650001	49670001	2.46823	0.215438	OL	HES5;PANK4
NC_056057.1	70105001	70125001	2.43333	0.236666	OL	HIBADH
NC_056060.1	10400001	10420001	2.34223	0.247549	OL	HOMER1
NC_056060.1	10405001	10425001	2.4613	0.278309	OL	HOMER1
NC_056060.1	10410001	10430001	3.62852	0.274207	OL	HOMER1
NC_056060.1	10415001	10435001	4.00603	0.253066	OL	HOMER1
NC_056060.1	10420001	10440001	4.94213	0.281207	OL	HOMER1
NC_056060.1	10425001	10445001	2.45894	0.258344	OL	HOMER1
NC_056070.1	69155001	69175001	3.65782	0.277226	OL	HORMAD2
NC_056070.1	69160001	69180001	9.18017	0.338125	OL	HORMAD2
NC_056070.1	69165001	69185001	13.973	0.371926	OL	HORMAD2
NC_056070.1	69170001	69190001	4.00587	0.257758	OL	HORMAD2
NC_056064.1	31555001	31575001	3.38224	0.268501	OL	HS3ST3A1
NC_056064.1	31560001	31580001	3.06139	0.296483	OL	HS3ST3A1
NC_056064.1	31565001	31585001	2.67621	0.28725	OL	HS3ST3A1
NC_056064.1	9090001	9110001	6.14648	0.473195	OL	HSF5

NC_056064.1	9095001	9115001	4.34444	0.39805	OL	HSF5
NC_056064.1	9100001	9120001	2.51961	0.295296	OL	HSF5
NC_056064.1	9070001	9090001	2.91072	0.301875	OL	HSF5;RNF43
NC_056064.1	9075001	9095001	5.6012	0.384563	OL	HSF5;RNF43
NC_056064.1	9080001	9100001	14.9714	0.464432	OL	HSF5;RNF43
NC_056075.1	36740001	36760001	2.34904	0.25842	OL	HSPA12A
NC_056075.1	36745001	36765001	3.30209	0.221734	OL	HSPA12A
NC_056054.1	112955001	112975001	10.4909	0.255942	OL	HSPA6
NC_056054.1	112950001	112970001	6.56404	0.247626	OL	HSPA6;LOC101106730
NC_056068.1	24150001	24170001	4.05389	0.336681	OL	HTR3B
NC_056055.1	90165001	90185001	3.74794	0.265338	OL	IFN1
NC_056055.1	90170001	90190001	2.56397	0.273354	OL	IFN1
NC_056055.1	242485001	242505001	7.12783	0.22417	OL	IFNLR1;IL22RA1
NC_056057.1	57485001	57505001	3.15516	0.343933	OL	IFRD1
NC_056057.1	57490001	57510001	4.4601	0.314405	OL	IFRD1
NC_056068.1	28925001	28945001	5.79519	0.324485	OL	IFT46;KMT2A;TMEM25;TTC36
NC_056068.1	28935001	28955001	2.48231	0.232798	OL	IFT46;TMEM25
NC_056068.1	28930001	28950001	4.28859	0.350269	OL	IFT46;TMEM25;TTC36
NC_056060.1	12235001	12255001	4.77004	0.346326	OL	IGDCC3
NC_056060.1	12240001	12260001	3.72693	0.40851	OL	IGDCC3
NC_056060.1	12245001	12265001	4.11054	0.379209	OL	IGDCC3
NC_056060.1	12250001	12270001	2.824	0.328879	OL	IGDCC3
NC_056074.1	23565001	23585001	2.45174	0.283141	OL	IGSF22;PTPN5
NC_056056.1	99530001	99550001	2.36733	0.28072	OL	IL1R1
NC_056056.1	99535001	99555001	2.5721	0.266825	OL	IL1R1
NC_056056.1	99540001	99560001	2.34393	0.273612	OL	IL1R1
NC_056056.1	99545001	99565001	2.44261	0.252309	OL	IL1R1
NC_056056.1	99565001	99585001	2.46608	0.340202	OL	IL1R1
NC_056056.1	99570001	99590001	2.82822	0.301819	OL	IL1R1
NC_056056.1	99575001	99595001	2.82817	0.335467	OL	IL1R1
NC_056056.1	99580001	99600001	4.33426	0.358543	OL	IL1R1
NC_056056.1	99585001	99605001	4.28134	0.331581	OL	IL1R1
NC_056056.1	99590001	99610001	4.25238	0.309038	OL	IL1R1
NC_056056.1	99595001	99615001	4.70841	0.213893	OL	IL1R1
NC_056055.1	242490001	242510001	11.7256	0.228318	OL	IL22RA1
NC_056058.1	9965001	9985001	2.9462	0.225181	OL	IL27RA
NC_056058.1	9955001	9975001	2.52922	0.27249	OL	IL27RA;PALM3
NC_056058.1	9960001	9980001	2.65997	0.247669	OL	IL27RA;PALM3
NC_056058.1	9970001	9990001	2.90542	0.231053	OL	IL27RA;RLN3
NC_056058.1	9975001	9995001	2.77104	0.221561	OL	IL27RA;RLN3
NC_056057.1	58705001	58725001	5.41057	0.281698	OL	IMMP2L
NC_056057.1	58710001	58730001	4.56394	0.266742	OL	IMMP2L
NC_056057.1	58715001	58735001	3.89209	0.262712	OL	IMMP2L
NC_056057.1	58720001	58740001	3.92773	0.262188	OL	IMMP2L
NC_056057.1	58725001	58745001	2.59533	0.230605	OL	IMMP2L
NC_056057.1	58730001	58750001	2.65473	0.251089	OL	IMMP2L
NC_056057.1	58735001	58755001	2.97967	0.270821	OL	IMMP2L
NC_056057.1	58740001	58760001	2.81026	0.257271	OL	IMMP2L
NC_056057.1	58745001	58765001	2.88317	0.274527	OL	IMMP2L
NC_056057.1	58750001	58770001	2.82128	0.280015	OL	IMMP2L
NC_056057.1	58755001	58775001	3.22203	0.223686	OL	IMMP2L
NC_056057.1	59105001	59125001	2.80762	0.35387	OL	IMMP2L
NC_056057.1	59110001	59130001	4.21032	0.419364	OL	IMMP2L
NC_056057.1	59115001	59135001	5.22919	0.350674	OL	IMMP2L
NC_056057.1	59120001	59140001	8.1464	0.338857	OL	IMMP2L
NC_056057.1	59125001	59145001	3.49121	0.285257	OL	IMMP2L
NC_056057.1	59235001	59255001	2.44427	0.218317	OL	IMMP2L
NC_056057.1	59240001	59260001	2.68272	0.217617	OL	IMMP2L
NC_056061.1	2755001	2775001	3.27227	0.355742	OL	IMPG1
NC_056054.1	166630001	166650001	2.36596	0.250237	OL	IMPG2
NC_056077.1	41790001	41810001	4.34574	0.356867	OL	INTS1;MAFK
NC_056077.1	41795001	41815001	3.84379	0.308923	OL	INTS1;MAFK
NC_056077.1	41800001	41820001	3.23237	0.254377	OL	INTS1;MAFK
NC_056056.1	212905001	212925001	10.4059	0.478529	OL	IQSEC3
NC_056056.1	212910001	212930001	2.78912	0.387663	OL	IQSEC3
NC_056080.1	126425001	126445001	2.38198	0.261014	OL	IRS4
NC_056060.1	86835001	86855001	24.0512	0.365054	OL	ISM2;SPTLC2
NC_056055.1	128320001	128340001	2.56	0.239207	OL	ITGA4
NC_056066.1	29515001	29535001	3.19833	0.242805	OL	ITGA8
NC_056080.1	63985001	64005001	3.3077	0.371178	OL	ITGB1BP2;NONO
NC_056056.1	188865001	188885001	5.64487	0.236515	OL	ITPR2
NC_056056.1	188870001	188890001	6.59027	0.230498	OL	ITPR2
NC_056057.1	66440001	66460001	5.03581	0.220945	OL	ITPRID1
NC_056057.1	66445001	66465001	2.97301	0.289189	OL	ITPRID1

NC_056057.1	66460001	66480001	2.44927	0.287196	OL	ITPRID1
NC_056057.1	66465001	66485001	2.66915	0.230555	OL	ITPRID1
NC_056057.1	66470001	66490001	2.63877	0.225648	OL	ITPRID1
NC_056057.1	66475001	66495001	3.0014	0.2246	OL	ITPRID1
NC_056057.1	66480001	66500001	3.09707	0.217241	OL	ITPRID1
NC_056057.1	66490001	66510001	4.49914	0.220196	OL	ITPRID1
NC_056070.1	29180001	29200001	10.6532	0.266234	OL	JADE1;SCLT1
NC_056066.1	3770001	3790001	2.33548	0.288338	OL	JAG1
NC_056066.1	3775001	3795001	2.42947	0.247358	OL	JAG1
NC_056066.1	3780001	3800001	2.67557	0.219567	OL	JAG1
NC_056066.1	3785001	3805001	2.52694	0.235774	OL	JAG1;LOC121816277
NC_056055.1	73335001	73355001	2.86275	0.222665	OL	JAK2
NC_056055.1	73340001	73360001	2.46078	0.306821	OL	JAK2
NC_056055.1	73345001	73365001	3.79503	0.4292	OL	JAK2
NC_056055.1	73350001	73370001	7.28573	0.508053	OL	JAK2
NC_056055.1	73380001	73400001	7.43165	0.617845	OL	JAK2
NC_056055.1	73385001	73405001	3.01204	0.502801	OL	JAK2
NC_056064.1	41350001	41370001	5.03694	0.275947	OL	K38;V15
NC_056064.1	41355001	41375001	3.19647	0.259653	OL	K38;V15
NC_056054.1	189790001	189810001	2.39592	0.290106	OL	KALRN
NC_056054.1	189795001	189815001	2.71222	0.28684	OL	KALRN
NC_056056.1	211075001	211095001	3.33643	0.232872	OL	KCNA1
NC_056056.1	211080001	211100001	3.125	0.259758	OL	KCNA1
NC_056056.1	211085001	211105001	2.67639	0.283655	OL	KCNA1
NC_056056.1	211090001	211110001	2.58486	0.350388	OL	KCNA1
NC_056056.1	211095001	211115001	2.52035	0.336572	OL	KCNA1
NC_056056.1	211100001	211120001	2.66615	0.300318	OL	KCNA1
NC_056059.1	41440001	41460001	4.77062	0.261197	OL	KCNIP4
NC_056059.1	41445001	41465001	13.8225	0.307117	OL	KCNIP4
NC_056059.1	41450001	41470001	15.2543	0.318964	OL	KCNIP4
NC_056059.1	41455001	41475001	8.30987	0.28614	OL	KCNIP4
NC_056059.1	41460001	41480001	3.48766	0.246935	OL	KCNIP4
NC_056059.1	41675001	41695001	4.71968	0.230934	OL	KCNIP4
NC_056060.1	3120001	3140001	3.4691	0.240407	OL	KCNN2
NC_056074.1	45460001	45480001	15.2625	0.568594	OL	KCNQ1
NC_056074.1	45465001	45485001	9.81081	0.458639	OL	KCNQ1
NC_056074.1	45470001	45490001	8.4069	0.417538	OL	KCNQ1
NC_056074.1	45475001	45495001	6.92364	0.317419	OL	KCNQ1
NC_056074.1	45480001	45500001	3.21787	0.213124	OL	KCNQ1
NC_056065.1	6310001	6330001	2.39719	0.212913	OL	KCNT2
NC_056055.1	74795001	74815001	5.06046	0.228625	OL	KDM4C
NC_056055.1	74800001	74820001	4.34884	0.212459	OL	KDM4C
NC_056080.1	44090001	44110001	10.4667	0.308244	OL	KDM6A
NC_056080.1	44095001	44115001	9.50002	0.270833	OL	KDM6A
NC_056080.1	44100001	44120001	8.3179	0.240413	OL	KDM6A
NC_056058.1	91375001	91395001	4.20123	0.241512	OL	KIAA0825
NC_056058.1	91380001	91400001	6.58312	0.257984	OL	KIAA0825
NC_056073.1	38785001	38805001	3.90188	0.238064	OL	KIF13A
NC_056073.1	38790001	38810001	4.90195	0.236109	OL	KIF13A
NC_056073.1	38880001	38900001	2.58977	0.280309	OL	KIF13A
NC_056073.1	38930001	38950001	2.92107	0.256973	OL	KIF13A
NC_056073.1	38935001	38955001	3.40399	0.231556	OL	KIF13A
NC_056073.1	38940001	38960001	5.64164	0.218181	OL	KIF13A
NC_056074.1	27605001	27625001	2.84025	0.231273	OL	KIRREL3
NC_056074.1	27610001	27630001	5.70727	0.225753	OL	KIRREL3
NC_056074.1	27615001	27635001	7.37631	0.251835	OL	KIRREL3
NC_056074.1	27635001	27655001	2.38355	0.224277	OL	KIRREL3
NC_056074.1	27640001	27660001	2.94425	0.34489	OL	KIRREL3
NC_056074.1	27645001	27665001	2.98051	0.379599	OL	KIRREL3
NC_056074.1	27650001	27670001	2.95313	0.366357	OL	KIRREL3
NC_056074.1	27655001	27675001	2.94696	0.369417	OL	KIRREL3
NC_056059.1	70935001	70955001	2.4171	0.249063	OL	KIT
NC_056059.1	70945001	70965001	3.08815	0.268796	OL	KIT
NC_056063.1	49460001	49480001	2.83346	0.2299	OL	KLF12
NC_056063.1	49465001	49485001	2.99413	0.223675	OL	KLF12
NC_056063.1	49470001	49490001	2.90401	0.22016	OL	KLF12
NC_056063.1	44570001	44590001	6.69525	0.307186	OL	KLHL1
NC_056063.1	44575001	44595001	6.9664	0.303363	OL	KLHL1
NC_056063.1	44580001	44600001	7.30951	0.246149	OL	KLHL1
NC_056063.1	44585001	44605001	10.2604	0.234558	OL	KLHL1
NC_056063.1	44700001	44720001	6.13417	0.241151	OL	KLHL1
NC_056063.1	44705001	44725001	11.2129	0.281225	OL	KLHL1
NC_056063.1	44710001	44730001	14.5911	0.299888	OL	KLHL1
NC_056063.1	44715001	44735001	10.6782	0.292957	OL	KLHL1

NC_056063.1	44720001	44740001	6.37637	0.298102	OL	KLHL1
NC_056063.1	44725001	44745001	2.95164	0.264461	OL	KLHL1
NC_056063.1	44755001	44775001	3.66843	0.230578	OL	KLHL1
NC_056063.1	44760001	44780001	6.3699	0.214502	OL	KLHL1
NC_056063.1	44765001	44785001	12.0567	0.221162	OL	KLHL1
NC_056063.1	44770001	44790001	14.4806	0.2228	OL	KLHL1
NC_056063.1	44795001	44815001	23.1434	0.250693	OL	KLHL1
NC_056063.1	44800001	44820001	21.0091	0.270916	OL	KLHL1
NC_056063.1	44805001	44825001	19.9638	0.298895	OL	KLHL1
NC_056063.1	44810001	44830001	26.9104	0.302595	OL	KLHL1
NC_056063.1	44815001	44835001	32.6498	0.298053	OL	KLHL1
NC_056063.1	44820001	44840001	46.5386	0.289956	OL	KLHL1
NC_056063.1	44825001	44845001	49.5	0.270302	OL	KLHL1
NC_056063.1	44830001	44850001	29.5097	0.260939	OL	KLHL1
NC_056063.1	44835001	44855001	18.2374	0.222019	OL	KLHL1
NC_056063.1	44855001	44875001	29.8627	0.215991	OL	KLHL1
NC_056063.1	44860001	44880001	16.1267	0.253359	OL	KLHL1
NC_056063.1	44865001	44885001	17.6118	0.281723	OL	KLHL1
NC_056063.1	44870001	44890001	18.6671	0.320428	OL	KLHL1
NC_056063.1	44875001	44895001	16.5726	0.35706	OL	KLHL1
NC_056063.1	44880001	44900001	14.7146	0.359881	OL	KLHL1
NC_056063.1	44885001	44905001	5.82697	0.278779	OL	KLHL1
NC_056063.1	45005001	45025001	2.36188	0.216761	OL	KLHL1
NC_056063.1	45010001	45030001	2.474	0.271993	OL	KLHL1
NC_056063.1	45045001	45065001	2.91922	0.22883	OL	KLHL1
NC_056063.1	45050001	45070001	3.93088	0.265608	OL	KLHL1
NC_056063.1	45055001	45075001	3.44067	0.293874	OL	KLHL1
NC_056063.1	45060001	45080001	2.78912	0.31603	OL	KLHL1
NC_056063.1	45065001	45085001	2.45984	0.302188	OL	KLHL1
NC_056063.1	45080001	45100001	2.33281	0.28852	OL	KLHL1
NC_056063.1	45085001	45105001	2.51751	0.288475	OL	KLHL1
NC_056063.1	45090001	45110001	2.80466	0.251964	OL	KLHL1
NC_056056.1	31180001	31200001	2.38519	0.234715	OL	KLHL29
NC_056054.1	3150001	3170001	3.06463	0.289407	OL	KLHL30
NC_056059.1	102815001	102835001	3.36	0.215486	OL	KLHL8
NC_056068.1	28840001	28860001	2.72832	0.27935	OL	KMT2A
NC_056068.1	28845001	28865001	5.30207	0.474234	OL	KMT2A
NC_056054.1	201260001	201280001	5.96354	0.341287	OL	KNG1
NC_056054.1	201265001	201285001	4.13008	0.245663	OL	KNG1
NC_056054.1	125770001	125790001	2.40849	0.431084	OL	KRTAP6-1
NC_056054.1	255340001	255360001	3.91876	0.249945	OL	KY
NC_056054.1	255345001	255365001	4.28087	0.312899	OL	KY
NC_056054.1	255350001	255370001	4.57306	0.326409	OL	KY
NC_056054.1	255355001	255375001	5.12467	0.266823	OL	KY
NC_056054.1	255360001	255380001	3.46788	0.221372	OL	KY
NC_056054.1	255365001	255385001	3.56665	0.215182	OL	KY
NC_056059.1	38065001	38085001	11.3	0.666422	OL	LCORL
NC_056059.1	38200001	38220001	4.04489	0.243119	OL	LCORL
NC_056059.1	38205001	38225001	3.31902	0.240467	OL	LCORL
NC_056059.1	38210001	38230001	3.07766	0.222712	OL	LCORL
NC_056059.1	38215001	38235001	2.67961	0.226169	OL	LCORL
NC_056068.1	56940001	56960001	4.29846	0.251503	OL	LGR4
NC_056068.1	56945001	56965001	7.73584	0.277397	OL	LGR4
NC_056056.1	107675001	107695001	2.83186	0.285963	OL	LGR5
NC_056065.1	79680001	79700001	2.88211	0.225847	OL	LGR6;PPP1R12B;UBE2T
NC_056060.1	9680001	9700001	8.64485	0.252097	OL	LHFPL2
NC_056060.1	9685001	9705001	27.1326	0.315538	OL	LHFPL2
NC_056060.1	9705001	9725001	9.87741	0.23616	OL	LHFPL2
NC_056060.1	9715001	9735001	4.84682	0.221465	OL	LHFPL2
NC_056067.1	55350001	55370001	2.53743	0.238344	OL	LIN7B;SNRNP70
NC_056068.1	56950001	56970001	12.523	0.274684	OL	LIN7C
NC_056068.1	56955001	56975001	7.36633	0.262821	OL	LIN7C
NC_056065.1	28540001	28560001	2.45084	0.296672	OL	LIN9
NC_056055.1	97530001	97550001	2.52509	0.223175	OL	LINGO2
NC_056055.1	97690001	97710001	3.64116	0.25185	OL	LINGO2
NC_056068.1	52540001	52560001	3.50448	0.245988	OL	LIPT2
NC_056056.1	189925001	189945001	2.46189	0.217467	OL	LMNTD1
NC_056056.1	198880001	198900001	2.44651	0.405603	OL	LMO3
NC_056064.1	41325001	41345001	2.60948	0.374277	OL	LOC100526781
NC_056064.1	41335001	41355001	2.54243	0.395023	OL	LOC100526781
NC_056064.1	41320001	41340001	3.47111	0.341365	OL	LOC100526781;LOC100526782
NC_056064.1	41340001	41360001	2.81151	0.316773	OL	LOC100526781;V15
NC_056064.1	41305001	41325001	3.57041	0.323583	OL	LOC100526782
NC_056064.1	41310001	41330001	4.1233	0.341274	OL	LOC100526782

NC_056064.1	41315001	41335001	3.65487	0.302686	OL	LOC100526782
NC_056077.1	37210001	37230001	2.50204	0.29484	OL	LOC101102366
NC_056077.1	37220001	37240001	2.42057	0.392094	OL	LOC101102366
NC_056077.1	37225001	37245001	2.8331	0.496758	OL	LOC101102366;ZSCAN25
NC_056077.1	37230001	37250001	3.94261	0.570768	OL	LOC101102366;ZSCAN25
NC_056077.1	37235001	37255001	12.1069	0.569868	OL	LOC101102366;ZSCAN25
NC_056055.1	64825001	64845001	3.32289	0.246327	OL	LOC101102454
NC_056061.1	57960001	57980001	4.37526	0.305026	OL	LOC101102492;LOC101118705
NC_056061.1	57955001	57975001	5.33205	0.343585	OL	LOC101102492;TAAR8
NC_056068.1	44470001	44490001	4.20919	0.212585	OL	LOC101104009
NC_056075.1	43590001	43610001	6.43332	0.408841	OL	LOC101104360
NC_056075.1	43595001	43615001	3.84775	0.364705	OL	LOC101104360
NC_056075.1	43600001	43620001	2.35938	0.329413	OL	LOC101104360
NC_056075.1	43580001	43600001	8.9187	0.345327	OL	LOC101104360;NKX1-2
NC_056058.1	8120001	8140001	4.13086	0.26868	OL	LOC101104488
NC_056058.1	8125001	8145001	5.24392	0.314168	OL	LOC101104488
NC_056058.1	39020001	39040001	2.38299	0.254026	OL	LOC101104974
NC_056077.1	4705001	4725001	3.59933	0.266698	OL	LOC101105047
NC_056077.1	4710001	4730001	3.73781	0.248569	OL	LOC101105047
NC_056077.1	4715001	4735001	3.19069	0.239168	OL	LOC101105047
NC_056076.1	37915001	37935001	8.10738	0.218639	OL	LOC101105123
NC_056076.1	37920001	37940001	12.1905	0.270162	OL	LOC101105123
NC_056077.1	1170001	1190001	10.401	0.349545	OL	LOC101105127;MSLN
NC_056077.1	1175001	1195001	13.6728	0.392484	OL	LOC101105127;MSLN
NC_056080.1	19350001	19370001	12.6397	0.213617	OL	LOC101105138
NC_056068.1	64205001	64225001	3.56278	0.261316	OL	LOC101105523
NC_056068.1	64210001	64230001	5.18681	0.362342	OL	LOC101105523
NC_056068.1	64215001	64235001	8.91207	0.406845	OL	LOC101105523
NC_056068.1	64220001	64240001	12.8742	0.406492	OL	LOC101105523;PDHX
NC_056068.1	64225001	64245001	13.2597	0.37297	OL	LOC101105523;PDHX
NC_056068.1	64230001	64250001	3.81362	0.307569	OL	LOC101105523;PDHX
NC_056074.1	35405001	35425001	13.0471	0.306108	OL	LOC101105540
NC_056063.1	20760001	20780001	5.71255	0.262204	OL	LOC101106088
NC_056063.1	20765001	20785001	3.49306	0.221892	OL	LOC101106088
NC_056054.1	21660001	21680001	3.90336	0.220733	OL	LOC101106121
NC_056054.1	21665001	21685001	5.04147	0.236396	OL	LOC101106121
NC_056080.1	64030001	64050001	9.83491	0.291037	OL	LOC101106743
NC_056080.1	64035001	64055001	7.98672	0.264885	OL	LOC101106743
NC_056072.1	55740001	55760001	2.35107	0.347816	OL	LOC101106751
NC_056072.1	55745001	55765001	2.62101	0.395465	OL	LOC101106751
NC_056072.1	55750001	55770001	2.42547	0.388323	OL	LOC101106751
NC_056072.1	55755001	55775001	2.56906	0.355859	OL	LOC101106751
NC_056056.1	200635001	200655001	2.8951	0.333905	OL	LOC101106925
NC_056068.1	53610001	53630001	12.2995	0.240869	OL	LOC101107135;LOC101107387
NC_056068.1	53615001	53635001	9.24537	0.292817	OL	LOC101107135;LOC101107387
NC_056068.1	53620001	53640001	6.9337	0.403573	OL	LOC101107135;LOC101107387
NC_056055.1	246405001	246425001	12.6444	0.255804	OL	LOC101107420
NC_056055.1	246410001	246430001	11.5426	0.297721	OL	LOC101107420
NC_056055.1	246415001	246435001	9.86538	0.303976	OL	LOC101107675
NC_056055.1	246420001	246440001	6.64803	0.295572	OL	LOC101107675
NC_056055.1	52575001	52595001	2.70685	0.235447	OL	LOC101108371;LOC114112800
NC_056058.1	38655001	38675001	6.25158	0.232107	OL	LOC101108746
NC_056058.1	38660001	38680001	7.58236	0.260662	OL	LOC101108746
NC_056058.1	38665001	38685001	7.60387	0.247463	OL	LOC101108746
NC_056058.1	38670001	38690001	4.13895	0.254437	OL	LOC101108746;LOC101116054
NC_056068.1	48390001	48410001	2.92356	0.249695	OL	LOC101108775
NC_056077.1	7640001	7660001	10.4411	0.225104	OL	LOC101109062
NC_056068.1	48400001	48420001	10.1364	0.358669	OL	LOC101109299
NC_056068.1	48405001	48425001	12.5096	0.364925	OL	LOC101109299
NC_056064.1	23970001	23990001	3.00209	0.254831	OL	LOC101109373
NC_056068.1	48420001	48440001	6.59952	0.288525	OL	LOC101109827;LOC114118490
NC_056057.1	106490001	106510001	4.72872	0.233217	OL	LOC101109961
NC_056057.1	106475001	106495001	5.33401	0.228482	OL	LOC101109961;PRSS37
NC_056057.1	106480001	106500001	6.14286	0.273792	OL	LOC101109961;PRSS37
NC_056057.1	106485001	106505001	5.19751	0.26002	OL	LOC101109961;PRSS37
NC_056074.1	15575001	15595001	10.1127	0.228014	OL	LOC101110189
NC_056074.1	15565001	15585001	2.86227	0.213654	OL	LOC101110189;NDUFC2
NC_056074.1	15570001	15590001	5.08333	0.251145	OL	LOC101110189;NDUFC2
NC_056077.1	28915001	28935001	3.11733	0.241233	OL	LOC101111335
NC_056077.1	28920001	28940001	3.20244	0.319779	OL	LOC101111335
NC_056054.1	107700001	107720001	4.81954	0.296393	OL	LOC101111337;LOC101111854
NC_056068.1	48500001	48520001	2.61161	0.47756	OL	LOC101111391
NC_056068.1	48505001	48525001	2.34406	0.468269	OL	LOC101111391
NC_056059.1	73080001	73100001	4.4375	0.217677	OL	LOC101111633;LOC114115373;POLR2B

NC_056059.1	73085001	73105001	5.33008	0.221864	OL	LOC101111633;LOC114115373;POLR2B
NC_056059.1	73095001	73115001	2.85777	0.220947	OL	LOC101111633;LOC114115373;POLR2B
NC_056068.1	48510001	48530001	2.42302	0.409284	OL	LOC101111904
NC_056068.1	48515001	48535001	2.57218	0.318352	OL	LOC101111904
NC_056068.1	48525001	48545001	4.4663	0.238504	OL	LOC101111904;LOC101112160
NC_056074.1	41890001	41910001	3.87375	0.251211	OL	LOC101111922
NC_056074.1	41880001	41900001	4.21987	0.277697	OL	LOC101111922;NUDT8;TBX10
NC_056074.1	41885001	41905001	5.1171	0.284759	OL	LOC101111922;TBX10
NC_056079.1	14815001	14835001	8.57793	0.21567	OL	LOC101112284
NC_056079.1	14820001	14840001	10.6891	0.221201	OL	LOC101112284
NC_056068.1	81345001	81365001	4.47984	0.507744	OL	LOC101112419
NC_056068.1	81350001	81370001	8.18489	0.585398	OL	LOC101112419
NC_056068.1	81360001	81380001	3.79688	0.46108	OL	LOC101112419
NC_056068.1	81365001	81385001	3.0979	0.435365	OL	LOC101112419
NC_056077.1	33195001	33215001	2.49114	0.232094	OL	LOC101112784
NC_056077.1	33200001	33220001	2.53583	0.230922	OL	LOC101112784
NC_056067.1	14285001	14305001	3.9331	0.449309	OL	LOC101113004;LOC101113264
NC_056077.1	19640001	19660001	2.50952	0.434383	OL	LOC101113819
NC_056078.1	40935001	40955001	2.42524	0.313879	OL	LOC101114082
NC_056078.1	40940001	40960001	2.90729	0.355814	OL	LOC101114082
NC_056078.1	40945001	40965001	3.24133	0.351731	OL	LOC101114082
NC_056078.1	40950001	40970001	5.09504	0.291161	OL	LOC101114082
NC_056078.1	40955001	40975001	7.23051	0.216503	OL	LOC101114082
NC_056057.1	106925001	106945001	11.796	0.300035	OL	LOC101114438;LOC121816042
NC_056057.1	106930001	106950001	21.1494	0.277819	OL	LOC101114438;LOC121816042
NC_056057.1	106935001	106955001	13.1657	0.239194	OL	LOC101114438;LOC121816042
NC_056065.1	4870001	4890001	3.72017	0.286521	OL	LOC101114456
NC_056061.1	50075001	50095001	2.49866	0.349782	OL	LOC101114528;ZNF292
NC_056067.1	62115001	62135001	8.06626	0.593494	OL	LOC101115398
NC_056058.1	9035001	9055001	34.0925	0.23224	OL	LOC101115538
NC_056058.1	9040001	9060001	32.5257	0.220302	OL	LOC101115538
NC_056058.1	9045001	9065001	29.2265	0.223124	OL	LOC101115538
NC_056058.1	38885001	38905001	2.49179	0.323054	OL	LOC101115539
NC_056060.1	86870001	86890001	8.74059	0.355208	OL	LOC101115626;SPTLC2
NC_056060.1	86875001	86895001	4.73697	0.257447	OL	LOC101115626;SPTLC2
NC_056080.1	34140001	34160001	16.6201	0.515843	OL	LOC101115943
NC_056080.1	34145001	34165001	12.0725	0.501211	OL	LOC101115943
NC_056080.1	34150001	34170001	10.6154	0.528362	OL	LOC101115943
NC_056080.1	34155001	34175001	10.5714	0.520604	OL	LOC101115943
NC_056058.1	9070001	9090001	21.2248	0.235912	OL	LOC101116053
NC_056058.1	9075001	9095001	23.2389	0.239513	OL	LOC101116053
NC_056058.1	9080001	9100001	20.8975	0.221003	OL	LOC101116053
NC_056063.1	19380001	19400001	3.81949	0.22143	OL	LOC101116064
NC_056075.1	44800001	44820001	2.59682	0.213709	OL	LOC101116189;UROS
NC_056075.1	44805001	44825001	2.97744	0.215371	OL	LOC101116189;UROS
NC_056065.1	55560001	55580001	2.64796	0.264	OL	LOC101116245
NC_056065.1	55550001	55570001	2.7341	0.216949	OL	LOC101116245;RABGAP1L
NC_056065.1	55555001	55575001	2.56667	0.234747	OL	LOC101116245;RABGAP1L
NC_056059.1	8845001	8865001	3.04279	0.239946	OL	LOC101116481
NC_056059.1	8850001	8870001	4.37458	0.272889	OL	LOC101116481
NC_056059.1	8855001	8875001	4.78113	0.243953	OL	LOC101116481
NC_056058.1	39050001	39070001	9.04325	0.312935	OL	LOC101117077
NC_056058.1	39055001	39075001	6.7248	0.291487	OL	LOC101117077
NC_056058.1	39060001	39080001	3.63465	0.264261	OL	LOC101117077
NC_056058.1	39065001	39085001	2.87301	0.332241	OL	LOC101117077;TRIM58
NC_056065.1	56820001	56840001	2.47984	0.219149	OL	LOC101117093
NC_056074.1	16620001	16640001	2.40652	0.230749	OL	LOC101117547
NC_056066.1	78185001	78205001	2.3494	0.226676	OL	LOC101117690
NC_056074.1	16750001	16770001	2.5066	0.215049	OL	LOC101117804
NC_056074.1	16840001	16860001	14.1067	0.255102	OL	LOC101117804
NC_056074.1	16845001	16865001	22.1199	0.318036	OL	LOC101117804
NC_056055.1	8320001	8340001	3.47832	0.216057	OL	LOC101118085
NC_056073.1	28645001	28665001	2.46189	0.259484	OL	LOC101118231
NC_056074.1	38025001	38045001	2.43808	0.291277	OL	LOC101118318
NC_056074.1	38030001	38050001	3.82781	0.377618	OL	LOC101118318
NC_056074.1	38035001	38055001	4.04616	0.358658	OL	LOC101118318
NC_056080.1	2755001	2775001	8.76775	0.246816	OL	LOC101118336
NC_056073.1	28785001	28805001	2.39932	0.214934	OL	LOC101119769
NC_056054.1	109675001	109695001	2.60038	0.253939	OL	LOC101119785
NC_056075.1	20675001	20695001	5.40001	0.305255	OL	LOC101120033
NC_056075.1	20680001	20700001	15.2102	0.377429	OL	LOC101120033
NC_056075.1	20685001	20705001	13.8865	0.462704	OL	LOC101120033
NC_056075.1	20700001	20720001	7.66667	0.336842	OL	LOC101120033
NC_056075.1	20705001	20725001	5.08787	0.238873	OL	LOC101120033

NC_056064.1	24045001	24065001	9.55343	0.303388	OL	LOC101120675
NC_056064.1	24040001	24060001	4.24198	0.271264	OL	LOC101120675;LOC114116981
NC_056068.1	53695001	53715001	11.6667	0.428262	OL	LOC101120816
NC_056068.1	53700001	53720001	9.14583	0.409604	OL	LOC101120816
NC_056068.1	53705001	53725001	7.06583	0.383843	OL	LOC101120816
NC_056068.1	53710001	53730001	5.72222	0.450645	OL	LOC101120816
NC_056068.1	53715001	53735001	7.32105	0.470932	OL	LOC101120816
NC_056068.1	53720001	53740001	7.36206	0.466647	OL	LOC101120816
NC_056068.1	53725001	53745001	7.28693	0.441189	OL	LOC101120816;LOC101122934
NC_056068.1	53730001	53750001	8.02174	0.416498	OL	LOC101120816;LOC101122934
NC_056068.1	53735001	53755001	7.10191	0.365441	OL	LOC101120816;LOC101122934
NC_056068.1	53740001	53760001	8.0435	0.303604	OL	LOC101120816;LOC101122934
NC_056064.1	24050001	24070001	5.38735	0.274897	OL	LOC101120929
NC_056064.1	24055001	24075001	4.02611	0.226201	OL	LOC101120929;SPATA22
NC_056060.1	25330001	25350001	20.4159	0.239236	OL	LOC101121100;LOC101121354
NC_056074.1	41870001	41890001	3.2595	0.243684	OL	LOC101121557;NDUFV1;NUDT8;TBX10
NC_056074.1	41875001	41895001	3.46793	0.286401	OL	LOC101121557;NUDT8;TBX10
NC_056058.1	49535001	49555001	4.74816	0.250334	OL	LOC101122108;LOC101122274;LOC105611202
NC_056058.1	49530001	49550001	3.96875	0.225876	OL	LOC101122108;LOC105611202
NC_056058.1	49635001	49655001	2.49235	0.276345	OL	LOC101122274;LOC105611202
NC_056058.1	49540001	49560001	3.72794	0.335627	OL	LOC101122274;LOC105611202;LOC105615366
NC_056058.1	49545001	49565001	4.01304	0.330351	OL	LOC101122274;LOC105611202;LOC105615366
NC_056058.1	49550001	49570001	4.14131	0.309838	OL	LOC101122274;LOC105611202;LOC105615366
NC_056058.1	49555001	49575001	4.21837	0.297675	OL	LOC101122274;LOC105611202;LOC105615366
NC_056058.1	49560001	49580001	3.10166	0.232477	OL	LOC101122274;LOC105611202;LOC105615366
NC_056058.1	49630001	49650001	3.71481	0.292541	OL	LOC101122274;LOC105611202;LOC106991193
NC_056072.1	52215001	52235001	8.01795	0.224567	OL	LOC101122312
NC_056072.1	52220001	52240001	6.9367	0.286653	OL	LOC101122312
NC_056072.1	52225001	52245001	4.13098	0.330471	OL	LOC101122312
NC_056056.1	206095001	206115001	2.57479	0.382256	OL	LOC101122428
NC_056058.1	39845001	39865001	2.39004	0.352156	OL	LOC101122438;LOC101122694
NC_056058.1	39850001	39870001	2.59893	0.236821	OL	LOC101122694
NC_056068.1	53745001	53765001	7.29333	0.291188	OL	LOC101122934
NC_056068.1	53750001	53770001	7.53434	0.264103	OL	LOC101122934
NC_056068.1	53755001	53775001	5.65853	0.239744	OL	LOC101122934
NC_056056.1	132990001	133010001	6.07767	0.458533	OL	LOC101123619;NPFF;TARBP2
NC_056078.1	40965001	40985001	5.25175	0.215111	OL	LOC105605095
NC_056078.1	40970001	40990001	2.67139	0.232095	OL	LOC105605095
NC_056071.1	65450001	65470001	6	0.276029	OL	LOC105605761;LOC114109056
NC_056071.1	65455001	65475001	4.2216	0.243538	OL	LOC105605761;LOC114109056
NC_056071.1	65420001	65440001	4.32501	0.257213	OL	LOC105605780
NC_056071.1	65425001	65445001	3.76445	0.253547	OL	LOC105605780
NC_056071.1	65430001	65450001	4.29084	0.263932	OL	LOC105605780
NC_056073.1	25575001	25595001	2.73191	0.284442	OL	LOC105605908
NC_056073.1	25985001	26005001	13.1599	0.242696	OL	LOC105605990
NC_056057.1	79960001	79980001	2.98003	0.231636	OL	LOC105606666
NC_056067.1	44755001	44775001	3.03013	0.288063	OL	LOC105607568;LOC114117976
NC_056068.1	37320001	37340001	3.02223	0.390107	OL	LOC105610137
NC_056057.1	6975001	6995001	22.103	0.233702	OL	LOC105614936
NC_056067.1	10440001	10460001	3.45342	0.309868	OL	LOC106991582;USP10
NC_056071.1	65440001	65460001	6.74158	0.291017	OL	LOC114109056
NC_056071.1	65445001	65465001	6.88739	0.290549	OL	LOC114109056
NC_056077.1	14170001	14190001	2.7653	0.221561	OL	LOC114110664
NC_056080.1	79045001	79065001	3.79162	0.309123	OL	LOC114111496
NC_056080.1	79050001	79070001	2.88968	0.269067	OL	LOC114111496
NC_056080.1	79055001	79075001	2.62531	0.241154	OL	LOC114111496
NC_056065.1	30225001	30245001	2.70679	0.275385	OL	LOC114112547;SMYD3
NC_056065.1	30230001	30250001	3.09805	0.259222	OL	LOC114112547;SMYD3
NC_056055.1	111030001	111050001	6.89048	0.369803	OL	LOC114113035
NC_056055.1	111035001	111055001	3.81159	0.333399	OL	LOC114113035
NC_056055.1	111040001	111060001	3.11368	0.287603	OL	LOC114113035
NC_056055.1	111045001	111065001	2.49691	0.294984	OL	LOC114113035
NC_056055.1	75170001	75190001	3.00872	0.231933	OL	LOC114113077
NC_056055.1	75175001	75195001	3.75512	0.263152	OL	LOC114113077
NC_056055.1	75180001	75200001	5.29959	0.277448	OL	LOC114113077
NC_056055.1	75185001	75205001	6.91537	0.283622	OL	LOC114113077
NC_056055.1	75190001	75210001	5.12172	0.234649	OL	LOC114113077
NC_056054.1	125795001	125815001	2.53997	0.466331	OL	LOC114113316
NC_056054.1	125830001	125850001	2.99011	0.292758	OL	LOC114113348
NC_056054.1	125835001	125855001	4.35568	0.299588	OL	LOC114113348
NC_056054.1	125840001	125860001	5.68832	0.293596	OL	LOC114113348
NC_056054.1	125845001	125865001	4.1214	0.283866	OL	LOC114113348
NC_056056.1	92180001	92200001	17.7586	0.363932	OL	LOC114113944
NC_056056.1	92185001	92205001	5.14624	0.440729	OL	LOC114113944

NC_056056.1	92190001	92210001	2.88491	0.461174	OL	LOC114113944
NC_056056.1	92175001	92195001	15.4484	0.292387	OL	LOC114113944;SRD5A2
NC_056058.1	38265001	38285001	8.00328	0.401043	OL	LOC114114935
NC_056058.1	38270001	38290001	4.04895	0.371113	OL	LOC114114935
NC_056064.1	23965001	23985001	2.52164	0.316589	OL	LOC114116984
NC_056065.1	42620001	42640001	2.94246	0.240735	OL	LOC114117226
NC_056065.1	42625001	42645001	5.23426	0.266763	OL	LOC114117226;UBE4B
NC_056065.1	55575001	55595001	3.65263	0.273862	OL	LOC114117245;MRPS14
NC_056065.1	55580001	55600001	3.03022	0.241526	OL	LOC114117245;MRPS14
NC_056065.1	61780001	61800001	18.4131	0.331903	OL	LOC114117249
NC_056065.1	61785001	61805001	23.5584	0.337607	OL	LOC114117249
NC_056065.1	61790001	61810001	17.4972	0.276935	OL	LOC114117249
NC_056065.1	61795001	61815001	20.5769	0.315501	OL	LOC114117249
NC_056067.1	44715001	44735001	2.59723	0.287227	OL	LOC114117974
NC_056068.1	35000001	35020001	2.40567	0.232749	OL	LOC114118401
NW_02459982	1415001	1435001	15.3095	0.29729	OL	LOC121816021
NW_02459982	1420001	1440001	11.5113	0.260357	OL	LOC121816021
NW_02459982	1425001	1445001	7.6488	0.238861	OL	LOC121816021
NW_02459982	1430001	1450001	6.08465	0.245214	OL	LOC121816021
NW_02459982	1435001	1455001	7.54933	0.236239	OL	LOC121816021
NW_02459982	1440001	1460001	9.60157	0.283382	OL	LOC121816021
NW_02459972	20001	40001	4.30081	0.259588	OL	LOC121816038
NW_02459972	25001	45001	5.23157	0.241232	OL	LOC121816038
NC_056057.1	106920001	106940001	7.83939	0.32244	OL	LOC121816042
NC_056069.1	18210001	18230001	2.66934	0.216073	OL	LOC121816801
NC_056069.1	18215001	18235001	2.59705	0.231591	OL	LOC121816801
NC_056076.1	24860001	24880001	2.49709	0.252576	OL	LOC121817702
NC_056076.1	24865001	24885001	4.9392	0.246021	OL	LOC121817702
NC_056076.1	24870001	24890001	10.9673	0.238412	OL	LOC121817702
NC_056076.1	24880001	24900001	3.52059	0.218494	OL	LOC121817702
NC_056076.1	24885001	24905001	3.22233	0.246854	OL	LOC121817702
NC_056080.1	81830001	81850001	4.31189	0.4556	OL	LOC121818145
NC_056080.1	81835001	81855001	3.14953	0.409147	OL	LOC121818145
NC_056080.1	131460001	131480001	5.55678	0.218378	OL	LOC121818354
NC_056054.1	116405001	116425001	4.33584	0.380345	OL	LOC121818668
NC_056054.1	116410001	116430001	7.16455	0.453955	OL	LOC121818668
NC_056054.1	116415001	116435001	10.3753	0.48565	OL	LOC121818668
NC_056054.1	116420001	116440001	8.27579	0.382045	OL	LOC121818668
NC_056054.1	116425001	116445001	3.38236	0.259636	OL	LOC121818668
NC_056065.1	12530001	12550001	2.78084	0.365983	OL	LOC121820762
NC_056065.1	12535001	12555001	4.05918	0.455869	OL	LOC121820762
NC_056063.1	16870001	16890001	2.77185	0.22037	OL	LRCH1
NC_056063.1	16875001	16895001	2.81184	0.224781	OL	LRCH1
NC_056063.1	16880001	16900001	2.84821	0.22549	OL	LRCH1
NC_056063.1	16885001	16905001	3.19458	0.240297	OL	LRCH1
NC_056063.1	16890001	16910001	2.5679	0.219849	OL	LRCH1
NC_056063.1	16895001	16915001	2.44724	0.251944	OL	LRCH1
NC_056063.1	16900001	16920001	2.63084	0.236033	OL	LRCH1
NC_056056.1	162410001	162430001	6.31677	0.357718	OL	LRP1
NC_056056.1	162395001	162415001	2.37128	0.404068	OL	LRP1;NXPH4
NC_056056.1	162400001	162420001	3.86214	0.44396	OL	LRP1;NXPH4
NC_056055.1	168320001	168340001	2.43715	0.40589	OL	LRP1B
NC_056055.1	168325001	168345001	2.85018	0.404429	OL	LRP1B
NC_056055.1	168375001	168395001	2.4901	0.239134	OL	LRP1B
NC_056056.1	80375001	80395001	2.68349	0.24446	OL	LRPPRC
NC_056056.1	80380001	80400001	3.74673	0.216397	OL	LRPPRC
NC_056060.1	17760001	17780001	2.54476	0.325346	OL	LRRC49
NC_056060.1	17840001	17860001	3.68786	0.225893	OL	LRRC49
NC_056060.1	17845001	17865001	3.47265	0.237743	OL	LRRC49
NC_056060.1	70255001	70275001	2.49443	0.233049	OL	LRRC9
NC_056060.1	70260001	70280001	2.3995	0.251159	OL	LRRC9
NC_056074.1	18125001	18145001	20.556	0.227704	OL	LUZP2
NC_056074.1	18130001	18150001	11.3194	0.236515	OL	LUZP2
NC_056060.1	4830001	4850001	2.40858	0.256095	OL	LVRN
NC_056066.1	7955001	7975001	2.56307	0.222014	OL	MACROD2
NC_056066.1	7960001	7980001	2.54489	0.260336	OL	MACROD2
NC_056066.1	7965001	7985001	2.60771	0.284021	OL	MACROD2
NC_056066.1	8365001	8385001	3.00505	0.249424	OL	MACROD2
NC_056066.1	8370001	8390001	2.73985	0.263643	OL	MACROD2
NC_056066.1	8375001	8395001	3.11746	0.233075	OL	MACROD2
NC_056066.1	9575001	9595001	3.97611	0.331459	OL	MACROD2
NC_056066.1	9650001	9670001	3.54234	0.353541	OL	MACROD2
NC_056066.1	9655001	9675001	6.10643	0.430387	OL	MACROD2
NC_056066.1	9660001	9680001	4.21176	0.475645	OL	MACROD2

NC_056077.1	41460001	41480001	2.62784	0.213831	OL	MAD1L1
NC_056077.1	41520001	41540001	23.2938	0.317236	OL	MAD1L1
NC_056077.1	41525001	41545001	21.3418	0.312752	OL	MAD1L1
NC_056077.1	41530001	41550001	18.7441	0.309089	OL	MAD1L1
NC_056077.1	41535001	41555001	14.2423	0.30441	OL	MAD1L1
NC_056077.1	41540001	41560001	6.62554	0.271274	OL	MAD1L1
NC_056077.1	41545001	41565001	5.97872	0.271042	OL	MAD1L1
NC_056077.1	41550001	41570001	4.58125	0.263631	OL	MAD1L1
NC_056077.1	41555001	41575001	5.97409	0.26058	OL	MAD1L1
NC_056077.1	41560001	41580001	7.73859	0.26664	OL	MAD1L1
NC_056077.1	41565001	41585001	4.64198	0.229447	OL	MAD1L1
NC_056077.1	41570001	41590001	5.44068	0.235891	OL	MAD1L1
NC_056077.1	41575001	41595001	4.66254	0.239516	OL	MAD1L1
NC_056077.1	41580001	41600001	4.86455	0.219685	OL	MAD1L1
NC_056077.1	41785001	41805001	6.57871	0.367647	OL	MAFK
NC_056077.1	41765001	41785001	11.9062	0.319124	OL	MAFK;PSMG3;TMEM184A
NC_056067.1	45285001	45305001	3.32159	0.29753	OL	MAG
NC_056067.1	45290001	45310001	2.89762	0.225505	OL	MAG
NC_056080.1	53685001	53705001	2.75107	0.241841	OL	MAGED1
NC_056080.1	68900001	68920001	5.48609	0.317284	OL	MAGT1
NC_056080.1	68905001	68925001	5.96581	0.27629	OL	MAGT1
NC_056076.1	58095001	58115001	2.37543	0.237569	OL	MALT1
NC_056055.1	241175001	241195001	4.06026	0.247611	OL	MAN1C1
NC_056055.1	241180001	241200001	8.59412	0.274158	OL	MAN1C1
NC_056056.1	225555001	225575001	2.53505	0.277469	OL	MAPK11;PLXNB2
NC_056074.1	38665001	38685001	3.7349	0.289362	OL	MARK2
NC_056074.1	38670001	38690001	5.91147	0.302306	OL	MARK2
NC_056074.1	38690001	38710001	9.96663	0.337926	OL	MARK2
NC_056074.1	38695001	38715001	8.28356	0.346506	OL	MARK2;RCOR2
NC_056074.1	38700001	38720001	2.72055	0.268984	OL	MARK2;RCOR2
NC_056078.1	34705001	34725001	6.87729	0.264069	OL	MAT1A
NC_056078.1	34680001	34700001	22.4587	0.359622	OL	MBLA;SFTPA1
NC_056078.1	34685001	34705001	20.4537	0.330799	OL	MBLA;SFTPA1
NC_056078.1	34690001	34710001	27.4799	0.333847	OL	MBLA;SFTPA1
NC_056078.1	34670001	34690001	8.21859	0.324392	OL	MBLA;SFTPD
NC_056078.1	34675001	34695001	21.5449	0.3529	OL	MBLA;SFTPD
NC_056067.1	14240001	14260001	2.44009	0.24875	OL	MC1R;TCF25
NC_056079.1	5555001	5575001	2.74721	0.22131	OL	MCPH1
NC_056079.1	5560001	5580001	4.05768	0.237719	OL	MCPH1
NC_056079.1	5565001	5585001	4.33957	0.221179	OL	MCPH1
NC_056079.1	5570001	5590001	5.42538	0.229172	OL	MCPH1
NC_056079.1	5575001	5595001	4.95877	0.228968	OL	MCPH1
NC_056079.1	5580001	5600001	2.85194	0.241114	OL	MCPH1
NC_056057.1	55040001	55060001	2.47029	0.327567	OL	MDFIC
NC_056057.1	55055001	55075001	2.56786	0.274987	OL	MDFIC
NC_056054.1	237705001	237725001	4.02784	0.216935	OL	MED12L
NC_056080.1	39960001	39980001	2.46268	0.244582	OL	MED14
NC_056066.1	28940001	28960001	20.8322	0.229319	OL	MEIG1
NC_056060.1	30900001	30920001	3.46797	0.226059	OL	MEIS2
NC_056060.1	30905001	30925001	3.58243	0.260366	OL	MEIS2
NC_056060.1	30910001	30930001	3.4881	0.280262	OL	MEIS2
NC_056059.1	37500001	37520001	15.1534	0.320177	OL	MEPE
NC_056059.1	37505001	37525001	16.7535	0.246981	OL	MEPE
NC_056059.1	37510001	37530001	19.8994	0.220797	OL	MEPE
NC_056057.1	53095001	53115001	2.83169	0.230752	OL	MET
NC_056057.1	53100001	53120001	2.91548	0.272228	OL	MET
NC_056057.1	53105001	53125001	2.89524	0.278936	OL	MET
NC_056057.1	53110001	53130001	3.05461	0.254919	OL	MET
NC_056057.1	53115001	53135001	3.14134	0.261257	OL	MET
NC_056057.1	53120001	53140001	3.75982	0.288439	OL	MET
NC_056057.1	53125001	53145001	5.5	0.273124	OL	MET
NC_056057.1	53150001	53170001	4.20329	0.233646	OL	MET
NC_056057.1	53155001	53175001	3.75746	0.278711	OL	MET
NC_056057.1	53160001	53180001	3.4078	0.31018	OL	MET
NC_056057.1	53165001	53185001	2.35699	0.245459	OL	MET
NC_056076.1	37305001	37325001	3.66549	0.3971	OL	METTL4
NC_056076.1	37310001	37330001	7.25194	0.46358	OL	METTL4
NC_056076.1	37315001	37335001	11.136	0.480775	OL	METTL4
NC_056076.1	37320001	37340001	12.5475	0.455692	OL	METTL4
NC_056076.1	37325001	37345001	12.0105	0.409547	OL	METTL4
NC_056076.1	37330001	37350001	7.04184	0.311331	OL	METTL4
NC_056056.1	122360001	122380001	2.39462	0.216755	OL	MGAT4C
NC_056056.1	123010001	123030001	4.09831	0.389643	OL	MGAT4C
NC_056068.1	40160001	40180001	2.60099	0.315133	OL	MICAL2

NC_056068.1	40165001	40185001	7.07575	0.335135	OL	MICAL2
NC_056056.1	225375001	225395001	5.02996	0.664563	OL	MLC1
NC_056056.1	225380001	225400001	5.00775	0.53384	OL	MLC1;MOV10L1
NC_056056.1	225385001	225405001	6.07274	0.412769	OL	MLC1;MOV10L1
NC_056056.1	225360001	225380001	12.2719	0.777415	OL	MLC1;TTLL8
NC_056056.1	225365001	225385001	8.56337	0.778178	OL	MLC1;TTLL8
NC_056056.1	225370001	225390001	5.33296	0.740814	OL	MLC1;TTLL8
NC_056060.1	51980001	52000001	2.55624	0.314564	OL	MNS1
NC_056074.1	46405001	46425001	3.54154	0.260105	OL	MOB2
NC_056074.1	46410001	46430001	3.04688	0.245747	OL	MOB2
NC_056074.1	46415001	46435001	3.0625	0.244149	OL	MOB2
NC_056074.1	46425001	46445001	3.8628	0.253228	OL	MOB2
NC_056056.1	225410001	225430001	6.52596	0.489381	OL	MOV10L1
NC_056056.1	225415001	225435001	6.4156	0.451445	OL	MOV10L1
NC_056056.1	225420001	225440001	6.88028	0.406363	OL	MOV10L1
NC_056077.1	690001	710001	7.54974	0.212872	OL	MPG;NPRL3
NC_056077.1	695001	715001	12.6894	0.275667	OL	MPG;NPRL3
NC_056056.1	62710001	62730001	2.85191	0.269305	OL	MRPL19
NC_056056.1	62715001	62735001	2.34044	0.284807	OL	MRPL19
NC_056065.1	55585001	55605001	3.85382	0.289107	OL	MRPS14
NC_056065.1	55590001	55610001	4.24324	0.274972	OL	MRPS14
NC_056065.1	55595001	55615001	3.03296	0.284017	OL	MRPS14
NC_056065.1	55600001	55620001	4.79509	0.301789	OL	MRPS14
NC_056063.1	22115001	22135001	12.3234	0.227462	OL	MRPS31
NC_056063.1	22120001	22140001	12	0.226684	OL	MRPS31
NC_056063.1	22125001	22145001	12.0407	0.268907	OL	MRPS31
NC_056063.1	22130001	22150001	4.51462	0.219047	OL	MRPS31
NC_056068.1	81895001	81915001	3.7602	0.219108	OL	MS4A13
NC_056074.1	34465001	34485001	2.58994	0.278682	OL	MS4A15;MS4A18
NC_056074.1	34470001	34490001	3.16982	0.244655	OL	MS4A15;MS4A18
NC_056054.1	52015001	52035001	5.9096	0.232309	OL	MSH4;RABGGTB
NC_056054.1	52020001	52040001	4.36595	0.216512	OL	MSH4;RABGGTB
NC_056060.1	74615001	74635001	4.70088	0.263355	OL	MTHFD1
NC_056060.1	74620001	74640001	3.02811	0.241634	OL	MTHFD1
NC_056065.1	41755001	41775001	2.33333	0.285244	OL	MTOR
NC_056063.1	31385001	31405001	3.313	0.219331	OL	MTUS2
NC_056063.1	31390001	31410001	2.71176	0.229487	OL	MTUS2
NC_056062.1	25720001	25740001	2.33209	0.222305	OL	MYC
NC_056064.1	27965001	27985001	3.6974	0.234325	OL	MYH10
NC_056064.1	27970001	27990001	3.80952	0.232174	OL	MYH10
NC_056077.1	35610001	35630001	2.62045	0.271003	OL	MYL10
NC_056077.1	35615001	35635001	2.69819	0.272989	OL	MYL10
NC_056077.1	35620001	35640001	2.82609	0.279412	OL	MYL10
NC_056077.1	35625001	35645001	3.2078	0.289474	OL	MYL10
NC_056067.1	14595001	14615001	2.44956	0.241806	OL	MYLK3
NC_056063.1	83325001	83345001	7.3481	0.214475	OL	MYO16
NC_056066.1	25855001	25875001	2.84869	0.306641	OL	MYO3A
NC_056066.1	25860001	25880001	2.53256	0.248064	OL	MYO3A
NC_056076.1	49860001	49880001	3.105	0.314948	OL	MYO5B
NC_056076.1	49865001	49885001	2.47187	0.317267	OL	MYO5B
NC_056076.1	49870001	49890001	3.1961	0.318681	OL	MYO5B
NC_056076.1	49875001	49895001	4.10156	0.320679	OL	MYO5B
NC_056076.1	49880001	49900001	9.44443	0.227213	OL	MYO5B
NC_056076.1	37805001	37825001	2.35757	0.247042	OL	MYOM1
NC_056076.1	37810001	37830001	3.83047	0.272275	OL	MYOM1
NC_056076.1	37815001	37835001	4.66358	0.238587	OL	MYOM1
NC_056056.1	112090001	112110001	3.23753	0.324742	OL	NAP1L1
NC_056056.1	112095001	112115001	7.66253	0.297273	OL	NAP1L1
NC_056056.1	112100001	112120001	10.5276	0.329668	OL	NAP1L1
NC_056056.1	112105001	112125001	16.0155	0.42248	OL	NAP1L1
NC_056056.1	112110001	112130001	14.6826	0.476219	OL	NAP1L1
NC_056056.1	112115001	112135001	13.319	0.504816	OL	NAP1L1
NC_056056.1	23605001	23625001	6.7344	0.329609	OL	NBAS
NC_056056.1	23610001	23630001	5.54687	0.289471	OL	NBAS
NC_056056.1	23615001	23635001	4.57938	0.26458	OL	NBAS
NC_056063.1	26580001	26600001	2.79018	0.256916	OL	NBEA
NC_056063.1	26585001	26605001	3.13808	0.308135	OL	NBEA
NC_056063.1	26590001	26610001	3.71547	0.290523	OL	NBEA
NC_056063.1	26595001	26615001	3.65217	0.289162	OL	NBEA
NC_056063.1	26600001	26620001	2.49606	0.28212	OL	NBEA
NC_056068.1	23215001	23235001	2.95813	0.328122	OL	NCAM1
NC_056068.1	23220001	23240001	4.18031	0.314225	OL	NCAM1
NC_056068.1	23225001	23245001	4.42799	0.271586	OL	NCAM1
NC_056059.1	38035001	38055001	10.5742	0.319894	OL	NCAPG

NC_056055.1	177505001	177525001	2.66133	0.261258	OL	NCKAP5
NC_056055.1	177510001	177530001	3.42064	0.247543	OL	NCKAP5
NC_056055.1	177515001	177535001	3.54937	0.264677	OL	NCKAP5
NC_056055.1	177605001	177625001	2.52702	0.366776	OL	NCKAP5
NC_056056.1	32220001	32240001	3.88541	0.223719	OL	NCOA1
NC_056056.1	32225001	32245001	6.15234	0.262648	OL	NCOA1
NC_056056.1	32230001	32250001	5	0.241249	OL	NCOA1
NC_056070.1	51245001	51265001	2.43478	0.218873	OL	NCOR2
NC_056070.1	51250001	51270001	2.63392	0.232144	OL	NCOR2
NC_056070.1	51255001	51275001	2.44914	0.255966	OL	NCOR2
NC_056070.1	51260001	51280001	2.35959	0.298463	OL	NCOR2
NC_056076.1	37360001	37380001	3.54992	0.246138	OL	NDC80
NC_056076.1	37365001	37385001	3.12801	0.24428	OL	NDC80
NC_056076.1	37370001	37390001	2.79078	0.262001	OL	NDC80
NC_056074.1	41850001	41870001	4.77126	0.215061	OL	NDUFV1
NC_056066.1	21660001	21680001	4.35525	0.221845	OL	NEBL
NC_056066.1	21665001	21685001	4.03294	0.220638	OL	NEBL
NC_056054.1	47815001	47835001	3.43413	0.225525	OL	NEGR1
NC_056054.1	47820001	47840001	6.52104	0.242007	OL	NEGR1
NC_056054.1	47825001	47845001	9.94361	0.261904	OL	NEGR1
NC_056054.1	47830001	47850001	6.71222	0.328694	OL	NEGR1
NC_056054.1	47835001	47855001	2.53429	0.237849	OL	NEGR1
NC_056055.1	111530001	111550001	3.24397	0.267481	OL	NEK1
NC_056055.1	111535001	111555001	3.52772	0.269292	OL	NEK1
NC_056055.1	111540001	111560001	3.25806	0.260053	OL	NEK1
NC_056055.1	111545001	111565001	2.98365	0.221762	OL	NEK1
NC_056055.1	111575001	111595001	2.54745	0.248044	OL	NEK1
NC_056055.1	111580001	111600001	3.64524	0.273358	OL	NEK1
NC_056055.1	111585001	111605001	5.49732	0.283771	OL	NEK1
NC_056055.1	111590001	111610001	4.97008	0.267446	OL	NEK1
NC_056072.1	1710001	1730001	3.23206	0.25432	OL	NEK10
NC_056072.1	1715001	1735001	5.8453	0.2631	OL	NEK10
NC_056072.1	1720001	1740001	8.11	0.271493	OL	NEK10
NC_056072.1	1725001	1745001	3.54456	0.287422	OL	NEK10
NC_056056.1	141815001	141835001	2.35927	0.252811	OL	NELL2
NC_056056.1	141820001	141840001	3.17529	0.341962	OL	NELL2
NC_056056.1	141825001	141845001	2.93104	0.337851	OL	NELL2
NC_056060.1	19715001	19735001	2.68501	0.356691	OL	NEO1
NC_056080.1	15625001	15645001	3.06145	0.216292	OL	NHS
NC_056080.1	15630001	15650001	4.30919	0.223811	OL	NHS
NC_056080.1	15735001	15755001	5.62264	0.422535	OL	NHS
NC_056080.1	15740001	15760001	8.65415	0.477248	OL	NHS
NC_056080.1	15745001	15765001	4.07877	0.475749	OL	NHS
NC_056080.1	15750001	15770001	2.47524	0.431871	OL	NHS
NC_056080.1	15770001	15790001	3.67421	0.300814	OL	NHS
NC_056060.1	41820001	41840001	2.43234	0.244692	OL	NIN
NC_056060.1	41825001	41845001	2.39662	0.218777	OL	NIN
NC_056061.1	13970001	13990001	2.54178	0.222659	OL	NKAIN2
NC_056061.1	13975001	13995001	2.50244	0.215024	OL	NKAIN2
NC_056061.1	14165001	14185001	2.94516	0.218424	OL	NKAIN2
NC_056061.1	14170001	14190001	2.81173	0.232413	OL	NKAIN2
NC_056061.1	14175001	14195001	2.76351	0.230098	OL	NKAIN2
NC_056061.1	14180001	14200001	2.74254	0.215192	OL	NKAIN2
NC_056061.1	14190001	14210001	3.1706	0.223231	OL	NKAIN2
NC_056061.1	14195001	14215001	4.01891	0.217265	OL	NKAIN2
NC_056061.1	14200001	14220001	5.70781	0.215867	OL	NKAIN2
NC_056061.1	14205001	14225001	5.44668	0.219473	OL	NKAIN2
NC_056062.1	40450001	40470001	7.57651	0.275025	OL	NKAIN3
NC_056062.1	40455001	40475001	13.2356	0.346451	OL	NKAIN3
NC_056062.1	40470001	40490001	4.05882	0.33757	OL	NKAIN3
NC_056062.1	40810001	40830001	3.0955	0.297605	OL	NKAIN3
NC_056062.1	40815001	40835001	2.89575	0.26908	OL	NKAIN3
NC_056062.1	40820001	40840001	3.34229	0.288145	OL	NKAIN3
NC_056062.1	40825001	40845001	2.50597	0.248817	OL	NKAIN3
NC_056062.1	40830001	40850001	2.5089	0.256245	OL	NKAIN3
NC_056054.1	213995001	214015001	2.72379	0.230049	OL	NLGN1
NC_056054.1	214005001	214025001	3.65843	0.232814	OL	NLGN1
NC_056080.1	3690001	3710001	6.16645	0.239881	OL	NLGN4X
NC_056080.1	3695001	3715001	10.8846	0.293485	OL	NLGN4X
NC_056080.1	3700001	3720001	43.2924	0.314499	OL	NLGN4X
NC_056080.1	3705001	3725001	23.083	0.280934	OL	NLGN4X
NC_056057.1	67365001	67385001	2.75249	0.246166	OL	NOD1
NC_056057.1	67385001	67405001	2.54662	0.231878	OL	NOD1
NC_056057.1	67420001	67440001	2.72789	0.305793	OL	NOD1

NC_056057.1	67425001	67445001	2.69157	0.237907	OL	NOD1
NC_056057.1	67430001	67450001	3.30119	0.247422	OL	NOD1
NC_056076.1	23180001	23200001	19.7148	0.302147	OL	NOL4
NC_056076.1	23185001	23205001	16.52	0.288639	OL	NOL4
NC_056076.1	23190001	23210001	3.83023	0.214348	OL	NOL4
NC_056076.1	23220001	23240001	4.34783	0.228547	OL	NOL4
NC_056076.1	23225001	23245001	10.7483	0.272203	OL	NOL4
NC_056076.1	23230001	23250001	12.5671	0.277116	OL	NOL4
NC_056076.1	23235001	23255001	18.4023	0.260719	OL	NOL4
NC_056076.1	23335001	23355001	2.65091	0.304487	OL	NOL4
NC_056076.1	23490001	23510001	3.02265	0.216942	OL	NOL4
NC_056076.1	23495001	23515001	3.26159	0.224466	OL	NOL4
NC_056071.1	34515001	34535001	3.21524	0.43287	OL	NOVA1
NC_056071.1	34520001	34540001	4.12321	0.466766	OL	NOVA1
NC_056071.1	34525001	34545001	3.32937	0.448275	OL	NOVA1
NC_056071.1	34530001	34550001	2.67864	0.416896	OL	NOVA1
NC_056060.1	86760001	86780001	2.65472	0.338736	OL	NOXRED1
NC_056060.1	86765001	86785001	4.35162	0.373857	OL	NOXRED1;VIPAS39
NC_056077.1	700001	720001	39.1263	0.303273	OL	NPRL3
NC_056077.1	715001	735001	28.9263	0.260683	OL	NPRL3
NC_056077.1	720001	740001	20.1783	0.23256	OL	NPRL3
NC_056057.1	73205001	73225001	4.05692	0.323947	OL	NPY
NC_056057.1	73210001	73230001	3.8596	0.322932	OL	NPY
NC_056057.1	50760001	50780001	2.45683	0.292101	OL	NRCAM
NC_056078.1	36145001	36165001	2.42544	0.422761	OL	NRG3
NC_056080.1	131595001	131615001	2.36519	0.224518	OL	NRK
NC_056056.1	73290001	73310001	5.68868	0.323486	OL	NRXN1
NC_056056.1	74060001	74080001	3.39041	0.230773	OL	NRXN1
NC_056056.1	74065001	74085001	3.73856	0.25933	OL	NRXN1
NC_056056.1	74070001	74090001	4.78481	0.27715	OL	NRXN1
NC_056056.1	74075001	74095001	4.83298	0.290669	OL	NRXN1
NC_056056.1	74080001	74100001	5.05252	0.257235	OL	NRXN1
NC_056056.1	74085001	74105001	4.2045	0.272351	OL	NRXN1
NC_056056.1	74090001	74110001	2.59603	0.241054	OL	NRXN1
NC_056074.1	39335001	39355001	4.94325	0.214691	OL	NRXN2
NC_056074.1	39340001	39360001	5.57407	0.217829	OL	NRXN2
NC_056060.1	88285001	88305001	4.31569	0.217226	OL	NRXN3
NC_056060.1	88290001	88310001	3.49105	0.213968	OL	NRXN3
NC_056060.1	88295001	88315001	2.99112	0.218948	OL	NRXN3
NC_056079.1	32510001	32530001	3.8896	0.226911	OL	NSD3;PLPP5
NC_056071.1	25975001	25995001	4.01289	0.429378	OL	NSMCE3
NC_056071.1	25980001	26000001	3.68714	0.424654	OL	NSMCE3
NC_056075.1	23485001	23505001	5.35989	0.252166	OL	NT5C2
NC_056075.1	23490001	23510001	13.7386	0.294284	OL	NT5C2
NC_056075.1	23495001	23515001	6.24542	0.282057	OL	NT5C2
NC_056077.1	14225001	14245001	2.7634	0.239904	OL	NTAN1;PDXDC1
NC_056077.1	14230001	14250001	2.6224	0.219482	OL	NTAN1;PDXDC1
NC_056077.1	14240001	14260001	2.43495	0.258427	OL	NTAN1;PDXDC1
NC_056077.1	14215001	14235001	3.33689	0.235009	OL	NTAN1;RRN3
NC_056077.1	14220001	14240001	3.21951	0.233249	OL	NTAN1;RRN3
NC_056065.1	37060001	37080001	3.13497	0.226425	OL	NTMT2
NC_056065.1	37065001	37085001	3.64612	0.234402	OL	NTMT2
NC_056065.1	37070001	37090001	3.35945	0.215164	OL	NTMT2
NC_056056.1	165630001	165650001	7.38494	0.226134	OL	NTN4
NC_056071.1	18635001	18655001	3.66025	0.340305	OL	NTRK3
NC_056071.1	18650001	18670001	7.78848	0.406373	OL	NTRK3
NC_056063.1	54350001	54370001	3.02576	0.308294	OL	OBI1;POU4F1
NC_056074.1	31360001	31380001	3.17758	0.212364	OL	OPCML
NC_056074.1	31595001	31615001	4.25759	0.215961	OL	OPCML
NC_056074.1	31600001	31620001	4.54741	0.215064	OL	OPCML
NC_056076.1	32810001	32830001	2.55088	0.223001	OL	OSBPL1A
NC_056076.1	32815001	32835001	2.71502	0.24303	OL	OSBPL1A
NC_056076.1	32820001	32840001	2.49221	0.215017	OL	OSBPL1A
NC_056057.1	72445001	72465001	2.39607	0.242377	OL	OSBPL3
NC_056057.1	72450001	72470001	2.81614	0.275183	OL	OSBPL3
NC_056056.1	116400001	116420001	2.36323	0.264494	OL	OTOGL
NC_056059.1	105555001	105575001	24.8926	0.272939	OL	OTOP1
NC_056059.1	105580001	105600001	4.66225	0.256499	OL	OTOP1
NC_056071.1	67420001	67440001	4.63721	0.512079	OL	PACS2;TEX22
NC_056055.1	248995001	249015001	7.00809	0.279525	OL	PADI1;PADI3
NC_056055.1	248960001	248980001	21.9252	0.288788	OL	PADI3
NC_056055.1	248965001	248985001	19.4166	0.303645	OL	PADI3
NC_056055.1	248970001	248990001	21.7333	0.304346	OL	PADI3
NC_056055.1	248975001	248995001	23.2475	0.29847	OL	PADI3

NC_056055.1	248980001	249000001	15.8333	0.297999	OL	PADI3
NC_056055.1	248955001	248975001	24.7929	0.257518	OL	PADI4
NW_02459982	1180001	1200001	3.6303	0.22639	OL	PAG3
NW_02459982	1185001	1205001	2.63964	0.212725	OL	PAG3
NC_056055.1	13390001	13410001	5.76341	0.212886	OL	PALM2AKAP2
NC_056055.1	13395001	13415001	3.97122	0.227768	OL	PALM2AKAP2
NC_056060.1	16210001	16230001	4.60409	0.282907	OL	PAQR5
NC_056060.1	16215001	16235001	4.22651	0.291361	OL	PAQR5
NC_056060.1	16220001	16240001	4.64725	0.316877	OL	PAQR5
NC_056054.1	37040001	37060001	13.9099	0.252129	OL	PATJ
NC_056054.1	37045001	37065001	15.2916	0.230053	OL	PATJ
NC_056054.1	37050001	37070001	5.03304	0.219701	OL	PATJ
NC_056056.1	115670001	115690001	3.08262	0.241146	OL	PAWR
NC_056056.1	115675001	115695001	3.20824	0.260948	OL	PAWR
NC_056056.1	115680001	115700001	2.68471	0.24086	OL	PAWR
NC_056058.1	43995001	44015001	6.08763	0.607378	OL	PCBD2
NC_056058.1	44000001	44020001	9.32772	0.640491	OL	PCBD2
NC_056058.1	44005001	44025001	5.41935	0.615153	OL	PCBD2
NC_056058.1	44010001	44030001	2.72699	0.488694	OL	PCBD2
NC_056054.1	266180001	266200001	3.50939	0.22203	OL	PCBP3
NC_056054.1	266185001	266205001	4.01348	0.238529	OL	PCBP3
NC_056054.1	266190001	266210001	4.46651	0.249569	OL	PCBP3
NC_056054.1	266195001	266215001	3.27579	0.225083	OL	PCBP3
NC_056054.1	266210001	266230001	3.13053	0.219357	OL	PCBP3
NC_056054.1	266215001	266235001	4.5285	0.255839	OL	PCBP3
NC_056054.1	266220001	266240001	6.0623	0.274269	OL	PCBP3
NC_056054.1	266225001	266245001	3.40813	0.221697	OL	PCBP3
NC_056054.1	266230001	266250001	3.21723	0.219411	OL	PCBP3
NC_056080.1	22865001	22885001	2.42932	0.225307	OL	PCYT1B
NC_056056.1	100870001	100890001	2.5602	0.232294	OL	PDCL3
NC_056056.1	100875001	100895001	2.51308	0.249915	OL	PDCL3
NC_056057.1	65755001	65775001	2.83163	0.243411	OL	PDE1C
NC_056057.1	65760001	65780001	2.94401	0.258048	OL	PDE1C
NC_056057.1	66120001	66140001	3.37242	0.219558	OL	PDE1C
NC_056057.1	66140001	66160001	3.82997	0.213448	OL	PDE1C
NC_056057.1	66145001	66165001	12.8413	0.35503	OL	PDE1C
NC_056057.1	66150001	66170001	18.4535	0.314286	OL	PDE1C
NC_056057.1	66155001	66175001	31.0081	0.312598	OL	PDE1C
NC_056057.1	66160001	66180001	40.4505	0.259129	OL	PDE1C
NC_056060.1	8535001	8555001	3.08903	0.592792	OL	PDE8B
NC_056060.1	8540001	8560001	2.49337	0.562063	OL	PDE8B
NC_056060.1	8560001	8580001	2.56313	0.566119	OL	PDE8B
NC_056054.1	263675001	263695001	3.13429	0.268291	OL	PDE9A
NC_056054.1	263680001	263700001	4.21136	0.256236	OL	PDE9A
NC_056068.1	4205001	4225001	4.46256	0.257747	OL	PDGFD
NC_056068.1	4210001	4230001	8.91369	0.279202	OL	PDGFD
NC_056068.1	4215001	4235001	7.2887	0.245925	OL	PDGFD
NC_056068.1	4220001	4240001	5.1817	0.259801	OL	PDGFD
NC_056068.1	4225001	4245001	3.81161	0.276492	OL	PDGFD
NC_056068.1	4230001	4250001	3.35301	0.249541	OL	PDGFD
NC_056068.1	4235001	4255001	3.3538	0.293565	OL	PDGFD
NC_056068.1	4240001	4260001	4.10944	0.262737	OL	PDGFD
NC_056056.1	145540001	145560001	2.99514	0.234809	OL	PDZRN4
NC_056056.1	145545001	145565001	9.94929	0.329642	OL	PDZRN4
NC_056056.1	145550001	145570001	8.69881	0.341758	OL	PDZRN4
NC_056056.1	145555001	145575001	3.62247	0.266629	OL	PDZRN4
NC_056060.1	66790001	66810001	10.0776	0.220905	OL	PELI2
NC_056061.1	68385001	68405001	5.24294	0.229233	OL	PHACTR2
NC_056061.1	68435001	68455001	5.66991	0.2373	OL	PHACTR2
NC_056057.1	20075001	20095001	2.96333	0.409231	OL	PHF14
NC_056055.1	2675001	2695001	3.15024	0.275893	OL	PHF19
NC_056055.1	2680001	2700001	3.23691	0.307495	OL	PHF19
NC_056055.1	2685001	2705001	4.24583	0.357192	OL	PHF19
NC_056055.1	27915001	27935001	3.08513	0.274433	OL	PHF2
NC_056055.1	27920001	27940001	4.49522	0.241091	OL	PHF2
NC_056055.1	27935001	27955001	4.88606	0.225284	OL	PHF2
NC_056055.1	27940001	27960001	5.37558	0.262835	OL	PHF2
NC_056055.1	27945001	27965001	8.10932	0.311429	OL	PHF2
NC_056055.1	27950001	27970001	6.67016	0.330916	OL	PHF2
NC_056054.1	97075001	97095001	2.5403	0.413388	OL	PHGDH
NC_056054.1	97085001	97105001	3.11014	0.423806	OL	PHGDH
NC_056054.1	97090001	97110001	2.72784	0.3967	OL	PHGDH
NC_056076.1	60870001	60890001	4.59048	0.232232	OL	PIGN
NC_056076.1	60875001	60895001	26.2689	0.280518	OL	PIGN

NC_056076.1	60880001	60900001	28.4782	0.281962	OL	PIGN
NC_056076.1	60895001	60915001	31.2318	0.316428	OL	PIGN
NC_056076.1	60900001	60920001	32.0472	0.349261	OL	PIGN;RELCH
NC_056076.1	60905001	60925001	30.8296	0.372036	OL	PIGN;RELCH
NC_056076.1	60915001	60935001	22.1961	0.336416	OL	PIGN;RELCH
NC_056076.1	14235001	14255001	15.6723	0.239168	OL	PIK3C3
NC_056076.1	14240001	14260001	16.4285	0.257101	OL	PIK3C3
NC_056057.1	49520001	49540001	2.54103	0.212826	OL	PIK3CG
NC_056067.1	7270001	7290001	8.73528	0.250694	OL	PKD1L2
NC_056067.1	7275001	7295001	14.5874	0.304489	OL	PKD1L2
NC_056067.1	7280001	7300001	18.2	0.307409	OL	PKD1L2
NC_056067.1	7290001	7310001	5.13702	0.238416	OL	PKD1L2
NC_056059.1	37305001	37325001	7.47711	0.215089	OL	PKD2
NC_056069.1	33370001	33390001	2.89605	0.227642	OL	PLCXD3
NC_056069.1	33375001	33395001	2.6824	0.23039	OL	PLCXD3
NC_056069.1	33380001	33400001	2.53854	0.21947	OL	PLCXD3
NC_056054.1	216415001	216435001	2.42508	0.276479	OL	PLD1
NC_056054.1	216440001	216460001	7.61273	0.216065	OL	PLD1
NC_056054.1	216445001	216465001	20.4026	0.251867	OL	PLD1
NC_056054.1	216460001	216480001	20.2035	0.315148	OL	PLD1
NC_056054.1	216465001	216485001	19.161	0.271328	OL	PLD1
NC_056056.1	196165001	196185001	2.4429	0.415589	OL	PLEKHA5
NC_056056.1	196170001	196190001	4.65663	0.39861	OL	PLEKHA5
NC_056056.1	196200001	196220001	2.36297	0.508127	OL	PLEKHA5
NC_056054.1	76670001	76690001	6.98324	0.25377	OL	PLPPR4
NC_056054.1	76675001	76695001	11.417	0.325048	OL	PLPPR4
NC_056054.1	76680001	76700001	16.8435	0.321493	OL	PLPPR4
NC_056054.1	76685001	76705001	18.8333	0.391667	OL	PLPPR4
NC_056054.1	76690001	76710001	17.528	0.31341	OL	PLPPR4
NC_056054.1	76705001	76725001	2.85941	0.270375	OL	PLPPR4
NC_056068.1	21335001	21355001	2.87535	0.241837	OL	POU2AF1
NC_056068.1	21340001	21360001	2.43334	0.234754	OL	POU2AF1
NC_056058.1	58930001	58950001	2.35752	0.253297	OL	PPARGC1B
NC_056056.1	117695001	117715001	3.05095	0.298275	OL	PPFIA2
NC_056056.1	117700001	117720001	6.13093	0.275362	OL	PPFIA2
NC_056056.1	115830001	115850001	5.29501	0.227197	OL	PPP1R12A
NC_056056.1	115835001	115855001	11.956	0.269278	OL	PPP1R12A
NC_056056.1	115840001	115860001	7.01133	0.25334	OL	PPP1R12A
NC_056056.1	115855001	115875001	2.61588	0.215994	OL	PPP1R12A
NC_056056.1	115860001	115880001	3.04068	0.217439	OL	PPP1R12A
NC_056065.1	79590001	79610001	3.20132	0.215402	OL	PPP1R12B
NC_056065.1	79595001	79615001	3.20714	0.254506	OL	PPP1R12B
NC_056065.1	79625001	79645001	2.42175	0.226078	OL	PPP1R12B
NC_056055.1	127660001	127680001	7.18117	0.237761	OL	PPP1R1C
NC_056055.1	127665001	127685001	7.11358	0.251198	OL	PPP1R1C
NC_056055.1	127670001	127690001	8.15242	0.271428	OL	PPP1R1C
NC_056055.1	127675001	127695001	7.25714	0.269894	OL	PPP1R1C
NC_056055.1	127680001	127700001	4.67947	0.249309	OL	PPP1R1C
NC_056055.1	127690001	127710001	3.35376	0.217856	OL	PPP1R1C
NC_056055.1	127695001	127715001	3.29775	0.229137	OL	PPP1R1C
NC_056059.1	5030001	5050001	2.49433	0.28089	OL	PRDM5
NC_056065.1	54285001	54305001	3.89894	0.250803	OL	PRDX6;SLC9C2
NC_056065.1	54290001	54310001	5.27868	0.284464	OL	PRDX6;SLC9C2
NC_056056.1	79980001	80000001	10.594	0.234734	OL	PREPL
NC_056056.1	79985001	80005001	3.26467	0.24859	OL	PREPL;SLC3A1
NC_056072.1	51180001	51200001	3.03954	0.432735	OL	PRKAR2A
NC_056056.1	78605001	78625001	4.97805	0.297474	OL	PRKCE
NC_056060.1	71810001	71830001	3.89321	0.231836	OL	PRKCH
NC_056075.1	7825001	7845001	3.68188	0.264464	OL	PRKG1
NC_056075.1	7830001	7850001	6.66313	0.273822	OL	PRKG1
NC_056075.1	7840001	7860001	32.193	0.26414	OL	PRKG1
NC_056075.1	7845001	7865001	25.2794	0.23422	OL	PRKG1
NC_056075.1	7850001	7870001	15.1665	0.222948	OL	PRKG1
NC_056061.1	85690001	85710001	3.42238	0.233619	OL	PRKN
NC_056061.1	85695001	85715001	3.75583	0.228422	OL	PRKN
NC_056059.1	112025001	112045001	3.11858	0.268602	OL	PROM1
NC_056059.1	112030001	112050001	7.94139	0.423676	OL	PROM1
NC_056059.1	112035001	112055001	15.1888	0.447671	OL	PROM1
NC_056059.1	112040001	112060001	17.3064	0.419847	OL	PROM1
NC_056059.1	112045001	112065001	12.617	0.306574	OL	PROM1
NC_056056.1	136935001	136955001	4.01701	0.255514	OL	PRPH
NC_056056.1	136940001	136960001	5.17339	0.247203	OL	PRPH
NC_056077.1	41750001	41770001	4.42501	0.292994	OL	PSMG3
NC_056077.1	41755001	41775001	4.49178	0.300629	OL	PSMG3;TMEM184A

NC_056077.1	41760001	41780001	4.10141	0.248659	OL	PSMG3;TMEM184A
NC_056071.1	29875001	29895001	3.36187	0.373705	OL	PSTPIP1
NC_056071.1	29880001	29900001	3.72618	0.391842	OL	PSTPIP1
NC_056071.1	29885001	29905001	2.86728	0.366761	OL	PSTPIP1
NC_056055.1	13875001	13895001	3.1523	0.354135	OL	PTPN3
NC_056055.1	78135001	78155001	3.48504	0.28848	OL	PTPRD
NC_056072.1	39215001	39235001	4.91712	0.420531	OL	PTPRG
NC_056072.1	39220001	39240001	4.01917	0.416875	OL	PTPRG
NC_056072.1	39525001	39545001	3.87457	0.361061	OL	PTPRG
NC_056072.1	39530001	39550001	6.32451	0.354989	OL	PTPRG
NC_056072.1	39535001	39555001	3.59615	0.280691	OL	PTPRG
NC_056055.1	71160001	71180001	2.70152	0.30349	OL	PUM3
NC_056057.1	48100001	48120001	5.01986	0.220763	OL	PUS7
NC_056056.1	194330001	194350001	3.69141	0.276298	OL	PYROXD1
NC_056064.1	43560001	43580001	7.96154	0.257993	OL	PYY
NC_056064.1	43565001	43585001	7.43403	0.253512	OL	PYY
NC_056059.1	4340001	4360001	3.15024	0.256857	OL	QRFPR
NC_056056.1	33375001	33395001	2.35862	0.273791	OL	RAB10
NC_056065.1	55460001	55480001	5.3502	0.264778	OL	RABGAP1L
NC_056065.1	55465001	55485001	6.99562	0.2152	OL	RABGAP1L
NC_056065.1	55505001	55525001	2.6093	0.282576	OL	RABGAP1L
NC_056065.1	55515001	55535001	2.46438	0.302181	OL	RABGAP1L
NC_056065.1	55520001	55540001	3.56146	0.260652	OL	RABGAP1L
NC_056057.1	78135001	78155001	2.64046	0.226109	OL	RAMP3
NC_056057.1	78140001	78160001	4.15301	0.266775	OL	RAMP3
NC_056057.1	78145001	78165001	6.69275	0.290619	OL	RAMP3
NC_056057.1	78150001	78170001	3.66083	0.278986	OL	RAMP3
NC_056058.1	20645001	20665001	2.37245	0.256451	OL	RAPGEF6
NC_056058.1	20650001	20670001	2.39011	0.253755	OL	RAPGEF6
NC_056079.1	39770001	39790001	7.09183	0.420307	OL	RARB
NC_056079.1	39775001	39795001	10.269	0.45681	OL	RARB
NC_056079.1	39780001	39800001	11.7329	0.434673	OL	RARB
NC_056079.1	39785001	39805001	15.3333	0.384549	OL	RARB
NC_056065.1	58935001	58955001	2.76255	0.235819	OL	RASAL2
NC_056065.1	58940001	58960001	4.30168	0.260128	OL	RASAL2
NC_056065.1	58945001	58965001	4.38117	0.257771	OL	RASAL2
NC_056065.1	58950001	58970001	2.56949	0.230951	OL	RASAL2
NC_056065.1	58990001	59010001	2.94687	0.223038	OL	RASAL2
NC_056065.1	58995001	59015001	3.1649	0.236106	OL	RASAL2
NC_056065.1	59000001	59020001	3.66219	0.242142	OL	RASAL2
NC_056077.1	6610001	6630001	4.32675	0.254112	OL	RBFOX1
NC_056077.1	6615001	6635001	4.78188	0.253563	OL	RBFOX1
NC_056077.1	6620001	6640001	4.46226	0.240848	OL	RBFOX1
NC_056077.1	6625001	6645001	4.07441	0.244902	OL	RBFOX1
NC_056073.1	39305001	39325001	2.34615	0.233793	OL	RBM24
NC_056073.1	39310001	39330001	2.46796	0.239404	OL	RBM24
NC_056072.1	3865001	3885001	2.51435	0.284422	OL	RBMS3
NC_056072.1	3870001	3890001	2.8431	0.241655	OL	RBMS3
NC_056072.1	4475001	4495001	2.78628	0.227095	OL	RBMS3
NC_056072.1	4480001	4500001	2.37534	0.240841	OL	RBMS3
NC_056072.1	4485001	4505001	2.71655	0.238553	OL	RBMS3
NC_056073.1	19550001	19570001	4.19759	0.2643	OL	RCAN2
NC_056073.1	19555001	19575001	2.55969	0.231762	OL	RCAN2
NC_056073.1	19670001	19690001	21.0476	0.212903	OL	RCAN2
NC_056071.1	29845001	29865001	8.51093	0.25046	OL	RCN2
NC_056055.1	52415001	52435001	3.32759	0.263855	OL	RECK
NC_056055.1	52420001	52440001	12.7736	0.342238	OL	RECK
NC_056076.1	60920001	60940001	16.307	0.277621	OL	RELCH
NC_056076.1	60925001	60945001	10.6454	0.217325	OL	RELCH
NC_056076.1	60945001	60965001	9.75212	0.233867	OL	RELCH
NC_056076.1	60950001	60970001	11.165	0.269754	OL	RELCH
NC_056076.1	61010001	61030001	4.7544	0.274824	OL	RELCH
NC_056076.1	61015001	61035001	2.62342	0.216877	OL	RELCH
NC_056057.1	46470001	46490001	3.02922	0.547101	OL	RELN
NC_056057.1	46480001	46500001	2.81932	0.428781	OL	RELN
NC_056057.1	46485001	46505001	2.91915	0.350466	OL	RELN
NC_056057.1	46490001	46510001	2.56897	0.240155	OL	RELN
NC_056065.1	44155001	44175001	3.58846	0.22194	OL	RERE
NC_056065.1	44160001	44180001	6.91379	0.254442	OL	RERE
NC_056065.1	44165001	44185001	7.83814	0.267327	OL	RERE
NC_056065.1	44170001	44190001	9.2778	0.263486	OL	RERE
NC_056065.1	44175001	44195001	8.10793	0.244951	OL	RERE
NC_056065.1	44180001	44200001	6.1976	0.213771	OL	RERE
NC_056056.1	100120001	100140001	20.562	0.434403	OL	RFX8

NC_056065.1	64380001	64400001	3.34649	0.237087	OL	RGL1
NC_056065.1	64390001	64410001	4.83457	0.220466	OL	RGL1
NC_056060.1	81585001	81605001	2.50284	0.317753	OL	RGS6
NC_056060.1	81590001	81610001	3.00565	0.383056	OL	RGS6
NC_056060.1	81595001	81615001	3.50363	0.451033	OL	RGS6
NC_056060.1	81600001	81620001	3.54377	0.430104	OL	RGS6
NC_056060.1	81625001	81645001	3.0787	0.365901	OL	RGS6
NC_056060.1	81630001	81650001	3.60402	0.39293	OL	RGS6
NC_056060.1	81635001	81655001	3.51005	0.401892	OL	RGS6
NC_056060.1	81640001	81660001	3.31441	0.398696	OL	RGS6
NC_056060.1	81645001	81665001	2.40047	0.341321	OL	RGS6
NC_056056.1	212700001	212720001	2.87559	0.276597	OL	RHNO1;TULP3
NC_056062.1	73880001	73900001	3.76958	0.252434	OL	RIMS2
NC_056062.1	73885001	73905001	3.33447	0.278523	OL	RIMS2
NC_056076.1	33575001	33595001	4.38462	0.240418	OL	RIOK3
NC_056063.1	20740001	20760001	3.64706	0.243545	OL	RNASEH2B
NC_056063.1	20745001	20765001	5.34532	0.261282	OL	RNASEH2B
NC_056058.1	68340001	68360001	2.61422	0.297534	OL	RNF145
NC_056058.1	68345001	68365001	2.76782	0.327294	OL	RNF145
NC_056058.1	68350001	68370001	3	0.353604	OL	RNF145
NC_056062.1	76990001	77010001	3.04106	0.432356	OL	RNF19A
NC_056062.1	76995001	77015001	3.99727	0.437071	OL	RNF19A
NC_056062.1	77000001	77020001	3.60308	0.400133	OL	RNF19A
NC_056062.1	77005001	77025001	2.93814	0.321728	OL	RNF19A
NC_056062.1	77010001	77030001	3.3649	0.287347	OL	RNF19A
NC_056062.1	77015001	77035001	3.08873	0.298447	OL	RNF19A
NC_056062.1	77020001	77040001	3.85035	0.296944	OL	RNF19A
NC_056062.1	77025001	77045001	2.37179	0.237635	OL	RNF19A
NC_056064.1	51715001	51735001	10.6554	0.314105	OL	RNF213
NC_056055.1	52105001	52125001	5.55844	0.383237	OL	RNF38
NC_056055.1	52125001	52145001	2.67204	0.45051	OL	RNF38
NC_056064.1	9060001	9080001	2.54378	0.300474	OL	RNF43
NC_056064.1	9065001	9085001	2.4898	0.283096	OL	RNF43
NC_056054.1	147740001	147760001	2.54275	0.218557	OL	ROBO1
NC_056054.1	147745001	147765001	4.61954	0.265594	OL	ROBO1
NC_056054.1	147750001	147770001	2.92075	0.236877	OL	ROBO1
NC_056054.1	147755001	147775001	2.41568	0.222094	OL	ROBO1
NC_056054.1	145375001	145395001	3.15492	0.214532	OL	ROBO2
NC_056054.1	39205001	39225001	2.51428	0.214273	OL	ROR1
NC_056060.1	47125001	47145001	4.43669	0.225553	OL	RORA
NC_056060.1	47130001	47150001	3.88811	0.335898	OL	RORA
NC_056060.1	47135001	47155001	3.51525	0.374516	OL	RORA
NC_056060.1	47140001	47160001	3.35069	0.481397	OL	RORA
NC_056060.1	47145001	47165001	2.84573	0.449506	OL	RORA
NC_056055.1	104515001	104535001	4.21054	0.287689	OL	RP1L1
NC_056055.1	104520001	104540001	7.82059	0.325933	OL	RP1L1
NC_056055.1	104525001	104545001	10.8514	0.32872	OL	RP1L1
NC_056055.1	104530001	104550001	12.4838	0.350677	OL	RP1L1
NC_056055.1	104535001	104555001	6.60627	0.290447	OL	RP1L1
NC_056080.1	37840001	37860001	2.91607	0.214052	OL	RPGR
NC_056077.1	14200001	14220001	3.13193	0.24528	OL	RRN3
NC_056077.1	14205001	14225001	3.61225	0.242755	OL	RRN3
NC_056066.1	30490001	30510001	3.62387	0.217637	OL	RSU1
NC_056066.1	30495001	30515001	8.65267	0.300032	OL	RSU1
NC_056066.1	30500001	30520001	4.41942	0.287636	OL	RSU1
NC_056066.1	30505001	30525001	3.42231	0.278701	OL	RSU1
NC_056066.1	30510001	30530001	2.95885	0.305123	OL	RSU1
NC_056066.1	30520001	30540001	2.36574	0.314723	OL	RSU1
NC_056064.1	43835001	43855001	3.07058	0.251903	OL	RUNDC3A
NC_056064.1	43840001	43860001	3.76988	0.257918	OL	RUNDC3A
NC_056054.1	267645001	267665001	2.81995	0.240817	OL	RUNX1
NC_056054.1	267660001	267680001	4.52795	0.259559	OL	RUNX1
NC_056060.1	8065001	8085001	2.47567	0.378926	OL	S100Z
NC_056060.1	8070001	8090001	3.42644	0.369195	OL	S100Z
NC_056060.1	8075001	8095001	5.63398	0.327096	OL	S100Z
NC_056060.1	8080001	8100001	7.04221	0.330499	OL	S100Z
NC_056060.1	8085001	8105001	3.20182	0.236832	OL	S100Z
NC_056054.1	143475001	143495001	3.28843	0.229983	OL	SAMSN1
NC_056054.1	143480001	143500001	2.44166	0.215331	OL	SAMSN1
NC_056055.1	117465001	117485001	2.61643	0.407832	OL	SAP130
NC_056055.1	117470001	117490001	2.35616	0.38182	OL	SAP130
NC_056055.1	117495001	117515001	2.38566	0.268357	OL	SAP130
NC_056055.1	117500001	117520001	3.17235	0.299427	OL	SAP130
NC_056055.1	117505001	117525001	2.47313	0.282733	OL	SAP130

NC_056055.1	117510001	117530001	2.41917	0.28288	OL	SAP130
NC_056058.1	43765001	43785001	2.387	0.352888	OL	SAR1B
NC_056058.1	43770001	43790001	2.41015	0.360732	OL	SAR1B
NC_056061.1	79310001	79330001	2.71951	0.344431	OL	SCAF8
NC_056061.1	79315001	79335001	2.70948	0.3347	OL	SCAF8
NC_056061.1	79320001	79340001	3.43599	0.307914	OL	SCAF8
NC_056063.1	53340001	53360001	2.48213	0.221852	OL	SCEL
NC_056070.1	29140001	29160001	2.78917	0.382837	OL	SCLT1
NC_056070.1	29145001	29165001	3.40039	0.454746	OL	SCLT1
NC_056070.1	29150001	29170001	3.10017	0.394317	OL	SCLT1
NC_056070.1	29155001	29175001	3.44985	0.369552	OL	SCLT1
NC_056070.1	29160001	29180001	3.67052	0.340757	OL	SCLT1
NC_056070.1	29165001	29185001	6.54797	0.321959	OL	SCLT1
NC_056070.1	29170001	29190001	9.05098	0.340183	OL	SCLT1
NC_056070.1	29175001	29195001	10.8841	0.330083	OL	SCLT1
NC_056054.1	15595001	15615001	2.52806	0.220358	OL	SCMH1
NC_056054.1	15600001	15620001	2.6008	0.285123	OL	SCMH1
NC_056054.1	15605001	15625001	2.63622	0.305953	OL	SCMH1
NC_056054.1	15610001	15630001	2.46378	0.264681	OL	SCMH1
NC_056054.1	15615001	15635001	2.33519	0.236959	OL	SCMH1
NC_056054.1	15535001	15555001	3.00926	0.394713	OL	SCMH1;SLFNL1
NC_056054.1	15540001	15560001	2.84616	0.382564	OL	SCMH1;SLFNL1
NC_056054.1	15545001	15565001	2.46629	0.283211	OL	SCMH1;SLFNL1
NC_056072.1	12135001	12155001	2.48241	0.251816	OL	SCN10A
NC_056059.1	16230001	16250001	3.22222	0.243514	OL	SEC24B
NC_056059.1	16235001	16255001	3.4499	0.232959	OL	SEC24B
NC_056059.1	16240001	16260001	5.77478	0.255384	OL	SEC24B
NC_056059.1	16245001	16265001	5.89842	0.25227	OL	SEC24B
NC_056056.1	225510001	225530001	6.008	0.376816	OL	SELENOO;TUBGCP6
NC_056067.1	48545001	48565001	2.52174	0.253933	OL	SELENOV
NC_056056.1	62410001	62430001	5.80401	0.393792	OL	SEPTIN10
NC_056056.1	62415001	62435001	5.12048	0.393223	OL	SEPTIN10
NC_056056.1	62420001	62440001	5.72858	0.364083	OL	SEPTIN10
NC_056056.1	62425001	62445001	5.81343	0.39008	OL	SEPTIN10
NC_056056.1	62430001	62450001	5.93924	0.385664	OL	SEPTIN10
NC_056056.1	62435001	62455001	5.8458	0.389407	OL	SEPTIN10
NC_056056.1	62440001	62460001	8.49462	0.423662	OL	SEPTIN10
NC_056056.1	62445001	62465001	10.8482	0.43623	OL	SEPTIN10
NC_056056.1	62450001	62470001	8.05556	0.419544	OL	SEPTIN10
NC_056056.1	62455001	62475001	6.44558	0.393331	OL	SEPTIN10
NC_056056.1	62405001	62425001	6.52336	0.386974	OL	SEPTIN10;SH3RF3
NC_056056.1	62460001	62480001	4.0254	0.34625	OL	SEPTIN10;SOWAHC
NC_056078.1	34665001	34685001	3.69857	0.27698	OL	SFTPD
NC_056061.1	59575001	59595001	4.68422	0.230182	OL	SGK1
NC_056055.1	86175001	86195001	2.56025	0.213247	OL	SH3GL2
NC_056080.1	18095001	18115001	3.98272	0.350353	OL	SH3KBP1
NC_056080.1	18115001	18135001	9.37502	0.236503	OL	SH3KBP1
NC_056080.1	18220001	18240001	3.90099	0.359065	OL	SH3KBP1
NC_056080.1	18225001	18245001	3.30232	0.325205	OL	SH3KBP1
NC_056080.1	18230001	18250001	3.16908	0.349307	OL	SH3KBP1
NC_056080.1	18235001	18255001	3.26398	0.331479	OL	SH3KBP1
NC_056080.1	18240001	18260001	3.5	0.342406	OL	SH3KBP1
NC_056080.1	18245001	18265001	9.44916	0.308916	OL	SH3KBP1
NC_056056.1	62375001	62395001	3.9972	0.225688	OL	SH3RF3
NC_056056.1	62380001	62400001	2.5347	0.238972	OL	SH3RF3
NC_056056.1	62390001	62410001	2.73846	0.281413	OL	SH3RF3
NC_056056.1	62395001	62415001	3.89087	0.331899	OL	SH3RF3
NC_056056.1	62400001	62420001	4.73947	0.354188	OL	SH3RF3
NC_056074.1	43965001	43985001	2.52557	0.228324	OL	SHANK2
NC_056074.1	43970001	43990001	2.3428	0.250217	OL	SHANK2
NC_056074.1	44055001	44075001	3.52892	0.277216	OL	SHANK2
NC_056074.1	44060001	44080001	4.65448	0.291635	OL	SHANK2
NC_056074.1	44065001	44085001	3.19589	0.310043	OL	SHANK2
NC_056074.1	44070001	44090001	2.38484	0.286264	OL	SHANK2
NC_056074.1	25595001	25615001	6.19049	0.33764	OL	SIAE;SPA17
NC_056067.1	56010001	56030001	2.45283	0.238265	OL	SIGLEC11;VRK3
NC_056068.1	27325001	27345001	2.49468	0.254017	OL	SIK3
NC_056068.1	27330001	27350001	2.86324	0.327483	OL	SIK3
NC_056068.1	27370001	27390001	3.57875	0.313363	OL	SIK3
NC_056068.1	27375001	27395001	5.53846	0.273177	OL	SIK3
NC_056068.1	27380001	27400001	8.07142	0.251417	OL	SIK3
NC_056068.1	27395001	27415001	4.60741	0.246378	OL	SIK3
NC_056068.1	27400001	27420001	2.75675	0.294933	OL	SIK3
NC_056068.1	27460001	27480001	3.87999	0.226644	OL	SIK3

NC_056060.1	5025001	5045001	2.4515	0.247682	OL	SIMC1
NC_056060.1	5030001	5050001	2.53855	0.274077	OL	SIMC1
NC_056060.1	5035001	5055001	2.61432	0.256794	OL	SIMC1
NC_056060.1	5040001	5060001	2.78752	0.235488	OL	SIMC1
NC_056060.1	5045001	5065001	5.14322	0.265191	OL	SIMC1
NC_056060.1	5050001	5070001	4.19401	0.253217	OL	SIMC1
NC_056060.1	5055001	5075001	5.28665	0.239859	OL	SIMC1
NC_056060.1	5060001	5080001	6.07317	0.229478	OL	SIMC1
NC_056060.1	5065001	5085001	6.03531	0.282804	OL	SIMC1
NC_056060.1	5070001	5090001	11.8606	0.335732	OL	SIMC1
NC_056060.1	5075001	5095001	9.43243	0.420104	OL	SIMC1
NC_056060.1	5080001	5100001	5.07472	0.384387	OL	SIMC1
NC_056060.1	5085001	5105001	3.23679	0.263864	OL	SIMC1
NC_056064.1	37950001	37970001	3.65158	0.257145	OL	SKAP1
NC_056064.1	37955001	37975001	4.71186	0.305512	OL	SKAP1
NC_056064.1	37960001	37980001	6.31465	0.338859	OL	SKAP1
NC_056064.1	37965001	37985001	5.4074	0.31335	OL	SKAP1
NC_056064.1	37970001	37990001	3.37024	0.228118	OL	SKAP1
NC_056064.1	38005001	38025001	4.61683	0.213555	OL	SKAP1
NC_056064.1	38010001	38030001	7.5255	0.256751	OL	SKAP1
NC_056064.1	38015001	38035001	9.17263	0.269044	OL	SKAP1
NC_056064.1	38025001	38045001	7.87366	0.304621	OL	SKAP1
NC_056064.1	38030001	38050001	4.75125	0.282783	OL	SKAP1
NC_056064.1	38035001	38055001	3.0106	0.260327	OL	SKAP1
NC_056062.1	21385001	21405001	4.23628	0.435716	OL	SLA;TG
NC_056057.1	89090001	89110001	3.12547	0.319399	OL	SLC13A1
NC_056076.1	45350001	45370001	3.03183	0.351074	OL	SLC14A2
NC_056076.1	45355001	45375001	4.41261	0.252768	OL	SLC14A2
NC_056054.1	187195001	187215001	2.36054	0.242202	OL	SLC15A2
NC_056056.1	199240001	199260001	3.82554	0.248302	OL	SLC15A5
NC_056056.1	199245001	199265001	5.79822	0.303273	OL	SLC15A5
NC_056056.1	199250001	199270001	5.06822	0.289035	OL	SLC15A5
NC_056056.1	199255001	199275001	6.16023	0.305963	OL	SLC15A5
NC_056056.1	199260001	199280001	4.9155	0.286793	OL	SLC15A5
NC_056080.1	66640001	66660001	4.48717	0.282887	OL	SLC16A2
NC_056080.1	66645001	66665001	2.7301	0.241392	OL	SLC16A2
NC_056066.1	46940001	46960001	2.33688	0.320842	OL	SLC23A2
NC_056055.1	88135001	88155001	2.76614	0.221707	OL	SLC24A2
NC_056072.1	51110001	51130001	2.73958	0.284654	OL	SLC25A20
NC_056074.1	40830001	40850001	2.63888	0.434537	OL	SLC29A2
NC_056074.1	40835001	40855001	2.98438	0.360479	OL	SLC29A2
NC_056054.1	17365001	17385001	3.29931	0.249131	OL	SLC2A1
NC_056054.1	17370001	17390001	2.52305	0.263499	OL	SLC2A1
NC_056056.1	147570001	147590001	10.5153	0.263878	OL	SLC2A13
NC_056056.1	147575001	147595001	15.7612	0.256721	OL	SLC2A13
NC_056056.1	147580001	147600001	23.114	0.258181	OL	SLC2A13
NC_056056.1	147585001	147605001	12.6576	0.222296	OL	SLC2A13
NC_056064.1	43815001	43835001	2.40389	0.215452	OL	SLC4A1
NC_056067.1	23375001	23395001	3.12245	0.218188	OL	SLC6A2
NC_056054.1	217620001	217640001	3.13658	0.283802	OL	SLC7A14
NC_056054.1	217625001	217645001	3.50639	0.29261	OL	SLC7A14
NC_056054.1	217630001	217650001	3.42094	0.241313	OL	SLC7A14
NC_056065.1	54295001	54315001	5.99999	0.24487	OL	SLC9C2
NC_056056.1	194335001	194355001	6.76032	0.300626	OL	SLCO1A2
NC_056056.1	194340001	194360001	5.30112	0.294742	OL	SLCO1A2
NC_056056.1	194345001	194365001	3.87069	0.298009	OL	SLCO1A2
NC_056080.1	51235001	51255001	2.38806	0.284051	OL	SMC1A
NC_056056.1	221640001	221660001	2.82629	0.267854	OL	SMC1B
NC_056056.1	221645001	221665001	2.37037	0.233914	OL	SMC1B
NC_056065.1	30605001	30625001	3.39434	0.258224	OL	SMYD3
NC_056080.1	63765001	63785001	2.41176	0.213131	OL	SNX12
NC_056062.1	93665001	93685001	8.11217	0.339431	OL	SNX16
NC_056062.1	93670001	93690001	4.33086	0.254553	OL	SNX16
NC_056058.1	28510001	28530001	2.70894	0.236574	OL	SNX24
NC_056079.1	14795001	14815001	2.39081	0.245642	OL	SNX25
NC_056056.1	77590001	77610001	2.86828	0.241165	OL	SOCS5
NC_056056.1	3610001	3630001	10.2778	0.446702	OL	SOHLH1
NC_056056.1	3615001	3635001	2.79493	0.263577	OL	SOHLH1
NC_056059.1	114330001	114350001	3.04809	0.255652	OL	SORCS2
NC_056056.1	191340001	191360001	3.07868	0.257091	OL	SOX5
NC_056056.1	191345001	191365001	11.0283	0.216957	OL	SOX5
NC_056056.1	191660001	191680001	2.33201	0.273385	OL	SOX5
NC_056056.1	191665001	191685001	2.73608	0.287102	OL	SOX5
NC_056056.1	191670001	191690001	3.54124	0.291306	OL	SOX5

NC_056056.1	191675001	191695001	4.87713	0.292982	OL	SOX5
NC_056056.1	191680001	191700001	7.9195	0.305347	OL	SOX5
NC_056056.1	191685001	191705001	19.0663	0.314106	OL	SOX5
NC_056056.1	191690001	191710001	7.70483	0.291887	OL	SOX5
NC_056056.1	191695001	191715001	3.57579	0.235707	OL	SOX5
NC_056056.1	192005001	192025001	2.59221	0.363014	OL	SOX5
NC_056056.1	192010001	192030001	3.98526	0.356336	OL	SOX5
NC_056056.1	192015001	192035001	6.01785	0.351622	OL	SOX5
NC_056056.1	192020001	192040001	7.84033	0.24927	OL	SOX5
NC_056064.1	58100001	58120001	2.52758	0.358517	OL	SOX9
NC_056069.1	1645001	1665001	6.21703	0.250935	OL	SPDL1
NC_056069.1	1650001	1670001	29.3846	0.286734	OL	SPDL1
NC_056069.1	1655001	1675001	47.6429	0.235258	OL	SPDL1
NC_056060.1	11870001	11890001	6.01858	0.284479	OL	SPG21
NC_056054.1	179140001	179160001	2.70809	0.21838	OL	SPICE1
NC_056060.1	86840001	86860001	28.3204	0.334065	OL	SPTLC2
NC_056066.1	6445001	6465001	7.76833	0.215982	OL	SPTLC3
NC_056066.1	6450001	6470001	8.34957	0.221773	OL	SPTLC3
NC_056070.1	56300001	56320001	26.2165	0.250314	OL	SRRM4
NC_056070.1	56315001	56335001	8.99999	0.243758	OL	SRRM4
NC_056070.1	56320001	56340001	4.34177	0.220775	OL	SRRM4
NC_056062.1	20955001	20975001	5.4628	0.240569	OL	ST3GAL1
NC_056062.1	20960001	20980001	11.9621	0.367203	OL	ST3GAL1
NC_056062.1	20995001	21015001	8.42587	0.382698	OL	ST3GAL1
NC_056062.1	21000001	21020001	5.18596	0.36977	OL	ST3GAL1
NC_056062.1	21005001	21025001	3.71126	0.366736	OL	ST3GAL1
NC_056062.1	21035001	21055001	2.3578	0.280073	OL	ST3GAL1
NC_056062.1	21040001	21060001	2.45259	0.265026	OL	ST3GAL1
NC_056056.1	193425001	193445001	5.5197	0.287115	OL	ST8SIA1
NC_056056.1	193430001	193450001	3.18955	0.27663	OL	ST8SIA1
NC_056056.1	193435001	193455001	3.27357	0.27819	OL	ST8SIA1
NC_056058.1	97700001	97720001	9.06719	0.25617	OL	ST8SIA4
NC_056057.1	51575001	51595001	2.50878	0.224666	OL	STARD3NL
NC_056057.1	51580001	51600001	2.6955	0.238606	OL	STARD3NL
NC_056080.1	61620001	61640001	3.78934	0.461144	OL	STARD8
NC_056064.1	42215001	42235001	3.11421	0.352092	OL	STAT3
NC_056058.1	56305001	56325001	4.56385	0.241173	OL	STK32A
NC_056055.1	38570001	38590001	4.20747	0.256167	OL	STMN4
NC_056055.1	38575001	38595001	2.98664	0.235319	OL	STMN4
NC_056079.1	13635001	13655001	8.9045	0.234983	OL	STOX2
NC_056056.1	77245001	77265001	2.81166	0.228678	OL	STPG4
NC_056070.1	46400001	46420001	3.72499	0.213397	OL	STX2
NC_056057.1	81985001	82005001	5.72668	0.215054	OL	SUGCT
NC_056057.1	82390001	82410001	3.78028	0.261896	OL	SUGCT
NC_056057.1	82395001	82415001	12.0381	0.390431	OL	SUGCT
NC_056057.1	82415001	82435001	9.96062	0.354651	OL	SUGCT
NC_056057.1	82420001	82440001	3.20584	0.307311	OL	SUGCT
NC_056057.1	82425001	82445001	2.81592	0.289341	OL	SUGCT
NC_056057.1	82510001	82530001	3.27917	0.317227	OL	SUGCT
NC_056057.1	82515001	82535001	3.87885	0.377436	OL	SUGCT
NC_056057.1	82545001	82565001	2.79869	0.552124	OL	SUGCT
NC_056057.1	82550001	82570001	3.3896	0.469383	OL	SUGCT
NC_056057.1	82555001	82575001	2.46863	0.319136	OL	SUGCT
NC_056062.1	46335001	46355001	13.3095	0.213543	OL	SULF1
NC_056062.1	46340001	46360001	7.6	0.246869	OL	SULF1
NC_056062.1	46345001	46365001	4.57875	0.216965	OL	SULF1
NC_056064.1	8990001	9010001	8.65036	0.268785	OL	SUPT4H1;TSPOAP1
NC_056056.1	115445001	115465001	3.28827	0.313164	OL	SYT1
NC_056056.1	115450001	115470001	2.94901	0.239715	OL	SYT1
NC_056056.1	182010001	182030001	3.14696	0.215531	OL	SYT10
NC_056056.1	182015001	182035001	3.09925	0.220529	OL	SYT10
NC_056080.1	37305001	37325001	2.36296	0.251087	OL	SYTL5
NC_056074.1	39790001	39810001	2.69269	0.224258	OL	SYVN1
NC_056061.1	57945001	57965001	3.45126	0.309659	OL	TAAR8
NC_056061.1	57950001	57970001	4.91882	0.33629	OL	TAAR8
NC_056062.1	94085001	94105001	2.42445	0.230266	OL	TAF2
NC_056062.1	94090001	94110001	4.25345	0.281131	OL	TAF2
NC_056062.1	94095001	94115001	7.88104	0.338318	OL	TAF2
NC_056062.1	94100001	94120001	8.58335	0.33359	OL	TAF2
NC_056062.1	94105001	94125001	10.2792	0.329908	OL	TAF2
NC_056062.1	94110001	94130001	14.5813	0.350786	OL	TAF2
NC_056062.1	94115001	94135001	8.54819	0.306804	OL	TAF2
NC_056062.1	94120001	94140001	9.57023	0.327983	OL	TAF2
NC_056062.1	94125001	94145001	8.0735	0.329541	OL	TAF2

NC_056062.1	94130001	94150001	7.09274	0.32662	OL	TAF2
NC_056062.1	94135001	94155001	7.02917	0.303468	OL	TAF2
NC_056062.1	94140001	94160001	5.45645	0.270589	OL	TAF2
NC_056062.1	94145001	94165001	5.31419	0.23834	OL	TAF2
NC_056062.1	94175001	94195001	6.65015	0.223021	OL	TAF2
NC_056064.1	47420001	47440001	2.58895	0.258062	OL	TANC2
NC_056064.1	47425001	47445001	2.70567	0.26882	OL	TANC2
NC_056059.1	46660001	46680001	2.77259	0.21443	OL	TBC1D19
NC_056060.1	51245001	51265001	2.49714	0.245657	OL	TCF12
NC_056060.1	51250001	51270001	2.3619	0.273467	OL	TCF12
NC_056060.1	51315001	51335001	2.45517	0.231506	OL	TCF12
NC_056060.1	51505001	51525001	2.44025	0.248935	OL	TCF12
NC_056060.1	51510001	51530001	3.32143	0.217157	OL	TCF12
NC_056058.1	41660001	41680001	2.77194	0.225783	OL	TCF3
NC_056058.1	41675001	41695001	2.8189	0.283324	OL	TCF3
NC_056058.1	41680001	41700001	2.86154	0.278207	OL	TCF3
NC_056058.1	41685001	41705001	2.76456	0.277396	OL	TCF3
NC_056076.1	55265001	55285001	5.73684	0.230338	OL	TCF4
NC_056076.1	55270001	55290001	4.31262	0.236209	OL	TCF4
NC_056076.1	55275001	55295001	2.75523	0.21291	OL	TCF4
NC_056055.1	50215001	50235001	20.9885	0.216416	OL	TDRD7
NC_056055.1	50250001	50270001	3.32379	0.444349	OL	TDRD7
NC_056059.1	80515001	80535001	2.71059	0.317829	OL	TECRL
NC_056059.1	80520001	80540001	7.13487	0.364121	OL	TECRL
NC_056059.1	80525001	80545001	7.60769	0.361795	OL	TECRL
NC_056059.1	80530001	80550001	7.80976	0.362829	OL	TECRL
NC_056059.1	80535001	80555001	5.86086	0.34567	OL	TECRL
NC_056059.1	80540001	80560001	3.92697	0.318726	OL	TECRL
NC_056059.1	80545001	80565001	3.14161	0.314893	OL	TECRL
NC_056080.1	108945001	108965001	2.55512	0.254642	OL	TENM1
NC_056080.1	108950001	108970001	2.57934	0.257628	OL	TENM1
NC_056080.1	108955001	108975001	2.92274	0.275358	OL	TENM1
NC_056060.1	10605001	10625001	3.75315	0.266795	OL	TENT2
NC_056060.1	10610001	10630001	4.24015	0.236144	OL	TENT2
NC_056060.1	10615001	10635001	4.59576	0.237063	OL	TENT2
NC_056060.1	10620001	10640001	4.78486	0.24844	OL	TENT2
NC_056078.1	24185001	24205001	3.19623	0.227733	OL	TET1
NC_056078.1	24200001	24220001	6.33888	0.301875	OL	TET1
NC_056078.1	24205001	24225001	4.47419	0.269274	OL	TET1
NC_056078.1	24210001	24230001	3.49824	0.240182	OL	TET1
NC_056078.1	24235001	24255001	2.34483	0.410353	OL	TET1
NC_056078.1	24240001	24260001	3.2644	0.406125	OL	TET1
NC_056078.1	24245001	24265001	5.92538	0.355318	OL	TET1
NC_056078.1	24250001	24270001	7.45003	0.347673	OL	TET1
NC_056080.1	63555001	63575001	2.82655	0.672179	OL	TEX11
NC_056055.1	233310001	233330001	3.8	0.352364	OL	TEX44
NC_056055.1	233315001	233335001	3.27875	0.399934	OL	TEX44
NC_056055.1	233320001	233340001	2.8522	0.387791	OL	TEX44
NC_056055.1	233325001	233345001	2.49236	0.371003	OL	TEX44
NC_056055.1	9115001	9135001	4.4744	0.267386	OL	TEX48
NC_056055.1	9120001	9140001	5.15636	0.290968	OL	TEX48
NC_056055.1	9125001	9145001	3.47422	0.240771	OL	TEX48;TMEM268
NC_056057.1	53875001	53895001	3.26307	0.274917	OL	TFEC
NC_056057.1	53880001	53900001	4.35841	0.332948	OL	TFEC
NC_056057.1	53885001	53905001	7.68672	0.359542	OL	TFEC
NC_056057.1	53890001	53910001	14.2415	0.379889	OL	TFEC
NC_056057.1	53895001	53915001	14.7639	0.368099	OL	TFEC
NC_056057.1	53905001	53925001	5.8653	0.278426	OL	TFEC
NC_056057.1	53910001	53930001	3.0929	0.233536	OL	TFEC
NC_056057.1	53955001	53975001	2.68768	0.240686	OL	TFEC
NC_056057.1	53960001	53980001	4.08807	0.253473	OL	TFEC
NC_056057.1	53965001	53985001	10.5895	0.263324	OL	TFEC
NC_056057.1	53970001	53990001	18.8174	0.251281	OL	TFEC
NC_056057.1	54000001	54020001	20.5643	0.215051	OL	TFEC
NC_056062.1	21295001	21315001	4.44279	0.226503	OL	TG
NC_056062.1	21300001	21320001	7.83385	0.418215	OL	TG
NC_056056.1	80900001	80920001	2.40171	0.27278	OL	THADA
NC_056056.1	80905001	80925001	3.8349	0.305503	OL	THADA
NC_056056.1	80910001	80930001	22.4857	0.361835	OL	THADA
NC_056056.1	80930001	80950001	10.8828	0.283203	OL	THADA
NC_056056.1	80935001	80955001	4.32771	0.243921	OL	THADA
NC_056056.1	80940001	80960001	3.00259	0.27114	OL	THADA
NC_056061.1	53180001	53200001	3.36085	0.229153	OL	THEMIS
NC_056061.1	53185001	53205001	2.65517	0.258568	OL	THEMIS

NC_056061.1	53355001	53375001	2.91148	0.27059	OL	THEMIS
NC_056061.1	53360001	53380001	2.44409	0.324158	OL	THEMIS
NC_056060.1	18465001	18485001	2.38477	0.284119	OL	THSD4
NC_056060.1	18470001	18490001	3.84675	0.323759	OL	THSD4
NC_056060.1	18475001	18495001	4.69527	0.354974	OL	THSD4
NC_056060.1	18480001	18500001	3.19454	0.347955	OL	THSD4
NC_056055.1	173330001	173350001	2.52628	0.315844	OL	THSD7B
NC_056054.1	124815001	124835001	2.42893	0.226288	OL	TIAM1
NC_056054.1	124820001	124840001	2.84914	0.234163	OL	TIAM1
NC_056054.1	124825001	124845001	2.86602	0.23433	OL	TIAM1
NC_056055.1	138610001	138630001	2.8338	0.218444	OL	TLK1
NC_056055.1	138615001	138635001	2.72607	0.229983	OL	TLK1
NC_056055.1	138620001	138640001	2.33732	0.228238	OL	TLK1
NC_056064.1	46655001	46675001	2.67418	0.227172	OL	TLK2
NC_056064.1	46660001	46680001	3.45672	0.258634	OL	TLK2
NC_056064.1	46665001	46685001	5.31337	0.281212	OL	TLK2
NC_056070.1	1830001	1850001	3.44444	0.242461	OL	TLL1
NC_056070.1	1835001	1855001	2.6503	0.241904	OL	TLL1
NC_056070.1	1920001	1940001	3.09045	0.214888	OL	TLL1
NC_056070.1	4185001	4205001	2.69776	0.333352	OL	TLR2
NC_056054.1	191745001	191765001	4.43803	0.244725	OL	TM4SF19;UBXN7
NC_056056.1	136545001	136565001	2.45283	0.225547	OL	TMBIM6
NC_056060.1	86700001	86720001	2.51021	0.278285	OL	TMED8
NC_056060.1	86705001	86725001	2.57468	0.3075	OL	TMED8
NC_056060.1	86710001	86730001	3.65217	0.337708	OL	TMED8
NC_056060.1	86715001	86735001	3.43104	0.326802	OL	TMED8
NC_056060.1	86720001	86740001	3.31598	0.281767	OL	TMED8
NC_056060.1	86725001	86745001	2.57906	0.223874	OL	TMED8
NC_056070.1	50725001	50745001	5.82309	0.251163	OL	TMEM132B
NC_056074.1	7975001	7995001	2.33405	0.390221	OL	TMEM135
NC_056056.1	84490001	84510001	2.37998	0.267382	OL	TMEM178A
NC_056056.1	84495001	84515001	2.52518	0.212686	OL	TMEM178A
NC_056056.1	98990001	99010001	2.90629	0.317551	OL	TMEM182
NC_056056.1	98995001	99015001	3.30464	0.266631	OL	TMEM182
NC_056056.1	99000001	99020001	3.47059	0.262525	OL	TMEM182
NC_056056.1	99005001	99025001	3.61004	0.234568	OL	TMEM182
NC_056057.1	29555001	29575001	2.33585	0.218661	OL	TMEM196
NC_056077.1	28770001	28790001	3.4757	0.218068	OL	TMEM248
NC_056055.1	9160001	9180001	2.36463	0.217291	OL	TMEM268
NC_056060.1	86540001	86560001	11.141	0.244222	OL	TMEM63C
NC_056062.1	21660001	21680001	3.71176	0.225595	OL	TMEM71
NC_056062.1	21665001	21685001	2.68464	0.246574	OL	TMEM71
NC_056055.1	50080001	50100001	5.62122	0.329924	OL	TMOD1
NC_056055.1	8660001	8680001	4.0874	0.221311	OL	TNC
NC_056055.1	8665001	8685001	3.2885	0.237688	OL	TNC
NC_056065.1	40070001	40090001	2.75351	0.283699	OL	TNFSF4
NC_056055.1	8875001	8895001	6.05808	0.219371	OL	TNFSF8
NC_056055.1	8880001	8900001	3.01788	0.220532	OL	TNFSF8
NC_056055.1	220115001	220135001	5.14414	0.470341	OL	TNS1
NC_056057.1	76375001	76395001	2.80215	0.212449	OL	TNS3
NC_056057.1	76395001	76415001	2.37362	0.244566	OL	TNS3
NC_056057.1	76415001	76435001	2.72174	0.218203	OL	TNS3
NC_056057.1	76420001	76440001	3.76755	0.259043	OL	TNS3
NC_056057.1	76425001	76445001	3.95833	0.259061	OL	TNS3
NC_056057.1	76430001	76450001	4.33516	0.236814	OL	TNS3
NC_056057.1	76435001	76455001	4.2744	0.219846	OL	TNS3
NC_056057.1	76490001	76510001	3.28821	0.22416	OL	TNS3
NC_056057.1	76495001	76515001	2.47289	0.240793	OL	TNS3
NC_056057.1	76540001	76560001	2.48275	0.236969	OL	TNS3
NC_056057.1	76545001	76565001	4.78087	0.304745	OL	TNS3
NC_056057.1	76550001	76570001	8.99361	0.36	OL	TNS3
NC_056057.1	76555001	76575001	9.77346	0.404886	OL	TNS3
NC_056057.1	76570001	76590001	4.72676	0.552092	OL	TNS3
NC_056073.1	26375001	26395001	2.3912	0.231611	OL	TNXB
NC_056056.1	107990001	108010001	12.5402	0.21429	OL	TPH2
NC_056056.1	107995001	108015001	19.2903	0.24373	OL	TPH2
NC_056056.1	108020001	108040001	5.06725	0.226836	OL	TPH2
NC_056056.1	108025001	108045001	4.15742	0.219093	OL	TPH2
NC_056057.1	108965001	108985001	22.5086	0.254744	OL	TPK1
NC_056057.1	108970001	108990001	7.73224	0.23716	OL	TPK1
NC_056054.1	2990001	3010001	2.38017	0.278782	OL	TRAF3IP1
NC_056054.1	2995001	3015001	2.56561	0.269195	OL	TRAF3IP1
NC_056073.1	15990001	16010001	2.4836	0.223796	OL	TRERF1
NC_056073.1	15995001	16015001	2.38842	0.222017	OL	TRERF1

NC_056056.1	108540001	108560001	4.63477	0.285072	OL	TRHDE
NC_056056.1	108545001	108565001	8.76015	0.304519	OL	TRHDE
NC_056056.1	108550001	108570001	8.99092	0.351586	OL	TRHDE
NC_056056.1	108555001	108575001	3.82167	0.286634	OL	TRHDE
NC_056080.1	44320001	44340001	19.2621	0.474872	OL	TRIM60
NC_056080.1	44325001	44345001	16.0069	0.519902	OL	TRIM60
NC_056080.1	44330001	44350001	9.56349	0.416213	OL	TRIM60
NC_056080.1	44335001	44355001	5.77717	0.330796	OL	TRIM60
NC_056062.1	61950001	61970001	5.17391	0.213868	OL	TRPS1
NC_056062.1	61965001	61985001	12.8286	0.216727	OL	TRPS1
NC_056062.1	61970001	61990001	6.50952	0.222526	OL	TRPS1
NC_056062.1	61975001	61995001	4.93061	0.231061	OL	TRPS1
NC_056062.1	61980001	62000001	3.75	0.22566	OL	TRPS1
NC_056059.1	26650001	26670001	2.50101	0.254943	OL	TSPAN5
NC_056059.1	26655001	26675001	3.60392	0.293614	OL	TSPAN5
NC_056059.1	26660001	26680001	3.06328	0.290298	OL	TSPAN5
NC_056059.1	26665001	26685001	2.77453	0.280165	OL	TSPAN5
NC_056080.1	38095001	38115001	3.35451	0.214835	OL	TSPAN7
NC_056080.1	38175001	38195001	5.44817	0.248872	OL	TSPAN7
NC_056064.1	8975001	8995001	8.78949	0.267624	OL	TSPOAP1
NC_056073.1	16850001	16870001	3.73729	0.228989	OL	TTBK1
NC_056073.1	16855001	16875001	4.83474	0.264558	OL	TTBK1
NC_056073.1	16860001	16880001	2.81498	0.243243	OL	TTBK1
NC_056056.1	91230001	91250001	2.75862	0.227346	OL	TTC27
NC_056056.1	91235001	91255001	2.73154	0.239283	OL	TTC27
NC_056056.1	91240001	91260001	2.62404	0.229764	OL	TTC27
NC_056070.1	11605001	11625001	3.24278	0.333139	OL	TTC29
NC_056070.1	11610001	11630001	4.33179	0.386189	OL	TTC29
NC_056070.1	11615001	11635001	3.72399	0.366952	OL	TTC29
NC_056070.1	11620001	11640001	3.2122	0.365919	OL	TTC29
NC_056070.1	11655001	11675001	2.86826	0.286657	OL	TTC29
NC_056070.1	11660001	11680001	3.63366	0.307632	OL	TTC29
NC_056058.1	92420001	92440001	3.3067	0.244856	OL	TTC37
NC_056058.1	92425001	92445001	3.18654	0.24219	OL	TTC37
NC_056058.1	92430001	92450001	2.93081	0.23201	OL	TTC37
NC_056058.1	92470001	92490001	2.73228	0.277038	OL	TTC37
NC_056058.1	92475001	92495001	4.53714	0.343629	OL	TTC37
NC_056058.1	92480001	92500001	5.42741	0.390168	OL	TTC37
NC_056058.1	92485001	92505001	4.02138	0.361403	OL	TTC37
NC_056056.1	212685001	212705001	5.92684	0.246234	OL	TULP3
NC_056055.1	13000001	13020001	16.3337	0.294806	OL	TXNDC8
NC_056055.1	13005001	13025001	25.219	0.305611	OL	TXNDC8
NC_056055.1	13010001	13030001	27.6904	0.344874	OL	TXNDC8
NC_056055.1	13015001	13035001	27.7478	0.370031	OL	TXNDC8
NC_056055.1	13030001	13050001	5.71781	0.306529	OL	TXNDC8
NC_056055.1	13035001	13055001	4.30705	0.276138	OL	TXNDC8
NC_056059.1	22350001	22370001	3.79669	0.227425	OL	UBE2D3
NC_056059.1	22355001	22375001	2.93141	0.21267	OL	UBE2D3
NC_056079.1	41415001	41435001	2.52495	0.257899	OL	UBE2E1
NC_056062.1	50055001	50075001	3.13402	0.295993	OL	UBE2W
NC_056062.1	50060001	50080001	2.60674	0.29115	OL	UBE2W
NC_056062.1	50075001	50095001	2.46902	0.302189	OL	UBE2W
NC_056062.1	50090001	50110001	2.57264	0.238074	OL	UBE2W
NC_056062.1	50095001	50115001	2.72728	0.246451	OL	UBE2W
NC_056062.1	50100001	50120001	2.53692	0.243766	OL	UBE2W
NC_056062.1	50105001	50125001	3.63303	0.32087	OL	UBE2W
NC_056062.1	50110001	50130001	3.51219	0.292558	OL	UBE2W
NC_056062.1	50115001	50135001	2.65625	0.31	OL	UBE2W
NC_056065.1	42630001	42650001	3.47777	0.30969	OL	UBE4B
NC_056065.1	11400001	11420001	7.09706	0.253946	OL	UCHL5
NC_056065.1	11420001	11440001	19.9302	0.270307	OL	UCHL5
NC_056065.1	11425001	11445001	21.6744	0.255815	OL	UCHL5
NC_056065.1	11430001	11450001	18.75	0.241256	OL	UCHL5
NC_056064.1	55110001	55130001	2.60819	0.220992	OL	UNC13D;UNK;WBP2
NC_056064.1	55105001	55125001	2.85093	0.239373	OL	UNC13D;WBP2
NC_056077.1	1615001	1635001	2.83441	0.272627	OL	UNKL
NC_056077.1	1620001	1640001	2.36071	0.257954	OL	UNKL
NC_056065.1	18845001	18865001	8.72917	0.241436	OL	USH2A
NC_056065.1	18850001	18870001	3.82136	0.254076	OL	USH2A
NC_056067.1	10410001	10430001	3.39547	0.301686	OL	USP10
NC_056067.1	10415001	10435001	3.17391	0.322108	OL	USP10
NC_056067.1	10420001	10440001	3.15426	0.369643	OL	USP10
NC_056054.1	208180001	208200001	2.83799	0.264726	OL	USP13
NC_056054.1	208185001	208205001	2.87139	0.253072	OL	USP13

NC_056054.1	208190001	208210001	3.76755	0.241287	OL	USP13
NC_056054.1	208195001	208215001	6.9313	0.336077	OL	USP13
NC_056054.1	208200001	208220001	8.67741	0.337973	OL	USP13
NC_056054.1	208205001	208225001	6.33923	0.29336	OL	USP13
NC_056054.1	208210001	208230001	3.52518	0.285398	OL	USP13
NC_056072.1	50810001	50830001	2.87024	0.351757	OL	USP4
NC_056072.1	50815001	50835001	3.36635	0.381664	OL	USP4
NC_056072.1	50820001	50840001	3.05912	0.333935	OL	USP4
NC_056072.1	50825001	50845001	4.29167	0.310961	OL	USP4
NC_056072.1	50830001	50850001	4.30666	0.219888	OL	USP4
NC_056080.1	40370001	40390001	3.91025	0.288054	OL	USP9X
NC_056080.1	40375001	40395001	5.86547	0.337796	OL	USP9X
NC_056080.1	40380001	40400001	6.35788	0.342148	OL	USP9X
NC_056080.1	40385001	40405001	6.8902	0.34022	OL	USP9X
NC_056080.1	40390001	40410001	6.10824	0.344923	OL	USP9X
NC_056080.1	40395001	40415001	3.45606	0.296778	OL	USP9X
NC_056061.1	73340001	73360001	2.46748	0.408663	OL	UST
NC_056064.1	41345001	41365001	3.15018	0.251642	OL	V15
NC_056080.1	82150001	82170001	2.89763	0.274747	OL	VAMP7
NC_056057.1	6735001	6755001	2.59124	0.247843	OL	VWC2
NC_056057.1	6740001	6760001	2.97426	0.312919	OL	VWC2
NC_056075.1	40405001	40425001	15.8027	0.237413	OL	WDR11
NC_056075.1	40410001	40430001	10.3917	0.233671	OL	WDR11
NC_056075.1	40415001	40435001	7.9547	0.225403	OL	WDR11
NC_056071.1	64655001	64675001	4.62865	0.36979	OL	WDR20
NC_056071.1	64660001	64680001	2.8669	0.28842	OL	WDR20
NC_056065.1	26790001	26810001	2.53261	0.242739	OL	WDR26
NC_056065.1	26795001	26815001	2.57287	0.236388	OL	WDR26
NC_056076.1	56400001	56420001	3.18382	0.248903	OL	WDR7
NC_056076.1	56405001	56425001	4.91473	0.219516	OL	WDR7
NC_056060.1	54690001	54710001	2.76416	0.536719	OL	WDR72
NC_056060.1	54695001	54715001	2.77184	0.544484	OL	WDR72
NC_056056.1	213900001	213920001	9.93153	0.229289	OL	WNT5B
NC_056080.1	7985001	8005001	2.46193	0.238921	OL	WWC3
NC_056080.1	7990001	8010001	3.06493	0.232146	OL	WWC3
NC_056080.1	7995001	8015001	2.73109	0.286303	OL	WWC3
NC_056056.1	92300001	92320001	4.20426	0.322391	OL	XDH
NC_056056.1	92305001	92325001	3.20915	0.331497	OL	XDH
NC_056062.1	35715001	35735001	5.31788	0.265366	OL	XKR4
NC_056062.1	35720001	35740001	28.1637	0.329386	OL	XKR4
NC_056062.1	35735001	35755001	9.01007	0.317395	OL	XKR4
NC_056062.1	35740001	35760001	2.75355	0.325311	OL	XKR4
NC_056062.1	35840001	35860001	2.79401	0.25263	OL	XKR4
NC_056055.1	49895001	49915001	2.35251	0.241468	OL	XPA
NC_056068.1	6335001	6355001	3.64538	0.295025	OL	YAP1
NC_056076.1	36085001	36105001	2.42857	0.276738	OL	YES1
NC_056060.1	8270001	8290001	3.06296	0.374104	OL	ZBED3
NC_056060.1	8275001	8295001	9.7973	0.523797	OL	ZBED3
NC_056068.1	24330001	24350001	2.92771	0.2276	OL	ZBTB16
NC_056068.1	24395001	24415001	2.44291	0.217336	OL	ZBTB16
NC_056068.1	24400001	24420001	2.53939	0.252575	OL	ZBTB16
NC_056068.1	24405001	24425001	2.45068	0.231035	OL	ZBTB16
NC_056068.1	24410001	24430001	2.51888	0.222645	OL	ZBTB16
NC_056068.1	24445001	24465001	3.51453	0.249045	OL	ZBTB16
NC_056068.1	24450001	24470001	2.72727	0.242462	OL	ZBTB16
NC_056068.1	24455001	24475001	4.32866	0.23719	OL	ZBTB16
NC_056068.1	24460001	24480001	4.65274	0.216934	OL	ZBTB16
NC_056061.1	81755001	81775001	3.8	0.22483	OL	ZDHHC14
NC_056061.1	81760001	81780001	4.5018	0.251507	OL	ZDHHC14
NC_056061.1	81765001	81785001	4.9191	0.291722	OL	ZDHHC14
NC_056061.1	81770001	81790001	3.28662	0.288861	OL	ZDHHC14
NC_056061.1	81775001	81795001	2.64953	0.252795	OL	ZDHHC14
NC_056060.1	86495001	86515001	3.27298	0.234299	OL	ZDHHC22
NC_056067.1	39120001	39140001	2.45947	0.239106	OL	ZNF19;ZNF23
NC_056078.1	12445001	12465001	4.1108	0.217388	OL	ZNF248
NC_056055.1	130600001	130620001	3.12391	0.310512	OL	ZNF385B
NC_056066.1	33640001	33660001	3.40202	0.22346	OL	ZNF438
NC_056067.1	13235001	13255001	2.45962	0.24769	OL	ZNF469
NC_056077.1	37290001	37310001	3.24138	0.295048	OL	ZNF655
NC_056077.1	37295001	37315001	2.53598	0.231978	OL	ZNF655
NC_056067.1	1845001	1865001	2.68902	0.227462	OL	ZNRF1
NC_056067.1	1850001	1870001	2.82978	0.253439	OL	ZNRF1
NC_056067.1	1855001	1875001	3.13856	0.23185	OL	ZNRF1
NC_056067.1	1860001	1880001	3.57825	0.220519	OL	ZNRF1

NC_056067.1	1865001	1885001	3.33483	0.232351	OL	ZNRF1
NC_056067.1	1875001	1895001	3.16059	0.269839	OL	ZNRF1
NC_056067.1	1880001	1900001	2.64213	0.294535	OL	ZNRF1
NC_056057.1	67480001	67500001	6.72826	0.239523	OL	ZNRF2
NC_056057.1	67555001	67575001	2.33561	0.221382	OL	ZNRF2
NC_056057.1	67560001	67580001	2.59449	0.218734	OL	ZNRF2
