# Supplementary material for: Genetic diversity and selection of Tibetan sheep breeds revealed by whole-genome resequencing
Source: Anim Biosci. 2023 May 2;36(7):991–1002. doi: 10.5713/ab.22.0432 (PMC10330983; doi:10.5713/ab.22.0432)
Supplement: Supplementary file 9 [file ab-22-0432-Supplementary-Table-9.pdf]

Supplementary Table9.Putative selection regions associated with coat color among different breeds

BD vs HZ							GY vs HZ		
CHROM	Start	end	Pi	Fst	Region	Gene	CHROM	Start	end
NC_056054.1	251645001	251665001	0.218182	0.284096	HZ	A4GNT;DZIP1L	NC_056055.1	175495001	175515001
NC_056074.1	15760001	15780001	0.190432	0.252225	HZ	AAMDC;RSF1	NC_056055.1	175500001	175520001
NC_056074.1	15765001	15785001	0.312169	0.28955	HZ	AAMDC;RSF1	NC_056054.1	46800001	46820001
NC_056054.1	71550001	71570001	0.22449	0.314845	HZ	ABCD3	NC_056054.1	46805001	46825001
NC_056054.1	71560001	71580001	0.181018	0.288245	HZ	ABCD3	NC_056054.1	46810001	46830001
NC_056054.1	71565001	71585001	0.166667	0.291426	HZ	ABCD3	NC_056077.1	27270001	27290001
NC_056054.1	71570001	71590001	0.093596	0.299641	HZ	ABCD3	NC_056056.1	104505001	104525001
NC_056054.1	71575001	71595001	0.0818406	0.368918	HZ	ABCD3	NC_056056.1	104510001	104530001
NC_056054.1	71580001	71600001	0.0669291	0.374419	HZ	ABCD3	NC_056080.1	57395001	57415001
NC_056054.1	71585001	71605001	0.200971	0.282023	HZ	ABCD3	NC_056080.1	57400001	57420001
NC_056056.1	80430001	80450001	0.243428	0.360927	HZ	ABCG5;ABCG8	NC_056080.1	57405001	57425001
NC_056056.1	80455001	80475001	0.0423776	0.328774	HZ	ABCG5;DYNC2LI1	NC_056056.1	104490001	104510001
NC_056056.1	80460001	80480001	0.0557338	0.251854	HZ	ABCG5;DYNC2LI1	NC_056056.1	104495001	104515001
NC_056070.1	29930001	29950001	0.328935	0.272922	HZ	ABHD18	NC_056056.1	104500001	104520001
NC_056059.1	115055001	115075001	0.0757356	0.241878	HZ	ABLIM2	NC_056067.1	57600001	57620001
NC_056059.1	115060001	115080001	0.0449493	0.257427	HZ	ABLIM2	NC_056062.1	29550001	29570001
NC_056059.1	115065001	115085001	0.0484843	0.255846	HZ	ABLIM2	NC_056062.1	29555001	29575001
NC_056059.1	115175001	115195001	0.296318	0.28773	HZ	ABLIM2	NC_056055.1	10665001	10685001
NC_056059.1	115180001	115200001	0.311808	0.322556	HZ	ABLIM2	NC_056055.1	10670001	10690001
NC_056054.1	257720001	257740001	0.16304	0.246581	HZ	ACAD11	NC_056056.1	107730001	107750001
NC_056054.1	257710001	257730001	0.147513	0.252227	HZ	ACAD11;ACKR4	NC_056062.1	20285001	20305001
NC_056054.1	257715001	257735001	0.140066	0.275934	HZ	ACAD11;ACKR4	NC_056062.1	20290001	20310001
NC_056061.1	83760001	83780001	0.169703	0.247724	HZ	ACAT2;LOC10111051	NC_056062.1	20295001	20315001
NC_056064.1	55015001	55035001	0.0647283	0.274238	HZ	ACOX1;TEN1	NC_056055.1	165610001	165630001
NC_056056.1	105175001	105195001	0.113217	0.249871	HZ	ACOXL	NC_056055.1	165615001	165635001
NC_056056.1	105180001	105200001	0.133232	0.294668	HZ	ACOXL	NC_056055.1	104865001	104885001
NC_056056.1	105185001	105205001	0.176285	0.332431	HZ	ACOXL	NC_056055.1	104870001	104890001
NC_056056.1	105190001	105210001	0.182385	0.402333	HZ	ACOXL	NC_056079.1	13345001	13365001
NC_056056.1	105195001	105215001	0.140674	0.43504	HZ	ACOXL	NC_056071.1	22395001	22415001
NC_056056.1	105200001	105220001	0.196428	0.406641	HZ	ACOXL	NC_056071.1	22400001	22420001
NC_056056.1	105205001	105225001	0.288421	0.388623	HZ	ACOXL	NC_056055.1	239735001	239755001
NC_056054.1	257990001	258010001	0.124299	0.253303	HZ	ACP3	NC_056055.1	239740001	239760001
NC_056054.1	258000001	258020001	0.174688	0.267945	HZ	ACP3	NC_056055.1	239745001	239765001

NC_056054.1	258005001	258025001	0.219917	0.287897	HZ	ACP3
NC_056054.1	258010001	258030001	0.204679	0.290282	HZ	ACP3
NC_056054.1	258015001	258035001	0.216398	0.295406	HZ	ACP3
NC_056054.1	258020001	258040001	0.263342	0.281393	HZ	ACP3
NC_056056.1	117285001	117305001	0.160947	0.28253	HZ	ACSS3
NC_056056.1	117290001	117310001	0.273425	0.344179	HZ	ACSS3
NC_056055.1	178990001	179010001	0.076923	0.257416	HZ	ACTR3
NC_056055.1	178995001	179015001	0.0926846	0.250992	HZ	ACTR3
NC_056072.1	36875001	36895001	0.197144	0.291038	HZ	ADAMTS9
NC_056072.1	36880001	36900001	0.26152	0.277314	HZ	ADAMTS9
NC_056072.1	36885001	36905001	0.292076	0.284986	HZ	ADAMTS9
NC_056072.1	36920001	36940001	0.295377	0.256439	HZ	ADAMTS9
NC_056072.1	36925001	36945001	0.292947	0.267103	HZ	ADAMTS9
NC_056072.1	36930001	36950001	0.309421	0.265489	HZ	ADAMTS9
NC_056055.1	86505001	86525001	0.259524	0.298495	HZ	ADAMTSL1
NC_056055.1	86510001	86530001	0.111258	0.379438	HZ	ADAMTSL1
NC_056055.1	86515001	86535001	0.049578	0.43335	HZ	ADAMTSL1
NC_056055.1	86520001	86540001	0.143047	0.405766	HZ	ADAMTSL1
NC_056055.1	86525001	86545001	0.295533	0.350082	HZ	ADAMTSL1
NC_056069.1	65765001	65785001	0.300033	0.24872	HZ	ADCY2
NC_056062.1	23080001	23100001	0.2943	0.255703	HZ	ADCY8
NC_056059.1	42550001	42570001	0.275565	0.302937	HZ	ADGRA3
NC_056062.1	5215001	5235001	0.34166	0.315397	HZ	ADGRB3
NC_056062.1	5220001	5240001	0.327955	0.309452	HZ	ADGRB3
NC_056062.1	5225001	5245001	0.331515	0.282549	HZ	ADGRB3
NC_056062.1	5230001	5250001	0.340342	0.284993	HZ	ADGRB3
NC_056058.1	88310001	88330001	0.09375	0.509237	HZ	ADGRV1
NC_056071.1	16390001	16410001	0.0765764	0.259285	HZ	AGBL1
NC_056071.1	16415001	16435001	0.0773021	0.241364	HZ	AGBL1
NC_056054.1	10915001	10935001	0.303251	0.437351	HZ	AGO3
NC_056054.1	10920001	10940001	0.251349	0.461279	HZ	AGO3
NC_056054.1	10925001	10945001	0.28835	0.49953	HZ	AGO3
NC_056054.1	40835001	40855001	0.293974	0.253229	HZ	AK4
NC_056071.1	15640001	15660001	0.1661	0.27633	HZ	AKAP13
NC_056071.1	40975001	40995001	0.173088	0.281995	HZ	AKAP6
NC_056068.1	16540001	16560001	0.268692	0.385421	HZ	ALKBH8

NC_056055.1	239750001	239770001
NC_056055.1	119915001	119935001
NC_056055.1	119920001	119940001
NC_056055.1	119925001	119945001
NC_056055.1	119930001	119950001
NC_056054.1	96625001	96645001
NC_056054.1	96630001	96650001
NC_056080.1	48170001	48190001
NC_056080.1	48195001	48215001
NC_056080.1	48200001	48220001
NC_056080.1	48205001	48225001
NC_056080.1	48210001	48230001
NC_056058.1	43315001	43335001
NC_056058.1	43320001	43340001
NC_056058.1	43325001	43345001
NC_056058.1	43330001	43350001
NC_056060.1	41255001	41275001
NC_056060.1	41260001	41280001
NC_056060.1	41265001	41285001
NC_056060.1	41270001	41290001
NC_056060.1	41275001	41295001
NC_056054.1	196905001	196925001
NC_056054.1	142045001	142065001
NC_056054.1	142050001	142070001
NC_056054.1	142055001	142075001
NC_056054.1	142060001	142080001
NC_056054.1	142065001	142085001
NC_056054.1	142070001	142090001
NC_056054.1	142075001	142095001
NC_056054.1	142080001	142100001
NC_056054.1	142085001	142105001
NC_056054.1	142090001	142110001
NC_056054.1	142095001	142115001
NC_056054.1	142115001	142135001
NC_056054.1	142120001	142140001
NC_056054.1	142125001	142145001

NC_056068.1	16545001	16565001	0.326835	0.365256	HZ	ALKBH8
NC_056056.1	95460001	95480001	0.182874	0.372235	HZ	ALMS1
NC_056056.1	95465001	95485001	0.0810473	0.354665	HZ	ALMS1
NC_056056.1	95470001	95490001	0.102305	0.30559	HZ	ALMS1
NC_056056.1	95475001	95495001	0.233961	0.265175	HZ	ALMS1
NC_056056.1	165820001	165840001	0.339164	0.254895	HZ	AMDHD1;HAL
NC_056068.1	15335001	15355001	0.278525	0.2559	HZ	AMOTL1
NC_056068.1	15340001	15360001	0.251133	0.278158	HZ	AMOTL1
NC_056054.1	255500001	255520001	0.270344	0.239841	HZ	ANAPC13
NC_056066.1	59855001	59875001	0.296545	0.283941	HZ	ANGPT4
NC_056066.1	59860001	59880001	0.33971	0.24119	HZ	ANGPT4
NC_056065.1	41690001	41710001	0.249293	0.296301	HZ	ANGPTL7;MTOR
NC_056065.1	41695001	41715001	0.312121	0.273893	HZ	ANGPTL7;MTOR
NC_056065.1	41700001	41720001	0.295918	0.298927	HZ	ANGPTL7;MTOR
NC_056056.1	170535001	170555001	0.236867	0.242049	HZ	ANO4
NC_056056.1	170540001	170560001	0.278727	0.253489	HZ	ANO4
NC_056056.1	170545001	170565001	0.336381	0.254303	HZ	ANO4
NC_056055.1	63500001	63520001	0.139931	0.421715	HZ	ANXA1
NC_056055.1	63505001	63525001	0.189545	0.427947	HZ	ANXA1
NC_056055.1	63510001	63530001	0.301406	0.424313	HZ	ANXA1
NC_056055.1	67030001	67050001	0.269842	0.273093	HZ	APBA1
NC_056059.1	60590001	60610001	0.139641	0.570545	HZ	APBB2
NC_056059.1	60600001	60620001	0.051875	0.387778	HZ	APBB2
NC_056070.1	5005001	5025001	0.339825	0.296397	HZ	ARFIP1
NC_056070.1	5010001	5030001	0.282145	0.320563	HZ	ARFIP1
NC_056070.1	5050001	5070001	0.0938947	0.239703	HZ	ARFIP1
NC_056070.1	5055001	5075001	0.148851	0.257855	HZ	ARFIP1
NC_056068.1	20420001	20440001	0.289762	0.247919	HZ	ARHGAP20
NC_056066.1	24920001	24940001	0.103668	0.25979	HZ	ARHGAP21
NC_056066.1	24930001	24950001	0.183477	0.271588	HZ	ARHGAP21
NC_056066.1	24935001	24955001	0.290295	0.246686	HZ	ARHGAP21
NC_056066.1	24965001	24985001	0.336385	0.307223	HZ	ARHGAP21
NC_056067.1	53735001	53755001	0.140209	0.26319	HZ	ARHGAP35
NC_056067.1	53740001	53760001	0.0590738	0.342259	HZ	ARHGAP35
NC_056067.1	53755001	53775001	0.144559	0.304951	HZ	ARHGAP35
NC_056054.1	106970001	106990001	0.153639	0.555745	HZ	ARHGEF11

NC_056076.1	35585001	35605001
NC_056076.1	35590001	35610001
NC_056076.1	35595001	35615001
NC_056076.1	35600001	35620001
NC_056067.1	10435001	10455001
NC_056054.1	184855001	184875001
NC_056054.1	184860001	184880001
NC_056054.1	184865001	184885001
NC_056074.1	41915001	41935001
NC_056079.1	30445001	30465001
NC_056079.1	30450001	30470001
NC_056079.1	30460001	30480001
NC_056059.1	29670001	29690001
NC_056058.1	35985001	36005001
NC_056058.1	35990001	36010001
NC_056058.1	35995001	36015001
NC_056058.1	36000001	36020001
NC_056058.1	36005001	36025001
NC_056058.1	36010001	36030001
NC_056058.1	36015001	36035001
NC_056058.1	36020001	36040001
NC_056058.1	36025001	36045001
NC_056072.1	13980001	14000001
NC_056072.1	13985001	14005001
NC_056072.1	13990001	14010001
NC_056072.1	13995001	14015001
NC_056072.1	14000001	14020001
NC_056058.1	36110001	36130001
NC_056058.1	36115001	36135001
NC_056058.1	36140001	36160001
NC_056058.1	36145001	36165001
NC_056058.1	36150001	36170001
NC_056058.1	36155001	36175001
NC_056058.1	36160001	36180001
NC_056058.1	36165001	36185001
NC_056063.1	51190001	51210001

NC_056068.1	30620001	30640001	0.322289	0.241455	HZ	ARHGEF12;POU2F3;T	NC_056063.1	51195001	51215001
NC_056061.1	81215001	81235001	0.278765	0.252085	HZ	ARID1B	NC_056063.1	51200001	51220001
NC_056061.1	81220001	81240001	0.247212	0.26807	HZ	ARID1B	NC_056057.1	120035001	120055001
NC_056060.1	55945001	55965001	0.212417	0.342334	HZ	ARPP19	NC_056057.1	120040001	120060001
NC_056060.1	55950001	55970001	0.178984	0.347318	HZ	ARPP19	NC_056057.1	120045001	120065001
NC_056060.1	55955001	55975001	0.281323	0.303325	HZ	ARPP19	NC_056057.1	120085001	120105001
NC_056060.1	55940001	55960001	0.234903	0.354681	HZ	ARPP19;FAM214A	NC_056057.1	120090001	120110001
NC_056056.1	200695001	200715001	0.249857	0.257947	HZ	ART4;C3H12orf60;SM	NC_056057.1	120095001	120115001
NC_056056.1	200700001	200720001	0.21688	0.267144	HZ	ART4;C3H12orf60;SM	NC_056057.1	120100001	120120001
NC_056056.1	200705001	200725001	0.275488	0.24539	HZ	ART4;C3H12orf60;SM	NC_056057.1	120105001	120125001
NC_056056.1	200690001	200710001	0.225584	0.248722	HZ	ART4;SMCO3	NC_056057.1	120110001	120130001
NC_056062.1	23455001	23475001	0.294307	0.261797	HZ	ASAP1	NC_056057.1	120115001	120135001
NC_056062.1	23460001	23480001	0.309109	0.260563	HZ	ASAP1	NC_056060.1	17625001	17645001
NC_056056.1	18615001	18635001	0.227713	0.267822	HZ	ASAP2	NC_056079.1	20695001	20715001
NC_056056.1	38405001	38425001	0.0665812	0.284011	HZ	ASPRV1	NC_056079.1	20700001	20720001
NC_056056.1	38410001	38430001	0.0783133	0.300081	HZ	ASPRV1	NC_056079.1	20705001	20725001
NC_056055.1	6680001	6700001	0.0707237	0.270841	HZ	ASTN2	NC_056079.1	20710001	20730001
NC_056055.1	7190001	7210001	0.322675	0.243394	HZ	ASTN2	NC_056079.1	20715001	20735001
NC_056055.1	7205001	7225001	0.0761326	0.241378	HZ	ASTN2	NC_056079.1	20720001	20740001
NC_056055.1	7210001	7230001	0.0462685	0.289741	HZ	ASTN2	NC_056079.1	20725001	20745001
NC_056056.1	33060001	33080001	0.333796	0.344879	HZ	ASXL2	NC_056061.1	82480001	82500001
NC_056056.1	33065001	33085001	0.210527	0.321144	HZ	ASXL2	NC_056061.1	82650001	82670001
NC_056056.1	33070001	33090001	0.295891	0.290695	HZ	ASXL2	NC_056061.1	82655001	82675001
NC_056058.1	79615001	79635001	0.0304333	0.257963	HZ	ATG10	NC_056061.1	82660001	82680001
NC_056058.1	79620001	79640001	0.0412392	0.252815	HZ	ATG10	NC_056061.1	82665001	82685001
NC_056058.1	79625001	79645001	0.0384598	0.260947	HZ	ATG10	NC_056061.1	82670001	82690001
NC_056063.1	36880001	36900001	0.205882	0.515918	HZ	ATP12A	NC_056061.1	6990001	7010001
NC_056063.1	36865001	36885001	0.125341	0.622659	HZ	ATP12A;RNF17	NC_056061.1	6995001	7015001
NC_056063.1	36870001	36890001	0.0965517	0.643494	HZ	ATP12A;RNF17	NC_056061.1	7000001	7020001
NC_056054.1	93715001	93735001	0.110017	0.258637	HZ	ATP1A1	NC_056061.1	7005001	7025001
NC_056054.1	259760001	259780001	0.268706	0.255632	HZ	ATP2C1	NC_056061.1	7010001	7030001
NC_056054.1	259765001	259785001	0.3361	0.336468	HZ	ATP2C1	NC_056061.1	7015001	7035001
NC_056056.1	19890001	19910001	0.256326	0.757269	HZ	ATP6V1C2	NC_056061.1	7020001	7040001
NC_056060.1	58285001	58305001	0.29471	0.310042	HZ	ATP8B4	NC_056061.1	7025001	7045001
NC_056060.1	58290001	58310001	0.295879	0.265941	HZ	ATP8B4	NC_056061.1	7030001	7050001
NC_056057.1	48310001	48330001	0.0404596	0.263045	HZ	ATXN7L1	NC_056061.1	7035001	7055001

NC_056064.1	43750001	43770001	0.29806	0.307432	HZ	ATXN7L3;UBTF
NC_056077.1	30870001	30890001	0.290857	0.285822	HZ	AUTS2
NC_056077.1	30875001	30895001	0.307606	0.276179	HZ	AUTS2
NC_056080.1	63110001	63130001	0.261456	0.296011	HZ	AWAT1
NC_056080.1	63115001	63135001	0.178691	0.262872	HZ	AWAT1
NC_056080.1	63120001	63140001	0.116742	0.241519	HZ	AWAT1
NC_056080.1	63130001	63150001	0.117647	0.242436	HZ	AWAT1;P2RY4
NC_056062.1	3110001	3130001	0.0478536	0.249628	HZ	B3GAT2
NC_056062.1	3195001	3215001	0.334129	0.244058	HZ	B3GAT2;SMAP1
NC_056056.1	35245001	35265001	0.311253	0.301325	HZ	BABAM2
NC_056056.1	35275001	35295001	0.23918	0.322368	HZ	BABAM2
NC_056056.1	35280001	35300001	0.216152	0.30746	HZ	BABAM2
NC_056056.1	35285001	35305001	0.163756	0.353038	HZ	BABAM2
NC_056072.1	51645001	51665001	0.103449	0.282146	HZ	BAC5
NC_056072.1	51650001	51670001	0.148438	0.271861	HZ	BAC5
NC_056057.1	64935001	64955001	0.321266	0.348765	HZ	BBS9
NC_056054.1	106605001	106625001	0.296228	0.313841	HZ	BCAN
NC_056064.1	11395001	11415001	0.306034	0.301572	HZ	BCAS3
NC_056071.1	61885001	61905001	0.313328	0.250226	HZ	BCL11B
NC_056071.1	61890001	61910001	0.301412	0.271133	HZ	BCL11B
NC_056071.1	61895001	61915001	0.32631	0.306625	HZ	BCL11B
NC_056080.1	16470001	16490001	0.330189	0.401263	HZ	BEND2
NC_056080.1	16490001	16510001	0.34507	0.262248	HZ	BEND2
NC_056080.1	16495001	16515001	0.311111	0.241678	HZ	BEND2
NC_056080.1	16500001	16520001	0.303868	0.279219	HZ	BEND2
NC_056066.1	37490001	37510001	0.228677	0.248108	HZ	BFSP1
NC_056055.1	28585001	28605001	0.182398	0.44293	HZ	BICD2
NC_056067.1	54325001	54345001	0.228738	0.374392	HZ	BICRA
NC_056067.1	54330001	54350001	0.161698	0.419124	HZ	BICRA
NC_056067.1	54335001	54355001	0.179002	0.417747	HZ	BICRA
NC_056055.1	118395001	118415001	0.27059	0.283356	HZ	BIN1
NC_056055.1	118400001	118420001	0.283801	0.274888	HZ	BIN1
NC_056055.1	118405001	118425001	0.313753	0.270278	HZ	BIN1
NC_056056.1	91520001	91540001	0.0485852	0.247008	HZ	BIRC6
NC_056080.1	13680001	13700001	0.296477	0.26224	HZ	BMX
NC_056054.1	178875001	178895001	0.131911	0.267554	HZ	BOC

NC_056058.1	92475001	92495001
NC_056058.1	92480001	92500001
NC_056058.1	92485001	92505001
NC_056055.1	143615001	143635001
NC_056055.1	143620001	143640001
NC_056055.1	143625001	143645001
NC_056078.1	3040001	3060001
NC_056078.1	3045001	3065001
NC_056078.1	3050001	3070001
NC_056078.1	3055001	3075001
NC_056078.1	3060001	3080001
NC_056078.1	3065001	3085001
NC_056078.1	3070001	3090001
NC_056078.1	3075001	3095001
NC_056066.1	81055001	81075001
NC_056066.1	81060001	81080001
NC_056069.1	58940001	58960001
NC_056069.1	58945001	58965001
NC_056068.1	29050001	29070001
NC_056067.1	34565001	34585001
NC_056073.1	38695001	38715001
NC_056073.1	38700001	38720001
NC_056056.1	35900001	35920001
NC_056064.1	40460001	40480001
NC_056080.1	134235001	134255001
NC_056080.1	134240001	134260001
NC_056080.1	134245001	134265001
NC_056080.1	134250001	134270001
NC_056063.1	13095001	13115001
NC_056063.1	13100001	13120001
NC_056058.1	32245001	32265001
NC_056058.1	32250001	32270001
NC_056058.1	32255001	32275001
NC_056058.1	32335001	32355001
NC_056058.1	32340001	32360001
NC_056056.1	118985001	119005001

NC_056054.1	178880001	178900001	0.0921303	0.308743	HZ	BOC	NC_056056.1	118990001	119010001
NC_056054.1	178885001	178905001	0.215368	0.317887	HZ	BOC	NC_056056.1	118995001	119015001
NC_056054.1	178890001	178910001	0.281632	0.324784	HZ	BOC	NC_056056.1	185555001	185575001
NC_056066.1	61985001	62005001	0.326573	0.244314	HZ	BPIFB2;BPIFB6	NC_056056.1	185560001	185580001
NC_056056.1	225125001	225145001	0.343332	0.27659	HZ	BRD1	NC_056068.1	28510001	28530001
NC_056056.1	225130001	225150001	0.300287	0.253594	HZ	BRD1	NC_056068.1	28515001	28535001
NC_056056.1	225135001	225155001	0.313491	0.263607	HZ	BRD1	NC_056062.1	68525001	68545001
NC_056074.1	46435001	46455001	0.310137	0.257095	HZ	BRSK2;MOB2	NC_056062.1	68530001	68550001
NC_056074.1	46440001	46460001	0.329144	0.258919	HZ	BRSK2;MOB2	NC_056062.1	68535001	68555001
NC_056072.1	57510001	57530001	0.272401	0.321766	HZ	C19H3orf20	NC_056072.1	57980001	58000001
NC_056072.1	57515001	57535001	0.223117	0.3263	HZ	C19H3orf20	NC_056072.1	57985001	58005001
NC_056072.1	57520001	57540001	0.1323	0.319746	HZ	C19H3orf20	NC_056061.1	55465001	55485001
NC_056072.1	57525001	57545001	0.0753342	0.34635	HZ	C19H3orf20;CCDC174	NC_056056.1	107780001	107800001
NC_056054.1	35095001	35115001	0.245175	0.244801	HZ	C1H1orf87	NC_056056.1	107785001	107805001
NC_056054.1	9155001	9175001	0.292054	0.243148	HZ	C1H1orf94	NC_056056.1	107790001	107810001
NC_056054.1	9160001	9180001	0.112792	0.325944	HZ	C1H1orf94	NC_056056.1	107805001	107825001
NC_056054.1	232620001	232640001	0.345124	0.245554	HZ	C1H3orf33	NC_056061.1	82750001	82770001
NC_056064.1	44405001	44425001	0.299437	0.347065	HZ	C1QL1	NC_056061.1	82755001	82775001
NC_056064.1	44375001	44395001	0.109383	0.270867	HZ	C1QL1;KIF18B	NC_056061.1	82760001	82780001
NC_056064.1	44380001	44400001	0.0535225	0.280387	HZ	C1QL1;KIF18B	NC_056061.1	82765001	82785001
NC_056073.1	44640001	44660001	0.297411	0.257878	HZ	C20H6orf52;LOC1011(	NC_056070.1	47790001	47810001
NC_056078.1	3395001	3415001	0.307012	0.281754	HZ	C25H1orf131;GNPAT	NC_056070.1	47795001	47815001
NC_056078.1	3400001	3420001	0.188885	0.31487	HZ	C25H1orf131;GNPAT	NC_056070.1	4205001	4225001
NC_056078.1	3405001	3425001	0.178711	0.248165	HZ	C25H1orf131;GNPAT	NC_056056.1	103270001	103290001
NC_056056.1	172620001	172640001	0.0294349	0.364113	HZ	C3H12orf42	NC_056056.1	103275001	103295001
NC_056064.1	140001	160001	0.283187	0.258055	HZ	CA10	NC_056056.1	103280001	103300001
NC_056064.1	145001	165001	0.284427	0.29791	HZ	CA10	NC_056056.1	103285001	103305001
NC_056064.1	150001	170001	0.318976	0.329536	HZ	CA10	NC_056056.1	103290001	103310001
NC_056072.1	47260001	47280001	0.201941	0.465944	HZ	CACNA1D	NC_056056.1	104105001	104125001
NC_056072.1	47265001	47285001	0.308542	0.385412	HZ	CACNA1D	NC_056054.1	256815001	256835001
NC_056072.1	47245001	47265001	0.301159	0.477499	HZ	CACNA1D;CHDH	NC_056054.1	256920001	256940001
NC_056072.1	47250001	47270001	0.127675	0.515457	HZ	CACNA1D;CHDH	NC_056054.1	257005001	257025001
NC_056072.1	47255001	47275001	0.132802	0.507613	HZ	CACNA1D;CHDH	NC_056077.1	17470001	17490001
NC_056072.1	38535001	38555001	0.142392	0.328809	HZ	CADPS	NC_056077.1	17475001	17495001
NC_056057.1	88685001	88705001	0.0786436	0.264499	HZ	CADPS2	NC_056077.1	17480001	17500001
NC_056058.1	59365001	59385001	0.124627	0.301215	HZ	CAMK2A	NC_056077.1	17545001	17565001

NC_056058.1	59370001	59390001	0.118922	0.307735	HZ	CAMK2A
NC_056058.1	59375001	59395001	0.105152	0.303123	HZ	CAMK2A
NC_056058.1	59380001	59400001	0.162716	0.254819	HZ	CAMK2A
NC_056056.1	79625001	79645001	0.251993	0.302513	HZ	CAMKMT
NC_056056.1	79630001	79650001	0.279056	0.304393	HZ	CAMKMT
NC_056056.1	79730001	79750001	0.310174	0.283323	HZ	CAMKMT
NC_056056.1	79735001	79755001	0.221533	0.318023	HZ	CAMKMT
NC_056056.1	79740001	79760001	0.184925	0.292405	HZ	CAMKMT
NC_056056.1	79745001	79765001	0.169553	0.251833	HZ	CAMKMT
NC_056056.1	79830001	79850001	0.105866	0.243513	HZ	CAMKMT
NC_056080.1	40720001	40740001	0.17891	0.284156	HZ	CASK
NC_056080.1	40725001	40745001	0.184937	0.310539	HZ	CASK
NC_056080.1	40730001	40750001	0.187099	0.319932	HZ	CASK
NC_056080.1	40735001	40755001	0.259306	0.382332	HZ	CASK
NC_056066.1	59185001	59205001	0.245205	0.239436	HZ	CASS4;CSTF1
NC_056072.1	51620001	51640001	0.104441	0.285775	HZ	CATHL1B;LOC105607
NC_056067.1	62665001	62685001	0.1834	0.356678	HZ	CCDC106;U2AF2;ZNF
NC_056054.1	189190001	189210001	0.336511	0.28795	HZ	CCDC14;ROPN1
NC_056080.1	100695001	100715001	0.237856	0.339795	HZ	CCDC160
NC_056055.1	84140001	84160001	0.127013	0.296474	HZ	CCDC171
NC_056056.1	1050001	1070001	0.27991	0.363889	HZ	CCDC183;RABL6;TM
NC_056056.1	1055001	1075001	0.27606	0.30832	HZ	CCDC183;TMEM141
NC_056054.1	114435001	114455001	0.18042	0.257361	HZ	CCDC190
NC_056058.1	23810001	23830001	0.315948	0.281216	HZ	CCDC192
NC_056058.1	23815001	23835001	0.233667	0.254064	HZ	CCDC192
NC_056058.1	23820001	23840001	0.170606	0.260689	HZ	CCDC192
NC_056058.1	23825001	23845001	0.251821	0.288884	HZ	CCDC192
NC_056058.1	23830001	23850001	0.320492	0.261932	HZ	CCDC192
NC_056058.1	23835001	23855001	0.307967	0.295766	HZ	CCDC192
NC_056058.1	23840001	23860001	0.30774	0.316135	HZ	CCDC192
NC_056058.1	23845001	23865001	0.340708	0.336839	HZ	CCDC192
NC_056058.1	14490001	14510001	0.275773	0.343991	HZ	CCL25
NC_056058.1	14495001	14515001	0.218388	0.356957	HZ	CCL25
NC_056058.1	14485001	14505001	0.3103	0.334923	HZ	CCL25;ELAVL1
NC_056058.1	14500001	14520001	0.330111	0.314693	HZ	CCL25;FBN3
NC_056060.1	53070001	53090001	0.344989	0.302463	HZ	CCPG1

NC_056075.1	17675001	17695001
NC_056075.1	17680001	17700001
NC_056075.1	17685001	17705001
NC_056075.1	17690001	17710001
NC_056075.1	17695001	17715001
NC_056070.1	4180001	4200001
NC_056060.1	45885001	45905001
NC_056060.1	45890001	45910001
NC_056073.1	6260001	6280001
NC_056073.1	6265001	6285001
NC_056054.1	124960001	124980001
NC_056060.1	17985001	18005001
NC_056056.1	107760001	107780001
NC_056056.1	107765001	107785001
NC_056055.1	186525001	186545001
NC_056055.1	186530001	186550001
NC_056055.1	186535001	186555001
NC_056055.1	186540001	186560001
NC_056055.1	186545001	186565001
NC_056054.1	256205001	256225001
NC_056054.1	256210001	256230001
NC_056054.1	256215001	256235001
NC_056054.1	256220001	256240001
NC_056056.1	59200001	59220001
NC_056056.1	59205001	59225001
NC_056080.1	63565001	63585001
NC_056080.1	63570001	63590001
NC_056080.1	63580001	63600001
NC_056080.1	63585001	63605001
NC_056080.1	63590001	63610001
NC_056054.1	95030001	95050001
NC_056054.1	95035001	95055001
NC_056055.1	239940001	239960001
NC_056055.1	239945001	239965001
NC_056074.1	14525001	14545001
NC_056074.1	14530001	14550001

NC_056060.1	53075001	53095001	0.247154	0.330716	HZ	CCPG1
NC_056060.1	53080001	53100001	0.111842	0.359239	HZ	CCPG1
NC_056060.1	53085001	53105001	0.133221	0.343611	HZ	CCPG1;PIGB
NC_056060.1	53090001	53110001	0.172043	0.328819	HZ	CCPG1;PIGB
NC_056060.1	53095001	53115001	0.312857	0.270479	HZ	CCPG1;PIGB
NC_056059.1	34330001	34350001	0.217782	0.363277	HZ	CCSER1
NC_056059.1	34335001	34355001	0.156729	0.378238	HZ	CCSER1
NC_056059.1	34340001	34360001	0.132122	0.336146	HZ	CCSER1
NC_056059.1	34400001	34420001	0.0474748	0.321975	HZ	CCSER1
NC_056059.1	34415001	34435001	0.0389373	0.257542	HZ	CCSER1
NC_056054.1	108940001	108960001	0.0427538	0.554137	HZ	CD1
NC_056054.1	108945001	108965001	0.0728747	0.512392	HZ	CD1
NC_056054.1	108950001	108970001	0.1158	0.41786	HZ	CD1
NC_056054.1	108955001	108975001	0.128364	0.307477	HZ	CD1
NC_056054.1	108960001	108980001	0.089755	0.287234	HZ	CD1
NC_056056.1	163715001	163735001	0.240794	0.270496	HZ	CD63;GDF11
NC_056056.1	163720001	163740001	0.258953	0.25424	HZ	CD63;RDH5
NC_056063.1	19205001	19225001	0.0898391	0.40232	HZ	CDADC1
NC_056063.1	19185001	19205001	0.056488	0.399571	HZ	CDADC1;MLNR
NC_056069.1	53760001	53780001	0.237108	0.371449	HZ	CDH18
NC_056069.1	53765001	53785001	0.252095	0.412348	HZ	CDH18
NC_056078.1	26865001	26885001	0.328758	0.294656	HZ	CDH23
NC_056078.1	26870001	26890001	0.286486	0.408739	HZ	CDH23
NC_056080.1	16690001	16710001	0.300388	0.388631	HZ	CDKL5
NC_056080.1	16695001	16715001	0.209425	0.375585	HZ	CDKL5
NC_056080.1	16700001	16720001	0.249118	0.288917	HZ	CDKL5
NC_056080.1	16705001	16725001	0.261566	0.314039	HZ	CDKL5
NC_056080.1	16710001	16730001	0.313901	0.327436	HZ	CDKL5
NC_056080.1	16715001	16735001	0.327103	0.307018	HZ	CDKL5
NC_056080.1	16720001	16740001	0.28401	0.351629	HZ	CDKL5
NC_056080.1	16795001	16815001	0.301075	0.25843	HZ	CDKL5
NC_056080.1	16800001	16820001	0.25329	0.257005	HZ	CDKL5
NC_056080.1	16825001	16845001	0.289941	0.270661	HZ	CDKL5
NC_056080.1	16830001	16850001	0.269231	0.262662	HZ	CDKL5
NC_056080.1	16835001	16855001	0.255125	0.257525	HZ	CDKL5
NC_056080.1	16840001	16860001	0.261846	0.24766	HZ	CDKL5

NC_056074.1	14535001	14555001
NC_056074.1	14540001	14560001
NC_056074.1	14545001	14565001
NC_056074.1	14550001	14570001
NC_056074.1	14555001	14575001
NC_056074.1	14560001	14580001
NC_056074.1	14985001	15005001
NC_056074.1	14990001	15010001
NC_056080.1	109165001	109185001
NC_056080.1	109170001	109190001
NC_056080.1	109175001	109195001
NC_056080.1	109180001	109200001
NC_056080.1	109185001	109205001
NC_056070.1	72835001	72855001
NC_056070.1	72840001	72860001
NC_056075.1	15525001	15545001
NC_056075.1	15530001	15550001
NC_056075.1	15535001	15555001
NC_056075.1	15540001	15560001
NC_056075.1	15545001	15565001
NC_056075.1	15550001	15570001
NC_056075.1	15555001	15575001
NC_056071.1	26620001	26640001
NC_056071.1	26630001	26650001
NC_056061.1	82825001	82845001
NC_056061.1	82830001	82850001
NC_056056.1	115000001	115020001
NC_056056.1	115030001	115050001
NC_056056.1	115035001	115055001
NC_056056.1	115040001	115060001
NC_056056.1	115045001	115065001
NC_056056.1	115050001	115070001
NC_056056.1	115055001	115075001
NC_056056.1	115060001	115080001
NC_056056.1	115065001	115085001
NC_056056.1	115070001	115090001

NC_056080.1	16850001	16870001	0.285266	0.279333	HZ	CDKL5	NC_056056.1	115280001	115300001
NC_056063.1	36740001	36760001	0.252046	0.370509	HZ	CENPJ;RNF17	NC_056054.1	100010001	100030001
NC_056063.1	36745001	36765001	0.246555	0.415211	HZ	CENPJ;RNF17	NC_056072.1	21975001	21995001
NC_056063.1	36750001	36770001	0.257143	0.397514	HZ	CENPJ;RNF17	NC_056059.1	86095001	86115001
NC_056063.1	36755001	36775001	0.238249	0.433904	HZ	CENPJ;RNF17	NC_056059.1	86100001	86120001
NC_056058.1	28040001	28060001	0.121401	0.247094	HZ	CEP120	NC_056057.1	81995001	82015001
NC_056060.1	59610001	59630001	0.0538049	0.288152	HZ	CEP152	NC_056057.1	82000001	82020001
NC_056060.1	59615001	59635001	0.0396376	0.332761	HZ	CEP152	NC_056057.1	82005001	82025001
NC_056056.1	130600001	130620001	0.147705	0.25048	HZ	CEP83	NC_056057.1	82015001	82035001
NC_056056.1	130605001	130625001	0.0552692	0.24134	HZ	CEP83	NC_056057.1	82020001	82040001
NC_056070.1	53365001	53385001	0.0636864	0.463326	HZ	CFAP251	NC_056054.1	236720001	236740001
NC_056054.1	179040001	179060001	0.0456262	0.240743	HZ	CFAP44	NC_056054.1	236725001	236745001
NC_056064.1	43415001	43435001	0.243244	0.301929	HZ	CFAP97D1;DUSP3;MP	NC_056054.1	236730001	236750001
NC_056064.1	43420001	43440001	0.262015	0.294294	HZ	CFAP97D1;MPP3	NC_056072.1	33925001	33945001
NC_056057.1	98420001	98440001	0.304231	0.271629	HZ	CHCHD3	NC_056061.1	71775001	71795001
NC_056057.1	98425001	98445001	0.275611	0.330247	HZ	CHCHD3	NC_056056.1	8545001	8565001
NC_056057.1	98430001	98450001	0.275348	0.324167	HZ	CHCHD3	NC_056056.1	8550001	8570001
NC_056057.1	98435001	98455001	0.186088	0.295288	HZ	CHCHD3	NC_056080.1	2695001	2715001
NC_056054.1	88660001	88680001	0.206457	0.256462	HZ	CHIA	NC_056080.1	2700001	2720001
NC_056072.1	26135001	26155001	0.263191	0.264563	HZ	CHL1	NC_056080.1	2705001	2725001
NC_056055.1	135065001	135085001	0.298597	0.261321	HZ	CHN1	NC_056055.1	242250001	242270001
NC_056078.1	11625001	11645001	0.116164	0.259769	HZ	CHRM3	NC_056055.1	242255001	242275001
NC_056078.1	11630001	11650001	0.169873	0.286033	HZ	CHRM3	NC_056055.1	242260001	242280001
NC_056078.1	11635001	11655001	0.246736	0.267204	HZ	CHRM3	NC_056055.1	242290001	242310001
NC_056078.1	11640001	11660001	0.307779	0.244312	HZ	CHRM3	NC_056062.1	49930001	49950001
NC_056055.1	234145001	234165001	0.188635	0.369048	HZ	CHRND;CHRNG;EIF4	NC_056062.1	49935001	49955001
NC_056056.1	104090001	104110001	0.227609	0.272716	HZ	CIAO1;SNRNP200;TM	NC_056062.1	49940001	49960001
NC_056056.1	104095001	104115001	0.232732	0.288036	HZ	CIAO1;TMEM127	NC_056062.1	49945001	49965001
NC_056075.1	360001	380001	0.140319	0.26361	HZ	CISD1;IPMK	NC_056062.1	49950001	49970001
NC_056075.1	370001	390001	0.137097	0.343239	HZ	CISD1;IPMK	NC_056062.1	49955001	49975001
NC_056074.1	15925001	15945001	0.209302	0.275314	HZ	CLNS1A	NC_056062.1	49960001	49980001
NC_056074.1	15930001	15950001	0.265632	0.286718	HZ	CLNS1A	NC_056062.1	49965001	49985001
NC_056074.1	15920001	15940001	0.213362	0.265957	HZ	CLNS1A;RSF1	NC_056057.1	51520001	51540001
NC_056054.1	10700001	10720001	0.344385	0.34931	HZ	CLSPN	NC_056063.1	28005001	28025001
NC_056054.1	248980001	249000001	0.272843	0.260832	HZ	CLSTN2	NC_056057.1	52715001	52735001
NC_056054.1	248985001	249005001	0.199842	0.290664	HZ	CLSTN2	NC_056057.1	52720001	52740001

NC_056054.1	249070001	249090001	0.30496	0.247715	HZ	CLSTN2
NC_056054.1	249075001	249095001	0.270577	0.293229	HZ	CLSTN2
NC_056054.1	249125001	249145001	0.301799	0.48262	HZ	CLSTN2
NC_056054.1	249130001	249150001	0.267654	0.506132	HZ	CLSTN2
NC_056054.1	249135001	249155001	0.294964	0.513857	HZ	CLSTN2
NC_056054.1	249275001	249295001	0.262612	0.274974	HZ	CLSTN2
NC_056072.1	2735001	2755001	0.170429	0.282673	HZ	CMC1;LOC114108644
NC_056072.1	2745001	2765001	0.174581	0.320337	HZ	CMC1;LOC114108644
NC_056067.1	33925001	33945001	0.267676	0.278722	HZ	CMTM3;CMTM4
NC_056062.1	88400001	88420001	0.338349	0.278487	HZ	CNBD1
NC_056062.1	88405001	88425001	0.253821	0.384694	HZ	CNBD1
NC_056062.1	88410001	88430001	0.115605	0.393603	HZ	CNBD1
NC_056062.1	88415001	88435001	0.155374	0.411609	HZ	CNBD1
NC_056062.1	88420001	88440001	0.211726	0.327112	HZ	CNBD1
NC_056057.1	100640001	100660001	0.323904	0.266753	HZ	CNOT4
NC_056058.1	63810001	63830001	0.131174	0.244743	HZ	CNOT8;FAXDC2
NC_056058.1	63830001	63850001	0.198957	0.280826	HZ	CNOT8;GEMIN5
NC_056058.1	63835001	63855001	0.170049	0.33998	HZ	CNOT8;GEMIN5
NC_056058.1	63840001	63860001	0.155324	0.346521	HZ	CNOT8;GEMIN5
NC_056055.1	36990001	37010001	0.217055	0.23987	HZ	CNTFR
NC_056055.1	36995001	37015001	0.344721	0.26323	HZ	CNTFR
NC_056072.1	27260001	27280001	0.342184	0.292108	HZ	CNTN3
NC_056072.1	27265001	27285001	0.260202	0.368547	HZ	CNTN3
NC_056068.1	8750001	8770001	0.147737	0.278925	HZ	CNTN5
NC_056068.1	8755001	8775001	0.0261117	0.312888	HZ	CNTN5
NC_056068.1	8760001	8780001	0.0164332	0.320132	HZ	CNTN5
NC_056068.1	8765001	8785001	0.0280703	0.317656	HZ	CNTN5
NC_056072.1	24975001	24995001	0.169098	0.239549	HZ	CNTN6
NC_056072.1	24980001	25000001	0.162304	0.2625	HZ	CNTN6
NC_056062.1	4220001	4240001	0.328493	0.301501	HZ	COL19A1
NC_056062.1	4225001	4245001	0.249625	0.261516	HZ	COL19A1
NC_056055.1	9520001	9540001	0.291051	0.265292	HZ	COL27A1
NC_056055.1	9525001	9545001	0.242383	0.278149	HZ	COL27A1
NC_056055.1	9530001	9550001	0.287325	0.273407	HZ	COL27A1
NC_056055.1	9535001	9555001	0.274765	0.274322	HZ	COL27A1
NC_056055.1	9560001	9580001	0.288626	0.279184	HZ	COL27A1

NC_056057.1	52725001	52745001
NC_056057.1	52730001	52750001
NC_056057.1	52735001	52755001
NC_056057.1	52755001	52775001
NC_056057.1	52760001	52780001
NC_056057.1	52765001	52785001
NC_056057.1	52785001	52805001
NC_056057.1	52790001	52810001
NC_056059.1	71525001	71545001
NC_056056.1	78740001	78760001
NC_056066.1	6545001	6565001
NC_056066.1	6550001	6570001
NC_056066.1	6555001	6575001
NC_056055.1	27080001	27100001
NC_056058.1	46125001	46145001
NC_056058.1	46130001	46150001
NC_056058.1	46135001	46155001
NC_056058.1	46140001	46160001
NC_056058.1	46145001	46165001
NC_056055.1	23180001	23200001
NC_056055.1	23185001	23205001
NC_056055.1	23190001	23210001
NC_056055.1	23195001	23215001
NC_056060.1	15760001	15780001
NC_056060.1	41280001	41300001
NC_056060.1	41285001	41305001
NC_056060.1	41290001	41310001
NC_056060.1	41295001	41315001
NC_056060.1	41300001	41320001
NC_056060.1	41305001	41325001
NC_056060.1	41310001	41330001
NC_056060.1	41315001	41335001
NC_056060.1	41320001	41340001
NC_056060.1	41325001	41345001
NC_056060.1	41330001	41350001
NC_056060.1	41335001	41355001

NC_056055.1	212210001	212230001	0.33208	0.252582	HZ	CPS1	NC_056060.1	41340001	41360001
NC_056065.1	75675001	75695001	0.256165	0.265379	HZ	CRB1	NC_056060.1	41345001	41365001
NC_056057.1	103125001	103145001	0.333333	0.241443	HZ	CREB3L2	NC_056056.1	129860001	129880001
NC_056057.1	103130001	103150001	0.299362	0.242208	HZ	CREB3L2	NC_056056.1	129865001	129885001
NC_056073.1	22180001	22200001	0.246454	0.24088	HZ	CRISP1	NC_056057.1	27295001	27315001
NC_056073.1	22245001	22265001	0.282829	0.270714	HZ	CRISP1	NC_056057.1	27300001	27320001
NC_056077.1	19545001	19565001	0.138204	0.245027	HZ	CRYM	NC_056057.1	27305001	27325001
NC_056077.1	19550001	19570001	0.236278	0.246195	HZ	CRYM	NC_056057.1	27310001	27330001
NC_056058.1	27795001	27815001	0.175727	0.323234	HZ	CSNK1G3	NC_056055.1	710001	730001
NC_056058.1	27800001	27820001	0.264386	0.275241	HZ	CSNK1G3	NC_056055.1	715001	735001
NC_056058.1	27805001	27825001	0.288383	0.256016	HZ	CSNK1G3	NC_056056.1	104070001	104090001
NC_056078.1	22425001	22445001	0.236267	0.470178	HZ	CTNNA3	NC_056072.1	15220001	15240001
NC_056078.1	22430001	22450001	0.157694	0.5156	HZ	CTNNA3	NC_056072.1	15225001	15245001
NC_056078.1	22435001	22455001	0.182362	0.50268	HZ	CTNNA3	NC_056074.1	940001	960001
NC_056078.1	22440001	22460001	0.241503	0.470321	HZ	CTNNA3	NC_056074.1	945001	965001
NC_056078.1	22475001	22495001	0.345026	0.342142	HZ	CTNNA3	NC_056074.1	950001	970001
NC_056078.1	22480001	22500001	0.260461	0.34938	HZ	CTNNA3	NC_056074.1	955001	975001
NC_056078.1	22485001	22505001	0.280736	0.303439	HZ	CTNNA3	NC_056076.1	50830001	50850001
NC_056078.1	23115001	23135001	0.0682848	0.398433	HZ	CTNNA3	NC_056076.1	50835001	50855001
NC_056078.1	23125001	23145001	0.113018	0.416499	HZ	CTNNA3	NC_056076.1	50840001	50860001
NC_056078.1	23130001	23150001	0.249435	0.319043	HZ	CTNNA3	NC_056076.1	50845001	50865001
NC_056058.1	24025001	24045001	0.261486	0.296618	HZ	CTXN3	NC_056076.1	50850001	50870001
NC_056067.1	24960001	24980001	0.287031	0.300651	HZ	CX3CL1	NC_056076.1	50855001	50875001
NC_056067.1	24965001	24985001	0.259601	0.331324	HZ	CX3CL1	NC_056076.1	50860001	50880001
NC_056067.1	24970001	24990001	0.238828	0.327035	HZ	CX3CL1	NC_056076.1	50865001	50885001
NC_056064.1	26490001	26510001	0.238602	0.350767	HZ	CXCL16;MED11;PELF	NC_056076.1	50870001	50890001
NC_056080.1	64265001	64285001	0.318037	0.303427	HZ	CXCR3;GCNA	NC_056076.1	50890001	50910001
NC_056064.1	47495001	47515001	0.118463	0.352258	HZ	CYB561	NC_056076.1	47870001	47890001
NC_056064.1	47485001	47505001	0.275348	0.377306	HZ	CYB561;TANC2	NC_056076.1	47875001	47895001
NC_056064.1	47490001	47510001	0.171537	0.355192	HZ	CYB561;TANC2	NC_056070.1	12785001	12805001
NC_056075.1	51420001	51440001	0.18046	0.245775	HZ	CYP2E1	NC_056070.1	12790001	12810001
NC_056075.1	51425001	51445001	0.144144	0.243355	HZ	CYP2E1	NC_056070.1	12795001	12815001
NC_056075.1	51430001	51450001	0.0951791	0.26496	HZ	CYP2E1	NC_056070.1	12800001	12820001
NC_056075.1	51435001	51455001	0.101709	0.28293	HZ	CYP2E1	NC_056070.1	12805001	12825001
NC_056075.1	51440001	51460001	0.0875911	0.288351	HZ	CYP2E1	NC_056070.1	12865001	12885001
NC_056075.1	51445001	51465001	0.0644939	0.304103	HZ	CYP2E1	NC_056070.1	12870001	12890001

NC_056060.1	82515001	82535001	0.030025	0.472262	HZ	DCAF4	NC_056070.1	12875001	12895001
NC_056060.1	82520001	82540001	0.106966	0.393639	HZ	DCAF4	NC_056060.1	49520001	49540001
NC_056076.1	52525001	52545001	0.263306	0.359332	HZ	DCC	NC_056060.1	49525001	49545001
NC_056076.1	52530001	52550001	0.301093	0.318324	HZ	DCC	NC_056060.1	49530001	49550001
NC_056072.1	47755001	47775001	0.280684	0.247883	HZ	DCP1A	NC_056063.1	61315001	61335001
NC_056074.1	36390001	36410001	0.0913983	0.32446	HZ	DDB1;TKFC	NC_056063.1	61320001	61340001
NC_056074.1	36395001	36415001	0.0541403	0.357391	HZ	DDB1;TKFC	NC_056063.1	61325001	61345001
NC_056054.1	114340001	114360001	0.142764	0.277722	HZ	DDR2;HSD17B7	NC_056063.1	61330001	61350001
NC_056054.1	114345001	114365001	0.100378	0.289955	HZ	DDR2;HSD17B7	NC_056059.1	40530001	40550001
NC_056070.1	70365001	70385001	0.120914	0.247239	HZ	DEPDC5	NC_056059.1	40535001	40555001
NC_056070.1	70375001	70395001	0.122642	0.274554	HZ	DEPDC5	NC_056059.1	40540001	40560001
NC_056080.1	63070001	63090001	0.319943	0.298607	HZ	DGAT2L6	NC_056059.1	40545001	40565001
NC_056080.1	63075001	63095001	0.269231	0.273656	HZ	DGAT2L6	NC_056059.1	40550001	40570001
NC_056080.1	63085001	63105001	0.223404	0.310104	HZ	DGAT2L6	NC_056059.1	40555001	40575001
NC_056070.1	72610001	72630001	0.198043	0.333895	HZ	DGCR2;ESS2;TSSK1B	NC_056059.1	40560001	40580001
NC_056070.1	72615001	72635001	0.0757962	0.376374	HZ	DGCR2;ESS2;TSSK1B	NC_056072.1	1880001	1900001
NC_056070.1	72605001	72625001	0.300038	0.302695	HZ	DGCR2;TSSK1B	NC_056055.1	148010001	148030001
NC_056063.1	12625001	12645001	0.289736	0.309183	HZ	DGKH	NC_056055.1	148015001	148035001
NC_056063.1	12630001	12650001	0.057289	0.417352	HZ	DGKH	NC_056055.1	148020001	148040001
NC_056063.1	12635001	12655001	0.0434328	0.438116	HZ	DGKH	NC_056055.1	148025001	148045001
NC_056063.1	12640001	12660001	0.106832	0.332719	HZ	DGKH	NC_056065.1	3275001	3295001
NC_056060.1	70405001	70425001	0.16426	0.276799	HZ	DHRS7	NC_056058.1	12945001	12965001
NC_056064.1	28775001	28795001	0.115502	0.295034	HZ	DHRS7C;GLP2R;GSG1	NC_056058.1	12950001	12970001
NC_056064.1	28770001	28790001	0.230257	0.313368	HZ	DHRS7C;GSG1L2	NC_056055.1	17670001	17690001
NC_056075.1	44835001	44855001	0.340369	0.252587	HZ	DHX32	NC_056055.1	17675001	17695001
NC_056056.1	135660001	135680001	0.291073	0.253127	HZ	DIP2B;LOC101117527	NC_056055.1	17680001	17700001
NC_056054.1	192405001	192425001	0.314102	0.252562	HZ	DLG1	NC_056055.1	17685001	17705001
NC_056054.1	192415001	192435001	0.213687	0.250645	HZ	DLG1	NC_056069.1	23675001	23695001
NC_056080.1	30995001	31015001	0.208249	0.276791	HZ	DMD	NC_056069.1	23680001	23700001
NC_056080.1	32015001	32035001	0.146657	0.252345	HZ	DMD	NC_056069.1	23685001	23705001
NC_056080.1	32020001	32040001	0.151373	0.260044	HZ	DMD	NC_056069.1	23690001	23710001
NC_056080.1	32025001	32045001	0.140503	0.254114	HZ	DMD	NC_056069.1	23695001	23715001
NC_056080.1	32055001	32075001	0.230822	0.25589	HZ	DMD	NC_056056.1	140135001	140155001
NC_056080.1	32060001	32080001	0.219264	0.271277	HZ	DMD	NC_056068.1	17100001	17120001
NC_056080.1	32065001	32085001	0.244843	0.283715	HZ	DMD	NC_056066.1	38780001	38800001
NC_056080.1	32505001	32525001	0.336899	0.438688	HZ	DMD	NC_056054.1	266050001	266070001

NC_056080.1	32510001	32530001	0.229437	0.444879	HZ	DMD	NC_056055.1	11785001	11805001
NC_056080.1	32530001	32550001	0.222367	0.317053	HZ	DMD	NC_056055.1	11790001	11810001
NC_056060.1	57035001	57055001	0.323568	0.26776	HZ	DMXL2	NC_056059.1	115190001	115210001
NC_056060.1	57040001	57060001	0.333181	0.276371	HZ	DMXL2	NC_056071.1	28285001	28305001
NC_056060.1	57045001	57065001	0.269443	0.258653	HZ	DMXL2	NC_056055.1	224590001	224610001
NC_056060.1	57050001	57070001	0.235938	0.255096	HZ	DMXL2	NC_056055.1	224595001	224615001
NC_056060.1	57055001	57075001	0.240095	0.266414	HZ	DMXL2	NC_056055.1	224600001	224620001
NC_056077.1	19200001	19220001	0.294062	0.314194	HZ	DNAH3;LYRM1	NC_056061.1	59585001	59605001
NC_056054.1	257915001	257935001	0.153136	0.255884	HZ	DNAJC13	NC_056061.1	59590001	59610001
NC_056054.1	257920001	257940001	0.166511	0.246375	HZ	DNAJC13	NC_056061.1	59595001	59615001
NC_056054.1	41020001	41040001	0.30299	0.467722	HZ	DNAJC6	NC_056061.1	59600001	59620001
NC_056054.1	18595001	18615001	0.30021	0.270808	HZ	DPH2;IPO13	NC_056054.1	42195001	42215001
NC_056054.1	105340001	105360001	0.115702	0.301171	HZ	DPM3;EFNA1;LOC114	NC_056063.1	34945001	34965001
NC_056068.1	80980001	81000001	0.341985	0.291007	HZ	DTX4	NC_056058.1	64735001	64755001
NC_056068.1	80990001	81010001	0.268974	0.256502	HZ	DTX4	NC_056058.1	64740001	64760001
NC_056068.1	80975001	80995001	0.33068	0.246929	HZ	DTX4;LOC121816730	NC_056058.1	64745001	64765001
NC_056064.1	50305001	50325001	0.208517	0.405592	HZ	DUS1L;GPS1;RFNG	NC_056058.1	65735001	65755001
NC_056064.1	50310001	50330001	0.205697	0.295533	HZ	DUS1L;GPS1;RFNG	NC_056060.1	61060001	61080001
NC_056068.1	4880001	4900001	0.24831	0.537167	HZ	DYNC2H1	NC_056060.1	61065001	61085001
NC_056068.1	4920001	4940001	0.181059	0.259682	HZ	DYNC2H1	NC_056056.1	219930001	219950001
NC_056068.1	4925001	4945001	0.081679	0.302358	HZ	DYNC2H1	NC_056072.1	11965001	11985001
NC_056068.1	4930001	4950001	0.058202	0.284836	HZ	DYNC2H1	NC_056056.1	10865001	10885001
NC_056068.1	4935001	4955001	0.0783853	0.2632	HZ	DYNC2H1	NC_056056.1	10870001	10890001
NC_056055.1	234090001	234110001	0.295101	0.303054	HZ	ECEL1	NC_056056.1	10875001	10895001
NC_056054.1	215450001	215470001	0.267158	0.315587	HZ	ECT2	NC_056056.1	10880001	10900001
NC_056054.1	215455001	215475001	0.295082	0.30433	HZ	ECT2	NC_056072.1	51660001	51680001
NC_056054.1	215460001	215480001	0.217553	0.268083	HZ	ECT2	NC_056072.1	51665001	51685001
NC_056058.1	81775001	81795001	0.219097	0.28783	HZ	EDIL3	NC_056068.1	42110001	42130001
NC_056058.1	81780001	81800001	0.255139	0.310373	HZ	EDIL3	NC_056068.1	42115001	42135001
NC_056058.1	81785001	81805001	0.277144	0.303197	HZ	EDIL3	NC_056068.1	42120001	42140001
NC_056058.1	81790001	81810001	0.248036	0.324582	HZ	EDIL3	NC_056068.1	42125001	42145001
NC_056065.1	31550001	31570001	0.233709	0.251596	HZ	EFCAB2	NC_056068.1	42130001	42150001
NC_056065.1	31555001	31575001	0.267873	0.256976	HZ	EFCAB2	NC_056056.1	220600001	220620001
NC_056058.1	104135001	104155001	0.0569734	0.247278	HZ	EFNA5	NC_056056.1	220605001	220625001
NC_056078.1	3520001	3540001	0.165949	0.291241	HZ	EGLN1;SPRTN	NC_056067.1	19045001	19065001
NC_056078.1	3525001	3545001	0.247847	0.255627	HZ	EGLN1;SPRTN	NC_056067.1	19050001	19070001

NC_056074.1	26430001	26450001	0.3125	0.271409	HZ	EI24
NC_056056.1	59300001	59320001	0.197259	0.267821	HZ	EIF2AK3
NC_056075.1	20410001	20430001	0.312546	0.28312	HZ	ENTPD7
NC_056075.1	20405001	20425001	0.304918	0.296048	HZ	ENTPD7;SLC25A28
NC_056055.1	238340001	238360001	0.257466	0.264888	HZ	EPB41
NC_056055.1	238345001	238365001	0.254149	0.26138	HZ	EPB41
NC_056055.1	160845001	160865001	0.22482	0.2636	HZ	EPC2
NC_056055.1	160850001	160870001	0.145434	0.296436	HZ	EPC2
NC_056055.1	160855001	160875001	0.151643	0.28714	HZ	EPC2
NC_056055.1	160860001	160880001	0.13973	0.311892	HZ	EPC2
NC_056055.1	160865001	160885001	0.116471	0.327571	HZ	EPC2
NC_056055.1	160870001	160890001	0.179127	0.311292	HZ	EPC2
NC_056055.1	160875001	160895001	0.202046	0.313504	HZ	EPC2
NC_056059.1	81715001	81735001	0.33994	0.347831	HZ	EPHA5
NC_056054.1	162770001	162790001	0.297642	0.354697	HZ	EPHA6
NC_056054.1	254760001	254780001	0.321972	0.2683	HZ	EPHB1
NC_056054.1	254780001	254800001	0.24593	0.322513	HZ	EPHB1
NC_056054.1	254785001	254805001	0.122449	0.25912	HZ	EPHB1
NC_056054.1	254850001	254870001	0.0953571	0.278292	HZ	EPHB1
NC_056067.1	62630001	62650001	0.307691	0.423572	HZ	EPN1
NC_056067.1	62635001	62655001	0.314146	0.474409	HZ	EPN1
NC_056067.1	62640001	62660001	0.301701	0.403895	HZ	EPN1;U2AF2
NC_056067.1	62645001	62665001	0.225191	0.40181	HZ	EPN1;U2AF2
NC_056067.1	62650001	62670001	0.209039	0.392457	HZ	EPN1;U2AF2
NC_056054.1	87095001	87115001	0.122415	0.294484	HZ	EPS8L3
NC_056054.1	87085001	87105001	0.203473	0.244499	HZ	EPS8L3;GSTM3
NC_056055.1	214380001	214400001	0.260851	0.267198	HZ	ERBB4
NC_056055.1	214385001	214405001	0.181017	0.268541	HZ	ERBB4
NC_056055.1	214390001	214410001	0.123541	0.269376	HZ	ERBB4
NC_056055.1	214395001	214415001	0.127299	0.260479	HZ	ERBB4
NC_056072.1	45025001	45045001	0.340836	0.3279	HZ	ERC2
NC_056069.1	4840001	4860001	0.303202	0.263996	HZ	ERGIC1
NC_056069.1	4845001	4865001	0.290082	0.266568	HZ	ERGIC1
NC_056066.1	7200001	7220001	0.0605475	0.350984	HZ	ESF1;NDUFAF5
NC_056054.1	3195001	3215001	0.198245	0.246951	HZ	ESPNL;SCLY
NC_056060.1	85985001	86005001	0.295376	0.420746	HZ	ESRRB

NC_056054.1	101940001	101960001
NC_056054.1	101945001	101965001
NC_056056.1	1875001	1895001
NC_056056.1	1880001	1900001
NC_056056.1	1885001	1905001
NC_056056.1	1890001	1910001
NC_056054.1	267650001	267670001
NC_056078.1	24000001	24020001
NC_056068.1	79550001	79570001
NC_056060.1	69910001	69930001
NC_056062.1	69450001	69470001
NC_056074.1	15845001	15865001
NC_056074.1	15850001	15870001
NC_056074.1	15855001	15875001
NC_056074.1	15860001	15880001
NC_056074.1	15865001	15885001
NC_056074.1	15870001	15890001
NC_056074.1	15900001	15920001
NC_056074.1	15905001	15925001
NC_056080.1	18620001	18640001
NC_056080.1	18625001	18645001
NC_056080.1	18630001	18650001
NC_056080.1	18635001	18655001
NC_056060.1	47060001	47080001
NC_056060.1	47065001	47085001
NC_056060.1	47250001	47270001
NC_056060.1	47255001	47275001
NC_056060.1	47260001	47280001
NC_056060.1	47265001	47285001
NC_056061.1	48595001	48615001
NC_056061.1	48600001	48620001
NC_056061.1	48605001	48625001
NC_056061.1	48610001	48630001
NC_056061.1	48615001	48635001
NC_056061.1	48620001	48640001
NC_056061.1	48625001	48645001

NC_056070.1	72625001	72645001	0.148191	0.280569	HZ	ESS2;GSC2;TSSK1B;T	NC_056061.1	48630001	48650001
NC_056054.1	251315001	251335001	0.299465	0.288951	HZ	ESYT3	NC_056061.1	48635001	48655001
NC_056056.1	40895001	40915001	0.288194	0.371918	HZ	ETAA1	NC_056061.1	48640001	48660001
NC_056057.1	98815001	98835001	0.344418	0.240279	HZ	EXOC4	NC_056061.1	48645001	48665001
NC_056057.1	98820001	98840001	0.32358	0.258822	HZ	EXOC4	NC_056063.1	36830001	36850001
NC_056057.1	98860001	98880001	0.212588	0.314229	HZ	EXOC4	NC_056063.1	36835001	36855001
NC_056057.1	98865001	98885001	0.222273	0.301976	HZ	EXOC4	NC_056063.1	36840001	36860001
NC_056057.1	98870001	98890001	0.198706	0.264719	HZ	EXOC4	NC_056063.1	36845001	36865001
NC_056057.1	98875001	98895001	0.322337	0.275333	HZ	EXOC4	NC_056063.1	36850001	36870001
NC_056057.1	99135001	99155001	0.0463919	0.357657	HZ	EXOC4	NC_056060.1	49365001	49385001
NC_056057.1	99140001	99160001	0.129917	0.298877	HZ	EXOC4	NC_056060.1	49370001	49390001
NC_056075.1	14050001	14070001	0.213814	0.267371	HZ	EXOC6	NC_056060.1	49375001	49395001
NC_056075.1	14055001	14075001	0.140435	0.257304	HZ	EXOC6	NC_056060.1	49380001	49400001
NC_056068.1	17895001	17915001	0.162555	0.251384	HZ	EXPH5	NC_056060.1	49385001	49405001
NC_056065.1	36420001	36440001	0.261636	0.450138	HZ	F5	NC_056060.1	49390001	49410001
NC_056065.1	36425001	36445001	0.245324	0.400458	HZ	F5	NC_056060.1	49395001	49415001
NC_056065.1	36430001	36450001	0.343242	0.306932	HZ	F5	NC_056060.1	49400001	49420001
NC_056063.1	20995001	21015001	0.143215	0.241876	HZ	FAM124A	NC_056080.1	128420001	128440001
NC_056062.1	17315001	17335001	0.16081	0.244639	HZ	FAM135B	NC_056080.1	128425001	128445001
NC_056060.1	55915001	55935001	0.303626	0.263407	HZ	FAM214A	NC_056062.1	1820001	1840001
NC_056060.1	55935001	55955001	0.289523	0.336912	HZ	FAM214A	NC_056062.1	1825001	1845001
NC_056055.1	37120001	37140001	0.345238	0.248214	HZ	FAM219A	NC_056062.1	1830001	1850001
NC_056068.1	38715001	38735001	0.125329	0.297544	HZ	FAR1	NC_056062.1	1835001	1855001
NC_056068.1	38720001	38740001	0.170314	0.276933	HZ	FAR1	NC_056062.1	1840001	1860001
NC_056068.1	38725001	38745001	0.227521	0.25591	HZ	FAR1	NC_056062.1	1845001	1865001
NC_056073.1	48425001	48445001	0.216155	0.267425	HZ	FARS2	NC_056062.1	1860001	1880001
NC_056073.1	48430001	48450001	0.212945	0.258291	HZ	FARS2	NC_056073.1	22020001	22040001
NC_056074.1	1740001	1760001	0.343636	0.24405	HZ	FAT3	NC_056073.1	22025001	22045001
NC_056064.1	55060001	55080001	0.0617785	0.305546	HZ	FBF1;MRPL38;TRIM6	NC_056065.1	63160001	63180001
NC_056065.1	51070001	51090001	0.308239	0.2794	HZ	FBLIM1	NC_056065.1	63165001	63185001
NC_056065.1	51075001	51095001	0.211431	0.252588	HZ	FBLIM1	NC_056060.1	52430001	52450001
NC_056065.1	51080001	51100001	0.246882	0.258537	HZ	FBLIM1	NC_056060.1	52435001	52455001
NC_056060.1	59785001	59805001	0.0944713	0.25348	HZ	FBN1	NC_056060.1	52440001	52460001
NC_056060.1	59790001	59810001	0.085389	0.306664	HZ	FBN1	NC_056060.1	52445001	52465001
NC_056060.1	59795001	59815001	0.0956021	0.357351	HZ	FBN1	NC_056055.1	71855001	71875001
NC_056060.1	59800001	59820001	0.0871155	0.376763	HZ	FBN1	NC_056055.1	71860001	71880001

NC_056060.1	59825001	59845001	0.0757813	0.328956	HZ	FBN1
NC_056060.1	59830001	59850001	0.208712	0.295302	HZ	FBN1
NC_056069.1	57500001	57520001	0.180532	0.330889	HZ	FBXL7
NC_056069.1	57505001	57525001	0.20117	0.337779	HZ	FBXL7
NC_056069.1	57510001	57530001	0.182976	0.321789	HZ	FBXL7
NC_056069.1	57515001	57535001	0.198316	0.267366	HZ	FBXL7
NC_056056.1	76560001	76580001	0.203615	0.259325	HZ	FBXO11
NC_056056.1	76625001	76645001	0.299765	0.35038	HZ	FBXO11
NC_056056.1	76630001	76650001	0.285354	0.33564	HZ	FBXO11
NC_056056.1	76615001	76635001	0.213115	0.28269	HZ	FBXO11;LOC1056069
NC_056056.1	76620001	76640001	0.245283	0.341708	HZ	FBXO11;LOC1056069
NC_056056.1	76645001	76665001	0.1856	0.333163	HZ	FBXO11;MSH6
NC_056056.1	76650001	76670001	0.139168	0.375482	HZ	FBXO11;MSH6
NC_056056.1	76655001	76675001	0.254901	0.397813	HZ	FBXO11;MSH6
NC_056069.1	8765001	8785001	0.0668607	0.263383	HZ	FCHO2
NC_056069.1	8770001	8790001	0.0550679	0.284965	HZ	FCHO2
NC_056069.1	8800001	8820001	0.246099	0.297259	HZ	FCHO2
NC_056058.1	105665001	105685001	0.314905	0.602719	HZ	FER
NC_056058.1	105670001	105690001	0.153473	0.603564	HZ	FER
NC_056058.1	105675001	105695001	0.0697754	0.546031	HZ	FER
NC_056058.1	105680001	105700001	0.0618418	0.451285	HZ	FER
NC_056058.1	105685001	105705001	0.0595702	0.345475	HZ	FER
NC_056058.1	105690001	105710001	0.0845615	0.318307	HZ	FER
NC_056058.1	105695001	105715001	0.149821	0.296066	HZ	FER
NC_056058.1	105700001	105720001	0.314581	0.242759	HZ	FER
NC_056069.1	30640001	30660001	0.255038	0.27932	HZ	FGF10
NC_056069.1	30645001	30665001	0.285714	0.319291	HZ	FGF10
NC_056069.1	30650001	30670001	0.331379	0.346228	HZ	FGF10
NC_056069.1	30660001	30680001	0.330978	0.362268	HZ	FGF10
NC_056069.1	30665001	30685001	0.242567	0.28854	HZ	FGF10
NC_056069.1	30670001	30690001	0.241789	0.292917	HZ	FGF10
NC_056069.1	30675001	30695001	0.290808	0.28347	HZ	FGF10
NC_056065.1	50345001	50365001	0.311565	0.290752	HZ	FNDC10
NC_056071.1	45485001	45505001	0.0698271	0.299048	HZ	FOXA1
NC_056071.1	45490001	45510001	0.198572	0.25806	HZ	FOXA1;LOC12181710
NC_056063.1	22155001	22175001	0.078137	0.311907	HZ	FOXO1

NC_056056.1	101870001	101890001
NC_056056.1	101875001	101895001
NC_056056.1	101880001	101900001
NC_056056.1	101885001	101905001
NC_056076.1	60920001	60940001
NC_056076.1	60925001	60945001
NC_056076.1	60930001	60950001
NC_056076.1	60935001	60955001
NC_056060.1	20160001	20180001
NC_056067.1	63135001	63155001
NC_056072.1	3985001	4005001
NC_056072.1	3990001	4010001
NC_056072.1	3995001	4015001
NC_056072.1	4000001	4020001
NC_056070.1	60745001	60765001
NC_056070.1	60750001	60770001
NC_056070.1	60810001	60830001
NC_056070.1	60815001	60835001
NC_056077.1	6480001	6500001
NC_056077.1	6485001	6505001
NC_056077.1	6490001	6510001
NC_056059.1	97325001	97345001
NC_056059.1	97330001	97350001
NC_056061.1	49755001	49775001
NC_056061.1	49760001	49780001
NC_056079.1	39765001	39785001
NC_056079.1	39770001	39790001
NC_056079.1	39775001	39795001
NC_056062.1	82765001	82785001
NC_056060.1	44965001	44985001
NC_056060.1	44970001	44990001
NC_056060.1	44975001	44995001
NC_056060.1	44980001	45000001
NC_056060.1	44985001	45005001
NC_056054.1	256095001	256115001
NC_056054.1	256100001	256120001

NC_056063.1	22160001	22180001	0.14912	0.270584	HZ	FOXO1
NC_056072.1	30360001	30380001	0.284849	0.448494	HZ	FOXP1
NC_056072.1	30365001	30385001	0.200346	0.485616	HZ	FOXP1
NC_056072.1	30370001	30390001	0.255102	0.459792	HZ	FOXP1
NC_056072.1	30375001	30395001	0.246881	0.534784	HZ	FOXP1
NC_056070.1	14385001	14405001	0.0774661	0.315366	HZ	FREM3
NC_056070.1	14390001	14410001	0.0439439	0.360819	HZ	FREM3
NC_056070.1	14395001	14415001	0.0706722	0.37979	HZ	FREM3
NC_056070.1	14400001	14420001	0.142163	0.318721	HZ	FREM3
NC_056072.1	32075001	32095001	0.0660378	0.247134	HZ	FRMD4B
NC_056072.1	32105001	32125001	0.339164	0.251195	HZ	FRMD4B
NC_056072.1	32160001	32180001	0.309103	0.302404	HZ	FRMD4B
NC_056072.1	32165001	32185001	0.283364	0.269363	HZ	FRMD4B
NC_056072.1	32170001	32190001	0.27192	0.261941	HZ	FRMD4B
NC_056072.1	32175001	32195001	0.275483	0.23969	HZ	FRMD4B
NC_056072.1	32410001	32430001	0.119522	0.401183	HZ	FRMD4B
NC_056072.1	32415001	32435001	0.194639	0.379778	HZ	FRMD4B
NC_056055.1	126875001	126895001	0.238285	0.262518	HZ	FRZB
NC_056071.1	52055001	52075001	0.111835	0.25157	HZ	FSCB;LOC101104431
NC_056057.1	93540001	93560001	0.291839	0.243853	HZ	FSCN3;PAX4
NC_056067.1	21880001	21900001	0.0539945	0.297376	HZ	FTO
NC_056060.1	75575001	75595001	0.307533	0.593673	HZ	FUT8
NC_056060.1	75580001	75600001	0.278092	0.573003	HZ	FUT8
NC_056060.1	75585001	75605001	0.313966	0.564955	HZ	FUT8
NC_056060.1	75590001	75610001	0.32041	0.557386	HZ	FUT8
NC_056060.1	75595001	75615001	0.288439	0.565057	HZ	FUT8
NC_056060.1	75605001	75625001	0.301436	0.574213	HZ	FUT8
NC_056060.1	75610001	75630001	0.343832	0.50665	HZ	FUT8
NC_056060.1	75615001	75635001	0.269175	0.372534	HZ	FUT8
NC_056060.1	75620001	75640001	0.204496	0.271334	HZ	FUT8
NC_056058.1	70930001	70950001	0.338996	0.269255	HZ	GABRA6
NC_056058.1	70935001	70955001	0.260191	0.29316	HZ	GABRA6
NC_056058.1	70940001	70960001	0.31611	0.26541	HZ	GABRA6
NC_056055.1	155555001	155575001	0.287561	0.300967	HZ	GALNT13
NC_056055.1	155560001	155580001	0.31817	0.291049	HZ	GALNT13
NC_056055.1	155675001	155695001	0.143904	0.244112	HZ	GALNT13

NC_056054.1	256105001	256125001
NC_056056.1	107830001	107850001
NC_056056.1	107835001	107855001
NC_056056.1	107840001	107860001
NC_056056.1	107845001	107865001
NC_056056.1	107850001	107870001
NC_056056.1	107855001	107875001
NC_056056.1	107860001	107880001
NC_056056.1	107865001	107885001
NC_056056.1	107870001	107890001
NC_056064.1	18290001	18310001
NC_056064.1	18295001	18315001
NC_056056.1	176525001	176545001
NC_056056.1	143000001	143020001
NC_056076.1	41145001	41165001
NC_056076.1	41150001	41170001
NC_056076.1	41155001	41175001
NC_056075.1	46935001	46955001
NC_056055.1	77120001	77140001
NC_056055.1	77125001	77145001
NC_056055.1	77130001	77150001
NC_056055.1	77135001	77155001
NC_056055.1	13955001	13975001
NC_056056.1	7300001	7320001
NC_056056.1	7305001	7325001
NC_056054.1	54955001	54975001
NC_056054.1	54960001	54980001
NC_056054.1	54965001	54985001
NC_056054.1	46795001	46815001
NC_056054.1	46785001	46805001
NC_056054.1	46790001	46810001
NC_056066.1	30345001	30365001
NC_056066.1	30350001	30370001
NC_056066.1	30355001	30375001
NC_056066.1	30360001	30380001
NC_056060.1	21630001	21650001

NC_056055.1	107495001	107515001	0.13001	0.240883	HZ	GALNT7	NC_056056.1	59775001	59795001
NC_056055.1	107500001	107520001	0.0922279	0.269733	HZ	GALNT7	NC_056056.1	59800001	59820001
NC_056076.1	25265001	25285001	0.330779	0.338913	HZ	GAREM1	NC_056079.1	38190001	38210001
NC_056076.1	25270001	25290001	0.298261	0.314341	HZ	GAREM1	NC_056079.1	38200001	38220001
NC_056056.1	8780001	8800001	0.304817	0.292157	HZ	GARNL3	NC_056079.1	38205001	38225001
NC_056056.1	8785001	8805001	0.309049	0.272974	HZ	GARNL3	NC_056055.1	59885001	59905001
NC_056056.1	163705001	163725001	0.230347	0.313213	HZ	GDF11	NC_056055.1	59890001	59910001
NC_056056.1	163710001	163730001	0.269973	0.288333	HZ	GDF11	NC_056055.1	59895001	59915001
NC_056056.1	163695001	163715001	0.279029	0.268027	HZ	GDF11;LOC101114432	NC_056055.1	59900001	59920001
NC_056056.1	163700001	163720001	0.224449	0.297504	HZ	GDF11;LOC101114432	NC_056055.1	59905001	59925001
NC_056058.1	63845001	63865001	0.181818	0.331484	HZ	GEMIN5	NC_056055.1	59910001	59930001
NC_056058.1	63850001	63870001	0.27304	0.261531	HZ	GEMIN5	NC_056080.1	36530001	36550001
NC_056054.1	260180001	260200001	0.272672	0.324377	HZ	GET1	NC_056056.1	221175001	221195001
NC_056054.1	260185001	260205001	0.122709	0.257511	HZ	GET1	NC_056056.1	221180001	221200001
NC_056054.1	260190001	260210001	0.0467323	0.251219	HZ	GET1	NC_056056.1	221185001	221205001
NC_056055.1	43895001	43915001	0.289444	0.302291	HZ	GFRA2	NC_056077.1	27275001	27295001
NC_056055.1	43900001	43920001	0.196428	0.31914	HZ	GFRA2	NC_056077.1	27280001	27300001
NC_056055.1	43905001	43925001	0.163737	0.319042	HZ	GFRA2	NC_056071.1	43155001	43175001
NC_056055.1	43910001	43930001	0.13591	0.320561	HZ	GFRA2	NC_056056.1	104425001	104445001
NC_056055.1	43915001	43935001	0.19064	0.294064	HZ	GFRA2	NC_056056.1	104430001	104450001
NC_056070.1	71485001	71505001	0.113261	0.337853	HZ	GGT5;LOC105603000	NC_056056.1	104435001	104455001
NC_056070.1	71490001	71510001	0.113464	0.434911	HZ	GGT5;LOC105603000	NC_056056.1	104440001	104460001
NC_056070.1	71495001	71515001	0.13223	0.437247	HZ	GGT5;LOC105603000	NC_056056.1	78135001	78155001
NC_056070.1	71500001	71520001	0.32425	0.313523	HZ	GGT5;LOC105603000	NC_056056.1	78230001	78250001
NC_056070.1	71480001	71500001	0.213268	0.316063	HZ	GGT5;SUSD2	NC_056056.1	78560001	78580001
NC_056057.1	114095001	114115001	0.207784	0.348463	HZ	GIMAP4	NC_056056.1	78565001	78585001
NC_056080.1	29905001	29925001	0.223437	0.291538	HZ	GK	NC_056056.1	78570001	78590001
NC_056054.1	247380001	247400001	0.232105	0.275153	HZ	GK5	NC_056056.1	78575001	78595001
NC_056054.1	247385001	247405001	0.130493	0.394005	HZ	GK5	NC_056056.1	78590001	78610001
NC_056054.1	247390001	247410001	0.081786	0.511373	HZ	GK5	NC_056057.1	49660001	49680001
NC_056054.1	247395001	247415001	0.124742	0.564878	HZ	GK5	NC_056057.1	49665001	49685001
NC_056054.1	247360001	247380001	0.231251	0.296859	HZ	GK5;LOC101110546	NC_056057.1	49670001	49690001
NC_056054.1	247365001	247385001	0.245061	0.291546	HZ	GK5;LOC101110546	NC_056057.1	49675001	49695001
NC_056054.1	247370001	247390001	0.250683	0.250576	HZ	GK5;LOC101110546	NC_056057.1	49680001	49700001
NC_056054.1	247375001	247395001	0.243565	0.243968	HZ	GK5;LOC101110546	NC_056057.1	49685001	49705001
NC_056054.1	247400001	247420001	0.16837	0.553939	HZ	GK5;TFDP2	NC_056072.1	51125001	51145001

NC_056054.1	247405001	247425001	0.298519	0.488128	HZ	GK5;TFDP2
NC_056057.1	80435001	80455001	0.253148	0.245778	HZ	GLI3
NC_056057.1	80445001	80465001	0.30349	0.249336	HZ	GLI3
NC_056057.1	80475001	80495001	0.115077	0.25069	HZ	GLI3
NC_056064.1	28790001	28810001	0.0637552	0.284185	HZ	GLP2R
NC_056064.1	28780001	28800001	0.068196	0.355273	HZ	GLP2R;GSG1L2
NC_056063.1	68985001	69005001	0.151009	0.301339	HZ	GPC6
NC_056063.1	68990001	69010001	0.269525	0.298993	HZ	GPC6
NC_056060.1	76595001	76615001	0.226202	0.252889	HZ	GPHN
NC_056060.1	76600001	76620001	0.248485	0.247308	HZ	GPHN
NC_056080.1	11955001	11975001	0.304543	0.246108	HZ	GPM6B
NC_056080.1	11960001	11980001	0.209356	0.303687	HZ	GPM6B
NC_056080.1	11965001	11985001	0.199525	0.310368	HZ	GPM6B
NC_056080.1	11970001	11990001	0.150307	0.339084	HZ	GPM6B
NC_056080.1	12045001	12065001	0.130081	0.304275	HZ	GPM6B
NC_056080.1	12050001	12070001	0.251497	0.288166	HZ	GPM6B
NC_056056.1	12695001	12715001	0.260394	0.262107	HZ	GPR21;RABGAP1
NC_056056.1	12700001	12720001	0.263441	0.28228	HZ	GPR21;RABGAP1
NC_056056.1	12705001	12725001	0.281734	0.293338	HZ	GPR21;RABGAP1
NC_056056.1	12710001	12730001	0.305178	0.26686	HZ	GPR21;RABGAP1
NC_056054.1	86760001	86780001	0.328687	0.24938	HZ	GPR61
NC_056059.1	36215001	36235001	0.24083	0.257215	HZ	GPRIN3
NC_056058.1	25190001	25210001	0.179301	0.253364	HZ	GRAMD2B
NC_056080.1	107250001	107270001	0.254818	0.284307	HZ	GRIA3
NC_056080.1	107255001	107275001	0.333588	0.284106	HZ	GRIA3
NC_056080.1	107370001	107390001	0.240928	0.252223	HZ	GRIA3
NC_056068.1	1615001	1635001	0.0726122	0.246455	HZ	GRIA4
NC_056068.1	1620001	1640001	0.0645076	0.24039	HZ	GRIA4
NC_056068.1	1665001	1685001	0.320477	0.283133	HZ	GRIA4
NC_056068.1	1670001	1690001	0.282449	0.265939	HZ	GRIA4
NC_056068.1	1675001	1695001	0.34089	0.27183	HZ	GRIA4
NC_056068.1	1680001	1700001	0.314793	0.258584	HZ	GRIA4
NC_056068.1	30935001	30955001	0.321735	0.252402	HZ	GRIK4
NC_056072.1	19200001	19220001	0.0649219	0.297134	HZ	GRM7
NC_056077.1	26025001	26045001	0.20853	0.261263	HZ	GSG1L
NC_056057.1	107855001	107875001	0.303844	0.249307	HZ	GSTK1

NC_056054.1	61105001	61125001
NC_056059.1	4905001	4925001
NC_056059.1	4910001	4930001
NC_056056.1	176545001	176565001
NC_056056.1	176550001	176570001
NC_056056.1	176555001	176575001
NC_056056.1	176560001	176580001
NC_056056.1	176565001	176585001
NC_056056.1	176570001	176590001
NC_056072.1	28790001	28810001
NC_056072.1	28795001	28815001
NC_056055.1	43160001	43180001
NC_056055.1	43165001	43185001
NC_056055.1	43170001	43190001
NC_056055.1	43175001	43195001
NC_056059.1	23995001	24015001
NC_056059.1	24000001	24020001
NC_056058.1	55760001	55780001
NC_056056.1	156990001	157010001
NC_056056.1	156995001	157015001
NC_056056.1	157000001	157020001
NC_056056.1	157005001	157025001
NC_056056.1	157010001	157030001
NC_056061.1	73825001	73845001
NC_056061.1	73830001	73850001
NC_056061.1	73835001	73855001
NC_056056.1	117475001	117495001
NC_056080.1	17010001	17030001
NC_056058.1	58905001	58925001
NC_056058.1	58910001	58930001
NC_056058.1	58920001	58940001
NC_056058.1	58925001	58945001
NC_056059.1	20115001	20135001
NC_056059.1	20120001	20140001
NC_056059.1	20125001	20145001
NC_056059.1	20130001	20150001

NC_056055.1	165770001	165790001	0.146766	0.415319	HZ	GTDC1	NC_056057.1	13360001	13380001
NC_056055.1	165790001	165810001	0.0941412	0.341411	HZ	GTDC1	NC_056057.1	13365001	13385001
NC_056054.1	16680001	16700001	0.184221	0.252951	HZ	GUCA2A;GUCA2B	NC_056054.1	99165001	99185001
NC_056055.1	120995001	121015001	0.151026	0.283838	HZ	GULP1	NC_056056.1	57970001	57990001
NC_056055.1	121025001	121045001	0.126733	0.24506	HZ	GULP1	NC_056054.1	186810001	186830001
NC_056055.1	121030001	121050001	0.0938047	0.24992	HZ	GULP1	NC_056054.1	186815001	186835001
NC_056054.1	239925001	239945001	0.0467851	0.295559	HZ	GYG1	NC_056055.1	220350001	220370001
NC_056068.1	47950001	47970001	0.0109887	0.313621	HZ	HBE1	NC_056055.1	220355001	220375001
NC_056064.1	43680001	43700001	0.221557	0.275058	HZ	HDAC5	NC_056056.1	130430001	130450001
NC_056064.1	43685001	43705001	0.179775	0.338604	HZ	HDAC5	NC_056056.1	130435001	130455001
NC_056064.1	43690001	43710001	0.218453	0.313987	HZ	HDAC5;HROB	NC_056072.1	51465001	51485001
NC_056080.1	65020001	65040001	0.193406	0.273852	HZ	HDAC8	NC_056072.1	51470001	51490001
NC_056080.1	65025001	65045001	0.128202	0.305883	HZ	HDAC8	NC_056055.1	73510001	73530001
NC_056080.1	65030001	65050001	0.168536	0.277022	HZ	HDAC8	NC_056055.1	73515001	73535001
NC_056054.1	106710001	106730001	0.317924	0.562659	HZ	HDGF;PRCC	NC_056055.1	73520001	73540001
NC_056060.1	83040001	83060001	0.334406	0.246247	HZ	HEATR4;LOC1011044	NC_056067.1	48455001	48475001
NC_056056.1	86995001	87015001	0.0889138	0.253702	HZ	HEATR5B	NC_056067.1	48460001	48480001
NC_056056.1	87000001	87020001	0.139247	0.247612	HZ	HEATR5B	NC_056065.1	1885001	1905001
NC_056056.1	87005001	87025001	0.207439	0.245559	HZ	HEATR5B	NC_056065.1	1890001	1910001
NC_056055.1	198680001	198700001	0.220459	0.255379	HZ	HECW2	NC_056065.1	1895001	1915001
NC_056055.1	198730001	198750001	0.324069	0.289147	HZ	HECW2	NC_056066.1	1000001	1020001
NC_056055.1	198735001	198755001	0.340077	0.30501	HZ	HECW2	NC_056066.1	1005001	1025001
NC_056075.1	15560001	15580001	0.0866873	0.312533	HZ	HELLS;TBC1D12	NC_056055.1	151205001	151225001
NC_056075.1	15565001	15585001	0.12259	0.298978	HZ	HELLS;TBC1D12	NC_056055.1	151210001	151230001
NC_056075.1	15570001	15590001	0.26937	0.257902	HZ	HELLS;TBC1D12	NC_056055.1	151215001	151235001
NC_056055.1	103455001	103475001	0.328583	0.274954	HZ	HMBOX1	NC_056056.1	7685001	7705001
NC_056055.1	103460001	103480001	0.233986	0.243685	HZ	HMBOX1	NC_056066.1	72915001	72935001
NC_056060.1	10465001	10485001	0.117985	0.358125	HZ	HOMER1	NC_056066.1	63925001	63945001
NC_056060.1	10490001	10510001	0.286386	0.319935	HZ	HOMER1	NC_056066.1	63995001	64015001
NC_056060.1	10495001	10515001	0.093115	0.447814	HZ	HOMER1	NC_056068.1	29030001	29050001
NC_056060.1	10505001	10525001	0.213444	0.361138	HZ	HOMER1	NC_056068.1	29035001	29055001
NC_056057.1	70445001	70465001	0.240404	0.243542	HZ	HOXA10;HOXA7;HOX	NC_056068.1	29040001	29060001
NC_056075.1	19480001	19500001	0.324193	0.444692	HZ	HPSE2	NC_056068.1	29045001	29065001
NC_056075.1	19485001	19505001	0.295258	0.464113	HZ	HPSE2	NC_056068.1	29025001	29045001
NC_056064.1	43695001	43715001	0.263415	0.286346	HZ	HROB	NC_056054.1	29740001	29760001
NC_056054.1	114350001	114370001	0.18941	0.239403	HZ	HSD17B7	NC_056080.1	20410001	20430001

NC_056054.1	96960001	96980001	0.0414938	0.27095	HZ	HSD3B1;ZNF697
NC_056080.1	119980001	120000001	0.148217	0.299805	HZ	HTR2C
NC_056080.1	119985001	120005001	0.0923567	0.313726	HZ	HTR2C
NC_056080.1	119990001	120010001	0.0696517	0.317483	HZ	HTR2C
NC_056080.1	119995001	120015001	0.149742	0.276178	HZ	HTR2C
NC_056080.1	120000001	120020001	0.221087	0.256733	HZ	HTR2C
NC_056057.1	118580001	118600001	0.313552	0.339876	HZ	HTR5A
NC_056057.1	118585001	118605001	0.344136	0.274646	HZ	HTR5A
NC_056057.1	118595001	118615001	0.326601	0.261548	HZ	HTR5A
NC_056075.1	12210001	12230001	0.300172	0.245036	HZ	HTR7
NC_056075.1	12215001	12235001	0.279489	0.268148	HZ	HTR7
NC_056075.1	12220001	12240001	0.297819	0.255348	HZ	HTR7
NC_056059.1	116365001	116385001	0.328304	0.384678	HZ	HTT
NC_056059.1	116370001	116390001	0.27502	0.376449	HZ	HTT
NC_056054.1	122840001	122860001	0.139931	0.264825	HZ	IFNAR1
NC_056054.1	122845001	122865001	0.194428	0.28508	HZ	IFNAR1
NC_056054.1	122850001	122870001	0.247571	0.291963	HZ	IFNAR1
NC_056054.1	122855001	122875001	0.256583	0.258387	HZ	IFNAR1
NC_056077.1	1705001	1725001	0.0937199	0.261758	HZ	IFT140;TELO2;TMEM
NC_056077.1	1710001	1730001	0.0549498	0.311054	HZ	IFT140;TMEM204
NC_056077.1	1720001	1740001	0.0683373	0.364978	HZ	IFT140;TMEM204
NC_056068.1	65825001	65845001	0.316771	0.361997	HZ	IFTAP;RAG1;RAG2
NC_056068.1	65830001	65850001	0.227968	0.379043	HZ	IFTAP;RAG2
NC_056054.1	122930001	122950001	0.272891	0.261429	HZ	IL10RB
NC_056054.1	122935001	122955001	0.330162	0.251022	HZ	IL10RB
NC_056080.1	28275001	28295001	0.149942	0.308862	HZ	IL1RAPL1
NC_056080.1	28280001	28300001	0.0736314	0.393378	HZ	IL1RAPL1
NC_056080.1	28285001	28305001	0.142758	0.403582	HZ	IL1RAPL1
NC_056080.1	28290001	28310001	0.250523	0.406502	HZ	IL1RAPL1
NC_056080.1	28295001	28315001	0.263815	0.420939	HZ	IL1RAPL1
NC_056080.1	28690001	28710001	0.250246	0.339328	HZ	IL1RAPL1
NC_056080.1	28695001	28715001	0.344705	0.361276	HZ	IL1RAPL1
NC_056056.1	59820001	59840001	0.29377	0.250163	HZ	IL1RN
NC_056054.1	252815001	252835001	0.152418	0.317016	HZ	IL20RB
NC_056054.1	252820001	252840001	0.126212	0.279575	HZ	IL20RB
NC_056054.1	252825001	252845001	0.126221	0.283811	HZ	IL20RB

NC_056080.1	20415001	20435001
NC_056080.1	20420001	20440001
NC_056080.1	20425001	20445001
NC_056080.1	20430001	20450001
NC_056056.1	206630001	206650001
NC_056056.1	206635001	206655001
NC_056056.1	206640001	206660001
NC_056055.1	68370001	68390001
NC_056055.1	68375001	68395001
NC_056055.1	68380001	68400001
NC_056055.1	68385001	68405001
NC_056055.1	68390001	68410001
NC_056055.1	68410001	68430001
NC_056055.1	68415001	68435001
NC_056055.1	68420001	68440001
NC_056055.1	68425001	68445001
NC_056060.1	66670001	66690001
NC_056056.1	44200001	44220001
NC_056063.1	28595001	28615001
NC_056063.1	28600001	28620001
NC_056063.1	28605001	28625001
NC_056080.1	22800001	22820001
NC_056068.1	3985001	4005001
NC_056068.1	3990001	4010001
NC_056060.1	8495001	8515001
NC_056060.1	8500001	8520001
NC_056060.1	8535001	8555001
NC_056058.1	58975001	58995001
NC_056058.1	58980001	59000001
NC_056058.1	58985001	59005001
NC_056058.1	58990001	59010001
NC_056058.1	58995001	59015001
NC_056058.1	59000001	59020001
NC_056058.1	59005001	59025001
NC_056058.1	59010001	59030001
NC_056058.1	59015001	59035001

NC_056054.1	252830001	252850001	0.278332	0.258536	HZ	IL20RB	NC_056058.1	59020001	59040001
NC_056054.1	252835001	252855001	0.286461	0.24201	HZ	IL20RB	NC_056058.1	59025001	59045001
NC_056054.1	252840001	252860001	0.341565	0.248219	HZ	IL20RB	NC_056058.1	59030001	59050001
NC_056061.1	62345001	62365001	0.255777	0.324882	HZ	IL22RA2	NC_056058.1	59035001	59055001
NC_056061.1	62350001	62370001	0.165887	0.279936	HZ	IL22RA2	NC_056058.1	59040001	59060001
NC_056061.1	62355001	62375001	0.199907	0.254724	HZ	IL22RA2	NC_056058.1	59045001	59065001
NC_056077.1	640001	660001	0.0672875	0.286432	HZ	IL9R;POLR3K	NC_056058.1	58970001	58990001
NC_056077.1	645001	665001	0.0793726	0.298543	HZ	IL9R;POLR3K;RHBDF	NC_056069.1	20450001	20470001
NC_056077.1	650001	670001	0.207274	0.305376	HZ	IL9R;POLR3K;RHBDF	NC_056069.1	20455001	20475001
NC_056064.1	22520001	22540001	0.280962	0.483452	HZ	INPP5K;PITPNA	NC_056059.1	71725001	71745001
NC_056064.1	22525001	22545001	0.297635	0.41566	HZ	INPP5K;PITPNA	NC_056059.1	71730001	71750001
NC_056064.1	22530001	22550001	0.300112	0.415334	HZ	INPP5K;PITPNA	NC_056057.1	39160001	39180001
NC_056064.1	22535001	22555001	0.280364	0.411832	HZ	INPP5K;PITPNA	NC_056057.1	39165001	39185001
NC_056068.1	37160001	37180001	0.345126	0.279355	HZ	INSC	NC_056057.1	39170001	39190001
NC_056068.1	37175001	37195001	0.272303	0.338665	HZ	INSC	NC_056057.1	39175001	39195001
NC_056068.1	37180001	37200001	0.270391	0.333594	HZ	INSC	NC_056059.1	50805001	50825001
NC_056068.1	37185001	37205001	0.276287	0.294058	HZ	INSC	NC_056059.1	50810001	50830001
NC_056068.1	37190001	37210001	0.284946	0.265747	HZ	INSC	NC_056056.1	35905001	35925001
NC_056072.1	49140001	49160001	0.145016	0.348792	HZ	IQCF6	NC_056059.1	17860001	17880001
NC_056054.1	228950001	228970001	0.338716	0.248112	HZ	IQCJ	NC_056059.1	17890001	17910001
NC_056060.1	7790001	7810001	0.310689	0.31963	HZ	IQGAP2	NC_056059.1	17895001	17915001
NC_056060.1	7795001	7815001	0.297424	0.295236	HZ	IQGAP2	NC_056055.1	7335001	7355001
NC_056055.1	128365001	128385001	0.288962	0.240188	HZ	ITGA4	NC_056068.1	64850001	64870001
NC_056055.1	128370001	128390001	0.238522	0.276824	HZ	ITGA4	NC_056068.1	64855001	64875001
NC_056056.1	163755001	163775001	0.0956341	0.436392	HZ	ITGA7	NC_056060.1	77205001	77225001
NC_056056.1	163760001	163780001	0.133704	0.374463	HZ	ITGA7;METTL7B	NC_056060.1	77210001	77230001
NC_056054.1	40540001	40560001	0.0962055	0.254528	HZ	JAK1	NC_056060.1	77215001	77235001
NC_056054.1	40545001	40565001	0.0802201	0.299535	HZ	JAK1	NC_056060.1	77220001	77240001
NC_056054.1	40575001	40595001	0.134789	0.279494	HZ	JAK1	NC_056060.1	77225001	77245001
NC_056074.1	30410001	30430001	0.311043	0.243783	HZ	JAM3	NC_056060.1	77230001	77250001
NC_056054.1	189395001	189415001	0.303674	0.306236	HZ	KALRN	NC_056060.1	77235001	77255001
NC_056065.1	52220001	52240001	0.326022	0.348277	HZ	KAZN	NC_056060.1	77240001	77260001
NC_056065.1	52225001	52245001	0.289997	0.320913	HZ	KAZN	NC_056060.1	77245001	77265001
NC_056065.1	52230001	52250001	0.25579	0.252269	HZ	KAZN	NC_056060.1	77250001	77270001
NC_056065.1	52235001	52255001	0.226636	0.251586	HZ	KAZN	NC_056060.1	77255001	77275001
NC_056065.1	52960001	52980001	0.283653	0.295238	HZ	KAZN	NC_056060.1	77260001	77280001

NC_056065.1	52965001	52985001	0.0558511	0.360794	HZ		KAZN
NC_056065.1	52970001	52990001	0.0441357	0.35324	HZ		KAZN
NC_056065.1	52980001	53000001	0.0435919	0.274834	HZ		KAZN
NC_056065.1	52985001	53005001	0.0507842	0.250524	HZ		KAZN
NC_056072.1	34555001	34575001	0.14067	0.320888	HZ		KBTBD8
NC_056072.1	34560001	34580001	0.15615	0.287302	HZ		KBTBD8
NC_056056.1	111010001	111030001	0.0644231	0.453656	HZ		KCNC2
NC_056056.1	111015001	111035001	0.0617477	0.430264	HZ		KCNC2
NC_056056.1	111020001	111040001	0.209088	0.298021	HZ		KCNC2
NC_056056.1	111025001	111045001	0.246774	0.243479	HZ		KCNC2
NC_056060.1	73240001	73260001	0.234563	0.304621	HZ		KCNH5
NC_056060.1	73245001	73265001	0.144696	0.250819	HZ		KCNH5
NC_056060.1	73250001	73270001	0.144801	0.247054	HZ		KCNH5
NC_056060.1	73350001	73370001	0.209967	0.282886	HZ		KCNH5
NC_056059.1	40865001	40885001	0.216736	0.326805	HZ		KCNIP4
NC_056059.1	40880001	40900001	0.220645	0.304194	HZ		KCNIP4
NC_056059.1	40885001	40905001	0.201834	0.322047	HZ		KCNIP4
NC_056059.1	40850001	40870001	0.315336	0.302582	HZ		KCNIP4;PACRGL
NC_056059.1	40855001	40875001	0.231821	0.310879	HZ		KCNIP4;PACRGL
NC_056059.1	40860001	40880001	0.169595	0.361702	HZ		KCNIP4;PACRGL
NC_056055.1	154665001	154685001	0.305397	0.276185	HZ		KCNJ3
NC_056055.1	154670001	154690001	0.314231	0.240605	HZ		KCNJ3
NC_056054.1	270705001	270725001	0.33282	0.280519	HZ		KCNJ6
NC_056062.1	16600001	16620001	0.30302	0.272272	HZ		KCNK9
NC_056054.1	209130001	209150001	0.305613	0.25822	HZ		KCNMB2
NC_056054.1	104945001	104965001	0.301092	0.247532	HZ		KCNN3
NC_056054.1	104950001	104970001	0.257828	0.253854	HZ		KCNN3
NC_056059.1	63640001	63660001	0.335454	0.244573	HZ		KCTD8
NC_056080.1	43950001	43970001	0.339474	0.40169	HZ		KDM6A
NC_056080.1	43955001	43975001	0.155902	0.39761	HZ		KDM6A
NC_056080.1	43960001	43980001	0.0931756	0.369896	HZ		KDM6A
NC_056070.1	65315001	65335001	0.119074	0.44429	HZ		KIAA1671
NC_056070.1	65320001	65340001	0.0725645	0.447518	HZ		KIAA1671
NC_056055.1	11105001	11125001	0.197219	0.242278	HZ		KIAA1958
NC_056055.1	11110001	11130001	0.180177	0.240321	HZ		KIAA1958
NC_056064.1	44365001	44385001	0.236642	0.302227	HZ		KIF18B

NC_056060.1	77265001	77285001
NC_056060.1	77270001	77290001
NC_056060.1	77275001	77295001
NC_056060.1	77280001	77300001
NC_056071.1	27965001	27985001
NC_056071.1	27970001	27990001
NC_056071.1	28010001	28030001
NC_056071.1	28015001	28035001
NC_056071.1	28020001	28040001
NC_056054.1	196915001	196935001
NC_056054.1	196920001	196940001
NC_056070.1	69675001	69695001
NC_056070.1	69680001	69700001
NC_056070.1	69685001	69705001
NC_056070.1	69690001	69710001
NC_056070.1	69695001	69715001
NC_056070.1	69700001	69720001
NC_056070.1	69705001	69725001
NC_056070.1	69710001	69730001
NC_056070.1	69715001	69735001
NC_056056.1	11065001	11085001
NC_056056.1	11070001	11090001
NC_056056.1	11075001	11095001
NC_056056.1	11090001	11110001
NC_056056.1	11095001	11115001
NC_056055.1	227625001	227645001
NC_056055.1	227630001	227650001
NC_056055.1	227850001	227870001
NC_056068.1	76480001	76500001
NC_056068.1	76485001	76505001
NC_056067.1	23685001	23705001
NC_056054.1	99170001	99190001
NC_056054.1	99175001	99195001
NC_056055.1	34985001	35005001
NC_056055.1	34990001	35010001
NC_056055.1	34995001	35015001

NC_056064.1	44370001	44390001	0.178572	0.312521	HZ	KIF18B	NC_056055.1	35000001	35020001
NC_056064.1	44355001	44375001	0.302179	0.305759	HZ	KIF18B;LOC443220	NC_056059.1	60390001	60410001
NC_056064.1	44360001	44380001	0.283774	0.322548	HZ	KIF18B;LOC443220	NC_056059.1	60395001	60415001
NC_056066.1	40025001	40045001	0.240113	0.257107	HZ	KIZ	NC_056059.1	105395001	105415001
NC_056076.1	24755001	24775001	0.312955	0.245214	HZ	KLHL14	NC_056064.1	45240001	45260001
NC_056076.1	24760001	24780001	0.331666	0.257231	HZ	KLHL14	NC_056064.1	45245001	45265001
NC_056071.1	52550001	52570001	0.207228	0.241259	HZ	KLHL28;TOGARAM1	NC_056060.1	87900001	87920001
NC_056080.1	118830001	118850001	0.101966	0.365473	HZ	KLHL4	NC_056060.1	87905001	87925001
NC_056080.1	118835001	118855001	0.240122	0.31739	HZ	KLHL4	NC_056056.1	73395001	73415001
NC_056057.1	116295001	116315001	0.248287	0.306244	HZ	KMT2C	NC_056056.1	73400001	73420001
NC_056056.1	134120001	134140001	0.0847628	0.250174	HZ	KRT7;LOC101112469;	NC_056056.1	73405001	73425001
NC_056057.1	50655001	50675001	0.276036	0.240652	HZ	LAMB4	NC_056055.1	207835001	207855001
NC_056057.1	50660001	50680001	0.218191	0.250578	HZ	LAMB4	NC_056055.1	207840001	207860001
NC_056056.1	177695001	177715001	0.219316	0.282548	HZ	LARGE1	NC_056080.1	131600001	131620001
NC_056056.1	177700001	177720001	0.0969214	0.390566	HZ	LARGE1	NC_056080.1	131605001	131625001
NC_056056.1	177705001	177725001	0.307316	0.335375	HZ	LARGE1	NC_056078.1	36995001	37015001
NC_056056.1	37325001	37345001	0.265285	0.246387	HZ	LCLAT1	NC_056078.1	37005001	37025001
NC_056076.1	49535001	49555001	0.180709	0.276897	HZ	LIPG	NC_056056.1	11100001	11120001
NC_056070.1	64205001	64225001	0.283394	0.241766	HZ	LOC101102091	NC_056056.1	11300001	11320001
NC_056068.1	47930001	47950001	0.0173544	0.275041	HZ	LOC101102421	NC_056056.1	11305001	11325001
NC_056068.1	47935001	47955001	0.0154479	0.346084	HZ	LOC101102421	NC_056056.1	11310001	11330001
NC_056068.1	48040001	48060001	0.0369255	0.293853	HZ	LOC101102671	NC_056056.1	11315001	11335001
NC_056068.1	48045001	48065001	0.0729601	0.281228	HZ	LOC101102671;LOC1(	NC_056056.1	11320001	11340001
NC_056068.1	48050001	48070001	0.0906764	0.257252	HZ	LOC101102671;LOC1(	NC_056056.1	11325001	11345001
NC_056064.1	9320001	9340001	0.115142	0.293268	HZ	LOC101102994;TEX14	NC_056065.1	41075001	41095001
NW_024599828	1200001	1220001	0.249821	0.250414	HZ	LOC101103023;LOC1(	NC_056059.1	19580001	19600001
NW_024599828	1205001	1225001	0.279401	0.250385	HZ	LOC101103023;LOC1(	NC_056059.1	19590001	19610001
NW_024599828	1210001	1230001	0.266717	0.254375	HZ	LOC101103023;LOC1(	NC_056059.1	19615001	19635001
NC_056054.1	224500001	224520001	0.103546	0.265078	HZ	LOC101103029	NC_056056.1	100650001	100670001
NC_056056.1	150520001	150540001	0.23134	0.262258	HZ	LOC101103222	NC_056056.1	100655001	100675001
NC_056056.1	150525001	150545001	0.282667	0.290978	HZ	LOC101103222	NC_056054.1	97360001	97380001
NC_056056.1	150530001	150550001	0.287533	0.260703	HZ	LOC101103222	NC_056054.1	97365001	97385001
NC_056056.1	150535001	150555001	0.257431	0.257108	HZ	LOC101103222	NC_056059.1	71760001	71780001
NC_056056.1	150540001	150560001	0.287745	0.275106	HZ	LOC101103222;LOC44	NC_056059.1	71765001	71785001
NW_024599828	1215001	1235001	0.268189	0.265569	HZ	LOC101103278;PAG3	NC_056059.1	71770001	71790001
NC_056071.1	52045001	52065001	0.306781	0.30705	HZ	LOC101104431	NC_056059.1	71775001	71795001

NC_056071.1	52050001	52070001	0.200112	0.257783	HZ	LOC101104431	NC_056059.1	71780001	71800001
NC_056064.1	49675001	49695001	0.338146	0.286689	HZ	LOC101104512;RAB4	NC_056059.1	71785001	71805001
NC_056064.1	49680001	49700001	0.232443	0.256291	HZ	LOC101104512;WDR4	NC_056065.1	63910001	63930001
NC_056066.1	67200001	67220001	0.257143	0.500635	HZ	LOC101104595	NC_056065.1	36145001	36165001
NC_056066.1	67205001	67225001	0.200619	0.427066	HZ	LOC101104595	NC_056065.1	36150001	36170001
NC_056066.1	67210001	67230001	0.196293	0.396388	HZ	LOC101104595	NC_056065.1	36155001	36175001
NC_056066.1	67215001	67235001	0.224786	0.299682	HZ	LOC101104595	NC_056064.1	19655001	19675001
NC_056057.1	94535001	94555001	0.184815	0.249233	HZ	LOC101105154	NC_056064.1	19660001	19680001
NC_056057.1	94540001	94560001	0.180619	0.257778	HZ	LOC101105154	NC_056064.1	19665001	19685001
NC_056057.1	94545001	94565001	0.227612	0.262128	HZ	LOC101105154	NC_056054.1	214225001	214245001
NC_056074.1	35430001	35450001	0.117164	0.25494	HZ	LOC101105540	NC_056061.1	13790001	13810001
NC_056068.1	48055001	48075001	0.100261	0.253279	HZ	LOC101105945	NC_056061.1	13795001	13815001
NC_056055.1	38195001	38215001	0.315509	0.246954	HZ	LOC101106139;LOC1	NC_056061.1	13805001	13825001
NC_056074.1	36410001	36430001	0.180668	0.294536	HZ	LOC101106637;TKFC;	NC_056061.1	13810001	13830001
NC_056075.1	15870001	15890001	0.125718	0.246692	HZ	LOC101106641	NC_056055.1	242240001	242260001
NC_056075.1	15875001	15895001	0.123139	0.268866	HZ	LOC101106641	NC_056080.1	15605001	15625001
NC_056075.1	15880001	15900001	0.158416	0.276947	HZ	LOC101106641	NC_056080.1	15610001	15630001
NC_056075.1	15885001	15905001	0.174451	0.28734	HZ	LOC101106641	NC_056080.1	15740001	15760001
NC_056058.1	11770001	11790001	0.203733	0.249032	HZ	LOC101107193;LOC1	NC_056080.1	66835001	66855001
NC_056064.1	36110001	36130001	0.285714	0.269068	HZ	LOC101107369;TMEM	NC_056080.1	66840001	66860001
NC_056058.1	11775001	11795001	0.163854	0.256953	HZ	LOC101107442	NC_056080.1	66845001	66865001
NC_056068.1	45445001	45465001	0.333455	0.245406	HZ	LOC101107549;LOC1	NC_056056.1	165415001	165435001
NC_056054.1	86965001	86985001	0.241249	0.246051	HZ	LOC101107831;LOC1	NC_056056.1	165420001	165440001
NC_056054.1	108005001	108025001	0.250731	0.324954	HZ	LOC101108789	NC_056056.1	165425001	165445001
NC_056066.1	3665001	3685001	0.342461	0.28868	HZ	LOC101109292	NC_056056.1	103945001	103965001
NC_056054.1	108140001	108160001	0.0261554	0.338861	HZ	LOC101109313	NC_056056.1	103950001	103970001
NC_056073.1	49750001	49770001	0.0609545	0.259264	HZ	LOC101109343;NQO2	NC_056054.1	7930001	7950001
NC_056073.1	49755001	49775001	0.0630488	0.267814	HZ	LOC101109343;NQO2	NC_056054.1	7935001	7955001
NC_056073.1	49760001	49780001	0.0836453	0.254615	HZ	LOC101109343;NQO2	NC_056060.1	19870001	19890001
NC_056057.1	114525001	114545001	0.196351	0.248615	HZ	LOC101109425;LOC11	NC_056060.1	19875001	19895001
NC_056056.1	12570001	12590001	0.310735	0.476407	HZ	LOC101109426	NC_056060.1	19880001	19900001
NC_056056.1	12580001	12600001	0.292383	0.45739	HZ	LOC101109426	NC_056060.1	19885001	19905001
NC_056056.1	12585001	12605001	0.300948	0.52573	HZ	LOC101109426	NC_056072.1	1695001	1715001
NC_056056.1	12590001	12610001	0.26789	0.501441	HZ	LOC101109426	NC_056072.1	1700001	1720001
NC_056056.1	12595001	12615001	0.282686	0.441893	HZ	LOC101109426	NC_056054.1	112535001	112555001
NC_056056.1	12600001	12620001	0.252232	0.446728	HZ	LOC101109426	NC_056069.1	25570001	25590001

NC_056056.1	12605001	12625001	0.318945	0.340955	HZ	LOC101109426	NC_056069.1	25575001	25595001
NC_056067.1	64270001	64290001	0.289461	0.247909	HZ	LOC101109603;LOC11	NC_056069.1	25580001	25600001
NC_056055.1	149840001	149860001	0.178383	0.249271	HZ	LOC101109941	NC_056069.1	25585001	25605001
NC_056055.1	149845001	149865001	0.15427	0.319817	HZ	LOC101109941	NC_056069.1	25590001	25610001
NC_056055.1	149850001	149870001	0.107345	0.36535	HZ	LOC101109941	NC_056069.1	25595001	25615001
NC_056055.1	149855001	149875001	0.0799696	0.407169	HZ	LOC101109941	NC_056056.1	86820001	86840001
NC_056055.1	149860001	149880001	0.0677732	0.300686	HZ	LOC101109941	NC_056056.1	32205001	32225001
NC_056055.1	149865001	149885001	0.104255	0.25966	HZ	LOC101109941	NC_056056.1	32210001	32230001
NC_056054.1	199300001	199320001	0.100584	0.283192	HZ	LOC101110107	NC_056054.1	215520001	215540001
NC_056054.1	199305001	199325001	0.082856	0.291136	HZ	LOC101110107	NC_056054.1	215525001	215545001
NC_056054.1	199310001	199330001	0.134128	0.282021	HZ	LOC101110107	NC_056054.1	215530001	215550001
NC_056054.1	199315001	199335001	0.181118	0.259478	HZ	LOC101110107	NC_056054.1	215535001	215555001
NC_056054.1	199985001	200005001	0.301488	0.258036	HZ	LOC101110107	NC_056054.1	215540001	215560001
NC_056054.1	199990001	200010001	0.137706	0.280547	HZ	LOC101110107	NC_056054.1	215545001	215565001
NC_056054.1	231665001	231685001	0.222592	0.248781	HZ	LOC101110289	NC_056054.1	215550001	215570001
NC_056061.1	83750001	83770001	0.195962	0.254043	HZ	LOC101110516	NC_056054.1	215555001	215575001
NC_056061.1	83755001	83775001	0.212337	0.257395	HZ	LOC101110516	NC_056054.1	215560001	215580001
NC_056080.1	119895001	119915001	0.0962555	0.309652	HZ	LOC101110741	NC_056054.1	215565001	215585001
NC_056068.1	48505001	48525001	0.0848683	0.2559	HZ	LOC101111391	NC_056062.1	86665001	86685001
NC_056070.1	71355001	71375001	0.163293	0.251572	HZ	LOC101111397;LOC1(	NC_056062.1	86670001	86690001
NC_056080.1	26425001	26445001	0.298651	0.287312	HZ	LOC101111610	NC_056062.1	86675001	86695001
NC_056056.1	164470001	164490001	0.235008	0.241251	HZ	LOC101111875	NC_056062.1	86680001	86700001
NC_056058.1	29340001	29360001	0.195927	0.240157	HZ	LOC101111962	NC_056074.1	15125001	15145001
NC_056056.1	134115001	134135001	0.0617111	0.296517	HZ	LOC101112469;LOC1(	NC_056057.1	45955001	45975001
NC_056056.1	134105001	134125001	0.196088	0.397844	HZ	LOC101112469;LOC1(	NC_056057.1	45960001	45980001
NC_056056.1	134110001	134130001	0.0849349	0.362576	HZ	LOC101112469;LOC12	NC_056057.1	45965001	45985001
NC_056080.1	90380001	90400001	0.122693	0.440601	HZ	LOC101112889	NC_056066.1	39135001	39155001
NC_056080.1	90385001	90405001	0.335887	0.390683	HZ	LOC101112889	NC_056066.1	39140001	39160001
NC_056054.1	108175001	108195001	0.0857433	0.313682	HZ	LOC101113122;LOC11	NC_056055.1	1220001	1240001
NC_056054.1	108180001	108200001	0.0905725	0.270625	HZ	LOC101113122;LOC11	NC_056059.1	6505001	6525001
NC_056054.1	108185001	108205001	0.0390469	0.244337	HZ	LOC101113122;LOC11	NC_056066.1	26000001	26020001
NC_056058.1	38450001	38470001	0.195843	0.343857	HZ	LOC101113495;NLRP3	NC_056066.1	26005001	26025001
NC_056058.1	38455001	38475001	0.297974	0.280078	HZ	LOC101113495;NLRP3	NC_056063.1	83630001	83650001
NC_056057.1	107720001	107740001	0.228453	0.275406	HZ	LOC101114011;LOC1(	NC_056063.1	83655001	83675001
NC_056077.1	19995001	20015001	0.0970339	0.251	HZ	LOC101114079	NC_056063.1	83660001	83680001
NC_056077.1	20000001	20020001	0.0892532	0.247338	HZ	LOC101114079	NC_056063.1	83665001	83685001

NC_056056.1	163690001	163710001	0.293796	0.273753	HZ	LOC101114432	NC_056063.1	83670001	83690001
NC_056065.1	53870001	53890001	0.0633733	0.293504	HZ	LOC101114795	NC_056063.1	83675001	83695001
NC_056065.1	53875001	53895001	0.0696981	0.290842	HZ	LOC101114795	NC_056063.1	83680001	83700001
NC_056065.1	53880001	53900001	0.0974265	0.267925	HZ	LOC101114795	NC_056063.1	83685001	83705001
NC_056080.1	56860001	56880001	0.115744	0.258455	HZ	LOC101114848	NC_056056.1	38425001	38445001
NC_056080.1	56865001	56885001	0.114682	0.264229	HZ	LOC101114848	NC_056056.1	38430001	38450001
NC_056080.1	56870001	56890001	0.11452	0.263852	HZ	LOC101114848	NC_056056.1	38435001	38455001
NC_056080.1	56875001	56895001	0.113861	0.261127	HZ	LOC101114848	NC_056056.1	9645001	9665001
NC_056057.1	114245001	114265001	0.0289501	0.245739	HZ	LOC101114852	NC_056056.1	9650001	9670001
NC_056057.1	114250001	114270001	0.0412788	0.248077	HZ	LOC101114852	NC_056056.1	9655001	9675001
NC_056057.1	114255001	114275001	0.0408754	0.26783	HZ	LOC101114852	NC_056054.1	191290001	191310001
NC_056057.1	114260001	114280001	0.0298144	0.253387	HZ	LOC101114852	NC_056054.1	191295001	191315001
NC_056054.1	220185001	220205001	0.264672	0.268976	HZ	LOC101114926	NC_056054.1	191300001	191320001
NC_056068.1	45710001	45730001	0.216342	0.486446	HZ	LOC101115307	NC_056080.1	84810001	84830001
NC_056068.1	45715001	45735001	0.139863	0.535431	HZ	LOC101115307;LOC1(	NC_056080.1	84815001	84835001
NC_056068.1	45720001	45740001	0.266552	0.458735	HZ	LOC101115567	NC_056080.1	84820001	84840001
NC_056057.1	114380001	114400001	0.143199	0.378536	HZ	LOC101116551	NC_056080.1	84825001	84845001
NC_056057.1	114385001	114405001	0.329016	0.271901	HZ	LOC101116551	NC_056056.1	154600001	154620001
NC_056067.1	2145001	2165001	0.271471	0.257245	HZ	LOC101116755	NC_056056.1	154635001	154655001
NC_056067.1	2150001	2170001	0.236612	0.291544	HZ	LOC101116755	NC_056056.1	154640001	154660001
NC_056067.1	2155001	2175001	0.207752	0.306277	HZ	LOC101116755	NC_056056.1	154645001	154665001
NC_056067.1	2160001	2180001	0.181644	0.261654	HZ	LOC101116755	NC_056056.1	154650001	154670001
NC_056080.1	5185001	5205001	0.276187	0.318883	HZ	LOC101117055	NC_056056.1	154675001	154695001
NC_056080.1	5190001	5210001	0.141894	0.354129	HZ	LOC101117055	NC_056056.1	154680001	154700001
NC_056054.1	256330001	256350001	0.204811	0.261184	HZ	LOC101117129	NC_056055.1	104355001	104375001
NC_056054.1	27460001	27480001	0.33821	0.243268	HZ	LOC101117285	NC_056080.1	48040001	48060001
NC_056056.1	165245001	165265001	0.191222	0.267907	HZ	LOC101117494	NC_056056.1	217615001	217635001
NC_056056.1	165250001	165270001	0.253371	0.242568	HZ	LOC101117494	NC_056056.1	217620001	217640001
NC_056064.1	19195001	19215001	0.160396	0.348705	HZ	LOC101118202	NC_056056.1	217625001	217645001
NC_056064.1	19200001	19220001	0.274554	0.281212	HZ	LOC101118202	NC_056056.1	217630001	217650001
NC_056080.1	2745001	2765001	0.13509	0.30824	HZ	LOC101118336	NC_056056.1	217635001	217655001
NC_056080.1	2750001	2770001	0.126399	0.336384	HZ	LOC101118336	NC_056056.1	217640001	217660001
NC_056080.1	2755001	2775001	0.0779268	0.379041	HZ	LOC101118336	NC_056056.1	217645001	217665001
NC_056080.1	2760001	2780001	0.133879	0.332184	HZ	LOC101118336	NC_056056.1	217650001	217670001
NC_056080.1	2765001	2785001	0.240163	0.293961	HZ	LOC101118336	NC_056056.1	217655001	217675001
NC_056080.1	2740001	2760001	0.295756	0.239649	HZ	LOC101118336;STS	NC_056056.1	217660001	217680001

NC_056055.1	38185001	38205001	0.344064	0.331238	HZ	LOC101118510	NC_056056.1	217680001	217700001
NC_056055.1	38190001	38210001	0.330855	0.357398	HZ	LOC101118510	NC_056056.1	104515001	104535001
NC_056055.1	118295001	118315001	0.0531434	0.305519	HZ	LOC101118856	NC_056056.1	102120001	102140001
NC_056062.1	14590001	14610001	0.216504	0.421254	HZ	LOC101119226	NC_056070.1	69720001	69740001
NC_056062.1	14595001	14615001	0.26983	0.372714	HZ	LOC101119226;LYPD2	NC_056070.1	69725001	69745001
NC_056064.1	15580001	15600001	0.323971	0.281808	HZ	LOC101119832	NC_056074.1	46410001	46430001
NC_056064.1	15585001	15605001	0.321853	0.310433	HZ	LOC101119832	NC_056074.1	46415001	46435001
NC_056064.1	15590001	15610001	0.313199	0.287777	HZ	LOC101119832	NC_056074.1	46425001	46445001
NC_056064.1	15595001	15615001	0.327103	0.295518	HZ	LOC101119832	NC_056073.1	21880001	21900001
NC_056064.1	15600001	15620001	0.234415	0.246273	HZ	LOC101119832	NC_056073.1	21885001	21905001
NC_056061.1	58175001	58195001	0.112513	0.344201	HZ	LOC101119994	NC_056073.1	21890001	21910001
NC_056054.1	99845001	99865001	0.125591	0.289695	HZ	LOC101120207	NC_056055.1	88815001	88835001
NC_056054.1	99850001	99870001	0.038806	0.359955	HZ	LOC101120207	NC_056055.1	88820001	88840001
NC_056054.1	99855001	99875001	0.179402	0.311851	HZ	LOC101120207	NC_056054.1	21170001	21190001
NC_056070.1	72690001	72710001	0.0750141	0.266134	HZ	LOC101120607	NC_056072.1	31765001	31785001
NC_056074.1	4845001	4865001	0.0684736	0.251261	HZ	LOC101120706	NC_056072.1	31770001	31790001
NC_056065.1	60480001	60500001	0.338558	0.291697	HZ	LOC101122123;TOR1A	NC_056072.1	31775001	31795001
NC_056080.1	53380001	53400001	0.234049	0.348388	HZ	LOC101122329	NC_056072.1	31795001	31815001
NC_056080.1	53385001	53405001	0.217528	0.320831	HZ	LOC101122329	NC_056072.1	31800001	31820001
NC_056080.1	53390001	53410001	0.24476	0.319267	HZ	LOC101122329	NC_056072.1	31805001	31825001
NC_056057.1	107725001	107745001	0.182152	0.355364	HZ	LOC101122595	NC_056072.1	31810001	31830001
NC_056057.1	107730001	107750001	0.25217	0.369893	HZ	LOC101122595	NC_056072.1	31815001	31835001
NC_056057.1	114105001	114125001	0.206714	0.305287	HZ	LOC101122778	NC_056072.1	31860001	31880001
NC_056057.1	114115001	114135001	0.0805912	0.290539	HZ	LOC101122778	NC_056072.1	31865001	31885001
NC_056057.1	114120001	114140001	0.0783863	0.285565	HZ	LOC101122778	NC_056072.1	31870001	31890001
NC_056057.1	114125001	114145001	0.0377832	0.321214	HZ	LOC101122778	NC_056072.1	31875001	31895001
NC_056072.1	56155001	56175001	0.338589	0.252097	HZ	LOC101122984;RHO	NC_056072.1	31880001	31900001
NC_056072.1	56160001	56180001	0.337575	0.294739	HZ	LOC101122984;RHO	NC_056072.1	31885001	31905001
NC_056054.1	260250001	260270001	0.0727792	0.250319	HZ	LOC101123341	NC_056072.1	31890001	31910001
NC_056054.1	247050001	247070001	0.122618	0.287681	HZ	LOC105601893;PLS1	NC_056072.1	31895001	31915001
NC_056054.1	196490001	196510001	0.0835322	0.447814	HZ	LOC105602566	NC_056056.1	102125001	102145001
NC_056054.1	196495001	196515001	0.10127	0.462244	HZ	LOC105602566	NC_056071.1	45360001	45380001
NC_056054.1	196500001	196520001	0.117946	0.460661	HZ	LOC105602566	NC_056071.1	45365001	45385001
NC_056054.1	196505001	196525001	0.124713	0.448491	HZ	LOC105602566	NC_056071.1	45370001	45390001
NC_056054.1	196510001	196530001	0.175884	0.406594	HZ	LOC105602566	NC_056071.1	45375001	45395001
NC_056078.1	41195001	41215001	0.10158	0.255875	HZ	LOC105605098	NC_056060.1	49560001	49580001

NC_056064.1	19150001	19170001	0.0817787	0.424561	HZ	LOC105605807	NC_056060.1	49565001	49585001
NC_056061.1	82540001	82560001	0.177541	0.383333	HZ	LOC105609000;TULP4	NC_056060.1	49570001	49590001
NC_056061.1	82545001	82565001	0.211009	0.346405	HZ	LOC105609000;TULP4	NC_056060.1	49575001	49595001
NC_056069.1	10760001	10780001	0.315102	0.283001	HZ	LOC105610637	NC_056060.1	49580001	49600001
NC_056064.1	43760001	43780001	0.268983	0.283418	HZ	LOC105612015;UBTF	NC_056060.1	49585001	49605001
NC_056056.1	176765001	176785001	0.247453	0.244402	HZ	LOC105614892;SYN3	NC_056060.1	49590001	49610001
NC_056056.1	176770001	176790001	0.163557	0.288995	HZ	LOC105614892;SYN3	NC_056060.1	49595001	49615001
NC_056056.1	176775001	176795001	0.141098	0.308323	HZ	LOC105614892;SYN3	NC_056060.1	49600001	49620001
NC_056056.1	176780001	176800001	0.134152	0.359973	HZ	LOC105614892;SYN3	NC_056060.1	49605001	49625001
NC_056056.1	176785001	176805001	0.137086	0.326041	HZ	LOC105614892;SYN3	NC_056060.1	49610001	49630001
NC_056064.1	12450001	12470001	0.193201	0.309981	HZ	LOC105616451;USP32	NC_056060.1	49615001	49635001
NC_056064.1	12455001	12475001	0.213598	0.279661	HZ	LOC105616451;USP32	NC_056060.1	49620001	49640001
NC_056064.1	12460001	12480001	0.337025	0.249714	HZ	LOC105616451;USP32	NC_056060.1	49625001	49645001
NC_056055.1	118140001	118160001	0.110791	0.314271	HZ	LOC106990950;MAP3I	NC_056060.1	49630001	49650001
NC_056058.1	48790001	48810001	0.291785	0.26524	HZ	LOC106991157;NRG2	NC_056060.1	49635001	49655001
NC_056072.1	34365001	34385001	0.233659	0.255692	HZ	LOC114109370	NC_056060.1	49640001	49660001
NC_056080.1	4715001	4735001	0.286735	0.266968	HZ	LOC114111162	NC_056080.1	8625001	8645001
NC_056080.1	4720001	4740001	0.300986	0.240483	HZ	LOC114111162	NC_056080.1	8630001	8650001
NC_056080.1	53620001	53640001	0.34202	0.321469	HZ	LOC114111483;MAGE	NC_056080.1	8635001	8655001
NC_056057.1	114515001	114535001	0.0403241	0.33638	HZ	LOC114114576	NC_056068.1	40320001	40340001
NC_056057.1	114520001	114540001	0.0205136	0.290533	HZ	LOC114114576	NC_056068.1	40340001	40360001
NC_056054.1	21490001	21510001	0.108612	0.612274	HZ	LOC114114831	NC_056076.1	34860001	34880001
NC_056054.1	21495001	21515001	0.0943626	0.515534	HZ	LOC114114831	NC_056076.1	34865001	34885001
NC_056054.1	21500001	21520001	0.142784	0.383537	HZ	LOC114114831	NC_056076.1	34870001	34890001
NC_056061.1	82495001	82515001	0.144246	0.38034	HZ	LOC114116165;TULP4	NC_056076.1	34875001	34895001
NC_056064.1	19010001	19030001	0.082426	0.288776	HZ	LOC114116901	NC_056076.1	34880001	34900001
NC_056064.1	19015001	19035001	0.0742541	0.293916	HZ	LOC114116901	NC_056076.1	34885001	34905001
NC_056064.1	19025001	19045001	0.0600747	0.327027	HZ	LOC114116901	NC_056076.1	34890001	34910001
NC_056064.1	19030001	19050001	0.0974393	0.300751	HZ	LOC114116901	NC_056056.1	122900001	122920001
NC_056064.1	19035001	19055001	0.118849	0.280734	HZ	LOC114116901	NC_056056.1	122905001	122925001
NC_056064.1	19165001	19185001	0.117284	0.340532	HZ	LOC114117017	NC_056056.1	122910001	122930001
NC_056064.1	19170001	19190001	0.0856453	0.387308	HZ	LOC114117017	NC_056056.1	122915001	122935001
NC_056064.1	19175001	19195001	0.0704714	0.387196	HZ	LOC114117017	NC_056056.1	181490001	181510001
NC_056064.1	19180001	19200001	0.0722248	0.386864	HZ	LOC114117017	NC_056079.1	23990001	24010001
NC_056064.1	19095001	19115001	0.206535	0.279461	HZ	LOC114117018	NC_056079.1	23995001	24015001
NC_056064.1	19100001	19120001	0.200755	0.330646	HZ	LOC114117018	NC_056079.1	24000001	24020001

NC_056064.1	19105001	19125001	0.206225	0.354124	HZ	LOC114117018
NC_056064.1	19110001	19130001	0.114228	0.413303	HZ	LOC114117018
NC_056064.1	19115001	19135001	0.174933	0.416193	HZ	LOC114117018
NC_056064.1	19120001	19140001	0.116667	0.453851	HZ	LOC114117018
NC_056065.1	50370001	50390001	0.240786	0.241143	HZ	LOC114117238;SSU72
NC_056065.1	50375001	50395001	0.312758	0.273018	HZ	LOC114117238;SSU72
NC_056054.1	108165001	108185001	0.111768	0.329307	HZ	LOC114117576;LOC11
NC_056054.1	108170001	108190001	0.0942339	0.327546	HZ	LOC114117576;LOC11
NC_056068.1	45495001	45515001	0.239229	0.244639	HZ	LOC114118262
NC_056068.1	79350001	79370001	0.312911	0.259035	HZ	LOC121816625;P2RX3
NC_056068.1	79345001	79365001	0.243528	0.381205	HZ	LOC121816625;SSRP1
NC_056079.1	26640001	26660001	0.237407	0.259784	HZ	LOC121818073
NC_056080.1	53395001	53415001	0.281429	0.299185	HZ	LOC121818202
NC_056080.1	53400001	53420001	0.328064	0.283931	HZ	LOC121818202
NC_056056.1	150500001	150520001	0.20452	0.257061	HZ	LOC121819182
NC_056056.1	150505001	150525001	0.0314869	0.279861	HZ	LOC121819182
NC_056056.1	150510001	150530001	0.0297292	0.299713	HZ	LOC121819182
NC_056054.1	61345001	61365001	0.214434	0.247415	HZ	LOC121819633;SAMD
NC_056054.1	61350001	61370001	0.128929	0.244286	HZ	LOC121819633;SAMD
NC_056059.1	14155001	14175001	0.330422	0.297481	HZ	LOC121819865
NC_056054.1	108970001	108990001	0.142998	0.274529	HZ	LOC443423
NC_056070.1	7005001	7025001	0.294915	0.246937	HZ	LRBA
NC_056070.1	7010001	7030001	0.326867	0.256633	HZ	LRBA
NC_056070.1	7275001	7295001	0.276913	0.268989	HZ	LRBA
NC_056070.1	7280001	7300001	0.303899	0.27827	HZ	LRBA
NC_056070.1	7285001	7305001	0.284273	0.302281	HZ	LRBA
NC_056070.1	7290001	7310001	0.2577	0.331498	HZ	LRBA
NC_056070.1	7295001	7315001	0.134838	0.272132	HZ	LRBA
NC_056070.1	7300001	7320001	0.221655	0.253198	HZ	LRBA
NC_056070.1	7585001	7605001	0.195113	0.242031	HZ	LRBA
NC_056054.1	88315001	88335001	0.299141	0.287359	HZ	LRIF1
NC_056054.1	88320001	88340001	0.19117	0.30207	HZ	LRIF1
NC_056054.1	88325001	88345001	0.0890625	0.322372	HZ	LRIF1
NC_056072.1	35160001	35180001	0.340101	0.254516	HZ	LRIG1
NC_056072.1	35165001	35185001	0.291891	0.280652	HZ	LRIG1
NC_056072.1	35170001	35190001	0.136323	0.298084	HZ	LRIG1

NC_056064.1	49315001	49335001
NC_056064.1	49330001	49350001
NC_056064.1	44140001	44160001
NC_056063.1	30300001	30320001
NC_056060.1	80300001	80320001
NC_056060.1	80305001	80325001
NC_056060.1	80310001	80330001
NC_056067.1	48450001	48470001
NC_056054.1	237420001	237440001
NC_056054.1	204635001	204655001
NC_056054.1	204640001	204660001
NC_056054.1	204645001	204665001
NC_056073.1	36975001	36995001
NC_056073.1	36980001	37000001
NC_056059.1	101565001	101585001
NC_056070.1	72040001	72060001
NC_056070.1	72045001	72065001
NC_056060.1	41530001	41550001
NC_056072.1	51880001	51900001
NC_056072.1	51885001	51905001
NC_056072.1	51890001	51910001
NC_056072.1	51895001	51915001
NC_056066.1	63915001	63935001
NC_056066.1	63920001	63940001
NC_056080.1	42880001	42900001
NC_056080.1	42885001	42905001
NC_056080.1	42890001	42910001
NC_056061.1	18920001	18940001
NC_056055.1	202305001	202325001
NC_056055.1	202310001	202330001
NC_056055.1	202315001	202335001
NC_056054.1	90905001	90925001
NC_056054.1	13970001	13990001
NC_056055.1	160085001	160105001
NC_056055.1	160090001	160110001
NC_056055.1	160095001	160115001

NC_056072.1	35175001	35195001	0.0810569	0.35623	HZ	LRIG1;SLC25A26	NC_056062.1	36135001	36155001
NC_056054.1	27785001	27805001	0.257011	0.401813	HZ	LRP8	NC_056062.1	36205001	36225001
NC_056054.1	27790001	27810001	0.171375	0.454513	HZ	LRP8	NC_056062.1	36210001	36230001
NC_056054.1	27795001	27815001	0.0716724	0.51289	HZ	LRP8	NC_056062.1	36215001	36235001
NC_056057.1	93925001	93945001	0.319907	0.350644	HZ	LRRC4;SND1	NC_056062.1	36220001	36240001
NC_056054.1	127825001	127845001	0.336127	0.566995	HZ	LTN1	NC_056062.1	36225001	36245001
NC_056074.1	17710001	17730001	0.16695	0.289898	HZ	LUZP2	NC_056062.1	36230001	36250001
NC_056074.1	17715001	17735001	0.218142	0.309339	HZ	LUZP2	NC_056062.1	36235001	36255001
NC_056074.1	17720001	17740001	0.268066	0.266315	HZ	LUZP2	NC_056056.1	102110001	102130001
NC_056055.1	160085001	160105001	0.286505	0.267626	HZ	LYPD6	NC_056056.1	102115001	102135001
NC_056055.1	160090001	160110001	0.158967	0.349619	HZ	LYPD6	NC_056056.1	102090001	102110001
NC_056055.1	160095001	160115001	0.134034	0.308834	HZ	LYPD6	NC_056056.1	102095001	102115001
NC_056077.1	19195001	19215001	0.283262	0.331049	HZ	LYRM1	NC_056056.1	102100001	102120001
NC_056077.1	41770001	41790001	0.221709	0.273008	HZ	MAFK;TMEM184A	NC_056056.1	102105001	102125001
NC_056077.1	41775001	41795001	0.128531	0.324075	HZ	MAFK;TMEM184A	NC_056056.1	102085001	102105001
NC_056080.1	53655001	53675001	0.345416	0.338889	HZ	MAGED1	NC_056056.1	121445001	121465001
NC_056072.1	35600001	35620001	0.0853137	0.247242	HZ	MAGI1	NC_056056.1	121450001	121470001
NC_056072.1	35605001	35625001	0.209292	0.251034	HZ	MAGI1	NC_056056.1	121455001	121475001
NC_056072.1	35760001	35780001	0.216112	0.288268	HZ	MAGI1	NC_056056.1	121460001	121480001
NC_056055.1	118110001	118130001	0.0841754	0.277742	HZ	MAP3K2	NC_056067.1	34550001	34570001
NC_056061.1	61905001	61925001	0.334045	0.257803	HZ	MAP3K5	NC_056067.1	34555001	34575001
NC_056059.1	101735001	101755001	0.330422	0.263535	HZ	MAPK10	NC_056067.1	34560001	34580001
NC_056076.1	50580001	50600001	0.285055	0.241229	HZ	MAPK4	NC_056055.1	170170001	170190001
NC_056076.1	50585001	50605001	0.0731851	0.27356	HZ	MAPK4	NC_056062.1	72970001	72990001
NC_056076.1	50590001	50610001	0.0274616	0.242273	HZ	MAPK4	NC_056062.1	72975001	72995001
NC_056054.1	200800001	200820001	0.107122	0.282561	HZ	MASP1	NC_056062.1	72980001	73000001
NC_056078.1	27945001	27965001	0.0920283	0.247885	HZ	MCU	NC_056078.1	31140001	31160001
NC_056061.1	10530001	10550001	0.222829	0.259131	HZ	ME1	NC_056078.1	31520001	31540001
NC_056061.1	10535001	10555001	0.110814	0.260436	HZ	ME1	NC_056071.1	49165001	49185001
NC_056063.1	30300001	30320001	0.109497	0.392346	HZ	MEDAG	NC_056071.1	49170001	49190001
NC_056063.1	30305001	30325001	0.193811	0.319442	HZ	MEDAG	NC_056070.1	7670001	7690001
NC_056058.1	24290001	24310001	0.27178	0.258046	HZ	MEGF10	NC_056060.1	41655001	41675001
NC_056058.1	24295001	24315001	0.231118	0.263975	HZ	MEGF10	NC_056059.1	57305001	57325001
NC_056058.1	24435001	24455001	0.246801	0.469942	HZ	MEGF10	NC_056059.1	57315001	57335001
NC_056058.1	24440001	24460001	0.175981	0.503701	HZ	MEGF10	NC_056059.1	57320001	57340001
NC_056058.1	24445001	24465001	0.20935	0.443369	HZ	MEGF10	NC_056057.1	118760001	118780001

NC_056058.1	24450001	24470001	0.195034	0.35927	HZ	MEGF10
NC_056056.1	206810001	206830001	0.33025	0.242107	HZ	MFAP5
NC_056054.1	208505001	208525001	0.19412	0.276466	HZ	MFN1
NC_056054.1	208510001	208530001	0.106454	0.313075	HZ	MFN1
NC_056054.1	208515001	208535001	0.0665692	0.299227	HZ	MFN1
NC_056054.1	237955001	237975001	0.318587	0.28032	HZ	MINDY4B
NC_056071.1	45445001	45465001	0.123839	0.276635	HZ	MIPOL1
NC_056072.1	31715001	31735001	0.251753	0.42011	HZ	MITF
NC_056072.1	31720001	31740001	0.0789165	0.483308	HZ	MITF
NC_056072.1	31895001	31915001	0.279411	0.429676	HZ	MITF
NC_056063.1	19180001	19200001	0.117081	0.418904	HZ	MLNR
NC_056080.1	99235001	99255001	0.206501	0.246609	HZ	MMGT1
NC_056054.1	174815001	174835001	0.0937362	0.272984	HZ	MORC1
NC_056063.1	36595001	36615001	0.0687276	0.304357	HZ	MPHOSPH8
NC_056063.1	36600001	36620001	0.0599377	0.309462	HZ	MPHOSPH8
NC_056054.1	251355001	251375001	0.290537	0.397559	HZ	MRAS
NC_056054.1	251360001	251380001	0.225679	0.415116	HZ	MRAS
NC_056054.1	251365001	251385001	0.0715961	0.375945	HZ	MRAS
NC_056054.1	251375001	251395001	0.257307	0.372718	HZ	MRAS
NC_056064.1	7910001	7930001	0.298043	0.264244	HZ	MSI2
NC_056064.1	7930001	7950001	0.25182	0.258299	HZ	MSI2
NC_056069.1	41525001	41545001	0.207578	0.273512	HZ	MTMR12
NC_056070.1	69020001	69040001	0.172029	0.289981	HZ	MTMR3
NC_056070.1	69035001	69055001	0.200778	0.316576	HZ	MTMR3
NC_056070.1	69040001	69060001	0.218926	0.297285	HZ	MTMR3
NC_056070.1	69045001	69065001	0.284092	0.295886	HZ	MTMR3
NC_056070.1	69050001	69070001	0.314201	0.293233	HZ	MTMR3
NC_056070.1	69065001	69085001	0.315407	0.251267	HZ	MTMR3
NC_056065.1	41650001	41670001	0.193268	0.372721	HZ	MTOR
NC_056065.1	41670001	41690001	0.168193	0.368962	HZ	MTOR
NC_056065.1	41675001	41695001	0.202232	0.362299	HZ	MTOR
NC_056065.1	41680001	41700001	0.203282	0.343415	HZ	MTOR
NC_056065.1	41685001	41705001	0.214026	0.322338	HZ	MTOR
NC_056055.1	133810001	133830001	0.18804	0.459473	HZ	MTX2
NC_056055.1	133815001	133835001	0.248058	0.393893	HZ	MTX2
NC_056055.1	133845001	133865001	0.259352	0.261269	HZ	MTX2

NC_056057.1	34020001	34040001
NC_056057.1	34025001	34045001
NC_056057.1	34030001	34050001
NC_056057.1	34035001	34055001
NC_056057.1	305001	325001
NC_056057.1	310001	330001
NC_056055.1	239695001	239715001
NC_056054.1	112895001	112915001
NC_056055.1	71235001	71255001
NC_056055.1	71240001	71260001
NC_056055.1	71245001	71265001
NW_024599801	15001	35001
NC_056077.1	27330001	27350001
NC_056067.1	51540001	51560001
NC_056067.1	60970001	60990001
NC_056056.1	104475001	104495001
NC_056056.1	104480001	104500001
NC_056056.1	104485001	104505001
NC_056056.1	104470001	104490001
NC_056068.1	48970001	48990001
NC_056067.1	63335001	63355001
NC_056056.1	104445001	104465001
NC_056056.1	104450001	104470001
NC_056056.1	104455001	104475001
NC_056056.1	104460001	104480001
NC_056056.1	104465001	104485001
NC_056054.1	112220001	112240001
NC_056054.1	112225001	112245001
NC_056054.1	101075001	101095001
NC_056055.1	89760001	89780001
NC_056055.1	89765001	89785001
NW_024599802	105001	125001
NW_024599802	110001	130001
NW_024599802	115001	135001
NC_056055.1	89745001	89765001
NC_056055.1	89750001	89770001

NC_056059.1	117075001	117095001	0.297456	0.294216	HZ	MXD4;ZFYVE28
NC_056075.1	14505001	14525001	0.122376	0.257314	HZ	MYOF
NC_056075.1	14510001	14530001	0.09609	0.344703	HZ	MYOF
NC_056075.1	14515001	14535001	0.0450941	0.412523	HZ	MYOF
NC_056055.1	1155001	1175001	0.0981829	0.251236	HZ	MYT1L
NC_056055.1	1160001	1180001	0.087644	0.301208	HZ	MYT1L
NC_056055.1	1165001	1185001	0.0398178	0.332013	HZ	MYT1L
NC_056055.1	1170001	1190001	0.0299847	0.281129	HZ	MYT1L
NC_056054.1	211990001	212010001	0.214594	0.249458	HZ	NAALADL2
NC_056054.1	211995001	212015001	0.211241	0.245776	HZ	NAALADL2
NC_056054.1	212000001	212020001	0.233824	0.244795	HZ	NAALADL2
NC_056054.1	212005001	212025001	0.265719	0.267867	HZ	NAALADL2
NC_056054.1	212570001	212590001	0.14097	0.250993	HZ	NAALADL2
NC_056054.1	212575001	212595001	0.134683	0.35006	HZ	NAALADL2
NC_056054.1	212580001	212600001	0.0583044	0.407782	HZ	NAALADL2
NC_056054.1	212585001	212605001	0.0545575	0.502942	HZ	NAALADL2
NC_056054.1	212590001	212610001	0.104365	0.46579	HZ	NAALADL2
NC_056054.1	212595001	212615001	0.143439	0.324743	HZ	NAALADL2
NC_056054.1	213320001	213340001	0.0824849	0.342831	HZ	NAALADL2
NC_056054.1	213325001	213345001	0.17796	0.283643	HZ	NAALADL2
NC_056054.1	213365001	213385001	0.258156	0.251834	HZ	NAALADL2
NC_056054.1	213385001	213405001	0.0215362	0.269453	HZ	NAALADL2
NC_056074.1	15060001	15080001	0.343386	0.264238	HZ	NARS2
NC_056074.1	15065001	15085001	0.338769	0.328944	HZ	NARS2
NC_056074.1	15095001	15115001	0.266171	0.386992	HZ	NARS2
NC_056074.1	15100001	15120001	0.175125	0.439996	HZ	NARS2
NC_056074.1	15105001	15125001	0.162393	0.408002	HZ	NARS2
NC_056074.1	15110001	15130001	0.120713	0.431368	HZ	NARS2
NC_056074.1	15115001	15135001	0.110169	0.436128	HZ	NARS2
NC_056074.1	15120001	15140001	0.0846348	0.43915	HZ	NARS2
NC_056074.1	15125001	15145001	0.0810462	0.46877	HZ	NARS2
NC_056074.1	15130001	15150001	0.101302	0.45518	HZ	NARS2
NC_056074.1	15135001	15155001	0.116133	0.409752	HZ	NARS2
NC_056074.1	15140001	15160001	0.150134	0.338948	HZ	NARS2
NC_056074.1	15145001	15165001	0.164706	0.250175	HZ	NARS2
NC_056074.1	15165001	15185001	0.150646	0.248981	HZ	NARS2

NC_056055.1	89755001	89775001
NC_056055.1	52565001	52585001
NC_056076.1	16735001	16755001
NC_056068.1	8180001	8200001
NC_056068.1	8185001	8205001
NC_056074.1	2055001	2075001
NC_056074.1	2060001	2080001
NC_056074.1	2065001	2085001
NC_056074.1	2070001	2090001
NC_056074.1	2075001	2095001
NC_056074.1	2080001	2100001
NC_056074.1	2085001	2105001
NC_056074.1	2090001	2110001
NC_056074.1	2105001	2125001
NC_056074.1	2110001	2130001
NC_056074.1	2135001	2155001
NC_056074.1	2145001	2165001
NC_056074.1	2150001	2170001
NC_056080.1	36370001	36390001
NC_056080.1	36375001	36395001
NC_056069.1	55870001	55890001
NC_056063.1	71345001	71365001
NC_056061.1	74790001	74810001
NC_056061.1	74795001	74815001
NC_056061.1	74800001	74820001
NC_056061.1	74805001	74825001
NC_056061.1	74810001	74830001
NC_056061.1	74815001	74835001
NC_056061.1	74820001	74840001
NC_056061.1	74825001	74845001
NC_056061.1	74830001	74850001
NC_056061.1	74835001	74855001
NC_056061.1	74840001	74860001
NC_056061.1	74845001	74865001
NC_056061.1	74850001	74870001
NC_056061.1	74855001	74875001

NC_056074.1	15170001	15190001	0.1698	0.269917	HZ	NARS2
NC_056074.1	15175001	15195001	0.196491	0.269973	HZ	NARS2
NC_056074.1	15205001	15225001	0.345022	0.274936	HZ	NARS2
NC_056056.1	23790001	23810001	0.266951	0.298952	HZ	NBAS
NC_056056.1	23795001	23815001	0.225762	0.242037	HZ	NBAS
NC_056056.1	23835001	23855001	0.238141	0.278634	HZ	NBAS
NC_056056.1	23840001	23860001	0.257956	0.27295	HZ	NBAS
NC_056056.1	23845001	23865001	0.28254	0.241795	HZ	NBAS
NC_056056.1	23850001	23870001	0.260016	0.240496	HZ	NBAS
NC_056054.1	136610001	136630001	0.201237	0.324733	HZ	NCAM2
NC_056054.1	136615001	136635001	0.102773	0.317045	HZ	NCAM2
NC_056054.1	136620001	136640001	0.13051	0.306687	HZ	NCAM2
NC_056057.1	120955001	120975001	0.321274	0.301121	HZ	NCAPG2
NC_056057.1	120960001	120980001	0.290411	0.276076	HZ	NCAPG2
NC_056057.1	120965001	120985001	0.261179	0.267767	HZ	NCAPG2
NC_056057.1	120970001	120990001	0.332919	0.259583	HZ	NCAPG2
NC_056055.1	177225001	177245001	0.241167	0.278791	HZ	NCKAP5
NC_056055.1	177245001	177265001	0.189159	0.439643	HZ	NCKAP5
NC_056055.1	177470001	177490001	0.210543	0.247467	HZ	NCKAP5
NC_056055.1	177535001	177555001	0.294366	0.270672	HZ	NCKAP5
NC_056055.1	177540001	177560001	0.265915	0.310471	HZ	NCKAP5
NC_056055.1	177765001	177785001	0.191924	0.25758	HZ	NCKAP5
NC_056055.1	177770001	177790001	0.153903	0.279298	HZ	NCKAP5
NC_056055.1	177775001	177795001	0.227337	0.250424	HZ	NCKAP5
NC_056062.1	46885001	46905001	0.340979	0.304831	HZ	NCOA2
NC_056062.1	46890001	46910001	0.191896	0.4132	HZ	NCOA2
NC_056062.1	46895001	46915001	0.26007	0.420168	HZ	NCOA2
NC_056062.1	46900001	46920001	0.278186	0.437528	HZ	NCOA2
NC_056066.1	74780001	74800001	0.343912	0.247219	HZ	NCOA5
NC_056059.1	7280001	7300001	0.220473	0.247535	HZ	NDST3
NC_056066.1	7205001	7225001	0.0559625	0.398513	HZ	NDUFAF5
NC_056066.1	7210001	7230001	0.0536515	0.390081	HZ	NDUFAF5
NC_056066.1	7215001	7235001	0.0698852	0.450474	HZ	NDUFAF5
NC_056066.1	7220001	7240001	0.0798668	0.446009	HZ	NDUFAF5
NC_056066.1	7225001	7245001	0.229843	0.394408	HZ	NDUFAF5
NC_056054.1	47575001	47595001	0.196847	0.280418	HZ	NEGR1

NC_056061.1	74895001	74915001
NC_056061.1	74900001	74920001
NC_056061.1	74905001	74925001
NC_056061.1	74910001	74930001
NC_056061.1	74915001	74935001
NC_056061.1	74920001	74940001
NC_056061.1	74925001	74945001
NC_056071.1	3155001	3175001
NC_056071.1	3160001	3180001
NC_056055.1	73475001	73495001
NC_056055.1	73480001	73500001
NC_056055.1	73485001	73505001
NC_056055.1	73490001	73510001
NC_056073.1	25945001	25965001
NC_056073.1	25950001	25970001
NC_056073.1	25955001	25975001
NC_056056.1	179885001	179905001
NC_056080.1	51365001	51385001
NC_056072.1	43495001	43515001
NC_056072.1	43500001	43520001
NC_056072.1	43505001	43525001
NC_056072.1	43510001	43530001
NC_056055.1	48360001	48380001
NC_056054.1	112335001	112355001
NC_056054.1	112340001	112360001
NC_056054.1	112345001	112365001
NC_056056.1	50480001	50500001
NC_056059.1	84845001	84865001
NC_056074.1	4835001	4855001
NC_056074.1	4840001	4860001
NC_056074.1	4845001	4865001
NC_056074.1	4850001	4870001
NC_056058.1	77300001	77320001
NC_056058.1	77305001	77325001
NC_056058.1	77310001	77330001
NC_056058.1	77425001	77445001

NC_056056.1	11510001	11530001	0.136313	0.267857	HZ	NEK6	NC_056058.1	77460001	77480001
NC_056056.1	141845001	141865001	0.0670699	0.290136	HZ	NELL2	NC_056058.1	77465001	77485001
NC_056056.1	141915001	141935001	0.302605	0.239991	HZ	NELL2	NC_056058.1	77470001	77490001
NC_056056.1	141940001	141960001	0.163211	0.2666	HZ	NELL2	NC_056058.1	77475001	77495001
NC_056056.1	141945001	141965001	0.175583	0.265558	HZ	NELL2	NC_056058.1	77480001	77500001
NC_056056.1	162525001	162545001	0.282103	0.257433	HZ	NEMP1	NC_056058.1	77485001	77505001
NC_056056.1	162530001	162550001	0.274877	0.278919	HZ	NEMP1	NC_056058.1	77490001	77510001
NC_056056.1	162535001	162555001	0.271186	0.299517	HZ	NEMP1	NC_056054.1	66445001	66465001
NC_056058.1	44640001	44660001	0.276622	0.241461	HZ	NEUROG1	NC_056055.1	232135001	232155001
NC_056056.1	213310001	213330001	0.161106	0.262276	HZ	NINJ2	NC_056054.1	50460001	50480001
NC_056059.1	117720001	117740001	0.28952	0.31503	HZ	NKX1-1	NC_056054.1	50465001	50485001
NC_056059.1	117725001	117745001	0.247503	0.431781	HZ	NKX1-1	NC_056054.1	50470001	50490001
NC_056059.1	117730001	117750001	0.219107	0.50761	HZ	NKX1-1	NC_056054.1	50475001	50495001
NC_056059.1	117735001	117755001	0.259924	0.480065	HZ	NKX1-1;UVSSA	NC_056054.1	50480001	50500001
NC_056075.1	20315001	20335001	0.278445	0.298101	HZ	NKX2-3	NC_056065.1	4800001	4820001
NC_056075.1	20320001	20340001	0.19838	0.241342	HZ	NKX2-3	NC_056055.1	38190001	38210001
NC_056075.1	20325001	20345001	0.335036	0.240576	HZ	NKX2-3	NC_056058.1	7305001	7325001
NC_056080.1	3740001	3760001	0.0330093	0.314579	HZ	NLGN4X	NC_056054.1	184870001	184890001
NC_056080.1	3745001	3765001	0.28279	0.261561	HZ	NLGN4X	NC_056074.1	16760001	16780001
NC_056064.1	19600001	19620001	0.234663	0.281793	HZ	NLK	NC_056066.1	78175001	78195001
NC_056064.1	19605001	19625001	0.200782	0.240399	HZ	NLK	NC_056066.1	78180001	78200001
NC_056064.1	19650001	19670001	0.129426	0.302786	HZ	NLK	NC_056066.1	78185001	78205001
NC_056064.1	19655001	19675001	0.0730028	0.318308	HZ	NLK	NC_056066.1	78190001	78210001
NC_056064.1	19660001	19680001	0.0786958	0.309203	HZ	NLK	NC_056066.1	78195001	78215001
NC_056058.1	38430001	38450001	0.0528609	0.39378	HZ	NLRP3	NC_056064.1	18865001	18885001
NC_056058.1	38445001	38465001	0.0781819	0.407089	HZ	NLRP3	NC_056056.1	165245001	165265001
NC_056076.1	23660001	23680001	0.127728	0.263234	HZ	NOL4	NC_056056.1	165250001	165270001
NC_056056.1	95230001	95250001	0.276401	0.249578	HZ	NOTO;PRADC1;SMYI	NC_056056.1	165255001	165275001
NC_056056.1	100650001	100670001	0.198872	0.272204	HZ	NPAS2	NC_056066.1	34850001	34870001
NC_056056.1	100655001	100675001	0.116737	0.301453	HZ	NPAS2	NC_056066.1	34855001	34875001
NC_056056.1	100660001	100680001	0.261135	0.270573	HZ	NPAS2	NC_056054.1	27330001	27350001
NC_056057.1	78530001	78550001	0.293534	0.311617	HZ	NPC1L1	NC_056056.1	165105001	165125001
NC_056057.1	78535001	78555001	0.203883	0.310042	HZ	NPC1L1	NC_056056.1	165110001	165130001
NC_056057.1	78540001	78560001	0.266311	0.261263	HZ	NPC1L1	NC_056056.1	165115001	165135001
NC_056054.1	257560001	257580001	0.2249	0.467413	HZ	NPHP3	NC_056080.1	56720001	56740001
NC_056054.1	257565001	257585001	0.269571	0.425732	HZ	NPHP3	NC_056054.1	220160001	220180001

NC_056073.1	49745001	49765001	0.0542577	0.33246	HZ	NQO2
NC_056058.1	48725001	48745001	0.26741	0.277195	HZ	NRG2
NC_056080.1	131595001	131615001	0.205007	0.255858	HZ	NRK
NC_056080.1	131600001	131620001	0.122575	0.327706	HZ	NRK
NC_056080.1	131605001	131625001	0.077465	0.370956	HZ	NRK
NC_056080.1	131610001	131630001	0.214849	0.273658	HZ	NRK
NC_056056.1	74135001	74155001	0.316871	0.305229	HZ	NRXN1
NC_056056.1	74185001	74205001	0.116532	0.243107	HZ	NRXN1
NC_056056.1	74190001	74210001	0.17609	0.243034	HZ	NRXN1
NC_056056.1	74245001	74265001	0.270137	0.252539	HZ	NRXN1
NC_056064.1	28345001	28365001	0.175304	0.257901	HZ	NTN1
NC_056054.1	84770001	84790001	0.148094	0.261006	HZ	NTNG1
NC_056054.1	84775001	84795001	0.189537	0.254969	HZ	NTNG1
NC_056055.1	171905001	171925001	0.220528	0.250001	HZ	NXPH2
NC_056055.1	171910001	171930001	0.286738	0.270643	HZ	NXPH2
NC_056058.1	2595001	2615001	0.210522	0.260125	HZ	OBSCN
NC_056058.1	2600001	2620001	0.0574244	0.331537	HZ	OBSCN
NC_056058.1	2605001	2625001	0.124633	0.31375	HZ	OBSCN
NC_056058.1	2610001	2630001	0.111834	0.302853	HZ	OBSCN
NC_056058.1	2615001	2635001	0.170642	0.247174	HZ	OBSCN
NC_056059.1	68150001	68170001	0.247321	0.27157	HZ	OCIAD1
NC_056080.1	11895001	11915001	0.334188	0.242438	HZ	OFD1
NC_056068.1	44875001	44895001	0.268998	0.248763	HZ	OLFML1
NC_056068.1	44880001	44900001	0.261415	0.250344	HZ	OLFML1
NC_056054.1	33210001	33230001	0.117415	0.241456	HZ	OMA1
NC_056055.1	203320001	203340001	0.310083	0.247604	HZ	ORC2
NC_056055.1	203325001	203345001	0.291947	0.240909	HZ	ORC2
NC_056057.1	72560001	72580001	0.303672	0.244359	HZ	OSBPL3
NC_056058.1	19980001	20000001	0.136775	0.287688	HZ	P4HA2
NC_056062.1	76540001	76560001	0.234139	0.335215	HZ	PABPC1
NC_056062.1	76545001	76565001	0.164261	0.319417	HZ	PABPC1
NW_024599828	1160001	1180001	0.140337	0.260873	HZ	PAG3
NW_024599828	1165001	1185001	0.0965859	0.274275	HZ	PAG3
NW_024599828	1170001	1190001	0.0480556	0.30021	HZ	PAG3
NW_024599828	1175001	1195001	0.101222	0.291824	HZ	PAG3
NW_024599828	1180001	1200001	0.16475	0.261316	HZ	PAG3

NC_056054.1	220170001	220190001
NC_056063.1	18995001	19015001
NC_056063.1	19000001	19020001
NC_056063.1	19005001	19025001
NC_056063.1	19010001	19030001
NC_056080.1	48770001	48790001
NC_056080.1	48780001	48800001
NC_056071.1	23735001	23755001
NC_056056.1	95645001	95665001
NC_056064.1	34685001	34705001
NC_056064.1	34735001	34755001
NC_056064.1	34740001	34760001
NC_056064.1	34745001	34765001
NC_056064.1	34755001	34775001
NC_056059.1	103800001	103820001
NC_056070.1	45375001	45395001
NC_056067.1	44370001	44390001
NC_056067.1	44375001	44395001
NC_056067.1	44380001	44400001
NC_056068.1	81655001	81675001
NC_056068.1	81660001	81680001
NC_056080.1	142080001	142100001
NC_056080.1	142085001	142105001
NC_056080.1	142100001	142120001
NC_056080.1	142105001	142125001
NC_056080.1	142110001	142130001
NC_056080.1	142115001	142135001
NC_056080.1	142120001	142140001
NC_056080.1	142125001	142145001
NC_056080.1	142130001	142150001
NC_056054.1	109155001	109175001
NC_056054.1	109160001	109180001
NC_056064.1	36720001	36740001
NC_056064.1	36725001	36745001
NC_056054.1	61660001	61680001
NC_056072.1	51430001	51450001

NC_056060.1	77230001	77250001	0.247778	0.305793	HZ	PALS1
NC_056060.1	77235001	77255001	0.200001	0.285233	HZ	PALS1
NC_056060.1	77240001	77260001	0.158014	0.287313	HZ	PALS1
NC_056060.1	77245001	77265001	0.164062	0.28169	HZ	PALS1
NC_056060.1	77250001	77270001	0.145038	0.291578	HZ	PALS1
NC_056060.1	77255001	77275001	0.135484	0.298646	HZ	PALS1
NC_056060.1	77260001	77280001	0.236804	0.259483	HZ	PALS1
NC_056060.1	77265001	77285001	0.256967	0.255951	HZ	PALS1
NC_056055.1	7500001	7520001	0.149304	0.346	HZ	PAPPA
NC_056055.1	7505001	7525001	0.0518584	0.387337	HZ	PAPPA
NC_056055.1	7510001	7530001	0.0398758	0.408308	HZ	PAPPA
NC_056055.1	7515001	7535001	0.0465256	0.404374	HZ	PAPPA
NC_056055.1	7520001	7540001	0.0777329	0.26527	HZ	PAPPA
NC_056055.1	7525001	7545001	0.166335	0.253868	HZ	PAPPA
NC_056078.1	42920001	42940001	0.299512	0.328181	HZ	PARG
NC_056069.1	28570001	28590001	0.257361	0.286987	HZ	PARP8
NC_056069.1	28575001	28595001	0.124053	0.321366	HZ	PARP8
NC_056069.1	28580001	28600001	0.0958026	0.376353	HZ	PARP8
NC_056069.1	28585001	28605001	0.149171	0.357852	HZ	PARP8
NC_056069.1	28590001	28610001	0.230801	0.285035	HZ	PARP8
NC_056068.1	40005001	40025001	0.301485	0.245411	HZ	PARVA
NC_056057.1	118530001	118550001	0.310979	0.239382	HZ	PAXIP1
NC_056054.1	116520001	116540001	0.333891	0.382624	HZ	PBX1
NC_056075.1	4735001	4755001	0.291009	0.38548	HZ	PCDH15
NC_056075.1	4740001	4760001	0.0450058	0.271016	HZ	PCDH15
NC_056075.1	4770001	4790001	0.080309	0.261149	HZ	PCDH15
NC_056060.1	80745001	80765001	0.121245	0.320672	HZ	PCNX1
NC_056060.1	80750001	80770001	0.225681	0.253913	HZ	PCNX1
NC_056054.1	246790001	246810001	0.0828299	0.281327	HZ	PCOLCE2
NC_056054.1	246795001	246815001	0.0393762	0.339603	HZ	PCOLCE2
NC_056055.1	73660001	73680001	0.309817	0.461998	HZ	PDCD1LG2
NC_056055.1	73665001	73685001	0.172171	0.441636	HZ	PDCD1LG2
NC_056055.1	73670001	73690001	0.213715	0.415736	HZ	PDCD1LG2
NC_056055.1	73675001	73695001	0.322738	0.339739	HZ	PDCD1LG2
NC_056055.1	73680001	73700001	0.292934	0.417448	HZ	PDCD1LG2
NC_056055.1	73685001	73705001	0.303238	0.516309	HZ	PDCD1LG2

NC_056058.1	37850001	37870001
NC_056058.1	37855001	37875001
NC_056058.1	37860001	37880001
NC_056054.1	250670001	250690001
NC_056068.1	48960001	48980001
NC_056068.1	48965001	48985001
NC_056055.1	188340001	188360001
NC_056056.1	180200001	180220001
NC_056056.1	180205001	180225001
NC_056056.1	180210001	180230001
NC_056056.1	180215001	180235001
NC_056058.1	57350001	57370001
NC_056058.1	57325001	57345001
NC_056058.1	57330001	57350001
NC_056058.1	57335001	57355001
NC_056058.1	57340001	57360001
NC_056058.1	57345001	57365001
NC_056055.1	52570001	52590001
NC_056055.1	52575001	52595001
NC_056067.1	46870001	46890001
NC_056067.1	46875001	46895001
NC_056067.1	46880001	46900001
NC_056080.1	116340001	116360001
NC_056080.1	116345001	116365001
NC_056080.1	116350001	116370001
NC_056080.1	116355001	116375001
NC_056080.1	116360001	116380001
NC_056080.1	116365001	116385001
NC_056054.1	228215001	228235001
NC_056074.1	24615001	24635001
NC_056063.1	70640001	70660001
NC_056063.1	70645001	70665001
NC_056063.1	70650001	70670001
NC_056063.1	70655001	70675001
NC_056063.1	70660001	70680001
NC_056063.1	70665001	70685001

NC_056055.1	73690001	73710001	0.323485	0.48987	HZ	PDCD1LG2
NC_056075.1	30935001	30955001	0.117822	0.253347	HZ	PDCD4
NC_056075.1	30940001	30960001	0.176058	0.260855	HZ	PDCD4
NC_056054.1	41665001	41685001	0.109958	0.250195	HZ	PDE4B
NC_056069.1	20445001	20465001	0.338407	0.273237	HZ	PDE4D
NC_056080.1	22800001	22820001	0.164719	0.268771	HZ	PDK3
NC_056080.1	22805001	22825001	0.191313	0.242445	HZ	PDK3
NC_056069.1	41640001	41660001	0.273207	0.278141	HZ	PDZD2
NC_056069.1	41645001	41665001	0.275935	0.293206	HZ	PDZD2
NC_056069.1	41650001	41670001	0.340037	0.317105	HZ	PDZD2
NC_056069.1	41825001	41845001	0.123815	0.256693	HZ	PDZD2
NC_056069.1	41830001	41850001	0.192273	0.321081	HZ	PDZD2
NC_056069.1	41835001	41855001	0.151777	0.349114	HZ	PDZD2
NC_056069.1	41840001	41860001	0.252101	0.287444	HZ	PDZD2
NC_056069.1	41905001	41925001	0.257088	0.328829	HZ	PDZD2
NC_056069.1	41910001	41930001	0.204969	0.341117	HZ	PDZD2
NC_056069.1	41915001	41935001	0.282805	0.315668	HZ	PDZD2
NC_056072.1	28480001	28500001	0.236028	0.295508	HZ	PDZRN3
NC_056072.1	28485001	28505001	0.207625	0.263945	HZ	PDZRN3
NC_056056.1	145630001	145650001	0.273507	0.284365	HZ	PDZRN4
NC_056056.1	145860001	145880001	0.262331	0.294076	HZ	PDZRN4
NC_056055.1	42875001	42895001	0.252326	0.250762	HZ	PEBP4
NC_056054.1	207955001	207975001	0.180733	0.240059	HZ	PEX5L
NC_056078.1	1895001	1915001	0.262355	0.295043	HZ	PGBD5
NC_056073.1	22170001	22190001	0.293537	0.241858	HZ	PGK2
NC_056068.1	7550001	7570001	0.30307	0.331306	HZ	PGR
NC_056058.1	24985001	25005001	0.160608	0.394029	HZ	PHAX;TEX43
NC_056058.1	24990001	25010001	0.22068	0.373726	HZ	PHAX;TEX43
NC_056080.1	20420001	20440001	0.219681	0.306122	HZ	PHEX
NC_056080.1	20425001	20445001	0.305073	0.298688	HZ	PHEX
NC_056055.1	2670001	2690001	0.244239	0.333148	HZ	PHF19
NC_056055.1	2675001	2695001	0.341728	0.256926	HZ	PHF19
NC_056067.1	15525001	15545001	0.256321	0.311707	HZ	PHKB
NC_056067.1	15530001	15550001	0.273072	0.257981	HZ	PHKB
NC_056070.1	67515001	67535001	0.28	0.312512	HZ	PITPNB
NC_056058.1	44090001	44110001	0.303266	0.362151	HZ	PITX1

NC_056054.1	264355001	264375001
NC_056054.1	264360001	264380001
NC_056074.1	24605001	24625001
NC_056074.1	24610001	24630001
NC_056074.1	24600001	24620001
NC_056058.1	8305001	8325001
NC_056056.1	7590001	7610001
NC_056073.1	27560001	27580001
NC_056080.1	83660001	83680001
NC_056080.1	83665001	83685001
NC_056057.1	94550001	94570001
NC_056057.1	94555001	94575001
NC_056057.1	94560001	94580001
NC_056079.1	6240001	6260001
NC_056068.1	17485001	17505001
NC_056066.1	67215001	67235001
NC_056066.1	67220001	67240001
NC_056066.1	67235001	67255001
NC_056056.1	199630001	199650001
NC_056054.1	163270001	163290001
NC_056054.1	163275001	163295001
NC_056067.1	33835001	33855001
NC_056067.1	33840001	33860001
NC_056067.1	63455001	63475001
NC_056067.1	63460001	63480001
NC_056062.1	73195001	73215001
NC_056062.1	73200001	73220001
NC_056062.1	73205001	73225001
NC_056062.1	73210001	73230001
NC_056057.1	108040001	108060001
NC_056057.1	108045001	108065001
NC_056056.1	180095001	180115001
NC_056056.1	180100001	180120001
NC_056056.1	180105001	180125001
NC_056056.1	180110001	180130001
NC_056056.1	180115001	180135001

NC_056054.1	185225001	185245001	0.308009	0.26359	HZ	PLA1A	NC_056056.1	180120001	180140001
NC_056054.1	185230001	185250001	0.295821	0.288875	HZ	PLA1A	NC_056056.1	180135001	180155001
NC_056054.1	185235001	185255001	0.31289	0.35444	HZ	PLA1A	NC_056057.1	89875001	89895001
NC_056066.1	2165001	2185001	0.25046	0.405958	HZ	PLCB4	NC_056054.1	112375001	112395001
NC_056066.1	2170001	2190001	0.230311	0.445635	HZ	PLCB4	NC_056054.1	112380001	112400001
NC_056066.1	2175001	2195001	0.270087	0.351502	HZ	PLCB4	NC_056054.1	112385001	112405001
NC_056054.1	232715001	232735001	0.314061	0.273172	HZ	PLCH1	NC_056054.1	112390001	112410001
NC_056054.1	232720001	232740001	0.272565	0.274905	HZ	PLCH1	NC_056054.1	117215001	117235001
NC_056054.1	232725001	232745001	0.225476	0.283726	HZ	PLCH1	NC_056054.1	117220001	117240001
NC_056065.1	1890001	1910001	0.165533	0.303729	HZ	PLEKHA6	NC_056075.1	10230001	10250001
NC_056065.1	1895001	1915001	0.146445	0.279354	HZ	PLEKHA6	NC_056058.1	19055001	19075001
NC_056065.1	1900001	1920001	0.11873	0.276693	HZ	PLEKHA6	NC_056055.1	117820001	117840001
NC_056060.1	77455001	77475001	0.232422	0.239591	HZ	PLEKHH1;TMEM229E	NC_056060.1	9665001	9685001
NC_056054.1	247020001	247040001	0.263615	0.26966	HZ	PLS1	NC_056060.1	9670001	9690001
NC_056057.1	97915001	97935001	0.284604	0.293751	HZ	PLXNA4	NC_056056.1	154695001	154715001
NC_056057.1	97920001	97940001	0.285772	0.311739	HZ	PLXNA4	NC_056056.1	154700001	154720001
NC_056055.1	119540001	119560001	0.0619991	0.255811	HZ	PMS1	NC_056056.1	154705001	154725001
NC_056055.1	119545001	119565001	0.0812218	0.336501	HZ	PMS1	NC_056056.1	154710001	154730001
NC_056055.1	119550001	119570001	0.180082	0.334442	HZ	PMS1	NC_056056.1	154715001	154735001
NC_056055.1	119555001	119575001	0.189015	0.316853	HZ	PMS1	NC_056056.1	154720001	154740001
NC_056057.1	90815001	90835001	0.119705	0.262865	HZ	POT1	NC_056056.1	154725001	154745001
NC_056068.1	30585001	30605001	0.194364	0.297855	HZ	POU2F3	NC_056068.1	74885001	74905001
NC_056068.1	30595001	30615001	0.0507464	0.343099	HZ	POU2F3	NC_056056.1	134210001	134230001
NC_056068.1	30600001	30620001	0.0571863	0.36562	HZ	POU2F3	NC_056061.1	21110001	21130001
NC_056068.1	30605001	30625001	0.0538462	0.378944	HZ	POU2F3	NC_056061.1	21115001	21135001
NC_056068.1	30610001	30630001	0.0687538	0.387352	HZ	POU2F3;TLCD5	NC_056057.1	116205001	116225001
NC_056068.1	30615001	30635001	0.162955	0.345133	HZ	POU2F3;TLCD5	NC_056057.1	116210001	116230001
NC_056061.1	73830001	73850001	0.27206	0.295076	HZ	PPIL4	NC_056057.1	116215001	116235001
NC_056054.1	227365001	227385001	0.324687	0.271546	HZ	PPM1L	NC_056057.1	116230001	116250001
NC_056054.1	227370001	227390001	0.156468	0.313286	HZ	PPM1L	NC_056057.1	116250001	116270001
NC_056054.1	227375001	227395001	0.141026	0.258814	HZ	PPM1L	NC_056057.1	116255001	116275001
NC_056055.1	239255001	239275001	0.0863061	0.252981	HZ	PPP1R8	NC_056057.1	116270001	116290001
NC_056055.1	239260001	239280001	0.0661539	0.247538	HZ	PPP1R8	NC_056065.1	34460001	34480001
NC_056055.1	239265001	239285001	0.0950923	0.243664	HZ	PPP1R8;STX12	NC_056070.1	525001	545001
NC_056054.1	253950001	253970001	0.190507	0.242756	HZ	PPP2R3A	NC_056070.1	530001	550001
NC_056054.1	253960001	253980001	0.337357	0.253131	HZ	PPP2R3A	NC_056070.1	535001	555001

NC_056054.1	106715001	106735001	0.136555	0.604005	HZ	PRCC
NC_056054.1	106725001	106745001	0.208566	0.537635	HZ	PRCC
NC_056066.1	76835001	76855001	0.214374	0.25306	HZ	PREX1
NC_056065.1	66835001	66855001	0.285584	0.29367	HZ	PRG4;TPR
NC_056065.1	66840001	66860001	0.165501	0.345915	HZ	PRG4;TPR
NC_056056.1	78635001	78655001	0.273438	0.279149	HZ	PRKCE
NC_056056.1	78640001	78660001	0.180842	0.347802	HZ	PRKCE
NC_056056.1	78680001	78700001	0.175887	0.329252	HZ	PRKCE
NC_056055.1	118080001	118100001	0.296996	0.267067	HZ	PROC
NC_056055.1	118085001	118105001	0.194519	0.332704	HZ	PROC
NC_056055.1	118090001	118110001	0.11872	0.349853	HZ	PROC
NC_056071.1	43175001	43195001	0.298495	0.451682	HZ	PRORP
NC_056071.1	43180001	43200001	0.17203	0.506056	HZ	PRORP
NC_056071.1	43185001	43205001	0.100472	0.546785	HZ	PRORP
NC_056071.1	43190001	43210001	0.103584	0.539813	HZ	PRORP
NC_056071.1	43195001	43215001	0.1697	0.463444	HZ	PRORP
NC_056067.1	55750001	55770001	0.206418	0.245845	HZ	PRR12
NC_056067.1	55755001	55775001	0.319664	0.249243	HZ	PRR12;RRAS
NC_056068.1	65730001	65750001	0.328942	0.276274	HZ	PRR5L
NC_056055.1	59860001	59880001	0.251174	0.297572	HZ	PRUNE2
NC_056055.1	59865001	59885001	0.194073	0.275989	HZ	PRUNE2
NC_056055.1	59875001	59895001	0.229698	0.253836	HZ	PRUNE2
NC_056058.1	48620001	48640001	0.178702	0.356341	HZ	PSD2
NC_056058.1	48625001	48645001	0.203108	0.273972	HZ	PSD2
NC_056056.1	59810001	59830001	0.310559	0.269446	HZ	PSD4
NC_056056.1	59815001	59835001	0.297631	0.291787	HZ	PSD4
NC_056071.1	43290001	43310001	0.343297	0.316043	HZ	PSMA6
NC_056071.1	43295001	43315001	0.151038	0.410413	HZ	PSMA6
NC_056071.1	43300001	43320001	0.113528	0.435419	HZ	PSMA6
NC_056071.1	43305001	43325001	0.148305	0.380906	HZ	PSMA6
NC_056071.1	43310001	43330001	0.262168	0.325409	HZ	PSMA6
NC_056056.1	11410001	11430001	0.118832	0.323646	HZ	PSMB7
NC_056055.1	185075001	185095001	0.315865	0.248199	HZ	PTPN4
NC_056072.1	39265001	39285001	0.184653	0.313115	HZ	PTPRG
NC_056072.1	39270001	39290001	0.190208	0.383408	HZ	PTPRG
NC_056072.1	39275001	39295001	0.17717	0.389652	HZ	PTPRG

NC_056054.1	108435001	108455001
NC_056054.1	108440001	108460001
NC_056065.1	31395001	31415001
NC_056065.1	31400001	31420001
NC_056065.1	31405001	31425001
NC_056065.1	31410001	31430001
NC_056064.1	56360001	56380001
NC_056064.1	56365001	56385001
NC_056055.1	11075001	11095001
NC_056055.1	11080001	11100001
NC_056055.1	11085001	11105001
NC_056055.1	11090001	11110001
NC_056055.1	11095001	11115001
NC_056055.1	11105001	11125001
NC_056055.1	11110001	11130001
NC_056055.1	11115001	11135001
NC_056055.1	11120001	11140001
NC_056066.1	24275001	24295001
NC_056066.1	24310001	24330001
NC_056066.1	24315001	24335001
NC_056070.1	35590001	35610001
NC_056058.1	91375001	91395001
NC_056058.1	91380001	91400001
NC_056058.1	91385001	91405001
NC_056058.1	91390001	91410001
NC_056058.1	91395001	91415001
NC_056055.1	74870001	74890001
NC_056073.1	38685001	38705001
NC_056073.1	38690001	38710001
NC_056079.1	31300001	31320001
NC_056079.1	31305001	31325001
NC_056079.1	31310001	31330001
NC_056062.1	680001	700001
NC_056062.1	685001	705001
NC_056062.1	690001	710001
NC_056054.1	104960001	104980001

NC_056072.1	39420001	39440001	0.302161	0.246548	HZ	PTPRG
NC_056072.1	39425001	39445001	0.258579	0.241712	HZ	PTPRG
NC_056072.1	39470001	39490001	0.30931	0.403636	HZ	PTPRG
NC_056072.1	39475001	39495001	0.244981	0.347723	HZ	PTPRG
NC_056066.1	71430001	71450001	0.0898278	0.460411	HZ	PTPRT
NC_056066.1	71435001	71455001	0.111608	0.325648	HZ	PTPRT
NC_056057.1	8580001	8600001	0.326918	0.264763	HZ	PTTG1IP2
NC_056060.1	52930001	52950001	0.246387	0.255763	HZ	PYGO1
NC_056060.1	52935001	52955001	0.329504	0.249022	HZ	PYGO1
NC_056075.1	37815001	37835001	0.224107	0.323563	HZ	RAB11FIP2
NC_056075.1	37820001	37840001	0.164716	0.360826	HZ	RAB11FIP2
NC_056054.1	256140001	256160001	0.233714	0.404387	HZ	RAB6B
NC_056054.1	256145001	256165001	0.271952	0.403203	HZ	RAB6B
NC_056054.1	256150001	256170001	0.300678	0.387969	HZ	RAB6B
NC_056054.1	256155001	256175001	0.339942	0.364893	HZ	RAB6B
NC_056054.1	256160001	256180001	0.335656	0.328643	HZ	RAB6B;TF
NC_056054.1	256165001	256185001	0.247092	0.392332	HZ	RAB6B;TF
NC_056054.1	256170001	256190001	0.184792	0.371476	HZ	RAB6B;TF
NC_056072.1	59080001	59100001	0.044776	0.284802	HZ	RAB7A
NC_056072.1	59085001	59105001	0.0431841	0.262377	HZ	RAB7A
NC_056056.1	12720001	12740001	0.344828	0.290302	HZ	RABGAP1
NC_056056.1	12725001	12745001	0.294736	0.283316	HZ	RABGAP1
NC_056056.1	12730001	12750001	0.300398	0.341917	HZ	RABGAP1
NC_056056.1	12735001	12755001	0.322493	0.319981	HZ	RABGAP1
NC_056056.1	12740001	12760001	0.311242	0.283952	HZ	RABGAP1
NC_056056.1	12785001	12805001	0.265896	0.239908	HZ	RABGAP1;ZBTB26
NC_056056.1	12790001	12810001	0.34375	0.272868	HZ	RABGAP1;ZBTB26
NC_056058.1	19795001	19815001	0.338379	0.250996	HZ	RAD50
NC_056056.1	9065001	9085001	0.240467	0.243429	HZ	RALGPS1
NC_056056.1	9070001	9090001	0.259988	0.271757	HZ	RALGPS1
NC_056056.1	9075001	9095001	0.320566	0.32258	HZ	RALGPS1
NC_056056.1	9080001	9100001	0.3091	0.35304	HZ	RALGPS1
NC_056056.1	9085001	9105001	0.325266	0.315998	HZ	RALGPS1
NC_056056.1	9090001	9110001	0.306601	0.306593	HZ	RALGPS1
NC_056080.1	102660001	102680001	0.124204	0.319863	HZ	RAP2C
NC_056054.1	229545001	229565001	0.321768	0.247845	HZ	RARRES1

NC_056054.1	104965001	104985001
NC_056054.1	104970001	104990001
NC_056054.1	104975001	104995001
NC_056054.1	104980001	105000001
NC_056054.1	104985001	105005001
NC_056054.1	104990001	105010001
NC_056054.1	104995001	105015001
NC_056054.1	105000001	105020001
NC_056054.1	208965001	208985001
NC_056054.1	208970001	208990001
NC_056054.1	208975001	208995001
NC_056078.1	32580001	32600001
NC_056067.1	47500001	47520001
NC_056054.1	270430001	270450001
NC_056054.1	270435001	270455001
NC_056059.1	41205001	41225001
NC_056059.1	41210001	41230001
NC_056056.1	104410001	104430001
NC_056056.1	104415001	104435001
NC_056056.1	104420001	104440001
NC_056056.1	104310001	104330001
NC_056056.1	104320001	104340001
NC_056056.1	104335001	104355001
NC_056056.1	104360001	104380001
NC_056056.1	104365001	104385001
NC_056056.1	104370001	104390001
NC_056056.1	104375001	104395001
NC_056056.1	104380001	104400001
NC_056056.1	104385001	104405001
NC_056056.1	104390001	104410001
NC_056056.1	104395001	104415001
NC_056056.1	104400001	104420001
NC_056056.1	104405001	104425001
NC_056065.1	71975001	71995001
NC_056065.1	71980001	72000001
NC_056065.1	71985001	72005001

NC_056054.1	247920001	247940001	0.213731	0.314352	HZ	RASA2
NC_056054.1	247925001	247945001	0.175207	0.413587	HZ	RASA2
NC_056055.1	36690001	36710001	0.25509	0.243367	HZ	RASEF
NC_056055.1	36695001	36715001	0.25696	0.290505	HZ	RASEF
NC_056055.1	36700001	36720001	0.2234	0.242901	HZ	RASEF
NC_056077.1	7125001	7145001	0.180091	0.348529	HZ	RBFOX1
NC_056077.1	7130001	7150001	0.0913009	0.350414	HZ	RBFOX1
NC_056077.1	7135001	7155001	0.0747171	0.316145	HZ	RBFOX1
NC_056064.1	52285001	52305001	0.202068	0.369509	HZ	RBFOX3
NC_056064.1	52295001	52315001	0.208804	0.267706	HZ	RBFOX3
NC_056064.1	52470001	52490001	0.301733	0.267093	HZ	RBFOX3
NC_056064.1	52475001	52495001	0.307043	0.259433	HZ	RBFOX3
NC_056064.1	52505001	52525001	0.339285	0.291377	HZ	RBFOX3
NC_056064.1	52510001	52530001	0.344165	0.284434	HZ	RBFOX3
NC_056064.1	52515001	52535001	0.267746	0.342016	HZ	RBFOX3
NC_056064.1	52520001	52540001	0.163613	0.426198	HZ	RBFOX3
NC_056064.1	52525001	52545001	0.315004	0.302502	HZ	RBFOX3
NC_056080.1	128200001	128220001	0.138718	0.295258	HZ	RBM41
NC_056080.1	128205001	128225001	0.244168	0.252854	HZ	RBM41
NC_056072.1	4055001	4075001	0.167285	0.296828	HZ	RBMS3
NC_056054.1	250155001	250175001	0.31692	0.327432	HZ	RBP1
NC_056054.1	250160001	250180001	0.247777	0.268948	HZ	RBP1
NC_056054.1	250165001	250185001	0.207474	0.249131	HZ	RBP1
NC_056054.1	250220001	250240001	0.287089	0.291681	HZ	RBP2
NC_056054.1	250225001	250245001	0.0752302	0.334408	HZ	RBP2
NC_056072.1	57090001	57110001	0.254887	0.28772	HZ	RBSN
NC_056072.1	57095001	57115001	0.136256	0.369941	HZ	RBSN
NC_056072.1	57100001	57120001	0.205759	0.327104	HZ	RBSN
NC_056072.1	57105001	57125001	0.161582	0.297118	HZ	RBSN
NC_056072.1	57110001	57130001	0.268639	0.264049	HZ	RBSN
NC_056060.1	77605001	77625001	0.167132	0.319084	HZ	RDH11;VTI1B
NC_056060.1	77610001	77630001	0.197202	0.376305	HZ	RDH11;VTI1B
NC_056056.1	200355001	200375001	0.196806	0.265664	HZ	RERG
NC_056056.1	200365001	200385001	0.103282	0.281098	HZ	RERG
NC_056056.1	175645001	175665001	0.327197	0.51719	HZ	RFX4
NC_056056.1	175650001	175670001	0.248032	0.473102	HZ	RFX4;RIC8B

NC_056055.1	225200001	225220001
NC_056055.1	225205001	225225001
NC_056055.1	225210001	225230001
NC_056056.1	111000001	111020001
NC_056056.1	111005001	111025001
NC_056072.1	59490001	59510001
NC_056072.1	59495001	59515001
NC_056072.1	59500001	59520001
NC_056072.1	59505001	59525001
NC_056056.1	103860001	103880001
NC_056055.1	211735001	211755001
NC_056055.1	211740001	211760001
NC_056055.1	211745001	211765001
NC_056064.1	41385001	41405001
NC_056067.1	12670001	12690001
NC_056060.1	84735001	84755001
NC_056060.1	84740001	84760001
NC_056055.1	73325001	73345001
NC_056055.1	73330001	73350001
NC_056055.1	73335001	73355001
NC_056055.1	73340001	73360001
NC_056055.1	73345001	73365001
NC_056055.1	73350001	73370001
NC_056055.1	73355001	73375001
NC_056055.1	73360001	73380001
NC_056055.1	73365001	73385001
NC_056055.1	73370001	73390001
NC_056055.1	73375001	73395001
NC_056055.1	73380001	73400001
NC_056055.1	73385001	73405001
NC_056055.1	73390001	73410001
NC_056055.1	73395001	73415001
NC_056055.1	73405001	73425001
NC_056055.1	73415001	73435001
NC_056055.1	73420001	73440001
NC_056055.1	73425001	73445001

NC_056056.1	175655001	175675001	0.219807	0.286288	HZ	RFX4;RIC8B
NC_056073.1	49670001	49690001	0.224359	0.300904	HZ	RIPK1
NC_056073.1	49675001	49695001	0.109925	0.240166	HZ	RIPK1
NC_056054.1	14775001	14795001	0.311644	0.455231	HZ	RLF
NC_056054.1	14780001	14800001	0.200106	0.437847	HZ	RLF
NC_056054.1	14815001	14835001	0.303965	0.396415	HZ	RLF
NC_056080.1	66760001	66780001	0.320001	0.303451	HZ	RLIM
NC_056060.1	49365001	49385001	0.322441	0.240545	HZ	RNF111
NC_056060.1	49370001	49390001	0.297922	0.272153	HZ	RNF111
NC_056060.1	49375001	49395001	0.206046	0.310161	HZ	RNF111
NC_056060.1	49380001	49400001	0.184524	0.344704	HZ	RNF111
NC_056060.1	49385001	49405001	0.217002	0.31344	HZ	RNF111
NC_056060.1	49390001	49410001	0.231593	0.318252	HZ	RNF111
NC_056070.1	16845001	16865001	0.263745	0.24522	HZ	RNF150
NC_056070.1	16850001	16870001	0.213576	0.308402	HZ	RNF150
NC_056063.1	36760001	36780001	0.248714	0.56518	HZ	RNF17
NC_056063.1	36765001	36785001	0.243698	0.565118	HZ	RNF17
NC_056063.1	36770001	36790001	0.275405	0.547483	HZ	RNF17
NC_056063.1	36775001	36795001	0.323485	0.502315	HZ	RNF17
NC_056063.1	36780001	36800001	0.330999	0.490365	HZ	RNF17
NC_056063.1	36785001	36805001	0.295928	0.506633	HZ	RNF17
NC_056063.1	36790001	36810001	0.253387	0.529149	HZ	RNF17
NC_056063.1	36795001	36815001	0.19966	0.586479	HZ	RNF17
NC_056063.1	36800001	36820001	0.20367	0.58258	HZ	RNF17
NC_056063.1	36805001	36825001	0.176935	0.605486	HZ	RNF17
NC_056063.1	36810001	36830001	0.174687	0.607685	HZ	RNF17
NC_056063.1	36815001	36835001	0.168269	0.604926	HZ	RNF17
NC_056063.1	36820001	36840001	0.152174	0.58717	HZ	RNF17
NC_056063.1	36825001	36845001	0.25	0.548437	HZ	RNF17
NC_056063.1	36830001	36850001	0.26783	0.522752	HZ	RNF17
NC_056063.1	36835001	36855001	0.22702	0.539049	HZ	RNF17
NC_056063.1	36840001	36860001	0.229843	0.560853	HZ	RNF17
NC_056063.1	36845001	36865001	0.182298	0.571579	HZ	RNF17
NC_056063.1	36850001	36870001	0.151961	0.596405	HZ	RNF17
NC_056063.1	36855001	36875001	0.225842	0.561389	HZ	RNF17
NC_056063.1	36860001	36880001	0.154132	0.604615	HZ	RNF17

NC_056055.1	73430001	73450001
NC_056055.1	73435001	73455001
NC_056055.1	73440001	73460001
NC_056054.1	40530001	40550001
NC_056054.1	40535001	40555001
NC_056054.1	40540001	40560001
NC_056054.1	40545001	40565001
NC_056054.1	40550001	40570001
NC_056054.1	40555001	40575001
NC_056054.1	40560001	40580001
NC_056054.1	40565001	40585001
NC_056054.1	40570001	40590001
NC_056054.1	40575001	40595001
NC_056057.1	66370001	66390001
NC_056057.1	66375001	66395001
NC_056054.1	112330001	112350001
NC_056069.1	26190001	26210001
NC_056060.1	15115001	15135001
NC_056060.1	15120001	15140001
NC_056060.1	15125001	15145001
NC_056060.1	15130001	15150001
NC_056060.1	15135001	15155001
NC_056060.1	15140001	15160001
NC_056066.1	6610001	6630001
NC_056066.1	6615001	6635001
NC_056066.1	6620001	6640001
NC_056071.1	32215001	32235001
NC_056071.1	32210001	32230001
NC_056067.1	11405001	11425001
NC_056070.1	8510001	8530001
NC_056055.1	73470001	73490001
NC_056055.1	73445001	73465001
NC_056055.1	73450001	73470001
NC_056055.1	73460001	73480001
NC_056055.1	73465001	73485001
NC_056054.1	42465001	42485001

NC_056061.1	48525001	48545001	0.157091	0.243131	HZ	RNGTT
NC_056061.1	48530001	48550001	0.132633	0.265993	HZ	RNGTT
NC_056061.1	48535001	48555001	0.169346	0.251164	HZ	RNGTT
NC_056061.1	48600001	48620001	0.103475	0.416279	HZ	RNGTT
NC_056061.1	48605001	48625001	0.103146	0.426798	HZ	RNGTT
NC_056054.1	147540001	147560001	0.116649	0.612319	HZ	ROBO1
NC_056054.1	147555001	147575001	0.0461716	0.423062	HZ	ROBO1
NC_056054.1	147560001	147580001	0.0750619	0.404595	HZ	ROBO1
NC_056054.1	147565001	147585001	0.268615	0.31494	HZ	ROBO1
NC_056054.1	147745001	147765001	0.236165	0.304298	HZ	ROBO1
NC_056054.1	147750001	147770001	0.275914	0.274528	HZ	ROBO1
NC_056054.1	147755001	147775001	0.333674	0.242196	HZ	ROBO1
NC_056054.1	189195001	189215001	0.155702	0.319377	HZ	ROPN1
NC_056054.1	189200001	189220001	0.235512	0.276027	HZ	ROPN1
NC_056055.1	26905001	26925001	0.226721	0.269841	HZ	ROR2
NC_056055.1	239185001	239205001	0.206492	0.306068	HZ	RPA2
NC_056055.1	239190001	239210001	0.213348	0.284201	HZ	RPA2;THEMIS2
NC_056055.1	239195001	239215001	0.314008	0.243296	HZ	RPA2;THEMIS2
NC_056065.1	69800001	69820001	0.0853898	0.277658	HZ	RPS6KC1
NC_056065.1	69805001	69825001	0.153699	0.311077	HZ	RPS6KC1
NC_056065.1	69810001	69830001	0.304652	0.32534	HZ	RPS6KC1
NC_056068.1	38110001	38130001	0.290843	0.2413	HZ	RRAS2
NC_056068.1	38115001	38135001	0.201265	0.320817	HZ	RRAS2
NC_056068.1	38120001	38140001	0.257585	0.448794	HZ	RRAS2
NC_056074.1	15800001	15820001	0.295745	0.336894	HZ	RSF1
NC_056074.1	15915001	15935001	0.221819	0.263948	HZ	RSF1
NC_056072.1	59395001	59415001	0.247949	0.498564	HZ	RUVBL1
NC_056072.1	59405001	59425001	0.311549	0.278288	HZ	RUVBL1
NC_056060.1	65335001	65355001	0.344828	0.300633	HZ	SAMD4A
NC_056060.1	65340001	65360001	0.287032	0.260075	HZ	SAMD4A
NC_056060.1	65350001	65370001	0.172454	0.249795	HZ	SAMD4A
NC_056060.1	65360001	65380001	0.223274	0.256559	HZ	SAMD4A
NC_056055.1	117435001	117455001	0.268697	0.257867	HZ	SAP130
NC_056061.1	79255001	79275001	0.195619	0.283553	HZ	SCAF8
NC_056070.1	28960001	28980001	0.303152	0.251414	HZ	SCLT1
NC_056070.1	28965001	28985001	0.209829	0.247719	HZ	SCLT1

NC_056068.1	37150001	37170001
NC_056068.1	37155001	37175001
NC_056068.1	37160001	37180001
NC_056068.1	37165001	37185001
NC_056068.1	37200001	37220001
NC_056068.1	37205001	37225001
NC_056070.1	15495001	15515001
NC_056070.1	15500001	15520001
NC_056070.1	15505001	15525001
NC_056070.1	15510001	15530001
NC_056070.1	15515001	15535001
NC_056070.1	15520001	15540001
NC_056061.1	2895001	2915001
NC_056061.1	2900001	2920001
NC_056057.1	58420001	58440001
NC_056057.1	58555001	58575001
NC_056057.1	58570001	58590001
NC_056057.1	58575001	58595001
NC_056057.1	58580001	58600001
NC_056057.1	58585001	58605001
NC_056057.1	58590001	58610001
NC_056057.1	58595001	58615001
NC_056057.1	58600001	58620001
NC_056057.1	58605001	58625001
NC_056067.1	48470001	48490001
NC_056067.1	48465001	48485001
NC_056065.1	4060001	4080001
NC_056072.1	50865001	50885001
NC_056072.1	50870001	50890001
NC_056060.1	85545001	85565001
NC_056056.1	7285001	7305001
NC_056056.1	7290001	7310001
NC_056055.1	11125001	11145001
NC_056058.1	32070001	32090001
NC_056077.1	21025001	21045001
NC_056060.1	5455001	5475001

NC_056070.1	28970001	28990001	0.169021	0.243828	HZ	SCLT1
NC_056054.1	3210001	3230001	0.325772	0.24331	HZ	SCLY
NC_056080.1	16585001	16605001	0.247956	0.342262	HZ	SCML2
NC_056080.1	16590001	16610001	0.22508	0.312925	HZ	SCML2
NC_056080.1	16630001	16650001	0.345134	0.32683	HZ	SCML2
NC_056080.1	16635001	16655001	0.343971	0.355111	HZ	SCML2
NC_056061.1	29650001	29670001	0.114731	0.292842	HZ	SCML4
NC_056061.1	29655001	29675001	0.284755	0.265742	HZ	SCML4
NC_056072.1	11965001	11985001	0.117292	0.247697	HZ	SCN5A
NC_056055.1	184800001	184820001	0.219991	0.623724	HZ	SCTR
NC_056068.1	43190001	43210001	0.281163	0.38048	HZ	SCUBE2
NC_056054.1	238230001	238250001	0.241969	0.283509	HZ	SELENOT
NC_056054.1	238235001	238255001	0.198857	0.353727	HZ	SELENOT
NC_056054.1	238240001	238260001	0.199853	0.394473	HZ	SELENOT
NC_056060.1	60865001	60885001	0.345161	0.264056	HZ	SEMA6D
NC_056060.1	60870001	60890001	0.299302	0.250341	HZ	SEMA6D
NC_056060.1	60890001	60910001	0.295556	0.2421	HZ	SEMA6D
NC_056060.1	60895001	60915001	0.243717	0.242371	HZ	SEMA6D
NC_056060.1	60900001	60920001	0.254201	0.240633	HZ	SEMA6D
NC_056060.1	60980001	61000001	0.183956	0.258994	HZ	SEMA6D
NC_056060.1	60985001	61005001	0.247207	0.259908	HZ	SEMA6D
NC_056060.1	61060001	61080001	0.256821	0.253857	HZ	SEMA6D
NC_056060.1	61065001	61085001	0.334957	0.311473	HZ	SEMA6D
NC_056060.1	61130001	61150001	0.300399	0.256702	HZ	SEMA6D
NC_056061.1	2515001	2535001	0.323394	0.250227	HZ	SENP6
NC_056061.1	2520001	2540001	0.309318	0.246747	HZ	SENP6
NC_056070.1	4065001	4085001	0.153369	0.311296	HZ	SFRP2
NC_056070.1	4070001	4090001	0.130781	0.308127	HZ	SFRP2
NW_024599827	1155001	1175001	0.218515	0.279148	HZ	SH3GL3
NW_024599827	1160001	1180001	0.225733	0.274814	HZ	SH3GL3
NW_024599827	1165001	1185001	0.17207	0.28455	HZ	SH3GL3
NW_024599827	1170001	1190001	0.167258	0.264883	HZ	SH3GL3
NC_056056.1	225900001	225920001	0.106141	0.241529	HZ	SHANK3
NC_056056.1	225905001	225925001	0.122764	0.242519	HZ	SHANK3
NC_056054.1	223010001	223030001	0.0251668	0.464011	HZ	SI
NC_056054.1	223025001	223045001	0.0402219	0.283222	HZ	SI

NC_056054.1	239770001	239790001
NC_056054.1	239775001	239795001
NC_056080.1	100550001	100570001
NC_056070.1	69120001	69140001
NC_056056.1	153975001	153995001
NC_056056.1	154010001	154030001
NC_056056.1	154015001	154035001
NC_056056.1	154025001	154045001
NC_056056.1	154030001	154050001
NC_056056.1	154035001	154055001
NC_056056.1	154040001	154060001
NC_056066.1	60790001	60810001
NC_056066.1	60795001	60815001
NC_056066.1	60800001	60820001
NC_056054.1	269785001	269805001
NC_056054.1	269790001	269810001
NC_056060.1	44375001	44395001
NC_056060.1	44380001	44400001
NC_056060.1	44385001	44405001
NC_056075.1	15560001	15580001
NC_056075.1	15565001	15585001
NC_056057.1	27810001	27830001
NC_056057.1	27815001	27835001
NC_056057.1	27820001	27840001
NC_056057.1	27845001	27865001
NC_056057.1	27850001	27870001
NC_056057.1	27855001	27875001
NC_056057.1	27860001	27880001
NC_056057.1	27865001	27885001
NC_056057.1	27870001	27890001
NC_056057.1	27875001	27895001
NC_056057.1	27880001	27900001
NC_056057.1	27900001	27920001
NC_056057.1	27905001	27925001
NC_056057.1	27910001	27930001
NC_056069.1	29410001	29430001

NC_056058.1	48005001	48025001	0.207912	0.392992	HZ	SIL1
NC_056058.1	48010001	48030001	0.147281	0.418224	HZ	SIL1
NC_056058.1	48015001	48035001	0.165833	0.42354	HZ	SIL1
NC_056058.1	48020001	48040001	0.170686	0.425214	HZ	SIL1
NC_056058.1	48025001	48045001	0.218978	0.395833	HZ	SIL1
NC_056060.1	81195001	81215001	0.249377	0.263166	HZ	SIPA1L1
NC_056060.1	81200001	81220001	0.229697	0.268226	HZ	SIPA1L1
NC_056060.1	81205001	81225001	0.178971	0.29826	HZ	SIPA1L1
NC_056060.1	81210001	81230001	0.201464	0.26205	HZ	SIPA1L1
NC_056060.1	81215001	81235001	0.28716	0.264098	HZ	SIPA1L1
NC_056060.1	81290001	81310001	0.230407	0.264453	HZ	SIPA1L1
NC_056057.1	70800001	70820001	0.24357	0.508794	HZ	SKAP2
NC_056057.1	70805001	70825001	0.294333	0.475788	HZ	SKAP2
NC_056060.1	60230001	60250001	0.240709	0.260697	HZ	SLC12A1
NC_056060.1	60270001	60290001	0.122742	0.305462	HZ	SLC12A1
NC_056060.1	60275001	60295001	0.238265	0.314333	HZ	SLC12A1
NC_056060.1	60280001	60300001	0.341947	0.273016	HZ	SLC12A1
NC_056058.1	19865001	19885001	0.252252	0.358332	HZ	SLC22A4
NC_056071.1	54965001	54985001	0.196721	0.299744	HZ	SLC24A4
NC_056071.1	54970001	54990001	0.249713	0.257748	HZ	SLC24A4
NC_056075.1	20375001	20395001	0.300546	0.319367	HZ	SLC25A28
NC_056075.1	20380001	20400001	0.289024	0.306551	HZ	SLC25A28
NC_056075.1	20385001	20405001	0.327103	0.325537	HZ	SLC25A28
NC_056075.1	20390001	20410001	0.313131	0.325059	HZ	SLC25A28
NC_056075.1	20395001	20415001	0.308626	0.321682	HZ	SLC25A28
NC_056075.1	20400001	20420001	0.227376	0.375128	HZ	SLC25A28
NC_056054.1	248585001	248605001	0.243392	0.241872	HZ	SLC25A36
NC_056060.1	63280001	63300001	0.206755	0.390643	HZ	SLC28A2
NC_056060.1	63285001	63305001	0.0699207	0.320585	HZ	SLC28A2
NC_056060.1	63290001	63310001	0.233118	0.26575	HZ	SLC28A2
NC_056070.1	71315001	71335001	0.130106	0.311675	HZ	SLC2A11
NC_056069.1	10660001	10680001	0.312978	0.362776	HZ	SLC30A5
NC_056054.1	252955001	252975001	0.327309	0.655835	HZ	SLC35G2
NC_056054.1	252960001	252980001	0.216967	0.664023	HZ	SLC35G2
NC_056055.1	148135001	148155001	0.253687	0.309649	HZ	SLC4A10
NC_056055.1	148140001	148160001	0.198623	0.316925	HZ	SLC4A10

NC_056072.1	28825001	28845001
NC_056072.1	28830001	28850001
NC_056072.1	28835001	28855001
NC_056072.1	28840001	28860001
NC_056072.1	28845001	28865001
NC_056072.1	28850001	28870001
NC_056072.1	28855001	28875001
NC_056072.1	28860001	28880001
NC_056072.1	28865001	28885001
NC_056072.1	28870001	28890001
NC_056072.1	28875001	28895001
NC_056080.1	125790001	125810001
NC_056080.1	125795001	125815001
NC_056080.1	125800001	125820001
NC_056080.1	125835001	125855001
NC_056077.1	11070001	11090001
NC_056077.1	11075001	11095001
NC_056077.1	11080001	11100001
NC_056077.1	11085001	11105001
NC_056077.1	11090001	11110001
NC_056077.1	11095001	11115001
NC_056077.1	11100001	11120001
NC_056077.1	11105001	11125001
NC_056059.1	114250001	114270001
NC_056074.1	6135001	6155001
NC_056074.1	6140001	6160001
NC_056056.1	153475001	153495001
NC_056056.1	153480001	153500001
NC_056077.1	9015001	9035001
NC_056077.1	9020001	9040001
NC_056077.1	9025001	9045001
NC_056077.1	9030001	9050001
NC_056077.1	9035001	9055001
NC_056077.1	9040001	9060001
NC_056057.1	5900001	5920001
NC_056057.1	5905001	5925001

NC_056055.1	148145001	148165001	0.24906	0.261914	HZ	SLC4A10
NC_056072.1	1880001	1900001	0.195212	0.255489	HZ	SLC4A7
NC_056072.1	1885001	1905001	0.179866	0.281157	HZ	SLC4A7
NC_056072.1	1890001	1910001	0.118563	0.34563	HZ	SLC4A7
NC_056056.1	121095001	121115001	0.0717642	0.277681	HZ	SLC6A15
NC_056054.1	245915001	245935001	0.218979	0.247091	HZ	SLC9A9
NC_056054.1	245920001	245940001	0.176786	0.256406	HZ	SLC9A9
NC_056054.1	245925001	245945001	0.132075	0.273098	HZ	SLC9A9
NC_056054.1	245930001	245950001	0.226684	0.315222	HZ	SLC9A9
NC_056059.1	40800001	40820001	0.141403	0.398681	HZ	SLIT2
NC_056058.1	45180001	45200001	0.13245	0.23933	HZ	SMAD5
NC_056058.1	45195001	45215001	0.137631	0.24388	HZ	SMAD5
NC_056058.1	45200001	45220001	0.105029	0.277669	HZ	SMAD5
NC_056058.1	45205001	45225001	0.0938526	0.301171	HZ	SMAD5
NC_056058.1	45255001	45275001	0.123008	0.258815	HZ	SMIM32
NC_056058.1	45260001	45280001	0.243505	0.29182	HZ	SMIM32;TRPC7
NC_056060.1	79830001	79850001	0.208627	0.363947	HZ	SMOC1
NC_056057.1	93915001	93935001	0.260743	0.305967	HZ	SND1
NC_056057.1	93920001	93940001	0.288119	0.327569	HZ	SND1
NC_056058.1	28570001	28590001	0.113763	0.322713	HZ	SNX24
NC_056077.1	11705001	11725001	0.314216	0.306531	HZ	SNX29
NC_056077.1	11715001	11735001	0.279871	0.245584	HZ	SNX29
NC_056077.1	11720001	11740001	0.302239	0.258818	HZ	SNX29
NC_056077.1	11725001	11745001	0.339514	0.285354	HZ	SNX29
NC_056077.1	11760001	11780001	0.327099	0.315328	HZ	SNX29
NC_056077.1	11765001	11785001	0.293483	0.306684	HZ	SNX29
NC_056075.1	27520001	27540001	0.224826	0.240487	HZ	SORCS1
NC_056064.1	43375001	43395001	0.224117	0.405441	HZ	SOST
NC_056064.1	43380001	43400001	0.321933	0.382179	HZ	SOST
NC_056064.1	33450001	33470001	0.331011	0.297566	HZ	SPECC1
NC_056068.1	38290001	38310001	0.203203	0.397724	HZ	SPON1
NC_056068.1	38295001	38315001	0.140703	0.392182	HZ	SPON1
NC_056068.1	38345001	38365001	0.289989	0.248086	HZ	SPON1
NC_056080.1	61540001	61560001	0.296902	0.240764	HZ	STARD8
NC_056080.1	61545001	61565001	0.210117	0.245962	HZ	STARD8
NC_056061.1	68730001	68750001	0.259564	0.333219	HZ	STX11

NC_056057.1	5910001	5930001
NC_056057.1	5915001	5935001
NC_056057.1	6030001	6050001
NC_056057.1	6035001	6055001
NC_056057.1	6040001	6060001
NC_056057.1	6045001	6065001
NC_056064.1	38725001	38745001
NC_056064.1	38730001	38750001
NC_056080.1	12055001	12075001
NC_056080.1	12060001	12080001
NC_056080.1	12065001	12085001
NC_056063.1	69200001	69220001
NC_056056.1	104270001	104290001
NC_056056.1	104275001	104295001
NC_056067.1	63140001	63160001
NC_056067.1	63145001	63165001
NC_056067.1	63150001	63170001
NC_056070.1	72885001	72905001
NC_056070.1	72875001	72895001
NC_056070.1	72880001	72900001
NC_056070.1	72865001	72885001
NC_056070.1	72870001	72890001
NC_056055.1	59260001	59280001
NC_056055.1	59300001	59320001
NC_056056.1	38490001	38510001
NC_056056.1	38495001	38515001
NC_056056.1	38500001	38520001
NC_056056.1	38505001	38525001
NC_056056.1	38510001	38530001
NC_056056.1	38515001	38535001
NC_056056.1	38520001	38540001
NC_056056.1	38525001	38545001
NC_056069.1	6960001	6980001
NC_056067.1	52525001	52545001
NC_056058.1	63845001	63865001
NC_056058.1	63850001	63870001

NC_056064.1	5050001	5070001	0.316329	0.396513	HZ	STXBP4
NC_056064.1	5055001	5075001	0.297363	0.354146	HZ	STXBP4
NC_056064.1	5060001	5080001	0.344445	0.276599	HZ	STXBP4
NC_056064.1	5080001	5100001	0.0737266	0.687667	HZ	STXBP4
NC_056064.1	5165001	5185001	0.0231041	0.482322	HZ	STXBP4
NC_056064.1	5170001	5190001	0.143939	0.479282	HZ	STXBP4
NC_056057.1	81935001	81955001	0.194912	0.42226	HZ	SUGCT
NC_056057.1	82005001	82025001	0.316712	0.450851	HZ	SUGCT
NC_056057.1	82010001	82030001	0.173261	0.479594	HZ	SUGCT
NC_056057.1	82015001	82035001	0.196485	0.441033	HZ	SUGCT
NC_056057.1	82095001	82115001	0.266216	0.324383	HZ	SUGCT
NC_056057.1	82100001	82120001	0.141562	0.363542	HZ	SUGCT
NC_056057.1	82105001	82125001	0.193277	0.33464	HZ	SUGCT
NC_056057.1	82110001	82130001	0.329232	0.321744	HZ	SUGCT
NC_056057.1	82135001	82155001	0.337871	0.325961	HZ	SUGCT
NC_056057.1	82140001	82160001	0.235507	0.349263	HZ	SUGCT
NC_056057.1	82145001	82165001	0.155327	0.421843	HZ	SUGCT
NC_056057.1	82160001	82180001	0.119954	0.397966	HZ	SUGCT
NC_056057.1	82170001	82190001	0.188429	0.350778	HZ	SUGCT
NC_056057.1	82175001	82195001	0.17742	0.324691	HZ	SUGCT
NC_056057.1	82205001	82225001	0.333332	0.323752	HZ	SUGCT
NC_056060.1	7370001	7390001	0.199064	0.384498	HZ	SV2C
NC_056060.1	7375001	7395001	0.113763	0.488779	HZ	SV2C
NC_056060.1	7380001	7400001	0.214167	0.466891	HZ	SV2C
NC_056060.1	7385001	7405001	0.307984	0.440973	HZ	SV2C
NC_056066.1	34055001	34075001	0.269519	0.239874	HZ	SVIL
NC_056066.1	34060001	34080001	0.18267	0.288939	HZ	SVIL
NC_056066.1	34065001	34085001	0.147713	0.266807	HZ	SVIL
NC_056057.1	103740001	103760001	0.0395991	0.38746	HZ	SVOPL
NC_056057.1	103745001	103765001	0.0362887	0.466257	HZ	SVOPL
NC_056057.1	103770001	103790001	0.197841	0.398096	HZ	SVOPL
NC_056075.1	51450001	51470001	0.0762101	0.3025	HZ	SYCE1
NC_056075.1	51455001	51475001	0.0731923	0.302743	HZ	SYCE1
NC_056075.1	51460001	51480001	0.0880781	0.301808	HZ	SYCE1
NC_056075.1	51465001	51485001	0.0753178	0.306975	HZ	SYCE1
NC_056075.1	51470001	51490001	0.0592692	0.306421	HZ	SYCE1

NC_056058.1	42100001	42120001
NC_056062.1	80960001	80980001
NC_056062.1	80965001	80985001
NC_056062.1	80970001	80990001
NC_056062.1	50590001	50610001
NC_056062.1	50595001	50615001
NC_056054.1	71040001	71060001
NC_056059.1	87740001	87760001
NC_056060.1	63225001	63245001
NC_056060.1	63230001	63250001
NC_056060.1	63235001	63255001
NC_056060.1	63240001	63260001
NC_056067.1	14330001	14350001
NC_056067.1	14335001	14355001
NC_056067.1	14340001	14360001
NC_056067.1	14345001	14365001
NC_056057.1	67265001	67285001
NC_056057.1	67270001	67290001
NC_056057.1	67285001	67305001
NC_056057.1	67290001	67310001
NC_056076.1	25260001	25280001
NC_056076.1	25265001	25285001
NC_056076.1	25270001	25290001
NC_056058.1	41470001	41490001
NC_056055.1	107705001	107725001
NC_056055.1	107810001	107830001
NC_056055.1	107520001	107540001
NC_056055.1	107525001	107545001
NC_056077.1	29970001	29990001
NC_056055.1	155525001	155545001
NC_056055.1	155530001	155550001
NC_056055.1	155535001	155555001
NC_056055.1	155540001	155560001
NC_056055.1	155545001	155565001
NC_056055.1	155550001	155570001
NC_056057.1	116185001	116205001

NC_056075.1	51475001	51495001	0.12104	0.282446	HZ	SYCE1
NC_056056.1	176790001	176810001	0.329621	0.27546	HZ	SYN3
NC_056056.1	176805001	176825001	0.322839	0.280851	HZ	SYN3
NC_056056.1	176810001	176830001	0.208889	0.259171	HZ	SYN3
NC_056056.1	176815001	176835001	0.147482	0.26417	HZ	SYN3
NC_056056.1	176835001	176855001	0.228031	0.282919	HZ	SYN3
NC_056056.1	176840001	176860001	0.248645	0.295085	HZ	SYN3
NC_056056.1	176925001	176945001	0.306175	0.290832	HZ	SYN3
NC_056056.1	176930001	176950001	0.297936	0.312497	HZ	SYN3
NC_056056.1	176935001	176955001	0.305035	0.283488	HZ	SYN3
NC_056056.1	176940001	176960001	0.262275	0.259752	HZ	SYN3
NC_056056.1	177060001	177080001	0.188837	0.251441	HZ	SYN3
NC_056056.1	176975001	176995001	0.123213	0.266757	HZ	SYN3;TIMP3
NC_056056.1	176980001	177000001	0.164983	0.274727	HZ	SYN3;TIMP3
NC_056056.1	176985001	177005001	0.254458	0.28466	HZ	SYN3;TIMP3
NC_056056.1	176990001	177010001	0.262812	0.260206	HZ	SYN3;TIMP3
NC_056056.1	177005001	177025001	0.332786	0.245439	HZ	SYN3;TIMP3
NC_056072.1	37930001	37950001	0.253777	0.28887	HZ	SYNPR
NC_056072.1	37935001	37955001	0.221953	0.284427	HZ	SYNPR
NC_056072.1	37940001	37960001	0.178349	0.246618	HZ	SYNPR
NC_056057.1	48645001	48665001	0.27257	0.342126	HZ	SYPL1
NC_056057.1	48650001	48670001	0.236001	0.348674	HZ	SYPL1
NC_056057.1	48655001	48675001	0.284871	0.291544	HZ	SYPL1
NC_056057.1	48660001	48680001	0.311278	0.247755	HZ	SYPL1
NC_056057.1	48665001	48685001	0.176363	0.276726	HZ	SYPL1
NC_056057.1	48670001	48690001	0.184792	0.287785	HZ	SYPL1
NC_056057.1	48675001	48695001	0.194958	0.242886	HZ	SYPL1
NC_056061.1	82810001	82830001	0.187382	0.441081	HZ	SYTL3
NC_056061.1	82815001	82835001	0.0670139	0.375731	HZ	SYTL3
NC_056061.1	82825001	82845001	0.129065	0.242452	HZ	SYTL3
NC_056069.1	40455001	40475001	0.16944	0.249067	HZ	TARS1
NC_056069.1	40460001	40480001	0.160151	0.253377	HZ	TARS1
NC_056080.1	29745001	29765001	0.333333	0.298474	HZ	TASL
NC_056080.1	29750001	29770001	0.319052	0.310144	HZ	TASL
NC_056066.1	6860001	6880001	0.0600431	0.319723	HZ	TASP1
NC_056066.1	6865001	6885001	0.0532891	0.326869	HZ	TASP1

NC_056057.1	116165001	116185001
NC_056057.1	116170001	116190001
NC_056057.1	116175001	116195001
NC_056057.1	116180001	116200001
NC_056080.1	83155001	83175001
NC_056080.1	83160001	83180001
NC_056080.1	83165001	83185001
NC_056080.1	83170001	83190001
NC_056055.1	209830001	209850001
NC_056055.1	209835001	209855001
NC_056057.1	9435001	9455001
NC_056057.1	9440001	9460001
NC_056055.1	123700001	123720001
NC_056055.1	123705001	123725001
NC_056071.1	22390001	22410001
NC_056055.1	51145001	51165001
NC_056055.1	36445001	36465001
NC_056055.1	36485001	36505001
NC_056055.1	36490001	36510001
NC_056055.1	36495001	36515001
NC_056055.1	83500001	83520001
NC_056055.1	83505001	83525001
NC_056055.1	83510001	83530001
NC_056055.1	68455001	68475001
NC_056054.1	98135001	98155001
NC_056054.1	98165001	98185001
NC_056054.1	98170001	98190001
NC_056054.1	98175001	98195001
NC_056060.1	28000001	28020001
NC_056060.1	28005001	28025001
NC_056060.1	28020001	28040001
NC_056060.1	28025001	28045001
NC_056060.1	28030001	28050001
NC_056060.1	28035001	28055001
NC_056060.1	28040001	28060001
NC_056060.1	28050001	28070001

NC_056066.1	6870001	6890001	0.149425	0.272994	HZ	TASP1
NC_056075.1	15510001	15530001	0.345273	0.283655	HZ	TBC1D12
NC_056075.1	15515001	15535001	0.310495	0.276562	HZ	TBC1D12
NC_056075.1	15520001	15540001	0.243016	0.27101	HZ	TBC1D12
NC_056075.1	15525001	15545001	0.214356	0.272317	HZ	TBC1D12
NC_056075.1	15530001	15550001	0.174104	0.287849	HZ	TBC1D12
NC_056075.1	15535001	15555001	0.135304	0.306915	HZ	TBC1D12
NC_056075.1	15540001	15560001	0.0847294	0.329065	HZ	TBC1D12
NC_056075.1	15545001	15565001	0.0736076	0.335017	HZ	TBC1D12
NC_056075.1	15550001	15570001	0.0711034	0.32534	HZ	TBC1D12
NC_056075.1	15555001	15575001	0.0737979	0.323605	HZ	TBC1D12
NC_056054.1	210660001	210680001	0.256685	0.258971	HZ	TBL1XR1
NC_056070.1	60175001	60195001	0.33939	0.303169	HZ	TBX3
NC_056076.1	55085001	55105001	0.32628	0.273214	HZ	TCF4
NC_056076.1	55090001	55110001	0.253324	0.300374	HZ	TCF4
NC_056076.1	55095001	55115001	0.182766	0.303758	HZ	TCF4
NC_056076.1	55100001	55120001	0.30363	0.273186	HZ	TCF4
NC_056055.1	50165001	50185001	0.339938	0.265079	HZ	TDRD7
NC_056055.1	50170001	50190001	0.338146	0.260559	HZ	TDRD7
NC_056074.1	14490001	14510001	0.262187	0.264646	HZ	TENM4
NC_056074.1	14740001	14760001	0.271587	0.336224	HZ	TENM4
NC_056074.1	14745001	14765001	0.223135	0.261004	HZ	TENM4
NC_056062.1	49350001	49370001	0.0773194	0.329489	HZ	TERF1
NC_056062.1	49355001	49375001	0.0501912	0.312207	HZ	TERF1
NC_056062.1	49365001	49385001	0.0531928	0.268362	HZ	TERF1
NC_056054.1	20070001	20090001	0.0885387	0.316393	HZ	TESK2
NC_056054.1	20075001	20095001	0.202186	0.264543	HZ	TESK2
NC_056064.1	9250001	9270001	0.256218	0.282705	HZ	TEX14
NC_056064.1	9255001	9275001	0.147002	0.255579	HZ	TEX14
NC_056063.1	30250001	30270001	0.171478	0.403804	HZ	TEX26
NC_056058.1	24980001	25000001	0.255662	0.270111	HZ	TEX43
NC_056054.1	256175001	256195001	0.174127	0.352996	HZ	TF
NC_056054.1	256180001	256200001	0.327848	0.334312	HZ	TF
NC_056055.1	121910001	121930001	0.180307	0.333956	HZ	TFPI
NC_056055.1	121915001	121935001	0.201072	0.338409	HZ	TFPI
NC_056056.1	97245001	97265001	0.301629	0.275984	HZ	TGFBRAP1

NC_056060.1	28055001	28075001
NC_056060.1	28060001	28080001
NC_056060.1	28065001	28085001
NC_056054.1	102305001	102325001
NC_056054.1	102310001	102330001
NC_056054.1	102300001	102320001
NC_056057.1	65395001	65415001
NC_056058.1	4440001	4460001
NC_056058.1	4445001	4465001
NC_056056.1	212775001	212795001
NC_056056.1	212780001	212800001
NC_056056.1	212785001	212805001
NC_056076.1	20755001	20775001
NC_056076.1	20760001	20780001
NC_056076.1	20765001	20785001
NC_056076.1	20770001	20790001
NC_056076.1	21085001	21105001
NC_056076.1	21090001	21110001
NC_056061.1	40430001	40450001
NC_056061.1	40435001	40455001
NC_056061.1	40440001	40460001
NC_056061.1	40445001	40465001
NC_056061.1	40450001	40470001
NC_056061.1	40455001	40475001
NC_056061.1	40460001	40480001
NC_056070.1	6270001	6290001
NC_056070.1	6275001	6295001
NC_056070.1	6280001	6300001
NC_056080.1	68535001	68555001
NC_056073.1	11000001	11020001
NC_056073.1	11005001	11025001
NC_056073.1	11010001	11030001
NC_056073.1	11015001	11035001
NC_056074.1	26370001	26390001
NC_056074.1	26375001	26395001
NC_056074.1	26380001	26400001

NC_056056.1	97250001	97270001	0.163107	0.427432	HZ	TGFBRAP1
NC_056056.1	97255001	97275001	0.130852	0.473545	HZ	TGFBRAP1
NC_056056.1	97260001	97280001	0.0502733	0.512427	HZ	TGFBRAP1
NC_056061.1	90845001	90865001	0.0977049	0.257732	HZ	THBS2
NC_056054.1	124650001	124670001	0.0375218	0.330662	HZ	TIAM1
NC_056054.1	124655001	124675001	0.0378966	0.332647	HZ	TIAM1
NC_056054.1	124660001	124680001	0.0458627	0.307989	HZ	TIAM1
NC_056054.1	124665001	124685001	0.0510531	0.25645	HZ	TIAM1
NC_056054.1	124850001	124870001	0.145564	0.257323	HZ	TIAM1
NC_056054.1	124965001	124985001	0.298878	0.288437	HZ	TIAM1
NC_056061.1	79400001	79420001	0.330304	0.260037	HZ	TIAM2
NC_056055.1	67475001	67495001	0.282012	0.276706	HZ	TJP2
NC_056055.1	67480001	67500001	0.217209	0.302377	HZ	TJP2
NC_056060.1	45430001	45450001	0.230477	0.346164	HZ	TLN2
NC_056060.1	45440001	45460001	0.102682	0.251367	HZ	TLN2
NC_056060.1	45890001	45910001	0.2736	0.269469	HZ	TLN2
NC_056075.1	21705001	21725001	0.2409	0.247857	HZ	TLX1
NC_056075.1	21710001	21730001	0.152124	0.35914	HZ	TLX1
NC_056075.1	21715001	21735001	0.262638	0.266616	HZ	TLX1
NC_056055.1	63840001	63860001	0.0505984	0.311284	HZ	TMC1
NC_056055.1	63860001	63880001	0.121354	0.261711	HZ	TMC1
NC_056054.1	256715001	256735001	0.309876	0.247803	HZ	TMEM108
NC_056054.1	256720001	256740001	0.231156	0.359739	HZ	TMEM108
NC_056054.1	256725001	256745001	0.108847	0.473309	HZ	TMEM108
NC_056054.1	256730001	256750001	0.0771946	0.491667	HZ	TMEM108
NC_056054.1	256735001	256755001	0.0929744	0.412431	HZ	TMEM108
NC_056054.1	256740001	256760001	0.0760713	0.241538	HZ	TMEM108
NC_056056.1	103275001	103295001	0.229363	0.247575	HZ	TMEM131
NC_056056.1	103280001	103300001	0.224293	0.253088	HZ	TMEM131
NC_056056.1	103285001	103305001	0.217262	0.27451	HZ	TMEM131
NC_056070.1	5185001	5205001	0.0553455	0.326803	HZ	TMEM154
NC_056061.1	82755001	82775001	0.045913	0.311295	HZ	TMEM181
NC_056061.1	82760001	82780001	0.102788	0.28356	HZ	TMEM181
NC_056061.1	82765001	82785001	0.16947	0.260303	HZ	TMEM181
NC_056060.1	86545001	86565001	0.136548	0.249101	HZ	TMEM63C
NC_056060.1	86550001	86570001	0.0963855	0.277938	HZ	TMEM63C

NC_056074.1	26385001	26405001
NC_056056.1	103845001	103865001
NC_056056.1	103850001	103870001
NC_056056.1	103855001	103875001
NC_056064.1	55880001	55900001
NC_056054.1	112975001	112995001
NC_056072.1	51500001	51520001
NC_056072.1	51505001	51525001
NC_056077.1	27285001	27305001
NC_056077.1	27290001	27310001
NC_056077.1	27295001	27315001
NC_056077.1	27300001	27320001
NC_056071.1	54445001	54465001
NC_056071.1	54450001	54470001
NC_056071.1	54455001	54475001
NC_056074.1	1635001	1655001
NC_056074.1	1645001	1665001
NC_056074.1	1650001	1670001
NC_056063.1	74605001	74625001
NC_056063.1	74610001	74630001
NC_056055.1	30995001	31015001
NC_056055.1	31000001	31020001
NC_056055.1	31005001	31025001
NC_056055.1	31010001	31030001
NC_056055.1	30945001	30965001
NC_056055.1	30950001	30970001
NC_056055.1	31045001	31065001
NC_056068.1	14125001	14145001
NC_056068.1	14130001	14150001
NC_056068.1	14135001	14155001
NC_056068.1	14140001	14160001
NC_056060.1	77165001	77185001
NC_056060.1	77170001	77190001
NC_056060.1	77175001	77195001
NC_056060.1	77180001	77200001
NC_056060.1	77190001	77210001

NC_056060.1	86555001	86575001	0.0496032	0.244497	HZ	TMEM63C
NC_056064.1	36105001	36125001	0.182733	0.34516	HZ	TMEM92
NC_056072.1	32565001	32585001	0.297981	0.24647	HZ	TMF1;UBA3
NC_056064.1	34860001	34880001	0.0873657	0.258023	HZ	TNFRSF13B;USP22
NC_056064.1	34865001	34885001	0.0837131	0.290326	HZ	TNFRSF13B;USP22
NC_056054.1	216790001	216810001	0.211574	0.278234	HZ	TNIK
NC_056054.1	216795001	216815001	0.20873	0.243678	HZ	TNIK
NC_056054.1	216800001	216820001	0.255211	0.24012	HZ	TNIK
NC_056054.1	216805001	216825001	0.316935	0.25817	HZ	TNIK
NC_056054.1	216810001	216830001	0.311553	0.243911	HZ	TNIK
NC_056057.1	94685001	94705001	0.12093	0.240884	HZ	TNPO3
NC_056054.1	256360001	256380001	0.300614	0.283015	HZ	TOPBP1
NC_056054.1	256365001	256385001	0.343396	0.303997	HZ	TOPBP1
NC_056054.1	256395001	256415001	0.331819	0.437159	HZ	TOPBP1
NC_056054.1	256400001	256420001	0.276144	0.459478	HZ	TOPBP1
NC_056054.1	256405001	256425001	0.298926	0.433205	HZ	TOPBP1
NC_056054.1	256410001	256430001	0.18323	0.386076	HZ	TOPBP1
NC_056054.1	256415001	256435001	0.178446	0.314488	HZ	TOPBP1
NC_056065.1	60490001	60510001	0.28651	0.319308	HZ	TOR1AIP1
NC_056065.1	60495001	60515001	0.207213	0.329036	HZ	TOR1AIP1
NC_056065.1	60500001	60520001	0.15074	0.305157	HZ	TOR1AIP1
NC_056054.1	198480001	198500001	0.328389	0.337916	HZ	TP63
NC_056054.1	198485001	198505001	0.231718	0.370872	HZ	TP63
NC_056054.1	198490001	198510001	0.20087	0.385713	HZ	TP63
NC_056054.1	198495001	198515001	0.283955	0.356549	HZ	TP63
NC_056060.1	45185001	45205001	0.293136	0.313978	HZ	TPM1
NC_056065.1	66845001	66865001	0.118334	0.363363	HZ	TPR
NC_056065.1	66850001	66870001	0.314636	0.351695	HZ	TPR
NC_056054.1	198935001	198955001	0.277486	0.257338	HZ	TPRG1
NC_056054.1	22345001	22365001	0.181535	0.240196	HZ	TRABD2B
NC_056054.1	22350001	22370001	0.170346	0.242098	HZ	TRABD2B
NC_056054.1	22355001	22375001	0.159603	0.241885	HZ	TRABD2B
NC_056054.1	264770001	264790001	0.277643	0.251505	HZ	TRAPPC10
NC_056064.1	21515001	21535001	0.207091	0.445581	HZ	TRARG1
NC_056064.1	21520001	21540001	0.165591	0.493035	HZ	TRARG1
NC_056064.1	21525001	21545001	0.131162	0.538899	HZ	TRARG1

NC_056060.1	58980001	59000001
NC_056060.1	58985001	59005001
NC_056060.1	58990001	59010001
NC_056060.1	58995001	59015001
NC_056060.1	59010001	59030001
NC_056060.1	59015001	59035001
NC_056060.1	59020001	59040001
NC_056060.1	59025001	59045001
NC_056055.1	37115001	37135001
NC_056055.1	37120001	37140001
NC_056055.1	37125001	37145001
NC_056055.1	37130001	37150001
NC_056055.1	37135001	37155001
NC_056055.1	37140001	37160001
NC_056055.1	37145001	37165001
NC_056060.1	55920001	55940001
NC_056060.1	55925001	55945001
NC_056060.1	55930001	55950001
NC_056060.1	55935001	55955001
NC_056059.1	36455001	36475001
NC_056059.1	36460001	36480001
NC_056059.1	36465001	36485001
NC_056059.1	36470001	36490001
NC_056059.1	36575001	36595001
NC_056059.1	36580001	36600001
NC_056059.1	36585001	36605001
NC_056057.1	32510001	32530001
NC_056057.1	32515001	32535001
NC_056055.1	28125001	28145001
NC_056072.1	42885001	42905001
NC_056056.1	104305001	104325001
NC_056056.1	104280001	104300001
NC_056056.1	104285001	104305001
NC_056056.1	104290001	104310001
NC_056056.1	104295001	104315001
NC_056067.1	1685001	1705001

NC_056078.1	3350001	3370001	0.267508	0.305563	HZ	TRIM67
NC_056055.1	66050001	66070001	0.340426	0.374675	HZ	TRPM3
NC_056055.1	66055001	66075001	0.254668	0.400595	HZ	TRPM3
NC_056062.1	14875001	14895001	0.144613	0.251524	HZ	TSNARE1
NC_056078.1	3045001	3065001	0.286958	0.319854	HZ	TTC13
NC_056078.1	3050001	3070001	0.257712	0.293235	HZ	TTC13
NC_056056.1	77305001	77325001	0.343537	0.259403	HZ	TTC7A
NC_056056.1	77365001	77385001	0.332594	0.343324	HZ	TTC7A
NC_056060.1	85125001	85145001	0.195246	0.397515	HZ	TTLL5
NC_056060.1	85130001	85150001	0.182128	0.400887	HZ	TTLL5
NC_056060.1	85135001	85155001	0.165316	0.400062	HZ	TTLL5
NC_056060.1	85140001	85160001	0.218664	0.367868	HZ	TTLL5
NC_056060.1	85145001	85165001	0.213992	0.370842	HZ	TTLL5
NC_056060.1	85150001	85170001	0.130406	0.399875	HZ	TTLL5
NC_056060.1	85155001	85175001	0.0864707	0.413085	HZ	TTLL5
NC_056060.1	85160001	85180001	0.204452	0.371025	HZ	TTLL5
NC_056060.1	85165001	85185001	0.324368	0.276938	HZ	TTLL5
NC_056060.1	85170001	85190001	0.331414	0.276804	HZ	TTLL5
NC_056060.1	85195001	85215001	0.261496	0.292232	HZ	TTLL5
NC_056056.1	225350001	225370001	0.333446	0.274917	HZ	TTLL8
NC_056061.1	82490001	82510001	0.29015	0.333942	HZ	TULP4
NC_056061.1	82550001	82570001	0.247589	0.32	HZ	TULP4
NC_056061.1	82600001	82620001	0.331915	0.300488	HZ	TULP4
NC_056061.1	82605001	82625001	0.292569	0.31705	HZ	TULP4
NC_056061.1	82610001	82630001	0.272091	0.328704	HZ	TULP4
NC_056055.1	13000001	13020001	0.180971	0.296469	HZ	TXNDC8
NC_056055.1	13005001	13025001	0.224937	0.303508	HZ	TXNDC8
NC_056055.1	13010001	13030001	0.224424	0.330198	HZ	TXNDC8
NC_056055.1	13015001	13035001	0.254125	0.339216	HZ	TXNDC8
NC_056055.1	13020001	13040001	0.340361	0.334765	HZ	TXNDC8
NC_056055.1	13025001	13045001	0.312417	0.330085	HZ	TXNDC8
NC_056068.1	33085001	33105001	0.278529	0.294104	HZ	UBASH3B
NC_056079.1	41665001	41685001	0.0807176	0.337615	HZ	UBE2E2
NC_056079.1	41670001	41690001	0.0961557	0.307252	HZ	UBE2E2
NC_056079.1	41675001	41695001	0.104379	0.280821	HZ	UBE2E2
NC_056057.1	120035001	120055001	0.227601	0.295464	HZ	UBE3C

NC_056073.1	47985001	48005001
NC_056073.1	47990001	48010001
NC_056068.1	17935001	17955001
NC_056068.1	17940001	17960001
NC_056068.1	17945001	17965001
NC_056068.1	17950001	17970001
NC_056068.1	17955001	17975001
NC_056068.1	17960001	17980001
NC_056057.1	99130001	99150001
NC_056057.1	99135001	99155001
NC_056071.1	62620001	62640001
NC_056054.1	69930001	69950001
NC_056054.1	69935001	69955001
NC_056056.1	40890001	40910001
NC_056056.1	40895001	40915001
NC_056056.1	40900001	40920001
NC_056056.1	40905001	40925001
NC_056060.1	85900001	85920001
NC_056060.1	85905001	85925001
NC_056060.1	85980001	86000001
NC_056060.1	85985001	86005001
NC_056054.1	271420001	271440001
NC_056054.1	271435001	271455001
NC_056072.1	44810001	44830001
NC_056072.1	44815001	44835001
NC_056072.1	44820001	44840001
NC_056072.1	44825001	44845001
NC_056069.1	13695001	13715001
NC_056069.1	13700001	13720001
NC_056069.1	13705001	13725001
NC_056069.1	13710001	13730001
NC_056054.1	87090001	87110001
NC_056054.1	87095001	87115001
NC_056077.1	36245001	36265001
NC_056077.1	36250001	36270001
NC_056077.1	36240001	36260001

NC_056057.1	120040001	120060001	0.1987	0.258584	HZ	UBE3C
NC_056057.1	120050001	120070001	0.126549	0.268011	HZ	UBE3C
NC_056057.1	120055001	120075001	0.11291	0.334216	HZ	UBE3C
NC_056057.1	120060001	120080001	0.113589	0.367003	HZ	UBE3C
NC_056057.1	120065001	120085001	0.170662	0.382216	HZ	UBE3C
NC_056057.1	120070001	120090001	0.198137	0.367707	HZ	UBE3C
NC_056057.1	120075001	120095001	0.241588	0.330676	HZ	UBE3C
NC_056057.1	120080001	120100001	0.256631	0.336005	HZ	UBE3C
NC_056057.1	120085001	120105001	0.220817	0.349926	HZ	UBE3C
NC_056057.1	120090001	120110001	0.167135	0.381834	HZ	UBE3C
NC_056057.1	120095001	120115001	0.161364	0.371452	HZ	UBE3C
NC_056057.1	120100001	120120001	0.166354	0.370874	HZ	UBE3C
NC_056057.1	120105001	120125001	0.164638	0.367164	HZ	UBE3C
NC_056057.1	120110001	120130001	0.204606	0.355684	HZ	UBE3C
NC_056057.1	120115001	120135001	0.201772	0.365959	HZ	UBE3C
NC_056057.1	120120001	120140001	0.168572	0.330731	HZ	UBE3C
NC_056057.1	120125001	120145001	0.155941	0.280654	HZ	UBE3C
NC_056057.1	120130001	120150001	0.185512	0.274502	HZ	UBE3C
NC_056057.1	120135001	120155001	0.184351	0.254726	HZ	UBE3C
NC_056057.1	120140001	120160001	0.229312	0.261864	HZ	UBE3C
NC_056057.1	120145001	120165001	0.316719	0.292392	HZ	UBE3C
NC_056057.1	120150001	120170001	0.338354	0.280794	HZ	UBE3C
NC_056064.1	43755001	43775001	0.254213	0.299968	HZ	UBTF
NC_056055.1	117290001	117310001	0.197574	0.240406	HZ	UGGT1
NC_056055.1	117305001	117325001	0.332977	0.35807	HZ	UGGT1
NC_056072.1	13985001	14005001	0.245166	0.368964	HZ	ULK4
NC_056072.1	13990001	14010001	0.0793793	0.441937	HZ	ULK4
NC_056072.1	13995001	14015001	0.142667	0.387198	HZ	ULK4
NC_056060.1	54045001	54065001	0.215121	0.240661	HZ	UNC13C
NC_056059.1	29765001	29785001	0.288398	0.253424	HZ	UNC5C
NC_056059.1	29770001	29790001	0.200791	0.266117	HZ	UNC5C
NC_056054.1	142040001	142060001	0.255173	0.359257	HZ	USP25
NC_056054.1	142045001	142065001	0.219905	0.38277	HZ	USP25
NC_056054.1	142050001	142070001	0.282427	0.363515	HZ	USP25
NC_056054.1	142090001	142110001	0.324749	0.268454	HZ	USP25
NC_056064.1	12440001	12460001	0.260745	0.258424	HZ	USP32

NC_056059.1	81540001	81560001
NC_056059.1	81545001	81565001
NC_056059.1	81610001	81630001
NC_056059.1	81615001	81635001
NC_056059.1	81620001	81640001
NC_056059.1	81625001	81645001
NC_056059.1	81650001	81670001
NC_056059.1	81655001	81675001
NC_056059.1	81660001	81680001
NC_056059.1	81665001	81685001
NC_056059.1	81670001	81690001
NC_056059.1	81895001	81915001
NC_056054.1	12520001	12540001
NC_056057.1	108020001	108040001
NC_056059.1	89995001	90015001
NC_056059.1	90000001	90020001
NC_056055.1	185350001	185370001
NC_056055.1	185355001	185375001
NC_056055.1	185360001	185380001
NC_056055.1	185365001	185385001
NC_056056.1	77970001	77990001
NC_056062.1	93985001	94005001
NC_056062.1	93990001	94010001
NC_056062.1	93995001	94015001
NC_056062.1	94000001	94020001
NC_056063.1	14195001	14215001
NC_056063.1	14200001	14220001
NC_056063.1	14205001	14225001
NC_056063.1	14210001	14230001
NC_056056.1	8380001	8400001
NC_056056.1	8385001	8405001
NC_056056.1	7600001	7620001
NC_056056.1	7595001	7615001
NC_056065.1	27840001	27860001
NC_056065.1	27845001	27865001
NC_056057.1	118795001	118815001

NC_056064.1	12445001	12465001	0.183815	0.296873	HZ	USP32
NC_056068.1	54000001	54020001	0.088285	0.279306	HZ	UVRAG
NC_056068.1	54005001	54025001	0.254955	0.262196	HZ	UVRAG
NC_056058.1	15450001	15470001	0.142475	0.294759	HZ	VAV1
NC_056054.1	85000001	85020001	0.151307	0.268822	HZ	VAV3
NC_056054.1	85005001	85025001	0.271035	0.26827	HZ	VAV3
NC_056072.1	55620001	55640001	0.335331	0.262474	HZ	VGLL4
NC_056055.1	59610001	59630001	0.337584	0.266039	HZ	VPS13A
NC_056054.1	203135001	203155001	0.268038	0.27059	HZ	VPS8
NC_056054.1	203140001	203160001	0.143759	0.285354	HZ	VPS8
NC_056054.1	203145001	203165001	0.141517	0.304431	HZ	VPS8
NC_056054.1	203150001	203170001	0.145143	0.297191	HZ	VPS8
NC_056054.1	203155001	203175001	0.28479	0.296586	HZ	VPS8
NC_056056.1	65975001	65995001	0.246475	0.335493	HZ	VRK2
NC_056078.1	40265001	40285001	0.292083	0.259404	HZ	WAPL
NC_056056.1	35800001	35820001	0.149367	0.254215	HZ	WDR43
NC_056056.1	35805001	35825001	0.233977	0.292858	HZ	WDR43
NC_056056.1	35810001	35830001	0.231444	0.289901	HZ	WDR43
NC_056056.1	35815001	35835001	0.212446	0.247593	HZ	WDR43
NC_056056.1	35820001	35840001	0.163593	0.250096	HZ	WDR43
NC_056080.1	116450001	116470001	0.265136	0.352486	HZ	WDR44
NC_056080.1	116455001	116475001	0.145475	0.309848	HZ	WDR44
NC_056080.1	116460001	116480001	0.164667	0.262886	HZ	WDR44
NC_056064.1	49685001	49705001	0.186441	0.28398	HZ	WDR45B
NC_056064.1	49690001	49710001	0.31268	0.250505	HZ	WDR45B
NC_056071.1	20440001	20460001	0.284032	0.288556	HZ	WDR93
NC_056055.1	239735001	239755001	0.178861	0.28545	HZ	WDTC1
NC_056055.1	239740001	239760001	0.0979795	0.402907	HZ	WDTC1
NC_056055.1	239745001	239765001	0.162476	0.336155	HZ	WDTC1
NC_056055.1	239750001	239770001	0.257196	0.293859	HZ	WDTC1
NC_056071.1	22395001	22415001	0.191001	0.273844	HZ	WHAMM
NC_056071.1	22400001	22420001	0.152228	0.244408	HZ	WHAMM
NC_056073.1	49950001	49970001	0.3036	0.290289	HZ	WRNIP1
NC_056073.1	49955001	49975001	0.28146	0.374343	HZ	WRNIP1
NC_056079.1	13215001	13235001	0.331803	0.255766	HZ	WWC2
NC_056067.1	5095001	5115001	0.274413	0.521094	HZ	WWOX

NC_056055.1	184150001	184170001
NC_056055.1	184155001	184175001
NC_056057.1	61675001	61695001
NC_056057.1	61680001	61700001
NC_056057.1	61685001	61705001
NC_056057.1	61690001	61710001
NC_056058.1	4450001	4470001
NC_056076.1	50775001	50795001
NC_056056.1	101940001	101960001
NC_056056.1	101945001	101965001
NC_056056.1	101950001	101970001
NC_056056.1	101955001	101975001
NC_056056.1	59215001	59235001
NC_056056.1	59220001	59240001
NC_056056.1	59225001	59245001
NC_056056.1	59230001	59250001
NC_056056.1	59235001	59255001
NC_056056.1	59240001	59260001
NC_056056.1	59245001	59265001
NC_056056.1	59250001	59270001
NC_056056.1	59255001	59275001
NC_056056.1	59260001	59280001
NC_056056.1	59265001	59285001
NC_056056.1	59270001	59290001
NC_056056.1	59275001	59295001
NC_056056.1	59280001	59300001
NC_056074.1	26430001	26450001
NC_056074.1	26435001	26455001
NC_056058.1	103970001	103990001
NC_056058.1	103975001	103995001
NC_056058.1	103980001	104000001
NC_056058.1	103985001	104005001
NC_056058.1	103990001	104010001
NC_056058.1	103995001	104015001
NC_056058.1	104010001	104030001
NC_056058.1	104015001	104035001

NC_056067.1	5100001	5120001	0.182115	0.515007	HZ	WWOX
NC_056067.1	5105001	5125001	0.120101	0.519492	HZ	WWOX
NC_056067.1	5110001	5130001	0.205999	0.389636	HZ	WWOX
NC_056067.1	5115001	5135001	0.246127	0.262666	HZ	WWOX
NC_056066.1	73225001	73245001	0.310769	0.242353	HZ	YWHAB
NC_056056.1	18895001	18915001	0.335111	0.331092	HZ	YWHAQ
NC_056056.1	18900001	18920001	0.227969	0.315242	HZ	YWHAQ
NC_056077.1	36270001	36290001	0.181818	0.338793	HZ	ZAN
NC_056068.1	24300001	24320001	0.303972	0.258657	HZ	ZBTB16
NC_056054.1	180210001	180230001	0.29139	0.396076	HZ	ZBTB20
NC_056054.1	180215001	180235001	0.309804	0.465354	HZ	ZBTB20
NC_056054.1	180220001	180240001	0.267447	0.415143	HZ	ZBTB20
NC_056054.1	180515001	180535001	0.237037	0.274779	HZ	ZBTB20
NC_056054.1	180520001	180540001	0.335441	0.249146	HZ	ZBTB20
NC_056065.1	75510001	75530001	0.300938	0.330519	HZ	ZBTB41
NC_056076.1	48190001	48210001	0.209341	0.239404	HZ	ZBTB7C
NC_056076.1	48195001	48215001	0.163912	0.257277	HZ	ZBTB7C
NC_056076.1	48250001	48270001	0.258082	0.339633	HZ	ZBTB7C
NC_056076.1	48255001	48275001	0.162379	0.337584	HZ	ZBTB7C
NC_056076.1	48260001	48280001	0.251762	0.265977	HZ	ZBTB7C
NC_056080.1	133510001	133530001	0.264706	0.240873	HZ	ZMAT1
NC_056060.1	51745001	51765001	0.291633	0.285387	HZ	ZNF280D
NC_056060.1	51750001	51770001	0.315183	0.274824	HZ	ZNF280D
NC_056067.1	13105001	13125001	0.255451	0.26354	HZ	ZNF469
NC_056067.1	13110001	13130001	0.174528	0.269924	HZ	ZNF469
NC_056067.1	13115001	13135001	0.180889	0.245959	HZ	ZNF469
NC_056067.1	13120001	13140001	0.200441	0.284031	HZ	ZNF469
NC_056067.1	13125001	13145001	0.272764	0.275338	HZ	ZNF469
NC_056055.1	29095001	29115001	0.28	0.284979	HZ	ZNF484
NC_056067.1	46595001	46615001	0.318992	0.300994	HZ	ZNF585B
NC_056054.1	157085001	157105001	0.107937	0.261612	HZ	ZNF654
NC_056054.1	157090001	157110001	0.0795137	0.277735	HZ	ZNF654
NC_056054.1	96975001	96995001	0.140604	0.259835	HZ	ZNF697
NC_056057.1	75010001	75030001	0.0739326	0.424756	HZ	ZNF804B
NC_056057.1	75015001	75035001	0.277936	0.340879	HZ	ZNF804B
NC_056057.1	75020001	75040001	0.339297	0.25823	HZ	ZNF804B

NC_056058.1	104020001	104040001
NC_056058.1	104085001	104105001
NC_056058.1	104090001	104110001
NC_056058.1	104095001	104115001
NC_056058.1	104100001	104120001
NC_056058.1	104180001	104200001
NC_056058.1	104185001	104205001
NC_056054.1	21225001	21245001
NC_056054.1	21230001	21250001
NC_056054.1	21235001	21255001
NC_056054.1	21240001	21260001
NC_056054.1	21245001	21265001
NC_056058.1	81790001	81810001
NC_056080.1	62830001	62850001
NC_056080.1	62835001	62855001
NC_056080.1	62900001	62920001
NC_056080.1	62905001	62925001
NC_056056.1	60905001	60925001
NC_056056.1	60910001	60930001
NC_056055.1	245165001	245185001
NC_056055.1	245170001	245190001
NC_056055.1	245175001	245195001
NC_056061.1	82770001	82790001
NC_056061.1	82775001	82795001
NC_056077.1	34920001	34940001
NC_056068.1	28060001	28080001
NC_056068.1	28065001	28085001
NC_056068.1	28070001	28090001
NC_056068.1	28075001	28095001
NC_056068.1	28080001	28100001
NC_056054.1	70540001	70560001
NC_056066.1	81985001	82005001
NC_056066.1	81990001	82010001
NC_056066.1	81995001	82015001
NC_056055.1	68555001	68575001
NC_056055.1	68560001	68580001

NC_056055.1	175495001	175515001	0.232001	0.240984	HZ	ZRANB3
NC_056055.1	175500001	175520001	0.241066	0.240125	HZ	ZRANB3
NC_056064.1	24525001	24545001	0.318639	0.25009	HZ	ZZEF1

NC_056055.1	68565001	68585001
NC_056055.1	68570001	68590001
NC_056055.1	68575001	68595001
NC_056055.1	68580001	68600001
NC_056055.1	68605001	68625001
NC_056055.1	68610001	68630001
NC_056055.1	68615001	68635001
NC_056055.1	68620001	68640001
NC_056055.1	68625001	68645001
NC_056054.1	71025001	71045001
NC_056054.1	71030001	71050001
NC_056054.1	71035001	71055001
NC_056054.1	71015001	71035001
NC_056054.1	71020001	71040001
NC_056056.1	8055001	8075001
NC_056055.1	37020001	37040001
NC_056055.1	37050001	37070001
NC_056055.1	37055001	37075001
NC_056055.1	37060001	37080001
NC_056055.1	37065001	37085001
NC_056055.1	37070001	37090001
NC_056055.1	37075001	37095001
NC_056064.1	30365001	30385001
NC_056064.1	30370001	30390001
NC_056077.1	19320001	19340001
NC_056077.1	19325001	19345001
NC_056065.1	27360001	27380001
NC_056065.1	27365001	27385001
NC_056065.1	27545001	27565001
NC_056065.1	27550001	27570001
NC_056065.1	27555001	27575001
NC_056065.1	27560001	27580001
NC_056058.1	32345001	32365001
NC_056058.1	32350001	32370001
NC_056058.1	32355001	32375001
NC_056058.1	32360001	32380001

NC_056058.1	32365001	32385001
NC_056080.1	31120001	31140001
NC_056080.1	32285001	32305001
NC_056079.1	23025001	23045001
NC_056079.1	23060001	23080001
NC_056079.1	23065001	23085001
NC_056079.1	23085001	23105001
NC_056063.1	48115001	48135001
NC_056057.1	102640001	102660001
NC_056057.1	102645001	102665001
NC_056062.1	29730001	29750001
NC_056067.1	14275001	14295001
NC_056067.1	14265001	14285001
NC_056067.1	14260001	14280001
NC_056062.1	86635001	86655001
NC_056062.1	86605001	86625001
NC_056062.1	86610001	86630001
NC_056062.1	86615001	86635001
NC_056062.1	86620001	86640001
NC_056062.1	86625001	86645001
NC_056062.1	86630001	86650001
NC_056064.1	13585001	13605001
NC_056064.1	13590001	13610001
NC_056064.1	13595001	13615001
NC_056064.1	13600001	13620001
NC_056056.1	212790001	212810001
NC_056059.1	24855001	24875001
NC_056076.1	52345001	52365001
NC_056076.1	52350001	52370001
NC_056056.1	134990001	135010001
NC_056061.1	90230001	90250001
NC_056061.1	90235001	90255001
NC_056063.1	46715001	46735001
NC_056063.1	46720001	46740001
NC_056054.1	31900001	31920001
NC_056067.1	18660001	18680001

NC_056067.1	18665001	18685001
NC_056067.1	18670001	18690001
NC_056076.1	50240001	50260001
NC_056069.1	14585001	14605001
NC_056069.1	14590001	14610001
NC_056077.1	35295001	35315001
NC_056077.1	35300001	35320001
NC_056077.1	35305001	35325001
NC_056077.1	35310001	35330001
NC_056077.1	35315001	35335001
NC_056080.1	104860001	104880001
NC_056066.1	30695001	30715001
NC_056066.1	30700001	30720001
NC_056065.1	42900001	42920001
NC_056065.1	42905001	42925001
NC_056078.1	22030001	22050001
NC_056078.1	22035001	22055001
NC_056078.1	22040001	22060001
NC_056076.1	48730001	48750001
NC_056059.1	86170001	86190001
NC_056066.1	39145001	39165001
NC_056057.1	103110001	103130001
NC_056057.1	103115001	103135001
NC_056057.1	103120001	103140001
NC_056057.1	103125001	103145001
NC_056057.1	103130001	103150001
NC_056057.1	103160001	103180001
NC_056065.1	75665001	75685001
NC_056065.1	75670001	75690001
NC_056065.1	75675001	75695001
NC_056065.1	75680001	75700001
NC_056069.1	5820001	5840001
NC_056069.1	5825001	5845001
NC_056055.1	49565001	49585001
NC_056055.1	49570001	49590001
NC_056054.1	14895001	14915001

NC_056054.1	14900001	14920001
NC_056054.1	14905001	14925001
NC_056054.1	14910001	14930001
NC_056055.1	229310001	229330001
NC_056055.1	229315001	229335001
NC_056055.1	229320001	229340001
NC_056055.1	229325001	229345001
NC_056057.1	5655001	5675001
NC_056055.1	189515001	189535001
NC_056057.1	111405001	111425001
NC_056057.1	111410001	111430001
NC_056057.1	112190001	112210001
NC_056057.1	112195001	112215001
NC_056057.1	112200001	112220001
NC_056057.1	112205001	112225001
NC_056057.1	112210001	112230001
NC_056057.1	112555001	112575001
NC_056057.1	112565001	112585001
NC_056068.1	9860001	9880001
NC_056068.1	9865001	9885001
NC_056068.1	9870001	9890001
NC_056068.1	9875001	9895001
NC_056072.1	23365001	23385001
NC_056072.1	23370001	23390001
NC_056058.1	63830001	63850001
NC_056058.1	63835001	63855001
NC_056058.1	63840001	63860001
NC_056056.1	103760001	103780001
NC_056054.1	71980001	72000001
NC_056054.1	71985001	72005001
NC_056054.1	71990001	72010001
NC_056073.1	11385001	11405001
NC_056059.1	71690001	71710001
NC_056059.1	71695001	71715001
NC_056059.1	71700001	71720001
NC_056059.1	71610001	71630001

NC_056059.1	71635001	71655001
NC_056059.1	71650001	71670001
NC_056059.1	71655001	71675001
NC_056059.1	71660001	71680001
NC_056059.1	71665001	71685001
NC_056059.1	71670001	71690001
NC_056059.1	71675001	71695001
NC_056059.1	71680001	71700001
NC_056059.1	71685001	71705001
NC_056071.1	57780001	57800001
NC_056071.1	57795001	57815001
NC_056071.1	57800001	57820001
NC_056071.1	57805001	57825001
NC_056056.1	35940001	35960001
NC_056056.1	35945001	35965001
NC_056070.1	53115001	53135001
NC_056070.1	53120001	53140001
NC_056056.1	207255001	207275001
NC_056056.1	207260001	207280001
NC_056077.1	10205001	10225001
NC_056077.1	10210001	10230001
NC_056079.1	13350001	13370001
NC_056079.1	13355001	13375001
NC_056056.1	60210001	60230001
NC_056063.1	21855001	21875001
NC_056063.1	21860001	21880001
NC_056063.1	21830001	21850001
NC_056063.1	21835001	21855001
NC_056063.1	21840001	21860001
NC_056063.1	21845001	21865001
NC_056063.1	21850001	21870001
NC_056064.1	39070001	39090001
NC_056064.1	39065001	39085001
NC_056071.1	28295001	28315001
NC_056056.1	104095001	104115001
NC_056056.1	104080001	104100001

NC_056056.1	104085001	104105001
NC_056056.1	104090001	104110001
NC_056056.1	104075001	104095001
NC_056055.1	135325001	135345001
NC_056055.1	135330001	135350001
NC_056055.1	135335001	135355001
NC_056078.1	11570001	11590001
NC_056078.1	11575001	11595001
NC_056078.1	11580001	11600001
NC_056078.1	11585001	11605001
NC_056078.1	11590001	11610001
NC_056078.1	11595001	11615001
NC_056078.1	11600001	11620001
NC_056078.1	11635001	11655001
NC_056078.1	11640001	11660001
NC_056078.1	11645001	11665001
NC_056078.1	11650001	11670001
NC_056078.1	11655001	11675001
NC_056078.1	11660001	11680001
NC_056065.1	34435001	34455001
NC_056072.1	57990001	58010001
NC_056066.1	39185001	39205001
NC_056066.1	39190001	39210001
NC_056066.1	39195001	39215001
NC_056066.1	39200001	39220001
NC_056066.1	39205001	39225001
NC_056066.1	39310001	39330001
NC_056066.1	39315001	39335001
NC_056055.1	184955001	184975001
NC_056055.1	184960001	184980001
NC_056055.1	184965001	184985001
NC_056055.1	184970001	184990001
NC_056068.1	14105001	14125001
NC_056068.1	14110001	14130001
NC_056068.1	14115001	14135001
NC_056068.1	14120001	14140001

NC_056068.1	14100001	14120001
NC_056065.1	60575001	60595001
NC_056066.1	64730001	64750001
NC_056068.1	27730001	27750001
NC_056068.1	27735001	27755001
NC_056068.1	27740001	27760001
NC_056060.1	90260001	90280001
NC_056073.1	21895001	21915001
NC_056080.1	132870001	132890001
NC_056080.1	132875001	132895001
NC_056080.1	132880001	132900001
NC_056066.1	15125001	15145001
NC_056056.1	134890001	134910001
NC_056060.1	41520001	41540001
NC_056060.1	41525001	41545001
NC_056060.1	21620001	21640001
NC_056060.1	21625001	21645001
NC_056054.1	12515001	12535001
NC_056072.1	51685001	51705001
NC_056072.1	51690001	51710001
NC_056056.1	209950001	209970001
NC_056055.1	209820001	209840001
NC_056066.1	17410001	17430001
NC_056066.1	17415001	17435001
NC_056066.1	17420001	17440001
NC_056066.1	17425001	17445001
NC_056066.1	17430001	17450001
NC_056066.1	17435001	17455001
NC_056066.1	17440001	17460001
NC_056066.1	17445001	17465001
NC_056066.1	17450001	17470001
NC_056066.1	17455001	17475001
NC_056066.1	17460001	17480001
NC_056066.1	17465001	17485001
NC_056066.1	17470001	17490001
NC_056058.1	84660001	84680001

NC_056060.1	49360001	49380001
NC_056055.1	36900001	36920001
NC_056055.1	36905001	36925001
NC_056077.1	34475001	34495001
NC_056055.1	36855001	36875001
NC_056055.1	229950001	229970001
NC_056056.1	68640001	68660001
NC_056056.1	68645001	68665001
NC_056056.1	68650001	68670001
NC_056056.1	68655001	68675001
NC_056056.1	68660001	68680001
NC_056056.1	68665001	68685001
NC_056056.1	68670001	68690001
NC_056056.1	68675001	68695001
NC_056056.1	68680001	68700001
NC_056056.1	68685001	68705001
NC_056056.1	68690001	68710001
NC_056056.1	68695001	68715001
NC_056070.1	52750001	52770001
NC_056072.1	51455001	51475001
NC_056072.1	51460001	51480001
NC_056060.1	69900001	69920001
NC_056060.1	69905001	69925001
NC_056060.1	69880001	69900001
NC_056060.1	69885001	69905001
NC_056060.1	69890001	69910001
NC_056060.1	69895001	69915001
NC_056064.1	50640001	50660001
NC_056057.1	33100001	33120001
NC_056057.1	33105001	33125001
NC_056076.1	8630001	8650001
NC_056076.1	8635001	8655001
NC_056055.1	68470001	68490001
NC_056055.1	68475001	68495001
NC_056055.1	68480001	68500001
NC_056055.1	68485001	68505001

NC_056055.1	68490001	68510001
NC_056055.1	68530001	68550001
NC_056055.1	68535001	68555001
NC_056055.1	68540001	68560001
NC_056067.1	47505001	47525001
NC_056072.1	51625001	51645001
NC_056072.1	51630001	51650001
NC_056072.1	51615001	51635001
NC_056072.1	51620001	51640001
NC_056080.1	40910001	40930001
NC_056059.1	12175001	12195001
NC_056059.1	12180001	12200001
NC_056059.1	12185001	12205001
NC_056059.1	12190001	12210001
NC_056059.1	12195001	12215001
NC_056059.1	12200001	12220001
NC_056059.1	12205001	12225001
NC_056059.1	12210001	12230001
NC_056058.1	59365001	59385001
NC_056058.1	59370001	59390001
NC_056058.1	59375001	59395001
NC_056057.1	88545001	88565001
NC_056057.1	88550001	88570001
NC_056057.1	88555001	88575001
NC_056057.1	88560001	88580001
NC_056072.1	46720001	46740001
NC_056056.1	95640001	95660001
NC_056075.1	45405001	45425001
NC_056066.1	30365001	30385001
NC_056054.1	113530001	113550001
NC_056054.1	113535001	113555001
NC_056054.1	113570001	113590001
NC_056054.1	113580001	113600001
NC_056054.1	113585001	113605001
NC_056072.1	50875001	50895001
NC_056072.1	50850001	50870001

NC_056072.1	50855001	50875001
NC_056068.1	74855001	74875001
NC_056068.1	74860001	74880001
NC_056068.1	74865001	74885001
NC_056066.1	64740001	64760001
NC_056065.1	64855001	64875001
NC_056065.1	64860001	64880001
NC_056065.1	64865001	64885001
NC_056064.1	56355001	56375001
NC_056058.1	5525001	5545001
NC_056058.1	5520001	5540001
NC_056074.1	46430001	46450001
NC_056074.1	46435001	46455001
NC_056074.1	46440001	46460001
NC_056074.1	46460001	46480001
NC_056074.1	46465001	46485001
NC_056074.1	46470001	46490001
NC_056074.1	46475001	46495001
NC_056074.1	46480001	46500001
NC_056074.1	46485001	46505001
NC_056074.1	46490001	46510001
NC_056057.1	99995001	100015001
NC_056057.1	100000001	100020001
NC_056057.1	100005001	100025001
NC_056063.1	48085001	48105001
NC_056063.1	48100001	48120001
NC_056063.1	48105001	48125001
NC_056063.1	48110001	48130001
NC_056063.1	48090001	48110001
NC_056063.1	48095001	48115001
NC_056054.1	100005001	100025001
NC_056075.1	50250001	50270001
NC_056064.1	21265001	21285001
NC_056056.1	91400001	91420001
NC_056056.1	91405001	91425001
NC_056056.1	91410001	91430001

NC_056056.1	91415001	91435001
NC_056056.1	91420001	91440001
NC_056056.1	91425001	91445001
NC_056056.1	91445001	91465001
NC_056056.1	91450001	91470001
NC_056056.1	91455001	91475001
NC_056056.1	91460001	91480001
NC_056054.1	200380001	200400001
NC_056054.1	200385001	200405001
NC_056054.1	200390001	200410001
NC_056054.1	200395001	200415001
NC_056067.1	55785001	55805001
NC_056067.1	55790001	55810001
NC_056067.1	55795001	55815001
NC_056067.1	55800001	55820001
NC_056057.1	64830001	64850001
NC_056057.1	64840001	64860001
NC_056057.1	64845001	64865001
NC_056057.1	64935001	64955001
NC_056057.1	65270001	65290001
NC_056057.1	65275001	65295001
NC_056059.1	23325001	23345001
NC_056059.1	23330001	23350001
NC_056059.1	23335001	23355001
NC_056059.1	23340001	23360001
NC_056059.1	23345001	23365001
NC_056077.1	38090001	38110001
NC_056077.1	38095001	38115001
NC_056077.1	38100001	38120001
NC_056077.1	38105001	38125001
NC_056077.1	38110001	38130001
NC_056077.1	38115001	38135001
NC_056077.1	38120001	38140001
NC_056077.1	38125001	38145001
NC_056079.1	32425001	32445001
NC_056061.1	47500001	47520001

NC_056061.1	47505001	47525001
NC_056072.1	51655001	51675001
NC_056072.1	51635001	51655001
NC_056072.1	51640001	51660001
NC_056072.1	51645001	51665001
NC_056072.1	51650001	51670001
NC_056056.1	35240001	35260001
NC_056056.1	35245001	35265001
NC_056056.1	35250001	35270001
NC_056056.1	35255001	35275001
NC_056056.1	35260001	35280001
NC_056056.1	35265001	35285001
NC_056064.1	49335001	49355001
NC_056054.1	204630001	204650001
NC_056062.1	3145001	3165001
NC_056062.1	3150001	3170001
NC_056055.1	142150001	142170001
NC_056055.1	142155001	142175001
NC_056080.1	63130001	63150001
NC_056080.1	63135001	63155001
NC_056080.1	63120001	63140001
NC_056080.1	63125001	63145001
NC_056060.1	26800001	26820001
NC_056054.1	21215001	21235001
NC_056054.1	21220001	21240001
NC_056054.1	21175001	21195001
NC_056054.1	21180001	21200001
NC_056054.1	21185001	21205001
NC_056054.1	21190001	21210001
NC_056054.1	21195001	21215001
NC_056054.1	21200001	21220001
NC_056060.1	77285001	77305001
NC_056060.1	77290001	77310001
NC_056060.1	77305001	77325001
NC_056060.1	77310001	77330001
NC_056072.1	54655001	54675001

NC_056072.1	54660001	54680001
NC_056063.1	85705001	85725001
NC_056058.1	79575001	79595001
NC_056058.1	79580001	79600001
NC_056056.1	38420001	38440001
NC_056056.1	38400001	38420001
NC_056056.1	38405001	38425001
NC_056056.1	38410001	38430001
NC_056056.1	38415001	38435001
NC_056057.1	13570001	13590001
NC_056057.1	13575001	13595001
NC_056057.1	13580001	13600001
NC_056058.1	92490001	92510001
NC_056060.1	55940001	55960001
NC_056077.1	37445001	37465001
NC_056077.1	37450001	37470001
NC_056077.1	37455001	37475001
NC_056077.1	37460001	37480001
NC_056077.1	37465001	37485001
NC_056077.1	37470001	37490001
NC_056056.1	188120001	188140001
NC_056059.1	72620001	72640001
NC_056059.1	72625001	72645001
NC_056059.1	72610001	72630001
NC_056054.1	162965001	162985001
NC_056054.1	162970001	162990001
NC_056054.1	162975001	162995001
NC_056054.1	162980001	163000001
NC_056054.1	162985001	163005001
NC_056054.1	162990001	163010001
NC_056072.1	51075001	51095001
NC_056072.1	51080001	51100001
NC_056072.1	51070001	51090001
NC_056056.1	103900001	103920001
NC_056056.1	103905001	103925001
NC_056056.1	103910001	103930001

NC_056056.1	103915001	103935001
NC_056061.1	81210001	81230001
NC_056061.1	81215001	81235001
NC_056061.1	81220001	81240001
NC_056061.1	81225001	81245001
NC_056072.1	44275001	44295001
NC_056080.1	9490001	9510001
NC_056080.1	9495001	9515001
NC_056061.1	55265001	55285001
NC_056061.1	55270001	55290001
NC_056061.1	55275001	55295001
NC_056061.1	55280001	55300001
NC_056061.1	55285001	55305001
NC_056061.1	55290001	55310001
NC_056061.1	55295001	55315001
NC_056060.1	28240001	28260001
NC_056060.1	28245001	28265001
NC_056060.1	28250001	28270001
NC_056060.1	28265001	28285001
NC_056060.1	28270001	28290001
NC_056060.1	41110001	41130001
NC_056068.1	27225001	27245001
NC_056068.1	27220001	27240001
NC_056060.1	44960001	44980001
NC_056058.1	41525001	41545001
NC_056074.1	46790001	46810001
NC_056074.1	46795001	46815001
NC_056074.1	46800001	46820001
NC_056074.1	46805001	46825001
NC_056058.1	12935001	12955001
NC_056056.1	38530001	38550001
NC_056058.1	5555001	5575001
NC_056058.1	5560001	5580001
NC_056058.1	5565001	5585001
NC_056056.1	170400001	170420001
NC_056056.1	170405001	170425001

NC_056062.1	70000001	70020001
NC_056062.1	70005001	70025001
NC_056062.1	70010001	70030001
NC_056062.1	70015001	70035001
NC_056054.1	92060001	92080001
NC_056054.1	72050001	72070001
NC_056054.1	72055001	72075001
NC_056054.1	72060001	72080001
NC_056055.1	50415001	50435001
NC_056055.1	50420001	50440001
NC_056055.1	50425001	50445001
NC_056055.1	50430001	50450001
NC_056060.1	50425001	50445001
NC_056060.1	50430001	50450001
NC_056060.1	50435001	50455001
NC_056060.1	50450001	50470001
NC_056060.1	50455001	50475001
NC_056067.1	49030001	49050001
NC_056067.1	49020001	49040001
NC_056067.1	49025001	49045001
NC_056067.1	49080001	49100001
NC_056067.1	49035001	49055001
NC_056067.1	49040001	49060001
NC_056067.1	49045001	49065001
NC_056067.1	49075001	49095001
NC_056061.1	49600001	49620001
NC_056061.1	49605001	49625001
NC_056061.1	49610001	49630001
NC_056054.1	5145001	5165001
NC_056054.1	5150001	5170001
NC_056080.1	86805001	86825001
NC_056080.1	86810001	86830001
NC_056080.1	86815001	86835001
NC_056080.1	86820001	86840001
NC_056080.1	86825001	86845001
NC_056065.1	32060001	32080001

NC_056065.1	32065001	32085001
NC_056065.1	32070001	32090001
NC_056056.1	104215001	104235001
NC_056067.1	55810001	55830001
NC_056067.1	55815001	55835001
NC_056067.1	55805001	55825001
NC_056058.1	87750001	87770001
NC_056058.1	87755001	87775001
NC_056058.1	87760001	87780001
NC_056058.1	87765001	87785001
NC_056058.1	87770001	87790001
NC_056059.1	78310001	78330001
NC_056059.1	78315001	78335001
NC_056061.1	67050001	67070001
NC_056061.1	67055001	67075001
NC_056073.1	20665001	20685001
NC_056062.1	5165001	5185001
NC_056062.1	5590001	5610001
NC_056062.1	5595001	5615001
NC_056062.1	5610001	5630001
NC_056075.1	30110001	30130001
NC_056075.1	30115001	30135001
NC_056075.1	30120001	30140001
NC_056075.1	30125001	30145001
NC_056075.1	30130001	30150001
NC_056075.1	30135001	30155001
NC_056075.1	30140001	30160001
NC_056075.1	30145001	30165001
NC_056075.1	30150001	30170001
NC_056075.1	30155001	30175001
NC_056077.1	3835001	3855001
NC_056077.1	3870001	3890001
NC_056077.1	3875001	3895001
NC_056054.1	188650001	188670001
NC_056054.1	188655001	188675001
NC_056054.1	188660001	188680001

NC_056054.1	188665001	188685001
NC_056054.1	188670001	188690001
NC_056056.1	32350001	32370001
NC_056069.1	65765001	65785001
NC_056069.1	65770001	65790001
NC_056072.1	36980001	37000001
NC_056072.1	36985001	37005001
NC_056072.1	36990001	37010001
NC_056069.1	14140001	14160001
NC_056069.1	14145001	14165001
NC_056069.1	14150001	14170001
NC_056069.1	14155001	14175001
NC_056069.1	14160001	14180001
NC_056069.1	14165001	14185001
NC_056069.1	14200001	14220001
NC_056069.1	14205001	14225001
NC_056069.1	14210001	14230001
NC_056075.1	45390001	45410001
NC_056066.1	67540001	67560001
NC_056056.1	104920001	104940001
NC_056056.1	104925001	104945001
NC_056056.1	104930001	104950001
NC_056056.1	104935001	104955001
NC_056056.1	104940001	104960001
NC_056056.1	104995001	105015001
NC_056056.1	105015001	105035001
NC_056056.1	105020001	105040001
NC_056056.1	105035001	105055001
NC_056056.1	105040001	105060001
NC_056056.1	105045001	105065001
NC_056056.1	105050001	105070001
NC_056056.1	105170001	105190001
NC_056056.1	105175001	105195001
NC_056056.1	105180001	105200001
NC_056056.1	105185001	105205001
NC_056056.1	105190001	105210001

NC_056056.1	105195001	105215001
NC_056056.1	105200001	105220001
NC_056056.1	105205001	105225001
NC_056056.1	105210001	105230001
NC_056056.1	105215001	105235001
NC_056056.1	105220001	105240001
NC_056056.1	105225001	105245001
NC_056056.1	105230001	105250001
NC_056056.1	105235001	105255001
NC_056065.1	28430001	28450001
NC_056065.1	28475001	28495001
NC_056057.1	8280001	8300001
NC_056064.1	51030001	51050001
NC_056064.1	51035001	51055001

Pi	Fst	Region	Gene	OL vs HZ<br>CHROM	Start	end	Pi	Fst	Region	Gene
0.440516	0.179088	HZ	ZRANB3	NC_056056.1	206655001	206675001	0.483584	0.158891	HZ	A2ML1
0.464265	0.171304	HZ	ZRANB3	NC_056056.1	5825001	5845001	0.384549	0.175479	HZ	ABL1
0.427595	0.15853	HZ	ZRANB2	NC_056068.1	63600001	63620001	0.352105	0.238162	HZ	ABTB2
0.317193	0.187023	HZ	ZRANB2	NC_056068.1	63605001	63625001	0.240185	0.22568	HZ	ABTB2
0.406737	0.178369	HZ	ZRANB2	NC_056068.1	63610001	63630001	0.239374	0.204795	HZ	ABTB2
0.514926	0.215024	HZ	ZNF689	NC_056063.1	52685001	52705001	0.483063	0.269189	HZ	ACOD1
0.43116	0.315365	HZ	ZNF514	NC_056056.1	104995001	105015001	0.28187	0.167861	HZ	ACOXL
0.511539	0.365183	HZ	ZNF514	NC_056056.1	105020001	105040001	0.44068	0.195846	HZ	ACOXL
0.314117	0.156187	HZ	ZNF41	NC_056056.1	105025001	105045001	0.426405	0.263694	HZ	ACOXL
0.150198	0.163599	HZ	ZNF41	NC_056056.1	105030001	105050001	0.356205	0.276873	HZ	ACOXL
0.147269	0.219294	HZ	ZNF41	NC_056056.1	105035001	105055001	0.249868	0.247186	HZ	ACOXL
0.347732	0.29659	HZ	ZNF2;ZNF514	NC_056056.1	105040001	105060001	0.145684	0.184434	HZ	ACOXL
0.379958	0.275043	HZ	ZNF2;ZNF514	NC_056056.1	105045001	105065001	0.137154	0.202631	HZ	ACOXL
0.415094	0.269167	HZ	ZNF2;ZNF514	NC_056056.1	105050001	105070001	0.179092	0.198862	HZ	ACOXL
0.4887	0.193522	HZ	ZNF175	NC_056056.1	105055001	105075001	0.25	0.227304	HZ	ACOXL
0.160919	0.143056	HZ	ZHX1	NC_056056.1	105060001	105080001	0.350048	0.285527	HZ	ACOXL
0.175365	0.149145	HZ	ZHX1	NC_056056.1	105065001	105085001	0.411155	0.287412	HZ	ACOXL
0.320129	0.200475	HZ	ZFP37	NC_056056.1	105070001	105090001	0.402877	0.313401	HZ	ACOXL
0.265425	0.225377	HZ	ZFP37	NC_056056.1	105075001	105095001	0.3162	0.292413	HZ	ACOXL
0.421927	0.249559	HZ	ZFC3H1	NC_056056.1	105080001	105100001	0.18744	0.273036	HZ	ACOXL
0.323044	0.162666	HZ	ZFAT	NC_056056.1	105085001	105105001	0.148945	0.233244	HZ	ACOXL
0.258336	0.198405	HZ	ZFAT	NC_056056.1	105090001	105110001	0.216367	0.171686	HZ	ACOXL
0.388019	0.153323	HZ	ZFAT	NC_056072.1	47120001	47140001	0.477115	0.473652	HZ	ACTR8;SELEN
0.372328	0.150794	HZ	ZEB2	NC_056066.1	45855001	45875001	0.491536	0.183657	HZ	ADARB2
0.398516	0.158602	HZ	ZEB2	NC_056067.1	2350001	2370001	0.432863	0.176718	HZ	ADAT1;GABA
0.278	0.164592	HZ	XKR6	NC_056059.1	42550001	42570001	0.297463	0.28465	HZ	ADGRA3
0.344537	0.144799	HZ	XKR6	NC_056059.1	42555001	42575001	0.447812	0.234964	HZ	ADGRA3
0.456373	0.206477	HZ	WWC2	NC_056059.1	77660001	77680001	0.342973	0.191376	HZ	ADGRL3
0.29682	0.202901	HZ	WHAMM	NC_056059.1	77665001	77685001	0.306458	0.185415	HZ	ADGRL3
0.34435	0.169951	HZ	WHAMM	NC_056059.1	77670001	77690001	0.37136	0.169927	HZ	ADGRL3
0.353657	0.244709	HZ	WDTC1	NC_056058.1	88300001	88320001	0.341574	0.356059	HZ	ADGRV1
0.222222	0.358708	HZ	WDTC1	NC_056058.1	88305001	88325001	0.193959	0.474252	HZ	ADGRV1
0.346873	0.26806	HZ	WDTC1	NC_056058.1	88310001	88330001	0.367188	0.387845	HZ	ADGRV1

0.473275	0.218657	HZ	WDTC1
0.336796	0.2035	HZ	WDR75
0.376085	0.217678	HZ	WDR75
0.475185	0.230573	HZ	WDR75
0.53052	0.219203	HZ	WDR75
0.49742	0.344035	HZ	WARS2
0.502262	0.358459	HZ	WARS2
0.535971	0.278047	HZ	VSIG4
0.519945	0.266337	HZ	VSIG4
0.464078	0.288815	HZ	VSIG4
0.524074	0.271583	HZ	VSIG4
0.537373	0.269437	HZ	VSIG4
0.29366	0.142444	HZ	VDAC1
0.177538	0.16076	HZ	VDAC1
0.269766	0.162882	HZ	VDAC1
0.358095	0.145492	HZ	VDAC1
0.313761	0.200479	HZ	VCPKMT
0.143802	0.260237	HZ	VCPKMT
0.0816854	0.271281	HZ	VCPKMT
0.120141	0.262665	HZ	VCPKMT
0.119689	0.269993	HZ	VCPKMT
0.525643	0.150262	HZ	UTS2B
0.501787	0.239185	HZ	USP25
0.452582	0.224619	HZ	USP25
0.335926	0.221561	HZ	USP25
0.324683	0.220306	HZ	USP25
0.35994	0.264441	HZ	USP25
0.385941	0.291131	HZ	USP25
0.428167	0.285198	HZ	USP25
0.450703	0.285376	HZ	USP25
0.440939	0.247637	HZ	USP25
0.457081	0.207271	HZ	USP25
0.494265	0.167061	HZ	USP25
0.511702	0.175742	HZ	USP25
0.440423	0.212717	HZ	USP25
0.48399	0.236548	HZ	USP25

NC_056059.1	115865001	115885001	0.0451349	0.319082	HZ	ADRA2C
NC_056059.1	115870001	115890001	0.261186	0.265635	HZ	ADRA2C
NC_056059.1	115875001	115895001	0.359873	0.201729	HZ	ADRA2C
NC_056071.1	16395001	16415001	0.452297	0.185141	HZ	AGBL1
NC_056061.1	60580001	60600001	0.341873	0.297229	HZ	AHI1
NC_056061.1	60585001	60605001	0.301871	0.291304	HZ	AHI1
NC_056061.1	60590001	60610001	0.260511	0.293775	HZ	AHI1
NC_056061.1	60595001	60615001	0.209121	0.240405	HZ	AHI1
NC_056061.1	60600001	60620001	0.247361	0.255587	HZ	AHI1
NC_056061.1	60605001	60625001	0.278009	0.237483	HZ	AHI1
NC_056061.1	60610001	60630001	0.359673	0.183267	HZ	AHI1
NC_056074.1	37345001	37365001	0.132242	0.285835	HZ	AHNAK
NC_056074.1	37350001	37370001	0.29682	0.347537	HZ	AHNAK
NC_056074.1	37355001	37375001	0.464226	0.309813	HZ	AHNAK
NC_056074.1	37330001	37350001	0.132327	0.207797	HZ	AHNAK;SCGB
NC_056064.1	33485001	33505001	0.405654	0.175161	HZ	AKAP10;SPEC
NC_056071.1	15640001	15660001	0.033997	0.235924	HZ	AKAP13
NC_056071.1	15645001	15665001	0.321746	0.163934	HZ	AKAP13
NC_056057.1	10020001	10040001	0.405368	0.266572	HZ	AKAP9
NC_056057.1	10025001	10045001	0.376141	0.172944	HZ	AKAP9
NC_056054.1	170980001	171000001	0.328089	0.160706	HZ	ALCAM
NC_056054.1	170985001	171005001	0.261012	0.158637	HZ	ALCAM
NC_056055.1	63775001	63795001	0.103146	0.183696	HZ	ALDH1A1
NC_056061.1	60230001	60250001	0.163743	0.185139	HZ	ALDH8A1
NC_056059.1	13575001	13595001	0.39061	0.171717	HZ	ALPK1
NC_056067.1	23695001	23715001	0.414577	0.163441	HZ	AMFR;NUDT2
NC_056067.1	23700001	23720001	0.39629	0.176715	HZ	AMFR;NUDT2
NC_056057.1	84045001	84065001	0.445634	0.291658	HZ	AMPH
NC_056057.1	84040001	84060001	0.478097	0.255854	HZ	AMPH;LOC10
NC_056065.1	41690001	41710001	0.211049	0.306173	HZ	ANGPTL7;MT
NC_056065.1	41695001	41715001	0.265152	0.297471	HZ	ANGPTL7;MT
NC_056065.1	41700001	41720001	0.284256	0.317118	HZ	ANGPTL7;MT
NC_056065.1	41705001	41725001	0.399665	0.294914	HZ	ANGPTL7;MT
NC_056065.1	41710001	41730001	0.478453	0.258287	HZ	ANGPTL7;MT
NC_056065.1	41715001	41735001	0.467844	0.266529	HZ	ANGPTL7;MT
NC_056076.1	33325001	33345001	0.356364	0.246851	HZ	ANKRD29

0.5301	0.173012	HZ	USP14	NC_056076.1	33330001	33350001	0.263359	0.257347	HZ	ANKRD29
0.53743	0.163855	HZ	USP14	NC_056076.1	33335001	33355001	0.208299	0.221189	HZ	ANKRD29
0.440308	0.190995	HZ	USP14	NC_056076.1	33340001	33360001	0.280477	0.214266	HZ	ANKRD29
0.525926	0.173026	HZ	USP14	NC_056076.1	33345001	33365001	0.262342	0.24252	HZ	ANKRD29
0.483272	0.145763	HZ	USP10	NC_056076.1	33350001	33370001	0.223404	0.261408	HZ	ANKRD29
0.538597	0.143726	HZ	UPK1B	NC_056060.1	6530001	6550001	0.293215	0.203237	HZ	ANKRD31;GC[illegible]
0.311484	0.215383	HZ	UPK1B	NC_056061.1	48060001	48080001	0.41175	0.290957	HZ	ANKRD6
0.352832	0.213492	HZ	UPK1B	NC_056061.1	48065001	48085001	0.3896	0.292928	HZ	ANKRD6
0.496708	0.141987	HZ	UNC93B1	NC_056061.1	48070001	48090001	0.355895	0.252971	HZ	ANKRD6
0.469781	0.157071	HZ	UNC5D	NC_056061.1	48075001	48095001	0.408777	0.16423	HZ	ANKRD6
0.489589	0.148504	HZ	UNC5D	NC_056056.1	168530001	168550001	0.4665	0.170264	HZ	ANKS1B
0.485315	0.166651	HZ	UNC5D	NC_056056.1	168535001	168555001	0.487243	0.205873	HZ	ANKS1B
0.470356	0.153091	HZ	UNC5C	NC_056056.1	168600001	168620001	0.110517	0.251512	HZ	ANKS1B
0.166139	0.148194	HZ	UNC5A	NC_056056.1	168605001	168625001	0.119384	0.226991	HZ	ANKS1B
0.228158	0.168067	HZ	UNC5A	NC_056056.1	168610001	168630001	0.25481	0.157676	HZ	ANKS1B
0.293227	0.209458	HZ	UNC5A	NC_056056.1	169385001	169405001	0.207704	0.162687	HZ	ANKS1B
0.299088	0.216751	HZ	UNC5A	NC_056056.1	169390001	169410001	0.210307	0.15855	HZ	ANKS1B
0.212602	0.252997	HZ	UNC5A	NC_056059.1	95385001	95405001	0.355669	0.171506	HZ	ANTXR2
0.12244	0.263395	HZ	UNC5A	NC_056059.1	95390001	95410001	0.292476	0.210137	HZ	ANTXR2
0.0530726	0.224376	HZ	UNC5A	NC_056059.1	95395001	95415001	0.278807	0.206191	HZ	ANTXR2
0.0502744	0.21875	HZ	UNC5A	NC_056059.1	95400001	95420001	0.355844	0.185256	HZ	ANTXR2
0.0463803	0.175368	HZ	UNC5A	NC_056057.1	62265001	62285001	0.43245	0.163288	HZ	AOAH
0.458621	0.262452	HZ	ULK4	NC_056057.1	62270001	62290001	0.364348	0.221934	HZ	AOAH
0.27358	0.352929	HZ	ULK4	NC_056060.1	44940001	44960001	0.16413	0.181156	HZ	APH1B
0.0710236	0.444219	HZ	ULK4	NC_056060.1	44945001	44965001	0.190977	0.184079	HZ	APH1B
0.0981045	0.411296	HZ	ULK4	NC_056060.1	44950001	44970001	0.176163	0.175549	HZ	APH1B
0.209504	0.279169	HZ	ULK4	NC_056060.1	44955001	44975001	0.229382	0.165701	HZ	APH1B;RAB8[illegible]
0.21831	0.147839	HZ	UIMC1	NC_056059.1	56365001	56385001	0.212698	0.197109	HZ	ARAP2
0.178191	0.144941	HZ	UIMC1	NC_056062.1	44365001	44385001	0.437384	0.167482	HZ	ARFGEF1
0.128902	0.199339	HZ	UIMC1	NC_056062.1	44370001	44390001	0.489249	0.174218	HZ	ARFGEF1
0.130848	0.206621	HZ	UIMC1	NC_056066.1	77105001	77125001	0.490772	0.166157	HZ	ARFGEF2
0.120421	0.230605	HZ	UIMC1	NC_056070.1	5025001	5045001	0.224891	0.233054	HZ	ARFIP1
0.125214	0.234826	HZ	UIMC1	NC_056070.1	5030001	5050001	0.391076	0.200458	HZ	ARFIP1
0.136594	0.214368	HZ	UIMC1	NC_056060.1	28265001	28285001	0.432343	0.161637	HZ	ARHGAP11A
0.176391	0.193681	HZ	UIMC1	NC_056060.1	28275001	28295001	0.452216	0.213417	HZ	ARHGAP11A
0.410959	0.16784	HZ	UCHL3	NC_056060.1	28245001	28265001	0.327596	0.176416	HZ	ARHGAP11A;[illegible]

0.405329	0.17264	HZ	UCHL3
0.436413	0.15619	HZ	UCHL3
0.402262	0.171442	HZ	UBE3C
0.401707	0.179789	HZ	UBE3C
0.416403	0.174815	HZ	UBE3C
0.515976	0.258517	HZ	UBE3C
0.421481	0.270971	HZ	UBE3C
0.387891	0.259425	HZ	UBE3C
0.373427	0.252724	HZ	UBE3C
0.360665	0.247059	HZ	UBE3C
0.420283	0.193485	HZ	UBE3C
0.485345	0.14653	HZ	UBE3C
0.476039	0.153608	HZ	UACA
0.197413	0.139916	HZ	TUSC3
0.20512	0.140921	HZ	TUSC3
0.214908	0.141826	HZ	TUSC3
0.222497	0.146163	HZ	TUSC3
0.215232	0.151535	HZ	TUSC3
0.212547	0.149728	HZ	TUSC3
0.247228	0.141486	HZ	TUSC3
0.279118	0.367886	HZ	TULP4
0.336881	0.2691	HZ	TULP4
0.345266	0.23561	HZ	TULP4
0.329546	0.196596	HZ	TULP4
0.355052	0.163204	HZ	TULP4
0.191896	0.153478	HZ	TULP4
0.403457	0.175348	HZ	TTK
0.47261	0.200611	HZ	TTK
0.488163	0.239851	HZ	TTK
0.476212	0.23277	HZ	TTK
0.452819	0.243893	HZ	TTK
0.452062	0.252737	HZ	TTK
0.447343	0.246285	HZ	TTK
0.467784	0.274913	HZ	TTK
0.462339	0.250482	HZ	TTK
0.460799	0.237639	HZ	TTK

NC_056060.1	28250001	28270001	0.350989	0.168851	HZ	ARHGAP11A;I
NC_056058.1	51610001	51630001	0.40348	0.181487	HZ	ARHGAP26
NC_056054.1	184940001	184960001	0.0494153	0.247453	HZ	ARHGAP31
NC_056054.1	184945001	184965001	0.0659945	0.259936	HZ	ARHGAP31
NC_056054.1	184950001	184970001	0.138314	0.234822	HZ	ARHGAP31
NC_056054.1	184970001	184990001	0.144406	0.164408	HZ	ARHGAP31
NC_056054.1	184975001	184995001	0.0806907	0.191267	HZ	ARHGAP31
NC_056054.1	184980001	185000001	0.0414434	0.194273	HZ	ARHGAP31
NC_056054.1	184985001	185005001	0.0406567	0.213484	HZ	ARHGAP31
NC_056054.1	184990001	185010001	0.159688	0.182837	HZ	ARHGAP31
NC_056064.1	31050001	31070001	0.2751	0.160006	HZ	ARHGAP44
NC_056054.1	106875001	106895001	0.490483	0.304258	HZ	ARHGEF11
NC_056054.1	106885001	106905001	0.47548	0.282536	HZ	ARHGEF11
NC_056054.1	106890001	106910001	0.411908	0.309953	HZ	ARHGEF11
NC_056054.1	106895001	106915001	0.340909	0.348606	HZ	ARHGEF11
NC_056054.1	106900001	106920001	0.312299	0.37322	HZ	ARHGEF11
NC_056054.1	106905001	106925001	0.227019	0.429155	HZ	ARHGEF11
NC_056054.1	106910001	106930001	0.339318	0.361041	HZ	ARHGEF11
NC_056054.1	106915001	106935001	0.481057	0.256005	HZ	ARHGEF11
NC_056069.1	7930001	7950001	0.379667	0.273664	HZ	ARHGEF28
NC_056069.1	7935001	7955001	0.422598	0.266352	HZ	ARHGEF28
NC_056058.1	58685001	58705001	0.486025	0.199097	HZ	ARHGEF37
NC_056055.1	114590001	114610001	0.107151	0.15807	HZ	ARHGEF4
NC_056055.1	114595001	114615001	0.0303136	0.164072	HZ	ARHGEF4
NC_056055.1	114600001	114620001	0.0229215	0.166114	HZ	ARHGEF4
NC_056055.1	114605001	114625001	0.0306429	0.162707	HZ	ARHGEF4
NC_056055.1	114615001	114635001	0.0445907	0.160925	HZ	ARHGEF4
NC_056055.1	114630001	114650001	0.0820668	0.158345	HZ	ARHGEF4
NC_056055.1	114635001	114655001	0.073667	0.161911	HZ	ARHGEF4
NC_056055.1	114640001	114660001	0.0751307	0.173473	HZ	ARHGEF4
NC_056055.1	114645001	114665001	0.0921945	0.180737	HZ	ARHGEF4
NC_056055.1	114650001	114670001	0.161378	0.196651	HZ	ARHGEF4
NC_056055.1	114655001	114675001	0.129832	0.188034	HZ	ARHGEF4
NC_056055.1	114660001	114680001	0.100096	0.173491	HZ	ARHGEF4
NC_056055.1	114665001	114685001	0.077381	0.173517	HZ	ARHGEF4
NC_056055.1	114670001	114690001	0.0469734	0.201902	HZ	ARHGEF4

0.235224	0.156188	HZ	TTC37	NC_056055.1	114675001	114695001	0.150704	0.218692	HZ	ARHGEF4
0.231436	0.192307	HZ	TTC37	NC_056055.1	114680001	114700001	0.300418	0.226686	HZ	ARHGEF4
0.308571	0.173412	HZ	TTC37	NC_056055.1	114685001	114705001	0.390579	0.236764	HZ	ARHGEF4
0.375498	0.186916	HZ	TTC21B	NC_056055.1	114810001	114830001	0.209192	0.15756	HZ	ARHGEF4
0.367258	0.155569	HZ	TTC21B	NC_056055.1	114820001	114840001	0.0992366	0.160884	HZ	ARHGEF4
0.325164	0.141457	HZ	TTC21B	NC_056055.1	114850001	114870001	0.14498	0.161669	HZ	ARHGEF4;FAM
0.420164	0.397053	HZ	TTC13	NC_056060.1	24360001	24380001	0.465487	0.169892	HZ	ARHGEF40
0.342082	0.297572	HZ	TTC13	NC_056061.1	81210001	81230001	0.366478	0.202038	HZ	ARID1B
0.30832	0.27131	HZ	TTC13	NC_056061.1	81215001	81235001	0.334892	0.239982	HZ	ARID1B
0.37044	0.198266	HZ	TTC13	NC_056061.1	81220001	81240001	0.272304	0.26887	HZ	ARID1B
0.376526	0.158715	HZ	TTC13	NC_056061.1	81225001	81245001	0.302139	0.250602	HZ	ARID1B
0.4032	0.194397	HZ	TTC13	NC_056054.1	162965001	162985001	0.360542	0.225438	HZ	ARL6
0.450704	0.18523	HZ	TTC13	NC_056054.1	162970001	162990001	0.269414	0.224884	HZ	ARL6
0.533172	0.169736	HZ	TTC13	NC_056054.1	162975001	162995001	0.353989	0.195543	HZ	ARL6
0.171917	0.208553	HZ	TSHZ2	NC_056054.1	162980001	163000001	0.415747	0.168777	HZ	ARL6
0.258454	0.154306	HZ	TSHZ2	NC_056061.1	28565001	28585001	0.200241	0.16089	HZ	ARMC2
0.513593	0.139947	HZ	TRIO	NC_056061.1	28570001	28590001	0.130338	0.17431	HZ	ARMC2
0.533974	0.167316	HZ	TRIO	NC_056060.1	55945001	55965001	0.305324	0.308112	HZ	ARPP19
0.509121	0.146014	HZ	TREH	NC_056060.1	55950001	55970001	0.264591	0.314653	HZ	ARPP19
0.465703	0.167496	HZ	TPPP3;ZDHHC1	NC_056060.1	55955001	55975001	0.452686	0.251414	HZ	ARPP19
0.328299	0.223645	HZ	TPMT	NC_056060.1	55940001	55960001	0.338786	0.312672	HZ	ARPP19;FAM2
0.301899	0.168926	HZ	TPMT	NC_056068.1	53195001	53215001	0.453851	0.157508	HZ	ARRB1
0.130181	0.198027	HZ	TOGARAM2	NC_056068.1	53200001	53220001	0.293256	0.218486	HZ	ARRB1
0.508903	0.209857	HZ	TNS4	NC_056068.1	53205001	53225001	0.163205	0.322718	HZ	ARRB1
0.424615	0.262685	HZ	TNMD	NC_056068.1	53210001	53230001	0.14542	0.331977	HZ	ARRB1
0.235778	0.257635	HZ	TNMD	NC_056068.1	53215001	53235001	0.157293	0.337191	HZ	ARRB1
0.169974	0.228847	HZ	TNMD	NC_056068.1	53220001	53240001	0.28934	0.294984	HZ	ARRB1
0.115384	0.18661	HZ	TNMD	NC_056068.1	53190001	53210001	0.359229	0.172011	HZ	ARRB1;TPBGI
0.329207	0.14407	HZ	TNFSF11	NC_056056.1	38405001	38425001	0.365771	0.159987	HZ	ASPRV1
0.283769	0.14031	HZ	TNFSF11	NC_056056.1	38410001	38430001	0.295181	0.216567	HZ	ASPRV1
0.399777	0.181665	HZ	TNFAIP8	NC_056065.1	57760001	57780001	0.408778	0.247814	HZ	ASTN1
0.185656	0.211941	HZ	TNFAIP8	NC_056055.1	7090001	7110001	0.480857	0.242459	HZ	ASTN2
0.223905	0.171716	HZ	TNFAIP8	NC_056055.1	7115001	7135001	0.328395	0.187706	HZ	ASTN2
0.465887	0.182291	HZ	TNFAIP8	NC_056055.1	7120001	7140001	0.272097	0.207727	HZ	ASTN2
0.468063	0.239038	HZ	TNFAIP8	NC_056055.1	7125001	7145001	0.161995	0.257672	HZ	ASTN2
0.368031	0.139938	HZ	TMTC2	NC_056055.1	7130001	7150001	0.279851	0.279419	HZ	ASTN2

0.322602	0.145948	HZ	TMTC2	NC_056055.1	7135001	7155001	0.408419	0.255316	HZ	ASTN2
0.283233	0.149668	HZ	TMTC2	NC_056055.1	7140001	7160001	0.341341	0.264735	HZ	ASTN2
0.448975	0.22869	HZ	TMTC1	NC_056055.1	7145001	7165001	0.327672	0.210971	HZ	ASTN2
0.497383	0.202562	HZ	TMTC1	NC_056055.1	7150001	7170001	0.291495	0.207669	HZ	ASTN2
0.534591	0.165537	HZ	TMPRSS4	NC_056055.1	7155001	7175001	0.308298	0.233393	HZ	ASTN2
0.481032	0.172249	HZ	TMPRSS4	NC_056055.1	7160001	7180001	0.451856	0.235134	HZ	ASTN2
0.52268	0.211486	HZ	TMEM74	NC_056055.1	7190001	7210001	0.361919	0.239989	HZ	ASTN2
0.4815	0.180158	HZ	TMEM74	NC_056062.1	29475001	29495001	0.271218	0.161845	HZ	ATAD2
0.48423	0.146144	HZ	TMEM74	NC_056058.1	70025001	70045001	0.311295	0.224589	HZ	ATP10B
0.373168	0.181333	HZ	TMEM43;XPC	NC_056058.1	70030001	70050001	0.306749	0.245438	HZ	ATP10B
0.364601	0.185349	HZ	TMEM43;XPC	NC_056058.1	70035001	70055001	0.46769	0.199857	HZ	ATP10B
0.418716	0.145076	HZ	TMEM244	NC_056058.1	70075001	70095001	0.447576	0.169936	HZ	ATP10B
0.370914	0.25744	HZ	TMEM19	NC_056080.1	95510001	95530001	0.282609	0.190598	HZ	ATP11C
0.231565	0.294862	HZ	TMEM19	NC_056080.1	95515001	95535001	0.257038	0.190335	HZ	ATP11C
0.172374	0.328303	HZ	TMEM19	NC_056080.1	95520001	95540001	0.321839	0.159152	HZ	ATP11C
0.472338	0.252554	HZ	TMEM19	NC_056063.1	36895001	36915001	0.443722	0.210923	HZ	ATP12A
0.337359	0.14843	HZ	TMEM181	NC_056075.1	35275001	35295001	0.39822	0.176607	HZ	ATRNL1
0.232695	0.211148	HZ	TMEM181	NC_056075.1	35280001	35300001	0.390208	0.165936	HZ	ATRNL1
0.189895	0.225298	HZ	TMEM181	NC_056080.1	68575001	68595001	0.351207	0.345556	HZ	ATRX
0.227923	0.206303	HZ	TMEM181	NC_056080.1	68580001	68600001	0.296875	0.405392	HZ	ATRX
0.383458	0.20174	HZ	TMEM132D	NC_056080.1	68625001	68645001	0.440828	0.349598	HZ	ATRX
0.280511	0.192576	HZ	TMEM132D	NC_056080.1	68680001	68700001	0.380711	0.408333	HZ	ATRX
0.0875001	0.38154	HZ	TMEM131L	NC_056080.1	68685001	68705001	0.380458	0.406019	HZ	ATRX
0.297436	0.178471	HZ	TMEM131	NC_056080.1	68690001	68710001	0.257679	0.460714	HZ	ATRX
0.247007	0.20531	HZ	TMEM131	NC_056080.1	68695001	68715001	0.371974	0.414015	HZ	ATRX
0.248715	0.209296	HZ	TMEM131	NC_056080.1	68700001	68720001	0.482569	0.411594	HZ	ATRX
0.248512	0.222731	HZ	TMEM131	NC_056057.1	48285001	48305001	0.0731925	0.184075	HZ	ATXN7L1
0.455989	0.146686	HZ	TMEM131	NC_056058.1	41070001	41090001	0.288777	0.167522	HZ	AZU1;ELANE;
0.374599	0.141569	HZ	TMEM127	NC_056054.1	184915001	184935001	0.20908	0.203799	HZ	B4GALT4
0.513102	0.245534	HZ	TMEM108	NC_056061.1	47505001	47525001	0.448231	0.260391	HZ	BACH2
0.508654	0.212738	HZ	TMEM108	NC_056061.1	47510001	47530001	0.349778	0.270846	HZ	BACH2
0.520392	0.149069	HZ	TMEM108	NC_056061.1	47515001	47535001	0.310215	0.246395	HZ	BACH2
0.466778	0.178755	HZ	TMC5	NC_056061.1	47520001	47540001	0.176274	0.266001	HZ	BACH2
0.429783	0.185776	HZ	TMC5	NC_056061.1	47525001	47545001	0.339769	0.192289	HZ	BACH2
0.529102	0.146286	HZ	TMC5	NC_056077.1	38100001	38120001	0.449364	0.210538	HZ	BAIAP2L1
0.441423	0.150874	HZ	TMC5	NC_056077.1	38105001	38125001	0.403581	0.263541	HZ	BAIAP2L1

0.215574	0.184031	HZ	TM9SF3	NC_056077.1	38110001	38130001	0.281376	0.344825	HZ	BAIAP2L1
0.213069	0.201809	HZ	TM9SF3	NC_056077.1	38115001	38135001	0.268893	0.365779	HZ	BAIAP2L1
0.13845	0.24397	HZ	TM9SF3	NC_056077.1	38120001	38140001	0.303779	0.351464	HZ	BAIAP2L1
0.130865	0.224224	HZ	TM9SF3	NC_056077.1	38125001	38145001	0.340821	0.301914	HZ	BAIAP2L1
0.233313	0.159974	HZ	TM9SF3	NC_056066.1	35405001	35425001	0.419009	0.195295	HZ	BAMBI
0.245772	0.169834	HZ	TLR2	NC_056059.1	23190001	23210001	0.20878	0.219256	HZ	BANK1
0.430686	0.21466	HZ	TLN2	NC_056059.1	23195001	23215001	0.154123	0.328043	HZ	BANK1
0.4436	0.243713	HZ	TLN2	NC_056059.1	23200001	23220001	0.0822628	0.327574	HZ	BANK1
0.510563	0.151601	HZ	TINAG	NC_056059.1	23205001	23225001	0.0525618	0.325171	HZ	BANK1
0.501825	0.165796	HZ	TINAG	NC_056059.1	23250001	23270001	0.101956	0.18189	HZ	BANK1
0.452157	0.146691	HZ	TIAM1	NC_056066.1	78800001	78820001	0.489186	0.256235	HZ	BCAS4
0.37931	0.205692	HZ	THSD4	NC_056054.1	98630001	98650001	0.412747	0.165828	HZ	BCL9
0.317962	0.236235	HZ	THAP2;ZFC3H1	NC_056077.1	38220001	38240001	0.402284	0.190038	HZ	BHLHA15;LM'
0.536233	0.198052	HZ	THAP2	NC_056077.1	38225001	38245001	0.325737	0.187198	HZ	BHLHA15;LM'
0.148421	0.140456	HZ	TFCP2L1	NC_056077.1	38210001	38230001	0.424458	0.195313	HZ	BHLHA15;TEC
0.167652	0.163781	HZ	TFCP2L1	NC_056054.1	99995001	100015001	0.0711383	0.255045	HZ	BOLA1;SV2A
0.197474	0.208069	HZ	TFCP2L1	NC_056054.1	100000001	100020001	0.0684211	0.310358	HZ	BOLA1;SV2A
0.256293	0.212611	HZ	TFCP2L1	NC_056054.1	100005001	100025001	0.0939412	0.260436	HZ	BOLA1;SV2A
0.350797	0.183196	HZ	TFCP2L1	NC_056058.1	41955001	41975001	0.331896	0.167408	HZ	BTBD2
0.533586	0.330134	HZ	TF	NC_056065.1	64855001	64875001	0.483372	0.169212	HZ	C12H1orf21
0.437868	0.393511	HZ	TF	NC_056075.1	36565001	36585001	0.416917	0.221422	HZ	C22H10orf82
0.453246	0.389987	HZ	TF	NC_056075.1	36570001	36590001	0.436202	0.225835	HZ	C22H10orf82
0.432297	0.36143	HZ	TF	NC_056060.1	14310001	14330001	0.453096	0.20583	HZ	C7H15orf61
0.504121	0.254242	HZ	TEX37	NC_056072.1	47255001	47275001	0.445644	0.327349	HZ	CACNA1D;CH
0.445236	0.266044	HZ	TEX37	NC_056054.1	154540001	154560001	0.386161	0.331376	HZ	CADM2
0.535385	0.215736	HZ	TEX11	NC_056054.1	154545001	154565001	0.404728	0.305585	HZ	CADM2
0.314319	0.278489	HZ	TEX11	NC_056062.1	86570001	86590001	0.470537	0.184264	HZ	CALB1
0.11733	0.264874	HZ	TEX11	NC_056062.1	86575001	86595001	0.407836	0.216372	HZ	CALB1;DECR1
0.128635	0.267936	HZ	TEX11	NC_056062.1	86580001	86600001	0.392064	0.269934	HZ	CALB1;DECR1
0.111697	0.18773	HZ	TEX11	NC_056066.1	16645001	16665001	0.382812	0.304567	HZ	CAMK1D
0.102675	0.184822	HZ	TENT5C	NC_056066.1	16650001	16670001	0.23346	0.330419	HZ	CAMK1D
0.450743	0.218109	HZ	TENT5C	NC_056066.1	16655001	16675001	0.208791	0.243445	HZ	CAMK1D
0.411068	0.185142	HZ	TENT5B;TRNP1	NC_056066.1	16660001	16680001	0.296251	0.256353	HZ	CAMK1D
0.503916	0.1521	HZ	TENT5B;TRNP1	NC_056066.1	16665001	16685001	0.336177	0.216934	HZ	CAMK1D
0.459966	0.196776	HZ	TENM4	NC_056066.1	16670001	16690001	0.477311	0.170613	HZ	CAMK1D
0.400652	0.199659	HZ	TENM4	NC_056064.1	26285001	26305001	0.309462	0.193659	HZ	CAMTA2;INC

0.437688	0.164582	HZ	TENM4	NC_056064.1	26290001	26310001	0.386635	0.16077	HZ	CAMTA2;INCA
0.446286	0.214586	HZ	TENM4	NC_056080.1	40970001	40990001	0.414042	0.368383	HZ	CASK
0.427704	0.240033	HZ	TENM4	NC_056080.1	40975001	40995001	0.483831	0.39328	HZ	CASK
0.406846	0.235299	HZ	TENM4	NC_056068.1	81595001	81615001	0.365152	0.19302	HZ	CBLIF
0.350625	0.221525	HZ	TENM4	NC_056068.1	81600001	81620001	0.200391	0.212361	HZ	CBLIF
0.369535	0.163286	HZ	TENM4	NC_056068.1	81605001	81625001	0.146024	0.163964	HZ	CBLIF;LOC114
0.387578	0.156502	HZ	TENM4	NC_056054.1	26740001	26760001	0.276173	0.217407	HZ	CC2D1B;ZFYV
0.436645	0.159072	HZ	TENM4	NC_056076.1	8430001	8450001	0.411594	0.177884	HZ	CCDC102B
0.48546	0.213217	HZ	TENM1	NC_056060.1	4105001	4125001	0.342138	0.189671	HZ	CCDC112
0.357061	0.311377	HZ	TENM1	NC_056060.1	4110001	4130001	0.291525	0.211696	HZ	CCDC112
0.169324	0.305576	HZ	TENM1	NC_056060.1	4115001	4135001	0.254384	0.187934	HZ	CCDC112
0.149758	0.265297	HZ	TENM1	NC_056060.1	4120001	4140001	0.25471	0.164461	HZ	CCDC112
0.194962	0.240642	HZ	TENM1	NC_056060.1	4125001	4145001	0.173293	0.217118	HZ	CCDC112
0.473373	0.150456	HZ	TBX1	NC_056060.1	4130001	4150001	0.148354	0.218048	HZ	CCDC112
0.406485	0.150181	HZ	TBX1	NC_056060.1	4100001	4120001	0.361027	0.177653	HZ	CCDC112;PGC
0.431928	0.16435	HZ	TBC1D12	NC_056060.1	79415001	79435001	0.37817	0.194002	HZ	CCDC177
0.397227	0.179694	HZ	TBC1D12	NC_056070.1	52750001	52770001	0.100506	0.209806	HZ	CCDC62
0.378654	0.183061	HZ	TBC1D12	NC_056064.1	23490001	23510001	0.4182	0.183932	HZ	CCDC92B
0.363724	0.193405	HZ	TBC1D12	NC_056064.1	23485001	23505001	0.483167	0.160276	HZ	CCDC92B;CLU
0.382626	0.186082	HZ	TBC1D12	NC_056077.1	34475001	34495001	0.474317	0.183446	HZ	CCL26
0.371279	0.184407	HZ	TBC1D12	NC_056077.1	17585001	17605001	0.371912	0.242634	HZ	CCP110;GDE1
0.37869	0.185976	HZ	TBC1D12	NC_056077.1	17590001	17610001	0.423712	0.233952	HZ	CCP110;GDE1
0.369874	0.14772	HZ	TARS3;TM2D3	NC_056060.1	53070001	53090001	0.409549	0.278891	HZ	CCPG1
0.462447	0.148022	HZ	TARS3;TM2D3	NC_056060.1	53075001	53095001	0.294039	0.310811	HZ	CCPG1
0.0945473	0.263438	HZ	SYTL3	NC_056060.1	53080001	53100001	0.125731	0.354911	HZ	CCPG1
0.362076	0.17793	HZ	SYTL3	NC_056060.1	53085001	53105001	0.17032	0.328794	HZ	CCPG1;PIGB
0.37054	0.302605	HZ	SYT1	NC_056060.1	53090001	53110001	0.22043	0.304926	HZ	CCPG1;PIGB
0.386709	0.320434	HZ	SYT1	NC_056060.1	53095001	53115001	0.368571	0.255968	HZ	CCPG1;PIGB
0.301206	0.346185	HZ	SYT1	NC_056059.1	34575001	34595001	0.108955	0.170081	HZ	CCSER1
0.313829	0.324296	HZ	SYT1	NC_056058.1	14690001	14710001	0.115856	0.218123	HZ	CD320;KANK3
0.313922	0.272895	HZ	SYT1	NC_056058.1	14685001	14705001	0.116984	0.193723	HZ	CD320;NDUFA
0.352698	0.25483	HZ	SYT1	NC_056055.1	240515001	240535001	0.404783	0.183405	HZ	CD52;CRYBG2
0.438582	0.192343	HZ	SYT1	NC_056055.1	240520001	240540001	0.32594	0.225554	HZ	CD52;CRYBG2
0.439011	0.188847	HZ	SYT1	NC_056055.1	240525001	240545001	0.357704	0.198	HZ	CD52;CRYBG2
0.413649	0.273217	HZ	SYT1	NC_056072.1	51685001	51705001	0.130861	0.197158	HZ	CDC25A
0.500273	0.242185	HZ	SYT1	NC_056072.1	51690001	51710001	0.300425	0.17091	HZ	CDC25A

0.389986	0.168345	HZ	SYT1	NC_056058.1	12790001	12810001	0.37452	0.544869	HZ	CDC37;LOC10
0.344773	0.282519	HZ	SV2A	NC_056061.1	27285001	27305001	0.286912	0.237511	HZ	CDC40
0.0690441	0.140408	HZ	SUMF1	NC_056061.1	27290001	27310001	0.0727034	0.319188	HZ	CDC40
0.367914	0.236076	HZ	SULT1E1	NC_056061.1	27295001	27315001	0.0893825	0.290593	HZ	CDC40
0.447861	0.237523	HZ	SULT1E1	NC_056061.1	27300001	27320001	0.0811139	0.304052	HZ	CDC40
0.276868	0.162163	HZ	SUGCT	NC_056061.1	27320001	27340001	0.150982	0.18986	HZ	CDC40
0.212663	0.250236	HZ	SUGCT	NC_056058.1	20930001	20950001	0.0427437	0.224147	HZ	CDC42SE2
0.125337	0.39073	HZ	SUGCT	NC_056057.1	31590001	31610001	0.427065	0.334714	HZ	CDCA7L;DNA
0.207667	0.380965	HZ	SUGCT	NC_056062.1	83100001	83120001	0.468535	0.343792	HZ	CDH17
0.293261	0.344237	HZ	SUGCT	NC_056066.1	54860001	54880001	0.119436	0.297939	HZ	CDH4
0.269289	0.227465	HZ	SUCNR1	NC_056066.1	54865001	54885001	0.222786	0.338541	HZ	CDH4
0.135271	0.232451	HZ	SUCNR1	NC_056066.1	54870001	54890001	0.264543	0.361385	HZ	CDH4
0.162657	0.207113	HZ	SUCNR1	NC_056066.1	54875001	54895001	0.351361	0.323985	HZ	CDH4
0.435328	0.250094	HZ	SUCLG2	NC_056066.1	54850001	54870001	0.23867	0.225141	HZ	CDH4;TAF4
0.499628	0.164135	HZ	STXBP5	NC_056066.1	54855001	54875001	0.185668	0.247945	HZ	CDH4;TAF4
0.442226	0.175059	HZ	STXBP1	NC_056064.1	61455001	61475001	0.246179	0.177164	HZ	CEP112
0.116146	0.191664	HZ	STXBP1	NC_056064.1	61550001	61570001	0.298942	0.217868	HZ	CEP112
0.28442	0.157766	HZ	STS	NC_056064.1	61555001	61575001	0.199993	0.272234	HZ	CEP112
0.281232	0.167598	HZ	STS	NC_056064.1	61560001	61580001	0.0767398	0.282559	HZ	CEP112
0.234719	0.168478	HZ	STS	NC_056064.1	61565001	61585001	0.212877	0.21956	HZ	CEP112
0.472004	0.248244	HZ	STPG1	NC_056060.1	90195001	90215001	0.29874	0.231444	HZ	CEP128
0.437056	0.21064	HZ	STPG1	NC_056060.1	90200001	90220001	0.271721	0.313652	HZ	CEP128
0.427601	0.175339	HZ	STPG1	NC_056060.1	90205001	90225001	0.24664	0.28895	HZ	CEP128
0.530564	0.146582	HZ	STPG1	NC_056060.1	90210001	90230001	0.262595	0.284239	HZ	CEP128
0.359161	0.195863	HZ	STAU2	NC_056060.1	90215001	90235001	0.468112	0.226478	HZ	CEP128
0.212471	0.282421	HZ	STAU2	NC_056055.1	58645001	58665001	0.451431	0.172107	HZ	CEP78
0.198342	0.316356	HZ	STAU2	NC_056056.1	130600001	130620001	0.398204	0.165112	HZ	CEP83
0.15767	0.33219	HZ	STAU2	NC_056072.1	42450001	42470001	0.46996	0.171558	HZ	CFAP20DC
0.0771914	0.350634	HZ	STAU2	NC_056072.1	42455001	42475001	0.297609	0.162042	HZ	CFAP20DC
0.100132	0.31412	HZ	STAU2	NC_056072.1	42460001	42480001	0.165399	0.173749	HZ	CFAP20DC
0.150934	0.270173	HZ	STAU2	NC_056072.1	42465001	42485001	0.175815	0.173416	HZ	CFAP20DC
0.293007	0.167863	HZ	STAU2	NC_056072.1	42470001	42490001	0.157643	0.169479	HZ	CFAP20DC
0.196838	0.141502	HZ	STARD3NL	NC_056072.1	42475001	42495001	0.181193	0.173558	HZ	CFAP20DC
0.108556	0.139218	HZ	STARD13	NC_056072.1	42480001	42500001	0.160589	0.184843	HZ	CFAP20DC
0.429958	0.189308	HZ	ST7	NC_056072.1	42485001	42505001	0.204628	0.184083	HZ	CFAP20DC
0.311627	0.208176	HZ	ST7	NC_056072.1	42490001	42510001	0.233859	0.18893	HZ	CFAP20DC

0.422938	0.30527	HZ	ST7	NC_056055.1	184960001	184980001	0.482445	0.207059	HZ	CFAP221
0.483312	0.343672	HZ	ST7	NC_056055.1	184965001	184985001	0.490587	0.218582	HZ	CFAP221
0.534386	0.341294	HZ	ST7	NC_056054.1	179030001	179050001	0.143306	0.168985	HZ	CFAP44
0.455269	0.148744	HZ	ST7	NC_056061.1	50150001	50170001	0.421091	0.219477	HZ	CGA
0.41725	0.160614	HZ	ST7	NC_056061.1	50155001	50175001	0.216291	0.351469	HZ	CGA
0.471851	0.152475	HZ	ST7	NC_056061.1	50160001	50180001	0.468814	0.260389	HZ	CGA
0.456989	0.163352	HZ	ST7	NC_056072.1	26010001	26030001	0.436946	0.163259	HZ	CHL1
0.415	0.141686	HZ	ST7	NC_056072.1	26135001	26155001	0.351351	0.243693	HZ	CHL1
0.414222	0.183592	HZ	SRD5A3	NC_056057.1	68290001	68310001	0.400001	0.209032	HZ	CHN2
0.437459	0.147885	HZ	SRBD1	NC_056057.1	68355001	68375001	0.287796	0.364206	HZ	CHN2
0.457633	0.150916	HZ	SPTLC3	NC_056057.1	68435001	68455001	0.294871	0.203387	HZ	CHN2
0.382709	0.178972	HZ	SPTLC3	NC_056079.1	36835001	36855001	0.289956	0.267152	HZ	CHRNA6
0.333212	0.163714	HZ	SPTLC3	NC_056079.1	36840001	36860001	0.173231	0.254849	HZ	CHRNA6
0.514741	0.161842	HZ	SPTLC1	NC_056079.1	36845001	36865001	0.275154	0.205498	HZ	CHRNA6
0.529807	0.183529	HZ	SPOCK1	NC_056079.1	36830001	36850001	0.398856	0.257928	HZ	CHRNA6;CHR
0.412327	0.190113	HZ	SPOCK1	NC_056061.1	64320001	64340001	0.359013	0.174249	HZ	CITED2
0.270369	0.247577	HZ	SPOCK1	NC_056057.1	8545001	8565001	0.404358	0.181109	HZ	CLDN12
0.382669	0.190591	HZ	SPOCK1	NC_056057.1	8550001	8570001	0.467879	0.195751	HZ	CLDN12
0.428359	0.14544	HZ	SPOCK1	NC_056079.1	1350001	1370001	0.480884	0.18022	HZ	CLN8
0.287723	0.152329	HZ	SPIN1	NC_056079.1	1355001	1375001	0.440213	0.225409	HZ	CLN8
0.281463	0.144982	HZ	SPIN1	NC_056074.1	15925001	15945001	0.305496	0.265621	HZ	CLNS1A
0.238591	0.155618	HZ	SPIN1	NC_056074.1	15930001	15950001	0.389687	0.260994	HZ	CLNS1A
0.209033	0.151985	HZ	SPIN1	NC_056074.1	15920001	15940001	0.302802	0.251818	HZ	CLNS1A;RSF1
0.424575	0.209592	HZ	SPESP1	NC_056060.1	12075001	12095001	0.095718	0.194497	HZ	CLPX
0.125104	0.253668	HZ	SOS2;VCPKMT	NC_056060.1	12080001	12100001	0.0670477	0.201187	HZ	CLPX
0.0690201	0.272239	HZ	SOS2;VCPKMT	NC_056060.1	12085001	12105001	0.0910988	0.194211	HZ	CLPX
0.0579306	0.24521	HZ	SOS2;VCPKMT	NC_056060.1	12090001	12110001	0.132631	0.17134	HZ	CLPX
0.083459	0.227958	HZ	SOS2;VCPKMT	NC_056064.1	23465001	23485001	0.233636	0.226265	HZ	CLUH
0.173272	0.244906	HZ	SOS2	NC_056064.1	23470001	23490001	0.335247	0.158876	HZ	CLUH
0.273825	0.262711	HZ	SOS2	NC_056064.1	23460001	23480001	0.33909	0.204417	HZ	CLUH;PAFAH
0.375386	0.274195	HZ	SOS2	NC_056067.1	33995001	34015001	0.345158	0.169138	HZ	CMTM4
0.376456	0.233506	HZ	SOS2	NC_056067.1	34000001	34020001	0.367278	0.162475	HZ	CMTM4
0.304523	0.231158	HZ	SOS2	NC_056067.1	34005001	34025001	0.41486	0.172448	HZ	CMTM4
0.258755	0.209993	HZ	SOS2	NC_056062.1	88545001	88565001	0.313583	0.166122	HZ	CNBD1
0.168046	0.198135	HZ	SOS2	NC_056062.1	88550001	88570001	0.288586	0.194287	HZ	CNBD1
0.0549828	0.240567	HZ	SOS2	NC_056062.1	88555001	88575001	0.318102	0.198604	HZ	CNBD1

0.0809668	0.217997	HZ	SOS2	NC_056062.1	88635001	88655001	0.492065	0.252608	HZ	CNBD1
0.0773876	0.15705	HZ	SOS2	NC_056062.1	88640001	88660001	0.470136	0.245524	HZ	CNBD1
0.502896	0.138675	HZ	SOCS2	NC_056063.1	11460001	11480001	0.39533	0.222574	HZ	CNMD
0.431432	0.180497	HZ	SOCS2	NC_056058.1	445001	465001	0.453462	0.235234	HZ	CNOT6
0.501767	0.142371	HZ	SNX13	NC_056058.1	450001	470001	0.434276	0.279587	HZ	CNOT6
0.508386	0.17161	HZ	SNX13	NC_056058.1	455001	475001	0.441931	0.315438	HZ	CNOT6
0.496048	0.2093	HZ	SNX13	NC_056058.1	460001	480001	0.467865	0.342186	HZ	CNOT6
0.498381	0.184155	HZ	SNX13	NC_056072.1	23315001	23335001	0.063882	0.170646	HZ	CNTN4
0.180183	0.194849	HZ	SNTG2	NC_056072.1	23325001	23345001	0.0365529	0.241231	HZ	CNTN4
0.268612	0.164806	HZ	SNTG2	NC_056072.1	23330001	23350001	0.0987553	0.221587	HZ	CNTN4
0.321737	0.158602	HZ	SNRNP200	NC_056072.1	23335001	23355001	0.185699	0.232555	HZ	CNTN4
0.344828	0.206389	HZ	SNRK	NC_056072.1	23340001	23360001	0.184185	0.19999	HZ	CNTN4
0.253102	0.245129	HZ	SNRK	NC_056072.1	23345001	23365001	0.20522	0.182425	HZ	CNTN4
0.442952	0.218665	HZ	SMCO4	NC_056072.1	23350001	23370001	0.166454	0.176426	HZ	CNTN4
0.372198	0.269473	HZ	SMCO4	NC_056057.1	112260001	112280001	0.390951	0.212957	HZ	CNTNAP2
0.368022	0.260576	HZ	SMCO4	NC_056078.1	25420001	25440001	0.204255	0.194131	HZ	COL13A1
0.474772	0.211013	HZ	SMCO4	NC_056078.1	25425001	25445001	0.183576	0.178858	HZ	COL13A1
0.271201	0.214889	HZ	SMAD4	NC_056060.1	59195001	59215001	0.29103	0.172104	HZ	COPS2;GALK2
0.181124	0.296939	HZ	SMAD4	NC_056060.1	59200001	59220001	0.289052	0.162752	HZ	COPS2;GALK2
0.186505	0.324117	HZ	SMAD4	NC_056070.1	62940001	62960001	0.357646	0.20724	HZ	COQ5;RNF10
0.303722	0.297037	HZ	SMAD4	NC_056070.1	62945001	62965001	0.358024	0.232748	HZ	COQ5;RNF10
0.43288	0.288055	HZ	SMAD4	NC_056069.1	37300001	37320001	0.490657	0.271054	HZ	CPLANE1
0.430455	0.300806	HZ	SMAD4	NC_056069.1	37305001	37325001	0.483119	0.263947	HZ	CPLANE1;NIP
0.433836	0.262316	HZ	SMAD4	NC_056056.1	151055001	151075001	0.449818	0.191872	HZ	CPM
0.450758	0.255253	HZ	SMAD4	NC_056062.1	80320001	80340001	0.417549	0.202422	HZ	CPQ
0.423729	0.184521	HZ	SMAD4	NC_056062.1	80325001	80345001	0.328408	0.235563	HZ	CPQ
0.218487	0.197912	HZ	SMAD4	NC_056062.1	80330001	80350001	0.405179	0.253279	HZ	CPQ
0.514596	0.170527	HZ	SMAD2	NC_056062.1	80335001	80355001	0.463662	0.255983	HZ	CPQ
0.515906	0.179756	HZ	SMAD2	NC_056057.1	103075001	103095001	0.35394	0.224386	HZ	CREB3L2
0.274331	0.172743	HZ	SMAD1	NC_056057.1	103080001	103100001	0.336255	0.276423	HZ	CREB3L2
0.335098	0.146433	HZ	SMAD1	NC_056057.1	103085001	103105001	0.338408	0.287209	HZ	CREB3L2
0.283928	0.180231	HZ	SMAD1	NC_056057.1	103090001	103110001	0.429865	0.276039	HZ	CREB3L2
0.319467	0.166309	HZ	SMAD1	NC_056057.1	103125001	103145001	0.430207	0.179087	HZ	CREB3L2
0.364128	0.149236	HZ	SMAD1	NC_056057.1	69115001	69135001	0.181916	0.17848	HZ	CREB5
0.489845	0.20283	HZ	SMAD1	NC_056057.1	69120001	69140001	0.220082	0.177769	HZ	CREB5
0.423955	0.238715	HZ	SMAD1	NC_056073.1	22180001	22200001	0.238832	0.231691	HZ	CRISP1

0.520437	0.246665	HZ	SMAD1
0.161844	0.165521	HZ	SLTM
0.19788	0.159527	HZ	SLTM
0.149631	0.166845	HZ	SLTM
0.456194	0.262251	HZ	SLITRK6
0.459054	0.184887	HZ	SLITRK6
0.400237	0.141406	HZ	SLITRK6
0.204907	0.197749	HZ	SLITRK6
0.246991	0.147976	HZ	SLIT2
0.179636	0.205175	HZ	SLIT2
0.164666	0.239728	HZ	SLIT2
0.230284	0.259892	HZ	SLIT2
0.278122	0.245848	HZ	SLIT2
0.326204	0.187156	HZ	SLIT2
0.387209	0.140884	HZ	SLIT2
0.486648	0.146728	HZ	SLC4A7
0.476652	0.144794	HZ	SLC4A10
0.364203	0.236183	HZ	SLC4A10
0.365416	0.231249	HZ	SLC4A10
0.383208	0.143305	HZ	SLC4A10
0.131993	0.139661	HZ	SLC45A3
0.28481	0.157673	HZ	SLC44A2
0.510371	0.153058	HZ	SLC44A2
0.45831	0.147198	HZ	SLC44A1
0.393214	0.191409	HZ	SLC44A1
0.415146	0.218719	HZ	SLC44A1
0.471705	0.195957	HZ	SLC44A1
0.364979	0.156092	HZ	SLC38A9
0.280607	0.176922	HZ	SLC38A9
0.175096	0.190978	HZ	SLC38A9
0.192987	0.168786	HZ	SLC38A9
0.345107	0.151229	HZ	SLC38A9
0.431643	0.140132	HZ	SLC38A2
0.0491838	0.174377	HZ	SLC35F2
0.482706	0.17961	HZ	SLC24A3
0.497769	0.146021	HZ	SLC19A1

NC_056073.1	22185001	22205001	0.293379	0.197884	HZ	CRISP1
NC_056056.1	175875001	175895001	0.273264	0.227656	HZ	CRY1
NC_056056.1	218460001	218480001	0.149782	0.174225	HZ	CSDC2;PMM1
NC_056056.1	218465001	218485001	0.109267	0.186794	HZ	CSDC2;PMM1
NC_056079.1	3635001	3655001	0.468614	0.166995	HZ	CSMD1
NC_056079.1	3640001	3660001	0.282473	0.162876	HZ	CSMD1
NC_056058.1	58675001	58695001	0.465624	0.171111	HZ	CSNK1A1
NC_056058.1	58680001	58700001	0.428168	0.229886	HZ	CSNK1A1
NC_056064.1	50095001	50115001	0.435073	0.215006	HZ	CSNK1D
NC_056060.1	43900001	43920001	0.325	0.191652	HZ	CSNK1G1
NC_056060.1	43890001	43910001	0.285428	0.216234	HZ	CSNK1G1;PCL
NC_056060.1	43895001	43915001	0.243394	0.210681	HZ	CSNK1G1;PCL
NC_056056.1	51655001	51675001	0.177235	0.174431	HZ	CTNNA2
NC_056056.1	51660001	51680001	0.154444	0.175133	HZ	CTNNA2
NC_056056.1	51665001	51685001	0.257801	0.160764	HZ	CTNNA2
NC_056056.1	51920001	51940001	0.28528	0.243573	HZ	CTNNA2
NC_056056.1	51925001	51945001	0.369696	0.254874	HZ	CTNNA2
NC_056056.1	51930001	51950001	0.427722	0.201918	HZ	CTNNA2
NC_056078.1	21665001	21685001	0.337161	0.163535	HZ	CTNNA3
NC_056078.1	23265001	23285001	0.407946	0.172707	HZ	CTNNA3
NC_056078.1	23375001	23395001	0.479395	0.174239	HZ	CTNNA3
NC_056078.1	23380001	23400001	0.458263	0.16548	HZ	CTNNA3
NC_056064.1	24230001	24250001	0.378472	0.219983	HZ	CTNS;EMC6;P
NC_056074.1	6945001	6965001	0.211952	0.166667	HZ	CTSC
NC_056054.1	89855001	89875001	0.447337	0.212949	HZ	CTTNBP2NL
NC_056069.1	14630001	14650001	0.240701	0.216373	HZ	CWC27
NC_056069.1	14635001	14655001	0.192727	0.260155	HZ	CWC27
NC_056069.1	14640001	14660001	0.364358	0.18706	HZ	CWC27
NC_056069.1	14645001	14665001	0.470493	0.160144	HZ	CWC27
NC_056059.1	68295001	68315001	0.25	0.190194	HZ	CWH43
NC_056059.1	68300001	68320001	0.251982	0.19511	HZ	CWH43
NC_056055.1	174710001	174730001	0.166424	0.241277	HZ	CXCR4
NC_056055.1	174715001	174735001	0.116667	0.235775	HZ	CXCR4
NC_056055.1	174720001	174740001	0.230033	0.178802	HZ	CXCR4
NC_056064.1	54560001	54580001	0.401779	0.163793	HZ	CYGB
NC_056063.1	46765001	46785001	0.167616	0.187925	HZ	DACH1

0.276771	0.183674	HZ	SHOC1
0.527004	0.145746	HZ	SHOC1
0.421819	0.165979	HZ	SH3TC1
0.46486	0.156322	HZ	SH2D7
0.49914	0.141962	HZ	SGPP2
0.508384	0.139928	HZ	SGPP2
0.445521	0.160017	HZ	SGPP2
0.469616	0.168928	HZ	SGK1
0.511357	0.161182	HZ	SGK1
0.405551	0.210529	HZ	SGK1
0.365675	0.210059	HZ	SGK1
0.461889	0.159004	HZ	SGIP1
0.526383	0.170889	HZ	SGCG
0.499838	0.249255	HZ	SGCD
0.2708	0.334131	HZ	SGCD
0.32732	0.308249	HZ	SGCD
0.529823	0.154666	HZ	SGCD
0.4205	0.202901	HZ	SEMA6D
0.508429	0.247971	HZ	SEMA6D
0.521873	0.157652	HZ	SCUBE1
0.468496	0.192964	HZ	SCN5A
0.258303	0.138507	HZ	SCAI
0.214756	0.18447	HZ	SCAI
0.286364	0.184684	HZ	SCAI
0.343558	0.166995	HZ	SCAI
0.180154	0.16319	HZ	SC5
0.0619419	0.20307	HZ	SC5
0.443097	0.182723	HZ	SBF2
0.250206	0.233934	HZ	SBF2
0.238061	0.222917	HZ	SBF2
0.298059	0.206054	HZ	SBF2
0.474752	0.163132	HZ	SBF2
0.415273	0.157578	HZ	SAMM50
0.441818	0.177931	HZ	SAMM50
0.434567	0.188687	HZ	SALL1
0.324873	0.168056	HZ	SALL1

NC_056063.1	46770001	46790001	0.128991	0.220327	HZ	DACH1
NC_056063.1	46775001	46795001	0.11293	0.219809	HZ	DACH1
NC_056063.1	46780001	46800001	0.232494	0.252001	HZ	DACH1
NC_056079.1	25465001	25485001	0.414536	0.291934	HZ	DCTN6
NC_056079.1	25470001	25490001	0.445985	0.241075	HZ	DCTN6
NC_056054.1	114285001	114305001	0.409557	0.169204	HZ	DDR2
NC_056054.1	114290001	114310001	0.345969	0.177666	HZ	DDR2
NC_056054.1	114295001	114315001	0.424816	0.17416	HZ	DDR2
NC_056054.1	114330001	114350001	0.41922	0.175056	HZ	DDR2
NC_056054.1	114335001	114355001	0.287998	0.182109	HZ	DDR2;HSD17E
NC_056054.1	114340001	114360001	0.0619016	0.264565	HZ	DDR2;HSD17E
NC_056054.1	114345001	114365001	0.0538709	0.285206	HZ	DDR2;HSD17E
NC_056062.1	86585001	86605001	0.341231	0.193065	HZ	DECR1
NC_056067.1	14260001	14280001	0.382718	0.325381	HZ	DEF8
NC_056067.1	14265001	14285001	0.173582	0.475951	HZ	DEF8;LOC101
NC_056067.1	14275001	14295001	0.370563	0.442301	HZ	DEF8;LOC101
NC_056054.1	91975001	91995001	0.162446	0.163909	HZ	DENND2C
NC_056054.1	91995001	92015001	0.0356482	0.18823	HZ	DENND2C
NC_056054.1	92000001	92020001	0.0927213	0.169183	HZ	DENND2C
NC_056070.1	72620001	72640001	0.134369	0.294816	HZ	DGCR2;ESS2;(
NC_056070.1	72610001	72630001	0.389592	0.251511	HZ	DGCR2;ESS2;T
NC_056070.1	72615001	72635001	0.244773	0.293991	HZ	DGCR2;ESS2;T
NC_056057.1	23325001	23345001	0.205826	0.209669	HZ	DGKB
NC_056057.1	23330001	23350001	0.0967537	0.276459	HZ	DGKB
NC_056057.1	23335001	23355001	0.14065	0.264422	HZ	DGKB
NC_056063.1	12600001	12620001	0.466279	0.159387	HZ	DGKH
NC_056063.1	12690001	12710001	0.454832	0.164551	HZ	DGKH
NC_056054.1	29260001	29280001	0.299283	0.179919	HZ	DHCR24;LEXM
NC_056059.1	44685001	44705001	0.375076	0.19605	HZ	DHX15
NC_056066.1	46260001	46280001	0.451835	0.173597	HZ	DIP2C
NC_056066.1	46265001	46285001	0.32361	0.192997	HZ	DIP2C
NC_056066.1	46270001	46290001	0.3365	0.198481	HZ	DIP2C
NC_056066.1	46275001	46295001	0.354676	0.178554	HZ	DIP2C
NC_056066.1	46280001	46300001	0.388384	0.173876	HZ	DIP2C
NC_056068.1	40460001	40480001	0.051655	0.312666	HZ	DKK3
NC_056068.1	40465001	40485001	0.0710824	0.319349	HZ	DKK3

0.35878	0.233579	HZ	S100A10	NC_056068.1	40470001	40490001	0.167736	0.305955	HZ	DKK3
0.501055	0.197	HZ	S100A10	NC_056068.1	40475001	40495001	0.208115	0.318825	HZ	DKK3
0.400565	0.188834	HZ	RXRA	NC_056068.1	40480001	40500001	0.381665	0.324186	HZ	DKK3
0.428419	0.192971	HZ	RXRA	NC_056068.1	40485001	40505001	0.483713	0.313594	HZ	DKK3;USP47
0.414041	0.196061	HZ	RXRA	NC_056074.1	11185001	11205001	0.414271	0.218839	HZ	DLG2
0.448048	0.206754	HZ	RXRA	NC_056074.1	11190001	11210001	0.268128	0.294475	HZ	DLG2
0.189504	0.147009	HZ	RUNX1	NC_056074.1	11195001	11215001	0.414369	0.21383	HZ	DLG2
0.39065	0.142415	HZ	RUFY2	NC_056060.1	56915001	56935001	0.238431	0.186933	HZ	DMXL2
0.529315	0.142494	HZ	RTN4RL2;SLC43A	NC_056060.1	56920001	56940001	0.200079	0.223501	HZ	DMXL2
0.405158	0.236318	HZ	RTN1	NC_056060.1	56925001	56945001	0.122995	0.18406	HZ	DMXL2
0.343935	0.147939	HZ	RSPO2	NC_056060.1	56985001	57005001	0.151652	0.237804	HZ	DMXL2
0.507957	0.143629	HZ	RSF1	NC_056060.1	56990001	57010001	0.0860349	0.242241	HZ	DMXL2
0.473353	0.154475	HZ	RSF1	NC_056060.1	57015001	57035001	0.0440551	0.21584	HZ	DMXL2
0.428775	0.196303	HZ	RSF1	NC_056060.1	57020001	57040001	0.0707378	0.196434	HZ	DMXL2
0.430638	0.147296	HZ	RSF1	NC_056060.1	57025001	57045001	0.157998	0.191834	HZ	DMXL2
0.432817	0.157951	HZ	RSF1	NC_056060.1	57030001	57050001	0.257905	0.233205	HZ	DMXL2
0.528676	0.156815	HZ	RSF1	NC_056060.1	57035001	57055001	0.324004	0.181888	HZ	DMXL2
0.522727	0.169752	HZ	RSF1	NC_056060.1	57040001	57060001	0.327213	0.179279	HZ	DMXL2
0.469602	0.183244	HZ	RSF1	NC_056060.1	57045001	57065001	0.276468	0.188718	HZ	DMXL2
0.336769	0.28925	HZ	RPS6KA3	NC_056060.1	57050001	57070001	0.170732	0.222998	HZ	DMXL2
0.360209	0.264708	HZ	RPS6KA3	NC_056060.1	57055001	57075001	0.0528302	0.271573	HZ	DMXL2
0.361194	0.233395	HZ	RPS6KA3	NC_056060.1	57060001	57080001	0.222142	0.298363	HZ	DMXL2
0.369756	0.194065	HZ	RPS6KA3	NC_056060.1	57065001	57085001	0.278652	0.308611	HZ	DMXL2
0.345988	0.354179	HZ	RORA	NC_056060.1	40915001	40935001	0.379747	0.162095	HZ	DNAAF2;POLI
0.388464	0.315966	HZ	RORA	NC_056057.1	31580001	31600001	0.488992	0.386587	HZ	DNAH11
0.434918	0.174278	HZ	RORA	NC_056057.1	31585001	31605001	0.418775	0.395452	HZ	DNAH11
0.476161	0.196681	HZ	RORA	NC_056065.1	27520001	27540001	0.260518	0.164064	HZ	DNAH14
0.462519	0.225838	HZ	RORA	NC_056065.1	27560001	27580001	0.359246	0.184356	HZ	DNAH14
0.503455	0.230313	HZ	RORA	NC_056065.1	27565001	27585001	0.464119	0.195575	HZ	DNAH14
0.495366	0.14114	HZ	RNGTT	NC_056065.1	27575001	27595001	0.475349	0.268581	HZ	DNAH14
0.300386	0.224084	HZ	RNGTT	NC_056065.1	27580001	27600001	0.432629	0.250007	HZ	DNAH14
0.235552	0.24806	HZ	RNGTT	NC_056065.1	27585001	27605001	0.338109	0.250681	HZ	DNAH14
0.224656	0.271365	HZ	RNGTT	NC_056065.1	27590001	27610001	0.209985	0.252115	HZ	DNAH14
0.243693	0.266237	HZ	RNGTT	NC_056064.1	30250001	30270001	0.492318	0.399957	HZ	DNAH9
0.311563	0.263826	HZ	RNGTT	NC_056064.1	30365001	30385001	0.336355	0.278524	HZ	DNAH9
0.338879	0.261688	HZ	RNGTT	NC_056064.1	30370001	30390001	0.312044	0.276343	HZ	DNAH9

0.361201	0.258879	HZ	RNGTT
0.423406	0.290624	HZ	RNGTT
0.461828	0.285444	HZ	RNGTT
0.536416	0.2343	HZ	RNGTT
0.47307	0.501722	HZ	RNF17
0.408079	0.497873	HZ	RNF17
0.417569	0.511552	HZ	RNF17
0.3963	0.537156	HZ	RNF17
0.339462	0.525687	HZ	RNF17
0.263972	0.268514	HZ	RNF111
0.267899	0.2958	HZ	RNF111
0.200478	0.324738	HZ	RNF111
0.193878	0.344913	HZ	RNF111
0.24944	0.336073	HZ	RNF111
0.250335	0.323232	HZ	RNF111
0.339105	0.298795	HZ	RNF111
0.522493	0.275649	HZ	RNF111
0.419157	0.222636	HZ	RIPPLY1
0.490395	0.241046	HZ	RIPPLY1
0.53099	0.152642	HZ	RIMS1
0.449889	0.166141	HZ	RIMS1
0.361067	0.185356	HZ	RIMS1
0.303393	0.168592	HZ	RIMS1
0.336975	0.155344	HZ	RIMS1
0.345039	0.141645	HZ	RIMS1
0.531204	0.161337	HZ	RIMS1
0.133659	0.178262	HZ	RHAG
0.161195	0.165159	HZ	RHAG
0.395091	0.25208	HZ	RGS8
0.172239	0.33506	HZ	RGS8
0.193175	0.138581	HZ	RFX7
0.162904	0.164542	HZ	RFX7
0.137019	0.194822	HZ	RFX7
0.133498	0.175705	HZ	RFX7
0.329032	0.175882	HZ	RFX3
0.440933	0.152414	HZ	RFX3

NC_056054.1	62125001	62145001	0.389171	0.177943	HZ	DNAI3
NC_056054.1	62130001	62150001	0.306633	0.193836	HZ	DNAI3
NC_056054.1	62135001	62155001	0.269957	0.202549	HZ	DNAI3
NC_056072.1	59240001	59260001	0.233574	0.252661	HZ	DNAJB8
NC_056072.1	59245001	59265001	0.150863	0.267029	HZ	DNAJB8
NC_056072.1	59250001	59270001	0.0922923	0.275632	HZ	DNAJB8
NC_056072.1	59235001	59255001	0.262228	0.25451	HZ	DNAJB8;GAT
NC_056075.1	45995001	46015001	0.347364	0.174045	HZ	DOCK1
NC_056075.1	46205001	46225001	0.439728	0.227513	HZ	DOCK1
NC_056075.1	46210001	46230001	0.393574	0.190366	HZ	DOCK1
NC_056072.1	49590001	49610001	0.469375	0.249537	HZ	DOCK3
NC_056060.1	82440001	82460001	0.133704	0.188506	HZ	DPF3
NC_056060.1	82445001	82465001	0.224019	0.173826	HZ	DPF3
NC_056055.1	180590001	180610001	0.479266	0.489121	HZ	DPP10
NC_056055.1	180990001	181010001	0.395847	0.244162	HZ	DPP10
NC_056055.1	180995001	181015001	0.4242	0.172141	HZ	DPP10
NC_056057.1	63660001	63680001	0.211066	0.227734	HZ	DPY19L1
NC_056057.1	63665001	63685001	0.232333	0.199934	HZ	DPY19L1
NC_056057.1	63670001	63690001	0.301116	0.203069	HZ	DPY19L1
NC_056057.1	63675001	63695001	0.383327	0.158043	HZ	DPY19L1
NC_056057.1	63685001	63705001	0.332257	0.163508	HZ	DPY19L1
NC_056068.1	27910001	27930001	0.438724	0.168964	HZ	DSCAML1
NC_056068.1	27915001	27935001	0.260768	0.236737	HZ	DSCAML1
NC_056068.1	27920001	27940001	0.0565959	0.272253	HZ	DSCAML1
NC_056068.1	27925001	27945001	0.173249	0.204855	HZ	DSCAML1
NC_056076.1	22570001	22590001	0.219823	0.167373	HZ	DTNA
NC_056064.1	50305001	50325001	0.484582	0.26708	HZ	DUS1L;GPS1;
NC_056064.1	50310001	50330001	0.259494	0.27726	HZ	DUS1L;GPS1;
NC_056064.1	50315001	50335001	0.208333	0.212197	HZ	DUS1L;GPS1;
NC_056075.1	30580001	30600001	0.372721	0.16407	HZ	DUSP5
NC_056080.1	62850001	62870001	0.191324	0.166797	HZ	EDA
NC_056080.1	62855001	62875001	0.287197	0.195486	HZ	EDA
NC_056080.1	62860001	62880001	0.381828	0.222244	HZ	EDA
NC_056080.1	62865001	62885001	0.417446	0.242314	HZ	EDA
NC_056080.1	62880001	62900001	0.465079	0.208812	HZ	EDA
NC_056072.1	59290001	59310001	0.155639	0.179188	HZ	EEFSEC

0.373121	0.179673	HZ	REV1	NC_056057.1	62620001	62640001	0.364515	0.167886	HZ	EEPD1
0.401316	0.216216	HZ	REV1	NC_056057.1	62625001	62645001	0.338153	0.159594	HZ	EEPD1
0.401396	0.253432	HZ	REV1	NC_056057.1	62630001	62650001	0.330433	0.162686	HZ	EEPD1
0.511181	0.236354	HZ	REV1	NC_056058.1	104180001	104200001	0.396431	0.309631	HZ	EFNA5
0.418975	0.14852	HZ	RELCH	NC_056058.1	104185001	104205001	0.17097	0.347769	HZ	EFNA5
0.288757	0.190265	HZ	RELCH	NC_056058.1	104190001	104210001	0.103008	0.353504	HZ	EFNA5
0.231338	0.200327	HZ	RELCH	NC_056058.1	104195001	104215001	0.110585	0.320204	HZ	EFNA5
0.351631	0.152849	HZ	RELCH	NC_056058.1	104200001	104220001	0.209597	0.246862	HZ	EFNA5
0.511235	0.222044	HZ	REC114	NC_056058.1	104205001	104225001	0.358306	0.211452	HZ	EFNA5
0.501803	0.169545	HZ	RDH13	NC_056069.1	36225001	36245001	0.43542	0.161081	HZ	EGFLAM
0.343321	0.154847	HZ	RBMS3	NC_056069.1	36230001	36250001	0.43226	0.161508	HZ	EGFLAM
0.201689	0.198402	HZ	RBMS3	NC_056069.1	36235001	36255001	0.438099	0.158418	HZ	EGFLAM
0.261086	0.192548	HZ	RBMS3	NC_056056.1	59240001	59260001	0.461916	0.169098	HZ	EIF2AK3
0.314729	0.154451	HZ	RBMS3	NC_056056.1	59245001	59265001	0.386218	0.205122	HZ	EIF2AK3
0.28331	0.179229	HZ	RBM19	NC_056056.1	59250001	59270001	0.355317	0.216772	HZ	EIF2AK3
0.0737474	0.215911	HZ	RBM19	NC_056056.1	59255001	59275001	0.460492	0.202348	HZ	EIF2AK3
0.0422862	0.260268	HZ	RBM19	NC_056056.1	59295001	59315001	0.393872	0.192954	HZ	EIF2AK3
0.043077	0.249539	HZ	RBM19	NC_056056.1	59300001	59320001	0.199423	0.275743	HZ	EIF2AK3
0.356779	0.195958	HZ	RBFOX1	NC_056056.1	59305001	59325001	0.272897	0.300483	HZ	EIF2AK3
0.420186	0.174901	HZ	RBFOX1	NC_056070.1	70105001	70125001	0.398164	0.178645	HZ	EIF4ENIF1
0.313239	0.172302	HZ	RBFOX1	NC_056063.1	11545001	11565001	0.288897	0.192883	HZ	ELF1
0.18613	0.187773	HZ	RASGEF1B	NC_056063.1	11550001	11570001	0.355625	0.198011	HZ	ELF1
0.226796	0.166606	HZ	RASGEF1B	NC_056063.1	11555001	11575001	0.335339	0.187402	HZ	ELF1
0.381127	0.169331	HZ	RARS2	NC_056063.1	11560001	11580001	0.361184	0.181126	HZ	ELF1
0.42245	0.15035	HZ	RARS2	NC_056063.1	11575001	11595001	0.331897	0.196672	HZ	ELF1
0.242004	0.183569	HZ	RARB	NC_056063.1	11580001	11600001	0.237683	0.222291	HZ	ELF1
0.263449	0.17423	HZ	RARB	NC_056063.1	11585001	11605001	0.109566	0.24849	HZ	ELF1
0.364377	0.149404	HZ	RARB	NC_056063.1	11590001	11610001	0.211087	0.186687	HZ	ELF1
0.431706	0.157007	HZ	RAD54B	NC_056077.1	41660001	41680001	0.415271	0.205964	HZ	ELFN1
0.155401	0.181091	HZ	RAB8B	NC_056077.1	41665001	41685001	0.321097	0.234019	HZ	ELFN1
0.139281	0.19967	HZ	RAB8B	NC_056077.1	41670001	41690001	0.259734	0.234695	HZ	ELFN1
0.216338	0.185376	HZ	RAB8B	NC_056077.1	41675001	41695001	0.373141	0.177674	HZ	ELFN1
0.247018	0.201943	HZ	RAB8B	NC_056058.1	4450001	4470001	0.233629	0.186784	HZ	ELL;FKBP8
0.401349	0.191922	HZ	RAB8B	NC_056058.1	4455001	4475001	0.286709	0.162529	HZ	ELL;FKBP8
0.324982	0.338764	HZ	RAB6B	NC_056061.1	6935001	6955001	0.459604	0.216653	HZ	ELOVL4
0.373842	0.33235	HZ	RAB6B	NC_056059.1	86735001	86755001	0.418462	0.218346	HZ	ENAM

0.472838	0.278936	HZ	RAB6B	NC_056063.1	14060001	14080001	0.185139	0.165694	HZ	ENOX1
0.222961	0.196756	HZ	RAB21	NC_056063.1	14065001	14085001	0.171936	0.165696	HZ	ENOX1
0.231354	0.17138	HZ	RAB21	NC_056056.1	540001	560001	0.426363	0.263823	HZ	ENTPD8;EXD3
0.200258	0.185454	HZ	RAB21	NC_056056.1	545001	565001	0.407592	0.238969	HZ	ENTPD8;EXD3
0.139356	0.195818	HZ	RAB21	NC_056056.1	530001	550001	0.458424	0.324098	HZ	ENTPD8;NOX/
0.135213	0.195794	HZ	RAB21	NC_056056.1	535001	555001	0.424348	0.288113	HZ	ENTPD8;NOX/
0.129112	0.194148	HZ	RAB21	NC_056054.1	158220001	158240001	0.335311	0.211454	HZ	EPHA3
0.146748	0.184447	HZ	RAB21	NC_056054.1	158225001	158245001	0.362541	0.230184	HZ	EPHA3
0.223723	0.172386	HZ	RAB21	NC_056059.1	81580001	81600001	0.453348	0.451732	HZ	EPHA5
0.308288	0.150966	HZ	RAB21	NC_056059.1	81585001	81605001	0.237988	0.557068	HZ	EPHA5
0.499776	0.151347	HZ	RAB11FIP4	NC_056059.1	81590001	81610001	0.347517	0.506457	HZ	EPHA5
0.50529	0.187435	HZ	RAB11FIP4	NC_056059.1	81595001	81615001	0.402681	0.473445	HZ	EPHA5
0.459157	0.151144	HZ	PWP1	NC_056059.1	81615001	81635001	0.40244	0.453136	HZ	EPHA5
0.43697	0.140044	HZ	PUS7L	NC_056059.1	81620001	81640001	0.391662	0.472464	HZ	EPHA5
0.526924	0.163644	HZ	PTPRM	NC_056059.1	81645001	81665001	0.414865	0.422535	HZ	EPHA5
0.489152	0.208405	HZ	PTPRM	NC_056059.1	81650001	81670001	0.129515	0.582779	HZ	EPHA5
0.499115	0.226476	HZ	PTPRM	NC_056059.1	81655001	81675001	0.213631	0.555061	HZ	EPHA5
0.112785	0.157584	HZ	PTPRE	NC_056059.1	81660001	81680001	0.430924	0.511868	HZ	EPHA5
0.398678	0.259559	HZ	PTPRD	NC_056059.1	81715001	81735001	0.354806	0.341786	HZ	EPHA5
0.253766	0.284707	HZ	PTPRD	NC_056059.1	81720001	81740001	0.220091	0.338515	HZ	EPHA5
0.188751	0.298322	HZ	PTPRD	NC_056059.1	81725001	81745001	0.236494	0.328164	HZ	EPHA5
0.418837	0.238002	HZ	PTPRD	NC_056059.1	81730001	81750001	0.258266	0.305622	HZ	EPHA5
0.113895	0.147732	HZ	PTPN3	NC_056059.1	81735001	81755001	0.402272	0.232493	HZ	EPHA5
0.342722	0.229432	HZ	PTPA	NC_056054.1	162040001	162060001	0.48677	0.158441	HZ	EPHA6
0.444007	0.179967	HZ	PTPA	NC_056054.1	162045001	162065001	0.373451	0.273718	HZ	EPHA6
0.394511	0.261298	HZ	PTGFR	NC_056054.1	162495001	162515001	0.438099	0.202987	HZ	EPHA6
0.34153	0.235187	HZ	PTGFR	NC_056054.1	162500001	162520001	0.480577	0.171595	HZ	EPHA6
0.503095	0.151562	HZ	PTGFR	NC_056065.1	28140001	28160001	0.354947	0.204874	HZ	EPHX1
0.496773	0.141722	HZ	PTGER3;ZRANB2	NC_056065.1	28145001	28165001	0.381242	0.242445	HZ	EPHX1;TMEM
0.392685	0.190005	HZ	PTGER3	NC_056065.1	28150001	28170001	0.353921	0.287044	HZ	EPHX1;TMEM
0.414247	0.163806	HZ	PTGER3	NC_056058.1	6430001	6450001	0.197755	0.173491	HZ	EPS15L1
0.167909	0.149034	HZ	PTER	NC_056058.1	6435001	6455001	0.0884246	0.257121	HZ	EPS15L1
0.0841502	0.183243	HZ	PTER	NC_056054.1	87095001	87115001	0.394633	0.173037	HZ	EPS8L3
0.0737447	0.185666	HZ	PTER	NC_056060.1	11520001	11540001	0.445811	0.226284	HZ	ERO1A;GPR13
0.0548357	0.188566	HZ	PTER	NC_056060.1	11525001	11545001	0.295122	0.263024	HZ	ERO1A;GPR13
0.452068	0.182115	HZ	PSMB11;PSMB5	NC_056060.1	11530001	11550001	0.14366	0.265507	HZ	ERO1A;GPR13

0.342525	0.160403	HZ	PSD4	NC_056070.1	72625001	72645001	0.288799	0.217527	HZ	ESS2;GSC2;TS
0.44093	0.196453	HZ	PSD4	NC_056067.1	51445001	51465001	0.113826	0.165257	HZ	ETHE1;ZNF57
0.154303	0.157982	HZ	PSD3	NC_056054.1	69885001	69905001	0.43142	0.251367	HZ	EVI5
0.0518054	0.173142	HZ	PSD3	NC_056054.1	69890001	69910001	0.480542	0.29476	HZ	EVI5
0.112472	0.187767	HZ	PSD3	NC_056056.1	560001	580001	0.409437	0.226932	HZ	EXD3
0.433134	0.161754	HZ	PRUNE2	NC_056056.1	565001	585001	0.388172	0.184988	HZ	EXD3
0.342375	0.25071	HZ	PRUNE2	NC_056056.1	570001	590001	0.41462	0.204887	HZ	EXD3
0.285534	0.263276	HZ	PRUNE2	NC_056056.1	575001	595001	0.488199	0.287873	HZ	EXD3
0.253534	0.309125	HZ	PRUNE2	NC_056056.1	610001	630001	0.483631	0.163826	HZ	EXD3
0.307644	0.276694	HZ	PRUNE2	NC_056056.1	550001	570001	0.419023	0.260229	HZ	EXD3;NOXA1
0.520379	0.147296	HZ	PRUNE2	NC_056056.1	555001	575001	0.416115	0.273331	HZ	EXD3;NOXA1
0.493617	0.144807	HZ	PRRG1	NC_056059.1	71955001	71975001	0.434014	0.161515	HZ	EXOC1L
0.188831	0.214062	HZ	PRR5	NC_056062.1	59445001	59465001	0.467286	0.200763	HZ	EXT1
0.324649	0.176353	HZ	PRR5	NC_056061.1	58800001	58820001	0.152322	0.190608	HZ	EYA4
0.443886	0.14461	HZ	PRR5	NC_056061.1	58805001	58825001	0.177901	0.180081	HZ	EYA4
0.450253	0.232417	HZ	PRR14;ZNF689	NC_056065.1	36420001	36440001	0.153954	0.472872	HZ	F5
0.433775	0.241023	HZ	PRR14	NC_056065.1	36425001	36445001	0.1467	0.428663	HZ	F5
0.477949	0.195795	HZ	PRORP	NC_056065.1	36430001	36450001	0.275324	0.3332	HZ	F5
0.430233	0.256836	HZ	PROM2	NC_056054.1	86010001	86030001	0.389769	0.159581	HZ	FAM102B;HEN
0.433696	0.273208	HZ	PROM2	NC_056060.1	55915001	55935001	0.413899	0.229132	HZ	FAM214A
0.415377	0.290536	HZ	PROM2	NC_056060.1	55935001	55955001	0.450952	0.25951	HZ	FAM214A
0.3958	0.315025	HZ	PROM2	NC_056055.1	52710001	52730001	0.305747	0.176763	HZ	FAM221B;TMI
0.499033	0.1625	HZ	PRKCE	NC_056055.1	29190001	29210001	0.102556	0.359201	HZ	FAM240B
0.45514	0.14632	HZ	PRKCE	NC_056055.1	29195001	29215001	0.0831636	0.370677	HZ	FAM240B
0.0503124	0.16789	HZ	PRKCE	NC_056055.1	29200001	29220001	0.10172	0.36613	HZ	FAM240B
0.0452511	0.191424	HZ	PRKCE	NC_056055.1	29205001	29225001	0.151737	0.351216	HZ	FAM240B
0.0419804	0.225971	HZ	PRKCE	NC_056055.1	29210001	29230001	0.237342	0.335399	HZ	FAM240B
0.0317602	0.239684	HZ	PRKCE	NC_056054.1	395001	415001	0.442179	0.168883	HZ	FARP2
0.171132	0.190335	HZ	PRKCE	NC_056054.1	400001	420001	0.446734	0.184301	HZ	FARP2
0.161972	0.449903	HZ	PRKAR2B	NC_056054.1	405001	425001	0.40401	0.206985	HZ	FARP2
0.331625	0.364871	HZ	PRKAR2B	NC_056054.1	410001	430001	0.371557	0.186758	HZ	FARP2
0.475806	0.294679	HZ	PRKAR2B	NC_056065.1	51075001	51095001	0.292651	0.187495	HZ	FBLIM1
0.489555	0.27753	HZ	PRKAR2B	NC_056065.1	51080001	51100001	0.324361	0.185227	HZ	FBLIM1
0.476735	0.282456	HZ	PRKAR2B	NC_056058.1	104680001	104700001	0.429612	0.204144	HZ	FBXL17
0.48987	0.271331	HZ	PRKAR2B	NC_056058.1	104685001	104705001	0.409838	0.209858	HZ	FBXL17
0.460565	0.364062	HZ	PRKAR2A;SLC25	NC_056058.1	104690001	104710001	0.434509	0.209206	HZ	FBXL17

0.235856	0.138642	HZ	PRKACB	NC_056058.1	104695001	104715001	0.485113	0.184015	HZ	FBXL17
0.518429	0.146189	HZ	PRDM5	NC_056064.1	39580001	39600001	0.414163	0.165979	HZ	FBXL20;MED1
0.504851	0.180279	HZ	PRDM5	NC_056071.1	47035001	47055001	0.432432	0.166626	HZ	FBXO33
0.38288	0.146408	HZ	PRDM4;PWP1	NC_056071.1	47040001	47060001	0.477321	0.181092	HZ	FBXO33
0.217201	0.212301	HZ	PRDM4	NC_056054.1	108050001	108070001	0.422992	0.290302	HZ	FCRL3
0.161836	0.237444	HZ	PRDM4	NC_056054.1	108055001	108075001	0.471348	0.231969	HZ	FCRL3
0.13951	0.243097	HZ	PRDM4	NC_056054.1	108015001	108035001	0.342603	0.197317	HZ	FCRL3;LOC10
0.218296	0.203292	HZ	PRDM4	NC_056064.1	55880001	55900001	0.398554	0.203344	HZ	FDXR;GRIN2C
0.412467	0.148243	HZ	PRDM4	NC_056080.1	68525001	68545001	0.237006	0.396574	HZ	FGF16
0.0706755	0.153124	HZ	PPP4R2	NC_056059.1	95690001	95710001	0.187997	0.179947	HZ	FGF5
0.0979496	0.150404	HZ	PPP4R2	NC_056056.1	188440001	188460001	0.191449	0.158605	HZ	FGFR1OP2
0.441315	0.161309	HZ	PPP3CC	NC_056061.1	27770001	27790001	0.436321	0.166817	HZ	FIG4
0.238627	0.159142	HZ	PPP3CC	NC_056058.1	4445001	4465001	0.29724	0.197129	HZ	FKBP8
0.210974	0.154164	HZ	PPP3CC	NC_056060.1	99260001	99280001	0.265663	0.203617	HZ	FOXN3
0.404784	0.144275	HZ	PPP3CC	NC_056060.1	99265001	99285001	0.376131	0.263093	HZ	FOXN3
0.421476	0.149649	HZ	PPP3CA	NC_056060.1	99270001	99290001	0.474291	0.260525	HZ	FOXN3
0.486357	0.148064	HZ	PPP3CA	NC_056072.1	30605001	30625001	0.351677	0.186374	HZ	FOXP1
0.494323	0.179716	HZ	PPP2R2B	NC_056072.1	30610001	30630001	0.361744	0.203637	HZ	FOXP1
0.0706401	0.192898	HZ	PPM1H	NC_056072.1	30615001	30635001	0.426449	0.282638	HZ	FOXP1
0.0718182	0.186618	HZ	PPM1H	NC_056072.1	30620001	30640001	0.449932	0.315561	HZ	FOXP1
0.0804763	0.159008	HZ	PPM1H	NC_056072.1	30625001	30645001	0.434324	0.298028	HZ	FOXP1
0.0788203	0.153037	HZ	PPM1H	NC_056072.1	30630001	30650001	0.469637	0.283823	HZ	FOXP1
0.117382	0.140497	HZ	PPM1H	NC_056057.1	55940001	55960001	0.341763	0.196209	HZ	FOXP2
0.405769	0.249274	HZ	PPIL4	NC_056057.1	55945001	55965001	0.360193	0.187137	HZ	FOXP2
0.293488	0.268678	HZ	PPIL4	NC_056057.1	55950001	55970001	0.398782	0.158741	HZ	FOXP2
0.516139	0.161322	HZ	PPIL4	NC_056061.1	21815001	21835001	0.388172	0.164597	HZ	FRK
0.44807	0.14771	HZ	PPFIA2	NC_056061.1	21820001	21840001	0.38542	0.165958	HZ	FRK
0.522501	0.271088	HZ	PPEF1	NC_056061.1	21825001	21845001	0.326524	0.18025	HZ	FRK
0.491676	0.167173	HZ	PPARGC1B	NC_056061.1	21830001	21850001	0.302409	0.238086	HZ	FRK
0.390908	0.14281	HZ	PPARGC1B	NC_056061.1	21835001	21855001	0.372407	0.208473	HZ	FRK
0.449854	0.158294	HZ	PPARGC1B	NC_056061.1	21840001	21860001	0.405019	0.187249	HZ	FRK
0.535767	0.181457	HZ	PPARGC1B	NC_056061.1	21890001	21910001	0.227469	0.172878	HZ	FRK
0.470884	0.157783	HZ	PPA2	NC_056061.1	21895001	21915001	0.246715	0.19663	HZ	FRK
0.458801	0.17066	HZ	PPA2	NC_056071.1	22390001	22410001	0.471565	0.184409	HZ	FSD2;WHAMM
0.455048	0.17287	HZ	PPA2	NC_056060.1	33530001	33550001	0.487894	0.163949	HZ	FSIP1
0.434002	0.142848	HZ	PPA2	NC_056070.1	37230001	37250001	0.424359	0.174512	HZ	FSTL5

0.0856599	0.153946	HZ	PON3	NC_056070.1	37235001	37255001	0.337968	0.193726	HZ	FSTL5
0.168751	0.147858	HZ	PON3	NC_056070.1	37240001	37260001	0.220813	0.20156	HZ	FSTL5
0.488253	0.210806	HZ	POLR3C;RNF115	NC_056060.1	75795001	75815001	0.432021	0.276275	HZ	FUT8
0.262254	0.218247	HZ	POLR1A	NC_056060.1	75800001	75820001	0.476623	0.26387	HZ	FUT8
0.297017	0.15002	HZ	POLQ	NC_056055.1	49235001	49255001	0.431842	0.18755	HZ	GABBR2
0.315238	0.152815	HZ	POLQ	NC_056055.1	49240001	49260001	0.412569	0.166288	HZ	GABBR2
0.239674	0.14592	HZ	PNKD	NC_056080.1	83360001	83380001	0.309504	0.206199	HZ	GABRA3
0.24471	0.153884	HZ	PNKD	NC_056080.1	83365001	83385001	0.312812	0.221103	HZ	GABRA3
0.0729579	0.174019	HZ	PLXNC1	NC_056080.1	83370001	83390001	0.283145	0.238549	HZ	GABRA3
0.167032	0.156814	HZ	PLXNC1	NC_056080.1	83375001	83395001	0.222885	0.248679	HZ	GABRA3
0.416379	0.194317	HZ	PLXNB1	NC_056080.1	83380001	83400001	0.150537	0.24983	HZ	GABRA3
0.459732	0.197879	HZ	PLXNB1	NC_056080.1	83385001	83405001	0.0709941	0.260551	HZ	GABRA3
0.465248	0.365655	HZ	PLGRKT	NC_056080.1	83390001	83410001	0.101031	0.241039	HZ	GABRA3
0.468133	0.362719	HZ	PLGRKT	NC_056080.1	83395001	83415001	0.224399	0.269122	HZ	GABRA3
0.467937	0.363477	HZ	PLGRKT	NC_056080.1	83400001	83420001	0.343782	0.280573	HZ	GABRA3
0.431776	0.295943	HZ	PLEKHG2;ZFP36	NC_056080.1	83405001	83425001	0.400387	0.273716	HZ	GABRA3
0.464455	0.299543	HZ	PLEKHG2;RPS16;[illegible]	NC_056080.1	83410001	83430001	0.404151	0.286123	HZ	GABRA3
0.451891	0.178107	HZ	PLEKHA6	NC_056080.1	83415001	83435001	0.337248	0.25594	HZ	GABRA3
0.396825	0.203451	HZ	PLEKHA6	NC_056080.1	83420001	83440001	0.309744	0.267051	HZ	GABRA3
0.475521	0.158223	HZ	PLEKHA6	NC_056080.1	83425001	83445001	0.356131	0.311768	HZ	GABRA3
0.0972543	0.147798	HZ	PLCB1	NC_056080.1	83430001	83450001	0.410612	0.358023	HZ	GABRA3
0.0416233	0.159448	HZ	PLCB1	NC_056080.1	83450001	83470001	0.467741	0.335831	HZ	GABRA3
0.441293	0.347775	HZ	PKP4	NC_056080.1	83455001	83475001	0.325411	0.273819	HZ	GABRA3
0.488504	0.259371	HZ	PKP4	NC_056080.1	83460001	83480001	0.269621	0.214451	HZ	GABRA3
0.537207	0.157338	HZ	PKP4	NC_056080.1	83465001	83485001	0.270883	0.202493	HZ	GABRA3
0.187154	0.141835	HZ	PKN3;SET	NC_056080.1	83470001	83490001	0.260289	0.177044	HZ	GABRA3
0.533465	0.140493	HZ	PKIG	NC_056080.1	83500001	83520001	0.213235	0.172574	HZ	GABRA3
0.425879	0.166505	HZ	PIGU	NC_056080.1	83505001	83525001	0.163663	0.242287	HZ	GABRA3
0.418014	0.146897	HZ	PIGU	NC_056080.1	83510001	83530001	0.124318	0.277825	HZ	GABRA3
0.36	0.160925	HZ	PHLDB1;TREH	NC_056078.1	1660001	1680001	0.0554568	0.167333	HZ	GALNT2
0.434411	0.152462	HZ	PHLDB1;TREH	NC_056078.1	1710001	1730001	0.220032	0.172123	HZ	GALNT2
0.460852	0.154337	HZ	PHLDB1;TREH	NC_056078.1	1715001	1735001	0.160575	0.180651	HZ	GALNT2
0.496735	0.143122	HZ	PHLDB1;TREH	NC_056078.1	1720001	1740001	0.261872	0.200577	HZ	GALNT2
0.341914	0.152534	HZ	PHLDB1	NC_056067.1	14340001	14360001	0.19677	0.43544	HZ	GAS8
0.413953	0.152499	HZ	PHF11	NC_056067.1	14345001	14365001	0.43686	0.35974	HZ	GAS8
0.476693	0.24998	HZ	PHEX	NC_056067.1	14330001	14350001	0.363251	0.362405	HZ	GAS8;LOC101

0.415198	0.273194	HZ	PHEX	NC_056067.1	14335001	14355001	0.226502	0.429245	HZ	GAS8;LOC101
0.209575	0.287944	HZ	PHEX	NC_056072.1	59225001	59245001	0.371246	0.172511	HZ	GATA2
0.283879	0.270567	HZ	PHEX	NC_056072.1	59230001	59250001	0.25436	0.268011	HZ	GATA2
0.356583	0.238119	HZ	PHEX	NC_056077.1	17580001	17600001	0.406952	0.200387	HZ	GDE1
0.41196	0.146758	HZ	PHC1	NC_056062.1	80965001	80985001	0.315083	0.206194	HZ	GDF6
0.258849	0.19165	HZ	PHC1	NC_056062.1	80970001	80990001	0.291506	0.247314	HZ	GDF6
0.302919	0.16551	HZ	PHC1	NC_056062.1	80975001	80995001	0.332387	0.209106	HZ	GDF6
0.182039	0.186552	HZ	PGM5	NC_056056.1	28525001	28545001	0.107408	0.19465	HZ	GDF7;LDAH
0.196262	0.163512	HZ	PGM5	NC_056056.1	28530001	28550001	0.0887228	0.193454	HZ	GDF7;LDAH
0.31128	0.143429	HZ	PGM5	NC_056054.1	260195001	260215001	0.254292	0.17082	HZ	GET1;LCA5L
0.315745	0.14118	HZ	PGM5	NC_056075.1	35975001	35995001	0.422924	0.165972	HZ	GFRA1
0.352053	0.144417	HZ	PGM5	NC_056063.1	76525001	76545001	0.204337	0.240751	HZ	GGACT
0.308099	0.183865	HZ	PGM5	NC_056063.1	76530001	76550001	0.112765	0.309778	HZ	GGACT
0.305367	0.182654	HZ	PGM5	NC_056063.1	76535001	76555001	0.078251	0.342	HZ	GGACT
0.330557	0.16602	HZ	PGM5	NC_056063.1	76540001	76560001	0.133462	0.288141	HZ	GGACT
0.376763	0.146517	HZ	PGM5	NC_056063.1	76545001	76565001	0.152986	0.25235	HZ	GGACT
0.511298	0.398058	HZ	PELI2	NC_056063.1	76550001	76570001	0.223435	0.242809	HZ	GGACT
0.501898	0.15958	HZ	PELI1	NC_056063.1	76555001	76575001	0.365495	0.203511	HZ	GGACT
0.33241	0.230047	HZ	PDS5B	NC_056063.1	76560001	76580001	0.412978	0.228131	HZ	GGACT
0.42857	0.19418	HZ	PDS5B	NC_056063.1	76565001	76585001	0.464807	0.211304	HZ	GGACT;TMTC
0.328571	0.170522	HZ	PDS5B	NC_056060.1	57115001	57135001	0.460605	0.159091	HZ	GLDN
0.406232	0.157521	HZ	PDK3	NC_056060.1	57130001	57150001	0.439937	0.157686	HZ	GLDN
0.475241	0.167352	HZ	PDGFD	NC_056060.1	57160001	57180001	0.46351	0.158184	HZ	GLDN
0.478143	0.152285	HZ	PDGFD	NC_056077.1	4060001	4080001	0.205697	0.171085	HZ	GLIS2;LOC101
0.489715	0.159541	HZ	PDE8B	NC_056064.1	21985001	22005001	0.224069	0.238761	HZ	GLOD4;LOC12
0.515833	0.157463	HZ	PDE8B	NC_056064.1	21990001	22010001	0.237812	0.234274	HZ	GLOD4;LOC12
0.386162	0.148379	HZ	PDE8B	NC_056064.1	21995001	22015001	0.379671	0.221749	HZ	GLOD4;LOC12
0.322405	0.267745	HZ	PDE6A;SLC26A2	NC_056064.1	21975001	21995001	0.404686	0.188016	HZ	GLOD4;MRM3
0.238258	0.303225	HZ	PDE6A;SLC26A2	NC_056064.1	21980001	22000001	0.190281	0.240562	HZ	GLOD4;MRM3
0.215535	0.293425	HZ	PDE6A;SLC26A2	NC_056056.1	38495001	38515001	0.26618	0.245992	HZ	GMCL1
0.170588	0.250458	HZ	PDE6A;SLC26A2	NC_056056.1	38500001	38520001	0.233436	0.257336	HZ	GMCL1
0.10479	0.196849	HZ	PDE6A;SLC26A2	NC_056056.1	38505001	38525001	0.314941	0.242295	HZ	GMCL1
0.123595	0.176331	HZ	PDE6A;SLC26A2	NC_056056.1	38510001	38530001	0.434253	0.229011	HZ	GMCL1
0.165579	0.184418	HZ	PDE6A;SLC26A2	NC_056055.1	59085001	59105001	0.244399	0.175637	HZ	GNAQ
0.175271	0.262452	HZ	PDE6A;SLC26A2	NC_056055.1	59090001	59110001	0.230589	0.21003	HZ	GNAQ
0.259875	0.291859	HZ	PDE6A;SLC26A2	NC_056055.1	59095001	59115001	0.275401	0.206371	HZ	GNAQ

0.293512	0.350838	HZ	PDE6A;SLC26A2	NC_056055.1	59100001	59120001	0.388199	0.158039	HZ	GNAQ
0.315364	0.393028	HZ	PDE6A;SLC26A2	NC_056078.1	7740001	7760001	0.427496	0.165082	HZ	GNG4;LYST
0.338831	0.365567	HZ	PDE6A;SLC26A2	NC_056063.1	66735001	66755001	0.42994	0.279196	HZ	GPC5
0.313471	0.316753	HZ	PDE6A;SLC26A2	NC_056063.1	66740001	66760001	0.426209	0.293662	HZ	GPC5
0.3125	0.247028	HZ	PDE6A;SLC26A2	NC_056063.1	66745001	66765001	0.399076	0.293748	HZ	GPC5
0.296422	0.172864	HZ	PDE6A;SLC26A2	NC_056063.1	68330001	68350001	0.343784	0.207465	HZ	GPC6
0.269268	0.173604	HZ	PDE6A	NC_056063.1	68335001	68355001	0.186674	0.294056	HZ	GPC6
0.466317	0.211045	HZ	PDE4D	NC_056063.1	68340001	68360001	0.103823	0.368955	HZ	GPC6
0.520428	0.167684	HZ	PDE4D	NC_056063.1	68345001	68365001	0.226982	0.389888	HZ	GPC6
0.394656	0.172966	HZ	PDCL2	NC_056063.1	68350001	68370001	0.479366	0.223215	HZ	GPC6
0.407694	0.163944	HZ	PDCL2	NC_056063.1	68470001	68490001	0.376989	0.183007	HZ	GPC6
0.494874	0.16383	HZ	PCLO	NC_056063.1	68960001	68980001	0.323529	0.158038	HZ	GPC6
0.510252	0.210452	HZ	PCLO	NC_056063.1	68965001	68985001	0.205576	0.182494	HZ	GPC6
0.526984	0.209259	HZ	PCLO	NC_056063.1	68970001	68990001	0.114912	0.198938	HZ	GPC6
0.501651	0.239766	HZ	PCLO	NC_056060.1	77030001	77050001	0.375771	0.172064	HZ	GPHN
0.041242	0.162881	HZ	PCDH7	NC_056060.1	77035001	77055001	0.433603	0.162105	HZ	GPHN
0.169186	0.154476	HZ	PCDH7	NC_056060.1	77130001	77150001	0.440631	0.270474	HZ	GPHN
0.0810568	0.222853	HZ	PCARE;TOGARA	NC_056060.1	77135001	77155001	0.463645	0.336049	HZ	GPHN
0.127329	0.194276	HZ	PAPSS1	NC_056060.1	77140001	77160001	0.460396	0.313832	HZ	GPHN
0.327738	0.225086	HZ	PAPSS1	NC_056060.1	77145001	77165001	0.453291	0.237729	HZ	GPHN
0.472765	0.176852	HZ	PAPSS1	NC_056060.1	77150001	77170001	0.461178	0.201081	HZ	GPHN
0.49597	0.147149	HZ	PAPPA	NC_056060.1	11535001	11555001	0.0745796	0.237365	HZ	GPR137C
0.42723	0.147524	HZ	PAMR1	NC_056060.1	11545001	11565001	0.0373491	0.181334	HZ	GPR137C
0.49465	0.143394	HZ	PAMR1	NC_056060.1	33690001	33710001	0.17747	0.17627	HZ	GPR176
0.299963	0.144842	HZ	PALS1	NC_056075.1	42950001	42970001	0.382779	0.163243	HZ	GPR26
0.30309	0.155266	HZ	PALS1	NC_056055.1	178595001	178615001	0.483489	0.197665	HZ	GPR39
0.364449	0.139532	HZ	PALS1	NC_056054.1	86240001	86260001	0.477051	0.236056	HZ	GPSM2
0.366421	0.183473	HZ	PALS1	NC_056068.1	33935001	33955001	0.299372	0.170477	HZ	GRAMD1B
0.302096	0.236687	HZ	PALS1	NC_056068.1	33940001	33960001	0.242145	0.220899	HZ	GRAMD1B
0.286667	0.239198	HZ	PALS1	NC_056068.1	33945001	33965001	0.346847	0.208903	HZ	GRAMD1B
0.208603	0.271066	HZ	PALS1	NC_056068.1	33950001	33970001	0.39798	0.179988	HZ	GRAMD1B
0.171558	0.282609	HZ	PALS1	NC_056077.1	9020001	9040001	0.221698	0.176547	HZ	GRIN2A
0.169643	0.28125	HZ	PALS1	NC_056077.1	9025001	9045001	0.176442	0.197198	HZ	GRIN2A
0.158125	0.284106	HZ	PALS1	NC_056077.1	9030001	9050001	0.136406	0.200082	HZ	GRIN2A
0.169032	0.280733	HZ	PALS1	NC_056064.1	55885001	55905001	0.470651	0.158006	HZ	GRIN2C;TME
0.265334	0.239807	HZ	PALS1	NC_056055.1	21440001	21460001	0.286368	0.182615	HZ	GRIN3A

0.298762	0.230468	HZ	PALS1	NC_056055.1	21445001	21465001	0.183989	0.253761	HZ	GRIN3A
0.411539	0.204885	HZ	PALS1	NC_056055.1	21450001	21470001	0.209566	0.192501	HZ	GRIN3A
0.438462	0.194477	HZ	PALS1	NC_056055.1	21455001	21475001	0.206971	0.18002	HZ	GRIN3A
0.409091	0.200654	HZ	PALS1	NC_056055.1	21460001	21480001	0.210506	0.160436	HZ	GRIN3A
0.42729	0.231498	HZ	OTUD7A	NC_056072.1	57655001	57675001	0.375024	0.167743	HZ	GRIP2
0.490084	0.231816	HZ	OTUD7A	NC_056072.1	57660001	57680001	0.317836	0.188538	HZ	GRIP2
0.330006	0.177763	HZ	OTUD7A	NC_056072.1	57665001	57685001	0.311717	0.214438	HZ	GRIP2
0.467554	0.201067	HZ	OTUD7A	NC_056072.1	57670001	57690001	0.347141	0.195683	HZ	GRIP2
0.462861	0.173599	HZ	OTUD7A	NC_056074.1	6210001	6230001	0.463645	0.228212	HZ	GRM5
0.507352	0.284339	HZ	OSTN;UTS2B	NC_056074.1	6215001	6235001	0.384076	0.277651	HZ	GRM5
0.522419	0.30387	HZ	OSTN;UTS2B	NC_056074.1	6220001	6240001	0.309073	0.331669	HZ	GRM5
0.513542	0.186681	HZ	OSBP2	NC_056074.1	6225001	6245001	0.30404	0.282556	HZ	GRM5
0.266358	0.222058	HZ	OSBP2	NC_056074.1	6230001	6250001	0.283026	0.201192	HZ	GRM5
0.28714	0.2452	HZ	OSBP2	NC_056074.1	6235001	6255001	0.379778	0.16953	HZ	GRM5
0.161654	0.370498	HZ	OSBP2	NC_056072.1	19205001	19225001	0.261199	0.169096	HZ	GRM7
0.117698	0.415473	HZ	OSBP2	NC_056072.1	19215001	19235001	0.150666	0.184259	HZ	GRM7
0.13012	0.431001	HZ	OSBP2	NC_056072.1	19235001	19255001	0.195163	0.163269	HZ	GRM7
0.150943	0.378692	HZ	OSBP2	NC_056072.1	19510001	19530001	0.456261	0.168968	HZ	GRM7
0.229911	0.30885	HZ	OSBP2	NC_056057.1	92400001	92420001	0.491324	0.178412	HZ	GRM8
0.25758	0.296635	HZ	OSBP2	NC_056057.1	92405001	92425001	0.490691	0.170452	HZ	GRM8
0.499172	0.281226	HZ	OLFML2A	NC_056077.1	26025001	26045001	0.203791	0.205927	HZ	GSG1L
0.526314	0.234655	HZ	OLFML2A	NC_056077.1	26030001	26050001	0.0622797	0.219483	HZ	GSG1L
0.452906	0.193186	HZ	OLFML2A	NC_056077.1	26035001	26055001	0.0429651	0.225957	HZ	GSG1L
0.400503	0.161637	HZ	OLFML2A	NC_056054.1	174690001	174710001	0.379168	0.163793	HZ	GUCA1C
0.394514	0.201938	HZ	OLFML2A	NC_056054.1	174695001	174715001	0.336355	0.205263	HZ	GUCA1C
0.477792	0.21811	HZ	NYAP2	NC_056054.1	174700001	174720001	0.333333	0.220915	HZ	GUCA1C
0.528827	0.177437	HZ	NYAP2	NC_056054.1	174705001	174725001	0.319971	0.22664	HZ	GUCA1C
0.462304	0.147235	HZ	NYAP2	NC_056054.1	174735001	174755001	0.345013	0.158051	HZ	GUCA1C;MOR
0.390949	0.172058	HZ	NUP160	NC_056080.1	125775001	125795001	0.276353	0.165668	HZ	GUCY2F
0.485682	0.220473	HZ	NUP160	NC_056080.1	125780001	125800001	0.356016	0.165818	HZ	GUCY2F
0.490567	0.144158	HZ	NUDT21;OGFOD1	NC_056056.1	165830001	165850001	0.305407	0.172819	HZ	HAL
0.453324	0.19828	HZ	NUDT17;PIAS3;PO	NC_056061.1	60300001	60320001	0.454043	0.170453	HZ	HBS1L
0.443992	0.172486	HZ	NUDT17;PIAS3;PO	NC_056056.1	173330001	173350001	0.311155	0.287977	HZ	HCFC2
0.401337	0.156006	HZ	NTRK2	NC_056056.1	173335001	173355001	0.198718	0.302355	HZ	HCFC2
0.41468	0.167474	HZ	NTRK2	NC_056056.1	173345001	173365001	0.431856	0.245205	HZ	HCFC2;LOC10
0.419806	0.16454	HZ	NTRK2	NC_056056.1	173360001	173380001	0.483993	0.261266	HZ	HCFC2;NFYB

0.442666	0.142701	HZ	NTRK2	NC_056057.1	27850001	27870001	0.285059	0.162139	HZ	HDAC9
0.211202	0.140517	HZ	NSUN7	NC_056057.1	27855001	27875001	0.307073	0.186378	HZ	HDAC9
0.145108	0.150426	HZ	NSUN7	NC_056057.1	27860001	27880001	0.287331	0.194807	HZ	HDAC9
0.374341	0.140046	HZ	NSG1	NC_056057.1	27865001	27885001	0.365995	0.229102	HZ	HDAC9
0.149466	0.156287	HZ	NSF	NC_056057.1	27870001	27890001	0.451512	0.237066	HZ	HDAC9
0.239513	0.143906	HZ	NSF	NC_056057.1	27875001	27895001	0.477798	0.173533	HZ	HDAC9
0.220413	0.171586	HZ	NRXN3	NC_056057.1	27905001	27925001	0.289664	0.310163	HZ	HDAC9
0.239689	0.165796	HZ	NRXN3	NC_056057.1	27910001	27930001	0.234637	0.368782	HZ	HDAC9
0.530371	0.237493	HZ	NRXN1	NC_056057.1	27915001	27935001	0.30837	0.359071	HZ	HDAC9
0.44624	0.206621	HZ	NRXN1	NC_056057.1	27920001	27940001	0.473081	0.399943	HZ	HDAC9
0.537824	0.149793	HZ	NRXN1	NC_056057.1	27925001	27945001	0.490995	0.320831	HZ	HDAC9
0.428295	0.146375	HZ	NRP2	NC_056057.1	27935001	27955001	0.49049	0.282577	HZ	HDAC9
0.47013	0.152944	HZ	NRP2	NC_056057.1	27940001	27960001	0.42974	0.239677	HZ	HDAC9
0.485648	0.221089	HZ	NRK	NC_056055.1	198710001	198730001	0.438131	0.159386	HZ	HECW2
0.446011	0.15935	HZ	NRK	NC_056055.1	198715001	198735001	0.401093	0.187076	HZ	HECW2
0.476918	0.194139	HZ	NRG3	NC_056055.1	198720001	198740001	0.40912	0.177925	HZ	HECW2
0.489952	0.181154	HZ	NRG3	NC_056075.1	15560001	15580001	0.3357	0.196319	HZ	HELLS;TBC1D
0.395022	0.219938	HZ	NR6A1;OLFML2A	NC_056075.1	15565001	15585001	0.377979	0.183349	HZ	HELLS;TBC1D
0.420225	0.202807	HZ	NR6A1	NC_056075.1	15570001	15590001	0.457746	0.162492	HZ	HELLS;TBC1D
0.336343	0.212539	HZ	NR6A1	NC_056060.1	44385001	44405001	0.482609	0.16565	HZ	HERC1
0.370203	0.177832	HZ	NR6A1	NC_056057.1	104520001	104540001	0.388552	0.376229	HZ	HIPK2
0.326316	0.170768	HZ	NR6A1	NC_056057.1	104525001	104545001	0.351731	0.435204	HZ	HIPK2
0.342921	0.155609	HZ	NR6A1	NC_056057.1	104530001	104550001	0.290804	0.461759	HZ	HIPK2
0.25586	0.152239	HZ	NR5A1;NR6A1	NC_056057.1	104535001	104555001	0.183299	0.495098	HZ	HIPK2
0.538264	0.164148	HZ	NPPB	NC_056057.1	104540001	104560001	0.191036	0.483492	HZ	HIPK2
0.376705	0.168147	HZ	NPNT	NC_056064.1	5345001	5365001	0.256114	0.167654	HZ	HLF
0.39535	0.152643	HZ	NPNT	NC_056064.1	5350001	5370001	0.198806	0.188814	HZ	HLF
0.44054	0.147389	HZ	NPNT	NC_056064.1	5355001	5375001	0.177261	0.202578	HZ	HLF
0.243794	0.228071	HZ	NPAS2	NC_056064.1	5360001	5380001	0.27672	0.205811	HZ	HLF
0.265939	0.216877	HZ	NPAS2	NC_056064.1	5365001	5385001	0.443397	0.159457	HZ	HLF
0.498001	0.138848	HZ	NOTCH2	NC_056064.1	5370001	5390001	0.413004	0.177748	HZ	HLF
0.308846	0.188867	HZ	NOTCH2	NC_056065.1	66125001	66145001	0.0845104	0.339957	HZ	HMCN1
0.309887	0.146863	HZ	NMU	NC_056065.1	66130001	66150001	0.102669	0.313627	HZ	HMCN1
0.472656	0.179734	HZ	NMU	NC_056065.1	66135001	66155001	0.0876866	0.308769	HZ	HMCN1
0.455492	0.259247	HZ	NMU	NC_056065.1	66140001	66160001	0.0648681	0.325297	HZ	HMCN1
0.431789	0.335878	HZ	NMU	NC_056065.1	66145001	66165001	0.053455	0.356459	HZ	HMCN1

0.426681	0.281191	HZ	NMU	NC_056065.1	66150001	66170001	0.180217	0.305933	HZ	HMCN1
0.378445	0.234784	HZ	NMU	NC_056065.1	66155001	66175001	0.234156	0.289856	HZ	HMCN1
0.53778	0.175242	HZ	NMNAT2	NC_056065.1	66160001	66180001	0.272702	0.247533	HZ	HMCN1
0.167919	0.161406	HZ	NME7	NC_056065.1	66165001	66185001	0.300608	0.235263	HZ	HMCN1
0.148472	0.178661	HZ	NME7	NC_056065.1	66170001	66190001	0.21225	0.275177	HZ	HMCN1
0.324859	0.160787	HZ	NME7	NC_056065.1	66175001	66195001	0.156738	0.24963	HZ	HMCN1
0.318181	0.191491	HZ	NLK	NC_056065.1	66180001	66200001	0.0827586	0.261087	HZ	HMCN1
0.275997	0.210381	HZ	NLK	NC_056062.1	51825001	51845001	0.317939	0.214261	HZ	HNF4G
0.479699	0.143105	HZ	NLK	NC_056056.1	19570001	19590001	0.145848	0.164238	HZ	HPCAL1
0.394468	0.21796	HZ	NLGN1	NC_056056.1	19585001	19605001	0.0655737	0.181946	HZ	HPCAL1;ODC
0.398299	0.17162	HZ	NKAIN2	NC_056055.1	106295001	106315001	0.10253	0.158811	HZ	HPGD
0.0454964	0.230663	HZ	NKAIN2	NC_056075.1	19695001	19715001	0.374719	0.158346	HZ	HPSE2
0.0810351	0.218974	HZ	NKAIN2	NC_056075.1	19720001	19740001	0.353902	0.166148	HZ	HPSE2
0.246656	0.16644	HZ	NKAIN2	NC_056075.1	19725001	19745001	0.470589	0.191236	HZ	HPSE2
0.49594	0.205978	HZ	NIPAL3;STPG1	NC_056060.1	5460001	5480001	0.439333	0.218008	HZ	HRH2
0.482883	0.189735	HZ	NHS	NC_056060.1	5465001	5485001	0.474556	0.197939	HZ	HRH2
0.42616	0.162086	HZ	NHS	NC_056060.1	5470001	5490001	0.478053	0.172847	HZ	HRH2
0.441424	0.168449	HZ	NHS	NC_056054.1	114350001	114370001	0.122507	0.252445	HZ	HSD17B7
0.440105	0.195855	HZ	NEXMIF	NC_056054.1	114355001	114375001	0.230258	0.206824	HZ	HSD17B7
0.40367	0.23834	HZ	NEXMIF	NC_056054.1	96955001	96975001	0.190332	0.180714	HZ	HSD3B1
0.531381	0.187634	HZ	NEXMIF	NC_056054.1	96960001	96980001	0.0497925	0.266544	HZ	HSD3B1;ZNF6
0.495376	0.158582	HZ	NEUROD4	NC_056054.1	96965001	96985001	0.0760729	0.277863	HZ	HSD3B1;ZNF6
0.429767	0.185042	HZ	NEUROD4	NC_056055.1	243385001	243405001	0.479785	0.28198	HZ	HTR1D;LOC11
0.42465	0.160028	HZ	NEUROD4	NC_056055.1	243390001	243410001	0.374953	0.366796	HZ	HTR1D;LOC11
0.482128	0.150259	HZ	NEURL3	NC_056054.1	124200001	124220001	0.240363	0.228151	HZ	HUNK
0.456174	0.158323	HZ	NEURL3	NC_056054.1	124205001	124225001	0.195023	0.28129	HZ	HUNK
0.427823	0.251677	HZ	NEU2	NC_056054.1	124210001	124230001	0.243692	0.236923	HZ	HUNK
0.411426	0.227763	HZ	NEU2	NC_056060.1	47920001	47940001	0.43772	0.172609	HZ	ICE2
0.216189	0.151156	HZ	NEO1	NC_056060.1	47925001	47945001	0.416896	0.198135	HZ	ICE2
0.0649415	0.236143	HZ	NEO1	NC_056060.1	47930001	47950001	0.437133	0.190204	HZ	ICE2
0.0767667	0.216261	HZ	NEO1	NC_056075.1	13695001	13715001	0.432446	0.187997	HZ	IDE
0.0909561	0.161735	HZ	NEO1	NC_056075.1	13700001	13720001	0.327823	0.190006	HZ	IDE
0.525607	0.215174	HZ	NEK10	NC_056056.1	7285001	7305001	0.269268	0.242127	HZ	IER5L
0.427158	0.246925	HZ	NEK10	NC_056056.1	7290001	7310001	0.247543	0.259957	HZ	IER5L
0.466813	0.160958	HZ	NECTIN4	NC_056056.1	225305001	225325001	0.466482	0.185848	HZ	IL17REL
0.168434	0.171458	HZ	NDUFS4	NC_056056.1	225310001	225330001	0.413069	0.214154	HZ	IL17REL

0.210034	0.22843	HZ	NDUFS4	NC_056073.1	8835001	8855001	0.357507	0.160796	HZ	ILRUN		
0.267959	0.242809	HZ	NDUFS4	NC_056057.1	58415001	58435001	0.326539	0.311819	HZ	IMMP2L		
0.388888	0.223051	HZ	NDUFS4	NC_056057.1	58420001	58440001	0.283203	0.294619	HZ	IMMP2L		
0.415728	0.196927	HZ	NDUFS4	NC_056057.1	58425001	58445001	0.453755	0.255831	HZ	IMMP2L		
0.417792	0.16878	HZ	NDUFS4	NC_056061.1	2880001	2900001	0.197685	0.197625	HZ	IMPG1		
0.37288	0.1423	HZ	NDUFAF7;PRKD3	NC_056061.1	2890001	2910001	0.112177	0.222351	HZ	IMPG1		
0.511271	0.160587	HZ	NCOA1	NC_056061.1	2895001	2915001	0.111019	0.253903	HZ	IMPG1		
0.531939	0.155756	HZ	NCOA1	NC_056061.1	2900001	2920001	0.105958	0.257134	HZ	IMPG1		
0.480279	0.155276	HZ	NCEH1	NC_056061.1	2905001	2925001	0.238864	0.222388	HZ	IMPG1		
0.431009	0.190854	HZ	NCEH1	NC_056055.1	73450001	73470001	0.471153	0.235728	HZ	INSL6		
0.30619	0.265796	HZ	NCEH1	NC_056055.1	73455001	73475001	0.455284	0.290859	HZ	INSL6		
0.230419	0.294394	HZ	NCEH1	NC_056055.1	73460001	73480001	0.481884	0.284689	HZ	INSL6		
0.206285	0.316297	HZ	NCEH1	NC_056055.1	73465001	73485001	0.478549	0.290205	HZ	INSL6		
0.207065	0.331499	HZ	NCEH1	NC_056055.1	73445001	73465001	0.485401	0.297709	HZ	INSL6;JAK2		
0.2919	0.327743	HZ	NCEH1	NC_056055.1	73470001	73490001	0.477722	0.342661	HZ	INSL6;LOC105		
0.356007	0.292135	HZ	NCEH1	NC_056072.1	51200001	51220001	0.427396	0.165531	HZ	IP6K2		
0.350142	0.285716	HZ	NCEH1	NC_056072.1	51205001	51225001	0.315825	0.207242	HZ	IP6K2		
0.486183	0.146546	HZ	NCEH1	NC_056072.1	51210001	51230001	0.317604	0.189137	HZ	IP6K2		
0.401433	0.14382	HZ	NBN	NC_056072.1	51215001	51235001	0.328659	0.176382	HZ	IP6K2		
0.283756	0.21046	HZ	NBN	NC_056061.1	78765001	78785001	0.253097	0.179167	HZ	IPCEF1		
0.307986	0.205882	HZ	NBN	NC_056069.1	17050001	17070001	0.207978	0.185589	HZ	IPO11		
0.520894	0.190861	HZ	NBN	NC_056069.1	17055001	17075001	0.0854152	0.220073	HZ	IPO11		
0.534305	0.232402	HZ	NARS2	NC_056069.1	17060001	17080001	0.147646	0.205512	HZ	IPO11		
0.537429	0.168019	HZ	NAPEPLD	NC_056069.1	17065001	17085001	0.206392	0.216032	HZ	IPO11		
0.487717	0.245592	HZ	NAPEPLD	NC_056069.1	17070001	17090001	0.407237	0.159658	HZ	IPO11		
0.530324	0.3843	HZ	NAPEPLD	NC_056071.1	21400001	21420001	0.464967	0.159576	HZ	IQGAP1		
0.473851	0.16099	HZ	NAA20	NC_056060.1	7770001	7790001	0.213666	0.186129	HZ	IQGAP2		
0.362227	0.17479	HZ	NAA20	NC_056060.1	7775001	7795001	0.160042	0.201384	HZ	IQGAP2		
0.35231	0.140734	HZ	MYT1L	NC_056060.1	7780001	7800001	0.240353	0.202117	HZ	IQGAP2		
0.525573	0.139642	HZ	MYOZ2	NC_056060.1	7785001	7805001	0.373643	0.186665	HZ	IQGAP2		
0.485495	0.156863	HZ	MYO3A	NC_056067.1	11360001	11380001	0.33681	0.170962	HZ	IRF8		
0.408415	0.146305	HZ	MYO3A	NC_056067.1	11365001	11385001	0.305635	0.186623	HZ	IRF8		
0.410655	0.151149	HZ	MYO16	NC_056067.1	11370001	11390001	0.263202	0.253523	HZ	IRF8		
0.405479	0.369464	HZ	MYO16	NC_056067.1	11375001	11395001	0.276493	0.238993	HZ	IRF8		
0.320862	0.435241	HZ	MYO16	NC_056067.1	11380001	11400001	0.481949	0.201993	HZ	IRF8		
0.185359	0.530172	HZ	MYO16	NC_056067.1	11385001	11405001	0.478489	0.19289	HZ	IRF8		

0.266895	0.457918	HZ	MYO16
0.38649	0.363533	HZ	MYO16
0.375956	0.299334	HZ	MYO16
0.476191	0.196528	HZ	MYO16
0.436515	0.159852	HZ	MXD1
0.404959	0.162862	HZ	MXD1
0.519894	0.210908	HZ	MXD1
0.4745	0.196592	HZ	MVB12B
0.459899	0.222196	HZ	MVB12B
0.489492	0.200286	HZ	MVB12B
0.447691	0.166306	HZ	MUC20
0.417923	0.168243	HZ	MUC20
0.432132	0.201949	HZ	MUC20
0.418982	0.17213	HZ	MTMR1
0.466708	0.21194	HZ	MTMR1
0.390378	0.267634	HZ	MTMR1
0.409315	0.154007	HZ	MTMR1
0.0905474	0.192058	HZ	MSRB3
0.143414	0.305368	HZ	MSRB3
0.311828	0.25043	HZ	MSRB3
0.3881	0.245086	HZ	MSRB3
0.5258	0.182443	HZ	MSRB3
0.383252	0.170558	HZ	MSRB3
0.295543	0.235445	HZ	MSRB3
0.508423	0.141822	HZ	MSRA
0.536382	0.278605	HZ	MSN
0.361813	0.16159	HZ	MRTFA
0.187551	0.274859	HZ	MRTFA
0.14242	0.314172	HZ	MRTFA
0.115362	0.281278	HZ	MRTFA
0.0998338	0.21701	HZ	MRTFA
0.0894645	0.200222	HZ	MRTFA
0.121258	0.208731	HZ	MRTFA
0.173233	0.202374	HZ	MRTFA
0.261682	0.222434	HZ	MRTFA
0.350327	0.20457	HZ	MRTFA

NC_056069.1	26210001	26230001	0.454803	0.168402	HZ	ITGA2
NC_056069.1	26215001	26235001	0.453215	0.175874	HZ	ITGA2
NC_056072.1	48185001	48205001	0.36913	0.205077	HZ	ITIH1
NC_056072.1	48190001	48210001	0.459249	0.258191	HZ	ITIH1
NC_056072.1	48170001	48190001	0.237087	0.180052	HZ	ITIH1;ITIH3
NC_056072.1	48175001	48195001	0.27291	0.203099	HZ	ITIH1;ITIH3
NC_056072.1	48180001	48200001	0.324599	0.196564	HZ	ITIH1;ITIH3
NC_056072.1	48165001	48185001	0.168855	0.227498	HZ	ITIH3
NC_056072.1	48150001	48170001	0.104044	0.371129	HZ	ITIH3;ITIH4
NC_056072.1	48155001	48175001	0.0738675	0.33385	HZ	ITIH3;ITIH4
NC_056072.1	48160001	48180001	0.0762226	0.302952	HZ	ITIH3;ITIH4
NC_056072.1	48140001	48160001	0.218055	0.218074	HZ	ITIH4;LOC101
NC_056072.1	48145001	48165001	0.112896	0.343195	HZ	ITIH4;MUSTN
NC_056055.1	73425001	73445001	0.489796	0.330007	HZ	JAK2
NC_056055.1	73430001	73450001	0.46875	0.346208	HZ	JAK2
NC_056055.1	73435001	73455001	0.478773	0.320519	HZ	JAK2
NC_056055.1	73440001	73460001	0.486486	0.294173	HZ	JAK2
NC_056058.1	56685001	56705001	0.444191	0.169835	HZ	JAKMIP2
NC_056057.1	69615001	69635001	0.292119	0.190975	HZ	JAZF1
NC_056057.1	69620001	69640001	0.253644	0.21654	HZ	JAZF1
NC_056057.1	69625001	69645001	0.257227	0.217695	HZ	JAZF1
NC_056057.1	69630001	69650001	0.309945	0.192248	HZ	JAZF1
NC_056057.1	69635001	69655001	0.19885	0.215746	HZ	JAZF1
NC_056057.1	69640001	69660001	0.15071	0.269159	HZ	JAZF1
NC_056057.1	69645001	69665001	0.099208	0.334595	HZ	JAZF1
NC_056057.1	69650001	69670001	0.0676141	0.384792	HZ	JAZF1
NC_056057.1	69655001	69675001	0.149196	0.389442	HZ	JAZF1
NC_056057.1	69660001	69680001	0.341395	0.295539	HZ	JAZF1
NC_056057.1	69675001	69695001	0.48087	0.180924	HZ	JAZF1
NC_056057.1	69680001	69700001	0.293526	0.259505	HZ	JAZF1
NC_056057.1	69685001	69705001	0.315457	0.219723	HZ	JAZF1
NC_056057.1	69690001	69710001	0.323887	0.219841	HZ	JAZF1
NC_056057.1	69695001	69715001	0.375204	0.219608	HZ	JAZF1
NC_056057.1	69700001	69720001	0.409952	0.21618	HZ	JAZF1
NC_056057.1	69705001	69725001	0.479373	0.213775	HZ	JAZF1
NC_056066.1	34425001	34445001	0.0810811	0.182682	HZ	JCAD

0.481336	0.154727	HZ	MRTFA	NC_056066.1	34430001	34450001	0.129586	0.20356	HZ	JCAD
0.429989	0.518078	HZ	MRPS5;ZNF514	NC_056066.1	34435001	34455001	0.220184	0.206979	HZ	JCAD
0.152981	0.270561	HZ	MRPL30	NC_056066.1	34440001	34460001	0.253732	0.191712	HZ	JCAD
0.331286	0.231517	HZ	MORC2	NC_056055.1	68930001	68950001	0.464507	0.234567	HZ	KANK1
0.426584	0.210423	HZ	MORC2	NC_056055.1	68935001	68955001	0.328402	0.304663	HZ	KANK1
0.528084	0.143089	HZ	MOB2	NC_056055.1	68940001	68960001	0.250999	0.342806	HZ	KANK1
0.502899	0.165631	HZ	MOB2	NC_056055.1	68945001	68965001	0.105374	0.417274	HZ	KANK1
0.396668	0.1977	HZ	MOB2	NC_056055.1	68950001	68970001	0.0893911	0.422428	HZ	KANK1
0.22336	0.167606	HZ	MMUT	NC_056055.1	68955001	68975001	0.0961053	0.400206	HZ	KANK1
0.325714	0.184397	HZ	MMUT	NC_056055.1	68960001	68980001	0.0835914	0.431739	HZ	KANK1
0.43246	0.168819	HZ	MMUT	NC_056055.1	68965001	68985001	0.0947192	0.46734	HZ	KANK1
0.410556	0.192902	HZ	MLLT3	NC_056055.1	68970001	68990001	0.182092	0.445832	HZ	KANK1
0.473499	0.143799	HZ	MLLT3	NC_056055.1	68975001	68995001	0.201102	0.405126	HZ	KANK1
0.473317	0.246906	HZ	MKNK1;MOB3C	NC_056055.1	68980001	69000001	0.208929	0.328206	HZ	KANK1
0.495059	0.171325	HZ	MITF	NC_056055.1	68985001	69005001	0.228176	0.251102	HZ	KANK1
0.499749	0.17306	HZ	MITF	NC_056055.1	68990001	69010001	0.182815	0.181512	HZ	KANK1
0.444142	0.149501	HZ	MITF	NC_056058.1	14695001	14715001	0.132303	0.215729	HZ	KANK3;NDUF
0.163921	0.23031	HZ	MITF	NC_056058.1	14700001	14720001	0.219238	0.180092	HZ	KANK3;NDUF
0.0824204	0.315518	HZ	MITF	NC_056077.1	25735001	25755001	0.133203	0.239437	HZ	KATNIP
0.0869884	0.38848	HZ	MITF	NC_056077.1	25740001	25760001	0.394389	0.17865	HZ	KATNIP
0.153637	0.368826	HZ	MITF	NC_056056.1	111155001	111175001	0.492159	0.16048	HZ	KCNC2
0.352853	0.309856	HZ	MITF	NC_056054.1	89280001	89300001	0.41884	0.17925	HZ	KCND3
0.308029	0.253346	HZ	MITF	NC_056056.1	20005001	20025001	0.391094	0.212863	HZ	KCNF1
0.291388	0.262961	HZ	MITF	NC_056056.1	20010001	20030001	0.408799	0.158793	HZ	KCNF1
0.303837	0.250221	HZ	MITF	NC_056059.1	40925001	40945001	0.396666	0.184616	HZ	KCNIP4
0.354452	0.227264	HZ	MITF	NC_056059.1	40930001	40950001	0.371829	0.159782	HZ	KCNIP4
0.383223	0.214241	HZ	MITF	NC_056059.1	40935001	40955001	0.315372	0.172904	HZ	KCNIP4
0.373157	0.251403	HZ	MITF	NC_056062.1	16595001	16615001	0.391596	0.199715	HZ	KCNK9
0.392524	0.305213	HZ	MITF	NC_056062.1	16600001	16620001	0.41221	0.281462	HZ	KCNK9
0.400326	0.441969	HZ	MITF	NC_056078.1	31885001	31905001	0.326381	0.176762	HZ	KCNMA1
0.262889	0.2387	HZ	MITD1;MRPL30	NC_056078.1	31890001	31910001	0.227288	0.177788	HZ	KCNMA1
0.35864	0.208828	HZ	MIPOL1	NC_056078.1	31895001	31915001	0.256814	0.162862	HZ	KCNMA1
0.33085	0.20416	HZ	MIPOL1	NC_056054.1	208970001	208990001	0.349762	0.187683	HZ	KCNMB2
0.339185	0.15967	HZ	MIPOL1	NC_056054.1	208975001	208995001	0.403647	0.187561	HZ	KCNMB2
0.415925	0.152324	HZ	MIPOL1	NC_056054.1	208605001	208625001	0.438333	0.198581	HZ	KCNMB3
0.193839	0.142714	HZ	MINDY2	NC_056054.1	208610001	208630001	0.429217	0.195882	HZ	KCNMB3

0.183891	0.160505	HZ	MINDY2
0.172238	0.174085	HZ	MINDY2
0.221898	0.16119	HZ	MINDY2
0.214122	0.18708	HZ	MINDY2
0.26159	0.181006	HZ	MINDY2
0.280984	0.180087	HZ	MINDY2
0.333334	0.175667	HZ	MINDY2
0.315621	0.173132	HZ	MINDY2
0.240385	0.180755	HZ	MINDY2
0.194876	0.193266	HZ	MINDY2
0.245043	0.202333	HZ	MINDY2
0.259141	0.211108	HZ	MINDY2
0.327132	0.210113	HZ	MINDY2
0.351227	0.199546	HZ	MINDY2
0.303862	0.19907	HZ	MINDY2
0.389494	0.18314	HZ	MINDY2
0.248415	0.161316	HZ	MID1
0.321954	0.160434	HZ	MID1
0.399101	0.15264	HZ	MID1
0.490869	0.215411	HZ	MICAL2
0.529257	0.174827	HZ	MICAL2
0.0997894	0.148879	HZ	MIB1
0.104008	0.180402	HZ	MIB1
0.0914321	0.207582	HZ	MIB1
0.149928	0.208203	HZ	MIB1
0.198499	0.191233	HZ	MIB1
0.256627	0.154995	HZ	MIB1
0.222618	0.151572	HZ	MIB1
0.483584	0.23853	HZ	MGAT4C
0.294215	0.252102	HZ	MGAT4C
0.303748	0.266994	HZ	MGAT4C
0.500853	0.231407	HZ	MGAT4C
0.444805	0.145387	HZ	MFNG
0.458442	0.204797	HZ	MFHAS1
0.356178	0.236086	HZ	MFHAS1
0.430543	0.192768	HZ	MFHAS1

NC_056054.1	208615001	208635001	0.454388	0.225372	HZ	KCNMB3
NC_056054.1	208620001	208640001	0.46834	0.225178	HZ	KCNMB3;PIK3
NC_056054.1	104920001	104940001	0.410141	0.239862	HZ	KCNN3
NC_056054.1	104925001	104945001	0.473686	0.281385	HZ	KCNN3
NC_056054.1	104940001	104960001	0.436612	0.280014	HZ	KCNN3
NC_056054.1	104945001	104965001	0.226191	0.284326	HZ	KCNN3
NC_056054.1	104950001	104970001	0.193738	0.27472	HZ	KCNN3
NC_056054.1	104955001	104975001	0.239544	0.252977	HZ	KCNN3
NC_056054.1	104960001	104980001	0.341463	0.21945	HZ	KCNN3
NC_056054.1	104965001	104985001	0.402249	0.1922	HZ	KCNN3
NC_056054.1	104970001	104990001	0.407946	0.213579	HZ	KCNN3
NC_056054.1	104975001	104995001	0.40359	0.232455	HZ	KCNN3
NC_056054.1	104980001	105000001	0.39057	0.232942	HZ	KCNN3
NC_056054.1	104985001	105005001	0.431117	0.24273	HZ	KCNN3
NC_056054.1	104990001	105010001	0.464896	0.230289	HZ	KCNN3
NC_056054.1	104995001	105015001	0.466903	0.180685	HZ	KCNN3
NC_056062.1	21930001	21950001	0.362376	0.309322	HZ	KCNQ3
NC_056062.1	21935001	21955001	0.312956	0.336992	HZ	KCNQ3
NC_056062.1	750001	770001	0.167215	0.192141	HZ	KCNQ5
NC_056062.1	755001	775001	0.19087	0.205604	HZ	KCNQ5
NC_056062.1	760001	780001	0.28258	0.181592	HZ	KCNQ5
NC_056062.1	1025001	1045001	0.477016	0.190257	HZ	KCNQ5
NC_056062.1	67395001	67415001	0.292419	0.205879	HZ	KCNV1
NC_056062.1	67400001	67420001	0.304156	0.213177	HZ	KCNV1
NC_056062.1	67405001	67425001	0.246789	0.21677	HZ	KCNV1
NC_056062.1	67410001	67430001	0.230873	0.263557	HZ	KCNV1
NC_056065.1	18035001	18055001	0.419757	0.228546	HZ	KCTD3
NC_056065.1	18040001	18060001	0.223548	0.25977	HZ	KCTD3
NC_056065.1	18045001	18065001	0.0632716	0.32656	HZ	KCTD3
NC_056065.1	18060001	18080001	0.082715	0.308693	HZ	KCTD3
NC_056065.1	18065001	18085001	0.230289	0.297695	HZ	KCTD3
NC_056065.1	18070001	18090001	0.354245	0.252571	HZ	KCTD3
NC_056065.1	18075001	18095001	0.421723	0.213186	HZ	KCTD3
NC_056065.1	18080001	18100001	0.414121	0.211819	HZ	KCTD3
NC_056065.1	18090001	18110001	0.36928	0.170276	HZ	KCTD3
NC_056065.1	18095001	18115001	0.36867	0.190131	HZ	KCTD3

0.51589	0.139523 HZ	METRNL	NC_056065.1	18100001	18120001	0.395404	0.189189 HZ	KCTD3;USH2A
0.101957	0.17303 HZ	METRNL	NC_056065.1	18105001	18125001	0.422244	0.194875 HZ	KCTD3;USH2A
0.492399	0.141464 HZ	MEIOC	NC_056065.1	18110001	18130001	0.433595	0.176127 HZ	KCTD3;USH2A
0.524022	0.191521 HZ	MEDAG	NC_056065.1	18115001	18135001	0.446571	0.186794 HZ	KCTD3;USH2A
0.342008	0.189602 HZ	MED6	NC_056073.1	465001	485001	0.452876	0.157887 HZ	KHDRBS2
0.269013	0.154243 HZ	MED6	NC_056070.1	35620001	35640001	0.465587	0.359478 HZ	KIAA1109
0.360413	0.168779 HZ	MED6	NC_056070.1	35625001	35645001	0.471624	0.370358 HZ	KIAA1109
0.487084	0.193919 HZ	MED29;PLEKHG2	NC_056070.1	35630001	35650001	0.416381	0.435397 HZ	KIAA1109
0.516187	0.173541 HZ	MED12L	NC_056070.1	35635001	35655001	0.347974	0.497431 HZ	KIAA1109
0.28322	0.235239 HZ	MCF2L2	NC_056070.1	35640001	35660001	0.358696	0.460195 HZ	KIAA1109
0.248665	0.247516 HZ	MCF2L2	NC_056070.1	35645001	35665001	0.361194	0.422827 HZ	KIAA1109
0.260266	0.222601 HZ	MCF2L2	NC_056070.1	35650001	35670001	0.469187	0.318557 HZ	KIAA1109
0.535756	0.170674 HZ	MBOAT1	NC_056070.1	35685001	35705001	0.477464	0.158322 HZ	KIAA1109
0.378992	0.200976 HZ	MBOAT1	NC_056070.1	35700001	35720001	0.446899	0.163382 HZ	KIAA1109
0.433346	0.142411 HZ	MAPK10	NC_056070.1	35705001	35725001	0.443808	0.160077 HZ	KIAA1109
0.322297	0.153228 HZ	MAPK1	NC_056066.1	24305001	24325001	0.25116	0.169843 HZ	KIAA1217
0.298716	0.155918 HZ	MAPK1	NC_056066.1	24310001	24330001	0.391167	0.17191 HZ	KIAA1217
0.491507	0.216341 HZ	MAP4K5	NC_056066.1	9890001	9910001	0.466404	0.187222 HZ	KIF16B
0.420411	0.148404 HZ	MAP4	NC_056066.1	9895001	9915001	0.261877	0.260976 HZ	KIF16B
0.343351	0.180266 HZ	MAP4	NC_056066.1	9900001	9920001	0.381957	0.172182 HZ	KIF16B
0.390599	0.180315 HZ	MAP4	NC_056080.1	63210001	63230001	0.37347	0.162388 HZ	KIF4A
0.46126	0.150479 HZ	MAP4	NC_056080.1	63215001	63235001	0.339623	0.165016 HZ	KIF4A
0.391049	0.138792 HZ	MAP1LC3A;PIGU	NC_056071.1	65960001	65980001	0.436731	0.192319 HZ	KLC1
0.425504	0.15604 HZ	MAP1LC3A;PIGU	NC_056058.1	6450001	6470001	0.140414	0.253829 HZ	KLF2
0.349337	0.158199 HZ	MAOB	NC_056057.1	116205001	116225001	0.361746	0.159738 HZ	KMT2C
0.199829	0.220661 HZ	MAOB	NC_056057.1	116220001	116240001	0.207497	0.160797 HZ	KMT2C
0.455205	0.147449 HZ	MAOB	NC_056057.1	116225001	116245001	0.23617	0.167265 HZ	KMT2C
0.438746	0.138817 HZ	MAN1A1	NC_056057.1	116250001	116270001	0.409638	0.187227 HZ	KMT2C
0.419221	0.152907 HZ	MAIP1	NC_056057.1	116255001	116275001	0.40421	0.197778 HZ	KMT2C
0.369501	0.177854 HZ	MAIP1	NC_056057.1	116315001	116335001	0.235107	0.170575 HZ	KMT2C
0.386393	0.199247 HZ	MAIP1	NC_056057.1	116320001	116340001	0.238044	0.176209 HZ	KMT2C
0.27381	0.171557 HZ	MAGI3	NC_056054.1	21110001	21130001	0.245028	0.164376 HZ	KNCN;MKNK1
0.422207	0.152429 HZ	MACF1	NC_056054.1	21115001	21135001	0.409512	0.196269 HZ	KNCN;MKNK1
0.49584	0.173353 HZ	LYPD6	NC_056064.1	40905001	40925001	0.466565	0.163713 HZ	KRT39
0.434675	0.194458 HZ	LYPD6	NC_056061.1	54890001	54910001	0.418654	0.204239 HZ	LAMA2
0.325886	0.191662 HZ	LYPD6	NC_056061.1	54895001	54915001	0.463996	0.176161 HZ	LAMA2

0.446513	0.222616 HZ	LYN	NC_056076.1	33260001	33280001	0.476536	0.198504 HZ	LAMA3
0.404877	0.15585 HZ	LYN	NC_056076.1	33265001	33285001	0.405573	0.200066 HZ	LAMA3
0.454346	0.257931 HZ	LYN	NC_056076.1	33270001	33290001	0.383815	0.184249 HZ	LAMA3
0.494346	0.29095 HZ	LYN	NC_056056.1	177425001	177445001	0.451759	0.201642 HZ	LARGE1
0.503963	0.338417 HZ	LYN	NC_056056.1	177430001	177450001	0.462457	0.166021 HZ	LARGE1
0.510534	0.352192 HZ	LYN	NC_056058.1	63715001	63735001	0.30504	0.164207 HZ	LARP1
0.489375	0.326017 HZ	LYN	NC_056058.1	63725001	63745001	0.366595	0.158279 HZ	LARP1
0.494428	0.313155 HZ	LYN	NC_056058.1	63765001	63785001	0.41358	0.181655 HZ	LARP1
0.287963	0.340024 HZ	LYG2;MRPL30	NC_056058.1	63770001	63790001	0.229242	0.240245 HZ	LARP1
0.255092	0.32372 HZ	LYG2;MRPL30	NC_056058.1	63775001	63795001	0.298508	0.187189 HZ	LARP1
0.441439	0.165087 HZ	LYG2	NC_056054.1	260200001	260220001	0.175421	0.199344 HZ	LCA5L
0.365768	0.20545 HZ	LYG2	NC_056054.1	260205001	260225001	0.199173	0.190495 HZ	LCA5L
0.405692	0.263925 HZ	LYG2	NC_056054.1	260210001	260230001	0.15808	0.193572 HZ	LCA5L
0.370742	0.291312 HZ	LYG2	NC_056054.1	260215001	260235001	0.180918	0.178597 HZ	LCA5L
0.515644	0.164514 HZ	LYG1;LYG2	NC_056054.1	260220001	260240001	0.146381	0.214212 HZ	LCA5L
0.366727	0.259288 HZ	LRRIQ1	NC_056054.1	260225001	260245001	0.103812	0.29836 HZ	LCA5L
0.216561	0.302305 HZ	LRRIQ1	NC_056054.1	260230001	260250001	0.188837	0.297767 HZ	LCA5L
0.174167	0.34359 HZ	LRRIQ1	NC_056054.1	260235001	260255001	0.37201	0.224434 HZ	LCA5L
0.23061	0.322993 HZ	LRRIQ1	NC_056055.1	175055001	175075001	0.19459	0.16206 HZ	LCT;UBXN4
0.500865	0.142627 HZ	LRRC36;TPPP3;ZI	NC_056054.1	29255001	29275001	0.202941	0.206982 HZ	LEXM
0.443112	0.179444 HZ	LRRC36;TPPP3;ZI	NC_056060.1	49825001	49845001	0.456166	0.240362 HZ	LIPC
0.407274	0.183409 HZ	LRRC36;TPPP3;ZI	NC_056060.1	49980001	50000001	0.193536	0.191911 HZ	LIPC
0.133712	0.138496 HZ	LRP1B	NC_056060.1	49985001	50005001	0.156589	0.21331 HZ	LIPC
0.412223	0.176523 HZ	LRP12	NC_056060.1	49990001	50010001	0.114943	0.219168 HZ	LIPC
0.372066	0.203559 HZ	LRP12	NC_056060.1	49995001	50015001	0.108045	0.214099 HZ	LIPC
0.370783	0.212937 HZ	LRP12	NC_056060.1	50000001	50020001	0.144304	0.167759 HZ	LIPC
0.476209	0.17375 HZ	LRMDA	NC_056060.1	50005001	50025001	0.16276	0.165506 HZ	LIPC
0.44498	0.148349 HZ	LRMDA	NC_056066.1	53470001	53490001	0.294117	0.230639 HZ	LKAAEAR1;NI
0.299456	0.183964 HZ	LRFN5	NC_056066.1	53475001	53495001	0.364905	0.185725 HZ	LKAAEAR1;NI
0.330404	0.180406 HZ	LRFN5	NC_056066.1	53480001	53500001	0.399793	0.167958 HZ	LKAAEAR1;OI
0.212401	0.150773 HZ	LRBA	NC_056056.1	189845001	189865001	0.389792	0.197623 HZ	LMNTD1
0.198356	0.148083 HZ	LOC121820086;M.	NC_056056.1	189850001	189870001	0.279591	0.181158 HZ	LMNTD1
0.538541	0.436995 HZ	LOC121819825	NC_056056.1	189855001	189875001	0.407839	0.15976 HZ	LMNTD1
0.530438	0.421613 HZ	LOC121819825	NC_056077.1	38230001	38250001	0.308558	0.189084 HZ	LMTK2
0.499063	0.371292 HZ	LOC121819825	NC_056077.1	38235001	38255001	0.143703	0.191008 HZ	LMTK2
0.533008	0.15385 HZ	LOC121819524	NC_056077.1	38240001	38260001	0.215384	0.170319 HZ	LMTK2

0.341869	0.172166	HZ	LOC121819507	NC_056077.1	4075001	4095001	0.137491	0.169567	HZ	LOC10110252[illegible]
0.313454	0.181507	HZ	LOC121819507	NC_056077.1	4080001	4100001	0.125	0.169199	HZ	LOC10110252[illegible]
0.300116	0.189373	HZ	LOC121819507	NC_056055.1	89850001	89870001	0.126846	0.225955	HZ	LOC10110296[illegible]
0.361263	0.159937	HZ	LOC121819507	NC_056055.1	89855001	89875001	0.0499177	0.294281	HZ	LOC10110296[illegible]
0.318174	0.141749	HZ	LOC121819374	NC_056075.1	36555001	36575001	0.45884	0.256648	HZ	LOC10110386[illegible]
0.31639	0.156199	HZ	LOC121819374	NC_056075.1	36560001	36580001	0.448212	0.263164	HZ	LOC10110386[illegible]
0.538948	0.139242	HZ	LOC121818843;W	NC_056067.1	33835001	33855001	0.111479	0.172417	HZ	LOC10110391[illegible]
0.201835	0.145159	HZ	LOC121818660	NC_056066.1	67210001	67230001	0.449188	0.29151	HZ	LOC10110459[illegible]
0.193159	0.163094	HZ	LOC121818643	NC_056066.1	67215001	67235001	0.403423	0.226297	HZ	LOC10110459[illegible]
0.0461401	0.208027	HZ	LOC121818643	NC_056054.1	1355001	1375001	0.486886	0.171277	HZ	LOC10110520[illegible]
0.114057	0.165823	HZ	LOC121818643	NC_056068.1	44550001	44570001	0.154625	0.184906	HZ	LOC10110527[illegible]
0.466194	0.162899	HZ	LOC121818469	NC_056068.1	44555001	44575001	0.202909	0.190506	HZ	LOC10110527[illegible]
0.445623	0.205001	HZ	LOC121817897	NC_056068.1	44545001	44565001	0.143129	0.16337	HZ	LOC10110527[illegible]
0.424743	0.226425	HZ	LOC121816523	NC_056058.1	50190001	50210001	0.0857143	0.193737	HZ	LOC10110549[illegible]
0.492889	0.156436	HZ	LOC121816444	NC_056058.1	50195001	50215001	0.139501	0.169466	HZ	LOC10110549[illegible]
0.322086	0.219892	HZ	LOC121815995;ZN	NC_056054.1	163695001	163715001	0.358317	0.173483	HZ	LOC10110586[illegible]
0.322581	0.25459	HZ	LOC121815995;ZN	NC_056054.1	163700001	163720001	0.335819	0.161113	HZ	LOC10110586[illegible]
0.318945	0.256673	HZ	LOC121815995;ZN	NC_056060.1	22155001	22175001	0.0994723	0.169492	HZ	LOC10110652[illegible]
0.379518	0.271718	HZ	LOC121815995	NC_056074.1	36405001	36425001	0.435878	0.159004	HZ	LOC10110663[illegible]
0.490783	0.214196	HZ	LOC114118273	NC_056074.1	36410001	36430001	0.433813	0.165741	HZ	LOC10110663[illegible]
0.537214	0.164844	HZ	LOC114118065	NC_056074.1	36415001	36435001	0.433696	0.161807	HZ	LOC10110663[illegible]
0.417178	0.334663	HZ	LOC114113736;LC	NC_056074.1	36420001	36440001	0.426426	0.16519	HZ	LOC10110663[illegible]
0.414049	0.337197	HZ	LOC114113736;LC	NC_056074.1	36425001	36445001	0.432398	0.160909	HZ	LOC10110663[illegible]
0.464808	0.363081	HZ	LOC114113736;LC	NC_056075.1	15880001	15900001	0.324375	0.187518	HZ	LOC101106641
0.492187	0.398937	HZ	LOC114113736;LC	NC_056075.1	15885001	15905001	0.298819	0.233476	HZ	LOC101106641
0.4375	0.348947	HZ	LOC114113736;LC	NC_056075.1	15890001	15910001	0.421415	0.191678	HZ	LOC101106641
0.411089	0.391589	HZ	LOC114113243	NC_056064.1	23805001	23825001	0.410141	0.165415	HZ	LOC10110678[illegible]
0.293988	0.430573	HZ	LOC114113243	NC_056080.1	43085001	43105001	0.487179	0.172327	HZ	LOC10110691[illegible]
0.5028	0.200056	HZ	LOC114113195;VI	NC_056056.1	164000001	164020001	0.389257	0.16424	HZ	LOC101107691
0.300827	0.179881	HZ	LOC114113016	NC_056056.1	164005001	164025001	0.362047	0.246093	HZ	LOC101107691
0.336704	0.150825	HZ	LOC114113016	NC_056056.1	164010001	164030001	0.440865	0.274992	HZ	LOC101107691
0.354876	0.150168	HZ	LOC114112914	NC_056054.1	86945001	86965001	0.481975	0.295425	HZ	LOC101107831
0.369407	0.148794	HZ	LOC114112914	NC_056054.1	86950001	86970001	0.472658	0.296993	HZ	LOC101107831
0.448613	0.179746	HZ	LOC114112914	NC_056072.1	48095001	48115001	0.25329	0.164403	HZ	LOC10110842[illegible]
0.376148	0.186519	HZ	LOC114112824	NC_056072.1	48100001	48120001	0.231643	0.168185	HZ	LOC10110842[illegible]
0.206652	0.234268	HZ	LOC114112824	NC_056054.1	38875001	38895001	0.460166	0.170423	HZ	LOC10110870[illegible]

0.297425	0.191759	HZ	LOC114112824
0.146132	0.155409	HZ	LOC114112799;LC
0.524481	0.170756	HZ	LOC114110488
0.359983	0.219955	HZ	LOC114110421
0.408374	0.22705	HZ	LOC114110421
0.20871	0.217711	HZ	LOC114110152
0.0649953	0.296294	HZ	LOC114110152
0.0576696	0.330792	HZ	LOC114110152
0.0772252	0.342954	HZ	LOC114110152
0.143081	0.335857	HZ	LOC114110152
0.140169	0.337294	HZ	LOC114110152
0.144991	0.318609	HZ	LOC114110152
0.11458	0.339226	HZ	LOC114110152
0.098554	0.31365	HZ	LOC114110152
0.208791	0.224473	HZ	LOC114110152
0.513029	0.209441	HZ	LOC114110152
0.479361	0.216576	HZ	LOC114110152
0.363147	0.148001	HZ	LOC114110152
0.103359	0.179788	HZ	LOC106991963
0.0776524	0.142134	HZ	LOC106991963
0.212288	0.158123	HZ	LOC106991650
0.526028	0.223295	HZ	LOC106991387
0.188499	0.141171	HZ	LOC106991302
0.151033	0.189457	HZ	LOC106991302
0.101161	0.217127	HZ	LOC106991302
0.201337	0.221821	HZ	LOC106991302
0.525125	0.182602	HZ	LOC106991302
0.526022	0.162079	HZ	LOC106991302
0.483821	0.16755	HZ	LOC106991302
0.39911	0.161517	HZ	LOC106991302
0.313805	0.220333	HZ	LOC106991302
0.209716	0.253796	HZ	LOC106991302
0.155157	0.276211	HZ	LOC106991302
0.0988649	0.303271	HZ	LOC106991302
0.146793	0.165653	HZ	LOC106991302
0.19056	0.139276	HZ	LOC106991302

NC_056054.1	38880001	38900001	0.490417	0.205863	HZ	LOC101108702
NC_056054.1	38885001	38905001	0.483315	0.219846	HZ	LOC101108702
NC_056054.1	108005001	108025001	0.24343	0.316395	HZ	LOC101108789
NC_056054.1	108010001	108030001	0.29284	0.256229	HZ	LOC101108789
NC_056054.1	166215001	166235001	0.12957	0.183488	HZ	LOC101108803
NC_056054.1	166220001	166240001	0.225015	0.181392	HZ	LOC101108803
NC_056054.1	166325001	166345001	0.229642	0.168876	HZ	LOC101108803
NC_056054.1	166330001	166350001	0.247217	0.169216	HZ	LOC101108803
NC_056054.1	166335001	166355001	0.252038	0.159343	HZ	LOC101108803
NC_056054.1	166345001	166365001	0.13558	0.157506	HZ	LOC101108803
NC_056054.1	166875001	166895001	0.414035	0.175226	HZ	LOC101109587
NC_056054.1	166890001	166910001	0.325769	0.193656	HZ	LOC101109854
NC_056068.1	81090001	81110001	0.450614	0.17412	HZ	LOC101110173
NC_056068.1	81095001	81115001	0.481963	0.240662	HZ	LOC101110173
NC_056068.1	81100001	81120001	0.369388	0.221372	HZ	LOC101110173
NC_056054.1	61660001	61680001	0.441303	0.182116	HZ	LOC101110888
NC_056054.1	61665001	61685001	0.333687	0.174423	HZ	LOC101110888
NC_056064.1	56235001	56255001	0.216166	0.232272	HZ	LOC101112832
NC_056064.1	56240001	56260001	0.119279	0.28083	HZ	LOC101112832
NC_056064.1	56245001	56265001	0.209427	0.236322	HZ	LOC101112832
NC_056064.1	56250001	56270001	0.166881	0.225102	HZ	LOC101112832
NC_056055.1	102020001	102040001	0.329083	0.162067	HZ	LOC101113728
NC_056055.1	102025001	102045001	0.438836	0.223236	HZ	LOC101113728
NC_056055.1	240970001	240990001	0.270177	0.305946	HZ	LOC101114173
NC_056055.1	240975001	240995001	0.206727	0.347237	HZ	LOC101114173
NC_056055.1	240965001	240985001	0.393118	0.241107	HZ	LOC101114173
NC_056055.1	240980001	241000001	0.335013	0.301214	HZ	LOC101114173
NC_056065.1	53865001	53885001	0.319829	0.232353	HZ	LOC101114795
NC_056065.1	53870001	53890001	0.438251	0.184297	HZ	LOC101114795
NC_056065.1	53875001	53895001	0.458051	0.175792	HZ	LOC101114795
NC_056057.1	41750001	41770001	0.0543933	0.186288	HZ	LOC101114861
NC_056063.1	33535001	33555001	0.381669	0.15803	HZ	LOC101115135
NC_056056.1	193640001	193660001	0.467276	0.162045	HZ	LOC101115359
NC_056080.1	67790001	67810001	0.204707	0.257371	HZ	LOC101116125
NC_056071.1	24445001	24465001	0.40729	0.158709	HZ	LOC101116178
NC_056056.1	219820001	219840001	0.171404	0.185186	HZ	LOC101116389

0.457387	0.14306	HZ	LOC106991302	NC_056056.1	219825001	219845001	0.137512	0.211004	HZ	LOC101116389
0.347793	0.150857	HZ	LOC106991302	NC_056056.1	165205001	165225001	0.394873	0.162213	HZ	LOC101117240
0.253589	0.169729	HZ	LOC106991302	NC_056058.1	12795001	12815001	0.19801	0.609272	HZ	LOC101117505
0.152908	0.192439	HZ	LOC106991302	NC_056058.1	12800001	12820001	0.255901	0.571039	HZ	LOC101117505
0.170916	0.144628	HZ	LOC106991302	NC_056058.1	12805001	12825001	0.402368	0.523626	HZ	LOC101117505
0.223332	0.228105	HZ	LOC106991302	NC_056068.1	79020001	79040001	0.30126	0.16539	HZ	LOC101118132
0.368457	0.171642	HZ	LOC106991302	NC_056058.1	6725001	6745001	0.485021	0.164446	HZ	LOC101118444
0.538263	0.18446	HZ	LOC106990481	NC_056068.1	79025001	79045001	0.234652	0.198092	HZ	LOC101118639
0.513445	0.1949	HZ	LOC106990481	NC_056068.1	79030001	79050001	0.132118	0.252499	HZ	LOC101118639
0.482649	0.341843	HZ	LOC105608024	NC_056068.1	79035001	79055001	0.0793652	0.283418	HZ	LOC101118639
0.477015	0.346863	HZ	LOC105608024	NC_056068.1	79040001	79060001	0.0719936	0.283207	HZ	LOC101118639
0.481452	0.348146	HZ	LOC105608024	NC_056068.1	79045001	79065001	0.0632382	0.288225	HZ	LOC101118639
0.462246	0.333685	HZ	LOC105608024	NC_056054.1	80905001	80925001	0.461249	0.162706	HZ	LOC101118831
0.513089	0.308741	HZ	LOC105605990	NC_056066.1	59120001	59140001	0.465788	0.268877	HZ	LOC101119068
0.531207	0.173089	HZ	LOC105605990	NC_056054.1	81220001	81240001	0.480735	0.206275	HZ	LOC101119090
0.531207	0.173089	HZ	LOC105605990	NC_056054.1	81225001	81245001	0.367886	0.26838	HZ	LOC101119090
0.157478	0.149445	HZ	LOC105605766	NC_056060.1	25890001	25910001	0.321629	0.158273	HZ	LOC101119311
0.318271	0.183671	HZ	LOC105605457	NC_056056.1	194370001	194390001	0.405351	0.196352	HZ	LOC101120402
0.475285	0.171275	HZ	LOC101123588	NC_056056.1	194375001	194395001	0.246267	0.254444	HZ	LOC101120402
0.466667	0.166174	HZ	LOC101123588	NC_056058.1	77430001	77450001	0.455097	0.222455	HZ	LOC101120408
0.498253	0.162987	HZ	LOC101123588	NC_056058.1	77435001	77455001	0.319965	0.221973	HZ	LOC101120408
0.490939	0.159638	HZ	LOC101123588	NC_056058.1	77440001	77460001	0.329887	0.202786	HZ	LOC101120408
0.379117	0.302823	HZ	LOC101123268	NC_056058.1	77445001	77465001	0.333485	0.165758	HZ	LOC101120408
0.457761	0.19876	HZ	LOC101122151	NC_056058.1	77450001	77470001	0.303533	0.187639	HZ	LOC101120408
0.433076	0.196455	HZ	LOC101122151	NC_056058.1	77455001	77475001	0.386067	0.179158	HZ	LOC101120408
0.516308	0.191782	HZ	LOC101122151	NC_056074.1	4835001	4855001	0.0446203	0.218538	HZ	LOC101120706
0.532097	0.16928	HZ	LOC101122016	NC_056074.1	4840001	4860001	0.0492555	0.239878	HZ	LOC101120706
0.467858	0.329011	HZ	LOC101121777	NC_056074.1	4845001	4865001	0.0449358	0.265573	HZ	LOC101120706
0.0433544	0.220208	HZ	LOC101120706	NC_056056.1	50480001	50500001	0.341485	0.227284	HZ	LOC101122016
0.0483964	0.243827	HZ	LOC101120706	NC_056056.1	50485001	50505001	0.454024	0.215898	HZ	LOC101122016
0.0485021	0.271926	HZ	LOC101120706	NC_056056.1	204445001	204465001	0.206368	0.19217	HZ	LOC101122269
0.421262	0.171094	HZ	LOC101120706	NC_056080.1	53385001	53405001	0.457705	0.218201	HZ	LOC101122329
0.423695	0.139397	HZ	LOC101120408	NC_056080.1	53390001	53410001	0.458739	0.225208	HZ	LOC101122329
0.371939	0.18243	HZ	LOC101120408	NC_056062.1	43965001	43985001	0.449345	0.171707	HZ	LOC101122444
0.347642	0.169474	HZ	LOC101120408	NC_056074.1	34880001	34900001	0.408058	0.228693	HZ	LOC101122563
0.311274	0.235718	HZ	LOC101120408	NC_056074.1	34885001	34905001	0.248077	0.27761	HZ	LOC101122563

0.0893745	0.290343	HZ	LOC101120408	NC_056057.1	107725001	107745001	0.174031	0.160698	HZ	LOC10112259
0.231083	0.205391	HZ	LOC101120408	NC_056057.1	107730001	107750001	0.208844	0.162517	HZ	LOC10112259
0.210549	0.183756	HZ	LOC101120408	NC_056057.1	84225001	84245001	0.255494	0.187172	HZ	LOC10112268
0.230835	0.177764	HZ	LOC101120408	NC_056057.1	84230001	84250001	0.205339	0.217221	HZ	LOC10112268
0.199918	0.195814	HZ	LOC101120408	NC_056057.1	84235001	84255001	0.229396	0.211886	HZ	LOC10112268
0.192073	0.226021	HZ	LOC101120408	NC_056057.1	84240001	84260001	0.282323	0.202102	HZ	LOC10112268
0.388889	0.176189	HZ	LOC101120408	NC_056055.1	48355001	48375001	0.233628	0.164954	HZ	LOC10112326
0.236672	0.151639	HZ	LOC101120367	NC_056055.1	48360001	48380001	0.166751	0.207471	HZ	LOC10112326
0.491477	0.14912	HZ	LOC101120225	NC_056068.1	81570001	81590001	0.171263	0.161673	HZ	LOC10560239
0.307195	0.143358	HZ	LOC101120030	NC_056065.1	6670001	6690001	0.201819	0.197889	HZ	LOC10560579
0.260011	0.180001	HZ	LOC101120030	NC_056055.1	73475001	73495001	0.471922	0.349014	HZ	LOC10560802
0.286089	0.177627	HZ	LOC101120030	NC_056055.1	73480001	73500001	0.46791	0.353	HZ	LOC10560802
0.327697	0.160227	HZ	LOC101120030	NC_056055.1	73485001	73505001	0.469884	0.355488	HZ	LOC10560802
0.360791	0.155929	HZ	LOC101120030	NC_056055.1	73490001	73510001	0.453741	0.339996	HZ	LOC10560802
0.524919	0.166066	HZ	LOC101119740	NC_056058.1	46970001	46990001	0.472476	0.189096	HZ	LOC105612521
0.444237	0.378617	HZ	LOC101118510	NC_056060.1	25895001	25915001	0.231545	0.186796	HZ	LOC10561333
0.515319	0.138487	HZ	LOC101118184	NC_056073.1	26850001	26870001	0.169901	0.178599	HZ	LOC10699011
0.446182	0.187059	HZ	LOC101117816;UI	NC_056073.1	26855001	26875001	0.118501	0.170299	HZ	LOC10699011
0.493359	0.253645	HZ	LOC101117804	NC_056056.1	150635001	150655001	0.423678	0.19498	HZ	LOC10699018
0.249485	0.156216	HZ	LOC101117690	NC_056059.1	80930001	80950001	0.0377091	0.207083	HZ	LOC10699122
0.23482	0.192508	HZ	LOC101117690	NC_056059.1	80935001	80955001	0.0311751	0.186594	HZ	LOC10699122
0.199427	0.198048	HZ	LOC101117690	NC_056059.1	80940001	80960001	0.044576	0.166914	HZ	LOC10699122
0.26394	0.24059	HZ	LOC101117690	NC_056059.1	80945001	80965001	0.0333124	0.228111	HZ	LOC10699122
0.418317	0.209846	HZ	LOC101117690	NC_056060.1	101105001	101125001	0.353865	0.17639	HZ	LOC11410860
0.516385	0.142215	HZ	LOC101117683	NC_056070.1	71105001	71125001	0.273605	0.202328	HZ	LOC114108841
0.283368	0.207964	HZ	LOC101117494	NC_056073.1	28115001	28135001	0.410343	0.164708	HZ	LOC11410956
0.343433	0.17539	HZ	LOC101117494	NC_056073.1	28120001	28140001	0.41087	0.19264	HZ	LOC11410956
0.415258	0.154454	HZ	LOC101117494	NC_056074.1	2060001	2080001	0.338904	0.1958	HZ	LOC11411015
0.219923	0.153878	HZ	LOC101117350	NC_056074.1	2065001	2085001	0.346802	0.224328	HZ	LOC11411015
0.191753	0.174957	HZ	LOC101117350	NC_056074.1	2070001	2090001	0.35013	0.235452	HZ	LOC11411015
0.400842	0.152422	HZ	LOC101117028	NC_056074.1	2075001	2095001	0.43865	0.214418	HZ	LOC11411015
0.423529	0.149828	HZ	LOC101116222	NC_056074.1	2080001	2100001	0.439355	0.211437	HZ	LOC11411015
0.351762	0.151269	HZ	LOC101116222	NC_056074.1	2085001	2105001	0.426954	0.197577	HZ	LOC11411015
0.313234	0.151374	HZ	LOC101116222	NC_056074.1	2090001	2110001	0.422744	0.202762	HZ	LOC11411015
0.188495	0.153408	HZ	LOC101115856;ZN	NC_056074.1	2095001	2115001	0.311615	0.208421	HZ	LOC11411015
0.367152	0.145112	HZ	LOC101114926	NC_056074.1	2100001	2120001	0.333203	0.207851	HZ	LOC11411015

0.488384	0.173922	HZ	LOC101114926	NC_056055.1	89545001	89565001	0.462589	0.174504	HZ	LOC11411282
0.443333	0.231151	HZ	LOC101114879	NC_056055.1	172945001	172965001	0.344331	0.316231	HZ	LOC11411287
0.433904	0.230977	HZ	LOC101114879	NC_056055.1	243395001	243415001	0.326658	0.366424	HZ	LOC11411291
0.440987	0.232364	HZ	LOC101114879	NC_056055.1	243400001	243420001	0.471468	0.276904	HZ	LOC11411291
0.452961	0.230836	HZ	LOC101114879	NC_056055.1	243405001	243425001	0.459843	0.222397	HZ	LOC11411291
0.53919	0.166025	HZ	LOC101114419	NC_056054.1	126005001	126025001	0.422081	0.159436	HZ	LOC11411336
0.523134	0.154154	HZ	LOC101114419	NC_056056.1	164820001	164840001	0.359755	0.34433	HZ	LOC11411378
0.155454	0.16793	HZ	LOC101114310	NC_056056.1	164825001	164845001	0.299713	0.281275	HZ	LOC11411378
0.408026	0.143338	HZ	LOC101114002	NC_056056.1	164830001	164850001	0.34036	0.226597	HZ	LOC11411378
0.228937	0.233948	HZ	LOC101113946;PL	NC_056056.1	164835001	164855001	0.389674	0.247549	HZ	LOC11411378
0.13624	0.265035	HZ	LOC101113946	NC_056056.1	182610001	182630001	0.4485	0.165225	HZ	LOC11411403
0.122128	0.265568	HZ	LOC101113946	NC_056056.1	218980001	219000001	0.367985	0.303071	HZ	LOC11411408
0.149739	0.229323	HZ	LOC101113946	NC_056059.1	45870001	45890001	0.484228	0.172311	HZ	LOC11411529
0.112285	0.165512	HZ	LOC101113946	NC_056054.1	81255001	81275001	0.12934	0.159053	HZ	LOC11411609
0.45678	0.154855	HZ	LOC101113239	NC_056061.1	74385001	74405001	0.0492295	0.169569	HZ	LOC11411613
0.0488566	0.187616	HZ	LOC101113101	NC_056062.1	69890001	69910001	0.217778	0.198829	HZ	LOC11411646
0.415503	0.211133	HZ	LOC101112921	NC_056062.1	69895001	69915001	0.280374	0.178257	HZ	LOC11411646
0.304709	0.23591	HZ	LOC101112921	NC_056063.1	18485001	18505001	0.492469	0.193086	HZ	LOC11411659
0.475251	0.143036	HZ	LOC101112921	NC_056063.1	27675001	27695001	0.19767	0.174379	HZ	LOC11411660
0.504431	0.152894	HZ	LOC101112257	NC_056063.1	36935001	36955001	0.1102	0.169293	HZ	LOC11411665
0.414291	0.150532	HZ	LOC101112257	NC_056070.1	21580001	21600001	0.277273	0.258432	HZ	LOC11411883
0.524787	0.175	HZ	LOC101112115	NC_056070.1	21585001	21605001	0.10042	0.294096	HZ	LOC11411883
0.453333	0.153186	HZ	LOC101112115	NC_056070.1	21590001	21610001	0.0249701	0.276081	HZ	LOC11411883
0.483861	0.178704	HZ	LOC101112115	NC_056065.1	53850001	53870001	0.450834	0.187252	HZ	LOC12181607
0.464183	0.218107	HZ	LOC101112115	NC_056065.1	53855001	53875001	0.333106	0.23801	HZ	LOC12181607
0.292004	0.191931	HZ	LOC101112115	NC_056065.1	53860001	53880001	0.33312	0.235264	HZ	LOC12181607
0.279925	0.205182	HZ	LOC101112115	NC_056067.1	61440001	61460001	0.0920998	0.247064	HZ	LOC12181655
0.258945	0.197738	HZ	LOC101112115	NC_056067.1	61445001	61465001	0.0920998	0.247064	HZ	LOC12181655
0.275636	0.156266	HZ	LOC101112115	NC_056067.1	61450001	61470001	0.0960189	0.251049	HZ	LOC12181655
0.178324	0.156707	HZ	LOC101112115	NC_056067.1	61455001	61475001	0.121432	0.22114	HZ	LOC12181655
0.171589	0.191622	HZ	LOC101111409	NC_056070.1	14135001	14155001	0.240893	0.232504	HZ	LOC12181691
0.189465	0.194991	HZ	LOC101111409	NC_056070.1	14140001	14160001	0.204497	0.21916	HZ	LOC12181691
0.319801	0.321145	HZ	LOC101111300	NC_056070.1	14145001	14165001	0.140349	0.19545	HZ	LOC12181691
0.353975	0.306444	HZ	LOC101111300	NC_056070.1	14150001	14170001	0.165826	0.203775	HZ	LOC12181691
0.436985	0.153528	HZ	LOC101110888	NC_056077.1	27550001	27570001	0.464235	0.201841	HZ	LOC12181791
0.512568	0.144222	HZ	LOC101110882	NC_056080.1	81850001	81870001	0.46	0.305958	HZ	LOC12181814

0.250297	0.208931	HZ	LOC101110674	NC_056080.1	81860001	81880001	0.210693	0.402094	HZ	LOC12181814[illegible]
0.18824	0.2336	HZ	LOC101110674	NC_056080.1	53395001	53415001	0.431429	0.223494	HZ	LOC12181820[illegible]
0.222432	0.215239	HZ	LOC101110674	NC_056080.1	53400001	53420001	0.420949	0.235862	HZ	LOC12181820[illegible]
0.171107	0.154331	HZ	LOC101110206	NC_056056.1	213405001	213425001	0.319001	0.280242	HZ	LOC12181922[illegible]
0.289792	0.199123	HZ	LOC101109826	NC_056056.1	213410001	213430001	0.326572	0.255306	HZ	LOC12181922[illegible]
0.457485	0.203657	HZ	LOC101109826	NC_056062.1	64825001	64845001	0.457408	0.1784	HZ	LOC12182031[illegible]
0.492896	0.152245	HZ	LOC101109675	NC_056062.1	64830001	64850001	0.416763	0.196344	HZ	LOC12182031[illegible]
0.0215894	0.183055	HZ	LOC101109077	NC_056062.1	64835001	64855001	0.42353	0.235545	HZ	LOC12182031[illegible]
0.0816295	0.192223	HZ	LOC101109077	NC_056062.1	64840001	64860001	0.439749	0.233256	HZ	LOC12182031[illegible]
0.109882	0.194765	HZ	LOC101109077	NC_056062.1	64015001	64035001	0.402626	0.19259	HZ	LOC12182034[illegible]
0.21676	0.143901	HZ	LOC101109077	NC_056062.1	64020001	64040001	0.438477	0.218772	HZ	LOC12182034[illegible]
0.163636	0.14001	HZ	LOC101108915;L[illegible]	NC_056062.1	64025001	64045001	0.452258	0.228358	HZ	LOC12182034[illegible]
0.256176	0.260474	HZ	LOC101108915	NC_056062.1	64030001	64050001	0.44698	0.226366	HZ	LOC12182034[illegible]
0.0688259	0.37602	HZ	LOC101108915	NC_056062.1	64035001	64055001	0.458152	0.228647	HZ	LOC12182034[illegible]
0.0716112	0.360678	HZ	LOC101108915	NC_056062.1	64040001	64060001	0.462068	0.225609	HZ	LOC12182034[illegible]
0.0565343	0.318661	HZ	LOC101108915	NC_056062.1	64045001	64065001	0.444412	0.194493	HZ	LOC12182034[illegible]
0.0641273	0.254246	HZ	LOC101108915	NC_056062.1	64050001	64070001	0.428113	0.160795	HZ	LOC12182034[illegible]
0.289656	0.146101	HZ	LOC101108371;L[illegible]	NC_056064.1	61445001	61465001	0.183126	0.224402	HZ	LOC12182068[illegible]
0.40776	0.155329	HZ	LOC101108371;L[illegible]	NC_056064.1	61450001	61470001	0.187812	0.220068	HZ	LOC12182068[illegible]
0.310973	0.200432	HZ	LOC101108155	NC_056054.1	108895001	108915001	0.477644	0.342113	HZ	LOC443441
0.219032	0.263358	HZ	LOC101108155	NC_056057.1	94135001	94155001	0.101955	0.164137	HZ	LOC443534
0.40439	0.164062	HZ	LOC101108155	NC_056057.1	94140001	94160001	0.111505	0.167635	HZ	LOC443534
0.377778	0.212457	HZ	LOC101108113;L[illegible]	NC_056057.1	94145001	94165001	0.120592	0.175635	HZ	LOC443534
0.24477	0.203093	HZ	LOC101108113	NC_056079.1	23390001	23410001	0.456393	0.198157	HZ	LONRF1
0.259036	0.191455	HZ	LOC101108113	NC_056070.1	7175001	7195001	0.450903	0.16176	HZ	LRBA
0.2	0.160655	HZ	LOC101108113	NC_056070.1	7285001	7305001	0.473685	0.247796	HZ	LRBA
0.340467	0.16287	HZ	LOC101108113	NC_056070.1	7290001	7310001	0.491228	0.257314	HZ	LRBA
0.49692	0.174773	HZ	LOC101108113	NC_056070.1	7295001	7315001	0.433681	0.168392	HZ	LRBA
0.505959	0.151276	HZ	LOC101107061	NC_056070.1	7300001	7320001	0.459143	0.172996	HZ	LRBA
0.491476	0.160948	HZ	LOC101106894	NC_056070.1	7480001	7500001	0.47111	0.19938	HZ	LRBA
0.347156	0.221258	HZ	LOC101106781	NC_056063.1	16980001	17000001	0.131422	0.160831	HZ	LRCH1
0.18909	0.316199	HZ	LOC101106781	NC_056054.1	88305001	88325001	0.414892	0.168107	HZ	LRIF1
0.208371	0.307774	HZ	LOC101106781	NC_056054.1	88310001	88330001	0.489743	0.184297	HZ	LRIF1
0.277553	0.285684	HZ	LOC101106781	NC_056072.1	35140001	35160001	0.475502	0.172474	HZ	LRIG1
0.430589	0.248994	HZ	LOC101106781	NC_056072.1	35180001	35200001	0.40582	0.237758	HZ	LRIG1;SLC25[illegible]
0.501996	0.219404	HZ	LOC101106781	NC_056072.1	35185001	35205001	0.439432	0.245977	HZ	LRIG1;SLC25[illegible]

0.270767	0.178435	HZ	LOC101106734	NC_056072.1	35190001	35210001	0.471633	0.278299	HZ	LRIG1;SLC25A
0.333334	0.172523	HZ	LOC101106734	NC_056062.1	72965001	72985001	0.406338	0.175764	HZ	LRP12
0.21896	0.235438	HZ	LOC101106638;LC	NC_056062.1	72970001	72990001	0.331797	0.207242	HZ	LRP12
0.361067	0.201289	HZ	LOC101106638;LC	NC_056062.1	72975001	72995001	0.373783	0.23778	HZ	LRP12
0.191797	0.21293	HZ	LOC101106638	NC_056062.1	72980001	73000001	0.458183	0.242749	HZ	LRP12
0.2783	0.143171	HZ	LOC101106524	NC_056055.1	168895001	168915001	0.434573	0.178973	HZ	LRP1B
0.228163	0.151776	HZ	LOC101106416;LC	NC_056055.1	168900001	168920001	0.428906	0.166602	HZ	LRP1B
0.484482	0.16937	HZ	LOC101106373;RI	NC_056056.1	203215001	203235001	0.441022	0.258773	HZ	LRP6
0.477522	0.197279	HZ	LOC101105892	NC_056056.1	203220001	203240001	0.210359	0.320145	HZ	LRP6
0.478794	0.170028	HZ	LOC101105892	NC_056056.1	203225001	203245001	0.143909	0.342613	HZ	LRP6
0.195123	0.164452	HZ	LOC101105154	NC_056056.1	203230001	203250001	0.128082	0.325871	HZ	LRP6
0.120645	0.162988	HZ	LOC101105154	NC_056056.1	203235001	203255001	0.126011	0.342703	HZ	LRP6
0.130591	0.148212	HZ	LOC101105154	NC_056056.1	203240001	203260001	0.208208	0.302152	HZ	LRP6
0.418594	0.162125	HZ	LOC101105133;LC	NC_056056.1	203245001	203265001	0.303714	0.226053	HZ	LRP6
0.320112	0.153033	HZ	LOC101104855	NC_056056.1	203250001	203270001	0.336584	0.221819	HZ	LRP6
0.49051	0.170133	HZ	LOC101104595	NC_056056.1	203255001	203275001	0.354077	0.191146	HZ	LRP6
0.359627	0.163759	HZ	LOC101104595	NC_056056.1	203260001	203280001	0.361004	0.160405	HZ	LRP6
0.531894	0.148868	HZ	LOC101104595	NC_056056.1	165880001	165900001	0.45158	0.205057	HZ	LTA4H
0.468005	0.191489	HZ	LOC101104225	NC_056056.1	165885001	165905001	0.427481	0.193649	HZ	LTA4H
0.274543	0.160834	HZ	LOC101103944	NC_056055.1	243410001	243430001	0.417266	0.248446	HZ	LUZP1
0.210301	0.184733	HZ	LOC101103944	NC_056055.1	243420001	243440001	0.471902	0.285463	HZ	LUZP1
0.514348	0.152361	HZ	LOC101103916;LC	NC_056055.1	243445001	243465001	0.297495	0.333293	HZ	LUZP1
0.52381	0.154762	HZ	LOC101103916;LC	NC_056055.1	243450001	243470001	0.141322	0.342635	HZ	LUZP1
0.359511	0.146328	HZ	LOC101103752	NC_056055.1	243455001	243475001	0.226632	0.274394	HZ	LUZP1
0.351244	0.146574	HZ	LOC101103752	NC_056074.1	17880001	17900001	0.180337	0.179212	HZ	LUZP2
0.491556	0.18758	HZ	LOC101102317	NC_056074.1	17890001	17910001	0.027564	0.212757	HZ	LUZP2
0.362782	0.21661	HZ	LOC101102317	NC_056074.1	17895001	17915001	0.0241028	0.27565	HZ	LUZP2
0.322828	0.189694	HZ	LOC101102317	NC_056056.1	102065001	102085001	0.0908405	0.214265	HZ	LYG1
0.32414	0.162244	HZ	LOC101102317	NC_056056.1	102070001	102090001	0.0653227	0.26474	HZ	LYG1
0.423771	0.201464	HZ	LOC101102056	NC_056056.1	102075001	102095001	0.0916668	0.307432	HZ	LYG1
0.466408	0.279162	HZ	LOC101102056	NC_056056.1	102080001	102100001	0.196561	0.298354	HZ	LYG1
0.1361	0.194547	HZ	LOC101102047	NC_056056.1	102085001	102105001	0.218189	0.289665	HZ	LYG1;LYG2
0.10059	0.214572	HZ	LOC101102047	NC_056056.1	102090001	102110001	0.219173	0.262167	HZ	LYG2
0.0502669	0.267656	HZ	LOC101102047	NC_056056.1	102095001	102115001	0.200064	0.282154	HZ	LYG2
0.0245975	0.273453	HZ	LOC101102047	NC_056056.1	102100001	102120001	0.150563	0.359731	HZ	LYG2
0.0176311	0.265903	HZ	LOC101102047	NC_056056.1	102105001	102125001	0.154412	0.362254	HZ	LYG2

0.0150686	0.258283	HZ	LOC101102047	NC_056056.1	102110001	102130001	0.149031	0.379009	HZ	LYG2;MRPL3(
0.0362433	0.16965	HZ	LOC101102047	NC_056056.1	102115001	102135001	0.128031	0.353442	HZ	LYG2;MRPL3(
0.474576	0.144171	HZ	LOC101101889	NC_056078.1	7745001	7765001	0.305649	0.171185	HZ	LYST
0.412075	0.212078	HZ	LOC100125357	NC_056072.1	14600001	14620001	0.245488	0.268895	HZ	LYZL4
0.413998	0.209291	HZ	LOC100125357	NC_056072.1	14605001	14625001	0.271444	0.258025	HZ	LYZL4
0.263159	0.227356	HZ	LOC100125357	NC_056072.1	14610001	14630001	0.316535	0.281718	HZ	LYZL4
0.303102	0.211175	HZ	LOC100125357	NC_056072.1	14615001	14635001	0.459876	0.263279	HZ	LYZL4
0.464802	0.186479	HZ	LMX1A	NC_056072.1	14620001	14640001	0.454763	0.302546	HZ	LYZL4
0.502992	0.145627	HZ	LMX1A	NC_056054.1	93415001	93435001	0.455659	0.17974	HZ	MAB21L3
0.534893	0.146903	HZ	LIPK	NC_056054.1	93420001	93440001	0.317203	0.198053	HZ	MAB21L3
0.518331	0.163288	HZ	LINGO3;PEAK3	NC_056054.1	93425001	93445001	0.195144	0.234184	HZ	MAB21L3
0.11875	0.139353	HZ	LIMS2	NC_056054.1	93430001	93450001	0.233534	0.226142	HZ	MAB21L3
0.233987	0.145458	HZ	LHFPL2	NC_056054.1	93435001	93455001	0.410804	0.197024	HZ	MAB21L3
0.206648	0.142312	HZ	LHFPL2	NC_056054.1	93440001	93460001	0.45045	0.191809	HZ	MAB21L3
0.413665	0.211428	HZ	LEMD3	NC_056066.1	8805001	8825001	0.352915	0.158253	HZ	MACROD2
0.427313	0.20573	HZ	LEMD3	NC_056066.1	8810001	8830001	0.264	0.165126	HZ	MACROD2
0.337772	0.236885	HZ	LEMD3	NC_056066.1	8815001	8835001	0.163295	0.162529	HZ	MACROD2
0.292952	0.216312	HZ	LEMD3	NC_056057.1	44115001	44135001	0.415729	0.158044	HZ	MAGI2
0.306346	0.213343	HZ	LEMD3	NC_056057.1	44120001	44140001	0.390081	0.180903	HZ	MAGI2
0.369004	0.186678	HZ	LEMD3	NC_056057.1	44125001	44145001	0.337957	0.192448	HZ	MAGI2
0.437051	0.140473	HZ	LEMD3	NC_056057.1	44130001	44150001	0.348502	0.188469	HZ	MAGI2
0.474559	0.170589	HZ	LARGE2;PHF21A	NC_056057.1	44255001	44275001	0.118059	0.247857	HZ	MAGI2
0.528336	0.15837	HZ	KRT80	NC_056057.1	44260001	44280001	0.192623	0.196581	HZ	MAGI2
0.519528	0.210147	HZ	KPNA5	NC_056057.1	44630001	44650001	0.420024	0.221349	HZ	MAGI2
0.484544	0.1982	HZ	KPNA5	NC_056057.1	44635001	44655001	0.224713	0.201451	HZ	MAGI2
0.313929	0.170275	HZ	KMT2C	NC_056057.1	44640001	44660001	0.224891	0.209987	HZ	MAGI2
0.15533	0.163537	HZ	KMT2C	NC_056061.1	18920001	18940001	0.367303	0.2718	HZ	MAN1A1
0.152059	0.162213	HZ	KMT2C	NC_056061.1	18925001	18945001	0.452503	0.241793	HZ	MAN1A1
0.501707	0.182029	HZ	KMT2C	NC_056061.1	19065001	19085001	0.381809	0.164151	HZ	MAN1A1
0.462651	0.171272	HZ	KMT2C	NC_056058.1	106385001	106405001	0.457737	0.202998	HZ	MAN2A1
0.421053	0.191419	HZ	KMT2C	NC_056058.1	106390001	106410001	0.472767	0.211901	HZ	MAN2A1
0.532582	0.22021	HZ	KMT2C	NC_056061.1	61970001	61990001	0.46221	0.215661	HZ	MAP3K5
0.364943	0.15713	HZ	KMO;OPN3	NC_056061.1	61975001	61995001	0.416967	0.249298	HZ	MAP3K5
0.475491	0.163363	HZ	KLHL2	NC_056061.1	61980001	62000001	0.273022	0.264838	HZ	MAP3K5
0.401771	0.181413	HZ	KLHL2	NC_056061.1	61985001	62005001	0.387951	0.255955	HZ	MAP3K5
0.348135	0.205163	HZ	KLHL2	NC_056061.1	61990001	62010001	0.483278	0.238411	HZ	MAP3K5

0.422734	0.141011	HZ	KIRREL1	NC_056060.1	41595001	41615001	0.387868	0.223996	HZ	MAP4K5
0.434226	0.162098	HZ	KIRREL1	NC_056060.1	41600001	41620001	0.378585	0.208044	HZ	MAP4K5
0.380508	0.209737	HZ	KIF26B	NC_056060.1	41605001	41625001	0.315898	0.210563	HZ	MAP4K5
0.377274	0.23128	HZ	KIF26B	NC_056060.1	41610001	41630001	0.322372	0.200077	HZ	MAP4K5
0.380575	0.212414	HZ	KIF26B	NC_056070.1	72040001	72060001	0.350935	0.170464	HZ	MAPK1
0.406229	0.222489	HZ	KIF26B	NC_056070.1	72045001	72065001	0.338918	0.162353	HZ	MAPK1
0.310646	0.19906	HZ	KIF19	NC_056076.1	50150001	50170001	0.485792	0.167304	HZ	MBD1
0.323509	0.194328	HZ	KIF19	NC_056080.1	102485001	102505001	0.362086	0.163343	HZ	MBNL3
0.230459	0.177918	HZ	KIAA1958	NC_056080.1	102490001	102510001	0.293233	0.16444	HZ	MBNL3
0.185326	0.231071	HZ	KIAA1958	NC_056073.1	36980001	37000001	0.490319	0.159745	HZ	MBOAT1
0.289712	0.228696	HZ	KIAA1958	NC_056056.1	219830001	219850001	0.123795	0.249745	HZ	MCAT
0.466887	0.205728	HZ	KIAA1958	NC_056056.1	219835001	219855001	0.100696	0.265962	HZ	MCAT
0.505257	0.207047	HZ	KIAA1958	NC_056056.1	219840001	219860001	0.102032	0.260674	HZ	MCAT;TSPO
0.50021	0.157017	HZ	KIAA1958	NC_056056.1	219845001	219865001	0.151853	0.220153	HZ	MCAT;TSPO
0.3748	0.190275	HZ	KIAA1958	NC_056060.1	38355001	38375001	0.15004	0.186302	HZ	MDGA2
0.390005	0.176827	HZ	KIAA1958	NC_056060.1	38360001	38380001	0.0581697	0.232735	HZ	MDGA2
0.373199	0.205299	HZ	KIAA1958	NC_056060.1	38365001	38385001	0.0541806	0.238907	HZ	MDGA2
0.500919	0.169849	HZ	KIAA1217	NC_056060.1	38370001	38390001	0.0428572	0.247	HZ	MDGA2
0.421523	0.15264	HZ	KIAA1217	NC_056060.1	38375001	38395001	0.0418806	0.213415	HZ	MDGA2
0.377132	0.142864	HZ	KIAA1217	NC_056060.1	38430001	38450001	0.229763	0.173166	HZ	MDGA2
0.538961	0.143316	HZ	KIAA1109	NC_056065.1	2095001	2115001	0.366887	0.160031	HZ	MDM4
0.253212	0.176476	HZ	KIAA0825	NC_056065.1	2100001	2120001	0.278976	0.203694	HZ	MDM4
0.166081	0.211042	HZ	KIAA0825	NC_056065.1	2105001	2125001	0.305075	0.207941	HZ	MDM4
0.264357	0.209867	HZ	KIAA0825	NC_056065.1	2110001	2130001	0.334395	0.205344	HZ	MDM4
0.305046	0.204955	HZ	KIAA0825	NC_056065.1	2115001	2135001	0.352718	0.223136	HZ	MDM4
0.377048	0.161518	HZ	KIAA0825	NC_056061.1	10520001	10540001	0.244493	0.16766	HZ	ME1
0.134199	0.14056	HZ	KDM4C	NC_056061.1	10525001	10545001	0.364993	0.172317	HZ	ME1
0.334845	0.210246	HZ	KDM1B;TPMT	NC_056061.1	10530001	10550001	0.396505	0.169494	HZ	ME1
0.356164	0.236909	HZ	KDM1B;TPMT	NC_056061.1	10535001	10555001	0.301291	0.178432	HZ	ME1
0.397613	0.196036	HZ	KCNU1	NC_056074.1	590001	610001	0.31579	0.242312	HZ	MED17
0.394736	0.192846	HZ	KCNU1	NC_056074.1	595001	615001	0.377299	0.225726	HZ	MED17
0.44617	0.197673	HZ	KCNU1	NC_056074.1	580001	600001	0.305318	0.294298	HZ	MED17;VSTM
0.0458716	0.185204	HZ	KCNQ5	NC_056074.1	585001	605001	0.175403	0.299467	HZ	MED17;VSTM
0.201279	0.171971	HZ	KCNQ5	NC_056056.1	4930001	4950001	0.337484	0.161699	HZ	MED27
0.370558	0.144322	HZ	KCNQ5	NC_056064.1	34570001	34590001	0.366765	0.304068	HZ	MED9
0.474594	0.159033	HZ	KCNN3	NC_056064.1	34575001	34595001	0.370414	0.269374	HZ	MED9

0.423674	0.190152	HZ	KCNN3	NC_056064.1	34565001	34585001	0.403399	0.285867	HZ	MED9;PEMT;F
0.374626	0.233618	HZ	KCNN3	NC_056063.1	30290001	30310001	0.442206	0.23105	HZ	MEDAG
0.245409	0.306378	HZ	KCNN3	NC_056063.1	30295001	30315001	0.441887	0.239506	HZ	MEDAG
0.205516	0.316786	HZ	KCNN3	NC_056063.1	30300001	30320001	0.468714	0.22265	HZ	MEDAG
0.195569	0.348448	HZ	KCNN3	NC_056058.1	24370001	24390001	0.0591437	0.18096	HZ	MEGF10
0.176551	0.360938	HZ	KCNN3	NC_056058.1	24375001	24395001	0.243273	0.170053	HZ	MEGF10
0.394367	0.252079	HZ	KCNN3	NC_056058.1	24380001	24400001	0.448342	0.16944	HZ	MEGF10
0.464113	0.2139	HZ	KCNN3	NC_056060.1	12765001	12785001	0.378825	0.219574	HZ	MEGF11
0.343997	0.149716	HZ	KCNMB2	NC_056060.1	12770001	12790001	0.411765	0.327222	HZ	MEGF11
0.222978	0.204479	HZ	KCNMB2	NC_056060.1	12775001	12795001	0.425957	0.344298	HZ	MEGF11
0.290159	0.181122	HZ	KCNMB2	NC_056060.1	12780001	12800001	0.430284	0.322387	HZ	MEGF11
0.483896	0.14824	HZ	KCNMA1	NC_056060.1	12785001	12805001	0.379934	0.267647	HZ	MEGF11
0.488398	0.163488	HZ	KCNK6;YIF1B	NC_056060.1	12790001	12810001	0.382107	0.243902	HZ	MEGF11
0.529143	0.164977	HZ	KCNJ6	NC_056060.1	12795001	12815001	0.390701	0.248607	HZ	MEGF11
0.507553	0.152246	HZ	KCNJ6	NC_056060.1	12800001	12820001	0.410875	0.202689	HZ	MEGF11
0.46724	0.181951	HZ	KCNIP4	NC_056065.1	48870001	48890001	0.430709	0.160081	HZ	MEGF6
0.41635	0.153781	HZ	KCNIP4	NC_056079.1	23995001	24015001	0.435217	0.21931	HZ	MFHAS1
0.321019	0.233385	HZ	KCNIP3;PROM2	NC_056057.1	106655001	106675001	0.244318	0.168576	HZ	MGAM
0.434427	0.261959	HZ	KCNIP3;PROM2	NC_056057.1	106660001	106680001	0.132509	0.228118	HZ	MGAM
0.432794	0.256447	HZ	KCNIP3;PROM2	NC_056057.1	106665001	106685001	0.159264	0.230817	HZ	MGAM
0.403413	0.14416	HZ	KCNIP3	NC_056057.1	106670001	106690001	0.243333	0.264256	HZ	MGAM
0.401724	0.141345	HZ	KCNIP3	NC_056068.1	40340001	40360001	0.302527	0.272954	HZ	MICAL2
0.509327	0.139889	HZ	KCNIP3	NC_056068.1	40345001	40365001	0.282023	0.346298	HZ	MICAL2
0.396666	0.162698	HZ	KCNIP3	NC_056068.1	40350001	40370001	0.38008	0.378767	HZ	MICAL2
0.440218	0.194124	HZ	KCNIP3	NC_056071.1	45255001	45275001	0.462439	0.201292	HZ	MIPOL1
0.434029	0.185911	HZ	KCNIP3	NC_056071.1	45260001	45280001	0.444486	0.207056	HZ	MIPOL1
0.428058	0.164578	HZ	KCNIP3	NC_056071.1	45265001	45285001	0.350665	0.218505	HZ	MIPOL1
0.180354	0.144801	HZ	KCNIP3	NC_056071.1	45270001	45290001	0.2751	0.241788	HZ	MIPOL1
0.138651	0.163742	HZ	KCNIP3	NC_056056.1	102125001	102145001	0.183003	0.262837	HZ	MITD1;MRPL3
0.157009	0.192859	HZ	KCNIP3	NC_056056.1	102130001	102150001	0.447156	0.177084	HZ	MITD1;MRPL3
0.150781	0.195324	HZ	KCNIP3	NC_056072.1	31670001	31690001	0.482291	0.297705	HZ	MITF
0.207399	0.219381	HZ	KCNIP3	NC_056072.1	31730001	31750001	0.409048	0.346341	HZ	MITF
0.253731	0.239103	HZ	KCNIP3	NC_056072.1	31735001	31755001	0.189583	0.319143	HZ	MITF
0.452681	0.233349	HZ	KCNH1	NC_056072.1	31740001	31760001	0.0969697	0.31062	HZ	MITF
0.396189	0.281664	HZ	KCNH1	NC_056072.1	31745001	31765001	0.181386	0.245266	HZ	MITF
0.357547	0.348742	HZ	KCNH1	NC_056072.1	31750001	31770001	0.226882	0.228341	HZ	MITF

0.35108	0.198374	HZ	KCNE4
0.35797	0.219769	HZ	KCNE4
0.338384	0.204752	HZ	KCNE4
0.516557	0.170082	HZ	KCNC2
0.433696	0.247373	HZ	KCNC2
0.42989	0.143388	HZ	KBTBD12
0.366543	0.181378	HZ	KBTBD12
0.301395	0.215523	HZ	KBTBD12
0.447879	0.17146	HZ	KBTBD12
0.480065	0.149169	HZ	KANSL3
0.478497	0.218625	HZ	KANSL1L
0.465348	0.21821	HZ	KANSL1L
0.527478	0.190612	HZ	KANSL1L
0.527572	0.147076	HZ	K38
0.383402	0.179306	HZ	JPH3
0.406971	0.162594	HZ	JDP2
0.286435	0.193358	HZ	JDP2
0.532111	0.347172	HZ	JAK2
0.255556	0.34965	HZ	JAK2
0.272957	0.366521	HZ	JAK2
0.355655	0.361441	HZ	JAK2
0.383962	0.353445	HZ	JAK2
0.424969	0.349394	HZ	JAK2
0.443069	0.341262	HZ	JAK2
0.48995	0.34933	HZ	JAK2
0.482114	0.356586	HZ	JAK2
0.480899	0.357496	HZ	JAK2
0.481062	0.355993	HZ	JAK2
0.469545	0.358604	HZ	JAK2
0.472391	0.34692	HZ	JAK2
0.458438	0.346279	HZ	JAK2
0.510122	0.306316	HZ	JAK2
0.509091	0.322556	HZ	JAK2
0.504807	0.298091	HZ	JAK2
0.469388	0.335333	HZ	JAK2
0.510204	0.316807	HZ	JAK2

NC_056072.1	31755001	31775001	0.238929	0.196183	HZ	MITF
NC_056072.1	31760001	31780001	0.178362	0.230281	HZ	MITF
NC_056072.1	31765001	31785001	0.114106	0.275881	HZ	MITF
NC_056072.1	31770001	31790001	0.10666	0.285711	HZ	MITF
NC_056072.1	31775001	31795001	0.194823	0.23209	HZ	MITF
NC_056072.1	31850001	31870001	0.15488	0.352132	HZ	MITF
NC_056072.1	31855001	31875001	0.165419	0.332307	HZ	MITF
NC_056072.1	31860001	31880001	0.119553	0.354727	HZ	MITF
NC_056072.1	31865001	31885001	0.115448	0.35926	HZ	MITF
NC_056072.1	31870001	31890001	0.0736026	0.372194	HZ	MITF
NC_056072.1	31885001	31905001	0.0868889	0.409199	HZ	MITF
NC_056072.1	31890001	31910001	0.226376	0.398585	HZ	MITF
NC_056072.1	31895001	31915001	0.482025	0.40811	HZ	MITF
NC_056054.1	21120001	21140001	0.341861	0.246918	HZ	MKNK1
NC_056054.1	21125001	21145001	0.153218	0.290707	HZ	MKNK1
NC_056054.1	21135001	21155001	0.0960156	0.231918	HZ	MKNK1
NC_056064.1	14315001	14335001	0.0961412	0.220434	HZ	MMP28
NC_056068.1	5830001	5850001	0.486738	0.175653	HZ	MMP7
NC_056059.1	35355001	35375001	0.437174	0.160694	HZ	MMRN1
NC_056056.1	157145001	157165001	0.101631	0.20979	HZ	MON2
NC_056056.1	157150001	157170001	0.159376	0.184698	HZ	MON2
NC_056056.1	157185001	157205001	0.0576718	0.165744	HZ	MON2
NC_056056.1	157190001	157210001	0.0408085	0.1646	HZ	MON2
NC_056066.1	35800001	35820001	0.338123	0.162403	HZ	MPP7
NC_056066.1	35810001	35830001	0.347608	0.182531	HZ	MPP7
NC_056066.1	35815001	35835001	0.367985	0.223472	HZ	MPP7
NC_056066.1	35820001	35840001	0.331835	0.207804	HZ	MPP7
NC_056066.1	35825001	35845001	0.381343	0.20797	HZ	MPP7
NC_056069.1	33640001	33660001	0.451705	0.168377	HZ	MROH2B
NC_056069.1	33645001	33665001	0.405064	0.203286	HZ	MROH2B
NC_056069.1	33650001	33670001	0.445119	0.214175	HZ	MROH2B
NC_056056.1	102120001	102140001	0.12486	0.279867	HZ	MRPL30
NC_056054.1	100020001	100040001	0.444008	0.345975	HZ	MTMR11;SF3[illegible]
NC_056065.1	41650001	41670001	0.308361	0.288907	HZ	MTOR
NC_056065.1	41665001	41685001	0.128586	0.356269	HZ	MTOR
NC_056065.1	41670001	41690001	0.147598	0.353566	HZ	MTOR

0.46875	0.346208	HZ	JAK2	NC_056065.1	41675001	41695001	0.171216	0.350795	HZ	MTOR
0.429245	0.34755	HZ	JAK2	NC_056065.1	41680001	41700001	0.157828	0.33883	HZ	MTOR
0.432432	0.317175	HZ	JAK2	NC_056065.1	41685001	41705001	0.166868	0.32565	HZ	MTOR
0.225137	0.162922	HZ	JAK1	NC_056056.1	9640001	9660001	0.199706	0.254917	HZ	MVB12B
0.185037	0.187654	HZ	JAK1	NC_056056.1	9645001	9665001	0.129046	0.324857	HZ	MVB12B
0.190272	0.214459	HZ	JAK1	NC_056056.1	9650001	9670001	0.107614	0.362016	HZ	MVB12B
0.27139	0.211524	HZ	JAK1	NC_056056.1	9655001	9675001	0.129425	0.359235	HZ	MVB12B
0.364573	0.184873	HZ	JAK1	NC_056064.1	29195001	29215001	0.144515	0.170263	HZ	MYH8
0.321855	0.191165	HZ	JAK1	NC_056063.1	83665001	83685001	0.401278	0.502838	HZ	MYO16
0.331103	0.176855	HZ	JAK1	NC_056063.1	83670001	83690001	0.286296	0.535981	HZ	MYO16
0.269307	0.197848	HZ	JAK1	NC_056057.1	78285001	78305001	0.395161	0.179581	HZ	MYO1G
0.271736	0.206127	HZ	JAK1	NC_056057.1	78290001	78310001	0.321429	0.166559	HZ	MYO1G
0.352635	0.168384	HZ	JAK1	NC_056054.1	212570001	212590001	0.391375	0.158954	HZ	NAALADL2
0.454446	0.145092	HZ	ITPRID1	NC_056054.1	212910001	212930001	0.433973	0.210288	HZ	NAALADL2
0.366689	0.156776	HZ	ITPRID1	NC_056054.1	212915001	212935001	0.313997	0.229244	HZ	NAALADL2
0.391964	0.204869	HZ	ITLN	NC_056054.1	212920001	212940001	0.350618	0.198165	HZ	NAALADL2
0.436902	0.149275	HZ	ITGA2	NC_056074.1	15070001	15090001	0.491615	0.329854	HZ	NARS2
0.406713	0.269649	HZ	ITGA11	NC_056074.1	15135001	15155001	0.465674	0.238494	HZ	NARS2
0.234265	0.253183	HZ	ITGA11	NC_056074.1	15140001	15160001	0.39075	0.234048	HZ	NARS2
0.306862	0.228187	HZ	ITGA11	NC_056074.1	15145001	15165001	0.292811	0.200616	HZ	NARS2
0.517469	0.178464	HZ	ITGA11	NC_056074.1	15150001	15170001	0.218533	0.192089	HZ	NARS2
0.507287	0.218673	HZ	ITGA11	NC_056074.1	15155001	15175001	0.213165	0.1896	HZ	NARS2
0.514396	0.228288	HZ	ITGA11	NC_056074.1	15160001	15180001	0.20866	0.195164	HZ	NARS2
0.385133	0.149818	HZ	ISM1	NC_056074.1	15165001	15185001	0.238642	0.213693	HZ	NARS2
0.418428	0.155593	HZ	ISM1	NC_056074.1	15170001	15190001	0.288054	0.223228	HZ	NARS2
0.523954	0.224544	HZ	ISM1	NC_056074.1	15175001	15195001	0.357895	0.210309	HZ	NARS2
0.369532	0.145469	HZ	ISLR2;PML	NC_056054.1	136395001	136415001	0.397834	0.221419	HZ	NCAM2
0.252934	0.169249	HZ	ISLR2	NC_056054.1	136470001	136490001	0.438675	0.173783	HZ	NCAM2
0.369467	0.188729	HZ	IRF8	NC_056055.1	177930001	177950001	0.382091	0.191369	HZ	NCKAP5
0.49055	0.142781	HZ	IQCM	NC_056055.1	177935001	177955001	0.385858	0.199257	HZ	NCKAP5
0.513613	0.319145	HZ	INSL6;LOC105608	NC_056069.1	18425001	18445001	0.256239	0.191586	HZ	NDUFAF2
0.459854	0.304511	HZ	INSL6;JAK2	NC_056069.1	18430001	18450001	0.297356	0.175591	HZ	NDUFAF2
0.437499	0.243665	HZ	INSL6	NC_056069.1	18435001	18455001	0.277285	0.173214	HZ	NDUFAF2
0.532609	0.260965	HZ	INSL6	NC_056060.1	19765001	19785001	0.290769	0.167363	HZ	NEO1
0.485149	0.27193	HZ	INSL6	NC_056060.1	19770001	19790001	0.22262	0.209348	HZ	NEO1
0.529499	0.168364	HZ	INSL5	NC_056060.1	19775001	19795001	0.219474	0.193495	HZ	NEO1

0.469629	0.145643	HZ	INSC	NC_056060.1	19780001	19800001	0.170888	0.184374	HZ	NEO1
0.34089	0.224062	HZ	INSC	NC_056076.1	5330001	5350001	0.329216	0.219485	HZ	NETO1
0.275814	0.307991	HZ	INSC	NC_056076.1	5335001	5355001	0.47143	0.191893	HZ	NETO1
0.215897	0.40003	HZ	INSC	NC_056055.1	82900001	82920001	0.311248	0.158248	HZ	NFIB
0.0909093	0.21459	HZ	INSC	NC_056055.1	26440001	26460001	0.370459	0.175554	HZ	NFIL3
0.310959	0.143166	HZ	INSC	NC_056080.1	15880001	15900001	0.385121	0.157566	HZ	NHS
0.448101	0.168664	HZ	INPP4B	NC_056080.1	15885001	15905001	0.344524	0.181942	HZ	NHS
0.37808	0.185551	HZ	INPP4B	NC_056080.1	15890001	15910001	0.26087	0.171681	HZ	NHS
0.298843	0.201607	HZ	INPP4B	NC_056080.1	15920001	15940001	0.36149	0.158988	HZ	NHS
0.192149	0.248018	HZ	INPP4B	NC_056080.1	64685001	64705001	0.333033	0.19973	HZ	NHSL2;RTL5
0.26158	0.204817	HZ	INPP4B	NC_056080.1	64690001	64710001	0.341002	0.196471	HZ	NHSL2;RTL5
0.283117	0.184071	HZ	INPP4B	NC_056062.1	78865001	78885001	0.435413	0.195723	HZ	NIPAL2
0.379934	0.143925	HZ	IMPG1	NC_056069.1	37380001	37400001	0.463734	0.239864	HZ	NIPBL
0.387119	0.141146	HZ	IMPG1	NC_056069.1	37385001	37405001	0.46337	0.237966	HZ	NIPBL
0.517253	0.194392	HZ	IMMP2L	NC_056069.1	37390001	37410001	0.467303	0.233365	HZ	NIPBL
0.499759	0.184316	HZ	IMMP2L	NC_056069.1	37395001	37415001	0.474642	0.230904	HZ	NIPBL
0.504557	0.225654	HZ	IMMP2L	NC_056062.1	40680001	40700001	0.425418	0.325968	HZ	NKAIN3
0.407146	0.258144	HZ	IMMP2L	NC_056062.1	40685001	40705001	0.359794	0.356924	HZ	NKAIN3
0.412663	0.264221	HZ	IMMP2L	NC_056062.1	40690001	40710001	0.316754	0.379108	HZ	NKAIN3
0.331042	0.218687	HZ	IMMP2L	NC_056062.1	40825001	40845001	0.385321	0.208051	HZ	NKAIN3
0.304419	0.215915	HZ	IMMP2L	NC_056055.1	42065001	42085001	0.483434	0.180777	HZ	NKX2-6
0.441259	0.216773	HZ	IMMP2L	NC_056059.1	99980001	100000001	0.13856	0.245623	HZ	NKX6-1
0.411397	0.20608	HZ	IMMP2L	NC_056064.1	19655001	19675001	0.222452	0.21646	HZ	NLK
0.440991	0.24689	HZ	IMMP2L	NC_056064.1	19660001	19680001	0.224845	0.220392	HZ	NLK
0.423143	0.270677	HZ	IL-15L;PLEKHG2;	NC_056059.1	71775001	71795001	0.11479	0.191757	HZ	NMU
0.439462	0.292377	HZ	IL-15L;PLEKHG2;	NC_056059.1	71780001	71800001	0.134835	0.198624	HZ	NMU
0.345557	0.144306	HZ	IKBKE;SRGAP2	NC_056059.1	71785001	71805001	0.289303	0.171832	HZ	NMU
0.259307	0.149394	HZ	IHO1	NC_056076.1	23655001	23675001	0.125433	0.243222	HZ	NOL4
0.190955	0.149283	HZ	IHO1	NC_056076.1	23660001	23680001	0.122593	0.268161	HZ	NOL4
0.511827	0.200568	HZ	IFT43	NC_056070.1	57885001	57905001	0.446036	0.205364	HZ	NOS1
0.477072	0.161632	HZ	IER5L	NC_056070.1	57890001	57910001	0.472574	0.247042	HZ	NOS1
0.421806	0.177842	HZ	IER5L	NC_056070.1	57895001	57915001	0.488778	0.283818	HZ	NOS1
0.455964	0.188082	HZ	HSDL2;KIAA1958	NC_056071.1	41690001	41710001	0.304979	0.165206	HZ	NPAS3
0.471556	0.150581	HZ	HSD17B4	NC_056071.1	41695001	41715001	0.306498	0.165609	HZ	NPAS3
0.523634	0.14118	HZ	HS3ST2	NC_056066.1	53465001	53485001	0.271872	0.254998	HZ	NPBWR2;OPR
0.52809	0.164897	HZ	HRH2	NC_056070.1	9925001	9945001	0.322003	0.185389	HZ	NR3C2

0.359938	0.16781	HZ	HPS3	NC_056057.1	50790001	50810001	0.464871	0.252992	HZ	NRCAM
0.397992	0.160267	HZ	HPS3	NC_056057.1	50795001	50815001	0.322765	0.191503	HZ	NRCAM
0.324175	0.239379	HZ	HPRT1	NC_056057.1	50800001	50820001	0.282652	0.195747	HZ	NRCAM
0.523198	0.144847	HZ	HORMAD2	NC_056060.1	99925001	99945001	0.48122	0.220728	HZ	NRDE2
0.488666	0.284514	HZ	HMGA2	NC_056060.1	99905001	99925001	0.471679	0.177602	HZ	NRDE2;PSMC
0.464696	0.239043	HZ	HMGA2	NC_056060.1	99910001	99930001	0.385751	0.187412	HZ	NRDE2;PSMC
0.531705	0.271303	HZ	HMGA2	NC_056060.1	99915001	99935001	0.319728	0.185419	HZ	NRDE2;PSMC
0.525376	0.228197	HZ	HMGA2	NC_056060.1	99920001	99940001	0.432653	0.205429	HZ	NRDE2;PSMC
0.492005	0.222208	HZ	HMGA2	NC_056055.1	207870001	207890001	0.394805	0.162312	HZ	NRP2
0.395443	0.166667	HZ	HMGA2	NC_056055.1	207880001	207900001	0.422857	0.224669	HZ	NRP2
0.401236	0.151282	HZ	HMGA2	NC_056055.1	207885001	207905001	0.44764	0.200608	HZ	NRP2
0.325044	0.139255	HZ	HM13	NC_056055.1	207890001	207910001	0.390162	0.208469	HZ	NRP2
0.304477	0.147972	HZ	HM13	NC_056055.1	207895001	207915001	0.28136	0.197392	HZ	NRP2
0.31181	0.142396	HZ	HM13	NC_056056.1	73300001	73320001	0.341651	0.196131	HZ	NRXN1
0.41501	0.141759	HZ	HLCS	NC_056056.1	73305001	73325001	0.27491	0.248665	HZ	NRXN1
0.392147	0.150503	HZ	HLCS	NC_056056.1	73310001	73330001	0.307143	0.277312	HZ	NRXN1
0.355263	0.150205	HZ	HERC1	NC_056056.1	73315001	73335001	0.433373	0.225079	HZ	NRXN1
0.425533	0.142803	HZ	HERC1	NC_056080.1	83030001	83050001	0.47311	0.289885	HZ	NSDHL
0.473913	0.147899	HZ	HERC1	NC_056080.1	83035001	83055001	0.491136	0.240224	HZ	NSDHL
0.371084	0.185416	HZ	HELLS;TBC1D12	NC_056056.1	173045001	173065001	0.390277	0.18266	HZ	NT5DC3;STAE
0.446096	0.166403	HZ	HELLS;TBC1D12	NC_056056.1	173050001	173070001	0.358661	0.178912	HZ	NT5DC3;STAE
0.458501	0.152436	HZ	HDAC9	NC_056055.1	35005001	35025001	0.464943	0.165314	HZ	NTRK2
0.512669	0.141636	HZ	HDAC9	NC_056056.1	175085001	175105001	0.394699	0.221458	HZ	NUAK1
0.426863	0.144959	HZ	HDAC9	NC_056056.1	175090001	175110001	0.404376	0.25855	HZ	NUAK1
0.334224	0.140718	HZ	HDAC9	NC_056056.1	175095001	175115001	0.370435	0.263411	HZ	NUAK1
0.259502	0.165896	HZ	HDAC9	NC_056056.1	175100001	175120001	0.335995	0.242707	HZ	NUAK1
0.277063	0.194013	HZ	HDAC9	NC_056056.1	175105001	175125001	0.448824	0.177997	HZ	NUAK1
0.268665	0.200517	HZ	HDAC9	NC_056080.1	128160001	128180001	0.209952	0.174098	HZ	NUP62CL;RBM
0.345822	0.238138	HZ	HDAC9	NC_056074.1	31260001	31280001	0.475674	0.217578	HZ	OPCML
0.419714	0.2481	HZ	HDAC9	NC_056074.1	31265001	31285001	0.32101	0.281128	HZ	OPCML
0.460925	0.174972	HZ	HDAC9	NC_056074.1	31270001	31290001	0.305043	0.289144	HZ	OPCML
0.480708	0.148698	HZ	HDAC9	NC_056074.1	31275001	31295001	0.199914	0.312426	HZ	OPCML
0.519317	0.30024	HZ	HDAC9	NC_056074.1	31280001	31300001	0.403457	0.257961	HZ	OPCML
0.318776	0.289377	HZ	HDAC9	NC_056055.1	119560001	119580001	0.192525	0.236033	HZ	ORMDL1;PMS
0.454376	0.284202	HZ	HDAC9	NC_056072.1	6475001	6495001	0.392813	0.163111	HZ	OSBPL10
0.511647	0.144048	HZ	HCN1	NC_056072.1	6480001	6500001	0.340281	0.1764	HZ	OSBPL10

0.495991	0.194601	HZ	GXYLT2	NC_056072.1	6485001	6505001	0.35913	0.185705	HZ	OSBPL10
0.222801	0.2758	HZ	GXYLT2	NC_056076.1	32830001	32850001	0.407592	0.175478	HZ	OSBPL1A
0.098955	0.306347	HZ	GXYLT2	NC_056076.1	32835001	32855001	0.111111	0.216211	HZ	OSBPL1A
0.267875	0.293221	HZ	GXYLT2	NC_056072.1	11725001	11745001	0.0990565	0.195686	HZ	OXSR1
0.358798	0.336457	HZ	GXYLT2	NC_056072.1	11730001	11750001	0.170732	0.17305	HZ	OXSR1
0.382174	0.374872	HZ	GXYLT2	NC_056058.1	19950001	19970001	0.216792	0.179061	HZ	P4HA2;PDLIM
0.443161	0.417948	HZ	GXYLT2	NC_056058.1	19955001	19975001	0.267459	0.177337	HZ	P4HA2;PDLIM
0.3434	0.441379	HZ	GXYLT2	NC_056062.1	57390001	57410001	0.476535	0.228184	HZ	PAG1
0.32998	0.436594	HZ	GXYLT2	NC_056080.1	123905001	123925001	0.220711	0.204683	HZ	PAK3
0.318426	0.417923	HZ	GXYLT2	NC_056080.1	123910001	123930001	0.177778	0.246506	HZ	PAK3
0.338214	0.281567	HZ	GXYLT2	NC_056080.1	123915001	123935001	0.12228	0.283342	HZ	PAK3
0.221683	0.254297	HZ	GUCY2F	NC_056080.1	123920001	123940001	0.216783	0.25544	HZ	PAK3
0.152568	0.201931	HZ	GUCY2F	NC_056080.1	123925001	123945001	0.272727	0.298415	HZ	PAK3
0.116359	0.339511	HZ	GUCY2F	NC_056080.1	123945001	123965001	0.258696	0.230451	HZ	PAK3
0.127986	0.247869	HZ	GUCY2F	NC_056080.1	123950001	123970001	0.237556	0.179574	HZ	PAK3
0.176845	0.21562	HZ	GSPT1;RSL1D1	NC_056080.1	123955001	123975001	0.237792	0.162472	HZ	PAK3
0.205223	0.184801	HZ	GSPT1	NC_056059.1	17835001	17855001	0.413498	0.207549	HZ	PAPSS1
0.183654	0.185948	HZ	GSPT1	NC_056060.1	12170001	12190001	0.391049	0.164071	HZ	PARP16
0.13176	0.203809	HZ	GSPT1	NC_056054.1	116745001	116765001	0.33331	0.480804	HZ	PBX1
0.121212	0.196479	HZ	GSPT1	NC_056054.1	116750001	116770001	0.294665	0.441085	HZ	PBX1
0.101434	0.186583	HZ	GSPT1	NC_056054.1	116755001	116775001	0.215752	0.458757	HZ	PBX1
0.103641	0.190263	HZ	GSPT1	NC_056075.1	5045001	5065001	0.393723	0.158202	HZ	PCDH15
0.151	0.161127	HZ	GSPT1	NC_056063.1	5450001	5470001	0.469377	0.159075	HZ	PCDH17
0.52927	0.346982	HZ	GRPEL1;LOC1218	NC_056059.1	50860001	50880001	0.276669	0.194015	HZ	PCDH7
0.508944	0.203688	HZ	GRM5	NC_056059.1	50865001	50885001	0.474486	0.178571	HZ	PCDH7
0.493044	0.188231	HZ	GRM5	NC_056060.1	43885001	43905001	0.357837	0.216052	HZ	PCLAF
0.392789	0.150371	HZ	GRIP1	NC_056071.1	26845001	26865001	0.416411	0.290531	HZ	PCSK6
0.446706	0.180026	HZ	GRIP1	NC_056071.1	26850001	26870001	0.414868	0.327178	HZ	PCSK6
0.292361	0.169068	HZ	GRIN2A	NC_056071.1	26855001	26875001	0.454153	0.30155	HZ	PCSK6
0.240042	0.166914	HZ	GRIN2A	NC_056055.1	73650001	73670001	0.334982	0.338562	HZ	PDCD1LG2
0.211827	0.183569	HZ	GRIN2A	NC_056055.1	73655001	73675001	0.191244	0.421832	HZ	PDCD1LG2
0.176443	0.185325	HZ	GRIN2A	NC_056055.1	73660001	73680001	0.23497	0.399673	HZ	PDCD1LG2
0.272617	0.161751	HZ	GRIN2A	NC_056055.1	73665001	73685001	0.285328	0.374308	HZ	PDCD1LG2
0.355077	0.147424	HZ	GRIN2A	NC_056055.1	73670001	73690001	0.488115	0.284356	HZ	PDCD1LG2
0.51269	0.151006	HZ	GRB10	NC_056056.1	195050001	195070001	0.0570589	0.158055	HZ	PDE3A
0.279462	0.222045	HZ	GRB10	NC_056054.1	41705001	41725001	0.31854	0.220986	HZ	PDE4B

0.242921	0.207777	HZ	GRB10	NC_056054.1	41710001	41730001	0.169868	0.326241	HZ	PDE4B
0.385318	0.174063	HZ	GRB10	NC_056054.1	41715001	41735001	0.139967	0.386741	HZ	PDE4B
0.154919	0.158329	HZ	GRB10	NC_056054.1	41720001	41740001	0.309607	0.317437	HZ	PDE4B
0.0652422	0.21081	HZ	GRB10	NC_056054.1	41725001	41745001	0.401657	0.281013	HZ	PDE4B
0.0866095	0.199318	HZ	GRB10	NC_056061.1	61380001	61400001	0.454888	0.183729	HZ	PDE7B
0.392281	0.151549	HZ	GRB10	NC_056061.1	61385001	61405001	0.352381	0.170236	HZ	PDE7B
0.510648	0.155074	HZ	GPR179;MRPL45	NC_056061.1	61390001	61410001	0.317186	0.206003	HZ	PDE7B
0.322896	0.158356	HZ	GPR179	NC_056061.1	61395001	61415001	0.278512	0.240143	HZ	PDE7B
0.517051	0.184137	HZ	GPM6B	NC_056061.1	61400001	61420001	0.396165	0.158661	HZ	PDE7B
0.534225	0.164952	HZ	GPM6B	NC_056061.1	61405001	61425001	0.382192	0.21878	HZ	PDE7B
0.51507	0.139306	HZ	GPM6B	NC_056061.1	61410001	61430001	0.37788	0.211839	HZ	PDE7B
0.482724	0.153355	HZ	GPC6	NC_056061.1	61415001	61435001	0.425234	0.205141	HZ	PDE7B
0.412151	0.179284	HZ	GPAT2	NC_056056.1	216470001	216490001	0.397132	0.178755	HZ	PDGFB
0.416229	0.178218	HZ	GPAT2	NC_056056.1	216475001	216495001	0.398504	0.195961	HZ	PDGFB
0.322752	0.232031	HZ	GP6	NC_056070.1	42430001	42450001	0.214446	0.217181	HZ	PDGFC
0.345354	0.176631	HZ	GP6	NC_056070.1	42435001	42455001	0.133797	0.355353	HZ	PDGFC
0.380373	0.140011	HZ	GP6	NC_056070.1	42440001	42460001	0.14871	0.361765	HZ	PDGFC
0.526353	0.173434	HZ	GNB1L;RTL10;TX	NC_056070.1	42445001	42465001	0.316115	0.237897	HZ	PDGFC
0.135135	0.399659	HZ	GNB1L;RTL10	NC_056079.1	18885001	18905001	0.457294	0.226552	HZ	PDGFRL;SLC7
0.152701	0.363332	HZ	GNB1L;RTL10	NC_056079.1	18890001	18910001	0.455155	0.220187	HZ	PDGFRL;SLC7
0.171953	0.246762	HZ	GNB1L	NC_056072.1	28270001	28290001	0.375406	0.174847	HZ	PDZRN3
0.11784	0.31637	HZ	GNB1L	NC_056072.1	28275001	28295001	0.396596	0.208411	HZ	PDZRN3
0.193599	0.198525	HZ	GNA14	NC_056064.1	34530001	34550001	0.199228	0.161208	HZ	PEMT
0.353687	0.187838	HZ	GNA14	NC_056064.1	34535001	34555001	0.407494	0.167783	HZ	PEMT
0.226451	0.191645	HZ	GMCL1	NC_056073.1	22165001	22185001	0.327708	0.200577	HZ	PGK2
0.102677	0.257679	HZ	GMCL1	NC_056073.1	22170001	22190001	0.309719	0.232669	HZ	PGK2
0.0917069	0.281179	HZ	GMCL1	NC_056061.1	68345001	68365001	0.111111	0.167838	HZ	PHACTR2
0.122853	0.26717	HZ	GMCL1	NC_056061.1	68355001	68375001	0.153921	0.202653	HZ	PHACTR2
0.126912	0.305951	HZ	GMCL1	NC_056061.1	68360001	68380001	0.279633	0.178626	HZ	PHACTR2
0.181384	0.268381	HZ	GMCL1	NC_056061.1	68365001	68385001	0.365586	0.157636	HZ	PHACTR2
0.112738	0.236114	HZ	GMCL1	NC_056061.1	68440001	68460001	0.360508	0.163964	HZ	PHACTR2
0.177143	0.21068	HZ	GMCL1	NC_056080.1	20405001	20425001	0.443669	0.163592	HZ	PHEX
0.519334	0.142732	HZ	GFM2;LOC101111	NC_056080.1	20410001	20430001	0.366278	0.217257	HZ	PHEX
0.502432	0.300843	HZ	GEMIN7;LOC1011	NC_056080.1	20415001	20435001	0.317195	0.23983	HZ	PHEX
0.269922	0.290939	HZ	GEMIN5	NC_056080.1	20420001	20440001	0.167021	0.271646	HZ	PHEX
0.384456	0.233328	HZ	GEMIN5	NC_056080.1	20425001	20445001	0.219653	0.245045	HZ	PHEX

0.0901577	0.140182	HZ	GDF9;LEAP2;LOC	NC_056080.1	20430001	20450001	0.260681	0.205531	HZ	PHEX
0.373814	0.14744	HZ	GDF6	NC_056080.1	65180001	65200001	0.39875	0.203419	HZ	PHKA1
0.385331	0.171325	HZ	GDF6	NC_056080.1	65185001	65205001	0.224644	0.240633	HZ	PHKA1
0.455598	0.170564	HZ	GDF6	NC_056080.1	65190001	65210001	0.187554	0.222405	HZ	PHKA1
0.506226	0.203741	HZ	GDAP1;JPH1	NC_056080.1	65195001	65215001	0.215277	0.206607	HZ	PHKA1
0.495703	0.172374	HZ	GDAP1	NC_056074.1	8900001	8920001	0.344592	0.172581	HZ	PICALM
0.520844	0.18023	HZ	GCLM	NC_056074.1	8905001	8925001	0.356979	0.164073	HZ	PICALM
0.136245	0.140082	HZ	GC	NC_056074.1	8990001	9010001	0.390071	0.162707	HZ	PICALM
0.38157	0.244468	HZ	GATM	NC_056074.1	9060001	9080001	0.275862	0.174086	HZ	PICALM
0.187365	0.370762	HZ	GATM	NC_056074.1	9065001	9085001	0.421053	0.164487	HZ	PICALM
0.115558	0.390605	HZ	GATM	NC_056055.1	231185001	231205001	0.0681541	0.223502	HZ	PID1
0.0386501	0.42531	HZ	GATM	NC_056055.1	231190001	231210001	0.043024	0.219278	HZ	PID1
0.393734	0.365355	HZ	GAS8;LOC101109	NC_056055.1	231195001	231215001	0.0247591	0.238035	HZ	PID1
0.247303	0.426701	HZ	GAS8;LOC101109	NC_056066.1	63930001	63950001	0.10705	0.298953	HZ	PIGU
0.215125	0.434362	HZ	GAS8	NC_056066.1	63935001	63955001	0.137816	0.298219	HZ	PIGU
0.472696	0.3572	HZ	GAS8	NC_056066.1	63940001	63960001	0.194707	0.286096	HZ	PIGU
0.391717	0.151249	HZ	GARS1	NC_056066.1	63945001	63965001	0.251717	0.286535	HZ	PIGU
0.521384	0.139382	HZ	GARS1	NC_056066.1	63950001	63970001	0.205882	0.303249	HZ	PIGU
0.407443	0.13961	HZ	GARS1	NC_056066.1	63955001	63975001	0.337349	0.265148	HZ	PIGU
0.235767	0.19045	HZ	GARS1	NC_056066.1	63990001	64010001	0.467451	0.170108	HZ	PIGU
0.511052	0.245296	HZ	GAREM1	NC_056056.1	196940001	196960001	0.306773	0.161437	HZ	PIK3C2G
0.427592	0.288151	HZ	GAREM1	NC_056056.1	196945001	196965001	0.225633	0.170287	HZ	PIK3C2G
0.363479	0.280615	HZ	GAREM1	NC_056056.1	196965001	196985001	0.12888	0.172249	HZ	PIK3C2G
0.51752	0.139852	HZ	GAMT;NDUFS7;P	NC_056056.1	196970001	196990001	0.1096	0.189523	HZ	PIK3C2G
0.412267	0.140886	HZ	GALNTL6	NC_056054.1	208695001	208715001	0.455143	0.165391	HZ	PIK3CA
0.109205	0.144604	HZ	GALNTL6	NC_056054.1	208720001	208740001	0.469474	0.176282	HZ	PIK3CA
0.421621	0.197774	HZ	GALNT7	NC_056057.1	49485001	49505001	0.488461	0.363983	HZ	PIK3CG
0.41874	0.174848	HZ	GALNT7	NC_056077.1	36570001	36590001	0.456035	0.247454	HZ	PILRA
0.361018	0.140403	HZ	GALNT17	NC_056077.1	36575001	36595001	0.435546	0.281218	HZ	PILRA
0.461325	0.218179	HZ	GALNT13	NC_056077.1	36580001	36600001	0.411114	0.272203	HZ	PILRA
0.433777	0.222078	HZ	GALNT13	NC_056077.1	36565001	36585001	0.445634	0.17082	HZ	PILRA;ZCWPW
0.325084	0.227302	HZ	GALNT13	NC_056056.1	182595001	182615001	0.39772	0.163197	HZ	PKP2
0.212851	0.257781	HZ	GALNT13	NC_056056.1	182600001	182620001	0.330618	0.194122	HZ	PKP2
0.31925	0.208958	HZ	GALNT13	NC_056055.1	246295001	246315001	0.472107	0.199405	HZ	PLA2G2D
0.464002	0.192935	HZ	GALNT13	NC_056055.1	246300001	246320001	0.383037	0.213928	HZ	PLA2G2D
0.482329	0.179209	HZ	GALNT11;KMT2C	NC_056055.1	246305001	246325001	0.327802	0.226026	HZ	PLA2G2D

0.477135	0.159453	HZ	GALNT11	NC_056056.1	200975001	200995001	0.360311	0.173633	HZ	PLBD1
0.411589	0.164988	HZ	GALNT11	NC_056066.1	1005001	1025001	0.0359474	0.164711	HZ	PLCB1
0.389412	0.18013	HZ	GALNT11	NC_056066.1	1010001	1030001	0.056005	0.162822	HZ	PLCB1
0.374212	0.207813	HZ	GALNT11	NC_056066.1	1110001	1130001	0.450218	0.18109	HZ	PLCB1
0.268005	0.248487	HZ	GABRQ	NC_056066.1	1255001	1275001	0.291366	0.174237	HZ	PLCB1
0.158261	0.265741	HZ	GABRQ	NC_056066.1	1265001	1285001	0.20859	0.162126	HZ	PLCB1
0.108696	0.268575	HZ	GABRQ	NC_056060.1	79375001	79395001	0.315983	0.162437	HZ	PLEKHD1
0.102985	0.250708	HZ	GABRQ	NC_056055.1	73510001	73530001	0.459931	0.368061	HZ	PLGRKT
0.385	0.329749	HZ	FZD5;LOC121818	NC_056055.1	73515001	73535001	0.458708	0.367996	HZ	PLGRKT
0.426828	0.338455	HZ	FZD5;LOC121818	NC_056055.1	73520001	73540001	0.459775	0.368053	HZ	PLGRKT
0.412797	0.195699	HZ	FZD1	NC_056055.1	73525001	73545001	0.462405	0.368444	HZ	PLGRKT
0.348579	0.238086	HZ	FZD1	NC_056055.1	73530001	73550001	0.483171	0.354746	HZ	PLGRKT
0.487146	0.187837	HZ	FSIP2	NC_056055.1	73535001	73555001	0.483446	0.348081	HZ	PLGRKT
0.49504	0.190273	HZ	FSIP2	NC_056055.1	73550001	73570001	0.480916	0.32964	HZ	PLGRKT
0.451122	0.18102	HZ	FSD2;WHAMM	NC_056054.1	242920001	242940001	0.252725	0.298262	HZ	PLOD2
0.342342	0.156574	HZ	FRMPD1	NC_056054.1	242925001	242945001	0.37702	0.258369	HZ	PLOD2
0.531637	0.182611	HZ	FRMD3	NC_056054.1	242930001	242950001	0.458781	0.207342	HZ	PLOD2
0.455724	0.19193	HZ	FRMD3	NC_056054.1	243035001	243055001	0.393302	0.211364	HZ	PLOD2
0.405633	0.170916	HZ	FRMD3	NC_056054.1	243040001	243060001	0.403344	0.259578	HZ	PLOD2
0.452016	0.170387	HZ	FRMD3	NC_056054.1	243045001	243065001	0.47287	0.326071	HZ	PLOD2
0.297404	0.15662	HZ	FREM1	NC_056054.1	243050001	243070001	0.418374	0.325506	HZ	PLOD2
0.346887	0.197968	HZ	FREM1	NC_056054.1	243055001	243075001	0.296896	0.361661	HZ	PLOD2
0.387695	0.245554	HZ	FREM1	NC_056065.1	74625001	74645001	0.45222	0.191704	HZ	PLXNA2
0.167974	0.245236	HZ	FOXD4	NC_056065.1	74630001	74650001	0.417297	0.172502	HZ	PLXNA2
0.460841	0.159569	HZ	FMO5;PRKAB2	NC_056065.1	74635001	74655001	0.45595	0.246164	HZ	PLXNA2
0.403413	0.237039	HZ	FMO5	NC_056065.1	74640001	74660001	0.406574	0.229842	HZ	PLXNA2
0.426829	0.220685	HZ	FMO5	NC_056064.1	32540001	32560001	0.360425	0.167758	HZ	PMP22
0.45199	0.196454	HZ	FMO5	NC_056064.1	32545001	32565001	0.318232	0.167927	HZ	PMP22
0.522851	0.504261	HZ	FMN1	NC_056064.1	32550001	32570001	0.338034	0.165705	HZ	PMP22
0.180145	0.629874	HZ	FMN1	NC_056055.1	119450001	119470001	0.328895	0.181159	HZ	PMS1
0.109576	0.607318	HZ	FMN1	NC_056055.1	119480001	119500001	0.076849	0.198147	HZ	PMS1
0.251237	0.525922	HZ	FMN1	NC_056055.1	119485001	119505001	0.107579	0.274553	HZ	PMS1
0.327541	0.488059	HZ	FMN1	NC_056055.1	119490001	119510001	0.087348	0.312581	HZ	PMS1
0.452438	0.456708	HZ	FMN1	NC_056055.1	119495001	119515001	0.0844371	0.290913	HZ	PMS1
0.503564	0.453348	HZ	FMN1	NC_056055.1	119500001	119520001	0.0740361	0.305291	HZ	PMS1
0.519611	0.417404	HZ	FMN1	NC_056055.1	119505001	119525001	0.0913671	0.29273	HZ	PMS1

0.450341	0.428921	HZ	FMN1	NC_056055.1	119510001	119530001	0.171233	0.25454	HZ	PMS1
0.487282	0.399464	HZ	FMN1	NC_056055.1	119515001	119535001	0.324212	0.186701	HZ	PMS1
0.51316	0.449936	HZ	FMN1	NC_056055.1	119545001	119565001	0.315169	0.256255	HZ	PMS1
0.480492	0.214129	HZ	FLG;LOC1056140'	NC_056055.1	119550001	119570001	0.168258	0.328931	HZ	PMS1
0.530098	0.18433	HZ	FLG;LOC1056140'	NC_056055.1	119555001	119575001	0.158832	0.332695	HZ	PMS1
0.507929	0.173246	HZ	FLG	NC_056061.1	48385001	48405001	0.4731	0.180556	HZ	PNRC1;SRSF1:
0.443548	0.181803	HZ	FKBP9	NC_056055.1	239250001	239270001	0.183311	0.212239	HZ	PPP1R8
0.470136	0.15237	HZ	FKBP8	NC_056055.1	239255001	239275001	0.0863061	0.261414	HZ	PPP1R8
0.286625	0.198274	HZ	FKBP8	NC_056054.1	253845001	253865001	0.354583	0.222416	HZ	PPP2R3A
0.220225	0.194215	HZ	FKBP4;ITFG2	NC_056054.1	253850001	253870001	0.286114	0.241025	HZ	PPP2R3A
0.379486	0.228537	HZ	FKBP4	NC_056054.1	253855001	253875001	0.291841	0.242498	HZ	PPP2R3A
0.369556	0.193741	HZ	FKBP4	NC_056054.1	253860001	253880001	0.299602	0.23175	HZ	PPP2R3A
0.274904	0.145444	HZ	FHOD3	NC_056054.1	253865001	253885001	0.249404	0.234454	HZ	PPP2R3A
0.10792	0.164926	HZ	FHOD3	NC_056054.1	253870001	253890001	0.295809	0.215965	HZ	PPP2R3A
0.0832639	0.173067	HZ	FHOD3	NC_056054.1	253875001	253895001	0.339918	0.187617	HZ	PPP2R3A
0.152347	0.157792	HZ	FHOD3	NC_056054.1	253880001	253900001	0.346296	0.183615	HZ	PPP2R3A
0.474144	0.158582	HZ	FHOD3	NC_056054.1	253885001	253905001	0.383463	0.172679	HZ	PPP2R3A
0.428403	0.202315	HZ	FHOD3	NC_056054.1	253925001	253945001	0.394785	0.257395	HZ	PPP2R3A
0.377032	0.225096	HZ	FHL5	NC_056054.1	253930001	253950001	0.397425	0.272161	HZ	PPP2R3A
0.260482	0.295784	HZ	FHL5	NC_056054.1	253935001	253955001	0.438743	0.252991	HZ	PPP2R3A
0.165158	0.339056	HZ	FHL5	NC_056054.1	253940001	253960001	0.465908	0.245044	HZ	PPP2R3A
0.120205	0.35901	HZ	FHL5	NC_056054.1	253950001	253970001	0.46055	0.256752	HZ	PPP2R3A
0.15406	0.317535	HZ	FHL5	NC_056056.1	225610001	225630001	0.431958	0.196732	HZ	PPP6R2
0.304917	0.258288	HZ	FHL5	NC_056061.1	31135001	31155001	0.290237	0.18497	HZ	PRDM1
0.479632	0.180409	HZ	FHL5	NC_056061.1	31140001	31160001	0.211496	0.162626	HZ	PRDM1
0.478768	0.149456	HZ	FHIP1A	NC_056061.1	31145001	31165001	0.190307	0.163376	HZ	PRDM1
0.47361	0.139272	HZ	FHIP1A	NC_056062.1	46740001	46760001	0.47255	0.208134	HZ	PRDM14
0.431259	0.159784	HZ	FHIP1A	NC_056062.1	46745001	46765001	0.395106	0.232525	HZ	PRDM14
0.537249	0.26654	HZ	FGF16	NC_056062.1	46750001	46770001	0.483736	0.198416	HZ	PRDM14
0.366395	0.208124	HZ	FGD2	NC_056058.1	28300001	28320001	0.427083	0.257378	HZ	PRDM6
0.141343	0.268023	HZ	FGD2	NC_056058.1	28305001	28325001	0.362813	0.255575	HZ	PRDM6
0.117797	0.263122	HZ	FGD2	NC_056058.1	28310001	28330001	0.420823	0.221489	HZ	PRDM6
0.297547	0.1823	HZ	FGD2	NC_056058.1	28315001	28335001	0.385314	0.185824	HZ	PRDM6
0.415804	0.141148	HZ	FEZ1	NC_056056.1	78155001	78175001	0.398819	0.189852	HZ	PRKCE
0.396112	0.174268	HZ	FEZ1	NC_056056.1	78160001	78180001	0.447217	0.203088	HZ	PRKCE
0.412321	0.18981	HZ	FEZ1	NC_056056.1	78170001	78190001	0.377227	0.313658	HZ	PRKCE

0.521463	0.200376	HZ	FEZ1	NC_056056.1	78175001	78195001	0.327156	0.305035	HZ	PRKCE
0.385354	0.183673	HZ	FER1L5;KANSL3	NC_056056.1	78180001	78200001	0.317308	0.261201	HZ	PRKCE
0.395641	0.195621	HZ	FER1L5;KANSL3	NC_056056.1	78185001	78205001	0.166184	0.19805	HZ	PRKCE
0.395918	0.196207	HZ	FER1L5;KANSL3	NC_056056.1	78190001	78210001	0.208975	0.162787	HZ	PRKCE
0.464158	0.20396	HZ	FDXR;GRIN2C	NC_056056.1	78640001	78660001	0.312139	0.329513	HZ	PRKCE
0.112572	0.195764	HZ	FCGR3A	NC_056056.1	78645001	78665001	0.214765	0.332639	HZ	PRKCE
0.274436	0.314219	HZ	FBXW12	NC_056056.1	78650001	78670001	0.292213	0.309524	HZ	PRKCE
0.059155	0.436777	HZ	FBXW12	NC_056056.1	78655001	78675001	0.339749	0.268612	HZ	PRKCE
0.417053	0.222235	HZ	FBRS;PRR14	NC_056056.1	78660001	78680001	0.392918	0.243492	HZ	PRKCE
0.528761	0.235409	HZ	FBRS;PRR14	NC_056075.1	7115001	7135001	0.404548	0.214876	HZ	PRKG1
0.532294	0.185071	HZ	FBRS;PRR14	NC_056075.1	7120001	7140001	0.341737	0.260551	HZ	PRKG1
0.529191	0.185901	HZ	FBRS	NC_056075.1	7125001	7145001	0.301896	0.25794	HZ	PRKG1
0.40969	0.223656	HZ	FBLN5	NC_056075.1	7130001	7150001	0.214647	0.295657	HZ	PRKG1
0.426452	0.21659	HZ	FBLN5	NC_056075.1	7135001	7155001	0.22966	0.294938	HZ	PRKG1
0.486962	0.21346	HZ	FBLN5	NC_056075.1	7140001	7160001	0.283176	0.265633	HZ	PRKG1
0.486358	0.165558	HZ	FAT3	NC_056075.1	7145001	7165001	0.318754	0.226325	HZ	PRKG1
0.178066	0.168911	HZ	FAT3	NC_056075.1	7150001	7170001	0.411379	0.198917	HZ	PRKG1
0.199264	0.163622	HZ	FAT3	NC_056075.1	7415001	7435001	0.40499	0.244184	HZ	PRKG1
0.452705	0.198526	HZ	FARP1	NC_056075.1	7420001	7440001	0.293284	0.236273	HZ	PRKG1
0.50752	0.160419	HZ	FARP1	NC_056075.1	7425001	7445001	0.324365	0.231635	HZ	PRKG1
0.338485	0.20981	HZ	FANCC;LOC1141	NC_056075.1	7430001	7450001	0.342697	0.173154	HZ	PRKG1
0.364609	0.221684	HZ	FANCC;LOC1141	NC_056061.1	84690001	84710001	0.18049	0.35784	HZ	PRKN
0.351993	0.214613	HZ	FANCC;LOC1141	NC_056061.1	84695001	84715001	0.294393	0.261971	HZ	PRKN
0.367397	0.162648	HZ	FANCC;LOC1141	NC_056061.1	84700001	84720001	0.343337	0.1876	HZ	PRKN
0.31484	0.149287	HZ	FANCC	NC_056067.1	55755001	55775001	0.226079	0.228848	HZ	PRR12;RRAS
0.466353	0.15049	HZ	FANCC	NC_056061.1	89015001	89035001	0.143219	0.175448	HZ	PRR18;SFT2D
0.371159	0.15071	HZ	FANCC	NC_056061.1	89020001	89040001	0.0835145	0.19856	HZ	PRR18;SFT2D
0.214063	0.314378	HZ	FAM76B	NC_056055.1	29460001	29480001	0.362299	0.17793	HZ	PRXL2C
0.27629	0.265701	HZ	FAM76B	NC_056076.1	31005001	31025001	0.471662	0.336027	HZ	PSMA8
0.255391	0.210431	HZ	FAM76B	NC_056076.1	31010001	31030001	0.475392	0.324421	HZ	PSMA8
0.317192	0.155855	HZ	FAM76B	NC_056076.1	31015001	31035001	0.462176	0.32662	HZ	PSMA8
0.350726	0.16306	HZ	FAM71D;GPHN	NC_056076.1	31020001	31040001	0.461432	0.332669	HZ	PSMA8
0.321688	0.170494	HZ	FAM71D	NC_056076.1	31025001	31045001	0.476708	0.332652	HZ	PSMA8
0.305121	0.16621	HZ	FAM71D	NC_056076.1	31030001	31050001	0.466794	0.341393	HZ	PSMA8;SS18
0.248118	0.202643	HZ	FAM71D	NC_056066.1	30350001	30370001	0.141333	0.173346	HZ	PTER
0.299223	0.140759	HZ	FAM71D	NC_056066.1	30355001	30375001	0.0966618	0.187103	HZ	PTER

0.220217	0.213235 HZ	FAM227B;FGF7	NC_056066.1	30360001	30380001	0.102193	0.183705	HZ	PTER
0.26859	0.191585 HZ	FAM227B;FGF7	NC_056054.1	54960001	54980001	0.439534	0.174442	HZ	PTGFR
0.32053	0.165836 HZ	FAM227B;FGF7	NC_056056.1	7300001	7320001	0.191549	0.280898	HZ	PTPA
0.364553	0.15084 HZ	FAM227B	NC_056056.1	7305001	7325001	0.420432	0.15915	HZ	PTPA
0.268531	0.201034 HZ	FAM227B	NC_056060.1	98260001	98280001	0.0943643	0.17054	HZ	PTPN21
0.225921	0.217884 HZ	FAM227B	NC_056060.1	98265001	98285001	0.16079	0.176954	HZ	PTPN21
0.134276	0.260385 HZ	FAM227B	NC_056055.1	76575001	76595001	0.101935	0.362322	HZ	PTPRD
0.255707	0.197027 HZ	FAM227B	NC_056055.1	76585001	76605001	0.108527	0.296433	HZ	PTPRD
0.42411	0.176927 HZ	FAM219A	NC_056055.1	76590001	76610001	0.142857	0.27707	HZ	PTPRD
0.399123	0.224481 HZ	FAM219A	NC_056055.1	76595001	76615001	0.156951	0.245023	HZ	PTPRD
0.433779	0.267151 HZ	FAM219A	NC_056055.1	76620001	76640001	0.175585	0.424669	HZ	PTPRD
0.4346	0.257157 HZ	FAM219A	NC_056072.1	39275001	39295001	0.399392	0.240069	HZ	PTPRG
0.444207	0.204272 HZ	FAM219A	NC_056072.1	39280001	39300001	0.456195	0.2311	HZ	PTPRG
0.448695	0.193629 HZ	FAM219A	NC_056068.1	62405001	62425001	0.325325	0.158226	HZ	QSER1
0.435517	0.149126 HZ	FAM219A	NC_056068.1	62410001	62430001	0.307124	0.173669	HZ	QSER1
0.510089	0.230215 HZ	FAM214A	NC_056068.1	62415001	62435001	0.271983	0.185228	HZ	QSER1
0.495328	0.256455 HZ	FAM214A	NC_056064.1	18275001	18295001	0.397924	0.169302	HZ	RAB11FIP4
0.479887	0.259243 HZ	FAM214A	NC_056064.1	18280001	18300001	0.25279	0.249068	HZ	RAB11FIP4
0.508571	0.218899 HZ	FAM214A	NC_056064.1	18285001	18305001	0.170507	0.304683	HZ	RAB11FIP4
0.510434	0.234802 HZ	FAM13A	NC_056064.1	18290001	18310001	0.0960252	0.36886	HZ	RAB11FIP4
0.36039	0.275264 HZ	FAM13A	NC_056064.1	18295001	18315001	0.0935253	0.408589	HZ	RAB11FIP4
0.347745	0.259633 HZ	FAM13A	NC_056064.1	18300001	18320001	0.195862	0.367964	HZ	RAB11FIP4
0.448626	0.222422 HZ	FAM13A	NC_056064.1	18305001	18325001	0.294389	0.310009	HZ	RAB11FIP4
0.446678	0.356628 HZ	FAM13A	NC_056064.1	18310001	18330001	0.352632	0.250743	HZ	RAB11FIP4
0.421358	0.322287 HZ	FAM13A	NC_056058.1	19805001	19825001	0.428918	0.190934	HZ	RAD50
0.533332	0.155873 HZ	FAM13A	NC_056056.1	8970001	8990001	0.294498	0.186287	HZ	RALGPS1
0.409519	0.203484 HZ	FAM126A	NC_056079.1	39630001	39650001	0.407971	0.174936	HZ	RARB
0.489147	0.173653 HZ	FAM126A	NC_056079.1	39720001	39740001	0.427091	0.277128	HZ	RARB
0.500408	0.148929 HZ	FAM120A	NC_056079.1	39725001	39745001	0.264621	0.205689	HZ	RARB
0.518405	0.170123 HZ	FAM107A	NC_056079.1	39730001	39750001	0.245419	0.175736	HZ	RARB
0.39705	0.148188 HZ	FAHD2A;KCNIP3	NC_056078.1	43450001	43470001	0.48455	0.229984	HZ	RASSF4
0.419595	0.179667 HZ	FAHD2A;GPAT2	NC_056078.1	43445001	43465001	0.481288	0.198267	HZ	RASSF4;ZNF2:
0.410846	0.173004 HZ	FAHD2A;GPAT2	NC_056077.1	22745001	22765001	0.486111	0.204777	HZ	RBBP6
0.38363	0.156405 HZ	FAHD2A;GPAT2	NC_056076.1	33925001	33945001	0.454656	0.239717	HZ	RBBP8
0.395031	0.159479 HZ	FAHD2A;GPAT2	NC_056076.1	33930001	33950001	0.201454	0.163164	HZ	RBBP8
0.0861537	0.142481 HZ	FA2H	NC_056077.1	6190001	6210001	0.487145	0.167759	HZ	RBFOX1

0.434288	0.165979	HZ	F13A1
0.392917	0.160922	HZ	F13A1
0.418285	0.146369	HZ	EXPH5
0.234992	0.227891	HZ	EXPH5
0.133417	0.274655	HZ	EXPH5
0.14352	0.252498	HZ	EXPH5
0.213693	0.220259	HZ	EXPH5
0.304014	0.178401	HZ	EXPH5
0.440951	0.170356	HZ	EXOC4
0.483743	0.141693	HZ	EXOC4
0.522027	0.252918	HZ	EVL
0.412711	0.214463	HZ	EVI5
0.284471	0.191934	HZ	EVI5
0.0975719	0.1767	HZ	ETAA1
0.0639881	0.210697	HZ	ETAA1
0.0622549	0.188639	HZ	ETAA1
0.0800855	0.177371	HZ	ETAA1
0.33618	0.152465	HZ	ESRRB
0.313818	0.153589	HZ	ESRRB
0.495841	0.260714	HZ	ESRRB
0.329194	0.340544	HZ	ESRRB
0.190581	0.30751	HZ	ERG
0.244918	0.2419	HZ	ERG
0.290657	0.167619	HZ	ERC2
0.199005	0.232008	HZ	ERC2
0.390184	0.195371	HZ	ERC2
0.509703	0.167548	HZ	ERC2
0.463768	0.15932	HZ	ERBIN
0.382581	0.217949	HZ	ERBIN
0.399206	0.22092	HZ	ERBIN
0.505919	0.200104	HZ	ERBIN
0.442946	0.159439	HZ	EPS8L3;GSTM3
0.39128	0.172876	HZ	EPS8L3
0.308372	0.183226	HZ	EPHB4;ZAN
0.497329	0.20276	HZ	EPHB4;ZAN
0.141141	0.14632	HZ	EPHB4

NC_056077.1	6195001	6215001	0.48559	0.16534	HZ	RBFOX1
NC_056077.1	6255001	6275001	0.21663	0.224794	HZ	RBFOX1
NC_056077.1	6260001	6280001	0.238928	0.241445	HZ	RBFOX1
NC_056077.1	6265001	6285001	0.235515	0.247678	HZ	RBFOX1
NC_056077.1	6270001	6290001	0.180913	0.198802	HZ	RBFOX1
NC_056077.1	6275001	6295001	0.25436	0.200353	HZ	RBFOX1
NC_056077.1	6280001	6300001	0.254675	0.257571	HZ	RBFOX1
NC_056077.1	6285001	6305001	0.305262	0.283219	HZ	RBFOX1
NC_056077.1	6290001	6310001	0.357443	0.333789	HZ	RBFOX1
NC_056077.1	6295001	6315001	0.354235	0.322314	HZ	RBFOX1
NC_056077.1	6300001	6320001	0.403431	0.278684	HZ	RBFOX1
NC_056077.1	6305001	6325001	0.482482	0.256645	HZ	RBFOX1
NC_056064.1	52520001	52540001	0.356675	0.394568	HZ	RBFOX3
NC_056064.1	52525001	52545001	0.474305	0.311538	HZ	RBFOX3
NC_056080.1	128230001	128250001	0.305354	0.223645	HZ	RBM41
NC_056072.1	4185001	4205001	0.343722	0.170199	HZ	RBMS3
NC_056072.1	4190001	4210001	0.397187	0.157743	HZ	RBMS3
NC_056072.1	4215001	4235001	0.453727	0.239634	HZ	RBMS3
NC_056072.1	4220001	4240001	0.471102	0.237661	HZ	RBMS3
NC_056078.1	18985001	19005001	0.343511	0.16566	HZ	REEP3
NC_056065.1	34870001	34890001	0.362828	0.189376	HZ	RGS7
NC_056065.1	34875001	34895001	0.164629	0.213185	HZ	RGS7
NC_056065.1	34880001	34900001	0.238008	0.210673	HZ	RGS7
NC_056065.1	34885001	34905001	0.324428	0.191985	HZ	RGS7
NC_056065.1	34890001	34910001	0.428297	0.196154	HZ	RGS7
NC_056065.1	35015001	35035001	0.0970577	0.159081	HZ	RGS7
NC_056058.1	92710001	92730001	0.438753	0.185383	HZ	RHOBTB3
NC_056062.1	73750001	73770001	0.245165	0.17252	HZ	RIMS2
NC_056062.1	73755001	73775001	0.229914	0.203971	HZ	RIMS2
NC_056062.1	73760001	73780001	0.353172	0.17249	HZ	RIMS2
NC_056066.1	39000001	39020001	0.373942	0.182866	HZ	RIN2
NC_056055.1	159080001	159100001	0.417372	0.157855	HZ	RND3
NC_056070.1	62950001	62970001	0.411905	0.273434	HZ	RNF10
NC_056070.1	62955001	62975001	0.390737	0.248565	HZ	RNF10
NC_056070.1	62960001	62980001	0.353313	0.190073	HZ	RNF10
NC_056060.1	49365001	49385001	0.349096	0.204346	HZ	RNF111

0.532873	0.436725	HZ	EPHA5	NC_056060.1	49370001	49390001	0.371055	0.22058	HZ	RNF111
0.532319	0.434075	HZ	EPHA5	NC_056060.1	49375001	49395001	0.281623	0.263511	HZ	RNF111
0.421546	0.449957	HZ	EPHA5	NC_056060.1	49380001	49400001	0.280612	0.280339	HZ	RNF111
0.289635	0.51846	HZ	EPHA5	NC_056060.1	49385001	49405001	0.346756	0.262779	HZ	RNF111
0.378636	0.487211	HZ	EPHA5	NC_056060.1	49390001	49410001	0.352075	0.251956	HZ	RNF111
0.498987	0.425178	HZ	EPHA5	NC_056055.1	234690001	234710001	0.469662	0.193643	HZ	RNF19B
0.388545	0.454091	HZ	EPHA5	NC_056055.1	52120001	52140001	0.169306	0.234355	HZ	RNF38
0.313326	0.492205	HZ	EPHA5	NC_056061.1	48520001	48540001	0.253927	0.192708	HZ	RNGTT
0.170365	0.576485	HZ	EPHA5	NC_056061.1	48525001	48545001	0.218133	0.222222	HZ	RNGTT
0.111179	0.62814	HZ	EPHA5	NC_056061.1	48530001	48550001	0.17271	0.228632	HZ	RNGTT
0.0620499	0.676181	HZ	EPHA5	NC_056061.1	48535001	48555001	0.21865	0.213615	HZ	RNGTT
0.409544	0.144069	HZ	EPHA5	NC_056054.1	147550001	147570001	0.468268	0.264321	HZ	ROBO1
0.416856	0.18106	HZ	EPHA10	NC_056054.1	148210001	148230001	0.443868	0.247518	HZ	ROBO1
0.40447	0.155575	HZ	EPHA1	NC_056054.1	148215001	148235001	0.479763	0.332868	HZ	ROBO1
0.256517	0.143427	HZ	EPGN	NC_056054.1	145690001	145710001	0.483073	0.163995	HZ	ROBO2
0.270328	0.145178	HZ	EPGN	NC_056060.1	47060001	47080001	0.492121	0.332204	HZ	RORA
0.496734	0.193188	HZ	EPB41L5	NC_056060.1	47065001	47085001	0.491094	0.300329	HZ	RORA
0.510805	0.18566	HZ	EPB41L5	NC_056060.1	47445001	47465001	0.397878	0.238565	HZ	RORA
0.482972	0.195695	HZ	EPB41L5	NC_056060.1	47450001	47470001	0.387717	0.254781	HZ	RORA
0.529644	0.183781	HZ	EPB41L5	NC_056060.1	47455001	47475001	0.351464	0.243579	HZ	RORA
0.337047	0.142598	HZ	EPAS1	NC_056060.1	47460001	47480001	0.391278	0.227179	HZ	RORA
0.485192	0.139812	HZ	ENPP2	NC_056060.1	47465001	47485001	0.453497	0.249233	HZ	RORA
0.321781	0.2044	HZ	ENPP2	NC_056060.1	47595001	47615001	0.408475	0.208246	HZ	RORA
0.294069	0.214147	HZ	ENPP2	NC_056060.1	47600001	47620001	0.212689	0.279604	HZ	RORA
0.455224	0.152787	HZ	ENPP2	NC_056060.1	47605001	47625001	0.150918	0.314649	HZ	RORA
0.523903	0.156491	HZ	ENOX1	NC_056060.1	47610001	47630001	0.0971328	0.330767	HZ	RORA
0.445866	0.171271	HZ	ENOX1	NC_056060.1	47615001	47635001	0.122932	0.310754	HZ	RORA
0.422416	0.176382	HZ	ENOX1	NC_056060.1	47620001	47640001	0.167807	0.279228	HZ	RORA
0.506427	0.147169	HZ	ENOX1	NC_056060.1	47625001	47645001	0.189849	0.260559	HZ	RORA
0.484918	0.161641	HZ	ENG;FPGS	NC_056060.1	47630001	47650001	0.208226	0.248987	HZ	RORA
0.508771	0.188805	HZ	ENG;FPGS	NC_056060.1	47635001	47655001	0.284405	0.249099	HZ	RORA
0.0779091	0.174618	HZ	ENDOG;LOC1011	NC_056060.1	47640001	47660001	0.364057	0.193284	HZ	RORA
0.131023	0.163501	HZ	ENDOG;LOC1011	NC_056070.1	61595001	61615001	0.3968	0.212682	HZ	RPH3A
0.53271	0.205037	HZ	ENAH	NC_056070.1	61600001	61620001	0.452956	0.229036	HZ	RPH3A
0.525777	0.18314	HZ	ENAH	NC_056070.1	61605001	61625001	0.346688	0.258368	HZ	RPH3A
0.28133	0.153184	HZ	EN2	NC_056070.1	61610001	61630001	0.33956	0.246791	HZ	RPH3A

0.527523	0.14445	HZ	EN1	NC_056070.1	61615001	61635001	0.300334	0.209135	HZ	RPH3A
0.523029	0.142786	HZ	EN1	NC_056070.1	61620001	61640001	0.258119	0.197215	HZ	RPH3A
0.465482	0.178767	HZ	ELMO1	NC_056080.1	64860001	64880001	0.433009	0.182327	HZ	RPS4X
0.390414	0.218028	HZ	ELMO1	NC_056061.1	89245001	89265001	0.389228	0.163852	HZ	RPS6KA2
0.414804	0.230603	HZ	ELMO1	NC_056080.1	18620001	18640001	0.450172	0.238978	HZ	RPS6KA3
0.467044	0.238026	HZ	ELMO1	NC_056080.1	18625001	18645001	0.467015	0.216169	HZ	RPS6KA3
0.266926	0.173684	HZ	ELL;FKBP8	NC_056060.1	100470001	100490001	0.361884	0.479003	HZ	RPS6KA5
0.459203	0.207202	HZ	ELAC1;ME2	NC_056060.1	100475001	100495001	0.308642	0.428725	HZ	RPS6KA5
0.475059	0.157276	HZ	EIF5B;REV1	NC_056060.1	100480001	100500001	0.424665	0.314667	HZ	RPS6KA5
0.424348	0.176784	HZ	EIF5B	NC_056061.1	48120001	48140001	0.227194	0.160952	HZ	RRAGD
0.385881	0.201849	HZ	EIF5B	NC_056061.1	48150001	48170001	0.177262	0.207474	HZ	RRAGD
0.514947	0.182713	HZ	EIF5B	NC_056061.1	48155001	48175001	0.155576	0.193899	HZ	RRAGD
0.21505	0.268738	HZ	EIF2AK3;LOC114	NC_056061.1	48160001	48180001	0.456479	0.23323	HZ	RRAGD;UBE2
0.185787	0.269566	HZ	EIF2AK3;LOC114	NC_056074.1	15915001	15935001	0.355152	0.225402	HZ	RSF1
0.15522	0.266962	HZ	EIF2AK3;LOC114	NC_056066.1	59125001	59145001	0.487296	0.283843	HZ	RTF2
0.218341	0.242839	HZ	EIF2AK3;LOC114	NC_056080.1	64680001	64700001	0.333096	0.197412	HZ	RTL5
0.339801	0.188095	HZ	EIF2AK3	NC_056054.1	78570001	78590001	0.112903	0.215604	HZ	S1PR1
0.437862	0.168105	HZ	EIF2AK3	NC_056054.1	78575001	78595001	0.276428	0.175423	HZ	S1PR1
0.494149	0.160267	HZ	EIF2AK3	NC_056060.1	23985001	24005001	0.320377	0.167033	HZ	SALL2
0.509689	0.155433	HZ	EIF2AK3	NC_056060.1	23990001	24010001	0.319271	0.182519	HZ	SALL2
0.397994	0.216966	HZ	EIF2AK3	NC_056060.1	65230001	65250001	0.348178	0.158231	HZ	SAMD4A
0.360017	0.245808	HZ	EIF2AK3	NC_056060.1	65240001	65260001	0.27006	0.16741	HZ	SAMD4A
0.311976	0.272384	HZ	EIF2AK3	NC_056072.1	51665001	51685001	0.117188	0.173461	HZ	SC5
0.32673	0.261834	HZ	EIF2AK3	NC_056056.1	140515001	140535001	0.420028	0.181902	HZ	SCAF11
0.421353	0.212899	HZ	EIF2AK3	NC_056056.1	140520001	140540001	0.439668	0.183118	HZ	SCAF11
0.475815	0.154595	HZ	EIF2AK3	NC_056056.1	140525001	140545001	0.463543	0.203732	HZ	SCAF11
0.288398	0.282143	HZ	EI24	NC_056054.1	124440001	124460001	0.138158	0.392562	HZ	SCAF4
0.508961	0.143236	HZ	EI24	NC_056054.1	124445001	124465001	0.253136	0.339449	HZ	SCAF4
0.32448	0.154236	HZ	EFNA5	NC_056054.1	124450001	124470001	0.357986	0.228475	HZ	SCAF4
0.338615	0.2067	HZ	EFNA5	NC_056061.1	79290001	79310001	0.473041	0.185346	HZ	SCAF8
0.331935	0.220991	HZ	EFNA5	NC_056071.1	29630001	29650001	0.266108	0.217717	HZ	SCAPER
0.315434	0.211722	HZ	EFNA5	NC_056071.1	29635001	29655001	0.331882	0.201674	HZ	SCAPER
0.301353	0.179591	HZ	EFNA5	NC_056071.1	29640001	29660001	0.413989	0.206866	HZ	SCAPER
0.32445	0.149425	HZ	EFNA5	NC_056064.1	57165001	57185001	0.44575	0.225073	HZ	SDK2
0.138081	0.13896	HZ	EFNA5	NC_056057.1	41420001	41440001	0.449899	0.189852	HZ	SEMA3C
0.0841529	0.143248	HZ	EFNA5	NC_056057.1	41425001	41445001	0.406314	0.226035	HZ	SEMA3C

0.122976	0.140405	HZ	EFNA5	NC_056057.1	41430001	41450001	0.487595	0.186749	HZ	SEMA3C
0.292823	0.158005	HZ	EFNA5	NC_056060.1	61190001	61210001	0.457616	0.240568	HZ	SEMA6D
0.209836	0.187353	HZ	EFNA5	NC_056060.1	61195001	61215001	0.199303	0.19273	HZ	SEMA6D
0.136449	0.244208	HZ	EFNA5	NC_056060.1	61210001	61230001	0.111224	0.160847	HZ	SEMA6D
0.28122	0.139192	HZ	EFNA5	NC_056060.1	61215001	61235001	0.157894	0.21411	HZ	SEMA6D
0.454616	0.177639	HZ	EFNA5	NC_056060.1	61220001	61240001	0.106051	0.220638	HZ	SEMA6D
0.50314	0.139517	HZ	EFNA5	NC_056060.1	61225001	61245001	0.457262	0.203213	HZ	SEMA6D
0.362301	0.172596	HZ	EFCAB14	NC_056054.1	166880001	166900001	0.323022	0.199879	HZ	SENP7;TRMT1
0.379857	0.18819	HZ	EFCAB14	NC_056054.1	166885001	166905001	0.232802	0.243813	HZ	SENP7;TRMT1
0.461101	0.293223	HZ	EFCAB14	NC_056054.1	100015001	100035001	0.32452	0.368031	HZ	SF3B4;SV2A
0.468179	0.321164	HZ	EFCAB14	NC_056061.1	89025001	89045001	0.103371	0.248058	HZ	SFT2D1
0.51131	0.368037	HZ	EFCAB14	NC_056061.1	89030001	89050001	0.205206	0.212385	HZ	SFT2D1
0.479243	0.228806	HZ	EDIL3	NC_056061.1	89035001	89055001	0.270595	0.197505	HZ	SFT2D1
0.460209	0.185721	HZ	EDA	NC_056058.1	64735001	64755001	0.456699	0.224343	HZ	SGCD
0.316017	0.164356	HZ	EDA	NC_056058.1	64740001	64760001	0.241888	0.342959	HZ	SGCD
0.304714	0.140383	HZ	EDA	NC_056058.1	64745001	64765001	0.211913	0.361623	HZ	SGCD
0.191621	0.142266	HZ	EDA	NC_056054.1	42185001	42205001	0.370197	0.165262	HZ	SGIP1
0.244373	0.183345	HZ	ECRG4	NC_056069.1	4350001	4370001	0.395509	0.165663	HZ	SH3PXD2B
0.185987	0.161746	HZ	ECRG4	NC_056069.1	4355001	4375001	0.182308	0.226585	HZ	SH3PXD2B
0.524126	0.15815	HZ	ECE1	NC_056069.1	4360001	4380001	0.236096	0.182949	HZ	SH3PXD2B
0.39778	0.150568	HZ	ECE1	NC_056055.1	23810001	23830001	0.440386	0.195797	HZ	SHC3
0.328286	0.151459	HZ	ECE1	NC_056055.1	23815001	23835001	0.372213	0.213726	HZ	SHC3
0.292101	0.170118	HZ	DYNLT1;TMEM18	NC_056064.1	29900001	29920001	0.365722	0.16813	HZ	SHISA6
0.328302	0.138652	HZ	DYNLT1;TMEM18	NC_056064.1	29905001	29925001	0.424269	0.160679	HZ	SHISA6
0.283333	0.138998	HZ	DTX2	NC_056064.1	30025001	30045001	0.312775	0.164572	HZ	SHISA6
0.438245	0.164667	HZ	DSCAML1	NC_056077.1	12295001	12315001	0.45684	0.210183	HZ	SHISA9
0.416041	0.19562	HZ	DSCAML1	NC_056077.1	12300001	12320001	0.393228	0.295251	HZ	SHISA9
0.384258	0.226101	HZ	DSCAML1	NC_056077.1	12305001	12325001	0.36455	0.291372	HZ	SHISA9
0.4144	0.222257	HZ	DSCAML1	NC_056077.1	12310001	12330001	0.293466	0.241841	HZ	SHISA9
0.494226	0.194243	HZ	DSCAML1	NC_056077.1	12315001	12335001	0.350691	0.243251	HZ	SHISA9
0.333909	0.1398	HZ	DR1	NC_056077.1	12325001	12345001	0.394321	0.175579	HZ	SHISA9
0.432793	0.144604	HZ	DOK5	NC_056063.1	16285001	16305001	0.444142	0.227409	HZ	SIAH3
0.376439	0.167473	HZ	DOK5	NC_056063.1	16290001	16310001	0.349782	0.184495	HZ	SIAH3
0.464228	0.146162	HZ	DOK5	NC_056064.1	9770001	9790001	0.435745	0.171622	HZ	SKA2;TRIM37
0.33033	0.168172	HZ	DOCK8	NC_056057.1	70800001	70820001	0.348012	0.377317	HZ	SKAP2
0.350066	0.148764	HZ	DOCK8	NC_056057.1	70805001	70825001	0.401736	0.339143	HZ	SKAP2

0.262684	0.181673	HZ	DOCK8	NC_056062.1	21395001	21415001	0.281069	0.282005	HZ	SLA;TG
0.292875	0.173313	HZ	DOCK8	NC_056059.1	102440001	102460001	0.38162	0.19512	HZ	SLC10A6
0.350915	0.15962	HZ	DOCK8	NC_056059.1	102445001	102465001	0.374871	0.210723	HZ	SLC10A6
0.387522	0.152297	HZ	DOCK8	NC_056059.1	102450001	102470001	0.427046	0.161317	HZ	SLC10A6
0.363128	0.160631	HZ	DOCK8	NC_056054.1	266050001	266070001	0.463181	0.15769	HZ	SLC19A1
0.271483	0.187644	HZ	DOCK8	NC_056055.1	229870001	229890001	0.354788	0.166857	HZ	SLC19A3
0.148879	0.242552	HZ	DOCK8	NC_056058.1	19895001	19915001	0.336058	0.163342	HZ	SLC22A4
0.0972647	0.261191	HZ	DOCK8	NC_056058.1	19900001	19920001	0.262308	0.161847	HZ	SLC22A4
0.520254	0.143584	HZ	DOCK8	NC_056058.1	19905001	19925001	0.275649	0.185224	HZ	SLC22A4
0.455409	0.222543	HZ	DNTTIP2;GCLM	NC_056058.1	19910001	19930001	0.299298	0.19638	HZ	SLC22A4
0.437896	0.238556	HZ	DNTTIP2;GCLM	NC_056058.1	19810001	19830001	0.383493	0.20837	HZ	SLC22A5
0.493863	0.22573	HZ	DNTTIP2;GCLM	NC_056058.1	19815001	19835001	0.48831	0.16815	HZ	SLC22A5
0.461356	0.269415	HZ	DNTTIP2	NC_056072.1	35205001	35225001	0.276094	0.314824	HZ	SLC25A26
0.454136	0.239797	HZ	DNTTIP2	NC_056072.1	35210001	35230001	0.265639	0.295641	HZ	SLC25A26
0.53211	0.146444	HZ	DNM1	NC_056072.1	35215001	35235001	0.164746	0.303975	HZ	SLC25A26
0.52	0.144046	HZ	DNAI1;ENHO	NC_056072.1	35220001	35240001	0.0932203	0.26866	HZ	SLC25A26
0.148936	0.223717	HZ	DNAI1	NC_056062.1	74015001	74035001	0.251492	0.186145	HZ	SLC25A32
0.291667	0.216311	HZ	DNAI1	NC_056062.1	85970001	85990001	0.349382	0.166251	HZ	SLC26A7
0.322368	0.199313	HZ	DNAI1	NC_056062.1	85975001	85995001	0.432173	0.195089	HZ	SLC26A7
0.335175	0.199933	HZ	DNAI1	NC_056065.1	3500001	3520001	0.203461	0.15889	HZ	SLC26A9
0.339896	0.167609	HZ	DNAI1	NC_056056.1	207040001	207060001	0.459155	0.203489	HZ	SLC2A3
0.289941	0.149715	HZ	DNAI1	NC_056054.1	232540001	232560001	0.377012	0.27773	HZ	SLC33A1
0.517289	0.175477	HZ	DNAH9	NC_056054.1	232545001	232565001	0.252457	0.25807	HZ	SLC33A1
0.520438	0.167876	HZ	DNAH9	NC_056054.1	232550001	232570001	0.330854	0.196115	HZ	SLC33A1
0.417206	0.257181	HZ	DNAH3	NC_056068.1	17045001	17065001	0.142163	0.273357	HZ	SLC35F2
0.502013	0.231779	HZ	DNAH3	NC_056068.1	17075001	17095001	0.0370699	0.362249	HZ	SLC35F2
0.503153	0.198558	HZ	DNAH14	NC_056068.1	17080001	17100001	0.203405	0.269589	HZ	SLC35F2
0.472726	0.207049	HZ	DNAH14	NC_056066.1	31755001	31775001	0.227012	0.292802	HZ	SLC39A12
0.481718	0.149736	HZ	DNAH14	NC_056066.1	31760001	31780001	0.335919	0.273244	HZ	SLC39A12
0.382165	0.178062	HZ	DNAH14	NC_056066.1	31765001	31785001	0.438822	0.286082	HZ	SLC39A12
0.400226	0.193043	HZ	DNAH14	NC_056055.1	148110001	148130001	0.430703	0.21266	HZ	SLC4A10
0.494582	0.173565	HZ	DNAH14	NC_056055.1	148115001	148135001	0.336647	0.299756	HZ	SLC4A10
0.454057	0.272344	HZ	DMXL1	NC_056055.1	148120001	148140001	0.321862	0.328983	HZ	SLC4A10
0.451926	0.298597	HZ	DMXL1	NC_056055.1	148125001	148145001	0.285611	0.3263	HZ	SLC4A10
0.379016	0.259003	HZ	DMXL1	NC_056055.1	148130001	148150001	0.442756	0.250931	HZ	SLC4A10
0.267062	0.210137	HZ	DMXL1	NC_056069.1	71195001	71215001	0.0448088	0.175861	HZ	SLC6A18;TER

0.222094	0.171534	HZ	DMXL1	NC_056069.1	71200001	71220001	0.0385809	0.200443	HZ	SLC6A18;TER
0.50775	0.158039	HZ	DMD	NC_056070.1	19680001	19700001	0.378049	0.196847	HZ	SLC7A11
0.41796	0.173518	HZ	DMD	NC_056070.1	19685001	19705001	0.379744	0.224298	HZ	SLC7A11
0.480849	0.15516	HZ	DLC1;LOC114111	NC_056070.1	19690001	19710001	0.478297	0.231723	HZ	SLC7A11
0.326882	0.163044	HZ	DLC1	NC_056054.1	245950001	245970001	0.172457	0.167095	HZ	SLC9A9
0.392034	0.168525	HZ	DLC1	NC_056054.1	245955001	245975001	0.326409	0.193503	HZ	SLC9A9
0.234127	0.157954	HZ	DLC1	NC_056054.1	245960001	245980001	0.474753	0.215778	HZ	SLC9A9
0.401525	0.183857	HZ	DIS3	NC_056056.1	194380001	194400001	0.111631	0.336483	HZ	SLCO1A2
0.445816	0.159921	HZ	DGKI	NC_056056.1	194385001	194405001	0.204927	0.31008	HZ	SLCO1A2
0.40419	0.163926	HZ	DGKI	NC_056056.1	194390001	194410001	0.281136	0.315721	HZ	SLCO1A2
0.493475	0.144239	HZ	DERL1	NC_056059.1	40800001	40820001	0.379862	0.295366	HZ	SLIT2
0.398269	0.419843	HZ	DEF8;LOC101113	NC_056063.1	61315001	61335001	0.463243	0.203218	HZ	SLITRK6
0.181765	0.495771	HZ	DEF8;LOC101113	NC_056063.1	61320001	61340001	0.299884	0.196921	HZ	SLITRK6
0.383191	0.427813	HZ	DEF8	NC_056063.1	61325001	61345001	0.117128	0.239716	HZ	SLITRK6
0.129324	0.17018	HZ	DECR1;NBN	NC_056060.1	49525001	49545001	0.273734	0.182029	HZ	SLTM
0.47254	0.148014	HZ	DECR1	NC_056060.1	49530001	49550001	0.198906	0.183714	HZ	SLTM
0.400625	0.197597	HZ	DECR1	NC_056060.1	13955001	13975001	0.342177	0.182444	HZ	SMAD3
0.322074	0.205081	HZ	DECR1	NC_056060.1	13960001	13980001	0.416223	0.190933	HZ	SMAD3
0.159861	0.203797	HZ	DECR1	NC_056080.1	113385001	113405001	0.208	0.306904	HZ	SMARCA1
0.112739	0.186981	HZ	DECR1	NC_056080.1	113390001	113410001	0.37931	0.198281	HZ	SMARCA1
0.119435	0.178193	HZ	DECR1	NC_056080.1	51260001	51280001	0.335312	0.160476	HZ	SMC1A
0.20761	0.164196	HZ	DDX52;SYNRG	NC_056074.1	875001	895001	0.272033	0.194265	HZ	SMCO4
0.111418	0.229335	HZ	DDX52;SYNRG	NC_056074.1	880001	900001	0.280587	0.161103	HZ	SMCO4
0.151383	0.225042	HZ	DDX52;SYNRG	NC_056060.1	79820001	79840001	0.489149	0.178937	HZ	SMOC1
0.293147	0.200231	HZ	DDX52	NC_056060.1	79825001	79845001	0.4693	0.195986	HZ	SMOC1
0.425465	0.147876	HZ	DDX11;FKBP4	NC_056073.1	8900001	8920001	0.086451	0.192701	HZ	SNRPC
0.301802	0.169776	HZ	DDIT4L	NC_056073.1	8920001	8940001	0.0507869	0.216599	HZ	SNRPC;UHRF
0.413295	0.265457	HZ	DCC	NC_056055.1	645001	665001	0.111582	0.175244	HZ	SNTG2
0.523471	0.189502	HZ	DCC	NC_056055.1	660001	680001	0.162455	0.157491	HZ	SNTG2
0.521606	0.138604	HZ	DAZAP2	NC_056063.1	25540001	25560001	0.408349	0.313983	HZ	SOHLH2
0.243451	0.222059	HZ	DACT2	NC_056063.1	25545001	25565001	0.476652	0.23089	HZ	SOHLH2
0.143994	0.285188	HZ	DACT2	NC_056063.1	25565001	25585001	0.435452	0.215205	HZ	SOHLH2
0.504778	0.271006	HZ	DACH1	NC_056063.1	25570001	25590001	0.348214	0.191525	HZ	SOHLH2
0.509451	0.227542	HZ	DACH1	NC_056075.1	27525001	27545001	0.436829	0.195985	HZ	SORCS1
0.533715	0.202571	HZ	DAB1	NC_056075.1	27530001	27550001	0.32216	0.262942	HZ	SORCS1
0.332172	0.143651	HZ	CYLD	NC_056075.1	27535001	27555001	0.179456	0.303122	HZ	SORCS1

0.205665	0.147738	HZ	CYLD	NC_056056.1	85160001	85180001	0.48892	0.320359	HZ	SOS1
0.245312	0.144296	HZ	CYLD	NC_056056.1	85165001	85185001	0.466301	0.293804	HZ	SOS1
0.51262	0.24206	HZ	CXXC1	NC_056056.1	191610001	191630001	0.441738	0.163056	HZ	SOX5
0.450511	0.179354	HZ	CWC27	NC_056060.1	98190001	98210001	0.398977	0.160988	HZ	SPATA7
0.483917	0.227567	HZ	CWC27	NC_056060.1	98195001	98215001	0.387639	0.318635	HZ	SPATA7
0.133254	0.160963	HZ	CUX1	NC_056064.1	33480001	33500001	0.332673	0.207505	HZ	SPECC1
0.129087	0.168951	HZ	CUX1	NC_056070.1	71710001	71730001	0.345836	0.20258	HZ	SPECC1L
0.133031	0.177488	HZ	CUX1	NC_056070.1	71715001	71735001	0.379423	0.228992	HZ	SPECC1L
0.156584	0.183751	HZ	CUX1	NC_056070.1	71720001	71740001	0.433486	0.210632	HZ	SPECC1L
0.320528	0.151761	HZ	CUX1	NC_056060.1	15745001	15765001	0.470195	0.170239	HZ	SPESP1
0.527778	0.14492	HZ	CUL4B	NC_056058.1	46130001	46150001	0.466093	0.16857	HZ	SPOCK1
0.516336	0.14378	HZ	CUBN	NC_056058.1	46135001	46155001	0.283857	0.241269	HZ	SPOCK1
0.526025	0.154678	HZ	CUBN	NC_056058.1	46140001	46160001	0.389687	0.195046	HZ	SPOCK1
0.451901	0.185434	HZ	CTNNBIP1	NC_056066.1	66275001	66295001	0.282691	0.321489	HZ	SRC
0.458672	0.190692	HZ	CTNNBIP1	NC_056066.1	66280001	66300001	0.395093	0.286896	HZ	SRC
0.496901	0.142447	HZ	CTNNA3	NC_056073.1	10590001	10610001	0.42679	0.162728	HZ	SRSF3
0.353079	0.222785	HZ	CTNNA3	NC_056077.1	34845001	34865001	0.394928	0.24	HZ	SSC4D
0.425144	0.250524	HZ	CTNNA3	NC_056062.1	34190001	34210001	0.393505	0.160954	HZ	ST18
0.492648	0.176932	HZ	CTIF	NC_056062.1	34195001	34215001	0.301485	0.161841	HZ	ST18
0.406651	0.161642	HZ	CSN1S1	NC_056076.1	46795001	46815001	0.451632	0.161331	HZ	ST8SIA5
0.417729	0.144611	HZ	CRNKL1;NAA20	NC_056076.1	46800001	46820001	0.443774	0.157775	HZ	ST8SIA5
0.498693	0.174433	HZ	CREB3L2	NC_056063.1	27800001	27820001	0.458505	0.200412	HZ	STARD13
0.415507	0.160299	HZ	CREB3L2	NC_056063.1	74840001	74860001	0.299106	0.184973	HZ	STK24
0.524344	0.143387	HZ	CREB3L2	NC_056063.1	74845001	74865001	0.163086	0.241984	HZ	STK24
0.437499	0.191465	HZ	CREB3L2	NC_056063.1	74850001	74870001	0.193075	0.226476	HZ	STK24
0.447133	0.148959	HZ	CREB3L2	NC_056066.1	73370001	73390001	0.145998	0.266684	HZ	STK4
0.263682	0.174036	HZ	CREB3L2	NC_056066.1	73385001	73405001	0.0748328	0.210535	HZ	STK4
0.375233	0.144614	HZ	CRB1	NC_056079.1	13810001	13830001	0.468302	0.212629	HZ	STOX2
0.378329	0.164702	HZ	CRB1	NC_056079.1	13815001	13835001	0.260249	0.246199	HZ	STOX2
0.411254	0.174938	HZ	CRB1	NC_056079.1	13820001	13840001	0.250967	0.262223	HZ	STOX2
0.383905	0.151807	HZ	CRB1	NC_056079.1	13825001	13845001	0.277097	0.263491	HZ	STOX2
0.417888	0.167932	HZ	CPEB4	NC_056079.1	13830001	13850001	0.269896	0.28789	HZ	STOX2
0.441666	0.1708	HZ	CPEB4	NC_056065.1	28820001	28840001	0.253513	0.167232	HZ	STUM
0.367667	0.150927	HZ	CORO2A	NC_056061.1	71765001	71785001	0.403357	0.258891	HZ	STXBP5
0.456127	0.161868	HZ	CORO2A	NC_056061.1	71770001	71790001	0.314286	0.226916	HZ	STXBP5
0.217724	0.147895	HZ	COL9A2;ZMPSTE	NC_056061.1	71775001	71795001	0.224611	0.172755	HZ	STXBP5

0.276371	0.163953	HZ	COL9A2;ZMPSTE	NC_056061.1	71780001	71800001	0.311017	0.159852	HZ	STXBP5
0.348711	0.198651	HZ	COL9A2	NC_056061.1	71835001	71855001	0.270845	0.163096	HZ	STXBP5
0.477573	0.173706	HZ	COL9A2	NC_056061.1	71840001	71860001	0.487207	0.171104	HZ	STXBP5
0.441931	0.182775	HZ	COL4A4	NC_056057.1	82540001	82560001	0.414514	0.181455	HZ	SUGCT
0.468153	0.189501	HZ	COL4A4	NC_056059.1	86095001	86115001	0.469635	0.160123	HZ	SULT1E1
0.474134	0.191631	HZ	COL4A4	NC_056054.1	100010001	100030001	0.253392	0.336211	HZ	SV2A
0.527366	0.172497	HZ	COL4A4	NC_056071.1	15450001	15470001	0.373322	0.158432	HZ	SV2B
0.460788	0.162801	HZ	COBL	NC_056057.1	103740001	103760001	0.395037	0.226613	HZ	SVOPL
0.418509	0.151027	HZ	CNTNAP5	NC_056057.1	103745001	103765001	0.28536	0.313061	HZ	SVOPL
0.491175	0.153323	HZ	CNTNAP2	NC_056057.1	103750001	103770001	0.191882	0.361227	HZ	SVOPL
0.513716	0.143547	HZ	CNTNAP2	NC_056057.1	103755001	103775001	0.140417	0.394449	HZ	SVOPL
0.520735	0.139382	HZ	CNTNAP2	NC_056057.1	103760001	103780001	0.252529	0.321594	HZ	SVOPL
0.355755	0.262562	HZ	CNTNAP2	NC_056057.1	103765001	103785001	0.3765	0.265653	HZ	SVOPL
0.289495	0.292978	HZ	CNTNAP2	NC_056056.1	114965001	114985001	0.478349	0.198024	HZ	SYT1
0.349266	0.258446	HZ	CNTNAP2	NC_056056.1	114970001	114990001	0.454242	0.214989	HZ	SYT1
0.383271	0.213494	HZ	CNTNAP2	NC_056056.1	114975001	114995001	0.474304	0.179416	HZ	SYT1
0.118653	0.140516	HZ	CNTNAP2	NC_056056.1	115065001	115085001	0.429089	0.247111	HZ	SYT1
0.0274606	0.13953	HZ	CNTNAP2	NC_056055.1	239665001	239685001	0.473716	0.212055	HZ	SYTL1;TMEM
0.463782	0.16171	HZ	CNTN5	NC_056055.1	239670001	239690001	0.416571	0.235547	HZ	SYTL1;TMEM
0.349279	0.202953	HZ	CNTN5	NC_056061.1	58120001	58140001	0.489844	0.169493	HZ	TAAR1;VNN1
0.308063	0.215938	HZ	CNTN5	NC_056066.1	54800001	54820001	0.405377	0.24123	HZ	TAF4
0.434942	0.154596	HZ	CNTN5	NC_056066.1	54805001	54825001	0.254615	0.226362	HZ	TAF4
0.0609587	0.147758	HZ	CNTN4	NC_056066.1	54810001	54830001	0.200925	0.248506	HZ	TAF4
0.0407697	0.151889	HZ	CNTN4	NC_056066.1	54815001	54835001	0.265136	0.239699	HZ	TAF4
0.398783	0.193043	HZ	CNOT8;GEMIN5	NC_056066.1	54820001	54840001	0.348478	0.220263	HZ	TAF4
0.296011	0.258043	HZ	CNOT8;GEMIN5	NC_056066.1	54825001	54845001	0.465691	0.202418	HZ	TAF4
0.260703	0.288022	HZ	CNOT8;GEMIN5	NC_056076.1	30900001	30920001	0.44411	0.269058	HZ	TAF4B
0.345679	0.146454	HZ	CNNM4	NC_056076.1	30905001	30925001	0.450494	0.2948	HZ	TAF4B
0.389364	0.185672	HZ	CNN3;SLC44A3	NC_056076.1	30910001	30930001	0.45539	0.314521	HZ	TAF4B
0.145478	0.298176	HZ	CNN3;SLC44A3	NC_056076.1	30915001	30935001	0.470799	0.329356	HZ	TAF4B
0.444502	0.202569	HZ	CNN3;SLC44A3	NC_056056.1	224355001	224375001	0.387283	0.208586	HZ	TAFA5
0.286619	0.138655	HZ	CMTR1	NC_056056.1	224360001	224380001	0.370748	0.265817	HZ	TAFA5
0.298126	0.2117	HZ	CLOCK;PDCL2	NC_056056.1	224365001	224385001	0.351849	0.290025	HZ	TAFA5
0.295646	0.152845	HZ	CLOCK;PDCL2	NC_056056.1	224370001	224390001	0.382669	0.285646	HZ	TAFA5
0.301898	0.14443	HZ	CLOCK;PDCL2	NC_056056.1	224375001	224395001	0.465792	0.297192	HZ	TAFA5
0.0784627	0.138887	HZ	CLOCK	NC_056056.1	204460001	204480001	0.124669	0.255131	HZ	TAS2R7;TAS2

0.170494	0.138772	HZ	CLOCK	NC_056056.1	204465001	204485001	0.209671	0.203816	HZ	TAS2R7;TAS2I
0.32003	0.169692	HZ	CLOCK	NC_056056.1	204455001	204475001	0.0647546	0.292242	HZ	TAS2R8;TAS2I
0.326611	0.161119	HZ	CLOCK	NC_056056.1	204450001	204470001	0.0452174	0.318165	HZ	TAS2R9
0.343813	0.162524	HZ	CLOCK	NC_056072.1	16585001	16605001	0.308929	0.209731	HZ	TATDN2
0.354011	0.159913	HZ	CLOCK	NC_056072.1	16590001	16610001	0.433286	0.18688	HZ	TATDN2
0.334533	0.141694	HZ	CLOCK	NC_056072.1	16595001	16615001	0.447911	0.200791	HZ	TATDN2
0.362893	0.186269	HZ	CLOCK	NC_056075.1	15525001	15545001	0.367236	0.168341	HZ	TBC1D12
0.407913	0.216368	HZ	CLOCK	NC_056075.1	15530001	15550001	0.326233	0.184954	HZ	TBC1D12
0.304664	0.23632	HZ	CLOCK	NC_056075.1	15535001	15555001	0.332677	0.187554	HZ	TBC1D12
0.314473	0.138945	HZ	CLMN	NC_056075.1	15540001	15560001	0.328285	0.19551	HZ	TBC1D12
0.445594	0.177679	HZ	CLMN	NC_056075.1	15545001	15565001	0.356764	0.186193	HZ	TBC1D12
0.373484	0.185482	HZ	CLMN	NC_056075.1	15550001	15570001	0.352365	0.18665	HZ	TBC1D12
0.482871	0.153544	HZ	CLMN	NC_056075.1	15555001	15575001	0.354462	0.190228	HZ	TBC1D12
0.341969	0.169876	HZ	CLIP4	NC_056061.1	52065001	52085001	0.348601	0.172527	HZ	TBX18
0.482948	0.156828	HZ	CLIP4	NC_056061.1	52070001	52090001	0.418606	0.202374	HZ	TBX18
0.503814	0.257524	HZ	CLIP1	NC_056061.1	52075001	52095001	0.429599	0.200691	HZ	TBX18
0.430079	0.260393	HZ	CLIP1	NC_056057.1	108185001	108205001	0.408608	0.217994	HZ	TCAF2
0.339415	0.140459	HZ	CLEC6A	NC_056063.1	2070001	2090001	0.259557	0.186108	HZ	TDRD3
0.217908	0.210414	HZ	CLEC6A	NC_056063.1	2075001	2095001	0.295881	0.188437	HZ	TDRD3
0.148301	0.151475	HZ	CLEC16A	NC_056063.1	2080001	2100001	0.343075	0.18212	HZ	TDRD3
0.151117	0.145077	HZ	CLEC16A	NC_056077.1	38205001	38225001	0.4105	0.175027	HZ	TECPR1
0.526653	0.193484	HZ	CLDN22;WWC2	NC_056059.1	80550001	80570001	0.393611	0.272073	HZ	TECRL
0.449249	0.191281	HZ	CLDN22;LOC1069	NC_056059.1	80555001	80575001	0.474946	0.256686	HZ	TECRL
0.403563	0.233521	HZ	CKAP2L;IL1A	NC_056059.1	80600001	80620001	0.413142	0.19783	HZ	TECRL
0.279036	0.194664	HZ	CKAP2;VPS36	NC_056059.1	80605001	80625001	0.198081	0.177589	HZ	TECRL
0.377531	0.142946	HZ	CKAP2;VPS36	NC_056059.1	80610001	80630001	0.19577	0.177291	HZ	TECRL
0.420649	0.201147	HZ	CKAP2;NEK3	NC_056059.1	80615001	80635001	0.236725	0.162617	HZ	TECRL
0.300122	0.243637	HZ	CKAP2;NEK3	NC_056059.1	80640001	80660001	0.441497	0.209842	HZ	TECRL
0.257872	0.235092	HZ	CKAP2	NC_056059.1	80645001	80665001	0.408638	0.237154	HZ	TECRL
0.23832	0.210188	HZ	CKAP2	NC_056080.1	108505001	108525001	0.279171	0.170312	HZ	TENM1
0.237879	0.199212	HZ	CKAP2	NC_056080.1	108510001	108530001	0.148284	0.157829	HZ	TENM1
0.265403	0.141041	HZ	CISD3;MLLT6;PC	NC_056069.1	66535001	66555001	0.490069	0.16006	HZ	TENT4A
0.236175	0.147553	HZ	CISD3;MLLT6	NC_056069.1	71175001	71195001	0.0844532	0.184703	HZ	TERT
0.535835	0.195309	HZ	CIB2;SH2D7	NC_056069.1	71180001	71200001	0.109688	0.160752	HZ	TERT
0.392659	0.163956	HZ	CIAO1;TMEM127	NC_056069.1	71185001	71205001	0.068783	0.173376	HZ	TERT
0.284988	0.167273	HZ	CIAO1;SNRNP200	NC_056080.1	63565001	63585001	0.467693	0.16825	HZ	TEX11

0.333633	0.166168	HZ	CIAO1;SNRNP200	NC_056055.1	186535001	186555001	0.321685	0.165931	HZ	TFCP2L1
0.382493	0.161866	HZ	CIAO1;SNRNP200	NC_056055.1	186540001	186560001	0.320939	0.192552	HZ	TFCP2L1
0.306306	0.157672	HZ	CIAO1;SNRNP200	NC_056055.1	186545001	186565001	0.261199	0.216581	HZ	TFCP2L1
0.366483	0.169285	HZ	CHRNA1	NC_056055.1	186550001	186570001	0.257915	0.157726	HZ	TFCP2L1
0.222343	0.22577	HZ	CHRNA1	NC_056054.1	263310001	263330001	0.121241	0.353175	HZ	TFF1
0.289182	0.231856	HZ	CHRNA1	NC_056054.1	263315001	263335001	0.101068	0.366362	HZ	TFF1
0.331992	0.289392	HZ	CHRM3	NC_056055.1	121955001	121975001	0.343582	0.177187	HZ	TFPI
0.161725	0.281552	HZ	CHRM3	NC_056062.1	21455001	21475001	0.07497	0.344704	HZ	TG
0.15901	0.307454	HZ	CHRM3	NC_056062.1	21460001	21480001	0.125066	0.316963	HZ	TG
0.146667	0.304227	HZ	CHRM3	NC_056062.1	21465001	21485001	0.271065	0.245788	HZ	TG
0.250197	0.262305	HZ	CHRM3	NC_056076.1	37995001	38015001	0.387555	0.202627	HZ	TGIF1
0.43483	0.218196	HZ	CHRM3	NC_056055.1	173375001	173395001	0.463984	0.169086	HZ	THSD7B
0.508949	0.178207	HZ	CHRM3	NC_056055.1	173380001	173400001	0.441606	0.185304	HZ	THSD7B
0.453982	0.170563	HZ	CHRM3	NC_056055.1	173385001	173405001	0.449122	0.213568	HZ	THSD7B
0.382453	0.204672	HZ	CHRM3	NC_056055.1	173390001	173410001	0.458924	0.219345	HZ	THSD7B
0.282988	0.216885	HZ	CHRM3	NC_056055.1	173800001	173820001	0.437529	0.204592	HZ	THSD7B
0.199204	0.190922	HZ	CHRM3	NC_056064.1	22695001	22715001	0.457542	0.160796	HZ	TLCD2;WDR8
0.155513	0.159105	HZ	CHRM3	NC_056060.1	45810001	45830001	0.307551	0.184217	HZ	TLN2
0.107087	0.140435	HZ	CHRM3	NC_056060.1	45815001	45835001	0.343767	0.179402	HZ	TLN2
0.363264	0.140552	HZ	CHML	NC_056060.1	45820001	45840001	0.383813	0.183819	HZ	TLN2
0.407536	0.178813	HZ	CHCHD4;TMEM4	NC_056060.1	45885001	45905001	0.402576	0.195844	HZ	TLN2
0.505131	0.144165	HZ	CFAP61	NC_056060.1	45890001	45910001	0.366809	0.227638	HZ	TLN2
0.269533	0.216396	HZ	CFAP61	NC_056054.1	36935001	36955001	0.0913431	0.160367	HZ	TM2D1
0.304565	0.204862	HZ	CFAP61	NC_056054.1	36940001	36960001	0.0893059	0.16086	HZ	TM2D1
0.359131	0.177327	HZ	CFAP61	NC_056054.1	36945001	36965001	0.0921581	0.16067	HZ	TM2D1
0.475563	0.139003	HZ	CFAP61	NC_056054.1	36950001	36970001	0.0900804	0.158977	HZ	TM2D1
0.27575	0.200491	HZ	CFAP61	NC_056077.1	17495001	17515001	0.2238	0.190504	HZ	TMC5
0.482774	0.147958	HZ	CFAP61	NC_056077.1	17500001	17520001	0.218905	0.218691	HZ	TMC5
0.454543	0.219852	HZ	CFAP221	NC_056077.1	17505001	17525001	0.280937	0.202375	HZ	TMC5
0.297848	0.286007	HZ	CFAP221	NC_056077.1	17510001	17530001	0.28046	0.196008	HZ	TMC5
0.314854	0.294365	HZ	CFAP221	NC_056077.1	17515001	17535001	0.260546	0.230155	HZ	TMC5
0.510426	0.230826	HZ	CFAP221	NC_056077.1	17520001	17540001	0.161078	0.277637	HZ	TMC5
0.264328	0.278223	HZ	CEP57;FAM76B	NC_056077.1	17525001	17545001	0.0770171	0.269997	HZ	TMC5
0.188305	0.357036	HZ	CEP57;FAM76B	NC_056077.1	17545001	17565001	0.0271845	0.274963	HZ	TMC5
0.181438	0.402865	HZ	CEP57;FAM76B	NC_056077.1	17550001	17570001	0.128172	0.202627	HZ	TMC5
0.174216	0.399011	HZ	CEP57;FAM76B	NC_056072.1	56030001	56050001	0.491766	0.273148	HZ	TMCC1

0.340457	0.165288	HZ	CEP57	NC_056072.1	56035001	56055001	0.411765	0.280762	HZ	TMCC1
0.532232	0.181908	HZ	CEP350	NC_056072.1	56040001	56060001	0.479042	0.240201	HZ	TMCC1
0.491531	0.191281	HZ	CEP250	NC_056056.1	131025001	131045001	0.40822	0.167645	HZ	TMCC3
0.444616	0.157279	HZ	CEP164	NC_056056.1	131030001	131050001	0.384681	0.251634	HZ	TMCC3
0.489185	0.283093	HZ	CEP164	NC_056056.1	131035001	131055001	0.357743	0.237506	HZ	TMCC3
0.522753	0.293821	HZ	CEP164	NC_056056.1	131040001	131060001	0.400385	0.196911	HZ	TMCC3
0.472902	0.141827	HZ	CEP128	NC_056056.1	131045001	131065001	0.473082	0.178267	HZ	TMCC3
0.502469	0.175684	HZ	CENPQ;MMUT	NC_056078.1	16550001	16570001	0.316494	0.157596	HZ	TMEM26
0.324323	0.270566	HZ	CENPI	NC_056078.1	16555001	16575001	0.263082	0.161358	HZ	TMEM26
0.199272	0.246734	HZ	CENPI	NC_056080.1	81865001	81885001	0.251381	0.360231	HZ	TMLHE
0.273694	0.153072	HZ	CENPI	NC_056080.1	81870001	81890001	0.363785	0.290226	HZ	TMLHE
0.512001	0.172121	HZ	CELF2	NC_056060.1	56640001	56660001	0.0692455	0.16624	HZ	TMOD3
0.496235	0.140628	HZ	CELA1;GALNT6	NC_056060.1	56645001	56665001	0.0835737	0.163712	HZ	TMOD3
0.493827	0.173753	HZ	CDKL1	NC_056054.1	262385001	262405001	0.484302	0.222433	HZ	TMPRSS2
0.488521	0.193652	HZ	CDKL1	NC_056054.1	262390001	262410001	0.463055	0.229804	HZ	TMPRSS2
0.508572	0.212158	HZ	CDH24;PSMB11;P	NC_056063.1	76595001	76615001	0.427282	0.226947	HZ	TMTC4
0.412182	0.183333	HZ	CDH24;PSMB11;P	NC_056063.1	76600001	76620001	0.176437	0.279873	HZ	TMTC4
0.446619	0.160285	HZ	CDCA8;EPHA10	NC_056063.1	76605001	76625001	0.0597921	0.263542	HZ	TMTC4
0.086774	0.180109	HZ	CDC25A	NC_056063.1	76610001	76630001	0.0419642	0.228124	HZ	TMTC4
0.194268	0.146595	HZ	CDC25A	NC_056063.1	76620001	76640001	0.0564862	0.268389	HZ	TMTC4
0.494544	0.138755	HZ	CD9	NC_056063.1	76625001	76645001	0.12477	0.310318	HZ	TMTC4
0.38224	0.304637	HZ	CCNYL1;FZD5;LC	NC_056063.1	76630001	76650001	0.312474	0.277795	HZ	TMTC4
0.34627	0.181264	HZ	CCNY;LOC12181(	NC_056063.1	76635001	76655001	0.419102	0.237068	HZ	TMTC4
0.204636	0.305579	HZ	CCNY;LOC12181(	NC_056065.1	55965001	55985001	0.464738	0.244873	HZ	TNR
0.273932	0.335177	HZ	CCNY;LOC12181(	NC_056065.1	55970001	55990001	0.43432	0.230109	HZ	TNR
0.431941	0.277786	HZ	CCNY	NC_056064.1	34340001	34360001	0.449001	0.225656	HZ	TOM1L2
0.486666	0.304547	HZ	CCNY	NC_056064.1	34345001	34365001	0.246739	0.351803	HZ	TOM1L2
0.486841	0.301452	HZ	CCNY	NC_056064.1	34350001	34370001	0.159496	0.369032	HZ	TOM1L2
0.469786	0.283122	HZ	CCNY	NC_056067.1	20410001	20430001	0.104322	0.223274	HZ	TOX3
0.471106	0.282572	HZ	CCNY	NC_056067.1	20415001	20435001	0.138765	0.265081	HZ	TOX3
0.382609	0.219369	HZ	CCNY	NC_056067.1	20420001	20440001	0.311746	0.165907	HZ	TOX3
0.368422	0.207359	HZ	CCNY	NC_056054.1	198450001	198470001	0.475971	0.232338	HZ	TP63
0.428735	0.237687	HZ	CCNY	NC_056054.1	198455001	198475001	0.449898	0.241346	HZ	TP63
0.474562	0.237103	HZ	CCNY	NC_056054.1	198460001	198480001	0.444816	0.24493	HZ	TP63
0.510696	0.237616	HZ	CCNY	NC_056054.1	198465001	198485001	0.455517	0.242977	HZ	TP63
0.481156	0.155711	HZ	CCNH	NC_056055.1	74385001	74405001	0.473699	0.238519	HZ	TPD52L3

0.317124	0.214247	HZ	CCNB2;RNF111	NC_056055.1	74390001	74410001	0.471849	0.238243	HZ	TPD52L3
0.415162	0.14079	HZ	CCL27;GALT;IL11	NC_056057.1	109010001	109030001	0.184076	0.16124	HZ	TPK1
0.505555	0.15898	HZ	CCL27;GALT;IL11	NC_056057.1	109015001	109035001	0.219873	0.167269	HZ	TPK1
0.491802	0.197308	HZ	CCL26	NC_056057.1	109020001	109040001	0.341406	0.173131	HZ	TPK1
0.482659	0.244869	HZ	CCL21	NC_056057.1	109025001	109045001	0.416411	0.217059	HZ	TPK1
0.48812	0.151504	HZ	CCL20	NC_056054.1	22340001	22360001	0.200763	0.220173	HZ	TRABD2B
0.436909	0.150964	HZ	CCDC88A	NC_056054.1	22345001	22365001	0.194333	0.217931	HZ	TRABD2B
0.418783	0.184328	HZ	CCDC88A	NC_056054.1	22350001	22370001	0.160795	0.227977	HZ	TRABD2B
0.419863	0.256524	HZ	CCDC88A	NC_056054.1	22355001	22375001	0.151191	0.229878	HZ	TRABD2B
0.422736	0.241186	HZ	CCDC88A	NC_056054.1	22360001	22380001	0.281785	0.1668	HZ	TRABD2B
0.42628	0.220791	HZ	CCDC88A	NC_056070.1	4680001	4700001	0.488444	0.314845	HZ	TRIM2
0.421822	0.204132	HZ	CCDC88A	NC_056070.1	4685001	4705001	0.487875	0.302815	HZ	TRIM2
0.416304	0.183916	HZ	CCDC88A	NC_056079.1	23195001	23215001	0.474026	0.204222	HZ	TRMT9B
0.329854	0.233911	HZ	CCDC88A	NC_056079.1	23200001	23220001	0.435366	0.193437	HZ	TRMT9B
0.210636	0.290403	HZ	CCDC88A	NC_056079.1	23205001	23225001	0.449024	0.169414	HZ	TRMT9B
0.234457	0.288674	HZ	CCDC88A	NC_056080.1	123110001	123130001	0.470547	0.211737	HZ	TRPC5
0.297178	0.27954	HZ	CCDC88A	NC_056080.1	123115001	123135001	0.407324	0.245217	HZ	TRPC5
0.438983	0.216467	HZ	CCDC88A	NC_056080.1	123120001	123140001	0.468273	0.214941	HZ	TRPC5
0.309735	0.147563	HZ	CCDC62	NC_056071.1	25315001	25335001	0.229978	0.222559	HZ	TRPM1
0.496266	0.180196	HZ	CCDC51;PLXNB1	NC_056071.1	25320001	25340001	0.235704	0.22833	HZ	TRPM1
0.441095	0.171672	HZ	CCDC51;PLXNB1	NC_056071.1	25325001	25345001	0.473264	0.242953	HZ	TRPM1
0.408208	0.217862	HZ	CCDC175;RTN1	NC_056071.1	25330001	25350001	0.447205	0.245553	HZ	TRPM1
0.433962	0.252301	HZ	CCDC175;RTN1	NC_056071.1	25335001	25355001	0.465416	0.274479	HZ	TRPM1
0.384165	0.154325	HZ	CCDC175	NC_056071.1	25340001	25360001	0.455006	0.256903	HZ	TRPM1
0.392031	0.171183	HZ	CCDC175	NC_056063.1	15190001	15210001	0.448819	0.159094	HZ	TSC22D1
0.408124	0.194618	HZ	CCDC175	NC_056080.1	127495001	127515001	0.161819	0.172996	HZ	TSC22D3
0.401372	0.209835	HZ	CCDC175	NC_056057.1	86910001	86930001	0.264706	0.241316	HZ	TSPAN12
0.432194	0.212669	HZ	CCDC137;OXLD1	NC_056057.1	86915001	86935001	0.434941	0.269237	HZ	TSPAN12
0.507205	0.202169	HZ	CCDC126	NC_056057.1	86945001	86965001	0.397919	0.160083	HZ	TSPAN12
0.470389	0.20069	HZ	CCDC126	NC_056057.1	86950001	86970001	0.473445	0.186173	HZ	TSPAN12
0.418412	0.212671	HZ	CCDC102B	NC_056057.1	86955001	86975001	0.488168	0.224417	HZ	TSPAN12
0.236078	0.262053	HZ	CCDC102B	NC_056078.1	3030001	3050001	0.482509	0.336838	HZ	TTC13
0.212766	0.209928	HZ	CBWD1;FOXD4	NC_056078.1	3035001	3055001	0.243105	0.487511	HZ	TTC13
0.220588	0.225522	HZ	CBWD1;FOXD4	NC_056078.1	3040001	3060001	0.0844186	0.514425	HZ	TTC13
0.0855512	0.266394	HZ	CBWD1	NC_056078.1	3045001	3065001	0.144357	0.372516	HZ	TTC13
0.233493	0.210978	HZ	CBWD1	NC_056078.1	3050001	3070001	0.219554	0.307326	HZ	TTC13

0.363127	0.17695	HZ	CBWD1	NC_056078.1	3055001	3075001	0.282331	0.223095	HZ	TTC13
0.359375	0.146561	HZ	CBWD1	NC_056078.1	3060001	3080001	0.306402	0.172291	HZ	TTC13
0.201708	0.221978	HZ	CBWD1	NC_056078.1	3065001	3085001	0.223025	0.280577	HZ	TTC13
0.142857	0.259243	HZ	CBWD1	NC_056078.1	3070001	3090001	0.129276	0.341667	HZ	TTC13
0.322544	0.189548	HZ	CATSPERG;KCNK	NC_056078.1	3075001	3095001	0.102179	0.36367	HZ	TTC13
0.28673	0.225544	HZ	CATHL3;LOC1056	NC_056078.1	3080001	3100001	0.255407	0.260262	HZ	TTC13
0.279142	0.226363	HZ	CATHL3;LOC1056	NC_056061.1	6990001	7010001	0.404189	0.192983	HZ	TTK
0.0738766	0.259363	HZ	CATHL1B;LOC10	NC_056061.1	6995001	7015001	0.387818	0.231347	HZ	TTK
0.109375	0.279646	HZ	CATHL1B;LOC10	NC_056061.1	7000001	7020001	0.454013	0.252073	HZ	TTK
0.536274	0.194037	HZ	CASK	NC_056061.1	7005001	7025001	0.446393	0.245868	HZ	TTK
0.1721	0.190145	HZ	CAMK2D	NC_056061.1	7010001	7030001	0.45816	0.248021	HZ	TTK
0.112125	0.225313	HZ	CAMK2D	NC_056061.1	7015001	7035001	0.453055	0.258205	HZ	TTK
0.170451	0.222838	HZ	CAMK2D	NC_056061.1	7020001	7040001	0.447343	0.24854	HZ	TTK
0.237557	0.212768	HZ	CAMK2D	NC_056061.1	7025001	7045001	0.484797	0.269531	HZ	TTK
0.275419	0.202973	HZ	CAMK2D	NC_056061.1	7030001	7050001	0.466878	0.249553	HZ	TTK
0.32104	0.186892	HZ	CAMK2D	NC_056061.1	7035001	7055001	0.44562	0.246066	HZ	TTK
0.357681	0.144342	HZ	CAMK2D	NC_056056.1	225320001	225340001	0.399696	0.209992	HZ	TTLL8
0.295357	0.140816	HZ	CAMK2D	NC_056079.1	20710001	20730001	0.176301	0.158494	HZ	TUSC3
0.434956	0.156398	HZ	CAMK2A	NC_056079.1	20715001	20735001	0.164667	0.166345	HZ	TUSC3
0.476743	0.156375	HZ	CAMK2A	NC_056079.1	20720001	20740001	0.154643	0.168624	HZ	TUSC3
0.456889	0.154808	HZ	CAMK2A	NC_056079.1	20725001	20745001	0.172346	0.161975	HZ	TUSC3
0.368242	0.140317	HZ	CADPS2	NC_056079.1	20730001	20750001	0.135711	0.170157	HZ	TUSC3
0.307582	0.153019	HZ	CADPS2	NC_056055.1	175060001	175080001	0.219909	0.181619	HZ	UBXN4
0.335818	0.151047	HZ	CADPS2	NC_056055.1	175065001	175085001	0.31672	0.19503	HZ	UBXN4
0.235955	0.190979	HZ	CADPS2	NC_056055.1	175070001	175090001	0.417521	0.210776	HZ	UBXN4
0.175503	0.141929	HZ	CACNA2D3	NC_056054.1	117910001	117930001	0.372753	0.181836	HZ	UCK2
0.367127	0.145524	HZ	C3H2orf78	NC_056054.1	117915001	117935001	0.391061	0.18645	HZ	UCK2
0.430359	0.188349	HZ	C22H10orf90	NC_056054.1	117920001	117940001	0.376238	0.18761	HZ	UCK2
0.0728309	0.150751	HZ	C1QL3;PTER	NC_056054.1	117925001	117945001	0.374436	0.168174	HZ	UCK2
0.421634	0.214482	HZ	C1H1orf226	NC_056055.1	117290001	117310001	0.291161	0.194154	HZ	UGGT1
0.454377	0.248502	HZ	C1H1orf226	NC_056073.1	8925001	8945001	0.135081	0.167356	HZ	UHRF1BP1
0.494352	0.153405	HZ	C1H1orf226	NC_056073.1	8940001	8960001	0.442943	0.182755	HZ	UHRF1BP1
0.444443	0.145364	HZ	C1H1orf226	NC_056058.1	36150001	36170001	0.266432	0.172221	HZ	UIMC1
0.524575	0.157938	HZ	C1H1orf226	NC_056058.1	36155001	36175001	0.223842	0.196425	HZ	UIMC1
0.145886	0.160779	HZ	C19H3orf84;IHO1	NC_056058.1	36160001	36180001	0.247892	0.183972	HZ	UIMC1
0.474899	0.158652	HZ	C19H3orf62;IHO1;	NC_056058.1	36165001	36185001	0.322027	0.162938	HZ	UIMC1

0.493087	0.139491	HZ	C19H3orf62;IHO1	NC_056072.1	13970001	13990001	0.487484	0.220855	HZ	ULK4
0.468455	0.149884	HZ	C15H11orf94;MAF	NC_056072.1	13975001	13995001	0.33434	0.306257	HZ	ULK4
0.441392	0.190233	HZ	C15H11orf94;MAF	NC_056072.1	13980001	14000001	0.27398	0.338371	HZ	ULK4
0.427948	0.164557	HZ	C15H11orf94;LAR	NC_056072.1	13985001	14005001	0.212654	0.394158	HZ	ULK4
0.429098	0.199249	HZ	C13H20orf173	NC_056072.1	13990001	14010001	0.0668457	0.450874	HZ	ULK4
0.520642	0.15252	HZ	C12H1orf21	NC_056072.1	13995001	14015001	0.0937811	0.418408	HZ	ULK4
0.514647	0.19215	HZ	C12H1orf21	NC_056072.1	14000001	14020001	0.275408	0.276891	HZ	ULK4
0.479704	0.221403	HZ	C12H1orf21	NC_056055.1	53230001	53250001	0.100605	0.16558	HZ	UNC13B
0.379577	0.195391	HZ	BTBD17;KIF19	NC_056055.1	53295001	53315001	0.328515	0.197292	HZ	UNC13B
0.181156	0.181255	HZ	BST-2A;CCDC194	NC_056059.1	29605001	29625001	0.433071	0.164094	HZ	UNC5C
0.102878	0.182485	HZ	BST-2A;CCDC194	NC_056065.1	18120001	18140001	0.442256	0.202589	HZ	USH2A
0.370347	0.210833	HZ	BRSK2;MOB2	NC_056076.1	35590001	35610001	0.406779	0.157675	HZ	USP14
0.365356	0.203198	HZ	BRSK2;MOB2	NC_056076.1	35595001	35615001	0.322104	0.196943	HZ	USP14
0.427338	0.19095	HZ	BRSK2;MOB2	NC_056076.1	35600001	35620001	0.362963	0.167318	HZ	USP14
0.294081	0.144644	HZ	BRSK2	NC_056054.1	141980001	142000001	0.369289	0.367767	HZ	USP25
0.221473	0.159535	HZ	BRSK2	NC_056054.1	141985001	142005001	0.348514	0.399908	HZ	USP25
0.191413	0.173305	HZ	BRSK2	NC_056054.1	141990001	142010001	0.331443	0.395344	HZ	USP25
0.150909	0.198106	HZ	BRSK2	NC_056054.1	141995001	142015001	0.464051	0.381922	HZ	USP25
0.123037	0.185813	HZ	BRSK2	NC_056054.1	142000001	142020001	0.45269	0.367943	HZ	USP25
0.113831	0.23373	HZ	BRSK2	NC_056054.1	142005001	142025001	0.472792	0.294767	HZ	USP25
0.357482	0.147151	HZ	BRSK2	NC_056054.1	142010001	142030001	0.46473	0.298182	HZ	USP25
0.425071	0.258838	HZ	BPGM	NC_056054.1	142015001	142035001	0.399193	0.298334	HZ	USP25
0.150694	0.262859	HZ	BPGM	NC_056054.1	142020001	142040001	0.420705	0.293333	HZ	USP25
0.174644	0.218885	HZ	BPGM	NC_056054.1	142025001	142045001	0.379121	0.354246	HZ	USP25
0.509742	0.151789	HZ	BORA;MZT1	NC_056054.1	142030001	142050001	0.27388	0.402439	HZ	USP25
0.306894	0.279953	HZ	BORA;DIS3	NC_056054.1	142035001	142055001	0.2305	0.426689	HZ	USP25
0.352003	0.279037	HZ	BORA;DIS3	NC_056054.1	142040001	142060001	0.138558	0.451366	HZ	USP25
0.357195	0.27339	HZ	BORA;DIS3	NC_056054.1	142045001	142065001	0.102503	0.454146	HZ	USP25
0.408447	0.192413	HZ	BORA	NC_056054.1	142050001	142070001	0.0955372	0.407326	HZ	USP25
0.330408	0.259191	HZ	BORA	NC_056054.1	142055001	142075001	0.0601133	0.352026	HZ	USP25
0.0522512	0.280258	HZ	BOLA1;SV2A	NC_056054.1	142060001	142080001	0.0512976	0.349289	HZ	USP25
0.358276	0.265818	HZ	BNIP3	NC_056054.1	142080001	142100001	0.0509208	0.446899	HZ	USP25
0.350395	0.139888	HZ	BLMH	NC_056054.1	142085001	142105001	0.0832218	0.403314	HZ	USP25
0.0893246	0.282733	HZ	BIRC6	NC_056054.1	142090001	142110001	0.148784	0.334747	HZ	USP25
0.100911	0.281091	HZ	BIRC6	NC_056054.1	142095001	142115001	0.20164	0.286278	HZ	USP25
0.10174	0.284171	HZ	BIRC6	NC_056080.1	40410001	40430001	0.486872	0.319181	HZ	USP9X

0.110298	0.272014	HZ	BIRC6	NC_056080.1	40415001	40435001	0.42515	0.352771	HZ	USP9X
0.160483	0.2409	HZ	BIRC6	NC_056056.1	60970001	60990001	0.43863	0.200013	HZ	UXS1
0.113852	0.256172	HZ	BIRC6	NC_056056.1	60975001	60995001	0.348125	0.165196	HZ	UXS1
0.16129	0.240338	HZ	BIRC6	NC_056060.1	41260001	41280001	0.295042	0.165974	HZ	VCPKMT
0.158298	0.221065	HZ	BIRC6	NC_056060.1	41265001	41285001	0.253226	0.230202	HZ	VCPKMT
0.175199	0.207729	HZ	BIRC6	NC_056072.1	14635001	14655001	0.428571	0.356563	HZ	VIPR1
0.192258	0.147727	HZ	BIRC6	NC_056072.1	14640001	14660001	0.455204	0.362169	HZ	VIPR1
0.259494	0.247329	HZ	BCL6	NC_056072.1	14645001	14665001	0.468367	0.346755	HZ	VIPR1
0.39304	0.23465	HZ	BCL6	NC_056072.1	14650001	14670001	0.420046	0.30572	HZ	VIPR1
0.424266	0.204922	HZ	BCL6	NC_056072.1	14655001	14675001	0.41588	0.238867	HZ	VIPR1
0.512365	0.171814	HZ	BCL6	NC_056072.1	630001	650001	0.458986	0.165661	HZ	VOPP1
0.237564	0.229282	HZ	BCL2L12;IRF3;SC	NC_056071.1	20960001	20980001	0.179706	0.206756	HZ	VPS33B
0.225262	0.28258	HZ	BCL2L12;IRF3;SC	NC_056071.1	20965001	20985001	0.0828754	0.213568	HZ	VPS33B
0.235594	0.329119	HZ	BCL2L12;IRF3;PR	NC_056057.1	83795001	83815001	0.2127	0.173844	HZ	VPS41
0.265912	0.340589	HZ	BCL2L12;IRF3;PR	NC_056057.1	83800001	83820001	0.207579	0.199063	HZ	VPS41
0.510372	0.157198	HZ	BBS9;LOC114108'	NC_056057.1	83805001	83825001	0.372988	0.221132	HZ	VPS41
0.501555	0.190426	HZ	BBS9	NC_056075.1	32545001	32565001	0.400555	0.161391	HZ	VTI1A
0.505189	0.157116	HZ	BBS9	NC_056075.1	32550001	32570001	0.366354	0.213459	HZ	VTI1A
0.515837	0.283983	HZ	BBS9	NC_056075.1	32555001	32575001	0.334837	0.203086	HZ	VTI1A
0.409208	0.156028	HZ	BBS9	NC_056056.1	171440001	171460001	0.128866	0.257609	HZ	WASHC3
0.445991	0.152486	HZ	BBS9	NC_056056.1	171445001	171465001	0.236934	0.199675	HZ	WASHC3
0.479448	0.149398	HZ	BANK1	NC_056063.1	21420001	21440001	0.228	0.186646	HZ	WDFY2
0.308746	0.171486	HZ	BANK1	NC_056063.1	21425001	21445001	0.189949	0.216023	HZ	WDFY2
0.264556	0.17913	HZ	BANK1	NC_056063.1	21430001	21450001	0.283309	0.197396	HZ	WDFY2
0.257018	0.174573	HZ	BANK1	NC_056080.1	116440001	116460001	0.474747	0.178205	HZ	WDR44
0.261109	0.15396	HZ	BANK1	NC_056080.1	116445001	116465001	0.228813	0.283333	HZ	WDR44
0.288994	0.246766	HZ	BAIAP2L1	NC_056080.1	116450001	116470001	0.267223	0.309756	HZ	WDR44
0.201884	0.286254	HZ	BAIAP2L1	NC_056080.1	116455001	116475001	0.176403	0.274906	HZ	WDR44
0.175695	0.319477	HZ	BAIAP2L1	NC_056080.1	116460001	116480001	0.176663	0.237102	HZ	WDR44
0.223018	0.33159	HZ	BAIAP2L1	NC_056080.1	116465001	116485001	0.292797	0.177067	HZ	WDR44
0.170165	0.389156	HZ	BAIAP2L1	NC_056055.1	239735001	239755001	0.318428	0.223561	HZ	WDTC1
0.216284	0.376775	HZ	BAIAP2L1	NC_056055.1	239740001	239760001	0.19899	0.345874	HZ	WDTC1
0.307656	0.327062	HZ	BAIAP2L1	NC_056055.1	239745001	239765001	0.368795	0.259695	HZ	WDTC1
0.345215	0.274166	HZ	BAIAP2L1	NC_056055.1	239750001	239770001	0.488806	0.213732	HZ	WDTC1
0.296096	0.155713	HZ	BAG4;LSM1	NC_056071.1	22395001	22415001	0.350264	0.186197	HZ	WHAMM
0.358473	0.303456	HZ	BACH2	NC_056071.1	22400001	22420001	0.350655	0.158134	HZ	WHAMM

0.505897	0.224656	HZ	BACH2	NC_056056.1	213415001	213435001	0.30196	0.257342	HZ	WNK1
0.272932	0.146912	HZ	BAC5;SC5	NC_056056.1	213420001	213440001	0.403722	0.19979	HZ	WNK1
0.304933	0.215502	HZ	BAC5;CATHL3;L(	NC_056056.1	213425001	213445001	0.401639	0.197455	HZ	WNK1
0.416113	0.173272	HZ	BAC5;CATHL3	NC_056056.1	213430001	213450001	0.309904	0.249488	HZ	WNK1
0.247267	0.185541	HZ	BAC5	NC_056056.1	213435001	213455001	0.255911	0.264727	HZ	WNK1
0.302084	0.153871	HZ	BAC5	NC_056056.1	213440001	213460001	0.23157	0.280465	HZ	WNK1
0.368554	0.263906	HZ	BABAM2	NC_056056.1	213445001	213465001	0.357541	0.219232	HZ	WNK1
0.294493	0.304901	HZ	BABAM2	NC_056056.1	222195001	222215001	0.384275	0.187903	HZ	WNT7B
0.327259	0.28961	HZ	BABAM2	NC_056073.1	49950001	49970001	0.458529	0.214633	HZ	WRNIP1
0.271951	0.321078	HZ	BABAM2	NC_056067.1	5000001	5020001	0.329705	0.170951	HZ	WWOX
0.382626	0.269792	HZ	BABAM2	NC_056067.1	5005001	5025001	0.429285	0.163668	HZ	WWOX
0.535055	0.2	HZ	BABAM2	NC_056067.1	5010001	5030001	0.446509	0.243387	HZ	WWOX
0.197022	0.152049	HZ	B3GNTL1	NC_056067.1	5015001	5035001	0.471571	0.216265	HZ	WWOX
0.400457	0.182857	HZ	B3GNT5;MCF2L2	NC_056067.1	5020001	5040001	0.463784	0.178998	HZ	WWOX
0.341382	0.139482	HZ	B3GAT2	NC_056067.1	5025001	5045001	0.464559	0.189494	HZ	WWOX
0.395478	0.146123	HZ	B3GAT2	NC_056055.1	43590001	43610001	0.408932	0.187993	HZ	XPO7
0.428944	0.15613	HZ	B3GALT1	NC_056055.1	43595001	43615001	0.366811	0.222695	HZ	XPO7
0.465228	0.148012	HZ	B3GALT1	NC_056057.1	116660001	116680001	0.34173	0.202779	HZ	XRCC2
0.290516	0.152026	HZ	AWAT1;P2RY4	NC_056057.1	116665001	116685001	0.321429	0.21321	HZ	XRCC2
0.343375	0.138512	HZ	AWAT1;P2RY4	NC_056054.1	193315001	193335001	0.469475	0.163988	HZ	XXYLT1
0.406336	0.150174	HZ	AWAT1	NC_056056.1	91560001	91580001	0.301038	0.159272	HZ	YIPF4
0.301933	0.179506	HZ	AWAT1	NC_056059.1	105450001	105470001	0.305211	0.213233	HZ	ZBTB49
0.502345	0.139122	HZ	AVEN	NC_056054.1	26715001	26735001	0.481049	0.172722	HZ	ZFYVE9
0.405693	0.145707	HZ	ATPAF1;EFCAB1	NC_056054.1	26720001	26740001	0.352987	0.23489	HZ	ZFYVE9
0.367031	0.164228	HZ	ATPAF1;EFCAB1	NC_056054.1	26725001	26745001	0.113821	0.34171	HZ	ZFYVE9
0.438834	0.265996	HZ	ATPAF1	NC_056054.1	26730001	26750001	0.245126	0.298291	HZ	ZFYVE9
0.423935	0.2624	HZ	ATPAF1	NC_056062.1	29885001	29905001	0.465425	0.158497	HZ	ZHX2
0.433415	0.272061	HZ	ATPAF1	NC_056062.1	29890001	29910001	0.454639	0.157482	HZ	ZHX2
0.381427	0.191468	HZ	ATPAF1	NC_056062.1	29895001	29915001	0.448808	0.157997	HZ	ZHX2
0.324685	0.150321	HZ	ATPAF1	NC_056062.1	29920001	29940001	0.343508	0.207636	HZ	ZHX2
0.38383	0.149741	HZ	ATPAF1	NC_056062.1	29925001	29945001	0.241499	0.230707	HZ	ZHX2
0.462242	0.171303	HZ	ATP6V1D;PALS1	NC_056062.1	29930001	29950001	0.157895	0.246371	HZ	ZHX2
0.428224	0.161095	HZ	ATP6V1D;PALS1	NC_056062.1	29955001	29975001	0.139978	0.256867	HZ	ZHX2
0.218183	0.152515	HZ	ATP6V1D;EIF2S1	NC_056080.1	57460001	57480001	0.335	0.180832	HZ	ZNF157
0.18711	0.155555	HZ	ATP6V1D;EIF2S1	NC_056080.1	57465001	57485001	0.45662	0.182586	HZ	ZNF157
0.477601	0.161615	HZ	ATP2B2	NC_056057.1	57695001	57715001	0.414948	0.165842	HZ	ZNF277

0.491453	0.160548	HZ	ATP2B2	NC_056057.1	57700001	57720001	0.386741	0.158646	HZ	ZNF277
0.530639	0.141151	HZ	ATP11A	NC_056057.1	57705001	57725001	0.411269	0.158764	HZ	ZNF277
0.432681	0.167212	HZ	ATG10	NC_056079.1	43585001	43605001	0.429749	0.183464	HZ	ZNF385D
0.318815	0.147846	HZ	ATG10	NC_056079.1	43590001	43610001	0.217882	0.16952	HZ	ZNF385D
0.428907	0.142722	HZ	ASPRV1;MXD1	NC_056055.1	29090001	29110001	0.177083	0.281992	HZ	ZNF484
0.0806203	0.238135	HZ	ASPRV1	NC_056055.1	29095001	29115001	0.148571	0.35631	HZ	ZNF484
0.140418	0.245802	HZ	ASPRV1	NC_056055.1	29100001	29120001	0.245339	0.312545	HZ	ZNF484
0.220154	0.240089	HZ	ASPRV1	NC_056067.1	46525001	46545001	0.418336	0.177102	HZ	ZNF568;ZNF82
0.384017	0.139771	HZ	ASPRV1	NC_056060.1	43785001	43805001	0.26033	0.181272	HZ	ZNF609
0.429453	0.154218	HZ	ASB4	NC_056060.1	43790001	43810001	0.254117	0.202968	HZ	ZNF609
0.361511	0.16522	HZ	ASB4	NC_056060.1	43795001	43815001	0.366796	0.194039	HZ	ZNF609
0.365257	0.143535	HZ	ASB4	NC_056054.1	96970001	96990001	0.101472	0.289404	HZ	ZNF697
0.307077	0.169563	HZ	ARSK;TTC37	NC_056054.1	96975001	96995001	0.182654	0.255801	HZ	ZNF697
0.514798	0.194818	HZ	ARPP19;FAM214A	NC_056054.1	97000001	97020001	0.148672	0.183041	HZ	ZNF697
0.465037	0.161539	HZ	ARPC1A;ARPC1B	NC_056057.1	75025001	75045001	0.312209	0.191101	HZ	ZNF804B
0.247391	0.213526	HZ	ARPC1A;ARPC1B	NC_056057.1	75030001	75050001	0.382502	0.228802	HZ	ZNF804B
0.208885	0.227511	HZ	ARPC1A;ARPC1B	NC_056075.1	43950001	43970001	0.164246	0.197343	HZ	ZRANB1
0.181281	0.238184	HZ	ARPC1A	NC_056075.1	43955001	43975001	0.184615	0.1883	HZ	ZRANB1
0.187955	0.261203	HZ	ARPC1A	NC_056075.1	43960001	43980001	0.205186	0.174578	HZ	ZRANB1
0.318181	0.210833	HZ	ARPC1A	NC_056075.1	43965001	43985001	0.408602	0.15849	HZ	ZRANB1
0.267815	0.145256	HZ	ARNTL2	NC_056075.1	43970001	43990001	0.381619	0.163623	HZ	ZRANB1
0.243655	0.139582	HZ	ARL9;THEGL	NC_056075.1	43975001	43995001	0.325581	0.158762	HZ	ZRANB1
0.191656	0.14753	HZ	ARL9;THEGL	NC_056075.1	43980001	44000001	0.333996	0.163494	HZ	ZRANB1
0.306749	0.140399	HZ	ARL9	NC_056075.1	43985001	44005001	0.29108	0.183045	HZ	ZRANB1
0.511661	0.179372	HZ	ARL6	NC_056075.1	43990001	44010001	0.278724	0.177738	HZ	ZRANB1
0.396653	0.206096	HZ	ARL6	NC_056075.1	43995001	44015001	0.33125	0.218744	HZ	ZRANB1
0.358975	0.221044	HZ	ARL6	NC_056075.1	44000001	44020001	0.388368	0.213181	HZ	ZRANB1
0.408661	0.213916	HZ	ARL6	NC_056055.1	175455001	175475001	0.123745	0.183286	HZ	ZRANB3
0.405537	0.198517	HZ	ARL6	NC_056055.1	175460001	175480001	0.151605	0.157659	HZ	ZRANB3
0.492078	0.185614	HZ	ARL6	NC_056055.1	175465001	175485001	0.256651	0.174374	HZ	ZRANB3
0.141727	0.615921	HZ	ARIH2;LOC10560	NC_056055.1	175470001	175490001	0.369811	0.160447	HZ	ZRANB3
0.352047	0.529433	HZ	ARIH2;LOC10560	NC_056055.1	175480001	175500001	0.476673	0.162026	HZ	ZRANB3
0.327965	0.531716	HZ	ARIH2	NC_056055.1	175485001	175505001	0.379651	0.181938	HZ	ZRANB3
0.467058	0.164562	HZ	ARID5A;KANSL3	NC_056055.1	175490001	175510001	0.290605	0.216784	HZ	ZRANB3
0.438332	0.168254	HZ	ARID5A;KANSL3	NC_056055.1	175495001	175515001	0.233548	0.240956	HZ	ZRANB3
0.493516	0.150301	HZ	ARID5A	NC_056055.1	175500001	175520001	0.1901	0.261418	HZ	ZRANB3

0.487113	0.149731	HZ	ARID5A
0.437501	0.246492	HZ	ARID1B
0.319925	0.271237	HZ	ARID1B
0.280669	0.285835	HZ	ARID1B
0.272728	0.259754	HZ	ARID1B
0.479492	0.142973	HZ	ARHGEF3
0.339673	0.164772	HZ	ARHGAP6
0.354347	0.139204	HZ	ARHGAP6
0.211134	0.171508	HZ	ARHGAP18
0.214476	0.165589	HZ	ARHGAP18
0.176043	0.207557	HZ	ARHGAP18
0.217831	0.201111	HZ	ARHGAP18
0.273237	0.169817	HZ	ARHGAP18
0.394387	0.167245	HZ	ARHGAP18
0.518914	0.15475	HZ	ARHGAP18
0.497545	0.159579	HZ	ARHGAP11A;LOC101102647
0.422808	0.174429	HZ	ARHGAP11A;LOC101102647
0.368644	0.168552	HZ	ARHGAP11A;LOC101102647
0.456861	0.153121	HZ	ARHGAP11A
0.497466	0.13898	HZ	ARHGAP11A
0.270984	0.221855	HZ	ARF6
0.49299	0.144906	HZ	APOA1;SIK3
0.361049	0.15277	HZ	APOA1;APOC3
0.335041	0.158972	HZ	APH1B;RAB8B
0.538647	0.150929	HZ	APC2
0.538288	0.221911	HZ	AP2A2;MUC6
0.488348	0.254444	HZ	AP2A2;MUC6
0.354037	0.303233	HZ	AP2A2;MUC6
0.483517	0.205857	HZ	AP2A2
0.383713	0.14901	HZ	AP1M2;SLC44A2
0.151485	0.163537	HZ	ANXA4;GMCL1
0.299934	0.196782	HZ	ANO8;GTPBP3;LOC101111878
0.369437	0.219453	HZ	ANO8;GTPBP3
0.475559	0.19999	HZ	ANO8;DDA1;GTPBP3
0.169069	0.146463	HZ	ANO4
0.158941	0.147432	HZ	ANO4

NC_056055.1	175505001	175525001	0.272865	0.266433	HZ	ZRANB3
NC_056055.1	175510001	175530001	0.407467	0.232092	HZ	ZRANB3

0.517964	0.150912	HZ	ANGPT1
0.514052	0.164586	HZ	ANGPT1
0.492899	0.184909	HZ	ANGPT1
0.524099	0.170892	HZ	ANGPT1
0.228085	0.164977	HZ	AMPD1
0.219836	0.155796	HZ	ALG14
0.199284	0.191389	HZ	ALG14
0.232044	0.229943	HZ	ALG14
0.454227	0.145024	HZ	ALDH1B1;IGFBPL1
0.329649	0.156867	HZ	ALDH1B1;IGFBPL1
0.225451	0.192217	HZ	ALDH1B1;IGFBPL1
0.3825	0.179033	HZ	ALDH1B1
0.507082	0.425018	HZ	ALDH1A2
0.393482	0.391928	HZ	ALDH1A2
0.159091	0.274331	HZ	ALDH1A2
0.0815731	0.27267	HZ	ALDH1A2
0.0591966	0.288921	HZ	ALDH1A2
0.303029	0.292252	HZ	AKT2;CCNP;TTC9B
0.514814	0.217234	HZ	AKT2;CCNP;MAP3K10;TTC9B
0.386634	0.268493	HZ	AKT2;CCNP;MAP3K10;TTC9B
0.440439	0.152488	HZ	AKT2;C14H19orf47
0.214348	0.314999	HZ	AKT2
0.28807	0.283355	HZ	AKT2
0.405254	0.266546	HZ	AKT2
0.44372	0.187312	HZ	AKT2
0.280156	0.226669	HZ	AKIRIN2
0.23934	0.198371	HZ	AKIRIN2
0.302096	0.174341	HZ	AKIRIN2
0.376696	0.150132	HZ	AGAP1
0.360046	0.157211	HZ	AGAP1
0.443785	0.144634	HZ	AFF2
0.461824	0.212296	HZ	AFF2
0.468998	0.263784	HZ	AFF2
0.438771	0.253989	HZ	AFF2
0.496793	0.206919	HZ	AFF2
0.538338	0.204652	HZ	ADSS2;C12H1orf100

0.417851	0.18513	HZ	ADSS2;C12H1orf100
0.394439	0.161942	HZ	ADSS2;C12H1orf100
0.396936	0.145714	HZ	ADRA2B
0.484195	0.361811	HZ	ADM5;CPT1C;PRMT1
0.529153	0.383991	HZ	ADM5;CPT1C;PRMT1
0.388838	0.303715	HZ	ADM5;BCL2L12;CPT1C;PRMT1
0.375698	0.256641	HZ	ADGRV1
0.215713	0.350092	HZ	ADGRV1
0.325688	0.29423	HZ	ADGRV1
0.327103	0.256832	HZ	ADGRV1
0.507104	0.139769	HZ	ADGRV1
0.527093	0.162871	HZ	ADGRL3
0.528369	0.165701	HZ	ADGRL3
0.193504	0.153168	HZ	ADGRG6
0.367419	0.139042	HZ	ADGRG6
0.302181	0.145231	HZ	ADGRF2
0.477144	0.161101	HZ	ADGRB3
0.134999	0.248309	HZ	ADGRB3
0.039007	0.319387	HZ	ADGRB3
0.070525	0.230108	HZ	ADGRB3
0.281507	0.169439	HZ	ADD3
0.162393	0.144218	HZ	ADD3
0.120246	0.139904	HZ	ADD3
0.091954	0.162076	HZ	ADD3
0.0961212	0.155713	HZ	ADD3
0.122051	0.160068	HZ	ADD3
0.0774709	0.196697	HZ	ADD3
0.191367	0.161507	HZ	ADD3
0.27022	0.17259	HZ	ADD3
0.2352	0.166849	HZ	ADD3
0.528558	0.426223	HZ	ADCY9
0.425104	0.250774	HZ	ADCY9
0.407285	0.278017	HZ	ADCY9
0.0631792	0.187233	HZ	ADCY5
0.0639878	0.192018	HZ	ADCY5
0.269126	0.210409	HZ	ADCY5

0.39426	0.216515	HZ	ADCY5
0.497949	0.19954	HZ	ADCY5
0.352333	0.142194	HZ	ADCY3
0.458319	0.170151	HZ	ADCY2
0.438239	0.168073	HZ	ADCY2
0.507403	0.22759	HZ	ADAMTS9
0.517766	0.206208	HZ	ADAMTS9
0.522728	0.181573	HZ	ADAMTS9
0.280992	0.154512	HZ	ADAMTS6;CENPK
0.27511	0.205705	HZ	ADAMTS6;CENPK
0.278724	0.239775	HZ	ADAMTS6
0.329501	0.267559	HZ	ADAMTS6
0.328472	0.254819	HZ	ADAMTS6
0.443573	0.214225	HZ	ADAMTS6
0.49871	0.166879	HZ	ADAMTS6
0.488373	0.178134	HZ	ADAMTS6
0.514562	0.153407	HZ	ADAMTS6
0.513237	0.215561	HZ	ADAM12
0.519284	0.139936	HZ	ACTR5;SLC32A1
0.128154	0.231314	HZ	ACOXL
0.0868557	0.295763	HZ	ACOXL
0.082817	0.293807	HZ	ACOXL
0.0841331	0.290427	HZ	ACOXL
0.122972	0.235939	HZ	ACOXL
0.381025	0.139291	HZ	ACOXL
0.229437	0.146278	HZ	ACOXL
0.413055	0.214167	HZ	ACOXL
0.409384	0.213601	HZ	ACOXL
0.262689	0.160632	HZ	ACOXL
0.337718	0.171863	HZ	ACOXL
0.42795	0.167723	HZ	ACOXL
0.251379	0.158367	HZ	ACOXL
0.2399	0.192142	HZ	ACOXL
0.29078	0.213858	HZ	ACOXL
0.336791	0.235453	HZ	ACOXL
0.426673	0.264968	HZ	ACOXL

0.431691	0.279228	HZ	ACOXL
0.440364	0.265086	HZ	ACOXL
0.445115	0.274382	HZ	ACOXL
0.434563	0.246933	HZ	ACOXL
0.410223	0.144357	HZ	ACOXL
0.377452	0.167059	HZ	ACOXL
0.370079	0.188292	HZ	ACOXL
0.365604	0.192043	HZ	ACOXL
0.52303	0.15589	HZ	ACOXL
0.365418	0.265536	HZ	ACBD3
0.503901	0.185784	HZ	ACBD3
0.524241	0.175555	HZ	ABCA13
0.418456	0.153467	HZ	AATK
0.474836	0.148587	HZ	AATK

ZK vs HZ						
CHROM	Start	end	Pi	Fst	Region	Gene
NC_056067.1	16065001	16085001	0.230769	0.230769	HZ	ABCC12
NC_056067.1	16070001	16090001	0.149063	0.247594	HZ	ABCC12
NC_056067.1	16075001	16095001	0.153643	0.213989	HZ	ABCC12
NC_056067.1	16080001	16100001	0.226905	0.161992	HZ	ABCC12
NC_056054.1	71520001	71540001	0.459264	0.332865	HZ	ABCD3
NC_056054.1	71525001	71545001	0.472702	0.350234	HZ	ABCD3
NC_056054.1	71530001	71550001	0.458187	0.326591	HZ	ABCD3
NC_056054.1	71535001	71555001	0.426927	0.29379	HZ	ABCD3
NC_056054.1	71540001	71560001	0.44249	0.282721	HZ	ABCD3
NC_056054.1	71545001	71565001	0.42506	0.248021	HZ	ABCD3
NC_056054.1	71550001	71570001	0.430029	0.238458	HZ	ABCD3
NC_056054.1	71555001	71575001	0.464726	0.237596	HZ	ABCD3
NC_056054.1	71570001	71590001	0.361248	0.194526	HZ	ABCD3
NC_056054.1	71575001	71595001	0.401059	0.237171	HZ	ABCD3
NC_056054.1	71580001	71600001	0.381102	0.238829	HZ	ABCD3
NC_056059.1	115170001	1.15E+08	0.359691	0.196634	HZ	ABLIM2
NC_056059.1	115175001	1.15E+08	0.293668	0.286655	HZ	ABLIM2
NC_056059.1	115180001	1.15E+08	0.345679	0.307642	HZ	ABLIM2
NC_056064.1	21625001	21645001	0.323246	0.156078	HZ	ABR
NC_056064.1	21630001	21650001	0.423172	0.15635	HZ	ABR
NC_056056.1	225945001	2.26E+08	0.16524	0.154732	HZ	ACR
NC_056056.1	117170001	1.17E+08	0.376835	0.149361	HZ	ACSS3
NC_056056.1	117175001	1.17E+08	0.214184	0.19069	HZ	ACSS3
NC_056070.1	35495001	35515001	0.2557	0.201834	HZ	ADAD1
NC_056070.1	35500001	35520001	0.324478	0.200985	HZ	ADAD1
NC_056079.1	34185001	34205001	0.47991	0.161492	HZ	ADAM18
NC_056058.1	1815001	1835001	0.135949	0.149769	HZ	ADAMTS2
NC_056058.1	1820001	1840001	0.072265	0.149312	HZ	ADAMTS2
NC_056058.1	1825001	1845001	0.053519	0.151865	HZ	ADAMTS2
NC_056058.1	1830001	1850001	0.053027	0.163825	HZ	ADAMTS2
NC_056058.1	1835001	1855001	0.137061	0.19319	HZ	ADAMTS2
NC_056058.1	1840001	1860001	0.229809	0.252753	HZ	ADAMTS2
NC_056058.1	1845001	1865001	0.371991	0.307988	HZ	ADAMTS2

NC_056058.1	1850001	1870001	0.466278	0.332681	HZ	ADAMTS2
NC_056058.1	1855001	1875001	0.462181	0.339439	HZ	ADAMTS2
NC_056058.1	1860001	1880001	0.481092	0.291336	HZ	ADAMTS2
NC_056058.1	1870001	1890001	0.460317	0.169722	HZ	ADAMTS2
NC_056058.1	1875001	1895001	0.463366	0.264268	HZ	ADAMTS2
NC_056058.1	1880001	1900001	0.444247	0.340542	HZ	ADAMTS2
NC_056058.1	1885001	1905001	0.449586	0.345675	HZ	ADAMTS2
NC_056058.1	1890001	1910001	0.471197	0.33104	HZ	ADAMTS2
NC_056058.1	1895001	1915001	0.470149	0.342429	HZ	ADAMTS2
NC_056058.1	1900001	1920001	0.473888	0.326989	HZ	ADAMTS2
NC_056058.1	1905001	1925001	0.476017	0.325277	HZ	ADAMTS2
NC_056054.1	130065001	1.3E+08	0.260889	0.165408	HZ	ADAMTS5
NC_056069.1	65765001	65785001	0.4554	0.180902	HZ	ADCY2
NC_056069.1	65770001	65790001	0.491957	0.160422	HZ	ADCY2
NC_056060.1	20660001	20680001	0.461538	0.161651	HZ	ADCY4;CIDEB;LTB4R;LTB4R2
NC_056060.1	20665001	20685001	0.469424	0.155055	HZ	ADCY4;CIDEB;LTB4R;LTB4R2;NOP9
NC_056061.1	71100001	71120001	0.404331	0.155146	HZ	ADGB
NC_056061.1	71105001	71125001	0.394898	0.166067	HZ	ADGB
NC_056061.1	71110001	71130001	0.341283	0.176993	HZ	ADGB
NC_056061.1	71115001	71135001	0.322663	0.177693	HZ	ADGB
NC_056061.1	71120001	71140001	0.305785	0.151324	HZ	ADGB
NC_056061.1	71125001	71145001	0.30094	0.147258	HZ	ADGB
NC_056061.1	71130001	71150001	0.382179	0.150965	HZ	ADGB
NC_056059.1	42550001	42570001	0.338551	0.278773	HZ	ADGRA3
NC_056059.1	42555001	42575001	0.486662	0.228396	HZ	ADGRA3
NC_056070.1	46115001	46135001	0.499416	0.247427	HZ	ADGRD1
NC_056070.1	46120001	46140001	0.466874	0.277714	HZ	ADGRD1
NC_056080.1	17245001	17265001	0.417583	0.159649	HZ	ADGRG2
NC_056058.1	87765001	87785001	0.461449	0.2412	HZ	ADGRV1
NC_056058.1	87770001	87790001	0.413034	0.194767	HZ	ADGRV1
NC_056058.1	87775001	87795001	0.292413	0.180437	HZ	ADGRV1
NC_056058.1	88015001	88035001	0.464581	0.2942	HZ	ADGRV1
NC_056056.1	104210001	1.04E+08	0.217151	0.179732	HZ	ADRA2B
NC_056056.1	104205001	1.04E+08	0.090709	0.152575	HZ	ADRA2B;ASTL
NC_056080.1	87125001	87145001	0.470134	0.170042	HZ	AFF2
NC_056080.1	87130001	87150001	0.387368	0.194953	HZ	AFF2

NC_056080.1	87135001	87155001	0.326531	0.178805	HZ	AFF2
NC_056056.1	101195001	1.01E+08	0.159492	0.150664	HZ	AFF3
NC_056057.1	26315001	26335001	0.386972	0.193637	HZ	AGR3
NC_056071.1	67090001	67110001	0.458832	0.153333	HZ	AHNAK2
NC_056080.1	113980001	1.14E+08	0.416636	0.183589	HZ	AIFM1;ELF4
NC_056058.1	7610001	7630001	0.229133	0.160961	HZ	AKAP8
NC_056072.1	48760001	48780001	0.338249	0.277837	HZ	ALAS1;POC1A
NC_056072.1	48765001	48785001	0.333716	0.203637	HZ	ALAS1;POC1A
NC_056054.1	170980001	1.71E+08	0.350561	0.152972	HZ	ALCAM
NC_056059.1	13570001	13590001	0.392506	0.264512	HZ	ALPK1
NC_056059.1	13575001	13595001	0.348357	0.214603	HZ	ALPK1
NC_056059.1	13580001	13600001	0.389746	0.18221	HZ	ALPK1
NC_056056.1	133075001	1.33E+08	0.19744	0.160139	HZ	AMHR2;SP1
NC_056066.1	59850001	59870001	0.454925	0.230979	HZ	ANGPT4
NC_056066.1	59855001	59875001	0.304222	0.263029	HZ	ANGPT4
NC_056066.1	59860001	59880001	0.491595	0.183907	HZ	ANGPT4
NC_056065.1	41690001	41710001	0.252125	0.265804	HZ	ANGPTL7;MTOR
NC_056065.1	41695001	41715001	0.309092	0.240612	HZ	ANGPTL7;MTOR
NC_056065.1	41700001	41720001	0.220117	0.267464	HZ	ANGPTL7;MTOR
NC_056065.1	41705001	41725001	0.321069	0.236242	HZ	ANGPTL7;MTOR
NC_056065.1	41710001	41730001	0.383356	0.22141	HZ	ANGPTL7;MTOR
NC_056065.1	41715001	41735001	0.307692	0.257227	HZ	ANGPTL7;MTOR
NC_056060.1	7040001	7060001	0.46255	0.194048	HZ	ANKDD1B
NC_056060.1	7045001	7065001	0.427832	0.236594	HZ	ANKDD1B
NC_056060.1	7050001	7070001	0.43467	0.22708	HZ	ANKDD1B
NC_056060.1	7055001	7075001	0.459109	0.229171	HZ	ANKDD1B
NC_056060.1	7060001	7080001	0.482221	0.192895	HZ	ANKDD1B
NC_056069.1	62695001	62715001	0.436392	0.232538	HZ	ANKRD33B
NC_056057.1	62440001	62460001	0.306731	0.176563	HZ	ANLN;KIAA0895
NC_056057.1	62445001	62465001	0.315412	0.178402	HZ	ANLN;KIAA0895
NC_056057.1	62450001	62470001	0.383354	0.207827	HZ	ANLN;KIAA0895
NC_056057.1	62455001	62475001	0.354943	0.220189	HZ	ANLN;KIAA0895
NC_056057.1	62460001	62480001	0.276006	0.193554	HZ	ANLN;KIAA0895
NC_056057.1	62465001	62485001	0.260884	0.163511	HZ	ANLN;KIAA0895
NC_056068.1	55675001	55695001	0.359123	0.191738	HZ	ANO3
NC_056068.1	55680001	55700001	0.20131	0.241541	HZ	ANO3

NC_056068.1	55685001	55705001	0.148777	0.24256	HZ	ANO3
NC_056068.1	55690001	55710001	0.228934	0.185792	HZ	ANO3
NC_056056.1	141135001	1.41E+08	0.10917	0.170617	HZ	ANO6
NC_056078.1	34340001	34360001	0.377982	0.15273	HZ	ANXA11;PLAC9
NC_056078.1	34345001	34365001	0.137288	0.17105	HZ	ANXA11;PLAC9
NC_056057.1	62265001	62285001	0.384811	0.165422	HZ	AOAH
NC_056057.1	62270001	62290001	0.358273	0.18872	HZ	AOAH
NC_056059.1	13660001	13680001	0.370754	0.190664	HZ	AP1AR
NC_056059.1	13665001	13685001	0.372409	0.179889	HZ	AP1AR
NC_056067.1	38780001	38800001	0.502027	0.189873	HZ	AP1G1
NC_056067.1	38785001	38805001	0.496913	0.213025	HZ	AP1G1
NC_056059.1	60505001	60525001	0.368882	0.153196	HZ	APBB2
NC_056076.1	42635001	42655001	0.24617	0.21304	HZ	APCDD1
NC_056076.1	42640001	42660001	0.196378	0.292324	HZ	APCDD1
NC_056076.1	42645001	42665001	0.184309	0.321606	HZ	APCDD1
NC_056076.1	42650001	42670001	0.156731	0.357143	HZ	APCDD1
NC_056076.1	42655001	42675001	0.162572	0.265643	HZ	APCDD1
NC_056076.1	42660001	42680001	0.11893	0.176822	HZ	APCDD1
NC_056076.1	42665001	42685001	0.068134	0.189817	HZ	APCDD1
NC_056076.1	42670001	42690001	0.058948	0.240709	HZ	APCDD1;NAPG
NC_056056.1	216365001	2.16E+08	0.079108	0.148	HZ	APOBEC3F
NC_056056.1	216370001	2.16E+08	0.08556	0.146767	HZ	APOBEC3F
NC_056056.1	216375001	2.16E+08	0.069368	0.149398	HZ	APOBEC3F
NC_056074.1	15960001	15980001	0.287872	0.291223	HZ	AQP11
NC_056074.1	15965001	15985001	0.33495	0.240432	HZ	AQP11
NC_056074.1	15970001	15990001	0.339655	0.182576	HZ	AQP11
NC_056074.1	15950001	15970001	0.259556	0.284805	HZ	AQP11;CLNS1A
NC_056074.1	15955001	15975001	0.280169	0.296062	HZ	AQP11;CLNS1A
NC_056070.1	5020001	5040001	0.422442	0.145598	HZ	ARFIP1
NC_056070.1	5025001	5045001	0.203785	0.242865	HZ	ARFIP1
NC_056070.1	5030001	5050001	0.176378	0.218009	HZ	ARFIP1
NC_056070.1	5035001	5055001	0.254382	0.181125	HZ	ARFIP1
NC_056055.1	166440001	1.66E+08	0.448684	0.149223	HZ	ARHGAP15
NC_056055.1	166570001	1.67E+08	0.301692	0.184751	HZ	ARHGAP15
NC_056055.1	166575001	1.67E+08	0.148481	0.290723	HZ	ARHGAP15
NC_056055.1	166640001	1.67E+08	0.094629	0.28094	HZ	ARHGAP15

NC_056055.1	166645001	1.67E+08	0.15875	0.250358	HZ	ARHGAP15
NC_056068.1	20400001	20420001	0.385031	0.147233	HZ	ARHGAP20
NC_056068.1	20405001	20425001	0.385915	0.174848	HZ	ARHGAP20
NC_056068.1	20410001	20430001	0.449046	0.186865	HZ	ARHGAP20
NC_056068.1	20415001	20435001	0.435561	0.194245	HZ	ARHGAP20
NC_056059.1	101445001	1.01E+08	0.40185	0.185213	HZ	ARHGAP24
NC_056059.1	101450001	1.01E+08	0.395251	0.207915	HZ	ARHGAP24
NC_056059.1	101455001	1.01E+08	0.370548	0.203334	HZ	ARHGAP24
NC_056059.1	101460001	1.01E+08	0.409125	0.200693	HZ	ARHGAP24
NC_056059.1	101465001	1.01E+08	0.443428	0.178902	HZ	ARHGAP24
NC_056064.1	31040001	31060001	0.444505	0.146052	HZ	ARHGAP44
NC_056064.1	31050001	31070001	0.280455	0.16053	HZ	ARHGAP44
NC_056069.1	7955001	7975001	0.385614	0.150253	HZ	ARHGEF28
NC_056061.1	81210001	81230001	0.279357	0.212284	HZ	ARID1B
NC_056061.1	81215001	81235001	0.250701	0.253006	HZ	ARID1B
NC_056061.1	81220001	81240001	0.254647	0.267914	HZ	ARID1B
NC_056061.1	81225001	81245001	0.296792	0.252462	HZ	ARID1B
NC_056056.1	140650001	1.41E+08	0.448728	0.165085	HZ	ARID2
NC_056056.1	140655001	1.41E+08	0.487449	0.194787	HZ	ARID2
NC_056060.1	19190001	19210001	0.411418	0.247293	HZ	ARIH1
NC_056060.1	19195001	19215001	0.341591	0.250316	HZ	ARIH1
NC_056060.1	19200001	19220001	0.14427	0.352981	HZ	ARIH1
NC_056060.1	19205001	19225001	0.149888	0.354608	HZ	ARIH1
NC_056060.1	19210001	19230001	0.332884	0.225253	HZ	ARIH1
NC_056060.1	19215001	19235001	0.360051	0.198959	HZ	ARIH1
NC_056060.1	19225001	19245001	0.458333	0.159793	HZ	ARIH1
NC_056060.1	19235001	19255001	0.460947	0.198662	HZ	ARIH1
NC_056060.1	19240001	19260001	0.4625	0.198292	HZ	ARIH1
NC_056060.1	19245001	19265001	0.450098	0.162003	HZ	ARIH1
NC_056060.1	19295001	19315001	0.452586	0.146222	HZ	ARIH1
NC_056060.1	19300001	19320001	0.487974	0.154846	HZ	ARIH1
NC_056060.1	19305001	19325001	0.499416	0.159808	HZ	ARIH1;BBS4
NC_056059.1	72610001	72630001	0.238651	0.182767	HZ	ARL9
NC_056059.1	72615001	72635001	0.098736	0.19635	HZ	ARL9;THEGL
NC_056059.1	72620001	72640001	0.070143	0.202262	HZ	ARL9;THEGL
NC_056059.1	72625001	72645001	0.057001	0.189979	HZ	ARL9;THEGL

NC_056054.1	19365001	19385001	0.285358	0.154747	HZ	ARMH1
NC_056054.1	19370001	19390001	0.144099	0.212017	HZ	ARMH1
NC_056054.1	19375001	19395001	0.124009	0.18699	HZ	ARMH1
NC_056061.1	76275001	76295001	0.156074	0.228685	HZ	ARMT1;RMND1
NC_056061.1	76280001	76300001	0.385807	0.146873	HZ	ARMT1;RMND1
NC_056079.1	32385001	32405001	0.421843	0.155436	HZ	ASH2L
NC_056079.1	32390001	32410001	0.477531	0.181481	HZ	ASH2L;STAR
NC_056055.1	7155001	7175001	0.4395	0.17748	HZ	ASTN2
NC_056056.1	135535001	1.36E+08	0.342881	0.244737	HZ	ATF1
NC_056056.1	135545001	1.36E+08	0.265072	0.214257	HZ	ATF1
NC_056056.1	135550001	1.36E+08	0.444063	0.166345	HZ	ATF1
NC_056068.1	75520001	75540001	0.380953	0.168915	HZ	ATG13
NC_056060.1	41665001	41685001	0.2265	0.156391	HZ	ATL1;LOC121820086
NC_056060.1	41660001	41680001	0.203703	0.145576	HZ	ATL1;LOC121820086;MAP4K5
NC_056063.1	36900001	36920001	0.197573	0.188143	HZ	ATP12A
NC_056072.1	54335001	54355001	0.119464	0.153239	HZ	ATP2B2
NC_056072.1	54650001	54670001	0.491545	0.209471	HZ	ATP2B2
NC_056072.1	54655001	54675001	0.363423	0.275326	HZ	ATP2B2
NC_056072.1	54660001	54680001	0.350757	0.300816	HZ	ATP2B2
NC_056072.1	54665001	54685001	0.415461	0.236433	HZ	ATP2B2
NC_056059.1	61995001	62015001	0.463446	0.255104	HZ	ATP8A1
NC_056059.1	62000001	62020001	0.328375	0.2253	HZ	ATP8A1
NC_056059.1	62005001	62025001	0.343291	0.184463	HZ	ATP8A1
NC_056076.1	57365001	57385001	0.123226	0.174112	HZ	ATP8B1
NC_056056.1	221945001	2.22E+08	0.247537	0.150198	HZ	ATXN10
NC_056056.1	221975001	2.22E+08	0.078842	0.199045	HZ	ATXN10
NC_056056.1	221980001	2.22E+08	0.186073	0.15914	HZ	ATXN10
NC_056056.1	221985001	2.22E+08	0.187807	0.158886	HZ	ATXN10
NC_056056.1	221995001	2.22E+08	0.252042	0.152588	HZ	ATXN10
NC_056056.1	222000001	2.22E+08	0.204485	0.17195	HZ	ATXN10
NC_056057.1	48285001	48305001	0.064043	0.187725	HZ	ATXN7L1
NC_056077.1	30865001	30885001	0.365509	0.294752	HZ	AUTS2
NC_056077.1	30870001	30890001	0.303428	0.287807	HZ	AUTS2
NC_056077.1	30875001	30895001	0.320468	0.271814	HZ	AUTS2
NC_056077.1	30880001	30900001	0.394269	0.249163	HZ	AUTS2
NC_056077.1	30885001	30905001	0.497969	0.212513	HZ	AUTS2

NC_056077.1	30890001	30910001	0.461249	0.186459	HZ	AUTS2
NC_056077.1	30895001	30915001	0.45852	0.175553	HZ	AUTS2
NC_056080.1	63130001	63150001	0.381753	0.176911	HZ	AWAT1;P2RY4
NC_056055.1	142145001	1.42E+08	0.499045	0.151051	HZ	B3GALT1
NC_056055.1	142150001	1.42E+08	0.304391	0.231565	HZ	B3GALT1
NC_056055.1	142155001	1.42E+08	0.385748	0.208863	HZ	B3GALT1
NC_056055.1	142160001	1.42E+08	0.445742	0.178425	HZ	B3GALT1
NC_056064.1	37170001	37190001	0.298032	0.165	HZ	B4GALNT2
NC_056054.1	184920001	1.85E+08	0.46423	0.172072	HZ	B4GALT4
NC_056072.1	51640001	51660001	0.382459	0.18473	HZ	BAC5;CATHL3
NC_056072.1	51635001	51655001	0.298206	0.216606	HZ	BAC5;CATHL3;LOC105607776
NC_056077.1	1585001	1605001	0.402698	0.270477	HZ	BAIAP3;GNPTG;TSR3
NC_056057.1	64845001	64865001	0.460545	0.233763	HZ	BBS9
NC_056070.1	62535001	62555001	0.312433	0.158308	HZ	BICDL1
NC_056070.1	62540001	62560001	0.365157	0.198865	HZ	BICDL1
NC_056070.1	62545001	62565001	0.391385	0.209928	HZ	BICDL1
NC_056070.1	62550001	62570001	0.383303	0.198573	HZ	BICDL1
NC_056070.1	62555001	62575001	0.373334	0.168192	HZ	BICDL1
NC_056070.1	62565001	62585001	0.401191	0.15756	HZ	BICDL1
NC_056070.1	62580001	62600001	0.364974	0.154971	HZ	BICDL1
NC_056070.1	62585001	62605001	0.367317	0.163293	HZ	BICDL1
NC_056070.1	62590001	62610001	0.403201	0.178994	HZ	BICDL1
NC_056070.1	62595001	62615001	0.425993	0.182359	HZ	BICDL1;RAB35
NC_056070.1	62600001	62620001	0.463008	0.200067	HZ	BICDL1;RAB35
NC_056070.1	62605001	62625001	0.464108	0.254334	HZ	BICDL1;RAB35
NC_056070.1	62610001	62630001	0.44587	0.286613	HZ	BICDL1;RAB35
NC_056055.1	85025001	85045001	0.19961	0.162522	HZ	BNC2
NC_056054.1	99995001	1E+08	0.303863	0.165872	HZ	BOLA1;SV2A
NC_056054.1	100000001	1E+08	0.224812	0.237162	HZ	BOLA1;SV2A
NC_056054.1	100005001	1E+08	0.17343	0.226001	HZ	BOLA1;SV2A
NC_056063.1	48090001	48110001	0.32623	0.248454	HZ	BORA
NC_056063.1	48095001	48115001	0.15738	0.351191	HZ	BORA
NC_056063.1	48100001	48120001	0.081836	0.392372	HZ	BORA;DIS3
NC_056063.1	48085001	48105001	0.493765	0.183434	HZ	BORA;MZT1
NC_056058.1	7615001	7635001	0.107635	0.178054	HZ	BRD4
NC_056054.1	29360001	29380001	0.367293	0.151287	HZ	BSND;TMEM61

NC_056055.1	10340001	10360001	0.421748	0.250276	HZ	BSPRY
NC_056055.1	10345001	10365001	0.396016	0.285246	HZ	BSPRY
NC_056055.1	10350001	10370001	0.465994	0.315389	HZ	BSPRY
NC_056054.1	232625001	2.33E+08	0.434489	0.158368	HZ	C1H3orf33
NC_056073.1	44620001	44640001	0.045174	0.149055	HZ	C20H6orf52;PAK1IP1
NC_056073.1	44625001	44645001	0.109631	0.153001	HZ	C20H6orf52;PAK1IP1
NC_056068.1	52170001	52190001	0.323359	0.157866	HZ	C2CD3
NC_056068.1	52175001	52195001	0.282093	0.223036	HZ	C2CD3
NC_056068.1	52180001	52200001	0.419738	0.20592	HZ	C2CD3
NC_056068.1	52185001	52205001	0.382352	0.186569	HZ	C2CD3
NC_056068.1	52190001	52210001	0.276182	0.153209	HZ	C2CD3
NC_056054.1	101770001	1.02E+08	0.350214	0.157504	HZ	C2CD4D;THEM5
NC_056072.1	47255001	47275001	0.485751	0.318792	HZ	CACNA1D;CHDH
NC_056057.1	39555001	39575001	0.169299	0.181419	HZ	CACNA2D1
NC_056057.1	39570001	39590001	0.292907	0.172297	HZ	CACNA2D1
NC_056057.1	39575001	39595001	0.456688	0.155416	HZ	CACNA2D1
NC_056054.1	154255001	1.54E+08	0.446577	0.165645	HZ	CADM2
NC_056054.1	154260001	1.54E+08	0.490726	0.164649	HZ	CADM2
NC_056057.1	88755001	88775001	0.496657	0.154438	HZ	CADPS2
NC_056057.1	88760001	88780001	0.47648	0.166015	HZ	CADPS2
NC_056057.1	88765001	88785001	0.468282	0.234329	HZ	CADPS2
NC_056057.1	88725001	88745001	0.49433	0.167606	HZ	CADPS2;RNF133
NC_056068.1	37265001	37285001	0.421466	0.213237	HZ	CALCA
NC_056059.1	12190001	12210001	0.356335	0.153061	HZ	CAMK2D
NC_056065.1	44985001	45005001	0.456484	0.16522	HZ	CAMTA1
NC_056065.1	44970001	44990001	0.498064	0.146566	HZ	CAMTA1;VAMP3
NC_056068.1	55230001	55250001	0.482413	0.167253	HZ	CAPN5;LOC101113008
NC_056080.1	41005001	41025001	0.493373	0.21903	HZ	CASK
NC_056080.1	41010001	41030001	0.455721	0.234339	HZ	CASK
NC_056072.1	51620001	51640001	0.09046	0.288655	HZ	CATHL1B;LOC105607776
NC_056072.1	51625001	51645001	0.269847	0.232351	HZ	CATHL3;LOC105607776
NC_056072.1	51630001	51650001	0.264571	0.231964	HZ	CATHL3;LOC105607776
NC_056055.1	131035001	1.31E+08	0.235196	0.166463	HZ	CCDC141
NC_056055.1	131040001	1.31E+08	0.141925	0.161633	HZ	CCDC141
NC_056055.1	131045001	1.31E+08	0.174531	0.151841	HZ	CCDC141
NC_056064.1	23490001	23510001	0.426426	0.180949	HZ	CCDC92B

NC_056064.1	23485001	23505001	0.49813	0.155846	HZ	CCDC92B;CLUH
NC_056064.1	15485001	15505001	0.37349	0.161542	HZ	CCL1
NC_056064.1	15490001	15510001	0.401277	0.204265	HZ	CCL1
NC_056064.1	15495001	15515001	0.462833	0.224305	HZ	CCL1
NC_056064.1	15500001	15520001	0.474804	0.317224	HZ	CCL1
NC_056069.1	10625001	10645001	0.457717	0.158009	HZ	CCNB1;SLC30A5
NC_056060.1	53080001	53100001	0.478799	0.1571	HZ	CCPG1
NC_056060.1	53085001	53105001	0.495783	0.15016	HZ	CCPG1;PIGB
NC_056056.1	95255001	95275001	0.479999	0.196639	HZ	CCT7;FBXO41
NC_056056.1	95250001	95270001	0.457625	0.219594	HZ	CCT7;PRADC1
NC_056056.1	95245001	95265001	0.471211	0.205928	HZ	CCT7;PRADC1;SMYD5
NC_056054.1	177265001	1.77E+08	0.383852	0.162893	HZ	CD96
NC_056054.1	177270001	1.77E+08	0.27635	0.192681	HZ	CD96
NC_056054.1	177275001	1.77E+08	0.409057	0.163445	HZ	CD96
NC_056072.1	51685001	51705001	0.149055	0.191103	HZ	CDC25A
NC_056072.1	51690001	51710001	0.334395	0.163102	HZ	CDC25A
NC_056074.1	39970001	39990001	0.404297	0.189101	HZ	CDC42EP2;DPF2
NC_056058.1	20840001	20860001	0.454544	0.301667	HZ	CDC42SE2
NC_056058.1	20845001	20865001	0.475675	0.299261	HZ	CDC42SE2
NC_056058.1	20855001	20875001	0.431006	0.274862	HZ	CDC42SE2
NC_056058.1	20860001	20880001	0.372711	0.218517	HZ	CDC42SE2
NC_056058.1	20865001	20885001	0.339317	0.150506	HZ	CDC42SE2
NC_056069.1	53870001	53890001	0.486954	0.151976	HZ	CDH18
NC_056069.1	54045001	54065001	0.287912	0.154684	HZ	CDH18
NC_056069.1	54115001	54135001	0.145236	0.174666	HZ	CDH18
NC_056069.1	54120001	54140001	0.231542	0.155806	HZ	CDH18
NC_056066.1	62855001	62875001	0.153846	0.146165	HZ	CDK5RAP1
NC_056066.1	62860001	62880001	0.149368	0.163554	HZ	CDK5RAP1
NC_056066.1	62865001	62885001	0.188794	0.15203	HZ	CDK5RAP1
NC_056060.1	41520001	41540001	0.490509	0.191469	HZ	CDKL1
NC_056054.1	25425001	25445001	0.206892	0.161271	HZ	CDKN2C;FAF1
NC_056060.1	65015001	65035001	0.480175	0.309302	HZ	CDKN3;CNIH1
NC_056077.1	20540001	20560001	0.053855	0.182284	HZ	CDR2;POLR3E
NC_056068.1	27730001	27750001	0.353214	0.174135	HZ	CEP164
NC_056068.1	27735001	27755001	0.443152	0.29319	HZ	CEP164
NC_056068.1	27740001	27760001	0.494708	0.308247	HZ	CEP164

NC_056066.1	64675001	64695001	0.404005	0.457536	HZ	CEP250
NC_056066.1	64670001	64690001	0.426155	0.432004	HZ	CEP250;GDF5
NC_056065.1	60575001	60595001	0.407033	0.246023	HZ	CEP350
NC_056065.1	60580001	60600001	0.373964	0.222539	HZ	CEP350
NC_056065.1	60585001	60605001	0.455411	0.177729	HZ	CEP350
NC_056057.1	95805001	95825001	0.36501	0.207361	HZ	CEP41
NC_056057.1	95810001	95830001	0.391905	0.186052	HZ	CEP41
NC_056057.1	95815001	95835001	0.405215	0.175195	HZ	CEP41
NC_056057.1	95820001	95840001	0.367161	0.173262	HZ	CEP41
NC_056057.1	95825001	95845001	0.299051	0.206516	HZ	CEP41
NC_056057.1	95830001	95850001	0.232425	0.217328	HZ	CEP41
NC_056057.1	95835001	95855001	0.203436	0.214758	HZ	CEP41
NC_056057.1	95840001	95860001	0.265487	0.260947	HZ	CEP41
NC_056057.1	95845001	95865001	0.314113	0.266175	HZ	CEP41
NC_056057.1	95850001	95870001	0.263599	0.340829	HZ	CEP41
NC_056057.1	95855001	95875001	0.198616	0.462197	HZ	CEP41
NC_056057.1	95790001	95810001	0.276812	0.248296	HZ	CEP41;CPA1
NC_056057.1	95795001	95815001	0.270098	0.241913	HZ	CEP41;CPA1
NC_056057.1	95800001	95820001	0.334507	0.221839	HZ	CEP41;CPA1
NC_056072.1	42695001	42715001	0.382326	0.169655	HZ	CFAP20DC
NC_056054.1	185315001	1.85E+08	0.273752	0.224309	HZ	CFAP91
NC_056054.1	185320001	1.85E+08	0.314952	0.245207	HZ	CFAP91
NC_056054.1	185325001	1.85E+08	0.333146	0.262071	HZ	CFAP91
NC_056054.1	185330001	1.85E+08	0.288337	0.189901	HZ	CFAP91
NC_056054.1	185340001	1.85E+08	0.334972	0.158851	HZ	CFAP91
NC_056054.1	185345001	1.85E+08	0.365199	0.176662	HZ	CFAP91
NC_056054.1	185350001	1.85E+08	0.49546	0.179729	HZ	CFAP91
NC_056064.1	43400001	43420001	0.29328	0.457379	HZ	CFAP97D1;DUSP3
NC_056064.1	43405001	43425001	0.256696	0.400413	HZ	CFAP97D1;DUSP3
NC_056064.1	43410001	43430001	0.351983	0.300525	HZ	CFAP97D1;DUSP3
NC_056059.1	116930001	1.17E+08	0.493871	0.2273	HZ	CFAP99;LOC114115254;RNF4
NC_056061.1	50155001	50175001	0.372041	0.25479	HZ	CGA
NC_056057.1	68245001	68265001	0.137333	0.160071	HZ	CHN2
NC_056057.1	68515001	68535001	0.349381	0.1472	HZ	CHN2
NC_056056.1	104090001	1.04E+08	0.405387	0.149458	HZ	CIAO1;SNRNP200;TMEM127
NC_056056.1	104095001	1.04E+08	0.409459	0.155307	HZ	CIAO1;TMEM127

NC_056064.1	39065001	39085001	0.164746	0.177148	HZ	CISD3;MLLT6
NC_056057.1	8545001	8565001	0.409723	0.158531	HZ	CLDN12
NC_056057.1	8550001	8570001	0.437254	0.194604	HZ	CLDN12
NC_056079.1	13355001	13375001	0.426691	0.181733	HZ	CLDN22;LOC106990148;WWC2
NC_056055.1	241885001	2.42E+08	0.19702	0.160851	HZ	CLIC4
NC_056055.1	241890001	2.42E+08	0.122152	0.182065	HZ	CLIC4
NC_056055.1	241895001	2.42E+08	0.142797	0.168564	HZ	CLIC4
NC_056074.1	15925001	15945001	0.354122	0.209679	HZ	CLNS1A
NC_056074.1	15930001	15950001	0.383413	0.228923	HZ	CLNS1A
NC_056074.1	15935001	15955001	0.470017	0.228534	HZ	CLNS1A
NC_056074.1	15940001	15960001	0.419617	0.213543	HZ	CLNS1A
NC_056074.1	15945001	15965001	0.336186	0.246609	HZ	CLNS1A
NC_056074.1	15920001	15940001	0.360992	0.205262	HZ	CLNS1A;RSF1
NC_056064.1	23465001	23485001	0.228601	0.224159	HZ	CLUH
NC_056064.1	23470001	23490001	0.329506	0.15696	HZ	CLUH
NC_056064.1	23475001	23495001	0.368582	0.152141	HZ	CLUH
NC_056064.1	23460001	23480001	0.330908	0.203833	HZ	CLUH;PAFAH1B1
NC_056067.1	34005001	34025001	0.458203	0.167204	HZ	CMTM4
NC_056067.1	34010001	34030001	0.448939	0.159317	HZ	CMTM4;DYNC1LI2
NC_056054.1	71985001	72005001	0.281468	0.227542	HZ	CNN3;SLC44A3
NC_056072.1	23370001	23390001	0.067797	0.148657	HZ	CNTN4
NC_056072.1	24975001	24995001	0.423013	0.180934	HZ	CNTN6
NC_056072.1	24980001	25000001	0.374584	0.202426	HZ	CNTN6
NC_056072.1	24985001	25005001	0.48354	0.172332	HZ	CNTN6
NC_056057.1	112195001	1.12E+08	0.371889	0.258383	HZ	CNTNAP2
NC_056057.1	112200001	1.12E+08	0.299145	0.284453	HZ	CNTNAP2
NC_056057.1	112205001	1.12E+08	0.394463	0.226187	HZ	CNTNAP2
NC_056057.1	112210001	1.12E+08	0.444772	0.180854	HZ	CNTNAP2
NC_056055.1	189510001	1.9E+08	0.25833	0.28166	HZ	CNTNAP5
NC_056055.1	189515001	1.9E+08	0.288433	0.181977	HZ	CNTNAP5
NC_056080.1	126475001	1.26E+08	0.333334	0.233743	HZ	COL4A5
NC_056054.1	14925001	14945001	0.450399	0.166173	HZ	COL9A2
NC_056058.1	35365001	35385001	0.418843	0.169583	HZ	COMMD10
NC_056058.1	35370001	35390001	0.29543	0.172375	HZ	COMMD10
NC_056057.1	96065001	96085001	0.338278	0.170635	HZ	COPG2
NC_056057.1	96080001	96100001	0.285404	0.201551	HZ	COPG2;TSGA13

NC_056057.1	96085001	96105001	0.294118	0.200343	HZ	COPG2;TSGA13
NC_056057.1	96090001	96110001	0.38619	0.224407	HZ	COPG2;TSGA13
NC_056057.1	95780001	95800001	0.333938	0.199074	HZ	CPA1;CPA5
NC_056057.1	95785001	95805001	0.295454	0.202346	HZ	CPA1;CPA5
NC_056057.1	95775001	95795001	0.354838	0.168185	HZ	CPA5
NC_056069.1	37205001	37225001	0.268524	0.191685	HZ	CPLANE1
NC_056069.1	37210001	37230001	0.343863	0.163915	HZ	CPLANE1
NC_056055.1	212195001	2.12E+08	0.348523	0.146338	HZ	CPS1
NC_056055.1	212200001	2.12E+08	0.246715	0.200322	HZ	CPS1
NC_056055.1	212205001	2.12E+08	0.317346	0.212451	HZ	CPS1
NC_056055.1	212210001	2.12E+08	0.429449	0.193651	HZ	CPS1
NC_056057.1	67195001	67215001	0.205105	0.196269	HZ	CRHR2
NC_056057.1	67200001	67220001	0.243733	0.191405	HZ	CRHR2
NC_056077.1	19545001	19565001	0.288972	0.188108	HZ	CRYM
NC_056077.1	19550001	19570001	0.275484	0.221499	HZ	CRYM
NC_056077.1	19555001	19575001	0.254349	0.232349	HZ	CRYM
NC_056079.1	2660001	2680001	0.493527	0.175188	HZ	CSMD1
NC_056079.1	2670001	2690001	0.47525	0.149699	HZ	CSMD1
NC_056079.1	2680001	2700001	0.46597	0.228803	HZ	CSMD1
NC_056079.1	2685001	2705001	0.412023	0.332912	HZ	CSMD1
NC_056079.1	2690001	2710001	0.492041	0.40561	HZ	CSMD1
NC_056079.1	2700001	2720001	0.49231	0.35199	HZ	CSMD1
NC_056079.1	2705001	2725001	0.426777	0.281608	HZ	CSMD1
NC_056079.1	2710001	2730001	0.329033	0.184217	HZ	CSMD1
NC_056054.1	8810001	8830001	0.482722	0.168653	HZ	CSMD2
NC_056064.1	50095001	50115001	0.362784	0.146379	HZ	CSNK1D
NC_056075.1	44040001	44060001	0.278284	0.154936	HZ	CTBP2
NC_056075.1	44100001	44120001	0.436653	0.162597	HZ	CTBP2
NC_056075.1	44035001	44055001	0.372785	0.150064	HZ	CTBP2;ZRANB1
NC_056062.1	74040001	74060001	0.446087	0.248633	HZ	CTHRC1
NC_056062.1	74045001	74065001	0.431673	0.285001	HZ	CTHRC1
NC_056078.1	22415001	22435001	0.442453	0.215034	HZ	CTNNA3
NC_056078.1	22420001	22440001	0.213059	0.408812	HZ	CTNNA3
NC_056078.1	22425001	22445001	0.129315	0.499516	HZ	CTNNA3
NC_056078.1	22430001	22450001	0.10089	0.530558	HZ	CTNNA3
NC_056078.1	22435001	22455001	0.111609	0.523796	HZ	CTNNA3

NC_056078.1	22440001	22460001	0.139547	0.498949	HZ	CTNNA3
NC_056078.1	22445001	22465001	0.303295	0.369698	HZ	CTNNA3
NC_056078.1	22450001	22470001	0.374106	0.338566	HZ	CTNNA3
NC_056078.1	22455001	22475001	0.300206	0.373746	HZ	CTNNA3
NC_056078.1	22460001	22480001	0.302314	0.38506	HZ	CTNNA3
NC_056078.1	22465001	22485001	0.203303	0.461029	HZ	CTNNA3
NC_056078.1	22470001	22490001	0.237883	0.426213	HZ	CTNNA3
NC_056078.1	22475001	22495001	0.195522	0.381595	HZ	CTNNA3
NC_056078.1	22480001	22500001	0.17114	0.365751	HZ	CTNNA3
NC_056078.1	22485001	22505001	0.224997	0.304399	HZ	CTNNA3
NC_056078.1	22490001	22510001	0.319379	0.174029	HZ	CTNNA3
NC_056072.1	13810001	13830001	0.407342	0.339087	HZ	CTNNB1
NC_056072.1	13815001	13835001	0.267457	0.225295	HZ	CTNNB1
NC_056072.1	13820001	13840001	0.307797	0.259669	HZ	CTNNB1
NC_056072.1	13825001	13845001	0.365576	0.264517	HZ	CTNNB1
NC_056072.1	13830001	13850001	0.44334	0.269142	HZ	CTNNB1
NC_056059.1	68295001	68315001	0.295401	0.155778	HZ	CWH43
NC_056059.1	68300001	68320001	0.280295	0.176631	HZ	CWH43
NC_056055.1	220195001	2.2E+08	0.420057	0.145524	HZ	CXCR2
NC_056075.1	15635001	15655001	0.337094	0.168837	HZ	CYP2C18
NC_056075.1	15640001	15660001	0.257797	0.149043	HZ	CYP2C18
NC_056063.1	46755001	46775001	0.333334	0.186615	HZ	DACH1
NC_056063.1	46760001	46780001	0.164468	0.188916	HZ	DACH1
NC_056063.1	46765001	46785001	0.124605	0.202141	HZ	DACH1
NC_056063.1	46770001	46790001	0.154534	0.208413	HZ	DACH1
NC_056063.1	47030001	47050001	0.497006	0.214399	HZ	DACH1
NC_056063.1	47035001	47055001	0.446114	0.216508	HZ	DACH1
NC_056063.1	47040001	47060001	0.319333	0.189294	HZ	DACH1
NC_056063.1	47045001	47065001	0.331878	0.167238	HZ	DACH1
NC_056060.1	22035001	22055001	0.347538	0.158384	HZ	DAD1
NC_056060.1	22040001	22060001	0.318891	0.16857	HZ	DAD1
NC_056060.1	22045001	22065001	0.314003	0.172252	HZ	DAD1
NC_056074.1	36680001	36700001	0.47858	0.196369	HZ	DAGLA
NC_056074.1	36685001	36705001	0.472923	0.185818	HZ	DAGLA
NC_056074.1	36690001	36710001	0.491107	0.191115	HZ	DAGLA
NC_056055.1	32015001	32035001	0.474124	0.192065	HZ	DAPK1

NC_056055.1	32020001	32040001	0.483511	0.235466	HZ	DAPK1
NC_056054.1	111275001	1.11E+08	0.222222	0.194568	HZ	DCAF8
NC_056054.1	111280001	1.11E+08	0.274845	0.195703	HZ	DCAF8
NC_056054.1	111285001	1.11E+08	0.289799	0.171934	HZ	DCAF8;PEX19
NC_056076.1	52630001	52650001	0.4401	0.221276	HZ	DCC
NC_056076.1	52635001	52655001	0.492689	0.241472	HZ	DCC
NC_056063.1	25980001	26000001	0.18718	0.212992	HZ	DCLK1
NC_056059.1	24840001	24860001	0.315894	0.1507	HZ	DDIT4L
NC_056056.1	215995001	2.16E+08	0.445704	0.202286	HZ	DDX17
NC_056056.1	216000001	2.16E+08	0.43387	0.198335	HZ	DDX17
NC_056056.1	216005001	2.16E+08	0.350939	0.21776	HZ	DDX17;DMC1
NC_056056.1	215975001	2.16E+08	0.314588	0.165175	HZ	DDX17;KDELR3
NC_056056.1	215980001	2.16E+08	0.348571	0.180829	HZ	DDX17;KDELR3
NC_056056.1	215985001	2.16E+08	0.384552	0.184892	HZ	DDX17;KDELR3
NC_056056.1	215990001	2.16E+08	0.419025	0.184016	HZ	DDX17;KDELR3
NC_056067.1	14255001	14275001	0.184163	0.352464	HZ	DEF8
NC_056067.1	14260001	14280001	0.114435	0.408257	HZ	DEF8
NC_056067.1	14265001	14285001	0.077148	0.502539	HZ	DEF8;LOC101113004
NC_056067.1	14275001	14295001	0.305628	0.48523	HZ	DEF8;LOC101113004;LOC101113264
NC_056067.1	14250001	14270001	0.281651	0.246958	HZ	DEF8;MC1R
NC_056065.1	76165001	76185001	0.380372	0.281869	HZ	DENND1B
NC_056065.1	76170001	76190001	0.338979	0.335807	HZ	DENND1B
NC_056065.1	76175001	76195001	0.29546	0.330645	HZ	DENND1B
NC_056065.1	76180001	76200001	0.409862	0.252712	HZ	DENND1B
NC_056080.1	54940001	54960001	0.3517	0.198637	HZ	DGKK
NC_056064.1	34925001	34945001	0.492907	0.165905	HZ	DHRS7B
NC_056080.1	137585001	1.38E+08	0.45903	0.160414	HZ	DIAPH2
NC_056080.1	137590001	1.38E+08	0.394413	0.176737	HZ	DIAPH2
NC_056080.1	137825001	1.38E+08	0.086583	0.156235	HZ	DIAPH2
NC_056080.1	137880001	1.38E+08	0.244281	0.160848	HZ	DIAPH2
NC_056066.1	46095001	46115001	0.414039	0.147619	HZ	DIP2C
NC_056063.1	48115001	48135001	0.17535	0.295496	HZ	DIS3
NC_056078.1	4080001	4100001	0.374571	0.150226	HZ	DISC1
NC_056078.1	4085001	4105001	0.474432	0.159926	HZ	DISC1
NC_056079.1	22760001	22780001	0.050676	0.152597	HZ	DLC1
NC_056079.1	22765001	22785001	0.272707	0.167938	HZ	DLC1

NC_056056.1	216010001	2.16E+08	0.281829	0.214104	HZ	DMC1
NC_056056.1	216015001	2.16E+08	0.220762	0.182675	HZ	DMC1
NC_056056.1	216020001	2.16E+08	0.169154	0.16359	HZ	DMC1
NC_056060.1	40915001	40935001	0.439042	0.150744	HZ	DNAAF2;POLE2
NC_056065.1	27555001	27575001	0.468638	0.157972	HZ	DNAH14
NC_056077.1	19220001	19240001	0.487499	0.30474	HZ	DNAH3
NC_056077.1	19200001	19220001	0.352969	0.314924	HZ	DNAH3;LYRM1
NC_056077.1	19205001	19225001	0.480898	0.307536	HZ	DNAH3;LYRM1
NC_056068.1	52105001	52125001	0.175769	0.156125	HZ	DNAJB13
NC_056068.1	52100001	52120001	0.23801	0.149077	HZ	DNAJB13;PAAF1
NC_056068.1	52110001	52130001	0.061207	0.170939	HZ	DNAJB13;UCP2
NC_056055.1	11870001	11890001	0.485811	0.263794	HZ	DNAJC25
NC_056065.1	38670001	38690001	0.484279	0.17812	HZ	DNM3
NC_056065.1	38740001	38760001	0.351701	0.210199	HZ	DNM3
NC_056065.1	38765001	38785001	0.443428	0.243289	HZ	DNM3
NC_056065.1	38770001	38790001	0.286178	0.162926	HZ	DNM3
NC_056075.1	46205001	46225001	0.443689	0.226347	HZ	DOCK1
NC_056075.1	46210001	46230001	0.384203	0.192701	HZ	DOCK1
NC_056075.1	46345001	46365001	0.418237	0.222052	HZ	DOCK1
NC_056075.1	46350001	46370001	0.432462	0.187673	HZ	DOCK1
NC_056063.1	75300001	75320001	0.450305	0.162956	HZ	DOCK9
NC_056063.1	75305001	75325001	0.465448	0.200445	HZ	DOCK9
NC_056063.1	75310001	75330001	0.185476	0.271604	HZ	DOCK9
NC_056063.1	75315001	75335001	0.21417	0.258137	HZ	DOCK9
NC_056063.1	75320001	75340001	0.243827	0.220898	HZ	DOCK9
NC_056063.1	75325001	75345001	0.196566	0.2396	HZ	DOCK9
NC_056063.1	75330001	75350001	0.316233	0.200184	HZ	DOCK9
NC_056063.1	75335001	75355001	0.309909	0.213372	HZ	DOCK9
NC_056063.1	75340001	75360001	0.348854	0.23396	HZ	DOCK9
NC_056063.1	75345001	75365001	0.311834	0.24271	HZ	DOCK9
NC_056074.1	39975001	39995001	0.346899	0.247384	HZ	DPF2
NC_056074.1	39980001	40000001	0.469575	0.20245	HZ	DPF2;TIGD3
NC_056056.1	171270001	1.71E+08	0.494273	0.178378	HZ	DRAM1
NC_056056.1	171275001	1.71E+08	0.305408	0.234356	HZ	DRAM1
NC_056056.1	171280001	1.71E+08	0.357326	0.243132	HZ	DRAM1
NC_056056.1	171285001	1.71E+08	0.358503	0.239821	HZ	DRAM1

NC_056054.1	261095001	2.61E+08	0.450271	0.173628	HZ	DSCAM
NC_056054.1	261100001	2.61E+08	0.230612	0.221959	HZ	DSCAM
NC_056054.1	261105001	2.61E+08	0.360084	0.157974	HZ	DSCAM
NC_056064.1	43395001	43415001	0.387374	0.45341	HZ	DUSP3;SOST
NC_056061.1	82770001	82790001	0.380282	0.157761	HZ	DYNLT1;TMEM181
NC_056054.1	251675001	2.52E+08	0.25821	0.193997	HZ	DZIP1L
NC_056054.1	187190001	1.87E+08	0.394044	0.226213	HZ	EAF2;SLC15A2
NC_056080.1	62670001	62690001	0.387921	0.345708	HZ	EDA
NC_056080.1	62675001	62695001	0.311231	0.492573	HZ	EDA
NC_056080.1	62680001	62700001	0.382701	0.463499	HZ	EDA
NC_056080.1	62685001	62705001	0.474314	0.434819	HZ	EDA
NC_056065.1	65180001	65200001	0.421921	0.150277	HZ	EDEM3
NC_056065.1	65185001	65205001	0.329071	0.221876	HZ	EDEM3
NC_056065.1	65190001	65210001	0.323829	0.253043	HZ	EDEM3
NC_056065.1	65195001	65215001	0.480569	0.305303	HZ	EDEM3
NC_056065.1	65220001	65240001	0.48474	0.17733	HZ	EDEM3
NC_056077.1	20500001	20520001	0.097849	0.183596	HZ	EEF2K;POLR3E
NC_056077.1	20505001	20525001	0.098539	0.178041	HZ	EEF2K;POLR3E
NC_056065.1	31525001	31545001	0.412817	0.222733	HZ	EFCAB2
NC_056065.1	31530001	31550001	0.282384	0.252361	HZ	EFCAB2
NC_056065.1	31535001	31555001	0.385073	0.239309	HZ	EFCAB2
NC_056062.1	22250001	22270001	0.047407	0.172167	HZ	EFR3A
NC_056072.1	29950001	29970001	0.283858	0.15426	HZ	EIF4E3
NC_056072.1	29940001	29960001	0.39418	0.162155	HZ	EIF4E3;GPR27
NC_056072.1	29945001	29965001	0.088781	0.250591	HZ	EIF4E3;GPR27
NC_056055.1	245555001	2.46E+08	0.095736	0.167434	HZ	EIF4G3
NC_056055.1	245560001	2.46E+08	0.086174	0.184818	HZ	EIF4G3
NC_056057.1	34790001	34810001	0.381548	0.161053	HZ	ELAPOR2
NC_056057.1	34795001	34815001	0.348709	0.189637	HZ	ELAPOR2
NC_056058.1	92870001	92890001	0.50259	0.162026	HZ	ELL2
NC_056055.1	102485001	1.03E+08	0.482392	0.150375	HZ	ELP3
NC_056055.1	102490001	1.03E+08	0.446034	0.184034	HZ	ELP3
NC_056072.1	52135001	52155001	0.06938	0.159823	HZ	ELP6
NC_056072.1	52140001	52160001	0.049975	0.174607	HZ	ELP6
NC_056056.1	7595001	7615001	0.114225	0.165994	HZ	ENDOG;LOC101106416;LOC114113869
NC_056056.1	7600001	7620001	0.072951	0.160572	HZ	ENDOG;LOC101106416;TBC1D13

NC_056080.1	104015001	1.04E+08	0.274097	0.184997	HZ	ENOX2
NC_056080.1	104020001	1.04E+08	0.315114	0.185986	HZ	ENOX2
NC_056080.1	104050001	1.04E+08	0.391343	0.274193	HZ	ENOX2
NC_056080.1	104055001	1.04E+08	0.413174	0.291591	HZ	ENOX2
NC_056080.1	104060001	1.04E+08	0.39303	0.263535	HZ	ENOX2
NC_056080.1	104070001	1.04E+08	0.420119	0.186417	HZ	ENOX2
NC_056080.1	104025001	1.04E+08	0.359315	0.22028	HZ	ENOX2;LOC105605606
NC_056080.1	104030001	1.04E+08	0.44924	0.271814	HZ	ENOX2;LOC105605606
NC_056080.1	104035001	1.04E+08	0.427447	0.248482	HZ	ENOX2;LOC105605606
NC_056080.1	104040001	1.04E+08	0.426217	0.278889	HZ	ENOX2;LOC105605606
NC_056080.1	104045001	1.04E+08	0.398649	0.28495	HZ	ENOX2;LOC105605606
NC_056060.1	1515001	1535001	0.065477	0.167825	HZ	EPB41L4A
NC_056060.1	1520001	1540001	0.041781	0.330821	HZ	EPB41L4A
NC_056060.1	1525001	1545001	0.070614	0.320151	HZ	EPB41L4A
NC_056060.1	1530001	1550001	0.071442	0.285124	HZ	EPB41L4A
NC_056060.1	1535001	1555001	0.08243	0.276908	HZ	EPB41L4A
NC_056061.1	44045001	44065001	0.486687	0.216257	HZ	EPHA7
NC_056058.1	6390001	6410001	0.162255	0.159196	HZ	EPS15L1
NC_056058.1	6425001	6445001	0.255761	0.149258	HZ	EPS15L1
NC_056054.1	87080001	87100001	0.396591	0.15005	HZ	EPS8L3;GSTM3
NC_056054.1	87075001	87095001	0.464043	0.155327	HZ	EPS8L3;GSTM3;LOC101108705
NC_056055.1	213900001	2.14E+08	0.356813	0.15269	HZ	ERBB4
NC_056069.1	13630001	13650001	0.399729	0.151476	HZ	ERBIN
NC_056072.1	44810001	44830001	0.212802	0.184646	HZ	ERC2
NC_056072.1	44815001	44835001	0.130597	0.250386	HZ	ERC2
NC_056072.1	44820001	44840001	0.271195	0.191616	HZ	ERC2
NC_056072.1	45445001	45465001	0.255671	0.162122	HZ	ERC2
NC_056054.1	271295001	2.71E+08	0.046055	0.162565	HZ	ERG
NC_056054.1	271300001	2.71E+08	0.061217	0.173922	HZ	ERG
NC_056054.1	271305001	2.71E+08	0.236212	0.151937	HZ	ERG
NC_056054.1	271380001	2.71E+08	0.258422	0.252619	HZ	ERG
NC_056054.1	271385001	2.71E+08	0.209505	0.237484	HZ	ERG
NC_056054.1	271390001	2.71E+08	0.16586	0.251833	HZ	ERG
NC_056054.1	271395001	2.71E+08	0.286602	0.227438	HZ	ERG
NC_056054.1	271400001	2.71E+08	0.503065	0.223447	HZ	ERG
NC_056079.1	595001	615001	0.045626	0.24172	HZ	ERICH1

NC_056079.1	600001	620001	0.044454	0.220828	HZ	ERICH1
NC_056079.1	610001	630001	0.067841	0.187391	HZ	ERICH1
NC_056079.1	615001	635001	0.104605	0.155898	HZ	ERICH1
NC_056060.1	74480001	74500001	0.475027	0.202865	HZ	ESR2
NC_056060.1	74475001	74495001	0.360019	0.174628	HZ	ESR2;SYNE2
NC_056065.1	19360001	19380001	0.381173	0.157684	HZ	ESRRG
NC_056065.1	19505001	19525001	0.215654	0.223365	HZ	ESRRG
NC_056065.1	19510001	19530001	0.200106	0.197243	HZ	ESRRG
NC_056065.1	19515001	19535001	0.265817	0.16983	HZ	ESRRG
NC_056059.1	104325001	1.04E+08	0.078037	0.228415	HZ	EVC2
NC_056059.1	104330001	1.04E+08	0.099716	0.26458	HZ	EVC2
NC_056059.1	104335001	1.04E+08	0.183673	0.251271	HZ	EVC2
NC_056059.1	104340001	1.04E+08	0.242682	0.223854	HZ	EVC2
NC_056054.1	69775001	69795001	0.489724	0.172511	HZ	EVI5
NC_056054.1	69780001	69800001	0.427447	0.174961	HZ	EVI5
NC_056054.1	69785001	69805001	0.371926	0.19154	HZ	EVI5
NC_056054.1	69790001	69810001	0.325064	0.176407	HZ	EVI5
NC_056054.1	69795001	69815001	0.306559	0.163113	HZ	EVI5
NC_056054.1	69800001	69820001	0.344033	0.157084	HZ	EVI5
NC_056054.1	69875001	69895001	0.347252	0.165652	HZ	EVI5
NC_056054.1	69880001	69900001	0.355102	0.215159	HZ	EVI5
NC_056054.1	69885001	69905001	0.362838	0.28004	HZ	EVI5
NC_056054.1	69890001	69910001	0.364919	0.346179	HZ	EVI5
NC_056054.1	69895001	69915001	0.279949	0.427532	HZ	EVI5
NC_056054.1	69900001	69920001	0.228861	0.44893	HZ	EVI5
NC_056054.1	69905001	69925001	0.154303	0.465001	HZ	EVI5
NC_056054.1	69910001	69930001	0.221135	0.425795	HZ	EVI5
NC_056054.1	69940001	69960001	0.338461	0.145417	HZ	EVI5
NC_056054.1	69945001	69965001	0.304136	0.156273	HZ	EVI5
NC_056054.1	69950001	69970001	0.388047	0.193487	HZ	EVI5
NC_056054.1	69955001	69975001	0.430832	0.20899	HZ	EVI5
NC_056057.1	98665001	98685001	0.401131	0.158806	HZ	EXOC4
NC_056057.1	98670001	98690001	0.457862	0.146419	HZ	EXOC4
NC_056057.1	99130001	99150001	0.440907	0.150382	HZ	EXOC4
NC_056067.1	1685001	1705001	0.061538	0.147294	HZ	FA2H
NC_056055.1	37115001	37135001	0.353527	0.248212	HZ	FAM219A

NC_056055.1	37120001	37140001	0.244987	0.239166	HZ	FAM219A
NC_056055.1	37125001	37145001	0.175871	0.250883	HZ	FAM219A
NC_056055.1	37130001	37150001	0.234412	0.23445	HZ	FAM219A
NC_056055.1	37135001	37155001	0.295425	0.26755	HZ	FAM219A
NC_056055.1	37140001	37160001	0.317282	0.262397	HZ	FAM219A
NC_056055.1	37145001	37165001	0.37814	0.269015	HZ	FAM219A
NC_056060.1	58960001	58980001	0.116473	0.249486	HZ	FAM227B;FGF7
NC_056059.1	13695001	13715001	0.242823	0.159997	HZ	FAM241A
NC_056059.1	13700001	13720001	0.160389	0.197337	HZ	FAM241A
NC_056059.1	13705001	13725001	0.243161	0.214071	HZ	FAM241A
NC_056059.1	13710001	13730001	0.330176	0.20813	HZ	FAM241A
NC_056059.1	13755001	13775001	0.447973	0.190207	HZ	FAM241A
NC_056054.1	193675001	1.94E+08	0.5	0.145955	HZ	FAM43A
NC_056054.1	193680001	1.94E+08	0.346226	0.208933	HZ	FAM43A
NC_056054.1	193685001	1.94E+08	0.385119	0.195841	HZ	FAM43A;LSG1
NC_056056.1	186000001	1.86E+08	0.465138	0.358575	HZ	FAR2
NC_056056.1	186005001	1.86E+08	0.483766	0.320721	HZ	FAR2
NC_056063.1	74790001	74810001	0.478803	0.199414	HZ	FARP1;STK24
NC_056060.1	59775001	59795001	0.268162	0.145303	HZ	FBN1
NC_056060.1	59780001	59800001	0.160742	0.200177	HZ	FBN1
NC_056060.1	59785001	59805001	0.214136	0.177427	HZ	FBN1
NC_056057.1	45780001	45800001	0.301436	0.156464	HZ	FBXL13
NC_056058.1	104900001	1.05E+08	0.084521	0.165179	HZ	FBXL17
NC_056058.1	104910001	1.05E+08	0.086242	0.153385	HZ	FBXL17
NC_056064.1	39585001	39605001	0.471464	0.146168	HZ	FBXL20;MED1
NC_056077.1	36385001	36405001	0.273134	0.151361	HZ	FBXO24;LRCH4;PCOLCE
NC_056077.1	36390001	36410001	0.245371	0.152192	HZ	FBXO24;LRCH4;PCOLCE
NC_056072.1	51515001	51535001	0.343104	0.28843	HZ	FBXW12
NC_056072.1	51520001	51540001	0.308591	0.305258	HZ	FBXW12
NC_056072.1	51525001	51545001	0.20185	0.369919	HZ	FBXW12
NC_056054.1	110245001	1.1E+08	0.314562	0.204256	HZ	FCER1A
NC_056054.1	110250001	1.1E+08	0.233103	0.279432	HZ	FCER1A;LOC101122073
NC_056054.1	110255001	1.1E+08	0.363554	0.237967	HZ	FCER1A;LOC101122073
NC_056054.1	110260001	1.1E+08	0.486999	0.17082	HZ	FCER1A;LOC101122073
NC_056068.1	51140001	51160001	0.192725	0.152497	HZ	FCHSD2
NC_056068.1	51145001	51165001	0.164203	0.15242	HZ	FCHSD2

NC_056068.1	51270001	51290001	0.380736	0.147792	HZ	FCHSD2
NC_056068.1	51275001	51295001	0.425177	0.172391	HZ	FCHSD2
NC_056068.1	51280001	51300001	0.429135	0.202833	HZ	FCHSD2
NC_056068.1	51285001	51305001	0.429699	0.188782	HZ	FCHSD2
NC_056056.1	182840001	1.83E+08	0.297865	0.148644	HZ	FGD4
NC_056069.1	30605001	30625001	0.487342	0.202929	HZ	FGF10
NC_056069.1	30610001	30630001	0.372172	0.298287	HZ	FGF10
NC_056069.1	30615001	30635001	0.313786	0.326925	HZ	FGF10
NC_056069.1	30620001	30640001	0.239752	0.337388	HZ	FGF10
NC_056069.1	30625001	30645001	0.433824	0.267778	HZ	FGF10
NC_056069.1	30630001	30650001	0.355372	0.183249	HZ	FGF10
NC_056069.1	30635001	30655001	0.274879	0.150352	HZ	FGF10
NC_056059.1	117440001	1.17E+08	0.275213	0.183972	HZ	FGFR3;LETM1
NC_056072.1	40465001	40485001	0.35619	0.227911	HZ	FHIT
NC_056072.1	40470001	40490001	0.291808	0.277206	HZ	FHIT
NC_056072.1	40475001	40495001	0.243635	0.321865	HZ	FHIT
NC_056072.1	40480001	40500001	0.433295	0.275706	HZ	FHIT
NC_056061.1	2305001	2325001	0.458309	0.155352	HZ	FILIP1
NC_056061.1	2310001	2330001	0.456712	0.215809	HZ	FILIP1
NC_056061.1	2315001	2335001	0.423883	0.257259	HZ	FILIP1
NC_056061.1	2320001	2340001	0.392506	0.232199	HZ	FILIP1
NC_056055.1	10535001	10555001	0.453806	0.15468	HZ	FKBP15
NC_056054.1	102370001	1.02E+08	0.061415	0.165194	HZ	FLG2
NC_056063.1	19060001	19080001	0.479676	0.199634	HZ	FNDC3A
NC_056063.1	19065001	19085001	0.454547	0.209004	HZ	FNDC3A
NC_056064.1	54885001	54905001	0.500213	0.190679	HZ	FOXJ1;RNF157
NC_056060.1	99260001	99280001	0.380767	0.15489	HZ	FOXN3
NC_056060.1	99265001	99285001	0.445266	0.23424	HZ	FOXN3
NC_056072.1	30595001	30615001	0.142602	0.18095	HZ	FOXP1
NC_056072.1	30635001	30655001	0.3	0.305743	HZ	FOXP1
NC_056057.1	55935001	55955001	0.490804	0.170139	HZ	FOXP2
NC_056057.1	55940001	55960001	0.240601	0.235958	HZ	FOXP2
NC_056057.1	55945001	55965001	0.279616	0.230982	HZ	FOXP2
NC_056057.1	55950001	55970001	0.317098	0.205248	HZ	FOXP2
NC_056057.1	55955001	55975001	0.489807	0.163489	HZ	FOXP2
NC_056078.1	41585001	41605001	0.488861	0.26288	HZ	FRMPD2

NC_056059.1	67870001	67890001	0.31319	0.181282	HZ	FRYL
NC_056059.1	67875001	67895001	0.265274	0.208443	HZ	FRYL
NC_056059.1	67880001	67900001	0.254288	0.208817	HZ	FRYL
NC_056077.1	27775001	27795001	0.270531	0.156984	HZ	FUS
NC_056077.1	27780001	27800001	0.370009	0.184005	HZ	FUS
NC_056077.1	27785001	27805001	0.427325	0.166827	HZ	FUS;LOC101105208
NC_056078.1	13100001	13120001	0.454088	0.231873	HZ	FXYD4
NC_056078.1	13105001	13125001	0.428741	0.2574	HZ	FXYD4
NC_056078.1	13110001	13130001	0.451999	0.238883	HZ	FXYD4
NC_056078.1	13115001	13135001	0.501564	0.236918	HZ	FXYD4;HNRNPF
NC_056055.1	103010001	1.03E+08	0.327496	0.170638	HZ	FZD3
NC_056055.1	103015001	1.03E+08	0.283396	0.191653	HZ	FZD3
NC_056055.1	103020001	1.03E+08	0.273455	0.202036	HZ	FZD3
NC_056055.1	103025001	1.03E+08	0.308642	0.166096	HZ	FZD3
NC_056059.1	66665001	66685001	0.45977	0.150855	HZ	GABRB1
NC_056059.1	66670001	66690001	0.472793	0.168219	HZ	GABRB1
NC_056059.1	66675001	66695001	0.307693	0.19893	HZ	GABRB1
NC_056059.1	66680001	66700001	0.236742	0.190921	HZ	GABRB1
NC_056061.1	48200001	48220001	0.341559	0.149628	HZ	GABRR2;UBE2J1
NC_056061.1	48205001	48225001	0.252724	0.156935	HZ	GABRR2;UBE2J1
NC_056060.1	59105001	59125001	0.312415	0.1566	HZ	GALK2
NC_056060.1	59110001	59130001	0.319599	0.157458	HZ	GALK2
NC_056060.1	59115001	59135001	0.332648	0.149279	HZ	GALK2
NC_056077.1	29965001	29985001	0.287906	0.16042	HZ	GALNT17
NC_056077.1	29970001	29990001	0.113898	0.210669	HZ	GALNT17
NC_056077.1	29975001	29995001	0.140292	0.218713	HZ	GALNT17
NC_056077.1	29980001	30000001	0.26322	0.146062	HZ	GALNT17
NC_056077.1	30000001	30020001	0.310254	0.158755	HZ	GALNT17
NC_056077.1	30005001	30025001	0.261971	0.190679	HZ	GALNT17
NC_056077.1	30010001	30030001	0.362675	0.182419	HZ	GALNT17
NC_056077.1	30015001	30035001	0.498122	0.157386	HZ	GALNT17
NC_056055.1	107510001	1.08E+08	0.470541	0.148509	HZ	GALNT7
NC_056055.1	107515001	1.08E+08	0.44661	0.1815	HZ	GALNT7
NC_056055.1	107520001	1.08E+08	0.41902	0.198857	HZ	GALNT7
NC_056055.1	107525001	1.08E+08	0.420813	0.175994	HZ	GALNT7
NC_056055.1	107810001	1.08E+08	0.07364	0.145893	HZ	GALNTL6

NC_056056.1	8760001	8780001	0.251336	0.236572	HZ	GARNL3
NC_056056.1	8765001	8785001	0.305857	0.259366	HZ	GARNL3
NC_056056.1	8770001	8790001	0.320455	0.272628	HZ	GARNL3
NC_056056.1	8775001	8795001	0.199152	0.305772	HZ	GARNL3
NC_056056.1	8865001	8885001	0.198413	0.324118	HZ	GARNL3
NC_056056.1	8870001	8890001	0.262963	0.317377	HZ	GARNL3
NC_056056.1	8875001	8895001	0.321123	0.257873	HZ	GARNL3
NC_056056.1	8880001	8900001	0.414932	0.228938	HZ	GARNL3
NC_056056.1	8885001	8905001	0.431399	0.199048	HZ	GARNL3
NC_056067.1	14340001	14360001	0.152717	0.45738	HZ	GAS8
NC_056067.1	14345001	14365001	0.347269	0.399055	HZ	GAS8
NC_056067.1	14325001	14345001	0.394052	0.339528	HZ	GAS8;LOC101109035
NC_056067.1	14330001	14350001	0.273497	0.401357	HZ	GAS8;LOC101109035
NC_056067.1	14335001	14355001	0.160247	0.463629	HZ	GAS8;LOC101109035
NC_056059.1	87740001	87760001	0.112355	0.145599	HZ	GC
NC_056059.1	87745001	87765001	0.095238	0.147394	HZ	GC
NC_056059.1	87750001	87770001	0.143626	0.151445	HZ	GC
NC_056070.1	62650001	62670001	0.464454	0.251754	HZ	GCN1
NC_056070.1	62655001	62675001	0.422495	0.243152	HZ	GCN1
NC_056070.1	62660001	62680001	0.402088	0.213797	HZ	GCN1
NC_056070.1	62665001	62685001	0.425586	0.170898	HZ	GCN1
NC_056070.1	62670001	62690001	0.442339	0.166876	HZ	GCN1
NC_056070.1	62675001	62695001	0.44155	0.1631	HZ	GCN1
NC_056070.1	62625001	62645001	0.451172	0.270248	HZ	GCN1;RAB35
NC_056066.1	72650001	72670001	0.333333	0.192164	HZ	GDAP1L1
NC_056062.1	80965001	80985001	0.396694	0.165569	HZ	GDF6
NC_056062.1	80970001	80990001	0.407336	0.197014	HZ	GDF6
NC_056062.1	80975001	80995001	0.468751	0.17192	HZ	GDF6
NC_056058.1	42100001	42120001	0.329075	0.147105	HZ	GDF9;LEAP2;LOC101121420
NC_056054.1	260180001	2.6E+08	0.45709	0.25455	HZ	GET1
NC_056054.1	260185001	2.6E+08	0.269322	0.213204	HZ	GET1
NC_056054.1	260190001	2.6E+08	0.136683	0.221779	HZ	GET1
NC_056054.1	260195001	2.6E+08	0.048283	0.252916	HZ	GET1;LCA5L
NC_056075.1	36130001	36150001	0.460193	0.151571	HZ	GFRA1
NC_056066.1	53280001	53300001	0.408754	0.225606	HZ	GINS1
NC_056066.1	53285001	53305001	0.460132	0.238603	HZ	GINS1

NC_056055.1	186040001	1.86E+08	0.461876	0.292005	HZ	GLI2
NC_056055.1	186045001	1.86E+08	0.430627	0.308373	HZ	GLI2
NC_056055.1	186050001	1.86E+08	0.433628	0.289556	HZ	GLI2
NC_056055.1	186055001	1.86E+08	0.450659	0.301702	HZ	GLI2
NC_056055.1	186060001	1.86E+08	0.455479	0.31371	HZ	GLI2
NC_056055.1	186065001	1.86E+08	0.435711	0.277984	HZ	GLI2
NC_056055.1	186070001	1.86E+08	0.390661	0.228894	HZ	GLI2
NC_056080.1	12895001	12915001	0.462809	0.162307	HZ	GLRA2
NC_056055.1	105965001	1.06E+08	0.477563	0.15081	HZ	GLRA3
NC_056055.1	105980001	1.06E+08	0.467363	0.285853	HZ	GLRA3
NC_056055.1	105985001	1.06E+08	0.329906	0.211257	HZ	GLRA3
NC_056056.1	3655001	3675001	0.274091	0.193167	HZ	GLT6D1;PAEP
NC_056067.1	63140001	63160001	0.47714	0.158575	HZ	GP6
NC_056080.1	101550001	1.02E+08	0.123195	0.175968	HZ	GPC4
NC_056063.1	67640001	67660001	0.223066	0.153003	HZ	GPC5
NC_056063.1	67645001	67665001	0.094587	0.17852	HZ	GPC5
NC_056071.1	67165001	67185001	0.306563	0.233009	HZ	GPR132
NC_056071.1	67170001	67190001	0.204158	0.2939	HZ	GPR132
NC_056071.1	67175001	67195001	0.190512	0.30333	HZ	GPR132
NC_056071.1	67180001	67200001	0.239281	0.282875	HZ	GPR132
NC_056068.1	33785001	33805001	0.361314	0.16493	HZ	GRAMD1B
NC_056056.1	20435001	20455001	0.31584	0.213265	HZ	GREB1
NC_056056.1	20440001	20460001	0.357486	0.183894	HZ	GREB1
NC_056056.1	20445001	20465001	0.428571	0.159016	HZ	GREB1
NC_056056.1	20465001	20485001	0.244783	0.248381	HZ	GREB1
NC_056056.1	20470001	20490001	0.137242	0.245008	HZ	GREB1
NC_056056.1	20475001	20495001	0.147475	0.22952	HZ	GREB1
NC_056062.1	75700001	75720001	0.315188	0.18001	HZ	GRHL2
NC_056062.1	75705001	75725001	0.254582	0.211946	HZ	GRHL2
NC_056062.1	75710001	75730001	0.305288	0.197938	HZ	GRHL2
NC_056062.1	75715001	75735001	0.29483	0.200076	HZ	GRHL2
NC_056062.1	75720001	75740001	0.388236	0.168632	HZ	GRHL2
NC_056062.1	75725001	75745001	0.470116	0.162284	HZ	GRHL2
NC_056062.1	75730001	75750001	0.470161	0.213556	HZ	GRHL2
NC_056062.1	75735001	75755001	0.474426	0.167378	HZ	GRHL2
NC_056062.1	75650001	75670001	0.269695	0.187345	HZ	GRHL2;NCALD

NC_056059.1	31910001	31930001	0.256005	0.177485	HZ	GRID2
NC_056059.1	31915001	31935001	0.284352	0.151051	HZ	GRID2
NC_056054.1	11665001	11685001	0.361083	0.171256	HZ	GRIK3
NC_056054.1	11670001	11690001	0.204943	0.221914	HZ	GRIK3
NC_056054.1	11675001	11695001	0.056014	0.252221	HZ	GRIK3
NC_056056.1	153465001	1.53E+08	0.265789	0.15452	HZ	GRIP1
NC_056056.1	153470001	1.53E+08	0.345073	0.153718	HZ	GRIP1
NC_056056.1	153475001	1.53E+08	0.385674	0.161969	HZ	GRIP1
NC_056056.1	153480001	1.54E+08	0.420758	0.182511	HZ	GRIP1
NC_056056.1	153495001	1.54E+08	0.475517	0.163435	HZ	GRIP1
NC_056070.1	65560001	65580001	0.363169	0.506317	HZ	GRK3
NC_056070.1	65565001	65585001	0.433733	0.594982	HZ	GRK3
NC_056070.1	65570001	65590001	0.422004	0.559855	HZ	GRK3
NC_056070.1	65595001	65615001	0.464561	0.392879	HZ	GRK3
NC_056070.1	65600001	65620001	0.412137	0.267191	HZ	GRK3
NC_056070.1	65605001	65625001	0.438035	0.23484	HZ	GRK3
NC_056057.1	93030001	93050001	0.113691	0.157367	HZ	GRM8
NC_056057.1	93035001	93055001	0.089888	0.166048	HZ	GRM8
NC_056057.1	93040001	93060001	0.274232	0.150132	HZ	GRM8
NC_056057.1	72710001	72730001	0.464802	0.208621	HZ	GSDME
NC_056057.1	72715001	72735001	0.461664	0.199152	HZ	GSDME
NC_056057.1	72720001	72740001	0.452739	0.183142	HZ	GSDME
NC_056059.1	19675001	19695001	0.367231	0.168093	HZ	GSTCD
NC_056057.1	107855001	1.08E+08	0.490393	0.195992	HZ	GSTK1
NC_056063.1	32475001	32495001	0.4583	0.156288	HZ	GSX1
NC_056063.1	32480001	32500001	0.462512	0.234652	HZ	GSX1
NC_056063.1	32485001	32505001	0.455025	0.261154	HZ	GSX1
NC_056063.1	32490001	32510001	0.456323	0.286677	HZ	GSX1
NC_056072.1	28840001	28860001	0.291541	0.278771	HZ	GXYLT2
NC_056072.1	28845001	28865001	0.313454	0.352254	HZ	GXYLT2
NC_056072.1	28850001	28870001	0.32291	0.398684	HZ	GXYLT2
NC_056072.1	28855001	28875001	0.38588	0.437607	HZ	GXYLT2
NC_056072.1	28865001	28885001	0.498994	0.353059	HZ	GXYLT2
NC_056072.1	28870001	28890001	0.468694	0.347546	HZ	GXYLT2
NC_056072.1	28875001	28895001	0.484592	0.215023	HZ	GXYLT2
NC_056055.1	107215001	1.07E+08	0.386159	0.28545	HZ	HAND2

NC_056068.1	48030001	48050001	0.034139	0.315237	HZ	HBB
NC_056068.1	48035001	48055001	0.096663	0.293364	HZ	HBB;LOC101102671
NC_056068.1	47950001	47970001	0.009132	0.31525	HZ	HBE1
NC_056068.1	47960001	47980001	0.008749	0.265277	HZ	HBE1;LOC101105185
NC_056054.1	2115001	2135001	0.379523	0.151355	HZ	HDAC4
NC_056054.1	2120001	2140001	0.410013	0.187557	HZ	HDAC4
NC_056057.1	27795001	27815001	0.293181	0.197367	HZ	HDAC9
NC_056057.1	27800001	27820001	0.373615	0.180384	HZ	HDAC9
NC_056057.1	27805001	27825001	0.429059	0.155542	HZ	HDAC9
NC_056057.1	27810001	27830001	0.465586	0.158144	HZ	HDAC9
NC_056057.1	27820001	27840001	0.395217	0.166765	HZ	HDAC9
NC_056057.1	27825001	27845001	0.410641	0.154753	HZ	HDAC9
NC_056057.1	27850001	27870001	0.277851	0.159376	HZ	HDAC9
NC_056057.1	27855001	27875001	0.297428	0.185428	HZ	HDAC9
NC_056057.1	27860001	27880001	0.282806	0.195572	HZ	HDAC9
NC_056057.1	27865001	27885001	0.355908	0.23394	HZ	HDAC9
NC_056057.1	27870001	27890001	0.425278	0.245905	HZ	HDAC9
NC_056057.1	27875001	27895001	0.340143	0.233901	HZ	HDAC9
NC_056057.1	27880001	27900001	0.356109	0.213811	HZ	HDAC9
NC_056057.1	27905001	27925001	0.429402	0.248648	HZ	HDAC9
NC_056057.1	27910001	27930001	0.495344	0.268154	HZ	HDAC9
NC_056070.1	62120001	62140001	0.420225	0.180939	HZ	HECTD4
NC_056070.1	62125001	62145001	0.465632	0.154174	HZ	HECTD4
NC_056055.1	198800001	1.99E+08	0.486101	0.146865	HZ	HECW2
NC_056055.1	198865001	1.99E+08	0.414185	0.162405	HZ	HECW2
NC_056055.1	198870001	1.99E+08	0.421186	0.207171	HZ	HECW2
NC_056055.1	198875001	1.99E+08	0.484798	0.197608	HZ	HECW2
NC_056055.1	198880001	1.99E+08	0.490036	0.175188	HZ	HECW2
NC_056054.1	174050001	1.74E+08	0.461538	0.187088	HZ	HHLA2
NC_056054.1	174120001	1.74E+08	0.380065	0.229957	HZ	HHLA2
NC_056054.1	174125001	1.74E+08	0.145709	0.243069	HZ	HHLA2
NC_056054.1	174130001	1.74E+08	0.210285	0.225354	HZ	HHLA2;MYH15
NC_056054.1	174135001	1.74E+08	0.336905	0.188932	HZ	HHLA2;MYH15
NC_056054.1	174140001	1.74E+08	0.47238	0.156043	HZ	HHLA2;MYH15
NC_056074.1	8715001	8735001	0.098263	0.148437	HZ	HIKESHI
NC_056074.1	8720001	8740001	0.079808	0.151481	HZ	HIKESHI

NC_056074.1	8725001	8745001	0.086032	0.159219	HZ	HIKESHI
NC_056074.1	8730001	8750001	0.179421	0.156106	HZ	HIKESHI
NC_056074.1	8735001	8755001	0.416733	0.148612	HZ	HIKESHI
NC_056059.1	115745001	1.16E+08	0.351364	0.183792	HZ	HMX1
NC_056059.1	115750001	1.16E+08	0.347626	0.199782	HZ	HMX1
NC_056070.1	63295001	63315001	0.167002	0.15619	HZ	HNF1A
NC_056056.1	132570001	1.33E+08	0.495593	0.283953	HZ	HOXC13
NC_056056.1	132445001	1.32E+08	0.444744	0.184179	HZ	HOXC4
NC_056055.1	245610001	2.46E+08	0.274568	0.177481	HZ	HP1BP3
NC_056075.1	19860001	19880001	0.478441	0.22039	HZ	HPSE2
NC_056075.1	19865001	19885001	0.40338	0.217937	HZ	HPSE2
NC_056075.1	19870001	19890001	0.300405	0.255536	HZ	HPSE2
NC_056075.1	19875001	19895001	0.210624	0.27357	HZ	HPSE2
NC_056075.1	19880001	19900001	0.168317	0.258931	HZ	HPSE2
NC_056075.1	19885001	19905001	0.111302	0.281362	HZ	HPSE2
NC_056075.1	19890001	19910001	0.205517	0.258427	HZ	HPSE2
NC_056075.1	19895001	19915001	0.458731	0.244278	HZ	HPSE2
NC_056077.1	23880001	23900001	0.401902	0.209155	HZ	HS3ST4
NC_056077.1	23885001	23905001	0.358228	0.196965	HZ	HS3ST4
NC_056077.1	23890001	23910001	0.352401	0.153833	HZ	HS3ST4
NC_056077.1	23895001	23915001	0.371428	0.145449	HZ	HS3ST4
NC_056063.1	72775001	72795001	0.497006	0.153448	HZ	HS6ST3
NC_056063.1	72780001	72800001	0.497905	0.175335	HZ	HS6ST3
NC_056067.1	55185001	55205001	0.136948	0.255695	HZ	HSD17B14;PLEKHA4
NC_056055.1	244665001	2.45E+08	0.460817	0.178207	HZ	HSPG2
NC_056055.1	244690001	2.45E+08	0.355463	0.166344	HZ	HSPG2;LDLRAD2
NC_056055.1	244695001	2.45E+08	0.328842	0.159773	HZ	HSPG2;LDLRAD2
NC_056080.1	119920001	1.2E+08	0.388619	0.168236	HZ	HTR2C
NC_056080.1	119940001	1.2E+08	0.346513	0.1772	HZ	HTR2C
NC_056080.1	119945001	1.2E+08	0.373105	0.163375	HZ	HTR2C
NC_056080.1	119980001	1.2E+08	0.482782	0.177161	HZ	HTR2C
NC_056080.1	119985001	1.2E+08	0.478163	0.162725	HZ	HTR2C
NC_056080.1	119990001	1.2E+08	0.443497	0.154724	HZ	HTR2C
NC_056054.1	122840001	1.23E+08	0.177474	0.261947	HZ	IFNAR1
NC_056054.1	122845001	1.23E+08	0.285548	0.248384	HZ	IFNAR1
NC_056054.1	122850001	1.23E+08	0.411582	0.219551	HZ	IFNAR1

NC_056054.1	122855001	1.23E+08	0.423131	0.197623	HZ	IFNAR1
NC_056054.1	227795001	2.28E+08	0.450128	0.259991	HZ	IFT80
NC_056054.1	227800001	2.28E+08	0.305716	0.319082	HZ	IFT80
NC_056054.1	227805001	2.28E+08	0.166815	0.319054	HZ	IFT80
NC_056054.1	227810001	2.28E+08	0.181355	0.286898	HZ	IFT80
NC_056054.1	227815001	2.28E+08	0.277191	0.223803	HZ	IFT80
NC_056054.1	227820001	2.28E+08	0.348383	0.150787	HZ	IFT80
NC_056064.1	40415001	40435001	0.473973	0.167816	HZ	IGFBP4
NC_056065.1	4060001	4080001	0.306402	0.153774	HZ	IKBKE;SRGAP2
NC_056070.1	35420001	35440001	0.461717	0.150869	HZ	IL2
NC_056065.1	70985001	71005001	0.314659	0.154533	HZ	INTS7
NC_056065.1	70990001	71010001	0.268261	0.187806	HZ	INTS7
NC_056065.1	70995001	71015001	0.357197	0.158072	HZ	INTS7
NC_056071.1	21460001	21480001	0.422535	0.176651	HZ	IQGAP1
NC_056067.1	11370001	11390001	0.432614	0.194054	HZ	IRF8
NC_056067.1	11375001	11395001	0.394509	0.196873	HZ	IRF8
NC_056067.1	11380001	11400001	0.493404	0.197744	HZ	IRF8
NC_056067.1	11385001	11405001	0.375598	0.234624	HZ	IRF8
NC_056067.1	11390001	11410001	0.263842	0.231368	HZ	IRF8
NC_056067.1	11395001	11415001	0.208882	0.25369	HZ	IRF8
NC_056067.1	11400001	11420001	0.201531	0.257906	HZ	IRF8
NC_056067.1	11405001	11425001	0.29313	0.244564	HZ	IRF8
NC_056066.1	6620001	6640001	0.47698	0.23989	HZ	ISM1
NC_056066.1	6650001	6670001	0.449781	0.211779	HZ	ISM1
NC_056066.1	6655001	6675001	0.41009	0.223103	HZ	ISM1
NC_056066.1	6660001	6680001	0.496441	0.216876	HZ	ISM1
NC_056069.1	26195001	26215001	0.220457	0.148425	HZ	ITGA2
NC_056069.1	26200001	26220001	0.361539	0.1467	HZ	ITGA2
NC_056069.1	26205001	26225001	0.475617	0.178402	HZ	ITGA2
NC_056069.1	26210001	26230001	0.407912	0.192354	HZ	ITGA2
NC_056069.1	26215001	26235001	0.436664	0.182	HZ	ITGA2
NC_056072.1	10995001	11015001	0.457628	0.285363	HZ	ITGA9
NC_056072.1	11000001	11020001	0.314059	0.189423	HZ	ITGA9
NC_056072.1	11005001	11025001	0.374333	0.27825	HZ	ITGA9
NC_056077.1	27880001	27900001	0.320442	0.151797	HZ	ITGAM
NC_056077.1	27885001	27905001	0.324324	0.149497	HZ	ITGAM

NC_056077.1	27890001	27910001	0.337465	0.160838	HZ	ITGAM
NC_056077.1	27895001	27915001	0.263811	0.241076	HZ	ITGAM
NC_056077.1	27900001	27920001	0.378871	0.191148	HZ	ITGAM
NC_056056.1	189000001	1.89E+08	0.398888	0.145308	HZ	ITPR2
NC_056056.1	189030001	1.89E+08	0.121842	0.173837	HZ	ITPR2
NC_056056.1	189035001	1.89E+08	0.114002	0.168558	HZ	ITPR2
NC_056056.1	189040001	1.89E+08	0.248781	0.148159	HZ	ITPR2
NC_056054.1	40510001	40530001	0.135429	0.149763	HZ	JAK1
NC_056054.1	40530001	40550001	0.092087	0.229898	HZ	JAK1
NC_056054.1	40535001	40555001	0.102818	0.227503	HZ	JAK1
NC_056054.1	40540001	40560001	0.094803	0.258357	HZ	JAK1
NC_056054.1	40545001	40565001	0.068113	0.304812	HZ	JAK1
NC_056054.1	40575001	40595001	0.24822	0.219142	HZ	JAK1
NC_056074.1	30400001	30420001	0.396888	0.194839	HZ	JAM3
NC_056074.1	30405001	30425001	0.451906	0.207708	HZ	JAM3
NC_056057.1	69640001	69660001	0.459003	0.185068	HZ	JAZF1
NC_056057.1	69645001	69665001	0.310547	0.285104	HZ	JAZF1
NC_056057.1	69650001	69670001	0.202412	0.35551	HZ	JAZF1
NC_056057.1	69655001	69675001	0.269625	0.391814	HZ	JAZF1
NC_056060.1	84730001	84750001	0.415985	0.303025	HZ	JDP2
NC_056060.1	84735001	84755001	0.224129	0.270102	HZ	JDP2
NC_056060.1	84740001	84760001	0.115457	0.270827	HZ	JDP2
NC_056066.1	72610001	72630001	0.366681	0.152622	HZ	JPH2;OSER1
NC_056066.1	72615001	72635001	0.240749	0.194736	HZ	JPH2;OSER1
NC_056077.1	25700001	25720001	0.411817	0.182054	HZ	KATNIP
NC_056077.1	25705001	25725001	0.313305	0.221684	HZ	KATNIP
NC_056077.1	25710001	25730001	0.223235	0.252386	HZ	KATNIP
NC_056077.1	25715001	25735001	0.262566	0.213588	HZ	KATNIP
NC_056077.1	25720001	25740001	0.210722	0.217058	HZ	KATNIP
NC_056077.1	25725001	25745001	0.274185	0.177087	HZ	KATNIP
NC_056072.1	34570001	34590001	0.102022	0.155543	HZ	KBTBD8
NC_056072.1	34575001	34595001	0.073401	0.195965	HZ	KBTBD8
NC_056056.1	110990001	1.11E+08	0.219226	0.187449	HZ	KCNC2
NC_056056.1	110995001	1.11E+08	0.15927	0.227614	HZ	KCNC2
NC_056056.1	111000001	1.11E+08	0.325607	0.229057	HZ	KCNC2
NC_056056.1	111005001	1.11E+08	0.467935	0.220311	HZ	KCNC2

NC_056055.1	225200001	2.25E+08	0.345678	0.18487	HZ	KCNE4
NC_056055.1	225205001	2.25E+08	0.17029	0.296898	HZ	KCNE4
NC_056055.1	225210001	2.25E+08	0.069445	0.316182	HZ	KCNE4
NC_056056.1	104370001	1.04E+08	0.498265	0.190217	HZ	KCNIP3
NC_056056.1	104375001	1.04E+08	0.462231	0.175951	HZ	KCNIP3
NC_056059.1	40865001	40885001	0.386611	0.236357	HZ	KCNIP4
NC_056059.1	40870001	40890001	0.300311	0.152061	HZ	KCNIP4
NC_056059.1	40875001	40895001	0.332052	0.161675	HZ	KCNIP4
NC_056059.1	40880001	40900001	0.354452	0.208292	HZ	KCNIP4
NC_056059.1	40885001	40905001	0.366414	0.218178	HZ	KCNIP4
NC_056059.1	40890001	40910001	0.429649	0.335751	HZ	KCNIP4
NC_056059.1	40895001	40915001	0.480431	0.425335	HZ	KCNIP4
NC_056059.1	40900001	40920001	0.355478	0.382423	HZ	KCNIP4
NC_056059.1	40905001	40925001	0.436604	0.368345	HZ	KCNIP4
NC_056059.1	40910001	40930001	0.504174	0.352031	HZ	KCNIP4
NC_056059.1	40915001	40935001	0.476999	0.255382	HZ	KCNIP4
NC_056059.1	40940001	40960001	0.375127	0.157379	HZ	KCNIP4
NC_056059.1	40945001	40965001	0.403999	0.176326	HZ	KCNIP4
NC_056059.1	40845001	40865001	0.376185	0.209873	HZ	KCNIP4;PACRGL
NC_056059.1	40850001	40870001	0.37797	0.218498	HZ	KCNIP4;PACRGL
NC_056059.1	40855001	40875001	0.36645	0.244518	HZ	KCNIP4;PACRGL
NC_056059.1	40860001	40880001	0.406417	0.257987	HZ	KCNIP4;PACRGL
NC_056054.1	270420001	2.7E+08	0.164212	0.152104	HZ	KCNJ6
NC_056054.1	270425001	2.7E+08	0.182762	0.192183	HZ	KCNJ6
NC_056054.1	270430001	2.7E+08	0.205669	0.219516	HZ	KCNJ6
NC_056054.1	270435001	2.7E+08	0.232866	0.20334	HZ	KCNJ6
NC_056065.1	67960001	67980001	0.161621	0.154609	HZ	KCNK2
NC_056065.1	67965001	67985001	0.075812	0.160433	HZ	KCNK2
NC_056065.1	67970001	67990001	0.087116	0.160197	HZ	KCNK2
NC_056054.1	208970001	2.09E+08	0.292317	0.170619	HZ	KCNMB2
NC_056054.1	208975001	2.09E+08	0.346117	0.156255	HZ	KCNMB2
NC_056074.1	45390001	45410001	0.413403	0.193698	HZ	KCNQ1
NC_056074.1	45395001	45415001	0.380477	0.227503	HZ	KCNQ1
NC_056074.1	45400001	45420001	0.3768	0.239385	HZ	KCNQ1
NC_056074.1	45405001	45425001	0.445732	0.258415	HZ	KCNQ1
NC_056062.1	21930001	21950001	0.493635	0.222444	HZ	KCNQ3

NC_056062.1	21935001	21955001	0.414233	0.230134	HZ	KCNQ3
NC_056062.1	21940001	21960001	0.440992	0.166526	HZ	KCNQ3
NC_056056.1	215970001	2.16E+08	0.278386	0.145977	HZ	KDELR3
NC_056056.1	58335001	58355001	0.075304	0.145786	HZ	KDM3A
NC_056080.1	43955001	43975001	0.409057	0.25068	HZ	KDM6A
NC_056080.1	43960001	43980001	0.371389	0.220667	HZ	KDM6A
NC_056080.1	43965001	43985001	0.253639	0.213281	HZ	KDM6A
NC_056080.1	43970001	43990001	0.256492	0.207447	HZ	KDM6A
NC_056080.1	43975001	43995001	0.242897	0.177996	HZ	KDM6A
NC_056080.1	43980001	44000001	0.175302	0.150666	HZ	KDM6A
NC_056056.1	127465001	1.27E+08	0.387495	0.20837	HZ	KERA
NC_056056.1	127470001	1.27E+08	0.259226	0.170235	HZ	KERA
NC_056070.1	35555001	35575001	0.471428	0.280944	HZ	KIAA1109
NC_056070.1	35560001	35580001	0.401174	0.237246	HZ	KIAA1109
NC_056070.1	35565001	35585001	0.418439	0.228259	HZ	KIAA1109
NC_056070.1	35570001	35590001	0.413186	0.23916	HZ	KIAA1109
NC_056070.1	35575001	35595001	0.371937	0.194715	HZ	KIAA1109
NC_056070.1	35580001	35600001	0.324444	0.175151	HZ	KIAA1109
NC_056070.1	35585001	35605001	0.324955	0.161559	HZ	KIAA1109
NC_056070.1	35590001	35610001	0.257576	0.162576	HZ	KIAA1109
NC_056070.1	35595001	35615001	0.299108	0.204542	HZ	KIAA1109
NC_056070.1	35600001	35620001	0.365123	0.277871	HZ	KIAA1109
NC_056070.1	35605001	35625001	0.346821	0.36955	HZ	KIAA1109
NC_056070.1	35610001	35630001	0.260443	0.413212	HZ	KIAA1109
NC_056070.1	35615001	35635001	0.191983	0.419271	HZ	KIAA1109
NC_056070.1	35620001	35640001	0.1417	0.427307	HZ	KIAA1109
NC_056070.1	35650001	35670001	0.142857	0.439828	HZ	KIAA1109
NC_056070.1	35655001	35675001	0.178117	0.375709	HZ	KIAA1109
NC_056070.1	35660001	35680001	0.233244	0.338173	HZ	KIAA1109
NC_056070.1	35665001	35685001	0.230392	0.324681	HZ	KIAA1109
NC_056070.1	35670001	35690001	0.265193	0.290555	HZ	KIAA1109
NC_056070.1	35675001	35695001	0.247962	0.239717	HZ	KIAA1109
NC_056070.1	35680001	35700001	0.196181	0.263905	HZ	KIAA1109
NC_056070.1	35685001	35705001	0.205633	0.227643	HZ	KIAA1109
NC_056070.1	35690001	35710001	0.187079	0.194707	HZ	KIAA1109
NC_056070.1	35695001	35715001	0.20797	0.212921	HZ	KIAA1109

NC_056070.1	35700001	35720001	0.202945	0.229027	HZ	KIAA1109
NC_056070.1	35705001	35725001	0.207569	0.22326	HZ	KIAA1109
NC_056070.1	35710001	35730001	0.260321	0.266736	HZ	KIAA1109
NC_056070.1	35715001	35735001	0.28932	0.319235	HZ	KIAA1109
NC_056070.1	35720001	35740001	0.342465	0.296575	HZ	KIAA1109
NC_056070.1	35725001	35745001	0.37037	0.339052	HZ	KIAA1109
NC_056070.1	35730001	35750001	0.409735	0.246313	HZ	KIAA1109
NC_056070.1	35735001	35755001	0.456512	0.258849	HZ	KIAA1109
NC_056066.1	24715001	24735001	0.499366	0.300064	HZ	KIAA1217
NC_056066.1	24720001	24740001	0.483853	0.302215	HZ	KIAA1217
NC_056076.1	20530001	20550001	0.44134	0.154486	HZ	KIAA1328
NC_056076.1	20535001	20555001	0.254975	0.174719	HZ	KIAA1328
NC_056076.1	20540001	20560001	0.220476	0.174162	HZ	KIAA1328
NC_056076.1	20545001	20565001	0.302112	0.17676	HZ	KIAA1328
NC_056074.1	27730001	27750001	0.217001	0.155977	HZ	KIRREL3
NC_056074.1	27735001	27755001	0.229358	0.164244	HZ	KIRREL3
NC_056074.1	27740001	27760001	0.245154	0.149628	HZ	KIRREL3
NC_056059.1	70850001	70870001	0.0657	0.256385	HZ	KIT
NC_056059.1	70875001	70895001	0.102236	0.210434	HZ	KIT
NC_056066.1	40025001	40045001	0.358757	0.166203	HZ	KIZ
NC_056066.1	40030001	40050001	0.370153	0.201752	HZ	KIZ
NC_056066.1	40035001	40055001	0.366959	0.184579	HZ	KIZ
NC_056057.1	96165001	96185001	0.435664	0.205314	HZ	KLF14
NC_056057.1	96170001	96190001	0.37763	0.196895	HZ	KLF14
NC_056057.1	96175001	96195001	0.487268	0.174366	HZ	KLF14
NC_056058.1	6450001	6470001	0.210621	0.24201	HZ	KLF2
NC_056055.1	247835001	2.48E+08	0.387969	0.323313	HZ	KLHDC7A
NC_056055.1	247840001	2.48E+08	0.333749	0.286221	HZ	KLHDC7A
NC_056055.1	247845001	2.48E+08	0.297511	0.24987	HZ	KLHDC7A
NC_056055.1	247850001	2.48E+08	0.340512	0.197855	HZ	KLHDC7A
NC_056063.1	44710001	44730001	0.429307	0.145551	HZ	KLHL1
NC_056070.1	580001	600001	0.464205	0.154406	HZ	KLHL2
NC_056070.1	585001	605001	0.482114	0.156157	HZ	KLHL2
NC_056080.1	118935001	1.19E+08	0.129304	0.152874	HZ	KLHL4;LOC101110213
NC_056057.1	116205001	1.16E+08	0.320166	0.18054	HZ	KMT2C
NC_056057.1	116210001	1.16E+08	0.222335	0.150573	HZ	KMT2C

NC_056057.1	116215001	1.16E+08	0.211194	0.149385	HZ	KMT2C
NC_056057.1	116220001	1.16E+08	0.210174	0.158839	HZ	KMT2C
NC_056057.1	116225001	1.16E+08	0.229787	0.1667	HZ	KMT2C
NC_056057.1	116230001	1.16E+08	0.450512	0.206622	HZ	KMT2C
NC_056057.1	116250001	1.16E+08	0.402409	0.166295	HZ	KMT2C
NC_056057.1	116255001	1.16E+08	0.414737	0.169573	HZ	KMT2C
NC_056077.1	37555001	37575001	0.240727	0.205966	HZ	KPNA7
NC_056077.1	37560001	37580001	0.19139	0.256897	HZ	KPNA7
NC_056077.1	37565001	37585001	0.28068	0.185617	HZ	KPNA7
NC_056057.1	50660001	50680001	0.43428	0.168156	HZ	LAMB4
NC_056057.1	50695001	50715001	0.40569	0.153629	HZ	LAMB4
NC_056057.1	50700001	50720001	0.315578	0.178268	HZ	LAMB4
NC_056080.1	36905001	36925001	0.297571	0.276802	HZ	LANCL3
NC_056080.1	36910001	36930001	0.389632	0.228855	HZ	LANCL3
NC_056080.1	36915001	36935001	0.442773	0.208501	HZ	LANCL3
NC_056054.1	260200001	2.6E+08	0.035441	0.258864	HZ	LCA5L
NC_056054.1	260205001	2.6E+08	0.030069	0.25853	HZ	LCA5L
NC_056054.1	260210001	2.6E+08	0.034529	0.242104	HZ	LCA5L
NC_056054.1	260215001	2.6E+08	0.048531	0.230705	HZ	LCA5L
NC_056054.1	260220001	2.6E+08	0.148821	0.210987	HZ	LCA5L
NC_056054.1	260225001	2.6E+08	0.220019	0.252569	HZ	LCA5L
NC_056054.1	260230001	2.6E+08	0.25635	0.277561	HZ	LCA5L
NC_056054.1	260235001	2.6E+08	0.451983	0.213609	HZ	LCA5L
NC_056059.1	38175001	38195001	0.467319	0.166658	HZ	LCORL
NC_056059.1	38180001	38200001	0.316667	0.172677	HZ	LCORL
NC_056059.1	38185001	38205001	0.259169	0.146698	HZ	LCORL
NC_056059.1	38210001	38230001	0.465169	0.204364	HZ	LCORL
NC_056059.1	38215001	38235001	0.432372	0.236551	HZ	LCORL
NC_056054.1	41215001	41235001	0.089958	0.179001	HZ	LEPR
NC_056059.1	117415001	1.17E+08	0.278316	0.173053	HZ	LETM1
NC_056059.1	117420001	1.17E+08	0.234792	0.210869	HZ	LETM1
NC_056059.1	117425001	1.17E+08	0.114644	0.245324	HZ	LETM1
NC_056059.1	117430001	1.17E+08	0.091354	0.262715	HZ	LETM1
NC_056074.1	38325001	38345001	0.326857	0.22336	HZ	LGALS12
NC_056074.1	38330001	38350001	0.312501	0.232504	HZ	LGALS12
NC_056074.1	38335001	38355001	0.31661	0.211097	HZ	LGALS12

NC_056074.1	38340001	38360001	0.43351	0.175337	HZ	LGALS12;LOC101120029
NC_056074.1	38315001	38335001	0.327377	0.193549	HZ	LGALS12;PLAAT5
NC_056074.1	38320001	38340001	0.368262	0.223999	HZ	LGALS12;PLAAT5
NC_056065.1	60800001	60820001	0.456842	0.216075	HZ	LHX4
NC_056065.1	60805001	60825001	0.441391	0.234724	HZ	LHX4
NC_056065.1	60810001	60830001	0.426923	0.229719	HZ	LHX4
NC_056056.1	13680001	13700001	0.438598	0.15081	HZ	LHX6;MORN5
NC_056056.1	13685001	13705001	0.437974	0.157847	HZ	LHX6;MORN5
NC_056056.1	189845001	1.9E+08	0.34419	0.188716	HZ	LMNTD1
NC_056056.1	189850001	1.9E+08	0.24064	0.185892	HZ	LMNTD1
NC_056056.1	189855001	1.9E+08	0.397389	0.155893	HZ	LMNTD1
NC_056055.1	134180001	1.34E+08	0.344635	0.158632	HZ	LNPK
NC_056055.1	134185001	1.34E+08	0.340087	0.202617	HZ	LNPK
NC_056055.1	134190001	1.34E+08	0.326955	0.2301	HZ	LNPK
NC_056055.1	134195001	1.34E+08	0.317392	0.216435	HZ	LNPK
NC_056055.1	134200001	1.34E+08	0.333911	0.233062	HZ	LNPK
NC_056055.1	134205001	1.34E+08	0.335538	0.224871	HZ	LNPK
NC_056055.1	134210001	1.34E+08	0.262059	0.207415	HZ	LNPK
NC_056055.1	134215001	1.34E+08	0.248722	0.188923	HZ	LNPK
NC_056055.1	134220001	1.34E+08	0.153118	0.156018	HZ	LNPK
NC_056055.1	134225001	1.34E+08	0.07156	0.151484	HZ	LNPK
NC_056068.1	47935001	47955001	0.014464	0.348658	HZ	LOC101102421
NC_056073.1	30460001	30480001	0.45377	0.303399	HZ	LOC101102521;ZNF322
NC_056073.1	29865001	29885001	0.229838	0.152374	HZ	LOC101102593
NC_056068.1	48040001	48060001	0.112877	0.243787	HZ	LOC101102671
NC_056068.1	48045001	48065001	0.191431	0.202157	HZ	LOC101102671;LOC101105945
NC_056068.1	48050001	48070001	0.207236	0.180943	HZ	LOC101102671;LOC101105945
NW_02459982	1190001	1210001	0.446064	0.16809	HZ	LOC101103023;PAG3
NC_056056.1	150530001	1.51E+08	0.441217	0.16526	HZ	LOC101103222
NC_056056.1	150535001	1.51E+08	0.323687	0.193755	HZ	LOC101103222
NC_056056.1	150540001	1.51E+08	0.386693	0.190313	HZ	LOC101103222;LOC443320
NC_056066.1	25650001	25670001	0.476109	0.1681	HZ	LOC101103383
NC_056068.1	48055001	48075001	0.18392	0.190224	HZ	LOC101105945
NC_056068.1	48060001	48080001	0.234235	0.152773	HZ	LOC101105945
NC_056068.1	53055001	53075001	0.37973	0.14716	HZ	LOC101106108
NC_056068.1	53060001	53080001	0.323156	0.153965	HZ	LOC101106108;LOC101106361

NC_056068.1	53065001	53085001	0.298647	0.147172	HZ	LOC101106108;LOC101106361
NC_056068.1	48065001	48085001	0.181531	0.169853	HZ	LOC101106199
NC_056068.1	48070001	48090001	0.161729	0.181813	HZ	LOC101106199
NC_056079.1	6275001	6295001	0.277101	0.149487	HZ	LOC101106394;LOC105605118
NC_056056.1	7590001	7610001	0.168774	0.157057	HZ	LOC101106416;LOC114113869
NC_056056.1	138375001	1.38E+08	0.480812	0.156197	HZ	LOC101106764
NC_056063.1	70645001	70665001	0.475228	0.164086	HZ	LOC101106781
NC_056080.1	116255001	1.16E+08	0.450348	0.157665	HZ	LOC101108113
NC_056080.1	116260001	1.16E+08	0.436672	0.158937	HZ	LOC101108113
NC_056080.1	116265001	1.16E+08	0.441607	0.161673	HZ	LOC101108113
NC_056080.1	116270001	1.16E+08	0.443162	0.159337	HZ	LOC101108113
NC_056080.1	116275001	1.16E+08	0.447326	0.159429	HZ	LOC101108113
NC_056080.1	116280001	1.16E+08	0.467306	0.153664	HZ	LOC101108113
NC_056054.1	108005001	1.08E+08	0.393439	0.255117	HZ	LOC101108789
NC_056054.1	265225001	2.65E+08	0.283577	0.191604	HZ	LOC101108798;LOC114110486;TSPEAR
NC_056054.1	265230001	2.65E+08	0.140933	0.194892	HZ	LOC101108798;TSPEAR
NC_056054.1	265235001	2.65E+08	0.159057	0.160717	HZ	LOC101108798;TSPEAR
NC_056054.1	166165001	1.66E+08	0.346041	0.170133	HZ	LOC101108803
NC_056054.1	166170001	1.66E+08	0.245379	0.227357	HZ	LOC101108803
NC_056054.1	166175001	1.66E+08	0.180274	0.257182	HZ	LOC101108803
NC_056054.1	166180001	1.66E+08	0.092159	0.288881	HZ	LOC101108803
NC_056054.1	166200001	1.66E+08	0.077296	0.196629	HZ	LOC101108803
NC_056055.1	149840001	1.5E+08	0.216169	0.240775	HZ	LOC101109941
NC_056055.1	149845001	1.5E+08	0.302112	0.268948	HZ	LOC101109941
NC_056055.1	149850001	1.5E+08	0.355932	0.281771	HZ	LOC101109941
NC_056055.1	149855001	1.5E+08	0.410511	0.294627	HZ	LOC101109941
NC_056055.1	149860001	1.5E+08	0.396958	0.179471	HZ	LOC101109941
NC_056055.1	149865001	1.5E+08	0.37234	0.164727	HZ	LOC101109941
NC_056055.1	149880001	1.5E+08	0.065782	0.172051	HZ	LOC101109941
NC_056055.1	149885001	1.5E+08	0.041934	0.172668	HZ	LOC101109941
NC_056055.1	149890001	1.5E+08	0.062593	0.161363	HZ	LOC101109941
NC_056055.1	149940001	1.5E+08	0.410975	0.147808	HZ	LOC101109941;MARCHF7
NC_056055.1	149945001	1.5E+08	0.272962	0.213313	HZ	LOC101109941;MARCHF7
NC_056055.1	149950001	1.5E+08	0.255135	0.201535	HZ	LOC101109941;MARCHF7
NC_056055.1	149955001	1.5E+08	0.255135	0.220253	HZ	LOC101109941;MARCHF7
NC_056072.1	51295001	51315001	0.488106	0.16117	HZ	LOC101110095;SLC26A6;TMEM89

NC_056054.1	199390001	1.99E+08	0.5	0.25413	HZ	LOC101110107
NC_056054.1	199395001	1.99E+08	0.340564	0.286534	HZ	LOC101110107
NC_056054.1	199400001	1.99E+08	0.294376	0.302558	HZ	LOC101110107
NC_056054.1	199405001	1.99E+08	0.275244	0.323412	HZ	LOC101110107
NC_056054.1	199410001	1.99E+08	0.345574	0.36316	HZ	LOC101110107
NC_056054.1	199415001	1.99E+08	0.341318	0.36118	HZ	LOC101110107
NC_056054.1	199420001	1.99E+08	0.394124	0.305192	HZ	LOC101110107
NC_056054.1	199425001	1.99E+08	0.398417	0.284488	HZ	LOC101110107
NC_056054.1	199430001	1.99E+08	0.397873	0.233196	HZ	LOC101110107
NC_056054.1	199435001	1.99E+08	0.391569	0.18394	HZ	LOC101110107
NC_056054.1	199440001	1.99E+08	0.396367	0.178006	HZ	LOC101110107
NC_056054.1	269915001	2.7E+08	0.053935	0.205705	HZ	LOC101110116;LOC101110385;TTC3
NC_056054.1	250670001	2.51E+08	0.223361	0.150976	HZ	LOC101110206
NC_056080.1	118940001	1.19E+08	0.120424	0.15908	HZ	LOC101110213
NC_056080.1	118945001	1.19E+08	0.120354	0.159499	HZ	LOC101110213
NC_056080.1	118950001	1.19E+08	0.172889	0.159084	HZ	LOC101110213
NC_056080.1	119880001	1.2E+08	0.446535	0.176938	HZ	LOC101110741
NC_056080.1	119885001	1.2E+08	0.464333	0.177491	HZ	LOC101110741
NC_056080.1	119890001	1.2E+08	0.440171	0.162047	HZ	LOC101110741
NC_056080.1	119895001	1.2E+08	0.433874	0.160884	HZ	LOC101110741
NC_056080.1	119900001	1.2E+08	0.371022	0.182486	HZ	LOC101110741
NC_056080.1	119905001	1.2E+08	0.266974	0.241562	HZ	LOC101110741
NC_056064.1	14040001	14060001	0.353043	0.155688	HZ	LOC101110777
NC_056080.1	26060001	26080001	0.393148	0.161804	HZ	LOC101110823
NC_056055.1	27740001	27760001	0.466755	0.15307	HZ	LOC101110824
NC_056067.1	61290001	61310001	0.439176	0.292328	HZ	LOC101111046
NC_056067.1	61295001	61315001	0.446193	0.321927	HZ	LOC101111046
NC_056055.1	149995001	1.5E+08	0.381285	0.174238	HZ	LOC101111351;LOC121818516;MARCHF7
NC_056055.1	52515001	52535001	0.421053	0.226251	HZ	LOC101111518
NC_056055.1	52520001	52540001	0.42155	0.213768	HZ	LOC101111518
NC_056055.1	52525001	52545001	0.432123	0.181184	HZ	LOC101111518
NC_056055.1	52530001	52550001	0.434734	0.159255	HZ	LOC101111518;LOC101111773
NC_056055.1	220770001	2.21E+08	0.172414	0.163084	HZ	LOC101111528
NC_056054.1	187245001	1.87E+08	0.048005	0.485847	HZ	LOC101111847
NC_056054.1	187250001	1.87E+08	0.095145	0.468239	HZ	LOC101111847
NC_056054.1	187255001	1.87E+08	0.14067	0.399063	HZ	LOC101111847

NC_056054.1	187260001	1.87E+08	0.479756	0.263786	HZ	LOC101111847
NC_056054.1	187225001	1.87E+08	0.103558	0.458633	HZ	LOC101111847;SLC15A2
NC_056054.1	187230001	1.87E+08	0.090656	0.461022	HZ	LOC101111847;SLC15A2
NC_056054.1	187235001	1.87E+08	0.07003	0.475994	HZ	LOC101111847;SLC15A2
NC_056054.1	187240001	1.87E+08	0.040134	0.490728	HZ	LOC101111847;SLC15A2
NC_056054.1	97210001	97230001	0.216511	0.158371	HZ	LOC101112189
NC_056054.1	97215001	97235001	0.276392	0.15612	HZ	LOC101112189
NC_056054.1	97220001	97240001	0.451851	0.149827	HZ	LOC101112189
NC_056054.1	97735001	97755001	0.329262	0.305619	HZ	LOC101112864
NC_056054.1	97760001	97780001	0.348916	0.350786	HZ	LOC101112864
NC_056054.1	97765001	97785001	0.316143	0.315473	HZ	LOC101112864
NC_056054.1	97770001	97790001	0.303636	0.267217	HZ	LOC101112864
NC_056054.1	97775001	97795001	0.390877	0.205033	HZ	LOC101112864
NC_056080.1	90365001	90385001	0.491151	0.282712	HZ	LOC101112889
NC_056056.1	13405001	13425001	0.462996	0.185741	HZ	LOC101112963;LOC101120070
NC_056070.1	45340001	45360001	0.136564	0.201037	HZ	LOC101113101
NC_056070.1	45345001	45365001	0.10247	0.209805	HZ	LOC101113101
NC_056070.1	45375001	45395001	0.086798	0.174595	HZ	LOC101113101
NC_056070.1	45380001	45400001	0.135758	0.19592	HZ	LOC101113101
NC_056070.1	45385001	45405001	0.209974	0.199638	HZ	LOC101113101
NC_056070.1	45390001	45410001	0.277493	0.190535	HZ	LOC101113101
NC_056059.1	103745001	1.04E+08	0.286036	0.159327	HZ	LOC101113239
NC_056059.1	103750001	1.04E+08	0.345286	0.149166	HZ	LOC101113239
NC_056059.1	103755001	1.04E+08	0.397885	0.16507	HZ	LOC101113239
NC_056059.1	103780001	1.04E+08	0.226812	0.21643	HZ	LOC101113239
NC_056059.1	103785001	1.04E+08	0.278085	0.194325	HZ	LOC101113239
NC_056059.1	103790001	1.04E+08	0.268244	0.197277	HZ	LOC101113239
NC_056059.1	103795001	1.04E+08	0.255109	0.205755	HZ	LOC101113239
NC_056059.1	103800001	1.04E+08	0.273989	0.240774	HZ	LOC101113239
NC_056059.1	103805001	1.04E+08	0.347996	0.27865	HZ	LOC101113239
NC_056059.1	103825001	1.04E+08	0.461739	0.194633	HZ	LOC101113239
NC_056059.1	103830001	1.04E+08	0.303074	0.211252	HZ	LOC101113239
NC_056059.1	103835001	1.04E+08	0.253109	0.222468	HZ	LOC101113239
NC_056059.1	103840001	1.04E+08	0.23852	0.20083	HZ	LOC101113239
NC_056059.1	103845001	1.04E+08	0.213372	0.201281	HZ	LOC101113239
NC_056059.1	103850001	1.04E+08	0.436735	0.14608	HZ	LOC101113239

NC_056059.1	103855001	1.04E+08	0.436652	0.189736	HZ	LOC101113239
NC_056059.1	103760001	1.04E+08	0.415987	0.146063	HZ	LOC101113239;LOC114115340
NC_056059.1	103770001	1.04E+08	0.305165	0.180563	HZ	LOC101113239;LOC114115340
NC_056059.1	103775001	1.04E+08	0.205107	0.217008	HZ	LOC101113239;LOC114115340
NC_056059.1	103860001	1.04E+08	0.493425	0.184597	HZ	LOC101113239;LOC121819790
NC_056073.1	28055001	28075001	0.246178	0.197085	HZ	LOC101113705
NC_056073.1	28060001	28080001	0.323119	0.181187	HZ	LOC101113705
NC_056073.1	28065001	28085001	0.336524	0.173351	HZ	LOC101113705
NC_056072.1	12715001	12735001	0.383735	0.217466	HZ	LOC101113800
NC_056072.1	12720001	12740001	0.314828	0.232302	HZ	LOC101113800
NC_056072.1	12725001	12745001	0.253747	0.232343	HZ	LOC101113800
NC_056072.1	12730001	12750001	0.19382	0.243763	HZ	LOC101113800
NC_056072.1	12735001	12755001	0.07305	0.277962	HZ	LOC101113800
NC_056072.1	12755001	12775001	0.121662	0.191108	HZ	LOC101113800
NC_056072.1	12760001	12780001	0.431472	0.158443	HZ	LOC101113800
NC_056057.1	107715001	1.08E+08	0.374956	0.146641	HZ	LOC101114011
NC_056057.1	107720001	1.08E+08	0.306319	0.196176	HZ	LOC101114011;LOC101122595
NC_056071.1	23735001	23755001	0.148233	0.175835	HZ	LOC101114310
NC_056061.1	50075001	50095001	0.353663	0.177761	HZ	LOC101114528;ZNF292
NC_056061.1	50080001	50100001	0.386986	0.17668	HZ	LOC101114528;ZNF292
NC_056061.1	50085001	50105001	0.480904	0.147643	HZ	LOC101114528;ZNF292
NC_056057.1	41750001	41770001	0.103081	0.160842	HZ	LOC101114861
NC_056063.1	18995001	19015001	0.44256	0.232633	HZ	LOC101114879
NC_056063.1	19000001	19020001	0.434624	0.231746	HZ	LOC101114879
NC_056063.1	19005001	19025001	0.436907	0.235491	HZ	LOC101114879
NC_056063.1	19010001	19030001	0.44657	0.231211	HZ	LOC101114879
NC_056067.1	65495001	65515001	0.388033	0.16736	HZ	LOC101115059
NC_056067.1	65500001	65520001	0.440426	0.204754	HZ	LOC101115059
NC_056067.1	65505001	65525001	0.438904	0.21227	HZ	LOC101115059
NC_056057.1	12865001	12885001	0.449648	0.149405	HZ	LOC101115117
NC_056057.1	12880001	12900001	0.324457	0.216682	HZ	LOC101115117
NC_056057.1	12885001	12905001	0.334773	0.206953	HZ	LOC101115117
NC_056054.1	109025001	1.09E+08	0.374193	0.158645	HZ	LOC101115176
NC_056054.1	109030001	1.09E+08	0.350424	0.176213	HZ	LOC101115176
NC_056067.1	62120001	62140001	0.352862	0.417233	HZ	LOC101115398
NC_056067.1	62125001	62145001	0.240094	0.327411	HZ	LOC101115398

NC_056067.1	62130001	62150001	0.317801	0.268065	HZ	LOC101115398
NC_056054.1	99945001	99965001	0.178011	0.15003	HZ	LOC101115753;LOC101121387;LOC101121643;LOC114113
NC_056057.1	108310001	1.08E+08	0.423327	0.287127	HZ	LOC101116648
NC_056080.1	68920001	68940001	0.479299	0.14896	HZ	LOC101116886;MAGT1
NC_056066.1	34850001	34870001	0.139463	0.163177	HZ	LOC101117350
NC_056066.1	34855001	34875001	0.129897	0.169348	HZ	LOC101117350
NC_056056.1	165245001	1.65E+08	0.216889	0.248257	HZ	LOC101117494
NC_056056.1	165250001	1.65E+08	0.286695	0.218406	HZ	LOC101117494
NC_056056.1	165255001	1.65E+08	0.35877	0.199668	HZ	LOC101117494
NC_056064.1	18980001	19000001	0.488784	0.150387	HZ	LOC101117683
NC_056072.1	53210001	53230001	0.449777	0.213636	HZ	LOC101117971
NC_056072.1	53215001	53235001	0.447039	0.205812	HZ	LOC101117971
NC_056074.1	38020001	38040001	0.301305	0.145549	HZ	LOC101118318
NC_056074.1	38025001	38045001	0.237113	0.184488	HZ	LOC101118318
NC_056074.1	38030001	38050001	0.197733	0.201338	HZ	LOC101118318
NC_056074.1	38035001	38055001	0.187345	0.195244	HZ	LOC101118318
NC_056080.1	2775001	2795001	0.344248	0.150523	HZ	LOC101118336
NC_056080.1	2780001	2800001	0.336242	0.160805	HZ	LOC101118336
NC_056068.1	79035001	79055001	0.484312	0.145327	HZ	LOC101118639
NC_056074.1	38275001	38295001	0.288877	0.292399	HZ	LOC101119087
NC_056074.1	38280001	38300001	0.38777	0.239921	HZ	LOC101119087
NC_056074.1	38245001	38265001	0.354143	0.147747	HZ	LOC101119087;LOC101119346
NC_056074.1	38250001	38270001	0.304885	0.194645	HZ	LOC101119087;LOC101119346
NC_056074.1	38255001	38275001	0.240325	0.257626	HZ	LOC101119087;LOC101119346;LOC121817564
NC_056074.1	38260001	38280001	0.190562	0.314451	HZ	LOC101119087;LOC101119346;LOC121817564
NC_056074.1	38265001	38285001	0.194496	0.318952	HZ	LOC101119087;LOC121817564
NC_056074.1	38270001	38290001	0.14389	0.3555	HZ	LOC101119087;LOC121817564
NC_056074.1	38285001	38305001	0.361963	0.220378	HZ	LOC101119087;PLAAT5
NC_056054.1	66245001	66265001	0.418597	0.154408	HZ	LOC101119517
NC_056054.1	66260001	66280001	0.167739	0.176766	HZ	LOC101119773
NC_056074.1	38345001	38365001	0.446359	0.193338	HZ	LOC101120029
NC_056074.1	38350001	38370001	0.460488	0.21712	HZ	LOC101120029
NC_056074.1	38355001	38375001	0.372587	0.243818	HZ	LOC101120029
NC_056074.1	38360001	38380001	0.324095	0.26393	HZ	LOC101120029
NC_056074.1	38365001	38385001	0.329482	0.255676	HZ	LOC101120029
NC_056074.1	38370001	38390001	0.250954	0.240577	HZ	LOC101120029

NC_056074.1	38375001	38395001	0.345558	0.193995	HZ	LOC101120029
NC_056074.1	38380001	38400001	0.230137	0.147992	HZ	LOC101120029
NC_056054.1	50465001	50485001	0.265496	0.178114	HZ	LOC101120030
NC_056054.1	50470001	50490001	0.29045	0.176089	HZ	LOC101120030
NC_056054.1	50475001	50495001	0.333445	0.15814	HZ	LOC101120030
NC_056054.1	50480001	50500001	0.364085	0.15499	HZ	LOC101120030
NC_056056.1	13410001	13430001	0.41871	0.225972	HZ	LOC101120070
NC_056059.1	85985001	86005001	0.468918	0.148628	HZ	LOC101120084
NC_056056.1	194515001	1.95E+08	0.480244	0.196919	HZ	LOC101120653
NC_056067.1	61785001	61805001	0.280406	0.257293	HZ	LOC101120904;LOC121816560
NC_056067.1	61795001	61815001	0.458647	0.272794	HZ	LOC101121159;LOC121816560
NC_056067.1	61800001	61820001	0.460914	0.297996	HZ	LOC101121159;LOC121816560
NC_056056.1	50480001	50500001	0.408515	0.205685	HZ	LOC101122016
NC_056065.1	60460001	60480001	0.489756	0.234054	HZ	LOC101122123;LOC101122372
NC_056065.1	60465001	60485001	0.360336	0.145883	HZ	LOC101122123;LOC101122372;TOR1AIP1
NC_056071.1	45655001	45675001	0.283301	0.185906	HZ	LOC101122138
NC_056071.1	45660001	45680001	0.318752	0.17097	HZ	LOC101122138
NC_056071.1	45665001	45685001	0.279607	0.156674	HZ	LOC101122138
NC_056063.1	22885001	22905001	0.296712	0.225445	HZ	LOC101122286
NC_056063.1	22890001	22910001	0.198425	0.223041	HZ	LOC101122286
NC_056063.1	22895001	22915001	0.304509	0.191136	HZ	LOC101122286
NC_056057.1	107690001	1.08E+08	0.072586	0.153989	HZ	LOC101122351
NC_056074.1	34885001	34905001	0.434595	0.202211	HZ	LOC101122563
NC_056057.1	107725001	1.08E+08	0.223212	0.265103	HZ	LOC101122595
NC_056057.1	107730001	1.08E+08	0.239562	0.268489	HZ	LOC101122595
NC_056057.1	107735001	1.08E+08	0.446701	0.30682	HZ	LOC101122595
NC_056073.1	49900001	49920001	0.406582	0.146329	HZ	LOC101123244
NC_056073.1	49895001	49915001	0.488427	0.171287	HZ	LOC101123244;LOC114109592
NC_056055.1	48360001	48380001	0.387229	0.248266	HZ	LOC101123268
NC_056072.1	43495001	43515001	0.466731	0.164554	HZ	LOC101123588
NC_056072.1	43500001	43520001	0.342308	0.228169	HZ	LOC101123588
NC_056072.1	43505001	43525001	0.303259	0.243282	HZ	LOC101123588
NC_056072.1	43510001	43530001	0.235548	0.278214	HZ	LOC101123588
NC_056072.1	43515001	43535001	0.231264	0.283115	HZ	LOC101123588
NC_056072.1	43520001	43540001	0.276986	0.262751	HZ	LOC101123588
NC_056072.1	43525001	43545001	0.237936	0.281915	HZ	LOC101123588

NC_056072.1	43530001	43550001	0.21988	0.284296	HZ	LOC101123588
NC_056072.1	43535001	43555001	0.240277	0.273999	HZ	LOC101123588
NC_056072.1	43540001	43560001	0.209258	0.259222	HZ	LOC101123588
NC_056072.1	43545001	43565001	0.171098	0.224773	HZ	LOC101123588
NC_056072.1	43550001	43570001	0.270379	0.170129	HZ	LOC101123588
NC_056068.1	81570001	81590001	0.173043	0.151509	HZ	LOC105602398;MRPL16;STX3
NC_056070.1	71515001	71535001	0.422192	0.177899	HZ	LOC105603000
NC_056054.1	265240001	2.65E+08	0.318879	0.161501	HZ	LOC105604734;TSPEAR
NC_056079.1	6260001	6280001	0.28432	0.150863	HZ	LOC105605118
NC_056079.1	6265001	6285001	0.274957	0.147815	HZ	LOC105605118
NC_056079.1	6270001	6290001	0.258427	0.162619	HZ	LOC105605118
NC_056064.1	19135001	19155001	0.453274	0.221532	HZ	LOC105605807
NC_056064.1	19140001	19160001	0.454128	0.220009	HZ	LOC105605807
NC_056064.1	19145001	19165001	0.475996	0.21176	HZ	LOC105605807
NC_056064.1	19150001	19170001	0.492827	0.203717	HZ	LOC105605807
NC_056055.1	73490001	73510001	0.468368	0.332101	HZ	LOC105608024
NC_056064.1	12450001	12470001	0.278753	0.258351	HZ	LOC105616451;USP32
NC_056064.1	12455001	12475001	0.360814	0.197669	HZ	LOC105616451;USP32
NC_056073.1	26850001	26870001	0.201188	0.178108	HZ	LOC106990117
NC_056073.1	26855001	26875001	0.108021	0.176622	HZ	LOC106990117
NC_056073.1	26860001	26880001	0.374077	0.146372	HZ	LOC106990117
NC_056059.1	80940001	80960001	0.041815	0.169693	HZ	LOC106991226
NC_056059.1	80945001	80965001	0.035512	0.229927	HZ	LOC106991226
NC_056073.1	47800001	47820001	0.279164	0.281007	HZ	LOC106991797
NC_056073.1	47805001	47825001	0.467419	0.204956	HZ	LOC106991797
NC_056067.1	48590001	48610001	0.103987	0.145533	HZ	LOC114108603
NC_056056.1	7535001	7555001	0.256594	0.300474	HZ	LOC114108697
NC_056056.1	7540001	7560001	0.394207	0.235977	HZ	LOC114108697
NC_056070.1	71105001	71125001	0.227542	0.185029	HZ	LOC114108841
NC_056073.1	28115001	28135001	0.111854	0.157063	HZ	LOC114109563
NC_056073.1	28120001	28140001	0.119669	0.183665	HZ	LOC114109563
NC_056073.1	28125001	28145001	0.479052	0.164917	HZ	LOC114109563;LOC114109564
NC_056080.1	90335001	90355001	0.502786	0.267849	HZ	LOC114111370
NC_056080.1	90340001	90360001	0.486603	0.265264	HZ	LOC114111370
NC_056080.1	90350001	90370001	0.501026	0.266267	HZ	LOC114111370
NC_056055.1	89530001	89550001	0.070237	0.147248	HZ	LOC114112818;LOC114112819

NC_056055.1	89525001	89545001	0.061423	0.150723	HZ	LOC114112818;LOC114112819;LOC114113084
NC_056055.1	89515001	89535001	0.04875	0.153817	HZ	LOC114112818;LOC114113084
NC_056055.1	89520001	89540001	0.047853	0.15486	HZ	LOC114112818;LOC114113084
NC_056055.1	172940001	1.73E+08	0.493679	0.272483	HZ	LOC114112873
NC_056055.1	172945001	1.73E+08	0.341616	0.340418	HZ	LOC114112873
NC_056056.1	13345001	13365001	0.482066	0.15379	HZ	LOC114113885
NC_056057.1	107550001	1.08E+08	0.079939	0.171758	HZ	LOC114114618
NC_056057.1	107555001	1.08E+08	0.084284	0.159037	HZ	LOC114114618
NC_056063.1	36930001	36950001	0.052783	0.200466	HZ	LOC114116652
NC_056063.1	36935001	36955001	0.066691	0.18906	HZ	LOC114116652
NC_056063.1	36940001	36960001	0.212961	0.154804	HZ	LOC114116652
NC_056064.1	19170001	19190001	0.487336	0.178469	HZ	LOC114117017
NC_056064.1	19175001	19195001	0.488337	0.178203	HZ	LOC114117017
NC_056064.1	19180001	19200001	0.49394	0.17723	HZ	LOC114117017
NC_056064.1	19185001	19205001	0.466355	0.213511	HZ	LOC114117017
NC_056064.1	19090001	19110001	0.461348	0.156319	HZ	LOC114117018
NC_056064.1	19095001	19115001	0.4417	0.185785	HZ	LOC114117018
NC_056064.1	19100001	19120001	0.46642	0.205906	HZ	LOC114117018
NC_056064.1	19105001	19125001	0.484603	0.209537	HZ	LOC114117018
NC_056064.1	19110001	19130001	0.451587	0.224339	HZ	LOC114117018
NC_056065.1	43970001	43990001	0.407206	0.216295	HZ	LOC114117228;RERE
NC_056065.1	7370001	7390001	0.459267	0.194716	HZ	LOC114117326
NC_056067.1	48630001	48650001	0.440618	0.163928	HZ	LOC114117809
NC_056067.1	48640001	48660001	0.428375	0.210766	HZ	LOC114117809
NC_056067.1	62480001	62500001	0.142373	0.167135	HZ	LOC114117836
NC_056065.1	53845001	53865001	0.49319	0.14586	HZ	LOC121816074
NC_056065.1	53850001	53870001	0.329009	0.227623	HZ	LOC121816074
NC_056065.1	53855001	53875001	0.308656	0.236481	HZ	LOC121816074
NC_056065.1	53860001	53880001	0.385064	0.19188	HZ	LOC121816074
NC_056067.1	61790001	61810001	0.354608	0.295621	HZ	LOC121816560
NC_056070.1	71650001	71670001	0.312565	0.183288	HZ	LOC121816897;SPECC1L
NC_056080.1	66455001	66475001	0.141039	0.15549	HZ	LOC121818183
NC_056080.1	14775001	14795001	0.449541	0.168181	HZ	LOC121818210
NC_056055.1	149990001	1.5E+08	0.289814	0.195803	HZ	LOC121818516;MARCHF7
NC_056059.1	103865001	1.04E+08	0.445033	0.170693	HZ	LOC121819790
NC_056062.1	64010001	64030001	0.352677	0.192389	HZ	LOC121820346

NC_056062.1	64015001	64035001	0.450335	0.170525	HZ	LOC121820346
NC_056062.1	64020001	64040001	0.445303	0.217734	HZ	LOC121820346
NC_056062.1	64025001	64045001	0.450918	0.230984	HZ	LOC121820346
NC_056062.1	64030001	64050001	0.446478	0.228188	HZ	LOC121820346
NC_056062.1	64035001	64055001	0.447914	0.236087	HZ	LOC121820346
NC_056062.1	64040001	64060001	0.448066	0.235037	HZ	LOC121820346
NC_056062.1	64045001	64065001	0.428338	0.204204	HZ	LOC121820346
NC_056062.1	64050001	64070001	0.403156	0.17392	HZ	LOC121820346
NC_056064.1	26420001	26440001	0.39514	0.235029	HZ	LOC121820578;PLD2
NC_056064.1	26425001	26445001	0.436544	0.226859	HZ	LOC121820578;PLD2;PSMB6
NC_056057.1	94150001	94170001	0.393776	0.15302	HZ	LOC443534
NC_056055.1	12485001	12505001	0.458464	0.305441	HZ	LPAR1
NC_056055.1	12495001	12515001	0.492436	0.174956	HZ	LPAR1
NC_056068.1	80645001	80665001	0.491687	0.189674	HZ	LPXN;ZFP91
NC_056070.1	7295001	7315001	0.377546	0.149406	HZ	LRBA
NC_056070.1	7635001	7655001	0.059398	0.172591	HZ	LRBA
NC_056070.1	7645001	7665001	0.117859	0.164293	HZ	LRBA
NC_056070.1	7650001	7670001	0.119623	0.17093	HZ	LRBA
NC_056070.1	7660001	7680001	0.192171	0.17079	HZ	LRBA
NC_056055.1	168345001	1.68E+08	0.344066	0.17245	HZ	LRP1B
NC_056055.1	168350001	1.68E+08	0.409805	0.160362	HZ	LRP1B
NC_056071.1	6775001	6795001	0.427699	0.165334	HZ	LRRC28
NC_056071.1	6780001	6800001	0.433296	0.212452	HZ	LRRC28
NC_056054.1	67270001	67290001	0.440141	0.153452	HZ	LRRC8D
NC_056056.1	121240001	1.21E+08	0.221489	0.159414	HZ	LRRIQ1;TSPAN19
NC_056056.1	127500001	1.28E+08	0.393323	0.166099	HZ	LUM
NC_056059.1	105490001	1.06E+08	0.436161	0.156411	HZ	LYAR;ZBTB49
NC_056077.1	19185001	19205001	0.460531	0.270444	HZ	LYRM1
NC_056077.1	19190001	19210001	0.438627	0.289437	HZ	LYRM1
NC_056077.1	19195001	19215001	0.356612	0.316425	HZ	LYRM1
NC_056072.1	53485001	53505001	0.452913	0.190009	HZ	LZTFL1;SLC6A20
NC_056080.1	53685001	53705001	0.175563	0.188626	HZ	MAGED1
NC_056080.1	53690001	53710001	0.416765	0.267584	HZ	MAGED1
NC_056057.1	44120001	44140001	0.418949	0.151784	HZ	MAGI2
NC_056057.1	44145001	44165001	0.431752	0.161896	HZ	MAGI2
NC_056057.1	44285001	44305001	0.257313	0.156743	HZ	MAGI2

NC_056057.1	44290001	44310001	0.128395	0.174779	HZ	MAGI2
NC_056080.1	68900001	68920001	0.486239	0.164318	HZ	MAGT1
NC_056080.1	68910001	68930001	0.431519	0.161217	HZ	MAGT1
NC_056080.1	68915001	68935001	0.468687	0.155623	HZ	MAGT1
NC_056080.1	42880001	42900001	0.319715	0.156803	HZ	MAOB
NC_056080.1	42885001	42905001	0.179245	0.22013	HZ	MAOB
NC_056069.1	9610001	9630001	0.500002	0.163716	HZ	MAP1B
NC_056055.1	211260001	2.11E+08	0.259555	0.233567	HZ	MAP2
NC_056054.1	202560001	2.03E+08	0.070252	0.165788	HZ	MAP3K13
NC_056054.1	202565001	2.03E+08	0.101297	0.191859	HZ	MAP3K13
NC_056054.1	202570001	2.03E+08	0.182902	0.170433	HZ	MAP3K13
NC_056055.1	118105001	1.18E+08	0.149282	0.252767	HZ	MAP3K2
NC_056055.1	118110001	1.18E+08	0.258137	0.21362	HZ	MAP3K2
NC_056055.1	118115001	1.18E+08	0.452407	0.150096	HZ	MAP3K2
NC_056055.1	239655001	2.4E+08	0.412594	0.203495	HZ	MAP3K6;SYTL1;TMEM222
NC_056055.1	239660001	2.4E+08	0.393734	0.216621	HZ	MAP3K6;SYTL1;TMEM222
NC_056056.1	99960001	99980001	0.350042	0.156173	HZ	MAP4K4
NC_056056.1	99965001	99985001	0.278765	0.189445	HZ	MAP4K4
NC_056056.1	99970001	99990001	0.28593	0.189932	HZ	MAP4K4
NC_056056.1	99975001	99995001	0.286874	0.187453	HZ	MAP4K4
NC_056073.1	10110001	10130001	0.38267	0.157867	HZ	MAPK14
NC_056073.1	10115001	10135001	0.38888	0.193798	HZ	MAPK14
NC_056073.1	10120001	10140001	0.422727	0.174967	HZ	MAPK14
NC_056076.1	50495001	50515001	0.19363	0.175251	HZ	MAPK4
NC_056076.1	50560001	50580001	0.02674	0.214737	HZ	MAPK4
NC_056076.1	50590001	50610001	0.039853	0.24273	HZ	MAPK4
NC_056076.1	50595001	50615001	0.244068	0.150744	HZ	MAPK4
NC_056076.1	50605001	50625001	0.248891	0.161911	HZ	MAPK4
NC_056064.1	45680001	45700001	0.303973	0.150358	HZ	MAPT
NC_056055.1	149960001	1.5E+08	0.254347	0.198871	HZ	MARCHF7
NC_056055.1	149965001	1.5E+08	0.297484	0.190695	HZ	MARCHF7
NC_056055.1	149970001	1.5E+08	0.307172	0.19413	HZ	MARCHF7
NC_056055.1	149975001	1.5E+08	0.297408	0.174906	HZ	MARCHF7
NC_056055.1	149980001	1.5E+08	0.271409	0.201943	HZ	MARCHF7
NC_056055.1	149985001	1.5E+08	0.259029	0.221822	HZ	MARCHF7
NC_056054.1	200795001	2.01E+08	0.132938	0.304047	HZ	MASP1

NC_056054.1	200800001	2.01E+08	0.402625	0.169469	HZ	MASP1
NC_056067.1	14235001	14255001	0.462612	0.185577	HZ	MC1R;TCF25
NC_056067.1	14240001	14260001	0.431308	0.255598	HZ	MC1R;TCF25
NC_056067.1	14245001	14265001	0.320666	0.266863	HZ	MC1R;TCF25
NC_056055.1	174980001	1.75E+08	0.184942	0.166711	HZ	MCM6
NC_056055.1	174985001	1.75E+08	0.391791	0.165261	HZ	MCM6
NC_056079.1	5770001	5790001	0.438858	0.21964	HZ	MCPH1
NC_056079.1	5775001	5795001	0.45076	0.190308	HZ	MCPH1
NC_056058.1	91710001	91730001	0.068908	0.14557	HZ	MCTP1
NC_056058.1	91975001	91995001	0.406325	0.342422	HZ	MCTP1
NC_056058.1	91980001	92000001	0.262987	0.418457	HZ	MCTP1
NC_056058.1	91985001	92005001	0.435654	0.352598	HZ	MCTP1
NC_056078.1	27945001	27965001	0.145891	0.232447	HZ	MCU
NC_056078.1	27950001	27970001	0.304133	0.171055	HZ	MCU
NC_056060.1	38315001	38335001	0.480011	0.236763	HZ	MDGA2
NC_056060.1	38320001	38340001	0.47726	0.241188	HZ	MDGA2
NC_056064.1	39590001	39610001	0.429234	0.159739	HZ	MED1
NC_056064.1	39595001	39615001	0.450397	0.16594	HZ	MED1
NC_056064.1	39600001	39620001	0.496469	0.161219	HZ	MED1
NC_056070.1	58865001	58885001	0.266436	0.28556	HZ	MED13L
NC_056070.1	58870001	58890001	0.336	0.203576	HZ	MED13L
NC_056070.1	58875001	58895001	0.340558	0.190226	HZ	MED13L
NC_056070.1	58880001	58900001	0.353847	0.192564	HZ	MED13L
NC_056070.1	58885001	58905001	0.349334	0.190985	HZ	MED13L
NC_056070.1	58890001	58910001	0.370845	0.205853	HZ	MED13L
NC_056070.1	58895001	58915001	0.360606	0.220279	HZ	MED13L
NC_056070.1	58900001	58920001	0.355932	0.186865	HZ	MED13L
NC_056070.1	58910001	58930001	0.384439	0.156091	HZ	MED13L
NC_056070.1	58915001	58935001	0.375566	0.15993	HZ	MED13L
NC_056070.1	58920001	58940001	0.384127	0.185809	HZ	MED13L
NC_056056.1	4750001	4770001	0.10757	0.162063	HZ	MED27
NC_056063.1	30290001	30310001	0.163748	0.352234	HZ	MEDAG
NC_056063.1	30295001	30315001	0.090855	0.406901	HZ	MEDAG
NC_056063.1	30305001	30325001	0.207926	0.33525	HZ	MEDAG
NC_056065.1	48870001	48890001	0.364414	0.191558	HZ	MEGF6
NC_056056.1	122985001	1.23E+08	0.495771	0.273618	HZ	MGAT4C

NC_056080.1	38300001	38320001	0.199755	0.15737	HZ	MID1IP1
NC_056071.1	45145001	45165001	0.368973	0.189163	HZ	MIPOL1
NC_056072.1	31725001	31745001	0.46079	0.330635	HZ	MITF
NC_056072.1	31730001	31750001	0.301016	0.368777	HZ	MITF
NC_056072.1	31735001	31755001	0.335416	0.26811	HZ	MITF
NC_056072.1	31740001	31760001	0.387879	0.208609	HZ	MITF
NC_056072.1	31745001	31765001	0.434103	0.165165	HZ	MITF
NC_056072.1	31750001	31770001	0.358261	0.18256	HZ	MITF
NC_056072.1	31860001	31880001	0.39046	0.22312	HZ	MITF
NC_056072.1	31865001	31885001	0.374933	0.230032	HZ	MITF
NC_056072.1	31870001	31890001	0.383508	0.216367	HZ	MITF
NC_056072.1	31875001	31895001	0.389951	0.214981	HZ	MITF
NC_056072.1	31880001	31900001	0.419432	0.201225	HZ	MITF
NC_056072.1	31885001	31905001	0.478667	0.196032	HZ	MITF
NC_056055.1	88815001	88835001	0.434999	0.204718	HZ	MLLT3
NC_056055.1	88820001	88840001	0.44778	0.18441	HZ	MLLT3
NC_056066.1	64485001	64505001	0.456329	0.148814	HZ	MMP24
NC_056066.1	64490001	64510001	0.404752	0.180016	HZ	MMP24
NC_056056.1	225445001	2.25E+08	0.4437	0.181231	HZ	MOV10L1
NC_056056.1	225450001	2.25E+08	0.365686	0.181654	HZ	MOV10L1;PANX2
NC_056056.1	225455001	2.25E+08	0.285978	0.187071	HZ	MOV10L1;PANX2
NC_056056.1	225460001	2.25E+08	0.350454	0.19269	HZ	MOV10L1;PANX2;TRABD
NC_056070.1	52425001	52445001	0.331688	0.16228	HZ	MPHOSPH9;PITPNM2
NC_056070.1	52430001	52450001	0.19656	0.197899	HZ	MPHOSPH9;PITPNM2
NC_056064.1	43435001	43455001	0.341916	0.149902	HZ	MPP3
NC_056066.1	35840001	35860001	0.306868	0.146369	HZ	MPP7
NC_056066.1	35845001	35865001	0.300339	0.15044	HZ	MPP7
NC_056066.1	66060001	66080001	0.380691	0.209459	HZ	MROH8
NC_056066.1	66065001	66085001	0.31139	0.216049	HZ	MROH8
NC_056066.1	66070001	66090001	0.403367	0.221053	HZ	MROH8
NC_056066.1	66055001	66075001	0.31987	0.196296	HZ	MROH8;RBL1
NC_056080.1	84810001	84830001	0.482033	0.167549	HZ	MTMR1
NC_056065.1	41650001	41670001	0.318133	0.305592	HZ	MTOR
NC_056065.1	41675001	41695001	0.130273	0.325739	HZ	MTOR
NC_056065.1	41680001	41700001	0.195707	0.302394	HZ	MTOR
NC_056065.1	41685001	41705001	0.220072	0.287944	HZ	MTOR

NC_056065.1	41720001	41740001	0.393444	0.225899	HZ	MTOR
NC_056065.1	41725001	41745001	0.364912	0.235739	HZ	MTOR
NC_056065.1	41730001	41750001	0.382765	0.223079	HZ	MTOR
NC_056065.1	41735001	41755001	0.476386	0.192198	HZ	MTOR
NC_056065.1	41740001	41760001	0.50334	0.181826	HZ	MTOR
NC_056065.1	41755001	41775001	0.480953	0.194801	HZ	MTOR
NC_056065.1	41760001	41780001	0.436938	0.214378	HZ	MTOR
NC_056065.1	41765001	41785001	0.454546	0.213131	HZ	MTOR
NC_056079.1	18605001	18625001	0.446947	0.188409	HZ	MTUS1
NC_056055.1	133845001	1.34E+08	0.410605	0.220737	HZ	MTX2
NC_056061.1	2720001	2740001	0.207243	0.165413	HZ	MYO6
NC_056054.1	179330001	1.79E+08	0.472957	0.145662	HZ	NAA50;USF3
NC_056054.1	212320001	2.12E+08	0.235658	0.198732	HZ	NAALADL2
NC_056054.1	212325001	2.12E+08	0.193182	0.257764	HZ	NAALADL2
NC_056054.1	212330001	2.12E+08	0.153578	0.314339	HZ	NAALADL2
NC_056054.1	212335001	2.12E+08	0.250854	0.267333	HZ	NAALADL2
NC_056054.1	212340001	2.12E+08	0.346644	0.229731	HZ	NAALADL2
NC_056054.1	212345001	2.12E+08	0.398701	0.1966	HZ	NAALADL2
NC_056054.1	212350001	2.12E+08	0.425538	0.197557	HZ	NAALADL2
NC_056054.1	212355001	2.12E+08	0.406786	0.212451	HZ	NAALADL2
NC_056054.1	212560001	2.13E+08	0.500001	0.172163	HZ	NAALADL2
NC_056054.1	212565001	2.13E+08	0.375604	0.158748	HZ	NAALADL2
NC_056054.1	212570001	2.13E+08	0.345553	0.189535	HZ	NAALADL2
NC_056054.1	212915001	2.13E+08	0.485374	0.162693	HZ	NAALADL2
NC_056076.1	42680001	42700001	0.079738	0.252143	HZ	NAPG
NC_056076.1	42685001	42705001	0.075254	0.232209	HZ	NAPG
NC_056076.1	42695001	42715001	0.136715	0.232103	HZ	NAPG;PIEZO2
NC_056076.1	42700001	42720001	0.411086	0.241377	HZ	NAPG;PIEZO2
NC_056074.1	22685001	22705001	0.383179	0.154841	HZ	NAV2
NC_056074.1	22690001	22710001	0.332808	0.155273	HZ	NAV2
NC_056074.1	22700001	22720001	0.221374	0.145202	HZ	NAV2
NC_056074.1	22715001	22735001	0.180382	0.151331	HZ	NAV2
NC_056074.1	22720001	22740001	0.275375	0.14801	HZ	NAV2
NC_056056.1	113965001	1.14E+08	0.484012	0.166209	HZ	NAV3
NC_056063.1	26105001	26125001	0.038336	0.296303	HZ	NBEA
NC_056063.1	26130001	26150001	0.26603	0.243373	HZ	NBEA

NC_056062.1	75640001	75660001	0.283932	0.206827	HZ	NCALD
NC_056062.1	75645001	75665001	0.237072	0.208719	HZ	NCALD
NC_056069.1	18445001	18465001	0.088335	0.162874	HZ	NDUFAF2
NC_056069.1	18450001	18470001	0.120311	0.15313	HZ	NDUFAF2
NC_056076.1	57490001	57510001	0.445118	0.147437	HZ	NEDD4L
NC_056073.1	44180001	44200001	0.457011	0.163863	HZ	NEDD9
NC_056073.1	44185001	44205001	0.285876	0.194086	HZ	NEDD9
NC_056073.1	44190001	44210001	0.217704	0.195695	HZ	NEDD9
NC_056073.1	44195001	44215001	0.108133	0.227103	HZ	NEDD9
NC_056054.1	259290001	2.59E+08	0.414089	0.145736	HZ	NEK11
NC_056054.1	259295001	2.59E+08	0.414696	0.183642	HZ	NEK11
NC_056054.1	259300001	2.59E+08	0.436251	0.181538	HZ	NEK11
NC_056063.1	21755001	21775001	0.463662	0.145325	HZ	NEK5
NC_056064.1	18590001	18610001	0.488235	0.150591	HZ	NF1
NC_056076.1	925001	945001	0.432288	0.172947	HZ	NFATC1
NC_056076.1	930001	950001	0.45679	0.169278	HZ	NFATC1
NC_056054.1	36310001	36330001	0.484895	0.27127	HZ	NFIA
NC_056054.1	36315001	36335001	0.371089	0.242854	HZ	NFIA
NC_056054.1	36350001	36370001	0.495845	0.168588	HZ	NFIA
NC_056060.1	43160001	43180001	0.477632	0.214704	HZ	NID2
NC_056061.1	14545001	14565001	0.129742	0.147306	HZ	NKAIN2
NC_056061.1	14550001	14570001	0.108466	0.151721	HZ	NKAIN2
NC_056054.1	97350001	97370001	0.461599	0.170767	HZ	NOTCH2
NC_056071.1	41135001	41155001	0.378728	0.307861	HZ	NPAS3
NC_056071.1	41140001	41160001	0.270361	0.362734	HZ	NPAS3
NC_056071.1	41145001	41165001	0.327813	0.307982	HZ	NPAS3
NC_056059.1	19605001	19625001	0.421653	0.163097	HZ	NPNT
NC_056059.1	19610001	19630001	0.458057	0.153908	HZ	NPNT
NC_056059.1	19615001	19635001	0.352702	0.156492	HZ	NPNT
NC_056070.1	9835001	9855001	0.390894	0.171666	HZ	NR3C2
NC_056070.1	9840001	9860001	0.184994	0.194878	HZ	NR3C2
NC_056070.1	9925001	9945001	0.497118	0.1607	HZ	NR3C2
NC_056056.1	74275001	74295001	0.458188	0.199438	HZ	NRXN1
NC_056056.1	74280001	74300001	0.396535	0.191172	HZ	NRXN1
NC_056056.1	74285001	74305001	0.362175	0.177812	HZ	NRXN1
NC_056056.1	74460001	74480001	0.312039	0.16018	HZ	NRXN1

NC_056056.1	74465001	74485001	0.315915	0.171738	HZ	NRXN1
NC_056056.1	74470001	74490001	0.433346	0.17871	HZ	NRXN1
NC_056056.1	74475001	74495001	0.497908	0.148808	HZ	NRXN1
NC_056060.1	89175001	89195001	0.3684	0.249041	HZ	NRXN3
NC_056060.1	89180001	89200001	0.426466	0.244037	HZ	NRXN3
NC_056054.1	259260001	2.59E+08	0.441359	0.1578	HZ	NUDT16
NC_056054.1	259265001	2.59E+08	0.385812	0.150269	HZ	NUDT16
NC_056057.1	100830001	1.01E+08	0.3309	0.211834	HZ	NUP205
NC_056057.1	100835001	1.01E+08	0.457404	0.233847	HZ	NUP205
NC_056055.1	227630001	2.28E+08	0.370385	0.157018	HZ	NYAP2
NC_056055.1	227635001	2.28E+08	0.334848	0.174186	HZ	NYAP2
NC_056055.1	227735001	2.28E+08	0.485948	0.146594	HZ	NYAP2
NC_056055.1	227740001	2.28E+08	0.435037	0.16178	HZ	NYAP2
NC_056055.1	227745001	2.28E+08	0.429882	0.189085	HZ	NYAP2
NC_056055.1	227750001	2.28E+08	0.415045	0.173969	HZ	NYAP2
NC_056054.1	26000001	26020001	0.200766	0.163423	HZ	OSBPL9
NC_056054.1	26005001	26025001	0.185115	0.240492	HZ	OSBPL9
NC_056054.1	26010001	26030001	0.289854	0.24858	HZ	OSBPL9
NC_056054.1	26015001	26035001	0.308544	0.202008	HZ	OSBPL9
NC_056054.1	26020001	26040001	0.398751	0.174204	HZ	OSBPL9
NC_056066.1	72620001	72640001	0.183018	0.188938	HZ	OSER1
NC_056066.1	72635001	72655001	0.336607	0.182759	HZ	OSER1
NC_056071.1	27670001	27690001	0.355014	0.252023	HZ	OTUD7A
NC_056069.1	32930001	32950001	0.378048	0.172496	HZ	OXCT1
NC_056069.1	32935001	32955001	0.386418	0.157119	HZ	OXCT1
NC_056068.1	52355001	52375001	0.177298	0.149984	HZ	P4HA3
NC_056068.1	52360001	52380001	0.190944	0.152802	HZ	P4HA3
NC_056068.1	52075001	52095001	0.372602	0.145531	HZ	PAAF1
NC_056059.1	40835001	40855001	0.377694	0.194402	HZ	PACRGL
NC_056059.1	40840001	40860001	0.373841	0.200339	HZ	PACRGL
NC_056056.1	3660001	3680001	0.185081	0.237384	HZ	PAEP
NC_056056.1	3665001	3685001	0.319177	0.209708	HZ	PAEP
NW_02459982	1155001	1175001	0.369085	0.188809	HZ	PAG3
NW_02459982	1160001	1180001	0.225076	0.246413	HZ	PAG3
NW_02459982	1165001	1185001	0.163116	0.266567	HZ	PAG3
NW_02459982	1170001	1190001	0.098056	0.277499	HZ	PAG3

NW_02459982	1175001	1195001	0.209133	0.238646	HZ	PAG3
NW_02459982	1180001	1200001	0.354367	0.196021	HZ	PAG3
NW_02459982	1185001	1205001	0.462581	0.166854	HZ	PAG3
NC_056073.1	44610001	44630001	0.108305	0.171377	HZ	PAK1IP1;TMEM14C
NC_056060.1	77235001	77255001	0.472044	0.220006	HZ	PALS1
NC_056060.1	77240001	77260001	0.391649	0.240385	HZ	PALS1
NC_056060.1	77245001	77265001	0.386161	0.247917	HZ	PALS1
NC_056060.1	77250001	77270001	0.38277	0.247537	HZ	PALS1
NC_056060.1	77255001	77275001	0.430968	0.249819	HZ	PALS1
NC_056063.1	32150001	32170001	0.435197	0.27459	HZ	PAN3
NC_056063.1	32155001	32175001	0.417661	0.268382	HZ	PAN3
NC_056063.1	32160001	32180001	0.410225	0.266414	HZ	PAN3
NC_056056.1	225465001	2.25E+08	0.390845	0.196148	HZ	PANX2;TRABD
NC_056056.1	225470001	2.25E+08	0.417866	0.205207	HZ	PANX2;TRABD
NC_056074.1	25545001	25565001	0.350974	0.15966	HZ	PANX3;TBRG1
NC_056074.1	25550001	25570001	0.46282	0.156458	HZ	PANX3;TBRG1
NC_056059.1	17835001	17855001	0.503068	0.146758	HZ	PAPSS1
NC_056054.1	37360001	37380001	0.150325	0.173254	HZ	PATJ
NC_056054.1	253645001	2.54E+08	0.488763	0.207897	HZ	PCCB
NC_056057.1	39170001	39190001	0.492063	0.24031	HZ	PCLO
NC_056054.1	112680001	1.13E+08	0.300107	0.23811	HZ	PCP4L1
NC_056054.1	112685001	1.13E+08	0.350976	0.218038	HZ	PCP4L1
NC_056054.1	112690001	1.13E+08	0.406823	0.17407	HZ	PCP4L1
NC_056075.1	30935001	30955001	0.370076	0.187179	HZ	PDCD4
NC_056075.1	30940001	30960001	0.331296	0.218509	HZ	PDCD4
NC_056075.1	30945001	30965001	0.44536	0.163865	HZ	PDCD4
NC_056068.1	50915001	50935001	0.434272	0.16159	HZ	PDE2A
NC_056068.1	50920001	50940001	0.474173	0.152115	HZ	PDE2A
NC_056070.1	42635001	42655001	0.473836	0.225754	HZ	PDGFC
NC_056068.1	4155001	4175001	0.16729	0.145526	HZ	PDGFD
NC_056068.1	4160001	4180001	0.188561	0.171007	HZ	PDGFD
NC_056068.1	4165001	4185001	0.150936	0.175525	HZ	PDGFD
NC_056068.1	4170001	4190001	0.209077	0.174549	HZ	PDGFD
NC_056068.1	4205001	4225001	0.269312	0.168255	HZ	PDGFD
NC_056077.1	18480001	18500001	0.37166	0.163709	HZ	PDILT;UMOD
NC_056080.1	22730001	22750001	0.416167	0.160237	HZ	PDK3

NC_056080.1	22735001	22755001	0.453084	0.178687	HZ	PDK3
NC_056080.1	22740001	22760001	0.368588	0.173904	HZ	PDK3
NC_056080.1	22745001	22765001	0.401099	0.182425	HZ	PDK3
NC_056080.1	22750001	22770001	0.214026	0.204551	HZ	PDK3
NC_056080.1	22755001	22775001	0.316436	0.228068	HZ	PDK3
NC_056080.1	22760001	22780001	0.301222	0.236383	HZ	PDK3
NC_056080.1	22765001	22785001	0.312018	0.23518	HZ	PDK3
NC_056080.1	22770001	22790001	0.325701	0.174777	HZ	PDK3
NC_056061.1	29905001	29925001	0.395759	0.234781	HZ	PDSS2
NC_056061.1	30155001	30175001	0.45799	0.313752	HZ	PDSS2
NC_056061.1	30160001	30180001	0.302808	0.310363	HZ	PDSS2
NC_056061.1	30165001	30185001	0.374709	0.263444	HZ	PDSS2
NC_056061.1	30170001	30190001	0.462326	0.287385	HZ	PDSS2
NC_056069.1	41910001	41930001	0.451642	0.148237	HZ	PDZD2
NC_056069.1	41915001	41935001	0.417043	0.149306	HZ	PDZD2
NC_056069.1	41920001	41940001	0.346007	0.171474	HZ	PDZD2
NC_056069.1	41925001	41945001	0.273345	0.168396	HZ	PDZD2
NC_056069.1	41930001	41950001	0.244024	0.180286	HZ	PDZD2
NC_056056.1	145780001	1.46E+08	0.485813	0.16648	HZ	PDZRN4
NC_056065.1	44955001	44975001	0.428229	0.148122	HZ	PER3;VAMP3
NC_056054.1	111290001	1.11E+08	0.356985	0.146804	HZ	PEX19
NC_056055.1	68265001	68285001	0.479306	0.230074	HZ	PGM5
NC_056061.1	68235001	68255001	0.333518	0.225414	HZ	PHACTR2
NC_056061.1	68240001	68260001	0.47199	0.242656	HZ	PHACTR2
NC_056061.1	68245001	68265001	0.395121	0.239962	HZ	PHACTR2
NC_056061.1	68250001	68270001	0.459447	0.189499	HZ	PHACTR2
NC_056080.1	20410001	20430001	0.147614	0.231121	HZ	PHEX
NC_056080.1	20435001	20455001	0.335371	0.215697	HZ	PHEX
NC_056080.1	20440001	20460001	0.441667	0.170586	HZ	PHEX
NC_056070.1	72240001	72260001	0.451268	0.156388	HZ	PI4KA;TMEM191C
NC_056055.1	231135001	2.31E+08	0.203281	0.15385	HZ	PID1
NC_056055.1	231140001	2.31E+08	0.387611	0.155007	HZ	PID1
NC_056055.1	231145001	2.31E+08	0.489669	0.148718	HZ	PID1
NC_056055.1	231170001	2.31E+08	0.488487	0.158222	HZ	PID1
NC_056055.1	231175001	2.31E+08	0.375959	0.158571	HZ	PID1
NC_056055.1	231180001	2.31E+08	0.212808	0.17832	HZ	PID1

NC_056055.1	231185001	2.31E+08	0.08089	0.229633	HZ	PID1
NC_056055.1	231190001	2.31E+08	0.059881	0.215789	HZ	PID1
NC_056055.1	231195001	2.31E+08	0.036764	0.234269	HZ	PID1
NC_056055.1	231200001	2.31E+08	0.021385	0.218652	HZ	PID1
NC_056076.1	42800001	42820001	0.209123	0.159452	HZ	PIEZO2
NC_056076.1	42805001	42825001	0.154229	0.162778	HZ	PIEZO2
NC_056076.1	42810001	42830001	0.124131	0.173013	HZ	PIEZO2
NC_056076.1	42815001	42835001	0.163082	0.15791	HZ	PIEZO2
NC_056076.1	42820001	42840001	0.202015	0.149724	HZ	PIEZO2
NC_056060.1	53120001	53140001	0.484904	0.260997	HZ	PIGB;PIGBOS1
NC_056070.1	52450001	52470001	0.185784	0.181717	HZ	PITPNM2
NC_056064.1	25230001	25250001	0.494308	0.152714	HZ	PITPNM3
NC_056058.1	106005001	1.06E+08	0.253547	0.166864	HZ	PJA2
NC_056055.1	246175001	2.46E+08	0.164308	0.231525	HZ	PLA2G2C;UBXN10
NC_056055.1	246180001	2.46E+08	0.169286	0.226829	HZ	PLA2G2C;UBXN10
NC_056055.1	246185001	2.46E+08	0.290533	0.148979	HZ	PLA2G2C;UBXN10
NC_056055.1	246295001	2.46E+08	0.429223	0.234382	HZ	PLA2G2D
NC_056055.1	246300001	2.46E+08	0.33114	0.239017	HZ	PLA2G2D
NC_056055.1	246305001	2.46E+08	0.318998	0.225793	HZ	PLA2G2D
NC_056074.1	38395001	38415001	0.178594	0.251114	HZ	PLAAT3
NC_056074.1	38400001	38420001	0.245154	0.264729	HZ	PLAAT3
NC_056074.1	38405001	38425001	0.37951	0.280201	HZ	PLAAT3
NC_056074.1	38290001	38310001	0.429829	0.181401	HZ	PLAAT5
NC_056074.1	38295001	38315001	0.380853	0.195779	HZ	PLAAT5
NC_056074.1	38300001	38320001	0.313894	0.166721	HZ	PLAAT5
NC_056074.1	38305001	38325001	0.353782	0.182466	HZ	PLAAT5
NC_056074.1	38310001	38330001	0.335487	0.199604	HZ	PLAAT5
NC_056065.1	33735001	33755001	0.47416	0.216887	HZ	PLD5
NC_056067.1	55190001	55210001	0.096001	0.288187	HZ	PLEKHA4
NC_056067.1	55200001	55220001	0.483293	0.332289	HZ	PLEKHA4;PPP1R15A;TULP2
NC_056055.1	73510001	73530001	0.486898	0.35058	HZ	PLGRKT
NC_056054.1	76280001	76300001	0.460756	0.165749	HZ	PLPPR5
NC_056054.1	76285001	76305001	0.492146	0.169596	HZ	PLPPR5
NC_056054.1	76315001	76335001	0.494023	0.336717	HZ	PLPPR5
NC_056054.1	76320001	76340001	0.499547	0.317116	HZ	PLPPR5
NC_056054.1	76325001	76345001	0.488244	0.320945	HZ	PLPPR5

NC_056054.1	76330001	76350001	0.417845	0.355927	HZ	PLPPR5
NC_056054.1	76335001	76355001	0.498918	0.358123	HZ	PLPPR5
NC_056060.1	50675001	50695001	0.342421	0.155346	HZ	POLR2M
NC_056077.1	20510001	20530001	0.105601	0.168341	HZ	POLR3E
NC_056077.1	20515001	20535001	0.06925	0.167534	HZ	POLR3E
NC_056054.1	253840001	2.54E+08	0.396977	0.220348	HZ	PPP2R3A
NC_056054.1	253845001	2.54E+08	0.259762	0.260041	HZ	PPP2R3A
NC_056054.1	253850001	2.54E+08	0.204418	0.271841	HZ	PPP2R3A
NC_056054.1	253855001	2.54E+08	0.216902	0.276679	HZ	PPP2R3A
NC_056054.1	253860001	2.54E+08	0.253439	0.260283	HZ	PPP2R3A
NC_056054.1	253865001	2.54E+08	0.226514	0.262741	HZ	PPP2R3A
NC_056054.1	253870001	2.54E+08	0.255848	0.252396	HZ	PPP2R3A
NC_056054.1	253875001	2.54E+08	0.299897	0.236731	HZ	PPP2R3A
NC_056054.1	253880001	2.54E+08	0.282898	0.234741	HZ	PPP2R3A
NC_056054.1	253885001	2.54E+08	0.31465	0.222197	HZ	PPP2R3A
NC_056054.1	253890001	2.54E+08	0.417969	0.189903	HZ	PPP2R3A
NC_056054.1	253895001	2.54E+08	0.41918	0.179272	HZ	PPP2R3A
NC_056054.1	253920001	2.54E+08	0.263316	0.219819	HZ	PPP2R3A
NC_056054.1	253925001	2.54E+08	0.142458	0.274633	HZ	PPP2R3A
NC_056054.1	253930001	2.54E+08	0.141674	0.282367	HZ	PPP2R3A
NC_056054.1	253935001	2.54E+08	0.172186	0.260416	HZ	PPP2R3A
NC_056054.1	253940001	2.54E+08	0.182576	0.269096	HZ	PPP2R3A
NC_056054.1	253945001	2.54E+08	0.169697	0.273096	HZ	PPP2R3A
NC_056054.1	253950001	2.54E+08	0.155228	0.278073	HZ	PPP2R3A
NC_056054.1	253955001	2.54E+08	0.261718	0.25959	HZ	PPP2R3A
NC_056054.1	253960001	2.54E+08	0.46061	0.183334	HZ	PPP2R3A
NC_056059.1	23745001	23765001	0.166917	0.244729	HZ	PPP3CA
NC_056071.1	38015001	38035001	0.424836	0.193371	HZ	PRKD1
NC_056071.1	38020001	38040001	0.178818	0.276928	HZ	PRKD1
NC_056071.1	38030001	38050001	0.180622	0.33477	HZ	PRKD1
NC_056071.1	38035001	38055001	0.342336	0.267943	HZ	PRKD1
NC_056071.1	38135001	38155001	0.421172	0.148262	HZ	PRKD1
NC_056071.1	38140001	38160001	0.38404	0.196721	HZ	PRKD1
NC_056062.1	32535001	32555001	0.036939	0.1454	HZ	PRKDC
NC_056062.1	32550001	32570001	0.064122	0.162952	HZ	PRKDC
NC_056075.1	7340001	7360001	0.423666	0.1496	HZ	PRKG1

NC_056075.1	7705001	7725001	0.16337	0.17951	HZ	PRKG1
NC_056075.1	7710001	7730001	0.051621	0.195276	HZ	PRKG1
NC_056075.1	7715001	7735001	0.060665	0.191599	HZ	PRKG1
NC_056075.1	7720001	7740001	0.054036	0.188602	HZ	PRKG1
NC_056075.1	7730001	7750001	0.130259	0.22306	HZ	PRKG1
NC_056054.1	267030001	2.67E+08	0.437555	0.179989	HZ	PRMT2
NC_056054.1	267035001	2.67E+08	0.428679	0.153896	HZ	PRMT2
NC_056055.1	118090001	1.18E+08	0.376628	0.213868	HZ	PROC
NC_056080.1	10885001	10905001	0.192053	0.178307	HZ	PRPS2
NC_056058.1	3050001	3070001	0.185927	0.149753	HZ	PRSS38
NC_056058.1	3060001	3080001	0.120612	0.153532	HZ	PRSS38
NC_056055.1	58580001	58600001	0.237218	0.190299	HZ	PSAT1
NC_056055.1	58595001	58615001	0.13002	0.234729	HZ	PSAT1
NC_056055.1	58600001	58620001	0.237358	0.227469	HZ	PSAT1
NC_056055.1	58605001	58625001	0.489184	0.153239	HZ	PSAT1
NC_056054.1	54955001	54975001	0.496017	0.206132	HZ	PTGFR
NC_056054.1	54960001	54980001	0.42509	0.18792	HZ	PTGFR
NC_056066.1	77630001	77650001	0.414407	0.168249	HZ	PTGIS
NC_056066.1	77645001	77665001	0.179436	0.195548	HZ	PTGIS
NC_056070.1	61895001	61915001	0.411765	0.184106	HZ	PTPN11
NC_056070.1	61900001	61920001	0.365671	0.243201	HZ	PTPN11
NC_056070.1	61905001	61925001	0.397352	0.299491	HZ	PTPN11
NC_056070.1	61910001	61930001	0.433365	0.258216	HZ	PTPN11
NC_056055.1	13955001	13975001	0.087699	0.155743	HZ	PTPN3
NC_056055.1	77125001	77145001	0.459589	0.176105	HZ	PTPRD
NC_056055.1	77130001	77150001	0.395298	0.209516	HZ	PTPRD
NC_056055.1	78295001	78315001	0.248708	0.198589	HZ	PTPRD
NC_056055.1	78300001	78320001	0.180677	0.24172	HZ	PTPRD
NC_056055.1	78305001	78325001	0.300982	0.225649	HZ	PTPRD
NC_056076.1	41315001	41335001	0.322868	0.159801	HZ	PTPRM
NC_056076.1	41320001	41340001	0.405159	0.182958	HZ	PTPRM
NC_056057.1	87945001	87965001	0.452494	0.182236	HZ	PTPRZ1
NC_056056.1	28180001	28200001	0.347032	0.146831	HZ	PUM2
NC_056056.1	28195001	28215001	0.317227	0.147995	HZ	PUM2
NC_056080.1	131820001	1.32E+08	0.377115	0.289902	HZ	PWWP3B
NC_056080.1	131825001	1.32E+08	0.412802	0.265037	HZ	PWWP3B

NC_056080.1	131830001	1.32E+08	0.504116	0.219635	HZ	PWWP3B
NC_056075.1	37810001	37830001	0.228013	0.221868	HZ	RAB11FIP2
NC_056075.1	37815001	37835001	0.125	0.341495	HZ	RAB11FIP2
NC_056075.1	37820001	37840001	0.089465	0.383067	HZ	RAB11FIP2
NC_056075.1	37825001	37845001	0.064914	0.362195	HZ	RAB11FIP2
NC_056076.1	42355001	42375001	0.500844	0.151861	HZ	RAB31
NC_056070.1	62615001	62635001	0.435796	0.296509	HZ	RAB35
NC_056070.1	62620001	62640001	0.444312	0.285653	HZ	RAB35
NC_056072.1	59065001	59085001	0.417712	0.166079	HZ	RAB7A
NC_056072.1	59070001	59090001	0.419081	0.195378	HZ	RAB7A
NC_056072.1	59075001	59095001	0.396544	0.17	HZ	RAB7A
NC_056072.1	59080001	59100001	0.386068	0.149095	HZ	RAB7A
NC_056080.1	11835001	11855001	0.309577	0.147393	HZ	RAB9A;TRAPPC2
NC_056065.1	55180001	55200001	0.315201	0.148351	HZ	RABGAP1L
NC_056056.1	225950001	2.26E+08	0.123703	0.152354	HZ	RABL2B
NC_056056.1	225955001	2.26E+08	0.221597	0.147495	HZ	RABL2B
NC_056060.1	34485001	34505001	0.411886	0.190463	HZ	RAD51;RMDN3
NC_056060.1	34490001	34510001	0.312406	0.2014	HZ	RAD51;RMDN3
NC_056060.1	34495001	34515001	0.310848	0.175274	HZ	RAD51;RMDN3
NC_056062.1	82755001	82775001	0.437132	0.14921	HZ	RAD54B
NC_056062.1	82770001	82790001	0.488487	0.173455	HZ	RAD54B
NC_056072.1	49290001	49310001	0.278319	0.151362	HZ	RAD54L2
NC_056056.1	8925001	8945001	0.087882	0.187489	HZ	RALGPS1
NC_056056.1	8965001	8985001	0.114433	0.211019	HZ	RALGPS1
NC_056056.1	8970001	8990001	0.180151	0.235633	HZ	RALGPS1
NC_056054.1	3345001	3365001	0.459459	0.181705	HZ	RAMP1
NC_056054.1	3350001	3370001	0.41112	0.150337	HZ	RAMP1
NC_056054.1	3355001	3375001	0.330342	0.153659	HZ	RAMP1
NC_056077.1	5635001	5655001	0.411324	0.18153	HZ	RBFOX1
NC_056077.1	6830001	6850001	0.35294	0.223124	HZ	RBFOX1
NC_056077.1	6835001	6855001	0.310234	0.220075	HZ	RBFOX1
NC_056077.1	6840001	6860001	0.355103	0.209809	HZ	RBFOX1
NC_056077.1	6845001	6865001	0.305468	0.187148	HZ	RBFOX1
NC_056064.1	52220001	52240001	0.167902	0.148865	HZ	RBFOX3
NC_056064.1	52240001	52260001	0.072311	0.150962	HZ	RBFOX3
NC_056064.1	52515001	52535001	0.463263	0.22511	HZ	RBFOX3

NC_056064.1	52520001	52540001	0.273561	0.394619	HZ	RBFOX3
NC_056064.1	52525001	52545001	0.389517	0.329358	HZ	RBFOX3
NC_056064.1	52530001	52550001	0.418945	0.314431	HZ	RBFOX3
NC_056070.1	60805001	60825001	0.402232	0.168535	HZ	RBM19
NC_056070.1	60810001	60830001	0.276953	0.176976	HZ	RBM19
NC_056070.1	60815001	60835001	0.212821	0.190334	HZ	RBM19
NC_056063.1	18590001	18610001	0.420142	0.218399	HZ	RCBTB2
NC_056063.1	18595001	18615001	0.227598	0.254102	HZ	RCBTB2
NC_056063.1	18600001	18620001	0.300905	0.360405	HZ	RCBTB2
NC_056055.1	52420001	52440001	0.443232	0.184669	HZ	RECK
NC_056055.1	52425001	52445001	0.435131	0.182316	HZ	RECK
NC_056055.1	52455001	52475001	0.467181	0.146683	HZ	RECK
NC_056055.1	52460001	52480001	0.455349	0.20658	HZ	RECK
NC_056055.1	52465001	52485001	0.443446	0.219417	HZ	RECK
NC_056055.1	52470001	52490001	0.418239	0.255052	HZ	RECK
NC_056055.1	52475001	52495001	0.448426	0.228496	HZ	RECK
NC_056055.1	52480001	52500001	0.422514	0.192053	HZ	RECK
NC_056055.1	52495001	52515001	0.419104	0.160669	HZ	RECK
NC_056055.1	52500001	52520001	0.432432	0.205164	HZ	RECK
NC_056055.1	52505001	52525001	0.417374	0.224128	HZ	RECK
NC_056055.1	52510001	52530001	0.412121	0.214878	HZ	RECK
NC_056056.1	200280001	2E+08	0.050677	0.171569	HZ	RERG
NC_056056.1	200290001	2E+08	0.075101	0.164994	HZ	RERG
NC_056064.1	42405001	42425001	0.143671	0.163753	HZ	RETREG3;TUBG1
NC_056055.1	71855001	71875001	0.335483	0.167679	HZ	RFX3
NC_056055.1	71860001	71880001	0.29451	0.185681	HZ	RFX3
NC_056056.1	175655001	1.76E+08	0.373189	0.214061	HZ	RFX4;RIC8B
NC_056060.1	52395001	52415001	0.284569	0.146181	HZ	RFX7
NC_056060.1	52425001	52445001	0.128015	0.154195	HZ	RFX7
NC_056060.1	52430001	52450001	0.079815	0.192043	HZ	RFX7
NC_056065.1	34690001	34710001	0.251801	0.146958	HZ	RGS7
NC_056065.1	34695001	34715001	0.293095	0.148604	HZ	RGS7
NC_056065.1	34785001	34805001	0.482657	0.30554	HZ	RGS7
NC_056073.1	22020001	22040001	0.131647	0.178954	HZ	RHAG
NC_056073.1	22025001	22045001	0.184065	0.157222	HZ	RHAG
NC_056071.1	20240001	20260001	0.477184	0.156718	HZ	RHCG

NC_056055.1	73755001	73775001	0.472004	0.254659	HZ	RIC1
NC_056062.1	1795001	1815001	0.350617	0.152612	HZ	RIMS1
NC_056062.1	73880001	73900001	0.498085	0.18249	HZ	RIMS2
NC_056062.1	73885001	73905001	0.427613	0.236481	HZ	RIMS2
NC_056061.1	76265001	76285001	0.302077	0.146503	HZ	RMND1
NC_056061.1	76270001	76290001	0.186536	0.199907	HZ	RMND1
NC_056060.1	49365001	49385001	0.390369	0.208713	HZ	RNF111
NC_056060.1	49370001	49390001	0.380293	0.238637	HZ	RNF111
NC_056060.1	49375001	49395001	0.27685	0.280694	HZ	RNF111
NC_056060.1	49380001	49400001	0.255952	0.308219	HZ	RNF111
NC_056060.1	49385001	49405001	0.322148	0.28721	HZ	RNF111
NC_056060.1	49390001	49410001	0.319946	0.286894	HZ	RNF111
NC_056060.1	49395001	49415001	0.473304	0.245409	HZ	RNF111
NC_056066.1	78020001	78040001	0.353825	0.216033	HZ	RNF114
NC_056066.1	78025001	78045001	0.198276	0.248694	HZ	RNF114
NC_056066.1	78030001	78050001	0.25341	0.279031	HZ	RNF114
NC_056066.1	78035001	78055001	0.303195	0.278238	HZ	RNF114;SNAI1
NC_056054.1	238995001	2.39E+08	0.046413	0.167414	HZ	RNF13
NC_056054.1	239000001	2.39E+08	0.038781	0.163958	HZ	RNF13
NC_056075.1	9920001	9940001	0.137427	0.194032	HZ	RNLS
NC_056066.1	66155001	66175001	0.297182	0.152887	HZ	RPN2
NC_056054.1	100460001	1E+08	0.492288	0.33316	HZ	RPRD2
NC_056054.1	100465001	1E+08	0.485665	0.31287	HZ	RPRD2
NC_056054.1	100470001	1E+08	0.412133	0.277172	HZ	RPRD2
NC_056054.1	100475001	1E+08	0.390603	0.250298	HZ	RPRD2
NC_056054.1	100480001	1.01E+08	0.417077	0.205248	HZ	RPRD2
NC_056054.1	100485001	1.01E+08	0.454281	0.180289	HZ	RPRD2
NC_056074.1	15845001	15865001	0.473474	0.147638	HZ	RSF1
NC_056074.1	15850001	15870001	0.460813	0.16201	HZ	RSF1
NC_056074.1	15855001	15875001	0.424502	0.191001	HZ	RSF1
NC_056074.1	15860001	15880001	0.402895	0.155638	HZ	RSF1
NC_056074.1	15865001	15885001	0.3912	0.160504	HZ	RSF1
NC_056074.1	15915001	15935001	0.361213	0.205967	HZ	RSF1
NC_056068.1	79540001	79560001	0.305625	0.193753	HZ	RTN4RL2;SLC43A1
NC_056068.1	79545001	79565001	0.149723	0.276271	HZ	RTN4RL2;SLC43A1
NC_056068.1	79550001	79570001	0.083966	0.293814	HZ	RTN4RL2;SLC43A1

NC_056079.1	13555001	13575001	0.379766	0.163456	HZ	RWDD4;TRAPPC11
NC_056079.1	13560001	13580001	0.410586	0.162443	HZ	RWDD4;TRAPPC11
NC_056079.1	13565001	13585001	0.391305	0.158396	HZ	RWDD4;TRAPPC11
NC_056054.1	78585001	78605001	0.196131	0.174933	HZ	S1PR1
NC_056054.1	78590001	78610001	0.213184	0.189187	HZ	S1PR1
NC_056054.1	124440001	1.24E+08	0.501096	0.241048	HZ	SCAF4
NC_056054.1	124465001	1.24E+08	0.294279	0.169251	HZ	SCAF4
NC_056054.1	124470001	1.24E+08	0.310042	0.20458	HZ	SCAF4
NC_056054.1	124475001	1.24E+08	0.313043	0.207818	HZ	SCAF4
NC_056054.1	124480001	1.25E+08	0.329182	0.206677	HZ	SCAF4
NC_056054.1	124485001	1.25E+08	0.335664	0.185884	HZ	SCAF4
NC_056054.1	124490001	1.25E+08	0.359244	0.16002	HZ	SCAF4
NC_056054.1	124495001	1.25E+08	0.401886	0.186268	HZ	SCAF4
NC_056054.1	124500001	1.25E+08	0.451681	0.266088	HZ	SCAF4;SOD1
NC_056054.1	124510001	1.25E+08	0.478049	0.326948	HZ	SCAF4;SOD1
NC_056054.1	124515001	1.25E+08	0.446377	0.298453	HZ	SCAF4;SOD1
NC_056071.1	29630001	29650001	0.455203	0.204864	HZ	SCAPER
NC_056070.1	29070001	29090001	0.324496	0.170513	HZ	SCLT1
NC_056070.1	29075001	29095001	0.25088	0.186576	HZ	SCLT1
NC_056055.1	236420001	2.36E+08	0.465952	0.199467	HZ	SDC3
NC_056055.1	236425001	2.36E+08	0.401624	0.298868	HZ	SDC3
NC_056055.1	236430001	2.36E+08	0.344545	0.284964	HZ	SDC3
NC_056055.1	236435001	2.36E+08	0.399172	0.21942	HZ	SDC3
NC_056077.1	39890001	39910001	0.292752	0.217648	HZ	SDK1
NC_056077.1	39895001	39915001	0.247037	0.26015	HZ	SDK1
NC_056077.1	39900001	39920001	0.385391	0.187142	HZ	SDK1
NC_056077.1	39910001	39930001	0.498093	0.180013	HZ	SDK1
NC_056077.1	39915001	39935001	0.485615	0.161465	HZ	SDK1
NC_056077.1	39920001	39940001	0.431424	0.182232	HZ	SDK1
NC_056077.1	39925001	39945001	0.268211	0.22973	HZ	SDK1
NC_056077.1	39930001	39950001	0.236987	0.223875	HZ	SDK1
NC_056077.1	39935001	39955001	0.381095	0.178099	HZ	SDK1
NC_056077.1	39940001	39960001	0.43956	0.167866	HZ	SDK1
NC_056062.1	36420001	36440001	0.402038	0.175509	HZ	SDR16C5
NC_056062.1	36425001	36445001	0.405797	0.184287	HZ	SDR16C5
NC_056062.1	36430001	36450001	0.400784	0.193317	HZ	SDR16C5

NC_056062.1	36435001	36455001	0.387755	0.16329	HZ	SDR16C5
NC_056054.1	188085001	1.88E+08	0.418801	0.285661	HZ	SEMA5B
NC_056054.1	188090001	1.88E+08	0.4201	0.248394	HZ	SEMA5B
NC_056060.1	61185001	61205001	0.460784	0.21425	HZ	SEMA6D
NC_056061.1	2460001	2480001	0.413342	0.165591	HZ	SENP6
NC_056061.1	2465001	2485001	0.379174	0.214113	HZ	SENP6
NC_056061.1	2470001	2490001	0.35294	0.228329	HZ	SENP6
NC_056061.1	2475001	2495001	0.338765	0.206808	HZ	SENP6
NC_056061.1	2480001	2500001	0.368645	0.17249	HZ	SENP6
NC_056061.1	2485001	2505001	0.40299	0.162651	HZ	SENP6
NC_056061.1	2500001	2520001	0.379333	0.164313	HZ	SENP6
NC_056061.1	2505001	2525001	0.328339	0.18221	HZ	SENP6
NC_056061.1	2510001	2530001	0.286974	0.209514	HZ	SENP6
NC_056061.1	2515001	2535001	0.292963	0.254136	HZ	SENP6
NC_056061.1	2520001	2540001	0.269931	0.253	HZ	SENP6
NC_056061.1	2525001	2545001	0.30838	0.198821	HZ	SENP6
NC_056073.1	49925001	49945001	0.443402	0.161117	HZ	SERPINB1
NC_056073.1	49930001	49950001	0.456011	0.184254	HZ	SERPINB1
NC_056070.1	66050001	66070001	0.486594	0.225074	HZ	SEZ6L
NC_056055.1	224590001	2.25E+08	0.483304	0.162142	HZ	SGPP2
NC_056055.1	224595001	2.25E+08	0.447036	0.164606	HZ	SGPP2
NC_056055.1	224600001	2.25E+08	0.431832	0.163763	HZ	SGPP2
NC_056071.1	28285001	28305001	0.476716	0.150144	HZ	SH2D7
NC_056059.1	115185001	1.15E+08	0.37224	0.286285	HZ	SH3TC1
NC_056077.1	12420001	12440001	0.35572	0.155318	HZ	SHISA9
NC_056077.1	12425001	12445001	0.354561	0.163839	HZ	SHISA9
NC_056077.1	12430001	12450001	0.387849	0.147416	HZ	SHISA9
NC_056061.1	70360001	70380001	0.406399	0.164945	HZ	SHPRH
NC_056061.1	70365001	70385001	0.388793	0.171759	HZ	SHPRH
NC_056061.1	70370001	70390001	0.306898	0.186621	HZ	SHPRH
NC_056061.1	70375001	70395001	0.254456	0.187053	HZ	SHPRH
NC_056061.1	70400001	70420001	0.446414	0.146258	HZ	SHPRH
NC_056061.1	70405001	70425001	0.482959	0.14748	HZ	SHPRH
NC_056054.1	222940001	2.23E+08	0.351917	0.193315	HZ	SI
NC_056054.1	222945001	2.23E+08	0.316156	0.20887	HZ	SI
NC_056054.1	222950001	2.23E+08	0.374507	0.21316	HZ	SI

NC_056054.1	222955001	2.23E+08	0.385241	0.234217	HZ	SI
NC_056060.1	80970001	80990001	0.334317	0.290726	HZ	SIPA1L1
NC_056060.1	80975001	80995001	0.394039	0.281024	HZ	SIPA1L1
NC_056060.1	81255001	81275001	0.197611	0.152446	HZ	SIPA1L1
NC_056060.1	81260001	81280001	0.257323	0.180371	HZ	SIPA1L1
NC_056057.1	70705001	70725001	0.402404	0.270214	HZ	SKAP2
NC_056057.1	70710001	70730001	0.413462	0.322955	HZ	SKAP2
NC_056057.1	70715001	70735001	0.422297	0.349317	HZ	SKAP2
NC_056057.1	70720001	70740001	0.458255	0.373913	HZ	SKAP2
NC_056057.1	70725001	70745001	0.377594	0.29854	HZ	SKAP2
NC_056057.1	70730001	70750001	0.319036	0.27319	HZ	SKAP2
NC_056057.1	70735001	70755001	0.335616	0.264197	HZ	SKAP2
NC_056057.1	70740001	70760001	0.330471	0.246677	HZ	SKAP2
NC_056057.1	70745001	70765001	0.333665	0.271543	HZ	SKAP2
NC_056057.1	70750001	70770001	0.411871	0.237022	HZ	SKAP2
NC_056057.1	70755001	70775001	0.447836	0.190768	HZ	SKAP2
NC_056057.1	70760001	70780001	0.484377	0.16295	HZ	SKAP2
NC_056057.1	70795001	70815001	0.445525	0.31517	HZ	SKAP2
NC_056057.1	70800001	70820001	0.335542	0.368937	HZ	SKAP2
NC_056057.1	70805001	70825001	0.393052	0.333113	HZ	SKAP2
NC_056070.1	12090001	12110001	0.114683	0.233841	HZ	SLC10A7
NC_056070.1	12095001	12115001	0.055226	0.295025	HZ	SLC10A7
NC_056070.1	12100001	12120001	0.113328	0.290208	HZ	SLC10A7
NC_056070.1	12105001	12125001	0.195473	0.265443	HZ	SLC10A7
NC_056070.1	12110001	12130001	0.341651	0.198004	HZ	SLC10A7
NC_056054.1	187195001	1.87E+08	0.175732	0.383301	HZ	SLC15A2
NC_056054.1	187220001	1.87E+08	0.087965	0.464938	HZ	SLC15A2
NC_056074.1	19950001	19970001	0.110345	0.250098	HZ	SLC17A6
NC_056066.1	38510001	38530001	0.337091	0.185185	HZ	SLC24A3
NC_056066.1	38515001	38535001	0.20181	0.193246	HZ	SLC24A3
NC_056066.1	38520001	38540001	0.19493	0.192913	HZ	SLC24A3
NC_056066.1	38525001	38545001	0.223077	0.180556	HZ	SLC24A3
NC_056066.1	38530001	38550001	0.470163	0.201099	HZ	SLC24A3
NC_056066.1	38545001	38565001	0.397598	0.25922	HZ	SLC24A3
NC_056066.1	38550001	38570001	0.26174	0.285285	HZ	SLC24A3
NC_056066.1	38555001	38575001	0.266735	0.284707	HZ	SLC24A3

NC_056066.1	38560001	38580001	0.324335	0.263114	HZ	SLC24A3
NC_056071.1	45140001	45160001	0.297302	0.19142	HZ	SLC25A21
NC_056062.1	74015001	74035001	0.308209	0.189116	HZ	SLC25A32
NC_056069.1	10630001	10650001	0.461953	0.198763	HZ	SLC30A5
NC_056069.1	10645001	10665001	0.401392	0.37375	HZ	SLC30A5
NC_056069.1	10650001	10670001	0.382392	0.377286	HZ	SLC30A5
NC_056069.1	10655001	10675001	0.352518	0.396364	HZ	SLC30A5
NC_056069.1	10660001	10680001	0.30044	0.354084	HZ	SLC30A5
NC_056069.1	10665001	10685001	0.464527	0.268849	HZ	SLC30A5
NC_056055.1	119795001	1.2E+08	0.499325	0.198262	HZ	SLC40A1
NC_056055.1	119800001	1.2E+08	0.416277	0.239924	HZ	SLC40A1
NC_056068.1	79555001	79575001	0.116444	0.279583	HZ	SLC43A1
NC_056068.1	79560001	79580001	0.138982	0.250336	HZ	SLC43A1
NC_056068.1	79565001	79585001	0.328656	0.213487	HZ	SLC43A1
NC_056055.1	148010001	1.48E+08	0.35476	0.18898	HZ	SLC4A10
NC_056055.1	148015001	1.48E+08	0.364773	0.256349	HZ	SLC4A10
NC_056055.1	148020001	1.48E+08	0.452418	0.271365	HZ	SLC4A10
NC_056055.1	148160001	1.48E+08	0.408174	0.145788	HZ	SLC4A10
NC_056072.1	1885001	1905001	0.387919	0.188468	HZ	SLC4A7
NC_056072.1	1890001	1910001	0.384248	0.209712	HZ	SLC4A7
NC_056072.1	53490001	53510001	0.303812	0.26428	HZ	SLC6A20
NC_056072.1	53495001	53515001	0.435478	0.201566	HZ	SLC6A20
NC_056080.1	99190001	99210001	0.428571	0.148498	HZ	SLC9A6
NC_056066.1	77905001	77925001	0.484243	0.269032	HZ	SLC9A8
NC_056054.1	245955001	2.46E+08	0.430283	0.149176	HZ	SLC9A9
NC_056071.1	14370001	14390001	0.30899	0.18541	HZ	SLCO3A1
NC_056071.1	14375001	14395001	0.371829	0.205652	HZ	SLCO3A1
NC_056071.1	14380001	14400001	0.460951	0.181828	HZ	SLCO3A1
NC_056059.1	40800001	40820001	0.476191	0.256655	HZ	SLIT2
NC_056076.1	50830001	50850001	0.501198	0.169858	HZ	SMAD4
NC_056076.1	50835001	50855001	0.47136	0.202278	HZ	SMAD4
NC_056077.1	17010001	17030001	0.354757	0.156146	HZ	SMG1
NC_056066.1	78040001	78060001	0.370755	0.312825	HZ	SNAI1
NC_056066.1	78045001	78065001	0.434713	0.328977	HZ	SNAI1
NC_056066.1	78050001	78070001	0.500807	0.282716	HZ	SNAI1
NC_056062.1	94870001	94890001	0.423401	0.289056	HZ	SNTB1

NC_056077.1	11385001	11405001	0.310093	0.202943	HZ	SNX29
NC_056077.1	11390001	11410001	0.190704	0.214203	HZ	SNX29
NC_056077.1	11395001	11415001	0.202096	0.176865	HZ	SNX29
NC_056077.1	11400001	11420001	0.272216	0.153165	HZ	SNX29
NC_056077.1	11410001	11430001	0.250869	0.153741	HZ	SNX29
NC_056077.1	11415001	11435001	0.284208	0.247986	HZ	SNX29
NC_056077.1	11420001	11440001	0.26948	0.305554	HZ	SNX29
NC_056077.1	11425001	11445001	0.355282	0.407171	HZ	SNX29
NC_056071.1	42745001	42765001	0.290433	0.171123	HZ	SNX6
NC_056071.1	42750001	42770001	0.144616	0.258555	HZ	SNX6
NC_056071.1	42755001	42775001	0.255213	0.202596	HZ	SNX6
NC_056071.1	42760001	42780001	0.313465	0.170314	HZ	SNX6
NC_056054.1	124520001	1.25E+08	0.452308	0.161852	HZ	SOD1
NC_056054.1	124525001	1.25E+08	0.246377	0.145259	HZ	SOD1
NC_056075.1	25250001	25270001	0.494611	0.195587	HZ	SORCS3
NC_056075.1	25415001	25435001	0.36926	0.17339	HZ	SORCS3
NC_056075.1	25420001	25440001	0.225564	0.227041	HZ	SORCS3
NC_056075.1	25425001	25445001	0.213023	0.231237	HZ	SORCS3
NC_056075.1	25430001	25450001	0.235893	0.217281	HZ	SORCS3
NC_056075.1	25435001	25455001	0.333163	0.174703	HZ	SORCS3
NC_056075.1	25505001	25525001	0.449896	0.158159	HZ	SORCS3
NC_056060.1	41300001	41320001	0.175576	0.241326	HZ	SOS2
NC_056060.1	41305001	41325001	0.260889	0.264503	HZ	SOS2
NC_056060.1	41310001	41330001	0.325617	0.293961	HZ	SOS2
NC_056060.1	41315001	41335001	0.335822	0.252649	HZ	SOS2
NC_056060.1	41320001	41340001	0.271967	0.247345	HZ	SOS2
NC_056060.1	41325001	41345001	0.247081	0.217538	HZ	SOS2
NC_056060.1	41330001	41350001	0.222268	0.178679	HZ	SOS2
NC_056060.1	41335001	41355001	0.14433	0.207006	HZ	SOS2
NC_056060.1	41340001	41360001	0.210272	0.173056	HZ	SOS2
NC_056060.1	41280001	41300001	0.11593	0.253538	HZ	SOS2;VCPKMT
NC_056060.1	41285001	41305001	0.0714	0.267188	HZ	SOS2;VCPKMT
NC_056060.1	41290001	41310001	0.055933	0.243939	HZ	SOS2;VCPKMT
NC_056060.1	41295001	41315001	0.094017	0.222281	HZ	SOS2;VCPKMT
NC_056056.1	191280001	1.91E+08	0.451592	0.398825	HZ	SOX5
NC_056056.1	191285001	1.91E+08	0.404448	0.40104	HZ	SOX5

NC_056056.1	191290001	1.91E+08	0.295825	0.367299	HZ	SOX5
NC_056056.1	191295001	1.91E+08	0.358125	0.278162	HZ	SOX5
NC_056068.1	35760001	35780001	0.209368	0.200025	HZ	SOX6
NC_056068.1	35765001	35785001	0.222064	0.188539	HZ	SOX6
NC_056068.1	35770001	35790001	0.250836	0.173516	HZ	SOX6
NC_056068.1	35775001	35795001	0.218086	0.206892	HZ	SOX6
NC_056068.1	35860001	35880001	0.066667	0.157165	HZ	SOX6
NC_056068.1	35865001	35885001	0.080769	0.185609	HZ	SOX6
NC_056056.1	91770001	91790001	0.21641	0.171466	HZ	SPAST
NC_056056.1	91775001	91795001	0.187786	0.196561	HZ	SPAST
NC_056056.1	91780001	91800001	0.216028	0.196797	HZ	SPAST
NC_056056.1	91785001	91805001	0.302586	0.158316	HZ	SPAST
NC_056070.1	71655001	71675001	0.333486	0.175244	HZ	SPECC1L
NC_056070.1	71660001	71680001	0.391855	0.158256	HZ	SPECC1L
NC_056060.1	15745001	15765001	0.337546	0.316528	HZ	SPESP1
NC_056060.1	15750001	15770001	0.207989	0.286025	HZ	SPESP1
NC_056060.1	15755001	15775001	0.15497	0.287423	HZ	SPESP1
NC_056060.1	15760001	15780001	0.142815	0.294909	HZ	SPESP1
NC_056060.1	15765001	15785001	0.218229	0.269882	HZ	SPESP1
NC_056060.1	15770001	15790001	0.420009	0.351618	HZ	SPESP1
NC_056062.1	32370001	32390001	0.107774	0.148427	HZ	SPIDR
NC_056066.1	66275001	66295001	0.50407	0.253509	HZ	SRC
NC_056066.1	66285001	66305001	0.422676	0.23508	HZ	SRC
NC_056066.1	66290001	66310001	0.408228	0.226896	HZ	SRC
NC_056066.1	66295001	66315001	0.470507	0.231361	HZ	SRC
NC_056066.1	66315001	66335001	0.474895	0.146359	HZ	SRC
NC_056069.1	13570001	13590001	0.352942	0.179435	HZ	SREK1
NC_056058.1	29550001	29570001	0.273784	0.151905	HZ	SRFBP1
NC_056058.1	29555001	29575001	0.217216	0.153167	HZ	SRFBP1
NC_056058.1	29560001	29580001	0.218886	0.157953	HZ	SRFBP1
NC_056054.1	28740001	28760001	0.469276	0.240835	HZ	SSBP3
NC_056054.1	28745001	28765001	0.452147	0.243458	HZ	SSBP3
NC_056054.1	28750001	28770001	0.439145	0.255379	HZ	SSBP3
NC_056054.1	28755001	28775001	0.424065	0.283112	HZ	SSBP3
NC_056054.1	28760001	28780001	0.366545	0.286382	HZ	SSBP3
NC_056054.1	28765001	28785001	0.364807	0.323602	HZ	SSBP3

NC_056054.1	28770001	28790001	0.350963	0.352824	HZ	SSBP3
NC_056054.1	28775001	28795001	0.334621	0.268426	HZ	SSBP3
NC_056054.1	28780001	28800001	0.451409	0.205427	HZ	SSBP3
NC_056054.1	28785001	28805001	0.44914	0.175364	HZ	SSBP3
NC_056064.1	20835001	20855001	0.173848	0.179917	HZ	SSH2
NC_056056.1	104145001	1.04E+08	0.255396	0.163742	HZ	STARD7
NC_056062.1	49830001	49850001	0.475669	0.168958	HZ	STAU2
NC_056062.1	49835001	49855001	0.471336	0.191875	HZ	STAU2
NC_056062.1	49840001	49860001	0.447481	0.212894	HZ	STAU2
NC_056062.1	49845001	49865001	0.435556	0.214204	HZ	STAU2
NC_056062.1	49930001	49950001	0.216955	0.252989	HZ	STAU2
NC_056062.1	49935001	49955001	0.100077	0.32781	HZ	STAU2
NC_056062.1	49940001	49960001	0.111901	0.357788	HZ	STAU2
NC_056062.1	49945001	49965001	0.075284	0.375266	HZ	STAU2
NC_056062.1	49950001	49970001	0.080244	0.349127	HZ	STAU2
NC_056062.1	49955001	49975001	0.102767	0.314829	HZ	STAU2
NC_056062.1	49960001	49980001	0.136677	0.276396	HZ	STAU2
NC_056062.1	49965001	49985001	0.262379	0.187131	HZ	STAU2
NC_056079.1	13635001	13655001	0.29447	0.197036	HZ	STOX2
NC_056079.1	13640001	13660001	0.273647	0.215162	HZ	STOX2
NC_056079.1	13645001	13665001	0.27971	0.204566	HZ	STOX2
NC_056079.1	13650001	13670001	0.214189	0.189326	HZ	STOX2
NC_056079.1	13655001	13675001	0.261921	0.167808	HZ	STOX2
NC_056079.1	13660001	13680001	0.289689	0.160359	HZ	STOX2
NC_056079.1	13710001	13730001	0.28125	0.149639	HZ	STOX2
NC_056079.1	13815001	13835001	0.421034	0.183784	HZ	STOX2
NC_056079.1	13820001	13840001	0.42837	0.197509	HZ	STOX2
NC_056079.1	13825001	13845001	0.438633	0.212491	HZ	STOX2
NC_056079.1	13830001	13850001	0.499998	0.220826	HZ	STOX2
NC_056064.1	28535001	28555001	0.183701	0.150696	HZ	STX8
NC_056064.1	28540001	28560001	0.192863	0.152892	HZ	STX8
NC_056064.1	28545001	28565001	0.182397	0.149669	HZ	STX8
NC_056064.1	28550001	28570001	0.230532	0.148608	HZ	STX8
NC_056061.1	71770001	71790001	0.479122	0.147171	HZ	STXBP5
NC_056054.1	236720001	2.37E+08	0.335854	0.202305	HZ	SUCNR1
NC_056054.1	236725001	2.37E+08	0.190527	0.205114	HZ	SUCNR1

NC_056054.1	236730001	2.37E+08	0.225659	0.184323	HZ	SUCNR1
NC_056054.1	100010001	1E+08	0.477254	0.243471	HZ	SV2A
NC_056065.1	65600001	65620001	0.355858	0.206387	HZ	SWT1
NC_056065.1	65605001	65625001	0.277392	0.260105	HZ	SWT1
NC_056065.1	65610001	65630001	0.28137	0.288559	HZ	SWT1
NC_056065.1	65615001	65635001	0.329528	0.29446	HZ	SWT1
NC_056065.1	65620001	65640001	0.469017	0.266583	HZ	SWT1
NC_056056.1	176830001	1.77E+08	0.383669	0.158859	HZ	SYN3
NC_056056.1	176835001	1.77E+08	0.444201	0.209213	HZ	SYN3
NC_056056.1	176840001	1.77E+08	0.468919	0.214091	HZ	SYN3
NC_056057.1	48665001	48685001	0.436363	0.194308	HZ	SYPL1
NC_056057.1	48670001	48690001	0.474802	0.168222	HZ	SYPL1
NC_056056.1	114970001	1.15E+08	0.461539	0.191633	HZ	SYT1
NC_056056.1	114975001	1.15E+08	0.399373	0.189365	HZ	SYT1
NC_056056.1	115000001	1.15E+08	0.363221	0.287617	HZ	SYT1
NC_056056.1	115035001	1.15E+08	0.434866	0.293294	HZ	SYT1
NC_056056.1	115040001	1.15E+08	0.483065	0.263664	HZ	SYT1
NC_056056.1	115045001	1.15E+08	0.490633	0.219979	HZ	SYT1
NC_056056.1	115065001	1.15E+08	0.455202	0.239382	HZ	SYT1
NC_056065.1	72770001	72790001	0.472705	0.215236	HZ	SYT14
NC_056065.1	72775001	72795001	0.456378	0.238005	HZ	SYT14
NC_056065.1	72795001	72815001	0.455539	0.207843	HZ	SYT14
NC_056065.1	72800001	72820001	0.301465	0.219802	HZ	SYT14
NC_056065.1	72805001	72825001	0.299298	0.180565	HZ	SYT14
NC_056065.1	72810001	72830001	0.267216	0.189921	HZ	SYT14
NC_056065.1	72815001	72835001	0.242844	0.224894	HZ	SYT14
NC_056065.1	72820001	72840001	0.298371	0.220974	HZ	SYT14
NC_056055.1	239665001	2.4E+08	0.38706	0.231117	HZ	SYTL1;TMEM222
NC_056055.1	239670001	2.4E+08	0.406143	0.240738	HZ	SYTL1;TMEM222
NC_056056.1	157500001	1.58E+08	0.449251	0.181048	HZ	TAFA2
NC_056056.1	157505001	1.58E+08	0.35938	0.183983	HZ	TAFA2
NC_056069.1	40460001	40480001	0.501731	0.322269	HZ	TARS1
NC_056057.1	107780001	1.08E+08	0.289573	0.164861	HZ	TAS2R39
NC_056057.1	107785001	1.08E+08	0.327112	0.153713	HZ	TAS2R39
NC_056057.1	107790001	1.08E+08	0.345401	0.145952	HZ	TAS2R39
NC_056064.1	51945001	51965001	0.205507	0.217593	HZ	TBC1D16

NC_056064.1	51950001	51970001	0.127659	0.245754	HZ	TBC1D16
NC_056064.1	51955001	51975001	0.133535	0.213617	HZ	TBC1D16
NC_056064.1	51960001	51980001	0.129197	0.164372	HZ	TBC1D16
NC_056063.1	51110001	51130001	0.103013	0.145784	HZ	TBC1D4
NC_056063.1	51120001	51140001	0.063492	0.237983	HZ	TBC1D4
NC_056054.1	28590001	28610001	0.395167	0.364956	HZ	TCEANC2
NC_056054.1	28595001	28615001	0.172797	0.404629	HZ	TCEANC2
NC_056054.1	28600001	28620001	0.145544	0.4082	HZ	TCEANC2
NC_056054.1	28605001	28625001	0.412484	0.254534	HZ	TCEANC2
NC_056060.1	51245001	51265001	0.315192	0.151515	HZ	TCF12
NC_056056.1	28925001	28945001	0.48069	0.168816	HZ	TDRD15
NC_056056.1	28930001	28950001	0.47367	0.170887	HZ	TDRD15
NC_056056.1	28935001	28955001	0.486605	0.159997	HZ	TDRD15
NC_056059.1	80535001	80555001	0.419564	0.150031	HZ	TECRL
NC_056059.1	80540001	80560001	0.26077	0.204849	HZ	TECRL
NC_056059.1	80545001	80565001	0.3199	0.174874	HZ	TECRL
NC_056059.1	80550001	80570001	0.177636	0.236004	HZ	TECRL
NC_056059.1	80555001	80575001	0.207889	0.204024	HZ	TECRL
NC_056059.1	80600001	80620001	0.214359	0.147056	HZ	TECRL
NC_056059.1	80605001	80625001	0.106565	0.163199	HZ	TECRL
NC_056059.1	80610001	80630001	0.101367	0.167761	HZ	TECRL
NC_056059.1	80615001	80635001	0.116879	0.151069	HZ	TECRL
NC_056059.1	80635001	80655001	0.236535	0.149892	HZ	TECRL
NC_056059.1	80640001	80660001	0.211225	0.177407	HZ	TECRL
NC_056059.1	80645001	80665001	0.193697	0.185081	HZ	TECRL
NC_056080.1	109165001	1.09E+08	0.493528	0.172729	HZ	TENM1
NC_056080.1	109170001	1.09E+08	0.370725	0.221157	HZ	TENM1
NC_056080.1	109175001	1.09E+08	0.166667	0.306346	HZ	TENM1
NC_056080.1	109180001	1.09E+08	0.091653	0.320773	HZ	TENM1
NC_056080.1	109185001	1.09E+08	0.123529	0.289187	HZ	TENM1
NC_056080.1	109190001	1.09E+08	0.131603	0.279862	HZ	TENM1
NC_056080.1	109195001	1.09E+08	0.365953	0.195008	HZ	TENM1
NC_056054.1	95035001	95055001	0.419145	0.217314	HZ	TENT5C
NC_056060.1	18250001	18270001	0.230045	0.170133	HZ	THSD4
NC_056060.1	18255001	18275001	0.11712	0.275486	HZ	THSD4
NC_056060.1	18260001	18280001	0.07741	0.340641	HZ	THSD4

NC_056060.1	18265001	18285001	0.079153	0.369153	HZ	THSD4
NC_056060.1	18270001	18290001	0.104976	0.34945	HZ	THSD4
NC_056060.1	18275001	18295001	0.188363	0.298496	HZ	THSD4
NC_056060.1	18280001	18300001	0.232883	0.247011	HZ	THSD4
NC_056060.1	18285001	18305001	0.441728	0.160013	HZ	THSD4
NC_056054.1	124840001	1.25E+08	0.475472	0.213154	HZ	TIAM1
NC_056054.1	124880001	1.25E+08	0.225534	0.178616	HZ	TIAM1
NC_056054.1	124885001	1.25E+08	0.214992	0.220322	HZ	TIAM1
NC_056054.1	124890001	1.25E+08	0.088271	0.201387	HZ	TIAM1
NC_056061.1	79495001	79515001	0.445896	0.157835	HZ	TIAM2
NC_056061.1	79545001	79565001	0.286699	0.167484	HZ	TIAM2
NC_056061.1	79550001	79570001	0.335641	0.180744	HZ	TIAM2
NC_056061.1	79555001	79575001	0.449691	0.211106	HZ	TIAM2
NC_056061.1	79560001	79580001	0.486161	0.179783	HZ	TIAM2
NC_056077.1	3270001	3290001	0.457256	0.299319	HZ	TIGD7;ZNF263;ZNF75A
NC_056077.1	3275001	3295001	0.46246	0.217941	HZ	TIGD7;ZNF263;ZNF75A
NC_056060.1	45885001	45905001	0.458805	0.231583	HZ	TLN2
NC_056060.1	45890001	45910001	0.339201	0.26486	HZ	TLN2
NC_056060.1	45895001	45915001	0.463122	0.238099	HZ	TLN2
NC_056055.1	194395001	1.94E+08	0.178073	0.205899	HZ	TMEFF2
NC_056055.1	194400001	1.94E+08	0.117912	0.241907	HZ	TMEFF2
NC_056055.1	194405001	1.94E+08	0.131368	0.221632	HZ	TMEFF2
NC_056055.1	194410001	1.94E+08	0.143786	0.207061	HZ	TMEFF2
NC_056055.1	194415001	1.94E+08	0.165794	0.160138	HZ	TMEFF2
NC_056055.1	194420001	1.94E+08	0.150805	0.150274	HZ	TMEFF2
NC_056056.1	142580001	1.43E+08	0.427699	0.202265	HZ	TMEM117
NC_056056.1	142585001	1.43E+08	0.364418	0.244877	HZ	TMEM117
NC_056056.1	142590001	1.43E+08	0.490211	0.233998	HZ	TMEM117
NC_056070.1	50490001	50510001	0.133515	0.199053	HZ	TMEM132B
NC_056070.1	50530001	50550001	0.133394	0.202055	HZ	TMEM132B
NC_056070.1	50535001	50555001	0.226969	0.269742	HZ	TMEM132B
NC_056070.1	50540001	50560001	0.295095	0.306376	HZ	TMEM132B
NC_056070.1	50545001	50565001	0.371113	0.326155	HZ	TMEM132B
NC_056070.1	50550001	50570001	0.414511	0.343427	HZ	TMEM132B
NC_056070.1	50555001	50575001	0.458915	0.255654	HZ	TMEM132B
NC_056070.1	50560001	50580001	0.423496	0.149018	HZ	TMEM132B

NC_056070.1	50580001	50600001	0.252619	0.182366	HZ	TMEM132B
NC_056070.1	50585001	50605001	0.20059	0.237117	HZ	TMEM132B
NC_056070.1	50590001	50610001	0.117004	0.236558	HZ	TMEM132B
NC_056070.1	50595001	50615001	0.101848	0.208632	HZ	TMEM132B
NC_056070.1	50600001	50620001	0.072007	0.176662	HZ	TMEM132B
NC_056070.1	50605001	50625001	0.048741	0.17928	HZ	TMEM132B
NC_056070.1	50610001	50630001	0.036842	0.171028	HZ	TMEM132B
NC_056070.1	50720001	50740001	0.10246	0.154944	HZ	TMEM132B
NC_056061.1	82750001	82770001	0.336968	0.183499	HZ	TMEM181
NC_056061.1	82755001	82775001	0.244869	0.225714	HZ	TMEM181
NC_056061.1	82760001	82780001	0.219512	0.228868	HZ	TMEM181
NC_056061.1	82765001	82785001	0.285112	0.201516	HZ	TMEM181
NC_056055.1	101875001	1.02E+08	0.1875	0.20927	HZ	TMEM215
NC_056055.1	101880001	1.02E+08	0.189545	0.204423	HZ	TMEM215
NC_056074.1	25980001	26000001	0.069798	0.154803	HZ	TMEM218
NC_056061.1	55465001	55485001	0.391608	0.24232	HZ	TMEM244
NC_056061.1	55470001	55490001	0.31175	0.255209	HZ	TMEM244
NC_056061.1	55475001	55495001	0.232633	0.271917	HZ	TMEM244
NC_056061.1	55480001	55500001	0.481295	0.204153	HZ	TMEM244
NC_056060.1	56640001	56660001	0.070678	0.169464	HZ	TMOD3
NC_056060.1	56645001	56665001	0.078386	0.168836	HZ	TMOD3
NC_056056.1	118835001	1.19E+08	0.337099	0.217609	HZ	TMTC2
NC_056056.1	118840001	1.19E+08	0.145911	0.264562	HZ	TMTC2
NC_056056.1	118845001	1.19E+08	0.168142	0.16815	HZ	TMTC2
NC_056056.1	118930001	1.19E+08	0.371731	0.147771	HZ	TMTC2
NC_056056.1	118980001	1.19E+08	0.327387	0.157615	HZ	TMTC2
NC_056056.1	118985001	1.19E+08	0.366834	0.14898	HZ	TMTC2
NC_056056.1	118990001	1.19E+08	0.330705	0.150879	HZ	TMTC2
NC_056065.1	56140001	56160001	0.373052	0.148259	HZ	TNR
NC_056065.1	56145001	56165001	0.277127	0.2175	HZ	TNR
NC_056065.1	56150001	56170001	0.265702	0.226477	HZ	TNR
NC_056065.1	56155001	56175001	0.301907	0.234333	HZ	TNR
NC_056065.1	56180001	56200001	0.35151	0.145931	HZ	TNR
NC_056065.1	56185001	56205001	0.270996	0.192999	HZ	TNR
NC_056065.1	56190001	56210001	0.411673	0.177473	HZ	TNR
NC_056065.1	56195001	56215001	0.484606	0.186068	HZ	TNR

NC_056077.1	22795001	22815001	0.488565	0.375608	HZ	TNRC6A
NC_056064.1	40435001	40455001	0.423822	0.155747	HZ	TNS4
NC_056064.1	40440001	40460001	0.39645	0.170513	HZ	TNS4
NC_056064.1	40445001	40465001	0.418503	0.191176	HZ	TNS4
NC_056064.1	40450001	40470001	0.377934	0.211111	HZ	TNS4
NC_056064.1	40455001	40475001	0.500001	0.188657	HZ	TNS4
NC_056064.1	34320001	34340001	0.323233	0.159135	HZ	TOM1L2
NC_056066.1	72375001	72395001	0.357481	0.162759	HZ	TOX2
NC_056054.1	198440001	1.98E+08	0.498447	0.186389	HZ	TP63
NC_056054.1	198445001	1.98E+08	0.492775	0.211719	HZ	TP63
NC_056054.1	198450001	1.98E+08	0.474257	0.225001	HZ	TP63
NC_056054.1	198455001	1.98E+08	0.460634	0.230953	HZ	TP63
NC_056054.1	198460001	1.98E+08	0.455267	0.237508	HZ	TP63
NC_056054.1	198465001	1.98E+08	0.465242	0.233826	HZ	TP63
NC_056057.1	108870001	1.09E+08	0.412627	0.149086	HZ	TPK1
NC_056057.1	108875001	1.09E+08	0.40034	0.169352	HZ	TPK1
NC_056057.1	108880001	1.09E+08	0.405525	0.145434	HZ	TPK1
NC_056057.1	108885001	1.09E+08	0.456372	0.147631	HZ	TPK1
NC_056057.1	108890001	1.09E+08	0.33184	0.202344	HZ	TPK1
NC_056057.1	108895001	1.09E+08	0.328704	0.220829	HZ	TPK1
NC_056057.1	108900001	1.09E+08	0.298898	0.241627	HZ	TPK1
NC_056057.1	108905001	1.09E+08	0.275207	0.215866	HZ	TPK1
NC_056057.1	108910001	1.09E+08	0.2838	0.205618	HZ	TPK1
NC_056057.1	108915001	1.09E+08	0.257153	0.190582	HZ	TPK1
NC_056057.1	108920001	1.09E+08	0.252779	0.22538	HZ	TPK1
NC_056057.1	108925001	1.09E+08	0.241623	0.264879	HZ	TPK1
NC_056057.1	108930001	1.09E+08	0.323959	0.225906	HZ	TPK1
NC_056057.1	108935001	1.09E+08	0.307957	0.234423	HZ	TPK1
NC_056057.1	108940001	1.09E+08	0.361646	0.181102	HZ	TPK1
NC_056057.1	108945001	1.09E+08	0.3704	0.164392	HZ	TPK1
NC_056057.1	108950001	1.09E+08	0.367746	0.147395	HZ	TPK1
NC_056079.1	13595001	13615001	0.329644	0.169403	HZ	TRAPPC11
NC_056079.1	13600001	13620001	0.204546	0.231499	HZ	TRAPPC11
NC_056079.1	13605001	13625001	0.365349	0.171673	HZ	TRAPPC11
NC_056073.1	29125001	29145001	0.433164	0.234081	HZ	TRIM27
NC_056073.1	29130001	29150001	0.21317	0.291881	HZ	TRIM27

NC_056077.1	27805001	27825001	0.398936	0.502688	HZ	TRIM72
NC_056077.1	27810001	27830001	0.279639	0.559368	HZ	TRIM72
NC_056077.1	27815001	27835001	0.183974	0.580505	HZ	TRIM72
NC_056077.1	27820001	27840001	0.30719	0.408228	HZ	TRIM72
NC_056060.1	42170001	42190001	0.46352	0.156001	HZ	TRIM9
NC_056060.1	42175001	42195001	0.354086	0.181446	HZ	TRIM9
NC_056060.1	42180001	42200001	0.403398	0.191119	HZ	TRIM9
NC_056069.1	58940001	58960001	0.382387	0.181342	HZ	TRIO
NC_056069.1	58945001	58965001	0.435945	0.17694	HZ	TRIO
NC_056079.1	23195001	23215001	0.451299	0.18123	HZ	TRMT9B
NC_056062.1	48590001	48610001	0.389664	0.205332	HZ	TRPA1
NC_056062.1	48595001	48615001	0.474013	0.181922	HZ	TRPA1
NC_056074.1	23750001	23770001	0.113583	0.14671	HZ	TSG101
NC_056057.1	96095001	96115001	0.425263	0.234292	HZ	TSGA13
NC_056057.1	86915001	86935001	0.460526	0.157891	HZ	TSPAN12
NC_056057.1	86950001	86970001	0.411734	0.181013	HZ	TSPAN12
NC_056057.1	86955001	86975001	0.441386	0.199347	HZ	TSPAN12
NC_056057.1	86960001	86980001	0.441409	0.245892	HZ	TSPAN12
NC_056057.1	26235001	26255001	0.425219	0.154595	HZ	TSPAN13
NC_056070.1	11550001	11570001	0.300974	0.184557	HZ	TTC29
NC_056070.1	11555001	11575001	0.384449	0.176369	HZ	TTC29
NC_056070.1	11560001	11580001	0.40289	0.179283	HZ	TTC29
NC_056060.1	98560001	98580001	0.356043	0.19458	HZ	TTC8
NC_056060.1	98565001	98585001	0.285005	0.302965	HZ	TTC8
NC_056060.1	98570001	98590001	0.424838	0.376669	HZ	TTC8
NC_056060.1	98590001	98610001	0.478879	0.242357	HZ	TTC8
NC_056056.1	60465001	60485001	0.459005	0.187784	HZ	TTL
NC_056056.1	60470001	60490001	0.340612	0.221616	HZ	TTL
NC_056056.1	60475001	60495001	0.38763	0.175758	HZ	TTL
NC_056060.1	85125001	85145001	0.36843	0.358888	HZ	TTLL5
NC_056060.1	85130001	85150001	0.344681	0.376602	HZ	TTLL5
NC_056060.1	85135001	85155001	0.301459	0.38708	HZ	TTLL5
NC_056060.1	85140001	85160001	0.404391	0.336016	HZ	TTLL5
NC_056060.1	85145001	85165001	0.386009	0.327455	HZ	TTLL5
NC_056060.1	85150001	85170001	0.25815	0.365616	HZ	TTLL5
NC_056060.1	85155001	85175001	0.20353	0.376729	HZ	TTLL5

NC_056060.1	85160001	85180001	0.144698	0.330913	HZ	TTLL5
NC_056060.1	85165001	85185001	0.198576	0.222684	HZ	TTLL5
NC_056060.1	85170001	85190001	0.226973	0.208994	HZ	TTLL5
NC_056060.1	85175001	85195001	0.286347	0.19127	HZ	TTLL5
NC_056060.1	85180001	85200001	0.399999	0.209128	HZ	TTLL5
NC_056056.1	225320001	2.25E+08	0.440898	0.201319	HZ	TTLL8
NC_056059.1	67485001	67505001	0.497027	0.371379	HZ	TXK
NC_056061.1	48175001	48195001	0.205148	0.205744	HZ	UBE2J1
NC_056061.1	48180001	48200001	0.151324	0.189474	HZ	UBE2J1
NC_056061.1	48185001	48205001	0.355226	0.167348	HZ	UBE2J1
NC_056061.1	48190001	48210001	0.410209	0.152544	HZ	UBE2J1
NC_056061.1	48195001	48215001	0.377709	0.146446	HZ	UBE2J1
NC_056055.1	246170001	2.46E+08	0.273132	0.188727	HZ	UBXN10
NC_056063.1	51190001	51210001	0.316729	0.181834	HZ	UCHL3
NC_056063.1	51195001	51215001	0.313035	0.182211	HZ	UCHL3
NC_056063.1	51200001	51220001	0.326624	0.170847	HZ	UCHL3
NC_056068.1	52135001	52155001	0.046429	0.152281	HZ	UCP3
NC_056068.1	52145001	52165001	0.082267	0.146749	HZ	UCP3
NC_056056.1	44370001	44390001	0.411085	0.14798	HZ	UGP2;VPS54
NC_056072.1	13965001	13985001	0.453624	0.196125	HZ	ULK4
NC_056072.1	13975001	13995001	0.419269	0.272003	HZ	ULK4
NC_056072.1	13980001	14000001	0.354232	0.311777	HZ	ULK4
NC_056072.1	13985001	14005001	0.274751	0.366252	HZ	ULK4
NC_056072.1	13990001	14010001	0.080871	0.444897	HZ	ULK4
NC_056072.1	13995001	14015001	0.146325	0.38715	HZ	ULK4
NC_056072.1	14000001	14020001	0.258412	0.247232	HZ	ULK4
NC_056059.1	29675001	29695001	0.480199	0.16101	HZ	UNC5C
NC_056059.1	29680001	29700001	0.452067	0.173169	HZ	UNC5C
NC_056079.1	30210001	30230001	0.497729	0.175891	HZ	UNC5D
NC_056079.1	30215001	30235001	0.480119	0.207524	HZ	UNC5D
NC_056066.1	16030001	16050001	0.457984	0.157995	HZ	UPF2
NC_056054.1	142090001	1.42E+08	0.477826	0.221366	HZ	USP25
NC_056054.1	142095001	1.42E+08	0.481146	0.19782	HZ	USP25
NC_056064.1	12425001	12445001	0.470162	0.167219	HZ	USP32
NC_056064.1	12430001	12450001	0.467238	0.165704	HZ	USP32
NC_056064.1	12435001	12455001	0.463507	0.175197	HZ	USP32

NC_056064.1	12440001	12460001	0.352434	0.223424	HZ	USP32
NC_056064.1	12445001	12465001	0.285549	0.247322	HZ	USP32
NC_056078.1	28715001	28735001	0.501706	0.198767	HZ	USP54
NC_056078.1	28720001	28740001	0.434397	0.182292	HZ	USP54
NC_056061.1	73175001	73195001	0.412343	0.24427	HZ	UST
NC_056061.1	73180001	73200001	0.488787	0.174727	HZ	UST
NC_056060.1	41260001	41280001	0.300534	0.161766	HZ	VCPKMT
NC_056060.1	41265001	41285001	0.117799	0.250501	HZ	VCPKMT
NC_056060.1	41270001	41290001	0.132067	0.252182	HZ	VCPKMT
NC_056060.1	41275001	41295001	0.122141	0.263724	HZ	VCPKMT
NC_056057.1	121265001	1.21E+08	0.435306	0.290353	HZ	VIPR2
NC_056057.1	121270001	1.21E+08	0.359219	0.277991	HZ	VIPR2
NC_056057.1	121275001	1.21E+08	0.403439	0.238999	HZ	VIPR2
NC_056062.1	82745001	82765001	0.495624	0.149807	HZ	VIRMA
NC_056062.1	82750001	82770001	0.356573	0.184561	HZ	VIRMA
NC_056062.1	77815001	77835001	0.465642	0.149935	HZ	VPS13B
NC_056062.1	77820001	77840001	0.35566	0.185057	HZ	VPS13B
NC_056062.1	77825001	77845001	0.303669	0.195109	HZ	VPS13B
NC_056062.1	77830001	77850001	0.326885	0.209444	HZ	VPS13B
NC_056062.1	77835001	77855001	0.448779	0.218	HZ	VPS13B
NC_056057.1	83665001	83685001	0.416386	0.16179	HZ	VPS41
NC_056056.1	44280001	44300001	0.458285	0.164146	HZ	VPS54
NC_056056.1	44285001	44305001	0.43554	0.175994	HZ	VPS54
NC_056056.1	44290001	44310001	0.459219	0.162988	HZ	VPS54
NC_056056.1	44295001	44315001	0.460878	0.168276	HZ	VPS54
NC_056056.1	44300001	44320001	0.472495	0.165236	HZ	VPS54
NC_056056.1	44305001	44325001	0.431176	0.17674	HZ	VPS54
NC_056056.1	44310001	44330001	0.366943	0.191968	HZ	VPS54
NC_056056.1	44315001	44335001	0.47546	0.168378	HZ	VPS54
NC_056059.1	100280001	1E+08	0.500651	0.20103	HZ	WDFY3
NC_056059.1	100285001	1E+08	0.502391	0.205863	HZ	WDFY3
NC_056059.1	100290001	1E+08	0.5028	0.180114	HZ	WDFY3
NC_056075.1	40390001	40410001	0.123071	0.149453	HZ	WDR11
NC_056075.1	40395001	40415001	0.114808	0.152668	HZ	WDR11
NC_056075.1	40400001	40420001	0.11071	0.151034	HZ	WDR11
NC_056075.1	40405001	40425001	0.079371	0.154076	HZ	WDR11

NC_056075.1	40410001	40430001	0.134588	0.147472	HZ	WDR11
NC_056080.1	116440001	1.16E+08	0.425686	0.193414	HZ	WDR44
NC_056080.1	116445001	1.16E+08	0.235593	0.285187	HZ	WDR44
NC_056080.1	116450001	1.16E+08	0.281837	0.303756	HZ	WDR44
NC_056080.1	116455001	1.16E+08	0.168385	0.277126	HZ	WDR44
NC_056080.1	116460001	1.16E+08	0.17012	0.235047	HZ	WDR44
NC_056080.1	116465001	1.16E+08	0.283442	0.162096	HZ	WDR44
NC_056065.1	34310001	34330001	0.372421	0.16888	HZ	WDR64
NC_056055.1	239735001	2.4E+08	0.378048	0.192207	HZ	WDTC1
NC_056055.1	239740001	2.4E+08	0.437373	0.248899	HZ	WDTC1
NC_056055.1	239745001	2.4E+08	0.29207	0.281792	HZ	WDTC1
NC_056055.1	239750001	2.4E+08	0.248515	0.30079	HZ	WDTC1
NC_056055.1	239755001	2.4E+08	0.220682	0.259632	HZ	WDTC1
NC_056055.1	239760001	2.4E+08	0.266348	0.230234	HZ	WDTC1
NC_056071.1	22395001	22415001	0.340226	0.168545	HZ	WHAMM
NC_056071.1	22400001	22420001	0.345949	0.152672	HZ	WHAMM
NC_056056.1	213430001	2.13E+08	0.474213	0.160315	HZ	WNK1
NC_056056.1	213435001	2.13E+08	0.366652	0.20326	HZ	WNK1
NC_056056.1	213440001	2.13E+08	0.338049	0.220413	HZ	WNK1
NC_056056.1	213445001	2.13E+08	0.419425	0.174918	HZ	WNK1
NC_056079.1	13345001	13365001	0.491204	0.170028	HZ	WWC2
NC_056062.1	35900001	35920001	0.440702	0.243419	HZ	XKR4
NC_056062.1	35905001	35925001	0.44086	0.243098	HZ	XKR4
NC_056057.1	116665001	1.17E+08	0.493656	0.15543	HZ	XRCC2
NC_056057.1	82940001	82960001	0.293969	0.179325	HZ	YAE1
NC_056057.1	82945001	82965001	0.296909	0.174831	HZ	YAE1
NC_056057.1	82950001	82970001	0.274062	0.18611	HZ	YAE1
NC_056054.1	179900001	1.8E+08	0.446655	0.238155	HZ	ZBTB20
NC_056054.1	179905001	1.8E+08	0.472636	0.231408	HZ	ZBTB20
NC_056077.1	10965001	10985001	0.162237	0.15873	HZ	ZC3H7A
NC_056077.1	10995001	11015001	0.222967	0.153649	HZ	ZC3H7A
NC_056059.1	45420001	45440001	0.360257	0.191606	HZ	ZCCHC4
NC_056059.1	45425001	45445001	0.378273	0.212675	HZ	ZCCHC4
NC_056059.1	45430001	45450001	0.39808	0.252866	HZ	ZCCHC4
NC_056059.1	45435001	45455001	0.42168	0.239199	HZ	ZCCHC4
NC_056059.1	45440001	45460001	0.45988	0.173104	HZ	ZCCHC4

NC_056062.1	20285001	20305001	0.305146	0.14852	HZ	ZFAT
NC_056062.1	20290001	20310001	0.249137	0.186983	HZ	ZFAT
NC_056067.1	13250001	13270001	0.482331	0.210637	HZ	ZFPM1;ZNF469
NC_056059.1	117020001	1.17E+08	0.270788	0.146791	HZ	ZFYVE28
NC_056059.1	117025001	1.17E+08	0.347676	0.155585	HZ	ZFYVE28
NC_056059.1	117030001	1.17E+08	0.349053	0.153986	HZ	ZFYVE28
NC_056079.1	35295001	35315001	0.503067	0.155938	HZ	ZMAT4
NC_056054.1	14850001	14870001	0.411675	0.176129	HZ	ZMPSTE24
NC_056054.1	14855001	14875001	0.319141	0.227978	HZ	ZMPSTE24
NC_056054.1	14860001	14880001	0.290421	0.215694	HZ	ZMPSTE24
NC_056054.1	14865001	14885001	0.314748	0.181871	HZ	ZMPSTE24
NC_056054.1	14870001	14890001	0.319403	0.170219	HZ	ZMPSTE24
NC_056061.1	50000001	50020001	0.435308	0.199054	HZ	ZNF292
NC_056061.1	50005001	50025001	0.336927	0.229981	HZ	ZNF292
NC_056061.1	50010001	50030001	0.214439	0.286259	HZ	ZNF292
NC_056061.1	50015001	50035001	0.083272	0.310657	HZ	ZNF292
NC_056061.1	50020001	50040001	0.078401	0.26057	HZ	ZNF292
NC_056061.1	50025001	50045001	0.243735	0.19519	HZ	ZNF292
NC_056073.1	30435001	30455001	0.260624	0.300037	HZ	ZNF322
NC_056073.1	30440001	30460001	0.226683	0.292583	HZ	ZNF322
NC_056073.1	30445001	30465001	0.22398	0.2735	HZ	ZNF322
NC_056073.1	30450001	30470001	0.291188	0.313341	HZ	ZNF322
NC_056073.1	30455001	30475001	0.413289	0.31247	HZ	ZNF322
NC_056079.1	43055001	43075001	0.479193	0.154451	HZ	ZNF385D
NC_056080.1	57375001	57395001	0.435838	0.149139	HZ	ZNF41
NC_056080.1	57400001	57420001	0.135376	0.17341	HZ	ZNF41
NC_056067.1	13085001	13105001	0.295842	0.370395	HZ	ZNF469
NC_056067.1	13090001	13110001	0.095808	0.435003	HZ	ZNF469
NC_056067.1	13240001	13260001	0.099384	0.462505	HZ	ZNF469
NC_056067.1	13245001	13265001	0.347066	0.258303	HZ	ZNF469
NC_056055.1	29390001	29410001	0.430128	0.17128	HZ	ZNF782
NC_056075.1	43995001	44015001	0.375	0.18548	HZ	ZRANB1
NC_056075.1	44000001	44020001	0.377111	0.191515	HZ	ZRANB1
NC_056075.1	44005001	44025001	0.272222	0.182497	HZ	ZRANB1
NC_056075.1	44010001	44030001	0.295773	0.198392	HZ	ZRANB1
NC_056075.1	44015001	44035001	0.323078	0.168247	HZ	ZRANB1

NC_056055.1	175495001	1.76E+08	0.357936	0.199926	HZ	ZRANB3
NC_056055.1	175500001	1.76E+08	0.363797	0.189958	HZ	ZRANB3
NC_056055.1	175505001	1.76E+08	0.48628	0.15594	HZ	ZRANB3
