# Supplementary material for: Genetic diversity and selection of Tibetan sheep breeds revealed by whole-genome resequencing
Source: Anim Biosci. 2023 May 2;36(7):991–1002. doi: 10.5713/ab.22.0432 (PMC10330983; doi:10.5713/ab.22.0432)
Supplement: Supplementary file 10 [file ab-22-0432-Supplementary-Table-10.pdf]

Supplementary Table10.Putative selection regions associated with wool traits among different breeds

OL vs GY

CHROM	Start	end	Pi	Fst	Region	Gene
NC_056068.	1180001	1200001	1.88552	0.307907	OL	AASDHPPT
NC_056068.	1185001	1205001	2.39952	0.354136	OL	AASDHPPT
NC_056068.	1190001	1210001	3.07207	0.374246	OL	AASDHPPT;KBTBD3
NC_056068.	1195001	1215001	3.48163	0.366194	OL	AASDHPPT;KBTBD3
NC_056068.	1200001	1220001	2.67955	0.29716	OL	AASDHPPT;KBTBD3
NC_056073.	16980001	17000001	2.87123	0.14394	OL	ABCC10
NC_056073.	16985001	17005001	3.21824	0.14846	OL	ABCC10
NC_056073.	16990001	17010001	2.66129	0.211714	OL	ABCC10;DLK2
NC_056073.	16995001	17015001	3.35196	0.244826	OL	ABCC10;DLK2
NC_056073.	17000001	17020001	3.07693	0.232305	OL	ABCC10;DLK2
NC_056073.	17005001	17025001	3.14337	0.251372	OL	ABCC10;DLK2
NC_056068.	34770001	34790001	10.0306	0.145139	OL	ABCC8
NC_056068.	34775001	34795001	17.9347	0.160907	OL	ABCC8
NC_056068.	34780001	34800001	16.2971	0.202367	OL	ABCC8
NC_056068.	34785001	34805001	16.4276	0.207817	OL	ABCC8
NC_056068.	34790001	34810001	21.5797	0.205606	OL	ABCC8
NC_056068.	34765001	34785001	2.59255	0.150045	OL	ABCC8;USH1C
NC_056058.	58285001	58305001	9.03604	0.188932	OL	ABLIM3
NC_056058.	58325001	58345001	20.7866	0.197786	OL	ABLIM3
NC_056058.	58330001	58350001	20.5466	0.197971	OL	ABLIM3
NC_056058.	58335001	58355001	17.2462	0.195475	OL	ABLIM3
NC_056054.	192965001	1.93E+08	9.25983	0.174332	OL	ACAP2
NC_056054.	192970001	1.93E+08	8.25161	0.145551	OL	ACAP2
NC_056054.	192975001	1.93E+08	7.03589	0.160659	OL	ACAP2
NC_056054.	192985001	1.93E+08	7.05761	0.142312	OL	ACAP2
NC_056054.	192990001	1.93E+08	4.93354	0.156065	OL	ACAP2
NC_056054.	192995001	1.93E+08	2.01617	0.159397	OL	ACAP2
NC_056065.	28430001	28450001	2.35646	0.319231	OL	ACBD3

OL vs ZK

CHROM	Start	end	Pi
NC_056066.1	65355001	65375001	1.95682
NC_056066.1	65360001	65380001	2.09816
NC_056073.1	16970001	16990001	16.6238
NC_056067.1	16035001	16055001	21.7895
NC_056067.1	16040001	16060001	5.44584
NC_056067.1	16045001	16065001	3.93412
NC_056067.1	16050001	16070001	2.98867
NC_056067.1	16055001	16075001	2.72374
NC_056067.1	16060001	16080001	2.91005
NC_056067.1	16065001	16085001	4.67008
NC_056067.1	16070001	16090001	7.08573
NC_056067.1	16075001	16095001	7.01731
NC_056054.1	71530001	71550001	2.32085
NC_056054.1	71535001	71555001	2.62033
NC_056054.1	71540001	71560001	2.67992
NC_056054.1	71545001	71565001	2.64457
NC_056054.1	71550001	71570001	2.31017
NC_056054.1	71555001	71575001	2.12049
NC_056054.1	71575001	71595001	2.29035
NC_056054.1	71580001	71600001	2.35537
NC_056059.1	37190001	37210001	2.40915
NC_056059.1	37195001	37215001	3.34052
NC_056058.1	58400001	58420001	3.0099
NC_056064.1	21620001	21640001	2.15964
NC_056064.1	21625001	21645001	2.19721
NC_056064.1	21670001	21690001	2.11389
NC_056059.1	98950001	98970001	3.21148
NC_056061.1	83760001	83780001	2.23246

NC_056067.	34765001	34785001	3.11344	0.361757	OL	ACD;C14H16orf86;CARM	NC_056067.1	34765001	34785001	3.87957
NC_056067.	34760001	34780001	4.26148	0.413338	OL	ACD;C14H16orf86;CARM	NC_056067.1	34760001	34780001	5.31277
NC_056067.	34770001	34790001	1.87053	0.27485	OL	ACD;C14H16orf86;ENKD	NC_056067.1	34770001	34790001	2.25269
NC_056067.	34750001	34770001	6.88649	0.457766	OL	ACD;CARMIL2	NC_056067.1	34750001	34770001	7.86419
NC_056067.	34755001	34775001	6.57642	0.441258	OL	ACD;CARMIL2;ENKD1;I	NC_056067.1	34755001	34775001	8.41342
NC_056058.	15980001	16000001	1.82108	0.142135	OL	ACER1	NC_056079.1	14370001	14390001	2.10325
NC_056068.	55075001	55095001	1.87468	0.175473	OL	ACER3	NC_056077.1	18755001	18775001	3.5618
NC_056068.	55080001	55100001	1.90258	0.196096	OL	ACER3	NC_056077.1	18760001	18780001	7.7381
NC_056068.	55085001	55105001	1.95665	0.211785	OL	ACER3	NC_056056.1	134345001	1.34E+08	2.26003
NC_056068.	55090001	55110001	1.8199	0.192558	OL	ACER3	NC_056056.1	134350001	1.34E+08	2.81324
NC_056060.	83140001	83160001	3.13305	0.20742	OL	ACOT6	NC_056056.1	134355001	1.34E+08	2.61924
NC_056060.	83155001	83175001	2.69719	0.201714	OL	ACOT6;DNAL1;LOC1011	NC_056060.1	84395001	84415001	2.96939
NC_056060.	83145001	83165001	3.6175	0.224188	OL	ACOT6;LOC101118736	NC_056064.1	44220001	44240001	4.60067
NC_056060.	83150001	83170001	3.57621	0.221837	OL	ACOT6;LOC101118736	NC_056064.1	44225001	44245001	3.71571
NC_056060.	83135001	83155001	2.67281	0.179657	OL	ACOT6;LOC106990150	NC_056064.1	44215001	44235001	5.0736
NC_056057.	116755001	1.17E+08	2.43412	0.159584	OL	ACTR3B	NC_056071.1	5920001	5940001	2.25956
NC_056057.	116760001	1.17E+08	3.50437	0.185605	OL	ACTR3B	NC_056058.1	1805001	1825001	1.97785
NC_056057.	116765001	1.17E+08	4.02097	0.161359	OL	ACTR3B	NC_056058.1	1810001	1830001	4.25203
NC_056064.	44220001	44240001	5.73663	0.234278	OL	ADAM11	NC_056058.1	1815001	1835001	9.2411
NC_056064.	44225001	44245001	4.59876	0.205417	OL	ADAM11	NC_056058.1	1820001	1840001	17.6198
NC_056064.	44205001	44225001	2.32359	0.172043	OL	ADAM11;DBF4B	NC_056058.1	1825001	1845001	23.9921
NC_056064.	44210001	44230001	5.48326	0.219893	OL	ADAM11;DBF4B	NC_056058.1	1830001	1850001	23.9083
NC_056064.	44215001	44235001	6.13614	0.247115	OL	ADAM11;DBF4B	NC_056058.1	1835001	1855001	9.12172
NC_056069.	14150001	14170001	2.97328	0.156429	OL	ADAMTS6	NC_056058.1	1840001	1860001	5.15334
NC_056069.	14155001	14175001	2.76453	0.151341	OL	ADAMTS6	NC_056058.1	1845001	1865001	2.91477
NC_056069.	14140001	14160001	2.28431	0.177891	OL	ADAMTS6;CENPK	NC_056058.1	1850001	1870001	2.22082
NC_056069.	14145001	14165001	2.73809	0.164179	OL	ADAMTS6;CENPK	NC_056058.1	1855001	1875001	2.13598
NC_056072.	37010001	37030001	1.94958	0.165298	OL	ADAMTS9	NC_056058.1	1860001	1880001	2.03712
NC_056072.	37015001	37035001	1.90455	0.15038	OL	ADAMTS9	NC_056058.1	1870001	1890001	1.96552
NC_056058.	41565001	41585001	2.52852	0.224889	OL	ADAMTSL5;PCSK4;REEI	NC_056058.1	1875001	1895001	2.04701
NC_056058.	41585001	41605001	2.93176	0.240601	OL	ADAMTSL5;PLK5	NC_056058.1	1880001	1900001	2.19721

NC_056066.	45775001	45795001	1.94041	0.156563	OL	ADARB2
NC_056060.	87080001	87100001	1.98941	0.21028	OL	ADCK1
NC_056060.	87085001	87105001	2.2068	0.195262	OL	ADCK1
NC_056054.	120025001	1.2E+08	2.14371	0.157135	OL	ADCY10
NC_056054.	120030001	1.2E+08	2.87379	0.163064	OL	ADCY10
NC_056054.	120035001	1.2E+08	3.49348	0.162516	OL	ADCY10
NC_056054.	120040001	1.2E+08	4.29495	0.159411	OL	ADCY10
NC_056054.	188655001	1.89E+08	11.7545	0.145002	OL	ADCY5
NC_056077.	3870001	3890001	2.24804	0.160657	OL	ADCY9
NC_056077.	3875001	3895001	2.17683	0.203185	OL	ADCY9
NC_056075.	30100001	30120001	1.93785	0.278446	OL	ADD3
NC_056075.	30105001	30125001	1.99032	0.296472	OL	ADD3
NC_056075.	30110001	30130001	2.22141	0.367484	OL	ADD3
NC_056075.	30115001	30135001	2.05263	0.300015	OL	ADD3
NC_056075.	30120001	30140001	3.0102	0.261107	OL	ADD3
NC_056075.	30125001	30145001	6.08332	0.316593	OL	ADD3
NC_056075.	30130001	30150001	8.60236	0.30693	OL	ADD3
NC_056075.	30135001	30155001	10.3	0.313212	OL	ADD3
NC_056075.	30140001	30160001	15.1839	0.342526	OL	ADD3
NC_056075.	30145001	30165001	5.66918	0.282298	OL	ADD3
NC_056075.	30150001	30170001	3.30612	0.281541	OL	ADD3
NC_056075.	30155001	30175001	3.70748	0.333278	OL	ADD3
NC_056075.	30160001	30180001	2.23529	0.226011	OL	ADD3
NC_056075.	30165001	30185001	2.27877	0.218579	OL	ADD3
NC_056075.	30170001	30190001	1.95669	0.155511	OL	ADD3
NC_056062.	5150001	5170001	2.38649	0.149855	OL	ADGRB3
NC_056062.	5160001	5180001	2.99007	0.175162	OL	ADGRB3
NC_056062.	5165001	5185001	2.61505	0.160009	OL	ADGRB3
NC_056062.	5170001	5190001	2.05562	0.171488	OL	ADGRB3
NC_056054.	55360001	55380001	3.57114	0.189124	OL	ADGRL4
NC_056054.	55365001	55385001	4.45731	0.196489	OL	ADGRL4

NC_056058.1	1885001	1905001	2.11367
NC_056058.1	1890001	1910001	2.37225
NC_056058.1	1895001	1915001	2.57341
NC_056058.1	1900001	1920001	3.18232
NC_056054.1	100560001	1.01E+08	2.84676
NC_056054.1	100565001	1.01E+08	2.32278
NC_056054.1	120025001	1.2E+08	9.29869
NC_056054.1	120030001	1.2E+08	9.83059
NC_056054.1	120035001	1.2E+08	9.70589
NC_056054.1	120040001	1.2E+08	9.39313
NC_056060.1	20670001	20690001	1.95518
NC_056060.1	20660001	20680001	3.15253
NC_056060.1	20665001	20685001	2.669
NC_056060.1	20655001	20675001	3.58198
NC_056075.1	30130001	30150001	2.20209
NC_056075.1	30135001	30155001	2.13988
NC_056075.1	30140001	30160001	2.14448
NC_056061.1	71100001	71120001	2.57513
NC_056061.1	71105001	71125001	2.42837
NC_056061.1	71110001	71130001	2.72826
NC_056054.1	55410001	55430001	2.19577
NC_056054.1	55415001	55435001	3.71901
NC_056054.1	55420001	55440001	7.87178
NC_056054.1	55425001	55445001	6.6618
NC_056054.1	55430001	55450001	4.41666
NC_056054.1	55435001	55455001	2.46355
NC_056058.1	88015001	88035001	2.34596
NC_056058.1	88020001	88040001	2.00222
NC_056078.1	29275001	29295001	2.26174
NC_056078.1	29280001	29300001	2.17078
NC_056078.1	29285001	29305001	2.1199

NC_056054.	55370001	55390001	3.26035	0.170952	OL	ADGRL4
NC_056054.	55425001	55445001	1.82818	0.293922	OL	ADGRL4
NC_056067.	55805001	55825001	2.5646	0.150562	OL	ADM5;BCL2L12;CPT1C;[illegible]
NC_056067.	55810001	55830001	2.29458	0.175186	OL	ADM5;CPT1C;PRMT1
NC_056067.	55815001	55835001	2.1841	0.18209	OL	ADM5;CPT1C;PRMT1
NC_056067.	55820001	55840001	2.03383	0.141309	OL	ADM5;CPT1C;PRMT1
NC_056080.	86810001	86830001	2.07662	0.177806	OL	AFF2
NC_056080.	86815001	86835001	1.88071	0.238514	OL	AFF2
NC_056080.	86820001	86840001	2.07231	0.278143	OL	AFF2
NC_056064.	53305001	53325001	2.52723	0.183564	OL	AFMID;SYNGR2;TK1
NC_056064.	53295001	53315001	2.40508	0.149801	OL	AFMID;TK1
NC_056064.	53300001	53320001	2.37852	0.167647	OL	AFMID;TK1
NC_056054.	5145001	5165001	1.86731	0.341098	OL	AGAP1
NC_056054.	5155001	5175001	1.86428	0.357955	OL	AGAP1
NC_056079.	5900001	5920001	3.09849	0.165815	OL	AGPAT5
NC_056079.	5925001	5945001	3.03156	0.170817	OL	AGPAT5
NC_056079.	5930001	5950001	4.30502	0.211562	OL	AGPAT5
NC_056079.	5935001	5955001	3.93177	0.200615	OL	AGPAT5
NC_056079.	5940001	5960001	2.84906	0.169002	OL	AGPAT5
NC_056069.	39315001	39335001	2.17449	0.184151	OL	AGXT2
NC_056069.	39320001	39340001	2.35947	0.203735	OL	AGXT2
NC_056069.	39325001	39345001	2.38355	0.262127	OL	AGXT2
NC_056054.	201370001	2.01E+08	2.15691	0.175813	OL	AHSG
NC_056054.	201375001	2.01E+08	2.03823	0.249025	OL	AHSG
NC_056056.	8340001	8360001	4.48333	0.172022	OL	AK1
NC_056056.	8345001	8365001	4.65355	0.196548	OL	AK1;ENG
NC_056056.	8350001	8370001	4.86755	0.290469	OL	AK1;ENG
NC_056071.	15735001	15755001	1.92432	0.161957	OL	AKAP13
NC_056061.	56655001	56675001	2.62207	0.153157	OL	AKAP7
NC_056061.	49600001	49620001	3.34374	0.201371	OL	AKIRIN2
NC_056061.	49605001	49625001	3.63563	0.197507	OL	AKIRIN2

NC_056078.1	29330001	29350001	3.15955
NC_056078.1	29335001	29355001	2.94085
NC_056078.1	29340001	29360001	3.37136
NC_056078.1	29345001	29365001	3.80122
NC_056078.1	29350001	29370001	6.9496
NC_056078.1	29355001	29375001	7.70716
NC_056078.1	29360001	29380001	9.01864
NC_056078.1	29365001	29385001	5.37877
NC_056078.1	29765001	29785001	2.28759
NC_056071.1	16980001	17000001	1.95458
NC_056079.1	5900001	5920001	3.56895
NC_056069.1	39310001	39330001	2.28593
NC_056069.1	39315001	39335001	3.1209
NC_056069.1	39320001	39340001	3.42558
NC_056069.1	39325001	39345001	4.65384
NC_056069.1	39330001	39350001	3.15854
NC_056069.1	39335001	39355001	1.95838
NC_056054.1	87280001	87300001	2.50205
NC_056054.1	87285001	87305001	2.38889
NC_056054.1	87310001	87330001	1.94253
NC_056065.1	47940001	47960001	2.74135
NC_056065.1	47945001	47965001	3.59245
NC_056065.1	47950001	47970001	3.88038
NC_056065.1	47955001	47975001	2.72061
NC_056055.1	234625001	2.35E+08	2.26316
NC_056061.1	76170001	76190001	2.31466
NC_056061.1	76175001	76195001	2.81757
NC_056061.1	76180001	76200001	2.06653
NC_056056.1	36170001	36190001	1.96826
NC_056056.1	133075001	1.33E+08	5.72222
NC_056056.1	133085001	1.33E+08	3.64286

NC_056061.	49610001	49630001	2.75546	0.174174	OL	AKIRIN2
NC_056060.	50360001	50380001	14.6785	0.144598	OL	ALDH1A2
NC_056060.	50365001	50385001	17.6667	0.147444	OL	ALDH1A2
NC_056060.	50370001	50390001	18.9286	0.148875	OL	ALDH1A2
NC_056060.	50375001	50395001	17.1633	0.146085	OL	ALDH1A2
NC_056060.	50410001	50430001	2.37534	0.150716	OL	ALDH1A2
NC_056060.	50415001	50435001	2.30434	0.18158	OL	ALDH1A2
NC_056060.	50420001	50440001	2.30351	0.204905	OL	ALDH1A2
NC_056060.	50425001	50445001	2.67287	0.249437	OL	ALDH1A2
NC_056060.	50430001	50450001	3.13131	0.255848	OL	ALDH1A2
NC_056060.	50435001	50455001	5.91304	0.261111	OL	ALDH1A2
NC_056060.	50450001	50470001	11.4821	0.22619	OL	ALDH1A2
NC_056060.	50455001	50475001	16.0803	0.230108	OL	ALDH1A2
NC_056054.	38800001	38820001	3.18007	0.165112	OL	ALG6
NC_056054.	38805001	38825001	3.32534	0.174665	OL	ALG6
NC_056054.	38810001	38830001	4.05739	0.163805	OL	ALG6
NC_056054.	38815001	38835001	3.12464	0.155177	OL	ALG6;LOC101108702
NC_056069.	39920001	39940001	2.78246	0.196814	OL	AMACR
NC_056069.	39925001	39945001	2.45934	0.143853	OL	AMACR;SLC45A2
NC_056054.	86725001	86745001	2.78214	0.20092	OL	AMIGO1;ATXN7L2;LOC
NC_056054.	86730001	86750001	2.12647	0.206899	OL	AMIGO1;ATXN7L2;LOC
NC_056072.	50515001	50535001	2.04184	0.159252	OL	AMIGO3;GMPPB;IP6K1;I
NC_056054.	92060001	92080001	4.60497	0.22614	OL	AMPD1
NC_056054.	92035001	92055001	3.73041	0.147496	OL	AMPD1;DENND2C
NC_056070.	53870001	53890001	7.72222	0.146119	OL	ANAPC5
NC_056065.	70060001	70080001	3.33094	0.155529	OL	ANGEL2;VASH2
NC_056065.	70065001	70085001	4.87857	0.207248	OL	ANGEL2;VASH2
NC_056055.	199620001	2E+08	2.83495	0.152297	OL	ANKRD44
NC_056055.	199625001	2E+08	2.88666	0.16356	OL	ANKRD44
NC_056056.	168885001	1.69E+08	1.84279	0.323366	OL	ANKS1B
NC_056077.	4375001	4395001	2.7986	0.163192	OL	ANKS3;DNAAF8

NC_056060.1	86220001	86240001	2.13258
NC_056060.1	86225001	86245001	6.07144
NC_056060.1	86230001	86250001	4.79999
NC_056065.1	70055001	70075001	4.32212
NC_056060.1	7015001	7035001	2.08726
NC_056060.1	7020001	7040001	2.13754
NC_056060.1	7025001	7045001	1.99519
NC_056060.1	7040001	7060001	1.94643
NC_056060.1	7045001	7065001	2.30951
NC_056060.1	7050001	7070001	2.344
NC_056060.1	7055001	7075001	2.18821
NC_056069.1	62695001	62715001	2.31945
NC_056055.1	199590001	2E+08	2.25294
NC_056055.1	199595001	2E+08	2.25756
NC_056061.1	48015001	48035001	2.02612
NC_056061.1	48020001	48040001	2.50281
NC_056061.1	48025001	48045001	3.19005
NC_056077.1	4370001	4390001	2.78989
NC_056077.1	4375001	4395001	4.40001
NC_056077.1	4380001	4400001	13.618
NC_056077.1	4350001	4370001	4.74444
NC_056077.1	4355001	4375001	2.60801
NC_056057.1	62435001	62455001	1.96334
NC_056057.1	62440001	62460001	2.92428
NC_056057.1	62445001	62465001	3.64318
NC_056057.1	62450001	62470001	2.91112
NC_056057.1	62455001	62475001	3.07634
NC_056057.1	62460001	62480001	3.76875
NC_056057.1	62465001	62485001	3.78542
NC_056057.1	62470001	62490001	3.50001
NC_056068.1	55680001	55700001	3.01838

NC_056077.	4380001	4400001	2.92049	0.180566	OL	ANKS3;DNAAF8;ZNF500
NC_056056.	170400001	1.7E+08	5.85151	0.166446	OL	ANO4
NC_056056.	170410001	1.7E+08	4.43307	0.164693	OL	ANO4
NC_056056.	170415001	1.7E+08	2.62069	0.150179	OL	ANO4
NC_056056.	38545001	38565001	4.63175	0.177135	OL	ANXA4
NC_056056.	38550001	38570001	3.81987	0.171194	OL	ANXA4
NC_056056.	38555001	38575001	3.43151	0.153846	OL	ANXA4
NC_056056.	38560001	38580001	3.83651	0.183061	OL	ANXA4
NC_056056.	38565001	38585001	3.65473	0.194895	OL	ANXA4
NC_056056.	38570001	38590001	2.44311	0.149548	OL	ANXA4
NC_056056.	38530001	38550001	6.19594	0.197932	OL	ANXA4;GMCL1
NC_056056.	38535001	38555001	5.90122	0.227719	OL	ANXA4;GMCL1
NC_056056.	38540001	38560001	3.82728	0.210993	OL	ANXA4;GMCL1
NC_056055.	31265001	31285001	3.13676	0.186306	OL	AOPEP
NC_056055.	31270001	31290001	2.14002	0.177819	OL	AOPEP
NC_056055.	31260001	31280001	2.30137	0.176739	OL	AOPEP;FANCC
NC_056058.	41525001	41545001	2.12556	0.185269	OL	APC2
NC_056058.	41530001	41550001	2.22831	0.1743	OL	APC2
NC_056058.	41535001	41555001	2.10732	0.152173	OL	APC2;C5H19orf25
NC_056058.	41520001	41540001	2.29386	0.174805	OL	APC2;RPS15
NC_056056.	219530001	2.2E+08	2.44631	0.198171	OL	ARFGAP3
NC_056056.	219535001	2.2E+08	3.88931	0.209081	OL	ARFGAP3
NC_056056.	219540001	2.2E+08	5.50332	0.217581	OL	ARFGAP3
NC_056056.	219545001	2.2E+08	7.11169	0.213681	OL	ARFGAP3
NC_056056.	219550001	2.2E+08	4.23995	0.189452	OL	ARFGAP3
NC_056056.	219555001	2.2E+08	2.55097	0.151367	OL	ARFGAP3
NC_056055.	166805001	1.67E+08	1.86425	0.143332	OL	ARHGAP15
NC_056078.	41890001	41910001	1.84407	0.180936	OL	ARHGAP22
NC_056078.	41895001	41915001	1.81011	0.182877	OL	ARHGAP22
NC_056068.	7855001	7875001	2.04868	0.142318	OL	ARHGAP42
NC_056068.	7860001	7880001	2.48359	0.196841	OL	ARHGAP42

NC_056068.1	55685001	55705001	3.95614
NC_056068.1	55690001	55710001	2.59771
NC_056068.1	55725001	55745001	2.33651
NC_056056.1	141135001	1.41E+08	7.53001
NC_056056.1	141155001	1.41E+08	5.49075
NC_056077.1	1155001	1175001	7.69389
NC_056077.1	1140001	1160001	5.27819
NC_056059.1	94010001	94030001	2.21631
NC_056059.1	94015001	94035001	2.09157
NC_056059.1	94005001	94025001	2.20058
NC_056059.1	13635001	13655001	3.19126
NC_056079.1	36360001	36380001	2.87889
NC_056079.1	36365001	36385001	2.51256
NC_056076.1	42635001	42655001	3.46059
NC_056076.1	42640001	42660001	5.2864
NC_056076.1	42645001	42665001	6.2513
NC_056076.1	42650001	42670001	8.78528
NC_056056.1	216355001	2.16E+08	4.53935
NC_056056.1	216365001	2.16E+08	15.4369
NC_056056.1	216370001	2.16E+08	14.2187
NC_056056.1	216375001	2.16E+08	17.5324
NC_056070.1	5030001	5050001	2.21727
NC_056055.1	166640001	1.67E+08	10.0202
NC_056055.1	166645001	1.67E+08	6.05513
NC_056055.1	166910001	1.67E+08	2.2334
NC_056055.1	166915001	1.67E+08	2.44934
NC_056064.1	38855001	38875001	2.28696
NC_056064.1	38860001	38880001	2.01206
NC_056061.1	81250001	81270001	2.45349
NC_056061.1	81280001	81300001	3.01481
NC_056061.1	81285001	81305001	2.08621

NC_056069.	25015001	25035001	3.71112	0.144166	OL	ARL15
NC_056069.	25020001	25040001	7.2417	0.217844	OL	ARL15
NC_056069.	25025001	25045001	3.19953	0.164862	OL	ARL15
NC_056064.	55645001	55665001	6.41748	0.147486	OL	ARMC7;JPT1;NT5C
NC_056055.	232945001	2.33E+08	2.20536	0.161804	OL	ARMC9
NC_056055.	233005001	2.33E+08	1.92762	0.183347	OL	ARMC9
NC_056055.	233010001	2.33E+08	1.87523	0.157846	OL	ARMC9
NC_056077.	37460001	37480001	5.78046	0.310032	OL	ARPC1A
NC_056077.	37465001	37485001	5.62581	0.369027	OL	ARPC1A
NC_056077.	37470001	37490001	3.39839	0.296733	OL	ARPC1A
NC_056077.	37475001	37495001	2.18334	0.175331	OL	ARPC1A
NC_056077.	37450001	37470001	4.42104	0.224956	OL	ARPC1A;ARPC1B
NC_056077.	37455001	37475001	5.08652	0.27478	OL	ARPC1A;ARPC1B
NC_056058.	92490001	92510001	3.23247	0.303677	OL	ARSK;TTC37
NC_056058.	92495001	92515001	2.29302	0.258123	OL	ARSK;TTC37
NC_056057.	13570001	13590001	2.13157	0.175742	OL	ASB4
NC_056057.	13575001	13595001	2.50487	0.208388	OL	ASB4
NC_056057.	13580001	13600001	2.40479	0.184478	OL	ASB4
NC_056060.	65900001	65920001	1.93333	0.163091	OL	ATG14
NC_056060.	41730001	41750001	2.33562	0.154527	OL	ATL1
NC_056060.	41735001	41755001	2.38614	0.169126	OL	ATL1
NC_056060.	41740001	41760001	2.69557	0.214553	OL	ATL1;SAV1
NC_056060.	41745001	41765001	3.46712	0.235261	OL	ATL1;SAV1
NC_056060.	41750001	41770001	4.38006	0.284717	OL	ATL1;SAV1
NC_056068.	17560001	17580001	8.33163	0.160185	OL	ATM
NC_056068.	17590001	17610001	3.17365	0.17478	OL	ATM
NC_056068.	17595001	17615001	3.91516	0.214556	OL	ATM
NC_056068.	17600001	17620001	5.89817	0.257755	OL	ATM
NC_056068.	17605001	17625001	7.91826	0.288417	OL	ATM
NC_056068.	17610001	17630001	5.53726	0.267549	OL	ATM
NC_056063.	85690001	85710001	1.95595	0.200857	OL	ATP11A

NC_056054.1	159280001	1.59E+08	5.94782
NC_056054.1	159285001	1.59E+08	9.14075
NC_056054.1	159290001	1.59E+08	7.82823
NC_056072.1	21245001	21265001	1.96208
NC_056073.1	9835001	9855001	2.73463
NC_056079.1	32360001	32380001	3.04023
NC_056079.1	32365001	32385001	2.48132
NC_056079.1	32370001	32390001	2.55817
NC_056079.1	32375001	32395001	2.59497
NC_056060.1	41715001	41735001	2.72256
NC_056060.1	41720001	41740001	2.69736
NC_056060.1	41725001	41745001	2.45951
NC_056060.1	41730001	41750001	2.17241
NC_056060.1	41735001	41755001	2.14858
NC_056060.1	41670001	41690001	2.5127
NC_056060.1	41740001	41760001	1.94282
NC_056060.1	41745001	41765001	2.03866
NC_056060.1	41750001	41770001	1.95007
NC_056054.1	93750001	93770001	2.17065
NC_056067.1	10170001	10190001	2.71789
NC_056067.1	10175001	10195001	2.22427
NC_056067.1	10180001	10200001	2.15309
NC_056056.1	221975001	2.22E+08	9.23418
NC_056077.1	30355001	30375001	2.2187
NC_056077.1	30360001	30380001	4.33423
NC_056077.1	30365001	30385001	3.85308
NC_056077.1	30370001	30390001	3.32608
NC_056060.1	26855001	26875001	2.1439
NC_056060.1	26860001	26880001	2.15434
NC_056054.1	227040001	2.27E+08	2.12884
NC_056054.1	227045001	2.27E+08	2.13712

NC_056063.	85695001	85715001	1.81811	0.200182	OL	ATP11A
NC_056060.	77315001	77335001	5.95596	0.159951	OL	ATP6V1D;EIF2S1
NC_056054.	21175001	21195001	2.03069	0.274828	OL	ATPAF1
NC_056054.	21180001	21200001	2.07772	0.279395	OL	ATPAF1
NC_056054.	21185001	21205001	2.05329	0.279395	OL	ATPAF1
NC_056054.	21190001	21210001	1.99598	0.259251	OL	ATPAF1
NC_056054.	21195001	21215001	2.03874	0.259875	OL	ATPAF1
NC_056054.	21200001	21220001	1.87321	0.239055	OL	ATPAF1
NC_056054.	21210001	21230001	1.81461	0.222852	OL	ATPAF1;EFCAB14;TEX3
NC_056054.	21215001	21235001	1.84211	0.234943	OL	ATPAF1;EFCAB14;TEX3
NC_056054.	21220001	21240001	2.02119	0.248271	OL	ATPAF1;EFCAB14;TEX3
NC_056054.	21205001	21225001	1.83854	0.228335	OL	ATPAF1;TEX38
NC_056056.	222060001	2.22E+08	2.2594	0.184199	OL	ATXN10
NC_056056.	222065001	2.22E+08	2.51854	0.158673	OL	ATXN10
NC_056054.	86720001	86740001	2.8339	0.173817	OL	ATXN7L2;LOC10110561
NC_056056.	35130001	35150001	2.64706	0.152222	OL	BABAM2
NC_056056.	35240001	35260001	3.06144	0.183899	OL	BABAM2
NC_056056.	35245001	35265001	3.6233	0.218287	OL	BABAM2
NC_056056.	35250001	35270001	3.33172	0.210961	OL	BABAM2
NC_056056.	35255001	35275001	4.00858	0.227365	OL	BABAM2
NC_056056.	35260001	35280001	3.07298	0.209209	OL	BABAM2
NC_056056.	35265001	35285001	2.73104	0.166499	OL	BABAM2
NC_056056.	35270001	35290001	3.66557	0.146716	OL	BABAM2
NC_056068.	27710001	27730001	2.41747	0.354403	OL	BACE1
NC_056068.	27715001	27735001	2.19956	0.352354	OL	BACE1;CEP164
NC_056068.	27720001	27740001	2.29284	0.314441	OL	BACE1;CEP164
NC_056068.	27725001	27745001	2.24054	0.343331	OL	BACE1;CEP164
NC_056068.	27685001	27705001	1.85013	0.326858	OL	BACE1;RNF214
NC_056068.	27690001	27710001	2.00358	0.365462	OL	BACE1;RNF214
NC_056068.	27695001	27715001	2.43627	0.339049	OL	BACE1;RNF214
NC_056068.	27700001	27720001	2.50621	0.369442	OL	BACE1;RNF214

NC_056054.1	227050001	2.27E+08	2.16655
NC_056054.1	227055001	2.27E+08	2.42305
NC_056068.1	27710001	27730001	2.21333
NC_056068.1	27715001	27735001	2.1948
NC_056068.1	27720001	27740001	2.02198
NC_056068.1	27725001	27745001	2.71159
NC_056068.1	27685001	27705001	4.97221
NC_056068.1	27690001	27710001	4.65833
NC_056068.1	27695001	27715001	2.71584
NC_056068.1	27700001	27720001	3.11583
NC_056068.1	27705001	27725001	2.31056
NC_056060.1	84820001	84840001	2.17008
NC_056057.1	64845001	64865001	2.05618
NC_056057.1	65065001	65085001	2.81616
NC_056055.1	85020001	85040001	3.48166
NC_056055.1	85025001	85045001	5.63844
NC_056055.1	85030001	85050001	2.27322
NC_056059.1	109440001	1.09E+08	2.61617
NC_056059.1	109445001	1.09E+08	2.66666
NC_056063.1	48095001	48115001	6.45341
NC_056063.1	48100001	48120001	12.8293
NC_056056.1	176640001	1.77E+08	3.831
NC_056055.1	4305001	4325001	2.31364
NC_056055.1	4310001	4330001	2.62355
NC_056055.1	4315001	4335001	2.73577
NC_056067.1	17370001	17390001	3.2954
NC_056067.1	17375001	17395001	2.88286
NC_056054.1	102650001	1.03E+08	2.69956
NC_056054.1	102655001	1.03E+08	2.28635
NC_056073.1	44620001	44640001	23.2649
NC_056073.1	44625001	44645001	9.93147

NC_056068.	27705001	27725001	2.54794	0.34738	OL	BACE1;RNF214
NC_056061.	47500001	47520001	2.05191	0.177778	OL	BACH2
NC_056060.	84790001	84810001	2.09342	0.18316	OL	BATF
NC_056057.	64930001	64950001	2.18759	0.169341	OL	BBS9
NC_056057.	64935001	64955001	2.23391	0.153975	OL	BBS9
NC_056057.	50155001	50175001	4.24075	0.256604	OL	BCAP29
NC_056071.	23770001	23790001	1.88106	0.172556	OL	BCL2A1;LOC101114310
NC_056067.	55795001	55815001	4.0072	0.192279	OL	BCL2L12;IRF3;PRMT1
NC_056067.	55800001	55820001	3.57713	0.188867	OL	BCL2L12;IRF3;PRMT1
NC_056067.	55790001	55810001	4.13214	0.163669	OL	BCL2L12;IRF3;SCAF1
NC_056054.	200380001	2E+08	3.27439	0.158642	OL	BCL6
NC_056054.	200385001	2E+08	2.17709	0.142235	OL	BCL6
NC_056056.	91350001	91370001	1.93662	0.193221	OL	BIRC6
NC_056056.	91360001	91380001	1.95122	0.174586	OL	BIRC6
NC_056056.	91400001	91420001	10.7723	0.300203	OL	BIRC6
NC_056056.	91405001	91425001	9.81248	0.2873	OL	BIRC6
NC_056056.	91410001	91430001	9.92105	0.280075	OL	BIRC6
NC_056056.	91415001	91435001	9.06123	0.27406	OL	BIRC6
NC_056056.	91420001	91440001	6.237	0.242712	OL	BIRC6
NC_056056.	91425001	91445001	8.66664	0.266043	OL	BIRC6
NC_056056.	91445001	91465001	5.86894	0.263441	OL	BIRC6
NC_056056.	91450001	91470001	6.33792	0.266169	OL	BIRC6
NC_056056.	91455001	91475001	5.88313	0.270202	OL	BIRC6
NC_056056.	91460001	91480001	5.84566	0.30914	OL	BIRC6
NC_056056.	91485001	91505001	2.03508	0.23636	OL	BIRC6
NC_056055.	204705001	2.05E+08	7.60158	0.143263	OL	BMPR2
NC_056055.	204710001	2.05E+08	7.35383	0.143407	OL	BMPR2
NC_056075.	50245001	50265001	1.95222	0.225242	OL	BNIP3
NC_056075.	50250001	50270001	3.21071	0.248774	OL	BNIP3
NC_056064.	48825001	48845001	1.86532	0.14584	OL	BPTF
NC_056065.	57830001	57850001	1.91244	0.143493	OL	BRINP2

NC_056073.1	44630001	44650001	3.74252
NC_056068.1	52200001	52220001	2.25634
NC_056068.1	52205001	52225001	2.20467
NC_056068.1	52210001	52230001	2.26092
NC_056068.1	52215001	52235001	2.29133
NC_056068.1	52220001	52240001	2.31978
NC_056068.1	52225001	52245001	2.3164
NC_056068.1	52230001	52250001	2.3563
NC_056068.1	52235001	52255001	2.37986
NC_056068.1	52240001	52260001	2.36965
NC_056068.1	52245001	52265001	2.38904
NC_056068.1	52250001	52270001	2.34259
NC_056060.1	34505001	34525001	2.36783
NC_056062.1	45395001	45415001	2.24491
NC_056062.1	49545001	49565001	5.78022
NC_056062.1	49550001	49570001	6.47252
NC_056062.1	49555001	49575001	6.13903
NC_056062.1	49560001	49580001	4.98021
NC_056062.1	49565001	49585001	3.92822
NC_056056.1	85001	105001	2.74733
NC_056056.1	90001	110001	2.40319
NC_056056.1	95001	115001	3.01605
NC_056056.1	100001	120001	3.01626
NC_056056.1	105001	125001	2.47324
NC_056056.1	110001	130001	2.83784
NC_056056.1	115001	135001	2.58065
NC_056056.1	120001	140001	2.64872
NC_056056.1	125001	145001	2.36667
NC_056056.1	130001	150001	2.91719
NC_056056.1	135001	155001	3.50496
NC_056056.1	140001	160001	2.76858

NC_056074.	46430001	46450001	2.57454	0.210034	OL	BRSK2;MOB2
NC_056074.	46435001	46455001	2.59213	0.204348	OL	BRSK2;MOB2
NC_056074.	46440001	46460001	2.31985	0.167465	OL	BRSK2;MOB2
NC_056058.	5520001	5540001	6.5893	0.21359	OL	BST-2A;CCDC194
NC_056058.	5525001	5545001	4.4799	0.207984	OL	BST-2A;CCDC194;LOC1(
NC_056068.	38965001	38985001	2.39712	0.150441	OL	BTBD10
NC_056068.	38970001	38990001	2.78448	0.173937	OL	BTBD10
NC_056068.	38975001	38995001	2.72268	0.177915	OL	BTBD10
NC_056068.	38980001	39000001	2.24115	0.150419	OL	BTBD10
NC_056066.	60385001	60405001	3.11509	0.282433	OL	C13H20orf96
NC_056066.	60390001	60410001	9.6928	0.41662	OL	C13H20orf96
NC_056066.	60395001	60415001	7.66054	0.407472	OL	C13H20orf96
NC_056066.	60400001	60420001	3.2694	0.340744	OL	C13H20orf96
NC_056066.	60405001	60425001	2.24661	0.293372	OL	C13H20orf96
NC_056066.	60380001	60400001	1.91111	0.171581	OL	C13H20orf96;ZCCHC3
NC_056068.	63120001	63140001	3.96836	0.205094	OL	C15H11orf91
NC_056068.	63125001	63145001	3.37255	0.191121	OL	C15H11orf91
NC_056068.	63130001	63150001	2.86003	0.196977	OL	C15H11orf91
NC_056068.	63135001	63155001	2.81517	0.194786	OL	C15H11orf91;CD59
NC_056068.	63140001	63160001	2.72621	0.194972	OL	C15H11orf91;CD59
NC_056068.	63145001	63165001	2.84701	0.185395	OL	C15H11orf91;CD59
NC_056068.	63150001	63170001	2.75051	0.168051	OL	C15H11orf91;CD59
NC_056054.	113530001	1.14E+08	2.43672	0.150061	OL	C1H1orf226
NC_056054.	113535001	1.14E+08	2.34179	0.167309	OL	C1H1orf226
NC_056055.	37150001	37170001	2.28407	0.289843	OL	C2H9orf24;FAM219A
NC_056055.	37155001	37175001	1.98961	0.208892	OL	C2H9orf24;FAM219A
NC_056055.	37160001	37180001	2.07534	0.181353	OL	C2H9orf24;FAM219A
NC_056055.	37165001	37185001	1.84416	0.142295	OL	C2H9orf24;FAM219A
NC_056058.	10680001	10700001	2.37405	0.159023	OL	CACNA1A
NC_056056.	60001	80001	2.91294	0.145862	OL	CACNA1B
NC_056056.	65001	85001	2.84431	0.232646	OL	CACNA1B

NC_056056.1	145001	165001	2.73415
NC_056056.1	150001	170001	2.1988
NC_056056.1	160001	180001	2.05726
NC_056056.1	165001	185001	2.06687
NC_056056.1	214155001	2.14E+08	4.2864
NC_056056.1	214160001	2.14E+08	2.55534
NC_056056.1	214165001	2.14E+08	1.94017
NC_056056.1	216790001	2.17E+08	2.56483
NC_056057.1	39555001	39575001	6.95626
NC_056072.1	46720001	46740001	6.33197
NC_056066.1	32085001	32105001	2.84751
NC_056066.1	32090001	32110001	3.86789
NC_056066.1	32095001	32115001	2.8494
NC_056068.1	25780001	25800001	2.04519
NC_056068.1	25785001	25805001	2.16837
NC_056054.1	154570001	1.55E+08	2.03859
NC_056054.1	154955001	1.55E+08	2.16632
NC_056054.1	154960001	1.55E+08	2.08733
NC_056054.1	154965001	1.55E+08	2.04883
NC_056057.1	88770001	88790001	2.03707
NC_056056.1	132810001	1.33E+08	3.20001
NC_056056.1	132815001	1.33E+08	3.83606
NC_056056.1	132820001	1.33E+08	3.39409
NC_056056.1	132825001	1.33E+08	2.6265
NC_056066.1	16600001	16620001	1.94509
NC_056078.1	28960001	28980001	2.14642
NC_056078.1	28965001	28985001	2.76502
NC_056056.1	3485001	3505001	4.19334
NC_056056.1	3490001	3510001	16.72
NC_056067.1	34735001	34755001	10.241
NC_056067.1	34740001	34760001	10.3277

NC_056056.	70001	90001	2.54938	0.240317	OL	CACNA1B
NC_056056.	75001	95001	2.40104	0.233876	OL	CACNA1B
NC_056056.	80001	100001	2.99297	0.256779	OL	CACNA1B
NC_056056.	85001	105001	4.57079	0.252535	OL	CACNA1B
NC_056056.	90001	110001	4.02675	0.238839	OL	CACNA1B
NC_056056.	95001	115001	5.22224	0.249245	OL	CACNA1B
NC_056056.	100001	120001	4.77683	0.240812	OL	CACNA1B
NC_056056.	105001	125001	4.10281	0.231479	OL	CACNA1B
NC_056056.	110001	130001	3.57954	0.219235	OL	CACNA1B
NC_056056.	115001	135001	2.59587	0.190974	OL	CACNA1B
NC_056056.	120001	140001	1.98936	0.148418	OL	CACNA1B
NC_056080.	55930001	55950001	1.89103	0.181569	OL	CACNA1F;LOC10111203
NC_056057.	39760001	39780001	1.84502	0.188724	OL	CACNA2D1
NC_056057.	39765001	39785001	4.00177	0.337215	OL	CACNA2D1
NC_056057.	39770001	39790001	3.88714	0.416075	OL	CACNA2D1
NC_056057.	39775001	39795001	3.38211	0.329686	OL	CACNA2D1
NC_056057.	39780001	39800001	2.72929	0.225751	OL	CACNA2D1
NC_056057.	39785001	39805001	3.13599	0.181427	OL	CACNA2D1
NC_056057.	39790001	39810001	5.35982	0.156452	OL	CACNA2D1
NC_056057.	39795001	39815001	3.64456	0.158695	OL	CACNA2D1
NC_056057.	39800001	39820001	2.68813	0.154653	OL	CACNA2D1
NC_056057.	39835001	39855001	2.51676	0.148603	OL	CACNA2D1
NC_056057.	39840001	39860001	2.88874	0.197898	OL	CACNA2D1
NC_056057.	39845001	39865001	2.12866	0.152733	OL	CACNA2D1
NC_056072.	46720001	46740001	4.66768	0.164815	OL	CACNA2D3
NC_056066.	32080001	32100001	2.17863	0.209903	OL	CACNB2
NC_056066.	32085001	32105001	3.45137	0.241056	OL	CACNB2
NC_056066.	32090001	32110001	4.54421	0.226424	OL	CACNB2
NC_056066.	32095001	32115001	4.04274	0.220613	OL	CACNB2
NC_056066.	32100001	32120001	2.32316	0.150639	OL	CACNB2
NC_056064.	62140001	62160001	2.02742	0.157574	OL	CACNG5

NC_056067.1	34745001	34765001	7.21604
NC_056054.1	171425001	1.71E+08	11.4135
NC_056054.1	171430001	1.71E+08	5.0376
NC_056057.1	71275001	71295001	2.88012
NC_056056.1	216400001	2.16E+08	19.0224
NC_056056.1	216405001	2.16E+08	5.17646
NC_056056.1	216410001	2.16E+08	2.08904
NC_056055.1	84145001	84165001	2.10248
NC_056055.1	84150001	84170001	2.10013
NC_056064.1	15485001	15505001	2.70906
NC_056064.1	15490001	15510001	2.51544
NC_056064.1	15495001	15515001	2.44417
NC_056064.1	15500001	15520001	2.56052
NC_056075.1	17255001	17275001	3.33703
NC_056075.1	17260001	17280001	4.8174
NC_056059.1	33685001	33705001	2.93969
NC_056059.1	33690001	33710001	5.58112
NC_056059.1	33695001	33715001	5.97368
NC_056059.1	33700001	33720001	6.47967
NC_056059.1	33845001	33865001	4.73852
NC_056059.1	33850001	33870001	8.31087
NC_056059.1	33855001	33875001	12.7992
NC_056059.1	33860001	33880001	12.0542
NC_056059.1	33865001	33885001	5.48768
NC_056059.1	34445001	34465001	2.3759
NC_056059.1	34450001	34470001	3.90551
NC_056059.1	34455001	34475001	4.30737
NC_056059.1	34460001	34480001	3.70994
NC_056059.1	34465001	34485001	2.26468
NC_056059.1	34470001	34490001	1.95315
NC_056056.1	46255001	46275001	3.25324

NC_056064.	62145001	62165001	2.01535	0.145595	OL	CACNG5
NC_056064.	62150001	62170001	2.03777	0.149541	OL	CACNG5
NC_056064.	62155001	62175001	2.03478	0.15109	OL	CACNG5
NC_056064.	62160001	62180001	2.0615	0.15076	OL	CACNG5
NC_056064.	62165001	62185001	1.99214	0.150031	OL	CACNG5
NC_056064.	62170001	62190001	1.92786	0.143649	OL	CACNG5
NC_056064.	62175001	62195001	2.10657	0.157127	OL	CACNG5
NC_056056.	132825001	1.33E+08	2.22061	0.14431	OL	CALCOCO1
NC_056057.	94380001	94400001	5.27514	0.155895	OL	CALU
NC_056065.	73155001	73175001	2.63479	0.160462	OL	CAMK1G;LAMB3
NC_056065.	73160001	73180001	2.08734	0.164389	OL	CAMK1G;LAMB3
NC_056054.	273260001	2.73E+08	2.36753	0.170539	OL	CAPN7
NC_056054.	273265001	2.73E+08	2.27129	0.191175	OL	CAPN7
NC_056054.	273270001	2.73E+08	2.15626	0.171809	OL	CAPN7
NC_056067.	34735001	34755001	8.75569	0.47704	OL	CARMIL2;CTCF
NC_056067.	34740001	34760001	8.47584	0.457069	OL	CARMIL2;CTCF
NC_056067.	34745001	34765001	6.31892	0.444143	OL	CARMIL2;CTCF
NC_056064.	40185001	40205001	2.52785	0.148493	OL	CASC3
NC_056064.	40190001	40210001	2.98193	0.174087	OL	CASC3;RAPGEFL1
NC_056064.	40195001	40215001	3.47422	0.201282	OL	CASC3;RAPGEFL1
NC_056054.	93035001	93055001	3.454	0.16334	OL	CASQ2
NC_056054.	93040001	93060001	6.24932	0.182053	OL	CASQ2
NC_056054.	93045001	93065001	5.22131	0.183402	OL	CASQ2
NC_056067.	47505001	47525001	5.93548	0.225808	OL	CATSPERG;KCNK6
NC_056067.	47510001	47530001	3.45239	0.189601	OL	CATSPERG;KCNK6
NC_056067.	47515001	47535001	1.99416	0.160618	OL	CATSPERG;KCNK6
NC_056054.	171375001	1.71E+08	2.27304	0.167499	OL	CBLB
NC_056054.	171400001	1.71E+08	2.01218	0.22445	OL	CBLB
NC_056054.	171405001	1.71E+08	2.08387	0.236579	OL	CBLB
NC_056054.	171410001	1.71E+08	1.98251	0.252683	OL	CBLB
NC_056054.	171425001	1.71E+08	2.04032	0.144271	OL	CBLB

NC_056054.1	177265001	1.77E+08	2.12936
NC_056054.1	177270001	1.77E+08	2.86045
NC_056055.1	40105001	40125001	2.61044
NC_056067.1	8770001	8790001	3.52667
NC_056069.1	53780001	53800001	2.10615
NC_056069.1	53785001	53805001	2.15263
NC_056069.1	53790001	53810001	2.18912
NC_056069.1	54045001	54065001	3.79171
NC_056069.1	54050001	54070001	4.26429
NC_056069.1	54055001	54075001	3.85115
NC_056069.1	54060001	54080001	2.44991
NC_056069.1	54065001	54085001	2.14917
NC_056076.1	29215001	29235001	3.53419
NC_056076.1	29220001	29240001	5.12574
NC_056076.1	60320001	60340001	3.03291
NC_056076.1	60325001	60345001	4.69748
NC_056076.1	60330001	60350001	4.7658
NC_056078.1	27180001	27200001	2.32044
NC_056078.1	27185001	27205001	2.00616
NC_056066.1	54910001	54930001	2.05496
NC_056066.1	54915001	54935001	1.96266
NC_056061.1	26940001	26960001	2.42191
NC_056060.1	41515001	41535001	2.07277
NC_056060.1	41520001	41540001	2.6058
NC_056060.1	65015001	65035001	2.34289
NC_056060.1	65020001	65040001	2.49336
NC_056060.1	65025001	65045001	2.40423
NC_056077.1	20540001	20560001	17.6743
NC_056060.1	90080001	90100001	2.04911
NC_056060.1	90085001	90105001	2.09664
NC_056060.1	90090001	90110001	2.0969

NC_056057.	71290001	71310001	1.99672	0.153074	OL	CBX3;HNRNPA2B1
NC_056076.	8630001	8650001	3.03545	0.301868	OL	CCDC102B
NC_056076.	8635001	8655001	5.26008	0.34717	OL	CCDC102B
NC_056060.	69880001	69900001	2.70424	0.193263	OL	CCDC175
NC_056060.	69885001	69905001	2.71549	0.194865	OL	CCDC175
NC_056060.	69890001	69910001	2.71532	0.177001	OL	CCDC175
NC_056060.	69895001	69915001	2.72141	0.204176	OL	CCDC175
NC_056060.	69900001	69920001	2.66534	0.1915	OL	CCDC175;RTN1
NC_056060.	69905001	69925001	2.52049	0.183361	OL	CCDC175;RTN1
NC_056058.	5530001	5550001	3.61661	0.173957	OL	CCDC194;LOC101111878
NC_056078.	14895001	14915001	1.84589	0.165969	OL	CCDC6
NC_056078.	14900001	14920001	3.15529	0.219372	OL	CCDC6
NC_056078.	14905001	14925001	3.41101	0.220543	OL	CCDC6
NC_056070.	55950001	55970001	3.18536	0.189587	OL	CCDC60
NC_056070.	55955001	55975001	4.7038	0.229101	OL	CCDC60
NC_056054.	178320001	1.78E+08	1.85029	0.171906	OL	CCDC80
NC_056054.	178325001	1.78E+08	2.252	0.194713	OL	CCDC80
NC_056056.	68680001	68700001	4.69059	0.214974	OL	CCDC88A
NC_056056.	68685001	68705001	4.2	0.219627	OL	CCDC88A
NC_056056.	68690001	68710001	3.25696	0.21712	OL	CCDC88A
NC_056080.	55130001	55150001	1.81383	0.364794	OL	CCNB3
NC_056075.	17260001	17280001	2.16971	0.178896	OL	CCNJ
NC_056066.	17425001	17445001	2.36554	0.240868	OL	CCNY
NC_056066.	17430001	17450001	2.23137	0.230496	OL	CCNY
NC_056066.	17435001	17455001	2.21781	0.23221	OL	CCNY
NC_056066.	17440001	17460001	2.18329	0.227273	OL	CCNY
NC_056066.	17445001	17465001	2.17718	0.225904	OL	CCNY
NC_056066.	17450001	17470001	2.29545	0.237319	OL	CCNY
NC_056066.	17455001	17475001	2.32234	0.230159	OL	CCNY
NC_056066.	17460001	17480001	2.23936	0.227679	OL	CCNY
NC_056066.	17465001	17485001	2.0896	0.217803	OL	CCNY

NC_056068.1	27730001	27750001	2.66885
NC_056068.1	27785001	27805001	2.77865
NC_056068.1	27790001	27810001	4.68019
NC_056068.1	27795001	27815001	3.76217
NC_056068.1	27800001	27820001	4.339
NC_056066.1	64675001	64695001	3.28571
NC_056066.1	64680001	64700001	2.04814
NC_056066.1	64685001	64705001	2.55453
NC_056066.1	64670001	64690001	3.08465
NC_056056.1	130630001	1.31E+08	2.00137
NC_056061.1	19665001	19685001	2.20099
NC_056061.1	19670001	19690001	2.1969
NC_056061.1	19675001	19695001	2.0526
NC_056061.1	19650001	19670001	2.46054
NC_056061.1	19655001	19675001	2.35308
NC_056061.1	19660001	19680001	2.21218
NC_056071.1	5495001	5515001	2.17738
NC_056071.1	5500001	5520001	2.19742
NC_056071.1	5510001	5530001	2.74817
NC_056071.1	5515001	5535001	3.02627
NC_056071.1	5520001	5540001	2.71791
NC_056060.1	6910001	6930001	3.19214
NC_056060.1	6915001	6935001	8.1263
NC_056059.1	95815001	95835001	3.25893
NC_056059.1	95820001	95840001	9.37096
NC_056059.1	95825001	95845001	6.66465
NC_056068.1	6350001	6370001	2.6133
NC_056080.1	35360001	35380001	2.0679
NC_056054.1	185335001	1.85E+08	3.74141
NC_056054.1	185340001	1.85E+08	3.26972
NC_056054.1	185345001	1.85E+08	2.60795

NC_056066.	17470001	17490001	1.97422	0.209333	OL	CCNY
NC_056066.	17475001	17495001	1.86319	0.201258	OL	CCNY
NC_056066.	17480001	17500001	1.83127	0.19494	OL	CCNY
NC_056066.	17410001	17430001	1.90894	0.144132	OL	CCNY;LOC121816286
NC_056066.	17415001	17435001	4.03236	0.211051	OL	CCNY;LOC121816286
NC_056066.	17420001	17440001	3.41398	0.21978	OL	CCNY;LOC121816286
NC_056055.	209820001	2.1E+08	3.81818	0.201604	OL	CCNYL1;FZD5;LOC1218
NC_056056.	46255001	46275001	2.29915	0.159507	OL	CCT4;COMMD1
NC_056056.	209475001	2.09E+08	2.60081	0.163503	OL	CD4
NC_056056.	209485001	2.1E+08	2.57028	0.159441	OL	CD4
NC_056056.	209490001	2.1E+08	2.46458	0.165936	OL	CD4;LAG3
NC_056054.	88200001	88220001	3.07814	0.213772	OL	CD53;LOC101105715
NC_056054.	88205001	88225001	3.01411	0.207575	OL	CD53;LOC101105715
NC_056054.	88210001	88230001	2.13526	0.155874	OL	CD53;LOC101105715
NC_056068.	63155001	63175001	3.46553	0.189961	OL	CD59
NC_056068.	63160001	63180001	2.89796	0.173141	OL	CD59
NC_056068.	63165001	63185001	3.13937	0.183022	OL	CD59
NC_056068.	63170001	63190001	2.59484	0.175677	OL	CD59;FBXO3
NC_056058.	47350001	47370001	2.03789	0.222681	OL	CDC25C;GFRA3
NC_056058.	47355001	47375001	1.81984	0.227836	OL	CDC25C;GFRA3
NC_056065.	29270001	29290001	2.02936	0.170295	OL	CDC42BPA
NC_056065.	29275001	29295001	2.31144	0.200154	OL	CDC42BPA
NC_056065.	29280001	29300001	3.16667	0.171002	OL	CDC42BPA
NC_056065.	29285001	29305001	4.24253	0.15718	OL	CDC42BPA
NC_056065.	29290001	29310001	5.38392	0.144265	OL	CDC42BPA
NC_056065.	29300001	29320001	3.25511	0.180494	OL	CDC42BPA
NC_056065.	29305001	29325001	2.7198	0.161469	OL	CDC42BPA
NC_056065.	29310001	29330001	3.17082	0.191322	OL	CDC42BPA
NC_056065.	29315001	29335001	4.92985	0.267307	OL	CDC42BPA
NC_056065.	29320001	29340001	6.43243	0.252658	OL	CDC42BPA
NC_056065.	29325001	29345001	9.08384	0.269048	OL	CDC42BPA

NC_056064.1	43400001	43420001	4.31537
NC_056060.1	50915001	50935001	2.02073
NC_056060.1	50945001	50965001	2.18352
NC_056060.1	50950001	50970001	3.29439
NC_056060.1	50955001	50975001	2.70364
NC_056060.1	50960001	50980001	2.71885
NC_056055.1	135210001	1.35E+08	2.19239
NC_056055.1	135215001	1.35E+08	1.97065
NC_056068.1	52730001	52750001	3.09367
NC_056077.1	1160001	1180001	4.3408
NC_056077.1	1165001	1185001	2.61951
NC_056055.1	186650001	1.87E+08	2.47033
NC_056055.1	186655001	1.87E+08	2.72864
NC_056055.1	186660001	1.87E+08	2.30172
NC_056055.1	186665001	1.87E+08	2.37584
NC_056080.1	8125001	8145001	5.80576
NC_056055.1	208295001	2.08E+08	9.59312
NC_056055.1	208300001	2.08E+08	5.80902
NC_056055.1	208275001	2.08E+08	23.4524
NC_056080.1	83975001	83995001	2.99999
NC_056080.1	83980001	84000001	4.80393
NC_056080.1	83985001	84005001	6.91506
NC_056080.1	83990001	84010001	10.6725
NC_056080.1	83995001	84015001	5.92491
NC_056054.1	71980001	72000001	2.14464
NC_056054.1	71985001	72005001	3.73258
NC_056055.1	85775001	85795001	2.25823
NC_056072.1	24050001	24070001	2.44419
NC_056068.1	8875001	8895001	1.95317
NC_056068.1	10040001	10060001	1.98433
NC_056068.1	10045001	10065001	2.52008

NC_056065.	29330001	29350001	9.0474	0.254065	OL	CDC42BPA
NC_056065.	29340001	29360001	6.33077	0.22659	OL	CDC42BPA
NC_056065.	29345001	29365001	9.70641	0.327093	OL	CDC42BPA
NC_056065.	29350001	29370001	11.8128	0.378269	OL	CDC42BPA
NC_056065.	29355001	29375001	9.89861	0.436281	OL	CDC42BPA
NC_056065.	29360001	29380001	6.43543	0.375571	OL	CDC42BPA
NC_056065.	29365001	29385001	4.47005	0.289922	OL	CDC42BPA
NC_056065.	29370001	29390001	3.5909	0.208084	OL	CDC42BPA
NC_056065.	29375001	29395001	5.49371	0.214234	OL	CDC42BPA
NC_056065.	29380001	29400001	4.60428	0.200604	OL	CDC42BPA
NC_056065.	29385001	29405001	3.46809	0.145797	OL	CDC42BPA
NC_056055.	136585001	1.37E+08	3.11674	0.149977	OL	CDCA7
NC_056055.	136590001	1.37E+08	3.62679	0.190099	OL	CDCA7
NC_056055.	136595001	1.37E+08	3.47163	0.154843	OL	CDCA7
NC_056067.	9610001	9630001	1.84401	0.26976	OL	CDH13
NC_056067.	9615001	9635001	1.82252	0.254942	OL	CDH13
NC_056076.	29165001	29185001	1.89775	0.230975	OL	CDH2
NC_056076.	29170001	29190001	1.90685	0.228923	OL	CDH2
NC_056076.	29175001	29195001	1.9201	0.233151	OL	CDH2
NC_056078.	27180001	27200001	3.05667	0.319049	OL	CDH23;PSAP
NC_056078.	27185001	27205001	3.99692	0.32393	OL	CDH23;PSAP
NC_056078.	27190001	27210001	2.12652	0.217651	OL	CDH23;PSAP
NC_056060.	21620001	21640001	1.86742	0.143816	OL	CDH24;PSMB11;PSMB5
NC_056056.	166160001	1.66E+08	1.86322	0.204755	OL	CDK17
NC_056056.	166165001	1.66E+08	1.81513	0.263056	OL	CDK17
NC_056056.	166170001	1.66E+08	1.80946	0.268806	OL	CDK17
NC_056060.	41525001	41545001	2.71664	0.144514	OL	CDKL1
NC_056063.	32370001	32390001	18.5795	0.23742	OL	CDX2
NC_056059.	84060001	84080001	3.97626	0.146954	OL	CENPC
NC_056059.	84065001	84085001	3.76294	0.156818	OL	CENPC
NC_056059.	84070001	84090001	3.72	0.161422	OL	CENPC

NC_056057.1	110555001	1.11E+08	3.62543
NC_056057.1	111705001	1.12E+08	1.9961
NC_056055.1	189690001	1.9E+08	2.13862
NC_056055.1	189695001	1.9E+08	3.78235
NC_056055.1	189700001	1.9E+08	4.10513
NC_056055.1	189705001	1.9E+08	2.40038
NC_056063.1	84465001	84485001	1.97729
NC_056063.1	84470001	84490001	2.45243
NC_056063.1	84475001	84495001	2.36454
NC_056063.1	84480001	84500001	2.61302
NC_056063.1	84485001	84505001	2.596
NC_056063.1	84490001	84510001	4.15696
NC_056063.1	84495001	84515001	3.6381
NC_056063.1	84500001	84520001	2.54413
NC_056063.1	84505001	84525001	2.38182
NC_056080.1	126475001	1.26E+08	2.14326
NC_056080.1	126680001	1.27E+08	1.98655
NC_056080.1	126685001	1.27E+08	2.76159
NC_056080.1	126690001	1.27E+08	2.88707
NC_056080.1	126695001	1.27E+08	2.32847
NC_056080.1	126805001	1.27E+08	2.93228
NC_056080.1	126810001	1.27E+08	2.25038
NC_056056.1	1680001	1700001	2.01734
NC_056056.1	46240001	46260001	2.66666
NC_056056.1	46245001	46265001	3.29348
NC_056056.1	46250001	46270001	3.48508
NC_056057.1	87195001	87215001	8.80132
NC_056057.1	87200001	87220001	3.64985
NC_056057.1	87095001	87115001	6.80673
NC_056057.1	87100001	87120001	7.74436
NC_056071.1	31500001	31520001	2.0775

NC_056059.	84075001	84095001	2.52917	0.156059	OL	CENPC
NC_056059.	84080001	84100001	2.10204	0.15957	OL	CENPC
NC_056080.	132865001	1.33E+08	1.89372	0.360578	OL	CENPI
NC_056080.	132870001	1.33E+08	3.30711	0.349692	OL	CENPI
NC_056080.	132875001	1.33E+08	5.27154	0.341638	OL	CENPI
NC_056080.	132880001	1.33E+08	3.85512	0.320688	OL	CENPI
NC_056080.	132885001	1.33E+08	1.858	0.237441	OL	CENPI
NC_056069.	14135001	14155001	2.28846	0.159333	OL	CENPK
NC_056073.	21895001	21915001	1.96864	0.148834	OL	CENPQ;MMUT
NC_056064.	61765001	61785001	1.87486	0.175032	OL	CEP112
NC_056064.	61770001	61790001	2.17696	0.233377	OL	CEP112
NC_056064.	61775001	61795001	2.0175	0.222665	OL	CEP112
NC_056068.	6445001	6465001	2.56005	0.183058	OL	CEP126
NC_056068.	6450001	6470001	2.68837	0.159373	OL	CEP126
NC_056068.	27730001	27750001	2.1202	0.395253	OL	CEP164
NC_056068.	27800001	27820001	2.44192	0.354404	OL	CEP164
NC_056068.	14115001	14135001	4.22981	0.171753	OL	CEP57;FAM76B
NC_056068.	14120001	14140001	5.48614	0.207018	OL	CEP57;FAM76B
NC_056055.	58670001	58690001	1.87285	0.187015	OL	CEP78
NC_056055.	58680001	58700001	1.93298	0.293537	OL	CEP78
NC_056055.	58685001	58705001	1.82036	0.284679	OL	CEP78
NC_056059.	96240001	96260001	2.14301	0.291144	OL	CFAP299
NC_056068.	6350001	6370001	2.7101	0.269313	OL	CFAP300
NC_056056.	68545001	68565001	1.95947	0.163014	OL	CFAP36
NC_056080.	35295001	35315001	2.47518	0.186193	OL	CFAP47
NC_056080.	35320001	35340001	2.57085	0.150558	OL	CFAP47
NC_056080.	35325001	35345001	3.19837	0.17503	OL	CFAP47
NC_056080.	35330001	35350001	2.66881	0.15569	OL	CFAP47
NC_056075.	24655001	24675001	2.31167	0.150622	OL	CFAP58
NC_056075.	24660001	24680001	2.77217	0.175332	OL	CFAP58
NC_056075.	24665001	24685001	4.45592	0.22186	OL	CFAP58

NC_056056.1	151090001	1.51E+08	2.61758
NC_056056.1	151095001	1.51E+08	3.64043
NC_056056.1	151100001	1.51E+08	3.64916
NC_056056.1	151105001	1.51E+08	2.8931
NC_056055.1	212195001	2.12E+08	2.16586
NC_056055.1	212200001	2.12E+08	3.14498
NC_056055.1	212205001	2.12E+08	2.7034
NC_056055.1	212210001	2.12E+08	2.31265
NC_056055.1	212235001	2.12E+08	2.45104
NC_056055.1	212240001	2.12E+08	3.38075
NC_056055.1	212245001	2.12E+08	3.85168
NC_056055.1	212250001	2.12E+08	4.34114
NC_056055.1	212255001	2.12E+08	4.12969
NC_056055.1	212260001	2.12E+08	2.31707
NC_056056.1	130135001	1.3E+08	4.30804
NC_056056.1	130140001	1.3E+08	8.25354
NC_056056.1	130145001	1.3E+08	8.74999
NC_056056.1	130150001	1.3E+08	3.69377
NC_056056.1	130155001	1.3E+08	3.09231
NC_056056.1	130160001	1.3E+08	2.51954
NC_056073.1	22070001	22090001	2.3808
NC_056073.1	22075001	22095001	2.01632
NC_056058.1	4390001	4410001	3.60556
NC_056057.1	25795001	25815001	2.29507
NC_056057.1	25800001	25820001	3.09971
NC_056057.1	25805001	25825001	3.69324
NC_056056.1	180960001	1.81E+08	6.15151
NC_056056.1	180965001	1.81E+08	6.73735
NC_056056.1	180970001	1.81E+08	4.58501
NC_056071.1	31530001	31550001	1.95822
NC_056071.1	31550001	31570001	1.99443

NC_056060.	50955001	50975001	2.07496	0.151185	OL	CGNL1
NC_056060.	50960001	50980001	2.07561	0.159201	OL	CGNL1
NC_056066.	47745001	47765001	2.49365	0.142343	OL	CHGB
NC_056057.	68420001	68440001	2.43109	0.502254	OL	CHN2
NC_056057.	68425001	68445001	5.13206	0.6057	OL	CHN2
NC_056057.	68430001	68450001	3.07624	0.500192	OL	CHN2
NC_056078.	11645001	11665001	2.13491	0.237297	OL	CHRM3
NC_056078.	11650001	11670001	2.31999	0.256978	OL	CHRM3
NC_056078.	11655001	11675001	2.44988	0.198652	OL	CHRM3
NC_056078.	11665001	11685001	2.06214	0.156639	OL	CHRM3
NC_056055.	135330001	1.35E+08	3.87965	0.1556	OL	CHRNA1
NC_056067.	43615001	43635001	2.57485	0.153263	OL	CHST8
NC_056067.	43625001	43645001	5.73435	0.197457	OL	CHST8
NC_056067.	43630001	43650001	5.31429	0.214586	OL	CHST8
NC_056067.	43635001	43655001	3.61337	0.16307	OL	CHST8
NC_056067.	43640001	43660001	3.17461	0.154026	OL	CHST8
NC_056067.	10735001	10755001	3.90756	0.201111	OL	CIBAR2
NC_056067.	10740001	10760001	3.21622	0.177451	OL	CIBAR2
NC_056056.	207255001	2.07E+08	2.86292	0.181456	OL	CLEC6A
NC_056056.	207260001	2.07E+08	4.53351	0.256785	OL	CLEC6A
NC_056056.	35940001	35960001	2.44697	0.228621	OL	CLIP4
NC_056059.	71595001	71615001	1.81913	0.142057	OL	CLOCK
NC_056059.	71600001	71620001	3.09474	0.208244	OL	CLOCK
NC_056059.	71605001	71625001	4.74027	0.253141	OL	CLOCK
NC_056059.	71610001	71630001	12.0714	0.334099	OL	CLOCK
NC_056059.	71615001	71635001	7.82469	0.328853	OL	CLOCK
NC_056059.	71620001	71640001	5.94582	0.31446	OL	CLOCK
NC_056059.	71625001	71645001	6.91429	0.305126	OL	CLOCK
NC_056059.	71630001	71650001	6.73529	0.27549	OL	CLOCK
NC_056059.	71635001	71655001	6.6653	0.281081	OL	CLOCK
NC_056059.	71640001	71660001	5.73928	0.270872	OL	CLOCK

NC_056054.1	8805001	8825001	2.23042
NC_056054.1	8810001	8830001	2.55145
NC_056075.1	44065001	44085001	2.70923
NC_056075.1	44070001	44090001	2.2031
NC_056067.1	34695001	34715001	4.05065
NC_056067.1	34700001	34720001	4.47546
NC_056067.1	34705001	34725001	5.09468
NC_056067.1	34710001	34730001	5.29238
NC_056067.1	34715001	34735001	5.42028
NC_056067.1	34720001	34740001	6.29084
NC_056067.1	34725001	34745001	8.14645
NC_056067.1	34730001	34750001	7.56807
NC_056067.1	34690001	34710001	3.81531
NC_056055.1	205840001	2.06E+08	2.62295
NC_056055.1	205845001	2.06E+08	2.16687
NC_056078.1	22495001	22515001	1.98141
NC_056071.1	22950001	22970001	2.21136
NC_056071.1	22955001	22975001	2.36015
NC_056071.1	22960001	22980001	2.45681
NC_056075.1	20480001	20500001	2.80935
NC_056075.1	20485001	20505001	3.91307
NC_056075.1	20490001	20510001	4.49855
NC_056075.1	20495001	20515001	4.02154
NC_056075.1	20500001	20520001	2.28885
NC_056075.1	15640001	15660001	2.21376
NC_056080.1	78045001	78065001	3.44952
NC_056080.1	78050001	78070001	4.58521
NC_056080.1	78055001	78075001	5.06809
NC_056080.1	78060001	78080001	4.14179
NC_056080.1	78065001	78085001	4.47908
NC_056080.1	78070001	78090001	4.52701

NC_056059.	71645001	71665001	4.68955	0.258431	OL	CLOCK
NC_056059.	71650001	71670001	3.32653	0.251365	OL	CLOCK
NC_056059.	71655001	71675001	3.08964	0.255298	OL	CLOCK
NC_056059.	71660001	71680001	2.9144	0.274439	OL	CLOCK
NC_056059.	71665001	71685001	2.48832	0.28771	OL	CLOCK
NC_056059.	71670001	71690001	2.61559	0.294512	OL	CLOCK
NC_056059.	71675001	71695001	2.35692	0.341057	OL	CLOCK
NC_056059.	71680001	71700001	1.93646	0.322834	OL	CLOCK
NC_056059.	71685001	71705001	2.89796	0.410103	OL	CLOCK
NC_056059.	71690001	71710001	2.98286	0.381337	OL	CLOCK;PDCL2
NC_056059.	71695001	71715001	2.85748	0.304525	OL	CLOCK;PDCL2
NC_056059.	71700001	71720001	2.77927	0.262049	OL	CLOCK;PDCL2
NC_056059.	71560001	71580001	1.875	0.22449	OL	CLOCK;TMEM165
NC_056054.	71980001	72000001	2.60033	0.284396	OL	CNN3;SLC44A3
NC_056054.	71985001	72005001	7.22174	0.379782	OL	CNN3;SLC44A3
NC_056054.	71990001	72010001	2.50546	0.190938	OL	CNN3;SLC44A3
NC_056058.	63840001	63860001	3.80422	0.146712	OL	CNOT8;GEMIN5
NC_056068.	9865001	9885001	2.3902	0.190583	OL	CNTN5
NC_056068.	9870001	9890001	2.77952	0.195457	OL	CNTN5
NC_056068.	9875001	9895001	1.80867	0.157526	OL	CNTN5
NC_056057.	112495001	1.13E+08	1.80935	0.221012	OL	CNTNAP2
NC_056057.	112500001	1.13E+08	1.97289	0.22009	OL	CNTNAP2
NC_056057.	112505001	1.13E+08	2.03465	0.203807	OL	CNTNAP2
NC_056067.	3045001	3065001	1.92062	0.227242	OL	CNTNAP4
NC_056055.	189690001	1.9E+08	2.67448	0.226074	OL	CNTNAP5
NC_056055.	189695001	1.9E+08	4.58255	0.286408	OL	CNTNAP5
NC_056055.	189700001	1.9E+08	4.15208	0.253136	OL	CNTNAP5
NC_056055.	189705001	1.9E+08	2.2193	0.17065	OL	CNTNAP5
NC_056054.	80245001	80265001	2.56628	0.194373	OL	COL11A1
NC_056054.	80250001	80270001	3.77788	0.227176	OL	COL11A1
NC_056054.	80255001	80275001	1.84448	0.26218	OL	COL11A1

NC_056080.1	78075001	78095001	3.69897
NC_056080.1	78080001	78100001	4.37751
NC_056080.1	78085001	78105001	3.48084
NC_056080.1	78090001	78110001	3.20243
NC_056080.1	78095001	78115001	3.71682
NC_056080.1	78100001	78120001	3.01145
NC_056080.1	78210001	78230001	4.61207
NC_056080.1	78215001	78235001	4.38329
NC_056080.1	78220001	78240001	5.3633
NC_056080.1	78225001	78245001	4.45887
NC_056080.1	78230001	78250001	3.93262
NC_056080.1	78235001	78255001	4.45355
NC_056080.1	78240001	78260001	4.03859
NC_056080.1	78245001	78265001	3.51898
NC_056080.1	78250001	78270001	3.81238
NC_056080.1	78255001	78275001	3.51445
NC_056080.1	78260001	78280001	3.93396
NC_056080.1	78265001	78285001	4.13481
NC_056080.1	78270001	78290001	3.76262
NC_056080.1	78275001	78295001	3.51732
NC_056080.1	78280001	78300001	3.19328
NC_056080.1	78285001	78305001	2.88214
NC_056080.1	78295001	78315001	2.97376
NC_056080.1	78300001	78320001	3.17972
NC_056080.1	78305001	78325001	3.05253
NC_056080.1	78310001	78330001	2.91588
NC_056055.1	174910001	1.75E+08	2.04324
NC_056055.1	174915001	1.75E+08	1.96821
NC_056055.1	174925001	1.75E+08	2.27419
NC_056076.1	52625001	52645001	3.45289
NC_056076.1	52630001	52650001	3.04922

NC_056077.	35635001	35655001	2.11563	0.211499	OL	COL26A1
NC_056077.	35640001	35660001	2.07789	0.203556	OL	COL26A1
NC_056077.	35645001	35665001	2.03303	0.20235	OL	COL26A1
NC_056077.	35650001	35670001	2.07555	0.205366	OL	COL26A1
NC_056077.	35655001	35675001	2.12996	0.165458	OL	COL26A1
NC_056063.	84410001	84430001	1.90849	0.178582	OL	COL4A2
NC_056063.	84465001	84485001	2.10318	0.231466	OL	COL4A2
NC_056063.	84470001	84490001	2.84038	0.22881	OL	COL4A2
NC_056063.	84475001	84495001	2.79195	0.228443	OL	COL4A2
NC_056063.	84480001	84500001	3.12749	0.231726	OL	COL4A2
NC_056063.	84485001	84505001	3.31305	0.221055	OL	COL4A2
NC_056063.	84490001	84510001	2.63468	0.149612	OL	COL4A2
NC_056062.	58180001	58200001	2.38504	0.144641	OL	COLEC10
NC_056055.	49570001	49590001	2.12355	0.144957	OL	CORO2A
NC_056071.	31420001	31440001	2.28097	0.224447	OL	COX5A
NC_056071.	31425001	31445001	2.31035	0.210378	OL	COX5A
NC_056071.	31430001	31450001	2.2	0.200837	OL	COX5A;FAM219B
NC_056071.	31435001	31455001	2.16327	0.209052	OL	COX5A;FAM219B
NC_056071.	31410001	31430001	2.27856	0.246096	OL	COX5A;RPP25
NC_056071.	31415001	31435001	2.25803	0.242073	OL	COX5A;RPP25
NC_056054.	239720001	2.4E+08	2.78518	0.158674	OL	CP
NC_056054.	239725001	2.4E+08	2.9465	0.15669	OL	CP
NC_056062.	44615001	44635001	1.84778	0.224236	OL	CPA6
NC_056057.	87165001	87185001	2.32956	0.149185	OL	CPED1
NC_056057.	87170001	87190001	2.42616	0.153928	OL	CPED1
NC_056057.	87175001	87195001	2.91854	0.169991	OL	CPED1
NC_056071.	31515001	31535001	2.39319	0.243074	OL	CPLX3;CSK;LMAN1L
NC_056071.	31510001	31530001	2.36916	0.24536	OL	CPLX3;LMAN1L
NC_056071.	31500001	31520001	2.27536	0.223058	OL	CPLX3;LMAN1L;ULK3
NC_056071.	31505001	31525001	2.29236	0.225855	OL	CPLX3;LMAN1L;ULK3
NC_056071.	31495001	31515001	2.18207	0.210526	OL	CPLX3;SCAMP2;ULK3

NC_056076.1	52635001	52655001	2.18598
NC_056066.1	28935001	28955001	3.14621
NC_056056.1	214145001	2.14E+08	2.28022
NC_056056.1	214150001	2.14E+08	2.46513
NC_056059.1	68430001	68450001	2.94203
NC_056059.1	68435001	68455001	4.62574
NC_056059.1	68440001	68460001	7.89566
NC_056059.1	68445001	68465001	21.4616
NC_056059.1	68450001	68470001	21.2184
NC_056054.1	62400001	62420001	2.22783
NC_056057.1	6185001	6205001	2.22824
NC_056056.1	215995001	2.16E+08	2.25625
NC_056056.1	216000001	2.16E+08	2.28727
NC_056056.1	216005001	2.16E+08	2.80082
NC_056056.1	215980001	2.16E+08	3.08198
NC_056056.1	215985001	2.16E+08	2.66522
NC_056056.1	215990001	2.16E+08	2.41088
NC_056067.1	35040001	35060001	2.28513
NC_056068.1	29100001	29120001	2.07692
NC_056068.1	29105001	29125001	2.29395
NC_056068.1	29110001	29130001	2.50888
NC_056065.1	76095001	76115001	2.02365
NC_056065.1	76165001	76185001	2.52445
NC_056065.1	76170001	76190001	2.86732
NC_056065.1	76175001	76195001	3.49147
NC_056065.1	76180001	76200001	2.64436
NC_056070.1	53175001	53195001	3.43651
NC_056070.1	53180001	53200001	3.52233
NC_056070.1	53185001	53205001	3.05548
NC_056080.1	137815001	1.38E+08	9.86231
NC_056080.1	137820001	1.38E+08	11.2088

NC_056062.	80410001	80430001	2.61714	0.187244	OL	CPQ
NC_056057.	68575001	68595001	2.14337	0.326135	OL	CPVL
NC_056057.	68580001	68600001	2.60637	0.330044	OL	CPVL
NC_056057.	68585001	68605001	2.90621	0.268681	OL	CPVL
NC_056057.	68590001	68610001	3.5114	0.250416	OL	CPVL
NC_056057.	68595001	68615001	3.20351	0.224937	OL	CPVL
NC_056057.	68600001	68620001	2.26695	0.274426	OL	CPVL
NC_056057.	68605001	68625001	2.01453	0.26845	OL	CPVL
NC_056057.	68705001	68725001	2.14725	0.226498	OL	CPVL
NC_056065.	75650001	75670001	1.86377	0.146409	OL	CRB1
NC_056065.	75655001	75675001	2.46338	0.175055	OL	CRB1
NC_056065.	75660001	75680001	2.95235	0.194543	OL	CRB1
NC_056065.	75665001	75685001	3.06488	0.164078	OL	CRB1
NC_056056.	180950001	1.81E+08	5.17552	0.16733	OL	CSF2RB
NC_056056.	180955001	1.81E+08	6.54188	0.195085	OL	CSF2RB
NC_056056.	180960001	1.81E+08	7.25	0.200529	OL	CSF2RB
NC_056056.	180965001	1.81E+08	9.94861	0.209053	OL	CSF2RB
NC_056056.	180970001	1.81E+08	33.4399	0.24152	OL	CSF2RB
NC_056056.	180975001	1.81E+08	33.7732	0.229163	OL	CSF2RB
NC_056071.	31530001	31550001	2.07869	0.202185	OL	CSK
NC_056071.	31535001	31555001	1.86626	0.183465	OL	CSK
NC_056071.	31540001	31560001	1.84788	0.186489	OL	CSK
NC_056071.	31545001	31565001	1.87529	0.1844	OL	CSK
NC_056071.	31550001	31570001	1.82412	0.184008	OL	CSK
NC_056071.	31520001	31540001	2.26617	0.231598	OL	CSK;LMAN1L
NC_056071.	31525001	31545001	2.20577	0.223191	OL	CSK;LMAN1L
NC_056079.	3165001	3185001	4.46099	0.203564	OL	CSMD1
NC_056079.	3170001	3190001	3.06904	0.197886	OL	CSMD1
NC_056080.	99840001	99860001	1.83707	0.164571	OL	CT55;LOC114111389
NC_056067.	34695001	34715001	3.42858	0.341669	OL	CTCF
NC_056067.	34700001	34720001	3.6475	0.38747	OL	CTCF

NC_056080.1	137825001	1.38E+08	12.8609
NC_056080.1	137830001	1.38E+08	14.5827
NC_056080.1	137835001	1.38E+08	8.05672
NC_056080.1	137840001	1.38E+08	4.85056
NC_056080.1	137845001	1.38E+08	3.35873
NC_056080.1	137850001	1.38E+08	2.87571
NC_056080.1	137855001	1.38E+08	2.99641
NC_056080.1	137860001	1.38E+08	2.40386
NC_056080.1	137865001	1.38E+08	2.44192
NC_056080.1	137870001	1.38E+08	2.40921
NC_056080.1	137875001	1.38E+08	2.56971
NC_056080.1	137880001	1.38E+08	4.45563
NC_056080.1	137885001	1.38E+08	3.74731
NC_056054.1	70120001	70140001	2.87665
NC_056054.1	70125001	70145001	2.39742
NC_056063.1	48115001	48135001	5.87318
NC_056079.1	22795001	22815001	2.51215
NC_056079.1	22800001	22820001	2.78509
NC_056079.1	22830001	22850001	2.22407
NC_056079.1	22835001	22855001	2.35067
NC_056079.1	22840001	22860001	2.08169
NC_056066.1	65540001	65560001	1.94622
NC_056060.1	65710001	65730001	2.35644
NC_056056.1	216010001	2.16E+08	3.58728
NC_056056.1	216015001	2.16E+08	4.71727
NC_056056.1	216020001	2.16E+08	6.44118
NC_056056.1	216025001	2.16E+08	5.3613
NC_056080.1	31695001	31715001	2.03733
NC_056080.1	31700001	31720001	1.96256
NC_056060.1	10040001	10060001	3.27158
NC_056060.1	10045001	10065001	3.79203

NC_056067.	34705001	34725001	4.05176	0.397213	OL	CTCF
NC_056067.	34710001	34730001	4.07183	0.407076	OL	CTCF
NC_056067.	34715001	34735001	4.24196	0.425516	OL	CTCF
NC_056067.	34720001	34740001	4.88579	0.445288	OL	CTCF
NC_056067.	34725001	34745001	6.14197	0.468334	OL	CTCF
NC_056067.	34730001	34750001	5.90476	0.45952	OL	CTCF
NC_056067.	34690001	34710001	3.27027	0.272596	OL	CTCF;RIPOR1
NC_056078.	22035001	22055001	2.69799	0.208749	OL	CTNNA3
NC_056078.	22040001	22060001	2.42355	0.204216	OL	CTNNA3
NC_056078.	22045001	22065001	1.84787	0.149732	OL	CTNNA3
NC_056071.	22965001	22985001	1.85728	0.175075	OL	CTSH
NC_056071.	22970001	22990001	1.94294	0.189864	OL	CTSH
NC_056071.	22985001	23005001	2.04173	0.188023	OL	CTSH
NC_056071.	22955001	22975001	2.59237	0.223234	OL	CTSH;MORF4L1
NC_056071.	22960001	22980001	2.53253	0.208451	OL	CTSH;MORF4L1
NC_056068.	17355001	17375001	2.99155	0.145683	OL	CUL5
NC_056078.	43900001	43920001	2.25719	0.164118	OL	CXCL12
NC_056078.	43905001	43925001	1.83274	0.162277	OL	CXCL12
NC_056059.	93015001	93035001	1.92065	0.240704	OL	CXCL13
NC_056054.	130510001	1.31E+08	1.81583	0.368734	OL	CYYR1
NC_056060.	69655001	69675001	2.01935	0.147284	OL	DAAM1;L3HYPDH
NC_056060.	69685001	69705001	2.2227	0.185371	OL	DAAM1;L3HYPDH
NC_056060.	69690001	69710001	2.4798	0.210394	OL	DAAM1;L3HYPDH
NC_056060.	69695001	69715001	2.00406	0.190914	OL	DAAM1;L3HYPDH
NC_056060.	69710001	69730001	1.94882	0.172697	OL	DAAM1;L3HYPDH
NC_056060.	44155001	44175001	1.87706	0.237905	OL	DAPK2
NC_056056.	134990001	1.35E+08	1.82268	0.232474	OL	DAZAP2
NC_056057.	33190001	33210001	2.14393	0.279371	OL	DBF4
NC_056057.	33195001	33215001	3.01737	0.34972	OL	DBF4
NC_056057.	33200001	33220001	3.05677	0.34595	OL	DBF4;SLC25A40
NC_056057.	33205001	33225001	2.51116	0.332222	OL	DBF4;SLC25A40

NC_056060.1	10050001	10070001	2.29782
NC_056062.1	21705001	21725001	2.46302
NC_056062.1	21710001	21730001	2.44239
NC_056077.1	4390001	4410001	4.2725
NC_056077.1	19200001	19220001	2.69382
NC_056077.1	19205001	19225001	2
NC_056067.1	14930001	14950001	3.28295
NC_056054.1	257775001	2.58E+08	2.29138
NC_056054.1	257780001	2.58E+08	4.56943
NC_056054.1	257785001	2.58E+08	3.48478
NC_056054.1	257790001	2.58E+08	2.00602
NC_056065.1	38590001	38610001	2.31687
NC_056065.1	38595001	38615001	2.98213
NC_056065.1	38600001	38620001	2.20265
NC_056065.1	38745001	38765001	2.4317
NC_056065.1	38750001	38770001	2.09158
NC_056065.1	38755001	38775001	2.1058
NC_056065.1	38760001	38780001	2.24357
NC_056065.1	38765001	38785001	2.30654
NC_056054.1	38040001	38060001	4.11267
NC_056054.1	38045001	38065001	10.3581
NC_056054.1	38050001	38070001	4.60995
NC_056054.1	38055001	38075001	2.96154
NC_056064.1	22950001	22970001	2.27148
NC_056062.1	82470001	82490001	2.0423
NC_056062.1	82475001	82495001	3.36803
NC_056062.1	82480001	82500001	3.79923
NC_056062.1	82485001	82505001	2.9606
NC_056062.1	82490001	82510001	2.16994
NC_056059.1	56460001	56480001	1.99471
NC_056067.1	35045001	35065001	3.22413

NC_056057.	33210001	33230001	2.26951	0.299098	OL	DBF4;SLC25A40
NC_056076.	52340001	52360001	1.93736	0.181465	OL	DCC
NC_056076.	52345001	52365001	2.57517	0.203689	OL	DCC
NC_056058.	59835001	59855001	2.25385	0.145044	OL	DCTN4
NC_056068.	18215001	18235001	2.48529	0.151674	OL	DDX10
NC_056068.	18220001	18240001	2.6032	0.155868	OL	DDX10
NC_056068.	18225001	18245001	2.48779	0.144231	OL	DDX10
NC_056064.	13600001	13620001	2.84788	0.257784	OL	DDX52
NC_056064.	13585001	13605001	4.27262	0.209797	OL	DDX52;SYNRG
NC_056064.	13590001	13610001	8.11428	0.285339	OL	DDX52;SYNRG
NC_056064.	13595001	13615001	5.39137	0.303789	OL	DDX52;SYNRG
NC_056075.	38810001	38830001	1.9059	0.143852	OL	DENND10
NC_056069.	18730001	18750001	2.60151	0.157587	OL	DEPDC1B
NC_056069.	18735001	18755001	3.38667	0.207663	OL	DEPDC1B
NC_056069.	18740001	18760001	5.06727	0.267543	OL	DEPDC1B
NC_056069.	18745001	18765001	6.04556	0.302819	OL	DEPDC1B
NC_056069.	18750001	18770001	9.06783	0.333591	OL	DEPDC1B
NC_056069.	18755001	18775001	9.91413	0.345241	OL	DEPDC1B
NC_056069.	18760001	18780001	4.28223	0.289919	OL	DEPDC1B
NC_056069.	18765001	18785001	2.41095	0.203953	OL	DEPDC1B
NC_056057.	102475001	1.02E+08	6.85169	0.162541	OL	DGKI
NC_056057.	102480001	1.03E+08	6.14147	0.162581	OL	DGKI
NC_056057.	102900001	1.03E+08	3.30929	0.168738	OL	DGKI
NC_056057.	102905001	1.03E+08	2.81987	0.189414	OL	DGKI
NC_056057.	102910001	1.03E+08	2.51613	0.225206	OL	DGKI
NC_056057.	102915001	1.03E+08	2.40817	0.310104	OL	DGKI
NC_056064.	10245001	10265001	2.45462	0.154029	OL	DHX40
NC_056064.	10250001	10270001	2.63227	0.153685	OL	DHX40
NC_056064.	10255001	10275001	2.33919	0.143949	OL	DHX40
NC_056065.	63340001	63360001	6.75472	0.174069	OL	DHX9
NC_056065.	63335001	63355001	5.42069	0.144665	OL	DHX9;NPL

NC_056067.1	35050001	35070001	2.48
NC_056067.1	35070001	35090001	2.40571
NC_056064.1	43390001	43410001	2.66445
NC_056064.1	43395001	43415001	3.70221
NC_056072.1	48890001	48910001	2.42553
NC_056068.1	4850001	4870001	2.23135
NC_056068.1	4880001	4900001	2.22577
NC_056068.1	4885001	4905001	1.9418
NC_056056.1	93355001	93375001	2.9255
NC_056056.1	93345001	93365001	2.60465
NC_056056.1	93350001	93370001	3.37205
NC_056054.1	187190001	1.87E+08	3.11556
NC_056055.1	245135001	2.45E+08	2.41323
NC_056055.1	245140001	2.45E+08	3.12062
NC_056055.1	245145001	2.45E+08	3.42542
NC_056055.1	245150001	2.45E+08	3.61528
NC_056067.1	47915001	47935001	4.23932
NC_056067.1	47920001	47940001	4.13461
NC_056067.1	47910001	47930001	6.87176
NC_056061.1	63890001	63910001	11.7671
NC_056061.1	63895001	63915001	8.29349
NC_056061.1	63900001	63920001	8.41176
NC_056061.1	63905001	63925001	12.9078
NC_056061.1	63925001	63945001	15.7066
NC_056080.1	62670001	62690001	2.61712
NC_056080.1	62675001	62695001	3.68841
NC_056080.1	62680001	62700001	3.03309
NC_056080.1	62685001	62705001	2.52194
NC_056065.1	65180001	65200001	2.90747
NC_056065.1	65185001	65205001	3.08833
NC_056065.1	65190001	65210001	3.0283

NC_056063.	2910001	2930001	2.12543	0.169986	OL	DIAPH3
NC_056063.	2915001	2935001	2.46881	0.194046	OL	DIAPH3
NC_056063.	2920001	2940001	3.1825	0.232134	OL	DIAPH3
NC_056063.	2925001	2945001	2.92297	0.216561	OL	DIAPH3
NC_056063.	2930001	2950001	2.56649	0.185788	OL	DIAPH3
NC_056063.	2970001	2990001	2.61502	0.17541	OL	DIAPH3
NC_056063.	2975001	2995001	2.76574	0.226344	OL	DIAPH3
NC_056063.	2980001	3000001	3.44124	0.252708	OL	DIAPH3
NC_056063.	2985001	3005001	2.6993	0.250629	OL	DIAPH3
NC_056063.	2990001	3010001	2.06287	0.162952	OL	DIAPH3
NC_056054.	70105001	70125001	1.89084	0.18781	OL	DIPK1A
NC_056054.	70110001	70130001	1.90777	0.155464	OL	DIPK1A
NC_056073.	17010001	17030001	3.63218	0.181693	OL	DLK2;TJAP1
NC_056080.	31895001	31915001	5.98261	0.14387	OL	DMD
NC_056058.	32345001	32365001	2.29748	0.158867	OL	DMXL1
NC_056058.	32350001	32370001	2.33069	0.15933	OL	DMXL1
NC_056056.	40180001	40200001	2.50486	0.143688	OL	DNAAF10
NC_056062.	21690001	21710001	1.88377	0.1977	OL	DNAAF11
NC_056062.	21695001	21715001	1.94883	0.205661	OL	DNAAF11
NC_056062.	21700001	21720001	2.4616	0.241615	OL	DNAAF11
NC_056062.	21705001	21725001	2.56736	0.184357	OL	DNAAF11
NC_056077.	4385001	4405001	2.67143	0.173859	OL	DNAAF8;ZNF500
NC_056065.	27665001	27685001	1.87716	0.164644	OL	DNAH14
NC_056055.	198130001	1.98E+08	2.65049	0.166667	OL	DNAH7
NC_056055.	198135001	1.98E+08	2.06533	0.193586	OL	DNAH7
NC_056055.	198150001	1.98E+08	4.17382	0.193162	OL	DNAH7
NC_056055.	198155001	1.98E+08	8.78755	0.25974	OL	DNAH7
NC_056055.	198160001	1.98E+08	11.8318	0.326693	OL	DNAH7
NC_056055.	198165001	1.98E+08	14.5987	0.319101	OL	DNAH7
NC_056055.	198170001	1.98E+08	7.30641	0.278606	OL	DNAH7
NC_056055.	198175001	1.98E+08	6.54347	0.254351	OL	DNAH7

NC_056065.1	65195001	65215001	2.04786
NC_056065.1	65210001	65230001	2.18025
NC_056065.1	65215001	65235001	1.93806
NC_056055.1	208265001	2.08E+08	18.8426
NC_056057.1	62670001	62690001	3.22505
NC_056077.1	41245001	41265001	2.16098
NC_056072.1	29945001	29965001	6.43183
NC_056055.1	92480001	92500001	2.07608
NC_056056.1	181435001	1.81E+08	2.10792
NC_056056.1	181440001	1.81E+08	2.3171
NC_056056.1	181445001	1.81E+08	2.31888
NC_056056.1	181450001	1.81E+08	2.30876
NC_056056.1	181455001	1.81E+08	2.33732
NC_056057.1	61530001	61550001	2.78572
NC_056072.1	52135001	52155001	14.1604
NC_056072.1	52140001	52160001	19.6717
NC_056072.1	52150001	52170001	4.64414
NC_056072.1	52155001	52175001	3.12852
NC_056072.1	52160001	52180001	2.33245
NC_056076.1	37570001	37590001	2.01811
NC_056060.1	98485001	98505001	3.62141
NC_056060.1	98490001	98510001	6.53652
NC_056060.1	98495001	98515001	4.34883
NC_056060.1	98500001	98520001	3.07822
NC_056056.1	69145001	69165001	7.49612
NC_056056.1	69150001	69170001	8.21923
NC_056056.1	69155001	69175001	8.15441
NC_056056.1	69165001	69185001	8.68201
NC_056056.1	69170001	69190001	6.53134
NC_056080.1	104050001	1.04E+08	2.52534
NC_056080.1	104055001	1.04E+08	2.54245

NC_056055.	198180001	1.98E+08	4.97821	0.211307	OL	DNAH7
NC_056055.	198185001	1.98E+08	5.58209	0.229211	OL	DNAH7
NC_056055.	198190001	1.98E+08	13.4286	0.276094	OL	DNAH7
NC_056055.	198195001	1.98E+08	12.2557	0.241507	OL	DNAH7
NC_056055.	198200001	1.98E+08	8.10752	0.215357	OL	DNAH7
NC_056055.	198205001	1.98E+08	3.76738	0.246291	OL	DNAH7
NC_056055.	198210001	1.98E+08	3.37733	0.197534	OL	DNAH7
NC_056055.	198215001	1.98E+08	2.89073	0.186031	OL	DNAH7
NC_056055.	198220001	1.98E+08	2.26764	0.164333	OL	DNAH7
NC_056055.	37095001	37115001	3.24224	0.15404	OL	DNAI1;FAM219A
NC_056055.	37100001	37120001	2.88341	0.194292	OL	DNAI1;FAM219A
NC_056055.	37105001	37125001	2.68845	0.215051	OL	DNAI1;FAM219A
NC_056054.	257835001	2.58E+08	2.63004	0.223333	OL	DNAJC13
NC_056054.	257840001	2.58E+08	2.41674	0.239575	OL	DNAJC13;LOC101119869
NC_056054.	257845001	2.58E+08	2.6565	0.293251	OL	DNAJC13;LOC101119869
NC_056054.	257850001	2.58E+08	2.67876	0.30364	OL	DNAJC13;LOC101119869
NC_056060.	83160001	83180001	1.96059	0.153088	OL	DNAL1;LOC101118736
NC_056065.	39000001	39020001	2.15625	0.159178	OL	DNM3
NC_056065.	39005001	39025001	2.3328	0.182999	OL	DNM3
NC_056065.	39010001	39030001	2.01117	0.172172	OL	DNM3
NC_056054.	71010001	71030001	1.82468	0.204351	OL	DNTTIP2
NC_056054.	71015001	71035001	2.21613	0.228347	OL	DNTTIP2
NC_056054.	71020001	71040001	2.22185	0.207739	OL	DNTTIP2
NC_056054.	71025001	71045001	2.13972	0.201071	OL	DNTTIP2;GCLM
NC_056054.	71030001	71050001	2.22304	0.212323	OL	DNTTIP2;GCLM
NC_056054.	71035001	71055001	2.06309	0.187978	OL	DNTTIP2;GCLM
NC_056063.	75050001	75070001	1.99585	0.21285	OL	DOCK9
NC_056061.	10315001	10335001	2.03494	0.148196	OL	DOP1A
NC_056061.	10215001	10235001	2.93103	0.193609	OL	DOP1A;UBE3D
NC_056061.	10225001	10245001	2.2884	0.146549	OL	DOP1A;UBE3D
NC_056064.	22950001	22970001	3.61203	0.276606	OL	DPH1;HIC1;OVCA2

NC_056080.1	104060001	1.04E+08	2.62691
NC_056080.1	104070001	1.04E+08	3.05987
NC_056080.1	104075001	1.04E+08	4.30701
NC_056080.1	104080001	1.04E+08	5.97342
NC_056080.1	104130001	1.04E+08	9.41667
NC_056080.1	104135001	1.04E+08	7.61046
NC_056080.1	104045001	1.04E+08	2.569
NC_056058.1	6355001	6375001	6.00862
NC_056058.1	6360001	6380001	11.4749
NC_056058.1	6365001	6385001	16.6373
NC_056058.1	6370001	6390001	29.8437
NC_056058.1	6375001	6395001	11.4081
NC_056056.1	199845001	2E+08	2.04544
NC_056056.1	199850001	2E+08	3.87498
NC_056056.1	199855001	2E+08	5.63298
NC_056056.1	199860001	2E+08	4.06804
NC_056056.1	199865001	2E+08	3.01636
NC_056056.1	199870001	2E+08	2.81797
NC_056056.1	199875001	2E+08	2.6514
NC_056056.1	199880001	2E+08	2.55468
NC_056056.1	199885001	2E+08	2.66023
NC_056056.1	199920001	2E+08	2.51153
NC_056056.1	199925001	2E+08	4.62093
NC_056056.1	199930001	2E+08	3.56043
NC_056054.1	87080001	87100001	2.47707
NC_056054.1	87075001	87095001	2.6273
NC_056055.1	213890001	2.14E+08	2.98346
NC_056055.1	213895001	2.14E+08	2.86239
NC_056055.1	213900001	2.14E+08	2.9013
NC_056056.1	213705001	2.14E+08	1.95495
NC_056056.1	213785001	2.14E+08	2.79555

NC_056064.	22955001	22975001	2.28937	0.244583	OL	DPH1;HIC1;OVCA2;SMG
NC_056064.	22945001	22965001	2.44094	0.267105	OL	DPH1;OVCA2
NC_056064.	22940001	22960001	1.82781	0.260032	OL	DPH1;OVCA2;RTN4RL1
NC_056080.	132970001	1.33E+08	1.98631	0.283452	OL	DRP2;TAF7L
NC_056068.	27810001	27830001	2.31163	0.261026	OL	DSCAML1
NC_056066.	38220001	38240001	1.98232	0.161187	OL	DTD1
NC_056055.	245165001	2.45E+08	2.36407	0.195533	OL	ECE1
NC_056055.	245170001	2.45E+08	2.70659	0.204816	OL	ECE1
NC_056055.	245175001	2.45E+08	3.13925	0.228327	OL	ECE1
NC_056055.	245180001	2.45E+08	2.57535	0.185386	OL	ECE1
NC_056055.	12075001	12095001	2.04661	0.193611	OL	ECPAS
NC_056055.	12080001	12100001	2.17128	0.198256	OL	ECPAS
NC_056055.	12105001	12125001	2.71809	0.180867	OL	ECPAS
NC_056055.	12110001	12130001	6.38848	0.368285	OL	ECPAS
NC_056055.	12115001	12135001	7.12605	0.376989	OL	ECPAS;LOC101112627
NC_056056.	60905001	60925001	2.79386	0.25763	OL	ECRG4
NC_056056.	60910001	60930001	3.26647	0.156644	OL	ECRG4
NC_056058.	81815001	81835001	3.36655	0.144716	OL	EDIL3
NC_056058.	81820001	81840001	3.03439	0.143912	OL	EDIL3
NC_056054.	21225001	21245001	2.0369	0.254847	OL	EFCAB14
NC_056054.	21230001	21250001	2.03409	0.253674	OL	EFCAB14
NC_056054.	21235001	21255001	2.05556	0.276773	OL	EFCAB14
NC_056054.	21240001	21260001	2.0689	0.286203	OL	EFCAB14
NC_056054.	21245001	21265001	2.05901	0.285266	OL	EFCAB14
NC_056054.	21250001	21270001	2.04338	0.283431	OL	EFCAB14
NC_056054.	21255001	21275001	1.98276	0.272799	OL	EFCAB14
NC_056054.	21260001	21280001	1.93561	0.252012	OL	EFCAB14
NC_056060.	33790001	33810001	2.84921	0.219508	OL	EIF2AK4
NC_056060.	33795001	33815001	3.32296	0.2448	OL	EIF2AK4
NC_056060.	33800001	33820001	3.51286	0.252689	OL	EIF2AK4
NC_056060.	33805001	33825001	3.15191	0.260083	OL	EIF2AK4

NC_056056.1	213805001	2.14E+08	2.23089
NC_056056.1	213810001	2.14E+08	3.85867
NC_056056.1	213815001	2.14E+08	2.28838
NC_056072.1	44815001	44835001	7.81904
NC_056072.1	45445001	45465001	3.69354
NC_056072.1	45450001	45470001	2.0641
NC_056072.1	45470001	45490001	2.24315
NC_056055.1	47750001	47770001	2.14425
NC_056060.1	74475001	74495001	2.60057
NC_056074.1	29335001	29355001	3.25095
NC_056059.1	104320001	1.04E+08	9.54946
NC_056059.1	104325001	1.04E+08	14.1288
NC_056065.1	41770001	41790001	2.1
NC_056065.1	41775001	41795001	2.02666
NC_056065.1	41780001	41800001	1.94323
NC_056062.1	59380001	59400001	1.9705
NC_056071.1	23900001	23920001	2.52654
NC_056080.1	140845001	1.41E+08	2.60515
NC_056080.1	140855001	1.41E+08	2.73053
NC_056080.1	140860001	1.41E+08	2.70213
NC_056080.1	140870001	1.41E+08	2.55556
NC_056080.1	140885001	1.41E+08	2.37947
NC_056058.1	90840001	90860001	3.01377
NC_056058.1	90845001	90865001	3.1865
NC_056055.1	67295001	67315001	1.94625
NC_056055.1	67300001	67320001	5.31178
NC_056055.1	67315001	67335001	34.4286
NC_056055.1	67320001	67340001	4.46829
NC_056071.1	31450001	31470001	2.00135
NC_056056.1	185995001	1.86E+08	1.9454
NC_056056.1	186000001	1.86E+08	2.45735

NC_056060.	77320001	77340001	4.90355	0.187886	OL	EIF2S1
NC_056060.	77325001	77345001	3.73121	0.252526	OL	EIF2S1;PLEK2
NC_056060.	77330001	77350001	3.35167	0.246619	OL	EIF2S1;PLEK2
NC_056060.	77335001	77355001	2.2754	0.175248	OL	EIF2S1;PLEK2
NC_056062.	69180001	69200001	1.90926	0.169031	OL	EIF3E
NC_056062.	69185001	69205001	2.07216	0.233061	OL	EIF3E
NC_056062.	69190001	69210001	1.8387	0.258663	OL	EIF3E
NC_056062.	60720001	60740001	3.02555	0.181636	OL	EIF3H
NC_056062.	60725001	60745001	3.15899	0.21115	OL	EIF3H
NC_056062.	60730001	60750001	3.36704	0.248871	OL	EIF3H
NC_056062.	60735001	60755001	2.01942	0.164202	OL	EIF3H
NC_056055.	184150001	1.84E+08	2.14783	0.147057	OL	EN1
NC_056055.	184155001	1.84E+08	2.10856	0.155838	OL	EN1
NC_056056.	8355001	8375001	4.66896	0.296372	OL	ENG
NC_056056.	8360001	8380001	2.05607	0.19663	OL	ENG
NC_056059.	97930001	97950001	2.15446	0.185685	OL	ENOPH1
NC_056059.	97935001	97955001	2.28152	0.240159	OL	ENOPH1
NC_056059.	97940001	97960001	2.82555	0.282527	OL	ENOPH1
NC_056059.	97945001	97965001	2.71608	0.306466	OL	ENOPH1
NC_056059.	97950001	97970001	2.71255	0.301991	OL	ENOPH1
NC_056059.	97955001	97975001	2.81177	0.30841	OL	ENOPH1
NC_056059.	97960001	97980001	2.17688	0.32209	OL	ENOPH1
NC_056059.	97915001	97935001	2.22525	0.175747	OL	ENOPH1;HNRNPDL
NC_056059.	97920001	97940001	2.22916	0.169056	OL	ENOPH1;HNRNPDL
NC_056059.	97925001	97945001	2.04724	0.15892	OL	ENOPH1;HNRNPDL
NC_056059.	97965001	97985001	2.10597	0.274324	OL	ENOPH1;TMEM150C
NC_056062.	93910001	93930001	1.81722	0.294056	OL	ENPP2
NC_056062.	93950001	93970001	1.93604	0.297029	OL	ENPP2
NC_056062.	93955001	93975001	2.05571	0.30595	OL	ENPP2
NC_056062.	93960001	93980001	2.59238	0.30778	OL	ENPP2
NC_056062.	93990001	94010001	2.71542	0.301136	OL	ENPP2

NC_056056.1	186005001	1.86E+08	2.35831
NC_056080.1	84000001	84020001	4.52631
NC_056080.1	84005001	84025001	3.48735
NC_056080.1	84015001	84035001	2.55721
NC_056058.1	104900001	1.05E+08	16.5302
NC_056058.1	104910001	1.05E+08	14.1429
NC_056058.1	104915001	1.05E+08	11.1191
NC_056058.1	104920001	1.05E+08	7.88889
NC_056058.1	104925001	1.05E+08	5.08
NC_056058.1	104930001	1.05E+08	3.84837
NC_056058.1	104935001	1.05E+08	2.80526
NC_056069.1	57585001	57605001	2.74384
NC_056069.1	57590001	57610001	2.85401
NC_056062.1	77145001	77165001	1.95072
NC_056070.1	5390001	5410001	2.07683
NC_056070.1	5395001	5415001	2.17407
NC_056054.1	110245001	1.1E+08	2.94408
NC_056054.1	110250001	1.1E+08	4.10045
NC_056054.1	110255001	1.1E+08	2.47995
NC_056080.1	52480001	52500001	2.37298
NC_056069.1	30605001	30625001	2.09524
NC_056069.1	30610001	30630001	3.00466
NC_056069.1	30615001	30635001	3.53536
NC_056069.1	30620001	30640001	4.53368
NC_056069.1	30625001	30645001	2.76553
NC_056070.1	35060001	35080001	10.042
NC_056075.1	41045001	41065001	2.23801
NC_056054.1	34635001	34655001	2.2552
NC_056076.1	20765001	20785001	2.17666
NC_056076.1	20770001	20790001	1.9438
NC_056076.1	21030001	21050001	2.18622

NC_056062.	93995001	94015001	3.06442	0.31675	OL	ENPP2
NC_056062.	94000001	94020001	1.8959	0.26989	OL	ENPP2
NC_056072.	2125001	2145001	1.84504	0.287402	OL	EOMES
NC_056077.	36235001	36255001	2.28735	0.194059	OL	EPHB4
NC_056077.	36240001	36260001	3.85115	0.412356	OL	EPHB4
NC_056077.	36245001	36265001	1.93217	0.377997	OL	EPHB4;ZAN
NC_056061.	70225001	70245001	2.07343	0.141323	OL	EPM2A
NC_056056.	199870001	2E+08	2.61325	0.16856	OL	EPS8
NC_056056.	199875001	2E+08	2.42566	0.166442	OL	EPS8
NC_056056.	199880001	2E+08	2.46304	0.176255	OL	EPS8
NC_056056.	199885001	2E+08	2.5363	0.177516	OL	EPS8
NC_056056.	199915001	2E+08	2.64963	0.174259	OL	EPS8
NC_056056.	199920001	2E+08	3.32316	0.194908	OL	EPS8
NC_056056.	199925001	2E+08	4.83662	0.227314	OL	EPS8
NC_056056.	199930001	2E+08	3.79549	0.210072	OL	EPS8
NC_056056.	199935001	2E+08	2.37276	0.167157	OL	EPS8
NC_056072.	44815001	44835001	5.13124	0.178208	OL	ERC2
NC_056072.	44820001	44840001	2.82338	0.150588	OL	ERC2
NC_056061.	76890001	76910001	3.30104	0.164122	OL	ESR1;SYNE1
NC_056056.	40890001	40910001	11.4378	0.288645	OL	ETAA1
NC_056056.	40895001	40915001	17.062	0.338992	OL	ETAA1
NC_056056.	40900001	40920001	18.2204	0.315374	OL	ETAA1
NC_056056.	40905001	40925001	14.1934	0.295324	OL	ETAA1
NC_056054.	123655001	1.24E+08	14.7989	0.19401	OL	EVA1C
NC_056054.	123660001	1.24E+08	14.0601	0.227253	OL	EVA1C
NC_056054.	123665001	1.24E+08	11.7381	0.244324	OL	EVA1C
NC_056054.	123670001	1.24E+08	8.92783	0.228138	OL	EVA1C
NC_056054.	123675001	1.24E+08	7.00393	0.193104	OL	EVA1C
NC_056054.	123755001	1.24E+08	8.30834	0.155864	OL	EVA1C
NC_056065.	41785001	41805001	2.84479	0.408954	OL	EXOSC10
NC_056065.	41770001	41790001	2.08981	0.424653	OL	EXOSC10;MTOR

NC_056061.1	2305001	2325001	2.41085
NC_056061.1	2310001	2330001	2.29967
NC_056061.1	2315001	2335001	2.4797
NC_056061.1	2320001	2340001	2.4463
NC_056066.1	72685001	72705001	2.06342
NC_056066.1	72690001	72710001	2.45342
NC_056064.1	34680001	34700001	2.72549
NC_056064.1	34675001	34695001	3.24009
NC_056063.1	32330001	32350001	18.9389
NC_056058.1	335001	355001	3.94189
NC_056058.1	340001	360001	3.72557
NC_056058.1	345001	365001	3.09278
NC_056058.1	350001	370001	2.74451
NC_056060.1	27815001	27835001	1.95945
NC_056055.1	157040001	1.57E+08	2.09297
NC_056055.1	157045001	1.57E+08	2.80386
NC_056061.1	83270001	83290001	2.24835
NC_056061.1	83275001	83295001	5.62131
NC_056061.1	83280001	83300001	9.06836
NC_056061.1	83285001	83305001	6.91697
NC_056061.1	83290001	83310001	4.03811
NC_056055.1	89310001	89330001	5.65192
NC_056055.1	89315001	89335001	3.29833
NC_056060.1	98960001	98980001	2.11701
NC_056072.1	30195001	30215001	2.68585
NC_056057.1	55315001	55335001	2.08361
NC_056057.1	55320001	55340001	2.25025
NC_056057.1	55325001	55345001	2.03372
NC_056057.1	55330001	55350001	1.93705
NC_056057.1	55545001	55565001	2.24969
NC_056057.1	55555001	55575001	3.15265

NC_056065.	41775001	41795001	3.59056	0.441105	OL	EXOSC10;MTOR
NC_056065.	41780001	41800001	4.21849	0.425531	OL	EXOSC10;MTOR
NC_056064.	55830001	55850001	2.37328	0.168034	OL	FADS6;USH1G
NC_056062.	37420001	37440001	3.86297	0.142015	OL	FAM110B
NC_056062.	37425001	37445001	3.61393	0.165661	OL	FAM110B
NC_056057.	32645001	32665001	1.85071	0.154314	OL	FAM126A
NC_056059.	36460001	36480001	2.98398	0.189552	OL	FAM13A
NC_056059.	36465001	36485001	2.90697	0.221046	OL	FAM13A
NC_056059.	36470001	36490001	2.4281	0.196645	OL	FAM13A
NC_056054.	17820001	17840001	9.54921	0.283783	OL	FAM183A
NC_056054.	17825001	17845001	5.40425	0.266477	OL	FAM183A
NC_056054.	17830001	17850001	1.90246	0.244756	OL	FAM183A
NC_056058.	36730001	36750001	2.64929	0.169757	OL	FAM193B
NC_056058.	36735001	36755001	3.90863	0.23264	OL	FAM193B
NC_056058.	36740001	36760001	4.11112	0.271294	OL	FAM193B
NC_056058.	36745001	36765001	4.66502	0.284556	OL	FAM193B
NC_056058.	36750001	36770001	3.44194	0.294109	OL	FAM193B
NC_056058.	36755001	36775001	4.85047	0.289253	OL	FAM193B
NC_056055.	37110001	37130001	2.31688	0.289727	OL	FAM219A
NC_056055.	37115001	37135001	2.16642	0.330737	OL	FAM219A
NC_056055.	37120001	37140001	2.05495	0.360368	OL	FAM219A
NC_056055.	37125001	37145001	1.86156	0.3961	OL	FAM219A
NC_056055.	37130001	37150001	1.92233	0.378993	OL	FAM219A
NC_056055.	37135001	37155001	2.01732	0.363752	OL	FAM219A
NC_056055.	37140001	37160001	2.04413	0.354809	OL	FAM219A
NC_056055.	37145001	37165001	2.17316	0.334494	OL	FAM219A
NC_056071.	31440001	31460001	2.13687	0.236136	OL	FAM219B;MPI
NC_056071.	31445001	31465001	2.03631	0.223361	OL	FAM219B;MPI
NC_056071.	31450001	31470001	2.07758	0.21668	OL	FAM219B;MPI
NC_056068.	14125001	14145001	4.19879	0.189274	OL	FAM76B
NC_056068.	14130001	14150001	3.34291	0.143362	OL	FAM76B

NC_056057.1	55560001	55580001	3.03291
NC_056059.1	93975001	93995001	3.3218
NC_056059.1	93980001	94000001	3.83442
NC_056059.1	93985001	94005001	5.06129
NC_056071.1	53320001	53340001	2.13849
NC_056071.1	53325001	53345001	2.11989
NC_056071.1	53330001	53350001	2.1705
NC_056071.1	53305001	53325001	2.22613
NC_056071.1	53310001	53330001	2.18738
NC_056071.1	53315001	53335001	2.22192
NC_056060.1	42805001	42825001	2.04293
NC_056063.1	29145001	29165001	2.26936
NC_056077.1	27765001	27785001	6.07371
NC_056077.1	27770001	27790001	8.83693
NC_056077.1	27775001	27795001	4.29563
NC_056077.1	27780001	27800001	3.11579
NC_056077.1	27785001	27805001	2.79456
NC_056077.1	27790001	27810001	2.30927
NC_056068.1	15595001	15615001	3.1182
NC_056055.1	103005001	1.03E+08	2.32839
NC_056055.1	103010001	1.03E+08	2.87238
NC_056071.1	3290001	3310001	9.60265
NC_056071.1	3295001	3315001	6.44851
NC_056061.1	48200001	48220001	2.55993
NC_056061.1	48205001	48225001	2.50069
NC_056061.1	48210001	48230001	2.36742
NC_056068.1	40880001	40900001	2.16807
NC_056068.1	40885001	40905001	1.99117
NC_056055.1	107405001	1.07E+08	2.23361
NC_056055.1	107410001	1.07E+08	2.17336
NC_056060.1	34500001	34520001	3.00951

NC_056056.	217160001	2.17E+08	1.99842	0.156864	OL	FAM83F
NC_056056.	217165001	2.17E+08	2.16028	0.159733	OL	FAM83F
NC_056056.	217170001	2.17E+08	2.05235	0.166836	OL	FAM83F
NC_056055.	30945001	30965001	2.8282	0.150585	OL	FANCC
NC_056077.	27300001	27320001	1.91459	0.225809	OL	FBRS
NC_056077.	27285001	27305001	3.10781	0.150964	OL	FBRS;PRR14
NC_056077.	27290001	27310001	1.95397	0.217452	OL	FBRS;PRR14
NC_056077.	27295001	27315001	1.97908	0.214548	OL	FBRS;PRR14
NC_056064.	39490001	39510001	1.89803	0.155063	OL	FBXL20
NC_056064.	39495001	39515001	2.39615	0.163994	OL	FBXL20
NC_056064.	39500001	39520001	2.11487	0.153253	OL	FBXL20
NC_056064.	39485001	39505001	2.10656	0.154737	OL	FBXL20;LOC101103335
NC_056061.	70255001	70275001	3.00577	0.170893	OL	FBXO30
NC_056058.	105360001	1.05E+08	1.85949	0.222909	OL	FER
NC_056058.	105365001	1.05E+08	2.18565	0.166819	OL	FER
NC_056056.	103845001	1.04E+08	2.37812	0.155981	OL	FER1L5;KANSL3
NC_056056.	103850001	1.04E+08	2.35593	0.155269	OL	FER1L5;KANSL3
NC_056056.	103855001	1.04E+08	2.35567	0.157407	OL	FER1L5;KANSL3
NC_056074.	26370001	26390001	2.19118	0.169437	OL	FEZ1
NC_056074.	26375001	26395001	2.37405	0.192978	OL	FEZ1
NC_056074.	26380001	26400001	2.40107	0.223507	OL	FEZ1
NC_056074.	26385001	26405001	1.9192	0.246151	OL	FEZ1
NC_056073.	11000001	11020001	2.94232	0.319814	OL	FGD2
NC_056073.	11005001	11025001	7.40714	0.422718	OL	FGD2
NC_056073.	11010001	11030001	8.88744	0.41818	OL	FGD2
NC_056073.	11015001	11035001	3.64259	0.3063	OL	FGD2
NC_056073.	11020001	11040001	1.98863	0.160154	OL	FGD2
NC_056054.	34190001	34210001	1.94678	0.270062	OL	FGGY
NC_056054.	34195001	34215001	2.2521	0.294186	OL	FGGY
NC_056054.	34200001	34220001	3.20998	0.335891	OL	FGGY
NC_056054.	34205001	34225001	4.57592	0.360968	OL	FGGY

NC_056056.1	34545001	34565001	2.05264
NC_056056.1	34550001	34570001	3.16588
NC_056056.1	34555001	34575001	2.85204
NC_056055.1	64415001	64435001	3
NC_056055.1	64420001	64440001	2.83779
NC_056066.1	72650001	72670001	2.98032
NC_056066.1	72655001	72675001	2.56262
NC_056066.1	72660001	72680001	2.78053
NC_056066.1	72665001	72685001	2.43766
NC_056066.1	72670001	72690001	1.9843
NC_056066.1	64665001	64685001	2.32258
NC_056064.1	40350001	40370001	2.10145
NC_056067.1	1460001	1480001	2.18512
NC_056055.1	186165001	1.86E+08	24.2024
NC_056070.1	42285001	42305001	5.33968
NC_056070.1	42290001	42310001	7.03997
NC_056070.1	42295001	42315001	11.8614
NC_056055.1	59170001	59190001	2.89394
NC_056055.1	59175001	59195001	2.54564
NC_056055.1	59240001	59260001	4.43666
NC_056055.1	59245001	59265001	3.97332
NC_056055.1	59250001	59270001	2.82298
NC_056054.1	86790001	86810001	2.68302
NC_056054.1	86795001	86815001	2.88291
NC_056054.1	86800001	86820001	3.02004
NC_056054.1	86805001	86825001	2.12441
NC_056055.1	59165001	59185001	2.86332
NC_056055.1	52275001	52295001	3.70235
NC_056055.1	52280001	52300001	3.35019
NC_056055.1	52285001	52305001	3.11237
NC_056055.1	52290001	52310001	2.4375

NC_056054.	34210001	34230001	3.50092	0.320498	OL	FGGY
NC_056054.	34215001	34235001	3.51823	0.271287	OL	FGGY
NC_056054.	34220001	34240001	2.73145	0.221972	OL	FGGY
NC_056054.	34225001	34245001	2.99033	0.193803	OL	FGGY
NC_056054.	34230001	34250001	2.82143	0.167264	OL	FGGY
NC_056080.	99045001	99065001	1.97845	0.144429	OL	FHL1
NC_056061.	40430001	40450001	2.66481	0.225303	OL	FHL5
NC_056061.	40435001	40455001	3.83377	0.279086	OL	FHL5
NC_056061.	40440001	40460001	6.10137	0.317099	OL	FHL5
NC_056061.	40445001	40465001	8.31054	0.350044	OL	FHL5
NC_056061.	40450001	40470001	6.2973	0.318114	OL	FHL5
NC_056061.	40455001	40475001	3.3063	0.229432	OL	FHL5
NC_056076.	20755001	20775001	3.43294	0.200471	OL	FHOD3
NC_056076.	20760001	20780001	10.2338	0.259919	OL	FHOD3
NC_056076.	20765001	20785001	13.06	0.264848	OL	FHOD3
NC_056076.	20770001	20790001	6.83721	0.245683	OL	FHOD3
NC_056076.	20775001	20795001	3.04868	0.167987	OL	FHOD3
NC_056054.	102305001	1.02E+08	2.09429	0.16825	OL	FLG;LOC105614079
NC_056063.	32310001	32330001	2.63515	0.146474	OL	FLT3
NC_056063.	32315001	32335001	3.06484	0.149086	OL	FLT3
NC_056063.	32320001	32340001	5.1685	0.142501	OL	FLT3
NC_056063.	32325001	32345001	11.5774	0.157765	OL	FLT3
NC_056060.	28005001	28025001	6.21532	0.159504	OL	FMN1
NC_056058.	20465001	20485001	1.91121	0.157691	OL	FNIP1;MEIKIN
NC_056055.	89300001	89320001	2.68031	0.176439	OL	FOCAD
NC_056055.	89305001	89325001	2.65249	0.152342	OL	FOCAD
NC_056073.	50685001	50705001	2.05947	0.237544	OL	FOXC1
NC_056073.	50680001	50700001	1.82159	0.235082	OL	FOXC1;GMDS
NC_056072.	30180001	30200001	1.83739	0.176749	OL	FOXP1
NC_056072.	30215001	30235001	1.91248	0.241736	OL	FOXP1
NC_056072.	30220001	30240001	2.21079	0.26489	OL	FOXP1

NC_056055.1	52295001	52315001	2.4557
NC_056055.1	52300001	52320001	3.07054
NC_056055.1	52305001	52325001	3.13333
NC_056075.1	18820001	18840001	2.18567
NC_056054.1	187050001	1.87E+08	2.40469
NC_056054.1	187055001	1.87E+08	1.98277
NC_056061.1	20405001	20425001	2.44573
NC_056061.1	20410001	20430001	2.14719
NC_056061.1	20395001	20415001	2.18865
NC_056061.1	20400001	20420001	2.53975
NC_056059.1	98975001	98995001	4.14902
NC_056059.1	98980001	99000001	3.69453
NC_056059.1	98985001	99005001	3.2032
NC_056065.1	20135001	20155001	2.24789
NC_056065.1	20140001	20160001	2.87284
NC_056065.1	20145001	20165001	2.23122
NC_056080.1	101550001	1.02E+08	7.11724
NC_056080.1	101555001	1.02E+08	5.57143
NC_056063.1	67635001	67655001	3.08586
NC_056063.1	67640001	67660001	5.07021
NC_056063.1	67645001	67665001	14.3333
NC_056063.1	67675001	67695001	8.08116
NC_056063.1	67680001	67700001	7.03794
NC_056063.1	67685001	67705001	6.98773
NC_056063.1	67710001	67730001	3.57695
NC_056063.1	67715001	67735001	3.28506
NC_056063.1	67720001	67740001	4.1718
NC_056063.1	67725001	67745001	6.3969
NC_056063.1	67730001	67750001	19.7238
NC_056063.1	67735001	67755001	23.3445
NC_056063.1	67740001	67760001	7.35979

NC_056072.	30225001	30245001	1.8858	0.203306	OL	FOXP1
NC_056057.	55755001	55775001	1.86018	0.18346	OL	FOXP2
NC_056057.	55760001	55780001	2.2173	0.16515	OL	FOXP2
NC_056057.	55765001	55785001	2.80965	0.148363	OL	FOXP2
NC_056057.	55770001	55790001	3.11245	0.141883	OL	FOXP2
NC_056055.	83495001	83515001	3.41664	0.152082	OL	FREM1
NC_056055.	83500001	83520001	3.52324	0.177008	OL	FREM1
NC_056055.	83505001	83525001	2.85497	0.194934	OL	FREM1
NC_056055.	83510001	83530001	2.53927	0.196773	OL	FREM1
NC_056063.	29145001	29165001	2.02402	0.15685	OL	FRY
NC_056063.	29185001	29205001	1.86861	0.144598	OL	FRY
NC_056060.	33605001	33625001	2.70069	0.185105	OL	FSIP1
NC_056060.	33610001	33630001	2.73281	0.264379	OL	FSIP1
NC_056055.	202225001	2.02E+08	2.50125	0.148999	OL	FTCDNL1
NC_056055.	202230001	2.02E+08	3.63173	0.159852	OL	FTCDNL1
NC_056055.	202235001	2.02E+08	5.69957	0.17269	OL	FTCDNL1
NC_056068.	15595001	15615001	3.00202	0.177078	OL	FUT4
NC_056068.	15600001	15620001	6.5658	0.173521	OL	FUT4
NC_056061.	26050001	26070001	1.8983	0.228103	OL	FYN
NC_056061.	26065001	26085001	1.82674	0.209021	OL	FYN
NC_056061.	26070001	26090001	1.99727	0.22205	OL	FYN
NC_056061.	26075001	26095001	2.27696	0.218819	OL	FYN
NC_056061.	26080001	26100001	1.88841	0.183168	OL	FYN
NC_056055.	209830001	2.1E+08	3.62338	0.227795	OL	FZD5;LOC121818740
NC_056055.	209835001	2.1E+08	2.49429	0.331642	OL	FZD5;LOC121818740
NC_056064.	42700001	42720001	1.8914	0.164933	OL	G6PC1
NC_056064.	42695001	42715001	2.14253	0.173462	OL	G6PC1;LOC101113086
NC_056061.	48310001	48330001	2.3483	0.141784	OL	GABRR1;PM20D2
NC_056061.	48230001	48250001	1.89271	0.392229	OL	GABRR2
NC_056061.	48240001	48260001	2.12396	0.204303	OL	GABRR2
NC_056061.	48245001	48265001	2.19286	0.207012	OL	GABRR2

NC_056054.1	86205001	86225001	2.02944
NC_056054.1	86210001	86230001	2.47462
NC_056056.1	20430001	20450001	2.10326
NC_056056.1	20435001	20455001	3.03891
NC_056056.1	20465001	20485001	4.38689
NC_056056.1	20470001	20490001	7.71362
NC_056062.1	75825001	75845001	2.12294
NC_056062.1	75830001	75850001	2.91369
NC_056062.1	75835001	75855001	2.89814
NC_056062.1	75840001	75860001	2.86115
NC_056080.1	107505001	1.08E+08	3.34711
NC_056068.1	1740001	1760001	7.32485
NC_056068.1	1745001	1765001	7.37484
NC_056068.1	1750001	1770001	7.25317
NC_056068.1	1755001	1775001	5.66166
NC_056054.1	126960001	1.27E+08	6.79637
NC_056054.1	11660001	11680001	2.17759
NC_056054.1	11665001	11685001	3.23057
NC_056054.1	11670001	11690001	6.46011
NC_056054.1	11675001	11695001	34.1052
NC_056056.1	153455001	1.53E+08	2.51241
NC_056056.1	153460001	1.53E+08	3.23914
NC_056056.1	153465001	1.53E+08	4.72985
NC_056056.1	153470001	1.53E+08	3.75001
NC_056056.1	153475001	1.53E+08	3.29274
NC_056056.1	153480001	1.54E+08	2.70873
NC_056056.1	153495001	1.54E+08	2.08436
NC_056070.1	65560001	65580001	3.78635
NC_056070.1	65565001	65585001	4.53471
NC_056060.1	90675001	90695001	10.1867
NC_056056.1	75880001	75900001	2.17272

NC_056061.	48250001	48270001	1.85553	0.189912	OL	GABRR2
NC_056061.	48255001	48275001	1.93888	0.205424	OL	GABRR2
NC_056068.	40865001	40885001	1.80863	0.152204	OL	GALNT18
NC_056056.	33390001	33410001	1.91971	0.311572	OL	GAREM2
NC_056054.	71040001	71060001	2.0504	0.146187	OL	GCLM
NC_056058.	63845001	63865001	3.6632	0.145461	OL	GEMIN5
NC_056069.	6960001	6980001	1.81894	0.28526	OL	GFM2;LOC101111906
NC_056055.	43860001	43880001	1.97008	0.190912	OL	GFRA2
NC_056057.	67335001	67355001	1.81831	0.259699	OL	GGCT
NC_056057.	67340001	67360001	2.1491	0.236662	OL	GGCT;NOD1
NC_056057.	67345001	67365001	2.57843	0.189844	OL	GGCT;NOD1
NC_056057.	67350001	67370001	2.83204	0.147664	OL	GGCT;NOD1
NC_056067.	47560001	47580001	4.87887	0.187539	OL	GGN;PSMD8;SPRED3
NC_056063.	36335001	36355001	2.22055	0.195829	OL	GJA3
NC_056063.	36340001	36360001	2.23881	0.243095	OL	GJA3
NC_056063.	36345001	36365001	2.12276	0.293624	OL	GJA3
NC_056063.	36275001	36295001	2.2639	0.154473	OL	GJB6
NC_056063.	36280001	36300001	2.37004	0.18598	OL	GJB6
NC_056064.	40340001	40360001	2.10833	0.168607	OL	GJD3;RARA
NC_056064.	40345001	40365001	3.41489	0.278656	OL	GJD3;RARA
NC_056055.	35915001	35935001	2.43219	0.189815	OL	GKAP1
NC_056055.	35920001	35940001	4.05756	0.209163	OL	GKAP1
NC_056055.	35925001	35945001	3.40188	0.197242	OL	GKAP1
NC_056055.	35930001	35950001	3.08035	0.205909	OL	GKAP1
NC_056055.	35935001	35955001	3.12355	0.190662	OL	GKAP1
NC_056055.	35940001	35960001	3.00253	0.193063	OL	GKAP1
NC_056055.	35945001	35965001	3.33497	0.203522	OL	GKAP1
NC_056055.	35950001	35970001	3.94326	0.20919	OL	GKAP1
NC_056055.	35955001	35975001	4.34371	0.224373	OL	GKAP1
NC_056055.	35960001	35980001	4.69765	0.234022	OL	GKAP1
NC_056055.	35965001	35985001	4.7426	0.239949	OL	GKAP1

NC_056072.1	28845001	28865001	3.58649
NC_056072.1	28850001	28870001	3.4899
NC_056072.1	28855001	28875001	3.04257
NC_056072.1	28860001	28880001	2.11154
NC_056072.1	28865001	28885001	2.07358
NC_056072.1	28870001	28890001	2.20324
NC_056068.1	48030001	48050001	26.5468
NC_056068.1	48035001	48055001	9.37746
NC_056068.1	47950001	47970001	94.9591
NC_056068.1	47960001	47980001	95.6288
NC_056056.1	225535001	2.26E+08	3.16743
NC_056056.1	225530001	2.26E+08	2.56392
NC_056061.1	23730001	23750001	1.93912
NC_056061.1	23735001	23755001	2.43857
NC_056061.1	23740001	23760001	2.57334
NC_056061.1	23745001	23765001	2.60982
NC_056061.1	23750001	23770001	2.53952
NC_056061.1	23755001	23775001	2.35801
NC_056061.1	23760001	23780001	2.29179
NC_056061.1	23765001	23785001	2.33654
NC_056054.1	2110001	2130001	2.12225
NC_056054.1	2115001	2135001	2.42649
NC_056054.1	2120001	2140001	2.42194
NC_056057.1	27585001	27605001	2.83014
NC_056057.1	27590001	27610001	5.25326
NC_056057.1	27595001	27615001	13.5
NC_056057.1	27615001	27635001	8.3801
NC_056057.1	27620001	27640001	5.22673
NC_056057.1	27625001	27645001	2.35063
NC_056057.1	27685001	27705001	2.76896
NC_056057.1	27690001	27710001	2.93881

NC_056055.	35970001	35990001	5.09214	0.245179	OL	GKAP1
NC_056055.	35975001	35995001	4.13526	0.225415	OL	GKAP1
NC_056055.	35980001	36000001	4.65659	0.226134	OL	GKAP1
NC_056055.	35985001	36005001	4.134	0.220551	OL	GKAP1
NC_056055.	35990001	36010001	3.40729	0.211685	OL	GKAP1
NC_056055.	35995001	36015001	3.0966	0.230202	OL	GKAP1
NC_056055.	36000001	36020001	2.64771	0.223226	OL	GKAP1
NC_056055.	72435001	72455001	1.85656	0.234395	OL	GLIS3
NC_056056.	38525001	38545001	4.94194	0.155568	OL	GMCL1
NC_056060.	65070001	65090001	2.21456	0.287013	OL	GMFB
NC_056060.	65075001	65095001	2.22382	0.306098	OL	GMFB
NC_056060.	65080001	65100001	2.27397	0.327794	OL	GMFB
NC_056055.	59170001	59190001	2.91285	0.146807	OL	GNA14
NC_056055.	59175001	59195001	2.7146	0.14884	OL	GNA14
NC_056055.	59255001	59275001	2.62353	0.187858	OL	GNA14
NC_056055.	59260001	59280001	4.82543	0.294921	OL	GNA14
NC_056055.	59300001	59320001	2.42223	0.266603	OL	GNA14
NC_056055.	59165001	59185001	2.88303	0.14987	OL	GNAQ
NC_056070.	72865001	72885001	4.07281	0.22492	OL	GNB1L
NC_056070.	72870001	72890001	7.03472	0.256847	OL	GNB1L
NC_056070.	72875001	72895001	7.11515	0.260788	OL	GNB1L;RTL10
NC_056070.	72880001	72900001	5.29743	0.245042	OL	GNB1L;RTL10
NC_056055.	52290001	52310001	3.28421	0.149667	OL	GNE
NC_056055.	52295001	52315001	4.64362	0.188115	OL	GNE
NC_056055.	52300001	52320001	5.22969	0.215125	OL	GNE
NC_056055.	52305001	52325001	3.74638	0.202824	OL	GNE
NC_056055.	52310001	52330001	2.57276	0.164658	OL	GNE
NC_056075.	18805001	18825001	2.15377	0.218881	OL	GOLGA7B
NC_056055.	135555001	1.36E+08	3.09011	0.433273	OL	GPR155
NC_056055.	135560001	1.36E+08	5.97986	0.496567	OL	GPR155
NC_056055.	135565001	1.36E+08	2.28538	0.377867	OL	GPR155

NC_056057.1	27695001	27715001	2.77652
NC_056057.1	27700001	27720001	2.09505
NC_056055.1	198775001	1.99E+08	2.47007
NC_056055.1	198780001	1.99E+08	3.35022
NC_056055.1	198785001	1.99E+08	2.68276
NC_056055.1	198790001	1.99E+08	2.45802
NC_056055.1	198795001	1.99E+08	2.1927
NC_056054.1	174050001	1.74E+08	2.19302
NC_056054.1	174120001	1.74E+08	2.60981
NC_056054.1	174125001	1.74E+08	6.80547
NC_056054.1	174130001	1.74E+08	4.65327
NC_056054.1	174135001	1.74E+08	2.90833
NC_056054.1	174140001	1.74E+08	2.03969
NC_056074.1	8715001	8735001	10.9681
NC_056074.1	8720001	8740001	13.7947
NC_056074.1	8725001	8745001	12.7968
NC_056074.1	8730001	8750001	6.15905
NC_056074.1	8735001	8755001	2.81351
NC_056074.1	8750001	8770001	1.96504
NC_056074.1	8755001	8775001	2.02044
NC_056074.1	8760001	8780001	2.32959
NC_056054.1	91255001	91275001	4.85281
NC_056054.1	91260001	91280001	9.65708
NC_056054.1	91265001	91285001	12.0273
NC_056054.1	91270001	91290001	12.3357
NC_056054.1	91275001	91295001	10.7041
NC_056055.1	103560001	1.04E+08	2.02031
NC_056055.1	103565001	1.04E+08	2.09612
NC_056060.1	6735001	6755001	1.95434
NC_056059.1	115730001	1.16E+08	10.1152
NC_056059.1	115735001	1.16E+08	24.302

NC_056057.	5905001	5925001	3.77242	0.217308	OL	GRB10
NC_056057.	5910001	5930001	4.86521	0.188293	OL	GRB10
NC_056057.	5915001	5935001	3.12255	0.146723	OL	GRB10
NC_056057.	6030001	6050001	3.975	0.291728	OL	GRB10
NC_056057.	6035001	6055001	10.4573	0.355147	OL	GRB10
NC_056057.	6040001	6060001	7.04407	0.336146	OL	GRB10
NC_056057.	6045001	6065001	2.22172	0.299304	OL	GRB10
NC_056062.	75735001	75755001	2.18919	0.175237	OL	GRHL2
NC_056055.	242295001	2.42E+08	2.23463	0.14844	OL	GRHL3;STPG1
NC_056059.	32515001	32535001	2.65566	0.202914	OL	GRID2
NC_056059.	32520001	32540001	4.73786	0.278455	OL	GRID2
NC_056059.	32525001	32545001	6.75953	0.312	OL	GRID2
NC_056059.	32530001	32550001	6.22717	0.304308	OL	GRID2
NC_056059.	32535001	32555001	3.83383	0.247149	OL	GRID2
NC_056059.	32540001	32560001	2.26099	0.164746	OL	GRID2
NC_056059.	32545001	32565001	2.16278	0.166162	OL	GRID2
NC_056059.	32550001	32570001	2.24566	0.173521	OL	GRID2
NC_056059.	33010001	33030001	1.92672	0.175538	OL	GRID2
NC_056059.	33045001	33065001	1.83477	0.223816	OL	GRID2
NC_056061.	35875001	35895001	2.66012	0.178304	OL	GRIK2
NC_056068.	31035001	31055001	2.99689	0.209085	OL	GRIK4
NC_056068.	31040001	31060001	6.19651	0.21452	OL	GRIK4
NC_056068.	31120001	31140001	2.08543	0.194471	OL	GRIK4
NC_056056.	153460001	1.53E+08	2.85629	0.15554	OL	GRIP1
NC_056056.	153465001	1.53E+08	4.77714	0.220388	OL	GRIP1
NC_056056.	153470001	1.53E+08	3.69491	0.195606	OL	GRIP1
NC_056056.	153475001	1.53E+08	3.2331	0.157934	OL	GRIP1
NC_056056.	153480001	1.54E+08	2.55139	0.161	OL	GRIP1
NC_056075.	38970001	38990001	2.41013	0.147282	OL	GRK5
NC_056075.	38975001	38995001	2.5543	0.141999	OL	GRK5
NC_056061.	70545001	70565001	1.90485	0.145588	OL	GRM1

NC_056059.1	115740001	1.16E+08	5.68669
NC_056059.1	115745001	1.16E+08	3.60382
NC_056059.1	115750001	1.16E+08	3.44452
NC_056067.1	47925001	47945001	4.83485
NC_056067.1	47930001	47950001	2.73111
NC_056056.1	132485001	1.33E+08	2.2199
NC_056056.1	132490001	1.33E+08	2.28236
NC_056056.1	132495001	1.33E+08	2.28038
NC_056056.1	132500001	1.33E+08	2.42857
NC_056061.1	23535001	23555001	2.14596
NC_056061.1	23540001	23560001	2.41359
NC_056061.1	23545001	23565001	3.41476
NC_056061.1	23550001	23570001	3.46145
NC_056073.1	17715001	17735001	3.15493
NC_056073.1	17720001	17740001	2.48966
NC_056073.1	17725001	17745001	2.28189
NC_056080.1	119920001	1.2E+08	2.04673
NC_056080.1	119935001	1.2E+08	2.26845
NC_056080.1	119940001	1.2E+08	2.60402
NC_056080.1	119945001	1.2E+08	2.55014
NC_056080.1	119950001	1.2E+08	2.042
NC_056080.1	119955001	1.2E+08	2.01973
NC_056058.1	57840001	57860001	3.41311
NC_056058.1	57845001	57865001	2.88017
NC_056058.1	57850001	57870001	2.69217
NC_056058.1	57855001	57875001	2.47312
NC_056057.1	118565001	1.19E+08	3.15217
NC_056057.1	118570001	1.19E+08	4.59598
NC_056057.1	118575001	1.19E+08	6.85872
NC_056057.1	118580001	1.19E+08	2.48105
NC_056064.1	40405001	40425001	2.10679

NC_056074.	6135001	6155001	1.85143	0.190456	OL	GRM5
NC_056074.	6140001	6160001	1.82676	0.174368	OL	GRM5
NC_056057.	93065001	93085001	1.84813	0.145998	OL	GRM8
NC_056067.	10785001	10805001	2.31541	0.162286	OL	GSE1
NC_056060.	86695001	86715001	2.28149	0.174505	OL	GSTZ1;TMED8
NC_056054.	66115001	66135001	2.52785	0.199215	OL	GTF2B
NC_056054.	66120001	66140001	2.3085	0.264866	OL	GTF2B
NC_056054.	66125001	66145001	2.27633	0.283405	OL	GTF2B
NC_056063.	32750001	32770001	3.29674	0.61214	OL	GTF3A;MTIF3
NC_056063.	32755001	32775001	1.84742	0.547669	OL	GTF3A;MTIF3
NC_056080.	125835001	1.26E+08	7.23324	0.168947	OL	GUCY2F
NC_056072.	28830001	28850001	3.66234	0.176872	OL	GXYLT2
NC_056072.	28835001	28855001	8.93169	0.150843	OL	GXYLT2
NC_056072.	28845001	28865001	3.13324	0.158017	OL	GXYLT2
NC_056072.	28850001	28870001	2.94872	0.192096	OL	GXYLT2
NC_056072.	28855001	28875001	2.6493	0.231763	OL	GXYLT2
NC_056072.	28860001	28880001	3.14184	0.27527	OL	GXYLT2
NC_056072.	28865001	28885001	3.13566	0.291509	OL	GXYLT2
NC_056072.	28870001	28890001	3.24297	0.270366	OL	GXYLT2
NC_056072.	28875001	28895001	2.95216	0.170923	OL	GXYLT2
NC_056073.	30895001	30915001	2.50559	0.165177	OL	H1-1;LOC101113369;LOC
NC_056073.	30900001	30920001	2.34352	0.200659	OL	H1-1;TRIM38
NC_056073.	30820001	30840001	2.01094	0.17398	OL	H1-6;LOC101105290;LOC
NC_056073.	30810001	30830001	2.64208	0.184079	OL	H1-6;LOC101111669;LOC
NC_056073.	30815001	30835001	2.25527	0.173289	OL	H1-6;LOC101111669;LOC
NC_056069.	29580001	29600001	6.5498	0.2	OL	HCN1
NC_056069.	29585001	29605001	8.53571	0.218756	OL	HCN1
NC_056069.	29590001	29610001	8.92064	0.220045	OL	HCN1
NC_056069.	29595001	29615001	8.59047	0.230987	OL	HCN1
NC_056069.	29600001	29620001	7.35874	0.173686	OL	HCN1
NC_056069.	29605001	29625001	8.22858	0.174864	OL	HCN1

NC_056064.1	40425001	40445001	1.94578
NC_056056.1	99455001	99475001	2.17558
NC_056056.1	99460001	99480001	2.222
NC_056056.1	99465001	99485001	2.47535
NC_056056.1	99470001	99490001	3.41981
NC_056056.1	99475001	99495001	5.03389
NC_056056.1	99480001	99500001	2.78399
NC_056061.1	2755001	2775001	2.07295
NC_056061.1	2760001	2780001	1.97645
NC_056054.1	166630001	1.67E+08	2.83457
NC_056057.1	87070001	87090001	4.07792
NC_056057.1	87075001	87095001	4.3908
NC_056057.1	87080001	87100001	4.11398
NC_056057.1	87085001	87105001	4.56085
NC_056057.1	87090001	87110001	6.36207
NC_056055.1	185655001	1.86E+08	3.26549
NC_056055.1	185660001	1.86E+08	2.31919
NC_056080.1	99390001	99410001	3.76568
NC_056055.1	103325001	1.03E+08	1.99123
NC_056055.1	103330001	1.03E+08	2.18181
NC_056055.1	47680001	47700001	3.86195
NC_056055.1	47685001	47705001	4.60479
NC_056055.1	47690001	47710001	4.01336
NC_056055.1	47695001	47715001	3.99999
NC_056055.1	47700001	47720001	3.21693
NC_056055.1	47705001	47725001	2.91668
NC_056055.1	47710001	47730001	2.10825
NC_056060.1	7875001	7895001	1.96859
NC_056072.1	16630001	16650001	2.02824
NC_056072.1	16635001	16655001	1.96475
NC_056072.1	16640001	16660001	2.40035

NC_056069.	29610001	29630001	3.52815	0.14297	OL	HCN1
NC_056057.	27580001	27600001	2.24359	0.160381	OL	HDAC9
NC_056061.	13200001	13220001	1.92165	0.143146	OL	HDDC2
NC_056061.	13205001	13225001	2.60488	0.176467	OL	HDDC2;TPD52L1
NC_056061.	13210001	13230001	4.07709	0.216606	OL	HDDC2;TPD52L1
NC_056061.	13215001	13235001	3.15718	0.222376	OL	HDDC2;TPD52L1
NC_056054.	174125001	1.74E+08	2.29575	0.16882	OL	HHLA2
NC_056057.	70085001	70105001	1.97013	0.164767	OL	HIBADH
NC_056057.	70090001	70110001	2.22326	0.182092	OL	HIBADH
NC_056057.	70095001	70115001	2.10483	0.216968	OL	HIBADH
NC_056064.	22960001	22980001	2.01333	0.220323	OL	HIC1;SMG6
NC_056057.	94330001	94350001	3.61931	0.194585	OL	HILPDA
NC_056057.	94335001	94355001	3.40541	0.163316	OL	HILPDA
NC_056054.	16320001	16340001	2.14281	0.173132	OL	HIVEP3
NC_056056.	153970001	1.54E+08	2.03738	0.199771	OL	HMGA2
NC_056056.	153975001	1.54E+08	2.23883	0.253984	OL	HMGA2
NC_056056.	154010001	1.54E+08	2.053	0.223985	OL	HMGA2
NC_056056.	154035001	1.54E+08	2.49492	0.437365	OL	HMGA2
NC_056056.	154040001	1.54E+08	2.51922	0.445972	OL	HMGA2
NC_056056.	154045001	1.54E+08	4.22414	0.418558	OL	HMGA2
NC_056056.	154050001	1.54E+08	4.23853	0.513959	OL	HMGA2
NC_056056.	154055001	1.54E+08	2.48947	0.566719	OL	HMGA2
NC_056056.	154060001	1.54E+08	1.93006	0.644566	OL	HMGA2
NC_056056.	154075001	1.54E+08	1.9145	0.688249	OL	HMGA2
NC_056078.	23980001	24000001	3.30459	0.152946	OL	HNRNPH3;RUFY2
NC_056078.	23985001	24005001	4.5433	0.167575	OL	HNRNPH3;RUFY2
NC_056078.	23990001	24010001	2.53905	0.196194	OL	HNRNPH3;RUFY2
NC_056060.	10420001	10440001	2.00847	0.158322	OL	HOMER1
NC_056057.	70525001	70545001	2.37303	0.153085	OL	HOXA1
NC_056063.	73285001	73305001	2.23511	0.151821	OL	HS6ST3
NC_056065.	73025001	73045001	3.943	0.155143	OL	HSD11B1;LAMB3

NC_056080.1	126420001	1.26E+08	2.30837
NC_056055.1	128300001	1.28E+08	2.10754
NC_056066.1	29445001	29465001	1.96408
NC_056066.1	29450001	29470001	2.02245
NC_056066.1	29455001	29475001	2.04162
NC_056072.1	10975001	10995001	3.06975
NC_056072.1	10980001	11000001	2.97783
NC_056077.1	27845001	27865001	3.2207
NC_056077.1	27850001	27870001	3.07349
NC_056077.1	27855001	27875001	2.37006
NC_056077.1	27860001	27880001	1.94576
NC_056077.1	27870001	27890001	2.14675
NC_056077.1	27875001	27895001	2.79497
NC_056077.1	27880001	27900001	3.24397
NC_056077.1	27885001	27905001	3.34523
NC_056077.1	27890001	27910001	3.22857
NC_056077.1	27895001	27915001	4.18353
NC_056077.1	27900001	27920001	3.25044
NC_056077.1	27905001	27925001	2.26497
NC_056054.1	112305001	1.12E+08	1.97518
NC_056054.1	112310001	1.12E+08	1.94935
NC_056057.1	66430001	66450001	3.59189
NC_056057.1	66435001	66455001	5.6576
NC_056057.1	66465001	66485001	1.99629
NC_056057.1	66470001	66490001	2.04533
NC_056057.1	66475001	66495001	3.16078
NC_056057.1	66480001	66500001	3.33332
NC_056057.1	66490001	66510001	4.89333
NC_056054.1	40510001	40530001	5.53125
NC_056058.1	56575001	56595001	2.56034
NC_056058.1	56580001	56600001	2.52987

NC_056065.	73030001	73050001	10.1913	0.213825	OL	HSD11B1;LAMB3
NC_056065.	73035001	73055001	9.207	0.209355	OL	HSD11B1;LAMB3
NC_056068.	65850001	65870001	1.82153	0.161852	OL	IFTAP
NC_056064.	39870001	39890001	2.70777	0.170426	OL	IKZF3
NC_056064.	39875001	39895001	1.96417	0.182435	OL	IKZF3
NC_056067.	48465001	48485001	2.06705	0.374478	OL	IL-15L;PLEKHG2;RPS16
NC_056067.	48470001	48490001	2.18095	0.352113	OL	IL-15L;PLEKHG2;RPS16;
NC_056067.	1010001	1030001	1.80735	0.175543	OL	IL34
NC_056067.	1015001	1035001	2.06113	0.164365	OL	IL34
NC_056067.	1020001	1040001	2.20923	0.172379	OL	IL34
NC_056067.	1025001	1045001	2.1469	0.154377	OL	IL34
NC_056067.	1030001	1050001	2.44721	0.167885	OL	IL34
NC_056067.	1035001	1055001	2.30785	0.164437	OL	IL34
NC_056067.	990001	1010001	2.55265	0.154971	OL	IL34;MTSS2
NC_056067.	995001	1015001	2.41466	0.164622	OL	IL34;MTSS2
NC_056067.	1000001	1020001	2.43872	0.167046	OL	IL34;MTSS2
NC_056067.	1005001	1025001	2.20965	0.190341	OL	IL34;MTSS2
NC_056057.	58550001	58570001	1.85435	0.244004	OL	IMMP2L
NC_056057.	58555001	58575001	2.00387	0.247843	OL	IMMP2L
NC_056057.	58560001	58580001	1.89087	0.263329	OL	IMMP2L
NC_056057.	58565001	58585001	1.84917	0.267753	OL	IMMP2L
NC_056057.	58570001	58590001	2.16609	0.307558	OL	IMMP2L
NC_056057.	58575001	58595001	2.54056	0.351811	OL	IMMP2L
NC_056057.	58580001	58600001	2.40179	0.35006	OL	IMMP2L
NC_056057.	58585001	58605001	2.68934	0.336282	OL	IMMP2L
NC_056057.	58590001	58610001	2.9	0.307029	OL	IMMP2L
NC_056057.	58600001	58620001	1.98008	0.25916	OL	IMMP2L
NC_056057.	58605001	58625001	2.08571	0.25242	OL	IMMP2L
NC_056070.	15495001	15515001	2.15496	0.162053	OL	INPP4B
NC_056070.	15500001	15520001	2.64582	0.183109	OL	INPP4B
NC_056070.	15505001	15525001	3.37145	0.209929	OL	INPP4B

NC_056074.1	30385001	30405001	2.08149
NC_056074.1	30390001	30410001	2.34528
NC_056074.1	30395001	30415001	2.4235
NC_056074.1	30400001	30420001	2.4242
NC_056060.1	84730001	84750001	2.62847
NC_056066.1	72610001	72630001	2.60126
NC_056066.1	72615001	72635001	4.00973
NC_056054.1	33475001	33495001	10.878
NC_056058.1	11020001	11040001	2.22897
NC_056078.1	29830001	29850001	6.01219
NC_056078.1	29835001	29855001	3.70504
NC_056078.1	29840001	29860001	2.05178
NC_056078.1	29900001	29920001	2.87045
NC_056078.1	29905001	29925001	2.80571
NC_056078.1	29910001	29930001	3.6266
NC_056078.1	29915001	29935001	2.49671
NC_056056.1	211075001	2.11E+08	2.23292
NC_056056.1	211080001	2.11E+08	2.04613
NC_056056.1	211090001	2.11E+08	2.2449
NC_056056.1	211095001	2.11E+08	2.03282
NC_056056.1	211100001	2.11E+08	2.97927
NC_056057.1	86650001	86670001	1.96108
NC_056054.1	89195001	89215001	3.30735
NC_056054.1	89200001	89220001	3.09131
NC_056054.1	89205001	89225001	2.6363
NC_056054.1	89210001	89230001	2.73519
NC_056054.1	89215001	89235001	2.98127
NC_056054.1	89220001	89240001	3.10595
NC_056054.1	89225001	89245001	3.67116
NC_056055.1	225200001	2.25E+08	2.9442
NC_056055.1	225205001	2.25E+08	5.51914

NC_056070.	15510001	15530001	5.22203	0.238037	OL	INPP4B
NC_056070.	15515001	15535001	3.76357	0.182326	OL	INPP4B
NC_056070.	15520001	15540001	3.391	0.150847	OL	INPP4B
NC_056070.	15550001	15570001	1.86846	0.142733	OL	INPP4B
NC_056070.	69875001	69895001	2.17616	0.154908	OL	INPP5J;SELENOM;SMTN
NC_056070.	69880001	69900001	2.23207	0.174802	OL	INPP5J;SELENOM;SMTN
NC_056054.	106765001	1.07E+08	2.41795	0.184706	OL	INSRR
NC_056054.	106770001	1.07E+08	8.53937	0.197856	OL	INSRR
NC_056056.	184425001	1.84E+08	2.32815	0.151185	OL	IPO8
NC_056060.	14075001	14095001	2.10233	0.143577	OL	IQCH
NC_056080.	126420001	1.26E+08	2.37104	0.18734	OL	IRS4
NC_056080.	126425001	1.26E+08	2.15116	0.220117	OL	IRS4
NC_056080.	126430001	1.26E+08	1.91233	0.231373	OL	IRS4
NC_056080.	126440001	1.26E+08	1.82549	0.183562	OL	IRS4
NC_056080.	126445001	1.26E+08	2.43015	0.170335	OL	IRS4
NC_056080.	126450001	1.26E+08	3.12209	0.157023	OL	IRS4
NC_056060.	15110001	15130001	1.86044	0.167547	OL	ITGA11
NC_056060.	15115001	15135001	2.85968	0.196322	OL	ITGA11
NC_056060.	15120001	15140001	4.43271	0.234315	OL	ITGA11
NC_056060.	15125001	15145001	3.64596	0.193528	OL	ITGA11
NC_056060.	15200001	15220001	1.94595	0.190961	OL	ITGA11
NC_056060.	15205001	15225001	1.93988	0.183834	OL	ITGA11
NC_056064.	24255001	24275001	1.9209	0.155069	OL	ITGAE;P2RX5
NC_056056.	188925001	1.89E+08	2.2337	0.142876	OL	ITPR2
NC_056057.	66475001	66495001	2.75799	0.163519	OL	ITPRID1
NC_056057.	66480001	66500001	3.20567	0.166143	OL	ITPRID1
NC_056054.	40510001	40530001	1.8114	0.172327	OL	JAK1
NC_056054.	40515001	40535001	2.22544	0.244421	OL	JAK1
NC_056054.	40520001	40540001	2.30272	0.251806	OL	JAK1
NC_056054.	40525001	40545001	2.28378	0.227773	OL	JAK1
NC_056067.	12670001	12690001	2.59612	0.150589	OL	JPH3

NC_056055.1	225210001	2.25E+08	13.4091
NC_056054.1	277365001	2.77E+08	2.1116
NC_056059.1	41730001	41750001	1.97259
NC_056060.1	99750001	99770001	6.94139
NC_056060.1	99755001	99775001	6.70079
NC_056060.1	99760001	99780001	4.07667
NC_056060.1	99765001	99785001	4.59355
NC_056060.1	99770001	99790001	3.33443
NC_056060.1	99775001	99795001	2.89934
NC_056065.1	67945001	67965001	3.09654
NC_056065.1	67950001	67970001	4.24771
NC_056065.1	67955001	67975001	5.6253
NC_056065.1	67960001	67980001	7.79684
NC_056065.1	67965001	67985001	20.2619
NC_056065.1	67970001	67990001	25.1933
NC_056065.1	67975001	67995001	3.65935
NC_056056.1	33870001	33890001	3.07692
NC_056065.1	61440001	61460001	2.82655
NC_056065.1	61445001	61465001	2.71375
NC_056055.1	247845001	2.48E+08	3.74533
NC_056055.1	247855001	2.48E+08	3.51602
NC_056063.1	44775001	44795001	2.61161
NC_056063.1	44780001	44800001	2.69601
NC_056063.1	44785001	44805001	2.44208
NC_056063.1	44790001	44810001	2.38453
NC_056063.1	44795001	44815001	2.33304
NC_056063.1	44800001	44820001	2.26464
NC_056063.1	44805001	44825001	2.37478
NC_056063.1	44810001	44830001	2.41349
NC_056063.1	44815001	44835001	2.39267
NC_056063.1	44820001	44840001	2.36478

NC_056054.	189790001	1.9E+08	1.89968	0.204122	OL	KALRN
NC_056054.	189795001	1.9E+08	2.42145	0.234743	OL	KALRN
NC_056055.	211740001	2.12E+08	2.16666	0.14369	OL	KANSL1L
NC_056066.	37930001	37950001	3.72377	0.149752	OL	KAT14
NC_056066.	37935001	37955001	3.89551	0.154473	OL	KAT14
NC_056078.	29830001	29850001	1.81028	0.154826	OL	KAT6B
NC_056078.	29950001	29970001	1.82138	0.232122	OL	KAT6B
NC_056078.	29955001	29975001	1.83945	0.244706	OL	KAT6B
NC_056054.	87905001	87925001	2.60236	0.220837	OL	KCNA2
NC_056054.	87910001	87930001	3.07277	0.246766	OL	KCNA2
NC_056054.	232130001	2.32E+08	2.19452	0.196697	OL	KCNAB1
NC_056054.	232135001	2.32E+08	2.07417	0.152072	OL	KCNAB1
NC_056066.	77495001	77515001	2.00173	0.162974	OL	KCNB1
NC_056066.	77500001	77520001	1.94927	0.149365	OL	KCNB1
NC_056066.	77510001	77530001	2.04155	0.164255	OL	KCNB1
NC_056055.	225195001	2.25E+08	2.36589	0.145602	OL	KCNE4
NC_056055.	225200001	2.25E+08	2.8989	0.254781	OL	KCNE4
NC_056055.	225205001	2.25E+08	2.6255	0.310834	OL	KCNE4
NC_056055.	225210001	2.25E+08	2.75187	0.335566	OL	KCNE4
NC_056065.	71985001	72005001	2.88259	0.147456	OL	KCNH1
NC_056069.	2575001	2595001	2.96182	0.158128	OL	KCNIP1
NC_056069.	2580001	2600001	7.05152	0.185627	OL	KCNIP1
NC_056069.	2585001	2605001	17.4405	0.228836	OL	KCNIP1
NC_056069.	2590001	2610001	4.43071	0.167588	OL	KCNIP1
NC_056060.	99760001	99780001	2.17787	0.157906	OL	KCNK13
NC_056060.	99765001	99785001	1.96307	0.192991	OL	KCNK13
NC_056065.	67965001	67985001	2.07056	0.181589	OL	KCNK2
NC_056065.	67970001	67990001	3.001	0.177673	OL	KCNK2
NC_056067.	47495001	47515001	2.51211	0.205227	OL	KCNK6;YIF1B
NC_056067.	47500001	47520001	3.96154	0.204577	OL	KCNK6;YIF1B
NC_056074.	45370001	45390001	1.92857	0.150237	OL	KCNQ1

NC_056063.1	44825001	44845001	2.32551
NC_056063.1	44830001	44850001	2.25496
NC_056063.1	44835001	44855001	2.14649
NC_056063.1	44840001	44860001	2.12238
NC_056063.1	44845001	44865001	2.10487
NC_056063.1	44850001	44870001	2.13824
NC_056063.1	44855001	44875001	2.15644
NC_056063.1	44860001	44880001	2.1822
NC_056063.1	44865001	44885001	2.19209
NC_056063.1	44870001	44890001	2.19459
NC_056063.1	44875001	44895001	2.23284
NC_056063.1	44880001	44900001	2.26379
NC_056071.1	15975001	15995001	6.09245
NC_056058.1	4355001	4375001	17.4147
NC_056057.1	104405001	1.04E+08	3.24608
NC_056057.1	104410001	1.04E+08	3.18566
NC_056056.1	133700001	1.34E+08	2.20896
NC_056056.1	134210001	1.34E+08	2.06935
NC_056056.1	134215001	1.34E+08	2.0664
NC_056061.1	55660001	55680001	2.00625
NC_056061.1	55665001	55685001	4.57586
NC_056061.1	55670001	55690001	8.08294
NC_056061.1	55675001	55695001	8.69407
NC_056061.1	55680001	55700001	11.0706
NC_056061.1	55685001	55705001	2.92561
NC_056076.1	33110001	33130001	2.27548
NC_056076.1	33115001	33135001	2.97388
NC_056076.1	33120001	33140001	2.81488
NC_056076.1	33125001	33145001	2.69126
NC_056076.1	33175001	33195001	2.8213
NC_056076.1	33180001	33200001	3.29471

NC_056074.	45375001	45395001	1.92724	0.165063	OL	KCNQ1
NC_056062.	595001	615001	1.95711	0.202524	OL	KCNQ5
NC_056062.	600001	620001	2.30213	0.205666	OL	KCNQ5
NC_056062.	605001	625001	2.43182	0.181913	OL	KCNQ5
NC_056073.	38680001	38700001	2.72566	0.192537	OL	KDM1B;TPMT
NC_056073.	38685001	38705001	3.62602	0.228211	OL	KDM1B;TPMT
NC_056073.	38690001	38710001	2.97692	0.224302	OL	KDM1B;TPMT
NC_056070.	53770001	53790001	2.52381	0.152941	OL	KDM2B
NC_056070.	53775001	53795001	2.24026	0.174359	OL	KDM2B
NC_056070.	53780001	53800001	2.2521	0.202193	OL	KDM2B
NC_056070.	53785001	53805001	2.1777	0.208712	OL	KDM2B;RNF34
NC_056070.	53790001	53810001	2.41667	0.230556	OL	KDM2B;RNF34
NC_056070.	53795001	53815001	2.98942	0.275641	OL	KDM2B;RNF34
NC_056055.	74730001	74750001	3.71578	0.216411	OL	KDM4C
NC_056055.	74735001	74755001	2.01633	0.143413	OL	KDM4C
NC_056055.	74780001	74800001	3.19603	0.141591	OL	KDM4C
NC_056055.	74785001	74805001	8.84103	0.215184	OL	KDM4C
NC_056058.	12860001	12880001	2.90176	0.144089	OL	KEAP1;S1PR5
NC_056058.	91370001	91390001	2.35556	0.173455	OL	KIAA0825
NC_056058.	91375001	91395001	3.84586	0.237177	OL	KIAA0825
NC_056058.	91380001	91400001	6.05647	0.25554	OL	KIAA0825
NC_056058.	91385001	91405001	3.81796	0.197021	OL	KIAA0825
NC_056058.	91390001	91410001	3.26134	0.170251	OL	KIAA0825
NC_056058.	91545001	91565001	1.86083	0.147487	OL	KIAA0825
NC_056058.	91550001	91570001	2.31363	0.202506	OL	KIAA0825
NC_056066.	24735001	24755001	1.81536	0.171301	OL	KIAA1217
NC_056068.	63090001	63110001	3.38462	0.213146	OL	KIAA1549L
NC_056068.	63095001	63115001	4.29019	0.20956	OL	KIAA1549L
NC_056068.	63100001	63120001	3.09102	0.173495	OL	KIAA1549L
NC_056068.	63105001	63125001	3.59005	0.191413	OL	KIAA1549L
NC_056068.	63110001	63130001	3.1899	0.170115	OL	KIAA1549L

NC_056076.1	33185001	33205001	2.6023
NC_056057.1	50675001	50695001	2.77339
NC_056057.1	50685001	50705001	2.60261
NC_056057.1	50690001	50710001	2.48946
NC_056057.1	50695001	50715001	2.67149
NC_056057.1	50700001	50720001	2.88731
NC_056065.1	63485001	63505001	2.04808
NC_056059.1	38175001	38195001	2.85665
NC_056059.1	38180001	38200001	4.76691
NC_056059.1	38185001	38205001	6.60376
NC_056059.1	38190001	38210001	7.47058
NC_056059.1	38195001	38215001	4.96946
NC_056059.1	38200001	38220001	3.17087
NC_056059.1	38205001	38225001	3.03933
NC_056059.1	38210001	38230001	3.06279
NC_056059.1	38215001	38235001	2.83077
NC_056059.1	38220001	38240001	2.13393
NC_056055.1	244715001	2.45E+08	7.26843
NC_056054.1	41180001	41200001	3.39872
NC_056054.1	41185001	41205001	7.83453
NC_056054.1	41190001	41210001	11.2023
NC_056054.1	41195001	41215001	12.4224
NC_056054.1	41200001	41220001	8.52096
NC_056054.1	41205001	41225001	3.96372
NC_056054.1	41210001	41230001	3.95857
NC_056054.1	41215001	41235001	8.06201
NC_056060.1	9685001	9705001	2.41891
NC_056060.1	9690001	9710001	2.78025
NC_056060.1	9695001	9715001	3.33389
NC_056060.1	9700001	9720001	2.92952
NC_056065.1	60810001	60830001	1.9568

NC_056068.	63115001	63135001	2.96672	0.169547	OL	KIAA1549L
NC_056055.	11070001	11090001	1.93505	0.149014	OL	KIAA1958
NC_056055.	11075001	11095001	3.91609	0.193643	OL	KIAA1958
NC_056055.	11080001	11100001	5.01632	0.204207	OL	KIAA1958
NC_056055.	11085001	11105001	3.57102	0.150759	OL	KIAA1958
NC_056055.	103780001	1.04E+08	2.63433	0.208094	OL	KIF13B
NC_056066.	9930001	9950001	1.93064	0.165794	OL	KIF16B
NC_056059.	70940001	70960001	1.91033	0.181101	OL	KIT
NC_056059.	70945001	70965001	2.21835	0.195899	OL	KIT
NC_056063.	28365001	28385001	2.01941	0.162392	OL	KL
NC_056070.	52235001	52255001	2.11945	0.146429	OL	KMT5A;RILPL2
NC_056070.	52240001	52260001	2.68987	0.175944	OL	KMT5A;RILPL2
NC_056070.	52245001	52265001	2.11342	0.171591	OL	KMT5A;RILPL2
NC_056064.	27775001	27795001	7.53247	0.148483	OL	KRBA2;ODF4
NC_056064.	27780001	27800001	9.58599	0.156018	OL	KRBA2;RPL26
NC_056064.	27785001	27805001	3.34	0.162911	OL	KRBA2;RPL26
NC_056056.	134210001	1.34E+08	1.91554	0.203574	OL	KRT80
NC_056056.	134215001	1.34E+08	2.01255	0.182616	OL	KRT80
NC_056054.	255350001	2.55E+08	2.25817	0.199954	OL	KY
NC_056054.	255355001	2.55E+08	2.21437	0.150503	OL	KY
NC_056061.	55645001	55665001	1.83858	0.193345	OL	L3MBTL3
NC_056061.	55650001	55670001	2.44644	0.244051	OL	L3MBTL3
NC_056061.	55655001	55675001	3.4507	0.280297	OL	L3MBTL3
NC_056061.	55660001	55680001	3.02253	0.22367	OL	L3MBTL3
NC_056061.	55665001	55685001	2.6019	0.168752	OL	L3MBTL3
NC_056061.	55670001	55690001	2.36701	0.144387	OL	L3MBTL3
NC_056056.	209495001	2.1E+08	2.27205	0.155488	OL	LAG3;PTMS
NC_056056.	209500001	2.1E+08	2.06258	0.167698	OL	LAG3;PTMS
NC_056065.	73150001	73170001	5.27607	0.142512	OL	LAMB3
NC_056065.	63485001	63505001	2.58426	0.154388	OL	LAMC1
NC_056056.	154695001	1.55E+08	2.34235	0.22774	OL	LEMD3

NC_056065.1	60815001	60835001	2.13475
NC_056070.1	69970001	69990001	2.0849
NC_056070.1	69975001	69995001	2.55212
NC_056070.1	69980001	70000001	3.28937
NC_056070.1	69985001	70005001	4.64077
NC_056055.1	98070001	98090001	2.90011
NC_056055.1	98075001	98095001	3.32772
NC_056055.1	98080001	98100001	2.54835
NC_056072.1	32490001	32510001	2.473
NC_056056.1	26560001	26580001	2.11367
NC_056068.1	47935001	47955001	61.1021
NC_056060.1	46070001	46090001	2.16611
NC_056073.1	30460001	30480001	2.33512
NC_056073.1	29865001	29885001	4.73201
NC_056073.1	29870001	29890001	4.078
NC_056073.1	29875001	29895001	2.50679
NC_056068.1	48040001	48060001	7.78733
NW_02459982	1190001	1210001	2.24491
NC_056073.1	29855001	29875001	2.96234
NC_056056.1	163925001	1.64E+08	3.92409
NC_056056.1	163940001	1.64E+08	8.68165
NC_056056.1	163945001	1.64E+08	8.26839
NC_056056.1	163950001	1.64E+08	3.55071
NC_056060.1	25775001	25795001	2.50159
NC_056060.1	25780001	25800001	2.81357
NC_056069.1	62605001	62625001	2.33095
NC_056069.1	62640001	62660001	2.63259
NC_056069.1	62645001	62665001	3.3254
NC_056069.1	62650001	62670001	3.85185
NC_056069.1	62655001	62675001	3.95324
NC_056069.1	62660001	62680001	3.56527

NC_056056.	154700001	1.55E+08	2.23024	0.229424	OL	LEMD3
NC_056056.	154705001	1.55E+08	2.96552	0.239788	OL	LEMD3
NC_056056.	154710001	1.55E+08	3.44361	0.218922	OL	LEMD3
NC_056056.	154715001	1.55E+08	3.26428	0.217568	OL	LEMD3
NC_056056.	154720001	1.55E+08	2.795	0.166418	OL	LEMD3
NC_056071.	55225001	55245001	1.99943	0.203608	OL	LGMN
NC_056071.	55230001	55250001	3.13183	0.192453	OL	LGMN
NC_056060.	9685001	9705001	1.95656	0.162993	OL	LHFPL2
NC_056070.	69970001	69990001	2.36111	0.142554	OL	LIMK2
NC_056070.	69975001	69995001	2.90385	0.165862	OL	LIMK2
NC_056070.	69980001	70000001	3.27737	0.155373	OL	LIMK2
NC_056070.	69985001	70005001	4.24888	0.156902	OL	LIMK2;PIK3IP1
NC_056054.	99255001	99275001	3.13008	0.168979	OL	LIX1L
NC_056054.	99250001	99270001	1.8995	0.146094	OL	LIX1L;RBM8A
NC_056054.	117215001	1.17E+08	2.30291	0.286587	OL	LMX1A
NC_056054.	117220001	1.17E+08	2.37968	0.231326	OL	LMX1A
NC_056054.	117225001	1.17E+08	2.03776	0.16916	OL	LMX1A
NC_056063.	32605001	32625001	5.1832	0.261635	OL	LNX2
NC_056063.	32610001	32630001	5.30613	0.255375	OL	LNX2
NC_056063.	32615001	32635001	4.25511	0.218509	OL	LNX2
NC_056063.	32620001	32640001	3.97803	0.174928	OL	LNX2
NC_056063.	32655001	32675001	3.17461	0.184007	OL	LNX2
NC_056063.	32660001	32680001	4.20408	0.244431	OL	LNX2
NC_056063.	32665001	32685001	4.62338	0.242211	OL	LNX2
NC_056063.	32670001	32690001	4.02954	0.232214	OL	LNX2
NC_056063.	32675001	32695001	5.16665	0.251932	OL	LNX2
NC_056063.	32680001	32700001	5.63173	0.25808	OL	LNX2
NC_056063.	32685001	32705001	4.65444	0.239684	OL	LNX2
NC_056063.	32690001	32710001	4.20578	0.219925	OL	LNX2
NC_056063.	32585001	32605001	2.6129	0.162249	OL	LNX2;LOC101113604;PO
NC_056063.	32600001	32620001	3.79084	0.239234	OL	LNX2;LOC101113604;PO

NC_056069.1	62665001	62685001	2.72063
NC_056069.1	62670001	62690001	2.56775
NC_056069.1	62680001	62700001	2.12755
NC_056069.1	62685001	62705001	2.21675
NC_056077.1	27795001	27815001	2.3078
NC_056077.1	27800001	27820001	2.82816
NC_056060.1	25785001	25805001	2.10801
NC_056068.1	48065001	48085001	4.81807
NC_056068.1	48070001	48090001	5.88704
NC_056067.1	58420001	58440001	3.04951
NC_056067.1	58425001	58445001	2.9665
NC_056057.1	100545001	1.01E+08	2.68377
NC_056057.1	100550001	1.01E+08	2.68045
NC_056057.1	100555001	1.01E+08	3.18586
NC_056057.1	100560001	1.01E+08	2.42745
NC_056057.1	100565001	1.01E+08	1.98101
NC_056057.1	100535001	1.01E+08	2.08748
NC_056080.1	116255001	1.16E+08	2.23913
NC_056080.1	116260001	1.16E+08	2.43074
NC_056080.1	116265001	1.16E+08	2.39875
NC_056080.1	116270001	1.16E+08	2.35688
NC_056080.1	116275001	1.16E+08	2.29891
NC_056080.1	116280001	1.16E+08	2.46417
NC_056080.1	116320001	1.16E+08	2.91429
NC_056058.1	8450001	8470001	2.16584
NC_056058.1	8445001	8465001	3.98834
NC_056054.1	265225001	2.65E+08	3.47463
NC_056054.1	265230001	2.65E+08	7.63483
NC_056054.1	265235001	2.65E+08	6.70913
NC_056056.1	102620001	1.03E+08	2.02167
NC_056058.1	15900001	15920001	5.02608

NC_056057.	108050001	1.08E+08	2.57517	0.17255	OL	LOC101102056;TAS2R60
NC_056057.	695001	715001	4.42902	0.180783	OL	LOC101102132
NC_056057.	700001	720001	3.92537	0.168407	OL	LOC101102132
NC_056062.	73200001	73220001	2.08463	0.36994	OL	LOC101102317
NC_056062.	73205001	73225001	2.20641	0.268242	OL	LOC101102317
NC_056062.	73210001	73230001	2.06166	0.210013	OL	LOC101102317
NC_056061.	57960001	57980001	3.17494	0.251992	OL	LOC101102492;LOC1011
NC_056061.	57955001	57975001	3.79654	0.295061	OL	LOC101102492;TAAR8
NC_056080.	96895001	96915001	2.95224	0.167358	OL	LOC101102539
NC_056080.	96900001	96920001	2.37219	0.154614	OL	LOC101102539
NC_056080.	96905001	96925001	1.90225	0.183361	OL	LOC101102539
NC_056054.	181455001	1.81E+08	2.57364	0.187618	OL	LOC101104028
NC_056054.	181460001	1.81E+08	2.26257	0.160184	OL	LOC101104028
NC_056068.	77265001	77285001	4.59031	0.163871	OL	LOC101104526
NC_056073.	43885001	43905001	2.05252	0.143227	OL	LOC101105035
NC_056056.	180010001	1.8E+08	2.06444	0.20625	OL	LOC101105069;LOC1011
NC_056056.	180015001	1.8E+08	1.97386	0.187126	OL	LOC101105069;LOC1011
NC_056079.	6230001	6250001	2.57453	0.142824	OL	LOC101105133;LOC1056
NC_056079.	6235001	6255001	2.98159	0.176195	OL	LOC101105133;LOC1056
NC_056079.	6240001	6260001	2.88275	0.184579	OL	LOC101105133;LOC1056
NC_056056.	180000001	1.8E+08	4.52874	0.311421	OL	LOC101105321
NC_056056.	180005001	1.8E+08	3.05975	0.26002	OL	LOC101105321
NC_056056.	179990001	1.8E+08	2.56553	0.248297	OL	LOC101105321;LOC1056
NC_056056.	179995001	1.8E+08	3.33556	0.282946	OL	LOC101105321;LOC1056
NC_056054.	17755001	17775001	1.90974	0.184703	OL	LOC101105378
NC_056054.	17760001	17780001	5.5722	0.171182	OL	LOC101105378
NC_056054.	17785001	17805001	12.8236	0.198503	OL	LOC101105621
NC_056054.	17790001	17810001	14.704	0.191523	OL	LOC101105621
NC_056058.	40535001	40555001	2.07909	0.143689	OL	LOC101105657
NC_056054.	88190001	88210001	2.22099	0.17139	OL	LOC101105715
NC_056054.	88195001	88215001	2.53629	0.194611	OL	LOC101105715

NC_056058.1	15905001	15925001	5.29631
NC_056064.1	49790001	49810001	2.12369
NC_056055.1	149880001	1.5E+08	13.5042
NC_056055.1	149885001	1.5E+08	20.1225
NC_056055.1	149890001	1.5E+08	14.2738
NC_056054.1	199405001	1.99E+08	3.47535
NC_056054.1	199410001	1.99E+08	2.91249
NC_056054.1	199450001	1.99E+08	3.13601
NC_056054.1	199455001	1.99E+08	2.84309
NC_056054.1	199460001	1.99E+08	2.99694
NC_056054.1	199465001	1.99E+08	2.32342
NC_056054.1	269915001	2.7E+08	21.1288
NC_056080.1	118945001	1.19E+08	8.06192
NC_056080.1	118950001	1.19E+08	5.19247
NC_056069.1	38335001	38355001	2.04502
NC_056080.1	119900001	1.2E+08	2.08012
NC_056080.1	119905001	1.2E+08	2.66543
NC_056080.1	29215001	29235001	2.38359
NC_056080.1	29220001	29240001	3.20077
NC_056080.1	29225001	29245001	2.88462
NC_056080.1	29230001	29250001	2.72669
NC_056080.1	29235001	29255001	3.05438
NC_056054.1	107655001	1.08E+08	4.30239
NC_056054.1	107660001	1.08E+08	4.75891
NC_056054.1	107665001	1.08E+08	4.88428
NC_056080.1	46875001	46895001	2.40554
NC_056080.1	46880001	46900001	2.66905
NC_056080.1	46885001	46905001	2.55738
NC_056055.1	52515001	52535001	2.37033
NC_056055.1	52520001	52540001	2.3694
NC_056055.1	52525001	52545001	2.309

NC_056063.	20755001	20775001	12.4181	0.305335	OL	LOC101106088
NC_056063.	20760001	20780001	4.56634	0.250687	OL	LOC101106088
NC_056063.	20765001	20785001	2.91595	0.206865	OL	LOC101106088
NC_056058.	8265001	8285001	2.09334	0.179355	OL	LOC101106259;LOC1056
NC_056058.	8270001	8290001	2.37805	0.142744	OL	LOC101106259;LOC1056
NC_056058.	8305001	8325001	3.22654	0.142342	OL	LOC101106524
NC_056074.	24595001	24615001	4.55708	0.154435	OL	LOC101106638
NC_056074.	24600001	24620001	5.12111	0.228389	OL	LOC101106638
NC_056074.	24605001	24625001	4.46165	0.230838	OL	LOC101106638;LOC1011
NC_056074.	24610001	24630001	2.74581	0.199368	OL	LOC101106638;LOC1011
NC_056063.	70645001	70665001	4.77788	0.161004	OL	LOC101106781
NC_056063.	70650001	70670001	4.30472	0.147604	OL	LOC101106781
NC_056074.	24615001	24635001	2.02433	0.157379	OL	LOC101106894
NC_056058.	36770001	36790001	5.40162	0.288429	OL	LOC101107521
NC_056058.	36775001	36795001	3.6254	0.27089	OL	LOC101107521
NC_056058.	36780001	36800001	4.42999	0.327278	OL	LOC101107521
NC_056080.	116345001	1.16E+08	3.74358	0.154905	OL	LOC101108113
NC_056080.	116350001	1.16E+08	3.51938	0.149038	OL	LOC101108113
NC_056080.	116340001	1.16E+08	2.60131	0.18062	OL	LOC101108113;LOC1056
NC_056054.	17770001	17790001	22.6068	0.177371	OL	LOC101108173
NC_056054.	17775001	17795001	17.226	0.19454	OL	LOC101108173
NC_056054.	17780001	17800001	16.1569	0.194931	OL	LOC101108173
NC_056058.	38660001	38680001	2.15183	0.180327	OL	LOC101108746
NC_056058.	38665001	38685001	2.26265	0.17575	OL	LOC101108746
NC_056058.	38670001	38690001	2.28723	0.19333	OL	LOC101108746;LOC1011
NC_056073.	30670001	30690001	2.78851	0.155407	OL	LOC101108868
NC_056058.	57325001	57345001	4.40462	0.256531	OL	LOC101108915
NC_056058.	57330001	57350001	14.6405	0.386722	OL	LOC101108915
NC_056058.	57335001	57355001	16.4107	0.315867	OL	LOC101108915
NC_056058.	57340001	57360001	22.9927	0.284954	OL	LOC101108915
NC_056058.	57345001	57365001	22.9549	0.253217	OL	LOC101108915

NC_056055.1	52530001	52550001	2.16499
NC_056054.1	187245001	1.87E+08	21.8513
NC_056054.1	187250001	1.87E+08	10.3356
NC_056054.1	187255001	1.87E+08	6.64117
NC_056054.1	187260001	1.87E+08	1.9519
NC_056054.1	187225001	1.87E+08	10.6564
NC_056054.1	187230001	1.87E+08	11.8769
NC_056054.1	187235001	1.87E+08	15.2512
NC_056054.1	187240001	1.87E+08	26.6667
NC_056077.1	41225001	41245001	2.12143
NC_056077.1	41235001	41255001	3.4241
NC_056077.1	41240001	41260001	2.38858
NC_056057.1	106800001	1.07E+08	2.72863
NC_056057.1	106805001	1.07E+08	5.4373
NC_056057.1	106810001	1.07E+08	4.15784
NC_056068.1	48635001	48655001	2.01403
NC_056072.1	12715001	12735001	2.57196
NC_056072.1	12720001	12740001	2.5504
NC_056054.1	110280001	1.1E+08	2.17873
NC_056077.1	19945001	19965001	2.43641
NC_056077.1	19950001	19970001	4.81234
NC_056077.1	19955001	19975001	5.83701
NC_056077.1	19960001	19980001	7.23276
NC_056077.1	19965001	19985001	4.37097
NC_056077.1	19970001	19990001	3.19702
NC_056077.1	20020001	20040001	3.13908
NC_056077.1	20025001	20045001	3.95771
NC_056077.1	20030001	20050001	3.02814
NC_056077.1	20035001	20055001	3.01729
NC_056077.1	20040001	20060001	3.50819
NC_056077.1	20045001	20065001	3.69667

NC_056058.	57350001	57370001	9.74194	0.18648	OL	LOC101108915;LOC1218	NC_056077.1	20050001	20070001	3.30673
NC_056055.	188335001	1.88E+08	2.05325	0.195662	OL	LOC101109675	NC_056077.1	20055001	20075001	2.74793
NC_056055.	188340001	1.88E+08	2.52055	0.217072	OL	LOC101109675	NC_056071.1	23750001	23770001	2.60318
NC_056073.	7405001	7425001	2.76271	0.14934	OL	LOC101109746;LOC1011	NC_056061.1	50075001	50095001	2.4853
NC_056068.	48960001	48980001	4.09043	0.147724	OL	LOC101109826	NC_056061.1	50080001	50100001	2.31726
NC_056068.	48965001	48985001	2.14269	0.197713	OL	LOC101109826	NC_056057.1	12880001	12900001	3.17589
NC_056067.	60960001	60980001	1.81727	0.226279	OL	LOC101109994;LOC1141	NC_056057.1	12885001	12905001	2.96703
NC_056054.	93125001	93145001	1.87553	0.198068	OL	LOC101110112	NC_056066.1	64385001	64405001	1.99399
NC_056057.	114155001	1.14E+08	2.43797	0.393571	OL	LOC101110230	NC_056066.1	64390001	64410001	2.04915
NC_056057.	114160001	1.14E+08	2.9434	0.454722	OL	LOC101110230	NC_056067.1	62115001	62135001	2.56686
NC_056057.	114165001	1.14E+08	2.41745	0.426716	OL	LOC101110230	NC_056067.1	62120001	62140001	2.37812
NC_056074.	5035001	5055001	5.88361	0.353234	OL	LOC101110373	NC_056074.1	16290001	16310001	5.95335
NC_056074.	5040001	5060001	6.37822	0.290932	OL	LOC101110373	NC_056074.1	16295001	16315001	4.32477
NC_056074.	5045001	5065001	3.17001	0.225096	OL	LOC101110373	NC_056074.1	16300001	16320001	4.96264
NC_056080.	65440001	65460001	1.97768	0.240544	OL	LOC101111256;LOC1218	NC_056074.1	16305001	16325001	3.25246
NC_056080.	65445001	65465001	2.0156	0.25336	OL	LOC101111256;LOC1218	NC_056074.1	16310001	16330001	2.65418
NC_056080.	65450001	65470001	2.46177	0.271358	OL	LOC101111256;LOC1218	NC_056074.1	16315001	16335001	2.34562
NC_056064.	36720001	36740001	3.1399	0.255612	OL	LOC101111300	NC_056074.1	16320001	16340001	2.31126
NC_056064.	36725001	36745001	2.85106	0.242687	OL	LOC101111300	NC_056074.1	16325001	16345001	2.81735
NC_056064.	36730001	36750001	1.81391	0.173351	OL	LOC101111300	NC_056074.1	16330001	16350001	3.71267
NC_056054.	107685001	1.08E+08	5.60482	0.26211	OL	LOC101111337	NC_056074.1	16335001	16355001	4.59391
NC_056054.	107690001	1.08E+08	3.14526	0.234981	OL	LOC101111337	NC_056074.1	16340001	16360001	4.75486
NC_056054.	107695001	1.08E+08	2.81501	0.205987	OL	LOC101111337	NC_056074.1	16345001	16365001	3.50153
NC_056054.	107700001	1.08E+08	2.64875	0.222997	OL	LOC101111337;LOC1011	NC_056074.1	16375001	16395001	3.14286
NC_056054.	107705001	1.08E+08	3.52193	0.156474	OL	LOC101111337;LOC1011	NC_056074.1	16380001	16400001	4.7336
NC_056054.	109155001	1.09E+08	6.14115	0.408126	OL	LOC101111409	NC_056074.1	16385001	16405001	4.67755
NC_056054.	109160001	1.09E+08	5.71162	0.398434	OL	LOC101111409	NC_056074.1	16390001	16410001	4.1866
NC_056056.	209560001	2.1E+08	2	0.256168	OL	LOC101111444	NC_056074.1	16395001	16415001	3.52727
NC_056056.	209565001	2.1E+08	2.68245	0.161796	OL	LOC101111444	NC_056074.1	16400001	16420001	3.1131
NC_056073.	30805001	30825001	2.60482	0.168544	OL	LOC101111669;LOC1011	NC_056074.1	16405001	16425001	2.63286
NC_056080.	142010001	1.42E+08	1.84449	0.216251	OL	LOC101112115	NC_056074.1	16410001	16430001	2.47831

NC_056080.	142015001	1.42E+08	2.03545	0.225314	OL	LOC101112115
NC_056080.	142020001	1.42E+08	2.09194	0.230785	OL	LOC101112115
NC_056080.	142025001	1.42E+08	1.92592	0.209002	OL	LOC101112115
NC_056080.	142130001	1.42E+08	5.20848	0.141809	OL	LOC101112115
NC_056080.	142135001	1.42E+08	6.64793	0.161683	OL	LOC101112115
NC_056080.	142140001	1.42E+08	7.59448	0.165183	OL	LOC101112115
NC_056054.	109415001	1.09E+08	3.06371	0.176647	OL	LOC101112179
NC_056055.	12120001	12140001	2.47418	0.285086	OL	LOC101112627
NC_056071.	9110001	9130001	1.89051	0.146943	OL	LOC101112928
NC_056071.	9115001	9135001	2.13715	0.175901	OL	LOC101112928
NC_056071.	9120001	9140001	2.15122	0.160644	OL	LOC101112928
NC_056071.	9125001	9145001	2.49481	0.141479	OL	LOC101112928
NC_056064.	42685001	42705001	1.96141	0.206038	OL	LOC101113086
NC_056064.	42690001	42710001	2.0131	0.182921	OL	LOC101113086
NC_056068.	46725001	46745001	2.2851	0.230199	OL	LOC101113528;LOC1011
NC_056063.	32575001	32595001	3.0119	0.185365	OL	LOC101113604
NC_056063.	32580001	32600001	2.70833	0.154442	OL	LOC101113604;POLR1D
NC_056068.	46730001	46750001	1.98357	0.214022	OL	LOC101113790;LOC1011
NC_056064.	34735001	34755001	8.08004	0.170863	OL	LOC101113946
NC_056064.	34740001	34760001	8.70296	0.17725	OL	LOC101113946
NC_056064.	34745001	34765001	7.31307	0.176941	OL	LOC101113946
NC_056064.	34755001	34775001	11.1882	0.177292	OL	LOC101113946
NC_056064.	34760001	34780001	10.8961	0.177841	OL	LOC101113946
NC_056064.	34765001	34785001	11.1429	0.173643	OL	LOC101113946
NC_056064.	34770001	34790001	8.91667	0.159922	OL	LOC101113946
NC_056064.	34780001	34800001	8.52379	0.183479	OL	LOC101113946
NC_056064.	34685001	34705001	5.88803	0.232225	OL	LOC101113946;PLD6
NC_056077.	19945001	19965001	3.23006	0.169213	OL	LOC101114079
NC_056077.	19950001	19970001	8.13914	0.245665	OL	LOC101114079
NC_056077.	19955001	19975001	8.73139	0.255158	OL	LOC101114079
NC_056077.	19960001	19980001	8.60514	0.262451	OL	LOC101114079

NC_056074.1	16415001	16435001	2.66538
NC_056074.1	16420001	16440001	2.94805
NC_056074.1	16425001	16445001	3.18887
NC_056074.1	16430001	16450001	3.05073
NC_056074.1	16435001	16455001	2.7196
NC_056074.1	16440001	16460001	2.38325
NC_056074.1	16445001	16465001	2.1189
NC_056074.1	16450001	16470001	2.14593
NC_056074.1	16455001	16475001	2.18709
NC_056074.1	16460001	16480001	2.15575
NC_056072.1	49295001	49315001	3.47611
NC_056072.1	49300001	49320001	2.62169
NC_056072.1	49305001	49325001	2.18669
NC_056074.1	16545001	16565001	4.87922
NC_056074.1	16550001	16570001	4.3149
NC_056074.1	16555001	16575001	2.4492
NC_056074.1	16560001	16580001	2.06797
NC_056074.1	16575001	16595001	2.37271
NC_056074.1	16580001	16600001	2.49564
NC_056074.1	16585001	16605001	3.22404
NC_056074.1	16590001	16610001	3.33277
NC_056074.1	16595001	16615001	3.0352
NC_056074.1	16600001	16620001	3.02375
NC_056074.1	16605001	16625001	3.03286
NC_056074.1	16610001	16630001	3.46784
NC_056074.1	16615001	16635001	3.67143
NC_056074.1	16620001	16640001	3.4023
NC_056074.1	16625001	16645001	2.61765
NC_056074.1	16630001	16650001	2.27177
NC_056074.1	16675001	16695001	2.33863
NC_056074.1	16730001	16750001	2.62964

NC_056077.	19965001	19985001	5.1132	0.237294	OL	LOC101114079
NC_056077.	19970001	19990001	4.08779	0.207496	OL	LOC101114079
NC_056077.	19975001	19995001	2.50395	0.141597	OL	LOC101114079
NC_056077.	20015001	20035001	2.18009	0.142281	OL	LOC101114079
NC_056077.	20020001	20040001	3.40192	0.185204	OL	LOC101114079
NC_056077.	20025001	20045001	4.28105	0.241995	OL	LOC101114079
NC_056077.	20030001	20050001	2.91626	0.185493	OL	LOC101114079
NC_056077.	20035001	20055001	2.85286	0.174864	OL	LOC101114079
NC_056077.	20040001	20060001	2.93955	0.178672	OL	LOC101114079
NC_056077.	20045001	20065001	2.69175	0.173778	OL	LOC101114079
NC_056077.	20050001	20070001	2.14442	0.158087	OL	LOC101114079
NC_056071.	23745001	23765001	3.65694	0.151405	OL	LOC101114310
NC_056071.	23750001	23770001	3.14113	0.174903	OL	LOC101114310
NC_056071.	23755001	23775001	2.46069	0.163508	OL	LOC101114310
NC_056071.	23760001	23780001	2.28839	0.166734	OL	LOC101114310
NC_056071.	23765001	23785001	2.13532	0.168651	OL	LOC101114310
NC_056061.	51195001	51215001	2.14395	0.288218	OL	LOC101115037
NC_056061.	51200001	51220001	2.14879	0.291379	OL	LOC101115037
NC_056061.	51205001	51225001	2.02669	0.279855	OL	LOC101115037
NC_056059.	84730001	84750001	4.91204	0.158241	OL	LOC101115964
NC_056059.	84735001	84755001	5.65315	0.218992	OL	LOC101115964
NC_056059.	84740001	84760001	7.32009	0.265647	OL	LOC101115964
NC_056059.	84745001	84765001	5.05557	0.247989	OL	LOC101115964;LOC1011
NC_056059.	84750001	84770001	2.40173	0.185388	OL	LOC101115964;LOC1011
NC_056066.	34115001	34135001	2.36959	0.167384	OL	LOC101116334;SVIL
NC_056066.	34120001	34140001	1.93458	0.158166	OL	LOC101116334;SVIL
NC_056064.	44135001	44155001	1.96285	0.167748	OL	LOC101116922;MEIOC
NC_056080.	98300001	98320001	3.12386	0.167385	OL	LOC101116968;LOC1141
NC_056054.	27330001	27350001	2.5979	0.191952	OL	LOC101117028
NC_056066.	78170001	78190001	2.46552	0.176098	OL	LOC101117690
NC_056066.	78175001	78195001	3.56907	0.254151	OL	LOC101117690

NC_056074.1	16740001	16760001	4.24519
NC_056074.1	16745001	16765001	4.75704
NC_056074.1	16750001	16770001	3.67505
NC_056074.1	16755001	16775001	2.71791
NC_056074.1	16845001	16865001	3.58315
NC_056056.1	201820001	2.02E+08	2.38376
NC_056056.1	201825001	2.02E+08	2.87468
NC_056056.1	201830001	2.02E+08	3.79293
NC_056056.1	201835001	2.02E+08	2.70683
NC_056056.1	201840001	2.02E+08	2.07476
NC_056074.1	38020001	38040001	3.33653
NC_056074.1	38025001	38045001	4.18993
NC_056074.1	38030001	38050001	4.96849
NC_056074.1	38035001	38055001	5.22517
NC_056074.1	38040001	38060001	2.53015
NC_056074.1	38045001	38065001	2.11258
NC_056074.1	38070001	38090001	2.19071
NC_056074.1	38185001	38205001	2.28886
NC_056074.1	38190001	38210001	3.19439
NC_056074.1	38195001	38215001	4.67192
NC_056074.1	38200001	38220001	3.65163
NC_056074.1	38205001	38225001	2.65731
NC_056074.1	38210001	38230001	2.08498
NC_056055.1	210205001	2.1E+08	2.13167
NC_056065.1	4995001	5015001	1.98085
NC_056065.1	5015001	5035001	2.60885
NC_056054.1	50445001	50465001	2.34643
NC_056054.1	50460001	50480001	3.15898
NC_056054.1	50465001	50485001	3.80992
NC_056054.1	50470001	50490001	3.56305
NC_056054.1	50475001	50495001	3.05477

NC_056066.	78180001	78200001	3.9697	0.29971	OL	LOC101117690
NC_056066.	78185001	78205001	4.90144	0.318756	OL	LOC101117690
NC_056066.	78190001	78210001	3.48528	0.374586	OL	LOC101117690
NC_056066.	78195001	78215001	2.07248	0.322633	OL	LOC101117690
NC_056055.	234055001	2.34E+08	2.43842	0.152813	OL	LOC101117831
NC_056067.	48900001	48920001	2.65096	0.17777	OL	LOC101118470;PSMC4
NC_056055.	38190001	38210001	2.35984	0.210024	OL	LOC101118510
NC_056073.	7960001	7980001	2.10281	0.143106	OL	LOC101118647
NC_056073.	7965001	7985001	2.20336	0.1764	OL	LOC101118647
NC_056074.	38200001	38220001	1.95571	0.229223	OL	LOC101119087;LOC1011
NC_056054.	50420001	50440001	2.27599	0.143404	OL	LOC101120030
NC_056054.	50445001	50465001	2.46005	0.168835	OL	LOC101120030
NC_056054.	50450001	50470001	2.22903	0.145865	OL	LOC101120030
NC_056054.	50455001	50475001	2.39154	0.14496	OL	LOC101120030
NC_056054.	50460001	50480001	3.21077	0.195834	OL	LOC101120030
NC_056054.	50465001	50485001	3.8903	0.239598	OL	LOC101120030
NC_056054.	50470001	50490001	3.61737	0.238548	OL	LOC101120030
NC_056054.	50475001	50495001	3.10835	0.223166	OL	LOC101120030
NC_056054.	50480001	50500001	2.79908	0.215916	OL	LOC101120030
NC_056054.	50485001	50505001	2.36332	0.181684	OL	LOC101120030
NC_056054.	50490001	50510001	2.42339	0.15408	OL	LOC101120030
NC_056058.	77300001	77320001	2.31279	0.183618	OL	LOC101120408
NC_056058.	77305001	77325001	2.43862	0.217835	OL	LOC101120408
NC_056058.	77310001	77330001	2.58079	0.202824	OL	LOC101120408
NC_056058.	77315001	77335001	2.0602	0.151564	OL	LOC101120408
NC_056060.	25345001	25365001	2.0452	0.161975	OL	LOC101121354
NC_056059.	66135001	66155001	9.13514	0.15075	OL	LOC101121518
NC_056059.	66140001	66160001	8.53916	0.1552	OL	LOC101121518
NC_056059.	66145001	66165001	7.89093	0.152567	OL	LOC101121518
NC_056059.	66150001	66170001	8.94522	0.160276	OL	LOC101121518
NC_056059.	66155001	66175001	7.12715	0.149047	OL	LOC101121518

NC_056054.1	50480001	50500001	2.77376
NC_056054.1	50485001	50505001	2.36942
NC_056054.1	50490001	50510001	2.3802
NC_056058.1	255001	275001	3.07955
NC_056058.1	260001	280001	3.21289
NC_056058.1	265001	285001	3.28467
NC_056058.1	270001	290001	3.38359
NC_056058.1	280001	300001	3.08546
NC_056058.1	285001	305001	2.92604
NC_056080.1	103210001	1.03E+08	1.97282
NC_056067.1	61785001	61805001	2.90245
NC_056067.1	61795001	61815001	2.15144
NC_056067.1	61800001	61820001	2.22453
NC_056071.1	45655001	45675001	3.40796
NC_056071.1	45660001	45680001	2.98776
NC_056063.1	22885001	22905001	2.67639
NC_056063.1	22890001	22910001	4.29762
NC_056063.1	22895001	22915001	2.81554
NC_056060.1	25405001	25425001	2.8179
NC_056067.1	58440001	58460001	2.15665
NC_056067.1	58445001	58465001	2.57267
NC_056067.1	58450001	58470001	2.71148
NC_056070.1	71515001	71535001	2.24769
NC_056054.1	265240001	2.65E+08	3.50803
NC_056055.1	53170001	53190001	2.2549
NC_056055.1	53175001	53195001	2.76908
NC_056055.1	53180001	53200001	2.76048
NC_056055.1	53185001	53205001	2.11619
NC_056060.1	21395001	21415001	2.0625
NC_056056.1	210990001	2.11E+08	2.31369
NC_056056.1	207400001	2.07E+08	3.74645

NC_056060.	25350001	25370001	1.91977	0.16756	OL	LOC101121608
NC_056059.	84845001	84865001	2.32154	0.154501	OL	LOC101121777
NC_056056.	132975001	1.33E+08	1.81073	0.235624	OL	LOC101123619
NC_056056.	132980001	1.33E+08	1.95677	0.294653	OL	LOC101123619
NC_056056.	132995001	1.33E+08	1.90865	0.209104	OL	LOC101123619;MAP3K1
NC_056056.	132985001	1.33E+08	1.83854	0.299671	OL	LOC101123619;NPFF
NC_056056.	132990001	1.33E+08	1.80925	0.289456	OL	LOC101123619;NPFF;TA
NC_056054.	218455001	2.18E+08	3.11413	0.240493	OL	LOC105605201;MECOM
NC_056056.	179880001	1.8E+08	5.12451	0.203332	OL	LOC105605766
NC_056056.	179885001	1.8E+08	5.599	0.142014	OL	LOC105605766
NC_056056.	179865001	1.8E+08	4.40755	0.252113	OL	LOC105605766;LOC1141
NC_056056.	179870001	1.8E+08	4.7939	0.261543	OL	LOC105605766;LOC1141
NC_056056.	179875001	1.8E+08	3.94172	0.238091	OL	LOC105605766;LOC1141
NC_056056.	179985001	1.8E+08	2.08216	0.212897	OL	LOC105605770
NC_056055.	53180001	53200001	2.72244	0.270741	OL	LOC105607745;UNC13B
NC_056055.	53185001	53205001	2.36887	0.239591	OL	LOC105607745;UNC13B
NC_056058.	8255001	8275001	2.07669	0.20382	OL	LOC105614131
NC_056058.	8260001	8280001	1.83928	0.203824	OL	LOC105614131
NC_056058.	7395001	7415001	1.87909	0.192492	OL	LOC106990140
NC_056058.	7400001	7420001	1.86792	0.186067	OL	LOC106990140
NC_056061.	74840001	74860001	2.0506	0.22881	OL	LOC106991302
NC_056073.	30485001	30505001	2.02746	0.178423	OL	LOC114109685
NC_056080.	99825001	99845001	1.8125	0.141775	OL	LOC114111389;ZNF75D
NC_056080.	79170001	79190001	2.45422	0.164	OL	LOC114111421
NC_056055.	89745001	89765001	2.65323	0.176274	OL	LOC114112824
NC_056055.	89750001	89770001	4.98802	0.218902	OL	LOC114112824
NC_056055.	89755001	89775001	3.38553	0.208312	OL	LOC114112824
NC_056055.	89760001	89780001	3.31517	0.251634	OL	LOC114113016
NC_056055.	89765001	89785001	2.84837	0.259245	OL	LOC114113016
NC_056055.	89770001	89790001	2.40746	0.253686	OL	LOC114113016
NC_056055.	89775001	89795001	2.15481	0.241298	OL	LOC114113016

NC_056056.1	207405001	2.07E+08	3.97557
NC_056056.1	207410001	2.07E+08	2.70103
NC_056056.1	13230001	13250001	2.42216
NC_056056.1	13235001	13255001	2.12986
NC_056064.1	12450001	12470001	3.61594
NC_056064.1	12455001	12475001	2.57573
NC_056080.1	25205001	25225001	5.30769
NC_056054.1	101080001	1.01E+08	2.07164
NC_056058.1	8440001	8460001	2.02356
NC_056059.1	70645001	70665001	1.98865
NC_056059.1	70650001	70670001	2.72605
NC_056060.1	43400001	43420001	2.02063
NC_056067.1	63700001	63720001	2.10835
NC_056068.1	47805001	47825001	12.4791
NC_056068.1	47810001	47830001	5.03207
NC_056063.1	25200001	25220001	2.98809
NC_056065.1	76895001	76915001	1.9966
NC_056065.1	76900001	76920001	2.1303
NC_056066.1	77710001	77730001	15.3762
NC_056066.1	77715001	77735001	17.4867
NC_056067.1	61790001	61810001	2.50148
NC_056071.1	4265001	4285001	2.42241
NC_056071.1	4270001	4290001	2.39339
NW_02459981.	35001	55001	2.01141
NW_02459981.	40001	60001	2.03529
NW_02459981.	70001	90001	1.98039
NC_056056.1	10620001	10640001	3.05041
NC_056054.1	37415001	37435001	3.69603
NC_056057.1	83390001	83410001	2.07329
NC_056059.1	57305001	57325001	3.13903
NC_056059.1	57310001	57330001	3.99485

NC_056055.	61425001	61445001	2.55844	0.251173	OL	LOC114113073	NC_056060.1	101130001	1.01E+08	2.77375
NC_056055.	61430001	61450001	2.66402	0.23552	OL	LOC114113073	NC_056055.1	12495001	12515001	2.26735
NC_056055.	61435001	61455001	2.0811	0.163188	OL	LOC114113073	NC_056055.1	12500001	12520001	2.02975
NC_056054.	125835001	1.26E+08	3.08135	0.176148	OL	LOC114113348	NC_056054.1	90475001	90495001	6.32737
NC_056054.	125840001	1.26E+08	3.31377	0.177252	OL	LOC114113348	NC_056054.1	90485001	90505001	15.2276
NC_056054.	125845001	1.26E+08	2.32916	0.144317	OL	LOC114113348	NC_056054.1	90490001	90510001	11.9451
NC_056056.	104465001	1.04E+08	2.67725	0.241099	OL	LOC114113736;LOC1218	NC_056054.1	90495001	90515001	2.68196
NC_056056.	104460001	1.04E+08	2.53571	0.263075	OL	LOC114113736;LOC1218	NC_056078.1	30680001	30700001	2.5922
NC_056056.	104450001	1.04E+08	2.99554	0.207687	OL	LOC114113736;LOC1218	NC_056078.1	30685001	30705001	3.21198
NC_056056.	104455001	1.04E+08	2.86	0.24887	OL	LOC114113736;LOC1218	NC_056078.1	30690001	30710001	2.45471
NC_056056.	104445001	1.04E+08	3.05042	0.193952	OL	LOC114113736;LOC1218	NC_056078.1	30700001	30720001	2.46252
NC_056056.	179840001	1.8E+08	1.95874	0.173062	OL	LOC114113800	NC_056078.1	30705001	30725001	3.0468
NC_056056.	179850001	1.8E+08	2.15189	0.200601	OL	LOC114113800	NC_056078.1	30710001	30730001	2.41841
NC_056056.	179855001	1.8E+08	6.01829	0.261029	OL	LOC114113800	NC_056078.1	30715001	30735001	2.29229
NC_056056.	179860001	1.8E+08	4.82505	0.243044	OL	LOC114113800	NC_056078.1	30720001	30740001	2.00445
NC_056056.	11355001	11375001	2.00302	0.318893	OL	LOC114113879;NR5A1	NC_056078.1	31155001	31175001	2.07371
NC_056056.	11360001	11380001	2.36154	0.213031	OL	LOC114113879;NR5A1	NC_056078.1	31305001	31325001	3.69392
NC_056059.	84335001	84355001	2.49116	0.172576	OL	LOC114115323	NC_056078.1	31310001	31330001	3.18433
NC_056059.	84340001	84360001	3.66056	0.211234	OL	LOC114115323	NC_056055.1	169310001	1.69E+08	2.63304
NC_056059.	84345001	84365001	2.46991	0.171965	OL	LOC114115323	NC_056055.1	169315001	1.69E+08	4.99367
NC_056068.	48970001	48990001	1.91151	0.224112	OL	LOC114118273	NC_056055.1	169320001	1.69E+08	3.5
NC_056056.	104470001	1.04E+08	3.33333	0.187517	OL	LOC121815995	NC_056055.1	169325001	1.69E+08	2.0555
NC_056067.	970001	990001	2.93526	0.160533	OL	LOC121816415;MTSS2;V	NC_056055.1	169500001	1.7E+08	3.00941
NC_056067.	980001	1000001	2.39163	0.144027	OL	LOC121816415;MTSS2;V	NC_056071.1	6745001	6765001	3.85135
NC_056067.	985001	1005001	2.58454	0.14708	OL	LOC121816415;MTSS2;V	NC_056054.1	45895001	45915001	4.46651
NC_056067.	60965001	60985001	1.88953	0.230134	OL	LOC121816443	NC_056054.1	45900001	45920001	4.89393
NC_056067.	60970001	60990001	1.91278	0.241254	OL	LOC121816444	NC_056054.1	45905001	45925001	3.74678
NC_056074.	26565001	26585001	2.43809	0.186856	OL	LOC121817574	NC_056077.1	19190001	19210001	2.19976
NC_056077.	27330001	27350001	2.103	0.164891	OL	LOC121817897	NC_056077.1	19195001	19215001	2.69713
NW_024599[illegible]	10001	30001	1.98596	0.155733	OL	LOC121818469	NC_056072.1	53485001	53505001	1.9857
NW_024599[illegible]	15001	35001	2.72958	0.207231	OL	LOC121818469	NC_056072.1	35580001	35600001	4.85382

NW_024599!	40001	60001	2.21795	0.213054	OL	LOC121818481
NW_024599!	70001	90001	1.94231	0.177688	OL	LOC121818481
NC_056057.	83390001	83410001	1.85915	0.189588	OL	LOC121819513
NC_056054.	71870001	71890001	4.9008	0.158242	OL	LOC121819811
NC_056054.	71875001	71895001	4.71336	0.145565	OL	LOC121819811
NC_056059.	57305001	57325001	2.29841	0.150706	OL	LOC121819825
NC_056059.	57310001	57330001	2.33708	0.151735	OL	LOC121819825
NC_056070.	7580001	7600001	2.01717	0.146785	OL	LRBA
NC_056078.	31140001	31160001	2.18497	0.235237	OL	LRMDA
NC_056078.	31145001	31165001	2.03535	0.157074	OL	LRMDA
NC_056078.	31150001	31170001	2.80971	0.176238	OL	LRMDA
NC_056056.	162435001	1.62E+08	3.32131	0.157251	OL	LRP1
NC_056055.	169365001	1.69E+08	2.66633	0.156944	OL	LRP1B
NC_056055.	169370001	1.69E+08	2.30781	0.144174	OL	LRP1B
NC_056055.	170010001	1.7E+08	3.33085	0.167326	OL	LRP1B
NC_056055.	170015001	1.7E+08	3.58571	0.144503	OL	LRP1B
NC_056055.	170160001	1.7E+08	2.86439	0.217118	OL	LRP1B
NC_056055.	170165001	1.7E+08	6.01768	0.318023	OL	LRP1B
NC_056055.	170170001	1.7E+08	11.7105	0.363865	OL	LRP1B
NC_056055.	170175001	1.7E+08	4.62979	0.317727	OL	LRP1B
NC_056055.	170180001	1.7E+08	2.50499	0.253822	OL	LRP1B
NC_056068.	70185001	70205001	1.88157	0.263189	OL	LRRC4C
NC_056068.	70190001	70210001	2.17284	0.281845	OL	LRRC4C
NC_056068.	70195001	70215001	2.1717	0.288062	OL	LRRC4C
NC_056068.	70200001	70220001	2.16315	0.28928	OL	LRRC4C
NC_056068.	70205001	70225001	2.13225	0.282689	OL	LRRC4C
NC_056068.	70210001	70230001	2.11023	0.287917	OL	LRRC4C
NC_056068.	70215001	70235001	2.06698	0.277085	OL	LRRC4C
NC_056068.	70220001	70240001	2.06007	0.271035	OL	LRRC4C
NC_056068.	70225001	70245001	2.07005	0.27402	OL	LRRC4C
NC_056068.	70230001	70250001	2.07052	0.271622	OL	LRRC4C

NC_056072.1	35585001	35605001	5.46212
NC_056072.1	35590001	35610001	2.22735
NC_056080.1	68900001	68920001	2.12937
NC_056080.1	68905001	68925001	2.07122
NC_056066.1	20235001	20255001	2.24859
NC_056070.1	17770001	17790001	2.01225
NC_056069.1	9610001	9630001	2.12532
NC_056054.1	202555001	2.03E+08	4.85331
NC_056054.1	202560001	2.03E+08	14.8078
NC_056054.1	202565001	2.03E+08	10.0548
NC_056054.1	202570001	2.03E+08	5.26359
NC_056054.1	202575001	2.03E+08	2.28881
NC_056056.1	225540001	2.26E+08	3.98149
NC_056056.1	225545001	2.26E+08	3.80791
NC_056056.1	10605001	10625001	2.6123
NC_056056.1	10610001	10630001	3.16302
NC_056056.1	10615001	10635001	3.11684
NC_056054.1	200795001	2.01E+08	6.12102
NC_056058.1	11010001	11030001	4.11174
NC_056058.1	11005001	11025001	2.86941
NC_056069.1	13000001	13020001	1.96919
NC_056055.1	161035001	1.61E+08	2.41821
NC_056055.1	174965001	1.75E+08	3.6
NC_056055.1	174970001	1.75E+08	3.90037
NC_056055.1	174975001	1.75E+08	4.79574
NC_056055.1	174980001	1.75E+08	5.69961
NC_056055.1	174985001	1.75E+08	2.63386
NC_056061.1	19415001	19435001	8.76595
NC_056061.1	19440001	19460001	26.6421
NC_056061.1	19445001	19465001	33.5157
NC_056061.1	19450001	19470001	16.4307

NC_056068.	70235001	70255001	2.19027	0.27378	OL	LRRC4C
NC_056068.	70240001	70260001	3.11741	0.242856	OL	LRRC4C
NC_056068.	70245001	70265001	3.92283	0.193177	OL	LRRC4C
NC_056068.	70250001	70270001	8.58374	0.166099	OL	LRRC4C
NC_056068.	70255001	70275001	14.0985	0.153894	OL	LRRC4C
NC_056056.	121445001	1.21E+08	2.18874	0.396416	OL	LRRIQ1
NC_056056.	121450001	1.21E+08	3.5905	0.472454	OL	LRRIQ1
NC_056056.	121455001	1.21E+08	4.69509	0.489796	OL	LRRIQ1
NC_056056.	121460001	1.21E+08	4.05157	0.383834	OL	LRRIQ1
NC_056072.	22465001	22485001	2.05556	0.170184	OL	LRRN1
NC_056072.	22470001	22490001	2.47577	0.201277	OL	LRRN1
NC_056072.	22475001	22495001	2.05797	0.17687	OL	LRRN1
NC_056072.	22480001	22500001	1.98639	0.174468	OL	LRRN1
NC_056062.	36135001	36155001	1.94376	0.291386	OL	LYN
NC_056062.	36210001	36230001	1.96596	0.312816	OL	LYN
NC_056062.	36215001	36235001	1.9327	0.30704	OL	LYN
NC_056062.	36220001	36240001	2.06946	0.308992	OL	LYN
NC_056062.	36225001	36245001	2.0449	0.318468	OL	LYN
NC_056062.	36230001	36250001	2.03393	0.316568	OL	LYN
NC_056062.	36235001	36255001	1.97082	0.318593	OL	LYN
NC_056058.	21000001	21020001	4.77239	0.247861	OL	LYRM7
NC_056054.	13965001	13985001	2.01138	0.220735	OL	MACF1
NC_056054.	13970001	13990001	2.09404	0.2712	OL	MACF1
NC_056054.	13975001	13995001	1.81061	0.230248	OL	MACF1
NC_056054.	13980001	14000001	1.87943	0.176045	OL	MACF1
NC_056066.	7775001	7795001	2.04376	0.188632	OL	MACROD2
NC_056066.	9040001	9060001	1.83078	0.161711	OL	MACROD2
NC_056066.	9100001	9120001	14.4265	0.153902	OL	MACROD2
NC_056066.	9105001	9125001	7.87981	0.144528	OL	MACROD2
NC_056068.	76095001	76115001	13.2138	0.185206	OL	MADD;MYBPC3
NC_056072.	35880001	35900001	1.99708	0.142142	OL	MAGI1

NC_056054.1	62025001	62045001	2.80976
NC_056054.1	62030001	62050001	2.75665
NC_056058.1	91690001	91710001	5.39575
NC_056058.1	91695001	91715001	5.75
NC_056058.1	91700001	91720001	7.4407
NC_056058.1	91705001	91725001	8.07801
NC_056078.1	27945001	27965001	6.88607
NC_056078.1	27950001	27970001	3.36684
NC_056078.1	27995001	28015001	2.44855
NC_056078.1	28000001	28020001	3.11763
NC_056078.1	28005001	28025001	2.00413
NC_056057.1	55035001	55055001	2.39788
NC_056057.1	55040001	55060001	2.52575
NC_056057.1	55045001	55065001	1.9735
NC_056057.1	55050001	55070001	2.29016
NC_056057.1	55055001	55075001	2.53616
NC_056057.1	55060001	55080001	3.02985
NC_056061.1	10385001	10405001	2.98326
NC_056061.1	10390001	10410001	4.31268
NC_056061.1	10395001	10415001	3.50113
NC_056061.1	10400001	10420001	2.5578
NC_056061.1	10405001	10425001	2.02869
NC_056061.1	10410001	10430001	2.04209
NC_056061.1	10445001	10465001	2.03521
NC_056061.1	10455001	10475001	2.28962
NC_056061.1	10460001	10480001	2.64547
NC_056061.1	10465001	10485001	2.32539
NC_056061.1	10470001	10490001	2.5122
NC_056058.1	20400001	20420001	2.30816
NC_056058.1	20405001	20425001	2.29373
NC_056058.1	20410001	20430001	2.28028

NC_056072.	35885001	35905001	2.57274	0.188225	OL	MAGI1
NC_056054.	94900001	94920001	1.97404	0.17345	OL	MAN1A2
NC_056054.	94905001	94925001	3.3563	0.193221	OL	MAN1A2
NC_056054.	94910001	94930001	4.20634	0.244737	OL	MAN1A2
NC_056054.	94915001	94935001	4.96475	0.337731	OL	MAN1A2
NC_056054.	94920001	94940001	5.33047	0.404029	OL	MAN1A2
NC_056054.	94925001	94945001	4.00274	0.413649	OL	MAN1A2
NC_056054.	94930001	94950001	3.76583	0.400716	OL	MAN1A2
NC_056054.	94935001	94955001	3.12321	0.37968	OL	MAN1A2
NC_056054.	94940001	94960001	2.05	0.33468	OL	MAN1A2
NC_056054.	94945001	94965001	2.36085	0.223732	OL	MAN1A2
NC_056054.	94950001	94970001	2.92045	0.272062	OL	MAN1A2
NC_056054.	94955001	94975001	2.73443	0.28208	OL	MAN1A2
NC_056054.	94960001	94980001	2.09542	0.256687	OL	MAN1A2
NC_056055.	241180001	2.41E+08	1.99864	0.17778	OL	MAN1C1
NC_056060.	41530001	41550001	2.27888	0.176542	OL	MAP4K5
NC_056060.	41535001	41555001	1.96627	0.17418	OL	MAP4K5
NC_056061.	37195001	37215001	2.47668	0.217756	OL	MCHR2
NC_056061.	37200001	37220001	2.43555	0.222819	OL	MCHR2
NC_056061.	37205001	37225001	2.49169	0.212048	OL	MCHR2
NC_056061.	37210001	37230001	2.10798	0.175191	OL	MCHR2
NC_056079.	5725001	5745001	1.98965	0.163458	OL	MCPH1
NC_056079.	5760001	5780001	1.95976	0.141434	OL	MCPH1
NC_056079.	5765001	5785001	1.82451	0.161991	OL	MCPH1
NC_056059.	16145001	16165001	2.54484	0.150123	OL	MCUB
NC_056059.	16150001	16170001	2.95455	0.190551	OL	MCUB
NC_056059.	16155001	16175001	2.71505	0.226615	OL	MCUB
NC_056054.	218460001	2.18E+08	4.50888	0.256825	OL	MECOM
NC_056054.	218465001	2.18E+08	3.67603	0.228009	OL	MECOM
NC_056054.	218470001	2.18E+08	2.70532	0.201772	OL	MECOM
NC_056067.	48450001	48470001	1.95455	0.25995	OL	MED29;PLEKHG2;ZFP36

NC_056058.1	20415001	20435001	2.26649
NC_056058.1	20420001	20440001	2.2612
NC_056058.1	20425001	20445001	2.2658
NC_056058.1	20430001	20450001	2.24368
NC_056058.1	20435001	20455001	2.21111
NC_056058.1	20440001	20460001	2.17798
NC_056058.1	20445001	20465001	2.16185
NC_056058.1	20450001	20470001	2.17
NC_056058.1	20455001	20475001	2.11937
NC_056058.1	20460001	20480001	2.14345
NC_056056.1	216675001	2.17E+08	2.16362
NC_056056.1	102590001	1.03E+08	2.74028
NC_056056.1	102595001	1.03E+08	2.44444
NC_056056.1	102600001	1.03E+08	2.27744
NC_056056.1	102605001	1.03E+08	1.95703
NC_056056.1	102615001	1.03E+08	2.29503
NC_056055.1	176490001	1.77E+08	2.12873
NC_056055.1	176495001	1.77E+08	2.03965
NC_056055.1	176500001	1.77E+08	2.01416
NC_056077.1	4335001	4355001	3.90997
NC_056077.1	4340001	4360001	5.09402
NC_056077.1	4345001	4365001	11
NC_056080.1	8605001	8625001	2.02816
NC_056080.1	8610001	8630001	2.01368
NC_056056.1	7395001	7415001	2.77462
NC_056056.1	7400001	7420001	2.80277
NC_056056.1	7405001	7425001	2.52164
NC_056056.1	7410001	7430001	2.0255
NC_056071.1	45145001	45165001	2.96557
NC_056071.1	45150001	45170001	2.12713
NC_056066.1	64480001	64500001	2.24522

NC_056060.	80300001	80320001	2.90224	0.185781	OL	MED6
NC_056056.	218590001	2.19E+08	5.00953	0.144231	OL	MEI1
NC_056058.	20395001	20415001	3.45517	0.163704	OL	MEIKIN
NC_056058.	20400001	20420001	3.16806	0.235764	OL	MEIKIN
NC_056058.	20405001	20425001	2.61094	0.209161	OL	MEIKIN
NC_056058.	20410001	20430001	2.37539	0.194859	OL	MEIKIN
NC_056058.	20415001	20435001	2.3915	0.192071	OL	MEIKIN
NC_056058.	20420001	20440001	1.97047	0.162494	OL	MEIKIN
NC_056058.	20425001	20445001	1.99093	0.167794	OL	MEIKIN
NC_056058.	20430001	20450001	2.07166	0.177761	OL	MEIKIN
NC_056058.	20440001	20460001	2.32745	0.193931	OL	MEIKIN
NC_056058.	20445001	20465001	2.48823	0.202899	OL	MEIKIN
NC_056058.	20450001	20470001	2.32085	0.189541	OL	MEIKIN
NC_056058.	20455001	20475001	2.39824	0.194643	OL	MEIKIN
NC_056058.	20460001	20480001	2.31166	0.188202	OL	MEIKIN
NC_056064.	44140001	44160001	2.43088	0.180998	OL	MEIOC
NC_056064.	44145001	44165001	2.75982	0.173603	OL	MEIOC
NC_056058.	41600001	41620001	1.98539	0.22204	OL	MEX3D;PLK5
NC_056056.	181495001	1.82E+08	4.03089	0.151201	OL	MFNG
NC_056056.	122555001	1.23E+08	1.87873	0.26928	OL	MGAT4C
NC_056056.	122560001	1.23E+08	2.01938	0.251074	OL	MGAT4C
NC_056056.	122565001	1.23E+08	2.22362	0.207437	OL	MGAT4C
NC_056056.	122570001	1.23E+08	2.22018	0.189204	OL	MGAT4C
NC_056056.	122575001	1.23E+08	2.04311	0.161755	OL	MGAT4C
NC_056056.	122695001	1.23E+08	1.91522	0.177847	OL	MGAT4C
NC_056056.	122700001	1.23E+08	2.24589	0.149116	OL	MGAT4C
NC_056070.	18110001	18130001	1.85237	0.178102	OL	MGST2
NC_056068.	40165001	40185001	1.97046	0.142435	OL	MICAL2
NC_056057.	66990001	67010001	1.88229	0.148971	OL	MINDY4
NC_056054.	21170001	21190001	1.8076	0.250817	OL	MKNK1;MOB3C
NC_056066.	36225001	36245001	1.99608	0.15236	OL	MKX

NC_056066.1	64485001	64505001	2.79195
NC_056066.1	64490001	64510001	3.51915
NC_056066.1	64495001	64515001	2.4907
NC_056071.1	22920001	22940001	2.28314
NC_056071.1	22925001	22945001	2.33258
NC_056071.1	22930001	22950001	2.24816
NC_056071.1	22935001	22955001	2.2338
NC_056071.1	22940001	22960001	2.27852
NC_056071.1	22945001	22965001	2.15455
NC_056071.1	31455001	31475001	2.0123
NC_056056.1	220145001	2.2E+08	1.975
NC_056068.1	28675001	28695001	3.16716
NC_056080.1	46670001	46690001	2.54412
NC_056080.1	46675001	46695001	3.01551
NC_056080.1	46745001	46765001	2.65482
NC_056080.1	46750001	46770001	3
NC_056080.1	46755001	46775001	3.55039
NC_056080.1	46795001	46815001	1.98667
NC_056080.1	46805001	46825001	1.98773
NC_056080.1	46810001	46830001	2.00649
NC_056080.1	46815001	46835001	2.03623
NC_056080.1	46835001	46855001	3.52702
NC_056080.1	46840001	46860001	3.05202
NC_056065.1	41765001	41785001	2.01333
NC_056079.1	18600001	18620001	2.05324
NC_056079.1	18605001	18625001	2.28397
NC_056063.1	31535001	31555001	2.20053
NC_056063.1	31540001	31560001	2.3988
NC_056063.1	31545001	31565001	2.39636
NC_056055.1	133845001	1.34E+08	2.27728
NC_056054.1	179325001	1.79E+08	2.21551

NC_056054.	233325001	2.33E+08	1.80901	0.1426	OL	MME
NC_056073.	21885001	21905001	3.16666	0.159819	OL	MMUT
NC_056073.	21890001	21910001	2.30786	0.143436	OL	MMUT
NC_056074.	46425001	46445001	2.27684	0.195244	OL	MOB2
NC_056070.	69720001	69740001	2.73064	0.141624	OL	MORC2
NC_056070.	69725001	69745001	2.13768	0.167684	OL	MORC2
NC_056071.	31455001	31475001	2.0123	0.212235	OL	MPI
NC_056071.	31460001	31480001	1.93494	0.196302	OL	MPI;SCAMP2
NC_056056.	104520001	1.05E+08	2.31372	0.314412	OL	MRPS5
NC_056056.	104515001	1.05E+08	3.00091	0.340465	OL	MRPS5;ZNF514
NC_056056.	13585001	13605001	2.27529	0.195624	OL	MRRF
NC_056056.	13590001	13610001	1.90488	0.206407	OL	MRRF
NC_056056.	217725001	2.18E+08	6.83333	0.200626	OL	MRTFA
NC_056056.	154595001	1.55E+08	6.38395	0.182387	OL	MSRB3
NC_056056.	154600001	1.55E+08	11.6484	0.242087	OL	MSRB3
NC_056056.	154635001	1.55E+08	6.8027	0.309524	OL	MSRB3
NC_056056.	154640001	1.55E+08	3.09606	0.251177	OL	MSRB3
NC_056056.	154645001	1.55E+08	2.5122	0.233169	OL	MSRB3
NC_056056.	154675001	1.55E+08	1.89706	0.159671	OL	MSRB3
NC_056056.	154680001	1.55E+08	2.79364	0.22922	OL	MSRB3
NC_056080.	84895001	84915001	2.36429	0.15749	OL	MTM1
NC_056080.	84900001	84920001	2.43168	0.159089	OL	MTM1
NC_056080.	84905001	84925001	2.46591	0.159482	OL	MTM1
NC_056079.	18730001	18750001	3.73522	0.174931	OL	MTUS1
NC_056079.	18735001	18755001	4.10789	0.219994	OL	MTUS1
NC_056079.	18740001	18760001	4.14732	0.233067	OL	MTUS1
NC_056079.	18745001	18765001	3.89892	0.228599	OL	MTUS1
NC_056079.	18750001	18770001	4.41089	0.153533	OL	MTUS1
NC_056079.	18755001	18775001	3.37066	0.165029	OL	MTUS1
NC_056079.	18760001	18780001	2.79145	0.242012	OL	MTUS1
NC_056079.	18765001	18785001	2.42421	0.179914	OL	MTUS1

NC_056054.1	179330001	1.79E+08	2.51063
NC_056054.1	212440001	2.12E+08	3.08236
NC_056054.1	212445001	2.12E+08	2.58701
NC_056054.1	212490001	2.13E+08	2.34065
NC_056054.1	213165001	2.13E+08	2.91227
NC_056054.1	213555001	2.14E+08	2.23331
NC_056054.1	213560001	2.14E+08	1.987
NC_056064.1	49780001	49800001	4.51163
NC_056064.1	49785001	49805001	4.24149
NC_056056.1	23675001	23695001	4.89735
NC_056056.1	23680001	23700001	6.15807
NC_056056.1	23685001	23705001	5.10714
NC_056056.1	23790001	23810001	2.54541
NC_056056.1	23795001	23815001	3.20818
NC_056056.1	23800001	23820001	4.51948
NC_056056.1	23805001	23825001	4.29634
NC_056056.1	23810001	23830001	3.25361
NC_056056.1	23815001	23835001	2.65018
NC_056054.1	136050001	1.36E+08	1.95781
NC_056054.1	136135001	1.36E+08	2.72304
NC_056054.1	136140001	1.36E+08	4.42266
NC_056054.1	136145001	1.36E+08	3.36682
NC_056054.1	136150001	1.36E+08	2.50863
NC_056054.1	136155001	1.36E+08	2.37834
NC_056056.1	60745001	60765001	2.87499
NC_056063.1	55175001	55195001	2.04824
NC_056055.1	208255001	2.08E+08	15.2706
NC_056076.1	57745001	57765001	2.30949
NC_056076.1	57750001	57770001	2.27307
NC_056073.1	44180001	44200001	2.02699
NC_056063.1	21755001	21775001	2.25204

NC_056079.	18770001	18790001	2.25838	0.183849	OL	MTUS1
NC_056079.	18775001	18795001	1.96282	0.141405	OL	MTUS1
NC_056063.	31380001	31400001	2.30427	0.191574	OL	MTUS2
NC_056063.	31385001	31405001	2.87126	0.212985	OL	MTUS2
NC_056063.	31390001	31410001	3.58005	0.238629	OL	MTUS2
NC_056063.	31395001	31415001	2.90137	0.198925	OL	MTUS2
NC_056063.	31400001	31420001	1.97933	0.156413	OL	MTUS2
NC_056067.	62245001	62265001	2.53898	0.158554	OL	MYADM
NC_056077.	35605001	35625001	2.12658	0.193508	OL	MYL10
NC_056077.	35610001	35630001	2.27106	0.243679	OL	MYL10
NC_056077.	35615001	35635001	2.29427	0.240666	OL	MYL10
NC_056077.	35620001	35640001	2.30088	0.239316	OL	MYL10
NC_056077.	35625001	35645001	2.32143	0.231481	OL	MYL10
NC_056054.	179325001	1.79E+08	2.00389	0.176716	OL	NAA50;USF3
NC_056054.	179330001	1.79E+08	2.11659	0.18541	OL	NAA50;USF3
NC_056054.	213235001	2.13E+08	1.91087	0.167405	OL	NAALADL2
NC_056054.	213240001	2.13E+08	2.26478	0.155483	OL	NAALADL2
NC_056054.	213245001	2.13E+08	2.47478	0.150783	OL	NAALADL2
NC_056064.	49775001	49795001	1.84475	0.193188	OL	NARF
NC_056064.	49780001	49800001	3.30406	0.202145	OL	NARF
NC_056064.	49785001	49805001	3.35777	0.204275	OL	NARF
NC_056056.	23790001	23810001	4.22026	0.24117	OL	NBAS
NC_056056.	23795001	23815001	5.31695	0.279362	OL	NBAS
NC_056056.	23800001	23820001	5.95718	0.293625	OL	NBAS
NC_056056.	23805001	23825001	3.85649	0.263568	OL	NBAS
NC_056054.	136135001	1.36E+08	2.54624	0.15908	OL	NCAM2
NC_056054.	136140001	1.36E+08	2.41698	0.153406	OL	NCAM2
NC_056054.	136145001	1.36E+08	2.37215	0.158832	OL	NCAM2
NC_056054.	136390001	1.36E+08	1.87687	0.313643	OL	NCAM2
NC_056054.	136460001	1.36E+08	1.86945	0.292959	OL	NCAM2
NC_056054.	136475001	1.36E+08	2.1111	0.325391	OL	NCAM2

NC_056065.1	76545001	76565001	6.75262
NC_056065.1	76550001	76570001	8.15042
NC_056065.1	76555001	76575001	11.8517
NC_056065.1	76560001	76580001	9.87025
NC_056060.1	84400001	84420001	3.13126
NC_056060.1	84405001	84425001	2.47116
NC_056074.1	21455001	21475001	2.3
NC_056056.1	141875001	1.42E+08	2.1415
NC_056056.1	141880001	1.42E+08	3.13146
NC_056076.1	925001	945001	2.7151
NC_056076.1	930001	950001	2.38675
NC_056076.1	935001	955001	2.14583
NC_056076.1	940001	960001	2.08447
NC_056067.1	35075001	35095001	3.08498
NC_056067.1	35080001	35100001	2.67248
NC_056067.1	35085001	35105001	2.38379
NC_056067.1	35095001	35115001	2.1801
NC_056067.1	35100001	35120001	2.23555
NC_056067.1	35105001	35125001	2.27123
NC_056067.1	35110001	35130001	2.29702
NC_056060.1	86625001	86645001	1.98635
NC_056060.1	86620001	86640001	2.34713
NC_056061.1	13795001	13815001	2.01422
NC_056061.1	13800001	13820001	2.07781
NC_056061.1	14115001	14135001	2.33364
NC_056061.1	14120001	14140001	3.52269
NC_056061.1	14125001	14145001	2.73371
NC_056061.1	14155001	14175001	3.27966
NC_056061.1	14160001	14180001	3.12394
NC_056061.1	14165001	14185001	3.02515
NC_056061.1	14170001	14190001	2.96458

NC_056054.	136480001	1.37E+08	2.35948	0.358372	OL	NCAM2
NC_056054.	136485001	1.37E+08	2.24472	0.348865	OL	NCAM2
NC_056054.	136490001	1.37E+08	2.24232	0.349626	OL	NCAM2
NC_056054.	136495001	1.37E+08	2.19696	0.335627	OL	NCAM2
NC_056054.	136500001	1.37E+08	2.10753	0.323814	OL	NCAM2
NC_056054.	136505001	1.37E+08	2.11362	0.328375	OL	NCAM2
NC_056054.	136510001	1.37E+08	2	0.322044	OL	NCAM2
NC_056054.	136630001	1.37E+08	2.0411	0.170423	OL	NCAM2
NC_056054.	136635001	1.37E+08	1.81818	0.143607	OL	NCAM2
NC_056054.	215530001	2.16E+08	3.23338	0.155044	OL	NCEH1
NC_056054.	215535001	2.16E+08	4.17985	0.17543	OL	NCEH1
NC_056054.	215540001	2.16E+08	4.48894	0.201239	OL	NCEH1
NC_056054.	215545001	2.16E+08	4.70853	0.2422	OL	NCEH1
NC_056054.	215550001	2.16E+08	3.63875	0.291735	OL	NCEH1
NC_056054.	215555001	2.16E+08	3.09677	0.247798	OL	NCEH1
NC_056054.	215560001	2.16E+08	3.29892	0.229548	OL	NCEH1
NC_056054.	215565001	2.16E+08	2.54316	0.149119	OL	NCEH1
NC_056055.	177540001	1.78E+08	1.86666	0.199163	OL	NCKAP5
NC_056055.	177605001	1.78E+08	2.02284	0.307633	OL	NCKAP5
NC_056056.	32215001	32235001	2.96861	0.238721	OL	NCOA1
NC_056056.	32220001	32240001	5.5396	0.266627	OL	NCOA1
NC_056056.	32225001	32245001	8.07693	0.291612	OL	NCOA1
NC_056056.	32230001	32250001	6.48649	0.286926	OL	NCOA1
NC_056056.	32235001	32255001	4.38082	0.231115	OL	NCOA1
NC_056056.	32240001	32260001	3.49241	0.208623	OL	NCOA1
NC_056056.	32245001	32265001	2.1615	0.150238	OL	NCOA1
NC_056056.	32255001	32275001	2.60256	0.157347	OL	NCOA1
NC_056066.	76045001	76065001	3.86154	0.153546	OL	NCOA3
NC_056058.	41465001	41485001	2.49677	0.351339	OL	NDUFS7;PWWP3A
NC_056054.	47610001	47630001	1.82558	0.267629	OL	NEGR1
NC_056072.	1695001	1715001	1.82441	0.331098	OL	NEK10

NC_056061.1	14175001	14195001	2.41299
NC_056061.1	14180001	14200001	2.15838
NC_056061.1	14185001	14205001	2.07018
NC_056061.1	14525001	14545001	3.20334
NC_056061.1	14530001	14550001	6.56585
NC_056061.1	14540001	14560001	6.01919
NC_056061.1	14560001	14580001	16.279
NC_056061.1	14565001	14585001	4.99642
NC_056080.1	3685001	3705001	2.38892
NC_056080.1	3690001	3710001	2.97622
NC_056080.1	3695001	3715001	3.1246
NC_056080.1	3700001	3720001	3.54637
NC_056080.1	3705001	3725001	3.67083
NC_056077.1	3490001	3510001	2.1757
NC_056069.1	31290001	31310001	2.28223
NC_056069.1	31295001	31315001	2.93198
NC_056069.1	31300001	31320001	2.31349
NC_056078.1	25845001	25865001	2.12588
NC_056078.1	25850001	25870001	2.44223
NC_056064.1	48885001	48905001	2.1114
NC_056054.1	97350001	97370001	2.21366
NC_056054.1	97355001	97375001	2.57739
NC_056054.1	97360001	97380001	2.40052
NC_056054.1	97365001	97385001	2.90448
NC_056054.1	97395001	97415001	2.85176
NC_056054.1	97400001	97420001	2.72112
NC_056054.1	97405001	97425001	2.66845
NC_056054.1	97410001	97430001	3.02382
NC_056054.1	97415001	97435001	4.30942
NC_056054.1	97420001	97440001	4.7076
NC_056054.1	97425001	97445001	3.57829

NC_056072.	1700001	1720001	2.07594	0.362694	OL	NEK10
NC_056072.	1705001	1725001	2.57032	0.263235	OL	NEK10
NC_056072.	1710001	1730001	2.45997	0.194486	OL	NEK10
NC_056072.	1715001	1735001	2.48941	0.145439	OL	NEK10
NC_056074.	20965001	20985001	1.94351	0.184818	OL	NELL1
NC_056074.	20970001	20990001	2.03645	0.21551	OL	NELL1
NC_056074.	20975001	20995001	2.25567	0.177454	OL	NELL1
NC_056074.	20980001	21000001	2.75539	0.157891	OL	NELL1
NC_056060.	19865001	19885001	2.13131	0.144777	OL	NEO1
NC_056060.	19870001	19890001	3.88813	0.156937	OL	NEO1
NC_056080.	66840001	66860001	2.23636	0.180628	OL	NEXMIF
NC_056054.	93170001	93190001	2.05028	0.164525	OL	NHLH2
NC_056054.	93175001	93195001	5.18585	0.180557	OL	NHLH2
NC_056073.	38720001	38740001	3.34963	0.144191	OL	NHLRC1
NC_056073.	38705001	38725001	2.67615	0.169217	OL	NHLRC1;TPMT
NC_056080.	15740001	15760001	1.81832	0.38876	OL	NHS
NC_056080.	15745001	15765001	1.84651	0.376022	OL	NHS
NC_056055.	242235001	2.42E+08	1.87694	0.141841	OL	NIPAL3;STPG1
NC_056055.	242240001	2.42E+08	1.84997	0.204944	OL	NIPAL3;STPG1
NC_056072.	48455001	48475001	3.9595	0.252599	OL	NISCH
NC_056072.	48460001	48480001	2.73809	0.191731	OL	NISCH
NC_056072.	48465001	48485001	2.14413	0.178271	OL	NISCH
NC_056072.	48440001	48460001	13.1693	0.244749	OL	NISCH;STAB1
NC_056072.	48445001	48465001	8.38396	0.246406	OL	NISCH;STAB1
NC_056072.	48450001	48470001	5.17636	0.238568	OL	NISCH;STAB1
NC_056061.	13790001	13810001	1.98474	0.289205	OL	NKAIN2
NC_056061.	13795001	13815001	17.1717	0.473911	OL	NKAIN2
NC_056061.	13805001	13825001	9.80252	0.457478	OL	NKAIN2
NC_056061.	13810001	13830001	3.22405	0.380544	OL	NKAIN2
NC_056061.	13815001	13835001	2.01994	0.288371	OL	NKAIN2
NC_056059.	117730001	1.18E+08	1.97116	0.212428	OL	NKX1-1

NC_056054.1	97435001	97455001	2.71429
NC_056054.1	97440001	97460001	2.67346
NC_056054.1	97445001	97465001	3.12858
NC_056054.1	97450001	97470001	3.29869
NC_056054.1	97455001	97475001	2.26493
NC_056054.1	97460001	97480001	2.14698
NC_056071.1	41140001	41160001	3.78148
NC_056071.1	41145001	41165001	2.97368
NC_056056.1	74460001	74480001	3.19396
NC_056056.1	74465001	74485001	3.22807
NC_056056.1	74470001	74490001	2.61157
NC_056056.1	74475001	74495001	2.23835
NC_056056.1	74485001	74505001	2.12649
NC_056060.1	89120001	89140001	2.08861
NC_056060.1	89125001	89145001	3.00744
NC_056060.1	89130001	89150001	5.2111
NC_056060.1	89135001	89155001	5.11616
NC_056060.1	89140001	89160001	3.10887
NC_056060.1	89150001	89170001	2.13803
NC_056060.1	89160001	89180001	1.99181
NC_056060.1	89175001	89195001	2.40464
NC_056060.1	89180001	89200001	2.05788
NC_056074.1	32265001	32285001	2.43334
NC_056074.1	32270001	32290001	3.86142
NC_056074.1	32275001	32295001	10.5767
NC_056074.1	32290001	32310001	2.21851
NC_056074.1	32395001	32415001	10.2395
NC_056054.1	84830001	84850001	2.03288
NC_056054.1	84835001	84855001	2.17973
NC_056054.1	84840001	84860001	4.37659
NC_056056.1	7415001	7435001	2.48889

NC_056054.	213995001	2.14E+08	1.92904	0.185888	OL	NLGN1
NC_056054.	214000001	2.14E+08	2.38941	0.197451	OL	NLGN1
NC_056054.	214005001	2.14E+08	2.54489	0.189011	OL	NLGN1
NC_056054.	214010001	2.14E+08	2.39005	0.163567	OL	NLGN1
NC_056054.	214225001	2.14E+08	2.25667	0.193498	OL	NLGN1
NC_056059.	71755001	71775001	2.68339	0.240242	OL	NMU
NC_056059.	71760001	71780001	2.20159	0.296225	OL	NMU
NC_056054.	97355001	97375001	2.44039	0.16194	OL	NOTCH2
NC_056054.	97360001	97380001	2.80544	0.152422	OL	NOTCH2
NC_056054.	97365001	97385001	5.30138	0.16926	OL	NOTCH2
NC_056071.	34460001	34480001	2.29862	0.293632	OL	NOVA1
NC_056071.	34465001	34485001	2.68656	0.225559	OL	NOVA1
NC_056071.	34470001	34490001	2.60493	0.211528	OL	NOVA1
NC_056071.	34475001	34495001	1.95984	0.185838	OL	NOVA1
NC_056060.	15880001	15900001	20.8555	0.175967	OL	NOX5
NC_056060.	15885001	15905001	9.14545	0.144814	OL	NOX5
NC_056056.	100505001	1.01E+08	1.82955	0.162199	OL	NPAS2
NC_056056.	100510001	1.01E+08	2.15994	0.198765	OL	NPAS2
NC_056056.	100515001	1.01E+08	2.27078	0.223491	OL	NPAS2
NC_056056.	100520001	1.01E+08	2.57071	0.237752	OL	NPAS2
NC_056056.	100525001	1.01E+08	3.44359	0.29021	OL	NPAS2
NC_056056.	100530001	1.01E+08	1.8612	0.320061	OL	NPAS2
NC_056056.	100610001	1.01E+08	2.71985	0.142835	OL	NPAS2
NC_056055.	47995001	48015001	1.95844	0.18295	OL	NR4A3
NC_056055.	48000001	48020001	2.64706	0.205721	OL	NR4A3
NC_056055.	48005001	48025001	4.56025	0.207152	OL	NR4A3
NC_056055.	48010001	48030001	3.10769	0.171549	OL	NR4A3
NC_056056.	11350001	11370001	1.86781	0.344013	OL	NR5A1
NC_056056.	11325001	11345001	6.61068	0.191701	OL	NR5A1;NR6A1
NC_056056.	11330001	11350001	3.07693	0.199207	OL	NR5A1;NR6A1
NC_056056.	11335001	11355001	2.16867	0.245767	OL	NR5A1;NR6A1

NC_056056.1	7420001	7440001	3.06665
NC_056067.1	24435001	24455001	2.32539
NC_056067.1	24440001	24460001	2.53007
NC_056067.1	24445001	24465001	2.51772
NC_056067.1	24450001	24470001	2.80679
NC_056067.1	24455001	24475001	4.952
NC_056067.1	24460001	24480001	5.52357
NC_056067.1	24465001	24485001	4.57049
NC_056067.1	24470001	24490001	3.28995
NC_056064.1	21890001	21910001	1.94876
NC_056072.1	6345001	6365001	2.05053
NC_056072.1	6350001	6370001	2.05802
NC_056072.1	6355001	6375001	2.18782
NC_056054.1	26000001	26020001	3.98093
NC_056054.1	26005001	26025001	4.58396
NC_056054.1	26010001	26030001	3.14816
NC_056054.1	26015001	26035001	3.14654
NC_056054.1	26020001	26040001	2.45065
NC_056054.1	26030001	26050001	1.96085
NC_056054.1	26035001	26055001	2.00327
NC_056066.1	72620001	72640001	5.38558
NC_056066.1	72630001	72650001	1.97555
NC_056066.1	72635001	72655001	3.15863
NC_056071.1	27670001	27690001	2.99428
NC_056073.1	25345001	25365001	6.38525
NC_056062.1	70860001	70880001	2.61916
NC_056062.1	70865001	70885001	3.15717
NC_056062.1	70870001	70890001	3.9057
NC_056062.1	70875001	70895001	2.94189
NC_056062.1	70880001	70900001	1.97494
NC_056062.1	70915001	70935001	2.17885

NC_056056.	11115001	11135001	2.82336	0.159414	OL	NR6A1
NC_056056.	11120001	11140001	3.408	0.157331	OL	NR6A1
NC_056056.	11125001	11145001	4.86516	0.157709	OL	NR6A1
NC_056056.	11130001	11150001	5.59814	0.152449	OL	NR6A1
NC_056056.	11165001	11185001	3.1822	0.146929	OL	NR6A1
NC_056056.	11170001	11190001	3.411	0.15828	OL	NR6A1
NC_056056.	11175001	11195001	3.37605	0.16214	OL	NR6A1
NC_056056.	11180001	11200001	3.99669	0.172136	OL	NR6A1
NC_056056.	11185001	11205001	3.31506	0.164398	OL	NR6A1
NC_056056.	11190001	11210001	3.09854	0.150113	OL	NR6A1
NC_056056.	11195001	11215001	2.55	0.144336	OL	NR6A1
NC_056056.	11210001	11230001	2.1807	0.148526	OL	NR6A1
NC_056056.	11215001	11235001	2.98724	0.152289	OL	NR6A1
NC_056056.	11220001	11240001	3.4875	0.141269	OL	NR6A1
NC_056056.	11225001	11245001	2.8724	0.145477	OL	NR6A1
NC_056056.	11230001	11250001	2.5026	0.143583	OL	NR6A1
NC_056056.	11255001	11275001	1.86528	0.156502	OL	NR6A1
NC_056056.	11260001	11280001	2.41589	0.14579	OL	NR6A1
NC_056056.	11265001	11285001	1.9711	0.150402	OL	NR6A1
NC_056056.	11285001	11305001	2.20177	0.162984	OL	NR6A1
NC_056056.	11290001	11310001	3.66276	0.17015	OL	NR6A1
NC_056056.	11295001	11315001	5.18072	0.166014	OL	NR6A1
NC_056056.	11300001	11320001	7.04812	0.17624	OL	NR6A1
NC_056056.	11305001	11325001	8.10069	0.176355	OL	NR6A1
NC_056056.	11310001	11330001	6.7256	0.180556	OL	NR6A1
NC_056056.	11315001	11335001	7.03869	0.184988	OL	NR6A1
NC_056056.	11320001	11340001	6.26451	0.180392	OL	NR6A1
NC_056056.	11100001	11120001	3.07672	0.213898	OL	NR6A1;OLFML2A
NC_056056.	11105001	11125001	2.58711	0.197651	OL	NR6A1;OLFML2A
NC_056056.	11110001	11130001	2.53554	0.175158	OL	NR6A1;OLFML2A
NC_056056.	73745001	73765001	2.35018	0.142721	OL	NRXN1

NC_056062.1	70920001	70940001	1.952
NC_056062.1	70925001	70945001	1.95148
NC_056074.1	40605001	40625001	3.5413
NC_056074.1	40610001	40630001	4.67312
NC_056074.1	40615001	40635001	4.90411
NC_056074.1	40620001	40640001	4.3371
NC_056055.1	248995001	2.49E+08	3.52186
NC_056055.1	249000001	2.49E+08	4.03237
NC_056055.1	249005001	2.49E+08	3.5836
NC_056055.1	248960001	2.49E+08	2.01816
NC_056055.1	248965001	2.49E+08	2.67599
NC_056055.1	248970001	2.49E+08	2.28361
NC_056055.1	248975001	2.49E+08	2.61893
NC_056055.1	248980001	2.49E+08	2.46574
NC_056055.1	248985001	2.49E+08	2.32329
NC_056055.1	248990001	2.49E+08	2.715
NW_02459982	1165001	1185001	5.51163
NW_02459982	1170001	1190001	9.11331
NW_02459982	1175001	1195001	4.39082
NW_02459982	1180001	1200001	2.77369
NW_02459982	1185001	1205001	2.14581
NC_056074.1	16250001	16270001	9.08164
NC_056074.1	16255001	16275001	5.63866
NC_056074.1	16260001	16280001	4.49246
NC_056074.1	16265001	16285001	3.39741
NC_056078.1	25865001	25885001	3.34228
NC_056078.1	25870001	25890001	4.99516
NC_056078.1	25875001	25895001	14.2366
NC_056078.1	25915001	25935001	14.3112
NC_056078.1	25920001	25940001	5.78205
NC_056078.1	25925001	25945001	2.89353

NC_056056.	73750001	73770001	2.48917	0.152304	OL	NRXN1
NC_056056.	73755001	73775001	2.56411	0.152517	OL	NRXN1
NC_056059.	105390001	1.05E+08	2.05218	0.223335	OL	NSG1
NC_056059.	105395001	1.05E+08	4.35056	0.212737	OL	NSG1
NC_056056.	165645001	1.66E+08	3.0459	0.154873	OL	NTN4
NC_056056.	165650001	1.66E+08	3.77272	0.166845	OL	NTN4
NC_056056.	165655001	1.66E+08	3.38969	0.192797	OL	NTN4
NC_056056.	165660001	1.66E+08	2.91078	0.187274	OL	NTN4
NC_056056.	165665001	1.66E+08	2.82676	0.208294	OL	NTN4
NC_056056.	165670001	1.66E+08	2.66071	0.201445	OL	NTN4
NC_056056.	165675001	1.66E+08	3.15241	0.237994	OL	NTN4
NC_056056.	165680001	1.66E+08	3.22543	0.22619	OL	NTN4
NC_056056.	165685001	1.66E+08	4.88691	0.259179	OL	NTN4
NC_056056.	165690001	1.66E+08	6.09524	0.266886	OL	NTN4
NC_056056.	165695001	1.66E+08	4.92664	0.215831	OL	NTN4
NC_056056.	165700001	1.66E+08	4.2363	0.224021	OL	NTN4
NC_056055.	34740001	34760001	2.10649	0.16605	OL	NTRK2
NC_056055.	34745001	34765001	2.51657	0.163435	OL	NTRK2
NC_056055.	34865001	34885001	1.94705	0.198329	OL	NTRK2
NC_056055.	34870001	34890001	1.98363	0.21596	OL	NTRK2
NC_056055.	34875001	34895001	2.49593	0.267046	OL	NTRK2
NC_056071.	39970001	39990001	2.14056	0.14854	OL	NUBPL
NC_056071.	39975001	39995001	3.59003	0.147055	OL	NUBPL
NC_056071.	39980001	40000001	3.64984	0.141545	OL	NUBPL
NC_056067.	24435001	24455001	2.13615	0.142101	OL	NUP93
NC_056067.	24440001	24460001	2.39076	0.150329	OL	NUP93
NC_056067.	24445001	24465001	2.16412	0.144899	OL	NUP93
NC_056067.	24450001	24470001	2.22184	0.142074	OL	NUP93
NC_056067.	24455001	24475001	3.2465	0.178765	OL	NUP93
NC_056067.	24460001	24480001	3.6254	0.191764	OL	NUP93
NC_056067.	24465001	24485001	3.13918	0.174264	OL	NUP93

NC_056055.1	13525001	13545001	2.06082
NC_056056.1	225470001	2.25E+08	2.16206
NC_056065.1	57150001	57170001	2.11639
NC_056065.1	57155001	57175001	2.29543
NC_056055.1	207555001	2.08E+08	3.1802
NC_056055.1	207560001	2.08E+08	2.72996
NC_056055.1	207565001	2.08E+08	1.99834
NC_056055.1	207670001	2.08E+08	2.65255
NC_056080.1	84055001	84075001	2.81525
NC_056080.1	84060001	84080001	2.80703
NC_056080.1	84065001	84085001	2.76658
NC_056080.1	84070001	84090001	2.83102
NC_056054.1	37360001	37380001	6.10412
NC_056056.1	10275001	10295001	3.14063
NC_056075.1	4465001	4485001	2.1522
NC_056075.1	4470001	4490001	2.26707
NC_056075.1	4475001	4495001	2.22932
NC_056075.1	4480001	4500001	1.99807
NC_056075.1	4620001	4640001	1.9443
NC_056063.1	40220001	40240001	3.11347
NC_056063.1	40225001	40245001	4.18168
NC_056063.1	40230001	40250001	3.76983
NC_056063.1	40235001	40255001	4.03172
NC_056057.1	66150001	66170001	1.96655
NC_056057.1	66155001	66175001	2.87631
NC_056057.1	66160001	66180001	2.83103
NC_056057.1	66165001	66185001	2.25969
NC_056070.1	42630001	42650001	2.55617
NC_056070.1	42635001	42655001	2.30588
NC_056068.1	4145001	4165001	2.15913
NC_056068.1	4150001	4170001	3.80259

NC_056067.	24470001	24490001	2.40435	0.154601	OL	NUP93
NC_056056.	11065001	11085001	2.40066	0.288923	OL	OLFML2A
NC_056056.	11070001	11090001	2.23334	0.260036	OL	OLFML2A
NC_056056.	11075001	11095001	2.53983	0.229512	OL	OLFML2A
NC_056056.	11080001	11100001	1.83794	0.172966	OL	OLFML2A
NC_056056.	11085001	11105001	2.08582	0.158668	OL	OLFML2A
NC_056056.	11090001	11110001	2.78931	0.195523	OL	OLFML2A
NC_056056.	11095001	11115001	3.00534	0.212134	OL	OLFML2A
NC_056070.	69690001	69710001	6.07301	0.212969	OL	OSBP2
NC_056070.	69695001	69715001	8.38083	0.233528	OL	OSBP2
NC_056070.	69700001	69720001	7.79015	0.240254	OL	OSBP2
NC_056070.	69705001	69725001	6.35326	0.202813	OL	OSBP2
NC_056070.	69710001	69730001	4.05101	0.182489	OL	OSBP2
NC_056070.	69715001	69735001	3.58316	0.177392	OL	OSBP2
NC_056072.	6350001	6370001	2.03489	0.160023	OL	OSBPL10
NC_056072.	6355001	6375001	2.23657	0.165043	OL	OSBPL10
NC_056054.	190820001	1.91E+08	3.40926	0.157423	OL	OSBPL11
NC_056054.	190825001	1.91E+08	4.35294	0.146099	OL	OSBPL11
NC_056064.	55815001	55835001	1.99068	0.145706	OL	OTOP2;OTOP3;USH1G
NC_056064.	55820001	55840001	2.14099	0.153449	OL	OTOP2;USH1G
NC_056064.	55825001	55845001	2.2714	0.160382	OL	OTOP2;USH1G
NC_056071.	27965001	27985001	2.40845	0.255719	OL	OTUD7A
NC_056071.	27970001	27990001	2.17655	0.236544	OL	OTUD7A
NC_056071.	28010001	28030001	2.248	0.307566	OL	OTUD7A
NC_056071.	28015001	28035001	1.84599	0.273755	OL	OTUD7A
NC_056061.	86450001	86470001	1.81008	0.148419	OL	PACRG
NC_056066.	2780001	2800001	2.10722	0.142352	OL	PAK5
NC_056071.	59110001	59130001	1.82064	0.144874	OL	PAPOLA
NC_056059.	17900001	17920001	2.07888	0.274603	OL	PAPSS1
NC_056066.	18210001	18230001	2.06876	0.191616	OL	PARD3
NC_056066.	18215001	18235001	2.40073	0.161695	OL	PARD3

NC_056068.1	4155001	4175001	8.84826
NC_056068.1	4160001	4180001	8.46348
NC_056068.1	4165001	4185001	10.4779
NC_056068.1	4170001	4190001	9.07625
NC_056068.1	4175001	4195001	6.36249
NC_056068.1	4180001	4200001	4.34101
NC_056068.1	4185001	4205001	2.04424
NC_056068.1	4200001	4220001	2.51168
NC_056068.1	4205001	4225001	6.16706
NC_056068.1	4210001	4230001	23.9465
NC_056068.1	4215001	4235001	18.8292
NC_056068.1	4220001	4240001	20.214
NC_056068.1	4225001	4245001	19.3333
NC_056068.1	4230001	4250001	10.2899
NC_056068.1	4235001	4255001	8.15584
NC_056068.1	4240001	4260001	4.13283
NC_056061.1	30035001	30055001	2.14052
NC_056061.1	30155001	30175001	2.53597
NC_056061.1	30160001	30180001	3.56451
NC_056054.1	21735001	21755001	2.33668
NC_056055.1	68400001	68420001	2.76911
NC_056055.1	68405001	68425001	2.76184
NC_056055.1	68410001	68430001	2.71338
NC_056055.1	68415001	68435001	2.56815
NC_056061.1	68170001	68190001	4.90722
NC_056061.1	68185001	68205001	4.32307
NC_056061.1	68190001	68210001	1.94692
NC_056061.1	68205001	68225001	2.30016
NC_056061.1	68210001	68230001	2.68567
NC_056061.1	68245001	68265001	2.42424
NC_056061.1	68250001	68270001	2.21528

NC_056078.	23930001	23950001	5.70969	0.158096	OL	PBLD
NC_056056.	35905001	35925001	10.4365	0.394316	OL	PCARE;TOGARAM2
NC_056054.	266280001	2.66E+08	2.50386	0.146028	OL	PCBP3
NC_056059.	50870001	50890001	1.85015	0.22113	OL	PCDH7
NC_056059.	50875001	50895001	2.71074	0.223519	OL	PCDH7
NC_056059.	50880001	50900001	2.60142	0.206944	OL	PCDH7
NC_056059.	50885001	50905001	2.4748	0.201144	OL	PCDH7
NC_056059.	50890001	50910001	2.43302	0.19811	OL	PCDH7
NC_056059.	50895001	50915001	2.42307	0.200324	OL	PCDH7
NC_056059.	50900001	50920001	2.46485	0.209472	OL	PCDH7
NC_056059.	50905001	50925001	3.03747	0.229891	OL	PCDH7
NC_056059.	50910001	50930001	2.1039	0.17936	OL	PCDH7
NC_056059.	50915001	50935001	2.24819	0.185853	OL	PCDH7
NC_056059.	50920001	50940001	2.23985	0.181795	OL	PCDH7
NC_056059.	50925001	50945001	2.09054	0.174944	OL	PCDH7
NC_056059.	50930001	50950001	2.28677	0.18486	OL	PCDH7
NC_056063.	40225001	40245001	2.05328	0.165468	OL	PCDH9
NC_056063.	40230001	40250001	2.16987	0.19749	OL	PCDH9
NC_056063.	40235001	40255001	2.13073	0.158907	OL	PCDH9
NC_056058.	41560001	41580001	2.42932	0.244618	OL	PCSK4;REEP6
NC_056069.	20455001	20475001	2.33617	0.168722	OL	PDE4D
NC_056060.	8535001	8555001	2.39639	0.53984	OL	PDE8B
NC_056060.	8540001	8560001	2.62888	0.512996	OL	PDE8B
NC_056068.	3985001	4005001	2.38425	0.173605	OL	PDGFD
NC_056068.	4220001	4240001	2.22398	0.165972	OL	PDGFD
NC_056068.	4225001	4245001	1.90894	0.177895	OL	PDGFD
NC_056075.	16600001	16620001	1.93698	0.165089	OL	PDLIM1
NC_056063.	28595001	28615001	2.98749	0.246967	OL	PDS5B
NC_056063.	28600001	28620001	2.40603	0.198179	OL	PDS5B
NC_056063.	28605001	28625001	2.39817	0.201015	OL	PDS5B
NC_056055.	68285001	68305001	3.08249	0.188977	OL	PGM5

NC_056080.1	20345001	20365001	5.34234
NC_056076.1	42755001	42775001	2.06005
NC_056076.1	42760001	42780001	1.9933
NC_056076.1	42765001	42785001	2.12109
NC_056076.1	42805001	42825001	6.31183
NC_056076.1	42810001	42830001	7.13598
NC_056076.1	42815001	42835001	5.29755
NC_056076.1	42820001	42840001	4.30446
NC_056076.1	42825001	42845001	3.60599
NC_056054.1	111040001	1.11E+08	5.06163
NC_056054.1	111045001	1.11E+08	4.01503
NC_056080.1	13570001	13590001	8.24698
NC_056080.1	13575001	13595001	8.72839
NC_056080.1	13580001	13600001	7.20731
NC_056080.1	13585001	13605001	3.92931
NC_056080.1	13590001	13610001	2.42074
NC_056080.1	13595001	13615001	2.55474
NC_056080.1	13600001	13620001	2.68935
NC_056080.1	13605001	13625001	2.77387
NC_056080.1	13615001	13635001	2.07131
NC_056080.1	13630001	13650001	3.40476
NC_056080.1	13635001	13655001	2.05826
NC_056080.1	13565001	13585001	6.32348
NC_056070.1	67500001	67520001	1.95745
NC_056070.1	67505001	67525001	1.97769
NC_056055.1	246175001	2.46E+08	5.93062
NC_056055.1	246180001	2.46E+08	5.89029
NC_056055.1	246185001	2.46E+08	3.52446
NC_056054.1	232670001	2.33E+08	2.37543
NC_056054.1	232675001	2.33E+08	1.95921
NC_056054.1	275120001	2.75E+08	2.07672

NC_056055.	68290001	68310001	3.97217	0.193804	OL	PGM5
NC_056055.	68295001	68315001	3.72336	0.174815	OL	PGM5
NC_056055.	68310001	68330001	4.10225	0.186983	OL	PGM5
NC_056055.	68315001	68335001	5.62601	0.233154	OL	PGM5
NC_056055.	68320001	68340001	11.1189	0.287586	OL	PGM5
NC_056055.	68325001	68345001	18.4032	0.243376	OL	PGM5
NC_056055.	68330001	68350001	17.2689	0.172216	OL	PGM5
NC_056055.	68335001	68355001	22.3675	0.171688	OL	PGM5
NC_056055.	68340001	68360001	22.783	0.166201	OL	PGM5
NC_056055.	68345001	68365001	17.2453	0.143575	OL	PGM5
NC_056055.	68370001	68390001	25.6932	0.211287	OL	PGM5
NC_056055.	68375001	68395001	20.8214	0.170901	OL	PGM5
NC_056055.	68400001	68420001	5.01523	0.205169	OL	PGM5
NC_056055.	68405001	68425001	5.09259	0.221437	OL	PGM5
NC_056055.	68410001	68430001	6.14	0.26694	OL	PGM5
NC_056055.	68415001	68435001	5.5495	0.258269	OL	PGM5
NC_056055.	68420001	68440001	3.65117	0.190366	OL	PGM5
NC_056080.	20305001	20325001	2.30473	0.282776	OL	PHEX
NC_056080.	20310001	20330001	2.88981	0.226121	OL	PHEX
NC_056080.	20315001	20335001	2.69982	0.258419	OL	PHEX
NC_056080.	20320001	20340001	1.98998	0.309598	OL	PHEX
NC_056080.	20340001	20360001	2.21591	0.180979	OL	PHEX
NC_056080.	20345001	20365001	5.09014	0.199551	OL	PHEX
NC_056080.	65330001	65350001	2.31335	0.14939	OL	PHKA1
NC_056068.	29015001	29035001	2.15166	0.271963	OL	PHLDB1
NC_056068.	29020001	29040001	2.38276	0.294908	OL	PHLDB1
NC_056068.	29025001	29045001	2.59887	0.314442	OL	PHLDB1
NC_056068.	29030001	29050001	2.42735	0.304246	OL	PHLDB1;TREH
NC_056068.	29035001	29055001	1.98375	0.266418	OL	PHLDB1;TREH
NC_056068.	29040001	29060001	1.96274	0.271194	OL	PHLDB1;TREH
NC_056055.	231245001	2.31E+08	2.51001	0.149992	OL	PID1

NC_056054.1	275125001	2.75E+08	1.97021
NC_056069.1	33390001	33410001	2.59029
NC_056065.1	33735001	33755001	2.25692
NC_056065.1	33765001	33785001	2.03534
NC_056065.1	33770001	33790001	2.15682
NC_056065.1	33855001	33875001	1.96441
NC_056056.1	80525001	80545001	2.03277
NC_056056.1	80535001	80555001	1.98028
NC_056056.1	80560001	80580001	1.95003
NC_056056.1	80565001	80585001	2.07977
NC_056055.1	209955001	2.1E+08	2.77586
NC_056055.1	209960001	2.1E+08	2.56018
NC_056055.1	209965001	2.1E+08	2.32868
NC_056055.1	209970001	2.1E+08	2.28067
NC_056055.1	209975001	2.1E+08	2.24653
NC_056055.1	209980001	2.1E+08	2.24542
NC_056055.1	209985001	2.1E+08	2.26853
NC_056055.1	209990001	2.1E+08	2.30548
NC_056055.1	209995001	2.1E+08	2.3228
NC_056055.1	210000001	2.1E+08	2.38203
NC_056055.1	210005001	2.1E+08	2.41646
NC_056055.1	210010001	2.1E+08	2.36624
NC_056055.1	210015001	2.1E+08	2.38108
NC_056055.1	210020001	2.1E+08	2.16666
NC_056072.1	48795001	48815001	2.78354
NC_056072.1	48800001	48820001	4.32529
NC_056060.1	7115001	7135001	2.16879
NC_056073.1	17065001	17085001	3.78341
NC_056077.1	20515001	20535001	16.3412
NC_056068.1	44745001	44765001	2.11962
NC_056068.1	44750001	44770001	2.32907

NC_056066.	72905001	72925001	1.83492	0.176948	OL	PKIG
NC_056066.	72910001	72930001	1.95926	0.233685	OL	PKIG
NC_056066.	72915001	72935001	2.03541	0.218799	OL	PKIG
NC_056060.	18985001	19005001	1.81415	0.291112	OL	PKM
NC_056066.	1430001	1450001	1.84615	0.244235	OL	PLCB1
NC_056054.	274955001	2.75E+08	2.04705	0.148181	OL	PLCL2
NC_056054.	274960001	2.75E+08	2.38038	0.157461	OL	PLCL2
NC_056054.	216605001	2.17E+08	2.18035	0.153434	OL	PLD1
NC_056067.	48460001	48480001	2.16327	0.319557	OL	PLEKHG2;RPS16;ZFP36
NC_056067.	48455001	48475001	2.34632	0.319488	OL	PLEKHG2;ZFP36
NC_056056.	80555001	80575001	1.88718	0.154839	OL	PLEKHH2
NC_056056.	80560001	80580001	1.9616	0.216898	OL	PLEKHH2
NC_056056.	80565001	80585001	2.13249	0.237507	OL	PLEKHH2
NC_056056.	80570001	80590001	2.36244	0.166606	OL	PLEKHH2
NC_056058.	41590001	41610001	3.36062	0.272991	OL	PLK5
NC_056058.	41595001	41615001	3.19513	0.27219	OL	PLK5
NC_056055.	22085001	22105001	1.82924	0.165418	OL	PLPPR1
NC_056055.	22095001	22115001	1.90765	0.17824	OL	PLPPR1
NC_056056.	130405001	1.3E+08	4.4281	0.417704	OL	PLXNC1
NC_056056.	130430001	1.3E+08	12.3044	0.558339	OL	PLXNC1
NC_056056.	130435001	1.3E+08	5.71817	0.472562	OL	PLXNC1
NC_056056.	130440001	1.3E+08	2.12627	0.373401	OL	PLXNC1
NC_056056.	130470001	1.3E+08	2.93678	0.170606	OL	PLXNC1
NC_056056.	130475001	1.3E+08	3.43365	0.196078	OL	PLXNC1
NC_056056.	130480001	1.31E+08	2.60099	0.188237	OL	PLXNC1
NC_056068.	21330001	21350001	3.59451	0.182397	OL	POU2AF1
NC_056056.	134995001	1.35E+08	1.9657	0.223265	OL	POU6F1
NC_056056.	135000001	1.35E+08	1.86819	0.164433	OL	POU6F1
NC_056056.	135040001	1.35E+08	2.21288	0.188812	OL	POU6F1;TFCP2
NC_056059.	44080001	44100001	2.42619	0.192375	OL	PPARGC1A
NC_056059.	44085001	44105001	4.16359	0.230655	OL	PPARGC1A

NC_056068.1	44755001	44775001	2.48697
NC_056068.1	44760001	44780001	2.15443
NC_056068.1	44765001	44785001	2.12608
NC_056068.1	44770001	44790001	2.28819
NC_056078.1	34150001	34170001	2.39337
NC_056078.1	34155001	34175001	2.38095
NC_056068.1	52300001	52320001	2.9624
NC_056065.1	79525001	79545001	4.15805
NC_056065.1	79530001	79550001	2.6553
NC_056065.1	79535001	79555001	2.40703
NC_056065.1	79590001	79610001	2.06293
NC_056065.1	79515001	79535001	2.81703
NC_056065.1	79520001	79540001	3.79124
NC_056054.1	253925001	2.54E+08	2.77125
NC_056054.1	253930001	2.54E+08	2.8052
NC_056054.1	253935001	2.54E+08	2.54808
NC_056054.1	253940001	2.54E+08	2.55186
NC_056054.1	253945001	2.54E+08	3.08928
NC_056054.1	253950001	2.54E+08	2.96693
NC_056054.1	253955001	2.54E+08	2.01493
NC_056059.1	23745001	23765001	5.62805
NC_056069.1	39230001	39250001	2.34834
NC_056069.1	39235001	39255001	2.81751
NC_056069.1	39240001	39260001	3.48692
NC_056069.1	39245001	39265001	2.41713
NC_056069.1	39250001	39270001	2.01778
NC_056058.1	11015001	11035001	3.42512
NC_056058.1	54710001	54730001	2.75032
NC_056058.1	54715001	54735001	2.48187
NC_056072.1	37295001	37315001	2.44477
NC_056073.1	3025001	3045001	2.05438

NC_056059.	44090001	44110001	3.92611	0.194814	OL	PPARGC1A
NC_056071.	31310001	31330001	2.20502	0.184879	OL	PPCDC
NC_056080.	17010001	17030001	2.10526	0.200167	OL	PPEF1
NC_056056.	144470001	1.44E+08	1.8857	0.177046	OL	PPHLN1
NC_056059.	37100001	37120001	5.35714	0.229905	OL	PPM1K
NC_056059.	37105001	37125001	4.40119	0.221165	OL	PPM1K
NC_056065.	79530001	79550001	1.97316	0.143699	OL	PPP1R12B
NC_056065.	79535001	79555001	3.06659	0.195614	OL	PPP1R12B
NC_056065.	79540001	79560001	4.86574	0.201636	OL	PPP1R12B
NC_056065.	79545001	79565001	10.8333	0.232225	OL	PPP1R12B
NC_056065.	79550001	79570001	14.7143	0.235815	OL	PPP1R12B
NC_056065.	79555001	79575001	8.62196	0.203904	OL	PPP1R12B
NC_056065.	79560001	79580001	3.78083	0.189671	OL	PPP1R12B
NC_056065.	79565001	79585001	2.61393	0.144046	OL	PPP1R12B
NC_056065.	79575001	79595001	2.73637	0.145079	OL	PPP1R12B
NC_056065.	79580001	79600001	3.49671	0.161686	OL	PPP1R12B
NC_056065.	79585001	79605001	4.13773	0.189382	OL	PPP1R12B
NC_056065.	79590001	79610001	3.03826	0.221162	OL	PPP1R12B
NC_056065.	79595001	79615001	3.41706	0.264167	OL	PPP1R12B
NC_056065.	79600001	79620001	2.40438	0.254872	OL	PPP1R12B
NC_056065.	79605001	79625001	2.27848	0.226534	OL	PPP1R12B
NC_056065.	79610001	79630001	2.37746	0.197157	OL	PPP1R12B
NC_056065.	79615001	79635001	2.35614	0.193935	OL	PPP1R12B
NC_056065.	79620001	79640001	2.4207	0.205488	OL	PPP1R12B
NC_056065.	79625001	79645001	2.36404	0.224136	OL	PPP1R12B
NC_056065.	79630001	79650001	1.98382	0.197896	OL	PPP1R12B
NC_056054.	192905001	1.93E+08	2.14526	0.201131	OL	PPP1R2
NC_056054.	192910001	1.93E+08	2.96113	0.200806	OL	PPP1R2
NC_056054.	192915001	1.93E+08	4.95134	0.200586	OL	PPP1R2
NC_056072.	28780001	28800001	3.93893	0.150564	OL	PPP4R2
NC_056072.	28785001	28805001	6.01436	0.189492	OL	PPP4R2

NC_056071.1	38030001	38050001	7.42715
NC_056071.1	38035001	38055001	4.2181
NC_056075.1	7705001	7725001	5.59386
NC_056075.1	7710001	7730001	15.7314
NC_056075.1	7715001	7735001	13.6483
NC_056075.1	7720001	7740001	16.4762
NC_056059.1	96680001	96700001	1.96028
NC_056059.1	96685001	96705001	2.0523
NC_056059.1	96695001	96715001	2.80884
NC_056059.1	96700001	96720001	3.95091
NC_056061.1	85680001	85700001	2.60402
NC_056061.1	85685001	85705001	3.5721
NC_056061.1	85690001	85710001	3.82587
NC_056061.1	85695001	85715001	2.58182
NC_056054.1	267035001	2.67E+08	1.94969
NC_056080.1	84020001	84040001	2.90839
NC_056080.1	84025001	84045001	2.13219
NC_056080.1	84030001	84050001	2.19753
NC_056077.1	27755001	27775001	3.43162
NC_056077.1	27760001	27780001	5.7252
NC_056058.1	3035001	3055001	2.26985
NC_056058.1	3040001	3060001	2.49536
NC_056058.1	3045001	3065001	3.46749
NC_056058.1	3050001	3070001	5.11111
NC_056058.1	3055001	3075001	5.55477
NC_056058.1	3060001	3080001	7.9249
NC_056058.1	3065001	3085001	4.12704
NC_056066.1	25050001	25070001	2.25862
NC_056066.1	25055001	25075001	3.06928
NC_056066.1	25060001	25080001	8.70199
NC_056066.1	25065001	25085001	3.66007

NC_056072.	28790001	28810001	17.1579	0.212411	OL	PPP4R2
NC_056072.	28795001	28815001	12.3023	0.188612	OL	PPP4R2
NC_056057.	49660001	49680001	7.52172	0.171697	OL	PRKAR2B
NC_056056.	78450001	78470001	5.13061	0.148094	OL	PRKCE
NC_056056.	78455001	78475001	5.99651	0.156359	OL	PRKCE
NC_056056.	78460001	78480001	4.45672	0.164106	OL	PRKCE
NC_056056.	78465001	78485001	3.99404	0.149794	OL	PRKCE
NC_056056.	78480001	78500001	6.32246	0.154799	OL	PRKCE
NC_056056.	78485001	78505001	8.69791	0.177684	OL	PRKCE
NC_056056.	78560001	78580001	24.5764	0.146955	OL	PRKCE
NC_056056.	39755001	39775001	1.99359	0.156995	OL	PROKR1
NC_056056.	104440001	1.04E+08	3.02041	0.156578	OL	PROM2
NC_056066.	27650001	27670001	3.03515	0.157088	OL	PRPF18
NC_056066.	27655001	27675001	2.91666	0.154572	OL	PRPF18
NC_056066.	27660001	27680001	2.76535	0.145302	OL	PRPF18
NC_056066.	27665001	27685001	2.85068	0.14499	OL	PRPF18
NC_056056.	221170001	2.21E+08	2.73407	0.232745	OL	PRR5
NC_056056.	221175001	2.21E+08	4.14315	0.399018	OL	PRR5
NC_056056.	221180001	2.21E+08	2.52381	0.290357	OL	PRR5
NC_056056.	221185001	2.21E+08	1.96691	0.217228	OL	PRR5
NC_056074.	8255001	8275001	1.94658	0.178443	OL	PRSS23
NC_056074.	8260001	8280001	1.81719	0.166161	OL	PRSS23
NC_056066.	25060001	25080001	1.90159	0.210118	OL	PRTFDC1
NC_056060.	11455001	11475001	2.2634	0.146055	OL	PSMC6
NC_056055.	232935001	2.33E+08	1.99843	0.163539	OL	PSMD1
NC_056055.	232940001	2.33E+08	2.12235	0.180927	OL	PSMD1
NC_056066.	77585001	77605001	1.85664	0.263059	OL	PTGIS
NC_056055.	13855001	13875001	9.38822	0.276203	OL	PTPN3
NC_056055.	13905001	13925001	3.6129	0.150707	OL	PTPN3
NC_056055.	77120001	77140001	2.73845	0.15149	OL	PTPRD
NC_056055.	77125001	77145001	4.33469	0.191234	OL	PTPRD

NC_056066.1	25070001	25090001	3.45291
NC_056068.1	37945001	37965001	3.2
NC_056068.1	37950001	37970001	2.19831
NC_056062.1	15855001	15875001	2.78069
NC_056062.1	15860001	15880001	3.39473
NC_056062.1	15875001	15895001	1.95671
NC_056062.1	15880001	15900001	1.9791
NC_056062.1	15930001	15950001	6.53849
NC_056070.1	61900001	61920001	1.97172
NC_056070.1	61905001	61925001	2.26693
NC_056070.1	61910001	61930001	2.43007
NC_056070.1	61915001	61935001	2.29078
NC_056055.1	76675001	76695001	2.0212
NC_056055.1	76680001	76700001	1.99533
NC_056055.1	78330001	78350001	2.04282
NC_056055.1	78365001	78385001	2.0313
NC_056055.1	78370001	78390001	2.00361
NC_056072.1	39300001	39320001	3.82539
NC_056072.1	39310001	39330001	4.31746
NC_056072.1	39315001	39335001	2.67669
NC_056072.1	39320001	39340001	2.47215
NC_056072.1	39325001	39345001	2.14105
NC_056072.1	39330001	39350001	2.11236
NC_056072.1	39335001	39355001	2.3125
NC_056072.1	39340001	39360001	2.10989
NC_056072.1	39345001	39365001	2.9374
NC_056072.1	39350001	39370001	3.01117
NC_056072.1	39355001	39375001	2.00526
NC_056057.1	120640001	1.21E+08	2.3485
NC_056057.1	120665001	1.21E+08	2.67337
NC_056057.1	120670001	1.21E+08	2.49999

NC_056055.	77130001	77150001	5.52191	0.188314	OL	PTPRD
NC_056075.	46930001	46950001	11.8734	0.173139	OL	PTPRE
NC_056075.	46935001	46955001	10.9321	0.209175	OL	PTPRE
NC_056075.	46940001	46960001	5.59504	0.146678	OL	PTPRE
NC_056072.	39230001	39250001	2.07956	0.222697	OL	PTPRG
NC_056072.	39235001	39255001	3.61174	0.280903	OL	PTPRG
NC_056072.	39240001	39260001	4.732	0.334386	OL	PTPRG
NC_056072.	39245001	39265001	5.2715	0.321704	OL	PTPRG
NC_056072.	39250001	39270001	5.15071	0.317922	OL	PTPRG
NC_056072.	39255001	39275001	5.74741	0.251673	OL	PTPRG
NC_056072.	39340001	39360001	2.59898	0.145169	OL	PTPRG
NC_056072.	39345001	39365001	2.80246	0.167943	OL	PTPRG
NC_056076.	41145001	41165001	2.18622	0.171521	OL	PTPRM
NC_056076.	41150001	41170001	2.45806	0.167297	OL	PTPRM
NC_056076.	41155001	41175001	2.54739	0.149631	OL	PTPRM
NC_056066.	71035001	71055001	2.43064	0.155105	OL	PTPRT
NC_056066.	71040001	71060001	3.88702	0.178534	OL	PTPRT
NC_056058.	41445001	41465001	2.47728	0.209382	OL	PWWP3A
NC_056058.	41450001	41470001	3.01307	0.279508	OL	PWWP3A
NC_056058.	41455001	41475001	2.94193	0.330218	OL	PWWP3A
NC_056058.	41460001	41480001	2.74193	0.338521	OL	PWWP3A
NC_056055.	175245001	1.75E+08	1.96221	0.235407	OL	R3HDM1
NC_056068.	17185001	17205001	7.62929	0.154136	OL	RAB39A
NC_056068.	17190001	17210001	15.2566	0.190371	OL	RAB39A
NC_056068.	17195001	17215001	12.6929	0.18689	OL	RAB39A
NC_056060.	44965001	44985001	2.65248	0.144144	OL	RAB8B
NC_056060.	44995001	45015001	3.00529	0.155445	OL	RAB8B
NC_056065.	55130001	55150001	1.8605	0.186524	OL	RABGAP1L
NC_056065.	55135001	55155001	2.46703	0.152659	OL	RABGAP1L
NC_056065.	55140001	55160001	3.57692	0.152982	OL	RABGAP1L
NC_056065.	55465001	55485001	1.94654	0.163251	OL	RABGAP1L

NC_056057.1	120675001	1.21E+08	2.12548
NC_056056.1	200225001	2E+08	3.28465
NC_056056.1	200230001	2E+08	4.15508
NC_056056.1	28105001	28125001	2.05664
NC_056056.1	28110001	28130001	2.15517
NC_056056.1	28150001	28170001	2.4976
NC_056056.1	28160001	28180001	2.89442
NC_056056.1	28175001	28195001	3.25377
NC_056056.1	28180001	28200001	2.81141
NC_056056.1	28185001	28205001	2.98652
NC_056056.1	28190001	28210001	2.45799
NC_056056.1	28195001	28215001	3.25165
NC_056060.1	53155001	53175001	2.57509
NC_056060.1	53160001	53180001	2.61956
NC_056076.1	42360001	42380001	2.21145
NC_056069.1	20780001	20800001	2.03824
NC_056069.1	20785001	20805001	2.28712
NC_056060.1	45005001	45025001	2.27982
NC_056060.1	45020001	45040001	2.01711
NC_056065.1	55175001	55195001	3.15019
NC_056065.1	55180001	55200001	2.84071
NC_056065.1	55185001	55205001	2.06498
NC_056065.1	55190001	55210001	2.04347
NC_056060.1	34485001	34505001	2.42026
NC_056060.1	34490001	34510001	3.5867
NC_056060.1	34495001	34515001	3.54691
NC_056060.1	78420001	78440001	2.45864
NC_056060.1	78425001	78445001	2.09865
NC_056072.1	49290001	49310001	3.36658
NC_056062.1	90890001	90910001	2.78722
NC_056062.1	90895001	90915001	3.30928

NC_056065.	55470001	55490001	2.89621	0.143925	OL	RABGAP1L
NC_056076.	42105001	42125001	2.35898	0.149556	OL	RALBP1
NC_056055.	137015001	1.37E+08	6.92306	0.200993	OL	RAPGEF4
NC_056079.	39765001	39785001	2.70782	0.339284	OL	RARB
NC_056079.	39770001	39790001	2.72905	0.345484	OL	RARB
NC_056079.	39775001	39795001	1.96599	0.344973	OL	RARB
NC_056059.	97320001	97340001	2.53708	0.203904	OL	RASGEF1B
NC_056059.	97325001	97345001	3.98243	0.290637	OL	RASGEF1B
NC_056059.	97330001	97350001	3.24754	0.262386	OL	RASGEF1B
NC_056054.	40280001	40300001	2.05034	0.165796	OL	RAVER2
NC_056054.	40285001	40305001	2.14924	0.162148	OL	RAVER2
NC_056077.	6450001	6470001	1.82353	0.16098	OL	RBFOX1
NC_056070.	60745001	60765001	3.23258	0.14196	OL	RBM19
NC_056070.	60750001	60770001	11.4928	0.184312	OL	RBM19
NC_056070.	60810001	60830001	22.967	0.294938	OL	RBM19
NC_056070.	60815001	60835001	23.75	0.256412	OL	RBM19
NC_056072.	3995001	4015001	3.04309	0.142394	OL	RBMS3
NC_056079.	25710001	25730001	1.90188	0.180687	OL	RBPMS
NC_056065.	54655001	54675001	2.58441	0.166082	OL	RC3H1
NC_056065.	54660001	54680001	2.03896	0.1874	OL	RC3H1
NC_056065.	54630001	54650001	1.87261	0.174594	OL	RC3H1;SERPINC1
NC_056059.	91080001	91100001	2.33943	0.206715	OL	RCHY1
NC_056059.	91085001	91105001	2.97313	0.240013	OL	RCHY1
NC_056059.	91090001	91110001	2.96412	0.256445	OL	RCHY1
NC_056059.	91095001	91115001	2.62145	0.277674	OL	RCHY1;THAP6
NC_056059.	91100001	91120001	2.15859	0.249195	OL	RCHY1;THAP6
NC_056056.	58225001	58245001	1.87532	0.142272	OL	REEP1
NC_056061.	63960001	63980001	5.16151	0.145951	OL	REPS1
NC_056056.	200325001	2E+08	1.89802	0.155958	OL	RERG
NC_056056.	200330001	2E+08	2.08144	0.204924	OL	RERG
NC_056056.	200335001	2E+08	1.84565	0.19997	OL	RERG

NC_056062.1	90900001	90920001	2.97183
NC_056062.1	90905001	90925001	3.14863
NC_056061.1	49775001	49795001	2.42224
NC_056061.1	49780001	49800001	3.1774
NC_056061.1	49785001	49805001	3.29407
NC_056061.1	49790001	49810001	2.59455
NC_056061.1	49795001	49815001	2.03127
NC_056059.1	89410001	89430001	2.34413
NC_056059.1	89415001	89435001	2.92248
NC_056059.1	89420001	89440001	2.84178
NC_056059.1	89425001	89445001	2.3201
NC_056077.1	7050001	7070001	1.96266
NC_056064.1	52220001	52240001	6.67824
NC_056064.1	52225001	52245001	10.7761
NC_056064.1	52235001	52255001	22.04
NC_056064.1	52240001	52260001	16.8658
NC_056070.1	60805001	60825001	2.47044
NC_056070.1	60810001	60830001	3.50669
NC_056070.1	60815001	60835001	4.80723
NC_056073.1	19555001	19575001	2.27984
NC_056073.1	19630001	19650001	3.55338
NC_056073.1	19635001	19655001	8.26279
NC_056063.1	18600001	18620001	4.06766
NC_056055.1	52420001	52440001	2.22332
NC_056055.1	52425001	52445001	2.28898
NC_056055.1	52460001	52480001	2.22439
NC_056055.1	52465001	52485001	2.26906
NC_056055.1	52470001	52490001	2.41729
NC_056055.1	52475001	52495001	2.25342
NC_056055.1	52480001	52500001	2.39793
NC_056055.1	52490001	52510001	2.23502

NC_056065.	64285001	64305001	1.90756	0.236896	OL	RGL1
NC_056065.	11615001	11635001	2.09855	0.183109	OL	RGS2
NC_056065.	63160001	63180001	2.65838	0.170455	OL	RGS8
NC_056065.	63165001	63185001	6.01177	0.236966	OL	RGS8
NC_056073.	22010001	22030001	4.22495	0.187182	OL	RHAG
NC_056073.	22015001	22035001	5.78034	0.216799	OL	RHAG
NC_056073.	22020001	22040001	6.6043	0.250924	OL	RHAG
NC_056073.	22025001	22045001	4.4325	0.247158	OL	RHAG
NC_056073.	22030001	22050001	2.52988	0.235517	OL	RHAG
NC_056073.	22035001	22055001	4.79572	0.363569	OL	RHAG
NC_056073.	22040001	22060001	4.16989	0.346455	OL	RHAG
NC_056073.	22045001	22065001	3.9821	0.373436	OL	RHAG
NC_056073.	22050001	22070001	3.29404	0.360753	OL	RHAG
NC_056062.	73515001	73535001	1.9918	0.212253	OL	RIMS2
NC_056062.	73520001	73540001	2.27023	0.230526	OL	RIMS2
NC_056062.	73525001	73545001	2.28841	0.249002	OL	RIMS2
NC_056062.	73530001	73550001	1.86447	0.26395	OL	RIMS2
NC_056067.	34670001	34690001	2.36776	0.182692	OL	RIPOR1
NC_056067.	34675001	34695001	2.38462	0.171461	OL	RIPOR1
NC_056067.	34680001	34700001	2.72754	0.168472	OL	RIPOR1
NC_056067.	34685001	34705001	2.89329	0.218105	OL	RIPOR1
NC_056063.	20745001	20765001	2.06103	0.242591	OL	RNASEH2B
NC_056063.	20750001	20770001	6.14509	0.266929	OL	RNASEH2B
NC_056070.	53800001	53820001	5.39286	0.340909	OL	RNF34
NC_056055.	52135001	52155001	2.35715	0.208123	OL	RNF38
NC_056055.	52220001	52240001	2.33082	0.144892	OL	RNF38
NC_056055.	52225001	52245001	2.39827	0.143357	OL	RNF38
NC_056061.	48600001	48620001	3.13368	0.163452	OL	RNGTT
NC_056061.	48605001	48625001	4.09007	0.187577	OL	RNGTT
NC_056061.	48610001	48630001	4.37048	0.202227	OL	RNGTT
NC_056061.	48615001	48635001	3.87885	0.17578	OL	RNGTT

NC_056055.1	52495001	52515001	2.33953
NC_056055.1	52500001	52520001	2.2986
NC_056055.1	52505001	52525001	2.37224
NC_056055.1	52510001	52530001	2.41054
NC_056057.1	46580001	46600001	2.93922
NC_056057.1	46585001	46605001	5.21248
NC_056057.1	46590001	46610001	4.13534
NC_056057.1	46610001	46630001	2.31856
NC_056057.1	46615001	46635001	2.16217
NC_056057.1	46620001	46640001	2.10379
NC_056065.1	44000001	44020001	16.5553
NC_056065.1	44005001	44025001	10.7687
NC_056065.1	44010001	44030001	10.7575
NC_056065.1	44015001	44035001	6.76815
NC_056065.1	44020001	44040001	5.46154
NC_056065.1	11615001	11635001	2.03459
NC_056073.1	22010001	22030001	5.33086
NC_056073.1	22015001	22035001	6.38877
NC_056073.1	22020001	22040001	6.70524
NC_056073.1	22025001	22045001	3.88177
NC_056073.1	22035001	22055001	3.07765
NC_056073.1	22040001	22060001	2.42526
NC_056073.1	22045001	22065001	2.45993
NC_056073.1	22050001	22070001	2.27989
NC_056064.1	54580001	54600001	2.01099
NC_056064.1	54585001	54605001	2.45711
NC_056062.1	73850001	73870001	2.02991
NC_056062.1	73880001	73900001	2.16025
NC_056062.1	73885001	73905001	2.38693
NC_056076.1	33575001	33595001	2.71429
NC_056067.1	34670001	34690001	3.23728

NC_056054.	39600001	39620001	2.87328	0.146611	OL	ROR1
NC_056054.	39605001	39625001	3.08516	0.225826	OL	ROR1
NC_056054.	39610001	39630001	2.00368	0.171536	OL	ROR1
NC_056060.	47250001	47270001	1.85531	0.303457	OL	RORA
NC_056071.	31400001	31420001	2.86038	0.260151	OL	RPP25
NC_056071.	31405001	31425001	2.46512	0.256954	OL	RPP25
NC_056060.	69910001	69930001	2.4809	0.185323	OL	RTN1
NC_056078.	23995001	24015001	1.96707	0.203821	OL	RUFY2
NC_056078.	24000001	24020001	2.4242	0.204756	OL	RUFY2
NC_056078.	24005001	24025001	1.96591	0.19262	OL	RUFY2
NC_056078.	24010001	24030001	1.96107	0.146409	OL	RUFY2
NC_056054.	267645001	2.68E+08	2.53234	0.216374	OL	RUNX1
NC_056054.	267650001	2.68E+08	5.73078	0.258237	OL	RUNX1
NC_056054.	267660001	2.68E+08	3.71939	0.231939	OL	RUNX1
NC_056054.	267665001	2.68E+08	2.42432	0.151763	OL	RUNX1
NC_056056.	1860001	1880001	5.6846	0.223925	OL	RXRA
NC_056056.	1865001	1885001	4.12026	0.176948	OL	RXRA
NC_056056.	1870001	1890001	3.09018	0.189627	OL	RXRA
NC_056056.	1875001	1895001	2.29479	0.242607	OL	RXRA
NC_056056.	1880001	1900001	2.05397	0.260476	OL	RXRA
NC_056056.	1885001	1905001	2.20285	0.266735	OL	RXRA
NC_056056.	1890001	1910001	2.13364	0.266671	OL	RXRA
NC_056056.	1895001	1915001	2.26281	0.223674	OL	RXRA
NC_056056.	1925001	1945001	2.06733	0.149696	OL	RXRA
NC_056056.	1930001	1950001	2.06206	0.221458	OL	RXRA
NC_056056.	1935001	1955001	3.00741	0.442535	OL	RXRA
NC_056056.	1940001	1960001	1.8739	0.294554	OL	RXRA
NC_056054.	101940001	1.02E+08	2.38428	0.238824	OL	S100A10
NC_056080.	113710001	1.14E+08	2.69444	0.207073	OL	SASH3
NC_056080.	113700001	1.14E+08	3.4965	0.178601	OL	SASH3;XPNPEP2
NC_056080.	113705001	1.14E+08	2.81028	0.155289	OL	SASH3;XPNPEP2

NC_056067.1	34675001	34695001	3.21117
NC_056067.1	34680001	34700001	3.67579
NC_056067.1	34685001	34705001	3.67829
NC_056054.1	269865001	2.7E+08	6.05278
NC_056063.1	20705001	20725001	3
NC_056066.1	78020001	78040001	2.88282
NC_056066.1	78025001	78045001	5.1884
NC_056066.1	78030001	78050001	4.03116
NC_056066.1	78035001	78055001	3.19058
NC_056054.1	239025001	2.39E+08	2.47561
NC_056054.1	19200001	19220001	2.37282
NC_056054.1	19205001	19225001	2.83027
NC_056055.1	52240001	52260001	5.00535
NC_056055.1	52245001	52265001	3.03413
NC_056055.1	52250001	52270001	3.01458
NC_056075.1	9920001	9940001	8.41277
NC_056075.1	9930001	9950001	9.43096
NC_056054.1	100460001	1E+08	1.96241
NC_056054.1	100465001	1E+08	1.98323
NC_056054.1	100470001	1E+08	2.19544
NC_056054.1	100475001	1E+08	2.22262
NC_056054.1	100480001	1.01E+08	2.10433
NC_056054.1	100485001	1.01E+08	1.9379
NC_056061.1	21165001	21185001	1.97629
NC_056061.1	21170001	21190001	2.09193
NC_056078.1	17605001	17625001	2.04869
NC_056078.1	17610001	17630001	2.24569
NC_056061.1	21345001	21365001	2.27281
NC_056054.1	104040001	1.04E+08	1.96811
NC_056054.1	104035001	1.04E+08	2.01017
NC_056060.1	65260001	65280001	2.1419

NC_056080.	113725001	1.14E+08	3.21177	0.173243	OL	SASH3;ZDHHC9
NC_056060.	41755001	41775001	5.13817	0.247488	OL	SAV1
NC_056060.	41760001	41780001	6.41693	0.229788	OL	SAV1
NC_056060.	41765001	41785001	6.04805	0.207547	OL	SAV1
NC_056060.	41770001	41790001	5.72598	0.167787	OL	SAV1
NC_056060.	41775001	41795001	7.92237	0.185787	OL	SAV1
NC_056060.	41780001	41800001	5.28737	0.151587	OL	SAV1
NC_056068.	42110001	42130001	2.39058	0.173774	OL	SBF2
NC_056068.	42115001	42135001	4.1525	0.228677	OL	SBF2
NC_056068.	42120001	42140001	4.44736	0.213332	OL	SBF2
NC_056068.	42125001	42145001	3.44812	0.205609	OL	SBF2
NC_056068.	42130001	42150001	2.15393	0.17369	OL	SBF2
NC_056056.	10860001	10880001	3.81493	0.171525	OL	SCAI
NC_056056.	10865001	10885001	4.01142	0.212252	OL	SCAI
NC_056056.	10870001	10890001	4.69728	0.243863	OL	SCAI
NC_056056.	10875001	10895001	3.50529	0.236558	OL	SCAI
NC_056056.	10880001	10900001	2.99107	0.204849	OL	SCAI
NC_056056.	10885001	10905001	2.74907	0.169442	OL	SCAI
NC_056071.	31465001	31485001	2.02467	0.204066	OL	SCAMP2
NC_056071.	31470001	31490001	2.08157	0.221663	OL	SCAMP2
NC_056071.	31475001	31495001	2.19315	0.238794	OL	SCAMP2
NC_056071.	31480001	31500001	2.24425	0.242425	OL	SCAMP2
NC_056071.	31485001	31505001	2.20349	0.243112	OL	SCAMP2;ULK3
NC_056071.	31490001	31510001	2.21978	0.228132	OL	SCAMP2;ULK3
NC_056056.	134700001	1.35E+08	1.91143	0.159408	OL	SCN8A
NC_056059.	16175001	16195001	2.46114	0.186688	OL	SEC24B
NC_056059.	16180001	16200001	3.81895	0.252021	OL	SEC24B
NC_056059.	16185001	16205001	3.94647	0.243684	OL	SEC24B
NC_056059.	16190001	16210001	1.89111	0.215497	OL	SEC24B
NC_056059.	45910001	45930001	2.17685	0.244936	OL	SEL1L3
NC_056059.	45915001	45935001	1.87804	0.217154	OL	SEL1L3

NC_056055.1	107355001	1.07E+08	2.18735
NC_056055.1	107360001	1.07E+08	2.1831
NC_056055.1	107365001	1.07E+08	2.12448
NC_056055.1	107375001	1.07E+08	2.1049
NC_056055.1	107350001	1.07E+08	2.14984
NC_056074.1	40505001	40525001	2.24704
NC_056061.1	72945001	72965001	2.49029
NC_056060.1	9520001	9540001	2.19448
NC_056060.1	9525001	9545001	2.33133
NC_056071.1	31465001	31485001	1.97228
NC_056071.1	31470001	31490001	2.06473
NC_056071.1	31475001	31495001	2.07671
NC_056071.1	31480001	31500001	1.94778
NC_056071.1	29440001	29460001	2.4322
NC_056071.1	29445001	29465001	2.83944
NC_056071.1	29450001	29470001	2.63481
NC_056071.1	29455001	29475001	2.05956
NC_056060.1	56835001	56855001	1.94345
NC_056077.1	39890001	39910001	2.87519
NC_056077.1	39895001	39915001	3.40012
NC_056077.1	39900001	39920001	2.34472
NC_056077.1	39925001	39945001	2.96966
NC_056077.1	39930001	39950001	3.45555
NC_056077.1	39935001	39955001	2.11522
NC_056077.1	40525001	40545001	2.22161
NC_056056.1	33545001	33565001	2.60578
NC_056054.1	188085001	1.88E+08	5.78204
NC_056054.1	188090001	1.88E+08	3.94557
NC_056054.1	188095001	1.88E+08	2.28852
NC_056061.1	2465001	2485001	2.39868
NC_056061.1	2470001	2490001	2.65756

NC_056070.	69865001	69885001	5.43776	0.232182	OL	SELENOM;SMTN
NC_056070.	69870001	69890001	2.99408	0.165523	OL	SELENOM;SMTN
NC_056057.	37640001	37660001	2.8549	0.146849	OL	SEMA3A
NC_056057.	37645001	37665001	3.50305	0.183266	OL	SEMA3A
NC_056057.	37650001	37670001	2.11706	0.148112	OL	SEMA3A
NC_056060.	60935001	60955001	1.97897	0.155091	OL	SEMA6D
NC_056071.	57010001	57030001	2.41267	0.17207	OL	SERPINA4
NC_056071.	57015001	57035001	2.75486	0.201479	OL	SERPINA4
NC_056071.	57020001	57040001	2.97868	0.205728	OL	SERPINA4
NC_056071.	57025001	57045001	3.15048	0.199093	OL	SERPINA4;SERPINA5
NC_056071.	57030001	57050001	5.2463	0.205835	OL	SERPINA4;SERPINA5
NC_056071.	57035001	57055001	4.79752	0.157408	OL	SERPINA5
NC_056076.	62260001	62280001	2.01037	0.218016	OL	SERPINB11
NC_056076.	62270001	62290001	1.9598	0.213036	OL	SERPINB7
NC_056056.	95060001	95080001	2.22451	0.147186	OL	SFXN5
NC_056056.	95065001	95085001	3.00817	0.17346	OL	SFXN5
NC_056056.	95070001	95090001	2.27986	0.143736	OL	SFXN5
NC_056061.	59570001	59590001	2.34715	0.178805	OL	SGK1
NC_056059.	17640001	17660001	2.45797	0.141952	OL	SGMS2
NC_056075.	24035001	24055001	2.7486	0.26851	OL	SH3PXD2A
NC_056075.	24040001	24060001	2.16952	0.246269	OL	SH3PXD2A
NC_056074.	44295001	44315001	2.2646	0.143054	OL	SHANK2
NC_056074.	44360001	44380001	1.88109	0.192724	OL	SHANK2
NC_056074.	44365001	44385001	1.91014	0.214295	OL	SHANK2
NC_056074.	44370001	44390001	2.31319	0.238379	OL	SHANK2
NC_056074.	44375001	44395001	2.9449	0.203686	OL	SHANK2
NC_056074.	44380001	44400001	2.02385	0.156273	OL	SHANK2
NC_056067.	47200001	47220001	2.36935	0.170275	OL	SIPA1L3
NC_056056.	79310001	79330001	2.48771	0.167055	OL	SIX2
NC_056056.	79315001	79335001	2.73631	0.155766	OL	SIX2
NC_056056.	79320001	79340001	2.40966	0.14921	OL	SIX2

NC_056061.1	2475001	2495001	2.74924
NC_056061.1	2480001	2500001	2.61477
NC_056061.1	2485001	2505001	2.48698
NC_056061.1	2500001	2520001	2.57382
NC_056061.1	2505001	2525001	2.82539
NC_056061.1	2510001	2530001	3.07114
NC_056061.1	2515001	2535001	3.10713
NC_056061.1	2520001	2540001	3.30931
NC_056061.1	2525001	2545001	2.78569
NC_056076.1	62260001	62280001	2.01037
NC_056063.1	25190001	25210001	2.84216
NC_056063.1	25195001	25215001	4.65903
NC_056056.1	4560001	4580001	2.96622
NC_056056.1	4565001	4585001	3.44846
NC_056056.1	4570001	4590001	2.15824
NC_056056.1	4575001	4595001	2.07035
NC_056054.1	42320001	42340001	2.62526
NC_056054.1	42325001	42345001	5.6965
NC_056054.1	42330001	42350001	4.30692
NC_056054.1	42335001	42355001	2.9242
NC_056074.1	44230001	44250001	2.56816
NC_056074.1	44235001	44255001	4.014
NC_056074.1	44240001	44260001	5.81148
NC_056074.1	44245001	44265001	7.10469
NC_056074.1	44250001	44270001	2.59021
NC_056055.1	23790001	23810001	1.93741
NC_056066.1	47610001	47630001	2.25877
NC_056078.1	40760001	40780001	1.99649
NC_056054.1	179145001	1.79E+08	3.13805
NC_056067.1	56010001	56030001	2.26305
NC_056067.1	56015001	56035001	2.03846

NC_056064.	37890001	37910001	2.06016	0.150152	OL	SKAP1
NC_056064.	37895001	37915001	2	0.156893	OL	SKAP1
NC_056064.	37900001	37920001	1.96926	0.150461	OL	SKAP1
NC_056059.	67760001	67780001	1.96212	0.187384	OL	SLAIN2
NC_056059.	67765001	67785001	2.65202	0.223103	OL	SLAIN2
NC_056059.	67770001	67790001	2.97903	0.233228	OL	SLAIN2
NC_056059.	67775001	67795001	2.33866	0.177877	OL	SLAIN2
NC_056057.	14400001	14420001	2.43622	0.176006	OL	SLC25A13
NC_056057.	14405001	14425001	2.13468	0.155143	OL	SLC25A13
NC_056069.	23680001	23700001	3.64905	0.144003	OL	SLC38A9
NC_056069.	23685001	23705001	5.75269	0.166752	OL	SLC38A9
NC_056069.	23690001	23710001	5.18465	0.151714	OL	SLC38A9
NC_056059.	87420001	87440001	1.93601	0.184252	OL	SLC4A4
NC_056056.	83745001	83765001	1.86643	0.189774	OL	SLC8A1
NC_056056.	83750001	83770001	1.99296	0.267514	OL	SLC8A1
NC_056056.	83755001	83775001	2.55155	0.250875	OL	SLC8A1
NC_056056.	83760001	83780001	1.85829	0.176829	OL	SLC8A1
NC_056080.	58075001	58095001	12.9407	0.222099	OL	SLC9A7
NC_056080.	58095001	58115001	13.85	0.187629	OL	SLC9A7
NC_056054.	245800001	2.46E+08	1.91879	0.142212	OL	SLC9A9
NC_056054.	246220001	2.46E+08	2.31868	0.165066	OL	SLC9A9
NC_056054.	246225001	2.46E+08	2.24749	0.155504	OL	SLC9A9
NC_056056.	194640001	1.95E+08	2.58333	0.147003	OL	SLCO1C1
NC_056056.	194645001	1.95E+08	3.02789	0.143726	OL	SLCO1C1
NC_056059.	40705001	40725001	2.41667	0.149851	OL	SLIT2
NC_056076.	47870001	47890001	1.99868	0.145425	OL	SMAD2
NC_056076.	47875001	47895001	2.05747	0.151265	OL	SMAD2
NC_056076.	50840001	50860001	4.64634	0.166693	OL	SMAD4
NC_056076.	50845001	50865001	3.12648	0.205005	OL	SMAD4
NC_056076.	50850001	50870001	2.34496	0.285412	OL	SMAD4
NC_056076.	50855001	50875001	2.2687	0.300561	OL	SMAD4

NC_056067.1	56020001	56040001	1.93707
NC_056063.1	53470001	53490001	2.00705
NC_056063.1	53475001	53495001	4.37873
NC_056063.1	53480001	53500001	4.13379
NC_056063.1	53485001	53505001	3.3293
NC_056063.1	53490001	53510001	4.51298
NC_056070.1	12090001	12110001	9.69696
NC_056070.1	12095001	12115001	21.3267
NC_056070.1	12100001	12120001	10.1455
NC_056070.1	12105001	12125001	7.75334
NC_056070.1	12110001	12130001	5.08818
NC_056070.1	12115001	12135001	1.97348
NC_056076.1	45355001	45375001	1.98039
NC_056054.1	187195001	1.87E+08	6.60952
NC_056054.1	187220001	1.87E+08	12.3988
NC_056074.1	34635001	34655001	2.1086
NC_056074.1	19950001	19970001	10.052
NC_056074.1	19980001	20000001	4.14597
NC_056074.1	19985001	20005001	3.8492
NC_056074.1	19990001	20010001	3.90296
NC_056074.1	19995001	20015001	4.09199
NC_056074.1	20000001	20020001	3.61047
NC_056066.1	38510001	38530001	3.03344
NC_056066.1	38515001	38535001	5.23319
NC_056066.1	38520001	38540001	5.2287
NC_056066.1	38525001	38545001	4.44828
NC_056066.1	38550001	38570001	2.68633
NC_056066.1	38555001	38575001	2.71736
NC_056066.1	38560001	38580001	2.40994
NC_056057.1	14285001	14305001	2.34872
NC_056071.1	44735001	44755001	1.98418

NC_056076.	50860001	50880001	2.35522	0.269623	OL	SMAD4
NC_056076.	50865001	50885001	2.43696	0.250527	OL	SMAD4
NC_056076.	50870001	50890001	2.70286	0.18592	OL	SMAD4
NC_056074.	945001	965001	1.88346	0.433874	OL	SMCO4
NC_056074.	950001	970001	1.87766	0.415227	OL	SMCO4
NC_056070.	69850001	69870001	2.68361	0.244823	OL	SMTN
NC_056070.	69855001	69875001	6.38442	0.234435	OL	SMTN
NC_056070.	69860001	69880001	5.87156	0.252544	OL	SMTN
NC_056065.	30180001	30200001	2.44128	0.160349	OL	SMYD3
NC_056065.	30185001	30205001	2.57289	0.174205	OL	SMYD3
NC_056065.	30190001	30210001	2.48437	0.173285	OL	SMYD3
NC_056066.	3370001	3390001	2.07491	0.192731	OL	SNAP25
NC_056066.	3375001	3395001	1.9975	0.195157	OL	SNAP25
NC_056072.	15225001	15245001	2.60392	0.50642	OL	SNRK
NC_056072.	15230001	15250001	1.83015	0.354546	OL	SNRK
NC_056061.	51255001	51275001	2.44263	0.197429	OL	SNX14
NC_056061.	51260001	51280001	2.14094	0.278119	OL	SNX14
NC_056071.	42755001	42775001	2.34702	0.145993	OL	SNX6
NC_056071.	42760001	42780001	2.35906	0.152892	OL	SNX6
NC_056071.	42765001	42785001	2.15458	0.157575	OL	SNX6
NC_056071.	42770001	42790001	2.29563	0.17436	OL	SNX6
NC_056071.	42775001	42795001	2.08364	0.147893	OL	SNX6
NC_056060.	41300001	41320001	5.51862	0.199965	OL	SOS2
NC_056060.	41330001	41350001	6.48523	0.156013	OL	SOS2
NC_056060.	41335001	41355001	22.1696	0.167794	OL	SOS2
NC_056060.	41345001	41365001	16.8723	0.18665	OL	SOS2
NC_056060.	41350001	41370001	11.6946	0.22671	OL	SOS2
NC_056060.	41355001	41375001	3.55047	0.182968	OL	SOS2
NC_056060.	41360001	41380001	2.62585	0.164622	OL	SOS2
NC_056060.	41280001	41300001	8.36	0.326661	OL	SOS2;VCPKMT
NC_056060.	41285001	41305001	15.4368	0.318314	OL	SOS2;VCPKMT

NC_056071.1	44740001	44760001	2.09127
NC_056071.1	44745001	44765001	2.21263
NC_056071.1	44755001	44775001	2.41918
NC_056071.1	44760001	44780001	2.25178
NC_056071.1	45140001	45160001	3.03692
NC_056071.1	62840001	62860001	2.24732
NC_056071.1	62845001	62865001	2.16011
NC_056071.1	62850001	62870001	2.04921
NC_056069.1	10650001	10670001	2.80412
NC_056069.1	10655001	10675001	2.85234
NC_056061.1	49800001	49820001	2.0773
NC_056064.1	57405001	57425001	4.45147
NC_056064.1	57410001	57430001	3.79793
NC_056059.1	87420001	87440001	2.04936
NC_056056.1	61740001	61760001	1.99485
NC_056072.1	53490001	53510001	3.0719
NC_056080.1	58070001	58090001	2.40774
NC_056080.1	58075001	58095001	10.1126
NC_056080.1	58080001	58100001	22.1568
NC_056080.1	58095001	58115001	13.5122
NC_056066.1	77935001	77955001	2.82357
NC_056066.1	77940001	77960001	4.74676
NC_056066.1	77985001	78005001	8.15596
NC_056059.1	40705001	40725001	1.95615
NC_056072.1	52000001	52020001	2.13296
NC_056072.1	52005001	52025001	2.45589
NC_056072.1	52010001	52030001	2.56993
NC_056072.1	52015001	52035001	3.21705
NC_056072.1	52020001	52040001	3.79039
NC_056072.1	52025001	52045001	4.60444
NC_056072.1	52030001	52050001	5.33498

NC_056060.	41290001	41310001	17.5931	0.27641	OL	SOS2;VCPKMT
NC_056060.	41295001	41315001	11.7891	0.229879	OL	SOS2;VCPKMT
NC_056054.	95565001	95585001	1.84847	0.200171	OL	SPAG17
NC_056054.	95570001	95590001	2.02249	0.183134	OL	SPAG17
NC_056054.	95395001	95415001	1.953	0.260683	OL	SPAG17;WDR3
NC_056059.	68610001	68630001	2.14912	0.167988	OL	SPATA18
NC_056055.	249375001	2.49E+08	2.37286	0.178154	OL	SPATA21
NC_056055.	249380001	2.49E+08	2.14245	0.206711	OL	SPATA21
NC_056055.	249385001	2.49E+08	2.029	0.187847	OL	SPATA21
NC_056055.	249390001	2.49E+08	1.88685	0.172163	OL	SPATA21;SZRD1
NC_056055.	72925001	72945001	1.90573	0.162547	OL	SPATA6L
NC_056055.	72935001	72955001	2.50618	0.171687	OL	SPATA6L
NC_056055.	72940001	72960001	2.14116	0.148392	OL	SPATA6L
NC_056062.	32415001	32435001	2.63746	0.162588	OL	SPIDR
NC_056062.	32420001	32440001	3.25179	0.146861	OL	SPIDR
NC_056054.	109580001	1.1E+08	2.2748	0.178068	OL	SPTA1
NC_056054.	109585001	1.1E+08	2.20609	0.176218	OL	SPTA1
NC_056054.	109590001	1.1E+08	1.93644	0.17066	OL	SPTA1
NC_056066.	6545001	6565001	1.95268	0.199725	OL	SPTLC3
NC_056066.	6550001	6570001	2.38091	0.192956	OL	SPTLC3
NC_056066.	6555001	6575001	2.64667	0.171758	OL	SPTLC3
NC_056059.	71525001	71545001	1.98495	0.269234	OL	SRD5A3
NC_056072.	17320001	17340001	1.85114	0.228255	OL	SRGAP3
NC_056058.	97695001	97715001	2.78657	0.147008	OL	ST8SIA4
NC_056058.	97700001	97720001	4.56972	0.208936	OL	ST8SIA4
NC_056058.	97705001	97725001	3.77823	0.193513	OL	ST8SIA4
NC_056058.	97710001	97730001	3.24004	0.154002	OL	ST8SIA4
NC_056063.	27815001	27835001	2.04207	0.225868	OL	STARD13
NC_056063.	27820001	27840001	3.21082	0.182053	OL	STARD13
NC_056063.	27825001	27845001	3.63928	0.143699	OL	STARD13
NC_056063.	27845001	27865001	10.7714	0.159127	OL	STARD13

NC_056072.1	52035001	52055001	5.3016
NC_056072.1	52040001	52060001	3.62934
NC_056072.1	52045001	52065001	2.55142
NC_056066.1	78040001	78060001	2.63738
NC_056066.1	78045001	78065001	2.25457
NC_056062.1	94845001	94865001	2.25731
NC_056062.1	94870001	94890001	3.71663
NC_056062.1	94875001	94895001	2.76267
NC_056062.1	94880001	94900001	2.22732
NC_056074.1	34300001	34320001	7.36476
NC_056077.1	11395001	11415001	3.75738
NC_056077.1	11400001	11420001	2.93046
NC_056077.1	11405001	11425001	2.77295
NC_056077.1	11410001	11430001	3.74734
NC_056077.1	11415001	11435001	3.71429
NC_056077.1	11420001	11440001	4.392
NC_056077.1	11425001	11445001	3.73636
NC_056077.1	11430001	11450001	2.53193
NC_056077.1	11435001	11455001	2.31117
NC_056077.1	11440001	11460001	1.94877
NC_056071.1	42735001	42755001	2.36917
NC_056071.1	42740001	42760001	2.46243
NC_056071.1	42745001	42765001	3.66468
NC_056071.1	42750001	42770001	7.29075
NC_056071.1	42755001	42775001	4.48693
NC_056071.1	42760001	42780001	3.7324
NC_056071.1	42765001	42785001	2.6033
NC_056075.1	25245001	25265001	2.13901
NC_056075.1	25250001	25270001	2.33866
NC_056075.1	25505001	25525001	2.15124
NC_056075.1	25510001	25530001	2.77952

NC_056063.	28040001	28060001	3.31598	0.16455	OL	STARD13
NC_056063.	28045001	28065001	4.3712	0.182406	OL	STARD13
NC_056063.	28050001	28070001	3.07598	0.162415	OL	STARD13
NC_056063.	28055001	28075001	3.01273	0.161055	OL	STARD13
NC_056063.	28265001	28285001	2.28918	0.163114	OL	STARD13
NC_056063.	28270001	28290001	2.1908	0.189167	OL	STARD13
NC_056063.	28275001	28295001	1.95698	0.204826	OL	STARD13
NC_056060.	36130001	36150001	3.39968	0.157383	OL	STARD9
NC_056060.	36135001	36155001	3.83474	0.18964	OL	STARD9
NC_056060.	36140001	36160001	3.44159	0.179403	OL	STARD9
NC_056060.	36145001	36165001	7.69119	0.224482	OL	STARD9
NC_056060.	36150001	36170001	4.12738	0.142959	OL	STARD9
NC_056062.	49930001	49950001	2.24112	0.299328	OL	STAU2
NC_056062.	49935001	49955001	4.44204	0.366587	OL	STAU2
NC_056062.	49940001	49960001	5.11045	0.365338	OL	STAU2
NC_056062.	49945001	49965001	6.39039	0.388563	OL	STAU2
NC_056062.	49950001	49970001	12.8701	0.437057	OL	STAU2
NC_056062.	49955001	49975001	9.99123	0.403581	OL	STAU2
NC_056062.	49960001	49980001	6.37459	0.370842	OL	STAU2
NC_056062.	49965001	49985001	3.22822	0.263153	OL	STAU2
NC_056056.	188225001	1.88E+08	1.91937	0.146442	OL	STK38L
NC_056055.	242250001	2.42E+08	2.23528	0.193535	OL	STPG1
NC_056055.	242275001	2.42E+08	2.67441	0.166889	OL	STPG1
NC_056055.	242280001	2.42E+08	3.24211	0.206357	OL	STPG1
NC_056055.	242285001	2.42E+08	2.82362	0.200687	OL	STPG1
NC_056055.	242290001	2.42E+08	2.35235	0.144121	OL	STPG1
NC_056056.	87135001	87155001	1.98122	0.158075	OL	STRN
NC_056056.	87140001	87160001	2.75579	0.181884	OL	STRN
NC_056056.	87145001	87165001	2.46667	0.22069	OL	STRN
NC_056056.	8550001	8570001	9.1044	0.153532	OL	STXBP1
NC_056057.	81970001	81990001	3.99783	0.152588	OL	SUGCT

NC_056075.1	25515001	25535001	2.13892
NC_056075.1	25520001	25540001	2.07141
NC_056060.1	41300001	41320001	5.4462
NC_056060.1	41335001	41355001	8.44558
NC_056060.1	41345001	41365001	8.55754
NC_056060.1	41350001	41370001	7.10779
NC_056060.1	41355001	41375001	6.77818
NC_056060.1	41360001	41380001	6.12698
NC_056060.1	41280001	41300001	9.02157
NC_056060.1	41285001	41305001	14.9222
NC_056060.1	41290001	41310001	18.2214
NC_056060.1	41295001	41315001	10.4652
NC_056056.1	191400001	1.91E+08	3.11644
NC_056056.1	191405001	1.91E+08	3.22754
NC_056056.1	192015001	1.92E+08	2.43761
NC_056056.1	192020001	1.92E+08	4.21219
NC_056056.1	192025001	1.92E+08	3.90613
NC_056068.1	35725001	35745001	2.01216
NC_056068.1	35730001	35750001	2.36437
NC_056068.1	35735001	35755001	2.30476
NC_056068.1	35740001	35760001	2.07109
NC_056068.1	35745001	35765001	2.26628
NC_056068.1	35750001	35770001	2.60163
NC_056068.1	35755001	35775001	2.59941
NC_056068.1	35760001	35780001	4.43805
NC_056068.1	35765001	35785001	4.13032
NC_056068.1	35770001	35790001	3.65697
NC_056068.1	35775001	35795001	3.9396
NC_056068.1	35780001	35800001	2.59954
NC_056068.1	35785001	35805001	2.41033
NC_056068.1	35790001	35810001	2.40565

NC_056057.	81975001	81995001	8.95506	0.21204	OL	SUGCT
NC_056057.	81980001	82000001	6.44312	0.218195	OL	SUGCT
NC_056057.	81985001	82005001	4.45078	0.218182	OL	SUGCT
NC_056057.	81990001	82010001	4.05231	0.212732	OL	SUGCT
NC_056057.	81995001	82015001	5.2763	0.252442	OL	SUGCT
NC_056057.	82000001	82020001	6.01524	0.269516	OL	SUGCT
NC_056057.	82005001	82025001	9.15054	0.316557	OL	SUGCT
NC_056057.	82015001	82035001	5.15385	0.318994	OL	SUGCT
NC_056057.	82020001	82040001	3.59005	0.318355	OL	SUGCT
NC_056072.	21935001	21955001	10.6074	0.141919	OL	SUMF1
NC_056072.	21975001	21995001	11.9755	0.144152	OL	SUMF1
NC_056061.	76895001	76915001	3.59724	0.168195	OL	SYNE1
NC_056061.	76900001	76920001	3.28538	0.145461	OL	SYNE1
NC_056064.	53310001	53330001	2.06709	0.152755	OL	SYNGR2;TK1
NC_056071.	7000001	7020001	1.94951	0.206806	OL	SYNM;TTC23
NC_056060.	72155001	72175001	1.9313	0.169672	OL	SYT16
NC_056061.	82825001	82845001	11.3863	0.182161	OL	SYTL3
NC_056061.	82830001	82850001	3.06399	0.150232	OL	SYTL3
NC_056061.	57945001	57965001	3.88694	0.280434	OL	TAAR8
NC_056061.	57950001	57970001	4.3504	0.299282	OL	TAAR8
NC_056061.	57940001	57960001	2.36376	0.187967	OL	TAAR8;TAAR9
NC_056062.	94100001	94120001	2.01531	0.183444	OL	TAF2
NC_056062.	94105001	94125001	2.22308	0.191565	OL	TAF2
NC_056062.	94110001	94130001	3.00843	0.241968	OL	TAF2
NC_056062.	94115001	94135001	2.74611	0.207929	OL	TAF2
NC_056062.	94120001	94140001	1.91408	0.163599	OL	TAF2
NC_056080.	132975001	1.33E+08	2.40894	0.240951	OL	TAF7L
NC_056072.	32790001	32810001	1.98354	0.147965	OL	TAFA4
NC_056072.	32795001	32815001	2.16772	0.217773	OL	TAFA4
NC_056072.	32800001	32820001	2.40516	0.238308	OL	TAFA4
NC_056072.	32805001	32825001	1.83125	0.198654	OL	TAFA4

NC_056068.1	35795001	35815001	2.43082
NC_056068.1	35800001	35820001	2.47601
NC_056068.1	35805001	35825001	2.65146
NC_056068.1	35810001	35830001	2.71912
NC_056068.1	35815001	35835001	2.44102
NC_056068.1	35820001	35840001	2.26135
NC_056068.1	35825001	35845001	2.18279
NC_056068.1	35830001	35850001	2.1202
NC_056068.1	35835001	35855001	2.41184
NC_056068.1	35840001	35860001	3.15198
NC_056068.1	35845001	35865001	4.75458
NC_056068.1	35850001	35870001	13.3109
NC_056068.1	35855001	35875001	15.7768
NC_056068.1	35860001	35880001	14.8
NC_056068.1	35865001	35885001	10.7143
NC_056068.1	35870001	35890001	5.80714
NC_056068.1	35875001	35895001	4.34694
NC_056068.1	35880001	35900001	3.85623
NC_056068.1	35885001	35905001	3.84013
NC_056068.1	35890001	35910001	4.78845
NC_056068.1	35895001	35915001	4.98379
NC_056068.1	35900001	35920001	4.06607
NC_056068.1	35905001	35925001	3.90455
NC_056068.1	35910001	35930001	3.14691
NC_056068.1	35915001	35935001	2.89447
NC_056068.1	35920001	35940001	3.52468
NC_056068.1	35925001	35945001	5.28144
NC_056064.1	58100001	58120001	2.10567
NC_056055.1	138960001	1.39E+08	3.83943
NC_056055.1	138965001	1.39E+08	5.03102
NC_056056.1	91765001	91785001	2.18566

NC_056064.	47395001	47415001	1.84615	0.30432	OL	TANC2
NC_056059.	58125001	58145001	2.06228	0.27168	OL	TBC1D1
NC_056061.	17190001	17210001	2.18726	0.17249	OL	TBC1D32
NC_056054.	275540001	2.76E+08	1.89678	0.179197	OL	TBC1D5
NC_056054.	275545001	2.76E+08	2.09907	0.203315	OL	TBC1D5
NC_056054.	275550001	2.76E+08	2.06533	0.204847	OL	TBC1D5
NC_056054.	275555001	2.76E+08	2.00758	0.207502	OL	TBC1D5
NC_056060.	99685001	99705001	3.33707	0.152876	OL	TDP1
NC_056080.	109195001	1.09E+08	1.82985	0.145056	OL	TENM1
NC_056080.	109200001	1.09E+08	1.8984	0.176988	OL	TENM1
NC_056074.	14540001	14560001	1.81896	0.141804	OL	TENM4
NC_056074.	14545001	14565001	1.93645	0.185812	OL	TENM4
NC_056074.	14550001	14570001	2.01125	0.193215	OL	TENM4
NC_056074.	14555001	14575001	2.1735	0.193669	OL	TENM4
NC_056074.	14560001	14580001	1.90797	0.154897	OL	TENM4
NC_056059.	20315001	20335001	3.23267	0.157564	OL	TET2
NC_056059.	20320001	20340001	4.15344	0.144484	OL	TET2
NC_056059.	20325001	20345001	6.26745	0.163434	OL	TET2
NC_056059.	20360001	20380001	2.84429	0.171768	OL	TET2
NC_056059.	20365001	20385001	2.84649	0.180326	OL	TET2
NC_056059.	20370001	20390001	3.27912	0.180974	OL	TET2
NC_056059.	20375001	20395001	3.12035	0.18001	OL	TET2
NC_056059.	20380001	20400001	4.24137	0.172526	OL	TET2
NC_056059.	20385001	20405001	3.37591	0.2106	OL	TET2
NC_056059.	20390001	20410001	2.30204	0.225626	OL	TET2
NC_056059.	20395001	20415001	2.09052	0.232926	OL	TET2
NC_056055.	9110001	9130001	2.04819	0.16194	OL	TEX48
NC_056055.	9120001	9140001	1.92112	0.164053	OL	TEX48
NC_056057.	53885001	53905001	1.84227	0.152168	OL	TFEC
NC_056057.	53890001	53910001	2.26507	0.193323	OL	TFEC
NC_056057.	53895001	53915001	2.28904	0.174167	OL	TFEC

NC_056056.1	91770001	91790001	3.77353
NC_056056.1	91775001	91795001	4.47696
NC_056069.1	1650001	1670001	2.56204
NC_056060.1	15765001	15785001	3.95293
NC_056060.1	15770001	15790001	2.31122
NC_056054.1	179125001	1.79E+08	2.9299
NC_056054.1	179130001	1.79E+08	2.57284
NC_056054.1	179135001	1.79E+08	3.34694
NC_056054.1	179140001	1.79E+08	3.01658
NC_056062.1	32415001	32435001	1.96486
NC_056062.1	32420001	32440001	2.23162
NC_056058.1	46420001	46440001	2.19951
NC_056058.1	46425001	46445001	2.89447
NC_056058.1	46430001	46450001	2.20576
NC_056066.1	66295001	66315001	2.38598
NC_056066.1	66300001	66320001	2.31951
NC_056066.1	66305001	66325001	2.09342
NC_056058.1	29550001	29570001	3.52381
NC_056058.1	29555001	29575001	4.38953
NC_056058.1	29560001	29580001	4.66203
NC_056058.1	29565001	29585001	2.18737
NC_056057.1	47900001	47920001	3.10979
NC_056057.1	47905001	47925001	3.82336
NC_056057.1	47910001	47930001	3.38664
NC_056057.1	47915001	47935001	3.0744
NC_056057.1	47955001	47975001	3.30726
NC_056057.1	47960001	47980001	3.00867
NC_056054.1	28760001	28780001	2.9916
NC_056054.1	28765001	28785001	4.41647
NC_056054.1	28770001	28790001	5.97261
NC_056054.1	28775001	28795001	6.63462

NC_056057.	53900001	53920001	2.34174	0.15007	OL	TFEC
NC_056072.	17215001	17235001	2.48352	0.142221	OL	THUMPD3
NC_056058.	17140001	17160001	6.38411	0.192384	OL	TICAM1
NC_056060.	16965001	16985001	1.9975	0.160782	OL	TLE3
NC_056070.	4180001	4200001	4.46654	0.290017	OL	TLR2
NC_056054.	239405001	2.39E+08	3.77809	0.146226	OL	TM4SF4
NC_056054.	239410001	2.39E+08	4.57394	0.148367	OL	TM4SF4
NC_056060.	86700001	86720001	2.51592	0.243901	OL	TMED8
NC_056060.	86705001	86725001	2.70478	0.255884	OL	TMED8
NC_056060.	86710001	86730001	2.11219	0.200921	OL	TMED8
NC_056060.	86715001	86735001	1.99001	0.182215	OL	TMED8
NC_056060.	86720001	86740001	1.95297	0.155594	OL	TMED8
NC_056055.	194470001	1.94E+08	1.95732	0.159426	OL	TMEFF2
NC_056055.	194475001	1.94E+08	2.2132	0.170692	OL	TMEFF2
NC_056070.	4205001	4225001	8.61432	0.531571	OL	TMEM131L
NC_056059.	97970001	97990001	2.2095	0.258733	OL	TMEM150C
NC_056059.	97975001	97995001	2.24688	0.249513	OL	TMEM150C
NC_056059.	97980001	98000001	2.37958	0.194346	OL	TMEM150C
NC_056059.	97985001	98005001	3.91144	0.159202	OL	TMEM150C
NC_056059.	97990001	98010001	6.40324	0.161386	OL	TMEM150C
NC_056059.	71530001	71550001	2.9135	0.288858	OL	TMEM165
NC_056059.	71535001	71555001	3.49062	0.246816	OL	TMEM165
NC_056059.	71540001	71560001	3.9758	0.252686	OL	TMEM165
NC_056059.	71545001	71565001	3.09248	0.270792	OL	TMEM165
NC_056059.	71550001	71570001	2.25656	0.239877	OL	TMEM165
NC_056059.	71555001	71575001	1.86538	0.225781	OL	TMEM165
NC_056076.	33660001	33680001	2.52732	0.190757	OL	TMEM241
NC_056076.	33665001	33685001	2.57725	0.186681	OL	TMEM241
NC_056076.	33670001	33690001	2.27873	0.185298	OL	TMEM241
NC_056062.	68550001	68570001	2.50644	0.192366	OL	TMEM74
NC_056058.	32335001	32355001	2.52655	0.14565	OL	TNFAIP8

NC_056062.1	20970001	20990001	2.21058
NC_056062.1	20975001	20995001	3.82992
NC_056062.1	20980001	21000001	11.658
NC_056062.1	20985001	21005001	15.3214
NC_056062.1	20995001	21015001	8.39937
NC_056062.1	21000001	21020001	5.96725
NC_056062.1	21005001	21025001	3.95866
NC_056062.1	21010001	21030001	2.41423
NC_056062.1	21025001	21045001	2.14167
NC_056062.1	21030001	21050001	3.21927
NC_056062.1	21035001	21055001	6.64723
NC_056062.1	21040001	21060001	4.91021
NC_056058.1	97695001	97715001	2.08138
NC_056063.1	28040001	28060001	3.21549
NC_056063.1	28045001	28065001	4.2271
NC_056063.1	28050001	28070001	2.10569
NC_056062.1	49930001	49950001	3.71009
NC_056062.1	49935001	49955001	9.43078
NC_056062.1	49940001	49960001	9.05819
NC_056062.1	49945001	49965001	13.3836
NC_056062.1	49950001	49970001	12.3805
NC_056062.1	49955001	49975001	9.73505
NC_056062.1	49960001	49980001	7.03956
NC_056062.1	49965001	49985001	3.60506
NC_056056.1	188225001	1.88E+08	3.22709
NC_056056.1	188230001	1.88E+08	2.08974
NC_056056.1	188235001	1.88E+08	2.65269
NC_056054.1	265395001	2.65E+08	2.79413
NC_056056.1	176760001	1.77E+08	1.95218
NC_056061.1	77025001	77045001	2.01456
NC_056061.1	77030001	77050001	3.23589

NC_056058.	32340001	32360001	2.42618	0.156286	OL	TNFAIP8
NC_056068.	79315001	79335001	2.08511	0.144418	OL	TNKS1BP1
NC_056068.	79320001	79340001	2.66724	0.200827	OL	TNKS1BP1
NC_056068.	79325001	79345001	2.45495	0.19386	OL	TNKS1BP1
NC_056068.	79330001	79350001	1.9568	0.191965	OL	TNKS1BP1
NC_056055.	220120001	2.2E+08	1.84768	0.247162	OL	TNS1
NC_056056.	35900001	35920001	6.77131	0.328987	OL	TOGARAM2
NC_056061.	13220001	13240001	2.37341	0.199955	OL	TPD52L1
NC_056076.	20615001	20635001	9.63679	0.160881	OL	TPGS2
NC_056076.	20620001	20640001	8.77655	0.146765	OL	TPGS2
NC_056073.	38695001	38715001	2.98305	0.225509	OL	TPMT
NC_056073.	38700001	38720001	3.18868	0.169391	OL	TPMT
NC_056073.	30905001	30925001	2.33956	0.207166	OL	TRIM38
NC_056073.	30910001	30930001	2.34861	0.201347	OL	TRIM38
NC_056072.	7275001	7295001	2.67116	0.185693	OL	TRIM71
NC_056069.	58940001	58960001	2.11047	0.148863	OL	TRIO
NC_056080.	132755001	1.33E+08	1.84284	0.191824	OL	TRMT2B
NC_056067.	41405001	41425001	5.827	0.178362	OL	TSHZ3
NC_056078.	25000001	25020001	2.26761	0.177671	OL	TSPAN15
NC_056078.	25005001	25025001	3.33079	0.257032	OL	TSPAN15
NC_056078.	25010001	25030001	4.75	0.343007	OL	TSPAN15
NC_056078.	25015001	25035001	3.16347	0.315903	OL	TSPAN15
NC_056078.	25020001	25040001	2.90135	0.304606	OL	TSPAN15
NC_056078.	25025001	25045001	2.40223	0.295291	OL	TSPAN15
NC_056055.	143615001	1.44E+08	2.56472	0.268711	OL	TTC21B
NC_056055.	143620001	1.44E+08	2.41605	0.26365	OL	TTC21B
NC_056055.	143625001	1.44E+08	2.73795	0.282968	OL	TTC21B
NC_056055.	143630001	1.44E+08	1.89487	0.214159	OL	TTC21B
NC_056058.	92455001	92475001	2.64811	0.254525	OL	TTC37
NC_056058.	92460001	92480001	3.46606	0.304896	OL	TTC37
NC_056058.	92465001	92485001	3.66072	0.312123	OL	TTC37

NC_056061.1	77035001	77055001	3.83238
NC_056061.1	77040001	77060001	3.18717
NC_056057.1	48665001	48685001	2.25625
NC_056066.1	73595001	73615001	2.44118
NC_056066.1	73600001	73620001	5.00555
NC_056066.1	73605001	73625001	5.71188
NC_056066.1	73610001	73630001	2.20332
NC_056065.1	72770001	72790001	2.41404
NC_056065.1	72775001	72795001	2.55979
NC_056065.1	72780001	72800001	2.48767
NC_056065.1	72785001	72805001	2.49155
NC_056065.1	72790001	72810001	2.40368
NC_056065.1	72795001	72815001	2.97165
NC_056065.1	72800001	72820001	3.92838
NC_056065.1	72805001	72825001	3.81666
NC_056065.1	72810001	72830001	4.04147
NC_056065.1	72815001	72835001	4.2706
NC_056065.1	72820001	72840001	3.82608
NC_056056.1	216670001	2.17E+08	2.13504
NC_056063.1	51110001	51130001	13.864
NC_056063.1	51120001	51140001	20.8333
NC_056063.1	51125001	51145001	3.6677
NC_056063.1	51130001	51150001	2.38174
NC_056080.1	128940001	1.29E+08	3.92818
NC_056059.1	67630001	67650001	3.12265
NC_056059.1	67635001	67655001	5.7751
NC_056059.1	67640001	67660001	8.23603
NC_056059.1	67645001	67665001	7.61782
NC_056059.1	67650001	67670001	2.5934
NC_056059.1	67655001	67675001	2.16009
NC_056059.1	67660001	67680001	1.9911

NC_056058.	92470001	92490001	3.49622	0.311271	OL	TTC37
NC_056058.	92475001	92495001	4.03045	0.329877	OL	TTC37
NC_056058.	92480001	92500001	4.32247	0.342424	OL	TTC37
NC_056058.	92485001	92505001	3.3002	0.308141	OL	TTC37
NC_056061.	82480001	82500001	4.10617	0.261856	OL	TULP4
NC_056061.	82650001	82670001	3.7228	0.261327	OL	TULP4
NC_056061.	82655001	82675001	3.31104	0.235267	OL	TULP4
NC_056061.	82660001	82680001	2.94252	0.218443	OL	TULP4
NC_056061.	82665001	82685001	2.44946	0.175205	OL	TULP4
NC_056061.	82670001	82690001	3.3913	0.190217	OL	TULP4
NC_056061.	82675001	82695001	2.56572	0.180549	OL	TULP4
NC_056061.	82680001	82700001	3.2971	0.183544	OL	TULP4
NC_056061.	82685001	82705001	3.58491	0.230187	OL	TULP4
NC_056061.	82690001	82710001	3.99998	0.235814	OL	TULP4
NC_056061.	82695001	82715001	3.19067	0.170912	OL	TULP4
NC_056060.	17625001	17645001	1.86832	0.186153	OL	UACA
NC_056066.	78125001	78145001	1.91255	0.226026	OL	UBE2V1
NC_056061.	10210001	10230001	2.06667	0.221364	OL	UBE3D
NC_056056.	44395001	44415001	5.22856	0.167094	OL	UGP2
NC_056056.	44400001	44420001	5.69387	0.181381	OL	UGP2
NC_056056.	44405001	44425001	5.3052	0.164547	OL	UGP2
NC_056056.	44410001	44430001	4.89116	0.175497	OL	UGP2
NC_056058.	36175001	36195001	2.14669	0.144529	OL	UIMC1
NC_056058.	36180001	36200001	2.1225	0.150334	OL	UIMC1
NC_056058.	36185001	36205001	2.16744	0.15061	OL	UIMC1
NC_056058.	36190001	36210001	2.14359	0.141486	OL	UIMC1
NC_056060.	54085001	54105001	2.26744	0.227872	OL	UNC13C
NC_056058.	35980001	36000001	10.0376	0.145753	OL	UNC5A
NC_056058.	35985001	36005001	5.36508	0.147439	OL	UNC5A
NC_056058.	36010001	36030001	8.10453	0.159067	OL	UNC5A
NC_056058.	36015001	36035001	17.7895	0.16652	OL	UNC5A

NC_056059.1	80485001	80505001	2.23615
NC_056059.1	80495001	80515001	2.13225
NC_056059.1	80500001	80520001	2.15011
NC_056059.1	80505001	80525001	2.02976
NC_056059.1	80510001	80530001	2.15487
NC_056059.1	80515001	80535001	2.14269
NC_056059.1	80520001	80540001	2.11241
NC_056059.1	80525001	80545001	2.19485
NC_056059.1	80530001	80550001	2.21212
NC_056059.1	80535001	80555001	2.21711
NC_056059.1	80540001	80560001	2.29344
NC_056059.1	80545001	80565001	2.27086
NC_056059.1	80560001	80580001	2.28261
NC_056059.1	80570001	80590001	2.1565
NC_056059.1	80580001	80600001	2.10395
NC_056059.1	80585001	80605001	2.13558
NC_056059.1	80590001	80610001	2.06346
NC_056059.1	80595001	80615001	2.00133
NC_056059.1	80630001	80650001	2.06139
NC_056059.1	80635001	80655001	2.10669
NC_056059.1	80640001	80660001	2.09018
NC_056080.1	108445001	1.08E+08	2.45421
NC_056080.1	109195001	1.09E+08	2.24542
NC_056072.1	5145001	5165001	2.05508
NC_056060.1	18565001	18585001	2.25158
NC_056054.1	124880001	1.25E+08	2.20893
NC_056054.1	124885001	1.25E+08	2.65325
NC_056054.1	124890001	1.25E+08	5.94979
NC_056061.1	79490001	79510001	2.10264
NC_056061.1	79495001	79515001	2.28414
NC_056061.1	79500001	79520001	2.01304

NC_056058.	36020001	36040001	18.7182	0.174732	OL	UNC5A
NC_056058.	36025001	36045001	19.6585	0.158419	OL	UNC5A
NC_056071.	55995001	56015001	1.86679	0.141423	OL	UNC79
NC_056054.	184860001	1.85E+08	3.1257	0.180412	OL	UPK1B
NC_056054.	184865001	1.85E+08	2.82361	0.142017	OL	UPK1B
NC_056065.	18190001	18210001	2.28169	0.180703	OL	USH2A
NC_056065.	18195001	18215001	2.75129	0.193659	OL	USH2A
NC_056065.	18200001	18220001	2.55058	0.152152	OL	USH2A
NC_056065.	18205001	18225001	2.63793	0.158594	OL	USH2A
NC_056065.	18210001	18230001	2.67325	0.151797	OL	USH2A
NC_056065.	18215001	18235001	2.45148	0.166245	OL	USH2A
NC_056065.	18220001	18240001	2.74148	0.198597	OL	USH2A
NC_056065.	18225001	18245001	2.03068	0.143074	OL	USH2A
NC_056067.	10400001	10420001	2.50804	0.175203	OL	USP10
NC_056067.	10405001	10425001	3.02967	0.194769	OL	USP10
NC_056067.	10410001	10430001	2.7824	0.155344	OL	USP10
NC_056067.	10430001	10450001	1.80738	0.222691	OL	USP10
NC_056067.	10435001	10455001	2.10769	0.241027	OL	USP10
NC_056063.	32980001	33000001	2.36624	0.152803	OL	USP12
NC_056063.	32985001	33005001	2.80368	0.189702	OL	USP12
NC_056063.	32990001	33010001	5.26719	0.271592	OL	USP12
NC_056068.	54090001	54110001	2.33691	0.2452	OL	UVRAG
NC_056068.	54095001	54115001	1.94155	0.151997	OL	UVRAG
NC_056067.	890001	910001	2.76934	0.280121	OL	VAC14
NC_056067.	895001	915001	2.85714	0.251263	OL	VAC14
NC_056067.	900001	920001	3.00236	0.258157	OL	VAC14
NC_056067.	905001	925001	3.2318	0.24348	OL	VAC14
NC_056067.	910001	930001	4.20759	0.272482	OL	VAC14
NC_056067.	915001	935001	4.5635	0.271578	OL	VAC14
NC_056067.	920001	940001	4.40225	0.195711	OL	VAC14
NC_056067.	950001	970001	3.39281	0.170227	OL	VAC14

NC_056061.1	79540001	79560001	8.21876
NC_056061.1	79545001	79565001	3.64664
NC_056061.1	79550001	79570001	3.05497
NC_056061.1	79555001	79575001	2.26405
NC_056061.1	79560001	79580001	2.05788
NC_056075.1	17545001	17565001	5.17284
NC_056054.1	239395001	2.39E+08	1.9674
NC_056055.1	194395001	1.94E+08	5.97864
NC_056055.1	194400001	1.94E+08	9.96175
NC_056055.1	194405001	1.94E+08	8.90476
NC_056055.1	194410001	1.94E+08	8.05651
NC_056055.1	194420001	1.94E+08	7.13593
NC_056055.1	194475001	1.94E+08	2.01452
NC_056070.1	50535001	50555001	5.49887
NC_056070.1	50540001	50560001	4.11253
NC_056070.1	50545001	50565001	3.31284
NC_056070.1	50550001	50570001	2.99824
NC_056070.1	50555001	50575001	2.68309
NC_056070.1	50585001	50605001	4.01091
NC_056070.1	50590001	50610001	5.51029
NC_056070.1	50595001	50615001	6.69436
NC_056070.1	50600001	50620001	10.2099
NC_056070.1	50605001	50625001	16.302
NC_056070.1	50610001	50630001	22.5462
NC_056070.1	50710001	50730001	2.01329
NC_056070.1	50715001	50735001	5.91694
NC_056070.1	50720001	50740001	9.60511
NC_056076.1	33650001	33670001	3.80883
NC_056076.1	33655001	33675001	3.99693
NC_056076.1	33660001	33680001	3.09028
NC_056076.1	33665001	33685001	3.49038

NC_056067.	955001	975001	4.23918	0.225807	OL	VAC14
NC_056067.	960001	980001	3.73292	0.202455	OL	VAC14
NC_056067.	965001	985001	3.10248	0.171582	OL	VAC14
NC_056065.	70070001	70090001	4.4803	0.212777	OL	VASH2
NC_056065.	70075001	70095001	4.32296	0.210589	OL	VASH2
NC_056065.	70080001	70100001	3.13001	0.177924	OL	VASH2
NC_056060.	41260001	41280001	2.05172	0.232917	OL	VCPKMT
NC_056060.	41265001	41285001	3.10001	0.299438	OL	VCPKMT
NC_056060.	41270001	41290001	5.02207	0.315395	OL	VCPKMT
NC_056060.	41275001	41295001	7.14335	0.347225	OL	VCPKMT
NC_056058.	43320001	43340001	5.85415	0.185064	OL	VDAC1
NC_056058.	43325001	43345001	3.84692	0.180392	OL	VDAC1
NC_056058.	43330001	43350001	2.88975	0.146564	OL	VDAC1
NC_056056.	138630001	1.39E+08	2.33494	0.223207	OL	VDR
NC_056056.	138635001	1.39E+08	2.14021	0.23153	OL	VDR
NC_056056.	138640001	1.39E+08	2.32348	0.246636	OL	VDR
NC_056055.	59550001	59570001	2.34115	0.17393	OL	VPS13A
NC_056055.	59555001	59575001	2.22526	0.190133	OL	VPS13A
NC_056055.	59560001	59580001	2.28505	0.20216	OL	VPS13A
NC_056055.	59565001	59585001	2.2383	0.193438	OL	VPS13A
NC_056055.	59570001	59590001	2.34865	0.197555	OL	VPS13A
NC_056055.	59575001	59595001	2.21591	0.190716	OL	VPS13A
NC_056055.	59580001	59600001	2.23776	0.185805	OL	VPS13A
NC_056055.	59585001	59605001	2.22452	0.187222	OL	VPS13A
NC_056055.	59590001	59610001	2.14705	0.19227	OL	VPS13A
NC_056055.	59595001	59615001	2.15092	0.195219	OL	VPS13A
NC_056054.	96620001	96640001	1.9781	0.216172	OL	WARS2
NC_056054.	96625001	96645001	2.24028	0.26663	OL	WARS2
NC_056054.	96630001	96650001	2.41196	0.285299	OL	WARS2
NC_056054.	96635001	96655001	1.98192	0.224909	OL	WARS2
NC_056054.	96640001	96660001	1.82298	0.150797	OL	WARS2

NC_056072.1	54245001	54265001	3.88696
NC_056072.1	54250001	54270001	4.03168
NC_056072.1	54240001	54260001	2.75812
NC_056058.1	4380001	4400001	13.1212
NC_056058.1	4385001	4405001	4.80987
NC_056060.1	86555001	86575001	3.07474
NC_056060.1	86560001	86580001	4.23947
NC_056060.1	86565001	86585001	5.22034
NC_056060.1	86570001	86590001	4.35431
NC_056054.1	263335001	2.63E+08	2.0912
NC_056056.1	118840001	1.19E+08	8.72894
NC_056056.1	118845001	1.19E+08	6.72933
NC_056056.1	118850001	1.19E+08	5.37007
NC_056056.1	119050001	1.19E+08	2.17395
NC_056056.1	119055001	1.19E+08	2.23185
NC_056056.1	119060001	1.19E+08	2.28695
NC_056056.1	119065001	1.19E+08	2.34446
NC_056056.1	119070001	1.19E+08	2.11887
NC_056056.1	119085001	1.19E+08	2.50771
NC_056056.1	119090001	1.19E+08	2.39354
NC_056056.1	119095001	1.19E+08	2.29917
NC_056056.1	119100001	1.19E+08	2.30703
NC_056065.1	40095001	40115001	2.07026
NC_056065.1	40100001	40120001	2.15262
NC_056065.1	56140001	56160001	2.37854
NC_056065.1	56145001	56165001	3.48888
NC_056065.1	56150001	56170001	3.58832
NC_056065.1	56155001	56175001	3.15189
NC_056065.1	56180001	56200001	3.37948
NC_056065.1	56185001	56205001	3.14862
NC_056064.1	40435001	40455001	1.96732

NC_056063.	21335001	21355001	7.57129	0.219608	OL	WDFY2
NC_056063.	21340001	21360001	9.88198	0.226868	OL	WDFY2
NC_056063.	21345001	21365001	5.96984	0.228123	OL	WDFY2
NC_056063.	21350001	21370001	6.80683	0.228435	OL	WDFY2
NC_056063.	21355001	21375001	4.65074	0.174381	OL	WDFY2
NC_056063.	21360001	21380001	4.14472	0.165358	OL	WDFY2
NC_056059.	105965001	1.06E+08	2.36557	0.200056	OL	WDR1
NC_056059.	105970001	1.06E+08	2.32601	0.208283	OL	WDR1
NC_056059.	105975001	1.06E+08	2.15249	0.182843	OL	WDR1
NC_056056.	11045001	11065001	1.8619	0.173987	OL	WDR38
NC_056060.	54690001	54710001	3.89963	0.578182	OL	WDR72
NC_056060.	54695001	54715001	4.74873	0.610288	OL	WDR72
NC_056060.	54700001	54720001	1.80732	0.450497	OL	WDR72
NC_056060.	54795001	54815001	1.99885	0.153032	OL	WDR72
NC_056056.	222125001	2.22E+08	1.97192	0.196101	OL	WNT7B
NC_056056.	222130001	2.22E+08	1.91851	0.19664	OL	WNT7B
NC_056068.	6330001	6350001	2.06058	0.218235	OL	YAP1
NC_056068.	6335001	6355001	3.2963	0.285828	OL	YAP1
NC_056054.	180455001	1.8E+08	13.1047	0.185836	OL	ZBTB20
NC_056054.	180460001	1.8E+08	7.92856	0.169188	OL	ZBTB20
NC_056068.	19865001	19885001	2.17351	0.186968	OL	ZC3H12C
NC_056068.	19870001	19890001	2.26444	0.223433	OL	ZC3H12C
NC_056068.	19875001	19895001	2.07347	0.168596	OL	ZC3H12C
NC_056068.	19880001	19900001	2.05011	0.153728	OL	ZC3H12C
NC_056080.	47015001	47035001	1.98413	0.201923	OL	ZC4H2
NC_056080.	47020001	47040001	1.87342	0.199495	OL	ZC4H2
NC_056080.	47025001	47045001	1.89441	0.141729	OL	ZC4H2
NC_056061.	81750001	81770001	2.67911	0.159749	OL	ZDHHC14
NC_056061.	81755001	81775001	3.47208	0.214267	OL	ZDHHC14
NC_056061.	81760001	81780001	5.13169	0.285945	OL	ZDHHC14
NC_056061.	81765001	81785001	3.89374	0.299533	OL	ZDHHC14

NC_056064.1	40445001	40465001	2.01579
NC_056064.1	40450001	40470001	2.2795
NC_056064.1	34320001	34340001	2.26901
NC_056064.1	34325001	34345001	2.16213
NC_056068.1	65750001	65770001	2.15771
NC_056068.1	65755001	65775001	2.28018
NC_056068.1	65760001	65780001	2.50691
NC_056068.1	65765001	65785001	3.41758
NC_056079.1	13600001	13620001	5.82608
NC_056079.1	13605001	13625001	6.53092
NC_056079.1	13610001	13630001	4.08128
NC_056073.1	29130001	29150001	4.06689
NC_056068.1	47485001	47505001	9.45931
NC_056068.1	47490001	47510001	5.02441
NC_056068.1	47495001	47515001	2.43493
NC_056072.1	7275001	7295001	3.25137
NC_056077.1	27805001	27825001	3.44
NC_056077.1	27810001	27830001	4.92232
NC_056077.1	27815001	27835001	6.96888
NC_056077.1	27820001	27840001	3.4766
NC_056060.1	42050001	42070001	3.23925
NC_056060.1	42065001	42085001	2.09206
NC_056060.1	42070001	42090001	2.35232
NC_056060.1	42075001	42095001	2.25294
NC_056060.1	42170001	42190001	1.9537
NC_056060.1	42175001	42195001	2.74725
NC_056060.1	42180001	42200001	2.42794
NC_056069.1	58935001	58955001	2.40784
NC_056069.1	58940001	58960001	2.83463
NC_056074.1	23740001	23760001	6.31801
NC_056074.1	23745001	23765001	9.55662

NC_056061.	81770001	81790001	2.17247	0.270834	OL	ZDHHC14
NC_056063.	36350001	36370001	2.28087	0.305991	OL	ZMYM2
NC_056063.	36355001	36375001	2.34402	0.288935	OL	ZMYM2
NC_056063.	36360001	36380001	2.30682	0.29147	OL	ZMYM2
NC_056063.	36365001	36385001	2.56862	0.293183	OL	ZMYM2
NC_056063.	36370001	36390001	2.53788	0.278115	OL	ZMYM2
NC_056063.	36375001	36395001	2.31734	0.304414	OL	ZMYM2
NC_056063.	36380001	36400001	2.41884	0.333608	OL	ZMYM2
NC_056063.	36385001	36405001	2.24207	0.313535	OL	ZMYM2
NC_056063.	36390001	36410001	2.67247	0.2828	OL	ZMYM2
NC_056063.	36395001	36415001	2.87415	0.296348	OL	ZMYM2
NC_056063.	36400001	36420001	2.83973	0.276023	OL	ZMYM2
NC_056063.	36405001	36425001	2.71258	0.265136	OL	ZMYM2
NC_056063.	36410001	36430001	2.20887	0.316683	OL	ZMYM2
NC_056063.	36415001	36435001	2.09859	0.305942	OL	ZMYM2
NC_056063.	36420001	36440001	2.10479	0.308227	OL	ZMYM2
NC_056063.	36430001	36450001	1.95767	0.2487	OL	ZMYM2
NC_056060.	36020001	36040001	3	0.155049	OL	ZNF106
NC_056060.	36025001	36045001	3.12658	0.148169	OL	ZNF106
NC_056079.	43740001	43760001	2.58884	0.143002	OL	ZNF385D
NC_056079.	43745001	43765001	2.62036	0.173274	OL	ZNF385D
NC_056079.	43750001	43770001	2.14031	0.182702	OL	ZNF385D
NC_056056.	104505001	1.05E+08	2.56304	0.177548	OL	ZNF514
NC_056056.	104510001	1.05E+08	2.30076	0.260069	OL	ZNF514
NC_056076.	32015001	32035001	1.91316	0.237873	OL	ZNF521
NC_056076.	32025001	32045001	1.82245	0.14159	OL	ZNF521
NC_056054.	208560001	2.09E+08	1.94015	0.14498	OL	ZNF639
NC_056062.	57000001	57020001	4.55451	0.220731	OL	ZNF704
NC_056062.	57005001	57025001	4.18568	0.223287	OL	ZNF704
NC_056062.	57010001	57030001	3.78682	0.208324	OL	ZNF704
NC_056057.	67480001	67500001	2.62287	0.148767	OL	ZNRF2

NC_056074.1	23750001	23770001	12.0206
NC_056074.1	23755001	23775001	10.3967
NC_056074.1	23760001	23780001	8.26457
NC_056074.1	23765001	23785001	5.35844
NC_056074.1	23770001	23790001	2.56438
NC_056074.1	23730001	23750001	2.87305
NC_056074.1	23735001	23755001	3.01769
NC_056060.1	98570001	98590001	4.28499
NC_056060.1	98575001	98595001	2.98489
NC_056060.1	98580001	98600001	2.38796
NC_056060.1	98590001	98610001	2.39461
NC_056060.1	85125001	85145001	2.86169
NC_056060.1	85130001	85150001	3.08148
NC_056060.1	85135001	85155001	3.44624
NC_056060.1	85140001	85160001	2.58597
NC_056060.1	85145001	85165001	2.68443
NC_056060.1	85150001	85170001	3.89691
NC_056060.1	85155001	85175001	4.91329
NC_056060.1	85160001	85180001	6.23888
NC_056060.1	85165001	85185001	3.77291
NC_056060.1	85170001	85190001	3.32971
NC_056060.1	85175001	85195001	2.60681
NC_056060.1	85180001	85200001	2.06818
NC_056061.1	48175001	48195001	4.79847
NC_056061.1	48180001	48200001	5.80417
NC_056061.1	48185001	48205001	2.57498
NC_056061.1	48190001	48210001	2.1902
NC_056061.1	48195001	48215001	2.26568
NC_056055.1	246170001	2.46E+08	3.34592
NC_056072.1	13880001	13900001	2.35486
NC_056072.1	13890001	13910001	2.26891

NC_056072.1	13895001	13915001	2.48854
NC_056072.1	13900001	13920001	2.69007
NC_056072.1	13905001	13925001	2.92722
NC_056072.1	13910001	13930001	2.52363
NC_056054.1	263000001	2.63E+08	2.46583
NC_056054.1	263005001	2.63E+08	4.52896
NC_056054.1	263010001	2.63E+08	3.74867
NC_056054.1	263015001	2.63E+08	2.84411
NC_056060.1	54080001	54100001	1.98736
NC_056060.1	54085001	54105001	2.51863
NC_056060.1	54090001	54110001	1.9738
NC_056060.1	54130001	54150001	2.16107
NC_056060.1	54135001	54155001	1.98174
NC_056060.1	54140001	54160001	2.15115
NC_056060.1	54155001	54175001	2.28663
NC_056060.1	54160001	54180001	2.41771
NC_056065.1	18505001	18525001	2.57522
NC_056064.1	34885001	34905001	2.41162
NC_056064.1	12440001	12460001	2.82603
NC_056064.1	12445001	12465001	3.45749
NC_056059.1	6450001	6470001	2.92961
NC_056061.1	73160001	73180001	2.09286
NC_056061.1	73165001	73185001	2.48276
NC_056061.1	73170001	73190001	2.28081
NC_056061.1	73175001	73195001	2.25032
NC_056061.1	73410001	73430001	2.01682
NC_056061.1	68930001	68950001	3.15643
NC_056061.1	68935001	68955001	3.48696
NC_056061.1	68940001	68960001	4.43673
NC_056061.1	68945001	68965001	4.03495
NC_056061.1	69390001	69410001	2.40999

NC_056067.1	890001	910001	2.5
NC_056067.1	895001	915001	2.80423
NC_056067.1	900001	920001	2.73147
NC_056067.1	905001	925001	2.80796
NC_056067.1	910001	930001	2.66619
NC_056067.1	915001	935001	2.51458
NC_056060.1	41265001	41285001	2.14964
NC_056060.1	41270001	41290001	4.56857
NC_056060.1	41275001	41295001	6.99999
NC_056057.1	121265001	1.21E+08	2.38335
NC_056057.1	121270001	1.21E+08	2.83364
NC_056057.1	121275001	1.21E+08	2.45726
NC_056062.1	82745001	82765001	1.99048
NC_056062.1	82750001	82770001	2.5419
NC_056062.1	77700001	77720001	2.68204
NC_056062.1	77705001	77725001	2.58778
NC_056062.1	77710001	77730001	2.16373
NC_056062.1	77815001	77835001	2.32292
NC_056062.1	77820001	77840001	3.14589
NC_056062.1	77825001	77845001	3.81572
NC_056062.1	77830001	77850001	3.57693
NC_056062.1	77835001	77855001	2.88044
NC_056062.1	77840001	77860001	2.29979
NC_056070.1	53165001	53185001	2.00125
NC_056070.1	53170001	53190001	2.63241
NC_056057.1	1185001	1205001	2.41928
NC_056057.1	1190001	1210001	2.93152
NC_056057.1	1195001	1215001	2.546
NC_056075.1	40395001	40415001	9.45223
NC_056075.1	40400001	40420001	10.9609
NC_056075.1	40405001	40425001	17.4456

NC_056075.1	40410001	40430001	10.5734
NC_056075.1	40415001	40435001	8.05199
NC_056075.1	40420001	40440001	3.6013
NC_056065.1	34300001	34320001	2.05541
NC_056065.1	34305001	34325001	2.23559
NC_056065.1	34310001	34330001	2.64054
NC_056071.1	53300001	53320001	2.25999
NC_056067.1	10080001	10100001	2.95645
NC_056068.1	6335001	6355001	3.03284
NC_056056.1	91605001	91625001	2.77231
NC_056054.1	180445001	1.8E+08	5.07144
NC_056054.1	180450001	1.8E+08	18.1714
NC_056054.1	180455001	1.8E+08	10.9206
NC_056054.1	180460001	1.8E+08	7.20778
NC_056054.1	262900001	2.63E+08	2.675
NC_056068.1	19855001	19875001	2.15098
NC_056068.1	19860001	19880001	2.96007
NC_056068.1	19865001	19885001	3.52291
NC_056068.1	19870001	19890001	3.20661
NC_056068.1	19875001	19895001	3.78385
NC_056068.1	19880001	19900001	4.13276
NC_056068.1	19885001	19905001	4.84879
NC_056057.1	104105001	1.04E+08	13.5357
NC_056057.1	104110001	1.04E+08	6.7619
NC_056057.1	104100001	1.04E+08	11.9603
NC_056080.1	46950001	46970001	3.06011
NC_056080.1	46955001	46975001	2.95628
NC_056080.1	46960001	46980001	3.27815
NC_056080.1	46995001	47015001	2.25735
NC_056080.1	47020001	47040001	1.96026
NC_056055.1	83250001	83270001	12.7903

NC_056055.1	83255001	83275001	5.02299
NC_056055.1	83260001	83280001	3.61792
NC_056067.1	37780001	37800001	2.70055
NC_056067.1	37785001	37805001	2.43889
NC_056067.1	13250001	13270001	2.60976
NC_056059.1	117005001	1.17E+08	2.65698
NC_056059.1	117010001	1.17E+08	4.24069
NC_056059.1	117015001	1.17E+08	4.09029
NC_056059.1	117020001	1.17E+08	2.38452
NC_056059.1	117040001	1.17E+08	3.59407
NC_056059.1	117045001	1.17E+08	3.50979
NC_056059.1	117050001	1.17E+08	2.67352
NC_056059.1	117055001	1.17E+08	2.84813
NC_056054.1	26640001	26660001	1.94396
NC_056054.1	26645001	26665001	1.94172
NC_056054.1	26650001	26670001	1.94163
NC_056054.1	26655001	26675001	2.36264
NC_056054.1	26665001	26685001	2.44
NC_056054.1	26670001	26690001	3.29721
NC_056054.1	26675001	26695001	3.36985
NC_056054.1	26680001	26700001	2.63275
NC_056078.1	34085001	34105001	2.12315
NC_056078.1	34090001	34110001	2.09352
NC_056054.1	14845001	14865001	2.05276
NC_056054.1	14850001	14870001	2.49506
NC_056054.1	14855001	14875001	3.33874
NC_056054.1	14860001	14880001	3.92647
NC_056070.1	44190001	44210001	2.86535
NC_056070.1	44195001	44215001	2.18206
NC_056061.1	50005001	50025001	2.40533
NC_056061.1	50010001	50030001	3.95

NC_056061.1	50015001	50035001	9.91232
NC_056061.1	50020001	50040001	9.9804
NC_056061.1	50025001	50045001	3.13396
NC_056073.1	30455001	30475001	2.57634
NC_056055.1	130600001	1.31E+08	2.44144
NC_056067.1	13085001	13105001	3.50921
NC_056067.1	13090001	13110001	10.0188
NC_056067.1	13240001	13260001	12.7519
NC_056067.1	13245001	13265001	3.80576
NC_056062.1	57010001	57030001	2.70062

			OL vs HZ						
Fst	Region	Gene	CHROM	Start	end	Pi	Fst	Region	Gene
0.195464	OL	AAR2	NC_056067.1	16235001	16255001	2.1801	0.189596	OL	ABCC11;LONP2
0.232015	OL	AAR2	NC_056058.1	58320001	58340001	5.751	0.159239	OL	ABLIM3
0.216526	OL	ABCC10	NC_056058.1	58325001	58345001	8.47284	0.169495	OL	ABLIM3
0.170473	OL	ABCC12	NC_056058.1	58330001	58350001	11.9458	0.183715	OL	ABLIM3
0.189105	OL	ABCC12	NC_056058.1	58335001	58355001	7.40588	0.177466	OL	ABLIM3
0.203555	OL	ABCC12	NC_056068.1	55080001	55100001	1.98946	0.201092	OL	ACER3
0.218335	OL	ABCC12	NC_056068.1	55085001	55105001	2.00422	0.211662	OL	ACER3
0.204809	OL	ABCC12	NC_056077.1	18740001	18760001	2.16335	0.173638	OL	ACSM1
0.225716	OL	ABCC12	NC_056077.1	18745001	18765001	2.42226	0.176339	OL	ACSM1
0.242519	OL	ABCC12	NC_056056.1	43115001	43135001	5.78345	0.162611	OL	ACTR2
0.2132	OL	ABCC12	NC_056056.1	214685001	214705001	2.84154	0.299977	OL	ADA2
0.175299	OL	ABCC12	NC_056056.1	214670001	214690001	2.76667	0.273317	OL	ADA2;HDHD5
0.205459	OL	ABCD3	NC_056056.1	214675001	214695001	2.95865	0.287925	OL	ADA2;HDHD5
0.270512	OL	ABCD3	NC_056056.1	214680001	214700001	2.72366	0.283922	OL	ADA2;HDHD5
0.339051	OL	ABCD3	NC_056060.1	49800001	49820001	2.20256	0.364234	OL	ADAM10
0.303747	OL	ABCD3	NC_056060.1	49805001	49825001	2.94324	0.445708	OL	ADAM10
0.243881	OL	ABCD3	NC_056060.1	49810001	49830001	2.06345	0.398328	OL	ADAM10
0.245138	OL	ABCD3	NC_056055.1	86890001	86910001	1.97522	0.205215	OL	ADAMTSL1
0.190724	OL	ABCD3	NW_02459982	870001	890001	4.18044	0.186975	OL	ADAMTSL3
0.222672	OL	ABCD3	NW_02459982	875001	895001	3.27296	0.203328	OL	ADAMTSL3
0.147593	OL	ABCG2	NW_02459982	880001	900001	2.84468	0.228675	OL	ADAMTSL3
0.153547	OL	ABCG2	NW_02459982	885001	905001	2.31763	0.259894	OL	ADAMTSL3
0.192694	OL	ABLIM3	NW_02459982	890001	910001	2.35631	0.235458	OL	ADAMTSL3
0.153775	OL	ABR	NW_02459982	895001	915001	2.40112	0.215673	OL	ADAMTSL3
0.196448	OL	ABR	NW_02459982	900001	920001	2.32085	0.190124	OL	ADAMTSL3
0.161152	OL	ABR	NW_02459982	905001	925001	2.38024	0.164139	OL	ADAMTSL3
0.192456	OL	ABRAXAS1	NC_056057.1	77870001	77890001	2.35496	0.162332	OL	ADCY1
0.183269	OL	ACAT2;LOC	NC_056057.1	77875001	77895001	2.24627	0.18838	OL	ADCY1

0.38376	OL	ACD;C14H1	NC_056054.1	120025001	120045001	17.4633	0.235198	OL	ADCY10
0.425713	OL	ACD;C14H1	NC_056054.1	120030001	120050001	21.939	0.232015	OL	ADCY10
0.308787	OL	ACD;C14H1	NC_056069.1	65925001	65945001	2.6587	0.219854	OL	ADCY2
0.449778	OL	ACD;CARM	NC_056062.1	14730001	14750001	3.01084	0.190738	OL	ADGRB1
0.452144	OL	ACD;CARM	NC_056058.1	9250001	9270001	2.22718	0.197694	OL	ADGRE2
0.235812	OL	ACSL1	NC_056058.1	9255001	9275001	2.72873	0.262651	OL	ADGRE2
0.153194	OL	ACSM1	NC_056058.1	9260001	9280001	2.93416	0.258479	OL	ADGRE2
0.21751	OL	ACSM1	NC_056058.1	9265001	9285001	2.87494	0.204277	OL	ADGRE2
0.284984	OL	ACVR1B	NC_056058.1	9270001	9290001	2.61582	0.159965	OL	ADGRE2
0.197515	OL	ACVR1B	NC_056061.1	66955001	66975001	2.24264	0.245859	OL	ADGRG6
0.181228	OL	ACVR1B	NC_056061.1	66960001	66980001	2.38187	0.224771	OL	ADGRG6
0.213927	OL	ACYP1;NEK	NC_056061.1	66965001	66985001	2.82506	0.227354	OL	ADGRG6
0.214201	OL	ADAM11	NC_056061.1	66970001	66990001	2.96803	0.226515	OL	ADGRG6
0.181959	OL	ADAM11	NC_056061.1	66975001	66995001	2.70464	0.208403	OL	ADGRG6
0.228679	OL	ADAM11;DI	NC_056061.1	66980001	67000001	2.13052	0.185056	OL	ADGRG6
0.145886	OL	ADAMTS17	NC_056058.1	87875001	87895001	3.5649	0.183884	OL	ADGRV1
0.187898	OL	ADAMTS2	NC_056058.1	87900001	87920001	2.04856	0.37358	OL	ADGRV1
0.321069	OL	ADAMTS2	NC_056056.1	214005001	214025001	1.99894	0.239567	OL	ADIPOR2
0.378512	OL	ADAMTS2	NC_056056.1	214015001	214035001	2.43144	0.164958	OL	ADIPOR2
0.381526	OL	ADAMTS2	NC_056055.1	38880001	38900001	5.12309	0.177721	OL	ADRA1A
0.388191	OL	ADAMTS2	NC_056055.1	38885001	38905001	6.1128	0.179807	OL	ADRA1A
0.393254	OL	ADAMTS2	NC_056055.1	38890001	38910001	6.17807	0.164335	OL	ADRA1A
0.344397	OL	ADAMTS2	NC_056055.1	38895001	38915001	4.87879	0.168495	OL	ADRA1A
0.340516	OL	ADAMTS2	NC_056059.1	114840001	114860001	3.16512	0.347941	OL	AFAP1
0.297221	OL	ADAMTS2	NC_056059.1	114845001	114865001	2.16873	0.353782	OL	AFAP1
0.260719	OL	ADAMTS2	NC_056059.1	114850001	114870001	2.27511	0.341263	OL	AFAP1
0.282627	OL	ADAMTS2	NC_056059.1	114855001	114875001	3.06601	0.35571	OL	AFAP1
0.241528	OL	ADAMTS2	NC_056059.1	114860001	114880001	3.27247	0.344934	OL	AFAP1
0.150543	OL	ADAMTS2	NC_056059.1	114865001	114885001	4.38866	0.360563	OL	AFAP1
0.229867	OL	ADAMTS2	NC_056059.1	114870001	114890001	5.2363	0.317333	OL	AFAP1
0.286785	OL	ADAMTS2	NC_056059.1	114875001	114895001	2.63016	0.205931	OL	AFAP1

0.317645	OL	ADAMTS2	NC_056059.1	114895001	114915001	2.35212	0.262647	OL	AFAP1
0.233494	OL	ADAMTS2	NC_056059.1	114900001	114920001	3.52608	0.309642	OL	AFAP1
0.217545	OL	ADAMTS2	NC_056059.1	114905001	114925001	2.82086	0.327478	OL	AFAP1
0.1615	OL	ADAMTS2	NC_056059.1	114910001	114930001	2.07465	0.265747	OL	AFAP1
0.165438	OL	ADAMTSL4	NC_056059.1	114835001	114855001	2.91467	0.256253	OL	AFAP1;SORCS2
0.167568	OL	ADAMTSL4	NC_056059.1	102635001	102655001	2.90811	0.168283	OL	AFF1
0.216598	OL	ADCY10	NC_056057.1	24875001	24895001	3.03437	0.228234	OL	AGMO
0.209299	OL	ADCY10	NC_056057.1	24880001	24900001	3.58052	0.245009	OL	AGMO
0.193684	OL	ADCY10	NC_056057.1	24885001	24905001	4.42379	0.230374	OL	AGMO
0.181912	OL	ADCY10	NC_056057.1	24890001	24910001	5.02798	0.212133	OL	AGMO
0.187145	OL	ADCY4;CID	NC_056069.1	39305001	39325001	1.99778	0.19009	OL	AGXT2
0.174358	OL	ADCY4;CID	NC_056069.1	39310001	39330001	2.96755	0.245746	OL	AGXT2
0.188628	OL	ADCY4;CID	NC_056069.1	39315001	39335001	3.54314	0.262429	OL	AGXT2
0.160342	OL	ADCY4;LTB	NC_056069.1	39320001	39340001	3.36661	0.262884	OL	AGXT2
0.216121	OL	ADD3	NC_056069.1	39325001	39345001	2.22138	0.258807	OL	AGXT2
0.210337	OL	ADD3	NC_056069.1	39335001	39355001	1.99153	0.272652	OL	AGXT2
0.192693	OL	ADD3	NC_056065.1	25375001	25395001	2.1201	0.166234	OL	AIDA;MIA3
0.149391	OL	ADGB	NC_056055.1	73050001	73070001	4.96406	0.204834	OL	AK3
0.153085	OL	ADGB	NC_056064.1	33540001	33560001	2.60858	0.258229	OL	AKAP10
0.149368	OL	ADGB	NC_056064.1	33545001	33565001	2.38902	0.257097	OL	AKAP10;ULK2
0.280361	OL	ADGRL4	NC_056061.1	76150001	76170001	2.46591	0.159497	OL	AKAP12
0.389861	OL	ADGRL4	NC_056061.1	76155001	76175001	2.24252	0.160926	OL	AKAP12
0.467105	OL	ADGRL4	NC_056061.1	76170001	76190001	2.69668	0.186386	OL	AKAP12
0.469661	OL	ADGRL4	NC_056055.1	247035001	247055001	9.94916	0.272387	OL	AKR7A2;SLC66A1
0.474466	OL	ADGRL4	NC_056055.1	247040001	247060001	3.9111	0.208318	OL	AKR7A2;SLC66A1
0.378462	OL	ADGRL4	NC_056055.1	247045001	247065001	2.91384	0.186291	OL	AKR7A2;SLC66A1
0.217046	OL	ADGRV1	NC_056056.1	36095001	36115001	2.99867	0.214151	OL	ALK
0.1923	OL	ADGRV1	NC_056056.1	36100001	36120001	4.50376	0.257979	OL	ALK
0.162775	OL	ADK	NC_056056.1	36105001	36125001	4.84925	0.261422	OL	ALK
0.184717	OL	ADK	NC_056056.1	36110001	36130001	3.62141	0.222033	OL	ALK
0.198091	OL	ADK	NC_056056.1	36115001	36135001	3.01827	0.168353	OL	ALK

0.195887	OL	ADK	NC_056069.1	39920001	39940001	4.12019	0.277819	OL		AMACR
0.219688	OL	ADK	NC_056069.1	39915001	39935001	1.97049	0.198581	OL		AMACR;C1QTNF3
0.218745	OL	ADK	NC_056069.1	39925001	39945001	5.49877	0.239135	OL		AMACR;SLC45A2
0.222463	OL	ADK	NC_056069.1	39930001	39950001	5.39785	0.246585	OL		AMACR;SLC45A2
0.167117	OL	ADK	NC_056069.1	39935001	39955001	4.43853	0.163619	OL		AMACR;SLC45A2
0.17204	OL	ADK	NC_056080.1	124940001	124960001	4.11429	0.303651	OL		AMMECR1
0.171449	OL	ADK	NC_056080.1	124945001	124965001	3.90358	0.308002	OL		AMMECR1
0.156657	OL	ADK	NC_056080.1	124950001	124970001	3.65283	0.297637	OL		AMMECR1
0.202689	OL	ADK	NC_056080.1	124955001	124975001	2.39345	0.353282	OL		AMMECR1
0.198952	OL	AGBL1	NC_056080.1	124960001	124980001	1.97129	0.338022	OL		AMMECR1
0.216991	OL	AGPAT5	NC_056080.1	124970001	124990001	2.02966	0.350524	OL		AMMECR1
0.215367	OL	AGXT2	NC_056080.1	124975001	124995001	2.07004	0.337299	OL		AMMECR1
0.254382	OL	AGXT2	NC_056071.1	65245001	65265001	3.64565	0.166833	OL		AMN;CDC42BPB
0.27072	OL	AGXT2	NC_056071.1	65250001	65270001	7.03549	0.232808	OL		AMN;CDC42BPB
0.352377	OL	AGXT2	NC_056071.1	65255001	65275001	8.9091	0.261121	OL		AMN;CDC42BPB
0.304091	OL	AGXT2	NC_056056.1	9025001	9045001	2.54107	0.254859	OL		ANGPTL2;RALGPS1
0.252237	OL	AGXT2	NC_056056.1	9030001	9050001	3.12567	0.259213	OL		ANGPTL2;RALGPS1
0.25498	OL	AHCYL1	NC_056056.1	9035001	9055001	2.43852	0.210636	OL		ANGPTL2;RALGPS1
0.183076	OL	AHCYL1	NC_056054.1	37985001	38005001	5.61905	0.195307	OL		ANGPTL3;DOCK7
0.150235	OL	AHCYL1	NC_056054.1	37990001	38010001	6.15705	0.23404	OL		ANGPTL3;DOCK7
0.172724	OL	AJAP1	NC_056059.1	12615001	12635001	2.00674	0.166716	OL		ANK2
0.220862	OL	AJAP1	NC_056059.1	12620001	12640001	1.97732	0.208592	OL		ANK2
0.228242	OL	AJAP1	NC_056062.1	76775001	76795001	1.96108	0.285132	OL		ANKRD46
0.208331	OL	AJAP1	NC_056073.1	9175001	9195001	2.00151	0.217217	OL		ANKS1A
0.173432	OL	AK2;LOC11	NC_056073.1	9180001	9200001	3.88252	0.305355	OL		ANKS1A
0.161112	OL	AKAP12	NC_056073.1	9185001	9205001	2.59999	0.274744	OL		ANKS1A
0.162693	OL	AKAP12	NC_056056.1	169425001	169445001	1.95269	0.248485	OL		ANKS1B
0.189262	OL	AKAP12	NC_056056.1	169430001	169450001	1.97491	0.251207	OL		ANKS1B
0.158777	OL	ALK	NC_056072.1	15425001	15445001	2.78571	0.637884	OL		ANO10
0.336452	OL	AMHR2;SP1	NC_056072.1	15430001	15450001	5.16081	0.634234	OL		ANO10
0.396387	OL	AMHR2;SP1	NC_056072.1	15435001	15455001	3.73088	0.57363	OL		ANO10

0.300925	OL	ANGEL1	NC_056072.1	15440001	15460001	4.3608	0.552605	OL	ANO10
0.251433	OL	ANGEL1	NC_056072.1	15445001	15465001	4.68638	0.489561	OL	ANO10
0.200172	OL	ANGEL1;LR	NC_056072.1	15450001	15470001	2.90015	0.41545	OL	ANO10
0.14893	OL	ANGEL2;VA	NC_056072.1	15455001	15475001	1.96846	0.376649	OL	ANO10
0.177728	OL	ANKDD1B	NC_056055.1	31265001	31285001	2.7003	0.172226	OL	AOPEP
0.186742	OL	ANKDD1B	NC_056055.1	31255001	31275001	2.3097	0.167079	OL	AOPEP;FANCC
0.202952	OL	ANKDD1B	NC_056055.1	31260001	31280001	2.43831	0.199623	OL	AOPEP;FANCC
0.258998	OL	ANKDD1B	NC_056070.1	68630001	68650001	1.96745	0.219017	OL	AP1B1
0.22075	OL	ANKDD1B	NC_056070.1	68635001	68655001	2.02806	0.218573	OL	AP1B1
0.181153	OL	ANKDD1B	NC_056071.1	25875001	25895001	2.55518	0.171775	OL	APBA2
0.147196	OL	ANKDD1B	NC_056055.1	166760001	166780001	2.61019	0.16317	OL	ARHGAP15
0.223195	OL	ANKRD33B	NC_056055.1	166765001	166785001	2.37517	0.158724	OL	ARHGAP15
0.163229	OL	ANKRD44	NC_056076.1	40265001	40285001	4.09934	0.17475	OL	ARHGAP28
0.17554	OL	ANKRD44	NC_056076.1	40280001	40300001	2.17722	0.201981	OL	ARHGAP28
0.190873	OL	ANKRD6	NC_056079.1	1485001	1505001	17.2667	0.162199	OL	ARHGEF10
0.21649	OL	ANKRD6	NC_056079.1	1490001	1510001	10.9492	0.160319	OL	ARHGEF10
0.226582	OL	ANKRD6	NC_056055.1	240185001	240205001	2.07048	0.207644	OL	ARID1A
0.162929	OL	ANKS3;DNA	NC_056069.1	24945001	24965001	2.20339	0.167572	OL	ARL15
0.219148	OL	ANKS3;DNA	NC_056069.1	24960001	24980001	2.27322	0.157474	OL	ARL15
0.292927	OL	ANKS3;DNA	NC_056069.1	24965001	24985001	2.14969	0.229591	OL	ARL15
0.222567	OL	ANKS3;MGI	NC_056071.1	24100001	24120001	6.51924	0.220983	OL	ARNT2
0.155645	OL	ANKS3;MGI	NC_056068.1	39045001	39065001	4.63929	0.17036	OL	ARNTL;BTBD10
0.285368	OL	ANLN;KIAA	NC_056070.1	68910001	68930001	2.1195	0.207155	OL	ASCC2
0.218513	OL	ANLN;KIAA	NC_056056.1	172305001	172325001	7.86846	0.165892	OL	ASCL1
0.197746	OL	ANLN;KIAA	NC_056057.1	15975001	15995001	2.96047	0.315146	OL	ASNS
0.229138	OL	ANLN;KIAA	NC_056057.1	15980001	16000001	2.25885	0.272924	OL	ASNS
0.240522	OL	ANLN;KIAA	NC_056056.1	31590001	31610001	1.97574	0.20254	OL	ATAD2B
0.239443	OL	ANLN;KIAA	NC_056056.1	31595001	31615001	2.7749	0.204729	OL	ATAD2B
0.221305	OL	ANLN;KIAA	NC_056056.1	31600001	31620001	2.59218	0.225278	OL	ATAD2B
0.166894	OL	ANLN;KIAA	NC_056056.1	31605001	31625001	2.15564	0.223226	OL	ATAD2B
0.51143	OL	ANO3	NC_056056.1	31610001	31630001	1.95401	0.220415	OL	ATAD2B

0.527646	OL	ANO3	NC_056054.1	113230001	113250001	2.20709	0.275574	OL	ATF6
0.461156	OL	ANO3	NC_056054.1	113235001	113255001	3.08571	0.323386	OL	ATF6
0.148178	OL	ANO3	NC_056054.1	113240001	113260001	2.82932	0.335136	OL	ATF6
0.337086	OL	ANO6	NC_056054.1	113245001	113265001	3.23456	0.385997	OL	ATF6
0.151366	OL	ANO6	NC_056054.1	113250001	113270001	2.18785	0.344435	OL	ATF6
0.244788	OL	ANTKMT;C	NC_056063.1	85635001	85655001	2.38103	0.266434	OL	ATP11A
0.160671	OL	ANTKMT;C	NC_056063.1	85640001	85660001	2.21522	0.276665	OL	ATP11A
0.175134	OL	ANXA3	NC_056063.1	85645001	85665001	2.07386	0.263025	OL	ATP11A
0.160525	OL	ANXA3	NC_056072.1	54375001	54395001	4.30115	0.221652	OL	ATP2B2
0.14918	OL	ANXA3;FRA	NC_056072.1	54380001	54400001	3.9318	0.213352	OL	ATP2B2
0.147278	OL	AP1AR	NC_056072.1	54385001	54405001	4.29011	0.211391	OL	ATP2B2
0.182367	OL	AP3M2	NC_056060.1	58350001	58370001	3.17048	0.168054	OL	ATP8B4
0.159618	OL	AP3M2;PLA	NC_056060.1	58355001	58375001	2.3403	0.17807	OL	ATP8B4
0.171278	OL	APCDD1	NC_056080.1	68765001	68785001	1.95817	0.368519	OL	ATRX
0.227303	OL	APCDD1	NC_056056.1	161825001	161845001	3.71309	0.259333	OL	AVIL;CTDSP2
0.227139	OL	APCDD1	NC_056056.1	161830001	161850001	3.80663	0.237492	OL	AVIL;CTDSP2
0.195188	OL	APCDD1	NC_056056.1	161845001	161865001	3.16347	0.259008	OL	AVIL;TSFM
0.17325	OL	APOBEC3A	NC_056055.1	234560001	234580001	2.06511	0.219061	OL	AZIN2
0.306011	OL	APOBEC3F	NC_056055.1	234570001	234590001	2.44831	0.164218	OL	AZIN2
0.305031	OL	APOBEC3F	NC_056054.1	260465001	260485001	2.11577	0.184777	OL	B3GALT5
0.31068	OL	APOBEC3F	NC_056066.1	77750001	77770001	8.97543	0.190744	OL	B4GALT5
0.163973	OL	ARFIP1	NC_056061.1	47470001	47490001	2.37956	0.376511	OL	BACH2
0.218934	OL	ARHGAP15	NC_056074.1	40530001	40550001	2.88485	0.176405	OL	BANF1;CATSPER1;CST6
0.183112	OL	ARHGAP15	NC_056074.1	40525001	40545001	2.02345	0.159827	OL	BANF1;CATSPER1;CST6;EIF1AD
0.165859	OL	ARHGAP15	NC_056056.1	112315001	112335001	3.01087	0.158977	OL	BBS10;OSBPL8
0.153724	OL	ARHGAP15	NC_056056.1	203390001	203410001	2.56141	0.218958	OL	BCL2L14
0.164821	OL	ARHGAP23	NC_056056.1	203395001	203415001	2.1607	0.203284	OL	BCL2L14
0.176255	OL	ARHGAP23	NC_056056.1	203400001	203420001	2.65749	0.199132	OL	BCL2L14
0.208865	OL	ARID1B	NC_056056.1	203405001	203425001	2.30598	0.18377	OL	BCL2L14
0.169801	OL	ARID1B	NC_056054.1	98575001	98595001	3.40667	0.268179	OL	BCL9
0.166428	OL	ARID1B	NC_056054.1	98580001	98600001	2.13889	0.20452	OL	BCL9

0.185869	OL	ARL13B	NC_056070.1	71820001	71840001	3.40092	0.187086	OL	BCR;RAB36
0.199672	OL	ARL13B;ST:	NC_056070.1	71825001	71845001	3.17011	0.175172	OL	BCR;RAB36
0.175816	OL	ARL13B;ST:	NC_056057.1	12120001	12140001	8.28831	0.174756	OL	BET1
0.207483	OL	ARL8B	NC_056073.1	16520001	16540001	2.01236	0.164274	OL	BICRAL
0.165565	OL	ARMC12	NC_056073.1	16525001	16545001	2.40707	0.164244	OL	BICRAL;RPL7L1
0.219724	OL	ASH2L	NC_056073.1	16530001	16550001	2.4177	0.1845	OL	BICRAL;RPL7L1
0.215049	OL	ASH2L	NC_056073.1	16535001	16555001	2.14416	0.212348	OL	BICRAL;RPL7L1
0.172011	OL	ASH2L	NC_056057.1	64460001	64480001	2.23352	0.372046	OL	BMPER
0.16299	OL	ASH2L	NC_056057.1	64465001	64485001	2.79927	0.362473	OL	BMPER
0.16115	OL	ATL1	NC_056057.1	64470001	64490001	2.8948	0.3707	OL	BMPER
0.171501	OL	ATL1	NC_056057.1	64475001	64495001	2.29508	0.327444	OL	BMPER
0.178415	OL	ATL1	NC_056069.1	5490001	5510001	6.08492	0.211301	OL	BOD1
0.159922	OL	ATL1	NC_056069.1	5495001	5515001	8.38968	0.222798	OL	BOD1
0.173038	OL	ATL1	NC_056069.1	5500001	5520001	7.96851	0.205861	OL	BOD1
0.148918	OL	ATL1;LOC1:	NC_056065.1	14320001	14340001	2.22888	0.181088	OL	BRINP3
0.179786	OL	ATL1;SAV1	NC_056068.1	38965001	38985001	3.52495	0.187817	OL	BTBD10
0.177898	OL	ATL1;SAV1	NC_056068.1	38985001	39005001	2.43314	0.170388	OL	BTBD10
0.190934	OL	ATL1;SAV1	NC_056068.1	38990001	39010001	2.49031	0.172724	OL	BTBD10
0.237755	OL	ATP1A1	NC_056068.1	38995001	39015001	2.60597	0.159154	OL	BTBD10
0.223833	OL	ATP2C2	NC_056068.1	38960001	38980001	5.31833	0.258138	OL	BTBD10;LOC101120269
0.171873	OL	ATP2C2	NC_056068.1	38955001	38975001	14.2696	0.273385	OL	BTBD10;LOC101120269;PTH
0.16746	OL	ATP2C2	NC_056068.1	63130001	63150001	2.73757	0.188435	OL	C15H11orf91
0.156642	OL	ATXN10	NC_056068.1	63135001	63155001	2.67267	0.182504	OL	C15H11orf91;CD59
0.178671	OL	AUTS2	NC_056068.1	63140001	63160001	2.57512	0.180995	OL	C15H11orf91;CD59
0.234475	OL	AUTS2	NC_056068.1	63145001	63165001	2.58103	0.166312	OL	C15H11orf91;CD59
0.236229	OL	AUTS2	NC_056080.1	104940001	104960001	2.13193	0.216221	OL	C1GALT1C1
0.232154	OL	AUTS2	NC_056080.1	104945001	104965001	2.30824	0.238977	OL	C1GALT1C1
0.177024	OL	AVEN	NC_056080.1	104950001	104970001	2.17588	0.21682	OL	C1GALT1C1
0.172982	OL	AVEN	NC_056055.1	203845001	203865001	2.82767	0.187911	OL	C2CD6
0.175475	OL	B3GALNT1	NC_056055.1	203850001	203870001	3.71123	0.204118	OL	C2CD6
0.180165	OL	B3GALNT1	NC_056055.1	203855001	203875001	4.27027	0.196095	OL	C2CD6

0.184051	OL	B3GALNT1	NC_056055.1	203860001	203880001	4.40091	0.188774	OL	C2CD6
0.198308	OL	B3GALNT1	NC_056055.1	203865001	203885001	5.33234	0.172623	OL	C2CD6
0.316385	OL	BACE1	NC_056055.1	203870001	203890001	5.69231	0.176369	OL	C2CD6
0.332884	OL	BACE1;CEP	NC_056055.1	203875001	203895001	5.59048	0.187103	OL	C2CD6
0.293939	OL	BACE1;CEP	NC_056055.1	203880001	203900001	6.48	0.222812	OL	C2CD6
0.360785	OL	BACE1;CEP	NC_056055.1	203885001	203905001	6.22165	0.235046	OL	C2CD6
0.451603	OL	BACE1;RNF	NC_056055.1	203890001	203910001	4.96034	0.217362	OL	C2CD6
0.4532	OL	BACE1;RNF	NC_056055.1	37170001	37190001	2.11572	0.169778	OL	C2H9orf24;MYORG
0.336004	OL	BACE1;RNF	NC_056055.1	64650001	64670001	2.3248	0.174617	OL	C2H9orf85
0.362988	OL	BACE1;RNF	NC_056055.1	64655001	64675001	3.68637	0.246296	OL	C2H9orf85
0.30444	OL	BACE1;RNF	NC_056055.1	64660001	64680001	4.6973	0.27406	OL	C2H9orf85
0.188732	OL	BATF	NC_056055.1	64665001	64685001	4.6712	0.277308	OL	C2H9orf85
0.175975	OL	BBS9	NC_056055.1	64670001	64690001	3.95494	0.264006	OL	C2H9orf85
0.178334	OL	BBS9	NC_056055.1	64675001	64695001	2.16773	0.162957	OL	C2H9orf85
0.211716	OL	BNC2	NC_056056.1	147790001	147810001	2.65974	0.281686	OL	C3H12orf40
0.305272	OL	BNC2	NC_056056.1	147795001	147815001	3.00881	0.272102	OL	C3H12orf40
0.175774	OL	BNC2	NC_056056.1	147800001	147820001	2.7631	0.246406	OL	C3H12orf40
0.194848	OL	BOD1L1	NC_056067.1	54060001	54080001	2.55448	0.203027	OL	C5AR1
0.234284	OL	BOD1L1	NC_056058.1	13140001	13160001	1.97271	0.15906	OL	C5H19orf38;CARM1
0.182752	OL	BORA	NC_056058.1	13135001	13155001	2.17693	0.165647	OL	C5H19orf38;DNM2;TMED1
0.19282	OL	BORA;DIS3	NC_056066.1	54550001	54570001	2.06421	0.210267	OL	CABLES2;RBBP8NL;RPS21
0.15412	OL	BPIFC	NC_056058.1	10605001	10625001	5.92064	0.159088	OL	CACNA1A
0.183004	OL	BRINP1	NC_056056.1	80001	100001	2.6006	0.249682	OL	CACNA1B
0.198074	OL	BRINP1	NC_056056.1	85001	105001	4.87264	0.270726	OL	CACNA1B
0.156094	OL	BRINP1	NC_056056.1	90001	110001	3.47963	0.238387	OL	CACNA1B
0.166626	OL	C14H16orf78	NC_056056.1	95001	115001	3.91652	0.237813	OL	CACNA1B
0.15899	OL	C14H16orf78	NC_056056.1	100001	120001	3.44569	0.223356	OL	CACNA1B
0.250241	OL	C1H1orf68	NC_056056.1	105001	125001	2.70978	0.197425	OL	CACNA1B
0.169623	OL	C1H1orf68	NC_056056.1	110001	130001	3.24742	0.205673	OL	CACNA1B
0.159603	OL	C20H6orf52;	NC_056056.1	115001	135001	2.97298	0.210281	OL	CACNA1B
0.170097	OL	C20H6orf52;	NC_056056.1	120001	140001	2.78273	0.21299	OL	CACNA1B

0.178462	OL	C20H6orf52;	NC_056056.1	125001	145001	2.39518	0.209835	OL		CACNA1B
0.155065	OL	C2CD3	NC_056056.1	130001	150001	2.88958	0.249208	OL		CACNA1B
0.15386	OL	C2CD3	NC_056056.1	135001	155001	3.06937	0.254599	OL		CACNA1B
0.161482	OL	C2CD3	NC_056056.1	140001	160001	2.5703	0.240093	OL		CACNA1B
0.16352	OL	C2CD3	NC_056056.1	145001	165001	2.50784	0.235119	OL		CACNA1B
0.158363	OL	C2CD3	NC_056056.1	150001	170001	2.00183	0.206829	OL		CACNA1B
0.150733	OL	C2CD3	NC_056056.1	160001	180001	2.08714	0.210972	OL		CACNA1B
0.151415	OL	C2CD3	NC_056056.1	165001	185001	2.15514	0.20736	OL		CACNA1B
0.148909	OL	C2CD3	NC_056056.1	170001	190001	2.09657	0.191542	OL		CACNA1B
0.148257	OL	C2CD3	NC_056080.1	55915001	55935001	2.27857	0.192087	OL		CACNA1F;CCDC22
0.153903	OL	C2CD3	NC_056056.1	216790001	216810001	3.50123	0.183407	OL		CACNA1I;LOC121819192
0.146375	OL	C2CD3	NC_056056.1	216795001	216815001	2.60676	0.173668	OL		CACNA1I;LOC121819192
0.180851	OL	C7H15orf62;	NC_056072.1	46560001	46580001	2.3764	0.164378	OL		CACNA2D3
0.160752	OL	C9H8orf34	NC_056072.1	46565001	46585001	2.48931	0.19141	OL		CACNA2D3
0.156755	OL	C9H8orf89	NC_056072.1	46570001	46590001	2.59318	0.20707	OL		CACNA2D3
0.173859	OL	C9H8orf89	NC_056072.1	46575001	46595001	2.04144	0.172952	OL		CACNA2D3
0.174689	OL	C9H8orf89	NC_056072.1	46830001	46850001	2.66548	0.168189	OL		CACNA2D3
0.158069	OL	C9H8orf89	NC_056072.1	46835001	46855001	5.57894	0.175367	OL		CACNA2D3
0.147131	OL	C9H8orf89;R	NC_056072.1	46840001	46860001	6.55073	0.170604	OL		CACNA2D3
0.197454	OL	CACNA1B	NC_056064.1	62140001	62160001	2.06222	0.167501	OL		CACNG5
0.183411	OL	CACNA1B	NC_056064.1	62160001	62180001	2.17134	0.163291	OL		CACNG5
0.201482	OL	CACNA1B	NC_056064.1	62165001	62185001	2.00522	0.167301	OL		CACNG5
0.197184	OL	CACNA1B	NC_056068.1	25510001	25530001	2.16377	0.195735	OL		CADM1
0.177109	OL	CACNA1B	NC_056054.1	154330001	154350001	4.66667	0.185467	OL		CADM2
0.184684	OL	CACNA1B	NC_056054.1	154570001	154590001	1.9823	0.270905	OL		CADM2
0.187739	OL	CACNA1B	NC_056054.1	154780001	154800001	3.0155	0.16612	OL		CADM2
0.195213	OL	CACNA1B	NC_056054.1	154785001	154805001	4.40906	0.208356	OL		CADM2
0.191993	OL	CACNA1B	NC_056054.1	154790001	154810001	2.87456	0.168766	OL		CADM2
0.225037	OL	CACNA1B	NC_056072.1	38735001	38755001	2.96621	0.280909	OL		CADPS
0.238247	OL	CACNA1B	NC_056072.1	38740001	38760001	2.46049	0.242856	OL		CADPS
0.220874	OL	CACNA1B	NC_056056.1	132825001	132845001	3.74151	0.208799	OL		CALCOCO1

0.220568	OL	CACNA1B	NC_056054.1	273245001	273265001	2.24624	0.167855	OL	CAPN7
0.199038	OL	CACNA1B	NC_056054.1	273250001	273270001	2.04311	0.186098	OL	CAPN7
0.195212	OL	CACNA1B	NC_056055.1	247030001	247050001	3.96416	0.190068	OL	CAPZB;SLC66A1
0.192945	OL	CACNA1B	NC_056067.1	34735001	34755001	1.95401	0.267008	OL	CARMIL2;CTCF
0.27781	OL	CACNA1C;I	NC_056067.1	34740001	34760001	1.98226	0.262806	OL	CARMIL2;CTCF
0.248023	OL	CACNA1C;I	NC_056080.1	40800001	40820001	2.95928	0.165227	OL	CASK
0.18146	OL	CACNA1C;I	NC_056079.1	14230001	14250001	2.1579	0.226159	OL	CASP3
0.158065	OL	CACNA1I;L(	NC_056057.1	33140001	33160001	3.14705	0.182701	OL	CCDC126;LOC106991101;LOC121819
0.173788	OL	CACNA2D1	NC_056076.1	24345001	24365001	5.1966	0.266297	OL	CCDC178
0.148927	OL	CACNA2D3	NC_056076.1	24350001	24370001	4.91469	0.251667	OL	CCDC178
0.201812	OL	CACNB2	NC_056076.1	24355001	24375001	4.27568	0.239274	OL	CCDC178
0.214965	OL	CACNB2	NC_056076.1	24360001	24380001	3.04842	0.208173	OL	CCDC178
0.204072	OL	CACNB2	NC_056077.1	27385001	27405001	2.29798	0.236111	OL	CCDC189;PHKG2;RNF40
0.147614	OL	CADM1	NC_056077.1	27390001	27410001	2.58854	0.237169	OL	CCDC189;PHKG2;RNF40
0.150247	OL	CADM1	NC_056077.1	27395001	27415001	3.21667	0.253588	OL	CCDC189;PHKG2;RNF40
0.266323	OL	CADM2	NC_056077.1	27400001	27420001	2.77404	0.25719	OL	CCDC189;RNF40;ZNF629
0.247147	OL	CADM2	NC_056074.1	34545001	34565001	2.72184	0.167249	OL	CCDC86;PTGDR2
0.203999	OL	CADM2	NC_056074.1	34550001	34570001	3.94911	0.200889	OL	CCDC86;PTGDR2
0.188941	OL	CADM2	NC_056074.1	34555001	34575001	3.37522	0.245789	OL	CCDC86;PTGDR2
0.178261	OL	CADPS2	NC_056060.1	49335001	49355001	3.19999	0.318621	OL	CCNB2
0.199008	OL	CALCOCO1	NC_056060.1	49340001	49360001	2.87559	0.314401	OL	CCNB2
0.210924	OL	CALCOCO1	NC_056060.1	49325001	49345001	4.57142	0.323038	OL	CCNB2;MYO1E
0.20757	OL	CALCOCO1	NC_056060.1	49330001	49350001	3.35715	0.321717	OL	CCNB2;MYO1E
0.177055	OL	CALCOCO1	NC_056072.1	7310001	7330001	4.20774	0.26726	OL	CCR4
0.17635	OL	CAMK1D	NC_056068.1	63155001	63175001	2.77881	0.164521	OL	CD59
0.172865	OL	CAMK2G	NC_056063.1	86345001	86365001	1.98398	0.167877	OL	CDC16;CFAP97D2
0.179226	OL	CAMK2G	NC_056063.1	86350001	86370001	2.01567	0.177973	OL	CDC16;CFAP97D2
0.219935	OL	CAMSAP1	NC_056065.1	29245001	29265001	2.17802	0.20642	OL	CDC42BPA
0.30686	OL	CAMSAP1	NC_056065.1	29250001	29270001	2.0428	0.239847	OL	CDC42BPA
0.471189	OL	CARMIL2;C	NC_056065.1	29255001	29275001	1.99849	0.26418	OL	CDC42BPA
0.459662	OL	CARMIL2;C	NC_056065.1	29260001	29280001	2.96482	0.369076	OL	CDC42BPA

0.441908	OL	CARMIL2;C	NC_056065.1	29265001	29285001	2.97752	0.343557	OL	CDC42BPA
0.265239	OL	CBLB	NC_056065.1	29270001	29290001	7.97694	0.343063	OL	CDC42BPA
0.149387	OL	CBLB	NC_056065.1	29315001	29335001	31.1693	0.344854	OL	CDC42BPA
0.146078	OL	CBX3	NC_056065.1	29320001	29340001	12.6212	0.286857	OL	CDC42BPA
0.409179	OL	CBX7	NC_056065.1	29325001	29345001	11.2934	0.280487	OL	CDC42BPA
0.388219	OL	CBX7	NC_056065.1	29330001	29350001	5.89606	0.23219	OL	CDC42BPA
0.298232	OL	CBX7	NC_056065.1	29345001	29365001	2.29253	0.195273	OL	CDC42BPA
0.198648	OL	CCDC171	NC_056065.1	29350001	29370001	2.28381	0.230347	OL	CDC42BPA
0.179449	OL	CCDC171	NC_056065.1	29355001	29375001	2.2076	0.28148	OL	CDC42BPA
0.258586	OL	CCL1	NC_056065.1	29360001	29380001	2.28572	0.250871	OL	CDC42BPA
0.324967	OL	CCL1	NC_056065.1	29365001	29385001	2.48007	0.209379	OL	CDC42BPA
0.2753	OL	CCL1	NC_056071.1	65260001	65280001	10.8292	0.262207	OL	CDC42BPB
0.220239	OL	CCL1	NC_056069.1	49165001	49185001	2.0185	0.165563	OL	CDH10
0.20103	OL	CCNJ	NC_056069.1	53510001	53530001	2.27951	0.166522	OL	CDH18
0.254514	OL	CCNJ	NC_056067.1	29245001	29265001	19.7723	0.169055	OL	CDH8
0.145717	OL	CCSER1	NC_056064.1	39625001	39645001	2.04964	0.200045	OL	CDK12
0.198757	OL	CCSER1	NC_056064.1	39630001	39650001	2.18466	0.18371	OL	CDK12
0.213548	OL	CCSER1	NC_056064.1	39635001	39655001	2.41981	0.167956	OL	CDK12
0.206481	OL	CCSER1	NC_056064.1	39640001	39660001	2.68262	0.162916	OL	CDK12
0.161676	OL	CCSER1	NC_056064.1	39650001	39670001	2.42442	0.171076	OL	CDK12
0.204173	OL	CCSER1	NC_056064.1	39660001	39680001	2.245	0.195467	OL	CDK12
0.229682	OL	CCSER1	NC_056064.1	39665001	39685001	2.27928	0.180004	OL	CDK12
0.222969	OL	CCSER1	NC_056064.1	39670001	39690001	2.23207	0.203699	OL	CDK12
0.170085	OL	CCSER1	NC_056064.1	39675001	39695001	2.0717	0.249657	OL	CDK12
0.187244	OL	CCSER1	NC_056064.1	39680001	39700001	1.96552	0.260381	OL	CDK12
0.242248	OL	CCSER1	NC_056080.1	57595001	57615001	2.68682	0.158286	OL	CDK16;UBA1
0.27467	OL	CCSER1	NC_056080.1	57600001	57620001	2.6014	0.161698	OL	CDK16;UBA1
0.229965	OL	CCSER1	NC_056056.1	166130001	166150001	2.9768	0.171733	OL	CDK17
0.170743	OL	CCSER1	NC_056056.1	166135001	166155001	4.48622	0.163998	OL	CDK17
0.152866	OL	CCSER1	NC_056056.1	166140001	166160001	8.42658	0.168803	OL	CDK17
0.198232	OL	CCT4;COMI	NC_056056.1	166145001	166165001	9.11888	0.174427	OL	CDK17

0.168221	OL	CD96	NC_056056.1	166150001	166170001	9.52444	0.231869	OL	CDK17
0.241447	OL	CD96	NC_056056.1	166155001	166175001	13.7582	0.297071	OL	CDK17
0.188661	OL	CDCA2;KCT	NC_056056.1	166160001	166180001	12.9735	0.389502	OL	CDK17
0.194678	OL	CDH13	NC_056056.1	166165001	166185001	14.2418	0.459624	OL	CDK17
0.194759	OL	CDH18	NC_056056.1	166170001	166190001	14.7143	0.471509	OL	CDK17
0.248455	OL	CDH18	NC_056056.1	166175001	166195001	12.1546	0.46561	OL	CDK17
0.282273	OL	CDH18	NC_056056.1	166180001	166200001	8.21391	0.452514	OL	CDK17
0.261306	OL	CDH18	NC_056056.1	166185001	166205001	5.63462	0.397285	OL	CDK17
0.240131	OL	CDH18	NC_056056.1	166190001	166210001	3	0.285903	OL	CDK17
0.220093	OL	CDH18	NC_056056.1	166195001	166215001	2.69963	0.228717	OL	CDK17
0.174856	OL	CDH18	NC_056056.1	166200001	166220001	2.65067	0.212902	OL	CDK17
0.157333	OL	CDH18	NC_056056.1	166205001	166225001	3.67791	0.233458	OL	CDK17
0.182307	OL	CDH2	NC_056056.1	166210001	166230001	6.31475	0.290951	OL	CDK17
0.179211	OL	CDH2	NC_056061.1	26775001	26795001	3.47313	0.179826	OL	CDK19
0.159091	OL	CDH20	NC_056061.1	26780001	26800001	3.38956	0.184392	OL	CDK19
0.191982	OL	CDH20	NC_056056.1	8490001	8510001	2.4039	0.167485	OL	CFAP157;PTRH1;STXBP1;TTC16
0.153584	OL	CDH20	NC_056056.1	8495001	8515001	2.57199	0.17153	OL	CFAP157;PTRH1;STXBP1;TTC16
0.263674	OL	CDH23;PSA	NC_056056.1	8480001	8500001	2.11111	0.164583	OL	CFAP157;PTRH1;TOR2A;TTC16
0.222962	OL	CDH23;PSA	NC_056056.1	8500001	8520001	2.6456	0.175299	OL	CFAP157;STXBP1
0.16802	OL	CDH4	NC_056056.1	8505001	8525001	2.61087	0.195709	OL	CFAP157;STXBP1
0.161962	OL	CDH4	NC_056075.1	24380001	24400001	9.25542	0.176393	OL	CFAP43;SFR1
0.157806	OL	CDK19	NC_056080.1	35355001	35375001	2.73803	0.166985	OL	CFAP47
0.163667	OL	CDKL1	NC_056080.1	35360001	35380001	5.79199	0.278418	OL	CFAP47
0.148603	OL	CDKL1	NC_056080.1	35365001	35385001	7.1056	0.266554	OL	CFAP47
0.14933	OL	CDKN3;CNI	NC_056080.1	35370001	35390001	13.3239	0.308479	OL	CFAP47
0.214951	OL	CDKN3;CNI	NC_056080.1	35375001	35395001	17.1722	0.344989	OL	CFAP47
0.216486	OL	CDKN3;CNI	NC_056080.1	35380001	35400001	18.3147	0.394605	OL	CFAP47
0.156806	OL	CDR2;POLR	NC_056080.1	35385001	35405001	21.4197	0.439597	OL	CFAP47
0.266258	OL	CEP128	NC_056080.1	35390001	35410001	20.4959	0.414124	OL	CFAP47
0.290789	OL	CEP128	NC_056080.1	35395001	35415001	18.8158	0.392808	OL	CFAP47
0.248455	OL	CEP128	NC_056080.1	35400001	35420001	15.3571	0.328286	OL	CFAP47

0.41891	OL	CEP164	NC_056080.1	35460001	35480001	15.3414	0.338195	OL	CFAP47
0.495522	OL	CEP164	NC_056080.1	35465001	35485001	14.1927	0.354371	OL	CFAP47
0.561659	OL	CEP164	NC_056080.1	35470001	35490001	11.9569	0.401556	OL	CFAP47
0.429903	OL	CEP164	NC_056080.1	35475001	35495001	3.75074	0.363497	OL	CFAP47
0.424184	OL	CEP164	NC_056080.1	35480001	35500001	2.56811	0.260308	OL	CFAP47
0.241909	OL	CEP250	NC_056080.1	35485001	35505001	2.21669	0.257066	OL	CFAP47
0.188172	OL	CEP250	NC_056072.1	47200001	47220001	1.99041	0.233151	OL	CHDH
0.217598	OL	CEP250	NC_056072.1	47205001	47225001	1.95427	0.320601	OL	CHDH
0.232301	OL	CEP250;GDI	NC_056072.1	47210001	47230001	1.9949	0.334025	OL	CHDH
0.233178	OL	CEP83	NC_056072.1	47215001	47235001	2.01105	0.335911	OL	CHDH
0.235498	OL	CEP85L	NC_056055.1	135210001	135230001	2.27088	0.34406	OL	CHN1
0.237717	OL	CEP85L	NC_056055.1	38340001	38360001	3.62745	0.210748	OL	CHRNA2
0.222524	OL	CEP85L	NC_056055.1	38345001	38365001	4.42193	0.216106	OL	CHRNA2;PTK2B
0.188566	OL	CEP85L;PLN	NC_056055.1	38350001	38370001	3.81399	0.191043	OL	CHRNA2;PTK2B
0.223578	OL	CEP85L;PLN	NC_056055.1	38355001	38375001	3.55763	0.172248	OL	CHRNA2;PTK2B
0.236823	OL	CEP85L;PLN	NC_056062.1	83485001	83505001	1.95682	0.392922	OL	CIBAR1
0.167089	OL	CERS3	NC_056056.1	60230001	60250001	2.16622	0.161584	OL	CKAP2L
0.17864	OL	CERS3	NC_056056.1	60235001	60255001	3.48232	0.184737	OL	CKAP2L;NT5DC4
0.229307	OL	CERS3	NC_056056.1	60240001	60260001	11.4752	0.225931	OL	CKAP2L;NT5DC4
0.214262	OL	CERS3	NC_056056.1	60245001	60265001	17.397	0.254589	OL	CKAP2L;NT5DC4
0.175334	OL	CERS3	NC_056054.1	197835001	197855001	11.1361	0.53544	OL	CLDN1
0.15467	OL	CERT1;POL	NC_056054.1	197840001	197860001	9.64788	0.521565	OL	CLDN1
0.163017	OL	CERT1;POL	NC_056054.1	197845001	197865001	6.76209	0.492962	OL	CLDN1
0.212269	OL	CFAP299	NC_056054.1	197850001	197870001	3.70714	0.394869	OL	CLDN1
0.269948	OL	CFAP299	NC_056054.1	197855001	197875001	2.32266	0.268235	OL	CLDN1
0.229411	OL	CFAP299	NC_056054.1	197860001	197880001	2.96021	0.232017	OL	CLDN1
0.245617	OL	CFAP300	NC_056054.1	197865001	197885001	2.62391	0.252576	OL	CLDN1
0.257837	OL	CFAP47	NC_056058.1	37410001	37430001	2.2508	0.268015	OL	CLK4
0.160045	OL	CFAP91	NC_056058.1	37415001	37435001	3.39335	0.292096	OL	CLK4
0.229278	OL	CFAP91	NC_056058.1	37420001	37440001	2.45357	0.230097	OL	CLK4
0.231174	OL	CFAP91	NC_056059.1	106360001	106380001	2.8453	0.165275	OL	CLNK

0.16888	OL	CFAP97D1;I	NC_056054.1	249215001	249235001	2.07308	0.257288	OL	CLSTN2
0.184417	OL	CGNL1	NC_056054.1	249220001	249240001	2.15884	0.262111	OL	CLSTN2
0.221443	OL	CGNL1	NC_056054.1	249225001	249245001	2.18627	0.230071	OL	CLSTN2
0.217572	OL	CGNL1	NC_056072.1	2700001	2720001	2.65937	0.168857	OL	CMC1;LOC114108644
0.185487	OL	CGNL1	NC_056072.1	2705001	2725001	4.02006	0.220496	OL	CMC1;LOC114108644
0.175719	OL	CGNL1	NC_056072.1	2710001	2730001	4.49405	0.231219	OL	CMC1;LOC114108644
0.336435	OL	CHN1	NC_056072.1	2715001	2735001	4.14004	0.222886	OL	CMC1;LOC114108644
0.345485	OL	CHN1	NC_056060.1	10710001	10730001	2.27402	0.160005	OL	CMYA5
0.156195	OL	CHRDL2	NC_056055.1	85790001	85810001	3.74341	0.193795	OL	CNTLN
0.208871	OL	CIAO3;HAG	NC_056072.1	23195001	23215001	2.09758	0.367904	OL	CNTN4;IL5RA
0.151919	OL	CIAO3;MSL	NC_056072.1	23200001	23220001	1.94686	0.270839	OL	CNTN4;IL5RA
0.189527	OL	CLASP1	NC_056068.1	10005001	10025001	2.31742	0.228309	OL	CNTN5
0.196759	OL	CLASP1	NC_056068.1	10010001	10030001	3.73405	0.214552	OL	CNTN5
0.18744	OL	CLASP1	NC_056068.1	10015001	10035001	6.14504	0.177963	OL	CNTN5
0.18925	OL	CLASP1	NC_056068.1	10020001	10040001	3.91483	0.165042	OL	CNTN5
0.154878	OL	CLCN4	NC_056054.1	80245001	80265001	2.50882	0.200177	OL	COL11A1
0.270394	OL	CMKLR2	NC_056054.1	80250001	80270001	3.21833	0.220977	OL	COL11A1
0.202714	OL	CMKLR2	NC_056054.1	80255001	80275001	2.23996	0.306428	OL	COL11A1
0.285879	OL	CMKLR2;EI	NC_056054.1	80260001	80280001	2.29263	0.326957	OL	COL11A1
0.176036	OL	CNGA2	NC_056058.1	37275001	37295001	3.15866	0.325358	OL	COL23A1
0.208294	OL	CNGA2	NC_056058.1	37280001	37300001	2.44503	0.296582	OL	COL23A1
0.248786	OL	CNGA2	NC_056058.1	37285001	37305001	1.95283	0.255125	OL	COL23A1
0.279088	OL	CNGA2	NC_056058.1	37290001	37310001	2.11463	0.269158	OL	COL23A1
0.238808	OL	CNGA2;FAT	NC_056058.1	37305001	37325001	2.3977	0.314661	OL	COL23A1
0.261888	OL	CNN3;SLC4	NC_056058.1	37310001	37330001	2.36919	0.292235	OL	COL23A1
0.311662	OL	CNN3;SLC4	NC_056058.1	37315001	37335001	2.6833	0.286069	OL	COL23A1
0.146769	OL	CNTLN	NC_056058.1	37320001	37340001	2.556	0.246103	OL	COL23A1
0.199717	OL	CNTN4	NC_056058.1	37325001	37345001	2.16599	0.179089	OL	COL23A1
0.183222	OL	CNTN5	NC_056055.1	120355001	120375001	4.0364	0.177891	OL	COL3A1
0.168422	OL	CNTN5	NC_056055.1	120360001	120380001	3.82984	0.214276	OL	COL3A1
0.197173	OL	CNTN5	NC_056055.1	120365001	120385001	2.50884	0.240854	OL	COL3A1

0.150919	OL	CNTNAP2	NC_056055.1	120370001	120390001	3.40495	0.243327	OL	COL3A1
0.158981	OL	CNTNAP2	NC_056055.1	120375001	120395001	2.78983	0.195426	OL	COL3A1
0.219147	OL	CNTNAP5	NC_056055.1	120380001	120400001	2.44015	0.17133	OL	COL3A1
0.281392	OL	CNTNAP5	NC_056063.1	84480001	84500001	2.3	0.176126	OL	COL4A2
0.25929	OL	CNTNAP5	NC_056063.1	84485001	84505001	3.1354	0.204901	OL	COL4A2
0.197775	OL	CNTNAP5	NC_056063.1	84490001	84510001	2.65572	0.161232	OL	COL4A2
0.214239	OL	COL4A2	NC_056080.1	126675001	126695001	2.01875	0.252614	OL	COL4A5
0.182828	OL	COL4A2	NC_056080.1	126680001	126700001	2.80569	0.283829	OL	COL4A5
0.176504	OL	COL4A2	NC_056080.1	126685001	126705001	3.48509	0.268846	OL	COL4A5
0.175046	OL	COL4A2	NC_056080.1	126690001	126710001	2.7762	0.210255	OL	COL4A5
0.159136	OL	COL4A2	NC_056080.1	126695001	126715001	2.2214	0.161853	OL	COL4A5
0.205948	OL	COL4A2	NC_056055.1	120190001	120210001	2.02345	0.176996	OL	COL5A2
0.205956	OL	COL4A2	NC_056054.1	164980001	165000001	9.65323	0.158322	OL	COL8A1
0.16184	OL	COL4A2	NC_056065.1	64395001	64415001	3.28439	0.217762	OL	COLGALT2;RGL1
0.151315	OL	COL4A2	NC_056056.1	46200001	46220001	2.02443	0.293237	OL	COMMD1
0.169114	OL	COL4A5	NC_056056.1	46220001	46240001	2.1948	0.26643	OL	COMMD1
0.240115	OL	COL4A5	NC_056054.1	250310001	250330001	2.28088	0.209597	OL	COPB2
0.244503	OL	COL4A5	NC_056056.1	209540001	209560001	3.3871	0.210275	OL	COPS7A
0.211954	OL	COL4A5	NC_056056.1	209545001	209565001	4.52154	0.318826	OL	COPS7A
0.169134	OL	COL4A5	NC_056056.1	209550001	209570001	5.71052	0.368466	OL	COPS7A
0.174076	OL	COL4A6	NC_056070.1	64445001	64465001	2.9339	0.160907	OL	CORO1C
0.197562	OL	COL4A6	NC_056071.1	31420001	31440001	2.30535	0.226787	OL	COX5A
0.260669	OL	COL5A1	NC_056071.1	31425001	31445001	2.31035	0.203763	OL	COX5A
0.176883	OL	COMMD1	NC_056071.1	31430001	31450001	2.1875	0.194179	OL	COX5A;FAM219B
0.192272	OL	COMMD1	NC_056071.1	31435001	31455001	2.19122	0.205196	OL	COX5A;FAM219B
0.202901	OL	COMMD1	NC_056071.1	31410001	31430001	2.35826	0.24833	OL	COX5A;RPP25
0.228835	OL	CPED1	NC_056071.1	31415001	31435001	2.37515	0.248939	OL	COX5A;RPP25
0.187559	OL	CPED1	NC_056059.1	110840001	110860001	2.32558	0.174118	OL	CPEB2
0.223412	OL	CPED1;ING3	NC_056059.1	110845001	110865001	2.47017	0.185051	OL	CPEB2
0.243801	OL	CPED1;ING3	NC_056059.1	110850001	110870001	2.66936	0.210814	OL	CPEB2
0.209031	OL	CPLX3;LMA	NC_056059.1	110855001	110875001	2.58036	0.229904	OL	CPEB2

0.18514	OL	CPM	NC_056059.1	110860001	110880001	2.31503	0.277032	OL	CPEB2
0.230541	OL	CPM	NC_056059.1	110865001	110885001	2.38983	0.29658	OL	CPEB2
0.275761	OL	CPM	NC_056059.1	110870001	110890001	2.06013	0.280535	OL	CPEB2
0.290158	OL	CPM	NC_056059.1	110875001	110895001	1.99723	0.265989	OL	CPEB2
0.172895	OL	CPS1	NC_056059.1	110900001	110920001	2.5464	0.168235	OL	CPEB2
0.250335	OL	CPS1	NC_056057.1	87120001	87140001	2.43767	0.159553	OL	CPED1
0.281977	OL	CPS1	NC_056057.1	87125001	87145001	2.03019	0.167605	OL	CPED1
0.264008	OL	CPS1	NC_056072.1	22975001	22995001	2.15602	0.17525	OL	CRBN
0.235938	OL	CPS1	NC_056072.1	22980001	23000001	2.71453	0.198248	OL	CRBN
0.256697	OL	CPS1	NC_056072.1	22985001	23005001	2.71538	0.212265	OL	CRBN
0.230607	OL	CPS1	NC_056072.1	22990001	23010001	2.84653	0.199745	OL	CRBN
0.192911	OL	CPS1	NC_056072.1	22995001	23015001	2.16629	0.159828	OL	CRBN
0.210154	OL	CPS1	NC_056057.1	25795001	25815001	2.1872	0.196701	OL	CRPPA
0.159494	OL	CPS1	NC_056058.1	4250001	4270001	3.01321	0.158443	OL	CRTC1;LOC101107864
0.45352	OL	CRADD	NC_056056.1	180955001	180975001	5.92857	0.158882	OL	CSF2RB
0.505992	OL	CRADD	NC_056056.1	180960001	180980001	6.44444	0.168059	OL	CSF2RB
0.455572	OL	CRADD	NC_056056.1	180965001	180985001	7.41813	0.1759	OL	CSF2RB
0.324974	OL	CRADD	NC_056056.1	180970001	180990001	9.32342	0.204936	OL	CSF2RB
0.278116	OL	CRADD	NC_056056.1	180975001	180995001	10.5983	0.198077	OL	CSF2RB
0.243368	OL	CRADD	NC_056056.1	180980001	181000001	7.23256	0.198124	OL	CSF2RB
0.200505	OL	CRISP2	NC_056056.1	180985001	181005001	5.35993	0.195248	OL	CSF2RB
0.224671	OL	CRISP2	NC_056056.1	180990001	181010001	4.10298	0.197941	OL	CSF2RB
0.231196	OL	CRLF1;TME	NC_056079.1	4115001	4135001	2.22727	0.176891	OL	CSMD1
0.23211	OL	CRPPA	NC_056079.1	4120001	4140001	1.97677	0.193041	OL	CSMD1
0.170011	OL	CRPPA	NC_056079.1	4125001	4145001	1.97817	0.188025	OL	CSMD1
0.149071	OL	CRPPA	NC_056072.1	12480001	12500001	2.67217	0.167279	OL	CSRNP1
0.155495	OL	CSF2RB	NC_056072.1	12475001	12495001	2.24616	0.193339	OL	CSRNP1;TTC21A
0.163895	OL	CSF2RB	NC_056072.1	12485001	12505001	2.43042	0.230526	OL	CSRNP1;XIRP1
0.152514	OL	CSF2RB	NC_056080.1	99840001	99860001	2.01755	0.223877	OL	CT55;LOC114111389
0.19146	OL	CSK	NC_056080.1	99845001	99865001	2.16545	0.196366	OL	CT55;LOC114111389
0.195744	OL	CSK	NC_056056.1	161815001	161835001	3.47992	0.265131	OL	CTDSP2

0.166324	OL	CSMD2	NC_056056.1	161820001	161840001	2.60688	0.257647	OL	CTDSP2
0.186744	OL	CSMD2	NC_056056.1	51195001	51215001	4.30373	0.194742	OL	CTNNA2
0.162239	OL	CTBP2	NC_056056.1	51200001	51220001	5.29554	0.180921	OL	CTNNA2
0.162471	OL	CTBP2	NC_056056.1	51310001	51330001	3.92302	0.158465	OL	CTNNA2
0.367776	OL	CTCF	NC_056080.1	104885001	104905001	2.11765	0.374662	OL	CUL4B
0.415859	OL	CTCF	NC_056080.1	104890001	104910001	2.29514	0.433411	OL	CUL4B
0.427528	OL	CTCF	NC_056075.1	20495001	20515001	3.77795	0.159814	OL	CUTC
0.431364	OL	CTCF	NC_056070.1	55120001	55140001	2.74828	0.158145	OL	CUX2;PHETA1
0.438155	OL	CTCF	NC_056070.1	55130001	55150001	2.17948	0.170294	OL	CUX2;PHETA1
0.452807	OL	CTCF	NC_056072.1	12550001	12570001	5.29508	0.272201	OL	CX3CR1
0.471385	OL	CTCF	NC_056072.1	12555001	12575001	2.65821	0.167777	OL	CX3CR1
0.465483	OL	CTCF	NC_056072.1	12560001	12580001	2.64477	0.211754	OL	CX3CR1
0.270065	OL	CTCF;RIPOI	NC_056072.1	12565001	12585001	2.54161	0.191438	OL	CX3CR1
0.327236	OL	CTLA4	NC_056072.1	12570001	12590001	2.90251	0.180787	OL	CX3CR1
0.232812	OL	CTLA4	NC_056072.1	12575001	12595001	2.88212	0.183181	OL	CX3CR1
0.174187	OL	CTNNA3	NC_056078.1	43895001	43915001	2.72893	0.187337	OL	CXCL12
0.190711	OL	CTSH;MORI	NC_056078.1	43900001	43920001	3.30001	0.197911	OL	CXCL12
0.195415	OL	CTSH;MORI	NC_056078.1	43905001	43925001	2.81559	0.229872	OL	CXCL12
0.190801	OL	CTSH;MORI	NC_056078.1	43910001	43930001	2.63841	0.230048	OL	CXCL12
0.158829	OL	CUTC	NC_056078.1	43915001	43935001	2.22638	0.205138	OL	CXCL12
0.163101	OL	CUTC	NC_056060.1	69635001	69655001	3.42379	0.233997	OL	DAAM1
0.194921	OL	CUTC	NC_056060.1	69640001	69660001	14.7584	0.378003	OL	DAAM1
0.211155	OL	CUTC	NC_056060.1	69645001	69665001	20.3529	0.369874	OL	DAAM1
0.155233	OL	CUTC	NC_056060.1	69680001	69700001	9.25597	0.161211	OL	DAAM1;L3HYPDH
0.180227	OL	CYP2C18	NC_056060.1	69685001	69705001	3.87564	0.180491	OL	DAAM1;L3HYPDH
0.183542	OL	DACH2	NC_056060.1	69690001	69710001	3.09476	0.185475	OL	DAAM1;L3HYPDH
0.215392	OL	DACH2	NC_056060.1	69710001	69730001	2.42786	0.252554	OL	DAAM1;L3HYPDH
0.214422	OL	DACH2	NC_056080.1	77875001	77895001	2.24436	0.164966	OL	DACH2
0.190809	OL	DACH2	NC_056080.1	77900001	77920001	2.16252	0.172191	OL	DACH2
0.19603	OL	DACH2	NC_056080.1	77905001	77925001	2.13297	0.172009	OL	DACH2
0.196919	OL	DACH2	NC_056080.1	77910001	77930001	1.98984	0.170922	OL	DACH2

0.180858	OL	DACH2	NC_056080.1	77915001	77935001	1.96202	0.165991	OL	DACH2
0.202078	OL	DACH2	NC_056080.1	77960001	77980001	2.31486	0.160278	OL	DACH2
0.170228	OL	DACH2	NC_056080.1	77965001	77985001	2.48576	0.172309	OL	DACH2
0.163741	OL	DACH2	NC_056080.1	77970001	77990001	2.54857	0.172477	OL	DACH2
0.191762	OL	DACH2	NC_056080.1	77975001	77995001	2.42384	0.162462	OL	DACH2
0.159046	OL	DACH2	NC_056080.1	78030001	78050001	2.44209	0.171232	OL	DACH2
0.20407	OL	DACH2	NC_056080.1	78035001	78055001	3.04853	0.205621	OL	DACH2
0.213057	OL	DACH2	NC_056080.1	78040001	78060001	3.74572	0.224015	OL	DACH2
0.23158	OL	DACH2	NC_056080.1	78045001	78065001	5.37453	0.255773	OL	DACH2
0.22045	OL	DACH2	NC_056080.1	78050001	78070001	6.99019	0.2635	OL	DACH2
0.205077	OL	DACH2	NC_056080.1	78055001	78075001	7.94002	0.255628	OL	DACH2
0.213195	OL	DACH2	NC_056080.1	78060001	78080001	7.2549	0.24256	OL	DACH2
0.204613	OL	DACH2	NC_056080.1	78065001	78085001	8.35459	0.239919	OL	DACH2
0.183707	OL	DACH2	NC_056080.1	78070001	78090001	8.04	0.246333	OL	DACH2
0.189567	OL	DACH2	NC_056080.1	78075001	78095001	6.68126	0.245995	OL	DACH2
0.181394	OL	DACH2	NC_056080.1	78080001	78100001	7.51723	0.256816	OL	DACH2
0.193164	OL	DACH2	NC_056080.1	78085001	78105001	5.92751	0.244878	OL	DACH2
0.196795	OL	DACH2	NC_056080.1	78090001	78110001	5.65	0.248402	OL	DACH2
0.190867	OL	DACH2	NC_056080.1	78095001	78115001	6.17647	0.256523	OL	DACH2
0.182685	OL	DACH2	NC_056080.1	78100001	78120001	5.02549	0.237496	OL	DACH2
0.172335	OL	DACH2	NC_056080.1	78105001	78125001	3.03439	0.245776	OL	DACH2
0.153053	OL	DACH2	NC_056080.1	78110001	78130001	2.47143	0.243851	OL	DACH2
0.151935	OL	DACH2	NC_056080.1	78115001	78135001	2.16515	0.235272	OL	DACH2
0.158776	OL	DACH2	NC_056080.1	78120001	78140001	2.06184	0.235103	OL	DACH2
0.158642	OL	DACH2	NC_056080.1	78125001	78145001	2.10594	0.234221	OL	DACH2
0.164723	OL	DACH2	NC_056080.1	78130001	78150001	2.11899	0.224224	OL	DACH2
0.178596	OL	DARS1	NC_056080.1	78135001	78155001	2.15044	0.21813	OL	DACH2
0.161005	OL	DARS1	NC_056080.1	78140001	78160001	2.13433	0.16763	OL	DACH2
0.160194	OL	DARS1	NC_056080.1	78200001	78220001	3.63022	0.19364	OL	DACH2
0.185818	OL	DCC	NC_056080.1	78205001	78225001	4.98344	0.228877	OL	DACH2
0.231054	OL	DCC	NC_056080.1	78210001	78230001	8.45176	0.258168	OL	DACH2

0.230564	OL	DCC	NC_056080.1	78215001	78235001	8.0399	0.268795	OL	DACH2
0.146026	OL	DCLRE1C;M	NC_056080.1	78220001	78240001	9.77031	0.278099	OL	DACH2
0.193738	OL	DCP1B	NC_056080.1	78225001	78245001	8.55641	0.282635	OL	DACH2
0.247361	OL	DCP1B	NC_056080.1	78230001	78250001	7.07509	0.273058	OL	DACH2
0.19964	OL	DCUN1D4	NC_056080.1	78235001	78255001	8.35405	0.275439	OL	DACH2
0.255315	OL	DCUN1D4	NC_056080.1	78240001	78260001	6.75122	0.269252	OL	DACH2
0.29513	OL	DCUN1D4	NC_056080.1	78245001	78265001	5.5814	0.251496	OL	DACH2
0.291249	OL	DCUN1D4	NC_056080.1	78250001	78270001	5.71858	0.248853	OL	DACH2
0.270901	OL	DCUN1D4	NC_056080.1	78255001	78275001	5.12512	0.241172	OL	DACH2
0.206413	OL	DDAH1	NC_056080.1	78260001	78280001	5.73928	0.241894	OL	DACH2
0.297893	OL	DDC	NC_056080.1	78265001	78285001	6.23204	0.244853	OL	DACH2
0.192922	OL	DDX17	NC_056080.1	78270001	78290001	6.12018	0.250276	OL	DACH2
0.191739	OL	DDX17	NC_056080.1	78275001	78295001	5.9456	0.249356	OL	DACH2
0.23127	OL	DDX17;DM(	NC_056080.1	78280001	78300001	5.44804	0.24796	OL	DACH2
0.149808	OL	DDX17;KDE	NC_056080.1	78285001	78305001	4.54647	0.222781	OL	DACH2
0.170232	OL	DDX17;KDE	NC_056080.1	78290001	78310001	3.68094	0.194586	OL	DACH2
0.171439	OL	DDX17;KDE	NC_056080.1	78295001	78315001	4.24825	0.206104	OL	DACH2
0.215992	OL	DDX28;DUS	NC_056080.1	78300001	78320001	4.45636	0.206465	OL	DACH2
0.249027	OL	DDX6	NC_056080.1	78305001	78325001	4.62833	0.216598	OL	DACH2
0.285244	OL	DDX6	NC_056080.1	78310001	78330001	5.57142	0.247999	OL	DACH2
0.301944	OL	DDX6	NC_056080.1	78315001	78335001	3.63889	0.24058	OL	DACH2
0.148911	OL	DENND1B	NC_056080.1	78320001	78340001	2.78817	0.22399	OL	DACH2
0.320198	OL	DENND1B	NC_056080.1	78325001	78345001	2.30159	0.203828	OL	DACH2
0.358086	OL	DENND1B	NC_056080.1	78330001	78350001	2.09091	0.21727	OL	DACH2
0.336964	OL	DENND1B	NC_056080.1	78350001	78370001	1.995	0.230634	OL	DACH2
0.276548	OL	DENND1B	NC_056080.1	78355001	78375001	2.31757	0.270018	OL	DACH2
0.208723	OL	DIABLO;VP	NC_056080.1	78360001	78380001	2.16154	0.228446	OL	DACH2
0.200947	OL	DIABLO;VP	NC_056080.1	78380001	78400001	2.10559	0.181107	OL	DACH2
0.167273	OL	DIABLO;VP	NC_056080.1	78385001	78405001	2.10945	0.171877	OL	DACH2
0.185528	OL	DIAPH2	NC_056080.1	78390001	78410001	2.13889	0.188687	OL	DACH2
0.196677	OL	DIAPH2	NC_056080.1	78395001	78415001	2.26011	0.15924	OL	DACH2

0.236773	OL	DIAPH2	NC_056080.1	78400001	78420001	1.9899	0.180234	OL	DACH2
0.184926	OL	DIAPH2	NC_056080.1	78410001	78430001	1.96018	0.173008	OL	DACH2
0.179023	OL	DIAPH2	NC_056080.1	78415001	78435001	2.06073	0.204656	OL	DACH2
0.168105	OL	DIAPH2	NC_056080.1	78420001	78440001	2.51973	0.211283	OL	DACH2
0.152619	OL	DIAPH2	NC_056080.1	78425001	78445001	2.56463	0.216473	OL	DACH2
0.162854	OL	DIAPH2	NC_056080.1	78430001	78450001	2.42957	0.232206	OL	DACH2
0.172722	OL	DIAPH2	NC_056080.1	78435001	78455001	2.35582	0.224433	OL	DACH2
0.153879	OL	DIAPH2	NC_056080.1	78440001	78460001	2.06344	0.194979	OL	DACH2
0.160785	OL	DIAPH2	NC_056060.1	78965001	78985001	2.15362	0.232177	OL	DCAF5
0.170708	OL	DIAPH2	NC_056074.1	36385001	36405001	2.10193	0.364421	OL	DDB1;TKFC
0.165268	OL	DIAPH2	NC_056057.1	6180001	6200001	2.3181	0.186462	OL	DDC
0.226991	OL	DIAPH2	NC_056057.1	6185001	6205001	2.26691	0.207732	OL	DDC
0.211301	OL	DIAPH2	NC_056056.1	4250001	4270001	2.50484	0.174063	OL	DDX31
0.168207	OL	DIPK1A	NC_056056.1	4255001	4275001	2.48926	0.182927	OL	DDX31
0.146883	OL	DIPK1A	NC_056056.1	4260001	4280001	2.37724	0.164212	OL	DDX31
0.161012	OL	DIS3	NC_056057.1	78505001	78525001	1.98237	0.268314	OL	DDX56;TMED4
0.159347	OL	DLC1	NC_056068.1	43485001	43505001	5.12745	0.29322	OL	DENND2B
0.189197	OL	DLC1	NC_056068.1	43490001	43510001	5.49483	0.280996	OL	DENND2B
0.165228	OL	DLC1	NC_056068.1	43495001	43515001	4.74227	0.282838	OL	DENND2B
0.166207	OL	DLC1	NC_056068.1	43505001	43525001	2.3969	0.180708	OL	DENND2B
0.155532	OL	DLC1	NC_056068.1	43510001	43530001	2.19062	0.223702	OL	DENND2B
0.274191	OL	DLGAP4	NC_056068.1	43515001	43535001	2.17623	0.216335	OL	DENND2B
0.173757	OL	DLGAP5	NC_056060.1	12525001	12545001	2.3721	0.171844	OL	DENND4A;SLC24A1
0.276138	OL	DMC1	NC_056062.1	94220001	94240001	2.68564	0.173347	OL	DEPTOR
0.286849	OL	DMC1	NC_056057.1	23665001	23685001	2.05193	0.16704	OL	DGKB
0.29059	OL	DMC1	NC_056057.1	23670001	23690001	2.35883	0.162139	OL	DGKB
0.1845	OL	DMC1	NC_056074.1	44655001	44675001	4.40909	0.181064	OL	DHCR7
0.200668	OL	DMD	NC_056067.1	38580001	38600001	2.26667	0.194617	OL	DHODH
0.168092	OL	DMD	NC_056067.1	38585001	38605001	2.54762	0.225203	OL	DHODH
0.363494	OL	DMGDH	NC_056067.1	38590001	38610001	2.58571	0.238313	OL	DHODH
0.39842	OL	DMGDH	NC_056067.1	38595001	38615001	2.38436	0.266937	OL	DHODH;PKD1L3

0.317269	OL	DMGDH	NC_056067.1	38600001	38620001	2.46205	0.282795	OL	DHODH;PKD1L3
0.179539	OL	DNAAF11	NC_056067.1	38605001	38625001	3.29724	0.241006	OL	DHODH;PKD1L3
0.152852	OL	DNAAF11	NC_056054.1	234080001	234100001	4.28355	0.165517	OL	DHX36;GPR149
0.196749	OL	DNAAF8;ZN	NC_056070.1	53175001	53195001	2.49747	0.168934	OL	DIABLO;VPS33A
0.175115	OL	DNAH3;LYF	NC_056080.1	137655001	137675001	2.39136	0.197065	OL	DIAPH2
0.175634	OL	DNAH3;LYF	NC_056080.1	137760001	137780001	2.80279	0.179768	OL	DIAPH2
0.218227	OL	DNAJA2	NC_056065.1	25665001	25685001	2.22966	0.197062	OL	DISP1
0.213051	OL	DNAJC13	NC_056065.1	25670001	25690001	2.64748	0.194209	OL	DISP1
0.296347	OL	DNAJC13	NC_056065.1	25675001	25695001	2.12402	0.169699	OL	DISP1
0.305267	OL	DNAJC13	NC_056079.1	22810001	22830001	2.12213	0.180853	OL	DLC1
0.273653	OL	DNAJC13	NC_056073.1	17025001	17045001	3.53295	0.159972	OL	DLK2;TJAP1
0.160489	OL	DNM3	NC_056080.1	31160001	31180001	2.15743	0.183366	OL	DMD
0.157826	OL	DNM3	NC_056080.1	31170001	31190001	2.28252	0.206209	OL	DMD
0.172612	OL	DNM3	NC_056080.1	31175001	31195001	2.2177	0.200205	OL	DMD
0.248737	OL	DNM3	NC_056080.1	31850001	31870001	2.36875	0.179331	OL	DMD
0.273747	OL	DNM3	NC_056055.1	69300001	69320001	3.08893	0.225818	OL	DMRT2
0.280917	OL	DNM3	NC_056055.1	69305001	69325001	2.95876	0.197377	OL	DMRT2
0.246314	OL	DNM3	NC_056055.1	43510001	43530001	2.19284	0.194019	OL	DMTN;FHIP2B
0.199442	OL	DNM3	NC_056055.1	43515001	43535001	1.99423	0.170952	OL	DMTN;FHIP2B
0.264778	OL	DOCK7	NC_056067.1	14930001	14950001	2.55115	0.179823	OL	DNAJA2
0.310498	OL	DOCK7	NC_056078.1	23540001	23560001	2.26523	0.170038	OL	DNAJC12
0.258652	OL	DOCK7	NC_056054.1	257845001	257865001	2.00209	0.161648	OL	DNAJC13;LOC101119869
0.19288	OL	DOCK7	NC_056069.1	39345001	39365001	3.36812	0.36988	OL	DNAJC21
0.212589	OL	DPH1;HIC1;	NC_056069.1	39350001	39370001	3.43088	0.382155	OL	DNAJC21
0.367239	OL	DPY19L4	NC_056069.1	39355001	39375001	2.17153	0.296519	OL	DNAJC21
0.443659	OL	DPY19L4	NC_056054.1	37995001	38015001	3.85019	0.209868	OL	DOCK7
0.462779	OL	DPY19L4	NC_056054.1	38000001	38020001	3.58128	0.215821	OL	DOCK7
0.402706	OL	DPY19L4	NC_056054.1	38035001	38055001	2.27006	0.192561	OL	DOCK7
0.313703	OL	DPY19L4	NC_056054.1	38040001	38060001	3.58282	0.239675	OL	DOCK7
0.267521	OL	DTHD1	NC_056054.1	38045001	38065001	9.64151	0.303423	OL	DOCK7
0.224027	OL	DUS2	NC_056054.1	38050001	38070001	7.1089	0.260645	OL	DOCK7

0.23032	OL	DUS2	NC_056054.1	38055001	38075001	5.48648	0.206585	OL	DOCK7
0.20486	OL	DUS2;NFAT	NC_056061.1	10230001	10250001	2.88303	0.224879	OL	DOP1A
0.190705	OL	DUSP3;SOS'	NC_056061.1	10235001	10255001	2.54666	0.204801	OL	DOP1A
0.179688	OL	DUSP3;SOS'	NC_056061.1	10240001	10260001	2.1284	0.24852	OL	DOP1A
0.152778	OL	DUSP7	NC_056061.1	10225001	10245001	2.05042	0.160817	OL	DOP1A;UBE3D
0.178146	OL	DYNC2H1	NC_056055.1	181100001	181120001	3.56693	0.378728	OL	DPP10
0.169217	OL	DYNC2H1	NC_056055.1	181105001	181125001	5.07857	0.408909	OL	DPP10
0.154767	OL	DYNC2H1	NC_056055.1	181110001	181130001	3.30294	0.352806	OL	DPP10
0.14806	OL	DYSF	NC_056055.1	181115001	181135001	2.41824	0.284566	OL	DPP10
0.147713	OL	DYSF;LOC1	NC_056056.1	815001	835001	2.16927	0.20599	OL	DPP7;LOC105605822
0.176145	OL	DYSF;LOC1	NC_056080.1	132925001	132945001	1.97471	0.17654	OL	DRP2
0.256699	OL	EAF2;SLC15	NC_056054.1	260845001	260865001	4.80245	0.173369	OL	DSCAM
0.147172	OL	ECE1	NC_056054.1	260850001	260870001	4.48982	0.198911	OL	DSCAM
0.196272	OL	ECE1	NC_056054.1	260855001	260875001	4.1451	0.216197	OL	DSCAM
0.182611	OL	ECE1	NC_056054.1	260860001	260880001	2.9184	0.182134	OL	DSCAM
0.163974	OL	ECE1	NC_056057.1	13785001	13805001	3.33324	0.253574	OL	DYNC1I1
0.171903	OL	ECH1;HNRN	NC_056057.1	13790001	13810001	3.13841	0.215042	OL	DYNC1I1
0.201345	OL	ECH1;HNRN	NC_056057.1	13795001	13815001	3.16572	0.18807	OL	DYNC1I1
0.153171	OL	ECH1;HNRN	NC_056057.1	13800001	13820001	3.08182	0.187197	OL	DYNC1I1
0.150459	OL	ECT2L	NC_056072.1	21160001	21180001	1.98798	0.205294	OL	EDEM1
0.199687	OL	ECT2L	NC_056056.1	995001	1015001	2.2196	0.300063	OL	EDF1;MAMDC4;TRAF2
0.243084	OL	ECT2L	NC_056056.1	990001	1010001	2.1411	0.30434	OL	EDF1;TRAF2
0.187161	OL	ECT2L	NW_02459982	660001	680001	7.56392	0.332234	OL	EFL1
0.154982	OL	ECT2L;REPS	NW_02459982	665001	685001	6.57892	0.31126	OL	EFL1;SAXO2
0.198068	OL	EDA	NW_02459982	670001	690001	7.89128	0.231962	OL	EFL1;SAXO2
0.327112	OL	EDA	NC_056056.1	38025001	38045001	2.30678	0.194331	OL	EHD3
0.301849	OL	EDA	NC_056056.1	38030001	38050001	2.44611	0.218867	OL	EHD3
0.287773	OL	EDA	NC_056057.1	61785001	61805001	1.97687	0.231358	OL	ELMO1
0.227684	OL	EDEM3	NC_056057.1	61795001	61815001	2	0.209618	OL	ELMO1
0.318943	OL	EDEM3	NC_056059.1	15545001	15565001	2.65614	0.15974	OL	ELOVL6
0.332415	OL	EDEM3	NC_056068.1	61330001	61350001	4.93846	0.174468	OL	ELP4

0.263102	OL	EDEM3	NC_056080.1	104085001	104105001	2.70797	0.161974	OL	ENOX2
0.206393	OL	EDEM3	NC_056080.1	104095001	104115001	3.21383	0.168389	OL	ENOX2
0.162879	OL	EDEM3	NC_056080.1	104100001	104120001	2.51282	0.194444	OL	ENOX2
0.259989	OL	EEF1B2;ND	NC_056080.1	104105001	104125001	2.6131	0.181832	OL	ENOX2
0.155213	OL	EEPD1	NC_056080.1	104110001	104130001	2.41609	0.160559	OL	ENOX2
0.173099	OL	EIF3B;LOC1	NC_056080.1	104115001	104135001	2.26963	0.176878	OL	ENOX2
0.152613	OL	EIF4E3;GPR	NC_056080.1	104120001	104140001	3.28784	0.167929	OL	ENOX2
0.16689	OL	ELAVL2	NC_056056.1	218140001	218160001	1.98678	0.163349	OL	EP300
0.164861	OL	ELFN2	NC_056056.1	218145001	218165001	2.19355	0.215913	OL	EP300
0.169563	OL	ELFN2	NC_056056.1	218150001	218170001	2.77249	0.238661	OL	EP300
0.166741	OL	ELFN2	NC_056056.1	218155001	218175001	4.05042	0.267677	OL	EP300
0.176692	OL	ELFN2	NC_056056.1	218160001	218180001	3.29586	0.249773	OL	EP300
0.175095	OL	ELFN2	NC_056066.1	65255001	65275001	2.2706	0.203768	OL	EPB41L1
0.147011	OL	ELMO1	NC_056066.1	65260001	65280001	2.09172	0.159343	OL	EPB41L1
0.246909	OL	ELP6	NC_056066.1	65265001	65285001	2.41044	0.158381	OL	EPB41L1
0.302112	OL	ELP6	NC_056066.1	65270001	65290001	2.47711	0.163806	OL	EPB41L1
0.304274	OL	ELP6	NC_056054.1	12560001	12580001	3.87979	0.169492	OL	EPHA10
0.237992	OL	ELP6;LOC1(	NC_056054.1	162090001	162110001	2.42757	0.18959	OL	EPHA6
0.162285	OL	ELP6;LOC1(	NC_056054.1	162755001	162775001	13.0235	0.16893	OL	EPHA6
0.195321	OL	EMILIN2	NC_056054.1	162760001	162780001	23.6452	0.175559	OL	EPHA6
0.181572	OL	EML5	NC_056061.1	44170001	44190001	2.72357	0.191371	OL	EPHA7
0.243388	OL	EML5	NC_056061.1	44175001	44195001	4.37482	0.19371	OL	EPHA7
0.210412	OL	EML5	NC_056058.1	6355001	6375001	14.5489	0.183447	OL	EPS15L1
0.187321	OL	EML5	NC_056058.1	6360001	6380001	9.74501	0.177823	OL	EPS15L1
0.157647	OL	EML6	NC_056056.1	199845001	199865001	2.27467	0.158082	OL	EPS8
0.156582	OL	EML6	NC_056056.1	199850001	199870001	4.25911	0.271538	OL	EPS8
0.151064	OL	EML6	NC_056056.1	199855001	199875001	6.51759	0.29159	OL	EPS8
0.147524	OL	EML6	NC_056056.1	199860001	199880001	4.39896	0.2708	OL	EPS8
0.146715	OL	EML6	NC_056056.1	199865001	199885001	2.68819	0.230276	OL	EPS8
0.190275	OL	ENOX2	NC_056056.1	199925001	199945001	2.53912	0.161204	OL	EPS8
0.188596	OL	ENOX2	NC_056055.1	214145001	214165001	2.26577	0.194984	OL	ERBB4

0.169201	OL	ENOX2	NC_056055.1	214150001	214170001	2.16568	0.28917	OL	ERBB4
0.197651	OL	ENOX2	NC_056055.1	214160001	214180001	2.1259	0.255222	OL	ERBB4
0.205577	OL	ENOX2	NC_056055.1	214165001	214185001	2.32221	0.212239	OL	ERBB4
0.235128	OL	ENOX2	NC_056055.1	214170001	214190001	2.30353	0.170251	OL	ERBB4
0.221644	OL	ENOX2	NC_056072.1	44975001	44995001	3.31148	0.160598	OL	ERC2
0.171681	OL	ENOX2	NC_056072.1	44985001	45005001	3.34615	0.221473	OL	ERC2
0.189387	OL	ENOX2;LOC	NC_056072.1	44990001	45010001	3.03949	0.249807	OL	ERC2
0.14858	OL	EPS15L1	NC_056072.1	45460001	45480001	3.57913	0.190538	OL	ERC2
0.176573	OL	EPS15L1	NC_056072.1	45465001	45485001	4.17235	0.205856	OL	ERC2
0.176323	OL	EPS15L1	NC_056069.1	18550001	18570001	5.3037	0.203611	OL	ERCC8
0.184098	OL	EPS15L1	NC_056069.1	18555001	18575001	4.09866	0.173027	OL	ERCC8
0.165444	OL	EPS15L1	NC_056069.1	18540001	18560001	2.81742	0.158884	OL	ERCC8;NDUFAF2
0.177897	OL	EPS8	NC_056069.1	18545001	18565001	3.76761	0.202509	OL	ERCC8;NDUFAF2
0.250388	OL	EPS8	NC_056054.1	50800001	50820001	2.69743	0.157468	OL	ERICH3
0.274206	OL	EPS8	NC_056056.1	133230001	133250001	1.95048	0.201494	OL	ESPL1;MFSD5
0.258309	OL	EPS8	NC_056061.1	76880001	76900001	3.2591	0.170857	OL	ESR1
0.243286	OL	EPS8	NC_056061.1	76890001	76910001	3.55113	0.172527	OL	ESR1;SYNE1
0.18966	OL	EPS8	NC_056074.1	29270001	29290001	2.02025	0.173857	OL	ETS1
0.188491	OL	EPS8	NC_056065.1	41785001	41805001	2.72978	0.398225	OL	EXOSC10
0.180439	OL	EPS8	NC_056065.1	41770001	41790001	2.15789	0.435213	OL	EXOSC10;MTOR
0.182078	OL	EPS8	NC_056065.1	41775001	41795001	3.4633	0.436426	OL	EXOSC10;MTOR
0.178635	OL	EPS8	NC_056065.1	41780001	41800001	4.18334	0.419836	OL	EXOSC10;MTOR
0.236756	OL	EPS8	NC_056062.1	59390001	59410001	2.10833	0.359189	OL	EXT1
0.205524	OL	EPS8	NC_056062.1	59395001	59415001	2.10586	0.277209	OL	EXT1
0.256382	OL	EPS8L3;GST	NC_056074.1	27060001	27080001	3.35295	0.171766	OL	FAM118B;FOXRED1;SRPRA
0.25373	OL	EPS8L3;GST	NC_056074.1	27065001	27085001	2.29006	0.225176	OL	FAM118B;FOXRED1;SRPRA
0.155274	OL	ERBB4	NC_056074.1	27055001	27075001	2.20624	0.157635	OL	FAM118B;SRPRA
0.151531	OL	ERBB4	NC_056066.1	29250001	29270001	1.97782	0.169223	OL	FAM171A1
0.16867	OL	ERBB4	NC_056055.1	122665001	122685001	2.2103	0.261271	OL	FAM171B
0.179688	OL	ERC1	NC_056055.1	122670001	122690001	2.48634	0.295559	OL	FAM171B
0.155969	OL	ERC1	NC_056055.1	122675001	122695001	2.57144	0.287417	OL	FAM171B;ITGAV

0.32323	OL	ERC1	NC_056055.1	122680001	122700001	2.36585	0.280803	OL	FAM171B;ITGAV
0.415032	OL	ERC1	NC_056055.1	122685001	122705001	2.15294	0.281241	OL	FAM171B;ITGAV
0.405462	OL	ERC1	NC_056071.1	31440001	31460001	2.20065	0.229013	OL	FAM219B;MPI
0.183923	OL	ERC2	NC_056071.1	31445001	31465001	2.07672	0.220481	OL	FAM219B;MPI
0.164041	OL	ERC2	NC_056071.1	31450001	31470001	1.9825	0.206819	OL	FAM219B;MPI
0.242243	OL	ERC2	NC_056078.1	25130001	25150001	2.22636	0.226059	OL	FAM241B
0.167971	OL	ERC2	NC_056078.1	25135001	25155001	2.31984	0.222159	OL	FAM241B
0.178169	OL	ERP44;INVS	NC_056078.1	25140001	25160001	2.34607	0.194364	OL	FAM241B
0.156903	OL	ESR2;SYNE	NC_056067.1	14190001	14210001	2.62	0.163488	OL	FANCA;SPIRE2
0.159418	OL	ETS1	NC_056080.1	13015001	13035001	2.15086	0.240813	OL	FANCB
0.147025	OL	EVC2	NC_056080.1	13020001	13040001	4.79999	0.24763	OL	FANCB
0.147148	OL	EVC2	NC_056080.1	13025001	13045001	5.75728	0.242605	OL	FANCB;MOSPD2
0.285965	OL	EXOSC10;M	NC_056080.1	13030001	13050001	6.00884	0.199398	OL	FANCB;MOSPD2
0.276668	OL	EXOSC10;M	NC_056055.1	31065001	31085001	2.88085	0.160554	OL	FANCC
0.265761	OL	EXOSC10;M	NC_056055.1	31070001	31090001	2.6681	0.170109	OL	FANCC
0.414006	OL	EXT1	NC_056068.1	38810001	38830001	5.18979	0.232566	OL	FAR1
0.153429	OL	FAH	NC_056056.1	106235001	106255001	2.05955	0.19246	OL	FBLN7
0.154717	OL	FAM133A	NC_056056.1	106260001	106280001	1.9537	0.177469	OL	FBLN7
0.149245	OL	FAM133A	NC_056058.1	12270001	12290001	2.37004	0.193304	OL	FBXL12;PIN1;UBL5
0.153309	OL	FAM133A	NC_056058.1	12260001	12280001	2.52244	0.201265	OL	FBXL12;UBL5
0.159132	OL	FAM133A	NC_056058.1	12265001	12285001	2.54897	0.197445	OL	FBXL12;UBL5
0.149962	OL	FAM133A;N	NC_056058.1	104715001	104735001	2.28571	0.209357	OL	FBXL17
0.150526	OL	FAM172A	NC_056058.1	104720001	104740001	2.04493	0.201922	OL	FBXL17
0.15006	OL	FAM172A	NC_056070.1	5380001	5400001	5.64287	0.319078	OL	FBXW7
0.364159	OL	FAM189A2	NC_056070.1	5385001	5405001	11.0884	0.378429	OL	FBXW7
0.478166	OL	FAM189A2	NC_056070.1	5405001	5425001	23.7174	0.318361	OL	FBXW7
0.280435	OL	FAM189A2	NC_056070.1	5410001	5430001	3.01901	0.201406	OL	FBXW7
0.183255	OL	FAM189A2	NC_056054.1	110815001	110835001	2.35264	0.168184	OL	FCRL6
0.20802	OL	FAM219B;M	NC_056058.1	105365001	105385001	1.94921	0.162417	OL	FER
0.177484	OL	FAR2	NC_056060.1	63665001	63685001	9.51514	0.163248	OL	FERMT2
0.209027	OL	FAR2	NC_056072.1	57395001	57415001	2.07241	0.158661	OL	FGD5

0.183275	OL	FAR2	NC_056070.1	4885001	4905001	1.98941	0.181086	OL	FHDC1
0.215951	OL	FATE1	NC_056056.1	97170001	97190001	2.87993	0.213073	OL	FHL2
0.184502	OL	FATE1	NC_056056.1	97175001	97195001	4.4488	0.19486	OL	FHL2
0.202916	OL	FATE1;PRR(	NC_056056.1	97180001	97200001	9.70086	0.184786	OL	FHL2
0.180018	OL	FBXL17	NC_056061.1	27795001	27815001	2.19299	0.168645	OL	FIG4
0.227475	OL	FBXL17	NC_056061.1	27800001	27820001	2.23831	0.183576	OL	FIG4
0.198753	OL	FBXL17	NC_056061.1	27805001	27825001	2.82774	0.251191	OL	FIG4
0.187883	OL	FBXL17	NC_056059.1	69660001	69680001	2.05706	0.29028	OL	FIP1L1
0.161763	OL	FBXL17	NC_056059.1	69665001	69685001	2.01688	0.289806	OL	FIP1L1
0.154219	OL	FBXL17	NC_056059.1	69670001	69690001	2.0038	0.294969	OL	FIP1L1;LNX1
0.153868	OL	FBXL17	NC_056059.1	69675001	69695001	2.09536	0.290807	OL	FIP1L1;LNX1
0.181544	OL	FBXL7	NC_056059.1	69680001	69700001	2.23929	0.292151	OL	FIP1L1;LNX1
0.162734	OL	FBXL7	NC_056055.1	157035001	157055001	2.30789	0.184417	OL	FMNL2
0.162671	OL	FBXO43;PO	NC_056055.1	157040001	157060001	2.47631	0.188671	OL	FMNL2
0.16087	OL	FBXW7	NC_056055.1	157045001	157065001	2.9852	0.209908	OL	FMNL2
0.147592	OL	FBXW7	NC_056080.1	87735001	87755001	3.52014	0.295449	OL	FMR1
0.205454	OL	FCER1A	NC_056058.1	20490001	20510001	2.02792	0.253673	OL	FNIP1
0.30472	OL	FCER1A;LO	NC_056058.1	20495001	20515001	2.22587	0.271749	OL	FNIP1
0.294289	OL	FCER1A;LO	NC_056058.1	20500001	20520001	2.83897	0.30833	OL	FNIP1
0.206481	OL	FGD1	NC_056058.1	20505001	20525001	3.57543	0.342272	OL	FNIP1
0.178243	OL	FGF10	NC_056058.1	20510001	20530001	3.8559	0.336962	OL	FNIP1
0.254988	OL	FGF10	NC_056058.1	20515001	20535001	3.49483	0.305175	OL	FNIP1
0.277244	OL	FGF10	NC_056058.1	20520001	20540001	2.6134	0.255149	OL	FNIP1
0.271961	OL	FGF10	NC_056058.1	20525001	20545001	4.13866	0.333316	OL	FNIP1
0.19037	OL	FGF10	NC_056058.1	20530001	20550001	6.59741	0.392892	OL	FNIP1
0.169554	OL	FGF2	NC_056058.1	20535001	20555001	6.71724	0.409536	OL	FNIP1
0.15171	OL	FGFR2	NC_056058.1	20540001	20560001	5.79603	0.404162	OL	FNIP1
0.161963	OL	FGGY	NC_056058.1	20545001	20565001	4.69879	0.396071	OL	FNIP1
0.148199	OL	FHOD3	NC_056058.1	20550001	20570001	2.57683	0.322351	OL	FNIP1
0.14672	OL	FHOD3	NC_056058.1	20470001	20490001	2.02368	0.205416	OL	FNIP1;MEIKIN
0.148366	OL	FHOD3	NC_056058.1	20475001	20495001	2.05623	0.232175	OL	FNIP1;MEIKIN

0.148825	OL	FILIP1	NC_056058.1	20480001	20500001	2.03434	0.246468	OL	FNIP1;MEIKIN
0.161769	OL	FILIP1	NC_056055.1	89300001	89320001	2.27722	0.173864	OL	FOCAD
0.20548	OL	FILIP1	NC_056060.1	99015001	99035001	2.13753	0.199545	OL	FOXN3
0.198521	OL	FILIP1	NC_056060.1	99020001	99040001	2.26146	0.18436	OL	FOXN3
0.164335	OL	FITM2;GDA	NC_056060.1	99025001	99045001	2.63715	0.232319	OL	FOXN3
0.165744	OL	FITM2;GDA	NC_056060.1	99030001	99050001	3.39363	0.27076	OL	FOXN3
0.154543	OL	FLCN;LOC1	NC_056060.1	99035001	99055001	2.55868	0.238043	OL	FOXN3
0.1517	OL	FLCN;PLD6	NC_056057.1	55750001	55770001	2.40311	0.252585	OL	FOXP2
0.147792	OL	FLT3;URAD	NC_056057.1	55755001	55775001	2.81569	0.260741	OL	FOXP2
0.18488	OL	FLT4	NC_056057.1	55870001	55890001	10.4811	0.225745	OL	FOXP2
0.193282	OL	FLT4	NC_056057.1	55875001	55895001	10.9754	0.205078	OL	FOXP2
0.180869	OL	FLT4	NC_056057.1	55880001	55900001	16.1619	0.291228	OL	FOXP2
0.185544	OL	FLT4	NC_056057.1	55885001	55905001	10.7824	0.29973	OL	FOXP2
0.209426	OL	FMN1	NC_056057.1	55890001	55910001	4.08058	0.207972	OL	FOXP2
0.147592	OL	FMNL2	NC_056057.1	55895001	55915001	2.95174	0.185987	OL	FOXP2
0.188836	OL	FMNL2	NC_056074.1	27070001	27090001	1.9598	0.2702	OL	FOXRED1;LOC101118490;SRPRA
0.194962	OL	FNDC1	NC_056059.1	93520001	93540001	1.9722	0.157506	OL	FRAS1
0.285077	OL	FNDC1	NC_056059.1	93525001	93545001	3.33572	0.238568	OL	FRAS1
0.298231	OL	FNDC1	NC_056059.1	93530001	93550001	4.65464	0.239353	OL	FRAS1
0.279294	OL	FNDC1	NC_056059.1	93535001	93555001	3.42491	0.215148	OL	FRAS1
0.211507	OL	FNDC1	NC_056059.1	93540001	93560001	2.19294	0.212296	OL	FRAS1
0.261822	OL	FOCAD	NC_056059.1	93565001	93585001	2.6448	0.170338	OL	FRAS1
0.221059	OL	FOCAD	NC_056066.1	27685001	27705001	1.94743	0.167244	OL	FRMD4A
0.226844	OL	FOXN3	NC_056072.1	32445001	32465001	2.98164	0.185821	OL	FRMD4B
0.178717	OL	FOXP1	NC_056071.1	53320001	53340001	5.05032	0.295443	OL	FRMD5
0.289154	OL	FOXP2	NC_056071.1	53325001	53345001	5.06061	0.298918	OL	FRMD5
0.306635	OL	FOXP2	NC_056071.1	53330001	53350001	3.21136	0.23682	OL	FRMD5
0.265222	OL	FOXP2	NC_056071.1	53335001	53355001	2.3209	0.172881	OL	FRMD5
0.231205	OL	FOXP2	NC_056071.1	53310001	53330001	2.48099	0.213849	OL	FRMD5;WDR76
0.154602	OL	FOXP2	NC_056071.1	53315001	53335001	3.7721	0.263648	OL	FRMD5;WDR76
0.156383	OL	FOXP2	NC_056055.1	202235001	202255001	4.13707	0.164809	OL	FTCDNL1

0.162788	OL	FOXP2	NC_056071.1	3280001	3300001	4.90516	0.159699	OL	GABRB3
0.155611	OL	FRAS1	NC_056071.1	3285001	3305001	13.4856	0.182403	OL	GABRB3
0.152308	OL	FRAS1	NC_056071.1	3295001	3315001	10.6137	0.176403	OL	GABRB3
0.150681	OL	FRAS1	NC_056058.1	71340001	71360001	8.84482	0.163927	OL	GABRG2
0.154657	OL	FRMD5	NC_056061.1	48295001	48315001	2.04718	0.211642	OL	GABRR1
0.155482	OL	FRMD5	NC_056061.1	48300001	48320001	2.68166	0.240641	OL	GABRR1
0.151042	OL	FRMD5	NC_056061.1	48305001	48325001	2.86565	0.256732	OL	GABRR1
0.169076	OL	FRMD5;WD	NC_056061.1	48310001	48330001	2.91417	0.243771	OL	GABRR1;PM20D2
0.162483	OL	FRMD5;WD	NC_056056.1	125880001	125900001	2.12084	0.162301	OL	GALNT4;POC1B
0.161571	OL	FRMD5;WD	NC_056056.1	125885001	125905001	2.46214	0.174216	OL	GALNT4;POC1B
0.21582	OL	FRMD6	NC_056056.1	211220001	211240001	1.97688	0.157494	OL	GALNT8
0.147174	OL	FRY	NC_056056.1	211225001	211245001	2.40963	0.212408	OL	GALNT8
0.199179	OL	FUS	NC_056056.1	211230001	211250001	2.50855	0.21249	OL	GALNT8
0.235761	OL	FUS	NC_056056.1	211235001	211255001	2.38889	0.233898	OL	GALNT8
0.200974	OL	FUS	NC_056055.1	108165001	108185001	7.77523	0.166382	OL	GALNTL6
0.223286	OL	FUS	NC_056056.1	33390001	33410001	2.04669	0.325716	OL	GAREM2
0.246277	OL	FUS;LOC101	NC_056074.1	19595001	19615001	5.86005	0.224437	OL	GAS2
0.250964	OL	FUS;LOC101	NC_056074.1	19600001	19620001	18.6141	0.267347	OL	GAS2
0.175906	OL	FUT4	NC_056074.1	19605001	19625001	9.10244	0.234705	OL	GAS2
0.158013	OL	FZD3	NC_056074.1	19610001	19630001	4.71313	0.229676	OL	GAS2
0.159701	OL	FZD3	NC_056054.1	150040001	150060001	5.47416	0.249978	OL	GBE1
0.163369	OL	GABRB3	NC_056054.1	150045001	150065001	22.2353	0.301423	OL	GBE1
0.15185	OL	GABRB3	NC_056054.1	150050001	150070001	12.0152	0.284972	OL	GBE1
0.217596	OL	GABRR2;UE	NC_056064.1	50550001	50570001	2.40734	0.158273	OL	GCGR;MCRIP1
0.215478	OL	GABRR2;UE	NC_056055.1	64415001	64435001	2.30378	0.21364	OL	GDA
0.230541	OL	GABRR2;UE	NC_056055.1	64420001	64440001	2.65987	0.222731	OL	GDA
0.262515	OL	GALNT18	NC_056067.1	34800001	34820001	2.08108	0.261981	OL	GFOD2
0.2429	OL	GALNT18	NC_056067.1	34805001	34825001	2.02016	0.241845	OL	GFOD2
0.151224	OL	GALNT7	NC_056067.1	34810001	34830001	2.08805	0.167711	OL	GFOD2;RANBP10
0.149901	OL	GALNT7	NC_056072.1	7345001	7365001	2.38542	0.175358	OL	GLB1
0.252694	OL	GCHFR;RMI	NC_056073.1	50595001	50615001	2.1	0.198864	OL	GMDS

0.205054	OL	GCKR	NC_056073.1	50600001	50620001	2.19075	0.225146	OL	GMDS
0.219713	OL	GCKR	NC_056073.1	50605001	50625001	2.21789	0.203704	OL	GMDS
0.17541	OL	GCKR	NC_056073.1	50610001	50630001	2.29434	0.190179	OL	GMDS
0.195828	OL	GDA	NC_056073.1	50615001	50635001	2.11323	0.196844	OL	GMDS
0.184788	OL	GDA	NC_056073.1	50625001	50645001	2.0209	0.173436	OL	GMDS
0.282418	OL	GDAP1L1	NC_056073.1	50630001	50650001	2.02433	0.179965	OL	GMDS
0.255125	OL	GDAP1L1	NC_056073.1	50635001	50655001	2.07541	0.180184	OL	GMDS
0.227477	OL	GDAP1L1	NC_056073.1	50640001	50660001	2.13944	0.193961	OL	GMDS
0.243186	OL	GDAP1L1	NC_056073.1	50645001	50665001	2.15175	0.195771	OL	GMDS
0.207273	OL	GDAP1L1	NC_056073.1	50650001	50670001	1.95915	0.17109	OL	GMDS
0.18964	OL	GDF5	NC_056057.1	12010001	12030001	2.40863	0.293065	OL	GNG11;GNGT1
0.201058	OL	GJD3	NC_056060.1	42905001	42925001	4.28524	0.17583	OL	GNG2
0.17874	OL	GLG1	NC_056060.1	42910001	42930001	3.42693	0.191364	OL	GNG2
0.198285	OL	GLI2	NC_056060.1	42915001	42935001	3.35027	0.175709	OL	GNG2
0.158734	OL	GLRB	NC_056060.1	42920001	42940001	3.61271	0.20052	OL	GNG2
0.148316	OL	GLRB	NC_056060.1	42930001	42950001	3.06868	0.157982	OL	GNG2
0.18356	OL	GLRB	NC_056059.1	98975001	98995001	3.63572	0.167824	OL	GPAT3
0.153408	OL	GNA14	NC_056059.1	98980001	99000001	3.42878	0.165128	OL	GPAT3
0.160189	OL	GNA14	NC_056059.1	98985001	99005001	2.95568	0.16196	OL	GPAT3
0.173326	OL	GNA14	NC_056067.1	42975001	42995001	2.07682	0.18207	OL	GPATCH1
0.217351	OL	GNA14	NC_056063.1	66930001	66950001	2.41898	0.182676	OL	GPC5
0.177053	OL	GNA14	NC_056063.1	67240001	67260001	1.97063	0.325288	OL	GPC5
0.258574	OL	GNAI3	NC_056055.1	153050001	153070001	2.91916	0.168777	OL	GPD2
0.309694	OL	GNAI3	NC_056055.1	153055001	153075001	3.38163	0.193635	OL	GPD2
0.295269	OL	GNAI3	NC_056055.1	153060001	153080001	3.10514	0.188559	OL	GPD2
0.260696	OL	GNAI3	NC_056055.1	153065001	153085001	2.72903	0.193695	OL	GPD2
0.149834	OL	GNAQ	NC_056055.1	153080001	153100001	2.26919	0.1844	OL	GPD2
0.194562	OL	GNE	NC_056055.1	153085001	153105001	2.13839	0.204867	OL	GPD2
0.169801	OL	GNE	NC_056062.1	14295001	14315001	1.95158	0.196034	OL	GPIHBP1
0.154285	OL	GNE	NC_056055.1	135555001	135575001	4.78017	0.492328	OL	GPR155
0.1657	OL	GNE	NC_056055.1	135560001	135580001	12.1224	0.541298	OL	GPR155

0.178595	OL	GNE	NC_056055.1	135565001	135585001	2.86338	0.418229	OL	GPR155
0.223317	OL	GNE	NC_056080.1	70345001	70365001	2.08812	0.319187	OL	GPR174
0.254929	OL	GNE	NC_056080.1	70350001	70370001	2.04247	0.298584	OL	GPR174
0.157303	OL	GOLGA7B	NC_056080.1	70355001	70375001	1.99187	0.302854	OL	GPR174
0.162562	OL	GOLGB1	NC_056067.1	14890001	14910001	5.00197	0.196767	OL	GPT2
0.202075	OL	GOLGB1	NC_056057.1	6065001	6085001	2.42829	0.250818	OL	GRB10
0.200061	OL	GOPC	NC_056062.1	75825001	75845001	2.03937	0.244346	OL	GRHL2
0.164317	OL	GOPC	NC_056062.1	75830001	75850001	2.34946	0.219855	OL	GRHL2
0.156093	OL	GOPC;LOC1	NC_056062.1	75835001	75855001	1.97846	0.202665	OL	GRHL2
0.189202	OL	GOPC;LOC1	NC_056062.1	75840001	75860001	2.11332	0.215685	OL	GRHL2
0.174662	OL	GPAT3	NC_056055.1	51315001	51335001	2.49351	0.252746	OL	GRHPR
0.169777	OL	GPAT3	NC_056055.1	51300001	51320001	3.06024	0.225711	OL	GRHPR;ZBTB5
0.172819	OL	GPAT3	NC_056055.1	51305001	51325001	2.95436	0.237931	OL	GRHPR;ZBTB5
0.233632	OL	GPATCH2	NC_056055.1	51310001	51330001	2.83092	0.240976	OL	GRHPR;ZBTB5
0.216731	OL	GPATCH2	NC_056059.1	32210001	32230001	2.79464	0.193981	OL	GRID2
0.163806	OL	GPATCH2	NC_056059.1	32215001	32235001	2.71769	0.175943	OL	GRID2
0.208323	OL	GPC4	NC_056059.1	32220001	32240001	2.71014	0.200737	OL	GRID2
0.172932	OL	GPC4	NC_056059.1	32225001	32245001	2.91243	0.245603	OL	GRID2
0.283356	OL	GPC5	NC_056059.1	32230001	32250001	3.95491	0.239413	OL	GRID2
0.261744	OL	GPC5	NC_056059.1	32235001	32255001	3.94005	0.22731	OL	GRID2
0.196517	OL	GPC5	NC_056059.1	32240001	32260001	2.97696	0.196363	OL	GRID2
0.156742	OL	GPC5	NC_056061.1	35435001	35455001	2.62918	0.262206	OL	GRIK2
0.156759	OL	GPC5	NC_056061.1	35440001	35460001	2.88401	0.270747	OL	GRIK2
0.149216	OL	GPC5	NC_056061.1	35445001	35465001	2.63097	0.228657	OL	GRIK2
0.147233	OL	GPC5	NC_056061.1	35450001	35470001	2.05309	0.16814	OL	GRIK2
0.153801	OL	GPC5	NC_056077.1	9105001	9125001	3.16874	0.17217	OL	GRIN2A
0.159041	OL	GPC5	NC_056077.1	9110001	9130001	4.65577	0.224971	OL	GRIN2A
0.150847	OL	GPC5	NC_056077.1	9115001	9135001	4.76951	0.232292	OL	GRIN2A
0.237962	OL	GPC5	NC_056077.1	9120001	9140001	3.09134	0.19806	OL	GRIN2A
0.247006	OL	GPC5	NC_056070.1	65575001	65595001	2.00421	0.188903	OL	GRK3
0.180612	OL	GPC5	NC_056070.1	65580001	65600001	2.16167	0.299535	OL	GRK3

0.182416	OL	GPSM2	NC_056066.1	64225001	64245001	2.5625	0.176108	OL	GSS
0.146134	OL	GPSM2	NC_056066.1	64230001	64250001	2.14149	0.224749	OL	GSS
0.200666	OL	GREB1	NC_056066.1	64245001	64265001	2.21591	0.324778	OL	GSS
0.157763	OL	GREB1	NC_056066.1	45940001	45960001	2.46932	0.340757	OL	GTPBP4
0.16057	OL	GREB1	NC_056070.1	43695001	43715001	2.2392	0.159576	OL	GUCY1A1
0.153549	OL	GREB1	NC_056056.1	144875001	144895001	2.46102	0.283196	OL	GXYLT1
0.189805	OL	GRHL2	NC_056056.1	144880001	144900001	2.5354	0.23664	OL	GXYLT1
0.204759	OL	GRHL2	NC_056056.1	144885001	144905001	3.34805	0.237522	OL	GXYLT1
0.226225	OL	GRHL2	NC_056058.1	63530001	63550001	9.58572	0.225146	OL	HAND1
0.2462	OL	GRHL2	NC_056058.1	63535001	63555001	8.015	0.173055	OL	HAND1
0.148888	OL	GRIA3	NC_056058.1	63525001	63545001	10.7293	0.247871	OL	HAND1;SAP30L
0.193904	OL	GRIA4	NC_056054.1	186980001	187000001	2.01404	0.168123	OL	HCLS1
0.209334	OL	GRIA4	NC_056069.1	29580001	29600001	3.42664	0.178557	OL	HCN1
0.186118	OL	GRIA4	NC_056069.1	29585001	29605001	5.61618	0.211498	OL	HCN1
0.155161	OL	GRIA4	NC_056069.1	29590001	29610001	7.46845	0.219182	OL	HCN1
0.164491	OL	GRIK1	NC_056069.1	29595001	29615001	8.35185	0.233644	OL	HCN1
0.166966	OL	GRIK3	NC_056069.1	29600001	29620001	6.75802	0.170439	OL	HCN1
0.204746	OL	GRIK3	NC_056069.1	29605001	29625001	7.48053	0.171465	OL	HCN1
0.241123	OL	GRIK3	NC_056061.1	23730001	23750001	2.42037	0.238938	OL	HDAC2
0.23996	OL	GRIK3	NC_056061.1	23735001	23755001	2.29575	0.250872	OL	HDAC2
0.217917	OL	GRIP1	NC_056057.1	27590001	27610001	4.62691	0.212962	OL	HDAC9
0.21258	OL	GRIP1	NC_056057.1	27595001	27615001	10.7285	0.274894	OL	HDAC9
0.244931	OL	GRIP1	NC_056057.1	27615001	27635001	9.01516	0.307782	OL	HDAC9
0.219284	OL	GRIP1	NC_056057.1	27620001	27640001	3.76333	0.249428	OL	HDAC9
0.173571	OL	GRIP1	NC_056064.1	62320001	62340001	1.95321	0.171001	OL	HELZ
0.172697	OL	GRIP1	NC_056064.1	62350001	62370001	2.32447	0.166076	OL	HELZ
0.155812	OL	GRIP1	NC_056064.1	62355001	62375001	2.9166	0.229355	OL	HELZ
0.289565	OL	GRK3	NC_056064.1	62360001	62380001	2.93586	0.245581	OL	HELZ
0.1926	OL	GRK3	NC_056064.1	62365001	62385001	2.95155	0.252145	OL	HELZ
0.172127	OL	GTF2A1	NC_056064.1	62370001	62390001	2.78017	0.24852	OL	HELZ
0.152016	OL	GTF2A1L	NC_056064.1	62375001	62395001	2.50769	0.232499	OL	HELZ

0.184143	OL	GXYLT2	NC_056064.1	62380001	62400001	2.60288	0.243686	OL	HELZ
0.221902	OL	GXYLT2	NC_056064.1	62385001	62405001	2.43293	0.233726	OL	HELZ
0.255314	OL	GXYLT2	NC_056064.1	62390001	62410001	2.92641	0.260549	OL	HELZ
0.205474	OL	GXYLT2	NC_056064.1	62395001	62415001	3.09948	0.254443	OL	HELZ
0.219284	OL	GXYLT2	NC_056064.1	62400001	62420001	3.30084	0.2417	OL	HELZ
0.211577	OL	GXYLT2	NC_056064.1	62405001	62425001	3.547	0.254479	OL	HELZ
0.246753	OL	HBB	NC_056064.1	62410001	62430001	2.31516	0.202324	OL	HELZ
0.209182	OL	HBB;LOC10	NC_056064.1	62415001	62435001	2.49999	0.232966	OL	HELZ
0.247664	OL	HBE1	NC_056064.1	62420001	62440001	2.65442	0.2461	OL	HELZ
0.204791	OL	HBE1;LOC1	NC_056064.1	62325001	62345001	2.02937	0.175704	OL	HELZ;LOC101118037
0.284848	OL	HDAC10;MA	NC_056064.1	62330001	62350001	2.46966	0.19935	OL	HELZ;LOC101118037
0.277299	OL	HDAC10;MA	NC_056064.1	62425001	62445001	2.86	0.248399	OL	HELZ;LOC106991442
0.161211	OL	HDAC2	NC_056064.1	62430001	62450001	3.07708	0.264478	OL	HELZ;LOC106991442
0.16925	OL	HDAC2	NC_056064.1	62435001	62455001	2.21479	0.21103	OL	HELZ;LOC106991442
0.148797	OL	HDAC2	NC_056064.1	62440001	62460001	2.03053	0.201882	OL	HELZ;LOC106991442
0.153104	OL	HDAC2	NC_056057.1	11330001	11350001	1.95691	0.16789	OL	HEPACAM2
0.161939	OL	HDAC2	NC_056065.1	49645001	49665001	2.33233	0.226643	OL	HES5;PANK4
0.173698	OL	HDAC2	NC_056065.1	49650001	49670001	2.20958	0.22494	OL	HES5;PANK4
0.17811	OL	HDAC2	NC_056057.1	70090001	70110001	2.06747	0.229962	OL	HIBADH
0.17142	OL	HDAC2	NC_056068.1	62715001	62735001	1.98455	0.18294	OL	HIPK3
0.186366	OL	HDAC4	NC_056054.1	99320001	99340001	2.21094	0.22264	OL	HJV
0.190091	OL	HDAC4	NC_056054.1	99325001	99345001	2.73177	0.256296	OL	HJV
0.195029	OL	HDAC4	NC_056054.1	99330001	99350001	2.5508	0.257975	OL	HJV
0.185456	OL	HDAC9	NC_056055.1	103355001	103375001	2.43788	0.194662	OL	HMBOX1;INTS9
0.225153	OL	HDAC9	NC_056055.1	103565001	103585001	1.986	0.223634	OL	HMBOX1;KIF13B
0.284768	OL	HDAC9	NC_056063.1	30515001	30535001	5.55055	0.165589	OL	HMGB1
0.25192	OL	HDAC9	NC_056063.1	30485001	30505001	2.25301	0.323724	OL	HMGB1;USPL1
0.22328	OL	HDAC9	NC_056063.1	30490001	30510001	3.6909	0.24056	OL	HMGB1;USPL1
0.155729	OL	HDAC9	NC_056055.1	132885001	132905001	2.01179	0.377624	OL	HNRNPA3;NFE2L2
0.160288	OL	HDAC9	NC_056056.1	132480001	132500001	2.11494	0.166667	OL	HOXC5;HOXC6
0.181057	OL	HDAC9	NC_056056.1	132490001	132510001	2.15555	0.164516	OL	HOXC6;HOXC8

0.213925	OL	HDAC9	NC_056056.1	28465001	28485001	2.21754	0.237865	OL	HS1BP3
0.202011	OL	HDAC9	NC_056056.1	28470001	28490001	3.25751	0.180026	OL	HS1BP3
0.283471	OL	HECW2	NC_056054.1	64180001	64200001	5.00982	0.165005	OL	HS2ST1
0.318099	OL	HECW2	NC_056054.1	64185001	64205001	5.05733	0.159329	OL	HS2ST1
0.325712	OL	HECW2	NC_056059.1	107170001	107190001	2.47643	0.160348	OL	HS3ST1
0.295791	OL	HECW2	NC_056059.1	107175001	107195001	2.3794	0.184725	OL	HS3ST1
0.285515	OL	HECW2	NC_056059.1	107180001	107200001	2.40229	0.182995	OL	HS3ST1
0.173699	OL	HHLA2	NC_056059.1	107185001	107205001	2.0339	0.219592	OL	HS3ST1
0.268958	OL	HHLA2	NC_056059.1	107195001	107215001	2.03065	0.210603	OL	HS3ST1
0.289902	OL	HHLA2	NC_056063.1	73030001	73050001	9.4717	0.16747	OL	HS6ST3
0.271305	OL	HHLA2;MY1	NC_056063.1	73215001	73235001	6.22842	0.228984	OL	HS6ST3
0.232257	OL	HHLA2;MY1	NC_056063.1	73220001	73240001	7.61176	0.246993	OL	HS6ST3
0.200282	OL	HHLA2;MY1	NC_056063.1	73225001	73245001	4.03529	0.189632	OL	HS6ST3
0.341685	OL	HIKESHI	NC_056067.1	8110001	8130001	3.0533	0.177092	OL	HSD17B2
0.366416	OL	HIKESHI	NC_056073.1	17715001	17735001	2.31724	0.186976	OL	HSP90AB1;SLC29A1
0.378876	OL	HIKESHI	NC_056075.1	13770001	13790001	1.9649	0.162581	OL	IDE
0.358997	OL	HIKESHI	NC_056080.1	63030001	63050001	3.248	0.229741	OL	IGBP1
0.255222	OL	HIKESHI	NC_056064.1	37255001	37275001	2.44118	0.263567	OL	IGF2BP1
0.170836	OL	HIKESHI	NC_056055.1	214920001	214940001	3.02447	0.257576	OL	IKZF2
0.183079	OL	HIKESHI	NC_056055.1	214925001	214945001	3.2212	0.27967	OL	IKZF2
0.204897	OL	HIKESHI	NC_056065.1	4430001	4450001	2.86194	0.161347	OL	IL19
0.175008	OL	HIPK1	NC_056065.1	4435001	4455001	3.01752	0.167161	OL	IL19
0.22422	OL	HIPK1	NC_056065.1	4440001	4460001	3.09938	0.177874	OL	IL19
0.237178	OL	HIPK1	NC_056065.1	4450001	4470001	2.02403	0.162097	OL	IL19
0.197836	OL	HIPK1	NC_056056.1	99470001	99490001	2.70041	0.279198	OL	IL1RL2
0.16381	OL	HIPK1	NC_056056.1	99475001	99495001	3.87746	0.282133	OL	IL1RL2
0.259651	OL	HMBOX1;K	NC_056056.1	59845001	59865001	2.0888	0.191247	OL	IL1RN
0.258237	OL	HMBOX1;K	NC_056056.1	59850001	59870001	3.473	0.189488	OL	IL1RN
0.149347	OL	HMGCR	NC_056067.1	1030001	1050001	3.85331	0.200999	OL	IL34
0.185974	OL	HMX1	NC_056060.1	34810001	34830001	2.52569	0.203552	OL	INO80
0.21869	OL	HMX1	NC_056060.1	34815001	34835001	2.60242	0.219637	OL	INO80

0.202178	OL	HMX1	NC_056060.1	34820001	34840001	2.75374	0.225252	OL	INO80
0.209476	OL	HMX1	NC_056060.1	34825001	34845001	2.3528	0.189762	OL	INO80
0.205137	OL	HMX1	NC_056070.1	15900001	15920001	2.40507	0.187377	OL	INPP4B
0.241841	OL	HNRNPL	NC_056070.1	15905001	15925001	2.16546	0.167034	OL	INPP4B
0.17077	OL	HNRNPL	NC_056056.1	188485001	188505001	12.1717	0.167252	OL	INTS13
0.178571	OL	HOXC6;HO	NC_056056.1	188490001	188510001	18.9271	0.173453	OL	INTS13
0.179487	OL	HOXC6;HO	NC_056056.1	184425001	184445001	2.24532	0.172197	OL	IPO8
0.186275	OL	HOXC6;HO	NC_056054.1	4750001	4770001	2.03064	0.192735	OL	IQCA1
0.150641	OL	HOXC8;HO	NC_056054.1	4755001	4775001	2.12283	0.262238	OL	IQCA1
0.214366	OL	HS3ST5	NC_056054.1	4760001	4780001	2.28605	0.318784	OL	IQCA1
0.239503	OL	HS3ST5	NC_056054.1	4765001	4785001	2.37554	0.275844	OL	IQCA1
0.246837	OL	HS3ST5	NC_056054.1	4770001	4790001	2.47634	0.259456	OL	IQCA1
0.249043	OL	HS3ST5	NC_056054.1	4775001	4795001	2.64193	0.243856	OL	IQCA1
0.218178	OL	HSP90AB1;S	NC_056054.1	4780001	4800001	2.77469	0.184101	OL	IQCA1
0.228605	OL	HSP90AB1;S	NC_056054.1	4785001	4805001	2.66348	0.181041	OL	IQCA1
0.242399	OL	HSP90AB1;S	NC_056054.1	4790001	4810001	2.70401	0.175692	OL	IQCA1
0.270996	OL	HTR2C	NC_056080.1	126420001	126440001	2.59406	0.262142	OL	IRS4
0.167798	OL	HTR2C	NC_056080.1	126425001	126445001	3.46875	0.311663	OL	IRS4
0.21577	OL	HTR2C	NC_056080.1	126430001	126450001	2.89627	0.319856	OL	IRS4
0.211219	OL	HTR2C	NC_056080.1	126435001	126455001	2.40959	0.277598	OL	IRS4
0.157109	OL	HTR2C	NC_056080.1	126440001	126460001	2.89036	0.264251	OL	IRS4
0.166738	OL	HTR2C	NC_056080.1	126445001	126465001	3.67223	0.237583	OL	IRS4
0.17449	OL	HTR4	NC_056067.1	15400001	15420001	7.928	0.266591	OL	ITFG1;PHKB
0.278324	OL	HTR4	NC_056067.1	15405001	15425001	3.84768	0.2126	OL	ITFG1;PHKB
0.293474	OL	HTR4	NC_056054.1	112310001	112330001	4.49648	0.171489	OL	ITLN
0.290415	OL	HTR4	NC_056080.1	70550001	70570001	2.04166	0.339932	OL	ITM2A
0.237554	OL	HTR5A	NC_056080.1	70555001	70575001	2.01321	0.331445	OL	ITM2A
0.268843	OL	HTR5A	NC_056080.1	70560001	70580001	1.95573	0.334771	OL	ITM2A
0.303209	OL	HTR5A	NC_056080.1	70585001	70605001	1.97623	0.349486	OL	ITM2A
0.24775	OL	HTR5A	NC_056071.1	55495001	55515001	2.23952	0.179267	OL	ITPK1
0.174837	OL	IGFBP4	NC_056071.1	55500001	55520001	2.42159	0.188382	OL	ITPK1

0.18125	OL	IGFBP4	NC_056056.1	56905001	56925001	2.40959	0.164051	OL	KCMF1
0.158612	OL	IL1RL2	NC_056056.1	56910001	56930001	2.21166	0.188809	OL	KCMF1
0.200498	OL	IL1RL2	NC_056056.1	56915001	56935001	2.23077	0.184123	OL	KCMF1
0.267676	OL	IL1RL2	NC_056054.1	87905001	87925001	2.04644	0.203827	OL	KCNA2
0.308384	OL	IL1RL2	NC_056054.1	87910001	87930001	2.50766	0.2275	OL	KCNA2
0.310964	OL	IL1RL2	NC_056062.1	49085001	49105001	2.26597	0.280107	OL	KCNB2
0.195825	OL	IL1RL2	NC_056057.1	86870001	86890001	3.16155	0.182782	OL	KCND2
0.297816	OL	IMPG1	NC_056059.1	41095001	41115001	2.96946	0.164823	OL	KCNIP4
0.272879	OL	IMPG1	NC_056059.1	41345001	41365001	2.07325	0.25158	OL	KCNIP4
0.263079	OL	IMPG2	NC_056059.1	41350001	41370001	2.93627	0.191012	OL	KCNIP4
0.171929	OL	ING3	NC_056060.1	99755001	99775001	6.67827	0.201248	OL	KCNK13
0.201853	OL	ING3	NC_056060.1	99760001	99780001	3.70374	0.276131	OL	KCNK13
0.181898	OL	ING3	NC_056060.1	99765001	99785001	3.79332	0.32923	OL	KCNK13
0.193584	OL	ING3	NC_056060.1	99770001	99790001	2.92109	0.380491	OL	KCNK13
0.2335	OL	ING3	NC_056080.1	44085001	44105001	2.0307	0.25577	OL	KDM6A
0.199218	OL	INHBB	NC_056080.1	44090001	44110001	2.259	0.293193	OL	KDM6A
0.180334	OL	INHBB	NC_056062.1	19435001	19455001	2.54529	0.373754	OL	KHDRBS3
0.153214	OL	INTS6L	NC_056062.1	19440001	19460001	3.33333	0.334864	OL	KHDRBS3
0.157122	OL	INTS9	NC_056062.1	19445001	19465001	2.8794	0.35195	OL	KHDRBS3
0.15701	OL	INTS9	NC_056062.1	19450001	19470001	3.19622	0.325269	OL	KHDRBS3
0.272645	OL	INVS	NC_056062.1	19455001	19475001	3.56344	0.290766	OL	KHDRBS3
0.266913	OL	INVS	NC_056062.1	19460001	19480001	3.00588	0.245752	OL	KHDRBS3
0.256917	OL	INVS	NC_056062.1	19465001	19485001	3.54071	0.208013	OL	KHDRBS3
0.229373	OL	INVS	NC_056062.1	19470001	19490001	3.77933	0.18169	OL	KHDRBS3
0.210073	OL	INVS	NC_056061.1	11095001	11115001	2.05042	0.163014	OL	KIAA0408;SOGA3
0.243303	OL	INVS	NC_056068.1	63095001	63115001	3.26567	0.169109	OL	KIAA1549L
0.227187	OL	INVS	NC_056068.1	63100001	63120001	3.4159	0.169137	OL	KIAA1549L
0.16118	OL	IQGAP2	NC_056068.1	63105001	63125001	3.51653	0.176295	OL	KIAA1549L
0.209501	OL	IRAK2	NC_056056.1	18225001	18245001	2.66136	0.16541	OL	KIDINS220
0.245923	OL	IRAK2	NC_056055.1	37195001	37215001	2.47191	0.193668	OL	KIF24;NUDT2
0.300872	OL	IRAK2	NC_056055.1	37200001	37220001	2.32052	0.181872	OL	KIF24;NUDT2

0.25	OL	IRS4	NC_056066.1	32730001	32750001	2.35491	0.173584	OL	KIF5B
0.147237	OL	ITGA4	NC_056066.1	32735001	32755001	2.24913	0.185613	OL	KIF5B
0.146604	OL	ITGA8	NC_056066.1	32740001	32760001	2.91183	0.182631	OL	KIF5B
0.173525	OL	ITGA8	NC_056062.1	74725001	74745001	1.96203	0.273706	OL	KLF10
0.186757	OL	ITGA8	NC_056060.1	69715001	69735001	2.23154	0.252975	OL	L3HYPDH
0.153731	OL	ITGA9	NC_056060.1	69720001	69740001	2.05057	0.22103	OL	L3HYPDH
0.162774	OL	ITGA9	NC_056061.1	55650001	55670001	2.1142	0.228942	OL	L3MBTL3
0.160888	OL	ITGAM	NC_056061.1	55655001	55675001	3.24811	0.276523	OL	L3MBTL3
0.187181	OL	ITGAM	NC_056061.1	55660001	55680001	4.1906	0.275501	OL	L3MBTL3
0.179098	OL	ITGAM	NC_056061.1	55665001	55685001	8.29375	0.284608	OL	L3MBTL3
0.169422	OL	ITGAM	NC_056061.1	55670001	55690001	14.6166	0.304628	OL	L3MBTL3
0.213586	OL	ITGAM	NC_056061.1	55675001	55695001	19.2323	0.309927	OL	L3MBTL3
0.226205	OL	ITGAM	NC_056061.1	55685001	55705001	22.125	0.308434	OL	L3MBTL3
0.204529	OL	ITGAM	NC_056061.1	55690001	55710001	6.44381	0.250973	OL	L3MBTL3
0.173155	OL	ITGAM	NC_056061.1	54850001	54870001	2.67678	0.160405	OL	LAMA2
0.173582	OL	ITGAM	NC_056061.1	54855001	54875001	1.98717	0.16812	OL	LAMA2
0.226152	OL	ITGAM	NC_056061.1	55050001	55070001	2.08156	0.163002	OL	LAMA2
0.183182	OL	ITGAM	NC_056063.1	16640001	16660001	2.67836	0.185981	OL	LCP1
0.172362	OL	ITGAM;ITG/	NC_056055.1	241230001	241250001	4.58576	0.160041	OL	LDLRAP1
0.285725	OL	ITLN	NC_056054.1	231305001	231325001	2.25487	0.170469	OL	LEKR1
0.228704	OL	ITLN	NC_056054.1	231310001	231330001	2.24494	0.159051	OL	LEKR1
0.167014	OL	ITPRID1	NC_056056.1	107630001	107650001	3.80244	0.316548	OL	LGR5
0.18589	OL	ITPRID1	NC_056056.1	107635001	107655001	5.98521	0.370006	OL	LGR5
0.153338	OL	ITPRID1	NC_056056.1	107640001	107660001	12.2526	0.34586	OL	LGR5
0.165139	OL	ITPRID1	NC_056056.1	107645001	107665001	12.5889	0.297461	OL	LGR5
0.181107	OL	ITPRID1	NC_056056.1	107650001	107670001	6.12163	0.173207	OL	LGR5
0.170073	OL	ITPRID1	NC_056056.1	107670001	107690001	2.74	0.161485	OL	LGR5
0.147928	OL	ITPRID1	NC_056056.1	107675001	107695001	2.62295	0.214204	OL	LGR5
0.363364	OL	JAK1	NC_056080.1	121985001	122005001	2.39029	0.158905	OL	LHFPL1
0.169438	OL	JAKMIP2	NC_056080.1	121990001	122010001	2.26246	0.176137	OL	LHFPL1
0.163437	OL	JAKMIP2	NC_056080.1	121995001	122015001	2.50562	0.26074	OL	LHFPL1

0.258866	OL	JAM3	NC_056080.1	122000001	122020001	2.5014	0.270036	OL	LHFPL1
0.265662	OL	JAM3	NC_056080.1	122005001	122025001	2.60758	0.257601	OL	LHFPL1
0.278497	OL	JAM3	NC_056080.1	122010001	122030001	2.76441	0.253393	OL	LHFPL1
0.30438	OL	JAM3	NC_056080.1	122015001	122035001	4.12441	0.271949	OL	LHFPL1
0.184787	OL	JDP2	NC_056080.1	122020001	122040001	4.96175	0.337353	OL	LHFPL1
0.167691	OL	JPH2;OSER1	NC_056080.1	122025001	122045001	3.13372	0.401614	OL	LHFPL1
0.220541	OL	JPH2;OSER1	NC_056080.1	122030001	122050001	3.5775	0.455835	OL	LHFPL1
0.146347	OL	JUN	NC_056080.1	122035001	122055001	2.7719	0.426747	OL	LHFPL1
0.155909	OL	JUNB;PRDX	NC_056080.1	122040001	122060001	2.75688	0.430598	OL	LHFPL1
0.329043	OL	KAT6B	NC_056080.1	122045001	122065001	3.08459	0.397531	OL	LHFPL1
0.286303	OL	KAT6B	NC_056080.1	122050001	122070001	2.1178	0.279136	OL	LHFPL1
0.211401	OL	KAT6B	NC_056065.1	76275001	76295001	3.45098	0.172122	OL	LHX9
0.166471	OL	KAT6B	NC_056055.1	98085001	98105001	3.19145	0.173159	OL	LINGO2
0.186127	OL	KAT6B	NC_056068.1	52540001	52560001	2.15955	0.158883	OL	LIPT2
0.188193	OL	KAT6B	NC_056059.1	69685001	69705001	2.06277	0.248211	OL	LNX1
0.167753	OL	KAT6B	NC_056059.1	69690001	69710001	2.44675	0.217803	OL	LNX1
0.164038	OL	KCNA1	NC_056059.1	69740001	69760001	4.71001	0.161047	OL	LNX1
0.170121	OL	KCNA1	NC_056078.1	12135001	12155001	3.19849	0.184288	OL	LOC100527962
0.244046	OL	KCNA1	NC_056078.1	12140001	12160001	3.16357	0.193591	OL	LOC100527962
0.222169	OL	KCNA1	NC_056078.1	12145001	12165001	3.20285	0.194678	OL	LOC100527962
0.251266	OL	KCNA1	NC_056078.1	12150001	12170001	3.10669	0.17581	OL	LOC100527962
0.201833	OL	KCND2	NC_056080.1	130965001	130985001	1.97756	0.20404	OL	LOC101101957
0.161333	OL	KCND3	NC_056080.1	130970001	130990001	2.01759	0.244724	OL	LOC101101957
0.186982	OL	KCND3	NC_056061.1	57960001	57980001	3.08864	0.242786	OL	LOC101102492;LOC101118705
0.221191	OL	KCND3	NC_056061.1	57955001	57975001	3.36729	0.2793	OL	LOC101102492;TAAR8
0.282867	OL	KCND3	NC_056060.1	21910001	21930001	2.13826	0.184669	OL	LOC101102649
0.24748	OL	KCND3	NC_056060.1	21915001	21935001	2.02612	0.186312	OL	LOC101102649
0.210069	OL	KCND3	NC_056054.1	123780001	123800001	2.00603	0.231598	OL	LOC101102772;URB1
0.148712	OL	KCND3	NC_056054.1	123785001	123805001	2.27425	0.213004	OL	LOC101102772;URB1
0.273482	OL	KCNE4	NC_056054.1	123790001	123810001	2.93457	0.195997	OL	LOC101102772;URB1
0.393989	OL	KCNE4	NC_056064.1	22645001	22665001	2.90876	0.171599	OL	LOC101103740;PRPF8

0.436562	OL	KCNE4	NC_056064.1	22635001	22655001	4.60151	0.206815	OL	LOC101103740;PRPF8;SCARF1
0.158724	OL	KCNH8	NC_056064.1	22640001	22660001	3.70859	0.212018	OL	LOC101103740;PRPF8;SCARF1
0.181836	OL	KCNIP4	NC_056056.1	163925001	163945001	3.66617	0.190181	OL	LOC101104213
0.154657	OL	KCNK13	NC_056056.1	163940001	163960001	7.8507	0.188953	OL	LOC101104465
0.18966	OL	KCNK13	NC_056056.1	163945001	163965001	4.85395	0.162894	OL	LOC101104465
0.245231	OL	KCNK13	NC_056056.1	57525001	57545001	5.21864	0.164129	OL	LOC101104728
0.303729	OL	KCNK13	NC_056056.1	57530001	57550001	11.3726	0.198003	OL	LOC101104728
0.342944	OL	KCNK13	NC_056067.1	65115001	65135001	1.99207	0.181086	OL	LOC101105018
0.318343	OL	KCNK13	NC_056067.1	65120001	65140001	2.09636	0.189852	OL	LOC101105018
0.181103	OL	KCNK2	NC_056067.1	65125001	65145001	2.27338	0.218659	OL	LOC101105018
0.252429	OL	KCNK2	NC_056067.1	65130001	65150001	2.04181	0.205355	OL	LOC101105018
0.314353	OL	KCNK2	NC_056068.1	44600001	44620001	2.07875	0.40215	OL	LOC101105776
0.344197	OL	KCNK2	NC_056062.1	34280001	34300001	3	0.286952	OL	LOC101106696;ST18
0.347155	OL	KCNK2	NC_056062.1	34290001	34310001	2.08889	0.18943	OL	LOC101106696;ST18
0.291256	OL	KCNK2	NC_056064.1	55150001	55170001	4.85858	0.18808	OL	LOC101108931;UNK
0.176914	OL	KCNK2	NC_056074.1	5000001	5020001	4.61918	0.202894	OL	LOC101109839
0.153544	OL	KCNK3	NC_056074.1	5005001	5025001	3.19191	0.173199	OL	LOC101109839
0.1479	OL	KIAA1614	NC_056058.1	8545001	8565001	2.64367	0.222981	OL	LOC101109878
0.148118	OL	KIAA1614	NC_056057.1	106490001	106510001	4.17371	0.210345	OL	LOC101109961
0.15133	OL	KLHDC7A	NC_056057.1	106475001	106495001	6.3421	0.231734	OL	LOC101109961;PRSS37
0.153385	OL	KLHDC7A	NC_056057.1	106480001	106500001	4.67545	0.248923	OL	LOC101109961;PRSS37
0.157646	OL	KLHL1	NC_056057.1	106485001	106505001	4.8356	0.245162	OL	LOC101109961;PRSS37
0.172853	OL	KLHL1	NC_056054.1	199815001	199835001	2.0619	0.237596	OL	LOC101110107
0.186019	OL	KLHL1	NC_056054.1	199820001	199840001	2.13394	0.246975	OL	LOC101110107
0.184105	OL	KLHL1	NC_056054.1	93120001	93140001	2.10276	0.162319	OL	LOC101110112
0.178302	OL	KLHL1	NC_056054.1	165750001	165770001	2.6674	0.171435	OL	LOC101110195
0.175721	OL	KLHL1	NC_056054.1	165755001	165775001	2.05075	0.160323	OL	LOC101110195
0.162287	OL	KLHL1	NC_056054.1	165870001	165890001	3.39253	0.207926	OL	LOC101110467
0.154358	OL	KLHL1	NC_056054.1	165875001	165895001	4.87015	0.244599	OL	LOC101110467
0.150589	OL	KLHL1	NC_056067.1	15460001	15480001	2.2637	0.20409	OL	LOC101110611;PHKB
0.158086	OL	KLHL1	NC_056067.1	60010001	60030001	2.90441	0.19358	OL	LOC101110828

0.177716	OL	KLHL1	NC_056067.1	60015001	60035001	2.19265	0.171692	OL	LOC101110828
0.196851	OL	KLHL1	NC_056054.1	107660001	107680001	8.27144	0.160001	OL	LOC101111069
0.224044	OL	KLHL1	NC_056054.1	107665001	107685001	8.63706	0.186963	OL	LOC101111069
0.250639	OL	KLHL1	NC_056054.1	107670001	107690001	5.64082	0.175896	OL	LOC101111337
0.233189	OL	KLHL1	NC_056054.1	107675001	107695001	4.79833	0.177435	OL	LOC101111337
0.23919	OL	KLHL1	NC_056054.1	107680001	107700001	4.28571	0.190886	OL	LOC101111337
0.252012	OL	KLHL1	NC_056054.1	107685001	107705001	3.00865	0.196283	OL	LOC101111337
0.213711	OL	KLHL1	NC_056056.1	209565001	209585001	6.73807	0.23661	OL	LOC101111444
0.203575	OL	KLHL1	NC_056065.1	41190001	41210001	3.11211	0.166905	OL	LOC101111567
0.181334	OL	KLHL1	NC_056065.1	41195001	41215001	3.8953	0.175484	OL	LOC101111567
0.152387	OL	KLHL1	NC_056080.1	56510001	56530001	2.11458	0.227565	OL	LOC101112029;PORCN
0.15333	OL	KLHL1	NC_056080.1	141980001	142000001	3.88112	0.165687	OL	LOC101112115
0.161542	OL	KLHL25	NC_056080.1	141985001	142005001	2.90842	0.194161	OL	LOC101112115
0.258407	OL	KLHL26	NC_056080.1	141990001	142010001	2.66449	0.220397	OL	LOC101112115
0.181145	OL	KLRG2	NC_056080.1	141995001	142015001	2.47648	0.253118	OL	LOC101112115
0.171594	OL	KLRG2	NC_056079.1	14800001	14820001	3.09776	0.181806	OL	LOC101112284;SNX25
0.208328	OL	KRT1	NC_056058.1	16560001	16580001	3.78613	0.162592	OL	LOC101112316
0.186928	OL	KRT80	NC_056077.1	33165001	33185001	2.18922	0.173928	OL	LOC101112784
0.165022	OL	KRT80	NC_056077.1	33170001	33190001	2.50916	0.180435	OL	LOC101112784
0.180067	OL	L3MBTL3	NC_056077.1	33175001	33195001	2.36715	0.17053	OL	LOC101112784
0.255146	OL	L3MBTL3	NC_056054.1	97795001	97815001	2.54511	0.185533	OL	LOC101112864
0.281176	OL	L3MBTL3	NC_056054.1	97800001	97820001	3.79845	0.26577	OL	LOC101112864
0.281821	OL	L3MBTL3	NC_056054.1	97805001	97825001	5.27393	0.306075	OL	LOC101112864
0.289822	OL	L3MBTL3	NC_056054.1	97810001	97830001	6.88842	0.315387	OL	LOC101112864
0.300015	OL	L3MBTL3	NC_056054.1	97815001	97835001	5.3133	0.285826	OL	LOC101112864
0.283784	OL	LAMA3	NC_056054.1	97820001	97840001	3.6448	0.241921	OL	LOC101112864
0.320853	OL	LAMA3	NC_056080.1	29430001	29450001	2.34403	0.16376	OL	LOC101112886;LOC114111481
0.313126	OL	LAMA3	NC_056068.1	46690001	46710001	2.56486	0.236639	OL	LOC101113010
0.314119	OL	LAMA3	NC_056068.1	46710001	46730001	4.3513	0.210343	OL	LOC101113528
0.218542	OL	LAMA3	NC_056068.1	46715001	46735001	3.58841	0.17659	OL	LOC101113528;LOC101119931
0.249282	OL	LAMA3	NC_056071.1	28100001	28120001	3.41288	0.158309	OL	LOC101113889

0.21881	OL	LAMA3	NC_056057.1	106925001	106945001	4.19907	0.210772	OL	LOC101114438;LOC121816042
0.148908	OL	LAMB4	NC_056057.1	106930001	106950001	3.94849	0.195389	OL	LOC101114438;LOC121816042
0.153222	OL	LAMB4	NC_056079.1	15150001	15170001	3.58904	0.161795	OL	LOC101114586
0.191432	OL	LAMB4	NC_056079.1	15155001	15175001	1.99651	0.196597	OL	LOC101114586
0.177556	OL	LAMB4	NC_056077.1	34600001	34620001	2.10234	0.190457	OL	LOC101115252
0.145784	OL	LAMB4	NC_056072.1	52655001	52675001	2.95881	0.223473	OL	LOC101115659
0.194001	OL	LAMC1	NC_056072.1	52660001	52680001	3.14325	0.213277	OL	LOC101115659
0.167886	OL	LCORL	NC_056072.1	52665001	52685001	3.21633	0.184902	OL	LOC101115659
0.24112	OL	LCORL	NC_056072.1	52670001	52690001	3.56631	0.178127	OL	LOC101115659
0.228363	OL	LCORL	NC_056072.1	52675001	52695001	3.74286	0.167037	OL	LOC101115659
0.234823	OL	LCORL	NC_056058.1	38910001	38930001	2.07839	0.176009	OL	LOC101115794
0.212789	OL	LCORL	NC_056058.1	38915001	38935001	2.84895	0.198774	OL	LOC101115794
0.183325	OL	LCORL	NC_056059.1	84730001	84750001	6.16067	0.160286	OL	LOC101115964
0.192804	OL	LCORL	NC_056059.1	84735001	84755001	6.27703	0.216996	OL	LOC101115964
0.185886	OL	LCORL	NC_056059.1	84740001	84760001	7.67892	0.254021	OL	LOC101115964
0.193331	OL	LCORL	NC_056059.1	84745001	84765001	5.04843	0.251932	OL	LOC101115964;LOC101116231
0.176485	OL	LCORL	NC_056059.1	84750001	84770001	2.84727	0.237321	OL	LOC101115964;LOC101116231
0.159527	OL	LDLRAD2	NC_056059.1	84755001	84775001	2.1642	0.227443	OL	LOC101116231
0.162249	OL	LEPR	NC_056080.1	98325001	98345001	2.98043	0.166176	OL	LOC101116968
0.198281	OL	LEPR	NC_056058.1	39050001	39070001	5.96511	0.30716	OL	LOC101117077
0.2092	OL	LEPR	NC_056058.1	39055001	39075001	5.27347	0.2862	OL	LOC101117077
0.210712	OL	LEPR	NC_056058.1	39060001	39080001	3.52495	0.259248	OL	LOC101117077
0.202105	OL	LEPR	NC_056058.1	39065001	39085001	2.83039	0.327723	OL	LOC101117077;TRIM58
0.166246	OL	LEPR	NC_056074.1	16290001	16310001	4.41216	0.180981	OL	LOC101117288
0.151926	OL	LEPR	NC_056074.1	16300001	16320001	4.79208	0.171239	OL	LOC101117288
0.158691	OL	LEPR	NC_056074.1	16305001	16325001	9.07318	0.250733	OL	LOC101117288
0.154746	OL	LHFPL2	NC_056074.1	16310001	16330001	25.605	0.293448	OL	LOC101117288
0.170646	OL	LHFPL2	NC_056074.1	16315001	16335001	37.1948	0.34829	OL	LOC101117288
0.189865	OL	LHFPL2	NC_056074.1	16325001	16345001	26.8857	0.434068	OL	LOC101117288
0.162675	OL	LHFPL2	NC_056074.1	16330001	16350001	15.5206	0.359083	OL	LOC101117288
0.462963	OL	LHX4	NC_056074.1	16335001	16355001	10.731	0.294444	OL	LOC101117288

0.436522	OL	LHX4	NC_056074.1	16340001	16360001	8.77823	0.249942	OL	LOC101117288
0.150536	OL	LIMK2	NC_056074.1	16345001	16365001	6.9101	0.216463	OL	LOC101117288
0.162247	OL	LIMK2	NC_056074.1	16350001	16370001	5.25048	0.217388	OL	LOC101117288
0.158875	OL	LIMK2	NC_056074.1	16355001	16375001	3.97059	0.229099	OL	LOC101117288
0.160048	OL	LIMK2;PIK3	NC_056074.1	16360001	16380001	3.17149	0.272642	OL	LOC101117288
0.172709	OL	LINGO2	NC_056074.1	16365001	16385001	2.82528	0.272668	OL	LOC101117288
0.210207	OL	LINGO2	NC_056074.1	16370001	16390001	3.55224	0.250801	OL	LOC101117288
0.192998	OL	LINGO2	NC_056074.1	16375001	16395001	4.51852	0.243239	OL	LOC101117288
0.232041	OL	LMOD3	NC_056074.1	16380001	16400001	4.55763	0.203833	OL	LOC101117288
0.209838	OL	LOC1011022	NC_056074.1	16385001	16405001	6.90361	0.206458	OL	LOC101117288
0.306311	OL	LOC1011024	NC_056074.1	16390001	16410001	5.00349	0.211981	OL	LOC101117288
0.250009	OL	LOC1011024	NC_056074.1	16395001	16415001	3.82535	0.234505	OL	LOC101117288
0.205393	OL	LOC1011025	NC_056074.1	16400001	16420001	3.18256	0.264968	OL	LOC101117288
0.30544	OL	LOC1011025	NC_056074.1	16405001	16425001	2.67488	0.295488	OL	LOC101117288
0.303887	OL	LOC1011025	NC_056074.1	16410001	16430001	2.42571	0.298083	OL	LOC101117288
0.212594	OL	LOC1011025	NC_056074.1	16415001	16435001	2.5411	0.283542	OL	LOC101117288
0.162484	OL	LOC1011026	NC_056074.1	16420001	16440001	2.90281	0.265547	OL	LOC101117288
0.158441	OL	LOC1011030	NC_056074.1	16425001	16445001	3.11631	0.240576	OL	LOC101117288
0.202981	OL	LOC1011033	NC_056074.1	16430001	16450001	2.91066	0.253822	OL	LOC101117288
0.188705	OL	LOC1011042	NC_056074.1	16435001	16455001	2.6923	0.276031	OL	LOC101117288
0.245543	OL	LOC1011044	NC_056074.1	16440001	16460001	2.31471	0.318537	OL	LOC101117288
0.244052	OL	LOC1011044	NC_056074.1	16445001	16465001	2.02327	0.359307	OL	LOC101117288
0.168791	OL	LOC1011044	NC_056074.1	16450001	16470001	2.31227	0.35246	OL	LOC101117288
0.27967	OL	LOC1011050	NC_056074.1	16455001	16475001	2.58269	0.327482	OL	LOC101117288
0.29105	OL	LOC1011050	NC_056074.1	16460001	16480001	2.84398	0.300474	OL	LOC101117288
0.151713	OL	LOC1011051	NC_056074.1	16545001	16565001	2.63359	0.176797	OL	LOC101117547
0.196993	OL	LOC1011051	NC_056074.1	16550001	16570001	2.6031	0.170968	OL	LOC101117547
0.197734	OL	LOC1011051	NC_056074.1	16600001	16620001	1.96065	0.183574	OL	LOC101117547
0.2137	OL	LOC1011051	NC_056074.1	16625001	16645001	3.14897	0.249422	OL	LOC101117547
0.206201	OL	LOC1011051	NC_056074.1	16630001	16650001	4.42021	0.251623	OL	LOC101117547
0.218938	OL	LOC1011051	NC_056074.1	16635001	16655001	4.94153	0.254911	OL	LOC101117547

0.227158	OL	LOC1011051	NC_056074.1	16640001	16660001	4.04067	0.217926	OL	LOC101117547
0.210448	OL	LOC1011051	NC_056074.1	16675001	16695001	2.13432	0.171042	OL	LOC101117547
0.185168	OL	LOC1011051	NC_056070.1	71035001	71055001	2.75505	0.183873	OL	LOC101117791;LOC101118053;LOC11
0.180298	OL	LOC1011051	NC_056070.1	71030001	71050001	2.763	0.207716	OL	LOC101118053;LOC114108841
0.264063	OL	LOC1011052	NC_056057.1	78345001	78365001	2.2139	0.162755	OL	LOC101118100
0.287002	OL	LOC1011052	NC_056057.1	78350001	78370001	2.5	0.176754	OL	LOC101118100
0.242379	OL	LOC1011055	NC_056066.1	16975001	16995001	2.26419	0.167703	OL	LOC101118373
0.145797	OL	LOC1011061	NC_056066.1	16980001	17000001	2.20561	0.164953	OL	LOC101118373
0.158074	OL	LOC1011061	NC_056066.1	16985001	17005001	2.13771	0.159687	OL	LOC101118373
0.153178	OL	LOC1011073	NC_056080.1	112100001	112120001	4.77294	0.266033	OL	LOC101119281
0.169018	OL	LOC1011073	NC_056080.1	112105001	112125001	5.14895	0.265254	OL	LOC101119281
0.153059	OL	LOC1011079	NC_056080.1	112110001	112130001	5.44623	0.272442	OL	LOC101119281
0.192575	OL	LOC1011079	NC_056080.1	112115001	112135001	5.29062	0.25778	OL	LOC101119281
0.264349	OL	LOC1011079	NC_056061.1	15260001	15280001	2.02309	0.234409	OL	LOC101119992
0.257447	OL	LOC1011079	NC_056064.1	44910001	44930001	2.54913	0.184153	OL	LOC101120000
0.234731	OL	LOC1011079	NC_056058.1	255001	275001	2.87044	0.16164	OL	LOC101120076
0.154795	OL	LOC1011079	NC_056058.1	285001	305001	2.49611	0.161559	OL	LOC101120076
0.230919	OL	LOC1011081	NC_056068.1	38940001	38960001	5.73852	0.179676	OL	LOC101120269;PTH
0.195964	OL	LOC1011081	NC_056068.1	38950001	38970001	10.7175	0.176037	OL	LOC101120269;PTH
0.199613	OL	LOC1011081	NC_056080.1	129285001	129305001	2.96825	0.208653	OL	LOC101120810
0.220077	OL	LOC1011081	NC_056080.1	129290001	129310001	2.49351	0.233539	OL	LOC101120810
0.238854	OL	LOC1011081	NC_056080.1	129295001	129315001	2.53572	0.27368	OL	LOC101120810
0.218348	OL	LOC1011081	NC_056080.1	129300001	129320001	3.14391	0.244582	OL	LOC101120810
0.188794	OL	LOC1011081	NC_056080.1	129305001	129325001	2.64023	0.175736	OL	LOC101120810
0.227387	OL	LOC1011083	NC_056059.1	66150001	66170001	9.03776	0.162632	OL	LOC101121518
0.317142	OL	LOC1011083	NC_056059.1	66155001	66175001	7.82193	0.158583	OL	LOC101121518
0.196768	OL	LOC1011087	NC_056064.1	6030001	6050001	2.16331	0.194811	OL	LOC101121870
0.220203	OL	LOC1011087	NC_056064.1	6035001	6055001	1.9849	0.161758	OL	LOC101121870
0.191783	OL	LOC1011087	NC_056058.1	49535001	49555001	2.91364	0.160033	OL	LOC101122108;LOC101122274;LOC10
0.215599	OL	LOC1011093	NC_056058.1	49540001	49560001	1.94751	0.197638	OL	LOC101122274;LOC105611202;LOC10
0.168668	OL	LOC1011094	NC_056058.1	49545001	49565001	2.0695	0.18828	OL	LOC101122274;LOC105611202;LOC10

0.178589	OL	LOC1011094	NC_056058.1	49550001	49570001	2.15254	0.179206	OL	LOC101122274;LOC105611202;LOC10
0.16495	OL	LOC1011098	NC_056058.1	49555001	49575001	2.03245	0.164084	OL	LOC101122274;LOC105611202;LOC10
0.174893	OL	LOC1011099	NC_056056.1	137690001	137710001	2.25536	0.161907	OL	LOC101123028
0.145762	OL	LOC1011099	NC_056056.1	137695001	137715001	2.16764	0.186963	OL	LOC101123028
0.152794	OL	LOC1011099	NC_056068.1	77130001	77150001	71.2251	0.194084	OL	LOC101123067
0.171767	OL	LOC1011101	NC_056054.1	102855001	102875001	2.19974	0.171621	OL	LOC101123167
0.155239	OL	LOC1011101	NC_056054.1	102860001	102880001	2.18845	0.198873	OL	LOC101123167
0.205635	OL	LOC1011101	NC_056056.1	132980001	133000001	1.9912	0.299739	OL	LOC101123619
0.159491	OL	LOC1011101	NC_056056.1	132995001	133015001	2.14595	0.209468	OL	LOC101123619;MAP3K12;NPFF;TARE
0.175904	OL	LOC1011101	NC_056067.1	22040001	22060001	2.66311	0.167342	OL	LOC105601845
0.229877	OL	LOC1011101	NC_056072.1	51090001	51110001	2.16289	0.309594	OL	LOC105603546;SLC25A20
0.396918	OL	LOC1011101	NC_056072.1	51095001	51115001	2.47605	0.250524	OL	LOC105603546;SLC25A20
0.161355	OL	LOC1011102	NC_056054.1	229905001	229925001	2.88607	0.220878	OL	LOC105603698
0.159916	OL	LOC1011102	NC_056054.1	229910001	229930001	2.84986	0.20424	OL	LOC105603698
0.159239	OL	LOC1011104	NC_056054.1	229915001	229935001	4.444	0.30261	OL	LOC105603698
0.176025	OL	LOC1011107	NC_056054.1	229920001	229940001	4.61728	0.296198	OL	LOC105603698
0.345179	OL	LOC1011107	NC_056054.1	229925001	229945001	5.61434	0.346221	OL	LOC105603698
0.206279	OL	LOC1011110	NC_056073.1	25830001	25850001	2.95186	0.191648	OL	LOC105603754
0.231537	OL	LOC1011110	NC_056073.1	25835001	25855001	3.50909	0.295067	OL	LOC105603754
0.221299	OL	LOC1011110	NC_056073.1	25840001	25860001	6.36924	0.350951	OL	LOC105603754
0.206246	OL	LOC1011110	NC_056073.1	25845001	25865001	3.76291	0.323552	OL	LOC105603754
0.20763	OL	LOC1011110	NC_056073.1	25850001	25870001	4.07578	0.330374	OL	LOC105603754
0.151566	OL	LOC1011110	NC_056073.1	25855001	25875001	3.65558	0.302217	OL	LOC105603754
0.159965	OL	LOC1011110	NC_056073.1	25860001	25880001	2.37453	0.258373	OL	LOC105603754
0.166772	OL	LOC1011110	NC_056073.1	25865001	25885001	2.01966	0.214248	OL	LOC105603754
0.176799	OL	LOC1011113	NC_056077.1	36745001	36765001	7.16818	0.164682	OL	LOC105604793
0.176517	OL	LOC1011113	NC_056054.1	218455001	218475001	2.28287	0.191548	OL	LOC105605201;MECOM
0.162829	OL	LOC1011113	NC_056056.1	795001	815001	2.01428	0.217357	OL	LOC105605821
0.23115	OL	LOC1011115	NC_056056.1	800001	820001	2	0.20179	OL	LOC105605821
0.212044	OL	LOC1011115	NC_056072.1	22030001	22050001	12.8915	0.160663	OL	LOC106991767
0.183664	OL	LOC1011115	NC_056072.1	22035001	22055001	11.9151	0.159259	OL	LOC106991767

0.163512	OL	LOC1011115	NC_056072.1	22040001	22060001	16.709	0.160675	OL	LOC106991767
0.454886	OL	LOC1011118	NC_056073.1	30485001	30505001	2.28481	0.232491	OL	LOC114109685
0.485107	OL	LOC1011118	NC_056078.1	415001	435001	2.24446	0.266662	OL	LOC114110808
0.46784	OL	LOC1011118	NC_056078.1	425001	445001	3.02977	0.287797	OL	LOC114110808
0.361181	OL	LOC1011118	NC_056078.1	430001	450001	3.57092	0.236108	OL	LOC114110808
0.379378	OL	LOC1011118	NC_056078.1	435001	455001	1.96839	0.181681	OL	LOC114110808
0.412176	OL	LOC1011118	NC_056080.1	99835001	99855001	2.02158	0.219118	OL	LOC114111389
0.442326	OL	LOC1011118	NC_056080.1	99825001	99845001	2.05917	0.196784	OL	LOC114111389;ZNF75D
0.439912	OL	LOC1011118	NC_056080.1	29435001	29455001	2.62439	0.245724	OL	LOC114111481
0.14605	OL	LOC1011126	NC_056080.1	29440001	29460001	3.79856	0.334971	OL	LOC114111481
0.145943	OL	LOC1011126	NC_056080.1	29445001	29465001	2.78947	0.332235	OL	LOC114111481
0.156023	OL	LOC1011126	NC_056080.1	29390001	29410001	2.10651	0.159639	OL	LOC114111721
0.421595	OL	LOC1011136	NC_056080.1	29395001	29415001	1.96753	0.174842	OL	LOC114111721
0.360669	OL	LOC1011136	NC_056080.1	29400001	29420001	2.33258	0.178132	OL	LOC114111721
0.252899	OL	LOC1011136	NC_056057.1	121425001	121445001	17.1062	0.231968	OL	LOC114114472
0.201132	OL	LOC1011136	NC_056057.1	121430001	121450001	10.2933	0.220984	OL	LOC114114472
0.221427	OL	LOC1011138	NC_056057.1	11220001	11240001	2.43842	0.232224	OL	LOC114114483;LOC114114485
0.217996	OL	LOC1011138	NC_056057.1	11225001	11245001	2.43279	0.177543	OL	LOC114114483;LOC114114485
0.208241	OL	LOC1011139	NC_056057.1	11230001	11250001	2.8028	0.181269	OL	LOC114114483;LOC114114485
0.311004	OL	LOC1011140	NC_056057.1	11210001	11230001	1.97914	0.192389	OL	LOC114114485
0.28516	OL	LOC1011140	NC_056057.1	11215001	11235001	2.2069	0.204251	OL	LOC114114485
0.294792	OL	LOC1011140	NC_056058.1	38265001	38285001	3.67492	0.321745	OL	LOC114114935
0.3104	OL	LOC1011140	NC_056058.1	38270001	38290001	2.32451	0.24972	OL	LOC114114935
0.284239	OL	LOC1011140	NC_056058.1	38920001	38940001	3.11704	0.176321	OL	LOC114115007
0.249826	OL	LOC1011140	NC_056058.1	38925001	38945001	3.13593	0.179754	OL	LOC114115007
0.332174	OL	LOC1011140	NC_056065.1	43980001	44000001	4.93699	0.159563	OL	LOC114117228;RERE
0.39618	OL	LOC1011140	NC_056068.1	21445001	21465001	2.29975	0.174811	OL	LOC114118245
0.341193	OL	LOC1011140	NC_056068.1	21450001	21470001	2.91448	0.162291	OL	LOC114118245
0.32609	OL	LOC1011140	NC_056068.1	78835001	78855001	3.80592	0.176399	OL	LOC114118302
0.332147	OL	LOC1011140	NC_056068.1	78840001	78860001	4.88858	0.209308	OL	LOC114118302
0.330528	OL	LOC1011140	NC_056068.1	78845001	78865001	2.96741	0.181903	OL	LOC114118302

0.344724	OL	LOC1011140	NC_056068.1	78850001	78870001	3.28048	0.210656	OL	LOC114118302
0.339656	OL	LOC1011140	NC_056063.1	25200001	25220001	2.35054	0.172053	OL	LOC121816025;SERTM1
0.152561	OL	LOC1011143	NC_056057.1	106920001	106940001	4.51643	0.228638	OL	LOC121816042
0.371724	OL	LOC1011145	NC_056069.1	71000001	71020001	2.32269	0.220711	OL	LOC121816830
0.370343	OL	LOC1011145	NC_056069.1	71005001	71025001	2.7114	0.26657	OL	LOC121816830
0.160749	OL	LOC1011151	NC_056069.1	71010001	71030001	2.71613	0.278635	OL	LOC121816830
0.175759	OL	LOC1011151	NC_056069.1	71015001	71035001	2.18552	0.252791	OL	LOC121816830
0.337244	OL	LOC1011153	NC_056069.1	71020001	71040001	2.86825	0.253384	OL	LOC121816830
0.3406	OL	LOC1011153	NC_056069.1	71025001	71045001	3.3272	0.281176	OL	LOC121816830
0.252944	OL	LOC1011153	NC_056069.1	71030001	71050001	2.49918	0.233486	OL	LOC121816830
0.360785	OL	LOC1011153	NC_056080.1	14790001	14810001	4.84176	0.432511	OL	LOC121818210
0.21071	OL	LOC1011172	NC_056080.1	14795001	14815001	2.37701	0.496345	OL	LOC121818210
0.188	OL	LOC1011172	NC_056080.1	14800001	14820001	2.86495	0.557741	OL	LOC121818210
0.174637	OL	LOC1011172	NC_056080.1	14805001	14825001	2.4183	0.539081	OL	LOC121818210
0.191809	OL	LOC1011172	NW_024599815	30001	50001	2.15265	0.166395	OL	LOC121818481
0.193195	OL	LOC1011172	NW_024599815	35001	55001	2.39717	0.201748	OL	LOC121818481
0.225344	OL	LOC1011172	NW_024599815	40001	60001	2.03529	0.189744	OL	LOC121818481
0.321725	OL	LOC1011172	NC_056054.1	98005001	98025001	12.5754	0.165436	OL	LOC121820159
0.324215	OL	LOC1011172	NC_056062.1	64355001	64375001	2.63366	0.191516	OL	LOC121820346
0.281631	OL	LOC1011172	NC_056062.1	64360001	64380001	3.86039	0.226153	OL	LOC121820346
0.243755	OL	LOC1011172	NC_056064.1	46385001	46405001	2.6911	0.158935	OL	LOC121820633
0.21098	OL	LOC1011172	NC_056064.1	46390001	46410001	3.73555	0.223938	OL	LOC121820633
0.169889	OL	LOC1011172	NC_056064.1	46410001	46430001	4.05815	0.317159	OL	LOC121820633
0.215332	OL	LOC1011172	NC_056064.1	46415001	46435001	3.29999	0.261569	OL	LOC121820633
0.210305	OL	LOC1011172	NC_056064.1	46420001	46440001	2.27737	0.220175	OL	LOC121820633
0.186817	OL	LOC1011172	NC_056067.1	16240001	16260001	2.38792	0.193485	OL	LONP2
0.201424	OL	LOC1011172	NC_056067.1	16245001	16265001	2.41935	0.200213	OL	LONP2
0.225761	OL	LOC1011172	NC_056067.1	16250001	16270001	2.43275	0.183756	OL	LONP2
0.259505	OL	LOC1011172	NC_056067.1	16255001	16275001	2.41486	0.176729	OL	LONP2
0.28824	OL	LOC1011172	NC_056067.1	16260001	16280001	2.40139	0.201715	OL	LONP2
0.297201	OL	LOC1011172	NC_056067.1	16265001	16285001	2.58164	0.182912	OL	LONP2

0.289518	OL	LOC1011172	NC_056067.1	16270001	16290001	2.52954	0.193918	OL	LONP2
0.266835	OL	LOC1011172	NC_056067.1	16275001	16295001	2.55228	0.19966	OL	LONP2
0.243381	OL	LOC1011172	NC_056067.1	16280001	16300001	2.62186	0.175669	OL	LONP2
0.256928	OL	LOC1011172	NC_056067.1	16285001	16305001	2.42829	0.188474	OL	LONP2
0.273696	OL	LOC1011172	NC_056067.1	16290001	16310001	2.42335	0.172475	OL	LONP2
0.319453	OL	LOC1011172	NC_056067.1	16295001	16315001	2.44081	0.171303	OL	LONP2
0.364303	OL	LOC1011172	NC_056067.1	16300001	16320001	2.37663	0.183032	OL	LONP2
0.337325	OL	LOC1011172	NC_056080.1	115475001	115495001	4.64491	0.198135	OL	LONRF3
0.300796	OL	LOC1011172	NC_056080.1	69935001	69955001	2.19526	0.168081	OL	LPAR4
0.259845	OL	LOC1011172	NC_056069.1	71035001	71055001	2.26382	0.202061	OL	LPCAT1
0.193542	OL	LOC1011175	NC_056063.1	16940001	16960001	6.68812	0.223119	OL	LRCH1
0.180795	OL	LOC1011175	NC_056063.1	16945001	16965001	5.59166	0.343677	OL	LRCH1
0.155649	OL	LOC1011175	NC_056063.1	16950001	16970001	3.04519	0.336467	OL	LRCH1
0.225884	OL	LOC1011175	NC_056063.1	16955001	16975001	2.6693	0.322254	OL	LRCH1
0.216635	OL	LOC1011175	NC_056063.1	16960001	16980001	2.51375	0.319777	OL	LRCH1
0.167115	OL	LOC1011175	NC_056072.1	35060001	35080001	2.22342	0.178071	OL	LRIG1
0.15572	OL	LOC1011175	NC_056055.1	169355001	169375001	2.97157	0.195069	OL	LRP1B
0.202759	OL	LOC1011175	NC_056055.1	169360001	169380001	5.12662	0.230657	OL	LRP1B
0.192599	OL	LOC1011175	NC_056055.1	169365001	169385001	6.21103	0.256604	OL	LRP1B
0.200697	OL	LOC1011175	NC_056055.1	169370001	169390001	5.57876	0.262729	OL	LRP1B
0.207157	OL	LOC1011175	NC_056055.1	169375001	169395001	4.14942	0.243549	OL	LRP1B
0.257023	OL	LOC1011175	NC_056055.1	169470001	169490001	2.85555	0.159376	OL	LRP1B
0.25616	OL	LOC1011175	NC_056055.1	169495001	169515001	2.32234	0.164715	OL	LRP1B
0.227508	OL	LOC1011175	NC_056055.1	169500001	169520001	2.95167	0.173459	OL	LRP1B
0.262432	OL	LOC1011175	NC_056055.1	169505001	169525001	2.48302	0.163641	OL	LRP1B
0.22873	OL	LOC1011175	NC_056056.1	203335001	203355001	2.14677	0.218944	OL	LRP6
0.222967	OL	LOC1011175	NC_056056.1	203340001	203360001	2.01588	0.178809	OL	LRP6
0.216193	OL	LOC1011175	NC_056064.1	38350001	38370001	2.07982	0.379454	OL	LRRC46;MRPL10;OSBPL7;SCRN2;SP(
0.158788	OL	LOC1011175	NC_056064.1	38345001	38365001	1.95764	0.339086	OL	LRRC46;MRPL10;SCRN2;SP6
0.196746	OL	LOC1011175	NC_056056.1	121380001	121400001	17.0748	0.189118	OL	LRRIQ1
0.154557	OL	LOC1011178	NC_056072.1	22440001	22460001	3.07976	0.246616	OL	LRRN1

0.192833	OL	LOC1011178	NC_056072.1	22445001	22465001	3.66749	0.252509	OL	LRRN1
0.205392	OL	LOC1011178	NC_056072.1	22450001	22470001	3.16862	0.242225	OL	LRRN1
0.233874	OL	LOC1011178	NC_056072.1	22455001	22475001	2.19594	0.186113	OL	LRRN1
0.283989	OL	LOC1011178	NC_056066.1	7680001	7700001	3.68855	0.186795	OL	MACROD2
0.222629	OL	LOC1011178	NC_056066.1	7685001	7705001	4.83333	0.176911	OL	MACROD2
0.255588	OL	LOC1011181	NC_056066.1	9265001	9285001	2.67272	0.160501	OL	MACROD2
0.298716	OL	LOC1011181	NC_056066.1	9270001	9290001	2.83101	0.165807	OL	MACROD2
0.321233	OL	LOC1011181	NC_056066.1	9275001	9295001	2.34092	0.164916	OL	MACROD2
0.341006	OL	LOC1011181	NC_056068.1	76095001	76115001	10.0048	0.181452	OL	MADD;MYBPC3
0.31452	OL	LOC1011181	NC_056072.1	35885001	35905001	1.98816	0.18221	OL	MAGI1
0.310126	OL	LOC1011183	NC_056072.1	35890001	35910001	2.08769	0.167346	OL	MAGI1
0.349194	OL	LOC1011183	NC_056061.1	41610001	41630001	2.7492	0.233845	OL	MANEA
0.383702	OL	LOC1011183	NC_056061.1	41615001	41635001	5.73481	0.292276	OL	MANEA
0.37982	OL	LOC1011183	NC_056061.1	41620001	41640001	4.28413	0.290014	OL	MANEA
0.225537	OL	LOC1011183	NC_056061.1	41625001	41645001	3.38685	0.29696	OL	MANEA
0.163663	OL	LOC1011183	NC_056064.1	30525001	30545001	2.2238	0.157877	OL	MAP2K4
0.155262	OL	LOC1011185	NC_056080.1	17905001	17925001	2.036	0.172779	OL	MAP3K15;PDHA1
0.170684	OL	LOC1011190	NC_056080.1	17910001	17930001	2.21067	0.169388	OL	MAP3K15;PDHA1
0.228694	OL	LOC1011190	NC_056062.1	79200001	79220001	2.04347	0.230305	OL	MATN2
0.301131	OL	LOC1011190	NC_056062.1	79210001	79230001	1.97412	0.397859	OL	MATN2
0.346461	OL	LOC1011190	NC_056062.1	79215001	79235001	2.37203	0.345786	OL	MATN2
0.346554	OL	LOC1011190	NC_056062.1	79220001	79240001	2.19092	0.326648	OL	MATN2
0.253205	OL	LOC1011190	NC_056062.1	79225001	79245001	1.97851	0.222621	OL	MATN2
0.18359	OL	LOC1011197	NC_056077.1	36750001	36770001	5.76894	0.159711	OL	MBLAC1
0.195066	OL	LOC1011200	NC_056057.1	55055001	55075001	2.18205	0.164921	OL	MDFIC
0.14901	OL	LOC1011200	NC_056054.1	218460001	218480001	3.20168	0.234821	OL	MECOM
0.161921	OL	LOC1011200	NC_056054.1	218465001	218485001	2.45599	0.196165	OL	MECOM
0.194131	OL	LOC1011200	NC_056054.1	218470001	218490001	1.96531	0.175386	OL	MECOM
0.237299	OL	LOC1011200	NC_056056.1	106110001	106130001	2.57143	0.165732	OL	MERTK
0.236696	OL	LOC1011200	NC_056077.1	4335001	4355001	4.60606	0.228268	OL	MGRN1
0.220552	OL	LOC1011200	NC_056077.1	4340001	4360001	6.30687	0.25938	OL	MGRN1

0.214412	OL	LOC101120(	NC_056070.1	18105001	18125001	2.98326	0.202513	OL		MGST2
0.182858	OL	LOC101120(	NC_056070.1	18110001	18130001	3.71589	0.252218	OL		MGST2
0.153039	OL	LOC101120(	NC_056070.1	18115001	18135001	3.72111	0.258812	OL		MGST2
0.15878	OL	LOC101120(	NC_056065.1	25350001	25370001	2.00533	0.319173	OL		MIA3
0.168317	OL	LOC101120(	NC_056065.1	25355001	25375001	2.18304	0.291661	OL		MIA3
0.149509	OL	LOC101120(	NC_056065.1	25360001	25380001	2.13455	0.299284	OL		MIA3
0.146995	OL	LOC101120(	NC_056065.1	25365001	25385001	2.20174	0.277494	OL		MIA3
0.147027	OL	LOC101120(	NC_056065.1	25370001	25390001	2.35282	0.200654	OL		MIA3
0.153999	OL	LOC101120(	NC_056065.1	25315001	25335001	2.02973	0.200377	OL		MIA3;TAF1A
0.18129	OL	LOC101120:	NC_056057.1	66990001	67010001	2.28112	0.187177	OL		MINDY4
0.280755	OL	LOC101120!	NC_056057.1	66995001	67015001	2.4907	0.169976	OL		MINDY4
0.296849	OL	LOC1011211	NC_056064.1	8840001	8860001	2.8813	0.235178	OL		MKS1
0.312767	OL	LOC1011211	NC_056080.1	99260001	99280001	2.23511	0.21937	OL		MMGT1
0.217412	OL	LOC1011221	NC_056080.1	99265001	99285001	2.69451	0.299092	OL		MMGT1
0.173738	OL	LOC1011221	NC_056060.1	51940001	51960001	2.8941	0.242452	OL		MNS1
0.418779	OL	LOC101122:	NC_056060.1	51945001	51965001	2.38046	0.244577	OL		MNS1
0.418834	OL	LOC101122:	NC_056060.1	51980001	52000001	1.9596	0.253713	OL		MNS1
0.36788	OL	LOC101122:	NC_056060.1	51985001	52005001	1.97625	0.239077	OL		MNS1
0.161586	OL	LOC101122:	NC_056060.1	51995001	52015001	2.20753	0.21439	OL		MNS1
0.169553	OL	LOC105601!	NC_056069.1	26110001	26130001	2.37787	0.159886	OL		MOCS2
0.223678	OL	LOC105601!	NC_056080.1	129245001	129265001	5.0268	0.183604	OL		MORF4L2
0.218618	OL	LOC105601!	NC_056080.1	129250001	129270001	4.62	0.211601	OL		MORF4L2
0.16298	OL	LOC105603(	NC_056080.1	129255001	129275001	4.1282	0.219961	OL		MORF4L2
0.175869	OL	LOC105604	NC_056080.1	129260001	129280001	3.88678	0.182093	OL		MORF4L2
0.252759	OL	LOC105607	NC_056056.1	220095001	220115001	2.37218	0.15769	OL		MPPED1
0.300773	OL	LOC105607	NC_056056.1	220100001	220120001	2.36635	0.198879	OL		MPPED1
0.293335	OL	LOC105607	NC_056064.1	38365001	38385001	2.72516	0.41422	OL		MRPL10;OSBPL7
0.262172	OL	LOC105607	NC_056077.1	13385001	13405001	2.44952	0.161886	OL		MRTFB
0.184127	OL	LOC105608:	NC_056070.1	62795001	62815001	3.01804	0.170214	OL		MSI1
0.150239	OL	LOC105608(	NC_056070.1	62800001	62820001	2.74178	0.183321	OL		MSI1
0.149284	OL	LOC105612:	NC_056070.1	62805001	62825001	2.0299	0.175365	OL		MSI1

0.181758	OL	LOC1056125	NC_056057.1	9975001	9995001	3.40377	0.169353	OL		MTERF1
0.193407	OL	LOC1056125	NC_056057.1	9980001	10000001	4.41633	0.205903	OL		MTERF1
0.201018	OL	LOC1056127	NC_056057.1	9985001	10005001	3.40251	0.215298	OL		MTERF1
0.180406	OL	LOC1056127	NC_056066.1	54715001	54735001	3.11154	0.317287	OL		MTG2;SS18L1
0.194582	OL	LOC1056164	NC_056079.1	19090001	19110001	2.56912	0.270429	OL		MTMR7
0.153426	OL	LOC1056164	NC_056079.1	19095001	19115001	4.14386	0.211345	OL		MTMR7
0.155548	OL	LOC1141115	NC_056079.1	19100001	19120001	3.90369	0.201892	OL		MTMR7
0.146846	OL	LOC1141131	NC_056079.1	19150001	19170001	2.75046	0.173455	OL		MTMR7;VPS37A
0.282948	OL	LOC1141147	NC_056066.1	64250001	64270001	3.52423	0.300526	OL		MYH7B
0.154135	OL	LOC1141154	NC_056066.1	64255001	64275001	5.21554	0.265578	OL		MYH7B
0.26162	OL	LOC1141154	NC_056066.1	64260001	64280001	3.19091	0.275402	OL		MYH7B
0.171674	OL	LOC1141156	NC_056066.1	64265001	64285001	2.00154	0.240014	OL		MYH7B
0.152959	OL	LOC1141180	NC_056067.1	14585001	14605001	2.17693	0.270763	OL		MYLK3
0.192303	OL	LOC1141182	NC_056067.1	14590001	14610001	1.98791	0.239375	OL		MYLK3
0.188837	OL	LOC1141182	NC_056069.1	56495001	56515001	3.02646	0.224178	OL		MYO10
0.189122	OL	LOC1218160	NC_056069.1	56500001	56520001	4.34332	0.233701	OL		MYO10
0.154651	OL	LOC1218160	NC_056069.1	56505001	56525001	3.36014	0.20501	OL		MYO10
0.182826	OL	LOC1218160	NC_056063.1	83200001	83220001	3.66666	0.20662	OL		MYO16
0.400692	OL	LOC1218162	NC_056063.1	83210001	83230001	7.28318	0.244472	OL		MYO16
0.40325	OL	LOC1218162	NC_056063.1	83215001	83235001	3.60321	0.182439	OL		MYO16
0.294667	OL	LOC1218165	NC_056060.1	49100001	49120001	2.06289	0.205622	OL		MYO1E
0.235815	OL	LOC1218171	NC_056060.1	49290001	49310001	16.5638	0.181689	OL		MYO1E
0.249204	OL	LOC1218171	NC_056060.1	56010001	56030001	2.15115	0.198424	OL		MYO5A
0.183632	OL	LOC1218184	NC_056076.1	49850001	49870001	2.44431	0.172674	OL		MYO5B
0.189744	OL	LOC1218184	NC_056076.1	49855001	49875001	2.25081	0.172714	OL		MYO5B
0.20453	OL	LOC1218184	NC_056068.1	34550001	34570001	2.65291	0.161112	OL		MYOD1
0.203289	OL	LOC1218191	NC_056055.1	37190001	37210001	2.14241	0.175549	OL		MYORG;NUDT2
0.147787	OL	LOC1218194	NC_056056.1	3325001	3345001	7.35187	0.173529	OL		NACC2
0.162988	OL	LOC1218195	NC_056056.1	3330001	3350001	9.38458	0.167014	OL		NACC2
0.274172	OL	LOC1218198	NC_056080.1	48905001	48925001	2.43403	0.157483	OL		NBDY
0.166835	OL	LOC1218198	NC_056080.1	48910001	48930001	4.96178	0.222659	OL		NBDY

0.155616	OL	LOC121820(	NC_056080.1	48915001	48935001	4.50932	0.20651	OL	NBDY
0.1488	OL	LPAR1	NC_056054.1	136140001	136160001	4.13045	0.206968	OL	NCAM2
0.191648	OL	LPAR1	NC_056054.1	136145001	136165001	3.36029	0.193916	OL	NCAM2
0.299503	OL	LRIG2	NC_056054.1	136150001	136170001	2.6934	0.170113	OL	NCAM2
0.343626	OL	LRIG2	NC_056055.1	126730001	126750001	2.34843	0.196406	OL	NCKAP1
0.343236	OL	LRIG2	NC_056055.1	126735001	126755001	2.00864	0.179182	OL	NCKAP1
0.282872	OL	LRIG2	NC_056062.1	46795001	46815001	2.1887	0.245407	OL	NCOA2
0.160776	OL	LRMDA	NC_056062.1	46800001	46820001	2.7192	0.260467	OL	NCOA2
0.245985	OL	LRMDA	NC_056076.1	37375001	37395001	2.65425	0.161255	OL	NDC80
0.202362	OL	LRMDA	NC_056076.1	37380001	37400001	3.04439	0.173031	OL	NDC80
0.233426	OL	LRMDA	NC_056076.1	37385001	37405001	3.24742	0.175857	OL	NDC80
0.241554	OL	LRMDA	NC_056056.1	218890001	218910001	4.7208	0.195204	OL	NDUFA6;PHETA2;SMDT1
0.2263	OL	LRMDA	NC_056056.1	218895001	218915001	2.71274	0.187091	OL	NDUFA6;PHETA2;SMDT1
0.237417	OL	LRMDA	NC_056080.1	57645001	57665001	2.46512	0.205175	OL	NDUFB11;RBM10
0.159432	OL	LRMDA	NC_056080.1	57650001	57670001	2.12315	0.229122	OL	NDUFB11;RBM10
0.160234	OL	LRMDA	NC_056065.1	76470001	76490001	2.46497	0.30814	OL	NEK7
0.160071	OL	LRMDA	NC_056065.1	76475001	76495001	6.59057	0.322346	OL	NEK7
0.147517	OL	LRMDA	NC_056065.1	76480001	76500001	7.55626	0.25538	OL	NEK7
0.262026	OL	LRP1B	NC_056065.1	76485001	76505001	7.50361	0.178131	OL	NEK7
0.238528	OL	LRP1B	NC_056056.1	141815001	141835001	2.10889	0.227269	OL	NELL2
0.211774	OL	LRP1B	NC_056060.1	41055001	41075001	2.14264	0.192479	OL	NEMF
0.178749	OL	LRP1B	NC_056060.1	41060001	41080001	2.6849	0.242269	OL	NEMF
0.149368	OL	LRP1B	NC_056060.1	41065001	41085001	3.24197	0.27004	OL	NEMF
0.150526	OL	LRRC28	NC_056060.1	41925001	41945001	2.03524	0.193082	OL	NIN
0.190001	OL	LRRC7	NC_056069.1	13885001	13905001	1.98154	0.166772	OL	NLN
0.176443	OL	LRRC7	NC_056054.1	97395001	97415001	3.27555	0.213293	OL	NOTCH2
0.157188	OL	LRRC7	NC_056054.1	97400001	97420001	2.66116	0.172682	OL	NOTCH2
0.156383	OL	LYRM1	NC_056067.1	53860001	53880001	3.61993	0.160725	OL	NPAS1;TMEM160
0.171425	OL	LYRM1	NC_056056.1	73735001	73755001	3.11374	0.200572	OL	NRXN1
0.169795	OL	LZTFL1;SLC	NC_056056.1	73740001	73760001	7.53037	0.271963	OL	NRXN1
0.20029	OL	MAGI1	NC_056056.1	73745001	73765001	14.8524	0.286024	OL	NRXN1

0.223466	OL	MAGI1	NC_056056.1	73750001	73770001	8.89677	0.26916	OL	NRXN1
0.183154	OL	MAGI1	NC_056056.1	73755001	73775001	4.97738	0.231201	OL	NRXN1
0.202499	OL	MAGT1	NC_056056.1	73760001	73780001	3.04313	0.179257	OL	NRXN1
0.152237	OL	MAGT1	NC_056060.1	87580001	87600001	2.36949	0.300968	OL	NRXN3
0.162896	OL	MALRD1	NC_056060.1	87585001	87605001	2.39414	0.319779	OL	NRXN3
0.180466	OL	MAML3	NC_056060.1	87590001	87610001	2.14686	0.254859	OL	NRXN3
0.166379	OL	MAP1B	NC_056060.1	89130001	89150001	3.54379	0.209273	OL	NRXN3
0.193368	OL	MAP3K13	NC_056060.1	89135001	89155001	3.93803	0.212167	OL	NRXN3
0.241557	OL	MAP3K13	NC_056060.1	89140001	89160001	3.12925	0.201078	OL	NRXN3
0.289622	OL	MAP3K13	NC_056060.1	89145001	89165001	2.52914	0.172617	OL	NRXN3
0.272698	OL	MAP3K13	NC_056060.1	89150001	89170001	2.87757	0.226935	OL	NRXN3
0.215007	OL	MAP3K13	NC_056060.1	89155001	89175001	2.37489	0.228619	OL	NRXN3
0.2927	OL	MAPK11;MA	NC_056071.1	25975001	25995001	2.32799	0.304175	OL	NSMCE3
0.265166	OL	MAPK11;MA	NC_056071.1	25980001	26000001	2.4	0.313283	OL	NSMCE3
0.163977	OL	MAPKAP1	NC_056056.1	60250001	60270001	9.93488	0.224603	OL	NT5DC4
0.215787	OL	MAPKAP1	NC_056056.1	60255001	60275001	5.22406	0.192722	OL	NT5DC4
0.229912	OL	MAPKAP1	NC_056061.1	51315001	51335001	3.4969	0.234799	OL	NT5E;SNX14
0.156408	OL	MASP1	NC_056056.1	165665001	165685001	2.4416	0.167879	OL	NTN4
0.202647	OL	MAST1;RNA	NC_056056.1	165670001	165690001	2.46585	0.172108	OL	NTN4
0.168336	OL	MAST1;RTB	NC_056056.1	165675001	165695001	2.3983	0.179196	OL	NTN4
0.239731	OL	MAST4	NC_056056.1	165680001	165700001	2.5078	0.163539	OL	NTN4
0.176688	OL	MBD5	NC_056054.1	84830001	84850001	2.03288	0.187719	OL	NTNG1
0.178528	OL	MCM6	NC_056054.1	84835001	84855001	2.09601	0.1916	OL	NTNG1
0.198354	OL	MCM6	NC_056054.1	84840001	84860001	2.63803	0.171997	OL	NTNG1
0.219422	OL	MCM6	NC_056071.1	18210001	18230001	2.47636	0.246165	OL	NTRK3
0.251832	OL	MCM6	NC_056071.1	18215001	18235001	2.15567	0.159233	OL	NTRK3
0.233075	OL	MCM6	NC_056057.1	78575001	78595001	2.22616	0.169377	OL	NUDCD3
0.217616	OL	MCM9	NC_056057.1	78580001	78600001	2.4723	0.183051	OL	NUDCD3
0.305979	OL	MCM9	NC_056057.1	78585001	78605001	2.01991	0.16231	OL	NUDCD3
0.309971	OL	MCM9	NC_056057.1	78590001	78610001	2.04479	0.164794	OL	NUDCD3
0.275562	OL	MCM9	NC_056069.1	10360001	10380001	20.3398	0.417688	OL	OCLN

0.218394	OL	MCOLN2;M	NC_056069.1	10365001	10385001	5.14747	0.344085	OL	OCLN
0.244984	OL	MCOLN3	NC_056069.1	10370001	10390001	2.9337	0.240495	OL	OCLN
0.172688	OL	MCTP1	NC_056057.1	78440001	78460001	2.75263	0.1576	OL	OGDH
0.175039	OL	MCTP1	NC_056057.1	78490001	78510001	2.82424	0.172457	OL	OGDH
0.156087	OL	MCTP1	NC_056057.1	78495001	78515001	2.21709	0.202688	OL	OGDH;TMED4
0.147833	OL	MCTP1	NC_056057.1	78500001	78520001	2.03714	0.237623	OL	OGDH;TMED4
0.23169	OL	MCU	NC_056072.1	6295001	6315001	2.01423	0.177326	OL	OSBPL10
0.157849	OL	MCU	NC_056072.1	6300001	6320001	3.29705	0.245318	OL	OSBPL10
0.290882	OL	MCU	NC_056072.1	6305001	6325001	3.92961	0.282441	OL	OSBPL10
0.270499	OL	MCU	NC_056072.1	6310001	6330001	3.58669	0.315828	OL	OSBPL10
0.194604	OL	MCU	NC_056072.1	6315001	6335001	3.32785	0.297383	OL	OSBPL10
0.277711	OL	MDFIC	NC_056072.1	6320001	6340001	2.88652	0.276568	OL	OSBPL10
0.32907	OL	MDFIC	NC_056072.1	6325001	6345001	2.61096	0.202497	OL	OSBPL10
0.414558	OL	MDFIC	NC_056064.1	38370001	38390001	3.17766	0.439043	OL	OSBPL7
0.36887	OL	MDFIC	NC_056064.1	38375001	38395001	3.40084	0.453431	OL	OSBPL7
0.273204	OL	MDFIC	NC_056064.1	38380001	38400001	2.73753	0.42725	OL	OSBPL7
0.208072	OL	MDFIC	NC_056056.1	112375001	112395001	2.54667	0.188445	OL	OSBPL8
0.280729	OL	ME1	NC_056056.1	112380001	112400001	2.84728	0.205373	OL	OSBPL8
0.387036	OL	ME1	NC_056056.1	112385001	112405001	2.1095	0.16238	OL	OSBPL8
0.361763	OL	ME1	NC_056056.1	112390001	112410001	2.14088	0.167014	OL	OSBPL8
0.335875	OL	ME1	NC_056056.1	112395001	112415001	2.14893	0.164454	OL	OSBPL8
0.329873	OL	ME1	NC_056056.1	112400001	112420001	2.02319	0.160018	OL	OSBPL8
0.290515	OL	ME1	NC_056056.1	112405001	112425001	2.18992	0.172399	OL	OSBPL8
0.326366	OL	ME1	NC_056056.1	112410001	112430001	2.44669	0.190063	OL	OSBPL8
0.364851	OL	ME1	NC_056056.1	112415001	112435001	2.67599	0.208151	OL	OSBPL8
0.368681	OL	ME1	NC_056056.1	112420001	112440001	3.3103	0.222222	OL	OSBPL8
0.323537	OL	ME1	NC_056056.1	112425001	112445001	4.58877	0.227969	OL	OSBPL8
0.27905	OL	ME1	NC_056056.1	112430001	112450001	10.5726	0.221933	OL	OSBPL8
0.163055	OL	MEIKIN	NC_056056.1	112435001	112455001	15.2708	0.256779	OL	OSBPL8
0.163491	OL	MEIKIN	NC_056056.1	112440001	112460001	9.31183	0.284902	OL	OSBPL8
0.163105	OL	MEIKIN	NC_056056.1	112445001	112465001	2.89303	0.194793	OL	OSBPL8

0.160256	OL	MEIKIN	NC_056054.1	274310001	274330001	2.03551	0.177198	OL	OXNAD1
0.158534	OL	MEIKIN	NC_056054.1	274330001	274350001	2.00135	0.219406	OL	OXNAD1
0.159973	OL	MEIKIN	NC_056080.1	70120001	70140001	1.94667	0.33829	OL	P2RY10
0.158054	OL	MEIKIN	NC_056080.1	70125001	70145001	1.95495	0.320285	OL	P2RY10
0.162715	OL	MEIKIN	NC_056080.1	70130001	70150001	1.98485	0.327886	OL	P2RY10
0.159681	OL	MEIKIN	NC_056080.1	70135001	70155001	2.03687	0.333882	OL	P2RY10
0.159162	OL	MEIKIN	NC_056080.1	70140001	70160001	1.98319	0.326316	OL	P2RY10
0.15873	OL	MEIKIN	NC_056080.1	100240001	100260001	2.66497	0.195633	OL	PABIR2
0.153846	OL	MEIKIN	NC_056080.1	142830001	142850001	2.16239	0.174879	OL	PABPC5
0.152174	OL	MEIKIN	NC_056080.1	142835001	142855001	2.29159	0.175995	OL	PABPC5
0.251021	OL	MGAT3;TAI	NC_056074.1	40665001	40685001	2.04757	0.20751	OL	PACS1
0.172128	OL	MGAT4A	NC_056074.1	40670001	40690001	2.2672	0.233496	OL	PACS1
0.165972	OL	MGAT4A	NC_056074.1	40675001	40695001	2.35213	0.240029	OL	PACS1
0.163222	OL	MGAT4A	NC_056074.1	40680001	40700001	2.08692	0.220491	OL	PACS1
0.147798	OL	MGAT4A	NC_056074.1	40710001	40730001	1.97202	0.191091	OL	PACS1
0.191311	OL	MGAT4A;UI	NW_02459982	425001	445001	3.01421	0.201185	OL	PAG3
0.185105	OL	MGAT5	NW_02459982	430001	450001	2.85369	0.194618	OL	PAG3
0.213739	OL	MGAT5	NW_02459982	435001	455001	2.67359	0.183381	OL	PAG3
0.177277	OL	MGAT5	NC_056074.1	16185001	16205001	4.25559	0.161773	OL	PAK1
0.212517	OL	MGRN1	NC_056074.1	16190001	16210001	4.80387	0.166105	OL	PAK1
0.243452	OL	MGRN1	NC_056074.1	16255001	16275001	3.14286	0.168475	OL	PAK1
0.275946	OL	MGRN1	NC_056074.1	16260001	16280001	3.06598	0.186567	OL	PAK1
0.286405	OL	MID1	NC_056074.1	16265001	16285001	4.18678	0.184822	OL	PAK1
0.340978	OL	MID1	NC_056067.1	48215001	48235001	4.0909	0.246216	OL	PAK4
0.166554	OL	MIGA2	NC_056065.1	49655001	49675001	2.14047	0.209788	OL	PANK4;PLCH2
0.166844	OL	MIGA2;SH3	NC_056055.1	207710001	207730001	1.95523	0.170473	OL	PARD3B
0.173951	OL	MIGA2;SH3	NC_056075.1	21375001	21395001	3.47839	0.165763	OL	PAX2
0.221366	OL	MIGA2;SH3	NC_056056.1	10200001	10220001	2.35437	0.226854	OL	PBX3
0.201862	OL	MIPOL1	NC_056056.1	10210001	10230001	2.51748	0.194212	OL	PBX3
0.150292	OL	MIPOL1	NC_056054.1	105160001	105180001	2.09266	0.273255	OL	PBXIP1
0.345248	OL	MMP24	NC_056054.1	105155001	105175001	3.26759	0.383523	OL	PBXIP1;PMVK

0.236018	OL	MMP24	NC_056054.1	253550001	253570001	2.30622	0.165338	OL	PCCB
0.21282	OL	MMP24	NC_056054.1	253555001	253575001	2.14321	0.262938	OL	PCCB
0.14678	OL	MMP24	NC_056054.1	253560001	253580001	3.38443	0.267173	OL	PCCB
0.222233	OL	MORF4L1	NC_056054.1	253565001	253585001	5.56811	0.228611	OL	PCCB
0.218605	OL	MORF4L1	NC_056054.1	253570001	253590001	6.03106	0.201159	OL	PCCB
0.216859	OL	MORF4L1	NC_056054.1	253575001	253595001	6.2549	0.20697	OL	PCCB
0.19974	OL	MORF4L1	NC_056054.1	253585001	253605001	3.29351	0.173846	OL	PCCB
0.207103	OL	MORF4L1	NC_056054.1	253590001	253610001	2.89273	0.18206	OL	PCCB
0.204762	OL	MORF4L1	NC_056075.1	4430001	4450001	2.27447	0.210926	OL	PCDH15
0.212235	OL	MPI	NC_056075.1	5060001	5080001	2.62597	0.215129	OL	PCDH15
0.183373	OL	MPPED1	NC_056075.1	5065001	5085001	2.69865	0.267431	OL	PCDH15
0.15947	OL	MPZL2;MPZ	NC_056075.1	5070001	5090001	2.26774	0.287961	OL	PCDH15
0.154085	OL	MTMR8	NC_056075.1	5080001	5100001	1.98593	0.241563	OL	PCDH15
0.146637	OL	MTMR8	NC_056075.1	5085001	5105001	2.1516	0.233271	OL	PCDH15
0.158333	OL	MTMR8	NC_056075.1	5090001	5110001	2.48663	0.22585	OL	PCDH15
0.170068	OL	MTMR8	NC_056075.1	5095001	5115001	5.30769	0.204975	OL	PCDH15
0.174074	OL	MTMR8	NC_056075.1	5100001	5120001	2.98509	0.187553	OL	PCDH15
0.169913	OL	MTMR8	NC_056063.1	40230001	40250001	3.19912	0.193987	OL	PCDH9
0.153509	OL	MTMR8	NC_056063.1	40235001	40255001	3.74656	0.188235	OL	PCDH9
0.185185	OL	MTMR8	NC_056069.1	20210001	20230001	1.95127	0.226586	OL	PDE4D
0.226562	OL	MTMR8	NC_056068.1	4175001	4195001	2.55779	0.181619	OL	PDGFD
0.168129	OL	MTMR8	NC_056068.1	4180001	4200001	2.51627	0.205124	OL	PDGFD
0.183642	OL	MTMR8	NC_056068.1	4185001	4205001	2.40055	0.217499	OL	PDGFD
0.245363	OL	MTOR	NC_056068.1	4190001	4210001	2.00398	0.17522	OL	PDGFD
0.181477	OL	MTUS1	NC_056068.1	4240001	4260001	2.31703	0.182289	OL	PDGFD
0.164293	OL	MTUS1	NC_056056.1	19920001	19940001	2.15	0.20131	OL	PDIA6
0.15688	OL	MTUS2	NC_056056.1	19925001	19945001	3.65646	0.237069	OL	PDIA6
0.168023	OL	MTUS2	NC_056056.1	19930001	19950001	3.93669	0.245175	OL	PDIA6
0.15391	OL	MTUS2	NC_056056.1	19935001	19955001	4.33652	0.26283	OL	PDIA6
0.187129	OL	MTX2	NC_056056.1	19940001	19960001	3.95317	0.240948	OL	PDIA6
0.202018	OL	NAA50;USF.	NC_056056.1	62950001	62970001	2.60836	0.222334	OL	PEX13

0.221924	OL	NAA50;USF	NC_056056.1	62955001	62975001	2.9403	0.217754	OL	PEX13
0.163242	OL	NAALADL2	NC_056056.1	62960001	62980001	2.36284	0.187725	OL	PEX13
0.167702	OL	NAALADL2	NC_056056.1	62965001	62985001	2.2496	0.192014	OL	PEX13
0.165532	OL	NAALADL2	NC_056056.1	62970001	62990001	3.02423	0.211175	OL	PEX13
0.160305	OL	NAALADL2	NC_056056.1	62975001	62995001	3.26061	0.237755	OL	PEX13
0.206899	OL	NAALADL2	NC_056056.1	62980001	63000001	3.12755	0.25015	OL	PEX13
0.192187	OL	NAALADL2	NC_056056.1	62985001	63005001	2.6868	0.237138	OL	PEX13;PUS10
0.235422	OL	NARF	NC_056056.1	62990001	63010001	1.97322	0.201063	OL	PEX13;PUS10
0.234239	OL	NARF	NC_056061.1	62155001	62175001	3.01133	0.162478	OL	PEX7
0.216356	OL	NBAS	NC_056055.1	199220001	199240001	4.5	0.16725	OL	PGAP1
0.221064	OL	NBAS	NC_056055.1	199225001	199245001	4.62981	0.171963	OL	PGAP1
0.171451	OL	NBAS	NC_056055.1	68360001	68380001	3.79446	0.176712	OL	PGM5
0.200668	OL	NBAS	NC_056055.1	68365001	68385001	3.96182	0.189848	OL	PGM5
0.237834	OL	NBAS	NC_056055.1	68370001	68390001	4.67718	0.19613	OL	PGM5
0.268532	OL	NBAS	NC_056055.1	68375001	68395001	4.08644	0.164873	OL	PGM5
0.261788	OL	NBAS	NC_056055.1	68400001	68420001	2.21291	0.179334	OL	PGM5
0.210729	OL	NBAS	NC_056055.1	68405001	68425001	2.40625	0.196186	OL	PGM5
0.219153	OL	NBAS	NC_056064.1	53150001	53170001	2.50851	0.16737	OL	PGS1
0.148147	OL	NCAM2	NC_056064.1	53155001	53175001	2.69477	0.174275	OL	PGS1;SOCS3
0.185578	OL	NCAM2	NC_056070.1	55135001	55155001	1.9808	0.171018	OL	PHETA1
0.225636	OL	NCAM2	NC_056080.1	65225001	65245001	2.74935	0.193914	OL	PHKA1
0.209346	OL	NCAM2	NC_056080.1	65230001	65250001	3.84257	0.245827	OL	PHKA1
0.174885	OL	NCAM2	NC_056080.1	65235001	65255001	3.21802	0.242833	OL	PHKA1
0.157244	OL	NCAM2	NC_056080.1	65240001	65260001	3.39037	0.265026	OL	PHKA1
0.151464	OL	NCK2	NC_056080.1	65245001	65265001	2.97637	0.263403	OL	PHKA1
0.15352	OL	NDFIP2	NC_056080.1	65250001	65270001	1.99653	0.299838	OL	PHKA1
0.189408	OL	NDUFS1	NC_056080.1	65255001	65275001	1.95574	0.3446	OL	PHKA1
0.171533	OL	NEDD4L	NC_056067.1	15465001	15485001	3.5405	0.28185	OL	PHKB
0.167151	OL	NEDD4L	NC_056067.1	15470001	15490001	3.89912	0.312008	OL	PHKB
0.167975	OL	NEDD9	NC_056067.1	15475001	15495001	3.83714	0.3248	OL	PHKB
0.188418	OL	NEK5	NC_056067.1	15480001	15500001	3.22857	0.300622	OL	PHKB

0.15403	OL	NEK7	NC_056067.1	15495001	15515001	1.96825	0.222996	OL	PHKB
0.165171	OL	NEK7	NC_056067.1	15500001	15520001	2.4949	0.24755	OL	PHKB
0.179667	OL	NEK7	NC_056067.1	15505001	15525001	7.87144	0.385294	OL	PHKB
0.167297	OL	NEK7	NC_056067.1	15510001	15530001	3.68621	0.310156	OL	PHKB
0.199173	OL	NEK9;ZC2H	NC_056067.1	15515001	15535001	2.11578	0.206473	OL	PHKB
0.172607	OL	NEK9;ZC2H	NC_056076.1	46905001	46925001	2.00277	0.159274	OL	PIAS2
0.175152	OL	NELL1	NC_056074.1	9145001	9165001	2.00971	0.255997	OL	PICALM
0.149081	OL	NELL2	NC_056055.1	231230001	231250001	2.14684	0.185212	OL	PID1
0.162342	OL	NELL2	NC_056055.1	231235001	231255001	2.35251	0.196444	OL	PID1
0.250474	OL	NFATC1	NC_056055.1	231240001	231260001	3.6754	0.22866	OL	PID1
0.27611	OL	NFATC1	NC_056055.1	231245001	231265001	4.99999	0.241066	OL	PID1
0.229403	OL	NFATC1	NC_056056.1	197055001	197075001	2.99345	0.17487	OL	PIK3C2G
0.150518	OL	NFATC1	NC_056056.1	197060001	197080001	2.68251	0.193596	OL	PIK3C2G
0.180387	OL	NFATC3	NC_056056.1	197065001	197085001	2.14423	0.243797	OL	PIK3C2G
0.185589	OL	NFATC3	NC_056056.1	197070001	197090001	2.02948	0.233121	OL	PIK3C2G
0.159775	OL	NFATC3	NC_056056.1	197075001	197095001	2.15158	0.200436	OL	PIK3C2G
0.14892	OL	NFATC3	NC_056054.1	250980001	251000001	2.27615	0.253938	OL	PIK3CB
0.148925	OL	NFATC3	NC_056054.1	250985001	251005001	2.55056	0.262081	OL	PIK3CB
0.15211	OL	NFATC3	NC_056054.1	250990001	251010001	3.49173	0.232111	OL	PIK3CB
0.151382	OL	NFATC3	NC_056054.1	250995001	251015001	3.95902	0.206871	OL	PIK3CB
0.200492	OL	NGB;POMT:	NC_056054.1	251000001	251020001	2.95377	0.178529	OL	PIK3CB
0.226098	OL	NGB;POMT:	NC_056054.1	251005001	251025001	2.1854	0.161887	OL	PIK3CB
0.275946	OL	NKAIN2	NC_056080.1	13630001	13650001	2.30645	0.20431	OL	PIR
0.275729	OL	NKAIN2	NC_056070.1	70225001	70245001	2.93113	0.180499	OL	PISD
0.207823	OL	NKAIN2	NC_056070.1	70200001	70220001	2.8494	0.211124	OL	PISD;SFI1
0.240288	OL	NKAIN2	NC_056070.1	70205001	70225001	3.25057	0.159932	OL	PISD;SFI1
0.208673	OL	NKAIN2	NC_056070.1	70215001	70235001	2.98185	0.160102	OL	PISD;SFI1
0.171251	OL	NKAIN2	NC_056070.1	46770001	46790001	3.64111	0.208533	OL	PIWIL1
0.195186	OL	NKAIN2	NC_056070.1	46775001	46795001	3.28276	0.211835	OL	PIWIL1
0.220722	OL	NKAIN2	NC_056070.1	46780001	46800001	2.45545	0.190154	OL	PIWIL1
0.23566	OL	NKAIN2	NC_056070.1	46765001	46785001	2.73307	0.16461	OL	PIWIL1;RIMBP2

0.208162	OL	NKAIN2	NC_056067.1	38610001	38630001	3.22905	0.205039	OL	PKD1L3
0.178902	OL	NKAIN2	NC_056067.1	38615001	38635001	3.77547	0.183704	OL	PKD1L3
0.154866	OL	NKAIN2	NC_056056.1	81995001	82015001	2.37332	0.199793	OL	PKDCC
0.161506	OL	NKAIN2	NC_056060.1	35755001	35775001	6.2751	0.1623	OL	PLA2G4F;VPS39
0.166571	OL	NKAIN2	NC_056060.1	35760001	35780001	3.68163	0.158868	OL	PLA2G4F;VPS39
0.162379	OL	NKAIN2	NC_056069.1	33345001	33365001	3.41646	0.188006	OL	PLCXD3
0.171995	OL	NKAIN2	NC_056056.1	196360001	196380001	3.40935	0.161735	OL	PLEKHA5
0.160162	OL	NKAIN2	NC_056056.1	196365001	196385001	5.43074	0.251639	OL	PLEKHA5
0.148022	OL	NLGN4X	NC_056056.1	196370001	196390001	7.20049	0.265954	OL	PLEKHA5
0.193214	OL	NLGN4X	NC_056056.1	196375001	196395001	7.91196	0.304732	OL	PLEKHA5
0.21715	OL	NLGN4X	NC_056056.1	196380001	196400001	3.57812	0.224182	OL	PLEKHA5
0.223172	OL	NLGN4X	NC_056066.1	20925001	20945001	2.59113	0.197013	OL	PLXDC2
0.196559	OL	NLGN4X	NC_056066.1	20930001	20950001	2.70705	0.220537	OL	PLXDC2
0.148336	OL	NLRC3	NC_056067.1	38460001	38480001	4.44133	0.218473	OL	PMFBP1
0.203403	OL	NNT	NC_056067.1	38465001	38485001	9.92553	0.254735	OL	PMFBP1
0.202781	OL	NNT	NC_056067.1	38480001	38500001	9.49342	0.264428	OL	PMFBP1
0.162435	OL	NNT	NC_056067.1	38485001	38505001	6.018	0.253729	OL	PMFBP1
0.266038	OL	NODAL	NC_056067.1	38490001	38510001	4.05112	0.202013	OL	PMFBP1
0.287122	OL	NODAL	NC_056054.1	105150001	105170001	2.83309	0.382217	OL	PMVK
0.165403	OL	NOL11	NC_056072.1	48795001	48815001	2.39925	0.184893	OL	POC1A
0.289337	OL	NOTCH2	NC_056072.1	48800001	48820001	3.45191	0.224535	OL	POC1A
0.198996	OL	NOTCH2	NC_056072.1	48810001	48830001	5.25661	0.256624	OL	POC1A
0.174337	OL	NOTCH2	NC_056072.1	48825001	48845001	3.17544	0.179567	OL	POC1A
0.157529	OL	NOTCH2	NC_056072.1	48830001	48850001	3.75848	0.191196	OL	POC1A
0.211538	OL	NOTCH2	NC_056072.1	48835001	48855001	3.37002	0.179908	OL	POC1A
0.203704	OL	NOTCH2	NC_056072.1	48840001	48860001	3.68132	0.198002	OL	POC1A
0.195802	OL	NOTCH2	NC_056056.1	125890001	125910001	2.35463	0.183734	OL	POC1B
0.209075	OL	NOTCH2	NC_056056.1	175355001	175375001	2.3038	0.166315	OL	POLR3B
0.208808	OL	NOTCH2	NC_056056.1	175360001	175380001	2.28538	0.185345	OL	POLR3B
0.196789	OL	NOTCH2	NC_056056.1	175365001	175385001	2.28843	0.191859	OL	POLR3B
0.160665	OL	NOTCH2	NC_056056.1	175370001	175390001	2.29426	0.183401	OL	POLR3B

0.162509	OL	NOTCH2	NC_056056.1	175375001	175395001	2.2967	0.167066	OL	POLR3B
0.190116	OL	NOTCH2	NC_056058.1	87670001	87690001	2.23643	0.168517	OL	POLR3G
0.231557	OL	NOTCH2	NC_056073.1	30220001	30240001	11.2199	0.264665	OL	POM121L2
0.228516	OL	NOTCH2	NC_056073.1	30225001	30245001	13.6769	0.257405	OL	POM121L2
0.207004	OL	NOTCH2	NC_056073.1	30230001	30250001	9.3913	0.231969	OL	POM121L2
0.199601	OL	NOTCH2	NC_056073.1	30235001	30255001	9.92362	0.187063	OL	POM121L2
0.16124	OL	NPAS3	NC_056059.1	44635001	44655001	2.44259	0.334314	OL	PPARGC1A
0.159751	OL	NPAS3	NC_056059.1	44640001	44660001	2.73935	0.346638	OL	PPARGC1A
0.181463	OL	NRXN1	NC_056071.1	31310001	31330001	2.86296	0.222402	OL	PPCDC
0.175571	OL	NRXN1	NC_056071.1	31315001	31335001	2.88764	0.165933	OL	PPCDC
0.191957	OL	NRXN1	NC_056068.1	44735001	44755001	2.84375	0.269339	OL	PPFIBP2
0.21981	OL	NRXN1	NC_056068.1	44740001	44760001	8.03755	0.348744	OL	PPFIBP2
0.155365	OL	NRXN1	NC_056068.1	44755001	44775001	29.7635	0.375414	OL	PPFIBP2
0.170434	OL	NRXN3	NC_056068.1	44760001	44780001	23.6389	0.374304	OL	PPFIBP2
0.175178	OL	NRXN3	NC_056068.1	44775001	44795001	20.798	0.220512	OL	PPFIBP2
0.21274	OL	NRXN3	NC_056077.1	4520001	4540001	1.97055	0.265509	OL	PPL
0.205384	OL	NRXN3	NC_056077.1	4525001	4545001	2.22415	0.295632	OL	PPL
0.167681	OL	NRXN3	NC_056077.1	4510001	4530001	2.17903	0.285712	OL	PPL;UBN1
0.154016	OL	NRXN3	NC_056077.1	4515001	4535001	1.96085	0.264784	OL	PPL;UBN1
0.149078	OL	NRXN3	NC_056059.1	37100001	37120001	3.37259	0.177631	OL	PPM1K
0.147925	OL	NRXN3	NC_056059.1	37105001	37125001	3.80613	0.2026	OL	PPM1K
0.183448	OL	NRXN3	NC_056059.1	37110001	37130001	3.97667	0.185248	OL	PPM1K
0.190347	OL	NTM	NC_056057.1	56085001	56105001	2.46541	0.272222	OL	PPP1R3A
0.231537	OL	NTM	NC_056057.1	56090001	56110001	2.09	0.267518	OL	PPP1R3A
0.2433	OL	NTM	NC_056069.1	39235001	39255001	2.66373	0.183961	OL	PR
0.156324	OL	NTM	NC_056058.1	28295001	28315001	2.27936	0.181041	OL	PRDM6
0.148191	OL	NTM	NC_056072.1	37225001	37245001	2.03038	0.209445	OL	PRICKLE2
0.200389	OL	NTNG1	NC_056072.1	37305001	37325001	2.24764	0.169878	OL	PRICKLE2
0.215312	OL	NTNG1	NC_056073.1	2930001	2950001	1.96913	0.282962	OL	PRIM2
0.253031	OL	NTNG1	NC_056073.1	2935001	2955001	1.98404	0.309374	OL	PRIM2
0.236357	OL	NUP188;SH3	NC_056073.1	3025001	3045001	2.3709	0.285613	OL	PRIM2

0.157197	OL	NUP188;SH3	NC_056061.1	85260001	85280001	6.36442	0.15878	OL	PRKN
0.153513	OL	NUP93	NC_056061.1	85410001	85430001	2.66357	0.171998	OL	PRKN
0.155691	OL	NUP93	NC_056061.1	85415001	85435001	3.01598	0.221764	OL	PRKN
0.153072	OL	NUP93	NC_056061.1	85420001	85440001	3.08027	0.240988	OL	PRKN
0.164303	OL	NUP93	NC_056061.1	85425001	85445001	3.23131	0.222605	OL	PRKN
0.210982	OL	NUP93	NC_056061.1	85430001	85450001	3.53695	0.244274	OL	PRKN
0.22261	OL	NUP93	NC_056061.1	85435001	85455001	2.85668	0.216151	OL	PRKN
0.209151	OL	NUP93	NC_056061.1	85440001	85460001	2.12969	0.179976	OL	PRKN
0.184415	OL	NUP93	NC_056063.1	23500001	23520001	2.14988	0.179908	OL	PROSER1;STOML3
0.185036	OL	NXN	NC_056074.1	34580001	34600001	2.78234	0.21425	OL	PRPF19
0.16233	OL	OSBPL10	NC_056058.1	24205001	24225001	3.69657	0.187835	OL	PRRC1
0.16431	OL	OSBPL10	NC_056079.1	37940001	37960001	2.05344	0.198636	OL	PSD3
0.164132	OL	OSBPL10	NC_056079.1	37945001	37965001	2.92529	0.263625	OL	PSD3
0.201111	OL	OSBPL9	NC_056079.1	37950001	37970001	2.60524	0.202681	OL	PSD3
0.200967	OL	OSBPL9	NC_056065.1	29155001	29175001	3.22201	0.244227	OL	PSEN2
0.217525	OL	OSBPL9	NC_056066.1	54730001	54750001	2.31123	0.255263	OL	PSMA7;SS18L1
0.208098	OL	OSBPL9	NC_056055.1	30820001	30840001	4.33428	0.170872	OL	PTCH1
0.188612	OL	OSBPL9	NC_056055.1	30825001	30845001	5.38723	0.187656	OL	PTCH1
0.17107	OL	OSBPL9	NC_056074.1	34560001	34580001	3.50578	0.220493	OL	PTGDR2
0.146716	OL	OSBPL9	NC_056054.1	46595001	46615001	2.86388	0.161742	OL	PTGER3
0.222378	OL	OSER1	NC_056065.1	67160001	67180001	2.71993	0.255582	OL	PTGS2
0.178919	OL	OSER1	NC_056065.1	67165001	67185001	2.14074	0.218036	OL	PTGS2
0.250631	OL	OSER1	NC_056068.1	38930001	38950001	5.89968	0.189742	OL	PTH
0.202237	OL	OTUD7A	NC_056068.1	38935001	38955001	5.07649	0.191126	OL	PTH
0.150529	OL	OVAR-DRB	NC_056059.1	102370001	102390001	2.17428	0.228479	OL	PTPN13
0.166178	OL	OXR1	NC_056059.1	102375001	102395001	3.23209	0.260911	OL	PTPN13
0.174581	OL	OXR1	NC_056059.1	102380001	102400001	4.57103	0.305066	OL	PTPN13
0.211416	OL	OXR1	NC_056059.1	102385001	102405001	3.33806	0.280839	OL	PTPN13
0.244322	OL	OXR1	NC_056059.1	102390001	102410001	2.10488	0.238131	OL	PTPN13
0.218191	OL	OXR1	NC_056055.1	76645001	76665001	2.35921	0.397579	OL	PTPRD
0.208884	OL	OXR1	NC_056055.1	76650001	76670001	4.1434	0.322847	OL	PTPRD

0.202637	OL	OXR1	NC_056055.1	76655001	76675001	5.95213	0.259493	OL	PTPRD
0.182946	OL	OXR1	NC_056055.1	76660001	76680001	4.53993	0.283249	OL	PTPRD
0.157648	OL	PACS1	NC_056055.1	76665001	76685001	3.53936	0.274874	OL	PTPRD
0.180043	OL	PACS1	NC_056055.1	77650001	77670001	2.27331	0.238206	OL	PTPRD
0.197522	OL	PACS1	NC_056055.1	77655001	77675001	2.48569	0.227965	OL	PTPRD
0.19194	OL	PACS1	NC_056055.1	77660001	77680001	2.78138	0.191027	OL	PTPRD
0.232686	OL	PADI1;PADI	NC_056055.1	77665001	77685001	3.28363	0.214768	OL	PTPRD
0.230498	OL	PADI1;PADI	NC_056055.1	77670001	77690001	4.17163	0.258587	OL	PTPRD
0.190986	OL	PADI1;PADI	NC_056055.1	77675001	77695001	6.9366	0.293037	OL	PTPRD
0.18344	OL	PADI3	NC_056055.1	77680001	77700001	18.546	0.331359	OL	PTPRD
0.214413	OL	PADI3	NC_056055.1	77685001	77705001	8.80171	0.330069	OL	PTPRD
0.203834	OL	PADI3	NC_056055.1	77690001	77710001	4.68128	0.373767	OL	PTPRD
0.211401	OL	PADI3	NC_056055.1	77695001	77715001	3.63157	0.356319	OL	PTPRD
0.19585	OL	PADI3	NC_056055.1	77700001	77720001	3.24743	0.352835	OL	PTPRD
0.184273	OL	PADI3	NC_056055.1	77705001	77725001	3.21316	0.349007	OL	PTPRD
0.202192	OL	PADI3	NC_056055.1	77710001	77730001	2.00846	0.264631	OL	PTPRD
0.170334	OL	PAG3	NC_056072.1	39230001	39250001	2.94051	0.294517	OL	PTPRG
0.204686	OL	PAG3	NC_056072.1	39235001	39255001	5.33196	0.336245	OL	PTPRG
0.188825	OL	PAG3	NC_056072.1	39240001	39260001	10.1619	0.389612	OL	PTPRG
0.178715	OL	PAG3	NC_056072.1	39245001	39265001	16.4107	0.392465	OL	PTPRG
0.151681	OL	PAG3	NC_056072.1	39250001	39270001	3.55867	0.335068	OL	PTPRG
0.210673	OL	PAK1	NC_056072.1	39255001	39275001	2.6449	0.249247	OL	PTPRG
0.214824	OL	PAK1	NC_056072.1	39525001	39545001	1.96391	0.215305	OL	PTPRG
0.222314	OL	PAK1	NC_056072.1	39530001	39550001	2.41773	0.216423	OL	PTPRG
0.174631	OL	PAK1	NC_056072.1	39535001	39555001	2.5614	0.180477	OL	PTPRG
0.327383	OL	PALD1	NC_056061.1	53535001	53555001	2.50492	0.200242	OL	PTPRK
0.32996	OL	PALD1	NC_056061.1	53540001	53560001	3.42954	0.191319	OL	PTPRK
0.268766	OL	PALD1	NC_056061.1	53545001	53565001	2.12746	0.171619	OL	PTPRK
0.402289	OL	PALD1	NC_056076.1	41080001	41100001	4.47125	0.20884	OL	PTPRM
0.310328	OL	PALD1	NC_056076.1	41085001	41105001	3.15606	0.30403	OL	PTPRM
0.193839	OL	PALD1	NC_056076.1	41090001	41110001	2.66948	0.362898	OL	PTPRM

0.170351	OL	PALM2AKA	NC_056076.1	41095001	41115001	2.50704	0.403398	OL	PTPRM
0.155287	OL	PANX2;TRA	NC_056057.1	120530001	120550001	9.38153	0.178702	OL	PTPRN2
0.163947	OL	PAPPA2	NC_056057.1	120535001	120555001	8.13184	0.21705	OL	PTPRN2
0.175624	OL	PAPPA2	NC_056057.1	120540001	120560001	6.77799	0.178929	OL	PTPRN2
0.285011	OL	PARD3B	NC_056057.1	120655001	120675001	3.27701	0.16144	OL	PTPRN2
0.284272	OL	PARD3B	NC_056057.1	120665001	120685001	5.05284	0.177264	OL	PTPRN2
0.24735	OL	PARD3B	NC_056057.1	120670001	120690001	4.25726	0.189733	OL	PTPRN2
0.156589	OL	PARD3B	NC_056057.1	120675001	120695001	3.59409	0.163402	OL	PTPRN2
0.263243	OL	PASD1	NC_056057.1	78340001	78360001	2.12946	0.158925	OL	PURB
0.26918	OL	PASD1	NC_056079.1	26215001	26235001	5.99704	0.168008	OL	PURG
0.268284	OL	PASD1	NC_056054.1	248280001	248300001	3.11111	0.540343	OL	PXYLP1
0.264566	OL	PASD1	NC_056054.1	248285001	248305001	6.31033	0.60179	OL	PXYLP1
0.159028	OL	PATJ	NC_056054.1	248290001	248310001	3.65873	0.528318	OL	PXYLP1
0.154293	OL	PBX3	NC_056054.1	248295001	248315001	2.31325	0.527469	OL	PXYLP1
0.260387	OL	PCDH15	NC_056060.1	45010001	45030001	3.37453	0.187222	OL	RAB8B
0.247579	OL	PCDH15	NC_056060.1	45015001	45035001	5.26639	0.229925	OL	RAB8B
0.253097	OL	PCDH15	NC_056060.1	45020001	45040001	5.79017	0.25997	OL	RAB8B
0.237579	OL	PCDH15	NC_056060.1	45025001	45045001	5.22456	0.258479	OL	RAB8B
0.155145	OL	PCDH15	NC_056060.1	45030001	45050001	3.65178	0.232671	OL	RAB8B
0.172839	OL	PCDH9	NC_056056.1	9210001	9230001	2.14233	0.183648	OL	RALGPS1
0.210658	OL	PCDH9	NC_056062.1	90890001	90910001	3.97361	0.176488	OL	RALYL
0.203545	OL	PCDH9	NC_056062.1	90895001	90915001	5.4	0.190601	OL	RALYL
0.18564	OL	PCDH9	NC_056062.1	90900001	90920001	4.99999	0.172989	OL	RALYL
0.171534	OL	PDE1C	NC_056062.1	90905001	90925001	5.21336	0.169941	OL	RALYL
0.205535	OL	PDE1C	NC_056058.1	20700001	20720001	1.98614	0.275995	OL	RAPGEF6
0.180566	OL	PDE1C	NC_056058.1	20705001	20725001	3.00602	0.349096	OL	RAPGEF6
0.145811	OL	PDE1C	NC_056058.1	20710001	20730001	4.34433	0.398943	OL	RAPGEF6
0.174115	OL	PDGFC	NC_056058.1	20715001	20735001	5.07462	0.406259	OL	RAPGEF6
0.178552	OL	PDGFC	NC_056058.1	20720001	20740001	4.21183	0.387065	OL	RAPGEF6
0.186931	OL	PDGFD	NC_056058.1	20725001	20745001	3.11016	0.333431	OL	RAPGEF6
0.26177	OL	PDGFD	NC_056063.1	32870001	32890001	2.72953	0.163178	OL	RASL11A

0.324014	OL	PDGFD	NC_056077.1	6570001	6590001	2.17003	0.166241	OL	RBFOX1
0.329219	OL	PDGFD	NC_056077.1	6575001	6595001	3.00578	0.179338	OL	RBFOX1
0.332558	OL	PDGFD	NC_056064.1	52335001	52355001	3.40958	0.173233	OL	RBFOX3
0.338203	OL	PDGFD	NC_056080.1	57635001	57655001	3.525	0.205121	OL	RBM10
0.258377	OL	PDGFD	NC_056080.1	57640001	57660001	2.44263	0.250549	OL	RBM10
0.252796	OL	PDGFD	NC_056054.1	250250001	250270001	2.07539	0.260037	OL	RBP2
0.221657	OL	PDGFD	NC_056054.1	250255001	250275001	2.16667	0.255978	OL	RBP2
0.266126	OL	PDGFD	NC_056073.1	19645001	19665001	4.54103	0.179585	OL	RCAN2
0.295711	OL	PDGFD	NC_056073.1	19650001	19670001	3.44061	0.160779	OL	RCAN2
0.309907	OL	PDGFD	NC_056065.1	44005001	44025001	8.39419	0.230082	OL	RERE
0.277771	OL	PDGFD	NC_056065.1	44010001	44030001	8.71438	0.211596	OL	RERE
0.309646	OL	PDGFD	NC_056065.1	44015001	44035001	5.11748	0.19424	OL	RERE
0.352163	OL	PDGFD	NC_056065.1	44020001	44040001	4.84879	0.177038	OL	RERE
0.320762	OL	PDGFD	NC_056056.1	175635001	175655001	1.9555	0.308891	OL	RFX4
0.363645	OL	PDGFD	NC_056065.1	64335001	64355001	3.04444	0.246838	OL	RGL1
0.276323	OL	PDGFD	NC_056065.1	64340001	64360001	4.18939	0.263238	OL	RGL1
0.158577	OL	PDSS2	NC_056065.1	64345001	64365001	4.72201	0.31732	OL	RGL1
0.160574	OL	PDSS2	NC_056065.1	64350001	64370001	4.2085	0.344987	OL	RGL1
0.157766	OL	PDSS2	NC_056065.1	64355001	64375001	2.39382	0.324073	OL	RGL1
0.2539	OL	PDZK1IP1	NC_056065.1	64360001	64380001	2.01553	0.350991	OL	RGL1
0.148035	OL	PGM5	NC_056065.1	64365001	64385001	2.51785	0.323578	OL	RGL1
0.169877	OL	PGM5	NC_056065.1	64370001	64390001	3.66818	0.312099	OL	RGL1
0.195043	OL	PGM5	NC_056065.1	64375001	64395001	6.92897	0.332914	OL	RGL1
0.188588	OL	PGM5	NC_056065.1	64380001	64400001	14.3962	0.326117	OL	RGL1
0.217308	OL	PHACTR2	NC_056080.1	128435001	128455001	3.51991	0.250923	OL	RIPPLY1
0.255519	OL	PHACTR2	NC_056080.1	128440001	128460001	13.2671	0.319967	OL	RIPPLY1
0.19127	OL	PHACTR2	NC_056056.1	16875001	16895001	2.08731	0.229019	OL	RNF144A
0.193482	OL	PHACTR2	NC_056064.1	51680001	51700001	19.7333	0.220235	OL	RNF213
0.218259	OL	PHACTR2	NC_056064.1	51685001	51705001	8.58035	0.188851	OL	RNF213
0.204592	OL	PHACTR2	NC_056064.1	51690001	51710001	4.39142	0.190306	OL	RNF213
0.215752	OL	PHACTR2	NC_056070.1	53800001	53820001	4.31429	0.314286	OL	RNF34

0.165337	OL	PHEX	NC_056070.1	53805001	53825001	2.57143	0.214583	OL	RNF34
0.180437	OL	PIEZO2	NC_056077.1	27405001	27425001	2.39109	0.214515	OL	RNF40;ZNF629
0.15405	OL	PIEZO2	NC_056054.1	147145001	147165001	2.50185	0.468172	OL	ROBO1
0.153502	OL	PIEZO2	NC_056054.1	147150001	147170001	3.82058	0.546709	OL	ROBO1
0.168557	OL	PIEZO2	NC_056054.1	147155001	147175001	4.37041	0.610413	OL	ROBO1
0.194571	OL	PIEZO2	NC_056054.1	147160001	147180001	2.83573	0.571855	OL	ROBO1
0.179744	OL	PIEZO2	NC_056054.1	147205001	147225001	1.94979	0.493339	OL	ROBO1
0.16767	OL	PIEZO2	NC_056054.1	147210001	147230001	2.16617	0.42347	OL	ROBO1
0.149335	OL	PIEZO2	NC_056054.1	147240001	147260001	2.57053	0.230609	OL	ROBO1
0.179459	OL	PIGM	NC_056054.1	147960001	147980001	1.95203	0.315671	OL	ROBO1
0.159626	OL	PIGM	NC_056054.1	147995001	148015001	3.20935	0.168466	OL	ROBO1
0.187074	OL	PIR	NC_056054.1	148000001	148020001	4.02285	0.17646	OL	ROBO1
0.20699	OL	PIR	NC_056056.1	20165001	20185001	2.07922	0.160792	OL	ROCK2;SLC66A3
0.220993	OL	PIR	NC_056055.1	26910001	26930001	2.7	0.177285	OL	ROR2
0.150458	OL	PIR	NC_056055.1	26915001	26935001	3.80426	0.2173	OL	ROR2
0.18847	OL	PIR	NC_056055.1	26920001	26940001	4.72617	0.235808	OL	ROR2
0.173883	OL	PIR	NC_056055.1	26925001	26945001	4.11094	0.215178	OL	ROR2
0.176873	OL	PIR	NC_056060.1	47485001	47505001	1.96126	0.262655	OL	RORA
0.167198	OL	PIR	NC_056060.1	47490001	47510001	2.40622	0.290152	OL	RORA
0.186235	OL	PIR	NC_056060.1	47495001	47515001	2.71195	0.333446	OL	RORA
0.322795	OL	PIR	NC_056060.1	47500001	47520001	3.06145	0.399098	OL	RORA
0.310876	OL	PIR	NC_056056.1	216565001	216585001	2.78252	0.16535	OL	RPL3
0.183776	OL	PIR;VEGFD	NC_056071.1	31400001	31420001	2.30025	0.21228	OL	RPP25
0.185356	OL	PITPNB	NC_056071.1	31405001	31425001	2.31152	0.242339	OL	RPP25
0.213435	OL	PITPNB	NC_056068.1	38135001	38155001	1.99461	0.189148	OL	RRAS2
0.261444	OL	PLA2G2C;U	NC_056068.1	38140001	38160001	2.04021	0.171361	OL	RRAS2
0.250186	OL	PLA2G2C;U	NC_056068.1	38145001	38165001	2.08915	0.179467	OL	RRAS2
0.175483	OL	PLA2G2C;U	NC_056068.1	38165001	38185001	2.57239	0.237726	OL	RRAS2
0.210286	OL	PLCH1	NC_056068.1	38170001	38190001	2.08637	0.255849	OL	RRAS2
0.194575	OL	PLCH1	NC_056054.1	264540001	264560001	5.11056	0.157782	OL	RRP1
0.158359	OL	PLCL2;TBC	NC_056066.1	30495001	30515001	1.97991	0.160944	OL	RSU1

0.162794	OL	PLCL2;TBC	NC_056060.1	70125001	70145001	11.1238	0.168798	OL	RTN1
0.166542	OL	PLCXD3	NC_056054.1	143475001	143495001	2.86035	0.188602	OL	SAMSN1
0.181933	OL	PLD5	NC_056054.1	143480001	143500001	2.96709	0.170753	OL	SAMSN1
0.195886	OL	PLD5	NC_056056.1	62940001	62960001	2.0018	0.214691	OL	SANBR
0.151321	OL	PLD5	NC_056058.1	63520001	63540001	7.2875	0.224208	OL	SAP30L
0.207493	OL	PLD5	NC_056074.1	40505001	40525001	2.32516	0.244274	OL	SART1
0.150048	OL	PLEKHH2	NC_056061.1	72945001	72965001	2.64433	0.203641	OL	SASH1
0.153212	OL	PLEKHH2	NC_056061.1	72950001	72970001	2.94872	0.158794	OL	SASH1
0.269209	OL	PLEKHH2	NC_056060.1	9525001	9545001	2.15	0.159975	OL	SCAMP1
0.312708	OL	PLEKHH2	NC_056060.1	9530001	9550001	2.30744	0.185568	OL	SCAMP1
0.150409	OL	PLEKHM3	NC_056060.1	9535001	9555001	2.22388	0.19501	OL	SCAMP1
0.171053	OL	PLEKHM3	NC_056071.1	31475001	31495001	1.98032	0.223148	OL	SCAMP2
0.197917	OL	PLEKHM3	NC_056071.1	29515001	29535001	4.9329	0.229331	OL	SCAPER
0.186082	OL	PLEKHM3	NC_056071.1	29520001	29540001	4.33749	0.221355	OL	SCAPER
0.168837	OL	PLEKHM3	NC_056071.1	29525001	29545001	2.22037	0.171443	OL	SCAPER
0.164038	OL	PLEKHM3	NC_056059.1	69580001	69600001	2.44444	0.313864	OL	SCFD2
0.177522	OL	PLEKHM3	NC_056059.1	69585001	69605001	2.32541	0.353553	OL	SCFD2
0.200495	OL	PLEKHM3	NC_056059.1	69590001	69610001	2.14687	0.353624	OL	SCFD2
0.22355	OL	PLEKHM3	NC_056073.1	31170001	31190001	2.90485	0.164956	OL	SCGN
0.245265	OL	PLEKHM3	NC_056054.1	15550001	15570001	2.40849	0.201212	OL	SCMH1
0.249323	OL	PLEKHM3	NC_056054.1	15555001	15575001	2.77917	0.1799	OL	SCMH1
0.218706	OL	PLEKHM3	NC_056054.1	15595001	15615001	2.01288	0.175008	OL	SCMH1
0.205493	OL	PLEKHM3	NC_056065.1	32910001	32930001	5.95877	0.200711	OL	SDCCAG8
0.148906	OL	PLEKHM3	NC_056065.1	32915001	32935001	2.84856	0.163676	OL	SDCCAG8
0.177126	OL	POC1A	NC_056065.1	33035001	33055001	2.88069	0.180987	OL	SDCCAG8
0.207121	OL	POC1A	NC_056065.1	33040001	33060001	2.79171	0.162795	OL	SDCCAG8
0.157163	OL	POC5	NC_056059.1	16230001	16250001	2.42857	0.174119	OL	SEC24B
0.182839	OL	POLR1C;XP	NC_056059.1	16235001	16255001	2.93643	0.189018	OL	SEC24B
0.157793	OL	POLR3E	NC_056059.1	16240001	16260001	8.70136	0.256244	OL	SEC24B
0.172281	OL	PPFIBP2	NC_056059.1	16245001	16265001	12.8138	0.277142	OL	SEC24B
0.182623	OL	PPFIBP2	NC_056060.1	90925001	90945001	7.41667	0.196663	OL	SEL1L

0.190181	OL	PPFIBP2	NC_056060.1	90930001	90950001	14.6148	0.22274	OL	SEL1L
0.16577	OL	PPFIBP2	NC_056060.1	90970001	90990001	10.843	0.251826	OL	SEL1L
0.167592	OL	PPFIBP2	NC_056060.1	90975001	90995001	4.8506	0.173963	OL	SEL1L
0.158591	OL	PPFIBP2	NC_056060.1	90985001	91005001	2.31398	0.224795	OL	SEL1L
0.178114	OL	PPIF;ZCCHC	NC_056060.1	90990001	91010001	2.01462	0.173	OL	SEL1L
0.176832	OL	PPIF;ZCCHC	NC_056070.1	69865001	69885001	3.31353	0.169544	OL	SELENOM;SMTN
0.145921	OL	PPME1	NC_056057.1	37625001	37645001	2.12473	0.182633	OL	SEMA3A
0.235334	OL	PPP1R12B	NC_056057.1	37630001	37650001	2.19032	0.172896	OL	SEMA3A
0.200949	OL	PPP1R12B	NC_056057.1	37635001	37655001	2.06866	0.161019	OL	SEMA3A
0.172906	OL	PPP1R12B	NC_056057.1	37640001	37660001	2.798	0.193078	OL	SEMA3A
0.169989	OL	PPP1R12B	NC_056057.1	37645001	37665001	4.14576	0.223071	OL	SEMA3A
0.189095	OL	PPP1R12B;S	NC_056057.1	37650001	37670001	2.56644	0.17725	OL	SEMA3A
0.224556	OL	PPP1R12B;S	NC_056072.1	50215001	50235001	3.48793	0.170777	OL	SEMA3F
0.246032	OL	PPP2R3A	NC_056069.1	64045001	64065001	2.11423	0.158551	OL	SEMA5A
0.24321	OL	PPP2R3A	NC_056069.1	64145001	64165001	2.96822	0.15821	OL	SEMA5A
0.222781	OL	PPP2R3A	NC_056060.1	61230001	61250001	1.95238	0.169442	OL	SEMA6D
0.218592	OL	PPP2R3A	NC_056080.1	114795001	114815001	3.10361	0.177961	OL	SEPTIN6
0.240363	OL	PPP2R3A	NC_056080.1	114800001	114820001	2.95885	0.186688	OL	SEPTIN6
0.239006	OL	PPP2R3A	NC_056080.1	114805001	114825001	3.62189	0.213002	OL	SEPTIN6
0.205275	OL	PPP2R3A	NC_056063.1	25195001	25215001	3.34869	0.186507	OL	SERTM1
0.360085	OL	PPP3CA	NC_056070.1	70170001	70190001	5.45148	0.157603	OL	SFI1
0.145846	OL	PR	NC_056070.1	70180001	70200001	3.10478	0.169933	OL	SFI1
0.241864	OL	PR	NC_056070.1	70185001	70205001	2.66906	0.208971	OL	SFI1
0.38895	OL	PR	NC_056070.1	70190001	70210001	2.80133	0.195681	OL	SFI1
0.401513	OL	PR	NC_056070.1	70195001	70215001	2.76953	0.215687	OL	SFI1
0.466037	OL	PR	NC_056058.1	65575001	65595001	2.86754	0.24954	OL	SGCD
0.189546	OL	PRDX2;RNA	NC_056058.1	65580001	65600001	2.55766	0.330117	OL	SGCD
0.183315	OL	PRELID2	NC_056058.1	65585001	65605001	3.10839	0.417047	OL	SGCD
0.146305	OL	PRELID2	NC_056058.1	65590001	65610001	3.50321	0.443777	OL	SGCD
0.157822	OL	PRICKLE2	NC_056058.1	65595001	65615001	2.85376	0.438917	OL	SGCD
0.252333	OL	PRIM2	NC_056058.1	65600001	65620001	5.00794	0.447532	OL	SGCD

0.195835	OL	PRKD1	NC_056058.1	65605001	65625001	5.43575	0.435825	OL	SGCD
0.162788	OL	PRKD1	NC_056058.1	65610001	65630001	2.2306	0.352387	OL	SGCD
0.153899	OL	PRKG1	NC_056058.1	65615001	65635001	2.26139	0.265567	OL	SGCD
0.169398	OL	PRKG1	NC_056061.1	59560001	59580001	2.47593	0.226341	OL	SGK1
0.162682	OL	PRKG1	NC_056061.1	59570001	59590001	2.88536	0.195506	OL	SGK1
0.1938	OL	PRKG1	NC_056066.1	72125001	72145001	2.45789	0.462799	OL	SGK2
0.206957	OL	PRKG2	NC_056066.1	72130001	72150001	2.56376	0.405218	OL	SGK2
0.244862	OL	PRKG2	NC_056056.1	8440001	8460001	2.77272	0.181479	OL	SH2D3C
0.35656	OL	PRKG2	NC_056056.1	8460001	8480001	7.38463	0.254163	OL	SH2D3C;TOR2A
0.327029	OL	PRKG2	NC_056056.1	8465001	8485001	4.55507	0.229832	OL	SH2D3C;TOR2A;TTC16
0.16821	OL	PRKN	NC_056056.1	8470001	8490001	3.67897	0.21759	OL	SH2D3C;TOR2A;TTC16
0.186055	OL	PRKN	NC_056058.1	58180001	58200001	10.2276	0.174481	OL	SH3TC2
0.16399	OL	PRKN	NC_056058.1	58185001	58205001	10.0892	0.177888	OL	SH3TC2
0.164638	OL	PRKN	NC_056058.1	58190001	58210001	8.20904	0.172447	OL	SH3TC2
0.218789	OL	PRMT2	NC_056058.1	58195001	58215001	9.04055	0.171172	OL	SH3TC2
0.238589	OL	PRRG3	NC_056058.1	58240001	58260001	9.63243	0.179262	OL	SH3TC2
0.231161	OL	PRRG3	NC_056064.1	29820001	29840001	2.12219	0.166512	OL	SHISA6
0.229395	OL	PRRG3	NC_056063.1	16275001	16295001	3.42486	0.205946	OL	SIAH3
0.165385	OL	PRSS36	NC_056058.1	48035001	48055001	2.25792	0.162179	OL	SIL1
0.186451	OL	PRSS36	NC_056058.1	48040001	48060001	2.85443	0.166489	OL	SIL1
0.248249	OL	PRSS38	NC_056064.1	37845001	37865001	2.30924	0.220101	OL	SKAP1
0.206741	OL	PRSS38	NC_056063.1	53480001	53500001	2.25249	0.319491	OL	SLAIN1
0.230591	OL	PRSS38	NC_056063.1	53490001	53510001	2.53649	0.218953	OL	SLAIN1
0.246547	OL	PRSS38	NC_056076.1	45830001	45850001	1.98148	0.171941	OL	SLC14A1
0.209097	OL	PRSS38	NC_056076.1	45320001	45340001	2.53757	0.274676	OL	SLC14A2
0.220471	OL	PRSS38	NC_056076.1	45325001	45345001	3.44736	0.297195	OL	SLC14A2
0.164826	OL	PRSS38	NC_056076.1	45330001	45350001	6.44591	0.366006	OL	SLC14A2
0.231617	OL	PRTFDC1	NC_056076.1	45335001	45355001	15.0774	0.408275	OL	SLC14A2
0.315369	OL	PRTFDC1	NC_056076.1	45340001	45360001	19.43	0.444522	OL	SLC14A2
0.414698	OL	PRTFDC1	NC_056076.1	45345001	45365001	14.0468	0.455603	OL	SLC14A2
0.321286	OL	PRTFDC1	NC_056076.1	45350001	45370001	4.5	0.418148	OL	SLC14A2

0.237184	OL	PRTFDC1	NC_056056.1	199235001	199255001	2.38662	0.190297	OL	SLC15A5
0.246095	OL	PSMA1	NC_056056.1	199240001	199260001	2.50704	0.183485	OL	SLC15A5
0.198967	OL	PSMA1	NC_056054.1	27660001	27680001	3.11407	0.248569	OL	SLC1A7
0.174535	OL	PTK2	NC_056054.1	27665001	27685001	4.29686	0.315341	OL	SLC1A7
0.199103	OL	PTK2	NC_056073.1	49530001	49550001	6.3877	0.163136	OL	SLC22A23
0.158505	OL	PTK2	NC_056060.1	12515001	12535001	3.71036	0.180153	OL	SLC24A1
0.148855	OL	PTK2	NC_056060.1	12520001	12540001	3.52044	0.195442	OL	SLC24A1
0.156423	OL	PTK2	NC_056072.1	51100001	51120001	2.05691	0.235627	OL	SLC25A20
0.148853	OL	PTPN11	NC_056055.1	35410001	35430001	13.1714	0.252561	OL	SLC28A3
0.183709	OL	PTPN11	NC_056055.1	35415001	35435001	17.22	0.309972	OL	SLC28A3
0.178007	OL	PTPN11	NC_056061.1	49800001	49820001	2.01997	0.205426	OL	SLC35A1
0.163137	OL	PTPN11	NC_056054.1	77365001	77385001	2.12575	0.191351	OL	SLC35A3
0.301869	OL	PTPRD	NC_056068.1	74770001	74790001	5.06997	0.165853	OL	SLC35C1
0.308648	OL	PTPRD	NC_056056.1	151215001	151235001	2.49554	0.217942	OL	SLC35E3
0.175799	OL	PTPRD	NC_056061.1	19965001	19985001	3.75624	0.15779	OL	SLC35F1
0.21392	OL	PTPRD	NC_056054.1	51575001	51595001	2.79825	0.19378	OL	SLC44A5
0.164046	OL	PTPRD	NC_056054.1	51580001	51600001	4.29267	0.208279	OL	SLC44A5
0.230583	OL	PTPRG	NC_056054.1	51595001	51615001	17.4516	0.159406	OL	SLC44A5
0.4642	OL	PTPRG	NC_056080.1	58075001	58095001	6.41598	0.193543	OL	SLC9A7
0.497539	OL	PTPRG	NC_056080.1	58080001	58100001	4.43137	0.192327	OL	SLC9A7
0.47303	OL	PTPRG	NC_056080.1	58085001	58105001	3.71557	0.159113	OL	SLC9A7
0.395158	OL	PTPRG	NC_056060.1	13515001	13535001	2.04826	0.159071	OL	SMAD6
0.265324	OL	PTPRG	NC_056060.1	13530001	13550001	2.26281	0.161541	OL	SMAD6
0.194845	OL	PTPRG	NC_056080.1	113430001	113450001	2.21918	0.158391	OL	SMARCA1
0.153255	OL	PTPRG	NC_056080.1	113435001	113455001	2.88163	0.181657	OL	SMARCA1
0.194663	OL	PTPRG	NC_056080.1	113440001	113460001	2.82832	0.173829	OL	SMARCA1
0.23862	OL	PTPRG	NC_056055.1	70370001	70390001	7.21025	0.174784	OL	SMARCA2
0.188052	OL	PTPRG	NC_056059.1	30990001	31010001	2.4435	0.19995	OL	SMARCAD1
0.186494	OL	PTPRN2	NC_056058.1	6145001	6165001	2.14085	0.17177	OL	SMIM7;TMEM38A
0.204483	OL	PTPRN2	NC_056061.1	15660001	15680001	1.97754	0.172751	OL	SMPDL3A
0.192658	OL	PTPRN2	NC_056080.1	20095001	20115001	3.88365	0.178568	OL	SMPX

0.151026	OL	PTPRN2	NC_056070.1	69855001	69875001	3.20429	0.160376	OL	SMTN
0.161306	OL	PTPRO	NC_056070.1	69860001	69880001	3.90244	0.195964	OL	SMTN
0.192158	OL	PTPRO	NC_056062.1	32935001	32955001	2.15995	0.203996	OL	SNAI2
0.151429	OL	PUM2	NC_056062.1	32940001	32960001	2.31919	0.173242	OL	SNAI2
0.1495	OL	PUM2	NC_056057.1	93575001	93595001	2.31489	0.286785	OL	SND1
0.1473	OL	PUM2	NC_056057.1	93580001	93600001	2.38406	0.196185	OL	SND1
0.14595	OL	PUM2	NC_056057.1	93585001	93605001	2.6528	0.210312	OL	SND1
0.146162	OL	PUM2	NC_056062.1	33655001	33675001	1.99639	0.206796	OL	SNTG1
0.149723	OL	PUM2	NC_056060.1	44080001	44100001	1.97304	0.190649	OL	SNX1
0.151052	OL	PUM2	NC_056061.1	51300001	51320001	3.19511	0.271348	OL	SNX14
0.155036	OL	PUM2	NC_056061.1	51305001	51325001	3.21818	0.314954	OL	SNX14
0.152143	OL	PUM2	NC_056061.1	51310001	51330001	2.93203	0.314113	OL	SNX14
0.155901	OL	RAB27A	NC_056079.1	14770001	14790001	3.90051	0.161285	OL	SNX25
0.166503	OL	RAB27A	NC_056079.1	14775001	14795001	2.29857	0.314834	OL	SNX25
0.203862	OL	RAB31	NC_056079.1	14795001	14815001	2.29976	0.246975	OL	SNX25
0.161652	OL	RAB3C	NC_056064.1	53160001	53180001	2.88516	0.186388	OL	SOCS3
0.148646	OL	RAB3C	NC_056064.1	53165001	53185001	2.44061	0.169453	OL	SOCS3
0.147126	OL	RAB8B	NC_056063.1	25580001	25600001	1.98969	0.342952	OL	SOHLH2
0.158905	OL	RAB8B	NC_056075.1	27035001	27055001	2.07091	0.16186	OL	SORCS1
0.152663	OL	RABGAP1L	NC_056075.1	27040001	27060001	2.02252	0.182162	OL	SORCS1
0.160718	OL	RABGAP1L	NC_056075.1	27140001	27160001	7.6268	0.325075	OL	SORCS1
0.219056	OL	RABGAP1L	NC_056075.1	27145001	27165001	13.6621	0.338701	OL	SORCS1
0.250701	OL	RABGAP1L	NC_056075.1	27150001	27170001	9.37263	0.255379	OL	SORCS1
0.317066	OL	RAD51;RMI	NC_056075.1	27155001	27175001	7.6974	0.167629	OL	SORCS1
0.333124	OL	RAD51;RMI	NC_056075.1	27170001	27190001	4.83806	0.181404	OL	SORCS1
0.294303	OL	RAD51;RMI	NC_056075.1	27175001	27195001	4.17148	0.255978	OL	SORCS1
0.155778	OL	RAD51B	NC_056075.1	27180001	27200001	3.50949	0.31941	OL	SORCS1
0.147414	OL	RAD51B	NC_056075.1	27185001	27205001	2.40038	0.294602	OL	SORCS1
0.149714	OL	RAD54L2	NC_056075.1	27190001	27210001	2.16303	0.244937	OL	SORCS1
0.182106	OL	RALYL	NC_056059.1	114330001	114350001	2.30437	0.315853	OL	SORCS2
0.190938	OL	RALYL	NC_056059.1	114830001	114850001	3.83394	0.182598	OL	SORCS2

0.167096	OL	RALYL	NC_056063.1	85350001	85370001	2.45176	0.239274	OL	SOX1
0.162136	OL	RALYL	NC_056063.1	85355001	85375001	3.22805	0.229959	OL	SOX1
0.234622	OL	RARS2	NC_056056.1	15855001	15875001	2.37788	0.309596	OL	SOX11
0.25856	OL	RARS2;SLC:	NC_056068.1	35640001	35660001	3.10358	0.165696	OL	SOX6
0.243917	OL	RARS2;SLC:	NC_056064.1	58080001	58100001	10.1589	0.619775	OL	SOX9
0.230475	OL	RARS2;SLC:	NC_056064.1	58095001	58115001	14.1058	0.549558	OL	SOX9
0.191261	OL	RARS2;SLC:	NC_056064.1	58100001	58120001	6.51659	0.469577	OL	SOX9
0.150658	OL	RASSF6	NC_056055.1	72925001	72945001	2.50674	0.167728	OL	SPATA6L
0.212687	OL	RASSF6	NC_056055.1	72930001	72950001	3.80314	0.179323	OL	SPATA6L
0.21031	OL	RASSF6	NC_056055.1	72935001	72955001	4.91689	0.225516	OL	SPATA6L
0.172411	OL	RASSF6	NC_056055.1	72940001	72960001	4.40666	0.218317	OL	SPATA6L
0.220152	OL	RBFOX1	NC_056055.1	72945001	72965001	2.52515	0.179594	OL	SPATA6L
0.180052	OL	RBFOX3	NC_056069.1	1635001	1655001	6.36913	0.208726	OL	SPDL1
0.189854	OL	RBFOX3	NC_056069.1	1640001	1660001	6.95532	0.218544	OL	SPDL1
0.171734	OL	RBFOX3	NC_056069.1	1645001	1665001	6.89	0.238443	OL	SPDL1
0.165365	OL	RBFOX3	NC_056069.1	1650001	1670001	5.2617	0.228227	OL	SPDL1
0.211157	OL	RBM19	NC_056069.1	1655001	1675001	3.27926	0.169254	OL	SPDL1
0.223869	OL	RBM19	NC_056067.1	14195001	14215001	2.42639	0.216294	OL	SPIRE2
0.2126	OL	RBM19	NC_056067.1	14200001	14220001	2.23488	0.291006	OL	SPIRE2
0.155816	OL	RCAN2	NC_056067.1	14205001	14225001	2.08707	0.316744	OL	SPIRE2
0.153192	OL	RCAN2	NC_056068.1	38210001	38230001	4.80596	0.166833	OL	SPON1
0.172067	OL	RCAN2	NC_056068.1	38215001	38235001	7.64567	0.164322	OL	SPON1
0.174763	OL	RCBTB2	NC_056068.1	38220001	38240001	8.96732	0.198023	OL	SPON1
0.194296	OL	RECK	NC_056068.1	38225001	38245001	4.19553	0.249985	OL	SPON1
0.18803	OL	RECK	NC_056068.1	38230001	38250001	3.19055	0.255576	OL	SPON1
0.200918	OL	RECK	NC_056068.1	38235001	38255001	2.17559	0.166079	OL	SPON1
0.215948	OL	RECK	NC_056054.1	109585001	109605001	2.04584	0.170645	OL	SPTA1
0.249847	OL	RECK	NC_056054.1	109590001	109610001	1.9712	0.171475	OL	SPTA1
0.227816	OL	RECK	NC_056066.1	54720001	54740001	4.57284	0.366667	OL	SS18L1
0.19422	OL	RECK	NC_056066.1	54725001	54745001	3.30392	0.314516	OL	SS18L1
0.151639	OL	RECK	NC_056070.1	64285001	64305001	1.96115	0.175875	OL	SSH1

0.168758	OL	RECK	NC_056070.1	64290001	64310001	2.31159	0.187066	OL	SSH1
0.216746	OL	RECK	NC_056070.1	64295001	64315001	2.53874	0.168552	OL	SSH1
0.232809	OL	RECK	NC_056062.1	34275001	34295001	2.11917	0.319156	OL	ST18
0.221966	OL	RECK	NC_056062.1	20965001	20985001	2.01003	0.240332	OL	ST3GAL1
0.223252	OL	RELN	NC_056062.1	20990001	21010001	2.13206	0.238914	OL	ST3GAL1
0.220121	OL	RELN	NC_056056.1	57790001	57810001	2.10625	0.195607	OL	ST3GAL5
0.188302	OL	RELN	NC_056056.1	57795001	57815001	2.04875	0.222158	OL	ST3GAL5
0.301801	OL	RELN	NC_056056.1	57800001	57820001	1.95281	0.217917	OL	ST3GAL5
0.326757	OL	RELN	NC_056054.1	253060001	253080001	2.89448	0.292142	OL	STAG1
0.330539	OL	RELN	NC_056054.1	253065001	253085001	2.34631	0.270629	OL	STAG1
0.237551	OL	RERE	NC_056054.1	253070001	253090001	2.75798	0.265941	OL	STAG1
0.230633	OL	RERE	NC_056054.1	253075001	253095001	3.17064	0.271229	OL	STAG1
0.211827	OL	RERE	NC_056054.1	253080001	253100001	2.71845	0.343591	OL	STAG1
0.203203	OL	RERE	NC_056054.1	253085001	253105001	3.00695	0.371073	OL	STAG1
0.168058	OL	RERE	NC_056054.1	253090001	253110001	2.9322	0.333255	OL	STAG1
0.172553	OL	RGS2	NC_056054.1	253095001	253115001	2.60429	0.330796	OL	STAG1
0.206802	OL	RHAG	NC_056054.1	253100001	253120001	2.78439	0.363246	OL	STAG1
0.222426	OL	RHAG	NC_056054.1	253105001	253125001	2.86641	0.329331	OL	STAG1
0.251952	OL	RHAG	NC_056054.1	253110001	253130001	2.51301	0.326069	OL	STAG1
0.23233	OL	RHAG	NC_056054.1	253115001	253135001	3.35715	0.319168	OL	STAG1
0.323359	OL	RHAG	NC_056054.1	253120001	253140001	3.05597	0.305851	OL	STAG1
0.302987	OL	RHAG	NC_056054.1	253125001	253145001	3.70115	0.379004	OL	STAG1
0.338782	OL	RHAG	NC_056054.1	253130001	253150001	3.7027	0.39516	OL	STAG1
0.340439	OL	RHAG	NC_056054.1	253135001	253155001	4.36428	0.404929	OL	STAG1
0.175302	OL	RHBDF2	NC_056054.1	253140001	253160001	5.87772	0.396267	OL	STAG1
0.179163	OL	RHBDF2	NC_056054.1	253145001	253165001	4.85714	0.358365	OL	STAG1
0.166376	OL	RIMS2	NC_056054.1	253150001	253170001	5.29048	0.34336	OL	STAG1
0.168864	OL	RIMS2	NC_056054.1	253155001	253175001	4.05819	0.306091	OL	STAG1
0.220106	OL	RIMS2	NC_056054.1	253160001	253180001	3.55776	0.284132	OL	STAG1
0.161262	OL	RIOK3	NC_056054.1	253165001	253185001	3.64536	0.296078	OL	STAG1
0.150482	OL	RIPOR1	NC_056054.1	253170001	253190001	3.49093	0.290089	OL	STAG1

0.15493	OL	RIPOR1	NC_056054.1	253175001	253195001	3.19126	0.298465	OL	STAG1
0.159158	OL	RIPOR1	NC_056054.1	253180001	253200001	3.22116	0.309262	OL	STAG1
0.221238	OL	RIPOR1	NC_056054.1	253185001	253205001	3.13952	0.313258	OL	STAG1
0.236929	OL	RIPPLY3	NC_056054.1	253190001	253210001	3.6403	0.36171	OL	STAG1
0.199316	OL	RNASEH2B	NC_056054.1	253195001	253215001	4.35065	0.363667	OL	STAG1
0.234231	OL	RNF114	NC_056054.1	253200001	253220001	4.28571	0.369056	OL	STAG1
0.314905	OL	RNF114	NC_056054.1	253205001	253225001	5.33976	0.368181	OL	STAG1
0.283974	OL	RNF114	NC_056054.1	253210001	253230001	4.81973	0.352456	OL	STAG1
0.22027	OL	RNF114;SN/	NC_056054.1	253215001	253235001	4.81318	0.368675	OL	STAG1
0.147255	OL	RNF13	NC_056054.1	253220001	253240001	4.61991	0.375053	OL	STAG1
0.167764	OL	RNF220	NC_056054.1	253225001	253245001	4.22765	0.372556	OL	STAG1
0.245238	OL	RNF220	NC_056054.1	253230001	253250001	4.22494	0.372412	OL	STAG1
0.24236	OL	RNF38	NC_056054.1	253235001	253255001	4.14321	0.387026	OL	STAG1
0.175526	OL	RNF38	NC_056054.1	253240001	253260001	4.30539	0.363484	OL	STAG1
0.173016	OL	RNF38	NC_056054.1	253245001	253265001	4.21403	0.35669	OL	STAG1
0.166892	OL	RNLS	NC_056054.1	253250001	253270001	4.63373	0.360116	OL	STAG1
0.188396	OL	RNLS	NC_056054.1	253255001	253275001	4.47619	0.354593	OL	STAG1
0.353253	OL	RPRD2	NC_056054.1	253260001	253280001	3.90476	0.381339	OL	STAG1
0.33474	OL	RPRD2	NC_056054.1	253265001	253285001	3.17333	0.387059	OL	STAG1
0.262875	OL	RPRD2	NC_056054.1	253270001	253290001	2.92683	0.363639	OL	STAG1
0.214557	OL	RPRD2	NC_056054.1	253275001	253295001	2.7602	0.346995	OL	STAG1
0.169684	OL	RPRD2	NC_056054.1	253280001	253300001	2.74826	0.331162	OL	STAG1
0.151954	OL	RPRD2	NC_056054.1	253285001	253305001	2.96135	0.315801	OL	STAG1
0.205793	OL	RSPH4A;ZU	NC_056054.1	253290001	253310001	2.81721	0.295664	OL	STAG1
0.197234	OL	RSPH4A;ZU	NC_056054.1	253295001	253315001	2.7539	0.317475	OL	STAG1
0.158897	OL	RTKN2	NC_056054.1	253300001	253320001	2.81701	0.272865	OL	STAG1
0.205706	OL	RTKN2	NC_056054.1	253305001	253325001	2.97904	0.288351	OL	STAG1
0.170278	OL	RWDD1	NC_056054.1	253310001	253330001	2.10911	0.233211	OL	STAG1
0.147735	OL	S100A14;S1(	NC_056054.1	253315001	253335001	2.13229	0.200359	OL	STAG1
0.168058	OL	S100A16	NC_056054.1	253320001	253340001	2.24555	0.239443	OL	STAG1
0.29235	OL	SAMD4A	NC_056054.1	253325001	253345001	2.07343	0.249308	OL	STAG1

0.15156	OL	SAP30	NC_056054.1	253330001	253350001	3.04298	0.324351	OL	STAG1
0.15381	OL	SAP30	NC_056054.1	253335001	253355001	3.22969	0.333721	OL	STAG1
0.151515	OL	SAP30	NC_056054.1	253340001	253360001	2.90727	0.311694	OL	STAG1
0.150997	OL	SAP30	NC_056054.1	253345001	253365001	3.41497	0.264027	OL	STAG1
0.150459	OL	SAP30;SCR(	NC_056054.1	253350001	253370001	4.02703	0.247177	OL	STAG1
0.224049	OL	SART1	NC_056054.1	253355001	253375001	4.25631	0.21709	OL	STAG1
0.15206	OL	SASH1	NC_056054.1	253360001	253380001	4.16791	0.202768	OL	STAG1
0.203745	OL	SCAMP1	NC_056054.1	253365001	253385001	4.23103	0.205792	OL	STAG1
0.149086	OL	SCAMP1	NC_056054.1	253370001	253390001	3.8689	0.201771	OL	STAG1
0.198624	OL	SCAMP2	NC_056054.1	253375001	253395001	3.58255	0.204085	OL	STAG1
0.219507	OL	SCAMP2	NC_056054.1	253380001	253400001	3.86268	0.213	OL	STAG1
0.225325	OL	SCAMP2	NC_056054.1	253385001	253405001	3.8522	0.224007	OL	STAG1
0.211287	OL	SCAMP2	NC_056054.1	253390001	253410001	3.88214	0.23627	OL	STAG1
0.168439	OL	SCAPER	NC_056054.1	253395001	253415001	4.40225	0.235235	OL	STAG1
0.175268	OL	SCAPER	NC_056054.1	253400001	253420001	4.47924	0.236481	OL	STAG1
0.183344	OL	SCAPER	NC_056054.1	253405001	253425001	5.65425	0.23382	OL	STAG1
0.168929	OL	SCAPER	NC_056054.1	253410001	253430001	6.06667	0.240994	OL	STAG1
0.187062	OL	SCG3	NC_056054.1	253415001	253435001	5.14285	0.228571	OL	STAG1
0.307998	OL	SDK1	NC_056054.1	253420001	253440001	5.04918	0.237551	OL	STAG1
0.379542	OL	SDK1	NC_056054.1	253425001	253445001	4.69035	0.231864	OL	STAG1
0.292559	OL	SDK1	NC_056054.1	253430001	253450001	4.65026	0.2171	OL	STAG1
0.366539	OL	SDK1	NC_056054.1	253435001	253455001	5.70131	0.223146	OL	STAG1
0.345193	OL	SDK1	NC_056054.1	253440001	253460001	5.59764	0.200864	OL	STAG1
0.261257	OL	SDK1	NC_056054.1	253445001	253465001	4.99452	0.204424	OL	STAG1
0.201011	OL	SDK1	NC_056054.1	253450001	253470001	4.18092	0.215389	OL	STAG1
0.148428	OL	SELENOI	NC_056054.1	253455001	253475001	4.06153	0.211481	OL	STAG1
0.16688	OL	SEMA5B	NC_056054.1	253460001	253480001	4.42308	0.227045	OL	STAG1
0.187803	OL	SEMA5B	NC_056054.1	253465001	253485001	3.70414	0.204503	OL	STAG1
0.204701	OL	SEMA5B	NC_056054.1	253490001	253510001	3.01776	0.177178	OL	STAG1
0.151239	OL	SENP6	NC_056054.1	253495001	253515001	2.60784	0.197463	OL	STAG1
0.162236	OL	SENP6	NC_056054.1	253500001	253520001	2.9977	0.207446	OL	STAG1

0.16497	OL	SENP6	NC_056054.1	253505001	253525001	2.93521	0.179943	OL	STAG1
0.148317	OL	SENP6	NC_056054.1	253510001	253530001	3.18526	0.192971	OL	STAG1
0.146257	OL	SENP6	NC_056054.1	253515001	253535001	3.4933	0.195006	OL	STAG1
0.150007	OL	SENP6	NC_056054.1	253520001	253540001	3.88018	0.202318	OL	STAG1
0.159242	OL	SENP6	NC_056054.1	253525001	253545001	4.25791	0.22623	OL	STAG1
0.169562	OL	SENP6	NC_056054.1	253530001	253550001	3.86551	0.194832	OL	STAG1
0.186485	OL	SENP6	NC_056064.1	42155001	42175001	3.0424	0.210832	OL	STAT3
0.190935	OL	SENP6	NC_056062.1	78375001	78395001	2.43877	0.16996	OL	STK3
0.158767	OL	SENP6	NC_056062.1	78380001	78400001	2.47864	0.168557	OL	STK3
0.207184	OL	SERPINB11	NC_056059.1	104615001	104635001	2.03486	0.234605	OL	STK32B
0.150442	OL	SERTM1	NC_056068.1	43650001	43670001	3.75806	0.186923	OL	STK33
0.216025	OL	SERTM1	NC_056068.1	43655001	43675001	4.29281	0.178545	OL	STK33
0.177641	OL	SETX	NC_056068.1	43660001	43680001	3.72212	0.191062	OL	STK33
0.155929	OL	SETX	NC_056068.1	43670001	43690001	2.29851	0.208792	OL	STK33
0.147381	OL	SETX	NC_056068.1	43675001	43695001	2.24041	0.231757	OL	STK33
0.147324	OL	SETX	NC_056068.1	43680001	43700001	2.08295	0.217375	OL	STK33
0.237832	OL	SGIP1	NC_056055.1	141265001	141285001	2.3957	0.223229	OL	STK39
0.282247	OL	SGIP1	NC_056055.1	141270001	141290001	2.04658	0.224662	OL	STK39
0.247076	OL	SGIP1	NC_056060.1	90795001	90815001	1.96133	0.169419	OL	STON2
0.238372	OL	SGIP1	NC_056060.1	90800001	90820001	2.204	0.162037	OL	STON2
0.174922	OL	SHANK2	NC_056060.1	90810001	90830001	2.3989	0.176529	OL	STON2
0.163544	OL	SHANK2	NC_056055.1	47930001	47950001	2.17256	0.166868	OL	STX17
0.177091	OL	SHANK2	NC_056055.1	47935001	47955001	2.10526	0.189673	OL	STX17
0.191569	OL	SHANK2	NC_056055.1	47940001	47960001	2.12293	0.208201	OL	STX17
0.21602	OL	SHANK2	NC_056070.1	46395001	46415001	9.87255	0.218285	OL	STX2
0.157392	OL	SHC3	NC_056070.1	46400001	46420001	8.61269	0.263024	OL	STX2
0.341586	OL	SHLD1	NC_056070.1	46405001	46425001	2.1009	0.185023	OL	STX2
0.176783	OL	SHLD2	NC_056065.1	61480001	61500001	2.5603	0.171787	OL	STX6
0.149451	OL	SIDT1;SPICI	NC_056064.1	5080001	5100001	2.3941	0.260653	OL	STXBP4
0.259831	OL	SIGLEC11;V	NC_056064.1	5085001	5105001	2.25985	0.252454	OL	STXBP4
0.351914	OL	SIGLEC11;V	NC_056065.1	39380001	39400001	3.84878	0.157524	OL	SUCO

0.389037	OL	SIGLEC11;V	NC_056057.1	82380001	82400001	2.23676	0.221634	OL	SUGCT
0.397913	OL	SLAIN1	NC_056057.1	82385001	82405001	3.85883	0.270935	OL	SUGCT
0.424905	OL	SLAIN1	NC_056057.1	82390001	82410001	2.71936	0.249574	OL	SUGCT
0.40702	OL	SLAIN1	NC_056057.1	82400001	82420001	1.99055	0.198314	OL	SUGCT
0.344569	OL	SLAIN1	NC_056056.1	220460001	220480001	2.4542	0.182254	OL	SULT4A1
0.276967	OL	SLAIN1	NC_056056.1	220465001	220485001	2.97623	0.190917	OL	SULT4A1
0.198274	OL	SLC10A7	NC_056056.1	220470001	220490001	3.94422	0.234174	OL	SULT4A1
0.243714	OL	SLC10A7	NC_056056.1	220475001	220495001	2.7551	0.185378	OL	SULT4A1
0.264941	OL	SLC10A7	NC_056063.1	24925001	24945001	2.07107	0.178591	OL	SUPT20H
0.235806	OL	SLC10A7	NC_056073.1	18475001	18495001	2.22395	0.191114	OL	SUPT3H
0.211711	OL	SLC10A7	NC_056073.1	18480001	18500001	2.271	0.259256	OL	SUPT3H
0.163968	OL	SLC10A7	NC_056073.1	18485001	18505001	2.27107	0.251664	OL	SUPT3H
0.166987	OL	SLC14A2	NC_056073.1	18490001	18510001	2.18729	0.246284	OL	SUPT3H
0.363134	OL	SLC15A2	NC_056073.1	18495001	18515001	2.12277	0.243048	OL	SUPT3H
0.394426	OL	SLC15A2	NC_056073.1	18500001	18520001	2.2751	0.191295	OL	SUPT3H
0.153656	OL	SLC15A3;TM	NC_056073.1	18505001	18525001	2.2097	0.218242	OL	SUPT3H
0.263335	OL	SLC17A6	NC_056073.1	18510001	18530001	2.09114	0.312621	OL	SUPT3H
0.1581	OL	SLC17A6	NC_056080.1	56380001	56400001	2.55065	0.175724	OL	SUV39H1
0.232068	OL	SLC17A6	NC_056080.1	56385001	56405001	2.45992	0.226558	OL	SUV39H1;WAS
0.237438	OL	SLC17A6	NC_056080.1	56390001	56410001	2.4329	0.208889	OL	SUV39H1;WAS
0.199296	OL	SLC17A6	NC_056061.1	76895001	76915001	3.37823	0.166146	OL	SYNE1
0.170265	OL	SLC17A6	NC_056061.1	76905001	76925001	2.99293	0.189684	OL	SYNE1
0.196896	OL	SLC24A3	NC_056061.1	76910001	76930001	2.54286	0.184299	OL	SYNE1
0.188161	OL	SLC24A3	NC_056061.1	76915001	76935001	2.10764	0.168831	OL	SYNE1
0.189579	OL	SLC24A3	NC_056061.1	76935001	76955001	2.89415	0.217704	OL	SYNE1
0.191105	OL	SLC24A3	NC_056061.1	76940001	76960001	2.10941	0.173254	OL	SYNE1
0.236369	OL	SLC24A3	NC_056061.1	77260001	77280001	2.18406	0.192403	OL	SYNE1
0.228881	OL	SLC24A3	NC_056060.1	74285001	74305001	2.43042	0.20289	OL	SYNE2
0.218057	OL	SLC24A3	NC_056060.1	74290001	74310001	2.18537	0.293988	OL	SYNE2
0.185354	OL	SLC25A13	NC_056059.1	6795001	6815001	1.98933	0.160694	OL	SYNPO2
0.335278	OL	SLC25A21	NC_056072.1	38055001	38075001	2.38598	0.163167	OL	SYNPR

0.349237	OL	SLC25A21	NC_056072.1	38060001	38080001	2.00458	0.234107	OL	SYNPR
0.274497	OL	SLC25A21	NC_056066.1	73595001	73615001	2.17541	0.240573	OL	SYS1
0.387172	OL	SLC25A21	NC_056066.1	73600001	73620001	2.27525	0.24646	OL	SYS1;TP53TG5
0.459917	OL	SLC25A21	NC_056066.1	73605001	73625001	2.61917	0.252883	OL	SYS1;TP53TG5
0.202261	OL	SLC25A21	NC_056080.1	37290001	37310001	2.45676	0.229301	OL	SYTL5
0.152925	OL	SLC25A29	NC_056080.1	37295001	37315001	2.51141	0.225703	OL	SYTL5
0.152382	OL	SLC25A29	NC_056080.1	37300001	37320001	2.63478	0.279213	OL	SYTL5
0.148902	OL	SLC25A29	NC_056080.1	37305001	37325001	2.46952	0.231556	OL	SYTL5
0.176116	OL	SLC30A5	NC_056080.1	37310001	37330001	2.34348	0.226787	OL	SYTL5
0.166151	OL	SLC30A5	NC_056080.1	37315001	37335001	2.23541	0.215935	OL	SYTL5
0.21189	OL	SLC35A1	NC_056061.1	57945001	57965001	3.29835	0.26792	OL	TAAR8
0.166318	OL	SLC39A11	NC_056061.1	57950001	57970001	3.69227	0.29022	OL	TAAR8
0.168872	OL	SLC39A11	NC_056061.1	57940001	57960001	2.12389	0.178429	OL	TAAR8;TAAR9
0.175573	OL	SLC4A4	NC_056072.1	33080001	33100001	9.50156	0.406859	OL	TAFA1
0.188516	OL	SLC5A7	NC_056072.1	33085001	33105001	9.60335	0.411235	OL	TAFA1
0.206709	OL	SLC6A20	NC_056072.1	33090001	33110001	8.18182	0.392597	OL	TAFA1
0.19053	OL	SLC9A7	NC_056072.1	33095001	33115001	4.92966	0.336067	OL	TAFA1
0.230342	OL	SLC9A7	NC_056072.1	33100001	33120001	3.64258	0.294123	OL	TAFA1
0.270384	OL	SLC9A7	NC_056072.1	33105001	33125001	2.54173	0.301955	OL	TAFA1
0.185756	OL	SLC9A7	NC_056055.1	148575001	148595001	2.27735	0.172188	OL	TANK
0.289156	OL	SLC9A8	NC_056055.1	148580001	148600001	2.30859	0.176476	OL	TANK
0.295599	OL	SLC9A8	NC_056055.1	148585001	148605001	2.28516	0.185044	OL	TANK
0.237067	OL	SLC9A8	NC_056055.1	148590001	148610001	2.27024	0.170028	OL	TANK
0.146046	OL	SLIT2	NC_056055.1	148600001	148620001	2.21787	0.169751	OL	TANK
0.202566	OL	SMARCC1	NC_056055.1	148605001	148625001	2.26835	0.177218	OL	TANK
0.209842	OL	SMARCC1	NC_056055.1	148610001	148630001	2.2681	0.19676	OL	TANK
0.22659	OL	SMARCC1	NC_056055.1	148615001	148635001	2.17316	0.211978	OL	TANK
0.208697	OL	SMARCC1	NC_056055.1	148620001	148640001	2.27099	0.210522	OL	TANK
0.203098	OL	SMARCC1	NC_056055.1	148625001	148645001	2.17795	0.206679	OL	TANK
0.199902	OL	SMARCC1	NC_056059.1	58110001	58130001	2.39893	0.174508	OL	TBC1D1
0.201808	OL	SMARCC1	NC_056059.1	58115001	58135001	3.59669	0.231936	OL	TBC1D1

0.203488	OL	SMARCC1	NC_056059.1	58120001	58140001	7.89014	0.330815	OL	TBC1D1
0.246562	OL	SMARCC1	NC_056059.1	58125001	58145001	11.6731	0.37749	OL	TBC1D1
0.318391	OL	SMARCC1	NC_056059.1	58130001	58150001	2.13393	0.223727	OL	TBC1D1
0.184906	OL	SNAI1	NC_056080.1	128455001	128475001	5.06665	0.181654	OL	TBC1D8B
0.162393	OL	SNAI1	NC_056056.1	219120001	219140001	3.30661	0.165093	OL	TCF20
0.157728	OL	SNTB1	NC_056056.1	219125001	219145001	3.69598	0.180083	OL	TCF20
0.169751	OL	SNTB1	NC_056056.1	219130001	219150001	3.86974	0.192967	OL	TCF20
0.186809	OL	SNTB1	NC_056056.1	219135001	219155001	3.65461	0.204667	OL	TCF20
0.159337	OL	SNTB1	NC_056056.1	219140001	219160001	3.04424	0.173523	OL	TCF20
0.177885	OL	SNX19	NC_056056.1	219145001	219165001	2.94493	0.16964	OL	TCF20
0.155306	OL	SNX29	NC_056054.1	104745001	104765001	2.30344	0.248068	OL	TDRD10
0.218111	OL	SNX29	NC_056054.1	104750001	104770001	3.02528	0.329518	OL	TDRD10
0.265328	OL	SNX29	NC_056054.1	104755001	104775001	2.63128	0.272282	OL	TDRD10
0.282057	OL	SNX29	NC_056054.1	104760001	104780001	2.29004	0.213304	OL	TDRD10
0.288852	OL	SNX29	NC_056059.1	67545001	67565001	2.12152	0.174536	OL	TEC
0.243599	OL	SNX29	NC_056059.1	80510001	80530001	2.15629	0.281424	OL	TECRL
0.269684	OL	SNX29	NC_056059.1	80515001	80535001	2.85864	0.30441	OL	TECRL
0.289688	OL	SNX29	NC_056059.1	80520001	80540001	7.26825	0.350105	OL	TECRL
0.292387	OL	SNX29	NC_056059.1	80525001	80545001	2.22748	0.356484	OL	TECRL
0.34586	OL	SNX29	NC_056079.1	12630001	12650001	2.75988	0.170064	OL	TENM3
0.151259	OL	SNX6	NC_056079.1	12635001	12655001	2.95094	0.173889	OL	TENM3
0.250271	OL	SNX6	NC_056079.1	12640001	12660001	2.77282	0.166348	OL	TENM3
0.258266	OL	SNX6	NC_056054.1	95055001	95075001	2.12342	0.300207	OL	TENT5C
0.296571	OL	SNX6	NC_056054.1	95060001	95080001	2.21025	0.322272	OL	TENT5C
0.25613	OL	SNX6	NC_056054.1	95065001	95085001	2.93381	0.307804	OL	TENT5C
0.232471	OL	SNX6	NC_056054.1	95070001	95090001	2.41014	0.279303	OL	TENT5C
0.194122	OL	SNX6	NC_056079.1	26105001	26125001	2.03326	0.320158	OL	TEX15
0.156401	OL	SORCS3	NC_056054.1	256240001	256260001	2.26938	0.209901	OL	TF
0.176881	OL	SORCS3	NC_056055.1	48690001	48710001	2.47749	0.280116	OL	TGFBR1
0.235411	OL	SORCS3	NC_056055.1	48695001	48715001	2.42658	0.263677	OL	TGFBR1
0.243671	OL	SORCS3	NC_056055.1	48700001	48720001	2.17466	0.239985	OL	TGFBR1

0.255513	OL	SORCS3	NC_056055.1	48705001	48725001	2.18905	0.223136	OL	TGFBR1
0.292136	OL	SORCS3	NC_056055.1	48710001	48730001	2.37267	0.217575	OL	TGFBR1
0.190488	OL	SOS2	NC_056055.1	48715001	48735001	2.43406	0.210925	OL	TGFBR1
0.14578	OL	SOS2	NC_056055.1	48720001	48740001	2.44539	0.224342	OL	TGFBR1
0.167446	OL	SOS2	NC_056055.1	48725001	48745001	2.28488	0.231612	OL	TGFBR1
0.204655	OL	SOS2	NC_056055.1	48730001	48750001	2.26717	0.237719	OL	TGFBR1
0.21675	OL	SOS2	NC_056055.1	48735001	48755001	2.18575	0.231375	OL	TGFBR1
0.226687	OL	SOS2	NC_056055.1	48740001	48760001	1.96858	0.169689	OL	TGFBR1
0.321011	OL	SOS2;VCPK	NC_056055.1	138610001	138630001	2.21099	0.165409	OL	TLK1
0.310587	OL	SOS2;VCPK	NC_056055.1	138615001	138635001	2.08334	0.177255	OL	TLK1
0.271139	OL	SOS2;VCPK	NC_056054.1	239385001	239405001	3.13532	0.178422	OL	TM4SF4
0.220028	OL	SOS2;VCPK	NC_056054.1	239390001	239410001	2.15132	0.209917	OL	TM4SF4
0.150799	OL	SOX5	NC_056060.1	86700001	86720001	2.52164	0.214044	OL	TMED8
0.184884	OL	SOX5	NC_056060.1	86705001	86725001	2.81717	0.240892	OL	TMED8
0.246094	OL	SOX5	NC_056060.1	86710001	86730001	2.21951	0.197611	OL	TMED8
0.203805	OL	SOX5	NC_056054.1	256875001	256895001	4.68582	0.167548	OL	TMEM108
0.17011	OL	SOX5	NC_056054.1	256880001	256900001	7.1875	0.288049	OL	TMEM108
0.222742	OL	SOX6	NC_056054.1	256885001	256905001	7.91498	0.347403	OL	TMEM108
0.252042	OL	SOX6	NC_056054.1	256890001	256910001	6.53079	0.353107	OL	TMEM108
0.254574	OL	SOX6	NC_056054.1	256895001	256915001	4.50814	0.385908	OL	TMEM108
0.324592	OL	SOX6	NC_056054.1	256900001	256920001	2.74822	0.331429	OL	TMEM108
0.294173	OL	SOX6	NC_056076.1	33660001	33680001	2.58431	0.193203	OL	TMEM241
0.261437	OL	SOX6	NC_056076.1	33665001	33685001	2.66308	0.192451	OL	TMEM241
0.291587	OL	SOX6	NC_056076.1	33670001	33690001	2.09203	0.183632	OL	TMEM241
0.212251	OL	SOX6	NC_056080.1	132810001	132830001	2.05787	0.159313	OL	TMEM35A
0.209792	OL	SOX6	NC_056058.1	6135001	6155001	3.21122	0.15949	OL	TMEM38A
0.217572	OL	SOX6	NC_056058.1	6140001	6160001	3.61844	0.206199	OL	TMEM38A
0.184351	OL	SOX6	NC_056080.1	81950001	81970001	2.65501	0.217764	OL	TMLHE
0.16194	OL	SOX6	NC_056056.1	118815001	118835001	3.39221	0.170748	OL	TMTC2
0.149042	OL	SOX6	NC_056068.1	79315001	79335001	2.10476	0.212211	OL	TNKS1BP1
0.151427	OL	SOX6	NC_056068.1	79320001	79340001	2.82072	0.235944	OL	TNKS1BP1

0.157109	OL	SOX6	NC_056068.1	79325001	79345001	2.65416	0.206406	OL	TNKS1BP1
0.174136	OL	SOX6	NC_056068.1	79330001	79350001	2.05022	0.170862	OL	TNKS1BP1
0.179861	OL	SOX6	NC_056057.1	76415001	76435001	2.18481	0.184854	OL	TNS3
0.201946	OL	SOX6	NC_056057.1	76420001	76440001	3.32946	0.241761	OL	TNS3
0.248508	OL	SOX6	NC_056057.1	76425001	76445001	3.9212	0.252757	OL	TNS3
0.177665	OL	SOX6	NC_056057.1	76430001	76450001	4.351	0.232798	OL	TNS3
0.179349	OL	SOX6	NC_056057.1	76435001	76455001	4.9021	0.231977	OL	TNS3
0.210939	OL	SOX6	NC_056057.1	76440001	76460001	3.69908	0.189382	OL	TNS3
0.191987	OL	SOX6	NC_056056.1	8475001	8495001	2.67488	0.186193	OL	TOR2A;TTC16
0.256079	OL	SOX6	NC_056056.1	980001	1000001	2.55391	0.31294	OL	TRAF2
0.232512	OL	SOX6	NC_056056.1	985001	1005001	2.24741	0.293961	OL	TRAF2
0.165442	OL	SOX6	NC_056058.1	39070001	39090001	2.23126	0.28471	OL	TRIM58
0.169555	OL	SOX6	NC_056068.1	43645001	43665001	2.68663	0.201244	OL	TRIM66
0.174363	OL	SOX6	NC_056078.1	3300001	3320001	1.97974	0.248627	OL	TRIM67
0.18204	OL	SOX6	NC_056078.1	3305001	3325001	3.41219	0.349986	OL	TRIM67
0.201508	OL	SOX6	NC_056078.1	3310001	3330001	7.84615	0.452998	OL	TRIM67
0.210238	OL	SOX6	NC_056078.1	3315001	3335001	8.00001	0.506505	OL	TRIM67
0.214275	OL	SOX6	NC_056078.1	3320001	3340001	4.67657	0.451253	OL	TRIM67
0.205601	OL	SOX6	NC_056078.1	3325001	3345001	3.91403	0.437388	OL	TRIM67
0.18793	OL	SOX6	NC_056078.1	3330001	3350001	3.54009	0.419364	OL	TRIM67
0.169378	OL	SOX6	NC_056078.1	3335001	3355001	2.73357	0.398801	OL	TRIM67
0.188	OL	SOX6	NC_056072.1	7260001	7280001	2.3559	0.158261	OL	TRIM71
0.199026	OL	SOX6	NC_056072.1	7265001	7285001	4.27027	0.185035	OL	TRIM71
0.212893	OL	SOX6	NC_056080.1	123040001	123060001	2.15216	0.190881	OL	TRPC5
0.214396	OL	SOX6	NC_056080.1	123045001	123065001	2.54653	0.204558	OL	TRPC5
0.178686	OL	SOX6	NC_056074.1	23730001	23750001	2.47227	0.18566	OL	TSG101;UEVLD
0.158158	OL	SOX6	NC_056074.1	23735001	23755001	2.65991	0.210276	OL	TSG101;UEVLD
0.3316	OL	SOX9	NC_056056.1	102235001	102255001	2.73458	0.160438	OL	TSGA10
0.157213	OL	SP5	NC_056056.1	102240001	102260001	3.15506	0.158507	OL	TSGA10
0.202075	OL	SP5	NC_056056.1	102250001	102270001	3.89237	0.158438	OL	TSGA10
0.21004	OL	SPAST	NC_056056.1	102255001	102275001	3.38194	0.164536	OL	TSGA10

0.183252	OL	SPAST	NC_056056.1	102260001	102280001	5.35065	0.164519	OL	TSGA10
0.152251	OL	SPAST	NC_056056.1	102265001	102285001	5.52121	0.163533	OL	TSGA10
0.146112	OL	SPDL1	NC_056056.1	102270001	102290001	7.72385	0.166746	OL	TSGA10
0.170159	OL	SPESP1	NC_056073.1	16845001	16865001	6.27414	0.193957	OL	TTBK1
0.281734	OL	SPESP1	NC_056073.1	16850001	16870001	6.09677	0.247597	OL	TTBK1
0.187542	OL	SPICE1	NC_056073.1	16855001	16875001	8.84497	0.284614	OL	TTBK1
0.198214	OL	SPICE1	NC_056073.1	16860001	16880001	3.89634	0.241949	OL	TTBK1
0.213112	OL	SPICE1	NC_056059.1	67435001	67455001	2.49895	0.333903	OL	TXK
0.205543	OL	SPICE1	NC_056058.1	12745001	12765001	2.03235	0.276202	OL	TYK2
0.153295	OL	SPIDR	NC_056058.1	12750001	12770001	2.9899	0.272872	OL	TYK2
0.157143	OL	SPIDR	NC_056058.1	12755001	12775001	2.41375	0.224108	OL	TYK2
0.148167	OL	SPOCK1	NC_056079.1	41470001	41490001	2.22362	0.168115	OL	UBE2E1
0.212813	OL	SPOCK1	NC_056062.1	74915001	74935001	1.99753	0.238701	OL	UBR5
0.170257	OL	SPOCK1	NC_056062.1	74920001	74940001	2.34859	0.252121	OL	UBR5
0.152509	OL	SRC	NC_056062.1	74925001	74945001	2.04554	0.165152	OL	UBR5
0.187788	OL	SRC	NC_056055.1	117320001	117340001	2.06868	0.289565	OL	UGGT1
0.152391	OL	SRC	NC_056055.1	117325001	117345001	1.98886	0.267623	OL	UGGT1
0.170114	OL	SRFBP1	NC_056059.1	85060001	85080001	8.59539	0.209841	OL	UGT2B7
0.176085	OL	SRFBP1	NC_056064.1	33550001	33570001	2.51206	0.268062	OL	ULK2
0.188909	OL	SRFBP1	NC_056064.1	33555001	33575001	2.57373	0.273045	OL	ULK2
0.155039	OL	SRFBP1	NC_056064.1	33605001	33625001	2.19872	0.204023	OL	ULK2
0.167218	OL	SRPK2	NC_056057.1	16680001	16700001	2.22951	0.194707	OL	UMAD1
0.156269	OL	SRPK2	NC_056054.1	262990001	263010001	3.04236	0.232906	OL	UMODL1
0.155172	OL	SRPK2	NC_056054.1	262995001	263015001	5.45701	0.273657	OL	UMODL1
0.150172	OL	SRPK2	NC_056054.1	263000001	263020001	9.1028	0.295643	OL	UMODL1
0.164283	OL	SRPK2	NC_056054.1	263020001	263040001	21.7364	0.278801	OL	UMODL1
0.155909	OL	SRPK2	NC_056054.1	263025001	263045001	6.87021	0.199363	OL	UMODL1
0.15463	OL	SSBP3	NC_056060.1	54080001	54100001	2.04914	0.267356	OL	UNC13C
0.16285	OL	SSBP3	NC_056060.1	54085001	54105001	2.98722	0.261378	OL	UNC13C
0.189089	OL	SSBP3	NC_056060.1	54380001	54400001	2.34982	0.168701	OL	UNC13C
0.172144	OL	SSBP3	NC_056060.1	54385001	54405001	2.13092	0.165509	OL	UNC13C

0.319416	OL	ST3GAL1	NC_056060.1	54495001	54515001	2.33113	0.280834	OL	UNC13C
0.365159	OL	ST3GAL1	NC_056054.1	123795001	123815001	3.88821	0.237556	OL	URB1
0.421936	OL	ST3GAL1	NC_056054.1	123800001	123820001	4.63836	0.238976	OL	URB1
0.394275	OL	ST3GAL1	NC_056054.1	123805001	123825001	4.1064	0.259219	OL	URB1
0.379029	OL	ST3GAL1	NC_056054.1	123810001	123830001	3.82679	0.249256	OL	URB1
0.375346	OL	ST3GAL1	NC_056054.1	123815001	123835001	2.85157	0.194258	OL	URB1
0.366559	OL	ST3GAL1	NC_056054.1	123820001	123840001	2.14403	0.167938	OL	URB1
0.339648	OL	ST3GAL1	NC_056065.1	18485001	18505001	3.60544	0.163675	OL	USH2A
0.275947	OL	ST3GAL1	NC_056065.1	18490001	18510001	3.76401	0.179636	OL	USH2A
0.323617	OL	ST3GAL1	NC_056065.1	18500001	18520001	5.7376	0.163859	OL	USH2A
0.370396	OL	ST3GAL1	NC_056065.1	18510001	18530001	2.06021	0.159438	OL	USH2A
0.326628	OL	ST3GAL1	NC_056059.1	6405001	6425001	4.23977	0.164232	OL	USP53
0.159212	OL	ST8SIA4	NC_056059.1	6410001	6430001	2.84677	0.205994	OL	USP53
0.16284	OL	STARD13	NC_056059.1	6415001	6435001	2.5483	0.201485	OL	USP53
0.180588	OL	STARD13	NC_056080.1	40370001	40390001	2.73542	0.235175	OL	USP9X
0.163685	OL	STARD13	NC_056080.1	40375001	40395001	3.64932	0.282563	OL	USP9X
0.355319	OL	STAU2	NC_056080.1	40380001	40400001	3.67545	0.292016	OL	USP9X
0.403814	OL	STAU2	NC_056080.1	40385001	40405001	4.19332	0.297026	OL	USP9X
0.392173	OL	STAU2	NC_056080.1	40390001	40410001	4.00338	0.315152	OL	USP9X
0.420167	OL	STAU2	NC_056080.1	40395001	40415001	2.36	0.249668	OL	USP9X
0.426664	OL	STAU2	NC_056064.1	17975001	17995001	6.97658	0.179691	OL	UTP6
0.393696	OL	STAU2	NC_056064.1	17980001	18000001	5.45014	0.166545	OL	UTP6
0.368261	OL	STAU2	NC_056067.1	890001	910001	2.00771	0.214055	OL	VAC14
0.265181	OL	STAU2	NC_056067.1	895001	915001	2.19461	0.201171	OL	VAC14
0.173047	OL	STK38L	NC_056067.1	900001	920001	2.40586	0.218523	OL	VAC14
0.149546	OL	STK38L	NC_056067.1	905001	925001	2.81126	0.220373	OL	VAC14
0.196047	OL	STK38L	NC_056067.1	910001	930001	4.08008	0.269853	OL	VAC14
0.147031	OL	SUMO3	NC_056067.1	915001	935001	4.42308	0.268769	OL	VAC14
0.149271	OL	SYN3	NC_056067.1	920001	940001	3.99659	0.189938	OL	VAC14
0.272121	OL	SYNE1	NC_056067.1	955001	975001	2.43602	0.164718	OL	VAC14
0.253862	OL	SYNE1	NC_056062.1	77565001	77585001	3.51798	0.160633	OL	VPS13B

0.263653	OL	SYNE1	NC_056062.1	77665001	77685001	7.34439	0.159888	OL	VPS13B
0.229997	OL	SYNE1	NC_056062.1	77700001	77720001	2.66973	0.177518	OL	VPS13B
0.156954	OL	SYPL1	NC_056062.1	77705001	77725001	3.13888	0.204947	OL	VPS13B
0.307672	OL	SYS1	NC_056062.1	77710001	77730001	2.84437	0.187446	OL	VPS13B
0.358503	OL	SYS1;TP53T	NC_056062.1	77715001	77735001	2.68678	0.173062	OL	VPS13B
0.336852	OL	SYS1;TP53T	NC_056078.1	24730001	24750001	2.19149	0.165639	OL	VPS26A
0.22644	OL	SYS1;TP53T	NC_056078.1	24735001	24755001	2.03002	0.194381	OL	VPS26A
0.148551	OL	SYT14	NC_056070.1	53170001	53190001	2.40086	0.174811	OL	VPS33A
0.186282	OL	SYT14	NC_056054.1	203195001	203215001	2.25048	0.168894	OL	VPS8
0.175976	OL	SYT14	NC_056074.1	535001	555001	3.65853	0.163839	OL	VSTM5
0.185482	OL	SYT14	NC_056061.1	66885001	66905001	3.57416	0.175967	OL	VTA1
0.178183	OL	SYT14	NC_056061.1	66890001	66910001	4.37474	0.174732	OL	VTA1
0.171036	OL	SYT14	NC_056061.1	66895001	66915001	5.12151	0.19431	OL	VTA1
0.21086	OL	SYT14	NC_056056.1	103010001	103030001	4.03704	0.179049	OL	VWA3B
0.218116	OL	SYT14	NC_056056.1	103015001	103035001	3.43032	0.196681	OL	VWA3B
0.216065	OL	SYT14	NC_056056.1	103020001	103040001	2.7112	0.190185	OL	VWA3B
0.235673	OL	SYT14	NC_056056.1	103025001	103045001	2.38572	0.1809	OL	VWA3B
0.197163	OL	SYT14	NC_056056.1	103035001	103055001	2.04536	0.166433	OL	VWA3B
0.28937	OL	TAB1	NC_056063.1	12420001	12440001	2.23293	0.176571	OL	VWA8
0.299745	OL	TBC1D4	NC_056074.1	36340001	36360001	1.97391	0.197039	OL	VWCE
0.378538	OL	TBC1D4	NC_056074.1	36345001	36365001	4.3431	0.321739	OL	VWCE
0.279259	OL	TBC1D4	NC_056056.1	210035001	210055001	3.98213	0.168161	OL	VWF
0.165904	OL	TBC1D4	NC_056056.1	210040001	210060001	2.55545	0.180528	OL	VWF
0.171067	OL	TCEAL9	NC_056057.1	89595001	89615001	3.65719	0.184358	OL	WASL
0.221178	OL	TEC	NC_056057.1	89600001	89620001	2.75365	0.158434	OL	WASL
0.277851	OL	TEC	NC_056078.1	42070001	42090001	2.34398	0.184772	OL	WDFY4
0.294408	OL	TEC	NC_056071.1	28505001	28525001	2.05755	0.197855	OL	WDR61
0.302993	OL	TEC	NC_056071.1	28510001	28530001	2.26817	0.178269	OL	WDR61
0.210874	OL	TEC	NC_056076.1	56575001	56595001	1.96646	0.162405	OL	WDR7
0.19954	OL	TEC	NC_056069.1	36920001	36940001	2.10872	0.218282	OL	WDR70
0.190804	OL	TEC	NC_056069.1	36925001	36945001	2.40822	0.215914	OL	WDR70

0.155639	OL	TECRL	NC_056060.1	54845001	54865001	3.86007	0.205288	OL	WDR72
0.146808	OL	TECRL	NC_056060.1	54850001	54870001	3.1598	0.195178	OL	WDR72
0.147938	OL	TECRL	NC_056060.1	54855001	54875001	2.93642	0.190801	OL	WDR72
0.1504	OL	TECRL	NC_056056.1	213955001	213975001	2.28886	0.199618	OL	WNT5B
0.161575	OL	TECRL	NC_056056.1	213960001	213980001	3.37021	0.298519	OL	WNT5B
0.159162	OL	TECRL	NC_056056.1	213965001	213985001	5.9086	0.413076	OL	WNT5B
0.15756	OL	TECRL	NC_056056.1	213970001	213990001	7.30278	0.463921	OL	WNT5B
0.155175	OL	TECRL	NC_056056.1	213975001	213995001	6.05796	0.43532	OL	WNT5B
0.154702	OL	TECRL	NC_056056.1	213980001	214000001	3.34794	0.348807	OL	WNT5B
0.153296	OL	TECRL	NC_056056.1	213985001	214005001	2.14804	0.20142	OL	WNT5B
0.153982	OL	TECRL	NC_056072.1	12490001	12510001	3.03214	0.261672	OL	XIRP1
0.148084	OL	TECRL	NC_056072.1	12495001	12515001	2.7463	0.210491	OL	XIRP1
0.147095	OL	TECRL	NC_056072.1	12505001	12525001	2.66729	0.19829	OL	XIRP1
0.152999	OL	TECRL	NC_056055.1	49885001	49905001	2.77165	0.15864	OL	XPA
0.146078	OL	TECRL	NC_056055.1	49890001	49910001	4.20376	0.206281	OL	XPA
0.152244	OL	TECRL	NC_056055.1	49895001	49915001	3.75149	0.193165	OL	XPA
0.147106	OL	TECRL	NC_056055.1	49900001	49920001	3.69552	0.186003	OL	XPA
0.152728	OL	TECRL	NC_056058.1	80565001	80585001	2.20498	0.160748	OL	XRCC4
0.148607	OL	TECRL	NC_056056.1	144730001	144750001	2.17122	0.205714	OL	YAF2
0.14999	OL	TECRL	NC_056056.1	144735001	144755001	2.07031	0.168406	OL	YAF2
0.148699	OL	TECRL	NC_056080.1	47655001	47675001	12.3571	0.379129	OL	ZC3H12B
0.147579	OL	TENM1	NC_056068.1	19830001	19850001	6.50486	0.379629	OL	ZC3H12C
0.16591	OL	TENM1	NC_056080.1	46950001	46970001	4.86957	0.187643	OL	ZC4H2
0.175461	OL	TGFBR2	NC_056080.1	46955001	46975001	4.25984	0.191396	OL	ZC4H2
0.160691	OL	THSD4	NC_056080.1	46960001	46980001	3.89763	0.164648	OL	ZC4H2
0.295242	OL	TIAM1	NC_056061.1	81755001	81775001	3.55019	0.217779	OL	ZDHHC14
0.307396	OL	TIAM1	NC_056061.1	81760001	81780001	5.17427	0.289626	OL	ZDHHC14
0.291667	OL	TIAM1	NC_056061.1	81765001	81785001	4.66992	0.315028	OL	ZDHHC14
0.29532	OL	TIAM2	NC_056061.1	81770001	81790001	3.58123	0.330718	OL	ZDHHC14
0.352585	OL	TIAM2	NC_056061.1	81775001	81795001	2.49838	0.270605	OL	ZDHHC14
0.331279	OL	TIAM2	NC_056080.1	67510001	67530001	3.71224	0.245833	OL	ZDHHC15

0.420952	OL	TIAM2	NC_056080.1	67525001	67545001	6.77069	0.195447	OL	ZDHHC15
0.333356	OL	TIAM2	NC_056080.1	67535001	67555001	6.37968	0.16246	OL	ZDHHC15
0.296621	OL	TIAM2	NC_056080.1	67540001	67560001	4.59482	0.177083	OL	ZDHHC15
0.260498	OL	TIAM2	NC_056080.1	67545001	67565001	5.07041	0.19133	OL	ZDHHC15
0.275501	OL	TIAM2	NC_056080.1	67550001	67570001	3.09345	0.202016	OL	ZDHHC15
0.178706	OL	TLL2	NC_056080.1	67555001	67575001	3.84568	0.186866	OL	ZDHHC15
0.15019	OL	TM4SF4	NC_056055.1	83245001	83265001	5.67122	0.219636	OL	ZDHHC21
0.147243	OL	TMEFF2	NC_056055.1	83250001	83270001	5.67532	0.204748	OL	ZDHHC21
0.191647	OL	TMEFF2	NC_056055.1	83255001	83275001	3.46275	0.166217	OL	ZDHHC21
0.163937	OL	TMEFF2	NC_056055.1	83260001	83280001	3.88039	0.182134	OL	ZDHHC21
0.155623	OL	TMEFF2	NC_056062.1	72180001	72200001	2.18506	0.294146	OL	ZFPM2
0.161217	OL	TMEFF2	NC_056062.1	72185001	72205001	2.24709	0.330096	OL	ZFPM2
0.153272	OL	TMEFF2	NC_056062.1	72190001	72210001	1.96916	0.325986	OL	ZFPM2
0.183721	OL	TMEM132B	NC_056055.1	27370001	27390001	6.16341	0.297465	OL	ZNF169
0.20421	OL	TMEM132B	NC_056055.1	27375001	27395001	4.64876	0.306618	OL	ZNF169
0.21525	OL	TMEM132B	NC_056055.1	27380001	27400001	3.18473	0.312111	OL	ZNF169
0.21665	OL	TMEM132B	NC_056055.1	130500001	130520001	2.12442	0.157515	OL	ZNF385B
0.16703	OL	TMEM132B	NC_056080.1	57425001	57445001	2.26476	0.210564	OL	ZNF41
0.179285	OL	TMEM132B	NC_056077.1	4395001	4415001	3.64	0.187843	OL	ZNF500
0.184464	OL	TMEM132B	NC_056055.1	9710001	9730001	4.30631	0.200397	OL	ZNF618
0.162099	OL	TMEM132B	NC_056079.1	32070001	32090001	2.66667	0.19519	OL	ZNF703
0.154767	OL	TMEM132B	NC_056057.1	74925001	74945001	5.14131	0.209894	OL	ZNF804B
0.158704	OL	TMEM132B	NC_056057.1	74930001	74950001	6.4021	0.208421	OL	ZNF804B
0.155632	OL	TMEM132B	NC_056057.1	74935001	74955001	2.92957	0.18008	OL	ZNF804B
0.178784	OL	TMEM132B							
0.264087	OL	TMEM132B							
0.291014	OL	TMEM132B							
0.199735	OL	TMEM241							
0.225471	OL	TMEM241							
0.235376	OL	TMEM241							
0.246826	OL	TMEM241							

0.221687	OL	TMEM42
0.235472	OL	TMEM42
0.175002	OL	TMEM42;ZDHHC3
0.331866	OL	TMEM59L
0.282072	OL	TMEM59L
0.159708	OL	TMEM63C
0.197426	OL	TMEM63C
0.213146	OL	TMEM63C
0.218036	OL	TMEM63C
0.190235	OL	TMPRSS3
0.15111	OL	TMTC2
0.158888	OL	TMTC2
0.197424	OL	TMTC2
0.178388	OL	TMTC2
0.228817	OL	TMTC2
0.214043	OL	TMTC2
0.180646	OL	TMTC2
0.163733	OL	TMTC2
0.182403	OL	TMTC2
0.185556	OL	TMTC2
0.175401	OL	TMTC2
0.189881	OL	TMTC2
0.180914	OL	TNFSF4
0.15017	OL	TNFSF4
0.213999	OL	TNR
0.174338	OL	TNR
0.166323	OL	TNR
0.178076	OL	TNR
0.153141	OL	TNR
0.187591	OL	TNR
0.181159	OL	TNS4

0.216049	OL	TNS4
0.243056	OL	TNS4
0.195478	OL	TOM1L2
0.21006	OL	TOM1L2
0.258765	OL	TRAF6
0.251633	OL	TRAF6
0.214336	OL	TRAF6
0.151361	OL	TRAF6
0.147054	OL	TRAPPC11
0.161948	OL	TRAPPC11
0.14696	OL	TRAPPC11
0.168384	OL	TRIM27
0.388248	OL	TRIM34
0.36345	OL	TRIM34
0.279246	OL	TRIM34
0.183053	OL	TRIM71
0.422997	OL	TRIM72
0.448249	OL	TRIM72
0.485309	OL	TRIM72
0.421259	OL	TRIM72
0.151933	OL	TRIM9
0.164832	OL	TRIM9
0.176736	OL	TRIM9
0.174352	OL	TRIM9
0.175202	OL	TRIM9
0.201437	OL	TRIM9
0.208349	OL	TRIM9
0.151651	OL	TRIO
0.173849	OL	TRIO
0.271791	OL	TSG101
0.289243	OL	TSG101

0.308588	OL	TSG101
0.301354	OL	TSG101
0.289379	OL	TSG101
0.274307	OL	TSG101
0.205009	OL	TSG101
0.212044	OL	TSG101;UEVLD
0.231649	OL	TSG101;UEVLD
0.168328	OL	TTC8
0.185692	OL	TTC8
0.152767	OL	TTC8
0.185538	OL	TTC8
0.206344	OL	TTLL5
0.214793	OL	TTLL5
0.225748	OL	TTLL5
0.189532	OL	TTLL5
0.188066	OL	TTLL5
0.221073	OL	TTLL5
0.231472	OL	TTLL5
0.250227	OL	TTLL5
0.232163	OL	TTLL5
0.267008	OL	TTLL5
0.291575	OL	TTLL5
0.26756	OL	TTLL5
0.308178	OL	UBE2J1
0.318819	OL	UBE2J1
0.220897	OL	UBE2J1
0.185949	OL	UBE2J1
0.193753	OL	UBE2J1
0.225468	OL	UBXN10
0.162903	OL	ULK4
0.154353	OL	ULK4

0.17912	OL	ULK4
0.187892	OL	ULK4
0.218993	OL	ULK4
0.189855	OL	ULK4
0.150083	OL	UMODL1
0.213907	OL	UMODL1
0.198175	OL	UMODL1
0.164109	OL	UMODL1
0.182237	OL	UNC13C
0.212715	OL	UNC13C
0.165527	OL	UNC13C
0.160087	OL	UNC13C
0.155465	OL	UNC13C
0.189676	OL	UNC13C
0.484306	OL	UNC13C
0.433647	OL	UNC13C
0.146761	OL	USH2A
0.168333	OL	USP22
0.164059	OL	USP32
0.190078	OL	USP32
0.182561	OL	USP53
0.184208	OL	UST
0.24079	OL	UST
0.245124	OL	UST
0.24573	OL	UST
0.148545	OL	UST
0.161576	OL	UTRN
0.151762	OL	UTRN
0.18612	OL	UTRN
0.177324	OL	UTRN
0.175644	OL	UTRN

0.2426	OL	VAC14
0.219173	OL	VAC14
0.215727	OL	VAC14
0.19184	OL	VAC14
0.189754	OL	VAC14
0.18486	OL	VAC14
0.226488	OL	VCPKMT
0.291374	OL	VCPKMT
0.332788	OL	VCPKMT
0.206853	OL	VIPR2
0.246811	OL	VIPR2
0.243195	OL	VIPR2
0.145909	OL	VIRMA
0.163897	OL	VIRMA
0.166176	OL	VPS13B
0.178748	OL	VPS13B
0.149759	OL	VPS13B
0.229675	OL	VPS13B
0.268724	OL	VPS13B
0.268403	OL	VPS13B
0.267541	OL	VPS13B
0.224586	OL	VPS13B
0.166998	OL	VPS13B
0.149211	OL	VPS33A
0.183403	OL	VPS33A
0.21813	OL	VSTM2A
0.278241	OL	VSTM2A
0.289713	OL	VSTM2A
0.169056	OL	WDR11
0.211568	OL	WDR11
0.252581	OL	WDR11

0.247649	OL	WDR11
0.248798	OL	WDR11
0.245617	OL	WDR11
0.198972	OL	WDR64
0.226903	OL	WDR64
0.241598	OL	WDR64
0.173529	OL	WDR76
0.159606	OL	WFDC1
0.262503	OL	YAP1
0.151281	OL	YIPF4
0.157505	OL	ZBTB20
0.186711	OL	ZBTB20
0.181004	OL	ZBTB20
0.165925	OL	ZBTB20
0.151155	OL	ZBTB21
0.188628	OL	ZC3H12C
0.243673	OL	ZC3H12C
0.286972	OL	ZC3H12C
0.312255	OL	ZC3H12C
0.28047	OL	ZC3H12C
0.270591	OL	ZC3H12C
0.243848	OL	ZC3H12C
0.213792	OL	ZC3HAV1
0.15914	OL	ZC3HAV1
0.186348	OL	ZC3HAV1;ZC3HAV1L
0.198355	OL	ZC4H2
0.202695	OL	ZC4H2
0.152915	OL	ZC4H2
0.213465	OL	ZC4H2
0.247947	OL	ZC4H2
0.254919	OL	ZDHHC21

0.211452	OL	ZDHHC21
0.185563	OL	ZDHHC21
0.147523	OL	ZFHX3
0.157155	OL	ZFHX3
0.154954	OL	ZFPM1;ZNF469
0.165654	OL	ZFYVE28
0.213828	OL	ZFYVE28
0.20544	OL	ZFYVE28
0.15417	OL	ZFYVE28
0.198244	OL	ZFYVE28
0.211972	OL	ZFYVE28
0.188031	OL	ZFYVE28
0.201114	OL	ZFYVE28
0.333843	OL	ZFYVE9
0.290415	OL	ZFYVE9
0.254519	OL	ZFYVE9
0.21399	OL	ZFYVE9
0.151797	OL	ZFYVE9
0.209133	OL	ZFYVE9
0.238624	OL	ZFYVE9
0.149851	OL	ZFYVE9
0.195203	OL	ZMIZ1
0.146668	OL	ZMIZ1
0.18642	OL	ZMPSTE24
0.199376	OL	ZMPSTE24
0.216616	OL	ZMPSTE24
0.177682	OL	ZMPSTE24
0.230555	OL	ZNF140
0.162736	OL	ZNF140
0.299451	OL	ZNF292
0.390716	OL	ZNF292

0.465543	OL	ZNF292
0.455035	OL	ZNF292
0.38296	OL	ZNF292
0.181141	OL	ZNF322
0.16575	OL	ZNF385B
0.150235	OL	ZNF469
0.216301	OL	ZNF469
0.297715	OL	ZNF469
0.180507	OL	ZNF469
0.167231	OL	ZNF704

395

4108841

5611202

5615366

5615366

)5615366

)5615366

3P2
