# Supplementary material for: Genetic diversity and selection of Tibetan sheep breeds revealed by whole-genome resequencing
Source: Anim Biosci. 2023 May 2;36(7):991–1002. doi: 10.5713/ab.22.0432 (PMC10330983; doi:10.5713/ab.22.0432)
Supplement: Supplementary file 11 [file ab-22-0432-Supplementary-Table-11.pdf]

Supplementary Table11.Putative selection regions associated with horn phenotypes among different breeds

OL vs SG1							SG1vs SG2		
CHROM	Start	end	Pi	Fst	Region	Gene	CHROM	Start	end
NC_056068.1	29460001	29480001	0.216667	0.276636	SG1	ABCG4;NLRX1	NC_056054.1	71130001	71150001
NC_056068.1	29465001	29485001	0.101015	0.247821	SG1	ABCG4;NLRX1	NC_056054.1	263215001	263235001
NC_056061.1	64050001	64070001	0.399738	0.214272	SG1	ABRACL	NC_056054.1	177700001	177720001
NC_056068.1	63600001	63620001	0.414554	0.377649	SG1	ABTB2	NC_056071.1	19905001	19925001
NC_056068.1	63605001	63625001	0.247739	0.444128	SG1	ABTB2	NC_056071.1	19910001	19930001
NC_056068.1	63610001	63630001	0.230405	0.424002	SG1	ABTB2	NC_056071.1	19915001	19935001
NC_056068.1	63615001	63635001	0.255218	0.386337	SG1	ABTB2	NC_056071.1	19920001	19940001
NC_056068.1	63620001	63640001	0.415094	0.374799	SG1	ABTB2	NC_056071.1	19925001	19945001
NC_056070.1	63105001	63125001	0.094065	0.232668	SG1	ACADS	NC_056071.1	19930001	19950001
NC_056070.1	63110001	63130001	0.243347	0.259242	SG1	ACADS	NC_056071.1	19950001	19970001
NC_056070.1	63115001	63135001	0.276162	0.254165	SG1	ACADS	NC_056071.1	19955001	19975001
NC_056070.1	63120001	63140001	0.393451	0.194612	SG1	ACADS	NC_056055.1	205385001	205405001
NC_056070.1	63100001	63120001	0.119612	0.219107	SG1	ACADS;UNC119B	NC_056075.1	34565001	34585001
NC_056068.1	17435001	17455001	0.418265	0.231746	SG1	ACAT1	NC_056075.1	34570001	34590001
NC_056068.1	17440001	17460001	0.377738	0.264167	SG1	ACAT1	NC_056059.1	115045001	115065001
NC_056058.1	78885001	78905001	0.392903	0.300174	SG1	ACOT12	NC_056059.1	115055001	115075001
NC_056058.1	78870001	78890001	0.192414	0.353278	SG1	ACOT12;ZCCHC9	NC_056059.1	115060001	115080001
NC_056058.1	78875001	78895001	0.230047	0.376247	SG1	ACOT12;ZCCHC9	NC_056059.1	115065001	115085001
NC_056058.1	78880001	78900001	0.281004	0.369155	SG1	ACOT12;ZCCHC9	NC_056059.1	115075001	115095001
NC_056056.1	105245001	1.05E+08	0.35794	0.19995	SG1	ACOXL	NC_056059.1	115080001	115100001
NC_056056.1	105250001	1.05E+08	0.362207	0.204645	SG1	ACOXL	NC_056059.1	115085001	115105001
NC_056056.1	105255001	1.05E+08	0.388499	0.205186	SG1	ACOXL	NC_056059.1	115170001	115190001
NC_056056.1	105260001	1.05E+08	0.304418	0.240449	SG1	ACOXL	NC_056059.1	115175001	115195001
NC_056056.1	105285001	1.05E+08	0.417666	0.196577	SG1	ACOXL;BCL2L11	NC_056059.1	115180001	115200001
NC_056067.1	48170001	48190001	0.418973	0.220983	SG1	ACP7	NC_056075.1	43890001	43910001
NC_056067.1	48175001	48195001	0.409941	0.191344	SG1	ACP7	NC_056066.1	17110001	17130001
NC_056058.1	66750001	66770001	0.377858	0.383604	SG1	ADAM19	NC_056066.1	17115001	17135001
NC_056058.1	66755001	66775001	0.453729	0.361277	SG1	ADAM19	NC_056066.1	17095001	17115001
NC_056056.1	143165001	1.43E+08	0.401615	0.19074	SG1	ADAMTS20	NC_056066.1	17100001	17120001

NC_056059.1	88200001	88220001	0.288732	0.186809	SG1	ADAMTS3	NC_056066.1	17105001	17125001
NC_056059.1	88205001	88225001	0.238277	0.243298	SG1	ADAMTS3	NC_056063.1	52665001	52685001
NC_056059.1	88210001	88230001	0.299156	0.262534	SG1	ADAMTS3	NC_056056.1	105105001	105125001
NC_056059.1	88215001	88235001	0.447741	0.226703	SG1	ADAMTS3	NC_056056.1	105110001	105130001
NC_056071.1	28160001	28180001	0.225479	0.219313	SG1	ADAMTS7	NC_056056.1	105115001	105135001
NC_056071.1	28165001	28185001	0.12771	0.199984	SG1	ADAMTS7	NC_056055.1	250001	270001
NC_056067.1	2350001	2370001	0.266783	0.207255	SG1	ADAT1;GABARAPL2	NC_056055.1	255001	275001
NC_056067.1	2360001	2380001	0.137888	0.209773	SG1	ADAT1;GABARAPL2	NC_056072.1	47140001	47160001
NC_056059.1	77480001	77500001	0.474693	0.219757	SG1	ADGRL3	NC_056056.1	18825001	18845001
NC_056059.1	77485001	77505001	0.423937	0.261616	SG1	ADGRL3	NC_056056.1	18830001	18850001
NC_056059.1	77490001	77510001	0.464002	0.257059	SG1	ADGRL3	NC_056056.1	18835001	18855001
NC_056059.1	77660001	77680001	0.473799	0.308401	SG1	ADGRL3	NC_056054.1	130175001	130195001
NC_056059.1	77665001	77685001	0.371076	0.359143	SG1	ADGRL3	NC_056054.1	130180001	130200001
NC_056059.1	77670001	77690001	0.321215	0.357311	SG1	ADGRL3	NC_056058.1	14905001	14925001
NC_056059.1	77675001	77695001	0.20213	0.294657	SG1	ADGRL3	NC_056058.1	14915001	14935001
NC_056059.1	77680001	77700001	0.254189	0.206301	SG1	ADGRL3	NC_056058.1	14920001	14940001
NC_056059.1	77685001	77705001	0.322578	0.187515	SG1	ADGRL3	NC_056058.1	14885001	14905001
NC_056059.1	77695001	77715001	0.329049	0.18942	SG1	ADGRL3	NC_056058.1	14890001	14910001
NC_056059.1	77770001	77790001	0.133686	0.183699	SG1	ADGRL3	NC_056058.1	14895001	14915001
NC_056058.1	88300001	88320001	0.096508	0.215323	SG1	ADGRV1	NC_056058.1	14900001	14920001
NC_056058.1	88305001	88325001	0.060366	0.240191	SG1	ADGRV1	NC_056069.1	40320001	40340001
NC_056058.1	88310001	88330001	0.145511	0.199471	SG1	ADGRV1	NC_056069.1	40325001	40345001
NC_056056.1	196075001	1.96E+08	0.433707	0.19615	SG1	AEBP2;LOC1069903	NC_056069.1	40330001	40350001
NC_056075.1	34365001	34385001	0.078886	0.226879	SG1	AFAP1L2	NC_056069.1	40335001	40355001
NC_056075.1	34370001	34390001	0.1842	0.24649	SG1	AFAP1L2	NC_056069.1	40355001	40375001
NC_056075.1	34375001	34395001	0.399538	0.281941	SG1	AFAP1L2	NC_056071.1	5810001	5830001
NC_056075.1	34285001	34305001	0.143406	0.290881	SG1	AFAP1L2;VWA2	NC_056071.1	6010001	6030001
NC_056071.1	16450001	16470001	0.362262	0.218431	SG1	AGBL1	NC_056071.1	6015001	6035001
NC_056071.1	16455001	16475001	0.315138	0.274998	SG1	AGBL1	NC_056071.1	6020001	6040001
NC_056071.1	16460001	16480001	0.326316	0.306379	SG1	AGBL1	NC_056071.1	6100001	6120001
NC_056069.1	39280001	39300001	0.430758	0.195179	SG1	AGXT2	NC_056071.1	6105001	6125001
NC_056069.1	39285001	39305001	0.328284	0.209299	SG1	AGXT2	NC_056054.1	265660001	265680001

NC_056069.1	39290001	39310001	0.26607	0.200123	SG1	AGXT2	NC_056054.1	265665001	265685001
NC_056069.1	39295001	39315001	0.23322	0.196966	SG1	AGXT2	NC_056054.1	265705001	265725001
NC_056061.1	60580001	60600001	0.436205	0.271288	SG1	AHI1	NC_056054.1	265710001	265730001
NC_056061.1	60585001	60605001	0.378186	0.296517	SG1	AHI1	NC_056054.1	265715001	265735001
NC_056061.1	60590001	60610001	0.314965	0.312043	SG1	AHI1	NC_056054.1	265720001	265740001
NC_056061.1	60595001	60615001	0.241004	0.282441	SG1	AHI1	NC_056054.1	265725001	265745001
NC_056061.1	60600001	60620001	0.272993	0.286672	SG1	AHI1	NC_056066.1	45740001	45760001
NC_056061.1	60605001	60625001	0.32683	0.251063	SG1	AHI1	NC_056066.1	45745001	45765001
NC_056061.1	60610001	60630001	0.474089	0.184561	SG1	AHI1	NC_056066.1	45750001	45770001
NC_056061.1	60690001	60710001	0.150243	0.192916	SG1	AHI1	NC_056066.1	45755001	45775001
NC_056074.1	37345001	37365001	0.144928	0.228913	SG1	AHNAK	NC_056066.1	45760001	45780001
NC_056074.1	37350001	37370001	0.292005	0.185008	SG1	AHNAK	NC_056066.1	45765001	45785001
NC_056074.1	37330001	37350001	0.148957	0.334934	SG1	AHNAK;SCGB1A1	NC_056066.1	45770001	45790001
NC_056080.1	114020001	1.14E+08	0.435256	0.206284	SG1	AIFM1;RAB33A	NC_056054.1	120050001	120070001
NC_056071.1	15610001	15630001	0.304449	0.252291	SG1	AKAP13	NC_056054.1	120055001	120075001
NC_056071.1	15615001	15635001	0.122957	0.341805	SG1	AKAP13	NC_056054.1	120060001	120080001
NC_056071.1	15620001	15640001	0.037149	0.382097	SG1	AKAP13	NC_056054.1	120065001	120085001
NC_056071.1	15640001	15660001	0.044081	0.38053	SG1	AKAP13	NC_056054.1	120070001	120090001
NC_056061.1	60235001	60255001	0.18851	0.208027	SG1	ALDH8A1	NC_056054.1	120075001	120095001
NC_056061.1	60240001	60260001	0.227383	0.221187	SG1	ALDH8A1	NC_056054.1	120080001	120100001
NC_056061.1	60245001	60265001	0.264744	0.235514	SG1	ALDH8A1	NC_056054.1	120085001	120105001
NC_056061.1	60250001	60270001	0.254771	0.290855	SG1	ALDH8A1	NC_056054.1	120090001	120110001
NC_056061.1	60255001	60275001	0.252495	0.30196	SG1	ALDH8A1	NC_056054.1	120095001	120115001
NC_056061.1	60260001	60280001	0.314545	0.28998	SG1	ALDH8A1;HBS1L	NC_056054.1	120100001	120120001
NC_056061.1	60265001	60285001	0.372855	0.267552	SG1	ALDH8A1;HBS1L	NC_056054.1	120105001	120125001
NC_056061.1	60270001	60290001	0.398308	0.239914	SG1	ALDH8A1;HBS1L	NC_056054.1	188545001	188565001
NC_056064.1	34170001	34190001	0.319392	0.189474	SG1	ALKBH5	NC_056057.1	66875001	66895001
NC_056064.1	34160001	34180001	0.454546	0.192846	SG1	ALKBH5;LLGL1	NC_056073.1	20165001	20185001
NC_056064.1	34175001	34195001	0.359684	0.274191	SG1	ALKBH5;MYO15A	NC_056073.1	20170001	20190001
NC_056064.1	34180001	34200001	0.344087	0.186779	SG1	ALKBH5;MYO15A	NC_056067.1	25125001	25145001
NC_056059.1	13575001	13595001	0.443444	0.186363	SG1	ALPK1	NC_056078.1	29440001	29460001
NC_056080.1	125030001	1.25E+08	0.432836	0.319972	SG1	AMMECR1	NC_056078.1	29445001	29465001

NC_056080.1	125035001	1.25E+08	0.336674	0.283499	SG1	AMMECR1	NC_056078.1	29450001	29470001
NC_056080.1	125040001	1.25E+08	0.366853	0.254003	SG1	AMMECR1	NC_056078.1	18165001	18185001
NC_056057.1	84045001	84065001	0.423013	0.234453	SG1	AMPH	NC_056078.1	18170001	18190001
NC_056057.1	84040001	84060001	0.458738	0.187521	SG1	AMPH;LOC10561323	NC_056078.1	18175001	18195001
NC_056076.1	33325001	33345001	0.350357	0.210194	SG1	ANKRD29	NC_056058.1	69130001	69150001
NC_056076.1	33345001	33365001	0.247602	0.207506	SG1	ANKRD29	NC_056058.1	69135001	69155001
NC_056076.1	33350001	33370001	0.211447	0.210623	SG1	ANKRD29	NC_056058.1	69140001	69160001
NC_056060.1	6670001	6690001	0.471709	0.192811	SG1	ANKRD31	NC_056075.1	31105001	31125001
NC_056060.1	6675001	6695001	0.434196	0.269505	SG1	ANKRD31	NC_056075.1	31110001	31130001
NC_056056.1	163290001	1.63E+08	0.40075	0.191659	SG1	ANKRD52	NC_056056.1	104200001	104220001
NC_056056.1	163310001	1.63E+08	0.350816	0.18381	SG1	ANKRD52;NABP2;RI	NC_056056.1	104205001	104225001
NC_056056.1	163295001	1.63E+08	0.378459	0.186592	SG1	ANKRD52;SLC39A5	NC_056054.1	240205001	240225001
NC_056061.1	48060001	48080001	0.465784	0.375327	SG1	ANKRD6	NC_056054.1	240210001	240230001
NC_056061.1	48065001	48085001	0.436981	0.363591	SG1	ANKRD6	NC_056054.1	240215001	240235001
NC_056061.1	48070001	48090001	0.369719	0.299573	SG1	ANKRD6	NC_056054.1	240250001	240270001
NC_056061.1	48075001	48095001	0.393838	0.196972	SG1	ANKRD6	NC_056065.1	47925001	47945001
NC_056056.1	168505001	1.69E+08	0.156863	0.199191	SG1	ANKS1B	NC_056065.1	47930001	47950001
NC_056056.1	168530001	1.69E+08	0.205989	0.256246	SG1	ANKS1B	NC_056065.1	47935001	47955001
NC_056056.1	168535001	1.69E+08	0.212064	0.262707	SG1	ANKS1B	NC_056065.1	47940001	47960001
NC_056056.1	168540001	1.69E+08	0.278542	0.232805	SG1	ANKS1B	NC_056065.1	47945001	47965001
NC_056056.1	168545001	1.69E+08	0.267103	0.236175	SG1	ANKS1B	NC_056065.1	47950001	47970001
NC_056055.1	202915001	2.03E+08	0.40819	0.203622	SG1	AOX1	NC_056065.1	47955001	47975001
NC_056080.1	13985001	14005001	0.438007	0.310934	SG1	AP1S2	NC_056071.1	15845001	15865001
NC_056080.1	13990001	14010001	0.439053	0.317892	SG1	AP1S2	NC_056071.1	15850001	15870001
NC_056080.1	13995001	14015001	0.411299	0.323406	SG1	AP1S2	NC_056057.1	84090001	84110001
NC_056080.1	14000001	14020001	0.442918	0.337486	SG1	AP1S2	NC_056058.1	13470001	13490001
NC_056080.1	14005001	14025001	0.419441	0.320756	SG1	AP1S2	NC_056058.1	13475001	13495001
NC_056080.1	14010001	14030001	0.417021	0.305126	SG1	AP1S2	NC_056057.1	10445001	10465001
NC_056080.1	13970001	13990001	0.470587	0.248146	SG1	AP1S2;ZRSR2	NC_056057.1	10450001	10470001
NC_056080.1	13980001	14000001	0.427874	0.297013	SG1	AP1S2;ZRSR2	NC_056057.1	10455001	10475001
NC_056064.1	14370001	14390001	0.432544	0.220187	SG1	AP2B1	NC_056057.1	10460001	10480001
NC_056064.1	14375001	14395001	0.417151	0.183242	SG1	AP2B1	NC_056057.1	10465001	10485001

NC_056064.1	14360001	14380001	0.346648	0.217701	SG1	AP2B1;RASL10B	NC_056057.1	10470001	10490001
NC_056064.1	14365001	14385001	0.396211	0.250905	SG1	AP2B1;RASL10B	NC_056075.1	10320001	10340001
NC_056067.1	53695001	53715001	0.398337	0.272748	SG1	AP2S1	NC_056065.1	50425001	50445001
NC_056067.1	53700001	53720001	0.363844	0.22886	SG1	AP2S1;ARHGAP35	NC_056057.1	84410001	84430001
NC_056060.1	9350001	9370001	0.44354	0.215741	SG1	AP3B1	NC_056057.1	84415001	84435001
NC_056060.1	9355001	9375001	0.399121	0.206553	SG1	AP3B1	NC_056057.1	84420001	84440001
NC_056059.1	56365001	56385001	0.386724	0.200236	SG1	ARAP2	NC_056057.1	84400001	84420001
NC_056066.1	32830001	32850001	0.445545	0.207582	SG1	ARHGAP12	NC_056057.1	84405001	84425001
NC_056066.1	32835001	32855001	0.227462	0.276332	SG1	ARHGAP12	NC_056057.1	62405001	62425001
NC_056068.1	20295001	20315001	0.441899	0.283181	SG1	ARHGAP20	NC_056057.1	62415001	62435001
NC_056058.1	51705001	51725001	0.470954	0.211259	SG1	ARHGAP26	NC_056068.1	55635001	55655001
NC_056058.1	51710001	51730001	0.340644	0.240489	SG1	ARHGAP26	NC_056057.1	62245001	62265001
NC_056058.1	51715001	51735001	0.228814	0.268602	SG1	ARHGAP26	NC_056057.1	62250001	62270001
NC_056058.1	51720001	51740001	0.285987	0.237919	SG1	ARHGAP26	NC_056057.1	62255001	62275001
NC_056058.1	51725001	51745001	0.354838	0.236795	SG1	ARHGAP26	NC_056057.1	62260001	62280001
NC_056058.1	51780001	51800001	0.315519	0.250826	SG1	ARHGAP26	NC_056057.1	62265001	62285001
NC_056054.1	184940001	1.85E+08	0.044703	0.236225	SG1	ARHGAP31	NC_056057.1	62270001	62290001
NC_056054.1	184945001	1.85E+08	0.066397	0.223378	SG1	ARHGAP31	NC_056057.1	62275001	62295001
NC_056054.1	184950001	1.85E+08	0.138255	0.192713	SG1	ARHGAP31	NC_056057.1	62280001	62300001
NC_056054.1	184985001	1.85E+08	0.105798	0.190421	SG1	ARHGAP31	NC_056057.1	62285001	62305001
NC_056058.1	58685001	58705001	0.454281	0.225982	SG1	ARHGEF37	NC_056057.1	62290001	62310001
NC_056080.1	98545001	98565001	0.366142	0.183281	SG1	ARHGEF6;CD40LG	NC_056057.1	62295001	62315001
NC_056080.1	46135001	46155001	0.348894	0.305432	SG1	ARHGEF9	NC_056074.1	46790001	46810001
NC_056080.1	46140001	46160001	0.255061	0.30695	SG1	ARHGEF9	NC_056074.1	46795001	46815001
NC_056080.1	46160001	46180001	0.325641	0.328001	SG1	ARHGEF9	NC_056074.1	46800001	46820001
NC_056080.1	46165001	46185001	0.396635	0.323529	SG1	ARHGEF9	NC_056071.1	22295001	22315001
NC_056080.1	46170001	46190001	0.359999	0.325794	SG1	ARHGEF9	NC_056071.1	22300001	22320001
NC_056080.1	46175001	46195001	0.297872	0.302827	SG1	ARHGEF9	NC_056071.1	22310001	22330001
NC_056080.1	46190001	46210001	0.2707	0.251948	SG1	ARHGEF9	NC_056071.1	39415001	39435001
NC_056080.1	46195001	46215001	0.379257	0.213308	SG1	ARHGEF9	NC_056071.1	39420001	39440001
NC_056080.1	46200001	46220001	0.454694	0.198608	SG1	ARHGEF9	NC_056056.1	174355001	174375001
NC_056061.1	81210001	81230001	0.361006	0.224091	SG1	ARID1B	NC_056056.1	174360001	174380001

NC_056061.1	81215001	81235001	0.329954	0.284808	SG1	ARID1B	NC_056057.1	66950001	66970001
NC_056061.1	81220001	81240001	0.273066	0.320473	SG1	ARID1B	NC_056068.1	20305001	20325001
NC_056061.1	81225001	81245001	0.317118	0.316845	SG1	ARID1B	NC_056068.1	20310001	20330001
NC_056061.1	81565001	81585001	0.448352	0.214508	SG1	ARID1B	NC_056078.1	41945001	41965001
NC_056069.1	25380001	25400001	0.468931	0.22056	SG1	ARL15	NC_056078.1	41950001	41970001
NC_056080.1	133270001	1.33E+08	0.417359	0.218832	SG1	ARMCX2	NC_056078.1	41955001	41975001
NC_056072.1	9700001	9720001	0.467912	0.198291	SG1	ARPP21	NC_056078.1	41960001	41980001
NC_056054.1	105520001	1.06E+08	0.366907	0.201451	SG1	ASH1L	NC_056078.1	41965001	41985001
NC_056057.1	15850001	15870001	0.262879	0.257468	SG1	ASNS	NC_056078.1	41970001	41990001
NC_056057.1	15855001	15875001	0.242492	0.247547	SG1	ASNS	NC_056078.1	41975001	41995001
NC_056057.1	15860001	15880001	0.308848	0.234921	SG1	ASNS	NC_056059.1	101410001	101430001
NC_056057.1	15865001	15885001	0.326819	0.247129	SG1	ASNS	NC_056059.1	101415001	101435001
NC_056065.1	57760001	57780001	0.396841	0.200165	SG1	ASTN1	NC_056059.1	101420001	101440001
NC_056055.1	6950001	6970001	0.054658	0.192645	SG1	ASTN2	NC_056059.1	101425001	101445001
NC_056072.1	55300001	55320001	0.413558	0.184098	SG1	ATG7	NC_056054.1	71355001	71375001
NC_056068.1	17630001	17650001	0.294092	0.199736	SG1	ATM	NC_056054.1	71360001	71380001
NC_056054.1	88875001	88895001	0.45123	0.282709	SG1	ATP5PB	NC_056054.1	71365001	71385001
NC_056054.1	88880001	88900001	0.46692	0.274009	SG1	ATP5PB;C1H1orf162	NC_056054.1	106965001	106985001
NC_056054.1	88870001	88890001	0.459257	0.247701	SG1	ATP5PB;WDR77	NC_056055.1	114730001	114750001
NC_056067.1	34640001	34660001	0.446237	0.189982	SG1	ATP6V0D1;LOC4432	NC_056055.1	114735001	114755001
NC_056067.1	34645001	34665001	0.462348	0.185909	SG1	ATP6V0D1;LOC4432	NC_056055.1	114740001	114760001
NC_056057.1	48325001	48345001	0.273662	0.201609	SG1	ATXN7L1	NC_056055.1	114745001	114765001
NC_056057.1	48330001	48350001	0.247035	0.218373	SG1	ATXN7L1	NC_056069.1	24955001	24975001
NC_056072.1	2770001	2790001	0.115775	0.328501	SG1	AZI2	NC_056069.1	24960001	24980001
NC_056072.1	2775001	2795001	0.139319	0.291328	SG1	AZI2	NC_056062.1	23600001	23620001
NC_056072.1	2780001	2800001	0.162837	0.27451	SG1	AZI2	NC_056080.1	6625001	6645001
NC_056072.1	2755001	2775001	0.382426	0.247895	SG1	AZI2;CMC1;LOC114	NC_056056.1	209350001	209370001
NC_056072.1	2760001	2780001	0.266242	0.262634	SG1	AZI2;CMC1;LOC114	NC_056056.1	209355001	209375001
NC_056072.1	2765001	2785001	0.187892	0.308765	SG1	AZI2;CMC1;LOC114	NC_056056.1	209360001	209380001
NC_056072.1	2785001	2805001	0.259947	0.224854	SG1	AZI2;ZCWPW2	NC_056056.1	209365001	209385001
NC_056058.1	41070001	41090001	0.338257	0.343884	SG1	AZU1;ELANE;PLPPR	NC_056056.1	209370001	209390001
NC_056058.1	41060001	41080001	0.433206	0.226538	SG1	AZU1;PLPPR3	NC_056056.1	209375001	209395001

NC_056058.1	41065001	41085001	0.373272	0.284135	SG1	AZU1;PLPPR3;PRTN	NC_056058.1	69885001	69905001
NC_056063.1	29920001	29940001	0.385462	0.184904	SG1	B3GLCT	NC_056063.1	36875001	36895001
NC_056063.1	29960001	29980001	0.340079	0.253918	SG1	B3GLCT	NC_056063.1	36880001	36900001
NC_056063.1	29965001	29985001	0.28631	0.31459	SG1	B3GLCT	NC_056063.1	36865001	36885001
NC_056063.1	29970001	29990001	0.390375	0.284303	SG1	B3GLCT	NC_056063.1	36870001	36890001
NC_056054.1	204610001	2.05E+08	0.466986	0.203039	SG1	B3GNT5;MCF2L2	NC_056056.1	125975001	125995001
NC_056067.1	50100001	50120001	0.458944	0.358127	SG1	B3GNT8;BCKDHA	NC_056056.1	125980001	126000001
NC_056067.1	50085001	50105001	0.210017	0.453321	SG1	B3GNT8;BCKDHA;E	NC_056056.1	125985001	126005001
NC_056067.1	50090001	50110001	0.256869	0.453641	SG1	B3GNT8;BCKDHA;E	NC_056054.1	122275001	122295001
NC_056067.1	50095001	50115001	0.473341	0.336894	SG1	B3GNT8;BCKDHA;E	NC_056056.1	19885001	19905001
NC_056067.1	50080001	50100001	0.15389	0.464863	SG1	B3GNT8;BCKDHA;E	NC_056056.1	19890001	19910001
NC_056067.1	50115001	50135001	0.447283	0.185228	SG1	B3GNT8;DMAC2	NC_056056.1	19895001	19915001
NC_056067.1	50070001	50090001	0.174852	0.308587	SG1	B9D2;EXOSC5;TMEI	NC_056056.1	19900001	19920001
NC_056067.1	50065001	50085001	0.258621	0.240456	SG1	B9D2;TMEM91	NC_056066.1	79435001	79455001
NC_056062.1	74260001	74280001	0.289546	0.226021	SG1	BAALC	NC_056066.1	79440001	79460001
NC_056062.1	74265001	74285001	0.255923	0.230959	SG1	BAALC	NC_056075.1	35655001	35675001
NC_056062.1	74275001	74295001	0.113241	0.249175	SG1	BAALC	NC_056077.1	31075001	31095001
NC_056062.1	74280001	74300001	0.224116	0.242149	SG1	BAALC	NC_056077.1	31080001	31100001
NC_056061.1	47520001	47540001	0.240867	0.212757	SG1	BACH2	NC_056077.1	31085001	31105001
NC_056061.1	47550001	47570001	0.380449	0.209257	SG1	BACH2	NC_056077.1	31090001	31110001
NC_056056.1	215675001	2.16E+08	0.283489	0.257377	SG1	BAIAP2L2;PICK1;SL	NC_056077.1	31095001	31115001
NC_056056.1	215680001	2.16E+08	0.407493	0.228706	SG1	BAIAP2L2;PICK1;SL	NC_056077.1	31100001	31120001
NC_056059.1	23075001	23095001	0.355421	0.195092	SG1	BANK1	NC_056077.1	31105001	31125001
NC_056059.1	23080001	23100001	0.38482	0.212797	SG1	BANK1	NC_056077.1	31110001	31130001
NC_056059.1	23085001	23105001	0.391345	0.209102	SG1	BANK1	NC_056077.1	31140001	31160001
NC_056059.1	23185001	23205001	0.190275	0.215944	SG1	BANK1	NC_056062.1	3145001	3165001
NC_056059.1	23190001	23210001	0.135684	0.192284	SG1	BANK1	NC_056062.1	3150001	3170001
NC_056059.1	23250001	23270001	0.108141	0.255805	SG1	BANK1	NC_056062.1	3155001	3175001
NC_056059.1	23255001	23275001	0.195122	0.185323	SG1	BANK1	NC_056062.1	3160001	3180001
NC_056056.1	190735001	1.91E+08	0.391983	0.364081	SG1	BCAT1	NC_056070.1	53190001	53210001
NC_056056.1	190740001	1.91E+08	0.342025	0.379095	SG1	BCAT1	NC_056070.1	53195001	53215001
NC_056056.1	190745001	1.91E+08	0.343316	0.342298	SG1	BCAT1	NC_056067.1	34360001	34380001

NC_056056.1	105290001	1.05E+08	0.419811	0.189936	SG1	BCL2L11	NC_056054.1	127410001	127430001
NC_056056.1	105295001	1.05E+08	0.417177	0.186461	SG1	BCL2L11	NC_056054.1	127415001	127435001
NC_056056.1	214945001	2.15E+08	0.424535	0.213093	SG1	BCL2L13	NC_056054.1	127420001	127440001
NC_056056.1	214950001	2.15E+08	0.439011	0.213311	SG1	BCL2L13;BID	NC_056056.1	215710001	215730001
NC_056054.1	192795001	1.93E+08	0.225913	0.185845	SG1	BDH1	NC_056056.1	215715001	215735001
NC_056078.1	13645001	13665001	0.347623	0.227074	SG1	BICC1	NC_056060.1	83455001	83475001
NC_056078.1	13650001	13670001	0.372321	0.274518	SG1	BICC1	NC_056060.1	83460001	83480001
NC_056075.1	17380001	17400001	0.380055	0.19379	SG1	BLNK	NC_056060.1	83465001	83485001
NC_056075.1	17385001	17405001	0.267587	0.20126	SG1	BLNK	NC_056060.1	83470001	83490001
NC_056075.1	17390001	17410001	0.249179	0.202782	SG1	BLNK	NC_056057.1	64845001	64865001
NC_056075.1	17400001	17420001	0.176444	0.227288	SG1	BLNK	NC_056057.1	65015001	65035001
NC_056075.1	17405001	17425001	0.214417	0.197141	SG1	BLNK	NC_056057.1	65020001	65040001
NC_056067.1	52600001	52620001	0.133896	0.248823	SG1	BLOC1S3;NKPD1;TR	NC_056057.1	65025001	65045001
NC_056067.1	52605001	52625001	0.171361	0.254357	SG1	BLOC1S3;NKPD1;TR	NC_056064.1	11865001	11885001
NC_056067.1	52610001	52630001	0.223575	0.215058	SG1	BLOC1S3;TRAPPC6/	NC_056064.1	11875001	11895001
NC_056067.1	52615001	52635001	0.280799	0.211719	SG1	BLOC1S3;TRAPPC6/	NC_056064.1	11880001	11900001
NW_02459982	1610001	1630001	0.441237	0.255817	SG1	BTBD1	NC_056065.1	34165001	34185001
NW_02459982	1615001	1635001	0.394756	0.24746	SG1	BTBD1	NC_056065.1	34170001	34190001
NC_056069.1	42100001	42120001	0.040698	0.307882	SG1	C16H5orf22	NC_056066.1	27505001	27525001
NC_056069.1	42115001	42135001	0.081509	0.475891	SG1	C16H5orf22;DROSH/	NC_056063.1	78410001	78430001
NC_056069.1	42120001	42140001	0.184119	0.442406	SG1	C16H5orf22;DROSH/	NC_056073.1	46595001	46615001
NC_056069.1	42125001	42145001	0.253108	0.371066	SG1	C16H5orf22;DROSH/	NC_056073.1	46600001	46620001
NC_056069.1	42130001	42150001	0.289091	0.282925	SG1	C16H5orf22;DROSH/	NC_056057.1	78980001	79000001
NC_056054.1	121715001	1.22E+08	0.433048	0.215518	SG1	C1H21orf140;SMIM1	NC_056057.1	78985001	79005001
NC_056060.1	46200001	46220001	0.353694	0.220776	SG1	C2CD4A	NC_056077.1	14355001	14375001
NC_056060.1	46205001	46225001	0.297699	0.282908	SG1	C2CD4A	NC_056077.1	14360001	14380001
NC_056060.1	46210001	46230001	0.369858	0.314663	SG1	C2CD4A	NC_056077.1	14365001	14385001
NC_056060.1	46215001	46235001	0.286818	0.331422	SG1	C2CD4A;VPS13C	NC_056077.1	14435001	14455001
NC_056055.1	199155001	1.99E+08	0.226443	0.208509	SG1	C2H2orf66	NC_056073.1	46795001	46815001
NC_056056.1	124320001	1.24E+08	0.44622	0.198818	SG1	C3H12orf29;C3H12or	NC_056073.1	46800001	46820001
NC_056054.1	31500001	31520001	0.320762	0.201571	SG1	C8A	NC_056073.1	46805001	46825001
NC_056054.1	31505001	31525001	0.328704	0.238779	SG1	C8A	NC_056057.1	64290001	64310001

NC_056054.1	31510001	31530001	0.45365	0.279387	SG1	C8A	NC_056078.1	40435001	40455001
NC_056054.1	31515001	31535001	0.449511	0.283713	SG1	C8A;C8B	NC_056063.1	28935001	28955001
NC_056054.1	31520001	31540001	0.466058	0.310072	SG1	C8A;C8B	NC_056063.1	28940001	28960001
NC_056054.1	31525001	31545001	0.426829	0.274882	SG1	C8A;C8B	NC_056063.1	28945001	28965001
NC_056054.1	31530001	31550001	0.410587	0.264012	SG1	C8A;C8B	NC_056063.1	28950001	28970001
NC_056054.1	31535001	31555001	0.424135	0.277229	SG1	C8B	NC_056063.1	28955001	28975001
NC_056054.1	31540001	31560001	0.385787	0.238181	SG1	C8B	NC_056063.1	28960001	28980001
NC_056054.1	31550001	31570001	0.447268	0.31409	SG1	C8B	NC_056063.1	28965001	28985001
NC_056054.1	31555001	31575001	0.472829	0.208427	SG1	C8B	NW_024599827.	1665001	1685001
NC_056080.1	13950001	13970001	0.345069	0.276399	SG1	CA5B;ZRSR2	NW_024599827.	1670001	1690001
NC_056080.1	13955001	13975001	0.330709	0.256963	SG1	CA5B;ZRSR2	NC_056054.1	273655001	273675001
NC_056070.1	71455001	71475001	0.443213	0.216339	SG1	CABIN1;SUSD2	NC_056067.1	49400001	49420001
NC_056072.1	47260001	47280001	0.463904	0.255619	SG1	CACNA1D	NC_056067.1	49405001	49425001
NC_056072.1	47255001	47275001	0.407476	0.296608	SG1	CACNA1D;CHDH	NC_056067.1	49410001	49430001
NC_056056.1	216855001	2.17E+08	0.468474	0.197875	SG1	CACNA1I	NC_056068.1	17705001	17725001
NC_056056.1	216860001	2.17E+08	0.384112	0.209591	SG1	CACNA1I	NC_056070.1	28915001	28935001
NC_056056.1	216865001	2.17E+08	0.343298	0.253594	SG1	CACNA1I	NC_056070.1	40550001	40570001
NC_056055.1	157510001	1.58E+08	0.282181	0.2129	SG1	CACNB4	NC_056070.1	40555001	40575001
NC_056054.1	154610001	1.55E+08	0.246551	0.193131	SG1	CADM2	NC_056071.1	58590001	58610001
NC_056054.1	154615001	1.55E+08	0.172483	0.275465	SG1	CADM2	NC_056071.1	58595001	58615001
NC_056054.1	154620001	1.55E+08	0.160979	0.311944	SG1	CADM2	NC_056071.1	58600001	58620001
NC_056054.1	154625001	1.55E+08	0.262202	0.250861	SG1	CADM2	NC_056054.1	113835001	113855001
NC_056054.1	154630001	1.55E+08	0.449676	0.236522	SG1	CADM2	NC_056054.1	113840001	113860001
NC_056062.1	86580001	86600001	0.387865	0.18337	SG1	CALB1;DECR1	NC_056054.1	113845001	113865001
NC_056057.1	11565001	11585001	0.135161	0.21063	SG1	CALCR	NC_056054.1	113850001	113870001
NC_056066.1	16470001	16490001	0.335583	0.285665	SG1	CAMK1D	NC_056073.1	26495001	26515001
NC_056066.1	16475001	16495001	0.275802	0.321491	SG1	CAMK1D	NC_056073.1	26500001	26520001
NC_056066.1	16480001	16500001	0.427218	0.242427	SG1	CAMK1D	NC_056073.1	26505001	26525001
NC_056066.1	16645001	16665001	0.432745	0.38739	SG1	CAMK1D	NC_056073.1	26490001	26510001
NC_056066.1	16650001	16670001	0.237432	0.359117	SG1	CAMK1D	NC_056073.1	26480001	26500001
NC_056066.1	16655001	16675001	0.188838	0.240739	SG1	CAMK1D	NC_056073.1	26485001	26505001
NC_056078.1	28935001	28955001	0.456048	0.201261	SG1	CAMK2G	NC_056075.1	42065001	42085001

NC_056055.1	247010001	2.47E+08	0.195137	0.186586	SG1	CAPZB	NC_056075.1	42070001	42090001
NC_056055.1	247015001	2.47E+08	0.155147	0.217835	SG1	CAPZB	NC_056075.1	42055001	42075001
NC_056055.1	247020001	2.47E+08	0.203144	0.241066	SG1	CAPZB;SLC66A1	NC_056075.1	42060001	42080001
NC_056055.1	247025001	2.47E+08	0.368384	0.203993	SG1	CAPZB;SLC66A1	NC_056077.1	4255001	4275001
NC_056058.1	93680001	93700001	0.298361	0.244626	SG1	CAST	NC_056077.1	4260001	4280001
NC_056058.1	93685001	93705001	0.395735	0.217392	SG1	CAST	NC_056078.1	2990001	3010001
NC_056058.1	16425001	16445001	0.309214	0.186876	SG1	CATSPERD;LONP1	NC_056056.1	96270001	96290001
NC_056054.1	189150001	1.89E+08	0.186724	0.186283	SG1	CCDC14	NC_056056.1	96275001	96295001
NC_056054.1	189155001	1.89E+08	0.15101	0.197943	SG1	CCDC14	NC_056056.1	96280001	96300001
NC_056054.1	189160001	1.89E+08	0.141896	0.207395	SG1	CCDC14	NC_056056.1	96285001	96305001
NC_056054.1	189165001	1.89E+08	0.130167	0.211477	SG1	CCDC14	NC_056076.1	33835001	33855001
NC_056067.1	53285001	53305001	0.384418	0.205114	SG1	CCDC61;PGLYRP1	NC_056066.1	54535001	54555001
NC_056056.1	67915001	67935001	0.452481	0.258275	SG1	CCDC85A	NC_056076.1	32885001	32905001
NC_056056.1	67920001	67940001	0.182584	0.257773	SG1	CCDC85A	NC_056076.1	32890001	32910001
NC_056056.1	67925001	67945001	0.09158	0.231975	SG1	CCDC85A	NC_056076.1	32895001	32915001
NC_056071.1	53985001	54005001	0.293452	0.24963	SG1	CCDC88C	NC_056056.1	216880001	216900001
NC_056071.1	53990001	54010001	0.137205	0.320445	SG1	CCDC88C	NC_056056.1	216885001	216905001
NC_056071.1	53995001	54015001	0.170114	0.337663	SG1	CCDC88C	NC_056056.1	216890001	216910001
NC_056071.1	54000001	54020001	0.260934	0.284174	SG1	CCDC88C	NC_056056.1	216895001	216915001
NC_056071.1	54005001	54025001	0.283133	0.281379	SG1	CCDC88C	NC_056057.1	39635001	39655001
NC_056060.1	36635001	36655001	0.186294	0.288142	SG1	CCNDBP1;EPB42;TM	NC_056057.1	39640001	39660001
NC_056060.1	36625001	36645001	0.327912	0.230657	SG1	CCNDBP1;TMEM62	NC_056057.1	39645001	39665001
NC_056060.1	36630001	36650001	0.263255	0.25302	SG1	CCNDBP1;TMEM62	NC_056057.1	39650001	39670001
NC_056077.1	17585001	17605001	0.394483	0.256591	SG1	CCP110;GDE1	NC_056072.1	50030001	50050001
NC_056077.1	17590001	17610001	0.459732	0.252663	SG1	CCP110;GDE1	NC_056072.1	50035001	50055001
NC_056059.1	34425001	34445001	0.385656	0.2001	SG1	CCSER1	NC_056072.1	50025001	50045001
NC_056059.1	34430001	34450001	0.380649	0.199756	SG1	CCSER1	NC_056066.1	31955001	31975001
NC_056054.1	119690001	1.2E+08	0.366816	0.217266	SG1	CD247	NC_056066.1	31960001	31980001
NC_056054.1	119695001	1.2E+08	0.246282	0.234946	SG1	CD247	NC_056066.1	31965001	31985001
NC_056054.1	119705001	1.2E+08	0.238776	0.189449	SG1	CD247	NC_056066.1	32055001	32075001
NC_056080.1	98550001	98570001	0.328657	0.189112	SG1	CD40LG	NC_056066.1	32060001	32080001
NC_056054.1	93895001	93915001	0.44661	0.307337	SG1	CD58	NC_056066.1	32065001	32085001

NC_056061.1	27290001	27310001	0.094833	0.232659	SG1	CDC40	NC_056066.1	32080001	32100001
NC_056061.1	27295001	27315001	0.109344	0.275776	SG1	CDC40	NC_056066.1	32090001	32110001
NC_056061.1	27300001	27320001	0.10789	0.246197	SG1	CDC40	NC_056066.1	32095001	32115001
NC_056066.1	54860001	54880001	0.12134	0.460148	SG1	CDH4	NC_056066.1	32100001	32120001
NC_056066.1	54865001	54885001	0.288752	0.547844	SG1	CDH4	NC_056066.1	32160001	32180001
NC_056066.1	54870001	54890001	0.358117	0.56229	SG1	CDH4	NC_056057.1	100230001	100250001
NC_056066.1	54850001	54870001	0.300366	0.34967	SG1	CDH4;TAF4	NC_056057.1	100235001	100255001
NC_056066.1	54855001	54875001	0.218298	0.388579	SG1	CDH4;TAF4	NC_056057.1	100240001	100260001
NC_056055.1	3040001	3060001	0.154046	0.198814	SG1	CDK5RAP2	NC_056057.1	100245001	100265001
NC_056055.1	3045001	3065001	0.165821	0.192711	SG1	CDK5RAP2	NC_056057.1	78735001	78755001
NC_056057.1	10935001	10955001	0.433075	0.218547	SG1	CDK6	NC_056057.1	78740001	78760001
NC_056063.1	32345001	32365001	0.20887	0.271255	SG1	CDX2;URAD	NC_056057.1	78745001	78765001
NC_056063.1	32350001	32370001	0.474139	0.231481	SG1	CDX2;URAD	NC_056057.1	78750001	78770001
NC_056060.1	19080001	19100001	0.433271	0.204395	SG1	CELF6	NC_056057.1	78755001	78775001
NC_056056.1	222585001	2.23E+08	0.356313	0.389613	SG1	CELSR1	NC_056057.1	78760001	78780001
NC_056056.1	222595001	2.23E+08	0.461093	0.296317	SG1	CELSR1	NC_056057.1	78765001	78785001
NC_056080.1	132845001	1.33E+08	0.354613	0.311802	SG1	CENPI	NC_056059.1	12230001	12250001
NC_056080.1	132850001	1.33E+08	0.307693	0.34371	SG1	CENPI	NC_056059.1	12235001	12255001
NC_056080.1	132855001	1.33E+08	0.377091	0.316881	SG1	CENPI	NC_056078.1	28960001	28980001
NC_056064.1	61455001	61475001	0.253601	0.319793	SG1	CEP112	NC_056078.1	28965001	28985001
NC_056064.1	61460001	61480001	0.468564	0.261159	SG1	CEP112	NC_056065.1	45305001	45325001
NC_056054.1	255425001	2.55E+08	0.4646	0.285772	SG1	CEP63	NC_056065.1	45310001	45330001
NC_056054.1	255435001	2.55E+08	0.458091	0.194282	SG1	CEP63	NC_056065.1	45315001	45335001
NC_056054.1	251215001	2.51E+08	0.359967	0.228864	SG1	CEP70	NC_056065.1	45320001	45340001
NC_056054.1	251220001	2.51E+08	0.299295	0.245214	SG1	CEP70	NC_056065.1	45325001	45345001
NC_056054.1	251225001	2.51E+08	0.356246	0.216296	SG1	CEP70	NC_056065.1	45330001	45350001
NC_056060.1	6845001	6865001	0.307207	0.257136	SG1	CERT1	NC_056065.1	26365001	26385001
NC_056060.1	6850001	6870001	0.115528	0.351941	SG1	CERT1	NC_056065.1	26370001	26390001
NC_056060.1	6870001	6890001	0.059094	0.374427	SG1	CERT1	NC_056067.1	34310001	34330001
NC_056060.1	6875001	6895001	0.05891	0.364043	SG1	CERT1	NC_056054.1	171300001	171320001
NC_056060.1	6880001	6900001	0.090647	0.303368	SG1	CERT1	NC_056054.1	171305001	171325001
NC_056060.1	6885001	6905001	0.183223	0.265862	SG1	CERT1	NC_056054.1	171310001	171330001

NC_056071.1	24735001	24755001	0.355844	0.250016	SG1	CFAP161	NC_056054.1	171315001	171335001
NC_056072.1	42450001	42470001	0.454545	0.194987	SG1	CFAP20DC	NC_056054.1	171320001	171340001
NC_056072.1	42455001	42475001	0.302853	0.245619	SG1	CFAP20DC	NC_056075.1	34145001	34165001
NC_056072.1	42460001	42480001	0.163996	0.301116	SG1	CFAP20DC	NC_056075.1	34150001	34170001
NC_056072.1	42465001	42485001	0.166261	0.291329	SG1	CFAP20DC	NC_056078.1	14905001	14925001
NC_056072.1	42470001	42490001	0.149209	0.299447	SG1	CFAP20DC	NC_056070.1	56045001	56065001
NC_056072.1	42475001	42495001	0.168803	0.289907	SG1	CFAP20DC	NC_056057.1	78255001	78275001
NC_056072.1	42480001	42500001	0.150932	0.304708	SG1	CFAP20DC	NC_056057.1	78260001	78280001
NC_056072.1	42485001	42505001	0.190701	0.291771	SG1	CFAP20DC	NC_056057.1	78265001	78285001
NC_056072.1	42490001	42510001	0.22027	0.294397	SG1	CFAP20DC	NC_056059.1	34400001	34420001
NC_056072.1	42495001	42515001	0.263199	0.249496	SG1	CFAP20DC	NC_056059.1	34405001	34425001
NC_056072.1	42630001	42650001	0.077656	0.189125	SG1	CFAP20DC	NC_056056.1	58680001	58700001
NC_056072.1	42635001	42655001	0.080613	0.191142	SG1	CFAP20DC	NC_056056.1	58685001	58705001
NC_056072.1	42640001	42660001	0.076275	0.189534	SG1	CFAP20DC	NC_056056.1	58690001	58710001
NC_056072.1	42645001	42665001	0.063259	0.19125	SG1	CFAP20DC	NC_056058.1	20865001	20885001
NC_056059.1	95825001	95845001	0.437201	0.30767	SG1	CFAP299	NC_056058.1	20870001	20890001
NC_056059.1	96295001	96315001	0.10341	0.183111	SG1	CFAP299	NC_056066.1	54880001	54900001
NC_056059.1	96300001	96320001	0.109157	0.192715	SG1	CFAP299	NC_056066.1	54905001	54925001
NC_056059.1	96350001	96370001	0.070175	0.184195	SG1	CFAP299	NC_056066.1	54910001	54930001
NC_056054.1	179025001	1.79E+08	0.127734	0.329278	SG1	CFAP44	NC_056066.1	54915001	54935001
NC_056054.1	179030001	1.79E+08	0.157459	0.33352	SG1	CFAP44	NC_056066.1	54920001	54940001
NC_056054.1	179035001	1.79E+08	0.267346	0.294496	SG1	CFAP44	NC_056066.1	54935001	54955001
NC_056054.1	179040001	1.79E+08	0.343628	0.269367	SG1	CFAP44	NC_056066.1	54940001	54960001
NC_056058.1	41085001	41105001	0.2625	0.334501	SG1	CFD;ELANE;MED16;	NC_056066.1	54945001	54965001
NC_056058.1	41075001	41095001	0.310298	0.378842	SG1	CFD;ELANE;MED16;	NC_056069.1	42385001	42405001
NC_056058.1	41080001	41100001	0.270651	0.344167	SG1	CFD;ELANE;MED16;	NC_056067.1	29150001	29170001
NC_056058.1	41090001	41110001	0.302326	0.283278	SG1	CFD;MED16;PLPPR3	NC_056067.1	29155001	29175001
NC_056057.1	52275001	52295001	0.454547	0.183301	SG1	CFTR	NC_056067.1	29160001	29180001
NC_056061.1	50150001	50170001	0.259061	0.186232	SG1	CGA	NC_056069.1	46760001	46780001
NC_056061.1	50160001	50180001	0.418125	0.205122	SG1	CGA	NC_056058.1	35740001	35760001
NC_056066.1	70100001	70120001	0.460909	0.221533	SG1	CHD6	NC_056058.1	35735001	35755001
NC_056057.1	68290001	68310001	0.438965	0.215218	SG1	CHN2	NC_056061.1	26950001	26970001

NC_056057.1	68355001	68375001	0.397985	0.475696	SG1	CHN2	NC_056061.1	26955001	26975001
NC_056057.1	68360001	68380001	0.445045	0.213336	SG1	CHN2	NC_056061.1	26960001	26980001
NC_056074.1	37745001	37765001	0.086331	0.255882	SG1	CHRM1	NC_056073.1	36195001	36215001
NC_056074.1	37750001	37770001	0.197855	0.232246	SG1	CHRM1	NC_056073.1	36200001	36220001
NC_056074.1	37755001	37775001	0.439811	0.317735	SG1	CHRM1	NC_056073.1	36205001	36225001
NC_056074.1	37760001	37780001	0.459736	0.311333	SG1	CHRM1;LOC1011127	NC_056056.1	202770001	202790001
NC_056071.1	28780001	28800001	0.053251	0.2465	SG1	CHRNA3;CHRNA5	NC_056056.1	202780001	202800001
NC_056071.1	28750001	28770001	0.380707	0.183169	SG1	CHRNA5	NC_056067.1	43295001	43315001
NC_056061.1	64315001	64335001	0.24682	0.184599	SG1	CITED2	NC_056067.1	43300001	43320001
NC_056061.1	64320001	64340001	0.29198	0.19022	SG1	CITED2	NC_056068.1	76205001	76225001
NC_056058.1	78840001	78860001	0.426958	0.201974	SG1	CKMT2	NC_056056.1	33910001	33930001
NC_056058.1	78845001	78865001	0.459176	0.260696	SG1	CKMT2	NC_056063.1	36700001	36720001
NC_056058.1	78810001	78830001	0.34949	0.224849	SG1	CKMT2;RASGRF2	NC_056063.1	36705001	36725001
NC_056058.1	78815001	78835001	0.411417	0.22494	SG1	CKMT2;RASGRF2	NC_056064.1	61695001	61715001
NC_056058.1	78820001	78840001	0.429695	0.186233	SG1	CKMT2;RASGRF2	NC_056060.1	90180001	90200001
NC_056058.1	78850001	78870001	0.460461	0.277793	SG1	CKMT2;ZCCHC9	NC_056060.1	90185001	90205001
NC_056071.1	57775001	57795001	0.377358	0.227416	SG1	CLMN	NC_056065.1	60700001	60720001
NC_056071.1	57780001	57800001	0.282409	0.270745	SG1	CLMN	NC_056054.1	255455001	255475001
NC_056071.1	57785001	57805001	0.421054	0.251036	SG1	CLMN	NC_056054.1	255460001	255480001
NC_056071.1	57840001	57860001	0.408932	0.299917	SG1	CLMN	NC_056054.1	255470001	255490001
NC_056071.1	57845001	57865001	0.400281	0.285141	SG1	CLMN	NC_056067.1	42850001	42870001
NC_056071.1	57850001	57870001	0.397553	0.304883	SG1	CLMN	NC_056067.1	42855001	42875001
NC_056071.1	57855001	57875001	0.417397	0.302139	SG1	CLMN	NC_056067.1	42860001	42880001
NC_056071.1	57860001	57880001	0.466081	0.253511	SG1	CLMN	NC_056067.1	42865001	42885001
NC_056059.1	71580001	71600001	0.191555	0.240515	SG1	CLOCK	NC_056067.1	42870001	42890001
NC_056059.1	71575001	71595001	0.244693	0.212024	SG1	CLOCK;TMEM165	NC_056067.1	42875001	42895001
NC_056060.1	12070001	12090001	0.228642	0.204835	SG1	CLPX	NC_056067.1	42880001	42900001
NC_056060.1	12075001	12095001	0.121951	0.200626	SG1	CLPX	NC_056064.1	48415001	48435001
NC_056060.1	12080001	12100001	0.086957	0.184359	SG1	CLPX	NC_056064.1	48420001	48440001
NC_056055.1	52350001	52370001	0.245566	0.235093	SG1	CLTA	NC_056064.1	48425001	48445001
NC_056055.1	52355001	52375001	0.196079	0.240439	SG1	CLTA	NC_056064.1	48430001	48450001
NC_056067.1	33995001	34015001	0.398229	0.186517	SG1	CMTM4	NC_056064.1	48435001	48455001

NC_056060.1	21355001	21375001	0.222222	0.26106	SG1	CMTM5;EFS;IL25	NC_056064.1	48440001	48460001
NC_056062.1	88520001	88540001	0.40222	0.211264	SG1	CNBD1	NC_056075.1	50760001	50780001
NC_056062.1	88525001	88545001	0.388335	0.239425	SG1	CNBD1	NC_056080.1	35420001	35440001
NC_056062.1	88535001	88555001	0.454764	0.20794	SG1	CNBD1	NC_056080.1	35425001	35445001
NC_056062.1	88540001	88560001	0.310029	0.184998	SG1	CNBD1	NC_056056.1	166530001	166550001
NC_056056.1	149425001	1.49E+08	0.397424	0.212226	SG1	CNOT2	NC_056056.1	166535001	166555001
NC_056056.1	149430001	1.49E+08	0.41757	0.211351	SG1	CNOT2	NC_056057.1	76045001	76065001
NC_056056.1	149460001	1.49E+08	0.341463	0.191503	SG1	CNOT2	NC_056057.1	76030001	76050001
NC_056056.1	149470001	1.49E+08	0.304771	0.19939	SG1	CNOT2	NC_056057.1	76035001	76055001
NC_056056.1	149475001	1.49E+08	0.237578	0.221276	SG1	CNOT2	NC_056057.1	76040001	76060001
NC_056056.1	149480001	1.5E+08	0.282362	0.18359	SG1	CNOT2	NC_056078.1	28500001	28520001
NC_056056.1	149485001	1.5E+08	0.230012	0.207968	SG1	CNOT2	NC_056059.1	116955001	116975001
NC_056056.1	149550001	1.5E+08	0.468443	0.204815	SG1	CNOT2	NC_056059.1	116960001	116980001
NC_056056.1	149555001	1.5E+08	0.306138	0.266561	SG1	CNOT2	NC_056074.1	40410001	40430001
NC_056058.1	450001	470001	0.474865	0.396818	SG1	CNOT6	NC_056074.1	40405001	40425001
NC_056058.1	455001	475001	0.468052	0.423132	SG1	CNOT6	NC_056074.1	40395001	40415001
NC_056059.1	93185001	93205001	0.432658	0.325477	SG1	CNOT6L	NC_056074.1	40400001	40420001
NC_056061.1	49120001	49140001	0.174763	0.203769	SG1	CNR1	NC_056074.1	40390001	40410001
NC_056068.1	9885001	9905001	0.40414	0.205755	SG1	CNTN5	NC_056056.1	60390001	60410001
NC_056068.1	9890001	9910001	0.253506	0.255749	SG1	CNTN5	NC_056056.1	60395001	60415001
NC_056068.1	9895001	9915001	0.197503	0.257477	SG1	CNTN5	NC_056056.1	60400001	60420001
NC_056068.1	9900001	9920001	0.186665	0.245966	SG1	CNTN5	NC_056056.1	60405001	60425001
NC_056068.1	9905001	9925001	0.157674	0.206676	SG1	CNTN5	NC_056062.1	36370001	36390001
NC_056057.1	5590001	5610001	0.435083	0.215227	SG1	COBL	NC_056062.1	39295001	39315001
NC_056057.1	5595001	5615001	0.447532	0.231419	SG1	COBL	NC_056062.1	39300001	39320001
NC_056062.1	17170001	17190001	0.424721	0.183623	SG1	COL22A1	NC_056054.1	88565001	88585001
NC_056056.1	46100001	46120001	0.436141	0.253572	SG1	COMMD1	NC_056054.1	88570001	88590001
NC_056060.1	59210001	59230001	0.16231	0.29046	SG1	COPS2	NC_056054.1	88575001	88595001
NC_056060.1	59215001	59235001	0.157738	0.255445	SG1	COPS2	NC_056054.1	88580001	88600001
NC_056060.1	59220001	59240001	0.124488	0.220194	SG1	COPS2	NC_056054.1	88585001	88605001
NC_056060.1	59225001	59245001	0.14983	0.191293	SG1	COPS2	NC_056054.1	88590001	88610001
NC_056060.1	59230001	59250001	0.141764	0.192436	SG1	COPS2	NC_056057.1	68210001	68230001

NC_056060.1	59190001	59210001	0.32763	0.297622	SG1	COPS2;GALK2	NC_056057.1	68215001	68235001
NC_056060.1	59195001	59215001	0.299405	0.312732	SG1	COPS2;GALK2	NC_056057.1	68245001	68265001
NC_056060.1	59200001	59220001	0.296088	0.294981	SG1	COPS2;GALK2	NC_056057.1	68270001	68290001
NC_056060.1	59205001	59225001	0.208308	0.303838	SG1	COPS2;GALK2	NC_056057.1	68310001	68330001
NC_056055.1	233490001	2.34E+08	0.142857	0.292683	SG1	COPS7B	NC_056057.1	68315001	68335001
NC_056055.1	233495001	2.34E+08	0.177829	0.264957	SG1	COPS7B	NC_056057.1	68320001	68340001
NC_056055.1	233500001	2.34E+08	0.425414	0.189922	SG1	COPS7B	NC_056057.1	68325001	68345001
NC_056059.1	98725001	98745001	0.335856	0.242362	SG1	COQ2	NC_056057.1	68330001	68350001
NC_056059.1	98730001	98750001	0.446309	0.231609	SG1	COQ2	NC_056057.1	68335001	68355001
NC_056070.1	62945001	62965001	0.336037	0.212891	SG1	COQ5;RNF10	NC_056057.1	68340001	68360001
NC_056073.1	10745001	10765001	0.240397	0.21314	SG1	CPNE5	NC_056057.1	68350001	68370001
NC_056073.1	10750001	10770001	0.30092	0.208804	SG1	CPNE5	NC_056060.1	86450001	86470001
NC_056073.1	10740001	10760001	0.349641	0.195631	SG1	CPNE5;LOC1011024[illegible]	NC_056060.1	86455001	86475001
NC_056073.1	10725001	10745001	0.405964	0.284687	SG1	CPNE5;LOC1011024[illegible]	NC_056060.1	86460001	86480001
NC_056073.1	10730001	10750001	0.438314	0.261035	SG1	CPNE5;LOC1011024[illegible]	NC_056060.1	86465001	86485001
NC_056073.1	10735001	10755001	0.402396	0.256076	SG1	CPNE5;LOC1011024[illegible]	NC_056060.1	86470001	86490001
NC_056056.1	102375001	1.02E+08	0.351995	0.241862	SG1	CRACDL	NC_056060.1	86475001	86495001
NC_056056.1	102380001	1.02E+08	0.361521	0.233361	SG1	CRACDL	NC_056080.1	8125001	8145001
NC_056056.1	102385001	1.02E+08	0.346257	0.236181	SG1	CRACDL	NC_056080.1	8150001	8170001
NC_056056.1	102390001	1.02E+08	0.303221	0.218347	SG1	CRACDL	NC_056080.1	8155001	8175001
NC_056056.1	102395001	1.02E+08	0.297445	0.207769	SG1	CRACDL	NC_056056.1	92870001	92890001
NC_056056.1	102400001	1.02E+08	0.457705	0.311673	SG1	CRACDL	NC_056070.1	17370001	17390001
NC_056056.1	102405001	1.02E+08	0.446639	0.326977	SG1	CRACDL	NC_056070.1	17375001	17395001
NC_056056.1	102410001	1.02E+08	0.450786	0.296558	SG1	CRACDL	NC_056070.1	17380001	17400001
NC_056056.1	102415001	1.02E+08	0.450076	0.255916	SG1	CRACDL	NC_056070.1	17385001	17405001
NC_056072.1	22855001	22875001	0.468365	0.192755	SG1	CRBN	NC_056070.1	17390001	17410001
NC_056057.1	103030001	1.03E+08	0.283343	0.269358	SG1	CREB3L2	NC_056070.1	17395001	17415001
NC_056057.1	103035001	1.03E+08	0.075361	0.354549	SG1	CREB3L2	NC_056054.1	121370001	121390001
NC_056057.1	103045001	1.03E+08	0.048893	0.284141	SG1	CREB3L2	NC_056054.1	121375001	121395001
NC_056057.1	103070001	1.03E+08	0.457994	0.226456	SG1	CREB3L2	NC_056071.1	57800001	57820001
NC_056057.1	103075001	1.03E+08	0.414773	0.296526	SG1	CREB3L2	NC_056062.1	88635001	88655001
NC_056057.1	69095001	69115001	0.068831	0.195114	SG1	CREB5	NC_056072.1	27430001	27450001

NC_056057.1	69105001	69125001	0.071687	0.237615	SG1	CREB5	NC_056072.1	27435001	27455001
NC_056064.1	45540001	45560001	0.34382	0.190211	SG1	CRHR1	NC_056072.1	27440001	27460001
NC_056056.1	175875001	1.76E+08	0.198604	0.19351	SG1	CRY1	NC_056068.1	9335001	9355001
NC_056061.1	30660001	30680001	0.422732	0.208209	SG1	CRYBG1;RTN4IP1	NC_056068.1	9535001	9555001
NC_056061.1	30665001	30685001	0.19483	0.28444	SG1	CRYBG1;RTN4IP1	NC_056068.1	9540001	9560001
NC_056061.1	30670001	30690001	0.254403	0.260741	SG1	CRYBG1;RTN4IP1	NC_056057.1	111015001	111035001
NC_056054.1	201435001	2.01E+08	0.452682	0.430806	SG1	CRYGS;TBCCD1	NC_056064.1	27435001	27455001
NC_056079.1	3660001	3680001	0.125866	0.226093	SG1	CSMD1	NC_056064.1	27440001	27460001
NC_056079.1	3665001	3685001	0.267448	0.213831	SG1	CSMD1	NC_056071.1	39320001	39340001
NC_056079.1	3670001	3690001	0.410164	0.229066	SG1	CSMD1	NC_056071.1	39325001	39345001
NC_056059.1	86250001	86270001	0.174022	0.193877	SG1	CSN1S2	NC_056055.1	229490001	229510001
NC_056059.1	86255001	86275001	0.170668	0.19251	SG1	CSN1S2	NC_056055.1	229495001	229515001
NC_056058.1	58680001	58700001	0.418732	0.256062	SG1	CSNK1A1	NC_056055.1	229500001	229520001
NC_056060.1	43900001	43920001	0.292135	0.184241	SG1	CSNK1G1	NC_056054.1	266555001	266575001
NC_056060.1	44005001	44025001	0.085454	0.185132	SG1	CSNK1G1	NC_056054.1	266560001	266580001
NC_056060.1	43890001	43910001	0.231767	0.199386	SG1	CSNK1G1;PCLAF	NC_056076.1	35720001	35740001
NC_056060.1	43895001	43915001	0.204713	0.20405	SG1	CSNK1G1;PCLAF	NC_056076.1	35725001	35745001
NC_056056.1	51920001	51940001	0.304719	0.326194	SG1	CTNNA2	NC_056076.1	35730001	35750001
NC_056056.1	51925001	51945001	0.40474	0.336804	SG1	CTNNA2	NC_056076.1	35735001	35755001
NC_056078.1	23260001	23280001	0.451806	0.259483	SG1	CTNNA3	NC_056076.1	35740001	35760001
NC_056078.1	23265001	23285001	0.443991	0.295729	SG1	CTNNA3	NC_056064.1	21445001	21465001
NC_056064.1	24230001	24250001	0.379791	0.270783	SG1	CTNS;EMC6;P2RX5;	NC_056057.1	87150001	87170001
NC_056057.1	52010001	52030001	0.079279	0.199683	SG1	CTTNBP2	NC_056057.1	87155001	87175001
NC_056057.1	52015001	52035001	0.082856	0.237839	SG1	CTTNBP2	NC_056057.1	87160001	87180001
NC_056054.1	89870001	89890001	0.291733	0.256248	SG1	CTTNBP2NL	NC_056057.1	87165001	87185001
NC_056054.1	89875001	89895001	0.160083	0.235906	SG1	CTTNBP2NL	NC_056057.1	87170001	87190001
NC_056054.1	89880001	89900001	0.210838	0.227523	SG1	CTTNBP2NL	NC_056057.1	87175001	87195001
NC_056066.1	30905001	30925001	0.437205	0.303487	SG1	CUBN	NC_056057.1	87180001	87200001
NC_056066.1	30910001	30930001	0.346104	0.357615	SG1	CUBN	NC_056057.1	87185001	87205001
NC_056066.1	30915001	30935001	0.288826	0.424193	SG1	CUBN	NC_056057.1	87190001	87210001
NC_056066.1	30920001	30940001	0.335097	0.421967	SG1	CUBN	NC_056057.1	87315001	87335001
NC_056069.1	14630001	14650001	0.264673	0.374805	SG1	CWC27	NC_056057.1	87320001	87340001

NC_056069.1	14635001	14655001	0.18996	0.41656	SG1	CWC27	NC_056057.1	87095001	87115001
NC_056069.1	14640001	14660001	0.363955	0.305519	SG1	CWC27	NC_056060.1	5260001	5280001
NC_056069.1	14645001	14665001	0.467942	0.245115	SG1	CWC27	NC_056066.1	64825001	64845001
NC_056067.1	50460001	50480001	0.416667	0.253843	SG1	CXCL17;LIPE	NC_056054.1	258790001	258810001
NC_056067.1	50465001	50485001	0.416736	0.201765	SG1	CXCL17;LIPE	NC_056054.1	258795001	258815001
NC_056055.1	174715001	1.75E+08	0.106682	0.185277	SG1	CXCR4	NC_056054.1	258800001	258820001
NC_056080.1	63800001	63820001	0.283506	0.407179	SG1	CXHXorf65;FOXO4	NC_056054.1	258825001	258845001
NC_056080.1	74755001	74775001	0.285715	0.27439	SG1	CYLC1	NC_056054.1	258830001	258850001
NC_056080.1	74780001	74800001	0.226138	0.290881	SG1	CYLC1	NC_056054.1	258835001	258855001
NC_056080.1	74785001	74805001	0.209635	0.289952	SG1	CYLC1	NC_056054.1	258840001	258860001
NC_056077.1	37180001	37200001	0.396805	0.428628	SG1	CYP3A24	NC_056054.1	258845001	258865001
NC_056077.1	37185001	37205001	0.31694	0.413218	SG1	CYP3A24	NC_056054.1	258850001	258870001
NC_056080.1	69405001	69425001	0.351255	0.197024	SG1	CYSLTR1	NC_056054.1	258855001	258875001
NC_056080.1	77730001	77750001	0.237399	0.294808	SG1	DACH2	NC_056054.1	258860001	258880001
NC_056080.1	77735001	77755001	0.212915	0.295985	SG1	DACH2	NC_056054.1	258865001	258885001
NC_056080.1	77740001	77760001	0.256972	0.298439	SG1	DACH2	NC_056054.1	258875001	258895001
NC_056080.1	77755001	77775001	0.284635	0.317066	SG1	DACH2	NC_056054.1	258910001	258930001
NC_056080.1	77760001	77780001	0.397933	0.306965	SG1	DACH2	NC_056054.1	258915001	258935001
NC_056080.1	77765001	77785001	0.462643	0.28752	SG1	DACH2	NC_056054.1	258920001	258940001
NC_056080.1	77770001	77790001	0.453521	0.270434	SG1	DACH2	NC_056054.1	258940001	258960001
NC_056060.1	78915001	78935001	0.132472	0.215359	SG1	DCAF5	NC_056054.1	258970001	258990001
NC_056068.1	60780001	60800001	0.228346	0.195995	SG1	DCDC1	NC_056054.1	258975001	258995001
NC_056063.1	25915001	25935001	0.436435	0.232294	SG1	DCLK1	NC_056054.1	258980001	259000001
NC_056063.1	25920001	25940001	0.455265	0.20638	SG1	DCLK1	NC_056054.1	258985001	259005001
NC_056063.1	25925001	25945001	0.370674	0.199521	SG1	DCLK1	NC_056054.1	259010001	259030001
NC_056079.1	13085001	13105001	0.396882	0.190303	SG1	DCTD	NC_056054.1	259015001	259035001
NC_056068.1	5170001	5190001	0.103879	0.210082	SG1	DCUN1D5	NC_056060.1	20860001	20880001
NC_056080.1	123710001	1.24E+08	0.130112	0.223131	SG1	DCX	NC_056057.1	68680001	68700001
NC_056080.1	123715001	1.24E+08	0.197183	0.220028	SG1	DCX	NC_056057.1	68685001	68705001
NC_056054.1	114275001	1.14E+08	0.368848	0.212487	SG1	DDR2	NC_056057.1	68690001	68710001
NC_056054.1	114280001	1.14E+08	0.465677	0.211248	SG1	DDR2	NC_056057.1	68695001	68715001
NC_056066.1	77310001	77330001	0.457898	0.234969	SG1	DDX27	NC_056075.1	43070001	43090001

NC_056066.1	77315001	77335001	0.387272	0.214643	SG1	DDX27	NC_056075.1	43105001	43125001
NC_056066.1	77320001	77340001	0.379252	0.203675	SG1	DDX27	NC_056075.1	43120001	43140001
NC_056055.1	101615001	1.02E+08	0.321239	0.185174	SG1	DDX58	NC_056075.1	43125001	43145001
NC_056054.1	91975001	91995001	0.233963	0.408983	SG1	DENND2C	NC_056054.1	104335001	104355001
NC_056054.1	91995001	92015001	0.073755	0.500461	SG1	DENND2C	NC_056054.1	104340001	104360001
NC_056054.1	92000001	92020001	0.19305	0.430906	SG1	DENND2C	NC_056054.1	104355001	104375001
NC_056054.1	92005001	92025001	0.432144	0.32441	SG1	DENND2C	NC_056054.1	104350001	104370001
NC_056068.1	43160001	43180001	0.284911	0.183366	SG1	DENND5A	NC_056054.1	104345001	104365001
NC_056068.1	43165001	43185001	0.310379	0.238493	SG1	DENND5A	NC_056056.1	202840001	202860001
NC_056068.1	43170001	43190001	0.412581	0.225313	SG1	DENND5A	NC_056056.1	202845001	202865001
NC_056070.1	72620001	72640001	0.140065	0.4451	SG1	DGCR2;ESS2;GSC2;T	NC_056056.1	202850001	202870001
NC_056070.1	72610001	72630001	0.395258	0.304298	SG1	DGCR2;ESS2;TSSK1	NC_056056.1	202855001	202875001
NC_056070.1	72615001	72635001	0.25251	0.395003	SG1	DGCR2;ESS2;TSSK1	NC_056056.1	202830001	202850001
NC_056057.1	103000001	1.03E+08	0.344371	0.216988	SG1	DGKI	NC_056056.1	202835001	202855001
NC_056080.1	54900001	54920001	0.363825	0.240838	SG1	DGKK	NC_056066.1	17565001	17585001
NC_056067.1	38575001	38595001	0.046038	0.312996	SG1	DHODH	NC_056066.1	17570001	17590001
NC_056067.1	38580001	38600001	0.107331	0.284517	SG1	DHODH	NC_056066.1	17625001	17645001
NC_056067.1	38585001	38605001	0.220618	0.244399	SG1	DHODH	NC_056064.1	22445001	22465001
NC_056067.1	38590001	38610001	0.330695	0.206181	SG1	DHODH	NC_056064.1	22450001	22470001
NC_056080.1	138345001	1.38E+08	0.368933	0.213314	SG1	DIAPH2	NC_056064.1	22455001	22475001
NC_056080.1	138350001	1.38E+08	0.25083	0.244862	SG1	DIAPH2	NC_056057.1	25575001	25595001
NC_056080.1	138355001	1.38E+08	0.244907	0.251186	SG1	DIAPH2	NC_056054.1	263990001	264010001
NC_056080.1	138360001	1.38E+08	0.252692	0.240632	SG1	DIAPH2	NC_056054.1	263995001	264015001
NC_056080.1	138365001	1.38E+08	0.19102	0.251027	SG1	DIAPH2	NC_056054.1	201455001	201475001
NC_056080.1	138370001	1.38E+08	0.252395	0.242498	SG1	DIAPH2	NC_056054.1	201445001	201465001
NC_056054.1	70045001	70065001	0.426544	0.266673	SG1	DIPK1A	NC_056054.1	201450001	201470001
NC_056054.1	70050001	70070001	0.457873	0.249692	SG1	DIPK1A	NC_056054.1	92100001	92120001
NC_056054.1	70030001	70050001	0.443478	0.366514	SG1	DIPK1A;RPL5	NC_056054.1	92105001	92125001
NC_056054.1	70035001	70055001	0.377804	0.332563	SG1	DIPK1A;RPL5	NC_056054.1	92090001	92110001
NC_056054.1	70040001	70060001	0.390473	0.299026	SG1	DIPK1A;RPL5	NC_056054.1	92095001	92115001
NC_056055.1	233645001	2.34E+08	0.382728	0.193904	SG1	DIS3L2	NC_056054.1	87245001	87265001
NC_056055.1	233650001	2.34E+08	0.398374	0.202006	SG1	DIS3L2	NC_056071.1	31535001	31555001

NC_056065.1	25540001	25560001	0.359161	0.212169	SG1	DISP1	NC_056071.1	31540001	31560001
NC_056065.1	25545001	25565001	0.197171	0.317132	SG1	DISP1	NC_056071.1	31545001	31565001
NC_056065.1	25550001	25570001	0.154351	0.308717	SG1	DISP1	NC_056071.1	31550001	31570001
NC_056065.1	25555001	25575001	0.234828	0.265278	SG1	DISP1	NC_056075.1	44085001	44105001
NC_056065.1	25560001	25580001	0.268913	0.229895	SG1	DISP1	NC_056075.1	44090001	44110001
NC_056060.1	34185001	34205001	0.313432	0.189056	SG1	DISP2	NC_056075.1	44200001	44220001
NC_056060.1	34190001	34210001	0.303318	0.198974	SG1	DISP2;KNSTRN	NC_056076.1	48700001	48720001
NC_056068.1	40460001	40480001	0.053484	0.334212	SG1	DKK3	NC_056076.1	48705001	48725001
NC_056068.1	40465001	40485001	0.071661	0.359665	SG1	DKK3	NC_056076.1	48710001	48730001
NC_056068.1	40470001	40490001	0.166515	0.342411	SG1	DKK3	NC_056076.1	48780001	48800001
NC_056068.1	40475001	40495001	0.207211	0.362078	SG1	DKK3	NC_056076.1	48785001	48805001
NC_056068.1	40480001	40500001	0.398125	0.286755	SG1	DKK3	NC_056076.1	48790001	48810001
NC_056057.1	50450001	50470001	0.336663	0.331459	SG1	DLD	NC_056076.1	48795001	48815001
NC_056057.1	50455001	50475001	0.204607	0.388036	SG1	DLD	NC_056069.1	61715001	61735001
NC_056057.1	50460001	50480001	0.144677	0.411305	SG1	DLD	NC_056069.1	61705001	61725001
NC_056057.1	50465001	50485001	0.281412	0.344535	SG1	DLD;LAMB1	NC_056069.1	61710001	61730001
NC_056074.1	11190001	11210001	0.340651	0.184232	SG1	DLG2	NC_056074.1	40415001	40435001
NC_056076.1	38310001	38330001	0.134657	0.252274	SG1	DLGAP1	NC_056066.1	17635001	17655001
NC_056076.1	38315001	38335001	0.159574	0.28581	SG1	DLGAP1	NC_056066.1	17665001	17685001
NC_056076.1	38320001	38340001	0.135105	0.322608	SG1	DLGAP1	NC_056066.1	17700001	17720001
NC_056076.1	38325001	38345001	0.119219	0.342524	SG1	DLGAP1	NC_056066.1	17705001	17725001
NC_056076.1	38330001	38350001	0.097187	0.290349	SG1	DLGAP1	NC_056066.1	17710001	17730001
NC_056076.1	38335001	38355001	0.152335	0.31731	SG1	DLGAP1	NC_056066.1	17715001	17735001
NC_056076.1	38340001	38360001	0.369637	0.265222	SG1	DLGAP1	NC_056066.1	17720001	17740001
NC_056080.1	31735001	31755001	0.331935	0.227899	SG1	DMD	NC_056066.1	17725001	17745001
NC_056080.1	31740001	31760001	0.277108	0.2365	SG1	DMD	NC_056054.1	140295001	140315001
NC_056080.1	31745001	31765001	0.382385	0.230056	SG1	DMD	NC_056054.1	140300001	140320001
NC_056060.1	57030001	57050001	0.293094	0.212187	SG1	DMXL2	NC_056054.1	140315001	140335001
NC_056060.1	57035001	57055001	0.380584	0.193035	SG1	DMXL2	NC_056071.1	31590001	31610001
NC_056060.1	57040001	57060001	0.369046	0.183801	SG1	DMXL2	NC_056071.1	31595001	31615001
NC_056060.1	57045001	57065001	0.312891	0.198207	SG1	DMXL2	NC_056071.1	31605001	31625001
NC_056060.1	57050001	57070001	0.182156	0.213089	SG1	DMXL2	NC_056071.1	31610001	31630001

NC_056060.1	57055001	57075001	0.05278	0.21827	SG1	DMXL2	NC_056071.1	31615001	31635001
NC_056060.1	57060001	57080001	0.22251	0.26927	SG1	DMXL2	NC_056058.1	49035001	49055001
NC_056060.1	57065001	57085001	0.278418	0.280795	SG1	DMXL2	NC_056058.1	49040001	49060001
NC_056057.1	31580001	31600001	0.411782	0.197643	SG1	DNAH11	NC_056058.1	49045001	49065001
NC_056057.1	31585001	31605001	0.360872	0.208764	SG1	DNAH11	NC_056058.1	49055001	49075001
NC_056077.1	19240001	19260001	0.313445	0.229853	SG1	DNAH3	NC_056080.1	77865001	77885001
NC_056077.1	19245001	19265001	0.164884	0.240404	SG1	DNAH3	NC_056080.1	77875001	77895001
NC_056077.1	19250001	19270001	0.36447	0.261885	SG1	DNAH3	NC_056080.1	77880001	77900001
NC_056055.1	198125001	1.98E+08	0.321839	0.266593	SG1	DNAH7	NC_056080.1	77900001	77920001
NC_056055.1	198130001	1.98E+08	0.179134	0.324113	SG1	DNAH7	NC_056080.1	77905001	77925001
NC_056055.1	198135001	1.98E+08	0.3	0.299771	SG1	DNAH7	NC_056080.1	77910001	77930001
NC_056055.1	198200001	1.98E+08	0.350866	0.350974	SG1	DNAH7	NC_056080.1	77915001	77935001
NC_056055.1	198205001	1.98E+08	0.439121	0.312575	SG1	DNAH7	NC_056080.1	77920001	77940001
NC_056054.1	62130001	62150001	0.340077	0.199699	SG1	DNAI3	NC_056080.1	77925001	77945001
NC_056054.1	62135001	62155001	0.303071	0.210325	SG1	DNAI3	NC_056080.1	77955001	77975001
NC_056056.1	190430001	1.9E+08	0.395636	0.316126	SG1	DNAI7	NC_056080.1	77960001	77980001
NC_056056.1	190435001	1.9E+08	0.370766	0.3029	SG1	DNAI7	NC_056080.1	77965001	77985001
NC_056056.1	190440001	1.9E+08	0.40484	0.337864	SG1	DNAI7	NC_056080.1	77995001	78015001
NC_056056.1	190445001	1.9E+08	0.387355	0.310392	SG1	DNAI7	NC_056080.1	78000001	78020001
NC_056056.1	190420001	1.9E+08	0.435714	0.349748	SG1	DNAI7;ETFRF1	NC_056080.1	78005001	78025001
NC_056056.1	190425001	1.9E+08	0.46309	0.374925	SG1	DNAI7;ETFRF1	NC_056080.1	78010001	78030001
NC_056056.1	190415001	1.9E+08	0.405951	0.324458	SG1	DNAI7;ETFRF1;KRA	NC_056080.1	78015001	78035001
NC_056056.1	190495001	1.91E+08	0.330959	0.189514	SG1	DNAI7;IRAG2	NC_056080.1	78020001	78040001
NC_056071.1	28465001	28485001	0.126274	0.214719	SG1	DNAJA4	NC_056080.1	78025001	78045001
NC_056071.1	28470001	28490001	0.213223	0.189925	SG1	DNAJA4	NC_056080.1	78110001	78130001
NC_056072.1	59240001	59260001	0.160064	0.240087	SG1	DNAJB8	NC_056080.1	78115001	78135001
NC_056072.1	59245001	59265001	0.109019	0.264485	SG1	DNAJB8	NC_056080.1	78120001	78140001
NC_056072.1	59250001	59270001	0.072626	0.226252	SG1	DNAJB8	NC_056080.1	78125001	78145001
NC_056072.1	59235001	59255001	0.17044	0.239688	SG1	DNAJB8;GATA2	NC_056080.1	78130001	78150001
NC_056066.1	22385001	22405001	0.393817	0.196733	SG1	DNAJC1	NC_056080.1	78135001	78155001
NC_056066.1	22390001	22410001	0.193086	0.271994	SG1	DNAJC1	NC_056080.1	78140001	78160001
NC_056066.1	22395001	22415001	0.194754	0.274018	SG1	DNAJC1	NC_056060.1	20835001	20855001

NC_056066.1	22400001	22420001	0.146165	0.280337	SG1	DNAJC1	NC_056060.1	20845001	20865001
NC_056066.1	22405001	22425001	0.183577	0.25252	SG1	DNAJC1	NC_056060.1	20840001	20860001
NC_056066.1	22410001	22430001	0.302358	0.184023	SG1	DNAJC1	NC_056054.1	120215001	120235001
NC_056066.1	22435001	22455001	0.456393	0.231945	SG1	DNAJC1	NC_056054.1	120220001	120240001
NC_056066.1	22440001	22460001	0.451913	0.230133	SG1	DNAJC1	NC_056054.1	120230001	120250001
NC_056066.1	22445001	22465001	0.46618	0.217923	SG1	DNAJC1	NC_056054.1	120240001	120260001
NC_056066.1	22525001	22545001	0.429921	0.224927	SG1	DNAJC1	NC_056054.1	120250001	120270001
NC_056066.1	22530001	22550001	0.238506	0.306559	SG1	DNAJC1	NC_056054.1	120255001	120275001
NC_056066.1	22535001	22555001	0.274095	0.323489	SG1	DNAJC1	NC_056054.1	120265001	120285001
NC_056066.1	22540001	22560001	0.39247	0.287861	SG1	DNAJC1	NC_056054.1	120270001	120290001
NC_056074.1	38985001	39005001	0.128588	0.188588	SG1	DNAJC4;FKBP2;PLC	NC_056054.1	120315001	120335001
NC_056055.1	227045001	2.27E+08	0.092795	0.195745	SG1	DOCK10	NC_056068.1	60565001	60585001
NC_056055.1	227050001	2.27E+08	0.129227	0.220044	SG1	DOCK10	NC_056068.1	60570001	60590001
NC_056055.1	227055001	2.27E+08	0.106241	0.254998	SG1	DOCK10	NC_056068.1	60575001	60595001
NC_056055.1	227060001	2.27E+08	0.142461	0.233069	SG1	DOCK10	NC_056068.1	60850001	60870001
NC_056055.1	227065001	2.27E+08	0.198441	0.190092	SG1	DOCK10	NC_056066.1	77335001	77355001
NC_056055.1	227070001	2.27E+08	0.164572	0.197621	SG1	DOCK10	NC_056066.1	77340001	77360001
NC_056060.1	82405001	82425001	0.224979	0.229108	SG1	DPF3	NC_056069.1	23600001	23620001
NC_056060.1	82440001	82460001	0.144491	0.357272	SG1	DPF3	NC_056069.1	23605001	23625001
NC_056060.1	82445001	82465001	0.245777	0.314961	SG1	DPF3	NC_056069.1	23610001	23630001
NC_056060.1	82450001	82470001	0.332994	0.246685	SG1	DPF3	NC_056069.1	23615001	23635001
NC_056054.1	75165001	75185001	0.396785	0.191305	SG1	DPYD	NC_056069.1	23620001	23640001
NC_056054.1	75170001	75190001	0.312068	0.222094	SG1	DPYD	NC_056065.1	26580001	26600001
NC_056054.1	75175001	75195001	0.212728	0.249366	SG1	DPYD	NC_056058.1	50640001	50660001
NC_056054.1	75180001	75200001	0.135647	0.282619	SG1	DPYD	NC_056070.1	70365001	70385001
NC_056078.1	42565001	42585001	0.44668	0.281747	SG1	DRGX	NC_056070.1	70370001	70390001
NC_056078.1	42570001	42590001	0.405252	0.333284	SG1	DRGX	NC_056070.1	70375001	70395001
NC_056078.1	42575001	42595001	0.222769	0.422308	SG1	DRGX	NC_056065.1	31825001	31845001
NC_056078.1	42580001	42600001	0.1843	0.384013	SG1	DRGX	NC_056065.1	31830001	31850001
NC_056069.1	42135001	42155001	0.24864	0.241893	SG1	DROSHA	NC_056065.1	31835001	31855001
NC_056054.1	260875001	2.61E+08	0.233617	0.186756	SG1	DSCAM	NC_056057.1	102735001	102755001
NC_056054.1	260880001	2.61E+08	0.307335	0.218167	SG1	DSCAM	NC_056057.1	102765001	102785001

NC_056059.1	56500001	56520001	0.408114	0.201585	SG1	DTHD1	NC_056057.1	102770001	102790001
NC_056059.1	56505001	56525001	0.357872	0.24966	SG1	DTHD1	NC_056057.1	102775001	102795001
NC_056059.1	56510001	56530001	0.325551	0.247153	SG1	DTHD1	NC_056065.1	40420001	40440001
NC_056076.1	22490001	22510001	0.172131	0.228129	SG1	DTNA	NC_056065.1	40425001	40445001
NC_056076.1	22565001	22585001	0.313808	0.187036	SG1	DTNA	NC_056066.1	46100001	46120001
NC_056075.1	30580001	30600001	0.350962	0.31037	SG1	DUSP5	NC_056066.1	46130001	46150001
NC_056075.1	30605001	30625001	0.397261	0.324817	SG1	DUSP5	NC_056066.1	46135001	46155001
NC_056075.1	30610001	30630001	0.183183	0.315381	SG1	DUSP5	NC_056066.1	46140001	46160001
NC_056068.1	5155001	5175001	0.398983	0.187601	SG1	DYNC2H1	NC_056066.1	46280001	46300001
NC_056068.1	5160001	5180001	0.271111	0.217644	SG1	DYNC2H1	NC_056066.1	46285001	46305001
NC_056061.1	82785001	82805001	0.238658	0.185542	SG1	DYNLT1;SYTL3;TMI	NC_056066.1	46290001	46310001
NC_056061.1	82775001	82795001	0.357064	0.211001	SG1	DYNLT1;TMEM181	NC_056066.1	46330001	46350001
NC_056061.1	82780001	82800001	0.335788	0.215043	SG1	DYNLT1;TMEM181	NC_056066.1	46335001	46355001
NC_056067.1	48770001	48790001	0.419408	0.199229	SG1	DYRK1B	NC_056066.1	46340001	46360001
NC_056067.1	48775001	48795001	0.46389	0.240407	SG1	DYRK1B;FBL	NC_056054.1	70165001	70185001
NC_056067.1	48780001	48800001	0.420382	0.210962	SG1	DYRK1B;FBL	NC_056060.1	13155001	13175001
NC_056058.1	68060001	68080001	0.445235	0.199413	SG1	EBF1	NC_056060.1	13160001	13180001
NC_056067.1	34925001	34945001	0.393221	0.194885	SG1	EDC4;NRN1L;NUTF2	NC_056060.1	13165001	13185001
NC_056067.1	34930001	34950001	0.389656	0.213962	SG1	EDC4;NRN1L;NUTF2	NC_056060.1	13170001	13190001
NC_056072.1	59290001	59310001	0.181885	0.211443	SG1	EEFSEC	NC_056060.1	13175001	13195001
NC_056072.1	56270001	56290001	0.448829	0.192366	SG1	EFCAB12;RPL32	NC_056078.1	3925001	3945001
NC_056060.1	21360001	21380001	0.374341	0.258658	SG1	EFS;IL25	NC_056065.1	25730001	25750001
NC_056060.1	21365001	21385001	0.419395	0.220681	SG1	EFS;IL25;SLC22A17	NC_056054.1	192320001	192340001
NC_056069.1	36210001	36230001	0.438886	0.19585	SG1	EGFLAM	NC_056054.1	192325001	192345001
NC_056069.1	36215001	36235001	0.309928	0.259999	SG1	EGFLAM	NC_056054.1	192330001	192350001
NC_056069.1	36220001	36240001	0.314202	0.272726	SG1	EGFLAM	NC_056054.1	192335001	192355001
NC_056069.1	36225001	36245001	0.348778	0.330324	SG1	EGFLAM	NC_056054.1	192340001	192360001
NC_056069.1	36230001	36250001	0.369009	0.363781	SG1	EGFLAM	NC_056054.1	192345001	192365001
NC_056069.1	36235001	36255001	0.43768	0.357796	SG1	EGFLAM	NC_056071.1	63255001	63275001
NC_056069.1	36240001	36260001	0.447829	0.35865	SG1	EGFLAM	NC_056071.1	63260001	63280001
NC_056069.1	36245001	36265001	0.392477	0.333809	SG1	EGFLAM	NC_056071.1	63265001	63285001
NC_056069.1	36250001	36270001	0.338121	0.30108	SG1	EGFLAM	NC_056071.1	63270001	63290001

NC_056069.1	36255001	36275001	0.240292	0.305346	SG1	EGFLAM	NC_056075.1	41985001	42005001	
NC_056069.1	36260001	36280001	0.184582	0.281602	SG1	EGFLAM	NC_056075.1	41990001	42010001	
NC_056069.1	36265001	36285001	0.183064	0.224891	SG1	EGFLAM	NC_056056.1	216015001	216035001	
NC_056069.1	36270001	36290001	0.236441	0.185068	SG1	EGFLAM	NC_056080.1	30915001	30935001	
NC_056069.1	36285001	36305001	0.289178	0.22246	SG1	EGFLAM	NC_056080.1	31335001	31355001	
NC_056069.1	36290001	36310001	0.195923	0.301973	SG1	EGFLAM	NC_056055.1	91150001	91170001	
NC_056069.1	36295001	36315001	0.177732	0.304304	SG1	EGFLAM	NC_056055.1	91160001	91180001	
NC_056069.1	36300001	36320001	0.158341	0.286482	SG1	EGFLAM	NC_056055.1	91165001	91185001	
NC_056069.1	36305001	36325001	0.200698	0.307503	SG1	EGFLAM	NC_056070.1	51935001	51955001	
NC_056078.1	3475001	3495001	0.175623	0.321974	SG1	EGLN1;SPRTN	NC_056070.1	51940001	51960001	
NC_056078.1	3480001	3500001	0.139387	0.378737	SG1	EGLN1;SPRTN	NC_056070.1	51945001	51965001	
NC_056078.1	3485001	3505001	0.128942	0.429875	SG1	EGLN1;SPRTN	NC_056070.1	51950001	51970001	
NC_056078.1	3490001	3510001	0.272545	0.412415	SG1	EGLN1;SPRTN	NC_056077.1	4160001	4180001	
NC_056078.1	3495001	3515001	0.420856	0.388867	SG1	EGLN1;SPRTN	NC_056077.1	4165001	4185001	
NC_056060.1	59465001	59485001	0.276707	0.25009	SG1	EID1;SHC4	NC_056077.1	4170001	4190001	
NC_056060.1	59470001	59490001	0.165586	0.272763	SG1	EID1;SHC4	NC_056078.1	28485001	28505001	
NC_056060.1	59475001	59495001	0.210526	0.3035	SG1	EID1;SHC4	NC_056078.1	28490001	28510001	
NC_056060.1	59480001	59500001	0.331081	0.253078	SG1	EID1;SHC4	NC_056078.1	28495001	28515001	
NC_056056.1	215485001	2.16E+08	0.116958	0.248222	SG1	EIF3L	NC_056078.1	28480001	28500001	
NC_056056.1	215490001	2.16E+08	0.312993	0.194094	SG1	EIF3L	NC_056075.1	17460001	17480001	
NC_056054.1	201220001	2.01E+08	0.431296	0.213364	SG1	EIF4A2;RFC4	NC_056075.1	17465001	17485001	
NC_056070.1	70105001	70125001	0.387674	0.243408	SG1	EIF4ENIF1	NC_056075.1	17470001	17490001	
NC_056070.1	70110001	70130001	0.406425	0.216819	SG1	EIF4ENIF1	NC_056075.1	17475001	17495001	
NC_056063.1	11555001	11575001	0.306721	0.197095	SG1	ELF1	NC_056069.1	2025001	2045001	
NC_056077.1	41670001	41690001	0.265534	0.241953	SG1	ELFN1	NC_056057.1	57910001	57930001	
NC_056077.1	41675001	41695001	0.375234	0.193016	SG1	ELFN1	NC_056057.1	57930001	57950001	
NC_056067.1	56335001	56355001	0.300656	0.258603	SG1	EMC10;FAM71E1;M'	NC_056054.1	261225001	261245001	
NC_056067.1	56340001	56360001	0.296298	0.260568	SG1	EMC10;FAM71E1;M'	NC_056054.1	261230001	261250001	
NC_056056.1	69020001	69040001	0.391469	0.202275	SG1	EML6;RTN4	NC_056054.1	261235001	261255001	
NC_056056.1	69025001	69045001	0.431002	0.188434	SG1	EML6;RTN4	NC_056069.1	4695001	4715001	
NC_056068.1	54670001	54690001	0.422111	0.215148	SG1	EMSY	NC_056064.1	13475001	13495001	
NC_056068.1	54675001	54695001	0.436046	0.224167	SG1	EMSY	NC_056064.1	13480001	13500001	

NC_056068.1	54680001	54700001	0.46519	0.221214	SG1	EMSY	NC_056064.1	13485001	13505001
NC_056068.1	54685001	54705001	0.418478	0.221148	SG1	EMSY	NC_056064.1	13470001	13490001
NC_056068.1	54690001	54710001	0.278547	0.280452	SG1	EMSY	NC_056065.1	4240001	4260001
NC_056068.1	54695001	54715001	0.300879	0.255997	SG1	EMSY	NC_056065.1	4245001	4265001
NC_056068.1	54700001	54720001	0.403333	0.214029	SG1	EMSY	NC_056054.1	174370001	174390001
NC_056076.1	36000001	36020001	0.418835	0.582043	SG1	ENOSF1;TYMS	NC_056054.1	174375001	174395001
NC_056063.1	14010001	14030001	0.277447	0.182997	SG1	ENOX1	NC_056055.1	12045001	12065001
NC_056056.1	535001	555001	0.425829	0.201516	SG1	ENTPD8;NOXA1	NC_056055.1	12050001	12070001
NC_056060.1	36655001	36675001	0.223724	0.314134	SG1	EPB42	NC_056058.1	81650001	81670001
NC_056054.1	158220001	1.58E+08	0.385757	0.383493	SG1	EPHA3	NC_056073.1	43345001	43365001
NC_056054.1	158225001	1.58E+08	0.444028	0.323538	SG1	EPHA3	NC_056073.1	43350001	43370001
NC_056059.1	81565001	81585001	0.352709	0.302416	SG1	EPHA5	NC_056073.1	46550001	46570001
NC_056059.1	81570001	81590001	0.25365	0.356622	SG1	EPHA5	NC_056073.1	46555001	46575001
NC_056059.1	81575001	81595001	0.207637	0.365638	SG1	EPHA5	NC_056073.1	46560001	46580001
NC_056059.1	81580001	81600001	0.209303	0.392251	SG1	EPHA5	NC_056073.1	46565001	46585001
NC_056059.1	81585001	81605001	0.146038	0.476606	SG1	EPHA5	NC_056057.1	62665001	62685001
NC_056059.1	81590001	81610001	0.262296	0.431656	SG1	EPHA5	NC_056057.1	62670001	62690001
NC_056059.1	81595001	81615001	0.315088	0.417888	SG1	EPHA5	NC_056065.1	31595001	31615001
NC_056059.1	81610001	81630001	0.446284	0.34103	SG1	EPHA5	NC_056056.1	220335001	220355001
NC_056059.1	81615001	81635001	0.353731	0.404426	SG1	EPHA5	NC_056080.1	61720001	61740001
NC_056059.1	81620001	81640001	0.315825	0.433921	SG1	EPHA5	NC_056080.1	61725001	61745001
NC_056059.1	81625001	81645001	0.34	0.413329	SG1	EPHA5	NC_056080.1	61745001	61765001
NC_056059.1	81630001	81650001	0.402655	0.35575	SG1	EPHA5	NC_056068.1	63980001	64000001
NC_056059.1	81635001	81655001	0.401721	0.303738	SG1	EPHA5	NC_056056.1	59295001	59315001
NC_056059.1	81640001	81660001	0.307051	0.278013	SG1	EPHA5	NC_056056.1	59300001	59320001
NC_056059.1	81645001	81665001	0.221053	0.273064	SG1	EPHA5	NC_056075.1	38785001	38805001
NC_056059.1	81650001	81670001	0.09176	0.307836	SG1	EPHA5	NC_056075.1	38790001	38810001
NC_056059.1	81655001	81675001	0.154753	0.312177	SG1	EPHA5	NC_056055.1	1780001	1800001
NC_056059.1	81660001	81680001	0.364186	0.417981	SG1	EPHA5	NC_056055.1	1785001	1805001
NC_056059.1	81665001	81685001	0.4254	0.460911	SG1	EPHA5	NC_056055.1	1790001	1810001
NC_056059.1	81680001	81700001	0.433472	0.394381	SG1	EPHA5	NC_056055.1	1795001	1815001
NC_056059.1	81685001	81705001	0.403415	0.335028	SG1	EPHA5	NC_056055.1	1800001	1820001

NC_056059.1	81690001	81710001	0.39536	0.288808	SG1	EPHA5	NC_056068.1	61145001	61165001
NC_056059.1	81695001	81715001	0.402962	0.266517	SG1	EPHA5	NC_056068.1	61150001	61170001
NC_056059.1	81700001	81720001	0.419089	0.265664	SG1	EPHA5	NC_056068.1	61155001	61175001
NC_056059.1	81705001	81725001	0.424632	0.277321	SG1	EPHA5	NC_056068.1	61160001	61180001
NC_056059.1	81710001	81730001	0.393804	0.285604	SG1	EPHA5	NC_056056.1	209320001	209340001
NC_056059.1	81715001	81735001	0.319073	0.279337	SG1	EPHA5	NC_056056.1	209325001	209345001
NC_056059.1	81720001	81740001	0.228436	0.261366	SG1	EPHA5	NC_056056.1	209330001	209350001
NC_056059.1	81725001	81745001	0.242598	0.232187	SG1	EPHA5	NC_056056.1	209335001	209355001
NC_056059.1	81730001	81750001	0.256357	0.227982	SG1	EPHA5	NC_056057.1	118810001	118830001
NC_056059.1	81735001	81755001	0.358489	0.219335	SG1	EPHA5	NC_056057.1	118815001	118835001
NC_056059.1	81745001	81765001	0.449102	0.27049	SG1	EPHA5	NC_056065.1	27950001	27970001
NC_056059.1	81755001	81775001	0.460474	0.246322	SG1	EPHA5	NC_056065.1	27955001	27975001
NC_056059.1	81760001	81780001	0.366666	0.215076	SG1	EPHA5	NC_056065.1	27960001	27980001
NC_056059.1	81765001	81785001	0.406308	0.188754	SG1	EPHA5	NC_056065.1	27965001	27985001
NC_056054.1	162045001	1.62E+08	0.359656	0.211604	SG1	EPHA6	NC_056075.1	36760001	36780001
NC_056054.1	162610001	1.63E+08	0.440346	0.207559	SG1	EPHA6	NC_056075.1	36765001	36785001
NC_056054.1	162615001	1.63E+08	0.459681	0.194599	SG1	EPHA6	NC_056075.1	36790001	36810001
NC_056054.1	254800001	2.55E+08	0.413553	0.216343	SG1	EPHB1	NC_056075.1	36795001	36815001
NC_056054.1	254805001	2.55E+08	0.385617	0.235353	SG1	EPHB1	NC_056079.1	13940001	13960001
NC_056054.1	254810001	2.55E+08	0.384583	0.21521	SG1	EPHB1	NC_056079.1	13945001	13965001
NC_056062.1	13895001	13915001	0.359664	0.24308	SG1	EPPK1	NC_056079.1	13950001	13970001
NC_056062.1	13900001	13920001	0.421122	0.24492	SG1	EPPK1;NRBP2	NC_056079.1	13955001	13975001
NC_056058.1	6435001	6455001	0.061145	0.217107	SG1	EPS15L1	NC_056060.1	83445001	83465001
NC_056054.1	3120001	3140001	0.161329	0.322928	SG1	ERFE;LOC101109322	NC_056060.1	83450001	83470001
NC_056054.1	3125001	3145001	0.083664	0.32062	SG1	ERFE;LOC101109322	NC_056066.1	32545001	32565001
NC_056054.1	3130001	3150001	0.242672	0.215113	SG1	ERFE;LOC101109322	NC_056066.1	32550001	32570001
NC_056069.1	4750001	4770001	0.165468	0.223684	SG1	ERGIC1	NC_056066.1	32555001	32575001
NC_056069.1	4755001	4775001	0.092037	0.195482	SG1	ERGIC1	NC_056066.1	32560001	32580001
NC_056069.1	4760001	4780001	0.090792	0.237851	SG1	ERGIC1	NC_056066.1	32565001	32585001
NC_056069.1	4765001	4785001	0.081272	0.23061	SG1	ERGIC1	NC_056066.1	32570001	32590001
NC_056069.1	4770001	4790001	0.074766	0.251611	SG1	ERGIC1	NC_056066.1	32575001	32595001
NC_056069.1	4775001	4795001	0.246154	0.218171	SG1	ERGIC1	NC_056076.1	45965001	45985001

NC_056061.1	76820001	76840001	0.461059	0.22682	SG1	ESR1	NC_056076.1	45970001	45990001
NC_056061.1	76825001	76845001	0.320763	0.242674	SG1	ESR1	NC_056076.1	45975001	45995001
NC_056061.1	76830001	76850001	0.300619	0.224239	SG1	ESR1	NC_056076.1	45980001	46000001
NC_056061.1	76835001	76855001	0.261102	0.218958	SG1	ESR1	NC_056076.1	45985001	46005001
NC_056070.1	72625001	72645001	0.280931	0.323672	SG1	ESS2;GSC2;TSSK1B;	NC_056057.1	108020001	108040001
NC_056067.1	51450001	51470001	0.094226	0.208168	SG1	ETHE1;XRCC1;ZNF5	NC_056054.1	255055001	255075001
NC_056067.1	51445001	51465001	0.102028	0.221235	SG1	ETHE1;ZNF575	NC_056054.1	255060001	255080001
NC_056056.1	203680001	2.04E+08	0.390538	0.190918	SG1	ETV6	NC_056054.1	255065001	255085001
NC_056056.1	203685001	2.04E+08	0.443787	0.191763	SG1	ETV6	NC_056054.1	255070001	255090001
NC_056078.1	3445001	3465001	0.338605	0.264598	SG1	EXOC8;GNPAT	NC_056065.1	22725001	22745001
NC_056078.1	3450001	3470001	0.466667	0.357262	SG1	EXOC8;SPRTN	NC_056065.1	22730001	22750001
NC_056067.1	50075001	50095001	0.081697	0.477707	SG1	EXOSC5;TMEM91	NC_056065.1	22735001	22755001
NC_056063.1	24955001	24975001	0.370807	0.352677	SG1	EXOSC8;SUPT20H	NC_056072.1	45145001	45165001
NC_056063.1	24960001	24980001	0.44287	0.367654	SG1	EXOSC8;SUPT20H	NC_056062.1	82550001	82570001
NC_056062.1	59445001	59465001	0.466813	0.187355	SG1	EXT1	NC_056062.1	82555001	82575001
NC_056061.1	58695001	58715001	0.283695	0.189577	SG1	EYA4	NC_056062.1	82560001	82580001
NC_056061.1	58705001	58725001	0.18254	0.220307	SG1	EYA4	NC_056062.1	82565001	82585001
NC_056061.1	58710001	58730001	0.244593	0.223782	SG1	EYA4	NC_056071.1	29305001	29325001
NC_056065.1	36420001	36440001	0.139688	0.281392	SG1	F5	NC_056071.1	29310001	29330001
NC_056065.1	36425001	36445001	0.162211	0.249309	SG1	F5	NC_056071.1	29315001	29335001
NC_056065.1	36430001	36450001	0.332656	0.201863	SG1	F5	NC_056071.1	29320001	29340001
NC_056080.1	81470001	81490001	0.248705	0.1906	SG1	F8	NC_056070.1	40540001	40560001
NC_056054.1	86010001	86030001	0.45326	0.315858	SG1	FAM102B;HENMT1;]	NC_056070.1	40545001	40565001
NC_056054.1	86015001	86035001	0.310177	0.326243	SG1	FAM102B;LOC12181	NC_056065.1	34230001	34250001
NC_056078.1	14235001	14255001	0.123616	0.242888	SG1	FAM13C	NC_056057.1	98605001	98625001
NC_056078.1	14240001	14260001	0.136424	0.214893	SG1	FAM13C	NC_056057.1	98610001	98630001
NC_056078.1	14225001	14245001	0.411933	0.225148	SG1	FAM13C;PHYHIPL	NC_056057.1	98615001	98635001
NC_056078.1	14230001	14250001	0.117788	0.321603	SG1	FAM13C;PHYHIPL	NC_056057.1	98620001	98640001
NC_056054.1	187680001	1.88E+08	0.235504	0.192677	SG1	FAM162A;WDR5B	NC_056056.1	94460001	94480001
NC_056068.1	51720001	51740001	0.451084	0.190377	SG1	FAM168A	NC_056061.1	58675001	58695001
NC_056068.1	51725001	51745001	0.333472	0.212322	SG1	FAM168A	NC_056061.1	58680001	58700001
NC_056068.1	51730001	51750001	0.282697	0.212654	SG1	FAM168A	NC_056061.1	58685001	58705001

NC_056068.1	51735001	51755001	0.298713	0.202018	SG1	FAM168A	NC_056072.1	42815001	42835001
NC_056055.1	52700001	52720001	0.303797	0.195811	SG1	FAM221B;TMEM8B	NC_056059.1	36585001	36605001
NC_056055.1	52705001	52725001	0.290514	0.210578	SG1	FAM221B;TMEM8B	NC_056055.1	122625001	122645001
NC_056055.1	52710001	52730001	0.255278	0.250802	SG1	FAM221B;TMEM8B	NC_056066.1	59230001	59250001
NC_056055.1	29210001	29230001	0.305797	0.192681	SG1	FAM240B	NC_056066.1	59240001	59260001
NC_056067.1	56325001	56345001	0.260244	0.198394	SG1	FAM71E1;MYBPC2	NC_056072.1	42800001	42820001
NC_056067.1	56330001	56350001	0.282169	0.215096	SG1	FAM71E1;MYBPC2	NC_056072.1	42805001	42825001
NC_056054.1	395001	415001	0.458639	0.256478	SG1	FARP2	NC_056072.1	42810001	42830001
NC_056054.1	400001	420001	0.463487	0.267023	SG1	FARP2	NC_056056.1	217190001	217210001
NC_056054.1	405001	425001	0.431227	0.300893	SG1	FARP2	NC_056056.1	217195001	217215001
NC_056054.1	410001	430001	0.391495	0.300913	SG1	FARP2	NC_056068.1	38730001	38750001
NC_056070.1	57920001	57940001	0.474064	0.300367	SG1	FBXO21	NC_056068.1	38735001	38755001
NC_056068.1	51120001	51140001	0.350725	0.224708	SG1	FCHSD2	NC_056068.1	38740001	38760001
NC_056068.1	51125001	51145001	0.191232	0.258022	SG1	FCHSD2	NC_056068.1	38745001	38765001
NC_056068.1	51130001	51150001	0.268233	0.271805	SG1	FCHSD2	NC_056068.1	38750001	38770001
NC_056068.1	51135001	51155001	0.321692	0.267188	SG1	FCHSD2	NC_056068.1	38755001	38775001
NC_056068.1	51140001	51160001	0.363489	0.272824	SG1	FCHSD2	NC_056068.1	38760001	38780001
NC_056068.1	51145001	51165001	0.422378	0.299791	SG1	FCHSD2	NC_056068.1	38765001	38785001
NC_056068.1	51150001	51170001	0.472738	0.353581	SG1	FCHSD2	NC_056068.1	38770001	38790001
NC_056054.1	108025001	1.08E+08	0.36595	0.231301	SG1	FCRL3	NC_056068.1	38775001	38795001
NC_056054.1	108050001	1.08E+08	0.403691	0.241833	SG1	FCRL3	NC_056068.1	38780001	38800001
NC_056054.1	108055001	1.08E+08	0.456954	0.214155	SG1	FCRL3	NC_056068.1	38795001	38815001
NC_056054.1	108020001	1.08E+08	0.437202	0.18411	SG1	FCRL3;LOC1011087{	NC_056068.1	38800001	38820001
NC_056071.1	21075001	21095001	0.12132	0.397371	SG1	FES;FURIN	NC_056074.1	1635001	1655001
NC_056071.1	21080001	21100001	0.233958	0.320798	SG1	FES;FURIN	NC_056074.1	1640001	1660001
NC_056067.1	45390001	45410001	0.449715	0.202705	SG1	FFAR2	NC_056074.1	1645001	1665001
NC_056059.1	95670001	95690001	0.397356	0.183807	SG1	FGF5	NC_056070.1	32635001	32655001
NC_056059.1	95675001	95695001	0.340191	0.220621	SG1	FGF5	NC_056070.1	32640001	32660001
NC_056059.1	95680001	95700001	0.301433	0.191007	SG1	FGF5	NC_056070.1	32645001	32665001
NC_056059.1	95685001	95705001	0.271438	0.218537	SG1	FGF5	NC_056070.1	32650001	32670001
NC_056059.1	95690001	95710001	0.168176	0.258655	SG1	FGF5	NC_056070.1	32655001	32675001
NC_056070.1	4880001	4900001	0.431247	0.198652	SG1	FHDC1	NC_056067.1	34370001	34390001

NC_056070.1	4885001	4905001	0.313476	0.225265	SG1	FHDC1	NC_056067.1	34365001	34385001
NC_056070.1	4890001	4910001	0.14718	0.270645	SG1	FHDC1	NC_056071.1	29010001	29030001
NC_056070.1	4895001	4915001	0.259961	0.224175	SG1	FHDC1	NC_056071.1	29015001	29035001
NC_056070.1	4900001	4920001	0.282635	0.212227	SG1	FHDC1	NC_056065.1	26540001	26560001
NC_056072.1	43295001	43315001	0.43165	0.200576	SG1	FLNB	NC_056065.1	26550001	26570001
NC_056066.1	7730001	7750001	0.404226	0.235775	SG1	FLRT3;MACROD2	NC_056065.1	26555001	26575001
NC_056066.1	7735001	7755001	0.348692	0.272458	SG1	FLRT3;MACROD2	NC_056065.1	26560001	26580001
NC_056066.1	7740001	7760001	0.245504	0.300185	SG1	FLRT3;MACROD2	NC_056068.1	51095001	51115001
NC_056066.1	7745001	7765001	0.195713	0.306818	SG1	FLRT3;MACROD2	NC_056068.1	51100001	51120001
NC_056063.1	31910001	31930001	0.111856	0.323014	SG1	FLT1	NC_056068.1	51105001	51125001
NC_056063.1	31915001	31935001	0.19984	0.202235	SG1	FLT1	NC_056060.1	63625001	63645001
NC_056067.1	55670001	55690001	0.260168	0.232847	SG1	FLT3LG;RPL13A;RPS	NC_056060.1	63630001	63650001
NC_056054.1	70605001	70625001	0.342981	0.189287	SG1	FNBP1L	NC_056060.1	63635001	63655001
NC_056054.1	70610001	70630001	0.189655	0.241026	SG1	FNBP1L	NC_056060.1	63640001	63660001
NC_056054.1	70620001	70640001	0.163792	0.270591	SG1	FNBP1L	NC_056060.1	63645001	63665001
NC_056054.1	70625001	70645001	0.192469	0.288872	SG1	FNBP1L	NC_056060.1	63650001	63670001
NC_056054.1	70630001	70650001	0.23489	0.269315	SG1	FNBP1L	NC_056060.1	63655001	63675001
NC_056054.1	70635001	70655001	0.331338	0.212691	SG1	FNBP1L	NC_056056.1	87430001	87450001
NC_056056.1	207140001	2.07E+08	0.248336	0.196126	SG1	FOXJ2	NC_056056.1	87435001	87455001
NC_056056.1	76110001	76130001	0.318301	0.199483	SG1	FOXN2	NC_056063.1	35610001	35630001
NC_056056.1	76115001	76135001	0.296652	0.198238	SG1	FOXN2	NC_056063.1	35615001	35635001
NC_056060.1	98880001	98900001	0.424971	0.304187	SG1	FOXN3	NC_056063.1	35620001	35640001
NC_056060.1	98885001	98905001	0.243168	0.325253	SG1	FOXN3	NC_056065.1	51455001	51475001
NC_056060.1	98890001	98910001	0.191077	0.289578	SG1	FOXN3	NC_056072.1	40670001	40690001
NC_056060.1	99065001	99085001	0.428488	0.22338	SG1	FOXN3	NC_056063.1	31980001	32000001
NC_056060.1	99070001	99090001	0.412286	0.255916	SG1	FOXN3	NC_056078.1	2635001	2655001
NC_056072.1	30615001	30635001	0.379378	0.222333	SG1	FOXP1	NC_056078.1	2640001	2660001
NC_056072.1	30620001	30640001	0.4072	0.249319	SG1	FOXP1	NC_056078.1	2645001	2665001
NC_056072.1	30625001	30645001	0.380993	0.202393	SG1	FOXP1	NC_056068.1	50505001	50525001
NC_056057.1	55455001	55475001	0.425625	0.222782	SG1	FOXP2	NC_056071.1	53440001	53460001
NC_056057.1	55460001	55480001	0.285036	0.263363	SG1	FOXP2	NC_056071.1	53445001	53465001
NC_056057.1	55465001	55485001	0.179993	0.283654	SG1	FOXP2	NC_056071.1	53465001	53485001

NC_056057.1	55470001	55490001	0.169453	0.285394	SG1	FOXP2	NC_056071.1	53470001	53490001
NC_056057.1	55475001	55495001	0.279178	0.27527	SG1	FOXP2	NC_056070.1	37170001	37190001
NC_056057.1	55670001	55690001	0.313168	0.19198	SG1	FOXP2	NC_056070.1	37175001	37195001
NC_056057.1	55675001	55695001	0.355083	0.202007	SG1	FOXP2	NC_056071.1	38955001	38975001
NC_056057.1	55680001	55700001	0.287865	0.195491	SG1	FOXP2	NC_056071.1	38960001	38980001
NC_056057.1	55685001	55705001	0.280957	0.201301	SG1	FOXP2	NC_056071.1	38965001	38985001
NC_056057.1	55810001	55830001	0.47161	0.240818	SG1	FOXP2	NC_056071.1	38970001	38990001
NC_056057.1	55815001	55835001	0.295103	0.279503	SG1	FOXP2	NC_056071.1	38975001	38995001
NC_056057.1	55820001	55840001	0.258997	0.289143	SG1	FOXP2	NC_056071.1	38995001	39015001
NC_056057.1	55825001	55845001	0.404153	0.286284	SG1	FOXP2	NC_056080.1	83375001	83395001
NC_056059.1	93840001	93860001	0.149517	0.183585	SG1	FRAS1	NC_056080.1	83380001	83400001
NC_056061.1	21830001	21850001	0.287172	0.208475	SG1	FRK	NC_056080.1	83385001	83405001
NC_056061.1	21890001	21910001	0.24017	0.200322	SG1	FRK	NC_056080.1	83390001	83410001
NC_056061.1	21895001	21915001	0.255659	0.214896	SG1	FRK	NC_056059.1	66690001	66710001
NC_056066.1	27720001	27740001	0.423224	0.302062	SG1	FRMD4A	NC_056078.1	1650001	1670001
NC_056066.1	27725001	27745001	0.357323	0.247059	SG1	FRMD4A	NC_056078.1	1655001	1675001
NC_056071.1	22390001	22410001	0.462373	0.259633	SG1	FSD2;WHAMM	NC_056078.1	1660001	1680001
NC_056060.1	33520001	33540001	0.165138	0.20319	SG1	FSIP1	NC_056055.1	107840001	107860001
NC_056060.1	33525001	33545001	0.338874	0.291199	SG1	FSIP1	NC_056055.1	107845001	107865001
NC_056070.1	37230001	37250001	0.404169	0.269962	SG1	FSTL5	NC_056055.1	107975001	107995001
NC_056070.1	37235001	37255001	0.34832	0.299238	SG1	FSTL5	NC_056055.1	107980001	108000001
NC_056070.1	37240001	37260001	0.230173	0.336202	SG1	FSTL5	NC_056055.1	107985001	108005001
NC_056070.1	37245001	37265001	0.281415	0.235814	SG1	FSTL5	NC_056055.1	107990001	108010001
NC_056060.1	75615001	75635001	0.374184	0.206093	SG1	FUT8	NC_056055.1	107995001	108015001
NC_056060.1	75620001	75640001	0.357873	0.248854	SG1	FUT8	NC_056055.1	108020001	108040001
NC_056060.1	75625001	75645001	0.413191	0.282586	SG1	FUT8	NC_056067.1	44255001	44275001
NC_056069.1	35465001	35485001	0.467328	0.229874	SG1	FYB1	NC_056067.1	44260001	44280001
NC_056069.1	35470001	35490001	0.378108	0.275754	SG1	FYB1	NC_056067.1	44165001	44185001
NC_056069.1	35475001	35495001	0.286617	0.300292	SG1	FYB1	NC_056054.1	264845001	264865001
NC_056069.1	35480001	35500001	0.361744	0.22943	SG1	FYB1	NC_056054.1	264830001	264850001
NC_056060.1	58110001	58130001	0.36777	0.221823	SG1	GABPB1	NC_056054.1	264835001	264855001
NC_056060.1	58115001	58135001	0.394837	0.188168	SG1	GABPB1	NC_056054.1	264840001	264860001

NC_056058.1	71170001	71190001	0.230897	0.197203	SG1	GABRA1	NC_056057.1	78770001	78790001
NC_056058.1	71175001	71195001	0.149355	0.290988	SG1	GABRA1	NC_056057.1	66940001	66960001
NC_056060.1	59130001	59150001	0.377849	0.203255	SG1	GALK2	NC_056057.1	66945001	66965001
NC_056060.1	59135001	59155001	0.239369	0.275075	SG1	GALK2	NC_056055.1	186125001	186145001
NC_056060.1	59140001	59160001	0.198586	0.269183	SG1	GALK2	NC_056055.1	186130001	186150001
NC_056060.1	59145001	59165001	0.313836	0.211756	SG1	GALK2	NC_056055.1	186135001	186155001
NC_056060.1	59150001	59170001	0.343483	0.183383	SG1	GALK2	NC_056055.1	186145001	186165001
NC_056060.1	59185001	59205001	0.423078	0.246077	SG1	GALK2	NC_056055.1	186150001	186170001
NC_056078.1	1710001	1730001	0.198862	0.202964	SG1	GALNT2	NC_056055.1	59200001	59220001
NC_056078.1	1715001	1735001	0.15158	0.214664	SG1	GALNT2	NC_056055.1	59245001	59265001
NC_056078.1	1720001	1740001	0.25836	0.244756	SG1	GALNT2	NC_056055.1	59250001	59270001
NC_056078.1	1725001	1745001	0.37499	0.264519	SG1	GALNT2	NC_056055.1	59255001	59275001
NC_056064.1	14355001	14375001	0.272295	0.184488	SG1	GAS2L2;RASL10B	NC_056055.1	59280001	59300001
NC_056072.1	59230001	59250001	0.164094	0.250612	SG1	GATA2	NC_056055.1	11820001	11840001
NC_056054.1	95315001	95335001	0.334235	0.292961	SG1	GDAP2	NC_056058.1	18750001	18770001
NC_056054.1	95320001	95340001	0.221547	0.455385	SG1	GDAP2	NC_056058.1	18880001	18900001
NC_056054.1	95325001	95345001	0.114757	0.470808	SG1	GDAP2	NC_056054.1	105845001	105865001
NC_056054.1	95330001	95350001	0.185558	0.426155	SG1	GDAP2	NC_056054.1	105850001	105870001
NC_056054.1	95335001	95355001	0.33483	0.324346	SG1	GDAP2	NC_056054.1	105865001	105885001
NC_056077.1	17570001	17590001	0.395336	0.225648	SG1	GDE1	NC_056063.1	66660001	66680001
NC_056077.1	17575001	17595001	0.411018	0.253345	SG1	GDE1	NC_056063.1	66665001	66685001
NC_056077.1	17580001	17600001	0.40717	0.259842	SG1	GDE1	NC_056063.1	66670001	66690001
NC_056077.1	17565001	17585001	0.370546	0.194692	SG1	GDE1;TMC5	NC_056063.1	66675001	66695001
NC_056056.1	28525001	28545001	0.122363	0.307524	SG1	GDF7;LDAH	NC_056063.1	66820001	66840001
NC_056056.1	28530001	28550001	0.136964	0.316446	SG1	GDF7;LDAH	NC_056063.1	66825001	66845001
NC_056063.1	76525001	76545001	0.208807	0.20723	SG1	GGACT	NC_056063.1	66830001	66850001
NC_056063.1	76530001	76550001	0.114612	0.272621	SG1	GGACT	NC_056063.1	66835001	66855001
NC_056063.1	76535001	76555001	0.082409	0.291068	SG1	GGACT	NC_056063.1	66840001	66860001
NC_056063.1	76540001	76560001	0.129638	0.261715	SG1	GGACT	NC_056063.1	67375001	67395001
NC_056063.1	76545001	76565001	0.147685	0.206197	SG1	GGACT	NC_056063.1	67380001	67400001
NC_056061.1	73905001	73925001	0.173653	0.195038	SG1	GINM1;KATNA1	NC_056063.1	67385001	67405001
NC_056057.1	42115001	42135001	0.20869	0.21486	SG1	GNAT3	NC_056063.1	67390001	67410001

NC_056057.1	42120001	42140001	0.144106	0.253181	SG1	GNAT3	NC_056060.1	76725001	76745001
NC_056078.1	3440001	3460001	0.261481	0.250483	SG1	GNPAT	NC_056067.1	44265001	44285001
NC_056072.1	10905001	10925001	0.345567	0.251105	SG1	GOLGA4	NC_056058.1	15510001	15530001
NC_056075.1	32255001	32275001	0.439667	0.302061	SG1	GPAM	NC_056066.1	25515001	25535001
NC_056075.1	32260001	32280001	0.227048	0.403749	SG1	GPAM	NC_056066.1	25520001	25540001
NC_056075.1	32265001	32285001	0.152174	0.441334	SG1	GPAM	NC_056066.1	25525001	25545001
NC_056075.1	32270001	32290001	0.191983	0.402991	SG1	GPAM	NC_056066.1	25565001	25585001
NC_056075.1	32275001	32295001	0.093573	0.429363	SG1	GPAM	NC_056054.1	120375001	120395001
NC_056075.1	32280001	32300001	0.118473	0.295878	SG1	GPAM	NC_056054.1	120380001	120400001
NC_056079.1	35875001	35895001	0.24985	0.191756	SG1	GPAT4	NC_056054.1	120385001	120405001
NC_056079.1	35880001	35900001	0.265713	0.203203	SG1	GPAT4;NKX6-3	NC_056054.1	120390001	120410001
NC_056063.1	66730001	66750001	0.366414	0.19538	SG1	GPC5	NC_056054.1	120395001	120415001
NC_056063.1	66735001	66755001	0.382871	0.266838	SG1	GPC5	NC_056054.1	120400001	120420001
NC_056063.1	66740001	66760001	0.371695	0.269856	SG1	GPC5	NC_056054.1	120405001	120425001
NC_056063.1	66745001	66765001	0.292457	0.244631	SG1	GPC5	NC_056056.1	202785001	202805001
NC_056063.1	68330001	68350001	0.367614	0.251668	SG1	GPC6	NC_056056.1	202790001	202810001
NC_056063.1	68335001	68355001	0.196362	0.334761	SG1	GPC6	NC_056056.1	202795001	202815001
NC_056063.1	68340001	68360001	0.106519	0.34146	SG1	GPC6	NC_056056.1	202800001	202820001
NC_056063.1	68345001	68365001	0.226837	0.29348	SG1	GPC6	NC_056061.1	40270001	40290001
NC_056063.1	68470001	68490001	0.231495	0.23096	SG1	GPC6	NC_056061.1	40275001	40295001
NC_056063.1	68475001	68495001	0.37037	0.200027	SG1	GPC6	NC_056061.1	40280001	40300001
NC_056060.1	77135001	77155001	0.43567	0.205447	SG1	GPHN	NC_056061.1	40285001	40305001
NC_056060.1	77140001	77160001	0.432559	0.200151	SG1	GPHN	NC_056058.1	60050001	60070001
NC_056080.1	70350001	70370001	0.464442	0.219116	SG1	GPR174	NC_056058.1	60055001	60075001
NC_056080.1	70355001	70375001	0.471607	0.211578	SG1	GPR174	NC_056058.1	60060001	60080001
NC_056060.1	33665001	33685001	0.471139	0.366291	SG1	GPR176	NC_056058.1	25200001	25220001
NC_056060.1	33670001	33690001	0.454852	0.318235	SG1	GPR176	NC_056058.1	25205001	25225001
NC_056060.1	33675001	33695001	0.320951	0.33571	SG1	GPR176	NC_056058.1	25210001	25230001
NC_056060.1	33680001	33700001	0.339923	0.266579	SG1	GPR176	NC_056058.1	25215001	25235001
NC_056060.1	33685001	33705001	0.339049	0.211623	SG1	GPR176	NC_056068.1	1740001	1760001
NC_056060.1	33690001	33710001	0.194372	0.301544	SG1	GPR176	NC_056068.1	1745001	1765001
NC_056060.1	33695001	33715001	0.22073	0.322823	SG1	GPR176	NC_056068.1	1750001	1770001

NC_056060.1	33700001	33720001	0.32106	0.316879	SG1	GPR176	NC_056068.1	1755001	1775001
NC_056075.1	42950001	42970001	0.37591	0.330172	SG1	GPR26	NC_056059.1	32575001	32595001
NC_056057.1	56945001	56965001	0.291966	0.187832	SG1	GPR85	NC_056059.1	32580001	32600001
NC_056058.1	62715001	62735001	0.212168	0.191603	SG1	GRIA1	NC_056059.1	32585001	32605001
NC_056059.1	32855001	32875001	0.39361	0.261491	SG1	GRID2	NC_056059.1	32665001	32685001
NC_056059.1	32860001	32880001	0.232109	0.368756	SG1	GRID2	NC_056059.1	32670001	32690001
NC_056059.1	32865001	32885001	0.153813	0.416835	SG1	GRID2	NC_056059.1	32675001	32695001
NC_056059.1	32870001	32890001	0.163644	0.432534	SG1	GRID2	NC_056059.1	32680001	32700001
NC_056059.1	32875001	32895001	0.277655	0.394139	SG1	GRID2	NC_056061.1	35875001	35895001
NC_056059.1	32880001	32900001	0.366361	0.367016	SG1	GRID2	NC_056059.1	116445001	116465001
NC_056059.1	32885001	32905001	0.460267	0.334675	SG1	GRID2	NC_056075.1	39120001	39140001
NC_056059.1	32965001	32985001	0.362371	0.418581	SG1	GRID2	NC_056072.1	19065001	19085001
NC_056059.1	32970001	32990001	0.343349	0.40764	SG1	GRID2	NC_056072.1	19070001	19090001
NC_056059.1	32975001	32995001	0.343577	0.390701	SG1	GRID2	NC_056072.1	19075001	19095001
NC_056059.1	32980001	33000001	0.381709	0.379496	SG1	GRID2	NC_056057.1	92750001	92770001
NC_056072.1	57665001	57685001	0.32427	0.189099	SG1	GRIP2	NC_056057.1	92790001	92810001
NC_056072.1	57670001	57690001	0.35152	0.201743	SG1	GRIP2	NC_056057.1	92795001	92815001
NC_056061.1	70730001	70750001	0.423734	0.227457	SG1	GRM1	NC_056080.1	53745001	53765001
NC_056061.1	70735001	70755001	0.335306	0.24576	SG1	GRM1	NC_056080.1	53750001	53770001
NC_056061.1	70740001	70760001	0.305405	0.238536	SG1	GRM1	NC_056080.1	53755001	53775001
NC_056061.1	70745001	70765001	0.192481	0.269686	SG1	GRM1	NC_056080.1	53760001	53780001
NC_056061.1	70750001	70770001	0.244555	0.244527	SG1	GRM1	NC_056080.1	53765001	53785001
NC_056074.1	6220001	6240001	0.278887	0.226904	SG1	GRM5	NC_056057.1	76170001	76190001
NC_056074.1	6225001	6245001	0.261013	0.22417	SG1	GRM5	NC_056072.1	28845001	28865001
NC_056074.1	6230001	6250001	0.259148	0.209445	SG1	GRM5	NC_056069.1	29655001	29675001
NC_056072.1	19210001	19230001	0.250904	0.189479	SG1	GRM7	NC_056069.1	29660001	29680001
NC_056072.1	19215001	19235001	0.178904	0.199169	SG1	GRM7	NC_056069.1	29665001	29685001
NC_056057.1	93135001	93155001	0.380405	0.232661	SG1	GRM8	NC_056069.1	29695001	29715001
NC_056057.1	93140001	93160001	0.428909	0.207847	SG1	GRM8	NC_056069.1	29705001	29725001
NC_056077.1	33435001	33455001	0.226604	0.212242	SG1	GTF2IRD1	NC_056054.1	2375001	2395001
NC_056077.1	33440001	33460001	0.121483	0.269686	SG1	GTF2IRD1	NC_056054.1	2380001	2400001
NC_056054.1	174680001	1.75E+08	0.352429	0.224815	SG1	GUCA1C	NC_056057.1	28370001	28390001

NC_056054.1	174685001	1.75E+08	0.3308	0.210381	SG1	GUCA1C	NC_056057.1	28375001	28395001
NC_056054.1	174690001	1.75E+08	0.31121	0.242428	SG1	GUCA1C	NC_056057.1	28380001	28400001
NC_056054.1	174695001	1.75E+08	0.269524	0.257176	SG1	GUCA1C	NC_056060.1	83000001	83020001
NC_056054.1	174700001	1.75E+08	0.244594	0.218665	SG1	GUCA1C	NC_056060.1	83005001	83025001
NC_056054.1	174705001	1.75E+08	0.202584	0.199121	SG1	GUCA1C	NC_056060.1	83010001	83030001
NC_056054.1	174710001	1.75E+08	0.255102	0.191016	SG1	GUCA1C	NC_056060.1	83015001	83035001
NC_056054.1	174720001	1.75E+08	0.410534	0.18387	SG1	GUCA1C	NC_056070.1	62010001	62030001
NC_056054.1	174725001	1.75E+08	0.433011	0.219132	SG1	GUCA1C	NC_056070.1	62025001	62045001
NC_056054.1	174730001	1.75E+08	0.321204	0.224698	SG1	GUCA1C;MORC1	NC_056057.1	79315001	79335001
NC_056054.1	174735001	1.75E+08	0.355555	0.278526	SG1	GUCA1C;MORC1	NC_056057.1	79320001	79340001
NC_056070.1	71545001	71565001	0.434949	0.197517	SG1	GUCD1;SNRPD3;UPl	NC_056057.1	79325001	79345001
NC_056068.1	16035001	16055001	0.398632	0.501551	SG1	GUCY1A2	NC_056057.1	79330001	79350001
NC_056056.1	165825001	1.66E+08	0.454959	0.243528	SG1	HAL	NC_056057.1	79485001	79505001
NC_056056.1	165830001	1.66E+08	0.316698	0.219932	SG1	HAL	NC_056057.1	79490001	79510001
NC_056066.1	50400001	50420001	0.107679	0.233245	SG1	HAO1	NC_056080.1	48385001	48405001
NC_056066.1	50405001	50425001	0.160939	0.235246	SG1	HAO1	NC_056080.1	48390001	48410001
NC_056066.1	50410001	50430001	0.197764	0.233516	SG1	HAO1	NC_056080.1	48395001	48415001
NC_056066.1	50415001	50435001	0.23124	0.215042	SG1	HAO1	NC_056062.1	56045001	56065001
NC_056066.1	50420001	50440001	0.209227	0.199629	SG1	HAO1	NC_056062.1	56050001	56070001
NC_056066.1	50425001	50445001	0.173709	0.190622	SG1	HAO1	NC_056054.1	99315001	99335001
NC_056061.1	60275001	60295001	0.457874	0.214558	SG1	HBS1L	NC_056077.1	4175001	4195001
NC_056061.1	60280001	60300001	0.472759	0.201515	SG1	HBS1L	NC_056077.1	4180001	4200001
NC_056056.1	173330001	1.73E+08	0.333334	0.270328	SG1	HCFC2	NC_056071.1	22415001	22435001
NC_056056.1	173335001	1.73E+08	0.201735	0.325381	SG1	HCFC2	NC_056071.1	22420001	22440001
NC_056056.1	173340001	1.73E+08	0.404617	0.193802	SG1	HCFC2;LOC1011124'	NC_056055.1	134030001	134050001
NC_056056.1	173345001	1.73E+08	0.377332	0.216127	SG1	HCFC2;LOC1011124'	NC_056075.1	36720001	36740001
NC_056066.1	61265001	61285001	0.313614	0.193174	SG1	HCK	NC_056075.1	36725001	36745001
NC_056071.1	21045001	21065001	0.213555	0.213165	SG1	HDDC3;MAN2A2	NC_056075.1	36730001	36750001
NC_056071.1	21040001	21060001	0.219251	0.185765	SG1	HDDC3;MAN2A2;UN	NC_056075.1	36735001	36755001
NC_056080.1	75410001	75430001	0.450866	0.184272	SG1	HDX	NC_056063.1	30075001	30095001
NC_056080.1	75420001	75440001	0.451613	0.217112	SG1	HDX	NC_056063.1	30080001	30100001
NC_056080.1	75455001	75475001	0.471875	0.1875	SG1	HDX	NC_056063.1	30085001	30105001

NC_056055.1	198655001	1.99E+08	0.324616	0.212473	SG1	HECW2	NC_056063.1	30090001	30110001
NC_056079.1	14555001	14575001	0.317258	0.211758	SG1	HELT	NC_056063.1	30095001	30115001
NC_056079.1	14560001	14580001	0.31748	0.221798	SG1	HELT	NC_056063.1	30100001	30120001
NC_056060.1	19085001	19105001	0.269277	0.308029	SG1	HEXA	NC_056063.1	30105001	30125001
NC_056060.1	19090001	19110001	0.403244	0.258027	SG1	HEXA	NC_056059.1	116440001	116460001
NC_056061.1	67500001	67520001	0.245225	0.197064	SG1	HIVEP2	NC_056057.1	76175001	76195001
NC_056061.1	67505001	67525001	0.213959	0.232682	SG1	HIVEP2	NC_056067.1	39380001	39400001
NC_056061.1	67510001	67530001	0.393502	0.188922	SG1	HIVEP2	NC_056067.1	39390001	39410001
NC_056061.1	67535001	67555001	0.390164	0.189117	SG1	HIVEP2	NC_056067.1	39395001	39415001
NC_056065.1	66125001	66145001	0.079059	0.249416	SG1	HMCN1	NC_056067.1	39400001	39420001
NC_056065.1	66130001	66150001	0.086956	0.242571	SG1	HMCN1	NC_056067.1	39405001	39425001
NC_056065.1	66135001	66155001	0.080895	0.309571	SG1	HMCN1	NC_056067.1	39445001	39465001
NC_056065.1	66140001	66160001	0.063129	0.356683	SG1	HMCN1	NC_056067.1	39450001	39470001
NC_056065.1	66145001	66165001	0.051572	0.401917	SG1	HMCN1	NC_056067.1	39455001	39475001
NC_056065.1	66150001	66170001	0.201444	0.395494	SG1	HMCN1	NC_056057.1	17115001	17135001
NC_056065.1	66155001	66175001	0.271368	0.386206	SG1	HMCN1	NC_056080.1	80485001	80505001
NC_056065.1	66160001	66180001	0.331961	0.36203	SG1	HMCN1	NC_056076.1	47190001	47210001
NC_056065.1	66165001	66185001	0.379363	0.344436	SG1	HMCN1	NC_056076.1	47195001	47215001
NC_056065.1	66170001	66190001	0.254202	0.399363	SG1	HMCN1	NC_056054.1	122960001	122980001
NC_056065.1	66175001	66195001	0.190042	0.383145	SG1	HMCN1	NC_056072.1	50110001	50130001
NC_056065.1	66180001	66200001	0.089619	0.330843	SG1	HMCN1	NC_056054.1	227860001	227880001
NC_056073.1	8445001	8465001	0.471236	0.22491	SG1	HMGA1	NC_056054.1	227865001	227885001
NC_056062.1	51700001	51720001	0.27889	0.229453	SG1	HNF4G	NC_056067.1	52170001	52190001
NC_056062.1	51705001	51725001	0.202804	0.195353	SG1	HNF4G	NC_056067.1	52180001	52200001
NC_056062.1	51825001	51845001	0.353426	0.188001	SG1	HNF4G	NC_056067.1	52185001	52205001
NC_056079.1	36915001	36935001	0.038462	0.267258	SG1	HOOK3;RNF170	NC_056071.1	24775001	24795001
NC_056056.1	19570001	19590001	0.153729	0.20516	SG1	HPCAL1	NC_056071.1	24780001	24800001
NC_056056.1	19585001	19605001	0.036613	0.209335	SG1	HPCAL1;ODC1	NC_056071.1	24785001	24805001
NC_056075.1	19685001	19705001	0.150238	0.206066	SG1	HPSE2	NC_056071.1	24790001	24810001
NC_056075.1	19690001	19710001	0.115766	0.250599	SG1	HPSE2	NC_056071.1	24795001	24815001
NC_056075.1	19695001	19715001	0.071552	0.267513	SG1	HPSE2	NC_056069.1	23490001	23510001
NC_056075.1	19700001	19720001	0.074515	0.25261	SG1	HPSE2	NC_056069.1	23350001	23370001

NC_056075.1	19705001	19725001	0.13751	0.230472	SG1	HPSE2	NC_056069.1	23355001	23375001
NC_056075.1	19710001	19730001	0.172636	0.215455	SG1	HPSE2	NC_056057.1	58930001	58950001
NC_056075.1	19715001	19735001	0.171428	0.191523	SG1	HPSE2	NC_056057.1	58935001	58955001
NC_056075.1	19720001	19740001	0.158408	0.209635	SG1	HPSE2	NC_056057.1	94260001	94280001
NC_056055.1	52600001	52620001	0.435691	0.258974	SG1	HRCT1	NC_056057.1	87075001	87095001
NC_056055.1	52605001	52625001	0.429779	0.213341	SG1	HRCT1;SPAAR	NC_056057.1	87080001	87100001
NC_056055.1	52610001	52630001	0.430491	0.224335	SG1	HRCT1;SPAAR	NC_056057.1	87085001	87105001
NC_056055.1	52615001	52635001	0.3861	0.18573	SG1	HRCT1;SPAAR	NC_056057.1	87090001	87110001
NC_056060.1	5460001	5480001	0.443578	0.270454	SG1	HRH2	NC_056054.1	4725001	4745001
NC_056060.1	5465001	5485001	0.473986	0.256773	SG1	HRH2	NC_056054.1	4730001	4750001
NC_056060.1	5470001	5490001	0.461989	0.220035	SG1	HRH2	NC_056054.1	4735001	4755001
NC_056068.1	72690001	72710001	0.419672	0.232252	SG1	HSD17B12	NC_056054.1	4790001	4810001
NC_056055.1	243390001	2.43E+08	0.319164	0.30213	SG1	HTR1D;LOC1141129	NC_056071.1	21450001	21470001
NC_056061.1	50230001	50250001	0.218272	0.219906	SG1	HTR1E	NC_056071.1	21520001	21540001
NC_056061.1	50235001	50255001	0.213563	0.255165	SG1	HTR1E	NC_056066.1	6700001	6720001
NC_056061.1	50240001	50260001	0.214842	0.209836	SG1	HTR1E	NC_056066.1	29405001	29425001
NC_056059.1	115310001	1.15E+08	0.464629	0.217241	SG1	HTRA3	NC_056066.1	29520001	29540001
NC_056054.1	124190001	1.24E+08	0.245849	0.195941	SG1	HUNK	NC_056066.1	29525001	29545001
NC_056054.1	124195001	1.24E+08	0.290922	0.196551	SG1	HUNK	NC_056066.1	29530001	29550001
NC_056054.1	124200001	1.24E+08	0.225053	0.261755	SG1	HUNK	NC_056066.1	29535001	29555001
NC_056054.1	124205001	1.24E+08	0.196177	0.259897	SG1	HUNK	NC_056057.1	66345001	66365001
NC_056054.1	124210001	1.24E+08	0.247771	0.23199	SG1	HUNK	NC_056057.1	66350001	66370001
NC_056056.1	18190001	18210001	0.452421	0.19083	SG1	ID2	NC_056057.1	66355001	66375001
NC_056056.1	34475001	34495001	0.396572	0.245725	SG1	IFT172;KRTCAP3;NF	NC_056057.1	66360001	66380001
NC_056056.1	34480001	34500001	0.315789	0.251451	SG1	IFT172;KRTCAP3;NF	NC_056057.1	66365001	66385001
NC_056056.1	34485001	34505001	0.354929	0.190937	SG1	IFT172;KRTCAP3;NF	NC_056057.1	69540001	69560001
NC_056070.1	54195001	54215001	0.178654	0.195437	SG1	IFT81	NC_056057.1	69605001	69625001
NC_056070.1	54200001	54220001	0.241008	0.192416	SG1	IFT81	NC_056057.1	69610001	69630001
NC_056054.1	237390001	2.37E+08	0.465888	0.268263	SG1	IGSF10;MED12L	NC_056057.1	69615001	69635001
NC_056054.1	237395001	2.37E+08	0.473769	0.203514	SG1	IGSF10;MED12L	NC_056078.1	18560001	18580001
NC_056068.1	22170001	22190001	0.363542	0.190502	SG1	IL18;TEX12	NC_056078.1	18555001	18575001
NC_056068.1	22175001	22195001	0.349937	0.193233	SG1	IL18;TEX12	NC_056057.1	65490001	65510001

NC_056069.1	23500001	23520001	0.314446	0.251227	SG1	IL31RA	NC_056056.1	210965001	210985001
NC_056069.1	23505001	23525001	0.269851	0.266477	SG1	IL31RA	NC_056056.1	210970001	210990001
NC_056069.1	23510001	23530001	0.274252	0.273716	SG1	IL31RA	NC_056056.1	211140001	211160001
NC_056058.1	19680001	19700001	0.372686	0.219423	SG1	IL5;RAD50	NC_056056.1	211145001	211165001
NC_056054.1	118975001	1.19E+08	0.437931	0.265546	SG1	ILDR2	NC_056056.1	211150001	211170001
NC_056054.1	118980001	1.19E+08	0.3829	0.298991	SG1	ILDR2	NC_056054.1	121745001	121765001
NC_056054.1	118985001	1.19E+08	0.463492	0.317106	SG1	ILDR2	NC_056054.1	121750001	121770001
NC_056054.1	118990001	1.19E+08	0.446977	0.303661	SG1	ILDR2	NC_056054.1	277370001	277390001
NC_056076.1	32610001	32630001	0.326577	0.341492	SG1	IMPACT	NC_056054.1	277375001	277395001
NC_056076.1	32615001	32635001	0.267826	0.369479	SG1	IMPACT	NC_056059.1	41470001	41490001
NC_056076.1	32620001	32640001	0.35147	0.296406	SG1	IMPACT	NC_056059.1	41475001	41495001
NC_056061.1	2870001	2890001	0.338519	0.232395	SG1	IMPG1	NC_056059.1	41480001	41500001
NC_056061.1	2875001	2895001	0.260761	0.229266	SG1	IMPG1	NC_056059.1	41485001	41505001
NC_056061.1	2880001	2900001	0.213764	0.214646	SG1	IMPG1	NC_056059.1	41495001	41515001
NC_056061.1	2885001	2905001	0.223999	0.202972	SG1	IMPG1	NC_056059.1	41500001	41520001
NC_056061.1	2890001	2910001	0.117235	0.302431	SG1	IMPG1	NC_056059.1	41505001	41525001
NC_056061.1	2895001	2915001	0.104651	0.344907	SG1	IMPG1	NC_056059.1	41510001	41530001
NC_056061.1	2900001	2920001	0.103872	0.310823	SG1	IMPG1	NC_056059.1	41515001	41535001
NC_056061.1	2905001	2925001	0.234634	0.243064	SG1	IMPG1	NC_056054.1	270740001	270760001
NC_056075.1	39400001	39420001	0.461245	0.213739	SG1	INPP5F	NC_056054.1	105085001	105105001
NC_056075.1	39405001	39425001	0.331408	0.282511	SG1	INPP5F	NC_056070.1	63865001	63885001
NC_056075.1	39410001	39430001	0.334595	0.272283	SG1	INPP5F	NC_056066.1	24645001	24665001
NC_056075.1	39415001	39435001	0.299432	0.254082	SG1	INPP5F	NC_056066.1	24650001	24670001
NC_056075.1	39420001	39440001	0.368806	0.254584	SG1	INPP5F	NC_056066.1	24655001	24675001
NC_056061.1	78765001	78785001	0.332559	0.318363	SG1	IPCEF1	NC_056066.1	24660001	24680001
NC_056069.1	16935001	16955001	0.296246	0.244095	SG1	IPO11	NC_056055.1	10980001	11000001
NC_056069.1	16940001	16960001	0.215839	0.212987	SG1	IPO11	NC_056055.1	74120001	74140001
NC_056069.1	16945001	16965001	0.263144	0.226769	SG1	IPO11	NC_056055.1	74125001	74145001
NC_056069.1	16950001	16970001	0.321083	0.190666	SG1	IPO11	NC_056055.1	74130001	74150001
NC_056069.1	17000001	17020001	0.475448	0.296994	SG1	IPO11	NC_056055.1	74135001	74155001
NC_056069.1	17005001	17025001	0.277677	0.360855	SG1	IPO11	NC_056055.1	74140001	74160001
NC_056069.1	17010001	17030001	0.360215	0.344803	SG1	IPO11	NC_056055.1	74150001	74170001

NC_056069.1	17015001	17035001	0.44003	0.309622	SG1	IPO11	NC_056055.1	74155001	74175001
NC_056069.1	17040001	17060001	0.4189	0.215868	SG1	IPO11	NC_056054.1	108395001	108415001
NC_056069.1	17045001	17065001	0.329372	0.256303	SG1	IPO11	NC_056054.1	108400001	108420001
NC_056069.1	17050001	17070001	0.251942	0.430733	SG1	IPO11	NC_056054.1	108405001	108425001
NC_056069.1	17055001	17075001	0.107071	0.524041	SG1	IPO11	NC_056074.1	27695001	27715001
NC_056069.1	17060001	17080001	0.184815	0.481325	SG1	IPO11	NC_056056.1	124745001	124765001
NC_056069.1	17065001	17085001	0.248796	0.457996	SG1	IPO11	NC_056056.1	124750001	124770001
NC_056069.1	17070001	17090001	0.434692	0.306682	SG1	IPO11	NC_056056.1	124755001	124775001
NC_056060.1	7770001	7790001	0.189588	0.350448	SG1	IQGAP2	NC_056056.1	124780001	124800001
NC_056060.1	7775001	7795001	0.141938	0.373964	SG1	IQGAP2	NC_056056.1	124785001	124805001
NC_056060.1	7780001	7800001	0.214098	0.356554	SG1	IQGAP2	NC_056056.1	124835001	124855001
NC_056060.1	7785001	7805001	0.33855	0.31086	SG1	IQGAP2	NC_056056.1	124840001	124860001
NC_056057.1	89360001	89380001	0.212472	0.243462	SG1	IQUB	NC_056056.1	124845001	124865001
NC_056057.1	89365001	89385001	0.117276	0.284164	SG1	IQUB	NC_056063.1	49575001	49595001
NC_056057.1	89370001	89390001	0.181273	0.275956	SG1	IQUB	NC_056063.1	49580001	49600001
NC_056057.1	89375001	89395001	0.203132	0.216017	SG1	IQUB	NC_056063.1	49585001	49605001
NC_056057.1	89380001	89400001	0.370312	0.184268	SG1	IQUB	NC_056063.1	49590001	49610001
NC_056057.1	89415001	89435001	0.299263	0.253436	SG1	IQUB	NC_056071.1	16000001	16020001
NC_056057.1	89420001	89440001	0.371528	0.282778	SG1	IQUB	NC_056071.1	16005001	16025001
NC_056058.1	19730001	19750001	0.265306	0.238076	SG1	IRF1;RAD50	NC_056071.1	16010001	16030001
NC_056058.1	19735001	19755001	0.167669	0.351039	SG1	IRF1;RAD50	NC_056071.1	52530001	52550001
NC_056072.1	48170001	48190001	0.197892	0.229815	SG1	ITIH1;ITIH3	NC_056061.1	40060001	40080001
NC_056072.1	48165001	48185001	0.14897	0.2538	SG1	ITIH3	NC_056061.1	40065001	40085001
NC_056072.1	48150001	48170001	0.118251	0.256125	SG1	ITIH3;ITIH4	NC_056061.1	40070001	40090001
NC_056072.1	48155001	48175001	0.080333	0.275646	SG1	ITIH3;ITIH4	NC_056056.1	205550001	205570001
NC_056072.1	48160001	48180001	0.072066	0.278442	SG1	ITIH3;ITIH4	NC_056056.1	205555001	205575001
NC_056072.1	48130001	48150001	0.201404	0.209634	SG1	ITIH4;LOC10110842[illegible]	NC_056056.1	205575001	205595001
NC_056072.1	48135001	48155001	0.208932	0.198344	SG1	ITIH4;LOC10110842[illegible]	NC_056054.1	125780001	125800001
NC_056072.1	48140001	48160001	0.232077	0.194491	SG1	ITIH4;LOC10110842[illegible]	NC_056054.1	125785001	125805001
NC_056072.1	48145001	48165001	0.126362	0.241111	SG1	ITIH4;MUSTN1	NC_056061.1	54815001	54835001
NC_056072.1	21525001	21545001	0.455822	0.186643	SG1	ITPR1	NC_056061.1	54820001	54840001
NC_056072.1	21530001	21550001	0.422756	0.236215	SG1	ITPR1	NC_056061.1	54825001	54845001

NC_056054.1	122440001	1.22E+08	0.415293	0.265468	SG1	ITSN1	NC_056061.1	54830001	54850001
NC_056054.1	122445001	1.22E+08	0.333334	0.233623	SG1	ITSN1	NC_056054.1	204740001	204760001
NC_056054.1	122465001	1.22E+08	0.455924	0.224257	SG1	ITSN1	NC_056054.1	204745001	204765001
NC_056054.1	122470001	1.22E+08	0.352355	0.253283	SG1	ITSN1	NC_056072.1	670001	690001
NC_056054.1	122475001	1.22E+08	0.420463	0.222189	SG1	ITSN1	NC_056072.1	675001	695001
NC_056057.1	69620001	69640001	0.42233	0.430439	SG1	JAZF1	NC_056072.1	680001	700001
NC_056057.1	69625001	69645001	0.417569	0.41212	SG1	JAZF1	NC_056056.1	3630001	3650001
NC_056057.1	69630001	69650001	0.449161	0.342037	SG1	JAZF1	NC_056059.1	112830001	112850001
NC_056057.1	69635001	69655001	0.262991	0.2966	SG1	JAZF1	NC_056059.1	112835001	112855001
NC_056057.1	69640001	69660001	0.203086	0.324919	SG1	JAZF1	NC_056059.1	112840001	112860001
NC_056057.1	69645001	69665001	0.140413	0.410207	SG1	JAZF1	NC_056059.1	112845001	112865001
NC_056057.1	69650001	69670001	0.088351	0.436186	SG1	JAZF1	NC_056059.1	112850001	112870001
NC_056057.1	69655001	69675001	0.165406	0.463615	SG1	JAZF1	NC_056068.1	65345001	65365001
NC_056057.1	69660001	69680001	0.279595	0.387666	SG1	JAZF1	NC_056068.1	65350001	65370001
NC_056057.1	69665001	69685001	0.335093	0.290053	SG1	JAZF1	NC_056060.1	83570001	83590001
NC_056057.1	69670001	69690001	0.420289	0.250523	SG1	JAZF1	NC_056060.1	83575001	83595001
NC_056066.1	34415001	34435001	0.386092	0.254869	SG1	JCAD	NC_056060.1	83580001	83600001
NC_056066.1	34420001	34440001	0.309388	0.288368	SG1	JCAD	NC_056065.1	28580001	28600001
NC_056066.1	34425001	34445001	0.105476	0.357824	SG1	JCAD	NC_056065.1	28585001	28605001
NC_056066.1	34430001	34450001	0.153066	0.332965	SG1	JCAD	NC_056065.1	28590001	28610001
NC_056066.1	34435001	34455001	0.243517	0.297309	SG1	JCAD	NC_056055.1	97110001	97130001
NC_056066.1	34440001	34460001	0.272839	0.272412	SG1	JCAD	NC_056057.1	119845001	119865001
NC_056066.1	34445001	34465001	0.422263	0.190524	SG1	JCAD	NC_056057.1	119850001	119870001
NC_056054.1	189400001	1.89E+08	0.390038	0.203929	SG1	KALRN	NC_056057.1	119855001	119875001
NC_056054.1	189405001	1.89E+08	0.352452	0.207555	SG1	KALRN	NC_056057.1	119860001	119880001
NC_056054.1	189410001	1.89E+08	0.333333	0.2002	SG1	KALRN	NC_056057.1	119885001	119905001
NC_056054.1	189445001	1.89E+08	0.169892	0.229076	SG1	KALRN	NC_056058.1	7810001	7830001
NC_056054.1	189450001	1.89E+08	0.097655	0.287637	SG1	KALRN	NC_056075.1	42110001	42130001
NC_056054.1	189455001	1.89E+08	0.109837	0.283201	SG1	KALRN	NC_056075.1	42115001	42135001
NC_056054.1	189460001	1.89E+08	0.188714	0.255098	SG1	KALRN	NC_056073.1	30460001	30480001
NC_056063.1	30790001	30810001	0.425974	0.229638	SG1	KATNAL1	NC_056073.1	30465001	30485001
NC_056063.1	30795001	30815001	0.335322	0.185844	SG1	KATNAL1	NC_056056.1	205545001	205565001

NC_056063.1	30800001	30820001	0.299223	0.187719	SG1	KATNAL1	NC_056056.1	205540001	205560001
NC_056063.1	30805001	30825001	0.301724	0.184572	SG1	KATNAL1	NC_056057.1	91980001	92000001
NC_056063.1	30815001	30835001	0.468505	0.183496	SG1	KATNAL1	NC_056057.1	91985001	92005001
NC_056077.1	25740001	25760001	0.470196	0.187359	SG1	KATNIP	NC_056071.1	20840001	20860001
NC_056054.1	121675001	1.22E+08	0.1915	0.195099	SG1	KCNE1	NC_056071.1	20845001	20865001
NC_056062.1	16580001	16600001	0.139	0.184712	SG1	KCNK9	NC_056071.1	20855001	20875001
NC_056062.1	16585001	16605001	0.267431	0.21849	SG1	KCNK9	NC_056055.1	89470001	89490001
NC_056062.1	16590001	16610001	0.399696	0.23307	SG1	KCNK9	NC_056055.1	89480001	89500001
NC_056062.1	21930001	21950001	0.359832	0.291514	SG1	KCNQ3	NC_056060.1	83070001	83090001
NC_056062.1	21935001	21955001	0.313052	0.321212	SG1	KCNQ3	NC_056060.1	83075001	83095001
NC_056063.1	19795001	19815001	0.384605	0.216696	SG1	KCNRG	NC_056067.1	33855001	33875001
NC_056058.1	53165001	53185001	0.464544	0.222885	SG1	KCTD16	NC_056067.1	33835001	33855001
NC_056058.1	53170001	53190001	0.38707	0.225271	SG1	KCTD16	NC_056067.1	33840001	33860001
NC_056058.1	53175001	53195001	0.400001	0.199139	SG1	KCTD16	NC_056067.1	33845001	33865001
NC_056058.1	53180001	53200001	0.376623	0.196612	SG1	KCTD16	NC_056067.1	33850001	33870001
NC_056065.1	18060001	18080001	0.091058	0.281234	SG1	KCTD3	NC_056071.1	65600001	65620001
NC_056065.1	18065001	18085001	0.261332	0.281489	SG1	KCTD3	NC_056071.1	65605001	65625001
NC_056065.1	18070001	18090001	0.424082	0.188266	SG1	KCTD3	NC_056071.1	65610001	65630001
NC_056073.1	955001	975001	0.275603	0.193457	SG1	KHDRBS2	NC_056071.1	65615001	65635001
NC_056073.1	960001	980001	0.333585	0.206539	SG1	KHDRBS2	NC_056060.1	83110001	83130001
NC_056073.1	965001	985001	0.375507	0.215401	SG1	KHDRBS2	NC_056060.1	83115001	83135001
NC_056073.1	970001	990001	0.446915	0.215473	SG1	KHDRBS2	NC_056060.1	83120001	83140001
NC_056065.1	55775001	55795001	0.372117	0.252193	SG1	KIAA0040	NC_056073.1	30245001	30265001
NC_056065.1	55780001	55800001	0.227791	0.345393	SG1	KIAA0040	NC_056073.1	30250001	30270001
NC_056065.1	55730001	55750001	0.436508	0.200618	SG1	KIAA0040;TNN	NC_056073.1	30255001	30275001
NC_056066.1	24280001	24300001	0.211717	0.196929	SG1	KIAA1217	NC_056057.1	89785001	89805001
NC_056066.1	24285001	24305001	0.098606	0.225883	SG1	KIAA1217	NC_056057.1	89790001	89810001
NC_056066.1	24290001	24310001	0.114512	0.217936	SG1	KIAA1217	NC_056054.1	264340001	264360001
NC_056066.1	24295001	24315001	0.165586	0.205158	SG1	KIAA1217	NC_056054.1	264345001	264365001
NC_056066.1	24310001	24330001	0.437603	0.204229	SG1	KIAA1217	NC_056057.1	89800001	89820001
NC_056066.1	24350001	24370001	0.314469	0.220853	SG1	KIAA1217	NC_056055.1	134105001	134125001
NC_056066.1	24355001	24375001	0.245168	0.237935	SG1	KIAA1217	NC_056058.1	11780001	11800001

NC_056066.1	24360001	24380001	0.144204	0.255545	SG1	KIAA1217	NC_056054.1	87025001	87045001
NC_056066.1	24365001	24385001	0.246785	0.224186	SG1	KIAA1217	NC_056054.1	87015001	87035001
NC_056068.1	63015001	63035001	0.473077	0.19572	SG1	KIAA1549L	NC_056054.1	87020001	87040001
NC_056070.1	65355001	65375001	0.442759	0.199209	SG1	KIAA1671	NC_056058.1	15515001	15535001
NC_056070.1	65400001	65420001	0.43372	0.218786	SG1	KIAA1671	NC_056071.1	31565001	31585001
NC_056066.1	9890001	9910001	0.420328	0.193302	SG1	KIF16B	NC_056071.1	31570001	31590001
NC_056066.1	9895001	9915001	0.252117	0.260408	SG1	KIF16B	NC_056071.1	31575001	31595001
NC_056066.1	9900001	9920001	0.385904	0.202438	SG1	KIF16B	NC_056071.1	31580001	31600001
NC_056058.1	6450001	6470001	0.074144	0.217255	SG1	KLF2	NC_056071.1	31585001	31605001
NC_056055.1	89890001	89910001	0.117845	0.19593	SG1	KLHL9	NC_056055.1	53020001	53040001
NC_056055.1	89895001	89915001	0.220671	0.221702	SG1	KLHL9	NC_056067.1	60770001	60790001
NC_056067.1	56765001	56785001	0.420596	0.203658	SG1	KLK14	NC_056067.1	60775001	60795001
NC_056067.1	56770001	56790001	0.42348	0.224654	SG1	KLK14	NC_056067.1	60790001	60810001
NC_056057.1	116315001	1.16E+08	0.204967	0.185498	SG1	KMT2C	NC_056067.1	60795001	60815001
NC_056057.1	116320001	1.16E+08	0.17129	0.197094	SG1	KMT2C	NC_056067.1	60800001	60820001
NC_056057.1	116325001	1.16E+08	0.22327	0.205067	SG1	KMT2C	NC_056067.1	60805001	60825001
NC_056057.1	116330001	1.16E+08	0.344299	0.198508	SG1	KMT2C	NC_056066.1	16945001	16965001
NC_056057.1	116360001	1.16E+08	0.431314	0.256281	SG1	KMT2C	NC_056054.1	199835001	199855001
NC_056057.1	116365001	1.16E+08	0.34885	0.272615	SG1	KMT2C	NC_056054.1	199840001	199860001
NC_056057.1	116370001	1.16E+08	0.305121	0.220206	SG1	KMT2C	NC_056054.1	199845001	199865001
NC_056054.1	21110001	21130001	0.230153	0.23877	SG1	KNCN;MKNK1	NC_056069.1	10280001	10300001
NC_056054.1	21115001	21135001	0.398869	0.211856	SG1	KNCN;MKNK1	NC_056069.1	10285001	10305001
NC_056064.1	40615001	40635001	0.318138	0.201063	SG1	KRT222	NC_056069.1	10290001	10310001
NC_056070.1	57305001	57325001	0.338494	0.215882	SG1	KSR2	NC_056055.1	10955001	10975001
NC_056061.1	54890001	54910001	0.397834	0.201263	SG1	LAMA2	NC_056055.1	10960001	10980001
NC_056061.1	54895001	54915001	0.447677	0.185583	SG1	LAMA2	NC_056055.1	10965001	10985001
NC_056076.1	33260001	33280001	0.453076	0.294548	SG1	LAMA3	NC_056055.1	10970001	10990001
NC_056076.1	33265001	33285001	0.378787	0.32523	SG1	LAMA3	NC_056055.1	10975001	10995001
NC_056076.1	33270001	33290001	0.366986	0.355049	SG1	LAMA3	NC_056063.1	29495001	29515001
NC_056076.1	33275001	33295001	0.467314	0.280988	SG1	LAMA3	NC_056063.1	29500001	29520001
NC_056056.1	177425001	1.77E+08	0.468787	0.287626	SG1	LARGE1	NC_056080.1	26050001	26070001
NC_056056.1	177435001	1.77E+08	0.451581	0.19645	SG1	LARGE1	NC_056080.1	26055001	26075001

NC_056058.1	63770001	63790001	0.306024	0.203152	SG1	LARP1	NC_056080.1	26065001	26085001
NC_056056.1	28535001	28555001	0.402662	0.216896	SG1	LDAH	NC_056060.1	75435001	75455001
NC_056059.1	112705001	1.13E+08	0.395562	0.236921	SG1	LDB2	NC_056080.1	26420001	26440001
NC_056056.1	194120001	1.94E+08	0.398169	0.185205	SG1	LDHB	NC_056080.1	26425001	26445001
NC_056056.1	194125001	1.94E+08	0.36773	0.202832	SG1	LDHB	NC_056080.1	26430001	26450001
NC_056057.1	47375001	47395001	0.395197	0.232065	SG1	LHFPL3	NC_056080.1	26445001	26465001
NC_056057.1	47380001	47400001	0.287688	0.261411	SG1	LHFPL3	NC_056080.1	26450001	26470001
NC_056057.1	47385001	47405001	0.229807	0.267019	SG1	LHFPL3	NC_056064.1	29265001	29285001
NC_056057.1	47390001	47410001	0.172414	0.233419	SG1	LHFPL3	NC_056064.1	29270001	29290001
NC_056057.1	47395001	47415001	0.258806	0.196108	SG1	LHFPL3	NC_056075.1	42205001	42225001
NC_056057.1	47455001	47475001	0.24082	0.198814	SG1	LHFPL3	NC_056075.1	42210001	42230001
NC_056057.1	47460001	47480001	0.198907	0.261858	SG1	LHFPL3	NC_056075.1	42215001	42235001
NC_056057.1	47465001	47485001	0.164955	0.2398	SG1	LHFPL3	NC_056080.1	90365001	90385001
NC_056057.1	47470001	47490001	0.151865	0.189824	SG1	LHFPL3	NC_056080.1	90370001	90390001
NC_056072.1	53645001	53665001	0.404299	0.200176	SG1	LIMD1	NC_056080.1	90375001	90395001
NC_056072.1	53650001	53670001	0.24362	0.258151	SG1	LIMD1	NC_056080.1	90380001	90400001
NC_056072.1	53655001	53675001	0.210097	0.225218	SG1	LIMD1	NC_056060.1	24585001	24605001
NC_056056.1	61990001	62010001	0.313364	0.230464	SG1	LIMS1	NC_056060.1	24590001	24610001
NC_056056.1	61995001	62015001	0.158609	0.327078	SG1	LIMS1	NC_056060.1	24605001	24625001
NC_056056.1	62000001	62020001	0.133394	0.380018	SG1	LIMS1	NC_056060.1	24610001	24630001
NC_056056.1	62005001	62025001	0.279043	0.398909	SG1	LIMS1	NC_056060.1	24635001	24655001
NC_056056.1	62010001	62030001	0.454986	0.317967	SG1	LIMS1	NC_056060.1	24640001	24660001
NC_056055.1	97220001	97240001	0.083342	0.406609	SG1	LINGO2	NC_056077.1	19720001	19740001
NC_056055.1	97225001	97245001	0.13366	0.404609	SG1	LINGO2	NC_056068.1	49350001	49370001
NC_056055.1	97230001	97250001	0.096816	0.260905	SG1	LINGO2	NC_056068.1	49355001	49375001
NC_056055.1	97235001	97255001	0.088723	0.225841	SG1	LINGO2	NC_056057.1	107715001	107735001
NC_056055.1	97250001	97270001	0.053002	0.330501	SG1	LINGO2	NC_056057.1	107720001	107740001
NC_056055.1	97255001	97275001	0.341151	0.236376	SG1	LINGO2	NC_056054.1	220160001	220180001
NW_02459982[illegible]	1095001	1115001	0.333248	0.191345	SG1	LOC101102275;PAG[illegible]	NC_056054.1	220165001	220185001
NW_02459982[illegible]	1100001	1120001	0.241063	0.237604	SG1	LOC101102275;PAG[illegible]	NC_056054.1	220170001	220190001
NW_02459982[illegible]	1105001	1125001	0.195839	0.261632	SG1	LOC101102275;PAG[illegible]	NC_056054.1	220175001	220195001
NC_056067.1	38560001	38580001	0.122396	0.270972	SG1	LOC101102413	NC_056065.1	5500001	5520001

NC_056068.1	47280001	47300001	0.275192	0.22007	SG1	LOC101102419	NC_056063.1	33555001	33575001
NC_056068.1	47285001	47305001	0.330897	0.309993	SG1	LOC101102419	NC_056063.1	33565001	33585001
NC_056067.1	50360001	50380001	0.435774	0.240389	SG1	LOC101103174	NC_056063.1	33570001	33590001
NC_056067.1	50365001	50385001	0.255874	0.338592	SG1	LOC101103174	NC_056063.1	33575001	33595001
NC_056067.1	50370001	50390001	0.212997	0.381533	SG1	LOC101103174	NC_056063.1	33580001	33600001
NC_056058.1	49945001	49965001	0.443749	0.206989	SG1	LOC101103233;LOC1	NC_056063.1	33585001	33605001
NC_056058.1	49935001	49955001	0.470085	0.185714	SG1	LOC101103233;LOC1	NC_056060.1	63565001	63585001
NC_056058.1	49940001	49960001	0.450658	0.192529	SG1	LOC101103233;LOC1	NC_056060.1	63570001	63590001
NC_056066.1	66405001	66425001	0.217948	0.200875	SG1	LOC101103339	NC_056060.1	63575001	63595001
NC_056066.1	66415001	66435001	0.155587	0.228448	SG1	LOC101103339	NC_056060.1	63580001	63600001
NC_056066.1	66420001	66440001	0.243285	0.190701	SG1	LOC101103339	NC_056060.1	63585001	63605001
NC_056066.1	66450001	66470001	0.443713	0.195553	SG1	LOC101103339	NC_056054.1	126090001	126110001
NC_056066.1	66455001	66475001	0.295138	0.234356	SG1	LOC101103339	NC_056066.1	62295001	62315001
NC_056067.1	50405001	50425001	0.366454	0.262029	SG1	LOC101103343	NC_056063.1	19430001	19450001
NC_056067.1	50410001	50430001	0.45411	0.2016	SG1	LOC101103343	NC_056080.1	24350001	24370001
NC_056061.1	74520001	74540001	0.442575	0.205194	SG1	LOC101103958	NC_056080.1	24355001	24375001
NC_056061.1	74525001	74545001	0.306456	0.244882	SG1	LOC101103958	NC_056056.1	204950001	204970001
NC_056058.1	50030001	50050001	0.268418	0.264936	SG1	LOC101104234	NC_056063.1	19440001	19460001
NC_056058.1	50035001	50055001	0.248555	0.283191	SG1	LOC101104234	NC_056063.1	19445001	19465001
NC_056058.1	50040001	50060001	0.335842	0.26339	SG1	LOC101104234;TAF7	NC_056063.1	19450001	19470001
NC_056055.1	90660001	90680001	0.444611	0.218534	SG1	LOC101104463	NC_056063.1	19455001	19475001
NC_056065.1	37960001	37980001	0.434857	0.258612	SG1	LOC101104591	NC_056061.1	52790001	52810001
NC_056065.1	37965001	37985001	0.385038	0.291264	SG1	LOC101104591	NC_056061.1	52795001	52815001
NC_056065.1	37970001	37990001	0.423651	0.291714	SG1	LOC101104591	NC_056061.1	52800001	52820001
NC_056058.1	50065001	50085001	0.458557	0.222486	SG1	LOC101104745;LOC1	NC_056064.1	19215001	19235001
NC_056068.1	17445001	17465001	0.405178	0.259211	SG1	LOC101104855	NC_056075.1	20650001	20670001
NC_056068.1	17450001	17470001	0.467863	0.232658	SG1	LOC101104855	NC_056075.1	20655001	20675001
NC_056056.1	180250001	1.8E+08	0.219895	0.2012	SG1	LOC101104893;LOC1	NC_056075.1	20660001	20680001
NC_056056.1	180255001	1.8E+08	0.24661	0.198951	SG1	LOC101104893;LOC1	NC_056075.1	20665001	20685001
NC_056056.1	180260001	1.8E+08	0.251607	0.194113	SG1	LOC101104893;LOC1	NC_056075.1	20715001	20735001
NC_056055.1	18530001	18550001	0.283712	0.209636	SG1	LOC101105139;LOC1	NC_056056.1	165325001	165345001
NC_056068.1	44550001	44570001	0.161612	0.233084	SG1	LOC101105275	NC_056064.1	16910001	16930001

NC_056068.1	44555001	44575001	0.212141	0.196646	SG1	LOC101105275	NC_056064.1	16915001	16935001
NC_056068.1	44540001	44560001	0.354663	0.185315	SG1	LOC101105275;LOC1	NC_056064.1	16920001	16940001
NC_056068.1	44545001	44565001	0.167129	0.245909	SG1	LOC101105275;LOC1	NC_056064.1	16925001	16945001
NC_056058.1	50140001	50160001	0.442895	0.227162	SG1	LOC101105495	NC_056058.1	77830001	77850001
NC_056058.1	50185001	50205001	0.168539	0.299024	SG1	LOC101105495	NC_056058.1	77835001	77855001
NC_056058.1	50190001	50210001	0.079606	0.421484	SG1	LOC101105495	NC_056056.1	205535001	205555001
NC_056058.1	50195001	50215001	0.128378	0.423261	SG1	LOC101105495	NC_056080.1	35025001	35045001
NC_056058.1	50200001	50220001	0.220519	0.353621	SG1	LOC101105495	NC_056080.1	35035001	35055001
NC_056058.1	50205001	50225001	0.366497	0.253148	SG1	LOC101105495	NC_056080.1	35040001	35060001
NC_056054.1	166730001	1.67E+08	0.43113	0.261151	SG1	LOC101105553;SENF	NC_056056.1	14090001	14110001
NC_056054.1	166735001	1.67E+08	0.349659	0.250077	SG1	LOC101105553;SENF	NC_056056.1	14095001	14115001
NC_056055.1	89920001	89940001	0.467694	0.256302	SG1	LOC101105632;LOC1	NC_056056.1	14100001	14120001
NC_056054.1	126065001	1.26E+08	0.342723	0.281695	SG1	LOC101106046;LOC1	NC_056079.1	21220001	21240001
NC_056080.1	69245001	69265001	0.184615	0.243674	SG1	LOC101106237	NC_056079.1	21225001	21245001
NC_056080.1	69250001	69270001	0.20806	0.216269	SG1	LOC101106237	NC_056079.1	21230001	21250001
NC_056080.1	69255001	69275001	0.217446	0.199185	SG1	LOC101106237	NC_056057.1	107725001	107745001
NC_056064.1	23805001	23825001	0.45098	0.209594	SG1	LOC101106784;LOC1	NC_056057.1	107730001	107750001
NC_056064.1	41555001	41575001	0.441078	0.213664	SG1	LOC101108147	NC_056072.1	13330001	13350001
NC_056055.1	52585001	52605001	0.372603	0.20752	SG1	LOC101108371	NC_056072.1	13335001	13355001
NC_056072.1	48090001	48110001	0.255841	0.206569	SG1	LOC101108428	NC_056072.1	13340001	13360001
NC_056072.1	48095001	48115001	0.22042	0.236662	SG1	LOC101108428	NC_056072.1	13345001	13365001
NC_056072.1	48100001	48120001	0.2	0.245064	SG1	LOC101108428	NC_056072.1	13305001	13325001
NC_056072.1	48105001	48125001	0.150877	0.250081	SG1	LOC101108428	NC_056072.1	13310001	13330001
NC_056072.1	48110001	48130001	0.145342	0.247616	SG1	LOC101108428	NC_056072.1	13315001	13335001
NC_056072.1	48115001	48135001	0.167907	0.232231	SG1	LOC101108428	NC_056071.1	65545001	65565001
NC_056072.1	48120001	48140001	0.165463	0.225722	SG1	LOC101108428	NC_056071.1	65550001	65570001
NC_056072.1	48125001	48145001	0.191971	0.211172	SG1	LOC101108428	NC_056071.1	65555001	65575001
NC_056066.1	44700001	44720001	0.237101	0.389993	SG1	LOC101108592	NC_056071.1	65560001	65580001
NC_056066.1	44705001	44725001	0.302194	0.361171	SG1	LOC101108592	NC_056071.1	65530001	65550001
NC_056066.1	44710001	44730001	0.381184	0.276421	SG1	LOC101108592	NC_056071.1	65535001	65555001
NC_056066.1	44715001	44735001	0.41405	0.22099	SG1	LOC101108592	NC_056071.1	65540001	65560001
NC_056066.1	44685001	44705001	0.415983	0.278069	SG1	LOC101108592;PITRI	NC_056072.1	56165001	56185001

NC_056066.1	44690001	44710001	0.262978	0.368471	SG1	LOC101108592;PITRI	NC_056072.1	56160001	56180001
NC_056066.1	44695001	44715001	0.163028	0.394305	SG1	LOC101108592;PITRI	NC_056068.1	49725001	49745001
NC_056054.1	166220001	1.66E+08	0.248513	0.21091	SG1	LOC101108803	NC_056056.1	205050001	205070001
NC_056054.1	166225001	1.66E+08	0.421053	0.292839	SG1	LOC101108803	NC_056056.1	205055001	205075001
NC_056054.1	166230001	1.66E+08	0.463424	0.28765	SG1	LOC101108803	NC_056058.1	79780001	79800001
NC_056054.1	166235001	1.66E+08	0.421801	0.206473	SG1	LOC101108803	NC_056058.1	79785001	79805001
NC_056054.1	166255001	1.66E+08	0.044147	0.275833	SG1	LOC101108803	NC_056058.1	79790001	79810001
NC_056054.1	166260001	1.66E+08	0.03642	0.389704	SG1	LOC101108803	NC_056058.1	79795001	79815001
NC_056054.1	166265001	1.66E+08	0.069454	0.395332	SG1	LOC101108803	NC_056067.1	58615001	58635001
NC_056054.1	166270001	1.66E+08	0.132492	0.375659	SG1	LOC101108803	NC_056067.1	58620001	58640001
NC_056054.1	166275001	1.66E+08	0.191632	0.366802	SG1	LOC101108803	NC_056067.1	58625001	58645001
NC_056054.1	166280001	1.66E+08	0.353126	0.299643	SG1	LOC101108803	NC_056067.1	58630001	58650001
NC_056054.1	166285001	1.66E+08	0.398569	0.406292	SG1	LOC101108803	NC_056067.1	58635001	58655001
NC_056054.1	166315001	1.66E+08	0.345013	0.246213	SG1	LOC101108803	NC_056067.1	58685001	58705001
NC_056054.1	166320001	1.66E+08	0.261129	0.284921	SG1	LOC101108803	NC_056067.1	58690001	58710001
NC_056054.1	166325001	1.66E+08	0.253357	0.299567	SG1	LOC101108803	NC_056067.1	58695001	58715001
NC_056054.1	166330001	1.66E+08	0.277677	0.296724	SG1	LOC101108803	NC_056067.1	58700001	58720001
NC_056054.1	166335001	1.66E+08	0.264057	0.230133	SG1	LOC101108803	NC_056067.1	58705001	58725001
NC_056054.1	166340001	1.66E+08	0.218113	0.288104	SG1	LOC101108803	NC_056067.1	58710001	58730001
NC_056054.1	166345001	1.66E+08	0.12	0.366682	SG1	LOC101108803	NC_056067.1	58715001	58735001
NC_056054.1	166350001	1.66E+08	0.090417	0.38936	SG1	LOC101108803	NC_056071.1	65620001	65640001
NC_056054.1	166355001	1.66E+08	0.110374	0.393998	SG1	LOC101108803	NC_056071.1	65625001	65645001
NC_056054.1	166360001	1.66E+08	0.207097	0.380615	SG1	LOC101108803	NC_056071.1	65630001	65650001
NC_056054.1	166365001	1.66E+08	0.286738	0.363372	SG1	LOC101108803	NC_056071.1	65635001	65655001
NC_056066.1	3510001	3530001	0.291447	0.216516	SG1	LOC101109032	NC_056071.1	65640001	65660001
NC_056066.1	3515001	3535001	0.176581	0.246831	SG1	LOC101109032	NC_056071.1	65645001	65665001
NC_056066.1	3520001	3540001	0.133865	0.256078	SG1	LOC101109032	NC_056071.1	65650001	65670001
NC_056066.1	3525001	3545001	0.216667	0.223801	SG1	LOC101109032;LOC1	NC_056071.1	65655001	65675001
NC_056056.1	180185001	1.8E+08	0.06683	0.217254	SG1	LOC101109077	NC_056072.1	13380001	13400001
NC_056056.1	180190001	1.8E+08	0.107935	0.185722	SG1	LOC101109077	NC_056054.1	230785001	230805001
NC_056054.1	3110001	3130001	0.341513	0.215701	SG1	LOC101109322	NC_056054.1	230790001	230810001
NC_056054.1	3115001	3135001	0.278424	0.232375	SG1	LOC101109322	NC_056071.1	65485001	65505001

NC_056080.1	72305001	72325001	0.195531	0.183862	SG1	LOC101109338	NC_056056.1	795001	815001
NC_056080.1	72310001	72330001	0.205993	0.189184	SG1	LOC101109338	NC_056056.1	800001	820001
NC_056055.1	53020001	53040001	0.40283	0.208492	SG1	LOC101109422	NC_056073.1	25640001	25660001
NC_056055.1	53045001	53065001	0.089804	0.229998	SG1	LOC101109422	NC_056068.1	62345001	62365001
NC_056055.1	53050001	53070001	0.1011	0.208725	SG1	LOC101109422	NC_056068.1	62350001	62370001
NC_056055.1	53055001	53075001	0.128524	0.186576	SG1	LOC101109422	NC_056068.1	62355001	62375001
NC_056055.1	52990001	53010001	0.443227	0.307348	SG1	LOC101109422;RUS(	NC_056068.1	62360001	62380001
NC_056054.1	166875001	1.67E+08	0.433804	0.326565	SG1	LOC101109587;SENI	NC_056066.1	17795001	17815001
NC_056063.1	27330001	27350001	0.050484	0.208681	SG1	LOC101109717	NC_056066.1	17800001	17820001
NC_056063.1	27335001	27355001	0.111605	0.231618	SG1	LOC101109717	NC_056066.1	17805001	17825001
NC_056066.1	16895001	16915001	0.440605	0.275599	SG1	LOC101109728	NC_056063.1	25085001	25105001
NC_056066.1	16925001	16945001	0.25026	0.354752	SG1	LOC101109728	NC_056063.1	25090001	25110001
NC_056054.1	166890001	1.67E+08	0.337321	0.317305	SG1	LOC101109854;SENI	NC_056063.1	25095001	25115001
NC_056054.1	166895001	1.67E+08	0.423175	0.27672	SG1	LOC101109854;TRM	NC_056056.1	13270001	13290001
NC_056056.1	164235001	1.64E+08	0.445672	0.286497	SG1	LOC101110319	NC_056060.1	81875001	81895001
NC_056056.1	164240001	1.64E+08	0.414896	0.252345	SG1	LOC101110319	NC_056059.1	111870001	111890001
NC_056056.1	164245001	1.64E+08	0.381224	0.250683	SG1	LOC101110319	NC_056059.1	111875001	111895001
NC_056068.1	55480001	55500001	0.23972	0.274542	SG1	LOC101110537	NC_056056.1	204955001	204975001
NC_056068.1	55485001	55505001	0.249999	0.284873	SG1	LOC101110537	NC_056056.1	204960001	204980001
NC_056068.1	55490001	55510001	0.275577	0.278275	SG1	LOC101110537	NC_056063.1	30130001	30150001
NC_056068.1	55495001	55515001	0.342776	0.265159	SG1	LOC101110537	NC_056063.1	30135001	30155001
NC_056054.1	10810001	10830001	0.348416	0.195942	SG1	LOC101111060	NC_056063.1	30140001	30160001
NC_056054.1	10815001	10835001	0.380061	0.183725	SG1	LOC101111060	NC_056063.1	30160001	30180001
NC_056068.1	31415001	31435001	0.431292	0.198165	SG1	LOC101111119	NC_056057.1	79585001	79605001
NC_056071.1	54070001	54090001	0.343883	0.338268	SG1	LOC101112168	NC_056057.1	79590001	79610001
NC_056071.1	54075001	54095001	0.368851	0.304207	SG1	LOC101112168	NC_056071.1	3160001	3180001
NC_056071.1	54080001	54100001	0.441029	0.286344	SG1	LOC101112168	NC_056062.1	13055001	13075001
NC_056071.1	54085001	54105001	0.448621	0.212759	SG1	LOC101112168	NC_056071.1	21555001	21575001
NC_056071.1	54050001	54070001	0.172192	0.310817	SG1	LOC101112168;PPP4	NC_056071.1	21560001	21580001
NC_056071.1	54055001	54075001	0.210746	0.322149	SG1	LOC101112168;PPP4	NC_056071.1	21535001	21555001
NC_056071.1	54060001	54080001	0.20438	0.318072	SG1	LOC101112168;PPP4	NC_056071.1	21540001	21560001
NC_056071.1	54065001	54085001	0.323306	0.33156	SG1	LOC101112168;PPP4	NC_056071.1	21545001	21565001

NC_056054.1	97210001	97230001	0.314559	0.492163	SG1	LOC101112189	NC_056071.1	21550001	21570001
NC_056054.1	97215001	97235001	0.400858	0.464556	SG1	LOC101112189	NC_056071.1	65515001	65535001
NC_056068.1	46640001	46660001	0.388453	0.219543	SG1	LOC101112258	NC_056071.1	65520001	65540001
NC_056068.1	46645001	46665001	0.372134	0.234374	SG1	LOC101112258	NC_056071.1	65525001	65545001
NC_056068.1	46650001	46670001	0.425187	0.198692	SG1	LOC101112258;LOC1	NC_056071.1	44045001	44065001
NC_056056.1	164780001	1.65E+08	0.379098	0.204945	SG1	LOC101113142	NC_056071.1	44050001	44070001
NC_056056.1	164785001	1.65E+08	0.339794	0.20195	SG1	LOC101113142	NC_056071.1	46700001	46720001
NC_056054.1	88790001	88810001	0.241401	0.275055	SG1	LOC101113807	NC_056071.1	46705001	46725001
NC_056055.1	240970001	2.41E+08	0.277413	0.322639	SG1	LOC101114173;MTFI	NC_056071.1	46710001	46730001
NC_056055.1	240975001	2.41E+08	0.211177	0.355439	SG1	LOC101114173;MTFI	NC_056071.1	46715001	46735001
NC_056055.1	240965001	2.41E+08	0.365303	0.253368	SG1	LOC101114173;MTFI	NC_056080.1	90335001	90355001
NC_056055.1	240980001	2.41E+08	0.335689	0.30836	SG1	LOC101114173;MTFI	NC_056080.1	90340001	90360001
NC_056054.1	66705001	66725001	0.172118	0.310031	SG1	LOC101114579	NC_056080.1	90345001	90365001
NC_056054.1	66710001	66730001	0.313831	0.205705	SG1	LOC101114579	NC_056080.1	90350001	90370001
NC_056062.1	35265001	35285001	0.394659	0.199949	SG1	LOC101114620	NC_056080.1	2100001	2120001
NC_056065.1	53865001	53885001	0.330745	0.335382	SG1	LOC101114795	NC_056080.1	2105001	2125001
NC_056065.1	53870001	53890001	0.465849	0.296203	SG1	LOC101114795	NC_056080.1	24895001	24915001
NC_056054.1	63625001	63645001	0.407908	0.329307	SG1	LOC101115586	NC_056080.1	24900001	24920001
NC_056054.1	63630001	63650001	0.396364	0.345531	SG1	LOC101115586	NC_056080.1	26455001	26475001
NC_056080.1	67790001	67810001	0.214518	0.420276	SG1	LOC101116125	NC_056080.1	26460001	26480001
NC_056056.1	219815001	2.2E+08	0.448608	0.237229	SG1	LOC101116389;MCA	NC_056054.1	125790001	125810001
NC_056056.1	219820001	2.2E+08	0.203615	0.3496	SG1	LOC101116389;MCA	NC_056054.1	125840001	125860001
NC_056056.1	219825001	2.2E+08	0.160868	0.368957	SG1	LOC101116389;MCA	NC_056067.1	58540001	58560001
NC_056054.1	223525001	2.24E+08	0.463645	0.394617	SG1	LOC101116449	NC_056068.1	76145001	76165001
NC_056054.1	223530001	2.24E+08	0.45144	0.381639	SG1	LOC101116449	NC_056069.1	41150001	41170001
NC_056067.1	65640001	65660001	0.121525	0.245453	SG1	LOC101116597;LOC1	NC_056069.1	41135001	41155001
NC_056062.1	65075001	65095001	0.468749	0.191401	SG1	LOC101116738	NC_056071.1	66855001	66875001
NC_056080.1	9235001	9255001	0.075893	0.334297	SG1	LOC101118248	NC_056071.1	66860001	66880001
NC_056080.1	9240001	9260001	0.133994	0.206542	SG1	LOC101118248	NC_056075.1	42005001	42025001
NC_056057.1	79010001	79030001	0.188941	0.195065	SG1	LOC101118606	NC_056080.1	54070001	54090001
NC_056068.1	47295001	47315001	0.474981	0.389174	SG1	LOC101118638	NC_056080.1	54075001	54095001
NC_056061.1	60205001	60225001	0.202495	0.270626	SG1	LOC101120251	NC_056055.1	141125001	141145001

NC_056056.1	194370001	1.94E+08	0.419636	0.321389	SG1	LOC101120402;SLCC	NC_056057.1	99460001	99480001
NC_056056.1	194375001	1.94E+08	0.247607	0.338855	SG1	LOC101120402;SLCC	NC_056072.1	10415001	10435001
NC_056068.1	49390001	49410001	0.239807	0.233413	SG1	LOC101120602	NC_056072.1	10420001	10440001
NC_056068.1	49395001	49415001	0.410272	0.186716	SG1	LOC101120602	NC_056072.1	10425001	10445001
NC_056074.1	4835001	4855001	0.032133	0.189749	SG1	LOC101120706	NC_056067.1	44095001	44115001
NC_056074.1	4840001	4860001	0.041148	0.183545	SG1	LOC101120706	NC_056067.1	44100001	44120001
NC_056080.1	103270001	1.03E+08	0.190184	0.23507	SG1	LOC101120813	NC_056067.1	44105001	44125001
NC_056080.1	103275001	1.03E+08	0.179587	0.251021	SG1	LOC101120813	NC_056067.1	44110001	44130001
NC_056080.1	103280001	1.03E+08	0.213653	0.211653	SG1	LOC101120813	NC_056057.1	84390001	84410001
NC_056054.1	66770001	66790001	0.460365	0.192069	SG1	LOC101120875	NC_056066.1	7650001	7670001
NC_056054.1	66775001	66795001	0.373737	0.237011	SG1	LOC101120875	NC_056066.1	7660001	7680001
NC_056054.1	66780001	66800001	0.397178	0.295767	SG1	LOC101120875	NC_056066.1	7665001	7685001
NC_056061.1	75055001	75075001	0.362613	0.223246	SG1	LOC101121838	NC_056068.1	76080001	76100001
NC_056061.1	75060001	75080001	0.371145	0.215481	SG1	LOC101121838	NC_056056.1	215790001	215810001
NC_056054.1	143730001	1.44E+08	0.422217	0.208199	SG1	LOC101122496	NC_056056.1	215795001	215815001
NC_056057.1	84230001	84250001	0.469925	0.218626	SG1	LOC101122689	NC_056057.1	44540001	44560001
NC_056057.1	84235001	84255001	0.411102	0.301616	SG1	LOC101122689	NC_056070.1	17965001	17985001
NC_056077.1	36065001	36085001	0.123255	0.325433	SG1	LOC101122753	NC_056070.1	17970001	17990001
NC_056077.1	36070001	36090001	0.208016	0.29823	SG1	LOC101122753	NC_056055.1	211415001	211435001
NC_056059.1	85690001	85710001	0.321894	0.187771	SG1	LOC101123042	NC_056055.1	211420001	211440001
NC_056059.1	85695001	85715001	0.296665	0.195597	SG1	LOC101123042	NC_056055.1	218255001	218275001
NC_056059.1	85700001	85720001	0.292241	0.191851	SG1	LOC101123042	NC_056069.1	12885001	12905001
NC_056059.1	85715001	85735001	0.35693	0.197012	SG1	LOC101123042	NC_056069.1	12890001	12910001
NC_056059.1	85720001	85740001	0.422416	0.193825	SG1	LOC101123042	NC_056069.1	12895001	12915001
NC_056059.1	85725001	85745001	0.372767	0.193362	SG1	LOC101123042	NC_056069.1	12900001	12920001
NC_056059.1	85730001	85750001	0.34955	0.217487	SG1	LOC101123042	NC_056069.1	12905001	12925001
NC_056055.1	48345001	48365001	0.248845	0.205534	SG1	LOC101123268	NC_056069.1	13145001	13165001
NC_056055.1	48350001	48370001	0.233571	0.204663	SG1	LOC101123268	NC_056066.1	17090001	17110001
NC_056055.1	89915001	89935001	0.403917	0.279315	SG1	LOC105601956	NC_056066.1	17065001	17085001
NC_056072.1	10505001	10525001	0.467455	0.184364	SG1	LOC105603612;MLH	NC_056066.1	17070001	17090001
NC_056072.1	10510001	10530001	0.290178	0.219219	SG1	LOC105603612;MLH	NC_056066.1	59285001	59305001
NC_056072.1	10515001	10535001	0.266641	0.216474	SG1	LOC105603612;MLH	NC_056066.1	59290001	59310001

NC_056072.1	10520001	10540001	0.335657	0.199282	SG1	LOC105603612;MLH	NC_056076.1	44000001	44020001
NC_056077.1	36720001	36740001	0.281206	0.18937	SG1	LOC105604793	NC_056076.1	44005001	44025001
NC_056065.1	6670001	6690001	0.198837	0.252773	SG1	LOC105605798	NC_056066.1	27215001	27235001
NC_056055.1	101390001	1.01E+08	0.382979	0.183997	SG1	LOC105608482	NC_056066.1	27220001	27240001
NC_056073.1	26850001	26870001	0.150526	0.23873	SG1	LOC106990117	NC_056066.1	27225001	27245001
NC_056073.1	26855001	26875001	0.108327	0.239514	SG1	LOC106990117	NC_056066.1	27230001	27250001
NC_056073.1	26860001	26880001	0.327223	0.187081	SG1	LOC106990117	NC_056066.1	27235001	27255001
NC_056061.1	74745001	74765001	0.28845	0.219041	SG1	LOC106991302	NC_056066.1	27240001	27260001
NC_056061.1	74770001	74790001	0.222204	0.194182	SG1	LOC106991302	NC_056066.1	27245001	27265001
NC_056061.1	74775001	74795001	0.165275	0.2702	SG1	LOC106991302	NC_056070.1	58930001	58950001
NC_056061.1	74780001	74800001	0.178199	0.233688	SG1	LOC106991302	NC_056056.1	4865001	4885001
NC_056061.1	74785001	74805001	0.170919	0.188909	SG1	LOC106991302	NC_056056.1	4870001	4890001
NC_056061.1	74860001	74880001	0.066576	0.256026	SG1	LOC106991302	NC_056056.1	4875001	4895001
NC_056061.1	74865001	74885001	0.027127	0.279153	SG1	LOC106991302	NC_056055.1	2880001	2900001
NC_056061.1	74870001	74890001	0.02322	0.29338	SG1	LOC106991302	NC_056055.1	2900001	2920001
NC_056061.1	74875001	74895001	0.030346	0.249195	SG1	LOC106991302	NC_056055.1	2905001	2925001
NC_056061.1	74725001	74745001	0.378358	0.188548	SG1	LOC106991302;LOC1	NC_056055.1	2910001	2930001
NC_056061.1	74730001	74750001	0.239898	0.242932	SG1	LOC106991302;LOC1	NC_056055.1	2915001	2935001
NC_056061.1	74735001	74755001	0.310675	0.230316	SG1	LOC106991302;LOC1	NC_056071.1	24630001	24650001
NC_056061.1	74740001	74760001	0.273329	0.242542	SG1	LOC106991302;LOC1	NC_056077.1	8055001	8075001
NC_056075.1	3870001	3890001	0.39367	0.191059	SG1	LOC106991845	NC_056077.1	8060001	8080001
NC_056080.1	36375001	36395001	0.465698	0.226069	SG1	LOC106991963	NC_056077.1	8065001	8085001
NC_056080.1	36380001	36400001	0.438571	0.192009	SG1	LOC106991963	NC_056054.1	117705001	117725001
NC_056054.1	88795001	88815001	0.248101	0.247177	SG1	LOC114108733	NC_056076.1	34850001	34870001
NC_056070.1	71100001	71120001	0.404718	0.383604	SG1	LOC114108841	NC_056076.1	34855001	34875001
NC_056055.1	127195001	1.27E+08	0.259282	0.191199	SG1	LOC114112858	NC_056076.1	34860001	34880001
NC_056055.1	243395001	2.43E+08	0.279776	0.278806	SG1	LOC114112910;LUZF	NC_056076.1	34865001	34885001
NC_056055.1	243400001	2.43E+08	0.387993	0.200315	SG1	LOC114112910;LUZF	NC_056076.1	34870001	34890001
NC_056056.1	137905001	1.38E+08	0.294898	0.185452	SG1	LOC114113757	NC_056076.1	34875001	34895001
NC_056056.1	137910001	1.38E+08	0.372034	0.191332	SG1	LOC114113757	NC_056076.1	34880001	34900001
NC_056056.1	137915001	1.38E+08	0.378807	0.219067	SG1	LOC114113757	NC_056076.1	34885001	34905001
NC_056056.1	133530001	1.34E+08	0.454469	0.19524	SG1	LOC114113973	NC_056076.1	34890001	34910001

NC_056056.1	218980001	2.19E+08	0.43381	0.31194	SG1	LOC114114084	NC_056068.1	40165001	40185001
NC_056058.1	19700001	19720001	0.414108	0.214146	SG1	LOC114114850;RAD:	NC_056080.1	8720001	8740001
NC_056058.1	19705001	19725001	0.421809	0.216169	SG1	LOC114114850;RAD:	NC_056080.1	8725001	8745001
NC_056058.1	78775001	78795001	0.342454	0.209031	SG1	LOC114114907;RAS(	NC_056080.1	8795001	8815001
NC_056054.1	66685001	66705001	0.136725	0.31321	SG1	LOC114115616	NC_056080.1	8845001	8865001
NC_056061.1	74485001	74505001	0.147914	0.213174	SG1	LOC114116078	NC_056080.1	8850001	8870001
NC_056061.1	74490001	74510001	0.14951	0.202879	SG1	LOC114116078	NC_056069.1	22455001	22475001
NC_056061.1	74185001	74205001	0.370602	0.183855	SG1	LOC114116085	NC_056069.1	22460001	22480001
NC_056061.1	74200001	74220001	0.441293	0.209963	SG1	LOC114116088	NC_056069.1	22465001	22485001
NC_056061.1	74385001	74405001	0.048204	0.315408	SG1	LOC114116134	NC_056069.1	22480001	22500001
NC_056061.1	74390001	74410001	0.180073	0.242585	SG1	LOC114116134	NC_056069.1	22485001	22505001
NC_056061.1	74395001	74415001	0.237636	0.203228	SG1	LOC114116134	NC_056065.1	28505001	28525001
NC_056061.1	74400001	74420001	0.217168	0.19968	SG1	LOC114116134	NC_056057.1	96895001	96915001
NC_056063.1	18490001	18510001	0.473483	0.189774	SG1	LOC114116599;RB1	NC_056061.1	57690001	57710001
NC_056063.1	27675001	27695001	0.214286	0.187204	SG1	LOC114116607;RFC3	NC_056061.1	57695001	57715001
NC_056064.1	6810001	6830001	0.426522	0.304767	SG1	LOC114116936	NC_056063.1	36595001	36615001
NC_056066.1	74650001	74670001	0.385106	0.197695	SG1	LOC114117487;PCIF:	NC_056063.1	36600001	36620001
NC_056066.1	74655001	74675001	0.375719	0.212708	SG1	LOC114117487;PCIF:	NC_056063.1	36605001	36625001
NC_056066.1	74660001	74680001	0.401721	0.216281	SG1	LOC114117487;PCIF:	NC_056063.1	36610001	36630001
NC_056067.1	58355001	58375001	0.449832	0.183653	SG1	LOC114117915	NC_056063.1	36615001	36635001
NC_056067.1	50310001	50330001	0.352248	0.221802	SG1	LOC114117997	NC_056063.1	36565001	36585001
NC_056067.1	50315001	50335001	0.305431	0.229627	SG1	LOC114117997	NC_056063.1	36570001	36590001
NC_056067.1	50320001	50340001	0.311623	0.220304	SG1	LOC114117997	NC_056063.1	36580001	36600001
NC_056067.1	50325001	50345001	0.257668	0.307571	SG1	LOC114117997	NC_056063.1	36585001	36605001
NC_056067.1	50330001	50350001	0.108594	0.398652	SG1	LOC114117997	NC_056063.1	36590001	36610001
NC_056067.1	50335001	50355001	0.119834	0.427464	SG1	LOC114117997	NC_056054.1	119940001	119960001
NC_056067.1	50340001	50360001	0.093023	0.449687	SG1	LOC114117997	NC_056054.1	119895001	119915001
NC_056067.1	62190001	62210001	0.373024	0.18432	SG1	LOC114118049	NC_056066.1	31700001	31720001
NC_056068.1	77570001	77590001	0.423149	0.250635	SG1	LOC114118440;LOC1	NC_056066.1	31705001	31725001
NC_056068.1	77575001	77595001	0.334419	0.22755	SG1	LOC114118440;LOC1	NC_056066.1	31710001	31730001
NC_056068.1	77580001	77600001	0.205841	0.203978	SG1	LOC114118440;LOC1	NC_056055.1	104065001	104085001
NC_056065.1	53850001	53870001	0.457505	0.261343	SG1	LOC121816074	NC_056079.1	19060001	19080001

NC_056065.1	53855001	53875001	0.33785	0.317529	SG1	LOC121816074	NC_056057.1	67705001	67725001
NC_056065.1	53860001	53880001	0.34209	0.328683	SG1	LOC121816074	NC_056064.1	29275001	29295001
NC_056067.1	54710001	54730001	0.445332	0.233592	SG1	LOC121816430;LOC1	NC_056064.1	29280001	29300001
NC_056070.1	14085001	14105001	0.180417	0.265728	SG1	LOC121816918	NC_056064.1	29285001	29305001
NC_056070.1	14135001	14155001	0.154152	0.205859	SG1	LOC121816918	NC_056064.1	29295001	29315001
NC_056070.1	14140001	14160001	0.138486	0.204791	SG1	LOC121816918	NC_056064.1	17140001	17160001
NC_056070.1	14145001	14165001	0.108223	0.258375	SG1	LOC121816918	NC_056064.1	17145001	17165001
NC_056070.1	14150001	14170001	0.127833	0.261618	SG1	LOC121816918	NC_056064.1	17425001	17445001
NC_056070.1	14155001	14175001	0.192075	0.273879	SG1	LOC121816918	NC_056057.1	78270001	78290001
NC_056070.1	14160001	14180001	0.223392	0.266581	SG1	LOC121816918	NC_056057.1	78275001	78295001
NC_056070.1	14165001	14185001	0.342403	0.215148	SG1	LOC121816918	NC_056057.1	78280001	78300001
NC_056071.1	31025001	31045001	0.288373	0.242857	SG1	LOC121817050;PTPN	NC_056080.1	62410001	62430001
NC_056071.1	31030001	31050001	0.38158	0.183564	SG1	LOC121817050;PTPN	NC_056080.1	62415001	62435001
NC_056071.1	4240001	4260001	0.223695	0.431482	SG1	LOC121817145	NC_056080.1	62420001	62440001
NC_056071.1	4245001	4265001	0.215204	0.435374	SG1	LOC121817145	NC_056080.1	62425001	62445001
NC_056071.1	4250001	4270001	0.325493	0.419219	SG1	LOC121817145	NC_056080.1	62430001	62450001
NC_056080.1	81860001	81880001	0.249071	0.381703	SG1	LOC121818145	NC_056074.1	22700001	22720001
NC_056080.1	14805001	14825001	0.468652	0.206044	SG1	LOC121818210	NC_056074.1	22705001	22725001
NC_056054.1	86020001	86040001	0.247787	0.271589	SG1	LOC121818611;PRPF	NC_056074.1	22710001	22730001
NC_056054.1	86025001	86045001	0.429945	0.389925	SG1	LOC121818611;PRPF	NC_056074.1	22715001	22735001
NC_056056.1	146660001	1.47E+08	0.341276	0.213843	SG1	LOC121819176;LOC1	NC_056056.1	23680001	23700001
NC_056056.1	146665001	1.47E+08	0.125538	0.319735	SG1	LOC121819176;LOC1	NC_056056.1	23790001	23810001
NC_056057.1	113760001	1.14E+08	0.462433	0.184238	SG1	LOC121819522;ZNF4	NC_056056.1	23795001	23815001
NC_056062.1	64830001	64850001	0.452187	0.210027	SG1	LOC121820313	NC_056056.1	23800001	23820001
NC_056062.1	64835001	64855001	0.459444	0.212512	SG1	LOC121820313	NC_056056.1	23805001	23825001
NC_056062.1	64840001	64860001	0.466102	0.205142	SG1	LOC121820313	NC_056063.1	26140001	26160001
NC_056054.1	107160001	1.07E+08	0.230992	0.223794	SG1	LOC121820608	NC_056063.1	26145001	26165001
NC_056054.1	107165001	1.07E+08	0.15451	0.240748	SG1	LOC121820608	NC_056063.1	26150001	26170001
NC_056054.1	107320001	1.07E+08	0.222558	0.197396	SG1	LOC121820612	NC_056063.1	26310001	26330001
NC_056054.1	107325001	1.07E+08	0.282717	0.231959	SG1	LOC121820612	NC_056063.1	26320001	26340001
NC_056064.1	61445001	61465001	0.147832	0.286927	SG1	LOC121820680	NC_056063.1	26325001	26345001
NC_056064.1	61450001	61470001	0.163843	0.303447	SG1	LOC121820680	NC_056063.1	26330001	26350001

NC_056066.1	36970001	36990001	0.375919	0.332932	SG1	LOC443027	NC_056063.1	26335001	26355001
NC_056066.1	36975001	36995001	0.391915	0.264683	SG1	LOC443027	NC_056063.1	26340001	26360001
NC_056066.1	36980001	37000001	0.432316	0.223988	SG1	LOC443027	NC_056063.1	26345001	26365001
NC_056066.1	36985001	37005001	0.416806	0.241552	SG1	LOC443027	NC_056063.1	26360001	26380001
NC_056066.1	36990001	37010001	0.431507	0.306851	SG1	LOC443027	NC_056063.1	26365001	26385001
NC_056066.1	36995001	37015001	0.399338	0.334347	SG1	LOC443027	NC_056063.1	26530001	26550001
NC_056066.1	37000001	37020001	0.376019	0.314406	SG1	LOC443027	NC_056063.1	26545001	26565001
NC_056066.1	37005001	37025001	0.34853	0.279326	SG1	LOC443027	NC_056063.1	26550001	26570001
NC_056074.1	36005001	36025001	0.20759	0.190047	SG1	LOC443348	NC_056063.1	26555001	26575001
NC_056058.1	16430001	16450001	0.227097	0.207066	SG1	LONP1;RPL36	NC_056063.1	26560001	26580001
NC_056067.1	23335001	23355001	0.350837	0.247089	SG1	LPCAT2	NC_056063.1	26565001	26585001
NC_056063.1	16980001	17000001	0.099503	0.318498	SG1	LRCH1	NC_056056.1	103985001	104005001
NC_056063.1	16985001	17005001	0.087988	0.389359	SG1	LRCH1	NC_056055.1	50015001	50035001
NC_056063.1	17000001	17020001	0.25606	0.225377	SG1	LRCH1	NC_056061.1	40125001	40145001
NC_056072.1	35190001	35210001	0.468861	0.254366	SG1	LRIG1;SLC25A26	NC_056061.1	40130001	40150001
NC_056073.1	6880001	6900001	0.380641	0.219459	SG1	LRRC1	NC_056061.1	40135001	40155001
NC_056068.1	70510001	70530001	0.441275	0.18513	SG1	LRRC4C	NC_056068.1	29925001	29945001
NC_056054.1	66910001	66930001	0.467074	0.228773	SG1	LRRC8B	NC_056068.1	29930001	29950001
NC_056054.1	66920001	66940001	0.312791	0.183209	SG1	LRRC8B	NC_056068.1	29935001	29955001
NC_056055.1	243440001	2.43E+08	0.376512	0.18543	SG1	LUZP1	NC_056064.1	18500001	18520001
NC_056055.1	243445001	2.43E+08	0.235446	0.194567	SG1	LUZP1	NC_056080.1	15850001	15870001
NC_056055.1	243450001	2.43E+08	0.101938	0.245978	SG1	LUZP1	NC_056080.1	15855001	15875001
NC_056055.1	243455001	2.43E+08	0.159603	0.189029	SG1	LUZP1	NC_056080.1	15860001	15880001
NC_056074.1	17870001	17890001	0.442657	0.310676	SG1	LUZP2	NC_056061.1	63565001	63585001
NC_056074.1	17875001	17895001	0.226712	0.404361	SG1	LUZP2	NC_056071.1	34620001	34640001
NC_056074.1	17880001	17900001	0.194357	0.454021	SG1	LUZP2	NC_056071.1	34625001	34645001
NC_056074.1	17885001	17905001	0.068418	0.393101	SG1	LUZP2	NC_056071.1	34630001	34650001
NC_056074.1	17890001	17910001	0.030011	0.38657	SG1	LUZP2	NC_056071.1	34635001	34655001
NC_056074.1	17895001	17915001	0.024825	0.349925	SG1	LUZP2	NC_056071.1	34640001	34660001
NC_056074.1	17965001	17985001	0.109081	0.271537	SG1	LUZP2	NC_056071.1	34645001	34665001
NC_056074.1	17970001	17990001	0.186188	0.212636	SG1	LUZP2	NC_056074.1	5690001	5710001
NC_056074.1	17975001	17995001	0.272338	0.219921	SG1	LUZP2	NC_056069.1	41095001	41115001

NC_056074.1	17990001	18010001	0.25505	0.214159	SG1	LUZP2	NC_056069.1	41100001	41120001
NC_056074.1	17995001	18015001	0.155011	0.269497	SG1	LUZP2	NC_056069.1	41105001	41125001
NC_056074.1	18000001	18020001	0.153846	0.262256	SG1	LUZP2	NC_056069.1	41110001	41130001
NC_056074.1	18005001	18025001	0.468511	0.191831	SG1	LUZP2	NC_056069.1	41115001	41135001
NC_056072.1	14600001	14620001	0.243074	0.320643	SG1	LYZL4	NC_056065.1	78285001	78305001
NC_056072.1	14605001	14625001	0.264431	0.301219	SG1	LYZL4	NC_056065.1	78290001	78310001
NC_056072.1	14610001	14630001	0.290843	0.259961	SG1	LYZL4	NC_056065.1	78295001	78315001
NC_056075.1	21580001	21600001	0.246575	0.186407	SG1	LZTS2;MRPL43;SEM	NC_056071.1	29040001	29060001
NC_056075.1	21585001	21605001	0.352446	0.204754	SG1	LZTS2;MRPL43;SEM	NC_056071.1	29045001	29065001
NC_056054.1	93425001	93445001	0.209646	0.183366	SG1	MAB21L3	NC_056071.1	29060001	29080001
NC_056054.1	93430001	93450001	0.243086	0.199737	SG1	MAB21L3	NC_056060.1	20850001	20870001
NC_056066.1	7555001	7575001	0.416378	0.407588	SG1	MACROD2	NC_056060.1	20855001	20875001
NC_056066.1	7560001	7580001	0.325512	0.431704	SG1	MACROD2	NC_056066.1	19080001	19100001
NC_056066.1	7565001	7585001	0.280025	0.420765	SG1	MACROD2	NC_056066.1	19085001	19105001
NC_056066.1	7570001	7590001	0.220935	0.404328	SG1	MACROD2	NC_056069.1	5940001	5960001
NC_056066.1	7575001	7595001	0.380759	0.393737	SG1	MACROD2	NC_056066.1	32450001	32470001
NC_056066.1	7580001	7600001	0.447496	0.391892	SG1	MACROD2	NC_056074.1	32185001	32205001
NC_056066.1	7750001	7770001	0.333333	0.219317	SG1	MACROD2	NC_056074.1	32190001	32210001
NC_056066.1	8250001	8270001	0.45376	0.2687	SG1	MACROD2	NC_056064.1	28410001	28430001
NC_056054.1	119060001	1.19E+08	0.469675	0.23901	SG1	MAEL	NC_056064.1	28415001	28435001
NC_056054.1	119065001	1.19E+08	0.408198	0.258576	SG1	MAEL	NC_056064.1	28420001	28440001
NC_056054.1	119070001	1.19E+08	0.190477	0.280223	SG1	MAEL	NC_056064.1	28425001	28445001
NC_056054.1	119075001	1.19E+08	0.190654	0.269511	SG1	MAEL	NC_056080.1	54015001	54035001
NC_056057.1	44630001	44650001	0.331726	0.22176	SG1	MAGI2	NC_056080.1	54025001	54045001
NC_056057.1	44635001	44655001	0.19183	0.236504	SG1	MAGI2	NC_056080.1	54030001	54050001
NC_056057.1	44640001	44660001	0.2	0.254321	SG1	MAGI2	NC_056057.1	17450001	17470001
NC_056061.1	18975001	18995001	0.291237	0.215321	SG1	MAN1A1	NC_056057.1	17455001	17475001
NC_056061.1	18980001	19000001	0.304422	0.237041	SG1	MAN1A1	NC_056057.1	17460001	17480001
NC_056061.1	18985001	19005001	0.453855	0.23333	SG1	MAN1A1	NC_056063.1	54375001	54395001
NC_056071.1	21050001	21070001	0.191997	0.226657	SG1	MAN2A2	NC_056054.1	123165001	123185001
NC_056071.1	21055001	21075001	0.156325	0.250803	SG1	MAN2A2	NC_056054.1	194585001	194605001
NC_056071.1	21060001	21080001	0.110475	0.332216	SG1	MAN2A2	NC_056054.1	194590001	194610001

NC_056071.1	31090001	31110001	0.334378	0.229616	SG1	MAN2C1;SIN3A	NC_056054.1	194595001	194615001
NC_056061.1	61975001	61995001	0.424233	0.202133	SG1	MAP3K5	NC_056054.1	194600001	194620001
NC_056061.1	61980001	62000001	0.266607	0.238152	SG1	MAP3K5	NC_056054.1	194605001	194625001
NC_056061.1	61985001	62005001	0.333062	0.223164	SG1	MAP3K5	NC_056054.1	194610001	194630001
NC_056061.1	61990001	62010001	0.38672	0.208928	SG1	MAP3K5	NC_056054.1	190835001	190855001
NC_056061.1	61995001	62015001	0.45953	0.21722	SG1	MAP3K5	NC_056054.1	190840001	190860001
NC_056060.1	41595001	41615001	0.394668	0.198284	SG1	MAP4K5	NC_056054.1	190845001	190865001
NC_056060.1	41600001	41620001	0.408006	0.189934	SG1	MAP4K5	NC_056054.1	190850001	190870001
NC_056060.1	41605001	41625001	0.379769	0.191252	SG1	MAP4K5	NC_056054.1	190855001	190875001
NC_056060.1	41610001	41630001	0.423349	0.187727	SG1	MAP4K5	NC_056057.1	72530001	72550001
NC_056056.1	219830001	2.2E+08	0.147365	0.418341	SG1	MCAT	NC_056057.1	72535001	72555001
NC_056056.1	219835001	2.2E+08	0.119874	0.432166	SG1	MCAT	NC_056057.1	72540001	72560001
NC_056056.1	219840001	2.2E+08	0.117504	0.424404	SG1	MCAT;TSPO	NC_056057.1	72545001	72565001
NC_056056.1	219845001	2.2E+08	0.153939	0.414744	SG1	MCAT;TSPO	NC_056057.1	72550001	72570001
NC_056060.1	38355001	38375001	0.20893	0.233915	SG1	MDGA2	NC_056057.1	72555001	72575001
NC_056060.1	38360001	38380001	0.077761	0.294305	SG1	MDGA2	NC_056057.1	72600001	72620001
NC_056060.1	38365001	38385001	0.066148	0.319722	SG1	MDGA2	NC_056056.1	112455001	112475001
NC_056060.1	38370001	38390001	0.050294	0.347905	SG1	MDGA2	NC_056056.1	112460001	112480001
NC_056060.1	38375001	38395001	0.048346	0.318512	SG1	MDGA2	NC_056056.1	112465001	112485001
NC_056065.1	2110001	2130001	0.360329	0.184462	SG1	MDM4	NC_056056.1	112470001	112490001
NC_056061.1	47795001	47815001	0.370308	0.183281	SG1	MDN1	NC_056056.1	112475001	112495001
NC_056061.1	47800001	47820001	0.3521	0.203889	SG1	MDN1	NC_056071.1	27870001	27890001
NC_056061.1	47805001	47825001	0.301971	0.191682	SG1	MDN1	NC_056055.1	7335001	7355001
NC_056054.1	218840001	2.19E+08	0.066745	0.195736	SG1	MECOM	NC_056055.1	7340001	7360001
NC_056080.1	63830001	63850001	0.353933	0.193353	SG1	MED12	NC_056055.1	7345001	7365001
NC_056080.1	63835001	63855001	0.375816	0.240594	SG1	MED12	NC_056055.1	7420001	7440001
NC_056058.1	41095001	41115001	0.319635	0.217756	SG1	MED16;PLPPR3	NC_056066.1	18050001	18070001
NC_056058.1	41100001	41120001	0.27027	0.291663	SG1	MED16;R3HDM4	NC_056066.1	18350001	18370001
NC_056074.1	585001	605001	0.154804	0.185087	SG1	MED17;VSTM5	NC_056066.1	18355001	18375001
NC_056056.1	4910001	4930001	0.302899	0.336927	SG1	MED27	NC_056066.1	18360001	18380001
NC_056056.1	4915001	4935001	0.331896	0.364485	SG1	MED27	NC_056054.1	204045001	204065001
NC_056056.1	4920001	4940001	0.392413	0.369786	SG1	MED27	NC_056054.1	204050001	204070001

NC_056056.1	4925001	4945001	0.290855	0.299192	SG1	MED27	NC_056077.1	14010001	14030001
NC_056056.1	4930001	4950001	0.362299	0.282817	SG1	MED27	NC_056077.1	14015001	14035001
NC_056056.1	4935001	4955001	0.381673	0.264217	SG1	MED27	NC_056063.1	36660001	36680001
NC_056056.1	4940001	4960001	0.385663	0.264307	SG1	MED27	NC_056063.1	36670001	36690001
NC_056056.1	4945001	4965001	0.398655	0.262375	SG1	MED27	NC_056063.1	36685001	36705001
NC_056056.1	4950001	4970001	0.355202	0.222957	SG1	MED27	NC_056063.1	36690001	36710001
NC_056056.1	4955001	4975001	0.381379	0.226306	SG1	MED27	NC_056063.1	36695001	36715001
NC_056056.1	4960001	4980001	0.400068	0.234109	SG1	MED27	NC_056056.1	10315001	10335001
NC_056063.1	30290001	30310001	0.205619	0.226739	SG1	MEDAG	NC_056054.1	266270001	266290001
NC_056063.1	30295001	30315001	0.235683	0.232916	SG1	MEDAG	NC_056054.1	266275001	266295001
NC_056063.1	30300001	30320001	0.303874	0.207347	SG1	MEDAG	NC_056054.1	266280001	266300001
NC_056063.1	30305001	30325001	0.423976	0.183658	SG1	MEDAG	NC_056054.1	266285001	266305001
NC_056063.1	30280001	30300001	0.351723	0.233114	SG1	MEDAG;TEX26	NC_056075.1	5215001	5235001
NC_056063.1	30285001	30305001	0.197483	0.263266	SG1	MEDAG;TEX26	NC_056063.1	5505001	5525001
NC_056058.1	24365001	24385001	0.215934	0.328544	SG1	MEGF10	NC_056063.1	5510001	5530001
NC_056058.1	24370001	24390001	0.081088	0.422812	SG1	MEGF10	NC_056055.1	60555001	60575001
NC_056058.1	24375001	24395001	0.296781	0.34557	SG1	MEGF10	NC_056071.1	26895001	26915001
NC_056060.1	12765001	12785001	0.460926	0.184045	SG1	MEGF11	NC_056071.1	26900001	26920001
NC_056060.1	12770001	12790001	0.439791	0.237107	SG1	MEGF11	NC_056071.1	26905001	26925001
NC_056060.1	12795001	12815001	0.415004	0.1836	SG1	MEGF11	NC_056071.1	26910001	26930001
NC_056060.1	12955001	12975001	0.369008	0.231857	SG1	MEGF11	NC_056071.1	26915001	26935001
NC_056060.1	12960001	12980001	0.44847	0.241609	SG1	MEGF11	NC_056080.1	22920001	22940001
NC_056060.1	12965001	12985001	0.382197	0.23489	SG1	MEGF11	NC_056080.1	22980001	23000001
NC_056060.1	30805001	30825001	0.370196	0.30527	SG1	MEIS2	NC_056080.1	22985001	23005001
NC_056060.1	30855001	30875001	0.314305	0.193535	SG1	MEIS2	NC_056057.1	65740001	65760001
NC_056064.1	43295001	43315001	0.440945	0.187113	SG1	MEOX1	NC_056057.1	65745001	65765001
NC_056060.1	23995001	24015001	0.365951	0.213948	SG1	METTL3;SALL2	NC_056057.1	65770001	65790001
NC_056054.1	14555001	14575001	0.460063	0.201496	SG1	MFSD2A	NC_056057.1	65775001	65795001
NC_056064.1	28105001	28125001	0.37637	0.204159	SG1	MFSD6L;PIK3R6	NC_056057.1	65780001	65800001
NC_056072.1	31720001	31740001	0.430228	0.205896	SG1	MITF	NC_056057.1	65785001	65805001
NC_056072.1	31725001	31745001	0.388199	0.223827	SG1	MITF	NC_056057.1	65790001	65810001
NC_056072.1	31730001	31750001	0.267673	0.230806	SG1	MITF	NC_056057.1	65795001	65815001

NC_056072.1	31735001	31755001	0.10814	0.223492	SG1	MITF	NC_056057.1	65850001	65870001
NC_056072.1	31740001	31760001	0.066866	0.334253	SG1	MITF	NC_056057.1	65855001	65875001
NC_056072.1	31745001	31765001	0.134645	0.305942	SG1	MITF	NC_056057.1	65860001	65880001
NC_056072.1	31750001	31770001	0.183265	0.31777	SG1	MITF	NC_056057.1	65865001	65885001
NC_056072.1	31755001	31775001	0.203509	0.320436	SG1	MITF	NC_056057.1	65870001	65890001
NC_056072.1	31760001	31780001	0.168975	0.305195	SG1	MITF	NC_056057.1	65875001	65895001
NC_056072.1	31765001	31785001	0.106588	0.293913	SG1	MITF	NC_056057.1	65880001	65900001
NC_056072.1	31770001	31790001	0.096642	0.248658	SG1	MITF	NC_056057.1	65890001	65910001
NC_056072.1	31775001	31795001	0.175604	0.203958	SG1	MITF	NC_056057.1	65895001	65915001
NC_056072.1	31830001	31850001	0.109706	0.23118	SG1	MITF	NC_056057.1	65900001	65920001
NC_056072.1	31835001	31855001	0.078054	0.244704	SG1	MITF	NC_056057.1	65905001	65925001
NC_056072.1	31840001	31860001	0.086008	0.204197	SG1	MITF	NC_056057.1	65965001	65985001
NC_056072.1	31900001	31920001	0.280822	0.187785	SG1	MITF	NC_056057.1	65970001	65990001
NC_056054.1	21120001	21140001	0.349169	0.274526	SG1	MKNK1	NC_056057.1	66055001	66075001
NC_056054.1	21125001	21145001	0.189155	0.370999	SG1	MKNK1	NC_056057.1	66060001	66080001
NC_056054.1	21135001	21155001	0.151703	0.337387	SG1	MKNK1	NC_056057.1	66065001	66085001
NC_056070.1	63060001	63080001	0.429188	0.31186	SG1	MLEC	NC_056057.1	66070001	66090001
NC_056070.1	63065001	63085001	0.431787	0.270379	SG1	MLEC	NC_056057.1	66075001	66095001
NC_056070.1	63070001	63090001	0.401487	0.231517	SG1	MLEC;UNC119B	NC_056057.1	66080001	66100001
NC_056070.1	63075001	63095001	0.312644	0.249833	SG1	MLEC;UNC119B	NC_056069.1	19560001	19580001
NC_056070.1	63080001	63100001	0.342075	0.205344	SG1	MLEC;UNC119B	NC_056069.1	19565001	19585001
NC_056055.1	88985001	89005001	0.467335	0.201667	SG1	MLLT3	NC_056069.1	19570001	19590001
NC_056064.1	14310001	14330001	0.373685	0.289272	SG1	MMP28	NC_056069.1	19680001	19700001
NC_056064.1	14315001	14335001	0.068853	0.345641	SG1	MMP28	NC_056069.1	19690001	19710001
NC_056054.1	174740001	1.75E+08	0.325699	0.337579	SG1	MORC1	NC_056069.1	20330001	20350001
NC_056054.1	174745001	1.75E+08	0.234293	0.469801	SG1	MORC1	NC_056069.1	20335001	20355001
NC_056054.1	174750001	1.75E+08	0.19054	0.622466	SG1	MORC1	NC_056069.1	20450001	20470001
NC_056054.1	174755001	1.75E+08	0.090674	0.657108	SG1	MORC1	NC_056061.1	61115001	61135001
NC_056054.1	174760001	1.75E+08	0.111465	0.547521	SG1	MORC1	NC_056061.1	61120001	61140001
NC_056054.1	174765001	1.75E+08	0.17707	0.389503	SG1	MORC1	NC_056061.1	61125001	61145001
NC_056061.1	89060001	89080001	0.408221	0.21926	SG1	MPC1	NC_056061.1	61130001	61150001
NC_056066.1	31745001	31765001	0.46257	0.199241	SG1	MRC1	NC_056061.1	61135001	61155001

NC_056059.1	104945001	1.05E+08	0.21208	0.194385	SG1	MSX1	NC_056061.1	61140001	61160001
NC_056071.1	67440001	67460001	0.138523	0.346591	SG1	MTA1	NC_056061.1	61145001	61165001
NC_056071.1	67445001	67465001	0.172296	0.284412	SG1	MTA1	NC_056061.1	61155001	61175001
NC_056071.1	67450001	67470001	0.199336	0.274144	SG1	MTA1	NC_056061.1	61160001	61180001
NC_056071.1	67455001	67475001	0.22042	0.280272	SG1	MTA1	NC_056059.1	30735001	30755001
NC_056071.1	67430001	67450001	0.353524	0.300346	SG1	MTA1;TEX22	NC_056059.1	30740001	30760001
NC_056071.1	67435001	67455001	0.249146	0.31293	SG1	MTA1;TEX22	NC_056071.1	30170001	30190001
NC_056056.1	146695001	1.47E+08	0.259343	0.261837	SG1	MUC19	NC_056071.1	30175001	30195001
NC_056056.1	146700001	1.47E+08	0.373766	0.202092	SG1	MUC19	NC_056071.1	30180001	30200001
NC_056067.1	56300001	56320001	0.273463	0.18831	SG1	MYBPC2;POLD1;SPI	NC_056067.1	43305001	43325001
NC_056067.1	56305001	56325001	0.323852	0.20283	SG1	MYBPC2;POLD1;SPI	NC_056071.1	7545001	7565001
NC_056054.1	189080001	1.89E+08	0.468812	0.185504	SG1	MYLK	NC_056071.1	7550001	7570001
NC_056054.1	212340001	2.12E+08	0.395331	0.205042	SG1	NAALADL2	NC_056071.1	7555001	7575001
NC_056056.1	163315001	1.63E+08	0.464512	0.199876	SG1	NABP2;RNF41;SLC3	NC_056071.1	7560001	7580001
NC_056056.1	23735001	23755001	0.442088	0.224211	SG1	NBAS	NC_056073.1	42950001	42970001
NC_056063.1	26070001	26090001	0.333333	0.216715	SG1	NBEA	NC_056073.1	42955001	42975001
NC_056063.1	26075001	26095001	0.369566	0.205094	SG1	NBEA	NC_056056.1	209340001	209360001
NC_056063.1	26080001	26100001	0.204211	0.25822	SG1	NBEA	NC_056064.1	20255001	20275001
NC_056063.1	26085001	26105001	0.263691	0.3197	SG1	NBEA	NC_056064.1	20260001	20280001
NC_056063.1	26090001	26110001	0.326456	0.331703	SG1	NBEA	NC_056080.1	65285001	65305001
NC_056063.1	26095001	26115001	0.401691	0.412295	SG1	NBEA	NC_056080.1	65290001	65310001
NC_056055.1	205125001	2.05E+08	0.462762	0.454673	SG1	NBEAL1	NC_056080.1	65295001	65315001
NC_056055.1	205130001	2.05E+08	0.471622	0.464105	SG1	NBEAL1	NC_056056.1	112045001	112065001
NC_056055.1	205140001	2.05E+08	0.388106	0.479544	SG1	NBEAL1	NC_056056.1	112050001	112070001
NC_056055.1	205145001	2.05E+08	0.273244	0.510088	SG1	NBEAL1	NC_056054.1	91055001	91075001
NC_056055.1	205150001	2.05E+08	0.309078	0.512548	SG1	NBEAL1	NC_056062.1	51160001	51180001
NC_056055.1	205155001	2.05E+08	0.353206	0.514646	SG1	NBEAL1	NC_056062.1	51165001	51185001
NC_056062.1	75315001	75335001	0.281734	0.186932	SG1	NCALD	NC_056063.1	48185001	48205001
NC_056054.1	136395001	1.36E+08	0.330724	0.198464	SG1	NCAM2	NC_056063.1	48190001	48210001
NC_056054.1	136465001	1.36E+08	0.415095	0.264289	SG1	NCAM2	NC_056063.1	48195001	48215001
NC_056054.1	136470001	1.36E+08	0.355688	0.321512	SG1	NCAM2	NC_056054.1	88680001	88700001
NC_056054.1	252870001	2.53E+08	0.341912	0.213047	SG1	NCK1	NC_056066.1	23045001	23065001

NC_056054.1	252875001	2.53E+08	0.301887	0.19565	SG1	NCK1	NC_056066.1	23050001	23070001
NC_056055.1	177900001	1.78E+08	0.470841	0.23369	SG1	NCKAP5	NC_056066.1	23055001	23075001
NC_056055.1	177920001	1.78E+08	0.465985	0.195361	SG1	NCKAP5	NC_056062.1	67795001	67815001
NC_056055.1	177925001	1.78E+08	0.39593	0.192827	SG1	NCKAP5	NC_056062.1	36315001	36335001
NC_056061.1	12640001	12660001	0.280749	0.201606	SG1	NCOA7	NC_056062.1	36320001	36340001
NC_056066.1	21640001	21660001	0.356814	0.21256	SG1	NEBL	NC_056062.1	36325001	36345001
NC_056054.1	176890001	1.77E+08	0.331734	0.194274	SG1	NECTIN3	NC_056062.1	36330001	36350001
NC_056054.1	176895001	1.77E+08	0.245131	0.218808	SG1	NECTIN3	NC_056062.1	36335001	36355001
NC_056054.1	176900001	1.77E+08	0.180934	0.233201	SG1	NECTIN3	NC_056062.1	36340001	36360001
NC_056054.1	176905001	1.77E+08	0.172314	0.227179	SG1	NECTIN3	NC_056066.1	1895001	1915001
NC_056054.1	176910001	1.77E+08	0.213908	0.205213	SG1	NECTIN3	NC_056066.1	1915001	1935001
NC_056054.1	176915001	1.77E+08	0.193137	0.211645	SG1	NECTIN3	NC_056066.1	1920001	1940001
NC_056054.1	176920001	1.77E+08	0.228663	0.205916	SG1	NECTIN3	NC_056055.1	209950001	209970001
NC_056054.1	176925001	1.77E+08	0.254237	0.206834	SG1	NECTIN3	NC_056054.1	242570001	242590001
NC_056054.1	176930001	1.77E+08	0.302657	0.210048	SG1	NECTIN3	NC_056066.1	20750001	20770001
NC_056054.1	176935001	1.77E+08	0.308501	0.196395	SG1	NECTIN3	NC_056066.1	20755001	20775001
NC_056054.1	176940001	1.77E+08	0.242869	0.192634	SG1	NECTIN3	NC_056066.1	20825001	20845001
NC_056054.1	176945001	1.77E+08	0.19304	0.19334	SG1	NECTIN3	NC_056066.1	20830001	20850001
NC_056054.1	176965001	1.77E+08	0.340118	0.202194	SG1	NECTIN3	NC_056066.1	20835001	20855001
NC_056076.1	57550001	57570001	0.459766	0.228552	SG1	NEDD4L	NC_056066.1	58185001	58205001
NC_056076.1	57555001	57575001	0.279041	0.243989	SG1	NEDD4L	NC_056066.1	58190001	58210001
NC_056076.1	57560001	57580001	0.217089	0.243725	SG1	NEDD4L	NC_056066.1	58195001	58215001
NC_056076.1	57565001	57585001	0.301632	0.193522	SG1	NEDD4L	NC_056066.1	58200001	58220001
NC_056054.1	47185001	47205001	0.429062	0.199046	SG1	NEGR1	NC_056057.1	96900001	96920001
NC_056072.1	1675001	1695001	0.411554	0.199362	SG1	NEK10	NC_056057.1	96905001	96925001
NC_056072.1	1680001	1700001	0.371662	0.22037	SG1	NEK10	NC_056057.1	96910001	96930001
NC_056072.1	1685001	1705001	0.425422	0.190295	SG1	NEK10	NC_056080.1	23040001	23060001
NC_056060.1	19765001	19785001	0.444707	0.486273	SG1	NEO1	NC_056080.1	23045001	23065001
NC_056060.1	19770001	19790001	0.286528	0.478459	SG1	NEO1	NC_056080.1	23050001	23070001
NC_056060.1	19775001	19795001	0.235441	0.372073	SG1	NEO1	NC_056059.1	117150001	117170001
NC_056060.1	19780001	19800001	0.169718	0.322143	SG1	NEO1	NC_056059.1	117155001	117175001
NC_056060.1	19785001	19805001	0.279243	0.207455	SG1	NEO1	NC_056059.1	117160001	117180001

NC_056054.1	36690001	36710001	0.413052	0.20058	SG1	NFIA	NC_056059.1	117165001	117185001
NC_056054.1	36695001	36715001	0.188907	0.210236	SG1	NFIA	NC_056059.1	117170001	117190001
NC_056055.1	82830001	82850001	0.390873	0.193396	SG1	NFIB	NC_056059.1	117175001	117195001
NC_056071.1	20955001	20975001	0.343689	0.208575	SG1	NGRN;VPS33B	NC_056059.1	117180001	117200001
NC_056080.1	15890001	15910001	0.224066	0.185644	SG1	NHS	NC_056059.1	117185001	117205001
NC_056080.1	15930001	15950001	0.408163	0.217878	SG1	NHS	NC_056059.1	117190001	117210001
NC_056080.1	15935001	15955001	0.376127	0.237353	SG1	NHS	NC_056059.1	117195001	117215001
NC_056080.1	64690001	64710001	0.413793	0.209002	SG1	NHSL2;RTL5	NC_056059.1	117200001	117220001
NC_056067.1	52590001	52610001	0.094903	0.186819	SG1	NKPD1;PPP1R37;TR/	NC_056059.1	117205001	117225001
NC_056067.1	52595001	52615001	0.10695	0.220027	SG1	NKPD1;TRAPPC6A	NC_056059.1	117210001	117230001
NC_056068.1	29470001	29490001	0.152597	0.217584	SG1	NLRX1	NC_056059.1	117215001	117235001
NC_056055.1	61725001	61745001	0.1656	0.18709	SG1	NMRK1	NC_056059.1	117230001	117250001
NC_056055.1	61730001	61750001	0.092361	0.243472	SG1	NMRK1	NC_056059.1	117235001	117255001
NC_056055.1	61735001	61755001	0.042424	0.264403	SG1	NMRK1	NC_056059.1	117240001	117260001
NC_056055.1	61740001	61760001	0.072333	0.236582	SG1	NMRK1	NC_056073.1	30220001	30240001
NC_056055.1	61745001	61765001	0.104701	0.189277	SG1	NMRK1	NC_056073.1	30225001	30245001
NC_056059.1	71770001	71790001	0.293394	0.246505	SG1	NMU	NC_056073.1	30230001	30250001
NC_056059.1	71775001	71795001	0.117012	0.353998	SG1	NMU	NC_056073.1	30235001	30255001
NC_056059.1	71780001	71800001	0.153815	0.331035	SG1	NMU	NC_056073.1	30240001	30260001
NC_056059.1	71785001	71805001	0.338709	0.258031	SG1	NMU	NC_056057.1	90710001	90730001
NC_056076.1	23565001	23585001	0.457976	0.239273	SG1	NOL4	NC_056054.1	12770001	12790001
NC_056076.1	23570001	23590001	0.423038	0.202505	SG1	NOL4	NC_056056.1	115790001	115810001
NC_056076.1	23650001	23670001	0.2145	0.190128	SG1	NOL4	NC_056056.1	115795001	115815001
NC_056076.1	23655001	23675001	0.132233	0.236847	SG1	NOL4	NC_056057.1	66320001	66340001
NC_056076.1	23660001	23680001	0.123465	0.229206	SG1	NOL4	NC_056058.1	55740001	55760001
NC_056054.1	257575001	2.58E+08	0.326516	0.25009	SG1	NPHP3	NC_056058.1	55745001	55765001
NC_056054.1	257580001	2.58E+08	0.279817	0.256685	SG1	NPHP3	NC_056058.1	55755001	55775001
NC_056054.1	257585001	2.58E+08	0.343364	0.230455	SG1	NPHP3;UBA5	NC_056058.1	56000001	56020001
NC_056054.1	257590001	2.58E+08	0.396747	0.187523	SG1	NPHP3;UBA5	NC_056058.1	56005001	56025001
NC_056055.1	233565001	2.34E+08	0.368897	0.18709	SG1	NPPC	NC_056065.1	70665001	70685001
NC_056055.1	233570001	2.34E+08	0.258752	0.232358	SG1	NPPC	NC_056065.1	70670001	70690001
NC_056055.1	52740001	52760001	0.418644	0.217118	SG1	NPR2	NC_056065.1	70675001	70695001

NC_056055.1	52745001	52765001	0.361982	0.203176	SG1	NPR2	NC_056065.1	70680001	70700001
NC_056055.1	52735001	52755001	0.435678	0.221906	SG1	NPR2;SPAG8	NC_056065.1	70685001	70705001
NC_056057.1	63785001	63805001	0.437195	0.321608	SG1	NPSR1	NC_056065.1	70690001	70710001
NC_056057.1	63790001	63810001	0.440402	0.283767	SG1	NPSR1	NC_056065.1	70695001	70715001
NC_056080.1	29495001	29515001	0.34903	0.185563	SG1	NR0B1	NC_056065.1	70700001	70720001
NC_056056.1	73300001	73320001	0.438423	0.206875	SG1	NRXN1	NC_056065.1	70705001	70725001
NC_056060.1	89195001	89215001	0.419961	0.231172	SG1	NRXN3	NC_056065.1	70715001	70735001
NC_056060.1	89200001	89220001	0.459818	0.227231	SG1	NRXN3	NC_056065.1	70720001	70740001
NC_056059.1	117345001	1.17E+08	0.227586	0.201706	SG1	NSD2	NC_056072.1	28745001	28765001
NC_056059.1	117360001	1.17E+08	0.236457	0.18462	SG1	NSD2	NC_056072.1	28750001	28770001
NC_056062.1	28025001	28045001	0.441011	0.231925	SG1	NSMCE2	NC_056072.1	28760001	28780001
NC_056062.1	28030001	28050001	0.131844	0.330583	SG1	NSMCE2	NC_056072.1	28765001	28785001
NC_056062.1	28050001	28070001	0.115071	0.325768	SG1	NSMCE2	NC_056065.1	49095001	49115001
NC_056062.1	28055001	28075001	0.246001	0.241967	SG1	NSMCE2	NC_056065.1	49245001	49265001
NC_056062.1	29435001	29455001	0.070715	0.187111	SG1	NTAQ1	NC_056065.1	49250001	49270001
NC_056062.1	29440001	29460001	0.036819	0.203794	SG1	NTAQ1	NC_056065.1	53070001	53090001
NC_056062.1	29445001	29465001	0.053767	0.195949	SG1	NTAQ1	NC_056071.1	37905001	37925001
NC_056065.1	3325001	3345001	0.398362	0.211606	SG1	NUCKS1	NC_056071.1	37910001	37930001
NC_056065.1	3330001	3350001	0.284341	0.235835	SG1	NUCKS1	NC_056071.1	37915001	37935001
NC_056065.1	3335001	3355001	0.190563	0.257362	SG1	NUCKS1	NC_056071.1	37920001	37940001
NC_056065.1	26705001	26725001	0.425224	0.201036	SG1	NVL	NC_056071.1	38235001	38255001
NC_056065.1	26710001	26730001	0.325311	0.215354	SG1	NVL	NC_056071.1	38240001	38260001
NC_056065.1	26715001	26735001	0.258193	0.221545	SG1	NVL	NC_056071.1	38245001	38265001
NC_056065.1	26720001	26740001	0.229807	0.208116	SG1	NVL	NC_056070.1	10615001	10635001
NC_056065.1	26725001	26745001	0.13359	0.233078	SG1	NVL	NC_056070.1	10620001	10640001
NC_056059.1	86335001	86355001	0.325023	0.190228	SG1	ODAM	NC_056070.1	10625001	10645001
NC_056059.1	86340001	86360001	0.215334	0.24775	SG1	ODAM	NC_056071.1	43185001	43205001
NC_056059.1	86345001	86365001	0.096275	0.335054	SG1	ODAM	NC_056071.1	43190001	43210001
NC_056059.1	86350001	86370001	0.120656	0.270537	SG1	ODAM	NC_056066.1	15870001	15890001
NC_056055.1	119560001	1.2E+08	0.216667	0.271839	SG1	ORMDL1;PMS1	NC_056066.1	15875001	15895001
NC_056072.1	6475001	6495001	0.379009	0.23761	SG1	OSBPL10	NC_056066.1	15880001	15900001
NC_056072.1	6480001	6500001	0.341353	0.240992	SG1	OSBPL10	NC_056056.1	221150001	221170001

NC_056072.1	6485001	6505001	0.403575	0.216647	SG1	OSBPL10	NC_056056.1	221155001	221175001
NC_056072.1	6495001	6515001	0.445373	0.192405	SG1	OSBPL10	NC_056056.1	221160001	221180001
NC_056068.1	34635001	34655001	0.370985	0.24347	SG1	OTOG	NC_056056.1	221165001	221185001
NC_056056.1	116350001	1.16E+08	0.418324	0.243311	SG1	OTOGL	NC_056068.1	62310001	62330001
NC_056080.1	62960001	62980001	0.090169	0.256941	SG1	OTUD6A	NC_056068.1	62315001	62335001
NC_056080.1	62965001	62985001	0.355826	0.262691	SG1	OTUD6A	NC_056068.1	62320001	62340001
NC_056071.1	27820001	27840001	0.32134	0.228159	SG1	OTUD7A	NC_056068.1	62325001	62345001
NC_056071.1	27825001	27845001	0.133365	0.278683	SG1	OTUD7A	NC_056068.1	62330001	62350001
NC_056071.1	27845001	27865001	0.292949	0.277352	SG1	OTUD7A	NC_056057.1	94250001	94270001
NC_056071.1	27895001	27915001	0.366448	0.210989	SG1	OTUD7A	NC_056057.1	94255001	94275001
NC_056071.1	27900001	27920001	0.41946	0.228888	SG1	OTUD7A	NC_056073.1	30310001	30330001
NC_056054.1	274310001	2.74E+08	0.447228	0.199463	SG1	OXNAD1	NC_056066.1	25045001	25065001
NC_056054.1	274315001	2.74E+08	0.253071	0.226662	SG1	OXNAD1	NC_056066.1	25050001	25070001
NC_056054.1	274320001	2.74E+08	0.286092	0.228339	SG1	OXNAD1	NC_056066.1	25055001	25075001
NC_056054.1	274325001	2.74E+08	0.379944	0.223034	SG1	OXNAD1	NC_056066.1	25060001	25080001
NC_056072.1	11725001	11745001	0.114629	0.234591	SG1	OXSR1	NC_056066.1	25065001	25085001
NC_056072.1	11730001	11750001	0.227324	0.192364	SG1	OXSR1	NC_056066.1	25110001	25130001
NC_056058.1	19950001	19970001	0.192223	0.214335	SG1	P4HA2;PDLIM4	NC_056066.1	25120001	25140001
NW_02459982ε	1110001	1130001	0.213064	0.243715	SG1	PAG3	NC_056066.1	25125001	25145001
NW_02459982ε	1115001	1135001	0.256449	0.200776	SG1	PAG3	NC_056066.1	25130001	25150001
NC_056080.1	123910001	1.24E+08	0.297668	0.193955	SG1	PAK3	NC_056066.1	25135001	25155001
NC_056080.1	123915001	1.24E+08	0.246218	0.221051	SG1	PAK3	NC_056059.1	114540001	114560001
NC_056080.1	123925001	1.24E+08	0.332571	0.205352	SG1	PAK3	NC_056059.1	114545001	114565001
NC_056080.1	123945001	1.24E+08	0.238477	0.234354	SG1	PAK3	NC_056059.1	114550001	114570001
NC_056080.1	123950001	1.24E+08	0.224839	0.230403	SG1	PAK3	NC_056059.1	114555001	114575001
NC_056080.1	123955001	1.24E+08	0.186666	0.207278	SG1	PAK3	NC_056079.1	37675001	37695001
NC_056080.1	123990001	1.24E+08	0.167208	0.193273	SG1	PAK3	NC_056079.1	37725001	37745001
NC_056066.1	17900001	17920001	0.403041	0.195446	SG1	PARD3	NC_056056.1	11415001	11435001
NC_056066.1	17905001	17925001	0.178043	0.26695	SG1	PARD3	NC_056056.1	11420001	11440001
NC_056066.1	17915001	17935001	0.055884	0.311039	SG1	PARD3	NC_056056.1	11425001	11445001
NC_056066.1	17920001	17940001	0.123398	0.287776	SG1	PARD3	NC_056056.1	11430001	11450001
NC_056060.1	12170001	12190001	0.43876	0.305347	SG1	PARP16	NC_056056.1	11435001	11455001

NC_056055.1	247670001	2.48E+08	0.338362	0.187539	SG1	PAX7	NC_056056.1	11440001	11460001
NC_056055.1	247675001	2.48E+08	0.164829	0.270962	SG1	PAX7	NC_056056.1	70085001	70105001
NC_056059.1	51020001	51040001	0.157284	0.19	SG1	PCDH7	NC_056056.1	70095001	70115001
NC_056063.1	11295001	11315001	0.220145	0.2192	SG1	PCDH8	NC_056063.1	36505001	36525001
NC_056063.1	11300001	11320001	0.240796	0.246647	SG1	PCDH8	NC_056063.1	36510001	36530001
NC_056063.1	11305001	11325001	0.42617	0.203203	SG1	PCDH8	NC_056063.1	36515001	36535001
NC_056063.1	39970001	39990001	0.275824	0.227094	SG1	PCDH9	NC_056063.1	36520001	36540001
NC_056063.1	39975001	39995001	0.347779	0.209514	SG1	PCDH9	NC_056063.1	36525001	36545001
NC_056063.1	39980001	40000001	0.388031	0.211061	SG1	PCDH9	NC_056063.1	36530001	36550001
NC_056063.1	40045001	40065001	0.464896	0.299094	SG1	PCDH9	NC_056063.1	36535001	36555001
NC_056057.1	39050001	39070001	0.37509	0.220472	SG1	PCLO	NC_056063.1	36540001	36560001
NC_056057.1	39055001	39075001	0.356818	0.215117	SG1	PCLO	NC_056063.1	36545001	36565001
NC_056057.1	39060001	39080001	0.355342	0.189375	SG1	PCLO	NC_056063.1	36550001	36570001
NC_056057.1	39095001	39115001	0.395138	0.192289	SG1	PCLO	NC_056063.1	36555001	36575001
NC_056057.1	39100001	39120001	0.197602	0.256949	SG1	PCLO	NC_056063.1	36560001	36580001
NC_056057.1	39105001	39125001	0.256845	0.221505	SG1	PCLO	NC_056080.1	21765001	21785001
NC_056056.1	194965001	1.95E+08	0.249582	0.198457	SG1	PDE3A	NC_056080.1	21770001	21790001
NC_056056.1	194975001	1.95E+08	0.284174	0.190409	SG1	PDE3A	NC_056070.1	61940001	61960001
NC_056056.1	194980001	1.95E+08	0.25555	0.203853	SG1	PDE3A	NC_056060.1	98220001	98240001
NC_056056.1	195035001	1.95E+08	0.164139	0.192595	SG1	PDE3A	NC_056060.1	98215001	98235001
NC_056056.1	195040001	1.95E+08	0.126921	0.205484	SG1	PDE3A	NC_056074.1	23530001	23550001
NC_056056.1	195045001	1.95E+08	0.080174	0.231939	SG1	PDE3A	NC_056057.1	120815001	120835001
NC_056056.1	195050001	1.95E+08	0.05012	0.249579	SG1	PDE3A	NC_056057.1	120820001	120840001
NC_056056.1	195055001	1.95E+08	0.121009	0.197508	SG1	PDE3A	NC_056057.1	120860001	120880001
NC_056055.1	233435001	2.33E+08	0.398564	0.186364	SG1	PDE6D	NC_056057.1	120865001	120885001
NC_056055.1	233440001	2.33E+08	0.297673	0.263097	SG1	PDE6D	NC_056057.1	120870001	120890001
NC_056055.1	233445001	2.33E+08	0.172567	0.347052	SG1	PDE6D	NC_056057.1	120875001	120895001
NC_056055.1	233450001	2.33E+08	0.185812	0.35435	SG1	PDE6D	NC_056057.1	120880001	120900001
NC_056055.1	233455001	2.33E+08	0.147748	0.370312	SG1	PDE6D	NC_056057.1	120885001	120905001
NC_056061.1	61190001	61210001	0.462277	0.319555	SG1	PDE7B	NC_056057.1	120890001	120910001
NC_056061.1	61195001	61215001	0.352003	0.35141	SG1	PDE7B	NC_056066.1	71540001	71560001
NC_056061.1	61200001	61220001	0.296218	0.30736	SG1	PDE7B	NC_056054.1	230850001	230870001

NC_056061.1	61205001	61225001	0.273326	0.31643	SG1	PDE7B	NC_056054.1	264815001	264835001
NC_056061.1	61210001	61230001	0.236713	0.314398	SG1	PDE7B	NC_056054.1	264820001	264840001
NC_056061.1	61215001	61235001	0.279838	0.25835	SG1	PDE7B	NC_056054.1	264825001	264845001
NC_056061.1	61220001	61240001	0.441682	0.249986	SG1	PDE7B	NC_056059.1	113575001	113595001
NC_056061.1	61230001	61250001	0.355387	0.296692	SG1	PDE7B	NC_056059.1	113580001	113600001
NC_056061.1	61235001	61255001	0.331365	0.24652	SG1	PDE7B	NC_056059.1	113585001	113605001
NC_056061.1	61240001	61260001	0.342583	0.231773	SG1	PDE7B	NC_056068.1	62365001	62385001
NC_056061.1	61245001	61265001	0.396689	0.192426	SG1	PDE7B	NC_056068.1	62375001	62395001
NC_056061.1	61385001	61405001	0.345737	0.242677	SG1	PDE7B	NC_056068.1	62380001	62400001
NC_056061.1	61390001	61410001	0.336703	0.254099	SG1	PDE7B	NC_056068.1	62420001	62440001
NC_056061.1	61395001	61415001	0.323608	0.295266	SG1	PDE7B	NC_056068.1	62425001	62445001
NC_056061.1	61400001	61420001	0.429354	0.278513	SG1	PDE7B	NC_056068.1	62430001	62450001
NC_056060.1	8500001	8520001	0.447341	0.296596	SG1	PDE8B	NC_056058.1	19745001	19765001
NC_056060.1	8515001	8535001	0.421232	0.472546	SG1	PDE8B	NC_056058.1	19750001	19770001
NC_056070.1	42430001	42450001	0.208092	0.198135	SG1	PDGFC	NC_056071.1	43595001	43615001
NC_056070.1	42435001	42455001	0.122957	0.258429	SG1	PDGFC	NC_056071.1	43600001	43620001
NC_056070.1	42440001	42460001	0.134523	0.185991	SG1	PDGFC	NC_056071.1	43605001	43625001
NC_056070.1	42530001	42550001	0.060999	0.238415	SG1	PDGFC	NC_056071.1	43610001	43630001
NC_056070.1	42535001	42555001	0.059345	0.233911	SG1	PDGFC	NC_056071.1	43615001	43635001
NC_056070.1	42540001	42560001	0.075176	0.226471	SG1	PDGFC	NC_056071.1	43620001	43640001
NC_056070.1	42545001	42565001	0.073147	0.216592	SG1	PDGFC	NC_056071.1	43625001	43645001
NC_056055.1	199165001	1.99E+08	0.349206	0.200174	SG1	PGAP1	NC_056071.1	43630001	43650001
NC_056061.1	68335001	68355001	0.457649	0.216692	SG1	PHACTR2	NC_056071.1	43635001	43655001
NC_056061.1	68340001	68360001	0.207792	0.239834	SG1	PHACTR2	NC_056071.1	43640001	43660001
NC_056061.1	68345001	68365001	0.074943	0.266912	SG1	PHACTR2	NC_056071.1	43645001	43665001
NC_056061.1	68355001	68375001	0.101739	0.274251	SG1	PHACTR2	NC_056071.1	43710001	43730001
NC_056061.1	68360001	68380001	0.201215	0.308838	SG1	PHACTR2	NC_056071.1	43715001	43735001
NC_056061.1	68365001	68385001	0.267971	0.299163	SG1	PHACTR2	NC_056071.1	43720001	43740001
NC_056061.1	68370001	68390001	0.39873	0.258237	SG1	PHACTR2	NC_056069.1	2955001	2975001
NC_056056.1	221305001	2.21E+08	0.432272	0.18552	SG1	PHF21B	NC_056069.1	3050001	3070001
NC_056056.1	221310001	2.21E+08	0.394264	0.188564	SG1	PHF21B	NC_056069.1	3055001	3075001
NC_056074.1	8900001	8920001	0.455776	0.257906	SG1	PICALM	NC_056069.1	3060001	3080001

NC_056056.1	215645001	2.16E+08	0.379881	0.256252	SG1	PICK1	NC_056069.1	3065001	3085001
NC_056056.1	215665001	2.16E+08	0.438852	0.262453	SG1	PICK1;SLC16A8	NC_056069.1	3070001	3090001
NC_056056.1	215670001	2.16E+08	0.320093	0.256548	SG1	PICK1;SLC16A8	NC_056069.1	3075001	3095001
NC_056055.1	231170001	2.31E+08	0.303947	0.187935	SG1	PID1	NC_056069.1	3080001	3100001
NC_056055.1	231175001	2.31E+08	0.228298	0.208564	SG1	PID1	NC_056069.1	3085001	3105001
NC_056055.1	231180001	2.31E+08	0.153988	0.278295	SG1	PID1	NC_056069.1	3090001	3110001
NC_056055.1	231185001	2.31E+08	0.065064	0.343409	SG1	PID1	NC_056069.1	3095001	3115001
NC_056055.1	231190001	2.31E+08	0.039729	0.389134	SG1	PID1	NC_056069.1	3100001	3120001
NC_056055.1	231195001	2.31E+08	0.023621	0.408087	SG1	PID1	NC_056069.1	3105001	3125001
NC_056055.1	231205001	2.31E+08	0.063628	0.318653	SG1	PID1	NC_056069.1	3155001	3175001
NC_056055.1	231210001	2.31E+08	0.141949	0.183579	SG1	PID1	NC_056069.1	3160001	3180001
NC_056066.1	63930001	63950001	0.118669	0.391435	SG1	PIGU	NC_056069.1	3165001	3185001
NC_056066.1	63935001	63955001	0.149909	0.366793	SG1	PIGU	NC_056069.1	3170001	3190001
NC_056066.1	63940001	63960001	0.1836	0.282289	SG1	PIGU	NC_056069.1	3175001	3195001
NC_056066.1	63945001	63965001	0.210325	0.236324	SG1	PIGU	NC_056064.1	23555001	23575001
NC_056066.1	63990001	64010001	0.427316	0.205439	SG1	PIGU	NC_056064.1	23560001	23580001
NC_056066.1	63995001	64015001	0.396246	0.207402	SG1	PIGU	NC_056064.1	23710001	23730001
NC_056057.1	49485001	49505001	0.324946	0.185991	SG1	PIK3CG	NC_056070.1	40085001	40105001
NC_056057.1	49490001	49510001	0.317631	0.207702	SG1	PIK3CG	NC_056070.1	40090001	40110001
NC_056057.1	49495001	49515001	0.293433	0.201517	SG1	PIK3CG	NC_056070.1	40095001	40115001
NC_056077.1	36575001	36595001	0.43309	0.373789	SG1	PILRA	NC_056070.1	40100001	40120001
NC_056077.1	36580001	36600001	0.360165	0.34007	SG1	PILRA	NC_056070.1	40105001	40125001
NC_056077.1	36565001	36585001	0.468015	0.314966	SG1	PILRA;ZCWPW1	NC_056070.1	40110001	40130001
NC_056075.1	20980001	21000001	0.442872	0.243606	SG1	PKD2L1	NC_056070.1	40115001	40135001
NC_056075.1	20985001	21005001	0.471203	0.233724	SG1	PKD2L1	NC_056066.1	54525001	54545001
NC_056054.1	65950001	65970001	0.24318	0.362283	SG1	PKN2	NC_056066.1	54530001	54550001
NC_056054.1	65955001	65975001	0.320398	0.320447	SG1	PKN2	NC_056065.1	54715001	54735001
NC_056054.1	65960001	65980001	0.420864	0.292865	SG1	PKN2	NC_056054.1	121580001	121600001
NC_056054.1	65965001	65985001	0.459047	0.258862	SG1	PKN2	NC_056054.1	121585001	121605001
NC_056074.1	26140001	26160001	0.269602	0.193111	SG1	PKNOX2	NC_056063.1	19435001	19455001
NC_056074.1	26145001	26165001	0.415788	0.187772	SG1	PKNOX2	NC_056056.1	197545001	197565001
NC_056066.1	895001	915001	0.468902	0.276575	SG1	PLCB1	NC_056056.1	197550001	197570001

NC_056066.1	900001	920001	0.455583	0.267708	SG1	PLCB1	NC_056064.1	14760001	14780001
NC_056066.1	995001	1015001	0.307246	0.264505	SG1	PLCB1	NC_056064.1	14770001	14790001
NC_056066.1	1000001	1020001	0.110115	0.363546	SG1	PLCB1	NC_056055.1	199880001	199900001
NC_056066.1	1005001	1025001	0.026748	0.406421	SG1	PLCB1	NC_056060.1	81990001	82010001
NC_056066.1	1010001	1030001	0.0392	0.343307	SG1	PLCB1	NC_056064.1	17600001	17620001
NC_056066.1	1015001	1035001	0.086982	0.28457	SG1	PLCB1	NC_056064.1	17610001	17630001
NC_056066.1	1020001	1040001	0.324933	0.185079	SG1	PLCB1	NC_056071.1	20260001	20280001
NC_056066.1	1255001	1275001	0.335173	0.264903	SG1	PLCB1	NC_056071.1	20265001	20285001
NC_056066.1	1260001	1280001	0.39576	0.256066	SG1	PLCB1	NC_056056.1	175735001	175755001
NC_056066.1	1265001	1285001	0.341488	0.205321	SG1	PLCB1	NC_056056.1	175740001	175760001
NC_056066.1	1270001	1290001	0.312837	0.229349	SG1	PLCB1	NC_056056.1	175745001	175765001
NC_056065.1	33980001	34000001	0.236522	0.194131	SG1	PLD5	NC_056069.1	35530001	35550001
NC_056067.1	55195001	55215001	0.311719	0.189009	SG1	PLEKHA4	NC_056069.1	35535001	35555001
NC_056067.1	55200001	55220001	0.292128	0.201781	SG1	PLEKHA4;PPP1R15A	NC_056069.1	35550001	35570001
NC_056056.1	196255001	1.96E+08	0.416573	0.262673	SG1	PLEKHA5	NC_056069.1	35555001	35575001
NC_056056.1	196300001	1.96E+08	0.279403	0.271037	SG1	PLEKHA5	NC_056062.1	1740001	1760001
NC_056054.1	31095001	31115001	0.253468	0.210309	SG1	PLPP3	NC_056062.1	1745001	1765001
NC_056054.1	31100001	31120001	0.233658	0.228714	SG1	PLPP3	NC_056062.1	1750001	1770001
NC_056054.1	31105001	31125001	0.317786	0.204887	SG1	PLPP3	NC_056056.1	58610001	58630001
NC_056058.1	41035001	41055001	0.265957	0.215725	SG1	PLPPR3;PTBP1	NC_056063.1	36785001	36805001
NC_056065.1	3435001	3455001	0.29661	0.196048	SG1	PM20D1	NC_056063.1	36790001	36810001
NC_056065.1	3440001	3460001	0.37707	0.196874	SG1	PM20D1	NC_056063.1	36795001	36815001
NC_056065.1	3450001	3470001	0.420679	0.197507	SG1	PM20D1	NC_056063.1	36800001	36820001
NC_056055.1	119475001	1.19E+08	0.051943	0.227335	SG1	PMS1	NC_056069.1	15400001	15420001
NC_056055.1	119480001	1.2E+08	0.071461	0.245702	SG1	PMS1	NC_056069.1	15405001	15425001
NC_056055.1	119485001	1.2E+08	0.115063	0.316129	SG1	PMS1	NC_056069.1	15410001	15430001
NC_056055.1	119490001	1.2E+08	0.079864	0.316334	SG1	PMS1	NC_056069.1	15415001	15435001
NC_056055.1	119495001	1.2E+08	0.067312	0.249295	SG1	PMS1	NC_056069.1	15420001	15440001
NC_056055.1	119500001	1.2E+08	0.057302	0.247901	SG1	PMS1	NC_056069.1	15425001	15445001
NC_056055.1	119545001	1.2E+08	0.459747	0.27736	SG1	PMS1	NC_056069.1	15430001	15450001
NC_056055.1	119550001	1.2E+08	0.18565	0.317179	SG1	PMS1	NC_056069.1	15435001	15455001
NC_056055.1	119555001	1.2E+08	0.170927	0.313108	SG1	PMS1	NC_056069.1	15440001	15460001

NC_056059.1	117130001	1.17E+08	0.471076	0.249968	SG1	POLN	NC_056069.1	15445001	15465001
NC_056067.1	50705001	50725001	0.186157	0.213983	SG1	POU2F2	NC_056069.1	15450001	15470001
NC_056064.1	9440001	9460001	0.251451	0.189296	SG1	PPM1E	NC_056069.1	15455001	15475001
NC_056072.1	48705001	48725001	0.353587	0.270883	SG1	PPM1M;WDR82	NC_056069.1	15460001	15480001
NC_056055.1	239255001	2.39E+08	0.086705	0.198236	SG1	PPP1R8	NC_056069.1	15465001	15485001
NC_056059.1	103455001	1.03E+08	0.413295	0.212791	SG1	PPP2R2C	NC_056069.1	15500001	15520001
NC_056059.1	103460001	1.03E+08	0.278345	0.223599	SG1	PPP2R2C	NC_056069.1	15505001	15525001
NC_056054.1	253845001	2.54E+08	0.332711	0.203301	SG1	PPP2R3A	NC_056076.1	43990001	44010001
NC_056054.1	253850001	2.54E+08	0.271096	0.224762	SG1	PPP2R3A	NC_056076.1	43995001	44015001
NC_056054.1	253855001	2.54E+08	0.277955	0.229907	SG1	PPP2R3A	NC_056054.1	147120001	147140001
NC_056054.1	253860001	2.54E+08	0.290448	0.221084	SG1	PPP2R3A	NC_056054.1	147125001	147145001
NC_056054.1	253865001	2.54E+08	0.246465	0.226661	SG1	PPP2R3A	NC_056054.1	147130001	147150001
NC_056054.1	253870001	2.54E+08	0.293947	0.208781	SG1	PPP2R3A	NC_056054.1	147135001	147155001
NC_056054.1	253875001	2.54E+08	0.344574	0.185071	SG1	PPP2R3A	NC_056054.1	147140001	147160001
NC_056071.1	54015001	54035001	0.217392	0.2475	SG1	PPP4R3A	NC_056054.1	147180001	147200001
NC_056071.1	54020001	54040001	0.160043	0.251226	SG1	PPP4R3A	NC_056054.1	147185001	147205001
NC_056071.1	54025001	54045001	0.161562	0.261677	SG1	PPP4R3A	NC_056054.1	147190001	147210001
NC_056071.1	54030001	54050001	0.262548	0.290916	SG1	PPP4R3A	NC_056054.1	147195001	147215001
NC_056071.1	54035001	54055001	0.193001	0.316636	SG1	PPP4R3A	NC_056054.1	147200001	147220001
NC_056071.1	54040001	54060001	0.164107	0.314223	SG1	PPP4R3A	NC_056054.1	147205001	147225001
NC_056071.1	54045001	54065001	0.115132	0.316692	SG1	PPP4R3A	NC_056064.1	22785001	22805001
NC_056056.1	78265001	78285001	0.194286	0.231309	SG1	PRKCE	NC_056056.1	59375001	59395001
NC_056056.1	78270001	78290001	0.113978	0.362155	SG1	PRKCE	NC_056056.1	59380001	59400001
NC_056056.1	78275001	78295001	0.21071	0.435701	SG1	PRKCE	NC_056056.1	59385001	59405001
NC_056056.1	78280001	78300001	0.268083	0.419124	SG1	PRKCE	NC_056056.1	59390001	59410001
NC_056056.1	78285001	78305001	0.409361	0.324138	SG1	PRKCE	NC_056072.1	13285001	13305001
NC_056075.1	7415001	7435001	0.447034	0.193223	SG1	PRKG1	NC_056072.1	13290001	13310001
NC_056059.1	96580001	96600001	0.07115	0.201687	SG1	PRKG2	NC_056072.1	13295001	13315001
NC_056059.1	96635001	96655001	0.366816	0.204148	SG1	PRKG2	NC_056072.1	13300001	13320001
NC_056059.1	96640001	96660001	0.204031	0.225532	SG1	PRKG2	NC_056071.1	63420001	63440001
NC_056059.1	96645001	96665001	0.195982	0.213444	SG1	PRKG2	NC_056071.1	63425001	63445001
NC_056059.1	96650001	96670001	0.212681	0.271895	SG1	PRKG2	NC_056070.1	40585001	40605001

NC_056059.1	96655001	96675001	0.298641	0.297223	SG1	PRKG2	NC_056070.1	40590001	40610001
NC_056061.1	84690001	84710001	0.156687	0.347663	SG1	PRKN	NC_056063.1	29505001	29525001
NC_056061.1	84695001	84715001	0.265288	0.285214	SG1	PRKN	NC_056063.1	29530001	29550001
NC_056061.1	84700001	84720001	0.307604	0.245172	SG1	PRKN	NC_056067.1	47685001	47705001
NC_056071.1	43220001	43240001	0.443627	0.269419	SG1	PRORP	NC_056067.1	47690001	47710001
NC_056071.1	43225001	43245001	0.433476	0.296462	SG1	PRORP	NC_056067.1	47695001	47715001
NC_056067.1	55755001	55775001	0.199814	0.227913	SG1	PRR12;RRAS	NC_056067.1	47700001	47720001
NC_056061.1	10720001	10740001	0.432401	0.346088	SG1	PRSS35	NC_056054.1	78580001	78600001
NC_056061.1	10725001	10745001	0.390388	0.338196	SG1	PRSS35	NC_056054.1	78585001	78605001
NC_056061.1	10730001	10750001	0.32052	0.256906	SG1	PRSS35	NC_056054.1	78590001	78610001
NC_056061.1	10735001	10755001	0.354258	0.25643	SG1	PRSS35;SNAP91	NC_056065.1	50845001	50865001
NC_056055.1	29460001	29480001	0.401425	0.25392	SG1	PRXL2C	NC_056065.1	50850001	50870001
NC_056055.1	29465001	29485001	0.390835	0.222532	SG1	PRXL2C	NC_056065.1	50855001	50875001
NC_056058.1	41025001	41045001	0.299294	0.241998	SG1	PTBP1	NC_056065.1	50860001	50880001
NC_056058.1	41030001	41050001	0.296363	0.211	SG1	PTBP1	NC_056071.1	29425001	29445001
NC_056054.1	55005001	55025001	0.426701	0.31158	SG1	PTGFR	NC_056071.1	29430001	29450001
NC_056060.1	98250001	98270001	0.139251	0.205404	SG1	PTPN21	NC_056070.1	17400001	17420001
NC_056071.1	31015001	31035001	0.412151	0.183144	SG1	PTPN9	NC_056070.1	17405001	17425001
NC_056071.1	31020001	31040001	0.375864	0.202998	SG1	PTPN9;SIN3A	NC_056070.1	17410001	17430001
NC_056065.1	77045001	77065001	0.448053	0.245904	SG1	PTPRC	NC_056056.1	219995001	220015001
NC_056065.1	77050001	77070001	0.465133	0.264255	SG1	PTPRC	NC_056056.1	220000001	220020001
NC_056055.1	76585001	76605001	0.116473	0.248848	SG1	PTPRD	NC_056056.1	220005001	220025001
NC_056055.1	76590001	76610001	0.164882	0.258741	SG1	PTPRD	NC_056068.1	43195001	43215001
NC_056055.1	76595001	76615001	0.175	0.248788	SG1	PTPRD	NC_056068.1	43200001	43220001
NC_056055.1	77770001	77790001	0.45476	0.206864	SG1	PTPRD	NC_056062.1	36400001	36420001
NC_056055.1	77775001	77795001	0.470951	0.214092	SG1	PTPRD	NC_056061.1	29450001	29470001
NC_056055.1	77945001	77965001	0.471144	0.276101	SG1	PTPRD	NC_056061.1	29455001	29475001
NC_056055.1	77950001	77970001	0.412579	0.307802	SG1	PTPRD	NC_056054.1	63965001	63985001
NC_056055.1	77955001	77975001	0.415708	0.322076	SG1	PTPRD	NC_056071.1	20875001	20895001
NC_056055.1	77960001	77980001	0.332033	0.262666	SG1	PTPRD	NC_056071.1	20885001	20905001
NC_056055.1	77965001	77985001	0.268272	0.210143	SG1	PTPRD	NC_056071.1	56965001	56985001
NC_056055.1	78310001	78330001	0.467202	0.203394	SG1	PTPRD	NC_056071.1	56970001	56990001

NC_056055.1	78315001	78335001	0.41298	0.195786	SG1	PTPRD	NC_056064.1	20265001	20285001
NC_056055.1	78320001	78340001	0.421414	0.190128	SG1	PTPRD	NC_056064.1	20270001	20290001
NC_056061.1	53835001	53855001	0.449325	0.261272	SG1	PTPRK	NC_056072.1	48060001	48080001
NC_056061.1	53850001	53870001	0.457657	0.287559	SG1	PTPRK	NC_056079.1	35565001	35585001
NC_056061.1	54020001	54040001	0.350482	0.186146	SG1	PTPRK	NC_056079.1	35570001	35590001
NC_056057.1	88005001	88025001	0.469972	0.315932	SG1	PTPRZ1	NC_056079.1	35575001	35595001
NC_056057.1	88010001	88030001	0.407831	0.242165	SG1	PTPRZ1	NC_056079.1	35580001	35600001
NC_056075.1	19095001	19115001	0.448041	0.282908	SG1	R3HCC1L	NC_056079.1	35585001	35605001
NC_056075.1	19100001	19120001	0.448011	0.341659	SG1	R3HCC1L	NC_056079.1	35590001	35610001
NC_056056.1	162215001	1.62E+08	0.369136	0.218337	SG1	R3HDM2	NC_056079.1	35595001	35615001
NC_056056.1	162220001	1.62E+08	0.373888	0.213527	SG1	R3HDM2	NC_056054.1	120565001	120585001
NC_056065.1	3365001	3385001	0.06445	0.441138	SG1	RAB29	NC_056058.1	64745001	64765001
NC_056065.1	3370001	3390001	0.13106	0.383867	SG1	RAB29;SLC41A1	NC_056059.1	115185001	115205001
NC_056080.1	114025001	1.14E+08	0.266766	0.304445	SG1	RAB33A	NC_056059.1	115190001	115210001
NC_056080.1	114030001	1.14E+08	0.269807	0.319431	SG1	RAB33A	NC_056059.1	115195001	115215001
NC_056080.1	114035001	1.14E+08	0.227867	0.330073	SG1	RAB33A;ZNF280C	NC_056059.1	115200001	115220001
NC_056074.1	7060001	7080001	0.225222	0.25966	SG1	RAB38	NC_056055.1	205001	225001
NC_056074.1	7065001	7085001	0.284918	0.223122	SG1	RAB38	NC_056055.1	210001	230001
NC_056073.1	10710001	10730001	0.412212	0.253921	SG1	RAB44	NC_056055.1	215001	235001
NC_056073.1	10715001	10735001	0.348541	0.298818	SG1	RAB44	NC_056055.1	220001	240001
NC_056073.1	10720001	10740001	0.368534	0.322674	SG1	RAB44	NC_056055.1	225001	245001
NC_056058.1	19685001	19705001	0.368699	0.19723	SG1	RAD50	NC_056066.1	47615001	47635001
NC_056058.1	19690001	19710001	0.394524	0.183135	SG1	RAD50	NC_056066.1	47620001	47640001
NC_056058.1	19740001	19760001	0.107415	0.453721	SG1	RAD50	NC_056066.1	47625001	47645001
NC_056058.1	19745001	19765001	0.136	0.533565	SG1	RAD50	NC_056066.1	47630001	47650001
NC_056058.1	19750001	19770001	0.240891	0.479131	SG1	RAD50	NC_056072.1	28975001	28995001
NC_056058.1	19755001	19775001	0.287016	0.463822	SG1	RAD50	NC_056075.1	36815001	36835001
NC_056058.1	19805001	19825001	0.430724	0.245999	SG1	RAD50	NC_056057.1	89080001	89100001
NC_056064.1	9360001	9380001	0.432894	0.207055	SG1	RAD51C	NC_056057.1	89085001	89105001
NC_056064.1	9365001	9385001	0.358646	0.196176	SG1	RAD51C	NC_056064.1	25050001	25070001
NC_056079.1	39725001	39745001	0.374563	0.441945	SG1	RARB	NC_056064.1	25055001	25075001
NC_056079.1	39730001	39750001	0.347714	0.429137	SG1	RARB	NC_056064.1	55540001	55560001

NC_056079.1	39735001	39755001	0.371633	0.380566	SG1	RARB	NC_056071.1	44790001	44810001
NC_056079.1	39740001	39760001	0.434689	0.293968	SG1	RARB	NC_056071.1	44795001	44815001
NC_056079.1	39745001	39765001	0.425615	0.256599	SG1	RARB	NC_056071.1	44800001	44820001
NC_056079.1	39750001	39770001	0.437672	0.240929	SG1	RARB	NC_056071.1	44840001	44860001
NC_056059.1	97165001	97185001	0.179821	0.210776	SG1	RASGEF1B	NC_056071.1	44845001	44865001
NC_056059.1	97170001	97190001	0.055599	0.22952	SG1	RASGEF1B	NC_056071.1	44880001	44900001
NC_056059.1	97175001	97195001	0.345724	0.192703	SG1	RASGEF1B	NC_056071.1	44885001	44905001
NC_056058.1	78745001	78765001	0.230948	0.18764	SG1	RASGRF2	NC_056071.1	44890001	44910001
NC_056058.1	78755001	78775001	0.099296	0.183299	SG1	RASGRF2	NC_056071.1	44895001	44915001
NC_056058.1	78760001	78780001	0.20112	0.21221	SG1	RASGRF2	NC_056071.1	44900001	44920001
NC_056058.1	78765001	78785001	0.235241	0.213796	SG1	RASGRF2	NC_056071.1	44905001	44925001
NC_056058.1	78770001	78790001	0.268549	0.229382	SG1	RASGRF2	NC_056071.1	44910001	44930001
NC_056076.1	33925001	33945001	0.425937	0.244425	SG1	RBBP8	NC_056071.1	44915001	44935001
NC_056077.1	5600001	5620001	0.362213	0.20751	SG1	RBFOX1	NC_056071.1	44920001	44940001
NC_056077.1	7135001	7155001	0.403638	0.201094	SG1	RBFOX1	NC_056071.1	44925001	44945001
NC_056054.1	143755001	1.44E+08	0.337935	0.187875	SG1	RBM11	NC_056071.1	44930001	44950001
NC_056078.1	18985001	19005001	0.383211	0.322267	SG1	REEP3	NC_056071.1	44935001	44955001
NC_056072.1	47920001	47940001	0.395472	0.185875	SG1	RFT1	NC_056071.1	44940001	44960001
NC_056072.1	47925001	47945001	0.412972	0.198031	SG1	RFT1	NC_056071.1	44945001	44965001
NC_056072.1	47930001	47950001	0.417562	0.228365	SG1	RFT1	NC_056071.1	44950001	44970001
NC_056055.1	71505001	71525001	0.249711	0.248169	SG1	RFX3	NC_056071.1	44955001	44975001
NC_056065.1	34870001	34890001	0.35963	0.287295	SG1	RGS7	NC_056071.1	44960001	44980001
NC_056065.1	34875001	34895001	0.156589	0.359665	SG1	RGS7	NC_056071.1	44965001	44985001
NC_056065.1	34880001	34900001	0.234802	0.278352	SG1	RGS7	NC_056071.1	44970001	44990001
NC_056065.1	34885001	34905001	0.306135	0.270018	SG1	RGS7	NC_056071.1	44975001	44995001
NC_056065.1	34890001	34910001	0.407663	0.234518	SG1	RGS7	NC_056071.1	44980001	45000001
NC_056055.1	229095001	2.29E+08	0.446997	0.247736	SG1	RHBDD1	NC_056071.1	44985001	45005001
NC_056062.1	73730001	73750001	0.441209	0.283437	SG1	RIMS2	NC_056071.1	44990001	45010001
NC_056062.1	73735001	73755001	0.260946	0.310619	SG1	RIMS2	NC_056062.1	85990001	86010001
NC_056062.1	73740001	73760001	0.253776	0.267673	SG1	RIMS2	NC_056062.1	85995001	86015001
NC_056070.1	62950001	62970001	0.384445	0.222846	SG1	RNF10	NC_056062.1	86000001	86020001
NC_056070.1	62955001	62975001	0.390272	0.209572	SG1	RNF10	NC_056062.1	86110001	86130001

NC_056070.1	62960001	62980001	0.372093	0.192589	SG1	RNF10	NC_056059.1	45765001	45785001
NC_056076.1	25405001	25425001	0.353211	0.18404	SG1	RNF138	NC_056059.1	45770001	45790001
NC_056076.1	25410001	25430001	0.379245	0.187405	SG1	RNF138	NC_056057.1	99630001	99650001
NC_056076.1	25415001	25435001	0.336969	0.204915	SG1	RNF138	NC_056069.1	23625001	23645001
NC_056076.1	25420001	25440001	0.369331	0.197677	SG1	RNF138	NC_056065.1	3270001	3290001
NC_056076.1	25425001	25445001	0.392821	0.183443	SG1	RNF138	NC_056065.1	3275001	3295001
NC_056076.1	25430001	25450001	0.390712	0.183605	SG1	RNF138	NC_056063.1	31700001	31720001
NC_056079.1	36900001	36920001	0.15472	0.247313	SG1	RNF170	NC_056063.1	31705001	31725001
NC_056055.1	52115001	52135001	0.425087	0.392118	SG1	RNF38	NC_056069.1	71240001	71260001
NC_056055.1	52120001	52140001	0.243006	0.609281	SG1	RNF38	NC_056069.1	71245001	71265001
NC_056054.1	148175001	1.48E+08	0.453804	0.224181	SG1	ROBO1	NC_056070.1	19535001	19555001
NC_056054.1	148180001	1.48E+08	0.399529	0.227618	SG1	ROBO1	NC_056070.1	19540001	19560001
NC_056054.1	148185001	1.48E+08	0.380907	0.213225	SG1	ROBO1	NC_056070.1	19545001	19565001
NC_056054.1	148210001	1.48E+08	0.358954	0.203753	SG1	ROBO1	NC_056070.1	19620001	19640001
NC_056054.1	148215001	1.48E+08	0.279195	0.290736	SG1	ROBO1	NC_056070.1	19625001	19645001
NC_056054.1	148220001	1.48E+08	0.378492	0.223665	SG1	ROBO1	NC_056070.1	19630001	19650001
NC_056060.1	47560001	47580001	0.285714	0.214897	SG1	RORA	NC_056070.1	19635001	19655001
NC_056060.1	47565001	47585001	0.365822	0.194632	SG1	RORA	NC_056070.1	19640001	19660001
NC_056070.1	61615001	61635001	0.330625	0.200715	SG1	RPH3A	NC_056070.1	19645001	19665001
NC_056070.1	61620001	61640001	0.281499	0.204355	SG1	RPH3A	NC_056079.1	18960001	18980001
NC_056080.1	74970001	74990001	0.405315	0.189371	SG1	RPS6KA6	NC_056079.1	18965001	18985001
NC_056080.1	74975001	74995001	0.404693	0.19255	SG1	RPS6KA6	NC_056054.1	245685001	245705001
NC_056080.1	75035001	75055001	0.375405	0.21131	SG1	RPS6KA6	NC_056054.1	245690001	245710001
NC_056080.1	75090001	75110001	0.439087	0.198016	SG1	RPS6KA6	NC_056054.1	245695001	245715001
NC_056080.1	75110001	75130001	0.320334	0.199693	SG1	RPS6KA6	NC_056054.1	245700001	245720001
NC_056080.1	75115001	75135001	0.340741	0.195918	SG1	RPS6KA6	NC_056054.1	245885001	245905001
NC_056080.1	75120001	75140001	0.412889	0.186508	SG1	RPS6KA6	NC_056054.1	245890001	245910001
NC_056080.1	75125001	75145001	0.380166	0.218391	SG1	RPS6KA6	NC_056054.1	245895001	245915001
NC_056080.1	75130001	75150001	0.418182	0.203704	SG1	RPS6KA6	NC_056054.1	245900001	245920001
NC_056060.1	35080001	35100001	0.296447	0.20235	SG1	RTF1	NC_056059.1	40385001	40405001
NC_056060.1	35085001	35105001	0.167028	0.242577	SG1	RTF1	NC_056059.1	40390001	40410001
NC_056060.1	35090001	35110001	0.252641	0.218217	SG1	RTF1	NC_056059.1	40395001	40415001

NC_056060.1	35095001	35115001	0.3125	0.207844	SG1	RTF1	NC_056059.1	40400001	40420001
NC_056060.1	35100001	35120001	0.367828	0.194697	SG1	RTF1	NC_056059.1	40405001	40425001
NC_056056.1	69010001	69030001	0.29914	0.203997	SG1	RTN4	NC_056058.1	45170001	45190001
NC_056056.1	69015001	69035001	0.32973	0.208024	SG1	RTN4	NC_056076.1	37470001	37490001
NC_056076.1	7410001	7430001	0.338177	0.220664	SG1	RTTN	NC_056076.1	37475001	37495001
NC_056076.1	7415001	7435001	0.306479	0.27189	SG1	RTTN	NC_056076.1	37480001	37500001
NC_056076.1	7420001	7440001	0.383556	0.264954	SG1	RTTN	NC_056076.1	37485001	37505001
NC_056076.1	7425001	7445001	0.378404	0.266677	SG1	RTTN	NC_056076.1	37490001	37510001
NC_056076.1	7430001	7450001	0.403366	0.259875	SG1	RTTN	NC_056076.1	37495001	37515001
NC_056076.1	7435001	7455001	0.287618	0.269253	SG1	RTTN	NC_056064.1	23045001	23065001
NC_056076.1	7440001	7460001	0.219795	0.227588	SG1	RTTN	NC_056064.1	23050001	23070001
NC_056076.1	7445001	7465001	0.134087	0.230233	SG1	RTTN	NC_056064.1	23055001	23075001
NC_056063.1	29540001	29560001	0.111843	0.267369	SG1	RXFP2	NC_056064.1	23060001	23080001
NC_056063.1	29545001	29565001	0.265858	0.347255	SG1	RXFP2	NC_056059.1	35670001	35690001
NC_056063.1	29550001	29570001	0.283375	0.324383	SG1	RXFP2	NC_056059.1	35675001	35695001
NC_056063.1	29555001	29575001	0.310728	0.317556	SG1	RXFP2	NC_056056.1	218555001	218575001
NC_056063.1	29560001	29580001	0.443514	0.275012	SG1	RXFP2	NC_056071.1	30915001	30935001
NC_056060.1	27455001	27475001	0.467879	0.194307	SG1	RYR3	NC_056071.1	30920001	30940001
NC_056054.1	61305001	61325001	0.399556	0.196601	SG1	SAMD13	NC_056071.1	30925001	30945001
NC_056054.1	61310001	61330001	0.28697	0.208345	SG1	SAMD13	NC_056061.1	51260001	51280001
NC_056060.1	65150001	65170001	0.379465	0.190723	SG1	SAMD4A	NC_056061.1	51265001	51285001
NC_056060.1	65155001	65175001	0.379369	0.206064	SG1	SAMD4A	NC_056061.1	51270001	51290001
NC_056060.1	65225001	65245001	0.368243	0.212458	SG1	SAMD4A	NC_056061.1	51275001	51295001
NC_056060.1	65230001	65250001	0.379572	0.193784	SG1	SAMD4A	NC_056061.1	51280001	51300001
NC_056054.1	124515001	1.25E+08	0.309936	0.206143	SG1	SCAF4;SOD1	NC_056061.1	51285001	51305001
NC_056061.1	79300001	79320001	0.461905	0.208494	SG1	SCAF8	NC_056077.1	11220001	11240001
NC_056071.1	29615001	29635001	0.43268	0.362428	SG1	SCAPER	NC_056077.1	11225001	11245001
NC_056055.1	143550001	1.44E+08	0.209108	0.232941	SG1	SCN1A	NC_056077.1	11230001	11250001
NC_056055.1	143555001	1.44E+08	0.092887	0.251113	SG1	SCN1A	NC_056077.1	11235001	11255001
NC_056055.1	143560001	1.44E+08	0.086141	0.265795	SG1	SCN1A	NC_056077.1	11590001	11610001
NC_056055.1	143565001	1.44E+08	0.112435	0.251357	SG1	SCN1A	NC_056074.1	40385001	40405001
NC_056055.1	143570001	1.44E+08	0.282931	0.197061	SG1	SCN1A	NC_056071.1	42735001	42755001

NC_056060.1	61215001	61235001	0.137996	0.209093	SG1	SEMA6D	NC_056071.1	42740001	42760001
NC_056060.1	61220001	61240001	0.09341	0.220954	SG1	SEMA6D	NC_056071.1	42745001	42765001
NC_056054.1	166880001	1.67E+08	0.329675	0.341487	SG1	SENP7;TRMT10C	NC_056059.1	114820001	114840001
NC_056054.1	166885001	1.67E+08	0.244876	0.378774	SG1	SENP7;TRMT10C	NC_056068.1	35595001	35615001
NC_056071.1	56960001	56980001	0.309728	0.222712	SG1	SERPINA12	NC_056060.1	98205001	98225001
NC_056055.1	130950001	1.31E+08	0.387378	0.214261	SG1	SESTD1	NC_056060.1	98210001	98230001
NC_056055.1	130960001	1.31E+08	0.225762	0.189552	SG1	SESTD1	NC_056058.1	57475001	57495001
NC_056072.1	47970001	47990001	0.121951	0.240701	SG1	SFMBT1	NC_056058.1	57480001	57500001
NC_056072.1	47975001	47995001	0.05845	0.306301	SG1	SFMBT1	NC_056066.1	72055001	72075001
NC_056072.1	47980001	48000001	0.087204	0.302867	SG1	SFMBT1	NC_056066.1	72060001	72080001
NC_056072.1	47985001	48005001	0.174913	0.28484	SG1	SFMBT1	NC_056058.1	79105001	79125001
NC_056061.1	89025001	89045001	0.088715	0.190486	SG1	SFT2D1	NC_056058.1	79110001	79130001
NC_056061.1	89030001	89050001	0.204981	0.261601	SG1	SFT2D1	NC_056058.1	79115001	79135001
NC_056061.1	89035001	89055001	0.282886	0.330874	SG1	SFT2D1	NC_056069.1	4060001	4080001
NC_056075.1	8595001	8615001	0.44194	0.18492	SG1	SGMS1	NC_056069.1	4065001	4085001
NC_056075.1	8600001	8620001	0.402238	0.184612	SG1	SGMS1	NC_056069.1	4070001	4090001
NC_056054.1	273340001	2.73E+08	0.450138	0.264979	SG1	SH3BP5	NC_056069.1	4075001	4095001
NC_056070.1	6750001	6770001	0.394384	0.275233	SG1	SH3D19	NC_056069.1	4080001	4100001
NC_056075.1	24075001	24095001	0.277124	0.1865	SG1	SH3PXD2A	NC_056069.1	4085001	4105001
NC_056075.1	24080001	24100001	0.153632	0.210222	SG1	SH3PXD2A	NC_056069.1	4090001	4110001
NC_056075.1	24085001	24105001	0.201447	0.203499	SG1	SH3PXD2A	NC_056069.1	4095001	4115001
NC_056075.1	24090001	24110001	0.361594	0.214827	SG1	SH3PXD2A	NC_056069.1	4100001	4120001
NC_056060.1	59460001	59480001	0.375	0.188827	SG1	SHC4	NC_056069.1	4105001	4125001
NC_056060.1	59485001	59505001	0.422797	0.219626	SG1	SHC4	NC_056069.1	4115001	4135001
NC_056056.1	218740001	2.19E+08	0.451614	0.309421	SG1	SHISA8;SREBF2	NC_056056.1	188210001	188230001
NC_056056.1	218745001	2.19E+08	0.329135	0.201627	SG1	SHISA8;SREBF2;TNI	NC_056071.1	32125001	32145001
NC_056077.1	12350001	12370001	0.457507	0.211847	SG1	SHISA9	NC_056071.1	32130001	32150001
NC_056077.1	12355001	12375001	0.415174	0.235992	SG1	SHISA9	NC_056071.1	39330001	39350001
NC_056077.1	12360001	12380001	0.412757	0.235151	SG1	SHISA9	NC_056071.1	39335001	39355001
NC_056077.1	12365001	12385001	0.439422	0.218737	SG1	SHISA9	NC_056071.1	39340001	39360001
NC_056056.1	220860001	2.21E+08	0.326756	0.208832	SG1	SHISAL1	NC_056071.1	39345001	39365001
NC_056056.1	220865001	2.21E+08	0.32592	0.203329	SG1	SHISAL1	NC_056071.1	39350001	39370001

NC_056080.1	54560001	54580001	0.43822	0.206279	SG1	SHROOM4	NC_056071.1	39355001	39375001
NC_056080.1	54565001	54585001	0.418803	0.215861	SG1	SHROOM4	NC_056071.1	39360001	39380001
NC_056080.1	54570001	54590001	0.408648	0.192468	SG1	SHROOM4	NC_056071.1	39365001	39385001
NC_056068.1	21665001	21685001	0.414296	0.285553	SG1	SIK2	NC_056071.1	39370001	39390001
NC_056068.1	21670001	21690001	0.345508	0.277572	SG1	SIK2	NC_056071.1	39375001	39395001
NC_056068.1	21675001	21695001	0.378357	0.295897	SG1	SIK2	NC_056071.1	39380001	39400001
NC_056071.1	31050001	31070001	0.37065	0.195476	SG1	SIN3A	NC_056071.1	39385001	39405001
NC_056071.1	31055001	31075001	0.341837	0.208307	SG1	SIN3A	NC_056071.1	39390001	39410001
NC_056071.1	31060001	31080001	0.331684	0.225532	SG1	SIN3A	NC_056070.1	46365001	46385001
NC_056071.1	31065001	31085001	0.206199	0.292623	SG1	SIN3A	NC_056070.1	46370001	46390001
NC_056071.1	31070001	31090001	0.144422	0.317656	SG1	SIN3A	NC_056070.1	46375001	46395001
NC_056071.1	31075001	31095001	0.111027	0.334289	SG1	SIN3A	NC_056070.1	46380001	46400001
NC_056071.1	31080001	31100001	0.114328	0.328481	SG1	SIN3A	NC_056070.1	46385001	46405001
NC_056071.1	31085001	31105001	0.22604	0.271551	SG1	SIN3A	NC_056070.1	46390001	46410001
NC_056059.1	102445001	1.02E+08	0.329795	0.190386	SG1	SLC10A6	NC_056054.1	186450001	186470001
NC_056068.1	64555001	64575001	0.128259	0.288889	SG1	SLC1A2	NC_056054.1	186455001	186475001
NC_056068.1	64560001	64580001	0.243445	0.269006	SG1	SLC1A2	NC_056054.1	186460001	186480001
NC_056058.1	19810001	19830001	0.381514	0.251826	SG1	SLC22A5	NC_056054.1	186465001	186485001
NC_056072.1	35205001	35225001	0.301194	0.210376	SG1	SLC25A26	NC_056054.1	186470001	186490001
NC_056066.1	75345001	75365001	0.361749	0.215207	SG1	SLC2A10;TP53RK	NC_056054.1	186655001	186675001
NC_056066.1	75350001	75370001	0.328687	0.214964	SG1	SLC2A10;TP53RK	NC_056054.1	186660001	186680001
NC_056056.1	207040001	2.07E+08	0.410063	0.316862	SG1	SLC2A3	NC_056054.1	186665001	186685001
NC_056054.1	78235001	78255001	0.24376	0.192921	SG1	SLC30A7	NC_056071.1	32805001	32825001
NC_056054.1	78240001	78260001	0.282735	0.216782	SG1	SLC30A7	NC_056071.1	32810001	32830001
NC_056054.1	77375001	77395001	0.3377	0.221301	SG1	SLC35A3	NC_056066.1	76180001	76200001
NC_056054.1	77380001	77400001	0.288416	0.250696	SG1	SLC35A3	NC_056055.1	11400001	11420001
NC_056054.1	77385001	77405001	0.334034	0.229352	SG1	SLC35A3	NC_056055.1	11405001	11425001
NC_056068.1	17040001	17060001	0.439442	0.318674	SG1	SLC35F2	NC_056055.1	12815001	12835001
NC_056068.1	17045001	17065001	0.141248	0.408747	SG1	SLC35F2	NC_056055.1	12820001	12840001
NC_056068.1	17075001	17095001	0.040141	0.431821	SG1	SLC35F2	NC_056062.1	67645001	67665001
NC_056068.1	17080001	17100001	0.206836	0.323125	SG1	SLC35F2	NC_056072.1	38050001	38070001
NC_056064.1	57655001	57675001	0.408889	0.185923	SG1	SLC39A11	NC_056064.1	13510001	13530001

NC_056064.1	57660001	57680001	0.408625	0.190287	SG1	SLC39A11	NC_056064.1	13515001	13535001
NC_056066.1	31755001	31775001	0.237237	0.236022	SG1	SLC39A12	NC_056064.1	13520001	13540001
NC_056066.1	31760001	31780001	0.369423	0.187083	SG1	SLC39A12	NC_056056.1	115190001	115210001
NC_056065.1	3375001	3395001	0.285234	0.338733	SG1	SLC41A1	NC_056056.1	115195001	115215001
NC_056065.1	3380001	3400001	0.467204	0.289003	SG1	SLC41A1	NC_056066.1	54800001	54820001
NC_056055.1	148120001	1.48E+08	0.298872	0.2279	SG1	SLC4A10	NC_056066.1	54805001	54825001
NC_056055.1	148125001	1.48E+08	0.258723	0.253605	SG1	SLC4A10	NC_056066.1	54810001	54830001
NC_056070.1	19675001	19695001	0.376807	0.194747	SG1	SLC7A11	NC_056066.1	54820001	54840001
NC_056070.1	19685001	19705001	0.380341	0.219065	SG1	SLC7A11	NC_056066.1	54825001	54845001
NC_056070.1	19690001	19710001	0.470057	0.191227	SG1	SLC7A11	NC_056066.1	54830001	54850001
NC_056070.1	19720001	19740001	0.405675	0.214565	SG1	SLC7A11	NC_056066.1	54835001	54855001
NC_056070.1	19725001	19745001	0.328586	0.223626	SG1	SLC7A11	NC_056066.1	54840001	54860001
NC_056070.1	19730001	19750001	0.320904	0.211894	SG1	SLC7A11	NC_056069.1	40475001	40495001
NC_056056.1	194380001	1.94E+08	0.117202	0.309138	SG1	SLCO1A2	NC_056072.1	44435001	44455001
NC_056056.1	194385001	1.94E+08	0.247063	0.234359	SG1	SLCO1A2	NC_056072.1	44440001	44460001
NC_056075.1	18275001	18295001	0.387754	0.217838	SG1	SLIT1	NC_056057.1	69940001	69960001
NC_056074.1	875001	895001	0.34762	0.20054	SG1	SMCO4	NC_056057.1	69945001	69965001
NC_056054.1	121705001	1.22E+08	0.381984	0.262052	SG1	SMIM11;SMIM34	NC_056054.1	275675001	275695001
NC_056054.1	121710001	1.22E+08	0.426551	0.236099	SG1	SMIM11;SMIM34	NC_056054.1	275685001	275705001
NC_056063.1	14785001	14805001	0.467302	0.192395	SG1	SMIM2	NC_056054.1	275690001	275710001
NC_056059.1	45960001	45980001	0.399501	0.245678	SG1	SMIM20	NC_056054.1	275695001	275715001
NC_056059.1	45965001	45985001	0.441678	0.233075	SG1	SMIM20	NC_056054.1	275700001	275720001
NC_056059.1	45970001	45990001	0.404494	0.227057	SG1	SMIM20	NC_056054.1	96540001	96560001
NC_056054.1	121690001	1.22E+08	0.091536	0.292908	SG1	SMIM34	NC_056054.1	96550001	96570001
NC_056054.1	121695001	1.22E+08	0.167852	0.297174	SG1	SMIM34	NC_056056.1	57045001	57065001
NC_056054.1	121700001	1.22E+08	0.278761	0.282445	SG1	SMIM34	NC_056056.1	57050001	57070001
NC_056066.1	50665001	50685001	0.110305	0.245267	SG1	SMOX	NC_056058.1	59455001	59475001
NC_056061.1	10740001	10760001	0.405987	0.211852	SG1	SNAP91	NC_056058.1	59460001	59480001
NC_056061.1	10795001	10815001	0.116266	0.225559	SG1	SNAP91	NC_056058.1	59465001	59485001
NC_056061.1	10800001	10820001	0.126949	0.240618	SG1	SNAP91	NC_056058.1	59470001	59490001
NC_056061.1	10805001	10825001	0.156372	0.223382	SG1	SNAP91	NC_056079.1	12420001	12440001
NC_056061.1	10810001	10830001	0.154209	0.222381	SG1	SNAP91	NC_056079.1	12425001	12445001

NC_056061.1	10815001	10835001	0.078834	0.321162	SG1	SNAP91	NC_056079.1	12430001	12450001
NC_056061.1	10820001	10840001	0.086242	0.313488	SG1	SNAP91	NC_056079.1	12435001	12455001
NC_056061.1	10825001	10845001	0.145722	0.315879	SG1	SNAP91	NC_056079.1	12440001	12460001
NC_056061.1	10830001	10850001	0.163275	0.324347	SG1	SNAP91	NC_056079.1	12455001	12475001
NC_056061.1	10835001	10855001	0.231762	0.219831	SG1	SNAP91	NC_056079.1	12460001	12480001
NC_056061.1	10840001	10860001	0.254445	0.206365	SG1	SNAP91	NC_056079.1	12775001	12795001
NC_056061.1	10845001	10865001	0.245496	0.212096	SG1	SNAP91	NC_056056.1	165455001	165475001
NC_056061.1	10850001	10870001	0.317263	0.24096	SG1	SNAP91	NC_056056.1	165460001	165480001
NC_056061.1	10855001	10875001	0.366858	0.262983	SG1	SNAP91	NC_056080.1	63570001	63590001
NC_056061.1	10860001	10880001	0.310592	0.337881	SG1	SNAP91	NC_056080.1	63590001	63610001
NC_056061.1	10865001	10885001	0.250817	0.336704	SG1	SNAP91	NC_056080.1	63595001	63615001
NC_056061.1	10870001	10890001	0.241085	0.311433	SG1	SNAP91	NC_056066.1	59010001	59030001
NC_056061.1	10875001	10895001	0.262843	0.301252	SG1	SNAP91	NC_056054.1	124885001	124905001
NC_056061.1	10880001	10900001	0.349675	0.259112	SG1	SNAP91	NC_056054.1	124890001	124910001
NC_056061.1	10885001	10905001	0.410748	0.263191	SG1	SNAP91	NC_056054.1	124895001	124915001
NC_056061.1	10890001	10910001	0.435746	0.256226	SG1	SNAP91	NC_056054.1	124900001	124920001
NC_056061.1	10895001	10915001	0.435575	0.230789	SG1	SNAP91	NC_056054.1	120445001	120465001
NC_056061.1	10925001	10945001	0.422408	0.396056	SG1	SNAP91	NC_056054.1	120455001	120475001
NC_056055.1	710001	730001	0.282416	0.194297	SG1	SNTG2	NC_056054.1	120460001	120480001
NC_056080.1	63775001	63795001	0.324841	0.347261	SG1	SNX12	NC_056054.1	120465001	120485001
NC_056054.1	124520001	1.25E+08	0.188473	0.247533	SG1	SOD1	NC_056054.1	120470001	120490001
NC_056054.1	124525001	1.25E+08	0.272656	0.210449	SG1	SOD1	NC_056054.1	120475001	120495001
NC_056063.1	25540001	25560001	0.415641	0.188116	SG1	SOHLH2	NC_056054.1	120480001	120500001
NC_056063.1	25545001	25565001	0.384916	0.193796	SG1	SOHLH2	NC_056054.1	120485001	120505001
NC_056063.1	25550001	25570001	0.279363	0.236472	SG1	SOHLH2	NC_056054.1	120490001	120510001
NC_056063.1	25555001	25575001	0.225558	0.251269	SG1	SOHLH2	NC_056054.1	120495001	120515001
NC_056063.1	25560001	25580001	0.201243	0.232964	SG1	SOHLH2	NC_056054.1	120525001	120545001
NC_056063.1	25565001	25585001	0.146849	0.268639	SG1	SOHLH2	NC_056054.1	120530001	120550001
NC_056063.1	25570001	25590001	0.232696	0.22464	SG1	SOHLH2	NC_056058.1	18565001	18585001
NC_056075.1	16655001	16675001	0.198676	0.294618	SG1	SORBS1	NC_056058.1	18570001	18590001
NC_056075.1	16660001	16680001	0.191005	0.318727	SG1	SORBS1	NC_056058.1	18575001	18595001
NC_056075.1	16665001	16685001	0.19201	0.329256	SG1	SORBS1	NC_056054.1	256800001	256820001

NC_056075.1	16670001	16690001	0.334653	0.316144	SG1	SORBS1	NC_056073.1	24715001	24735001
NC_056075.1	16675001	16695001	0.468407	0.2634	SG1	SORBS1	NC_056073.1	24720001	24740001
NC_056075.1	16865001	16885001	0.434782	0.212978	SG1	SORBS1	NC_056057.1	105850001	105870001
NC_056075.1	16870001	16890001	0.366127	0.304932	SG1	SORBS1	NC_056056.1	215815001	215835001
NC_056075.1	16875001	16895001	0.449871	0.280838	SG1	SORBS1	NC_056056.1	215820001	215840001
NC_056075.1	27530001	27550001	0.15649	0.240334	SG1	SORCS1	NC_056056.1	215825001	215845001
NC_056075.1	27535001	27555001	0.098573	0.293907	SG1	SORCS1	NC_056056.1	215830001	215850001
NC_056059.1	114410001	1.14E+08	0.379405	0.218239	SG1	SORCS2	NC_056056.1	215835001	215855001
NC_056059.1	114415001	1.14E+08	0.361092	0.21174	SG1	SORCS2	NC_056056.1	215840001	215860001
NC_056068.1	31830001	31850001	0.280598	0.203487	SG1	SORL1	NC_056070.1	10630001	10650001
NC_056068.1	31835001	31855001	0.316248	0.201862	SG1	SORL1	NC_056076.1	33640001	33660001
NC_056054.1	95430001	95450001	0.454776	0.243345	SG1	SPAG17	NC_056076.1	33645001	33665001
NC_056054.1	95435001	95455001	0.366237	0.283224	SG1	SPAG17	NC_056076.1	33650001	33670001
NC_056055.1	202635001	2.03E+08	0.432933	0.229088	SG1	SPATS2L	NC_056076.1	33655001	33675001
NC_056055.1	202645001	2.03E+08	0.468525	0.310035	SG1	SPATS2L	NC_056076.1	33660001	33680001
NC_056064.1	33480001	33500001	0.353701	0.295204	SG1	SPECC1	NC_056076.1	33665001	33685001
NC_056070.1	71710001	71730001	0.3766	0.344155	SG1	SPECC1L	NC_056076.1	33705001	33725001
NC_056070.1	71715001	71735001	0.413498	0.330129	SG1	SPECC1L	NC_056076.1	33710001	33730001
NC_056054.1	179100001	1.79E+08	0.465524	0.311659	SG1	SPICE1	NC_056076.1	33715001	33735001
NC_056055.1	23205001	23225001	0.309442	0.192068	SG1	SPIN1	NC_056071.1	29155001	29175001
NC_056055.1	23210001	23230001	0.277684	0.189345	SG1	SPIN1	NC_056071.1	29160001	29180001
NC_056055.1	23215001	23235001	0.295738	0.196779	SG1	SPIN1	NC_056071.1	29165001	29185001
NC_056055.1	23230001	23250001	0.398983	0.20138	SG1	SPIN1	NC_056071.1	29170001	29190001
NC_056055.1	23235001	23255001	0.358893	0.201557	SG1	SPIN1	NC_056071.1	29175001	29195001
NC_056055.1	23240001	23260001	0.31587	0.207755	SG1	SPIN1	NC_056080.1	11000001	11020001
NC_056055.1	23245001	23265001	0.323005	0.20361	SG1	SPIN1	NC_056080.1	11005001	11025001
NC_056055.1	23250001	23270001	0.319906	0.208982	SG1	SPIN1	NC_056058.1	32250001	32270001
NC_056055.1	23255001	23275001	0.34058	0.216876	SG1	SPIN1	NC_056058.1	32255001	32275001
NC_056055.1	23260001	23280001	0.369463	0.224893	SG1	SPIN1	NC_056060.1	57465001	57485001
NC_056055.1	23265001	23285001	0.36121	0.224568	SG1	SPIN1	NC_056060.1	57470001	57490001
NC_056055.1	23270001	23290001	0.331056	0.198584	SG1	SPIN1	NC_056060.1	57475001	57495001
NC_056068.1	38305001	38325001	0.396585	0.190913	SG1	SPON1	NC_056055.1	8865001	8885001

NC_056068.1	38310001	38330001	0.43391	0.191061	SG1	SPON1	NC_056054.1	216910001	216930001
NC_056064.1	45585001	45605001	0.471247	0.247279	SG1	SPPL2C	NC_056055.1	220045001	220065001
NC_056070.1	63170001	63190001	0.377477	0.200321	SG1	SPPL3	NC_056071.1	52565001	52585001
NC_056070.1	63175001	63195001	0.327182	0.224279	SG1	SPPL3	NC_056071.1	52570001	52590001
NC_056070.1	63180001	63200001	0.274448	0.242713	SG1	SPPL3	NC_056071.1	52575001	52595001
NC_056070.1	63185001	63205001	0.312653	0.21177	SG1	SPPL3	NC_056065.1	26375001	26395001
NC_056070.1	63230001	63250001	0.216764	0.205133	SG1	SPPL3	NC_056065.1	26380001	26400001
NC_056070.1	63235001	63255001	0.247735	0.264014	SG1	SPPL3	NC_056057.1	109215001	109235001
NC_056070.1	63240001	63260001	0.227147	0.268966	SG1	SPPL3	NC_056068.1	65755001	65775001
NC_056070.1	63245001	63265001	0.255584	0.19901	SG1	SPPL3	NC_056054.1	264735001	264755001
NC_056078.1	3470001	3490001	0.393024	0.258512	SG1	SPRTN	NC_056054.1	264740001	264760001
NC_056056.1	69370001	69390001	0.444375	0.188882	SG1	SPTBN1	NC_056054.1	264745001	264765001
NC_056056.1	69375001	69395001	0.261116	0.231956	SG1	SPTBN1	NC_056054.1	264790001	264810001
NC_056056.1	69380001	69400001	0.222028	0.238402	SG1	SPTBN1	NC_056054.1	264795001	264815001
NC_056056.1	69385001	69405001	0.250942	0.235723	SG1	SPTBN1	NC_056054.1	264800001	264820001
NC_056056.1	69390001	69410001	0.301428	0.216858	SG1	SPTBN1	NC_056054.1	264805001	264825001
NC_056056.1	172950001	1.73E+08	0.426841	0.204028	SG1	STAB2	NC_056054.1	264810001	264830001
NC_056056.1	172955001	1.73E+08	0.409815	0.186639	SG1	STAB2	NC_056054.1	91770001	91790001
NC_056057.1	51555001	51575001	0.169719	0.189005	SG1	STARD3NL	NC_056054.1	91775001	91795001
NC_056057.1	51560001	51580001	0.412799	0.192603	SG1	STARD3NL	NC_056071.1	54475001	54495001
NC_056055.1	41920001	41940001	0.378001	0.248217	SG1	STC1	NC_056071.1	54480001	54500001
NC_056055.1	41925001	41945001	0.371627	0.249666	SG1	STC1	NC_056080.1	122870001	122890001
NC_056055.1	41930001	41950001	0.397669	0.249906	SG1	STC1	NC_056058.1	45265001	45285001
NC_056055.1	41935001	41955001	0.355348	0.271065	SG1	STC1	NC_056058.1	45275001	45295001
NC_056075.1	24120001	24140001	0.391451	0.209302	SG1	STN1	NC_056058.1	45280001	45300001
NC_056075.1	24125001	24145001	0.44605	0.197012	SG1	STN1	NC_056058.1	45425001	45445001
NC_056065.1	28880001	28900001	0.121895	0.190975	SG1	STUM	NC_056057.1	26215001	26235001
NC_056057.1	82535001	82555001	0.201798	0.189036	SG1	SUGCT	NC_056057.1	26220001	26240001
NC_056071.1	15250001	15270001	0.425988	0.305547	SG1	SV2B	NC_056080.1	38210001	38230001
NC_056057.1	103740001	1.04E+08	0.337821	0.220039	SG1	SVOPL	NC_056080.1	38215001	38235001
NC_056057.1	103745001	1.04E+08	0.25237	0.263492	SG1	SVOPL	NC_056078.1	3035001	3055001
NC_056057.1	103750001	1.04E+08	0.175808	0.300282	SG1	SVOPL	NC_056078.1	3040001	3060001

NC_056057.1	103755001	1.04E+08	0.134531	0.324215	SG1	SVOPL	NC_056078.1	3045001	3065001
NC_056057.1	103760001	1.04E+08	0.271435	0.256844	SG1	SVOPL	NC_056078.1	3060001	3080001
NC_056057.1	103765001	1.04E+08	0.368272	0.217054	SG1	SVOPL	NC_056078.1	3065001	3085001
NC_056056.1	224360001	2.24E+08	0.326662	0.253625	SG1	TAFA5	NC_056078.1	3070001	3090001
NC_056056.1	224365001	2.24E+08	0.30577	0.279534	SG1	TAFA5	NC_056078.1	3075001	3095001
NC_056056.1	224370001	2.24E+08	0.336822	0.228906	SG1	TAFA5	NC_056078.1	3080001	3100001
NC_056056.1	204465001	2.04E+08	0.245248	0.19172	SG1	TAS2R7;TAS2R8;TA!	NC_056060.1	85275001	85295001
NC_056056.1	222985001	2.23E+08	0.16968	0.327201	SG1	TBC1D22A	NC_056054.1	60860001	60880001
NC_056056.1	222990001	2.23E+08	0.219318	0.227902	SG1	TBC1D22A	NC_056061.1	64250001	64270001
NC_056056.1	155100001	1.55E+08	0.322513	0.279586	SG1	TBC1D30	NC_056074.1	5830001	5850001
NC_056056.1	155105001	1.55E+08	0.415277	0.250697	SG1	TBC1D30	NC_056074.1	5835001	5855001
NC_056060.1	8935001	8955001	0.31306	0.387134	SG1	TBCA	NC_056074.1	5840001	5860001
NC_056054.1	201425001	2.01E+08	0.403383	0.402512	SG1	TBCCD1	NC_056074.1	5950001	5970001
NC_056054.1	201430001	2.01E+08	0.389341	0.435653	SG1	TBCCD1	NC_056074.1	5955001	5975001
NC_056080.1	128970001	1.29E+08	0.408965	0.197256	SG1	TCEAL7	NC_056066.1	78130001	78150001
NC_056056.1	219095001	2.19E+08	0.421712	0.361487	SG1	TCF20	NC_056073.1	16355001	16375001
NC_056076.1	55125001	55145001	0.152459	0.227838	SG1	TCF4	NC_056073.1	16360001	16380001
NC_056063.1	1980001	2000001	0.284606	0.189976	SG1	TDRD3	NC_056073.1	16365001	16385001
NC_056063.1	1985001	2005001	0.223541	0.206128	SG1	TDRD3	NC_056073.1	16370001	16390001
NC_056063.1	1990001	2010001	0.228915	0.184854	SG1	TDRD3	NC_056054.1	117950001	117970001
NC_056063.1	2060001	2080001	0.359671	0.192045	SG1	TDRD3	NC_056072.1	14365001	14385001
NC_056063.1	2065001	2085001	0.30292	0.273467	SG1	TDRD3	NC_056055.1	53190001	53210001
NC_056063.1	2070001	2090001	0.25823	0.295544	SG1	TDRD3	NC_056055.1	53235001	53255001
NC_056063.1	2075001	2095001	0.300571	0.29524	SG1	TDRD3	NC_056071.1	55840001	55860001
NC_056063.1	2080001	2100001	0.334816	0.263191	SG1	TDRD3	NC_056071.1	55845001	55865001
NC_056063.1	2085001	2105001	0.414368	0.184994	SG1	TDRD3	NC_056071.1	55850001	55870001
NC_056063.1	2120001	2140001	0.268143	0.276754	SG1	TDRD3	NC_056071.1	55855001	55875001
NC_056059.1	80595001	80615001	0.463387	0.191615	SG1	TECRL	NC_056071.1	55860001	55880001
NC_056059.1	80600001	80620001	0.343527	0.204798	SG1	TECRL	NC_056071.1	55865001	55885001
NC_056059.1	80605001	80625001	0.188467	0.235525	SG1	TECRL	NC_056071.1	55870001	55890001
NC_056059.1	80610001	80630001	0.19937	0.213719	SG1	TECRL	NC_056054.1	29555001	29575001
NC_056080.1	108500001	1.09E+08	0.327218	0.215254	SG1	TENM1	NC_056054.1	29560001	29580001

NC_056080.1	108505001	1.09E+08	0.175463	0.31604	SG1	TENM1	NC_056054.1	29565001	29585001
NC_056080.1	108510001	1.09E+08	0.096414	0.329046	SG1	TENM1	NC_056054.1	29570001	29590001
NC_056080.1	108515001	1.09E+08	0.154845	0.222233	SG1	TENM1	NC_056054.1	141960001	141980001
NC_056074.1	14765001	14785001	0.462373	0.190715	SG1	TENM4	NC_056054.1	141965001	141985001
NC_056064.1	9200001	9220001	0.293544	0.194187	SG1	TEX14	NC_056054.1	141970001	141990001
NC_056064.1	9280001	9300001	0.102967	0.208216	SG1	TEX14	NC_056054.1	142065001	142085001
NC_056064.1	9285001	9305001	0.099219	0.209888	SG1	TEX14	NC_056054.1	142070001	142090001
NC_056064.1	9300001	9320001	0.142672	0.198639	SG1	TEX14	NC_056080.1	40410001	40430001
NC_056063.1	30245001	30265001	0.353669	0.214678	SG1	TEX26	NC_056080.1	40415001	40435001
NC_056062.1	21455001	21475001	0.042466	0.301119	SG1	TG	NC_056080.1	40420001	40440001
NC_056062.1	21460001	21480001	0.07266	0.225464	SG1	TG	NC_056068.1	54030001	54050001
NC_056066.1	41405001	41425001	0.366227	0.245082	SG1	THBD	NC_056054.1	230775001	230795001
NC_056060.1	18310001	18330001	0.434827	0.237766	SG1	THSD4	NC_056054.1	230780001	230800001
NC_056060.1	18315001	18335001	0.450313	0.234242	SG1	THSD4	NC_056054.1	230840001	230860001
NC_056060.1	18320001	18340001	0.460817	0.233025	SG1	THSD4	NC_056065.1	40475001	40495001
NC_056054.1	124940001	1.25E+08	0.449244	0.286494	SG1	TIAM1	NC_056065.1	40480001	40500001
NC_056054.1	36935001	36955001	0.076353	0.198615	SG1	TM2D1	NC_056065.1	40670001	40690001
NC_056054.1	36940001	36960001	0.077916	0.198172	SG1	TM2D1	NC_056065.1	40675001	40695001
NC_056054.1	36945001	36965001	0.084603	0.195773	SG1	TM2D1	NC_056065.1	40680001	40700001
NW_02459982	1555001	1575001	0.311355	0.243137	SG1	TM6SF1	NC_056065.1	40685001	40705001
NC_056066.1	61280001	61300001	0.452779	0.186915	SG1	TM9SF4	NC_056065.1	40690001	40710001
NC_056077.1	17490001	17510001	0.331495	0.209318	SG1	TMC5	NC_056065.1	40695001	40715001
NC_056077.1	17495001	17515001	0.206856	0.219865	SG1	TMC5	NC_056065.1	40700001	40720001
NC_056077.1	17500001	17520001	0.216005	0.22235	SG1	TMC5	NC_056065.1	40705001	40725001
NC_056077.1	17505001	17525001	0.262873	0.202937	SG1	TMC5	NC_056065.1	40710001	40730001
NC_056077.1	17510001	17530001	0.267796	0.190263	SG1	TMC5	NC_056075.1	34255001	34275001
NC_056077.1	17515001	17535001	0.245245	0.213251	SG1	TMC5	NC_056075.1	34260001	34280001
NC_056077.1	17520001	17540001	0.157102	0.212543	SG1	TMC5	NC_056075.1	34265001	34285001
NC_056077.1	17525001	17545001	0.097712	0.240837	SG1	TMC5	NC_056075.1	34270001	34290001
NC_056070.1	47875001	47895001	0.391238	0.239063	SG1	TMEM132D	NC_056063.1	12215001	12235001
NC_056070.1	47880001	47900001	0.194508	0.319906	SG1	TMEM132D	NC_056063.1	12220001	12240001
NC_056070.1	47885001	47905001	0.188573	0.306473	SG1	TMEM132D	NC_056063.1	12225001	12245001

NC_056070.1	47890001	47910001	0.356359	0.268728	SG1	TMEM132D	NC_056063.1	12230001	12250001
NC_056057.1	105890001	1.06E+08	0.423599	0.256763	SG1	TMEM178B	NC_056063.1	12235001	12255001
NC_056057.1	105895001	1.06E+08	0.398683	0.248368	SG1	TMEM178B	NC_056063.1	12275001	12295001
NC_056057.1	105900001	1.06E+08	0.42432	0.248506	SG1	TMEM178B	NC_056063.1	12280001	12300001
NC_056057.1	105905001	1.06E+08	0.439764	0.24871	SG1	TMEM178B	NC_056063.1	12390001	12410001
NC_056057.1	105910001	1.06E+08	0.418021	0.249464	SG1	TMEM178B	NC_056063.1	12395001	12415001
NC_056057.1	105915001	1.06E+08	0.309275	0.286924	SG1	TMEM178B	NC_056063.1	12400001	12420001
NC_056057.1	105920001	1.06E+08	0.290209	0.233864	SG1	TMEM178B	NC_056063.1	12405001	12425001
NC_056078.1	16550001	16570001	0.272716	0.212503	SG1	TMEM26	NC_056056.1	210040001	210060001
NC_056078.1	16555001	16575001	0.215443	0.212959	SG1	TMEM26	NC_056056.1	210045001	210065001
NC_056078.1	16560001	16580001	0.292189	0.200471	SG1	TMEM26	NC_056056.1	210050001	210070001
NC_056068.1	42995001	43015001	0.295359	0.18537	SG1	TMEM41B	NC_056056.1	210105001	210125001
NC_056060.1	36610001	36630001	0.392336	0.197712	SG1	TMEM62	NC_056056.1	210110001	210130001
NC_056060.1	36615001	36635001	0.367864	0.210728	SG1	TMEM62	NC_056056.1	210115001	210135001
NC_056055.1	52695001	52715001	0.237464	0.191663	SG1	TMEM8B	NC_056056.1	210120001	210140001
NC_056080.1	81865001	81885001	0.311112	0.344443	SG1	TMLHE	NC_056078.1	41995001	42015001
NC_056080.1	81870001	81890001	0.459794	0.22759	SG1	TMLHE	NC_056078.1	42005001	42025001
NC_056054.1	262390001	2.62E+08	0.456337	0.19638	SG1	TMPRSS2	NC_056071.1	62945001	62965001
NC_056056.1	118930001	1.19E+08	0.322301	0.241712	SG1	TMTC2	NC_056071.1	62950001	62970001
NC_056056.1	118935001	1.19E+08	0.152024	0.28575	SG1	TMTC2	NC_056071.1	62955001	62975001
NC_056056.1	118940001	1.19E+08	0.199952	0.275449	SG1	TMTC2	NC_056061.1	91070001	91090001
NC_056056.1	118945001	1.19E+08	0.262411	0.239773	SG1	TMTC2	NC_056061.1	91075001	91095001
NC_056066.1	510001	530001	0.444562	0.199245	SG1	TMX4	NC_056061.1	91080001	91100001
NC_056066.1	515001	535001	0.395551	0.23323	SG1	TMX4	NC_056061.1	91085001	91105001
NC_056066.1	520001	540001	0.307057	0.264427	SG1	TMX4	NC_056071.1	22410001	22430001
NC_056066.1	525001	545001	0.231646	0.290379	SG1	TMX4	NC_056057.1	67975001	67995001
NC_056066.1	530001	550001	0.183432	0.292133	SG1	TMX4	NC_056057.1	67980001	68000001
NC_056066.1	535001	555001	0.182718	0.270669	SG1	TMX4	NC_056057.1	67985001	68005001
NC_056066.1	540001	560001	0.242215	0.242015	SG1	TMX4	NC_056080.1	37035001	37055001
NC_056066.1	545001	565001	0.316797	0.204555	SG1	TMX4	NC_056080.1	37040001	37060001
NC_056063.1	34675001	34695001	0.318181	0.191024	SG1	TNFRSF19	NC_056080.1	37045001	37065001
NC_056063.1	34680001	34700001	0.347482	0.189707	SG1	TNFRSF19	NC_056057.1	116655001	116675001

NC_056063.1	34685001	34705001	0.382922	0.210929	SG1	TNFRSF19	NC_056057.1	116660001	116680001
NC_056063.1	34690001	34710001	0.423374	0.26495	SG1	TNFRSF19	NC_056066.1	17060001	17080001
NC_056063.1	34695001	34715001	0.430663	0.249506	SG1	TNFRSF19	NC_056064.1	22420001	22440001
NC_056055.1	218850001	2.19E+08	0.102629	0.279253	SG1	TNP1	NC_056063.1	28970001	28990001
NC_056055.1	218855001	2.19E+08	0.093656	0.279051	SG1	TNP1	NC_056063.1	28975001	28995001
NC_056065.1	55965001	55985001	0.448815	0.205474	SG1	TNR	NC_056063.1	28980001	29000001
NC_056065.1	55970001	55990001	0.418513	0.207986	SG1	TNR	NC_056063.1	28985001	29005001
NC_056077.1	39330001	39350001	0.423557	0.189524	SG1	TNRC18	NC_056063.1	28990001	29010001
NC_056077.1	22785001	22805001	0.410948	0.195912	SG1	TNRC6A	NC_056063.1	28995001	29015001
NC_056064.1	34350001	34370001	0.290077	0.206349	SG1	TOM1L2	NC_056054.1	180055001	180075001
NC_056062.1	37995001	38015001	0.411766	0.1859	SG1	TOX	NC_056068.1	19830001	19850001
NC_056062.1	38000001	38020001	0.408642	0.193033	SG1	TOX	NC_056061.1	81800001	81820001
NC_056062.1	38005001	38025001	0.424375	0.199241	SG1	TOX	NC_056061.1	81805001	81825001
NC_056062.1	38010001	38030001	0.435187	0.205594	SG1	TOX	NC_056061.1	81810001	81830001
NC_056062.1	38015001	38035001	0.439335	0.204597	SG1	TOX	NC_056061.1	81815001	81835001
NC_056062.1	38020001	38040001	0.421831	0.189956	SG1	TOX	NC_056060.1	86480001	86500001
NC_056062.1	38170001	38190001	0.079876	0.189572	SG1	TOX	NC_056060.1	86485001	86505001
NC_056055.1	74385001	74405001	0.474758	0.196194	SG1	TPD52L3	NC_056062.1	93785001	93805001
NC_056057.1	108985001	1.09E+08	0.17599	0.205931	SG1	TPK1	NC_056062.1	93790001	93810001
NC_056057.1	108990001	1.09E+08	0.207132	0.205384	SG1	TPK1	NC_056067.1	37770001	37790001
NC_056057.1	108995001	1.09E+08	0.213649	0.232223	SG1	TPK1	NC_056067.1	37790001	37810001
NC_056057.1	109000001	1.09E+08	0.226642	0.203059	SG1	TPK1	NC_056067.1	37795001	37815001
NC_056057.1	109005001	1.09E+08	0.230681	0.226289	SG1	TPK1	NC_056067.1	37800001	37820001
NC_056057.1	109010001	1.09E+08	0.176521	0.273891	SG1	TPK1	NC_056067.1	37805001	37825001
NC_056057.1	109015001	1.09E+08	0.208955	0.272742	SG1	TPK1	NC_056067.1	37810001	37830001
NC_056057.1	109020001	1.09E+08	0.313006	0.291393	SG1	TPK1	NC_056067.1	37815001	37835001
NC_056057.1	109025001	1.09E+08	0.382118	0.269885	SG1	TPK1	NC_056067.1	37820001	37840001
NC_056072.1	10635001	10655001	0.295143	0.221164	SG1	TRANK1	NC_056067.1	37825001	37845001
NC_056072.1	10640001	10660001	0.344412	0.209586	SG1	TRANK1	NC_056066.1	69880001	69900001
NC_056072.1	10645001	10665001	0.331028	0.200021	SG1	TRANK1	NC_056066.1	69885001	69905001
NC_056056.1	108755001	1.09E+08	0.332529	0.210976	SG1	TRHDE	NC_056066.1	69890001	69910001
NC_056056.1	108760001	1.09E+08	0.191804	0.277855	SG1	TRHDE	NC_056066.1	69895001	69915001

NC_056077.1	36055001	36075001	0.271293	0.218038	SG1	TRIM56	NC_056066.1	69900001	69920001
NC_056059.1	115435001	1.15E+08	0.433787	0.507094	SG1	TRMT44	NC_056080.1	97660001	97680001
NC_056056.1	219850001	2.2E+08	0.40327	0.257395	SG1	TSPO	NC_056080.1	97665001	97685001
NC_056060.1	36345001	36365001	0.347431	0.253462	SG1	TTBK2	NC_056080.1	97680001	97700001
NC_056060.1	36350001	36370001	0.322425	0.253854	SG1	TTBK2	NC_056080.1	97685001	97705001
NC_056060.1	36355001	36375001	0.317904	0.225634	SG1	TTBK2	NC_056080.1	97690001	97710001
NC_056060.1	36360001	36380001	0.325001	0.248087	SG1	TTBK2	NC_056078.1	12340001	12360001
NC_056060.1	36365001	36385001	0.394919	0.235237	SG1	TTBK2	NC_056058.1	60010001	60030001
NC_056060.1	36370001	36390001	0.421626	0.238041	SG1	TTBK2	NC_056058.1	60015001	60035001
NC_056060.1	36375001	36395001	0.303935	0.280808	SG1	TTBK2	NC_056058.1	36255001	36275001
NC_056060.1	36380001	36400001	0.142345	0.294141	SG1	TTBK2	NC_056073.1	30210001	30230001
NC_056060.1	36385001	36405001	0.082793	0.308699	SG1	TTBK2	NC_056067.1	42355001	42375001
NC_056055.1	143670001	1.44E+08	0.144037	0.224641	SG1	TTC21B	NC_056067.1	42360001	42380001
NC_056055.1	143675001	1.44E+08	0.094871	0.252381	SG1	TTC21B	NC_056067.1	42365001	42385001
NC_056055.1	143685001	1.44E+08	0.028915	0.307432	SG1	TTC21B	NC_056067.1	42370001	42390001
NC_056055.1	143690001	1.44E+08	0.026162	0.323436	SG1	TTC21B	NC_056067.1	42375001	42395001
NC_056055.1	143700001	1.44E+08	0.032931	0.332004	SG1	TTC21B	NC_056067.1	40480001	40500001
NC_056055.1	143705001	1.44E+08	0.138801	0.265353	SG1	TTC21B	NC_056067.1	40485001	40505001
NC_056070.1	67730001	67750001	0.21395	0.232701	SG1	TTC28	NC_056067.1	40490001	40510001
NC_056070.1	67735001	67755001	0.375583	0.29652	SG1	TTC28	NC_056067.1	40495001	40515001
NC_056070.1	67740001	67760001	0.423339	0.29215	SG1	TTC28	NC_056067.1	40530001	40550001
NC_056070.1	67745001	67765001	0.442666	0.29419	SG1	TTC28	NC_056054.1	97010001	97030001
NC_056070.1	67750001	67770001	0.242793	0.254222	SG1	TTC28	NC_056071.1	21530001	21550001
NC_056070.1	67755001	67775001	0.092316	0.191071	SG1	TTC28	NC_056058.1	12205001	12225001
NC_056070.1	67760001	67780001	0.076519	0.235504	SG1	TTC28	NC_056066.1	77350001	77370001
NC_056070.1	67765001	67785001	0.101655	0.236767	SG1	TTC28	NC_056054.1	62685001	62705001
NC_056070.1	67770001	67790001	0.191223	0.197623	SG1	TTC28	NC_056054.1	62690001	62710001
NC_056070.1	67800001	67820001	0.384246	0.193461	SG1	TTC28	NC_056058.1	16630001	16650001
NC_056070.1	67850001	67870001	0.262103	0.195188	SG1	TTC28	NC_056058.1	16635001	16655001
NC_056070.1	67855001	67875001	0.211712	0.235465	SG1	TTC28	NC_056058.1	16640001	16660001
NC_056070.1	67860001	67880001	0.326389	0.216272	SG1	TTC28	NC_056058.1	16645001	16665001
NC_056056.1	173540001	1.74E+08	0.431606	0.209728	SG1	TXNRD1	NC_056058.1	16650001	16670001

NC_056056.1	173545001	1.74E+08	0.400544	0.245015	SG1	TXNRD1	NC_056069.1	18115001	18135001
NC_056056.1	173550001	1.74E+08	0.424227	0.336544	SG1	TXNRD1	NC_056069.1	18120001	18140001
NC_056056.1	173555001	1.74E+08	0.461154	0.373544	SG1	TXNRD1	NC_056069.1	18125001	18145001
NC_056055.1	81165001	81185001	0.293078	0.246423	SG1	TYRP1	NC_056069.1	18130001	18150001
NC_056055.1	81170001	81190001	0.321073	0.306015	SG1	TYRP1	NC_056069.1	18135001	18155001
NC_056055.1	81175001	81195001	0.193281	0.266177	SG1	TYRP1	NC_056069.1	18140001	18160001
NC_056055.1	81180001	81200001	0.14	0.271908	SG1	TYRP1	NC_056069.1	18145001	18165001
NC_056055.1	81185001	81205001	0.058001	0.26944	SG1	TYRP1	NC_056069.1	18150001	18170001
NC_056055.1	81190001	81210001	0.060057	0.289993	SG1	TYRP1	NC_056069.1	18155001	18175001
NC_056055.1	81195001	81215001	0.150164	0.344623	SG1	TYRP1			
NC_056055.1	81200001	81220001	0.270773	0.385859	SG1	TYRP1			
NC_056060.1	36565001	36585001	0.154432	0.195571	SG1	UBR1			
NC_056054.1	117950001	1.18E+08	0.229692	0.214996	SG1	UCK2			
NC_056055.1	117285001	1.17E+08	0.445173	0.205256	SG1	UGGT1			
NC_056055.1	117290001	1.17E+08	0.300537	0.271286	SG1	UGGT1			
NC_056055.1	117295001	1.17E+08	0.195794	0.317589	SG1	UGGT1			
NC_056055.1	117300001	1.17E+08	0.277943	0.282582	SG1	UGGT1			
NC_056072.1	13970001	13990001	0.444445	0.200207	SG1	ULK4			
NC_056072.1	13975001	13995001	0.286289	0.248295	SG1	ULK4			
NC_056072.1	13980001	14000001	0.24177	0.275259	SG1	ULK4			
NC_056072.1	13985001	14005001	0.176901	0.311018	SG1	ULK4			
NC_056072.1	13990001	14010001	0.053769	0.311743	SG1	ULK4			
NC_056072.1	13995001	14015001	0.07599	0.289516	SG1	ULK4			
NC_056072.1	14000001	14020001	0.215996	0.200482	SG1	ULK4			
NC_056059.1	29605001	29625001	0.403423	0.220833	SG1	UNC5C			
NC_056068.1	34725001	34745001	0.334643	0.183098	SG1	USH1C			
NC_056068.1	34750001	34770001	0.081088	0.224418	SG1	USH1C			
NC_056068.1	34755001	34775001	0.201471	0.222839	SG1	USH1C			
NC_056072.1	630001	650001	0.444239	0.242179	SG1	VOPP1			
NC_056072.1	635001	655001	0.419438	0.210902	SG1	VOPP1			
NC_056072.1	640001	660001	0.448241	0.192887	SG1	VOPP1			
NC_056071.1	20960001	20980001	0.266902	0.2675	SG1	VPS33B			

NC_056071.1	20965001	20985001	0.161896	0.314024	SG1	VPS33B
NC_056071.1	20970001	20990001	0.436418	0.238792	SG1	VPS33B
NC_056064.1	22065001	22085001	0.347677	0.239526	SG1	VPS53
NC_056075.1	32540001	32560001	0.452191	0.30793	SG1	VTI1A
NC_056075.1	32545001	32565001	0.437766	0.302553	SG1	VTI1A
NC_056075.1	32550001	32570001	0.470487	0.373879	SG1	VTI1A
NC_056075.1	34280001	34300001	0.297671	0.254776	SG1	VWA2
NC_056063.1	12340001	12360001	0.407663	0.221401	SG1	VWA8
NC_056063.1	12345001	12365001	0.44855	0.289318	SG1	VWA8
NC_056063.1	12350001	12370001	0.438415	0.274589	SG1	VWA8
NC_056063.1	12355001	12375001	0.411882	0.281379	SG1	VWA8
NC_056063.1	33260001	33280001	0.428267	0.213993	SG1	WASF3
NC_056063.1	33270001	33290001	0.395952	0.220645	SG1	WASF3
NC_056063.1	21420001	21440001	0.291241	0.214633	SG1	WDFY2
NC_056063.1	21425001	21445001	0.209424	0.242443	SG1	WDFY2
NC_056063.1	21430001	21450001	0.311909	0.205533	SG1	WDFY2
NC_056063.1	21435001	21455001	0.384687	0.1842	SG1	WDFY2
NC_056061.1	91055001	91075001	0.284638	0.512302	SG1	WDR27
NC_056057.1	115575001	1.16E+08	0.393917	0.189741	SG1	WDR86
NC_056055.1	239740001	2.4E+08	0.242312	0.200757	SG1	WDTC1
NC_056071.1	22395001	22415001	0.354579	0.248871	SG1	WHAMM
NC_056071.1	22400001	22420001	0.400478	0.228035	SG1	WHAMM
NC_056056.1	213430001	2.13E+08	0.356805	0.334709	SG1	WNK1
NC_056056.1	213435001	2.13E+08	0.292695	0.413833	SG1	WNK1
NC_056056.1	213440001	2.13E+08	0.26423	0.430999	SG1	WNK1
NC_056056.1	213445001	2.13E+08	0.41188	0.35714	SG1	WNK1
NC_056056.1	222195001	2.22E+08	0.402935	0.237804	SG1	WNT7B
NC_056055.1	43590001	43610001	0.425429	0.233696	SG1	XPO7
NC_056055.1	43595001	43615001	0.373332	0.250232	SG1	XPO7
NC_056067.1	51455001	51475001	0.139099	0.223803	SG1	XRCC1;ZNF575
NC_056067.1	51460001	51480001	0.200357	0.211938	SG1	XRCC1;ZNF575
NC_056057.1	95490001	95510001	0.441224	0.412278	SG1	ZC3HC1

NC_056057.1	95495001	95515001	0.355587	0.42122	SG1	ZC3HC1
NC_056057.1	95500001	95520001	0.375533	0.403233	SG1	ZC3HC1
NC_056058.1	78855001	78875001	0.45642	0.278305	SG1	ZCCHC9
NC_056058.1	78860001	78880001	0.445624	0.276993	SG1	ZCCHC9
NC_056058.1	78865001	78885001	0.252311	0.309814	SG1	ZCCHC9
NC_056072.1	2910001	2930001	0.45709	0.246486	SG1	ZCWPW2
NC_056072.1	2915001	2935001	0.434099	0.298991	SG1	ZCWPW2
NC_056072.1	2920001	2940001	0.4487	0.308728	SG1	ZCWPW2
NC_056072.1	2925001	2945001	0.4547	0.331917	SG1	ZCWPW2
NC_056080.1	114040001	1.14E+08	0.310053	0.26731	SG1	ZNF280C
NC_056060.1	51800001	51820001	0.409233	0.183075	SG1	ZNF280D
NC_056060.1	51845001	51865001	0.33515	0.185125	SG1	ZNF280D
NC_056060.1	51850001	51870001	0.285964	0.191096	SG1	ZNF280D
NC_056079.1	43590001	43610001	0.26139	0.452199	SG1	ZNF385D
NC_056079.1	43595001	43615001	0.369582	0.385029	SG1	ZNF385D
NC_056066.1	33720001	33740001	0.474748	0.198084	SG1	ZNF438
NC_056066.1	33760001	33780001	0.450307	0.25607	SG1	ZNF438
NC_056066.1	33765001	33785001	0.440577	0.258586	SG1	ZNF438
NC_056066.1	33770001	33790001	0.411387	0.257012	SG1	ZNF438
NC_056066.1	33775001	33795001	0.272638	0.29451	SG1	ZNF438
NC_056066.1	33780001	33800001	0.158211	0.304851	SG1	ZNF438
NC_056066.1	33785001	33805001	0.138393	0.305669	SG1	ZNF438
NC_056066.1	33790001	33810001	0.153216	0.305048	SG1	ZNF438
NC_056066.1	33795001	33815001	0.182779	0.290685	SG1	ZNF438
NC_056066.1	33800001	33820001	0.245772	0.266999	SG1	ZNF438
NC_056066.1	33805001	33825001	0.283372	0.235935	SG1	ZNF438
NC_056066.1	33810001	33830001	0.254612	0.243214	SG1	ZNF438
NC_056066.1	33815001	33835001	0.323208	0.218037	SG1	ZNF438
NC_056057.1	113755001	1.14E+08	0.330641	0.201055	SG1	ZNF467
NC_056070.1	12465001	12485001	0.392419	0.192675	SG1	ZNF827
NC_056055.1	175455001	1.75E+08	0.086924	0.195421	SG1	ZRANB3
NC_056055.1	175460001	1.75E+08	0.107537	0.191792	SG1	ZRANB3

NC_056055.1	175465001	1.75E+08	0.182865	0.196988	SG1	ZRANB3
NC_056080.1	13960001	13980001	0.359863	0.263146	SG1	ZRSR2
NC_056080.1	13965001	13985001	0.420184	0.25489	SG1	ZRSR2
NC_056064.1	24480001	24500001	0.232709	0.192037	SG1	ZZEF1
NC_056064.1	24510001	24530001	0.141773	0.387857	SG1	ZZEF1
NC_056064.1	24515001	24535001	0.327177	0.26826	SG1	ZZEF1

Pi	Fst	Region	Gene	ZK vs SG1<br>CHROM	Start	end	Pi	Fst	Region	Gene
0.26282	0.135004	SG2	ABCA4	NC_056067.1	16035001	16055001	0.0390304	0.260881	SG1	ABCC12
0.454383	0.143481	SG2	ABCG1	NC_056067.1	16040001	16060001	0.17991	0.272082	SG1	ABCC12
0.46019	0.168901	SG2	ABHD10	NC_056067.1	16045001	16065001	0.26043	0.283392	SG1	ABCC12
0.202643	0.188893	SG2	ABHD2	NC_056067.1	16050001	16070001	0.36521	0.270694	SG1	ABCC12
0.173228	0.241321	SG2	ABHD2	NC_056067.1	16055001	16075001	0.409462	0.257962	SG1	ABCC12
0.226957	0.238205	SG2	ABHD2	NC_056067.1	16060001	16080001	0.365806	0.28066	SG1	ABCC12
0.197346	0.25812	SG2	ABHD2	NC_056067.1	16065001	16085001	0.228794	0.347701	SG1	ABCC12
0.264627	0.196273	SG2	ABHD2	NC_056067.1	16070001	16090001	0.159964	0.409192	SG1	ABCC12
0.359761	0.144477	SG2	ABHD2	NC_056067.1	16075001	16095001	0.174477	0.442834	SG1	ABCC12
0.207246	0.201935	SG2	ABHD2	NC_056067.1	16080001	16100001	0.272374	0.451862	SG1	ABCC12
0.327299	0.147729	SG2	ABHD2	NC_056067.1	16085001	16105001	0.484323	0.392017	SG1	ABCC12
0.165337	0.136448	SG2	ABI2	NC_056075.1	20570001	20590001	0.257282	0.175949	SG1	ABCC2
0.485387	0.136209	SG2	ABLIM1	NC_056075.1	20575001	20595001	0.155306	0.183196	SG1	ABCC2
0.38117	0.22517	SG2	ABLIM1	NC_056075.1	20580001	20600001	0.0862759	0.178212	SG1	ABCC2
0.113495	0.186539	SG2	ABLIM2	NC_056064.1	37085001	37105001	0.330363	0.182149	SG1	ABI3;GNGT2
0.047361	0.184029	SG2	ABLIM2	NC_056064.1	37075001	37095001	0.360618	0.194139	SG1	ABI3;GNGT2;[illegible]
0.052513	0.195265	SG2	ABLIM2	NC_056064.1	37080001	37100001	0.360526	0.177537	SG1	ABI3;GNGT2;[illegible]
0.060953	0.21378	SG2	ABLIM2	NC_056064.1	37070001	37090001	0.429228	0.192872	SG1	ABI3;PHOSPH[illegible]
0.099687	0.283188	SG2	ABLIM2	NC_056072.1	11600001	11620001	0.300044	0.234949	SG1	ACAA1;DLEC[illegible]
0.206536	0.220213	SG2	ABLIM2	NC_056070.1	63110001	63130001	0.410714	0.177593	SG1	ACADS
0.300161	0.157753	SG2	ABLIM2	NC_056070.1	63100001	63120001	0.219805	0.192269	SG1	ACADS;UNC1
0.442375	0.141189	SG2	ABLIM2	NC_056056.1	105285001	105305001	0.412968	0.193449	SG1	ACOXL;BCL2[illegible]
0.452847	0.181927	SG2	ABLIM2	NC_056056.1	225940001	225960001	0.256665	0.322821	SG1	ACR
0.462043	0.205036	SG2	ABLIM2	NC_056056.1	225945001	225965001	0.129315	0.417151	SG1	ACR
0.554095	0.190513	SG2	ABRAXAS2	NC_056056.1	225935001	225955001	0.41167	0.219019	SG1	ACR;SHANK3
0.548756	0.163452	SG2	ACBD5	NC_056077.1	18985001	19005001	0.204211	0.176796	SG1	ACSM3;ERI2
0.513926	0.179416	SG2	ACBD5	NC_056056.1	134350001	134370001	0.386301	0.31089	SG1	ACVR1B
0.543557	0.143787	SG2	ACBD5;MASTL	NC_056056.1	134355001	134375001	0.383627	0.260875	SG1	ACVR1B
0.42236	0.190376	SG2	ACBD5;MASTL	NC_056058.1	66745001	66765001	0.470344	0.315826	SG1	ADAM19

0.413462	0.18302	SG2	ACBD5;MASTL	NC_056058.1	66750001	66770001	0.351984	0.384639	SG1	ADAM19
0.558362	0.168011	SG2	ACOD1	NC_056058.1	66755001	66775001	0.404937	0.371745	SG1	ADAM19
0.40102	0.170977	SG2	ACOXL	NC_056054.1	130165001	130185001	0.418441	0.173513	SG1	ADAMTS1
0.473312	0.187285	SG2	ACOXL	NC_056058.1	1810001	1830001	0.223433	0.30034	SG1	ADAMTS2
0.519582	0.185471	SG2	ACOXL	NC_056058.1	1815001	1835001	0.105329	0.369811	SG1	ADAMTS2
0.121507	0.144542	SG2	ACP1;ALKAL2	NC_056058.1	1820001	1840001	0.0552528	0.379194	SG1	ADAMTS2
0.223067	0.137216	SG2	ACP1;ALKAL2	NC_056058.1	1825001	1845001	0.0407836	0.385091	SG1	ADAMTS2
0.500415	0.13822	SG2	ACTR8;IL17RB	NC_056058.1	1830001	1850001	0.0411241	0.388044	SG1	ADAMTS2
0.414794	0.167754	SG2	ADAM17;CPSF3;IA	NC_056058.1	1835001	1855001	0.110345	0.33792	SG1	ADAMTS2
0.452242	0.171528	SG2	ADAM17;CPSF3;IA	NC_056058.1	1840001	1860001	0.194109	0.297217	SG1	ADAMTS2
0.550315	0.158673	SG2	ADAM17;IAH1	NC_056058.1	1845001	1865001	0.340559	0.219178	SG1	ADAMTS2
0.552524	0.195247	SG2	ADAMTS1	NC_056054.1	130015001	130035001	0.314306	0.199573	SG1	ADAMTS5
0.387205	0.213025	SG2	ADAMTS1	NC_056054.1	130060001	130080001	0.33447	0.183182	SG1	ADAMTS5
0.493711	0.178732	SG2	ADAMTS10	NC_056054.1	130065001	130085001	0.273325	0.205864	SG1	ADAMTS5
0.381563	0.159551	SG2	ADAMTS10	NC_056054.1	130070001	130090001	0.230135	0.206178	SG1	ADAMTS5
0.206645	0.232281	SG2	ADAMTS10	NC_056054.1	130075001	130095001	0.206915	0.212827	SG1	ADAMTS5
0.491716	0.260412	SG2	ADAMTS10;MYO1	NC_056060.1	20660001	20680001	0.295263	0.227892	SG1	ADCY4;CIDEE
0.467147	0.300779	SG2	ADAMTS10;MYO1	NC_056060.1	20665001	20685001	0.351052	0.220891	SG1	ADCY4;CIDEE
0.46736	0.284467	SG2	ADAMTS10;MYO1	NC_056060.1	20655001	20675001	0.249081	0.237939	SG1	ADCY4;LTB4R
0.420621	0.248205	SG2	ADAMTS10;MYO1	NC_056060.1	20650001	20670001	0.261295	0.185688	SG1	ADCY4;RIPK3
0.495858	0.194767	SG2	ADAMTS12	NC_056062.1	22975001	22995001	0.480284	0.195522	SG1	ADCY8
0.405469	0.212991	SG2	ADAMTS12	NC_056062.1	22980001	23000001	0.232341	0.214866	SG1	ADCY8
0.278739	0.233443	SG2	ADAMTS12	NC_056062.1	22985001	23005001	0.239074	0.203897	SG1	ADCY8
0.553073	0.194691	SG2	ADAMTS12	NC_056070.1	46115001	46135001	0.293553	0.218493	SG1	ADGRD1
0.51348	0.278175	SG2	ADAMTS12	NC_056070.1	46120001	46140001	0.433654	0.200835	SG1	ADGRD1
0.347039	0.138001	SG2	ADAMTS17	NC_056059.1	77490001	77510001	0.483804	0.190172	SG1	ADGRL3
0.310977	0.152198	SG2	ADAMTS17	NC_056058.1	87775001	87795001	0.286378	0.217333	SG1	ADGRV1
0.120466	0.14679	SG2	ADAMTS17	NC_056058.1	87780001	87800001	0.421699	0.216207	SG1	ADGRV1
0.073246	0.150527	SG2	ADAMTS17	NC_056078.1	29280001	29300001	0.425197	0.203968	SG1	ADK
0.319459	0.182469	SG2	ADAMTS17	NC_056078.1	29285001	29305001	0.439117	0.178415	SG1	ADK
0.410068	0.16084	SG2	ADAMTS17	NC_056078.1	29765001	29785001	0.434077	0.332763	SG1	ADK
0.382354	0.140896	SG2	ADARB1	NC_056066.1	50630001	50650001	0.464906	0.191928	SG1	ADRA1D

0.168831	0.147095	SG2	ADARB1	NC_056066.1	50640001	50660001	0.47853	0.188306	SG1	ADRA1D
0.147019	0.187023	SG2	ADARB1	NC_056075.1	34310001	34330001	0.235372	0.191652	SG1	AFAP1L2
0.087139	0.22726	SG2	ADARB1	NC_056075.1	34315001	34335001	0.221733	0.237661	SG1	AFAP1L2
0.128408	0.26809	SG2	ADARB1	NC_056075.1	34320001	34340001	0.222096	0.213151	SG1	AFAP1L2
0.181992	0.237291	SG2	ADARB1	NC_056075.1	34325001	34345001	0.220101	0.21032	SG1	AFAP1L2
0.25618	0.211542	SG2	ADARB1	NC_056075.1	34335001	34355001	0.360633	0.185753	SG1	AFAP1L2
0.403168	0.162922	SG2	ADARB2	NC_056075.1	34340001	34360001	0.410057	0.223343	SG1	AFAP1L2
0.272124	0.222199	SG2	ADARB2	NC_056075.1	34345001	34365001	0.415936	0.211798	SG1	AFAP1L2
0.326531	0.20217	SG2	ADARB2	NC_056075.1	34285001	34305001	0.254518	0.264853	SG1	AFAP1L2;VW/
0.361738	0.224888	SG2	ADARB2	NC_056075.1	34290001	34310001	0.0950225	0.231201	SG1	AFAP1L2;VW/
0.382064	0.229109	SG2	ADARB2	NC_056075.1	34295001	34315001	0.0613485	0.18605	SG1	AFAP1L2;VW/
0.458231	0.208308	SG2	ADARB2	NC_056054.1	77285001	77305001	0.468377	0.215336	SG1	AGL
0.545867	0.166688	SG2	ADARB2	NC_056060.1	86965001	86985001	0.371566	0.198501	SG1	ALKBH1
0.463891	0.251399	SG2	ADCY10	NC_056056.1	133075001	133095001	0.210526	0.436552	SG1	AMHR2;SP1
0.268427	0.325181	SG2	ADCY10	NC_056056.1	133085001	133105001	0.285068	0.396918	SG1	AMHR2;SP1
0.145833	0.401462	SG2	ADCY10	NC_056070.1	53870001	53890001	0.169653	0.203871	SG1	ANAPC5
0.127	0.408469	SG2	ADCY10	NC_056070.1	53875001	53895001	0.240223	0.18366	SG1	ANAPC5
0.163648	0.379487	SG2	ADCY10	NC_056060.1	7045001	7065001	0.409601	0.187239	SG1	ANKDD1B
0.180361	0.377201	SG2	ADCY10	NC_056060.1	7050001	7070001	0.448223	0.194346	SG1	ANKDD1B
0.159645	0.388696	SG2	ADCY10	NC_056055.1	199295001	199315001	0.283058	0.251858	SG1	ANKRD44
0.119353	0.416547	SG2	ADCY10	NC_056056.1	168620001	168640001	0.264051	0.191464	SG1	ANKS1B
0.078547	0.439889	SG2	ADCY10	NC_056056.1	168625001	168645001	0.374346	0.179037	SG1	ANKS1B
0.107738	0.417186	SG2	ADCY10	NC_056054.1	239080001	239100001	0.155374	0.192546	SG1	ANKUB1
0.343849	0.295989	SG2	ADCY10	NC_056054.1	239085001	239105001	0.120164	0.187563	SG1	ANKUB1
0.562868	0.221364	SG2	ADCY10	NC_056054.1	239075001	239095001	0.14639	0.212933	SG1	ANKUB1;RNF
0.314707	0.147572	SG2	ADCY5	NC_056056.1	141135001	141155001	0.103235	0.225741	SG1	ANO6
0.546921	0.203385	SG2	ADCYAP1R1	NC_056080.1	1425001	1445001	0.284952	0.18283	SG1	ANOS1
0.103301	0.142186	SG2	ADGRF1	NC_056078.1	34340001	34360001	0.290141	0.310305	SG1	ANXA11;PLA(
0.122402	0.142315	SG2	ADGRF1	NC_056078.1	34345001	34365001	0.108241	0.238473	SG1	ANXA11;PLA(
0.466793	0.155989	SG2	ADGRG5	NC_056059.1	13635001	13655001	0.35557	0.199374	SG1	AP1AR
0.516744	0.206644	SG2	ADK	NC_056059.1	13640001	13660001	0.352164	0.178427	SG1	AP1AR
0.479658	0.200723	SG2	ADK	NC_056059.1	13645001	13665001	0.346092	0.204152	SG1	AP1AR

0.507371	0.185629	SG2	ADK	NC_056059.1	13650001	13670001	0.356247	0.243532	SG1	AP1AR
0.215628	0.182282	SG2	ADO	NC_056059.1	13655001	13675001	0.354375	0.244232	SG1	AP1AR
0.473829	0.163128	SG2	ADO;EGR2	NC_056059.1	13660001	13680001	0.333174	0.305874	SG1	AP1AR
0.53995	0.150603	SG2	ADO;EGR2	NC_056059.1	13665001	13685001	0.346572	0.264876	SG1	AP1AR
0.415271	0.285618	SG2	ADRA1B	NC_056059.1	13630001	13650001	0.4332	0.189385	SG1	AP1AR;TIFA
0.314815	0.19389	SG2	ADRA1B	NC_056056.1	216335001	216355001	0.252775	0.248885	SG1	APOBEC3A
0.419737	0.138885	SG2	ADRA1B	NC_056056.1	216340001	216360001	0.183405	0.274443	SG1	APOBEC3A
0.390195	0.189066	SG2	ADRA2A	NC_056056.1	216345001	216365001	0.239347	0.272041	SG1	APOBEC3A
0.315042	0.163841	SG2	ADRA2A	NC_056056.1	216350001	216370001	0.174708	0.305532	SG1	APOBEC3A
0.092486	0.15637	SG2	ADRA2B;ASTL	NC_056056.1	216355001	216375001	0.13758	0.30755	SG1	APOBEC3A
0.41288	0.150861	SG2	ADRA2B;ASTL	NC_056056.1	216365001	216385001	0.0461885	0.354348	SG1	APOBEC3F
0.180467	0.1395	SG2	AGTR1	NC_056056.1	216370001	216390001	0.0526028	0.354057	SG1	APOBEC3F
0.292045	0.167209	SG2	AGTR1	NC_056056.1	216375001	216395001	0.0487034	0.366667	SG1	APOBEC3F
0.562591	0.14727	SG2	AGTR1	NC_056055.1	166440001	166460001	0.433705	0.173307	SG1	ARHGAP15
0.407643	0.142632	SG2	AGTR1	NC_056055.1	166570001	166590001	0.25081	0.248169	SG1	ARHGAP15
0.44733	0.180529	SG2	AJAP1	NC_056055.1	166575001	166595001	0.121547	0.312267	SG1	ARHGAP15
0.386912	0.231147	SG2	AJAP1	NC_056055.1	166640001	166660001	0.0816327	0.267806	SG1	ARHGAP15
0.328973	0.305191	SG2	AJAP1	NC_056055.1	166645001	166665001	0.138798	0.218048	SG1	ARHGAP15
0.550261	0.274771	SG2	AJAP1	NC_056068.1	20405001	20425001	0.450658	0.235725	SG1	ARHGAP20
0.554864	0.289927	SG2	AJAP1	NC_056059.1	101440001	101460001	0.313981	0.184652	SG1	ARHGAP24
0.548846	0.29339	SG2	AJAP1	NC_056058.1	51700001	51720001	0.479452	0.333333	SG1	ARHGAP26
0.562544	0.26864	SG2	AJAP1	NC_056058.1	51705001	51725001	0.230291	0.324366	SG1	ARHGAP26
0.398965	0.142269	SG2	AKAP13	NC_056058.1	51710001	51730001	0.152159	0.327326	SG1	ARHGAP26
0.384087	0.146611	SG2	AKAP13	NC_056058.1	51715001	51735001	0.0916314	0.329976	SG1	ARHGAP26
0.480222	0.221445	SG2	AMPH	NC_056058.1	51720001	51740001	0.0424215	0.329912	SG1	ARHGAP26
0.474791	0.202059	SG2	ANGPTL8;DOCK6	NC_056067.1	53720001	53740001	0.38558	0.186426	SG1	ARHGAP35
0.231445	0.270559	SG2	ANGPTL8;DOCK6	NC_056054.1	106930001	106950001	0.395698	0.262652	SG1	ARHGEF11
0.343142	0.17219	SG2	ANKIB1	NC_056054.1	106935001	106955001	0.442457	0.219366	SG1	ARHGEF11
0.237147	0.22606	SG2	ANKIB1	NC_056054.1	234190001	234210001	0.315765	0.188437	SG1	ARHGEF26
0.156274	0.257265	SG2	ANKIB1	NC_056054.1	234210001	234230001	0.210938	0.18092	SG1	ARHGEF26
0.117161	0.263299	SG2	ANKIB1	NC_056054.1	234215001	234235001	0.164905	0.20607	SG1	ARHGEF26
0.206531	0.246357	SG2	ANKIB1	NC_056054.1	234220001	234240001	0.122057	0.213129	SG1	ARHGEF26

0.310947	0.208336	SG2	ANKIB1	NC_056054.1	234225001	234245001	0.0483872	0.244686	SG1	ARHGEF26
0.540251	0.141821	SG2	ANKRD22;LOC101	NC_056061.1	81210001	81230001	0.275186	0.244964	SG1	ARID1B
0.538169	0.139597	SG2	ANKRD65;TMEM8	NC_056061.1	81215001	81235001	0.247004	0.314894	SG1	ARID1B
0.212025	0.205207	SG2	ANKRD7	NC_056061.1	81220001	81240001	0.255359	0.327252	SG1	ARID1B
0.205316	0.204269	SG2	ANKRD7	NC_056061.1	81225001	81245001	0.311506	0.329669	SG1	ARID1B
0.247864	0.170743	SG2	ANKRD7	NC_056061.1	81285001	81305001	0.454138	0.183564	SG1	ARID1B
0.356031	0.187771	SG2	ANKRD7;LSM8	NC_056069.1	25375001	25395001	0.304573	0.337885	SG1	ARL15
0.250261	0.25904	SG2	ANKRD7;LSM8	NC_056069.1	25380001	25400001	0.205925	0.324819	SG1	ARL15
0.551257	0.350015	SG2	ANLN	NC_056069.1	25385001	25405001	0.266852	0.331499	SG1	ARL15
0.46084	0.288612	SG2	ANLN;KIAA0895	NC_056069.1	25390001	25410001	0.248342	0.315524	SG1	ARL15
0.456362	0.182016	SG2	ANO3	NC_056069.1	25395001	25415001	0.302443	0.278197	SG1	ARL15
0.366727	0.161374	SG2	AOAH	NC_056069.1	25400001	25420001	0.370726	0.228426	SG1	ARL15
0.328349	0.180625	SG2	AOAH	NC_056069.1	25405001	25425001	0.324966	0.174266	SG1	ARL15
0.258141	0.221134	SG2	AOAH	NC_056061.1	76275001	76295001	0.314424	0.214298	SG1	ARMT1;RMNI
0.236437	0.267974	SG2	AOAH	NC_056071.1	24060001	24080001	0.485062	0.190471	SG1	ARNT2
0.21471	0.32489	SG2	AOAH	NC_056071.1	24180001	24200001	0.371415	0.200509	SG1	ARNT2
0.226621	0.36252	SG2	AOAH	NC_056071.1	24185001	24205001	0.289925	0.200528	SG1	ARNT2
0.378786	0.352095	SG2	AOAH	NC_056071.1	24190001	24210001	0.328602	0.216391	SG1	ARNT2
0.479522	0.31473	SG2	AOAH	NC_056071.1	24195001	24215001	0.285627	0.193355	SG1	ARNT2
0.467649	0.265197	SG2	AOAH	NC_056061.1	19395001	19415001	0.33765	0.244654	SG1	ASF1A;MCM9
0.448949	0.244033	SG2	AOAH	NC_056061.1	19400001	19420001	0.365613	0.234617	SG1	ASF1A;MCM9
0.450403	0.244307	SG2	AOAH	NC_056061.1	19405001	19425001	0.291649	0.256439	SG1	ASF1A;MCM9
0.515104	0.236807	SG2	AP2A2;MUC6	NC_056077.1	26865001	26885001	0.387167	0.192272	SG1	ASPHD1;KCTI
0.521054	0.236449	SG2	AP2A2;MUC6	NC_056077.1	26870001	26890001	0.329777	0.217354	SG1	ASPHD1;KCTI
0.522291	0.227235	SG2	AP2A2;MUC6	NC_056054.1	259585001	259605001	0.421503	0.18296	SG1	ASTE1;ATP2C
0.545806	0.194761	SG2	AP3B2	NC_056054.1	259590001	259610001	0.417839	0.202789	SG1	ASTE1;ATP2C
0.55286	0.234647	SG2	AP3B2	NC_056054.1	259595001	259615001	0.372104	0.235222	SG1	ASTE1;ATP2C
0.557159	0.299721	SG2	AP3B2	NC_056068.1	17520001	17540001	0.482442	0.17966	SG1	ATM
0.468139	0.136324	SG2	AP4S1;STRN3	NC_056068.1	17630001	17650001	0.271063	0.2119	SG1	ATM
0.5391	0.140504	SG2	AP4S1;STRN3	NC_056068.1	17635001	17655001	0.193715	0.190888	SG1	ATM
0.254614	0.172899	SG2	APPL2	NC_056068.1	17640001	17660001	0.230539	0.189976	SG1	ATM
0.164387	0.150558	SG2	APPL2	NC_056068.1	17645001	17665001	0.306569	0.180226	SG1	ATM

0.572829	0.154541	SG2	AQP1	NC_056054.1	259600001	259620001	0.447576	0.222679	SG1	ATP2C1
0.438446	0.187908	SG2	ARHGAP20	NC_056056.1	221975001	221995001	0.0740047	0.310278	SG1	ATXN10
0.451871	0.19769	SG2	ARHGAP20	NC_056056.1	221980001	222000001	0.183627	0.294061	SG1	ATXN10
0.534031	0.150487	SG2	ARHGAP22	NC_056056.1	221985001	222005001	0.188921	0.289251	SG1	ATXN10
0.49782	0.18247	SG2	ARHGAP22	NC_056056.1	221990001	222010001	0.271228	0.256489	SG1	ATXN10
0.555491	0.198455	SG2	ARHGAP22	NC_056056.1	221995001	222015001	0.250707	0.267564	SG1	ATXN10
0.28374	0.255516	SG2	ARHGAP22	NC_056056.1	222000001	222020001	0.197576	0.282487	SG1	ATXN10
0.23265	0.281303	SG2	ARHGAP22	NC_056056.1	222005001	222025001	0.329209	0.235153	SG1	ATXN10
0.353235	0.194152	SG2	ARHGAP22	NC_056056.1	222010001	222030001	0.381481	0.213175	SG1	ATXN10
0.370835	0.174008	SG2	ARHGAP22	NC_056057.1	48215001	48235001	0.483272	0.310172	SG1	ATXN7L1
0.337442	0.177026	SG2	ARHGAP24	NC_056057.1	48220001	48240001	0.470587	0.316853	SG1	ATXN7L1
0.338547	0.22095	SG2	ARHGAP24	NC_056057.1	48225001	48245001	0.300354	0.367153	SG1	ATXN7L1
0.42422	0.196148	SG2	ARHGAP24	NC_056057.1	48325001	48345001	0.263374	0.224644	SG1	ATXN7L1
0.406628	0.205694	SG2	ARHGAP24	NC_056057.1	48330001	48350001	0.249613	0.252381	SG1	ATXN7L1
0.164005	0.140933	SG2	ARHGAP29	NC_056057.1	48335001	48355001	0.335771	0.224725	SG1	ATXN7L1
0.142439	0.164977	SG2	ARHGAP29	NC_056077.1	30360001	30380001	0.28894	0.25201	SG1	AUTS2
0.173317	0.156192	SG2	ARHGAP29	NC_056077.1	30365001	30385001	0.321646	0.21905	SG1	AUTS2
0.32309	0.135283	SG2	ARHGEF11	NC_056077.1	30370001	30390001	0.345865	0.198826	SG1	AUTS2
0.408418	0.145103	SG2	ARHGEF4	NC_056054.1	227055001	227075001	0.413161	0.177966	SG1	B3GALNT1
0.36256	0.156845	SG2	ARHGEF4	NC_056055.1	141690001	141710001	0.487401	0.208577	SG1	B3GALT1
0.338899	0.17345	SG2	ARHGEF4	NC_056055.1	141695001	141715001	0.477755	0.207937	SG1	B3GALT1
0.389299	0.148667	SG2	ARHGEF4	NC_056063.1	29920001	29940001	0.401982	0.186862	SG1	B3GLCT
0.212872	0.146871	SG2	ARL15	NC_056063.1	29925001	29945001	0.339481	0.182841	SG1	B3GLCT
0.188163	0.147822	SG2	ARL15	NC_056067.1	50110001	50130001	0.302924	0.295916	SG1	B3GNT8;BCKI
0.474609	0.162161	SG2	ASAP1	NC_056067.1	50115001	50135001	0.133031	0.341011	SG1	B3GNT8;DMA
0.394626	0.139208	SG2	ASMTL;SLC25A6	NC_056066.1	77825001	77845001	0.187172	0.24432	SG1	B4GALT5
0.364979	0.136942	SG2	ATN1;C3H12orf57;	NC_056066.1	77830001	77850001	0.245752	0.329958	SG1	B4GALT5
0.414254	0.176867	SG2	ATN1;C3H12orf57;	NC_056066.1	77835001	77855001	0.191837	0.351048	SG1	B4GALT5
0.427126	0.179623	SG2	ATN1;C3H12orf57;	NC_056068.1	27710001	27730001	0.435203	0.223772	SG1	BACE1
0.459366	0.174059	SG2	ATN1;ENO2	NC_056068.1	27715001	27735001	0.430569	0.252519	SG1	BACE1;CEP16
0.466778	0.187816	SG2	ATN1;ENO2;LRRC	NC_056068.1	27725001	27745001	0.360193	0.287019	SG1	BACE1;CEP16
0.552974	0.141202	SG2	ATN1;ENO2;LRRC	NC_056068.1	27685001	27705001	0.178882	0.337931	SG1	BACE1;RNF21

0.411819	0.137315	SG2	ATP10B	NC_056068.1	27690001	27710001	0.185471	0.325174	SG1	BACE1;RNF21
0.151253	0.164828	SG2	ATP12A	NC_056068.1	27695001	27715001	0.356031	0.233291	SG1	BACE1;RNF21
0.277125	0.13654	SG2	ATP12A	NC_056068.1	27700001	27720001	0.308702	0.263409	SG1	BACE1;RNF21
0.16034	0.148384	SG2	ATP12A;RNF17	NC_056068.1	27705001	27725001	0.409148	0.201517	SG1	BACE1;RNF21
0.135776	0.160794	SG2	ATP12A;RNF17	NC_056074.1	39025001	39045001	0.249008	0.235671	SG1	BAD;GPR137;I
0.469158	0.173155	SG2	ATP2B1	NC_056074.1	39030001	39050001	0.264901	0.29821	SG1	BAD;GPR137;I
0.477655	0.190849	SG2	ATP2B1	NC_056074.1	39020001	39040001	0.278769	0.204087	SG1	BAD;GPR137;I
0.559444	0.141153	SG2	ATP2B1	NC_056074.1	39015001	39035001	0.331988	0.181111	SG1	BAD;PLCB3
0.393638	0.140368	SG2	ATP5PO	NC_056056.1	215675001	215695001	0.299443	0.230287	SG1	BAIAP2L2;PIC
0.43261	0.397746	SG2	ATP6V1C2	NC_056056.1	215680001	215700001	0.424395	0.193583	SG1	BAIAP2L2;PIC
0.438064	0.41508	SG2	ATP6V1C2	NC_056075.1	30965001	30985001	0.195718	0.311672	SG1	BBIP1;PDCD4
0.426087	0.446581	SG2	ATP6V1C2	NC_056075.1	30970001	30990001	0.245954	0.313322	SG1	BBIP1;PDCD4
0.555976	0.334279	SG2	ATP6V1C2;PDIA6	NC_056075.1	30975001	30995001	0.253432	0.279756	SG1	BBIP1;PDCD4
0.243136	0.147131	SG2	ATP9A	NC_056075.1	30980001	31000001	0.408163	0.276391	SG1	BBIP1;PDCD4;
0.503446	0.13535	SG2	ATP9A	NC_056077.1	27720001	27740001	0.208868	0.329573	SG1	BCKDK;KAT8
0.317716	0.137387	SG2	ATRNL1	NC_056077.1	27725001	27745001	0.302574	0.313299	SG1	BCKDK;KAT8
0.487872	0.141737	SG2	AUTS2	NC_056077.1	27730001	27750001	0.427819	0.319225	SG1	BCKDK;KAT8
0.401143	0.158937	SG2	AUTS2	NC_056077.1	27715001	27735001	0.204784	0.316758	SG1	BCKDK;PRSS!
0.362869	0.158343	SG2	AUTS2	NC_056077.1	27710001	27730001	0.314748	0.290121	SG1	BCKDK;PRSS!
0.527912	0.14861	SG2	AUTS2	NC_056056.1	105290001	105310001	0.408395	0.190772	SG1	BCL2L11
0.467463	0.154129	SG2	AUTS2	NC_056056.1	105295001	105315001	0.405928	0.187359	SG1	BCL2L11
0.481518	0.202332	SG2	AUTS2	NC_056056.1	105300001	105320001	0.422622	0.174142	SG1	BCL2L11
0.534437	0.230184	SG2	AUTS2	NC_056056.1	214940001	214960001	0.46542	0.228947	SG1	BCL2L13
0.543018	0.242406	SG2	AUTS2	NC_056056.1	214945001	214965001	0.37836	0.255728	SG1	BCL2L13
0.322351	0.135364	SG2	AUTS2	NC_056056.1	214950001	214970001	0.370541	0.256127	SG1	BCL2L13;BID
0.276467	0.192913	SG2	B3GAT2	NC_056056.1	214955001	214975001	0.442652	0.237793	SG1	BCL2L13;BID
0.378769	0.176256	SG2	B3GAT2	NC_056056.1	214965001	214985001	0.473977	0.191253	SG1	BCL2L13;BID
0.465086	0.180529	SG2	B3GAT2	NC_056080.1	128930001	128950001	0.428505	0.252445	SG1	BEX3
0.479196	0.186084	SG2	B3GAT2	NC_056070.1	62575001	62595001	0.303797	0.187325	SG1	BICDL1
0.39128	0.162311	SG2	B3GNT4;DIABLO	NC_056070.1	62580001	62600001	0.295134	0.212047	SG1	BICDL1
0.234593	0.16011	SG2	B3GNT4;DIABLO;I	NC_056070.1	62585001	62605001	0.268935	0.216884	SG1	BICDL1
0.47339	0.14652	SG2	B3GNT9;FBXL8;H!	NC_056070.1	62590001	62610001	0.29133	0.213484	SG1	BICDL1

0.465695	0.147962	SG2	BACH1	NC_056070.1	62595001	62615001	0.257643	0.221037	SG1	BICDL1;RAB3
0.551849	0.150988	SG2	BACH1	NC_056070.1	62600001	62620001	0.224798	0.201212	SG1	BICDL1;RAB3
0.522133	0.144907	SG2	BACH1	NC_056070.1	62605001	62625001	0.241949	0.208981	SG1	BICDL1;RAB3
0.120235	0.185879	SG2	BAIAP2L2;PLA2G6	NC_056070.1	62610001	62630001	0.260833	0.207535	SG1	BICDL1;RAB3
0.102265	0.186866	SG2	BAIAP2L2;PLA2G6	NC_056056.1	214970001	214990001	0.429335	0.18613	SG1	BID
0.139658	0.289683	SG2	BBOF1;ENTPD5	NC_056056.1	214975001	214995001	0.195538	0.223634	SG1	BID;MICAL3
0.256801	0.247952	SG2	BBOF1;ENTPD5	NC_056056.1	214985001	215005001	0.210661	0.222297	SG1	BID;MICAL3
0.299814	0.222212	SG2	BBOF1;ENTPD5	NC_056071.1	21215001	21235001	0.418246	0.281472	SG1	BLM
0.407628	0.170181	SG2	BBOF1;ENTPD5	NC_056075.1	17400001	17420001	0.416443	0.186633	SG1	BLNK
0.520055	0.352773	SG2	BBS9	NC_056073.1	4785001	4805001	0.455993	0.221692	SG1	BMP5
0.390834	0.187414	SG2	BBS9	NC_056073.1	4790001	4810001	0.469102	0.204459	SG1	BMP5
0.385192	0.226026	SG2	BBS9	NC_056073.1	4795001	4815001	0.417997	0.194128	SG1	BMP5
0.552337	0.147303	SG2	BBS9	NC_056063.1	48090001	48110001	0.361447	0.349853	SG1	BORA
0.569819	0.326116	SG2	BCAS3	NC_056063.1	48095001	48115001	0.176535	0.45117	SG1	BORA
0.545576	0.365404	SG2	BCAS3	NC_056063.1	48100001	48120001	0.0943069	0.50737	SG1	BORA;DIS3
0.550165	0.343679	SG2	BCAS3	NC_056066.1	62735001	62755001	0.359355	0.235631	SG1	BPIFA1;BPIFB
0.206504	0.24526	SG2	BECN2	NC_056066.1	62740001	62760001	0.371251	0.223149	SG1	BPIFA1;BPIFB
0.469083	0.183183	SG2	BECN2	NC_056066.1	62745001	62765001	0.272454	0.231132	SG1	BPIFB1
0.454482	0.14658	SG2	BEND7	NC_056066.1	62750001	62770001	0.220206	0.185496	SG1	BPIFB1
0.440166	0.140451	SG2	BIVM;ERCC5	NC_056066.1	62755001	62775001	0.263367	0.194277	SG1	BPIFB1
0.491877	0.333782	SG2	BLOC1S5	NC_056066.1	62760001	62780001	0.342961	0.17898	SG1	BPIFB1
0.395348	0.193963	SG2	BLOC1S5	NC_056058.1	7615001	7635001	0.377193	0.198628	SG1	BRD4
0.497493	0.180602	SG2	BLVRA	NC_056054.1	69230001	69250001	0.345956	0.174091	SG1	BRDT
0.47747	0.182859	SG2	BLVRA	NC_056054.1	69235001	69255001	0.290464	0.201724	SG1	BRDT
0.550412	0.147173	SG2	BMERB1	NC_056054.1	69240001	69260001	0.242535	0.213534	SG1	BRDT
0.213367	0.245196	SG2	BMERB1	NC_056055.1	4310001	4330001	0.382867	0.178652	SG1	BRINP1
0.196055	0.249415	SG2	BMERB1	NW_024599827.	1610001	1630001	0.481352	0.18724	SG1	BTBD1
0.525919	0.146798	SG2	BMERB1	NC_056068.1	39005001	39025001	0.381497	0.220633	SG1	BTBD10
0.332728	0.206862	SG2	BMP6	NC_056068.1	39010001	39030001	0.308383	0.240775	SG1	BTBD10
0.31491	0.235365	SG2	BMP6	NC_056068.1	39015001	39035001	0.216862	0.256711	SG1	BTBD10
0.488084	0.236096	SG2	BMP6	NC_056068.1	39020001	39040001	0.173643	0.26288	SG1	BTBD10
0.475794	0.149057	SG2	BMPER	NC_056068.1	39025001	39045001	0.245464	0.239293	SG1	BTBD10

0.555983	0.136904	SG2	BMPR1A	NC_056068.1	39030001	39050001	0.28777	0.210952	SG1	BTBD10
0.437583	0.262564	SG2	BRCA2	NC_056075.1	41625001	41645001	0.421864	0.279058	SG1	BTBD16;TACC
0.337954	0.276051	SG2	BRCA2	NC_056075.1	41630001	41650001	0.435205	0.280762	SG1	BTBD16;TACC
0.323273	0.284958	SG2	BRCA2	NC_056067.1	17355001	17375001	0.42047	0.237686	SG1	C14H16orf78
0.442222	0.230397	SG2	BRCA2;ZAR1L	NC_056067.1	17360001	17380001	0.27012	0.214557	SG1	C14H16orf78
0.442953	0.210926	SG2	BRCA2;ZAR1L	NC_056067.1	17375001	17395001	0.482093	0.182568	SG1	C14H16orf78
0.379254	0.221261	SG2	BRCA2;ZAR1L	NC_056069.1	42105001	42125001	0.478095	0.310872	SG1	C16H5orf22
0.372436	0.204604	SG2	BRCA2;ZAR1L	NC_056069.1	42110001	42130001	0.438447	0.327703	SG1	C16H5orf22
0.540839	0.295703	SG2	BTBD1	NC_056069.1	42115001	42135001	0.427574	0.332932	SG1	C16H5orf22;DI
0.423077	0.378922	SG2	BTBD1	NC_056069.1	42120001	42140001	0.438215	0.298052	SG1	C16H5orf22;DI
0.1	0.143316	SG2	BTD	NC_056069.1	42125001	42145001	0.443505	0.269867	SG1	C16H5orf22;DI
0.428294	0.135861	SG2	C14H19orf54;COQ8	NC_056069.1	42130001	42150001	0.347271	0.22302	SG1	C16H5orf22;DI
0.449506	0.161128	SG2	C14H19orf54;ITPK(	NC_056072.1	37745001	37765001	0.12616	0.194033	SG1	C19H3orf49;TI
0.450592	0.14205	SG2	C14H19orf54;ITPK(	NC_056054.1	25565001	25585001	0.0960489	0.398142	SG1	C1H1orf185
0.432396	0.141681	SG2	C15H11orf65	NC_056054.1	25570001	25590001	0.154162	0.335014	SG1	C1H1orf185
0.371212	0.160009	SG2	C17H4orf33;SCLT1	NC_056054.1	25575001	25595001	0.245599	0.251073	SG1	C1H1orf185
0.320043	0.251956	SG2	C17H4orf46;ETFDI	NC_056068.1	52150001	52170001	0.167263	0.22778	SG1	C2CD3;UCP3
0.37969	0.20808	SG2	C17H4orf46;ETFDI	NC_056068.1	52155001	52175001	0.248237	0.198255	SG1	C2CD3;UCP3
0.067742	0.154021	SG2	C18H14orf132	NC_056055.1	10275001	10295001	0.344766	0.174482	SG1	C2H9orf43;RG
0.055585	0.154483	SG2	C18H14orf132	NC_056072.1	47255001	47275001	0.444149	0.245734	SG1	CACNA1D;CH
0.082292	0.148513	SG2	C18H14orf132	NC_056072.1	46440001	46460001	0.258879	0.204764	SG1	CACNA2D3
0.483871	0.245025	SG2	C1H1orf226	NC_056072.1	46445001	46465001	0.142314	0.190253	SG1	CACNA2D3
0.382418	0.26188	SG2	C1H1orf226	NC_056072.1	46450001	46470001	0.207869	0.182293	SG1	CACNA2D3
0.413759	0.247677	SG2	C1H1orf226	NC_056072.1	46455001	46475001	0.248282	0.191375	SG1	CACNA2D3
0.431349	0.246816	SG2	C1H1orf226	NC_056072.1	46460001	46480001	0.229166	0.187729	SG1	CACNA2D3
0.116228	0.274603	SG2	C2	NC_056072.1	46465001	46485001	0.262582	0.22838	SG1	CACNA2D3
0.17341	0.257086	SG2	C2	NC_056072.1	46470001	46490001	0.154545	0.205181	SG1	CACNA2D3
0.227273	0.22339	SG2	C2	NC_056054.1	154970001	154990001	0.429765	0.197096	SG1	CADM2
0.133634	0.270249	SG2	C2;CFB	NC_056054.1	154985001	155005001	0.418781	0.278675	SG1	CADM2
0.248202	0.145833	SG2	C2;CFB;LOC10111	NC_056054.1	154995001	155015001	0.481347	0.277062	SG1	CADM2
0.15423	0.220952	SG2	C2;CFB;NELFE	NC_056066.1	16465001	16485001	0.388002	0.265653	SG1	CAMK1D
0.153676	0.22201	SG2	C22H10orf120	NC_056066.1	16470001	16490001	0.249308	0.330769	SG1	CAMK1D

0.202737	0.251356	SG2	C22H10orf120	NC_056066.1	16475001	16495001	0.10971	0.384026	SG1	CAMK1D
0.129505	0.192065	SG2	C22H10orf120;LOC	NC_056066.1	16480001	16500001	0.186908	0.332592	SG1	CAMK1D
0.104273	0.190046	SG2	C22H10orf120;LOC	NC_056066.1	16485001	16505001	0.254267	0.308357	SG1	CAMK1D
0.529867	0.171	SG2	C24H16orf96	NC_056066.1	16490001	16510001	0.3808	0.21447	SG1	CAMK1D
0.538718	0.221243	SG2	C24H16orf96	NC_056066.1	16585001	16605001	0.480302	0.206079	SG1	CAMK1D
0.464125	0.164243	SG2	C25H1orf198	NC_056066.1	16590001	16610001	0.39906	0.206779	SG1	CAMK1D
0.514479	0.151619	SG2	C3H2orf81;LOC101	NC_056066.1	16595001	16615001	0.324633	0.208515	SG1	CAMK1D
0.483186	0.187327	SG2	C3H2orf81;LOC101	NC_056066.1	16605001	16625001	0.39275	0.179032	SG1	CAMK1D
0.358351	0.213643	SG2	C3H2orf81;LOC101	NC_056070.1	53915001	53935001	0.383228	0.178643	SG1	CAMKK2
0.285288	0.174652	SG2	C3H2orf81;RTKN;\	NC_056070.1	53920001	53940001	0.313344	0.190868	SG1	CAMKK2
0.540199	0.389063	SG2	CABLES1	NC_056070.1	53925001	53945001	0.295827	0.227483	SG1	CAMKK2
0.515842	0.174209	SG2	CABLES2;RBBP8N	NC_056070.1	53930001	53950001	0.198581	0.242949	SG1	CAMKK2
0.419435	0.194524	SG2	CABYR;OSBPL1A	NC_056070.1	53935001	53955001	0.159817	0.247021	SG1	CAMKK2
0.407725	0.203373	SG2	CABYR;OSBPL1A	NC_056070.1	53940001	53960001	0.142534	0.256159	SG1	CAMKK2
0.551173	0.180307	SG2	CABYR;OSBPL1A	NC_056070.1	53950001	53970001	0.117925	0.260237	SG1	CAMKK2;P2R
0.558023	0.185755	SG2	CACNA1I	NC_056056.1	3490001	3510001	0.0631091	0.224874	SG1	CAMSAP1
0.394845	0.20203	SG2	CACNA1I	NC_056064.1	26280001	26300001	0.307228	0.176878	SG1	CAMTA2;INC
0.254194	0.253001	SG2	CACNA1I	NC_056064.1	26285001	26305001	0.311609	0.173705	SG1	CAMTA2;INC
0.322564	0.216416	SG2	CACNA1I	NC_056064.1	26290001	26310001	0.32998	0.197123	SG1	CAMTA2;INC
0.190445	0.172639	SG2	CACNA2D1	NC_056064.1	26295001	26315001	0.356569	0.179634	SG1	CAMTA2;INC
0.161062	0.177379	SG2	CACNA2D1	NC_056069.1	38390001	38410001	0.476398	0.195582	SG1	CAPSL
0.22019	0.16033	SG2	CACNA2D1	NC_056069.1	38395001	38415001	0.420668	0.213792	SG1	CAPSL
0.300648	0.136106	SG2	CACNA2D1	NC_056069.1	38400001	38420001	0.427113	0.178871	SG1	CAPSL
0.504133	0.2213	SG2	CACNA2D2;LOC1(	NC_056069.1	38405001	38425001	0.479314	0.175064	SG1	CAPSL
0.571429	0.209381	SG2	CACNA2D2;LOC1(	NC_056056.1	181525001	181545001	0.403056	0.225232	SG1	CARD10
0.525105	0.232252	SG2	CACNA2D2;TMEM	NC_056056.1	181530001	181550001	0.407744	0.214143	SG1	CARD10
0.554129	0.151871	SG2	CACNB2	NC_056056.1	181510001	181530001	0.211501	0.278791	SG1	CARD10;MFN
0.519212	0.15907	SG2	CACNB2	NC_056056.1	181515001	181535001	0.385675	0.214819	SG1	CARD10;MFN
0.405964	0.200194	SG2	CACNB2	NC_056056.1	181520001	181540001	0.36178	0.251581	SG1	CARD10;MFN
0.547979	0.158951	SG2	CACNB2	NC_056058.1	93680001	93700001	0.423353	0.17812	SG1	CAST
0.537926	0.19337	SG2	CACNB2	NC_056058.1	16425001	16445001	0.315432	0.213785	SG1	CATSPERD;L(
0.51749	0.211732	SG2	CACNB2	NC_056054.1	171425001	171445001	0.110741	0.179826	SG1	CBLB

0.228571	0.243051	SG2	CACNB2	NC_056056.1	216400001	216420001	0.0521996	0.220977	SG1	CBX7
0.1254	0.255517	SG2	CACNB2	NC_056054.1	26750001	26770001	0.350427	0.177981	SG1	CC2D1B;ORC[illegible]
0.132294	0.239159	SG2	CACNB2	NC_056055.1	131030001	131050001	0.351492	0.227383	SG1	CCDC141
0.253968	0.194304	SG2	CACNB2	NC_056055.1	131035001	131055001	0.211059	0.272765	SG1	CCDC141
0.543513	0.137237	SG2	CACNB2	NC_056055.1	131040001	131060001	0.119753	0.35955	SG1	CCDC141
0.340143	0.144122	SG2	CALD1	NC_056055.1	131045001	131065001	0.159676	0.348622	SG1	CCDC141
0.103704	0.189013	SG2	CALD1	NC_056055.1	131050001	131070001	0.242717	0.287568	SG1	CCDC141
0.111624	0.184909	SG2	CALD1	NC_056055.1	131055001	131075001	0.305818	0.252945	SG1	CCDC141
0.102016	0.205911	SG2	CALD1	NC_056055.1	131060001	131080001	0.325194	0.228564	SG1	CCDC141
0.42397	0.140796	SG2	CAMK2B	NC_056055.1	131065001	131085001	0.36462	0.20001	SG1	CCDC141
0.258837	0.194101	SG2	CAMK2B	NC_056055.1	131090001	131110001	0.446984	0.189311	SG1	CCDC141
0.178698	0.196765	SG2	CAMK2B	NC_056055.1	131095001	131115001	0.255626	0.190523	SG1	CCDC141
0.165315	0.167589	SG2	CAMK2B	NC_056056.1	67915001	67935001	0.33471	0.180956	SG1	CCDC85A
0.220184	0.17062	SG2	CAMK2B;YKT6	NC_056056.1	67920001	67940001	0.141386	0.245048	SG1	CCDC85A
0.222311	0.177329	SG2	CAMK2B;YKT6	NC_056056.1	67925001	67945001	0.087149	0.223987	SG1	CCDC85A
0.180317	0.165062	SG2	CAMK2B;YKT6	NC_056064.1	15485001	15505001	0.376419	0.278703	SG1	CCL1
0.207156	0.135597	SG2	CAMK2D	NC_056064.1	15490001	15510001	0.412901	0.365333	SG1	CCL1
0.253888	0.138775	SG2	CAMK2D	NC_056064.1	15495001	15515001	0.47983	0.385345	SG1	CCL1
0.461949	0.144305	SG2	CAMK2G	NC_056064.1	15500001	15520001	0.446999	0.34139	SG1	CCL1
0.191604	0.229032	SG2	CAMK2G	NC_056059.1	34155001	34175001	0.433119	0.190001	SG1	CCSER1
0.441035	0.143701	SG2	CAMTA1	NC_056059.1	34445001	34465001	0.379533	0.301231	SG1	CCSER1
0.312132	0.188881	SG2	CAMTA1	NC_056059.1	34450001	34470001	0.257522	0.378603	SG1	CCSER1
0.237867	0.20234	SG2	CAMTA1	NC_056059.1	34455001	34475001	0.210993	0.394571	SG1	CCSER1
0.192262	0.196056	SG2	CAMTA1	NC_056059.1	34460001	34480001	0.209754	0.335201	SG1	CCSER1
0.174271	0.179186	SG2	CAMTA1	NC_056059.1	34465001	34485001	0.381761	0.377312	SG1	CCSER1
0.171617	0.143172	SG2	CAMTA1	NC_056059.1	34470001	34490001	0.441888	0.363078	SG1	CCSER1
0.513006	0.229424	SG2	CAPN2;TP53BP2	NC_056059.1	34475001	34495001	0.478259	0.309123	SG1	CCSER1
0.472465	0.252185	SG2	CAPN2;TP53BP2	NC_056059.1	34670001	34690001	0.332328	0.207818	SG1	CCSER1
0.39313	0.137525	SG2	CBFB;PHAF1	NC_056059.1	34675001	34695001	0.260282	0.245811	SG1	CCSER1
0.142553	0.154527	SG2	CBLB	NC_056059.1	34680001	34700001	0.155372	0.281027	SG1	CCSER1
0.143625	0.137474	SG2	CBLB	NC_056059.1	34685001	34705001	0.15916	0.290046	SG1	CCSER1
0.077634	0.149849	SG2	CBLB	NC_056059.1	34690001	34710001	0.139043	0.297798	SG1	CCSER1

0.077691	0.142991	SG2	CBLB	NC_056059.1	34695001	34715001	0.139629	0.284565	SG1	CCSER1
0.119579	0.140336	SG2	CBLB	NC_056059.1	34700001	34720001	0.155642	0.270395	SG1	CCSER1
0.507445	0.173038	SG2	CCDC186	NC_056059.1	34705001	34725001	0.135401	0.279562	SG1	CCSER1
0.405927	0.224475	SG2	CCDC186	NC_056059.1	34710001	34730001	0.161791	0.288122	SG1	CCSER1
0.16472	0.144793	SG2	CCDC6	NC_056059.1	34715001	34735001	0.194876	0.300987	SG1	CCSER1
0.251542	0.155635	SG2	CCDC60	NC_056059.1	34720001	34740001	0.345209	0.296055	SG1	CCSER1
0.555556	0.206126	SG2	CCM2	NC_056054.1	178020001	178040001	0.403668	0.183069	SG1	CD200
0.363917	0.222128	SG2	CCM2	NC_056054.1	178025001	178045001	0.463074	0.1828	SG1	CD200
0.303013	0.236669	SG2	CCM2	NC_056054.1	178050001	178070001	0.399678	0.217999	SG1	CD200
0.345673	0.214427	SG2	CCSER1	NC_056054.1	178055001	178075001	0.356224	0.214161	SG1	CD200
0.400018	0.187423	SG2	CCSER1	NC_056054.1	178060001	178080001	0.418182	0.20195	SG1	CD200
0.421792	0.145267	SG2	CD8B	NC_056054.1	178065001	178085001	0.436438	0.244539	SG1	CD200
0.419004	0.167187	SG2	CD8B	NC_056054.1	178070001	178090001	0.447586	0.245075	SG1	CD200
0.40433	0.193006	SG2	CD8B	NC_056054.1	178075001	178095001	0.46799	0.221254	SG1	CD200
0.547824	0.137899	SG2	CDC42SE2	NC_056054.1	112070001	112090001	0.234326	0.189731	SG1	CD244
0.54058	0.163883	SG2	CDC42SE2	NC_056054.1	112075001	112095001	0.140789	0.207439	SG1	CD244
0.470117	0.198986	SG2	CDH4	NC_056054.1	119690001	119710001	0.399103	0.212098	SG1	CD247
0.409728	0.154408	SG2	CDH4	NC_056054.1	119695001	119715001	0.335502	0.203105	SG1	CD247
0.301966	0.177134	SG2	CDH4	NC_056054.1	119620001	119640001	0.37277	0.28965	SG1	CD247;POU2F
0.383591	0.153389	SG2	CDH4	NC_056056.1	205850001	205870001	0.274827	0.222857	SG1	CD69
0.415202	0.135076	SG2	CDH4	NC_056056.1	205855001	205875001	0.140114	0.260997	SG1	CD69
0.462206	0.186589	SG2	CDH4	NC_056056.1	205860001	205880001	0.162082	0.218269	SG1	CD69;LOC101
0.554513	0.222535	SG2	CDH4	NC_056065.1	29300001	29320001	0.450501	0.19938	SG1	CDC42BPA
0.472961	0.273967	SG2	CDH4	NC_056071.1	65330001	65350001	0.164557	0.177283	SG1	CDC42BPB
0.566666	0.198762	SG2	CDH6	NC_056074.1	39970001	39990001	0.473143	0.312232	SG1	CDC42EP2;DP
0.520723	0.145125	SG2	CDH8	NC_056058.1	20835001	20855001	0.477306	0.287328	SG1	CDC42SE2
0.452527	0.145298	SG2	CDH8	NC_056058.1	20840001	20860001	0.45681	0.289039	SG1	CDC42SE2
0.482657	0.137382	SG2	CDH8	NC_056058.1	20845001	20865001	0.454193	0.2674	SG1	CDC42SE2
0.545383	0.236712	SG2	CDH9	NC_056058.1	20850001	20870001	0.468549	0.286943	SG1	CDC42SE2
0.555757	0.196112	SG2	CDHR2	NC_056058.1	20855001	20875001	0.439491	0.255712	SG1	CDC42SE2
0.530654	0.177347	SG2	CDHR2;RNF44	NC_056058.1	20860001	20880001	0.39001	0.204343	SG1	CDC42SE2
0.459765	0.168158	SG2	CDK19	NC_056058.1	20830001	20850001	0.482166	0.284311	SG1	CDC42SE2;RA

0.359203	0.214321	SG2	CDK19	NC_056076.1	60310001	60330001	0.354212	0.178007	SG1	CDH20
0.322293	0.269242	SG2	CDK19	NC_056076.1	60320001	60340001	0.353359	0.184697	SG1	CDH20
0.437583	0.140912	SG2	CDKAL1	NC_056076.1	60410001	60430001	0.269011	0.173096	SG1	CDH20
0.413259	0.16119	SG2	CDKAL1	NC_056067.1	29220001	29240001	0.461235	0.260907	SG1	CDH8
0.362613	0.142056	SG2	CDKAL1	NC_056067.1	29225001	29245001	0.225905	0.269376	SG1	CDH8
0.525806	0.17237	SG2	CDKN1B	NC_056067.1	29230001	29250001	0.206065	0.198732	SG1	CDH8
0.507788	0.321429	SG2	CDKN1B	NC_056057.1	9090001	9110001	0.38691	0.246245	SG1	CDK14
0.381338	0.170685	SG2	CEBPG;PEPD	NC_056057.1	9095001	9115001	0.358659	0.25536	SG1	CDK14
0.406981	0.163349	SG2	CEBPG;PEPD	NC_056057.1	9100001	9120001	0.415958	0.206124	SG1	CDK14
0.437198	0.15381	SG2	CELF1	NC_056057.1	9115001	9135001	0.438343	0.199578	SG1	CDK14
0.456735	0.150745	SG2	CENPA;SLC35F6	NC_056057.1	9120001	9140001	0.39384	0.208792	SG1	CDK14
0.44834	0.161855	SG2	CENPJ;PARP4	NC_056057.1	9125001	9145001	0.321	0.229794	SG1	CDK14
0.488105	0.140873	SG2	CENPJ;PARP4	NC_056057.1	9130001	9150001	0.291346	0.241836	SG1	CDK14
0.178873	0.142844	SG2	CEP112	NC_056057.1	9135001	9155001	0.259372	0.235613	SG1	CDK14
0.48102	0.146581	SG2	CEP128	NC_056057.1	9140001	9160001	0.4015	0.209049	SG1	CDK14
0.517205	0.15883	SG2	CEP128	NC_056066.1	62880001	62900001	0.201439	0.19252	SG1	CDK5RAP1;SN
0.540527	0.140144	SG2	CEP350	NC_056066.1	62885001	62905001	0.203997	0.201347	SG1	CDK5RAP1;SN
0.446318	0.171262	SG2	CEP63	NC_056077.1	20580001	20600001	0.479549	0.181313	SG1	CDR2;LOC105
0.500369	0.188537	SG2	CEP63	NC_056077.1	20540001	20560001	0.0604118	0.248672	SG1	CDR2;POLR3E
0.568262	0.191513	SG2	CEP63	NC_056077.1	20545001	20565001	0.138124	0.265723	SG1	CDR2;POLR3E
0.259109	0.186434	SG2	CEP89	NC_056077.1	20550001	20570001	0.221732	0.185237	SG1	CDR2;POLR3E
0.295384	0.189326	SG2	CEP89	NC_056054.1	256480001	256500001	0.449585	0.175839	SG1	CDV3;LOC121
0.378703	0.164006	SG2	CEP89	NC_056063.1	32350001	32370001	0.272415	0.211348	SG1	CDX2;URAD
0.272678	0.210184	SG2	CEP89	NC_056080.1	65760001	65780001	0.424242	0.225862	SG1	CDX4
0.276437	0.210476	SG2	CEP89	NC_056056.1	222695001	222715001	0.371192	0.181144	SG1	CELSR1
0.373284	0.169477	SG2	CEP89	NC_056056.1	222700001	222720001	0.326826	0.211601	SG1	CELSR1;LOC1
0.406878	0.142434	SG2	CEP89;FAAP24	NC_056056.1	218755001	218775001	0.405351	0.179897	SG1	CENPM;SHISA
0.512385	0.210503	SG2	CEP95	NC_056060.1	59530001	59550001	0.129132	0.357862	SG1	CEP152
0.48902	0.215027	SG2	CEP95	NC_056060.1	59520001	59540001	0.264257	0.326701	SG1	CEP152;SHC4
0.470038	0.225154	SG2	CEP95	NC_056060.1	59525001	59545001	0.180715	0.367242	SG1	CEP152;SHC4
0.477011	0.219047	SG2	CEP95	NC_056068.1	27730001	27750001	0.333333	0.309575	SG1	CEP164
0.505546	0.203052	SG2	CEP95	NC_056068.1	27735001	27755001	0.38469	0.310599	SG1	CEP164

0.537951	0.195079	SG2	CEP95;SMURF2	NC_056068.1	27740001	27760001	0.426356	0.297614	SG1	CEP164
0.450531	0.146435	SG2	CFAP46;LOC12181	NC_056068.1	27785001	27805001	0.247984	0.353608	SG1	CEP164
0.514062	0.320608	SG2	CFAP47	NC_056068.1	27790001	27810001	0.139558	0.408192	SG1	CEP164
0.486505	0.341371	SG2	CFAP47	NC_056068.1	27795001	27815001	0.20751	0.368152	SG1	CEP164
0.276524	0.138153	SG2	CFAP54	NC_056068.1	27800001	27820001	0.192348	0.388776	SG1	CEP164
0.245154	0.150239	SG2	CFAP54	NC_056066.1	64675001	64695001	0.241209	0.227466	SG1	CEP250
0.504414	0.204582	SG2	CFAP69	NC_056066.1	64685001	64705001	0.287544	0.183679	SG1	CEP250
0.471678	0.240452	SG2	CFAP69;STEAP2	NC_056066.1	64670001	64690001	0.252168	0.212259	SG1	CEP250;GDF5
0.405931	0.258588	SG2	CFAP69;STEAP2	NC_056061.1	19625001	19645001	0.230487	0.177032	SG1	CEP85L
0.364554	0.270156	SG2	CFAP69;STEAP2	NC_056061.1	19725001	19745001	0.189456	0.195391	SG1	CEP85L
0.537881	0.19964	SG2	CFAP70;DNAJC9;F	NC_056061.1	19630001	19650001	0.29383	0.18718	SG1	CEP85L;PLN
0.508035	0.237241	SG2	CFAP99;LOC11411	NC_056061.1	19635001	19655001	0.328358	0.215298	SG1	CEP85L;PLN
0.529959	0.242355	SG2	CFAP99;LOC11411	NC_056061.1	19640001	19660001	0.277406	0.24481	SG1	CEP85L;PLN
0.485063	0.214966	SG2	CFL1;EFEMP2;MU	NC_056061.1	19645001	19665001	0.339143	0.248462	SG1	CEP85L;PLN
0.412373	0.164481	SG2	CFL1;EFEMP2;MU	NC_056061.1	19650001	19670001	0.363207	0.232822	SG1	CEP85L;PLN
0.43038	0.156674	SG2	CFL1;MUS81;SNX:	NC_056061.1	19655001	19675001	0.404985	0.202735	SG1	CEP85L;PLN
0.424108	0.159226	SG2	CFL1;MUS81;SNX:	NC_056060.1	6850001	6870001	0.445343	0.17405	SG1	CERT1
0.409867	0.142157	SG2	CFL1;SNX32	NC_056060.1	6855001	6875001	0.409657	0.200505	SG1	CERT1
0.188755	0.255174	SG2	CHCHD5	NC_056060.1	6860001	6880001	0.416461	0.224487	SG1	CERT1
0.185714	0.229634	SG2	CHCHD5	NC_056060.1	6865001	6885001	0.407384	0.21328	SG1	CERT1
0.228931	0.173557	SG2	CHCHD5	NC_056060.1	6870001	6890001	0.408404	0.199047	SG1	CERT1
0.269697	0.149633	SG2	CHCHD5;POLR1B	NC_056060.1	6875001	6895001	0.40648	0.189358	SG1	CERT1
0.211513	0.1405	SG2	CHCHD7	NC_056059.1	95815001	95835001	0.251591	0.224395	SG1	CFAP299
0.567337	0.219155	SG2	CHD7	NC_056059.1	95820001	95840001	0.0646957	0.339557	SG1	CFAP299
0.555673	0.199062	SG2	CHD7	NC_056059.1	95825001	95845001	0.0655999	0.383104	SG1	CFAP299
0.513286	0.156879	SG2	CHI3L2	NC_056059.1	95830001	95850001	0.30021	0.266546	SG1	CFAP299
0.509508	0.177179	SG2	CHI3L2	NC_056059.1	96305001	96325001	0.124054	0.200856	SG1	CFAP299
0.569492	0.20714	SG2	CHI3L2	NC_056059.1	96310001	96330001	0.226502	0.190456	SG1	CFAP299
0.451598	0.204745	SG2	CHI3L2	NC_056059.1	96350001	96370001	0.0536063	0.177148	SG1	CFAP299
0.538328	0.230117	SG2	CHI3L2	NC_056080.1	35530001	35550001	0.429319	0.184696	SG1	CFAP47
0.47432	0.204373	SG2	CHI3L2	NC_056080.1	35535001	35555001	0.334525	0.223868	SG1	CFAP47
0.318061	0.177828	SG2	CHN2	NC_056080.1	35540001	35560001	0.190232	0.205501	SG1	CFAP47

0.105488	0.202808	SG2	CHN2	NC_056080.1	35545001	35565001	0.271389	0.193661	SG1	CFAP47
0.247897	0.174469	SG2	CHN2	NC_056054.1	185340001	185360001	0.296356	0.20972	SG1	CFAP91
0.495742	0.175753	SG2	CHN2	NC_056064.1	43400001	43420001	0.224576	0.198292	SG1	CFAP97D1;DU
0.45819	0.241522	SG2	CHN2	NC_056080.1	77385001	77405001	0.201944	0.385937	SG1	CHM
0.405568	0.273245	SG2	CHN2	NC_056080.1	77390001	77410001	0.270736	0.435124	SG1	CHM
0.305494	0.243326	SG2	CHN2	NC_056080.1	77430001	77450001	0.486405	0.49596	SG1	CHM
0.24512	0.266246	SG2	CHN2	NC_056080.1	77450001	77470001	0.488152	0.435572	SG1	CHM
0.289234	0.240943	SG2	CHN2	NC_056080.1	77455001	77475001	0.469461	0.461765	SG1	CHM
0.284706	0.225048	SG2	CHN2	NC_056080.1	77460001	77480001	0.431211	0.453703	SG1	CHM
0.222614	0.243181	SG2	CHN2	NC_056080.1	77465001	77485001	0.433884	0.427341	SG1	CHM
0.346456	0.213232	SG2	CHN2	NC_056080.1	77470001	77490001	0.486352	0.438386	SG1	CHM
0.505237	0.16908	SG2	CIPC	NC_056080.1	77495001	77515001	0.468907	0.464128	SG1	CHM
0.495098	0.174884	SG2	CIPC	NC_056080.1	77500001	77520001	0.403588	0.46224	SG1	CHM
0.398683	0.19641	SG2	CIPC	NC_056080.1	77505001	77525001	0.434783	0.518696	SG1	CHM
0.408551	0.184681	SG2	CIPC	NC_056080.1	77510001	77530001	0.347394	0.457857	SG1	CHM
0.5639	0.151794	SG2	CIPC	NC_056080.1	77515001	77535001	0.32439	0.419763	SG1	CHM
0.417541	0.173612	SG2	CIPC;ZDHHC22	NC_056080.1	77520001	77540001	0.342553	0.394008	SG1	CHM
0.473191	0.174332	SG2	CLCN4	NC_056080.1	77525001	77545001	0.374761	0.379628	SG1	CHM
0.465355	0.254786	SG2	CLCN4	NC_056080.1	77530001	77550001	0.403846	0.397617	SG1	CHM
0.395757	0.244056	SG2	CLCN4	NC_056080.1	77535001	77555001	0.461334	0.396843	SG1	CHM
0.53433	0.162407	SG2	CLEC4F;FIGLA	NC_056080.1	77555001	77575001	0.48072	0.350041	SG1	CHM
0.510955	0.412614	SG2	CLGN	NC_056080.1	77565001	77585001	0.453191	0.422881	SG1	CHM
0.461769	0.376822	SG2	CLGN	NC_056080.1	77580001	77600001	0.481817	0.401678	SG1	CHM
0.416973	0.333934	SG2	CLGN	NC_056080.1	77625001	77645001	0.42	0.449708	SG1	CHM
0.418278	0.330375	SG2	CLGN;SCOC	NC_056080.1	77630001	77650001	0.447029	0.440937	SG1	CHM
0.459746	0.32959	SG2	CLGN;SCOC	NC_056080.1	77635001	77655001	0.487913	0.514117	SG1	CHM
0.512978	0.391751	SG2	CLGN;SCOC	NC_056068.1	52715001	52735001	0.30179	0.194484	SG1	CHRDL2
0.368853	0.1875	SG2	CLIC6	NC_056068.1	52720001	52740001	0.27379	0.201878	SG1	CHRDL2
0.456455	0.154827	SG2	CLIC6	NC_056068.1	52725001	52745001	0.322148	0.226432	SG1	CHRDL2
0.529982	0.163739	SG2	CLMN	NC_056068.1	52730001	52750001	0.400609	0.22513	SG1	CHRDL2
0.543707	0.210807	SG2	CNBD1	NC_056057.1	102035001	102055001	0.334017	0.174929	SG1	CHRM2
0.528484	0.265207	SG2	CNTN3	NC_056057.1	102045001	102065001	0.228713	0.177272	SG1	CHRM2

0.426715	0.288465	SG2	CNTN3	NC_056057.1	102050001	102070001	0.175041	0.257347	SG1	CHRM2
0.329875	0.275223	SG2	CNTN3	NC_056057.1	102055001	102075001	0.186562	0.314569	SG1	CHRM2
0.435753	0.141415	SG2	CNTN5	NC_056057.1	102060001	102080001	0.265801	0.292892	SG1	CHRM2
0.353081	0.175084	SG2	CNTN5	NC_056057.1	102065001	102085001	0.318505	0.27841	SG1	CHRM2
0.440643	0.165971	SG2	CNTN5	NC_056078.1	11695001	11715001	0.466402	0.192682	SG1	CHRM3
0.553544	0.150471	SG2	CNTNAP2	NC_056071.1	27465001	27485001	0.395807	0.181396	SG1	CHRNA7
0.480392	0.139536	SG2	CNTROB	NC_056054.1	63590001	63610001	0.416481	0.254158	SG1	CLCA1
0.425532	0.175562	SG2	CNTROB	NC_056054.1	63595001	63615001	0.366529	0.338333	SG1	CLCA1
0.433437	0.157333	SG2	COCH;STRN3	NC_056077.1	10285001	10305001	0.424111	0.213177	SG1	CLEC16A
0.379779	0.231844	SG2	COCH;STRN3	NC_056077.1	10290001	10310001	0.30865	0.231744	SG1	CLEC16A
0.55269	0.145837	SG2	COL4A3	NC_056077.1	10295001	10315001	0.246662	0.255329	SG1	CLEC16A
0.549671	0.146473	SG2	COL4A3	NC_056077.1	10300001	10320001	0.218117	0.24893	SG1	CLEC16A
0.518137	0.154178	SG2	COL4A3	NC_056077.1	10305001	10325001	0.27342	0.262422	SG1	CLEC16A
0.362134	0.150434	SG2	COL6A2	NC_056077.1	10310001	10330001	0.376253	0.177644	SG1	CLEC16A
0.500841	0.164815	SG2	COL6A2	NC_056055.1	241885001	241905001	0.216758	0.224219	SG1	CLIC4
0.470018	0.199208	SG2	COLEC12	NC_056055.1	241890001	241910001	0.133971	0.265093	SG1	CLIC4
0.217802	0.245949	SG2	COLEC12	NC_056055.1	241895001	241915001	0.164899	0.263317	SG1	CLIC4
0.256831	0.196412	SG2	COLEC12	NC_056055.1	241900001	241920001	0.273996	0.234384	SG1	CLIC4
0.310757	0.186221	SG2	COLEC12	NC_056055.1	241905001	241925001	0.423077	0.219245	SG1	CLIC4
0.341543	0.178672	SG2	COLEC12	NC_056055.1	241930001	241950001	0.135229	0.229804	SG1	CLIC4;LOC101
0.546406	0.146568	SG2	CPD	NC_056055.1	241935001	241955001	0.165293	0.206085	SG1	CLIC4;LOC101
0.21394	0.225883	SG2	CPED1	NC_056055.1	241910001	241930001	0.48487	0.177465	SG1	CLIC4;LOC114
0.339709	0.200105	SG2	CPED1	NC_056055.1	241920001	241940001	0.286043	0.209478	SG1	CLIC4;LOC114
0.308138	0.213159	SG2	CPED1	NC_056055.1	241925001	241945001	0.255845	0.202995	SG1	CLIC4;LOC114
0.284494	0.223168	SG2	CPED1	NC_056059.1	71580001	71600001	0.223481	0.218772	SG1	CLOCK
0.29088	0.195404	SG2	CPED1	NC_056059.1	71575001	71595001	0.272626	0.183343	SG1	CLOCK;TMEM
0.238437	0.212224	SG2	CPED1	NC_056072.1	27505001	27525001	0.384084	0.207777	SG1	CNTN3
0.285138	0.200425	SG2	CPED1	NC_056068.1	10040001	10060001	0.483251	0.238368	SG1	CNTN5
0.340451	0.17399	SG2	CPED1	NC_056068.1	10045001	10065001	0.356056	0.249084	SG1	CNTN5
0.405165	0.157737	SG2	CPED1	NC_056057.1	110555001	110575001	0.270766	0.213008	SG1	CNTNAP2
0.43536	0.13988	SG2	CPED1	NC_056057.1	110560001	110580001	0.164162	0.243714	SG1	CNTNAP2
0.435127	0.137963	SG2	CPED1	NC_056057.1	110565001	110585001	0.177289	0.250899	SG1	CNTNAP2

0.386641	0.160954	SG2	CPED1;ING3	NC_056057.1	110570001	110590001	0.213318	0.252304	SG1	CNTNAP2
0.494035	0.135347	SG2	CPLX2	NC_056057.1	110575001	110595001	0.256607	0.21189	SG1	CNTNAP2
0.416958	0.275597	SG2	CPNE1;SPAG4	NC_056057.1	110580001	110600001	0.254418	0.207471	SG1	CNTNAP2
0.568421	0.207165	SG2	CPNE4	NC_056057.1	110585001	110605001	0.339254	0.205334	SG1	CNTNAP2
0.437613	0.161903	SG2	CPNE4	NC_056057.1	111230001	111250001	0.417985	0.192685	SG1	CNTNAP2
0.442856	0.137429	SG2	CPNE4	NC_056057.1	111500001	111520001	0.375924	0.201333	SG1	CNTNAP2
0.276924	0.164417	SG2	CPNE4	NC_056057.1	111505001	111525001	0.374258	0.260217	SG1	CNTNAP2
0.185969	0.204015	SG2	CPNE4	NC_056057.1	111510001	111530001	0.374857	0.258364	SG1	CNTNAP2
0.181332	0.20296	SG2	CPNE4	NC_056057.1	111515001	111535001	0.256411	0.284152	SG1	CNTNAP2
0.248014	0.199887	SG2	CPNE4	NC_056057.1	111520001	111540001	0.304085	0.216966	SG1	CNTNAP2
0.452039	0.183821	SG2	CPNE4	NC_056057.1	111540001	111560001	0.151652	0.185905	SG1	CNTNAP2
0.526935	0.167459	SG2	CPNE4	NC_056057.1	111545001	111565001	0.191702	0.186914	SG1	CNTNAP2
0.534274	0.155336	SG2	CPNE4	NC_056057.1	111550001	111570001	0.246639	0.181389	SG1	CNTNAP2
0.374027	0.151447	SG2	CPNE4	NC_056054.1	272940001	272960001	0.347355	0.205608	SG1	COL6A5
0.326084	0.145848	SG2	CPNE4	NC_056054.1	272945001	272965001	0.20972	0.234629	SG1	COL6A5
0.428968	0.184151	SG2	CPNE4	NC_056054.1	272950001	272970001	0.0871658	0.291484	SG1	COL6A5
0.472282	0.197295	SG2	CPNE4	NC_056054.1	272955001	272975001	0.229105	0.248453	SG1	COL6A5
0.371171	0.181152	SG2	CPNE4	NC_056057.1	96065001	96085001	0.425373	0.408081	SG1	COPG2
0.351832	0.144443	SG2	CPNE4	NC_056057.1	96080001	96100001	0.400612	0.536348	SG1	COPG2;TSGA1
0.562073	0.145002	SG2	CPNE4	NC_056057.1	96085001	96105001	0.425926	0.532828	SG1	COPG2;TSGA1
0.566276	0.243478	SG2	CPNE4	NC_056065.1	29185001	29205001	0.465361	0.226691	SG1	COQ8A
0.558987	0.232403	SG2	CPNE4	NC_056058.1	5890001	5910001	0.0448841	0.191938	SG1	CPAMD8
0.528068	0.229116	SG2	CPNE4	NC_056058.1	5905001	5925001	0.0348577	0.199995	SG1	CPAMD8
0.512941	0.175366	SG2	CPNE4	NC_056055.1	212200001	212220001	0.286683	0.174291	SG1	CPS1
0.389038	0.164095	SG2	CPNE4	NC_056055.1	212205001	212225001	0.361991	0.174448	SG1	CPS1
0.525687	0.170497	SG2	CPNE4	NC_056055.1	212255001	212275001	0.215997	0.188262	SG1	CPS1
0.222418	0.165077	SG2	CPNE6;NRL;PCK2	NC_056056.1	212115001	212135001	0.441989	0.217431	SG1	CRACR2A
0.533178	0.280855	SG2	CPVL	NC_056056.1	212120001	212140001	0.307907	0.24887	SG1	CRACR2A
0.4365	0.262461	SG2	CPVL	NC_056056.1	212125001	212145001	0.217954	0.257117	SG1	CRACR2A
0.386229	0.240448	SG2	CPVL	NC_056056.1	212130001	212150001	0.256795	0.240432	SG1	CRACR2A
0.553549	0.251838	SG2	CPVL	NC_056057.1	103030001	103050001	0.476244	0.299472	SG1	CREB3L2
0.570598	0.193223	SG2	CPXM2	NC_056057.1	103035001	103055001	0.0848375	0.351691	SG1	CREB3L2

0.560906	0.185529	SG2	CPXM2	NC_056057.1	103050001	103070001	0.208438	0.210174	SG1	CREB3L2
0.418815	0.186617	SG2	CPXM2	NC_056057.1	103055001	103075001	0.333333	0.179774	SG1	CREB3L2
0.461958	0.162598	SG2	CPXM2	NC_056057.1	103060001	103080001	0.419077	0.18385	SG1	CREB3L2
0.408388	0.161616	SG2	CREB3L4;CRTC2;J	NC_056057.1	67195001	67215001	0.25939	0.222508	SG1	CRHR2
0.28961	0.206302	SG2	CREB3L4;CRTC2;J	NC_056057.1	67200001	67220001	0.357229	0.249135	SG1	CRHR2
0.288043	0.143626	SG2	CREB3L4;JTB;NUI	NC_056058.1	4390001	4410001	0.286396	0.221356	SG1	CRLF1;TMEM
0.228528	0.171604	SG2	CREB3L4;JTB;RAI	NC_056054.1	201435001	201455001	0.485836	0.384981	SG1	CRYGS;TBCC
0.226597	0.185653	SG2	CREB3L4;JTB;RAI	NC_056079.1	2660001	2680001	0.447979	0.173776	SG1	CSMD1
0.308479	0.211458	SG2	CREBL2	NC_056079.1	2670001	2690001	0.427927	0.176205	SG1	CSMD1
0.277632	0.198617	SG2	CREBL2	NC_056079.1	2675001	2695001	0.408676	0.212559	SG1	CSMD1
0.352313	0.160841	SG2	CREBL2	NC_056079.1	2680001	2700001	0.413654	0.247805	SG1	CSMD1
0.396964	0.16262	SG2	CREBL2	NC_056079.1	2685001	2705001	0.330633	0.262243	SG1	CSMD1
0.347826	0.15369	SG2	CREBL2;GPR19	NC_056079.1	2690001	2710001	0.389536	0.279463	SG1	CSMD1
0.328238	0.174483	SG2	CREBL2;GPR19	NC_056079.1	2695001	2715001	0.403963	0.257384	SG1	CSMD1
0.508957	0.246348	SG2	CREM	NC_056079.1	2700001	2720001	0.415203	0.246992	SG1	CSMD1
0.560239	0.241123	SG2	CREM	NC_056079.1	2705001	2725001	0.40396	0.235409	SG1	CSMD1
0.508922	0.212284	SG2	CREM	NC_056079.1	2710001	2730001	0.314509	0.223654	SG1	CSMD1
0.296753	0.170205	SG2	CRK	NC_056079.1	2715001	2735001	0.258056	0.181078	SG1	CSMD1
0.321818	0.169841	SG2	CRK	NC_056055.1	205840001	205860001	0.442742	0.324654	SG1	CTLA4
0.420091	0.149802	SG2	CRK	NC_056072.1	13810001	13830001	0.356303	0.275514	SG1	CTNNB1
0.531351	0.164427	SG2	CRPPA	NC_056072.1	13815001	13835001	0.263636	0.338707	SG1	CTNNB1
0.285392	0.140136	SG2	CRYAA	NC_056072.1	13820001	13840001	0.301609	0.301195	SG1	CTNNB1
0.146047	0.137477	SG2	CRYAA	NC_056072.1	13825001	13845001	0.360955	0.311541	SG1	CTNNB1
0.412734	0.137437	SG2	CRYGS	NC_056072.1	13830001	13850001	0.445109	0.239882	SG1	CTNNB1
0.388922	0.137694	SG2	CRYGS;TBCCD1	NC_056072.1	13855001	13875001	0.333051	0.329542	SG1	CTNNB1;ULK
0.230681	0.134922	SG2	CRYGS;TBCCD1	NC_056072.1	13860001	13880001	0.273409	0.370274	SG1	CTNNB1;ULK
0.487655	0.231877	SG2	CSDE1	NC_056072.1	13865001	13885001	0.24572	0.36537	SG1	CTNNB1;ULK
0.524752	0.215784	SG2	CSDE1	NC_056065.1	42935001	42955001	0.445783	0.205983	SG1	CTNNBIP1
0.547017	0.201834	SG2	CSDE1;NRAS	NC_056069.1	62290001	62310001	0.460463	0.202751	SG1	CTNND2
0.490919	0.223685	SG2	CSDE1;NRAS	NC_056069.1	62350001	62370001	0.414145	0.214295	SG1	CTNND2
0.522549	0.136872	SG2	CSF1	NC_056069.1	62355001	62375001	0.0792221	0.222415	SG1	CTNND2
0.390092	0.13929	SG2	CSK	NC_056057.1	113145001	113165001	0.457944	0.175189	SG1	CUL1;EZH2

0.369848	0.147745	SG2	CSK	NC_056059.1	68345001	68365001	0.479292	0.242096	SG1	CWH43
0.281655	0.146955	SG2	CSK	NC_056080.1	63800001	63820001	0.296392	0.328161	SG1	CXHXorf65;FC
0.277829	0.166893	SG2	CSK	NC_056080.1	74755001	74775001	0.285715	0.256234	SG1	CYLC1
0.497207	0.161601	SG2	CTBP2	NC_056080.1	74760001	74780001	0.244216	0.276482	SG1	CYLC1
0.483287	0.173204	SG2	CTBP2	NC_056077.1	37180001	37200001	0.344874	0.411889	SG1	CYP3A24
0.527648	0.314375	SG2	CTBP2	NC_056077.1	37185001	37205001	0.292653	0.195914	SG1	CYP3A24
0.480053	0.222393	SG2	CTIF	NC_056054.1	31795001	31815001	0.468956	0.203147	SG1	DAB1
0.478605	0.220544	SG2	CTIF	NC_056054.1	31800001	31820001	0.422466	0.2375	SG1	DAB1
0.550657	0.153833	SG2	CTIF	NC_056063.1	46680001	46700001	0.387155	0.28114	SG1	DACH1
0.227862	0.170276	SG2	CTIF	NC_056063.1	46685001	46705001	0.439129	0.30188	SG1	DACH1
0.131472	0.207305	SG2	CTIF	NC_056080.1	77735001	77755001	0.469461	0.176218	SG1	DACH2
0.063448	0.216348	SG2	CTIF	NC_056080.1	77760001	77780001	0.472869	0.231382	SG1	DACH2
0.056067	0.200864	SG2	CTIF	NC_056080.1	78045001	78065001	0.340147	0.472896	SG1	DACH2
0.480304	0.159773	SG2	CTNND2	NC_056080.1	78050001	78070001	0.244305	0.477749	SG1	DACH2
0.382992	0.141854	SG2	CTNND2;LOC1141	NC_056080.1	78055001	78075001	0.220657	0.442739	SG1	DACH2
0.402597	0.159335	SG2	CTNND2;LOC1141	NC_056080.1	78060001	78080001	0.2643	0.430004	SG1	DACH2
0.473772	0.210163	SG2	CTSW;EFEMP2;MU	NC_056080.1	78065001	78085001	0.225945	0.432476	SG1	DACH2
0.432548	0.180408	SG2	CUL2	NC_056080.1	78070001	78090001	0.219151	0.428693	SG1	DACH2
0.374396	0.147682	SG2	CUL2	NC_056080.1	78075001	78095001	0.279227	0.465493	SG1	DACH2
0.315061	0.169681	SG2	CUL2	NC_056080.1	78080001	78100001	0.241513	0.46258	SG1	DACH2
0.356522	0.143832	SG2	CUL2	NC_056080.1	78085001	78105001	0.340581	0.47313	SG1	DACH2
0.329244	0.191223	SG2	CUL2	NC_056080.1	78090001	78110001	0.392063	0.486499	SG1	DACH2
0.338542	0.190566	SG2	CUL2	NC_056080.1	78095001	78115001	0.316971	0.452102	SG1	DACH2
0.379757	0.136047	SG2	CUL2	NC_056080.1	78100001	78120001	0.404322	0.474085	SG1	DACH2
0.375502	0.141329	SG2	CUL2	NC_056080.1	78205001	78225001	0.398934	0.468105	SG1	DACH2
0.213836	0.136886	SG2	CXADR	NC_056080.1	78210001	78230001	0.227049	0.445009	SG1	DACH2
0.21532	0.138193	SG2	CXADR	NC_056080.1	78215001	78235001	0.237806	0.460097	SG1	DACH2
0.260156	0.139513	SG2	CXADR	NC_056080.1	78220001	78240001	0.192092	0.469229	SG1	DACH2
0.308307	0.183099	SG2	CYP1A1	NC_056080.1	78225001	78245001	0.247064	0.452734	SG1	DACH2
0.236364	0.263466	SG2	CYP1A1	NC_056080.1	78230001	78250001	0.282812	0.474224	SG1	DACH2
0.278482	0.191343	SG2	CYP1A1	NC_056080.1	78235001	78255001	0.248157	0.475811	SG1	DACH2
0.418088	0.195722	SG2	CYP1A1	NC_056080.1	78240001	78260001	0.27655	0.44602	SG1	DACH2

0.458362	0.188976	SG2	CYP1A1	NC_056080.1	78245001	78265001	0.313426	0.443404	SG1	DACH2
0.080847	0.140527	SG2	CYSTM1	NC_056080.1	78250001	78270001	0.286111	0.441852	SG1	DACH2
0.074608	0.139642	SG2	CYSTM1	NC_056080.1	78255001	78275001	0.317317	0.435714	SG1	DACH2
0.062457	0.143936	SG2	CYSTM1	NC_056080.1	78260001	78280001	0.273304	0.43857	SG1	DACH2
0.072116	0.141209	SG2	CYSTM1	NC_056080.1	78265001	78285001	0.256369	0.443744	SG1	DACH2
0.557116	0.247332	SG2	DACH2	NC_056080.1	78270001	78290001	0.293344	0.450972	SG1	DACH2
0.541408	0.340197	SG2	DACH2	NC_056080.1	78275001	78295001	0.329526	0.448768	SG1	DACH2
0.542234	0.3368	SG2	DACH2	NC_056080.1	78280001	78300001	0.372166	0.401199	SG1	DACH2
0.537233	0.3016	SG2	DACH2	NC_056080.1	78285001	78305001	0.418534	0.375753	SG1	DACH2
0.51739	0.312865	SG2	DACH2	NC_056080.1	78295001	78315001	0.409948	0.394431	SG1	DACH2
0.474201	0.321744	SG2	DACH2	NC_056080.1	78300001	78320001	0.379217	0.400015	SG1	DACH2
0.49709	0.317413	SG2	DACH2	NC_056080.1	78305001	78325001	0.389689	0.388945	SG1	DACH2
0.56313	0.315456	SG2	DACH2	NC_056080.1	78310001	78330001	0.37511	0.279789	SG1	DACH2
0.562098	0.322208	SG2	DACH2	NC_056060.1	22035001	22055001	0.373132	0.198342	SG1	DAD1
0.54604	0.319141	SG2	DACH2	NC_056060.1	22040001	22060001	0.329263	0.194648	SG1	DAD1
0.51724	0.320406	SG2	DACH2	NC_056060.1	22045001	22065001	0.323752	0.200409	SG1	DAD1
0.559442	0.285387	SG2	DACH2	NC_056060.1	22050001	22070001	0.268018	0.193569	SG1	DAD1
0.541584	0.300002	SG2	DACH2	NC_056059.1	87030001	87050001	0.314093	0.245377	SG1	DCK;MOB1B
0.536961	0.309212	SG2	DACH2	NC_056059.1	87035001	87055001	0.376004	0.215674	SG1	DCK;MOB1B
0.490706	0.344581	SG2	DACH2	NC_056059.1	87040001	87060001	0.42885	0.176399	SG1	DCK;MOB1B
0.476314	0.351446	SG2	DACH2	NC_056063.1	25925001	25945001	0.385187	0.199294	SG1	DCLK1
0.506285	0.334638	SG2	DACH2	NC_056063.1	25975001	25995001	0.270763	0.217876	SG1	DCLK1
0.514043	0.331935	SG2	DACH2	NC_056063.1	25980001	26000001	0.148073	0.246122	SG1	DCLK1
0.527466	0.324254	SG2	DACH2	NC_056060.1	930001	950001	0.380682	0.223184	SG1	DCP2
0.497595	0.427945	SG2	DACH2	NC_056060.1	925001	945001	0.321445	0.228478	SG1	DCP2;LOC101
0.357847	0.44564	SG2	DACH2	NC_056059.1	68430001	68450001	0.322806	0.251447	SG1	DCUN1D4
0.30493	0.445619	SG2	DACH2	NC_056059.1	68435001	68455001	0.218118	0.331957	SG1	DCUN1D4
0.292695	0.452039	SG2	DACH2	NC_056059.1	68440001	68460001	0.132176	0.398888	SG1	DCUN1D4
0.32994	0.432219	SG2	DACH2	NC_056059.1	68445001	68465001	0.0498498	0.424847	SG1	DCUN1D4
0.358547	0.416026	SG2	DACH2	NC_056059.1	68450001	68470001	0.056333	0.329846	SG1	DCUN1D4
0.533695	0.342895	SG2	DACH2	NC_056080.1	123690001	123710001	0.315694	0.233084	SG1	DCX
0.338575	0.167316	SG2	DCAF11;FITM1;PC	NC_056080.1	123715001	123735001	0.164319	0.210136	SG1	DCX

0.221723	0.244289	SG2	DCAF11;NRL;PCK	NC_056067.1	14255001	14275001	0.14089	0.185325	SG1	DEF8
0.307622	0.211934	SG2	DCAF11;PCK2	NC_056067.1	14260001	14280001	0.0970991	0.199114	SG1	DEF8
0.541573	0.257248	SG2	DCAF6	NC_056067.1	14265001	14285001	0.0648648	0.195179	SG1	DEF8;LOC101
0.563889	0.244076	SG2	DCAF6	NC_056067.1	14275001	14295001	0.264024	0.198151	SG1	DEF8;LOC101
0.452685	0.282118	SG2	DCAF6	NC_056065.1	76015001	76035001	0.288779	0.201047	SG1	DENND1B
0.547987	0.250997	SG2	DCAF6	NC_056065.1	76020001	76040001	0.329591	0.232532	SG1	DENND1B
0.560371	0.230667	SG2	DCAF6	NC_056065.1	76025001	76045001	0.388225	0.290665	SG1	DENND1B
0.525252	0.228075	SG2	DCAF6	NC_056065.1	76030001	76050001	0.405236	0.297156	SG1	DENND1B
0.457236	0.24224	SG2	DCAF6	NC_056065.1	76035001	76055001	0.405342	0.271879	SG1	DENND1B
0.511029	0.235531	SG2	DCAF6	NC_056065.1	76040001	76060001	0.374179	0.263866	SG1	DENND1B
0.547987	0.160243	SG2	DCAF6;GPR161	NC_056065.1	76045001	76065001	0.273248	0.207039	SG1	DENND1B
0.518886	0.244646	SG2	DCDC1	NC_056065.1	76050001	76070001	0.378497	0.213124	SG1	DENND1B
0.495548	0.271915	SG2	DCDC1	NC_056065.1	76055001	76075001	0.299786	0.272553	SG1	DENND1B
0.444168	0.305429	SG2	DCDC1	NC_056065.1	76060001	76080001	0.268042	0.36565	SG1	DENND1B
0.305701	0.136335	SG2	DCDC1	NC_056065.1	76085001	76105001	0.301138	0.433838	SG1	DENND1B
0.511605	0.182492	SG2	DDX27;ZNFX1	NC_056065.1	76165001	76185001	0.368438	0.206379	SG1	DENND1B
0.408823	0.191704	SG2	DDX27;ZNFX1	NC_056065.1	76170001	76190001	0.317721	0.22235	SG1	DENND1B
0.499423	0.15148	SG2	DDX4	NC_056065.1	76175001	76195001	0.267741	0.206323	SG1	DENND1B
0.405904	0.175558	SG2	DDX4	NC_056065.1	76065001	76085001	0.291824	0.411703	SG1	DENND1B;LO
0.23055	0.261441	SG2	DDX4	NC_056065.1	76070001	76090001	0.215904	0.466373	SG1	DENND1B;LO
0.118186	0.310763	SG2	DDX4;SLC38A9	NC_056065.1	76075001	76095001	0.336427	0.431979	SG1	DENND1B;LO
0.276385	0.286298	SG2	DDX4;SLC38A9	NC_056065.1	76080001	76100001	0.336195	0.434577	SG1	DENND1B;LO
0.408512	0.1495	SG2	DEGS1	NC_056062.1	29730001	29750001	0.468072	0.226698	SG1	DERL1
0.556467	0.194481	SG2	DELE1	NC_056062.1	29725001	29745001	0.364714	0.192012	SG1	DERL1;TBC1D
0.413282	0.297409	SG2	DEPDC5	NC_056054.1	201665001	201685001	0.42153	0.177309	SG1	DGKG
0.395683	0.258441	SG2	DEPDC5	NC_056054.1	201670001	201690001	0.282477	0.201573	SG1	DGKG
0.511362	0.218174	SG2	DEPDC5	NC_056054.1	201675001	201695001	0.123995	0.257135	SG1	DGKG
0.450886	0.266422	SG2	DESI2	NC_056080.1	54940001	54960001	0.338534	0.177039	SG1	DGKK
0.458335	0.254951	SG2	DESI2	NC_056067.1	38575001	38595001	0.050736	0.311147	SG1	DHODH
0.566389	0.189479	SG2	DESI2	NC_056067.1	38580001	38600001	0.113294	0.279684	SG1	DHODH
0.50655	0.197327	SG2	DGKI	NC_056067.1	38585001	38605001	0.221443	0.239146	SG1	DHODH
0.524712	0.13697	SG2	DGKI	NC_056067.1	38590001	38610001	0.33374	0.201205	SG1	DHODH

0.546832	0.159497	SG2	DGKI	NC_056066.1	16050001	16070001	0.299358	0.197988	SG1	DHTKD1
0.565252	0.226677	SG2	DGKI	NC_056066.1	16055001	16075001	0.10772	0.285311	SG1	DHTKD1
0.51626	0.138129	SG2	DHRS3	NC_056070.1	53175001	53195001	0.477725	0.225209	SG1	DIABLO;VPS3
0.499144	0.136322	SG2	DHRS3	NC_056080.1	137805001	137825001	0.239619	0.289753	SG1	DIAPH2
0.503875	0.155734	SG2	DIP2C	NC_056080.1	137810001	137830001	0.145238	0.321188	SG1	DIAPH2
0.488093	0.201777	SG2	DIP2C	NC_056080.1	137815001	137835001	0.0864807	0.319006	SG1	DIAPH2
0.492975	0.203016	SG2	DIP2C	NC_056080.1	137820001	137840001	0.0808607	0.281065	SG1	DIAPH2
0.492244	0.219598	SG2	DIP2C	NC_056080.1	138315001	138335001	0.436592	0.214435	SG1	DIAPH2
0.572167	0.175198	SG2	DIP2C	NC_056080.1	138320001	138340001	0.279071	0.218042	SG1	DIAPH2
0.527333	0.174152	SG2	DIP2C	NC_056080.1	138325001	138345001	0.305904	0.177713	SG1	DIAPH2
0.552954	0.179846	SG2	DIP2C	NC_056056.1	135615001	135635001	0.427975	0.19078	SG1	DIP2B
0.472082	0.137121	SG2	DIP2C	NC_056056.1	135620001	135640001	0.418437	0.22094	SG1	DIP2B
0.436757	0.173577	SG2	DIP2C	NC_056056.1	135625001	135645001	0.373533	0.247755	SG1	DIP2B
0.483588	0.13657	SG2	DIP2C	NC_056054.1	70120001	70140001	0.485349	0.211069	SG1	DIPK1A
0.504622	0.161472	SG2	DIPK1A	NC_056054.1	70030001	70050001	0.48323	0.350892	SG1	DIPK1A;RPL5
0.5	0.326485	SG2	DIS3L	NC_056054.1	70035001	70055001	0.442019	0.284617	SG1	DIPK1A;RPL5
0.462909	0.353368	SG2	DIS3L	NC_056054.1	70040001	70060001	0.466605	0.24711	SG1	DIPK1A;RPL5
0.459503	0.355983	SG2	DIS3L	NC_056063.1	48115001	48135001	0.184739	0.297798	SG1	DIS3
0.425874	0.290666	SG2	DIS3L	NC_056055.1	233665001	233685001	0.247975	0.186435	SG1	DIS3L2
0.334376	0.160045	SG2	DIS3L	NC_056072.1	11560001	11580001	0.317904	0.186208	SG1	DLEC1
0.545381	0.158905	SG2	DISC1	NC_056072.1	11570001	11590001	0.256411	0.185648	SG1	DLEC1
0.485954	0.152437	SG2	DISP1	NC_056060.1	84205001	84225001	0.259259	0.226849	SG1	DLST
0.139872	0.182179	SG2	DLG1	NC_056060.1	84210001	84230001	0.223128	0.213757	SG1	DLST
0.117961	0.235352	SG2	DLG1	NC_056060.1	84200001	84220001	0.36121	0.237902	SG1	DLST;PROX2
0.164642	0.229865	SG2	DLG1	NC_056060.1	84230001	84250001	0.121372	0.19293	SG1	DLST;RPS6KL
0.193629	0.232677	SG2	DLG1	NC_056067.1	50120001	50140001	0.118102	0.330866	SG1	DMAC2
0.324228	0.197895	SG2	DLG1	NC_056067.1	50125001	50145001	0.231574	0.268646	SG1	DMAC2
0.44685	0.15971	SG2	DLG1	NC_056057.1	31395001	31415001	0.369037	0.17552	SG1	DNAH11
0.318114	0.163592	SG2	DLK1	NC_056077.1	19220001	19240001	0.381729	0.302	SG1	DNAH3
0.385396	0.196019	SG2	DLK1	NC_056077.1	19235001	19255001	0.38275	0.289619	SG1	DNAH3
0.365708	0.242118	SG2	DLK1	NC_056077.1	19240001	19260001	0.328572	0.278894	SG1	DNAH3
0.345319	0.207542	SG2	DLK1	NC_056077.1	19245001	19265001	0.156606	0.33077	SG1	DNAH3

0.234221	0.200241	SG2	DMBT1	NC_056077.1	19250001	19270001	0.301213	0.355802	SG1	DNAH3
0.119816	0.258467	SG2	DMBT1	NC_056077.1	19215001	19235001	0.478689	0.256011	SG1	DNAH3;LYRM
0.543874	0.144167	SG2	DMC1	NC_056068.1	52110001	52130001	0.0641952	0.185231	SG1	DNAJB13;UCP
0.550485	0.215237	SG2	DMD	NC_056066.1	22400001	22420001	0.338158	0.178732	SG1	DNAJC1
0.487822	0.173037	SG2	DMD	NC_056066.1	22405001	22425001	0.311265	0.179887	SG1	DNAJC1
0.10678	0.173926	SG2	DMRTA1	NC_056066.1	22435001	22455001	0.459975	0.216954	SG1	DNAJC1
0.074605	0.192255	SG2	DMRTA1	NC_056066.1	22440001	22460001	0.444808	0.222488	SG1	DNAJC1
0.103209	0.179997	SG2	DMRTA1	NC_056066.1	22445001	22465001	0.437089	0.208341	SG1	DNAJC1
0.324401	0.135841	SG2	DNAH10	NC_056065.1	38585001	38605001	0.439438	0.188914	SG1	DNM3
0.254489	0.181563	SG2	DNAH10	NC_056065.1	38590001	38610001	0.347716	0.198825	SG1	DNM3
0.092844	0.300989	SG2	DNAH10	NC_056065.1	38595001	38615001	0.271784	0.195939	SG1	DNM3
0.394405	0.317531	SG2	DNAH10	NC_056065.1	38735001	38755001	0.147657	0.236786	SG1	DNM3
0.386935	0.226918	SG2	DNAJA3;NMRAL1	NC_056065.1	38740001	38760001	0.231481	0.355752	SG1	DNM3
0.3	0.173044	SG2	DNAJA3;NMRAL1	NC_056065.1	38745001	38765001	0.408253	0.406002	SG1	DNM3
0.388164	0.19009	SG2	DNAJA3;NMRAL1	NC_056065.1	38760001	38780001	0.423271	0.34287	SG1	DNM3
0.33225	0.246103	SG2	DNAJC9;FAM149B	NC_056065.1	38765001	38785001	0.334573	0.250208	SG1	DNM3
0.388964	0.23831	SG2	DNAJC9;FAM149B	NC_056065.1	38770001	38790001	0.199433	0.191437	SG1	DNM3
0.472142	0.224793	SG2	DNAJC9;FAM149B	NC_056076.1	7615001	7635001	0.314092	0.371771	SG1	DOK6
0.412028	0.191243	SG2	DNAJC9;FAM149B	NC_056076.1	7620001	7640001	0.303839	0.344458	SG1	DOK6
0.553673	0.136984	SG2	DNTT	NC_056076.1	7625001	7645001	0.343668	0.327831	SG1	DOK6
0.532707	0.199308	SG2	DNTT	NC_056076.1	7630001	7650001	0.360892	0.227662	SG1	DOK6
0.542594	0.200889	SG2	DNTT	NC_056074.1	39975001	39995001	0.404519	0.268266	SG1	DPF2
0.550016	0.197175	SG2	DNTT	NC_056058.1	56370001	56390001	0.476067	0.182586	SG1	DPYSL3
0.433839	0.170625	SG2	DOCK2;INSYN2B	NC_056078.1	42570001	42590001	0.457135	0.417841	SG1	DRGX
0.233813	0.149593	SG2	DOCK4	NC_056078.1	42575001	42595001	0.266175	0.401769	SG1	DRGX
0.084755	0.181134	SG2	DOCK4	NC_056078.1	42580001	42600001	0.183731	0.229934	SG1	DRGX
0.46042	0.203298	SG2	DSCAM	NC_056064.1	43390001	43410001	0.372975	0.185432	SG1	DUSP3;SOST
0.522002	0.281693	SG2	DSCAM	NC_056064.1	43395001	43415001	0.268794	0.205691	SG1	DUSP3;SOST
0.490874	0.296228	SG2	DSCAM	NC_056075.1	30610001	30630001	0.425425	0.253079	SG1	DUSP5
0.565082	0.171539	SG2	DUSP1	NC_056061.1	82770001	82790001	0.390566	0.177458	SG1	DYNLT1;TME
0.37085	0.167155	SG2	DUSP14	NC_056061.1	82775001	82795001	0.409638	0.185617	SG1	DYNLT1;TME
0.408565	0.153925	SG2	DUSP14	NC_056061.1	82780001	82800001	0.428572	0.176054	SG1	DYNLT1;TME

0.477045	0.139058	SG2	DUSP14	NC_056067.1	48770001	48790001	0.466557	0.181866	SG1	DYRK1B
0.439999	0.150616	SG2	DUSP14;TADA2A	NC_056061.1	63890001	63910001	0.0587762	0.277646	SG1	ECT2L
0.43672	0.139053	SG2	DYRK3	NC_056061.1	63895001	63915001	0.0874939	0.234772	SG1	ECT2L
0.397557	0.136318	SG2	DYRK3	NC_056061.1	63900001	63920001	0.095031	0.219526	SG1	ECT2L
0.504061	0.138732	SG2	DZIP3	NC_056061.1	63905001	63925001	0.111375	0.199148	SG1	ECT2L
0.544407	0.150389	SG2	DZIP3	NC_056061.1	63925001	63945001	0.164836	0.184653	SG1	ECT2L;REPS1
0.44161	0.161926	SG2	ECPAS	NC_056065.1	65215001	65235001	0.454536	0.187186	SG1	EDEM3
0.345229	0.190885	SG2	ECPAS	NC_056065.1	65220001	65240001	0.406822	0.234607	SG1	EDEM3
0.363005	0.168166	SG2	EDIL3	NC_056077.1	20495001	20515001	0.381843	0.275363	SG1	EEF2K
0.393245	0.202193	SG2	EDN1	NC_056077.1	20500001	20520001	0.100897	0.214037	SG1	EEF2K;POLR3
0.538167	0.204475	SG2	EDN1	NC_056065.1	31525001	31545001	0.309152	0.186411	SG1	EFCAB2
0.293323	0.253752	SG2	EEF1E1	NC_056065.1	31530001	31550001	0.265045	0.194985	SG1	EFCAB2
0.084605	0.280525	SG2	EEF1E1	NC_056056.1	220195001	220215001	0.373187	0.177808	SG1	EFCAB6
0.221087	0.232421	SG2	EEF1E1	NC_056069.1	36210001	36230001	0.46962	0.200676	SG1	EGFLAM
0.480037	0.165997	SG2	EEF1E1	NC_056069.1	36215001	36235001	0.457627	0.20277	SG1	EGFLAM
0.441489	0.146851	SG2	EEPD1	NC_056069.1	36250001	36270001	0.456353	0.219225	SG1	EGFLAM
0.465224	0.149242	SG2	EEPD1	NC_056069.1	36255001	36275001	0.465049	0.194527	SG1	EGFLAM
0.476461	0.179203	SG2	EFCAB2	NC_056069.1	36290001	36310001	0.440545	0.185917	SG1	EGFLAM
0.528283	0.143697	SG2	EFCAB6	NC_056060.1	59470001	59490001	0.408929	0.189216	SG1	EID1;SHC4
0.523992	0.300971	SG2	EFNB1	NC_056060.1	59475001	59495001	0.401756	0.23247	SG1	EID1;SHC4
0.526096	0.309315	SG2	EFNB1	NC_056060.1	59480001	59500001	0.402591	0.224471	SG1	EID1;SHC4
0.559471	0.295581	SG2	EFNB1	NC_056062.1	69160001	69180001	0.330082	0.204721	SG1	EIF3E
0.180569	0.355718	SG2	EHF	NC_056056.1	215490001	215510001	0.199569	0.26014	SG1	EIF3L
0.470023	0.195257	SG2	EIF2AK3	NC_056056.1	215495001	215515001	0.0687173	0.347694	SG1	EIF3L
0.257491	0.215614	SG2	EIF2AK3	NC_056057.1	61530001	61550001	0.365096	0.226504	SG1	ELMO1
0.533475	0.176943	SG2	EIF3A	NC_056057.1	61535001	61555001	0.343047	0.203365	SG1	ELMO1
0.499082	0.159806	SG2	EIF3A	NC_056057.1	61540001	61560001	0.323378	0.180374	SG1	ELMO1
0.493176	0.169879	SG2	EIPR1	NC_056057.1	61545001	61565001	0.441438	0.193697	SG1	ELMO1
0.466092	0.200975	SG2	EIPR1	NC_056055.1	102480001	102500001	0.464026	0.193655	SG1	ELP3
0.42016	0.226584	SG2	EIPR1	NC_056055.1	102485001	102505001	0.453101	0.220883	SG1	ELP3
0.420673	0.230382	SG2	EIPR1	NC_056055.1	102490001	102510001	0.412656	0.217043	SG1	ELP3
0.497068	0.21125	SG2	EIPR1	NC_056068.1	61135001	61155001	0.28203	0.435808	SG1	ELP4

0.460125	0.255789	SG2	ELP4	NC_056068.1	61140001	61160001	0.369839	0.415485	SG1	ELP4
0.428864	0.209635	SG2	ELP4	NC_056068.1	61125001	61145001	0.463621	0.380577	SG1	ELP4;IMMP1L
0.311665	0.244745	SG2	ELP4	NC_056068.1	61130001	61150001	0.327109	0.481171	SG1	ELP4;IMMP1L
0.477935	0.164394	SG2	ELP4	NC_056072.1	52135001	52155001	0.0613315	0.272731	SG1	ELP6
0.516322	0.218636	SG2	EMG1;LPCAT3;PH	NC_056072.1	52140001	52160001	0.0426432	0.189574	SG1	ELP6
0.403249	0.26139	SG2	EMG1;LPCAT3;PH	NC_056072.1	52155001	52175001	0.271953	0.173429	SG1	ELP6;LOC1011
0.338248	0.252181	SG2	EMG1;PHB2;PTPN	NC_056072.1	52160001	52180001	0.333333	0.191924	SG1	ELP6;LOC1011
0.300672	0.238391	SG2	EMG1;PHB2;PTPN	NC_056056.1	7595001	7615001	0.0862942	0.29913	SG1	ENDOG;LOC1(
0.183214	0.189743	SG2	EN2	NC_056056.1	7600001	7620001	0.0583241	0.341617	SG1	ENDOG;LOC1(
0.450483	0.137041	SG2	EN2	NC_056063.1	13955001	13975001	0.384308	0.174733	SG1	ENOX1
0.342763	0.160806	SG2	ENAH	NC_056063.1	13960001	13980001	0.428704	0.217724	SG1	ENOX1
0.247013	0.214432	SG2	ENAH	NC_056063.1	13965001	13985001	0.453067	0.268436	SG1	ENOX1
0.200899	0.254614	SG2	ENAH	NC_056080.1	104070001	104090001	0.438273	0.260572	SG1	ENOX2
0.174837	0.257065	SG2	ENAH	NC_056080.1	104075001	104095001	0.393782	0.333261	SG1	ENOX2
0.320104	0.229501	SG2	ENO4;HSPA12A	NC_056080.1	104080001	104100001	0.309211	0.387663	SG1	ENOX2
0.527261	0.251805	SG2	ENO4;HSPA12A	NC_056080.1	104130001	104150001	0.10338	0.331125	SG1	ENOX2
0.326236	0.166733	SG2	ENO4;SHTN1	NC_056080.1	104135001	104155001	0.144903	0.197583	SG1	ENOX2
0.435063	0.223923	SG2	ENO4;SHTN1	NC_056060.1	1505001	1525001	0.408845	0.200339	SG1	EPB41L4A
0.513048	0.182472	SG2	ENPP6	NC_056060.1	1510001	1530001	0.22741	0.229465	SG1	EPB41L4A
0.33551	0.197495	SG2	ENPP6	NC_056060.1	1515001	1535001	0.0578542	0.203804	SG1	EPB41L4A
0.341873	0.236708	SG2	ENPP6	NC_056057.1	51255001	51275001	0.155718	0.174178	SG1	EPDR1
0.423413	0.225004	SG2	ENPP6	NC_056057.1	51260001	51280001	0.128163	0.184529	SG1	EPDR1
0.347306	0.171536	SG2	ENTPD5	NC_056057.1	51265001	51285001	0.119921	0.185073	SG1	EPDR1
0.202899	0.254353	SG2	ENTPD5	NC_056057.1	51270001	51290001	0.107001	0.185198	SG1	EPDR1
0.361724	0.162813	SG2	EPC1	NC_056057.1	51275001	51295001	0.0787737	0.188522	SG1	EPDR1
0.257041	0.21505	SG2	EPC1	NC_056059.1	89990001	90010001	0.129802	0.239574	SG1	EPGN
0.214495	0.238594	SG2	EPC1	NC_056059.1	89995001	90015001	0.191489	0.196317	SG1	EPGN
0.187356	0.252081	SG2	EPC1	NC_056059.1	90000001	90020001	0.214427	0.19228	SG1	EPGN
0.25	0.2302	SG2	EPC1	NC_056059.1	89985001	90005001	0.259259	0.199922	SG1	EPGN;MTHFD
0.430134	0.194641	SG2	EPC1	NC_056069.1	13630001	13650001	0.230109	0.23635	SG1	ERBIN
0.440456	0.203449	SG2	EPC1	NC_056069.1	13635001	13655001	0.285274	0.180879	SG1	ERBIN
0.413332	0.145022	SG2	EPG5	NC_056069.1	13735001	13755001	0.117001	0.202206	SG1	ERBIN

0.303095	0.1868	SG2	EPG5	NC_056056.1	213805001	213825001	0.40412	0.180967	SG1	ERC1
0.279931	0.198543	SG2	EPG5	NC_056056.1	213810001	213830001	0.2207	0.226582	SG1	ERC1
0.26013	0.211819	SG2	EPG5	NC_056056.1	213815001	213835001	0.330393	0.220145	SG1	ERC1
0.531232	0.175504	SG2	EPG5	NC_056054.1	271285001	271305001	0.300049	0.174932	SG1	ERG
0.381064	0.147137	SG2	EPHA1	NC_056054.1	271290001	271310001	0.111475	0.296999	SG1	ERG
0.550723	0.174472	SG2	EPHB1	NC_056054.1	271295001	271315001	0.0475715	0.337819	SG1	ERG
0.433841	0.200972	SG2	EPHB1	NC_056054.1	271300001	271320001	0.0520079	0.377854	SG1	ERG
0.377687	0.19694	SG2	EPHB1	NC_056054.1	271305001	271325001	0.184358	0.313174	SG1	ERG
0.552604	0.150189	SG2	EPHB1	NC_056054.1	271310001	271330001	0.323403	0.225214	SG1	ERG
0.202132	0.167309	SG2	EPRS1	NC_056054.1	271315001	271335001	0.438419	0.185837	SG1	ERG
0.179409	0.180311	SG2	EPRS1	NC_056054.1	271335001	271355001	0.481787	0.254203	SG1	ERG
0.284147	0.146789	SG2	EPRS1	NC_056054.1	271340001	271360001	0.402251	0.244162	SG1	ERG
0.374799	0.16191	SG2	ERC2	NC_056054.1	271380001	271400001	0.192409	0.234588	SG1	ERG
0.407261	0.172316	SG2	ESRP1	NC_056054.1	271385001	271405001	0.190112	0.263486	SG1	ERG
0.26084	0.228578	SG2	ESRP1	NC_056054.1	271390001	271410001	0.154401	0.289686	SG1	ERG
0.287007	0.216832	SG2	ESRP1	NC_056054.1	271395001	271415001	0.288392	0.231177	SG1	ERG
0.516096	0.14335	SG2	ESRP1	NC_056056.1	185765001	185785001	0.0519842	0.282088	SG1	ERGIC2
0.341105	0.343507	SG2	ETFA	NC_056056.1	185770001	185790001	0.0638581	0.267354	SG1	ERGIC2
0.305846	0.327392	SG2	ETFA	NC_056056.1	185775001	185795001	0.0955221	0.227181	SG1	ERGIC2
0.310309	0.31058	SG2	ETFA	NC_056061.1	76830001	76850001	0.395287	0.174368	SG1	ESR1
0.460669	0.195847	SG2	ETFA	NC_056061.1	76835001	76855001	0.227385	0.244628	SG1	ESR1
0.519939	0.203114	SG2	ETFDH	NC_056061.1	76840001	76860001	0.0778975	0.316004	SG1	ESR1
0.492098	0.221154	SG2	ETFDH	NC_056061.1	76845001	76865001	0.265389	0.234615	SG1	ESR1
0.500756	0.282322	SG2	EXO1	NC_056054.1	69780001	69800001	0.479411	0.319558	SG1	EVI5
0.333333	0.2045	SG2	EXOC4	NC_056054.1	69785001	69805001	0.434661	0.326903	SG1	EVI5
0.25492	0.226416	SG2	EXOC4	NC_056054.1	69790001	69810001	0.363184	0.277902	SG1	EVI5
0.28218	0.232021	SG2	EXOC4	NC_056054.1	69795001	69815001	0.322979	0.255922	SG1	EVI5
0.321575	0.201241	SG2	EXOC4	NC_056054.1	69800001	69820001	0.360425	0.244284	SG1	EVI5
0.349229	0.146955	SG2	EXOC6B	NC_056054.1	69870001	69890001	0.396355	0.300737	SG1	EVI5
0.318778	0.213793	SG2	EYA4	NC_056054.1	69875001	69895001	0.334391	0.247107	SG1	EVI5
0.360131	0.196863	SG2	EYA4	NC_056054.1	69880001	69900001	0.332272	0.202284	SG1	EVI5
0.402274	0.166195	SG2	EYA4	NC_056054.1	69895001	69915001	0.26209	0.202897	SG1	EVI5

0.367678	0.136258	SG2	FAM107A;FAM3D	NC_056054.1	69900001	69920001	0.220452	0.223293	SG1	EVI5
0.533672	0.182823	SG2	FAM13A	NC_056054.1	69905001	69925001	0.152851	0.276623	SG1	EVI5
0.143039	0.140962	SG2	FAM171B	NC_056054.1	69910001	69930001	0.225699	0.267698	SG1	EVI5
0.318472	0.142679	SG2	FAM210B	NC_056054.1	69940001	69960001	0.302891	0.244347	SG1	EVI5
0.2819	0.15378	SG2	FAM210B	NC_056054.1	69945001	69965001	0.232861	0.289358	SG1	EVI5
0.368625	0.13835	SG2	FAM3D	NC_056054.1	69950001	69970001	0.290323	0.338484	SG1	EVI5
0.287732	0.14237	SG2	FAM3D	NC_056054.1	69955001	69975001	0.295105	0.388455	SG1	EVI5
0.227954	0.156696	SG2	FAM3D	NC_056054.1	69960001	69980001	0.458885	0.306865	SG1	EVI5
0.36139	0.136586	SG2	FAM83F	NC_056054.1	24990001	25010001	0.321519	0.194324	SG1	FAF1
0.376279	0.138844	SG2	FAM83F	NC_056054.1	24995001	25015001	0.274315	0.203855	SG1	FAF1
0.328174	0.137305	SG2	FAR1	NC_056054.1	25005001	25025001	0.375	0.190196	SG1	FAF1
0.515924	0.370152	SG2	FAR1	NC_056054.1	25010001	25030001	0.440172	0.19048	SG1	FAF1
0.561744	0.409915	SG2	FAR1	NC_056062.1	17500001	17520001	0.424501	0.203924	SG1	FAM135B
0.571121	0.410471	SG2	FAR1	NC_056062.1	17505001	17525001	0.365471	0.259875	SG1	FAM135B
0.546817	0.415384	SG2	FAR1	NC_056062.1	17510001	17530001	0.462121	0.239997	SG1	FAM135B
0.546169	0.413579	SG2	FAR1	NC_056059.1	13695001	13715001	0.261935	0.182389	SG1	FAM241A
0.529557	0.415048	SG2	FAR1	NC_056063.1	74790001	74810001	0.407354	0.206116	SG1	FARP1;STK24
0.523908	0.432695	SG2	FAR1	NC_056063.1	74795001	74815001	0.426444	0.194712	SG1	FARP1;STK24
0.548841	0.467746	SG2	FAR1	NC_056075.1	10525001	10545001	0.475927	0.258548	SG1	FAS
0.558764	0.458739	SG2	FAR1	NC_056075.1	10530001	10550001	0.438967	0.281287	SG1	FAS
0.564327	0.465644	SG2	FAR1	NC_056075.1	10535001	10555001	0.482658	0.280795	SG1	FAS
0.562797	0.423943	SG2	FAR1	NC_056058.1	60540001	60560001	0.427659	0.175006	SG1	FAT2
0.567378	0.34331	SG2	FAR1	NC_056058.1	104960001	104980001	0.40201	0.236145	SG1	FBXL17
0.480302	0.138422	SG2	FAT3	NC_056058.1	104965001	104985001	0.454664	0.253192	SG1	FBXL17
0.300553	0.166	SG2	FAT3	NC_056054.1	110245001	110265001	0.32547	0.203305	SG1	FCER1A
0.18921	0.151147	SG2	FAT3	NC_056054.1	110250001	110270001	0.275471	0.323263	SG1	FCER1A;LOC1
0.324409	0.248501	SG2	FAT4	NC_056054.1	110255001	110275001	0.38588	0.30364	SG1	FCER1A;LOC1
0.254294	0.27588	SG2	FAT4	NC_056068.1	51130001	51150001	0.259912	0.271054	SG1	FCHSD2
0.262384	0.253142	SG2	FAT4	NC_056068.1	51135001	51155001	0.191043	0.331557	SG1	FCHSD2
0.268058	0.239625	SG2	FAT4	NC_056068.1	51140001	51160001	0.144662	0.388042	SG1	FCHSD2
0.403571	0.177395	SG2	FAT4	NC_056068.1	51145001	51165001	0.144988	0.44114	SG1	FCHSD2
0.502794	0.13633	SG2	FBXL8;HSF4;KIAA	NC_056068.1	51150001	51170001	0.238785	0.464255	SG1	FCHSD2

0.311111	0.188034	SG2	FBXL8;HSF4;KIAA	NC_056068.1	51255001	51275001	0.0943668	0.17536	SG1	FCHSD2
0.452727	0.210146	SG2	FBXO22;NRG4	NC_056069.1	30605001	30625001	0.358696	0.213527	SG1	FGF10
0.486375	0.193434	SG2	FBXO22;NRG4	NC_056069.1	30610001	30630001	0.280104	0.232979	SG1	FGF10
0.528967	0.159081	SG2	FBXO28	NC_056069.1	30615001	30635001	0.246269	0.266219	SG1	FGF10
0.37396	0.171135	SG2	FBXO28	NC_056069.1	30620001	30640001	0.206196	0.201136	SG1	FGF10
0.254009	0.221112	SG2	FBXO28	NC_056069.1	30625001	30645001	0.327778	0.264049	SG1	FGF10
0.383938	0.183776	SG2	FBXO28	NC_056069.1	30630001	30650001	0.26598	0.323207	SG1	FGF10
0.406276	0.15448	SG2	FCHSD2	NC_056069.1	30635001	30655001	0.203317	0.285837	SG1	FGF10
0.288621	0.182677	SG2	FCHSD2	NC_056069.1	30640001	30660001	0.290454	0.25873	SG1	FGF10
0.465154	0.188569	SG2	FCHSD2	NC_056069.1	30645001	30665001	0.37579	0.196212	SG1	FGF10
0.292212	0.19256	SG2	FERMT2	NC_056069.1	30665001	30685001	0.240588	0.225889	SG1	FGF10
0.348485	0.17599	SG2	FERMT2	NC_056069.1	30670001	30690001	0.352694	0.274017	SG1	FGF10
0.277778	0.199073	SG2	FERMT2	NC_056069.1	30675001	30695001	0.367187	0.259715	SG1	FGF10
0.28113	0.196983	SG2	FERMT2	NC_056069.1	30680001	30700001	0.454154	0.272124	SG1	FGF10
0.173987	0.236427	SG2	FERMT2	NC_056069.1	30685001	30705001	0.451632	0.262509	SG1	FGF10
0.114189	0.260849	SG2	FERMT2	NC_056072.1	40345001	40365001	0.0623389	0.178245	SG1	FHIT
0.28681	0.19743	SG2	FERMT2	NC_056063.1	32270001	32290001	0.452865	0.300909	SG1	FLT3
0.474859	0.140895	SG2	FEZ2	NC_056063.1	32325001	32345001	0.239854	0.20135	SG1	FLT3
0.542803	0.136872	SG2	FEZ2	NC_056063.1	32330001	32350001	0.0443724	0.306254	SG1	FLT3;URAD
0.424232	0.140518	SG2	FGF9	NC_056058.1	20545001	20565001	0.486766	0.280142	SG1	FNIP1
0.423361	0.155545	SG2	FGF9	NC_056058.1	20590001	20610001	0.479533	0.27987	SG1	FNIP1
0.44366	0.150737	SG2	FGF9	NC_056058.1	20595001	20615001	0.451613	0.279311	SG1	FNIP1;RAPGEI
0.564169	0.136321	SG2	FHAD1	NC_056058.1	20600001	20620001	0.453395	0.281463	SG1	FNIP1;RAPGEI
0.407217	0.222412	SG2	FHIT	NC_056055.1	89255001	89275001	0.200483	0.254736	SG1	FOCAD
0.082725	0.136075	SG2	FLT1	NC_056055.1	89310001	89330001	0.186781	0.329402	SG1	FOCAD
0.185813	0.15508	SG2	FMN2	NC_056055.1	89315001	89335001	0.325572	0.275694	SG1	FOCAD
0.242843	0.171265	SG2	FMN2	NC_056056.1	76110001	76130001	0.287798	0.22926	SG1	FOXN2
0.437689	0.167039	SG2	FMN2	NC_056056.1	76115001	76135001	0.287449	0.220925	SG1	FOXN2
0.146821	0.176704	SG2	FOLR1;FOLR3	NC_056056.1	76120001	76140001	0.322799	0.200284	SG1	FOXN2
0.506787	0.146962	SG2	FRMD5	NC_056072.1	30240001	30260001	0.41878	0.255156	SG1	FOXP1
0.479323	0.142486	SG2	FRMD5	NC_056057.1	55810001	55830001	0.388144	0.211855	SG1	FOXP2
0.428375	0.146834	SG2	FRMD5	NC_056057.1	55815001	55835001	0.303492	0.253196	SG1	FOXP2

0.404737	0.171695	SG2	FRMD5	NC_056057.1	55820001	55840001	0.210469	0.278566	SG1	FOXP2
0.484789	0.156374	SG2	FSTL5	NC_056057.1	55825001	55845001	0.346712	0.263241	SG1	FOXP2
0.561009	0.162033	SG2	FSTL5	NC_056059.1	93840001	93860001	0.134817	0.184315	SG1	FRAS1
0.572292	0.138566	SG2	G2E3	NC_056063.1	29145001	29165001	0.474441	0.218998	SG1	FRY
0.460756	0.234924	SG2	G2E3	NC_056059.1	67865001	67885001	0.391238	0.176845	SG1	FRYL
0.459791	0.242919	SG2	G2E3	NC_056059.1	67870001	67890001	0.280437	0.201716	SG1	FRYL
0.426959	0.195032	SG2	G2E3	NC_056059.1	67875001	67895001	0.225065	0.193542	SG1	FRYL
0.379257	0.162182	SG2	G2E3	NC_056059.1	67880001	67900001	0.203826	0.187984	SG1	FRYL
0.472175	0.13945	SG2	G2E3	NC_056059.1	67905001	67925001	0.219704	0.183291	SG1	FRYL
0.444207	0.179635	SG2	GABRA3	NC_056059.1	67915001	67935001	0.374321	0.176243	SG1	FRYL
0.369503	0.210339	SG2	GABRA3	NC_056071.1	22390001	22410001	0.452647	0.224535	SG1	FSD2;WHAMM
0.313206	0.221131	SG2	GABRA3	NC_056077.1	27765001	27785001	0.21609	0.333912	SG1	FUS
0.366921	0.208772	SG2	GABRA3	NC_056077.1	27770001	27790001	0.143208	0.323204	SG1	FUS
0.441963	0.188448	SG2	GABRB1	NC_056077.1	27775001	27795001	0.290657	0.340577	SG1	FUS
0.326458	0.136346	SG2	GALNT2	NC_056077.1	27780001	27800001	0.366617	0.304662	SG1	FUS
0.347365	0.194885	SG2	GALNT2	NC_056077.1	27785001	27805001	0.45995	0.272031	SG1	FUS;LOC1011(
0.454356	0.215524	SG2	GALNT2	NC_056058.1	70925001	70945001	0.436199	0.280087	SG1	GABRA6
0.072893	0.144389	SG2	GALNTL6	NC_056059.1	66675001	66695001	0.36806	0.175877	SG1	GABRB1
0.067086	0.145334	SG2	GALNTL6	NC_056059.1	66680001	66700001	0.299043	0.181826	SG1	GABRB1
0.043281	0.155981	SG2	GALNTL6	NC_056061.1	48215001	48235001	0.372981	0.202698	SG1	GABRR2
0.083333	0.144323	SG2	GALNTL6	NC_056061.1	48200001	48220001	0.336697	0.323029	SG1	GABRR2;UBE
0.093842	0.141588	SG2	GALNTL6	NC_056061.1	48205001	48225001	0.242496	0.291412	SG1	GABRR2;UBE
0.080514	0.144634	SG2	GALNTL6	NC_056061.1	48210001	48230001	0.252464	0.267598	SG1	GABRR2;UBE
0.059392	0.149922	SG2	GALNTL6	NC_056072.1	5240001	5260001	0.437127	0.227492	SG1	GADL1
0.078117	0.147955	SG2	GALNTL6	NC_056054.1	181270001	181290001	0.487921	0.209795	SG1	GAP43
0.517389	0.190169	SG2	GARRE1;GPI	NC_056054.1	181275001	181295001	0.388283	0.216915	SG1	GAP43
0.473302	0.208155	SG2	GARRE1;GPI	NC_056054.1	181280001	181300001	0.437079	0.200538	SG1	GAP43
0.544833	0.181785	SG2	GARRE1;LOC1011	NC_056066.1	72650001	72670001	0.329988	0.176404	SG1	GDAP1L1
0.423603	0.14153	SG2	GATD3	NC_056066.1	53280001	53300001	0.460988	0.252405	SG1	GINS1
0.448676	0.160969	SG2	GATD3;PWP2	NC_056055.1	52395001	52415001	0.33935	0.317588	SG1	GLIPR2
0.455906	0.200852	SG2	GATD3;PWP2	NC_056080.1	12895001	12915001	0.373748	0.201971	SG1	GLRA2
0.388609	0.163532	SG2	GATD3;PWP2	NC_056055.1	105970001	105990001	0.472406	0.209605	SG1	GLRA3

0.215739	0.158798	SG2	GCK;YKT6	NC_056055.1	105975001	105995001	0.475228	0.181514	SG1	GLRA3
0.507794	0.182843	SG2	GHRHR	NC_056075.1	48910001	48930001	0.460432	0.271131	SG1	GLRX3
0.523105	0.177767	SG2	GHRHR	NC_056054.1	86790001	86810001	0.3764	0.252564	SG1	GNAI3
0.490683	0.139999	SG2	GLI2	NC_056054.1	86795001	86815001	0.352742	0.298679	SG1	GNAI3
0.404162	0.157033	SG2	GLI2	NC_056054.1	86800001	86820001	0.333147	0.29303	SG1	GNAI3
0.281623	0.192005	SG2	GLI2	NC_056054.1	86805001	86825001	0.485846	0.270308	SG1	GNAI3
0.255682	0.224737	SG2	GLI2	NC_056058.1	50710001	50730001	0.336674	0.193675	SG1	GNPDA1
0.408939	0.176112	SG2	GLI2	NC_056058.1	50715001	50735001	0.429575	0.311112	SG1	GNPDA1
0.272408	0.149047	SG2	GNA14	NC_056058.1	50720001	50740001	0.458665	0.349176	SG1	GNPDA1
0.066374	0.161315	SG2	GNA14	NC_056058.1	50725001	50745001	0.416022	0.353839	SG1	GNPDA1
0.087791	0.169762	SG2	GNA14	NC_056058.1	50705001	50725001	0.418326	0.187938	SG1	GNPDA1;RNF1
0.11082	0.165698	SG2	GNA14	NC_056072.1	10905001	10925001	0.391056	0.202964	SG1	GOLGA4
0.075844	0.139562	SG2	GNA14	NC_056072.1	10910001	10930001	0.335756	0.195539	SG1	GOLGA4
0.435097	0.135069	SG2	GNG10;SHOC1	NC_056075.1	32255001	32275001	0.453996	0.399789	SG1	GPAM
0.449129	0.139447	SG2	GNG7	NC_056075.1	32260001	32280001	0.241891	0.440771	SG1	GPAM
0.563481	0.156371	SG2	GNG7	NC_056075.1	32265001	32285001	0.158696	0.407453	SG1	GPAM
0.463094	0.224796	SG2	GON4L	NC_056075.1	32270001	32290001	0.192511	0.440394	SG1	GPAM
0.215268	0.301928	SG2	GON4L;YY1AP1	NC_056075.1	32275001	32295001	0.0868896	0.300215	SG1	GPAM
0.188172	0.31838	SG2	GON4L;YY1AP1	NC_056059.1	98975001	98995001	0.210223	0.250113	SG1	GPAT3
0.44899	0.223746	SG2	GPC5	NC_056059.1	98980001	99000001	0.281076	0.273908	SG1	GPAT3
0.259947	0.286239	SG2	GPC5	NC_056059.1	98985001	99005001	0.339607	0.28125	SG1	GPAT3
0.3233	0.208241	SG2	GPC5	NC_056059.1	98990001	99010001	0.418423	0.291659	SG1	GPAT3
0.424829	0.147417	SG2	GPC5	NC_056059.1	98995001	99015001	0.452099	0.24451	SG1	GPAT3
0.387349	0.14836	SG2	GPC5	NC_056059.1	99000001	99020001	0.378252	0.276853	SG1	GPAT3
0.336006	0.172393	SG2	GPC5	NC_056059.1	99005001	99025001	0.385321	0.249763	SG1	GPAT3
0.350911	0.176112	SG2	GPC5	NC_056079.1	35865001	35885001	0.38332	0.185151	SG1	GPAT4
0.450766	0.147601	SG2	GPC5	NC_056079.1	35870001	35890001	0.299796	0.206338	SG1	GPAT4
0.534007	0.142588	SG2	GPC5	NC_056079.1	35875001	35895001	0.229479	0.2254	SG1	GPAT4
0.164706	0.140143	SG2	GPC5	NC_056079.1	35880001	35900001	0.202864	0.181208	SG1	GPAT4;NKX6-
0.087889	0.141364	SG2	GPC5	NC_056080.1	101485001	101505001	0.470588	0.282761	SG1	GPC4
0.088218	0.139501	SG2	GPC5	NC_056080.1	101550001	101570001	0.137833	0.210551	SG1	GPC4
0.050776	0.158572	SG2	GPC5	NC_056063.1	66695001	66715001	0.310357	0.182867	SG1	GPC5

0.569818	0.176184	SG2	GPHN	NC_056063.1	66700001	66720001	0.306651	0.194708	SG1	GPC5
0.394335	0.156314	SG2	GPI	NC_056063.1	67540001	67560001	0.452398	0.210932	SG1	GPC5
0.546108	0.145675	SG2	GPR108;LOC10110	NC_056066.1	25475001	25495001	0.171512	0.183401	SG1	GPR158
0.473953	0.19402	SG2	GPR158	NC_056066.1	25480001	25500001	0.127625	0.200564	SG1	GPR158
0.365214	0.211112	SG2	GPR158	NC_056062.1	75700001	75720001	0.380244	0.188253	SG1	GRHL2
0.380872	0.217942	SG2	GPR158	NC_056062.1	75705001	75725001	0.292466	0.228448	SG1	GRHL2
0.465368	0.137043	SG2	GPR158	NC_056062.1	75710001	75730001	0.322334	0.213656	SG1	GRHL2
0.380805	0.221849	SG2	GPR161	NC_056062.1	75715001	75735001	0.317529	0.238374	SG1	GRHL2
0.289473	0.235932	SG2	GPR161	NC_056062.1	75720001	75740001	0.433467	0.22082	SG1	GRHL2
0.270229	0.219797	SG2	GPR161	NC_056062.1	75830001	75850001	0.272078	0.20796	SG1	GRHL2
0.256097	0.198797	SG2	GPR161	NC_056062.1	75835001	75855001	0.301339	0.190199	SG1	GRHL2
0.23586	0.198705	SG2	GPR161	NC_056062.1	75650001	75670001	0.259013	0.21342	SG1	GRHL2;NCALI
0.216825	0.229158	SG2	GPR161	NC_056054.1	126875001	126895001	0.136646	0.340148	SG1	GRIK1
0.158113	0.238894	SG2	GPR161	NC_056054.1	126955001	126975001	0.116564	0.174214	SG1	GRIK1
0.458874	0.29257	SG2	GPR19	NC_056054.1	126960001	126980001	0.152524	0.211098	SG1	GRIK1
0.398633	0.245994	SG2	GPR19	NC_056056.1	153455001	153475001	0.403892	0.210106	SG1	GRIP1
0.330472	0.210642	SG2	GPR19	NC_056056.1	153460001	153480001	0.30554	0.193846	SG1	GRIP1
0.290323	0.161572	SG2	GPR19	NC_056056.1	153465001	153485001	0.205822	0.209978	SG1	GRIP1
0.480388	0.164341	SG2	GPR63	NC_056056.1	153470001	153490001	0.2822	0.190527	SG1	GRIP1
0.439496	0.177599	SG2	GPR63	NC_056070.1	65610001	65630001	0.456389	0.236648	SG1	GRK3
0.416011	0.180833	SG2	GPR63	NC_056060.1	90675001	90695001	0.0833337	0.306456	SG1	GTF2A1
0.444509	0.169475	SG2	GPR63	NC_056060.1	90680001	90700001	0.254961	0.22803	SG1	GTF2A1
0.473505	0.168498	SG2	GPX3	NC_056077.1	33355001	33375001	0.200844	0.207821	SG1	GTF2IRD1
0.435134	0.181178	SG2	GPX3	NC_056077.1	33360001	33380001	0.152312	0.226333	SG1	GTF2IRD1
0.371134	0.211181	SG2	GPX3;TNIP1	NC_056077.1	33365001	33385001	0.214068	0.208218	SG1	GTF2IRD1
0.457306	0.17345	SG2	GRAMD2B	NC_056077.1	33370001	33390001	0.280116	0.244903	SG1	GTF2IRD1
0.442204	0.192474	SG2	GRAMD2B	NC_056077.1	33375001	33395001	0.318349	0.275966	SG1	GTF2IRD1
0.476891	0.182529	SG2	GRAMD2B	NC_056077.1	33380001	33400001	0.302906	0.224885	SG1	GTF2IRD1
0.502864	0.168515	SG2	GRAMD2B	NC_056077.1	33385001	33405001	0.323477	0.184323	SG1	GTF2IRD1
0.461938	0.144648	SG2	GRIA4	NC_056068.1	16265001	16285001	0.198905	0.179574	SG1	GUCY1A2
0.454744	0.16486	SG2	GRIA4	NC_056055.1	29690001	29710001	0.407111	0.174429	SG1	HABP4
0.440984	0.182641	SG2	GRIA4	NC_056055.1	29695001	29715001	0.41894	0.19506	SG1	HABP4

0.480437	0.138758	SG2	GRIA4	NC_056055.1	29700001	29720001	0.422285	0.176944	SG1	HABP4
0.539121	0.167578	SG2	GRID2	NC_056066.1	61255001	61275001	0.194986	0.184214	SG1	HCK
0.510889	0.161651	SG2	GRID2	NC_056066.1	61260001	61280001	0.090211	0.216577	SG1	HCK
0.491761	0.154697	SG2	GRID2	NC_056066.1	61265001	61285001	0.200642	0.236281	SG1	HCK
0.452083	0.135791	SG2	GRID2	NC_056066.1	61270001	61290001	0.28897	0.214433	SG1	HCK;TM9SF4
0.413514	0.140768	SG2	GRID2	NC_056066.1	61275001	61295001	0.398631	0.189011	SG1	HCK;TM9SF4
0.354343	0.156436	SG2	GRID2	NC_056080.1	75380001	75400001	0.427999	0.228771	SG1	HDX
0.362543	0.145564	SG2	GRID2	NC_056080.1	75390001	75410001	0.439718	0.226379	SG1	HDX
0.408488	0.203554	SG2	GRIK2	NC_056080.1	75405001	75425001	0.328859	0.206762	SG1	HDX
0.458492	0.250435	SG2	GRK4;HTT	NC_056080.1	75410001	75430001	0.421965	0.210604	SG1	HDX
0.420591	0.212572	SG2	GRK5	NC_056080.1	75420001	75440001	0.409678	0.204875	SG1	HDX
0.478142	0.179136	SG2	GRM7	NC_056080.1	75440001	75460001	0.456928	0.207879	SG1	HDX
0.514681	0.198572	SG2	GRM7	NC_056080.1	75445001	75465001	0.360656	0.233902	SG1	HDX
0.560626	0.181703	SG2	GRM7	NC_056080.1	75455001	75475001	0.359374	0.194637	SG1	HDX
0.142857	0.138295	SG2	GRM8	NC_056080.1	75470001	75490001	0.444915	0.183964	SG1	HDX
0.280983	0.14201	SG2	GRM8	NC_056070.1	62120001	62140001	0.396187	0.202442	SG1	HECTD4
0.323467	0.15381	SG2	GRM8	NC_056055.1	198780001	198800001	0.281463	0.212254	SG1	HECW2
0.37249	0.259284	SG2	GSPT2	NC_056056.1	153695001	153715001	0.286803	0.187021	SG1	HELB
0.382819	0.2656	SG2	GSPT2	NC_056074.1	8715001	8735001	0.0946501	0.465842	SG1	HIKESHI
0.424185	0.317691	SG2	GSPT2	NC_056074.1	8720001	8740001	0.0751678	0.486288	SG1	HIKESHI
0.552025	0.319753	SG2	GSPT2	NC_056074.1	8725001	8745001	0.0823477	0.462165	SG1	HIKESHI
0.553031	0.32807	SG2	GSPT2	NC_056074.1	8730001	8750001	0.17538	0.365774	SG1	HIKESHI
0.413131	0.197263	SG2	GTPBP10;HUS1	NC_056074.1	8735001	8755001	0.413285	0.298108	SG1	HIKESHI
0.534168	0.154506	SG2	GXYLT2	NC_056074.1	8760001	8780001	0.422301	0.208933	SG1	HIKESHI
0.489986	0.163616	SG2	HCN1	NC_056078.1	24785001	24805001	0.38308	0.322365	SG1	HKDC1
0.461427	0.189727	SG2	HCN1	NC_056078.1	24790001	24810001	0.3548	0.423293	SG1	HKDC1
0.455989	0.1902	SG2	HCN1	NC_056078.1	24795001	24815001	0.329602	0.349679	SG1	HKDC1
0.409041	0.176397	SG2	HCN1	NC_056078.1	24780001	24800001	0.484343	0.255497	SG1	HKDC1;SUPV[illegible]
0.137539	0.188424	SG2	HCN1	NC_056059.1	115750001	115770001	0.357639	0.180805	SG1	HMX1
0.427405	0.315048	SG2	HDAC4	NC_056055.1	243240001	243260001	0.240064	0.179427	SG1	HNRNPR
0.499776	0.27691	SG2	HDAC4	NC_056055.1	243265001	243285001	0.109948	0.188829	SG1	HNRNPR
0.460881	0.152229	SG2	HDAC9	NC_056055.1	243270001	243290001	0.122029	0.265654	SG1	HNRNPR

0.43068	0.165526	SG2	HDAC9	NC_056057.1	70525001	70545001	0.133217	0.362755	SG1	HOXA1
0.551272	0.173299	SG2	HDAC9	NC_056057.1	70515001	70535001	0.27154	0.309692	SG1	HOXA1;HOXA
0.469615	0.215565	SG2	HEATR4	NC_056057.1	70520001	70540001	0.164557	0.369606	SG1	HOXA1;HOXA
0.306659	0.206743	SG2	HEATR4;RIOX1	NC_056056.1	132565001	132585001	0.46834	0.237717	SG1	HOXC13
0.27157	0.202642	SG2	HEATR4;RIOX1	NC_056056.1	132570001	132590001	0.462729	0.233824	SG1	HOXC13
0.325366	0.148555	SG2	HEATR4;RIOX1	NC_056075.1	19685001	19705001	0.343004	0.236186	SG1	HPSE2
0.546436	0.148426	SG2	HECTD4	NC_056075.1	19690001	19710001	0.243661	0.241519	SG1	HPSE2
0.56316	0.149347	SG2	HECTD4	NC_056075.1	19695001	19715001	0.187069	0.238306	SG1	HPSE2
0.382772	0.318402	SG2	HECW1	NC_056075.1	19700001	19720001	0.20288	0.204228	SG1	HPSE2
0.178932	0.358027	SG2	HECW1	NC_056075.1	19870001	19890001	0.344854	0.178363	SG1	HPSE2
0.310214	0.29038	SG2	HECW1	NC_056077.1	23875001	23895001	0.430912	0.218601	SG1	HS3ST4
0.424109	0.266997	SG2	HECW1	NC_056077.1	23880001	23900001	0.33045	0.192077	SG1	HS3ST4
0.489221	0.141365	SG2	HECW1	NC_056058.1	16435001	16455001	0.313472	0.204873	SG1	HSD11B1L;LO
0.526909	0.181113	SG2	HECW1	NC_056067.1	55185001	55205001	0.113914	0.249266	SG1	HSD17B14;PLI
0.196683	0.239206	SG2	HEPH	NC_056061.1	50210001	50230001	0.366416	0.215338	SG1	HTR1E
0.393041	0.153337	SG2	HEPH	NC_056061.1	50225001	50245001	0.287975	0.199405	SG1	HTR1E
0.365168	0.160145	SG2	HEPH	NC_056061.1	50230001	50250001	0.197912	0.245817	SG1	HTR1E
0.546376	0.191659	SG2	HEY1	NC_056061.1	50235001	50255001	0.163313	0.265345	SG1	HTR1E
0.55427	0.179291	SG2	HEY1	NC_056061.1	50240001	50260001	0.177922	0.208901	SG1	HTR1E
0.501215	0.200439	SG2	HJV	NC_056058.1	57840001	57860001	0.355623	0.229009	SG1	HTR4
0.489592	0.209338	SG2	HMOX2;NMRAL1	NC_056058.1	57845001	57865001	0.379311	0.319822	SG1	HTR4
0.533429	0.198562	SG2	HMOX2;NMRAL1	NC_056058.1	57850001	57870001	0.407875	0.338865	SG1	HTR4
0.479982	0.227556	SG2	HOMER2;WHAMM	NC_056058.1	57855001	57875001	0.465776	0.372964	SG1	HTR4
0.439726	0.215256	SG2	HOMER2;WHAMM	NC_056057.1	118565001	118585001	0.39129	0.289408	SG1	HTR5A
0.23125	0.166667	SG2	HOXD11;HOXD12;	NC_056057.1	118570001	118590001	0.266835	0.306206	SG1	HTR5A
0.450154	0.160214	SG2	HSPA12A	NC_056057.1	118575001	118595001	0.178782	0.357007	SG1	HTR5A
0.473887	0.147652	SG2	HSPA12A	NC_056057.1	118580001	118600001	0.465527	0.289946	SG1	HTR5A
0.460744	0.153338	SG2	HSPA12A	NC_056061.1	9320001	9340001	0.425899	0.194136	SG1	IBTK
0.494839	0.145303	SG2	HSPA12A	NC_056055.1	239395001	239415001	0.459372	0.183761	SG1	IFI6
0.244334	0.173226	SG2	HSPH1	NC_056072.1	56200001	56220001	0.255291	0.178222	SG1	IFT122
0.318308	0.176758	SG2	HSPH1	NC_056057.1	77775001	77795001	0.0953322	0.19577	SG1	IGFBP1;IGFBP
0.361711	0.146003	SG2	HSPH1	NC_056057.1	77780001	77800001	0.0780002	0.185873	SG1	IGFBP1;IGFBP

0.306569	0.156469	SG2	HSPH1	NC_056057.1	77785001	77805001	0.110213	0.174355	SG1	IGFBP1;IGFBP
0.186704	0.171291	SG2	HSPH1	NC_056057.1	77760001	77780001	0.409961	0.190409	SG1	IGFBP3
0.081841	0.202093	SG2	HSPH1	NC_056057.1	77765001	77785001	0.299626	0.221719	SG1	IGFBP3
0.04311	0.241138	SG2	HSPH1	NC_056057.1	77770001	77790001	0.16489	0.255124	SG1	IGFBP3
0.493764	0.222195	SG2	HTT	NC_056056.1	99445001	99465001	0.469879	0.184831	SG1	IL1RL2
0.446671	0.247016	SG2	HUS1	NC_056056.1	99450001	99470001	0.410266	0.20855	SG1	IL1RL2
0.297298	0.153071	SG2	HYDIN	NC_056056.1	99455001	99475001	0.414328	0.183477	SG1	IL1RL2
0.229723	0.176817	SG2	HYDIN	NC_056054.1	118975001	118995001	0.408047	0.284026	SG1	ILDR2
0.206575	0.216128	SG2	HYDIN	NC_056054.1	118980001	119000001	0.391574	0.299636	SG1	ILDR2
0.206317	0.138888	SG2	HYDIN	NC_056054.1	118985001	119005001	0.473004	0.328718	SG1	ILDR2
0.32273	0.138354	SG2	HYDIN	NC_056054.1	118990001	119010001	0.451922	0.309294	SG1	ILDR2
0.359225	0.151103	SG2	HYDIN	NC_056057.1	58515001	58535001	0.487319	0.176874	SG1	IMMP2L
0.204537	0.210357	SG2	HYDIN	NC_056064.1	26275001	26295001	0.278181	0.185984	SG1	INCA1;KIF1C
0.339703	0.193945	SG2	HYDIN	NC_056070.1	15065001	15085001	0.446575	0.297623	SG1	INPP4B
0.53861	0.180372	SG2	ICA1;LOC1218194	NC_056070.1	15070001	15090001	0.346572	0.30349	SG1	INPP4B
0.502165	0.265229	SG2	IDH3G;PLXNB3;SF	NC_056070.1	15075001	15095001	0.390486	0.289391	SG1	INPP4B
0.528905	0.136675	SG2	IER3IP1	NC_056070.1	15080001	15100001	0.442489	0.269339	SG1	INPP4B
0.508393	0.14874	SG2	IER3IP1	NC_056075.1	39415001	39435001	0.378445	0.25314	SG1	INPP5F
0.391626	0.143944	SG2	IFNAR2;IL10RB	NC_056075.1	39420001	39440001	0.404127	0.258031	SG1	INPP5F
0.191443	0.141326	SG2	IFRD2;LSMEM2	NC_056055.1	47680001	47700001	0.264182	0.276021	SG1	INVS
0.56166	0.159256	SG2	IFT80	NC_056055.1	47685001	47705001	0.211392	0.268989	SG1	INVS
0.500237	0.154596	SG2	IFT80	NC_056055.1	47690001	47710001	0.249881	0.278509	SG1	INVS
0.370196	0.152065	SG2	IGSF23	NC_056055.1	47695001	47715001	0.237246	0.246988	SG1	INVS
0.376366	0.136789	SG2	IGSF23	NC_056055.1	47700001	47720001	0.301276	0.252574	SG1	INVS
0.360499	0.137897	SG2	IGSF23	NC_056055.1	47705001	47725001	0.35803	0.296478	SG1	INVS
0.461447	0.210717	SG2	IL16	NC_056073.1	7995001	8015001	0.461472	0.173074	SG1	IP6K3
0.365682	0.243424	SG2	IL16	NC_056066.1	6615001	6635001	0.434396	0.186236	SG1	ISM1
0.292809	0.258974	SG2	IL16	NC_056066.1	6650001	6670001	0.436297	0.221508	SG1	ISM1
0.272186	0.267664	SG2	IL16	NC_056066.1	6655001	6675001	0.405369	0.225433	SG1	ISM1
0.395119	0.190248	SG2	IL16	NC_056072.1	10950001	10970001	0.482375	0.254517	SG1	ITGA9
0.570144	0.164374	SG2	IL31RA	NC_056072.1	10955001	10975001	0.388443	0.260216	SG1	ITGA9
0.327854	0.19533	SG2	IL6ST;LOC1141187	NC_056072.1	10960001	10980001	0.385423	0.286958	SG1	ITGA9

0.355287	0.164909	SG2	IL6ST;LOC1141187	NC_056072.1	10965001	10985001	0.365701	0.313154	SG1	ITGA9
0.548742	0.223329	SG2	IMMP2L	NC_056072.1	10970001	10990001	0.357822	0.374294	SG1	ITGA9
0.549325	0.222586	SG2	IMMP2L	NC_056072.1	10975001	10995001	0.375999	0.387013	SG1	ITGA9
0.214463	0.198968	SG2	IMPDH1;LOC1218	NC_056072.1	10980001	11000001	0.396098	0.325736	SG1	ITGA9
0.525278	0.140092	SG2	ING3	NC_056072.1	10985001	11005001	0.413492	0.306899	SG1	ITGA9
0.411181	0.159187	SG2	ING3	NC_056072.1	10990001	11010001	0.394064	0.291858	SG1	ITGA9
0.3151	0.191152	SG2	ING3	NC_056072.1	10995001	11015001	0.341263	0.25865	SG1	ITGA9
0.338056	0.192789	SG2	ING3	NC_056072.1	11000001	11020001	0.199856	0.247102	SG1	ITGA9
0.529763	0.25918	SG2	IQCA1	NC_056072.1	11005001	11025001	0.230263	0.32151	SG1	ITGA9
0.343784	0.285554	SG2	IQCA1	NC_056072.1	11010001	11030001	0.324637	0.24335	SG1	ITGA9
0.156483	0.322573	SG2	IQCA1	NC_056072.1	11015001	11035001	0.444065	0.190269	SG1	ITGA9
0.054234	0.22395	SG2	IQCA1	NC_056077.1	27845001	27865001	0.308212	0.179745	SG1	ITGAM
0.436235	0.173339	SG2	IQGAP1	NC_056077.1	27870001	27890001	0.481798	0.236564	SG1	ITGAM
0.567568	0.171568	SG2	IQGAP1	NC_056077.1	27875001	27895001	0.39218	0.263952	SG1	ITGAM
0.084169	0.144907	SG2	ISM1	NC_056077.1	27880001	27900001	0.37454	0.246919	SG1	ITGAM
0.473685	0.175156	SG2	ITGA8	NC_056077.1	27885001	27905001	0.345916	0.237681	SG1	ITGAM
0.17547	0.263361	SG2	ITGA8	NC_056077.1	27890001	27910001	0.342897	0.280802	SG1	ITGAM
0.348745	0.225476	SG2	ITGA8	NC_056077.1	27895001	27915001	0.239302	0.249994	SG1	ITGAM
0.493802	0.199535	SG2	ITGA8	NC_056077.1	27900001	27920001	0.26848	0.178187	SG1	ITGAM
0.564816	0.187804	SG2	ITGA8	NC_056080.1	63970001	63990001	0.211823	0.349874	SG1	ITGB1BP2;NO
0.349581	0.206721	SG2	ITPRID1	NC_056080.1	63975001	63995001	0.184977	0.303751	SG1	ITGB1BP2;NO
0.279661	0.210618	SG2	ITPRID1	NC_056058.1	56575001	56595001	0.397614	0.252502	SG1	JAKMIP2
0.101167	0.268554	SG2	ITPRID1	NC_056058.1	56580001	56600001	0.414637	0.244661	SG1	JAKMIP2
0.175624	0.201652	SG2	ITPRID1	NC_056057.1	69645001	69665001	0.439528	0.365421	SG1	JAZF1
0.336235	0.149206	SG2	ITPRID1	NC_056057.1	69650001	69670001	0.264491	0.405455	SG1	JAZF1
0.501767	0.186748	SG2	JAZF1	NC_056057.1	69655001	69675001	0.298919	0.440593	SG1	JAZF1
0.483376	0.249685	SG2	JAZF1	NC_056064.1	54395001	54415001	0.262045	0.317007	SG1	JMJD6;MXRA
0.416667	0.295472	SG2	JAZF1	NC_056064.1	54400001	54420001	0.322715	0.276289	SG1	JMJD6;MXRA
0.493197	0.246456	SG2	JAZF1	NC_056054.1	33475001	33495001	0.0685333	0.298265	SG1	JUN
0.466366	0.149504	SG2	JMJD1C	NC_056054.1	33480001	33500001	0.100796	0.302168	SG1	JUN
0.451751	0.140589	SG2	JMJD1C;NRBF2	NC_056054.1	33485001	33505001	0.188482	0.196837	SG1	JUN
0.483996	0.286726	SG2	KBTBD2	NC_056054.1	189435001	189455001	0.206522	0.213477	SG1	KALRN

0.245126	0.224234	SG2	KCNA5	NC_056054.1	189440001	189460001	0.18257	0.229819	SG1	KALRN
0.500239	0.166059	SG2	KCNA5	NC_056054.1	189445001	189465001	0.164683	0.238463	SG1	KALRN
0.335004	0.170797	SG2	KCNA6	NC_056054.1	189450001	189470001	0.0956801	0.296119	SG1	KALRN
0.433676	0.194544	SG2	KCNA6	NC_056054.1	189455001	189475001	0.107253	0.276424	SG1	KALRN
0.48324	0.154699	SG2	KCNA6	NC_056054.1	189460001	189480001	0.157732	0.25858	SG1	KALRN
0.137037	0.154883	SG2	KCNE2;SMIM11	NC_056054.1	189465001	189485001	0.404935	0.194182	SG1	KALRN
0.211925	0.140964	SG2	KCNE2;SMIM11	NC_056077.1	27735001	27755001	0.46357	0.217002	SG1	KAT8;PRSS36;
0.440955	0.197191	SG2	KCNH8	NC_056055.1	225205001	225225001	0.145331	0.264327	SG1	KCNE4
0.444325	0.261471	SG2	KCNH8	NC_056055.1	225210001	225230001	0.0559228	0.296452	SG1	KCNE4
0.185366	0.146038	SG2	KCNIP4	NC_056054.1	277625001	277645001	0.13204	0.203388	SG1	KCNH8
0.150401	0.159812	SG2	KCNIP4	NC_056054.1	277630001	277650001	0.0818997	0.219033	SG1	KCNH8
0.128093	0.169245	SG2	KCNIP4	NC_056054.1	277635001	277655001	0.113003	0.21989	SG1	KCNH8
0.155604	0.158223	SG2	KCNIP4	NC_056054.1	277640001	277660001	0.171994	0.216617	SG1	KCNH8
0.260387	0.141452	SG2	KCNIP4	NC_056054.1	277645001	277665001	0.230306	0.207225	SG1	KCNH8
0.24063	0.16528	SG2	KCNIP4	NC_056054.1	277650001	277670001	0.257748	0.189552	SG1	KCNH8
0.222354	0.182071	SG2	KCNIP4	NC_056054.1	277655001	277675001	0.261034	0.184378	SG1	KCNH8
0.218033	0.1942	SG2	KCNIP4	NC_056054.1	277670001	277690001	0.169574	0.199044	SG1	KCNH8
0.311283	0.168713	SG2	KCNIP4	NC_056054.1	277675001	277695001	0.127869	0.24101	SG1	KCNH8
0.364083	0.140893	SG2	KCNJ6	NC_056054.1	277680001	277700001	0.305795	0.195146	SG1	KCNH8
0.508578	0.152874	SG2	KCNN3	NC_056065.1	67910001	67930001	0.413473	0.262591	SG1	KCNK2
0.308172	0.147816	SG2	KCTD10;MYO1H	NC_056065.1	67915001	67935001	0.302637	0.281395	SG1	KCNK2
0.530287	0.145266	SG2	KIAA1217	NC_056065.1	67920001	67940001	0.38361	0.289365	SG1	KCNK2
0.366963	0.199521	SG2	KIAA1217	NC_056065.1	67925001	67945001	0.38698	0.282968	SG1	KCNK2
0.23331	0.300891	SG2	KIAA1217	NC_056065.1	67930001	67950001	0.421888	0.274564	SG1	KCNK2
0.316841	0.242087	SG2	KIAA1217	NC_056065.1	67935001	67955001	0.452324	0.25427	SG1	KCNK2
0.4451	0.136919	SG2	KIAA1958;LOC101	NC_056065.1	67940001	67960001	0.345804	0.238181	SG1	KCNK2
0.221145	0.140901	SG2	KIAA2026	NC_056065.1	67945001	67965001	0.397825	0.199775	SG1	KCNK2
0.155803	0.161382	SG2	KIAA2026	NC_056074.1	45390001	45410001	0.423938	0.235105	SG1	KCNQ1
0.130259	0.170359	SG2	KIAA2026	NC_056074.1	45395001	45415001	0.382536	0.238605	SG1	KCNQ1
0.0556	0.192404	SG2	KIAA2026	NC_056074.1	45400001	45420001	0.370283	0.232582	SG1	KCNQ1
0.060822	0.189329	SG2	KIAA2026	NC_056074.1	45405001	45425001	0.38773	0.178358	SG1	KCNQ1
0.108006	0.174314	SG2	KIAA2026	NC_056062.1	21935001	21955001	0.414359	0.204528	SG1	KCNQ3

0.145757	0.161134	SG2	KIAA2026;RANBP(	NC_056062.1	21940001	21960001	0.451835	0.192253	SG1	KCNQ3
0.410397	0.151859	SG2	KIRREL1	NC_056062.1	21945001	21965001	0.428036	0.241293	SG1	KCNQ3
0.232899	0.188308	SG2	KIRREL1	NC_056063.1	19775001	19795001	0.345435	0.201615	SG1	KCNRG;TRIM
0.316306	0.16277	SG2	KIRREL1	NC_056056.1	127470001	127490001	0.254696	0.219251	SG1	KERA
0.202391	0.1523	SG2	KIRREL3	NC_056066.1	24350001	24370001	0.443728	0.18936	SG1	KIAA1217
0.156963	0.222825	SG2	KITLG	NC_056066.1	24355001	24375001	0.329603	0.230824	SG1	KIAA1217
0.186063	0.21097	SG2	KITLG	NC_056066.1	24360001	24380001	0.194676	0.265816	SG1	KIAA1217
0.176471	0.160027	SG2	KITLG	NC_056066.1	24365001	24385001	0.301898	0.227984	SG1	KIAA1217
0.349039	0.146676	SG2	KITLG	NC_056065.1	61445001	61465001	0.414099	0.180082	SG1	KIAA1614
0.441261	0.154704	SG2	KITLG	NC_056058.1	6450001	6470001	0.111217	0.213774	SG1	KLF2
0.419939	0.16631	SG2	KITLG	NC_056063.1	44635001	44655001	0.341643	0.203132	SG1	KLHL1
0.220657	0.195674	SG2	KITLG	NC_056063.1	44640001	44660001	0.391835	0.243931	SG1	KLHL1
0.224306	0.160881	SG2	KITLG	NC_056063.1	44645001	44665001	0.421167	0.263153	SG1	KLHL1
0.52212	0.175361	SG2	KLF12	NC_056063.1	44650001	44670001	0.384765	0.224709	SG1	KLHL1
0.300195	0.207063	SG2	KLF12	NC_056063.1	44655001	44675001	0.350921	0.190927	SG1	KLHL1
0.113497	0.216619	SG2	KLF12	NC_056063.1	44665001	44685001	0.375898	0.208668	SG1	KLHL1
0.114806	0.178878	SG2	KLF12	NC_056063.1	44670001	44690001	0.434806	0.273163	SG1	KLHL1
0.560056	0.205552	SG2	KLHL25	NC_056063.1	44675001	44695001	0.447158	0.314378	SG1	KLHL1
0.54902	0.198507	SG2	KLHL25	NC_056063.1	44680001	44700001	0.472211	0.320174	SG1	KLHL1
0.535964	0.161646	SG2	KLHL25	NC_056063.1	44685001	44705001	0.448551	0.288954	SG1	KLHL1
0.541852	0.152086	SG2	KLHL28	NC_056063.1	44690001	44710001	0.422041	0.279784	SG1	KLHL1
0.527414	0.190342	SG2	KLHL32	NC_056063.1	44695001	44715001	0.457005	0.291719	SG1	KLHL1
0.504576	0.197681	SG2	KLHL32	NC_056063.1	44725001	44745001	0.463581	0.278401	SG1	KLHL1
0.522638	0.192446	SG2	KLHL32	NC_056063.1	44750001	44770001	0.409914	0.219517	SG1	KLHL1
0.355707	0.15759	SG2	KLRF2;LOC101102	NC_056063.1	44755001	44775001	0.471833	0.328651	SG1	KLHL1
0.557145	0.136166	SG2	KLRF2;LOC101102	NC_056063.1	44775001	44795001	0.467812	0.313398	SG1	KLHL1
0.535499	0.137539	SG2	KLRF2;LOC101120	NC_056063.1	44780001	44800001	0.442321	0.314407	SG1	KLHL1
0.414857	0.593588	SG2	KRTAP6-1;LOC114	NC_056063.1	44785001	44805001	0.475326	0.328507	SG1	KLHL1
0.416753	0.557387	SG2	KRTAP6-1;LOC114	NC_056063.1	44795001	44815001	0.485375	0.343762	SG1	KLHL1
0.549101	0.170326	SG2	LAMA2	NC_056063.1	44805001	44825001	0.474444	0.334959	SG1	KLHL1
0.442989	0.18289	SG2	LAMA2	NC_056063.1	44810001	44830001	0.472681	0.336462	SG1	KLHL1
0.494733	0.156551	SG2	LAMA2	NC_056063.1	44815001	44835001	0.481247	0.334825	SG1	KLHL1

0.503865	0.15509	SG2	LAMA2	NC_056063.1	44820001	44840001	0.47736	0.338204	SG1	KLHL1
0.378378	0.25188	SG2	LAMP3	NC_056063.1	44825001	44845001	0.47619	0.341454	SG1	KLHL1
0.50505	0.19341	SG2	LAMP3	NC_056063.1	44830001	44850001	0.473721	0.336081	SG1	KLHL1
0.347039	0.189076	SG2	LANCL2	NC_056063.1	44835001	44855001	0.480668	0.322144	SG1	KLHL1
0.4375	0.168923	SG2	LANCL2	NC_056063.1	44840001	44860001	0.478031	0.322888	SG1	KLHL1
0.55581	0.161075	SG2	LANCL2	NC_056063.1	44845001	44865001	0.488154	0.309139	SG1	KLHL1
0.487555	0.161958	SG2	LCN9	NC_056063.1	44850001	44870001	0.485682	0.315041	SG1	KLHL1
0.522367	0.171137	SG2	LDB2	NC_056063.1	44855001	44875001	0.473549	0.327669	SG1	KLHL1
0.400187	0.261068	SG2	LDB2	NC_056063.1	44860001	44880001	0.479673	0.318457	SG1	KLHL1
0.407894	0.250064	SG2	LDB2	NC_056063.1	44865001	44885001	0.477418	0.322823	SG1	KLHL1
0.388627	0.232122	SG2	LDB2	NC_056063.1	44870001	44890001	0.480983	0.322861	SG1	KLHL1
0.469875	0.159468	SG2	LDB2	NC_056063.1	44875001	44895001	0.482833	0.32421	SG1	KLHL1
0.486135	0.281697	SG2	LDLRAD3	NC_056063.1	44880001	44900001	0.475534	0.342135	SG1	KLHL1
0.403771	0.248263	SG2	LDLRAD3	NC_056063.1	44885001	44905001	0.432936	0.275471	SG1	KLHL1
0.468084	0.195281	SG2	LIN52	NC_056063.1	44890001	44910001	0.39283	0.213292	SG1	KLHL1
0.409143	0.207221	SG2	LIN52	NC_056080.1	118925001	118945001	0.432788	0.394579	SG1	KLHL4
0.57065	0.177373	SG2	LIN52	NC_056080.1	118930001	118950001	0.174333	0.401786	SG1	KLHL4
0.419834	0.161755	SG2	LIN9	NC_056080.1	118935001	118955001	0.115934	0.382886	SG1	KLHL4;LOC10
0.342106	0.237071	SG2	LIN9	NC_056057.1	104400001	104420001	0.338292	0.261368	SG1	KLRG2
0.40408	0.208774	SG2	LIN9	NC_056057.1	104405001	104425001	0.39706	0.32459	SG1	KLRG2
0.544217	0.148438	SG2	LINGO2	NC_056057.1	104410001	104430001	0.412704	0.327291	SG1	KLRG2
0.11715	0.144424	SG2	LMBR1	NC_056077.1	37550001	37570001	0.421689	0.19631	SG1	KPNA7
0.211528	0.149151	SG2	LMBR1	NC_056077.1	37555001	37575001	0.353039	0.219503	SG1	KPNA7
0.2265	0.14	SG2	LMBR1	NC_056061.1	81675001	81695001	0.370715	0.21807	SG1	LDHAL6B;TM
0.236988	0.15609	SG2	LMBR1	NC_056061.1	81680001	81700001	0.356655	0.209392	SG1	LDHAL6B;TM
0.275158	0.173411	SG2	LMBR1	NC_056074.1	38330001	38350001	0.37255	0.18278	SG1	LGALS12
0.558312	0.179476	SG2	LOC101101981	NC_056074.1	38340001	38360001	0.347548	0.185793	SG1	LGALS12;LOC
0.366323	0.173438	SG2	LOC101102109	NC_056074.1	38315001	38335001	0.368677	0.197532	SG1	LGALS12;PLA
0.35851	0.171363	SG2	LOC101102109	NC_056067.1	48705001	48725001	0.321682	0.247865	SG1	LGALS15
0.150433	0.137817	SG2	LOC101102521;ZN	NC_056067.1	48710001	48730001	0.0562826	0.361749	SG1	LGALS15
0.116133	0.141436	SG2	LOC101102521;ZN	NC_056057.1	47460001	47480001	0.198454	0.251235	SG1	LHFPL3
0.168686	0.186052	SG2	LOC101102719	NC_056057.1	47465001	47485001	0.129909	0.246057	SG1	LHFPL3

0.125732	0.186688	SG2	LOC101102719;LO	NC_056057.1	47480001	47500001	0.108507	0.195069	SG1	LHFPL3
0.372569	0.141533	SG2	LOC101103150	NC_056057.1	47485001	47505001	0.238965	0.210656	SG1	LHFPL3
0.490483	0.203542	SG2	LOC101103150	NC_056057.1	47490001	47510001	0.349208	0.230614	SG1	LHFPL3
0.176411	0.205691	SG2	LOC101103352	NC_056057.1	47495001	47515001	0.44372	0.228216	SG1	LHFPL3
0.239216	0.161232	SG2	LOC101103352	NC_056057.1	47500001	47520001	0.45939	0.220029	SG1	LHFPL3
0.241986	0.145363	SG2	LOC101103352	NC_056057.1	47505001	47525001	0.466306	0.233004	SG1	LHFPL3
0.073572	0.149732	SG2	LOC101103376	NC_056057.1	47510001	47530001	0.460304	0.273491	SG1	LHFPL3
0.151274	0.135878	SG2	LOC101103376	NC_056057.1	47515001	47535001	0.472573	0.313784	SG1	LHFPL3
0.539574	0.14985	SG2	LOC101103726	NC_056057.1	47520001	47540001	0.485716	0.334288	SG1	LHFPL3
0.516715	0.155715	SG2	LOC101103726	NC_056057.1	47525001	47545001	0.472987	0.31197	SG1	LHFPL3
0.305935	0.169864	SG2	LOC101103916;LO	NC_056065.1	60800001	60820001	0.482861	0.241059	SG1	LHX4
0.368197	0.159289	SG2	LOC101103916;LO	NC_056065.1	60805001	60825001	0.45529	0.263071	SG1	LHX4
0.308926	0.183301	SG2	LOC101103916;LO	NC_056065.1	60810001	60830001	0.433932	0.26051	SG1	LHX4
0.252094	0.200244	SG2	LOC101103916;LO	NC_056056.1	62000001	62020001	0.481873	0.2373	SG1	LIMS1
0.219877	0.213138	SG2	LOC101103916;LO	NC_056055.1	97170001	97190001	0.271034	0.199009	SG1	LINGO2
0.442869	0.221595	SG2	LOC101104530	NC_056055.1	97175001	97195001	0.428469	0.317099	SG1	LINGO2
0.455079	0.223868	SG2	LOC101104530	NC_056055.1	97180001	97200001	0.367186	0.323324	SG1	LINGO2
0.444101	0.242708	SG2	LOC101104530	NC_056055.1	97205001	97225001	0.294378	0.367121	SG1	LINGO2
0.43533	0.24878	SG2	LOC101104530	NC_056055.1	97210001	97230001	0.232795	0.302184	SG1	LINGO2
0.387507	0.191016	SG2	LOC101104669	NC_056055.1	97215001	97235001	0.269886	0.330342	SG1	LINGO2
0.405479	0.184682	SG2	LOC101104669;LO	NC_056055.1	97220001	97240001	0.41326	0.332554	SG1	LINGO2
0.391552	0.185003	SG2	LOC101104669;LO	NC_056055.1	97230001	97250001	0.410243	0.202901	SG1	LINGO2
0.155863	0.266735	SG2	LOC101105537	NC_056055.1	97235001	97255001	0.342442	0.178172	SG1	LINGO2
0.144108	0.264871	SG2	LOC101105537	NC_056055.1	97240001	97260001	0.330056	0.207557	SG1	LINGO2
0.17697	0.248109	SG2	LOC101105537	NC_056055.1	97245001	97265001	0.275182	0.221573	SG1	LINGO2
0.24563	0.152164	SG2	LOC101106519	NC_056055.1	97250001	97270001	0.316386	0.263669	SG1	LINGO2
0.168416	0.197293	SG2	LOC101106519	NC_056055.1	97255001	97275001	0.437953	0.181316	SG1	LINGO2
0.434541	0.170935	SG2	LOC101106734	NC_056054.1	117155001	117175001	0.471474	0.200638	SG1	LMX1A
0.094937	0.138211	SG2	LOC101106734	NC_056060.1	32320001	32340001	0.465228	0.317117	SG1	LOC10024071(
0.335628	0.169332	SG2	LOC101107188	NC_056060.1	32325001	32345001	0.299146	0.300794	SG1	LOC10024071(
0.426703	0.143156	SG2	LOC101107331	NC_056060.1	32330001	32350001	0.266851	0.263519	SG1	LOC10024071(
0.411486	0.141697	SG2	LOC101107442	NC_056075.1	42160001	42180001	0.254761	0.235128	SG1	LOC10110210(

0.56272	0.13532	SG2	LOC101108092	NC_056075.1	42165001	42185001	0.194532	0.188676	SG1	LOC101102109
0.466467	0.176288	SG2	LOC101108092;LO	NC_056057.1	89940001	89960001	0.321365	0.208917	SG1	LOC101102143
0.385179	0.183643	SG2	LOC101108092;LO	NC_056057.1	89945001	89965001	0.446328	0.17835	SG1	LOC101102143
0.539216	0.160224	SG2	LOC101108131	NC_056067.1	38560001	38580001	0.111979	0.280894	SG1	LOC101102413
0.157392	0.277716	SG2	LOC101109214	NC_056067.1	38565001	38585001	0.063341	0.307392	SG1	LOC101102413
0.15076	0.270369	SG2	LOC101109214	NC_056067.1	38555001	38575001	0.366266	0.184449	SG1	LOC101102413
0.221015	0.22675	SG2	LOC101109214	NC_056073.1	29865001	29885001	0.220162	0.272246	SG1	LOC101102593
0.230672	0.2246	SG2	LOC101109214	NC_056073.1	29870001	29890001	0.24827	0.209265	SG1	LOC101102593
0.297454	0.196084	SG2	LOC101109214	NC_056072.1	20185001	20205001	0.451134	0.173614	SG1	LOC101102845
0.437114	0.137445	SG2	LOC101109422	NC_056072.1	20190001	20210001	0.442122	0.213147	SG1	LOC101102845
0.281155	0.334094	SG2	LOC101109476	NC_056072.1	20195001	20215001	0.438568	0.230075	SG1	LOC101102845
0.112683	0.421098	SG2	LOC101109476	NC_056072.1	20200001	20220001	0.46735	0.247744	SG1	LOC101102845
0.040541	0.453918	SG2	LOC101109476;LO	NC_056067.1	50375001	50395001	0.264211	0.375648	SG1	LOC101103174
0.178448	0.360283	SG2	LOC101109476;LO	NC_056067.1	50380001	50400001	0.310755	0.348633	SG1	LOC101103174
0.30016	0.264217	SG2	LOC101109476;LO	NC_056056.1	150530001	150550001	0.434771	0.197364	SG1	LOC101103222
0.479775	0.150906	SG2	LOC101109476;LO	NC_056056.1	150535001	150555001	0.321543	0.254704	SG1	LOC101103222
0.417577	0.175046	SG2	LOC101109728	NC_056056.1	150540001	150560001	0.395809	0.269518	SG1	LOC101103222
0.379341	0.13681	SG2	LOC101110107	NC_056066.1	66415001	66435001	0.0990099	0.234195	SG1	LOC101103339
0.34082	0.183361	SG2	LOC101110107	NC_056066.1	66450001	66470001	0.464137	0.210778	SG1	LOC101103339
0.362016	0.171496	SG2	LOC101110107	NC_056066.1	66455001	66475001	0.315079	0.254801	SG1	LOC101103339
0.441514	0.179953	SG2	LOC101110450	NC_056066.1	66425001	66445001	0.198291	0.198246	SG1	LOC101103339
0.368622	0.157187	SG2	LOC101110450	NC_056067.1	50385001	50405001	0.289902	0.341347	SG1	LOC101103343
0.374736	0.149801	SG2	LOC101110450	NC_056067.1	50390001	50410001	0.309243	0.331431	SG1	LOC101103343
0.347262	0.145462	SG2	LOC101110478	NC_056067.1	50410001	50430001	0.220841	0.275586	SG1	LOC101103343
0.234443	0.154905	SG2	LOC101110478	NC_056073.1	29855001	29875001	0.349075	0.266991	SG1	LOC101103354
0.132889	0.146466	SG2	LOC101110478	NC_056072.1	53185001	53205001	0.455043	0.195666	SG1	LOC101103766
0.07702	0.164062	SG2	LOC101110478	NC_056073.1	28955001	28975001	0.308936	0.187343	SG1	LOC101104643
0.227799	0.179448	SG2	LOC101110478	NC_056059.1	89810001	89830001	0.309138	0.22407	SG1	LOC101104661
0.395192	0.243283	SG2	LOC101110773;RX	NC_056069.1	62635001	62655001	0.391335	0.187963	SG1	LOC101105188
0.310606	0.278133	SG2	LOC101110773;RX	NC_056069.1	62640001	62660001	0.28549	0.214526	SG1	LOC101105188
0.498475	0.170341	SG2	LOC101110823	NC_056069.1	62645001	62665001	0.237646	0.230349	SG1	LOC101105188
0.517605	0.141899	SG2	LOC101110823	NC_056069.1	62650001	62670001	0.253918	0.273322	SG1	LOC101105188

0.477753	0.151186	SG2	LOC101110823	NC_056069.1	62655001	62675001	0.309813	0.291411	SG1	LOC101105188
0.495972	0.171202	SG2	LOC101111101	NC_056069.1	62660001	62680001	0.339209	0.273029	SG1	LOC101105188
0.498556	0.191743	SG2	LOC101111610	NC_056069.1	62665001	62685001	0.395395	0.238891	SG1	LOC101105188
0.353884	0.241869	SG2	LOC101111610	NC_056055.1	89920001	89940001	0.440855	0.276311	SG1	LOC101105632
0.522513	0.186541	SG2	LOC101111610;LO	NC_056055.1	38215001	38235001	0.345395	0.25084	SG1	LOC101106139
0.487664	0.135505	SG2	LOC101111863;LO	NC_056055.1	38205001	38225001	0.235385	0.246752	SG1	LOC101106139
0.458106	0.16375	SG2	LOC101111863;LO	NC_056055.1	38210001	38230001	0.263087	0.263277	SG1	LOC101106139
0.184069	0.145458	SG2	LOC101111980;MY	NC_056055.1	38220001	38240001	0.472348	0.245143	SG1	LOC101106395
0.34997	0.166944	SG2	LOC101111980;MY	NC_056056.1	7585001	7605001	0.157546	0.2791	SG1	LOC101106416
0.419723	0.356223	SG2	LOC101112274;LO	NC_056056.1	7590001	7610001	0.130458	0.285611	SG1	LOC101106416
0.395833	0.300203	SG2	LOC101112274;LO	NC_056055.1	147600001	147620001	0.429799	0.218199	SG1	LOC101106492
0.483348	0.199625	SG2	LOC101112526	NC_056055.1	147605001	147625001	0.428387	0.19201	SG1	LOC101106492
0.331566	0.195721	SG2	LOC101112889	NC_056060.1	22145001	22165001	0.204884	0.179238	SG1	LOC101106528
0.382232	0.158654	SG2	LOC101112889	NC_056072.1	55735001	55755001	0.329166	0.437654	SG1	LOC101106751
0.421052	0.169445	SG2	LOC101112889	NC_056072.1	55740001	55760001	0.336957	0.462019	SG1	LOC101106751
0.505444	0.146838	SG2	LOC101112889	NC_056072.1	55745001	55765001	0.316394	0.433802	SG1	LOC101106751
0.081266	0.139519	SG2	LOC101113073;LO	NC_056072.1	55750001	55770001	0.334035	0.373914	SG1	LOC101106751
0.059001	0.140563	SG2	LOC101113073;LO	NC_056072.1	55755001	55775001	0.437074	0.254204	SG1	LOC101106751
0.043042	0.159003	SG2	LOC101113335	NC_056072.1	55775001	55795001	0.447848	0.18552	SG1	LOC101106751
0.036461	0.161861	SG2	LOC101113335	NC_056072.1	55780001	55800001	0.468821	0.262947	SG1	LOC101106751
0.036514	0.155991	SG2	LOC101113761	NC_056072.1	55785001	55805001	0.476281	0.285863	SG1	LOC101106751
0.073088	0.148789	SG2	LOC101113761	NC_056072.1	55730001	55750001	0.402321	0.343627	SG1	LOC101106751
0.477646	0.166098	SG2	LOC101113819	NC_056067.1	58410001	58430001	0.483292	0.267966	SG1	LOC101107382
0.22193	0.216365	SG2	LOC101113881	NC_056067.1	58415001	58435001	0.414881	0.343298	SG1	LOC101107382
0.306985	0.197645	SG2	LOC101113881	NC_056067.1	58420001	58440001	0.398937	0.394945	SG1	LOC101107382
0.53127	0.178925	SG2	LOC101114011	NC_056056.1	163980001	164000001	0.391679	0.207798	SG1	LOC101107433
0.336662	0.191654	SG2	LOC101114011;LO	NC_056056.1	163985001	164005001	0.368897	0.235422	SG1	LOC101107433
0.359127	0.229552	SG2	LOC101114926	NC_056056.1	163990001	164010001	0.331531	0.205319	SG1	LOC101107433
0.413637	0.216253	SG2	LOC101114926	NC_056063.1	21970001	21990001	0.427439	0.220923	SG1	LOC101107613
0.426335	0.238446	SG2	LOC101114926	NC_056080.1	116255001	116275001	0.472835	0.343631	SG1	LOC101108113
0.472729	0.209886	SG2	LOC101114926	NC_056080.1	116260001	116280001	0.462927	0.342633	SG1	LOC101108113
0.055413	0.264268	SG2	LOC101114941	NC_056080.1	116265001	116285001	0.467634	0.340168	SG1	LOC101108113

0.544464	0.140002	SG2	LOC101115135	NC_056080.1	116270001	116290001	0.465397	0.353801	SG1	LOC101108113
0.236569	0.176167	SG2	LOC101115135	NC_056080.1	116275001	116295001	0.473819	0.337789	SG1	LOC101108113
0.2083	0.187609	SG2	LOC101115135	NC_056080.1	116315001	116335001	0.487015	0.279802	SG1	LOC101108113
0.25621	0.210156	SG2	LOC101115135	NC_056080.1	116320001	116340001	0.440251	0.173468	SG1	LOC101108113
0.293268	0.221408	SG2	LOC101115135	NC_056058.1	8450001	8470001	0.420016	0.220279	SG1	LOC101108306
0.488964	0.140858	SG2	LOC101115135	NC_056058.1	8445001	8465001	0.228684	0.249836	SG1	LOC101108306
0.132138	0.267684	SG2	LOC101115542	NC_056056.1	217575001	217595001	0.391727	0.192261	SG1	LOC101108561
0.178293	0.245862	SG2	LOC101115542	NC_056054.1	166160001	166180001	0.235408	0.203547	SG1	LOC101108803
0.134498	0.264779	SG2	LOC101115542	NC_056054.1	166165001	166185001	0.272877	0.273228	SG1	LOC101108803
0.078243	0.291475	SG2	LOC101115542	NC_056054.1	166170001	166190001	0.234828	0.335669	SG1	LOC101108803
0.085396	0.288521	SG2	LOC101115542	NC_056054.1	166175001	166195001	0.175186	0.353463	SG1	LOC101108803
0.5536	0.245319	SG2	LOC101115849	NC_056054.1	166180001	166200001	0.0988391	0.380981	SG1	LOC101108803
0.541851	0.298311	SG2	LOC101115983	NC_056054.1	166200001	166220001	0.0690219	0.204857	SG1	LOC101108803
0.433784	0.165476	SG2	LOC101116064;RC	NC_056054.1	166235001	166255001	0.4296	0.201819	SG1	LOC101108803
0.174433	0.13914	SG2	LOC101116122	NC_056054.1	166260001	166280001	0.269163	0.305085	SG1	LOC101108803
0.167082	0.146411	SG2	LOC101116122	NC_056054.1	166265001	166285001	0.199149	0.333122	SG1	LOC101108803
0.440365	0.174164	SG2	LOC101116641;LO	NC_056054.1	166270001	166290001	0.283912	0.327976	SG1	LOC101108803
0.475424	0.136729	SG2	LOC101116843;RC	NC_056066.1	3510001	3530001	0.355861	0.232574	SG1	LOC101109032
0.485668	0.135847	SG2	LOC101116843;RC	NC_056066.1	3515001	3535001	0.192861	0.262	SG1	LOC101109032
0.474459	0.150745	SG2	LOC101116843;RC	NC_056066.1	3520001	3540001	0.127216	0.26816	SG1	LOC101109032
0.470444	0.153267	SG2	LOC101116843;RC	NC_056066.1	3525001	3545001	0.173404	0.245825	SG1	LOC101109032
0.451885	0.151176	SG2	LOC101117851	NC_056066.1	3530001	3550001	0.162379	0.215183	SG1	LOC101109032
0.395272	0.161214	SG2	LOC101117851	NC_056080.1	72330001	72350001	0.214679	0.183257	SG1	LOC101109338
0.475248	0.15581	SG2	LOC101117851	NC_056076.1	2415001	2435001	0.482759	0.321423	SG1	LOC101109407
0.373698	0.184339	SG2	LOC101118202	NC_056076.1	2420001	2440001	0.433321	0.310271	SG1	LOC101109407
0.415941	0.22292	SG2	LOC101120033	NC_056055.1	149840001	149860001	0.31579	0.194347	SG1	LOC101109941
0.291764	0.269922	SG2	LOC101120033	NC_056055.1	149845001	149865001	0.351495	0.185548	SG1	LOC101109941
0.364529	0.276174	SG2	LOC101120033	NC_056055.1	149855001	149875001	0.421095	0.183096	SG1	LOC101109941
0.493888	0.275951	SG2	LOC101120033	NC_056055.1	149860001	149880001	0.4429	0.174868	SG1	LOC101109941
0.525525	0.184776	SG2	LOC101120033	NC_056055.1	149865001	149885001	0.427524	0.192051	SG1	LOC101109941
0.545939	0.139184	SG2	LOC101120067	NC_056055.1	149870001	149890001	0.418239	0.190772	SG1	LOC101109941
0.552611	0.229531	SG2	LOC101120341	NC_056055.1	149875001	149895001	0.268333	0.177406	SG1	LOC101109941

0.316411	0.212018	SG2	LOC101120341	NC_056055.1	149880001	149900001	0.10625	0.189053	SG1	LOC101109941
0.308737	0.178249	SG2	LOC101120341	NC_056055.1	149885001	149905001	0.0900734	0.178683	SG1	LOC101109941
0.51574	0.143305	SG2	LOC101120341	NC_056055.1	149890001	149910001	0.115385	0.1907	SG1	LOC101109941
0.518286	0.135437	SG2	LOC101120408	NC_056055.1	149895001	149915001	0.189642	0.221304	SG1	LOC101109941
0.554479	0.13522	SG2	LOC101120408	NC_056055.1	149900001	149920001	0.321453	0.236609	SG1	LOC101109941
0.229838	0.170741	SG2	LOC101120482	NC_056055.1	149905001	149925001	0.354226	0.232704	SG1	LOC101109941
0.479009	0.148701	SG2	LOC101121400	NC_056055.1	149910001	149930001	0.406596	0.23878	SG1	LOC101109941
0.369016	0.165888	SG2	LOC101121400	NC_056055.1	149915001	149935001	0.419201	0.228619	SG1	LOC101109941
0.322342	0.175804	SG2	LOC101121400	NC_056055.1	149920001	149940001	0.414197	0.218839	SG1	LOC101109941
0.107277	0.144501	SG2	LOC101122097	NC_056055.1	149925001	149945001	0.412519	0.211055	SG1	LOC101109941
0.070738	0.150517	SG2	LOC101122097	NC_056055.1	149930001	149950001	0.427835	0.205507	SG1	LOC101109941
0.070211	0.15348	SG2	LOC101122097	NC_056055.1	149935001	149955001	0.442802	0.212432	SG1	LOC101109941
0.246436	0.433138	SG2	LOC101122163	NC_056055.1	149940001	149960001	0.441098	0.226046	SG1	LOC101109941
0.295973	0.424499	SG2	LOC101122163	NC_056055.1	149945001	149965001	0.444211	0.262348	SG1	LOC101109941
0.551501	0.350669	SG2	LOC101122163	NC_056055.1	149950001	149970001	0.45648	0.245389	SG1	LOC101109941
0.127702	0.179464	SG2	LOC101122595	NC_056055.1	149955001	149975001	0.425993	0.218843	SG1	LOC101109941
0.084644	0.153745	SG2	LOC101122595	NC_056067.1	60895001	60915001	0.480747	0.188825	SG1	LOC10110999
0.47486	0.153609	SG2	LOC101122932	NC_056054.1	269915001	269935001	0.0738982	0.282488	SG1	LOC10111011
0.567283	0.137106	SG2	LOC101122932	NC_056080.1	118940001	118960001	0.110479	0.35031	SG1	LOC10111021
0.530343	0.14294	SG2	LOC101122932	NC_056080.1	118945001	118965001	0.109252	0.266287	SG1	LOC10111021
0.560655	0.140156	SG2	LOC101122932	NC_056080.1	118950001	118970001	0.18552	0.237979	SG1	LOC10111021
0.25766	0.229352	SG2	LOC101122932;RP	NC_056056.1	185750001	185770001	0.170128	0.184109	SG1	LOC10111022
0.324715	0.20503	SG2	LOC101122932;ZN	NC_056056.1	185755001	185775001	0.124235	0.262975	SG1	LOC10111022
0.507264	0.160813	SG2	LOC101122932;ZN	NC_056056.1	185760001	185780001	0.0673845	0.279309	SG1	LOC10111022
0.501428	0.156939	SG2	LOC101122983	NC_056058.1	39550001	39570001	0.370504	0.257111	SG1	LOC10111058
0.511715	0.173175	SG2	LOC101122983	NC_056080.1	119880001	119900001	0.430548	0.42845	SG1	LOC101110741
0.529083	0.152459	SG2	LOC101122983	NC_056080.1	119885001	119905001	0.434263	0.401406	SG1	LOC101110741
0.466425	0.225225	SG2	LOC101122983	NC_056080.1	119890001	119910001	0.463247	0.31109	SG1	LOC101110741
0.435081	0.196716	SG2	LOC101122983;LO	NC_056080.1	119895001	119915001	0.446913	0.178663	SG1	LOC101110741
0.449026	0.204511	SG2	LOC101122983;LO	NC_056058.1	37870001	37890001	0.452237	0.185502	SG1	LOC10111093
0.505852	0.150455	SG2	LOC101122983;LO	NC_056067.1	61290001	61310001	0.487298	0.211937	SG1	LOC10111104
0.561571	0.166461	SG2	LOC101122984;LO	NC_056054.1	10810001	10830001	0.324786	0.210699	SG1	LOC10111106

0.478767	0.18079	SG2	LOC101122984;RH	NC_056054.1	97210001	97230001	0.240902	0.549929	SG1	LOC101112189
0.062149	0.145964	SG2	LOC101123149;LO	NC_056054.1	97215001	97235001	0.308682	0.52388	SG1	LOC101112189
0.393413	0.292446	SG2	LOC101123288	NC_056054.1	97220001	97240001	0.473479	0.438394	SG1	LOC101112189
0.367839	0.255983	SG2	LOC101123288	NC_056054.1	97730001	97750001	0.472972	0.188542	SG1	LOC101112864
0.518067	0.139794	SG2	LOC101123629	NC_056054.1	97735001	97755001	0.248951	0.215798	SG1	LOC101112864
0.430027	0.146067	SG2	LOC101123629	NC_056080.1	77355001	77375001	0.341066	0.436313	SG1	LOC101112957
0.329714	0.136364	SG2	LOC101123629	NC_056080.1	77360001	77380001	0.270554	0.382251	SG1	LOC101112957
0.382505	0.141414	SG2	LOC101123629	NC_056080.1	77365001	77385001	0.204724	0.396833	SG1	LOC101112957
0.316917	0.163152	SG2	LOC105601990;LO	NC_056080.1	77370001	77390001	0.190514	0.36592	SG1	LOC101112957
0.188961	0.202823	SG2	LOC105601990;LO	NC_056080.1	77375001	77395001	0.205218	0.408044	SG1	LOC101112957
0.178172	0.210232	SG2	LOC105601990;LO	NC_056056.1	164780001	164800001	0.452058	0.217183	SG1	LOC101113142
0.187036	0.189665	SG2	LOC105601990;LO	NC_056056.1	164785001	164805001	0.384063	0.207076	SG1	LOC101113142
0.252796	0.156499	SG2	LOC105601990;LO	NC_056059.1	103720001	103740001	0.260319	0.202002	SG1	LOC101113239
0.263354	0.161554	SG2	LOC105601990;LO	NC_056059.1	103725001	103745001	0.30534	0.217045	SG1	LOC101113239
0.339432	0.151484	SG2	LOC105601990;LO	NC_056070.1	63530001	63550001	0.361192	0.187851	SG1	LOC101113276
0.386548	0.150612	SG2	LOC105601990;LO	NC_056070.1	63535001	63555001	0.404922	0.268043	SG1	LOC101113276
0.493435	0.141607	SG2	LOC105601990;LO	NC_056057.1	106795001	106815001	0.483352	0.361291	SG1	LOC101113672
0.447346	0.154079	SG2	LOC105601990;LO	NC_056057.1	106800001	106820001	0.327993	0.318556	SG1	LOC101113672
0.447405	0.158048	SG2	LOC105601990;LO	NC_056057.1	106805001	106825001	0.151459	0.189779	SG1	LOC101113672
0.45177	0.147608	SG2	LOC105601990;LO	NC_056072.1	12720001	12740001	0.477385	0.298002	SG1	LOC101113800
0.439277	0.243878	SG2	LOC105603310	NC_056072.1	12725001	12745001	0.334626	0.29726	SG1	LOC101113800
0.439159	0.248562	SG2	LOC105603310	NC_056072.1	12730001	12750001	0.199835	0.302394	SG1	LOC101113800
0.438322	0.248632	SG2	LOC105603310	NC_056072.1	12735001	12755001	0.0695708	0.183161	SG1	LOC101113800
0.43824	0.248766	SG2	LOC105603310	NC_056072.1	12755001	12775001	0.128125	0.201826	SG1	LOC101113800
0.437029	0.251278	SG2	LOC105603310	NC_056077.1	19950001	19970001	0.196366	0.173447	SG1	LOC101114079
0.438221	0.247346	SG2	LOC105603310	NC_056077.1	19955001	19975001	0.161463	0.192996	SG1	LOC101114079
0.439613	0.245729	SG2	LOC105603310	NC_056077.1	20070001	20090001	0.429179	0.173544	SG1	LOC101114079
0.535387	0.181903	SG2	LOC105603310	NC_056068.1	24850001	24870001	0.36942	0.174613	SG1	LOC101114973
0.234043	0.152447	SG2	LOC105603383	NC_056068.1	24855001	24875001	0.355058	0.18803	SG1	LOC101114973
0.040369	0.186061	SG2	LOC105605206;VE	NC_056067.1	62120001	62140001	0.403611	0.298828	SG1	LOC101115398
0.090064	0.159338	SG2	LOC105605206;VE	NC_056067.1	62125001	62145001	0.320634	0.228223	SG1	LOC101115398
0.334817	0.142229	SG2	LOC105605761	NC_056067.1	62130001	62150001	0.385435	0.216708	SG1	LOC101115398

0.490843	0.20354	SG2	LOC105605821	NC_056067.1	62200001	62220001	0.21427	0.194902	SG1	LOC10111616
0.516128	0.183268	SG2	LOC105605821	NC_056067.1	62195001	62215001	0.267857	0.212131	SG1	LOC10111616
0.536558	0.218275	SG2	LOC105605908	NC_056066.1	62650001	62670001	0.382161	0.39361	SG1	LOC10111624
0.392475	0.186482	SG2	LOC105610665;PR	NC_056066.1	62655001	62675001	0.448129	0.339681	SG1	LOC10111624
0.347772	0.184821	SG2	LOC105610665;QS	NC_056066.1	62660001	62680001	0.420833	0.320127	SG1	LOC10111624
0.128547	0.147835	SG2	LOC105610665;QS	NC_056054.1	63790001	63810001	0.298295	0.211532	SG1	LOC10111626
0.155153	0.151126	SG2	LOC105610665;QS	NC_056054.1	63795001	63815001	0.429825	0.209624	SG1	LOC10111626
0.411457	0.153719	SG2	LOC105611265	NC_056067.1	65640001	65660001	0.165211	0.232946	SG1	LOC10111659
0.381137	0.149148	SG2	LOC105611265	NC_056056.1	164210001	164230001	0.1885	0.224951	SG1	LOC10111697
0.394821	0.139415	SG2	LOC105611265;PA	NC_056056.1	164215001	164235001	0.165092	0.281095	SG1	LOC10111697
0.211907	0.164558	SG2	LOC105612445	NC_056057.1	108445001	108465001	0.0960496	0.234756	SG1	LOC10111741
0.223255	0.161822	SG2	LOC105612445	NC_056059.1	85400001	85420001	0.389318	0.212419	SG1	LOC10111818
0.177815	0.174567	SG2	LOC105612445	NC_056059.1	85405001	85425001	0.292539	0.335738	SG1	LOC10111818
0.488729	0.157968	SG2	LOC105612728	NC_056059.1	85410001	85430001	0.308351	0.3711	SG1	LOC10111818
0.570596	0.172791	SG2	LOC105612852;RG	NC_056059.1	85415001	85435001	0.334607	0.353857	SG1	LOC10111818
0.414174	0.169618	SG2	LOC105614340	NC_056074.1	38190001	38210001	0.308686	0.181941	SG1	LOC10111908
0.396781	0.169852	SG2	LOC105614340	NC_056074.1	38195001	38215001	0.23386	0.215091	SG1	LOC10111908
0.220345	0.253426	SG2	LOC105614844	NC_056074.1	38200001	38220001	0.298095	0.233015	SG1	LOC10111908
0.367617	0.179656	SG2	LOC105614844	NC_056074.1	38205001	38225001	0.35234	0.214601	SG1	LOC10111908
0.057151	0.313485	SG2	LOC105616258	NC_056068.1	47560001	47580001	0.338341	0.188653	SG1	LOC10111941
0.144965	0.252858	SG2	LOC105616258	NC_056077.1	26910001	26930001	0.417008	0.28764	SG1	LOC10111943
0.327618	0.189108	SG2	LOC105616258	NC_056054.1	66245001	66265001	0.403965	0.221645	SG1	LOC10111951
0.513122	0.226988	SG2	LOC105616258	NC_056054.1	66250001	66270001	0.435573	0.229335	SG1	LOC10111951
0.570162	0.147714	SG2	LOC106990358	NC_056054.1	66260001	66280001	0.184842	0.296848	SG1	LOC10111977
0.403818	0.198718	SG2	LOC106990358	NC_056054.1	66265001	66285001	0.329166	0.180675	SG1	LOC10111977
0.296023	0.135726	SG2	LOC106990481	NC_056074.1	38345001	38365001	0.383855	0.256753	SG1	LOC10112002
0.389928	0.166366	SG2	LOC106991342	NC_056074.1	38350001	38370001	0.415423	0.292887	SG1	LOC10112002
0.497736	0.243429	SG2	LOC114109004	NC_056074.1	38355001	38375001	0.351375	0.307809	SG1	LOC10112002
0.533632	0.296783	SG2	LOC114109004	NC_056074.1	38360001	38380001	0.31974	0.300963	SG1	LOC10112002
0.27128	0.289416	SG2	LOC114109004;ZN	NC_056074.1	38365001	38385001	0.326252	0.273281	SG1	LOC10112002
0.233734	0.25235	SG2	LOC114109004;ZN	NC_056074.1	38370001	38390001	0.231527	0.204605	SG1	LOC10112002
0.324091	0.194642	SG2	LOC114109004;ZN	NC_056074.1	38375001	38395001	0.174253	0.191265	SG1	LOC10112002

0.365543	0.266975	SG2	LOC114109004;ZN	NC_056074.1	38380001	38400001	0.0909091	0.219415	SG1	LOC101120029
0.431052	0.20573	SG2	LOC114109057	NC_056068.1	38950001	38970001	0.47629	0.362932	SG1	LOC101120269
0.426052	0.209624	SG2	LOC114109057	NC_056080.1	111440001	111460001	0.383616	0.279412	SG1	LOC101120496
0.436277	0.194189	SG2	LOC114109057	NC_056080.1	111445001	111465001	0.212163	0.342995	SG1	LOC101120496
0.281334	0.143234	SG2	LOC114109112	NC_056058.1	39555001	39575001	0.347724	0.259283	SG1	LOC101120664
0.299088	0.155735	SG2	LOC114109112	NC_056058.1	39560001	39580001	0.30871	0.261239	SG1	LOC101120664
0.350302	0.262926	SG2	LOC114109119	NC_056058.1	39565001	39585001	0.243587	0.229085	SG1	LOC101120664
0.338068	0.277632	SG2	LOC114109119	NC_056058.1	39570001	39590001	0.267916	0.174617	SG1	LOC101120664
0.396964	0.245627	SG2	LOC114109119	NC_056080.1	103265001	103285001	0.0752476	0.273782	SG1	LOC101120813
0.327689	0.253977	SG2	LOC114109119	NC_056080.1	103270001	103290001	0.0707568	0.284898	SG1	LOC101120813
0.433926	0.244465	SG2	LOC114111370	NC_056080.1	103275001	103295001	0.076966	0.241205	SG1	LOC101120813
0.365546	0.251981	SG2	LOC114111370	NC_056080.1	103280001	103300001	0.112516	0.242821	SG1	LOC101120813
0.316967	0.237967	SG2	LOC114111370	NC_056055.1	82340001	82360001	0.361316	0.275865	SG1	LOC101120890
0.27908	0.252447	SG2	LOC114111370	NC_056055.1	82345001	82365001	0.230442	0.277842	SG1	LOC101120890
0.3943	0.213253	SG2	LOC114111378	NC_056055.1	82350001	82370001	0.124002	0.24326	SG1	LOC101120890
0.457109	0.206483	SG2	LOC114111378	NC_056055.1	82355001	82375001	0.3772	0.208623	SG1	LOC101120890
0.541254	0.138435	SG2	LOC114111425	NC_056067.1	61785001	61805001	0.380752	0.420647	SG1	LOC101120904
0.520635	0.139719	SG2	LOC114111425	NC_056073.1	28930001	28950001	0.401761	0.183044	SG1	LOC101121555
0.431745	0.165702	SG2	LOC114111502	NC_056073.1	28935001	28955001	0.366184	0.180686	SG1	LOC101121810
0.491528	0.17825	SG2	LOC114111502	NC_056073.1	28940001	28960001	0.378408	0.201368	SG1	LOC101121810
0.504515	0.525219	SG2	LOC114113316	NC_056073.1	28945001	28965001	0.295718	0.208386	SG1	LOC101121810
0.526365	0.50219	SG2	LOC114113348	NC_056057.1	107615001	107635001	0.284953	0.239244	SG1	LOC101122103
0.552174	0.152629	SG2	LOC114118023	NC_056057.1	107620001	107640001	0.1162	0.283461	SG1	LOC101122103
0.542752	0.138304	SG2	LOC121816623	NC_056071.1	45655001	45675001	0.386188	0.351031	SG1	LOC101122138
0.537117	0.258049	SG2	LOC121816819	NC_056071.1	45660001	45680001	0.379688	0.291427	SG1	LOC101122138
0.555113	0.145203	SG2	LOC121816819;NP	NC_056071.1	45665001	45685001	0.294314	0.224505	SG1	LOC101122138
0.079888	0.157418	SG2	LOC121817141	NC_056071.1	45670001	45690001	0.385356	0.185643	SG1	LOC101122138
0.066773	0.153175	SG2	LOC121817141	NC_056072.1	55720001	55740001	0.335851	0.271618	SG1	LOC101122224
0.327354	0.187147	SG2	LOC121817630	NC_056072.1	55725001	55745001	0.332284	0.283374	SG1	LOC101122224
0.493409	0.200078	SG2	LOC121818333	NC_056063.1	22885001	22905001	0.301134	0.186306	SG1	LOC101122286
0.47892	0.182409	SG2	LOC121818333	NC_056063.1	22890001	22910001	0.20208	0.180081	SG1	LOC101122286
0.142415	0.137201	SG2	LOC121818807;STI	NC_056072.1	52165001	52185001	0.372702	0.247203	SG1	LOC101122312

0.489362	0.366037	SG2	LRGUK	NC_056072.1	52170001	52190001	0.236673	0.231634	SG1	LOC101122312
0.481013	0.191572	SG2	LRRFIP2	NC_056057.1	107690001	107710001	0.0625324	0.183794	SG1	LOC101122351
0.519204	0.190696	SG2	LRRFIP2	NC_056059.1	85555001	85575001	0.436409	0.369618	SG1	LOC101122788
0.511674	0.147565	SG2	LRRFIP2	NC_056059.1	85560001	85580001	0.326669	0.322051	SG1	LOC101122788
0.475915	0.150084	SG2	LSM14A	NC_056059.1	85705001	85725001	0.330827	0.193262	SG1	LOC101123042
0.497435	0.144842	SG2	LSM14A	NC_056059.1	85710001	85730001	0.271614	0.212631	SG1	LOC101123042
0.509017	0.145045	SG2	LSM14A	NC_056059.1	85715001	85735001	0.283151	0.210278	SG1	LOC101123042
0.548562	0.138639	SG2	LSM14A	NC_056059.1	85720001	85740001	0.372415	0.194009	SG1	LOC101123042
0.41545	0.138698	SG2	LSM8	NC_056059.1	85725001	85745001	0.252232	0.232393	SG1	LOC101123042
0.117534	0.139053	SG2	MACROD2	NC_056061.1	12830001	12850001	0.425001	0.182684	SG1	LOC101123128
0.129799	0.143102	SG2	MACROD2	NC_056080.1	54715001	54735001	0.401666	0.178571	SG1	LOC101123606
0.136391	0.143313	SG2	MACROD2	NC_056080.1	54720001	54740001	0.466387	0.185374	SG1	LOC101123606
0.569589	0.146166	SG2	MADD	NC_056080.1	54725001	54745001	0.480493	0.177551	SG1	LOC101123606
0.202462	0.170635	SG2	MAFF;TMEM184B	NC_056055.1	89915001	89935001	0.409718	0.277161	SG1	LOC105601956
0.367906	0.160629	SG2	MAFF;TMEM184B	NC_056067.1	58445001	58465001	0.482873	0.347252	SG1	LOC105601988
0.268258	0.147811	SG2	MAGI2	NC_056067.1	58450001	58470001	0.437499	0.29513	SG1	LOC105601988
0.225434	0.143595	SG2	MAML3	NC_056054.1	218435001	218455001	0.430045	0.209507	SG1	LOC105605201
0.224342	0.142774	SG2	MAML3	NC_056054.1	218440001	218460001	0.386397	0.204883	SG1	LOC105605201
0.505695	0.207757	SG2	MAP2	NC_056054.1	218445001	218465001	0.308197	0.203523	SG1	LOC105605201
0.562964	0.167749	SG2	MAP2	NC_056067.1	61740001	61760001	0.387631	0.42887	SG1	LOC105605833
0.089403	0.178727	SG2	MARCHF4	NC_056054.1	17995001	18015001	0.127079	0.198989	SG1	LOC105607154
0.267267	0.240902	SG2	MAST4	NC_056055.1	101390001	101410001	0.409321	0.190251	SG1	LOC105608482
0.235542	0.270707	SG2	MAST4	NC_056055.1	101395001	101415001	0.449429	0.179094	SG1	LOC105608482
0.189831	0.279476	SG2	MAST4	NC_056054.1	25995001	26015001	0.394174	0.236285	SG1	LOC105609515
0.198432	0.255374	SG2	MAST4	NC_056061.1	55960001	55980001	0.472614	0.189462	SG1	LOC105610726
0.502518	0.175123	SG2	MAST4	NC_056056.1	207395001	207415001	0.404302	0.1777	SG1	LOC105612575
0.545246	0.23227	SG2	MAST4	NC_056056.1	207405001	207425001	0.255682	0.180571	SG1	LOC105612575
0.52782	0.161649	SG2	MASTL	NC_056073.1	34360001	34380001	0.484127	0.187514	SG1	LOC105613815
0.226811	0.33225	SG2	MASTL;YME1L1	NC_056073.1	34365001	34385001	0.469636	0.209064	SG1	LOC105613815
0.503209	0.232167	SG2	MASTL;YME1L1	NC_056073.1	26850001	26870001	0.178245	0.247774	SG1	LOC106990117
0.334829	0.164286	SG2	MC3R	NC_056073.1	26855001	26875001	0.0987471	0.230135	SG1	LOC106990117
0.431084	0.139456	SG2	MC3R	NC_056059.1	89815001	89835001	0.44901	0.251449	SG1	LOC114108665

0.148387	0.146262	SG2	MC5R;RNMT	NC_056059.1	89820001	89840001	0.454931	0.27919	SG1	LOC11410866[illegible]
0.121552	0.144974	SG2	MC5R;RNMT	NC_056056.1	7535001	7555001	0.168351	0.386248	SG1	LOC11410869[illegible]
0.323153	0.406215	SG2	MCM10	NC_056056.1	7540001	7560001	0.283364	0.310033	SG1	LOC11410869[illegible]
0.114764	0.435828	SG2	MCM10	NC_056070.1	70750001	70770001	0.331398	0.275852	SG1	LOC114108841
0.100787	0.445199	SG2	MCM10	NC_056070.1	70755001	70775001	0.486379	0.290958	SG1	LOC114108841
0.152194	0.43523	SG2	MCM10	NC_056071.1	50735001	50755001	0.467656	0.173962	SG1	LOC11410912[illegible]
0.132218	0.434945	SG2	MCM10	NC_056071.1	50740001	50760001	0.462468	0.191006	SG1	LOC11410912[illegible]
0.221654	0.393367	SG2	MCM10	NC_056073.1	28115001	28135001	0.346739	0.219516	SG1	LOC11410956[illegible]
0.484581	0.255889	SG2	MCM10	NC_056080.1	25200001	25220001	0.367829	0.389814	SG1	LOC11411155[illegible]
0.500266	0.150695	SG2	MED13L	NC_056080.1	25205001	25225001	0.191176	0.374738	SG1	LOC11411155[illegible]
0.486434	0.188266	SG2	MED27	NC_056080.1	25210001	25230001	0.275794	0.322516	SG1	LOC11411155[illegible]
0.361203	0.181344	SG2	MED27	NC_056080.1	25215001	25235001	0.442244	0.27192	SG1	LOC11411155[illegible]
0.372919	0.158618	SG2	MED27	NC_056056.1	222705001	222725001	0.436934	0.193588	SG1	LOC11411383[illegible]
0.563179	0.135815	SG2	MEGF9	NC_056056.1	7575001	7595001	0.17488	0.256161	SG1	LOC11411386[illegible]
0.47463	0.223618	SG2	MEGF9	NC_056056.1	7580001	7600001	0.173413	0.269767	SG1	LOC11411386[illegible]
0.204288	0.17242	SG2	MEGF9	NC_056056.1	7565001	7585001	0.35802	0.186287	SG1	LOC11411386[illegible]
0.182562	0.15481	SG2	MEGF9	NC_056056.1	7570001	7590001	0.28207	0.208619	SG1	LOC11411386[illegible]
0.096045	0.15769	SG2	MEGF9	NC_056057.1	107550001	107570001	0.0509929	0.196551	SG1	LOC11411461[illegible]
0.515037	0.136653	SG2	MESD;TLNRD1	NC_056057.1	107555001	107575001	0.0568239	0.19508	SG1	LOC11411461[illegible]
0.57095	0.155854	SG2	METTL22	NC_056057.1	107610001	107630001	0.428066	0.174755	SG1	LOC114114621
0.437338	0.192755	SG2	METTL22	NC_056058.1	38285001	38305001	0.355094	0.177119	SG1	LOC11411493[illegible]
0.454422	0.198435	SG2	METTL22	NC_056059.1	92260001	92280001	0.424738	0.21449	SG1	LOC11411533[illegible]
0.564202	0.173406	SG2	MGST3	NC_056067.1	50310001	50330001	0.245182	0.276289	SG1	LOC11411799[illegible]
0.30659	0.220371	SG2	MIB1	NC_056067.1	50315001	50335001	0.203918	0.278622	SG1	LOC11411799[illegible]
0.151771	0.270471	SG2	MIB1	NC_056067.1	50320001	50340001	0.201789	0.272515	SG1	LOC11411799[illegible]
0.17111	0.249275	SG2	MIB1	NC_056067.1	50325001	50345001	0.239263	0.308178	SG1	LOC11411799[illegible]
0.194671	0.233628	SG2	MIB1	NC_056067.1	50335001	50355001	0.0785121	0.446361	SG1	LOC11411799[illegible]
0.151171	0.240262	SG2	MIB1	NC_056067.1	50340001	50360001	0.0677907	0.458397	SG1	LOC11411799[illegible]
0.218382	0.215199	SG2	MIB1	NC_056067.1	62190001	62210001	0.449228	0.221421	SG1	LOC11411804[illegible]
0.257462	0.216947	SG2	MIB1	NC_056065.1	53850001	53870001	0.333878	0.291797	SG1	LOC12181607[illegible]
0.34091	0.183551	SG2	MIB1	NC_056065.1	53855001	53875001	0.313052	0.31666	SG1	LOC12181607[illegible]
0.430108	0.16205	SG2	MIB1	NC_056065.1	53860001	53880001	0.395433	0.283988	SG1	LOC12181607[illegible]

0.40303	0.145569	SG2	MICAL2	NC_056067.1	60985001	61005001	0.463067	0.174012	SG1	LOC12181644
0.35033	0.175373	SG2	MID1	NC_056067.1	61790001	61810001	0.45619	0.427317	SG1	LOC12181656
0.277108	0.200254	SG2	MID1	NC_056071.1	15210001	15230001	0.395984	0.211427	SG1	LOC12181707
0.480701	0.154223	SG2	MID1	NC_056071.1	15215001	15235001	0.47763	0.216035	SG1	LOC12181707
0.339984	0.175275	SG2	MID1	NC_056080.1	66445001	66465001	0.474859	0.262622	SG1	LOC12181818
0.384976	0.174805	SG2	MID1	NC_056080.1	66450001	66470001	0.223837	0.30831	SG1	LOC12181818
0.558984	0.207554	SG2	MIER3	NC_056080.1	66455001	66475001	0.154472	0.2445	SG1	LOC12181818
0.537084	0.243613	SG2	MIER3	NC_056054.1	256475001	256495001	0.413503	0.179028	SG1	LOC12181857
0.540888	0.242849	SG2	MIER3	NC_056058.1	38320001	38340001	0.474951	0.225831	SG1	LOC12181969
0.464516	0.138661	SG2	MIER3;SETD9	NC_056059.1	57305001	57325001	0.340095	0.18342	SG1	LOC12181982
0.539618	0.14562	SG2	MIER3;SETD9	NC_056054.1	107160001	107180001	0.383001	0.187363	SG1	LOC12182060
0.563214	0.193013	SG2	MIXL1	NC_056054.1	107165001	107185001	0.360259	0.180255	SG1	LOC12182060
0.085175	0.153407	SG2	MKLN1;PODXL	NC_056058.1	16430001	16450001	0.230472	0.224562	SG1	LONP1;RPL36
0.569855	0.218178	SG2	MOXD1	NC_056080.1	119585001	119605001	0.053211	0.288936	SG1	LRCH2
0.48531	0.261989	SG2	MOXD1	NC_056080.1	119590001	119610001	0.0588837	0.275847	SG1	LRCH2
0.45923	0.161479	SG2	MPHOSPH8	NC_056080.1	119595001	119615001	0.0872745	0.249465	SG1	LRCH2
0.46363	0.15745	SG2	MPHOSPH8	NC_056080.1	119600001	119620001	0.118989	0.185068	SG1	LRCH2
0.45399	0.153733	SG2	MPHOSPH8	NC_056078.1	30640001	30660001	0.471393	0.238576	SG1	LRMDA
0.468255	0.144505	SG2	MPHOSPH8	NC_056078.1	30645001	30665001	0.414104	0.202649	SG1	LRMDA
0.49177	0.142713	SG2	MPHOSPH8;PARP	NC_056078.1	30650001	30670001	0.393908	0.188914	SG1	LRMDA
0.495206	0.147174	SG2	MPHOSPH8;PSPC1	NC_056078.1	31325001	31345001	0.39403	0.276855	SG1	LRMDA
0.488269	0.141616	SG2	MPHOSPH8;PSPC1	NC_056078.1	31330001	31350001	0.388548	0.298509	SG1	LRMDA
0.456423	0.137625	SG2	MPHOSPH8;PSPC1	NC_056078.1	31335001	31355001	0.423157	0.266966	SG1	LRMDA
0.467877	0.145742	SG2	MPHOSPH8;PSPC1	NC_056078.1	31340001	31360001	0.450279	0.207949	SG1	LRMDA
0.457731	0.150713	SG2	MPHOSPH8;PSPC1	NC_056055.1	169305001	169325001	0.391614	0.199005	SG1	LRP1B
0.537211	0.180044	SG2	MPZL1	NC_056055.1	169310001	169330001	0.233098	0.256359	SG1	LRP1B
0.564695	0.234388	SG2	MPZL1;RCSD1	NC_056055.1	169315001	169335001	0.134468	0.28086	SG1	LRP1B
0.500296	0.166399	SG2	MRC1	NC_056055.1	169320001	169340001	0.18772	0.246553	SG1	LRP1B
0.414603	0.229553	SG2	MRC1	NC_056068.1	70465001	70485001	0.374685	0.247615	SG1	LRRC4C
0.519926	0.172691	SG2	MRC1	NC_056068.1	70470001	70490001	0.359232	0.296057	SG1	LRRC4C
0.236185	0.148316	SG2	MSRA	NC_056068.1	70475001	70495001	0.459183	0.2386	SG1	LRRC4C
0.559965	0.146191	SG2	MTMR7	NC_056054.1	45725001	45745001	0.389442	0.233577	SG1	LRRC7

0.480369	0.142958	SG2	MTURN	NC_056056.1	7495001	7515001	0.100051	0.248501	SG1	LRRC8A
0.441	0.165848	SG2	MYH1	NC_056056.1	7480001	7500001	0.188302	0.220676	SG1	LRRC8A;PHYI
0.456281	0.167498	SG2	MYH1	NC_056056.1	7485001	7505001	0.182365	0.209405	SG1	LRRC8A;PHYI
0.475941	0.160204	SG2	MYH1	NC_056056.1	7490001	7510001	0.154744	0.242431	SG1	LRRC8A;PHYI
0.487301	0.137096	SG2	MYH1;MYH2	NC_056054.1	66955001	66975001	0.39434	0.196797	SG1	LRRC8B
0.424788	0.158984	SG2	MYO1D	NC_056054.1	66960001	66980001	0.386809	0.175591	SG1	LRRC8B
0.500202	0.141229	SG2	MYO1D	NC_056067.1	49305001	49325001	0.202648	0.280481	SG1	LTBP4
0.236516	0.136209	SG2	MYO1D	NC_056067.1	49310001	49330001	0.40656	0.187349	SG1	LTBP4
0.245459	0.254665	SG2	MYO1G	NC_056067.1	49300001	49320001	0.145643	0.19954	SG1	LTBP4;SHKBP
0.213327	0.260967	SG2	MYO1G	NC_056072.1	52950001	52970001	0.437959	0.174152	SG1	LTF
0.178896	0.282092	SG2	MYO1G	NC_056072.1	52955001	52975001	0.410287	0.176354	SG1	LTF
0.513547	0.271328	SG2	NALF2	NC_056056.1	127490001	127510001	0.116915	0.260273	SG1	LUM
0.299735	0.300249	SG2	NALF2	NC_056056.1	127495001	127515001	0.189144	0.19985	SG1	LUM
0.221913	0.280742	SG2	NALF2	NC_056056.1	127500001	127520001	0.324688	0.191599	SG1	LUM
0.275449	0.25609	SG2	NALF2	NC_056066.1	7540001	7560001	0.247365	0.249968	SG1	MACROD2
0.459319	0.181935	SG2	NALF2	NC_056066.1	7545001	7565001	0.171166	0.351639	SG1	MACROD2
0.555371	0.136336	SG2	NAV2	NC_056066.1	7550001	7570001	0.0840921	0.498171	SG1	MACROD2
0.544908	0.143282	SG2	NAV2	NC_056066.1	7555001	7575001	0.0641976	0.550005	SG1	MACROD2
0.502762	0.154509	SG2	NAV2	NC_056066.1	7560001	7580001	0.0527177	0.546694	SG1	MACROD2
0.520784	0.145724	SG2	NAV2	NC_056066.1	7565001	7585001	0.126994	0.482323	SG1	MACROD2
0.408141	0.154792	SG2	NBAS	NC_056066.1	7570001	7590001	0.215757	0.401456	SG1	MACROD2
0.250055	0.173716	SG2	NBAS	NC_056066.1	7575001	7595001	0.218158	0.431142	SG1	MACROD2
0.195275	0.204904	SG2	NBAS	NC_056066.1	7580001	7600001	0.205216	0.462735	SG1	MACROD2
0.182851	0.210151	SG2	NBAS	NC_056066.1	7585001	7605001	0.0816804	0.591699	SG1	MACROD2
0.31119	0.177043	SG2	NBAS	NC_056066.1	7600001	7620001	0.168377	0.574146	SG1	MACROD2
0.46597	0.231934	SG2	NBEA	NC_056054.1	119060001	119080001	0.469675	0.269207	SG1	MAEL
0.510345	0.239652	SG2	NBEA	NC_056054.1	119065001	119085001	0.404633	0.282972	SG1	MAEL
0.517166	0.241308	SG2	NBEA	NC_056054.1	119070001	119090001	0.187404	0.294596	SG1	MAEL
0.298412	0.29032	SG2	NBEA	NC_056072.1	35575001	35595001	0.356547	0.187679	SG1	MAGI1
0.121282	0.402833	SG2	NBEA	NC_056072.1	35580001	35600001	0.215	0.180327	SG1	MAGI1
0.11831	0.412597	SG2	NBEA	NC_056057.1	44590001	44610001	0.297538	0.349932	SG1	MAGI2
0.17302	0.390357	SG2	NBEA	NC_056057.1	44595001	44615001	0.476222	0.342313	SG1	MAGI2

0.162537	0.391604	SG2	NBEA	NC_056054.1	202530001	202550001	0.321105	0.234677	SG1	MAP3K13
0.136276	0.406644	SG2	NBEA	NC_056054.1	202535001	202555001	0.20598	0.309801	SG1	MAP3K13
0.104991	0.418279	SG2	NBEA	NC_056054.1	202540001	202560001	0.232604	0.321158	SG1	MAP3K13
0.187123	0.35271	SG2	NBEA	NC_056054.1	202545001	202565001	0.205277	0.316559	SG1	MAP3K13
0.340581	0.27746	SG2	NBEA	NC_056054.1	202550001	202570001	0.240529	0.33124	SG1	MAP3K13
0.554382	0.245806	SG2	NBEA	NC_056054.1	202555001	202575001	0.19602	0.37697	SG1	MAP3K13
0.550187	0.176667	SG2	NBEA	NC_056054.1	202560001	202580001	0.0693159	0.413996	SG1	MAP3K13
0.486591	0.204082	SG2	NBEA	NC_056054.1	202565001	202585001	0.11022	0.423829	SG1	MAP3K13
0.485508	0.220933	SG2	NBEA	NC_056054.1	202570001	202590001	0.185204	0.35898	SG1	MAP3K13
0.444915	0.244805	SG2	NBEA	NC_056054.1	202575001	202595001	0.380629	0.21027	SG1	MAP3K13
0.536587	0.217669	SG2	NBEA	NC_056080.1	18520001	18540001	0.137976	0.231288	SG1	MAP7D2
0.111349	0.138655	SG2	NCAPH	NC_056055.1	149960001	149980001	0.417857	0.183794	SG1	MARCHF7
0.442409	0.151161	SG2	NCBP1;TSTD2	NC_056069.1	12595001	12615001	0.415692	0.20779	SG1	MAST4
0.463245	0.190736	SG2	NDUFAF4	NC_056069.1	12600001	12620001	0.223782	0.187319	SG1	MAST4
0.32144	0.227909	SG2	NDUFAF4	NC_056055.1	161035001	161055001	0.435276	0.200719	SG1	MBD5
0.501247	0.166903	SG2	NDUFAF4	NC_056062.1	32555001	32575001	0.107307	0.232265	SG1	MCM4;PRKDC
0.525698	0.194995	SG2	NECTIN1	NC_056061.1	19410001	19430001	0.177948	0.305127	SG1	MCM9
0.518461	0.18887	SG2	NECTIN1	NC_056061.1	19415001	19435001	0.102935	0.314556	SG1	MCM9
0.516829	0.178827	SG2	NECTIN1	NC_056061.1	19440001	19460001	0.0323349	0.331871	SG1	MCM9
0.501416	0.20394	SG2	NF1	NC_056061.1	19445001	19465001	0.0255652	0.330345	SG1	MCM9
0.411428	0.154286	SG2	NHS	NC_056061.1	19450001	19470001	0.0523859	0.285666	SG1	MCM9
0.39279	0.141837	SG2	NHS	NC_056079.1	5765001	5785001	0.450696	0.288274	SG1	MCPH1
0.320707	0.145833	SG2	NHS	NC_056079.1	5770001	5790001	0.324996	0.244934	SG1	MCPH1
0.181027	0.138236	SG2	NHSL1	NC_056079.1	5775001	5795001	0.39292	0.212553	SG1	MCPH1
0.457274	0.256397	SG2	NOVA1	NC_056058.1	91690001	91710001	0.171283	0.221233	SG1	MCTP1
0.393315	0.250406	SG2	NOVA1	NC_056058.1	91695001	91715001	0.13869	0.219996	SG1	MCTP1
0.266394	0.196802	SG2	NOVA1	NC_056058.1	91700001	91720001	0.109394	0.222008	SG1	MCTP1
0.388481	0.211546	SG2	NOVA1	NC_056058.1	91705001	91725001	0.0835804	0.22185	SG1	MCTP1
0.485168	0.167464	SG2	NOVA1	NC_056078.1	27945001	27965001	0.136246	0.258648	SG1	MCU
0.533253	0.156288	SG2	NOVA1	NC_056057.1	55035001	55055001	0.36598	0.178902	SG1	MDFIC
0.249055	0.148078	SG2	NOX4	NC_056057.1	55040001	55060001	0.337045	0.208913	SG1	MDFIC
0.282608	0.1423	SG2	NPR3	NC_056057.1	55045001	55065001	0.410985	0.235103	SG1	MDFIC

0.274726	0.201539	SG2	NPR3	NC_056057.1	55050001	55070001	0.360758	0.214137	SG1	MDFIC
0.39708	0.199758	SG2	NPR3	NC_056060.1	38255001	38275001	0.400683	0.173683	SG1	MDGA2
0.408221	0.188842	SG2	NPR3	NC_056061.1	47800001	47820001	0.272266	0.183348	SG1	MDN1
0.568016	0.157102	SG2	NPR3	NC_056061.1	47805001	47825001	0.287186	0.17321	SG1	MDN1
0.233459	0.161223	SG2	NR5A2	NC_056061.1	10385001	10405001	0.415507	0.309753	SG1	ME1
0.18559	0.17581	SG2	NR5A2	NC_056061.1	10390001	10410001	0.243819	0.385076	SG1	ME1
0.11828	0.193231	SG2	NR5A2	NC_056061.1	10395001	10415001	0.292472	0.35839	SG1	ME1
0.547997	0.165021	SG2	NRG4	NC_056061.1	10400001	10420001	0.368878	0.297594	SG1	ME1
0.492632	0.183079	SG2	NRG4	NC_056061.1	10405001	10425001	0.413082	0.246695	SG1	ME1
0.571429	0.167489	SG2	NRG4	NC_056061.1	10410001	10430001	0.384981	0.197614	SG1	ME1
0.332884	0.182527	SG2	NRL;PCK2	NC_056061.1	10415001	10435001	0.445462	0.17857	SG1	ME1
0.251689	0.189841	SG2	NRL;PCK2	NC_056054.1	218430001	218450001	0.480627	0.181072	SG1	MECOM
0.361545	0.156126	SG2	NRP1	NC_056054.1	218900001	218920001	0.282361	0.173563	SG1	MECOM
0.42552	0.14003	SG2	NRP1	NC_056056.1	4750001	4770001	0.0715799	0.193115	SG1	MED27
0.495319	0.14892	SG2	NSG2	NC_056063.1	30290001	30310001	0.0761402	0.278948	SG1	MEDAG
0.41061	0.152864	SG2	NSUN6	NC_056063.1	30295001	30315001	0.0484581	0.310023	SG1	MEDAG
0.566923	0.237021	SG2	NTM	NC_056063.1	30305001	30325001	0.146687	0.271193	SG1	MEDAG
0.482999	0.238106	SG2	NTM	NC_056063.1	30285001	30305001	0.190919	0.231964	SG1	MEDAG;TEX2
0.30181	0.17743	SG2	NTN1	NC_056060.1	30845001	30865001	0.316182	0.182536	SG1	MEIS2
0.228723	0.258243	SG2	NTN1	NC_056060.1	30850001	30870001	0.403925	0.216104	SG1	MEIS2
0.321875	0.223613	SG2	NTN1	NC_056060.1	30855001	30875001	0.486233	0.217691	SG1	MEIS2
0.449847	0.17464	SG2	NTN1	NC_056056.1	106125001	106145001	0.432339	0.193646	SG1	MERTK
0.274074	0.249783	SG2	NUDT11	NC_056056.1	106130001	106150001	0.281088	0.203414	SG1	MERTK
0.404255	0.213051	SG2	NUDT11	NC_056056.1	106135001	106155001	0.116244	0.192559	SG1	MERTK
0.453925	0.228795	SG2	NUDT11	NC_056064.1	23270001	23290001	0.365417	0.273557	SG1	METTL16
0.386667	0.144226	SG2	NXPH1	NC_056064.1	23275001	23295001	0.359184	0.306156	SG1	METTL16
0.372504	0.188221	SG2	NXPH1	NC_056064.1	23280001	23300001	0.362013	0.283555	SG1	METTL16
0.565649	0.139184	SG2	NXPH1	NC_056064.1	23285001	23305001	0.375828	0.247328	SG1	METTL16
0.546706	0.222433	SG2	OBI1	NW_024599827.	475001	495001	0.402368	0.1825	SG1	MEX3B
0.559585	0.153102	SG2	OLIG1	NW_024599827.	480001	500001	0.445255	0.200487	SG1	MEX3B
0.524729	0.215769	SG2	OPA1	NC_056056.1	181490001	181510001	0.208282	0.352957	SG1	MFNG
0.409342	0.212157	SG2	OPA1	NC_056056.1	181495001	181515001	0.136679	0.343139	SG1	MFNG

0.457395	0.186578	SG2	OPA1	NC_056056.1	181500001	181520001	0.143646	0.330059	SG1	MFNG
0.494574	0.194512	SG2	OPA1	NC_056056.1	181505001	181525001	0.10261	0.344141	SG1	MFNG
0.37867	0.233975	SG2	OPA1	NC_056064.1	28105001	28125001	0.356025	0.195795	SG1	MFSD6L;PIK3l
0.477229	0.243036	SG2	OPA1	NC_056064.1	28110001	28130001	0.471417	0.188726	SG1	MFSD6L;PIK3l
0.326252	0.189505	SG2	OSBPL11	NC_056056.1	122975001	122995001	0.416057	0.21155	SG1	MGAT4C
0.315917	0.253694	SG2	OSBPL11	NC_056056.1	122980001	123000001	0.33757	0.205748	SG1	MGAT4C
0.358679	0.263128	SG2	OSBPL11	NC_056056.1	122985001	123005001	0.368099	0.192924	SG1	MGAT4C
0.346611	0.231373	SG2	OSBPL11	NC_056056.1	214990001	215010001	0.192984	0.249808	SG1	MICAL3
0.34044	0.188471	SG2	OSBPL11	NC_056056.1	214995001	215015001	0.192989	0.24324	SG1	MICAL3
0.563941	0.208196	SG2	OSBPL3	NC_056056.1	215000001	215020001	0.170745	0.279464	SG1	MICAL3
0.41499	0.21009	SG2	OSBPL3	NC_056056.1	215005001	215025001	0.12692	0.347165	SG1	MICAL3
0.353794	0.188234	SG2	OSBPL3	NC_056056.1	215010001	215030001	0.146843	0.360229	SG1	MICAL3
0.232124	0.148622	SG2	OSBPL3	NC_056056.1	215015001	215035001	0.247544	0.329427	SG1	MICAL3
0.223275	0.141344	SG2	OSBPL3	NC_056056.1	215020001	215040001	0.230101	0.324958	SG1	MICAL3
0.415055	0.140646	SG2	OSBPL3	NC_056056.1	215025001	215045001	0.30111	0.294338	SG1	MICAL3
0.565318	0.270085	SG2	OSBPL3	NC_056056.1	215030001	215050001	0.32591	0.288721	SG1	MICAL3
0.380254	0.257186	SG2	OSBPL8	NC_056056.1	215035001	215055001	0.38878	0.280786	SG1	MICAL3
0.328125	0.276535	SG2	OSBPL8	NC_056056.1	215525001	215545001	0.100671	0.19486	SG1	MICALL1
0.307012	0.253186	SG2	OSBPL8	NC_056056.1	7395001	7415001	0.418751	0.24512	SG1	MIGA2
0.374422	0.218123	SG2	OSBPL8	NC_056056.1	7410001	7430001	0.466313	0.230774	SG1	MIGA2;SH3GL
0.457064	0.207886	SG2	OSBPL8	NC_056072.1	31720001	31740001	0.394589	0.254666	SG1	MITF
0.314222	0.138256	SG2	OTUD7A	NC_056072.1	31725001	31745001	0.3292	0.266323	SG1	MITF
0.530347	0.174088	SG2	PAPPA	NC_056072.1	31730001	31750001	0.196979	0.193287	SG1	MITF
0.484501	0.169489	SG2	PAPPA	NC_056072.1	31735001	31755001	0.191325	0.26299	SG1	MITF
0.459091	0.14915	SG2	PAPPA	NC_056072.1	31740001	31760001	0.267463	0.243579	SG1	MITF
0.310611	0.140536	SG2	PAPPA	NC_056072.1	31745001	31765001	0.322239	0.280807	SG1	MITF
0.492322	0.229978	SG2	PARD3	NC_056072.1	31750001	31770001	0.289387	0.263226	SG1	MITF
0.494167	0.212655	SG2	PARD3	NC_056072.1	31755001	31775001	0.387719	0.213273	SG1	MITF
0.485712	0.219096	SG2	PARD3	NC_056068.1	5735001	5755001	0.272917	0.17812	SG1	MMP20
0.55621	0.199731	SG2	PARD3	NC_056068.1	5740001	5760001	0.223295	0.186602	SG1	MMP20
0.439905	0.198661	SG2	PARL	NC_056068.1	5745001	5765001	0.259823	0.203859	SG1	MMP20
0.394603	0.213266	SG2	PARL	NC_056068.1	5750001	5770001	0.254634	0.183399	SG1	MMP20

0.287289	0.174354	SG2	PARN	NC_056075.1	18515001	18535001	0.363925	0.211571	SG1	MMS19
0.368662	0.136169	SG2	PARN	NC_056059.1	87020001	87040001	0.453318	0.242488	SG1	MOB1B
0.462266	0.137188	SG2	PARP4	NC_056059.1	87025001	87045001	0.342886	0.256197	SG1	MOB1B
0.490672	0.135139	SG2	PARP4	NC_056055.1	96115001	96135001	0.382226	0.246225	SG1	MOB3B
0.477306	0.154525	SG2	PARP4	NC_056055.1	96120001	96140001	0.353217	0.266673	SG1	MOB3B
0.459524	0.1573	SG2	PARP4	NC_056055.1	96125001	96145001	0.440935	0.2453	SG1	MOB3B
0.45984	0.153638	SG2	PARP4	NC_056066.1	66130001	66150001	0.416379	0.184663	SG1	MROH8;RPN2
0.561166	0.149246	SG2	PBX3	NC_056070.1	63735001	63755001	0.425979	0.208764	SG1	MVK
0.566502	0.167284	SG2	PCBP3	NC_056070.1	63740001	63760001	0.328447	0.177748	SG1	MVK
0.548499	0.189188	SG2	PCBP3	NC_056064.1	54405001	54425001	0.271565	0.2951	SG1	MXRA7
0.368477	0.2224	SG2	PCBP3	NC_056064.1	54410001	54430001	0.446707	0.203507	SG1	MXRA7
0.408609	0.20294	SG2	PCBP3	NC_056064.1	17340001	17360001	0.468125	0.196415	SG1	MYO1D
0.164623	0.139249	SG2	PCDH15	NC_056064.1	17345001	17365001	0.44937	0.190632	SG1	MYO1D
0.12313	0.161215	SG2	PCDH17	NC_056054.1	212295001	212315001	0.260265	0.209325	SG1	NAALADL2
0.191818	0.148936	SG2	PCDH17	NC_056054.1	212300001	212320001	0.295302	0.256334	SG1	NAALADL2
0.516529	0.150729	SG2	PCSK5	NC_056054.1	212305001	212325001	0.323908	0.245881	SG1	NAALADL2
0.536316	0.165841	SG2	PCSK6	NC_056054.1	212310001	212330001	0.451491	0.324372	SG1	NAALADL2
0.421183	0.181934	SG2	PCSK6	NC_056054.1	212315001	212335001	0.366888	0.325602	SG1	NAALADL2
0.326722	0.192617	SG2	PCSK6	NC_056054.1	212320001	212340001	0.249777	0.309266	SG1	NAALADL2
0.280418	0.199358	SG2	PCSK6	NC_056054.1	212325001	212345001	0.185975	0.34572	SG1	NAALADL2
0.239093	0.150005	SG2	PCSK6	NC_056054.1	212330001	212350001	0.110775	0.261436	SG1	NAALADL2
0.330232	0.223036	SG2	PCYT1B	NC_056054.1	212335001	212355001	0.179716	0.226891	SG1	NAALADL2
0.202573	0.223622	SG2	PCYT1B;POLA1	NC_056054.1	212340001	212360001	0.283082	0.241234	SG1	NAALADL2
0.309123	0.17458	SG2	PCYT1B;POLA1	NC_056054.1	212345001	212365001	0.382351	0.251656	SG1	NAALADL2
0.51721	0.156395	SG2	PDE1C	NC_056054.1	212425001	212445001	0.277253	0.211592	SG1	NAALADL2
0.566989	0.283284	SG2	PDE1C	NC_056054.1	212430001	212450001	0.210055	0.267259	SG1	NAALADL2
0.535116	0.327055	SG2	PDE1C	NC_056054.1	212435001	212455001	0.176867	0.27547	SG1	NAALADL2
0.403176	0.248503	SG2	PDE1C	NC_056054.1	212440001	212460001	0.252822	0.249743	SG1	NAALADL2
0.336559	0.233154	SG2	PDE1C	NC_056054.1	212445001	212465001	0.348099	0.225855	SG1	NAALADL2
0.32389	0.229558	SG2	PDE1C	NC_056054.1	212500001	212520001	0.283315	0.181211	SG1	NAALADL2
0.420421	0.239321	SG2	PDE1C	NC_056063.1	26105001	26125001	0.0477856	0.523332	SG1	NBEA
0.445252	0.242013	SG2	PDE1C	NC_056063.1	26130001	26150001	0.284672	0.376192	SG1	NBEA

0.356491	0.316951	SG2	PDE1C	NC_056055.1	205220001	205240001	0.421231	0.176387	SG1	NBEAL1
0.307197	0.305473	SG2	PDE1C	NC_056055.1	205225001	205245001	0.260144	0.213129	SG1	NBEAL1
0.18448	0.288473	SG2	PDE1C	NC_056055.1	205230001	205250001	0.404936	0.180384	SG1	NBEAL1
0.224953	0.267021	SG2	PDE1C	NC_056062.1	75640001	75660001	0.292295	0.196431	SG1	NCALD
0.431374	0.246744	SG2	PDE1C	NC_056062.1	75645001	75665001	0.238596	0.227108	SG1	NCALD
0.420219	0.232499	SG2	PDE1C	NC_056055.1	177185001	177205001	0.46041	0.182037	SG1	NCKAP5
0.567611	0.231738	SG2	PDE1C	NC_056055.1	177190001	177210001	0.418848	0.181164	SG1	NCKAP5
0.423253	0.274202	SG2	PDE1C	NC_056055.1	177195001	177215001	0.359098	0.179726	SG1	NCKAP5
0.299134	0.329872	SG2	PDE1C	NC_056055.1	177915001	177935001	0.472572	0.19311	SG1	NCKAP5
0.401123	0.314919	SG2	PDE1C	NC_056055.1	177920001	177940001	0.417725	0.184984	SG1	NCKAP5
0.462746	0.350409	SG2	PDE1C	NC_056063.1	55175001	55195001	0.350911	0.19785	SG1	NDFIP2
0.531588	0.173046	SG2	PDE1C	NC_056069.1	18440001	18460001	0.299005	0.227698	SG1	NDUFAF2
0.483155	0.161988	SG2	PDE1C	NC_056069.1	18445001	18465001	0.0737614	0.216882	SG1	NDUFAF2
0.567002	0.166181	SG2	PDE1C	NC_056069.1	18450001	18470001	0.108349	0.190066	SG1	NDUFAF2
0.568162	0.165183	SG2	PDE1C	NC_056066.1	21595001	21615001	0.392692	0.174817	SG1	NEBL
0.561732	0.173207	SG2	PDE1C	NC_056066.1	21600001	21620001	0.344422	0.179487	SG1	NEBL
0.539613	0.183219	SG2	PDE1C	NC_056066.1	21605001	21625001	0.296771	0.18754	SG1	NEBL
0.530288	0.201332	SG2	PDE1C	NC_056066.1	21635001	21655001	0.307376	0.181849	SG1	NEBL
0.514955	0.198387	SG2	PDE1C	NC_056066.1	21640001	21660001	0.222515	0.212243	SG1	NEBL
0.489498	0.184194	SG2	PDE4D	NC_056066.1	21645001	21665001	0.347147	0.174202	SG1	NEBL
0.543695	0.203579	SG2	PDE4D	NC_056060.1	52565001	52585001	0.427863	0.200802	SG1	NEDD4
0.555194	0.176171	SG2	PDE4D	NC_056076.1	57550001	57570001	0.378516	0.217842	SG1	NEDD4L
0.400318	0.142592	SG2	PDE4D	NC_056076.1	57555001	57575001	0.273746	0.223613	SG1	NEDD4L
0.562309	0.142842	SG2	PDE4D	NC_056076.1	57560001	57580001	0.241848	0.193727	SG1	NEDD4L
0.506249	0.150696	SG2	PDE4D	NC_056054.1	259280001	259300001	0.180049	0.38084	SG1	NEK11
0.49902	0.161437	SG2	PDE4D	NC_056054.1	259285001	259305001	0.154683	0.399782	SG1	NEK11
0.373938	0.15045	SG2	PDE4D	NC_056054.1	259290001	259310001	0.202237	0.371216	SG1	NEK11
0.283892	0.253175	SG2	PDE7B	NC_056054.1	259295001	259315001	0.280695	0.288733	SG1	NEK11
0.527529	0.16498	SG2	PDE7B	NC_056054.1	259300001	259320001	0.392887	0.233252	SG1	NEK11
0.559878	0.156397	SG2	PDE7B	NC_056054.1	259365001	259385001	0.436726	0.202149	SG1	NEK11
0.537957	0.166324	SG2	PDE7B	NC_056054.1	259370001	259390001	0.365902	0.220301	SG1	NEK11
0.393598	0.141362	SG2	PDE7B	NC_056054.1	259375001	259395001	0.458226	0.200779	SG1	NEK11

0.305128	0.150355	SG2	PDE7B	NC_056054.1	259275001	259295001	0.236492	0.330883	SG1	NEK11;NUDT1
0.276471	0.138826	SG2	PDE7B	NC_056065.1	76550001	76570001	0.166972	0.176541	SG1	NEK7
0.371508	0.184656	SG2	PDE7B	NC_056065.1	76555001	76575001	0.114678	0.183812	SG1	NEK7
0.47694	0.17103	SG2	PDE7B	NC_056065.1	76560001	76580001	0.120302	0.173726	SG1	NEK7
0.540363	0.20964	SG2	PDLIM5	NC_056065.1	76610001	76630001	0.30226	0.188075	SG1	NEK7
0.475368	0.22192	SG2	PDLIM5	NC_056065.1	76615001	76635001	0.304626	0.213895	SG1	NEK7
0.533154	0.153212	SG2	PEAK1	NC_056074.1	21470001	21490001	0.347222	0.173085	SG1	NELL1
0.502727	0.138001	SG2	PEAK1	NC_056074.1	21475001	21495001	0.345466	0.205712	SG1	NELL1
0.475741	0.136248	SG2	PEAK1	NC_056074.1	21480001	21500001	0.338883	0.245186	SG1	NELL1
0.437841	0.154001	SG2	PEPD	NC_056074.1	21485001	21505001	0.314268	0.219263	SG1	NELL1
0.431113	0.265183	SG2	PGPEP1L	NC_056076.1	925001	945001	0.330603	0.300965	SG1	NFATC1
0.347272	0.285203	SG2	PGPEP1L	NC_056076.1	930001	950001	0.353416	0.290945	SG1	NFATC1
0.305249	0.269287	SG2	PGPEP1L	NC_056076.1	935001	955001	0.467022	0.211239	SG1	NFATC1
0.367337	0.196819	SG2	PGPEP1L	NC_056080.1	15730001	15750001	0.486486	0.198157	SG1	NHS
0.567648	0.300943	SG2	PHACTR1	NC_056080.1	15735001	15755001	0.418623	0.213018	SG1	NHS
0.543128	0.296474	SG2	PHACTR1	NC_056078.1	8150001	8170001	0.448298	0.359327	SG1	NID1
0.504004	0.166015	SG2	PHB2;PTPN6	NC_056078.1	8155001	8175001	0.40896	0.27776	SG1	NID1
0.377359	0.206118	SG2	PHF12;SEZ6	NC_056061.1	13795001	13815001	0.434379	0.312578	SG1	NKAIN2
0.234759	0.252297	SG2	PHF12;SEZ6	NC_056061.1	13800001	13820001	0.369786	0.321855	SG1	NKAIN2
0.513606	0.135217	SG2	PHKA1	NC_056061.1	13805001	13825001	0.462524	0.339303	SG1	NKAIN2
0.425603	0.147599	SG2	PHKA1	NC_056061.1	14530001	14550001	0.179588	0.204585	SG1	NKAIN2
0.325424	0.184071	SG2	PHKA1	NC_056061.1	14535001	14555001	0.227198	0.174511	SG1	NKAIN2
0.532299	0.214785	SG2	PHLDA1	NC_056061.1	14540001	14560001	0.170607	0.179554	SG1	NKAIN2
0.487617	0.191654	SG2	PHLDA1	NC_056061.1	14560001	14580001	0.0520197	0.194536	SG1	NKAIN2
0.396923	0.144776	SG2	PHTF1	NC_056061.1	14565001	14585001	0.201293	0.178457	SG1	NKAIN2
0.47276	0.154931	SG2	PI15	NC_056054.1	214585001	214605001	0.455572	0.253236	SG1	NLGN1
0.416119	0.18443	SG2	PI15	NC_056069.1	31290001	31310001	0.435625	0.219419	SG1	NNT
0.325956	0.136932	SG2	PIBF1	NC_056069.1	31295001	31315001	0.268493	0.1796	SG1	NNT
0.352866	0.152596	SG2	PIBF1	NC_056080.1	63965001	63985001	0.330014	0.346487	SG1	NONO
0.350059	0.138484	SG2	PIBF1	NC_056070.1	57740001	57760001	0.454422	0.205558	SG1	NOS1
0.560792	0.143224	SG2	PIFO	NC_056070.1	57745001	57765001	0.303385	0.205907	SG1	NOS1
0.402291	0.273173	SG2	PIP4K2A	NC_056070.1	57750001	57770001	0.367169	0.214373	SG1	NOS1

0.405544	0.249924	SG2	PIP4K2A	NC_056070.1	57755001	57775001	0.422546	0.240505	SG1	NOS1
0.425876	0.226523	SG2	PIP4K2A	NC_056060.1	15875001	15895001	0.143482	0.347934	SG1	NOX5
0.542309	0.156468	SG2	PKHD1L1	NC_056071.1	41135001	41155001	0.37044	0.329023	SG1	NPAS3
0.408488	0.158497	SG2	PLAG1	NC_056071.1	41140001	41160001	0.265836	0.286259	SG1	NPAS3
0.250001	0.160648	SG2	PLAG1	NC_056071.1	41145001	41165001	0.321598	0.20976	SG1	NPAS3
0.240059	0.153844	SG2	PLAG1	NC_056054.1	257575001	257595001	0.405316	0.247177	SG1	NPHP3
0.210074	0.148001	SG2	PLAG1	NC_056054.1	257580001	257600001	0.33798	0.252699	SG1	NPHP3
0.230661	0.14355	SG2	PLAG1	NC_056054.1	257585001	257605001	0.427147	0.224219	SG1	NPHP3;UBA5
0.334464	0.136031	SG2	PLAG1	NC_056054.1	257590001	257610001	0.45085	0.200329	SG1	NPHP3;UBA5
0.55831	0.189757	SG2	PLCB4	NC_056055.1	233565001	233585001	0.341772	0.192413	SG1	NPPC
0.473892	0.151482	SG2	PLCB4	NC_056055.1	233570001	233590001	0.232877	0.238291	SG1	NPPC
0.419739	0.15015	SG2	PLCB4	NC_056057.1	95175001	95195001	0.449888	0.254271	SG1	NRF1
0.525688	0.142012	SG2	PLEKHM3	NC_056056.1	73500001	73520001	0.41306	0.220723	SG1	NRXN1
0.25771	0.166763	SG2	PLSCR1	NC_056056.1	74460001	74480001	0.466899	0.182455	SG1	NRXN1
0.534818	0.180424	SG2	PLXDC2	NC_056056.1	74465001	74485001	0.451759	0.189002	SG1	NRXN1
0.519529	0.176519	SG2	PLXDC2	NC_056060.1	88305001	88325001	0.352157	0.193311	SG1	NRXN3
0.278781	0.361211	SG2	PLXDC2	NC_056060.1	88310001	88330001	0.414415	0.176335	SG1	NRXN3
0.193487	0.391823	SG2	PLXDC2	NC_056060.1	88765001	88785001	0.260612	0.233781	SG1	NRXN3
0.469978	0.319319	SG2	PLXDC2	NC_056060.1	88770001	88790001	0.336171	0.189546	SG1	NRXN3
0.374487	0.15066	SG2	PMEPA1	NC_056060.1	88775001	88795001	0.367359	0.177998	SG1	NRXN3
0.315323	0.180036	SG2	PMEPA1	NC_056060.1	89130001	89150001	0.21675	0.202116	SG1	NRXN3
0.291445	0.171886	SG2	PMEPA1	NC_056060.1	89135001	89155001	0.222345	0.215889	SG1	NRXN3
0.278899	0.154096	SG2	PMEPA1	NC_056060.1	89140001	89160001	0.36274	0.201835	SG1	NRXN3
0.111109	0.153413	SG2	PODXL	NC_056060.1	89145001	89165001	0.475483	0.181204	SG1	NRXN3
0.124434	0.162423	SG2	PODXL	NC_056060.1	89175001	89195001	0.4618	0.237106	SG1	NRXN3
0.236905	0.157612	SG2	PODXL	NC_056059.1	117350001	117370001	0.294155	0.185433	SG1	NSD2
0.417583	0.168008	SG2	POLA1	NC_056059.1	117355001	117375001	0.273304	0.194013	SG1	NSD2
0.38562	0.192438	SG2	POLA1	NC_056059.1	117360001	117380001	0.249634	0.203586	SG1	NSD2
0.338889	0.223424	SG2	POLA1	NC_056074.1	32255001	32275001	0.420744	0.187871	SG1	NTM
0.359636	0.325012	SG2	POLN	NC_056074.1	32260001	32280001	0.387845	0.216693	SG1	NTM
0.294322	0.35528	SG2	POLN	NC_056074.1	32265001	32285001	0.357883	0.245556	SG1	NTM
0.152575	0.419498	SG2	POLN	NC_056074.1	32270001	32290001	0.278851	0.269132	SG1	NTM

0.050411	0.439255	SG2	POLN	NC_056074.1	32275001	32295001	0.102822	0.174359	SG1	NTM
0.047918	0.43805	SG2	POLN	NC_056064.1	28320001	28340001	0.383697	0.212479	SG1	NTN1
0.183035	0.374006	SG2	POLN	NC_056064.1	28325001	28345001	0.379209	0.25972	SG1	NTN1
0.320665	0.305259	SG2	POLN	NC_056064.1	28330001	28350001	0.364894	0.319438	SG1	NTN1
0.332411	0.290195	SG2	POLN	NC_056065.1	3310001	3330001	0.35255	0.24841	SG1	NUCKS1
0.344441	0.23178	SG2	POLN	NC_056065.1	3315001	3335001	0.222469	0.29848	SG1	NUCKS1
0.188353	0.241857	SG2	POLN	NC_056065.1	3320001	3340001	0.255866	0.288739	SG1	NUCKS1
0.035975	0.288363	SG2	POLN	NC_056065.1	3325001	3345001	0.326229	0.244382	SG1	NUCKS1
0.02615	0.277528	SG2	POLN	NC_056065.1	3330001	3350001	0.406445	0.192777	SG1	NUCKS1
0.025523	0.314804	SG2	POLN	NC_056054.1	259260001	259280001	0.405099	0.234943	SG1	NUDT16
0.022481	0.376046	SG2	POLN	NC_056054.1	259265001	259285001	0.384566	0.251559	SG1	NUDT16
0.04724	0.41695	SG2	POLN	NC_056054.1	259270001	259290001	0.317545	0.264769	SG1	NUDT16
0.097983	0.312338	SG2	POLN	NC_056055.1	227765001	227785001	0.438677	0.238256	SG1	NYAP2
0.211494	0.218629	SG2	POLN	NC_056055.1	227770001	227790001	0.436484	0.226259	SG1	NYAP2
0.167952	0.317089	SG2	POM121L2	NC_056055.1	227815001	227835001	0.37888	0.181906	SG1	NYAP2
0.175342	0.27052	SG2	POM121L2	NC_056066.1	36140001	36160001	0.419173	0.283588	SG1	ODAD2
0.319291	0.194325	SG2	POM121L2	NC_056066.1	36145001	36165001	0.443163	0.237801	SG1	ODAD2
0.200261	0.246883	SG2	POM121L2	NC_056066.1	36150001	36170001	0.487357	0.194983	SG1	ODAD2
0.19697	0.244644	SG2	POM121L2	NC_056059.1	86350001	86370001	0.290057	0.233663	SG1	ODAM
0.370479	0.144889	SG2	POT1	NC_056055.1	135870001	135890001	0.359823	0.265861	SG1	OLA1
0.234947	0.171187	SG2	POU3F1	NC_056055.1	135875001	135895001	0.332954	0.273838	SG1	OLA1
0.401741	0.299751	SG2	PPP1R12A	NC_056055.1	135880001	135900001	0.460703	0.233435	SG1	OLA1
0.564931	0.286133	SG2	PPP1R12A	NC_056055.1	135885001	135905001	0.488003	0.223863	SG1	OLA1
0.470978	0.254269	SG2	PPP1R17	NC_056055.1	135900001	135920001	0.453944	0.24631	SG1	OLA1
0.139943	0.14027	SG2	PPP2R2B	NC_056054.1	26000001	26020001	0.246972	0.306054	SG1	OSBPL9
0.087591	0.148148	SG2	PPP2R2B	NC_056054.1	26005001	26025001	0.20681	0.355485	SG1	OSBPL9
0.12	0.136893	SG2	PPP2R2B	NC_056054.1	26010001	26030001	0.295304	0.339434	SG1	OSBPL9
0.417597	0.158384	SG2	PPP2R2B	NC_056054.1	26015001	26035001	0.307099	0.338291	SG1	OSBPL9
0.409137	0.156914	SG2	PPP2R2B	NC_056054.1	26020001	26040001	0.403957	0.277919	SG1	OSBPL9
0.553191	0.279386	SG2	PPP2R5A	NC_056066.1	72620001	72640001	0.170771	0.19043	SG1	OSER1
0.35006	0.28629	SG2	PPP2R5A	NC_056066.1	72635001	72655001	0.295906	0.17667	SG1	OSER1
0.345269	0.283898	SG2	PPP2R5A	NC_056073.1	25345001	25365001	0.171045	0.18056	SG1	OVAR-DRB1

0.329744	0.290037	SG2	PPP2R5A	NC_056069.1	32930001	32950001	0.417742	0.265203	SG1	OXCT1
0.310344	0.296724	SG2	PPP2R5A	NC_056069.1	32935001	32955001	0.43415	0.252787	SG1	OXCT1
0.343851	0.291667	SG2	PPP2R5A	NC_056062.1	70860001	70880001	0.40377	0.349483	SG1	OXR1
0.43219	0.258443	SG2	PPP2R5A	NC_056062.1	70865001	70885001	0.327612	0.36884	SG1	OXR1
0.526644	0.224757	SG2	PPP2R5A	NC_056062.1	70870001	70890001	0.266045	0.421486	SG1	OXR1
0.561681	0.193681	SG2	PPP2R5A	NC_056062.1	70875001	70895001	0.326838	0.391162	SG1	OXR1
0.306238	0.254167	SG2	PPP2R5A	NC_056062.1	70880001	70900001	0.460893	0.311943	SG1	OXR1
0.402495	0.140173	SG2	PPP2R5A	NC_056072.1	11685001	11705001	0.301383	0.261857	SG1	OXSR1
0.147587	0.142035	SG2	PPP4R2	NC_056072.1	11690001	11710001	0.139553	0.281272	SG1	OXSR1
0.110092	0.172554	SG2	PPP4R2	NC_056072.1	11695001	11715001	0.122247	0.291236	SG1	OXSR1
0.091584	0.174761	SG2	PPP4R2	NC_056072.1	11700001	11720001	0.109504	0.317287	SG1	OXSR1
0.218807	0.144173	SG2	PPP4R2	NC_056072.1	11705001	11725001	0.0898391	0.265971	SG1	OXSR1
0.571664	0.403305	SG2	PRDM16	NC_056072.1	11710001	11730001	0.132812	0.22355	SG1	OXSR1
0.560075	0.452375	SG2	PRDM16	NC_056072.1	11715001	11735001	0.183918	0.184898	SG1	OXSR1
0.567507	0.46554	SG2	PRDM16	NC_056055.1	67920001	67940001	0.291688	0.178533	SG1	PABIR1;PIP5K
0.391604	0.135217	SG2	PRDM2	NC_056074.1	16145001	16165001	0.151643	0.174415	SG1	PAK1
0.36507	0.179623	SG2	PRKD1	NC_056074.1	16150001	16170001	0.168052	0.191667	SG1	PAK1
0.411329	0.193838	SG2	PRKD1	NC_056063.1	32150001	32170001	0.430501	0.280093	SG1	PAN3
0.403924	0.215731	SG2	PRKD1	NC_056063.1	32155001	32175001	0.413713	0.273217	SG1	PAN3
0.524859	0.149276	SG2	PRKD1	NC_056063.1	32160001	32180001	0.405172	0.284615	SG1	PAN3
0.476682	0.1551	SG2	PRKD1	NC_056063.1	32225001	32245001	0.274336	0.271253	SG1	PAN3
0.412585	0.199606	SG2	PRKD1	NC_056063.1	32230001	32250001	0.215116	0.250197	SG1	PAN3
0.387592	0.236854	SG2	PRKD1	NC_056059.1	94390001	94410001	0.37328	0.176636	SG1	PAQR3
0.556286	0.251139	SG2	PRMT9;TMEM184	NC_056059.1	94395001	94415001	0.434214	0.194677	SG1	PAQR3
0.568933	0.20442	SG2	PRMT9;TMEM184	NC_056066.1	17900001	17920001	0.308407	0.184505	SG1	PARD3
0.530881	0.181903	SG2	PRMT9;TMEM184	NC_056066.1	17905001	17925001	0.0534606	0.308126	SG1	PARD3
0.220238	0.142069	SG2	PRORP	NC_056066.1	17915001	17935001	0.084155	0.298427	SG1	PARD3
0.173029	0.146883	SG2	PRORP	NC_056066.1	17920001	17940001	0.161859	0.270711	SG1	PARD3
0.209014	0.212811	SG2	PROSER2	NC_056054.1	37160001	37180001	0.436311	0.254621	SG1	PATJ
0.196667	0.224626	SG2	PROSER2	NC_056055.1	247685001	247705001	0.302385	0.17317	SG1	PAX7
0.097222	0.272129	SG2	PROSER2	NC_056058.1	44015001	44035001	0.480095	0.231415	SG1	PCBD2
0.363446	0.15865	SG2	PRR5	NC_056063.1	40220001	40240001	0.43619	0.464357	SG1	PCDH9

0.32382	0.162013	SG2	PRR5	NC_056063.1	40225001	40245001	0.303064	0.507954	SG1	PCDH9
0.332287	0.155927	SG2	PRR5	NC_056063.1	40230001	40250001	0.340833	0.496379	SG1	PCDH9
0.436866	0.15014	SG2	PRR5	NC_056063.1	40235001	40255001	0.320678	0.48341	SG1	PCDH9
0.565046	0.151872	SG2	PRRG4	NC_056054.1	112680001	112700001	0.233249	0.195762	SG1	PCP4L1
0.434165	0.16876	SG2	PRRG4	NC_056075.1	30935001	30955001	0.400304	0.296485	SG1	PDCD4
0.436095	0.170722	SG2	PRRG4	NC_056075.1	30940001	30960001	0.315164	0.292942	SG1	PDCD4
0.492209	0.174464	SG2	PRRG4	NC_056075.1	30945001	30965001	0.381962	0.301258	SG1	PDCD4
0.539726	0.167347	SG2	PRRG4	NC_056075.1	30950001	30970001	0.330677	0.350029	SG1	PDCD4
0.303748	0.172966	SG2	PRRT4	NC_056075.1	30955001	30975001	0.202039	0.371624	SG1	PDCD4
0.30621	0.146335	SG2	PRRT4	NC_056075.1	30960001	30980001	0.177942	0.35033	SG1	PDCD4
0.506536	0.135827	SG2	PRSS16	NC_056057.1	66155001	66175001	0.362296	0.225248	SG1	PDE1C
0.284961	0.251695	SG2	PRTFDC1	NC_056057.1	66160001	66180001	0.367044	0.197978	SG1	PDE1C
0.245148	0.271284	SG2	PRTFDC1	NC_056056.1	194865001	194885001	0.483298	0.212886	SG1	PDE3A
0.172112	0.348538	SG2	PRTFDC1	NC_056056.1	194935001	194955001	0.453364	0.237521	SG1	PDE3A
0.099634	0.409109	SG2	PRTFDC1	NC_056056.1	194940001	194960001	0.486964	0.248542	SG1	PDE3A
0.300619	0.329233	SG2	PRTFDC1	NC_056054.1	41860001	41880001	0.0591368	0.238522	SG1	PDE4B
0.572751	0.189353	SG2	PRTFDC1	NC_056061.1	61445001	61465001	0.412116	0.254344	SG1	PDE7B
0.555633	0.228123	SG2	PRTFDC1	NC_056061.1	61450001	61470001	0.347461	0.27798	SG1	PDE7B
0.476091	0.214222	SG2	PRTFDC1	NC_056061.1	61455001	61475001	0.411957	0.230039	SG1	PDE7B
0.498588	0.192897	SG2	PRTFDC1	NC_056071.1	22050001	22070001	0.484731	0.224326	SG1	PDE8A
0.514494	0.188462	SG2	PRTFDC1	NC_056070.1	42630001	42650001	0.450561	0.267262	SG1	PDGFC
0.486234	0.165074	SG2	PSAPL1;SORCS2	NC_056070.1	42635001	42655001	0.434829	0.239057	SG1	PDGFC
0.44434	0.202196	SG2	PSAPL1;SORCS2	NC_056068.1	4175001	4195001	0.312348	0.205019	SG1	PDGFD
0.440542	0.177506	SG2	PSAPL1;SORCS2	NC_056068.1	4180001	4200001	0.381449	0.277901	SG1	PDGFD
0.418648	0.15124	SG2	PSAPL1;SORCS2	NC_056068.1	4200001	4220001	0.446974	0.350594	SG1	PDGFD
0.501884	0.151665	SG2	PSD3	NC_056068.1	4205001	4225001	0.191532	0.439419	SG1	PDGFD
0.567276	0.17105	SG2	PSD3	NC_056068.1	4210001	4230001	0.0500812	0.476763	SG1	PDGFD
0.467421	0.198931	SG2	PSMB7	NC_056068.1	4215001	4235001	0.0677965	0.462302	SG1	PDGFD
0.472019	0.273578	SG2	PSMB7	NC_056068.1	4220001	4240001	0.0620355	0.464368	SG1	PDGFD
0.498653	0.302056	SG2	PSMB7	NC_056068.1	4225001	4245001	0.0655023	0.456906	SG1	PDGFD
0.500605	0.267522	SG2	PSMB7	NC_056068.1	4230001	4250001	0.127778	0.406735	SG1	PDGFD
0.466285	0.230511	SG2	PSMB7	NC_056068.1	4235001	4255001	0.157787	0.370617	SG1	PDGFD

0.477111	0.154614	SG2	PSMB7	NC_056068.1	4240001	4260001	0.381883	0.247316	SG1	PDGFD
0.553533	0.168752	SG2	PSME4	NC_056080.1	22770001	22790001	0.259259	0.195714	SG1	PDK3
0.510146	0.142572	SG2	PSME4	NC_056080.1	22775001	22795001	0.173209	0.184945	SG1	PDK3
0.453856	0.137113	SG2	PSPC1	NC_056061.1	30155001	30175001	0.401155	0.250787	SG1	PDSS2
0.460255	0.147965	SG2	PSPC1	NC_056061.1	30160001	30180001	0.278026	0.251128	SG1	PDSS2
0.462775	0.147466	SG2	PSPC1	NC_056061.1	30165001	30185001	0.343251	0.213554	SG1	PDSS2
0.457241	0.151508	SG2	PSPC1	NC_056061.1	30170001	30190001	0.385928	0.218744	SG1	PDSS2
0.455055	0.162118	SG2	PSPC1	NC_056069.1	41905001	41925001	0.403239	0.179986	SG1	PDZD2
0.443716	0.16062	SG2	PSPC1	NC_056069.1	41910001	41930001	0.354456	0.18384	SG1	PDZD2
0.474644	0.160161	SG2	PSPC1	NC_056069.1	41915001	41935001	0.348676	0.180311	SG1	PDZD2
0.498135	0.148552	SG2	PSPC1	NC_056069.1	41920001	41940001	0.342534	0.190762	SG1	PDZD2
0.515367	0.151137	SG2	PSPC1	NC_056069.1	41925001	41945001	0.304636	0.175593	SG1	PDZD2
0.534216	0.153334	SG2	PSPC1	NC_056056.1	145610001	145630001	0.27461	0.188365	SG1	PDZRN4
0.511111	0.159435	SG2	PSPC1	NC_056054.1	207880001	207900001	0.477635	0.402046	SG1	PEX5L
0.516068	0.15562	SG2	PSPC1	NC_056054.1	207890001	207910001	0.467084	0.329162	SG1	PEX5L
0.483871	0.245874	SG2	PTCHD1	NC_056054.1	238915001	238935001	0.179952	0.263497	SG1	PFN2
0.542239	0.254275	SG2	PTCHD1	NC_056054.1	238920001	238940001	0.167096	0.304236	SG1	PFN2
0.550668	0.213679	SG2	PTPN11	NC_056054.1	238925001	238945001	0.193802	0.277395	SG1	PFN2;RNF13
0.410782	0.234888	SG2	PTPN21	NC_056054.1	238930001	238950001	0.387074	0.206814	SG1	PFN2;RNF13
0.292943	0.249924	SG2	PTPN21;SPATA7	NC_056055.1	68270001	68290001	0.463013	0.182289	SG1	PGM5
0.518272	0.151347	SG2	PTPN5	NC_056061.1	68195001	68215001	0.459687	0.173362	SG1	PHACTR2
0.569811	0.172564	SG2	PTPRN2	NC_056061.1	68200001	68220001	0.461468	0.173123	SG1	PHACTR2
0.154444	0.158639	SG2	PTPRN2	NC_056061.1	68235001	68255001	0.351226	0.25912	SG1	PHACTR2
0.102362	0.13688	SG2	PTPRN2	NC_056061.1	68240001	68260001	0.474649	0.232805	SG1	PHACTR2
0.107598	0.195411	SG2	PTPRN2	NC_056061.1	68245001	68265001	0.377863	0.233075	SG1	PHACTR2
0.129593	0.292768	SG2	PTPRN2	NC_056061.1	68250001	68270001	0.419413	0.220025	SG1	PHACTR2
0.145389	0.330002	SG2	PTPRN2	NC_056062.1	21590001	21610001	0.409508	0.203397	SG1	PHF20L1
0.153124	0.326601	SG2	PTPRN2	NC_056062.1	21595001	21615001	0.361645	0.257372	SG1	PHF20L1
0.258272	0.314954	SG2	PTPRN2	NC_056062.1	21600001	21620001	0.44163	0.322716	SG1	PHF20L1
0.518093	0.400946	SG2	PTPRN2	NC_056062.1	21605001	21625001	0.475682	0.308093	SG1	PHF20L1
0.244806	0.159392	SG2	PTPRT	NC_056062.1	21610001	21630001	0.472705	0.331438	SG1	PHF20L1
0.072284	0.182776	SG2	PTX3;VEPH1	NC_056062.1	21615001	21635001	0.397916	0.251284	SG1	PHF20L1

0.237277	0.219744	SG2	PWP2;TRAPPC10	NC_056054.1	91075001	91095001	0.449726	0.173553	SG1	PHTF1;RSBN1
0.332216	0.196643	SG2	PWP2;TRAPPC10	NC_056070.1	72245001	72265001	0.412372	0.205353	SG1	PI4KA
0.407521	0.192282	SG2	PWP2;TRAPPC10	NC_056070.1	72250001	72270001	0.291017	0.224338	SG1	PI4KA;SERPIN
0.367847	0.170521	SG2	QDPR	NC_056070.1	72255001	72275001	0.233902	0.221837	SG1	PI4KA;SERPIN
0.432443	0.185566	SG2	QDPR	NC_056070.1	72260001	72280001	0.286755	0.195606	SG1	PI4KA;SERPIN
0.493716	0.160763	SG2	QDPR	NC_056070.1	72240001	72260001	0.431122	0.178125	SG1	PI4KA;TMEM1
0.219512	0.145736	SG2	QSER1	NC_056056.1	215645001	215665001	0.38716	0.226533	SG1	PICK1
0.165898	0.153571	SG2	QSER1	NC_056056.1	215665001	215685001	0.459358	0.21301	SG1	PICK1;SLC16A
0.168487	0.145253	SG2	QSER1	NC_056056.1	215670001	215690001	0.333894	0.225845	SG1	PICK1;SLC16A
0.07733	0.156815	SG2	QSER1	NC_056055.1	231175001	231195001	0.281071	0.183972	SG1	PID1
0.089655	0.15017	SG2	QSER1	NC_056055.1	231180001	231200001	0.184243	0.262353	SG1	PID1
0.201323	0.139589	SG2	QSER1	NC_056055.1	231185001	231205001	0.0772229	0.339678	SG1	PID1
0.423442	0.189617	SG2	RAD50	NC_056055.1	231190001	231210001	0.0552948	0.380327	SG1	PID1
0.368932	0.168168	SG2	RAD50	NC_056055.1	231195001	231215001	0.0350735	0.405825	SG1	PID1
0.471234	0.146631	SG2	RALGAPA1	NC_056055.1	231200001	231220001	0.0208546	0.376589	SG1	PID1
0.442437	0.159008	SG2	RALGAPA1	NC_056055.1	231205001	231225001	0.125615	0.285956	SG1	PID1
0.441613	0.164359	SG2	RALGAPA1	NC_056076.1	14115001	14135001	0.4359	0.175066	SG1	PIK3C3
0.449817	0.160177	SG2	RALGAPA1	NC_056055.1	67905001	67925001	0.443022	0.252671	SG1	PIP5K1B
0.466506	0.146418	SG2	RALGAPA1	NC_056055.1	67910001	67930001	0.267543	0.249696	SG1	PIP5K1B
0.452738	0.150192	SG2	RALGAPA1	NC_056055.1	67915001	67935001	0.204892	0.21994	SG1	PIP5K1B
0.459211	0.149238	SG2	RALGAPA1	NC_056080.1	13615001	13635001	0.455269	0.180686	SG1	PIR
0.466368	0.141339	SG2	RALGAPA1	NC_056080.1	13620001	13640001	0.454221	0.178843	SG1	PIR
0.405481	0.165934	SG2	RALGAPA1	NC_056080.1	13625001	13645001	0.473513	0.179853	SG1	PIR
0.287574	0.202318	SG2	RALGAPA1	NC_056080.1	62065001	62085001	0.410023	0.334309	SG1	PJA1
0.115008	0.249706	SG2	RALGAPA1	NC_056080.1	62070001	62090001	0.368421	0.355014	SG1	PJA1
0.045042	0.372496	SG2	RALGAPA1	NC_056080.1	62075001	62095001	0.358574	0.280407	SG1	PJA1
0.107466	0.273134	SG2	RALGAPA1	NC_056054.1	65950001	65970001	0.353858	0.290905	SG1	PKN2
0.143984	0.180173	SG2	RALGAPA1	NC_056054.1	65955001	65975001	0.425993	0.251933	SG1	PKN2
0.55035	0.140239	SG2	RANBP17	NC_056054.1	65960001	65980001	0.356116	0.281409	SG1	PKN2
0.413555	0.147473	SG2	RANBP17	NC_056054.1	65965001	65985001	0.28381	0.313405	SG1	PKN2
0.356903	0.185274	SG2	RANBP17	NC_056056.1	182560001	182580001	0.431326	0.178543	SG1	PKP2
0.299726	0.208547	SG2	RANBP17	NC_056056.1	182565001	182585001	0.445605	0.261507	SG1	PKP2

0.467499	0.181439	SG2	RANBP17	NC_056055.1	246180001	246200001	0.179071	0.193642	SG1	PLA2G2C;UBX
0.523268	0.176989	SG2	RANBP17	NC_056055.1	246185001	246205001	0.283506	0.173394	SG1	PLA2G2C;UBX
0.470004	0.186529	SG2	RANBP17	NC_056074.1	38395001	38415001	0.137233	0.180531	SG1	PLAAT3
0.492224	0.182578	SG2	RANBP17	NC_056074.1	38295001	38315001	0.337692	0.182969	SG1	PLAAT5
0.405393	0.209345	SG2	RANBP17	NC_056074.1	38300001	38320001	0.317533	0.229098	SG1	PLAAT5
0.360112	0.227001	SG2	RANBP17	NC_056074.1	38305001	38325001	0.367033	0.209369	SG1	PLAAT5
0.448468	0.197435	SG2	RANBP17	NC_056074.1	38310001	38330001	0.372145	0.190393	SG1	PLAAT5
0.449407	0.187767	SG2	RANBP17	NC_056066.1	880001	900001	0.409792	0.268437	SG1	PLCB1
0.532425	0.163798	SG2	RANBP17	NC_056066.1	885001	905001	0.413683	0.274294	SG1	PLCB1
0.562746	0.158539	SG2	RANBP17	NC_056066.1	890001	910001	0.445575	0.275415	SG1	PLCB1
0.363663	0.216414	SG2	RANBP17	NC_056066.1	895001	915001	0.445173	0.259684	SG1	PLCB1
0.288352	0.228675	SG2	RANBP17	NC_056066.1	900001	920001	0.416463	0.252306	SG1	PLCB1
0.108917	0.313341	SG2	RANBP17	NC_056066.1	905001	925001	0.413333	0.272851	SG1	PLCB1
0.429068	0.250899	SG2	RANBP17	NC_056066.1	910001	930001	0.460827	0.343614	SG1	PLCB1
0.439382	0.147687	SG2	RAP1GAP2	NC_056066.1	915001	935001	0.486535	0.382644	SG1	PLCB1
0.517369	0.135552	SG2	RAP1GAP2	NC_056066.1	930001	950001	0.478113	0.298381	SG1	PLCB1
0.487068	0.175362	SG2	RAP1GAP2	NC_056066.1	935001	955001	0.457929	0.245311	SG1	PLCB1
0.500297	0.180222	SG2	RAPGEF2	NC_056066.1	940001	960001	0.44915	0.231631	SG1	PLCB1
0.339887	0.219984	SG2	RAPGEF2	NC_056066.1	945001	965001	0.412479	0.201733	SG1	PLCB1
0.328125	0.211451	SG2	RAPGEF2	NC_056065.1	33765001	33785001	0.437901	0.18985	SG1	PLD5
0.286988	0.213805	SG2	RAPGEF2	NC_056065.1	33855001	33875001	0.468102	0.236888	SG1	PLD5
0.333891	0.198375	SG2	RAPGEF2	NC_056062.1	13815001	13835001	0.435831	0.187826	SG1	PLEC
0.271805	0.216735	SG2	RAPGEF2	NC_056062.1	13835001	13855001	0.443832	0.207363	SG1	PLEC
0.29861	0.226275	SG2	RAPGEF2	NC_056067.1	55190001	55210001	0.0693144	0.265782	SG1	PLEKHA4
0.45251	0.154663	SG2	RBBP8NL	NC_056067.1	55200001	55220001	0.249078	0.228625	SG1	PLEKHA4;PPP
0.414901	0.16803	SG2	RBBP8NL	NC_056056.1	80565001	80585001	0.481864	0.211609	SG1	PLEKHH2
0.319679	0.190052	SG2	RC3H1	NC_056055.1	209975001	209995001	0.351952	0.239322	SG1	PLEKHM3
0.464401	0.142073	SG2	RCAN1	NC_056055.1	209980001	210000001	0.365625	0.291794	SG1	PLEKHM3
0.519184	0.152522	SG2	RCAN1	NC_056055.1	209985001	210005001	0.396002	0.358365	SG1	PLEKHM3
0.470445	0.14488	SG2	RCBTB1	NC_056055.1	209990001	210010001	0.461634	0.411797	SG1	PLEKHM3
0.435158	0.155882	SG2	RERGL	NC_056055.1	209995001	210015001	0.462558	0.395481	SG1	PLEKHM3
0.495283	0.143314	SG2	RERGL	NC_056055.1	210000001	210020001	0.448628	0.37825	SG1	PLEKHM3

0.571638	0.154901	SG2	RFFL	NC_056055.1	210005001	210025001	0.405419	0.344045	SG1	PLEKHM3
0.560882	0.174266	SG2	RFFL	NC_056055.1	210010001	210030001	0.392697	0.29005	SG1	PLEKHM3
0.09901	0.136202	SG2	RFTN2	NC_056055.1	210015001	210035001	0.319698	0.256988	SG1	PLEKHM3
0.545141	0.213274	SG2	RGS6	NC_056055.1	210020001	210040001	0.317423	0.226346	SG1	PLEKHM3
0.539938	0.22787	SG2	RHBDL3	NC_056055.1	210025001	210045001	0.334742	0.223656	SG1	PLEKHM3
0.527673	0.208299	SG2	RHBDL3	NC_056055.1	210030001	210050001	0.264563	0.212162	SG1	PLEKHM3
0.516629	0.193149	SG2	RHCG	NC_056055.1	210035001	210055001	0.456	0.249975	SG1	PLEKHM3
0.490416	0.201585	SG2	RHCG	NC_056067.1	53425001	53445001	0.475621	0.272148	SG1	PNMA8A
0.563278	0.155865	SG2	RIC8B	NC_056055.1	102780001	102800001	0.438134	0.305728	SG1	PNOC;ZNF395
0.540496	0.159672	SG2	RIC8B	NC_056055.1	102785001	102805001	0.364361	0.260996	SG1	PNOC;ZNF395
0.477019	0.162337	SG2	RIC8B	NC_056077.1	20515001	20535001	0.0977181	0.262948	SG1	POLR3E
0.139046	0.163877	SG2	RICTOR	NC_056054.1	119610001	119630001	0.443492	0.271116	SG1	POU2F1
0.099841	0.187687	SG2	RICTOR	NC_056054.1	119615001	119635001	0.39911	0.279028	SG1	POU2F1
0.191457	0.178858	SG2	RICTOR	NC_056067.1	50705001	50725001	0.169451	0.212828	SG1	POU2F2
0.335581	0.157686	SG2	RICTOR	NC_056059.1	20150001	20170001	0.20846	0.20611	SG1	PPA2
0.463826	0.165496	SG2	RIMS1	NC_056068.1	44785001	44805001	0.48617	0.181388	SG1	PPFIBP2
0.443345	0.17196	SG2	RIMS1	NC_056064.1	9435001	9455001	0.284504	0.176356	SG1	PPM1E
0.410345	0.155232	SG2	RIMS1	NC_056064.1	9440001	9460001	0.273221	0.180327	SG1	PPM1E
0.415812	0.152726	SG2	RMND5A	NC_056056.1	75985001	76005001	0.084308	0.193868	SG1	PPP1R21
0.171721	0.140922	SG2	RNF17	NC_056056.1	76045001	76065001	0.181208	0.20312	SG1	PPP1R21
0.183799	0.149728	SG2	RNF17	NC_056054.1	253840001	253860001	0.359981	0.200613	SG1	PPP2R3A
0.181382	0.138213	SG2	RNF17	NC_056054.1	253845001	253865001	0.243739	0.245334	SG1	PPP2R3A
0.185217	0.139489	SG2	RNF17	NC_056054.1	253850001	253870001	0.193687	0.258711	SG1	PPP2R3A
0.502615	0.149503	SG2	RNF180	NC_056054.1	253855001	253875001	0.206582	0.265949	SG1	PPP2R3A
0.34959	0.223656	SG2	RNF180	NC_056054.1	253860001	253880001	0.245696	0.254124	SG1	PPP2R3A
0.283727	0.246105	SG2	RNF180	NC_056054.1	253865001	253885001	0.223845	0.258429	SG1	PPP2R3A
0.168023	0.276971	SG2	RNF180	NC_056054.1	253870001	253890001	0.254237	0.249306	SG1	PPP2R3A
0.153846	0.281134	SG2	RNF180	NC_056054.1	253875001	253895001	0.304005	0.238211	SG1	PPP2R3A
0.126561	0.269562	SG2	RNF180	NC_056054.1	253880001	253900001	0.283351	0.235288	SG1	PPP2R3A
0.14425	0.267421	SG2	RNF180	NC_056054.1	253885001	253905001	0.312053	0.227743	SG1	PPP2R3A
0.147813	0.249799	SG2	RNF180	NC_056054.1	253890001	253910001	0.418787	0.195171	SG1	PPP2R3A
0.20627	0.21842	SG2	RNF180	NC_056054.1	253895001	253915001	0.411541	0.179912	SG1	PPP2R3A

0.235132	0.215565	SG2	RNF180	NC_056071.1	54040001	54060001	0.394503	0.246362	SG1	PPP4R3A
0.269015	0.213369	SG2	RNF180	NC_056071.1	54045001	54065001	0.260965	0.192011	SG1	PPP4R3A
0.319018	0.196216	SG2	RNF180	NC_056069.1	39240001	39260001	0.293066	0.216834	SG1	PR
0.352089	0.15901	SG2	RNF180	NC_056069.1	39245001	39265001	0.375898	0.217142	SG1	PR
0.390554	0.13926	SG2	RNF180	NC_056069.1	39250001	39270001	0.38713	0.231383	SG1	PR
0.450238	0.158552	SG2	RNF180	NC_056071.1	38020001	38040001	0.177419	0.302632	SG1	PRKD1
0.566138	0.160989	SG2	RNF180	NC_056071.1	38030001	38050001	0.121334	0.257811	SG1	PRKD1
0.334728	0.147752	SG2	RNMT	NC_056062.1	32490001	32510001	0.15113	0.177561	SG1	PRKDC
0.17594	0.152332	SG2	RNMT	NC_056062.1	32495001	32515001	0.0697301	0.219289	SG1	PRKDC
0.498057	0.136743	SG2	ROBO1	NC_056062.1	32500001	32520001	0.0567647	0.211072	SG1	PRKDC
0.337648	0.191157	SG2	ROBO1	NC_056062.1	32505001	32525001	0.0612566	0.211516	SG1	PRKDC
0.409037	0.159901	SG2	ROBO1	NC_056062.1	32510001	32530001	0.0631155	0.20602	SG1	PRKDC
0.429759	0.156015	SG2	ROBO1	NC_056062.1	32515001	32535001	0.0502577	0.195369	SG1	PRKDC
0.445176	0.15141	SG2	ROBO1	NC_056062.1	32520001	32540001	0.051157	0.20147	SG1	PRKDC
0.485776	0.135584	SG2	ROBO1	NC_056062.1	32525001	32545001	0.027709	0.206418	SG1	PRKDC
0.512134	0.157692	SG2	ROBO1	NC_056062.1	32530001	32550001	0.019474	0.214628	SG1	PRKDC
0.401777	0.194011	SG2	ROBO1	NC_056062.1	32535001	32555001	0.0248491	0.249187	SG1	PRKDC
0.368049	0.223704	SG2	ROBO1	NC_056062.1	32550001	32570001	0.0480911	0.289725	SG1	PRKDC
0.527786	0.229582	SG2	ROBO1	NC_056059.1	96680001	96700001	0.478283	0.216598	SG1	PRKG2
0.518661	0.230383	SG2	ROBO1	NC_056059.1	96685001	96705001	0.462953	0.253755	SG1	PRKG2
0.46424	0.146006	SG2	RPA1;SMYD4	NC_056059.1	96695001	96715001	0.353805	0.324252	SG1	PRKG2
0.109795	0.158847	SG2	RPIA	NC_056059.1	96700001	96720001	0.242032	0.272515	SG1	PRKG2
0.092915	0.157592	SG2	RPIA	NC_056061.1	84690001	84710001	0.331316	0.212219	SG1	PRKN
0.074695	0.160167	SG2	RPIA	NC_056061.1	84695001	84715001	0.376456	0.17459	SG1	PRKN
0.296438	0.13623	SG2	RPIA	NC_056061.1	85680001	85700001	0.384037	0.245314	SG1	PRKN
0.397933	0.15564	SG2	RPL14	NC_056061.1	85685001	85705001	0.286299	0.228332	SG1	PRKN
0.347485	0.175706	SG2	RPL14	NC_056060.1	84175001	84195001	0.296261	0.192466	SG1	PROX2
0.259665	0.222492	SG2	RPL14;ZNF619	NC_056060.1	84190001	84210001	0.416066	0.185931	SG1	PROX2
0.227582	0.235113	SG2	RPL14;ZNF619	NC_056060.1	84195001	84215001	0.425934	0.23054	SG1	PROX2
0.497345	0.138342	SG2	RTL1	NC_056074.1	34590001	34610001	0.339082	0.213715	SG1	PRPF19
0.482887	0.162508	SG2	RTL1	NC_056074.1	34595001	34615001	0.252288	0.215268	SG1	PRPF19
0.390879	0.168412	SG2	RXFP1	NC_056074.1	34600001	34620001	0.185339	0.189692	SG1	PRPF19

0.469338	0.223919	SG2	RXFP1	NC_056074.1	34605001	34625001	0.218949	0.186934	SG1	PRPF19;TMEM
0.418533	0.223685	SG2	RXFP2	NC_056077.1	27755001	27775001	0.421419	0.180922	SG1	PRSS36
0.568606	0.166353	SG2	RXFP2	NC_056077.1	27760001	27780001	0.258162	0.259755	SG1	PRSS36
0.349134	0.188601	SG2	RYR1	NC_056058.1	3015001	3035001	0.260469	0.1809	SG1	PRSS38
0.310509	0.186195	SG2	RYR1	NC_056058.1	3020001	3040001	0.368688	0.172913	SG1	PRSS38
0.294549	0.192094	SG2	RYR1	NC_056058.1	3025001	3045001	0.421728	0.193477	SG1	PRSS38
0.323423	0.143085	SG2	RYR1	NC_056058.1	3030001	3050001	0.408854	0.185628	SG1	PRSS38
0.530645	0.179361	SG2	S1PR1	NC_056058.1	3035001	3055001	0.215164	0.217812	SG1	PRSS38
0.53223	0.176336	SG2	S1PR1	NC_056058.1	3040001	3060001	0.229567	0.214303	SG1	PRSS38
0.551107	0.159382	SG2	S1PR1	NC_056058.1	3045001	3065001	0.244142	0.324169	SG1	PRSS38
0.444635	0.156525	SG2	SAMD11	NC_056058.1	3050001	3070001	0.1875	0.385132	SG1	PRSS38
0.385404	0.174556	SG2	SAMD11	NC_056058.1	3055001	3075001	0.164822	0.356755	SG1	PRSS38
0.259445	0.24315	SG2	SAMD11	NC_056058.1	3060001	3080001	0.113904	0.393463	SG1	PRSS38
0.184252	0.264409	SG2	SAMD11	NC_056058.1	3065001	3085001	0.219455	0.31668	SG1	PRSS38
0.350269	0.273677	SG2	SCAPER	NC_056058.1	3070001	3090001	0.345367	0.223227	SG1	PRSS38
0.36649	0.273166	SG2	SCAPER	NC_056077.1	27705001	27725001	0.47034	0.25717	SG1	PRSS53;VKOR
0.492027	0.347688	SG2	SCOC	NC_056077.1	27700001	27720001	0.483871	0.175952	SG1	PRSS53;VKOR
0.522275	0.373999	SG2	SCOC	NC_056066.1	25070001	25090001	0.357975	0.264099	SG1	PRTFDC1
0.516021	0.361827	SG2	SCOC	NC_056076.1	31045001	31065001	0.435087	0.181592	SG1	PSMA8;SS18
0.5009	0.136865	SG2	SCUBE1	NC_056054.1	73875001	73895001	0.457901	0.199812	SG1	PTBP2
0.476491	0.144721	SG2	SCUBE1	NC_056054.1	73880001	73900001	0.397899	0.218952	SG1	PTBP2
0.493091	0.137903	SG2	SCUBE1	NC_056054.1	73885001	73905001	0.435766	0.208608	SG1	PTBP2
0.493797	0.14307	SG2	SCUBE2	NC_056062.1	15925001	15945001	0.232591	0.199338	SG1	PTK2
0.539064	0.153302	SG2	SCUBE2	NC_056062.1	15930001	15950001	0.121353	0.262105	SG1	PTK2
0.288122	0.162533	SG2	SDR16C5	NC_056062.1	15955001	15975001	0.341475	0.216602	SG1	PTK2
0.366225	0.138079	SG2	SEC63	NC_056055.1	78290001	78310001	0.451272	0.185635	SG1	PTPRD
0.202879	0.147987	SG2	SEC63	NC_056055.1	78295001	78315001	0.266369	0.222148	SG1	PTPRD
0.499998	0.150184	SG2	SELENOF	NC_056055.1	78300001	78320001	0.186887	0.216822	SG1	PTPRD
0.307525	0.138095	SG2	SEMA4B	NC_056055.1	78305001	78325001	0.276025	0.222259	SG1	PTPRD
0.443155	0.182456	SG2	SEMA4B	NC_056055.1	78310001	78330001	0.430197	0.197501	SG1	PTPRD
0.225735	0.180673	SG2	SERPINA12	NC_056075.1	46795001	46815001	0.475673	0.271719	SG1	PTPRE
0.129012	0.172974	SG2	SERPINA12	NC_056075.1	46800001	46820001	0.234329	0.213346	SG1	PTPRE

0.351128	0.229687	SG2	SEZ6	NC_056072.1	39610001	39630001	0.444376	0.212035	SG1	PTPRG
0.423869	0.20188	SG2	SEZ6	NC_056072.1	39640001	39660001	0.237354	0.205561	SG1	PTPRG
0.192118	0.142241	SG2	SFMBT1	NC_056072.1	39645001	39665001	0.178755	0.217115	SG1	PTPRG
0.381571	0.153452	SG2	SFRP1	NC_056072.1	39650001	39670001	0.139857	0.230785	SG1	PTPRG
0.37802	0.153949	SG2	SFRP1	NC_056072.1	39655001	39675001	0.0460875	0.248836	SG1	PTPRG
0.40147	0.174327	SG2	SFRP1	NC_056072.1	39665001	39685001	0.125395	0.180892	SG1	PTPRG
0.427786	0.18996	SG2	SFRP1	NC_056056.1	200155001	200175001	0.476126	0.200866	SG1	PTPRO
0.4687	0.234967	SG2	SFRP1	NC_056056.1	200160001	200180001	0.458196	0.183738	SG1	PTPRO
0.477919	0.254987	SG2	SFRP1	NC_056056.1	200165001	200185001	0.414812	0.19032	SG1	PTPRO
0.462922	0.229993	SG2	SFRP1	NC_056056.1	200190001	200210001	0.420415	0.178415	SG1	PTPRO
0.326291	0.142601	SG2	SFT2D2	NC_056056.1	200230001	200250001	0.3255	0.192439	SG1	PTPRO
0.245831	0.150888	SG2	SGCD	NC_056080.1	131820001	131840001	0.359447	0.243752	SG1	PWWP3B
0.464022	0.203242	SG2	SH3TC1	NC_056080.1	131825001	131845001	0.414725	0.230866	SG1	PWWP3B
0.348433	0.174633	SG2	SH3TC1	NC_056061.1	86720001	86740001	0.476949	0.317041	SG1	QKI
0.167086	0.184063	SG2	SH3TC1	NC_056076.1	42360001	42380001	0.466085	0.25305	SG1	RAB31
0.092405	0.207873	SG2	SH3TC1	NC_056070.1	62615001	62635001	0.301615	0.177867	SG1	RAB35
0.151603	0.142963	SG2	SH3YL1	NC_056070.1	62620001	62640001	0.34576	0.173203	SG1	RAB35
0.079844	0.166366	SG2	SH3YL1	NC_056072.1	59065001	59085001	0.451428	0.25185	SG1	RAB7A
0.080527	0.160374	SG2	SH3YL1	NC_056072.1	59070001	59090001	0.428572	0.247282	SG1	RAB7A
0.067886	0.155902	SG2	SH3YL1	NC_056072.1	59075001	59095001	0.419624	0.234159	SG1	RAB7A
0.078003	0.152781	SG2	SH3YL1	NC_056072.1	59080001	59100001	0.402071	0.206565	SG1	RAB7A
0.388353	0.145543	SG2	SHLD1	NC_056065.1	55155001	55175001	0.173049	0.234744	SG1	RABGAP1L
0.415423	0.160919	SG2	SHLD1	NC_056065.1	55160001	55180001	0.182847	0.236341	SG1	RABGAP1L
0.429262	0.152793	SG2	SHLD1	NC_056065.1	55165001	55185001	0.157216	0.25381	SG1	RABGAP1L
0.42102	0.1381	SG2	SHLD1	NC_056065.1	55170001	55190001	0.188352	0.254132	SG1	RABGAP1L
0.084401	0.140994	SG2	SHQ1	NC_056065.1	55175001	55195001	0.250495	0.259974	SG1	RABGAP1L
0.569434	0.151272	SG2	SHTN1	NC_056065.1	55180001	55200001	0.284994	0.236333	SG1	RABGAP1L
0.335726	0.23387	SG2	SLC13A1	NC_056065.1	55185001	55205001	0.470287	0.184691	SG1	RABGAP1L
0.392205	0.192996	SG2	SLC13A1	NC_056065.1	55190001	55210001	0.46792	0.188491	SG1	RABGAP1L
0.07577	0.179074	SG2	SLC13A5	NC_056056.1	225950001	225970001	0.0881059	0.433575	SG1	RABL2B
0.059933	0.179352	SG2	SLC13A5	NC_056056.1	225955001	225975001	0.1581	0.408227	SG1	RABL2B
0.130659	0.136864	SG2	SLC25A19	NC_056056.1	225960001	225980001	0.326492	0.30001	SG1	RABL2B

0.403727	0.175861	SG2	SLC25A21	NC_056056.1	225980001	226000001	0.485714	0.210727	SG1	RABL2B
0.24968	0.23423	SG2	SLC25A21	NC_056060.1	78400001	78420001	0.345668	0.232909	SG1	RAD51B
0.122616	0.31604	SG2	SLC25A21	NC_056060.1	78405001	78425001	0.385417	0.258086	SG1	RAD51B
0.127872	0.223526	SG2	SLC25A21	NC_056060.1	78410001	78430001	0.391999	0.255875	SG1	RAD51B
0.297025	0.139599	SG2	SLC25A21	NC_056060.1	78415001	78435001	0.397908	0.222358	SG1	RAD51B
0.392837	0.266038	SG2	SLC25A21	NC_056064.1	9350001	9370001	0.458393	0.25259	SG1	RAD51C
0.314349	0.22216	SG2	SLC25A21	NC_056064.1	9355001	9375001	0.350111	0.253401	SG1	RAD51C
0.394066	0.150433	SG2	SLC25A21	NC_056064.1	9360001	9380001	0.321621	0.28252	SG1	RAD51C
0.376926	0.160542	SG2	SLC25A21	NC_056064.1	9365001	9385001	0.257009	0.302112	SG1	RAD51C
0.325234	0.196217	SG2	SLC25A21	NC_056064.1	9370001	9390001	0.343131	0.200899	SG1	RAD51C
0.282467	0.225463	SG2	SLC25A21	NC_056058.1	20605001	20625001	0.458114	0.272495	SG1	RAPGEF6
0.196794	0.240152	SG2	SLC25A21	NC_056058.1	20610001	20630001	0.466667	0.283065	SG1	RAPGEF6
0.185393	0.238457	SG2	SLC25A21	NC_056058.1	20615001	20635001	0.463975	0.267896	SG1	RAPGEF6
0.177508	0.215502	SG2	SLC25A21	NC_056058.1	20620001	20640001	0.461004	0.258614	SG1	RAPGEF6
0.159711	0.180091	SG2	SLC25A21	NC_056058.1	20660001	20680001	0.483461	0.26391	SG1	RAPGEF6
0.170143	0.184083	SG2	SLC25A21	NC_056058.1	20665001	20685001	0.452116	0.279004	SG1	RAPGEF6
0.256908	0.206738	SG2	SLC25A21	NC_056058.1	20670001	20690001	0.467583	0.273392	SG1	RAPGEF6
0.360473	0.219997	SG2	SLC25A21	NC_056058.1	20695001	20715001	0.482229	0.272165	SG1	RAPGEF6
0.436609	0.27681	SG2	SLC25A21	NC_056058.1	20700001	20720001	0.483071	0.266915	SG1	RAPGEF6
0.397109	0.305248	SG2	SLC25A21	NC_056058.1	20710001	20730001	0.47521	0.281986	SG1	RAPGEF6
0.377716	0.219199	SG2	SLC25A21	NC_056058.1	20715001	20735001	0.475598	0.28443	SG1	RAPGEF6
0.300691	0.210808	SG2	SLC25A21	NC_056058.1	20720001	20740001	0.472	0.283879	SG1	RAPGEF6
0.426295	0.190115	SG2	SLC25A21	NC_056058.1	20725001	20745001	0.445455	0.268194	SG1	RAPGEF6
0.556249	0.157237	SG2	SLC25A21	NC_056058.1	20730001	20750001	0.463292	0.252571	SG1	RAPGEF6
0.530985	0.189128	SG2	SLC25A21	NC_056058.1	20760001	20780001	0.486264	0.258702	SG1	RAPGEF6
0.560933	0.176946	SG2	SLC25A21	NC_056058.1	20785001	20805001	0.463957	0.287055	SG1	RAPGEF6
0.485493	0.147785	SG2	SLC25A21	NC_056058.1	20790001	20810001	0.464353	0.276868	SG1	RAPGEF6
0.331035	0.161896	SG2	SLC25A21	NC_056058.1	20795001	20815001	0.446809	0.253594	SG1	RAPGEF6
0.143302	0.364152	SG2	SLC26A7	NC_056058.1	20800001	20820001	0.420206	0.218662	SG1	RAPGEF6
0.306844	0.300148	SG2	SLC26A7	NC_056058.1	20805001	20825001	0.411765	0.223351	SG1	RAPGEF6
0.495504	0.212127	SG2	SLC26A7	NC_056058.1	20810001	20830001	0.408207	0.239303	SG1	RAPGEF6
0.550327	0.203939	SG2	SLC26A7	NC_056058.1	20815001	20835001	0.428854	0.247615	SG1	RAPGEF6

0.16403	0.140523	SG2	SLC34A2	NC_056058.1	20820001	20840001	0.479565	0.281976	SG1	RAPGEF6
0.228711	0.14106	SG2	SLC34A2	NC_056058.1	20825001	20845001	0.477777	0.283811	SG1	RAPGEF6
0.438891	0.165321	SG2	SLC35B4	NC_056065.1	59020001	59040001	0.432478	0.199467	SG1	RASAL2
0.49135	0.194559	SG2	SLC38A9	NC_056065.1	59025001	59045001	0.43361	0.19537	SG1	RASAL2
0.167164	0.22409	SG2	SLC45A3	NC_056065.1	59030001	59050001	0.48069	0.185756	SG1	RASAL2
0.16153	0.219293	SG2	SLC45A3	NC_056060.1	32335001	32355001	0.426965	0.199952	SG1	RASGRP1
0.227478	0.144175	SG2	SLC46A3	NC_056066.1	60250001	60270001	0.274139	0.190477	SG1	RBCK1;TBC1I
0.098856	0.168125	SG2	SLC46A3	NC_056066.1	60255001	60275001	0.285456	0.182615	SG1	RBCK1;TBC1I
0.129067	0.144683	SG2	SLC6A19	NC_056077.1	7050001	7070001	0.47805	0.214877	SG1	RBFOX1
0.099697	0.149206	SG2	SLC6A19	NC_056064.1	52220001	52240001	0.183981	0.210117	SG1	RBFOX3
0.36113	0.16767	SG2	SLC7A11	NC_056064.1	52225001	52245001	0.096821	0.260165	SG1	RBFOX3
0.404273	0.270861	SG2	SLC7A11	NC_056064.1	52235001	52255001	0.0494074	0.256958	SG1	RBFOX3
0.422784	0.304308	SG2	SLC7A11	NC_056064.1	52240001	52260001	0.0677128	0.210533	SG1	RBFOX3
0.482521	0.311562	SG2	SLC7A11	NC_056066.1	66015001	66035001	0.475248	0.29462	SG1	RBL1
0.392061	0.305179	SG2	SLC7A11	NC_056066.1	66020001	66040001	0.281218	0.317583	SG1	RBL1
0.327362	0.297272	SG2	SLC7A11	NC_056066.1	66025001	66045001	0.174438	0.355691	SG1	RBL1
0.335032	0.231935	SG2	SLC7A11	NC_056066.1	66045001	66065001	0.275316	0.274905	SG1	RBL1
0.395007	0.177789	SG2	SLC7A11	NC_056066.1	66050001	66070001	0.322851	0.186964	SG1	RBL1
0.495282	0.140373	SG2	SLC7A11	NC_056055.1	149375001	149395001	0.434372	0.342105	SG1	RBMS1
0.4074	0.166927	SG2	SLC7A2	NC_056055.1	149380001	149400001	0.414479	0.322191	SG1	RBMS1
0.515789	0.166581	SG2	SLC7A2	NC_056055.1	149385001	149405001	0.385523	0.329881	SG1	RBMS1
0.333128	0.199552	SG2	SLC9A9	NC_056055.1	149390001	149410001	0.482946	0.277723	SG1	RBMS1
0.215708	0.224123	SG2	SLC9A9	NC_056055.1	149465001	149485001	0.447557	0.405647	SG1	RBMS1
0.27664	0.20596	SG2	SLC9A9	NC_056056.1	217965001	217985001	0.199351	0.188995	SG1	RBX1;XPNPEI
0.422732	0.157061	SG2	SLC9A9	NC_056068.1	20000001	20020001	0.471304	0.200953	SG1	RDX
0.484384	0.17914	SG2	SLC9A9	NC_056068.1	20005001	20025001	0.293415	0.26012	SG1	RDX
0.445246	0.228234	SG2	SLC9A9	NC_056068.1	20010001	20030001	0.250936	0.28894	SG1	RDX
0.540482	0.190657	SG2	SLC9A9	NC_056068.1	20015001	20035001	0.296868	0.275988	SG1	RDX
0.569469	0.17957	SG2	SLC9A9	NC_056057.1	46585001	46605001	0.205799	0.215654	SG1	RELN
0.359124	0.167825	SG2	SLIT2	NC_056057.1	46590001	46610001	0.235086	0.24777	SG1	RELN
0.215754	0.17842	SG2	SLIT2	NC_056057.1	46595001	46615001	0.355765	0.288414	SG1	RELN
0.18488	0.18141	SG2	SLIT2	NC_056057.1	46600001	46620001	0.42536	0.223029	SG1	RELN

0.183033	0.184537	SG2	SLIT2	NC_056061.1	63995001	64015001	0.437823	0.186447	SG1	REPS1
0.303468	0.144148	SG2	SLIT2	NC_056061.1	64000001	64020001	0.425842	0.201284	SG1	REPS1
0.18224	0.151498	SG2	SMAD5	NC_056061.1	64010001	64030001	0.313664	0.235365	SG1	REPS1
0.45667	0.138571	SG2	SMCHD1	NC_056061.1	64015001	64035001	0.249206	0.173978	SG1	REPS1
0.386489	0.17322	SG2	SMCHD1	NC_056064.1	42405001	42425001	0.140722	0.238464	SG1	RETREG3;TUI
0.416666	0.170351	SG2	SMCHD1	NC_056062.1	1495001	1515001	0.417677	0.212002	SG1	RIMS1
0.467642	0.162551	SG2	SMCHD1	NC_056054.1	269865001	269885001	0.284486	0.21182	SG1	RIPPLY3
0.511675	0.152587	SG2	SMCHD1	NC_056061.1	76270001	76290001	0.351386	0.200044	SG1	RMND1
0.525359	0.144646	SG2	SMCHD1	NC_056063.1	20695001	20715001	0.318152	0.41975	SG1	RNASEH2B
0.504587	0.137081	SG2	SMG6	NC_056063.1	20700001	20720001	0.222222	0.446017	SG1	RNASEH2B
0.478914	0.145238	SG2	SMG6	NC_056063.1	20705001	20725001	0.307643	0.259012	SG1	RNASEH2B
0.44096	0.174332	SG2	SMG6	NC_056054.1	239070001	239090001	0.331169	0.176675	SG1	RNF13
0.443012	0.212764	SG2	SMG6	NC_056068.1	27680001	27700001	0.435805	0.275737	SG1	RNF214
0.437424	0.262498	SG2	SNCA	NC_056054.1	19205001	19225001	0.391827	0.203964	SG1	RNF220
0.477376	0.234212	SG2	SNCA	NC_056075.1	9915001	9935001	0.352205	0.259131	SG1	RNLS
0.536377	0.256254	SG2	SNU13	NC_056075.1	9920001	9940001	0.132693	0.24306	SG1	RNLS
0.350697	0.136383	SG2	SNUPN	NC_056056.1	20170001	20190001	0.410912	0.269451	SG1	ROCK2;SLC66
0.211949	0.148549	SG2	SNUPN	NC_056066.1	66135001	66155001	0.0994766	0.303258	SG1	RPN2
0.129781	0.151134	SG2	SNUPN	NC_056066.1	66140001	66160001	0.0687062	0.301471	SG1	RPN2
0.101063	0.152534	SG2	SNX14	NC_056066.1	66145001	66165001	0.130678	0.273914	SG1	RPN2
0.088775	0.136536	SG2	SNX14	NC_056066.1	66150001	66170001	0.238987	0.225461	SG1	RPN2
0.088873	0.144031	SG2	SNX14	NC_056066.1	66155001	66175001	0.419782	0.201379	SG1	RPN2
0.105874	0.167998	SG2	SNX14	NC_056061.1	89270001	89290001	0.396343	0.174677	SG1	RPS6KA2
0.25435	0.149654	SG2	SNX14	NC_056061.1	89275001	89295001	0.40633	0.184432	SG1	RPS6KA2
0.377313	0.148352	SG2	SNX14	NC_056080.1	74955001	74975001	0.353699	0.187457	SG1	RPS6KA6
0.435746	0.162528	SG2	SNX29	NC_056080.1	74970001	74990001	0.299003	0.214701	SG1	RPS6KA6
0.436902	0.16629	SG2	SNX29	NC_056080.1	74975001	74995001	0.278592	0.197624	SG1	RPS6KA6
0.385705	0.173133	SG2	SNX29	NC_056080.1	74980001	75000001	0.35514	0.264915	SG1	RPS6KA6
0.365846	0.156687	SG2	SNX29	NC_056080.1	74985001	75005001	0.282485	0.26256	SG1	RPS6KA6
0.557206	0.180405	SG2	SNX29	NC_056080.1	74990001	75010001	0.230946	0.258527	SG1	RPS6KA6
0.528436	0.206357	SG2	SNX32	NC_056080.1	75080001	75100001	0.41769	0.26443	SG1	RPS6KA6
0.18225	0.226755	SG2	SNX6	NC_056080.1	75085001	75105001	0.387268	0.228727	SG1	RPS6KA6

0.198008	0.168988	SG2	SNX6	NC_056080.1	75090001	75110001	0.375635	0.213037	SG1	RPS6KA6
0.243183	0.142815	SG2	SNX6	NC_056080.1	75105001	75125001	0.462732	0.187033	SG1	RPS6KA6
0.51872	0.138475	SG2	SORCS2	NC_056080.1	75110001	75130001	0.381616	0.187085	SG1	RPS6KA6
0.47208	0.136302	SG2	SOX6	NC_056080.1	75115001	75135001	0.385185	0.190795	SG1	RPS6KA6
0.23688	0.152719	SG2	SPATA7	NC_056080.1	75120001	75140001	0.400955	0.245298	SG1	RPS6KA6
0.274439	0.200925	SG2	SPATA7	NC_056080.1	75125001	75145001	0.289256	0.270763	SG1	RPS6KA6
0.044	0.14566	SG2	SPINK7	NC_056054.1	91080001	91100001	0.411007	0.203002	SG1	RSBN1
0.029574	0.149331	SG2	SPINK7	NC_056054.1	91085001	91105001	0.434332	0.185649	SG1	RSBN1
0.488193	0.175899	SG2	SRSF6	NC_056061.1	21165001	21185001	0.417831	0.179823	SG1	RSPH4A;ZUP1
0.472354	0.186461	SG2	SRSF6	NC_056061.1	21170001	21190001	0.421757	0.185106	SG1	RSPH4A;ZUP1
0.418945	0.14093	SG2	SSBP2	NC_056060.1	35075001	35095001	0.413869	0.224358	SG1	RTF1
0.49009	0.170624	SG2	SSBP2	NC_056060.1	35080001	35100001	0.254822	0.240965	SG1	RTF1
0.511628	0.158421	SG2	SSBP2	NC_056060.1	35085001	35105001	0.193059	0.251602	SG1	RTF1
0.202323	0.159567	SG2	STK10	NC_056060.1	35090001	35110001	0.277011	0.215098	SG1	RTF1
0.141329	0.231126	SG2	STK10	NC_056060.1	35095001	35115001	0.324653	0.212263	SG1	RTF1
0.047548	0.275889	SG2	STK10	NC_056060.1	35100001	35120001	0.393228	0.196341	SG1	RTF1
0.019201	0.282562	SG2	STK10	NC_056068.1	79535001	79555001	0.42529	0.172963	SG1	RTN4RL2
0.023172	0.268616	SG2	STK10	NC_056068.1	79540001	79560001	0.307268	0.188637	SG1	RTN4RL2;SLC
0.083333	0.214558	SG2	STK10	NC_056068.1	79545001	79565001	0.152865	0.195737	SG1	RTN4RL2;SLC
0.162777	0.21371	SG2	STK10	NC_056068.1	79550001	79570001	0.0969047	0.176393	SG1	RTN4RL2;SLC
0.169026	0.18693	SG2	STK10	NC_056063.1	29540001	29560001	0.197369	0.217706	SG1	RXFP2
0.250578	0.142725	SG2	STK10	NC_056060.1	65150001	65170001	0.39375	0.198738	SG1	SAMD4A
0.244684	0.141577	SG2	STK10	NC_056060.1	65155001	65175001	0.463579	0.182154	SG1	SAMD4A
0.225312	0.146184	SG2	STK10	NC_056064.1	19805001	19825001	0.475222	0.240612	SG1	SARM1
0.247557	0.139648	SG2	STK38L	NC_056064.1	19815001	19835001	0.395418	0.23767	SG1	SARM1;SLC46
0.423711	0.21645	SG2	STRA6	NC_056064.1	19820001	19840001	0.367153	0.191901	SG1	SARM1;SLC46
0.459578	0.225136	SG2	STRA6	NC_056074.1	40505001	40525001	0.455445	0.274296	SG1	SART1
0.336076	0.271637	SG2	STRN3	NC_056054.1	276550001	276570001	0.471212	0.290018	SG1	SATB1
0.401529	0.300318	SG2	STRN3	NC_056054.1	276560001	276580001	0.462899	0.280223	SG1	SATB1
0.242424	0.228086	SG2	STRN3	NC_056055.1	201765001	201785001	0.378195	0.197744	SG1	SATB2
0.336033	0.221961	SG2	STRN3	NC_056055.1	201770001	201790001	0.213592	0.222817	SG1	SATB2
0.326415	0.216168	SG2	STRN3	NC_056055.1	201775001	201795001	0.246753	0.183935	SG1	SATB2

0.253497	0.196375	SG2	STRN3	NC_056054.1	124505001	124525001	0.321629	0.199243	SG1	SCAF4;SOD1
0.278583	0.188628	SG2	STRN3	NC_056054.1	124510001	124530001	0.212813	0.246032	SG1	SCAF4;SOD1
0.189189	0.191816	SG2	STRN3	NC_056054.1	124515001	124535001	0.140383	0.266705	SG1	SCAF4;SOD1
0.210095	0.18819	SG2	STRN3	NC_056071.1	29440001	29460001	0.487604	0.213371	SG1	SCAPER
0.297821	0.162302	SG2	STRN3	NC_056071.1	29445001	29465001	0.38313	0.192566	SG1	SCAPER
0.32334	0.154677	SG2	STRN3	NC_056055.1	236420001	236440001	0.464176	0.225908	SG1	SDC3
0.402253	0.167529	SG2	STRN3	NC_056055.1	236425001	236445001	0.418604	0.267576	SG1	SDC3
0.421589	0.140312	SG2	STRN3	NC_056055.1	236430001	236450001	0.375556	0.264283	SG1	SDC3
0.341815	0.180651	SG2	STX2	NC_056055.1	236435001	236455001	0.480697	0.237315	SG1	SDC3
0.261259	0.234155	SG2	STX2	NC_056077.1	39890001	39910001	0.324965	0.318066	SG1	SDK1
0.273629	0.259528	SG2	STX2	NC_056077.1	39895001	39915001	0.282634	0.246781	SG1	SDK1
0.235938	0.290936	SG2	STX2	NC_056077.1	39900001	39920001	0.427799	0.188075	SG1	SDK1
0.367453	0.191266	SG2	STX2	NC_056077.1	39920001	39940001	0.46703	0.236782	SG1	SDK1
0.449785	0.146253	SG2	STX2	NC_056077.1	39925001	39945001	0.322783	0.2511	SG1	SDK1
0.395272	0.140069	SG2	STXBP5L	NC_056077.1	39930001	39950001	0.282268	0.195687	SG1	SDK1
0.311305	0.193174	SG2	STXBP5L	NC_056077.1	39935001	39955001	0.411119	0.179851	SG1	SDK1
0.26412	0.19911	SG2	STXBP5L	NC_056062.1	36415001	36435001	0.450098	0.213879	SG1	SDR16C5
0.283161	0.19837	SG2	STXBP5L	NC_056062.1	36420001	36440001	0.394008	0.261111	SG1	SDR16C5
0.384691	0.16616	SG2	STXBP5L	NC_056062.1	36425001	36445001	0.404563	0.245359	SG1	SDR16C5
0.51917	0.170033	SG2	STXBP5L	NC_056062.1	36430001	36450001	0.392322	0.267978	SG1	SDR16C5
0.485279	0.175449	SG2	STXBP5L	NC_056062.1	36435001	36455001	0.387755	0.242323	SG1	SDR16C5
0.50131	0.14887	SG2	STXBP5L	NC_056080.1	131750001	131770001	0.471764	0.186396	SG1	SERPINA7
0.466401	0.152237	SG2	STXBP6	NC_056073.1	49910001	49930001	0.408563	0.203621	SG1	SERPINB1
0.428507	0.151453	SG2	STXBP6	NC_056073.1	49915001	49935001	0.4455	0.20654	SG1	SERPINB1
0.198372	0.151164	SG2	SULF2	NC_056073.1	49920001	49940001	0.418711	0.203461	SG1	SERPINB1
0.262306	0.168188	SG2	SUSD1	NC_056073.1	49925001	49945001	0.40043	0.201942	SG1	SERPINB1
0.338837	0.156936	SG2	SUSD1	NC_056076.1	44940001	44960001	0.422024	0.187935	SG1	SETBP1
0.398873	0.13841	SG2	SVEP1	NC_056056.1	62245001	62265001	0.360971	0.20342	SG1	SH3RF3
0.420308	0.141687	SG2	SVEP1	NC_056056.1	62250001	62270001	0.344982	0.21495	SG1	SH3RF3
0.497244	0.137336	SG2	SYBU	NC_056056.1	62255001	62275001	0.410721	0.193269	SG1	SH3RF3
0.565927	0.139126	SG2	SYNPR	NC_056056.1	62265001	62285001	0.414547	0.184629	SG1	SH3RF3
0.412669	0.143085	SG2	SYNRG	NC_056056.1	62270001	62290001	0.481976	0.179666	SG1	SH3RF3

0.411354	0.147693	SG2	SYNRG	NC_056060.1	59485001	59505001	0.380829	0.2323	SG1	SHC4
0.330189	0.191935	SG2	SYNRG	NC_056060.1	59490001	59510001	0.353063	0.216071	SG1	SHC4
0.519517	0.189725	SG2	SYT1	NC_056060.1	59495001	59515001	0.319375	0.189583	SG1	SHC4
0.436634	0.183003	SG2	SYT1	NC_056060.1	59500001	59520001	0.293454	0.175672	SG1	SHC4
0.258065	0.207839	SG2	TAF4	NC_056060.1	59505001	59525001	0.316335	0.186044	SG1	SHC4
0.200521	0.254432	SG2	TAF4	NC_056060.1	59510001	59530001	0.371312	0.219425	SG1	SHC4
0.099929	0.308219	SG2	TAF4	NC_056060.1	59515001	59535001	0.380504	0.266	SG1	SHC4
0.052247	0.367175	SG2	TAF4	NC_056056.1	218745001	218765001	0.483626	0.177758	SG1	SHISA8;SREBI
0.055555	0.383116	SG2	TAF4	NC_056056.1	220860001	220880001	0.370165	0.209568	SG1	SHISAL1
0.095694	0.330692	SG2	TAF4	NC_056056.1	220865001	220885001	0.332819	0.229376	SG1	SHISAL1
0.226381	0.254916	SG2	TAF4	NC_056075.1	31020001	31040001	0.443914	0.297137	SG1	SHOC2
0.266331	0.167766	SG2	TAF4	NC_056075.1	31025001	31045001	0.393112	0.309258	SG1	SHOC2
0.516698	0.2247	SG2	TARS1	NC_056075.1	31030001	31050001	0.342189	0.30079	SG1	SHOC2
0.359629	0.152862	SG2	TASOR	NC_056075.1	31035001	31055001	0.333651	0.318456	SG1	SHOC2
0.37826	0.158653	SG2	TASOR	NC_056075.1	31040001	31060001	0.351094	0.287344	SG1	SHOC2
0.567752	0.181931	SG2	TAX1BP1	NC_056075.1	31045001	31065001	0.385564	0.274069	SG1	SHOC2
0.571303	0.173314	SG2	TAX1BP1	NC_056075.1	31050001	31070001	0.425397	0.224067	SG1	SHOC2
0.564516	0.196483	SG2	TBC1D5	NC_056061.1	70370001	70390001	0.421801	0.191458	SG1	SHPRH
0.467628	0.22651	SG2	TBC1D5	NC_056061.1	70375001	70395001	0.297348	0.289043	SG1	SHPRH
0.401112	0.252981	SG2	TBC1D5	NC_056061.1	70380001	70400001	0.373207	0.255887	SG1	SHPRH
0.436817	0.236617	SG2	TBC1D5	NC_056054.1	222940001	222960001	0.344215	0.198276	SG1	SI
0.505331	0.225837	SG2	TBC1D5	NC_056054.1	222945001	222965001	0.326707	0.199485	SG1	SI
0.213567	0.149894	SG2	TBX15	NC_056054.1	222950001	222970001	0.390432	0.205397	SG1	SI
0.106505	0.266667	SG2	TBX15	NC_056054.1	222955001	222975001	0.40917	0.250522	SG1	SI
0.552567	0.135172	SG2	TCF7L1	NC_056057.1	70705001	70725001	0.387472	0.174583	SG1	SKAP2
0.505577	0.135128	SG2	TCF7L1	NC_056057.1	70710001	70730001	0.388585	0.205051	SG1	SKAP2
0.231092	0.269196	SG2	TCOF1	NC_056057.1	70715001	70735001	0.379081	0.199178	SG1	SKAP2
0.24486	0.258642	SG2	TCOF1	NC_056057.1	70720001	70740001	0.399174	0.203512	SG1	SKAP2
0.299902	0.233541	SG2	TCOF1	NC_056074.1	19950001	19970001	0.0857913	0.196422	SG1	SLC17A6
0.526408	0.142097	SG2	TCOF1	NC_056074.1	19970001	19990001	0.144661	0.18444	SG1	SLC17A6
0.556013	0.190903	SG2	TENM3	NC_056066.1	38545001	38565001	0.413817	0.289467	SG1	SLC24A3
0.33229	0.231083	SG2	TENM3	NC_056066.1	38550001	38570001	0.293014	0.233549	SG1	SLC24A3

0.244972	0.305254	SG2	TENM3	NC_056066.1	38555001	38575001	0.289967	0.194139	SG1	SLC24A3
0.154784	0.279727	SG2	TENM3	NC_056056.1	217870001	217890001	0.241984	0.267276	SG1	SLC25A17
0.0807	0.291444	SG2	TENM3	NC_056056.1	217875001	217895001	0.05375	0.417954	SG1	SLC25A17
0.12744	0.215878	SG2	TENM3	NC_056071.1	44855001	44875001	0.449575	0.173347	SG1	SLC25A21
0.31612	0.166773	SG2	TENM3	NC_056066.1	75350001	75370001	0.459543	0.174044	SG1	SLC2A10;TP53
0.55197	0.177477	SG2	TENM3	NC_056061.1	59435001	59455001	0.4	0.21173	SG1	SLC2A12
0.181961	0.15842	SG2	TESPA1	NC_056061.1	59440001	59460001	0.421384	0.207174	SG1	SLC2A12
0.303083	0.138202	SG2	TESPA1	NC_056061.1	59445001	59465001	0.253937	0.315219	SG1	SLC2A12
0.410334	0.308929	SG2	TEX11	NC_056061.1	59450001	59470001	0.19263	0.359473	SG1	SLC2A12
0.120482	0.299678	SG2	TEX11	NC_056061.1	59455001	59475001	0.177195	0.376249	SG1	SLC2A12
0.43043	0.246975	SG2	TEX11	NC_056061.1	59485001	59505001	0.17865	0.33631	SG1	SLC2A12
0.56782	0.195762	SG2	TFAP2C	NC_056061.1	59490001	59510001	0.20078	0.314059	SG1	SLC2A12
0.3639	0.139164	SG2	TIAM1	NC_056062.1	60185001	60205001	0.326975	0.182819	SG1	SLC30A8
0.294336	0.139415	SG2	TIAM1	NC_056062.1	60190001	60210001	0.351105	0.180311	SG1	SLC30A8
0.427648	0.172019	SG2	TIAM1	NC_056054.1	77370001	77390001	0.388029	0.244762	SG1	SLC35A3
0.565619	0.181108	SG2	TIAM1	NC_056054.1	77375001	77395001	0.468705	0.218195	SG1	SLC35A3
0.377451	0.214227	SG2	TIPRL	NC_056054.1	77380001	77400001	0.47833	0.211407	SG1	SLC35A3
0.438596	0.229169	SG2	TIPRL	NC_056068.1	79555001	79575001	0.13662	0.181947	SG1	SLC43A1
0.286472	0.174386	SG2	TIPRL	NC_056072.1	1885001	1905001	0.475719	0.218141	SG1	SLC4A7
0.256608	0.141591	SG2	TIPRL	NC_056063.1	31255001	31275001	0.29824	0.190103	SG1	SLC7A1
0.196424	0.134937	SG2	TIPRL	NC_056063.1	31260001	31280001	0.296274	0.216505	SG1	SLC7A1
0.161946	0.139246	SG2	TIPRL	NC_056063.1	31265001	31285001	0.432178	0.1773	SG1	SLC7A1
0.154124	0.141565	SG2	TIPRL	NC_056070.1	19720001	19740001	0.388869	0.202487	SG1	SLC7A11
0.088959	0.154226	SG2	TIPRL	NC_056070.1	19725001	19745001	0.359006	0.197292	SG1	SLC7A11
0.080274	0.158401	SG2	TIPRL	NC_056080.1	99180001	99200001	0.488126	0.183499	SG1	SLC9A6
0.076421	0.159052	SG2	TIPRL	NC_056066.1	77905001	77925001	0.406149	0.179672	SG1	SLC9A8
0.081445	0.154923	SG2	TIPRL	NC_056054.1	177965001	177985001	0.291667	0.233196	SG1	SLC9C1
0.105824	0.157944	SG2	TIPRL	NC_056054.1	177970001	177990001	0.298357	0.239748	SG1	SLC9C1
0.488019	0.144117	SG2	TLE2;TLE6	NC_056054.1	177975001	177995001	0.244202	0.260361	SG1	SLC9C1
0.353837	0.191539	SG2	TLE6	NC_056054.1	177980001	178000001	0.32078	0.229132	SG1	SLC9C1
0.531766	0.140425	SG2	TLE6	NC_056054.1	177985001	178005001	0.2804	0.240052	SG1	SLC9C1
0.200654	0.136684	SG2	TMEM108	NC_056054.1	177990001	178010001	0.232616	0.233376	SG1	SLC9C1

0.523129	0.216783	SG2	TMEM14A	NC_056054.1	177995001	178015001	0.268542	0.192651	SG1	SLC9C1
0.524576	0.211285	SG2	TMEM14A	NC_056072.1	51995001	52015001	0.406073	0.186726	SG1	SMARCC1
0.543343	0.207599	SG2	TMEM178B	NC_056072.1	52000001	52020001	0.325226	0.20961	SG1	SMARCC1
0.325545	0.217403	SG2	TMEM184B	NC_056072.1	52005001	52025001	0.263056	0.221864	SG1	SMARCC1
0.301533	0.179154	SG2	TMEM184B	NC_056072.1	52010001	52030001	0.248264	0.214737	SG1	SMARCC1
0.251582	0.18884	SG2	TMEM184B	NC_056072.1	52015001	52035001	0.250485	0.230246	SG1	SMARCC1
0.242677	0.193624	SG2	TMEM184B	NC_056072.1	52020001	52040001	0.223633	0.228919	SG1	SMARCC1
0.288444	0.15641	SG2	TMEM184B	NC_056072.1	52025001	52045001	0.234375	0.220415	SG1	SMARCC1
0.219124	0.236057	SG2	TMEM184B	NC_056072.1	52030001	52050001	0.219697	0.214727	SG1	SMARCC1
0.443113	0.173881	SG2	TMEM184C	NC_056072.1	52035001	52055001	0.211883	0.216166	SG1	SMARCC1
0.435435	0.13603	SG2	TMEM241	NC_056072.1	52040001	52060001	0.339896	0.19538	SG1	SMARCC1
0.413025	0.192833	SG2	TMEM241	NC_056072.1	52045001	52065001	0.359342	0.202292	SG1	SMARCC1
0.419366	0.298195	SG2	TMEM241	NC_056072.1	52050001	52070001	0.401042	0.207119	SG1	SMARCC1
0.410716	0.363342	SG2	TMEM241	NC_056072.1	52055001	52075001	0.446505	0.200759	SG1	SMARCC1
0.463168	0.36755	SG2	TMEM241	NC_056061.1	90470001	90490001	0.155441	0.242473	SG1	SMOC2
0.49511	0.38424	SG2	TMEM241	NC_056061.1	90475001	90495001	0.224219	0.197003	SG1	SMOC2
0.48128	0.145515	SG2	TMEM241	NC_056066.1	50665001	50685001	0.186503	0.209507	SG1	SMOX
0.419381	0.160278	SG2	TMEM241	NC_056066.1	50670001	50690001	0.217687	0.221356	SG1	SMOX
0.491378	0.135444	SG2	TMEM241	NC_056066.1	50675001	50695001	0.224719	0.213404	SG1	SMOX
0.465057	0.16666	SG2	TMEM266	NC_056066.1	50680001	50700001	0.223675	0.205228	SG1	SMOX
0.462159	0.182214	SG2	TMEM266	NC_056066.1	50685001	50705001	0.242903	0.18817	SG1	SMOX
0.342558	0.209571	SG2	TMEM266	NC_056066.1	50690001	50710001	0.441491	0.234255	SG1	SMOX
0.400942	0.229924	SG2	TMEM266	NC_056066.1	3340001	3360001	0.318639	0.203783	SG1	SNAP25
0.563352	0.256062	SG2	TMEM266	NC_056080.1	63775001	63795001	0.280255	0.380645	SG1	SNX12
0.561913	0.155757	SG2	TMSB4X	NC_056074.1	34295001	34315001	0.216128	0.236847	SG1	SNX19
0.521528	0.167201	SG2	TMSB4X	NC_056077.1	11385001	11405001	0.277576	0.327751	SG1	SNX29
0.271311	0.157548	SG2	TNFAIP8	NC_056077.1	11390001	11410001	0.174703	0.281566	SG1	SNX29
0.267507	0.145677	SG2	TNFAIP8	NC_056077.1	11395001	11415001	0.208205	0.194579	SG1	SNX29
0.40231	0.154392	SG2	TNFAIP8L3	NC_056054.1	124520001	124540001	0.114486	0.267864	SG1	SOD1
0.40681	0.184663	SG2	TNFAIP8L3	NC_056054.1	124525001	124545001	0.0937747	0.231213	SG1	SOD1
0.42772	0.160107	SG2	TNFAIP8L3	NC_056075.1	16655001	16675001	0.439845	0.201308	SG1	SORBS1
0.45229	0.146837	SG2	TNFSF8	NC_056059.1	114410001	114430001	0.446796	0.18182	SG1	SORCS2

0.156153	0.135308	SG2	TNIK	NC_056075.1	25250001	25270001	0.466383	0.189919	SG1	SORCS3
0.527615	0.141706	SG2	TNS1	NC_056075.1	25410001	25430001	0.483586	0.294727	SG1	SORCS3
0.252101	0.176553	SG2	TOGARAM1	NC_056075.1	25415001	25435001	0.328299	0.331221	SG1	SORCS3
0.230135	0.192045	SG2	TOGARAM1	NC_056075.1	25420001	25440001	0.192946	0.363791	SG1	SORCS3
0.323069	0.175756	SG2	TOGARAM1	NC_056075.1	25425001	25445001	0.195776	0.29228	SG1	SORCS3
0.504242	0.24116	SG2	TP53BP2	NC_056075.1	25430001	25450001	0.220111	0.262178	SG1	SORCS3
0.566548	0.206466	SG2	TP53BP2	NC_056075.1	25435001	25455001	0.33064	0.232597	SG1	SORCS3
0.471519	0.135487	SG2	TPK1	NC_056075.1	25440001	25460001	0.385222	0.186592	SG1	SORCS3
0.489099	0.136996	SG2	TRAF6	NC_056075.1	25460001	25480001	0.472452	0.188583	SG1	SORCS3
0.369796	0.229856	SG2	TRAPPC10	NC_056075.1	25465001	25485001	0.421415	0.184115	SG1	SORCS3
0.392871	0.237582	SG2	TRAPPC10	NC_056075.1	25475001	25495001	0.438665	0.235727	SG1	SORCS3
0.556097	0.2001	SG2	TRAPPC10	NC_056060.1	41335001	41355001	0.162881	0.211242	SG1	SOS2
0.380639	0.144133	SG2	TRAPPC10	NC_056068.1	35775001	35795001	0.375241	0.195697	SG1	SOX6
0.259424	0.218485	SG2	TRAPPC10	NC_056068.1	35780001	35800001	0.418532	0.220447	SG1	SOX6
0.137761	0.28574	SG2	TRAPPC10	NC_056068.1	35785001	35805001	0.452857	0.246696	SG1	SOX6
0.134286	0.283055	SG2	TRAPPC10	NC_056068.1	35790001	35810001	0.456356	0.255343	SG1	SOX6
0.214971	0.295492	SG2	TRAPPC10	NC_056068.1	35795001	35815001	0.453571	0.25066	SG1	SOX6
0.303968	0.184572	SG2	TRIM33	NC_056068.1	35800001	35820001	0.441719	0.236203	SG1	SOX6
0.327934	0.174536	SG2	TRIM33	NC_056068.1	35805001	35825001	0.437634	0.228922	SG1	SOX6
0.494626	0.149291	SG2	TRIP11	NC_056068.1	35810001	35830001	0.433881	0.220532	SG1	SOX6
0.534639	0.138741	SG2	TRIP11	NC_056068.1	35815001	35835001	0.458823	0.211696	SG1	SOX6
0.501279	0.141419	SG2	TRPC5	NC_056068.1	35820001	35840001	0.466025	0.231291	SG1	SOX6
0.036082	0.409076	SG2	TRPC7	NC_056068.1	35825001	35845001	0.463189	0.243364	SG1	SOX6
0.153311	0.354126	SG2	TRPC7	NC_056068.1	35830001	35850001	0.466105	0.262186	SG1	SOX6
0.274793	0.216899	SG2	TRPC7	NC_056068.1	35835001	35855001	0.449759	0.261269	SG1	SOX6
0.403618	0.152585	SG2	TRPC7	NC_056068.1	35840001	35860001	0.37429	0.225376	SG1	SOX6
0.17587	0.178081	SG2	TSPAN13	NC_056068.1	35905001	35925001	0.422265	0.181018	SG1	SOX6
0.079872	0.144396	SG2	TSPAN13	NC_056055.1	138965001	138985001	0.208523	0.179153	SG1	SP5
0.35915	0.20352	SG2	TSPAN7	NC_056055.1	202630001	202650001	0.287195	0.191429	SG1	SPATS2L
0.143527	0.280646	SG2	TSPAN7	NC_056055.1	202635001	202655001	0.403125	0.238252	SG1	SPATS2L
0.491395	0.135981	SG2	TTC13	NC_056055.1	202640001	202660001	0.463797	0.323091	SG1	SPATS2L
0.54279	0.164109	SG2	TTC13	NC_056069.1	38635001	38655001	0.119886	0.259159	SG1	SPEF2

0.494667	0.157072	SG2	TTC13	NC_056069.1	38640001	38660001	0.190238	0.269388	SG1	SPEF2
0.468957	0.155024	SG2	TTC13	NC_056069.1	38645001	38665001	0.173765	0.24805	SG1	SPEF2
0.429532	0.191818	SG2	TTC13	NC_056069.1	38650001	38670001	0.224534	0.220507	SG1	SPEF2
0.392684	0.206787	SG2	TTC13	NC_056060.1	15740001	15760001	0.449492	0.218272	SG1	SPESP1
0.524147	0.187075	SG2	TTC13	NC_056060.1	15745001	15765001	0.281343	0.22576	SG1	SPESP1
0.572424	0.162144	SG2	TTC13	NC_056060.1	15750001	15770001	0.173793	0.199275	SG1	SPESP1
0.425349	0.135626	SG2	TTLL5	NC_056060.1	15755001	15775001	0.129195	0.202107	SG1	SPESP1
0.213213	0.136758	SG2	TTLL7	NC_056060.1	15760001	15780001	0.121917	0.212786	SG1	SPESP1
0.466105	0.17101	SG2	TXLNB	NC_056060.1	15765001	15785001	0.18294	0.188725	SG1	SPESP1
0.29512	0.190471	SG2	TYR	NC_056060.1	15770001	15790001	0.319476	0.233748	SG1	SPESP1
0.278427	0.248304	SG2	TYR	NC_056054.1	179100001	179120001	0.426487	0.276179	SG1	SPICE1
0.537963	0.170097	SG2	TYR	NC_056054.1	179105001	179125001	0.340535	0.269524	SG1	SPICE1
0.567927	0.228957	SG2	TYR	NC_056054.1	179110001	179130001	0.4658	0.197217	SG1	SPICE1
0.520814	0.24066	SG2	TYR	NC_056054.1	179120001	179140001	0.415859	0.19425	SG1	SPICE1
0.346917	0.144594	SG2	UBE2V1	NC_056062.1	32370001	32390001	0.106601	0.225887	SG1	SPIDR
0.413523	0.153349	SG2	UBR2	NC_056062.1	32375001	32395001	0.2324	0.206256	SG1	SPIDR
0.509242	0.168397	SG2	UBR2	NC_056069.1	13565001	13585001	0.421981	0.207423	SG1	SREK1
0.530414	0.175027	SG2	UBR2	NC_056069.1	13570001	13590001	0.300669	0.245349	SG1	SREK1
0.532913	0.186992	SG2	UBR2	NC_056069.1	13575001	13595001	0.277591	0.242896	SG1	SREK1
0.559999	0.142162	SG2	UCK2	NC_056069.1	13580001	13600001	0.274795	0.210152	SG1	SREK1
0.125	0.144035	SG2	ULK4	NC_056069.1	13585001	13605001	0.365339	0.185597	SG1	SREK1
0.548291	0.303807	SG2	UNC13B	NC_056080.1	37655001	37675001	0.342732	0.195661	SG1	SRPX
0.183453	0.140067	SG2	UNC13B	NC_056080.1	37660001	37680001	0.387978	0.175855	SG1	SRPX
0.341648	0.143709	SG2	UNC79	NC_056076.1	31050001	31070001	0.454722	0.259939	SG1	SS18
0.293403	0.153114	SG2	UNC79	NC_056076.1	31055001	31075001	0.335897	0.196464	SG1	SS18
0.310924	0.145273	SG2	UNC79	NC_056054.1	28740001	28760001	0.4823	0.26831	SG1	SSBP3
0.252174	0.145554	SG2	UNC79	NC_056054.1	28745001	28765001	0.461353	0.264993	SG1	SSBP3
0.269446	0.150485	SG2	UNC79	NC_056054.1	28750001	28770001	0.447837	0.265667	SG1	SSBP3
0.4555	0.189036	SG2	UNC79	NC_056054.1	28755001	28775001	0.420138	0.271271	SG1	SSBP3
0.547014	0.168219	SG2	UNC79	NC_056054.1	28760001	28780001	0.365421	0.23692	SG1	SSBP3
0.485519	0.206861	SG2	USP24	NC_056054.1	28765001	28785001	0.36017	0.246296	SG1	SSBP3
0.300792	0.2577	SG2	USP24	NC_056054.1	28770001	28790001	0.33069	0.2555	SG1	SSBP3

0.323261	0.234577	SG2	USP24	NC_056054.1	163975001	163995001	0.108898	0.184101	SG1	ST3GAL6
0.443586	0.1943	SG2	USP24	NC_056054.1	52945001	52965001	0.425962	0.272371	SG1	ST6GALNAC3
0.268126	0.148813	SG2	USP25	NC_056054.1	52950001	52970001	0.407609	0.287518	SG1	ST6GALNAC3
0.226348	0.172106	SG2	USP25	NC_056063.1	74800001	74820001	0.415273	0.211604	SG1	STK24
0.244519	0.181354	SG2	USP25	NC_056063.1	24940001	24960001	0.396053	0.179487	SG1	SUPT20H
0.318958	0.156114	SG2	USP25	NC_056063.1	24945001	24965001	0.383335	0.173045	SG1	SUPT20H
0.341463	0.156891	SG2	USP25	NC_056066.1	33985001	34005001	0.305152	0.192628	SG1	SVIL
0.489486	0.210327	SG2	USP9X	NC_056065.1	65600001	65620001	0.377318	0.175318	SG1	SWT1
0.401974	0.212133	SG2	USP9X	NC_056065.1	65605001	65625001	0.285338	0.212715	SG1	SWT1
0.54075	0.177776	SG2	USP9X	NC_056065.1	65610001	65630001	0.282555	0.225309	SG1	SWT1
0.420764	0.136484	SG2	UVRAG	NC_056065.1	65615001	65635001	0.325564	0.219028	SG1	SWT1
0.149514	0.162417	SG2	VEPH1	NC_056065.1	65620001	65640001	0.44086	0.182436	SG1	SWT1
0.059322	0.184172	SG2	VEPH1	NC_056061.1	77030001	77050001	0.429065	0.467025	SG1	SYNE1
0.044768	0.185123	SG2	VEPH1	NC_056061.1	77035001	77055001	0.354491	0.512363	SG1	SYNE1
0.481416	0.135159	SG2	VPS13D	NC_056061.1	77040001	77060001	0.417876	0.39214	SG1	SYNE1
0.489858	0.142953	SG2	VPS13D	NC_056057.1	48665001	48685001	0.461539	0.277853	SG1	SYPL1
0.515641	0.147065	SG2	VPS13D	NC_056054.1	177735001	177755001	0.359953	0.228423	SG1	TAGLN3
0.536476	0.158213	SG2	VPS13D	NC_056069.1	40460001	40480001	0.481378	0.244754	SG1	TARS1
0.535499	0.158993	SG2	VPS13D	NC_056057.1	107775001	107795001	0.450272	0.21243	SG1	TAS2R39
0.458203	0.172999	SG2	VPS13D	NC_056057.1	107780001	107800001	0.307016	0.234629	SG1	TAS2R39
0.398304	0.185345	SG2	VPS13D	NC_056057.1	107785001	107805001	0.362765	0.198968	SG1	TAS2R39
0.273783	0.218167	SG2	VPS13D	NC_056057.1	107790001	107810001	0.419158	0.176271	SG1	TAS2R39
0.281574	0.218726	SG2	VPS13D	NC_056064.1	51945001	51965001	0.233588	0.194463	SG1	TBC1D16
0.388464	0.18493	SG2	VPS13D	NC_056064.1	51950001	51970001	0.128523	0.217991	SG1	TBC1D16
0.547788	0.139288	SG2	VPS13D	NC_056064.1	51955001	51975001	0.128863	0.220921	SG1	TBC1D16
0.535906	0.344023	SG2	VWA2	NC_056064.1	51960001	51980001	0.113389	0.186316	SG1	TBC1D16
0.31242	0.402713	SG2	VWA2	NC_056056.1	222965001	222985001	0.224334	0.175985	SG1	TBC1D22A
0.195349	0.427805	SG2	VWA2	NC_056056.1	222970001	222990001	0.117889	0.290891	SG1	TBC1D22A
0.324742	0.36588	SG2	VWA2	NC_056056.1	222975001	222995001	0.0733775	0.352825	SG1	TBC1D22A
0.527411	0.182538	SG2	VWA8	NC_056056.1	222990001	223010001	0.0665838	0.27303	SG1	TBC1D22A
0.43432	0.183257	SG2	VWA8	NC_056063.1	51110001	51130001	0.051291	0.270118	SG1	TBC1D4
0.335843	0.204911	SG2	VWA8	NC_056063.1	51120001	51140001	0.0454858	0.326819	SG1	TBC1D4

0.402564	0.179492	SG2	VWA8	NC_056063.1	51125001	51145001	0.28182	0.243938	SG1	TBC1D4
0.477953	0.181697	SG2	VWA8	NC_056054.1	201425001	201445001	0.471016	0.364311	SG1	TBCCD1
0.407504	0.158027	SG2	VWA8	NC_056054.1	201430001	201450001	0.432844	0.398572	SG1	TBCCD1
0.406034	0.16316	SG2	VWA8	NC_056055.1	243180001	243200001	0.32964	0.191422	SG1	TCEA3
0.375338	0.202471	SG2	VWA8	NC_056055.1	243185001	243205001	0.244145	0.24196	SG1	TCEA3
0.464763	0.229393	SG2	VWA8	NC_056055.1	243190001	243210001	0.327265	0.191118	SG1	TCEA3
0.527971	0.23009	SG2	VWA8	NC_056080.1	128970001	128990001	0.325322	0.190486	SG1	TCEAL7
0.542243	0.236727	SG2	VWA8	NC_056080.1	128935001	128955001	0.411403	0.27662	SG1	TCEAL9
0.443928	0.170534	SG2	VWF	NC_056080.1	128940001	128960001	0.270388	0.224653	SG1	TCEAL9
0.438621	0.22724	SG2	VWF	NC_056060.1	51175001	51195001	0.476415	0.237508	SG1	TCF12
0.462279	0.226289	SG2	VWF	NC_056060.1	51190001	51210001	0.399528	0.246527	SG1	TCF12
0.076506	0.26214	SG2	VWF	NC_056060.1	51220001	51240001	0.481098	0.324582	SG1	TCF12
0.088567	0.274043	SG2	VWF	NC_056060.1	51245001	51265001	0.427692	0.496459	SG1	TCF12
0.163833	0.224727	SG2	VWF	NC_056060.1	51250001	51270001	0.360314	0.530082	SG1	TCF12
0.335312	0.136202	SG2	VWF	NC_056060.1	51265001	51285001	0.439527	0.577512	SG1	TCF12
0.19576	0.139111	SG2	WDFY4	NC_056060.1	51285001	51305001	0.468703	0.538463	SG1	TCF12
0.130023	0.154563	SG2	WDFY4	NC_056060.1	51290001	51310001	0.317925	0.59932	SG1	TCF12
0.490742	0.148101	SG2	WDR25	NC_056060.1	51295001	51315001	0.386335	0.571602	SG1	TCF12
0.425808	0.16897	SG2	WDR25	NC_056060.1	51300001	51320001	0.418698	0.544631	SG1	TCF12
0.449118	0.142763	SG2	WDR25	NC_056060.1	51485001	51505001	0.462745	0.444538	SG1	TCF12
0.393872	0.152883	SG2	WDR27	NC_056060.1	51490001	51510001	0.476703	0.447013	SG1	TCF12
0.400237	0.1837	SG2	WDR27	NC_056060.1	51495001	51515001	0.465035	0.433044	SG1	TCF12
0.394954	0.2037	SG2	WDR27	NC_056076.1	55125001	55145001	0.362295	0.173459	SG1	TCF4
0.466166	0.160224	SG2	WDR27	NC_056076.1	55130001	55150001	0.366125	0.188096	SG1	TCF4
0.434968	0.215608	SG2	WHAMM	NC_056068.1	39700001	39720001	0.300203	0.177939	SG1	TEAD1
0.530822	0.267058	SG2	WIPF3	NC_056059.1	80490001	80510001	0.415533	0.232217	SG1	TECRL
0.157326	0.24019	SG2	WIPF3	NC_056059.1	80495001	80515001	0.418631	0.244263	SG1	TECRL
0.31511	0.199633	SG2	WIPF3	NC_056059.1	80500001	80520001	0.36127	0.247631	SG1	TECRL
0.323404	0.177833	SG2	XK	NC_056059.1	80600001	80620001	0.178239	0.18243	SG1	TECRL
0.35432	0.170128	SG2	XK	NC_056059.1	80605001	80625001	0.101393	0.224847	SG1	TECRL
0.332313	0.179146	SG2	XK	NC_056059.1	80610001	80630001	0.103231	0.21395	SG1	TECRL
0.108007	0.207619	SG2	XRCC2	NC_056059.1	80615001	80635001	0.119105	0.184964	SG1	TECRL

0.059137	0.203844	SG2	XRCC2	NC_056080.1	109165001	109185001	0.334049	0.183231	SG1	TENM1
0.113365	0.391017	SG2	YME1L1	NC_056080.1	109170001	109190001	0.291071	0.261348	SG1	TENM1
0.370275	0.148148	SG2	YWHAE	NC_056080.1	109175001	109195001	0.147636	0.27635	SG1	TENM1
0.316006	0.223941	SG2	ZAR1L	NC_056080.1	109180001	109200001	0.0956357	0.225001	SG1	TENM1
0.330521	0.221406	SG2	ZAR1L	NC_056080.1	109185001	109205001	0.145243	0.173913	SG1	TENM1
0.314355	0.222397	SG2	ZAR1L	NC_056064.1	9280001	9300001	0.114074	0.195875	SG1	TEX14
0.315675	0.225655	SG2	ZAR1L	NC_056064.1	9285001	9305001	0.107066	0.200478	SG1	TEX14
0.410648	0.203864	SG2	ZAR1L	NC_056064.1	9300001	9320001	0.190662	0.181019	SG1	TEX14
0.563896	0.198297	SG2	ZAR1L	NC_056063.1	30245001	30265001	0.255456	0.238259	SG1	TEX26
0.567721	0.2221	SG2	ZBTB20	NC_056057.1	53920001	53940001	0.37983	0.220288	SG1	TFEC
0.230059	0.152201	SG2	ZC3H12C	NC_056062.1	21490001	21510001	0.415555	0.186682	SG1	TG
0.355915	0.151046	SG2	ZDHHC14	NC_056062.1	21495001	21515001	0.321389	0.232269	SG1	TG
0.335362	0.190919	SG2	ZDHHC14	NC_056062.1	21500001	21520001	0.318438	0.240667	SG1	TG
0.354915	0.204652	SG2	ZDHHC14	NC_056062.1	21505001	21525001	0.338655	0.234789	SG1	TG
0.422837	0.148059	SG2	ZDHHC14	NC_056062.1	21510001	21530001	0.450056	0.217674	SG1	TG
0.458492	0.16453	SG2	ZDHHC22	NC_056056.1	92575001	92595001	0.279511	0.205566	SG1	TGFA
0.446414	0.181507	SG2	ZDHHC22	NC_056066.1	66985001	67005001	0.449497	0.244609	SG1	TGM2
0.546857	0.158707	SG2	ZFAND1	NC_056066.1	66990001	67010001	0.275886	0.221704	SG1	TGM2
0.570837	0.167304	SG2	ZFAND1	NC_056066.1	66995001	67015001	0.293873	0.216183	SG1	TGM2
0.433706	0.142591	SG2	ZFHX3	NC_056066.1	67000001	67020001	0.391386	0.201549	SG1	TGM2
0.452803	0.142191	SG2	ZFHX3	NC_056066.1	67025001	67045001	0.345595	0.229008	SG1	TGM2
0.425798	0.184707	SG2	ZFHX3	NC_056071.1	52810001	52830001	0.259821	0.197594	SG1	TGM5
0.417293	0.198549	SG2	ZFHX3	NC_056056.1	80970001	80990001	0.436782	0.223933	SG1	THADA;ZFP36
0.445715	0.185458	SG2	ZFHX3	NC_056055.1	194395001	194415001	0.192334	0.408282	SG1	TMEFF2
0.441335	0.18603	SG2	ZFHX3	NC_056055.1	194400001	194420001	0.12315	0.454642	SG1	TMEFF2
0.450007	0.173122	SG2	ZFHX3	NC_056055.1	194405001	194425001	0.137255	0.419016	SG1	TMEFF2
0.519248	0.144822	SG2	ZFHX3	NC_056055.1	194410001	194430001	0.151411	0.400799	SG1	TMEFF2
0.546178	0.135652	SG2	ZFHX3	NC_056055.1	194415001	194435001	0.185366	0.35671	SG1	TMEFF2
0.499017	0.153268	SG2	ZHX3	NC_056055.1	194420001	194440001	0.160938	0.336575	SG1	TMEFF2
0.420849	0.141273	SG2	ZHX3	NC_056055.1	194425001	194445001	0.459016	0.253108	SG1	TMEFF2
0.369085	0.164278	SG2	ZHX3	NC_056055.1	194485001	194505001	0.351768	0.179339	SG1	TMEFF2
0.335233	0.178769	SG2	ZHX3	NC_056055.1	194490001	194510001	0.301775	0.189125	SG1	TMEFF2

0.458002	0.175648	SG2	ZHX3	NC_056055.1	194500001	194520001	0.292241	0.193468	SG1	TMEFF2
0.456	0.169238	SG2	ZIC3	NC_056070.1	50580001	50600001	0.422008	0.19089	SG1	TMEM132B
0.561056	0.192981	SG2	ZIC3	NC_056070.1	50585001	50605001	0.276558	0.235149	SG1	TMEM132B
0.54673	0.267676	SG2	ZIC3	NC_056070.1	50590001	50610001	0.168357	0.256744	SG1	TMEM132B
0.47649	0.31496	SG2	ZIC3	NC_056070.1	50595001	50615001	0.135803	0.279803	SG1	TMEM132B
0.432773	0.337078	SG2	ZIC3	NC_056070.1	50600001	50620001	0.0842774	0.31873	SG1	TMEM132B
0.461836	0.135314	SG2	ZNF25	NC_056070.1	50605001	50625001	0.051808	0.326096	SG1	TMEM132B
0.42599	0.211014	SG2	ZNF300	NC_056070.1	50610001	50630001	0.0366492	0.316386	SG1	TMEM132B
0.318783	0.245813	SG2	ZNF300	NC_056070.1	50625001	50645001	0.0640702	0.210468	SG1	TMEM132B
0.284302	0.137096	SG2	ZNF346	NC_056057.1	105905001	105925001	0.485599	0.213046	SG1	TMEM178B
0.516529	0.187686	SG2	ZNF391	NC_056061.1	82765001	82785001	0.298941	0.173689	SG1	TMEM181
0.417076	0.190313	SG2	ZNF507	NC_056055.1	101875001	101895001	0.187784	0.244907	SG1	TMEM215
0.273837	0.226506	SG2	ZNF507	NC_056055.1	101880001	101900001	0.207044	0.264605	SG1	TMEM215
0.326087	0.208253	SG2	ZNF507	NC_056055.1	101885001	101905001	0.307549	0.194479	SG1	TMEM215
0.349607	0.198462	SG2	ZNF507	NC_056061.1	81685001	81705001	0.40016	0.202812	SG1	TMEM242
0.492222	0.141923	SG2	ZNF507	NC_056061.1	81690001	81710001	0.429693	0.202448	SG1	TMEM242
0.532569	0.149191	SG2	ZNF536	NC_056072.1	54245001	54265001	0.291539	0.259166	SG1	TMEM42
0.439387	0.173198	SG2	ZNF536	NC_056072.1	54250001	54270001	0.282609	0.251379	SG1	TMEM42
0.479192	0.158834	SG2	ZNF536	NC_056072.1	54230001	54250001	0.364228	0.186032	SG1	TMEM42;ZDH
0.568437	0.135216	SG2	ZNF536	NC_056072.1	54235001	54255001	0.423842	0.220521	SG1	TMEM42;ZDH
0.538717	0.170476	SG2	ZNF536	NC_056072.1	54240001	54260001	0.392527	0.256523	SG1	TMEM42;ZDH
0.256788	0.148786	SG2	ZNF697	NC_056058.1	4380001	4400001	0.0693278	0.236541	SG1	TMEM59L
0.39723	0.253693	SG2	ZNF774	NC_056058.1	4385001	4405001	0.192412	0.219537	SG1	TMEM59L
0.189097	0.151188	SG2	ZNF846	NC_056056.1	118835001	118855001	0.271199	0.243355	SG1	TMTC2
0.475435	0.153531	SG2	ZNFX1	NC_056056.1	118840001	118860001	0.104718	0.260194	SG1	TMTC2
0.241	0.165274	SG2	ZNHIT6	NC_056056.1	118845001	118865001	0.118433	0.215403	SG1	TMTC2
0.289473	0.166445	SG2	ZNHIT6	NC_056056.1	118925001	118945001	0.329947	0.200552	SG1	TMTC2
0.303266	0.219445	SG2	ZNRF4	NC_056056.1	118930001	118950001	0.169023	0.304223	SG1	TMTC2
0.260186	0.234385	SG2	ZNRF4	NC_056056.1	118935001	118955001	0.121467	0.294926	SG1	TMTC2
0.313965	0.214574	SG2	ZNRF4	NC_056056.1	118940001	118960001	0.160806	0.285292	SG1	TMTC2
0.328058	0.20854	SG2	ZNRF4	NC_056056.1	118945001	118965001	0.213224	0.246286	SG1	TMTC2
0.510879	0.140197	SG2	ZNRF4	NC_056056.1	118950001	118970001	0.427325	0.199631	SG1	TMTC2

0.133403	0.137387	SG2	ZSWIM6	NC_056056.1	118970001	118990001	0.403012	0.233486	SG1	TMTC2
0.154665	0.175025	SG2	ZSWIM6	NC_056056.1	118975001	118995001	0.252697	0.230114	SG1	TMTC2
0.195038	0.162389	SG2	ZSWIM6	NC_056056.1	119000001	119020001	0.238309	0.174559	SG1	TMTC2
0.276327	0.138326	SG2	ZSWIM6	NC_056056.1	119005001	119025001	0.116028	0.244014	SG1	TMTC2
0.224885	0.152442	SG2	ZSWIM6	NC_056056.1	119010001	119030001	0.183876	0.184234	SG1	TMTC2
0.131715	0.179809	SG2	ZSWIM6	NC_056065.1	39915001	39935001	0.126933	0.400909	SG1	TNFSF18
0.145747	0.177667	SG2	ZSWIM6	NC_056065.1	39920001	39940001	0.118272	0.397616	SG1	TNFSF18
0.153846	0.175166	SG2	ZSWIM6	NC_056065.1	39925001	39945001	0.0471486	0.384502	SG1	TNFSF18
0.224954	0.154119	SG2	ZSWIM6	NC_056074.1	45940001	45960001	0.392857	0.327728	SG1	TNNT3
				NC_056074.1	45945001	45965001	0.389563	0.261101	SG1	TNNT3
				NC_056055.1	218850001	218870001	0.153763	0.253245	SG1	TNP1
				NC_056055.1	218855001	218875001	0.0785498	0.286859	SG1	TNP1
				NC_056055.1	218860001	218880001	0.38766	0.192407	SG1	TNP1
				NC_056077.1	22785001	22805001	0.412583	0.189632	SG1	TNRC6A
				NC_056064.1	34320001	34340001	0.429156	0.325945	SG1	TOM1L2
				NC_056054.1	256430001	256450001	0.268163	0.20045	SG1	TOPBP1
				NC_056054.1	256435001	256455001	0.35707	0.19311	SG1	TOPBP1
				NC_056062.1	37995001	38015001	0.41984	0.18304	SG1	TOX
				NC_056062.1	38000001	38020001	0.408642	0.193033	SG1	TOX
				NC_056062.1	38005001	38025001	0.425002	0.20034	SG1	TOX
				NC_056062.1	38010001	38030001	0.442266	0.203162	SG1	TOX
				NC_056062.1	38015001	38035001	0.448	0.201633	SG1	TOX
				NC_056062.1	38020001	38040001	0.433857	0.186016	SG1	TOX
				NC_056057.1	108985001	109005001	0.168675	0.212899	SG1	TPK1
				NC_056057.1	108990001	109010001	0.252276	0.195618	SG1	TPK1
				NC_056057.1	108995001	109015001	0.298698	0.205983	SG1	TPK1
				NC_056072.1	14520001	14540001	0.441403	0.274472	SG1	TRAK1
				NC_056072.1	14525001	14545001	0.098223	0.310322	SG1	TRAK1
				NC_056072.1	14530001	14550001	0.0903638	0.303644	SG1	TRAK1
				NC_056072.1	14535001	14555001	0.136502	0.219287	SG1	TRAK1
				NC_056072.1	55820001	55840001	0.374516	0.233095	SG1	TRH
				NC_056073.1	29125001	29145001	0.420567	0.223289	SG1	TRIM27

NC_056073.1	29130001	29150001	0.194548	0.214043	SG1	TRIM27
NC_056077.1	36050001	36070001	0.194049	0.297571	SG1	TRIM56
NC_056080.1	44320001	44340001	0.380785	0.212498	SG1	TRIM60
NC_056077.1	27805001	27825001	0.314183	0.231196	SG1	TRIM72
NC_056077.1	27810001	27830001	0.210778	0.270549	SG1	TRIM72
NC_056077.1	27815001	27835001	0.139839	0.250436	SG1	TRIM72
NC_056077.1	27820001	27840001	0.240164	0.2477	SG1	TRIM72
NC_056062.1	84215001	84235001	0.188804	0.1807	SG1	TRIQK
NC_056080.1	38145001	38165001	0.322259	0.242377	SG1	TSPAN7
NC_056054.1	270025001	270045001	0.31791	0.268182	SG1	TTC3
NC_056060.1	98570001	98590001	0.301836	0.175104	SG1	TTC8
NC_056061.1	48170001	48190001	0.331248	0.18688	SG1	UBE2J1
NC_056061.1	48175001	48195001	0.138713	0.266296	SG1	UBE2J1
NC_056061.1	48180001	48200001	0.118636	0.320036	SG1	UBE2J1
NC_056061.1	48185001	48205001	0.316894	0.308846	SG1	UBE2J1
NC_056061.1	48190001	48210001	0.396838	0.30005	SG1	UBE2J1
NC_056061.1	48195001	48215001	0.374549	0.30199	SG1	UBE2J1
NC_056060.1	36395001	36415001	0.321381	0.182817	SG1	UBR1
NC_056068.1	52135001	52155001	0.0587476	0.289075	SG1	UCP3
NC_056068.1	52140001	52160001	0.0722087	0.270996	SG1	UCP3
NC_056068.1	52145001	52165001	0.0898205	0.260218	SG1	UCP3
NC_056072.1	13870001	13890001	0.27412	0.331674	SG1	ULK4
NC_056072.1	13875001	13895001	0.360015	0.264807	SG1	ULK4
NC_056072.1	13880001	13900001	0.430189	0.222306	SG1	ULK4
NC_056072.1	13910001	13930001	0.307262	0.182769	SG1	ULK4
NC_056072.1	13975001	13995001	0.359012	0.235675	SG1	ULK4
NC_056072.1	13980001	14000001	0.312587	0.273728	SG1	ULK4
NC_056072.1	13985001	14005001	0.228558	0.301206	SG1	ULK4
NC_056072.1	13990001	14010001	0.0650504	0.268654	SG1	ULK4
NC_056072.1	13995001	14015001	0.118566	0.188581	SG1	ULK4
NC_056054.1	262995001	263015001	0.299439	0.233049	SG1	UMODL1
NC_056054.1	263000001	263020001	0.250316	0.265453	SG1	UMODL1

NC_056054.1	263005001	263025001	0.150581	0.289722	SG1	UMODL1
NC_056054.1	263010001	263030001	0.207294	0.289991	SG1	UMODL1
NC_056054.1	263015001	263035001	0.279507	0.273771	SG1	UMODL1
NC_056054.1	263020001	263040001	0.356205	0.239703	SG1	UMODL1
NC_056071.1	55990001	56010001	0.47938	0.200739	SG1	UNC79
NC_056080.1	55615001	55635001	0.104469	0.173233	SG1	USP27X
NC_056059.1	6450001	6470001	0.35206	0.288034	SG1	USP53
NC_056056.1	87340001	87360001	0.442703	0.204087	SG1	VIT
NC_056062.1	77815001	77835001	0.453542	0.21808	SG1	VPS13B
NC_056062.1	77820001	77840001	0.343352	0.253309	SG1	VPS13B
NC_056062.1	77825001	77845001	0.27956	0.247649	SG1	VPS13B
NC_056062.1	77830001	77850001	0.299645	0.219787	SG1	VPS13B
NC_056055.1	245975001	245995001	0.275672	0.195633	SG1	VWA5B1
NC_056055.1	245980001	246000001	0.123092	0.261218	SG1	VWA5B1
NC_056055.1	245985001	246005001	0.050312	0.303584	SG1	VWA5B1
NC_056063.1	12340001	12360001	0.326005	0.24968	SG1	VWA8
NC_056063.1	12345001	12365001	0.363678	0.316121	SG1	VWA8
NC_056063.1	12350001	12370001	0.366592	0.299107	SG1	VWA8
NC_056063.1	12355001	12375001	0.34025	0.307085	SG1	VWA8
NC_056063.1	12360001	12380001	0.261886	0.242999	SG1	VWA8
NC_056063.1	12365001	12385001	0.17884	0.2125	SG1	VWA8
NC_056063.1	12370001	12390001	0.168029	0.182667	SG1	VWA8
NC_056057.1	89650001	89670001	0.48246	0.278525	SG1	WASL
NC_056078.1	42240001	42260001	0.353784	0.202962	SG1	WDFY4
NC_056078.1	42245001	42265001	0.246777	0.198311	SG1	WDFY4
NC_056061.1	91140001	91160001	0.310808	0.180574	SG1	WDR27
NC_056071.1	22395001	22415001	0.344417	0.214426	SG1	WHAMM
NC_056071.1	22400001	22420001	0.395103	0.174445	SG1	WHAMM
NC_056056.1	213435001	213455001	0.419354	0.322573	SG1	WNK1
NC_056056.1	213440001	213460001	0.385727	0.343295	SG1	WNK1
NC_056056.1	213445001	213465001	0.483169	0.292933	SG1	WNK1
NC_056056.1	222155001	222175001	0.44708	0.190164	SG1	WNT7B

NC_056056.1	222160001	222180001	0.383167	0.192554	SG1	WNT7B
NC_056054.1	239255001	239275001	0.335908	0.22467	SG1	WWTR1
NC_056054.1	239260001	239280001	0.303468	0.256502	SG1	WWTR1
NC_056054.1	239265001	239285001	0.397259	0.247804	SG1	WWTR1
NC_056054.1	239270001	239290001	0.443052	0.245805	SG1	WWTR1
NC_056056.1	217955001	217975001	0.0496203	0.252359	SG1	XPNPEP3
NC_056056.1	217960001	217980001	0.0518622	0.257233	SG1	XPNPEP3
NC_056056.1	91600001	91620001	0.387362	0.22632	SG1	YIPF4
NC_056056.1	91605001	91625001	0.356431	0.271963	SG1	YIPF4
NC_056056.1	91610001	91630001	0.408871	0.251755	SG1	YIPF4
NC_056060.1	84155001	84175001	0.0854887	0.212995	SG1	YLPM1
NC_056060.1	84160001	84180001	0.116093	0.243423	SG1	YLPM1
NC_056060.1	84165001	84185001	0.100719	0.277706	SG1	YLPM1
NC_056054.1	262905001	262925001	0.249008	0.186911	SG1	ZBTB21
NC_056054.1	262910001	262930001	0.217717	0.225248	SG1	ZBTB21
NC_056054.1	262915001	262935001	0.275049	0.213694	SG1	ZBTB21
NC_056057.1	104105001	104125001	0.0751174	0.373262	SG1	ZC3HAV1
NC_056057.1	104110001	104130001	0.138462	0.302828	SG1	ZC3HAV1
NC_056057.1	104115001	104135001	0.166345	0.303679	SG1	ZC3HAV1
NC_056057.1	104120001	104140001	0.217855	0.290919	SG1	ZC3HAV1
NC_056057.1	104125001	104145001	0.375155	0.22166	SG1	ZC3HAV1
NC_056057.1	104090001	104110001	0.171087	0.393225	SG1	ZC3HAV1;ZC3
NC_056057.1	104095001	104115001	0.103222	0.39697	SG1	ZC3HAV1;ZC3
NC_056057.1	104100001	104120001	0.0840559	0.368666	SG1	ZC3HAV1;ZC3
NC_056057.1	95505001	95525001	0.457072	0.287755	SG1	ZC3HC1
NC_056079.1	19210001	19230001	0.457916	0.239541	SG1	ZDHHC2
NC_056072.1	54215001	54235001	0.40457	0.228442	SG1	ZDHHC3
NC_056072.1	54220001	54240001	0.420414	0.211023	SG1	ZDHHC3
NC_056072.1	54225001	54245001	0.387691	0.193427	SG1	ZDHHC3
NC_056062.1	20225001	20245001	0.28338	0.380294	SG1	ZFAT
NC_056062.1	20230001	20250001	0.191133	0.35715	SG1	ZFAT
NC_056062.1	20235001	20255001	0.423311	0.202941	SG1	ZFAT

NC_056062.1	20280001	20300001	0.480197	0.221188	SG1	ZFAT
NC_056062.1	20285001	20305001	0.33863	0.278475	SG1	ZFAT
NC_056062.1	20290001	20310001	0.278372	0.340923	SG1	ZFAT
NC_056062.1	20295001	20315001	0.410899	0.284578	SG1	ZFAT
NC_056062.1	52825001	52845001	0.303704	0.175414	SG1	ZFHX4
NC_056062.1	52830001	52850001	0.292978	0.202246	SG1	ZFHX4
NC_056062.1	52835001	52855001	0.215718	0.305556	SG1	ZFHX4
NC_056062.1	52840001	52860001	0.212547	0.32186	SG1	ZFHX4
NC_056062.1	52845001	52865001	0.145744	0.282328	SG1	ZFHX4
NC_056062.1	52850001	52870001	0.303364	0.232449	SG1	ZFHX4
NC_056059.1	117005001	117025001	0.363288	0.293828	SG1	ZFYVE28
NC_056059.1	117010001	117030001	0.231177	0.343081	SG1	ZFYVE28
NC_056059.1	117015001	117035001	0.240238	0.351328	SG1	ZFYVE28
NC_056059.1	117020001	117040001	0.420078	0.286246	SG1	ZFYVE28
NC_056059.1	117030001	117050001	0.444444	0.283371	SG1	ZFYVE28
NC_056059.1	117035001	117055001	0.481266	0.268481	SG1	ZFYVE28
NC_056059.1	117040001	117060001	0.28118	0.372534	SG1	ZFYVE28
NC_056059.1	117045001	117065001	0.29014	0.364779	SG1	ZFYVE28
NC_056059.1	117050001	117070001	0.385503	0.315887	SG1	ZFYVE28
NC_056059.1	117055001	117075001	0.360169	0.310747	SG1	ZFYVE28
NC_056054.1	26655001	26675001	0.440796	0.377503	SG1	ZFYVE9
NC_056054.1	26660001	26680001	0.359375	0.342251	SG1	ZFYVE9
NC_056054.1	26665001	26685001	0.438597	0.327887	SG1	ZFYVE9
NC_056054.1	26670001	26690001	0.288306	0.345551	SG1	ZFYVE9
NC_056054.1	26675001	26695001	0.286556	0.354675	SG1	ZFYVE9
NC_056054.1	26680001	26700001	0.409137	0.272516	SG1	ZFYVE9
NC_056078.1	34085001	34105001	0.46347	0.302528	SG1	ZMIZ1
NC_056054.1	190615001	190635001	0.432691	0.234296	SG1	ZNF148
NC_056057.1	113480001	113500001	0.195251	0.18118	SG1	ZNF212
NC_056057.1	113490001	113510001	0.216015	0.17967	SG1	ZNF212;ZNF7[illegible]
NC_056060.1	51845001	51865001	0.273388	0.183948	SG1	ZNF280D
NC_056060.1	51850001	51870001	0.216217	0.180845	SG1	ZNF280D

NC_056060.1	51855001	51875001	0.267051	0.177677	SG1	ZNF280D
NC_056061.1	50010001	50030001	0.246914	0.228751	SG1	ZNF292
NC_056061.1	50015001	50035001	0.099044	0.288787	SG1	ZNF292
NC_056061.1	50020001	50040001	0.0973282	0.279183	SG1	ZNF292
NC_056061.1	50025001	50045001	0.288929	0.230304	SG1	ZNF292
NC_056079.1	43060001	43080001	0.484465	0.19416	SG1	ZNF385D
NC_056058.1	38325001	38345001	0.330218	0.256252	SG1	ZNF496
NC_056058.1	38330001	38350001	0.288905	0.24776	SG1	ZNF496
NC_056058.1	38335001	38355001	0.219617	0.235168	SG1	ZNF496
NC_056058.1	38340001	38360001	0.178261	0.185249	SG1	ZNF496
NC_056058.1	38345001	38365001	0.159908	0.179178	SG1	ZNF496
NC_056054.1	68280001	68300001	0.372327	0.327422	SG1	ZNF644
NC_056054.1	68285001	68305001	0.22554	0.409294	SG1	ZNF644
NC_056054.1	68290001	68310001	0.197747	0.453165	SG1	ZNF644
NC_056054.1	68295001	68315001	0.180519	0.477366	SG1	ZNF644
NC_056054.1	68300001	68320001	0.217245	0.462327	SG1	ZNF644
NC_056054.1	68305001	68325001	0.268356	0.431819	SG1	ZNF644
NC_056054.1	68310001	68330001	0.380749	0.373616	SG1	ZNF644
NC_056055.1	124975001	124995001	0.482615	0.184657	SG1	ZNF804A

);LOC101106395;LOC101118764

);LOC101106395;LOC101118764
