# Supplementary material for: Genetic diversity and selection of Tibetan sheep breeds revealed by whole-genome resequencing
Source: Anim Biosci. 2023 May 2;36(7):991–1002. doi: 10.5713/ab.22.0432 (PMC10330983; doi:10.5713/ab.22.0432)
Supplement: Supplementary file 12 [file ab-22-0432-Supplementary-Table-12.pdf]

Supplementary Table12. Lists of putative co-selected genes among different breeds

Hypoxic Adaptability	Coat Color	Wool Traits	Horn Phenotypes
RRN3	MITF	IRS4	GPC5
ERBB4	ULK4	RAB8B	ULK4
LCORL	RBFOX1	EPS8	VWA8
MET	LRBA	TM4SF4	CCSER1
SUGCT	NRXN1	RBFOX1	MED27
MYOM1	ARID1B	FAM219B;MPI	CRYGS;TBCCD1
GRM8	WDTC1	CACNA1B	SORCS2
SLC23A2	TLN2	ARHGAP15	KIAA1217
DACH1	UNC5C	HDAC9	CNTN5
TRIM60	SEMA6D	GRHL2	FCHSD2
DSCC1	RNF111	FOCAD	SLC7A11
ATMIN;CENPN	PHEX	OSBPL10	JAZF1
SLC2A13	MIPOL1	SLC9A7	PDE7B
MEPE	KCNC2	TMEM241	DACH2
NCOR2	WHAMM	PDGFD	ARHGAP20
PACS2;TEX22	SLIT2	EXOSC10;MTOR	TMEM178B
RBFOX1	RSF1	LRP1B	SCAPER
DBX2	KMT2C	USH2A	NBEA
THADA	MEDAG	NOTCH2	MAGI2
TSPAN7	ZRANB3	CARMIL2;CTCF	BTBD1
KDM6A	USP25	CNTN5	LINGO2
EXOSC10	CTNNA3	ERC2	TPK1
TCF12	SLC4A10	NRXN1	WDR27
PLD1	KCNIP4	AGXT2	DIPK1A
LOC101111335	KCNMB2	ZC3H12C	NHS
RARB	ADGRV1	MAGI1	WHAMM
IGDCC3	CASK	ADCY10	ARL15
MTOR		CPED1	MACROD2
PKD1L2		CACNA2D3	PARD3

LOC101116053	VAC14	RXFP2
TENM1	COL4A2	
LOC101115398	ZC4H2	
DIXDC1	CFAP47	
CPVL	FOXP2	
PAWR	LOC121818481	
SH3KBP1	PTPRD	
FHIT	NCAM2	
LOC100526781	PTPRG	
MAD1L1	SYNE1	
CLIP2	TRIM71	
FOXN2	KCNK13	
NPY	PCDH9	
KLF12	UNC13C	
LOC121818354	SCAMP2	
CNTN5	DMD	
LOC101106743	PGM5	
ERC2	L3MBTL3	
NDC80	ABLIM3	
GLI3	CALCOCO1	
ITPR2	CSF2RB	
NRXN1		
RFX8		
LOC101120816		
IFRD1		
TSPAN5		
GABPA		
KCNQ1		
FAM13A		
CYTH4		
GSG1L		

CAMKMT

DLG2

LOC101112419

NOL4

AFF2

RORA

FAM184B

TNFSF4

TG

TMEM182

FLOT1;MDC1;TUBB

EFL1

LOC121818668

CCAR1;TET1

EDA

LINGO2

COMMD10

DDHD2;NSD3;PLPP5

TNFSF8

ERO1A

PTPRG

ELMO1

LOC100526781;LOC100526782

C15H11orf97

UBE4B

LOC105605990

PROM1

KCNIP4

PRPH

LOC101118336

KIF13A

LOC100526782

B4GAT1;BRMS1

NHS

RBMS3

SLC29A2

FLOT1;IER3;MDC1;TUBB

NT5C2

METTL4

IMMP2L

RASAL2

KIRREL3

GPD2

TET1

DMD

DEPTOR;DSCC1

LOC101120816;LOC101122934

SIK3

TDRD7

CSN2

GMPS

ADAM22

GRIA4

RELN

TRPS1

XKR4

CWF19L2

LOC100526781;V15

ERC1

SAR1B

HECW1
