# Supplementary material for: Genetic diversity and selection of Tibetan sheep breeds revealed by whole-genome resequencing
Source: Anim Biosci. 2023 May 2;36(7):991–1002. doi: 10.5713/ab.22.0432 (PMC10330983; doi:10.5713/ab.22.0432)
Supplement: Supplementary file 13 [file ab-22-0432-Supplementary-Table-13.pdf]

Supplementary Table 13. KEGG analysis of putative selected genes compared with SG2 breed

BD vs SG2				
PathwayID	Pathway	Level1	Level2	list_number
oas00512	Mucin type O-glycan biosynthesis	Metabolism	Glycan biosynthesis and metal	11
oas04724	Glutamatergic synapse	Organismal Systems	Nervous system	19
oas05031	Amphetamine addiction	Human Diseases	Substance dependence	13
oas04977	Vitamin digestion and absorption	Organismal Systems	Digestive system	7
oas04728	Dopaminergic synapse	Organismal Systems	Nervous system	20
oas00514	Other types of O-glycan biosynthesis	Metabolism	Glycan biosynthesis and metal	10
oas04512	ECM-receptor interaction	Environmental Information Processing	Signaling molecules and intera	15
oas04666	Fc gamma R-mediated phagocytosis	Organismal Systems	Immune system	15
oas03018	RNA degradation	Genetic Information Processing	Folding, sorting and degradati	13
oas04072	Phospholipase D signaling pathway	Environmental Information Processing	Signal transduction	21
oas05412	Arrhythmogenic right ventricular cardiomyop	Human Diseases	Cardiovascular disease	12
oas04713	Circadian entrainment	Organismal Systems	Environmental adaptation	14
oas05221	Acute myeloid leukemia	Human Diseases	Cancer: specific types	11
oas05206	MicroRNAs in cancer	Human Diseases	Cancer: overview	22
GY vs SG2				
oas04012	ErbB signaling pathway	Environmental Information Processing	Signal transduction	17
oas04070	Phosphatidylinositol signaling system	Environmental Information Processing	Signal transduction	18
oas04724	Glutamatergic synapse	Organismal Systems	Nervous system	19
oas04072	Phospholipase D signaling pathway	Environmental Information Processing	Signal transduction	24
oas04974	Protein digestion and absorption	Organismal Systems	Digestive system	21
oas01521	EGFR tyrosine kinase inhibitor resistance	Human Diseases	Drug resistance: antineoplastic	15
oas05225	Hepatocellular carcinoma	Human Diseases	Cancer: specific types	26
oas05223	Non-small cell lung cancer	Human Diseases	Cancer: specific types	13
oas00512	Mucin type O-glycan biosynthesis	Metabolism	Glycan biosynthesis and metal	8
oas00230	Purine metabolism	Metabolism	Nucleotide metabolism	20
oas04310	Wnt signaling pathway	Environmental Information Processing	Signal transduction	24
oas04728	Dopaminergic synapse	Organismal Systems	Nervous system	19
oas04151	PI3K-Akt signaling pathway	Environmental Information Processing	Signal transduction	46
oas05200	Pathways in cancer	Human Diseases	Cancer: overview	63
oas04935	Growth hormone synthesis, secretion and acti	Organismal Systems	Endocrine system	18

oas00514	Other types of O-glycan biosynthesis	Metabolism	Glycan biosynthesis and metab	9
oas05206	MicroRNAs in cancer	Human Diseases	Cancer: overview	23
oas05231	Choline metabolism in cancer	Human Diseases	Cancer: overview	15
oas05226	Gastric cancer	Human Diseases	Cancer: specific types	21
oas04971	Gastric acid secretion	Organismal Systems	Digestive system	12
oas00232	Caffeine metabolism	Metabolism	Biosynthesis of other secondar	2
oas00515	Mannose type O-glycan biosynthesis	Metabolism	Glycan biosynthesis and metab	5
HZ vs SG2				
oas04728	Dopaminergic synapse	Organismal Systems	Nervous system	22
oas04974	Protein digestion and absorption	Organismal Systems	Digestive system	21
oas04512	ECM-receptor interaction	Environmental Information Processing	Signaling molecules and intera	16
oas04724	Glutamatergic synapse	Organismal Systems	Nervous system	18
oas04071	Sphingolipid signaling pathway	Environmental Information Processing	Signal transduction	19
oas00561	Glycerolipid metabolism	Metabolism	Lipid metabolism	12
oas05132	Salmonella infection	Human Diseases	Infectious disease: bacterial	34
oas04713	Circadian entrainment	Organismal Systems	Environmental adaptation	15
oas04930	Type II diabetes mellitus	Human Diseases	Endocrine and metabolic disea	9
oas04933	AGE-RAGE signaling pathway in diabetic co	Human Diseases	Endocrine and metabolic disea	16
oas04920	Adipocytokine signaling pathway	Organismal Systems	Endocrine system	12
oas05200	Pathways in cancer	Human Diseases	Cancer: overview	63
oas04670	Leukocyte transendothelial migration	Organismal Systems	Immune system	17
oas00564	Glycerophospholipid metabolism	Metabolism	Lipid metabolism	15
oas04012	ErbB signaling pathway	Environmental Information Processing	Signal transduction	13
oas04926	Relaxin signaling pathway	Organismal Systems	Endocrine system	18
OL vs SG2				
oas04919	Thyroid hormone signaling pathway	Organismal Systems	Endocrine system	21
oas04724	Glutamatergic synapse	Organismal Systems	Nervous system	18
oas05206	MicroRNAs in cancer	Human Diseases	Cancer: overview	25
oas03018	RNA degradation	Genetic Information Processing	Folding, sorting and degradati	14
oas00230	Purine metabolism	Metabolism	Nucleotide metabolism	20
oas04072	Phospholipase D signaling pathway	Environmental Information Processing	Signal transduction	22
oas03450	Non-homologous end-joining	Genetic Information Processing	Replication and repair	4
oas05230	Central carbon metabolism in cancer	Human Diseases	Cancer: overview	12

oas05235	PD-L1 expression and PD-1 checkpoint pathw	Human Diseases	Cancer: overview	14
oas05220	Chronic myeloid leukemia	Human Diseases	Cancer: specific types	13
oas04510	Focal adhesion	Cellular Processes	Cellular community - eukaryot	27
oas05215	Prostate cancer	Human Diseases	Cancer: specific types	15
oas00232	Caffeine metabolism	Metabolism	Biosynthesis of other secondar	2
oas00785	Lipoic acid metabolism	Metabolism	Metabolism of cofactors and v	2

total_number	Pvalue	FDR
36	0.000188932	0.057813339
109	0.003138168	0.423779449
68	0.006085525	0.423779449
26	0.006143422	0.423779449
127	0.008024417	0.423779449
48	0.008309401	0.423779449
90	0.012213932	0.533923311
95	0.019485083	0.706887423
79	0.020790807	0.706887423
150	0.023726033	0.726016606
76	0.034315764	0.891176645
94	0.036728021	0.891176645
69	0.039146715	0.891176645
168	0.040772788	0.891176645
84	0.001332344	0.355287338
97	0.002711342	0.355287338
109	0.004342299	0.355287338
150	0.004644279	0.355287338
129	0.006366503	0.386213056
83	0.007572805	0.386213056
179	0.011744761	0.46308878
73	0.013982556	0.46308878
36	0.014043189	0.46308878
131	0.01513362	0.46308878
168	0.01815505	0.505040491
127	0.021400525	0.507615544
373	0.021565366	0.507615544
541	0.025590468	0.528365772
121	0.025953692	0.528365772

48	0.027626968	0.528365772
168	0.031809868	0.572577623
100	0.037163807	0.618516484
154	0.040356813	0.618516484
76	0.041657023	0.618516484
4	0.044020523	0.618516484
22	0.044468505	0.618516484
127	0.00283403	0.800610733
129	0.007276965	0.800610733
90	0.007798156	0.800610733
109	0.01070531	0.814459064
120	0.013749447	0.814459064
67	0.018586766	0.814459064
257	0.020596531	0.814459064
94	0.024933829	0.814459064
47	0.026094905	0.814459064
104	0.028697667	0.814459064
72	0.03120219	0.814459064
541	0.031732171	0.814459064
115	0.03477577	0.823918252
100	0.040656554	0.874857795
84	0.043702257	0.874857795
129	0.049841947	0.874857795
123	0.005003657	0.999994076
109	0.012494171	0.999994076
168	0.013796417	0.999994076
79	0.014432796	0.999994076
131	0.019942036	0.999994076
150	0.023227286	0.999994076
13	0.027644351	0.999994076
71	0.031531761	0.999994076

89	0.037109678	0.999994076
81	0.037765196	0.999994076
203	0.040233156	0.999994076
99	0.042415326	0.999994076
4	0.046390226	0.999994076
4	0.046390226	0.999994076

List
GALNT18(GALNT18),GALNTL6(GALNTL6),GALNT2(GALNT2),C1GALT1C1(C1GALT1C1),ST3GAL1(ST3GAL1),GALNT7(GALNT7),GALNT5(
PPP3CA(PPP3CA),DLG4(DLG4),HOMER1(HOMER1),GRIN2A(GRIN2A),SHANK3(SHANK3),GRM8(GRM8),PLD1(PLD1),GNG14(GNG14),GRIA
PPP3CA(PPP3CA),GRIN2A(GRIN2A),GRIA4(GRIA4),ADCY5(ADCY5),GRIA3(GRIA3),CAMK2A(CAMK2A),CREB5(CREB5),PRKCA(PRKCA),L
LOC101107957(LOC101107957),LMBRD1(LMBRD1),ABCC1(ABCC1),RBP2(RBP2),CUBN(CUBN),LOC101103610(LOC101103610),APOB(APO
PPP3CA(PPP3CA),PPP2R5E(PPP2R5E),GRIN2A(GRIN2A),GNG14(GNG14),GRIA4(GRIA4),ITPR2(ITPR2),ARNTL(ARNTL),ADCY5(ADCY5),GN
GALNT18(GALNT18),GALNTL6(GALNTL6),GALNT2(GALNT2),C1GALT1C1(C1GALT1C1),POGLUT1(POGLUT1),GALNT7(GALNT7),GALNT5
SV2C(SV2C),COL4A6(COL4A6),LAMA1(LAMA1),HSPG2(HSPG2),SV2B(SV2B),RELN(RELN),COL6A3(COL6A3),HMMR(HMMR),COL4A2(CO
VAV1(VAV1),ASAP1(ASAP1),ACTR3(ACTR3),GAB2(GAB2),PLD1(PLD1),CFL1(CFL1),FCGR3A(FCGR3A),DNM2(DNM2),VAV3(VAV3),PRKC
EXOSC10(EXOSC10),XRN2(XRN2),LSM1(LSM1),EXOSC5(EXOSC5),XRN1(XRN1),MTREX(MTREX),PABPC1(PABPC1),TTC37(TTC37),CNO
DGKH(DGKH),KIT(KIT),GAB2(GAB2),MTOR(MTOR),GRM8(GRM8),PLD1(PLD1),LOC114110058(LOC114110058),AGPAT5(AGPAT5),DNM2(
LAMA1(LAMA1),CTNNA3(CTNNA3),ATP2A3(ATP2A3),LEF1(LEF1),RYR2(RYR2),CTNNA2(CTNNA2),DSC2(DSC2),LAMA2(LAMA2),CACNA
GRIN2A(GRIN2A),GNG14(GNG14),NOS1(NOS1),GRIA4(GRIA4),ADCY5(ADCY5),GNG7(GNG7),GRIA3(GRIA3),CAMK2A(CAMK2A),RYR2(R
KIT(KIT),MTOR(MTOR),EIF4EBP1(EIF4EBP1),LOC114110058(LOC114110058),STAT3(STAT3),BAD(BAD),LEF1(LEF1),CCND1(CCND1),MPO
BCL2L11(BCL2L11),TP63(TP63),ZEB1(ZEB1),MTOR(MTOR),BMPR2(BMPR2),EFNA5(EFNA5),ABCC1(ABCC1),LOC114110058(LOC11411005
SOS2(SOS2),ERBB4(ERBB4),MTOR(MTOR),MAPK8(MAPK8),PAK3(PAK3),LOC114110058(LOC114110058),NRG3(NRG3),AKT3(AKT3),CAM
INPP4B(INPP4B),BPNT2(BPNT2),DGKZ(DGKZ),ITPR2(ITPR2),PIP4K2A(PIP4K2A),PLCB1(PLCB1),SYNJ1(SYNJ1),PIP4K2B(PIP4K2B),PI4K2B(
PPP3CA(PPP3CA),HOMER1(HOMER1),ADCY9(ADCY9),GRM8(GRM8),PLD1(PLD1),GRIA4(GRIA4),ITPR2(ITPR2),PLCB1(PLCB1),GRM7(GR
SOS2(SOS2),MTOR(MTOR),AGPAT3(AGPAT3),AGPAT2(AGPAT2),ADCY9(ADCY9),GRM8(GRM8),PLD1(PLD1),DGKZ(DGKZ),LOC11411005
COL4A6(COL4A6),ACE2(ACE2),COL24A1(COL24A1),LOC101105540(LOC101105540),KCNQ1(KCNQ1),SLC6A19(SLC6A19),LOC101118398(
SOS2(SOS2),BCL2L11(BCL2L11),MTOR(MTOR),JAK2(JAK2),LOC114110058(LOC114110058),AKT3(AKT3),MET(MET),PRKCA(PRKCA),HGF
SOS2(SOS2),MGST2(MGST2),WNT8B(WNT8B),MTOR(MTOR),NQO1(NQO1),LOC114110058(LOC114110058),AKT3(AKT3),LOC101106976(L
SOS2(SOS2),FHIT(FHIT),RXRA(RXRA),LOC114110058(LOC114110058),AKT3(AKT3),MET(MET),PRKCA(PRKCA),RARB(RARB),POLK(POLK
GALNT18(GALNT18),GALNTL6(GALNTL6),GALNT2(GALNT2),C1GALT1C1(C1GALT1C1),GALNT13(GALNT13),GALNT7(GALNT7),GALNT9
ADSS2(ADSS2),NT5C2(NT5C2),PDE5A(PDE5A),FHIT(FHIT),PDE6A(PDE6A),ADCY9(ADCY9),HDDC2(HDDC2),GMPS(GMPS),XDH(XDH),NM
PPP3CA(PPP3CA),TLE4(TLE4),LOC101116245(LOC101116245),NLK(NLK),WNT8B(WNT8B),NOTUM(NOTUM),CTNNBIP1(CTNNBIP1),MAPK
PPP3CA(PPP3CA),CLOCK(CLOCK),PPP2R5E(PPP2R5E),MAPK8(MAPK8),GRIA4(GRIA4),ITPR2(ITPR2),AKT3(AKT3),PLCB1(PLCB1),PPP2R5
LOC101105632(LOC101105632),COL4A6(COL4A6),SOS2(SOS2),BDNF(BDNF),BCL2L11(BCL2L11),RPTOR(RPTOR),MAGI1(MAGI1),ANGPT1
LOC101105632(LOC101105632),IL6ST(IL6ST),COL4A6(COL4A6),SOS2(SOS2),BCL2L11(BCL2L11),MGST2(MGST2),WNT8B(WNT8B),JAG2(J
SOS2(SOS2),GHR(GHR),MTOR(MTOR),MAPK8(MAPK8),ADCY9(ADCY9),JAK2(JAK2),LOC114110058(LOC114110058),ITPR2(ITPR2),AKT3(

GALNT18(GALNT18),B3GLCT(B3GLCT),GALNTL6(GALNTL6),GALNT2(GALNT2),C1GALT1C1(C1GALT1C1),GALNT13(GALNT13),GALNT7
SOS2(SOS2),BCL2L11(BCL2L11),RPTOR(RPTOR),PRKCE(PRKCE),MTOR(MTOR),ROCK1(ROCK1),TNC(TNC),EFNA5(EFNA5),HMGA2(HM
SOS2(SOS2),MTOR(MTOR),MAPK8(MAPK8),PCYT1B(PCYT1B),PLD1(PLD1),DGKZ(DGKZ),LOC114110058(LOC114110058),AKT3(AKT3),SL
SOS2(SOS2),WNT8B(WNT8B),CTNNA3(CTNNA3),MTOR(MTOR),RXRA(RXRA),LOC114110058(LOC114110058),AKT3(AKT3),MET(MET),T
CFTR(CFTR),ADCY9(ADCY9),KCNQ1(KCNQ1),LOC101118398(LOC101118398),ITPR2(ITPR2),KCNK10(KCNK10),PLCB1(PLCB1),CHRM3(C
XDH(XDH),LOC101109214(LOC101109214)
LOC101117098(LOC101117098),B4GAT1(B4GAT1),LARGE1(LARGE1),POMK(POMK),B3GALNT2(B3GALNT2)
PPP3CA(PPP3CA),LOC101118179(LOC101118179),FOS(FOS),GRIN2A(GRIN2A),PPP2R2C(PPP2R2C),CAMK2D(CAMK2D),GNGT1(GNGT1),G
PAG3(PAG3),COL4A6(COL4A6),COL4A5(COL4A5),ACE2(ACE2),COL4A4(COL4A4),SLC15A1(SLC15A1),LOC101105540(LOC101105540),CO
SV2C(SV2C),COL4A6(COL4A6),LAMA1(LAMA1),COL4A5(COL4A5),COL4A4(COL4A4),ITGA11(ITGA11),HSPG2(HSPG2),TNC(TNC),RELN(R
PPP3CA(PPP3CA),LOC101118179(LOC101118179),HOMER1(HOMER1),GRK3(GRK3),GRIN2A(GRIN2A),GRM8(GRM8),PLD1(PLD1),GNGT1(
ACER2(ACER2),SGMS1(SGMS1),TRAF2(TRAF2),PRKCE(PRKCE),ASAH1(ASAH1),ROCK1(ROCK1),ABCC1(ABCC1),PLD1(PLD1),PPP2R2C(
DGKH(DGKH),PLPP5(PLPP5),LOC101107135(LOC101107135),TKFC(TKFC),LOC101107387(LOC101107387),AGPAT2(AGPAT2),LOC1011229
LOC101103740(LOC101103740),TRAF2(TRAF2),MYL7(MYL7),EXOC7(EXOC7),FOS(FOS),PAK3(PAK3),TUBB(TUBB),AHNAK(AHNAK),ARP
CACNA1I(CACNA1I),LOC101118179(LOC101118179),FOS(FOS),ADCYAP1(ADCYAP1),GRIN2A(GRIN2A),NOS1(NOS1),CAMK2D(CAMK2D),
GCK(GCK),PRKCE(PRKCE),MTOR(MTOR),CACNA1C(CACNA1C),SOCS3(SOCS3),LOC101102503(LOC101102503),PIK3CB(PIK3CB),MAPK1
CYBB(CYBB),COL4A6(COL4A6),MMP2(MMP2),COL4A5(COL4A5),PRKCE(PRKCE),COL4A4(COL4A4),TGFB1(TGFB1),JAK2(JAK2),AKT3(A
TRAF2(TRAF2),MTOR(MTOR),ADIPOR2(ADIPOR2),JAK2(JAK2),PPARGC1A(PPARGC1A),AKT3(AKT3),NPY(NPY),LOC101102230(LOC1011
TPM3(TPM3),LOC101105632(LOC101105632),COL4A6(COL4A6),TRAF2(TRAF2),MMP2(MMP2),LAMA1(LAMA1),BCL2L11(BCL2L11),COL4
CYBB(CYBB),MMP2(MMP2),NCF1(NCF1),SIPA1(SIPA1),MYL7(MYL7),CTNNA3(CTNNA3),ROCK1(ROCK1),CLDN1(CLDN1),ROCK2(ROCK
DGKH(DGKH),PLB1(PLB1),PLPP5(PLPP5),PHOSPHO1(PHOSPHO1),LPCAT1(LPCAT1),AGPAT2(AGPAT2),PLD1(PLD1),DGKZ(DGKZ),MBOA
ERBB4(ERBB4),MTOR(MTOR),BRAF(BRAF),PAK3(PAK3),CAMK2D(CAMK2D),NRG3(NRG3),AKT3(AKT3),ABL1(ABL1),NRG2(NRG2),PLC
COL4A6(COL4A6),RLN3(RLN3),MMP2(MMP2),COL4A5(COL4A5),COL4A4(COL4A4),TGFB1(TGFB1),FOS(FOS),NOS1(NOS1),GNGT1(GNGT
HDAC1(HDAC1),MED17(MED17),ATP2A1(ATP2A1),KRAS(KRAS),SLC2A1(SLC2A1),MTOR(MTOR),RCAN2(RCAN2),NCOA1(NCOA1),AKT3
PPP3CA(PPP3CA),HOMER1(HOMER1),GRM8(GRM8),PLD1(PLD1),GRIA4(GRIA4),ITPR2(ITPR2),PLCB1(PLCB1),GRM1(GRM1),GRM7(GRM7
HDAC1(HDAC1),BCL2L11(BCL2L11),PRKCE(PRKCE),KRAS(KRAS),TNN(TNN),MTOR(MTOR),TNC(TNC),NFKB1(NFKB1),HMGA2(HMGA2)
EXOSC10(EXOSC10),CNOT3(CNOT3),EXOSC5(EXOSC5),PARN(PARN),EXOSC8(EXOSC8),ZCCHC7(ZCCHC7),TTC37(TTC37),CNOT6L(CN
GUCY2C(GUCY2C),NT5C2(NT5C2),GUCY1A2(GUCY1A2),LOC101117505(LOC101117505),FHIT(FHIT),ENPP4(ENPP4),GMPS(GMPS),XDH(X
KIT(KIT),PIK3CG(PIK3CG),PDGFC(PDGFC),KRAS(KRAS),MTOR(MTOR),AGPAT2(AGPAT2),PTGFR(PTGFR),GRM8(GRM8),PLD1(PLD1),PD
RAD50(RAD50),DCLRE1C(DCLRE1C),PRKDC(PRKDC),XRCC6(XRCC6)
KIT(KIT),GCK(GCK),KRAS(KRAS),SLC2A1(SLC2A1),MTOR(MTOR),PDGFRA(PDGFRA),AKT3(AKT3),MET(MET),MYC(MYC),LDHB(LDHB)

PPP3CA(PPP3CA),KRAS(KRAS),MTOR(MTOR),NFKB1(NFKB1),JAK2(JAK2),CHUK(CHUK),AKT3(AKT3),STAT3(STAT3),CD247(CD247),ALK
HDAC1(HDAC1),RB1(RB1),KRAS(KRAS),NFKB1(NFKB1),CHUK(CHUK),BAK1(BAK1),AKT3(AKT3),MYC(MYC),SHC4(SHC4),CCND1(CCNI
PDGFC(PDGFC),TNN(TNN),MYL7(MYL7),TNC(TNC),RELN(RELN),PAK3(PAK3),PDGFRA(PDGFRA),PPP1R12B(PPP1R12B),PDGFD(PDGFD),
RB1(RB1),PDGFC(PDGFC),SRD5A2(SRD5A2),KRAS(KRAS),ERG(ERG),MTOR(MTOR),NFKB1(NFKB1),CHUK(CHUK),PDGFRA(PDGFRA),PD
XDH(XDH),LOC101109214(LOC101109214)
ACSM1(ACSM1),LIPT2(LIPT2)

(GALNT5),GCNT1(GCNT1),GALNTL5(GALNTL5),GALNT16(GALNT16),GALNT11(GALNT11)								
A4(GRIA4),ITPR2(ITPR2),GRM7(GRM7),ADCY5(ADCY5),GNG7(GNG7),GRIA3(GRIA3),GRIK1(GRIK1),PRKCA(PRKCA),GRIN3A(								
OC101105840(LOC101105840),GRIN3A(GRIN3A),ATF2(ATF2),DDC(DDC),TH(TH)								
DB)								
G7(GNG7),GRIA3(GRIA3),CAMK2A(CAMK2A),CREB5(CREB5),PRKCA(PRKCA),PPP2R2B(PPP2R2B),LOC101105840(LOC10110								
5(GALNT5),GALNTL5(GALNTL5),GALNT16(GALNT16),GALNT11(GALNT11)								
DL4A2),LAMA2(LAMA2),GP1BA(GP1BA),FRAS1(FRAS1),CHAD(CHAD),ITGA4(ITGA4),LAMB4(LAMB4)								
A(PRKCA),PLPP1(PLPP1),ARF6(ARF6),CRK(CRK),PLCG2(PLCG2),MYO10(MYO10)								
T2(CNOT2),CNOT8(CNOT8),DDX6(DDX6),LOC101108592(LOC101108592),DIS3L(DIS3L)								
DNM2),MRAS(MRAS),GRM7(GRM7),ADCY5(ADCY5),PRKCA(PRKCA),PLPP1(PLPP1),ARF6(ARF6),CYTH4(CYTH4),DNM3(DNM								
A2D3(CACNA2D3),CACNA2D2(CACNA2D2),SGCD(SGCD),ITGA4(ITGA4)								
YR2),PRKCA(PRKCA),ADCY10(ADCY10),LOC101105840(LOC101105840),C1H1orf226(C1H1orf226),GNGT2(GNGT2)								
(MPO),ZBTB16(ZBTB16),GRB2(GRB2)								
8),MET(MET),STAT3(STAT3),RECK(RECK),FOXP1(FOXP1),MMP16(MMP16),CCND1(CCND1),FSCN1(FSCN1),PRKCA(PRKCA),								
http://www.kegg.jp/kegg-bin/show_pathway?map04012/K06619%09%23FFFFFF,red/K08774%09%23FFFFFF,red/K05456%09%23FFFI								
http://www.kegg.jp/kegg-bin/show_pathway?map04070/K04959%09%23FFFFFF,red/K01109%09%23FFFFFF,red/K05859%09%23FFFI								
http://www.kegg.jp/kegg-bin/show_pathway?map04724/K04959%09%23FFFFFF,red/K05858%09%23FFFFFF,red/K05213%09%23FFFI								
http://www.kegg.jp/kegg-bin/show_pathway?map04072/K05461%09%23FFFFFF,red/K05859%09%23FFFFFF,red/K07941%09%23FFFI								
http://www.kegg.jp/kegg-bin/show_pathway?map04974/K06237%09%23FFFFFF,red/K13781%09%23FFFFFF,red/K24340%09%23FFFI								
http://www.kegg.jp/kegg-bin/show_pathway?map01521/K08774%09%23FFFFFF,red/K05099%09%23FFFFFF,red/K04447%09%23FFFI								
http://www.kegg.jp/kegg-bin/show_pathway?map05225/K08957%09%23FFFFFF,red/K02376%09%23FFFFFF,red/K08774%09%23FFFI								
http://www.kegg.jp/kegg-bin/show_pathway?map05223/K01522%09%23FFFFFF,red/K08774%09%23FFFFFF,red/K05099%09%23FFFI								
http://www.kegg.jp/kegg-bin/show_pathway?map00512/K09663%09%23FFFFFF,red/K00710%09%23FFFFFF,red/K09653%09%23FFFI								
http://www.kegg.jp/kegg-bin/show_pathway?map00230/K01939%09%23FFFFFF,red/K19970%09%23FFFFFF,red/K13293%09%23FFFI								
http://www.kegg.jp/kegg-bin/show_pathway?map04310/K03362%09%23FFFFFF,red/K08957%09%23FFFFFF,red/K02376%09%23FFFI								
http://www.kegg.jp/kegg-bin/show_pathway?map04728/K04959%09%23FFFFFF,red/K05858%09%23FFFFFF,red/K04354%09%23FFFI								
http://www.kegg.jp/kegg-bin/show_pathway?map04151/K05461%09%23FFFFFF,red/K05099%09%23FFFFFF,red/K12230%09%23FFFI								
http://www.kegg.jp/kegg-bin/show_pathway?map05200/K02376%09%23FFFFFF,red/K05461%09%23FFFFFF,red/K05099%09%23FFFI								
http://www.kegg.jp/kegg-bin/show_pathway?map04935/K04959%09%23FFFFFF,red/K05859%09%23FFFFFF,red/K04447%09%23FFFI								

http://www.kegg.jp/kegg-bin/show_pathway?map00514/K18134%09%23FFFFFF,red/K13675%09%23FFFFFF,red/K00710%09%23FFF								
http://www.kegg.jp/kegg-bin/show_pathway?map05206/K06619%09%23FFFFFF,red/K04514%09%23FFFFFF,red/K05099%09%23FFF								
http://www.kegg.jp/kegg-bin/show_pathway?map05231/K15377%09%23FFFFFF,red/K00901%09%23FFFFFF,red/K05089%09%23FFF								
http://www.kegg.jp/kegg-bin/show_pathway?map05226/K08957%09%23FFFFFF,red/K02376%09%23FFFFFF,red/K05099%09%23FFF								
http://www.kegg.jp/kegg-bin/show_pathway?map04971/K04959%09%23FFFFFF,red/K01539%09%23FFFFFF,red/K04926%09%23FFF								
http://www.kegg.jp/kegg-bin/show_pathway?map00232/K07409%09%23FFFFFF,red/K00106%09%23FFFFFF,red/								
http://www.kegg.jp/kegg-bin/show_pathway?map00515/K09654%09%23FFFFFF,red/K21032%09%23FFFFFF,red/K17547%09%23FFF								
http://www.kegg.jp/kegg-bin/show_pathway?map04728/K04959%09%23FFFFFF,red/K04379%09%23FFFFFF,red/K04850%09%23FFF								
http://www.kegg.jp/kegg-bin/show_pathway?map04974/K06237%09%23FFFFFF,red/K08135%09%23FFFFFF,red/K24340%09%23FFF								
http://www.kegg.jp/kegg-bin/show_pathway?map04512/K06245%09%23FFFFFF,red/K06237%09%23FFFFFF,red/K06252%09%23FFF								
http://www.kegg.jp/kegg-bin/show_pathway?map04724/K04959%09%23FFFFFF,red/K04603%09%23FFFFFF,red/K04850%09%23FFF								
http://www.kegg.jp/kegg-bin/show_pathway?map04071/K04514%09%23FFFFFF,red/K18050%09%23FFFFFF,red/K05665%09%23FFF								
http://www.kegg.jp/kegg-bin/show_pathway?map00561/K14458%09%23FFFFFF,red/K00901%09%23FFFFFF,red/K13506%09%23FFF								
http://www.kegg.jp/kegg-bin/show_pathway?map05132/K02187%09%23FFFFFF,red/K07863%09%23FFFFFF,red/K20183%09%23FFF								
http://www.kegg.jp/kegg-bin/show_pathway?map04713/K04379%09%23FFFFFF,red/K05200%09%23FFFFFF,red/K05210%09%23FFF								
http://www.kegg.jp/kegg-bin/show_pathway?map04930/K18050%09%23FFFFFF,red/K12407%09%23FFFFFF,red/K07203%09%23FFF								
http://www.kegg.jp/kegg-bin/show_pathway?map04933/K21421%09%23FFFFFF,red/K02187%09%23FFFFFF,red/K06237%09%23FFF								
http://www.kegg.jp/kegg-bin/show_pathway?map04920/K05232%09%23FFFFFF,red/K04447%09%23FFFFFF,red/K06259%09%23FFF								
http://www.kegg.jp/kegg-bin/show_pathway?map05200/K02187%09%23FFFFFF,red/K05099%09%23FFFFFF,red/K04447%09%23FFF								
http://www.kegg.jp/kegg-bin/show_pathway?map04670/K04514%09%23FFFFFF,red/K05859%09%23FFFFFF,red/K06088%09%23FFF								
http://www.kegg.jp/kegg-bin/show_pathway?map00564/K14621%09%23FFFFFF,red/K14676%09%23FFFFFF,red/K00901%09%23FFF								
http://www.kegg.jp/kegg-bin/show_pathway?map04012/K06619%09%23FFFFFF,red/K05456%09%23FFFFFF,red/K05859%09%23FFF								
http://www.kegg.jp/kegg-bin/show_pathway?map04926/K06237%09%23FFFFFF,red/K13375%09%23FFFFFF,red/K04379%09%23FFF								
http://www.kegg.jp/kegg-bin/show_pathway?map04919/K17903%09%23FFFFFF,red/K04503%09%23FFFFFF,red/K04377%09%23FFF								
http://www.kegg.jp/kegg-bin/show_pathway?map04724/K04959%09%23FFFFFF,red/K04603%09%23FFFFFF,red/K05858%09%23FFF								
http://www.kegg.jp/kegg-bin/show_pathway?map05206/K06619%09%23FFFFFF,red/K05099%09%23FFFFFF,red/K18050%09%23FFF								
http://www.kegg.jp/kegg-bin/show_pathway?map03018/K12580%09%23FFFFFF,red/K12590%09%23FFFFFF,red/K12610%09%23FFF								
http://www.kegg.jp/kegg-bin/show_pathway?map00230/K12320%09%23FFFFFF,red/K13293%09%23FFFFFF,red/K01081%09%23FFF								
http://www.kegg.jp/kegg-bin/show_pathway?map04072/K05461%09%23FFFFFF,red/K04603%09%23FFFFFF,red/K07941%09%23FFF								
http://www.kegg.jp/kegg-bin/show_pathway?map03450/K10887%09%23FFFFFF,red/K06642%09%23FFFFFF,red/K10866%09%23FFF								
http://www.kegg.jp/kegg-bin/show_pathway?map05230/K04363%09%23FFFFFF,red/K05099%09%23FFFFFF,red/K04377%09%23FFF								

http://www.kegg.jp/kegg-bin/show_pathway?map05235/K04447%09%23FFFFFF,red/K10785%09%23FFFFFF,red/K10159%09%23FFF	
http://www.kegg.jp/kegg-bin/show_pathway?map05220/K06619%09%23FFFFFF,red/K14021%09%23FFFFFF,red/K04503%09%23FFF	
http://www.kegg.jp/kegg-bin/show_pathway?map04510/K05099%09%23FFFFFF,red/K05096%09%23FFFFFF,red/K04503%09%23FFF	
http://www.kegg.jp/kegg-bin/show_pathway?map05215/K04363%09%23FFFFFF,red/K04503%09%23FFFFFF,red/K09435%09%23FFF	
http://www.kegg.jp/kegg-bin/show_pathway?map00232/K07409%09%23FFFFFF,red/K00106%09%23FFFFFF,red/	
http://www.kegg.jp/kegg-bin/show_pathway?map00785/K23756%09%23FFFFFF,red/K23735%09%23FFFFFF,red/	

FFF,red/K05859%09%23FFFFFF,red/K05457%09%23FFFFFF,red/K05085%09%23FFFFFF,red/K07203%09%23FFFFFF,red/K05733%0
FFF,red/K07756%09%23FFFFFF,red/K18082%09%23FFFFFF,red/K15759%09%23FFFFFF,red/K00920%09%23FFFFFF,red/K05858%0
FFF,red/K05205%09%23FFFFFF,red/K04958%09%23FFFFFF,red/K08049%09%23FFFFFF,red/K05197%09%23FFFFFF,red/K04609%0
FFF,red/K07203%09%23FFFFFF,red/K05858%09%23FFFFFF,red/K18441%09%23FFFFFF,red/K04351%09%23FFFFFF,red/K08049%0
FFF,red/K14209%09%23FFFFFF,red/K01539%09%23FFFFFF,red/K04926%09%23FFFFFF,red/K09708%09%23FFFFFF,red/K19721%0
FFF,red/K05456%09%23FFFFFF,red/K05859%09%23FFFFFF,red/K05460%09%23FFFFFF,red/K05089%09%23FFFFFF,red/K07203%0
FFF,red/K05099%09%23FFFFFF,red/K05859%09%23FFFFFF,red/K07203%09%23FFFFFF,red/K00444%09%23FFFFFF,red/K03511%0
FFF,red/K05859%09%23FFFFFF,red/K05460%09%23FFFFFF,red/K03511%09%23FFFFFF,red/K08528%09%23FFFFFF,red/K04364%0
FFF,red/K01081%09%23FFFFFF,red/K11265%09%23FFFFFF,red/K13755%09%23FFFFFF,red/K00940%09%23FFFFFF,red/K07127%0
FFF,red/K04468%09%23FFFFFF,red/K05858%09%23FFFFFF,red/K00444%09%23FFFFFF,red/K23097%09%23FFFFFF,red/K04493%0
FFF,red/K04958%09%23FFFFFF,red/K04515%09%23FFFFFF,red/K04145%09%23FFFFFF,red/K05197%09%23FFFFFF,red/K04348%0
FFF,red/K04447%09%23FFFFFF,red/K07204%09%23FFFFFF,red/K05085%09%23FFFFFF,red/K13302%09%23FFFFFF,red/K04360%0
FFF,red/K04447%09%23FFFFFF,red/K09095%09%23FFFFFF,red/K05858%09%23FFFFFF,red/K03511%09%23FFFFFF,red/K06230%0
FFF,red/K07203%09%23FFFFFF,red/K05858%09%23FFFFFF,red/K11265%09%23FFFFFF,red/K04218%09%23FFFFFF,red/K04958%0

FFF,red/K05859%09%23FFFFFF,red/K18050%09%23FFFFFF,red/K07204%09%23FFFFFF,red/K07203%09%23FFFFFF,red/K17442%0

FFF,red/K00968%09%23FFFFFF,red/K01080%09%23FFFFFF,red/K07203%09%23FFFFFF,red/K04440%09%23FFFFFF,red/K04364%0

FFF,red/K07203%09%23FFFFFF,red/K00444%09%23FFFFFF,red/K03511%09%23FFFFFF,red/K11126%09%23FFFFFF,red/K08524%0

FFF,red/K04920%09%23FFFFFF,red/K05858%09%23FFFFFF,red/K04218%09%23FFFFFF,red/K04131%09%23FFFFFF,red/K05031%0

FFF,red/K04546%09%23FFFFFF,red/K04354%09%23FFFFFF,red/K04515%09%23FFFFFF,red/K04348%09%23FFFFFF,red/K00274%0

FFF,red/K04926%09%23FFFFFF,red/K09708%09%23FFFFFF,red/K24356%09%23FFFFFF,red/K19721%09%23FFFFFF,red/K06002%0

FFF,red/K06259%09%23FFFFFF,red/K06255%09%23FFFFFF,red/K23328%09%23FFFFFF,red/K06258%09%23FFFFFF,red/K06249%0

FFF,red/K04546%09%23FFFFFF,red/K04609%09%23FFFFFF,red/K04348%09%23FFFFFF,red/K04549%09%23FFFFFF,red/K04610%0

FFF,red/K04354%09%23FFFFFF,red/K00922%09%23FFFFFF,red/K04714%09%23FFFFFF,red/K03898%09%23FFFFFF,red/K04456%0

FFF,red/K13509%09%23FFFFFF,red/K22283%09%23FFFFFF,red/K14457%09%23FFFFFF,red/K00863%09%23FFFFFF,red/K18693%0

FFF,red/K21954%09%23FFFFFF,red/K18441%09%23FFFFFF,red/K23612%09%23FFFFFF,red/K23934%09%23FFFFFF,red/K14021%0

FFF,red/K05209%09%23FFFFFF,red/K04850%09%23FFFFFF,red/K11265%09%23FFFFFF,red/K07376%09%23FFFFFF,red/K04546%0

FFF,red/K04850%09%23FFFFFF,red/K04440%09%23FFFFFF,red/K00922%09%23FFFFFF,red/K07594%09%23FFFFFF,red/K04696%0

FFF,red/K13375%09%23FFFFFF,red/K05859%09%23FFFFFF,red/K18050%09%23FFFFFF,red/K04447%09%23FFFFFF,red/K05449%0

FFF,red/K01897%09%23FFFFFF,red/K07297%09%23FFFFFF,red/K07203%09%23FFFFFF,red/K04440%09%23FFFFFF,red/K07202%0

FFF,red/K01398%09%23FFFFFF,red/K06230%09%23FFFFFF,red/K04546%09%23FFFFFF,red/K09290%09%23FFFFFF,red/K00799%0

FFF,red/K01398%09%23FFFFFF,red/K06087%09%23FFFFFF,red/K00922%09%23FFFFFF,red/K08016%09%23FFFFFF,red/K05763%0

FFF,red/K00995%09%23FFFFFF,red/K13510%09%23FFFFFF,red/K13506%09%23FFFFFF,red/K13509%09%23FFFFFF,red/K06124%0

FFF,red/K05457%09%23FFFFFF,red/K07203%09%23FFFFFF,red/K05085%09%23FFFFFF,red/K04688%09%23FFFFFF,red/K04440%0

FFF,red/K05449%09%23FFFFFF,red/K04440%09%23FFFFFF,red/K01398%09%23FFFFFF,red/K04546%09%23FFFFFF,red/K00922%0

FFF,red/K15162%09%23FFFFFF,red/K06067%09%23FFFFFF,red/K15159%09%23FFFFFF,red/K07203%09%23FFFFFF,red/K05858%0

FFF,red/K05213%09%23FFFFFF,red/K05204%09%23FFFFFF,red/K05197%09%23FFFFFF,red/K04609%09%23FFFFFF,red/K04348%0

FFF,red/K04503%09%23FFFFFF,red/K04377%09%23FFFFFF,red/K06067%09%23FFFFFF,red/K02580%09%23FFFFFF,red/K17460%0

FFF,red/K12591%09%23FFFFFF,red/K12614%09%23FFFFFF,red/K12586%09%23FFFFFF,red/K18681%09%23FFFFFF,red/K12597%0

FFF,red/K18436%09%23FFFFFF,red/K11265%09%23FFFFFF,red/K13755%09%23FFFFFF,red/K07127%09%23FFFFFF,red/K18424%0

FFF,red/K07203%09%23FFFFFF,red/K05858%09%23FFFFFF,red/K18441%09%23FFFFFF,red/K04609%09%23FFFFFF,red/K05450%0

FFF,red/K05091%09%23FFFFFF,red/K12407%09%23FFFFFF,red/K07827%09%23FFFFFF,red/K07203%09%23FFFFFF,red/K05101%0

FFF,red/K05119%09%23FFFFFF,red/K02580%09%23FFFFFF,red/K07827%09%23FFFFFF,red/K07203%09%23FFFFFF,red/K04467%0

FFF,red/K04377%09%23FFFFFF,red/K04462%09%23FFFFFF,red/K06067%09%23FFFFFF,red/K02580%09%23FFFFFF,red/K06618%0

FFF,red/K12329%09%23FFFFFF,red/K06249%09%23FFFFFF,red/K05733%09%23FFFFFF,red/K05450%09%23FFFFFF,red/K06483%0

FFF,red/K02580%09%23FFFFFF,red/K06618%09%23FFFFFF,red/K12344%09%23FFFFFF,red/K07827%09%23FFFFFF,red/K07203%0

9%23FFFFFF,red/K04515%09%23FFFFFF,red/K02677%09%23FFFFFF,red/K04456%09%23FFFFFF,red/K04438%09%23FFFFFF,red/

9%23FFFFFF,red/K04958%09%23FFFFFF,red/K02677%09%23FFFFFF,red/K00901%09%23FFFFFF,red/K18081%09%23FFFFFF,red/

9%23FFFFFF,red/K04348%09%23FFFFFF,red/K02677%09%23FFFFFF,red/K04610%09%23FFFFFF,red/K04549%09%23FFFFFF,red/

9%23FFFFFF,red/K04609%09%23FFFFFF,red/K02677%09%23FFFFFF,red/K13523%09%23FFFFFF,red/K04610%09%23FFFFFF,red/

9%23FFFFFF,red/K05334%09%23FFFFFF,red/K19720%09%23FFFFFF,red/K05849%09%23FFFFFF,red/K06002%09%23FFFFFF,red/

9%23FFFFFF,red/K16341%09%23FFFFFF,red/K11217%09%23FFFFFF,red/K04364%09%23FFFFFF,red/K05083%09%23FFFFFF,red/

9%23FFFFFF,red/K11757%09%23FFFFFF,red/K11126%09%23FFFFFF,red/K00799%09%23FFFFFF,red/K00714%09%23FFFFFF,red/

9%23FFFFFF,red/K08524%09%23FFFFFF,red/K05083%09%23FFFFFF,red/K02677%09%23FFFFFF,red/K03099%09%23FFFFFF,red/

9%23FFFFFF,red/K08049%09%23FFFFFF,red/K00106%09%23FFFFFF,red/K01514%09%23FFFFFF,red/K01522%09%23FFFFFF,red/

9%23FFFFFF,red/K00714%09%23FFFFFF,red/K04515%09%23FFFFFF,red/K04348%09%23FFFFFF,red/K02166%09%23FFFFFF,red/

9%23FFFFFF,red/K09047%09%23FFFFFF,red/K02677%09%23FFFFFF,red/K00274%09%23FFFFFF,red/K04456%09%23FFFFFF,red/

9%23FFFFFF,red/K11217%09%23FFFFFF,red/K05431%09%23FFFFFF,red/K06249%09%23FFFFFF,red/K05414%09%23FFFFFF,red/

9%23FFFFFF,red/K11126%09%23FFFFFF,red/K11217%09%23FFFFFF,red/K05431%09%23FFFFFF,red/K00799%09%23FFFFFF,red/

9%23FFFFFF,red/K08049%09%23FFFFFF,red/K02677%09%23FFFFFF,red/K09047%09%23FFFFFF,red/K04456%09%23FFFFFF,red/

9%23FFFFFF,red/K05462%09%23FFFFFF,red/K23560%09%23FFFFFF,red/K15379%09%23FFFFFF,red/K02677%09%23FFFFFF,red/								
9%23FFFFFF,red/K01115%09%23FFFFFF,red/K02677%09%23FFFFFF,red/K08200%09%23FFFFFF,red/K04456%09%23FFFFFF,red/								
9%23FFFFFF,red/K00714%09%23FFFFFF,red/K04456%09%23FFFFFF,red/K05691%09%23FFFFFF,red/K05460%09%23FFFFFF,red/								
9%23FFFFFF,red/K04958%09%23FFFFFF,red/K08049%09%23FFFFFF,red/K04515%09%23FFFFFF,red/K02677%09%23FFFFFF,red/								
9%23FFFFFF,red/K09047%09%23FFFFFF,red/K04456%09%23FFFFFF,red/K04549%09%23FFFFFF,red/K05200%09%23FFFFFF,red/								
9%23FFFFFF,red/K06238%09%23FFFFFF,red/K16617%09%23FFFFFF,red/K14206%09%23FFFFFF,red/K24358%09%23FFFFFF,red/								
9%23FFFFFF,red/K05637%09%23FFFFFF,red/K06587%09%23FFFFFF,red/K06238%09%23FFFFFF,red/K06584%09%23FFFFFF,red/								
9%23FFFFFF,red/K05200%09%23FFFFFF,red/K15010%09%23FFFFFF,red/K05210%09%23FFFFFF,red/K05209%09%23FFFFFF,red/								
9%23FFFFFF,red/K04268%09%23FFFFFF,red/K12348%09%23FFFFFF,red/K23727%09%23FFFFFF,red/K04440%09%23FFFFFF,red/								
9%23FFFFFF,red/K12754%09%23FFFFFF,red/K08727%09%23FFFFFF,red/K12366%09%23FFFFFF,red/K04440%09%23FFFFFF,red/								
9%23FFFFFF,red/K04856%09%23FFFFFF,red/K04515%09%23FFFFFF,red/K05262%09%23FFFFFF,red/K13240%09%23FFFFFF,red/								
9%23FFFFFF,red/K04440%09%23FFFFFF,red/K01398%09%23FFFFFF,red/K00922%09%23FFFFFF,red/K04441%09%23FFFFFF,red/								
9%23FFFFFF,red/K05637%09%23FFFFFF,red/K05414%09%23FFFFFF,red/K03898%09%23FFFFFF,red/K13375%09%23FFFFFF,red/								
9%23FFFFFF,red/K21421%09%23FFFFFF,red/K05691%09%23FFFFFF,red/K12754%09%23FFFFFF,red/K12757%09%23FFFFFF,red/								
9%23FFFFFF,red/K18693%09%23FFFFFF,red/K00111%09%23FFFFFF,red/K01115%09%23FFFFFF,red/K13516%09%23FFFFFF,red/								
9%23FFFFFF,red/K05733%09%23FFFFFF,red/K00922%09%23FFFFFF,red/K04515%09%23FFFFFF,red/K04365%09%23FFFFFF,red/								
9%23FFFFFF,red/K04441%09%23FFFFFF,red/K13240%09%23FFFFFF,red/K09047%09%23FFFFFF,red/K04456%09%23FFFFFF,red/								
9%23FFFFFF,red/K15156%09%23FFFFFF,red/K07201%09%23FFFFFF,red/K08550%09%23FFFFFF,red/K09101%09%23FFFFFF,red/								
9%23FFFFFF,red/K04610%09%23FFFFFF,red/K04549%09%23FFFFFF,red/K05200%09%23FFFFFF,red/K15010%09%23FFFFFF,red/								
9%23FFFFFF,red/K07203%09%23FFFFFF,red/K06626%09%23FFFFFF,red/K00558%09%23FFFFFF,red/K10127%09%23FFFFFF,red/								
9%23FFFFFF,red/K12581%09%23FFFFFF,red/K12603%09%23FFFFFF,red/K12600%09%23FFFFFF,red/K00850%09%23FFFFFF,red/								
9%23FFFFFF,red/K13758%09%23FFFFFF,red/K00856%09%23FFFFFF,red/K00106%09%23FFFFFF,red/K01522%09%23FFFFFF,red/								
9%23FFFFFF,red/K04610%09%23FFFFFF,red/K04456%09%23FFFFFF,red/K04363%09%23FFFFFF,red/K21289%09%23FFFFFF,red/								
9%23FFFFFF,red/K00016%09%23FFFFFF,red/K00850%09%23FFFFFF,red/K07299%09%23FFFFFF,red/K04456%09%23FFFFFF,red/								

09%23FFFFFF,red/K06453%09%23FFFFFF,red/K04692%09%23FFFFFF,red/K03115%09%23FFFFFF,red/K04441%09%23FFFFFF,red/K

09%23FFFFFF,red/K07827%09%23FFFFFF,red/K17449%09%23FFFFFF,red/K08367%09%23FFFFFF,red/K04467%09%23FFFFFF,red/K

09%23FFFFFF,red/K04456%09%23FFFFFF,red/K12756%09%23FFFFFF,red/K06270%09%23FFFFFF,red/K05717%09%23FFFFFF,red/K

09%23FFFFFF,red/K04467%09%23FFFFFF,red/K06626%09%23FFFFFF,red/K05450%09%23FFFFFF,red/K07201%09%23FFFFFF,red/K

K05202%09%23FFFFFF,red/K05200%09%23FFFFFF,red/K15010%09%23FFFFFF,red/K15009%09%23FFFFFF,red/K04604%09%23FF

K04456%09%23FFFFFF,red/K05703%09%23FFFFFF,red/K00901%09%23FFFFFF,red/K13509%09%23FFFFFF,red/K05089%09%23FF

K02677%09%23FFFFFF,red/K04456%09%23FFFFFF,red/K11653%09%23FFFFFF,red/K05460%09%23FFFFFF,red/K04490%09%23FF

K13762%09%23FFFFFF,red/K00364%09%23FFFFFF,red/K01951%09%23FFFFFF,red/K07023%09%23FFFFFF,red/K18437%09%23FF

K02677%09%23FFFFFF,red/K23491%09%23FFFFFF,red/K04507%09%23FFFFFF,red/K04309%09%23FFFFFF,red/K04497%09%23FF

K04549%09%23FFFFFF,red/K05200%09%23FFFFFF,red/K04440%09%23FFFFFF,red/K11584%09%23FFFFFF,red/K02223%09%23FF

K09047%09%23FFFFFF,red/K06237%09%23FFFFFF,red/K06252%09%23FFFFFF,red/K05080%09%23FFFFFF,red/K23692%09%23FF

K08049%09%23FFFFFF,red/K00714%09%23FFFFFF,red/K05414%09%23FFFFFF,red/K07201%09%23FFFFFF,red/K06237%09%23FF

K05080%09%23FFFFFF,red/K04438%09%23FFFFFF,red/K04440%09%23FFFFFF,red/K04364%09%23FFFFFF,red/K03099%09%23FF

K04438%09%23FFFFFF,red/K06252%09%23FFFFFF,red/K09283%09%23FFFFFF,red/K05089%09%23FFFFFF,red/K07996%09%23FF

K04490%09%23FFFFFF,red/K08528%09%23FFFFFF,red/K02353%09%23FFFFFF,red/K04358%09%23FFFFFF,red/K04364%09%23FF

K05210%09%23FFFFFF,red/K04146%09%23FFFFFF,red/K05209%09%23FFFFFF,red/K11583%09%23FFFFFF,red/K04440%09%23FF

K15009%09%23FFFFFF,red/K00910%09%23FFFFFF,red/K01115%09%23FFFFFF,red/K05201%09%23FFFFFF,red/K15008%09%23FF

K11583%09%23FFFFFF,red/K17388%09%23FFFFFF,red/K01115%09%23FFFFFF,red/K01441%09%23FFFFFF,red/K04441%09%23FF

K04437%09%23FFFFFF,red/K17388%09%23FFFFFF,red/K10396%09%23FFFFFF,red/K03173%09%23FFFFFF,red/K16060%09%23FF

K06237%09%23FFFFFF,red/K09455%09%23FFFFFF,red/K14021%09%23FFFFFF,red/K05691%09%23FFFFFF,red/K04498%09%23FF

K01540%09%23FFFFFF,red/K04456%09%23FFFFFF,red/K05853%09%23FFFFFF,red/K15133%09%23FFFFFF,red/K07827%09%23FF

K01915%09%23FFFFFF,red/K15009%09%23FFFFFF,red/K04604%09%23FFFFFF,red/K01115%09%23FFFFFF,red/K05201%09%23FF

K04692%09%23FFFFFF,red/K17461%09%23FFFFFF,red/K04363%09%23FFFFFF,red/K14021%09%23FFFFFF,red/K06252%09%23FF

K13761%09%23FFFFFF,red/K12318%09%23FFFFFF,red/K00364%09%23FFFFFF,red/K01951%09%23FFFFFF,red/K19021%09%23FF

K00901%09%23FFFFFF,red/K05091%09%23FFFFFF,red/K13509%09%23FFFFFF,red/K07827%09%23FFFFFF,red/K17449%09%23FF

[illegible]04363%09%23FFFFFF,red/K12757%09%23FFFFFF,red/K12754%09%23FFFFFF,red/K06252%09%23FFFFFF,red/K17449%09%23FF

'FFFF,red/K01080%09%23FFFFFF,red/K21290%09%23FFFFFF,red/K04364%09%23FFFFFF,red/K04604%09%23FFFFFF,red/K01115%

'FFFF,red/K02353%09%23FFFFFF,red/K04364%09%23FFFFFF,red/K22198%09%23FFFFFF,red/K00355%09%23FFFFFF,red/K03099%

'FFFF,red/K19882%09%23FFFFFF,red/K04440%09%23FFFFFF,red/K04490%09%23FFFFFF,red/K02353%09%23FFFFFF,red/K04512%

'FFFF,red/K04355%09%23FFFFFF,red/K11584%09%23FFFFFF,red/K04358%09%23FFFFFF,red/K05083%09%23FFFFFF,red/K03099%

'FFFF,red/K05691%09%23FFFFFF,red/K09455%09%23FFFFFF,red/K10055%09%23FFFFFF,red/K09291%09%23FFFFFF,red/K04440%

'FFFF,red/K16341%09%23FFFFFF,red/K04364%09%23FFFFFF,red/K23582%09%23FFFFFF,red/K04728%09%23FFFFFF,red/K05083%

'FFFF,red/K10396%09%23FFFFFF,red/K04441%09%23FFFFFF,red/K04548%09%23FFFFFF,red/K04833%09%23FFFFFF,red/

'FFFF,red/K17260%09%23FFFFFF,red/K12800%09%23FFFFFF,red/K04379%09%23FFFFFF,red/K07375%09%23FFFFFF,red/K07195%

'FFFF,red/K09291%09%23FFFFFF,red/K04440%09%23FFFFFF,red/K17388%09%23FFFFFF,red/K01115%09%23FFFFFF,red/K10053%

'FFFF,red/K09283%09%23FFFFFF,red/K07827%09%23FFFFFF,red/K16341%09%23FFFFFF,red/K17449%09%23FFFFFF,red/K23582%

'FFFF,red/K04262%09%23FFFFFF,red/K21290%09%23FFFFFF,red/K04604%09%23FFFFFF,red/K01115%09%23FFFFFF,red/

FFFF,red/K00907%09%23FFFFFF,red/K06238%09%23FFFFFF,red/K05636%09%23FFFFFF,red/K06584%09%23FFFFFF,red/K06271%

%09%23FFFFFF,red/K06250%09%23FFFFFF,red/K08774%09%23FFFFFF,red/K07203%09%23FFFFFF,red/K05462%09%23FFFFFF,red

%09%23FFFFFF,red/K02353%09%23FFFFFF,red/K04358%09%23FFFFFF,red/K01115%09%23FFFFFF,red/K05083%09%23FFFFFF,red

%09%23FFFFFF,red/K00923%09%23FFFFFF,red/K13883%09%23FFFFFF,red/K05733%09%23FFFFFF,red/K15348%09%23FFFFFF,red

%09%23FFFFFF,red/K03173%09%23FFFFFF,red/K04365%09%23FFFFFF,red/K04548%09%23FFFFFF,red/K06245%09%23FFFFFF,red

l/K04354%09%23FFFFFF,red/K08524%09%23FFFFFF,red/K02677%09%23FFFFFF,red/K04549%09%23FFFFFF,red/K04456%09%23F

l/K00355%09%23FFFFFF,red/K03099%09%23FFFFFF,red/K08774%09%23FFFFFF,red/K06619%09%23FFFFFF,red/K04514%09%23F

l/K10417%09%23FFFFFF,red/K00922%09%23FFFFFF,red/K04456%09%23FFFFFF,red/K12757%09%23FFFFFF,red/K04490%09%23F

l/K06619%09%23FFFFFF,red/K16060%09%23FFFFFF,red/K04514%09%23FFFFFF,red/K05859%09%23FFFFFF,red/K04379%09%23F

FFFFF,red/K07198%09%23FFFFFF,red/K05631%09%23FFFFFF,red/K05460%09%23FFFFFF,red/K05089%09%23FFFFFF,red/K0541[illegible]

FFFFF,red/K05859%09%23FFFFFF,red/K06051%09%23FFFFFF,red/K21635%09%23FFFFFF,red/K07203%09%23FFFFFF,red/K0044[illegible]

FFFFFF,red/K03176%09%23FFFFFF,red/K21635%09%23FFFFFF,red/K07203%09%23FFFFFF,red/K00444%09%23FFFFFF,red/K0435[illegible]

5%09%23FFFFFF,red/K05465%09%23FFFFFF,red/K16341%09%23FFFFFF,red/K21290%09%23FFFFFF,red/K04364%09%23FFFFFF,r
4%09%23FFFFFF,red/K08524%09%23FFFFFF,red/K04515%09%23FFFFFF,red/K02677%09%23FFFFFF,red/K04456%09%23FFFFFF,r

)%09%23FFFFFF,red/K04688%09%23FFFFFF,red/K00922%09%23FFFFFF,red/K05437%09%23FFFFFF,red/K04515%09%23FFFFFF,r

ed/K04549%09%23FFFFFF,red/K04438%09%23FFFFFF,red/K04462%0

red/K04456%09%23FFFFFF,red/K04549%09%23FFFFFF,red/K09091%0
