# Supplementary material for: Genetic diversity and selection of Tibetan sheep breeds revealed by whole-genome resequencing
Source: Anim Biosci. 2023 May 2;36(7):991–1002. doi: 10.5713/ab.22.0432 (PMC10330983; doi:10.5713/ab.22.0432)
Supplement: Supplementary file 14 [file ab-22-0432-Supplementary-Table-14.pdf]

Supplementary Table 14. KEGG analysis of putative selected genes compared with HZ breed

BD vs HZ

PathwayID	Pathway	Level1	Level2
oas04360	Axon guidance	Organismal Systems	Development and regeneration
oas04970	Salivary secretion	Organismal Systems	Digestive system
oas04211	Longevity regulating pathway	Organismal Systems	Aging
oas04137	Mitophagy - animal	Cellular Processes	Transport and catabolism
oas04024	cAMP signaling pathway	Environmental Information P	Signal transduction
oas04530	Tight junction	Cellular Processes	Cellular community - eukaryotes
oas04072	Phospholipase D signaling pathway	Environmental Information P	Signal transduction
oas04713	Circadian entrainment	Organismal Systems	Environmental adaptation
oas04261	Adrenergic signaling in cardiomyocytes	Organismal Systems	Circulatory system
oas04066	HIF-1 signaling pathway	Environmental Information P	Signal transduction
oas04911	Insulin secretion	Organismal Systems	Endocrine system
oas04925	Aldosterone synthesis and secretion	Organismal Systems	Endocrine system
oas04750	Inflammatory mediator regulation of TRP channels	Organismal Systems	Sensory system
oas04724	Glutamatergic synapse	Organismal Systems	Nervous system
oas04725	Cholinergic synapse	Organismal Systems	Nervous system

GY vs HZ

PathwayID	Pathway	Level1	Level2
oas04911	Insulin secretion	Organismal Systems	Endocrine system
oas00514	Other types of O-glycan biosynthesis	Metabolism	Glycan biosynthesis and metabolism
oas04970	Salivary secretion	Organismal Systems	Digestive system
oas04020	Calcium signaling pathway	Environmental Information P	Signal transduction
oas04713	Circadian entrainment	Organismal Systems	Environmental adaptation
oas04725	Cholinergic synapse	Organismal Systems	Nervous system
oas04922	Glucagon signaling pathway	Organismal Systems	Endocrine system
oas00512	Mucin type O-glycan biosynthesis	Metabolism	Glycan biosynthesis and metabolism
oas04926	Relaxin signaling pathway	Organismal Systems	Endocrine system
oas04724	Glutamatergic synapse	Organismal Systems	Nervous system
oas04728	Dopaminergic synapse	Organismal Systems	Nervous system

oas04360	Axon guidance	Organismal Systems	Development and regeneration
oas04022	cGMP-PKG signaling pathway	Environmental Information P	Signal transduction
oas04520	Adherens junction	Cellular Processes	Cellular community - eukaryotes
oas03420	Nucleotide excision repair	Genetic Information Processi	Replication and repair
oas04927	Cortisol synthesis and secretion	Organismal Systems	Endocrine system
oas04720	Long-term potentiation	Organismal Systems	Nervous system
oas00230	Purine metabolism	Metabolism	Nucleotide metabolism
oas04371	Apelin signaling pathway	Environmental Information P	Signal transduction
oas04727	GABAergic synapse	Organismal Systems	Nervous system
oas04925	Aldosterone synthesis and secretion	Organismal Systems	Endocrine system
oas04723	Retrograde endocannabinoid signaling	Organismal Systems	Nervous system
oas04512	ECM-receptor interaction	Environmental Information P	Signaling molecules and interaction
oas04912	GnRH signaling pathway	Organismal Systems	Endocrine system
oas04916	Melanogenesis	Organismal Systems	Endocrine system
oas04621	NOD-like receptor signaling pathway	Organismal Systems	Immune system
oas00330	Arginine and proline metabolism	Metabolism	Amino acid metabolism
oas04024	cAMP signaling pathway	Environmental Information P	Signal transduction
oas04062	Chemokine signaling pathway	Organismal Systems	Immune system
oas04530	Tight junction	Cellular Processes	Cellular community - eukaryotes
oas04261	Adrenergic signaling in cardiomyocytes	Organismal Systems	Circulatory system
oas04611	Platelet activation	Organismal Systems	Immune system
oas04921	Oxytocin signaling pathway	Organismal Systems	Endocrine system
oas04072	Phospholipase D signaling pathway	Environmental Information P	Signal transduction
oas04810	Regulation of actin cytoskeleton	Cellular Processes	Cell motility
oas04915	Estrogen signaling pathway	Organismal Systems	Endocrine system
oas00511	Other glycan degradation	Metabolism	Glycan biosynthesis and metabolism
oas03460	Fanconi anemia pathway	Genetic Information Processi	Replication and repair
oas04971	Gastric acid secretion	Organismal Systems	Digestive system
oas04310	Wnt signaling pathway	Environmental Information P	Signal transduction
oas04914	Progesterone-mediated oocyte maturation	Organismal Systems	Endocrine system
ZK vs HZ			

oas04925	Aldosterone synthesis and secretion	Organismal Systems	Endocrine system
oas04927	Cortisol synthesis and secretion	Organismal Systems	Endocrine system
oas04010	MAPK signaling pathway	Environmental Information P	Signal transduction
oas04934	Cushing syndrome	Human Diseases	Endocrine and metabolic disease
oas05412	Arrhythmogenic right ventricular cardiomyopathy	Human Diseases	Cardiovascular disease
oas05205	Proteoglycans in cancer	Human Diseases	Cancer: overview
oas04970	Salivary secretion	Organismal Systems	Digestive system
oas04072	Phospholipase D signaling pathway	Environmental Information P	Signal transduction
oas04512	ECM-receptor interaction	Environmental Information P	Signaling molecules and interaction
oas04725	Cholinergic synapse	Organismal Systems	Nervous system
oas04912	GnRH signaling pathway	Organismal Systems	Endocrine system
oas00512	Mucin type O-glycan biosynthesis	Metabolism	Glycan biosynthesis and metabolism
oas05031	Amphetamine addiction	Human Diseases	Substance dependence
oas04020	Calcium signaling pathway	Environmental Information P	Signal transduction
OL vs HZ			
oas04360	Axon guidance	Organismal Systems	Development and regeneration
oas04725	Cholinergic synapse	Organismal Systems	Nervous system
oas04520	Adherens junction	Cellular Processes	Cellular community - eukaryotes
oas04926	Relaxin signaling pathway	Organismal Systems	Endocrine system
oas04020	Calcium signaling pathway	Environmental Information P	Signal transduction
oas04727	GABAergic synapse	Organismal Systems	Nervous system
oas04929	GnRH secretion	Organismal Systems	Endocrine system
oas04724	Glutamatergic synapse	Organismal Systems	Nervous system
oas04713	Circadian entrainment	Organismal Systems	Environmental adaptation
oas04072	Phospholipase D signaling pathway	Environmental Information P	Signal transduction
oas04935	Growth hormone synthesis, secretion and action	Organismal Systems	Endocrine system
oas04726	Serotonergic synapse	Organismal Systems	Nervous system
oas04062	Chemokine signaling pathway	Organismal Systems	Immune system
oas04810	Regulation of actin cytoskeleton	Cellular Processes	Cell motility
oas00604	Glycosphingolipid biosynthesis - ganglio series	Metabolism	Glycan biosynthesis and metabolism
oas04080	Neuroactive ligand-receptor interaction	Environmental Information P	Signaling molecules and interaction

oas00512	Mucin type O-glycan biosynthesis	Metabolism	Glycan biosynthesis and metabolism
oas04371	Apelin signaling pathway	Environmental Information P	Signal transduction
oas04010	MAPK signaling pathway	Environmental Information P	Signal transduction
oas04723	Retrograde endocannabinoid signaling	Organismal Systems	Nervous system
oas04022	cGMP-PKG signaling pathway	Environmental Information P	Signal transduction
oas00514	Other types of O-glycan biosynthesis	Metabolism	Glycan biosynthesis and metabolism
oas04611	Platelet activation	Organismal Systems	Immune system
oas04540	Gap junction	Cellular Processes	Cellular community - eukaryotes
oas04024	cAMP signaling pathway	Environmental Information P	Signal transduction
oas04141	Protein processing in endoplasmic reticulum	Genetic Information Processi	Folding, sorting and degradation
oas04270	Vascular smooth muscle contraction	Organismal Systems	Circulatory system
oas04927	Cortisol synthesis and secretion	Organismal Systems	Endocrine system
oas04012	ErbB signaling pathway	Environmental Information P	Signal transduction
oas04068	FoxO signaling pathway	Environmental Information P	Signal transduction
oas00100	Steroid biosynthesis	Metabolism	Lipid metabolism
oas00785	Lipoic acid metabolism	Metabolism	Metabolism of cofactors and vitamins
oas00230	Purine metabolism	Metabolism	Nucleotide metabolism
oas04911	Insulin secretion	Organismal Systems	Endocrine system
oas04925	Aldosterone synthesis and secretion	Organismal Systems	Endocrine system
oas04928	Parathyroid hormone synthesis, secretion and actio	Organismal Systems	Endocrine system

list_number	total_number	Pvalue	FDR	List
27	180	0.002635829	0.597287098	EPHB1(EPHB1
15	89	0.007710027	0.597287098	LOC10560777
15	90	0.008545734	0.597287098	PRKAB2(PRK
13	74	0.008959032	0.597287098	ATG5(ATG5),
30	226	0.009602686	0.597287098	VAV1(VAV1),
24	175	0.01317495	0.682901578	PRKAB2(PRK
21	150	0.015742921	0.69943549	DGKH(DGKH
14	94	0.027139467	0.752830498	ADCY8(ADCY
20	149	0.027321144	0.752830498	SCN5A(SCN5
16	113	0.029288547	0.752830498	ARNT(ARNT),
13	87	0.031698322	0.752830498	ADCY8(ADCY
14	96	0.031869378	0.752830498	HSD3B1(HSD
15	106	0.034223548	0.752830498	TRPM8(TRPM
15	109	0.042373972	0.752830498	HOMER1(HO
15	109	0.042373972	0.752830498	ADCY8(ADCY

list_number	total_number	Pvalue	FDR	List
19	87	0.0001932	0.022797446	KCNMB4(KC
13	48	0.000209949	0.022797446	GALNT18(GA
19	89	0.000263907	0.022797446	LOC10560777
38	238	0.000294161	0.022797446	PPP3CA(PPP3
19	94	0.000546982	0.029110455	RYR3(RYR3),
21	109	0.000563428	0.029110455	CACNA1F(CA
20	105	0.000876551	0.03367414	PPP3CA(PPP3
10	36	0.000899261	0.03367414	GALNT18(GA
23	129	0.000977636	0.03367414	COL4A6(COL
20	109	0.001418423	0.043971103	PPP3CA(PPP3
22	127	0.001831476	0.051614331	PPP3CA(PPP3

28	180	0.002573068	0.061357781	PPP3CA(PPP3(
26	165	0.002998431	0.066393835	PPP3CA(PPP3(
14	73	0.004641717	0.089933269	NLK(NLK),CT
10	45	0.005407579	0.091121053	ERCC8(ERCC:
13	67	0.005646465	0.091121053	CACNA1F(CA
13	67	0.005646465	0.091121053	PPP3CA(PPP3(
21	131	0.005878778	0.091121053	AK8(AK8),AD
21	133	0.007002243	0.102939959	PRKAB2(PRK.
15	85	0.007696791	0.103739351	CACNA1F(CA
16	96	0.010460213	0.135111079	CAMK1D(CAI
22	147	0.01092729	0.135498401	CACNA1F(CA
15	90	0.012917784	0.154019727	COL4A6(COL4
15	92	0.015648885	0.171958692	SOS2(SOS2),C
16	101	0.016635529	0.171958692	FZD1(FZD1),A
25	180	0.017245368	0.171958692	ERBIN(ERBIN
10	53	0.017293466	0.171958692	L3HYPDH(L3I
30	226	0.017750575	0.171958692	PTGER3(PTGI
26	191	0.019479767	0.182991747	SOS2(SOS2),L
24	175	0.022213506	0.201005999	PRKAB2(PRK.
21	149	0.023662094	0.201005999	SCN5A(SCN5/
18	123	0.024574373	0.201005999	TLN2(TLN2),L
21	150	0.025287852	0.201005999	PPP3CA(PPP3(
21	150	0.025287852	0.201005999	SOS2(SOS2),L
28	218	0.031419045	0.243497596	SOS2(SOS2),F
19	136	0.032801274	0.24689958	SOS2(SOS2),N
5	21	0.033981221	0.24689958	LOC10111784(
9	51	0.034614592	0.24689958	FANCB(FANC
12	76	0.035840262	0.24689958	ADCY9(ADCY
22	168	0.043325625	0.285764759	PPP3CA(PPP3(
13	88	0.047599791	0.293817541	RPS6KA3(RPS

17	96	0.006291595	0.938522456	ATF1(ATF1),C
13	67	0.007369242	0.938522456	CACNA1F(CA
38	292	0.018401964	0.938522456	PPP3CA(PPP3(
23	159	0.019658783	0.938522456	CACNA1F(CA
13	76	0.020586594	0.938522456	CACNA2D1(C
28	207	0.024925557	0.938522456	HPSE2(HPSE2
14	89	0.032345286	0.938522456	LOC10560777(
21	150	0.034316787	0.938522456	KIT(KIT),PDG
14	90	0.035184928	0.938522456	COL4A6(COL4
16	109	0.041084968	0.938522456	CACNA1F(CA
14	92	0.041394575	0.938522456	SOS2(SOS2),C
7	36	0.043107917	0.938522456	GALNTL6(GA
11	68	0.045269458	0.938522456	PPP3CA(PPP3(
30	238	0.047707999	0.938522456	PPP3CA(PPP3(

34	180	2.13E-05	0.006716448	SEMA3C(SEM
21	109	0.000579809	0.046660003	CACNA1A(CA
16	73	0.000592508	0.046660003	NLK(NLK),CT
23	129	0.001006863	0.047097382	COL4A5(COL4
36	238	0.001203106	0.047097382	CACNA1A(CA
17	85	0.001215969	0.047097382	CACNA1A(CA
14	64	0.001303156	0.047097382	GABBR2(GAB
20	109	0.0014563	0.047097382	CACNA1A(CA
18	94	0.001495155	0.047097382	CACNA1I(CA(
25	150	0.001685367	0.04826277	DGKH(DGKH)
21	121	0.00230418	0.05184406	CACNA1F(CA
19	114	0.005724793	0.119436934	CACNA1A(CA
28	191	0.006173306	0.119436934	JAK2(JAK2),G
31	218	0.006445803	0.119436934	FGF5(FGF5),P
5	15	0.007911735	0.124609823	ST8SIA5(ST8S
45	355	0.010470419	0.157056282	PTGER3(PTGE

8	36	0.012479313	0.17091233	GALNTL6(GA[illegible]
20	133	0.014359114	0.188463372	PIK3CG(PIK3[illegible]
37	292	0.019005604	0.239470604	CACNA1A(CA
21	147	0.021102768	0.249089919	CACNA1A(CA
23	165	0.021350564	0.249089919	PIK3CG(PIK3[illegible]
9	48	0.024516043	0.262712213	GALNTL6(GA[illegible]
18	123	0.025052666	0.262712213	TLN2(TLN2),P
14	89	0.025854218	0.262712213	PLCB1(PLCB1
29	226	0.030005747	0.28548838	PTGER3(PTG[illegible]
23	171	0.03094746	0.28548838	UGGT1(UGGT
19	136	0.033435853	0.289256013	PRKCE(PRKC[illegible]
11	67	0.034207191	0.289256013	CACNA1I(CA[illegible]
13	84	0.034894376	0.289256013	ERBB4(ERBB[illegible]
18	130	0.040675033	0.316401891	NLK(NLK),TG
5	22	0.041182468	0.316401891	NSDHL(NSDH
2	4	0.042335756	0.317518171	ACSM1(ACS[illegible]
18	131	0.0433806	0.317788119	LOC10110903[illegible]
13	87	0.044655717	0.319033851	KCNMA1(KC[illegible]
14	96	0.045576264	0.319033851	CACNA1I(CA[illegible]
15	106	0.049484421	0.331650909	CREB3L2(CR[illegible]

URL

http://www.kegg.jp/kegg-bin/show_pathway?map04360/K06619%09%23FFFFFF,red/K24609%09%23FFFFFF,red/K07532%09%23FFFFFF,

http://www.kegg.jp/kegg-bin/show_pathway?map04970/K08042%09%23FFFFFF,red/K12318%09%23FFFFFF,red/K01539%09%23FFFFFF,

http://www.kegg.jp/kegg-bin/show_pathway?map04211/K08042%09%23FFFFFF,red/K07199%09%23FFFFFF,red/K09048%09%23FFFFFF,

http://www.kegg.jp/kegg-bin/show_pathway?map04137/K07830%09%23FFFFFF,red/K09455%09%23FFFFFF,red/K04556%09%23FFFFFF,

http://www.kegg.jp/kegg-bin/show_pathway?map04024/K07830%09%23FFFFFF,red/K04379%09%23FFFFFF,red/K04249%09%23FFFFFF,

http://www.kegg.jp/kegg-bin/show_pathway?map04530/K12076%09%23FFFFFF,red/K17260%09%23FFFFFF,red/K18050%09%23FFFFFF,

http://www.kegg.jp/kegg-bin/show_pathway?map04072/K07830%09%23FFFFFF,red/K05859%09%23FFFFFF,red/K07831%09%23FFFFFF,

http://www.kegg.jp/kegg-bin/show_pathway?map04713/K08042%09%23FFFFFF,red/K05199%09%23FFFFFF,red/K05200%09%23FFFFFF,

http://www.kegg.jp/kegg-bin/show_pathway?map04261/K05858%09%23FFFFFF,red/K04851%09%23FFFFFF,red/K04135%09%23FFFFFF,

http://www.kegg.jp/kegg-bin/show_pathway?map04066/K02991%09%23FFFFFF,red/K05859%09%23FFFFFF,red/K03259%09%23FFFFFF,

http://www.kegg.jp/kegg-bin/show_pathway?map04911/K08042%09%23FFFFFF,red/K09048%09%23FFFFFF,red/K01539%09%23FFFFFF,

http://www.kegg.jp/kegg-bin/show_pathway?map04925/K08042%09%23FFFFFF,red/K06070%09%23FFFFFF,red/K00070%09%23FFFFFF,

http://www.kegg.jp/kegg-bin/show_pathway?map04750/K08042%09%23FFFFFF,red/K07413%09%23FFFFFF,red/K18051%09%23FFFFFF,

http://www.kegg.jp/kegg-bin/show_pathway?map04724/K08042%09%23FFFFFF,red/K05199%09%23FFFFFF,red/K15010%09%23FFFFFF,

http://www.kegg.jp/kegg-bin/show_pathway?map04725/K08042%09%23FFFFFF,red/K09048%09%23FFFFFF,red/K04379%09%23FFFFFF,

URL

http://www.kegg.jp/kegg-bin/show_pathway?map04911/K04853%09%23FFFFFF,red/K04936%09%23FFFFFF,red/K05858%09%23FFFFFF,

http://www.kegg.jp/kegg-bin/show_pathway?map00514/K13676%09%23FFFFFF,red/K18134%09%23FFFFFF,red/K00731%09%23FFFFFF,

http://www.kegg.jp/kegg-bin/show_pathway?map04970/K08042%09%23FFFFFF,red/K08045%09%23FFFFFF,red/K04963%09%23FFFFFF,

http://www.kegg.jp/kegg-bin/show_pathway?map04020/K04853%09%23FFFFFF,red/K04636%09%23FFFFFF,red/K05858%09%23FFFFFF,

http://www.kegg.jp/kegg-bin/show_pathway?map04713/K05000%09%23FFFFFF,red/K05858%09%23FFFFFF,red/K11265%09%23FFFFFF,

http://www.kegg.jp/kegg-bin/show_pathway?map04725/K04447%09%23FFFFFF,red/K05000%09%23FFFFFF,red/K04853%09%23FFFFFF,

http://www.kegg.jp/kegg-bin/show_pathway?map04922/K11262%09%23FFFFFF,red/K00871%09%23FFFFFF,red/K19524%09%23FFFFFF,

http://www.kegg.jp/kegg-bin/show_pathway?map00512/K00731%09%23FFFFFF,red/K00710%09%23FFFFFF,red/

http://www.kegg.jp/kegg-bin/show_pathway?map04926/K22001%09%23FFFFFF,red/K19720%09%23FFFFFF,red/K05858%09%23FFFFFF,

http://www.kegg.jp/kegg-bin/show_pathway?map04724/K14207%09%23FFFFFF,red/K05858%09%23FFFFFF,red/K04546%09%23FFFFFF,

http://www.kegg.jp/kegg-bin/show_pathway?map04728/K05000%09%23FFFFFF,red/K05858%09%23FFFFFF,red/K04546%09%23FFFFFF,

http://www.kegg.jp/kegg-bin/show_pathway?map04360/K06765%09%23FFFFFF,red/K07526%09%23FFFFFF,red/K00444%09%23FFFFFF,

http://www.kegg.jp/kegg-bin/show_pathway?map04022/K18050%09%23FFFFFF,red/K04853%09%23FFFFFF,red/K12335%09%23FFFFFF,

http://www.kegg.jp/kegg-bin/show_pathway?map04520/K04427%09%23FFFFFF,red/K05703%09%23FFFFFF,red/K04371%09%23FFFFFF,

http://www.kegg.jp/kegg-bin/show_pathway?map03420/K10847%09%23FFFFFF,red/K10838%09%23FFFFFF,red/K06634%09%23FFFFFF,

http://www.kegg.jp/kegg-bin/show_pathway?map04927/K08042%09%23FFFFFF,red/K09048%09%23FFFFFF,red/K08045%09%23FFFFFF,

http://www.kegg.jp/kegg-bin/show_pathway?map04720/K04371%09%23FFFFFF,red/K05209%09%23FFFFFF,red/K04373%09%23FFFFFF,

http://www.kegg.jp/kegg-bin/show_pathway?map00230/K01939%09%23FFFFFF,red/K00944%09%23FFFFFF,red/K00939%09%23FFFFFF,

http://www.kegg.jp/kegg-bin/show_pathway?map04371/K18050%09%23FFFFFF,red/K10435%09%23FFFFFF,red/K05858%09%23FFFFFF,

http://www.kegg.jp/kegg-bin/show_pathway?map04727/K08042%09%23FFFFFF,red/K08045%09%23FFFFFF,red/K05000%09%23FFFFFF,

http://www.kegg.jp/kegg-bin/show_pathway?map04925/K08042%09%23FFFFFF,red/K06070%09%23FFFFFF,red/K09048%09%23FFFFFF,

http://www.kegg.jp/kegg-bin/show_pathway?map04723/K03940%09%23FFFFFF,red/K05000%09%23FFFFFF,red/K04853%09%23FFFFFF,

http://www.kegg.jp/kegg-bin/show_pathway?map04512/K23380%09%23FFFFFF,red/K06237%09%23FFFFFF,red/K06264%09%23FFFFFF,

http://www.kegg.jp/kegg-bin/show_pathway?map04912/K08042%09%23FFFFFF,red/K04371%09%23FFFFFF,red/K08045%09%23FFFFFF,

http://www.kegg.jp/kegg-bin/show_pathway?map04916/K08042%09%23FFFFFF,red/K09048%09%23FFFFFF,red/K04371%09%23FFFFFF,

http://www.kegg.jp/kegg-bin/show_pathway?map04621/K04427%09%23FFFFFF,red/K13916%09%23FFFFFF,red/K10435%09%23FFFFFF,

http://www.kegg.jp/kegg-bin/show_pathway?map00330/K00613%09%23FFFFFF,red/K13242%09%23FFFFFF,red/K00472%09%23FFFFFF,

http://www.kegg.jp/kegg-bin/show_pathway?map04024/K04249%09%23FFFFFF,red/K04853%09%23FFFFFF,red/K05730%09%23FFFFFF,

http://www.kegg.jp/kegg-bin/show_pathway?map04062/K16062%09%23FFFFFF,red/K04447%09%23FFFFFF,red/K05730%09%23FFFFFF,

http://www.kegg.jp/kegg-bin/show_pathway?map04530/K10352%09%23FFFFFF,red/K12076%09%23FFFFFF,red/K18050%09%23FFFFFF,

http://www.kegg.jp/kegg-bin/show_pathway?map04261/K04853%09%23FFFFFF,red/K05858%09%23FFFFFF,red/K04374%09%23FFFFFF,

http://www.kegg.jp/kegg-bin/show_pathway?map04611/K06264%09%23FFFFFF,red/K04350%09%23FFFFFF,red/K19720%09%23FFFFFF,

http://www.kegg.jp/kegg-bin/show_pathway?map04921/K05000%09%23FFFFFF,red/K04853%09%23FFFFFF,red/K11987%09%23FFFFFF,

http://www.kegg.jp/kegg-bin/show_pathway?map04072/K07941%09%23FFFFFF,red/K05858%09%23FFFFFF,red/K05871%09%23FFFFFF,

http://www.kegg.jp/kegg-bin/show_pathway?map04810/K10352%09%23FFFFFF,red/K02085%09%23FFFFFF,red/K05730%09%23FFFFFF,

http://www.kegg.jp/kegg-bin/show_pathway?map04915/K05000%09%23FFFFFF,red/K05858%09%23FFFFFF,red/K11255%09%23FFFFFF,

http://www.kegg.jp/kegg-bin/show_pathway?map00511/K12373%09%23FFFFFF,red/K12312%09%23FFFFFF,red/K01206%09%23FFFFFF,

http://www.kegg.jp/kegg-bin/show_pathway?map03460/K03515%09%23FFFFFF,red/K10892%09%23FFFFFF,red/K10888%09%23FFFFFF,

http://www.kegg.jp/kegg-bin/show_pathway?map04971/K08042%09%23FFFFFF,red/K04150%09%23FFFFFF,red/K08045%09%23FFFFFF,

http://www.kegg.jp/kegg-bin/show_pathway?map04310/K04427%09%23FFFFFF,red/K04468%09%23FFFFFF,red/K02085%09%23FFFFFF,

http://www.kegg.jp/kegg-bin/show_pathway?map04914/K08042%09%23FFFFFF,red/K04371%09%23FFFFFF,red/K08045%09%23FFFFFF,

http://www.kegg.jp/kegg-bin/show_pathway?map04925/K04959%09%23FFFFFF,red/K06070%09%23FFFFFF,red/K04853%09%23FFFFFF,

http://www.kegg.jp/kegg-bin/show_pathway?map04927/K08042%09%23FFFFFF,red/K04959%09%23FFFFFF,red/K04684%09%23FFFFFF,

http://www.kegg.jp/kegg-bin/show_pathway?map04010/K02187%09%23FFFFFF,red/K05461%09%23FFFFFF,red/K07830%09%23FFFFFF,

http://www.kegg.jp/kegg-bin/show_pathway?map04934/K04959%09%23FFFFFF,red/K14964%09%23FFFFFF,red/K04853%09%23FFFFFF,

http://www.kegg.jp/kegg-bin/show_pathway?map05412/K12563%09%23FFFFFF,red/K04860%09%23FFFFFF,red/K05691%09%23FFFFFF,

http://www.kegg.jp/kegg-bin/show_pathway?map05205/K07293%09%23FFFFFF,red/K04959%09%23FFFFFF,red/K02187%09%23FFFFFF,

http://www.kegg.jp/kegg-bin/show_pathway?map04970/K08042%09%23FFFFFF,red/K04959%09%23FFFFFF,red/K12318%09%23FFFFFF,

http://www.kegg.jp/kegg-bin/show_pathway?map04072/K07293%09%23FFFFFF,red/K05461%09%23FFFFFF,red/K07830%09%23FFFFFF,

http://www.kegg.jp/kegg-bin/show_pathway?map04512/K06245%09%23FFFFFF,red/K06237%09%23FFFFFF,red/K06252%09%23FFFFFF,

http://www.kegg.jp/kegg-bin/show_pathway?map04725/K04959%09%23FFFFFF,red/K04447%09%23FFFFFF,red/K05000%09%23FFFFFF,

http://www.kegg.jp/kegg-bin/show_pathway?map04912/K08042%09%23FFFFFF,red/K04959%09%23FFFFFF,red/K04853%09%23FFFFFF,

http://www.kegg.jp/kegg-bin/show_pathway?map00512/K00710%09%23FFFFFF,red/K09653%09%23FFFFFF,red/K03373%09%23FFFFFF,

http://www.kegg.jp/kegg-bin/show_pathway?map05031/K04560%09%23FFFFFF,red/K09048%09%23FFFFFF,red/K05209%09%23FFFFFF,

http://www.kegg.jp/kegg-bin/show_pathway?map04020/K04959%09%23FFFFFF,red/K05096%09%23FFFFFF,red/K04853%09%23FFFFFF,

http://www.kegg.jp/kegg-bin/show_pathway?map04360/K06619%09%23FFFFFF,red/K06840%09%23FFFFFF,red/K05766%09%23FFFFFF,

http://www.kegg.jp/kegg-bin/show_pathway?map04725/K04447%09%23FFFFFF,red/K04849%09%23FFFFFF,red/K04928%09%23FFFFFF,

http://www.kegg.jp/kegg-bin/show_pathway?map04520/K05706%09%23FFFFFF,red/K05691%09%23FFFFFF,red/K04371%09%23FFFFFF,

http://www.kegg.jp/kegg-bin/show_pathway?map04926/K17448%09%23FFFFFF,red/K04674%09%23FFFFFF,red/K19720%09%23FFFFFF,

http://www.kegg.jp/kegg-bin/show_pathway?map04020/K10785%09%23FFFFFF,red/K04853%09%23FFFFFF,red/K05085%09%23FFFFFF,

http://www.kegg.jp/kegg-bin/show_pathway?map04727/K04849%09%23FFFFFF,red/K04853%09%23FFFFFF,red/K08041%09%23FFFFFF,

http://www.kegg.jp/kegg-bin/show_pathway?map04929/K04954%09%23FFFFFF,red/K04371%09%23FFFFFF,red/K04853%09%23FFFFFF,

http://www.kegg.jp/kegg-bin/show_pathway?map04724/K16342%09%23FFFFFF,red/K05858%09%23FFFFFF,red/K05213%09%23FFFFFF,

http://www.kegg.jp/kegg-bin/show_pathway?map04713/K05858%09%23FFFFFF,red/K08041%09%23FFFFFF,red/K11265%09%23FFFFFF,

http://www.kegg.jp/kegg-bin/show_pathway?map04072/K17386%09%23FFFFFF,red/K17448%09%23FFFFFF,red/K07830%09%23FFFFFF,

http://www.kegg.jp/kegg-bin/show_pathway?map04935/K17448%09%23FFFFFF,red/K04447%09%23FFFFFF,red/K04853%09%23FFFFFF,

http://www.kegg.jp/kegg-bin/show_pathway?map04726/K07413%09%23FFFFFF,red/K02187%09%23FFFFFF,red/K04849%09%23FFFFFF,

http://www.kegg.jp/kegg-bin/show_pathway?map04062/K17448%09%23FFFFFF,red/K04447%09%23FFFFFF,red/K05747%09%23FFFFFF,

http://www.kegg.jp/kegg-bin/show_pathway?map04810/K17386%09%23FFFFFF,red/K17260%09%23FFFFFF,red/K07830%09%23FFFFFF,

http://www.kegg.jp/kegg-bin/show_pathway?map00604/K03369%09%23FFFFFF,red/K03372%09%23FFFFFF,red/K03370%09%23FFFFFF,

http://www.kegg.jp/kegg-bin/show_pathway?map04080/K05213%09%23FFFFFF,red/K05424%09%23FFFFFF,red/K08522%09%23FFFFFF,

http://www.kegg.jp/kegg-bin/show_pathway?map00512/K09905%09%23FFFFFF,red/K09663%09%23FFFFFF,red/K00710%09%23FFFFFF,

http://www.kegg.jp/kegg-bin/show_pathway?map04371/K17845%09%23FFFFFF,red/K07830%09%23FFFFFF,red/K18050%09%23FFFFFF,

http://www.kegg.jp/kegg-bin/show_pathway?map04010/K04423%09%23FFFFFF,red/K02187%09%23FFFFFF,red/K07830%09%23FFFFFF,

http://www.kegg.jp/kegg-bin/show_pathway?map04723/K04849%09%23FFFFFF,red/K04853%09%23FFFFFF,red/K03951%09%23FFFFFF,

http://www.kegg.jp/kegg-bin/show_pathway?map04022/K18050%09%23FFFFFF,red/K04853%09%23FFFFFF,red/K04936%09%23FFFFFF,

http://www.kegg.jp/kegg-bin/show_pathway?map00514/K13676%09%23FFFFFF,red/K05948%09%23FFFFFF,red/K11703%09%23FFFFFF,

http://www.kegg.jp/kegg-bin/show_pathway?map04611/K16342%09%23FFFFFF,red/K19720%09%23FFFFFF,red/K05858%09%23FFFFFF,

http://www.kegg.jp/kegg-bin/show_pathway?map04540/K17386%09%23FFFFFF,red/K08042%09%23FFFFFF,red/K04371%09%23FFFFFF,

http://www.kegg.jp/kegg-bin/show_pathway?map04024/K07830%09%23FFFFFF,red/K04153%09%23FFFFFF,red/K04853%09%23FFFFFF,

http://www.kegg.jp/kegg-bin/show_pathway?map04141/K04426%09%23FFFFFF,red/K10950%09%23FFFFFF,red/K23741%09%23FFFFFF,

http://www.kegg.jp/kegg-bin/show_pathway?map04270/K18050%09%23FFFFFF,red/K04853%09%23FFFFFF,red/K16342%09%23FFFFFF,

http://www.kegg.jp/kegg-bin/show_pathway?map04927/K08042%09%23FFFFFF,red/K09048%09%23FFFFFF,red/K00070%09%23FFFFFF,

http://www.kegg.jp/kegg-bin/show_pathway?map04012/K06619%09%23FFFFFF,red/K17448%09%23FFFFFF,red/K04371%09%23FFFFFF,

http://www.kegg.jp/kegg-bin/show_pathway?map04068/K17845%09%23FFFFFF,red/K13304%09%23FFFFFF,red/K04468%09%23FFFFFF,

http://www.kegg.jp/kegg-bin/show_pathway?map00100/K00213%09%23FFFFFF,red/K01824%09%23FFFFFF,red/K07748%09%23FFFFFF,

http://www.kegg.jp/kegg-bin/show_pathway?map00785/K23756%09%23FFFFFF,red/K23735%09%23FFFFFF,red/

http://www.kegg.jp/kegg-bin/show_pathway?map00230/K01487%09%23FFFFFF,red/K00944%09%23FFFFFF,red/K19970%09%23FFFFFF,

http://www.kegg.jp/kegg-bin/show_pathway?map04911/K08042%09%23FFFFFF,red/K04939%09%23FFFFFF,red/K09048%09%23FFFFFF,

http://www.kegg.jp/kegg-bin/show_pathway?map04925/K08042%09%23FFFFFF,red/K09048%09%23FFFFFF,red/K00070%09%23FFFFFF,

http://www.kegg.jp/kegg-bin/show_pathway?map04928/K03068%09%23FFFFFF,red/K08042%09%23FFFFFF,red/K09048%09%23FFFFFF,

red/K05859%09%23FFFFFF,red/K06765%09%23FFFFFF,red/K04237%09%23FFFFFF,red/K07520%09%23FFFFFF,red/K00444%09%23FFF

red/K13916%09%23FFFFFF,red/K08048%09%23FFFFFF,red/K05742%09%23FFFFFF,red/K05858%09%23FFFFFF,red/K07376%09%23FFF

red/K08048%09%23FFFFFF,red/K03259%09%23FFFFFF,red/K02580%09%23FFFFFF,red/K08339%09%23FFFFFF,red/K07203%09%23FFF

red/K21356%09%23FFFFFF,red/K07831%09%23FFFFFF,red/K08339%09%23FFFFFF,red/K21347%09%23FFFFFF,red/K04440%09%23FFF

red/K05730%09%23FFFFFF,red/K02580%09%23FFFFFF,red/K13293%09%23FFFFFF,red/K06230%09%23FFFFFF,red/K11265%09%23FFF

red/K04426%09%23FFFFFF,red/K04237%09%23FFFFFF,red/K05747%09%23FFFFFF,red/K06088%09%23FFFFFF,red/K06087%09%23FFF

red/K04603%09%23FFFFFF,red/K16342%09%23FFFFFF,red/K07203%09%23FFFFFF,red/K05858%09%23FFFFFF,red/K05871%09%23FFF

red/K12318%09%23FFFFFF,red/K04379%09%23FFFFFF,red/K05000%09%23FFFFFF,red/K08048%09%23FFFFFF,red/K05858%09%23FFF

red/K04515%09%23FFFFFF,red/K10373%09%23FFFFFF,red/K04894%09%23FFFFFF,red/K09047%09%23FFFFFF,red/K04962%09%23FFF

red/K14736%09%23FFFFFF,red/K02580%09%23FFFFFF,red/K00844%09%23FFFFFF,red/K07203%09%23FFFFFF,red/K00927%09%23FFF

red/K08048%09%23FFFFFF,red/K05858%09%23FFFFFF,red/K04851%09%23FFFFFF,red/K04131%09%23FFFFFF,red/K04944%09%23FFF

red/K09048%09%23FFFFFF,red/K18050%09%23FFFFFF,red/K01539%09%23FFFFFF,red/K08048%09%23FFFFFF,red/K05858%09%23FFF

red/K18050%09%23FFFFFF,red/K05859%09%23FFFFFF,red/K08048%09%23FFFFFF,red/K16342%09%23FFFFFF,red/K05858%09%23FFF

red/K05200%09%23FFFFFF,red/K08048%09%23FFFFFF,red/K04603%09%23FFFFFF,red/K16342%09%23FFFFFF,red/K05858%09%23FFF

red/K05000%09%23FFFFFF,red/K08048%09%23FFFFFF,red/K05858%09%23FFFFFF,red/K04809%09%23FFFFFF,red/K04851%09%23FFF

red/K04131%09%23FFFFFF,red/K04374%09%23FFFFFF,red/K04944%09%23FFFFFF,red/K08043%09%23FFFFFF,red/K08049%09%23FFF

red/K13916%09%23FFFFFF,red/K04936%09%23FFFFFF,red/K05858%09%23FFFFFF,red/K04131%09%23FFFFFF,red/K05850%09%23FFF

red/K04131%09%23FFFFFF,red/K04360%09%23FFFFFF,red/K08043%09%23FFFFFF,red/K08049%09%23FFFFFF,red/K05098%09%23FFF

red/K04546%09%23FFFFFF,red/K08043%09%23FFFFFF,red/K08049%09%23FFFFFF,red/K04515%09%23FFFFFF,red/K05211%09%23FFF

red/K05858%09%23FFFFFF,red/K04131%09%23FFFFFF,red/K04546%09%23FFFFFF,red/K04374%09%23FFFFFF,red/K08049%09%23FFF

red/K05858%09%23FFFFFF,red/K11434%09%23FFFFFF,red/K04374%09%23FFFFFF,red/K16311%09%23FFFFFF,red/K04515%09%23FFF

red/K04546%09%23FFFFFF,red/K04374%09%23FFFFFF,red/K13242%09%23FFFFFF,red/K04361%09%23FFFFFF,red/K08043%09%23FFF

red/K08043%09%23FFFFFF,red/K08049%09%23FFFFFF,red/K05211%09%23FFFFFF,red/K04348%09%23FFFFFF,red/K08042%09%23FFF

red/K04374%09%23FFFFFF,red/K04354%09%23FFFFFF,red/K04515%09%23FFFFFF,red/K00274%09%23FFFFFF,red/K04348%09%23FFF

red/K05462%09%23FFFFFF,red/K05113%09%23FFFFFF,red/K05733%09%23FFFFFF,red/K06841%09%23FFFFFF,red/K04515%09%23FFF

red/K04936%09%23FFFFFF,red/K05858%09%23FFFFFF,red/K05862%09%23FFFFFF,red/K04374%09%23FFFFFF,red/K04135%09%23FFF

red/K04468%09%23FFFFFF,red/K05691%09%23FFFFFF,red/K04491%09%23FFFFFF,red/K06593%09%23FFFFFF,red/K23612%09%23FFF

red/K10843%09%23FFFFFF,red/K10844%09%23FFFFFF,red/K10849%09%23FFFFFF,red/K10842%09%23FFFFFF,red/K10570%09%23FFF

red/K04853%09%23FFFFFF,red/K14349%09%23FFFFFF,red/K05858%09%23FFFFFF,red/K08560%09%23FFFFFF,red/K04374%09%23FFF

red/K05858%09%23FFFFFF,red/K04374%09%23FFFFFF,red/K08050%09%23FFFFFF,red/K04604%09%23FFFFFF,red/K04515%09%23FFF

red/K13759%09%23FFFFFF,red/K13293%09%23FFFFFF,red/K18436%09%23FFFFFF,red/K00940%09%23FFFFFF,red/K11265%09%23FFF

red/K11265%09%23FFFFFF,red/K04546%09%23FFFFFF,red/K13242%09%23FFFFFF,red/K08043%09%23FFFFFF,red/K08049%09%23FFF

red/K14207%09%23FFFFFF,red/K04853%09%23FFFFFF,red/K05192%09%23FFFFFF,red/K15015%09%23FFFFFF,red/K04539%09%23FFF

red/K08045%09%23FFFFFF,red/K18050%09%23FFFFFF,red/K04853%09%23FFFFFF,red/K05858%09%23FFFFFF,red/K04374%09%23FFF

red/K11987%09%23FFFFFF,red/K05858%09%23FFFFFF,red/K15015%09%23FFFFFF,red/K04546%09%23FFFFFF,red/K08049%09%23FFF

red/K06591%09%23FFFFFF,red/K06585%09%23FFFFFF,red/K23379%09%23FFFFFF,red/K16338%09%23FFFFFF,red/K06258%09%23FFF

red/K04853%09%23FFFFFF,red/K05858%09%23FFFFFF,red/K05871%09%23FFFFFF,red/K04440%09%23FFFFFF,red/K04374%09%23FFF

red/K02432%09%23FFFFFF,red/K08045%09%23FFFFFF,red/K09455%09%23FFFFFF,red/K00444%09%23FFFFFF,red/K05858%09%23FFF

red/K05858%09%23FFFFFF,red/K05862%09%23FFFFFF,red/K11217%09%23FFFFFF,red/K10160%09%23FFFFFF,red/K03671%09%23FFF

red/K00933%09%23FFFFFF,red/K00128%09%23FFFFFF,red/K00274%09%23FFFFFF,red/K18384%09%23FFFFFF,red/K00542%09%23FFF

red/K13293%09%23FFFFFF,red/K11265%09%23FFFFFF,red/K10042%09%23FFFFFF,red/K08043%09%23FFFFFF,red/K08049%09%23FFF

red/K14625%09%23FFFFFF,red/K05858%09%23FFFFFF,red/K05871%09%23FFFFFF,red/K04546%09%23FFFFFF,red/K16598%09%23FFF

red/K06088%09%23FFFFFF,red/K06091%09%23FFFFFF,red/K08367%09%23FFFFFF,red/K06087%09%23FFFFFF,red/K04354%09%23FFF

red/K04135%09%23FFFFFF,red/K04354%09%23FFFFFF,red/K08043%09%23FFFFFF,red/K08049%09%23FFFFFF,red/K04515%09%23FFF

red/K05858%09%23FFFFFF,red/K04298%09%23FFFFFF,red/K13242%09%23FFFFFF,red/K06481%09%23FFFFFF,red/K08043%09%23FFF

red/K05858%09%23FFFFFF,red/K13242%09%23FFFFFF,red/K04361%09%23FFFFFF,red/K08043%09%23FFFFFF,red/K08049%09%23FFF

red/K01528%09%23FFFFFF,red/K04361%09%23FFFFFF,red/K08043%09%23FFFFFF,red/K08049%09%23FFFFFF,red/K05450%09%23FFF

red/K04131%09%23FFFFFF,red/K06487%09%23FFFFFF,red/K23612%09%23FFFFFF,red/K06481%09%23FFFFFF,red/K04361%09%23FFF

red/K04374%09%23FFFFFF,red/K09571%09%23FFFFFF,red/K13242%09%23FFFFFF,red/K04361%09%23FFFFFF,red/K08043%09%23FFF

red/K16618%09%23FFFFFF,red/K10905%09%23FFFFFF,red/K06640%09%23FFFFFF,red/K10849%09%23FFFFFF,red/K10890%09%23FFF

red/K05858%09%23FFFFFF,red/K04131%09%23FFFFFF,red/K00907%09%23FFFFFF,red/K08043%09%23FFFFFF,red/K08049%09%23FFF

red/K00444%09%23FFFFFF,red/K05858%09%23FFFFFF,red/K23097%09%23FFFFFF,red/K04493%09%23FFFFFF,red/K04515%09%23FFF

red/K02602%09%23FFFFFF,red/K04373%09%23FFFFFF,red/K04440%09%23FFFFFF,red/K21771%09%23FFFFFF,red/K08043%09%23FFF

red/K08044%09%23FFFFFF,red/K04851%09%23FFFFFF,red/K04374%09%23FFFFFF,red/K04515%09%23FFFFFF,red/K18283%09%23FFF

red/K09048%09%23FFFFFF,red/K04853%09%23FFFFFF,red/K08044%09%23FFFFFF,red/K04851%09%23FFFFFF,red/K04374%09%23FFF

red/K05096%09%23FFFFFF,red/K08052%09%23FFFFFF,red/K04853%09%23FFFFFF,red/K04407%09%23FFFFFF,red/K05085%09%23FFF

red/K02329%09%23FFFFFF,red/K08044%09%23FFFFFF,red/K00444%09%23FFFFFF,red/K04851%09%23FFFFFF,red/K04374%09%23FFF

red/K04858%09%23FFFFFF,red/K04853%09%23FFFFFF,red/K02105%09%23FFFFFF,red/K06585%09%23FFFFFF,red/K04490%09%23FFF

red/K02991%09%23FFFFFF,red/K07830%09%23FFFFFF,red/K16865%09%23FFFFFF,red/K07965%09%23FFFFFF,red/K06236%09%23FFF

red/K13916%09%23FFFFFF,red/K08044%09%23FFFFFF,red/K07376%09%23FFFFFF,red/K05850%09%23FFFFFF,red/K04135%09%23FFF

red/K05050%09%23FFFFFF,red/K07203%09%23FFFFFF,red/K08044%09%23FFFFFF,red/K01528%09%23FFFFFF,red/K04289%09%23FFF

red/K06259%09%23FFFFFF,red/K06236%09%23FFFFFF,red/K06264%09%23FFFFFF,red/K06585%09%23FFFFFF,red/K06255%09%23FFF

red/K04928%09%23FFFFFF,red/K04853%09%23FFFFFF,red/K04926%09%23FFFFFF,red/K08044%09%23FFFFFF,red/K04851%09%23FFF

red/K08044%09%23FFFFFF,red/K04440%09%23FFFFFF,red/K04851%09%23FFFFFF,red/K05704%09%23FFFFFF,red/K04420%09%23FFF

red/K04851%09%23FFFFFF,red/K04374%09%23FFFFFF,red/K04515%09%23FFFFFF,red/K06269%09%23FFFFFF,red/K00274%09%23FFF

red/K08044%09%23FFFFFF,red/K05085%09%23FFFFFF,red/K05862%09%23FFFFFF,red/K04851%09%23FFFFFF,red/K04135%09%23FFF

red/K07520%09%23FFFFFF,red/K04968%09%23FFFFFF,red/K00444%09%23FFFFFF,red/K05462%09%23FFFFFF,red/K04189%09%23FFF

red/K04853%09%23FFFFFF,red/K05858%09%23FFFFFF,red/K08041%09%23FFFFFF,red/K04851%09%23FFFFFF,red/K04546%09%23FFF

red/K04468%09%23FFFFFF,red/K08889%09%23FFFFFF,red/K04498%09%23FFFFFF,red/K05747%09%23FFFFFF,red/K04674%09%23FFF

red/K05858%09%23FFFFFF,red/K04439%09%23FFFFFF,red/K08041%09%23FFFFFF,red/K04546%09%23FFFFFF,red/K00922%09%23FFF

red/K05858%09%23FFFFFF,red/K04851%09%23FFFFFF,red/K04360%09%23FFFFFF,red/K05101%09%23FFFFFF,red/K08794%09%23FFF

red/K05190%09%23FFFFFF,red/K04851%09%23FFFFFF,red/K04546%09%23FFFFFF,red/K08341%09%23FFFFFF,red/K05181%09%23FFF

red/K04968%09%23FFFFFF,red/K05858%09%23FFFFFF,red/K04439%09%23FFFFFF,red/K04851%09%23FFFFFF,red/K04944%09%23FFF

red/K08041%09%23FFFFFF,red/K04851%09%23FFFFFF,red/K04546%09%23FFFFFF,red/K04634%09%23FFFFFF,red/K05211%09%23FFF

red/K04851%09%23FFFFFF,red/K04546%09%23FFFFFF,red/K04634%09%23FFFFFF,red/K05211%09%23FFFFFF,red/K13240%09%23FFF

red/K16342%09%23FFFFFF,red/K07203%09%23FFFFFF,red/K05858%09%23FFFFFF,red/K05871%09%23FFFFFF,red/K08041%09%23FFF

red/K07203%09%23FFFFFF,red/K05858%09%23FFFFFF,red/K08041%09%23FFFFFF,red/K11265%09%23FFFFFF,red/K04851%09%23FFF

red/K01593%09%23FFFFFF,red/K04153%09%23FFFFFF,red/K04853%09%23FFFFFF,red/K16342%09%23FFFFFF,red/K11987%09%23FFF

red/K05858%09%23FFFFFF,red/K05871%09%23FFFFFF,red/K08041%09%23FFFFFF,red/K04439%09%23FFFFFF,red/K04189%09%23FFF

red/K05766%09%23FFFFFF,red/K04189%09%23FFFFFF,red/K06487%09%23FFFFFF,red/K23612%09%23FFFFFF,red/K06481%09%23FFF

red/K04610%09%23FFFFFF,red/K05202%09%23FFFFFF,red/K05051%09%23FFFFFF,red/K05186%09%23FFFFFF,red/K04804%09%23FFF

red/K04674%09%23FFFFFF,red/K07203%09%23FFFFFF,red/K05858%09%23FFFFFF,red/K08041%09%23FFFFFF,red/K11265%09%23FFF

red/K04426%09%23FFFFFF,red/K04853%09%23FFFFFF,red/K05085%09%23FFFFFF,red/K04360%09%23FFFFFF,red/K04439%09%23FFF

red/K11987%09%23FFFFFF,red/K05858%09%23FFFFFF,red/K08041%09%23FFFFFF,red/K05190%09%23FFFFFF,red/K04851%09%23FFF

red/K05858%09%23FFFFFF,red/K08041%09%23FFFFFF,red/K04851%09%23FFFFFF,red/K04135%09%23FFFFFF,red/K04634%09%23FFF

red/K08041%09%23FFFFFF,red/K06481%09%23FFFFFF,red/K04634%09%23FFFFFF,red/K00922%09%23FFFFFF,red/K08042%09%23FFF

red/K12318%09%23FFFFFF,red/K05858%09%23FFFFFF,red/K05704%09%23FFFFFF,red/K07376%09%23FFFFFF,red/K08041%09%23FFF

red/K13293%09%23FFFFFF,red/K05213%09%23FFFFFF,red/K08041%09%23FFFFFF,red/K11265%09%23FFFFFF,red/K04851%09%23FFF

red/K12669%09%23FFFFFF,red/K12666%09%23FFFFFF,red/K01230%09%23FFFFFF,red/K10636%09%23FFFFFF,red/K24348%09%23FFF

red/K04936%09%23FFFFFF,red/K05858%09%23FFFFFF,red/K08041%09%23FFFFFF,red/K04851%09%23FFFFFF,red/K04135%09%23FFF

red/K04853%09%23FFFFFF,red/K09355%09%23FFFFFF,red/K05858%09%23FFFFFF,red/K08041%09%23FFFFFF,red/K04851%09%23FFF

red/K05734%09%23FFFFFF,red/K07203%09%23FFFFFF,red/K04430%09%23FFFFFF,red/K05085%09%23FFFFFF,red/K04409%09%23FFF

red/K04674%09%23FFFFFF,red/K13303%09%23FFFFFF,red/K13302%09%23FFFFFF,red/K00922%09%23FFFFFF,red/K08341%09%23FFF

red/K13293%09%23FFFFFF,red/K18436%09%23FFFFFF,red/K11265%09%23FFFFFF,red/K08041%09%23FFFFFF,red/K07127%09%23FFF

red/K04853%09%23FFFFFF,red/K15297%09%23FFFFFF,red/K04936%09%23FFFFFF,red/K05858%09%23FFFFFF,red/K08041%09%23FFF

red/K18050%09%23FFFFFF,red/K04853%09%23FFFFFF,red/K05858%09%23FFFFFF,red/K08041%09%23FFFFFF,red/K04851%09%23FFF

red/K04371%09%23FFFFFF,red/K05858%09%23FFFFFF,red/K13293%09%23FFFFFF,red/K05261%09%23FFFFFF,red/K04439%09%23FFF

FFF,red/K20020%09%23FFFFFF,red/K05462%09%23FFFFFF,red/K05733%09%23FFFFFF,red/K00922%09%23FFFFFF,red/K05110%09%23

FFF,red/K07202%09%23FFFFFF,red/K07206%09%23FFFFFF,red/K04450%09%23FFFFFF,red/K00922%09%23FFFFFF,red/K07201%09%23

FFF,red/K15485%09%23FFFFFF,red/K07897%09%23FFFFFF,red/K07829%09%23FFFFFF,red/K08860%09%23FFFFFF,red/K02833%09%23

FFF,red/K04851%09%23FFFFFF,red/K00232%09%23FFFFFF,red/K00922%09%23FFFFFF,red/K04515%09%23FFFFFF,red/K07829%09%23

FFF,red/K04851%09%23FFFFFF,red/K06091%09%23FFFFFF,red/K06448%09%23FFFFFF,red/K05762%09%23FFFFFF,red/K13305%09%23

FFF,red/K01528%09%23FFFFFF,red/K07206%09%23FFFFFF,red/K00922%09%23FFFFFF,red/K07829%09%23FFFFFF,red/K04609%09%23

FFF,red/K07376%09%23FFFFFF,red/K11265%09%23FFFFFF,red/K04851%09%23FFFFFF,red/K04997%09%23FFFFFF,red/K04515%09%23

FFF,red/K08042%09%23FFFFFF,red/K04860%09%23FFFFFF,red/K09048%09%23FFFFFF,red/K01539%09%23FFFFFF,red/K05742%09%23

FFF,red/K00161%09%23FFFFFF,red/K09097%09%23FFFFFF,red/K00922%09%23FFFFFF,red/K04515%09%23FFFFFF,red/K05467%09%23

FFF,red/K04450%09%23FFFFFF,red/K04515%09%23FFFFFF,red/K04938%09%23FFFFFF,red/K09047%09%23FFFFFF,red/K04962%09%23

FFF,red/K04851%09%23FFFFFF,red/K05850%09%23FFFFFF,red/K04450%09%23FFFFFF,red/K04515%09%23FFFFFF,red/K09047%09%23

FFF,red/K04440%09%23FFFFFF,red/K07425%09%23FFFFFF,red/K04157%09%23FFFFFF,red/K00922%09%23FFFFFF,red/K04515%09%23

FFF,red/K15009%09%23FFFFFF,red/K04851%09%23FFFFFF,red/K05204%09%23FFFFFF,red/K04997%09%23FFFFFF,red/K05201%09%23

FFF,red/K04131%09%23FFFFFF,red/K21290%09%23FFFFFF,red/K00922%09%23FFFFFF,red/K04997%09%23FFFFFF,red/K04515%09%23

FFF,red/K04515%09%23FFFFFF,red/K04938%09%23FFFFFF,red/K07594%09%23FFFFFF,red/K05274%09%23FFFFFF,red/K08042%09%23

FFF,red/K04135%09%23FFFFFF,red/K08043%09%23FFFFFF,red/K08049%09%23FFFFFF,red/K13915%09%23FFFFFF,red/K04345%09%23

FFF,red/K08794%09%23FFFFFF,red/K04348%09%23FFFFFF,red/K08042%09%23FFFFFF,red/K00907%09%23FFFFFF,red/K04262%09%23

FFF,red/K16513%09%23FFFFFF,red/K08042%09%23FFFFFF,red/K08045%09%23FFFFFF,red/K05200%09%23FFFFFF,red/K04371%09%23

FFF,red/K08043%09%23FFFFFF,red/K04515%09%23FFFFFF,red/K04456%09%23FFFFFF,red/K08042%09%23FFFFFF,red/K04930%09%23

FFF,red/K04348%09%23FFFFFF,red/K04456%09%23FFFFFF,red/K08042%09%23FFFFFF,red/K09048%09%23FFFFFF,red/K07199%09%23

FFF,red/K08049%09%23FFFFFF,red/K04500%09%23FFFFFF,red/K04456%09%23FFFFFF,red/K21998%09%23FFFFFF,red/K08042%09%23

FFF,red/K08045%09%23FFFFFF,red/K05200%09%23FFFFFF,red/K04371%09%23FFFFFF,red/K05209%09%23FFFFFF,red/K04539%09%23

FFF,red/K04456%09%23FFFFFF,red/K08045%09%23FFFFFF,red/K05200%09%23FFFFFF,red/K09048%09%23FFFFFF,red/K05209%09%23

FFF,red/K06842%09%23FFFFFF,red/K06839%09%23FFFFFF,red/K05463%09%23FFFFFF,red/K07521%09%23FFFFFF,red/K04348%09%23

FFF,red/K13242%09%23FFFFFF,red/K08043%09%23FFFFFF,red/K08049%09%23FFFFFF,red/K04938%09%23FFFFFF,red/K03898%09%23

FFF,red/K04361%09%23FFFFFF,red/K05083%09%23FFFFFF,red/K05693%09%23FFFFFF,red/K06085%09%23FFFFFF,red/K04501%09%23

FFF,red/K18437%09%23FFFFFF,red/K08043%09%23FFFFFF,red/K08049%09%23FFFFFF,red/K22398%09%23FFFFFF,red/K04345%09%23

FFF,red/K13755%09%23FFFFFF,red/K07127%09%23FFFFFF,red/K12322%09%23FFFFFF,red/K08043%09%23FFFFFF,red/K08049%09%23

FFF,red/K04500%09%23FFFFFF,red/K04456%09%23FFFFFF,red/K08042%09%23FFFFFF,red/K07199%09%23FFFFFF,red/K04371%09%23

FFF,red/K04546%09%23FFFFFF,red/K15376%09%23FFFFFF,red/K08043%09%23FFFFFF,red/K08049%09%23FFFFFF,red/K06027%09%23

FFF,red/K05850%09%23FFFFFF,red/K08043%09%23FFFFFF,red/K08049%09%23FFFFFF,red/K04515%09%23FFFFFF,red/K08794%09%23

FFF,red/K08043%09%23FFFFFF,red/K15291%09%23FFFFFF,red/K08042%09%23FFFFFF,red/K04371%09%23FFFFFF,red/K03937%09%23

FFF,red/K06487%09%23FFFFFF,red/K08131%09%23FFFFFF,red/K06587%09%23FFFFFF,red/K06481%09%23FFFFFF,red/K05636%09%23

FFF,red/K04361%09%23FFFFFF,red/K08043%09%23FFFFFF,red/K08049%09%23FFFFFF,red/K04515%09%23FFFFFF,red/K03099%09%23

FFF,red/K04491%09%23FFFFFF,red/K08043%09%23FFFFFF,red/K08049%09%23FFFFFF,red/K00505%09%23FFFFFF,red/K04515%09%23

FFF,red/K04371%09%23FFFFFF,red/K20876%09%23FFFFFF,red/K12796%09%23FFFFFF,red/K20915%09%23FFFFFF,red/K01165%09%23

FFF,red/K04515%09%23FFFFFF,red/K05211%09%23FFFFFF,red/K04456%09%23FFFFFF,red/K08042%09%23FFFFFF,red/K06225%09%23

FFF,red/K08043%09%23FFFFFF,red/K08049%09%23FFFFFF,red/K21096%09%23FFFFFF,red/K04456%09%23FFFFFF,red/K05854%09%23

FFF,red/K06448%09%23FFFFFF,red/K13305%09%23FFFFFF,red/K05763%09%23FFFFFF,red/K07902%09%23FFFFFF,red/K07199%09%23

FFF,red/K04456%09%23FFFFFF,red/K08042%09%23FFFFFF,red/K04860%09%23FFFFFF,red/K09048%09%23FFFFFF,red/K04371%09%23

FFF,red/K08049%09%23FFFFFF,red/K04456%09%23FFFFFF,red/K08042%09%23FFFFFF,red/K05854%09%23FFFFFF,red/K05703%09%23

FFF,red/K04515%09%23FFFFFF,red/K08794%09%23FFFFFF,red/K04348%09%23FFFFFF,red/K08042%09%23FFFFFF,red/K04860%09%23

FFF,red/K13523%09%23FFFFFF,red/K04456%09%23FFFFFF,red/K08042%09%23FFFFFF,red/K05703%09%23FFFFFF,red/K08045%09%23

FFF,red/K05733%09%23FFFFFF,red/K05450%09%23FFFFFF,red/K03898%09%23FFFFFF,red/K05763%09%23FFFFFF,red/K04371%09%23

FFF,red/K08049%09%23FFFFFF,red/K09101%09%23FFFFFF,red/K04456%09%23FFFFFF,red/K08042%09%23FFFFFF,red/K11256%09%23

FFF,red/K04348%09%23FFFFFF,red/K02432%09%23FFFFFF,red/K04308%09%23FFFFFF,red/K04497%09%23FFFFFF,red/K04491%09%23

FFF,red/K08049%09%23FFFFFF,red/K06645%09%23FFFFFF,red/K21770%09%23FFFFFF,red/K04456%09%23FFFFFF,red/K04345%09%23

FFF,red/K09047%09%23FFFFFF,red/K16931%09%23FFFFFF,red/K08042%09%23FFFFFF,red/K09048%09%23FFFFFF,red/K09053%09%23
FFF,red/K04855%09%23FFFFFF,red/K22398%09%23FFFFFF,red/K09047%09%23FFFFFF,red/K04913%09%23FFFFFF,red/K16931%09%23
FFF,red/K04851%09%23FFFFFF,red/K04374%09%23FFFFFF,red/K04425%09%23FFFFFF,red/K04348%09%23FFFFFF,red/K04852%09%23
FFF,red/K04515%09%23FFFFFF,red/K09047%09%23FFFFFF,red/K16931%09%23FFFFFF,red/K08042%09%23FFFFFF,red/K09048%09%23

FFF,red/K02329%09%23FFFFFF,red/K06255%09%23FFFFFF,red/K07203%09%23FFFFFF,red/K00444%09%23FFFFFF,red/K05085%09%23

FFF,red/K07206%09%23FFFFFF,red/K05450%09%23FFFFFF,red/K08089%09%23FFFFFF,red/K04610%09%23FFFFFF,red/K08042%09%23
FFF,red/K06258%09%23FFFFFF,red/K08131%09%23FFFFFF,red/K06587%09%23FFFFFF,red/K06481%09%23FFFFFF,red/K06824%09%23
FFF,red/K04374%09%23FFFFFF,red/K04515%09%23FFFFFF,red/K09047%09%23FFFFFF,red/K04130%09%23FFFFFF,red/K08042%09%23
FFF,red/K04374%09%23FFFFFF,red/K01115%09%23FFFFFF,red/K04515%09%23FFFFFF,red/K04441%09%23FFFFFF,red/K08522%09%23

FFF,red/K04515%09%23FFFFFF,red/K20858%09%23FFFFFF,red/K05450%09%23FFFFFF,red/K04348%09%23FFFFFF,red/K04130%09%23

FFF,red/K06754%09%23FFFFFF,red/K05733%09%23FFFFFF,red/K06841%09%23FFFFFF,red/K00922%09%23FFFFFF,red/K20013%09%23
FFF,red/K04808%09%23FFFFFF,red/K04634%09%23FFFFFF,red/K00922%09%23FFFFFF,red/K09047%09%23FFFFFF,red/K08042%09%23
FFF,red/K06082%09%23FFFFFF,red/K23605%09%23FFFFFF,red/K05704%09%23FFFFFF,red/K23612%09%23FFFFFF,red/K05693%09%23
FFF,red/K13240%09%23FFFFFF,red/K09047%09%23FFFFFF,red/K21998%09%23FFFFFF,red/K08042%09%23FFFFFF,red/K06237%09%23
FFF,red/K13240%09%23FFFFFF,red/K08042%09%23FFFFFF,red/K05219%09%23FFFFFF,red/K00907%09%23FFFFFF,red/K04262%09%23
FFF,red/K08042%09%23FFFFFF,red/K05186%09%23FFFFFF,red/K04541%09%23FFFFFF,red/K05704%09%23FFFFFF,red/K05175%09%23
FFF,red/K04856%09%23FFFFFF,red/K04634%09%23FFFFFF,red/K00922%09%23FFFFFF,red/K08522%09%23FFFFFF,red/K04615%09%23
FFF,red/K04609%09%23FFFFFF,red/K04610%09%23FFFFFF,red/K05202%09%23FFFFFF,red/K08042%09%23FFFFFF,red/K04371%09%23
FFF,red/K08042%09%23FFFFFF,red/K12318%09%23FFFFFF,red/K04371%09%23FFFFFF,red/K07843%09%23FFFFFF,red/K05209%09%23
FFF,red/K00922%09%23FFFFFF,red/K07829%09%23FFFFFF,red/K05450%09%23FFFFFF,red/K04609%09%23FFFFFF,red/K23484%09%23
FFF,red/K04634%09%23FFFFFF,red/K00922%09%23FFFFFF,red/K04692%09%23FFFFFF,red/K09047%09%23FFFFFF,red/K08042%09%23
FFF,red/K05858%09%23FFFFFF,red/K04851%09%23FFFFFF,red/K04546%09%23FFFFFF,red/K04634%09%23FFFFFF,red/K05181%09%23
FFF,red/K04546%09%23FFFFFF,red/K04634%09%23FFFFFF,red/K00922%09%23FFFFFF,red/K04692%09%23FFFFFF,red/K10031%09%23
FFF,red/K05733%09%23FFFFFF,red/K00922%09%23FFFFFF,red/K07829%09%23FFFFFF,red/K05450%09%23FFFFFF,red/K10031%09%23

FFF,red/K05219%09%23FFFFFF,red/K04262%09%23FFFFFF,red/K04260%09%23FFFFFF,red/K04604%09%23FFFFFF,red/K04010%09%23

FFF,red/K04546%09%23FFFFFF,red/K04634%09%23FFFFFF,red/K07829%09%23FFFFFF,red/K13240%09%23FFFFFF,red/K08042%09%23

FFF,red/K04851%09%23FFFFFF,red/K04459%09%23FFFFFF,red/K04860%09%23FFFFFF,red/K04445%09%23FFFFFF,red/K04373%09%23

FFF,red/K04546%09%23FFFFFF,red/K04634%09%23FFFFFF,red/K11351%09%23FFFFFF,red/K05181%09%23FFFFFF,red/K08042%09%23

FFF,red/K04938%09%23FFFFFF,red/K09047%09%23FFFFFF,red/K08042%09%23FFFFFF,red/K21289%09%23FFFFFF,red/K04939%09%23

FFF,red/K21289%09%23FFFFFF,red/K04371%09%23FFFFFF,red/K12318%09%23FFFFFF,red/K03900%09%23FFFFFF,red/K05704%09%23

FFF,red/K04604%09%23FFFFFF,red/K04634%09%23FFFFFF,red/K08959%09%23FFFFFF,red/K05450%09%23FFFFFF,red/K03099%09%23

FFF,red/K00922%09%23FFFFFF,red/K07829%09%23FFFFFF,red/K05211%09%23FFFFFF,red/K09047%09%23FFFFFF,red/K08522%09%23

FFF,red/K10084%09%23FFFFFF,red/K09503%09%23FFFFFF,red/K11718%09%23FFFFFF,red/K09584%09%23FFFFFF,red/K04556%09%23

FFF,red/K04634%09%23FFFFFF,red/K12331%09%23FFFFFF,red/K04938%09%23FFFFFF,red/K01047%09%23FFFFFF,red/K08042%09%23

FFF,red/K04692%09%23FFFFFF,red/K21770%09%23FFFFFF,red/K04371%09%23FFFFFF,red/K04498%09%23FFFFFF,red/K23605%09%23

FFF,red/K12322%09%23FFFFFF,red/K08042%09%23FFFFFF,red/K12318%09%23FFFFFF,red/K01518%09%23FFFFFF,red/K19572%09%23

FFF,red/K04851%09%23FFFFFF,red/K04944%09%23FFFFFF,red/K04634%09%23FFFFFF,red/K04938%09%23FFFFFF,red/K09047%09%23

FFF,red/K04856%09%23FFFFFF,red/K05850%09%23FFFFFF,red/K04634%09%23FFFFFF,red/K08794%09%23FFFFFF,red/K09047%09%23

FFF,red/K08041%09%23FFFFFF,red/K04634%09%23FFFFFF,red/K12331%09%23FFFFFF,red/K09047%09%23FFFFFF,red/K16529%09%23

FFFFFF,red/K04515%09%23FFFFFF,red/K07829%09%23FFFFFF,red/K05463%09%23FFFFFF,red/K06842%09%23FFFFFF,red/K06839%09

FFFFFF,red/K04578%09%23FFFFFF,red/K09047%09%23FFFFFF,red/K04962%09%23FFFFFF,red/K08042%09%23FFFFFF,red/K05199%09

FFFFFF,red/K05631%09%23FFFFFF,red/K07199%09%23FFFFFF,red/K05731%09%23FFFFFF,red/K18584%09%23FFFFFF,red/K06104%09

FFFFFF,red/K04610%09%23FFFFFF,red/K08042%09%23FFFFFF,red/K00901%09%23FFFFFF,red/K08048%09%23FFFFFF,red/K01080%09

FFFFFF,red/K08048%09%23FFFFFF,red/K11583%09%23FFFFFF,red/K12314%09%23FFFFFF,red/K21290%09%23FFFFFF,red/K05850%09

FFFFFF,red/K09048%09%23FFFFFF,red/K08045%09%23FFFFFF,red/K16882%09%23FFFFFF,red/K04941%09%23FFFFFF,red/K04345%09

FFFFFF,red/K04358%09%23FFFFFF,red/K04260%09%23FFFFFF,red/K05850%09%23FFFFFF,red/K07190%09%23FFFFFF,red/K04604%09

FFFFFF,red/K04963%09%23FFFFFF,red/K05209%09%23FFFFFF,red/K04539%09%23FFFFFF,red/K04345%09%23FFFFFF,red/K04548%09

FFFFFF,red/K09048%09%23FFFFFF,red/K05703%09%23FFFFFF,red/K04371%09%23FFFFFF,red/K08045%09%23FFFFFF,red/K04539%09

FFFFFF,red/K07202%09%23FFFFFF,red/K04583%09%23FFFFFF,red/K07190%09%23FFFFFF,red/K04345%09%23FFFFFF,red/

FFFFFF,red/K06237%09%23FFFFFF,red/K09048%09%23FFFFFF,red/K04371%09%23FFFFFF,red/K08045%09%23FFFFFF,red/K04440%09

FFFFFF,red/K00910%09%23FFFFFF,red/K04604%09%23FFFFFF,red/K05203%09%23FFFFFF,red/K15008%09%23FFFFFF,red/K04345%09

FFFFFF,red/K04440%09%23FFFFFF,red/K04539%09%23FFFFFF,red/K02223%09%23FFFFFF,red/K10396%09%23FFFFFF,red/K04345%09

FFFFFF,red/K06572%09%23FFFFFF,red/K06225%09%23FFFFFF,red/K05703%09%23FFFFFF,red/K04371%09%23FFFFFF,red/K06753%09
FFFFFF,red/K04348%09%23FFFFFF,red/K04456%09%23FFFFFF,red/K05274%09%23FFFFFF,red/K08042%09%23FFFFFF,red/K09048%09
FFFFFF,red/K00760%09%23FFFFFF,red/K08042%09%23FFFFFF,red/K08045%09%23FFFFFF,red/K01490%09%23FFFFFF,red/K18437%09
FFFFFF,red/K08045%09%23FFFFFF,red/K04963%09%23FFFFFF,red/K04539%09%23FFFFFF,red/K00907%09%23FFFFFF,red/K07202%09
FFFFFF,red/K08045%09%23FFFFFF,red/K05200%09%23FFFFFF,red/K05192%09%23FFFFFF,red/K04440%09%23FFFFFF,red/K04539%09
FFFFFF,red/K05415%09%23FFFFFF,red/K04440%09%23FFFFFF,red/K20898%09%23FFFFFF,red/K07211%09%23FFFFFF,red/K05411%09
FFFFFF,red/K09048%09%23FFFFFF,red/K04371%09%23FFFFFF,red/K05673%09%23FFFFFF,red/K08045%09%23FFFFFF,red/K05200%09
FFFFFF,red/K08042%09%23FFFFFF,red/K04371%09%23FFFFFF,red/K08045%09%23FFFFFF,red/K05731%09%23FFFFFF,red/K12366%09
FFFFFF,red/K05731%09%23FFFFFF,red/K04440%09%23FFFFFF,red/K20479%09%23FFFFFF,red/K12075%09%23FFFFFF,red/K05083%09
FFFFFF,red/K08045%09%23FFFFFF,red/K08050%09%23FFFFFF,red/K05850%09%23FFFFFF,red/K04838%09%23FFFFFF,red/K04345%09
FFFFFF,red/K04371%09%23FFFFFF,red/K08045%09%23FFFFFF,red/K00907%09%23FFFFFF,red/K04345%09%23FFFFFF,red/K06271%09
FFFFFF,red/K07199%09%23FFFFFF,red/K04371%09%23FFFFFF,red/K08045%09%23FFFFFF,red/K04963%09%23FFFFFF,red/K00907%09
FFFFFF,red/K04371%09%23FFFFFF,red/K00901%09%23FFFFFF,red/K04262%09%23FFFFFF,red/K04604%09%23FFFFFF,red/K04275%09
FFFFFF,red/K05731%09%23FFFFFF,red/K06585%09%23FFFFFF,red/K06591%09%23FFFFFF,red/K04409%09%23FFFFFF,red/K00907%09
FFFFFF,red/K09048%09%23FFFFFF,red/K04371%09%23FFFFFF,red/K08045%09%23FFFFFF,red/K07604%09%23FFFFFF,red/K04345%09
FFFFFF,red/K04440%09%23FFFFFF,red/K04511%09%23FFFFFF,red/K04512%09%23FFFFFF,red/K02375%09%23FFFFFF,red/K04501%09

FFFFFF,red/K05850%09%23FFFFFF,red/K04855%09%23FFFFFF,red/K13806%09%23FFFFFF,red/K05869%09%23FFFFFF,red/

FFFFFF,red/K04860%09%23FFFFFF,red/K04457%09%23FFFFFF,red/K04440%09%23FFFFFF,red/K04358%09%23FFFFFF,red/K04446%09

FFFFFF,red/K04684%09%23FFFFFF,red/K02105%09%23FFFFFF,red/K04490%09%23FFFFFF,red/K22499%09%23FFFFFF,red/K04579%09

FFFFFF,red/K06481%09%23FFFFFF,red/K04515%09%23FFFFFF,red/K04500%09%23FFFFFF,red/K04692%09%23FFFFFF,red/K08550%09

FFFFFF,red/K00901%09%23FFFFFF,red/K05091%09%23FFFFFF,red/K04262%09%23FFFFFF,red/K01115%09%23FFFFFF,red/K04275%09

FFFFFF,red/K04852%09%23FFFFFF,red/K08042%09%23FFFFFF,red/K04297%09%23FFFFFF,red/K04150%09%23FFFFFF,red/K05209%09

FFFFFF,red/K07829%09%23FFFFFF,red/K06842%09%23FFFFFF,red/K06839%09%23FFFFFF,red/K10031%09%23FFFFFF,red/K07521%09

FFFFFF,red/K21289%09%23FFFFFF,red/K04930%09%23FFFFFF,red/K09048%09%23FFFFFF,red/K04371%09%23FFFFFF,red/K04344%09

FFFFFF,red/K04371%09%23FFFFFF,red/K09048%09%23FFFFFF,red/K04430%09%23FFFFFF,red/K04541%09%23FFFFFF,red/K05704%09

FFFFFF,red/K04260%09%23FFFFFF,red/K04358%09%23FFFFFF,red/K07190%09%23FFFFFF,red/K04856%09%23FFFFFF,red/K05850%09

FFFFFF,red/K04344%09%23FFFFFF,red/K15376%09%23FFFFFF,red/K04615%09%23FFFFFF,red/K04548%09%23FFFFFF,red/

FFFFFF,red/K05209%09%23FFFFFF,red/K04344%09%23FFFFFF,red/K00910%09%23FFFFFF,red/K04541%09%23FFFFFF,red/K04604%09

FFFFFF,red/K04445%09%23FFFFFF,red/K04541%09%23FFFFFF,red/K07376%09%23FFFFFF,red/K04856%09%23FFFFFF,red/K04548%09

FFFFFF,red/K04610%09%23FFFFFF,red/K08042%09%23FFFFFF,red/K21289%09%23FFFFFF,red/K04371%09%23FFFFFF,red/K00901%09

FFFFFF,red/K09048%09%23FFFFFF,red/K04371%09%23FFFFFF,red/K04498%09%23FFFFFF,red/K04430%09%23FFFFFF,red/K17446%09

FFFFFF,red/K04371%09%23FFFFFF,red/K04892%09%23FFFFFF,red/K04344%09%23FFFFFF,red/K04541%09%23FFFFFF,red/K07414%09

FFFFFF,red/K21096%09%23FFFFFF,red/K08042%09%23FFFFFF,red/K21289%09%23FFFFFF,red/K04371%09%23FFFFFF,red/K04192%09

FFFFFF,red/K16848%09%23FFFFFF,red/K05750%09%23FFFFFF,red/K05769%09%23FFFFFF,red/K05767%09%23FFFFFF,red/K04371%09

FFFFFF,red/K04288%09%23FFFFFF,red/K04814%09%23FFFFFF,red/K04216%09%23FFFFFF,red/K08377%09%23FFFFFF,red/K04153%09

FFFFFF,red/K21289%09%23FFFFFF,red/K04371%09%23FFFFFF,red/K23605%09%23FFFFFF,red/K04541%09%23FFFFFF,red/K00907%09

FFFFFF,red/K04430%09%23FFFFFF,red/K04358%09%23FFFFFF,red/K04856%09%23FFFFFF,red/K03173%09%23FFFFFF,red/K03099%09

FFFFFF,red/K04371%09%23FFFFFF,red/K05186%09%23FFFFFF,red/K04344%09%23FFFFFF,red/K05175%09%23FFFFFF,red/K04541%09

FFFFFF,red/K09048%09%23FFFFFF,red/K04371%09%23FFFFFF,red/K12318%09%23FFFFFF,red/K19021%09%23FFFFFF,red/K04140%09

FFFFFF,red/K07376%09%23FFFFFF,red/K00907%09%23FFFFFF,red/K17388%09%23FFFFFF,red/K06271%09%23FFFFFF,red/

FFFFFF,red/K08042%09%23FFFFFF,red/K06225%09%23FFFFFF,red/K09048%09%23FFFFFF,red/K04371%09%23FFFFFF,red/K04498%09

FFFFFF,red/K04079%09%23FFFFFF,red/K10578%09%23FFFFFF,red/K14001%09%23FFFFFF,red/K05638%09%23FFFFFF,red/K14007%09

FFFFFF,red/K04939%09%23FFFFFF,red/K04371%09%23FFFFFF,red/K12318%09%23FFFFFF,red/K07376%09%23FFFFFF,red/K00907%09

FFFFFF,red/K04411%09%23FFFFFF,red/K17446%09%23FFFFFF,red/K04288%09%23FFFFFF,red/K03099%09%23FFFFFF,red/

%23FFFFFF,red/K06843%09%23FFFFFF,red/K07521%09%23FFFFFF,red/K05107%09%23FFFFFF,red/K06753%09%23FFFFFF,red/K16351

%23FFFFFF,red/K05200%09%23FFFFFF,red/K09048%09%23FFFFFF,red/K01539%09%23FFFFFF,red/K08048%09%23FFFFFF,red/K05731

%23FFFFFF,red/K04440%09%23FFFFFF,red/K07839%09%23FFFFFF,red/K20479%09%23FFFFFF,red/K06785%09%23FFFFFF,red/K06098

%23FFFFFF,red/K05083%09%23FFFFFF,red/K04345%09%23FFFFFF,red/K00871%09%23FFFFFF,red/K05871%09%23FFFFFF,red/K05862

%23FFFFFF,red/K04539%09%23FFFFFF,red/K04345%09%23FFFFFF,red/K04548%09%23FFFFFF,red/K03099%09%23FFFFFF,red/

%23FFFFFF,red/K06766%09%23FFFFFF,red/K04409%09%23FFFFFF,red/K05746%09%23FFFFFF,red/K06821%09%23FFFFFF,red/K05102

%23FFFFFF,red/K04371%09%23FFFFFF,red/K08045%09%23FFFFFF,red/K04139%09%23FFFFFF,red/K00907%09%23FFFFFF,red/K05850

%23FFFFFF,red/K04604%09%23FFFFFF,red/K13985%09%23FFFFFF,red/K04548%09%23FFFFFF,red/K04345%09%23FFFFFF,red/

%23FFFFFF,red/K05731%09%23FFFFFF,red/K05209%09%23FFFFFF,red/K04440%09%23FFFFFF,red/K04409%09%23FFFFFF,red/K04260

%23FFFFFF,red/K04539%09%23FFFFFF,red/K00910%09%23FFFFFF,red/K04409%09%23FFFFFF,red/K04179%09%23FFFFFF,red/K04345

%23FFFFFF,red/K04358%09%23FFFFFF,red/K05746%09%23FFFFFF,red/K13708%09%23FFFFFF,red/K06587%09%23FFFFFF,red/K05757

%23FFFFFF,red/K04420%09%23FFFFFF,red/K03171%09%23FFFFFF,red/K04855%09%23FFFFFF,red/K05467%09%23FFFFFF,red/K03099
%23FFFFFF,red/K04855%09%23FFFFFF,red/K06622%09%23FFFFFF,red/K22398%09%23FFFFFF,red/K04913%09%23FFFFFF,red/K02375
%23FFFFFF,red/K05763%09%23FFFFFF,red/K16848%09%23FFFFFF,red/K02105%09%23FFFFFF,red/K05731%09%23FFFFFF,red/K08122
%23FFFFFF,red/K05449%09%23FFFFFF,red/K04992%09%23FFFFFF,red/K05094%09%23FFFFFF,red/K04157%09%23FFFFFF,red/K04262
%23FFFFFF,red/K05107%09%23FFFFFF,red/K04371%09%23FFFFFF,red/K06225%09%23FFFFFF,red/K06766%09%23FFFFFF,red/K06753
%23FFFFFF,red/K04604%09%23FFFFFF,red/K17386%09%23FFFFFF,red/K04849%09%23FFFFFF,red/K00871%09%23FFFFFF,red/K05871
%23FFFFFF,red/K04262%09%23FFFFFF,red/K04604%09%23FFFFFF,red/K04275%09%23FFFFFF,red/K03099%09%23FFFFFF,red/
%23FFFFFF,red/K12366%09%23FFFFFF,red/K04409%09%23FFFFFF,red/K00910%09%23FFFFFF,red/K05704%09%23FFFFFF,red/K04541
%23FFFFFF,red/K05734%09%23FFFFFF,red/K04409%09%23FFFFFF,red/K05704%09%23FFFFFF,red/K04358%09%23FFFFFF,red/K00907
%23FFFFFF,red/K04274%09%23FFFFFF,red/K05261%09%23FFFFFF,red/K05190%09%23FFFFFF,red/K04808%09%23FFFFFF,red/K04135

%23FFFFFF,red/K17386%09%23FFFFFF,red/K04372%09%23FFFFFF,red/K04468%09%23FFFFFF,red/K04849%09%23FFFFFF,red/K04412

%23FFFFFF,red/K07376%09%23FFFFFF,red/K00907%09%23FFFFFF,red/K17388%09%23FFFFFF,red/K05850%09%23FFFFFF,red/K17446

%23FFFFFF,red/K05209%09%23FFFFFF,red/K19021%09%23FFFFFF,red/K04409%09%23FFFFFF,red/K04260%09%23FFFFFF,red/K17388

%23FFFFFF,red/K09054%09%23FFFFFF,red/K08860%09%23FFFFFF,red/K14026%09%23FFFFFF,red/K03173%09%23FFFFFF,red/

%09%23FFFFFF,red/K05742%09%23FFFFFF,red/K04440%09%23FFFFFF,red/K04265%09%23FFFFFF,red/K05850%09%23FFFFFF,red/K1[illegible]

%09%23FFFFFF,red/K13755%09%23FFFFFF,red/K04135%09%23FFFFFF,red/K13242%09%23FFFFFF,red/K04361%09%23FFFFFF,red/K0[illegible]

%09%23FFFFFF,red/K04380%09%23FFFFFF,red/K04858%09%23FFFFFF,red/K04412%09%23FFFFFF,red/K04674%09%23FFFFFF,red/K0

%09%23FFFFFF,red/K05704%09%23FFFFFF,red/K04441%09%23FFFFFF,red/K06269%09%23FFFFFF,red/K02375%09%23FFFFFF,red/K0

%09%23FFFFFF,red/K04358%09%23FFFFFF,red/K07190%09%23FFFFFF,red/K04855%09%23FFFFFF,red/K05850%09%23FFFFFF,red/K0

%09%23FFFFFF,red/K05734%09%23FFFFFF,red/K05704%09%23FFFFFF,red/K04409%09%23FFFFFF,red/K06845%09%23FFFFFF,red/K0

%09%23FFFFFF,red/K08041%09%23FFFFFF,red/K04189%09%23FFFFFF,red/K04135%09%23FFFFFF,red/K04634%09%23FFFFFF,red/K0

%09%23FFFFFF,red/K17388%09%23FFFFFF,red/K04179%09%23FFFFFF,red/K03099%09%23FFFFFF,red/K04548%09%23FFFFFF,red/

%09%23FFFFFF,red/K17388%09%23FFFFFF,red/K13708%09%23FFFFFF,red/K05741%09%23FFFFFF,red/K04275%09%23FFFFFF,red/K0

%09%23FFFFFF,red/K04589%09%23FFFFFF,red/K04609%09%23FFFFFF,red/K05211%09%23FFFFFF,red/K05249%09%23FFFFFF,red/K0

%09%23FFFFFF,red/K16342%09%23FFFFFF,red/K04674%09%23FFFFFF,red/K05462%09%23FFFFFF,red/K07829%09%23FFFFFF,red/K0[illegible]

4515%09%23FFFFFF,red/K05211%09%23FFFFFF,red/K05450%09%23FFFFFF,red/K04150%09%23FFFFFF,red/K04963%09%23FFFFFF,red

4859%09%23FFFFFF,red/K17614%09%23FFFFFF,red/K05450%09%23FFFFFF,red/K05091%09%23FFFFFF,red/K04462%09%23FFFFFF,red

5450%09%23FFFFFF,red/K05211%09%23FFFFFF,red/K04150%09%23FFFFFF,red/K05209%09%23FFFFFF,red/K23449%09%23FFFFFF,red

5770%09%23FFFFFF,red/K05181%09%23FFFFFF,red/K21998%09%23FFFFFF,red/K04150%09%23FFFFFF,red/K05247%09%23FFFFFF,red

5450%09%23FFFFFF,red/K04371%09%23FFFFFF,red/K04870%09%23FFFFFF,red/K04462%09%23FFFFFF,red/K04344%09%23FFFFFF,red

l/K05209%09%23FFFFFF,red/K05175%09%23FFFFFF,red/K04140%09%23FFFFFF,red/K08039%09%23FFFFFF,red/K04583%09%23FFFFFI

F,red/K08397%09%23FFFFFF,red/K05207%09%23FFFFFF,red/K04275%09%23FFFFFF,red/K04615%09%23FFFFFF,red/
