# Supplementary material for: Genetic diversity and selection of Tibetan sheep breeds revealed by whole-genome resequencing
Source: Anim Biosci. 2023 May 2;36(7):991–1002. doi: 10.5713/ab.22.0432 (PMC10330983; doi:10.5713/ab.22.0432)
Supplement: Supplementary file 15 [file ab-22-0432-Supplementary-Table-15.pdf]

Supplementary Table 15. KEGG analysis of putative selected genes compared with OL breed

GY vs OL

PathwayID	Pathway	Level1	Level2	list_number	total_number
oas04927	Cortisol synthesis and secretion	Organismal Systems	Endocrine system	17	67
oas04928	Parathyroid hormone synthesis, sec	Organismal Systems	Endocrine system	22	106
oas04724	Glutamatergic synapse	Organismal Systems	Nervous system	22	109
oas04725	Cholinergic synapse	Organismal Systems	Nervous system	22	109
oas04911	Insulin secretion	Organismal Systems	Endocrine system	18	87
oas00230	Purine metabolism	Metabolism	Nucleotide metabolism	23	131
oas04022	cGMP-PKG signaling pathway	Environmental Information Proc	Signal transduction	27	165
oas04922	Glucagon signaling pathway	Organismal Systems	Endocrine system	19	105
oas04918	Thyroid hormone synthesis	Organismal Systems	Endocrine system	15	76
oas04360	Axon guidance	Organismal Systems	Development and regeneration	28	180
oas04961	Endocrine and other factor-regulat	Organismal Systems	Excretory system	11	51
oas04925	Aldosterone synthesis and secretio	Organismal Systems	Endocrine system	17	96
oas04730	Long-term depression	Organismal Systems	Nervous system	12	59
oas04971	Gastric acid secretion	Organismal Systems	Digestive system	14	76
oas04921	Oxytocin signaling pathway	Organismal Systems	Endocrine system	23	150
oas04540	Gap junction	Cellular Processes	Cellular community - eukaryotes	15	89
oas04020	Calcium signaling pathway	Environmental Information Proc	Signal transduction	32	238
oas04261	Adrenergic signaling in cardiomyo	Organismal Systems	Circulatory system	22	149
oas00520	Amino sugar and nucleotide sugar	Metabolism	Carbohydrate metabolism	10	52
oas04728	Dopaminergic synapse	Organismal Systems	Nervous system	19	127
oas04270	Vascular smooth muscle contractio	Organismal Systems	Circulatory system	20	136
oas04713	Circadian entrainment	Organismal Systems	Environmental adaptation	15	94
oas04723	Retrograde endocannabinoid signa	Organismal Systems	Nervous system	21	147
oas04390	Hippo signaling pathway	Environmental Information Proc	Signal transduction	22	156
oas04935	Growth hormone synthesis, secreti	Organismal Systems	Endocrine system	18	121
oas04924	Renin secretion	Organismal Systems	Endocrine system	12	71
oas04970	Salivary secretion	Organismal Systems	Digestive system	14	89
oas04072	Phospholipase D signaling pathway	Environmental Information Proc	Signal transduction	21	150
oas00513	Various types of N-glycan biosyntl	Metabolism	Glycan biosynthesis and metabolism	8	42

oas04392	Hippo signaling pathway - multiple	Environmental Information Proc	Signal transduction	6	28
oas04720	Long-term potentiation	Organismal Systems	Nervous system	11	67
oas00510	N-Glycan biosynthesis	Metabolism	Glycan biosynthesis and metabolism	9	52
ZK vs OL					
oas04020	Calcium signaling pathway	Environmental Information Proc	Signal transduction	43	238
oas04724	Glutamatergic synapse	Organismal Systems	Nervous system	25	109
oas04713	Circadian entrainment	Organismal Systems	Environmental adaptation	21	94
oas05031	Amphetamine addiction	Human Diseases	Substance dependence	17	68
oas04510	Focal adhesion	Cellular Processes	Cellular community - eukaryotes	35	203
oas04360	Axon guidance	Organismal Systems	Development and regeneration	32	180
oas05033	Nicotine addiction	Human Diseases	Substance dependence	12	40
oas04022	cGMP-PKG signaling pathway	Environmental Information Proc	Signal transduction	29	165
oas04810	Regulation of actin cytoskeleton	Cellular Processes	Cell motility	35	218
oas04015	Rap1 signaling pathway	Environmental Information Proc	Signal transduction	34	212
oas04010	MAPK signaling pathway	Environmental Information Proc	Signal transduction	43	292
oas04512	ECM-receptor interaction	Environmental Information Proc	Signaling molecules and interaction	18	90
oas00230	Purine metabolism	Metabolism	Nucleotide metabolism	23	131
oas04728	Dopaminergic synapse	Organismal Systems	Nervous system	22	127
oas04540	Gap junction	Cellular Processes	Cellular community - eukaryotes	17	89
HZ vs OL					
oas04360	Axon guidance	Organismal Systems	Development and regeneration	34	180
oas04725	Cholinergic synapse	Organismal Systems	Nervous system	21	109
oas04520	Adherens junction	Cellular Processes	Cellular community - eukaryotes	16	73
oas04926	Relaxin signaling pathway	Organismal Systems	Endocrine system	23	129
oas04020	Calcium signaling pathway	Environmental Information Proc	Signal transduction	36	238
oas04727	GABAergic synapse	Organismal Systems	Nervous system	17	85
oas04929	GnRH secretion	Organismal Systems	Endocrine system	14	64
oas04724	Glutamatergic synapse	Organismal Systems	Nervous system	20	109
oas04713	Circadian entrainment	Organismal Systems	Environmental adaptation	18	94
oas04072	Phospholipase D signaling pathway	Environmental Information Proc	Signal transduction	25	150
oas04935	Growth hormone synthesis, secretio	Organismal Systems	Endocrine system	21	121
oas04726	Serotonergic synapse	Organismal Systems	Nervous system	19	114

oas04062	Chemokine signaling pathway	Organismal Systems	Immune system	28	191
oas04810	Regulation of actin cytoskeleton	Cellular Processes	Cell motility	31	218
oas00604	Glycosphingolipid biosynthesis - g	Metabolism	Glycan biosynthesis and metabolism	5	15
oas04080	Neuroactive ligand-receptor interac	Environmental Information Proc	Signaling molecules and interaction	45	355
oas05200	Pathways in cancer	Human Diseases	Cancer: overview	64	541
oas00512	Mucin type O-glycan biosynthesis	Metabolism	Glycan biosynthesis and metabolism	8	36
oas04371	Apelin signaling pathway	Environmental Information Proc	Signal transduction	20	133
oas04010	MAPK signaling pathway	Environmental Information Proc	Signal transduction	37	292
oas04723	Retrograde endocannabinoid signa	Organismal Systems	Nervous system	21	147
oas04022	cGMP-PKG signaling pathway	Environmental Information Proc	Signal transduction	23	165
oas00514	Other types of O-glycan biosynthes	Metabolism	Glycan biosynthesis and metabolism	9	48
oas04611	Platelet activation	Organismal Systems	Immune system	18	123
oas04540	Gap junction	Cellular Processes	Cellular community - eukaryotes	14	89
oas04024	cAMP signaling pathway	Environmental Information Proc	Signal transduction	29	226
oas04141	Protein processing in endoplasmic	Genetic Information Processing	Folding, sorting and degradation	23	171
oas04270	Vascular smooth muscle contractio	Organismal Systems	Circulatory system	19	136
oas04927	Cortisol synthesis and secretion	Organismal Systems	Endocrine system	11	67
oas04012	ErbB signaling pathway	Environmental Information Proc	Signal transduction	13	84
oas04068	FoxO signaling pathway	Environmental Information Proc	Signal transduction	18	130
oas00100	Steroid biosynthesis	Metabolism	Lipid metabolism	5	22
oas00785	Lipoic acid metabolism	Metabolism	Metabolism of cofactors and vitamin	2	4
oas00230	Purine metabolism	Metabolism	Nucleotide metabolism	18	131
oas04911	Insulin secretion	Organismal Systems	Endocrine system	13	87
oas04925	Aldosterone synthesis and secretioı	Organismal Systems	Endocrine system	14	96
oas04928	Parathyroid hormone synthesis, sec	Organismal Systems	Endocrine system	15	106

Pvalue	FDR	List
6.48E-05	0.019110924	HSD3B1(HSD
0.000157115	0.019110924	VDR(VDR),A
0.000239635	0.019110924	CACNA1A(CA
0.000239635	0.019110924	CACNA1A(CA
0.00062713	0.040010899	CACNA1F(CA
0.001361613	0.062050635	AK1(AK1),GU
0.001636955	0.06527357	PPP1CB(PPP1
0.002404438	0.084473767	G6PC1(G6PC
0.002810307	0.084473767	CGA(CGA),PI
0.002912889	0.084473767	PARD6A(PAR
0.004909044	0.122263973	VDR(VDR),PI
0.004982544	0.122263973	CAMK1D(CA
0.005571332	0.126946776	CACNA1A(CA
0.007241635	0.154005442	SLC9A4(SLC
0.007806663	0.155645353	PPP1CB(PPP1
0.012603068	0.234115438	SOS2(SOS2),C
0.013890118	0.234115438	CACNA1A(CA
0.01394418	0.234115438	PPP1CB(PPP1
0.016087357	0.244374618	GALE(GALE),
0.018710625	0.257554042	PPP1CB(PPP1
0.019202304	0.257554042	PPP1CB(PPP1
0.020184486	0.257554042	GUCY1A2(GU
0.022500899	0.259615331	CACNA1A(CA
0.02271681	0.259615331	YAP1(YAP1),
0.022858481	0.259615331	SOS2(SOS2),C
0.023601394	0.259615331	CACNA1F(CA
0.027154795	0.274064469	KCNMA1(KC
0.02749236	0.274064469	KIT(KIT),PIK3
0.031354751	0.294181341	MAN1C1(MA

0.035227603	0.315763443	YAP1(YAP1),
0.035634746	0.315763443	PPP1CB(PPP1
0.040552975	0.349632404	MAN1C1(MA
6.75E-06	0.001480622	FLT4(FLT4),P
9.20E-06	0.001480622	PPP3CA(PPP3
7.04E-05	0.006169976	CACNA1I(CA
7.72E-05	0.006169976	PPP3CA(PPP3
0.000125088	0.006169976	FLT4(FLT4),C
0.000133621	0.006169976	EPHB1(EPHB
0.00013413	0.006169976	SLC17A6(SLC
0.00032946	0.013260762	PPP3CA(PPP3
0.000507738	0.018165741	ARHGEF6(AR
0.000618131	0.019903826	FLT4(FLT4),M
0.000819473	0.023988201	FLT4(FLT4),M
0.000930684	0.024973352	COL4A6(COL
0.001323039	0.032770658	PAPSS2(PAPS
0.001988565	0.045737004	PPP3CA(PPP3
0.002151384	0.046183046	PRKCB(PRKC
2.13E-05	0.006716448	SEMA3C(SEM
0.000579809	0.046660003	CACNA1A(CA
0.000592508	0.046660003	NLK(NLK),CT
0.001006863	0.047097382	COL4A5(COL
0.001203106	0.047097382	CACNA1A(CA
0.001215969	0.047097382	CACNA1A(CA
0.001303156	0.047097382	GABBR2(GAI
0.0014563	0.047097382	CACNA1A(CA
0.001495155	0.047097382	CACNA1I(CA
0.001685367	0.04826277	DGKH(DGKH
0.00230418	0.05184406	CACNA1F(CA
0.005724793	0.119436934	CACNA1A(CA

0.006173306	0.119436934	JAK2(JAK2),[illegible]
0.006445803	0.119436934	FGF5(FGF5),P[illegible]
0.007911735	0.124609823	ST8SIA5(ST8[illegible]
0.010470419	0.157056282	PTGER3(PTG[illegible]
0.012094613	0.17091233	PTGER3(PTG[illegible]
0.012479313	0.17091233	GALNTL6(GA[illegible]
0.014359114	0.188463372	PIK3CG(PIK3[illegible]
0.019005604	0.239470604	CACNA1A(C[illegible]
0.021102768	0.249089919	CACNA1A(C[illegible]
0.021350564	0.249089919	PIK3CG(PIK3[illegible]
0.024516043	0.262712213	GALNTL6(GA[illegible]
0.025052666	0.262712213	TLN2(TLN2),[illegible]
0.025854218	0.262712213	PLCB1(PLCB[illegible]
0.030005747	0.28548838	PTGER3(PTG[illegible]
0.03094746	0.28548838	UGGT1(UGG[illegible]
0.033435853	0.289256013	PRKCE(PRKC[illegible]
0.034207191	0.289256013	CACNA1I(CA[illegible]
0.034894376	0.289256013	ERBB4(ERBB[illegible]
0.040675033	0.316401891	NLK(NLK),T[illegible]
0.041182468	0.316401891	NSDHL(NSD[illegible]
0.042335756	0.317518171	ACSM1(ACS[illegible]
0.0433806	0.317788119	LOC10110903[illegible]
0.044655717	0.319033851	KCNMA1(KC[illegible]
0.045576264	0.319033851	CACNA1I(CA[illegible]
0.049484421	0.331650909	CREB3L2(CR[illegible]

URL

http://www.kegg.jp/kegg-bin/show_pathway?map04927/K04959%09%23FFFFFF,red/K04853%09%23FFFFFF,red/K09355%09%23FFFFFF,red/K143

http://www.kegg.jp/kegg-bin/show_pathway?map04928/K04959%09%23FFFFFF,red/K05858%09%23FFFFFF,red/K13293%09%23FFFFFF,red/K044

http://www.kegg.jp/kegg-bin/show_pathway?map04724/K04959%09%23FFFFFF,red/K04603%09%23FFFFFF,red/K05858%09%23FFFFFF,red/K052

http://www.kegg.jp/kegg-bin/show_pathway?map04725/K04959%09%23FFFFFF,red/K04849%09%23FFFFFF,red/K04928%09%23FFFFFF,red/K048

http://www.kegg.jp/kegg-bin/show_pathway?map04911/K04853%09%23FFFFFF,red/K15297%09%23FFFFFF,red/K04936%09%23FFFFFF,red/K058

http://www.kegg.jp/kegg-bin/show_pathway?map00230/K00764%09%23FFFFFF,red/K00939%09%23FFFFFF,red/K13293%09%23FFFFFF,red/K184

http://www.kegg.jp/kegg-bin/show_pathway?map04022/K04959%09%23FFFFFF,red/K18050%09%23FFFFFF,red/K04853%09%23FFFFFF,red/K049

http://www.kegg.jp/kegg-bin/show_pathway?map04922/K04959%09%23FFFFFF,red/K19524%09%23FFFFFF,red/K05858%09%23FFFFFF,red/K114

http://www.kegg.jp/kegg-bin/show_pathway?map04918/K04959%09%23FFFFFF,red/K09048%09%23FFFFFF,red/K08045%09%23FFFFFF,red/K004

http://www.kegg.jp/kegg-bin/show_pathway?map04360/K05099%09%23FFFFFF,red/K06765%09%23FFFFFF,red/K04237%09%23FFFFFF,red/K068

http://www.kegg.jp/kegg-bin/show_pathway?map04961/K05858%09%23FFFFFF,red/K01528%09%23FFFFFF,red/K04634%09%23FFFFFF,red/K080

http://www.kegg.jp/kegg-bin/show_pathway?map04925/K04959%09%23FFFFFF,red/K06070%09%23FFFFFF,red/K18050%09%23FFFFFF,red/K048

http://www.kegg.jp/kegg-bin/show_pathway?map04730/K05854%09%23FFFFFF,red/K04959%09%23FFFFFF,red/K12318%09%23FFFFFF,red/K046

http://www.kegg.jp/kegg-bin/show_pathway?map04971/K04959%09%23FFFFFF,red/K08045%09%23FFFFFF,red/K13961%09%23FFFFFF,red/K049

http://www.kegg.jp/kegg-bin/show_pathway?map04921/K04959%09%23FFFFFF,red/K04858%09%23FFFFFF,red/K04853%09%23FFFFFF,red/K048

http://www.kegg.jp/kegg-bin/show_pathway?map04540/K04959%09%23FFFFFF,red/K12318%09%23FFFFFF,red/K08045%09%23FFFFFF,red/K046

http://www.kegg.jp/kegg-bin/show_pathway?map04020/K04959%09%23FFFFFF,red/K05099%09%23FFFFFF,red/K05096%09%23FFFFFF,red/K048

http://www.kegg.jp/kegg-bin/show_pathway?map04261/K04858%09%23FFFFFF,red/K04853%09%23FFFFFF,red/K04863%09%23FFFFFF,red/K049

http://www.kegg.jp/kegg-bin/show_pathway?map00520/K01784%09%23FFFFFF,red/K12373%09%23FFFFFF,red/K01711%09%23FFFFFF,red/K124

http://www.kegg.jp/kegg-bin/show_pathway?map04728/K04959%09%23FFFFFF,red/K04849%09%23FFFFFF,red/K05858%09%23FFFFFF,red/K044

http://www.kegg.jp/kegg-bin/show_pathway?map04270/K04959%09%23FFFFFF,red/K18050%09%23FFFFFF,red/K04853%09%23FFFFFF,red/K049

http://www.kegg.jp/kegg-bin/show_pathway?map04713/K16513%09%23FFFFFF,red/K12318%09%23FFFFFF,red/K05200%09%23FFFFFF,red/K080

http://www.kegg.jp/kegg-bin/show_pathway?map04723/K04959%09%23FFFFFF,red/K03940%09%23FFFFFF,red/K04849%09%23FFFFFF,red/K048

http://www.kegg.jp/kegg-bin/show_pathway?map04390/K03362%09%23FFFFFF,red/K16687%09%23FFFFFF,red/K02085%09%23FFFFFF,red/K042

http://www.kegg.jp/kegg-bin/show_pathway?map04935/K04959%09%23FFFFFF,red/K17448%09%23FFFFFF,red/K04853%09%23FFFFFF,red/K072

http://www.kegg.jp/kegg-bin/show_pathway?map04924/K04959%09%23FFFFFF,red/K12318%09%23FFFFFF,red/K08045%09%23FFFFFF,red/K048

http://www.kegg.jp/kegg-bin/show_pathway?map04970/K04959%09%23FFFFFF,red/K12318%09%23FFFFFF,red/K08045%09%23FFFFFF,red/K049

http://www.kegg.jp/kegg-bin/show_pathway?map04072/K17448%09%23FFFFFF,red/K04603%09%23FFFFFF,red/K07203%09%23FFFFFF,red/K058

http://www.kegg.jp/kegg-bin/show_pathway?map00513/K07441%09%23FFFFFF,red/K12373%09%23FFFFFF,red/K01230%09%23FFFFFF,red/K007

http://www.kegg.jp/kegg-bin/show_pathway?map04392/K16682%09%23FFFFFF,red/K08791%09%23FFFFFF,red/K16685%09%23FFFFFF,red/K166

http://www.kegg.jp/kegg-bin/show_pathway?map04720/K04959%09%23FFFFFF,red/K04373%09%23FFFFFF,red/K04603%09%23FFFFFF,red/K058

http://www.kegg.jp/kegg-bin/show_pathway?map00510/K12345%09%23FFFFFF,red/K03850%09%23FFFFFF,red/K12666%09%23FFFFFF,red/K074

http://www.kegg.jp/kegg-bin/show_pathway?map04020/K05096%09%23FFFFFF,red/K04603%09%23FFFFFF,red/K04636%09%23FFFFFF,red/K050

http://www.kegg.jp/kegg-bin/show_pathway?map04724/K04630%09%23FFFFFF,red/K04606%09%23FFFFFF,red/K04603%09%23FFFFFF,red/K080

http://www.kegg.jp/kegg-bin/show_pathway?map04713/K04630%09%23FFFFFF,red/K08044%09%23FFFFFF,red/K04850%09%23FFFFFF,red/K112

http://www.kegg.jp/kegg-bin/show_pathway?map05031/K01593%09%23FFFFFF,red/K06067%09%23FFFFFF,red/K04448%09%23FFFFFF,red/K048

http://www.kegg.jp/kegg-bin/show_pathway?map04510/K06245%09%23FFFFFF,red/K17448%09%23FFFFFF,red/K05096%09%23FFFFFF,red/K064

http://www.kegg.jp/kegg-bin/show_pathway?map04360/K06619%09%23FFFFFF,red/K07293%09%23FFFFFF,red/K24609%09%23FFFFFF,red/K046

http://www.kegg.jp/kegg-bin/show_pathway?map05033/K05199%09%23FFFFFF,red/K04849%09%23FFFFFF,red/K05200%09%23FFFFFF,red/K052

http://www.kegg.jp/kegg-bin/show_pathway?map04022/K04630%09%23FFFFFF,red/K17333%09%23FFFFFF,red/K18050%09%23FFFFFF,red/K080

http://www.kegg.jp/kegg-bin/show_pathway?map04810/K05720%09%23FFFFFF,red/K06461%09%23FFFFFF,red/K04289%09%23FFFFFF,red/K064

http://www.kegg.jp/kegg-bin/show_pathway?map04015/K04630%09%23FFFFFF,red/K06070%09%23FFFFFF,red/K05096%09%23FFFFFF,red/K064

http://www.kegg.jp/kegg-bin/show_pathway?map04010/K05096%09%23FFFFFF,red/K04863%09%23FFFFFF,red/K04448%09%23FFFFFF,red/K050

http://www.kegg.jp/kegg-bin/show_pathway?map04512/K23380%09%23FFFFFF,red/K06245%09%23FFFFFF,red/K06237%09%23FFFFFF,red/K062

http://www.kegg.jp/kegg-bin/show_pathway?map00230/K01487%09%23FFFFFF,red/K00939%09%23FFFFFF,red/K08044%09%23FFFFFF,red/K132

http://www.kegg.jp/kegg-bin/show_pathway?map04728/K04630%09%23FFFFFF,red/K04849%09%23FFFFFF,red/K01593%09%23FFFFFF,red/K048

http://www.kegg.jp/kegg-bin/show_pathway?map04540/K04630%09%23FFFFFF,red/K04603%09%23FFFFFF,red/K08044%09%23FFFFFF,red/K042

http://www.kegg.jp/kegg-bin/show_pathway?map04360/K06619%09%23FFFFFF,red/K06840%09%23FFFFFF,red/K05766%09%23FFFFFF,red/K075

http://www.kegg.jp/kegg-bin/show_pathway?map04725/K04447%09%23FFFFFF,red/K04849%09%23FFFFFF,red/K04928%09%23FFFFFF,red/K048

http://www.kegg.jp/kegg-bin/show_pathway?map04520/K05706%09%23FFFFFF,red/K05691%09%23FFFFFF,red/K04371%09%23FFFFFF,red/K044

http://www.kegg.jp/kegg-bin/show_pathway?map04926/K17448%09%23FFFFFF,red/K04674%09%23FFFFFF,red/K19720%09%23FFFFFF,red/K058

http://www.kegg.jp/kegg-bin/show_pathway?map04020/K10785%09%23FFFFFF,red/K04853%09%23FFFFFF,red/K05085%09%23FFFFFF,red/K058

http://www.kegg.jp/kegg-bin/show_pathway?map04727/K04849%09%23FFFFFF,red/K04853%09%23FFFFFF,red/K08041%09%23FFFFFF,red/K051

http://www.kegg.jp/kegg-bin/show_pathway?map04929/K04954%09%23FFFFFF,red/K04371%09%23FFFFFF,red/K04853%09%23FFFFFF,red/K049

http://www.kegg.jp/kegg-bin/show_pathway?map04724/K16342%09%23FFFFFF,red/K05858%09%23FFFFFF,red/K05213%09%23FFFFFF,red/K080

http://www.kegg.jp/kegg-bin/show_pathway?map04713/K05858%09%23FFFFFF,red/K08041%09%23FFFFFF,red/K11265%09%23FFFFFF,red/K048

http://www.kegg.jp/kegg-bin/show_pathway?map04072/K17386%09%23FFFFFF,red/K17448%09%23FFFFFF,red/K07830%09%23FFFFFF,red/K163

http://www.kegg.jp/kegg-bin/show_pathway?map04935/K17448%09%23FFFFFF,red/K04447%09%23FFFFFF,red/K04853%09%23FFFFFF,red/K072

http://www.kegg.jp/kegg-bin/show_pathway?map04726/K07413%09%23FFFFFF,red/K02187%09%23FFFFFF,red/K04849%09%23FFFFFF,red/K015

http://www.kegg.jp/kegg-bin/show_pathway?map04062/K17448%09%23FFFFFF,red/K04447%09%23FFFFFF,red/K05747%09%23FFFFFF,red/K058

http://www.kegg.jp/kegg-bin/show_pathway?map04810/K17386%09%23FFFFFF,red/K17260%09%23FFFFFF,red/K07830%09%23FFFFFF,red/K057

http://www.kegg.jp/kegg-bin/show_pathway?map00604/K03369%09%23FFFFFF,red/K03372%09%23FFFFFF,red/K03370%09%23FFFFFF,red/K007

http://www.kegg.jp/kegg-bin/show_pathway?map04080/K05213%09%23FFFFFF,red/K05424%09%23FFFFFF,red/K08522%09%23FFFFFF,red/K046

http://www.kegg.jp/kegg-bin/show_pathway?map05200/K02187%09%23FFFFFF,red/K04447%09%23FFFFFF,red/K06067%09%23FFFFFF,red/K119

http://www.kegg.jp/kegg-bin/show_pathway?map00512/K09905%09%23FFFFFF,red/K09663%09%23FFFFFF,red/K00710%09%23FFFFFF,red/K096

http://www.kegg.jp/kegg-bin/show_pathway?map04371/K17845%09%23FFFFFF,red/K07830%09%23FFFFFF,red/K18050%09%23FFFFFF,red/K046

http://www.kegg.jp/kegg-bin/show_pathway?map04010/K04423%09%23FFFFFF,red/K02187%09%23FFFFFF,red/K07830%09%23FFFFFF,red/K044

http://www.kegg.jp/kegg-bin/show_pathway?map04723/K04849%09%23FFFFFF,red/K04853%09%23FFFFFF,red/K03951%09%23FFFFFF,red/K119

http://www.kegg.jp/kegg-bin/show_pathway?map04022/K18050%09%23FFFFFF,red/K04853%09%23FFFFFF,red/K04936%09%23FFFFFF,red/K058

http://www.kegg.jp/kegg-bin/show_pathway?map00514/K13676%09%23FFFFFF,red/K05948%09%23FFFFFF,red/K11703%09%23FFFFFF,red/K007

http://www.kegg.jp/kegg-bin/show_pathway?map04611/K16342%09%23FFFFFF,red/K19720%09%23FFFFFF,red/K05858%09%23FFFFFF,red/K080

http://www.kegg.jp/kegg-bin/show_pathway?map04540/K17386%09%23FFFFFF,red/K08042%09%23FFFFFF,red/K04371%09%23FFFFFF,red/K123

http://www.kegg.jp/kegg-bin/show_pathway?map04024/K07830%09%23FFFFFF,red/K04153%09%23FFFFFF,red/K04853%09%23FFFFFF,red/K132

http://www.kegg.jp/kegg-bin/show_pathway?map04141/K04426%09%23FFFFFF,red/K10950%09%23FFFFFF,red/K23741%09%23FFFFFF,red/K126

http://www.kegg.jp/kegg-bin/show_pathway?map04270/K18050%09%23FFFFFF,red/K04853%09%23FFFFFF,red/K16342%09%23FFFFFF,red/K049

http://www.kegg.jp/kegg-bin/show_pathway?map04927/K08042%09%23FFFFFF,red/K09048%09%23FFFFFF,red/K00070%09%23FFFFFF,red/K048

http://www.kegg.jp/kegg-bin/show_pathway?map04012/K06619%09%23FFFFFF,red/K17448%09%23FFFFFF,red/K04371%09%23FFFFFF,red/K057

http://www.kegg.jp/kegg-bin/show_pathway?map04068/K17845%09%23FFFFFF,red/K13304%09%23FFFFFF,red/K04468%09%23FFFFFF,red/K046

http://www.kegg.jp/kegg-bin/show_pathway?map00100/K00213%09%23FFFFFF,red/K01824%09%23FFFFFF,red/K07748%09%23FFFFFF,red/K098

http://www.kegg.jp/kegg-bin/show_pathway?map00785/K23756%09%23FFFFFF,red/K23735%09%23FFFFFF,red/

http://www.kegg.jp/kegg-bin/show_pathway?map00230/K01487%09%23FFFFFF,red/K00944%09%23FFFFFF,red/K19970%09%23FFFFFF,red/K132

http://www.kegg.jp/kegg-bin/show_pathway?map04911/K08042%09%23FFFFFF,red/K04939%09%23FFFFFF,red/K09048%09%23FFFFFF,red/K048

http://www.kegg.jp/kegg-bin/show_pathway?map04925/K08042%09%23FFFFFF,red/K09048%09%23FFFFFF,red/K00070%09%23FFFFFF,red/K180

http://www.kegg.jp/kegg-bin/show_pathway?map04928/K03068%09%23FFFFFF,red/K08042%09%23FFFFFF,red/K09048%09%23FFFFFF,red/K043

49%09%23FFFFFF,red/K05858%09%23FFFFFF,red/K04958%09%23FFFFFF,red/K04634%09%23FFFFFF,red/K08049%09%23FFFFFF
39%09%23FFFFFF,red/K08049%09%23FFFFFF,red/K04958%09%23FFFFFF,red/K12331%09%23FFFFFF,red/K04634%09%23FFFFFF
04%09%23FFFFFF,red/K04958%09%23FFFFFF,red/K04634%09%23FFFFFF,red/K08049%09%23FFFFFF,red/K04609%09%23FFFFFF
53%09%23FFFFFF,red/K04815%09%23FFFFFF,red/K04926%09%23FFFFFF,red/K05858%09%23FFFFFF,red/K04131%09%23FFFFFF
58%09%23FFFFFF,red/K04131%09%23FFFFFF,red/K04351%09%23FFFFFF,red/K04634%09%23FFFFFF,red/K08049%09%23FFFFFF
36%09%23FFFFFF,red/K01081%09%23FFFFFF,red/K11265%09%23FFFFFF,red/K13758%09%23FFFFFF,red/K12322%09%23FFFFFF
36%09%23FFFFFF,red/K05858%09%23FFFFFF,red/K05862%09%23FFFFFF,red/K18154%09%23FFFFFF,red/K08049%09%23FFFFFF
34%09%23FFFFFF,red/K00016%09%23FFFFFF,red/K16311%09%23FFFFFF,red/K04958%09%23FFFFFF,red/K04634%09%23FFFFFF
32%09%23FFFFFF,red/K04249%09%23FFFFFF,red/K05858%09%23FFFFFF,red/K04958%09%23FFFFFF,red/K04634%09%23FFFFFF
40%09%23FFFFFF,red/K07520%09%23FFFFFF,red/K07526%09%23FFFFFF,red/K20020%09%23FFFFFF,red/K04671%09%23FFFFFF
49%09%23FFFFFF,red/K05849%09%23FFFFFF,red/K08550%09%23FFFFFF,red/K19662%09%23FFFFFF,red/K08539%09%23FFFFFF
53%09%23FFFFFF,red/K05858%09%23FFFFFF,red/K04958%09%23FFFFFF,red/K04634%09%23FFFFFF,red/K08049%09%23FFFFFF
03%09%23FFFFFF,red/K05858%09%23FFFFFF,red/K04344%09%23FFFFFF,red/K07376%09%23FFFFFF,red/K04958%09%23FFFFFF
26%09%23FFFFFF,red/K05858%09%23FFFFFF,red/K04131%09%23FFFFFF,red/K00907%09%23FFFFFF,red/K04958%09%23FFFFFF
63%09%23FFFFFF,red/K05858%09%23FFFFFF,red/K12329%09%23FFFFFF,red/K04859%09%23FFFFFF,red/K18154%09%23FFFFFF
03%09%23FFFFFF,red/K05858%09%23FFFFFF,red/K07376%09%23FFFFFF,red/K04604%09%23FFFFFF,red/K04958%09%23FFFFFF
49%09%23FFFFFF,red/K04853%09%23FFFFFF,red/K04603%09%23FFFFFF,red/K04636%09%23FFFFFF,red/K05858%09%23FFFFFF
26%09%23FFFFFF,red/K05858%09%23FFFFFF,red/K04859%09%23FFFFFF,red/K04351%09%23FFFFFF,red/K08049%09%23FFFFFF
09%09%23FFFFFF,red/K00966%09%23FFFFFF,red/K18674%09%23FFFFFF,red/K00621%09%23FFFFFF,red/K01809%09%23FFFFFF
39%09%23FFFFFF,red/K04147%09%23FFFFFF,red/K04958%09%23FFFFFF,red/K04634%09%23FFFFFF,red/K05036%09%23FFFFFF
36%09%23FFFFFF,red/K05858%09%23FFFFFF,red/K12329%09%23FFFFFF,red/K08447%09%23FFFFFF,red/K08049%09%23FFFFFF
45%09%23FFFFFF,red/K07843%09%23FFFFFF,red/K04445%09%23FFFFFF,red/K05858%09%23FFFFFF,red/K07376%09%23FFFFFF
53%09%23FFFFFF,red/K04603%09%23FFFFFF,red/K05858%09%23FFFFFF,red/K05190%09%23FFFFFF,red/K04277%09%23FFFFFF
37%09%23FFFFFF,red/K16681%09%23FFFFFF,red/K04671%09%23FFFFFF,red/K00572%09%23FFFFFF,red/K08791%09%23FFFFFF
03%09%23FFFFFF,red/K05858%09%23FFFFFF,red/K11265%09%23FFFFFF,red/K08049%09%23FFFFFF,red/K04958%09%23FFFFFF
53%09%23FFFFFF,red/K04936%09%23FFFFFF,red/K05858%09%23FFFFFF,red/K19021%09%23FFFFFF,red/K04958%09%23FFFFFF
36%09%23FFFFFF,red/K05858%09%23FFFFFF,red/K07376%09%23FFFFFF,red/K04131%09%23FFFFFF,red/K04958%09%23FFFFFF
58%09%23FFFFFF,red/K01528%09%23FFFFFF,red/K04351%09%23FFFFFF,red/K08049%09%23FFFFFF,red/K05450%09%23FFFFFF
17%09%23FFFFFF,red/K13748%09%23FFFFFF,red/K12666%09%23FFFFFF,red/K09672%09%23FFFFFF,red/

87%09%23FFFFFF,red/K16822%09%23FFFFFF,red/K16686%09%23FFFFFF,red/

58%09%23FFFFFF,red/K04604%09%23FFFFFF,red/K04958%09%23FFFFFF,red/K04634%09%23FFFFFF,red/K06269%09%23FFFFFF

41%09%23FFFFFF,red/K03848%09%23FFFFFF,red/K00717%09%23FFFFFF,red/K01230%09%23FFFFFF,red/K13748%09%23FFFFFF

85%09%23FFFFFF,red/K04850%09%23FFFFFF,red/K09565%09%23FFFFFF,red/K04360%09%23FFFFFF,red/K08794%09%23FFFFFF

44%09%23FFFFFF,red/K04850%09%23FFFFFF,red/K04634%09%23FFFFFF,red/K05211%09%23FFFFFF,red/K04609%09%23FFFFFF

65%09%23FFFFFF,red/K04634%09%23FFFFFF,red/K04515%09%23FFFFFF,red/K05211%09%23FFFFFF,red/K05197%09%23FFFFFF

50%09%23FFFFFF,red/K04515%09%23FFFFFF,red/K05211%09%23FFFFFF,red/K05197%09%23FFFFFF,red/K05036%09%23FFFFFF

84%09%23FFFFFF,red/K04392%09%23FFFFFF,red/K04448%09%23FFFFFF,red/K12329%09%23FFFFFF,red/K05635%09%23FFFFFF

30%09%23FFFFFF,red/K17333%09%23FFFFFF,red/K06765%09%23FFFFFF,red/K07520%09%23FFFFFF,red/K02329%09%23FFFFFF

10%09%23FFFFFF,red/K05209%09%23FFFFFF,red/K05175%09%23FFFFFF,red/K05190%09%23FFFFFF,red/K04808%09%23FFFFFF

44%09%23FFFFFF,red/K04850%09%23FFFFFF,red/K09565%09%23FFFFFF,red/K18154%09%23FFFFFF,red/K04135%09%23FFFFFF

83%09%23FFFFFF,red/K05763%09%23FFFFFF,red/K05769%09%23FFFFFF,red/K05748%09%23FFFFFF,red/K05767%09%23FFFFFF

61%09%23FFFFFF,red/K04392%09%23FFFFFF,red/K08044%09%23FFFFFF,red/K05462%09%23FFFFFF,red/K04277%09%23FFFFFF

85%09%23FFFFFF,red/K04850%09%23FFFFFF,red/K04388%09%23FFFFFF,red/K04360%09%23FFFFFF,red/K04459%09%23FFFFFF

52%09%23FFFFFF,red/K06484%09%23FFFFFF,red/K06585%09%23FFFFFF,red/K23379%09%23FFFFFF,red/K05635%09%23FFFFFF

93%09%23FFFFFF,red/K01081%09%23FFFFFF,red/K18436%09%23FFFFFF,red/K11265%09%23FFFFFF,red/K13755%09%23FFFFFF

50%09%23FFFFFF,red/K04147%09%23FFFFFF,red/K04634%09%23FFFFFF,red/K04515%09%23FFFFFF,red/K05197%09%23FFFFFF

89%09%23FFFFFF,red/K04634%09%23FFFFFF,red/K05450%09%23FFFFFF,red/K19477%09%23FFFFFF,red/K08042%09%23FFFFFF

20%09%23FFFFFF,red/K04968%09%23FFFFFF,red/K00444%09%23FFFFFF,red/K05462%09%23FFFFFF,red/K04189%09%23FFFFFF

53%09%23FFFFFF,red/K05858%09%23FFFFFF,red/K08041%09%23FFFFFF,red/K04851%09%23FFFFFF,red/K04546%09%23FFFFFF

68%09%23FFFFFF,red/K08889%09%23FFFFFF,red/K04498%09%23FFFFFF,red/K05747%09%23FFFFFF,red/K04674%09%23FFFFFF

58%09%23FFFFFF,red/K04439%09%23FFFFFF,red/K08041%09%23FFFFFF,red/K04546%09%23FFFFFF,red/K00922%09%23FFFFFF

58%09%23FFFFFF,red/K04851%09%23FFFFFF,red/K04360%09%23FFFFFF,red/K05101%09%23FFFFFF,red/K08794%09%23FFFFFF

90%09%23FFFFFF,red/K04851%09%23FFFFFF,red/K04546%09%23FFFFFF,red/K08341%09%23FFFFFF,red/K05181%09%23FFFFFF

68%09%23FFFFFF,red/K05858%09%23FFFFFF,red/K04439%09%23FFFFFF,red/K04851%09%23FFFFFF,red/K04944%09%23FFFFFF

41%09%23FFFFFF,red/K04851%09%23FFFFFF,red/K04546%09%23FFFFFF,red/K04634%09%23FFFFFF,red/K05211%09%23FFFFFF

51%09%23FFFFFF,red/K04546%09%23FFFFFF,red/K04634%09%23FFFFFF,red/K05211%09%23FFFFFF,red/K13240%09%23FFFFFF

42%09%23FFFFFF,red/K07203%09%23FFFFFF,red/K05858%09%23FFFFFF,red/K05871%09%23FFFFFF,red/K08041%09%23FFFFFF

03%09%23FFFFFF,red/K05858%09%23FFFFFF,red/K08041%09%23FFFFFF,red/K11265%09%23FFFFFF,red/K04851%09%23FFFFFF

93%09%23FFFFFF,red/K04153%09%23FFFFFF,red/K04853%09%23FFFFFF,red/K16342%09%23FFFFFF,red/K11987%09%23FFFFFF

58%09%23FFFFFF,red/K05871%09%23FFFFFF,red/K08041%09%23FFFFFF,red/K04439%09%23FFFFFF,red/K04189%09%23FFFFFF

66%09%23FFFFFF,red/K04189%09%23FFFFFF,red/K06487%09%23FFFFFF,red/K23612%09%23FFFFFF,red/K06481%09%23FFFFFF

80%09%23FFFFFF,red/K12309%09%23FFFFFF,red/

10%09%23FFFFFF,red/K05202%09%23FFFFFF,red/K05051%09%23FFFFFF,red/K05186%09%23FFFFFF,red/K04804%09%23FFFFFF

87%09%23FFFFFF,red/K06618%09%23FFFFFF,red/K05858%09%23FFFFFF,red/K11126%09%23FFFFFF,red/K04546%09%23FFFFFF

53%09%23FFFFFF,red/K00780%09%23FFFFFF,red/

74%09%23FFFFFF,red/K07203%09%23FFFFFF,red/K05858%09%23FFFFFF,red/K08041%09%23FFFFFF,red/K11265%09%23FFFFFF

26%09%23FFFFFF,red/K04853%09%23FFFFFF,red/K05085%09%23FFFFFF,red/K04360%09%23FFFFFF,red/K04439%09%23FFFFFF

87%09%23FFFFFF,red/K05858%09%23FFFFFF,red/K08041%09%23FFFFFF,red/K05190%09%23FFFFFF,red/K04851%09%23FFFFFF

58%09%23FFFFFF,red/K08041%09%23FFFFFF,red/K04851%09%23FFFFFF,red/K04135%09%23FFFFFF,red/K04634%09%23FFFFFF

10%09%23FFFFFF,red/K09653%09%23FFFFFF,red/K23800%09%23FFFFFF,red/

41%09%23FFFFFF,red/K06481%09%23FFFFFF,red/K04634%09%23FFFFFF,red/K00922%09%23FFFFFF,red/K08042%09%23FFFFFF

18%09%23FFFFFF,red/K05858%09%23FFFFFF,red/K05704%09%23FFFFFF,red/K07376%09%23FFFFFF,red/K08041%09%23FFFFFF

93%09%23FFFFFF,red/K05213%09%23FFFFFF,red/K08041%09%23FFFFFF,red/K11265%09%23FFFFFF,red/K04851%09%23FFFFFF

69%09%23FFFFFF,red/K12666%09%23FFFFFF,red/K01230%09%23FFFFFF,red/K10636%09%23FFFFFF,red/K24348%09%23FFFFFF

36%09%23FFFFFF,red/K05858%09%23FFFFFF,red/K08041%09%23FFFFFF,red/K04851%09%23FFFFFF,red/K04135%09%23FFFFFF

53%09%23FFFFFF,red/K09355%09%23FFFFFF,red/K05858%09%23FFFFFF,red/K08041%09%23FFFFFF,red/K04851%09%23FFFFFF

34%09%23FFFFFF,red/K07203%09%23FFFFFF,red/K04430%09%23FFFFFF,red/K05085%09%23FFFFFF,red/K04409%09%23FFFFFF

74%09%23FFFFFF,red/K13303%09%23FFFFFF,red/K13302%09%23FFFFFF,red/K00922%09%23FFFFFF,red/K08341%09%23FFFFFF

28%09%23FFFFFF,red/K13373%09%23FFFFFF,red/

93%09%23FFFFFF,red/K18436%09%23FFFFFF,red/K11265%09%23FFFFFF,red/K08041%09%23FFFFFF,red/K07127%09%23FFFFFF

53%09%23FFFFFF,red/K15297%09%23FFFFFF,red/K04936%09%23FFFFFF,red/K05858%09%23FFFFFF,red/K08041%09%23FFFFFF

50%09%23FFFFFF,red/K04853%09%23FFFFFF,red/K05858%09%23FFFFFF,red/K08041%09%23FFFFFF,red/K04851%09%23FFFFFF

71%09%23FFFFFF,red/K05858%09%23FFFFFF,red/K13293%09%23FFFFFF,red/K05261%09%23FFFFFF,red/K04439%09%23FFFFFF

F,red/K09047%09%23FFFFFF,red/K00070%09%23FFFFFF,red/K09048%09%23FFFFFF,red/K08045%09%23FFFFFF,red/K08560%09%
F,red/K08524%09%23FFFFFF,red/K09047%09%23FFFFFF,red/K03068%09%23FFFFFF,red/K08045%09%23FFFFFF,red/K09048%09%
F,red/K04348%09%23FFFFFF,red/K04610%09%23FFFFFF,red/K05202%09%23FFFFFF,red/K08045%09%23FFFFFF,red/K05200%09%
F,red/K08049%09%23FFFFFF,red/K04634%09%23FFFFFF,red/K04958%09%23FFFFFF,red/K09047%09%23FFFFFF,red/K04930%09%
F,red/K09047%09%23FFFFFF,red/K01540%09%23FFFFFF,red/K08045%09%23FFFFFF,red/K09048%09%23FFFFFF,red/K18211%09%
F,red/K08049%09%23FFFFFF,red/K18438%09%23FFFFFF,red/K08045%09%23FFFFFF,red/K12318%09%23FFFFFF,red/K19021%09%
F,red/K04634%09%23FFFFFF,red/K04958%09%23FFFFFF,red/K09047%09%23FFFFFF,red/K04348%09%23FFFFFF,red/K01540%09%
F,red/K09047%09%23FFFFFF,red/K04348%09%23FFFFFF,red/K00873%09%23FFFFFF,red/K09048%09%23FFFFFF,red/K03841%09%
F,red/K08049%09%23FFFFFF,red/K19662%09%23FFFFFF,red/K08522%09%23FFFFFF,red/K01540%09%23FFFFFF,red/K09047%09%
F,red/K05113%09%23FFFFFF,red/K04189%09%23FFFFFF,red/K20013%09%23FFFFFF,red/K06842%09%23FFFFFF,red/K06839%09%

F,red/K08794%09%23FFFFFF,red/K09047%09%23FFFFFF,red/K01540%09%23FFFFFF,red/K08045%09%23FFFFFF,red/K09048%09%
F,red/K04634%09%23FFFFFF,red/K19662%09%23FFFFFF,red/K05207%09%23FFFFFF,red/K19477%09%23FFFFFF,red/
F,red/K04634%09%23FFFFFF,red/K08049%09%23FFFFFF,red/K19662%09%23FFFFFF,red/K01540%09%23FFFFFF,red/K04913%09%
F,red/K08049%09%23FFFFFF,red/K04634%09%23FFFFFF,red/K04958%09%23FFFFFF,red/K08794%09%23FFFFFF,red/K04348%09%
F,red/K04634%09%23FFFFFF,red/K08049%09%23FFFFFF,red/K19662%09%23FFFFFF,red/K05450%09%23FFFFFF,red/K03099%09%
F,red/K04360%09%23FFFFFF,red/K04163%09%23FFFFFF,red/K04131%09%23FFFFFF,red/K05862%09%23FFFFFF,red/K04189%09%
F,red/K04634%09%23FFFFFF,red/K09047%09%23FFFFFF,red/K01540%09%23FFFFFF,red/K04860%09%23FFFFFF,red/K21289%09%

F,red/K09047%09%23FFFFFF,red/K04348%09%23FFFFFF,red/K08045%09%23FFFFFF,red/K09048%09%23FFFFFF,red/K05200%09%
F,red/K04634%09%23FFFFFF,red/K12331%09%23FFFFFF,red/K04958%09%23FFFFFF,red/K01047%09%23FFFFFF,red/K08045%09%
F,red/K11265%09%23FFFFFF,red/K04958%09%23FFFFFF,red/K04634%09%23FFFFFF,red/K08049%09%23FFFFFF,red/K19662%09%
F,red/K08049%09%23FFFFFF,red/K04634%09%23FFFFFF,red/K04958%09%23FFFFFF,red/K03964%09%23FFFFFF,red/K08045%09%
F,red/K20013%09%23FFFFFF,red/K00714%09%23FFFFFF,red/K04500%09%23FFFFFF,red/K16686%09%23FFFFFF,red/K03982%09%
F,red/K04634%09%23FFFFFF,red/K09047%09%23FFFFFF,red/K08045%09%23FFFFFF,red/K09048%09%23FFFFFF,red/K17449%09%
F,red/K04634%09%23FFFFFF,red/K04348%09%23FFFFFF,red/K04345%09%23FFFFFF,red/K19477%09%23FFFFFF,red/
F,red/K04634%09%23FFFFFF,red/K08049%09%23FFFFFF,red/K19662%09%23FFFFFF,red/K01540%09%23FFFFFF,red/K04345%09%
F,red/K04609%09%23FFFFFF,red/K04610%09%23FFFFFF,red/K21289%09%23FFFFFF,red/K08045%09%23FFFFFF,red/K05703%09%

,red/K04348%09%23FFFFFF,red/K08042%09%23FFFFFF,red/K05097%09%23FFFFFF,red/K05093%09%23FFFFFF,red/K04358%09%
,red/K05197%09%23FFFFFF,red/K04348%09%23FFFFFF,red/K05199%09%23FFFFFF,red/K08042%09%23FFFFFF,red/K04371%09%
,red/K19477%09%23FFFFFF,red/K05199%09%23FFFFFF,red/K08042%09%23FFFFFF,red/K12318%09%23FFFFFF,red/K04371%09%
,red/K09047%09%23FFFFFF,red/K04348%09%23FFFFFF,red/K05199%09%23FFFFFF,red/K05200%09%23FFFFFF,red/K09048%09%
,red/K06249%09%23FFFFFF,red/K05450%09%23FFFFFF,red/K06483%09%23FFFFFF,red/K06237%09%23FFFFFF,red/K04371%09%
,red/K04392%09%23FFFFFF,red/K05462%09%23FFFFFF,red/K04189%09%23FFFFFF,red/K06841%09%23FFFFFF,red/K05110%09%
,red/K12302%09%23FFFFFF,red/K05211%09%23FFFFFF,red/K05197%09%23FFFFFF,red/K05181%09%23FFFFFF,red/
,red/K04634%09%23FFFFFF,red/K09047%09%23FFFFFF,red/K04348%09%23FFFFFF,red/K19477%09%23FFFFFF,red/K08042%09%
,red/K06462%09%23FFFFFF,red/K06585%09%23FFFFFF,red/K05744%09%23FFFFFF,red/K05704%09%23FFFFFF,red/K05093%09%
,red/K04289%09%23FFFFFF,red/K04634%09%23FFFFFF,red/K05450%09%23FFFFFF,red/K08042%09%23FFFFFF,red/K05631%09%
,red/K04348%09%23FFFFFF,red/K04860%09%23FFFFFF,red/K04403%09%23FFFFFF,red/K04457%09%23FFFFFF,red/K05097%09%
,red/K16338%09%23FFFFFF,red/K06249%09%23FFFFFF,red/K06587%09%23FFFFFF,red/K06238%09%23FFFFFF,red/K06240%09%
,red/K13758%09%23FFFFFF,red/K00856%09%23FFFFFF,red/K13485%09%23FFFFFF,red/K08042%09%23FFFFFF,red/K12305%09%
,red/K05036%09%23FFFFFF,red/K09047%09%23FFFFFF,red/K04348%09%23FFFFFF,red/K05199%09%23FFFFFF,red/K05200%09%
,red/K12318%09%23FFFFFF,red/K04371%09%23FFFFFF,red/K07376%09%23FFFFFF,red/K05704%09%23FFFFFF,red/K04157%09%

,red/K06754%09%23FFFFFF,red/K05733%09%23FFFFFF,red/K06841%09%23FFFFFF,red/K00922%09%23FFFFFF,red/K20013%09%
,red/K04808%09%23FFFFFF,red/K04634%09%23FFFFFF,red/K00922%09%23FFFFFF,red/K09047%09%23FFFFFF,red/K08042%09%
,red/K06082%09%23FFFFFF,red/K23605%09%23FFFFFF,red/K05704%09%23FFFFFF,red/K23612%09%23FFFFFF,red/K05693%09%
,red/K13240%09%23FFFFFF,red/K09047%09%23FFFFFF,red/K21998%09%23FFFFFF,red/K08042%09%23FFFFFF,red/K06237%09%
,red/K13240%09%23FFFFFF,red/K08042%09%23FFFFFF,red/K05219%09%23FFFFFF,red/K00907%09%23FFFFFF,red/K04262%09%
,red/K08042%09%23FFFFFF,red/K05186%09%23FFFFFF,red/K04541%09%23FFFFFF,red/K05704%09%23FFFFFF,red/K05175%09%
,red/K04856%09%23FFFFFF,red/K04634%09%23FFFFFF,red/K00922%09%23FFFFFF,red/K08522%09%23FFFFFF,red/K04615%09%
,red/K04609%09%23FFFFFF,red/K04610%09%23FFFFFF,red/K05202%09%23FFFFFF,red/K08042%09%23FFFFFF,red/K04371%09%
,red/K08042%09%23FFFFFF,red/K12318%09%23FFFFFF,red/K04371%09%23FFFFFF,red/K07843%09%23FFFFFF,red/K05209%09%
,red/K00922%09%23FFFFFF,red/K07829%09%23FFFFFF,red/K05450%09%23FFFFFF,red/K04609%09%23FFFFFF,red/K23484%09%
,red/K04634%09%23FFFFFF,red/K00922%09%23FFFFFF,red/K04692%09%23FFFFFF,red/K09047%09%23FFFFFF,red/K08042%09%
,red/K05858%09%23FFFFFF,red/K04851%09%23FFFFFF,red/K04546%09%23FFFFFF,red/K04634%09%23FFFFFF,red/K05181%09%

,red/K04546%09%23FFFFFF,red/K04634%09%23FFFFFF,red/K00922%09%23FFFFFF,red/K04692%09%23FFFFFF,red/K10031%09%

,red/K05733%09%23FFFFFF,red/K00922%09%23FFFFFF,red/K07829%09%23FFFFFF,red/K05450%09%23FFFFFF,red/K10031%09%

,red/K05219%09%23FFFFFF,red/K04262%09%23FFFFFF,red/K04260%09%23FFFFFF,red/K04604%09%23FFFFFF,red/K04010%09%

,red/K00572%09%23FFFFFF,red/K06487%09%23FFFFFF,red/K05637%09%23FFFFFF,red/K00799%09%23FFFFFF,red/K12331%09%

,red/K04546%09%23FFFFFF,red/K04634%09%23FFFFFF,red/K07829%09%23FFFFFF,red/K13240%09%23FFFFFF,red/K08042%09%

,red/K04851%09%23FFFFFF,red/K04459%09%23FFFFFF,red/K04860%09%23FFFFFF,red/K04445%09%23FFFFFF,red/K04373%09%

,red/K04546%09%23FFFFFF,red/K04634%09%23FFFFFF,red/K11351%09%23FFFFFF,red/K05181%09%23FFFFFF,red/K08042%09%

,red/K04938%09%23FFFFFF,red/K09047%09%23FFFFFF,red/K08042%09%23FFFFFF,red/K21289%09%23FFFFFF,red/K04939%09%

,red/K21289%09%23FFFFFF,red/K04371%09%23FFFFFF,red/K12318%09%23FFFFFF,red/K03900%09%23FFFFFF,red/K05704%09%

,red/K04604%09%23FFFFFF,red/K04634%09%23FFFFFF,red/K08959%09%23FFFFFF,red/K05450%09%23FFFFFF,red/K03099%09%

,red/K00922%09%23FFFFFF,red/K07829%09%23FFFFFF,red/K05211%09%23FFFFFF,red/K09047%09%23FFFFFF,red/K08522%09%

,red/K10084%09%23FFFFFF,red/K09503%09%23FFFFFF,red/K11718%09%23FFFFFF,red/K09584%09%23FFFFFF,red/K04556%09%

,red/K04634%09%23FFFFFF,red/K12331%09%23FFFFFF,red/K04938%09%23FFFFFF,red/K01047%09%23FFFFFF,red/K08042%09%

,red/K05704%09%23FFFFFF,red/K00922%09%23FFFFFF,red/K05733%09%23FFFFFF,red/K03099%09%23FFFFFF,red/

,red/K04692%09%23FFFFFF,red/K21770%09%23FFFFFF,red/K04371%09%23FFFFFF,red/K04498%09%23FFFFFF,red/K23605%09%

,red/K12322%09%23FFFFFF,red/K08042%09%23FFFFFF,red/K12318%09%23FFFFFF,red/K01518%09%23FFFFFF,red/K19572%09%

,red/K04851%09%23FFFFFF,red/K04944%09%23FFFFFF,red/K04634%09%23FFFFFF,red/K04938%09%23FFFFFF,red/K09047%09%

,red/K04856%09%23FFFFFF,red/K05850%09%23FFFFFF,red/K04634%09%23FFFFFF,red/K08794%09%23FFFFFF,red/K09047%09%

,red/K08041%09%23FFFFFF,red/K04634%09%23FFFFFF,red/K12331%09%23FFFFFF,red/K09047%09%23FFFFFF,red/K16529%09%

23FFFFFF,red/K01115%09%23FFFFFF,red/K19662%09%23FFFFFF,red/K08539%09%23FFFFFF,red/K16529%09%23FFFFFF,red/K1

23FFFFFF,red/K15010%09%23FFFFFF,red/K15009%09%23FFFFFF,red/K04344%09%23FFFFFF,red/K04604%09%23FFFFFF,red/K01

23FFFFFF,red/K21289%09%23FFFFFF,red/K09048%09%23FFFFFF,red/K08045%09%23FFFFFF,red/K05703%09%23FFFFFF,red/K0

23FFFFFF,red/K16882%09%23FFFFFF,red/K05032%09%23FFFFFF,red/K19662%09%23FFFFFF,red/K04345%09%23FFFFFF,red/

23FFFFFF,red/K01490%09%23FFFFFF,red/K01510%09%23FFFFFF,red/K07023%09%23FFFFFF,red/K01587%09%23FFFFFF,red/K1

23FFFFFF,red/K19477%09%23FFFFFF,red/K21289%09%23FFFFFF,red/K12318%09%23FFFFFF,red/K09048%09%23FFFFFF,red/K0

23FFFFFF,red/K07202%09%23FFFFFF,red/K07190%09%23FFFFFF,red/K01084%09%23FFFFFF,red/K04345%09%23FFFFFF,red/

23FFFFFF,red/K10031%09%23FFFFFF,red/K07521%09%23FFFFFF,red/K04348%09%23FFFFFF,red/K06572%09%23FFFFFF,red/K0

23FFFFFF,red/K04860%09%23FFFFFF,red/K21289%09%23FFFFFF,red/K12318%09%23FFFFFF,red/K08045%09%23FFFFFF,red/K0

23FFFFFF,red/K08049%09%23FFFFFF,red/K04634%09%23FFFFFF,red/K04958%09%23FFFFFF,red/K05098%09%23FFFFFF,red/K0

23FFFFFF,red/K08045%09%23FFFFFF,red/K09048%09%23FFFFFF,red/K09052%09%23FFFFFF,red/K04870%09%23FFFFFF,red/K0

23FFFFFF,red/K04344%09%23FFFFFF,red/K06269%09%23FFFFFF,red/K19662%09%23FFFFFF,red/K02223%09%23FFFFFF,red/K0

23FFFFFF,red/K12318%09%23FFFFFF,red/K07376%09%23FFFFFF,red/K00907%09%23FFFFFF,red/K12324%09%23FFFFFF,red/K0

23FFFFFF,red/K05200%09%23FFFFFF,red/K05175%09%23FFFFFF,red/K04344%09%23FFFFFF,red/K04604%09%23FFFFFF,red/K1

23FFFFFF,red/K05691%09%23FFFFFF,red/K06093%09%23FFFFFF,red/K16822%09%23FFFFFF,red/K16682%09%23FFFFFF,red/K1

23FFFFFF,red/K17446%09%23FFFFFF,red/K19662%09%23FFFFFF,red/K03099%09%23FFFFFF,red/K04345%09%23FFFFFF,red/

23FFFFFF,red/K00901%09%23FFFFFF,red/K05091%09%23FFFFFF,red/K19007%09%23FFFFFF,red/K17449%09%23FFFFFF,red/K0

23FFFFFF,red/K00907%09%23FFFFFF,red/K04260%09%23FFFFFF,red/K04856%09%23FFFFFF,red/K04604%09%23FFFFFF,red/K0
23FFFFFF,red/K05200%09%23FFFFFF,red/K05210%09%23FFFFFF,red/K05209%09%23FFFFFF,red/K00910%09%23FFFFFF,red/K1
23FFFFFF,red/K05200%09%23FFFFFF,red/K05210%09%23FFFFFF,red/K07843%09%23FFFFFF,red/K05209%09%23FFFFFF,red/K0

23FFFFFF,red/K05725%09%23FFFFFF,red/K06252%09%23FFFFFF,red/K05097%09%23FFFFFF,red/K06585%09%23FFFFFF,red/K0
23FFFFFF,red/K04515%09%23FFFFFF,red/K20013%09%23FFFFFF,red/K06839%09%23FFFFFF,red/K04348%09%23FFFFFF,red/K0

23FFFFFF,red/K21289%09%23FFFFFF,red/K12318%09%23FFFFFF,red/K09048%09%23FFFFFF,red/K04371%09%23FFFFFF,red/K0
23FFFFFF,red/K04358%09%23FFFFFF,red/K00907%09%23FFFFFF,red/K13708%09%23FFFFFF,red/K05729%09%23FFFFFF,red/K0
23FFFFFF,red/K06093%09%23FFFFFF,red/K04371%09%23FFFFFF,red/K05210%09%23FFFFFF,red/K05097%09%23FFFFFF,red/K0
23FFFFFF,red/K04445%09%23FFFFFF,red/K04446%09%23FFFFFF,red/K05093%09%23FFFFFF,red/K04358%09%23FFFFFF,red/K0

23FFFFFF,red/K12318%09%23FFFFFF,red/K13762%09%23FFFFFF,red/K19021%09%23FFFFFF,red/K01510%09%23FFFFFF,red/K1
23FFFFFF,red/K09048%09%23FFFFFF,red/K05210%09%23FFFFFF,red/K05209%09%23FFFFFF,red/K03456%09%23FFFFFF,red/K1

23FFFFFF,red/K07829%09%23FFFFFF,red/K06842%09%23FFFFFF,red/K06839%09%23FFFFFF,red/K10031%09%23FFFFFF,red/K0
23FFFFFF,red/K21289%09%23FFFFFF,red/K04930%09%23FFFFFF,red/K09048%09%23FFFFFF,red/K04371%09%23FFFFFF,red/K0

23FFFFFF,red/K04371%09%23FFFFFF,red/K09048%09%23FFFFFF,red/K04430%09%23FFFFFF,red/K04541%09%23FFFFFF,red/K0
23FFFFFF,red/K04260%09%23FFFFFF,red/K04358%09%23FFFFFF,red/K07190%09%23FFFFFF,red/K04856%09%23FFFFFF,red/K0
23FFFFFF,red/K04344%09%23FFFFFF,red/K15376%09%23FFFFFF,red/K04615%09%23FFFFFF,red/K04548%09%23FFFFFF,red/

23FFFFFF,red/K05209%09%23FFFFFF,red/K04344%09%23FFFFFF,red/K00910%09%23FFFFFF,red/K04541%09%23FFFFFF,red/K0
23FFFFFF,red/K04445%09%23FFFFFF,red/K04541%09%23FFFFFF,red/K07376%09%23FFFFFF,red/K04856%09%23FFFFFF,red/K0
23FFFFFF,red/K04610%09%23FFFFFF,red/K08042%09%23FFFFFF,red/K21289%09%23FFFFFF,red/K04371%09%23FFFFFF,red/K0
23FFFFFF,red/K09048%09%23FFFFFF,red/K04371%09%23FFFFFF,red/K04498%09%23FFFFFF,red/K04430%09%23FFFFFF,red/K1
23FFFFFF,red/K04371%09%23FFFFFF,red/K04892%09%23FFFFFF,red/K04344%09%23FFFFFF,red/K04541%09%23FFFFFF,red/K0

23FFFFFF,red/K21096%09%23FFFFFF,red/K08042%09%23FFFFFF,red/K21289%09%23FFFFFF,red/K04371%09%23FFFFFF,red/K0

23FFFFFF,red/K16848%09%23FFFFFF,red/K05750%09%23FFFFFF,red/K05769%09%23FFFFFF,red/K05767%09%23FFFFFF,red/K0

23FFFFFF,red/K04288%09%23FFFFFF,red/K04814%09%23FFFFFF,red/K04216%09%23FFFFFF,red/K08377%09%23FFFFFF,red/K0

23FFFFFF,red/K04692%09%23FFFFFF,red/K05414%09%23FFFFFF,red/K08550%09%23FFFFFF,red/K08042%09%23FFFFFF,red/K0

23FFFFFF,red/K21289%09%23FFFFFF,red/K04371%09%23FFFFFF,red/K23605%09%23FFFFFF,red/K04541%09%23FFFFFF,red/K0

23FFFFFF,red/K04430%09%23FFFFFF,red/K04358%09%23FFFFFF,red/K04856%09%23FFFFFF,red/K03173%09%23FFFFFF,red/K0

23FFFFFF,red/K04371%09%23FFFFFF,red/K05186%09%23FFFFFF,red/K04344%09%23FFFFFF,red/K05175%09%23FFFFFF,red/K0

23FFFFFF,red/K09048%09%23FFFFFF,red/K04371%09%23FFFFFF,red/K12318%09%23FFFFFF,red/K19021%09%23FFFFFF,red/K0

23FFFFFF,red/K07376%09%23FFFFFF,red/K00907%09%23FFFFFF,red/K17388%09%23FFFFFF,red/K06271%09%23FFFFFF,red/

23FFFFFF,red/K08042%09%23FFFFFF,red/K06225%09%23FFFFFF,red/K09048%09%23FFFFFF,red/K04371%09%23FFFFFF,red/K0

23FFFFFF,red/K04079%09%23FFFFFF,red/K10578%09%23FFFFFF,red/K14001%09%23FFFFFF,red/K05638%09%23FFFFFF,red/K1

23FFFFFF,red/K04939%09%23FFFFFF,red/K04371%09%23FFFFFF,red/K12318%09%23FFFFFF,red/K07376%09%23FFFFFF,red/K0

23FFFFFF,red/K04411%09%23FFFFFF,red/K17446%09%23FFFFFF,red/K04288%09%23FFFFFF,red/K03099%09%23FFFFFF,red/

1115%09%23FFFFFF,red/K05201%09%23FFFFFF,red/K19662%09%23FFFFFF,red/K15008%09%23FFFFFF,red/K04345%09%23FFFF

045%09%23FFFFFF,red/K19021%09%23FFFFFF,red/K04140%09%23FFFFFF,red/K07376%09%23FFFFFF,red/K00907%09%23FFFF

521%09%23FFFFFF,red/K05107%09%23FFFFFF,red/K06093%09%23FFFFFF,red/K05703%09%23FFFFFF,red/K06766%09%23FFFF

870%09%23FFFFFF,red/K00907%09%23FFFFFF,red/K12324%09%23FFFFFF,red/K06269%09%23FFFFFF,red/K19662%09%23FFFF

794%09%23FFFFFF,red/K05450%09%23FFFFFF,red/K04348%09%23FFFFFF,red/K04852%09%23FFFFFF,red/K04344%09%23FFFF

4849%09%23FFFFFF,red/K08044%09%23FFFFFF,red/K13755%09%23FFFFFF,red/K04161%09%23FFFFFF,red/K04994%09%23FFFF

5009%09%23FFFFFF,red/K04604%09%23FFFFFF,red/K05201%09%23FFFFFF,red/K05203%09%23FFFFFF,red/K12302%09%23FFFF

5449%09%23FFFFFF,red/K00889%09%23FFFFFF,red/K04409%09%23FFFFFF,red/K17449%09%23FFFFFF,red/K05704%09%23FFFF

5572%09%23FFFFFF,red/K19862%09%23FFFFFF,red/K06093%09%23FFFFFF,red/K04371%09%23FFFFFF,red/K05725%09%23FFFF

1539%09%23FFFFFF,red/K13762%09%23FFFFFF,red/K19021%09%23FFFFFF,red/K04140%09%23FFFFFF,red/K07376%09%23FFFF

5759%09%23FFFFFF,red/K06584%09%23FFFFFF,red/K03099%09%23FFFFFF,red/K06484%09%23FFFFFF,red/K04392%09%23FFFF

5731%09%23FFFFFF,red/K06069%09%23FFFFFF,red/K05209%09%23FFFFFF,red/K05629%09%23FFFFFF,red/K05449%09%23FFFF

4856%09%23FFFFFF,red/K03171%09%23FFFFFF,red/K03099%09%23FFFFFF,red/K05092%09%23FFFFFF,red/K04372%09%23FFFF

7521%09%23FFFFFF,red/K05107%09%23FFFFFF,red/K04371%09%23FFFFFF,red/K06225%09%23FFFFFF,red/K06766%09%23FFFF

5850%09%23FFFFFF,red/K04604%09%23FFFFFF,red/K17386%09%23FFFFFF,red/K04849%09%23FFFFFF,red/K00871%09%23FFFF

0901%09%23FFFFFF,red/K04262%09%23FFFFFF,red/K04604%09%23FFFFFF,red/K04275%09%23FFFFFF,red/K03099%09%23FFFF

4192%09%23FFFFFF,red/K12366%09%23FFFFFF,red/K04409%09%23FFFFFF,red/K00910%09%23FFFFFF,red/K05704%09%23FFFF

4371%09%23FFFFFF,red/K05734%09%23FFFFFF,red/K04409%09%23FFFFFF,red/K05704%09%23FFFFFF,red/K04358%09%23FFFF

4153%09%23FFFFFF,red/K04274%09%23FFFFFF,red/K05261%09%23FFFFFF,red/K05190%09%23FFFFFF,red/K04808%09%23FFFF

5237%09%23FFFFFF,red/K20994%09%23FFFFFF,red/K05691%09%23FFFFFF,red/K09455%09%23FFFFFF,red/K06055%09%23FFFF

3099%09%23FFFFFF,red/K17386%09%23FFFFFF,red/K04372%09%23FFFFFF,red/K04468%09%23FFFFFF,red/K04849%09%23FFFF

4140%09%23FFFFFF,red/K07376%09%23FFFFFF,red/K00907%09%23FFFFFF,red/K17388%09%23FFFFFF,red/K05850%09%23FFFF

4498%09%23FFFFFF,red/K05209%09%23FFFFFF,red/K19021%09%23FFFFFF,red/K04409%09%23FFFFFF,red/K04260%09%23FFFF

4007%09%23FFFFFF,red/K09054%09%23FFFFFF,red/K08860%09%23FFFFFF,red/K14026%09%23FFFFFF,red/K03173%09%23FFFF

FF,red/K05849%09%23FFFFFF,red/K17446%09%23FFFFFF,red/K12324%09%23FFFFFF,red/K06269%09%23FFFFFF,red/

FF,red/K06753%09%23FFFFFF,red/K05744%09%23FFFFFF,red/K06845%09%23FFFFFF,red/K06820%09%23FFFFFF,red/K05736%09

FF,red/K05219%09%23FFFFFF,red/K04358%09%23FFFFFF,red/K00907%09%23FFFFFF,red/K07190%09%23FFFFFF,red/K23445%09

FF,red/K04189%09%23FFFFFF,red/K04135%09%23FFFFFF,red/K04634%09%23FFFFFF,red/K04515%09%23FFFFFF,red/K20858%09

FF,red/K00907%09%23FFFFFF,red/K13708%09%23FFFFFF,red/K06587%09%23FFFFFF,red/K06238%09%23FFFFFF,red/K06240%09

FF,red/K06753%09%23FFFFFF,red/K06766%09%23FFFFFF,red/K05744%09%23FFFFFF,red/K04409%09%23FFFFFF,red/K05704%09

FF,red/K04265%09%23FFFFFF,red/K04446%09%23FFFFFF,red/K00907%09%23FFFFFF,red/K05852%09%23FFFFFF,red/K17446%09

FF,red/K12329%09%23FFFFFF,red/K04189%09%23FFFFFF,red/K05450%09%23FFFFFF,red/K04371%09%23FFFFFF,red/K05725%09

FF,red/K06082%09%23FFFFFF,red/K05704%09%23FFFFFF,red/K05093%09%23FFFFFF,red/K04358%09%23FFFFFF,red/K18497%09

FF,red/K17333%09%23FFFFFF,red/K04849%09%23FFFFFF,red/K04858%09%23FFFFFF,red/K04392%09%23FFFFFF,red/K17614%09

FF,red/K06753%09%23FFFFFF,red/K05734%09%23FFFFFF,red/K05704%09%23FFFFFF,red/K04409%09%23FFFFFF,red/K06845%09

FF,red/K05871%09%23FFFFFF,red/K08041%09%23FFFFFF,red/K04189%09%23FFFFFF,red/K04135%09%23FFFFFF,red/K04634%09

FF,red/K04541%09%23FFFFFF,red/K17388%09%23FFFFFF,red/K04179%09%23FFFFFF,red/K03099%09%23FFFFFF,red/K04548%09

FF,red/K00907%09%23FFFFFF,red/K17388%09%23FFFFFF,red/K13708%09%23FFFFFF,red/K05741%09%23FFFFFF,red/K04275%09

FF,red/K04135%09%23FFFFFF,red/K04589%09%23FFFFFF,red/K04609%09%23FFFFFF,red/K05211%09%23FFFFFF,red/K05249%09

FF,red/K04498%09%23FFFFFF,red/K04445%09%23FFFFFF,red/K04079%09%23FFFFFF,red/K23605%09%23FFFFFF,red/K04541%09

FF,red/K04412%09%23FFFFFF,red/K16342%09%23FFFFFF,red/K04674%09%23FFFFFF,red/K05462%09%23FFFFFF,red/K07829%09

FF,red/K17388%09%23FFFFFF,red/K05850%09%23FFFFFF,red/K18435%09%23FFFFFF,red/K04615%09%23FFFFFF,red/

)%23FFFFFF,red/K04604%09%23FFFFFF,red/K05849%09%23FFFFFF,red/K19662%09%23FFFFFF,red/K04345%09%23FFFFFF,red/

)%23FFFFFF,red/K05450%09%23FFFFFF,red/K05211%09%23FFFFFF,red/K04297%09%23FFFFFF,red/K04150%09%23FFFFFF,red/K

)%23FFFFFF,red/K19662%09%23FFFFFF,red/K06584%09%23FFFFFF,red/K06271%09%23FFFFFF,red/K03099%09%23FFFFFF,red/

)%23FFFFFF,red/K05731%09%23FFFFFF,red/K00889%09%23FFFFFF,red/K04409%09%23FFFFFF,red/K18497%09%23FFFFFF,red/K

)%23FFFFFF,red/K04441%09%23FFFFFF,red/K19662%09%23FFFFFF,red/K05759%09%23FFFFFF,red/K06271%09%23FFFFFF,red/

)%23FFFFFF,red/K05462%09%23FFFFFF,red/K03175%09%23FFFFFF,red/K05450%09%23FFFFFF,red/K04443%09%23FFFFFF,red/K

)%23FFFFFF,red/K06820%09%23FFFFFF,red/K17388%09%23FFFFFF,red/K07522%09%23FFFFFF,red/K05108%09%23FFFFFF,red/

)%23FFFFFF,red/K05450%09%23FFFFFF,red/K05211%09%23FFFFFF,red/K04150%09%23FFFFFF,red/K05209%09%23FFFFFF,red/K

)%23FFFFFF,red/K05770%09%23FFFFFF,red/K05181%09%23FFFFFF,red/K21998%09%23FFFFFF,red/K04150%09%23FFFFFF,red/K

)%23FFFFFF,red/K04260%09%23FFFFFF,red/K04358%09%23FFFFFF,red/K17388%09%23FFFFFF,red/K04738%09%23FFFFFF,red/K

)%23FFFFFF,red/K05450%09%23FFFFFF,red/K04371%09%23FFFFFF,red/K04870%09%23FFFFFF,red/K04462%09%23FFFFFF,red/K

05209%09%23FFFFFF,red/K05449%09%23FFFFFF,red/K04993%09%23FFFFFF,red/K04157%09%23FFFFFF,red/K05852%09%23FFF

04371%09%23FFFFFF,red/K04422%09%23FFFFFF,red/K05449%09%23FFFFFF,red/K04409%09%23FFFFFF,red/K04411%09%23FFF

05247%09%23FFFFFF,red/K05209%09%23FFFFFF,red/K05175%09%23FFFFFF,red/K04140%09%23FFFFFF,red/K08039%09%23FFF

03173%09%23FFFFFF,red/K04548%09%23FFFFFF,red/K03099%09%23FFFFFF,red/K06619%09%23FFFFFF,red/K17386%09%23FFF

'FFF,red/K18497%09%23FFFFFF,red/K04169%09%23FFFFFF,red/K04160%09%23FFFFFF,red/K19662%09%23FFFFFF,red/

'FFF,red/K04583%09%23FFFFFF,red/K08397%09%23FFFFFF,red/K05207%09%23FFFFFF,red/K04275%09%23FFFFFF,red/K04615%(

'FFF,red/K04674%09%23FFFFFF,red/K07203%09%23FFFFFF,red/K00444%09%23FFFFFF,red/K08041%09%23FFFFFF,red/K04189%(

)09%23FFFFFF,red/K04634%09%23FFFFFF,red/K00922%0
