# Supplementary material for: Genetic diversity and selection of Tibetan sheep breeds revealed by whole-genome resequencing
Source: Anim Biosci. 2023 May 2;36(7):991–1002. doi: 10.5713/ab.22.0432 (PMC10330983; doi:10.5713/ab.22.0432)
Supplement: Supplementary file 16 [file ab-22-0432-Supplementary-Table-16.pdf]

Supplementary Table 16.KEGG analysis of putative selected genes compared with SG1 breed

SG1 vs ZK

PathwayID	Pathway	Level1	Level2	list_number
oas04928	Parathyroid hormone synthesis, secretioı	Organismal Systems	Endocrine system	21
oas04725	Cholinergic synapse	Organismal Systems	Nervous system	20
oas04022	cGMP-PKG signaling pathway	Environmental Information Proc	Signal transduction	26
oas04062	Chemokine signaling pathway	Organismal Systems	Immune system	29
oas04810	Regulation of actin cytoskeleton	Cellular Processes	Cell motility	32
oas04261	Adrenergic signaling in cardiomyocytes	Organismal Systems	Circulatory system	24
oas04921	Oxytocin signaling pathway	Organismal Systems	Endocrine system	24
oas04713	Circadian entrainment	Organismal Systems	Environmental adaptation	17
oas04024	cAMP signaling pathway	Environmental Information Proc	Signal transduction	32
oas04970	Salivary secretion	Organismal Systems	Digestive system	16
oas04927	Cortisol synthesis and secretion	Organismal Systems	Endocrine system	13
oas04973	Carbohydrate digestion and absorption	Organismal Systems	Digestive system	10
oas04724	Glutamatergic synapse	Organismal Systems	Nervous system	18
oas04924	Renin secretion	Organismal Systems	Endocrine system	13
oas04020	Calcium signaling pathway	Environmental Information Proc	Signal transduction	32
oas04510	Focal adhesion	Cellular Processes	Cellular community - eukaryotes	28
oas04742	Taste transduction	Organismal Systems	Sensory system	14
oas04012	ErbB signaling pathway	Environmental Information Proc	Signal transduction	14
oas05100	Bacterial invasion of epithelial cells	Human Diseases	Infectious disease: bacterial	13
oas04935	Growth hormone synthesis, secretion an	Organismal Systems	Endocrine system	18
oas00230	Purine metabolism	Metabolism	Nucleotide metabolism	19
oas04015	Rap1 signaling pathway	Environmental Information Proc	Signal transduction	27
oas04350	TGF-beta signaling pathway	Environmental Information Proc	Signal transduction	14
oas04730	Long-term depression	Organismal Systems	Nervous system	10
oas04972	Pancreatic secretion	Organismal Systems	Digestive system	15
oas04611	Platelet activation	Organismal Systems	Immune system	17
oas04911	Insulin secretion	Organismal Systems	Endocrine system	13

oas04925	Aldosterone synthesis and secretion	Organismal Systems	Endocrine system	14
oas04070	Phosphatidylinositol signaling system	Environmental Information Proc	Signal transduction	14
oas04010	MAPK signaling pathway	Environmental Information Proc	Signal transduction	34
oas04270	Vascular smooth muscle contraction	Organismal Systems	Circulatory system	18
oas04710	Circadian rhythm	Organismal Systems	Environmental adaptation	6
oas04926	Relaxin signaling pathway	Organismal Systems	Endocrine system	17
oas00562	Inositol phosphate metabolism	Metabolism	Carbohydrate metabolism	11
SG1 vs OL				
oas04360	Axon guidance	Organismal Systems	Development and regeneration	28
oas04724	Glutamatergic synapse	Organismal Systems	Nervous system	19
oas04742	Taste transduction	Organismal Systems	Sensory system	15
oas04072	Phospholipase D signaling pathway	Environmental Information Proc	Signal transduction	23
oas04929	GnRH secretion	Organismal Systems	Endocrine system	12
oas00513	Various types of N-glycan biosynthesis	Metabolism	Glycan biosynthesis and metabolism	9
oas04961	Endocrine and other factor-regulated cal	Organismal Systems	Excretory system	10
oas04730	Long-term depression	Organismal Systems	Nervous system	11
oas04725	Cholinergic synapse	Organismal Systems	Nervous system	17
oas04020	Calcium signaling pathway	Environmental Information Proc	Signal transduction	31
oas04721	Synaptic vesicle cycle	Organismal Systems	Nervous system	13
oas04924	Renin secretion	Organismal Systems	Endocrine system	12
oas04512	ECM-receptor interaction	Environmental Information Proc	Signaling molecules and interaction	14
oas05100	Bacterial invasion of epithelial cells	Human Diseases	Infectious disease: bacterial	12
oas04925	Aldosterone synthesis and secretion	Organismal Systems	Endocrine system	14
oas03018	RNA degradation	Genetic Information Processing	Folding, sorting and degradation	12
SG1 vs SG2				
oas04360	Axon guidance	Organismal Systems	Development and regeneration	32
oas04713	Circadian entrainment	Organismal Systems	Environmental adaptation	19
oas04020	Calcium signaling pathway	Environmental Information Proc	Signal transduction	35
oas04725	Cholinergic synapse	Organismal Systems	Nervous system	19
oas04911	Insulin secretion	Organismal Systems	Endocrine system	16

oas04926	Relaxin signaling pathway	Organismal Systems	Endocrine system	21
oas04922	Glucagon signaling pathway	Organismal Systems	Endocrine system	18
oas04371	Apelin signaling pathway	Environmental Information Proc	Signal transduction	21
oas04916	Melanogenesis	Organismal Systems	Endocrine system	17
oas03440	Homologous recombination	Genetic Information Processing	Replication and repair	9
oas04728	Dopaminergic synapse	Organismal Systems	Nervous system	20
oas04925	Aldosterone synthesis and secretion	Organismal Systems	Endocrine system	16
oas04726	Serotonergic synapse	Organismal Systems	Nervous system	18
oas04730	Long-term depression	Organismal Systems	Nervous system	11
oas04921	Oxytocin signaling pathway	Organismal Systems	Endocrine system	22
oas04971	Gastric acid secretion	Organismal Systems	Digestive system	13
oas04724	Glutamatergic synapse	Organismal Systems	Nervous system	17
oas04270	Vascular smooth muscle contraction	Organismal Systems	Circulatory system	20
oas03450	Non-homologous end-joining	Genetic Information Processing	Replication and repair	4
oas04924	Renin secretion	Organismal Systems	Endocrine system	12
oas04929	GnRH secretion	Organismal Systems	Endocrine system	11
oas00860	Porphyrin metabolism	Metabolism	Metabolism of cofactors and vitami	8
oas04012	ErbB signaling pathway	Environmental Information Proc	Signal transduction	13
oas04071	Sphingolipid signaling pathway	Environmental Information Proc	Signal transduction	17
oas04022	cGMP-PKG signaling pathway	Environmental Information Proc	Signal transduction	22

total_number	Pvalue	FDR	List
106	0.00021659	0.068442356	VDR(VDR),A[illegible]
109	0.000851386	0.079935723	PIK3CD(PIK3[illegible]
165	0.001687772	0.079935723	ATP2B1(ATP[illegible]
191	0.001701135	0.079935723	CCL24(CCL2[illegible]
218	0.001780733	0.079935723	PFN2(PFN2),[illegible]
149	0.001845793	0.079935723	CACNA2D1([illegible]
150	0.002023689	0.079935723	CACNA2D1([illegible]
94	0.002371373	0.083261531	CACNA1I(CA[illegible]
226	0.003172963	0.087994199	PIK3CD(PIK3[illegible]
89	0.003341552	0.087994199	ATP2B1(ATP[illegible]
67	0.003973603	0.090815703	CACNA1I(CA[illegible]
45	0.004023481	0.090815703	PIK3CD(PIK3[illegible]
109	0.004961831	0.097996163	SLC17A6(SL[illegible]
71	0.006623214	0.121823833	CLCA1(CLCA[illegible]
238	0.006939332	0.121823833	PDGFC(PDGF[illegible]
203	0.007897611	0.131349737	PDGFC(PDGF[illegible]
81	0.008315715	0.131388298	TAS2R5(TAS[illegible]
84	0.01140427	0.171607116	SOS2(SOS2),[illegible]
77	0.013029959	0.187157587	PIK3CD(PIK3[illegible]
121	0.0144173	0.19808116	SOS2(SOS2),[illegible]
131	0.015696705	0.206673288	ADSS2(ADSS[illegible]
212	0.0237248	0.288347571	PFN2(PFN2),[illegible]
93	0.026153745	0.292495407	GDF5(GDF5),[illegible]
59	0.026809633	0.292495407	GUCY1A2(G[illegible]
103	0.028600778	0.292495407	CLCA1(CLCA[illegible]
123	0.032639574	0.292495407	PRKCI(PRKC[illegible]
87	0.033079615	0.292495407	ADCY8(ADC[illegible]

96	0.033322262	0.292495407	CACNA1I(CA
97	0.036000728	0.307465676	PIK3C3(PIK3(
292	0.040058199	0.314734311	RPS6KA6(RPS
136	0.041456701	0.314734311	ADCY8(ADC'
31	0.044633765	0.32800627	CLOCK(CLO(
129	0.048184455	0.344230548	SOS2(SOS2),F
74	0.049020173	0.344230548	PIK3C3(PIK3(
180	0.002365941	0.495205664	EPHB1(EPHB
109	0.003163774	0.495205664	SLC1A7(SLC1
81	0.004613717	0.495205664	GABRA1(GAI
150	0.006587003	0.53025377	PLPP3(PLPP3)
64	0.009668825	0.537785325	HCN1(HCN1)
42	0.010020845	0.537785325	LOC10560582
51	0.012815686	0.542915527	VDR(VDR),AI
59	0.013488585	0.542915527	KRAS(KRAS)
109	0.015212716	0.54427718	PIK3CG(PIK3(
238	0.019176194	0.572164706	FGF5(FGF5),S
79	0.020903766	0.572164706	ATP6V1H(AT
71	0.021322908	0.572164706	GUCY1A2(GU
90	0.026546492	0.65753618	CD44(CD44),I
77	0.037685419	0.866764628	PIK3CD(PIK3(
96	0.043078655	0.899744912	CACNA1I(CA
79	0.044707822	0.899744912	PFKL(PFKL),I
180	0.00018325	0.058823269	EPHB1(EPHB
94	0.000731586	0.117419493	GNG10(GNG1
238	0.003235267	0.261223051	SLC25A6(SLC
109	0.004375907	0.261223051	CREB3L4(CR
87	0.005013412	0.261223051	CREB3L4(CR

129	0.006417312	0.261223051	CREB3L4(CR
105	0.006510232	0.261223051	CREB3L4(CR
133	0.009081503	0.294801394	GNG10(GNG1
101	0.00964419	0.294801394	CREB3L4(CR
41	0.01027494	0.294801394	POLD4(POLD
127	0.011020613	0.294801394	CREB3L4(CR
96	0.012934407	0.319380345	CREB3L4(CR
114	0.014957439	0.320089203	GNG10(GNG1
59	0.016541388	0.331583873	NRAS(NRAS)
150	0.017560517	0.331583873	CACNA2D1(C
76	0.019324995	0.334464309	KCNE2(KCNE
109	0.019796953	0.334464309	GNG10(GNG1
136	0.022195229	0.35623342	LOC10110767
13	0.025256472	0.38206274	RAD50(RAD5
71	0.026184985	0.38206274	AGTR1(AGTR
64	0.029013795	0.404931659	HCN1(HCN1)
42	0.033895711	0.453355137	LOC10111824
84	0.040476022	0.519712119	NRAS(NRAS)
120	0.045066164	0.520033042	CTSD(CTSD),
165	0.045513433	0.520033042	CREB3L4(CR

URL

http://www.kegg.jp/kegg-bin/show_pathway?map04928/K04630%09%23FFFFFF,red/K17895%09%23FFFFFF,red/K08044%09%23FFFFFF,red/K058

http://www.kegg.jp/kegg-bin/show_pathway?map04725/K04630%09%23FFFFFF,red/K04928%09%23FFFFFF,red/K04926%09%23FFFFFF,red/K041

http://www.kegg.jp/kegg-bin/show_pathway?map04022/K04630%09%23FFFFFF,red/K04137%09%23FFFFFF,red/K02158%09%23FFFFFF,red/K050

http://www.kegg.jp/kegg-bin/show_pathway?map04062/K04630%09%23FFFFFF,red/K04237%09%23FFFFFF,red/K02158%09%23FFFFFF,red/K080

http://www.kegg.jp/kegg-bin/show_pathway?map04810/K10352%09%23FFFFFF,red/K05751%09%23FFFFFF,red/K06589%09%23FFFFFF,red/K064

http://www.kegg.jp/kegg-bin/show_pathway?map04261/K04630%09%23FFFFFF,red/K04137%09%23FFFFFF,red/K04858%09%23FFFFFF,red/K048

http://www.kegg.jp/kegg-bin/show_pathway?map04921/K07359%09%23FFFFFF,red/K04630%09%23FFFFFF,red/K04858%09%23FFFFFF,red/K048

http://www.kegg.jp/kegg-bin/show_pathway?map04713/K04630%09%23FFFFFF,red/K04961%09%23FFFFFF,red/K08044%09%23FFFFFF,red/K048

http://www.kegg.jp/kegg-bin/show_pathway?map04024/K04630%09%23FFFFFF,red/K04153%09%23FFFFFF,red/K02158%09%23FFFFFF,red/K044

http://www.kegg.jp/kegg-bin/show_pathway?map04970/K04137%09%23FFFFFF,red/K12318%09%23FFFFFF,red/K08045%09%23FFFFFF,red/K015

http://www.kegg.jp/kegg-bin/show_pathway?map04927/K04684%09%23FFFFFF,red/K09048%09%23FFFFFF,red/K08045%09%23FFFFFF,red/K080

http://www.kegg.jp/kegg-bin/show_pathway?map04973/K12047%09%23FFFFFF,red/K01539%09%23FFFFFF,red/K00844%09%23FFFFFF,red/K058

http://www.kegg.jp/kegg-bin/show_pathway?map04724/K04630%09%23FFFFFF,red/K08044%09%23FFFFFF,red/K04850%09%23FFFFFF,red/K058

http://www.kegg.jp/kegg-bin/show_pathway?map04924/K04630%09%23FFFFFF,red/K12318%09%23FFFFFF,red/K08045%09%23FFFFFF,red/K048

http://www.kegg.jp/kegg-bin/show_pathway?map04020/K05099%09%23FFFFFF,red/K04137%09%23FFFFFF,red/K05096%09%23FFFFFF,red/K041

http://www.kegg.jp/kegg-bin/show_pathway?map04510/K05099%09%23FFFFFF,red/K05096%09%23FFFFFF,red/K06589%09%23FFFFFF,red/K021

http://www.kegg.jp/kegg-bin/show_pathway?map04742/K04836%09%23FFFFFF,red/K04153%09%23FFFFFF,red/K08048%09%23FFFFFF,red/K080

http://www.kegg.jp/kegg-bin/show_pathway?map04012/K08774%09%23FFFFFF,red/K05725%09%23FFFFFF,red/K05457%09%23FFFFFF,red/K021

http://www.kegg.jp/kegg-bin/show_pathway?map05100/K16938%09%23FFFFFF,red/K05099%09%23FFFFFF,red/K05691%09%23FFFFFF,red/K137

http://www.kegg.jp/kegg-bin/show_pathway?map04935/K04630%09%23FFFFFF,red/K08044%09%23FFFFFF,red/K04850%09%23FFFFFF,red/K058

http://www.kegg.jp/kegg-bin/show_pathway?map00230/K01939%09%23FFFFFF,red/K00939%09%23FFFFFF,red/K00893%09%23FFFFFF,red/K080

http://www.kegg.jp/kegg-bin/show_pathway?map04015/K04630%09%23FFFFFF,red/K06070%09%23FFFFFF,red/K05099%09%23FFFFFF,red/K050

http://www.kegg.jp/kegg-bin/show_pathway?map04350/K04673%09%23FFFFFF,red/K04684%09%23FFFFFF,red/K04672%09%23FFFFFF,red/K046

http://www.kegg.jp/kegg-bin/show_pathway?map04730/K04630%09%23FFFFFF,red/K12318%09%23FFFFFF,red/K04961%09%23FFFFFF,red/K058

http://www.kegg.jp/kegg-bin/show_pathway?map04972/K04964%09%23FFFFFF,red/K08045%09%23FFFFFF,red/K01539%09%23FFFFFF,red/K049

http://www.kegg.jp/kegg-bin/show_pathway?map04611/K04630%09%23FFFFFF,red/K08044%09%23FFFFFF,red/K05858%09%23FFFFFF,red/K043

http://www.kegg.jp/kegg-bin/show_pathway?map04911/K09048%09%23FFFFFF,red/K08045%09%23FFFFFF,red/K01539%09%23FFFFFF,red/K080

http://www.kegg.jp/kegg-bin/show_pathway?map04925/K06070%09%23FFFFFF,red/K09048%09%23FFFFFF,red/K08045%09%23FFFFFF,red/K015

http://www.kegg.jp/kegg-bin/show_pathway?map04070/K01109%09%23FFFFFF,red/K07756%09%23FFFFFF,red/K00901%09%23FFFFFF,red/K009

http://www.kegg.jp/kegg-bin/show_pathway?map04010/K05099%09%23FFFFFF,red/K05096%09%23FFFFFF,red/K04863%09%23FFFFFF,red/K044

http://www.kegg.jp/kegg-bin/show_pathway?map04270/K10352%09%23FFFFFF,red/K04137%09%23FFFFFF,red/K08044%09%23FFFFFF,red/K048

http://www.kegg.jp/kegg-bin/show_pathway?map04710/K07198%09%23FFFFFF,red/K03347%09%23FFFFFF,red/K03094%09%23FFFFFF,red/K038

http://www.kegg.jp/kegg-bin/show_pathway?map04926/K04630%09%23FFFFFF,red/K04448%09%23FFFFFF,red/K08044%09%23FFFFFF,red/K058

http://www.kegg.jp/kegg-bin/show_pathway?map00562/K01109%09%23FFFFFF,red/K00914%09%23FFFFFF,red/K00888%09%23FFFFFF,red/K058

http://www.kegg.jp/kegg-bin/show_pathway?map04360/K06838%09%23FFFFFF,red/K05099%09%23FFFFFF,red/K04237%09%23FFFFFF,red/K068

http://www.kegg.jp/kegg-bin/show_pathway?map04724/K04603%09%23FFFFFF,red/K04850%09%23FFFFFF,red/K05858%09%23FFFFFF,red/K048

http://www.kegg.jp/kegg-bin/show_pathway?map04742/K04153%09%23FFFFFF,red/K19729%09%23FFFFFF,red/K04603%09%23FFFFFF,red/K048

http://www.kegg.jp/kegg-bin/show_pathway?map04072/K08091%09%23FFFFFF,red/K04603%09%23FFFFFF,red/K07941%09%23FFFFFF,red/K058

http://www.kegg.jp/kegg-bin/show_pathway?map04929/K04954%09%23FFFFFF,red/K04964%09%23FFFFFF,red/K07827%09%23FFFFFF,red/K048

http://www.kegg.jp/kegg-bin/show_pathway?map00513/K09656%09%23FFFFFF,red/K12373%09%23FFFFFF,red/K01231%09%23FFFFFF,red/K237

http://www.kegg.jp/kegg-bin/show_pathway?map04961/K04644%09%23FFFFFF,red/K14757%09%23FFFFFF,red/K01539%09%23FFFFFF,red/K118

http://www.kegg.jp/kegg-bin/show_pathway?map04730/K12318%09%23FFFFFF,red/K04603%09%23FFFFFF,red/K07827%09%23FFFFFF,red/K058

http://www.kegg.jp/kegg-bin/show_pathway?map04725/K04129%09%23FFFFFF,red/K04928%09%23FFFFFF,red/K04850%09%23FFFFFF,red/K058

http://www.kegg.jp/kegg-bin/show_pathway?map04020/K05099%09%23FFFFFF,red/K05096%09%23FFFFFF,red/K04129%09%23FFFFFF,red/K046

http://www.kegg.jp/kegg-bin/show_pathway?map04721/K04644%09%23FFFFFF,red/K15290%09%23FFFFFF,red/K11827%09%23FFFFFF,red/K021

http://www.kegg.jp/kegg-bin/show_pathway?map04924/K12318%09%23FFFFFF,red/K04850%09%23FFFFFF,red/K05858%09%23FFFFFF,red/K190

http://www.kegg.jp/kegg-bin/show_pathway?map04512/K23380%09%23FFFFFF,red/K06256%09%23FFFFFF,red/K06252%09%23FFFFFF,red/K233

http://www.kegg.jp/kegg-bin/show_pathway?map05100/K16938%09%23FFFFFF,red/K05099%09%23FFFFFF,red/K04644%09%23FFFFFF,red/K056

http://www.kegg.jp/kegg-bin/show_pathway?map04925/K09048%09%23FFFFFF,red/K18050%09%23FFFFFF,red/K01539%09%23FFFFFF,red/K048

http://www.kegg.jp/kegg-bin/show_pathway?map03018/K12590%09%23FFFFFF,red/K12605%09%23FFFFFF,red/K12586%09%23FFFFFF,red/K035

http://www.kegg.jp/kegg-bin/show_pathway?map04360/K07293%09%23FFFFFF,red/K04514%09%23FFFFFF,red/K05099%09%23FFFFFF,red/K067

http://www.kegg.jp/kegg-bin/show_pathway?map04713/K16513%09%23FFFFFF,red/K12318%09%23FFFFFF,red/K05200%09%23FFFFFF,red/K080

http://www.kegg.jp/kegg-bin/show_pathway?map04020/K05099%09%23FFFFFF,red/K05096%09%23FFFFFF,red/K10785%09%23FFFFFF,red/K052

http://www.kegg.jp/kegg-bin/show_pathway?map04725/K09048%09%23FFFFFF,red/K07828%09%23FFFFFF,red/K08045%09%23FFFFFF,red/K044

http://www.kegg.jp/kegg-bin/show_pathway?map04911/K04939%09%23FFFFFF,red/K09048%09%23FFFFFF,red/K08045%09%23FFFFFF,red/K015

http://www.kegg.jp/kegg-bin/show_pathway?map04926/K19720%09%23FFFFFF,red/K05858%09%23FFFFFF,red/K16366%09%23FFFFFF,red/K009

http://www.kegg.jp/kegg-bin/show_pathway?map04922/K09048%09%23FFFFFF,red/K11262%09%23FFFFFF,red/K12407%09%23FFFFFF,red/K015

http://www.kegg.jp/kegg-bin/show_pathway?map04371/K18050%09%23FFFFFF,red/K04503%09%23FFFFFF,red/K04961%09%23FFFFFF,red/K092

http://www.kegg.jp/kegg-bin/show_pathway?map04916/K05461%09%23FFFFFF,red/K09048%09%23FFFFFF,red/K07828%09%23FFFFFF,red/K080

http://www.kegg.jp/kegg-bin/show_pathway?map03440/K10879%09%23FFFFFF,red/K10866%09%23FFFFFF,red/K08775%09%23FFFFFF,red/K108

http://www.kegg.jp/kegg-bin/show_pathway?map04728/K05000%09%23FFFFFF,red/K05858%09%23FFFFFF,red/K00501%09%23FFFFFF,red/K043

http://www.kegg.jp/kegg-bin/show_pathway?map04925/K04166%09%23FFFFFF,red/K06070%09%23FFFFFF,red/K09048%09%23FFFFFF,red/K080

http://www.kegg.jp/kegg-bin/show_pathway?map04726/K05000%09%23FFFFFF,red/K04162%09%23FFFFFF,red/K04153%09%23FFFFFF,red/K163

http://www.kegg.jp/kegg-bin/show_pathway?map04730/K07828%09%23FFFFFF,red/K12318%09%23FFFFFF,red/K08067%09%23FFFFFF,red/K049

http://www.kegg.jp/kegg-bin/show_pathway?map04921/K04514%09%23FFFFFF,red/K05000%09%23FFFFFF,red/K04503%09%23FFFFFF,red/K048

http://www.kegg.jp/kegg-bin/show_pathway?map04971/K05008%09%23FFFFFF,red/K04896%09%23FFFFFF,red/K08045%09%23FFFFFF,red/K015

http://www.kegg.jp/kegg-bin/show_pathway?map04724/K05202%09%23FFFFFF,red/K15010%09%23FFFFFF,red/K05200%09%23FFFFFF,red/K080

http://www.kegg.jp/kegg-bin/show_pathway?map04270/K04514%09%23FFFFFF,red/K18050%09%23FFFFFF,red/K16342%09%23FFFFFF,red/K058

http://www.kegg.jp/kegg-bin/show_pathway?map03450/K10887%09%23FFFFFF,red/K06642%09%23FFFFFF,red/K10866%09%23FFFFFF,red/K009

http://www.kegg.jp/kegg-bin/show_pathway?map04924/K04166%09%23FFFFFF,red/K12318%09%23FFFFFF,red/K08045%09%23FFFFFF,red/K132

http://www.kegg.jp/kegg-bin/show_pathway?map04929/K04954%09%23FFFFFF,red/K04955%09%23FFFFFF,red/K07828%09%23FFFFFF,red/K050

http://www.kegg.jp/kegg-bin/show_pathway?map00860/K14163%09%23FFFFFF,red/K01764%09%23FFFFFF,red/K21418%09%23FFFFFF,red/K147

http://www.kegg.jp/kegg-bin/show_pathway?map04012/K07828%09%23FFFFFF,red/K04438%09%23FFFFFF,red/K05085%09%23FFFFFF,red/K072

http://www.kegg.jp/kegg-bin/show_pathway?map04071/K01379%09%23FFFFFF,red/K04514%09%23FFFFFF,red/K07828%09%23FFFFFF,red/K180

http://www.kegg.jp/kegg-bin/show_pathway?map04022/K04514%09%23FFFFFF,red/K18050%09%23FFFFFF,red/K09260%09%23FFFFFF,red/K058

58%09%23FFFFFF,red/K13293%09%23FFFFFF,red/K16834%09%23FFFFFF,red/K05261%09%23FFFFFF,red/K12330%09%23FFFFFF

33%09%23FFFFFF,red/K08044%09%23FFFFFF,red/K04850%09%23FFFFFF,red/K05858%09%23FFFFFF,red/K04131%09%23FFFFFF

01%09%23FFFFFF,red/K08044%09%23FFFFFF,red/K04850%09%23FFFFFF,red/K05858%09%23FFFFFF,red/K03121%09%23FFFFFF

44%09%23FFFFFF,red/K05858%09%23FFFFFF,red/K04176%09%23FFFFFF,red/K00922%09%23FFFFFF,red/K08893%09%23FFFFFF

61%09%23FFFFFF,red/K04133%09%23FFFFFF,red/K05766%09%23FFFFFF,red/K04131%09%23FFFFFF,red/K05732%09%23FFFFFF

63%09%23FFFFFF,red/K04926%09%23FFFFFF,red/K08044%09%23FFFFFF,red/K04850%09%23FFFFFF,red/K05858%09%23FFFFFF

63%09%23FFFFFF,red/K04961%09%23FFFFFF,red/K04448%09%23FFFFFF,red/K08044%09%23FFFFFF,red/K04850%09%23FFFFFF

50%09%23FFFFFF,red/K05858%09%23FFFFFF,red/K04851%09%23FFFFFF,red/K04997%09%23FFFFFF,red/K13240%09%23FFFFFF

48%09%23FFFFFF,red/K08044%09%23FFFFFF,red/K04850%09%23FFFFFF,red/K13293%09%23FFFFFF,red/K04851%09%23FFFFFF

39%09%23FFFFFF,red/K08048%09%23FFFFFF,red/K05742%09%23FFFFFF,red/K08044%09%23FFFFFF,red/K05858%09%23FFFFFF

48%09%23FFFFFF,red/K08044%09%23FFFFFF,red/K04850%09%23FFFFFF,red/K05858%09%23FFFFFF,red/K04851%09%23FFFFFF

58%09%23FFFFFF,red/K04851%09%23FFFFFF,red/K14158%09%23FFFFFF,red/K00922%09%23FFFFFF,red/K01203%09%23FFFFFF

58%09%23FFFFFF,red/K05205%09%23FFFFFF,red/K04851%09%23FFFFFF,red/K04997%09%23FFFFFF,red/K04610%09%23FFFFFF

50%09%23FFFFFF,red/K05858%09%23FFFFFF,red/K19021%09%23FFFFFF,red/K05030%09%23FFFFFF,red/K04851%09%23FFFFFF

33%09%23FFFFFF,red/K04961%09%23FFFFFF,red/K08044%09%23FFFFFF,red/K04850%09%23FFFFFF,red/K05858%09%23FFFFFF

58%09%23FFFFFF,red/K04448%09%23FFFFFF,red/K05732%09%23FFFFFF,red/K06249%09%23FFFFFF,red/K00922%09%23FFFFFF

44%09%23FFFFFF,red/K04850%09%23FFFFFF,red/K05858%09%23FFFFFF,red/K05175%09%23FFFFFF,red/K04131%09%23FFFFFF

58%09%23FFFFFF,red/K04448%09%23FFFFFF,red/K04440%09%23FFFFFF,red/K22517%09%23FFFFFF,red/K17449%09%23FFFFFF

44%09%23FFFFFF,red/K05725%09%23FFFFFF,red/K02105%09%23FFFFFF,red/K12366%09%23FFFFFF,red/K17449%09%23FFFFFF

58%09%23FFFFFF,red/K04851%09%23FFFFFF,red/K00922%09%23FFFFFF,red/K09047%09%23FFFFFF,red/K08045%09%23FFFFFF

44%09%23FFFFFF,red/K13293%09%23FFFFFF,red/K18436%09%23FFFFFF,red/K13755%09%23FFFFFF,red/K00856%09%23FFFFFF

96%09%23FFFFFF,red/K06461%09%23FFFFFF,red/K04237%09%23FFFFFF,red/K17697%09%23FFFFFF,red/K08044%09%23FFFFFF

79%09%23FFFFFF,red/K13567%09%23FFFFFF,red/K06766%09%23FFFFFF,red/K03868%09%23FFFFFF,red/K04668%09%23FFFFFF

58%09%23FFFFFF,red/K07376%09%23FFFFFF,red/K13240%09%23FFFFFF,red/K04365%09%23FFFFFF,red/K05207%09%23FFFFFF

26%09%23FFFFFF,red/K08048%09%23FFFFFF,red/K05742%09%23FFFFFF,red/K08044%09%23FFFFFF,red/K05858%09%23FFFFFF

50%09%23FFFFFF,red/K12330%09%23FFFFFF,red/K05732%09%23FFFFFF,red/K00922%09%23FFFFFF,red/K19477%09%23FFFFFF

48%09%23FFFFFF,red/K18211%09%23FFFFFF,red/K08044%09%23FFFFFF,red/K04850%09%23FFFFFF,red/K05858%09%23FFFFFF

39%09%23FFFFFF,red/K08048%09%23FFFFFF,red/K08044%09%23FFFFFF,red/K04850%09%23FFFFFF,red/K05858%09%23FFFFFF

14%09%23FFFFFF,red/K00888%09%23FFFFFF,red/K05858%09%23FFFFFF,red/K00889%09%23FFFFFF,red/K00923%09%23FFFFFF

07%09%23FFFFFF,red/K04448%09%23FFFFFF,red/K04850%09%23FFFFFF,red/K04851%09%23FFFFFF,red/K04360%09%23FFFFFF

50%09%23FFFFFF,red/K05858%09%23FFFFFF,red/K04851%09%23FFFFFF,red/K12330%09%23FFFFFF,red/K12331%09%23FFFFFF

68%09%23FFFFFF,red/K02223%09%23FFFFFF,red/K08532%09%23FFFFFF,red/

58%09%23FFFFFF,red/K00922%09%23FFFFFF,red/K13240%09%23FFFFFF,red/K09047%09%23FFFFFF,red/K04549%09%23FFFFFF

58%09%23FFFFFF,red/K00889%09%23FFFFFF,red/K19006%09%23FFFFFF,red/K00923%09%23FFFFFF,red/K21798%09%23FFFFFF

40%09%23FFFFFF,red/K07520%09%23FFFFFF,red/K06754%09%23FFFFFF,red/K04189%09%23FFFFFF,red/K00922%09%23FFFFFF

51%09%23FFFFFF,red/K04958%09%23FFFFFF,red/K04997%09%23FFFFFF,red/K05197%09%23FFFFFF,red/K04609%09%23FFFFFF

50%09%23FFFFFF,red/K05858%09%23FFFFFF,red/K05175%09%23FFFFFF,red/K13755%09%23FFFFFF,red/K04990%09%23FFFFFF

58%09%23FFFFFF,red/K01528%09%23FFFFFF,red/K00922%09%23FFFFFF,red/K07829%09%23FFFFFF,red/K05450%09%23FFFFFF

50%09%23FFFFFF,red/K05858%09%23FFFFFF,red/K04851%09%23FFFFFF,red/K04856%09%23FFFFFF,red/K04997%09%23FFFFFF

41%09%23FFFFFF,red/K12666%09%23FFFFFF,red/K01230%09%23FFFFFF,red/K00717%09%23FFFFFF,red/K13748%09%23FFFFFF

27%09%23FFFFFF,red/K05858%09%23FFFFFF,red/K01528%09%23FFFFFF,red/K08550%09%23FFFFFF,red/K08539%09%23FFFFFF

58%09%23FFFFFF,red/K07376%09%23FFFFFF,red/K04958%09%23FFFFFF,red/K05197%09%23FFFFFF,red/K04578%09%23FFFFFF

58%09%23FFFFFF,red/K04851%09%23FFFFFF,red/K04958%09%23FFFFFF,red/K04997%09%23FFFFFF,red/K00922%09%23FFFFFF

03%09%23FFFFFF,red/K04636%09%23FFFFFF,red/K05085%09%23FFFFFF,red/K04850%09%23FFFFFF,red/K05858%09%23FFFFFF

54%09%23FFFFFF,red/K01528%09%23FFFFFF,red/K08486%09%23FFFFFF,red/K12302%09%23FFFFFF,red/K05613%09%23FFFFFF

21%09%23FFFFFF,red/K05030%09%23FFFFFF,red/K09864%09%23FFFFFF,red/K04851%09%23FFFFFF,red/K13755%09%23FFFFFF

79%09%23FFFFFF,red/K03900%09%23FFFFFF,red/K19719%09%23FFFFFF,red/K06258%09%23FFFFFF,red/K05637%09%23FFFFFF

91%09%23FFFFFF,red/K07863%09%23FFFFFF,red/K17449%09%23FFFFFF,red/K01528%09%23FFFFFF,red/K16939%09%23FFFFFF

50%09%23FFFFFF,red/K05858%09%23FFFFFF,red/K04851%09%23FFFFFF,red/K04856%09%23FFFFFF,red/K04958%09%23FFFFFF

14%09%23FFFFFF,red/K12603%09%23FFFFFF,red/K12600%09%23FFFFFF,red/K12616%09%23FFFFFF,red/K00850%09%23FFFFFF

65%09%23FFFFFF,red/K04237%09%23FFFFFF,red/K06840%09%23FFFFFF,red/K07520%09%23FFFFFF,red/K04968%09%23FFFFFF

45%09%23FFFFFF,red/K05000%09%23FFFFFF,red/K04961%09%23FFFFFF,red/K05858%09%23FFFFFF,red/K11265%09%23FFFFFF

15%09%23FFFFFF,red/K04162%09%23FFFFFF,red/K04961%09%23FFFFFF,red/K04636%09%23FFFFFF,red/K05085%09%23FFFFFF

47%09%23FFFFFF,red/K05000%09%23FFFFFF,red/K04926%09%23FFFFFF,red/K05858%09%23FFFFFF,red/K04131%09%23FFFFFF

39%09%23FFFFFF,red/K12407%09%23FFFFFF,red/K18211%09%23FFFFFF,red/K05858%09%23FFFFFF,red/K04131%09%23FFFFFF

22%09%23FFFFFF,red/K13240%09%23FFFFFF,red/K09047%09%23FFFFFF,red/K22000%09%23FFFFFF,red/K06237%09%23FFFFFF

96%09%23FFFFFF,red/K17491%09%23FFFFFF,red/K13296%09%23FFFFFF,red/K05858%09%23FFFFFF,red/K16333%09%23FFFFFF

60%09%23FFFFFF,red/K07203%09%23FFFFFF,red/K06052%09%23FFFFFF,red/K05858%09%23FFFFFF,red/K11265%09%23FFFFFF

45%09%23FFFFFF,red/K05858%09%23FFFFFF,red/K00444%09%23FFFFFF,red/K04490%09%23FFFFFF,red/K16366%09%23FFFFFF

67%09%23FFFFFF,red/K07466%09%23FFFFFF,red/K03505%09%23FFFFFF,red/K10869%09%23FFFFFF,red/K20773%09%23FFFFFF

54%09%23FFFFFF,red/K04515%09%23FFFFFF,red/K00274%09%23FFFFFF,red/K09047%09%23FFFFFF,red/K08045%09%23FFFFFF

45%09%23FFFFFF,red/K18050%09%23FFFFFF,red/K01539%09%23FFFFFF,red/K05858%09%23FFFFFF,red/K04856%09%23FFFFFF

42%09%23FFFFFF,red/K05858%09%23FFFFFF,red/K21278%09%23FFFFFF,red/K00274%09%23FFFFFF,red/K05181%09%23FFFFFF

61%09%23FFFFFF,red/K16342%09%23FFFFFF,red/K05858%09%23FFFFFF,red/K13240%09%23FFFFFF,red/K04365%09%23FFFFFF

58%09%23FFFFFF,red/K04863%09%23FFFFFF,red/K04961%09%23FFFFFF,red/K16342%09%23FFFFFF,red/K05858%09%23FFFFFF

39%09%23FFFFFF,red/K04926%09%23FFFFFF,red/K05858%09%23FFFFFF,red/K04131%09%23FFFFFF,red/K04515%09%23FFFFFF

45%09%23FFFFFF,red/K16342%09%23FFFFFF,red/K05858%09%23FFFFFF,red/K15009%09%23FFFFFF,red/K04536%09%23FFFFFF

58%09%23FFFFFF,red/K04136%09%23FFFFFF,red/K16366%09%23FFFFFF,red/K12331%09%23FFFFFF,red/K01047%09%23FFFFFF

77%09%23FFFFFF,red/

96%09%23FFFFFF,red/K05858%09%23FFFFFF,red/K09864%09%23FFFFFF,red/K01363%09%23FFFFFF,red/K13755%09%23FFFFFF

00%09%23FFFFFF,red/K05858%09%23FFFFFF,red/K04968%09%23FFFFFF,red/K04856%09%23FFFFFF,red/K04944%09%23FFFFFF

35%09%23FFFFFF,red/K00214%09%23FFFFFF,red/K00699%09%23FFFFFF,red/

03%09%23FFFFFF,red/K04440%09%23FFFFFF,red/K06624%09%23FFFFFF,red/K22517%09%23FFFFFF,red/K04409%09%23FFFFFF

50%09%23FFFFFF,red/K05665%09%23FFFFFF,red/K05858%09%23FFFFFF,red/K04440%09%23FFFFFF,red/K11584%09%23FFFFFF

58%09%23FFFFFF,red/K04136%09%23FFFFFF,red/K09047%09%23FFFFFF,red/K04138%09%23FFFFFF,red/K06270%09%23FFFFFF

,red/K12331%09%23FFFFFF,red/K09047%09%23FFFFFF,red/K04684%09%23FFFFFF,red/K09048%09%23FFFFFF,red/K08045%09%

,red/K04851%09%23FFFFFF,red/K04809%09%23FFFFFF,red/K00922%09%23FFFFFF,red/K04997%09%23FFFFFF,red/K09047%09%

,red/K04851%09%23FFFFFF,red/K12336%09%23FFFFFF,red/K09047%09%23FFFFFF,red/K19477%09%23FFFFFF,red/K08045%09%

,red/K21096%09%23FFFFFF,red/K04549%09%23FFFFFF,red/K05505%09%23FFFFFF,red/K08045%09%23FFFFFF,red/K05514%09%

,red/K05745%09%23FFFFFF,red/K12330%09%23FFFFFF,red/K23612%09%23FFFFFF,red/K00922%09%23FFFFFF,red/K05733%09%

,red/K04859%09%23FFFFFF,red/K04851%09%23FFFFFF,red/K04354%09%23FFFFFF,red/K09047%09%23FFFFFF,red/K04962%09%

,red/K05858%09%23FFFFFF,red/K04859%09%23FFFFFF,red/K04851%09%23FFFFFF,red/K08292%09%23FFFFFF,red/K04997%09%

,red/K04962%09%23FFFFFF,red/K19477%09%23FFFFFF,red/K04549%09%23FFFFFF,red/K08045%09%23FFFFFF,red/K12318%09%

,red/K00922%09%23FFFFFF,red/K09047%09%23FFFFFF,red/K04130%09%23FFFFFF,red/K04962%09%23FFFFFF,red/K05673%09%

,red/K07376%09%23FFFFFF,red/K04131%09%23FFFFFF,red/K05850%09%23FFFFFF,red/K13915%09%23FFFFFF,red/K13240%09%

,red/K04856%09%23FFFFFF,red/K18437%09%23FFFFFF,red/K09047%09%23FFFFFF,red/K04913%09%23FFFFFF,red/

,red/K04549%09%23FFFFFF,red/K04964%09%23FFFFFF,red/K08045%09%23FFFFFF,red/K08048%09%23FFFFFF,red/K15009%09%

,red/K13755%09%23FFFFFF,red/K05027%09%23FFFFFF,red/K01283%09%23FFFFFF,red/K19477%09%23FFFFFF,red/

,red/K04360%09%23FFFFFF,red/K13755%09%23FFFFFF,red/K04131%09%23FFFFFF,red/K04851%09%23FFFFFF,red/K04809%09%

,red/K05733%09%23FFFFFF,red/K05450%09%23FFFFFF,red/K10151%09%23FFFFFF,red/K06252%09%23FFFFFF,red/K05725%09%

,red/K13755%09%23FFFFFF,red/K08474%09%23FFFFFF,red/K04824%09%23FFFFFF,red/K04626%09%23FFFFFF,red/

,red/K04409%09%23FFFFFF,red/K05733%09%23FFFFFF,red/K00922%09%23FFFFFF,red/K04365%09%23FFFFFF,red/K03099%09%

,red/K01528%09%23FFFFFF,red/K23612%09%23FFFFFF,red/K00922%09%23FFFFFF,red/K05757%09%23FFFFFF,red/

,red/K09048%09%23FFFFFF,red/K10138%09%23FFFFFF,red/K05725%09%23FFFFFF,red/K08048%09%23FFFFFF,red/K04440%09%

,red/K16855%09%23FFFFFF,red/K13485%09%23FFFFFF,red/K01522%09%23FFFFFF,red/K08045%09%23FFFFFF,red/K12318%09%

,red/K05858%09%23FFFFFF,red/K00922%09%23FFFFFF,red/K05450%09%23FFFFFF,red/K05631%09%23FFFFFF,red/K08020%09%

,red/K04663%09%23FFFFFF,red/K03094%09%23FFFFFF,red/K03347%09%23FFFFFF,red/K04664%09%23FFFFFF,red/K16790%09%

,red/K05030%09%23FFFFFF,red/K04131%09%23FFFFFF,red/K05027%09%23FFFFFF,red/K05850%09%23FFFFFF,red/K01047%09%

,red/K08045%09%23FFFFFF,red/K12318%09%23FFFFFF,red/K08048%09%23FFFFFF,red/K06069%09%23FFFFFF,red/K03900%09%

,red/K04851%09%23FFFFFF,red/K04131%09%23FFFFFF,red/K09047%09%23FFFFFF,red/K04962%09%23FFFFFF,red/

F,red/K04851%09%23FFFFFF,red/K04856%09%23FFFFFF,red/K05850%09%23FFFFFF,red/K08794%09%23FFFFFF,red/K09047%09%

F,red/K04459%09%23FFFFFF,red/K03158%09%23FFFFFF,red/K04860%09%23FFFFFF,red/K04373%09%23FFFFFF,red/K04440%09%

F,red/K12336%09%23FFFFFF,red/K01047%09%23FFFFFF,red/K08045%09%23FFFFFF,red/K12318%09%23FFFFFF,red/K08048%09%

F,red/K08045%09%23FFFFFF,red/K09048%09%23FFFFFF,red/K08048%09%23FFFFFF,red/K04440%09%23FFFFFF,red/K17449%09%

F,red/K05733%09%23FFFFFF,red/K20013%09%23FFFFFF,red/K05110%09%23FFFFFF,red/K07829%09%23FFFFFF,red/K04515%09%

F,red/K13576%09%23FFFFFF,red/K04610%09%23FFFFFF,red/K04964%09%23FFFFFF,red/K04604%09%23FFFFFF,red/K01115%09%

F,red/K04609%09%23FFFFFF,red/K04610%09%23FFFFFF,red/K21289%09%23FFFFFF,red/K00901%09%23FFFFFF,red/K07827%09%

F,red/K04515%09%23FFFFFF,red/K09047%09%23FFFFFF,red/K04930%09%23FFFFFF,red/K21289%09%23FFFFFF,red/K09048%09%

F,red/K13755%09%23FFFFFF,red/K04851%09%23FFFFFF,red/K04189%09%23FFFFFF,red/K05101%09%23FFFFFF,red/K04958%09%

F,red/K02146%09%23FFFFFF,red/K05618%09%23FFFFFF,red/K15295%09%23FFFFFF,red/K11825%09%23FFFFFF,red/K02144%09%

F,red/K06249%09%23FFFFFF,red/K05636%09%23FFFFFF,red/K06240%09%23FFFFFF,red/K06584%09%23FFFFFF,red/

F,red/K04515%09%23FFFFFF,red/K08794%09%23FFFFFF,red/K07188%09%23FFFFFF,red/K09047%09%23FFFFFF,red/K04919%09%

F,red/K00444%09%23FFFFFF,red/K00922%09%23FFFFFF,red/K05110%09%23FFFFFF,red/K04515%09%23FFFFFF,red/K05463%09%

F,red/K04856%09%23FFFFFF,red/K04536%09%23FFFFFF,red/K04543%09%23FFFFFF,red/K04587%09%23FFFFFF,red/K04515%09%

F,red/K05858%09%23FFFFFF,red/K04136%09%23FFFFFF,red/K13755%09%23FFFFFF,red/K04131%09%23FFFFFF,red/K05101%09%

F,red/K04536%09%23FFFFFF,red/K00922%09%23FFFFFF,red/K04543%09%23FFFFFF,red/K04515%09%23FFFFFF,red/K04545%09%

F,red/K04944%09%23FFFFFF,red/K04587%09%23FFFFFF,red/K04515%09%23FFFFFF,red/K09047%09%23FFFFFF,red/

F,red/K08045%09%23FFFFFF,red/K07828%09%23FFFFFF,red/K09048%09%23FFFFFF,red/K04306%09%23FFFFFF,red/K16858%09%

F,red/K07190%09%23FFFFFF,red/K16311%09%23FFFFFF,red/K04515%09%23FFFFFF,red/K09047%09%23FFFFFF,red/

F,red/K13240%09%23FFFFFF,red/K11406%09%23FFFFFF,red/K04166%09%23FFFFFF,red/K08045%09%23FFFFFF,red/K07828%09%

F,red/K00505%09%23FFFFFF,red/K00182%09%23FFFFFF,red/K04515%09%23FFFFFF,red/K04198%09%23FFFFFF,red/K00506%09%

F,red/K05200%09%23FFFFFF,red/K09048%09%23FFFFFF,red/K11584%09%23FFFFFF,red/K04440%09%23FFFFFF,red/K04536%09%

F,red/K05850%09%23FFFFFF,red/K04515%09%23FFFFFF,red/K05869%09%23FFFFFF,red/K09047%09%23FFFFFF,red/

F,red/K08045%09%23FFFFFF,red/K07828%09%23FFFFFF,red/K04819%09%23FFFFFF,red/K04892%09%23FFFFFF,red/K04536%09%

F,red/K04859%09%23FFFFFF,red/K17901%09%23FFFFFF,red/K04515%09%23FFFFFF,red/K06270%09%23FFFFFF,red/K08045%09%

F,red/K04543%09%23FFFFFF,red/K05201%09%23FFFFFF,red/K04545%09%23FFFFFF,red/K15008%09%23FFFFFF,red/K04609%09%

F,red/K06270%09%23FFFFFF,red/K04166%09%23FFFFFF,red/K04939%09%23FFFFFF,red/K08045%09%23FFFFFF,red/K12318%09%

F,red/K07983%09%23FFFFFF,red/K03171%09%23FFFFFF,red/K04354%09%23FFFFFF,red/K00922%09%23FFFFFF,red/K00654%09%

F,red/K04166%09%23FFFFFF,red/K04939%09%23FFFFFF,red/K08045%09%23FFFFFF,red/K12318%09%23FFFFFF,red/K09048%09%

23FFFFFF,red/K08048%09%23FFFFFF,red/K07996%09%23FFFFFF,red/K01115%09%23FFFFFF,red/K08539%09%23FFFFFF,red/K0

23FFFFFF,red/K04130%09%23FFFFFF,red/K04549%09%23FFFFFF,red/K09048%09%23FFFFFF,red/K08045%09%23FFFFFF,red/K0

23FFFFFF,red/K09048%09%23FFFFFF,red/K12318%09%23FFFFFF,red/K01539%09%23FFFFFF,red/K08048%09%23FFFFFF,red/K1

23FFFFFF,red/K05725%09%23FFFFFF,red/K05731%09%23FFFFFF,red/K08048%09%23FFFFFF,red/K21097%09%23FFFFFF,red/K1

23FFFFFF,red/K05762%09%23FFFFFF,red/K05450%09%23FFFFFF,red/K04130%09%23FFFFFF,red/K05725%09%23FFFFFF,red/K0

23FFFFFF,red/K04860%09%23FFFFFF,red/K08045%09%23FFFFFF,red/K09048%09%23FFFFFF,red/K01539%09%23FFFFFF,red/K0

23FFFFFF,red/K08794%09%23FFFFFF,red/K04962%09%23FFFFFF,red/K07198%09%23FFFFFF,red/K04860%09%23FFFFFF,red/K1

23FFFFFF,red/K08045%09%23FFFFFF,red/K09048%09%23FFFFFF,red/K01539%09%23FFFFFF,red/K08048%09%23FFFFFF,red/K0

23FFFFFF,red/K00910%09%23FFFFFF,red/K01115%09%23FFFFFF,red/K12302%09%23FFFFFF,red/K05201%09%23FFFFFF,red/

23FFFFFF,red/K04161%09%23FFFFFF,red/K05101%09%23FFFFFF,red/K20858%09%23FFFFFF,red/K05450%09%23FFFFFF,red/K0

23FFFFFF,red/K06585%09%23FFFFFF,red/K02105%09%23FFFFFF,red/K05460%09%23FFFFFF,red/K03900%09%23FFFFFF,red/K0

23FFFFFF,red/K17449%09%23FFFFFF,red/K11220%09%23FFFFFF,red/K04433%09%23FFFFFF,red/K03099%09%23FFFFFF,red/

23FFFFFF,red/K08048%09%23FFFFFF,red/K19021%09%23FFFFFF,red/K01510%09%23FFFFFF,red/K18437%09%23FFFFFF,red/K1

23FFFFFF,red/K08045%09%23FFFFFF,red/K08048%09%23FFFFFF,red/K05731%09%23FFFFFF,red/K02105%09%23FFFFFF,red/K0

23FFFFFF,red/K04446%09%23FFFFFF,red/K04358%09%23FFFFFF,red/K04856%09%23FFFFFF,red/K03171%09%23FFFFFF,red/K0

23FFFFFF,red/K12327%09%23FFFFFF,red/K07376%09%23FFFFFF,red/K17388%09%23FFFFFF,red/K04365%09%23FFFFFF,red/

23FFFFFF,red/K06842%09%23FFFFFF,red/K05463%09%23FFFFFF,red/K07521%09%23FFFFFF,red/K06572%09%23FFFFFF,red/K0

23FFFFFF,red/K05613%09%23FFFFFF,red/K12302%09%23FFFFFF,red/K05203%09%23FFFFFF,red/K15008%09%23FFFFFF,red/K0

23FFFFFF,red/K01080%09%23FFFFFF,red/K19007%09%23FFFFFF,red/K17449%09%23FFFFFF,red/K04262%09%23FFFFFF,red/K2

23FFFFFF,red/K04515%09%23FFFFFF,red/K05450%09%23FFFFFF,red/K08794%09%23FFFFFF,red/K04150%09%23FFFFFF,red/K0

23FFFFFF,red/K06839%09%23FFFFFF,red/K10031%09%23FFFFFF,red/K06843%09%23FFFFFF,red/K06724%09%23FFFFFF,red/K0

23FFFFFF,red/K04515%09%23FFFFFF,red/K20858%09%23FFFFFF,red/K05450%09%23FFFFFF,red/K13240%09%23FFFFFF,red/K0

[illegible]23FFFFFF,red/K04440%09%23FFFFFF,red/K04307%09%23FFFFFF,red/K04536%09%23FFFFFF,red/K04543%09%23FFFFFF,red/K0[illegible]

[illegible]23FFFFFF,red/K13296%09%23FFFFFF,red/K04536%09%23FFFFFF,red/K08334%09%23FFFFFF,red/K04543%09%23FFFFFF,red/K0[illegible]

[illegible]23FFFFFF,red/K12318%09%23FFFFFF,red/K07828%09%23FFFFFF,red/K01242%09%23FFFFFF,red/K17332%09%23FFFFFF,red/K0[illegible]

[illegible]23FFFFFF,red/K08449%09%23FFFFFF,red/K16343%09%23FFFFFF,red/K12327%09%23FFFFFF,red/K04365%09%23FFFFFF,red/

[illegible]23FFFFFF,red/K01539%09%23FFFFFF,red/K04139%09%23FFFFFF,red/K13296%09%23FFFFFF,red/K05863%09%23FFFFFF,red/K0[illegible]

9021%09%23FFFFFF,red/K07376%09%23FFFFFF,red/K04446%09%23FFFFFF,red/K21290%09%23FFFFFF,red/K05850%09%23FFFFF

2366%09%23FFFFFF,red/K00910%09%23FFFFFF,red/K17449%09%23FFFFFF,red/K04409%09%23FFFFFF,red/K21290%09%23FFFFF

5731%09%23FFFFFF,red/K05742%09%23FFFFFF,red/K06585%09%23FFFFFF,red/K00889%09%23FFFFFF,red/K04409%09%23FFFFF

8048%09%23FFFFFF,red/K05742%09%23FFFFFF,red/K11583%09%23FFFFFF,red/K21290%09%23FFFFFF,red/K05850%09%23FFFFF

2318%09%23FFFFFF,red/K08045%09%23FFFFFF,red/K08048%09%23FFFFFF,red/K04446%09%23FFFFFF,red/K21290%09%23FFFFF

5731%09%23FFFFFF,red/K05742%09%23FFFFFF,red/K04440%09%23FFFFFF,red/K19021%09%23FFFFFF,red/K04409%09%23FFFFF

8794%09%23FFFFFF,red/K13240%09%23FFFFFF,red/K04130%09%23FFFFFF,red/K04962%09%23FFFFFF,red/K04297%09%23FFFFF

4440%09%23FFFFFF,red/K00889%09%23FFFFFF,red/K17449%09%23FFFFFF,red/K04409%09%23FFFFFF,red/K19719%09%23FFFFF

6069%09%23FFFFFF,red/K05629%09%23FFFFFF,red/K05460%09%23FFFFFF,red/K04358%09%23FFFFFF,red/K05759%09%23FFFFF

4365%09%23FFFFFF,red/K04433%09%23FFFFFF,red/K03099%09%23FFFFFF,red/K05092%09%23FFFFFF,red/K08774%09%23FFFF

5521%09%23FFFFFF,red/K04964%09%23FFFFFF,red/K05107%09%23FFFFFF,red/K07365%09%23FFFFFF,red/K06766%09%23FFFF

4963%09%23FFFFFF,red/K16858%09%23FFFFFF,red/K05863%09%23FFFFFF,red/K05219%09%23FFFFFF,red/K04322%09%23FFFF

5572%09%23FFFFFF,red/K06521%09%23FFFFFF,red/K07828%09%23FFFFFF,red/K12757%09%23FFFFFF,red/K06753%09%23FFFF

4166%09%23FFFFFF,red/K16858%09%23FFFFFF,red/K00911%09%23FFFFFF,red/K05863%09%23FFFFFF,red/K01242%09%23FFFF

FF,red/K17388%09%23FFFFFF,red/K11220%09%23FFFFFF,red/K08011%09%23FFFFFF,red/K04365%09%23FFFFFF,red/K03099%09

FF,red/K04358%09%23FFFFFF,red/K17388%09%23FFFFFF,red/K05759%09%23FFFFFF,red/K04365%09%23FFFFFF,red/K05757%09

FF,red/K04446%09%23FFFFFF,red/K04579%09%23FFFFFF,red/K05850%09%23FFFFFF,red/K17388%09%23FFFFFF,red/K01115%09

FF,red/K08048%09%23FFFFFF,red/K05460%09%23FFFFFF,red/K04358%09%23FFFFFF,red/K04856%09%23FFFFFF,red/K05850%09

FF,red/K17388%09%23FFFFFF,red/K06238%09%23FFFFFF,red/K04365%09%23FFFFFF,red/K03099%09%23FFFFFF,red/K05736%09

FF,red/K04858%09%23FFFFFF,red/K04350%09%23FFFFFF,red/K04859%09%23FFFFFF,red/K17614%09%23FFFFFF,red/K05450%09

FF,red/K06753%09%23FFFFFF,red/K07827%09%23FFFFFF,red/K16351%09%23FFFFFF,red/K06845%09%23FFFFFF,red/K05108%09

FF,red/K04358%09%23FFFFFF,red/K04262%09%23FFFFFF,red/K00907%09%23FFFFFF,red/K04856%09%23FFFFFF,red/K04604%09

FF,red/K04409%09%23FFFFFF,red/K05765%09%23FFFFFF,red/K17332%09%23FFFFFF,red/K06821%09%23FFFFFF,red/K06529%09

FF,red/K04358%09%23FFFFFF,red/K07190%09%23FFFFFF,red/K04856%09%23FFFFFF,red/K05850%09%23FFFFFF,red/K23468%09

)%23FFFFFF,red/K05852%09%23FFFFFF,red/K04160%09%23FFFFFF,red/K04365%09%23FFFFFF,red/K12490%09%23FFFFFF,red/

[illegible]%23FFFFFF,red/K04462%09%23FFFFFF,red/K05460%09%23FFFFFF,red/K04422%09%23FFFFFF,red/K04409%09%23FFFFFF,red/K

04390%09%23FFFFFF,red/
