# Supplementary material for: Genetic diversity and selection of Tibetan sheep breeds revealed by whole-genome resequencing
Source: Anim Biosci. 2023 May 2;36(7):991–1002. doi: 10.5713/ab.22.0432 (PMC10330983; doi:10.5713/ab.22.0432)
Supplement: Supplementary file 17 [file ab-22-0432-Supplementary-Table-17.pdf]

Supplementary Table17. Primer information of Sanger sequencing

| Genes | Chr | position | Reference allele | Alternative allele | Mutation | Sequences(5' - 3') |
|---|---|---|---|---|---|---|
| RXFP2 | ch10 | 29481646 | A | G | exon10:c.A49G:p.A16A | F:ATTGTGAATTAACTGCAGGG |
| | | | | | | R: ACACCGTCCCTAAAGTGG |
| | ch10 | 29469024 | T | C | exon17:c.T25C:p.P9P | F:GACAGTTTACGCTAGAGCATC |
| | | | | | | R:GATCGACAAAGAAAACAATG |
| | ch10 | 29462010 | C | T | exon17:c.C144T:p.V48M | F:GTATTCTGGCCTCGTCAAAC |
| | | | | | | R:GCTGGCTTTCCTCATCATTG |
| | ch10 | 29461968 | C | T | exon17:c.C186T:p.E62K | F:GTATTCTGGCCTCGTCAAAC |
| | | | | | | R:GCTGGCTTTCCTCATCATTG |

| Fragment size (bp) |
|---|
| 157 |
| |
| 167 |
| |
| 309 |
| |
| 309 |
| |
