# Supplementary material for: Genetic diversity and selection of Tibetan sheep breeds revealed by whole-genome resequencing
Source: Anim Biosci. 2023 May 2;36(7):991–1002. doi: 10.5713/ab.22.0432 (PMC10330983; doi:10.5713/ab.22.0432)
Supplement: Supplementary file 18 [file ab-22-0432-Supplementary-Table-18.pdf]

Supplementary Table18. Mutations in exons of gene

Gene Name	SNPs	Mutation type	Transcript ID	Mutation	Chr	position	Reference allele
RXFP2	RXFP2-CDS09_SNP-49	synonymous variant	ENSOARE00000113128	exon9:c.A49G:p.A16A	chr10	29481646	A
RXFP2	RXFP2-CDS14_SNP-25	synonymous variant	ENSOARE00000113156	exon17:c.T25C:p.P9P	chr10	29469024	T
RXFP2	RXFP2-CDS17_SNP-144	missense variant	ENSOARE00000113166	exon17:c.C144T:p.V48M	chr10	29462010	C
RXFP2	RXFP2-CDS17_SNP-186	missense variant	ENSOARE00000113166	exon17:c.C186T:p.E62K	chr10	29461968	C

Alternative allele
G
C
T
T
