# Supplementary figures and images for: Genetic diversity and selection of Tibetan sheep breeds revealed by whole-genome resequencing
Source: Anim Biosci. 2023 May 2;36(7):991–1002. doi: 10.5713/ab.22.0432 (PMC10330983; doi:10.5713/ab.22.0432)

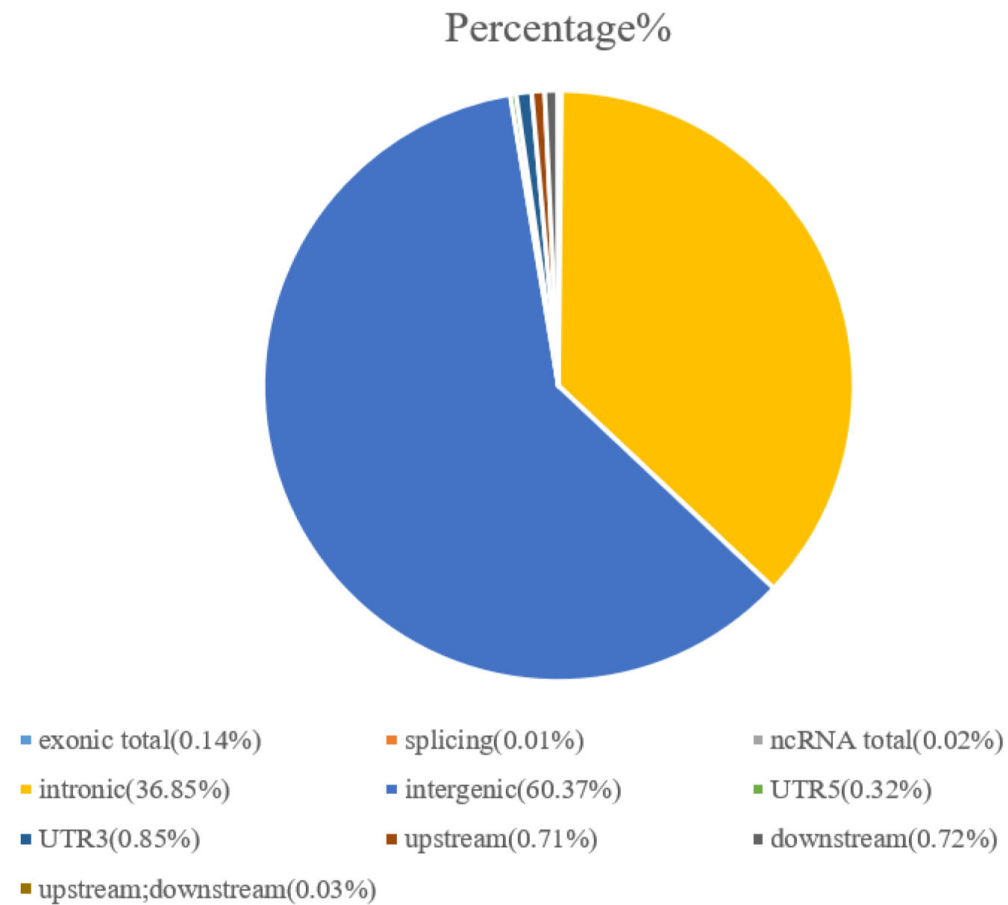

**Fig.s1** The pie plot shows annotated SNPs.

Supplement: Supplementary file 19 [file ab-22-0432-Supplementary-Fig-1.pdf]

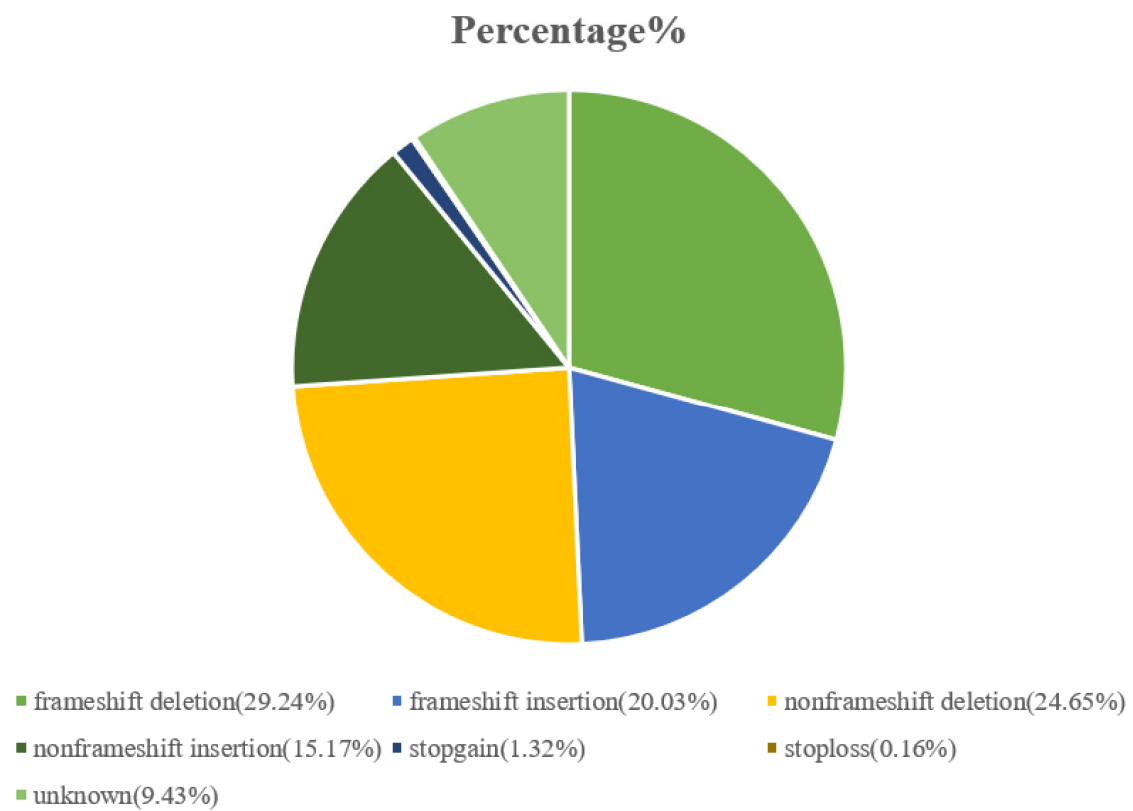

**Fig.s2** The pie plot shows annotated SNPs at exonic regions.

Supplement: Supplementary file 20 [file ab-22-0432-Supplementary-Fig-2.pdf]

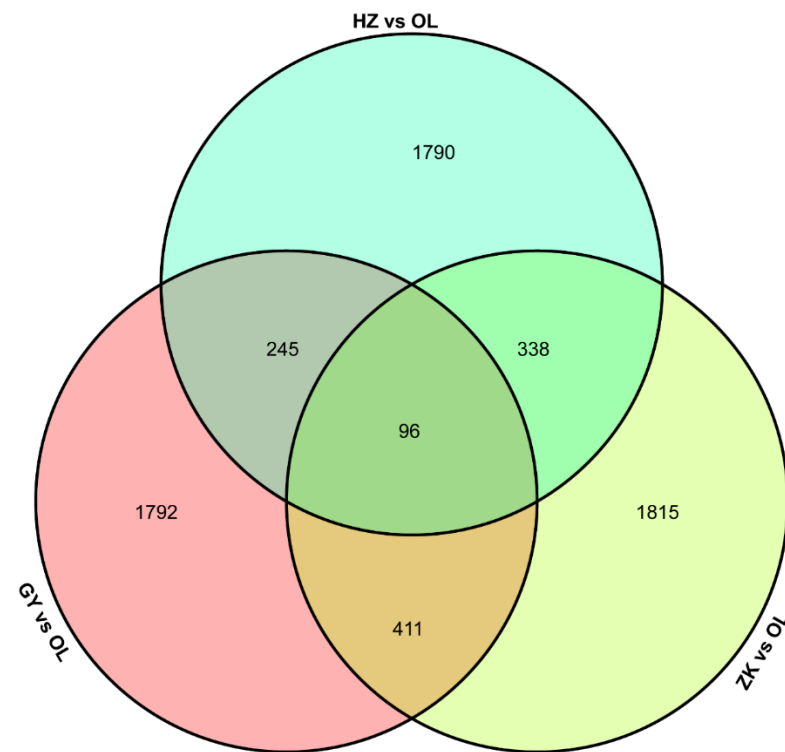

**Fig.s3** Venn diagrams of common selected regions for wool traits.

Supplement: Supplementary file 21 [file ab-22-0432-Supplementary-Fig-3.pdf]

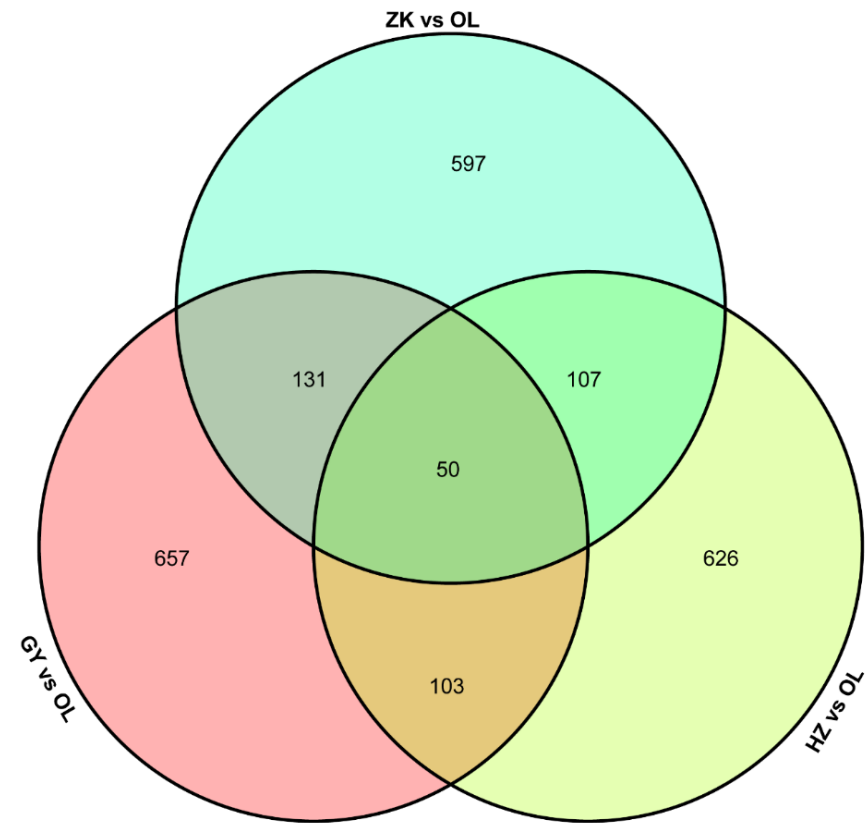

**Fig.s4** Venn diagrams of common selected genes for wool traits.

Supplement: Supplementary file 22 [file ab-22-0432-Supplementary-Fig-4.pdf]

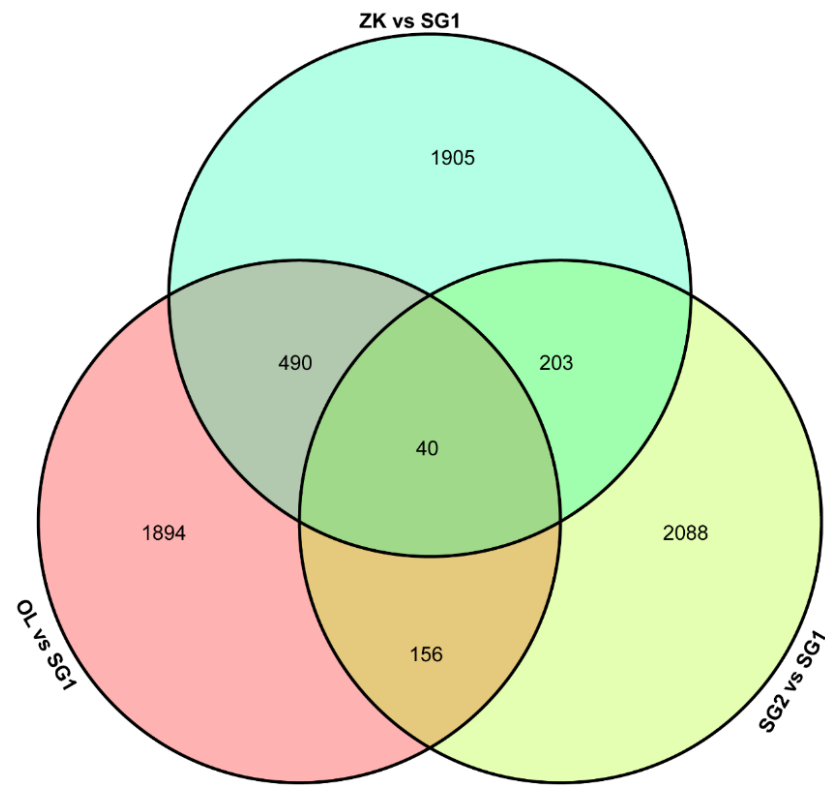

**Fig.s5** Venn diagrams of common selected regions for horn phenotypes.

Supplement: Supplementary file 23 [file ab-22-0432-Supplementary-Fig-5.pdf]

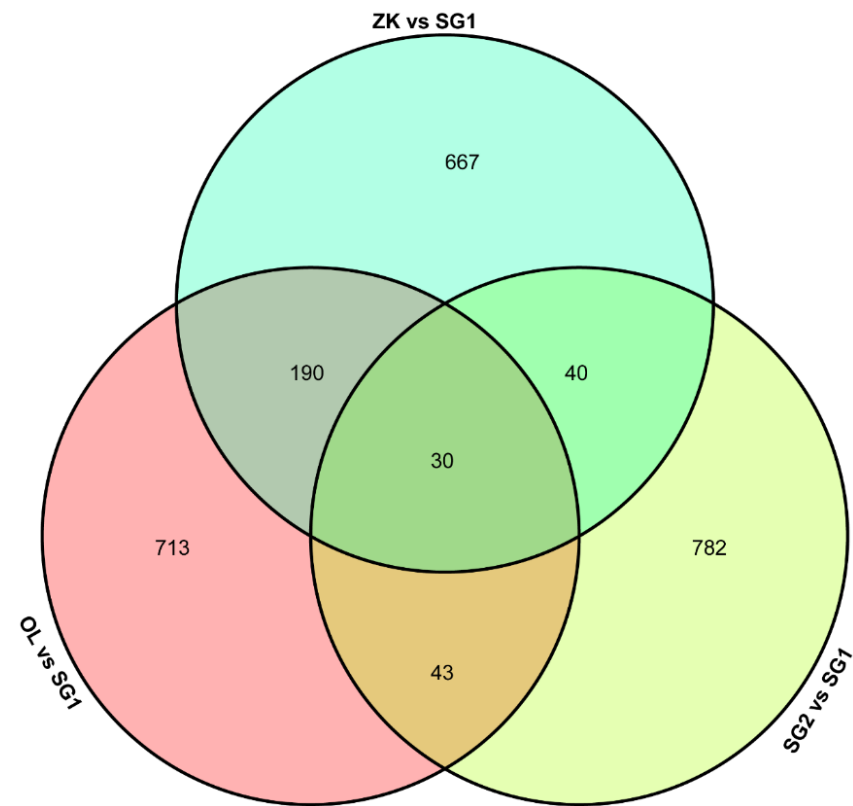

**Fig.s6** Venn diagrams of common selected genes for horn phenotypes.

Supplement: Supplementary file 24 [file ab-22-0432-Supplementary-Fig-6.pdf]
